# Inflation vs. Exhaustion of Antiviral CD8+ T-Cell Populations in Persistent Infections: Two Sides of the Same Coin?

**DOI:** 10.3389/fimmu.2019.00197

**Published:** 2019-03-06

**Authors:** Emanuele Marchi, Lian Ni Lee, Paul Klenerman

**Affiliations:** ^1^Peter Medawar Building for Pathogen Research, Nuffield Department of Medicine, University of Oxford, Oxford, United Kingdom; ^2^Translational Gastroenterology Unit, John Radcliffe Hospital, Oxford, United Kingdom

**Keywords:** exhaustion, inflation, bioinformatics, LCMV (lymphocytic choriomeningitis virus), CMV (cytomegalovirus)

## Abstract

Persistent virus infection can drive CD8+ T-cell responses which are markedly divergent in terms of frequency, phenotype, function, and distribution. On the one hand viruses such as Lymphocytic Choriomeningitis Virus (LCMV) Clone 13 can drive T-cell “exhaustion”, associated with upregulation of checkpoint molecules, loss of effector functions, and diminished control of viral replication. On the other, low-level persistence of viruses such as Cytomegalovirus and Adenoviral vaccines can drive memory “inflation,” associated with sustained populations of CD8+ T-cells over time, with maintained effector functions and a distinct phenotype. Underpinning these divergent memory pools are distinct transcriptional patterns—we aimed to compare these to explore the regulation of CD8+ T-cell memory against persistent viruses at the level of molecular networks and address whether dysregulation of specific modules may account for the phenotype observed. By exploring in parallel and also merging existing datasets derived from different investigators we attempted to develop a combined model of inflation vs. exhaustion and investigate the gene expression networks that are shared in these memory pools. In such comparisons, co-ordination of a critical module of genes driven by Tbx21 is markedly different between the two memory types. These exploratory data highlight both the molecular similarities as well as the differences between inflation and exhaustion and we hypothesize that co-ordinated regulation of a key genetic module may underpin the markedly different resultant functions and phenotypes *in vivo*—an idea which could be tested directly in future experiments.

## Introduction

CD8+ T cell responses play a critical role in control of many virus infections. In the case of Lymphocytic Choriomeningitis (LCMV) it has been extensively modeled in the mouse and the dynamics, specificity, and function of antiviral CD8+ T cell responses are well-understood. One feature of this infection is the development of CD8+ T cell exhaustion, a feature first described in the pre-tetramer era as loss of function and finally deletion in the presence of persisting viruses such as DOCILE strains (1), and subsequently investigated at a molecular level using LCMV Clone 13, which shows similar features (2). Mapping the transcriptional underpinning of CD8+ T cell exhaustion was crucial in defining its mechanisms—key of which include expression of checkpoint molecules such as PD-1 (3). These discoveries have been very influential in understanding both antiviral and also anti-cancer responses and have driven the development of new checkpoint blockade therapies.

In contrast, persistent infection with cytomegaloviruses (CMVs)—human and murine CMV (MCMV)—is linked with the development of memory “inflation” (4). This is marked by the late expansion and maintenance of a number of CD8+ T cell pools directed at a subset of peptides (5). Their phenotype lacks the expression of checkpoint molecules, rather showing acquisition of markers of cellular differentiation over time. Importantly, in contrast to the exhausted phenotype, these cells retain strong effector functions. The transcriptional underpinning of this—and of the related model of memory inflation driven by adenoviral vectors (6)—has also been explored, and this is also highly distinct from the development of conventional “central” long-lived memory cells (7).

Both models are associated with viral persistence—high level viremia in the case of LCMV, and very low level local reactivation in the case of MCMV. How is the development of these two apparently very distinct forms of CD8+ T cell memory driven? To address this we aimed to compare the gene expression profiles and gene networks in these different settings. Such datasets are complex to generate and require highly reproducible and well-established models, coupled with adequate T cell numbers specific for individual epitopes. Thus, currently such data are valuable and remain an important resource to explore. Typically, even with newer techniques such as RNA-Seq, such studies only focus on immune responses to a single pathogen rather than comparing diverse pathogens.

To achieve the comparison we sought, distinct datasets generated from different platforms by two different laboratories were merged, and an integrated model created and subjected to validation. We explored whether two data sets generated from comparable experimental designs were sufficiently suitable to be merged, despite differences in array platforms, mouse suppliers and viruses, and whether this could provide some new insights into the relationship between these different T cell populations.

**Figure 1:**
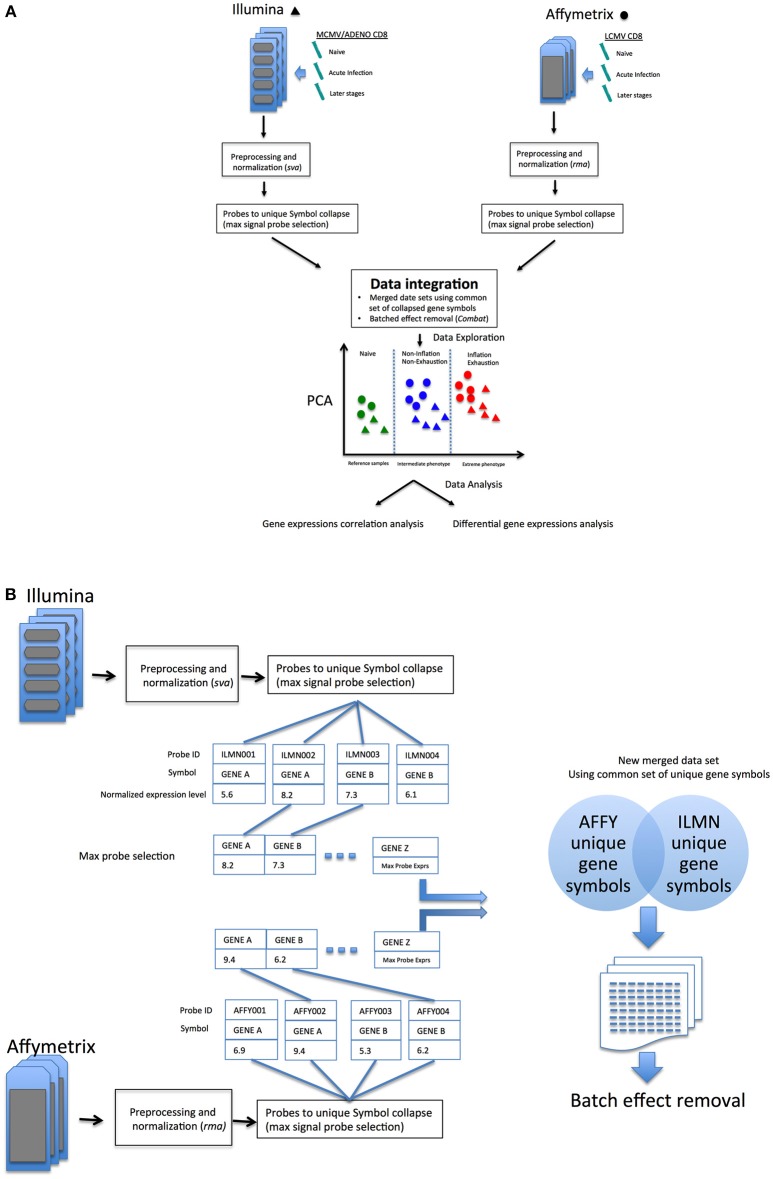
**(A)** Overview of the data integration process and analysis. **(B)** Overview of probes-to-gene collapse method and data sets merge.

As outlined in Figure 1A, data sets comparing memory Inflation against conventional memory (7) and CD8 T cell Exhaustion vs. conventional memory (8) studies include comparable or analogous samples in which CD8 transcriptomics from early and later stages of viral infections where referred to Naïve cells. In our model, samples from these experiments were expected to be assimilated to three broad categories, the *common reference* samples (Naïve CD8 T cells, the most comparable expression profiles), the *intermediate phenotype* (CD8 T cells from not Inflating or Exhausted samples) and an *Extreme phenotype* (CD8 T cells Inflating or Exhausted, hence expected to diverge significantly from the rest of samples and hypothetically from each other. The reliability of integration could be evaluated from the expected distribution of expression profiles in exploratory analysis (i.e. principal component analysis, PCA) and further data mining could explore the validity of the model and hypotheses generated (Figure 1A).

Via such an integrative and comparative bioinformatics analysis of expression profiles from inflation and exhaustion murine models, we describe similarities and dissimilarities of the phenomena at a transcriptomic level. We observe some predicted and also some unexpected features, with some possible practical implications for future experimental design. The results presented here explore the use of such a workflow as one approach to integrate valuable existing datasets from different platforms. While data from such *in silico* comparisons cannot reach the quality and accuracy of those derived from a single unified *in vivo* experiment, there is an opportunity that existing, publicly available data can be studied further in order to address questions not originally anticipated at the time datasets were generated, and to help develop new ideas for the field.

## Results and Discussion

### PCA of Expression Data From *Inflation* and *Exhaustion* Models Reveals Comparable Events Between the Phenomena

We first addressed the overall transcriptomic similarities between memory inflation and exhaustion by re-exploring a previously generated dataset, GSE73314 (7). This dataset was derived from mice infected with MCMV, tracking one inflationary response, M38, and one conventional memory response (M45) at both early (acute, d7) and late (memory, d50) timepoints.

**Figure 2:**
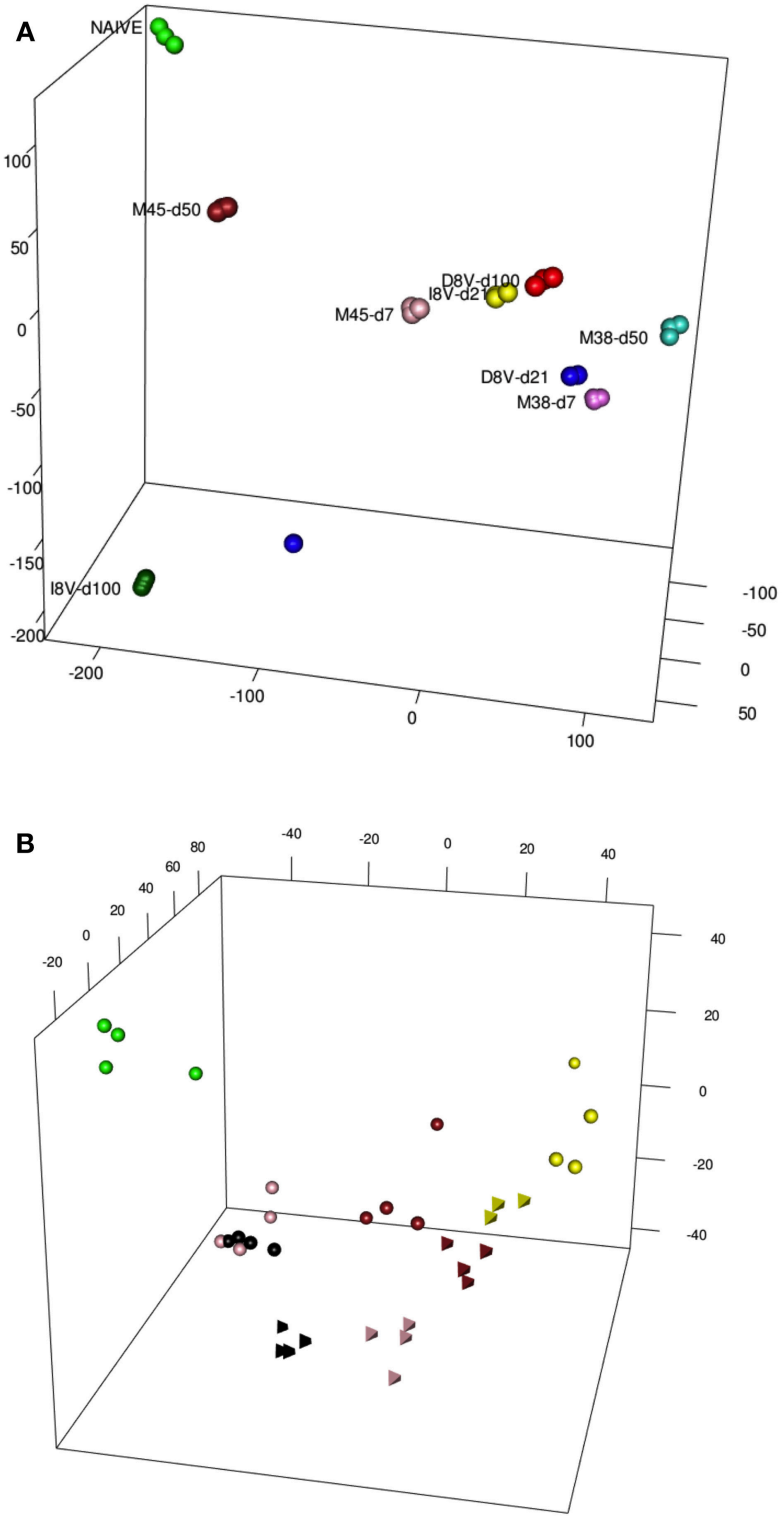
**(A)** A PCA of Inflating/non-Inflating CD8 T cells. 3D PCA showing distribution of transcription profiles of two independent models of Inflating samples (M38, D8V) and non-Inflating Samples (M45,I8V), at acute stages (days 7 or 21) and later stages (days 50 or 100), and naive samples. **(B)** PCA of Exhausted/non-Exhausted CD8 T cells. 3D PCA showing distribution of transcription profiles of a model of Exhaustion (Cl13,Tetrahedrons), with non-Exhaustive samples (Arm, spheres) at different stages, and naive samples. Stages: 6 days (yellow), 8 days (brown), 15 days (pink), 30 days (black), naive (green).

Antigen-specific T cell populations were FACS-sorted following tetramer staining and gene expression analyzed using microarray. In parallel inflationary and conventional responses to beta-galactosidase expressed in a replication-deficient human Adenovirus serotype 5 construct (HuAd5-lacZ) were studied following HuAd5-lacZ immunization. Responses to the inflationary epitope βgal_96_ (referred to as D8V in this paper) and the conventional epitope βgal_497_ (referred to as I8V in this paper) were analyzed again at the peak acute (d21) and late memory (d100) time points. The analysis of this dataset has been previously described, including principal components analysis based on all informative genes or subsets of transcription factors, and importantly the close relationships between these expression profiles and those of human “inflationary” populations derived from studies of CMV were confirmed (7). Data from Figure 2A from the paper are reproduced here [previously published as Figure S4 from (7)] which depicts the first 3 principal components of these data prior to addition of the integrated exhaustion dataset. A clustering of data derived from the acute timepoint for the relevant tetramers [D8V d21 (blue), I8V d21 (yellow), M38 d7 (magenta), and M45 d7 (pink)] is observed. However, with reference to the naive (green) dataset, the inflationary populations at the late timepoints [D8V d100 (red), M38 d50 (turquoise)] sit slightly further segregated than the acute samples, while the conventional memory pools at the late timepoints (M45 d50) have shifted in the opposite direction, with some re-expression of profiles (M45 d50) linked to resting naive cells. I8V at d100 are slightly divergent from M45 at d50, possibly reflecting subtle intrinsic differences (e.g., tissue tropism, antigen levels during infection) between the two infection models, especially as MCMV is a replicating virus with periodic episodes of viral reactivation while AdHu5 is a non-replicating vector which does not reactivate. Nonetheless the expression of both groups of conventional memory cells at the early phase of infection (M45 d7 and I8V d21) show reduced divergence, suggestive of a conserved response between acute and conventional cells at the molecular level.

The same type of descriptive analysis was next performed on the exhaustion data set, GSE41867 (8). The data points were derived from acutely-resolved lymphocytic choriomeningitis (LCMV) infection (Arm) or chronic LCMV infection (Arm Cl13) groups at days 6, 8, 15, and 30 post-infection and were generated on the Affymatrix platform. In this study, gene-expression profiles of exhausted CD8+ T-cells from mice infected with chronic LCMV (Arm C13) were compared with functional CD8+ cells, from mice infected with the Armstrong strain of the virus. The analysis revealed a comparable layout of sample distribution (Figure 2B), with groups' relative distances denoting a good concordance with the experimental outcomes. Dysfunctional clone-13 specific CD8+ T-cells (tetrahedron) (15 and 30 days, pink and black, respectively), appear to drift away at late time points and do not cluster around conventional CD8+ T-cells (spheres, pink, and black). In contrast, as was previously observed in the inflation model, the gene expression profiles of cells at the early timepoints [day 6 (pink) and 8 (brown) post-infection] from acutely resolving (spheres) and chronic LCMV (tetrahedron) are seen to cluster together.

Data set integration, when it is possible and successfully achieved, should allow more direct comparisons between samples generated in independent experiments. An alternative perspective of the differences or similarities between samples, without redesigning an entirely new experiment (which could become quite large, complex and very costly) could allow researchers to investigate new hypotheses and improve experimental design. In an attempt to improve the comparative analysis of the two models, the two data sets were merged (schematic in Figure 1A), following a pipeline aimed to reduce batch effects from multiple sources (see methods) as much as possible, which would confound accurate detection of gene expression signals (Schematic in Figure 1B).

**Figure 3:**
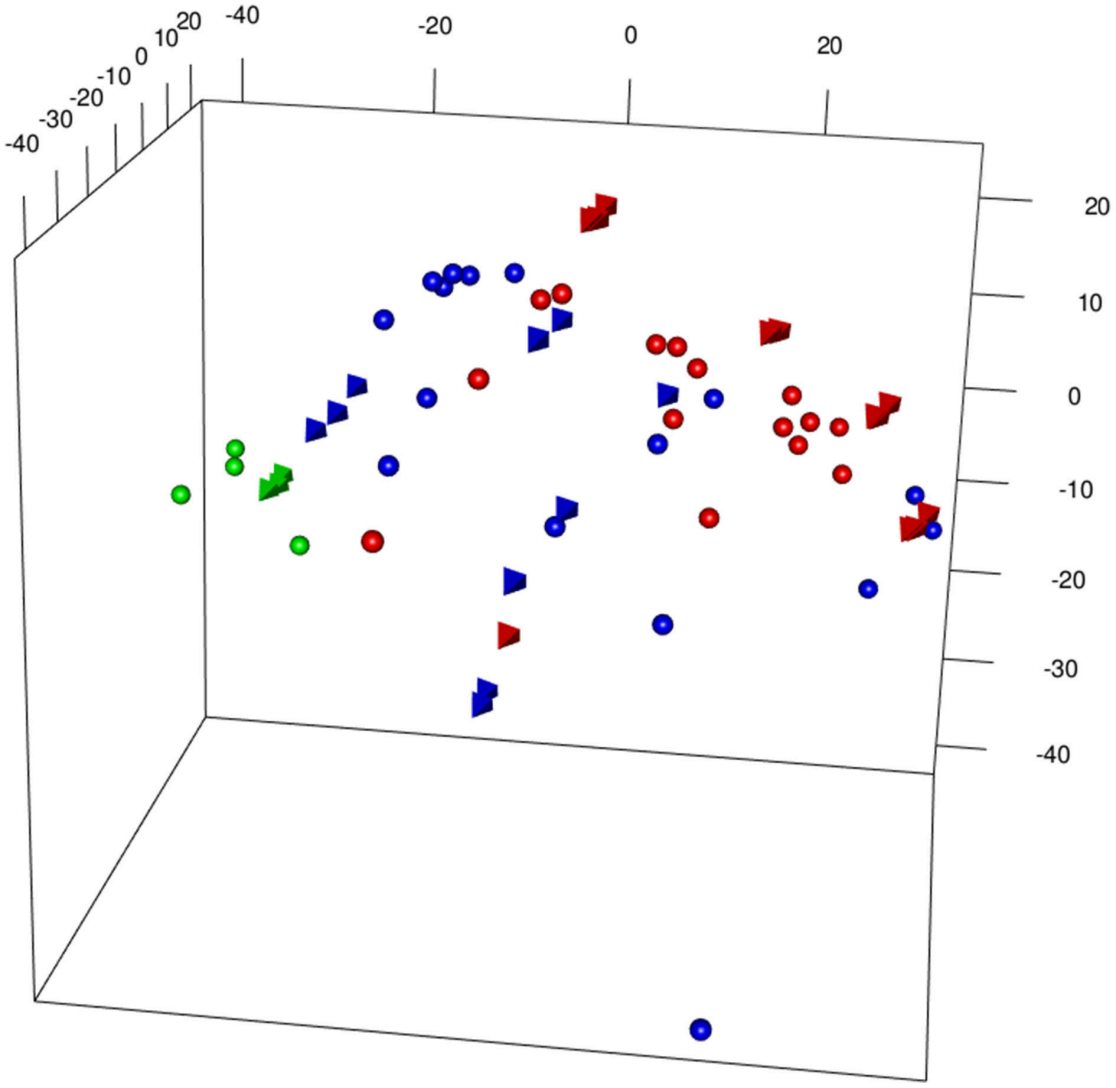
3DPCA of merged samples from Inflating and Exhausted models' data sets following batch effects removal between different microarray platforms (spheres: Affymetrix, Exhaustion study, tetrahedron: Illumina, Inflation study). The plot is showing overall distribution of Naive (green), Non-Inflating and Non-exhausting (blue), and Inflating and Exhausting (red) samples using the total common set of expressed genes (~14,000).

Figure 3 shows a 3D PCA of the two merged data sets [from Bolinger et al. (7), (B) and Doering et al. (8) (D)], generated with different microarray platform (Illumina and Affymetrix), plotting together Inflating and Exhausting samples (in red) with the respective functional counterparts (in blue). Projections of the first three principal components capture most of the variability (>50%) of the total set of common expressed genes between the two platforms (~14,000). The results are also shown as a dendogram in Figure S1A. In order to check the robustness of the results, 5 outliers were removed and the merged dataset reprocessed (without extra normalization after ComBat processing) and the most variable genes were selected, with genes filtered by variance (interquartile range (IQR) > 0.5, *n* = 1,660) (9). As shown in Figures S1A–C, the sample distribution is in good agreement with the original 3D PCA (Figure 3).

The appropriateness of the data integration is highlighted by the fact that naive samples [in green, spheres: Affymetrix, (8); tetrahedron: Illumina, (7)], which are the most comparable samples between the two data sets cluster in close proximity to each other. Interestingly, late timepoint Inflation and Exhausted samples (in red), other than having the tendency to cluster relatively close to each other, diverge at most from naive and non-Inflation and non-Exhausted samples (in blue), which instead occupy an intermediate position in the overall plot (Figure 3). In accordance with their extremely differentiated phenotype, Inflating samples are placed at the furthest distance from naive samples, immediately followed by the exhausted cells.

Batch effects may still play a role in this type of data analysis, and this was investigated by *pvca* (10). Prior to ComBat processing, “platform” factor (Affymetrix, Illumina) was assessed as the major source of batch effect; following batch effect removal the “State” factor (Naive, Resolving, Inflating_Exhausting, as batch describing three simplified categories of samples: reference sample, intermediate phenotype, and extreme phenotype) represent the major source of variability (Figure S7). This initial, descriptive statistical approach suggests that immune responses demonstrating Inflation and Exhaustion share some common features at molecular level—despite the divergence that is observed at phenotypic level.

It is reasonable to postulate the existence of a common set of genes behind these immune responses; a pathway, in which the behavior and the intrinsic dynamics of its components could determine the fate of T-cells toward either Inflation or Exhaustion.

### Weighted Gene Co-expression Network Analysis of Inflating Samples

In order to test the hypothesis of a shared set of genes behind opposite immune responses, yet highly related at the origin, we performed Weighted Gene Co-Expression Network Analysis [WGCNA, (11)] on the Inflation data set only (i.e., without any potential artifacts generated through merging), prior filtering the genes by variance (*n* = 2,231), with the intention of detecting a module of genes characteristic of the phenomenon.

**Figure 4:**
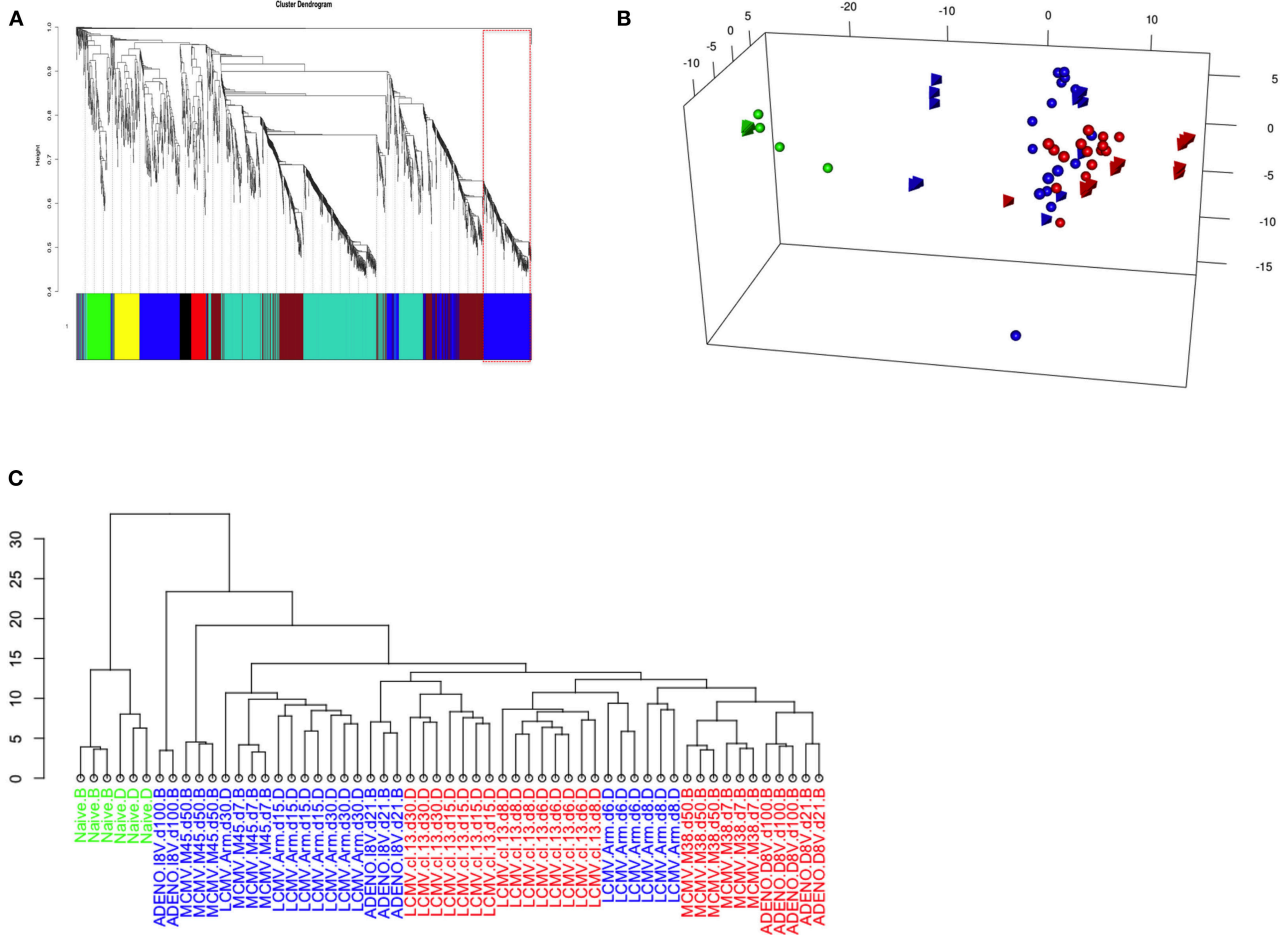
**(A)** Weighted Gene Co-expression Network Analysis of Inflating samples. Gene co-expression network analysis detected 6 gene modules (merging distance = 0.25, soft-thresholding power β = 9); Blue module (highlighted) genes are enriched with immune relevant GO categories (Table S3A) and contains relevant genes such as Tbx21, Eomes, Zeb2, and E2f2 (Table S5A). **(B)** PCA of Inflating/Exhausted samples based on Blue module genes. PCA plot using the first three principal components and based on a gene set of 588 genes, detected as blue module in Gene co-expression network analysis of Inflating samples only (Figure 3A). The plot shows distribution of Naïve (green), Non-inflating and Non-exhausting (blue), and Inflating and Exhausting (red) samples (spheres: Exhaustion study; tetrahedron: Inflation study). **(C)** Hierarchical clustering of Inflating/Exhausted samples based on Blue module genes. Dendogram plot showing sample clustering analysis (Euclidian distance) on Inflating-Exhausted merged sets, based on a gene set of 469 genes, detected as blue module in a repeated Gene co-expression network analysis of Inflating samples after removing outliers (Soft-thresholding power β = 20).

The dendogram in Figure 4A shows gene hierarchical clustering highlighting modules of genes with high interconnectivity based on TOM similarity (Topological Overlap Measure, a robust measure of network proximity). The second largest detected module (blue) was found enriched with immune relevant genes, after checking for the most statistically represented GO category (*GOenrichmentAnalysis* in WGCNA package, Tables S1A,B). Reactome pathway enrichment analysis (12) confirmed the overrepresentation of immune relevant pathways within blue module (Figure S2A). Genes such as Tbx21 and Eomes, known to have pivotal roles in T-cell differentiation, are contained in the blue module, along with other genes, such as E2f2, involved in cell proliferation and consistent with the Inflation phenotype.

We therefore repeated PCA on the whole integrated data set, using the 588 genes assigned to the blue module, to test if sample distribution in respect to these genes was unaltered, improved or changed (Figure 4B); it was observed, in fact, that the sample layout was conserved: independent naive samples (in green, spheres, and tetrahedrons), that could function as calibrating samples, were clustered even closer, while inflating samples (red, tetrahedrons) were separated further compared to the PCA plot using the whole gene set (Figure 3). In general, the rest of Inflating and Exhausted samples appear to cluster tighter in the context of this subset of genes (blue module genes).

It is important to note that the same analysis performed using other gene modules, e.g. the turquoise module, can disrupt the PCA sample layout we observe using all genes (or blue module genes) and it generates a less informative PCA plot or hierarchical clustering dendogram. The clustering analysis using genes from the Turquoise module (not enriched in immune relevant GO terms, Tables S1A,B or Reactome pathways, Figure S2B) produced a sample layout (Figure S3) that is noticeably different from the layout produced when employing blue module genes. Module detection, clustering analysis, GO terms and Reactome pathway enrichment (Figures S2A,B) were consistent after removing outliers and changing parameters to fit best a scale free topology (more appropriate soft-thresholding power, β = 20). The equivalent approach of hierarchical sample clustering analysis, employing again blue module genes, shows clearly that Inflating and Exhausted samples cluster together (in red), along with recently activated samples at acute phases from both data sets (Figure 4C).

### Graphical Representation of Gene Networks Obtained in Inflating and Exhausted Data Sets

**Figure 5:**
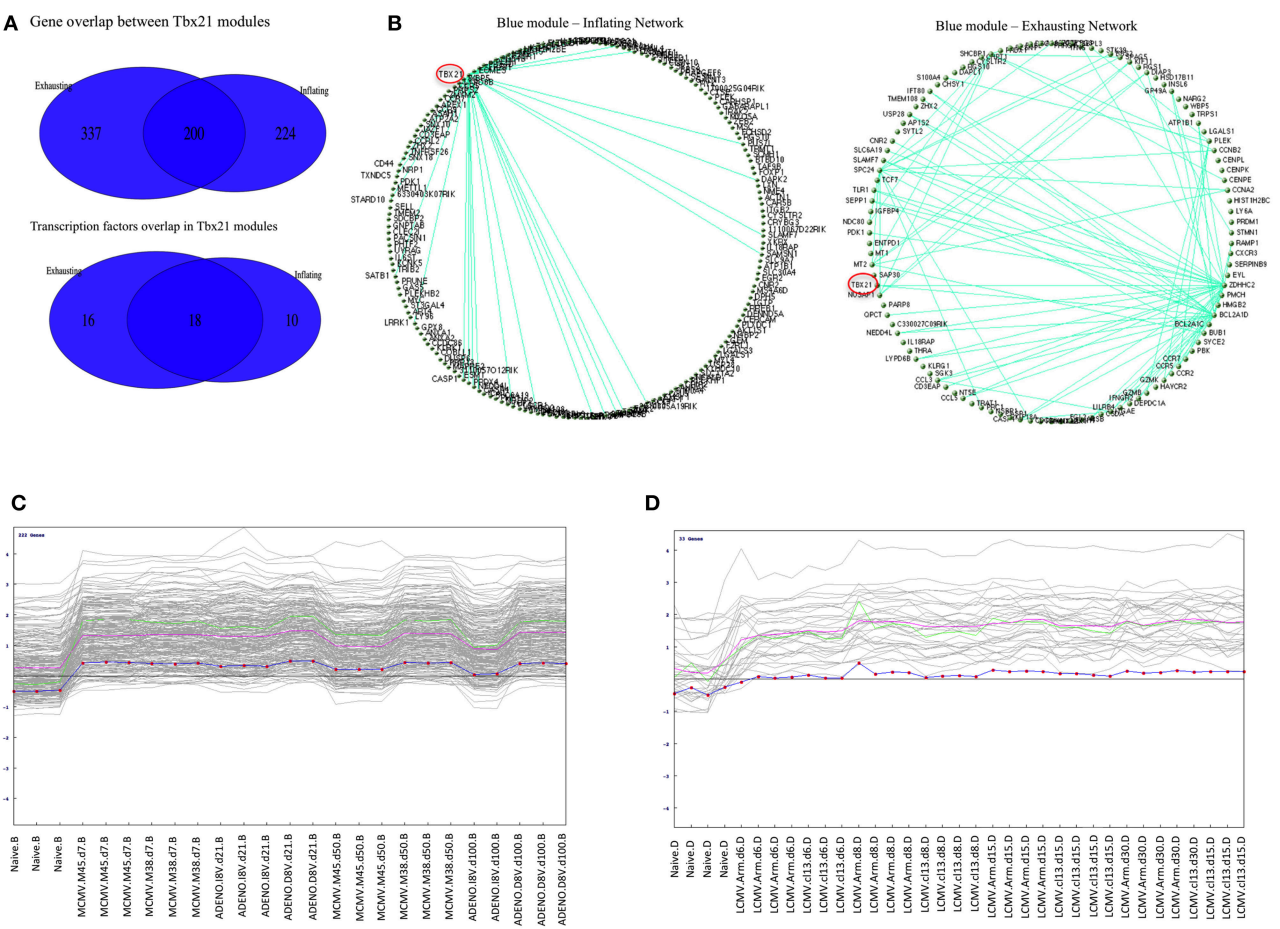
**(A)** Overlap between Inflation and Exhausted gene modules. Venn diagrams showing gene content overlap between modules containing Tbx21 detected when WGCNA was performed independently on Inflating and Exhausted data sets. **(B)** Graphical representation of Blue modules networks. Circular topology imposed to blue modules (containing Tbx21) detected in Inflating and Exhausting data sets visualize nodes (genes) with highest connectivity. Only edges with a *weight* >0.41 (for Inflating) and >54 were plotted. Tbx21 (highlighted in red) is the main hub in Inflating blue module, being the gene with the highest number of connection with other genes. In the Exhausted blue module, although highly expressed, Tbx21 loses the property of being the only hub. **(C)** Pavlidis Template test in Inflating data Set. Tbx21 used as template for detecting genes with matching expression trajectory across time points. In the Inflating data set 222 genes match the Tbx21 expression pattern (*R* > 0.8); The green line is the template (Tbx21), the violet line represents average expression, and the dotted line is the centralized (*z*-score) template expression. **(D)** Pavlidis Template test in Exhausted data Set. Tbx21 used as template for a method designed to retrieve other genes with a similar expression trajectory across time points. In Exhausting data set 33 genes match Tbx21 expression pattern (*R* > 0.8); The red line is the template (Tbx21), while the violet line represents average expression, and the dotted line is the centralized (*z*-score) template expression.

A separate WCGNA was executed on Exhaustion data set, matching the criteria utilized for the Inflation network inference, and similarly a module containing Tbx21 was detected with a gene composition significantly enriched in immunological pathways. Indeed, there is a considerable gene overlap between the two modules that is even more evident within the context of transcription factor genes where the overlap reaches the 41% of the features (Figure 5A).

To have a further insight in the two related Tbx21 modules a graphical representation of the nodes connectivity was produced. A circular topology was imposed in both gene modules to make the networks comparable and observe whether the gene hubs (nodes with the highest connectivity) were conserved or changed. Edges between genes reflect their direct correlations and a similar correlation with “third party” genes, measured in the TOM matrix.

We discovered that in the module network generated using the Inflation data set, Tbx21 appeared to be a dominant hub, connecting with the vast majority of genes in the module, emphasized clearly when only edges with a *weight* > 0.41 were plotted (Figure 5B). Tbx21 has been previously described to be pivotal in controlling CD8 T cell activation (13) and this finding would extend the role of Tbx21 in maintaining functionality and effector status of the T cells in a subset of long-term memory populations.

In contrast, in the corresponding graphical representation of the analogous module in the *Exhaustion* data set, Tbx21 does not appear anymore to be the unique hub, losing its central role. Even lowering the cut off (*weight* < 0.54) of plotted edges shows evidently that other genes are more likely to function as hubs (Figure 5B). The Tables S2A,C matrices represent the TOMs (topological overlay measures) used as input to visualize blue (Tbx21) and turquoise modules networks in Cytoscape and imposing a circular topology (Figure 5B). In order to corroborate this finding, we analyzed the data with an alternative approach, employing a method that searches for genes following a similar trajectory to Tbx21 across the time points of the two experiments (Figures 5C,D). We applied the Pavlidis template matching test (14, 15) using Tbx21 as template, equally for Inflating and Exhausted modules. Consistent with network analysis we observe that in Exhaustion data there are only 33 genes that match significantly the expression trajectory of Tbx21 (Figure 5D), while in *Inflating* data module over 200 genes display expression patterns strongly matching the one of Tbx21 (Figure 5C).

In order to further explore the hypothesis that Tbx21 is the main master regulator driving the inflation and directly interacting with the genes in its module, we analyzed ChIP-Seq data publicly available (16) where antibodies against Tbx21 where used to analyze transcription factor-DNA binding in Cytotoxic T Lymphocytes from mice infected with LCMV (P14 mice). A Tbx21 binding profile was obtained running the *macs2* peak caller algorithm (17) on wild type sample (SRR2075567, Tbx21 ChIP-seq on effector P14 CD8+ T cells) and using an input control (SRR2075584) (*q* < 0.01). Following gene annotation of peaks region [Homer bioinformatics tools, homer.ucsd.edu/homer, (18)], 383 out of 588 genes in Tbx21 module obtained with Inflating samples were found with binding sites for Tbx21 (in proximity, < 1 kbp); 414 genes if we include peaks within a distance of 2 kbp. A Fisher test was performed to demonstrate that Tbx21 module was significantly enriched with peaks: highly significant peaks (macs2 *peak score* >50) mapping to blue module (63/588) were significantly more enriched (*p* = 0.0003) than turquoise module peaks (44/805).

We can formulate the hypothesis that in the case of an Inflationary response there is the presence of a “homogenous” genetic program, mainly coordinated by the transcription factor Tbx21. We postulated that a significant difference between Inflating and Exhausted populations could be more evident from differential correlations in expression between genes, rather than absolute changes in expression levels of the same genes between models. The strongest correlations that Tbx21 shows with its putative target genes in the Inflation data, highlight its fully functional role as a master regulator, while in the Exhaustion data its task could be mitigated or disrupted by other coexisting transcriptional pathways. In order to elucidate the strongest level of correlation between Tbx21 and genes within its module, it could be informative to examine the expression levels across the time points of Tbx21 and one representative gene. E2f2 is a transcription factor involved in cell growth and proliferation. The relationship between Tbx21 and E2f2 was analyzed separately in the Exhaustion and Inflation datasets (Figure S4). In the Inflation time course, E2f2 shows an expression pattern that matches well to that of Tbx21, especially at later stages of infection (beyond the dashed line), while in the Exhaustion time series the pattern of expression is poorly correlated.

In support of this observation, inflating samples at the late timepoints are enriched with Reactome pathways involved in cell division and proliferation. The late timepoints of the inflation and exhausted samples within the integrated dataset were checked for enrichment of 674 curated Reactome gene sets [MSigDB Collections, (19) c2.cp.reactome.v6.2.symbols.gmt]. Direct comparison between inflated (M38, day 50) vs. exhausted cell populations (Arm Cl13 day 30) was analyzed by GSEA [Gene Set Enrichment Analysis, (20)] and it was noted that the top most enriched gene sets in Inflation are homogenous and belong to categories of cell division and DNA replication (Table S3, Figure S5). Conversely, the most significant Reactome pathways enriched in exhausted samples are quite heterogeneous (Table S4, Figure S4B). Repeating this analysis with inflating cells from the Adenovirus model (Day 50) vs. exhausted samples (Arm Cl13 day 30) yielded consistent results in spite of the presence of a clear outlier in the inflating samples (S11 D8V), which would have reduced the statistical power (data not shown). Taken together, these findings lend credence to the idea that a key difference between inflation and exhausted cells at the later timepoints is that regulation of the former's proliferative capacities is retained, akin to those of activated and highly proliferating effector and memory CD8 T cells after acute Arm infection (8).

Finally, we further explored the new potential insights gained from data integration using GSEA of Immunological signatures from MsigDB (4,872 gene sets, c7.all.v6.2.symbols.gmt). In the same comparison, between Inflation vs. Exhaustion at late timepoints (MCMV M38 Days 50 vs. LCMV Cl13 days 30); significantly enriched gene sets either in Inflation or Exhaustion populations (FDR < 0.25, based on gene sets permutations) from comparable studies were found to be biologically interpretable. Data and relevant examples consistent with experimental data (21) are shown in Table S5 and Figure S6.

## Conclusions

This analysis of previously published transcriptional data was aimed to address a simple but important question: what is the relationship between memory inflation and immune exhaustion? Both types of memory are distinct from conventional central memory development where populations contract following an acute expansion. In the one case the cells remain functional (inflation) and in the other they lose function (exhaustion)—associated with distinct phenotypes. We hypothesized that dysregulation of a key module of genes might account for the phenotypic and functional differences seen between these T cell types.

Although it is possible to address this question using parallel analyses of existing data -and we have done this here—there is additional power in the merging of datasets, although great care must be taken to avoid artifacts due to batch and platform effects. Clearly a repeat set of experiments with mice treated with the different infections and vaccines in parallel, with a conserved pipeline for gene expression measurement and downstream data analysis would be ideal, but also impractical and costly given constraints on animal models and animal welfare in different settings (for example in the UK, LCMV Arm Cl13 remains a biosafety group 3 pathogen). We therefore think approaches of data integration—using a range of appropriate tools—from the increasing number of datasets publicly available will have important potential for future studies. Indeed this potential should only increase over time with convergence of sequencing approaches toward high-throughput RNA-Seq methods and accrual of datasets.

We observed two features of note from this analysis. Firstly, using a merged dataset, some transcriptional features of exhaustion and inflation appear to be shared, and cluster broadly closer to those derived from responses analyzed at an acute timepoint than to naive T cells or conventional memory. This is perhaps not a surprise since both inflationary and exhaustive memory are dependent on persistent/repetitive antigen stimulation. While the nature and/or the intensity of this antigen re-encounter may differ between the settings, in both cases, TCR triggering occurs and it is the response to this triggering which distinguishes the populations. While the populations clearly differ in expression of inhibitory receptors such as PD-1 and TIM-3, these represent only a relatively small set of genes within the total number up- and down-regulated in these populations compared to naive cells—and may overall contribute little to the PCA and hierarchical methods used to compare these subsets. This is not to say that such features are not critical and distinctive, but simply that other shared features may be worth exploration in future.

The second feature of interest relates to the relative connectivity of Tbx21 amongst genes within the same module—this analysis was initially performed using the primary non-merged datasets. All such genes in each setting (inflation or exhaustion) show a degree of correlation—however the role of Tbx21 as a central master transcription factor amongst these genes is fundamentally different in the inflationary pool. This fits with a well-demonstrated type 1 responsiveness driven by Tbx21 and associated to these populations, functional control of virus, and also with a differentiated phenotype which depends on functional interactions between Tbx21 and Zeb2 (16, 22). The lack of connectivity of Tbx21 in exhaustion has been previously described and is reproduced in this comparative analysis—however the cause of this has yet to be fully elucidated.

It is interesting to note that in the absence of Eomes, a transcription factor prominent in exhausted (8) but not in inflating T cells, memory CD8 T cells display a phenotype which strikingly resembles that of inflating memory cells, being IL-2 low and high in Granzyme B, Klrg-1, and Perforin (23). However, these memory cells were not able to proliferate properly in the recall response. This does not appear to be the case in inflating memory cells, and underscores the importance of the co-ordinated cell cycling module present in inflating cells which is not observed in exhausted cells. Furthermore, retention of their proliferative capacity would also explain their numerical superiority.

Infection with CMV drives not only memory inflation and conventional memory, but also is associated with the development of “peripheral memory” and tissue resident memory. Peripheral memory is linked to an intermediate expression of the chemokine receptor CX3CR1 (fractalkine receptor) and with the potential to proliferate *in vivo* as well as differentiate to both CX3CR1 high and low subsets, depending on the exposure to antigen (24, 25). Tissue resident cells have lost CXC3R1 expression like central memory cells, but have evolved tissue associated phenotypes such as expression of CD69 and CD103 (26). It is likely the combination of these multiple distinct memory types are responsible for long term virus control, especially the maintenance of inflationary pools being dependent on longer-lived central and peripheral memory cells. Further transcriptional analyses of peripheral and tissue-resident memory within the MCMV and adenovirus model vs. the LCMV model for example using such an integrated approach as shown here will be of value in defining their key distinguishing characteristics and how they are inter-related. Overall this will certainly contribute to our understanding of the host responses to chronic viral infection and hopefully to the development of novel vaccines.

In conclusion, we have tried to address an important immunological question by a deeper analysis of valuable experimentally derived datasets, and in doing so generate new ideas about the causes of exhaustion and the mechanisms involved in robust memory after vaccination. Such studies can be examined in parallel but the tools available to interrogate the data as one integrated group are attractive and this area will doubtless be explored further in future. The major issues include not only potential batch effects, but also fundamental differences in mouse strains used, housing and handling, all of which can lead to artifacts. For immunologic experiments, the presence of very well-defined/conserved cell populations (in this case naive and conventional memory CD8+ T cells) provides some important internal references and controls following dataset integration. Further mathematical tools should also be applied to assess the quality of the integration and avoid biases (e.g., the PVCA-R package). As gene expression studies develop and cross-referencing of RNA-Seq datasets becomes both more attractive and more powerful this issue of normalization and integration will become even more important, for example in initiatives such as the Human Cell Atlas (https://humancellatlas.org). Further, however well-integrated mathematically, such data ultimately demand experimental validation—in this case a deeper analysis of the role of Tbx21 (and associated genes in the module) in inflationary memory would be valuable.

## Methods

PCA of expression profiles were performed in R (27) using *princomp/pricomp* functions. Three-dimensional plots employing the first three principal components were generated using R package *rgl* (28), providing high-level functions for 3D interactive graphics.

The merging of data sets from different microarray platforms (Illumina, Affymetrix) is summarized in the following steps:

1. Independent arrays platform preprocessing and normalization of Affymetrix and Illumina data (*rma* function (*oligo*) and *lumiN(method* = ”*vsn)* function (*Lumi*), respectively).
2. Probes to gene collapse: in cases where multiple probes were designed to a gene, the unique best detected features for each gene symbol was selected (max signal probe) in Affymetrix and Illumina data (similarly to max probe collapse in GSEA algorithm, Figure S2).
3. Common gene intersection: a common set of collapsed symbols between Affymetrix and Illumina arrays were taken to build a merged data set. (14113 unique symbols x 62 array samples); in-house R code was written to perform probes collapse and unique gene symbol intersection between the two array platform (Figure S2).
4. Removal of platform batch affect: biases due by different arrays platform employed (Affymetrix and Illumina) using *ComBat* R function [s*va* package (29)] on the new merged data set using a common set of annotated gene symbols.
5. (Optional) Normalization of the new merged and batch effect corrected data set was performed to remove residual biases and make arrays more comparable [methods: “scale,” “quantile”(30), or ”vsn”(31)].

Weighted Correlation Network Analysis [WGCNA, (32)] was executed using R package *WGCNA* (11) on a subset of highly variable genes (IQR > 0.5, 2231 features).

Hierarchical clustering analysis of samples using gene modules was performed using edited functions in *flashClust* R package (33).

Graphical visualization of gene module networks was generate on Cytoscape (34), with gene edges based on Topological Overlap Measures (TOM). Pavlidis matching template analysis (14) using Tbx21 gene as reference for expression pattern to match was performed on *MeV* java-based application (15).

Peak calling on Tbx21 binding profile data (16) was performed using *macs2* algorithm to identify genome-wide locations of transcription factor binding from ChIP-seq data (17). Tbx21 peaks were annotated using Homer bioinformatics tools [http://homer.ucsd.edu/homer/ngs/annotation.html (18)].

Effectiveness of batch value correction was performed using R package *pvca*, (10).

The versions of all the packages used is provided in the Supplementary Materials and Methods.

## Author Contributions

EM conceived the bioinformatic model, develop the idea, performed the bioinformatic analysis, and wrote the article. PK conceived the model, reviewed and wrote the article, and gave final approval. LNL contributed to the interpretation and design of the bioinformatics analysis and writing of the article. LNL and PK contributed equally.

### Conflict of Interest Statement

The authors declare that the research was conducted in the absence of any commercial or financial relationships that could be construed as a potential conflict of interest. The handling editor declared a past co-authorship with the authors EM and PK.

## Supplementary Material

Figure S1 **(A)** Clustering analysis of merged samples from Inflating and Exhausted models. Dendogram showing samples clustering (equivalent to analysis in Figure 3) after removing outliers and filtering gene by expression variance (IQR > 0.5). **(B)** PCA analysis of merged samples from Inflating and Exhausted models. Samples PCA using first two principal components (equivalent to analysis in Figure 3) after removing outliers and filtering gene by expression variance (IQR > 0.5). **(C)** PCA analysis of merged samples from Inflating and Exhausted models. Samples PCA using first three principal components (equivalent to analysis in Figure 3) after removing outliers and filtering gene by expression variance (IQR > 0.5).

Figure S2 **(A,B)** Reactome pathways enrichment analysis. Most represented pathways in blue module **(A)** and in turquoise module **(B)**. Gene ratio is the proportion of pathways genes in the total number of module genes.

Figure S3 Hierarchical clustering of Inflating/Exhausted samples based on turquoise module genes. Dendogram plot showing sample clustering analysis (Euclidian distance) on Inflating-Exhausted merged sets, based on a gene set of 692 genes, detected as turquoise module in a repeated Gene co-expression network analysis of Inflating samples after removing outliers (Soft-thresholding power β = 20).

Figure S4 Tbx21 and E2f2 Expression graphs. Normalized level of expressions of Tbx21 and E2f2 (in blue) across time points in Exhaustion and Inflation model experiments. Dash line marks the limit between early and late stage of infection.

Figure S5 **(A,B)** GSEA enrichment plots of Reactome gene sets. First 9 top enriched gene sets pathways in Inflating samples **(A)** and in Exhausting samples **(B)** at late stages (IFNL: M38, 50 Days; EXHA: Cl13, 30 Days).

Figure S6 **(A,B)** Representative GSEA enrichment plots of Inflation vs. Exhaustion. Illustrative enrichments of a CD8 effector signature (exact source: GSE30962_1570_200_UP) in Inflation samples **(A)** and a CD8 memory signature (exact source: GSE1000002_1582_200_DN) in Exhaustion samples **(B)**.

Figure S7 Assessment of batch effect contribution prior and after ComBat processing. An analysis by pvca R package was performed to estimate the variability of experimental effects.

Table S1 **(A,B)** GO term enrichment analysis of WGCNA modules. GO categories enrichment analysis in modules detected including all Inflating samples (**A**, β = 9) and excluding possible outliers (**B**, β = 20).

Table S2 **(A–C)** TOM matrices of blue and turquoise modules. Table matrices with topological overlay measures (TOM) of blue modules detected including all samples **(A)** or excluding possible outliers **(B)**; TOM matrix of turquoise module including all inflating samples **(C)**.

Table S3 GSEA report of Reactome gene sets enriched in Inflation. GSEA report of Reactome curated pathways found enriched (FDR < 0.25) in Inflating samples (M38, days 50) vs. Exhausting samples (Cl13, days 30).

Table S4 GSEA report of Reactome gene sets enriched in Exhaustion. GSEA report of Reactome curated pathways found enriched (FDR < 0.25) in Exhausting samples (Cl13, days 30) vs. Inflating samples (M38, days 50).

Table S5 **(A,B)** GSEA reports of MsigDB Immunological Signatures gene sets in Inflation and Exhaustion. GSEA reports of immunological signatures found enriched (FDR < 0.25) in Inflating samples **(A)** (M38, days 50) vs. Exhausting samples **(B)** (Cl13, days 30).

Supplementary Materials and Methods Relevant R and Bioconductor packages and versions used.
