# Supplementary figures and images for: Inflation vs. Exhaustion of Antiviral CD8+ T-Cell Populations in Persistent Infections: Two Sides of the Same Coin?
Source: Front Immunol. 2019 Mar 6;10:197. doi: 10.3389/fimmu.2019.00197 (PMC6414785; doi:10.3389/fimmu.2019.00197)

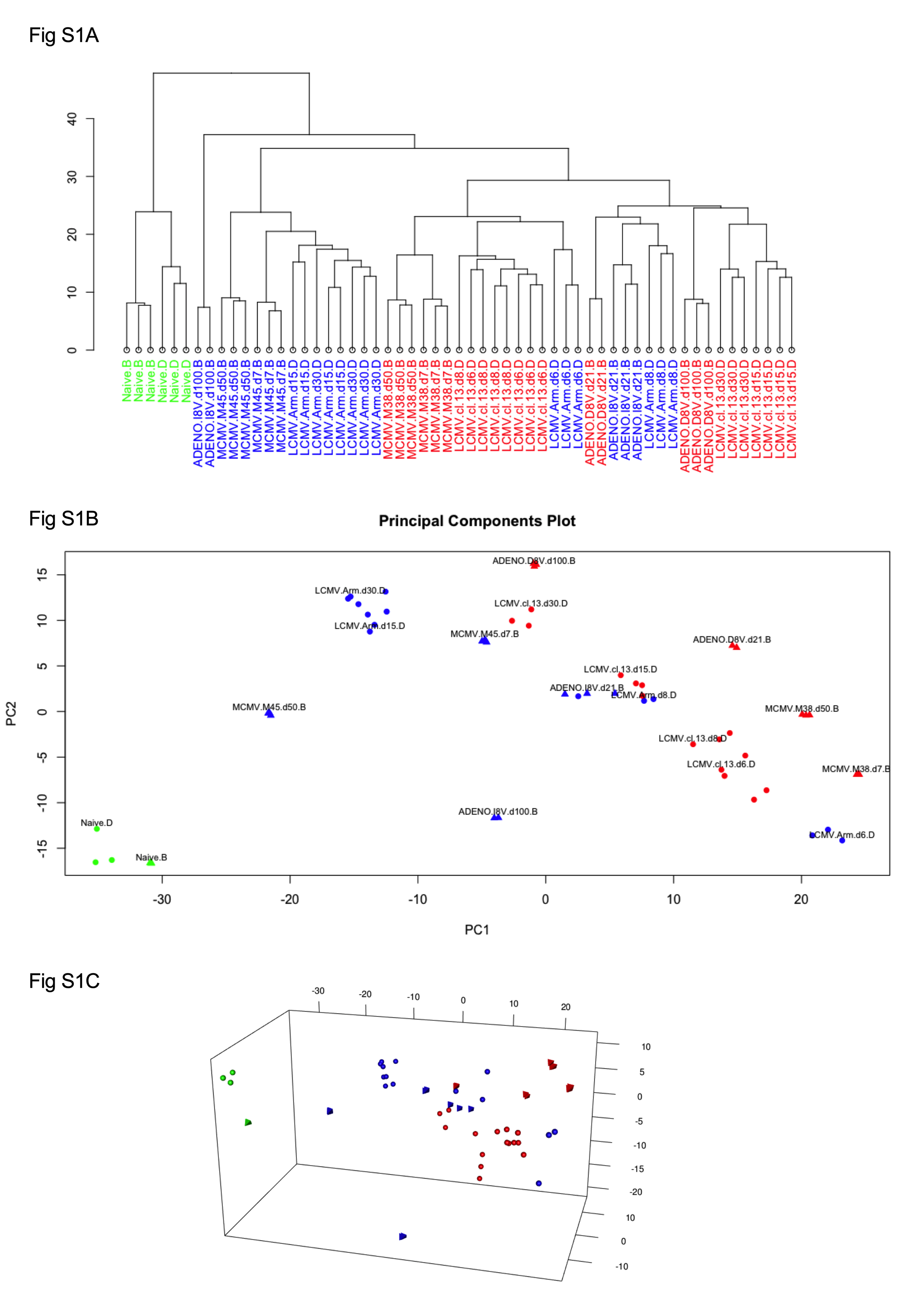

Supplement: Figure S1 — (A) Clustering analysis of merged samples from Inflating and Exhausted models. Dendogram showing samples clustering (equivalent to analysis in Figure 3) after removing outliers and filtering gene by expression variance (IQR > 0.5). (B) PCA analysis of merged samples from Inflating and Exhausted models. Samples PCA using first two principal components (equivalent to analysis in Figure 3) after removing outliers and filtering gene by expression variance (IQR > 0.5). (C) PCA analysis of merged samples from Inflating and Exhausted models. Samples PCA using first three principal components (equivalent to analysis in Figure 3) after removing outliers and filtering gene by expression variance (IQR > 0.5). [file Image_1.tiff]

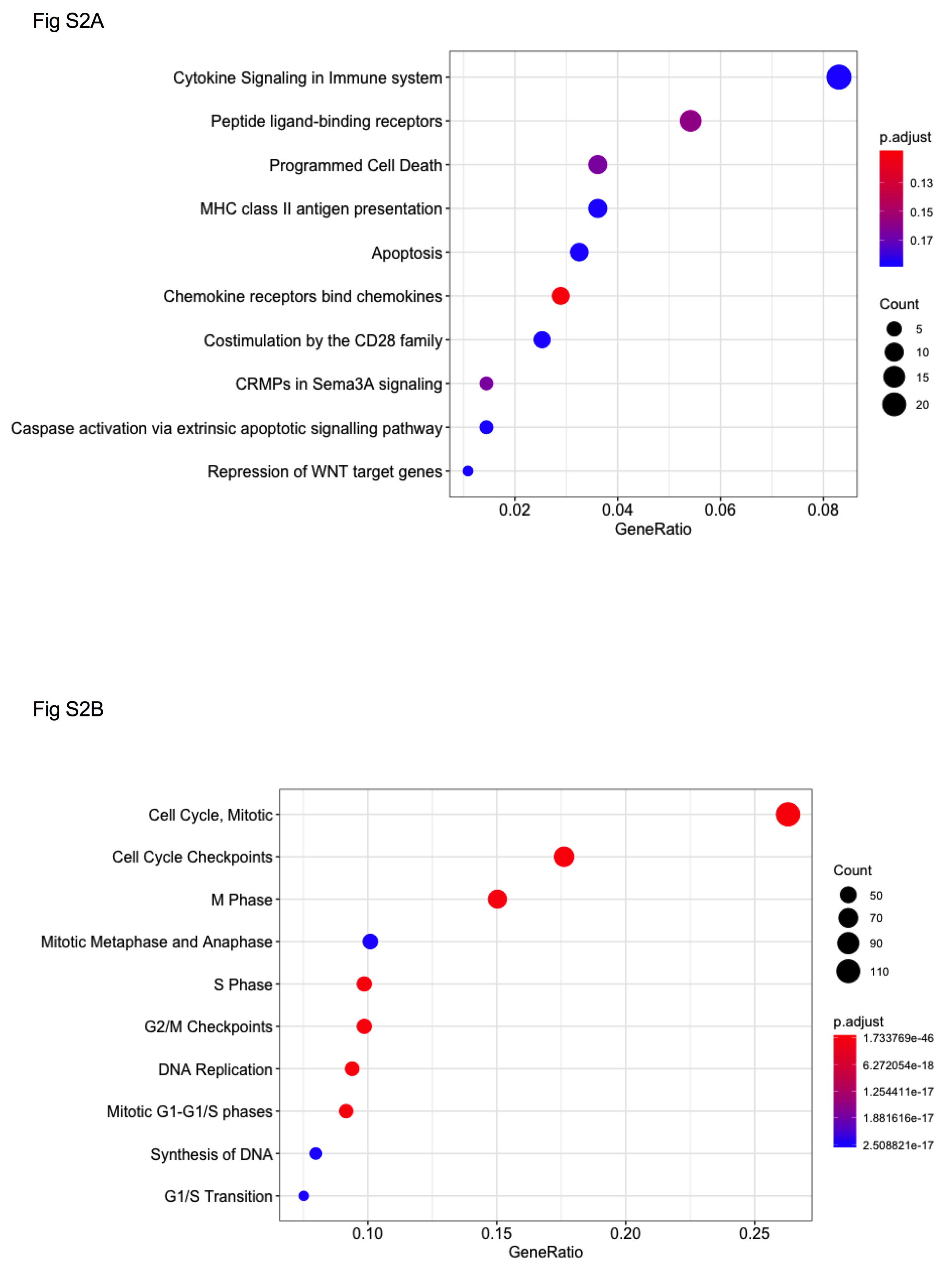

Supplement: Figure S2 — (A,B) Reactome pathways enrichment analysis. Most represented pathways in blue module (A) and in turquoise module (B). Gene ratio is the proportion of pathways genes in the total number of module genes. [file Image_2.TIFF]

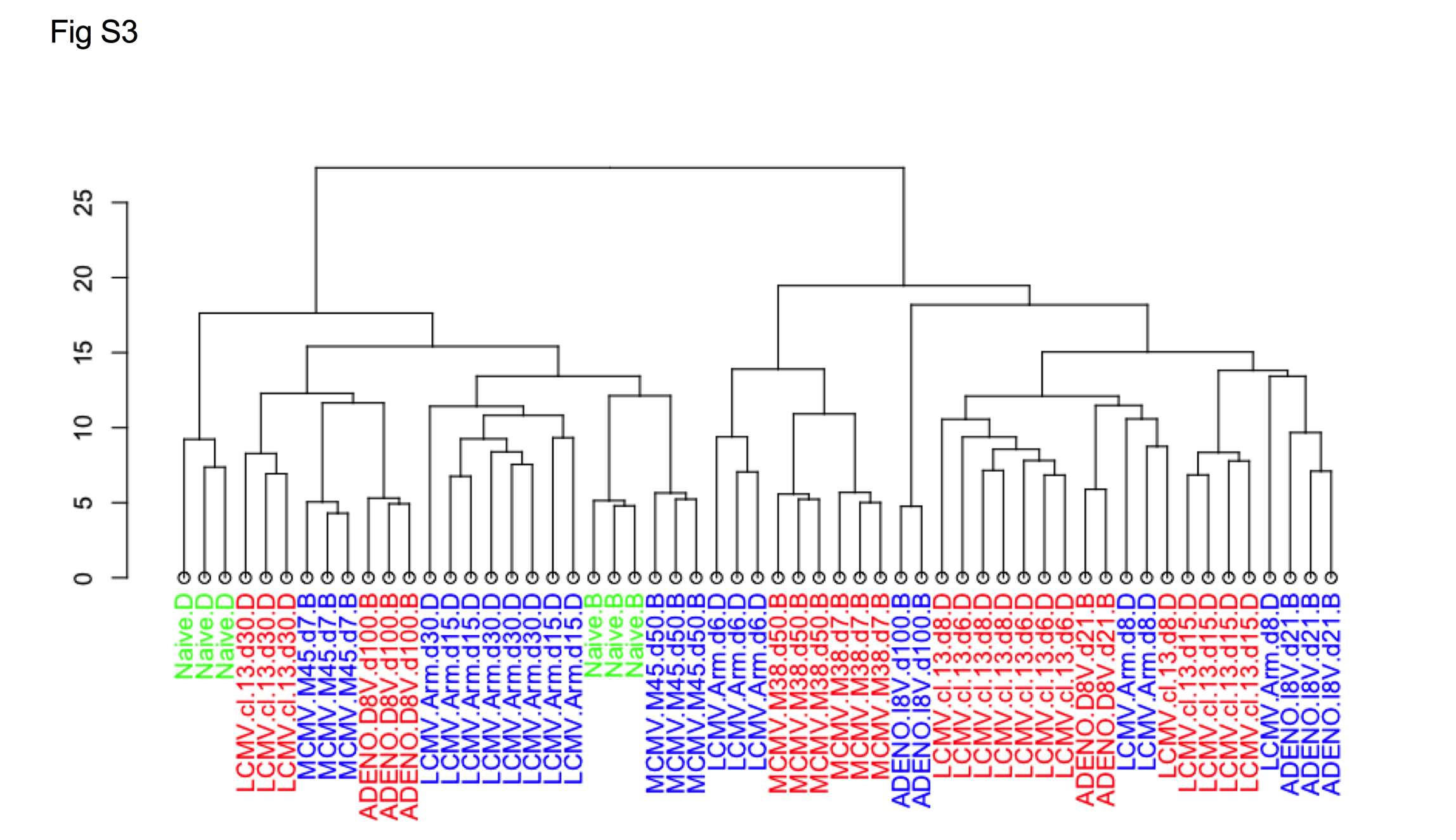

Supplement: Figure S3 — Hierarchical clustering of Inflating/Exhausted samples based on turquoise module genes. Dendogram plot showing sample clustering analysis (Euclidian distance) on Inflating-Exhausted merged sets, based on a gene set of 692 genes, detected as turquoise module in a repeated Gene co-expression network analysis of Inflating samples after removing outliers (Soft-thresholding power β = 20). [file Image_3.TIFF]

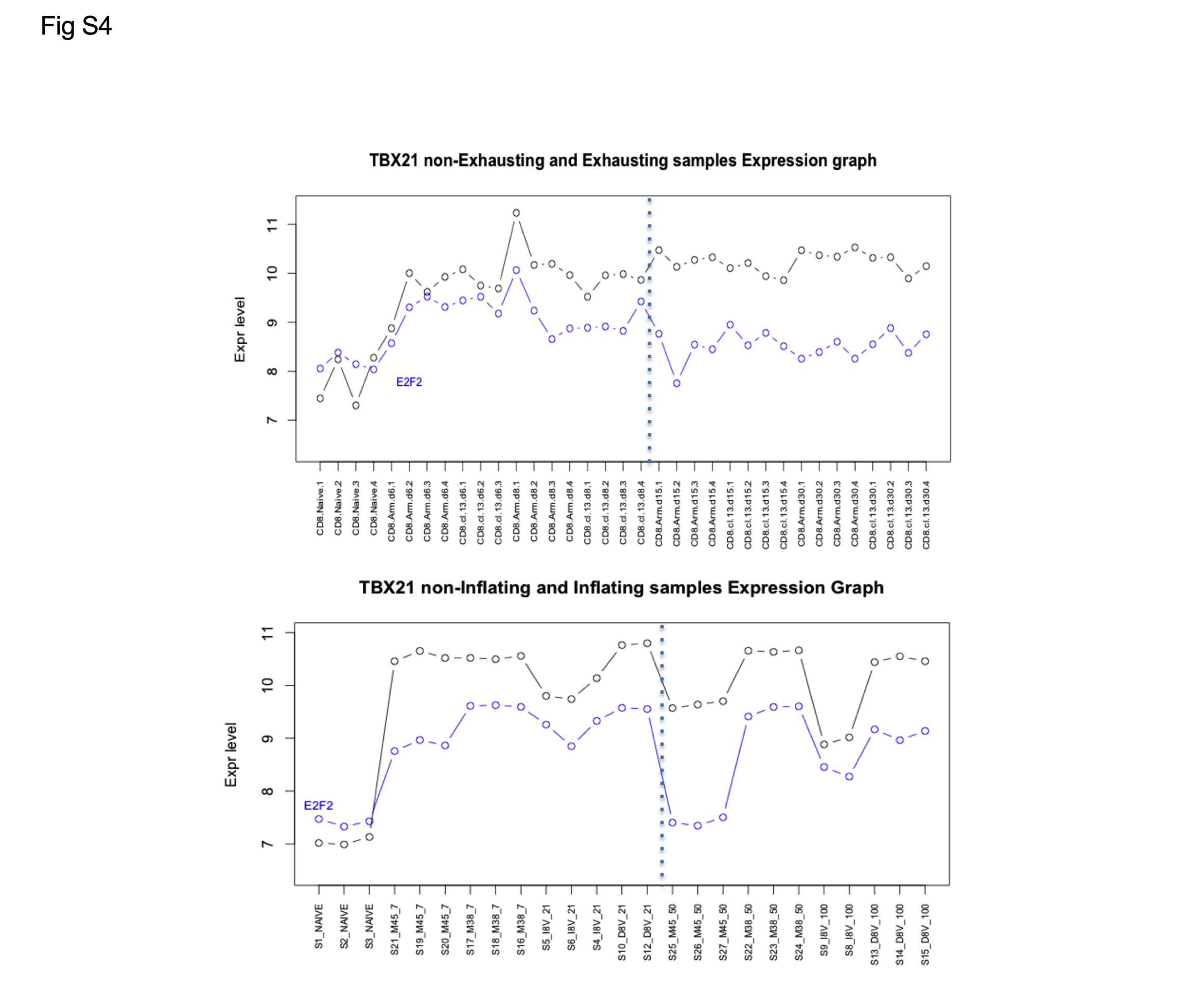

Supplement: Figure S4 — Tbx21 and E2f2 Expression graphs. Normalized level of expressions of Tbx21 and E2f2 (in blue) across time points in Exhaustion and Inflation model experiments. Dash line marks the limit between early and late stage of infection. [file Image_4.tiff]

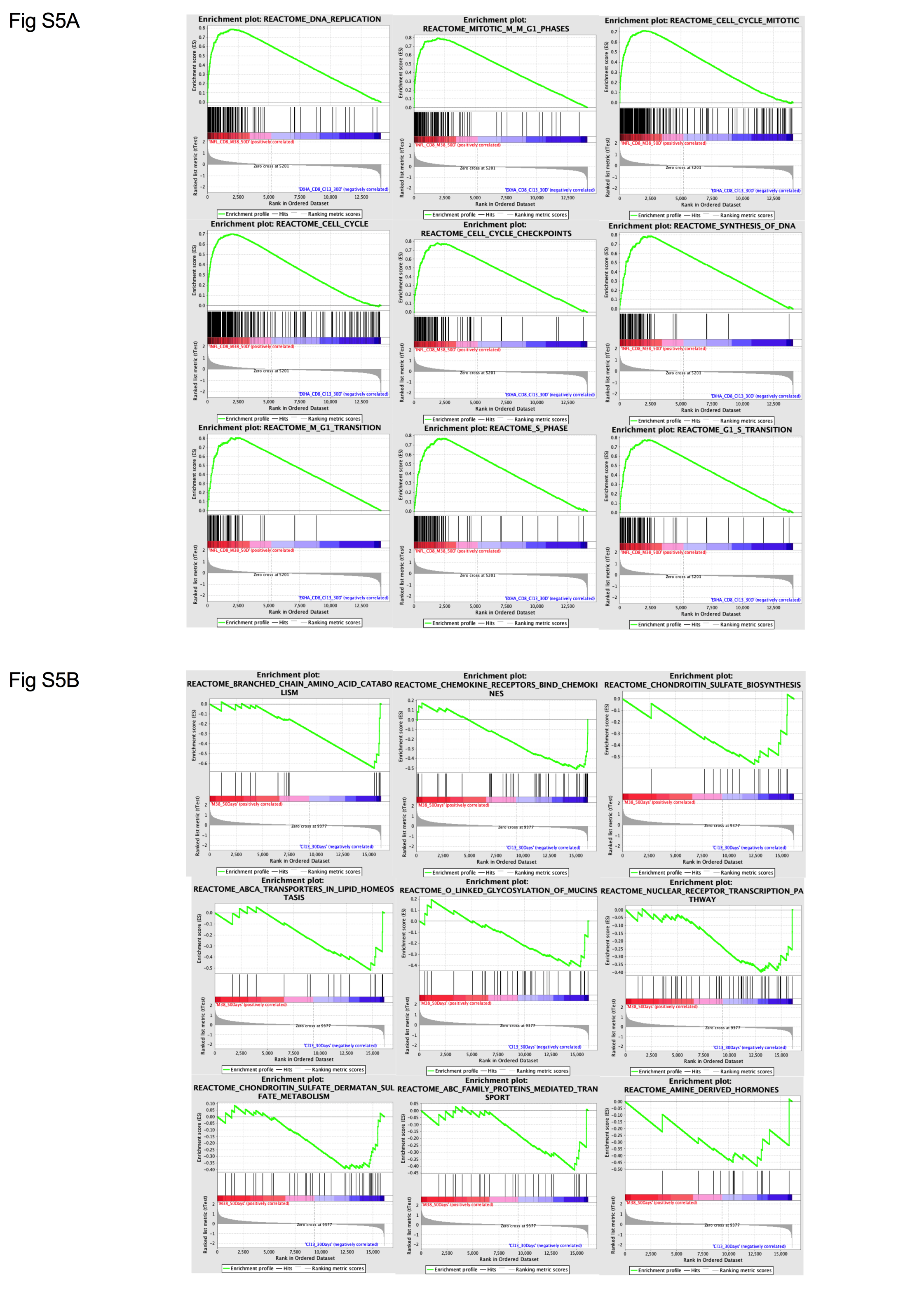

Supplement: Figure S5 — (A,B) GSEA enrichment plots of Reactome gene sets. First 9 top enriched gene sets pathways in Inflating samples (A) and in Exhausting samples (B) at late stages (IFNL: M38, 50 Days; EXHA: Cl13, 30 Days). [file Image_5.TIFF]

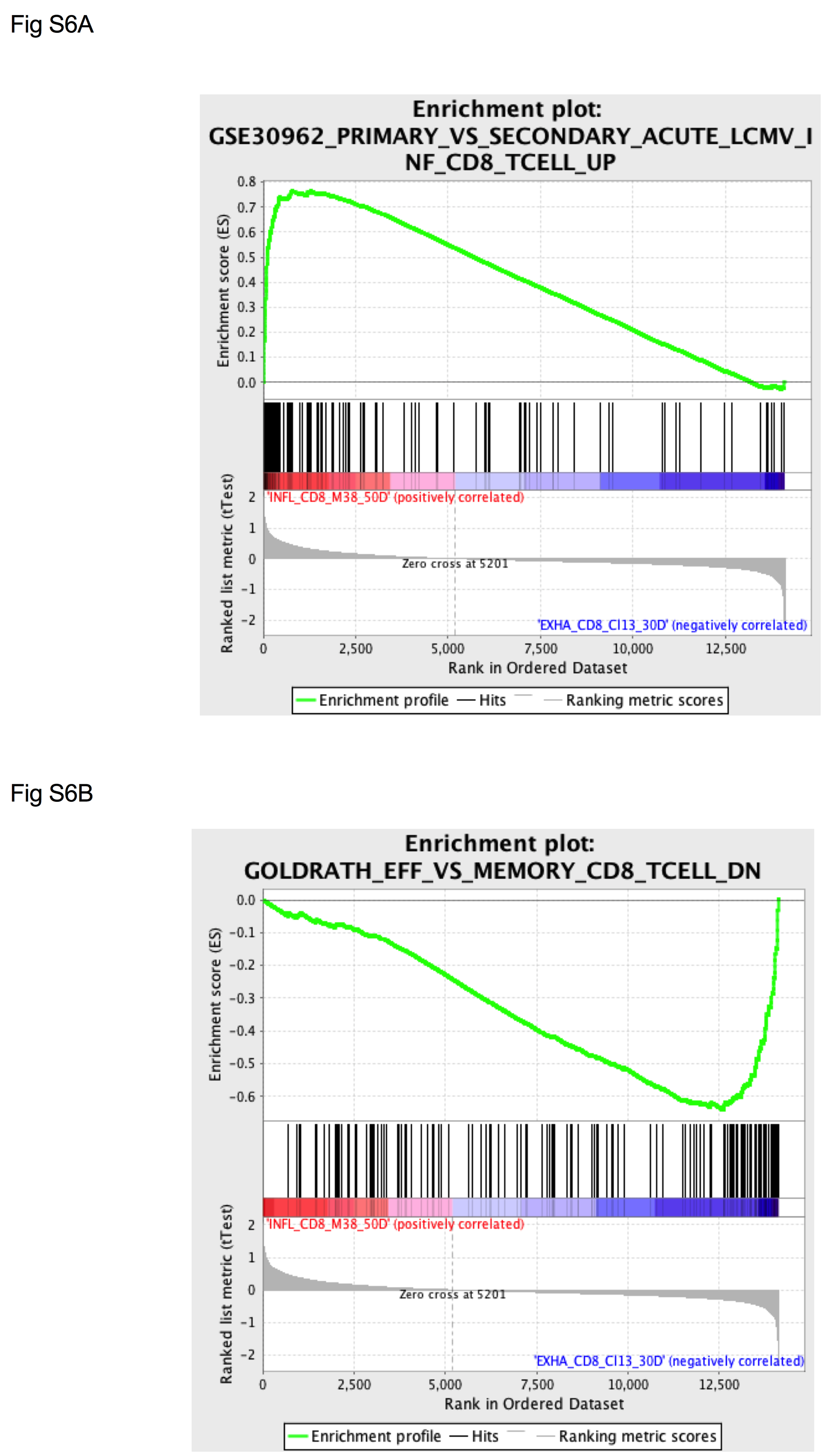

Supplement: Figure S6 — (A,B) Representative GSEA enrichment plots of Inflation vs. Exhaustion. Illustrative enrichments of a CD8 effector signature (exact source: GSE30962_1570_200_UP) in Inflation samples (A) and a CD8 memory signature (exact source: GSE1000002_1582_200_DN) in Exhaustion samples (B). [file Image_6.TIFF]

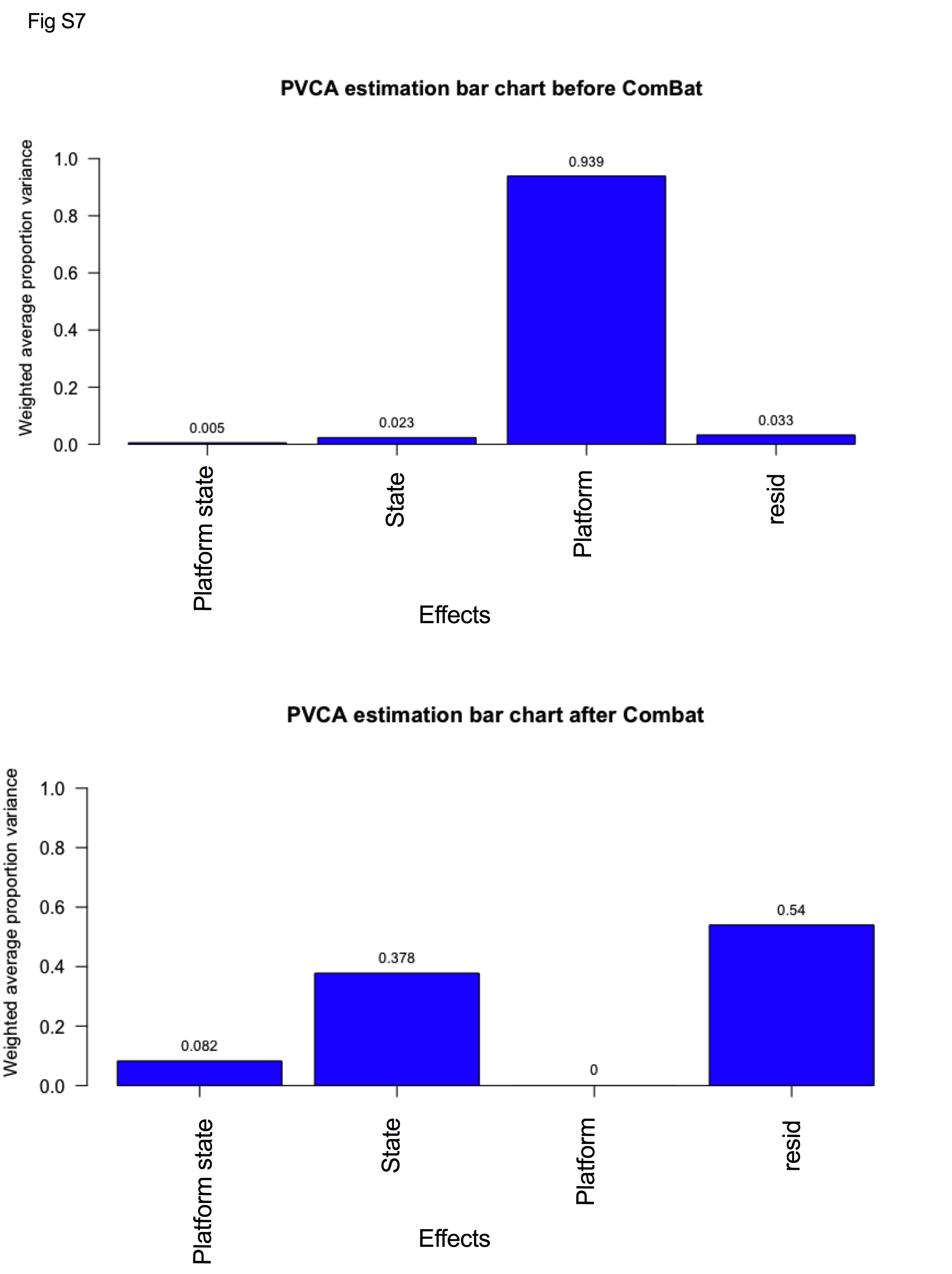

Supplement: Figure S7 — Assessment of batch effect contribution prior and after ComBat processing. An analysis by pvca R package was performed to estimate the variability of experimental effects. [file Image_7.TIFF]
