# Supplementary material for: Inflation vs. Exhaustion of Antiviral CD8+ T-Cell Populations in Persistent Infections: Two Sides of the Same Coin?
Source: Front Immunol. 2019 Mar 6;10:197. doi: 10.3389/fimmu.2019.00197 (PMC6414785; doi:10.3389/fimmu.2019.00197)
Supplement: Table S1 — (A,B) GO term enrichment analysis of WGCNA modules. GO categories enrichment analysis in modules detected including all Inflating samples (A, β = 9) and excluding possible outliers (B, β = 20). [file Table_1.pdf]

Table S1A
"module" "modSize" "bkgrModSize" "rank" "enrichmentP"
"BonferoniP" "nModGenesInTerm" "fracOfBkgrModSize"
"fracOfBkgrTermSize" "bkgrTermSize" "termID" "termOntology"
"termName" "termDefinition"
"black" 55 55 1 0.00102027033004189 1
10 0.181818181818182 0.0869565217391304
115 "GO:0003700" "MF" "DNA binding transcription factor
activity" "Interacting selectively and non-covalently with a
specific DNA sequence (sometimes referred to as a motif) within the
regulatory region of a gene in order to modulate transcription."
"black" 55 55 2 0.00228327036383225 1
2 0.0363636363636364 0.666666666666667
3 "GO:0030858" "BP" "positive regulation of epithelial
cell differentiation" "Any process that activates or increases
the frequency, rate or extent of epithelial cell differentiation."
"black" 55 55 3 0.00228327036383225 1
2 0.0363636363636364 0.666666666666667
3 "GO:1902235" "BP" "regulation of endoplasmic
reticulum stress-induced intrinsic apoptotic signaling
pathway" "Any process that modulates the frequency, rate or
extent of an endoplasmic reticulum stress-induced intrinsic
apoptotic signaling pathway."
"black" 55 55 4 0.0034724101754248 1
13 0.236363636363636 0.0631067961165049
206 "GO:0006366" "BP" "transcription by RNA polymerase
II" "The synthesis of RNA from a DNA template by RNA polymerase
II, originating at an RNA polymerase II promoter. Includes
transcription of messenger RNA (mRNA) and certain small nuclear RNAs
(snRNAs)."
"black" 55 55 5 0.00385682654148793 1
3 0.0545454545454545 0.25 12 "GO:
0002028" "BP" "regulation of sodium ion transport" "Any
process that modulates the frequency, rate or extent of the directed
movement of sodium ions (Na+) into, out of or within a cell, or
between cells, by means of some agent such as a transporter or
pore."
"black" 55 55 6 0.00422124135195175 1
7 0.127272727272727 0.0933333333333333
75 "GO:0000977" "MF" "RNA polymerase II regulatory
region sequence-specific DNA binding" "Interacting selectively
and non-covalently with a specific sequence of DNA that is part of a
regulatory region that controls the transcription of a gene or
cistron by RNA polymerase II."
"black" 55 55 7 0.00454914803360826 1
7 0.127272727272727 0.0921052631578947
76 "GO:0001012" "MF" "RNA polymerase II regulatory
region DNA binding" "Interacting selectively and non-
covalently with a DNA region that controls the transcription of a
region of DNA by RNA polymerase II. Binding may occur as a sequence
specific interaction or as an interaction observed only once a
factor has been recruited to the DNA by other factors."
"black" 55 55 8 0.00478800741696137 1
8 0.145454545454545 0.0824742268041237

97	"GO:0044212"	"MF"	"transcription regulatory region DNA binding"	"Interacting selectively and non-covalently with a DNA region that regulates the transcription of a region of DNA, which may be a gene, cistron, or operon. Binding may occur as a sequence specific interaction or as an interaction observed only once a factor has been recruited to the DNA by other factors."
"black"	55	55	9	0.00658913669227423	1
19	0.345454545454545	0.0490956072351421
387	"GO:0051252"	"BP"	"regulation of RNA metabolic process"	"Any process that modulates the frequency, rate or extent of the chemical reactions and pathways involving RNA."
"black"	55	55	10	0.00681839673136198	1
12	0.218181818181818	0.0609137055837563
197	"GO:0006357"	"BP"	"regulation of transcription by RNA polymerase II"	"Any process that modulates the frequency, rate or extent of transcription mediated by RNA polymerase II."
"blue"	517	516	1	3.59704861339132e-08
0.00063527475561104	267	0.517441860465116
0.328009828009828	814	"GO:
0016020"	"CC"	"membrane"	"A lipid bilayer along with all the proteins and protein complexes embedded in it an attached to it."
"blue"	517	516	2	8.90002243191713e-08
0.00157183296170088	159	0.308139534883721
0.363844393592677	437	"GO:
0031224"	"CC"	"intrinsic component of membrane"	"The component of a membrane consisting of the gene products having some covalently attached portion, for example part of a peptide sequence or some other covalently attached group such as a GPI anchor, which spans or is embedded in one or both leaflets of the membrane."
"blue"	517	516	3	9.22164233608937e-08
0.00162863425297674	153	0.296511627906977
0.366906474820144	417	"GO:
0016021"	"CC"	"integral component of membrane"	"The component of a membrane consisting of the gene products and protein complexes having at least some part of their peptide sequence embedded in the hydrophobic region of the membrane."
"blue"	517	516	4	1.10328444081307e-06
0.0194851065091996	168	0.325581395348837
0.348547717842324	482	"GO:0005886"	"CC"	"plasma membrane"	"The membrane surrounding a cell that separates the cell from its external environment. It consists of a phospholipid bilayer and associated proteins."
"blue"	517	516	5	1.3334439646557e-06
0.0235499538597843	172	0.333333333333333
0.346076458752515	497	"GO:0071944"	"CC"	"cell periphery"	"The part of a cell encompassing the cell cortex, the plasma membrane, and any external encapsulating structures."
"blue"	517	516	6	1.44106247756729e-06
0.025450604416316	78	0.151162790697674
0.414893617021277	188	"GO:
0040011"	"BP"	"locomotion"	"Self-propelled movement of a cell or organism from one location to another."
"blue"	517	516	7	1.61565774517377e-06

0.0285341314375139 129 0.25 0.366477272727273
352 "GO:0023051" "BP" "regulation of signaling" "Any
process that modulates the frequency, rate or extent of a signaling
process."
"blue" 517 516 8 1.68658243694586e-06
0.0297867324189008 193 0.374031007751938
0.337412587412587 572 "GO:
0023052" "BP" "signaling" "The entirety of a process in
which information is transmitted within a biological system. This
process begins with an active signal and ends when a cellular
response has been triggered."
"blue" 517 516 9 1.81593596026381e-06
0.0320712449942192 95 0.184108527131783
0.392561983471074 242 "GO:
0009653" "BP" "anatomical structure morphogenesis" "The
process in which anatomical structures are generated and organized.
Morphogenesis pertains to the creation of form."
"blue" 517 516 10 1.92415344064214e-06
0.0339824739151808 99 0.191860465116279
0.388235294117647 255 "GO:0006955" "BP" "immune
response" "Any immune system process that functions in the
calibrated response of an organism to a potential internal or
invasive threat."
"brown" 395 389 1 7.26397695625327e-05 1
29 0.0745501285347044 0.391891891891892
74 "GO:1903706" "BP" "regulation of
hemopoiesis" "Any process that modulates the frequency, rate
or extent of hemopoiesis."
"brown" 395 389 2 0.00153872309165422 1
4 0.0102827763496144 1 4 "GO:
0051591" "BP" "response to cAMP" "Any process that results
in a change in state or activity of a cell or an organism (in terms
of movement, secretion, enzyme production, gene expression, etc.) as
a result of a cAMP (cyclic AMP, adenosine 3',5'-cyclophosphate)
stimulus."
"brown" 395 389 3 0.00153872309165422 1
4 0.0102827763496144 1 4 "GO:
0005507" "MF" "copper ion binding" "Interacting selectively
and non-covalently with copper (Cu) ions."
"brown" 395 389 4 0.00157079038783707 1
50 0.12853470437018 0.290697674418605 172 "GO:
0002520" "BP" "immune system development" "The process
whose specific outcome is the progression of an organismal system
whose objective is to provide calibrated responses by an organism to
a potential internal or invasive threat, over time, from its
formation to the mature structure. A system is a regularly
interacting or interdependent group of organs or tissues that work
together to carry out a given biological process."
"brown" 395 389 5 0.00202878256972319 1
40 0.102827763496144 0.303030303030303
132 "GO:0005730" "CC" "nucleolus" "A small, dense
body one or more of which are present in the nucleus of eukaryotic
cells. It is rich in RNA and protein, is not bounded by a limiting
membrane, and is not seen during mitosis. Its prime function is the

transcription of the nucleolar DNA into 45S ribosomal-precursor RNA, the processing of this RNA into 5.8S, 18S, and 28S components of ribosomal RNA, and the association of these components with 5S RNA and proteins synthesized outside the nucleolus. This association results in the formation of ribonucleoprotein precursors; these pass into the cytoplasm and mature into the 40S and 60S subunits of the ribosome."
"brown" 395 389 6 0.00338550039299209 1
11 0.0282776349614396 0.458333333333333
24 "GO:0001935" "BP" "endothelial cell proliferation" "The multiplication or reproduction of endothelial cells, resulting in the expansion of a cell population. Endothelial cells are thin flattened cells which line the inside surfaces of body cavities, blood vessels, and lymph vessels, making up the endothelium."
"brown" 395 389 7 0.00357282369479165 1
46 0.118251928020566 0.285714285714286
161 "GO:0048534" "BP" "hematopoietic or lymphoid organ development" "The process whose specific outcome is the progression of any organ involved in hematopoiesis (also known as hemopoiesis) or lymphoid cell activation over time, from its formation to the mature structure. Such development includes differentiation of resident cell types (stromal cells) and of migratory cell types dependent on the unique microenvironment afforded by the organ for their proper differentiation."
"brown" 395 389 8 0.00368161376659798 1
10 0.025706940874036 0.476190476190476
21 "GO:0001936" "BP" "regulation of endothelial cell proliferation" "Any process that modulates the frequency, rate, or extent of endothelial cell proliferation."
"brown" 395 389 9 0.00388694662242634 1
9 0.0231362467866324 0.5 18 "GO:0007179" "BP" "transforming growth factor beta receptor signaling pathway" "A series of molecular signals initiated by the binding of an extracellular ligand to a transforming growth factor beta receptor on the surface of a target cell, and ending with regulation of a downstream cellular process, e.g. transcription."
"brown" 395 389 10 0.00445578440918749 1
5 0.012853470437018 0.714285714285714
7 "GO:0030512" "BP" "negative regulation of transforming growth factor beta receptor signaling pathway" "Any process that stops, prevents, or reduces the frequency, rate or extent of any TGF-beta receptor signaling pathway."
"green" 103 100 1 0.000129446081022539 1
3 0.03 1 3 "GO:0060441" "BP" "epithelial tube branching involved in lung morphogenesis" "The process in which a highly ordered sequence of patterning events generates the branched epithelial tubes of the lung, consisting of reiterated combinations of bud outgrowth, elongation, and dichotomous subdivision of terminal units."
"green" 103 100 2 0.000498516390802402 1
3 0.03 0.75 4 "GO:0060425" "BP" "lung morphogenesis" "The process in which the anatomical structures of the lung are generated and organized."

"green" 103 100 3 0.000662590827701156 1
4 0.04 0.444444444444444 9 "GO:
0006367" "BP" "transcription initiation from RNA polymerase II
promoter" "Any process involved in the assembly of the RNA
polymerase II preinitiation complex (PIC) at an RNA polymerase II
promoter region of a DNA template, resulting in the subsequent
synthesis of RNA from that promoter. The initiation phase includes
PIC assembly and the formation of the first few bonds in the RNA
chain, including abortive initiation, which occurs when the first
few nucleotides are repeatedly synthesized and then released.
Promoter clearance, or release, is the transition between the
initiation and elongation phases of transcription."
"green" 103 100 4 0.000875944128420456 1
5 0.05 0.3125 16 "GO:
0035456" "BP" "response to interferon-beta" "Any process that
results in a change in state or activity of a cell or an organism
(in terms of movement, secretion, enzyme production, gene
expression, etc.) as a result of an interferon-beta stimulus.
Interferon-beta is a type I interferon."
"green" 103 100 5 0.00120001441051704 1
3 0.03 0.6 5 "GO:0036041" "MF" "long-
chain fatty acid binding" "Interacting selectively and non-
covalently with a long-chain fatty acid. A long-chain fatty acid is
a fatty acid with a chain length between C13 and C22."
"green" 103 100 6 0.00130783749389977 1
10 0.1 0.153846153846154 65 "GO:
0009615" "BP" "response to virus" "Any process that results
in a change in state or activity of a cell or an organism (in terms
of movement, secretion, enzyme production, gene expression, etc.) as
a result of a stimulus from a virus."
"green" 103 100 7 0.00150446227638271 1
24 0.24 0.0941176470588235 255 "GO:
0006955" "BP" "immune response" "Any immune system
process that functions in the calibrated response of an organism to
a potential internal or invasive threat."
"green" 103 100 8 0.00158301461058731 1
5 0.05 0.277777777777778 18 "GO:
0060541" "BP" "respiratory system development" "The progression
of the respiratory system over time from its formation to its mature
structure. The respiratory system carries out respiratory gaseous
exchange."
"green" 103 100 9 0.0016025792515915 1
4 0.04 0.363636363636364 11 "GO:
0035455" "BP" "response to interferon-alpha" "Any process that
results in a change in state or activity of a cell or an organism
(in terms of movement, secretion, enzyme production, gene
expression, etc.) as a result of an interferon-alpha stimulus.
Interferon-alpha is a type I interferon."
"green" 103 100 10 0.00193746081524262 1
11 0.11 0.1375 80 "GO:0002764" "BP" "immune
response-regulating signaling pathway" "The cascade of processes
by which a signal interacts with a receptor, causing a change in the
level or activity of a second messenger or other downstream target,
and ultimately leading to the activation, perpetuation, or

inhibition of an immune response."
"grey" 12 12 1 0.00219730353393327 1
2 0.166666666666667 0.166666666666667
12 "GO:0004867" "MF" "serine-type endopeptidase inhibitor activity" "Stops, prevents or reduces the activity of serine-type endopeptidases, enzymes that catalyze the hydrolysis of nonterminal peptide bonds in a polypeptide chain; a serine residue (and a histidine residue) are at the active center of the enzyme."
"grey" 12 12 2 0.00612870275791622 1
1 0.0833333333333333 1 1 "GO:
0070054" "BP" "mRNA splicing, via endonucleolytic cleavage and ligation" "Splicing of mRNA substrates via recognition of the folded RNA structure that brings the 5' and 3' splice sites into proximity and cleavage of the RNA at both the 3' and 5' splice sites by an endonucleolytic mechanism, followed by ligation of the exons."
"grey" 12 12 3 0.00612870275791622 1
1 0.0833333333333333 1 1 "GO:
0070055" "BP" "mRNA endonucleolytic cleavage involved in unfolded protein response" "The endonucleolytic cleavage of a mRNA containing an HAC1-type intron at the 5' and 3' splice sites. The cleavage step is part of unconventional mRNA splicing, and contributes to the endoplasmic reticulum unfolded protein response."
"grey" 12 12 4 0.00612870275791622 1
1 0.0833333333333333 1 1 "GO:
1901142" "BP" "insulin metabolic process" "The chemical reactions and pathways involving insulin."
"grey" 12 12 5 0.00612870275791622 1
1 0.0833333333333333 1 1 "GO:
1990579" "BP" "peptidyl-serine trans-autophosphorylation" "The phosphorylation of a peptidyl-serine to form peptidyl-O-phospho-L-serine on an identical protein. For example, phosphorylation by the other kinase within a homodimer."
"grey" 12 12 6 0.00612870275791622 1
1 0.0833333333333333 1 1 "GO:
0005581" "CC" "collagen trimer" "A protein complex consisting of three collagen chains assembled into a left-handed triple helix. These trimers typically assemble into higher order structures."
"grey" 12 12 7 0.00612870275791622 1
1 0.0833333333333333 1 1 "GO:
0031372" "CC" "UBC13-MMS2 complex" "A heterodimeric ubiquitin conjugating enzyme complex that catalyzes assembly of K63-linked polyubiquitin chains, which act as a signal to promote error-free DNA postreplication repair; in Saccharomyces the complex comprises Ubc13p and Mms2p."
"grey" 12 12 8 0.00612870275791622 1
1 0.0833333333333333 1 1 "GO:
0035370" "CC" "UBC13-UEV1A complex" "A heterodimeric ubiquitin conjugating enzyme complex that catalyzes assembly of K63-linked polyubiquitin chains and is involved in NF-kappaB activation. In humans at least, the complex comprises the ubiquitin-conjugating enzyme UBC13 and ubiquitin-conjugating enzyme variant 1A (UEV1A)."

"grey"	12	12	9	0.00612870275791622	1
1	0.0833333333333333	1	1	"GO:
1990597"	"CC"	"AIP1-IRE1 complex"	"A protein complex
consisting of IRE1 (inositol-requiring enzyme-1) bound to AIP1
(ASK1-interacting protein 1/DAB2-interacting protein)."
"grey"	12	12	10	0.00612870275791622	1
1	0.0833333333333333	1	1	"GO:
1990604"	"CC"	"IRE1-TRAF2-ASK1 complex"	"A protein complex of the
endoplasmic reticulum membrane that consists of IRE1 (Inositol-
requiring enzyme-1), TRAF2 (TNF receptor-associated factor 2) and
ASK1 (Apoptosis signal-regulating kinase 1, a MAP3K)."
"red"	62	61	1	0.000236243073463681	1
6	0.0983606557377049	0.2	30	"GO:
0045732"	"BP"	"positive regulation of protein catabolic
process"	"Any process that activates or increases the frequency,
rate or extent of the chemical reactions and pathways resulting in
the breakdown of a protein by the destruction of the native, active
configuration, with or without the hydrolysis of peptide bonds."
"red"	62	61	2	0.000353830763853935	1
8	0.131147540983607	0.135593220338983
59	"GO:0042176"	"BP"	"regulation of protein catabolic
process"	"Any process that modulates the frequency, rate or extent
of the chemical reactions and pathways resulting in the breakdown of
a protein by the destruction of the native, active configuration,
with or without the hydrolysis of peptide bonds."
"red"	62	61	3	0.000676083034389494	1
4	0.0655737704918033	0.285714285714286
14	"GO:1903052"	"BP"	"positive regulation of
proteolysis involved in cellular protein catabolic process"	"Any
process that activates or increases the frequency, rate or extent of
proteolysis involved in cellular protein catabolic process."
"red"	62	61	4	0.000955163178929205	1
2	0.0327868852459016	1	2	"GO:
0060292"	"BP"	"long term synaptic depression"	"A process that
modulates synaptic plasticity such that synapses are changed
resulting in the decrease in the rate, or frequency of synaptic
transmission at the synapse."
"red"	62	61	5	0.00149825320565425	1
4	0.0655737704918033	0.235294117647059
17	"GO:0043524"	"BP"	"negative regulation of neuron
apoptotic process"	"Any process that stops, prevents, or
reduces the frequency, rate or extent of cell death by apoptotic
process in neurons."
"red"	62	61	6	0.00149825320565425	1
4	0.0655737704918033	0.235294117647059
17	"GO:1903364"	"BP"	"positive regulation of cellular
protein catabolic process"	"Any process that activates or
increases the frequency, rate or extent of cellular protein
catabolic process."
"red"	62	61	7	0.00172390875816899	1
11	0.180327868852459	0.0846153846153846
130	"GO:0030163"	"BP"	"protein catabolic
process"	"The chemical reactions and pathways resulting in the
breakdown of a protein by the destruction of the native, active

configuration, with or without the hydrolysis of peptide bonds."
"red" 62 61 8 0.00224893978687442 1
6 0.0983606557377049 0.133333333333333
45 "GO:0046390" "BP" "ribose phosphate biosynthetic process" "The chemical reactions and pathways resulting in the formation of ribose phosphate, any phosphorylated ribose sugar."
"red" 62 61 9 0.00226446448323347 1
5 0.0819672131147541 0.161290322580645
31 "GO:1903362" "BP" "regulation of cellular protein catabolic process" "Any process that modulates the frequency, rate or extent of cellular protein catabolic process."
"red" 62 61 10 0.00233249153178043 1
9 0.147540983606557 0.09375 96 "GO:0051603" "BP" "proteolysis involved in cellular protein catabolic process" "The hydrolysis of a peptide bond or bonds within a protein as part of the chemical reactions and pathways resulting in the breakdown of a protein by individual cells."
"turquoise" 718 716 1 1.22363233871473e-16
2.16105707340409e-12 70 0.0977653631284916
0.786516853932584 89 "GO:0000280" "BP" "nuclear division" "The division of a cell nucleus into two nuclei, with DNA and other nuclear contents distributed between the daughter nuclei."
"turquoise" 718 716 2 2.06928432447053e-16
3.65456304544741e-12 72 0.100558659217877
0.774193548387097 93 "GO:0048285" "BP" "organelle fission" "The creation of two or more organelles by division of one organelle."
"turquoise" 718 716 3 2.17231301665199e-16
3.83652201870908e-12 115 0.160614525139665
0.657142857142857 175 "GO:0000278" "BP" "mitotic cell cycle" "Progression through the phases of the mitotic cell cycle, the most common eukaryotic cell cycle, which canonically comprises four successive phases called G1, S, G2, and M and includes replication of the genome and the subsequent segregation of chromosomes into daughter cells. In some variant cell cycles nuclear replication or nuclear division may not be followed by cell division, or G1 and G2 phases may be absent."
"turquoise" 718 716 4 7.28470232118828e-15
1.28655127694506e-10 173 0.241620111731844
0.565359477124183 306 "GO:0007049" "BP" "cell cycle" "The progression of biochemical and morphological phases and events that occur in a cell during successive cell replication or nuclear replication events. Canonically, the cell cycle comprises the replication and segregation of genetic material followed by the division of the cell, but in endocycles or syncytial cells nuclear replication or nuclear division may not be followed by cell division."
"turquoise" 718 716 5 1.09740062203727e-13
1.93811923858003e-09 59 0.0824022346368715
0.776315789473684 76 "GO:0007059" "BP" "chromosome segregation" "The process in which genetic material, in the form of chromosomes, is organized into specific structures and then physically separated and apportioned to

two or more sets. In eukaryotes, chromosome segregation begins with the condensation of chromosomes, includes chromosome separation, and ends when chromosomes have completed movement to the spindle poles."
"turquoise" 718 716 6 1.3093518689316e-13
2.31244633572009e-09 127 0.177374301675978
0.601895734597156 211 "GO:
0005694" "CC" "chromosome" "A structure composed of a very long molecule of DNA and associated proteins (e.g. histones) that carries hereditary information."
"turquoise" 718 716 7 3.26833620653413e-11
5.77220857435992e-07 41 0.0572625698324022 0.82
50 "GO:0000819" "BP" "sister chromatid
segregation" "The cell cycle process in which sister chromatids are organized and then physically separated and apportioned to two or more sets."
"turquoise" 718 716 8 1.9425856731341e-10
3.43080055732214e-06 82 0.114525139664804
0.630769230769231 130 "GO:0051301" "BP" "cell
division" "The process resulting in division and partitioning of components of a cell to form more cells; may or may not be accompanied by the physical separation of a cell into distinct, individually membrane-bounded daughter cells."
"turquoise" 718 716 9 4.03367757081188e-10
7.12387795781086e-06 38 0.053072625698324
0.808510638297872 47 "GO:0000070" "BP" "mitotic
sister chromatid segregation" "The cell cycle process in which replicated homologous chromosomes are organized and then physically separated and apportioned to two sets during the mitotic cell cycle. Each replicated chromosome, composed of two sister chromatids, aligns at the cell equator, paired with its homologous partner. One homolog of each morphologic type goes into each of the resulting chromosome sets."
"turquoise" 718 716 10 7.75731067505495e-10
1.37001863832145e-05 62 0.0865921787709497
0.673913043478261 92 "GO:0006281" "BP" "DNA
repair" "The process of restoring DNA after damage. Genomes are subject to damage by chemical and physical agents in the environment (e.g. UV and ionizing radiations, chemical mutagens, fungal and bacterial toxins, etc.) and by free radicals or alkylating agents endogenously generated in metabolism. DNA is also damaged because of errors during its replication. A variety of different DNA repair pathways have been reported that include direct reversal, base excision repair, nucleotide excision repair, photoreactivation, bypass, double-strand break repair pathway, and mismatch repair pathway."
"yellow" 109 109 1 0.00028249302839376 1
5 0.0458715596330275 0.416666666666667
12 "GO:0008135" "MF" "translation factor activity, RNA
binding" "Functions during translation by interacting selectively and non-covalently with RNA during polypeptide synthesis at the ribosome."
"yellow" 109 109 2 0.000922281647246977 1
4 0.036697247706422 0.444444444444444
9 "GO:0003743" "MF" "translation initiation factor

activity"   "Functions in the initiation of ribosome-mediated translation of mRNA into a polypeptide."
"yellow" 109   109   3   0.00159698293449057   1
13   0.119266055045872   0.138297872340426
94   "GO:0043604"   "BP"   "amide biosynthetic process" "The chemical reactions and pathways resulting in the formation of an amide, any derivative of an oxoacid in which an acidic hydroxy group has been replaced by an amino or substituted amino group."
"yellow" 109   109   4   0.00176251899598819   1
5   0.0458715596330275   0.294117647058824
17   "GO:0051087"   "MF"   "chaperone binding" "Interacting selectively and non-covalently with a chaperone protein, a class of proteins that bind to nascent or unfolded polypeptides and ensure correct folding or transport."
"yellow" 109   109   5   0.00183000872612569   1
12   0.110091743119266   0.142857142857143
84   "GO:0043043"   "BP"   "peptide biosynthetic process" "The chemical reactions and pathways resulting in the formation of peptides, compounds of 2 or more (but usually less than 100) amino acids where the alpha carboxyl group of one is bound to the alpha amino group of another. This may include the translation of a precursor protein and its subsequent processing into a functional peptide."
"yellow" 109   109   6   0.00227958067996459   1
21   0.192660550458716   0.105527638190955
199   "GO:0098772"   "MF"   "molecular function regulator"   "A molecular function that modulates the activity of a gene product or complex. Examples include enzyme regulators and channel regulators."
"yellow" 109   109   7   0.00307218058534279   1
2   0.018348623853211   1   2   "GO:0033363" "BP"   "secretory granule organization" "A process that is carried out at the cellular level which results in the assembly, arrangement of constituent parts, or disassembly of a secretory granule. A secretory granule is a small subcellular vesicle, surrounded by a membrane, that is formed from the Golgi apparatus and contains a highly concentrated protein destined for secretion."
"yellow" 109   109   8   0.00307218058534279   1
2   0.018348623853211   1   2   "GO:0035162" "BP"   "embryonic hemopoiesis" "The stages of blood cell formation that take place within the embryo."
"yellow" 109   109   9   0.00307218058534279   1
2   0.018348623853211   1   2   "GO:0001405" "CC"   "presequence translocase-associated import motor"   "Protein complex located on the matrix side of the mitochondrial inner membrane and associated with the presequence translocase complex; hydrolyzes ATP to provide the force to drive import of proteins into the mitochondrial matrix."
"yellow" 109   109   10   0.00307218058534279   1
2   0.018348623853211   1   2   "GO:0005851" "CC"   "eukaryotic translation initiation factor 2B complex" "A multisubunit guanine nucleotide exchange factor which catalyzes the exchange of GDP bound to initiation factor eIF2 for

GTP, generating active eIF2-GTP. In humans, it is composed of five subunits, alpha, beta, delta, gamma and epsilon."

Table S1B

"module"	"modSize"	"bkgrModSize"	"rank"	"enrichmentP"	"BonferoniP"	"nModGenesInTerm"	"fracOfBkgrModSize"	"fracOfBkgrTermSize"	"bkgrTermSize"	"termID"	"termOntology"	"termName"	"termDefinition"
"blue"	453	452	1	0.00288106655992114	1	8	0.0176991150442478	0.727272727272727	11	"GO:0035282"	"BP"	"segmentation"	"The regionalization process that divides an organism or part of an organism into a series of semi-repetitive parts, or segments, often arranged along a longitudinal axis."
"blue"	453	452	2	0.00288106655992114	1	8	0.0176991150442478	0.727272727272727	11	"GO:2000779"	"BP"	"regulation of double-strand break repair"	"Any process that modulates the frequency, rate or extent of double-strand break repair."
"blue"	453	452	3	0.00417208635173806	1	20	0.0442477876106195	0.48780487804878	41	"GO:0033218"	"MF"	"amide binding"	"Interacting selectively and non-covalently with an amide, any derivative of an oxoacid in which an acidic hydroxy group has been replaced by an amino or substituted amino group."
"blue"	453	452	4	0.00532822279855509	1	14	0.0309734513274336	0.538461538461538	26	"GO:0002224"	"BP"	"toll-like receptor signaling pathway"	"Any series of molecular signals generated as a consequence of binding to a toll-like receptor. Toll-like receptors directly bind pattern motifs from a variety of microbial sources to initiate innate immune response."
"blue"	453	452	5	0.00533917707527118	1	83	0.183628318584071	0.357758620689655	232	"GO:0006955"	"BP"	"immune response"	"Any immune system process that functions in the calibrated response of an organism to a potential internal or invasive threat."
"blue"	453	452	6	0.0054652527882605	1	9	0.0199115044247788	0.642857142857143	14	"GO:0032088"	"BP"	"negative regulation of NF-kappaB transcription factor activity"	"Any process that stops, prevents, or reduces the frequency, rate or extent of the activity of the transcription factor NF-kappaB."
"blue"	453	452	7	0.00650150584143631	1	4	0.00884955752212389	1	4	"GO:0018027"	"BP"	"peptidyl-lysine dimethylation"	"The methylation of peptidyl-lysine to form peptidyl-N6,N6-dimethyl-L-lysine."
"blue"	453	452	8	0.00650150584143631	1	4	0.00884955752212389	1	4	"GO:0036124"	"BP"	"histone H3-K9 trimethylation"	"The modification of histone H3 by addition of three methyl groups to lysine at position 9 of the histone."
"blue"	453	452	9	0.00650150584143631	1	4	0.00884955752212389	1	4	"GO:0045953"	"BP"	"negative regulation of natural killer cell mediated cytotoxicity"	"Any process that stops, prevents, or reduces the rate of natural killer mediated cytotoxicity."

"blue" 453   452   10   0.00650150584143631   1
4   0.00884955752212389   1   4   "GO:
0051496" "BP"   "positive regulation of stress fiber
assembly"   "Any process that activates or increases the
frequency, rate or extent of the assembly of a stress fiber, a
bundle of microfilaments and other proteins found in fibroblasts."
"brown" 202   202   1   0.00114784471337333   1
4   0.0198019801980198   0.8   5   "GO:
0046654" "BP"   "tetrahydrofolate biosynthetic process"   "The
chemical reactions and pathways resulting in the formation of
tetrahydrofolate, 5,6,7,8-tetrahydrofolic acid, a folate derivative
bearing additional hydrogens on the pterin group."
"brown" 202   202   2   0.00114784471337333   1
4   0.0198019801980198   0.8   5   "GO:
0071480" "BP"   "cellular response to gamma radiation"   "Any
process that results in a change in state or activity of a cell (in
terms of movement, secretion, enzyme production, gene expression,
etc.) as a result of a gamma radiation stimulus. Gamma radiation is
a form of electromagnetic radiation (EMR) or light emission of a
specific frequency produced from sub-atomic particle interaction,
such as electron-positron annihilation and radioactive decay. Gamma
rays are generally characterized as EMR having the highest frequency
and energy, and also the shortest wavelength, within the
electromagnetic radiation spectrum."
"brown" 202   202   3   0.00259780601442417   1
5   0.0247524752475248   0.555555555555556
9   "GO:0030261"   "BP"   "chromosome condensation" "The
progressive compaction of dispersed interphase chromatin into
threadlike chromosomes prior to mitotic or meiotic nuclear division,
or during apoptosis, in eukaryotic cells."
"brown" 202   202   4   0.00278233163730142   1
11   0.0544554455445545   0.314285714285714
35   "GO:0016607"   "CC"   "nuclear speck" "A discrete
extra-nucleolar subnuclear domain, 20-50 in number, in which
splicing factors are seen to be localized by immunofluorescence
microscopy."
"brown" 202   202   5   0.00310059871354082   1
4   0.0198019801980198   0.666666666666667
6   "GO:0009396"   "BP"   "folic acid-containing compound
biosynthetic process"   "The chemical reactions and pathways
resulting in the formation of folic acid and its derivatives."
"brown" 202   202   6   0.00310059871354082   1
4   0.0198019801980198   0.666666666666667
6   "GO:0042559"   "BP"   "pteridine-containing compound
biosynthetic process"   "The chemical reactions and pathways
resulting in the formation of any compound containing pteridine
(pyrazino(2,3-dipyrimidine)), e.g. pteroic acid, xanthopterin and
folic acid."
"brown" 202   202   7   0.0058755113578508   1
18   0.0891089108910891   0.233766233766234
77   "GO:0016604"   "CC"   "nuclear body"   "Extra-nucleolar
nuclear domains usually visualized by confocal microscopy and
fluorescent antibodies to specific proteins."
"brown" 202   202   8   0.00651730103494638   1

4 0.0198019801980198 0.571428571428571
7 "GO:0007076" "BP" "mitotic chromosome condensation" "The cell cycle process in which chromatin structure is compacted prior to and during mitosis in eukaryotic cells."
"brown" 202 202 9 0.00729480443214277 1
6 0.0297029702970297 0.4 15 "GO:0071479" "BP" "cellular response to ionizing radiation" "Any process that results in a change in state or activity of a cell (in terms of movement, secretion, enzyme production, gene expression, etc.) as a result of a ionizing radiation stimulus. Ionizing radiation is radiation with sufficient energy to remove electrons from atoms and may arise from spontaneous decay of unstable isotopes, resulting in alpha and beta particles and gamma rays. Ionizing radiation also includes X-rays."
"brown" 202 202 10 0.00736133131089119 1
3 0.0148514851485149 0.75 4 "GO:0003151" "BP" "outflow tract morphogenesis" "The process in which the anatomical structures of the outflow tract are generated and organized. The outflow tract is the portion of the heart through which blood flows into the arteries."
"grey" 129 129 1 0.000549199072322138 1
4 0.0310077519379845 0.666666666666667
6 "GO:0051383" "BP" "kinetochore organization" "A process that is carried out at the cellular level which results in the assembly, arrangement of constituent parts, or disassembly of the kinetochore, a multisubunit complex that is located at the centromeric region of DNA and provides an attachment point for the spindle microtubules."
"grey" 129 129 2 0.00120093830306354 1
4 0.0310077519379845 0.571428571428571
7 "GO:0032930" "BP" "positive regulation of superoxide anion generation" "Any process that activates or increases the frequency, rate or extent of enzymatic generation of superoxide by a cell."
"grey" 129 129 3 0.00133952348435357 1
15 0.116279069767442 0.1875 80 "GO:0055082" "BP" "cellular chemical homeostasis" "Any biological process involved in the maintenance of an internal steady state of a chemical at the level of the cell."
"grey" 129 129 4 0.00174458730862561 1
15 0.116279069767442 0.182926829268293
82 "GO:0046982" "MF" "protein heterodimerization activity" "Interacting selectively and non-covalently with a nonidentical protein to form a heterodimer."
"grey" 129 129 5 0.00197349258162666 1
3 0.0232558139534884 0.75 4 "GO:0051382" "BP" "kinetochore assembly" "The aggregation, arrangement and bonding together of a set of components to form the kinetochore, a multisubunit complex that is located at the centromeric region of DNA and provides an attachment point for the spindle microtubules."
"grey" 129 129 6 0.00197349258162666 1
3 0.0232558139534884 0.75 4 "GO:

0005249" "MF" "voltage-gated potassium channel activity" "Enables the transmembrane transfer of a potassium ion by a voltage-gated channel. A voltage-gated channel is a channel whose open state is dependent on the voltage across the membrane in which it is embedded."
"grey" 129 129 7 0.00202061653626245 1
25 0.193798449612403 0.144508670520231
173 "GO:0051276" "BP" "chromosome organization" "A process that is carried out at the cellular level that results in the assembly, arrangement of constituent parts, or disassembly of chromosomes, structures composed of a very long molecule of DNA and associated proteins that carries hereditary information. This term covers covalent modifications at the molecular level as well as spatial relationships among the major components of a chromosome."
"grey" 129 129 8 0.00225130614615595 1
4 0.0310077519379845 0.5 8 "GO:
0032928" "BP" "regulation of superoxide anion
generation" "Any process that modulates the frequency, rate or extent of enzymatic generation of superoxide by a cell."
"grey" 129 129 9 0.00225130614615595 1
4 0.0310077519379845 0.5 8 "GO:
0034508" "BP" "centromere complex assembly" "The aggregation, arrangement and bonding together of proteins and centromeric DNA molecules to form a centromeric protein-DNA complex. Includes the formation of the chromatin structures which form a platform for the kinetochore, and assembly of the kinetochore onto this specialized chromatin. In fission yeast and higher eukaryotes this process also includes the formation of heterochromatin at the outer repeat (pericentric) regions of the centromere."
"grey" 129 129 10 0.00225130614615595 1
4 0.0310077519379845 0.5 8 "GO:
2000027" "BP" "regulation of organ morphogenesis" "Any process that modulates the frequency, rate or extent of animal organ morphogenesis."
"turquoise" 656 654 1 0.00119598850717356
1 29 0.0443425076452599 0.644444444444444
45 "GO:0001882" "MF" "nucleoside
binding" "Interacting selectively and non-covalently with a nucleoside, a compound consisting of a purine or pyrimidine nitrogenous base linked either to ribose or deoxyribose."
"turquoise" 656 654 2 0.00186938264948625
1 27 0.0412844036697248 0.642857142857143
42 "GO:0005525" "MF" "GTP binding" "Interacting selectively and non-covalently with GTP, guanosine triphosphate."
"turquoise" 656 654 3 0.00208623827327965
1 9 0.0137614678899083 0.9 10 "GO:
0005164" "MF" "tumor necrosis factor receptor
binding" "Interacting selectively and non-covalently with the tumor necrosis factor receptor."
"turquoise" 656 654 4 0.00309343548986666
1 27 0.0412844036697248 0.627906976744186
43 "GO:0001883" "MF" "purine nucleoside
binding" "Interacting selectively and non-covalently with a purine nucleoside, a compound consisting of a purine base linked

either to ribose or deoxyribose."
"turquoise"	656	654	5	0.00309343548986666
1	27	0.0412844036697248	0.627906976744186
43	"GO:0032549"	"MF"	"ribonucleoside
binding"	"Interacting selectively and non-covalently with a
ribonucleoside, a compound consisting of a purine or pyrimidine
nitrogenous base linked to ribose."
"turquoise"	656	654	6	0.00352796220090091
1	10	0.0152905198776758	0.833333333333333
12	"GO:0032813"	"MF"	"tumor necrosis factor receptor
superfamily binding"	"Interacting selectively and non-
covalently with any member of the tumor necrosis factor receptor
superfamily."
"turquoise"	656	654	7	0.00462285207280264
1	8	0.0122324159021407	0.888888888888889
9	"GO:0010765"	"BP"	"positive regulation of sodium ion
transport"	"Any process that increases the frequency, rate or
extent of the directed movement of sodium ions (Na+) into, out of or
within a cell, or between cells, by means of some agent such as a
transporter or pore."
"turquoise"	656	654	8	0.00760384761242237
1	27	0.0412844036697248	0.6	45	"GO:
0019001"	"MF"	"guanyl nucleotide binding"	"Interacting
selectively and non-covalently with guanyl nucleotides, any compound
consisting of guanosine esterified with (ortho)phosphate."
"turquoise"	656	654	9	0.00760384761242237
1	27	0.0412844036697248	0.6	45	"GO:
0032561"	"MF"	"guanyl ribonucleotide binding"	"Interacting
selectively and non-covalently with a guanyl ribonucleotide, any
compound consisting of guanosine esterified with (ortho)phosphate or
an oligophosphate at any hydroxyl group on the ribose moiety."
"turquoise"	656	654	10	0.0101256007445482
1	7	0.0107033639143731	0.875	8	"GO:
0030216"	"BP"	"keratinocyte differentiation"	"The process in
which a relatively unspecialized cell acquires specialized features
of a keratinocyte."
"yellow"	151	151	1	0.000233024404374016	1
15	0.0993377483443709	0.254237288135593
59	"GO:0071900"	"BP"	"regulation of protein serine/
threonine kinase activity"	"Any process that modulates the
rate, frequency, or extent of protein serine/threonine kinase
activity."
"yellow"	151	151	2	0.00163473400425846	1
10	0.0662251655629139	0.27027027027027	37	"GO:
0043405"	"BP"	"regulation of MAP kinase activity"	"Any
process that modulates the frequency, rate or extent of MAP kinase
activity."
"yellow"	151	151	3	0.00207215916107485	1
9	0.0596026490066225	0.28125	32	"GO:
0000082"	"BP"	"G1/S transition of mitotic cell cycle"	"The
mitotic cell cycle transition by which a cell in G1 commits to S
phase. The process begins with the build up of G1 cyclin-dependent
kinase (G1 CDK), resulting in the activation of transcription of G1
cyclins. The process ends with the positive feedback of the G1

cyclins on the G1 CDK which commits the cell to S phase, in which DNA replication is initiated."
"yellow" 151 151 4 0.00207215916107485 1
9 0.0596026490066225 0.28125 32 "GO:
0005743" "CC" "mitochondrial inner membrane" "The inner, i.e. lumen-facing, lipid bilayer of the mitochondrial envelope. It is highly folded to form cristae."
"yellow" 151 151 5 0.00226483868588603 1
7 0.0463576158940397 0.333333333333333
21 "GO:0007281" "BP" "germ cell development" "The process whose specific outcome is the progression of an immature germ cell over time, from its formation to the mature structure (gamete). A germ cell is any reproductive cell in a multicellular organism."
"yellow" 151 151 6 0.00252256767850981 1
10 0.0662251655629139 0.256410256410256
39 "GO:0019866" "CC" "organelle inner
membrane" "The inner, i.e. lumen-facing, lipid bilayer of an organelle envelope; usually highly selective to most ions and metabolites."
"yellow" 151 151 7 0.00327978936704289 1
9 0.0596026490066225 0.264705882352941
34 "GO:0044843" "BP" "cell cycle G1/S phase transition"
"The cell cycle process by which a cell in G1 phase commits to S phase."
"yellow" 151 151 8 0.00332186158669777 1
5 0.033112582781457 0.416666666666667
12 "GO:2000403" "BP" "positive regulation of lymphocyte migration" "Any process that activates or increases the frequency, rate or extent of lymphocyte migration."
"yellow" 151 151 9 0.00341669431456101 1
6 0.0397350993377483 0.352941176470588
17 "GO:0072678" "BP" "T cell migration" "The movement of a T cell within or between different tissues and organs of the body."
"yellow" 151 151 10 0.00405434524122959 1
7 0.0463576158940397 0.304347826086957
23 "GO:0043406" "BP" "positive regulation of MAP kinase activity" "Any process that activates or increases the frequency, rate or extent of MAP kinase activity."
