# Supplementary material for: Inflation vs. Exhaustion of Antiviral CD8+ T-Cell Populations in Persistent Infections: Two Sides of the Same Coin?
Source: Front Immunol. 2019 Mar 6;10:197. doi: 10.3389/fimmu.2019.00197 (PMC6414785; doi:10.3389/fimmu.2019.00197)
Supplement: Table S3 — GSEA report of Reactome gene sets enriched in Inflation. GSEA report of Reactome curated pathways found enriched (FDR < 0.25) in Inflating samples (M38, days 50) vs. Exhausting samples (Cl13, days 30). [file Table_3.pdf]

Table S3

NAME	GS<br>follow link to MSigDB	GS DETAILS	SIZE	ES	NES	NOM p-val	FDR q-val	FWER p-val	RANK AT MAX	LEADING EDGE
REACTOME_DNA_REPLICATION	REACTOME_DNA_REPLICATION	Details ...	161	0.78869563	2.5848584	0	0	0	2765	"tags=83%, list=17%, signal=99%"
REACTOME_MITOTIC_M_M_G1_PHASES	REACTOME_MITOTIC_M_M_G1_PHASES	Details ...	143	0.78841156	2.520427	0	0	0	2765	"tags=82%, list=17%, signal=98%"
REACTOME_CELL_CYCLE_MITOTIC	REACTOME_CELL_CYCLE_MITOTIC	Details ...	260	0.7375567	2.4846542	0	0	0	2765	"tags=70%, list=17%, signal=83%"
REACTOME_G1_S_TRANSITION	REACTOME_G1_S_TRANSITION	Details ...	92	0.7910559	2.455121	0	0	0	2738	"tags=85%, list=17%, signal=102%"
REACTOME_CELL_CYCLE	REACTOME_CELL_CYCLE	Details ...	316	0.7305762	2.451121	0	0	0	2706	"tags=66%, list=17%, signal=78%"
REACTOME_S_PHASE	REACTOME_S_PHASE	Details ...	91	0.7886546	2.4393938	0	0	0	2690	"tags=85%, list=17%, signal=101%"
REACTOME_CELL_CYCLE_CHECKPOINTS	REACTOME_CELL_CYCLE_CHECKPOINTS	Details ...	96	0.77978736	2.4209056	0	0	0	2668	"tags=79%, list=17%, signal=94%"
REACTOME_SYNTHESIS_OF_DNA	REACTOME_SYNTHESIS_OF_DNA	Details ...	78	0.80050945	2.4191465	0	0	0	2690	"tags=88%, list=17%, signal=106%"
REACTOME_M_G1_TRANSITION	REACTOME_M_G1_TRANSITION	Details ...	67	0.81085366	2.4167511	0	0	0	2668	"tags=90%, list=17%, signal=107%"
REACTOME_MITOTIC_G1_G1_S_PHASES	REACTOME_MITOTIC_G1_G1_S_PHASES	Details ...	112	0.7584441	2.4039187	0	0	0	2738	"tags=77%, list=17%, signal=92%"
REACTOME_MITOTIC_PROMETAPHASE	REACTOME_MITOTIC_PROMETAPHASE	Details ...	73	0.78409654	2.379741	0	0	0	1954	"tags=67%, list=12%, signal=76%"
REACTOME_REGULATION_OF_MITOTIC_CELL_CYCLE	REACTOME_REGULATION_OF_MITOTIC_CELL_CYCLE	Details ...	71	0.7838498	2.3530934	0	0	0	2668	"tags=76%, list=17%, signal=91%"
REACTOME_HOST_INTERACTIONS_OF_HIV_FACTORS	REACTOME_HOST_INTERACTIONS_OF_HIV_FACTORS	Details ...	107	0.73496544	2.3394303	0	0	0	3093	"tags=71%, list=19%, signal=87%"
REACTOME_ASSEMBLY_OF_THE_PRE_REPLICATIVE_COMPLEX	REACTOME_ASSEMBLY_OF_THE_PRE_REPLICATIVE_COMPLEX	Details ...	54	0.802842	2.3383176	0	0	0	2668	"tags=87%, list=17%, signal=104%"
REACTOME_MEIOTIC_RECOMBINATION	REACTOME_MEIOTIC_RECOMBINATION	Details ...	50	0.8093249	2.3278651	0	0	0	1003	"tags=58%, list=6%, signal=62%"
REACTOME_CHROMOSOME_MAINTENANCE	REACTOME_CHROMOSOME_MAINTENANCE	Details ...	83	0.7562414	2.3254275	0				

0	0	2249	"tags=66%, list=14%, signal=77%"
REACTOME_APC_C_CDC20_MEDIATED_DEGRADATION_OF_MITOTIC_PROTEINS
REACTOME_APC_C_CDC20_MEDIATED_DEGRADATION_OF_MITOTIC_PROTEINS
Details ...	61	0.7811348	2.3230066	0
0	0	2668	"tags=79%, list=17%, signal=94%"
REACTOME_TELOMERE_MAINTENANCE	REACTOME_TELOMERE_MAINTENANCE
Details ...	51	0.80506754	2.3171136	0
0	0	2249	"tags=80%, list=14%, signal=93%"
REACTOME_DEPOSITION_OF_NEW_CENPA_CONTAINING_NUCLEOSOMES_AT_THE_CENTR
OMERE
REACTOME_DEPOSITION_OF_NEW_CENPA_CONTAINING_NUCLEOSOMES_AT_THE_CENTR
OMERE	Details ...	34	0.8541647	2.3165529
0	0	0	998	"tags=71%, list=6%, signal=75%"
REACTOME_RESPIRATORY_ELECTRON_TRANSPORT_ATP_SYNTHESIS_BY_CHEMIOSMOTI
C_COUPLING_AND_HEAT_PRODUCTION_BY_UNCOUPLING_PROTEINS_
REACTOME_RESPIRATORY_ELECTRON_TRANSPORT_ATP_SYNTHESIS_BY_CHEMIOSMOTI
C_COUPLING_AND_HEAT_PRODUCTION_BY_UNCOUPLING_PROTEINS_
Details ...	68	0.77703553	2.3080008	0
0	0	2924	"tags=85%, list=18%, signal=104%"
REACTOME_RNA_POL_I_PROMOTER_OPENING
REACTOME_RNA_POL_I_PROMOTER_OPENING		30
0.8706158	2.298471	0	0	0
1003	"tags=73%, list=6%, signal=78%"
REACTOME_TCA_CYCLE_AND_RESPIRATORY_ELECTRON_TRANSPORT
REACTOME_TCA_CYCLE_AND_RESPIRATORY_ELECTRON_TRANSPORT
98	0.7268342	2.2933068	0	0	0
2973	"tags=78%, list=18%, signal=95%"
REACTOME_ER_PHAGOSOME_PATHWAY	REACTOME_ER_PHAGOSOME_PATHWAY
	50	0.7840883	2.2895174	0	0
0	2668	"tags=82%, list=17%, signal=98%"
REACTOME_ORC1_REMOVAL_FROM_CHROMATIN
REACTOME_ORC1_REMOVAL_FROM_CHROMATIN		56
0.7865059	2.286647	0	0	0
2668	"tags=84%, list=17%, signal=100%"
REACTOME_APC_C_CDH1_MEDIATED_DEGRADATION_OF_CDC20_AND_OTHER_APC_C_CD
H1_TARGETED_PROTEINS_IN_LATE_MITOSIS_EARLY_G1
REACTOME_APC_C_CDH1_MEDIATED_DEGRADATION_OF_CDC20_AND_OTHER_APC_C_CD
H1_TARGETED_PROTEINS_IN_LATE_MITOSIS_EARLY_G1		60
0.7776883	2.2833645	0	0	0
2668	"tags=78%, list=17%, signal=94%"
REACTOME_CDK_MEDIATED_PHOSPHORYLATION_AND_REMOVAL_OF_CDC6
REACTOME_CDK_MEDIATED_PHOSPHORYLATION_AND_REMOVAL_OF_CDC6
43	0.7909794	2.2752893	0	0	0
2668	"tags=86%, list=17%, signal=103%"
REACTOME_RESPIRATORY_ELECTRON_TRANSPORT
REACTOME_RESPIRATORY_ELECTRON_TRANSPORT		54
0.7702804	2.2692463	0	0	0
2924	"tags=85%, list=18%, signal=104%"
REACTOME_CDT1_ASSOCIATION_WITH_THE_CDC6_ORC_ORIGIN_COMPLEX
REACTOME_CDT1_ASSOCIATION_WITH_THE_CDC6_ORC_ORIGIN_COMPLEX
45	0.78918034	2.2586434	0	0	0
2668	"tags=84%, list=17%, signal=101%"
REACTOME_CYCLIN_E_ASSOCIATED_EVENTS_DURING_G1_S_TRANSITION_
REACTOME_CYCLIN_E_ASSOCIATED_EVENTS_DURING_G1_S_TRANSITION_

58	0.76881486	2.2541435	0	0	0
2668	"tags=79%, list=17%, signal=95%"
REACTOME_SCF_BETA_TRCP_MEDIATED_DEGRADATION_OF_EMI1
REACTOME_SCF_BETA_TRCP_MEDIATED_DEGRADATION_OF_EMI1
45	0.7882718	2.2519166	0	0	0
2668	"tags=82%, list=17%, signal=98%"
REACTOME_SCFSKP2_MEDIATED_DEGRADATION_OF_P27_P21
REACTOME_SCFSKP2_MEDIATED_DEGRADATION_OF_P27_P21	50
0.77751833	2.2404475	0	0	0
2668	"tags=82%, list=17%, signal=98%"
REACTOME_G2_M_CHECKPOINTS REACTOME_G2_M_CHECKPOINTS	30
0.8458865	2.2397203	0	0	0
1538	"tags=80%, list=10%, signal=88%"
REACTOME_AUTODEGRADATION_OF_CDH1_BY_CDH1_APC_C
REACTOME_AUTODEGRADATION_OF_CDH1_BY_CDH1_APC_C	54
0.77187735	2.2342322	0	0	0
2668	"tags=78%, list=17%, signal=93%"
REACTOME_DNA_STRAND_ELONGATION	REACTOME_DNA_STRAND_ELONGATION
27	0.84076446	2.2288375	0	0
0	2249	"tags=93%, list=14%, signal=107%"
REACTOME_AMYLOIDS	REACTOME_AMYLOIDS	46
0.79445565	2.2211552	0	0	0
1003	"tags=46%, list=6%, signal=49%"
REACTOME_SIGNALING_BY_WNT REACTOME_SIGNALING_BY_WNT	56
0.75162977	2.206472 0	0	0
3053	"tags=80%, list=19%, signal=99%"
REACTOME_P53_DEPENDENT_G1_DNA_DAMAGE_RESPONSE
REACTOME_P53_DEPENDENT_G1_DNA_DAMAGE_RESPONSE	49
0.75394356	2.2016776	0	0	0
2668	"tags=80%, list=17%, signal=95%"
REACTOME_REGULATION_OF_APOPTOSIS	REACTOME_REGULATION_OF_APOPTOSIS
51	0.76376444	2.201272 0	0	0
2668	"tags=75%, list=17%, signal=89%"
REACTOME_DESTABILIZATION_OF_MRNA_BY_AUF1_HNRNP_D0
REACTOME_DESTABILIZATION_OF_MRNA_BY_AUF1_HNRNP_D0	46
0.77340597	2.1961904	0	0	0
2668	"tags=80%, list=17%, signal=96%"
REACTOME_P53_INDEPENDENT_G1_S_DNA_DAMAGE_CHECKPOINT
REACTOME_P53_INDEPENDENT_G1_S_DNA_DAMAGE_CHECKPOINT
45	0.77980363	2.1932652	0	0	0
2668	"tags=84%, list=17%, signal=101%"
REACTOME_HIV_INFECTION	REACTOME_HIV_INFECTION	167
0.6647168	2.1860485	0	0	0
2668	"tags=57%, list=17%, signal=68%"
REACTOME_ACTIVATION_OF_ATR_IN_RESPONSE_TO_REPLICATION_STRESS
REACTOME_ACTIVATION_OF_ATR_IN_RESPONSE_TO_REPLICATION_STRESS
25	0.8510356	2.1837802	0	0
0	2087	"tags=88%, list=13%, signal=101%"
REACTOME_CROSS_PRESENTATION_OF_SOLUBLE_EXOGENOUS_ANTIGENS_ENDOSOMES
REACTOME_CROSS_PRESENTATION_OF_SOLUBLE_EXOGENOUS_ANTIGENS_ENDOSOMES
43	0.787741 2.1805656	0	0	0
2668	"tags=81%, list=17%, signal=97%"
REACTOME_RNA_POL_I_TRANSCRIPTION	REACTOME_RNA_POL_I_TRANSCRIPTION
50	0.76578796	2.1791706	0	0

0	1003	"tags=48%, list=6%, signal=51%"
REACTOME_PACKAGING_OF_TELOMERE_ENDS
REACTOME_PACKAGING_OF_TELOMERE_ENDS		26
0.8334472	2.1748927	0	0	0
914	"tags=65%, list=6%, signal=69%"
REACTOME_MEIOSIS REACTOME_MEIOSIS	74	0.72017777
2.1692438	0	0	0	2189	"tags=54%,
list=14%, signal=62%"
REACTOME_ANTIGEN_PROCESSING_CROSS_PRESENTATION
REACTOME_ANTIGEN_PROCESSING_CROSS_PRESENTATION		62
0.72450024	2.1686313	0	0	0
2668	"tags=69%, list=17%, signal=83%"
REACTOME_REGULATION_OF_ORNITHINE_DECARBOXYLASE_ODC
REACTOME_REGULATION_OF_ORNITHINE_DECARBOXYLASE_ODC	45
0.7665451	2.1684968	0	0	0
2668	"tags=78%, list=17%, signal=93%"
REACTOME_VIF_MEDIATED_DEGRADATION_OF_APOBEC3G
REACTOME_VIF_MEDIATED_DEGRADATION_OF_APOBEC3G		45
0.7728417	2.1652725	0	0	0
3270	"tags=89%, list=20%, signal=111%"
REACTOME_REGULATION_OF_MRNA_STABILITY_BY_PROTEINS_THAT_BIND_AU_RICH_
ELEMENTS
REACTOME_REGULATION_OF_MRNA_STABILITY_BY_PROTEINS_THAT_BIND_AU_RICH_
ELEMENTS	72	0.7167441	2.1609774	0
0	0	3296	"tags=71%, list=20%, signal=89%"
REACTOME_ACTIVATION_OF_NF_KAPPAB_IN_B_CELLS
REACTOME_ACTIVATION_OF_NF_KAPPAB_IN_B_CELLS		57
0.73404765	2.1494226	0	0	0
2668	"tags=68%, list=17%, signal=82%"
REACTOME_AUTODEGRADATION_OF_THE_E3_UBIQUITIN_LIGASE_COP1
REACTOME_AUTODEGRADATION_OF_THE_E3_UBIQUITIN_LIGASE_COP1
43	0.7685827	2.1427708	0	0	0
3071	"tags=86%, list=19%, signal=106%"
REACTOME_ACTIVATION_OF_THE_PRE_REPLICATIVE_COMPLEX
REACTOME_ACTIVATION_OF_THE_PRE_REPLICATIVE_COMPLEX	22
0.8516214	2.1190932	0	0	0
2349	"tags=95%, list=15%, signal=112%"
REACTOME_EXTENSION_OF_TELOMERES	REACTOME_EXTENSION_OF_TELOMERES
	25	0.8280412	2.1181946	0	0
0	2249	"tags=88%, list=14%, signal=102%"
REACTOME_METABOLISM_OF_RNA	REACTOME_METABOLISM_OF_RNA
	209	0.6318977	2.0889328	0	0
0	3131	"tags=54%, list=19%, signal=66%"
REACTOME_METABOLISM_OF_MRNA	REACTOME_METABOLISM_OF_MRNA
	168	0.6350364	2.0792878	0	0
0	3545	"tags=58%, list=22%, signal=74%"
REACTOME_E2F_MEDIATED_REGULATION_OF_DNA_REPLICATION
REACTOME_E2F_MEDIATED_REGULATION_OF_DNA_REPLICATION
24	0.8109448	2.0652668	0	0	0
2349	"tags=83%, list=15%, signal=97%"
REACTOME_DOWNSTREAM_SIGNALING_EVENTS_OF_B_CELL_RECEPTOR_BCR
REACTOME_DOWNSTREAM_SIGNALING_EVENTS_OF_B_CELL_RECEPTOR_BCR
83	0.6694285	2.0540242	0	0	0
2668	"tags=57%, list=17%, signal=68%"

REACTOME_TRANSCRIPTION	REACTOME_TRANSCRIPTION		142
0.6363671	2.0534878	0	0	0
2541	"tags=47%, list=16%, signal=56%"
REACTOME_MEIOTIC_SYNAPSIS REACTOME_MEIOTIC_SYNAPSIS	46
0.7230101	2.043081 0	0	0
1726	"tags=52%, list=11%, signal=58%"
REACTOME_LAGGING_STRAND_SYNTHESIS REACTOME_LAGGING_STRAND_SYNTHESIS
	19	0.8339135	2.0228088	0	0
0	2690	"tags=100%, list=17%, signal=120%"
REACTOME_RNA_POL_I_RNA_POL_III_AND_MITOCHONDRIAL_TRANSCRIPTION
REACTOME_RNA_POL_I_RNA_POL_III_AND_MITOCHONDRIAL_TRANSCRIPTION
	79	0.6645122	2.0082498	0	0
0	2153	"tags=43%, list=13%, signal=49%"
REACTOME_PROCESSING_OF_CAPPED_INTRON_CONTAINING_PRE_MRNA
REACTOME_PROCESSING_OF_CAPPED_INTRON_CONTAINING_PRE_MRNA
101	0.63119835	1.9826515	0	1.65E-05 0.001
3083	"tags=55%, list=19%, signal=68%"
REACTOME_DNA_REPAIR	REACTOME_DNA_REPAIR		90
0.629968 1.9553496	0	1.62E-05 0.001
2690	"tags=49%, list=17%, signal=58%"
REACTOME_SIGNALING_BY_THE_B_CELL_RECEPTOR_BCR
REACTOME_SIGNALING_BY_THE_B_CELL_RECEPTOR_BCR		110
0.61634815	1.9544188	0	1.60E-05 0.001
3347	"tags=58%, list=21%, signal=73%"
REACTOME_APOPTOSIS	REACTOME_APOPTOSIS		127
0.6065087	1.9460149	0	3.13E-05 0.002
3053	"tags=59%, list=19%, signal=72%"
REACTOME_MRNA_SPLICING	REACTOME_MRNA_SPLICING		74
0.63119173	1.9337256	0	4.73E-05 0.003
3625	"tags=64%, list=23%, signal=82%"
REACTOME_TRANSLATION	REACTOME_TRANSLATION		111
0.6085699	1.9254034	0	6.25E-05 0.004
4860	"tags=73%, list=30%, signal=104%"
REACTOME_MRNA_PROCESSING	REACTOME_MRNA_PROCESSING		117
0.6059184	1.9190344	0	6.16E-05 0.004
4097	"tags=63%, list=25%, signal=84%"
REACTOME_MHC_CLASS_II_ANTIGEN_PRESENTATION
REACTOME_MHC_CLASS_II_ANTIGEN_PRESENTATION	65
0.6381284	1.9057755	0	1.21E-04 0.008
2943	"tags=52%, list=18%, signal=64%"
REACTOME_PROCESSIVE_SYNTHESIS_ON_THE_LAGGING_STRAND
REACTOME_PROCESSIVE_SYNTHESIS_ON_THE_LAGGING_STRAND
15	0.83370626	1.9022654	0	1.50E-04 0.01
2690	"tags=100%, list=17%, signal=120%"
REACTOME_INFLUENZA_LIFE_CYCLE	REACTOME_INFLUENZA_LIFE_CYCLE
	100	0.6054846	1.8994455	0	1.63E-04
0.011	4452	"tags=64%, list=28%, signal=88%"
REACTOME_INHIBITION_OF_THE_PROTEOLYTIC_ACTIVITY_OF_APC_C_REQUIRED_FO
R_THE_ONSET_OF_ANAPHASE_BY_MITOTIC_SPINDLE_CHECKPOINT_COMPONENTS
REACTOME_INHIBITION_OF_THE_PROTEOLYTIC_ACTIVITY_OF_APC_C_REQUIRED_FO
R_THE_ONSET_OF_ANAPHASE_BY_MITOTIC_SPINDLE_CHECKPOINT_COMPONENTS
	18	0.77951956	1.8872938	0	2.05E-04
0.014	2529	"tags=67%, list=16%, signal=79%"
REACTOME_MITOTIC_G2_G2_M_PHASES	REACTOME_MITOTIC_G2_G2_M_PHASES

62 0.63001895 1.8832685 0 2.31E-04
0.016 1802 "tags=45%, list=11%, signal=51%"
REACTOME_CLASS_I_MHC_MEDIATED_ANTIGEN_PROCESSING_PRESENTATION
REACTOME_CLASS_I_MHC_MEDIATED_ANTIGEN_PROCESSING_PRESENTATION
209 0.5615058 1.8732063 0 3.12E-04
0.022 2668 "tags=43%, list=17%, signal=51%"
REACTOME_METABOLISM_OF_NON_CODING_RNA
REACTOME_METABOLISM_OF_NON_CODING_RNA 42
0.66467977 1.8494879 0 5.31E-04 0.038
2566 "tags=52%, list=16%, signal=62%"
REACTOME_KINESINS REACTOME_KINESINS 17
0.7696647 1.8409367 0 6.20E-04 0.044
1004 "tags=47%, list=6%, signal=50%"
REACTOME_ANTIGEN_PROCESSING_UBIQUITINATION_PROTEASOME_DEGRADATION
REACTOME_ANTIGEN_PROCESSING_UBIQUITINATION_PROTEASOME_DEGRADATION
180 0.56208265 1.8390976 0 6.39E-04
0.046 3417 "tags=49%, list=21%, signal=62%"
REACTOME_SRP_DEPENDENT_COTRANSLATIONAL_PROTEIN_TARGETING_TO_MEMBRANE

REACTOME_SRP_DEPENDENT_COTRANSLATIONAL_PROTEIN_TARGETING_TO_MEMBRANE
80 0.6074608 1.8380127 0
6.31E-04 0.046 5170 "tags=76%, list=32%, signal=112%"
REACTOME_FORMATION_OF_THE_TERNARY_COMPLEX_AND_SUBSEQUENTLY_THE_43S_C
OMPLEX
REACTOME_FORMATION_OF_THE_TERNARY_COMPLEX_AND_SUBSEQUENTLY_THE_43S_C
OMPLEX 34 0.6693575 1.8376149 0
6.23E-04 0.046 5011 "tags=85%, list=31%, signal=124%"
REACTOME_INTERACTIONS_OF_VPR_WITH_HOST_CELLULAR_PROTEINS
REACTOME_INTERACTIONS_OF_VPR_WITH_HOST_CELLULAR_PROTEINS
29 0.7054716 1.8346659 0 6.93E-04 0.051
2566 "tags=52%, list=16%, signal=61%"
REACTOME_THE_ROLE_OF_NEF_IN_HIV1_REPLICATION_AND_DISEASE_PATHOGENESI
S
REACTOME_THE_ROLE_OF_NEF_IN_HIV1_REPLICATION_AND_DISEASE_PATHOGENESI
S 22 0.72063744 1.8282096 0
7.62E-04 0.057 2388 "tags=59%, list=15%, signal=69%"
REACTOME_SIGNAL_TRANSDUCTION_BY_L1
REACTOME_SIGNAL_TRANSDUCTION_BY_L1 32
0.6843626 1.8276379 0.001257862 7.53E-04 0.057
3168 "tags=56%, list=20%, signal=70%"
REACTOME_3_UTR_MEDIATED_TRANSLATIONAL_REGULATION
REACTOME_3_UTR_MEDIATED_TRANSLATIONAL_REGULATION 73
0.5974011 1.8265032 0 7.69E-04 0.059
5170 "tags=77%, list=32%, signal=112%"
REACTOME_SYNTHESIS_AND_INTERCONVERSION_OF_NUCLEOTIDE_DI_AND_TRIPHOSP
HATES
REACTOME_SYNTHESIS_AND_INTERCONVERSION_OF_NUCLEOTIDE_DI_AND_TRIPHOSP
HATES 15 0.77967 1.8250481 0 7.73E-04
0.06 1305 "tags=53%, list=8%, signal=58%"
REACTOME_SMOOTH_MUSCLE_CONTRACTION
REACTOME_SMOOTH_MUSCLE_CONTRACTION 21
0.73312557 1.8187292 0 9.10E-04 0.072
2757 "tags=57%, list=17%, signal=69%"
REACTOME_PHOSPHORYLATION_OF_THE_APC_C

REACTOME_PHOSPHORYLATION_OF_THE_APC_C	16
0.77748173	1.8156877	0	9.60E-04	0.076
2529	"tags=63%, list=16%, signal=74%"
REACTOME_TRANSPORT_OF_RIBONUCLEOPROTEINS_INTO_THE_HOST_NUCLEUS
REACTOME_TRANSPORT_OF_RIBONUCLEOPROTEINS_INTO_THE_HOST_NUCLEUS
	25	0.71044284	1.8108827	0	9.97E-04
0.08	2566	"tags=52%, list=16%, signal=62%"
REACTOME_DOUBLE_STRAND_BREAK_REPAIR
REACTOME_DOUBLE_STRAND_BREAK_REPAIR	20
0.7331169	1.8094913	0.002624672	9.98E-04	0.081
3217	"tags=75%, list=20%, signal=94%"
REACTOME_APC_CDC20_MEDIATED_DEGRADATION_OF_NEK2A
REACTOME_APC_CDC20_MEDIATED_DEGRADATION_OF_NEK2A	20
0.7370271	1.8091898	0	9.87E-04	0.081
2529	"tags=60%, list=16%, signal=71%"
REACTOME_HIV_LIFE_CYCLE	REACTOME_HIV_LIFE_CYCLE	98
0.5751513	1.8084722	0	9.76E-04	0.081
2884	"tags=49%, list=18%, signal=59%"
REACTOME_G0_AND_EARLY_G1	REACTOME_G0_AND_EARLY_G1	20
0.7407984	1.8070996	0	9.66E-04	0.081
1960	"tags=60%, list=12%, signal=68%"
REACTOME_ADAPTIVE_IMMUNE_SYSTEM	REACTOME_ADAPTIVE_IMMUNE_SYSTEM
	428	0.5322155	1.80087	0	0.0010237
0.087	3349	"tags=46%, list=21%, signal=56%"
REACTOME_INFLUENZA_VIRAL_RNA_TRANSCRIPTION_AND_REPLICATION
REACTOME_INFLUENZA_VIRAL_RNA_TRANSCRIPTION_AND_REPLICATION
70	0.59453005	1.797866	0	0.001068719	0.092
5170	"tags=74%, list=32%, signal=109%"
REACTOME_TRANSPORT_OF_MATURE_MRNA_DERIVED_FROM_AN_INTRONLESS_TRANSCR
IPT
REACTOME_TRANSPORT_OF_MATURE_MRNA_DERIVED_FROM_AN_INTRONLESS_TRANSCR
IPT	30	0.6769309	1.7917185
0.001243781	0.001190095	0.102	2566	"tags=50%,
list=16%, signal=59%"
REACTOME_NUCLEOTIDE_EXCISION_REPAIR
REACTOME_NUCLEOTIDE_EXCISION_REPAIR	43
0.6357211	1.7853262	0	0.001320466	0.115
2087	"tags=49%, list=13%, signal=56%"
REACTOME_ACTIVATION_OF_THE_MRNA_UPON_BINDING_OF_THE_CAP_BINDING_COMP
LEX_AND_EIFS_AND_SUBSEQUENT_BINDING_TO_43S
REACTOME_ACTIVATION_OF_THE_MRNA_UPON_BINDING_OF_THE_CAP_BINDING_COMP
LEX_AND_EIFS_AND_SUBSEQUENT_BINDING_TO_43S	42
0.6423143	1.7824532	0	0.001394577	0.122
4050	"tags=71%, list=25%, signal=95%"
REACTOME_L1CAM_INTERACTIONS	REACTOME_L1CAM_INTERACTIONS
	66	0.59871936	1.7823344	0
0.001390955	0.122	3210	"tags=41%, list=20%, signal=51%"
REACTOME_RECYCLING_PATHWAY_OF_L1	REACTOME_RECYCLING_PATHWAY_OF_L1
	20	0.7276558	1.7787886	0
0.001516169	0.135	3046	"tags=65%, list=19%, signal=80%"
REACTOME_APC_C_CDC20_MEDIATED_DEGRADATION_OF_CYCLIN_B
REACTOME_APC_C_CDC20_MEDIATED_DEGRADATION_OF_CYCLIN_B
17	0.746631	1.7735586	0	0.001722047	0.152
2529	"tags=59%, list=16%, signal=70%"

REACTOME_GLOBAL_GENOMIC_NER_GG_NER
REACTOME_GLOBAL_GENOMIC_NER_GG_NER 31
0.6602998 1.7729254 0 0.001715832 0.152
2087 "tags=52%, list=13%, signal=59%"
REACTOME_CYTOSOLIC_TRNA_AMINOACYLATION
REACTOME_CYTOSOLIC_TRNA_AMINOACYLATION 22
0.6983128 1.7600849 0.002621232 0.002215929
0.194 2930 "tags=64%, list=18%, signal=78%"
REACTOME_TRANSPORT_OF_MATURE_TRANSCRIPT_TO_CYTOPLASM
REACTOME_TRANSPORT_OF_MATURE_TRANSCRIPT_TO_CYTOPLASM
39 0.64466804 1.7580438 0.001212121
0.002245039 0.199 2566 "tags=49%, list=16%, signal=58%"
REACTOME_NEP_NS2_INTERACTS_WITH_THE_CELLULAR_EXPORT_MACHINERY
REACTOME_NEP_NS2_INTERACTS_WITH_THE_CELLULAR_EXPORT_MACHINERY
25 0.67900044 1.7579929 0.003816794
0.002223453 0.199 2647 "tags=52%, list=16%, signal=62%"
REACTOME_LATE_PHASE_OF_HIV_LIFE_CYCLE
REACTOME_LATE_PHASE_OF_HIV_LIFE_CYCLE 85
0.5708293 1.7497482 0 0.002552712 0.224
4097 "tags=61%, list=25%, signal=82%"
REACTOME_RNA_POL_II_TRANSCRIPTION REACTOME_RNA_POL_II_TRANSCRIPTION
75 0.5697471 1.7480543 0
0.002608493 0.229 4834 "tags=68%, list=30%, signal=97%"
REACTOME_REGULATION_OF_GLUCOKINASE_BY_GLUCOKINASE_REGULATORY_PROTEIN

REACTOME_REGULATION_OF_GLUCOKINASE_BY_GLUCOKINASE_REGULATORY_PROTEIN
25 0.6814263 1.7390118
0.001290323 0.002958851 0.254 2566 "tags=48%,
list=16%, signal=57%"
REACTOME_SEMAPHORIN_INTERACTIONS REACTOME_SEMAPHORIN_INTERACTIONS
54 0.6040439 1.7382782 0
0.002950968 0.256 3168 "tags=48%, list=20%, signal=60%"
REACTOME_DEADENYLATION_DEPENDENT_MRNA_DECAY
REACTOME_DEADENYLATION_DEPENDENT_MRNA_DECAY 39
0.6275189 1.7357222 0 0.003048554 0.265
3951 "tags=56%, list=25%, signal=75%"
REACTOME_ELONGATION_ARREST_AND_RECOVERY
REACTOME_ELONGATION_ARREST_AND_RECOVERY 24
0.67978466 1.730744 0 0.003337209 0.29
3093 "tags=67%, list=19%, signal=82%"
REACTOME_TRANSCRIPTION_COUPLED_NER_TC_NER
REACTOME_TRANSCRIPTION_COUPLED_NER_TC_NER 38
0.6377384 1.7296275 0 0.003344868 0.294
2087 "tags=50%, list=13%, signal=57%"
REACTOME_PEPTIDE_CHAIN_ELONGATION REACTOME_PEPTIDE_CHAIN_ELONGATION
58 0.59065306 1.7290201 0
0.003315003 0.294 5170 "tags=72%, list=32%, signal=106%"
REACTOME_NONSENSE_MEDIATED_DECAY_ENHANCED_BY_THE_EXON_JUNCTION_COMPL
EX
REACTOME_NONSENSE_MEDIATED_DECAY_ENHANCED_BY_THE_EXON_JUNCTION_COMPL
EX 74 0.5738943 1.7268243 0
0.003425381 0.304 5170 "tags=70%, list=32%, signal=103%"
REACTOME_FACTORS_INVOLVED_IN_MEGAKARYOCYTE_DEVELOPMENT_AND_PLATELET_
PRODUCTION

REACTOME_FACTORS_INVOLVED_IN_MEGAKARYOCYTE_DEVELOPMENT_AND_PLATELET_
PRODUCTION 92 0.5572926 1.7246474
0 0.003515385 0.313 1935 "tags=30%, list=12%,
signal=34%"
REACTOME_PYRUVATE_METABOLISM_AND_CITRIC_ACID_TCA_CYCLE
REACTOME_PYRUVATE_METABOLISM_AND_CITRIC_ACID_TCA_CYCLE
33 0.64319754 1.7216444 0.002484472
0.00365842 0.328 3143 "tags=67%, list=20%, signal=83%"
REACTOME_SIGNALLING_BY_NGF REACTOME_SIGNALLING_BY_NGF
177 0.5259908 1.7189999 0
0.003772554 0.339 3286 "tags=41%, list=20%, signal=51%"
REACTOME_GLUCOSE_TRANSPORT REACTOME_GLUCOSE_TRANSPORT
35 0.63752085 1.7161608 0
0.004000464 0.363 2566 "tags=40%, list=16%, signal=47%"
REACTOME_CITRIC_ACID_CYCLE_TCA_CYCLE
REACTOME_CITRIC_ACID_CYCLE_TCA_CYCLE 17
0.7196686 1.7127221 0 0.004280001 0.387
3820 "tags=94%, list=24%, signal=123%"
REACTOME_NEF_MEDIATES_DOWN_MODULATION_OF_CELL_SURFACE_RECEPTORS_BY_R
ECRUITING_THEM_TO_CLATHRIN_ADAPTERS
REACTOME_NEF_MEDIATES_DOWN_MODULATION_OF_CELL_SURFACE_RECEPTORS_BY_R
ECRUITING_THEM_TO_CLATHRIN_ADAPTERS 17 0.726079
1.7105254 0 0.004367189 0.396
2915 "tags=65%, list=18%, signal=79%"
REACTOME_NGF_SIGNALLING_VIA_TRKA_FROM_THE_PLASMA_MEMBRANE
REACTOME_NGF_SIGNALLING_VIA_TRKA_FROM_THE_PLASMA_MEMBRANE
114 0.5340791 1.7057137 0 0.004741552
0.426 3051 "tags=40%, list=19%, signal=49%"
REACTOME_RNA_POL_II_PRE_TRANSCRIPTION_EVENTS
REACTOME_RNA_POL_II_PRE_TRANSCRIPTION_EVENTS 46
0.59376645 1.7052727 0.00120773 0.004755336
0.43 4754 "tags=72%, list=30%, signal=101%"
REACTOME_CONVERSION_FROM_APC_C_CDC20_TO_APC_C_CDH1_IN_LATE_ANAPHASE
REACTOME_CONVERSION_FROM_APC_C_CDC20_TO_APC_C_CDH1_IN_LATE_ANAPHASE
16 0.72515655 1.7014331 0.004149378
0.005148049 0.461 2529 "tags=56%, list=16%, signal=67%"
REACTOME_FORMATION_OF_RNA_POL_II_ELONGATION_COMPLEX_
REACTOME_FORMATION_OF_RNA_POL_II_ELONGATION_COMPLEX_
33 0.63125914 1.6975124 0 0.005481726
0.482 4651 "tags=76%, list=29%, signal=106%"
REACTOME_SEMA4D_IN_SEMAPHORIN_SIGNALING
REACTOME_SEMA4D_IN_SEMAPHORIN_SIGNALING 21
0.6668347 1.6743152 0.003963012 0.007788757
0.622 2060 "tags=57%, list=13%, signal=65%"
REACTOME_TRAFFICKING_OF_AMPA_RECEPTORS
REACTOME_TRAFFICKING_OF_AMPA_RECEPTORS 23
0.66755944 1.671353 0.002670227 0.008139056 0.635
2388 "tags=39%, list=15%, signal=46%"
REACTOME_METABOLISM_OF_NUCLEOTIDES
REACTOME_METABOLISM_OF_NUCLEOTIDES 62
0.56756264 1.670183 0 0.008191384 0.642
1341 "tags=31%, list=8%, signal=33%"
REACTOME_SEMA4D_INDUCED_CELL_MIGRATION_AND_GROWTH_CONE_COLLAPSE
REACTOME_SEMA4D_INDUCED_CELL_MIGRATION_AND_GROWTH_CONE_COLLAPSE

18	0.6948419	1.6645529	0.00538358
0.008839243	0.68	2060	"tags=61%, list=13%, signal=70%"
REACTOME_METABOLISM_OF_PROTEINS	REACTOME_METABOLISM_OF_PROTEINS
335	0.4903597	1.6538304	0
0.010253902	0.734	4064	"tags=48%, list=25%, signal=63%"
REACTOME_MRNA_SPLICING_MINOR_PATHWAY
REACTOME_MRNA_SPLICING_MINOR_PATHWAY	28
0.62721676	1.6496663	0.003841229	0.010663147
0.756	4834	"tags=82%, list=30%, signal=117%"
REACTOME_SIGNALING_BY_EGFR_IN_CANCER
REACTOME_SIGNALING_BY_EGFR_IN_CANCER	93
0.5283671	1.6400547	0	0.012001592	0.795
2829	"tags=45%, list=18%, signal=54%"
REACTOME_RECRUITMENT_OF_MITOTIC_CENTROSOME_PROTEINS_AND_COMPLEXES
REACTOME_RECRUITMENT_OF_MITOTIC_CENTROSOME_PROTEINS_AND_COMPLEXES
48	0.5665597	1.6382773	0
0.012255388	0.805	2395	"tags=46%, list=15%, signal=54%"
REACTOME_GLUCONEOGENESIS	REACTOME_GLUCONEOGENESIS	27
0.62301564	1.6316622	0.007731959	0.013247914
0.83	1979	"tags=44%, list=12%, signal=51%"
REACTOME_G1_PHASE	REACTOME_G1_PHASE	29
0.61381996	1.6301979	0.005134788	0.013423311
0.832	2269	"tags=55%, list=14%, signal=64%"
REACTOME_RESPONSE_TO_ELEVATED_PLATELET_CYTOSOLIC_CA2_
REACTOME_RESPONSE_TO_ELEVATED_PLATELET_CYTOSOLIC_CA2_
68	0.5463355	1.6299331	0.001140251
0.013338902	0.832	2772	"tags=35%, list=17%, signal=42%"
REACTOME_COSTIMULATION_BY_THE_CD28_FAMILY
REACTOME_COSTIMULATION_BY_THE_CD28_FAMILY	47	0.573619
1.629841	0.003631961	0.013263412	0.833
2738	"tags=49%, list=17%, signal=59%"
REACTOME_ANTIVIRAL_MECHANISM_BY_IFN_STIMULATED_GENES
REACTOME_ANTIVIRAL_MECHANISM_BY_IFN_STIMULATED_GENES
56	0.559681	1.629356	0.002301496	0.013258281	0.833
2661	"tags=48%, list=17%, signal=58%"
REACTOME_GLUCOSE_METABOLISM	REACTOME_GLUCOSE_METABOLISM
57	0.55505055	1.6275308	0.00238379
0.013414376	0.835	2948	"tags=47%, list=18%, signal=58%"
REACTOME_ACTIVATION_OF_CHAPERONE_GENES_BY_XBP1S
REACTOME_ACTIVATION_OF_CHAPERONE_GENES_BY_XBP1S	37
0.59127975	1.6179823	0.002528445	0.015110023
0.864	4175	"tags=62%, list=26%, signal=84%"
REACTOME_FORMATION_OF_THE_HIV1_EARLY_ELONGATION_COMPLEX
REACTOME_FORMATION_OF_THE_HIV1_EARLY_ELONGATION_COMPLEX
25	0.6322273	1.6164488	0.010309278
0.015214064	0.87	4651	"tags=76%, list=29%, signal=107%"
REACTOME_INTERFERON_SIGNALING	REACTOME_INTERFERON_SIGNALING
117	0.5084619	1.6137779	0
0.01568209	0.886	3379	"tags=47%, list=21%, signal=59%"
REACTOME_MITOCHONDRIAL_PROTEIN_IMPORT
REACTOME_MITOCHONDRIAL_PROTEIN_IMPORT	43	0.572403
1.6049218	0.001182033	0.01740689	0.914
3298	"tags=56%, list=20%, signal=70%"
REACTOME_AXON_GUIDANCE	REACTOME_AXON_GUIDANCE	197

0.48736268	1.6045227	0	0.017365336	0.919
3210	"tags=32%, list=20%, signal=40%"
REACTOME_ABORTIVE_ELONGATION_OF_HIV1_TRANSCRIPT_IN_THE_ABSENCE_OF_TA
T
REACTOME_ABORTIVE_ELONGATION_OF_HIV1_TRANSCRIPT_IN_THE_ABSENCE_OF_TA
T		17	0.6862599	1.60001	0.009320905
0.018202595	0.929	4366	"tags=82%, list=27%, signal=113%"
REACTOME_ASPARAGINE_N_LINKED_GLYCOSYLATION
REACTOME_ASPARAGINE_N_LINKED_GLYCOSYLATION	70
0.5320143	1.5989769	0.002290951	0.018324966
0.93	4187	"tags=56%, list=26%, signal=75%"
REACTOME_UNFOLDED_PROTEIN_RESPONSE
REACTOME_UNFOLDED_PROTEIN_RESPONSE	63
0.5472108	1.596561	0	0.018824704	0.936
3533	"tags=49%, list=22%, signal=63%"
REACTOME_PYRIMIDINE_METABOLISM	REACTOME_PYRIMIDINE_METABOLISM
	21	0.63343203	1.5837096	0.013245033
0.021954898	0.962	948	"tags=29%, list=6%, signal=30%"
REACTOME_DARPP_32_EVENTS	REACTOME_DARPP_32_EVENTS	23
0.621281	1.5816603	0.009174312	0.022206824	0.962
3286	"tags=70%, list=20%, signal=87%"
REACTOME_HEMOSTASIS	REACTOME_HEMOSTASIS	383
0.46375072	1.5812035	0	0.022205468	0.963
3806	"tags=38%, list=24%, signal=48%"
REACTOME_TGF_BETA_RECEPTOR_SIGNALING_ACTIVATES_SMADS
REACTOME_TGF_BETA_RECEPTOR_SIGNALING_ACTIVATES_SMADS
19	0.6596114	1.5732145	0.006648936
0.024259897	0.974	3725	"tags=63%, list=23%, signal=82%"
REACTOME_CELL_DEATH_SIGNALLING_VIA_NRAGE_NRIF_AND_NADE
REACTOME_CELL_DEATH_SIGNALLING_VIA_NRAGE_NRIF_AND_NADE
46	0.5498246	1.5692052	0.004716981
0.025370775	0.977	2764	"tags=37%, list=17%, signal=44%"
REACTOME_MRNA_CAPPING	REACTOME_MRNA_CAPPING	22
0.6229884	1.5673361	0.01615074	0.025755115
0.977	4651	"tags=73%, list=29%, signal=102%"
REACTOME_ERK_MAPK_TARGETS	REACTOME_ERK_MAPK_TARGETS	18
0.6541157	1.563207	0.02	0.026880778	0.983
3051	"tags=61%, list=19%, signal=75%"
REACTOME_MUSCLE_CONTRACTION	REACTOME_MUSCLE_CONTRACTION
	40	0.56232274	1.5617294	0.007185629
0.027248725	0.983	3435	"tags=43%, list=21%, signal=54%"
REACTOME_P75_NTR_RECEPTOR_MEDIATED_SIGNALLING
REACTOME_P75_NTR_RECEPTOR_MEDIATED_SIGNALLING	65
0.5221838	1.5613767	0	0.02718797	0.984
3270	"tags=40%, list=20%, signal=50%"
REACTOME_LOSS_OF_NLP_FROM_MITOTIC_CENTROSOMES
REACTOME_LOSS_OF_NLP_FROM_MITOTIC_CENTROSOMES	43
0.5452198	1.5569755	0.012135922	0.028316831
0.986	2069	"tags=44%, list=13%, signal=51%"
REACTOME_NUCLEAR_EVENTS_KINASE_AND_TRANSCRIPTION_FACTOR_ACTIVATION
REACTOME_NUCLEAR_EVENTS_KINASE_AND_TRANSCRIPTION_FACTOR_ACTIVATION
	21	0.6214711	1.5562999	0.022280471
0.028317956	0.986	3051	"tags=57%, list=19%, signal=70%"
REACTOME_PRE_NOTCH_PROCESSING_IN_GOLGI

REACTOME_PRE_NOTCH_PROCESSING_IN_GOLGI 15
0.67220306 1.5553675 0.023709903 0.028438674
0.986 2121 "tags=53%, list=13%, signal=61%"
REACTOME_BIOSYNTHESIS_OF_THE_N_GLYCAN_PRECURSOR_DOLICHOL_LIPID_LINKE
D_OLIGOSACCHARIDE_LLO_AND_TRANSFER_TO_A_NASCENT_PROTEIN
REACTOME_BIOSYNTHESIS_OF_THE_N_GLYCAN_PRECURSOR_DOLICHOL_LIPID_LINKE
D_OLIGOSACCHARIDE_LLO_AND_TRANSFER_TO_A_NASCENT_PROTEIN
26 0.59594065 1.5455664 0.014138818
0.03163929 0.989 2832 "tags=42%, list=18%, signal=51%"
REACTOME_GLYCOLYSIS REACTOME_GLYCOLYSIS 26
0.60091716 1.5445992 0.015645372 0.03180582
0.99 1979 "tags=50%, list=12%, signal=57%"
REACTOME_INTEGRIN_ALPHAIIB_BETA3_SIGNALING
REACTOME_INTEGRIN_ALPHAIIB_BETA3_SIGNALING 26
0.58810294 1.5427231 0.013192612 0.03231226
0.99 1958 "tags=35%, list=12%, signal=39%"
REACTOME_DEADENYLATION_OF_MRNA REACTOME_DEADENYLATION_OF_MRNA
17 0.6533003 1.5318488 0.024423338
0.035981923 0.995 3951 "tags=71%, list=25%, signal=93%"
REACTOME_GENERATION_OF_SECOND_MESSENGER_MOLECULES
REACTOME_GENERATION_OF_SECOND_MESSENGER_MOLECULES 19
0.6289413 1.531593 0.02631579 0.03585712 0.995
3347 "tags=53%, list=21%, signal=66%"
REACTOME_RESOLUTION_OF_AP_SITES_VIA_THE_MULTIPLE_NUCLEOTIDE_PATCH_RE
PLACEMENT_PATHWAY
REACTOME_RESOLUTION_OF_AP_SITES_VIA_THE_MULTIPLE_NUCLEOTIDE_PATCH_RE
PLACEMENT_PATHWAY 17 0.64834094
1.5262007 0.026760563 0.037770435 0.996
2690 "tags=53%, list=17%, signal=63%"
REACTOME_PLATELET_ACTIVATION_SIGNALING_AND_AGGREGATION
REACTOME_PLATELET_ACTIVATION_SIGNALING_AND_AGGREGATION
178 0.46318376 1.5216906 0 0.03939347
0.997 2052 "tags=29%, list=13%, signal=32%"
REACTOME_LYSOSOME_VESICLE_BIOGENESIS
REACTOME_LYSOSOME_VESICLE_BIOGENESIS 18
0.6262256 1.5216589 0.027061855 0.03916722
0.997 2915 "tags=56%, list=18%, signal=68%"
REACTOME_BASE_EXCISION_REPAIR REACTOME_BASE_EXCISION_REPAIR
19 0.6275396 1.5186992 0.034574468
0.040160246 0.998 2690 "tags=47%, list=17%, signal=57%"
REACTOME_OTHER_SEMAPHORIN_INTERACTIONS
REACTOME_OTHER_SEMAPHORIN_INTERACTIONS 15
0.65096515 1.5168 0.035063114 0.040771037 0.998
3168 "tags=47%, list=20%, signal=58%"
REACTOME_APOPTOTIC_EXECUTION_PHASE
REACTOME_APOPTOTIC_EXECUTION_PHASE 45
0.5342928 1.5164152 0.014234875 0.04065423
0.998 3681 "tags=56%, list=23%, signal=72%"
REACTOME_INSULIN_RECEPTOR_RECYCLING
REACTOME_INSULIN_RECEPTOR_RECYCLING 19
0.6197653 1.5160776 0.038356163 0.04058078
0.998 4179 "tags=58%, list=26%, signal=78%"
REACTOME_DIABETES_PATHWAYS REACTOME_DIABETES_PATHWAYS
103 0.48451638 1.51457 0.003282276

0.04104693	0.998	3533	"tags=40%, list=22%, signal=51%"
REACTOME_CTLA4_INHIBITORY_SIGNALING
REACTOME_CTLA4_INHIBITORY_SIGNALING	18
0.6183529	1.5137483	0.026279392	0.041229915
0.998	1988	"tags=61%, list=12%, signal=70%"
REACTOME_SIGNALING_BY_ERBB4	REACTOME_SIGNALING_BY_ERBB4
72	0.4966521	1.5028979	0.01849711
0.045822594	0.998	2986	"tags=33%, list=19%, signal=41%"
REACTOME_CHOLESTEROL_BIOSYNTHESIS REACTOME_CHOLESTEROL_BIOSYNTHESIS
15	0.6607252	1.5009083	0.030054646
0.046495333	0.998	2491	"tags=47%, list=15%, signal=55%"
REACTOME_EGFR_DOWNREGULATION	REACTOME_EGFR_DOWNREGULATION
21	0.6057746	1.5001383	0.047557842
0.046629004	0.998	2559	"tags=62%, list=16%, signal=74%"
REACTOME_RNA_POL_II_TRANSCRIPTION_PRE_INITIATION_AND_PROMOTER_OPENIN
G
REACTOME_RNA_POL_II_TRANSCRIPTION_PRE_INITIATION_AND_PROMOTER_OPENIN
G	31	0.5594787	1.4969417
0.038961038	0.04776846	0.998	4754	"tags=68%,
list=30%, signal=96%"
REACTOME_SHC1_EVENTS_IN_ERBB4_SIGNALING
REACTOME_SHC1_EVENTS_IN_ERBB4_SIGNALING	16
0.6376965	1.4928368	0.044058744	0.04953755
1	5284	"tags=81%, list=33%, signal=121%"
REACTOME_LATENT_INFECTION_OF_HOMO_SAPIENS_WITH_MYCOBACTERIUM_TUBERCU
LOSIS
REACTOME_LATENT_INFECTION_OF_HOMO_SAPIENS_WITH_MYCOBACTERIUM_TUBERCU
LOSIS	28	0.56258905	1.4894764
0.03937008	0.051070616	1	2115	"tags=32%,
list=13%, signal=37%"
REACTOME_CLEAVAGE_OF_GROWING_TRANSCRIPT_IN_THE_TERMINATION_REGION_
REACTOME_CLEAVAGE_OF_GROWING_TRANSCRIPT_IN_THE_TERMINATION_REGION_
28	0.5705338	1.4870299	0.035714287
0.051901374	1	3733	"tags=57%, list=23%, signal=74%"
REACTOME_SIGNALING_BY_INSULIN_RECEPTOR
REACTOME_SIGNALING_BY_INSULIN_RECEPTOR	90
0.48083267	1.4869583	0.001106195	0.05163475
1	4179	"tags=39%, list=26%, signal=52%"
REACTOME_REGULATION_OF_HYPOXIA_INDUCIBLE_FACTOR_HIF_BY_OXYGEN
REACTOME_REGULATION_OF_HYPOXIA_INDUCIBLE_FACTOR_HIF_BY_OXYGEN
21	0.5862116	1.486153 0.031128405
0.051741984	1	3270	"tags=48%, list=20%, signal=60%"
REACTOME_TRANS_GOLGI_NETWORK_VESICLE_BUDDING
REACTOME_TRANS_GOLGI_NETWORK_VESICLE_BUDDING	48
0.50925297	1.4786589	0.03206651	0.05534111
1	2915	"tags=38%, list=18%, signal=46%"
REACTOME_DOWNREGULATION_OF_TGF_BETA_RECEPTOR_SIGNALING
REACTOME_DOWNREGULATION_OF_TGF_BETA_RECEPTOR_SIGNALING
17	0.6280016	1.4781026	0.040221915
0.05528069	1	3725	"tags=59%, list=23%, signal=76%"
REACTOME_METABOLISM_OF_AMINO_ACIDS_AND_DERIVATIVES
REACTOME_METABOLISM_OF_AMINO_ACIDS_AND_DERIVATIVES	169
0.44886824	1.4695334	0.003125 0.059624087	1
3075	"tags=34%, list=19%, signal=41%"

REACTOME_SIGNALING_BY_SCF_KIT	REACTOME_SIGNALING_BY_SCF_KIT
	64	0.49729866	1.4662116	0.017381229
0.06099129	1	2020	"tags=33%, list=13%, signal=37%"
REACTOME_SIGNALING_BY_NOTCH	REACTOME_SIGNALING_BY_NOTCH
	90	0.4654709	1.4477421	0.010952903
0.07214959	1	3410	"tags=38%, list=21%, signal=48%"
REACTOME_GOLGI_ASSOCIATED_VESICLE_BIOGENESIS
REACTOME_GOLGI_ASSOCIATED_VESICLE_BIOGENESIS	43
0.5124902	1.4454365	0.02166065	0.07329529
1	2915	"tags=37%, list=18%, signal=45%"
REACTOME_IL_2_SIGNALING	REACTOME_IL_2_SIGNALING	38
0.5272081	1.4430958	0.024125453	0.07451256
1	2788	"tags=39%, list=17%, signal=48%"
REACTOME_SIGNALING_BY_RHO_GTPASES REACTOME_SIGNALING_BY_RHO_GTPASES
	81	0.46811026	1.4415308	0.023359288
0.075124696	1	1951	"tags=22%, list=12%, signal=25%"
REACTOME_MAP_KINASE_ACTIVATION_IN_TLR_CASCADE
REACTOME_MAP_KINASE_ACTIVATION_IN_TLR_CASCADE	42
0.51065904	1.4413218	0.033573143	0.07486099
1	3051	"tags=40%, list=19%, signal=50%"
REACTOME_MEMBRANE_TRAFFICKING	REACTOME_MEMBRANE_TRAFFICKING
	106	0.45771813	1.4372097	0.011956521
0.07731212	1	2975	"tags=34%, list=18%, signal=41%"
REACTOME_TRNA_AMINOACYLATION	REACTOME_TRNA_AMINOACYLATION
	37	0.51880366	1.4305457	0.041198503
0.081851564	1	2930	"tags=46%, list=18%, signal=56%"
REACTOME_SIGNALING_BY_ERBB2	REACTOME_SIGNALING_BY_ERBB2
	81	0.46478215	1.4232733	0.012208657
0.08668588	1	3286	"tags=41%, list=20%, signal=51%"
REACTOME_CYTOKINE_SIGNALING_IN_IMMUNE_SYSTEM
REACTOME_CYTOKINE_SIGNALING_IN_IMMUNE_SYSTEM	213
0.42438123	1.4231553	0	0.08638959	1
3379	"tags=40%, list=21%, signal=50%"
REACTOME_PKB_MEDIATED_EVENTS	REACTOME_PKB_MEDIATED_EVENTS
	22	0.57329684	1.4224935	0.052700922
0.08639353	1	3898	"tags=55%, list=24%, signal=72%"
REACTOME_PROCESSING_OF_CAPPED_INTRONLESS_PRE_MRNA
REACTOME_PROCESSING_OF_CAPPED_INTRONLESS_PRE_MRNA	19
0.5811632	1.4044877	0.071614586	0.10044746
1	2541	"tags=47%, list=16%, signal=56%"
REACTOME_G_ALPHA1213_SIGNALLING_EVENTS
REACTOME_G_ALPHA1213_SIGNALLING_EVENTS	62
0.4705548	1.4008491	0.03207331	0.10297428
1	2988	"tags=32%, list=19%, signal=39%"
REACTOME_LIPID_DIGESTION_MOBILIZATION_AND_TRANSPORT
REACTOME_LIPID_DIGESTION_MOBILIZATION_AND_TRANSPORT
37	0.5067276	1.3982203	0.058894232
0.10484053	1	1717	"tags=22%, list=11%, signal=24%"
REACTOME_INSULIN_RECEPTOR_SIGNALLING_CASCADE
REACTOME_INSULIN_RECEPTOR_SIGNALLING_CASCADE	72
0.45837775	1.3944067	0.03644647	0.107554585
1	3114	"tags=29%, list=19%, signal=36%"
REACTOME_TRANSFERRIN_ENDOCYTOSIS_AND_RECYCLING
REACTOME_TRANSFERRIN_ENDOCYTOSIS_AND_RECYCLING	21

0.5689315	1.3941731	0.07919463	0.10718353
1	4179	"tags=48%, list=26%, signal=64%"
REACTOME_DEVELOPMENTAL_BIOLOGY	REACTOME_DEVELOPMENTAL_BIOLOGY
	320	0.41610628	1.3940388	0
0.10676829	1	3225	"tags=28%, list=20%, signal=34%"
REACTOME_PLATELET_AGGREGATION_PLUG_FORMATION
REACTOME_PLATELET_AGGREGATION_PLUG_FORMATION		32
0.5189374	1.3908336	0.07033248	0.10905019
1	1958	"tags=28%, list=12%, signal=32%"
REACTOME_IRON_UPTAKE_AND_TRANSPORT
REACTOME_IRON_UPTAKE_AND_TRANSPORT		30
0.5132028	1.3858503	0.086185046	0.11278215
1	4179	"tags=43%, list=26%, signal=58%"
REACTOME_NOTCH1_INTRACELLULAR_DOMAIN_REGULATES_TRANSCRIPTION
REACTOME_NOTCH1_INTRACELLULAR_DOMAIN_REGULATES_TRANSCRIPTION
	38	0.50318706	1.3858489	0.067065865
0.11222657	1	3270	"tags=37%, list=20%, signal=46%"
REACTOME_DOWNSTREAM_SIGNALING_OF_ACTIVATED_FGFR
REACTOME_DOWNSTREAM_SIGNALING_OF_ACTIVATED_FGFR		87
0.44470233	1.3835279	0.028792912	0.113802545
1	3004	"tags=31%, list=19%, signal=38%"
REACTOME_OXYGEN_DEPENDENT_PROLINE_HYDROXYLATION_OF_HYPOXIA_INDUCIBLE
_FACTOR_ALPHA
REACTOME_OXYGEN_DEPENDENT_PROLINE_HYDROXYLATION_OF_HYPOXIA_INDUCIBLE
_FACTOR_ALPHA		15	0.6024506	1.3800757
0.092769444	0.11653545	1	3270	"tags=47%,
list=20%, signal=58%"
REACTOME_SIGNALING_BY_NOTCH1	REACTOME_SIGNALING_BY_NOTCH1
	61	0.46326584	1.3768913	0.048642535
0.11886141	1	3270	"tags=34%, list=20%, signal=43%"
REACTOME_PI3K_AKT_ACTIVATION	REACTOME_PI3K_AKT_ACTIVATION
	30	0.51620746	1.3754896	0.08483291
0.11967617	1	2126	"tags=33%, list=13%, signal=38%"
REACTOME_MAPK_TARGETS_NUCLEAR_EVENTS_MEDIATED_BY_MAP_KINASES
REACTOME_MAPK_TARGETS_NUCLEAR_EVENTS_MEDIATED_BY_MAP_KINASES
	27	0.52466226	1.3697917	0.080729164
0.124757424	1	3051	"tags=48%, list=19%, signal=59%"
REACTOME_SIGNALING_BY_CONSTITUTIVELY_ACTIVE_EGFR
REACTOME_SIGNALING_BY_CONSTITUTIVELY_ACTIVE_EGFR		15
0.594575	1.3693995	0.09324324	0.12455175	1
4849	"tags=67%, list=30%, signal=95%"
REACTOME_METABOLISM_OF_CARBOHYDRATES
REACTOME_METABOLISM_OF_CARBOHYDRATES		202
0.41117728	1.369196	0.005138746	0.124184676	1
2948	"tags=28%, list=18%, signal=34%"
REACTOME_GAB1_SIGNALOSOME	REACTOME_GAB1_SIGNALOSOME	32
0.51564765	1.3673989	0.0797546	0.12526618
1	2006	"tags=31%, list=12%, signal=36%"
REACTOME_CA_DEPENDENT_EVENTS	REACTOME_CA_DEPENDENT_EVENTS
	26	0.5409789	1.367129	0.098445594
0.12494292	1	3927	"tags=58%, list=24%, signal=76%"
REACTOME_OPIOID_SIGNALLING	REACTOME_OPIOID_SIGNALLING
	72	0.451319	1.3653657	0.04214123
0.12600851	1	3286	"tags=42%, list=20%, signal=52%"

REACTOME_SIGNALING_BY_FGFR	REACTOME_SIGNALING_BY_FGFR
	96	0.4349534	1.365025	0.035869565
0.1257539	1	3286	"tags=34%, list=20%, signal=43%"
REACTOME_SIGNALLING_TO_ERKS	REACTOME_SIGNALLING_TO_ERKS
	29	0.51831084	1.3638144	0.09147095
0.12634799	1	4739	"tags=48%, list=29%, signal=68%"
REACTOME_DOWNSTREAM_SIGNAL_TRANSDUCTION
REACTOME_DOWNSTREAM_SIGNAL_TRANSDUCTION	81
0.43959624	1.3521423	0.056369785	0.13755772
1	3347	"tags=36%, list=21%, signal=45%"
REACTOME_PRE_NOTCH_EXPRESSION_AND_PROCESSING
REACTOME_PRE_NOTCH_EXPRESSION_AND_PROCESSING	37
0.49640688	1.3504195	0.079404466	0.13866757
1	2413	"tags=41%, list=15%, signal=48%"
REACTOME_SIGNALLING_TO_RAS	REACTOME_SIGNALLING_TO_RAS
	22	0.5413619	1.3473772	0.10396717
0.1411887	1	4739	"tags=55%, list=29%, signal=77%"
REACTOME_CD28_CO_STIMULATION	REACTOME_CD28_CO_STIMULATION
	25	0.52811235	1.3440611	0.0931436
0.1441658	1	1988	"tags=40%, list=12%, signal=46%"
REACTOME_SIGNALING_BY_FGFR_IN_DISEASE
REACTOME_SIGNALING_BY_FGFR_IN_DISEASE	107
0.41856766	1.3422688	0.04756757	0.14523934
1	3385	"tags=34%, list=21%, signal=42%"
REACTOME_PIP3_ACTIVATES_AKT_SIGNALING
REACTOME_PIP3_ACTIVATES_AKT_SIGNALING	23
0.53431517	1.34047	0.10381077	0.14651957	1
2006	"tags=35%, list=12%, signal=40%"
REACTOME_GRB2_EVENTS_IN_ERBB2_SIGNALING
REACTOME_GRB2_EVENTS_IN_ERBB2_SIGNALING	17
0.56314963	1.3373914	0.113513514	0.14908119
1	5284	"tags=71%, list=33%, signal=105%"
REACTOME_TRANSPORT_OF_VITAMINS_NUCLEOSIDES_AND_RELATED_MOLECULES
REACTOME_TRANSPORT_OF_VITAMINS_NUCLEOSIDES_AND_RELATED_MOLECULES
	26	0.51263314	1.336087	0.090561226
0.15000911	1	2810	"tags=35%, list=17%, signal=42%"
REACTOME_PROTEIN_FOLDING	REACTOME_PROTEIN_FOLDING	41
0.48034793	1.3313626	0.103831895	0.15435883
1	3014	"tags=37%, list=19%, signal=45%"
REACTOME_RNA_POL_III_TRANSCRIPTION_INITIATION_FROM_TYPE_2_PROMOTER
REACTOME_RNA_POL_III_TRANSCRIPTION_INITIATION_FROM_TYPE_2_PROMOTER
	19	0.54879355	1.3250803	0.125855
0.16066085	1	2514	"tags=42%, list=16%, signal=50%"
REACTOME_INTERFERON_GAMMA_SIGNALING
REACTOME_INTERFERON_GAMMA_SIGNALING	41
0.46971253	1.3242685	0.103785105	0.16082138
1	3927	"tags=49%, list=24%, signal=64%"
REACTOME_INTRINSIC_PATHWAY_FOR_APOPTOSIS
REACTOME_INTRINSIC_PATHWAY_FOR_APOPTOSIS	27
0.5078573	1.3212428	0.114173226	0.16370907
1	3594	"tags=63%, list=22%, signal=81%"
REACTOME_TCR_SIGNALING	REACTOME_TCR_SIGNALING	42
0.47395182	1.3120363	0.09951457	0.17386088
1	3347	"tags=40%, list=21%, signal=51%"

REACTOME_IL_3_5_AND_GM_CSF_SIGNALING
REACTOME_IL_3_5_AND_GM_CSF_SIGNALING 39
0.4736197 1.3057634 0.117788464 0.1808304
1 2958 "tags=38%, list=18%, signal=47%"
REACTOME_PEROXISOMAL_LIPID_METABOLISM
REACTOME_PEROXISOMAL_LIPID_METABOLISM 19
0.5458747 1.3052243 0.12689655 0.18071304
1 3576 "tags=47%, list=22%, signal=61%"
REACTOME_NRAGE_SIGNALS_DEATH_THROUGH_JNK
REACTOME_NRAGE_SIGNALS_DEATH_THROUGH_JNK 32
0.48870337 1.3050854 0.10745891 0.18008006
1 2625 "tags=28%, list=16%, signal=34%"
REACTOME_IL_RECEPTOR_SHC_SIGNALING
REACTOME_IL_RECEPTOR_SHC_SIGNALING 25
0.5037273 1.2890283 0.12631579 0.19898382
1 2700 "tags=36%, list=17%, signal=43%"
REACTOME_INTEGRIN_CELL_SURFACE_INTERACTIONS
REACTOME_INTEGRIN_CELL_SURFACE_INTERACTIONS 72
0.42530087 1.2887005 0.09572072 0.19861762
1 1958 "tags=19%, list=12%, signal=22%"
REACTOME_GLUCAGON_SIGNALING_IN_METABOLIC_REGULATION
REACTOME_GLUCAGON_SIGNALING_IN_METABOLIC_REGULATION
30 0.4889806 1.288609 0.13071066 0.19787735
1 2014 "tags=30%, list=13%, signal=34%"
REACTOME_APOPTOTIC_CLEAVAGE_OF_CELLULAR_PROTEINS
REACTOME_APOPTOTIC_CLEAVAGE_OF_CELLULAR_PROTEINS 32
0.47866452 1.2817689 0.1425 0.20617211 1
3927 "tags=56%, list=24%, signal=74%"
REACTOME_PI3K_EVENTS_IN_ERBB2_SIGNALING
REACTOME_PI3K_EVENTS_IN_ERBB2_SIGNALING 34
0.4691399 1.2685235 0.14727722 0.22331908
1 2006 "tags=26%, list=12%, signal=30%"
REACTOME_SIGNALING_BY_PDGF REACTOME_SIGNALING_BY_PDGF
107 0.4016076 1.2673794 0.09836066
0.22397995 1 3004 "tags=27%, list=19%, signal=33%"
REACTOME_INSULIN_SYNTHESIS_AND_PROCESSING
REACTOME_INSULIN_SYNTHESIS_AND_PROCESSING 15
0.54891026 1.2645928 0.17331499 0.22710156
1 4386 "tags=53%, list=27%, signal=73%"
REACTOME_TRIF_MEDIATED_TLR3_SIGNALING
REACTOME_TRIF_MEDIATED_TLR3_SIGNALING 63
0.4281533 1.2639191 0.10477299 0.22706622
1 3270 "tags=35%, list=20%, signal=44%"
REACTOME_DOWNSTREAM_TCR_SIGNALING REACTOME_DOWNSTREAM_TCR_SIGNALING
26 0.48458752 1.2637196 0.15625
0.22637773 1 2642 "tags=38%, list=16%, signal=46%"
REACTOME_PI3K_EVENTS_IN_ERBB4_SIGNALING
REACTOME_PI3K_EVENTS_IN_ERBB4_SIGNALING 30
0.47562042 1.2615726 0.15861215 0.22865681
1 2006 "tags=27%, list=12%, signal=30%"
REACTOME_POST_TRANSLATIONAL_PROTEIN_MODIFICATION
REACTOME_POST_TRANSLATIONAL_PROTEIN_MODIFICATION 143
0.39191654 1.2592033 0.078306876 0.23111582
1 3798 "tags=37%, list=24%, signal=48%"

REACTOME_MYD88_MAL_CASCADE_INITIATED_ON_PLASMA_MEMBRANE
REACTOME_MYD88_MAL_CASCADE_INITIATED_ON_PLASMA_MEMBRANE
71 0.4187655 1.2549387 0.12472406
0.23637374 1 3051 "tags=34%, list=19%, signal=42%"
REACTOME_THROMBIN_SIGNALLING_THROUGH_PROTEINASE_ACTIVATED_RECEPTORS_PARS
REACTOME_THROMBIN_SIGNALLING_THROUGH_PROTEINASE_ACTIVATED_RECEPTORS_PARS 31 0.47291595 1.2535733
0.17980295 0.237506 1 2014 "tags=35%, list=13%, signal=40%"
REACTOME_DAG_AND_IP3_SIGNALING REACTOME_DAG_AND_IP3_SIGNALING
28 0.4750497 1.2531904 0.16709183
0.23716757 1 3286 "tags=46%, list=20%, signal=58%"
REACTOME_MICRORNA_MIRNA_BIOGENESIS
REACTOME_MICRORNA_MIRNA_BIOGENESIS 17
0.52341795 1.2525779 0.1808367 0.23708503
1 4097 "tags=59%, list=25%, signal=79%"
REACTOME_ACTIVATED_NOTCH1_TRANSMITS_SIGNAL_TO_THE_NUCLEUS
REACTOME_ACTIVATED_NOTCH1_TRANSMITS_SIGNAL_TO_THE_NUCLEUS
24 0.4840843 1.2495642 0.20026526
0.24035953 1 1709 "tags=29%, list=11%, signal=33%"
REACTOME_MRNA_3_END_PROCESSING REACTOME_MRNA_3_END_PROCESSING
21 0.4987793 1.2361149 0.2
0.25918123 1 3552 "tags=48%, list=22%, signal=61%"
REACTOME_PLC_BETA_MEDIATED_EVENTS REACTOME_PLC_BETA_MEDIATED_EVENTS
38 0.44891536 1.2355055 0.16479401
0.25918177 1 3927 "tags=47%, list=24%, signal=62%"
REACTOME_ANTIGEN_ACTIVATES_B_CELL_RECEPTOR_LEADING_TO_GENERATION_OF_SECOND_MESSENGERS
REACTOME_ANTIGEN_ACTIVATES_B_CELL_RECEPTOR_LEADING_TO_GENERATION_OF_SECOND_MESSENGERS 27 0.48082128
1.2348659 0.16494845 0.25911748 1
3347 "tags=52%, list=21%, signal=65%"
REACTOME_SYNTHESIS_SECRETION_AND_INACTIVATION_OF_GLP1
REACTOME_SYNTHESIS_SECRETION_AND_INACTIVATION_OF_GLP1
17 0.5144637 1.2325749 0.22252375
0.2617169 1 5459 "tags=65%, list=34%, signal=98%"
REACTOME_TRAF6_MEDIATED_INDUCTION_OF_NFKB_AND_MAP_KINASES_UPON_TLR7_8_OR_9_ACTIVATION
REACTOME_TRAF6_MEDIATED_INDUCTION_OF_NFKB_AND_MAP_KINASES_UPON_TLR7_8_OR_9_ACTIVATION 64 0.41094047 1.232489
0.15940367 0.26081252 1 3051 "tags=36%, list=19%, signal=44%"
REACTOME_POST_NMDA_RECEPTOR_ACTIVATION_EVENTS
REACTOME_POST_NMDA_RECEPTOR_ACTIVATION_EVENTS 29
0.46766838 1.2324373 0.18607596 0.25984812
1 2829 "tags=38%, list=18%, signal=46%"
REACTOME_ACYL_CHAIN_REMODELLING_OF_PS
REACTOME_ACYL_CHAIN_REMODELLING_OF_PS 15
0.5272505 1.228565 0.22115384 0.26482457 1
1773 "tags=27%, list=11%, signal=30%"
REACTOME_PI3K_CASCADE REACTOME_PI3K_CASCADE 59
0.4183384 1.2266499 0.1616279 0.266687 1
3406 "tags=27%, list=21%, signal=34%"

REACTOME_CREB_PHOSPHORYLATION_THROUGH_THE_ACTIVATION_OF_RAS
REACTOME_CREB_PHOSPHORYLATION_THROUGH_THE_ACTIVATION_OF_RAS
24 0.48146838 1.226241 0.20316623 0.26623464
1 2829 "tags=38%, list=18%, signal=45%"
REACTOME_METABOLISM_OF_LIPIDS_AND_LIPOPROTEINS
REACTOME_METABOLISM_OF_LIPIDS_AND_LIPOPROTEINS 395
0.35708523 1.2203437 0.034136545 0.27484 1
3648 "tags=29%, list=23%, signal=36%"
REACTOME_SIGNALING_BY_TGF_BETA_RECEPTOR_COMPLEX
REACTOME_SIGNALING_BY_TGF_BETA_RECEPTOR_COMPLEX 50
0.424907 1.2199463 0.18072289 0.2743251 1
2357 "tags=34%, list=15%, signal=40%"
REACTOME_NUCLEAR_SIGNALING_BY_ERBB4
REACTOME_NUCLEAR_SIGNALING_BY_ERBB4 31
0.45640323 1.2179049 0.2244389 0.27646014
1 5284 "tags=45%, list=33%, signal=67%"
REACTOME_ACTIVATION_OF_NMDA_RECEPTOR_UPON_GLUTAMATE_BINDING_AND_POST
SYNAPTIC_EVENTS
REACTOME_ACTIVATION_OF_NMDA_RECEPTOR_UPON_GLUTAMATE_BINDING_AND_POST
SYNAPTIC_EVENTS 33 0.45321473 1.2165005
0.20828106 0.27746862 1 2829 "tags=36%,
list=18%, signal=44%"
REACTOME_RNA_POL_I_TRANSCRIPTION_INITIATION
REACTOME_RNA_POL_I_TRANSCRIPTION_INITIATION 19
0.49992546 1.2123783 0.21646747 0.28259194
1 4651 "tags=58%, list=29%, signal=81%"
REACTOME_RNA_POL_III_TRANSCRIPTION
REACTOME_RNA_POL_III_TRANSCRIPTION 28
0.45576313 1.2084124 0.23126616 0.2880629
1 2514 "tags=32%, list=16%, signal=38%"
REACTOME_FATTY_ACID_TRIACYLGLYCEROL_AND_KETONE_BODY_METABOLISM
REACTOME_FATTY_ACID_TRIACYLGLYCEROL_AND_KETONE_BODY_METABOLISM
143 0.37382826 1.2049018 0.1256656
0.29250348 1 3453 "tags=30%, list=21%, signal=38%"
REACTOME_CD28_DEPENDENT_PI3K_AKT_SIGNALING
REACTOME_CD28_DEPENDENT_PI3K_AKT_SIGNALING 18 0.505297
1.2038263 0.24696356 0.29305497 1
1988 "tags=39%, list=12%, signal=44%"
REACTOME_CELL_SURFACE_INTERACTIONS_AT_THE_VASCULAR_WALL
REACTOME_CELL_SURFACE_INTERACTIONS_AT_THE_VASCULAR_WALL
72 0.39667407 1.1961315 0.1804009
0.30438137 1 4014 "tags=36%, list=25%, signal=48%"
REACTOME_INTEGRATION_OF_ENERGY_METABOLISM
REACTOME_INTEGRATION_OF_ENERGY_METABOLISM 104
0.37871853 1.1923518 0.15098469 0.30969298
1 3741 "tags=37%, list=23%, signal=47%"
REACTOME_SPHINGOLIPID_METABOLISM REACTOME_SPHINGOLIPID_METABOLISM
50 0.4094894 1.1910682 0.20567375
0.31079105 1 5195 "tags=50%, list=32%, signal=74%"
REACTOME_SIGNAL_AMPLIFICATION REACTOME_SIGNAL_AMPLIFICATION
29 0.44635376 1.1779573 0.24234694
0.33209726 1 3679 "tags=41%, list=23%, signal=54%"
REACTOME_FORMATION_OF_TUBULIN_FOLDING_INTERMEDIATES_BY_CCT_TRIC
REACTOME_FORMATION_OF_TUBULIN_FOLDING_INTERMEDIATES_BY_CCT_TRIC

16	0.4988859	1.1650507	0.25306123
0.3535536	1	2917	"tags=44%, list=18%, signal=53%"
REACTOME_G_BETA_GAMMA_SIGNALLING_THROUGH_PI3KGAMMA
REACTOME_G_BETA_GAMMA_SIGNALLING_THROUGH_PI3KGAMMA	24
0.45623758	1.1627133	0.24664879	0.35645515
1	2876	"tags=46%, list=18%, signal=56%"
REACTOME_FATTY_ACYL_COA_BIOSYNTHESIS
REACTOME_FATTY_ACYL_COA_BIOSYNTHESIS	16
0.49761885	1.1615182	0.29655173	0.3572304
1	2687	"tags=44%, list=17%, signal=52%"
REACTOME_STRIATED_MUSCLE_CONTRACTION
REACTOME_STRIATED_MUSCLE_CONTRACTION	23
0.46054882	1.1596441	0.2763685	0.35940903
1	3435	"tags=35%, list=21%, signal=44%"
REACTOME_TRIGLYCERIDE_BIOSYNTHESIS
REACTOME_TRIGLYCERIDE_BIOSYNTHESIS	32
0.42701283	1.1578246	0.27376425	0.3612767
1	2734	"tags=34%, list=17%, signal=41%"
REACTOME_INCRETIN_SYNTHESIS_SECRETION_AND_INACTIVATION
REACTOME_INCRETIN_SYNTHESIS_SECRETION_AND_INACTIVATION
20	0.47777152	1.154294	0.28820375	0.36637452
1	5459	"tags=65%, list=34%, signal=98%"
REACTOME_GLYCOSPHINGOLIPID_METABOLISM
REACTOME_GLYCOSPHINGOLIPID_METABOLISM	31
0.43511721	1.1508249	0.26349613	0.37142828
1	5080	"tags=55%, list=32%, signal=80%"
REACTOME_SIGNALING_BY_ROBO_RECEPTOR
REACTOME_SIGNALING_BY_ROBO_RECEPTOR	21
0.46765122	1.1505439	0.2931507	0.37065455
1	1935	"tags=29%, list=12%, signal=32%"
REACTOME_PREFOLDIN_MEDIATED_TRANSFER_OF_SUBSTRATE_TO_CCT_TRIC
REACTOME_PREFOLDIN_MEDIATED_TRANSFER_OF_SUBSTRATE_TO_CCT_TRIC
21	0.46521753	1.1465011	0.30352303
0.37661865	1	2917	"tags=43%, list=18%, signal=52%"
REACTOME_NEUROTRANSMITTER_RECEPTOR_BINDING_AND_DOWNSTREAM_TRANSMISSI
ON_IN_THE_POSTSYNAPTIC_CELL
REACTOME_NEUROTRANSMITTER_RECEPTOR_BINDING_AND_DOWNSTREAM_TRANSMISSI
ON_IN_THE_POSTSYNAPTIC_CELL	119	0.36100492
1.1462749	0.21794872	0.3757098	1
2829	"tags=24%, list=18%, signal=28%"
REACTOME_GPVI_MEDIATED_ACTIVATION_CASCADE
REACTOME_GPVI_MEDIATED_ACTIVATION_CASCADE	30
0.43010035	1.1409236	0.27701864	0.38436112
1	2876	"tags=40%, list=18%, signal=49%"
REACTOME_PPARA_ACTIVATES_GENE_EXPRESSION
REACTOME_PPARA_ACTIVATES_GENE_EXPRESSION	89
0.3651034	1.1374336	0.25336322	0.38950095
1	3894	"tags=31%, list=24%, signal=41%"
REACTOME_PURINE_METABOLISM	REACTOME_PURINE_METABOLISM
29	0.43587914	1.1349047	0.30472636
0.39282483	1	4301	"tags=59%, list=27%, signal=80%"
REACTOME_SIGNALING_BY_ILS	REACTOME_SIGNALING_BY_ILS	95
0.3601196	1.1321762	0.25248072	0.39662513
1	2986	"tags=34%, list=19%, signal=41%"

REACTOME_PI_3K_CASCADE	REACTOME_PI_3K_CASCADE		49
0.3922303	1.1321337	0.28211284	0.3952573
1	2006	"tags=18%, list=12%, signal=21%"
REACTOME_ENDOSOMAL_SORTING_COMPLEX_REQUIRED_FOR_TRANSPORT_ESCRT
REACTOME_ENDOSOMAL_SORTING_COMPLEX_REQUIRED_FOR_TRANSPORT_ESCRT
	21	0.45573264	1.1272072	0.31571993
0.40333015	1	3982	"tags=57%, list=25%, signal=76%"
REACTOME_NFKB_AND_MAP_KINASES_ACTIVATION_MEDIATED_BY_TLR4_SIGNALING_
REPERTOIRE
REACTOME_NFKB_AND_MAP_KINASES_ACTIVATION_MEDIATED_BY_TLR4_SIGNALING_
REPERTOIRE		61	0.37830347	1.1234835
0.2848837	0.4087796	1	3571	"tags=36%,
list=22%, signal=46%"
REACTOME_G_ALPHA_Z_SIGNALLING_EVENTS
REACTOME_G_ALPHA_Z_SIGNALLING_EVENTS		40
0.39743912	1.1206797	0.30184048	0.41269934
1	2014	"tags=25%, list=13%, signal=29%"
REACTOME_G_PROTEIN_BETA_GAMMA_SIGNALLING
REACTOME_G_PROTEIN_BETA_GAMMA_SIGNALLING		27
0.4200036	1.1033555	0.31906614	0.4447794
1	2876	"tags=41%, list=18%, signal=50%"
REACTOME_ENOS_ACTIVATION_AND_REGULATION
REACTOME_ENOS_ACTIVATION_AND_REGULATION		18
0.4563841	1.1031727	0.3430851	0.44361335
1	1628	"tags=39%, list=10%, signal=43%"
REACTOME_TIE2_SIGNALING	REACTOME_TIE2_SIGNALING		16
0.4641082	1.0965275	0.3452055	0.45526227
1	1977	"tags=25%, list=12%, signal=28%"
REACTOME_NEPHRIN_INTERACTIONS	REACTOME_NEPHRIN_INTERACTIONS
	15	0.47646946	1.0902483	0.3657534
0.46624845	1	3347	"tags=40%, list=21%, signal=50%"
REACTOME_ADP_SIGNALLING_THROUGH_P2RY12
REACTOME_ADP_SIGNALLING_THROUGH_P2RY12		19
0.4456618	1.0875566	0.36806494	0.46996665
1	2763	"tags=37%, list=17%, signal=44%"
REACTOME_IL1_SIGNALING	REACTOME_IL1_SIGNALING		34
0.39861977	1.0753284	0.38131315	0.49327073
1	2986	"tags=32%, list=19%, signal=40%"
REACTOME_ACTIVATED_TLR4_SIGNALLING
REACTOME_ACTIVATED_TLR4_SIGNALLING		81
0.34826952	1.0674802	0.36141908	0.5074716
1	2829	"tags=28%, list=18%, signal=34%"
REACTOME_FORMATION_OF_INCISION_COMPLEX_IN_GG_NER
REACTOME_FORMATION_OF_INCISION_COMPLEX_IN_GG_NER		19
0.4433623	1.0641192	0.40104848	0.51242733
1	4651	"tags=53%, list=29%, signal=74%"
REACTOME_GABA_SYNTHESIS_RELEASE_REUPTAKE_AND_DEGRADATION
REACTOME_GABA_SYNTHESIS_RELEASE_REUPTAKE_AND_DEGRADATION
17	0.44204354	1.0592654	0.39550266
0.52082014	1	14	"tags=6%, list=0%, signal=6%"
REACTOME_RNA_POL_III_TRANSCRIPTION_TERMINATION
REACTOME_RNA_POL_III_TRANSCRIPTION_TERMINATION		15
0.45738745	1.0563631	0.40189445	0.52506113
1	2362	"tags=33%, list=15%, signal=39%"

REACTOME_PHOSPHOLIPID_METABOLISM	REACTOME_PHOSPHOLIPID_METABOLISM
	159	0.32667845	1.0560291	0.3788038
0.5239411	1	5080	"tags=40%, list=32%, signal=57%"
REACTOME_NEGATIVE_REGULATION_OF_FGFR_SIGNALING
REACTOME_NEGATIVE_REGULATION_OF_FGFR_SIGNALING		33
0.39328125	1.0539385	0.40076336	0.52655345
1	1958	"tags=21%, list=12%, signal=24%"
REACTOME_REGULATION_OF_WATER_BALANCE_BY_RENAL_AQUAPORINS
REACTOME_REGULATION_OF_WATER_BALANCE_BY_RENAL_AQUAPORINS
40	0.3781114	1.0498848	0.40243903
0.5329458	1	2014	"tags=25%, list=13%, signal=29%"
REACTOME_PLATELET_HOMEOSTASIS	REACTOME_PLATELET_HOMEOSTASIS
	71	0.34320095	1.0437512	0.42519686
0.54382837	1	3743	"tags=28%, list=23%, signal=37%"
REACTOME_TRANSMISSION_ACROSS_CHEMICAL_SYNAPSES
REACTOME_TRANSMISSION_ACROSS_CHEMICAL_SYNAPSES		166
0.32143307	1.0426464	0.40226337	0.5442828
1	2909	"tags=19%, list=18%, signal=23%"
REACTOME_FORMATION_OF_TRANSCRIPTION_COUPLED_NER_TC_NER_REPAIR_COMPLE
X
REACTOME_FORMATION_OF_TRANSCRIPTION_COUPLED_NER_TC_NER_REPAIR_COMPLE
X		23	0.40521893	1.0410866
0.42302543	0.54568195	1	4651	"tags=52%,
list=29%, signal=73%"
REACTOME_RNA_POL_III_TRANSCRIPTION_INITIATION_FROM_TYPE_3_PROMOTER
REACTOME_RNA_POL_III_TRANSCRIPTION_INITIATION_FROM_TYPE_3_PROMOTER
	21	0.41680393	1.0372066	0.44625852
0.5522046	1	2514	"tags=33%, list=16%, signal=39%"
REACTOME_RNA_POL_I_TRANSCRIPTION_TERMINATION
REACTOME_RNA_POL_I_TRANSCRIPTION_TERMINATION		16
0.43861082	1.0372064	0.43539324	0.55038816
1	4651	"tags=50%, list=29%, signal=70%"
REACTOME_INNATE_IMMUNE_SYSTEM	REACTOME_INNATE_IMMUNE_SYSTEM
	190	0.31323275	1.03396	0.4376931
0.55528086	1	2829	"tags=26%, list=18%, signal=32%"
REACTOME_REGULATION_OF_SIGNALING_BY_CBL
REACTOME_REGULATION_OF_SIGNALING_BY_CBL		16
0.4428787	1.0334655	0.44459644	0.55443305
1	2958	"tags=31%, list=18%, signal=38%"
REACTOME_PRE_NOTCH_TRANSCRIPTION_AND_TRANSLATION
REACTOME_PRE_NOTCH_TRANSCRIPTION_AND_TRANSLATION		24
0.401224	1.0327336	0.42444152	0.5540947	1
4062	"tags=50%, list=25%, signal=67%"
REACTOME_SIGNALING_BY_HIPPO	REACTOME_SIGNALING_BY_HIPPO
	16	0.43369883	1.0196912	0.4480432
0.5785788	1	4940	"tags=50%, list=31%, signal=72%"
REACTOME_ACTIVATION_OF_KAINATE_RECEPTORS_UPON_GLUTAMATE_BINDING
REACTOME_ACTIVATION_OF_KAINATE_RECEPTORS_UPON_GLUTAMATE_BINDING
	29	0.38892907	1.01944	0.47409326
0.57720757	1	2763	"tags=31%, list=17%, signal=37%"
REACTOME_SIGNALING_BY_FGFR1_FUSION_MUTANTS
REACTOME_SIGNALING_BY_FGFR1_FUSION_MUTANTS		15
0.43399853	1.017579	0.4488712	0.57907397	1
5630	"tags=60%, list=35%, signal=92%"

REACTOME_THROMBOXANE_SIGNALLING_THROUGH_TP_RECEPTOR
REACTOME_THROMBOXANE_SIGNALLING_THROUGH_TP_RECEPTOR
22 0.40807655 1.0146043 0.4814815 0.583477
1 2014 "tags=27%, list=13%, signal=31%"
REACTOME_SULFUR_AMINO_ACID_METABOLISM
REACTOME_SULFUR_AMINO_ACID_METABOLISM 21
0.40635842 1.0111812 0.46866485 0.5887128
1 5281 "tags=57%, list=33%, signal=85%"
REACTOME_SLC_MEDIATED_TRANSMEMBRANE_TRANSPORT
REACTOME_SLC_MEDIATED_TRANSMEMBRANE_TRANSPORT 211
0.30356 1.0059482 0.491353 0.59726053 1
4224 "tags=26%, list=26%, signal=35%"
REACTOME_PHOSPHOLIPASE_C_MEDIATED_CASCADE
REACTOME_PHOSPHOLIPASE_C_MEDIATED_CASCADE 49
0.34682494 1.0021098 0.48739496 0.6030427
1 3286 "tags=27%, list=20%, signal=33%"
REACTOME_ASSOCIATION_OF_TRIC_CCT_WITH_TARGET_PROTEINS_DURING_BIOSYNT
HESIS
REACTOME_ASSOCIATION_OF_TRIC_CCT_WITH_TARGET_PROTEINS_DURING_BIOSYNT
HESIS 23 0.3932797 1.0008059
0.48021108 0.60367227 1 5068 "tags=48%,
list=31%, signal=70%"
REACTOME_GLYCEROPHOSPHOLIPID_BIOSYNTHESIS
REACTOME_GLYCEROPHOSPHOLIPID_BIOSYNTHESIS 70
0.33080915 1.0003648 0.48350397 0.6025836
1 5356 "tags=39%, list=33%, signal=58%"
REACTOME_ADP_SIGNALLING_THROUGH_P2RY1
REACTOME_ADP_SIGNALLING_THROUGH_P2RY1 24
0.39727604 1.00033 0.48687664 0.6007422 1
3051 "tags=33%, list=19%, signal=41%"
REACTOME_PERK_REGULATED_GENE_EXPRESSION
REACTOME_PERK_REGULATED_GENE_EXPRESSION 21
0.39879778 0.99224234 0.4812903 0.61535025
1 3487 "tags=38%, list=22%, signal=49%"
REACTOME_PKA_MEDIATED_PHOSPHORYLATION_OF_CREB
REACTOME_PKA_MEDIATED_PHOSPHORYLATION_OF_CREB 15
0.43633595 0.9911224 0.5155807 0.615563 1
3286 "tags=40%, list=20%, signal=50%"
REACTOME_TOLL_RECEPTOR_CASCADES REACTOME_TOLL_RECEPTOR_CASCADES
98 0.31087464 0.99026185 0.4977827
0.6153422 1 2829 "tags=27%, list=18%, signal=32%"
REACTOME_SYNTHESIS_OF_PA REACTOME_SYNTHESIS_OF_PA 24
0.38889664 0.9887992 0.48914433 0.6163913
1 3619 "tags=33%, list=22%, signal=43%"
REACTOME_EFFECTS_OF_PIP2_HYDROLYSIS
REACTOME_EFFECTS_OF_PIP2_HYDROLYSIS 22
0.39202818 0.98717725 0.5152318 0.6176327
1 3927 "tags=36%, list=24%, signal=48%"
REACTOME_TRANSPORT_TO_THE_GOLGI_AND_SUBSEQUENT_MODIFICATION
REACTOME_TRANSPORT_TO_THE_GOLGI_AND_SUBSEQUENT_MODIFICATION
30 0.37257278 0.98187286 0.50125 0.62634236
1 3608 "tags=40%, list=22%, signal=51%"
REACTOME_AQUAPORIN_MEDIATED_TRANSPORT
REACTOME_AQUAPORIN_MEDIATED_TRANSPORT 46

0.34712666	0.975205	0.5356711	0.6374958	1
2014	"tags=22%, list=13%, signal=25%"
REACTOME_METAL_ION_SLC_TRANSPORTERS
REACTOME_METAL_ION_SLC_TRANSPORTERS	19
0.40363133	0.9745841	0.52005535	0.63671607
1	4675	"tags=42%, list=29%, signal=59%"
REACTOME_TRANSMEMBRANE_TRANSPORT_OF_SMALL_MOLECULES
REACTOME_TRANSMEMBRANE_TRANSPORT_OF_SMALL_MOLECULES
356	0.28757602	0.9745736	0.5780463
0.63478553	1	4288	"tags=27%, list=27%, signal=36%"
REACTOME_PROSTACYCLIN_SIGNALLING_THROUGH_PROSTACYCLIN_RECEPTOR
REACTOME_PROSTACYCLIN_SIGNALLING_THROUGH_PROSTACYCLIN_RECEPTOR
18	0.407628	0.9711098	0.51424694
0.6396208	1	2763	"tags=33%, list=17%, signal=40%"
REACTOME_DESTABILIZATION_OF_MRNA_BY_TRISTETRAPROLIN_TTP
REACTOME_DESTABILIZATION_OF_MRNA_BY_TRISTETRAPROLIN_TTP
15	0.41718024	0.96045655	0.5246132
0.65844065	1	3487	"tags=33%, list=22%, signal=43%"
REACTOME_MITOCHONDRIAL_TRNA_AMINOACYLATION
REACTOME_MITOCHONDRIAL_TRNA_AMINOACYLATION	18
0.3924648	0.9499798	0.5331492	0.67670494
1	1800	"tags=28%, list=11%, signal=31%"
REACTOME_REGULATION_OF_INSULIN_SECRETION
REACTOME_REGULATION_OF_INSULIN_SECRETION	80
0.3093804	0.9483786	0.5709459	0.67753243
1	3741	"tags=31%, list=23%, signal=41%"
REACTOME_NEGATIVE_REGULATORS_OF_RIG_I_MDA5_SIGNALING
REACTOME_NEGATIVE_REGULATORS_OF_RIG_I_MDA5_SIGNALING
24	0.3742998	0.94706905	0.5561358
0.67812914	1	3947	"tags=38%, list=25%, signal=50%"
REACTOME_LIPOPROTEIN_METABOLISM	REACTOME_LIPOPROTEIN_METABOLISM
22	0.37931678	0.94561166	0.54392767
0.67891866	1	3738	"tags=27%, list=23%, signal=35%"
REACTOME_REGULATORY_RNA_PATHWAYS	REACTOME_REGULATORY_RNA_PATHWAYS
20	0.3853273	0.94507325	0.5572917
0.6778869	1	4097	"tags=50%, list=25%, signal=67%"
REACTOME_G_PROTEIN_ACTIVATION	REACTOME_G_PROTEIN_ACTIVATION
25	0.370242	0.93932855	0.5716163
0.68680036	1	2763	"tags=28%, list=17%, signal=34%"
REACTOME_NETRIN1_SIGNALING	REACTOME_NETRIN1_SIGNALING
33	0.34604663	0.93743914	0.56511056
0.68826306	1	3917	"tags=27%, list=24%, signal=36%"
REACTOME_GROWTH_HORMONE_RECEPTOR_SIGNALING
REACTOME_GROWTH_HORMONE_RECEPTOR_SIGNALING	20
0.38019186	0.93008614	0.56568366	0.699521	1
5035	"tags=55%, list=31%, signal=80%"
REACTOME_ACYL_CHAIN_REMODELLING_OF_PE
REACTOME_ACYL_CHAIN_REMODELLING_OF_PE	20
0.3824634	0.92929184	0.56901044	0.6989235
1	1773	"tags=20%, list=11%, signal=22%"
REACTOME_FRS2_MEDIATED_CASCADE	REACTOME_FRS2_MEDIATED_CASCADE
33	0.34336466	0.92478293	0.60831237
0.7053286	1	4666	"tags=30%, list=29%, signal=43%"
REACTOME_SMAD2_SMAD3_SMAD4_HETEROTRIMER_REGULATES_TRANSCRIPTION

REACTOME_SMAD2_SMAD3_SMAD4_HETEROTRIMER_REGULATES_TRANSCRIPTION
21 0.37260628 0.9216183 0.59031415
0.70909363 1 3389 "tags=43%, list=21%, signal=54%"
REACTOME_PHASE_II_CONJUGATION REACTOME_PHASE_II_CONJUGATION
44 0.32906285 0.9212346 0.61660564
0.70773405 1 5243 "tags=39%, list=33%, signal=57%"
REACTOME_SPHINGOLIPID_DE_NOVO_BIOSYNTHESIS
REACTOME_SPHINGOLIPID_DE_NOVO_BIOSYNTHESIS 19
0.37807366 0.91877854 0.5953361 0.710276 1
5533 "tags=47%, list=34%, signal=72%"
REACTOME_NCAM_SIGNALING_FOR_NEURITE_OUT_GROWTH
REACTOME_NCAM_SIGNALING_FOR_NEURITE_OUT_GROWTH 51
0.3165836 0.9146666 0.61117715 0.7157686
1 2829 "tags=22%, list=18%, signal=26%"
REACTOME_RIP_MEDIATED_NFKB_ACTIVATION_VIA_DAI
REACTOME_RIP_MEDIATED_NFKB_ACTIVATION_VIA_DAI 17
0.38333574 0.90554047 0.6179625 0.7302101
1 1805 "tags=24%, list=11%, signal=26%"
REACTOME_DESTABILIZATION_OF_MRNA_BY_BRF1
REACTOME_DESTABILIZATION_OF_MRNA_BY_BRF1 15
0.39177057 0.90506816 0.614654 0.7288746 1
3487 "tags=33%, list=22%, signal=43%"
REACTOME_CELL_CELL_COMMUNICATION REACTOME_CELL_CELL_COMMUNICATION
98 0.28731665 0.89969385 0.6794311
0.7362808 1 3454 "tags=23%, list=21%, signal=30%"
REACTOME_ION_TRANSPORT_BY_P_TYPE_ATPASES
REACTOME_ION_TRANSPORT_BY_P_TYPE_ATPASES 28
0.34385026 0.89566666 0.62627554 0.7412477
1 2081 "tags=21%, list=13%, signal=25%"
REACTOME_INTERFERON_ALPHA_BETA_SIGNALING
REACTOME_INTERFERON_ALPHA_BETA_SIGNALING 45
0.31525612 0.8876015 0.6598311 0.75348085
1 3776 "tags=40%, list=23%, signal=52%"
REACTOME_IMMUNOREGULATORY_INTERACTIONS_BETWEEN_A_LYMPHOID_AND_A_NON_
LYMPHOID_CELL
REACTOME_IMMUNOREGULATORY_INTERACTIONS_BETWEEN_A_LYMPHOID_AND_A_NON_
LYMPHOID_CELL 38 0.32090333 0.8851272
0.64683545 0.7556571 1 4261 "tags=42%,
list=26%, signal=57%"
REACTOME_NEUROTRANSMITTER_RELEASE_CYCLE
REACTOME_NEUROTRANSMITTER_RELEASE_CYCLE 33
0.3308476 0.88227636 0.65473145 0.7583941
1 14 "tags=3%, list=0%, signal=3%"
REACTOME_TRANSCRIPTIONAL_REGULATION_OF_WHITE_ADIPOCYTE_DIFFERENTIATI
ON
REACTOME_TRANSCRIPTIONAL_REGULATION_OF_WHITE_ADIPOCYTE_DIFFERENTIATI
ON 62 0.2924625 0.8729698
0.6913146 0.772256 1 3225 "tags=24%, list=20%,
signal=30%"
REACTOME_PTM_GAMMA_CARBOXYLATION_HYPUSINE_FORMATION_AND_ARYLSULFATAS
E_ACTIVATION
REACTOME_PTM_GAMMA_CARBOXYLATION_HYPUSINE_FORMATION_AND_ARYLSULFATAS
E_ACTIVATION 21 0.35109916 0.86015105
0.6716016 0.79150736 1 1186 "tags=19%,

list=7%, signal=21%"
REACTOME_BASIGIN_INTERACTIONS	REACTOME_BASIGIN_INTERACTIONS
	22	0.33642042	0.84969556	0.68175584
0.8063928	1	3985	"tags=41%, list=25%, signal=54%"
REACTOME_ACTIVATION_OF_GENES_BY_ATF4
REACTOME_ACTIVATION_OF_GENES_BY_ATF4		19
0.34159008	0.8422937	0.70872486	0.8162125
1	5433	"tags=53%, list=34%, signal=79%"
REACTOME_RIG_I_MDA5_MEDIATED_INDUCTION_OF_IFN_ALPHA_BETA_PATHWAYS
REACTOME_RIG_I_MDA5_MEDIATED_INDUCTION_OF_IFN_ALPHA_BETA_PATHWAYS
	58	0.28638497	0.8354551	0.7361751
0.8249568	1	3270	"tags=26%, list=20%, signal=32%"
REACTOME_INHIBITION_OF_VOLTAGE_GATED_CA2_CHANNELS_VIA_GBETA_GAMMA_SU
BUNITS
REACTOME_INHIBITION_OF_VOLTAGE_GATED_CA2_CHANNELS_VIA_GBETA_GAMMA_SU
BUNITS		22	0.33302817	0.8295597
0.7168597	0.8319584	1	2763	"tags=23%,
list=17%, signal=27%"
REACTOME_PI_METABOLISM	REACTOME_PI_METABOLISM		40
0.2889518	0.8118715	0.77926826	0.85643053
1	4479	"tags=40%, list=28%, signal=55%"
REACTOME_GABA_B_RECEPTOR_ACTIVATION
REACTOME_GABA_B_RECEPTOR_ACTIVATION		33
0.29824442	0.80482125	0.75930524	0.8642466
1	2014	"tags=18%, list=13%, signal=21%"
REACTOME_CREB_PHOSPHORYLATION_THROUGH_THE_ACTIVATION_OF_CAMKII
REACTOME_CREB_PHOSPHORYLATION_THROUGH_THE_ACTIVATION_OF_CAMKII
	15	0.34690943	0.79947126	0.75137365
0.8696085	1	2829	"tags=27%, list=18%, signal=32%"
REACTOME_SIGNALING_BY_GPCR	REACTOME_SIGNALING_BY_GPCR
	496	0.23503175	0.79673827	0.966967
0.8711433	1	2992	"tags=17%, list=19%, signal=20%"
REACTOME_MYOGENESIS	REACTOME_MYOGENESIS		23
0.31608462	0.79618317	0.77380955	0.8694966
1	3051	"tags=22%, list=19%, signal=27%"
REACTOME_SIGNALING_BY_FGFR1_MUTANTS
REACTOME_SIGNALING_BY_FGFR1_MUTANTS		26
0.3035993	0.7928136	0.78210115	0.87184393
1	6876	"tags=54%, list=43%, signal=94%"
REACTOME_REGULATION_OF_GENE_EXPRESSION_IN_BETA_CELLS
REACTOME_REGULATION_OF_GENE_EXPRESSION_IN_BETA_CELLS
18	0.32683465	0.7886911	0.7480315
0.87529975	1	3475	"tags=22%, list=22%, signal=28%"
REACTOME_REGULATION_OF_INSULIN_SECRETION_BY_GLUCAGON_LIKE_PEPTIDE1
REACTOME_REGULATION_OF_INSULIN_SECRETION_BY_GLUCAGON_LIKE_PEPTIDE1
	37	0.28737226	0.7884001	0.79551125
0.87325954	1	2763	"tags=27%, list=17%, signal=33%"
REACTOME_SHC_MEDIATED_CASCADE	REACTOME_SHC_MEDIATED_CASCADE
	26	0.3032395	0.7789972	0.7911803
0.8838116	1	1958	"tags=12%, list=12%, signal=13%"
REACTOME_TRANSCRIPTIONAL_ACTIVITY_OF_SMAD2_SMAD3_SMAD4_HETEROTRIMER
REACTOME_TRANSCRIPTIONAL_ACTIVITY_OF_SMAD2_SMAD3_SMAD4_HETEROTRIMER
	29	0.29475316	0.7788836	0.7989556
0.8815507	1	3389	"tags=34%, list=21%, signal=44%"

REACTOME_NEURONAL_SYSTEM REACTOME_NEURONAL_SYSTEM 243
0.23390295 0.7780947 0.92740285 0.8802297
1 2909 "tags=16%, list=18%, signal=19%"
REACTOME_GLUTATHIONE_CONJUGATION REACTOME_GLUTATHIONE_CONJUGATION
21 0.31055108 0.77331996 0.7629139
0.8843205 1 4258 "tags=43%, list=26%, signal=58%"
REACTOME_ACYL_CHAIN_REMODELLING_OF_PC
REACTOME_ACYL_CHAIN_REMODELLING_OF_PC 20
0.3193281 0.77125436 0.79360855 0.8845448
1 1773 "tags=15%, list=11%, signal=17%"
REACTOME_ION_CHANNEL_TRANSPORT REACTOME_ION_CHANNEL_TRANSPORT
46 0.26774627 0.7548999 0.8424821
0.9026321 1 2081 "tags=13%, list=13%, signal=15%"
REACTOME_CIRCADIAN_REPRESSION_OF_EXPRESSION_BY_REV_ERBA
REACTOME_CIRCADIAN_REPRESSION_OF_EXPRESSION_BY_REV_ERBA
21 0.30232504 0.74939084 0.8337731
0.9067431 1 4336 "tags=38%, list=27%, signal=52%"
REACTOME_SIGNALING_BY_FGFR_MUTANTS
REACTOME_SIGNALING_BY_FGFR_MUTANTS 39
0.26977748 0.74206233 0.8626506 0.91293716
1 6876 "tags=51%, list=43%, signal=89%"
REACTOME_YAP1_AND_WWTR1_TAZ_STIMULATED_GENE_EXPRESSION
REACTOME_YAP1_AND_WWTR1_TAZ_STIMULATED_GENE_EXPRESSION
22 0.2998875 0.7358706 0.8599476
0.9178227 1 6114 "tags=45%, list=38%, signal=73%"
REACTOME_RORA_ACTIVATES_CIRCADIAN_EXPRESSION
REACTOME_RORA_ACTIVATES_CIRCADIAN_EXPRESSION 22
0.29237083 0.72619385 0.8326586 0.92630196
1 4336 "tags=36%, list=27%, signal=50%"
REACTOME_STEROID_HORMONES REACTOME_STEROID_HORMONES 26
0.27913076 0.7251906 0.8565841 0.92500573
1 1272 "tags=12%, list=8%, signal=13%"
REACTOME_G_BETA_GAMMA_SIGNALLING_THROUGH_PLC_BETA
REACTOME_G_BETA_GAMMA_SIGNALLING_THROUGH_PLC_BETA 19
0.29685807 0.7239235 0.8379814 0.9238416
1 2763 "tags=26%, list=17%, signal=32%"
REACTOME_REGULATION_OF_IFNA_SIGNALING
REACTOME_REGULATION_OF_IFNA_SIGNALING 19
0.29616362 0.7205028 0.8426667 0.9250849
1 4272 "tags=32%, list=27%, signal=43%"
REACTOME_GABA_RECEPTOR_ACTIVATION REACTOME_GABA_RECEPTOR_ACTIVATION
45 0.25154182 0.70793205 0.90311
0.9350785 1 2014 "tags=13%, list=13%, signal=15%"
REACTOME_METABOLISM_OF_STEROID_HORMONES_AND_VITAMINS_A_AND_D
REACTOME_METABOLISM_OF_STEROID_HORMONES_AND_VITAMINS_A_AND_D
32 0.26271537 0.70653045 0.8852868
0.9339455 1 2354 "tags=16%, list=15%, signal=18%"
REACTOME_INHIBITION_OF_INSULIN_SECRETION_BY_ADRENALINE_NORADRENALINE

REACTOME_INHIBITION_OF_INSULIN_SECRETION_BY_ADRENALINE_NORADRENALINE
23 0.28021055 0.705044 0.8558201
0.9329754 1 2014 "tags=22%, list=13%, signal=25%"
REACTOME_TRAF6_MEDIATED_IRF7_ACTIVATION
REACTOME_TRAF6_MEDIATED_IRF7_ACTIVATION 23

0.27642128 0.69903994 0.85064936 0.93617606
1 3221 "tags=30%, list=20%, signal=38%"
REACTOME_CELL_JUNCTION_ORGANIZATION
REACTOME_CELL_JUNCTION_ORGANIZATION 65
0.23175707 0.69037074 0.9266589 0.9416039
1 3680 "tags=20%, list=23%, signal=26%"
REACTOME_BIOLOGICAL_OXIDATIONS REACTOME_BIOLOGICAL_OXIDATIONS
84 0.2209486 0.6837002 0.9609811
0.9449094 1 5003 "tags=29%, list=31%, signal=41%"
REACTOME_SYNTHESIS_OF_BILE_ACIDS_AND_BILE_SALTS
REACTOME_SYNTHESIS_OF_BILE_ACIDS_AND_BILE_SALTS 18
0.27103505 0.6612517 0.8800558 0.9604295
1 3433 "tags=22%, list=21%, signal=28%"
REACTOME_TAK1_ACTIVATES_NFKB_BY_PHOSPHORYLATION_AND_ACTIVATION_OF_IK
KS_COMPLEX
REACTOME_TAK1_ACTIVATES_NFKB_BY_PHOSPHORYLATION_AND_ACTIVATION_OF_IK
KS_COMPLEX 19 0.2753058 0.65421236
0.9092123 0.9629734 1 1805 "tags=16%,
list=11%, signal=18%"
REACTOME_GASTRIN_CREB_SIGNALLING_PATHWAY_VIA_PKC_AND_MAPK
REACTOME_GASTRIN_CREB_SIGNALLING_PATHWAY_VIA_PKC_AND_MAPK
172 0.19884135 0.6470578 0.9907407
0.9651953 1 5481 "tags=34%, list=34%, signal=51%"
REACTOME_ACYL_CHAIN_REMODELLING_OF_PG
REACTOME_ACYL_CHAIN_REMODELLING_OF_PG 15
0.2709748 0.62259144 0.9212707 0.977045 1
6357 "tags=47%, list=39%, signal=77%"
REACTOME_AMINE_LIGAND_BINDING_RECEPTORS
REACTOME_AMINE_LIGAND_BINDING_RECEPTORS 34
0.23039414 0.618259 0.94199246 0.9766749 1
5919 "tags=44%, list=37%, signal=70%"
REACTOME_TRANSPORT_OF_GLUCOSE_AND_OTHER_SUGARS_BILE_SALTS_AND_ORGANI
C_ACIDS_METAL_IONS_AND_AMINE_COMPOUNDS
REACTOME_TRANSPORT_OF_GLUCOSE_AND_OTHER_SUGARS_BILE_SALTS_AND_ORGANI
C_ACIDS_METAL_IONS_AND_AMINE_COMPOUNDS 77
0.19574025 0.5972136 0.9889625 0.9839573
1 4769 "tags=23%, list=30%, signal=33%"
REACTOME_AMINO_ACID_TRANSPORT_ACROSS_THE_PLASMA_MEMBRANE
REACTOME_AMINO_ACID_TRANSPORT_ACROSS_THE_PLASMA_MEMBRANE
27 0.21992444 0.5768872 0.9670051
0.9891975 1 6400 "tags=52%, list=40%, signal=86%"
REACTOME_ADHERENS_JUNCTIONS_INTERACTIONS
REACTOME_ADHERENS_JUNCTIONS_INTERACTIONS 22
0.22494422 0.55687165 0.96925133 0.9928596
1 451 "tags=5%, list=3%, signal=5%"
REACTOME_SYNTHESIS_OF_PIPS_AT_THE_PLASMA_MEMBRANE
REACTOME_SYNTHESIS_OF_PIPS_AT_THE_PLASMA_MEMBRANE 26
0.21720828 0.55668706 0.97800773 0.9903575
1 2876 "tags=23%, list=18%, signal=28%"
REACTOME_AMINO_ACID_AND_OLIGOPEPTIDE_SLC_TRANSPORTERS
REACTOME_AMINO_ACID_AND_OLIGOPEPTIDE_SLC_TRANSPORTERS
45 0.18233047 0.51824576 0.9963592
0.99573106 1 6754 "tags=47%, list=42%, signal=80%"
REACTOME_BILE_ACID_AND_BILE_SALT_METABOLISM

REACTOME_BILE_ACID_AND_BILE_SALT_METABOLISM 23
0.19808148 0.504981 0.9894459 0.9951909 1
6300 "tags=43%, list=39%, signal=71%"
REACTOME_GAP_JUNCTION_TRAFFICKING REACTOME_GAP_JUNCTION_TRAFFICKING
21 0.19025835 0.47218207 0.992053
0.9962331 1 6616 "tags=48%, list=41%, signal=81%"
