# Supplementary material for: Inflation vs. Exhaustion of Antiviral CD8+ T-Cell Populations in Persistent Infections: Two Sides of the Same Coin?
Source: Front Immunol. 2019 Mar 6;10:197. doi: 10.3389/fimmu.2019.00197 (PMC6414785; doi:10.3389/fimmu.2019.00197)
Supplement: Table S4 — GSEA report of Reactome gene sets enriched in Exhaustion. GSEA report of Reactome curated pathways found enriched (FDR < 0.25) in Exhausting samples (Cl13, days 30) vs. Inflating samples (M38, days 50). [file Table_4.pdf]

Table S4

NAME	GS<br>follow link to MSigDB	GS DETAILS	SIZE	ES	NES	NOM p-val	FDR q-val	FWER p-val	RANK AT MAX	LEADING EDGE
REACTOME_BRANCHED_CHAIN_AMINO_ACID_CATABOLISM	REACTOME_BRANCHED_CHAIN_AMINO_ACID_CATABOLISM	Details ...	16	−0.6489923	−1.7977923	0.007490637	0.27548754	0.29	607	"tags=38%, list=4%, signal=39%"
REACTOME_CHEMOKINE_RECEPTORS_BIND_CHEMOKINES	REACTOME_CHEMOKINE_RECEPTORS_BIND_CHEMOKINES	Details ...	43	−0.5107791	−1.7853953	0	0.14803281	0.308	1052	"tags=23%, list=7%, signal=25%"
REACTOME_CHONDROITIN_SULFATE_BIOSYNTHESIS	REACTOME_CHONDROITIN_SULFATE_BIOSYNTHESIS	Details ...	15	−0.5678584	−1.568396	0.027303753	0.51064867	0.86	3705	"tags=53%, list=23%, signal=69%"
REACTOME_ABCA_TRANSPORTERS_IN_LIPID_HOMEOSTASIS	REACTOME_ABCA_TRANSPORTERS_IN_LIPID_HOMEOSTASIS	Details ...	16	−0.51873195	−1.5050563	0.055762082	0.57423794	0.955	1295	"tags=31%, list=8%, signal=34%"
REACTOME_O_LINKED_GLYCOSYLATION_OF_MUCINS	REACTOME_O_LINKED_GLYCOSYLATION_OF_MUCINS	Details ...	35	−0.4127908	−1.4414263	0.01904762	0.66052645	0.988	764	"tags=17%, list=5%, signal=18%"
REACTOME_NUCLEAR_RECEPTOR_TRANSCRIPTION_PATHWAY	REACTOME_NUCLEAR_RECEPTOR_TRANSCRIPTION_PATHWAY	Details ...	45	−0.39875722	−1.4389706	0.039106146	0.55885756	0.989	3025	"tags=31%, list=19%, signal=38%"
REACTOME_CHONDROITIN_SULFATE_DERMATAN_SULFATE_METABOLISM	REACTOME_CHONDROITIN_SULFATE_DERMATAN_SULFATE_METABOLISM	Details ...	40	−0.39552835	−1.4061259	0.046242774	0.57626593	0.995	3330	"tags=45%, list=21%, signal=57%"
REACTOME_ABC_FAMILY_PROTEINS_MEDIATED_TRANSPORT	REACTOME_ABC_FAMILY_PROTEINS_MEDIATED_TRANSPORT	Details ...	27	−0.42959142	−1.3560073	0.106796116	0.6582773	0.999	1295	"tags=22%, list=8%, signal=24%"
REACTOME_AMINE_DERIVED_HORMONES	REACTOME_AMINE_DERIVED_HORMONES	Details ...	15	−0.47977668	−1.3064651	0.14539006	0.76858824	1	3343	"tags=33%, list=21%, signal=42%"
REACTOME_HS_GAG_DEGRADATION	REACTOME_HS_GAG_DEGRADATION	Details ...	18	−0.45053902	−1.3058697	0.12177122	0.693516	1	1386	"tags=39%, list=9%, signal=43%"
REACTOME_G_ALPHA_S_SIGNALLING_EVENTS	REACTOME_G_ALPHA_S_SIGNALLING_EVENTS	Details ...	106	−0.30575708	−1.299223	0.071428575	0.6534717	1	2649	"tags=26%, list=16%, signal=31%"
REACTOME_NUCLEOTIDE_BINDING_DOMAIN_LEUCINE_RICH_REPEAT_CONTAINING_RECEPTOR_NLR_SIGNALING_PATHWAYS	REACTOME_NUCLEOTIDE_BINDING_DOMAIN_LEUCINE_RICH_REPEAT_CONTAINING_RECEPTOR_NLR_SIGNALING_PATHWAYS	Details ...	37	−0.36239654	−1.2961794	0.10638298				

0.60876477	1	1061	"tags=22%, list=7%, signal=23%"
REACTOME_SIGNALING_BY_BMP	REACTOME_SIGNALING_BY_BMP	Details ...
19	-0.43093348	-1.2810671	0.11904762
0.6089994	1	407	"tags=11%, list=3%, signal=11%"
REACTOME_CGMP_EFFECTS	REACTOME_CGMP_EFFECTS	Details ...
17	-0.43923298	-1.2535503	0.18217054
0.6501979	1	4315	"tags=41%, list=27%, signal=56%"
REACTOME_CLASS_B_2_SECRETIN_FAMILY_RECEPTORS
REACTOME_CLASS_B_2_SECRETIN_FAMILY_RECEPTORS	Details ...
80	-0.30857426	-1.2418737	0.074074075
0.6414273	1	2086	"tags=19%, list=13%, signal=21%"
REACTOME_GPCR_LIGAND_BINDING	REACTOME_GPCR_LIGAND_BINDING
Details ...	336	-0.2607	-1.2400212	0
0.6062522	1	1328	"tags=10%, list=8%, signal=11%"
REACTOME_NITRIC_OXIDE_STIMULATES_GUANYLATE_CYCLASE
REACTOME_NITRIC_OXIDE_STIMULATES_GUANYLATE_CYCLASE	Details ...
23	-0.3951604	-1.2335496	0.1779661
0.58966094	1	3721	"tags=30%, list=23%, signal=40%"
REACTOME_TIGHT_JUNCTION_INTERACTIONS
REACTOME_TIGHT_JUNCTION_INTERACTIONS	Details ...
27	-0.37647226	-1.2297101	0.19313304
0.56707436	1	1546	"tags=19%, list=10%, signal=20%"
REACTOME_HEPARAN_SULFATE_HEPARIN_HS_GAG_METABOLISM
REACTOME_HEPARAN_SULFATE_HEPARIN_HS_GAG_METABOLISM	Details ...
44	-0.33875406	-1.2174972	0.15204678
0.57009435	1	2552	"tags=32%, list=16%, signal=38%"
REACTOME_GLYCOSAMINOGLYCAN_METABOLISM
REACTOME_GLYCOSAMINOGLYCAN_METABOLISM	Details ...
93	-0.28849828	-1.2140415	0.09090909
0.5513953	1	2235	"tags=27%, list=14%, signal=31%"
REACTOME_PEPTIDE_LIGAND_BINDING_RECEPTORS
REACTOME_PEPTIDE_LIGAND_BINDING_RECEPTORS
152	-0.27405497	-1.1982422	0.083333336
0.56597656	1	1061	"tags=8%, list=7%, signal=8%"
REACTOME_A_TETRASACCHARIDE_LINKER_SEQUENCE_IS_REQUIRED_FOR_GAG_SYNTH
ESIS
REACTOME_A_TETRASACCHARIDE_LINKER_SEQUENCE_IS_REQUIRED_FOR_GAG_SYNTH
ESIS	22	-0.37047687	-1.1668662
0.2532189	0.62896675	1	2142	"tags=32%,
list=13%, signal=37%"
REACTOME_SYNTHESIS_OF_GLYCOSYLPHOSPHATIDYLINOSITOL_GPI
REACTOME_SYNTHESIS_OF_GLYCOSYLPHOSPHATIDYLINOSITOL_GPI
16	-0.42373523	-1.1648282	0.25925925
0.6072135	1	548	"tags=13%, list=3%, signal=13%"
REACTOME_CLASS_A1_RHODOPSIN_LIKE_RECEPTORS
REACTOME_CLASS_A1_RHODOPSIN_LIKE_RECEPTORS
244	-0.2503815	-1.1372054	0	0.66193956
1	1328	"tags=9%, list=8%, signal=10%"
REACTOME_GENERIC_TRANSCRIPTION_PATHWAY
REACTOME_GENERIC_TRANSCRIPTION_PATHWAY
126	-0.26103568	-1.1192234	0.16949153	0.689175
1	1170	"tags=13%, list=7%, signal=14%"
REACTOME_UNBLOCKING_OF_NMDA_RECEPTOR_GLUTAMATE_BINDING_AND_ACTIVATIO
N

REACTOME_UNBLOCKING_OF_NMDA_RECEPTOR_GLUTAMATE_BINDING_AND_ACTIVATIO
N	15	-0.41929132	-1.1156546
0.33082706	0.67291105	1	3619	"tags=40%,
list=22%, signal=52%"
REACTOME_N_GLYCAN_ANTENNAE_ELONGATION_IN_THE_MEDIAL_TRANS_GOLGI
REACTOME_N_GLYCAN_ANTENNAE_ELONGATION_IN_THE_MEDIAL_TRANS_GOLGI
	17	-0.39525217	-1.1143262	0.3283582
0.6518005	1	376	"tags=18%, list=2%, signal=18%"
REACTOME_KERATAN_SULFATE_BIOSYNTHESIS
REACTOME_KERATAN_SULFATE_BIOSYNTHESIS
23	-0.367459	-1.1018753	0.26506025
0.66482574	1	1461	"tags=22%, list=9%, signal=24%"
REACTOME_KERATAN_SULFATE_KERATIN_METABOLISM
REACTOME_KERATAN_SULFATE_KERATIN_METABOLISM
27	-0.34299007	-1.0915431	0.2962963
0.67344934	1	2014	"tags=26%, list=13%, signal=30%"
REACTOME_CYTOCHROME_P450_ARRANGED_BY_SUBSTRATE_TYPE
REACTOME_CYTOCHROME_P450_ARRANGED_BY_SUBSTRATE_TYPE
26	-0.34240884	-1.0733625	0.34246576
0.7056716	1	3539	"tags=35%, list=22%, signal=44%"
REACTOME_CELL_CELL_JUNCTION_ORGANIZATION
REACTOME_CELL_CELL_JUNCTION_ORGANIZATION
49	-0.28193632	-1.0441118	0.37583894
0.77484345	1	1546	"tags=14%, list=10%, signal=16%"
REACTOME_BMAL1_CLOCK_NPAS2_ACTIVATES_CIRCADIAN_EXPRESSION
REACTOME_BMAL1_CLOCK_NPAS2_ACTIVATES_CIRCADIAN_EXPRESSION
32	-0.31686595	-1.0367054	0.4278846
0.7741386	1	1700	"tags=25%, list=11%, signal=28%"
REACTOME_INWARDLY_RECTIFYING_K_CHANNELS
REACTOME_INWARDLY_RECTIFYING_K_CHANNELS
27	-0.31654474	-1.0158521	0.42460316
0.81804544	1	1478	"tags=15%, list=9%, signal=16%"
REACTOME_METABOLISM_OF_VITAMINS_AND_COFACTORS
REACTOME_METABOLISM_OF_VITAMINS_AND_COFACTORS
47	-0.2855358	-1.0141844	0.40782124
0.79937935	1	1228	"tags=21%, list=8%, signal=23%"
REACTOME_AMINE_COMPOUND_SLC_TRANSPORTERS
REACTOME_AMINE_COMPOUND_SLC_TRANSPORTERS
25	-0.32119498	-1.013522	0.4159664
0.7786651	1	1528	"tags=12%, list=9%, signal=13%"
REACTOME_FGFR_LIGAND_BINDING_AND_ACTIVATION
REACTOME_FGFR_LIGAND_BINDING_AND_ACTIVATION
21	-0.3327589	-1.004576	0.44871795
0.7841729	1	4842	"tags=43%, list=30%, signal=61%"
REACTOME_G_ALPHA_Q_SIGNALLING_EVENTS
REACTOME_G_ALPHA_Q_SIGNALLING_EVENTS
154	-0.22736567	-0.9964397	0.45	0.7893129
1	1306	"tags=11%, list=8%, signal=12%"
REACTOME_DEGRADATION_OF_THE_EXTRACELLULAR_MATRIX
REACTOME_DEGRADATION_OF_THE_EXTRACELLULAR_MATRIX
22	-0.3380446	-0.9959557	0.46724892
0.7696134	1	1343	"tags=23%, list=8%, signal=25%"
REACTOME_ACTIVATION_OF_BH3_ONLY_PROTEINS
REACTOME_ACTIVATION_OF_BH3_ONLY_PROTEINS

15	-0.35888064	-0.98521763	0.45692885
0.78170377	1	472	"tags=20%, list=3%, signal=21%"
REACTOME_G_ALPHA_I_SIGNALLING_EVENTS
REACTOME_G_ALPHA_I_SIGNALLING_EVENTS
160	-0.22020432	-0.96978223	0.4848485
0.8069782	1	1061	"tags=9%, list=7%, signal=10%"
REACTOME_NOD1_2_SIGNALING_PATHWAY REACTOME_NOD1_2_SIGNALING_PATHWAY
25	-0.31413874	-0.96706086	0.47234043
0.795189 1	2072	"tags=28%, list=13%, signal=32%"
REACTOME_GLUCAGON_TYPE_LIGAND_RECEPTORS
REACTOME_GLUCAGON_TYPE_LIGAND_RECEPTORS
30	-0.29616773	-0.96622574	0.5225225
0.7784876	1	2086	"tags=27%, list=13%, signal=31%"
REACTOME_EXTRACELLULAR_MATRIX_ORGANIZATION
REACTOME_EXTRACELLULAR_MATRIX_ORGANIZATION
74	-0.23627485	-0.94526154	0.57575756
0.81976277	1	1014	"tags=7%, list=6%, signal=7%"
REACTOME_POTASSIUM_CHANNELS	REACTOME_POTASSIUM_CHANNELS
86	-0.23787256	-0.9313706	0.6018519
0.8387527	1	2165	"tags=15%, list=13%, signal=17%"
REACTOME_GPCR_DOWNSTREAM_SIGNALING
REACTOME_GPCR_DOWNSTREAM_SIGNALING
399	-0.20538066	-0.931024	0.6666667
0.82112175	1	1328	"tags=11%, list=8%, signal=12%"
REACTOME_CIRCADIAN_CLOCK	REACTOME_CIRCADIAN_CLOCK
45	-0.2600521	-0.92666036	0.6193182	0.813837
1	1700	"tags=22%, list=11%, signal=25%"
REACTOME_PLATELET_CALCIUM_HOMEOSTASIS
REACTOME_PLATELET_CALCIUM_HOMEOSTASIS
16	-0.3349629	-0.9229411	0.53386456
0.8051865	1	518	"tags=13%, list=3%, signal=13%"
REACTOME_POST_TRANSLATIONAL_MODIFICATION_SYNTHESIS_OF_GPI_ANCHORED_P
ROTEINS
REACTOME_POST_TRANSLATIONAL_MODIFICATION_SYNTHESIS_OF_GPI_ANCHORED_P
ROTEINS	22	-0.28982884	-0.9046209
0.5921569	0.83320713	1	548	"tags=9%,
list=3%, signal=9%"
REACTOME_NA_CL_DEPENDENT_NEUROTRANSMITTER_TRANSPORTERS
REACTOME_NA_CL_DEPENDENT_NEUROTRANSMITTER_TRANSPORTERS
16	-0.31856427	-0.89613193	0.6070175
0.83681494	1	1528	"tags=13%, list=9%, signal=14%"
REACTOME_SYNTHESIS_OF_PC	REACTOME_SYNTHESIS_OF_PC
17	-0.3113741	-0.8745453	0.63837636
0.8705006	1	3835	"tags=35%, list=24%, signal=46%"
REACTOME_LIGAND_GATED_ION_CHANNEL_TRANSPORT
REACTOME_LIGAND_GATED_ION_CHANNEL_TRANSPORT
18	-0.30542606	-0.8708247	0.65909094
0.8614056	1	11194	"tags=100%, list=69%, signal=327%"
REACTOME_VOLTAGE_GATED_POTASSIUM_CHANNELS
REACTOME_VOLTAGE_GATED_POTASSIUM_CHANNELS
39	-0.250812	-0.86196625	0.70238096
0.8637342	1	3176	"tags=23%, list=20%, signal=29%"
REACTOME_NCAM1_INTERACTIONS	REACTOME_NCAM1_INTERACTIONS
35	-0.24883743	-0.8564386	0.69945353

0.8592211 1 2230 "tags=20%, list=14%, signal=23%"
REACTOME_REGULATION_OF_BETA_CELL_DEVELOPMENT
REACTOME_REGULATION_OF_BETA_CELL_DEVELOPMENT
27 -0.25832987 -0.8389582 0.7212389
0.8796693 1 449 "tags=7%, list=3%, signal=8%"
REACTOME_TRAF6_MEDIATED_NFKB_ACTIVATION
REACTOME_TRAF6_MEDIATED_NFKB_ACTIVATION
19 -0.27839863 -0.8304289 0.70124483
0.8805764 1 1816 "tags=26%, list=11%, signal=30%"
REACTOME_ACTIVATED_POINT_MUTANTS_OF_FGFR2
REACTOME_ACTIVATED_POINT_MUTANTS_OF_FGFR2
16 -0.29289395 -0.827075 0.71375465
0.8713698 1 4842 "tags=44%, list=30%, signal=62%"
REACTOME_HS_GAG_BIOSYNTHESIS REACTOME_HS_GAG_BIOSYNTHESIS
26 -0.2567796 -0.82390505 0.7372881
0.8617031 1 2552 "tags=27%, list=16%, signal=32%"
REACTOME_COMPLEMENT_CASCADE REACTOME_COMPLEMENT_CASCADE
23 -0.26168847 -0.8217021 0.72540987
0.8507769 1 2515 "tags=22%, list=16%, signal=26%"
REACTOME_COLLAGEN_FORMATION REACTOME_COLLAGEN_FORMATION
52 -0.21823767 -0.80191046 0.86624205
0.86886394 1 24 "tags=2%, list=0%, signal=2%"
REACTOME_FORMATION_OF_FIBRIN_CLOT_CLOTTING_CASCADE
REACTOME_FORMATION_OF_FIBRIN_CLOT_CLOTTING_CASCADE
26 -0.22654064 -0.73532605 0.87603307
0.94426924 1 12465 "tags=100%, list=77%, signal=442%"
REACTOME_RAS_ACTIVATION_UOPN_CA2_INFUX_THROUGH_NMDA_RECEPTOR
REACTOME_RAS_ACTIVATION_UOPN_CA2_INFUX_THROUGH_NMDA_RECEPTOR
16 -0.25505435 -0.73126334 0.8759124
0.9332839 1 3619 "tags=38%, list=22%, signal=48%"
REACTOME_TRANSPORT_OF_INORGANIC_CATIONS_ANIONS_AND_AMINO_ACIDS_OLIGO
PEPTIDES
REACTOME_TRANSPORT_OF_INORGANIC_CATIONS_ANIONS_AND_AMINO_ACIDS_OLIGO
PEPTIDES 83 -0.17395541 -0.6868997
0.9782609 0.95774716 1 1786 "tags=11%,
list=11%, signal=12%"
REACTOME_PHASE1_FUNCTIONALIZATION_OF_COMPOUNDS
REACTOME_PHASE1_FUNCTIONALIZATION_OF_COMPOUNDS
41 -0.18800858 -0.6642104 0.96276593
0.95834196 1 3539 "tags=29%, list=22%, signal=37%"
REACTOME_GAP_JUNCTION_ASSEMBLY REACTOME_GAP_JUNCTION_ASSEMBLY
15 -0.19885658 -0.54589146 0.97445256
0.9893459 1 12908 "tags=100%, list=80%, signal=503%"
