# Supplementary material for: Inflation vs. Exhaustion of Antiviral CD8+ T-Cell Populations in Persistent Infections: Two Sides of the Same Coin?
Source: Front Immunol. 2019 Mar 6;10:197. doi: 10.3389/fimmu.2019.00197 (PMC6414785; doi:10.3389/fimmu.2019.00197)
Supplement: Table S5 — (A,B) GSEA reports of MsigDB Immunological Signatures gene sets in Inflation and Exhaustion. GSEA reports of immunological signatures found enriched (FDR < 0.25) in Inflating samples (A) (M38, days 50) vs. Exhausting samples (B) (Cl13, days 30). [file Table_5.pdf]

Table S5A

NAME	GS<br>follow link to MSigDB	GS DETAILS	SIZE	ES	NES	NOM p-val	FDR q-val	FWER p-val	RANK AT MAX	LEADING EDGE
GSE14415_TCONV_VS_FOXP3_KO_INDUCED_TREG_DN	GSE14415_TCONV_VS_FOXP3_KO_INDUCED_TREG_DN	Details ...	152	0.8512679	3.5359576	0	0	0	1397	"tags=76%, list=10%, signal=84%"
GSE13547_CTRL_VS_ANTI_IGM_STIM_BCELL_12H_UP	GSE13547_CTRL_VS_ANTI_IGM_STIM_BCELL_12H_UP	Details ...	145	0.8570669	3.5275145	0	0	0	808	"tags=69%, list=6%, signal=72%"
GSE15750_DAY6_VS_DAY10_TRAF6KO_EFF_CD8_TCELL_UP	GSE15750_DAY6_VS_DAY10_TRAF6KO_EFF_CD8_TCELL_UP	Details ...	143	0.84650564	3.5108736	0	0	0	870	"tags=64%, list=6%, signal=68%"
GSE15750_DAY6_VS_DAY10_EFF_CD8_TCELL_UP	GSE15750_DAY6_VS_DAY10_EFF_CD8_TCELL_UP	Details ...	144	0.8506729	3.505754	0	0	0	1305	"tags=72%, list=9%, signal=78%"
GSE14415_INDUCED_VS_NATURAL_TREG_DN	GSE14415_INDUCED_VS_NATURAL_TREG_DN	Details ...	151	0.85111314	3.5001361	0	0	0	1085	"tags=70%, list=8%, signal=75%"
GSE14415_INDUCED_TREG_VS_TCONV_UP	GSE14415_INDUCED_TREG_VS_TCONV_UP	Details ...	139	0.8412118	3.4431365	0	0	0	1680	"tags=81%, list=12%, signal=91%"
GSE14415_NATURAL_TREG_VS_TCONV_DN	GSE14415_NATURAL_TREG_VS_TCONV_DN	Details ...	145	0.83156735	3.4202006	0	0	0	1098	"tags=65%, list=8%, signal=70%"
GSE13547_CTRL_VS_ANTI_IGM_STIM_ZFX_KO_BCELL_2H_UP	GSE13547_CTRL_VS_ANTI_IGM_STIM_ZFX_KO_BCELL_2H_UP	Details ...	133	0.8219061	3.407093	0	0	0	1587	"tags=76%, list=11%, signal=85%"
GSE33162_UNTREATED_VS_4H_LPS_STIM_HDAC3_KO_MACROPHAGE_DN	GSE33162_UNTREATED_VS_4H_LPS_STIM_HDAC3_KO_MACROPHAGE_DN	Details ...	159	0.8052403	3.350093	0	0	0	1703	"tags=70%, list=12%, signal=79%"
GSE11386_NAIVE_VS_MEMORY_BCELL_UP	GSE11386_NAIVE_VS_MEMORY_BCELL_UP	Details ...	153	0.7917242	3.3050165	0	0	0	2003	"tags=71%, list=14%, signal=82%"
GSE13547_2H_VS_12_H_ANTI_IGM_STIM_BCELL_DN	GSE13547_2H_VS_12_H_ANTI_IGM_STIM_BCELL_DN	Details ...	143	0.8038657	3.3014963	0	0	0	1439	"tags=67%, list=10%, signal=74%"
GOLDRATH_EFF_VS_MEMORY_CD8_TCELL_UP	GOLDRATH_EFF_VS_MEMORY_CD8_TCELL_UP	Details ...	158	0.7837205	3.2980487	0	0	0	1277	"tags=66%, list=9%, signal=72%"
GSE27241_WT_VS_RORGT_KO_TH17_POLARIZED_CD4_TCELL_UP	GSE27241_WT_VS_RORGT_KO_TH17_POLARIZED_CD4_TCELL_UP	Details ...	130	0.813163	3.2881105	0	0	0	840	"tags=54%, list=6%, signal=57%"
KAECH_DAY8_EFF_VS_MEMORY_CD8_TCELL_UP										

KAECH_DAY8_EFF_VS_MEMORY_CD8_TCELL_UP	Details ...	151
0.77719486	3.2687707	0	0	0
1732	"tags=69%, list=12%, signal=78%"
GSE36476_CTRL_VS_TSST_ACT_72H_MEMORY_CD4_TCELL_YOUNG_DN
GSE36476_CTRL_VS_TSST_ACT_72H_MEMORY_CD4_TCELL_YOUNG_DN
Details ...	147	0.7711256	3.251116	0	0
0	1321	"tags=55%, list=9%, signal=60%"
KAECH_DAY8_EFF_VS_DAY15_EFF_CD8_TCELL_UP
KAECH_DAY8_EFF_VS_DAY15_EFF_CD8_TCELL_UP	Details ...	154
0.78424054	3.2422447	0	0	0
2110	"tags=71%, list=15%, signal=82%"
GSE36476_CTRL_VS_TSST_ACT_72H_MEMORY_CD4_TCELL_OLD_DN
GSE36476_CTRL_VS_TSST_ACT_72H_MEMORY_CD4_TCELL_OLD_DN
Details ...	142	0.7718059	3.192243	0	0
0	1299	"tags=55%, list=9%, signal=60%"
GSE30962_PRIMARY_VS_SECONDARY_ACUTE_LCMV_INF_CD8_TCELL_UP
GSE30962_PRIMARY_VS_SECONDARY_ACUTE_LCMV_INF_CD8_TCELL_UP
Details ...	147	0.7653149	3.1840854	0
0	0	773	"tags=51%, list=5%, signal=53%"
KAECH_NAIVE_VS_DAY8_EFF_CD8_TCELL_DN
KAECH_NAIVE_VS_DAY8_EFF_CD8_TCELL_DN	Details ...	158
0.7613454	3.1713278	0	0	0
1701	"tags=66%, list=12%, signal=75%"
GSE15930_NAIVE_VS_48H_IN_VITRO_STIM_CD8_TCELL_DN
GSE15930_NAIVE_VS_48H_IN_VITRO_STIM_CD8_TCELL_DN	Details ...
167	0.74816865	3.1370368	0	0	0
2115	"tags=63%, list=15%, signal=73%"
GSE19941_LPS_VS_LPS_AND_IL10_STIM_IL10_KO_MACROPHAGE_UP
GSE19941_LPS_VS_LPS_AND_IL10_STIM_IL10_KO_MACROPHAGE_UP
143	0.75656354	3.1358154	0	0	0
2154	"tags=67%, list=15%, signal=78%"
GSE13547_2H_VS_12_H_ANTI_IGM_STIM_ZFX_KO_BCELL_DN
GSE13547_2H_VS_12_H_ANTI_IGM_STIM_ZFX_KO_BCELL_DN	131
0.78582615	3.135025	0	0	0
1633	"tags=66%, list=12%, signal=74%"
GSE10239_NAIVE_VS_DAY4.5_EFF_CD8_TCELL_DN
GSE10239_NAIVE_VS_DAY4.5_EFF_CD8_TCELL_DN	144
0.75621414	3.134018	0	0	0
1425	"tags=59%, list=10%, signal=65%"
GSE13547_WT_VS_ZFX_KO_BCELL_ANTI_IGM_STIM_2H_UP
GSE13547_WT_VS_ZFX_KO_BCELL_ANTI_IGM_STIM_2H_UP	140
0.76360744	3.1273365	0	0	0
1819	"tags=64%, list=13%, signal=73%"
GOLDRATH_NAIVE_VS_EFF_CD8_TCELL_DN
GOLDRATH_NAIVE_VS_EFF_CD8_TCELL_DN	154
0.74207205	3.1188004	0	0	0
1321	"tags=60%, list=9%, signal=66%"
GSE39556_CD8A_DC_VS_NK_CELL_MOUSE_3H_POST_POLYIC_INJ_UP
GSE39556_CD8A_DC_VS_NK_CELL_MOUSE_3H_POST_POLYIC_INJ_UP
158	0.7508221	3.1139772	0	0	0
1270	"tags=56%, list=9%, signal=61%"
GSE24634_TEFF_VS_TCONV_DAY7_IN_CULTURE_UP
GSE24634_TEFF_VS_TCONV_DAY7_IN_CULTURE_UP	158
0.75740373	3.1012747	0	0	0

1273 "tags=53%, list=9%, signal=57%"
GSE24634_TREG_VS_TCONV_POST_DAY7_IL4_CONVERSION_UP
GSE24634_TREG_VS_TCONV_POST_DAY7_IL4_CONVERSION_UP 158
0.7399121 3.0886056 0 0 0
1306 "tags=51%, list=9%, signal=56%"
GSE36476_CTRL_VS_TSST_ACT_40H_MEMORY_CD4_TCELL_OLD_DN
GSE36476_CTRL_VS_TSST_ACT_40H_MEMORY_CD4_TCELL_OLD_DN
152 0.7380422 3.0856833 0 0 0
1299 "tags=52%, list=9%, signal=57%"
GSE33162_UNTREATED_VS_4H_LPS_STIM_HDAC3_KO_MACROPHAGE_UP
GSE33162_UNTREATED_VS_4H_LPS_STIM_HDAC3_KO_MACROPHAGE_UP
157 0.73611486 3.0789392 0 0 0
1948 "tags=68%, list=14%, signal=78%"
GSE9650_EFFECTOR_VS_MEMORY_CD8_TCELL_UP
GSE9650_EFFECTOR_VS_MEMORY_CD8_TCELL_UP 159
0.73577434 3.0645502 0 0 0
2223 "tags=65%, list=16%, signal=77%"
GSE36476_CTRL_VS_TSST_ACT_40H_MEMORY_CD4_TCELL_YOUNG_DN
GSE36476_CTRL_VS_TSST_ACT_40H_MEMORY_CD4_TCELL_YOUNG_DN
146 0.7446198 3.063678 0 0 0
1270 "tags=53%, list=9%, signal=57%"
GSE21063_WT_VS_NFATC1_KO_8H_ANTI_IGM_STIM_BCELL_UP
GSE21063_WT_VS_NFATC1_KO_8H_ANTI_IGM_STIM_BCELL_UP 138
0.7326221 3.0585988 0 0 0
1418 "tags=56%, list=10%, signal=61%"
GSE5679_CTRL_VS_PPARG_LIGAND_ROSIGLITAZONE_TREATED_DC_UP
GSE5679_CTRL_VS_PPARG_LIGAND_ROSIGLITAZONE_TREATED_DC_UP
157 0.72627586 3.053774 0 0 0
1935 "tags=61%, list=14%, signal=70%"
GSE7568_IL4_VS_IL4_AND_DEXAMETHASONE_TREATED_MACROPHAGE_UP
GSE7568_IL4_VS_IL4_AND_DEXAMETHASONE_TREATED_MACROPHAGE_UP
131 0.75964916 3.045681 0 0 0
2154 "tags=73%, list=15%, signal=85%"
GSE18893_TCONV_VS_TREG_24H_TNF_STIM_UP
GSE18893_TCONV_VS_TREG_24H_TNF_STIM_UP 145
0.73635024 3.0437188 0 0 0
1939 "tags=61%, list=14%, signal=70%"
GSE10239_NAIVE_VS_KLRG1HIGH_EFF_CD8_TCELL_DN
GSE10239_NAIVE_VS_KLRG1HIGH_EFF_CD8_TCELL_DN 143
0.74177957 3.0405846 0 0 0
1269 "tags=49%, list=9%, signal=53%"
GSE14415_INDUCED_TREG_VS_FAILED_INDUCED_TREG_UP
GSE14415_INDUCED_TREG_VS_FAILED_INDUCED_TREG_UP 146
0.7289072 3.037504 0 0 0
1973 "tags=58%, list=14%, signal=67%"
GSE5679_CTRL_VS_RARA_AGONIST_AM580_TREATED_DC_UP
GSE5679_CTRL_VS_RARA_AGONIST_AM580_TREATED_DC_UP 142
0.7266131 3.0057685 0 0 0
1677 "tags=57%, list=12%, signal=64%"
GSE39110_DAY3_VS_DAY6_POST_IMMUNIZATION_CD8_TCELL_DN
GSE39110_DAY3_VS_DAY6_POST_IMMUNIZATION_CD8_TCELL_DN
147 0.7231883 2.9937875 0 0 0
1256 "tags=56%, list=9%, signal=61%"
GSE2405_S_AUREUS_VS_UNTREATED_NEUTROPHIL_DN

GSE2405_S_AUREUS_VS_UNTREATED_NEUTROPHIL_DN 150
0.714343 2.9510558 0 0 0
992 "tags=41%, list=7%, signal=43%"
GSE37532_WT_VS_PPARG_KO_VISCERAL_ADIPOSE_TISSUE_TREG_UP
GSE37532_WT_VS_PPARG_KO_VISCERAL_ADIPOSE_TISSUE_TREG_UP
141 0.71816593 2.9393973 0 0 0
1241 "tags=46%, list=9%, signal=50%"
GSE24574_BCL6_LOW_TFH_VS_TCONV_CD4_TCELL_DN
GSE24574_BCL6_LOW_TFH_VS_TCONV_CD4_TCELL_DN 151
0.7147146 2.9319491 0 0 0
1940 "tags=56%, list=14%, signal=65%"
GSE35543_IN_VITRO_ITREG_VS_CONVERTED_EX_ITREG_UP
GSE35543_IN_VITRO_ITREG_VS_CONVERTED_EX_ITREG_UP 143
0.7019634 2.9309633 0 0 0
1947 "tags=53%, list=14%, signal=61%"
GSE23568_CTRL_VS_ID3_TRANSDUCED_CD8_TCELL_DN
GSE23568_CTRL_VS_ID3_TRANSDUCED_CD8_TCELL_DN 143
0.71250176 2.9301596 0 0 0
1811 "tags=57%, list=13%, signal=64%"
GSE15930_NAIVE_VS_48H_IN_VITRO_STIM_IL12_CD8_TCELL_DN
GSE15930_NAIVE_VS_48H_IN_VITRO_STIM_IL12_CD8_TCELL_DN
153 0.70310175 2.9180515 0 0 0
2114 "tags=56%, list=15%, signal=65%"
GSE26156_DOUBLE_POSITIVE_VS_CD4_SINGLE_POSITIVE_THYMOCYTE_DN
GSE26156_DOUBLE_POSITIVE_VS_CD4_SINGLE_POSITIVE_THYMOCYTE_DN
165 0.6959177 2.908654 0 0 0
2018 "tags=57%, list=14%, signal=66%"
GSE28726_NAIVE_VS_ACTIVATED_CD4_TCELL_DN
GSE28726_NAIVE_VS_ACTIVATED_CD4_TCELL_DN 156
0.6989041 2.9029412 0 0 0
1309 "tags=49%, list=9%, signal=53%"
GSE40274_CTRL_VS_FOXP3_TRANSDUCED_ACTIVATED_CD4_TCELL_UP
GSE40274_CTRL_VS_FOXP3_TRANSDUCED_ACTIVATED_CD4_TCELL_UP
143 0.70318794 2.9018831 0 0 0
843 "tags=46%, list=6%, signal=49%"
GSE33292_WT_VS_TCF1_KO_DN3_THYMOCYTE_DN
GSE33292_WT_VS_TCF1_KO_DN3_THYMOCYTE_DN 157
0.69059426 2.8903832 0 0 0
1270 "tags=47%, list=9%, signal=51%"
GSE2128_C57BL6_VS_NOD_THYMOCYTE_UP
GSE2128_C57BL6_VS_NOD_THYMOCYTE_UP 154
0.6925558 2.8820584 0 0 0
1763 "tags=51%, list=12%, signal=57%"
GSE23568_ID3_KO_VS_WT_CD8_TCELL_UP
GSE23568_ID3_KO_VS_WT_CD8_TCELL_UP 158 0.680959
2.8684742 0 0 0 1775 "tags=54%,
list=13%, signal=62%"
GSE9650_NAIVE_VS_EFF_CD8_TCELL_DN GSE9650_NAIVE_VS_EFF_CD8_TCELL_DN
157 0.68651515 2.8648112 0 0
0 1665 "tags=57%, list=12%, signal=64%"
GSE24634_IL4_VS_CTRL_TREATED_NAIVE_CD4_TCELL_DAY7_UP
GSE24634_IL4_VS_CTRL_TREATED_NAIVE_CD4_TCELL_DAY7_UP
147 0.6981343 2.8582373 0 0 0
1503 "tags=45%, list=11%, signal=50%"

GSE24671_BAKIMULC_VS_SENDAI_VIRUS_INFECTED_MOUSE_SPLENOCYTES_UP
GSE24671_BAKIMULC_VS_SENDAI_VIRUS_INFECTED_MOUSE_SPLENOCYTES_UP
142 0.68038505 2.8564417 0 0
0 1973 "tags=45%, list=14%, signal=52%"
GSE24634_TEFF_VS_TCONV_DAY5_IN_CULTURE_UP
GSE24634_TEFF_VS_TCONV_DAY5_IN_CULTURE_UP 152
0.68927395 2.8559809 0 0 0
2110 "tags=55%, list=15%, signal=64%"
GSE15330_HSC_VS_LYMPHOID_PRIMED_MULTIPOTENT_PROGENITOR_DN
GSE15330_HSC_VS_LYMPHOID_PRIMED_MULTIPOTENT_PROGENITOR_DN
144 0.6898951 2.844418 0 0 0
2358 "tags=67%, list=17%, signal=79%"
GSE14699_DELETIONAL_TOLERANCE_VS_ACTIVATED_CD8_TCELL_DN
GSE14699_DELETIONAL_TOLERANCE_VS_ACTIVATED_CD8_TCELL_DN
131 0.6994229 2.8431845 0 0 0
1701 "tags=59%, list=12%, signal=66%"
GSE28726_NAIVE_CD4_TCELL_VS_NAIVE_VA24NEG_NKTCELL_UP
GSE28726_NAIVE_CD4_TCELL_VS_NAIVE_VA24NEG_NKTCELL_UP
152 0.68672854 2.8428822 0 0 0
1496 "tags=44%, list=11%, signal=49%"
GSE13547_WT_VS_ZFX_KO_BCELL_DN GSE13547_WT_VS_ZFX_KO_BCELL_DN
136 0.68299216 2.841029 0 0 0
2116 "tags=62%, list=15%, signal=72%"
GSE39110_DAY3_VS_DAY6_POST_IMMUNIZATION_CD8_TCELL_WITH_IL2_TREATMENT_UP
GSE39110_DAY3_VS_DAY6_POST_IMMUNIZATION_CD8_TCELL_WITH_IL2_TREATMENT_UP 145 0.68458974 2.8325279 0
0 0 1297 "tags=48%, list=9%, signal=53%"
GSE24634_NAIVE_CD4_TCELL_VS_DAY7_IL4_CONV_TREG_DN
GSE24634_NAIVE_CD4_TCELL_VS_DAY7_IL4_CONV_TREG_DN 161
0.66085863 2.8270183 0 0 0
1895 "tags=52%, list=13%, signal=60%"
GSE25088_WT_VS_STAT6_KO_MACROPHAGE_IL4_STIM_DN
GSE25088_WT_VS_STAT6_KO_MACROPHAGE_IL4_STIM_DN 137
0.687397 2.823852 0 0 0 1645 "tags=45%,
list=12%, signal=51%"
GSE15930_NAIVE_VS_72H_IN_VITRO_STIM_IFNAB_CD8_TCELL_DN
GSE15930_NAIVE_VS_72H_IN_VITRO_STIM_IFNAB_CD8_TCELL_DN
155 0.672546 2.8218887 0 0 0
1862 "tags=55%, list=13%, signal=62%"
GSE9650_EFFECTOR_VS_EXHAUSTED_CD8_TCELL_UP
GSE9650_EFFECTOR_VS_EXHAUSTED_CD8_TCELL_UP 161
0.6758747 2.818339 0 0 0
1810 "tags=53%, list=13%, signal=60%"
GSE43863_TH1_VS_LY6C_LOW_CXCR5NEG_EFFECTOR_CD4_TCELL_UP
GSE43863_TH1_VS_LY6C_LOW_CXCR5NEG_EFFECTOR_CD4_TCELL_UP
147 0.6727956 2.8137655 0 0 0
1472 "tags=50%, list=10%, signal=55%"
GSE25085_FETAL_LIVER_VS_ADULT_BM_SP4_THYMIC_IMPLANT_DN
GSE25085_FETAL_LIVER_VS_ADULT_BM_SP4_THYMIC_IMPLANT_DN
142 0.6784559 2.80778 0 0 0
1703 "tags=52%, list=12%, signal=59%"
GSE43863_TH1_VS_LY6C_INT_CXCR5POS_EFFECTOR_CD4_TCELL_UP
GSE43863_TH1_VS_LY6C_INT_CXCR5POS_EFFECTOR_CD4_TCELL_UP

152 0.67223275 2.7963166 0 0 0
2621 "tags=69%, list=19%, signal=84%"
GSE40274_CTRL_VS_EOS_TRANSDUCED_ACTIVATED_CD4_TCELL_UP
GSE40274_CTRL_VS_EOS_TRANSDUCED_ACTIVATED_CD4_TCELL_UP
138 0.6852847 2.796253 0 0 0
1407 "tags=51%, list=10%, signal=57%"
GSE12845_IGD_POS_BLOOD_VS_PRE_GC_TONSIL_BCELL_DN
GSE12845_IGD_POS_BLOOD_VS_PRE_GC_TONSIL_BCELL_DN 139
0.6762841 2.7710586 0 0 0
1580 "tags=47%, list=11%, signal=53%"
GSE12392_CD8A_POS_VS_NEG_SPLEEN_DC_DN
GSE12392_CD8A_POS_VS_NEG_SPLEEN_DC_DN 147
0.65894884 2.7640219 0 0 0
1375 "tags=43%, list=10%, signal=47%"
GSE5142_HTERT_TRANSDUCED_VS_CTRL_CD8_TCELL_LATE_PASSAGE_CLONE_UP
GSE5142_HTERT_TRANSDUCED_VS_CTRL_CD8_TCELL_LATE_PASSAGE_CLONE_UP
139 0.66726935 2.761134 0 0 0
2307 "tags=55%, list=16%, signal=66%"
GSE15930_NAIVE_VS_72H_IN_VITRO_STIM_CD8_TCELL_DN
GSE15930_NAIVE_VS_72H_IN_VITRO_STIM_CD8_TCELL_DN 149
0.6609955 2.751783 0 0 0
2209 "tags=60%, list=16%, signal=70%"
GSE22886_UNSTIM_VS_IL15_STIM_NKCELL_DN
GSE22886_UNSTIM_VS_IL15_STIM_NKCELL_DN 160
0.6542361 2.751199 0 0 0
2586 "tags=64%, list=18%, signal=77%"
GSE40274_CTRL_VS_FOXP3_AND_HELIOS_TRANSDUCED_ACTIVATED_CD4_TCELL_DN
GSE40274_CTRL_VS_FOXP3_AND_HELIOS_TRANSDUCED_ACTIVATED_CD4_TCELL_DN
152 0.6581813 2.748779 0 0 0
1896 "tags=48%, list=13%, signal=55%"
GSE13547_CTRL_VS_ANTI_IGM_STIM_BCELL_2H_UP
GSE13547_CTRL_VS_ANTI_IGM_STIM_BCELL_2H_UP 138
0.6655232 2.73739 0 0 0
915 "tags=56%, list=6%, signal=59%"
GSE7852_LN_VS_THYMUS_TCONV_DN GSE7852_LN_VS_THYMUS_TCONV_DN
143 0.65680146 2.7257123 0 0
0 740 "tags=35%, list=5%, signal=37%"
GSE10239_NAIVE_VS_KLRG1INT_EFF_CD8_TCELL_DN
GSE10239_NAIVE_VS_KLRG1INT_EFF_CD8_TCELL_DN 142
0.6571187 2.7239554 0 0 0
1190 "tags=40%, list=8%, signal=43%"
GSE19825_NAIVE_VS_DAY3_EFF_CD8_TCELL_DN
GSE19825_NAIVE_VS_DAY3_EFF_CD8_TCELL_DN 145
0.6642833 2.7201946 0 0 0
2295 "tags=54%, list=16%, signal=64%"
GSE14699_DELETIONAL_TOLERANCE_VS_ACTIVATED_CD8_TCELL_UP
GSE14699_DELETIONAL_TOLERANCE_VS_ACTIVATED_CD8_TCELL_UP
151 0.65125775 2.7186508 0 0 0
2274 "tags=60%, list=16%, signal=71%"
GSE22886_UNSTIM_VS_IL2_STIM_NKCELL_DN
GSE22886_UNSTIM_VS_IL2_STIM_NKCELL_DN 157 0.644924
2.7181265 0 0 0 2474 "tags=61%,
list=18%, signal=73%"
GSE22886_NAIVE_CD4_TCELL_VS_48H_ACT_TH2_DN

GSE22886_NAIVE_CD4_TCELL_VS_48H_ACT_TH2_DN 154
0.64639074 2.708443 0 0 0
2420 "tags=51%, list=17%, signal=61%"
GSE15930_NAIVE_VS_72H_IN_VITRO_STIM_TRICHOSTATINA_CD8_TCELL_DN
GSE15930_NAIVE_VS_72H_IN_VITRO_STIM_TRICHOSTATINA_CD8_TCELL_DN
151 0.65504014 2.7083886 0 0
0 1910 "tags=57%, list=14%, signal=65%"
GSE22886_NAIVE_CD4_TCELL_VS_48H_ACT_TH1_DN
GSE22886_NAIVE_CD4_TCELL_VS_48H_ACT_TH1_DN 156
0.6451833 2.7043798 0 0 0
2548 "tags=62%, list=18%, signal=74%"
GSE39110_UNTREATED_VS_IL2_TREATED_CD8_TCELL_DAY3_POST_IMMUNIZATION_D
N
GSE39110_UNTREATED_VS_IL2_TREATED_CD8_TCELL_DAY3_POST_IMMUNIZATION_D
N 145 0.651958 2.6996682 0 0
0 1058 "tags=44%, list=7%, signal=47%"
GSE24634_TEFF_VS_TCONV_DAY10_IN_CULTURE_UP
GSE24634_TEFF_VS_TCONV_DAY10_IN_CULTURE_UP 147
0.6512839 2.696317 0 0 0
1324 "tags=39%, list=9%, signal=43%"
GSE21063_CTRL_VS_ANTI_IGM_STIM_BCELL_NFATC1_KO_8H_DN
GSE21063_CTRL_VS_ANTI_IGM_STIM_BCELL_NFATC1_KO_8H_DN
141 0.6554793 2.685251 0 0 0
1301 "tags=40%, list=9%, signal=43%"
GSE37532_VISCERAL_ADIPOSE_TISSUE_VS_LN_DERIVED_PPARG_KO_TCONV_CD4_TC
ELL_UP
GSE37532_VISCERAL_ADIPOSE_TISSUE_VS_LN_DERIVED_PPARG_KO_TCONV_CD4_TC
ELL_UP 138 0.6539664 2.6825902 0
0 0 2295 "tags=54%, list=16%, signal=63%"
GSE36826_WT_VS_IL1R_KO_SKIN_STAPH_AUREUS_INF_UP
GSE36826_WT_VS_IL1R_KO_SKIN_STAPH_AUREUS_INF_UP 158
0.636575 2.6781924 0 0 0
2276 "tags=54%, list=16%, signal=64%"
GSE37532_WT_VS_PPARG_KO_LN_TCONV_DN
GSE37532_WT_VS_PPARG_KO_LN_TCONV_DN 136
0.64824706 2.6738932 0 0 0
1653 "tags=51%, list=12%, signal=58%"
GSE7764_IL15_TREATED_VS_CTRL_NK_CELL_24H_UP
GSE7764_IL15_TREATED_VS_CTRL_NK_CELL_24H_UP 146
0.65088737 2.668331 0 0 0
736 "tags=33%, list=5%, signal=34%"
GSE15930_NAIVE_VS_48H_IN_VITRO_STIM_IFNAB_CD8_TCELL_DN
GSE15930_NAIVE_VS_48H_IN_VITRO_STIM_IFNAB_CD8_TCELL_DN
163 0.64458597 2.6674898 0 0 0
2115 "tags=52%, list=15%, signal=60%"
GSE9006_HEALTHY_VS_TYPE_2_DIABETES_PBMC_AT_DX_UP
GSE9006_HEALTHY_VS_TYPE_2_DIABETES_PBMC_AT_DX_UP 142
0.65568805 2.6616974 0 0 0
2270 "tags=56%, list=16%, signal=66%"
GSE24574_BCL6_HIGH_VS_LOW_TFH_CD4_TCELL_DN
GSE24574_BCL6_HIGH_VS_LOW_TFH_CD4_TCELL_DN 148
0.6368096 2.6602654 0 0 0
2293 "tags=51%, list=16%, signal=61%"
GSE22601_DOUBLE_POSITIVE_VS_CD4_SINGLE_POSITIVE_THYMOCYTE_DN

GSE22601_DOUBLE_POSITIVE_VS_CD4_SINGLE_POSITIVE_THYMOCYTE_DN
146 0.6492296 2.6563344 0 0
0 1615 "tags=47%, list=11%, signal=52%"
GSE32986_GMCSF_VS_GMCSF_AND_CURDLAN_LOWDOSE_STIM_DC_UP
GSE32986_GMCSF_VS_GMCSF_AND_CURDLAN_LOWDOSE_STIM_DC_UP
150 0.64130694 2.6480868 0 0 0
1922 "tags=51%, list=14%, signal=58%"
GSE3982_NKCELL_VS_TH2_DN GSE3982_NKCELL_VS_TH2_DN 151
0.6327275 2.643352 0 0 0
1977 "tags=45%, list=14%, signal=52%"
GSE40274_FOXP3_VS_FOXP3_AND_EOS_TRANSDUCED_ACTIVATED_CD4_TCELL_DN
GSE40274_FOXP3_VS_FOXP3_AND_EOS_TRANSDUCED_ACTIVATED_CD4_TCELL_DN
129 0.6619322 2.6429143 0 0
0 776 "tags=40%, list=5%, signal=41%"
GSE29614_CTRL_VS_DAY7_TIV_FLU_VACCINE_PBMC_DN
GSE29614_CTRL_VS_DAY7_TIV_FLU_VACCINE_PBMC_DN 125
0.65756404 2.63784 0 0 0
1273 "tags=37%, list=9%, signal=40%"
GSE45365_HEALTHY_VS_MCMV_INFECTION_CD11B_DC_DN
GSE45365_HEALTHY_VS_MCMV_INFECTION_CD11B_DC_DN 113
0.6569679 2.6200056 0 0 0
552 "tags=27%, list=4%, signal=28%"
GSE3982_CENT_MEMORY_CD4_TCELL_VS_TH1_DN
GSE3982_CENT_MEMORY_CD4_TCELL_VS_TH1_DN 148
0.62779796 2.6080918 0 0 0
1798 "tags=49%, list=13%, signal=55%"
GSE3982_MEMORY_CD4_TCELL_VS_TH2_DN
GSE3982_MEMORY_CD4_TCELL_VS_TH2_DN 150
0.62820786 2.6036837 0 0 0
1270 "tags=39%, list=9%, signal=42%"
GSE28237_FOLLICULAR_VS_EARLY_GC_BCELL_DN
GSE28237_FOLLICULAR_VS_EARLY_GC_BCELL_DN 152
0.62275946 2.6014333 0 0 0
1310 "tags=35%, list=9%, signal=38%"
GSE21546_ELK1_KO_VS_SAP1A_KO_AND_ELK1_KO_DP_THYMOCYTES_DN
GSE21546_ELK1_KO_VS_SAP1A_KO_AND_ELK1_KO_DP_THYMOCYTES_DN
138 0.64299214 2.598992 0 0 0
2313 "tags=49%, list=16%, signal=57%"
GSE40068_CXCR5NEG_BCL6NEG_CD4_TCELL_VS_CXCR5POS_BCL6NEG_TFH_UP
GSE40068_CXCR5NEG_BCL6NEG_CD4_TCELL_VS_CXCR5POS_BCL6NEG_TFH_UP
146 0.6240541 2.5859892 0 0
0 2257 "tags=53%, list=16%, signal=62%"
GSE34205_HEALTHY_VS_RSV_INF_INFANT_PBMC_DN
GSE34205_HEALTHY_VS_RSV_INF_INFANT_PBMC_DN 139
0.6198443 2.5831923 0 0 0
1751 "tags=39%, list=12%, signal=44%"
GSE12845_NAIVE_VS_PRE_GC_TONSIL_BCELL_DN
GSE12845_NAIVE_VS_PRE_GC_TONSIL_BCELL_DN 154
0.61578417 2.582643 0 0 0
2442 "tags=55%, list=17%, signal=65%"
GSE9006_TYPE_1_VS_TYPE_2_DIABETES_PBMC_AT_DX_UP
GSE9006_TYPE_1_VS_TYPE_2_DIABETES_PBMC_AT_DX_UP 154
0.6236098 2.5805986 0 0 0
2047 "tags=45%, list=15%, signal=53%"

GSE16451_CTRL_VS_WEST_EQUINE_ENC_VIRUS_IMMATURE_NEURON_CELL_LINE_DN
GSE16451_CTRL_VS_WEST_EQUINE_ENC_VIRUS_IMMATURE_NEURON_CELL_LINE_DN
144 0.6183405 2.579624 0 0 0
1597 "tags=39%, list=11%, signal=43%"
GSE21927_SPLEEN_C57BL6_VS_EL4_TUMOR_BALBC_MONOCYTES_DN
GSE21927_SPLEEN_C57BL6_VS_EL4_TUMOR_BALBC_MONOCYTES_DN
159 0.61917 2.5737414 0 0 0
2697 "tags=56%, list=19%, signal=68%"
GSE45365_WT_VS_IFNAR_KO_BCELL_DN GSE45365_WT_VS_IFNAR_KO_BCELL_DN
109 0.65398026 2.5721624 0 0
0 776 "tags=30%, list=5%, signal=32%"
GSE22313_HEALTHY_VS_SLE_MOUSE_CD4_TCELL_DN
GSE22313_HEALTHY_VS_SLE_MOUSE_CD4_TCELL_DN 140
0.6116361 2.551419 0 0 0
1258 "tags=35%, list=9%, signal=38%"
GSE45365_WT_VS_IFNAR_KO_CD11B_DC_MCMV_INFECTION_DN
GSE45365_WT_VS_IFNAR_KO_CD11B_DC_MCMV_INFECTION_DN 108
0.6481348 2.5478394 0 0 0
709 "tags=25%, list=5%, signal=26%"
GSE9601_UNTREATED_VS_PI3K_INHIBITOR_TREATED_HCMV_INF_MONOCYTE_UP
GSE9601_UNTREATED_VS_PI3K_INHIBITOR_TREATED_HCMV_INF_MONOCYTE_UP
139 0.62458324 2.5412438 0 0
0 2156 "tags=54%, list=15%, signal=63%"
GSE32986_UNSTIM_VS_CURDLAN_LOWDOSE_STIM_DC_DN
GSE32986_UNSTIM_VS_CURDLAN_LOWDOSE_STIM_DC_DN 151
0.61258316 2.5386395 0 0 0
1639 "tags=43%, list=12%, signal=48%"
GSE21670_IL6_VS_TGFB_AND_IL6_TREATED_STAT3_KO_CD4_TCELL_UP
GSE21670_IL6_VS_TGFB_AND_IL6_TREATED_STAT3_KO_CD4_TCELL_UP
141 0.61036044 2.5382388 0 0 0
2275 "tags=51%, list=16%, signal=60%"
GSE2405_0H_VS_9H_A_PHAGOCYTOPHILUM_STIM_NEUTROPHIL_DN
GSE2405_0H_VS_9H_A_PHAGOCYTOPHILUM_STIM_NEUTROPHIL_DN
116 0.63601685 2.536018 0 0 0
2676 "tags=60%, list=19%, signal=74%"
GSE32164_ALTERNATIVELY_ACT_M2_VS_CMYC_INHIBITED_MACROPHAGE_DN
GSE32164_ALTERNATIVELY_ACT_M2_VS_CMYC_INHIBITED_MACROPHAGE_DN
153 0.6063723 2.5346525 0 0
0 1804 "tags=46%, list=13%, signal=53%"
GSE15930_NAIVE_VS_24H_IN_VITRO_STIM_CD8_TCELL_DN
GSE15930_NAIVE_VS_24H_IN_VITRO_STIM_CD8_TCELL_DN 155
0.61407876 2.5330005 0 0 0
1935 "tags=52%, list=14%, signal=59%"
GSE25088_WT_VS_STAT6_KO_MACROPHAGE_DN
GSE25088_WT_VS_STAT6_KO_MACROPHAGE_DN 140
0.61719376 2.5300953 0 0 0
1928 "tags=43%, list=14%, signal=49%"
GSE37301_HEMATOPOIETIC_STEM_CELL_VS_CD4_TCELL_UP
GSE37301_HEMATOPOIETIC_STEM_CELL_VS_CD4_TCELL_UP 139
0.6217151 2.528301 0 0 0
1871 "tags=45%, list=13%, signal=52%"
GSE5589_WT_VS_IL6_KO_LPS_AND_IL10_STIM_MACROPHAGE_45MIN_UP
GSE5589_WT_VS_IL6_KO_LPS_AND_IL10_STIM_MACROPHAGE_45MIN_UP
147 0.6089877 2.5249362 0 0 0

1905 "tags=39%, list=13%, signal=45%"
GSE24634_TREG_VS_TCONV_POST_DAY3_IL4_CONVERSION_UP
GSE24634_TREG_VS_TCONV_POST_DAY3_IL4_CONVERSION_UP 149
0.612257 2.523744 0 0 0 2645 "tags=55%,
list=19%, signal=67%"
GSE3982_EFF_MEMORY_CD4_TCELL_VS_TH2_DN
GSE3982_EFF_MEMORY_CD4_TCELL_VS_TH2_DN 153
0.60296255 2.5182736 0 0 0
2242 "tags=50%, list=16%, signal=58%"
GSE18203_CTRL_VS_INTRATUMORAL_CPG_INJ_MC38_TUMOR_DN
GSE18203_CTRL_VS_INTRATUMORAL_CPG_INJ_MC38_TUMOR_DN
155 0.5954051 2.5139892 0 0 0
1705 "tags=50%, list=12%, signal=57%"
GSE15930_NAIVE_VS_24H_IN_VITRO_STIM_IL12_CD8_TCELL_DN
GSE15930_NAIVE_VS_24H_IN_VITRO_STIM_IL12_CD8_TCELL_DN
155 0.60220075 2.511217 0 0 0
2243 "tags=54%, list=16%, signal=64%"
GSE6259_FLT3L_INDUCED_DEC205_POS_DC_VS_BCELL_DN
GSE6259_FLT3L_INDUCED_DEC205_POS_DC_VS_BCELL_DN 129
0.6202054 2.5049446 0 0 0
1569 "tags=43%, list=11%, signal=48%"
GSE22886_UNSTIM_VS_STIM_MEMORY_TCELL_DN
GSE22886_UNSTIM_VS_STIM_MEMORY_TCELL_DN 157
0.59870696 2.5047152 0 0 0
2389 "tags=49%, list=17%, signal=58%"
GSE33425_CD8_ALPHAALPHA_VS_ALPHABETA_CD161_HIGH_TCELL_DN
GSE33425_CD8_ALPHAALPHA_VS_ALPHABETA_CD161_HIGH_TCELL_DN
161 0.5986556 2.503509 0 0 0
1804 "tags=49%, list=13%, signal=56%"
GSE15930_NAIVE_VS_24H_IN_VITRO_STIM_INFAB_CD8_TCELL_DN
GSE15930_NAIVE_VS_24H_IN_VITRO_STIM_INFAB_CD8_TCELL_DN
156 0.6025294 2.5033822 0 0 0
1935 "tags=51%, list=14%, signal=59%"
GSE14415_ACT_VS_CTRL_NATURAL_TREG_UP
GSE14415_ACT_VS_CTRL_NATURAL_TREG_UP 135
0.6068627 2.4954836 0 0 0
2281 "tags=50%, list=16%, signal=60%"
GSE19923_WT_VS_HEB_AND_E2A_KO_DP_THYMOCYTE_UP
GSE19923_WT_VS_HEB_AND_E2A_KO_DP_THYMOCYTE_UP 157
0.5998507 2.4954605 0 0 0
1819 "tags=40%, list=13%, signal=46%"
GSE2770_UNTREATED_VS_IL4_TREATED_ACT_CD4_TCELL_48H_DN
GSE2770_UNTREATED_VS_IL4_TREATED_ACT_CD4_TCELL_48H_DN
140 0.6124492 2.4894364 0 0 0
2313 "tags=56%, list=16%, signal=67%"
GSE3720_UNSTIM_VS_PMA_STIM_VD2_GAMMADELTA_TCELL_DN
GSE3720_UNSTIM_VS_PMA_STIM_VD2_GAMMADELTA_TCELL_DN 146
0.6001136 2.4880924 0 0 0
2332 "tags=51%, list=17%, signal=61%"
GSE28408_LY6G_POS_VS_NEG_DC_DN GSE28408_LY6G_POS_VS_NEG_DC_DN
146 0.6030568 2.4864986 0 0
0 1673 "tags=47%, list=12%, signal=53%"
GSE3982_CENT_MEMORY_CD4_TCELL_VS_TH2_DN
GSE3982_CENT_MEMORY_CD4_TCELL_VS_TH2_DN 157 0.589623

2.4837284	0	0	0	1735	"tags=39%, list=12%, signal=44%"
GSE9509_LPS_VS_LPS_AND_IL10_STIM_IL10_KO_MACROPHAGE_20MIN_DN
GSE9509_LPS_VS_LPS_AND_IL10_STIM_IL10_KO_MACROPHAGE_20MIN_DN
	146	0.6001523	2.4812777	0	0
0	2026	"tags=47%, list=14%, signal=54%"
GSE3982_NKCELL_VS_TH1_DN	GSE3982_NKCELL_VS_TH1_DN	155
0.592321	2.4807045	0	0	0
1694	"tags=37%, list=12%, signal=42%"
GSE40068_BCL6_POS_VS_NEG_CXCR5_POS_TFH_UP
GSE40068_BCL6_POS_VS_NEG_CXCR5_POS_TFH_UP	149
0.59915423	2.473783	0	0	0
1816	"tags=47%, list=13%, signal=53%"
GSE20727_CTRL_VS_ROS_INH_AND_DNFB_ALLERGEN_TREATED_DC_DN
GSE20727_CTRL_VS_ROS_INH_AND_DNFB_ALLERGEN_TREATED_DC_DN
133	0.6005652	2.4727142	0	0	0
2362	"tags=44%, list=17%, signal=53%"
GSE7509_UNSTIM_VS_IFNA_STIM_IMMATURE_DC_UP
GSE7509_UNSTIM_VS_IFNA_STIM_IMMATURE_DC_UP	133
0.6010014	2.471666	0	0	0
1599	"tags=36%, list=11%, signal=40%"
GSE25146_UNSTIM_VS_HELIOBACTER_PYLORI_LPS_STIM_AGS_CELL_UP
GSE25146_UNSTIM_VS_HELIOBACTER_PYLORI_LPS_STIM_AGS_CELL_UP
142	0.59852684	2.4711654	0	0	0
1947	"tags=44%, list=14%, signal=51%"
GSE17301_CTRL_VS_48H_ACD3_ACD28_IFNA2_STIM_CD8_TCELL_DN
GSE17301_CTRL_VS_48H_ACD3_ACD28_IFNA2_STIM_CD8_TCELL_DN
138	0.58892107	2.4610934	0	0	0
904	"tags=25%, list=6%, signal=27%"
GSE14415_TCONV_VS_FOXP3_KO_INDUCED_TREG_UP
GSE14415_TCONV_VS_FOXP3_KO_INDUCED_TREG_UP	133
0.5951013	2.45899	0	0	0
2583	"tags=60%, list=18%, signal=73%"
GSE411_WT_VS_SOCS3_KO_MACROPHAGE_IL6_STIM_100MIN_UP
GSE411_WT_VS_SOCS3_KO_MACROPHAGE_IL6_STIM_100MIN_UP
154	0.5887366	2.457361	0	0	0
2253	"tags=46%, list=16%, signal=54%"
GSE2770_IL12_AND_TGFB_VS_IL4_TREATED_ACT_CD4_TCELL_6H_UP
GSE2770_IL12_AND_TGFB_VS_IL4_TREATED_ACT_CD4_TCELL_6H_UP
149	0.5988964	2.4569511	0	0	0
1817	"tags=35%, list=13%, signal=40%"
GSE17301_ACD3_ACD28_VS_ACD3_ACD28_AND_IFNA5_STIM_CD8_TCELL_UP
GSE17301_ACD3_ACD28_VS_ACD3_ACD28_AND_IFNA5_STIM_CD8_TCELL_UP
	136	0.6052465	2.4567094	0	0
0	1241	"tags=31%, list=9%, signal=34%"
GSE24634_TREG_VS_TCONV_POST_DAY10_IL4_CONVERSION_UP
GSE24634_TREG_VS_TCONV_POST_DAY10_IL4_CONVERSION_UP
157	0.5883456	2.453566	0	0	0
1901	"tags=46%, list=13%, signal=52%"
GSE24634_TREG_VS_TCONV_POST_DAY5_IL4_CONVERSION_UP
GSE24634_TREG_VS_TCONV_POST_DAY5_IL4_CONVERSION_UP	147
0.5905059	2.4500937	0	0	0
2089	"tags=48%, list=15%, signal=56%"
GSE40666_WT_VS_STAT1_KO_CD8_TCELL_WITH_IFNA_STIM_90MIN_DN

GSE40666_WT_VS_STAT1_KO_CD8_TCELL_WITH_IFNA_STIM_90MIN_DN
160 0.5840697 2.449145 0 0 0
1049 "tags=36%, list=7%, signal=38%"
GSE32901_NAIVE_VS_TH17_NEG_CD4_TCELL_UP
GSE32901_NAIVE_VS_TH17_NEG_CD4_TCELL_UP 136
0.5981389 2.4468088 0 0 0
1297 "tags=36%, list=9%, signal=39%"
GSE40225_WT_VS_RIP_B7X_DIABETIC_MOUSE_PANCREATIC_CD8_TCELL_DN
GSE40225_WT_VS_RIP_B7X_DIABETIC_MOUSE_PANCREATIC_CD8_TCELL_DN
148 0.58486927 2.4460025 0 0
0 2564 "tags=49%, list=18%, signal=59%"
GSE21670_UNTREATED_VS_TGFB_IL6_TREATED_CD4_TCELL_UP
GSE21670_UNTREATED_VS_TGFB_IL6_TREATED_CD4_TCELL_UP
125 0.60750455 2.4431272 0 0 0
1246 "tags=38%, list=9%, signal=42%"
GSE3982_BCELL_VS_TH2_DN GSE3982_BCELL_VS_TH2_DN 143
0.5883848 2.4357803 0 0 0
1275 "tags=28%, list=9%, signal=30%"
GSE12845_IGD_NEG_BLOOD_VS_NAIVE_TONSIL_BCELL_UP
GSE12845_IGD_NEG_BLOOD_VS_NAIVE_TONSIL_BCELL_UP 151
0.5853255 2.4330666 0 0 0
2617 "tags=51%, list=19%, signal=62%"
GSE15930_NAIVE_VS_72H_IN_VITRO_STIM_IL12_CD8_TCELL_DN
GSE15930_NAIVE_VS_72H_IN_VITRO_STIM_IL12_CD8_TCELL_DN
162 0.5859038 2.4315937 0 0 0
1553 "tags=44%, list=11%, signal=49%"
GSE17974_CTRL_VS_ACT_IL4_AND_ANTI_IL12_24H_CD4_TCELL_DN
GSE17974_CTRL_VS_ACT_IL4_AND_ANTI_IL12_24H_CD4_TCELL_DN
142 0.58871824 2.4286215 0 0 0
2035 "tags=46%, list=14%, signal=53%"
GSE12963_ENV_NEF_VS_ENV_NEF_AND_VPR_DEFICIENT_HIV1_INF_CD4_TCELL_DN
GSE12963_ENV_NEF_VS_ENV_NEF_AND_VPR_DEFICIENT_HIV1_INF_CD4_TCELL_DN
117 0.5991131 2.421048 0 0 0
1065 "tags=26%, list=8%, signal=28%"
GSE45837_WT_VS_GFI1_KO_PDC_DN GSE45837_WT_VS_GFI1_KO_PDC_DN
156 0.58249253 2.4199922 0 0
0 2724 "tags=60%, list=19%, signal=73%"
GSE20727_DNFB_ALLERGEN_VS_ROS_INH_AND_DNFB_ALLERGEN_TREATED_DC_DN
GSE20727_DNFB_ALLERGEN_VS_ROS_INH_AND_DNFB_ALLERGEN_TREATED_DC_DN
143 0.582463 2.4180481 0 0 0
1263 "tags=35%, list=9%, signal=38%"
GSE12366_GC_VS_MEMORY_BCELL_UP GSE12366_GC_VS_MEMORY_BCELL_UP
134 0.5935658 2.4136615 0 0
0 1949 "tags=44%, list=14%, signal=51%"
GSE12366_GC_VS_NAIVE_BCELL_UP GSE12366_GC_VS_NAIVE_BCELL_UP
149 0.58573806 2.413124 0 0 0
2216 "tags=46%, list=16%, signal=54%"
GSE3039_B2_VS_B1_BCELL_DN GSE3039_B2_VS_B1_BCELL_DN 145
0.5860225 2.412156 0 0 0
1981 "tags=41%, list=14%, signal=48%"
GSE3720_VD1_VS_VD2_GAMMADELTA_TCELL_WITH_LPS_STIM_DN
GSE3720_VD1_VS_VD2_GAMMADELTA_TCELL_WITH_LPS_STIM_DN
141 0.5859902 2.4104795 0 0 0
2218 "tags=48%, list=16%, signal=57%"

GSE20727_CTRL_VS_DNFB_ALLERGEN_TREATED_DC_UP
GSE20727_CTRL_VS_DNFB_ALLERGEN_TREATED_DC_UP 149
0.58292454 2.4083626 0 0 0
1358 "tags=34%, list=10%, signal=37%"
GSE19941_UNSTIM_VS_LPS_AND_IL10_STIM_IL10_KO_MACROPHAGE_DN
GSE19941_UNSTIM_VS_LPS_AND_IL10_STIM_IL10_KO_MACROPHAGE_DN
138 0.5903348 2.4073522 0 0 0
2075 "tags=41%, list=15%, signal=48%"
GSE19941_IL10_KO_VS_IL10_KO_AND_NFKBP50_KO_LPS_AND_IL10_STIM_MACROPH
AGE_DN
GSE19941_IL10_KO_VS_IL10_KO_AND_NFKBP50_KO_LPS_AND_IL10_STIM_MACROPH
AGE_DN 136 0.5899889 2.4072094 0
0 0 1713 "tags=44%, list=12%, signal=50%"
GSE24026_PD1_LIGATION_VS_CTRL_IN_ACT_TCELL_LINE_DN
GSE24026_PD1_LIGATION_VS_CTRL_IN_ACT_TCELL_LINE_DN 143
0.5783402 2.406726 0 0 0
2529 "tags=45%, list=18%, signal=54%"
GSE10239_MEMORY_VS_KLRG1INT_EFF_CD8_TCELL_DN
GSE10239_MEMORY_VS_KLRG1INT_EFF_CD8_TCELL_DN 146
0.58496135 2.4038494 0 0 0
1984 "tags=38%, list=14%, signal=44%"
GSE40274_CTRL_VS_XBP1_TRANSDUCED_ACTIVATED_CD4_TCELL_DN
GSE40274_CTRL_VS_XBP1_TRANSDUCED_ACTIVATED_CD4_TCELL_DN
133 0.5900242 2.3938165 0 0 0
1953 "tags=41%, list=14%, signal=48%"
GSE10500_ARTHRITIC_SYNOVIAL_FLUID_VS_HEALTHY_MACROPHAGE_UP
GSE10500_ARTHRITIC_SYNOVIAL_FLUID_VS_HEALTHY_MACROPHAGE_UP
117 0.59644145 2.392941 0 0 0
1939 "tags=38%, list=14%, signal=44%"
GSE45365_WT_VS_IFNAR_KO_BCELL_MCMV_INFECTION_DN
GSE45365_WT_VS_IFNAR_KO_BCELL_MCMV_INFECTION_DN 116
0.5948199 2.3926413 0 0 0
552 "tags=24%, list=4%, signal=25%"
GSE22886_NAIVE_CD4_TCELL_VS_12H_ACT_TH2_DN
GSE22886_NAIVE_CD4_TCELL_VS_12H_ACT_TH2_DN 155
0.5689719 2.3917584 0 0 0
2548 "tags=52%, list=18%, signal=63%"
GSE21670_TGFB_VS_TGFB_AND_IL6_TREATED_STAT3_KO_CD4_TCELL_DN
GSE21670_TGFB_VS_TGFB_AND_IL6_TREATED_STAT3_KO_CD4_TCELL_DN
152 0.57494336 2.3911855 0 0 0
2257 "tags=47%, list=16%, signal=56%"
GSE17974_0.5H_VS_72H_UNTREATED_IN_VITRO_CD4_TCELL_DN
GSE17974_0.5H_VS_72H_UNTREATED_IN_VITRO_CD4_TCELL_DN
145 0.5720194 2.3894746 0 0 0
2658 "tags=51%, list=19%, signal=62%"
GSE20727_CTRL_VS_ROS_INHIBITOR_TREATED_DC_DN
GSE20727_CTRL_VS_ROS_INHIBITOR_TREATED_DC_DN 142
0.5799407 2.3881369 0 0 0
2449 "tags=52%, list=17%, signal=62%"
GSE2405_0H_VS_12H_A_PHAGOCYTOPHILUM_STIM_NEUTROPHIL_DN
GSE2405_0H_VS_12H_A_PHAGOCYTOPHILUM_STIM_NEUTROPHIL_DN
149 0.57084703 2.3853555 0 0 0
896 "tags=24%, list=6%, signal=26%"
GSE1460_INTRATHYMIC_T_PROGENITOR_VS_NAIVE_CD4_TCELL_ADULT_BLOOD_UP

GSE1460_INTRATHYMIC_T_PROGENITOR_VS_NAIVE_CD4_TCELL_ADULT_BLOOD_UP
163 0.57414716 2.3840444 0 0
0 1946 "tags=45%, list=14%, signal=52%"
GSE17301_IFNA2_VS_IFNA5_STIM_ACD3_ACD28_ACT_CD8_TCELL_DN
GSE17301_IFNA2_VS_IFNA5_STIM_ACD3_ACD28_ACT_CD8_TCELL_DN
145 0.57936716 2.3838136 0 0 0
2225 "tags=37%, list=16%, signal=44%"
GSE21546_UNSTIM_VS_ANTI_CD3_STIM_DP_THYMOCYTES_DN
GSE21546_UNSTIM_VS_ANTI_CD3_STIM_DP_THYMOCYTES_DN 139
0.57870984 2.3776212 0 0 0
1726 "tags=35%, list=12%, signal=40%"
GSE3982_MAC_VS_TH1_DN GSE3982_MAC_VS_TH1_DN 143
0.5742005 2.3757503 0 0 0
1672 "tags=36%, list=12%, signal=41%"
GSE26669_CTRL_VS_COSTIM_BLOCK_MLR_CD8_TCELL_UP
GSE26669_CTRL_VS_COSTIM_BLOCK_MLR_CD8_TCELL_UP 147
0.5722038 2.3755436 0 0 0
2388 "tags=48%, list=17%, signal=58%"
GSE19888_CTRL_VS_A3R_INHIBITOR_TREATED_MAST_CELL_UP
GSE19888_CTRL_VS_A3R_INHIBITOR_TREATED_MAST_CELL_UP
131 0.5878837 2.3749745 0 0 0
1613 "tags=40%, list=11%, signal=44%"
GSE40274_CTRL_VS_FOXP3_AND_XBP1_TRANSDUCED_ACTIVATED_CD4_TCELL_UP
GSE40274_CTRL_VS_FOXP3_AND_XBP1_TRANSDUCED_ACTIVATED_CD4_TCELL_UP
123 0.58335656 2.3714917 0 0
0 1531 "tags=41%, list=11%, signal=46%"
GSE5542_IFNA_VS_IFNA_AND_IFNG_TREATED_EPITHELIAL_CELLS_24H_DN
GSE5542_IFNA_VS_IFNA_AND_IFNG_TREATED_EPITHELIAL_CELLS_24H_DN
136 0.574008 2.3699253 0 0 0
1453 "tags=34%, list=10%, signal=37%"
GSE40273_GATA1_KO_VS_WT_TREG_UP GSE40273_GATA1_KO_VS_WT_TREG_UP
149 0.5645756 2.3672912 0 0
0 2236 "tags=44%, list=16%, signal=51%"
GSE32986_UNSTIM_VS_GMCSF_AND_CURDLAN_HIGHDOSE_STIM_DC_UP
GSE32986_UNSTIM_VS_GMCSF_AND_CURDLAN_HIGHDOSE_STIM_DC_UP
142 0.5775169 2.366511 0 0 0
2444 "tags=55%, list=17%, signal=66%"
GSE45365_HEALTHY_VS_MCMV_INFECTION_CD11B_DC_IFNAR_KO_DN
GSE45365_HEALTHY_VS_MCMV_INFECTION_CD11B_DC_IFNAR_KO_DN
119 0.5853258 2.36568 0 0 0
1554 "tags=29%, list=11%, signal=32%"
GSE22443_IL2_VS_IL12_TREATED_ACT_CD8_TCELL_DN
GSE22443_IL2_VS_IL12_TREATED_ACT_CD8_TCELL_DN 159
0.56583136 2.3653517 0 0 0
1950 "tags=43%, list=14%, signal=49%"
GSE42021_CD24LO_TREG_VS_CD24LO_TCONV_THYMUS_DN
GSE42021_CD24LO_TREG_VS_CD24LO_TCONV_THYMUS_DN 158
0.5672348 2.3639624 0 0 0
2848 "tags=54%, list=20%, signal=67%"
GSE32986_UNSTIM_VS_CURDLAN_HIGHDOSE_STIM_DC_UP
GSE32986_UNSTIM_VS_CURDLAN_HIGHDOSE_STIM_DC_UP 156
0.56331444 2.3626049 0 0 0
1893 "tags=43%, list=13%, signal=49%"
GSE37301_COMMON_LYMPHOID_PROGENITOR_VS_PRO_BCELL_DN

GSE37301_COMMON_LYMPHOID_PROGENITOR_VS_PRO_BCELL_DN
149 0.5640636 2.356952 0 0 0
2450 "tags=46%, list=17%, signal=55%"
GSE24634_TEFF_VS_TCONV_DAY3_IN_CULTURE_UP
GSE24634_TEFF_VS_TCONV_DAY3_IN_CULTURE_UP 152
0.5655341 2.3514094 0 0 0
1928 "tags=44%, list=14%, signal=51%"
GSE21670_UNTREATED_VS_TGFB_IL6_TREATED_STAT3_KO_CD4_TCELL_UP
GSE21670_UNTREATED_VS_TGFB_IL6_TREATED_STAT3_KO_CD4_TCELL_UP
149 0.5602451 2.3513467 0 0
0 1597 "tags=38%, list=11%, signal=42%"
GSE24634_NAIVE_CD4_TCELL_VS_DAY3_IL4_CONV_TREG_DN
GSE24634_NAIVE_CD4_TCELL_VS_DAY3_IL4_CONV_TREG_DN 162
0.5606628 2.3478334 0 0 0
2315 "tags=49%, list=16%, signal=58%"
GSE20715_0H_VS_48H_OZONE_LUNG_DN GSE20715_0H_VS_48H_OZONE_LUNG_DN
173 0.5526912 2.346801 0 0 0
1262 "tags=29%, list=9%, signal=31%"
GSE17974_CTRL_VS_ACT_IL4_AND_ANTI_IL12_48H_CD4_TCELL_DN
GSE17974_CTRL_VS_ACT_IL4_AND_ANTI_IL12_48H_CD4_TCELL_DN
150 0.5594226 2.3413372 0 0 0
2064 "tags=40%, list=15%, signal=46%"
GSE3337_CTRL_VS_16H_IFNG_IN_CD8POS_DC_DN
GSE3337_CTRL_VS_16H_IFNG_IN_CD8POS_DC_DN 154
0.5592881 2.3381767 0 0 0
2320 "tags=44%, list=16%, signal=51%"
GSE23505_IL6_IL1_IL23_VS_IL6_IL1_TGFB_TREATED_CD4_TCELL_DN
GSE23505_IL6_IL1_IL23_VS_IL6_IL1_TGFB_TREATED_CD4_TCELL_DN
146 0.569462 2.3366556 0 0 0
2271 "tags=48%, list=16%, signal=57%"
GSE3982_BCELL_VS_TH1_DN GSE3982_BCELL_VS_TH1_DN 153
0.5631584 2.33178 0 0 0
1275 "tags=29%, list=9%, signal=31%"
GSE7460_CTRL_VS_TGFB_TREATED_ACT_TREG_UP
GSE7460_CTRL_VS_TGFB_TREATED_ACT_TREG_UP 139 0.569759
2.3307245 0 0 0 2009 "tags=43%,
list=14%, signal=50%"
GSE22033_UNTREATED_VS_MRL24_TREATED_MEF_UP
GSE22033_UNTREATED_VS_MRL24_TREATED_MEF_UP 150
0.55236536 2.3229797 0 0 0
2671 "tags=43%, list=19%, signal=53%"
GSE21927_GMCSF_IL6_VS_GMCSF_GCSF_TREATED_BONE_MARROW_DN
GSE21927_GMCSF_IL6_VS_GMCSF_GCSF_TREATED_BONE_MARROW_DN
152 0.5591084 2.3213499 0 0 0
2697 "tags=55%, list=19%, signal=67%"
GSE21063_CTRL_VS_ANTI_IGM_STIM_BCELL_16H_UP
GSE21063_CTRL_VS_ANTI_IGM_STIM_BCELL_16H_UP 146
0.56337243 2.3184814 0 0 0
1584 "tags=32%, list=11%, signal=36%"
GSE32986_UNSTIM_VS_GMCSF_AND_CURDLAN_LOWDOSE_STIM_DC_UP
GSE32986_UNSTIM_VS_GMCSF_AND_CURDLAN_LOWDOSE_STIM_DC_UP
154 0.553301 2.3155882 0 0 0
2128 "tags=43%, list=15%, signal=50%"
GSE23505_IL6_IL1_VS_IL6_IL1_IL23_TREATED_CD4_TCELL_UP

GSE23505_IL6_IL1_VS_IL6_IL1_IL23_TREATED_CD4_TCELL_UP
150	0.5554595	2.3117833	0	0	0
2011	"tags=43%, list=14%, signal=49%"
GSE10239_MEMORY_VS_DAY4.5_EFF_CD8_TCELL_DN
GSE10239_MEMORY_VS_DAY4.5_EFF_CD8_TCELL_DN	140
0.5600948	2.3105702	0	0	0
2610	"tags=46%, list=18%, signal=56%"
GSE24210_CTRL_VS_IL35_TREATED_TCONV_CD4_TCELL_DN
GSE24210_CTRL_VS_IL35_TREATED_TCONV_CD4_TCELL_DN	153
0.55258685	2.3096368	0	0	0
1939	"tags=41%, list=14%, signal=46%"
GSE32164_RESTING_DIFFERENTIATED_VS_ALTERNATIVELY_ACT_M2_MACROPHAGE_UP
GSE32164_RESTING_DIFFERENTIATED_VS_ALTERNATIVELY_ACT_M2_MACROPHAGE_UP	157	0.55507356	2.3078055	0
0	0	1023	"tags=29%, list=7%, signal=31%"
GSE20366_EX_VIVO_VS_HOMEOSTATIC_CONVERSION_TREG_DN
GSE20366_EX_VIVO_VS_HOMEOSTATIC_CONVERSION_TREG_DN	145
0.56721026	2.3076932	0	0	0
1564	"tags=36%, list=11%, signal=40%"
GSE15330_LYMPHOID_MULTIPOTENT_VS_MEGAKARYOCYTE_ERYTHROID_PROGENITOR_IKAROS_KO_DN
GSE15330_LYMPHOID_MULTIPOTENT_VS_MEGAKARYOCYTE_ERYTHROID_PROGENITOR_IKAROS_KO_DN	146	0.5606066	2.3069382
0	0	0	2332	"tags=55%, list=17%, signal=65%"
GSE3920_UNTREATED_VS_IFNB_TREATED_ENDOTHELIAL_CELL_DN
GSE3920_UNTREATED_VS_IFNB_TREATED_ENDOTHELIAL_CELL_DN
125	0.5768284	2.3048594	0	0	0
2020	"tags=40%, list=14%, signal=46%"
GSE36078_WT_VS_IL1R_KO_LUNG_DC_AFTER_AD5_T425A_HEXON_INF_UP
GSE36078_WT_VS_IL1R_KO_LUNG_DC_AFTER_AD5_T425A_HEXON_INF_UP
142	0.5621406	2.3036826	0	0	0
2307	"tags=44%, list=16%, signal=52%"
GSE24634_NAIVE_CD4_TCELL_VS_DAY10_IL4_CONV_TREG_DN
GSE24634_NAIVE_CD4_TCELL_VS_DAY10_IL4_CONV_TREG_DN	154
0.5597548	2.3036602	0	0	0
2234	"tags=49%, list=16%, signal=58%"
GSE3982_EFF_MEMORY_CD4_TCELL_VS_TH1_DN
GSE3982_EFF_MEMORY_CD4_TCELL_VS_TH1_DN	153
0.5565747	2.3021371	0	0	0
1351	"tags=35%, list=10%, signal=39%"
GSE26030_TH1_VS_TH17_RESTIMULATED_DAY5_POST_POLARIZATION_UP
GSE26030_TH1_VS_TH17_RESTIMULATED_DAY5_POST_POLARIZATION_UP
145	0.55980027	2.3019583	0	0	0
2873	"tags=49%, list=20%, signal=61%"
GSE29617_CTRL_VS_TIV_FLU_VACCINE_PBMC_2008_DN
GSE29617_CTRL_VS_TIV_FLU_VACCINE_PBMC_2008_DN	134
0.5591789	2.3012352	0	0	0
2409	"tags=49%, list=17%, signal=58%"
GSE29614_DAY3_VS_DAY7_TIV_FLU_VACCINE_PBMC_DN
GSE29614_DAY3_VS_DAY7_TIV_FLU_VACCINE_PBMC_DN	121
0.5652389	2.3002796	0	0	0
2329	"tags=45%, list=17%, signal=54%"
GSE26030_TH1_VS_TH17_RESTIMULATED_DAY15_POST_POLARIZATION_UP

GSE26030_TH1_VS_TH17_RESTIMULATED_DAY15_POST_POLARIZATION_UP
143 0.55522096 2.2984738 0 0
0 1966 "tags=41%, list=14%, signal=47%"
GSE17812_WT_VS_THPOK_KO_MEMORY_CD8_TCELL_DN
GSE17812_WT_VS_THPOK_KO_MEMORY_CD8_TCELL_DN 154
0.54874915 2.2980886 0 0 0
1706 "tags=40%, list=12%, signal=45%"
GSE1460_INTRATHYMIC_T_PROGENITOR_VS_NAIVE_CD4_TCELL_CORD_BLOOD_UP
GSE1460_INTRATHYMIC_T_PROGENITOR_VS_NAIVE_CD4_TCELL_CORD_BLOOD_UP
154 0.5517947 2.2971683 0 0
0 2474 "tags=50%, list=18%, signal=60%"
GSE2770_IL4_ACT_VS_ACT_CD4_TCELL_2H_UP
GSE2770_IL4_ACT_VS_ACT_CD4_TCELL_2H_UP 147 0.553723
2.2951944 0 0 0 2589 "tags=53%,
list=18%, signal=64%"
GSE22432_MULTIPOTENT_PROGENITOR_VS_CDC_DN
GSE22432_MULTIPOTENT_PROGENITOR_VS_CDC_DN 143
0.5472776 2.2895255 0 0 0
1960 "tags=38%, list=14%, signal=43%"
GSE2770_UNTREATED_VS_ACT_CD4_TCELL_6H_UP
GSE2770_UNTREATED_VS_ACT_CD4_TCELL_6H_UP 148
0.5502999 2.2886496 0 0 0
2823 "tags=52%, list=20%, signal=64%"
GSE2405_HEAT_KILLED_LYSATE_VS_LIVE_A_PHAGOCYTOPHILUM_STIM_NEUTROPHIL
_9H_UP
GSE2405_HEAT_KILLED_LYSATE_VS_LIVE_A_PHAGOCYTOPHILUM_STIM_NEUTROPHIL
_9H_UP 148 0.5494499 2.2885482 0
0 0 843 "tags=25%, list=6%, signal=26%"
GSE36078_UNTREATED_VS_AD5_INF_MOUSE_LUNG_DC_DN
GSE36078_UNTREATED_VS_AD5_INF_MOUSE_LUNG_DC_DN 145
0.5554156 2.2863178 0 0 0
2478 "tags=54%, list=18%, signal=65%"
GSE40274_CTRL_VS_FOXP3_AND_LEF1_TRANSDUCED_ACTIVATED_CD4_TCELL_DN
GSE40274_CTRL_VS_FOXP3_AND_LEF1_TRANSDUCED_ACTIVATED_CD4_TCELL_DN
131 0.5583967 2.2834182 0 0
0 2018 "tags=38%, list=14%, signal=44%"
GSE21546_WT_VS_ELK1_KO_ANTI_CD3_STIM_DP_THYMOCYTES_DN
GSE21546_WT_VS_ELK1_KO_ANTI_CD3_STIM_DP_THYMOCYTES_DN
128 0.5644414 2.2810006 0 0 0
1750 "tags=38%, list=12%, signal=42%"
GSE16266_CTRL_VS_HEATSHOCK_AND_LPS_STIM_MEF_UP
GSE16266_CTRL_VS_HEATSHOCK_AND_LPS_STIM_MEF_UP 144
0.5472693 2.2762184 0 0 0
2414 "tags=50%, list=17%, signal=60%"
GSE10239_MEMORY_VS_KLRG1HIGH_EFF_CD8_TCELL_DN
GSE10239_MEMORY_VS_KLRG1HIGH_EFF_CD8_TCELL_DN 145
0.5454946 2.2734468 0 0 0
2447 "tags=42%, list=17%, signal=50%"
GSE15624_CTRL_VS_3H_HALOFUGINONE_TREATED_CD4_TCELL_DN
GSE15624_CTRL_VS_3H_HALOFUGINONE_TREATED_CD4_TCELL_DN
130 0.55617434 2.273216 0 0 0
1987 "tags=39%, list=14%, signal=45%"
GSE6259_33D1_POS_DC_VS_CD4_TCELL_UP
GSE6259_33D1_POS_DC_VS_CD4_TCELL_UP 138

0.54855824	2.2724836	0	0	0
1795	"tags=42%, list=13%, signal=48%"
GSE7509_UNSTIM_VS_FCGRIIB_STIM_MONOCYTE_UP
GSE7509_UNSTIM_VS_FCGRIIB_STIM_MONOCYTE_UP	131
0.56053907	2.271753	0	0	0
2474	"tags=49%, list=18%, signal=59%"
GSE37532_TREG_VS_TCONV_CD4_TCELL_FROM_VISCERAL_ADIPOSE_TISSUE_DN
GSE37532_TREG_VS_TCONV_CD4_TCELL_FROM_VISCERAL_ADIPOSE_TISSUE_DN
	123	0.5616505	2.2696273	0	0
0	1545	"tags=37%, list=11%, signal=42%"
GSE3982_MEMORY_CD4_TCELL_VS_TH1_DN
GSE3982_MEMORY_CD4_TCELL_VS_TH1_DN	154
0.54445636	2.2690523	0	0	0
1342	"tags=36%, list=10%, signal=39%"
GSE41867_NAIVE_VS_EFFECTOR_CD8_TCELL_UP
GSE41867_NAIVE_VS_EFFECTOR_CD8_TCELL_UP	146
0.5458973	2.2680647	0	0	0
2071	"tags=40%, list=15%, signal=46%"
GSE26030_TH1_VS_TH17_RESTIMULATED_DAY15_POST_POLARIZATION_DN
GSE26030_TH1_VS_TH17_RESTIMULATED_DAY15_POST_POLARIZATION_DN
	139	0.5516829	2.2667253	0	0
0	2977	"tags=53%, list=21%, signal=67%"
GSE6259_DEC205_POS_DC_VS_CD8_TCELL_UP
GSE6259_DEC205_POS_DC_VS_CD8_TCELL_UP	146
0.5448889	2.2646937	0	0	0
1614	"tags=38%, list=11%, signal=42%"
GSE33425_CD161_HIGH_VS_INT_CD8_TCELL_DN
GSE33425_CD161_HIGH_VS_INT_CD8_TCELL_DN	154
0.5390908	2.2645366	0	0	0
2103	"tags=49%, list=15%, signal=57%"
GSE17580_UNINFECTED_VS_S_MANSONI_INF_TREG_DN
GSE17580_UNINFECTED_VS_S_MANSONI_INF_TREG_DN	154
0.54731363	2.2636428	0	0	0
1998	"tags=48%, list=14%, signal=55%"
GSE41867_DAY8_VS_DAY15_LCMV_CLONE13_EFFECTOR_CD8_TCELL_DN
GSE41867_DAY8_VS_DAY15_LCMV_CLONE13_EFFECTOR_CD8_TCELL_DN
147	0.54263335	2.261878	0	0	0
1977	"tags=43%, list=14%, signal=49%"
GSE42021_CD24HI_TREG_VS_CD24HI_TCONV_THYMUS_UP
GSE42021_CD24HI_TREG_VS_CD24HI_TCONV_THYMUS_UP	156
0.54344827	2.2591293	0	0	0
2792	"tags=50%, list=20%, signal=62%"
GSE14415_ACT_TCONV_VS_ACT_NATURAL_TREG_DN
GSE14415_ACT_TCONV_VS_ACT_NATURAL_TREG_DN	144
0.5400034	2.2580976	0	0	0
1679	"tags=39%, list=12%, signal=44%"
GSE3982_EOSINOPHIL_VS_TH1_DN	GSE3982_EOSINOPHIL_VS_TH1_DN
	139	0.54390264	2.254577	0	0	0
2131	"tags=42%, list=15%, signal=49%"
GSE41867_NAIVE_VS_DAY30_LCMV_ARMSTRONG_MEMORY_CD8_TCELL_DN
GSE41867_NAIVE_VS_DAY30_LCMV_ARMSTRONG_MEMORY_CD8_TCELL_DN
138	0.5497242	2.251938	0	0	0
2366	"tags=39%, list=17%, signal=47%"
GSE37534_UNTREATED_VS_GW1929_TREATED_CD4_TCELL_PPARG1_AND_FOXP3_TRAS

DUCED_DN
GSE37534_UNTREATED_VS_GW1929_TREATED_CD4_TCELL_PPARG1_AND_FOXP3_TRASDUCED_DN 153 0.5453345 2.251743 0 0
0 1570 "tags=35%, list=11%, signal=39%"
GSE19888_ADENOSINE_A3R_ACT_VS_A3R_ACT_WITH_A3R_INH_PRETREATMENT_IN_MAST_CELL_UP
GSE19888_ADENOSINE_A3R_ACT_VS_A3R_ACT_WITH_A3R_INH_PRETREATMENT_IN_MAST_CELL_UP 124 0.5559117 2.2510467
0 0 0 1613 "tags=40%, list=11%, signal=44%"
GSE17974_2H_VS_72H_UNTREATED_IN_VITRO_CD4_TCELL_DN
GSE17974_2H_VS_72H_UNTREATED_IN_VITRO_CD4_TCELL_DN 141
0.5491321 2.250649 0 0 0
1639 "tags=36%, list=12%, signal=41%"
GSE22886_NAIVE_VS_IGG_IGA_MEMORY_BCELL_DN
GSE22886_NAIVE_VS_IGG_IGA_MEMORY_BCELL_DN 144
0.5427474 2.2505906 0 0 0
2022 "tags=44%, list=14%, signal=51%"
GSE33162_HDAC3_KO_VS_HDAC3_KO_MACROPHAGE_DN
GSE33162_HDAC3_KO_VS_HDAC3_KO_MACROPHAGE_DN 146
0.54358447 2.240989 0 0 0
1679 "tags=42%, list=12%, signal=47%"
GSE7831_1H_VS_4H_CPG_STIM_PDC_DN GSE7831_1H_VS_4H_CPG_STIM_PDC_DN
147 0.5427485 2.2401748 0 0
0 2154 "tags=37%, list=15%, signal=44%"
GSE17974_2.5H_VS_72H_IL4_AND_ANTI_IL12_ACT_CD4_TCELL_DN
GSE17974_2.5H_VS_72H_IL4_AND_ANTI_IL12_ACT_CD4_TCELL_DN
125 0.5541741 2.2391198 0 0 0
1928 "tags=47%, list=14%, signal=54%"
GSE17974_0H_VS_24H_IN_VITRO_ACT_CD4_TCELL_DN
GSE17974_0H_VS_24H_IN_VITRO_ACT_CD4_TCELL_DN 137
0.5457666 2.2386508 0 0 0
1439 "tags=38%, list=10%, signal=42%"
GSE15930_STIM_VS_STIM_AND_IFNAB_48H_CD8_T_CELL_UP
GSE15930_STIM_VS_STIM_AND_IFNAB_48H_CD8_T_CELL_UP 156
0.535355 2.2359908 0 0 0
1804 "tags=44%, list=13%, signal=49%"
GSE25085_FETAL_LIVER_VS_FETAL_BM_SP4_THYMIC_IMPLANT_UP
GSE25085_FETAL_LIVER_VS_FETAL_BM_SP4_THYMIC_IMPLANT_UP
142 0.5436143 2.2351072 0 0 0
2023 "tags=42%, list=14%, signal=49%"
GSE17974_1H_VS_72H_UNTREATED_IN_VITRO_CD4_TCELL_DN
GSE17974_1H_VS_72H_UNTREATED_IN_VITRO_CD4_TCELL_DN 146
0.5465373 2.2328959 0 0 0
2519 "tags=47%, list=18%, signal=56%"
GSE4984_UNTREATED_VS_LPS_TREATED_DC_DN
GSE4984_UNTREATED_VS_LPS_TREATED_DC_DN 135
0.5445905 2.2319064 0 0 0
2331 "tags=47%, list=17%, signal=56%"
GSE40666_UNTREATED_VS_IFNA_STIM_EFFECTOR_CD8_TCELL_90MIN_UP
GSE40666_UNTREATED_VS_IFNA_STIM_EFFECTOR_CD8_TCELL_90MIN_UP
132 0.5486251 2.2283988 0 0 0
2036 "tags=41%, list=14%, signal=47%"
GSE26351_UNSTIM_VS_BMP_PATHWAY_STIM_HEMATOPOIETIC_PROGENITORS_UP
GSE26351_UNSTIM_VS_BMP_PATHWAY_STIM_HEMATOPOIETIC_PROGENITORS_UP

147	0.53666365	2.2271132	0	0
0	2226	"tags=41%, list=16%, signal=49%"
GSE6674_CPG_VS_CPG_AND_ANTI_IGM_STIM_BCELL_UP
GSE6674_CPG_VS_CPG_AND_ANTI_IGM_STIM_BCELL_UP	141
0.5437195	2.2256455	0	0	0
2324	"tags=48%, list=16%, signal=57%"
GSE9878_CTRL_VS_EBF_TRANSDUCED_PAX5_KO_PRO_BCELL_DN
GSE9878_CTRL_VS_EBF_TRANSDUCED_PAX5_KO_PRO_BCELL_DN
156	0.5357059	2.224478	0	0	0
2204	"tags=38%, list=16%, signal=44%"
GSE33424_CD161_HIGH_VS_NEG_CD8_TCELL_DN
GSE33424_CD161_HIGH_VS_NEG_CD8_TCELL_DN	155
0.5324388	2.2243009	0	0	0
1811	"tags=39%, list=13%, signal=44%"
GSE9239_CTRL_VS_TNF_INHIBITOR_TREATED_DC_DN
GSE9239_CTRL_VS_TNF_INHIBITOR_TREATED_DC_DN	143
0.5439436	2.223532	0	0	0
1534	"tags=31%, list=11%, signal=34%"
GSE12845_IGD_POS_VS_NEG_BLOOD_BCELL_DN
GSE12845_IGD_POS_VS_NEG_BLOOD_BCELL_DN	141
0.5416371	2.2194946	0	0	0
2088	"tags=42%, list=15%, signal=49%"
GSE21670_IL6_VS_TGFB_AND_IL6_TREATED_CD4_TCELL_DN
GSE21670_IL6_VS_TGFB_AND_IL6_TREATED_CD4_TCELL_DN	142
0.53406113	2.2190666	0	0	0
1967	"tags=30%, list=14%, signal=34%"
GSE10240_CTRL_VS_IL17_AND_IL22_STIM_PRIMARY_BRONCHIAL_EPITHELIAL_CEL
LS_DN
GSE10240_CTRL_VS_IL17_AND_IL22_STIM_PRIMARY_BRONCHIAL_EPITHELIAL_CEL
LS_DN	149	0.53610736	2.2181544	0
0	0	924	"tags=21%, list=7%, signal=22%"
GSE3920_IFNB_VS_IFNG_TREATED_ENDOTHELIAL_CELL_DN
GSE3920_IFNB_VS_IFNG_TREATED_ENDOTHELIAL_CELL_DN	132
0.5461136	2.2173955	0	0	0
2012	"tags=39%, list=14%, signal=45%"
GSE22886_NAIVE_BCELL_VS_BLOOD_PLASMA_CELL_DN
GSE22886_NAIVE_BCELL_VS_BLOOD_PLASMA_CELL_DN	149
0.5327127	2.2167175	0	0	0
2215	"tags=42%, list=16%, signal=50%"
GSE40274_IRF4_VS_FOXP3_AND_IRF4_TRANSDUCED_ACTIVATED_CD4_TCELL_UP
GSE40274_IRF4_VS_FOXP3_AND_IRF4_TRANSDUCED_ACTIVATED_CD4_TCELL_UP
140	0.54104716	2.2151437	0	0
0	2586	"tags=47%, list=18%, signal=57%"
GSE36888_STAT5_AB_KNOCKIN_VS_WT_TCELL_IL2_TREATED_6H_DN
GSE36888_STAT5_AB_KNOCKIN_VS_WT_TCELL_IL2_TREATED_6H_DN
145	0.53584856	2.2140415	0	0	0
2029	"tags=41%, list=14%, signal=48%"
GSE20500_CTRL_VS_RETINOIC_ACID_TREATED_CD4_TCELL_UP
GSE20500_CTRL_VS_RETINOIC_ACID_TREATED_CD4_TCELL_UP
147	0.5295427	2.2120469	0	0	0
1999	"tags=35%, list=14%, signal=40%"
GSE20727_CTRL_VS_ROS_INHIBITOR_TREATED_DC_UP
GSE20727_CTRL_VS_ROS_INHIBITOR_TREATED_DC_UP	142
0.5298261	2.2112331	0	0	0

2225	"tags=40%, list=16%, signal=47%"
GSE36078_UNTREATED_VS_AD5_T425A_HEXON_INF_MOUSE_LUNG_DC_DN
GSE36078_UNTREATED_VS_AD5_T425A_HEXON_INF_MOUSE_LUNG_DC_DN
151	0.5372375	2.2105522	0	0	0
2351	"tags=44%, list=17%, signal=52%"
GSE41867_NAIVE_VS_DAY30_LCMV_ARMSTRONG_MEMORY_CD8_TCELL_UP
GSE41867_NAIVE_VS_DAY30_LCMV_ARMSTRONG_MEMORY_CD8_TCELL_UP
144	0.5327052	2.209408 0	0	0
2725	"tags=51%, list=19%, signal=63%"
GSE13547_CTRL_VS_ANTI_IGM_STIM_BCELL_12H_DN
GSE13547_CTRL_VS_ANTI_IGM_STIM_BCELL_12H_DN	140
0.5430119	2.2037249	0	0	0
2294	"tags=49%, list=16%, signal=57%"
GSE9509_LPS_VS_LPS_AND_IL10_STIM_IL10_KO_MACROPHAGE_30MIN_DN
GSE9509_LPS_VS_LPS_AND_IL10_STIM_IL10_KO_MACROPHAGE_30MIN_DN
129	0.54299396	2.1998923	0	0
0	1300	"tags=29%, list=9%, signal=31%"
GSE2770_UNTREATED_VS_IL4_TREATED_ACT_CD4_TCELL_2H_DN
GSE2770_UNTREATED_VS_IL4_TREATED_ACT_CD4_TCELL_2H_DN
145	0.53576267	2.1980212	0	0	0
2424	"tags=44%, list=17%, signal=53%"
GSE2405_0H_VS_24H_A_PHAGOCYTOPHILUM_STIM_NEUTROPHIL_UP
GSE2405_0H_VS_24H_A_PHAGOCYTOPHILUM_STIM_NEUTROPHIL_UP
118	0.545167 2.197927 0	0	0
2676	"tags=53%, list=19%, signal=64%"
GSE3982_BASOPHIL_VS_TH1_DN	GSE3982_BASOPHIL_VS_TH1_DN
157	0.5258279	2.1977947	0	0
0	1619	"tags=31%, list=11%, signal=35%"
GSE23502_WT_VS_HDC_KO_MYELOID_DERIVED_SUPPRESSOR_CELL_COLON_TUMOR_UP

GSE23502_WT_VS_HDC_KO_MYELOID_DERIVED_SUPPRESSOR_CELL_COLON_TUMOR_UP
148	0.5324888	2.1968784	0
0	0	1064	"tags=27%, list=8%, signal=29%"
GSE19941_UNSTIM_VS_LPS_STIM_IL10_KO_NFKBP50_KO_MACROPHAGE_UP
GSE19941_UNSTIM_VS_LPS_STIM_IL10_KO_NFKBP50_KO_MACROPHAGE_UP
148	0.5286393	2.1952672	0	0
0	1300	"tags=30%, list=9%, signal=32%"
GSE19825_NAIVE_VS_IL2RAHIGH_DAY3_EFF_CD8_TCELL_DN
GSE19825_NAIVE_VS_IL2RAHIGH_DAY3_EFF_CD8_TCELL_DN	141
0.5280405	2.194101 0	0	0
2525	"tags=49%, list=18%, signal=59%"
GSE12845_IGD_POS_BLOOD_VS_DARKZONE_GC_TONSIL_BCELL_DN
GSE12845_IGD_POS_BLOOD_VS_DARKZONE_GC_TONSIL_BCELL_DN
139	0.5316551	2.1935863	0	0	0
2102	"tags=45%, list=15%, signal=52%"
GSE1460_DP_THYMOCYTE_VS_NAIVE_CD4_TCELL_ADULT_BLOOD_UP
GSE1460_DP_THYMOCYTE_VS_NAIVE_CD4_TCELL_ADULT_BLOOD_UP
149	0.52605176	2.1863234	0	0	0
1833	"tags=40%, list=13%, signal=46%"
GSE39556_UNTREATED_VS_3H_POLYIC_INJ_MOUSE_NK_CELL_UP
GSE39556_UNTREATED_VS_3H_POLYIC_INJ_MOUSE_NK_CELL_UP
156	0.5257982	2.186302 0	0	0
1251	"tags=31%, list=9%, signal=34%"
GSE33424_CD161_HIGH_VS_INT_CD8_TCELL_DN

GSE33424_CD161_HIGH_VS_INT_CD8_TCELL_DN 147
0.5290912 2.1833203 0 0 0
1944 "tags=44%, list=14%, signal=51%"
GSE10240_CTRL_VS_IL17_AND_IL22_STIM_PRIMARY_BRONCHIAL_EPITHELIAL_CELLS_UP
GSE10240_CTRL_VS_IL17_AND_IL22_STIM_PRIMARY_BRONCHIAL_EPITHELIAL_CELLS_UP 140 0.5264703 2.1803515 0
0 0 1600 "tags=36%, list=11%, signal=40%"
GSE21927_BALBC_VS_C57BL6_MONOCYTE_TUMOR_UP
GSE21927_BALBC_VS_C57BL6_MONOCYTE_TUMOR_UP 154
0.52483696 2.178264 0 0 0
2034 "tags=40%, list=14%, signal=47%"
GSE3039_CD4_TCELL_VS_ALPHAALPHA_CD8_TCELL_DN
GSE3039_CD4_TCELL_VS_ALPHAALPHA_CD8_TCELL_DN 145
0.5252378 2.176375 0 0 0
2237 "tags=38%, list=16%, signal=45%"
GSE22432_CDC_VS_COMMON_DC_PROGENITOR_DN
GSE22432_CDC_VS_COMMON_DC_PROGENITOR_DN 150
0.5218988 2.175259 0 0 0
1726 "tags=31%, list=12%, signal=35%"
GSE22432_CONVENTIONAL_CDC_VS_PLASMACYTOID_PDC_UP
GSE22432_CONVENTIONAL_CDC_VS_PLASMACYTOID_PDC_UP 153
0.51942015 2.1718476 0 0 0
2273 "tags=41%, list=16%, signal=49%"
GSE17974_0H_VS_72H_IN_VITRO_ACT_CD4_TCELL_DN
GSE17974_0H_VS_72H_IN_VITRO_ACT_CD4_TCELL_DN 149
0.51885384 2.1715784 0 0 0
1471 "tags=33%, list=10%, signal=36%"
GSE5589_WT_VS_IL10_KO_LPS_STIM_MACROPHAGE_45MIN_UP
GSE5589_WT_VS_IL10_KO_LPS_STIM_MACROPHAGE_45MIN_UP 123
0.5368559 2.1658745 0 0 0
1865 "tags=36%, list=13%, signal=41%"
GSE26351_WNT_VS_BMP_PATHWAY_STIM_HEMATOPOIETIC_PROGENITORS_UP
GSE26351_WNT_VS_BMP_PATHWAY_STIM_HEMATOPOIETIC_PROGENITORS_UP
140 0.5329395 2.1638386 0 0
0 2136 "tags=34%, list=15%, signal=40%"
GSE17186_CD21LOW_VS_CD21HIGH_TRANSITIONAL_BCELL_DN
GSE17186_CD21LOW_VS_CD21HIGH_TRANSITIONAL_BCELL_DN 151
0.5177239 2.1625907 0 0 0
2036 "tags=38%, list=14%, signal=44%"
GSE22886_NAIVE_CD4_TCELL_VS_12H_ACT_TH1_DN
GSE22886_NAIVE_CD4_TCELL_VS_12H_ACT_TH1_DN 158
0.51691353 2.16165 0 0 0
2548 "tags=46%, list=18%, signal=56%"
GSE22432_PDC_VS_TGFB1_TREATEDCOMMON_DC_PROGENITOR_UP
GSE22432_PDC_VS_TGFB1_TREATEDCOMMON_DC_PROGENITOR_UP
142 0.5248854 2.1591027 0 0 0
2866 "tags=52%, list=20%, signal=65%"
GSE39022_LN_VS_SPLEEN_DC_UP GSE39022_LN_VS_SPLEEN_DC_UP
148 0.5169322 2.1582758 0 0
0 2449 "tags=39%, list=17%, signal=47%"
GSE17721_PAM3CSK4_VS_CPG_6H_BMDC_UP
GSE17721_PAM3CSK4_VS_CPG_6H_BMDC_UP 145
0.5206435 2.1563447 0 0 0

2166 "tags=38%, list=15%, signal=44%"
GSE21063_3H_VS_16H_ANTI_IGM_STIM_NFATC1_KOBCELL_DN
GSE21063_3H_VS_16H_ANTI_IGM_STIM_NFATC1_KOBCELL_DN 144
0.51956546 2.1529787 0 0 0
1820 "tags=31%, list=13%, signal=36%"
GSE17186_NAIVE_VS_CD21LOW_TRANSITIONAL_BCELL_CORD_BLOOD_DN
GSE17186_NAIVE_VS_CD21LOW_TRANSITIONAL_BCELL_CORD_BLOOD_DN
152 0.5172316 2.1524143 0 0 0
1949 "tags=39%, list=14%, signal=45%"
GSE40685_NAIVE_CD4_TCELL_VS_FOXP3_KO_TREG_PRECURSOR_UP
GSE40685_NAIVE_CD4_TCELL_VS_FOXP3_KO_TREG_PRECURSOR_UP
147 0.5196199 2.1516664 0 0 0
2718 "tags=50%, list=19%, signal=61%"
GSE24634_NAIVE_CD4_TCELL_VS_DAY5_IL4_CONV_TREG_DN
GSE24634_NAIVE_CD4_TCELL_VS_DAY5_IL4_CONV_TREG_DN 165
0.5117713 2.1508162 0 0 0
2474 "tags=47%, list=18%, signal=56%"
GSE17974_CTRL_VS_ACT_IL4_AND_ANTI_IL12_72H_CD4_TCELL_DN
GSE17974_CTRL_VS_ACT_IL4_AND_ANTI_IL12_72H_CD4_TCELL_DN
142 0.5215968 2.149899 0 0 0
1394 "tags=34%, list=10%, signal=37%"
GSE2585_CD80_HIGH_VS_LOW_AIRE_KO_MTEC_DN
GSE2585_CD80_HIGH_VS_LOW_AIRE_KO_MTEC_DN 144
0.5208317 2.1469872 0 0 0
1172 "tags=20%, list=8%, signal=22%"
GSE32164_RESTING_DIFFERENTIATED_VS_CMYC_INHIBITED_MACROPHAGE_DN
GSE32164_RESTING_DIFFERENTIATED_VS_CMYC_INHIBITED_MACROPHAGE_DN
156 0.51315606 2.1439319 0 0
0 2447 "tags=46%, list=17%, signal=55%"
GSE27434_WT_VS_DNMT1_KO_TREG_UP GSE27434_WT_VS_DNMT1_KO_TREG_UP
154 0.51116353 2.1433609 0 0
0 2792 "tags=43%, list=20%, signal=53%"
GSE19825_CD24LOW_VS_IL2RA_HIGH_DAY3_EFF_CD8_TCELL_DN
GSE19825_CD24LOW_VS_IL2RA_HIGH_DAY3_EFF_CD8_TCELL_DN
149 0.5149353 2.1431124 0 0 0
2410 "tags=42%, list=17%, signal=50%"
GSE15330_MEGAKARYOCYTE_ERYTHROID_VS_GRANULOCYTE_MONOCYTE_PROGENITOR_
UP
GSE15330_MEGAKARYOCYTE_ERYTHROID_VS_GRANULOCYTE_MONOCYTE_PROGENITOR_
UP 139 0.51957047 2.1420717 0
0 0 2332 "tags=49%, list=17%, signal=58%"
GSE8685_IL2_STARVED_VS_IL2_ACT_IL2_STARVED_CD4_TCELL_DN
GSE8685_IL2_STARVED_VS_IL2_ACT_IL2_STARVED_CD4_TCELL_DN
136 0.51619583 2.1417727 0 0 0
1054 "tags=24%, list=7%, signal=25%"
GSE17186_CD21LOW_VS_CD21HIGH_TRANSITIONAL_BCELL_UP
GSE17186_CD21LOW_VS_CD21HIGH_TRANSITIONAL_BCELL_UP 139
0.52077127 2.1412473 0 0 0
1468 "tags=32%, list=10%, signal=36%"
GSE37532_TREG_VS_TCONV_PPARG_KO_CD4_TCELL_FROM_LN_UP
GSE37532_TREG_VS_TCONV_PPARG_KO_CD4_TCELL_FROM_LN_UP
144 0.5179443 2.1398559 0 0 0
2476 "tags=47%, list=18%, signal=56%"
GSE22601_CD4_SINGLE_POSITIVE_VS_CD8_SINGLE_POSITIVE_THYMOCYTE_UP

GSE22601_CD4_SINGLE_POSITIVE_VS_CD8_SINGLE_POSITIVE_THYMOCYTE_UP
146 0.5137974 2.1387684 0 0
0 1691 "tags=38%, list=12%, signal=43%"
GSE40666_UNTREATED_VS_IFNA_STIM_STAT4_KO_EFFECTOR_CD8_TCELL_90MIN_UP

GSE40666_UNTREATED_VS_IFNA_STIM_STAT4_KO_EFFECTOR_CD8_TCELL_90MIN_UP
147 0.52040356 2.138689 0 0
0 2041 "tags=40%, list=14%, signal=46%"
GSE22103_UNSTIM_VS_GMCSF_AND_IFNG_STIM_NEUTROPHIL_DN
GSE22103_UNSTIM_VS_GMCSF_AND_IFNG_STIM_NEUTROPHIL_DN
148 0.5241436 2.1373804 0 0 0
2046 "tags=34%, list=14%, signal=40%"
GSE5589_LPS_AND_IL10_VS_LPS_AND_IL6_STIM_MACROPHAGE_45MIN_DN
GSE5589_LPS_AND_IL10_VS_LPS_AND_IL6_STIM_MACROPHAGE_45MIN_DN
151 0.51875544 2.1346383 0 0
0 1905 "tags=40%, list=13%, signal=46%"
GSE3982_MAC_VS_TH2_DN GSE3982_MAC_VS_TH2_DN 134
0.5219401 2.1333134 0 0 0
1980 "tags=34%, list=14%, signal=40%"
GSE22229_RENAL_TRANSPLANT_VS_HEALTHY_PBMC_UP
GSE22229_RENAL_TRANSPLANT_VS_HEALTHY_PBMC_UP 144
0.5123896 2.126435 0 0 0
2174 "tags=42%, list=15%, signal=49%"
GSE12963_UNINF_VS_ENV_AND_NEF_DEFICIENT_HIV1_INF_CD4_TCELL_DN
GSE12963_UNINF_VS_ENV_AND_NEF_DEFICIENT_HIV1_INF_CD4_TCELL_DN
111 0.5339042 2.124058 0 0 0
806 "tags=23%, list=6%, signal=25%"
GSE6259_33D1_POS_DC_VS_BCELL_DN GSE6259_33D1_POS_DC_VS_BCELL_DN
116 0.52345395 2.1235642 0 0
0 1899 "tags=41%, list=13%, signal=46%"
GSE21033_3H_VS_24H_POLYIC_STIM_DC_DN
GSE21033_3H_VS_24H_POLYIC_STIM_DC_DN 99
0.54400355 2.122539 0 0 0
1273 "tags=34%, list=9%, signal=37%"
KAECH_NAIVE_VS_DAY15_EFF_CD8_TCELL_DN
KAECH_NAIVE_VS_DAY15_EFF_CD8_TCELL_DN 159
0.5102029 2.1212902 0 0 0
2226 "tags=50%, list=16%, signal=58%"
GSE29164_UNTREATED_VS_CD8_TCELL_TREATED_MELANOMA_DAY7_UP
GSE29164_UNTREATED_VS_CD8_TCELL_TREATED_MELANOMA_DAY7_UP
165 0.5134444 2.1180294 0 0 0
1802 "tags=39%, list=13%, signal=44%"
GSE30962_ACUTE_VS_CHRONIC_LCMV_SECONDARY_INF_CD8_TCELL_DN
GSE30962_ACUTE_VS_CHRONIC_LCMV_SECONDARY_INF_CD8_TCELL_DN
158 0.50065804 2.1124005 0 3.54E-06 0.001
1935 "tags=39%, list=14%, signal=45%"
GSE13411_NAIVE_VS_IGM_MEMORY_BCELL_DN
GSE13411_NAIVE_VS_IGM_MEMORY_BCELL_DN 149
0.5103834 2.1120665 0 3.53E-06 0.001
2463 "tags=43%, list=17%, signal=51%"
GSE41867_DAY8_EFFECTOR_VS_DAY30_MEMORY_CD8_TCELL_LCMV_ARMSTRONG_UP
GSE41867_DAY8_EFFECTOR_VS_DAY30_MEMORY_CD8_TCELL_LCMV_ARMSTRONG_UP
156 0.50589275 2.1090655 0 3.51E-06
0.001 2294 "tags=43%, list=16%, signal=51%"

GSE3039_CD4_TCELL_VS_NKT_CELL_DN GSE3039_CD4_TCELL_VS_NKT_CELL_DN
143 0.50824356 2.1080186 0 3.50E-06
0.001 2158 "tags=36%, list=15%, signal=42%"
GSE37532_VISCERAL_ADIPOSE_TISSUE_VS_LN_DERIVED_PPARG_KO_TREG_CD4_TCE
LL_UP
GSE37532_VISCERAL_ADIPOSE_TISSUE_VS_LN_DERIVED_PPARG_KO_TREG_CD4_TCE
LL_UP 133 0.5157549 2.1059484 0
3.49E-06 0.001 1804 "tags=37%, list=13%, signal=42%"
GSE5542_IFNG_VS_IFNA_TREATED_EPITHELIAL_CELLS_24H_UP
GSE5542_IFNG_VS_IFNA_TREATED_EPITHELIAL_CELLS_24H_UP
141 0.5107079 2.1022608 0 3.48E-06 0.001
2418 "tags=52%, list=17%, signal=62%"
GSE31082_DN_VS_DP_THYMOCYTE_UP GSE31082_DN_VS_DP_THYMOCYTE_UP
139 0.5104441 2.1020784 0 3.47E-06
0.001 2455 "tags=40%, list=17%, signal=48%"
GSE21670_STAT3_KO_VS_WT_CD4_TCELL_TGFB_IL6_TREATED_UP
GSE21670_STAT3_KO_VS_WT_CD4_TCELL_TGFB_IL6_TREATED_UP
138 0.5167893 2.0983474 0 3.46E-06 0.001
2443 "tags=49%, list=17%, signal=58%"
GSE20500_CTRL_VS_RARA_ANTAGONIST_TREATED_CD4_TCELL_DN
GSE20500_CTRL_VS_RARA_ANTAGONIST_TREATED_CD4_TCELL_DN
132 0.51516014 2.0982623 0 3.45E-06 0.001
1905 "tags=35%, list=13%, signal=40%"
GSE6674_ANTI_IGM_VS_PL2_3_STIM_BCELL_UP
GSE6674_ANTI_IGM_VS_PL2_3_STIM_BCELL_UP 138
0.50840986 2.0961843 0 3.44E-06 0.001
2361 "tags=41%, list=17%, signal=48%"
GSE11961_UNSTIM_VS_ANTI_IGM_AND_CD40_STIM_6H_FOLLICULAR_BCELL_UP
GSE11961_UNSTIM_VS_ANTI_IGM_AND_CD40_STIM_6H_FOLLICULAR_BCELL_UP
150 0.5085082 2.0957932 0 3.43E-06
0.001 1737 "tags=35%, list=12%, signal=39%"
GSE9006_HEALTHY_VS_TYPE_1_DIABETES_PBMC_1MONTH_POST_DX_UP
GSE9006_HEALTHY_VS_TYPE_1_DIABETES_PBMC_1MONTH_POST_DX_UP
144 0.51012397 2.0940366 0 3.42E-06 0.001
2391 "tags=47%, list=17%, signal=56%"
GSE5542_IFNG_VS_IFNA_TREATED_EPITHELIAL_CELLS_6H_UP
GSE5542_IFNG_VS_IFNA_TREATED_EPITHELIAL_CELLS_6H_UP
123 0.5216831 2.0929298 0 3.41E-06 0.001
2154 "tags=41%, list=15%, signal=48%"
GSE17721_POLYIC_VS_PAM3CSK4_12H_BMDC_DN
GSE17721_POLYIC_VS_PAM3CSK4_12H_BMDC_DN 148
0.5060083 2.0919604 0 3.40E-06 0.001
2476 "tags=39%, list=18%, signal=47%"
GSE14769_20MIN_VS_360MIN_LPS_BMDM_UP
GSE14769_20MIN_VS_360MIN_LPS_BMDM_UP 153
0.5010999 2.0899942 0 3.39E-06 0.001
2243 "tags=37%, list=16%, signal=44%"
GSE22886_NAIVE_CD4_TCELL_VS_DC_DN GSE22886_NAIVE_CD4_TCELL_VS_DC_DN
154 0.49985042 2.0873966 0 3.38E-06
0.001 2519 "tags=45%, list=18%, signal=55%"
GSE24142_ADULT_VS_FETAL_EARLY_THYMIC_PROGENITOR_DN
GSE24142_ADULT_VS_FETAL_EARLY_THYMIC_PROGENITOR_DN 158
0.5034771 2.0842319 0 3.37E-06 0.001
2258 "tags=39%, list=16%, signal=46%"

GSE7831_CPG_VS_INFLUENZA_STIM_PDC_4H_DN
GSE7831_CPG_VS_INFLUENZA_STIM_PDC_4H_DN 148
0.49993294 2.083176 0 3.36E-06 0.001
2158 "tags=34%, list=15%, signal=39%"
GSE12366_GC_BCELL_VS_PLASMA_CELL_UP
GSE12366_GC_BCELL_VS_PLASMA_CELL_UP 129
0.5086994 2.0817206 0 3.35E-06 0.001
1898 "tags=36%, list=13%, signal=42%"
GSE9650_NAIVE_VS_MEMORY_CD8_TCELL_DN
GSE9650_NAIVE_VS_MEMORY_CD8_TCELL_DN 162
0.4981238 2.0812778 0 3.34E-06 0.001
1678 "tags=44%, list=12%, signal=50%"
GSE22601_DOUBLE_POSITIVE_VS_CD8_SINGLE_POSITIVE_THYMOCYTE_DN
GSE22601_DOUBLE_POSITIVE_VS_CD8_SINGLE_POSITIVE_THYMOCYTE_DN
148 0.4952345 2.0774455 0 3.33E-06
0.001 2274 "tags=40%, list=16%, signal=47%"
GSE7218_IGM_VS_IGG_SIGNAL_THGOUGH_ANTIGEN_BCELL_UP
GSE7218_IGM_VS_IGG_SIGNAL_THGOUGH_ANTIGEN_BCELL_UP 151
0.4944139 2.0767467 0 3.32E-06 0.001
1657 "tags=29%, list=12%, signal=33%"
GSE12003_4D_VS_8D_CULTURE_MIR223_KO_BM_PROGENITOR_DN
GSE12003_4D_VS_8D_CULTURE_MIR223_KO_BM_PROGENITOR_DN
134 0.50532705 2.075477 0 3.31E-06 0.001
2331 "tags=40%, list=17%, signal=47%"
GSE10273_LOW_IL7_VS_HIGH_IL7_AND_IRF4_IN_IRF4_8_NULL_PRE_BCELL_UP
GSE10273_LOW_IL7_VS_HIGH_IL7_AND_IRF4_IN_IRF4_8_NULL_PRE_BCELL_UP
144 0.503825 2.0749407 0 3.30E-06 0.001
2710 "tags=39%, list=19%, signal=48%"
KAECH_NAIVE_VS_MEMORY_CD8_TCELL_DN
KAECH_NAIVE_VS_MEMORY_CD8_TCELL_DN 162
0.49505186 2.0744474 0 3.29E-06 0.001
1678 "tags=45%, list=12%, signal=51%"
GSE29164_CD8_TCELL_VS_CD8_TCELL_AND_IL12_TREATED_MELANOMA_DAY7_DN
GSE29164_CD8_TCELL_VS_CD8_TCELL_AND_IL12_TREATED_MELANOMA_DAY7_DN
121 0.51388854 2.0743377 0 3.28E-06
0.001 1912 "tags=33%, list=14%, signal=38%"
GSE13411_PLASMA_CELL_VS_MEMORY_BCELL_UP
GSE13411_PLASMA_CELL_VS_MEMORY_BCELL_UP 129 0.507841
2.0740986 0 3.27E-06 0.001 1046 "tags=25%,
list=7%, signal=27%"
GSE8921_UNSTIM_0H_VS_TLR1_2_STIM_MONOCYTE_12H_UP
GSE8921_UNSTIM_0H_VS_TLR1_2_STIM_MONOCYTE_12H_UP 138
0.50668305 2.0737097 0 3.26E-06 0.001
1740 "tags=33%, list=12%, signal=37%"
GSE10273_HIGH_IL7_VS_HIGH_IL7_AND_IRF4_IN_IRF4_8_NULL_PRE_BCELL_DN
GSE10273_HIGH_IL7_VS_HIGH_IL7_AND_IRF4_IN_IRF4_8_NULL_PRE_BCELL_DN
151 0.50547004 2.0730858 0 3.26E-06
0.001 2190 "tags=37%, list=16%, signal=43%"
GSE37563_WT_VS_CTLA4_KO_CD4_TCELL_D4_POST_IMMUNIZATION_DN
GSE37563_WT_VS_CTLA4_KO_CD4_TCELL_D4_POST_IMMUNIZATION_DN
150 0.49893996 2.072852 0 3.25E-06 0.001
2349 "tags=45%, list=17%, signal=54%"
GSE24142_EARLY_THYMIC_PROGENITOR_VS_DN2_THYMOCYTE_ADULT_DN
GSE24142_EARLY_THYMIC_PROGENITOR_VS_DN2_THYMOCYTE_ADULT_DN

156	0.49549127	2.070647	0	3.24E-06	0.001
1682	"tags=32%, list=12%, signal=36%"
GSE1460_DP_THYMOCYTE_VS_NAIVE_CD4_TCELL_CORD_BLOOD_UP
GSE1460_DP_THYMOCYTE_VS_NAIVE_CD4_TCELL_CORD_BLOOD_UP
141	0.50345904	2.0701437	0	3.23E-06	0.001
1556	"tags=34%, list=11%, signal=38%"
GSE17721_POLYIC_VS_PAM3CSK4_24H_BMDC_DN
GSE17721_POLYIC_VS_PAM3CSK4_24H_BMDC_DN	152
0.5042449	2.0684428	0	3.22E-06	0.001
1995	"tags=39%, list=14%, signal=45%"
GSE27786_CD4_TCELL_VS_NKTCELL_DN	GSE27786_CD4_TCELL_VS_NKTCELL_DN
139	0.5004093	2.060685	0	3.21E-06	0.001
2293	"tags=38%, list=16%, signal=45%"
GSE7460_CD8_TCELL_VS_TREG_ACT_DN	GSE7460_CD8_TCELL_VS_TREG_ACT_DN
141	0.49475738	2.059832	0	3.20E-06	0.001
1422	"tags=33%, list=10%, signal=37%"
GSE14908_ATOPIC_VS_NONATOPIC_PATIENT_RESTING_CD4_TCELL_DN
GSE14908_ATOPIC_VS_NONATOPIC_PATIENT_RESTING_CD4_TCELL_DN
153	0.49209467	2.0582695	0	3.19E-06	0.001
2804	"tags=52%, list=20%, signal=65%"
GSE6674_CPG_VS_PL2_3_STIM_BCELL_UP
GSE6674_CPG_VS_PL2_3_STIM_BCELL_UP	132
0.5027897	2.0576847	0	3.18E-06	0.001
1813	"tags=37%, list=13%, signal=42%"
GSE36078_UNTREATED_VS_AD5_INF_IL1R_KO_MOUSE_LUNG_DC_DN
GSE36078_UNTREATED_VS_AD5_INF_IL1R_KO_MOUSE_LUNG_DC_DN
140	0.49696085	2.0571835	0	3.17E-06	0.001
1662	"tags=28%, list=12%, signal=31%"
GSE15271_CXCR4_POS_VS_NEG_GC_BCELL_UP
GSE15271_CXCR4_POS_VS_NEG_GC_BCELL_UP	142	0.516498
2.05691	0	3.17E-06	0.001	2519	"tags=43%, list=18%, signal=52%"
GSE11057_NAIVE_VS_MEMORY_CD4_TCELL_DN
GSE11057_NAIVE_VS_MEMORY_CD4_TCELL_DN	138
0.49733818	2.0562015	0	3.16E-06	0.001
2311	"tags=46%, list=16%, signal=55%"
GSE11961_GERMINAL_CENTER_BCELL_DAY7_VS_GERMINAL_CENTER_BCELL_DAY40_UP
GSE11961_GERMINAL_CENTER_BCELL_DAY7_VS_GERMINAL_CENTER_BCELL_DAY40_UP	148	0.4925212	2.0552225	0
3.15E-06	0.001	1805	"tags=35%, list=13%, signal=40%"
GSE411_WT_VS_SOCS3_KO_MACROPHAGE_UP
GSE411_WT_VS_SOCS3_KO_MACROPHAGE_UP	148
0.49825507	2.0546517	0	3.14E-06	0.001
2331	"tags=45%, list=17%, signal=53%"
GSE13522_CTRL_VS_T_CRUZI_Y_STRAIN_INF_SKIN_IFNG_KO_UP
GSE13522_CTRL_VS_T_CRUZI_Y_STRAIN_INF_SKIN_IFNG_KO_UP
128	0.50487	2.054015	0	3.13E-06	0.001
2697	"tags=46%, list=19%, signal=56%"
GSE25085_FETAL_LIVER_VS_ADULT_BM_SP4_THYMIC_IMPLANT_UP
GSE25085_FETAL_LIVER_VS_ADULT_BM_SP4_THYMIC_IMPLANT_UP
131	0.5004592	2.0528085	0	3.12E-06	0.001
2175	"tags=41%, list=15%, signal=48%"
GSE18791_UNSTIM_VS_NEWCATSLE_VIRUS_DC_18H_UP

GSE18791_UNSTIM_VS_NEWCATSLE_VIRUS_DC_18H_UP 149
0.49735165 2.0524538 0 3.11E-06 0.001
2311 "tags=42%, list=16%, signal=49%"
GSE16522_ANTI_CD3CD28_STIM_VS_UNSTIM_NAIVE_CD8_TCELL_DN
GSE16522_ANTI_CD3CD28_STIM_VS_UNSTIM_NAIVE_CD8_TCELL_DN
152 0.49089074 2.0502486 0 3.11E-06 0.001
2094 "tags=38%, list=15%, signal=44%"
GSE27859_DC_VS_CD11C_INT_F480_HI_MACROPHAGE_DN
GSE27859_DC_VS_CD11C_INT_F480_HI_MACROPHAGE_DN 119
0.50708723 2.0474823 0 6.23E-06 0.002
2661 "tags=37%, list=19%, signal=45%"
GSE27859_DC_VS_CD11C_INT_F480_INT_DC_DN
GSE27859_DC_VS_CD11C_INT_F480_INT_DC_DN 147
0.49372488 2.0465655 0 6.22E-06 0.002
2726 "tags=46%, list=19%, signal=57%"
GSE2405_HEAT_KILLED_VS_LIVE_A_PHAGOCYTOPHILUM_STIM_NEUTROPHIL_9H_UP
GSE2405_HEAT_KILLED_VS_LIVE_A_PHAGOCYTOPHILUM_STIM_NEUTROPHIL_9H_UP
147 0.49182975 2.0459692 0 6.20E-06
0.002 2500 "tags=45%, list=18%, signal=54%"
GSE39110_UNTREATED_VS_IL2_TREATED_CD8_TCELL_DAY6_POST_IMMUNIZATION_U
P
GSE39110_UNTREATED_VS_IL2_TREATED_CD8_TCELL_DAY6_POST_IMMUNIZATION_U
P 163 0.4848007 2.045462 0 6.18E-06
0.002 1715 "tags=41%, list=12%, signal=46%"
GSE9988_ANTI_TREM1_VS_ANTI_TREM1_AND_LPS_MONOCYTE_UP
GSE9988_ANTI_TREM1_VS_ANTI_TREM1_AND_LPS_MONOCYTE_UP
147 0.49255547 2.0445137 0 6.17E-06 0.002
2851 "tags=48%, list=20%, signal=59%"
GSE7509_UNSTIM_VS_FCGRIIB_STIM_DC_UP
GSE7509_UNSTIM_VS_FCGRIIB_STIM_DC_UP 137
0.50276047 2.0410087 0 6.15E-06 0.002
1268 "tags=19%, list=9%, signal=21%"
GSE22886_TCELL_VS_BCELL_NAIVE_UP GSE22886_TCELL_VS_BCELL_NAIVE_UP
152 0.48639363 2.040706 0 6.13E-06 0.002
2358 "tags=45%, list=17%, signal=54%"
GSE6269_HEALTHY_VS_FLU_INF_PBMC_DN
GSE6269_HEALTHY_VS_FLU_INF_PBMC_DN 109
0.52277243 2.0386143 0 6.12E-06 0.002
2442 "tags=51%, list=17%, signal=62%"
GSE19923_HEB_KO_VS_HEB_AND_E2A_KO_DP_THYMOCYTE_UP
GSE19923_HEB_KO_VS_HEB_AND_E2A_KO_DP_THYMOCYTE_UP 157
0.48304236 2.0367749 0 6.10E-06 0.002
1615 "tags=32%, list=11%, signal=36%"
GSE36095_WT_VS_HDAC9_KO_TREG_UP GSE36095_WT_VS_HDAC9_KO_TREG_UP
148 0.48932877 2.0362551 0 6.09E-06
0.002 1960 "tags=37%, list=14%, signal=43%"
GSE39820_TGFBETA1_IL6_VS_TGFBETA1_IL6_IL23A_TREATED_CD4_TCELL_DN
GSE39820_TGFBETA1_IL6_VS_TGFBETA1_IL6_IL23A_TREATED_CD4_TCELL_DN
139 0.49961647 2.0360928 0 6.07E-06
0.002 2291 "tags=37%, list=16%, signal=44%"
GSE19923_E2A_KO_VS_E2A_AND_HEB_KO_DP_THYMOCYTE_UP
GSE19923_E2A_KO_VS_E2A_AND_HEB_KO_DP_THYMOCYTE_UP 151
0.48960713 2.0349267 0 6.05E-06 0.002
2085 "tags=38%, list=15%, signal=44%"

GSE9006_TYPE_1_DIABETES_AT_DX_VS_1MONTH_POST_DX_PBMC_UP
GSE9006_TYPE_1_DIABETES_AT_DX_VS_1MONTH_POST_DX_PBMC_UP
157 0.48892224 2.0346835 0 6.04E-06 0.002
1790 "tags=36%, list=13%, signal=40%"
GSE12003_MIR223_KO_VS_WT_BM_PROGENITOR_8D_CULTURE_DN
GSE12003_MIR223_KO_VS_WT_BM_PROGENITOR_8D_CULTURE_DN
116 0.5164812 2.0343766 0 6.02E-06 0.002
1765 "tags=29%, list=13%, signal=33%"
GSE21546_SAP1A_KO_VS_SAP1A_KO_AND_ELK1_KO_DP_THYMOCYTES_DN
GSE21546_SAP1A_KO_VS_SAP1A_KO_AND_ELK1_KO_DP_THYMOCYTES_DN
148 0.4943078 2.0327182 0 9.12E-06 0.003
2344 "tags=40%, list=17%, signal=47%"
GSE339_EX_VIVO_VS_IN_CULTURE_CD4POS_DC_UP
GSE339_EX_VIVO_VS_IN_CULTURE_CD4POS_DC_UP 149
0.49584392 2.0312757 0 9.10E-06 0.003
2450 "tags=44%, list=17%, signal=53%"
GSE43260_BTLA_POS_VS_NEG_INTRATUMORAL_CD8_TCELL_UP
GSE43260_BTLA_POS_VS_NEG_INTRATUMORAL_CD8_TCELL_UP 148
0.49057636 2.0311332 0 9.08E-06 0.003
2222 "tags=41%, list=16%, signal=48%"
GSE11057_NAIVE_VS_EFF_MEMORY_CD4_TCELL_DN
GSE11057_NAIVE_VS_EFF_MEMORY_CD4_TCELL_DN 126
0.49262792 2.0291781 0 9.05E-06 0.003
1738 "tags=37%, list=12%, signal=42%"
GSE16450_IMMATURE_VS_MATURE_NEURON_CELL_LINE_DN
GSE16450_IMMATURE_VS_MATURE_NEURON_CELL_LINE_DN 144
0.49062783 2.027763 0 1.21E-05 0.004
1726 "tags=34%, list=12%, signal=38%"
GSE21360_SECONDARY_VS_TERTIARY_MEMORY_CD8_TCELL_UP
GSE21360_SECONDARY_VS_TERTIARY_MEMORY_CD8_TCELL_UP 153
0.48096925 2.0249505 0 1.20E-05 0.004
3033 "tags=52%, list=21%, signal=66%"
GSE7218_UNSTIM_VS_ANTIGEN_STIM_THROUGH_IGG_BCELL_UP
GSE7218_UNSTIM_VS_ANTIGEN_STIM_THROUGH_IGG_BCELL_UP
140 0.4967213 2.024783 0 1.20E-05 0.004
2188 "tags=33%, list=16%, signal=39%"
GSE17301_ACD3_ACD28_VS_ACD3_ACD28_AND_IFNA2_STIM_CD8_TCELL_UP
GSE17301_ACD3_ACD28_VS_ACD3_ACD28_AND_IFNA2_STIM_CD8_TCELL_UP
142 0.48896873 2.0244975 0 1.20E-05
0.004 1448 "tags=32%, list=10%, signal=36%"
GSE44955_MCSF_VS_MCSF_AND_IL27_STIM_MACROPHAGE_DN
GSE44955_MCSF_VS_MCSF_AND_IL27_STIM_MACROPHAGE_DN 135
0.48963037 2.024221 0 1.19E-05 0.004
1594 "tags=29%, list=11%, signal=32%"
GSE25088_IL4_VS_IL4_AND_ROSIGLITAZONE_STIM_STAT6_KO_MACROPHAGE_DAY10_UP
GSE25088_IL4_VS_IL4_AND_ROSIGLITAZONE_STIM_STAT6_KO_MACROPHAGE_DAY10_UP 128 0.50062853 2.0238788 0
1.19E-05 0.004 2132 "tags=38%, list=15%, signal=44%"
GSE15624_CTRL_VS_6H_HALOFUGINONE_TREATED_CD4_TCELL_DN
GSE15624_CTRL_VS_6H_HALOFUGINONE_TREATED_CD4_TCELL_DN
124 0.49607667 2.0223908 0 1.19E-05 0.004
2668 "tags=42%, list=19%, signal=51%"
GSE21360_SECONDARY_VS_TERTIARY_MEMORY_CD8_TCELL_DN

GSE21360_SECONDARY_VS_TERTIARY_MEMORY_CD8_TCELL_DN 130
0.49336416 2.0223882 0 1.18E-05 0.004
1435 "tags=29%, list=10%, signal=32%"
GSE22601_IMMATURE_CD4_SINGLE_POSITIVE_VS_CD4_SINGLE_POSITIVE_THYMOCYTE_UP
GSE22601_IMMATURE_CD4_SINGLE_POSITIVE_VS_CD4_SINGLE_POSITIVE_THYMOCYTE_UP 161 0.48816898 2.0218642 0
1.18E-05 0.004 2167 "tags=42%, list=15%, signal=49%"
GSE41867_DAY8_EFFECTOR_VS_DAY30_EXHAUSTED_CD8_TCELL_LCMV_CLONE13_UP
GSE41867_DAY8_EFFECTOR_VS_DAY30_EXHAUSTED_CD8_TCELL_LCMV_CLONE13_UP
147 0.49063227 2.0218525 0 1.18E-05
0.004 2428 "tags=44%, list=17%, signal=52%"
GSE20754_WT_VS_TCF1_KO_MEMORY_CD8_TCELL_DN
GSE20754_WT_VS_TCF1_KO_MEMORY_CD8_TCELL_DN 132
0.49546292 2.0210056 0 1.18E-05 0.004
1844 "tags=38%, list=13%, signal=43%"
GSE9037_WT_VS_IRAK4_KO_LPS_4H_STIM_BMDM_DN
GSE9037_WT_VS_IRAK4_KO_LPS_4H_STIM_BMDM_DN 152
0.4858284 2.0206285 0 1.17E-05 0.004
2066 "tags=33%, list=15%, signal=38%"
GSE557_CIITA_KO_VS_I_AB_KO_DC_DN GSE557_CIITA_KO_VS_I_AB_KO_DC_DN
152 0.4850563 2.0194204 0 1.17E-05
0.004 2581 "tags=49%, list=18%, signal=59%"
GSE411_UNSTIM_VS_400MIN_IL6_STIM_SOCS3_KO_MACROPHAGE_DN
GSE411_UNSTIM_VS_400MIN_IL6_STIM_SOCS3_KO_MACROPHAGE_DN
148 0.48347297 2.0181472 0 1.17E-05 0.004
2302 "tags=41%, list=16%, signal=48%"
GSE7831_UNSTIM_VS_CPG_STIM_PDC_4H_UP
GSE7831_UNSTIM_VS_CPG_STIM_PDC_4H_UP 159
0.48228884 2.018097 0 1.16E-05 0.004
1355 "tags=33%, list=10%, signal=36%"
GSE22033_UNTREATED_VS_ROSIGLITAZONE_TREATED_MEF_UP
GSE22033_UNTREATED_VS_ROSIGLITAZONE_TREATED_MEF_UP 142
0.48947307 2.0168073 0 1.16E-05 0.004
2127 "tags=37%, list=15%, signal=43%"
GSE39864_WT_VS_GATA3_KO_TREG_DN GSE39864_WT_VS_GATA3_KO_TREG_DN
139 0.48643276 2.016549 0 1.16E-05 0.004
2669 "tags=45%, list=19%, signal=54%"
GSE5589_UNSTIM_VS_180MIN_LPS_STIM_MACROPHAGE_DN
GSE5589_UNSTIM_VS_180MIN_LPS_STIM_MACROPHAGE_DN 139
0.4901306 2.016224 0 1.45E-05 0.005
1893 "tags=37%, list=13%, signal=43%"
GSE36078_WT_VS_IL1R_KO_LUNG_DC_AFTER_AD5_INF_UP
GSE36078_WT_VS_IL1R_KO_LUNG_DC_AFTER_AD5_INF_UP 162
0.4788029 2.01499 0 1.45E-05 0.005
1715 "tags=39%, list=12%, signal=44%"
GSE17721_POLYIC_VS_PAM3CSK4_8H_BMDC_DN
GSE17721_POLYIC_VS_PAM3CSK4_8H_BMDC_DN 148
0.4841246 2.0139976 0 1.44E-05 0.005
1916 "tags=36%, list=14%, signal=42%"
GSE17721_LPS_VS_POLYIC_16H_BMDC_UP
GSE17721_LPS_VS_POLYIC_16H_BMDC_UP 155
0.48370448 2.0062797 0 1.44E-05 0.005
1901 "tags=35%, list=13%, signal=40%"

GSE7768_OVA_ALONE_VS_OVA_WITH_MPL_IMMUNIZED_MOUSE_WHOLE_SPLEEN_6H_UP

GSE7768_OVA_ALONE_VS_OVA_WITH_MPL_IMMUNIZED_MOUSE_WHOLE_SPLEEN_6H_UP
146 0.48715582 2.0031958 0
1.73E-05 0.006 1097 "tags=23%, list=8%, signal=24%"
GSE339_EX_VIVO_VS_IN_CULTURE_CD4CD8DN_DC_UP
GSE339_EX_VIVO_VS_IN_CULTURE_CD4CD8DN_DC_UP 151
0.48493993 2.0021853 0 1.72E-05 0.006
2204 "tags=39%, list=16%, signal=46%"
GSE13411_NAIVE_VS_MEMORY_BCELL_DN GSE13411_NAIVE_VS_MEMORY_BCELL_DN
153 0.47950014 2.0016074 0 1.72E-05
0.006 2354 "tags=39%, list=17%, signal=46%"
GSE3920_UNTREATED_VS_IFNA_TREATED_ENDOTHELIAL_CELL_DN
GSE3920_UNTREATED_VS_IFNA_TREATED_ENDOTHELIAL_CELL_DN
137 0.48943377 2.0011213 0 1.72E-05 0.006
2103 "tags=39%, list=15%, signal=46%"
GSE9650_GP33_VS_GP276_LCMV_SPECIFIC_EXHAUSTED_CD8_TCELL_DN
GSE9650_GP33_VS_GP276_LCMV_SPECIFIC_EXHAUSTED_CD8_TCELL_DN
151 0.48188674 1.9985553 0 1.71E-05 0.006
2570 "tags=43%, list=18%, signal=52%"
GSE18893_CTRL_VS_TNF_TREATED_TCONV_2H_UP
GSE18893_CTRL_VS_TNF_TREATED_TCONV_2H_UP 142
0.48163432 1.998345 0 1.71E-05 0.006
2464 "tags=42%, list=17%, signal=50%"
GSE29614_CTRL_VS_TIV_FLU_VACCINE_PBMC_2007_DN
GSE29614_CTRL_VS_TIV_FLU_VACCINE_PBMC_2007_DN 101
0.5077185 1.9981614 0 1.70E-05 0.006
1413 "tags=26%, list=10%, signal=28%"
GSE22886_NAIVE_VS_IGM_MEMORY_BCELL_DN
GSE22886_NAIVE_VS_IGM_MEMORY_BCELL_DN 145
0.48775253 1.9978532 0 1.70E-05 0.006
2022 "tags=39%, list=14%, signal=45%"
GSE360_CTRL_VS_L_DONOVANI_MAC_UP GSE360_CTRL_VS_L_DONOVANI_MAC_UP
151 0.47973397 1.9976256 0 1.70E-05
0.006 1964 "tags=36%, list=14%, signal=42%"
GSE17974_0.5H_VS_72H_IL4_AND_ANTI_IL12_ACT_CD4_TCELL_DN
GSE17974_0.5H_VS_72H_IL4_AND_ANTI_IL12_ACT_CD4_TCELL_DN
129 0.48641834 1.996479 0 1.69E-05 0.006
1819 "tags=32%, list=13%, signal=36%"
GSE14350_TREG_VS_TEFF_IN_IL2RB_KO_DN
GSE14350_TREG_VS_TEFF_IN_IL2RB_KO_DN 141
0.4791688 1.9938678 0 1.69E-05 0.006
2402 "tags=32%, list=17%, signal=38%"
GSE23505_IL6_IL1_VS_IL6_IL1_TGFB_TREATED_CD4_TCELL_DN
GSE23505_IL6_IL1_VS_IL6_IL1_TGFB_TREATED_CD4_TCELL_DN
140 0.4827984 1.9920083 0 2.24E-05 0.008
1816 "tags=39%, list=13%, signal=45%"
GSE41867_NAIVE_VS_DAY15_LCMV_EFFECTOR_CD8_TCELL_UP
GSE41867_NAIVE_VS_DAY15_LCMV_EFFECTOR_CD8_TCELL_UP 148
0.48113543 1.9898667 0 2.23E-05 0.008
2462 "tags=44%, list=17%, signal=53%"
GSE34205_HEALTHY_VS_FLU_INF_INFANT_PBMC_DN
GSE34205_HEALTHY_VS_FLU_INF_INFANT_PBMC_DN 134
0.48467776 1.9895285 0 2.23E-05 0.008

2358	"tags=43%, list=17%, signal=51%"
GSE39556_CD8A_DC_VS_NK_CELL_UP	GSE39556_CD8A_DC_VS_NK_CELL_UP
	159	0.47178733	1.9863707	0	2.22E-05
0.008	1313	"tags=25%, list=9%, signal=27%"
GSE27786_NEUTROPHIL_VS_MONO_MAC_DN
GSE27786_NEUTROPHIL_VS_MONO_MAC_DN			133
0.48653585	1.981576	0	2.22E-05	0.008
2266	"tags=38%, list=16%, signal=44%"
GSE12845_IGD_NEG_BLOOD_VS_PRE_GC_TONSIL_BCELL_DN
GSE12845_IGD_NEG_BLOOD_VS_PRE_GC_TONSIL_BCELL_DN		140
0.47552994	1.9807467	0	2.21E-05	0.008
1260	"tags=31%, list=9%, signal=33%"
GSE7219_WT_VS_NIK_NFKB2_KO_LPS_AND_ANTI_CD40_STIM_DC_UP
GSE7219_WT_VS_NIK_NFKB2_KO_LPS_AND_ANTI_CD40_STIM_DC_UP
156	0.48008284	1.9794075	0	2.21E-05	0.008
1822	"tags=28%, list=13%, signal=32%"
GSE24726_WT_VS_E2_22_KO_PDC_DAY4_POST_DELETION_DN
GSE24726_WT_VS_E2_22_KO_PDC_DAY4_POST_DELETION_DN		148
0.47669807	1.9783413	0	2.20E-05	0.008
2444	"tags=44%, list=17%, signal=53%"
GSE17186_MEMORY_VS_CD21LOW_TRANSITIONAL_BCELL_UP
GSE17186_MEMORY_VS_CD21LOW_TRANSITIONAL_BCELL_UP		151
0.46858168	1.9782135	0	2.20E-05	0.008
2151	"tags=38%, list=15%, signal=44%"
GSE3982_MAST_CELL_VS_TH1_DN	GSE3982_MAST_CELL_VS_TH1_DN
	152	0.47912443	1.9777949	0	2.19E-05
0.008	1837	"tags=35%, list=13%, signal=40%"
GSE40655_FOXO1_KO_VS_WT_NTREG_DN	GSE40655_FOXO1_KO_VS_WT_NTREG_DN
	139	0.47817516	1.9740409	0	2.19E-05
0.008	1365	"tags=29%, list=10%, signal=32%"
GSE1925_3H_VS_24H_IFNG_STIM_IFNG_PRIMED_MACROPHAGE_DN
GSE1925_3H_VS_24H_IFNG_STIM_IFNG_PRIMED_MACROPHAGE_DN
162	0.47149497	1.973137	0	2.18E-05	0.008
2350	"tags=36%, list=17%, signal=42%"
GSE34006_UNTREATED_VS_A2AR_AGONIST_TREATED_TREG_UP
GSE34006_UNTREATED_VS_A2AR_AGONIST_TREATED_TREG_UP		146
0.4719086	1.9711292	0	2.18E-05	0.008
2291	"tags=40%, list=16%, signal=48%"
GSE369_SOCS3_KO_VS_IFNG_KO_LIVER_DN
GSE369_SOCS3_KO_VS_IFNG_KO_LIVER_DN			134
0.48681444	1.9705576	0	2.17E-05	0.008
1453	"tags=29%, list=10%, signal=32%"
GSE3982_NEUTROPHIL_VS_TH1_DN	GSE3982_NEUTROPHIL_VS_TH1_DN
	150	0.47286713	1.9699202	0	2.17E-05
0.008	2016	"tags=37%, list=14%, signal=43%"
GSE27786_LIN_NEG_VS_NKTCELL_UP	GSE27786_LIN_NEG_VS_NKTCELL_UP
	150	0.47406873	1.9679489	0	2.16E-05
0.008	1485	"tags=23%, list=11%, signal=25%"
GSE5503_MLN_DC_VS_PLN_DC_ACTIVATED_ALLOGENIC_TCELL_UP
GSE5503_MLN_DC_VS_PLN_DC_ACTIVATED_ALLOGENIC_TCELL_UP
146	0.47841263	1.9665279	0	2.42E-05	0.009
1490	"tags=27%, list=11%, signal=30%"
GSE17974_0H_VS_48H_IN_VITRO_ACT_CD4_TCELL_DN
GSE17974_0H_VS_48H_IN_VITRO_ACT_CD4_TCELL_DN			132

0.48724815	1.9660674	0	2.42E-05 0.009
1942	"tags=36%, list=14%, signal=42%"
GSE5679_PPARG_LIGAND_ROSIGLITAZONE_VS_RARA_AGONIST_AM580_TREATED_DC_
UP
GSE5679_PPARG_LIGAND_ROSIGLITAZONE_VS_RARA_AGONIST_AM580_TREATED_DC_
UP		140	0.47542232	1.9646393	0
2.41E-05 0.009	1990	"tags=33%, list=14%, signal=38%"
GSE6269_FLU_VS_E_COLI_INF_PBMC_UP GSE6269_FLU_VS_E_COLI_INF_PBMC_UP
	108	0.50288916	1.9643987	0	2.40E-05
0.009	2481	"tags=49%, list=18%, signal=59%"
GSE3982_EOSINOPHIL_VS_TH2_DN	GSE3982_EOSINOPHIL_VS_TH2_DN
	145	0.47642544	1.9635074	0	2.40E-05
0.009	2016	"tags=39%, list=14%, signal=45%"
GSE360_DC_VS_MAC_B_MALAYI_LOW_DOSE_UP
GSE360_DC_VS_MAC_B_MALAYI_LOW_DOSE_UP		152	0.469002
1.9632349	0	2.39E-05 0.009	2542	"tags=39%,
list=18%, signal=47%"
GSE22886_IGM_MEMORY_BCELL_VS_BLOOD_PLASMA_CELL_DN
GSE22886_IGM_MEMORY_BCELL_VS_BLOOD_PLASMA_CELL_DN		156
0.469131 1.9625113	0	2.39E-05 0.009
1813	"tags=37%, list=13%, signal=41%"
GSE26912_TUMORICIDAL_VS_CTRL_MACROPHAGE_DN
GSE26912_TUMORICIDAL_VS_CTRL_MACROPHAGE_DN	161
0.4631704	1.9603925	0	2.38E-05 0.009
1270	"tags=24%, list=9%, signal=26%"
GSE14908_RESTING_VS_HDM_STIM_CD4_TCELL_ATOPIC_PATIENT_UP
GSE14908_RESTING_VS_HDM_STIM_CD4_TCELL_ATOPIC_PATIENT_UP
143	0.47228324	1.9594052	0	2.38E-05 0.009
2548	"tags=43%, list=18%, signal=52%"
GSE12845_NAIVE_VS_DARKZONE_GC_TONSIL_BCELL_DN
GSE12845_NAIVE_VS_DARKZONE_GC_TONSIL_BCELL_DN		145
0.47399792	1.9593478	0	2.37E-05 0.009
2744	"tags=48%, list=19%, signal=59%"
GSE29618_BCELL_VS_MDC_DN GSE29618_BCELL_VS_MDC_DN		148
0.47520313	1.9591899	0	2.37E-05 0.009
1775	"tags=37%, list=13%, signal=42%"
GSE13547_2H_VS_12_H_ANTI_IGM_STIM_BCELL_UP
GSE13547_2H_VS_12_H_ANTI_IGM_STIM_BCELL_UP	137
0.4743393	1.9556162	0	2.88E-05 0.011
456	"tags=32%, list=3%, signal=33%"
GSE8685_IL2_ACT_IL2_STARVED_VS_IL21_ACT_IL2_STARVED_CD4_TCELL_UP
GSE8685_IL2_ACT_IL2_STARVED_VS_IL21_ACT_IL2_STARVED_CD4_TCELL_UP
	133	0.48011276	1.9552706	0	2.87E-05
0.011	1880	"tags=41%, list=13%, signal=46%"
GSE7568_IL4_VS_IL4_AND_TGFB_TREATED_MACROPHAGE_24H_DN
GSE7568_IL4_VS_IL4_AND_TGFB_TREATED_MACROPHAGE_24H_DN
145	0.470789 1.9544473	0	2.86E-05 0.011
2483	"tags=43%, list=18%, signal=51%"
GSE6674_PL2_3_VS_ANTI_IGM_AND_CPG_STIM_BCELL_UP
GSE6674_PL2_3_VS_ANTI_IGM_AND_CPG_STIM_BCELL_UP		154
0.4687588	1.9533278	0	3.11E-05 0.012
2672	"tags=48%, list=19%, signal=59%"
GSE6259_FLT3L_INDUCED_DEC205_POS_DC_VS_CD8_TCELL_DN
GSE6259_FLT3L_INDUCED_DEC205_POS_DC_VS_CD8_TCELL_DN

137	0.47334504	1.952782	0	3.10E-05	0.012
2320	"tags=39%, list=16%, signal=46%"
GSE29164_UNTREATED_VS_CD8_TCELL_AND_IL12_TREATED_MELANOMA_DAY3_DN
GSE29164_UNTREATED_VS_CD8_TCELL_AND_IL12_TREATED_MELANOMA_DAY3_DN
	145	0.47741687	1.9527074	0	3.10E-05
0.012	2331	"tags=41%, list=17%, signal=49%"
GSE9650_EXHAUSTED_VS_MEMORY_CD8_TCELL_DN
GSE9650_EXHAUSTED_VS_MEMORY_CD8_TCELL_DN	149
0.46819085	1.9525769	0	3.09E-05	0.012
1835	"tags=40%, list=13%, signal=46%"
GSE21360_NAIVE_VS_TERTIARY_MEMORY_CD8_TCELL_DN
GSE21360_NAIVE_VS_TERTIARY_MEMORY_CD8_TCELL_DN	140
0.47581404	1.9519706	0	3.08E-05	0.012
1786	"tags=29%, list=13%, signal=32%"
GSE7852_THYMUS_VS_FAT_TCONV_UP	GSE7852_THYMUS_VS_FAT_TCONV_UP
	132	0.48363778	1.9517785	0	3.08E-05
0.012	2132	"tags=35%, list=15%, signal=41%"
GSE6259_FLT3L_INDUCED_33D1_POS_DC_VS_CD4_TCELL_DN
GSE6259_FLT3L_INDUCED_33D1_POS_DC_VS_CD4_TCELL_DN	132
0.48140943	1.9495919	0	3.33E-05	0.013
2015	"tags=37%, list=14%, signal=43%"
GSE25147_UNSTIM_VS_HELIOBACTER_PYLORI_LPS_STIM_MKN45_CELL_UP
GSE25147_UNSTIM_VS_HELIOBACTER_PYLORI_LPS_STIM_MKN45_CELL_UP
	131	0.47547337	1.9482507	0	3.33E-05
0.013	2828	"tags=40%, list=20%, signal=49%"
GSE1925_CTRL_VS_IFNG_PRIMED_MACROPHAGE_UP
GSE1925_CTRL_VS_IFNG_PRIMED_MACROPHAGE_UP	159
0.47242197	1.9468716	0	3.32E-05	0.013
1513	"tags=26%, list=11%, signal=29%"
GSE2770_IL12_VS_IL4_TREATED_ACT_CD4_TCELL_48H_UP
GSE2770_IL12_VS_IL4_TREATED_ACT_CD4_TCELL_48H_UP	148
0.4702994	1.9462967	0	3.31E-05	0.013
1491	"tags=29%, list=11%, signal=32%"
GSE22611_UNSTIM_VS_2H_MDP_STIM_MUTANT_NOD2_TRANSDUCED_HEK293T_CELL_D
N
GSE22611_UNSTIM_VS_2H_MDP_STIM_MUTANT_NOD2_TRANSDUCED_HEK293T_CELL_D
N	149	0.46538877	1.9459195	0
3.30E-05	0.013	2391	"tags=42%, list=17%, signal=50%"
GSE41867_NAIVE_VS_DAY8_LCMV_EFFECTOR_CD8_TCELL_UP
GSE41867_NAIVE_VS_DAY8_LCMV_EFFECTOR_CD8_TCELL_UP	134
0.4686185	1.9443641	0	3.30E-05	0.013
2697	"tags=46%, list=19%, signal=56%"
GSE22886_NEUTROPHIL_VS_MONOCYTE_DN
GSE22886_NEUTROPHIL_VS_MONOCYTE_DN	143
0.46916577	1.9415542	0	3.80E-05	0.015
2594	"tags=50%, list=18%, signal=60%"
GSE17974_1.5H_VS_72H_IL4_AND_ANTI_IL12_ACT_CD4_TCELL_DN
GSE17974_1.5H_VS_72H_IL4_AND_ANTI_IL12_ACT_CD4_TCELL_DN
127	0.47808987	1.9400413	0	3.79E-05	0.015
2189	"tags=41%, list=16%, signal=48%"
GSE44649_NAIVE_VS_ACTIVATED_CD8_TCELL_DN
GSE44649_NAIVE_VS_ACTIVATED_CD8_TCELL_DN	129
0.47784173	1.9374014	0	4.04E-05	0.016
2194	"tags=35%, list=16%, signal=41%"

GSE16450_CTRL_VS_IFNA_12H_STIM_MATURE_NEURON_CELL_LINE_DN
GSE16450_CTRL_VS_IFNA_12H_STIM_MATURE_NEURON_CELL_LINE_DN
149 0.46561405 1.9370401 0 4.03E-05 0.016
2630 "tags=43%, list=19%, signal=52%"
GSE557_WT_VS_I_AB_KO_DC_DN GSE557_WT_VS_I_AB_KO_DC_DN
133 0.48189187 1.9364685 0 4.03E-05
0.016 2424 "tags=41%, list=17%, signal=49%"
GSE3920_UNTREATED_VS_IFNG_TREATED_ENDOTHELIAL_CELL_DN
GSE3920_UNTREATED_VS_IFNG_TREATED_ENDOTHELIAL_CELL_DN
142 0.47080314 1.9356892 0 4.02E-05 0.016
2337 "tags=36%, list=17%, signal=43%"
GSE40274_CTRL_VS_FOXP3_AND_GATA1_TRANSDUCED_ACTIVATED_CD4_TCELL_DN
GSE40274_CTRL_VS_FOXP3_AND_GATA1_TRANSDUCED_ACTIVATED_CD4_TCELL_DN
119 0.4738535 1.9348025 0 4.26E-05
0.017 2482 "tags=34%, list=18%, signal=40%"
GSE17186_BLOOD_VS_CORD_BLOOD_CD21HIGH_TRANSITIONAL_BCELL_UP
GSE17186_BLOOD_VS_CORD_BLOOD_CD21HIGH_TRANSITIONAL_BCELL_UP
156 0.46032012 1.9336427 0 4.25E-05 0.017
2325 "tags=42%, list=16%, signal=50%"
GSE15735_2H_VS_12H_HDAC_INHIBITOR_TREATED_CD4_TCELL_DN
GSE15735_2H_VS_12H_HDAC_INHIBITOR_TREATED_CD4_TCELL_DN
150 0.46636367 1.9324269 0 4.24E-05 0.017
1893 "tags=35%, list=13%, signal=40%"
GSE15330_LYMPHOID_MULTIPOTENT_VS_GRANULOCYTE_MONOCYTE_PROGENITOR_DN
GSE15330_LYMPHOID_MULTIPOTENT_VS_GRANULOCYTE_MONOCYTE_PROGENITOR_DN
146 0.46480972 1.9309987 0 4.48E-05
0.018 2605 "tags=44%, list=18%, signal=53%"
GSE22886_NAIVE_TCELL_VS_NEUTROPHIL_UP
GSE22886_NAIVE_TCELL_VS_NEUTROPHIL_UP 147 0.465985
1.9301603 0 4.47E-05 0.018 1982 "tags=41%,
list=14%, signal=47%"
GSE3982_DC_VS_TH2_DN GSE3982_DC_VS_TH2_DN 138
0.47501326 1.929265 0 4.46E-05 0.018
1465 "tags=32%, list=10%, signal=35%"
GSE3720_UNSTIM_VS_PMA_STIM_VD1_GAMMADELTA_TCELL_UP
GSE3720_UNSTIM_VS_PMA_STIM_VD1_GAMMADELTA_TCELL_UP 129
0.47741753 1.9262205 0 4.45E-05 0.018
2090 "tags=43%, list=15%, signal=50%"
GSE22045_TREG_VS_TCONV_UP GSE22045_TREG_VS_TCONV_UP 129
0.46884978 1.9257694 0 4.69E-05 0.019
766 "tags=17%, list=5%, signal=18%"
GSE41978_KLRG1_HIGH_VS_LOW_EFFECTOR_CD8_TCELL_DN
GSE41978_KLRG1_HIGH_VS_LOW_EFFECTOR_CD8_TCELL_DN 135
0.47220197 1.9250108 0 4.93E-05 0.02
1893 "tags=33%, list=13%, signal=37%"
GSE11057_NAIVE_VS_CENT_MEMORY_CD4_TCELL_DN
GSE11057_NAIVE_VS_CENT_MEMORY_CD4_TCELL_DN 149
0.4626717 1.9242226 0 4.92E-05 0.02
2311 "tags=43%, list=16%, signal=51%"
GSE7509_FCGRIIB_VS_TNFA_IL1B_IL6_PGE_STIM_DC_UP
GSE7509_FCGRIIB_VS_TNFA_IL1B_IL6_PGE_STIM_DC_UP 145
0.47323352 1.9240338 0 4.91E-05 0.02
2345 "tags=46%, list=17%, signal=54%"
GSE5503_MLN_DC_VS_SPLEEN_DC_ACTIVATED_ALLOGENIC_TCELL_DN

GSE5503_MLN_DC_VS_SPLEEN_DC_ACTIVATED_ALLOGENIC_TCELL_DN
148	0.46036765	1.9239113	0	4.90E-05	0.02
2309	"tags=37%, list=16%, signal=44%"
GSE18281_SUBCAPSULAR_CORTICAL_REGION_VS_WHOLE_MEDULLA_THYMUS_UP
GSE18281_SUBCAPSULAR_CORTICAL_REGION_VS_WHOLE_MEDULLA_THYMUS_UP
	144	0.46403998	1.9230773	0	5.14E-05
0.021	2163	"tags=26%, list=15%, signal=31%"
GSE11961_FOLLICULAR_BCELL_VS_GERMINAL_CENTER_BCELL_DAY40_DN
GSE11961_FOLLICULAR_BCELL_VS_GERMINAL_CENTER_BCELL_DAY40_DN
145	0.46503294	1.9209561	0	5.37E-05	0.022
2106	"tags=36%, list=15%, signal=42%"
GSE17186_NAIVE_VS_CD21HIGH_TRANSITIONAL_BCELL_DN
GSE17186_NAIVE_VS_CD21HIGH_TRANSITIONAL_BCELL_DN	151
0.45976335	1.9198984	0	5.36E-05	0.022
1742	"tags=30%, list=12%, signal=34%"
GSE360_CTRL_VS_M_TUBERCULOSIS_MAC_UP
GSE360_CTRL_VS_M_TUBERCULOSIS_MAC_UP	139
0.4656569	1.9193932	0	5.35E-05	0.022
1942	"tags=35%, list=14%, signal=40%"
GSE34006_UNTREATED_VS_A2AR_AGONIST_TREATED_TREG_DN
GSE34006_UNTREATED_VS_A2AR_AGONIST_TREATED_TREG_DN	163
0.45688596	1.9167888	0	5.58E-05	0.023
2000	"tags=37%, list=14%, signal=42%"
GSE17721_LPS_VS_GARDIQUIMOD_24H_BMDC_DN
GSE17721_LPS_VS_GARDIQUIMOD_24H_BMDC_DN	159
0.45729503	1.9146867	0	5.56E-05	0.023
1974	"tags=33%, list=14%, signal=38%"
GSE22886_NAIVE_BCELL_VS_MONOCYTE_DN
GSE22886_NAIVE_BCELL_VS_MONOCYTE_DN	155
0.46326554	1.9140435	0	5.55E-05	0.023
2085	"tags=41%, list=15%, signal=47%"
GSE14308_TH2_VS_TH17_UP	GSE14308_TH2_VS_TH17_UP	138
0.47186217	1.9133608	0	5.78E-05	0.024
2392	"tags=38%, list=17%, signal=46%"
GSE15330_MEGAKARYOCYTE_ERYTHROID_VS_GRANULOCYTE_MONOCYTE_PROGENITOR_IKAROS_KO_UP
GSE15330_MEGAKARYOCYTE_ERYTHROID_VS_GRANULOCYTE_MONOCYTE_PROGENITOR_IKAROS_KO_UP	146	0.46162105	1.9114032
0	6.25E-05	0.026	2173	"tags=40%, list=15%, signal=46%"
GSE22443_NAIVE_VS_ACT_AND_IL12_TREATED_CD8_TCELL_UP
GSE22443_NAIVE_VS_ACT_AND_IL12_TREATED_CD8_TCELL_UP
161	0.4610749	1.9094074	0	6.24E-05	0.026
2104	"tags=34%, list=15%, signal=39%"
GSE16450_CTRL_VS_IFNA_6H_STIM_MATURE_NEURON_CELL_LINE_DN
GSE16450_CTRL_VS_IFNA_6H_STIM_MATURE_NEURON_CELL_LINE_DN
144	0.4597654	1.9087653	0	6.23E-05	0.026
2072	"tags=37%, list=15%, signal=43%"
GSE21380_TFH_VS_GERMINAL_CENTER_TFH_CD4_TCELL_DN
GSE21380_TFH_VS_GERMINAL_CENTER_TFH_CD4_TCELL_DN	137
0.46341085	1.9077842	0	6.21E-05	0.026
1613	"tags=24%, list=11%, signal=27%"
GSE31082_DN_VS_CD4_SP_THYMOCYTE_UP
GSE31082_DN_VS_CD4_SP_THYMOCYTE_UP	133
0.47421497	1.9070151	0	6.20E-05	0.026

2221 "tags=38%, list=16%, signal=45%"
GSE40273_EOS_KO_VS_WT_TREG_UP GSE40273_EOS_KO_VS_WT_TREG_UP
154 0.45678967 1.9060503 0 6.19E-05
0.026 2740 "tags=50%, list=19%, signal=61%"
GSE17301_CTRL_VS_48H_ACD3_ACD28_IFNA5_STIM_CD8_TCELL_DN
GSE17301_CTRL_VS_48H_ACD3_ACD28_IFNA5_STIM_CD8_TCELL_DN
151 0.4631745 1.9043828 0 6.89E-05 0.029
792 "tags=23%, list=6%, signal=24%"
GSE3982_DC_VS_TH1_DN GSE3982_DC_VS_TH1_DN 156
0.45577127 1.9037493 0 7.11E-05 0.03
1672 "tags=33%, list=12%, signal=37%"
GSE15330_MEGAKARYOCYTE_ERYTHROID_PROGENITOR_VS_PRO_BCELL_UP
GSE15330_MEGAKARYOCYTE_ERYTHROID_PROGENITOR_VS_PRO_BCELL_UP
157 0.44891495 1.9028845 0 7.10E-05 0.03
1699 "tags=30%, list=12%, signal=34%"
GSE15330_HSC_VS_PRO_BCELL_UP GSE15330_HSC_VS_PRO_BCELL_UP
151 0.4543195 1.902401 0 7.08E-05 0.03
2630 "tags=43%, list=19%, signal=52%"
GSE21670_STAT3_KO_VS_WT_CD4_TCELL_TGFB_TREATED_UP
GSE21670_STAT3_KO_VS_WT_CD4_TCELL_TGFB_TREATED_UP 152
0.4551819 1.9019326 0 7.07E-05 0.03
2000 "tags=34%, list=14%, signal=39%"
GSE14308_TH1_VS_TH17_UP GSE14308_TH1_VS_TH17_UP 143
0.46207556 1.9016725 0 7.06E-05 0.03
1770 "tags=28%, list=13%, signal=32%"
GSE25123_WT_VS_PPARG_KO_MACROPHAGE_DN
GSE25123_WT_VS_PPARG_KO_MACROPHAGE_DN 123
0.46863955 1.899728 0 7.04E-05 0.03
2951 "tags=50%, list=21%, signal=63%"
GSE27786_LSK_VS_LIN_NEG_CELL_DN GSE27786_LSK_VS_LIN_NEG_CELL_DN
145 0.45280775 1.8985767 0 7.26E-05
0.031 2427 "tags=38%, list=17%, signal=45%"
GSE43863_TH1_VS_LY6C_LOW_CXCR5NEG_EFFECTOR_CD4_TCELL_DN
GSE43863_TH1_VS_LY6C_LOW_CXCR5NEG_EFFECTOR_CD4_TCELL_DN
149 0.45343548 1.8983035 0 7.25E-05 0.031
1817 "tags=35%, list=13%, signal=40%"
GSE14769_UNSTIM_VS_360MIN_LPS_BMDM_UP
GSE14769_UNSTIM_VS_360MIN_LPS_BMDM_UP 154
0.4512494 1.8977765 0 7.47E-05 0.032
2483 "tags=42%, list=18%, signal=50%"
GSE37416_0H_VS_12H_F_TULARENSIS_LVS_NEUTROPHIL_UP
GSE37416_0H_VS_12H_F_TULARENSIS_LVS_NEUTROPHIL_UP 128
0.4704533 1.8965629 0 7.91E-05 0.034
2712 "tags=43%, list=19%, signal=53%"
GSE17721_POLYIC_VS_PAM3CSK4_16H_BMDC_DN
GSE17721_POLYIC_VS_PAM3CSK4_16H_BMDC_DN 150
0.45362055 1.8962232 0 7.90E-05 0.034
2199 "tags=37%, list=16%, signal=44%"
GSE24671_BAKIMULC_VS_SENDAI_VIRUS_INFECTED_MOUSE_SPLENOCYTES_DN
GSE24671_BAKIMULC_VS_SENDAI_VIRUS_INFECTED_MOUSE_SPLENOCYTES_DN
135 0.4595001 1.891713 0 8.58E-05 0.037
2170 "tags=33%, list=15%, signal=39%"
GSE3039_NKT_CELL_VS_ALPHAALPHA_CD8_TCELL_DN
GSE3039_NKT_CELL_VS_ALPHAALPHA_CD8_TCELL_DN 149

0.46058038 1.8911915 0 8.56E-05 0.037
2048 "tags=35%, list=15%, signal=40%"
GSE5463_CTRL_VS_DEXAMETHASONE_TREATED_THYMOCYTE_UP
GSE5463_CTRL_VS_DEXAMETHASONE_TREATED_THYMOCYTE_UP 151
0.4558007 1.8911594 0 8.54E-05 0.037
2524 "tags=44%, list=18%, signal=53%"
GSE23321_CD8_STEM_CELL_MEMORY_VS_NAIVE_CD8_TCELL_DN
GSE23321_CD8_STEM_CELL_MEMORY_VS_NAIVE_CD8_TCELL_DN
126 0.457118 1.8902236 0 8.77E-05 0.038
2292 "tags=41%, list=16%, signal=49%"
GSE27786_NKCELL_VS_NKTCELL_UP GSE27786_NKCELL_VS_NKTCELL_UP
142 0.4563652 1.8899655 0 8.75E-05
0.038 1917 "tags=27%, list=14%, signal=31%"
GSE5589_IL6_KO_VS_IL10_KO_LPS_AND_IL6_STIM_MACROPHAGE_45MIN_UP
GSE5589_IL6_KO_VS_IL10_KO_LPS_AND_IL6_STIM_MACROPHAGE_45MIN_UP
144 0.45427904 1.8885311 0 9.19E-05
0.04 1568 "tags=29%, list=11%, signal=32%"
GSE21033_CTRL_VS_POLYIC_STIM_DC_24H_DN
GSE21033_CTRL_VS_POLYIC_STIM_DC_24H_DN 115
0.4735898 1.8871876 0 9.40E-05 0.041
1940 "tags=34%, list=14%, signal=39%"
GSE27786_CD4_TCELL_VS_MONO_MAC_DN GSE27786_CD4_TCELL_VS_MONO_MAC_DN
145 0.45096034 1.8857496 0 9.85E-05
0.043 1388 "tags=23%, list=10%, signal=25%"
GSE27859_MACROPHAGE_VS_CD11C_INT_F480_HI_MACROPHAGE_DN
GSE27859_MACROPHAGE_VS_CD11C_INT_F480_HI_MACROPHAGE_DN
134 0.45860192 1.884948 0 9.83E-05 0.043
2373 "tags=42%, list=17%, signal=50%"
GSE22589_HEALTHY_VS_HIV_AND_SIV_INFECTED_DC_UP
GSE22589_HEALTHY_VS_HIV_AND_SIV_INFECTED_DC_UP 133
0.463828 1.8840568 0 1.00E-04 0.044
2052 "tags=36%, list=15%, signal=42%"
GSE6259_BCELL_VS_CD4_TCELL_UP GSE6259_BCELL_VS_CD4_TCELL_UP
123 0.46475846 1.8831203 0 1.00E-04
0.044 2060 "tags=31%, list=15%, signal=36%"
GSE8921_UNSTIM_VS_TLR1_2_STIM_MONOCYTE_6H_DN
GSE8921_UNSTIM_VS_TLR1_2_STIM_MONOCYTE_6H_DN 135
0.46636385 1.8800867 0 1.04E-04 0.046
1873 "tags=31%, list=13%, signal=36%"
GSE23114_PERITONEAL_CAVITY_B1A_BCELL_VS_SPLEEN_BCELL_DN
GSE23114_PERITONEAL_CAVITY_B1A_BCELL_VS_SPLEEN_BCELL_DN
150 0.4513794 1.8793433 0 1.07E-04 0.047
2246 "tags=41%, list=16%, signal=49%"
GSE27241_CTRL_VS_DIGOXIN_TREATED_RORGT_KO_CD4_TCELL_IN_TH17_POLARIZI
NG_CONDITIONS_UP
GSE27241_CTRL_VS_DIGOXIN_TREATED_RORGT_KO_CD4_TCELL_IN_TH17_POLARIZI
NG_CONDITIONS_UP 127 0.45520577 1.8757708
0 1.09E-04 0.048 1204 "tags=19%, list=9%, signal=20%"
GSE10239_NAIVE_VS_MEMORY_CD8_TCELL_DN
GSE10239_NAIVE_VS_MEMORY_CD8_TCELL_DN 141
0.45610774 1.8754203 0 1.08E-04 0.048
2138 "tags=31%, list=15%, signal=36%"
GSE20715_0H_VS_48H_OZONE_TLR4_KO_LUNG_DN
GSE20715_0H_VS_48H_OZONE_TLR4_KO_LUNG_DN 162

0.4428539	1.8739543	0	1.13E-04 0.05
2474	"tags=39%, list=18%, signal=47%"
GSE17721_CTRL_VS_POLYIC_1H_BMDC_UP
GSE17721_CTRL_VS_POLYIC_1H_BMDC_UP	147
0.44744065	1.8707279	0	1.24E-04 0.055
1483	"tags=28%, list=11%, signal=31%"
GSE32423_IL7_VS_IL4_MEMORY_CD8_TCELL_DN
GSE32423_IL7_VS_IL4_MEMORY_CD8_TCELL_DN	139
0.45248365	1.8706776	0	1.24E-04 0.055
1472	"tags=27%, list=10%, signal=30%"
GSE22886_NAIVE_BCELL_VS_DC_DN	GSE22886_NAIVE_BCELL_VS_DC_DN
166	0.44226098	1.8704512	0	1.23E-04
0.055	2121	"tags=38%, list=15%, signal=44%"
GSE42724_NAIVE_VS_B1_BCELL_DN	GSE42724_NAIVE_VS_B1_BCELL_DN
123	0.4584758	1.8699183	0	1.23E-04
0.055	2686	"tags=41%, list=19%, signal=50%"
GSE3039_NKT_CELL_VS_ALPHABETA_CD8_TCELL_UP
GSE3039_NKT_CELL_VS_ALPHABETA_CD8_TCELL_UP	154
0.44461027	1.869419 0	1.23E-04 0.055
2301	"tags=36%, list=16%, signal=43%"
GSE24726_WT_VS_E2_2_KO_PDC_DAY6_POST_DELETION_UP
GSE24726_WT_VS_E2_2_KO_PDC_DAY6_POST_DELETION_UP	136
0.46062633	1.868541 0	1.25E-04 0.056
1309	"tags=29%, list=9%, signal=32%"
GSE37532_TREG_VS_TCONV_PPARG_KO_CD4_TCELL_FROM_VISCERAL_ADIPOSE_TISS
UE_UP
GSE37532_TREG_VS_TCONV_PPARG_KO_CD4_TCELL_FROM_VISCERAL_ADIPOSE_TISS
UE_UP	145	0.44671726	1.8663074	0
1.27E-04 0.057	2520	"tags=43%, list=18%, signal=52%"
GSE22886_NEUTROPHIL_VS_DC_DN	GSE22886_NEUTROPHIL_VS_DC_DN
156	0.44634882	1.8651099	0.001996008
1.29E-04 0.058	1858	"tags=34%, list=13%, signal=39%"
GSE13411_IGM_MEMORY_BCELL_VS_PLASMA_CELL_DN
GSE13411_IGM_MEMORY_BCELL_VS_PLASMA_CELL_DN	142
0.44788185	1.8642664	0	1.31E-04 0.059
1307	"tags=22%, list=9%, signal=24%"
GSE3982_BASOPHIL_VS_TH2_DN	GSE3982_BASOPHIL_VS_TH2_DN
154	0.4456335	1.8614124	0	1.35E-04
0.061	2211	"tags=33%, list=16%, signal=39%"
GSE1448_CTRL_VS_ANTI_VALPHA2_DP_THYMOCYTE_UP
GSE1448_CTRL_VS_ANTI_VALPHA2_DP_THYMOCYTE_UP	158
0.4447446	1.8612792	0	1.35E-04 0.061
2151	"tags=31%, list=15%, signal=36%"
GSE28726_ACT_CD4_TCELL_VS_ACT_VA24NEG_NKTCELL_UP
GSE28726_ACT_CD4_TCELL_VS_ACT_VA24NEG_NKTCELL_UP	150
0.44627836	1.8608606	0	1.35E-04 0.061
2726	"tags=50%, list=19%, signal=61%"
GSE10239_KLRG1INT_VS_KLRG1HIGH_EFF_CD8_TCELL_DN
GSE10239_KLRG1INT_VS_KLRG1HIGH_EFF_CD8_TCELL_DN	145
0.44902363	1.8594803	0	1.37E-04 0.062
1982	"tags=28%, list=14%, signal=32%"
GSE23321_CD8_STEM_CELL_MEMORY_VS_EFFECTOR_MEMORY_CD8_TCELL_DN
GSE23321_CD8_STEM_CELL_MEMORY_VS_EFFECTOR_MEMORY_CD8_TCELL_DN
154	0.44414863	1.859353 0	1.36E-04 0.062

2121 "tags=33%, list=15%, signal=39%"
GSE411_100MIN_VS_400MIN_IL6_STIM_SOCS3_KO_MACROPHAGE_UP
GSE411_100MIN_VS_400MIN_IL6_STIM_SOCS3_KO_MACROPHAGE_UP
168 0.44191712 1.8574313 0 1.38E-04 0.063
2320 "tags=37%, list=16%, signal=44%"
GSE7509_UNSTIM_VS_TNFA_IL1B_IL6_PGE_STIM_DC_UP
GSE7509_UNSTIM_VS_TNFA_IL1B_IL6_PGE_STIM_DC_UP 161
0.45016292 1.8538127 0 1.53E-04 0.069
2262 "tags=34%, list=16%, signal=40%"
GSE21033_3H_VS_12H_POLYIC_STIM_DC_UP
GSE21033_3H_VS_12H_POLYIC_STIM_DC_UP 122
0.4577377 1.8530618 0 1.55E-04 0.07
1948 "tags=30%, list=14%, signal=35%"
GSE8835_HEALTHY_VS_CLL_CD8_TCELL_UP
GSE8835_HEALTHY_VS_CLL_CD8_TCELL_UP 141
0.4524522 1.8518717 0 1.57E-04 0.071
1433 "tags=29%, list=10%, signal=32%"
GSE8921_UNSTIM_VS_TLR1_2_STIM_MONOCYTE_24H_DN
GSE8921_UNSTIM_VS_TLR1_2_STIM_MONOCYTE_24H_DN 151
0.44719702 1.8516437 0 1.57E-04 0.071
2327 "tags=34%, list=16%, signal=41%"
GSE28726_ACT_CD4_TCELL_VS_ACT_NKTCELL_UP
GSE28726_ACT_CD4_TCELL_VS_ACT_NKTCELL_UP 146
0.44185716 1.8515272 0 1.57E-04 0.071
2202 "tags=42%, list=16%, signal=50%"
GSE1925_CTRL_VS_24H_IFNG_STIM_IFNG_PRIMED_MACROPHAGE_DN
GSE1925_CTRL_VS_24H_IFNG_STIM_IFNG_PRIMED_MACROPHAGE_DN
158 0.44033217 1.8505459 0 1.56E-04 0.071
1665 "tags=31%, list=12%, signal=35%"
GSE17721_PAM3CSK4_VS_GADIQUIMOD_6H_BMDC_UP
GSE17721_PAM3CSK4_VS_GADIQUIMOD_6H_BMDC_UP 143
0.4528801 1.8492497 0 1.58E-04 0.072
2107 "tags=34%, list=15%, signal=40%"
GSE11864_CSF1_VS_CSF1_IFNG_IN_MAC_DN
GSE11864_CSF1_VS_CSF1_IFNG_IN_MAC_DN 125
0.45601463 1.8487524 0 1.60E-04 0.072
2283 "tags=33%, list=16%, signal=39%"
GSE24574_NAIVE_VS_TCONV_CD4_TCELL_DN
GSE24574_NAIVE_VS_TCONV_CD4_TCELL_DN 145
0.4480858 1.8485037 0 1.60E-04 0.072
2202 "tags=41%, list=16%, signal=49%"
GSE13485_DAY1_VS_DAY21_YF17D_VACCINE_PBMC_DN
GSE13485_DAY1_VS_DAY21_YF17D_VACCINE_PBMC_DN 129
0.4554276 1.8479152 0 1.59E-04 0.072
2427 "tags=44%, list=17%, signal=53%"
GSE12839_CTRL_VS_IL12_TREATED_PBMC_UP
GSE12839_CTRL_VS_IL12_TREATED_PBMC_UP 115 0.460717
1.8476256 0 1.59E-04 0.072 1639 "tags=27%,
list=12%, signal=30%"
GSE2770_IL12_ACT_VS_ACT_CD4_TCELL_48H_DN
GSE2770_IL12_ACT_VS_ACT_CD4_TCELL_48H_DN 138
0.45599824 1.8475797 0 1.59E-04 0.072
2005 "tags=33%, list=14%, signal=38%"
GSE22432_MULTIPOTENT_VS_COMMON_DC_PROGENITOR_UP

GSE22432_MULTIPOTENT_VS_COMMON_DC_PROGENITOR_UP 143
0.4467142 1.8473736 0 1.58E-04 0.072
2595 "tags=38%, list=18%, signal=46%"
GSE27786_NKTCELL_VS_MONO_MAC_DN GSE27786_NKTCELL_VS_MONO_MAC_DN
145 0.44482738 1.8472195 0 1.58E-04
0.072 1106 "tags=19%, list=8%, signal=20%"
GSE7831_UNSTIM_VS_CPG_STIM_PDC_1H_UP
GSE7831_UNSTIM_VS_CPG_STIM_PDC_1H_UP 148
0.44166803 1.846844 0 1.58E-04 0.072
2163 "tags=41%, list=15%, signal=47%"
GSE17721_LPS_VS_PAM3CSK4_1H_BMDC_UP
GSE17721_LPS_VS_PAM3CSK4_1H_BMDC_UP 141
0.45064592 1.8466896 0 1.58E-04 0.072
2416 "tags=35%, list=17%, signal=42%"
GSE360_CTRL_VS_B_MALAYI_HIGH_DOSE_MAC_UP
GSE360_CTRL_VS_B_MALAYI_HIGH_DOSE_MAC_UP 147
0.4459504 1.8455501 0.00203666 1.62E-04 0.074
3085 "tags=40%, list=22%, signal=51%"
GSE41978_ID2_KO_VS_ID2_KO_AND_BIM_KO_KLRG1_LOW_EFFECTOR_CD8_TCELL_DN

GSE41978_ID2_KO_VS_ID2_KO_AND_BIM_KO_KLRG1_LOW_EFFECTOR_CD8_TCELL_DN
132 0.4527199 1.8448008 0
1.65E-04 0.076 2855 "tags=45%, list=20%, signal=56%"
GSE5679_PPARG_LIGAND_ROSIGLITAZONE_VS_ROSIGLITAZONE_AND_RARA_AGONIST
_AM580_TREATED_DC_UP
GSE5679_PPARG_LIGAND_ROSIGLITAZONE_VS_ROSIGLITAZONE_AND_RARA_AGONIST
_AM580_TREATED_DC_UP 151 0.44471213
1.8445544 0 1.65E-04 0.076 2128 "tags=38%,
list=15%, signal=45%"
GSE17721_CTRL_VS_POLYIC_12H_BMDC_UP
GSE17721_CTRL_VS_POLYIC_12H_BMDC_UP 149
0.44482696 1.8443191 0 1.69E-04 0.078
2347 "tags=37%, list=17%, signal=44%"
GSE21670_STAT3_KO_VS_WT_CD4_TCELL_TGFB_TREATED_DN
GSE21670_STAT3_KO_VS_WT_CD4_TCELL_TGFB_TREATED_DN 141
0.4516246 1.8426715 0 1.75E-04 0.081
1439 "tags=16%, list=10%, signal=17%"
GSE22432_MULTIPOTENT_PROGENITOR_VS_PDC_DN
GSE22432_MULTIPOTENT_PROGENITOR_VS_PDC_DN 146
0.4403504 1.8420082 0 1.77E-04 0.082
1821 "tags=30%, list=13%, signal=34%"
GSE19401_PAM2CSK4_VS_RETINOIC_ACID_STIM_FOLLICULAR_DC_UP
GSE19401_PAM2CSK4_VS_RETINOIC_ACID_STIM_FOLLICULAR_DC_UP
135 0.45551133 1.8418813 0 1.77E-04 0.082
2089 "tags=33%, list=15%, signal=38%"
GSE2770_UNTREATED_VS_IL4_TREATED_ACT_CD4_TCELL_2H_UP
GSE2770_UNTREATED_VS_IL4_TREATED_ACT_CD4_TCELL_2H_UP
151 0.4436434 1.839876 0 1.81E-04 0.084
895 "tags=23%, list=6%, signal=24%"
GSE2770_IL12_AND_TGFB_ACT_VS_ACT_CD4_TCELL_6H_DN
GSE2770_IL12_AND_TGFB_ACT_VS_ACT_CD4_TCELL_6H_DN 154
0.44413495 1.8386917 0 1.84E-04 0.086
1784 "tags=38%, list=13%, signal=43%"
GSE12845_IGD_NEG_BLOOD_VS_DARKZONE_GC_TONSIL_BCELL_DN

GSE12845_IGD_NEG_BLOOD_VS_DARKZONE_GC_TONSIL_BCELL_DN
143 0.44413707 1.8385892 0 1.84E-04 0.086
2047 "tags=37%, list=15%, signal=43%"
GSE24142_EARLY_THYMIC_PROGENITOR_VS_DN2_THYMOCYTE_DN
GSE24142_EARLY_THYMIC_PROGENITOR_VS_DN2_THYMOCYTE_DN
166 0.44034836 1.8383491 0 1.84E-04 0.086
1195 "tags=25%, list=8%, signal=27%"
GSE360_CTRL_VS_L_MAJOR_MAC_UP GSE360_CTRL_VS_L_MAJOR_MAC_UP
156 0.43786082 1.8377931 0 1.86E-04
0.087 2532 "tags=37%, list=18%, signal=45%"
GSE1925_CTRL_VS_IFNG_PRIMED_MACROPHAGE_24H_IFNG_STIM_DN
GSE1925_CTRL_VS_IFNG_PRIMED_MACROPHAGE_24H_IFNG_STIM_DN
149 0.45009485 1.8374546 0 1.85E-04 0.087
1861 "tags=33%, list=13%, signal=37%"
GSE15330_MEGAKARYOCYTE_ERYTHROID_PROGENITOR_VS_PRO_BCELL_DN
GSE15330_MEGAKARYOCYTE_ERYTHROID_PROGENITOR_VS_PRO_BCELL_DN
147 0.44783896 1.836799 0 1.85E-04 0.087
2401 "tags=41%, list=17%, signal=49%"
GSE21063_WT_VS_NFATC1_KO_BCELL_DN GSE21063_WT_VS_NFATC1_KO_BCELL_DN
142 0.44781038 1.8340623 0 1.93E-04
0.091 1738 "tags=28%, list=12%, signal=32%"
GSE11961_MARGINAL_ZONE_BCELL_VS_MEMORY_BCELL_DAY40_DN
GSE11961_MARGINAL_ZONE_BCELL_VS_MEMORY_BCELL_DAY40_DN
133 0.44608954 1.8319278 0 1.99E-04 0.094
1556 "tags=30%, list=11%, signal=33%"
GSE18791_CTRL_VS_NEWCASTLE_VIRUS_DC_18H_UP
GSE18791_CTRL_VS_NEWCASTLE_VIRUS_DC_18H_UP 133
0.44612595 1.8316334 0 2.02E-04 0.096
1601 "tags=30%, list=11%, signal=34%"
GSE29618_PRE_VS_DAY7_POST_LAIV_FLU_VACCINE_PDC_DN
GSE29618_PRE_VS_DAY7_POST_LAIV_FLU_VACCINE_PDC_DN 140
0.44974366 1.8316293 0 2.02E-04 0.096
2714 "tags=44%, list=19%, signal=54%"
GSE3982_MAST_CELL_VS_TH2_DN GSE3982_MAST_CELL_VS_TH2_DN
148 0.4425076 1.8315998 0 2.02E-04
0.096 1493 "tags=31%, list=11%, signal=34%"
GSE360_CTRL_VS_L_MAJOR_DC_UP GSE360_CTRL_VS_L_MAJOR_DC_UP
150 0.44467452 1.8315698 0 2.01E-04
0.096 2716 "tags=43%, list=19%, signal=52%"
GSE6875_WT_VS_FOXP3_KO_TREG_DN GSE6875_WT_VS_FOXP3_KO_TREG_DN
126 0.44933307 1.8314185 0 2.01E-04
0.096 1873 "tags=27%, list=13%, signal=31%"
GSE37301_MULTIPOTENT_PROGENITOR_VS_RAG2_KO_NK_CELL_DN
GSE37301_MULTIPOTENT_PROGENITOR_VS_RAG2_KO_NK_CELL_DN
158 0.4372485 1.8311113 0 2.05E-04 0.098
2402 "tags=35%, list=17%, signal=42%"
GSE2585_CTEC_VS_THYMIC_MACROPHAGE_DN
GSE2585_CTEC_VS_THYMIC_MACROPHAGE_DN 149
0.44537422 1.8293625 0 2.17E-04 0.104
2322 "tags=38%, list=16%, signal=45%"
GSE17721_PAM3CSK4_VS_GADIQUIMOD_0.5H_BMDC_DN
GSE17721_PAM3CSK4_VS_GADIQUIMOD_0.5H_BMDC_DN 162
0.4379374 1.8290575 0 2.16E-04 0.104
2046 "tags=30%, list=14%, signal=34%"

GSE10325_BCELL_VS_LUPUS_BCELL_DN GSE10325_BCELL_VS_LUPUS_BCELL_DN
147 0.44312975 1.8289301 0 2.18E-04
0.104 1728 "tags=33%, list=12%, signal=38%"
GSE19941_IL10_KO_VS_IL10_KO_AND_NFKBP50_KO_LPS_STIM_MACROPHAGE_DN
GSE19941_IL10_KO_VS_IL10_KO_AND_NFKBP50_KO_LPS_STIM_MACROPHAGE_DN
148 0.44473726 1.8286531 0 2.17E-04
0.104 2294 "tags=39%, list=16%, signal=46%"
GSE2405_0H_VS_1.5H_A_PHAGOCYTOPHILUM_STIM_NEUTROPHIL_DN
GSE2405_0H_VS_1.5H_A_PHAGOCYTOPHILUM_STIM_NEUTROPHIL_DN
159 0.43524632 1.8281847 0 2.17E-04 0.104
2479 "tags=36%, list=18%, signal=43%"
GSE5099_MONOCYTE_VS_CLASSICAL_M1_MACROPHAGE_DN
GSE5099_MONOCYTE_VS_CLASSICAL_M1_MACROPHAGE_DN 138
0.44145438 1.8273174 0 2.19E-04 0.105
1956 "tags=33%, list=14%, signal=38%"
GSE17186_BLOOD_VS_CORD_BLOOD_NAIVE_BCELL_UP
GSE17186_BLOOD_VS_CORD_BLOOD_NAIVE_BCELL_UP 155
0.4409953 1.8263149 0 2.20E-04 0.106
1900 "tags=33%, list=13%, signal=38%"
GSE22140_GERMFREE_VS_SPF_ARTHRITIC_MOUSE_CD4_TCELL_DN
GSE22140_GERMFREE_VS_SPF_ARTHRITIC_MOUSE_CD4_TCELL_DN
141 0.44361293 1.8255975 0 2.22E-04 0.107
1905 "tags=33%, list=13%, signal=37%"
GSE17721_LPS_VS_PAM3CSK4_6H_BMDC_DN
GSE17721_LPS_VS_PAM3CSK4_6H_BMDC_DN 150
0.44160414 1.825571 0 2.22E-04 0.107
1939 "tags=32%, list=14%, signal=37%"
GSE4984_GALECTIN1_VS_VEHICLE_CTRL_TREATED_DC_DN
GSE4984_GALECTIN1_VS_VEHICLE_CTRL_TREATED_DC_DN 149
0.4431308 1.8252054 0 2.21E-04 0.107
2583 "tags=41%, list=18%, signal=50%"
GSE27786_LIN_NEG_VS_CD8_TCELL_UP GSE27786_LIN_NEG_VS_CD8_TCELL_UP
151 0.43614054 1.8242353 0 2.25E-04
0.108 2052 "tags=33%, list=15%, signal=38%"
GSE32423_IL7_VS_IL7_IL4_MEMORY_CD8_TCELL_DN
GSE32423_IL7_VS_IL7_IL4_MEMORY_CD8_TCELL_DN 141
0.44049683 1.8240587 0 2.27E-04 0.109
2324 "tags=34%, list=16%, signal=40%"
GSE19401_RETINOIC_ACID_VS_RETINOIC_ACID_AND_PAM2CSK4_STIM_FOLLICULAR_DC_DN
GSE19401_RETINOIC_ACID_VS_RETINOIC_ACID_AND_PAM2CSK4_STIM_FOLLICULAR_DC_DN 150 0.44155565 1.8233085 0
2.28E-04 0.11 2455 "tags=37%, list=17%, signal=45%"
GSE15330_GRANULOCYTE_MONOCYTE_PROGENITOR_VS_PRO_BCELL_UP
GSE15330_GRANULOCYTE_MONOCYTE_PROGENITOR_VS_PRO_BCELL_UP
144 0.43788972 1.8232919 0 2.28E-04 0.11
3114 "tags=51%, list=22%, signal=64%"
GSE15330_MEGAKARYOCYTE_ERYTHROID_VS_GRANULOCYTE_MONOCYTE_PROGENITOR_IKAROS_KO_DN
GSE15330_MEGAKARYOCYTE_ERYTHROID_VS_GRANULOCYTE_MONOCYTE_PROGENITOR_IKAROS_KO_DN 153 0.44329423 1.8230177
0 2.27E-04 0.11 1282 "tags=27%, list=9%, signal=29%"
GSE41978_WT_VS_ID2_KO_AND_BIM_KO_KLRG1_LOW_EFFECTOR_CD8_TCELL_UP
GSE41978_WT_VS_ID2_KO_AND_BIM_KO_KLRG1_LOW_EFFECTOR_CD8_TCELL_UP

148 0.4381092 1.8225901 0 2.35E-04
0.114 1679 "tags=27%, list=12%, signal=30%"
GSE3720_LPS_VS_PMA_STIM_VD1_GAMMADELTA_TCELL_DN
GSE3720_LPS_VS_PMA_STIM_VD1_GAMMADELTA_TCELL_DN 129
0.44639358 1.8201995 0 2.42E-04 0.117
2090 "tags=39%, list=15%, signal=45%"
GSE2770_UNTREATED_VS_IL4_TREATED_ACT_CD4_TCELL_6H_DN
GSE2770_UNTREATED_VS_IL4_TREATED_ACT_CD4_TCELL_6H_DN
145 0.44200164 1.8200208 0 2.42E-04 0.117
1506 "tags=25%, list=11%, signal=28%"
GSE45365_NK_CELL_VS_CD8A_DC_UP GSE45365_NK_CELL_VS_CD8A_DC_UP
155 0.43967015 1.8200033 0 2.42E-04
0.117 2487 "tags=39%, list=18%, signal=47%"
GSE23505_UNTREATED_VS_4DAY_IL6_IL1_TREATED_CD4_TCELL_UP
GSE23505_UNTREATED_VS_4DAY_IL6_IL1_TREATED_CD4_TCELL_UP
152 0.4415413 1.8191745 0 2.47E-04 0.12
2771 "tags=44%, list=20%, signal=54%"
GSE45365_HEALTHY_VS_MCMV_INFECTION_BCELL_IFNAR_KO_UP
GSE45365_HEALTHY_VS_MCMV_INFECTION_BCELL_IFNAR_KO_UP
140 0.44050345 1.8186941 0 2.51E-04 0.121
1744 "tags=26%, list=12%, signal=30%"
GSE9006_HEALTHY_VS_TYPE_1_DIABETES_PBMC_4MONTH_POST_DX_UP
GSE9006_HEALTHY_VS_TYPE_1_DIABETES_PBMC_4MONTH_POST_DX_UP
145 0.43985856 1.8168923 0 2.52E-04 0.122
1859 "tags=36%, list=13%, signal=41%"
GSE37416_0H_VS_24H_F_TULARENSIS_LVS_NEUTROPHIL_UP
GSE37416_0H_VS_24H_F_TULARENSIS_LVS_NEUTROPHIL_UP 135
0.44517478 1.8165883 0 2.54E-04 0.123
1932 "tags=32%, list=14%, signal=37%"
GSE9988_ANTI_TREM1_AND_LPS_VS_VEHICLE_TREATED_MONOCYTES_DN
GSE9988_ANTI_TREM1_AND_LPS_VS_VEHICLE_TREATED_MONOCYTES_DN
145 0.4390374 1.8162836 0 2.53E-04 0.123
2083 "tags=36%, list=15%, signal=42%"
GSE41867_DAY6_VS_DAY8_LCMV_ARMSTRONG_EFFECTOR_CD8_TCELL_DN
GSE41867_DAY6_VS_DAY8_LCMV_ARMSTRONG_EFFECTOR_CD8_TCELL_DN
144 0.44380397 1.8153663 0 2.55E-04 0.124
1972 "tags=34%, list=14%, signal=39%"
GSE7831_CPG_VS_INFLUENZA_STIM_PDC_1H_UP
GSE7831_CPG_VS_INFLUENZA_STIM_PDC_1H_UP 155
0.4311617 1.8149781 0 2.54E-04 0.124
2805 "tags=43%, list=20%, signal=53%"
GSE17721_PAM3CSK4_VS_GADIQUIMOD_1H_BMDC_DN
GSE17721_PAM3CSK4_VS_GADIQUIMOD_1H_BMDC_DN 159
0.4353567 1.8148464 0 2.56E-04 0.125
2656 "tags=40%, list=19%, signal=49%"
GSE27859_DC_VS_CD11C_INT_F480_HI_MACROPHAGE_UP
GSE27859_DC_VS_CD11C_INT_F480_HI_MACROPHAGE_UP 134
0.44530737 1.8144146 0 2.56E-04 0.125
2329 "tags=35%, list=17%, signal=42%"
GSE27786_CD8_TCELL_VS_NKTCELL_DN GSE27786_CD8_TCELL_VS_NKTCELL_DN
142 0.44398817 1.814366 0 2.55E-04 0.125
2394 "tags=39%, list=17%, signal=47%"
GSE339_CD4POS_VS_CD8POS_DC_IN_CULTURE_DN
GSE339_CD4POS_VS_CD8POS_DC_IN_CULTURE_DN 147

0.44050986	1.812706	0	2.62E-04	0.128
2722	"tags=43%, list=19%, signal=53%"
GSE32901_NAIVE_VS_TH1_CD4_TCELL_DN
GSE32901_NAIVE_VS_TH1_CD4_TCELL_DN	142	0.440842
1.8122282	0	2.62E-04	0.128	2043	"tags=36%,
list=14%, signal=42%"
GSE15330_WT_VS_IKAROS_KO_HSC_DN	GSE15330_WT_VS_IKAROS_KO_HSC_DN
149	0.43497294	1.8120302	0	2.62E-04
0.128	2122	"tags=36%, list=15%, signal=42%"
GSE16385_UNTREATED_VS_12H_ROSIGLITAZONE_IL4_TREATED_MACROPHAGE_UP
GSE16385_UNTREATED_VS_12H_ROSIGLITAZONE_IL4_TREATED_MACROPHAGE_UP
153	0.4362719	1.8117858	0	2.61E-04
0.128	2245	"tags=33%, list=16%, signal=39%"
GSE9509_10MIN_VS_30MIN_LPS_AND_IL10_STIM_IL10_KO_MACROPHAGE_UP
GSE9509_10MIN_VS_30MIN_LPS_AND_IL10_STIM_IL10_KO_MACROPHAGE_UP
136	0.44522944	1.8100517	0	2.65E-04
0.13	1602	"tags=27%, list=11%, signal=30%"
GSE17721_CTRL_VS_POLYIC_6H_BMDC_UP
GSE17721_CTRL_VS_POLYIC_6H_BMDC_UP	148
0.43668297	1.8097641	0	2.68E-04	0.131
1811	"tags=34%, list=13%, signal=39%"
GSE40274_SATB1_VS_FOXP3_AND_SATB1_TRANSDUCED_ACTIVATED_CD4_TCELL_UP
GSE40274_SATB1_VS_FOXP3_AND_SATB1_TRANSDUCED_ACTIVATED_CD4_TCELL_UP
134	0.44008708	1.8093016	0	2.68E-04
0.131	2196	"tags=31%, list=16%, signal=36%"
GSE31082_DP_VS_CD8_SP_THYMOCYTE_UP
GSE31082_DP_VS_CD8_SP_THYMOCYTE_UP	144
0.43669805	1.8035158	0	2.80E-04	0.138
2607	"tags=39%, list=18%, signal=47%"
GSE24210_RESTING_TREG_VS_TCONV_DN	GSE24210_RESTING_TREG_VS_TCONV_DN
145	0.43810517	1.8025763	0	2.84E-04
0.14	2668	"tags=42%, list=19%, signal=51%"
GSE37416_0H_VS_48H_F_TULARENSIS_LVS_NEUTROPHIL_UP
GSE37416_0H_VS_48H_F_TULARENSIS_LVS_NEUTROPHIL_UP	138
0.43992847	1.801921	0	2.87E-04	0.142
1893	"tags=34%, list=13%, signal=39%"
GSE4984_UNTREATED_VS_VEHICLE_CTRL_TREATED_DC_DN
GSE4984_UNTREATED_VS_VEHICLE_CTRL_TREATED_DC_DN	132
0.44587076	1.8007334	0	2.89E-04	0.143
2365	"tags=35%, list=17%, signal=41%"
GSE41867_LCMV_ARMSTRONG_VS_CLONE13_DAY6_EFFECTOR_CD8_TCELL_UP
GSE41867_LCMV_ARMSTRONG_VS_CLONE13_DAY6_EFFECTOR_CD8_TCELL_UP
140	0.43596417	1.8006501	0	2.88E-04
0.143	2685	"tags=44%, list=19%, signal=53%"
GSE2770_TGFB_AND_IL4_ACT_VS_ACT_CD4_TCELL_48H_DN
GSE2770_TGFB_AND_IL4_ACT_VS_ACT_CD4_TCELL_48H_DN	123
0.45024836	1.8000166	0	2.92E-04	0.145
1892	"tags=32%, list=13%, signal=36%"
GSE3982_EOSINOPHIL_VS_MAST_CELL_DN
GSE3982_EOSINOPHIL_VS_MAST_CELL_DN	140
0.44067213	1.7997121	0	2.95E-04	0.147
1750	"tags=29%, list=12%, signal=33%"
GSE2770_UNTREATED_VS_TGFB_AND_IL12_TREATED_ACT_CD4_TCELL_2H_DN
GSE2770_UNTREATED_VS_TGFB_AND_IL12_TREATED_ACT_CD4_TCELL_2H_DN

148	0.43161914	1.7991141	0	2.94E-04
0.147	969	"tags=20%, list=7%, signal=21%"
GSE5542_IFNA_VS_IFNA_AND_IFNG_TREATED_EPITHELIAL_CELLS_6H_UP
GSE5542_IFNA_VS_IFNA_AND_IFNG_TREATED_EPITHELIAL_CELLS_6H_UP
137	0.4383007	1.7970691	0	3.11E-04
0.155	2769	"tags=34%, list=20%, signal=41%"
GSE30962_ACUTE_VS_CHRONIC_LCMV_PRIMARY_INF_CD8_TCELL_DN
GSE30962_ACUTE_VS_CHRONIC_LCMV_PRIMARY_INF_CD8_TCELL_DN
153	0.43493527	1.7952961	0	3.18E-04	0.159
1415	"tags=35%, list=10%, signal=38%"
GSE25123_ROSIGLITAZONE_VS_IL4_AND_ROSIGLITAZONE_STIM_PPARG_KO_MACROP
HAGE_DAY10_UP
GSE25123_ROSIGLITAZONE_VS_IL4_AND_ROSIGLITAZONE_STIM_PPARG_KO_MACROP
HAGE_DAY10_UP	137	0.44289637	1.7935758
0	3.27E-04	0.163	1355	"tags=24%, list=10%, signal=26%"
GSE17721_ALL_VS_24H_PAM3CSK4_BMDC_UP
GSE17721_ALL_VS_24H_PAM3CSK4_BMDC_UP	154
0.43411595	1.7929428	0	3.28E-04	0.163
1847	"tags=35%, list=13%, signal=40%"
GSE31082_DN_VS_CD8_SP_THYMOCYTE_UP
GSE31082_DN_VS_CD8_SP_THYMOCYTE_UP	146
0.43095478	1.7928044	0	3.28E-04	0.163
2575	"tags=36%, list=18%, signal=44%"
GSE8678_IL7R_LOW_VS_HIGH_EFF_CD8_TCELL_UP
GSE8678_IL7R_LOW_VS_HIGH_EFF_CD8_TCELL_UP	146
0.43026802	1.7920961	0	3.35E-04	0.166
2539	"tags=47%, list=18%, signal=57%"
GSE37416_0H_VS_3H_F_TULARENSIS_LVS_NEUTROPHIL_UP
GSE37416_0H_VS_3H_F_TULARENSIS_LVS_NEUTROPHIL_UP	126
0.44024122	1.7913734	0	3.34E-04	0.166
2566	"tags=42%, list=18%, signal=51%"
GSE360_HIGH_VS_LOW_DOSE_B_MALAYI_DC_DN
GSE360_HIGH_VS_LOW_DOSE_B_MALAYI_DC_DN	145
0.43779114	1.7909219	0	3.35E-04	0.167
1022	"tags=21%, list=7%, signal=23%"
GSE17721_CTRL_VS_POLYIC_8H_BMDC_UP
GSE17721_CTRL_VS_POLYIC_8H_BMDC_UP	141
0.43198097	1.7904916	0	3.35E-04	0.167
1990	"tags=27%, list=14%, signal=31%"
GSE15930_STIM_VS_STIM_AND_IL12_72H_CD8_T_CELL_UP
GSE15930_STIM_VS_STIM_AND_IL12_72H_CD8_T_CELL_UP	145
0.43187767	1.7902601	0	3.36E-04	0.168
2226	"tags=40%, list=16%, signal=47%"
GSE27786_ERYTHROBLAST_VS_MONO_MAC_UP
GSE27786_ERYTHROBLAST_VS_MONO_MAC_UP	143
0.4339295	1.7898177	0	3.39E-04	0.17
2237	"tags=27%, list=16%, signal=31%"
GSE24142_EARLY_THYMIC_PROGENITOR_VS_DN3_THYMOCYTE_ADULT_DN
GSE24142_EARLY_THYMIC_PROGENITOR_VS_DN3_THYMOCYTE_ADULT_DN
147	0.4359033	1.7890652	0	3.46E-04	0.173
2481	"tags=39%, list=18%, signal=47%"
GSE19401_UNSTIM_VS_RETINOIC_ACID_STIM_FOLLICULAR_DC_UP
GSE19401_UNSTIM_VS_RETINOIC_ACID_STIM_FOLLICULAR_DC_UP
148	0.43538824	1.7877792	0	3.53E-04	0.177

1741 "tags=36%, list=12%, signal=40%"
GSE30962_PRIMARY_VS_SECONDARY_CHRONIC_LCMV_INF_CD8_TCELL_UP
GSE30962_PRIMARY_VS_SECONDARY_CHRONIC_LCMV_INF_CD8_TCELL_UP
152 0.43261304 1.7872978 0 3.54E-04 0.178
1704 "tags=36%, list=12%, signal=40%"
GSE17721_0.5H_VS_24H_PAM3CSK4_BMDC_UP
GSE17721_0.5H_VS_24H_PAM3CSK4_BMDC_UP 162
0.42659667 1.7863164 0 3.58E-04 0.18
2416 "tags=40%, list=17%, signal=47%"
GSE20152_SPHK1_KO_VS_HTNFA_OVEREXPRESS_ANKLE_DN
GSE20152_SPHK1_KO_VS_HTNFA_OVEREXPRESS_ANKLE_DN 134
0.4380871 1.7856404 0 3.57E-04 0.18
2154 "tags=34%, list=15%, signal=40%"
GSE22033_WT_VS_PPARG_KO_MEF_DN GSE22033_WT_VS_PPARG_KO_MEF_DN
139 0.4385107 1.7854531 0 3.57E-04
0.18 1949 "tags=27%, list=14%, signal=31%"
GSE3982_EOSINOPHIL_VS_BCELL_DN GSE3982_EOSINOPHIL_VS_BCELL_DN
131 0.44351444 1.7848167 0 3.60E-04
0.182 2473 "tags=37%, list=18%, signal=44%"
GSE15330_LYMPHOID_MULTIPOTENT_VS_GRANULOCYTE_MONOCYTE_PROGENITOR_UP
GSE15330_LYMPHOID_MULTIPOTENT_VS_GRANULOCYTE_MONOCYTE_PROGENITOR_UP
155 0.4244996 1.7847722 0 3.61E-04
0.183 2243 "tags=34%, list=16%, signal=40%"
GSE6092_UNSTIM_VS_IFNG_STIM_AND_B_BURGDORFERI_INF_ENDOTHELIAL_CELL_U
P
GSE6092_UNSTIM_VS_IFNG_STIM_AND_B_BURGDORFERI_INF_ENDOTHELIAL_CELL_U
P 132 0.43435517 1.7820905 0
3.69E-04 0.186 1912 "tags=26%, list=14%, signal=30%"
GSE31082_CD4_VS_CD8_SP_THYMOCYTE_DN
GSE31082_CD4_VS_CD8_SP_THYMOCYTE_DN 138
0.43607605 1.7817247 0 3.69E-04 0.186
2397 "tags=36%, list=17%, signal=43%"
GSE3982_MAC_VS_CENT_MEMORY_CD4_TCELL_UP
GSE3982_MAC_VS_CENT_MEMORY_CD4_TCELL_UP 150
0.42798847 1.7814873 0 3.70E-04 0.187
2393 "tags=39%, list=17%, signal=46%"
GSE39820_CTRL_VS_TGFBETA1_IL6_IL23A_CD4_TCELL_DN
GSE39820_CTRL_VS_TGFBETA1_IL6_IL23A_CD4_TCELL_DN 146
0.42870703 1.7807761 0 3.77E-04 0.19
1977 "tags=32%, list=14%, signal=36%"
GSE32423_CTRL_VS_IL7_IL4_MEMORY_CD8_TCELL_DN
GSE32423_CTRL_VS_IL7_IL4_MEMORY_CD8_TCELL_DN 140
0.43685302 1.7807232 0 3.78E-04 0.191
1980 "tags=31%, list=14%, signal=36%"
GSE15330_WT_VS_IKAROS_KO_MEGAKARYOCYTE_ERYTHROID_PROGENITOR_UP
GSE15330_WT_VS_IKAROS_KO_MEGAKARYOCYTE_ERYTHROID_PROGENITOR_UP
147 0.43042612 1.7806168 0 3.78E-04
0.191 2186 "tags=33%, list=15%, signal=39%"
GSE10211_UV_INACT_SENDAI_VS_LIVE_SENDAI_VIRUS_TRACHEAL_EPITHELIAL_CE
LLS_DN
GSE10211_UV_INACT_SENDAI_VS_LIVE_SENDAI_VIRUS_TRACHEAL_EPITHELIAL_CE
LLS_DN 132 0.4355403 1.7805637 0
3.77E-04 0.191 1018 "tags=21%, list=7%, signal=23%"
GSE42021_CD24INT_TREG_VS_CD24INT_TCONV_THYMUS_UP

GSE42021_CD24INT_TREG_VS_CD24INT_TCONV_THYMUS_UP 160
0.42754906 1.779352 0 3.87E-04 0.195
2792 "tags=47%, list=20%, signal=58%"
GSE32533_MIR17_KO_VS_MIR17_OVEREXPRESS_ACT_CD4_TCELL_UP
GSE32533_MIR17_KO_VS_MIR17_OVEREXPRESS_ACT_CD4_TCELL_UP
145 0.43123898 1.7761041 0 4.03E-04 0.199
2132 "tags=32%, list=15%, signal=37%"
GSE7852_TREG_VS_TCONV_FAT_DN GSE7852_TREG_VS_TCONV_FAT_DN
147 0.4332224 1.7757585 0 4.04E-04
0.2 2274 "tags=38%, list=16%, signal=45%"
GSE17974_0H_VS_12H_IN_VITRO_ACT_CD4_TCELL_DN
GSE17974_0H_VS_12H_IN_VITRO_ACT_CD4_TCELL_DN 133
0.4369197 1.7753143 0 4.09E-04 0.203
1737 "tags=31%, list=12%, signal=35%"
GSE17721_CTRL_VS_PAM3CSK4_1H_BMDC_UP
GSE17721_CTRL_VS_PAM3CSK4_1H_BMDC_UP 147
0.43132934 1.77516 0 4.14E-04 0.206
2118 "tags=33%, list=15%, signal=38%"
GSE12001_MIR223_KO_VS_WT_NEUTROPHIL_UP
GSE12001_MIR223_KO_VS_WT_NEUTROPHIL_UP 141
0.43108836 1.7744284 0 4.13E-04 0.206
1827 "tags=34%, list=13%, signal=39%"
GSE25123_CTRL_VS_IL4_STIM_MACROPHAGE_UP
GSE25123_CTRL_VS_IL4_STIM_MACROPHAGE_UP 128
0.4317647 1.7725366 0 4.29E-04 0.213
2267 "tags=32%, list=16%, signal=38%"
GSE26030_TH1_VS_TH17_RESTIMULATED_DAY5_POST_POLARIZATION_DN
GSE26030_TH1_VS_TH17_RESTIMULATED_DAY5_POST_POLARIZATION_DN
142 0.42819628 1.772156 0 4.30E-04 0.214
2428 "tags=35%, list=17%, signal=42%"
GSE3982_EOSINOPHIL_VS_CENT_MEMORY_CD4_TCELL_DN
GSE3982_EOSINOPHIL_VS_CENT_MEMORY_CD4_TCELL_DN 139
0.4318371 1.7720853 0 4.29E-04 0.214
1061 "tags=22%, list=8%, signal=23%"
GSE27786_LIN_NEG_VS_BCELL_UP GSE27786_LIN_NEG_VS_BCELL_UP
149 0.42488584 1.7711746 0 4.34E-04
0.216 2331 "tags=32%, list=17%, signal=37%"
GSE6269_FLU_VS_STREP_PNEUMO_INF_PBMC_UP
GSE6269_FLU_VS_STREP_PNEUMO_INF_PBMC_UP 107
0.44458088 1.7688656 0 4.48E-04 0.222
2358 "tags=43%, list=17%, signal=51%"
GSE18804_SPLEEN_MACROPHAGE_VS_COLON_TUMORAL_MACROPHAGE_UP
GSE18804_SPLEEN_MACROPHAGE_VS_COLON_TUMORAL_MACROPHAGE_UP
153 0.42156157 1.7686585 0 4.47E-04 0.222
2255 "tags=33%, list=16%, signal=39%"
GSE40277_EOS_AND_LEF1_TRANSDUCED_VS_GATA1_AND_SATB1_TRANSDUCED_CD4_T
CELL_DN
GSE40277_EOS_AND_LEF1_TRANSDUCED_VS_GATA1_AND_SATB1_TRANSDUCED_CD4_T
CELL_DN 156 0.42418292 1.7686331 0
4.46E-04 0.222 1863 "tags=31%, list=13%, signal=35%"
GSE21927_C26GM_VS_4T1_TUMOR_MONOCYTE_BALBC_UP
GSE21927_C26GM_VS_4T1_TUMOR_MONOCYTE_BALBC_UP 147
0.4301312 1.7673826 0 4.51E-04 0.225
2052 "tags=32%, list=15%, signal=37%"

GSE3982_DC_VS_NEUTROPHIL_LPS_STIM_UP
GSE3982_DC_VS_NEUTROPHIL_LPS_STIM_UP 145
0.42488116 1.7669698 0 4.52E-04 0.226
2375 "tags=37%, list=17%, signal=43%"
GSE40184_HEALTHY_VS_HCV_INFECTED_DONOR_PBMC_DN
GSE40184_HEALTHY_VS_HCV_INFECTED_DONOR_PBMC_DN 160
0.42028594 1.766343 0 4.58E-04 0.23
2039 "tags=31%, list=14%, signal=36%"
GSE17721_CTRL_VS_POLYIC_4H_BMDC_UP
GSE17721_CTRL_VS_POLYIC_4H_BMDC_UP 147
0.4284545 1.7646879 0 4.68E-04 0.234
1973 "tags=29%, list=14%, signal=33%"
GSE7831_1H_VS_4H_INFLUENZA_STIM_PDC_DN
GSE7831_1H_VS_4H_INFLUENZA_STIM_PDC_DN 143
0.43074784 1.7640945 0 4.73E-04 0.236
1720 "tags=27%, list=12%, signal=30%"
GSE8921_UNSTIM_VS_TLR1_2_STIM_MONOCYTE_3H_UP
GSE8921_UNSTIM_VS_TLR1_2_STIM_MONOCYTE_3H_UP 141
0.4313486 1.7640874 0.002222222 4.72E-04 0.236
1480 "tags=26%, list=10%, signal=28%"
GSE27786_LSK_VS_NKCELL_DN GSE27786_LSK_VS_NKCELL_DN 155
0.42547494 1.7638425 0 4.73E-04 0.237
2614 "tags=41%, list=19%, signal=50%"
GSE29617_CTRL_VS_DAY3_TIV_FLU_VACCINE_PBMC_2008_DN
GSE29617_CTRL_VS_DAY3_TIV_FLU_VACCINE_PBMC_2008_DN 141
0.43542567 1.7632787 0 4.83E-04 0.242
2391 "tags=40%, list=17%, signal=48%"
GSE17721_CTRL_VS_CPG_1H_BMDC_UP GSE17721_CTRL_VS_CPG_1H_BMDC_UP
149 0.42491594 1.7632246 0 4.82E-04
0.242 2505 "tags=40%, list=18%, signal=48%"
GSE6875_TCONV_VS_TREG_DN GSE6875_TCONV_VS_TREG_DN 139
0.42574328 1.7630719 0 4.82E-04 0.242
2249 "tags=27%, list=16%, signal=31%"
GSE22140_HEALTHY_VS_ARTHRITIC_MOUSE_CD4_TCELL_UP
GSE22140_HEALTHY_VS_ARTHRITIC_MOUSE_CD4_TCELL_UP 151
0.42270714 1.7618855 0 4.99E-04 0.251
1920 "tags=30%, list=14%, signal=34%"
GSE27241_WT_VS_RORGT_KO_TH17_POLARIZED_CD4_TCELL_TREATED_WITH_DIGOXIN_UP
GSE27241_WT_VS_RORGT_KO_TH17_POLARIZED_CD4_TCELL_TREATED_WITH_DIGOXIN_UP 132 0.43551698 1.7597723 0
5.14E-04 0.257 1820 "tags=26%, list=13%, signal=29%"
GSE33374_CD8_ALPHAALPHA_VS_ALPHABETA_CD161_HIGH_TCELL_DN
GSE33374_CD8_ALPHAALPHA_VS_ALPHABETA_CD161_HIGH_TCELL_DN
162 0.42137134 1.7593415 0 5.13E-04 0.257
1982 "tags=36%, list=14%, signal=42%"
GSE14415_INDUCED_TREG_VS_FAILED_INDUCED_TREG_DN
GSE14415_INDUCED_TREG_VS_FAILED_INDUCED_TREG_DN 141
0.43422303 1.7588556 0 5.14E-04 0.257
1679 "tags=35%, list=12%, signal=40%"
GSE17721_0.5H_VS_12H_PAM3CSK4_BMDC_DN
GSE17721_0.5H_VS_12H_PAM3CSK4_BMDC_DN 152
0.4292747 1.758024 0.002136752 5.22E-04 0.261
2053 "tags=31%, list=15%, signal=36%"

GSE37605_FOXP3_FUSION_GFP_VS_IRES_GFP_TREG_NOD_UP
GSE37605_FOXP3_FUSION_GFP_VS_IRES_GFP_TREG_NOD_UP 152
0.4241378 1.756834 0 5.33E-04 0.268
2327 "tags=39%, list=16%, signal=46%"
GSE17721_0.5H_VS_24H_GARDIQUIMOD_BMDC_UP
GSE17721_0.5H_VS_24H_GARDIQUIMOD_BMDC_UP 154
0.42084014 1.755841 0 5.43E-04 0.273
2648 "tags=39%, list=19%, signal=47%"
GSE17721_CTRL_VS_PAM3CSK4_0.5H_BMDC_UP
GSE17721_CTRL_VS_PAM3CSK4_0.5H_BMDC_UP 146
0.42249876 1.7553325 0 5.46E-04 0.275
2539 "tags=38%, list=18%, signal=45%"
GSE5589_WT_VS_IL10_KO_LPS_AND_IL10_STIM_MACROPHAGE_45MIN_UP
GSE5589_WT_VS_IL10_KO_LPS_AND_IL10_STIM_MACROPHAGE_45MIN_UP
145 0.42597806 1.7553233 0 5.45E-04 0.275
1582 "tags=26%, list=11%, signal=29%"
GSE14908_ATOPIC_VS_NONATOPIC_PATIENT_HDM_STIM_CD4_TCELL_DN
GSE14908_ATOPIC_VS_NONATOPIC_PATIENT_HDM_STIM_CD4_TCELL_DN
137 0.42379722 1.7543087 0 5.53E-04 0.28
1559 "tags=28%, list=11%, signal=31%"
GSE37301_MULTIPOTENT_PROGENITOR_VS_COMMON_LYMPHOID_PROGENITOR_DN
GSE37301_MULTIPOTENT_PROGENITOR_VS_COMMON_LYMPHOID_PROGENITOR_DN
162 0.41851452 1.7535444 0 5.57E-04
0.283 2383 "tags=33%, list=17%, signal=40%"
GSE5503_LIVER_DC_VS_MLN_DC_ACTIVATED_ALLOGENIC_TCELL_UP
GSE5503_LIVER_DC_VS_MLN_DC_ACTIVATED_ALLOGENIC_TCELL_UP
150 0.4187717 1.752091 0 5.75E-04 0.293
1442 "tags=20%, list=10%, signal=22%"
GSE31082_DP_VS_CD4_SP_THYMOCYTE_UP
GSE31082_DP_VS_CD4_SP_THYMOCYTE_UP 128
0.42663565 1.7495794 0 5.97E-04 0.302
2166 "tags=40%, list=15%, signal=47%"
GSE27786_LSK_VS_NKTCELL_UP GSE27786_LSK_VS_NKTCELL_UP
145 0.42344677 1.7472581 0 6.24E-04
0.316 2268 "tags=31%, list=16%, signal=37%"
GSE2770_UNTREATED_VS_IL12_TREATED_ACT_CD4_TCELL_2H_UP
GSE2770_UNTREATED_VS_IL12_TREATED_ACT_CD4_TCELL_2H_UP
141 0.42423594 1.7472312 0 6.23E-04 0.316
1742 "tags=33%, list=12%, signal=38%"
GSE24081_CONTROLLER_VS_PROGRESSOR_HIV_SPECIFIC_CD8_TCELL_DN
GSE24081_CONTROLLER_VS_PROGRESSOR_HIV_SPECIFIC_CD8_TCELL_DN
123 0.43681067 1.746241 0 6.25E-04 0.317
2068 "tags=31%, list=15%, signal=36%"
GSE14415_INDUCED_TREG_VS_FOXP3_KO_INDUCED_TREG_DN
GSE14415_INDUCED_TREG_VS_FOXP3_KO_INDUCED_TREG_DN 153
0.4197917 1.7446338 0 6.47E-04 0.326
2431 "tags=46%, list=17%, signal=55%"
GSE44732_UNSTIM_VS_IL27_STIM_IMATURE_DC_DN
GSE44732_UNSTIM_VS_IL27_STIM_IMATURE_DC_DN 139
0.42537054 1.7437774 0 6.52E-04 0.328
1846 "tags=30%, list=13%, signal=34%"
GSE3920_IFNA_VS_IFNG_TREATED_ENDOTHELIAL_CELL_DN
GSE3920_IFNA_VS_IFNG_TREATED_ENDOTHELIAL_CELL_DN 135
0.42776456 1.7432312 0 6.62E-04 0.333

1365 "tags=23%, list=10%, signal=25%"
GSE45365_WT_VS_IFNAR_KO_CD11B_DC_DN
GSE45365_WT_VS_IFNAR_KO_CD11B_DC_DN 121
0.4275737 1.7423879 0 6.71E-04 0.337
1073 "tags=18%, list=8%, signal=20%"
GSE17721_LPS_VS_CPG_24H_BMDC_DN GSE17721_LPS_VS_CPG_24H_BMDC_DN
155 0.41541517 1.7421826 0 6.72E-04
0.338 1949 "tags=26%, list=14%, signal=30%"
GSE3982_MAC_VS_NEUTROPHIL_LPS_STIM_UP
GSE3982_MAC_VS_NEUTROPHIL_LPS_STIM_UP 143
0.4228622 1.7418915 0 6.74E-04 0.34
2122 "tags=30%, list=15%, signal=35%"
GSE17721_LPS_VS_CPG_16H_BMDC_DN GSE17721_LPS_VS_CPG_16H_BMDC_DN
155 0.41438875 1.7414981 0 6.75E-04
0.341 2205 "tags=28%, list=16%, signal=33%"
GSE19401_UNSTIM_VS_RETINOIC_ACID_AND_PAM2CSK4_STIM_FOLLICULAR_DC_UP
GSE19401_UNSTIM_VS_RETINOIC_ACID_AND_PAM2CSK4_STIM_FOLLICULAR_DC_UP
145 0.4196221 1.7408631 0 6.81E-04
0.343 1910 "tags=31%, list=14%, signal=36%"
GSE7831_UNSTIM_VS_INFLUENZA_STIM_PDC_1H_UP
GSE7831_UNSTIM_VS_INFLUENZA_STIM_PDC_1H_UP 148
0.42010945 1.7403877 0 6.85E-04 0.344
2106 "tags=34%, list=15%, signal=39%"
GSE36476_CTRL_VS_TSST_ACT_16H_MEMORY_CD4_TCELL_OLD_DN
GSE36476_CTRL_VS_TSST_ACT_16H_MEMORY_CD4_TCELL_OLD_DN
144 0.41983214 1.7402912 0 6.84E-04 0.344
2378 "tags=39%, list=17%, signal=46%"
GSE17721_CTRL_VS_GARDIQUIMOD_1H_BMDC_UP
GSE17721_CTRL_VS_GARDIQUIMOD_1H_BMDC_UP 152
0.4203001 1.740276 0.001934236 6.83E-04 0.344
1787 "tags=28%, list=13%, signal=32%"
GSE16385_IFNG_TNF_VS_IL4_STIM_MACROPHAGE_ROSIGLITAZONE_TREATED_DN
GSE16385_IFNG_TNF_VS_IL4_STIM_MACROPHAGE_ROSIGLITAZONE_TREATED_DN
141 0.4199341 1.7390686 0 6.94E-04
0.348 1717 "tags=30%, list=12%, signal=34%"
GSE14308_INDUCED_VS_NATURAL_TREG_UP
GSE14308_INDUCED_VS_NATURAL_TREG_UP 137
0.42338184 1.7387793 0 6.98E-04 0.348
2028 "tags=33%, list=14%, signal=38%"
GSE17721_0.5H_VS_12H_CPG_BMDC_UP GSE17721_0.5H_VS_12H_CPG_BMDC_UP
151 0.4169375 1.7387627 0 6.97E-04
0.348 2379 "tags=36%, list=17%, signal=43%"
GSE29617_CTRL_VS_DAY7_TIV_FLU_VACCINE_PBMC_2008_DN
GSE29617_CTRL_VS_DAY7_TIV_FLU_VACCINE_PBMC_2008_DN 121
0.43073142 1.738061 0 6.99E-04 0.349
2072 "tags=34%, list=15%, signal=39%"
GSE11961_MARGINAL_ZONE_BCELL_VS_GERMINAL_CENTER_BCELL_DAY7_DN
GSE11961_MARGINAL_ZONE_BCELL_VS_GERMINAL_CENTER_BCELL_DAY7_DN
137 0.4296988 1.7380229 0 6.98E-04
0.349 1007 "tags=23%, list=7%, signal=24%"
GSE6259_DEC205_POS_DC_VS_BCELL_DN GSE6259_DEC205_POS_DC_VS_BCELL_DN
132 0.42634425 1.7375631 0.002087683
7.01E-04 0.351 2248 "tags=39%, list=16%, signal=46%"
GSE7831_UNSTIM_VS_INFLUENZA_STIM_PDC_4H_DN

GSE7831_UNSTIM_VS_INFLUENZA_STIM_PDC_4H_DN 147
0.41918156 1.7360234 0 7.11E-04 0.355
2103 "tags=36%, list=15%, signal=42%"
GSE7768_OVA_WITH_LPS_VS_OVA_WITH_MPL_IMMUNIZED_MOUSE_WHOLE_SPLEEN_6H_UP
GSE7768_OVA_WITH_LPS_VS_OVA_WITH_MPL_IMMUNIZED_MOUSE_WHOLE_SPLEEN_6H_UP 133 0.42204043 1.7356408 0
7.15E-04 0.356 1482 "tags=25%, list=11%, signal=27%"
GSE17721_PAM3CSK4_VS_GADIQUIMOD_12H_BMDC_UP
GSE17721_PAM3CSK4_VS_GADIQUIMOD_12H_BMDC_UP 155
0.4159739 1.7331204 0 7.49E-04 0.366
2879 "tags=43%, list=20%, signal=54%"
GSE36392_EOSINOPHIL_VS_NEUTROPHIL_IL25_TREATED_LUNG_DN
GSE36392_EOSINOPHIL_VS_NEUTROPHIL_IL25_TREATED_LUNG_DN
150 0.4172725 1.7321897 0 7.58E-04 0.369
2864 "tags=46%, list=20%, signal=57%"
GSE360_L_MAJOR_VS_B_MALAYI_HIGH_DOSE_MAC_DN
GSE360_L_MAJOR_VS_B_MALAYI_HIGH_DOSE_MAC_DN 152
0.41429982 1.7314386 0 7.70E-04 0.374
2686 "tags=36%, list=19%, signal=43%"
GSE40666_STAT1_KO_VS_STAT4_KO_CD8_TCELL_UP
GSE40666_STAT1_KO_VS_STAT4_KO_CD8_TCELL_UP 137
0.42552128 1.7308556 0 7.74E-04 0.376
2766 "tags=47%, list=20%, signal=58%"
GSE17721_CPG_VS_GARDIQUIMOD_1H_BMDC_DN
GSE17721_CPG_VS_GARDIQUIMOD_1H_BMDC_DN 154
0.41598752 1.7291394 0 7.96E-04 0.384
2297 "tags=37%, list=16%, signal=44%"
GSE17721_LPS_VS_CPG_4H_BMDC_DN GSE17721_LPS_VS_CPG_4H_BMDC_DN
143 0.42161602 1.7275916 0 8.12E-04
0.389 2536 "tags=37%, list=18%, signal=45%"
GSE17721_LPS_VS_PAM3CSK4_12H_BMDC_DN
GSE17721_LPS_VS_PAM3CSK4_12H_BMDC_DN 154
0.4163272 1.7265486 0 8.24E-04 0.393
2063 "tags=31%, list=15%, signal=36%"
GSE13738_RESTING_VS_BYSTANDER_ACTIVATED_CD4_TCELL_DN
GSE13738_RESTING_VS_BYSTANDER_ACTIVATED_CD4_TCELL_DN
147 0.41752592 1.7246742 0 8.49E-04 0.403
1976 "tags=32%, list=14%, signal=37%"
GSE17721_0.5H_VS_8H_GARDIQUIMOD_BMDC_UP
GSE17721_0.5H_VS_8H_GARDIQUIMOD_BMDC_UP 147
0.41658503 1.7245843 0 8.48E-04 0.403
1977 "tags=35%, list=14%, signal=40%"
GSE9037_WT_VS_IRAK4_KO_BMDM_DN GSE9037_WT_VS_IRAK4_KO_BMDM_DN
142 0.4213122 1.7245767 0 8.47E-04
0.403 2050 "tags=25%, list=15%, signal=29%"
GSE9509_10MIN_VS_30MIN_LPS_STIM_IL10_KO_MACROPHAGE_UP
GSE9509_10MIN_VS_30MIN_LPS_STIM_IL10_KO_MACROPHAGE_UP
144 0.41400373 1.7244364 0 8.47E-04 0.404
1935 "tags=36%, list=14%, signal=41%"
GSE23321_CD8_STEM_CELL_MEMORY_VS_CENTRAL_MEMORY_CD8_TCELL_DN
GSE23321_CD8_STEM_CELL_MEMORY_VS_CENTRAL_MEMORY_CD8_TCELL_DN
125 0.42727464 1.7230366 0 8.59E-04
0.409 2503 "tags=38%, list=18%, signal=45%"

GSE360_DC_VS_MAC_DN	GSE360_DC_VS_MAC_DN		147
0.41546208	1.7227689	0	8.58E-04 0.409
2025	"tags=32%, list=14%, signal=37%"
GSE32533_WT_VS_MIR17_KO_ACT_CD4_TCELL_DN
GSE32533_WT_VS_MIR17_KO_ACT_CD4_TCELL_DN		139
0.4209421	1.7224561	0.002057613	8.59E-04 0.409
2892	"tags=44%, list=20%, signal=55%"
GSE339_CD4POS_VS_CD4CD8DN_DC_IN_CULTURE_DN
GSE339_CD4POS_VS_CD4CD8DN_DC_IN_CULTURE_DN	155
0.4111531	1.7214234	0	8.70E-04 0.415
2190	"tags=28%, list=16%, signal=33%"
GSE19941_UNSTIM_VS_LPS_AND_IL10_STIM_IL10_KO_NFKBP50_KO_MACROPHAGE_D
N
GSE19941_UNSTIM_VS_LPS_AND_IL10_STIM_IL10_KO_NFKBP50_KO_MACROPHAGE_D
N	147	0.41555882	1.7213494	0
8.69E-04 0.415	1784	"tags=24%, list=13%, signal=28%"
GSE22886_IGG_IGA_MEMORY_BCELL_VS_BLOOD_PLASMA_CELL_DN
GSE22886_IGG_IGA_MEMORY_BCELL_VS_BLOOD_PLASMA_CELL_DN
142	0.41560692	1.7213423	0	8.68E-04 0.415
2052	"tags=32%, list=15%, signal=37%"
GSE27786_CD4_TCELL_VS_NKCELL_DN	GSE27786_CD4_TCELL_VS_NKCELL_DN
	148	0.41428375	1.7208519	0	8.78E-04
0.419	3160	"tags=46%, list=22%, signal=59%"
GSE3720_UNSTIM_VS_LPS_STIM_VD1_GAMMADELTA_TCELL_UP
GSE3720_UNSTIM_VS_LPS_STIM_VD1_GAMMADELTA_TCELL_UP		135
0.4149178	1.7204264	0	8.83E-04 0.422
2688	"tags=37%, list=19%, signal=45%"
GSE17721_PAM3CSK4_VS_CPG_0.5H_BMDC_DN
GSE17721_PAM3CSK4_VS_CPG_0.5H_BMDC_DN		150
0.41680804	1.7188841	0	9.00E-04 0.429
1824	"tags=23%, list=13%, signal=26%"
GSE2770_IL12_ACT_VS_ACT_CD4_TCELL_6H_UP
GSE2770_IL12_ACT_VS_ACT_CD4_TCELL_6H_UP		133
0.42106563	1.7178504	0.002020202	9.09E-04 0.432
1886	"tags=32%, list=13%, signal=37%"
GSE15659_NAIVE_CD4_TCELL_VS_ACTIVATED_TREG_DN
GSE15659_NAIVE_CD4_TCELL_VS_ACTIVATED_TREG_DN		131
0.42269352	1.7178104	0	9.07E-04 0.432
1702	"tags=21%, list=12%, signal=24%"
GSE2585_AIRE_KO_VS_WT_CD80_HIGH_MTEC_UP
GSE2585_AIRE_KO_VS_WT_CD80_HIGH_MTEC_UP		151
0.41240042	1.7174169	0	9.08E-04 0.432
2280	"tags=32%, list=16%, signal=38%"
GSE16385_ROSIGLITAZONE_IL4_VS_ROSIGLITAZONE_ALONE_STIM_MACROPHAGE_DN

GSE16385_ROSIGLITAZONE_IL4_VS_ROSIGLITAZONE_ALONE_STIM_MACROPHAGE_DN
	156	0.4126134	1.71704	0	9.13E-04
0.436	1483	"tags=21%, list=11%, signal=23%"
GSE27786_BCELL_VS_MONO_MAC_DN	GSE27786_BCELL_VS_MONO_MAC_DN
	149	0.4127313	1.7166439	0.001980198
9.13E-04 0.437	2311	"tags=29%, list=16%, signal=34%"
GSE3982_NEUTROPHIL_VS_EFF_MEMORY_CD4_TCELL_DN
GSE3982_NEUTROPHIL_VS_EFF_MEMORY_CD4_TCELL_DN		135
0.42241246	1.7165817	0	9.12E-04 0.437

1791 "tags=29%, list=13%, signal=33%"
GSE21670_UNTREATED_VS_TGFB_IL6_TREATED_CD4_TCELL_DN
GSE21670_UNTREATED_VS_TGFB_IL6_TREATED_CD4_TCELL_DN
105 0.43140125 1.715659 0 9.24E-04 0.443
2647 "tags=39%, list=19%, signal=48%"
GSE28726_NAIVE_VS_ACTIVATED_NKTCELL_DN
GSE28726_NAIVE_VS_ACTIVATED_NKTCELL_DN 153
0.40469941 1.7141031 0 9.48E-04 0.448
2420 "tags=41%, list=17%, signal=49%"
GSE40068_CXCR5NEG_BCL6NEG_CD4_TCELL_VS_CXCR5POS_BCL6NEG_TFH_DN
GSE40068_CXCR5NEG_BCL6NEG_CD4_TCELL_VS_CXCR5POS_BCL6NEG_TFH_DN
161 0.40582362 1.7140617 0 9.47E-04
0.448 1702 "tags=35%, list=12%, signal=40%"
GSE26669_CTRL_VS_COSTIM_BLOCK_MLR_CD4_TCELL_UP
GSE26669_CTRL_VS_COSTIM_BLOCK_MLR_CD4_TCELL_UP 140
0.4149378 1.713751 0 9.52E-04 0.449
2389 "tags=40%, list=17%, signal=48%"
GSE40274_CTRL_VS_FOXP3_AND_PBX1_TRANSDUCED_ACTIVATED_CD4_TCELL_DN
GSE40274_CTRL_VS_FOXP3_AND_PBX1_TRANSDUCED_ACTIVATED_CD4_TCELL_DN
121 0.42815414 1.7120692 0.00209205
9.73E-04 0.458 2791 "tags=38%, list=20%, signal=47%"
GSE5960_TH1_VS_ANERGIC_TH1_DN GSE5960_TH1_VS_ANERGIC_TH1_DN
167 0.40794784 1.711562 0 9.78E-04 0.46
2228 "tags=35%, list=16%, signal=41%"
GSE41867_NAIVE_VS_EFFECTOR_CD8_TCELL_DN
GSE41867_NAIVE_VS_EFFECTOR_CD8_TCELL_DN 142
0.42142835 1.7108586 0 9.91E-04 0.464
2420 "tags=35%, list=17%, signal=42%"
GSE25677_R848_VS_MPL_AND_R848_STIM_BCELL_DN
GSE25677_R848_VS_MPL_AND_R848_STIM_BCELL_DN 126
0.41822153 1.7097559 0.002132196 0.001010808
0.471 2091 "tags=35%, list=15%, signal=41%"
GSE360_L_DONOVANI_VS_B_MALAYI_LOW_DOSE_DC_DN
GSE360_L_DONOVANI_VS_B_MALAYI_LOW_DOSE_DC_DN 153
0.4152448 1.7095271 0 0.001011038 0.471
2319 "tags=37%, list=16%, signal=43%"
GSE20152_SPHK1_KO_VS_WT_HTNFA_OVERXPRESS_ANKLE_DN
GSE20152_SPHK1_KO_VS_WT_HTNFA_OVERXPRESS_ANKLE_DN 130
0.42045686 1.7063305 0.002227172 0.00106538
0.488 2008 "tags=32%, list=14%, signal=37%"
GSE6269_HEALTHY_VS_STAPH_PNEUMO_INF_PBMC_UP
GSE6269_HEALTHY_VS_STAPH_PNEUMO_INF_PBMC_UP 130
0.4177004 1.7059225 0 0.00106867 0.49
1854 "tags=32%, list=13%, signal=36%"
GSE3720_VD1_VS_VD2_GAMMADELTA_TCELL_UP
GSE3720_VD1_VS_VD2_GAMMADELTA_TCELL_UP 156
0.40701628 1.7044055 0 0.001100511 0.504
1841 "tags=27%, list=13%, signal=31%"
GSE5542_UNTREATED_VS_IFNA_AND_IFNG_TREATED_EPITHELIAL_CELLS_24H_UP
GSE5542_UNTREATED_VS_IFNA_AND_IFNG_TREATED_EPITHELIAL_CELLS_24H_UP
130 0.4137904 1.7041712 0
0.001099025 0.504 1726 "tags=32%, list=12%, signal=36%"
GSE17721_CTRL_VS_PAM3CSK4_8H_BMDC_UP
GSE17721_CTRL_VS_PAM3CSK4_8H_BMDC_UP 146

0.41120285	1.7039543	0	0.001099095	0.504
1243	"tags=25%, list=9%, signal=27%"
GSE14308_TH17_VS_NAIVE_CD4_TCELL_UP
GSE14308_TH17_VS_NAIVE_CD4_TCELL_UP		141
0.40761074	1.703338 0	0.001102394	0.506
2271	"tags=33%, list=16%, signal=39%"
GSE15330_HSC_VS_PRO_BCELL_DN	GSE15330_HSC_VS_PRO_BCELL_DN
	145	0.40987214	1.7028477	0
0.00110404	0.507	1857	"tags=30%, list=13%, signal=35%"
GSE24726_WT_VS_E2_2_KO_PDC_UP	GSE24726_WT_VS_E2_2_KO_PDC_UP
	141	0.4171976	1.7018771	0
0.001123105	0.513	1241	"tags=27%, list=9%, signal=29%"
GSE2770_IL4_ACT_VS_ACT_CD4_TCELL_6H_UP
GSE2770_IL4_ACT_VS_ACT_CD4_TCELL_6H_UP		126
0.41571927	1.6989745	0	0.001170409	0.522
2094	"tags=37%, list=15%, signal=42%"
GSE22886_NAIVE_CD4_TCELL_VS_NKCELL_DN
GSE22886_NAIVE_CD4_TCELL_VS_NKCELL_DN		146
0.40728027	1.69886 0	0.001170462	0.522
2531	"tags=40%, list=18%, signal=49%"
GSE29618_BCELL_VS_MONOCYTE_DN	GSE29618_BCELL_VS_MONOCYTE_DN
	160	0.40227136	1.6973538	0
0.001192484	0.529	1847	"tags=32%, list=13%, signal=36%"
GSE37301_COMMON_LYMPHOID_PROGENITOR_VS_CD4_TCELL_DN
GSE37301_COMMON_LYMPHOID_PROGENITOR_VS_CD4_TCELL_DN
152	0.40515268	1.6971345	0	0.0011987
0.53	1912	"tags=30%, list=14%, signal=35%"
GSE22886_NAIVE_VS_MEMORY_TCELL_DN GSE22886_NAIVE_VS_MEMORY_TCELL_DN
	154	0.4034129	1.6969836	0
0.001198699	0.531	1348	"tags=23%, list=10%, signal=26%"
GSE5455_EX_VIVO_VS_POST_24H_INCUBATION_MONOCYTES_FROM_TUMOR_BEARING_
MOUSE_UP
GSE5455_EX_VIVO_VS_POST_24H_INCUBATION_MONOCYTES_FROM_TUMOR_BEARING_
MOUSE_UP	135	0.41636324	1.6967914	0
0.001200212	0.532	2232	"tags=33%, list=16%, signal=38%"
GSE3982_DC_VS_MAC_DN	GSE3982_DC_VS_MAC_DN		144
0.4063687	1.6965706	0	0.001201723	0.532
956	"tags=17%, list=7%, signal=18%"
GSE22432_MULTIPOTENT_VS_COMMON_DC_PROGENITOR_UNTREATED_UP
GSE22432_MULTIPOTENT_VS_COMMON_DC_PROGENITOR_UNTREATED_UP
146	0.4156539	1.6963916	0	0.001206378
0.535	2505	"tags=40%, list=18%, signal=48%"
GSE2585_THYMIC_MACROPHAGE_VS_MTEC_UP
GSE2585_THYMIC_MACROPHAGE_VS_MTEC_UP		147
0.40821102	1.6957787	0	0.001220504	0.539
1490	"tags=22%, list=11%, signal=25%"
GSE39820_TGFBETA1_VS_TGFBETA3_IN_IL6_TREATED_CD4_TCELL_DN
GSE39820_TGFBETA1_VS_TGFBETA3_IN_IL6_TREATED_CD4_TCELL_DN
137	0.41552153	1.6952335	0	0.001232887
0.543	2481	"tags=32%, list=18%, signal=39%"
GSE11057_NAIVE_CD4_VS_PBMC_CD4_TCELL_DN
GSE11057_NAIVE_CD4_VS_PBMC_CD4_TCELL_DN		147
0.4120574	1.6950207	0	0.001234341	0.543
2444	"tags=33%, list=17%, signal=40%"

GSE41867_NAIVE_VS_DAY15_LCMV_ARMSTRONG_EFFECTOR_CD8_TCELL_UP
GSE41867_NAIVE_VS_DAY15_LCMV_ARMSTRONG_EFFECTOR_CD8_TCELL_UP
149 0.40734735 1.6946855 0
0.001238889 0.546 2412 "tags=38%, list=17%, signal=45%"
GSE10325_MYELOID_VS_LUPUS_MYELOID_DN
GSE10325_MYELOID_VS_LUPUS_MYELOID_DN 137
0.4170317 1.6945504 0 0.001238838 0.546
1924 "tags=36%, list=14%, signal=42%"
GSE39152_BRAIN_VS_SPLEEN_CD103_NEG_MEMORY_CD8_TCELL_DN
GSE39152_BRAIN_VS_SPLEEN_CD103_NEG_MEMORY_CD8_TCELL_DN
159 0.40436935 1.6932917 0 0.001257313
0.554 2115 "tags=36%, list=15%, signal=42%"
GSE12198_NK_VS_NK_ACT_EXPANSION_SYSTEM_DERIVED_NK_CELL_DN
GSE12198_NK_VS_NK_ACT_EXPANSION_SYSTEM_DERIVED_NK_CELL_DN
164 0.39815804 1.6919252 0 0.001286472
0.562 1889 "tags=34%, list=13%, signal=38%"
GSE22611_NOD2_TRANSDUCED_VS_CTRL_HEK293T_STIMULATED_WITH_MDP_2H_DN
GSE22611_NOD2_TRANSDUCED_VS_CTRL_HEK293T_STIMULATED_WITH_MDP_2H_DN
152 0.4111575 1.6919045 0
0.001284782 0.562 2064 "tags=32%, list=15%, signal=37%"
GSE360_CTRL_VS_T_GONDII_MAC_UP GSE360_CTRL_VS_T_GONDII_MAC_UP
154 0.40866825 1.6910763 0
0.001296969 0.568 1751 "tags=28%, list=12%, signal=32%"
GOLDRATH_NAIVE_VS_MEMORY_CD8_TCELL_DN
GOLDRATH_NAIVE_VS_MEMORY_CD8_TCELL_DN 167
0.40099472 1.6907716 0 0.00130304 0.57
1682 "tags=34%, list=12%, signal=38%"
GSE3337_CTRL_VS_4H_IFNG_IN_CD8POS_DC_DN
GSE3337_CTRL_VS_4H_IFNG_IN_CD8POS_DC_DN 162 0.400072
1.6902888 0 0.001315129 0.572
2067 "tags=37%, list=15%, signal=43%"
GSE17974_IL4_AND_ANTI_IL12_VS_UNTREATED_0.5H_ACT_CD4_TCELL_UP
GSE17974_IL4_AND_ANTI_IL12_VS_UNTREATED_0.5H_ACT_CD4_TCELL_UP
105 0.42640665 1.6902071 0.002057613
0.00131341 0.572 2657 "tags=35%, list=19%, signal=43%"
GSE3565_CTRL_VS_LPS_INJECTED_DUSP1_KO_SPLENOCYTES_UP
GSE3565_CTRL_VS_LPS_INJECTED_DUSP1_KO_SPLENOCYTES_UP
153 0.4052992 1.6900929 0 0.001313265
0.572 1737 "tags=39%, list=12%, signal=43%"
GSE3982_MAC_VS_NEUTROPHIL_UP GSE3982_MAC_VS_NEUTROPHIL_UP
148 0.4063195 1.6886318 0
0.001348353 0.587 2656 "tags=39%, list=19%, signal=48%"
GSE32901_NAIVE_VS_TH17_NEG_CD4_TCELL_DN
GSE32901_NAIVE_VS_TH17_NEG_CD4_TCELL_DN 99
0.43831342 1.6872908 0.002061856 0.001386461
0.594 1297 "tags=19%, list=9%, signal=21%"
GSE17301_CTRL_VS_48H_ACD3_ACD28_IFNA2_STIM_CD8_TCELL_UP
GSE17301_CTRL_VS_48H_ACD3_ACD28_IFNA2_STIM_CD8_TCELL_UP
150 0.40834108 1.6868436 0 0.001398416
0.599 1961 "tags=33%, list=14%, signal=38%"
GSE18791_CTRL_VS_NEWCASTLE_VIRUS_DC_16H_UP
GSE18791_CTRL_VS_NEWCASTLE_VIRUS_DC_16H_UP 130
0.41598248 1.6859671 0 0.001411968 0.604
2378 "tags=34%, list=17%, signal=40%"

GSE17721_CTRL_VS_LPS_4H_BMDC_UP GSE17721_CTRL_VS_LPS_4H_BMDC_UP
151 0.4019444 1.6858487 0
0.001417813 0.605 1961 "tags=31%, list=14%, signal=36%"
GSE2770_IL12_VS_TGFB_AND_IL12_TREATED_ACT_CD4_TCELL_6H_DN
GSE2770_IL12_VS_TGFB_AND_IL12_TREATED_ACT_CD4_TCELL_6H_DN
131 0.4153464 1.6855081 0 0.001432687
0.61 2612 "tags=40%, list=19%, signal=48%"
GSE6269_E_COLI_VS_STAPH_AUREUS_INF_PBMC_DN
GSE6269_E_COLI_VS_STAPH_AUREUS_INF_PBMC_DN 138
0.4080738 1.6845616 0 0.001458181 0.62
1988 "tags=33%, list=14%, signal=38%"
GSE43957_UNTREATED_VS_NACL_TREATED_ANTI_CD3_CD28_STIM_CD4_TCELL_DN
GSE43957_UNTREATED_VS_NACL_TREATED_ANTI_CD3_CD28_STIM_CD4_TCELL_DN
155 0.40393364 1.6844286 0
0.001460856 0.621 2423 "tags=34%, list=17%, signal=41%"
GSE41087_WT_VS_FOXP3_MUT_ANTI_CD3_CD28_STIM_CD4_TCELL_DN
GSE41087_WT_VS_FOXP3_MUT_ANTI_CD3_CD28_STIM_CD4_TCELL_DN
141 0.40659484 1.6802456 0 0.001551224
0.642 2197 "tags=36%, list=16%, signal=42%"
GSE19198_CTRL_VS_IL21_TREATED_TCELL_6H_UP
GSE19198_CTRL_VS_IL21_TREATED_TCELL_6H_UP 158
0.39947432 1.6795741 0 0.001565946 0.646
1974 "tags=32%, list=14%, signal=37%"
GSE30971_CTRL_VS_LPS_STIM_MACROPHAGE_WBP7_HET_4H_DN
GSE30971_CTRL_VS_LPS_STIM_MACROPHAGE_WBP7_HET_4H_DN
145 0.40680167 1.6770166 0.00210084
0.001616901 0.653 2360 "tags=35%, list=17%, signal=42%"
GSE34205_RSV_VS_FLU_INF_INFANT_PBMC_DN
GSE34205_RSV_VS_FLU_INF_INFANT_PBMC_DN 126
0.41490388 1.6757274 0 0.001640515 0.656
2696 "tags=36%, list=19%, signal=44%"
GSE7460_CTRL_VS_FOXP3_OVEREXPR_TCONV_1_UP
GSE7460_CTRL_VS_FOXP3_OVEREXPR_TCONV_1_UP 146
0.4060927 1.6755712 0 0.001638409 0.656
1633 "tags=21%, list=12%, signal=24%"
GSE25088_WT_VS_STAT6_KO_MACROPHAGE_ROSIGLITAZONE_AND_IL4_STIM_UP
GSE25088_WT_VS_STAT6_KO_MACROPHAGE_ROSIGLITAZONE_AND_IL4_STIM_UP
149 0.40876377 1.6750623 0
0.001663272 0.664 2140 "tags=30%, list=15%, signal=34%"
GSE7568_IL4_TGFB_DEXAMETHASONE_VS_IL4_TGFB_TREATED_MACROPHAGE_UP
GSE7568_IL4_TGFB_DEXAMETHASONE_VS_IL4_TGFB_TREATED_MACROPHAGE_UP
138 0.40995824 1.6746371 0
0.001674631 0.667 1755 "tags=30%, list=12%, signal=34%"
GSE23114_WT_VS_SLE2C1_MOUSE_SPLEEN_B1A_BCELL_UP
GSE23114_WT_VS_SLE2C1_MOUSE_SPLEEN_B1A_BCELL_UP 151
0.40657526 1.6743749 0 0.001681556 0.67
2598 "tags=42%, list=18%, signal=51%"
GSE27670_CTRL_VS_LMP1_TRANSDUCED_GC_BCELL_UP
GSE27670_CTRL_VS_LMP1_TRANSDUCED_GC_BCELL_UP 158
0.39813933 1.674329 0 0.001680867 0.67
1718 "tags=27%, list=12%, signal=30%"
GSE7218_UNSTIM_VS_ANTIGEN_STIM_THROUGH_IGG_BCELL_DN
GSE7218_UNSTIM_VS_ANTIGEN_STIM_THROUGH_IGG_BCELL_DN
135 0.40755746 1.673412 0 0.001698241 0.672

2374 "tags=39%, list=17%, signal=47%"
GSE1460_CORD_VS_ADULT_BLOOD_NAIVE_CD4_TCELL_DN
GSE1460_CORD_VS_ADULT_BLOOD_NAIVE_CD4_TCELL_DN 141
0.40478355 1.6728038 0 0.001703589 0.673
2625 "tags=38%, list=19%, signal=46%"
GSE339_CD8POS_VS_CD4CD8DN_DC_IN_CULTURE_UP
GSE339_CD8POS_VS_CD4CD8DN_DC_IN_CULTURE_UP 158
0.39681807 1.6715391 0 0.001731159 0.678
2215 "tags=34%, list=16%, signal=40%"
GSE13485_DAY3_VS_DAY7_YF17D_VACCINE_PBMC_DN
GSE13485_DAY3_VS_DAY7_YF17D_VACCINE_PBMC_DN 122
0.41400284 1.6713579 0 0.001734905 0.68
1256 "tags=25%, list=9%, signal=27%"
GSE29618_PRE_VS_DAY7_FLU_VACCINE_PDC_DN
GSE29618_PRE_VS_DAY7_FLU_VACCINE_PDC_DN 157
0.39555502 1.6694753 0 0.001784771 0.687
2082 "tags=32%, list=15%, signal=38%"
GSE21360_NAIVE_VS_TERTIARY_MEMORY_CD8_TCELL_UP
GSE21360_NAIVE_VS_TERTIARY_MEMORY_CD8_TCELL_UP 143
0.403636 1.6670755 0 0.001850933 0.697
1938 "tags=31%, list=14%, signal=35%"
GSE21033_CTRL_VS_POLYIC_STIM_DC_3H_UP
GSE21033_CTRL_VS_POLYIC_STIM_DC_3H_UP 137
0.40547243 1.6653861 0 0.00188874 0.706
1571 "tags=25%, list=11%, signal=28%"
GSE42088_2H_VS_24H_LEISHMANIA_INF_DC_UP
GSE42088_2H_VS_24H_LEISHMANIA_INF_DC_UP 140
0.40692842 1.6648823 0 0.001905659 0.71
1800 "tags=35%, list=13%, signal=40%"
GSE17721_12H_VS_24H_LPS_BMDC_DN GSE17721_12H_VS_24H_LPS_BMDC_DN
148 0.39953494 1.6645244 0
0.001916613 0.713 2455 "tags=36%, list=17%, signal=43%"
GSE17721_POLYIC_VS_CPG_8H_BMDC_DN GSE17721_POLYIC_VS_CPG_8H_BMDC_DN
145 0.4028562 1.6640104 0
0.0019319 0.718 2287 "tags=36%, list=16%, signal=42%"
GSE9988_LOW_LPS_VS_VEHICLE_TREATED_MONOCYTE_DN
GSE9988_LOW_LPS_VS_VEHICLE_TREATED_MONOCYTE_DN 142
0.40699273 1.6637946 0 0.0019354 0.719
2038 "tags=32%, list=14%, signal=37%"
GSE39152_CD103_NEG_VS_POS_MEMORY_CD8_TCELL_DN
GSE39152_CD103_NEG_VS_POS_MEMORY_CD8_TCELL_DN 167
0.39314175 1.6635929 0 0.001934464 0.72
2319 "tags=36%, list=16%, signal=42%"
GSE17721_POLYIC_VS_PAM3CSK4_12H_BMDC_UP
GSE17721_POLYIC_VS_PAM3CSK4_12H_BMDC_UP 149
0.39869526 1.6613894 0 0.002005899 0.73
2774 "tags=36%, list=20%, signal=45%"
GSE17721_CTRL_VS_CPG_12H_BMDC_UP GSE17721_CTRL_VS_CPG_12H_BMDC_UP
147 0.40156487 1.660895 0 0.002019652
0.732 1967 "tags=33%, list=14%, signal=38%"
GSE360_T_GONDII_VS_B_MALAYI_LOW_DOSE_MAC_DN
GSE360_T_GONDII_VS_B_MALAYI_LOW_DOSE_MAC_DN 156
0.40168065 1.6601593 0 0.002043698 0.738
2517 "tags=36%, list=18%, signal=43%"

GSE4142_PLASMA_CELL_VS_MEMORY_BCELL_DN
GSE4142_PLASMA_CELL_VS_MEMORY_BCELL_DN 127
0.4093514 1.6601583 0 0.00204114 0.738
1961 "tags=26%, list=14%, signal=30%"
GSE40666_UNTREATED_VS_IFNA_STIM_EFFECTOR_CD8_TCELL_90MIN_DN
GSE40666_UNTREATED_VS_IFNA_STIM_EFFECTOR_CD8_TCELL_90MIN_DN
139 0.40393838 1.6597878 0 0.00205335
0.741 2569 "tags=44%, list=18%, signal=53%"
GSE15930_STIM_VS_STIM_AND_IL12_24H_CD8_T_CELL_UP
GSE15930_STIM_VS_STIM_AND_IL12_24H_CD8_T_CELL_UP 163
0.39543566 1.6593474 0 0.002062532 0.742
1819 "tags=27%, list=13%, signal=31%"
GSE5589_LPS_VS_LPS_AND_IL10_STIM_IL6_KO_MACROPHAGE_45MIN_DN
GSE5589_LPS_VS_LPS_AND_IL10_STIM_IL6_KO_MACROPHAGE_45MIN_DN
148 0.40374503 1.658756 0 0.002087705 0.747
2470 "tags=36%, list=18%, signal=43%"
GSE14769_40MIN_VS_360MIN_LPS_BMDM_UP
GSE14769_40MIN_VS_360MIN_LPS_BMDM_UP 149
0.4036747 1.6572652 0 0.002134848 0.755
2243 "tags=35%, list=16%, signal=41%"
GSE3982_NEUTROPHIL_VS_BCELL_DN GSE3982_NEUTROPHIL_VS_BCELL_DN
144 0.39491642 1.6571921 0
0.002135076 0.755 1942 "tags=28%, list=14%, signal=33%"
GSE22886_CTRL_VS_LPS_24H_DC_UP GSE22886_CTRL_VS_LPS_24H_DC_UP
149 0.39823627 1.6560014 0
0.002164385 0.762 2695 "tags=46%, list=19%, signal=56%"
GSE15139_GMCSF_TREATED_VS_UNTREATED_NEUTROPHILS_DN
GSE15139_GMCSF_TREATED_VS_UNTREATED_NEUTROPHILS_DN 141
0.40373963 1.6559271 0 0.00216314 0.762
1559 "tags=26%, list=11%, signal=29%"
GSE16451_IMMATURE_VS_MATURE_NEURON_CELL_LINE_WEST_EQUINE_ENC_VIRUS_U
P
GSE16451_IMMATURE_VS_MATURE_NEURON_CELL_LINE_WEST_EQUINE_ENC_VIRUS_U
P 154 0.39963052 1.6545024 0
0.002198177 0.767 2113 "tags=36%, list=15%, signal=42%"
GSE4748_CTRL_VS_CYANOBACTERIUM_LPSLIKE_STIM_DC_3H_DN
GSE4748_CTRL_VS_CYANOBACTERIUM_LPSLIKE_STIM_DC_3H_DN
152 0.40034512 1.6544784 0 0.002198324
0.767 1944 "tags=34%, list=14%, signal=39%"
GSE27786_CD8_TCELL_VS_NEUTROPHIL_DN
GSE27786_CD8_TCELL_VS_NEUTROPHIL_DN 137
0.40690175 1.6532074 0 0.002236068 0.771
1594 "tags=23%, list=11%, signal=25%"
GSE27241_WT_VS_RORGT_KO_TH17_POLARIZED_CD4_TCELL_DN
GSE27241_WT_VS_RORGT_KO_TH17_POLARIZED_CD4_TCELL_DN
137 0.40092307 1.6522043 0 0.002272544
0.774 2630 "tags=38%, list=19%, signal=46%"
GSE41867_LCMV_ARMSTRONG_VS_CLONE13_DAY6_EFFECTOR_CD8_TCELL_DN
GSE41867_LCMV_ARMSTRONG_VS_CLONE13_DAY6_EFFECTOR_CD8_TCELL_DN
157 0.39017788 1.6510258 0
0.002311709 0.779 2354 "tags=38%, list=17%, signal=45%"
GSE5099_UNSTIM_VS_MCSF_TREATED_MONOCYTE_DAY3_DN
GSE5099_UNSTIM_VS_MCSF_TREATED_MONOCYTE_DAY3_DN 138
0.40406725 1.650897 0.002173913 0.002311713 0.779

2136	"tags=36%, list=15%, signal=41%"
GSE16266_CTRL_VS_HEATSHOCK_AND_LPS_STIM_MEF_DN
GSE16266_CTRL_VS_HEATSHOCK_AND_LPS_STIM_MEF_DN		160
0.39039952	1.6508685	0.002188184	0.00230887
0.779	2533	"tags=41%, list=18%, signal=49%"
GSE3982_NEUTROPHIL_VS_TH2_DN	GSE3982_NEUTROPHIL_VS_TH2_DN
	146	0.39831328	1.6505935	0
0.002323301	0.781	2115	"tags=32%, list=15%, signal=37%"
GSE27859_MACROPHAGE_VS_CD11C_INT_F480_INT_DC_DN
GSE27859_MACROPHAGE_VS_CD11C_INT_F480_INT_DC_DN		144
0.39851877	1.6497011	0	0.00233915	0.785
2035	"tags=26%, list=14%, signal=31%"
GSE21360_PRIMARY_VS_TERTIARY_MEMORY_CD8_TCELL_UP
GSE21360_PRIMARY_VS_TERTIARY_MEMORY_CD8_TCELL_UP		155
0.39697072	1.6495892	0	0.002337684	0.785
1726	"tags=28%, list=12%, signal=32%"
GSE3982_MAC_VS_EFF_MEMORY_CD4_TCELL_UP
GSE3982_MAC_VS_EFF_MEMORY_CD4_TCELL_UP		149
0.39675793	1.6491838	0	0.002353412	0.789
2079	"tags=32%, list=15%, signal=37%"
GSE6674_ANTI_IGM_VS_PL2_3_STIM_BCELL_DN
GSE6674_ANTI_IGM_VS_PL2_3_STIM_BCELL_DN		152
0.3978387	1.6491628	0	0.002350535	0.789
2420	"tags=36%, list=17%, signal=43%"
GSE17721_12H_VS_24H_CPG_BMDC_DN	GSE17721_12H_VS_24H_CPG_BMDC_DN
	167	0.39008898	1.6485603	0
0.002366343	0.791	2424	"tags=35%, list=17%, signal=41%"
GSE8621_LPS_STIM_VS_LPS_PRIMED_AND_LPS_STIM_MACROPHAGE_DN
GSE8621_LPS_STIM_VS_LPS_PRIMED_AND_LPS_STIM_MACROPHAGE_DN
150	0.3964257	1.6479819	0.002118644
0.002377595	0.793	1849	"tags=29%, list=13%, signal=33%"
GSE13411_NAIVE_BCELL_VS_PLASMA_CELL_DN
GSE13411_NAIVE_BCELL_VS_PLASMA_CELL_DN		137
0.40354884	1.646439	0	0.002433263	0.802
1896	"tags=28%, list=13%, signal=32%"
GSE16386_IL4_VS_IL4_AND_ROSIGLITAZONE_STIM_MACROPHAGE_6H_UP
GSE16386_IL4_VS_IL4_AND_ROSIGLITAZONE_STIM_MACROPHAGE_6H_UP
147	0.39909658	1.6438304	0	0.002544415
0.821	1893	"tags=28%, list=13%, signal=32%"
GSE28237_FOLLICULAR_VS_LATE_GC_BCELL_DN
GSE28237_FOLLICULAR_VS_LATE_GC_BCELL_DN		136
0.40245262	1.6436623	0	0.002547042	0.822
2332	"tags=34%, list=17%, signal=40%"
GSE16385_MONOCYTE_VS_12H_ROSIGLITAZONE_TREATED_MACROPHAGE_DN
GSE16385_MONOCYTE_VS_12H_ROSIGLITAZONE_TREATED_MACROPHAGE_DN
	139	0.3934993	1.6428907	0
0.002588044	0.823	2268	"tags=32%, list=16%, signal=37%"
GSE36826_NORMAL_VS_STAPH_AUREUS_INF_SKIN_UP
GSE36826_NORMAL_VS_STAPH_AUREUS_INF_SKIN_UP		145
0.39588204	1.6423053	0	0.002604928	0.826
2251	"tags=37%, list=16%, signal=44%"
GSE3337_4H_VS_16H_IFNG_IN_CD8POS_DC_UP
GSE3337_4H_VS_16H_IFNG_IN_CD8POS_DC_UP		164
0.38953173	1.6418883	0	0.002608912	0.827

2692 "tags=42%, list=19%, signal=51%"
GSE17721_PAM3CSK4_VS_GADIQUIMOD_8H_BMDC_UP
GSE17721_PAM3CSK4_VS_GADIQUIMOD_8H_BMDC_UP 154
0.39136842 1.6409181 0 0.002649803 0.836
2450 "tags=34%, list=17%, signal=41%"
GSE2770_UNTREATED_VS_ACT_CD4_TCELL_48H_UP
GSE2770_UNTREATED_VS_ACT_CD4_TCELL_48H_UP 139
0.40148538 1.640356 0 0.002663592 0.838
2520 "tags=35%, list=18%, signal=42%"
GSE32034_LY6C_HIGH_VS_LOW_MONOCYTE_UP
GSE32034_LY6C_HIGH_VS_LOW_MONOCYTE_UP 165
0.39113885 1.6400716 0 0.002674405 0.839
2100 "tags=27%, list=15%, signal=32%"
GSE24634_IL4_VS_CTRL_TREATED_NAIVE_CD4_TCELL_DAY10_UP
GSE24634_IL4_VS_CTRL_TREATED_NAIVE_CD4_TCELL_DAY10_UP
143 0.39477932 1.640054 0.002123142 0.002671183
0.839 1432 "tags=24%, list=10%, signal=27%"
GSE2770_TGFB_AND_IL4_ACT_VS_ACT_CD4_TCELL_6H_UP
GSE2770_TGFB_AND_IL4_ACT_VS_ACT_CD4_TCELL_6H_UP 129
0.40277904 1.6396914 0.00209205 0.002680708
0.841 2535 "tags=40%, list=18%, signal=48%"
GSE2770_TGFB_AND_IL4_VS_IL4_TREATED_ACT_CD4_TCELL_48H_DN
GSE2770_TGFB_AND_IL4_VS_IL4_TREATED_ACT_CD4_TCELL_48H_DN
137 0.39950135 1.6392814 0 0.002690102
0.844 2267 "tags=32%, list=16%, signal=38%"
GSE36392_TYPE_2_MYELOID_VS_NEUTROPHIL_IL25_TREATED_LUNG_DN
GSE36392_TYPE_2_MYELOID_VS_NEUTROPHIL_IL25_TREATED_LUNG_DN
148 0.39337125 1.6384743 0.002061856
0.00272073 0.852 1742 "tags=29%, list=12%, signal=33%"
GSE3920_UNTREATED_VS_IFNG_TREATED_FIBROBLAST_UP
GSE3920_UNTREATED_VS_IFNG_TREATED_FIBROBLAST_UP 127
0.3985585 1.6379663 0.002232143 0.00273276
0.856 3116 "tags=43%, list=22%, signal=55%"
GSE7460_CTRL_VS_TGFB_TREATED_ACT_CD8_TCELL_UP
GSE7460_CTRL_VS_TGFB_TREATED_ACT_CD8_TCELL_UP 159
0.3912897 1.6363578 0 0.002806778 0.863
1529 "tags=28%, list=11%, signal=31%"
GSE15659_RESTING_VS_ACTIVATED_TREG_DN
GSE15659_RESTING_VS_ACTIVATED_TREG_DN 138
0.3991642 1.6360871 0.002188184 0.002811827
0.864 1969 "tags=25%, list=14%, signal=29%"
GSE22611_NOD2_VS_CTRL_TRANSDUCED_HEK293T_CELL_UP
GSE22611_NOD2_VS_CTRL_TRANSDUCED_HEK293T_CELL_UP 132
0.40087894 1.6354123 0 0.002844962 0.867
2059 "tags=31%, list=15%, signal=36%"
GSE339_CD4POS_VS_CD4CD8DN_DC_DN GSE339_CD4POS_VS_CD4CD8DN_DC_DN
149 0.39151677 1.6353862 0
0.002841567 0.867 1991 "tags=33%, list=14%, signal=38%"
GSE15139_GMCSF_TREATED_VS_UNTREATED_NEUTROPHILS_UP
GSE15139_GMCSF_TREATED_VS_UNTREATED_NEUTROPHILS_UP 139
0.40574816 1.633755 0.002169197 0.002902563 0.871
2502 "tags=37%, list=18%, signal=44%"
GSE37416_CTRL_VS_12H_F_TULARENSIS_LVS_NEUTROPHIL_UP
GSE37416_CTRL_VS_12H_F_TULARENSIS_LVS_NEUTROPHIL_UP

134	0.3994707	1.6336013	0	0.002904693
0.871	2345	"tags=39%, list=17%, signal=46%"
GSE19888_ADENOSINE_A3R_INH_VS_ACT_IN_MAST_CELL_UP
GSE19888_ADENOSINE_A3R_INH_VS_ACT_IN_MAST_CELL_UP	128
0.40717515	1.6334791	0	0.00291519	0.872
2658	"tags=36%, list=19%, signal=44%"
GSE17186_MEMORY_VS_NAIVE_BCELL_DN GSE17186_MEMORY_VS_NAIVE_BCELL_DN
144	0.3997855	1.6332204	0
0.002927192	0.874	2040	"tags=33%, list=14%, signal=38%"
GSE2197_IMMUNOSUPPRESSIVE_DNA_VS_UNTREATEDIN_DC_DN
GSE2197_IMMUNOSUPPRESSIVE_DNA_VS_UNTREATEDIN_DC_DN	150
0.39348012	1.6330047	0	0.002932109	0.874
1423	"tags=24%, list=10%, signal=26%"
GSE2770_UNTREATED_VS_IL12_TREATED_ACT_CD4_TCELL_2H_DN
GSE2770_UNTREATED_VS_IL12_TREATED_ACT_CD4_TCELL_2H_DN
145	0.39449406	1.6322846	0.002083333
0.002968958	0.876	1416	"tags=24%, list=10%, signal=27%"
GSE22886_DAY0_VS_DAY7_MONOCYTE_IN_CULTURE_DN
GSE22886_DAY0_VS_DAY7_MONOCYTE_IN_CULTURE_DN	148
0.39493406	1.6311857	0	0.003018114	0.879
2400	"tags=37%, list=17%, signal=44%"
GSE360_CTRL_VS_B_MALAYI_HIGH_DOSE_DC_UP
GSE360_CTRL_VS_B_MALAYI_HIGH_DOSE_DC_UP	141
0.39197615	1.6311277	0	0.003017334	0.879
2407	"tags=31%, list=17%, signal=37%"
GSE22935_24H_VS_48H_MBOVIS_BCG_STIM_MACROPHAGE_DN
GSE22935_24H_VS_48H_MBOVIS_BCG_STIM_MACROPHAGE_DN	145
0.3953061	1.6309007	0.006097561	0.003020702
0.88	1833	"tags=30%, list=13%, signal=34%"
GSE10147_IL3_VS_IL3_AND_CPG_STIM_PDC_UP
GSE10147_IL3_VS_IL3_AND_CPG_STIM_PDC_UP	105
0.41394234	1.6299044	0	0.003061413	0.882
1892	"tags=23%, list=13%, signal=26%"
GSE17721_CTRL_VS_GARDIQUIMOD_8H_BMDC_UP
GSE17721_CTRL_VS_GARDIQUIMOD_8H_BMDC_UP	149
0.3934003	1.6293012	0	0.0030814	0.882
1636	"tags=26%, list=12%, signal=29%"
GSE17721_CTRL_VS_LPS_0.5H_BMDC_UP GSE17721_CTRL_VS_LPS_0.5H_BMDC_UP
152	0.3958024	1.6278028	0.002079002
0.003153861	0.886	2251	"tags=32%, list=16%, signal=38%"
GSE2770_TGFB_AND_IL4_VS_IL12_TREATED_ACT_CD4_TCELL_48H_UP
GSE2770_TGFB_AND_IL4_VS_IL12_TREATED_ACT_CD4_TCELL_48H_UP
148	0.39111575	1.626573	0	0.0032219	0.895
2065	"tags=29%, list=15%, signal=34%"
GSE411_100MIN_VS_400MIN_IL6_STIM_MACROPHAGE_DN
GSE411_100MIN_VS_400MIN_IL6_STIM_MACROPHAGE_DN	140
0.40027145	1.6262382	0.002132196	0.003242768
0.896	1976	"tags=34%, list=14%, signal=39%"
GSE12963_UNINF_VS_ENV_AND_NEF_AND_VPR_DEFICIENT_HIV1_INF_CD4_TCELL_D
N
GSE12963_UNINF_VS_ENV_AND_NEF_AND_VPR_DEFICIENT_HIV1_INF_CD4_TCELL_D
N	109	0.4120101	1.6254516
0.006147541	0.003273259	0.902	965	"tags=17%,
list=7%, signal=18%"

GSE21927_SPLEEN_VS_BONE_MARROW_MONOCYTE_BALBC_DN
GSE21927_SPLEEN_VS_BONE_MARROW_MONOCYTE_BALBC_DN 136
0.39359236 1.6249193 0 0.003298145 0.906
1806 "tags=26%, list=13%, signal=29%"
GSE13484_UNSTIM_VS_YF17D_VACCINE_STIM_PBMC_UP
GSE13484_UNSTIM_VS_YF17D_VACCINE_STIM_PBMC_UP 151
0.39350766 1.6246483 0 0.003303936 0.906
2169 "tags=36%, list=15%, signal=43%"
GSE11973_MIR223_KOVS_WT_BONE_MARROW_NEUTROPHIL_DN
GSE11973_MIR223_KOVS_WT_BONE_MARROW_NEUTROPHIL_DN 132
0.3991157 1.6235609 0 0.003345289 0.908
2132 "tags=34%, list=15%, signal=40%"
GSE11924_TFH_VS_TH17_CD4_TCELL_DN GSE11924_TFH_VS_TH17_CD4_TCELL_DN
147 0.39395916 1.6219261 0
0.003423609 0.913 1957 "tags=29%, list=14%, signal=33%"
GSE17721_LPS_VS_CPG_8H_BMDC_DN GSE17721_LPS_VS_CPG_8H_BMDC_DN
148 0.38957724 1.6216499 0
0.003425057 0.913 2414 "tags=34%, list=17%, signal=40%"
GSE4811_CLASSSICALY_ACTIVATED_VS_TYPE_2_ACTIVATED_MACROPHAGE_UP
GSE4811_CLASSSICALY_ACTIVATED_VS_TYPE_2_ACTIVATED_MACROPHAGE_UP
127 0.40103006 1.6213571 0
0.003431873 0.913 2061 "tags=24%, list=15%, signal=28%"
GSE43955_1H_VS_42H_ACT_CD4_TCELL_UP
GSE43955_1H_VS_42H_ACT_CD4_TCELL_UP 157
0.38821235 1.620982 0 0.003447009 0.914
1616 "tags=27%, list=11%, signal=31%"
GSE37532_TREG_VS_TCONV_PPARG_KO_CD4_TCELL_FROM_LN_DN
GSE37532_TREG_VS_TCONV_PPARG_KO_CD4_TCELL_FROM_LN_DN
143 0.39377517 1.6205546 0.004115226
0.003460759 0.916 2238 "tags=34%, list=16%, signal=39%"
GSE3039_CD4_TCELL_VS_B1_BCELL_UP GSE3039_CD4_TCELL_VS_B1_BCELL_UP
133 0.39526033 1.6200204 0
0.00348394 0.918 1153 "tags=23%, list=8%, signal=24%"
GSE27786_BCELL_VS_CD8_TCELL_UP GSE27786_BCELL_VS_CD8_TCELL_UP
155 0.38549417 1.6198395 0
0.003490862 0.919 2119 "tags=33%, list=15%, signal=38%"
GSE45365_WT_VS_IFNAR_KO_CD8A_DC_UP
GSE45365_WT_VS_IFNAR_KO_CD8A_DC_UP 144
0.3905321 1.6195624 0 0.003503158 0.92
2292 "tags=31%, list=16%, signal=37%"
GSE17721_0.5H_VS_4H_LPS_BMDC_UP GSE17721_0.5H_VS_4H_LPS_BMDC_UP
145 0.39064533 1.6182834 0
0.003565554 0.926 1961 "tags=31%, list=14%, signal=36%"
GSE27786_LSK_VS_MONO_MAC_DN GSE27786_LSK_VS_MONO_MAC_DN
141 0.3953705 1.6181072 0.002118644
0.003576368 0.926 2479 "tags=30%, list=18%, signal=37%"
GSE3982_MAST_CELL_VS_NEUTROPHIL_UP
GSE3982_MAST_CELL_VS_NEUTROPHIL_UP 148
0.3888645 1.6178743 0.002159827 0.003582984
0.926 2416 "tags=33%, list=17%, signal=40%"
GSE3039_ALPHAALPHA_CD8_TCELL_VS_B1_BCELL_DN
GSE3039_ALPHAALPHA_CD8_TCELL_VS_B1_BCELL_DN 156
0.38607946 1.6157236 0 0.003721132 0.932
2224 "tags=31%, list=16%, signal=36%"

GSE2405_S_AUREUS_VS_UNTREATED_NEUTROPHIL_UP
GSE2405_S_AUREUS_VS_UNTREATED_NEUTROPHIL_UP 143
0.39132708 1.6144985 0.00204499 0.003777804
0.937 1613 "tags=29%, list=11%, signal=33%"
GSE18281_PERIMEDULLARY_CORTICAL_REGION_VS_WHOLE_CORTEX_THYMUS_UP
GSE18281_PERIMEDULLARY_CORTICAL_REGION_VS_WHOLE_CORTEX_THYMUS_UP
148 0.38769075 1.6141427 0.004545454
0.003785686 0.937 1556 "tags=20%, list=11%, signal=23%"
GSE2706_UNSTIM_VS_8H_LPS_AND_R848_DC_UP
GSE2706_UNSTIM_VS_8H_LPS_AND_R848_DC_UP 141
0.39732048 1.614061 0.002087683 0.003782659 0.937
1470 "tags=27%, list=10%, signal=30%"
GSE17721_CTRL_VS_LPS_12H_BMDC_UP GSE17721_CTRL_VS_LPS_12H_BMDC_UP
134 0.3948602 1.613763 0.002132196
0.003790489 0.938 1864 "tags=31%, list=13%, signal=35%"
GSE27786_LSK_VS_CD8_TCELL_DN GSE27786_LSK_VS_CD8_TCELL_DN
147 0.3883621 1.6137587 0.002024292
0.003786147 0.938 2638 "tags=37%, list=19%, signal=45%"
GSE16385_IFNG_TNF_VS_ROSIGLITAZONE_STIM_MACROPHAGE_UP
GSE16385_IFNG_TNF_VS_ROSIGLITAZONE_STIM_MACROPHAGE_UP
104 0.41094086 1.6134864 0 0.00379389
0.938 1322 "tags=21%, list=9%, signal=23%"
GSE25087_FETAL_VS_ADULT_TREG_UP GSE25087_FETAL_VS_ADULT_TREG_UP
146 0.39203328 1.613439 0 0.003793564
0.938 1798 "tags=30%, list=13%, signal=34%"
GSE17721_LPS_VS_POLYIC_4H_BMDC_DN GSE17721_LPS_VS_POLYIC_4H_BMDC_DN
147 0.3894305 1.6131132 0
0.003812082 0.938 2315 "tags=32%, list=16%, signal=38%"
GSE17721_4H_VS_24H_POLYIC_BMDC_UP GSE17721_4H_VS_24H_POLYIC_BMDC_UP
152 0.38548696 1.612087 0.00203666
0.003857312 0.941 2122 "tags=29%, list=15%, signal=34%"
GSE13484_UNSTIM_VS_3H_YF17D_VACCINE_STIM_PBMC_UP
GSE13484_UNSTIM_VS_3H_YF17D_VACCINE_STIM_PBMC_UP 143
0.3907611 1.6116444 0.004291846 0.003866295
0.942 2466 "tags=38%, list=17%, signal=45%"
GSE28726_NAIVE_VS_ACTIVATED_VA24NEG_NKTCELL_UP
GSE28726_NAIVE_VS_ACTIVATED_VA24NEG_NKTCELL_UP 148
0.38965556 1.6111498 0 0.003879169 0.943
2424 "tags=36%, list=17%, signal=44%"
GSE12845_IGD_NEG_BLOOD_VS_DARKZONE_GC_TONSIL_BCELL_UP
GSE12845_IGD_NEG_BLOOD_VS_DARKZONE_GC_TONSIL_BCELL_UP
131 0.393278 1.6109782 0.002105263 0.003892046
0.943 2511 "tags=34%, list=18%, signal=41%"
GSE17721_POLYIC_VS_GARDIQUIMOD_16H_BMDC_DN
GSE17721_POLYIC_VS_GARDIQUIMOD_16H_BMDC_DN 145
0.38626373 1.6108457 0 0.003900915 0.943
2767 "tags=41%, list=20%, signal=51%"
GSE20366_TREG_VS_NAIVE_CD4_TCELL_HOMEOSTATIC_CONVERSION_UP
GSE20366_TREG_VS_NAIVE_CD4_TCELL_HOMEOSTATIC_CONVERSION_UP
136 0.39033934 1.6097414 0 0.003969482
0.947 1538 "tags=25%, list=11%, signal=28%"
GSE360_CTRL_VS_B_MALAYI_LOW_DOSE_MAC_DN
GSE360_CTRL_VS_B_MALAYI_LOW_DOSE_MAC_DN 146
0.38921034 1.6095587 0 0.003976833 0.947

2228	"tags=29%, list=16%, signal=35%"
GSE12366_PLASMA_CELL_VS_NAIVE_BCELL_DN
GSE12366_PLASMA_CELL_VS_NAIVE_BCELL_DN	137
0.39313376	1.6084342	0	0.004029412	0.95
2409	"tags=37%, list=17%, signal=44%"
GSE2770_IL12_AND_TGFB_ACT_VS_ACT_CD4_TCELL_48H_DN
GSE2770_IL12_AND_TGFB_ACT_VS_ACT_CD4_TCELL_48H_DN	142
0.38925847	1.6083995	0.006289308	0.004027526
0.95	2159	"tags=30%, list=15%, signal=35%"
GSE9988_LPS_VS_VEHICLE_TREATED_MONOCYTE_DN
GSE9988_LPS_VS_VEHICLE_TREATED_MONOCYTE_DN	144	0.389405
1.6079671	0	0.004052085	0.95
2068	"tags=31%, list=15%, signal=36%"
GSE20366_EX_VIVO_VS_HOMEOSTATIC_CONVERSION_NAIVE_CD4_TCELL_DN
GSE20366_EX_VIVO_VS_HOMEOSTATIC_CONVERSION_NAIVE_CD4_TCELL_DN
139	0.39268816	1.6078452	0
0.004051467	0.95	2150	"tags=27%, list=15%, signal=32%"
GSE3982_CTRL_VS_PMA_STIM_EOSINOPHIL_UP
GSE3982_CTRL_VS_PMA_STIM_EOSINOPHIL_UP	140
0.39297825	1.6070946	0	0.004081287	0.95
2421	"tags=31%, list=17%, signal=37%"
GSE17721_POLYIC_VS_PAM3CSK4_1H_BMDC_UP
GSE17721_POLYIC_VS_PAM3CSK4_1H_BMDC_UP	159
0.38778433	1.6065366	0	0.004108289	0.95
2411	"tags=33%, list=17%, signal=40%"
GSE36476_CTRL_VS_TSST_ACT_16H_MEMORY_CD4_TCELL_YOUNG_DN
GSE36476_CTRL_VS_TSST_ACT_16H_MEMORY_CD4_TCELL_YOUNG_DN
143	0.3904821	1.6065135	0	0.004105007
0.95	2546	"tags=37%, list=18%, signal=45%"
GSE2826_XID_VS_BTK_KO_BCELL_DN	GSE2826_XID_VS_BTK_KO_BCELL_DN
162	0.3865051	1.6058632	0
0.004147855	0.951	2355	"tags=34%, list=17%, signal=40%"
GSE39152_BRAIN_VS_SPLEEN_CD103_NEG_MEMORY_CD8_TCELL_UP
GSE39152_BRAIN_VS_SPLEEN_CD103_NEG_MEMORY_CD8_TCELL_UP
147	0.38968107	1.6056709	0	0.0041525
0.951	2300	"tags=40%, list=16%, signal=47%"
GSE13522_WT_VS_IFNAR_KO_SKIN_DN	GSE13522_WT_VS_IFNAR_KO_SKIN_DN
137	0.39430356	1.6050111	0.004264392
0.004181945	0.952	2269	"tags=42%, list=16%, signal=49%"
GSE7548_DAY7_VS_DAY28_PCC_IMMUNIZATION_CD4_TCELL_DN
GSE7548_DAY7_VS_DAY28_PCC_IMMUNIZATION_CD4_TCELL_DN
146	0.39147007	1.6045696	0	0.004202084
0.953	1190	"tags=23%, list=8%, signal=24%"
GSE22527_ANTI_CD3_INVIVO_VS_UNTREATED_MOUSE_TREG_DN
GSE22527_ANTI_CD3_INVIVO_VS_UNTREATED_MOUSE_TREG_DN
142	0.39081922	1.6041591	0.00409836
0.004225025	0.954	2157	"tags=31%, list=15%, signal=36%"
GSE22886_CD8_TCELL_VS_BCELL_NAIVE_UP
GSE22886_CD8_TCELL_VS_BCELL_NAIVE_UP	159
0.3843783	1.6038197	0.002012072	0.00425811
0.955	2046	"tags=37%, list=14%, signal=43%"
GSE8685_IL15_ACT_IL2_STARVED_VS_IL21_ACT_IL2_STARVED_CD4_TCELL_UP
GSE8685_IL15_ACT_IL2_STARVED_VS_IL21_ACT_IL2_STARVED_CD4_TCELL_UP
134	0.39265716	1.6036698	0

0.00426918	0.955	2330	"tags=31%, list=17%, signal=36%"
GSE360_L_DONOVANI_VS_B_MALAYI_HIGH_DOSE_MAC_DN
GSE360_L_DONOVANI_VS_B_MALAYI_HIGH_DOSE_MAC_DN	163
0.3808678	1.603564	0	0.004269626	0.955
2792	"tags=39%, list=20%, signal=48%"
GSE22886_NAIVE_TCELL_VS_NKCELL_DN GSE22886_NAIVE_TCELL_VS_NKCELL_DN
131	0.390333	1.6034628	0	0.004271439
0.955	1880	"tags=33%, list=13%, signal=38%"
GSE33292_DN3_THYMOCYTE_VS_TCELL_LYMPHOMA_FROM_TCF1_KO_DN
GSE33292_DN3_THYMOCYTE_VS_TCELL_LYMPHOMA_FROM_TCF1_KO_DN
148	0.38594416	1.603093	0	0.004294045	0.955
1525	"tags=21%, list=11%, signal=23%"
GSE8685_IL2_STARVED_VS_IL15_ACT_IL2_STARVED_CD4_TCELL_DN
GSE8685_IL2_STARVED_VS_IL15_ACT_IL2_STARVED_CD4_TCELL_DN
140	0.38873756	1.6030415	0.002096436
0.004290573	0.955	2492	"tags=38%, list=18%, signal=46%"
GSE7400_CTRL_VS_CSF3_IN_VIVO_TREATED_PBMC_DN
GSE7400_CTRL_VS_CSF3_IN_VIVO_TREATED_PBMC_DN	131
0.39456117	1.6011939	0.002118644	0.004426486
0.959	2239	"tags=31%, list=16%, signal=37%"
GSE27786_CD8_TCELL_VS_NKCELL_DN	GSE27786_CD8_TCELL_VS_NKCELL_DN
146	0.38462892	1.6000823	0
0.00449174	0.959	1285	"tags=21%, list=9%, signal=23%"
GSE339_EX_VIVO_VS_IN_CULTURE_CD8POS_DC_UP
GSE339_EX_VIVO_VS_IN_CULTURE_CD8POS_DC_UP	156
0.38510028	1.5999358	0	0.004495896	0.959
2195	"tags=34%, list=16%, signal=40%"
GSE12392_CD8A_POS_VS_NEG_SPLEEN_IFNB_KO_DC_UP
GSE12392_CD8A_POS_VS_NEG_SPLEEN_IFNB_KO_DC_UP	160
0.3808445	1.5997697	0	0.004503829	0.959
2726	"tags=39%, list=19%, signal=47%"
GSE29618_LAIV_VS_TIV_FLU_VACCINE_DAY7_MONOCYTE_DN
GSE29618_LAIV_VS_TIV_FLU_VACCINE_DAY7_MONOCYTE_DN	143
0.39169702	1.5996859	0.002008032	0.004502777
0.959	2071	"tags=28%, list=15%, signal=32%"
GSE17721_POLYIC_VS_CPG_16H_BMDC_DN
GSE17721_POLYIC_VS_CPG_16H_BMDC_DN	137
0.3906073	1.5992836	0.002109705	0.004521107
0.96	1953	"tags=28%, list=14%, signal=32%"
GSE17721_PAM3CSK4_VS_GADIQUIMOD_24H_BMDC_DN
GSE17721_PAM3CSK4_VS_GADIQUIMOD_24H_BMDC_DN	147
0.385283	1.5986396	0.00209205	0.004563886	0.963
2268	"tags=30%, list=16%, signal=35%"
GSE17721_0.5H_VS_8H_LPS_BMDC_UP	GSE17721_0.5H_VS_8H_LPS_BMDC_UP
150	0.3853624	1.5986173	0
0.00456016	0.963	2347	"tags=31%, list=17%, signal=37%"
GSE24142_DN2_VS_DN3_THYMOCYTE_ADULT_UP
GSE24142_DN2_VS_DN3_THYMOCYTE_ADULT_UP	157
0.38121897	1.5983201	0.002314815	0.004579496
0.964	2494	"tags=31%, list=18%, signal=37%"
GSE22140_HEALTHY_VS_ARTHRITIC_GERMFREE_MOUSE_CD4_TCELL_UP
GSE22140_HEALTHY_VS_ARTHRITIC_GERMFREE_MOUSE_CD4_TCELL_UP
152	0.38465515	1.5969428	0	0.004666001
0.965	1921	"tags=32%, list=14%, signal=36%"

GSE12507_PDC_CELL_LINE_VS_IMMATUE_T_CELL_LINE_UP
GSE12507_PDC_CELL_LINE_VS_IMMATUE_T_CELL_LINE_UP 118
0.39940605 1.5967777 0.004273505 0.004675003
0.965 1424 "tags=25%, list=10%, signal=28%"
GSE13484_12H_UNSTIM_VS_YF17D_VACCINE_STIM_PBMC_UP
GSE13484_12H_UNSTIM_VS_YF17D_VACCINE_STIM_PBMC_UP 134
0.39345315 1.5966138 0 0.004678856 0.965
2416 "tags=41%, list=17%, signal=49%"
GSE25123_CTRL_VS_IL4_STIM_PPARG_KO_MACROPHAGE_UP
GSE25123_CTRL_VS_IL4_STIM_PPARG_KO_MACROPHAGE_UP 156
0.38226065 1.5963068 0 0.004711011 0.967
1546 "tags=19%, list=11%, signal=21%"
GSE46606_UNSTIM_VS_CD40L_IL2_IL5_DAY1_STIMULATED_BCELL_DN
GSE46606_UNSTIM_VS_CD40L_IL2_IL5_DAY1_STIMULATED_BCELL_DN
133 0.38900936 1.596053 0.002169197 0.00473153
0.968 1234 "tags=20%, list=9%, signal=22%"
GSE22886_NAIVE_CD4_TCELL_VS_MEMORY_TCELL_DN
GSE22886_NAIVE_CD4_TCELL_VS_MEMORY_TCELL_DN 147
0.38117412 1.5955465 0 0.004759602 0.969
2099 "tags=34%, list=15%, signal=40%"
GSE39820_CTRL_VS_IL1B_IL6_IL23A_CD4_TCELL_UP
GSE39820_CTRL_VS_IL1B_IL6_IL23A_CD4_TCELL_UP 148
0.38344482 1.5949152 0 0.004804542 0.969
1797 "tags=30%, list=13%, signal=34%"
GSE41867_MEMORY_VS_EXHAUSTED_CD8_TCELL_DAY30_LCMV_DN
GSE41867_MEMORY_VS_EXHAUSTED_CD8_TCELL_DAY30_LCMV_DN
136 0.3852811 1.5938218 0.002136752
0.004886044 0.971 1907 "tags=29%, list=14%, signal=33%"
GSE17301_CTRL_VS_48H_IFNA2_STIM_CD8_TCELL_DN
GSE17301_CTRL_VS_48H_IFNA2_STIM_CD8_TCELL_DN 144
0.3889694 1.5936329 0.002083333 0.004895967
0.972 1664 "tags=21%, list=12%, signal=23%"
GSE36826_WT_VS_IL1R_KO_SKIN_DN GSE36826_WT_VS_IL1R_KO_SKIN_DN
152 0.3869395 1.5935509 0
0.004894456 0.972 2625 "tags=39%, list=19%, signal=47%"
GSE21360_PRIMARY_VS_SECONDARY_MEMORY_CD8_TCELL_UP
GSE21360_PRIMARY_VS_SECONDARY_MEMORY_CD8_TCELL_UP 128
0.39472872 1.5934988 0.002083333 0.004900598
0.972 2199 "tags=27%, list=16%, signal=32%"
GSE6674_UNSTIM_VS_ANTI_IGM_AND_CPG_STIM_BCELL_UP
GSE6674_UNSTIM_VS_ANTI_IGM_AND_CPG_STIM_BCELL_UP 141
0.39088312 1.5932059 0 0.00490932 0.973
1418 "tags=26%, list=10%, signal=29%"
GSE9988_ANTI_TREM1_AND_LPS_VS_CTRL_TREATED_MONOCYTES_DN
GSE9988_ANTI_TREM1_AND_LPS_VS_CTRL_TREATED_MONOCYTES_DN
145 0.38992128 1.5931343 0.002079002
0.004907883 0.973 2609 "tags=37%, list=18%, signal=44%"
GSE7764_IL15_NK_CELL_24H_VS_SPLENOCYTE_UP
GSE7764_IL15_NK_CELL_24H_VS_SPLENOCYTE_UP 143
0.38620278 1.5921409 0.004264392 0.004987654
0.975 2583 "tags=41%, list=18%, signal=50%"
GSE45739_NRAS_KO_VS_WT_UNSTIM_CD4_TCELL_UP
GSE45739_NRAS_KO_VS_WT_UNSTIM_CD4_TCELL_UP 143
0.38299277 1.5914867 0.002016129 0.005034281

0.977	2389	"tags=31%, list=17%, signal=37%"
GSE43955_10H_VS_60H_ACT_CD4_TCELL_WITH_TGFB_IL6_UP
GSE43955_10H_VS_60H_ACT_CD4_TCELL_WITH_TGFB_IL6_UP	165
0.37606284	1.5907758	0	0.005079547	0.977
2783	"tags=37%, list=20%, signal=46%"
GSE29164_DAY3_VS_DAY7_CD8_TCELL_TREATED_MELANOMA_DN
GSE29164_DAY3_VS_DAY7_CD8_TCELL_TREATED_MELANOMA_DN
143	0.38524735	1.5906137	0	0.005085501
0.977	2033	"tags=28%, list=14%, signal=32%"
GSE8685_IL2_ACT_IL2_STARVED_VS_IL15_ACT_IL2_STARVED_CD4_TCELL_DN
GSE8685_IL2_ACT_IL2_STARVED_VS_IL15_ACT_IL2_STARVED_CD4_TCELL_DN
	140	0.3823031	1.5901346	0
0.005110466	0.977	2499	"tags=31%, list=18%, signal=38%"
KAECH_DAY15_EFF_VS_MEMORY_CD8_TCELL_UP
KAECH_DAY15_EFF_VS_MEMORY_CD8_TCELL_UP	155
0.3838409	1.5895411	0	0.005145267	0.977
2053	"tags=34%, list=15%, signal=40%"
GSE36009_WT_VS_NLRP10_KO_DC_LPS_STIM_UP
GSE36009_WT_VS_NLRP10_KO_DC_LPS_STIM_UP	148
0.38849485	1.5892544	0.00610998	0.005152336
0.977	2630	"tags=40%, list=19%, signal=48%"
GSE17721_CTRL_VS_GARDIQUIMOD_4H_BMDC_UP
GSE17721_CTRL_VS_GARDIQUIMOD_4H_BMDC_UP	144
0.3796222	1.5890347	0.002114165	0.005161915
0.978	1870	"tags=28%, list=13%, signal=32%"
GSE1460_NAIVE_CD4_TCELL_ADULT_BLOOD_VS_THYMIC_STROMAL_CELL_DN
GSE1460_NAIVE_CD4_TCELL_ADULT_BLOOD_VS_THYMIC_STROMAL_CELL_DN
	147	0.3839672	1.5885272	0.002145923
0.005186557	0.979	1101	"tags=16%, list=8%, signal=18%"
GSE17721_POLYIC_VS_CPG_12H_BMDC_DN
GSE17721_POLYIC_VS_CPG_12H_BMDC_DN	143
0.38114217	1.5884601	0.004081633	0.005186027
0.979	2704	"tags=32%, list=19%, signal=39%"
GSE25123_CTRL_VS_ROSIGLITAZONE_STIM_MACROPHAGE_DN
GSE25123_CTRL_VS_ROSIGLITAZONE_STIM_MACROPHAGE_DN	133
0.3907722	1.5881926	0.006302521	0.005186847
0.979	2835	"tags=35%, list=20%, signal=44%"
GSE42021_CD24HI_VS_CD24INT_TREG_THYMUS_UP
GSE42021_CD24HI_VS_CD24INT_TREG_THYMUS_UP	150
0.38327366	1.5878483	0	0.0052063	0.979
2642	"tags=38%, list=19%, signal=46%"
GSE19888_CTRL_VS_A3R_ACTIVATION_MAST_CELL_UP
GSE19888_CTRL_VS_A3R_ACTIVATION_MAST_CELL_UP	127
0.389427	1.5874484	0	0.005235951	0.979
2487	"tags=34%, list=18%, signal=41%"
GSE411_UNSTIM_VS_100MIN_IL6_STIM_SOCS3_KO_MACROPHAGE_DN
GSE411_UNSTIM_VS_100MIN_IL6_STIM_SOCS3_KO_MACROPHAGE_DN
150	0.38052827	1.5869014	0	0.005281528
0.98	1608	"tags=27%, list=11%, signal=31%"
GSE30971_2H_VS_4H_LPS_STIM_MACROPHAGE_WBP7_HET_UP
GSE30971_2H_VS_4H_LPS_STIM_MACROPHAGE_WBP7_HET_UP	112
0.40213192	1.5853658	0	0.005399816	0.981
2311	"tags=37%, list=16%, signal=43%"
GSE17721_CTRL_VS_LPS_1H_BMDC_UP	GSE17721_CTRL_VS_LPS_1H_BMDC_UP

142	0.38674363	1.5849984	0
0.005420202	0.981	1556	"tags=24%, list=11%, signal=27%"
GSE29618_MONOCYTE_VS_MDC_DAY7_FLU_VACCINE_DN
GSE29618_MONOCYTE_VS_MDC_DAY7_FLU_VACCINE_DN		126
0.38910568	1.5848082	0.002	0.005431998	0.981
1936	"tags=31%, list=14%, signal=36%"
GSE2770_TGFB_AND_IL4_VS_TGFB_AND_IL12_TREATED_ACT_CD4_TCELL_48H_DN
GSE2770_TGFB_AND_IL4_VS_TGFB_AND_IL12_TREATED_ACT_CD4_TCELL_48H_DN
137	0.38649428	1.5840622	0.004024145
0.00548368	0.981	2060	"tags=31%, list=15%, signal=36%"
GSE14908_ATOPIC_VS_NONATOPIC_PATIENT_HDM_STIM_CD4_TCELL_UP
GSE14908_ATOPIC_VS_NONATOPIC_PATIENT_HDM_STIM_CD4_TCELL_UP
146	0.38182434	1.5828446	0	0.005578857
0.983	2468	"tags=37%, list=17%, signal=44%"
GSE19923_WT_VS_HEB_KO_DP_THYMOCYTE_DN
GSE19923_WT_VS_HEB_KO_DP_THYMOCYTE_DN		169
0.37783706	1.5823272	0	0.005610202	0.983
2779	"tags=38%, list=20%, signal=47%"
GSE1460_NAIVE_CD4_TCELL_CORD_BLOOD_VS_THYMIC_STROMAL_CELL_DN
GSE1460_NAIVE_CD4_TCELL_CORD_BLOOD_VS_THYMIC_STROMAL_CELL_DN
143	0.38451198	1.5818074	0
0.00563914	0.983	931	"tags=20%, list=7%, signal=21%"
GSE17301_CTRL_VS_48H_ACD3_ACD28_IFNA5_STIM_CD8_TCELL_UP
GSE17301_CTRL_VS_48H_ACD3_ACD28_IFNA5_STIM_CD8_TCELL_UP
147	0.37964272	1.5812371	0.004048583
0.005671784	0.984	2102	"tags=31%, list=15%, signal=36%"
GSE1791_CTRL_VS_NEUROMEDINU_IN_T_CELL_LINE_12H_DN
GSE1791_CTRL_VS_NEUROMEDINU_IN_T_CELL_LINE_12H_DN		97
0.40616202	1.5797745	0.006302521	0.005771175
0.988	1265	"tags=19%, list=9%, signal=20%"
GSE11973_MIR223_KOVS_WT_BONE_MARROW_NEUTROPHIL_UP
GSE11973_MIR223_KOVS_WT_BONE_MARROW_NEUTROPHIL_UP		163
0.37336558	1.5793257	0	0.005806068	0.989
2272	"tags=39%, list=16%, signal=46%"
GSE21033_CTRL_VS_POLYIC_STIM_DC_3H_DN
GSE21033_CTRL_VS_POLYIC_STIM_DC_3H_DN		137
0.38614276	1.5783644	0.002123142	0.005890215
0.99	2213	"tags=35%, list=16%, signal=41%"
GSE37416_CTRL_VS_48H_F_TULARENSIS_LVS_NEUTROPHIL_DN
GSE37416_CTRL_VS_48H_F_TULARENSIS_LVS_NEUTROPHIL_DN
140	0.38511878	1.5781811	0.002237137
0.005898831	0.991	2519	"tags=33%, list=18%, signal=40%"
GSE6092_CTRL_VS_BORRELIA_BIRGDOFERI_INF_ENDOTHELIAL_CELL_UP
GSE6092_CTRL_VS_BORRELIA_BIRGDOFERI_INF_ENDOTHELIAL_CELL_UP
125	0.38693178	1.5780327	0.002188184
0.005906199	0.991	2389	"tags=38%, list=17%, signal=46%"
GSE43955_1H_VS_20H_ACT_CD4_TCELL_WITH_TGFB_IL6_UP
GSE43955_1H_VS_20H_ACT_CD4_TCELL_WITH_TGFB_IL6_UP		162
0.3757371	1.5777214	0	0.005930858	0.992
2384	"tags=33%, list=17%, signal=39%"
GSE2770_UNTREATED_VS_ACT_CD4_TCELL_2H_DN
GSE2770_UNTREATED_VS_ACT_CD4_TCELL_2H_DN		139
0.38897678	1.5773599	0	0.005946823	0.992
1973	"tags=29%, list=14%, signal=33%"

GSE9988_ANTI_TREM1_VS_VEHICLE_TREATED_MONOCYTES_DN
GSE9988_ANTI_TREM1_VS_VEHICLE_TREATED_MONOCYTES_DN 153
0.37940896 1.5773253 0 0.005941814 0.992
2620 "tags=37%, list=19%, signal=45%"
GSE36078_WT_VS_IL1R_KO_LUNG_DC_DN GSE36078_WT_VS_IL1R_KO_LUNG_DC_DN
153 0.37930226 1.5765328 0.001930502
0.005994745 0.993 2350 "tags=42%, list=17%, signal=50%"
GSE40274_CTRL_VS_FOXP3_AND_SATB1_TRANSDUCED_ACTIVATED_CD4_TCELL_UP
GSE40274_CTRL_VS_FOXP3_AND_SATB1_TRANSDUCED_ACTIVATED_CD4_TCELL_UP
126 0.39283505 1.5759503 0.004464286
0.006038946 0.993 2418 "tags=37%, list=17%, signal=45%"
GSE21379_TFH_VS_NON_TFH_SAP_KO_CD4_TCELL_UP
GSE21379_TFH_VS_NON_TFH_SAP_KO_CD4_TCELL_UP 143
0.37983334 1.5750705 0.002053388 0.006108584
0.993 2474 "tags=32%, list=18%, signal=39%"
GSE45365_WT_VS_IFNAR_KO_BCELL_UP GSE45365_WT_VS_IFNAR_KO_BCELL_UP
122 0.39029208 1.5745846 0.006263048
0.006140214 0.993 1544 "tags=23%, list=11%, signal=26%"
GSE41867_DAY6_VS_DAY8_LCMV_ARMSTRONG_EFFECTOR_CD8_TCELL_UP
GSE41867_DAY6_VS_DAY8_LCMV_ARMSTRONG_EFFECTOR_CD8_TCELL_UP
158 0.37927407 1.5743902 0 0.006149754
0.993 2862 "tags=41%, list=20%, signal=51%"
GSE3982_NEUTROPHIL_VS_NKCELL_DN GSE3982_NEUTROPHIL_VS_NKCELL_DN
143 0.37975395 1.5734797 0.002164502
0.006209578 0.993 1622 "tags=27%, list=11%, signal=31%"
GSE27092_WT_VS_HDAC7_PHOSPHO_DEFICIENT_CD8_TCELL_DN
GSE27092_WT_VS_HDAC7_PHOSPHO_DEFICIENT_CD8_TCELL_DN
161 0.38044155 1.5731794 0 0.006229879
0.993 2012 "tags=27%, list=14%, signal=31%"
GSE15930_STIM_VS_STIM_AND_IFNAB_72H_CD8_T_CELL_DN
GSE15930_STIM_VS_STIM_AND_IFNAB_72H_CD8_T_CELL_DN 162
0.3745099 1.5718237 0 0.006346912 0.993
1819 "tags=28%, list=13%, signal=32%"
GSE2706_2H_VS_8H_R848_AND_LPS_STIM_DC_UP
GSE2706_2H_VS_8H_R848_AND_LPS_STIM_DC_UP 147
0.38364092 1.5717628 0.004201681 0.006346325
0.993 1858 "tags=33%, list=13%, signal=37%"
GSE17580_UNINFECTED_VS_S_MANSONI_INF_TEFF_DN
GSE17580_UNINFECTED_VS_S_MANSONI_INF_TEFF_DN 152
0.37687066 1.5717559 0.002040816 0.006339734
0.993 1621 "tags=26%, list=11%, signal=29%"
GSE17721_0.5H_VS_12H_GARDIQUIMOD_BMDC_UP
GSE17721_0.5H_VS_12H_GARDIQUIMOD_BMDC_UP 144
0.37859666 1.5710974 0.004115226 0.006384241
0.993 2511 "tags=38%, list=18%, signal=45%"
GSE9988_LPS_VS_CTRL_TREATED_MONOCYTE_DN
GSE9988_LPS_VS_CTRL_TREATED_MONOCYTE_DN 143
0.37834626 1.568886 0.00209205 0.006553038 0.993
1916 "tags=27%, list=14%, signal=30%"
GSE10856_CTRL_VS_TNFRSF6B_IN_MACROPHAGE_DN
GSE10856_CTRL_VS_TNFRSF6B_IN_MACROPHAGE_DN 123
0.3848313 1.5686076 0.006550218 0.006574097
0.993 2486 "tags=33%, list=18%, signal=39%"
GSE3565_CTRL_VS_LPS_INJECTED_SPLENOCYTES_UP

GSE3565_CTRL_VS_LPS_INJECTED_SPLENOCYTES_UP 148
0.3768584 1.5669492 0 0.006741728 0.993
2274 "tags=43%, list=16%, signal=50%"
GSE22935_WT_VS_MYD88_KO_MACROPHAGE_UP
GSE22935_WT_VS_MYD88_KO_MACROPHAGE_UP 169
0.37043935 1.5663316 0 0.006784463 0.993
1712 "tags=25%, list=12%, signal=29%"
GSE15930_STIM_VS_STIM_AND_TRICHOSTATINA_48H_CD8_T_CELL_UP
GSE15930_STIM_VS_STIM_AND_TRICHOSTATINA_48H_CD8_T_CELL_UP
154 0.3749493 1.5658001 0.00209205
0.006830721 0.993 2204 "tags=36%, list=16%, signal=43%"
GSE17721_0.5H_VS_8H_POLYIC_BMDC_UP
GSE17721_0.5H_VS_8H_POLYIC_BMDC_UP 161
0.37683246 1.5655977 0 0.00683943 0.993
1798 "tags=26%, list=13%, signal=30%"
GSE3982_NEUTROPHIL_VS_CENT_MEMORY_CD4_TCELL_DN
GSE3982_NEUTROPHIL_VS_CENT_MEMORY_CD4_TCELL_DN 128
0.3835098 1.5639539 0 0.006972677 0.993
1978 "tags=31%, list=14%, signal=36%"
GSE13485_CTRL_VS_DAY1_YF17D_VACCINE_PBMC_UP
GSE13485_CTRL_VS_DAY1_YF17D_VACCINE_PBMC_UP 130
0.38039348 1.5635759 0.004132231 0.006995781
0.993 2173 "tags=33%, list=15%, signal=39%"
GSE17721_CPG_VS_GARDIQUIMOD_8H_BMDC_UP
GSE17721_CPG_VS_GARDIQUIMOD_8H_BMDC_UP 154
0.37813148 1.5630114 0.00204918 0.007029606
0.993 1724 "tags=25%, list=12%, signal=28%"
GSE5589_LPS_VS_LPS_AND_IL10_STIM_IL10_KO_MACROPHAGE_45MIN_DN
GSE5589_LPS_VS_LPS_AND_IL10_STIM_IL10_KO_MACROPHAGE_45MIN_DN
143 0.3789538 1.5621743 0.004494382
0.007099373 0.993 2350 "tags=32%, list=17%, signal=38%"
GSE29164_UNTREATED_VS_CD8_TCELL_AND_IL12_TREATED_MELANOMA_DAY7_UP
GSE29164_UNTREATED_VS_CD8_TCELL_AND_IL12_TREATED_MELANOMA_DAY7_UP
137 0.3804969 1.5617772 0
0.007126852 0.995 2391 "tags=34%, list=17%, signal=40%"
GSE18791_CTRL_VS_NEWCASTLE_VIRUS_DC_14H_UP
GSE18791_CTRL_VS_NEWCASTLE_VIRUS_DC_14H_UP 147
0.37490973 1.5614542 0.002105263 0.007155654
0.995 2695 "tags=40%, list=19%, signal=49%"
GSE23308_WT_VS_MINERALCORTICOID_REC_KO_MACROPHAGE_UP
GSE23308_WT_VS_MINERALCORTICOID_REC_KO_MACROPHAGE_UP
139 0.3735309 1.5613453 0.002053388
0.007157912 0.995 1816 "tags=25%, list=13%, signal=29%"
GSE28726_NAIVE_CD4_TCELL_VS_NAIVE_NKTCELL_UP
GSE28726_NAIVE_CD4_TCELL_VS_NAIVE_NKTCELL_UP 148
0.37773594 1.560384 0 0.007244274 0.995
2490 "tags=43%, list=18%, signal=52%"
GSE14308_TH1_VS_NATURAL_TREG_UP GSE14308_TH1_VS_NATURAL_TREG_UP
148 0.37856612 1.5599284 0.004376368
0.007280101 0.995 2632 "tags=39%, list=19%, signal=48%"
GSE15930_STIM_VS_STIM_AND_TRICHOSTATINA_24H_CD8_T_CELL_DN
GSE15930_STIM_VS_STIM_AND_TRICHOSTATINA_24H_CD8_T_CELL_DN
151 0.37342262 1.5599151 0 0.007272671
0.995 2267 "tags=33%, list=16%, signal=39%"

GSE360_HIGH_VS_LOW_DOSE_B_MALAYI_MAC_UP
GSE360_HIGH_VS_LOW_DOSE_B_MALAYI_MAC_UP 140
0.37524796 1.5598589 0.002227172 0.007274865
0.995 1984 "tags=30%, list=14%, signal=35%"
GSE5542_UNTREATED_VS_IFNA_TREATED_EPITHELIAL_CELLS_6H_UP
GSE5542_UNTREATED_VS_IFNA_TREATED_EPITHELIAL_CELLS_6H_UP
141 0.37912384 1.5590767 0.004264392
0.007347642 0.995 1738 "tags=27%, list=12%, signal=30%"
GSE360_L_DONOVANI_VS_L_MAJOR_MAC_UP
GSE360_L_DONOVANI_VS_L_MAJOR_MAC_UP 148
0.3789573 1.5590358 0.00409836 0.007341375
0.995 2672 "tags=34%, list=19%, signal=42%"
GSE360_L_DONOVANI_VS_B_MALAYI_LOW_DOSE_MAC_DN
GSE360_L_DONOVANI_VS_B_MALAYI_LOW_DOSE_MAC_DN 150
0.36967456 1.5587559 0.002057613 0.00736124
0.995 1798 "tags=27%, list=13%, signal=31%"
GSE360_T_GONDII_VS_B_MALAYI_HIGH_DOSE_MAC_DN
GSE360_T_GONDII_VS_B_MALAYI_HIGH_DOSE_MAC_DN 157
0.3689763 1.5581068 0 0.00741452 0.995
2517 "tags=32%, list=18%, signal=38%"
GSE3982_EOSINOPHIL_VS_NKCELL_DN GSE3982_EOSINOPHIL_VS_NKCELL_DN
141 0.38176426 1.5577323 0.004140787
0.007453491 0.995 1870 "tags=24%, list=13%, signal=28%"
GSE37416_0H_VS_6H_F_TULARENSIS_LVS_NEUTROPHIL_UP
GSE37416_0H_VS_6H_F_TULARENSIS_LVS_NEUTROPHIL_UP 138
0.37907043 1.5575377 0 0.007463802 0.996
2223 "tags=30%, list=16%, signal=35%"
GSE360_L_DONOVANI_VS_B_MALAYI_HIGH_DOSE_DC_DN
GSE360_L_DONOVANI_VS_B_MALAYI_HIGH_DOSE_DC_DN 148
0.37574542 1.5554687 0.002079002 0.0076559
0.996 2389 "tags=32%, list=17%, signal=38%"
GSE411_UNSTIM_VS_100MIN_IL6_STIM_MACROPHAGE_UP
GSE411_UNSTIM_VS_100MIN_IL6_STIM_MACROPHAGE_UP 140
0.3742847 1.554416 0 0.007759846 0.998
2157 "tags=34%, list=15%, signal=39%"
GSE43955_10H_VS_60H_ACT_CD4_TCELL_UP
GSE43955_10H_VS_60H_ACT_CD4_TCELL_UP 161
0.37436202 1.5539938 0.003944773 0.007793457
0.998 2131 "tags=29%, list=15%, signal=34%"
GSE17721_0.5H_VS_8H_CPG_BMDC_UP GSE17721_0.5H_VS_8H_CPG_BMDC_UP
158 0.3726927 1.5532751 0.001996008
0.007862594 0.998 2209 "tags=32%, list=16%, signal=38%"
GSE13411_NAIVE_VS_SWITCHED_MEMORY_BCELL_DN
GSE13411_NAIVE_VS_SWITCHED_MEMORY_BCELL_DN 141
0.37606376 1.5530369 0.002173913 0.007874813
0.998 2089 "tags=30%, list=15%, signal=35%"
GSE16385_MONOCYTE_VS_12H_ROSIGLITAZONE_IL4_TREATED_MACROPHAGE_DN
GSE16385_MONOCYTE_VS_12H_ROSIGLITAZONE_IL4_TREATED_MACROPHAGE_DN
149 0.37263688 1.5530071 0.002141328
0.007869279 0.998 2082 "tags=34%, list=15%, signal=39%"
GSE27786_LIN_NEG_VS_ERYTHROBLAST_DN
GSE27786_LIN_NEG_VS_ERYTHROBLAST_DN 138
0.3800726 1.5527018 0.00845666 0.007892117
0.998 1084 "tags=12%, list=8%, signal=13%"

GSE25123_ROSIGLITAZONE_VS_IL4_AND_ROSIGLITAZONE_STIM_MACROPHAGE_DAY10_UP
GSE25123_ROSIGLITAZONE_VS_IL4_AND_ROSIGLITAZONE_STIM_MACROPHAGE_DAY10_UP 148 0.37477902 1.5522867 0.002109705 0.007928835 0.998 2390 "tags=36%, list=17%, signal=43%"
GSE3982_NEUTROPHIL_VS_CENT_MEMORY_CD4_TCELL_UP
GSE3982_NEUTROPHIL_VS_CENT_MEMORY_CD4_TCELL_UP 147 0.3798275 1.5521216 0.001992032 0.00794204 0.998 1840 "tags=29%, list=13%, signal=33%"
GSE3982_CTRL_VS_IGE_STIM_MAST_CELL_DN
GSE3982_CTRL_VS_IGE_STIM_MAST_CELL_DN 136 0.37961325 1.5520834 0 0.007940001 0.998 1290 "tags=21%, list=9%, signal=22%"
GSE42021_TREG_PLN_VS_CD24HI_TREG_THYMUS_DN
GSE42021_TREG_PLN_VS_CD24HI_TREG_THYMUS_DN 159 0.3752082 1.5520757 0.006423983 0.007932045 0.998 2792 "tags=43%, list=20%, signal=53%"
GSE42021_TCONV_PLN_VS_CD24INT_TCONV_THYMUS_DN
GSE42021_TCONV_PLN_VS_CD24INT_TCONV_THYMUS_DN 159 0.36964738 1.5519804 0.002118644 0.007935791 0.998 1715 "tags=23%, list=12%, signal=26%"
GSE20366_EX_VIVO_VS_DEC205_CONVERSION_NAIVE_CD4_TCELL_UP
GSE20366_EX_VIVO_VS_DEC205_CONVERSION_NAIVE_CD4_TCELL_UP 148 0.37309518 1.5519408 0.0041841 0.007931364 0.998 1011 "tags=20%, list=7%, signal=22%"
GSE24814_STAT5_KO_VS_WT_PRE_BCELL_DN
GSE24814_STAT5_KO_VS_WT_PRE_BCELL_DN 153 0.36801586 1.5518837 0.002212389 0.007930446 0.998 1753 "tags=27%, list=12%, signal=30%"
GSE20500_CTRL_VS_RARA_ANTAGONIST_TREATED_CD4_TCELL_UP
GSE20500_CTRL_VS_RARA_ANTAGONIST_TREATED_CD4_TCELL_UP 145 0.3766786 1.5518379 0.002145923 0.007926029 0.998 2660 "tags=32%, list=19%, signal=39%"
GSE8621_LPS_PRIMED_UNSTIM_VS_LPS_PRIMED_AND_LPS_STIM_MACROPHAGE_DN
GSE8621_LPS_PRIMED_UNSTIM_VS_LPS_PRIMED_AND_LPS_STIM_MACROPHAGE_DN 141 0.37880388 1.5508274 0.004 0.008019932 0.998 2407 "tags=33%, list=17%, signal=39%"
GSE17721_LPS_VS_POLYIC_6H_BMDC_DN GSE17721_LPS_VS_POLYIC_6H_BMDC_DN 148 0.37539217 1.5502843 0.004255319 0.008092624 0.998 2100 "tags=30%, list=15%, signal=35%"
GSE37301_MULTIPOTENT_PROGENITOR_VS_CD4_TCELL_DN
GSE37301_MULTIPOTENT_PROGENITOR_VS_CD4_TCELL_DN 144 0.3729083 1.5501258 0 0.008097325 0.998 1905 "tags=30%, list=13%, signal=34%"
GSE8621_UNSTIM_VS_LPS_PRIMED_UNSTIM_MACROPHAGE_DN
GSE8621_UNSTIM_VS_LPS_PRIMED_UNSTIM_MACROPHAGE_DN 136 0.3798228 1.549745 0.00210084 0.008134862 0.998 1919 "tags=30%, list=14%, signal=35%"
GSE5589_UNSTIM_VS_180MIN_LPS_AND_IL10_STIM_MACROPHAGE_DN
GSE5589_UNSTIM_VS_180MIN_LPS_AND_IL10_STIM_MACROPHAGE_DN 143 0.37601638 1.5495478 0.008948546 0.008152536 0.998 2947 "tags=42%, list=21%, signal=52%"
GSE17721_POLYIC_VS_PAM3CSK4_4H_BMDC_DN

GSE17721_POLYIC_VS_PAM3CSK4_4H_BMDC_DN 143
0.3784309 1.5486141 0.006355932 0.008256048
0.998 2277 "tags=33%, list=16%, signal=39%"
GSE17186_BLOOD_VS_CORD_BLOOD_CD21LOW_TRANSITIONAL_BCELL_DN
GSE17186_BLOOD_VS_CORD_BLOOD_CD21LOW_TRANSITIONAL_BCELL_DN
157 0.37286922 1.5482264 0 0.008280407
0.998 2215 "tags=30%, list=16%, signal=35%"
GSE21670_UNTREATED_VS_TGFB_IL6_TREATED_STAT3_KO_CD4_TCELL_DN
GSE21670_UNTREATED_VS_TGFB_IL6_TREATED_STAT3_KO_CD4_TCELL_DN
142 0.37130615 1.5477564 0.002028398
0.008336156 0.998 2374 "tags=42%, list=17%, signal=49%"
GSE6092_UNSTIM_VS_IFNG_STIM_ENDOTHELIAL_CELL_DN
GSE6092_UNSTIM_VS_IFNG_STIM_ENDOTHELIAL_CELL_DN 142
0.3718413 1.547162 0 0.008404573 0.999
2132 "tags=37%, list=15%, signal=43%"
GSE44732_UNSTIM_VS_IL27_STIM_IMATURE_DC_UP
GSE44732_UNSTIM_VS_IL27_STIM_IMATURE_DC_UP 142
0.37091085 1.5450885 0 0.008610868 1
1983 "tags=29%, list=14%, signal=33%"
GSE23568_ID3_TRANSDUCED_VS_ID3_KO_CD8_TCELL_UP
GSE23568_ID3_TRANSDUCED_VS_ID3_KO_CD8_TCELL_UP 156
0.3732626 1.5450217 0.003968254 0.008605845
1 1991 "tags=37%, list=14%, signal=43%"
GSE26030_UNSTIM_VS_RESTIM_TH17_DAY15_POST_POLARIZATION_DN
GSE26030_UNSTIM_VS_RESTIM_TH17_DAY15_POST_POLARIZATION_DN
147 0.3777152 1.5442342 0.00617284
0.008675061 1 2176 "tags=30%, list=15%, signal=35%"
GSE40274_CTRL_VS_IRF4_TRANSDUCED_ACTIVATED_CD4_TCELL_UP
GSE40274_CTRL_VS_IRF4_TRANSDUCED_ACTIVATED_CD4_TCELL_UP
128 0.3808835 1.5411342 0.002028398
0.009025077 1 2148 "tags=30%, list=15%, signal=36%"
GSE17186_NAIVE_VS_CD21LOW_TRANSITIONAL_BCELL_UP
GSE17186_NAIVE_VS_CD21LOW_TRANSITIONAL_BCELL_UP 154
0.36875102 1.5406781 0.002155172 0.009062348
1 2046 "tags=34%, list=14%, signal=39%"
GSE6269_E_COLI_VS_STREP_PNEUMO_INF_PBMC_DN
GSE6269_E_COLI_VS_STREP_PNEUMO_INF_PBMC_DN 120
0.38358757 1.5402412 0.002096436 0.009102103
1 1530 "tags=27%, list=11%, signal=30%"
GSE45365_HEALTHY_VS_MCMV_INFECTION_CD8A_DC_UP
GSE45365_HEALTHY_VS_MCMV_INFECTION_CD8A_DC_UP 146
0.37278587 1.5400091 0.002169197 0.009117327
1 1921 "tags=28%, list=14%, signal=32%"
GSE24574_BCL6_HIGH_TFH_VS_NAIVE_CD4_TCELL_DN
GSE24574_BCL6_HIGH_TFH_VS_NAIVE_CD4_TCELL_DN 153
0.36675882 1.5399213 0 0.00912096 1
2598 "tags=46%, list=18%, signal=56%"
GSE32986_UNSTIM_VS_CURDLAN_LOWDOSE_STIM_DC_UP
GSE32986_UNSTIM_VS_CURDLAN_LOWDOSE_STIM_DC_UP 153
0.36561763 1.5397973 0.00212766 0.009120126
1 2706 "tags=37%, list=19%, signal=46%"
GSE17721_POLYIC_VS_GARDIQUIMOD_24H_BMDC_DN
GSE17721_POLYIC_VS_GARDIQUIMOD_24H_BMDC_DN 152
0.36812282 1.5396802 0.003960396 0.009126199

1	2057	"tags=32%, list=15%, signal=37%"
GSE17721_LPS_VS_GARDIQUIMOD_16H_BMDC_DN
GSE17721_LPS_VS_GARDIQUIMOD_16H_BMDC_DN		152
0.37192938	1.5396099	0	0.009126428	1
2383	"tags=32%, list=17%, signal=38%"
GSE369_PRE_VS_POST_IL6_INJECTION_SOCS3_KO_LIVER_DN
GSE369_PRE_VS_POST_IL6_INJECTION_SOCS3_KO_LIVER_DN		158
0.36601362	1.5395738	0.002061856	0.009121003
1	2506	"tags=39%, list=18%, signal=47%"
GSE16522_ANTI_CD3CD28_STIM_VS_UNSTIM_MEMORY_CD8_TCELL_DN
GSE16522_ANTI_CD3CD28_STIM_VS_UNSTIM_MEMORY_CD8_TCELL_DN
139	0.37023184	1.5395465	0.010799136
0.009113243	1	2059	"tags=35%, list=15%, signal=40%"
GSE34515_CD16_NEG_VS_POS_MONOCYTE_UP
GSE34515_CD16_NEG_VS_POS_MONOCYTE_UP		145
0.37409547	1.5393674	0	0.009120512	1
1995	"tags=27%, list=14%, signal=31%"
GSE35685_CD34POS_CD10NEG_CD62LPOS_VS_CD34POS_CD10POS_BONE_MARROW_UP
GSE35685_CD34POS_CD10NEG_CD62LPOS_VS_CD34POS_CD10POS_BONE_MARROW_UP
	162	0.36905855	1.5390228	0
0.009147118	1	3002	"tags=41%, list=21%, signal=51%"
GSE21927_SPLEEN_VS_4T1_TUMOR_MONOCYTE_BALBC_DN
GSE21927_SPLEEN_VS_4T1_TUMOR_MONOCYTE_BALBC_DN		131
0.37885886	1.5380511	0	0.009267369	1
2588	"tags=34%, list=18%, signal=41%"
GSE30962_ACUTE_VS_CHRONIC_LCMV_PRIMARY_INF_CD8_TCELL_UP
GSE30962_ACUTE_VS_CHRONIC_LCMV_PRIMARY_INF_CD8_TCELL_UP
140	0.37366277	1.5379957	0.002164502
0.009266393	1	2126	"tags=34%, list=15%, signal=40%"
GSE5589_LPS_AND_IL10_VS_LPS_AND_IL6_STIM_IL6_KO_MACROPHAGE_45MIN_DN
GSE5589_LPS_AND_IL10_VS_LPS_AND_IL6_STIM_IL6_KO_MACROPHAGE_45MIN_DN
	145	0.36791486	1.5376871	0.002145923
0.009291806	1	3047	"tags=39%, list=22%, signal=50%"
GSE26030_UNSTIM_VS_RESTIM_TH1_DAY5_POST_POLARIZATION_DN
GSE26030_UNSTIM_VS_RESTIM_TH1_DAY5_POST_POLARIZATION_DN
154	0.3704244	1.5375109	0.002141328
0.00930221	1	1965	"tags=28%, list=14%, signal=32%"
GSE17721_0.5H_VS_12H_POLYIC_BMDC_UP
GSE17721_0.5H_VS_12H_POLYIC_BMDC_UP		144
0.36634168	1.5374558	0.002155172	0.009302324
1	1978	"tags=25%, list=14%, signal=29%"
GSE21927_SPLEEN_VS_TUMOR_MONOCYTE_BALBC_DN
GSE21927_SPLEEN_VS_TUMOR_MONOCYTE_BALBC_DN	129
0.37908295	1.5369778	0.008474576	0.009369599
1	2055	"tags=33%, list=15%, signal=39%"
GSE3039_NKT_CELL_VS_B1_BCELL_DN	GSE3039_NKT_CELL_VS_B1_BCELL_DN
	134	0.38108516	1.5354729	0.002227172
0.009540831	1	1901	"tags=26%, list=13%, signal=30%"
GSE16385_ROSIGLITAZONE_VS_UNTREATED_IFNG_TNF_STIM_MACROPHAGE_DN
GSE16385_ROSIGLITAZONE_VS_UNTREATED_IFNG_TNF_STIM_MACROPHAGE_DN
	143	0.37292537	1.5349915	0.002024292
0.009584006	1	2479	"tags=34%, list=18%, signal=41%"
GSE30971_CTRL_VS_LPS_STIM_MACROPHAGE_WBP7_KO_2H_DN
GSE30971_CTRL_VS_LPS_STIM_MACROPHAGE_WBP7_KO_2H_DN		134

0.37650427 1.5336496 0.006048387 0.009764202
1 2118 "tags=31%, list=15%, signal=36%"
GSE17721_CPG_VS_GARDIQUIMOD_6H_BMDC_DN
GSE17721_CPG_VS_GARDIQUIMOD_6H_BMDC_DN 156
0.3654791 1.5327011 0.002118644 0.009862682
1 1733 "tags=26%, list=12%, signal=30%"
GSE39820_CTRL_VS_TGFBETA3_IL6_IL23A_CD4_TCELL_DN
GSE39820_CTRL_VS_TGFBETA3_IL6_IL23A_CD4_TCELL_DN 153
0.3719422 1.532474 0.006651885 0.00988504 1
2204 "tags=30%, list=16%, signal=35%"
GSE17721_POLYIC_VS_CPG_0.5H_BMDC_DN
GSE17721_POLYIC_VS_CPG_0.5H_BMDC_DN 147
0.3720376 1.5320238 0 0.009938914 1
2209 "tags=23%, list=16%, signal=27%"
GSE41176_WT_VS_TAK1_KO_ANTI_IGM_STIM_BCELL_3H_DN
GSE41176_WT_VS_TAK1_KO_ANTI_IGM_STIM_BCELL_3H_DN 154
0.36923182 1.5317096 0.002136752 0.009975651
1 1402 "tags=23%, list=10%, signal=25%"
GSE7509_UNSTIM_VS_FCGRIIB_STIM_DC_DN
GSE7509_UNSTIM_VS_FCGRIIB_STIM_DC_DN 144 0.36788
1.5305678 0.007984032 0.01012488 1
2358 "tags=36%, list=17%, signal=43%"
GSE36891_UNSTIM_VS_PAM_TLR2_STIM_PERITONEAL_MACROPHAGE_DN
GSE36891_UNSTIM_VS_PAM_TLR2_STIM_PERITONEAL_MACROPHAGE_DN
122 0.38212347 1.5299678 0.002105263
0.010211033 1 2138 "tags=33%, list=15%, signal=38%"
GSE10273_LOW_IL7_VS_HIGH_IL7_AND_IRF4_IN_IRF4_8_NULL_PRE_BCELL_DN
GSE10273_LOW_IL7_VS_HIGH_IL7_AND_IRF4_IN_IRF4_8_NULL_PRE_BCELL_DN
123 0.3760292 1.529665 0.0041841
0.010248644 1 1608 "tags=23%, list=11%, signal=25%"
GSE42021_CD24HI_VS_CD24LOW_TCONV_THYMUS_UP
GSE42021_CD24HI_VS_CD24LOW_TCONV_THYMUS_UP 149
0.36993143 1.5296364 0.002114165 0.010247793
1 1809 "tags=23%, list=13%, signal=26%"
GSE27786_NKTCELL_VS_NEUTROPHIL_DN GSE27786_NKTCELL_VS_NEUTROPHIL_DN
144 0.3691845 1.5295577 0.008385744
0.01024803 1 1725 "tags=22%, list=12%, signal=24%"
GSE17721_CTRL_VS_GARDIQUIMOD_12H_BMDC_UP
GSE17721_CTRL_VS_GARDIQUIMOD_12H_BMDC_UP 150
0.36779213 1.5292392 0.006237006 0.010284326
1 1667 "tags=25%, list=12%, signal=28%"
GSE17721_0.5H_VS_4H_POLYIC_BMDC_UP
GSE17721_0.5H_VS_4H_POLYIC_BMDC_UP 154
0.36569053 1.5289106 0.002004008 0.010321557
1 1854 "tags=23%, list=13%, signal=27%"
GSE17721_0.5H_VS_24H_POLYIC_BMDC_UP
GSE17721_0.5H_VS_24H_POLYIC_BMDC_UP 143
0.37063235 1.5286438 0.004338395 0.010337502
1 1864 "tags=26%, list=13%, signal=30%"
GSE34179_THPOK_KO_VS_WT_VA14I_NKTCELL_DN
GSE34179_THPOK_KO_VS_WT_VA14I_NKTCELL_DN 148
0.37425154 1.5279394 0 0.010422776 1
1828 "tags=23%, list=13%, signal=26%"
GSE8384_CTRL_VS_B_ABORTUS_4H_MAC_CELL_LINE_UP

GSE8384_CTRL_VS_B_ABORTUS_4H_MAC_CELL_LINE_UP 153
0.36688617 1.5277096 0.002070393 0.010453215
1 1848 "tags=27%, list=13%, signal=31%"
GSE14769_UNSTIM_VS_240MIN_LPS_BMDM_UP
GSE14769_UNSTIM_VS_240MIN_LPS_BMDM_UP 150
0.3667797 1.5274115 0 0.010486809 1
2271 "tags=31%, list=16%, signal=36%"
GSE21546_WT_VS_SAP1A_KO_AND_ELK1_KO_ANTI_CD3_STIM_DP_THYMOCYTES_DN
GSE21546_WT_VS_SAP1A_KO_AND_ELK1_KO_ANTI_CD3_STIM_DP_THYMOCYTES_DN
149 0.36729962 1.5273368 0.006342495
0.010485784 1 1511 "tags=24%, list=11%, signal=27%"
GSE25123_IL4_VS_IL4_AND_ROSIGLITAZONE_STIM_MACROPHAGE_DAY10_UP
GSE25123_IL4_VS_IL4_AND_ROSIGLITAZONE_STIM_MACROPHAGE_DAY10_UP
156 0.3674501 1.526009 0 0.010665714
1 1802 "tags=28%, list=13%, signal=31%"
GSE3720_VD1_VS_VD2_GAMMADELTA_TCELL_DN
GSE3720_VD1_VS_VD2_GAMMADELTA_TCELL_DN 149
0.36688685 1.5259145 0.004366812 0.010665628
1 1270 "tags=19%, list=9%, signal=21%"
GSE33425_CD161_INT_VS_NEG_CD8_TCELL_UP
GSE33425_CD161_INT_VS_NEG_CD8_TCELL_UP 157
0.36634162 1.5258094 0.004192872 0.01067675
1 2157 "tags=35%, list=15%, signal=41%"
GSE11961_PLASMA_CELL_DAY7_VS_MEMORY_BCELL_DAY40_UP
GSE11961_PLASMA_CELL_DAY7_VS_MEMORY_BCELL_DAY40_UP 155
0.36909673 1.5257293 0 0.010677742 1
2742 "tags=34%, list=19%, signal=42%"
GSE2770_IL12_AND_TGFB_ACT_VS_ACT_CD4_TCELL_2H_DN
GSE2770_IL12_AND_TGFB_ACT_VS_ACT_CD4_TCELL_2H_DN 129
0.37709308 1.5250784 0.006329114 0.010756509
1 2155 "tags=33%, list=15%, signal=39%"
GSE14308_TH1_VS_NAIVE_CD4_TCELL_UP
GSE14308_TH1_VS_NAIVE_CD4_TCELL_UP 140
0.37181637 1.5249889 0 0.010760752 1
2817 "tags=41%, list=20%, signal=50%"
GSE6090_UNSTIM_VS_DC_SIGN_STIM_DC_UP
GSE6090_UNSTIM_VS_DC_SIGN_STIM_DC_UP 132
0.37502748 1.5247959 0 0.01078276 1
2393 "tags=29%, list=17%, signal=34%"
GSE11864_UNTREATED_VS_CSF1_PAM3CYS_IN_MAC_DN
GSE11864_UNTREATED_VS_CSF1_PAM3CYS_IN_MAC_DN 131
0.37270728 1.5247604 0.004056795 0.010777049
1 2329 "tags=27%, list=17%, signal=32%"
GSE21927_UNTREATED_VS_GMCSF_IL6_TREATED_BONE_MARROW_UP
GSE21927_UNTREATED_VS_GMCSF_IL6_TREATED_BONE_MARROW_UP
113 0.38207603 1.5245348 0.004 0.010795672
1 1810 "tags=23%, list=13%, signal=26%"
GSE29618_PDC_VS_MDC_DAY7_FLU_VACCINE_DN
GSE29618_PDC_VS_MDC_DAY7_FLU_VACCINE_DN 152
0.3640018 1.5244014 0.002020202 0.010800011
1 1682 "tags=30%, list=12%, signal=34%"
GSE5589_WT_VS_IL10_KO_LPS_STIM_MACROPHAGE_180MIN_UP
GSE5589_WT_VS_IL10_KO_LPS_STIM_MACROPHAGE_180MIN_UP
122 0.37819663 1.5242573 0.014861995

0.010814182 1 2564 "tags=38%, list=18%, signal=46%"
GSE23308_WT_VS_MINERALCORTICOID_REC_KO_MACROPHAGE_CORTICOSTERONE_TREATED_DN
GSE23308_WT_VS_MINERALCORTICOID_REC_KO_MACROPHAGE_CORTICOSTERONE_TREATED_DN 156 0.3658038 1.5239948 0
0.010834836 1 2051 "tags=30%, list=15%, signal=35%"
GSE27291_6H_VS_7D_STIM_GAMMADELTA_TCELL_UP
GSE27291_6H_VS_7D_STIM_GAMMADELTA_TCELL_UP 137
0.37548038 1.5239619 0.008298756 0.010829106
1 1842 "tags=26%, list=13%, signal=29%"
GSE18791_CTRL_VS_NEWCASTLE_VIRUS_DC_10H_UP
GSE18791_CTRL_VS_NEWCASTLE_VIRUS_DC_10H_UP 134
0.37094074 1.5231556 0.002070393 0.010934717
1 2191 "tags=28%, list=16%, signal=33%"
GSE12198_CTRL_VS_LOW_IL2_STIM_NK_CELL_DN
GSE12198_CTRL_VS_LOW_IL2_STIM_NK_CELL_DN 153
0.37123618 1.5230862 0.002109705 0.010934319
1 1508 "tags=24%, list=11%, signal=26%"
GSE17721_POLYIC_VS_PAM3CSK4_6H_BMDC_DN
GSE17721_POLYIC_VS_PAM3CSK4_6H_BMDC_DN 156
0.35964826 1.5219603 0.002109705 0.011088129
1 2548 "tags=35%, list=18%, signal=42%"
GSE45365_CD8A_DC_VS_CD11B_DC_UP GSE45365_CD8A_DC_VS_CD11B_DC_UP
135 0.37494016 1.5210376 0.004237288
0.011179043 1 2033 "tags=22%, list=14%, signal=26%"
GSE8621_UNSTIM_VS_LPS_PRIMED_AND_LPS_STIM_MACROPHAGE_UP
GSE8621_UNSTIM_VS_LPS_PRIMED_AND_LPS_STIM_MACROPHAGE_UP
157 0.3588075 1.5197031 0.004255319
0.011372644 1 1490 "tags=17%, list=11%, signal=18%"
GSE4984_GALECTIN1_VS_LPS_STIM_DC_DN
GSE4984_GALECTIN1_VS_LPS_STIM_DC_DN 128
0.37306765 1.5197017 0.002118644 0.011362015
1 1997 "tags=28%, list=14%, signal=32%"
GSE37416_CTRL_VS_6H_F_TULARENSIS_LVS_NEUTROPHIL_UP
GSE37416_CTRL_VS_6H_F_TULARENSIS_LVS_NEUTROPHIL_UP 128
0.37767392 1.5194628 0.00210084 0.011378785
1 2223 "tags=34%, list=16%, signal=40%"
GSE13306_TREG_VS_TCONV_DN GSE13306_TREG_VS_TCONV_DN 141
0.36446953 1.5191413 0.004301075 0.011405407
1 2189 "tags=33%, list=16%, signal=38%"
GSE22886_NAIVE_TCELL_VS_NKCELL_UP GSE22886_NAIVE_TCELL_VS_NKCELL_UP
138 0.37112415 1.5191166 0.006134969
0.011395874 1 1694 "tags=30%, list=12%, signal=33%"
GSE13485_DAY1_VS_DAY7_YF17D_VACCINE_PBMC_DN
GSE13485_DAY1_VS_DAY7_YF17D_VACCINE_PBMC_DN 130
0.3801409 1.5189819 0.00877193 0.011404933
1 1671 "tags=34%, list=12%, signal=38%"
GSE46606_UNSTIM_VS_CD40L_IL2_IL5_DAY3_STIMULATED_BCELL_DN
GSE46606_UNSTIM_VS_CD40L_IL2_IL5_DAY3_STIMULATED_BCELL_DN
136 0.3686041 1.5187749 0 0.011419357
1 2586 "tags=36%, list=18%, signal=44%"
GSE17721_LPS_VS_GARDIQUIMOD_1H_BMDC_DN
GSE17721_LPS_VS_GARDIQUIMOD_1H_BMDC_DN 153
0.3598188 1.5185156 0.004166667 0.011434947

1 2200 "tags=28%, list=16%, signal=33%"
GSE11961_MARGINAL_ZONE_BCELL_VS_PLASMA_CELL_DAY7_DN
GSE11961_MARGINAL_ZONE_BCELL_VS_PLASMA_CELL_DAY7_DN
136 0.36942083 1.5183568 0.010570824
0.011450494 1 2252 "tags=32%, list=16%, signal=37%"
GSE1112_OT1_VS_HY_CD8AB_THYMOCYTE_RTOC_CULTURE_UP
GSE1112_OT1_VS_HY_CD8AB_THYMOCYTE_RTOC_CULTURE_UP 138
0.37454724 1.518053 0.002141328 0.011483422 1
2361 "tags=32%, list=17%, signal=38%"
GSE22886_IL2_VS_IL15_STIM_NKCELL_DN
GSE22886_IL2_VS_IL15_STIM_NKCELL_DN 139
0.36867785 1.5180469 0.006535948 0.011473859
1 2474 "tags=34%, list=18%, signal=41%"
GSE22229_RENAL_TRANSPLANT_IMMUNOSUPP_THERAPY_VS_HEALTHY_PBMC_DN
GSE22229_RENAL_TRANSPLANT_IMMUNOSUPP_THERAPY_VS_HEALTHY_PBMC_DN
152 0.36462244 1.5176132 0.006696429
0.011535997 1 1766 "tags=25%, list=13%, signal=28%"
GSE360_HIGH_DOSE_B_MALAYI_VS_M_TUBERCULOSIS_MAC_UP
GSE360_HIGH_DOSE_B_MALAYI_VS_M_TUBERCULOSIS_MAC_UP 142
0.37037966 1.5156311 0.006507592 0.011823176
1 2506 "tags=33%, list=18%, signal=40%"
GSE10273_HIGH_IL7_VS_HIGH_IL7_AND_IRF4_IN_IRF4_8_NULL_PRE_BCELL_UP
GSE10273_HIGH_IL7_VS_HIGH_IL7_AND_IRF4_IN_IRF4_8_NULL_PRE_BCELL_UP
149 0.36688414 1.5145198 0.003992016
0.011968679 1 2345 "tags=30%, list=17%, signal=35%"
GSE14308_TH17_VS_NATURAL_TREG_UP GSE14308_TH17_VS_NATURAL_TREG_UP
146 0.3695463 1.5141283 0.012219959
0.012007459 1 1819 "tags=29%, list=13%, signal=33%"
GSE17721_LPS_VS_PAM3CSK4_4H_BMDC_DN
GSE17721_LPS_VS_PAM3CSK4_4H_BMDC_DN 142
0.36624876 1.5136788 0.006048387 0.012060397
1 2343 "tags=32%, list=17%, signal=38%"
GSE17721_0.5H_VS_12H_LPS_BMDC_UP GSE17721_0.5H_VS_12H_LPS_BMDC_UP
135 0.36798492 1.5135751 0
0.012064413 1 1864 "tags=28%, list=13%, signal=32%"
GSE22103_LPS_VS_GMCSF_AND_IFNG_STIM_NEUTROPHIL_UP
GSE22103_LPS_VS_GMCSF_AND_IFNG_STIM_NEUTROPHIL_UP 132
0.36782497 1.5133684 0.004395605 0.012083578
1 2722 "tags=42%, list=19%, signal=51%"
GSE2128_C57BL6_VS_NOD_THYMOCYTE_MIMETOPE_NEGATIVE_SELECTION_DN
GSE2128_C57BL6_VS_NOD_THYMOCYTE_MIMETOPE_NEGATIVE_SELECTION_DN
148 0.36532962 1.513077 0.002087683
0.012117856 1 2424 "tags=34%, list=17%, signal=41%"
GSE17721_POLYIC_VS_GARDIQUIMOD_6H_BMDC_DN
GSE17721_POLYIC_VS_GARDIQUIMOD_6H_BMDC_DN 154
0.36062643 1.5127738 0.002164502 0.012165108
1 2611 "tags=36%, list=19%, signal=43%"
GSE21379_WT_VS_SAP_KO_CD4_TCELL_DN
GSE21379_WT_VS_SAP_KO_CD4_TCELL_DN 149
0.36236355 1.5122517 0.006024096 0.012229487
1 1515 "tags=23%, list=11%, signal=26%"
GSE25123_IL4_VS_IL4_AND_ROSIGLITAZONE_STIM_PPARG_KO_MACROPHAGE_DAY10_UP
GSE25123_IL4_VS_IL4_AND_ROSIGLITAZONE_STIM_PPARG_KO_MACROPHAGE_DAY10

_UP 107 0.3781922 1.5109183
0.005928854 0.012440501 1 2180 "tags=29%,
list=15%, signal=34%"
GSE11924_TFH_VS_TH2_CD4_TCELL_DN GSE11924_TFH_VS_TH2_CD4_TCELL_DN
144 0.36461774 1.5109007 0.004175365
0.012432341 1 2381 "tags=30%, list=17%, signal=36%"
GSE32901_TH1_VS_TH17_NEG_CD4_TCELL_DN
GSE32901_TH1_VS_TH17_NEG_CD4_TCELL_DN 93
0.39116958 1.5106761 0.00203666 0.01246173
1 764 "tags=17%, list=5%, signal=18%"
GSE4142_PLASMA_CELL_VS_GC_BCELL_DN
GSE4142_PLASMA_CELL_VS_GC_BCELL_DN 138
0.37292242 1.5105668 0.00678733 0.012465359
1 1972 "tags=31%, list=14%, signal=36%"
GSE5589_UNSTIM_VS_45MIN_LPS_STIM_MACROPHAGE_UP
GSE5589_UNSTIM_VS_45MIN_LPS_STIM_MACROPHAGE_UP 153
0.36573938 1.5103135 0.002087683 0.01248944
1 2744 "tags=36%, list=19%, signal=44%"
GSE17974_CTRL_VS_ACT_IL4_AND_ANTI_IL12_12H_CD4_TCELL_DN
GSE17974_CTRL_VS_ACT_IL4_AND_ANTI_IL12_12H_CD4_TCELL_DN
143 0.3680829 1.5100245 0.00589391
0.012539035 1 1750 "tags=31%, list=12%, signal=36%"
GSE44955_MCSF_VS_MCSF_AND_IL27_STIM_MACROPHAGE_UP
GSE44955_MCSF_VS_MCSF_AND_IL27_STIM_MACROPHAGE_UP 144
0.36341053 1.5100145 0.009009009 0.012528664
1 2339 "tags=31%, list=17%, signal=37%"
GSE18893_CTRL_VS_TNF_TREATED_TCONV_2H_DN
GSE18893_CTRL_VS_TNF_TREATED_TCONV_2H_DN 139
0.36628503 1.5091122 0.004175365 0.012640331
1 2345 "tags=29%, list=17%, signal=35%"
GSE369_IFNG_KO_VS_WT_LIVER_DN GSE369_IFNG_KO_VS_WT_LIVER_DN
141 0.36458054 1.5078237 0.003929273
0.01284928 1 2660 "tags=36%, list=19%, signal=44%"
GSE32423_CTRL_VS_IL4_MEMORY_CD8_TCELL_DN
GSE32423_CTRL_VS_IL4_MEMORY_CD8_TCELL_DN 139
0.36687726 1.5073799 0.00204918 0.012916703
1 2336 "tags=32%, list=17%, signal=38%"
GSE22196_HEALTHY_VS_OBESE_MOUSE_SKIN_GAMMADELTA_TCELL_UP
GSE22196_HEALTHY_VS_OBESE_MOUSE_SKIN_GAMMADELTA_TCELL_UP
142 0.36083168 1.5071192 0.00212766
0.012952987 1 1521 "tags=25%, list=11%, signal=28%"
GSE23568_CTRL_TRANSDUCED_VS_WT_CD8_TCELL_DN
GSE23568_CTRL_TRANSDUCED_VS_WT_CD8_TCELL_DN 165
0.35887402 1.5068821 0.004115226 0.012980676
1 2275 "tags=38%, list=16%, signal=44%"
GSE18791_CTRL_VS_NEWCASTLE_VIRUS_DC_4H_UP
GSE18791_CTRL_VS_NEWCASTLE_VIRUS_DC_4H_UP 115
0.37582988 1.5066642 0.010638298 0.013002979
1 1920 "tags=27%, list=14%, signal=31%"
GSE5589_UNSTIM_VS_45MIN_LPS_AND_IL6_STIM_MACROPHAGE_DN
GSE5589_UNSTIM_VS_45MIN_LPS_AND_IL6_STIM_MACROPHAGE_DN
144 0.36176 1.5058805 0.00631579 0.013113609
1 2586 "tags=32%, list=18%, signal=39%"
GSE40274_FOXP3_VS_FOXP3_AND_PBX1_TRANSDUCED_ACTIVATED_CD4_TCELL_UP

GSE40274_FOXP3_VS_FOXP3_AND_PBX1_TRANSDUCED_ACTIVATED_CD4_TCELL_UP
144 0.3629832 1.5044116 0.002173913
0.01334692 1 2003 "tags=31%, list=14%, signal=35%"
GSE18893_TCONV_VS_TREG_2H_CULTURE_DN
GSE18893_TCONV_VS_TREG_2H_CULTURE_DN 153
0.36407697 1.5038337 0.00409836 0.013426553
1 2274 "tags=36%, list=16%, signal=42%"
GSE21546_WT_VS_SAP1A_KO_ANTI_CD3_STIM_DP_THYMOCYTES_UP
GSE21546_WT_VS_SAP1A_KO_ANTI_CD3_STIM_DP_THYMOCYTES_UP
137 0.3650449 1.5037044 0.006451613
0.01344082 1 1725 "tags=25%, list=12%, signal=28%"
GSE43955_TH0_VS_TGFB_IL6_TH17_ACT_CD4_TCELL_42H_UP
GSE43955_TH0_VS_TGFB_IL6_TH17_ACT_CD4_TCELL_42H_UP 156
0.35822067 1.5034692 0.00660793 0.013461581
1 2706 "tags=37%, list=19%, signal=45%"
GSE1460_CD4_THYMOCYTE_VS_NAIVE_CD4_TCELL_CORD_BLOOD_UP
GSE1460_CD4_THYMOCYTE_VS_NAIVE_CD4_TCELL_CORD_BLOOD_UP
159 0.36587507 1.5018126 0.006036217
0.013721551 1 2462 "tags=38%, list=17%, signal=45%"
GSE13484_UNSTIM_VS_12H_YF17D_VACCINE_STIM_PBMC_UP
GSE13484_UNSTIM_VS_12H_YF17D_VACCINE_STIM_PBMC_UP 138
0.36564752 1.501612 0.010638298 0.013744262 1
2679 "tags=33%, list=19%, signal=40%"
GSE17721_LPS_VS_PAM3CSK4_24H_BMDC_DN
GSE17721_LPS_VS_PAM3CSK4_24H_BMDC_DN 146
0.36712527 1.5013404 0.004444445 0.013789982
1 2533 "tags=35%, list=18%, signal=42%"
GSE40274_CTRL_VS_SATB1_TRANSDUCED_ACTIVATED_CD4_TCELL_UP
GSE40274_CTRL_VS_SATB1_TRANSDUCED_ACTIVATED_CD4_TCELL_UP
138 0.3624638 1.5012419 0.008528785
0.013793511 1 1592 "tags=23%, list=11%, signal=26%"
GSE23984_CTRL_VS_HYPOCALEMIC_VITAMIND_ANALOG_TCELL_UP
GSE23984_CTRL_VS_HYPOCALEMIC_VITAMIND_ANALOG_TCELL_UP
149 0.36444494 1.5008947 0.004219409
0.013846589 1 2616 "tags=40%, list=19%, signal=48%"
GSE5542_UNTREATED_VS_IFNG_TREATED_EPITHELIAL_CELLS_24H_UP
GSE5542_UNTREATED_VS_IFNG_TREATED_EPITHELIAL_CELLS_24H_UP
151 0.3638981 1.5006057 0.004024145
0.013891164 1 2030 "tags=32%, list=14%, signal=37%"
GSE6259_FLT3L_INDUCED_33D1_POS_DC_VS_BCELL_DN
GSE6259_FLT3L_INDUCED_33D1_POS_DC_VS_BCELL_DN 148
0.36309522 1.5005317 0.004048583 0.013896558
1 2422 "tags=36%, list=17%, signal=44%"
GSE13522_CTRL_VS_T_CRUZI_Y_STRAIN_INF_SKIN_BALBC_MOUSE_UP
GSE13522_CTRL_VS_T_CRUZI_Y_STRAIN_INF_SKIN_BALBC_MOUSE_UP
103 0.37946093 1.5000446 0.010799136
0.013953796 1 1829 "tags=26%, list=13%, signal=30%"
GSE17721_CTRL_VS_POLYIC_0.5H_BMDC_UP
GSE17721_CTRL_VS_POLYIC_0.5H_BMDC_UP 159
0.35997882 1.4996624 0.002109705 0.014014769
1 2421 "tags=31%, list=17%, signal=37%"
GSE29618_PDC_VS_MDC_DN GSE29618_PDC_VS_MDC_DN 141
0.36473653 1.4991477 0.013916501 0.014092472
1 1809 "tags=31%, list=13%, signal=35%"

GSE1460_INTRATHYMIC_T_PROGENITOR_VS_CD4_THYMOCYTE_UP
GSE1460_INTRATHYMIC_T_PROGENITOR_VS_CD4_THYMOCYTE_UP
151 0.3555643 1.4990509 0.00409836
0.014099769 1 1239 "tags=26%, list=9%, signal=28%"
GSE23321_EFFECTOR_MEMORY_VS_NAIVE_CD8_TCELL_DN
GSE23321_EFFECTOR_MEMORY_VS_NAIVE_CD8_TCELL_DN 129
0.36852705 1.498598 0.004329004 0.014182856 1
2222 "tags=29%, list=16%, signal=35%"
GSE20198_IL12_VS_IFNA_TREATED_ACT_CD4_TCELL_UP
GSE20198_IL12_VS_IFNA_TREATED_ACT_CD4_TCELL_UP 136
0.36766633 1.498243 0.004454343 0.014242408 1
2892 "tags=36%, list=20%, signal=45%"
GSE18791_CTRL_VS_NEWCASTLE_VIRUS_DC_12H_UP
GSE18791_CTRL_VS_NEWCASTLE_VIRUS_DC_12H_UP 129
0.36639905 1.4979451 0.006289308 0.014283114
1 2282 "tags=29%, list=16%, signal=34%"
GSE9037_WT_VS_IRAK4_KO_LPS_1H_STIM_BMDM_DN
GSE9037_WT_VS_IRAK4_KO_LPS_1H_STIM_BMDM_DN 135
0.36754328 1.4977012 0.006437768 0.014315377
1 703 "tags=13%, list=5%, signal=13%"
GSE17721_0.5H_VS_24H_CPG_BMDC_UP GSE17721_0.5H_VS_24H_CPG_BMDC_UP
149 0.35797822 1.4972688 0.004273505
0.014367272 1 1581 "tags=23%, list=11%, signal=26%"
GSE22886_NAIVE_CD8_TCELL_VS_DC_DN GSE22886_NAIVE_CD8_TCELL_VS_DC_DN
156 0.3575303 1.496768 0.00203666
0.014444572 1 2213 "tags=33%, list=16%, signal=39%"
GSE42021_TCONV_PLN_VS_CD24LO_TCONV_THYMUS_DN
GSE42021_TCONV_PLN_VS_CD24LO_TCONV_THYMUS_DN 151
0.35764948 1.4964699 0.00625 0.014488023 1
1701 "tags=23%, list=12%, signal=25%"
GSE29618_BCELL_VS_PDC_DN GSE29618_BCELL_VS_PDC_DN 156
0.3564622 1.4963185 0.002053388 0.01450218
1 1844 "tags=27%, list=13%, signal=31%"
GSE17721_POLYIC_VS_GARDIQUIMOD_4H_BMDC_UP
GSE17721_POLYIC_VS_GARDIQUIMOD_4H_BMDC_UP 152
0.35700363 1.4953816 0.004106776 0.014645564
1 2382 "tags=28%, list=17%, signal=33%"
GSE46242_CTRL_VS_EGR2_DELETED_ANERGIC_TH1_CD4_TCELL_UP
GSE46242_CTRL_VS_EGR2_DELETED_ANERGIC_TH1_CD4_TCELL_UP
134 0.36757842 1.4945228 0.008368201
0.014814527 1 1388 "tags=25%, list=10%, signal=28%"
GSE19923_E2A_KO_VS_HEB_AND_E2A_KO_DP_THYMOCYTE_UP
GSE19923_E2A_KO_VS_HEB_AND_E2A_KO_DP_THYMOCYTE_UP 153
0.35769972 1.4943154 0.00390625 0.014836695
1 1719 "tags=27%, list=12%, signal=31%"
GSE8921_UNSTIM_0H_VS_TLR1_2_STIM_MONOCYTE_6H_UP
GSE8921_UNSTIM_0H_VS_TLR1_2_STIM_MONOCYTE_6H_UP 145
0.36488733 1.4942787 0.008421052 0.014833999
1 2114 "tags=28%, list=15%, signal=33%"
GSE27786_NKCELL_VS_MONO_MAC_DN GSE27786_NKCELL_VS_MONO_MAC_DN
143 0.3660644 1.4940943 0.006382979
0.014855096 1 1924 "tags=24%, list=14%, signal=28%"
GSE26351_UNSTIM_VS_BMP_PATHWAY_STIM_HEMATOPOIETIC_PROGENITORS_DN
GSE26351_UNSTIM_VS_BMP_PATHWAY_STIM_HEMATOPOIETIC_PROGENITORS_DN

133	0.37019682	1.494075	0.012195122
0.014847125	1	2539	"tags=34%, list=18%, signal=41%"
GSE1460_DP_VS_CD4_THYMOCYTE_UP	GSE1460_DP_VS_CD4_THYMOCYTE_UP
157	0.35876453	1.4937514	0.004237288
0.014892099	1	1882	"tags=27%, list=13%, signal=31%"
GSE360_LOW_DOSE_B_MALAYI_VS_M_TUBERCULOSIS_MAC_UP
GSE360_LOW_DOSE_B_MALAYI_VS_M_TUBERCULOSIS_MAC_UP	149
0.36161983	1.4927943	0.004048583	0.015047675
1	1880	"tags=27%, list=13%, signal=31%"
GSE17721_CPG_VS_GARDIQUIMOD_24H_BMDC_UP
GSE17721_CPG_VS_GARDIQUIMOD_24H_BMDC_UP	154
0.35722667	1.4919659	0.00862069	0.015207166
1	1580	"tags=20%, list=11%, signal=22%"
GSE17974_0H_VS_6H_IN_VITRO_ACT_CD4_TCELL_DN
GSE17974_0H_VS_6H_IN_VITRO_ACT_CD4_TCELL_DN	147
0.36211604	1.4917401	0.004705882	0.015239004
1	1426	"tags=27%, list=10%, signal=29%"
GSE34515_CD16_NEG_MONOCYTE_VS_DC_DN
GSE34515_CD16_NEG_MONOCYTE_VS_DC_DN	145
0.36819366	1.4914463	0.008948546	0.015286383
1	1498	"tags=24%, list=11%, signal=27%"
GSE17721_LPS_VS_CPG_6H_BMDC_DN	GSE17721_LPS_VS_CPG_6H_BMDC_DN
145	0.3584711	1.4913207	0.002173913
0.015292518	1	2533	"tags=33%, list=18%, signal=40%"
GSE17721_0.5H_VS_8H_PAM3CSK4_BMDC_UP
GSE17721_0.5H_VS_8H_PAM3CSK4_BMDC_UP	150
0.3607117	1.4912603	0.00209205	0.015292488
1	2767	"tags=41%, list=20%, signal=50%"
GSE17721_0.5H_VS_4H_CPG_BMDC_UP	GSE17721_0.5H_VS_4H_CPG_BMDC_UP
151	0.3572927	1.491119	0.002053388
0.015305852	1	1722	"tags=27%, list=12%, signal=31%"
GSE17721_CTRL_VS_PAM3CSK4_12H_BMDC_UP
GSE17721_CTRL_VS_PAM3CSK4_12H_BMDC_UP	151
0.35581696	1.4906	0.010183299	0.015398362	1
1371	"tags=19%, list=10%, signal=21%"
GSE4142_NAIVE_VS_GC_BCELL_DN	GSE4142_NAIVE_VS_GC_BCELL_DN
132	0.3658771	1.4902703	0.004237288
0.015446419	1	2135	"tags=27%, list=15%, signal=32%"
GSE36826_NORMAL_VS_STAPH_AUREUS_INF_IL1R_KO_SKIN_DN
GSE36826_NORMAL_VS_STAPH_AUREUS_INF_IL1R_KO_SKIN_DN
143	0.3651143	1.4899192	0.008016032
0.015507878	1	1966	"tags=33%, list=14%, signal=38%"
GSE17186_MEMORY_VS_CD21HIGH_TRANSITIONAL_BCELL_DN
GSE17186_MEMORY_VS_CD21HIGH_TRANSITIONAL_BCELL_DN	161
0.36083525	1.4895058	0	0.015564142	1
2229	"tags=35%, list=16%, signal=42%"
GSE22886_NAIVE_TCELL_VS_DC_DN	GSE22886_NAIVE_TCELL_VS_DC_DN
164	0.35179883	1.4863182	0.006198347
0.01616872	1	2181	"tags=34%, list=15%, signal=39%"
GSE42021_TCONV_PLN_VS_CD24HI_TCONV_THYMUS_DN
GSE42021_TCONV_PLN_VS_CD24HI_TCONV_THYMUS_DN	141
0.36258808	1.4857934	0.008350731	0.016274525
1	1637	"tags=23%, list=12%, signal=26%"
GSE22935_UNSTIM_VS_12H_MBOVIS_BCG_STIM_MYD88_KO_MACROPHAGE_DN

GSE22935_UNSTIM_VS_12H_MBOVIS_BCG_STIM_MYD88_KO_MACROPHAGE_DN
131 0.36799937 1.4855201 0.006185567
0.01631467 1 2355 "tags=34%, list=17%, signal=41%"
GSE22886_NAIVE_CD8_TCELL_VS_NEUTROPHIL_UP
GSE22886_NAIVE_CD8_TCELL_VS_NEUTROPHIL_UP 141
0.3572779 1.4850193 0.006263048 0.016406735
1 2739 "tags=40%, list=19%, signal=49%"
GSE40666_WT_VS_STAT4_KO_CD8_TCELL_DN
GSE40666_WT_VS_STAT4_KO_CD8_TCELL_DN 123
0.3679055 1.4842137 0.008298756 0.016548593
1 1691 "tags=28%, list=12%, signal=32%"
GSE360_L_MAJOR_VS_T_GONDII_MAC_DN GSE360_L_MAJOR_VS_T_GONDII_MAC_DN
147 0.3568946 1.4837985 0.006263048
0.016630929 1 1740 "tags=25%, list=12%, signal=28%"
GSE26495_NAIVE_VS_PD1LOW_CD8_TCELL_DN
GSE26495_NAIVE_VS_PD1LOW_CD8_TCELL_DN 141
0.3619825 1.4830662 0.010893246 0.016759332
1 2274 "tags=32%, list=16%, signal=38%"
GSE17721_PAM3CSK4_VS_GADIQUIMOD_4H_BMDC_UP
GSE17721_PAM3CSK4_VS_GADIQUIMOD_4H_BMDC_UP 159
0.35315955 1.4830421 0.012903226 0.016748898
1 1640 "tags=23%, list=12%, signal=25%"
GSE3982_NEUTROPHIL_VS_NKCELL_UP GSE3982_NEUTROPHIL_VS_NKCELL_UP
149 0.3549093 1.4823753 0.01814059
0.016889129 1 2027 "tags=28%, list=14%, signal=33%"
GSE19888_NO_PRETREAT_VS_ADENOSINE_A3R_INHIBITOR_PRETREATED_MAST_CELL_TCELL_MEMBRANES_ACT_UP
GSE19888_NO_PRETREAT_VS_ADENOSINE_A3R_INHIBITOR_PRETREATED_MAST_CELL_TCELL_MEMBRANES_ACT_UP 139 0.35694954
1.4820433 0.010822511 0.016943432 1
2636 "tags=35%, list=19%, signal=42%"
GSE32128_INOS_DEPENDENT_VS_INOS_INDEPENDENT_ACTIVATED_TCELL_UP
GSE32128_INOS_DEPENDENT_VS_INOS_INDEPENDENT_ACTIVATED_TCELL_UP
162 0.35074562 1.4820291 0.004048583
0.016930759 1 2840 "tags=36%, list=20%, signal=45%"
GSE11864_CSF1_IFNG_VS_CSF1_PAM3CYS_IN_MAC_UP
GSE11864_CSF1_IFNG_VS_CSF1_PAM3CYS_IN_MAC_UP 136
0.35838184 1.4818418 0.010162601 0.016951606
1 1705 "tags=28%, list=12%, signal=31%"
GSE21927_SPLEEN_C57BL6_VS_4T1_TUMOR_BALBC_MONOCYTES_UP
GSE21927_SPLEEN_C57BL6_VS_4T1_TUMOR_BALBC_MONOCYTES_UP
129 0.36709934 1.4815247 0.012396694
0.0169967 1 1796 "tags=25%, list=13%, signal=28%"
GSE360_CTRL_VS_B_MALAYI_LOW_DOSE_MAC_UP
GSE360_CTRL_VS_B_MALAYI_LOW_DOSE_MAC_UP 152
0.3597263 1.48116 0.006085193 0.017059112 1
2370 "tags=33%, list=17%, signal=39%"
GSE29618_LAIV_VS_TIV_FLU_VACCINE_DAY7_MONOCYTE_UP
GSE29618_LAIV_VS_TIV_FLU_VACCINE_DAY7_MONOCYTE_UP 137
0.36171207 1.4810128 0.008333334 0.017076856
1 1371 "tags=17%, list=10%, signal=18%"
GSE339_CD8POS_VS_CD4CD8DN_DC_IN_CULTURE_DN
GSE339_CD8POS_VS_CD4CD8DN_DC_IN_CULTURE_DN 154
0.36006603 1.4809526 0.004056795 0.01707734

1 2094 "tags=33%, list=15%, signal=38%"
GSE22886_CD4_TCELL_VS_BCELL_NAIVE_UP
GSE22886_CD4_TCELL_VS_BCELL_NAIVE_UP 156
0.35573202 1.4808233 0.012987013 0.017093018
1 2265 "tags=38%, list=16%, signal=45%"
GSE17721_CTRL_VS_PAM3CSK4_6H_BMDC_DN
GSE17721_CTRL_VS_PAM3CSK4_6H_BMDC_DN 148
0.35939032 1.4800344 0.00862069 0.017246848
1 1626 "tags=24%, list=12%, signal=26%"
GSE13485_PRE_VS_POST_YF17D_VACCINATION_PBMC_DN
GSE13485_PRE_VS_POST_YF17D_VACCINATION_PBMC_DN 141
0.36370072 1.4796779 0.006493507 0.017313674
1 2383 "tags=36%, list=17%, signal=43%"
GSE17721_CTRL_VS_GARDIQUIMOD_0.5H_BMDC_UP
GSE17721_CTRL_VS_GARDIQUIMOD_0.5H_BMDC_UP 150
0.35339156 1.4787512 0.008385744 0.017504988
1 2349 "tags=34%, list=17%, signal=40%"
GSE10147_IL3_AND_HIVP17_VS_IL3_AND_CPG_STIM_PDC_DN
GSE10147_IL3_AND_HIVP17_VS_IL3_AND_CPG_STIM_PDC_DN 120
0.3693053 1.4784214 0.008438818 0.017550407
1 2008 "tags=29%, list=14%, signal=34%"
GSE22886_DAY1_VS_DAY7_MONOCYTE_IN_CULTURE_DN
GSE22886_DAY1_VS_DAY7_MONOCYTE_IN_CULTURE_DN 161
0.35478818 1.4768791 0.002114165 0.017859615
1 2430 "tags=39%, list=17%, signal=46%"
GSE43955_1H_VS_10H_ACT_CD4_TCELL_UP
GSE43955_1H_VS_10H_ACT_CD4_TCELL_UP 152
0.35244438 1.4766557 0.013245033 0.017884742
1 2996 "tags=39%, list=21%, signal=50%"
GSE3565_DUSP1_VS_WT_SPLENOCYTES_POST_LPS_INJECTION_DN
GSE3565_DUSP1_VS_WT_SPLENOCYTES_POST_LPS_INJECTION_DN
117 0.36598387 1.4766338 0.010080645
0.017872443 1 1964 "tags=26%, list=14%, signal=30%"
GSE15330_LYMPHOID_MULTIPOTENT_VS_MEGAKARYOCYTE_ERYTHROID_PROGENITOR_
UP
GSE15330_LYMPHOID_MULTIPOTENT_VS_MEGAKARYOCYTE_ERYTHROID_PROGENITOR_
UP 142 0.35490802 1.4750662 0.00625
0.01819752 1 2355 "tags=28%, list=17%, signal=33%"
GSE46242_CTRL_VS_EGR2_DELETED_TH1_CD4_TCELL_UP
GSE46242_CTRL_VS_EGR2_DELETED_TH1_CD4_TCELL_UP 137
0.35880777 1.4749937 0.00409836 0.018191969
1 2102 "tags=28%, list=15%, signal=33%"
GSE17721_LPS_VS_GARDIQUIMOD_6H_BMDC_DN
GSE17721_LPS_VS_GARDIQUIMOD_6H_BMDC_DN 148
0.35589507 1.4748696 0.004347826 0.018208563
1 1965 "tags=33%, list=14%, signal=38%"
GSE16385_UNTREATED_VS_12H_IFNG_TNF_TREATED_MACROPHAGE_DN
GSE16385_UNTREATED_VS_12H_IFNG_TNF_TREATED_MACROPHAGE_DN
125 0.36587164 1.4745218 0.014830508
0.01826505 1 2735 "tags=38%, list=19%, signal=46%"
GSE37605_C57BL6_VS_NOD_FOXP3_FUSION_GFP_TCONV_DN
GSE37605_C57BL6_VS_NOD_FOXP3_FUSION_GFP_TCONV_DN 136
0.36033913 1.4736106 0.006369427 0.018473325
1 2270 "tags=38%, list=16%, signal=45%"

GSE18791_UNSTIM_VS_NEWCATSLE_VIRUS_DC_10H_UP
GSE18791_UNSTIM_VS_NEWCATSLE_VIRUS_DC_10H_UP 133
0.36223662 1.4733183 0.004192872 0.01852239
1 2591 "tags=35%, list=18%, signal=43%"
GSE360_CTRL_VS_B_MALAYI_HIGH_DOSE_MAC_DN
GSE360_CTRL_VS_B_MALAYI_HIGH_DOSE_MAC_DN 159
0.34684184 1.4732127 0.008333334 0.018527582
1 1788 "tags=23%, list=13%, signal=26%"
GSE17721_LPS_VS_CPG_1H_BMDC_UP GSE17721_LPS_VS_CPG_1H_BMDC_UP
152 0.35160086 1.4726863 0.012048192
0.018637631 1 2275 "tags=29%, list=16%, signal=34%"
GSE2770_IL12_VS_IL4_TREATED_ACT_CD4_TCELL_2H_UP
GSE2770_IL12_VS_IL4_TREATED_ACT_CD4_TCELL_2H_UP 100
0.37682807 1.4717438 0.024242423 0.018833032
1 1616 "tags=24%, list=11%, signal=27%"
GSE5542_IFNG_VS_IFNA_AND_IFNG_TREATED_EPITHELIAL_CELLS_6H_UP
GSE5542_IFNG_VS_IFNA_AND_IFNG_TREATED_EPITHELIAL_CELLS_6H_UP
144 0.35115516 1.4716948 0.013422819
0.018824983 1 3038 "tags=39%, list=22%, signal=49%"
GSE7218_IGM_VS_IGG_SIGNAL_THGOUGH_ANTIGEN_BCELL_DN
GSE7218_IGM_VS_IGG_SIGNAL_THGOUGH_ANTIGEN_BCELL_DN 144
0.3545311 1.4704123 0.008213553 0.019096632
1 1795 "tags=28%, list=13%, signal=32%"
GSE17721_PAM3CSK4_VS_GADIQUIMOD_16H_BMDC_DN
GSE17721_PAM3CSK4_VS_GADIQUIMOD_16H_BMDC_DN 153
0.35526425 1.4699004 0.007766991 0.019181903
1 2011 "tags=27%, list=14%, signal=31%"
GSE411_UNSTIM_VS_400MIN_IL6_STIM_MACROPHAGE_DN
GSE411_UNSTIM_VS_400MIN_IL6_STIM_MACROPHAGE_DN 133
0.35936946 1.4679258 0.014227643 0.019610068
1 2019 "tags=31%, list=14%, signal=36%"
GSE46606_UNSTIM_VS_CD40L_IL2_IL5_1DAY_STIMULATED_IRF4_KO_BCELL_UP
GSE46606_UNSTIM_VS_CD40L_IL2_IL5_1DAY_STIMULATED_IRF4_KO_BCELL_UP
142 0.35256198 1.466178 0.006048387
0.019998442 1 2225 "tags=29%, list=16%, signal=34%"
GSE9006_HEALTHY_VS_TYPE_1_DIABETES_PBMC_AT_DX_UP
GSE9006_HEALTHY_VS_TYPE_1_DIABETES_PBMC_AT_DX_UP 145
0.35428342 1.4649734 0.006036217 0.020258578
1 2854 "tags=39%, list=20%, signal=48%"
GSE360_L_DONOVANI_VS_T_GONDII_DC_DN
GSE360_L_DONOVANI_VS_T_GONDII_DC_DN 139
0.36141226 1.4646372 0.00625 0.020315917 1
2170 "tags=27%, list=15%, signal=31%"
GSE6259_DEC205_POS_DC_VS_CD4_TCELL_DN
GSE6259_DEC205_POS_DC_VS_CD4_TCELL_DN 151
0.3530683 1.462541 0.008333334 0.020785274 1
2004 "tags=25%, list=14%, signal=29%"
GSE10240_IL17_VS_IL17_AND_IL22_STIM_PRIMARY_BRONCHIAL_EPITHELIAL_CEL
LS_UP
GSE10240_IL17_VS_IL17_AND_IL22_STIM_PRIMARY_BRONCHIAL_EPITHELIAL_CEL
LS_UP 128 0.3618985 1.460562 0.01048218
0.02122843 1 2297 "tags=30%, list=16%, signal=36%"
GSE39152_SPLEEN_CD103_NEG_VS_BRAIN_CD103_POS_MEMORY_CD8_TCELL_UP
GSE39152_SPLEEN_CD103_NEG_VS_BRAIN_CD103_POS_MEMORY_CD8_TCELL_UP

164 0.35126683 1.4603083 0.004201681
0.021285959 1 2185 "tags=27%, list=15%, signal=32%"
GSE45365_CD8A_DC_VS_CD11B_DC_IFNAR_KO_MCMV_INFECTION_UP
GSE45365_CD8A_DC_VS_CD11B_DC_IFNAR_KO_MCMV_INFECTION_UP
120 0.3593219 1.4600825 0.011286682
0.021328265 1 2260 "tags=28%, list=16%, signal=32%"
GSE26030_UNSTIM_VS_RESTIM_TH1_DAY15_POST_POLARIZATION_DN
GSE26030_UNSTIM_VS_RESTIM_TH1_DAY15_POST_POLARIZATION_DN
152 0.35053137 1.4599504 0.00617284
0.021350738 1 2107 "tags=30%, list=15%, signal=35%"
GSE16385_MONOCYTE_VS_12H_IL4_TREATED_MACROPHAGE_DN
GSE16385_MONOCYTE_VS_12H_IL4_TREATED_MACROPHAGE_DN 152
0.34990954 1.4596828 0.008492569 0.021404628
1 2472 "tags=33%, list=18%, signal=39%"
GSE17721_CTRL_VS_LPS_2H_BMDC_UP GSE17721_CTRL_VS_LPS_2H_BMDC_UP
140 0.35287187 1.4596177 0.01026694
0.02140731 1 2006 "tags=23%, list=14%, signal=26%"
GSE3982_MAST_CELL_VS_CENT_MEMORY_CD4_TCELL_UP
GSE3982_MAST_CELL_VS_CENT_MEMORY_CD4_TCELL_UP 151
0.35222676 1.4592925 0.006369427 0.021470051
1 2422 "tags=37%, list=17%, signal=44%"
GSE17721_POLYIC_VS_PAM3CSK4_0.5H_BMDC_UP
GSE17721_POLYIC_VS_PAM3CSK4_0.5H_BMDC_UP 159
0.3445719 1.4582264 0 0.021717666 1
1795 "tags=17%, list=13%, signal=19%"
GSE15733_BM_VS_SPLEEN_MEMORY_CD4_TCELL_UP
GSE15733_BM_VS_SPLEEN_MEMORY_CD4_TCELL_UP 149
0.35350916 1.4575337 0.008264462 0.021884497
1 1277 "tags=17%, list=9%, signal=19%"
GSE43955_TH0_VS_TGFB_IL6_TH17_ACT_CD4_TCELL_10H_DN
GSE43955_TH0_VS_TGFB_IL6_TH17_ACT_CD4_TCELL_10H_DN 162
0.34255785 1.4568901 0.012448133 0.022016317
1 1615 "tags=17%, list=11%, signal=19%"
GSE21670_TGFB_VS_TGFB_AND_IL6_TREATED_CD4_TCELL_UP
GSE21670_TGFB_VS_TGFB_AND_IL6_TREATED_CD4_TCELL_UP 144
0.3512145 1.4561107 0.012875536 0.022233585
1 1522 "tags=24%, list=11%, signal=27%"
GSE2770_TGFB_AND_IL4_VS_TGFB_AND_IL12_TREATED_ACT_CD4_TCELL_2H_DN
GSE2770_TGFB_AND_IL4_VS_TGFB_AND_IL12_TREATED_ACT_CD4_TCELL_2H_DN
130 0.3601114 1.455808 0.013071896
0.022289386 1 2560 "tags=35%, list=18%, signal=43%"
GSE3982_EOSINOPHIL_VS_EFF_MEMORY_CD4_TCELL_DN
GSE3982_EOSINOPHIL_VS_EFF_MEMORY_CD4_TCELL_DN 140
0.35015765 1.4557377 0.008733625 0.022283562
1 1454 "tags=19%, list=10%, signal=20%"
GSE2585_THYMIC_DC_VS_THYMIC_MACROPHAGE_DN
GSE2585_THYMIC_DC_VS_THYMIC_MACROPHAGE_DN 129
0.36084664 1.4556785 0.012422361 0.022277612
1 1090 "tags=19%, list=8%, signal=20%"
GSE4748_CYANOBACTERIUM_LPSLIKE_VS_LPS_AND_CYANOBACTERIUM_LPSLIKE_STI
M_DC_3H_DN
GSE4748_CYANOBACTERIUM_LPSLIKE_VS_LPS_AND_CYANOBACTERIUM_LPSLIKE_STI
M_DC_3H_DN 121 0.36017346 1.4542108
0.017738359 0.022655513 1 1774 "tags=20%,

list=13%, signal=22%"
GSE13484_12H_VS_3H_YF17D_VACCINE_STIM_PBMC_UP
GSE13484_12H_VS_3H_YF17D_VACCINE_STIM_PBMC_UP 135
0.3521266 1.4534103 0.012738854 0.02283608
1 2611 "tags=38%, list=19%, signal=46%"
GSE6259_FLT3L_INDUCED_33D1_POS_DC_VS_CD8_TCELL_UP
GSE6259_FLT3L_INDUCED_33D1_POS_DC_VS_CD8_TCELL_UP 115
0.36653754 1.4531695 0.014644352 0.022872834
1 1378 "tags=22%, list=10%, signal=24%"
GSE23398_WT_VS_IL2_KO_CD4_TCELL_SCURFY_MOUSE_UP
GSE23398_WT_VS_IL2_KO_CD4_TCELL_SCURFY_MOUSE_UP 143
0.3538237 1.4529009 0.010729614 0.022935824
1 2286 "tags=31%, list=16%, signal=36%"
GSE3982_DC_VS_NEUTROPHIL_UP GSE3982_DC_VS_NEUTROPHIL_UP
153 0.3471607 1.4525373 0.004366812
0.023005601 1 2200 "tags=29%, list=16%, signal=34%"
GSE27786_ERYTHROBLAST_VS_NEUTROPHIL_UP
GSE27786_ERYTHROBLAST_VS_NEUTROPHIL_UP 137 0.356136
1.4523054 0.016771488 0.023051716 1
2677 "tags=29%, list=19%, signal=36%"
GSE43863_TH1_VS_LY6C_INT_CXCR5POS_MEMORY_CD4_TCELL_UP
GSE43863_TH1_VS_LY6C_INT_CXCR5POS_MEMORY_CD4_TCELL_UP
141 0.3528322 1.4521438 0.010683761
0.02307356 1 2492 "tags=31%, list=18%, signal=38%"
GSE35825_IFNA_VS_IFNG_STIM_MACROPHAGE_DN
GSE35825_IFNA_VS_IFNG_STIM_MACROPHAGE_DN 143
0.35346794 1.4519889 0.008810572 0.02308571
1 1889 "tags=26%, list=13%, signal=30%"
GSE17322_CD103_POS_VS_CD11B_HIGH_LUNG_DC_UP
GSE17322_CD103_POS_VS_CD11B_HIGH_LUNG_DC_UP 127
0.35717598 1.4519657 0.010845987 0.023075342
1 2428 "tags=38%, list=17%, signal=45%"
GSE17721_CTRL_VS_CPG_24H_BMDC_DN GSE17721_CTRL_VS_CPG_24H_BMDC_DN
155 0.34585392 1.4518347 0.00409836
0.023089282 1 1828 "tags=23%, list=13%, signal=26%"
GSE7219_UNSTIM_VS_LPS_AND_ANTI_CD40_STIM_NIK_NFKB2_KO_DC_UP
GSE7219_UNSTIM_VS_LPS_AND_ANTI_CD40_STIM_NIK_NFKB2_KO_DC_UP
150 0.34952685 1.4511362 0.015283843
0.023246743 1 2311 "tags=39%, list=16%, signal=47%"
GSE6269_FLU_VS_STAPH_AUREUS_INF_PBMC_DN
GSE6269_FLU_VS_STAPH_AUREUS_INF_PBMC_DN 124
0.35862318 1.450861 0.008333334 0.023311978 1
2083 "tags=33%, list=15%, signal=38%"
GSE45365_HEALTHY_VS_MCMV_INFECTION_CD8A_DC_IFNAR_KO_UP
GSE45365_HEALTHY_VS_MCMV_INFECTION_CD8A_DC_IFNAR_KO_UP
132 0.3617188 1.4501892 0.012448133
0.023468167 1 1882 "tags=26%, list=13%, signal=29%"
GSE25123_CTRL_VS_IL4_AND_ROSIGLITAZONE_STIM_MACROPHAGE_UP
GSE25123_CTRL_VS_IL4_AND_ROSIGLITAZONE_STIM_MACROPHAGE_UP
156 0.34882382 1.4500169 0.010416667
0.02350013 1 2082 "tags=30%, list=15%, signal=35%"
GSE11961_GERMINAL_CENTER_BCELL_DAY7_VS_PLASMA_CELL_DAY7_UP
GSE11961_GERMINAL_CENTER_BCELL_DAY7_VS_PLASMA_CELL_DAY7_UP
157 0.3494847 1.4499125 0.006423983

0.023508644 1 1741 "tags=22%, list=12%, signal=24%"
GSE9946_IMMATURE_VS_PROSTAGLANDINE2_TREATED_MATURE_DC_DN
GSE9946_IMMATURE_VS_PROSTAGLANDINE2_TREATED_MATURE_DC_DN
117 0.36919984 1.4494753 0.017857144
0.023606358 1 1964 "tags=21%, list=14%, signal=24%"
GSE4984_UNTREATED_VS_GALECTIN1_TREATED_DC_UP
GSE4984_UNTREATED_VS_GALECTIN1_TREATED_DC_UP 126
0.35819548 1.4492921 0.004048583 0.023627292
1 1501 "tags=21%, list=11%, signal=23%"
GSE17721_POLYIC_VS_CPG_6H_BMDC_DN GSE17721_POLYIC_VS_CPG_6H_BMDC_DN
148 0.35096148 1.4491891 0.010752688
0.023634903 1 1702 "tags=26%, list=12%, signal=29%"
GSE42021_TREG_VS_TCONV_PLN_DN GSE42021_TREG_VS_TCONV_PLN_DN
153 0.34922633 1.4487231 0.008888889
0.023748675 1 1679 "tags=22%, list=12%, signal=25%"
GSE21033_1H_VS_24H_POLYIC_STIM_DC_DN
GSE21033_1H_VS_24H_POLYIC_STIM_DC_DN 121
0.36183408 1.4482374 0.023255814 0.023861941
1 2291 "tags=36%, list=16%, signal=42%"
GSE22601_DOUBLE_NEGATIVE_VS_CD4_SINGLE_POSITIVE_THYMOCYTE_DN
GSE22601_DOUBLE_NEGATIVE_VS_CD4_SINGLE_POSITIVE_THYMOCYTE_DN
153 0.351722 1.4475894 0.012269938
0.024041504 1 1976 "tags=29%, list=14%, signal=33%"
GSE27786_CD8_TCELL_VS_MONO_MAC_DN GSE27786_CD8_TCELL_VS_MONO_MAC_DN
136 0.35119826 1.4470317 0.006507592
0.024187155 1 1160 "tags=20%, list=8%, signal=21%"
GSE22935_WT_VS_MYD88_KO_MACROPHAGE_12H_MBOVIS_BCG_STIM_DN
GSE22935_WT_VS_MYD88_KO_MACROPHAGE_12H_MBOVIS_BCG_STIM_DN
155 0.34733018 1.4467771 0.014830508
0.024236524 1 1949 "tags=26%, list=14%, signal=30%"
GSE1460_INTRATHYMIC_T_PROGENITOR_VS_THYMIC_STROMAL_CELL_UP
GSE1460_INTRATHYMIC_T_PROGENITOR_VS_THYMIC_STROMAL_CELL_UP
145 0.35103208 1.4464875 0.015118791
0.024288666 1 1725 "tags=26%, list=12%, signal=30%"
GSE37605_TREG_VS_TCONV_C57BL6_FOXP3_IRES_GFP_DN
GSE37605_TREG_VS_TCONV_C57BL6_FOXP3_IRES_GFP_DN 119
0.359218 1.4463407 0.028761063 0.024304284 1
2259 "tags=34%, list=16%, signal=41%"
GSE32986_CURDLAN_LOWDOSE_VS_GMCSF_AND_CURDLAN_LOWDOSE_STIM_DC_UP
GSE32986_CURDLAN_LOWDOSE_VS_GMCSF_AND_CURDLAN_LOWDOSE_STIM_DC_UP
134 0.3538945 1.4453471 0.00408998
0.024596553 1 2191 "tags=33%, list=16%, signal=39%"
GSE10240_CTRL_VS_IL17_STIM_PRIMARY_BRONCHIAL_EPITHELIAL_CELLS_DN
GSE10240_CTRL_VS_IL17_STIM_PRIMARY_BRONCHIAL_EPITHELIAL_CELLS_DN
155 0.34317774 1.4442364 0.010504202
0.024911316 1 2926 "tags=34%, list=21%, signal=43%"
GSE38304_MYC_NEG_VS_POS_GC_BCELL_DN
GSE38304_MYC_NEG_VS_POS_GC_BCELL_DN 159
0.34642395 1.4441979 0.008130081 0.024904488
1 1806 "tags=32%, list=13%, signal=36%"
GSE6259_FLT3L_INDUCED_VS_WT_SPLENIC_DC_33D1_POS_DN
GSE6259_FLT3L_INDUCED_VS_WT_SPLENIC_DC_33D1_POS_DN 124
0.35763645 1.4437176 0.006329114 0.025027473
1 1603 "tags=17%, list=11%, signal=19%"

GSE22229_RENAL_TRANSPLANT_IMMUNOSUPP_THERAPY_VS_HEALTHY_PBMC_UP
GSE22229_RENAL_TRANSPLANT_IMMUNOSUPP_THERAPY_VS_HEALTHY_PBMC_UP
151 0.34648806 1.4432821 0.010752688
0.025122358 1 1782 "tags=26%, list=13%, signal=29%"
GSE41867_LCMV_ARMSTRONG_VS_CLONE13_DAY8_EFFECTOR_CD8_TCELL_DN
GSE41867_LCMV_ARMSTRONG_VS_CLONE13_DAY8_EFFECTOR_CD8_TCELL_DN
139 0.35215405 1.443237 0.022869023
0.0251105 1 1640 "tags=26%, list=12%, signal=29%"
GSE43955_1H_VS_42H_ACT_CD4_TCELL_WITH_TGFB_IL6_UP
GSE43955_1H_VS_42H_ACT_CD4_TCELL_WITH_TGFB_IL6_UP 157
0.34498182 1.4430797 0.002188184 0.025126344
1 1461 "tags=25%, list=10%, signal=28%"
GSE11924_TFH_VS_TH1_CD4_TCELL_DN GSE11924_TFH_VS_TH1_CD4_TCELL_DN
141 0.3519067 1.4405198 0.014403292
0.025857134 1 2253 "tags=26%, list=16%, signal=30%"
GSE17721_4_VS_24H_GARDIQUIMOD_BMDC_DN
GSE17721_4_VS_24H_GARDIQUIMOD_BMDC_DN 148
0.35017365 1.4398848 0.004366812 0.026009353
1 2092 "tags=30%, list=15%, signal=35%"
GSE12392_CD8A_POS_VS_NEG_SPLEEN_IFNB_KO_DC_DN
GSE12392_CD8A_POS_VS_NEG_SPLEEN_IFNB_KO_DC_DN 155
0.3487442 1.439651 0.013574661 0.02604623 1
1898 "tags=30%, list=13%, signal=34%"
GSE13493_DP_VS_CD8POS_THYMOCYTE_DN
GSE13493_DP_VS_CD8POS_THYMOCYTE_DN 154
0.34110498 1.43892 0.010504202 0.026222639 1
2361 "tags=32%, list=17%, signal=39%"
GSE29618_BCELL_VS_PDC_DAY7_FLU_VACCINE_DN
GSE29618_BCELL_VS_PDC_DAY7_FLU_VACCINE_DN 161
0.34061545 1.4388523 0.01010101 0.026221404
1 1307 "tags=20%, list=9%, signal=22%"
GSE25088_CTRL_VS_ROSIGLITAZONE_STIM_STAT6_KO_MACROPHAGE_DN
GSE25088_CTRL_VS_ROSIGLITAZONE_STIM_STAT6_KO_MACROPHAGE_DN
142 0.34655887 1.4387652 0.012345679
0.02622973 1 2841 "tags=42%, list=20%, signal=52%"
GSE46468_LUNG_INNATE_LYMPHOID_CELL_VS_SPLEEN_CD4_TCELL_DN
GSE46468_LUNG_INNATE_LYMPHOID_CELL_VS_SPLEEN_CD4_TCELL_DN
145 0.34805444 1.4382179 0.012422361
0.026387926 1 2539 "tags=34%, list=18%, signal=42%"
GSE27291_0H_VS_7D_STIM_GAMMADELTA_TCELL_DN
GSE27291_0H_VS_7D_STIM_GAMMADELTA_TCELL_DN 126
0.35525885 1.4372115 0.008264462 0.026697505
1 1616 "tags=21%, list=11%, signal=24%"
GSE45365_NK_CELL_VS_CD8_TCELL_DN GSE45365_NK_CELL_VS_CD8_TCELL_DN
93 0.36929286 1.4369292 0.02296451
0.026755486 1 1962 "tags=24%, list=14%, signal=27%"
GSE22611_UNSTIM_VS_2H_MDP_STIM_MUTANT_NOD2_TRANSDUCED_HEK293T_CELL_U
P
GSE22611_UNSTIM_VS_2H_MDP_STIM_MUTANT_NOD2_TRANSDUCED_HEK293T_CELL_U
P 142 0.35020563 1.4365904
0.008639309 0.02682378 1 1749 "tags=25%,
list=12%, signal=29%"
GSE11864_UNTREATED_VS_CSF1_IFNG_IN_MAC_DN
GSE11864_UNTREATED_VS_CSF1_IFNG_IN_MAC_DN 141

0.35374302	1.436522	0.014112903	0.026819281	1
2572	"tags=37%, list=18%, signal=45%"
GSE7219_WT_VS_NIK_NFKB2_KO_DC_DN	GSE7219_WT_VS_NIK_NFKB2_KO_DC_DN
	138	0.35222447	1.4360505	0.016528925
0.02696305	1	2431	"tags=36%, list=17%, signal=42%"
GSE37532_TREG_VS_TCONV_CD4_TCELL_FROM_LN_UP
GSE37532_TREG_VS_TCONV_CD4_TCELL_FROM_LN_UP		144
0.3427324	1.4352227	0.00877193	0.027189426
1	1480	"tags=19%, list=10%, signal=22%"
GSE369_PRE_VS_POST_IL6_INJECTION_IFNG_KO_LIVER_UP
GSE369_PRE_VS_POST_IL6_INJECTION_IFNG_KO_LIVER_UP		140
0.34828407	1.4348234	0.018789144	0.027283493
1	2617	"tags=41%, list=19%, signal=49%"
GSE46606_IRF4_KO_VS_WT_CD40L_IL2_IL5_3DAY_STIMULATED_BCELL_DN
GSE46606_IRF4_KO_VS_WT_CD40L_IL2_IL5_3DAY_STIMULATED_BCELL_DN
	118	0.35844505	1.4345894	0.01735358
0.027335158	1	2139	"tags=29%, list=15%, signal=34%"
GSE10240_IL22_VS_IL17_STIM_PRIMARY_BRONCHIAL_EPITHELIAL_CELLS_DN
GSE10240_IL22_VS_IL17_STIM_PRIMARY_BRONCHIAL_EPITHELIAL_CELLS_DN
	131	0.3477207	1.4341408	0.010869565
0.027463757	1	2182	"tags=29%, list=15%, signal=34%"
GSE27670_BLIMP1_VS_LMP1_TRANSDUCED_GC_BCELL_DN
GSE27670_BLIMP1_VS_LMP1_TRANSDUCED_GC_BCELL_DN		155
0.34614044	1.433827	0.007968128	0.027567806	1
1692	"tags=25%, list=12%, signal=28%"
GSE7768_OVA_ALONE_VS_OVA_WITH_LPS_IMMUNIZED_MOUSE_WHOLE_SPLEEN_6H_UP

GSE7768_OVA_ALONE_VS_OVA_WITH_LPS_IMMUNIZED_MOUSE_WHOLE_SPLEEN_6H_UP
		124	0.3517274	1.4332644
0.026915114	0.0276972	1	1835	"tags=24%,
list=13%, signal=28%"
GSE8868_SPLEEN_VS_INTESTINE_CD11B_POS_CD11C_NEG_DC_UP
GSE8868_SPLEEN_VS_INTESTINE_CD11B_POS_CD11C_NEG_DC_UP
128	0.34821796	1.4326161	0.01923077
0.027886381	1	1804	"tags=23%, list=13%, signal=27%"
GSE8621_UNSTIM_VS_LPS_STIM_MACROPHAGE_DN
GSE8621_UNSTIM_VS_LPS_STIM_MACROPHAGE_DN		140
0.3497448	1.4315584	0.004415011	0.028208062
1	2291	"tags=34%, list=16%, signal=40%"
GSE1448_CTRL_VS_ANTI_VBETA5_DP_THYMOCYTE_UP
GSE1448_CTRL_VS_ANTI_VBETA5_DP_THYMOCYTE_UP		150
0.3473443	1.4311996	0.014056225	0.028299833
1	2166	"tags=29%, list=15%, signal=34%"
GSE13484_3H_UNSTIM_VS_YF17D_VACCINE_STIM_PBMC_UP
GSE13484_3H_UNSTIM_VS_YF17D_VACCINE_STIM_PBMC_UP		147
0.34751022	1.4308907	0.00617284	0.028370764
1	2296	"tags=33%, list=16%, signal=39%"
GSE19888_CTRL_VS_T_CELL_MEMBRANES_ACT_MAST_CELL_UP
GSE19888_CTRL_VS_T_CELL_MEMBRANES_ACT_MAST_CELL_UP		143
0.34790266	1.4308584	0.010615711	0.028365018
1	2836	"tags=38%, list=20%, signal=47%"
GSE12366_PLASMA_CELL_VS_MEMORY_BCELL_DN
GSE12366_PLASMA_CELL_VS_MEMORY_BCELL_DN		134
0.3461858	1.4303765	0.011389522	0.028473359

1	2380	"tags=35%, list=17%, signal=42%"
GSE5542_IFNG_VS_IFNA_AND_IFNG_TREATED_EPITHELIAL_CELLS_24H_UP
GSE5542_IFNG_VS_IFNA_AND_IFNG_TREATED_EPITHELIAL_CELLS_24H_UP
	138	0.35056928	1.4299575	0.006237006
0.02858228	1	2130	"tags=29%, list=15%, signal=34%"
GSE20198_UNTREATED_VS_IFNA_TREATED_ACT_CD4_TCELL_DN
GSE20198_UNTREATED_VS_IFNA_TREATED_ACT_CD4_TCELL_DN
138	0.34430844	1.4297241	0.002118644
0.028646423	1	2571	"tags=34%, list=18%, signal=41%"
GSE2770_IL12_AND_TGFB_ACT_VS_ACT_CD4_TCELL_2H_UP
GSE2770_IL12_AND_TGFB_ACT_VS_ACT_CD4_TCELL_2H_UP	136
0.35025558	1.4291006	0.006396588	0.028834132
1	2106	"tags=26%, list=15%, signal=30%"
GSE9650_NAIVE_VS_EXHAUSTED_CD8_TCELL_UP
GSE9650_NAIVE_VS_EXHAUSTED_CD8_TCELL_UP	160
0.34292242	1.4290776	0.013422819	0.02881589
1	1762	"tags=28%, list=12%, signal=32%"
GSE360_CTRL_VS_L_DONOVANI_DC_UP	GSE360_CTRL_VS_L_DONOVANI_DC_UP
	144	0.34480733	1.4282838	0.016736401
0.029078625	1	2696	"tags=35%, list=19%, signal=42%"
GSE25088_CTRL_VS_ROSIGLITAZONE_STIM_MACROPHAGE_DN
GSE25088_CTRL_VS_ROSIGLITAZONE_STIM_MACROPHAGE_DN	105
0.3652165	1.4281843	0.025210084	0.029096639
1	1857	"tags=20%, list=13%, signal=23%"
GSE25088_ROSIGLITAZONE_VS_IL4_AND_ROSIGLITAZONE_STIM_STAT6_KO_MACROP
HAGE_DAY10_DN
GSE25088_ROSIGLITAZONE_VS_IL4_AND_ROSIGLITAZONE_STIM_STAT6_KO_MACROP
HAGE_DAY10_DN	136	0.34466854	1.4276167
0.019027485	0.029236242	1	2569	"tags=38%,
list=18%, signal=46%"
GSE21063_WT_VS_NFATC1_KO_3H_ANTI_IGM_STIM_BCELL_DN
GSE21063_WT_VS_NFATC1_KO_3H_ANTI_IGM_STIM_BCELL_DN	143
0.3439998	1.4274976	0.015184382	0.029255103
1	1839	"tags=27%, list=13%, signal=30%"
GSE7596_AKT_TRANSD_VS_CTRL_CD4_TCONV_WITH_TGFB_UP
GSE7596_AKT_TRANSD_VS_CTRL_CD4_TCONV_WITH_TGFB_UP	125
0.35247964	1.4274412	0.015384615	0.029254248
1	1681	"tags=28%, list=12%, signal=32%"
GSE9006_TYPE_1_DIABETES_AT_DX_VS_4MONTH_POST_DX_PBMC_UP
GSE9006_TYPE_1_DIABETES_AT_DX_VS_4MONTH_POST_DX_PBMC_UP
151	0.3464713	1.4270977	0.008510638
0.029356264	1	1966	"tags=32%, list=14%, signal=37%"
GSE3920_UNTREATED_VS_IFNB_TREATED_ENDOTHELIAL_CELL_UP
GSE3920_UNTREATED_VS_IFNB_TREATED_ENDOTHELIAL_CELL_UP
135	0.34750852	1.4266887	0.00990099
0.02946481	1	2293	"tags=30%, list=16%, signal=35%"
GSE3982_NEUTROPHIL_VS_EFF_MEMORY_CD4_TCELL_UP
GSE3982_NEUTROPHIL_VS_EFF_MEMORY_CD4_TCELL_UP	141
0.34384272	1.4266574	0.013972056	0.029455414
1	2157	"tags=28%, list=15%, signal=32%"
GSE2405_HEAT_KILLED_LYSATE_VS_LIVE_A_PHAGOCYTOPHILUM_STIM_NEUTROPHIL
_9H_DN
GSE2405_HEAT_KILLED_LYSATE_VS_LIVE_A_PHAGOCYTOPHILUM_STIM_NEUTROPHIL
_9H_DN	151	0.34347123	1.4265954

0.001984127 0.02945265 1 1742 "tags=28%,
list=12%, signal=32%"
GSE27859_CD11C_INT_F480_HI_MACROPHAGE_VS_CD11C_ING_F480_INT_DC_UP
GSE27859_CD11C_INT_F480_HI_MACROPHAGE_VS_CD11C_ING_F480_INT_DC_UP
134 0.34979874 1.4264842 0.014675053
0.029468307 1 2550 "tags=34%, list=18%, signal=41%"
GSE17721_CTRL_VS_GARDIQUIMOD_0.5H_BMDC_DN
GSE17721_CTRL_VS_GARDIQUIMOD_0.5H_BMDC_DN 147
0.34543496 1.4260037 0.012578616 0.029604949
1 1677 "tags=24%, list=12%, signal=27%"
GSE41867_NAIVE_VS_DAY6_LCMV_EFFECTOR_CD8_TCELL_UP
GSE41867_NAIVE_VS_DAY6_LCMV_EFFECTOR_CD8_TCELL_UP 142
0.34406906 1.4258446 0.016771488 0.029645452
1 1949 "tags=30%, list=14%, signal=35%"
GSE20715_0H_VS_6H_OZONE_TLR4_KO_LUNG_UP
GSE20715_0H_VS_6H_OZONE_TLR4_KO_LUNG_UP 156
0.3414325 1.4245924 0.008298756 0.030035637
1 1917 "tags=23%, list=14%, signal=26%"
GSE3720_UNSTIM_VS_LPS_STIM_VD2_GAMMADELTA_TCELL_UP
GSE3720_UNSTIM_VS_LPS_STIM_VD2_GAMMADELTA_TCELL_UP 120
0.35836494 1.4240446 0.021153847 0.030198097
1 2695 "tags=41%, list=19%, signal=50%"
GSE41867_NAIVE_VS_DAY8_LCMV_CLONE13_EFFECTOR_CD8_TCELL_UP
GSE41867_NAIVE_VS_DAY8_LCMV_CLONE13_EFFECTOR_CD8_TCELL_UP
147 0.34182072 1.4240342 0.010893246
0.030179001 1 1140 "tags=20%, list=8%, signal=21%"
GSE2770_TGFB_AND_IL4_VS_IL12_TREATED_ACT_CD4_TCELL_6H_DN
GSE2770_TGFB_AND_IL4_VS_IL12_TREATED_ACT_CD4_TCELL_6H_DN
128 0.3448729 1.4237615 0.006369427
0.030248575 1 2371 "tags=33%, list=17%, signal=39%"
GSE27786_LSK_VS_CD8_TCELL_UP GSE27786_LSK_VS_CD8_TCELL_UP
136 0.35065103 1.4235804 0.010775862
0.030292109 1 1646 "tags=24%, list=12%, signal=26%"
GSE17721_LPS_VS_CPG_16H_BMDC_UP GSE17721_LPS_VS_CPG_16H_BMDC_UP
148 0.34472454 1.4229622 0.009861933
0.030473195 1 1313 "tags=20%, list=9%, signal=22%"
GSE20715_0H_VS_6H_OZONE_LUNG_UP GSE20715_0H_VS_6H_OZONE_LUNG_UP
154 0.33975503 1.4229286 0.012396694
0.030463923 1 1992 "tags=25%, list=14%, signal=28%"
GSE3039_ALPHAALPHA_CD8_TCELL_VS_B2_BCELL_UP
GSE3039_ALPHAALPHA_CD8_TCELL_VS_B2_BCELL_UP 145
0.34259424 1.4224343 0.012658228 0.030636558
1 1725 "tags=26%, list=12%, signal=29%"
GSE18893_TCONV_VS_TREG_24H_CULTURE_DN
GSE18893_TCONV_VS_TREG_24H_CULTURE_DN 145
0.34222096 1.4220245 0.016460905 0.030766591
1 1082 "tags=19%, list=8%, signal=21%"
GSE25088_IL4_VS_IL4_AND_ROSIGLITAZONE_STIM_MACROPHAGE_DAY10_UP
GSE25088_IL4_VS_IL4_AND_ROSIGLITAZONE_STIM_MACROPHAGE_DAY10_UP
142 0.3452639 1.4211321 0.024742268
0.031027587 1 2172 "tags=29%, list=15%, signal=34%"
GSE21033_CTRL_VS_POLYIC_STIM_DC_12H_UP
GSE21033_CTRL_VS_POLYIC_STIM_DC_12H_UP 122
0.35269615 1.4208068 0.026694044 0.031124953

1	2347	"tags=39%, list=17%, signal=46%"
GSE17721_LPS_VS_PAM3CSK4_16H_BMDC_DN
GSE17721_LPS_VS_PAM3CSK4_16H_BMDC_DN		148	0.346213
1.420596	0.014767933	0.031168435	1
2180	"tags=26%, list=15%, signal=30%"
GSE22886_IGM_MEMORY_BCELL_VS_BM_PLASMA_CELL_UP
GSE22886_IGM_MEMORY_BCELL_VS_BM_PLASMA_CELL_UP		127
0.34655792	1.4202837	0.00856531	0.031246623
1	2199	"tags=31%, list=16%, signal=37%"
GSE37301_LYMPHOID_PRIMED_MPP_VS_GRAN_MONO_PROGENITOR_DN
GSE37301_LYMPHOID_PRIMED_MPP_VS_GRAN_MONO_PROGENITOR_DN
141	0.34860066	1.419784	0.009195402	0.03139299
1	1900	"tags=31%, list=13%, signal=36%"
GSE22611_UNSTIM_VS_2H_MDP_STIM_NOD2_TRANSDUCED_HEK293T_CELL_UP
GSE22611_UNSTIM_VS_2H_MDP_STIM_NOD2_TRANSDUCED_HEK293T_CELL_UP
	139	0.34175113	1.4193983	0.004246285
0.031500887	1	2611	"tags=31%, list=19%, signal=38%"
GSE37301_MULTIPOTENT_PROGENITOR_VS_RAG2_KO_NK_CELL_UP
GSE37301_MULTIPOTENT_PROGENITOR_VS_RAG2_KO_NK_CELL_UP
156	0.34145784	1.41869	0.012195122	0.031716537
1	2285	"tags=26%, list=16%, signal=31%"
GSE27786_BCELL_VS_ERYTHROBLAST_UP GSE27786_BCELL_VS_ERYTHROBLAST_UP
	139	0.34245026	1.4179732	0.014256619
0.03195337	1	2638	"tags=35%, list=19%, signal=42%"
GSE42724_NAIVE_BCELL_VS_PLASMABLAST_DN
GSE42724_NAIVE_BCELL_VS_PLASMABLAST_DN		124
0.35133195	1.4176179	0.030368764	0.03204232
1	1829	"tags=22%, list=13%, signal=25%"
GSE32901_TH1_VS_TH17_ENRICHED_CD4_TCELL_DN
GSE32901_TH1_VS_TH17_ENRICHED_CD4_TCELL_DN		116
0.3541023	1.4173598	0.0256917	0.032114714
1	2112	"tags=35%, list=15%, signal=41%"
GSE8685_IL2_STARVED_VS_IL21_ACT_IL2_STARVED_CD4_TCELL_DN
GSE8685_IL2_STARVED_VS_IL21_ACT_IL2_STARVED_CD4_TCELL_DN
135	0.34188616	1.4173359	0.024742268
0.032101657	1	1882	"tags=26%, list=13%, signal=30%"
GSE15330_WT_VS_IKAROS_KO_GRANULOCYTE_MONOCYTE_PROGENITOR_DN
GSE15330_WT_VS_IKAROS_KO_GRANULOCYTE_MONOCYTE_PROGENITOR_DN
163	0.33593228	1.4170309	0.018480493
0.032163188	1	1847	"tags=28%, list=13%, signal=31%"
GSE17721_PAM3CSK4_VS_CPG_12H_BMDC_UP
GSE17721_PAM3CSK4_VS_CPG_12H_BMDC_UP		144
0.34560135	1.4168056	0.020876827	0.032233845
1	2299	"tags=35%, list=16%, signal=41%"
GSE34156_NOD2_LIGAND_VS_TLR1_TLR2_LIGAND_6H_TREATED_MONOCYTE_DN
GSE34156_NOD2_LIGAND_VS_TLR1_TLR2_LIGAND_6H_TREATED_MONOCYTE_DN
	133	0.34616598	1.416692	0.013779528
0.0322489	1	1388	"tags=20%, list=10%, signal=22%"
GSE15330_LYMPHOID_MULTIPOTENT_VS_PRO_BCELL_DN
GSE15330_LYMPHOID_MULTIPOTENT_VS_PRO_BCELL_DN		153
0.34201938	1.4166219	0.012738854	0.032259367
1	1722	"tags=25%, list=12%, signal=29%"
GSE19401_PLN_VS_PEYERS_PATCH_FOLLICULAR_DC_DN
GSE19401_PLN_VS_PEYERS_PATCH_FOLLICULAR_DC_DN		156

0.3393369	1.4164999	0.006160164	0.032289956
1	2612	"tags=37%, list=19%, signal=44%"
GSE13946_CTRL_VS_DSS_COLITIS_GD_TCELL_FROM_COLON_UP
GSE13946_CTRL_VS_DSS_COLITIS_GD_TCELL_FROM_COLON_UP
144	0.34511012	1.4163253	0.012024048
0.032328513	1	2274	"tags=37%, list=16%, signal=43%"
GSE17721_CTRL_VS_LPS_8H_BMDC_UP	GSE17721_CTRL_VS_LPS_8H_BMDC_UP
	150	0.34119853	1.4159331	0.012552301
0.03247454	1	1705	"tags=21%, list=12%, signal=23%"
GSE22886_NAIVE_CD8_TCELL_VS_DC_UP GSE22886_NAIVE_CD8_TCELL_VS_DC_UP
	128	0.3477366	1.4156828	0.021551725
0.032531805	1	1810	"tags=28%, list=13%, signal=32%"
GSE15930_STIM_VS_STIM_AND_IL12_48H_CD8_T_CELL_DN
GSE15930_STIM_VS_STIM_AND_IL12_48H_CD8_T_CELL_DN	174
0.33133787	1.4154423	0.006479482	0.032597832
1	1874	"tags=30%, list=13%, signal=35%"
GSE16385_MONOCYTE_VS_12H_IFNG_TNF_TREATED_MACROPHAGE_UP
GSE16385_MONOCYTE_VS_12H_IFNG_TNF_TREATED_MACROPHAGE_UP
146	0.34183878	1.4153435	0.006342495
0.032604426	1	2586	"tags=36%, list=18%, signal=43%"
GSE43955_1H_VS_10H_ACT_CD4_TCELL_DN
GSE43955_1H_VS_10H_ACT_CD4_TCELL_DN	156
0.34293613	1.4142985	0.016241299	0.032965668
1	2206	"tags=28%, list=16%, signal=32%"
GSE32128_INOS_DEPENDENT_VS_INOS_INDEPENDENT_ACTIVATED_TCELL_DN
GSE32128_INOS_DEPENDENT_VS_INOS_INDEPENDENT_ACTIVATED_TCELL_DN
	147	0.338495 1.4139928	0.006185567
0.03304662	1	2587	"tags=31%, list=18%, signal=37%"
GSE16522_MEMORY_VS_NAIVE_ANTI_CD3CD28_STIM_CD8_TCELL_DN
GSE16522_MEMORY_VS_NAIVE_ANTI_CD3CD28_STIM_CD8_TCELL_DN
157	0.3405737	1.413838 0.008196721	0.033086147
1	2229	"tags=32%, list=16%, signal=38%"
GSE27786_LIN_NEG_VS_NKCELL_UP	GSE27786_LIN_NEG_VS_NKCELL_UP
	138	0.3406941	1.4129027	0.014553014
0.03341467	1	2663	"tags=31%, list=19%, signal=38%"
GSE14308_TH17_VS_INDUCED_TREG_DN	GSE14308_TH17_VS_INDUCED_TREG_DN
	135	0.3474869	1.412272 0.02385686
0.033653263	1	1974	"tags=27%, list=14%, signal=31%"
GSE28237_EARLY_VS_LATE_GC_BCELL_DN
GSE28237_EARLY_VS_LATE_GC_BCELL_DN	143
0.3406609	1.4119033	0.010438413	0.033778485
1	2344	"tags=34%, list=17%, signal=40%"
GSE35685_CD34POS_CD38NEG_VS_CD34POS_CD10NEG_CD62LPOS_BONE_MARROW_UP
GSE35685_CD34POS_CD38NEG_VS_CD34POS_CD10NEG_CD62LPOS_BONE_MARROW_UP
	155	0.33499697	1.4117225	0.014553014
0.03381435	1	2414	"tags=35%, list=17%, signal=42%"
GSE8921_UNSTIM_VS_TLR1_2_STIM_MONOCYTE_12H_DN
GSE8921_UNSTIM_VS_TLR1_2_STIM_MONOCYTE_12H_DN	137
0.3377336	1.4112087	0.015810277	0.033987854
1	2199	"tags=29%, list=16%, signal=34%"
GSE26023_PHD3_KO_VS_WT_NEUTROPHIL_HYPOXIA_DN
GSE26023_PHD3_KO_VS_WT_NEUTROPHIL_HYPOXIA_DN	145
0.3353287	1.411163 0.015659954	0.033978008	1
2616	"tags=28%, list=19%, signal=34%"

GSE3565_DUSP1_VS_WT_SPLENOCYTES_UP
GSE3565_DUSP1_VS_WT_SPLENOCYTES_UP 134
0.34306857 1.4111477 0.021186441 0.033955626
1 1737 "tags=33%, list=12%, signal=37%"
GSE3982_BCELL_VS_EFF_MEMORY_CD4_TCELL_DN
GSE3982_BCELL_VS_EFF_MEMORY_CD4_TCELL_DN 132
0.34307286 1.4110223 0.020283977 0.033972852
1 1739 "tags=17%, list=12%, signal=20%"
GSE14308_TH2_VS_NAIVE_CD4_TCELL_UP
GSE14308_TH2_VS_NAIVE_CD4_TCELL_UP 148
0.34050435 1.4104013 0.006060606 0.03418186
1 2771 "tags=39%, list=20%, signal=48%"
GSE24142_DN2_VS_DN3_THYMOCYTE_UP GSE24142_DN2_VS_DN3_THYMOCYTE_UP
159 0.33565846 1.4101714 0.01268499
0.0342281 1 1539 "tags=23%, list=11%, signal=26%"
GSE22601_CD4_SINGLE_POSITIVE_VS_CD8_SINGLE_POSITIVE_THYMOCYTE_DN
GSE22601_CD4_SINGLE_POSITIVE_VS_CD8_SINGLE_POSITIVE_THYMOCYTE_DN
140 0.3434384 1.4098854 0.021231422
0.034319293 1 2427 "tags=31%, list=17%, signal=37%"
GSE17721_POLYIC_VS_CPG_24H_BMDC_DN
GSE17721_POLYIC_VS_CPG_24H_BMDC_DN 141
0.34349248 1.4098451 0.010373444 0.034306582
1 2367 "tags=32%, list=17%, signal=38%"
GSE9037_CTRL_VS_LPS_4H_STIM_BMDM_UP
GSE9037_CTRL_VS_LPS_4H_STIM_BMDM_UP 147
0.34264365 1.409062 0.012958963 0.034593735 1
1910 "tags=29%, list=14%, signal=33%"
GSE29618_BCELL_VS_MDC_DAY7_FLU_VACCINE_DN
GSE29618_BCELL_VS_MDC_DAY7_FLU_VACCINE_DN 154
0.33848158 1.4088464 0.004319654 0.034639817
1 2081 "tags=31%, list=15%, signal=35%"
GSE17186_BLOOD_VS_CORD_BLOOD_CD21LOW_TRANSITIONAL_BCELL_UP
GSE17186_BLOOD_VS_CORD_BLOOD_CD21LOW_TRANSITIONAL_BCELL_UP
141 0.3426527 1.4085003 0.024175825
0.03473268 1 2103 "tags=30%, list=15%, signal=35%"
GSE2706_LPS_VS_R848_AND_LPS_8H_STIM_DC_UP
GSE2706_LPS_VS_R848_AND_LPS_8H_STIM_DC_UP 132
0.3465431 1.4081255 0.012875536 0.034855574
1 1517 "tags=23%, list=11%, signal=25%"
GSE39820_CTRL_VS_TGFBETA3_IL6_IL23A_CD4_TCELL_UP
GSE39820_CTRL_VS_TGFBETA3_IL6_IL23A_CD4_TCELL_UP 138
0.34509954 1.408024 0.014613778 0.034866426 1
2020 "tags=26%, list=14%, signal=30%"
GSE45382_UNTREATED_VS_TGFB_TREATED_MACROPHAGES_UP
GSE45382_UNTREATED_VS_TGFB_TREATED_MACROPHAGES_UP 150
0.34173238 1.4078345 0.01871102 0.034907542
1 2257 "tags=33%, list=16%, signal=38%"
GSE40274_FOXP3_VS_FOXP3_AND_SATB1_TRANSDUCED_ACTIVATED_CD4_TCELL_UP
GSE40274_FOXP3_VS_FOXP3_AND_SATB1_TRANSDUCED_ACTIVATED_CD4_TCELL_UP
90 0.3675994 1.4071459 0.01863354
0.03512968 1 100 "tags=7%, list=1%, signal=7%"
GSE19888_ADENOSINE_A3R_ACT_VS_TCELL_MEMBRANES_ACT_IN_MAST_CELL_UP
GSE19888_ADENOSINE_A3R_ACT_VS_TCELL_MEMBRANES_ACT_IN_MAST_CELL_UP
155 0.3376209 1.407074 0.016701462

0.035131466	1	2193	"tags=30%, list=16%, signal=36%"
GSE7831_UNSTIM_VS_INFLUENZA_STIM_PDC_4H_UP
GSE7831_UNSTIM_VS_INFLUENZA_STIM_PDC_4H_UP	157
0.33537552	1.4067993	0.014767933	0.035225447
1	1932	"tags=27%, list=14%, signal=31%"
GSE360_L_DONOVANI_VS_B_MALAYI_LOW_DOSE_MAC_UP
GSE360_L_DONOVANI_VS_B_MALAYI_LOW_DOSE_MAC_UP	144
0.33712494	1.4065666	0.015555556	0.03527763
1	2519	"tags=31%, list=18%, signal=38%"
GSE21379_WT_VS_SAP_KO_CD4_TCELL_UP
GSE21379_WT_VS_SAP_KO_CD4_TCELL_UP	150
0.33845338	1.4065573	0.014285714	0.0352555
1	1805	"tags=21%, list=13%, signal=24%"
GSE3982_DC_VS_MAC_LPS_STIM_UP	GSE3982_DC_VS_MAC_LPS_STIM_UP
141	0.34018457	1.4051862	0.016701462
0.03575574	1	2170	"tags=27%, list=15%, signal=32%"
GSE5542_UNTREATED_VS_IFNG_TREATED_EPITHELIAL_CELLS_6H_DN
GSE5542_UNTREATED_VS_IFNG_TREATED_EPITHELIAL_CELLS_6H_DN
143	0.3379244	1.4051636	0.014767933
0.03573849	1	2093	"tags=29%, list=15%, signal=34%"
GSE17721_LPS_VS_POLYIC_8H_BMDC_UP GSE17721_LPS_VS_POLYIC_8H_BMDC_UP
150	0.3390479	1.4048691	0.014553014
0.0358211	1	1420	"tags=21%, list=10%, signal=23%"
GSE22140_GERMFREE_VS_SPF_MOUSE_CD4_TCELL_DN
GSE22140_GERMFREE_VS_SPF_MOUSE_CD4_TCELL_DN	161
0.33239752	1.4046822	0.017660044	0.035858586
1	1707	"tags=25%, list=12%, signal=29%"
GSE29618_PRE_VS_DAY7_POST_TIV_FLU_VACCINE_BCELL_DN
GSE29618_PRE_VS_DAY7_POST_TIV_FLU_VACCINE_BCELL_DN	133
0.34297642	1.4042671	0.014583333	0.035990268
1	2842	"tags=40%, list=20%, signal=49%"
GSE26727_WT_VS_KLF2_KO_LPS_STIM_MACROPHAGE_DN
GSE26727_WT_VS_KLF2_KO_LPS_STIM_MACROPHAGE_DN	146
0.34219515	1.403429 0.01443299	0.036308207	1
2462	"tags=41%, list=17%, signal=49%"
GSE360_L_MAJOR_VS_B_MALAYI_LOW_DOSE_MAC_DN
GSE360_L_MAJOR_VS_B_MALAYI_LOW_DOSE_MAC_DN	149
0.33946672	1.4032813	0.01091703	0.036347654
1	2380	"tags=34%, list=17%, signal=41%"
GSE21927_SPLEEN_C57BL6_VS_4T1_TUMOR_BALBC_MONOCYTES_DN
GSE21927_SPLEEN_C57BL6_VS_4T1_TUMOR_BALBC_MONOCYTES_DN
129	0.34637305	1.4032652	0.017821781
0.03632483	1	2419	"tags=36%, list=17%, signal=43%"
GSE29618_PRE_VS_DAY7_POST_TIV_FLU_VACCINE_PDC_DN
GSE29618_PRE_VS_DAY7_POST_TIV_FLU_VACCINE_PDC_DN	147
0.33879182	1.4029981	0.012371134	0.036397062
1	2313	"tags=31%, list=16%, signal=37%"
GSE5455_HEALTHY_VS_TUMOR_BEARING_MOUSE_SPLEEN_MONOCYTE_24H_INCUBATIO
N_DN
GSE5455_HEALTHY_VS_TUMOR_BEARING_MOUSE_SPLEEN_MONOCYTE_24H_INCUBATIO
N_DN	152	0.3411842	1.4028797
0.00589391	0.0364145	1	1309	"tags=23%,
list=9%, signal=25%"
GSE17721_LPS_VS_CPG_8H_BMDC_UP	GSE17721_LPS_VS_CPG_8H_BMDC_UP

158	0.33542103	1.4025724	0.011605416
0.036500502	1	2132	"tags=27%, list=15%, signal=31%"
GSE21063_CTRL_VS_ANTI_IGM_STIM_BCELL_NFATC1_KO_3H_UP
GSE21063_CTRL_VS_ANTI_IGM_STIM_BCELL_NFATC1_KO_3H_UP
157	0.33628944	1.4021496	0.011650485
0.036621958	1	2168	"tags=30%, list=15%, signal=35%"
GSE17721_LPS_VS_PAM3CSK4_8H_BMDC_DN
GSE17721_LPS_VS_PAM3CSK4_8H_BMDC_DN	143
0.33498502	1.401679 0.016	0.03679511	1
2972	"tags=38%, list=21%, signal=48%"
GSE17721_POLYIC_VS_GARDIQUIMOD_1H_BMDC_DN
GSE17721_POLYIC_VS_GARDIQUIMOD_1H_BMDC_DN	158
0.33490193	1.4013244	0.019396551	0.036903117
1	1465	"tags=24%, list=10%, signal=27%"
GSE3982_NEUTROPHIL_VS_BCELL_UP	GSE3982_NEUTROPHIL_VS_BCELL_UP
139	0.34309933	1.3998234	0.024489796
0.037458327	1	2496	"tags=28%, list=18%, signal=34%"
GSE7460_TREG_VS_TCONV_ACT_WITH_TGFB_DN
GSE7460_TREG_VS_TCONV_ACT_WITH_TGFB_DN	142
0.33448642	1.3994392	0.017021276	0.037608758
1	2046	"tags=29%, list=14%, signal=33%"
GSE2770_IL4_ACT_VS_ACT_CD4_TCELL_6H_DN
GSE2770_IL4_ACT_VS_ACT_CD4_TCELL_6H_DN	140
0.34262535	1.3991958	0.016597511	0.037689064
1	1659	"tags=25%, list=12%, signal=28%"
GSE2706_UNSTIM_VS_8H_LPS_DC_UP	GSE2706_UNSTIM_VS_8H_LPS_DC_UP
140	0.3440418	1.3991438	0.022222223
0.03768023	1	1898	"tags=26%, list=13%, signal=30%"
GSE10325_CD4_TCELL_VS_BCELL_UP	GSE10325_CD4_TCELL_VS_BCELL_UP
161	0.3309688	1.3988627	0.016877636
0.037750836	1	1579	"tags=27%, list=11%, signal=30%"
GSE360_LOW_DOSE_B_MALAYI_VS_M_TUBERCULOSIS_DC_UP
GSE360_LOW_DOSE_B_MALAYI_VS_M_TUBERCULOSIS_DC_UP	150
0.33678564	1.3986201	0.022088353	0.03781181
1	2036	"tags=28%, list=14%, signal=32%"
GSE14000_TRANSLATED_RNA_VS_MRNA_DC_UP
GSE14000_TRANSLATED_RNA_VS_MRNA_DC_UP	151
0.3395965	1.3982787	0.006410257	0.03792812
1	2586	"tags=35%, list=18%, signal=43%"
GSE25087_FETAL_VS_ADULT_TCONV_UP	GSE25087_FETAL_VS_ADULT_TCONV_UP
141	0.3398353	1.3972744	0.018556701
0.03835172	1	2330	"tags=35%, list=17%, signal=42%"
GSE25123_WT_VS_PPARG_KO_MACROPHAGE_IL4_AND_ROSIGLITAZONE_STIM_UP
GSE25123_WT_VS_PPARG_KO_MACROPHAGE_IL4_AND_ROSIGLITAZONE_STIM_UP
121	0.3501819	1.3969603	0.023012552
0.03845466	1	1475	"tags=20%, list=10%, signal=22%"
GSE14308_TH1_VS_INDUCED_TREG_DN	GSE14308_TH1_VS_INDUCED_TREG_DN
136	0.34486866	1.3959593	0.020408163
0.03890199	1	2882	"tags=36%, list=20%, signal=45%"
GSE360_L_DONOVANI_VS_T_GONDII_MAC_DN
GSE360_L_DONOVANI_VS_T_GONDII_MAC_DN	161
0.33073947	1.3958327	0.008830022	0.03892176
1	2282	"tags=29%, list=16%, signal=34%"
GSE42088_UNINF_VS_LEISHMANIA_INF_DC_24H_UP

GSE42088_UNINF_VS_LEISHMANIA_INF_DC_24H_UP	156
0.33429688	1.3954582	0.01026694	0.03905075
1	1703	"tags=26%, list=12%, signal=30%"
GSE17721_CTRL_VS_LPS_24H_BMDC_DN	GSE17721_CTRL_VS_LPS_24H_BMDC_DN
	150	0.33561727	1.3951318	0.013544018
0.039172206	1	2482	"tags=35%, list=18%, signal=42%"
GSE17721_POLYIC_VS_PAM3CSK4_4H_BMDC_UP
GSE17721_POLYIC_VS_PAM3CSK4_4H_BMDC_UP	158
0.3341658	1.3947215	0.012	0.039309863	1
2224	"tags=27%, list=16%, signal=32%"
GSE360_LOW_DOSE_B_MALAYI_VS_M_TUBERCULOSIS_DC_DN
GSE360_LOW_DOSE_B_MALAYI_VS_M_TUBERCULOSIS_DC_DN	154
0.33398944	1.3942761	0.013215859	0.039463557
1	1567	"tags=26%, list=11%, signal=29%"
GSE2935_UV_INACTIVATED_VS_LIVE_SENDAI_VIRUS_INF_MACROPHAGE_DN
GSE2935_UV_INACTIVATED_VS_LIVE_SENDAI_VIRUS_INF_MACROPHAGE_DN
	151	0.33648756	1.3937734	0.019693654
0.039622054	1	2274	"tags=39%, list=16%, signal=46%"
GSE15330_HSC_VS_LYMPHOID_PRIMED_MULTIPOTENT_PROGENITOR_IKAROS_KO_DN
GSE15330_HSC_VS_LYMPHOID_PRIMED_MULTIPOTENT_PROGENITOR_IKAROS_KO_DN
	152	0.3355929	1.3937125	0.02631579
0.03961711	1	2238	"tags=30%, list=16%, signal=35%"
GSE17721_CTRL_VS_CPG_0.5H_BMDC_UP GSE17721_CTRL_VS_CPG_0.5H_BMDC_UP
	155	0.33116376	1.3931608	0.014799154
0.039818138	1	2041	"tags=25%, list=14%, signal=29%"
GSE43863_NAIVE_VS_TFH_CD4_EFF_TCELL_D6_LCMV_UP
GSE43863_NAIVE_VS_TFH_CD4_EFF_TCELL_D6_LCMV_UP	139
0.34123015	1.3930883	0.010183299	0.0398182
1	1383	"tags=19%, list=10%, signal=21%"
GSE2770_TGFB_AND_IL4_VS_IL12_TREATED_ACT_CD4_TCELL_48H_DN
GSE2770_TGFB_AND_IL4_VS_IL12_TREATED_ACT_CD4_TCELL_48H_DN
136	0.3386931	1.3923677	0.027149322
0.040111605	1	2389	"tags=35%, list=17%, signal=41%"
GSE1432_CTRL_VS_IFNG_6H_MICROGLIA_UP
GSE1432_CTRL_VS_IFNG_6H_MICROGLIA_UP	154
0.33442512	1.3916321	0.016096579	0.040408086
1	1896	"tags=23%, list=13%, signal=27%"
GSE17721_POLYIC_VS_GARDIQUIMOD_8H_BMDC_DN
GSE17721_POLYIC_VS_GARDIQUIMOD_8H_BMDC_DN	141
0.33848706	1.3914398	0.017621145	0.040466327
1	2747	"tags=35%, list=19%, signal=44%"
GSE5589_WT_VS_IL10_KO_LPS_AND_IL10_STIM_MACROPHAGE_180MIN_UP
GSE5589_WT_VS_IL10_KO_LPS_AND_IL10_STIM_MACROPHAGE_180MIN_UP
	124	0.34765333	1.3911204	0.02079002
0.040568534	1	2474	"tags=31%, list=18%, signal=37%"
GSE6674_UNSTIM_VS_PL2_3_STIM_BCELL_UP
GSE6674_UNSTIM_VS_PL2_3_STIM_BCELL_UP	112
0.3512485	1.3902308	0.023454158	0.04094233
1	1071	"tags=16%, list=8%, signal=17%"
GSE10240_CTRL_VS_IL22_STIM_PRIMARY_BRONCHIAL_EPITHELIAL_CELLS_DN
GSE10240_CTRL_VS_IL22_STIM_PRIMARY_BRONCHIAL_EPITHELIAL_CELLS_DN
	132	0.34219512	1.3901358	0.008658009
0.040963892	1	2298	"tags=31%, list=16%, signal=37%"
GSE2770_UNTREATED_VS_TGFB_AND_IL4_TREATED_ACT_CD4_TCELL_48H_DN

GSE2770_UNTREATED_VS_TGFB_AND_IL4_TREATED_ACT_CD4_TCELL_48H_DN
151 0.33427837 1.389877 0.022869023
0.041058406 1 1903 "tags=27%, list=13%, signal=31%"
GSE13411_SWITCHED_MEMORY_BCELL_VS_PLASMA_CELL_DN
GSE13411_SWITCHED_MEMORY_BCELL_VS_PLASMA_CELL_DN 131
0.3432248 1.3892637 0.019189766 0.041302152
1 1377 "tags=16%, list=10%, signal=18%"
GSE15324_ELF4_KO_VS_WT_NAIVE_CD8_TCELL_UP
GSE15324_ELF4_KO_VS_WT_NAIVE_CD8_TCELL_UP 144
0.3392387 1.3887253 0.02783726 0.04152361
1 1171 "tags=17%, list=8%, signal=19%"
GSE36527_CD62L_HIGH_VS_CD62L_LOW_TREG_CD69_NEG_KLRG1_NEG_UP
GSE36527_CD62L_HIGH_VS_CD62L_LOW_TREG_CD69_NEG_KLRG1_NEG_UP
154 0.33481163 1.3883681 0.010729614
0.041652866 1 2099 "tags=32%, list=15%, signal=37%"
GSE37534_PIOGLITAZONE_VS_ROSIGLITAZONE_TREATED_CD4_TCELL_PPARG1_FOXP3_TRANSDUCED_UP
GSE37534_PIOGLITAZONE_VS_ROSIGLITAZONE_TREATED_CD4_TCELL_PPARG1_FOXP3_TRANSDUCED_UP 135 0.33729854 1.3883384
0.023305085 0.04163108 1 2238 "tags=30%,
list=16%, signal=35%"
GSE22886_UNSTIM_VS_STIM_MEMORY_TCELL_UP
GSE22886_UNSTIM_VS_STIM_MEMORY_TCELL_UP 137
0.3391121 1.3882533 0.008264462 0.041627258
1 1610 "tags=21%, list=11%, signal=24%"
GSE41978_WT_VS_ID2_KO_AND_BIM_KO_KLRG1_LOW_EFFECTOR_CD8_TCELL_DN
GSE41978_WT_VS_ID2_KO_AND_BIM_KO_KLRG1_LOW_EFFECTOR_CD8_TCELL_DN
158 0.32924893 1.3877099 0.021786492
0.04182269 1 1691 "tags=23%, list=12%, signal=26%"
GSE1925_CTRL_VS_IFNG_PRIMED_MACROPHAGE_DN
GSE1925_CTRL_VS_IFNG_PRIMED_MACROPHAGE_DN 145
0.33722755 1.3873905 0.025229357 0.041938208
1 1278 "tags=21%, list=9%, signal=23%"
GSE28737_WT_VS_BCL6_KO_FOLLICULAR_BCELL_DN
GSE28737_WT_VS_BCL6_KO_FOLLICULAR_BCELL_DN 158
0.33067888 1.387219 0.016 0.04199135 1
2361 "tags=32%, list=17%, signal=38%"
GSE40273_XBP1_KO_VS_WT_TREG_UP GSE40273_XBP1_KO_VS_WT_TREG_UP
147 0.33438513 1.3855922 0.026804123
0.042718273 1 2629 "tags=35%, list=19%, signal=42%"
GSE3720_UNSTIM_VS_PMA_STIM_VD2_GAMMADELTA_TCELL_UP
GSE3720_UNSTIM_VS_PMA_STIM_VD2_GAMMADELTA_TCELL_UP 122
0.34246653 1.3853277 0.034042552 0.042802956
1 1824 "tags=34%, list=13%, signal=38%"
GSE17974_0H_VS_4H_IN_VITRO_ACT_CD4_TCELL_DN
GSE17974_0H_VS_4H_IN_VITRO_ACT_CD4_TCELL_DN 130
0.33969608 1.3852597 0.022680413 0.042800732
1 1794 "tags=25%, list=13%, signal=29%"
GSE40274_FOXP3_VS_FOXP3_AND_IRF4_TRANSDUCED_ACTIVATED_CD4_TCELL_DN
GSE40274_FOXP3_VS_FOXP3_AND_IRF4_TRANSDUCED_ACTIVATED_CD4_TCELL_DN
144 0.33367875 1.3850665 0.026666667
0.042858243 1 1871 "tags=26%, list=13%, signal=29%"
GSE37534_GW1929_VS_ROSIGLITAZONE_TREATED_CD4_TCELL_PPARG1_FOXP3_TRANSDUCED_UP

GSE37534_GW1929_VS_ROSIGLITAZONE_TREATED_CD4_TCELL_PPARG1_FOXP3_TRANSDUCED_UP 118 0.34808236 1.3838658 0.025 0.043380775 1 1678 "tags=25%, list=12%, signal=28%"
GSE40277_EOS_AND_LEF1_TRANSDUCED_VS_CTRL_CD4_TCELL_DN GSE40277_EOS_AND_LEF1_TRANSDUCED_VS_CTRL_CD4_TCELL_DN 152 0.3310863 1.3832537 0.018867925 0.04363762 1 2463 "tags=30%, list=17%, signal=35%"
GSE6259_FLT3L_INDUCED_33D1_POS_DC_VS_CD8_TCELL_DN GSE6259_FLT3L_INDUCED_33D1_POS_DC_VS_CD8_TCELL_DN 121 0.34243894 1.3827293 0.020920502 0.043864828 1 2036 "tags=26%, list=14%, signal=30%"
GSE2770_TGFB_AND_IL4_VS_IL4_TREATED_ACT_CD4_TCELL_2H_DN GSE2770_TGFB_AND_IL4_VS_IL4_TREATED_ACT_CD4_TCELL_2H_DN 135 0.337614 1.3808137 0.028688524 0.044769876 1 1752 "tags=21%, list=12%, signal=24%"
GSE16385_MONOCYTE_VS_12H_ROSIGLITAZONE_IFNG_TNF_TREATED_MACROPHAGE_DN GSE16385_MONOCYTE_VS_12H_ROSIGLITAZONE_IFNG_TNF_TREATED_MACROPHAGE_DN 148 0.3323705 1.3797415 0.020876827 0.04523537 1 1833 "tags=22%, list=13%, signal=25%"
GSE41176_UNSTIM_VS_ANTI_IGM_STIM_TAK1_KO_BCELL_1H_UP GSE41176_UNSTIM_VS_ANTI_IGM_STIM_TAK1_KO_BCELL_1H_UP 136 0.33934814 1.379581 0.010660981 0.04528139 1 2594 "tags=38%, list=18%, signal=46%"
GSE37301_PRO_BCELL_VS_RAG2_KO_NK_CELL_UP GSE37301_PRO_BCELL_VS_RAG2_KO_NK_CELL_UP 150 0.33070293 1.3794683 0.032989692 0.045308832 1 2437 "tags=35%, list=17%, signal=42%"
GSE339_CD4POS_VS_CD8POS_DC_DN GSE339_CD4POS_VS_CD8POS_DC_DN 160 0.32945955 1.3779445 0.026915114 0.04603111 1 2234 "tags=31%, list=16%, signal=37%"
GSE43955_1H_VS_60H_ACT_CD4_TCELL_WITH_TGFB_IL6_UP GSE43955_1H_VS_60H_ACT_CD4_TCELL_WITH_TGFB_IL6_UP 154 0.33105534 1.3779131 0.01923077 0.046008036 1 1900 "tags=26%, list=13%, signal=30%"
GSE17721_PAM3CSK4_VS_GADIQUIMOD_2H_BMDC_DN GSE17721_PAM3CSK4_VS_GADIQUIMOD_2H_BMDC_DN 133 0.33279788 1.3778656 0.026530612 0.045994308 1 2297 "tags=32%, list=16%, signal=38%"
GSE37416_CTRL_VS_24H_F_TULARENSIS_LVS_NEUTROPHIL_UP GSE37416_CTRL_VS_24H_F_TULARENSIS_LVS_NEUTROPHIL_UP 133 0.33442408 1.3777274 0.020833334 0.046032272 1 2413 "tags=35%, list=17%, signal=41%"
GSE2826_WT_VS_BTK_KO_BCELL_DN GSE2826_WT_VS_BTK_KO_BCELL_DN 154 0.3277732 1.3777077 0.026156941 0.046006776 1 1227 "tags=21%, list=9%, signal=23%"
GSE17721_CTRL_VS_PAM3CSK4_4H_BMDC_UP GSE17721_CTRL_VS_PAM3CSK4_4H_BMDC_UP 135 0.33503106 1.3776114 0.022680413 0.04602511 1 1697 "tags=25%, list=12%, signal=28%"
GSE45739_NRAS_KO_VS_WT_ACD3_ACD28_STIM_CD4_TCELL_UP GSE45739_NRAS_KO_VS_WT_ACD3_ACD28_STIM_CD4_TCELL_UP

138 0.3396444 1.377424 0.022727273 0.04608983
1 2010 "tags=26%, list=14%, signal=30%"
GSE43955_TGFB_IL6_VS_TGFB_IL6_IL23_TH17_ACT_CD4_TCELL_52H_UP
GSE43955_TGFB_IL6_VS_TGFB_IL6_IL23_TH17_ACT_CD4_TCELL_52H_UP
157 0.33007202 1.3772811 0.016949153
0.04614956 1 3023 "tags=34%, list=21%, signal=43%"
GSE24574_NAIVE_VS_TCONV_CD4_TCELL_UP
GSE24574_NAIVE_VS_TCONV_CD4_TCELL_UP 144
0.33458874 1.3766952 0.019693654 0.046396688
1 1725 "tags=19%, list=12%, signal=22%"
GSE2770_UNTREATED_VS_TGFB_AND_IL4_TREATED_ACT_CD4_TCELL_2H_DN
GSE2770_UNTREATED_VS_TGFB_AND_IL4_TREATED_ACT_CD4_TCELL_2H_DN
143 0.33356354 1.3766865 0.014644352
0.046366815 1 2010 "tags=28%, list=14%, signal=32%"
GSE17721_LPS_VS_GARDIQUIMOD_0.5H_BMDC_DN
GSE17721_LPS_VS_GARDIQUIMOD_0.5H_BMDC_DN 148
0.33376893 1.3763909 0.016736401 0.046473052
1 2404 "tags=28%, list=17%, signal=33%"
GSE43955_TH0_VS_TGFB_IL6_TH17_ACT_CD4_TCELL_4H_DN
GSE43955_TH0_VS_TGFB_IL6_TH17_ACT_CD4_TCELL_4H_DN 161
0.32869762 1.3761804 0.018404908 0.04653433
1 2093 "tags=28%, list=15%, signal=32%"
GSE17721_CTRL_VS_GARDIQUIMOD_24H_BMDC_DN
GSE17721_CTRL_VS_GARDIQUIMOD_24H_BMDC_DN 145
0.33206147 1.3751239 0.025531914 0.0470147
1 2610 "tags=34%, list=18%, signal=42%"
GSE21546_SAP1A_KO_VS_SAP1A_KO_AND_ELK1_KO_ANTI_CD3_STIM_DP_THYMOCYTES_UP
GSE21546_SAP1A_KO_VS_SAP1A_KO_AND_ELK1_KO_ANTI_CD3_STIM_DP_THYMOCYTES_UP 143 0.3343307 1.3751041
0.010373444 0.04699196 1 2349 "tags=28%, list=17%, signal=33%"
GSE5589_LPS_AND_IL10_VS_LPS_AND_IL6_STIM_IL6_KO_MACROPHAGE_45MIN_UP
GSE5589_LPS_AND_IL10_VS_LPS_AND_IL6_STIM_IL6_KO_MACROPHAGE_45MIN_UP
144 0.33157808 1.3749838 0.026422765
0.047013003 1 2298 "tags=36%, list=16%, signal=43%"
GSE14000_UNSTIM_VS_4H_LPS_DC_UP GSE14000_UNSTIM_VS_4H_LPS_DC_UP
123 0.34276205 1.3749394 0.022540983
0.04700298 1 2499 "tags=37%, list=18%, signal=44%"
GSE10240_IL22_VS_IL17_STIM_PRIMARY_BRONCHIAL_EPITHELIAL_CELLS_UP
GSE10240_IL22_VS_IL17_STIM_PRIMARY_BRONCHIAL_EPITHELIAL_CELLS_UP
137 0.33859935 1.374659 0.030364372
0.04708584 1 1860 "tags=25%, list=13%, signal=28%"
GSE22611_NOD2_VS_CTRL_TRANSDUCED_HEK293T_CELL_DN
GSE22611_NOD2_VS_CTRL_TRANSDUCED_HEK293T_CELL_DN 138
0.33321515 1.3745447 0.016771488 0.047114883
1 2036 "tags=30%, list=14%, signal=34%"
GSE22935_WT_VS_MYD88_KO_MACROPHAGE_48H_MBOVIS_BCG_STIM_DN
GSE22935_WT_VS_MYD88_KO_MACROPHAGE_48H_MBOVIS_BCG_STIM_DN
147 0.33448866 1.3741434 0.019480519
0.047292035 1 1820 "tags=30%, list=13%, signal=34%"
GSE17322_CD103_POS_VS_CD11B_HIGH_LUNG_DC_DN
GSE17322_CD103_POS_VS_CD11B_HIGH_LUNG_DC_DN 146
0.337035 1.3733633 0.025157232 0.047656737 1

2697 "tags=37%, list=19%, signal=45%"
GSE22140_HEALTHY_VS_ARTHRITIC_MOUSE_CD4_TCELL_DN
GSE22140_HEALTHY_VS_ARTHRITIC_MOUSE_CD4_TCELL_DN 160
0.32920596 1.3726326 0.016913319 0.0479882
1 2654 "tags=35%, list=19%, signal=43%"
GSE26495_NAIVE_VS_PD1HIGH_CD8_TCELL_DN
GSE26495_NAIVE_VS_PD1HIGH_CD8_TCELL_DN 139
0.3343104 1.3726003 0.037815128 0.047976613
1 2000 "tags=28%, list=14%, signal=32%"
GSE18281_MEDULLARY_THYMOCYTE_VS_WHOLE_MEDULLA_THYMUS_DN
GSE18281_MEDULLARY_THYMOCYTE_VS_WHOLE_MEDULLA_THYMUS_DN
121 0.34077215 1.3723476 0.034334764
0.048061494 1 2641 "tags=37%, list=19%, signal=45%"
GSE46606_IRF4MID_VS_WT_CD40L_IL2_IL5_DAY3_STIMULATED_BCELL_DN
GSE46606_IRF4MID_VS_WT_CD40L_IL2_IL5_DAY3_STIMULATED_BCELL_DN
147 0.33095777 1.372324 0.028077753
0.048042327 1 1840 "tags=31%, list=13%, signal=35%"
GSE17721_LPS_VS_GARDIQUIMOD_12H_BMDC_UP
GSE17721_LPS_VS_GARDIQUIMOD_12H_BMDC_UP 155
0.33218908 1.3719788 0.023904383 0.048182674
1 2320 "tags=27%, list=16%, signal=32%"
GSE22886_NAIVE_CD8_TCELL_VS_NKCELL_UP
GSE22886_NAIVE_CD8_TCELL_VS_NKCELL_UP 139
0.3326063 1.3702738 0.027542373 0.04900312
1 1873 "tags=28%, list=13%, signal=32%"
GSE6674_UNSTIM_VS_CPG_STIM_BCELL_UP
GSE6674_UNSTIM_VS_CPG_STIM_BCELL_UP 117
0.33995515 1.3702277 0.02826087 0.048994996
1 2081 "tags=26%, list=15%, signal=30%"
GSE3982_EOSINOPHIL_VS_MAC_DN GSE3982_EOSINOPHIL_VS_MAC_DN
155 0.32971582 1.3684393 0.022680413
0.049878817 1 2643 "tags=37%, list=19%, signal=46%"
GSE17721_CTRL_VS_GARDIQUIMOD_6H_BMDC_UP
GSE17721_CTRL_VS_GARDIQUIMOD_6H_BMDC_UP 146
0.3313284 1.3671515 0.020703934 0.05050982
1 1901 "tags=25%, list=13%, signal=28%"
GSE43955_TGFB_IL6_VS_TGFB_IL6_IL23_TH17_ACT_CD4_TCELL_60H_DN
GSE43955_TGFB_IL6_VS_TGFB_IL6_IL23_TH17_ACT_CD4_TCELL_60H_DN
165 0.32419232 1.3668686 0.018947368
0.050615784 1 1389 "tags=17%, list=10%, signal=19%"
GSE32164_ALTERNATIVELY_ACT_M2_VS_CMYC_INHIBITED_MACROPHAGE_UP
GSE32164_ALTERNATIVELY_ACT_M2_VS_CMYC_INHIBITED_MACROPHAGE_UP
154 0.32693687 1.3661343 0.01875
0.05099047 1 2698 "tags=36%, list=19%, signal=44%"
GSE25088_CTRL_VS_IL4_AND_ROSIGLITAZONE_STIM_MACROPHAGE_DN
GSE25088_CTRL_VS_IL4_AND_ROSIGLITAZONE_STIM_MACROPHAGE_DN
117 0.34213945 1.3655833 0.031446543
0.05124437 1 2525 "tags=34%, list=18%, signal=41%"
GSE40666_NAIVE_VS_EFFECTOR_CD8_TCELL_WITH_IFNA_STIM_90MIN_DN
GSE40666_NAIVE_VS_EFFECTOR_CD8_TCELL_WITH_IFNA_STIM_90MIN_DN
148 0.32896096 1.3650702 0.0186722
0.05150691 1 1015 "tags=18%, list=7%, signal=19%"
GSE1432_CTRL_VS_IFNG_24H_MICROGLIA_DN
GSE1432_CTRL_VS_IFNG_24H_MICROGLIA_DN 137

0.33248118	1.3650376	0.027196653	0.051488005
1	2444	"tags=39%, list=17%, signal=46%"
GSE42724_B1_BCELL_VS_PLASMABLAST_UP
GSE42724_B1_BCELL_VS_PLASMABLAST_UP		151
0.32729986	1.3648282	0.024048096	0.05156416
1	2041	"tags=25%, list=14%, signal=28%"
GSE37605_TREG_VS_TCONV_C57BL6_FOXP3_FUSION_GFP_DN
GSE37605_TREG_VS_TCONV_C57BL6_FOXP3_FUSION_GFP_DN		137
0.33238152	1.3646587	0.030120483	0.05162879
1	2390	"tags=37%, list=17%, signal=44%"
GSE37416_CTRL_VS_48H_F_TULARENSIS_LVS_NEUTROPHIL_UP
GSE37416_CTRL_VS_48H_F_TULARENSIS_LVS_NEUTROPHIL_UP
114	0.3388693	1.36413	0.026804123	0.051867224
1	1399	"tags=22%, list=10%, signal=24%"
GSE17721_LPS_VS_POLYIC_12H_BMDC_UP
GSE17721_LPS_VS_POLYIC_12H_BMDC_UP		148
0.3274653	1.3637168	0.034907598	0.052026916
1	2490	"tags=28%, list=18%, signal=33%"
GSE17721_PAM3CSK4_VS_CPG_6H_BMDC_DN
GSE17721_PAM3CSK4_VS_CPG_6H_BMDC_DN		155
0.3265965	1.3635514	0.023504274	0.052073732
1	2579	"tags=27%, list=18%, signal=33%"
GSE360_CTRL_VS_M_TUBERCULOSIS_DC_UP
GSE360_CTRL_VS_M_TUBERCULOSIS_DC_UP		147
0.3280979	1.3628621	0.029288704	0.05240941
1	2531	"tags=34%, list=18%, signal=41%"
GSE16451_CTRL_VS_WEST_EQUINE_ENC_VIRUS_MATURE_NEURON_CELL_LINE_DN
GSE16451_CTRL_VS_WEST_EQUINE_ENC_VIRUS_MATURE_NEURON_CELL_LINE_DN
	148	0.32547897	1.3624645	0.021231422
0.052589484	1	1234	"tags=22%, list=9%, signal=23%"
GSE42021_CD24LO_TREG_VS_CD24LO_TCONV_THYMUS_UP
GSE42021_CD24LO_TREG_VS_CD24LO_TCONV_THYMUS_UP		151
0.32850134	1.3615006	0.03640257	0.053057343
1	2063	"tags=26%, list=15%, signal=31%"
GSE17186_BLOOD_VS_CORD_BLOOD_NAIVE_BCELL_DN
GSE17186_BLOOD_VS_CORD_BLOOD_NAIVE_BCELL_DN		128
0.3328828	1.3607996	0.022680413	0.053425502
1	2060	"tags=30%, list=15%, signal=34%"
GSE17721_PAM3CSK4_VS_CPG_8H_BMDC_DN
GSE17721_PAM3CSK4_VS_CPG_8H_BMDC_DN		164
0.32673454	1.3605654	0.030991735	0.05354087
1	2059	"tags=27%, list=15%, signal=32%"
GSE3203_WT_VS_IFNAR1_KO_INFLUENZA_INFECTED_LN_BCELL_DN
GSE3203_WT_VS_IFNAR1_KO_INFLUENZA_INFECTED_LN_BCELL_DN
132	0.33416665	1.3602413	0.029473685
0.05367336	1	2249	"tags=24%, list=16%, signal=29%"
GSE15930_STIM_VS_STIM_AND_TRICHOSTATINA_72H_CD8_T_CELL_UP
GSE15930_STIM_VS_STIM_AND_TRICHOSTATINA_72H_CD8_T_CELL_UP
154	0.32461724	1.3596879	0.023554605
0.053962216	1	1598	"tags=22%, list=11%, signal=25%"
GSE34392_ST2_KO_VS_WT_DAY8_LCMV_EFFECTOR_CD8_TCELL_UP
GSE34392_ST2_KO_VS_WT_DAY8_LCMV_EFFECTOR_CD8_TCELL_UP
138	0.335405	1.3595425	0.03797468	0.054005828
1	2091	"tags=31%, list=15%, signal=36%"

GSE20198_IL12_IL18_VS_IFNA_TREATED_ACT_CD4_TCELL_UP
GSE20198_IL12_IL18_VS_IFNA_TREATED_ACT_CD4_TCELL_UP
131 0.33548936 1.3594977 0.015217391
0.0539903 1 2364 "tags=32%, list=17%, signal=38%"
GSE43863_DAY6_EFF_VS_DAY150_MEM_TH1_CD4_TCELL_DN
GSE43863_DAY6_EFF_VS_DAY150_MEM_TH1_CD4_TCELL_DN 148
0.32677513 1.3592889 0.024844721 0.054063875
1 1573 "tags=27%, list=11%, signal=30%"
GSE21670_STAT3_KO_VS_WT_CD4_TCELL_IL6_TREATED_UP
GSE21670_STAT3_KO_VS_WT_CD4_TCELL_IL6_TREATED_UP 146
0.32942787 1.3591871 0.01863354 0.054092336
1 2850 "tags=34%, list=20%, signal=42%"
GSE17186_CD21LOW_VS_CD21HIGH_TRANSITIONAL_BCELL_CORD_BLOOD_UP
GSE17186_CD21LOW_VS_CD21HIGH_TRANSITIONAL_BCELL_CORD_BLOOD_UP
145 0.32908243 1.3587582 0.037894737
0.05427899 1 2521 "tags=36%, list=18%, signal=43%"
GSE5589_LPS_VS_LPS_AND_IL10_STIM_IL6_KO_MACROPHAGE_180MIN_DN
GSE5589_LPS_VS_LPS_AND_IL10_STIM_IL6_KO_MACROPHAGE_180MIN_DN
138 0.3315438 1.3587383 0.028508771
0.05425105 1 2539 "tags=28%, list=18%, signal=34%"
GSE6259_BCELL_VS_CD4_TCELL_DN GSE6259_BCELL_VS_CD4_TCELL_DN
137 0.33098966 1.358071 0.024017468
0.054595172 1 2122 "tags=27%, list=15%, signal=31%"
GSE32901_NAIVE_VS_TH17_ENRICHED_CD4_TCELL_DN
GSE32901_NAIVE_VS_TH17_ENRICHED_CD4_TCELL_DN 138
0.3335725 1.3579834 0.035051547 0.054603674
1 2392 "tags=35%, list=17%, signal=41%"
GSE37301_LYMPHOID_PRIMED_MPP_VS_COMMON_LYMPHOID_PROGENITOR_DN
GSE37301_LYMPHOID_PRIMED_MPP_VS_COMMON_LYMPHOID_PROGENITOR_DN
162 0.32218552 1.3578827 0.014
0.054622885 1 2116 "tags=30%, list=15%, signal=34%"
GSE7460_CTRL_VS_TGFB_TREATED_ACT_TCONV_UP
GSE7460_CTRL_VS_TGFB_TREATED_ACT_TCONV_UP 162
0.3239413 1.3574786 0.012711864 0.0548174
1 1947 "tags=26%, list=14%, signal=30%"
GSE43863_NAIVE_VS_MEMORY_LY6C_INT_CXCR5POS_CD4_TCELL_D150_LCMV_DN
GSE43863_NAIVE_VS_MEMORY_LY6C_INT_CXCR5POS_CD4_TCELL_D150_LCMV_DN
138 0.33103305 1.3574286 0.037117902
0.054803662 1 1710 "tags=24%, list=12%, signal=27%"
GSE12392_WT_VS_IFNB_KO_CD8A_NEG_SPLEEN_DC_UP
GSE12392_WT_VS_IFNB_KO_CD8A_NEG_SPLEEN_DC_UP 149
0.32296073 1.3567711 0.02296451 0.055159815
1 2205 "tags=30%, list=16%, signal=35%"
GSE17721_LPS_VS_PAM3CSK4_12H_BMDC_UP
GSE17721_LPS_VS_PAM3CSK4_12H_BMDC_UP 159
0.32418385 1.3565427 0.024096385 0.05524931
1 1470 "tags=23%, list=10%, signal=26%"
GSE41176_WT_VS_TAK1_KO_ANTI_IGM_STIM_BCELL_24H_UP
GSE41176_WT_VS_TAK1_KO_ANTI_IGM_STIM_BCELL_24H_UP 146
0.32918394 1.3561167 0.032467533 0.055448707
1 2040 "tags=29%, list=14%, signal=33%"
GSE20198_IL12_IL18_VS_IFNA_TREATED_ACT_CD4_TCELL_DN
GSE20198_IL12_IL18_VS_IFNA_TREATED_ACT_CD4_TCELL_DN
142 0.32766336 1.355965 0.03411514 0.05550629

1	1243	"tags=17%, list=9%, signal=18%"
GSE15330_HSC_VS_MEGAKARYOCYTE_ERYTHROID_PROGENITOR_IKAROS_KO_UP
GSE15330_HSC_VS_MEGAKARYOCYTE_ERYTHROID_PROGENITOR_IKAROS_KO_UP
	156	0.3271561	1.3556265	0.027484143
0.05567598	1	1648	"tags=26%, list=12%, signal=29%"
GSE22886_IGM_MEMORY_BCELL_VS_BM_PLASMA_CELL_DN
GSE22886_IGM_MEMORY_BCELL_VS_BM_PLASMA_CELL_DN	151
0.32747534	1.3549557	0.014799154	0.05602567
1	1862	"tags=28%, list=13%, signal=32%"
GSE27786_LIN_NEG_VS_ERYTHROBLAST_UP
GSE27786_LIN_NEG_VS_ERYTHROBLAST_UP	139	0.329311
1.3543998	0.04025424	0.056279205	1
2798	"tags=39%, list=20%, signal=48%"
GSE45365_WT_VS_IFNAR_KO_CD8A_DC_MCMV_INFECTION_UP
GSE45365_WT_VS_IFNAR_KO_CD8A_DC_MCMV_INFECTION_UP	134
0.33047175	1.3542986	0.03655914	0.05630764
1	2613	"tags=33%, list=19%, signal=40%"
GSE37416_CTRL_VS_3H_F_TULARENSIS_LVS_NEUTROPHIL_UP
GSE37416_CTRL_VS_3H_F_TULARENSIS_LVS_NEUTROPHIL_UP	119
0.33714828	1.3539906	0.04255319	0.056432918
1	2486	"tags=34%, list=18%, signal=40%"
GSE9509_LPS_VS_LPS_AND_IL10_STIM_IL10_KO_MACROPHAGE_10MIN_DN
GSE9509_LPS_VS_LPS_AND_IL10_STIM_IL10_KO_MACROPHAGE_10MIN_DN
	143	0.32900727	1.3534561	0.03773585
0.0567041	1	2963	"tags=40%, list=21%, signal=50%"
GSE7460_CTRL_VS_TGFB_TREATED_ACT_FOXP3_HET_TCONV_UP
GSE7460_CTRL_VS_TGFB_TREATED_ACT_FOXP3_HET_TCONV_UP
145	0.3292112	1.3530362	0.020283977
0.056902472	1	2768	"tags=34%, list=20%, signal=42%"
GSE1460_CORD_VS_ADULT_BLOOD_NAIVE_CD4_TCELL_UP
GSE1460_CORD_VS_ADULT_BLOOD_NAIVE_CD4_TCELL_UP	130
0.33259735	1.3524603	0.025477707	0.057202414
1	1579	"tags=23%, list=11%, signal=26%"
GSE26030_TH1_VS_TH17_DAY5_POST_POLARIZATION_DN
GSE26030_TH1_VS_TH17_DAY5_POST_POLARIZATION_DN	151
0.32401037	1.3514868	0.023206752	0.057724886
1	1082	"tags=18%, list=8%, signal=19%"
GSE26495_PD1HIGH_VS_PD1LOW_CD8_TCELL_DN
GSE26495_PD1HIGH_VS_PD1LOW_CD8_TCELL_DN	110
0.34212673	1.3513185	0.052516412	0.057787463
1	817	"tags=14%, list=6%, signal=14%"
GSE11864_CSF1_IFNG_VS_CSF1_IFNG_PAM3CYS_IN_MAC_UP
GSE11864_CSF1_IFNG_VS_CSF1_IFNG_PAM3CYS_IN_MAC_UP	137
0.32576686	1.3512118	0.034764826	0.05782659
1	2093	"tags=28%, list=15%, signal=33%"
GSE37533_PPARG2_FOXP3_VS_FOXP3_TRANSDUCED_CD4_TCELL_PIOGLITAZONE_TRE
ATED_DN
GSE37533_PPARG2_FOXP3_VS_FOXP3_TRANSDUCED_CD4_TCELL_PIOGLITAZONE_TRE
ATED_DN	143	0.33030432	1.350874 0.030800821
0.057986528	1	2108	"tags=27%, list=15%, signal=31%"
GSE339_CD4POS_VS_CD4CD8DN_DC_UP	GSE339_CD4POS_VS_CD4CD8DN_DC_UP
	157	0.32743293	1.3504169	0.025104603
0.058199912	1	2090	"tags=24%, list=15%, signal=28%"
GSE37301_LYMPHOID_PRIMED_MPP_VS_PRO_BCELL_UP

GSE37301_LYMPHOID_PRIMED_MPP_VS_PRO_BCELL_UP 145
0.3223583 1.3494142 0.01923077 0.058778796
1 2369 "tags=32%, list=17%, signal=39%"
GSE9988_LOW_LPS_VS_CTRL_TREATED_MONOCYTE_DN
GSE9988_LOW_LPS_VS_CTRL_TREATED_MONOCYTE_DN 134
0.33654246 1.3493314 0.023504274 0.058787245
1 2638 "tags=33%, list=19%, signal=40%"
GSE32255_UNSTIM_VS_4H_LPS_STIM_DC_DN
GSE32255_UNSTIM_VS_4H_LPS_STIM_DC_DN 112
0.34137234 1.3483356 0.03550296 0.059358902
1 2783 "tags=33%, list=20%, signal=41%"
GSE19825_NAIVE_VS_IL2RALOW_DAY3_EFF_CD8_TCELL_DN
GSE19825_NAIVE_VS_IL2RALOW_DAY3_EFF_CD8_TCELL_DN 136
0.329733 1.3474052 0.036659878 0.059883427 1
2325 "tags=31%, list=16%, signal=37%"
GSE12845_IGD_NEG_BLOOD_VS_PRE_GC_TONSIL_BCELL_UP
GSE12845_IGD_NEG_BLOOD_VS_PRE_GC_TONSIL_BCELL_UP 145
0.32562575 1.3473967 0.021929825 0.059846524
1 2143 "tags=30%, list=15%, signal=35%"
GSE24634_IL4_VS_CTRL_TREATED_NAIVE_CD4_TCELL_DAY10_DN
GSE24634_IL4_VS_CTRL_TREATED_NAIVE_CD4_TCELL_DAY10_DN
154 0.32683527 1.3473072 0.029748283
0.05986008 1 1903 "tags=23%, list=13%, signal=27%"
GSE32423_IL7_VS_IL4_MEMORY_CD8_TCELL_UP
GSE32423_IL7_VS_IL4_MEMORY_CD8_TCELL_UP 136
0.33185485 1.3471044 0.03846154 0.059927493
1 1805 "tags=24%, list=13%, signal=27%"
GSE27786_LSK_VS_CD4_TCELL_UP GSE27786_LSK_VS_CD4_TCELL_UP
140 0.3274854 1.3466609 0.041753653
0.060158458 1 2074 "tags=22%, list=15%, signal=26%"
GSE339_CD8POS_VS_CD4CD8DN_DC_UP GSE339_CD8POS_VS_CD4CD8DN_DC_UP
159 0.32100523 1.346603 0.024793388
0.060151808 1 2322 "tags=36%, list=16%, signal=42%"
GSE20151_CTRL_VS_FUSOBACT_NUCLEATUM_NEUTROPHIL_DN
GSE20151_CTRL_VS_FUSOBACT_NUCLEATUM_NEUTROPHIL_DN 146
0.32870185 1.3455671 0.023952097 0.06074213
1 2416 "tags=33%, list=17%, signal=39%"
GSE1432_1H_VS_6H_IFNG_MICROGLIA_UP
GSE1432_1H_VS_6H_IFNG_MICROGLIA_UP 153
0.32653353 1.3454 0.03131524 0.06079966 1
2047 "tags=24%, list=15%, signal=28%"
GSE5589_LPS_VS_LPS_AND_IL6_STIM_IL6_KO_MACROPHAGE_45MIN_DN
GSE5589_LPS_VS_LPS_AND_IL6_STIM_IL6_KO_MACROPHAGE_45MIN_DN
155 0.320341 1.3452244 0.019650655 0.060850855
1 1665 "tags=25%, list=12%, signal=27%"
GSE21927_BALBC_VS_C57BL6_MONOCYTE_TUMOR_DN
GSE21927_BALBC_VS_C57BL6_MONOCYTE_TUMOR_DN 104
0.33877364 1.3449508 0.035051547 0.060972206
1 2404 "tags=30%, list=17%, signal=36%"
GSE23114_WT_VS_SLE2C1_MOUSE_SPLEEN_B1A_BCELL_DN
GSE23114_WT_VS_SLE2C1_MOUSE_SPLEEN_B1A_BCELL_DN 153
0.32389227 1.3447039 0.02603037 0.06109978
1 2205 "tags=27%, list=16%, signal=31%"
GSE19941_IL10_KO_VS_IL10_KO_AND_NFKBP50_KO_LPS_STIM_MACROPHAGE_UP

GSE19941_IL10_KO_VS_IL10_KO_AND_NFKBP50_KO_LPS_STIM_MACROPHAGE_UP
154 0.323937 1.3446598 0.034632035
0.061086934 1 2572 "tags=29%, list=18%, signal=35%"
GSE42724_MEMORY_BCELL_VS_PLASMABLAST_DN
GSE42724_MEMORY_BCELL_VS_PLASMABLAST_DN 146
0.31985825 1.3444692 0.035940804 0.06117921
1 2402 "tags=34%, list=17%, signal=40%"
GSE36009_UNSTIM_VS_LPS_STIM_NLRP10_KO_DC_UP
GSE36009_UNSTIM_VS_LPS_STIM_NLRP10_KO_DC_UP 148
0.3186754 1.3444192 0.026923077 0.061175875
1 1593 "tags=22%, list=11%, signal=24%"
GSE11864_CSF1_IFNG_VS_CSF1_IFNG_PAM3CYS_IN_MAC_DN
GSE11864_CSF1_IFNG_VS_CSF1_IFNG_PAM3CYS_IN_MAC_DN 142
0.32716218 1.3434968 0.023655914 0.06169967
1 2269 "tags=25%, list=16%, signal=30%"
GSE17721_PAM3CSK4_VS_CPG_8H_BMDC_UP
GSE17721_PAM3CSK4_VS_CPG_8H_BMDC_UP 145
0.32675532 1.3433678 0.038961038 0.061731566
1 2347 "tags=31%, list=17%, signal=37%"
GSE22886_NAIVE_CD8_TCELL_VS_MEMORY_TCELL_DN
GSE22886_NAIVE_CD8_TCELL_VS_MEMORY_TCELL_DN 154
0.32122913 1.3425856 0.016913319 0.06216778
1 1313 "tags=21%, list=9%, signal=23%"
GSE17721_0.5H_VS_8H_GARDIQUIMOD_BMDC_DN
GSE17721_0.5H_VS_8H_GARDIQUIMOD_BMDC_DN 158 0.327884
1.3421534 0.037656903 0.062421605 1
2754 "tags=37%, list=20%, signal=45%"
GSE41176_WT_VS_TAK1_KO_ANTI_IGM_STIM_BCELL_24H_DN
GSE41176_WT_VS_TAK1_KO_ANTI_IGM_STIM_BCELL_24H_DN 130
0.3299337 1.341442 0.04821803 0.06286242 1
2250 "tags=30%, list=16%, signal=35%"
GSE22919_RESTING_VS_IL2_IL12_IL15_STIM_NK_CELL_UP
GSE22919_RESTING_VS_IL2_IL12_IL15_STIM_NK_CELL_UP 146
0.3249997 1.3397154 0.03050109 0.06396166
1 2654 "tags=36%, list=19%, signal=43%"
GSE13485_CTRL_VS_DAY7_YF17D_VACCINE_PBMC_DN
GSE13485_CTRL_VS_DAY7_YF17D_VACCINE_PBMC_DN 138
0.33014372 1.3396363 0.035051547 0.06396996
1 1208 "tags=22%, list=9%, signal=24%"
GSE36527_CD69_NEG_VS_POS_TREG_CD62L_LOS_KLRG1_NEG_DN
GSE36527_CD69_NEG_VS_POS_TREG_CD62L_LOS_KLRG1_NEG_DN
148 0.32367718 1.3396263 0.040816326
0.063932374 1 1429 "tags=23%, list=10%, signal=25%"
GSE26488_CTRL_VS_PEPTIDE_INJECTION_HDAC7_DELTAP_TG_OT2_THYMOCYTE_UP
GSE26488_CTRL_VS_PEPTIDE_INJECTION_HDAC7_DELTAP_TG_OT2_THYMOCYTE_UP
165 0.31741253 1.3395619 0.022222223
0.06393821 1 1995 "tags=19%, list=14%, signal=22%"
GSE17721_CTRL_VS_CPG_4H_BMDC_UP GSE17721_CTRL_VS_CPG_4H_BMDC_UP
153 0.32136935 1.3390026 0.02631579
0.06425539 1 2242 "tags=33%, list=16%, signal=39%"
GSE17721_0.5H_VS_4H_PAM3CSK4_BMDC_UP
GSE17721_0.5H_VS_4H_PAM3CSK4_BMDC_UP 153
0.3208677 1.3383684 0.022522522 0.064599425
1 2801 "tags=35%, list=20%, signal=43%"

GSE17721_12H_VS_24H_GARDIQUIMOD_BMDC_DN
GSE17721_12H_VS_24H_GARDIQUIMOD_BMDC_DN 146
0.32517618 1.33834 0.018329939 0.06457574 1
1395 "tags=24%, list=10%, signal=26%"
GSE10239_KLRG1INT_VS_KLRG1HIGH_EFF_CD8_TCELL_UP
GSE10239_KLRG1INT_VS_KLRG1HIGH_EFF_CD8_TCELL_UP 140
0.32633278 1.3382632 0.04192872 0.06457811
1 2620 "tags=32%, list=19%, signal=39%"
GSE25088_WT_VS_STAT6_KO_MACROPHAGE_IL4_STIM_UP
GSE25088_WT_VS_STAT6_KO_MACROPHAGE_IL4_STIM_UP 140
0.33023283 1.3380494 0.033472802 0.0646584
1 1820 "tags=21%, list=13%, signal=24%"
GSE5542_UNTREATED_VS_IFNG_TREATED_EPITHELIAL_CELLS_6H_UP
GSE5542_UNTREATED_VS_IFNG_TREATED_EPITHELIAL_CELLS_6H_UP
128 0.33235756 1.3378824 0.029535865
0.06472117 1 2108 "tags=24%, list=15%, signal=28%"
GSE40274_CTRL_VS_FOXP3_AND_EOS_TRANSDUCED_ACTIVATED_CD4_TCELL_UP
GSE40274_CTRL_VS_FOXP3_AND_EOS_TRANSDUCED_ACTIVATED_CD4_TCELL_UP
135 0.32905814 1.3377693 0.032258064
0.06474312 1 2395 "tags=28%, list=17%, signal=34%"
GSE7568_IL4_TGFB_DEXAMETHASONE_VS_IL4_TGFB_TREATED_MACROPHAGE_DN
GSE7568_IL4_TGFB_DEXAMETHASONE_VS_IL4_TGFB_TREATED_MACROPHAGE_DN
139 0.32299304 1.3376864 0.041152265
0.064750224 1 2555 "tags=35%, list=18%, signal=43%"
GSE15659_CD45RA_NEG_CD4_TCELL_VS_ACTIVATED_TREG_DN
GSE15659_CD45RA_NEG_CD4_TCELL_VS_ACTIVATED_TREG_DN 134
0.32938588 1.3370812 0.038306452 0.06506899
1 1717 "tags=20%, list=12%, signal=23%"
GSE22589_HEALTHY_VS_HIV_AND_SIV_INFECTED_DC_DN
GSE22589_HEALTHY_VS_HIV_AND_SIV_INFECTED_DC_DN 144
0.32692632 1.3368928 0.03305785 0.065152206
1 1901 "tags=25%, list=13%, signal=29%"
GSE12845_IGD_POS_BLOOD_VS_NAIVE_TONSIL_BCELL_DN
GSE12845_IGD_POS_BLOOD_VS_NAIVE_TONSIL_BCELL_DN 132
0.3282069 1.3367176 0.03930131 0.065231614
1 1296 "tags=22%, list=9%, signal=24%"
GSE17580_TREG_VS_TEFF_S_MANSONI_INF_UP
GSE17580_TREG_VS_TEFF_S_MANSONI_INF_UP 168
0.3156776 1.3358655 0.030487806 0.06574245
1 1580 "tags=26%, list=11%, signal=29%"
GSE16451_IMMATURE_VS_MATURE_NEURON_CELL_LINE_WEST_EQUINE_ENC_VIRUS_D
N
GSE16451_IMMATURE_VS_MATURE_NEURON_CELL_LINE_WEST_EQUINE_ENC_VIRUS_D
N 160 0.32306728 1.3357538
0.021782178 0.06576041 1 1596 "tags=23%,
list=11%, signal=26%"
GSE27786_LSK_VS_ERYTHROBLAST_DN GSE27786_LSK_VS_ERYTHROBLAST_DN
133 0.3247654 1.3356694 0.02834008
0.0657737 1 1668 "tags=18%, list=12%, signal=20%"
GSE369_SOCS3_KO_VS_WT_LIVER_UP GSE369_SOCS3_KO_VS_WT_LIVER_UP
146 0.3221498 1.3354968 0.041431263
0.06583534 1 2253 "tags=32%, list=16%, signal=38%"
GSE45365_NK_CELL_VS_CD11B_DC_MCMV_INFECTION_UP
GSE45365_NK_CELL_VS_CD11B_DC_MCMV_INFECTION_UP 146

0.31965244	1.3345338	0.032128513	0.06640196
1	2870	"tags=30%, list=20%, signal=37%"
GSE2706_LPS_VS_R848_AND_LPS_2H_STIM_DC_UP
GSE2706_LPS_VS_R848_AND_LPS_2H_STIM_DC_UP	110
0.33429787	1.3339987	0.042735044	0.06672602
1	1799	"tags=19%, list=13%, signal=22%"
GSE22103_UNSTIM_VS_LPS_STIM_NEUTROPHIL_UP
GSE22103_UNSTIM_VS_LPS_STIM_NEUTROPHIL_UP	151
0.3176817	1.333893	0.035643563	0.066751696	1
2149	"tags=30%, list=15%, signal=36%"
GSE3982_NEUTROPHIL_VS_BASOPHIL_DN GSE3982_NEUTROPHIL_VS_BASOPHIL_DN
138	0.3240411	1.3335754	0.03245436
0.066914715	1	2416	"tags=31%, list=17%, signal=37%"
GSE43955_TGFB_IL6_VS_TGFB_IL6_IL23_TH17_ACT_CD4_TCELL_60H_UP
GSE43955_TGFB_IL6_VS_TGFB_IL6_IL23_TH17_ACT_CD4_TCELL_60H_UP
156	0.32075116	1.3332634	0.03125
0.0670709	1	1964	"tags=24%, list=14%, signal=28%"
GSE46606_IRF4HIGH_VS_WT_CD40L_IL2_IL5_DAY3_STIMULATED_BCELL_DN
GSE46606_IRF4HIGH_VS_WT_CD40L_IL2_IL5_DAY3_STIMULATED_BCELL_DN
144	0.32361206	1.3330529	0.0397351
0.06715485	1	1873	"tags=24%, list=13%, signal=28%"
GSE43955_1H_VS_20H_ACT_CD4_TCELL_UP
GSE43955_1H_VS_20H_ACT_CD4_TCELL_UP	160
0.31758428	1.3330088	0.04208417	0.0671415
1	2394	"tags=32%, list=17%, signal=38%"
GSE20715_WT_VS_TLR4_KO_LUNG_UP	GSE20715_WT_VS_TLR4_KO_LUNG_UP
156	0.31944925	1.3329473	0.042918455
0.06714135	1	2321	"tags=29%, list=16%, signal=34%"
GSE43260_BTLA_POS_VS_NEG_INTRATUMORAL_CD8_TCELL_DN
GSE43260_BTLA_POS_VS_NEG_INTRATUMORAL_CD8_TCELL_DN	135
0.3286797	1.3315938	0.029288704	0.06803087
1	2789	"tags=34%, list=20%, signal=42%"
GSE6674_ANTI_IGM_VS_ANTI_IGM_AND_CPG_STIM_BCELL_UP
GSE6674_ANTI_IGM_VS_ANTI_IGM_AND_CPG_STIM_BCELL_UP	132
0.32839447	1.331122	0.027484143	0.06828075	1
2059	"tags=31%, list=15%, signal=36%"
GSE5503_LIVER_DC_VS_SPLEEN_DC_ACTIVATED_ALLOGENIC_TCELL_DN
GSE5503_LIVER_DC_VS_SPLEEN_DC_ACTIVATED_ALLOGENIC_TCELL_DN
144	0.32013753	1.3307545	0.048523206
0.068474144	1	2408	"tags=30%, list=17%, signal=36%"
GSE29618_MONOCYTE_VS_PDC_UP	GSE29618_MONOCYTE_VS_PDC_UP
147	0.32282168	1.3306482	0.036324788
0.06851205	1	1428	"tags=23%, list=10%, signal=25%"
GSE26890_CXCR1_NEG_VS_POS_EFFECTOR_CD8_TCELL_DN
GSE26890_CXCR1_NEG_VS_POS_EFFECTOR_CD8_TCELL_DN	134
0.3230367	1.3302798	0.04233871	0.06871426
1	595	"tags=15%, list=4%, signal=15%"
GSE43955_1H_VS_10H_ACT_CD4_TCELL_WITH_TGFB_IL6_UP
GSE43955_1H_VS_10H_ACT_CD4_TCELL_WITH_TGFB_IL6_UP	153
0.32000703	1.3295982	0.0385439	0.069115415
1	2676	"tags=33%, list=19%, signal=41%"
GSE15930_STIM_VS_STIM_AND_IFNAB_24H_CD8_T_CELL_UP
GSE15930_STIM_VS_STIM_AND_IFNAB_24H_CD8_T_CELL_UP	165
0.31232354	1.3287348	0.023758098	0.06965859

1 1865 "tags=25%, list=13%, signal=28%"
GSE17721_0.5H_VS_8H_LPS_BMDC_DN GSE17721_0.5H_VS_8H_LPS_BMDC_DN
150 0.32105055 1.3277823 0.054393306
0.07020864 1 1618 "tags=22%, list=11%, signal=25%"
GSE17721_PAM3CSK4_VS_CPG_24H_BMDC_DN
GSE17721_PAM3CSK4_VS_CPG_24H_BMDC_DN 153
0.31596816 1.3272134 0.04106776 0.07053794
1 2612 "tags=33%, list=19%, signal=40%"
GSE25677_MPL_VS_R848_STIM_BCELL_DN
GSE25677_MPL_VS_R848_STIM_BCELL_DN 142
0.3203891 1.3270655 0.036659878 0.07059817
1 2374 "tags=35%, list=17%, signal=42%"
GSE9006_HEALTHY_VS_TYPE_1_DIABETES_PBMC_AT_DX_DN
GSE9006_HEALTHY_VS_TYPE_1_DIABETES_PBMC_AT_DX_DN 140
0.32179484 1.3269922 0.0332681 0.07060811
1 1223 "tags=18%, list=9%, signal=19%"
GSE360_T_GONDII_VS_B_MALAYI_LOW_DOSE_DC_DN
GSE360_T_GONDII_VS_B_MALAYI_LOW_DOSE_DC_DN 152
0.31687838 1.3261728 0.037037037 0.07112269
1 1883 "tags=25%, list=13%, signal=29%"
GSE16385_MONOCYTE_VS_MACROPHAGE_UP
GSE16385_MONOCYTE_VS_MACROPHAGE_UP 142
0.32356608 1.326063 0.021186441 0.07114911 1
1265 "tags=16%, list=9%, signal=18%"
GSE40666_WT_VS_STAT4_KO_CD8_TCELL_WITH_IFNA_STIM_90MIN_DN
GSE40666_WT_VS_STAT4_KO_CD8_TCELL_WITH_IFNA_STIM_90MIN_DN
155 0.3152129 1.3260213 0.036679536
0.071128584 1 984 "tags=18%, list=7%, signal=19%"
GSE43863_NAIVE_VS_MEMORY_TFH_CD4_TCELL_D150_LCMV_DN
GSE43863_NAIVE_VS_MEMORY_TFH_CD4_TCELL_D150_LCMV_DN
147 0.31659108 1.3258444 0.036217302
0.0711999 1 2229 "tags=28%, list=16%, signal=33%"
GSE39820_TGFBETA1_VS_TGFBETA3_IN_IL6_IL23A_TREATED_CD4_TCELL_UP
GSE39820_TGFBETA1_VS_TGFBETA3_IN_IL6_IL23A_TREATED_CD4_TCELL_UP
135 0.3275108 1.3257753 0.028688524
0.071196385 1 2278 "tags=29%, list=16%, signal=34%"
GSE9988_ANTI_TREM1_VS_CTRL_TREATED_MONOCYTES_DN
GSE9988_ANTI_TREM1_VS_CTRL_TREATED_MONOCYTES_DN 138
0.32518667 1.3250923 0.054166667 0.071626954
1 2520 "tags=34%, list=18%, signal=41%"
GSE18791_CTRL_VS_NEWCASTLE_VIRUS_DC_6H_UP
GSE18791_CTRL_VS_NEWCASTLE_VIRUS_DC_6H_UP 123
0.32851017 1.3248644 0.051282052 0.071737155
1 2282 "tags=28%, list=16%, signal=34%"
GSE45739_UNSTIM_VS_ACD3_ACD28_STIM_WT_CD4_TCELL_DN
GSE45739_UNSTIM_VS_ACD3_ACD28_STIM_WT_CD4_TCELL_DN 145
0.31921512 1.3247539 0.042735044 0.071763985
1 2041 "tags=31%, list=14%, signal=36%"
GSE27786_LIN_NEG_VS_CD4_TCELL_UP GSE27786_LIN_NEG_VS_CD4_TCELL_UP
143 0.3205618 1.3239543 0.041753653
0.07225501 1 1972 "tags=22%, list=14%, signal=25%"
GSE17721_POLYIC_VS_GARDIQUIMOD_12H_BMDC_DN
GSE17721_POLYIC_VS_GARDIQUIMOD_12H_BMDC_DN 145
0.32096112 1.3239036 0.037254903 0.07224305

1	2710	"tags=35%, list=19%, signal=43%"
GSE17721_0.5H_VS_8H_POLYIC_BMDC_DN
GSE17721_0.5H_VS_8H_POLYIC_BMDC_DN	160
0.31585094	1.323781 0.034552846	0.07227489	1
2132	"tags=28%, list=15%, signal=32%"
GSE29618_PDC_VS_MDC_DAY7_FLU_VACCINE_UP
GSE29618_PDC_VS_MDC_DAY7_FLU_VACCINE_UP	148
0.31413987	1.3228993	0.042168673	0.07282924
1	2232	"tags=28%, list=16%, signal=33%"
GSE1432_CTRL_VS_IFNG_6H_MICROGLIA_DN
GSE1432_CTRL_VS_IFNG_6H_MICROGLIA_DN	139
0.3246325	1.32243 0.032967035	0.07310999	1
2614	"tags=36%, list=19%, signal=44%"
GSE2770_UNTREATED_VS_TGFB_AND_IL4_TREATED_ACT_CD4_TCELL_48H_UP
GSE2770_UNTREATED_VS_TGFB_AND_IL4_TREATED_ACT_CD4_TCELL_48H_UP
112	0.3357545	1.3223479	0.038383838
0.0731181	1	1651	"tags=18%, list=12%, signal=20%"
GSE17721_PAM3CSK4_VS_CPG_12H_BMDC_DN
GSE17721_PAM3CSK4_VS_CPG_12H_BMDC_DN	164
0.3177448	1.322309 0.044680852	0.073097214	1
2156	"tags=26%, list=15%, signal=30%"
GSE43955_1H_VS_60H_ACT_CD4_TCELL_UP
GSE43955_1H_VS_60H_ACT_CD4_TCELL_UP	161
0.32062256	1.3220758	0.03448276	0.073197216
1	2394	"tags=32%, list=17%, signal=38%"
GSE29618_PRE_VS_DAY7_POST_LAIV_FLU_VACCINE_MONOCYTE_UP
GSE29618_PRE_VS_DAY7_POST_LAIV_FLU_VACCINE_MONOCYTE_UP
135	0.32599837	1.3216289	0.045833334
0.073458135	1	2071	"tags=26%, list=15%, signal=30%"
GSE28783_CTRL_ANTI_MIR_VS_UNTREATED_ATHEROSCLEROSIS_MACROPHAGE_UP
GSE28783_CTRL_ANTI_MIR_VS_UNTREATED_ATHEROSCLEROSIS_MACROPHAGE_UP
139	0.3241074	1.3211768	0.0583501
0.07371965	1	1637	"tags=20%, list=12%, signal=23%"
GSE41176_UNSTIM_VS_ANTI_IGM_STIM_TAK1_KO_BCELL_24H_UP
GSE41176_UNSTIM_VS_ANTI_IGM_STIM_TAK1_KO_BCELL_24H_UP
141	0.3222698	1.3207942	0.025210084
0.07394556	1	1796	"tags=28%, list=13%, signal=31%"
GSE37605_C57BL6_VS_NOD_FOXP3_IRES_GFP_TCONV_DN
GSE37605_C57BL6_VS_NOD_FOXP3_IRES_GFP_TCONV_DN	127
0.32363608	1.3201622	0.039915968	0.07434732
1	1737	"tags=20%, list=12%, signal=23%"
GSE19772_HCMV_INFL_VS_HCMV_INF_MONOCYTES_AND_PI3K_INHIBITION_DN
GSE19772_HCMV_INFL_VS_HCMV_INF_MONOCYTES_AND_PI3K_INHIBITION_DN
153	0.31643015	1.3197954	0.043209877
0.07459453	1	2143	"tags=33%, list=15%, signal=38%"
GSE43955_TH0_VS_TGFB_IL6_TH17_ACT_CD4_TCELL_60H_DN
GSE43955_TH0_VS_TGFB_IL6_TH17_ACT_CD4_TCELL_60H_DN	164
0.3136029	1.319405 0.041322313	0.07482415	1
2027	"tags=22%, list=14%, signal=25%"
GSE42021_TCONV_PLN_VS_CD24LO_TCONV_THYMUS_UP
GSE42021_TCONV_PLN_VS_CD24LO_TCONV_THYMUS_UP	159
0.314141 1.3192858	0.039915968	0.07485551	1
1929	"tags=23%, list=14%, signal=26%"
GSE13484_UNSTIM_VS_12H_YF17D_VACCINE_STIM_PBMC_DN

GSE13484_UNSTIM_VS_12H_YF17D_VACCINE_STIM_PBMC_DN 152
0.320721 1.3184274 0.039130434 0.07545567 1
2393 "tags=32%, list=17%, signal=38%"
GSE32986_CURDLAN_LOWDOSE_VS_CURDLAN_HIGHDOSE_STIM_DC_UP
GSE32986_CURDLAN_LOWDOSE_VS_CURDLAN_HIGHDOSE_STIM_DC_UP
136 0.31981775 1.3183976 0.034782607
0.07542888 1 1368 "tags=21%, list=10%, signal=23%"
GSE22196_HEALTHY_VS_OBESE_MOUSE_SKIN_GAMMADELTA_TCELL_DN
GSE22196_HEALTHY_VS_OBESE_MOUSE_SKIN_GAMMADELTA_TCELL_DN
160 0.31530002 1.3182495 0.043824703
0.07547832 1 2027 "tags=28%, list=14%, signal=32%"
GSE360_T_GONDII_VS_M_TUBERCULOSIS_MAC_UP
GSE360_T_GONDII_VS_M_TUBERCULOSIS_MAC_UP 149
0.31970763 1.3176582 0.03726708 0.07588036
1 2547 "tags=28%, list=18%, signal=34%"
GSE14308_NAIVE_CD4_TCELL_VS_INDUCED_TREG_DN
GSE14308_NAIVE_CD4_TCELL_VS_INDUCED_TREG_DN 141
0.32114962 1.3176068 0.045643155 0.07586552
1 2717 "tags=37%, list=19%, signal=45%"
GSE3039_ALPHABETA_CD8_TCELL_VS_B2_BCELL_DN
GSE3039_ALPHABETA_CD8_TCELL_VS_B2_BCELL_DN 155 0.315535
1.3173726 0.034858387 0.07597894 1
1951 "tags=23%, list=14%, signal=27%"
GSE17721_CTRL_VS_POLYIC_24H_BMDC_UP
GSE17721_CTRL_VS_POLYIC_24H_BMDC_UP 146
0.31760776 1.3172615 0.040339705 0.07600107
1 1794 "tags=24%, list=13%, signal=27%"
GSE17721_LPS_VS_PAM3CSK4_16H_BMDC_UP
GSE17721_LPS_VS_PAM3CSK4_16H_BMDC_UP 161
0.31736776 1.3172364 0.034 0.075967245 1
2055 "tags=25%, list=15%, signal=29%"
GSE1460_CD4_THYMOCYTE_VS_THYMIC_STROMAL_CELL_UP
GSE1460_CD4_THYMOCYTE_VS_THYMIC_STROMAL_CELL_UP 134
0.32258844 1.3164968 0.04 0.0764487 1
1858 "tags=22%, list=13%, signal=25%"
GSE17721_LPS_VS_GARDIQUIMOD_2H_BMDC_DN
GSE17721_LPS_VS_GARDIQUIMOD_2H_BMDC_DN 146
0.31793508 1.3159491 0.03426124 0.076808296
1 1835 "tags=22%, list=13%, signal=25%"
GSE13887_RESTING_VS_ACT_CD4_TCELL_DN
GSE13887_RESTING_VS_ACT_CD4_TCELL_DN 100
0.33836028 1.31591 0.05364807 0.07679139 1
2595 "tags=33%, list=18%, signal=40%"
GSE21670_UNTREATED_VS_IL6_TREATED_CD4_TCELL_UP
GSE21670_UNTREATED_VS_IL6_TREATED_CD4_TCELL_UP 132
0.32273528 1.315792 0.032258064 0.07682419 1
2718 "tags=33%, list=19%, signal=41%"
GSE41176_UNSTIM_VS_ANTI_IGM_STIM_BCELL_6H_UP
GSE41176_UNSTIM_VS_ANTI_IGM_STIM_BCELL_6H_UP 144
0.31702933 1.3155463 0.056092843 0.07693763
1 2204 "tags=28%, list=16%, signal=33%"
GSE17721_LPS_VS_POLYIC_24H_BMDC_UP
GSE17721_LPS_VS_POLYIC_24H_BMDC_UP 147
0.31736284 1.315088 0.042510122 0.07725332 1

2707 "tags=35%, list=19%, signal=42%"
GSE26495_PD1HIGH_VS_PD1LOW_CD8_TCELL_UP
GSE26495_PD1HIGH_VS_PD1LOW_CD8_TCELL_UP 105
0.33313945 1.3150246 0.060085837 0.07724528
1 2376 "tags=29%, list=17%, signal=34%"
GSE2585_THYMIC_MACROPHAGE_VS_MTEC_DN
GSE2585_THYMIC_MACROPHAGE_VS_MTEC_DN 131
0.32356238 1.3149663 0.04821803 0.077233404
1 1711 "tags=22%, list=12%, signal=25%"
GSE369_SOCS3_KO_VS_WT_LIVER_DN GSE369_SOCS3_KO_VS_WT_LIVER_DN
132 0.3234081 1.3148158 0.038135592
0.07729361 1 1611 "tags=20%, list=11%, signal=22%"
GSE7852_LN_VS_THYMUS_TREG_DN GSE7852_LN_VS_THYMUS_TREG_DN
153 0.3177659 1.3141017 0.024439918
0.07776506 1 2150 "tags=30%, list=15%, signal=35%"
GSE7460_CD8_TCELL_VS_TREG_ACT_UP GSE7460_CD8_TCELL_VS_TREG_ACT_UP
151 0.3169884 1.3135772 0.02892562
0.07810457 1 1355 "tags=22%, list=10%, signal=24%"
GSE17721_CTRL_VS_LPS_12H_BMDC_DN GSE17721_CTRL_VS_LPS_12H_BMDC_DN
162 0.3138808 1.3132867 0.04183267
0.078273185 1 2575 "tags=27%, list=18%, signal=33%"
GSE17721_POLYIC_VS_GARDIQUIMOD_1H_BMDC_UP
GSE17721_POLYIC_VS_GARDIQUIMOD_1H_BMDC_UP 147
0.31544197 1.313043 0.051224943 0.078411065 1
1997 "tags=22%, list=14%, signal=25%"
GSE5589_LPS_VS_LPS_AND_IL10_STIM_MACROPHAGE_45MIN_UP
GSE5589_LPS_VS_LPS_AND_IL10_STIM_MACROPHAGE_45MIN_UP
145 0.31486025 1.313042 0.038793102 0.07836246
1 1586 "tags=23%, list=11%, signal=26%"
GSE15330_HSC_VS_LYMPHOID_PRIMED_MULTIPOTENT_PROGENITOR_IKAROS_KO_UP
GSE15330_HSC_VS_LYMPHOID_PRIMED_MULTIPOTENT_PROGENITOR_IKAROS_KO_UP
148 0.31790006 1.3125247 0.044397462
0.07868503 1 2065 "tags=27%, list=15%, signal=31%"
GSE17721_12H_VS_24H_GARDIQUIMOD_BMDC_UP
GSE17721_12H_VS_24H_GARDIQUIMOD_BMDC_UP 158
0.31349364 1.3125193 0.04385965 0.07864159
1 2311 "tags=28%, list=16%, signal=34%"
GSE2770_UNTREATED_VS_TGFB_AND_IL4_TREATED_ACT_CD4_TCELL_2H_UP
GSE2770_UNTREATED_VS_TGFB_AND_IL4_TREATED_ACT_CD4_TCELL_2H_UP
143 0.31786084 1.311686 0.034979425
0.079208955 1 3034 "tags=38%, list=21%, signal=48%"
GSE33425_CD161_HIGH_VS_NEG_CD8_TCELL_DN
GSE33425_CD161_HIGH_VS_NEG_CD8_TCELL_DN 156
0.3128206 1.3116456 0.03837953 0.07918918
1 1595 "tags=22%, list=11%, signal=24%"
GSE339_EX_VIVO_VS_IN_CULTURE_CD8POS_DC_DN
GSE339_EX_VIVO_VS_IN_CULTURE_CD8POS_DC_DN 166 0.312652
1.3116417 0.029045643 0.07914479 1
2224 "tags=31%, list=16%, signal=36%"
GSE43863_NAIVE_VS_TH1_EFF_CD4_TCELL_D6_LCMV_UP
GSE43863_NAIVE_VS_TH1_EFF_CD4_TCELL_D6_LCMV_UP 125
0.32283527 1.3115429 0.04845815 0.07916998
1 2339 "tags=25%, list=17%, signal=29%"
GSE23695_CD57_POS_VS_NEG_NK_CELL_DN

GSE23695_CD57_POS_VS_NEG_NK_CELL_DN		148
0.31562257	1.3100882	0.049792532	0.0802093
1	2305	"tags=30%, list=16%, signal=36%"
GSE6259_DEC205_POS_DC_VS_CD4_TCELL_UP
GSE6259_DEC205_POS_DC_VS_CD4_TCELL_UP		142
0.31657532	1.3095775	0.049484536	0.08057151
1	2243	"tags=28%, list=16%, signal=33%"
GSE360_T_GONDII_VS_B_MALAYI_HIGH_DOSE_MAC_UP
GSE360_T_GONDII_VS_B_MALAYI_HIGH_DOSE_MAC_UP			146
0.31379354	1.3093644	0.05498982	0.08068812
1	1419	"tags=22%, list=10%, signal=24%"
GSE25123_CTRL_VS_ROSIGLITAZONE_STIM_MACROPHAGE_UP
GSE25123_CTRL_VS_ROSIGLITAZONE_STIM_MACROPHAGE_UP		148
0.31844893	1.3089005	0.045081966	0.08097464
1	1683	"tags=26%, list=12%, signal=29%"
GSE21927_SPLENIC_C26GM_TUMOROUS_VS_4T1_TUMOR_MONOCYTES_UP
GSE21927_SPLENIC_C26GM_TUMOROUS_VS_4T1_TUMOR_MONOCYTES_UP
109	0.32754812	1.3086289	0.048780486
0.081124865	1	2279	"tags=25%, list=16%, signal=29%"
GSE17721_POLYIC_VS_GARDIQUIMOD_0.5H_BMDC_DN
GSE17721_POLYIC_VS_GARDIQUIMOD_0.5H_BMDC_DN			142
0.3235781	1.3084811	0.04831933	0.08118652
1	2365	"tags=30%, list=17%, signal=35%"
GSE24972_WT_VS_IRF8_KO_SPLEEN_FOLLICULAR_BCELL_UP
GSE24972_WT_VS_IRF8_KO_SPLEEN_FOLLICULAR_BCELL_UP		137
0.32368383	1.3080581	0.05543237	0.081456564
1	2003	"tags=20%, list=14%, signal=23%"
GSE13306_LAMINA_PROPRIA_VS_SPLEEN_TREG_DN
GSE13306_LAMINA_PROPRIA_VS_SPLEEN_TREG_DN		133
0.3189793	1.3078959	0.059322033	0.081558675
1	1680	"tags=22%, list=12%, signal=25%"
GSE24972_MARGINAL_ZONE_BCELL_VS_FOLLICULAR_BCELL_IRF8_KO_UP
GSE24972_MARGINAL_ZONE_BCELL_VS_FOLLICULAR_BCELL_IRF8_KO_UP
140	0.31662956	1.3077406	0.056133058
0.081621416	1	2177	"tags=21%, list=15%, signal=25%"
GSE3982_EOSINOPHIL_VS_NEUTROPHIL_UP
GSE3982_EOSINOPHIL_VS_NEUTROPHIL_UP		131
0.3236977	1.3074589	0.055084746	0.08176671
1	1835	"tags=27%, list=13%, signal=30%"
GSE25088_WT_VS_STAT6_KO_MACROPHAGE_ROSIGLITAZONE_STIM_UP
GSE25088_WT_VS_STAT6_KO_MACROPHAGE_ROSIGLITAZONE_STIM_UP
128	0.3218735	1.3072692	0.045081966
0.08186877	1	2011	"tags=25%, list=14%, signal=29%"
GSE16385_IFNG_TNF_VS_IL4_STIM_MACROPHAGE_UP
GSE16385_IFNG_TNF_VS_IL4_STIM_MACROPHAGE_UP			150
0.310826	1.3071352	0.033826638	0.08192457	1
2124	"tags=28%, list=15%, signal=33%"
GSE46468_LUNG_INNATE_LYMPHOID_CELL_VS_SPLEEN_CD4_TCELL_UP
GSE46468_LUNG_INNATE_LYMPHOID_CELL_VS_SPLEEN_CD4_TCELL_UP
116	0.33121368	1.3055011	0.067510545
0.08312365	1	1840	"tags=27%, list=13%, signal=30%"
GSE22601_DOUBLE_NEGATIVE_VS_IMMATURE_CD4_SP_THYMOCYTE_UP
GSE22601_DOUBLE_NEGATIVE_VS_IMMATURE_CD4_SP_THYMOCYTE_UP
126	0.32107142	1.3048822	0.04821803

0.083591305	1	2221	"tags=32%, list=16%, signal=37%"
GSE15930_STIM_VS_STIM_AND_IL12_48H_CD8_T_CELL_UP
GSE15930_STIM_VS_STIM_AND_IL12_48H_CD8_T_CELL_UP	152
0.31449682	1.3045473	0.052734375	0.083789445
1	2301	"tags=30%, list=16%, signal=36%"
GSE26928_NAIVE_VS_CENT_MEMORY_CD4_TCELL_DN
GSE26928_NAIVE_VS_CENT_MEMORY_CD4_TCELL_DN	140
0.31747323	1.3035113	0.06431536	0.08453651
1	1604	"tags=25%, list=11%, signal=28%"
GSE17721_LPS_VS_GARDIQUIMOD_12H_BMDC_DN
GSE17721_LPS_VS_GARDIQUIMOD_12H_BMDC_DN	155
0.31195506	1.3034778	0.04831933	0.084504634
1	1977	"tags=23%, list=14%, signal=27%"
GSE360_HIGH_DOSE_B_MALAYI_VS_M_TUBERCULOSIS_DC_UP
GSE360_HIGH_DOSE_B_MALAYI_VS_M_TUBERCULOSIS_DC_UP	156
0.30970418	1.3034589	0.03409091	0.08446772
1	2325	"tags=31%, list=16%, signal=37%"
GSE43955_1H_VS_60H_ACT_CD4_TCELL_DN
GSE43955_1H_VS_60H_ACT_CD4_TCELL_DN	155
0.31328613	1.3033848	0.048523206	0.084487714
1	1283	"tags=16%, list=9%, signal=18%"
GSE17721_CTRL_VS_LPS_6H_BMDC_UP	GSE17721_CTRL_VS_LPS_6H_BMDC_UP
	147	0.31622607	1.3033352	0.047034767
0.08447949	1	1889	"tags=28%, list=13%, signal=32%"
GSE1432_1H_VS_6H_IFNG_MICROGLIA_DN
GSE1432_1H_VS_6H_IFNG_MICROGLIA_DN	145
0.31505615	1.3030558	0.05882353	0.08466807
1	2444	"tags=32%, list=17%, signal=39%"
GSE1448_CTRL_VS_ANTI_VBETA5_DP_THYMOCYTE_DN
GSE1448_CTRL_VS_ANTI_VBETA5_DP_THYMOCYTE_DN	161
0.3117354	1.3022718	0.040948275	0.08523737
1	1841	"tags=26%, list=13%, signal=30%"
GSE24026_PD1_LIGATION_VS_CTRL_IN_ACT_TCELL_LINE_UP
GSE24026_PD1_LIGATION_VS_CTRL_IN_ACT_TCELL_LINE_UP	144
0.31532395	1.3022494	0.038709678	0.08520227
1	2623	"tags=36%, list=19%, signal=44%"
GSE27786_LSK_VS_BCELL_DN	GSE27786_LSK_VS_BCELL_DN	144
0.31129968	1.300397	0.05944798	0.08656825	1
2671	"tags=35%, list=19%, signal=43%"
GSE17721_12H_VS_24H_POLYIC_BMDC_DN
GSE17721_12H_VS_24H_POLYIC_BMDC_DN	145
0.31636027	1.2993301	0.058577407	0.08738757
1	1689	"tags=21%, list=12%, signal=24%"
GSE46606_IRF4HIGH_VS_WT_CD40L_IL2_IL5_DAY3_STIMULATED_BCELL_UP
GSE46606_IRF4HIGH_VS_WT_CD40L_IL2_IL5_DAY3_STIMULATED_BCELL_UP
	142	0.3133011	1.2989092	0.06734694
0.087717004	1	2828	"tags=37%, list=20%, signal=46%"
GSE43955_TH0_VS_TGFB_IL6_IL23_TH17_ACT_CD4_TCELL_52H_UP
GSE43955_TH0_VS_TGFB_IL6_IL23_TH17_ACT_CD4_TCELL_52H_UP
158	0.31184307	1.2975789	0.045454547
0.088725016	1	2072	"tags=27%, list=15%, signal=31%"
GSE20715_WT_VS_TLR4_KO_48H_OZONE_LUNG_DN
GSE20715_WT_VS_TLR4_KO_48H_OZONE_LUNG_DN	159
0.31103495	1.2972482	0.04892368	0.08893417

1 2452 "tags=31%, list=17%, signal=38%"
GSE6092_B_BURGDOFERI_VS_B_BURGDORFERI_AND_IFNG_STIM_ENDOTHELIAL_CELL_DN
GSE6092_B_BURGDOFERI_VS_B_BURGDORFERI_AND_IFNG_STIM_ENDOTHELIAL_CELL_DN 149 0.31057057 1.2971178
0.0491453 0.088988386 1 2228 "tags=24%, list=16%, signal=28%"
GSE3337_4H_VS_16H_IFNG_IN_CD8POS_DC_DN
GSE3337_4H_VS_16H_IFNG_IN_CD8POS_DC_DN 152
0.31331614 1.2967666 0.05732484 0.08923763
1 1948 "tags=28%, list=14%, signal=32%"
GSE26343_WT_VS_NFAT5_KO_MACROPHAGE_LPS_STIM_DN
GSE26343_WT_VS_NFAT5_KO_MACROPHAGE_LPS_STIM_DN 140
0.30840355 1.2966542 0.04411765 0.08926405
1 1044 "tags=16%, list=7%, signal=18%"
GSE7509_DC_VS_MONOCYTE_DN GSE7509_DC_VS_MONOCYTE_DN 143
0.31607586 1.2965219 0.036956523 0.089328624
1 1839 "tags=25%, list=13%, signal=29%"
GSE11057_CD4_EFF_MEM_VS_PBMC_DN GSE11057_CD4_EFF_MEM_VS_PBMC_DN
136 0.315034 1.2964967 0.054507338
0.08929488 1 1847 "tags=20%, list=13%, signal=23%"
GSE2770_IL12_VS_IL4_TREATED_ACT_CD4_TCELL_6H_DN
GSE2770_IL12_VS_IL4_TREATED_ACT_CD4_TCELL_6H_DN 149
0.31612778 1.2958899 0.05567452 0.08972693
1 2720 "tags=38%, list=19%, signal=46%"
GSE3691_CONVENTIONAL_VS_PLASMACYTOID_DC_SPLEEN_DN
GSE3691_CONVENTIONAL_VS_PLASMACYTOID_DC_SPLEEN_DN 149
0.31236413 1.295818 0.052516412 0.0897266 1
2124 "tags=19%, list=15%, signal=23%"
GSE32986_GMCSF_AND_CURDLAN_LOWDOSE_VS_GMCSF_AND_CURDLAN_HIGHDOSE_STIM_DC_UP
GSE32986_GMCSF_AND_CURDLAN_LOWDOSE_VS_GMCSF_AND_CURDLAN_HIGHDOSE_STIM_DC_UP 145 0.31565467 1.2953982
0.058333334 0.09001183 1 2371 "tags=23%, list=17%, signal=27%"
GSE26343_WT_VS_NFAT5_KO_MACROPHAGE_UP
GSE26343_WT_VS_NFAT5_KO_MACROPHAGE_UP 152
0.31286305 1.2947401 0.055679288 0.09054236
1 1584 "tags=24%, list=11%, signal=27%"
GSE360_DC_VS_MAC_B_MALAYI_HIGH_DOSE_UP
GSE360_DC_VS_MAC_B_MALAYI_HIGH_DOSE_UP 156
0.31275123 1.2933846 0.05 0.09163373 1
2428 "tags=32%, list=17%, signal=38%"
GSE30971_CTRL_VS_LPS_STIM_MACROPHAGE_WBP7_HET_2H_DN
GSE30971_CTRL_VS_LPS_STIM_MACROPHAGE_WBP7_HET_2H_DN
132 0.31671444 1.2923868 0.052953158
0.09245727 1 1399 "tags=19%, list=10%, signal=21%"
GSE34006_A2AR_KO_VS_A2AR_AGONIST_TREATED_TREG_DN
GSE34006_A2AR_KO_VS_A2AR_AGONIST_TREATED_TREG_DN 140
0.3100655 1.2919112 0.06680585 0.09279899
1 2222 "tags=26%, list=16%, signal=31%"
GSE18791_CTRL_VS_NEWCASTLE_VIRUS_DC_8H_UP
GSE18791_CTRL_VS_NEWCASTLE_VIRUS_DC_8H_UP 124
0.31991875 1.2916536 0.06212425 0.092959434

1	2695	"tags=33%, list=19%, signal=41%"
GSE28783_ANTI_MIR33_VS_UNTREATED_ATHEROSCLEROSIS_MACROPHAGE_UP
GSE28783_ANTI_MIR33_VS_UNTREATED_ATHEROSCLEROSIS_MACROPHAGE_UP
	147	0.31168237	1.2914311	0.047413792
0.09309706	1	1882	"tags=23%, list=13%, signal=26%"
GSE360_CTRL_VS_T_GONDII_MAC_DN	GSE360_CTRL_VS_T_GONDII_MAC_DN
	152	0.30785725	1.2914027	0.045908183
0.093063295	1	2286	"tags=28%, list=16%, signal=33%"
GSE15659_RESTING_TREG_VS_NONSUPPRESSIVE_TCELL_DN
GSE15659_RESTING_TREG_VS_NONSUPPRESSIVE_TCELL_DN	148
0.30990604	1.2913224	0.05732484	0.09307483
1	1919	"tags=22%, list=14%, signal=25%"
GSE26030_TH1_VS_TH17_DAY15_POST_POLARIZATION_DN
GSE26030_TH1_VS_TH17_DAY15_POST_POLARIZATION_DN	138
0.3145141	1.2913152	0.05106383	0.09302311
1	2449	"tags=33%, list=17%, signal=40%"
GSE17721_ALL_VS_24H_PAM3CSK4_BMDC_DN
GSE17721_ALL_VS_24H_PAM3CSK4_BMDC_DN	151
0.30987653	1.2912883	0.04347826	0.09299534
1	1674	"tags=24%, list=12%, signal=27%"
GSE6269_HEALTHY_VS_STAPH_AUREUS_INF_PBMC_UP
GSE6269_HEALTHY_VS_STAPH_AUREUS_INF_PBMC_UP	112
0.3296434	1.2907643	0.055670105	0.093389936
1	2385	"tags=36%, list=17%, signal=43%"
GSE15659_NONSUPPRESSIVE_TCELL_VS_ACTIVATED_TREG_DN
GSE15659_NONSUPPRESSIVE_TCELL_VS_ACTIVATED_TREG_DN	135
0.31722283	1.290595	0.057312254	0.093471296	1
1527	"tags=18%, list=11%, signal=20%"
GSE34156_UNTREATED_VS_24H_NOD2_LIGAND_TREATED_MONOCYTE_UP
GSE34156_UNTREATED_VS_24H_NOD2_LIGAND_TREATED_MONOCYTE_UP
111	0.32521632	1.2904387	0.067653276
0.0935681	1	1559	"tags=18%, list=11%, signal=20%"
GSE17721_CTRL_VS_CPG_6H_BMDC_UP	GSE17721_CTRL_VS_CPG_6H_BMDC_UP
	154	0.3048646	1.2899737	0.052419353
0.093910344	1	2464	"tags=31%, list=17%, signal=37%"
GSE17721_0.5H_VS_8H_PAM3CSK4_BMDC_DN
GSE17721_0.5H_VS_8H_PAM3CSK4_BMDC_DN	155
0.30787683	1.2898743	0.045454547	0.093945675
1	1594	"tags=18%, list=11%, signal=20%"
GSE17721_CTRL_VS_LPS_6H_BMDC_DN	GSE17721_CTRL_VS_LPS_6H_BMDC_DN
	161	0.30693528	1.289502	0.04761905	0.094197
1	1218	"tags=17%, list=9%, signal=19%"
GSE27786_BCELL_VS_CD4_TCELL_UP	GSE27786_BCELL_VS_CD4_TCELL_UP
	139	0.31890324	1.2890517	0.057259712
0.09452127	1	2140	"tags=28%, list=15%, signal=33%"
GSE18281_CORTICAL_VS_MEDULLARY_THYMOCYTE_UP
GSE18281_CORTICAL_VS_MEDULLARY_THYMOCYTE_UP	152
0.3120152	1.2885498	0.06652807	0.09490139
1	2511	"tags=37%, list=18%, signal=44%"
GSE1432_6H_VS_24H_IFNG_MICROGLIA_DN
GSE1432_6H_VS_24H_IFNG_MICROGLIA_DN	148
0.31493294	1.2884029	0.0524109	0.09495494
1	1910	"tags=26%, list=14%, signal=30%"
GSE17721_12H_VS_24H_POLYIC_BMDC_UP

GSE17721_12H_VS_24H_POLYIC_BMDC_UP 159
0.3060229 1.2883681 0.05394191 0.09492551
1 2142 "tags=28%, list=15%, signal=33%"
GSE17721_CTRL_VS_GARDIQUIMOD_24H_BMDC_UP
GSE17721_CTRL_VS_GARDIQUIMOD_24H_BMDC_UP 145
0.30965865 1.288236 0.058951966 0.09499554 1
2795 "tags=33%, list=20%, signal=41%"
GSE22886_NAIVE_CD4_TCELL_VS_MONOCYTE_DN
GSE22886_NAIVE_CD4_TCELL_VS_MONOCYTE_DN 155
0.3062105 1.2874305 0.04809619 0.09567556
1 2083 "tags=30%, list=15%, signal=35%"
GSE26343_UNSTIM_VS_LPS_STIM_MACROPHAGE_UP
GSE26343_UNSTIM_VS_LPS_STIM_MACROPHAGE_UP 152
0.31079462 1.2873605 0.050314464 0.09567004
1 2215 "tags=30%, list=16%, signal=35%"
GSE360_DC_VS_MAC_L_DONOVANI_DN GSE360_DC_VS_MAC_L_DONOVANI_DN
164 0.3054931 1.2869413 0.046709128
0.095957525 1 1840 "tags=22%, list=13%, signal=25%"
GSE26488_WT_VS_HDAC7_KO_DOUBLE_POSITIVE_THYMOCYTE_UP
GSE26488_WT_VS_HDAC7_KO_DOUBLE_POSITIVE_THYMOCYTE_UP
116 0.32122326 1.2864608 0.06437768
0.096365675 1 1396 "tags=20%, list=10%, signal=22%"
GSE14308_TH2_VS_TH1_UP GSE14308_TH2_VS_TH1_UP 143
0.3104531 1.2858962 0.050301813 0.096783325
1 2524 "tags=30%, list=18%, signal=36%"
GSE43863_DAY6_EFF_VS_DAY150_MEM_LY6C_INT_CXCR5POS_CD4_TCELL_DN
GSE43863_DAY6_EFF_VS_DAY150_MEM_LY6C_INT_CXCR5POS_CD4_TCELL_DN
146 0.31349042 1.2853615 0.055785123
0.09719815 1 1860 "tags=27%, list=13%, signal=31%"
GSE23321_CD8_STEM_CELL_MEMORY_VS_NAIVE_CD8_TCELL_UP
GSE23321_CD8_STEM_CELL_MEMORY_VS_NAIVE_CD8_TCELL_UP
129 0.31787503 1.2848557 0.058951966
0.09759305 1 1861 "tags=27%, list=13%, signal=31%"
GSE22611_NOD2_VS_MUTANT_NOD2_TRANSDUCED_HEK293T_CELL_UP
GSE22611_NOD2_VS_MUTANT_NOD2_TRANSDUCED_HEK293T_CELL_UP
138 0.31266966 1.2848433 0.055555556
0.09754558 1 2226 "tags=28%, list=16%, signal=33%"
GSE9988_LPS_VS_LOW_LPS_MONOCYTE_UP
GSE9988_LPS_VS_LOW_LPS_MONOCYTE_UP 89
0.33304673 1.2845179 0.08315098 0.097748175
1 1670 "tags=16%, list=12%, signal=18%"
GSE17721_LPS_VS_POLYIC_16H_BMDC_DN
GSE17721_LPS_VS_POLYIC_16H_BMDC_DN 157
0.30735993 1.2844105 0.055009823 0.097776756
1 1424 "tags=18%, list=10%, signal=20%"
GSE21670_TGFB_VS_TGFB_AND_IL6_TREATED_CD4_TCELL_DN
GSE21670_TGFB_VS_TGFB_AND_IL6_TREATED_CD4_TCELL_DN 141
0.30699223 1.2841984 0.04140787 0.09786819
1 2065 "tags=31%, list=15%, signal=36%"
GSE6259_FLT3L_INDUCED_DEC205_POS_DC_VS_CD4_TCELL_UP
GSE6259_FLT3L_INDUCED_DEC205_POS_DC_VS_CD4_TCELL_UP
128 0.31947982 1.2841705 0.054968286
0.097828485 1 2594 "tags=36%, list=18%, signal=44%"
GSE42088_UNINF_VS_LEISHMANIA_INF_DC_4H_DN

GSE42088_UNINF_VS_LEISHMANIA_INF_DC_4H_DN	118
0.3150066	1.2841388	0.07172996	0.09780032
1	1725	"tags=25%, list=12%, signal=29%"
GSE42021_CD24HI_VS_CD24INT_TREG_THYMUS_DN
GSE42021_CD24HI_VS_CD24INT_TREG_THYMUS_DN	135
0.3139351	1.2839139	0.043659043	0.09793933
1	2358	"tags=34%, list=17%, signal=41%"
GSE36392_TYPE_2_MYELOID_VS_MAC_IL25_TREATED_LUNG_DN
GSE36392_TYPE_2_MYELOID_VS_MAC_IL25_TREATED_LUNG_DN
136	0.31037706	1.2834569	0.0498008
0.09832921	1	2486	"tags=29%, list=18%, signal=34%"
GSE22886_NAIVE_BCELL_VS_BM_PLASMA_CELL_UP
GSE22886_NAIVE_BCELL_VS_BM_PLASMA_CELL_UP	139
0.31335035	1.2829624	0.06772009	0.09870289
1	2194	"tags=30%, list=16%, signal=35%"
GSE40666_NAIVE_VS_EFFECTOR_CD8_TCELL_WITH_IFNA_STIM_90MIN_UP
GSE40666_NAIVE_VS_EFFECTOR_CD8_TCELL_WITH_IFNA_STIM_90MIN_UP
	149	0.30876228	1.2828958	0.057971016
0.098698504	1	2349	"tags=33%, list=17%, signal=39%"
GSE40274_CTRL_VS_IRF4_TRANSDUCED_ACTIVATED_CD4_TCELL_DN
GSE40274_CTRL_VS_IRF4_TRANSDUCED_ACTIVATED_CD4_TCELL_DN
137	0.31247514	1.2828587	0.056640625
0.09866205	1	1678	"tags=23%, list=12%, signal=25%"
GSE44649_NAIVE_VS_ACTIVATED_CD8_TCELL_MIR155_KO_DN
GSE44649_NAIVE_VS_ACTIVATED_CD8_TCELL_MIR155_KO_DN	135
0.31249774	1.2822431	0.052854124	0.09915458
1	2215	"tags=30%, list=16%, signal=36%"
GSE13306_TREG_VS_TCONV_SPLEEN_DN	GSE13306_TREG_VS_TCONV_SPLEEN_DN
	148	0.3118044	1.2821782	0.05144033
0.099158496	1	1680	"tags=24%, list=12%, signal=27%"
GSE22886_NAIVE_CD8_TCELL_VS_MEMORY_TCELL_UP
GSE22886_NAIVE_CD8_TCELL_VS_MEMORY_TCELL_UP	139
0.31053042	1.282039	0.046653144	0.099239185	1
2588	"tags=31%, list=18%, signal=38%"
GSE22611_UNSTIM_VS_6H_MDP_STIM_MUTANT_NOD2_TRANSDUCED_HEK293T_CELL_U
P
GSE22611_UNSTIM_VS_6H_MDP_STIM_MUTANT_NOD2_TRANSDUCED_HEK293T_CELL_U
P	139	0.3118703	1.2815424
0.08024691	0.09963718	1	2204	"tags=27%,
list=16%, signal=31%"
GSE360_L_DONOVANI_VS_M_TUBERCULOSIS_DC_UP
GSE360_L_DONOVANI_VS_M_TUBERCULOSIS_DC_UP	154
0.3072704	1.2810888	0.06903765	0.09998977
1	2007	"tags=25%, list=14%, signal=28%"
GSE5503_PLN_DC_VS_SPLEEN_DC_ACTIVATED_ALLOGENIC_TCELL_DN
GSE5503_PLN_DC_VS_SPLEEN_DC_ACTIVATED_ALLOGENIC_TCELL_DN
145	0.3124793	1.2792999	0.060796645
0.10152703	1	2223	"tags=31%, list=16%, signal=36%"
GSE11864_UNTREATED_VS_CSF1_IN_MAC_DN
GSE11864_UNTREATED_VS_CSF1_IN_MAC_DN	116
0.31989363	1.2791822	0.09131403	0.10156936
1	2093	"tags=29%, list=15%, signal=34%"
GSE37416_CTRL_VS_6H_F_TULARENSIS_LVS_NEUTROPHIL_DN
GSE37416_CTRL_VS_6H_F_TULARENSIS_LVS_NEUTROPHIL_DN	127

0.3173269	1.2789466	0.074157305	0.10173296
1	2836	"tags=34%, list=20%, signal=42%"
GSE12505_WT_VS_E2_2_HET_PDC_DN	GSE12505_WT_VS_E2_2_HET_PDC_DN
	115	0.3244711	1.2788435	0.062240664
0.10175317	1	1536	"tags=20%, list=11%, signal=22%"
GSE12484_HEALTHY_VS_PERIDONTITIS_NEUTROPHILS_DN
GSE12484_HEALTHY_VS_PERIDONTITIS_NEUTROPHILS_DN		117
0.31931335	1.2782909	0.060165975	0.10220934
1	2333	"tags=29%, list=17%, signal=35%"
GSE27786_BCELL_VS_NEUTROPHIL_DN	GSE27786_BCELL_VS_NEUTROPHIL_DN
	148	0.31201857	1.2782125	0.06962025
0.10221567	1	2360	"tags=21%, list=17%, signal=25%"
GSE4748_CTRL_VS_LPS_AND_CYANOBACTERIUM_LPSLIKE_STIM_DC_3H_DN
GSE4748_CTRL_VS_LPS_AND_CYANOBACTERIUM_LPSLIKE_STIM_DC_3H_DN
	167	0.3003566	1.2782089	0.046025105
0.10216045	1	2292	"tags=24%, list=16%, signal=28%"
GSE360_HIGH_VS_LOW_DOSE_B_MALAYI_MAC_DN
GSE360_HIGH_VS_LOW_DOSE_B_MALAYI_MAC_DN		154
0.31028458	1.2779235	0.042035397	0.10235351
1	2081	"tags=27%, list=15%, signal=31%"
GSE22432_CDC_VS_COMMON_DC_PROGENITOR_UP
GSE22432_CDC_VS_COMMON_DC_PROGENITOR_UP		147
0.3060167	1.2772461	0.040358745	0.10291855
1	2172	"tags=28%, list=15%, signal=33%"
GSE15330_LYMPHOID_MULTIPOTENT_VS_GRANULOCYTE_MONOCYTE_PROGENITOR_IKAROS_KO_DN
GSE15330_LYMPHOID_MULTIPOTENT_VS_GRANULOCYTE_MONOCYTE_PROGENITOR_IKAROS_KO_DN		160	0.30914572	1.2771609
0.051229507	0.102925904	1	1762	"tags=27%, list=12%, signal=30%"
GSE3039_NKT_CELL_VS_B2_BCELL_UP	GSE3039_NKT_CELL_VS_B2_BCELL_UP
	146	0.30764076	1.2771553	0.068085104
0.1028675	1	1860	"tags=24%, list=13%, signal=27%"
GSE22432_UNTREATED_VS_TGFB1_TREATED_COMMON_DC_PROGENITOR_DN
GSE22432_UNTREATED_VS_TGFB1_TREATED_COMMON_DC_PROGENITOR_DN
140	0.3101202	1.2767028	0.05986696
0.103213355	1	1795	"tags=24%, list=13%, signal=27%"
GSE10325_LUPUS_BCELL_VS_LUPUS_MYELOID_UP
GSE10325_LUPUS_BCELL_VS_LUPUS_MYELOID_UP		130
0.31401864	1.2764221	0.057569295	0.103435226
1	2094	"tags=28%, list=15%, signal=32%"
GSE35685_CD34POS_CD38NEG_VS_CD34POS_CD10NEG_CD62LPOS_BONE_MARROW_DN
GSE35685_CD34POS_CD38NEG_VS_CD34POS_CD10NEG_CD62LPOS_BONE_MARROW_DN
	164	0.3029894	1.276321	0.05785124
0.10346833	1	2473	"tags=28%, list=18%, signal=34%"
GSE12845_PRE_GC_VS_DARKZONE_GC_TONSIL_BCELL_DN
GSE12845_PRE_GC_VS_DARKZONE_GC_TONSIL_BCELL_DN		139
0.30990356	1.2759354	0.048387095	0.10376527
1	2412	"tags=35%, list=17%, signal=42%"
GSE20366_EX_VIVO_VS_DEC205_CONVERSION_UP
GSE20366_EX_VIVO_VS_DEC205_CONVERSION_UP		138
0.31005624	1.2756896	0.058212057	0.103941746
1	1780	"tags=25%, list=13%, signal=28%"
GSE1432_6H_VS_24H_IFNG_MICROGLIA_UP

GSE1432_6H_VS_24H_IFNG_MICROGLIA_UP 155
0.30563143 1.2750304 0.0554371 0.10451878
1 2445 "tags=32%, list=17%, signal=38%"
GSE360_DC_VS_MAC_B_MALAYI_HIGH_DOSE_DN
GSE360_DC_VS_MAC_B_MALAYI_HIGH_DOSE_DN 143
0.30976674 1.2747774 0.064 0.104691274 1
1793 "tags=24%, list=13%, signal=28%"
GSE33292_DN3_THYMOCYTE_VS_TCELL_LYMPHOMA_FROM_TCF1_KO_UP
GSE33292_DN3_THYMOCYTE_VS_TCELL_LYMPHOMA_FROM_TCF1_KO_UP
135 0.31267434 1.2743694 0.06147541
0.10499059 1 2062 "tags=30%, list=15%, signal=34%"
GSE360_L_MAJOR_VS_M_TUBERCULOSIS_MAC_DN
GSE360_L_MAJOR_VS_M_TUBERCULOSIS_MAC_DN 136
0.3084623 1.2735618 0.0524109 0.10568838
1 1850 "tags=25%, list=13%, signal=28%"
GSE16522_MEMORY_VS_NAIVE_CD8_TCELL_UP
GSE16522_MEMORY_VS_NAIVE_CD8_TCELL_UP 152
0.3093631 1.2734454 0.04684318 0.10573403
1 1951 "tags=32%, list=14%, signal=36%"
GSE17721_PAM3CSK4_VS_CPG_16H_BMDC_DN
GSE17721_PAM3CSK4_VS_CPG_16H_BMDC_DN 143
0.30803388 1.2734245 0.052313883 0.105687626
1 2011 "tags=25%, list=14%, signal=29%"
GSE3982_MEMORY_CD4_TCELL_VS_BCELL_UP
GSE3982_MEMORY_CD4_TCELL_VS_BCELL_UP 132
0.31190082 1.2733029 0.067073174 0.10573933
1 1673 "tags=22%, list=12%, signal=25%"
GSE17721_LPS_VS_POLYIC_8H_BMDC_DN GSE17721_LPS_VS_POLYIC_8H_BMDC_DN
163 0.30559996 1.2730832 0.06567796
0.10588484 1 1913 "tags=24%, list=14%, signal=27%"
GSE3982_MAST_CELL_VS_DC_UP GSE3982_MAST_CELL_VS_DC_UP
126 0.31453332 1.2722466 0.074152544
0.106586695 1 1879 "tags=26%, list=13%, signal=30%"
GSE2826_WT_VS_XID_BCELL_UP GSE2826_WT_VS_XID_BCELL_UP
154 0.30135828 1.2720548 0.06595745
0.10668832 1 1525 "tags=20%, list=11%, signal=22%"
GSE15330_HSC_VS_GRANULOCYTE_MONOCYTE_PROGENITOR_IKAROS_KO_DN
GSE15330_HSC_VS_GRANULOCYTE_MONOCYTE_PROGENITOR_IKAROS_KO_DN
156 0.30459565 1.2718836 0.061965812
0.106779754 1 1987 "tags=26%, list=14%, signal=30%"
GSE2770_UNTREATED_VS_TGFB_AND_IL12_TREATED_ACT_CD4_TCELL_48H_UP
GSE2770_UNTREATED_VS_TGFB_AND_IL12_TREATED_ACT_CD4_TCELL_48H_UP
143 0.30991292 1.2714207 0.072463766
0.10715537 1 2234 "tags=26%, list=16%, signal=30%"
GSE33425_CD161_HIGH_VS_NEG_CD8_TCELL_UP
GSE33425_CD161_HIGH_VS_NEG_CD8_TCELL_UP 163
0.30419898 1.2708783 0.05942623 0.107617125
1 2317 "tags=31%, list=16%, signal=36%"
GSE34156_UNTREATED_VS_24H_NOD2_LIGAND_TREATED_MONOCYTE_DN
GSE34156_UNTREATED_VS_24H_NOD2_LIGAND_TREATED_MONOCYTE_DN
139 0.308764 1.270331 0.06324111 0.108090125 1
1258 "tags=19%, list=9%, signal=20%"
GSE27786_CD4_TCELL_VS_NEUTROPHIL_DN
GSE27786_CD4_TCELL_VS_NEUTROPHIL_DN 143

0.30974343 1.2700667 0.07581967 0.10828474
1 1736 "tags=17%, list=12%, signal=19%"
GSE23308_CTRL_VS_CORTICOSTERONE_TREATED_MACROPHAGE_MINERALCORTICOID_
REC_KO_DN
GSE23308_CTRL_VS_CORTICOSTERONE_TREATED_MACROPHAGE_MINERALCORTICOID_
REC_KO_DN 140 0.3071835 1.2699479
0.06625259 0.10833896 1 2485 "tags=32%,
list=18%, signal=39%"
GSE41867_NAIVE_VS_DAY30_LCMV_CLONE13_EXHAUSTED_CD8_TCELL_DN
GSE41867_NAIVE_VS_DAY30_LCMV_CLONE13_EXHAUSTED_CD8_TCELL_DN
147 0.30834192 1.269232 0.06236559 0.10897534
1 2033 "tags=26%, list=14%, signal=30%"
GSE20198_IL12_VS_IFNA_TREATED_ACT_CD4_TCELL_DN
GSE20198_IL12_VS_IFNA_TREATED_ACT_CD4_TCELL_DN 127
0.31277403 1.2691878 0.06860707 0.10895387
1 1433 "tags=20%, list=10%, signal=23%"
GSE5142_CTRL_VS_HTERT_TRANSDUCED_CD8_TCELL_EARLY_PASSAGE_CLONE_DN
GSE5142_CTRL_VS_HTERT_TRANSDUCED_CD8_TCELL_EARLY_PASSAGE_CLONE_DN
145 0.30721593 1.2689874 0.07070707
0.10909777 1 2347 "tags=29%, list=17%, signal=34%"
GSE22103_UNSTIM_VS_LPS_STIM_NEUTROPHIL_DN
GSE22103_UNSTIM_VS_LPS_STIM_NEUTROPHIL_DN 154
0.30512923 1.2685648 0.07112971 0.109457225
1 672 "tags=12%, list=5%, signal=12%"
GSE17721_PAM3CSK4_VS_CPG_24H_BMDC_UP
GSE17721_PAM3CSK4_VS_CPG_24H_BMDC_UP 152
0.30941516 1.2671353 0.06904232 0.11078211
1 1824 "tags=23%, list=13%, signal=26%"
GSE46242_TH1_VS_ANERGIC_TH1_CD4_TCELL_UP
GSE46242_TH1_VS_ANERGIC_TH1_CD4_TCELL_UP 131
0.30822605 1.2665051 0.07838983 0.11134148
1 2083 "tags=29%, list=15%, signal=34%"
GSE17721_LPS_VS_GARDIQUIMOD_4H_BMDC_DN
GSE17721_LPS_VS_GARDIQUIMOD_4H_BMDC_DN 136
0.30860782 1.2664291 0.072765075 0.11135275
1 2107 "tags=29%, list=15%, signal=33%"
GSE34156_NOD2_LIGAND_VS_TLR1_TLR2_LIGAND_24H_TREATED_MONOCYTE_UP
GSE34156_NOD2_LIGAND_VS_TLR1_TLR2_LIGAND_24H_TREATED_MONOCYTE_UP
110 0.31904843 1.2656043 0.08742005
0.11210091 1 1886 "tags=22%, list=13%, signal=25%"
GSE43700_UNTREATED_VS_IL10_TREATED_PBMC_DN
GSE43700_UNTREATED_VS_IL10_TREATED_PBMC_DN 160
0.30314296 1.2654026 0.055984557 0.112227306
1 2560 "tags=32%, list=18%, signal=38%"
GSE17721_PAM3CSK4_VS_CPG_1H_BMDC_UP
GSE17721_PAM3CSK4_VS_CPG_1H_BMDC_UP 153
0.3060283 1.2649444 0.064449064 0.11263059
1 1628 "tags=22%, list=12%, signal=25%"
GSE5589_IL6_KO_VS_IL10_KO_LPS_AND_IL10_STIM_MACROPHAGE_180MIN_UP
GSE5589_IL6_KO_VS_IL10_KO_LPS_AND_IL10_STIM_MACROPHAGE_180MIN_UP
141 0.30351478 1.2636676 0.060165975
0.11387212 1 1446 "tags=20%, list=10%, signal=22%"
GSE7852_LN_VS_FAT_TREG_UP GSE7852_LN_VS_FAT_TREG_UP 139
0.30873787 1.263374 0.07974138 0.11410907 1

2639	"tags=29%, list=19%, signal=35%"
GSE5589_UNSTIM_VS_45MIN_LPS_STIM_MACROPHAGE_DN
GSE5589_UNSTIM_VS_45MIN_LPS_STIM_MACROPHAGE_DN	142
0.30721974	1.2631581	0.05672269	0.114252605
1	2371	"tags=27%, list=17%, signal=33%"
GSE2405_0H_VS_9H_A_PHAGOCYTOPHILUM_STIM_NEUTROPHIL_UP
GSE2405_0H_VS_9H_A_PHAGOCYTOPHILUM_STIM_NEUTROPHIL_UP
133	0.3068376	1.2630084	0.076595746
0.11433119	1	2379	"tags=29%, list=17%, signal=35%"
GSE29618_MONOCYTE_VS_MDC_DN	GSE29618_MONOCYTE_VS_MDC_DN
	121	0.31469095	1.2628857	0.0751073
0.11439596	1	2004	"tags=29%, list=14%, signal=33%"
GSE37416_12H_VS_24H_F_TULARENSIS_LVS_NEUTROPHIL_UP
GSE37416_12H_VS_24H_F_TULARENSIS_LVS_NEUTROPHIL_UP	142
0.30509815	1.2627902	0.048565123	0.11441769
1	2091	"tags=28%, list=15%, signal=33%"
GSE28726_NAIVE_CD4_TCELL_VS_NAIVE_NKTCELL_DN
GSE28726_NAIVE_CD4_TCELL_VS_NAIVE_NKTCELL_DN	161
0.30421472	1.2622681	0.05957447	0.114857666
1	1579	"tags=18%, list=11%, signal=20%"
GSE1432_1H_VS_24H_IFNG_MICROGLIA_DN
GSE1432_1H_VS_24H_IFNG_MICROGLIA_DN	132
0.3105812	1.2621629	0.08547009	0.11490662
1	2444	"tags=33%, list=17%, signal=39%"
GSE17721_LPS_VS_CPG_0.5H_BMDC_DN	GSE17721_LPS_VS_CPG_0.5H_BMDC_DN
	144	0.30538273	1.26188	0.069196425
0.11513579	1	1924	"tags=20%, list=14%, signal=23%"
GSE9960_HEALTHY_VS_GRAM_POS_SEPSIS_PBMC_UP
GSE9960_HEALTHY_VS_GRAM_POS_SEPSIS_PBMC_UP	113
0.3183103	1.2614979	0.08588957	0.11544856
1	2887	"tags=36%, list=20%, signal=45%"
GSE9988_LPS_VS_LOW_LPS_MONOCYTE_DN
GSE9988_LPS_VS_LOW_LPS_MONOCYTE_DN	106
0.32016954	1.2612611	0.08245243	0.11562196
1	181	"tags=7%, list=1%, signal=7%"
GSE17721_12H_VS_24H_PAM3CSK4_BMDC_DN
GSE17721_12H_VS_24H_PAM3CSK4_BMDC_DN	162
0.29721433	1.2612222	0.07905983	0.1156001
1	1571	"tags=22%, list=11%, signal=24%"
GSE15324_ELF4_KO_VS_WT_ACTIVATED_CD8_TCELL_DN
GSE15324_ELF4_KO_VS_WT_ACTIVATED_CD8_TCELL_DN	145
0.31100363	1.2606258	0.075	0.11614752	1
2734	"tags=35%, list=19%, signal=43%"
GSE26928_EFF_MEM_VS_CENTR_MEM_CD4_TCELL_UP
GSE26928_EFF_MEM_VS_CENTR_MEM_CD4_TCELL_UP	126
0.30946037	1.260119	0.082969435	0.11660748	1
1226	"tags=19%, list=9%, signal=21%"
GSE3691_IFN_PRODUCING_KILLER_DC_VS_PLASMACYTOID_DC_SPLEEN_DN
GSE3691_IFN_PRODUCING_KILLER_DC_VS_PLASMACYTOID_DC_SPLEEN_DN
	137	0.30789816	1.2600249	0.065789476
0.11664205	1	1023	"tags=14%, list=7%, signal=15%"
GSE21670_UNTREATED_VS_IL6_TREATED_STAT3_KO_CD4_TCELL_UP
GSE21670_UNTREATED_VS_IL6_TREATED_STAT3_KO_CD4_TCELL_UP
152	0.30289346	1.2597669	0.07777778

0.116853125	1	2060	"tags=30%, list=15%, signal=34%"
GSE360_T_GONDII_VS_B_MALAYI_LOW_DOSE_MAC_UP
GSE360_T_GONDII_VS_B_MALAYI_LOW_DOSE_MAC_UP		143
0.30781972	1.2590877	0.064989515	0.11748422
1	1536	"tags=21%, list=11%, signal=23%"
GSE17721_0.5H_VS_4H_PAM3CSK4_BMDC_DN
GSE17721_0.5H_VS_4H_PAM3CSK4_BMDC_DN		141
0.3065217	1.2587904	0.1026694	0.11771931
1	2128	"tags=27%, list=15%, signal=31%"
GSE46606_IRF4_KO_VS_WT_CD40L_IL2_IL5_3DAY_STIMULATED_BCELL_UP
GSE46606_IRF4_KO_VS_WT_CD40L_IL2_IL5_3DAY_STIMULATED_BCELL_UP
	141	0.30264828	1.258705	0.06326531
0.1177446	1	1706	"tags=19%, list=12%, signal=22%"
GSE12392_WT_VS_IFNB_KO_CD8A_POS_SPLEEN_DC_UP
GSE12392_WT_VS_IFNB_KO_CD8A_POS_SPLEEN_DC_UP		148
0.30469173	1.2583443	0.09307359	0.11803787
1	2296	"tags=31%, list=16%, signal=37%"
GSE37301_LYMPHOID_PRIMED_MPP_VS_COMMON_LYMPHOID_PROGENITOR_UP
GSE37301_LYMPHOID_PRIMED_MPP_VS_COMMON_LYMPHOID_PROGENITOR_UP
	160	0.29953298	1.25819	0.056962024
0.11813621	1	1211	"tags=14%, list=9%, signal=16%"
GSE22443_NAIVE_VS_ACT_AND_IL12_TREATED_CD8_TCELL_DN
GSE22443_NAIVE_VS_ACT_AND_IL12_TREATED_CD8_TCELL_DN
135	0.30376035	1.2581364	0.067391306
0.118136406	1	1657	"tags=20%, list=12%, signal=22%"
GSE27786_LIN_NEG_VS_NKTCELL_DN	GSE27786_LIN_NEG_VS_NKTCELL_DN
	154	0.30515537	1.2576265	0.059196617
0.11860613	1	2045	"tags=27%, list=14%, signal=31%"
GSE2770_TGFB_AND_IL4_ACT_VS_ACT_CD4_TCELL_2H_UP
GSE2770_TGFB_AND_IL4_ACT_VS_ACT_CD4_TCELL_2H_UP		119
0.31435746	1.2575773	0.08559499	0.11858156
1	1811	"tags=26%, list=13%, signal=30%"
GSE17721_POLYIC_VS_GARDIQUIMOD_6H_BMDC_UP
GSE17721_POLYIC_VS_GARDIQUIMOD_6H_BMDC_UP	159
0.30135015	1.2569587	0.07597536	0.119183466
1	1863	"tags=24%, list=13%, signal=27%"
GSE19941_UNSTIM_VS_LPS_STIM_IL10_KO_MACROPHAGE_DN
GSE19941_UNSTIM_VS_LPS_STIM_IL10_KO_MACROPHAGE_DN		136
0.3058174	1.256589	0.07608695	0.11953114	1
2311	"tags=30%, list=16%, signal=36%"
GSE40277_GATA1_AND_SATB1_TRANSDUCED_VS_CTRL_CD4_TCELL_UP
GSE40277_GATA1_AND_SATB1_TRANSDUCED_VS_CTRL_CD4_TCELL_UP
144	0.30309635	1.2558273	0.058455113
0.12026809	1	2129	"tags=28%, list=15%, signal=32%"
GSE3982_NEUTROPHIL_VS_TH1_UP	GSE3982_NEUTROPHIL_VS_TH1_UP
	136	0.3049756	1.2552007	0.09913793
0.120846555	1	2145	"tags=24%, list=15%, signal=27%"
GSE360_LOW_DOSE_B_MALAYI_VS_M_TUBERCULOSIS_MAC_DN
GSE360_LOW_DOSE_B_MALAYI_VS_M_TUBERCULOSIS_MAC_DN		154
0.29780456	1.2547917	0.061023623	0.12122628
1	2266	"tags=27%, list=16%, signal=32%"
GSE14769_UNSTIM_VS_60MIN_LPS_BMDM_UP
GSE14769_UNSTIM_VS_60MIN_LPS_BMDM_UP		138
0.30711362	1.2545704	0.07966457	0.12139047

1	2060	"tags=30%, list=15%, signal=35%"
GSE11864_UNTREATED_VS_CSF1_IN_MAC_UP
GSE11864_UNTREATED_VS_CSF1_IN_MAC_UP	124
0.31452087	1.2540379	0.09127789	0.12188452
1	2548	"tags=31%, list=18%, signal=37%"
GSE42088_2H_VS_24H_LEISHMANIA_INF_DC_DN
GSE42088_2H_VS_24H_LEISHMANIA_INF_DC_DN	138
0.30870667	1.2535607	0.08035714	0.122329935
1	2121	"tags=18%, list=15%, signal=21%"
GSE3982_CTRL_VS_IGE_STIM_MAST_CELL_UP
GSE3982_CTRL_VS_IGE_STIM_MAST_CELL_UP	133
0.3097727	1.2531627	0.085020244	0.122683905
1	2641	"tags=31%, list=19%, signal=38%"
GSE2128_CTRL_VS_MIMETOPE_NEGATIVE_SELECTION_DP_THYMOCYTE_C57BL6_DN
GSE2128_CTRL_VS_MIMETOPE_NEGATIVE_SELECTION_DP_THYMOCYTE_C57BL6_DN
149	0.30509323	1.2530224	0.06396589
0.12277252	1	1868	"tags=19%, list=13%, signal=22%"
GSE29164_DAY3_VS_DAY7_CD8_TCELL_TREATED_MELANOMA_UP
GSE29164_DAY3_VS_DAY7_CD8_TCELL_TREATED_MELANOMA_UP
131	0.30644405	1.2528428	0.079166666
0.12289235	1	2002	"tags=23%, list=14%, signal=26%"
GSE45365_NK_CELL_VS_CD8_TCELL_MCMV_INFECTION_UP
GSE45365_NK_CELL_VS_CD8_TCELL_MCMV_INFECTION_UP	153
0.30018988	1.2527524	0.09014675	0.12293052
1	2350	"tags=35%, list=17%, signal=42%"
GSE5589_LPS_VS_LPS_AND_IL6_STIM_MACROPHAGE_45MIN_DN
GSE5589_LPS_VS_LPS_AND_IL6_STIM_MACROPHAGE_45MIN_DN
154	0.30234823	1.252668	0.061965812	0.12294022
1	2546	"tags=34%, list=18%, signal=41%"
GSE45365_NK_CELL_VS_CD11B_DC_MCMV_INFECTION_DN
GSE45365_NK_CELL_VS_CD11B_DC_MCMV_INFECTION_DN	155
0.29981837	1.2521265	0.08367347	0.12342656
1	1840	"tags=23%, list=13%, signal=26%"
GSE34156_UNTREATED_VS_6H_NOD2_AND_TLR1_TLR2_LIGAND_TREATED_MONOCYTE_
DN
GSE34156_UNTREATED_VS_6H_NOD2_AND_TLR1_TLR2_LIGAND_TREATED_MONOCYTE_
DN	113	0.31787646	1.25183	0.10526316
0.123636656	1	2354	"tags=38%, list=17%, signal=45%"
GSE17721_CTRL_VS_CPG_6H_BMDC_DN	GSE17721_CTRL_VS_CPG_6H_BMDC_DN
165	0.29566315	1.25173	0.072210066
0.1236771	1	2420	"tags=25%, list=17%, signal=30%"
GSE30083_SP2_VS_SP3_THYMOCYTE_DN	GSE30083_SP2_VS_SP3_THYMOCYTE_DN
152	0.3014977	1.2517059	0.067940556
0.123627454	1	1539	"tags=21%, list=11%, signal=23%"
GSE20198_UNTREATED_VS_IL12_TREATED_ACT_CD4_TCELL_UP
GSE20198_UNTREATED_VS_IL12_TREATED_ACT_CD4_TCELL_UP
137	0.29981616	1.2509464	0.07112971
0.1243985	1	2641	"tags=31%, list=19%, signal=38%"
GSE19923_E2A_KO_VS_E2A_AND_HEB_KO_DP_THYMOCYTE_DN
GSE19923_E2A_KO_VS_E2A_AND_HEB_KO_DP_THYMOCYTE_DN	165
0.2988532	1.250161	0.05773196	0.12519836	1
1293	"tags=22%, list=9%, signal=24%"
GSE17721_LPS_VS_POLYIC_0.5H_BMDC_UP
GSE17721_LPS_VS_POLYIC_0.5H_BMDC_UP	166

0.29562762	1.249752 0.07064018	0.12557548	1
2250	"tags=25%, list=16%, signal=30%"
GSE36392_EOSINOPHIL_VS_MAC_IL25_TREATED_LUNG_DN
GSE36392_EOSINOPHIL_VS_MAC_IL25_TREATED_LUNG_DN	136
0.30557117	1.2491279	0.06958251	0.1261739
1	2148	"tags=26%, list=15%, signal=30%"
GSE4590_SMALL_VS_VPREB_POS_LARGE_PRE_BCELL_DN
GSE4590_SMALL_VS_VPREB_POS_LARGE_PRE_BCELL_DN	124
0.3098576	1.2488581	0.08713693	0.12638737
1	2646	"tags=30%, list=19%, signal=36%"
GSE38681_WT_VS_LYL1_KO_LYMPHOID_PRIMED_MULTIPOTENT_PROGENITOR_DN
GSE38681_WT_VS_LYL1_KO_LYMPHOID_PRIMED_MULTIPOTENT_PROGENITOR_DN
157	0.29785642	1.2486587	0.07186858
0.1265299	1	2229	"tags=34%, list=16%, signal=40%"
GSE17721_CTRL_VS_PAM3CSK4_24H_BMDC_DN
GSE17721_CTRL_VS_PAM3CSK4_24H_BMDC_DN	155
0.29558572	1.2478787	0.074324325	0.1272679
1	1855	"tags=24%, list=13%, signal=27%"
GSE16522_MEMORY_VS_NAIVE_ANTI_CD3CD28_STIM_CD8_TCELL_UP
GSE16522_MEMORY_VS_NAIVE_ANTI_CD3CD28_STIM_CD8_TCELL_UP
158	0.29808548	1.247467 0.058189657	0.12765089
1	1684	"tags=25%, list=12%, signal=28%"
GSE13484_UNSTIM_VS_3H_YF17D_VACCINE_STIM_PBMC_DN
GSE13484_UNSTIM_VS_3H_YF17D_VACCINE_STIM_PBMC_DN	152
0.30010766	1.2473054	0.08008658	0.12774876
1	2608	"tags=33%, list=18%, signal=40%"
GSE17721_4_VS_24H_GARDIQUIMOD_BMDC_UP
GSE17721_4_VS_24H_GARDIQUIMOD_BMDC_UP	158
0.2972959	1.2468934	0.05	0.12810615	1
2177	"tags=28%, list=15%, signal=33%"
GSE42021_CD24HI_VS_CD24LOW_TREG_THYMUS_UP
GSE42021_CD24HI_VS_CD24LOW_TREG_THYMUS_UP	140
0.30240145	1.2467796	0.06736842	0.12815925
1	2400	"tags=29%, list=17%, signal=34%"
GSE3039_ALPHABETA_CD8_TCELL_VS_B1_BCELL_DN
GSE3039_ALPHABETA_CD8_TCELL_VS_B1_BCELL_DN	146
0.30144924	1.2466831	0.063318774	0.1281774
1	2786	"tags=32%, list=20%, signal=39%"
GSE28449_WT_VS_LRF_KO_GERMINAL_CENTER_BCELL_DN
GSE28449_WT_VS_LRF_KO_GERMINAL_CENTER_BCELL_DN	135
0.3048122	1.2464917	0.08154506	0.12829345
1	1845	"tags=21%, list=13%, signal=24%"
GSE27786_CD4_TCELL_VS_ERYTHTROBLAST_DN
GSE27786_CD4_TCELL_VS_ERYTHTROBLAST_DN	135
0.30339715	1.2464799	0.11060948	0.1282342
1	1409	"tags=12%, list=10%, signal=13%"
GSE11961_FOLLICULAR_BCELL_VS_MEMORY_BCELL_DAY7_DN
GSE11961_FOLLICULAR_BCELL_VS_MEMORY_BCELL_DAY7_DN	134
0.30599478	1.246181 0.09128631	0.12847501	1
2742	"tags=31%, list=19%, signal=39%"
GSE19888_ADENOSINE_A3R_INH_VS_TCELL_MEMBRANES_ACT_MAST_CELL_DN
GSE19888_ADENOSINE_A3R_INH_VS_TCELL_MEMBRANES_ACT_MAST_CELL_DN
138	0.3009759	1.2461386	0.079229124
0.12845317	1	2002	"tags=20%, list=14%, signal=23%"

GSE17721_CTRL_VS_CPG_2H_BMDC_UP GSE17721_CTRL_VS_CPG_2H_BMDC_UP
141 0.3042394 1.245865 0.06048387
0.1286665 1 1967 "tags=23%, list=14%, signal=27%"
GSE17721_0.5H_VS_24H_PAM3CSK4_BMDC_DN
GSE17721_0.5H_VS_24H_PAM3CSK4_BMDC_DN 151
0.2986845 1.2456787 0.070866145 0.12879272
1 2011 "tags=22%, list=14%, signal=25%"
GSE17721_12H_VS_24H_PAM3CSK4_BMDC_UP
GSE17721_12H_VS_24H_PAM3CSK4_BMDC_UP 146
0.29977813 1.2454828 0.08722109 0.12893474
1 2372 "tags=30%, list=17%, signal=36%"
GSE25088_IL4_VS_IL4_AND_ROSIGLITAZONE_STIM_MACROPHAGE_DAY10_DN
GSE25088_IL4_VS_IL4_AND_ROSIGLITAZONE_STIM_MACROPHAGE_DAY10_DN
152 0.29831204 1.2450984 0.07709251
0.12930436 1 1737 "tags=21%, list=12%, signal=24%"
GSE3982_MAST_CELL_VS_MAC_DN GSE3982_MAST_CELL_VS_MAC_DN
151 0.29714355 1.244904 0.072765075
0.12944597 1 1908 "tags=21%, list=14%, signal=24%"
GSE25087_TREG_VS_TCONV_ADULT_UP GSE25087_TREG_VS_TCONV_ADULT_UP
147 0.3005185 1.2443322 0.08113591
0.12998757 1 1738 "tags=25%, list=12%, signal=28%"
GSE26928_EFF_MEMORY_VS_CXCR5_POS_CD4_TCELL_UP
GSE26928_EFF_MEMORY_VS_CXCR5_POS_CD4_TCELL_UP 119
0.31056473 1.244236 0.10204082 0.13002643 1
2575 "tags=24%, list=18%, signal=30%"
GSE24972_WT_VS_IRF8_KO_SPLEEN_FOLLICULAR_BCELL_DN
GSE24972_WT_VS_IRF8_KO_SPLEEN_FOLLICULAR_BCELL_DN 134
0.3049223 1.2441516 0.07709251 0.13004404
1 2290 "tags=31%, list=16%, signal=36%"
GSE46242_TH1_VS_ANERGIC_TH1_CD4_TCELL_WITH_EGR2_DELETED_DN
GSE46242_TH1_VS_ANERGIC_TH1_CD4_TCELL_WITH_EGR2_DELETED_DN
130 0.3052497 1.2441272 0.0952381
0.13000007 1 1375 "tags=18%, list=10%, signal=20%"
GSE17721_CTRL_VS_PAM3CSK4_2H_BMDC_UP
GSE17721_CTRL_VS_PAM3CSK4_2H_BMDC_UP 140
0.3053586 1.2440169 0.08583691 0.13003331
1 2217 "tags=28%, list=16%, signal=33%"
GSE32164_RESTING_DIFFERENTIATED_VS_ALTERNATIVELY_ACT_M2_MACROPHAGE_D
N
GSE32164_RESTING_DIFFERENTIATED_VS_ALTERNATIVELY_ACT_M2_MACROPHAGE_D
N 149 0.30036974 1.2440118
0.06732673 0.12996745 1 1621 "tags=21%,
list=11%, signal=24%"
GSE2770_IL12_VS_IL4_TREATED_ACT_CD4_TCELL_6H_UP
GSE2770_IL12_VS_IL4_TREATED_ACT_CD4_TCELL_6H_UP 148
0.3009552 1.2439266 0.063917525 0.12998423
1 2970 "tags=30%, list=21%, signal=38%"
GSE19401_UNSTIM_VS_PAM2CSK4_STIM_FOLLICULAR_DC_DN
GSE19401_UNSTIM_VS_PAM2CSK4_STIM_FOLLICULAR_DC_DN 146
0.2998678 1.2438575 0.077568136 0.12998784
1 2462 "tags=34%, list=17%, signal=41%"
GSE3039_CD4_TCELL_VS_ALPHABETA_CD8_TCELL_DN
GSE3039_CD4_TCELL_VS_ALPHABETA_CD8_TCELL_DN 146
0.296295 1.2434665 0.07777778 0.1303532 1

1852 "tags=25%, list=13%, signal=28%"
GSE37301_COMMON_LYMPHOID_PROGENITOR_VS_GRAN_MONO_PROGENITOR_UP
GSE37301_COMMON_LYMPHOID_PROGENITOR_VS_GRAN_MONO_PROGENITOR_UP
145 0.29909742 1.2434641 0.07494646
0.130282 1 2089 "tags=26%, list=15%, signal=30%"
GSE15330_WT_VS_IKAROS_KO_LYMPHOID_MULTIPOTENT_PROGENITOR_UP
GSE15330_WT_VS_IKAROS_KO_LYMPHOID_MULTIPOTENT_PROGENITOR_UP
152 0.29768902 1.2430798 0.07882883
0.13061579 1 2421 "tags=34%, list=17%, signal=40%"
GSE44649_NAIVE_VS_ACTIVATED_CD8_TCELL_UP
GSE44649_NAIVE_VS_ACTIVATED_CD8_TCELL_UP 158
0.29443446 1.242721 0.07645875 0.13091867 1
2589 "tags=30%, list=18%, signal=36%"
GSE13738_RESTING_VS_TCR_ACTIVATED_CD4_TCELL_DN
GSE13738_RESTING_VS_TCR_ACTIVATED_CD4_TCELL_DN 144
0.30584282 1.2427206 0.097510375 0.13084461
1 2337 "tags=31%, list=17%, signal=36%"
GSE2770_UNTREATED_VS_IL12_TREATED_ACT_CD4_TCELL_6H_DN
GSE2770_UNTREATED_VS_IL12_TREATED_ACT_CD4_TCELL_6H_DN
146 0.30150855 1.241811 0.09259259 0.13176602
1 2530 "tags=30%, list=18%, signal=36%"
GSE27786_LSK_VS_CD4_TCELL_DN GSE27786_LSK_VS_CD4_TCELL_DN
128 0.3088986 1.2408834 0.09775967
0.13277262 1 2528 "tags=30%, list=18%, signal=37%"
GSE43955_1H_VS_20H_ACT_CD4_TCELL_DN
GSE43955_1H_VS_20H_ACT_CD4_TCELL_DN 156
0.29612648 1.2406758 0.08225108 0.13291739
1 2222 "tags=22%, list=16%, signal=26%"
GSE27786_CD4_TCELL_VS_NKTCELL_UP GSE27786_CD4_TCELL_VS_NKTCELL_UP
145 0.3060528 1.2396097 0.095744684
0.13402985 1 2021 "tags=23%, list=14%, signal=27%"
GSE37534_GW1929_VS_PIOGLITAZONE_TREATED_CD4_TCELL_PPARG1_FOXP3_TRANSDUCED_DN
GSE37534_GW1929_VS_PIOGLITAZONE_TREATED_CD4_TCELL_PPARG1_FOXP3_TRANSDUCED_DN 147 0.3023118 1.2395074
0.062240664 0.13407354 1 2080 "tags=27%, list=15%, signal=32%"
GSE3039_ALPHAALPHA_VS_ALPHABETA_CD8_TCELL_UP
GSE3039_ALPHAALPHA_VS_ALPHABETA_CD8_TCELL_UP 146
0.30263585 1.2394418 0.093023255 0.13407357
1 2301 "tags=33%, list=16%, signal=39%"
GSE12392_WT_VS_IFNAR_KO_CD8A_NEG_SPLEEN_DC_UP
GSE12392_WT_VS_IFNAR_KO_CD8A_NEG_SPLEEN_DC_UP 150
0.30215776 1.2393837 0.08742005 0.13405763
1 2489 "tags=31%, list=18%, signal=38%"
GSE2770_TGFB_AND_IL4_VS_IL4_TREATED_ACT_CD4_TCELL_6H_DN
GSE2770_TGFB_AND_IL4_VS_IL4_TREATED_ACT_CD4_TCELL_6H_DN
137 0.3025419 1.2386403 0.07974138
0.13483968 1 2520 "tags=30%, list=18%, signal=36%"
GSE24671_CTRL_VS_BAKIMULC_INFECTED_MOUSE_SPLENOCYTES_UP
GSE24671_CTRL_VS_BAKIMULC_INFECTED_MOUSE_SPLENOCYTES_UP
138 0.30371603 1.2383717 0.086065575
0.13505921 1 2351 "tags=33%, list=17%, signal=40%"
GSE411_WT_VS_SOCS3_KO_MACROPHAGE_IL6_STIM_100MIN_DN

GSE411_WT_VS_SOCS3_KO_MACROPHAGE_IL6_STIM_100MIN_DN
132 0.30011842 1.2374406 0.07272727
0.13603435 1 2175 "tags=28%, list=15%, signal=33%"
GSE14350_IL2RB_KO_VS_WT_TEFF_DN GSE14350_IL2RB_KO_VS_WT_TEFF_DN
135 0.30393648 1.2371274 0.08982036
0.136336 1 2382 "tags=31%, list=17%, signal=37%"
GSE45365_NK_CELL_VS_BCELL_MCMV_INFECTION_DN
GSE45365_NK_CELL_VS_BCELL_MCMV_INFECTION_DN 144
0.29701552 1.2370043 0.106299214 0.13639721
1 2354 "tags=29%, list=17%, signal=35%"
GSE17721_POLYIC_VS_GARDIQUIMOD_4H_BMDC_DN
GSE17721_POLYIC_VS_GARDIQUIMOD_4H_BMDC_DN 149
0.29915923 1.2367744 0.08050848 0.13658273
1 2053 "tags=25%, list=15%, signal=29%"
GSE5463_CTRL_VS_DEXAMETHASONE_TREATED_THYMOCYTE_DN
GSE5463_CTRL_VS_DEXAMETHASONE_TREATED_THYMOCYTE_DN 151
0.29871643 1.2366251 0.07021277 0.1366808
1 2424 "tags=32%, list=17%, signal=38%"
GSE22611_UNSTIM_VS_2H_MDP_STIM_NOD2_TRANSDUCED_HEK293T_CELL_DN
GSE22611_UNSTIM_VS_2H_MDP_STIM_NOD2_TRANSDUCED_HEK293T_CELL_DN
133 0.30307648 1.2364395 0.092402466
0.13681372 1 1694 "tags=20%, list=12%, signal=22%"
GSE27786_NKCELL_VS_MONO_MAC_UP GSE27786_NKCELL_VS_MONO_MAC_UP
144 0.30142772 1.236104 0.08595388
0.13710654 1 1771 "tags=25%, list=13%, signal=28%"
GSE19401_PAM2CSK4_VS_RETINOIC_ACID_AND_PAM2CSK4_STIM_FOLLICULAR_DC_D
N
GSE19401_PAM2CSK4_VS_RETINOIC_ACID_AND_PAM2CSK4_STIM_FOLLICULAR_DC_D
N 149 0.2960576 1.2357358
0.08730159 0.1374633 1 1933 "tags=26%,
list=14%, signal=30%"
GSE4535_BM_DERIVED_DC_VS_FOLLICULAR_DC_UP
GSE4535_BM_DERIVED_DC_VS_FOLLICULAR_DC_UP 153
0.30113474 1.2349526 0.08742005 0.13833347
1 2128 "tags=29%, list=15%, signal=33%"
GSE14350_TREG_VS_TEFF_DN GSE14350_TREG_VS_TEFF_DN 153
0.29583308 1.2347782 0.08991228 0.1384491
1 2036 "tags=18%, list=14%, signal=20%"
GSE24210_TCONV_VS_TREG_DN GSE24210_TCONV_VS_TREG_DN 146
0.30005744 1.2344582 0.07658643 0.13873412
1 806 "tags=10%, list=6%, signal=10%"
GSE33292_DN3_THYMOCYTE_VS_TCF1_KO_TCELL_LYMPHOMA_DN
GSE33292_DN3_THYMOCYTE_VS_TCF1_KO_TCELL_LYMPHOMA_DN
117 0.31105584 1.2343646 0.111788616 0.13877
1 2047 "tags=21%, list=15%, signal=24%"
GSE6269_FLU_VS_E_COLI_INF_PBMC_DN GSE6269_FLU_VS_E_COLI_INF_PBMC_DN
104 0.3161322 1.2341621 0.120162934
0.1389254 1 2475 "tags=21%, list=18%, signal=25%"
GSE18893_CTRL_VS_TNF_TREATED_TREG_24H_DN
GSE18893_CTRL_VS_TNF_TREATED_TREG_24H_DN 141
0.3023956 1.2338763 0.08951965 0.1391678
1 1881 "tags=22%, list=13%, signal=25%"
GSE42088_UNINF_VS_LEISHMANIA_INF_DC_2H_DN
GSE42088_UNINF_VS_LEISHMANIA_INF_DC_2H_DN 120

0.30004832	1.2335987	0.0809628	0.13942868
1	2419	"tags=28%, list=17%, signal=34%"
GSE17721_LPS_VS_PAM3CSK4_8H_BMDC_UP
GSE17721_LPS_VS_PAM3CSK4_8H_BMDC_UP		156
0.29346886	1.2332711	0.0754717	0.13974519
1	1264	"tags=17%, list=9%, signal=19%"
GSE4748_LPS_VS_LPS_AND_CYANOBACTERIUM_LPSLIKE_STIM_DC_3H_DN
GSE4748_LPS_VS_LPS_AND_CYANOBACTERIUM_LPSLIKE_STIM_DC_3H_DN
102	0.3124662	1.233095	0.10526316	0.13985848
1	1590	"tags=17%, list=11%, signal=19%"
GSE17721_0.5H_VS_12H_LPS_BMDC_DN	GSE17721_0.5H_VS_12H_LPS_BMDC_DN
	157	0.2945279	1.2328018	0.08730159
0.1401346	1	2588	"tags=31%, list=18%, signal=37%"
GSE37416_CTRL_VS_12H_F_TULARENSIS_LVS_NEUTROPHIL_DN
GSE37416_CTRL_VS_12H_F_TULARENSIS_LVS_NEUTROPHIL_DN
126	0.30408686	1.232685	0.09034908	0.14019914
1	2536	"tags=32%, list=18%, signal=38%"
GSE29618_PRE_VS_DAY7_FLU_VACCINE_BCELL_UP
GSE29618_PRE_VS_DAY7_FLU_VACCINE_BCELL_UP		142
0.3012322	1.2325612	0.1039823	0.14026345
1	2078	"tags=18%, list=15%, signal=21%"
GSE1460_CD4_THYMOCYTE_VS_NAIVE_CD4_TCELL_ADULT_BLOOD_UP
GSE1460_CD4_THYMOCYTE_VS_NAIVE_CD4_TCELL_ADULT_BLOOD_UP
157	0.29942238	1.2323734	0.08074534
0.14041853	1	2272	"tags=32%, list=16%, signal=38%"
GSE29618_LAIV_VS_TIV_FLU_VACCINE_DAY7_MDC_UP
GSE29618_LAIV_VS_TIV_FLU_VACCINE_DAY7_MDC_UP		142
0.2959893	1.2319812	0.08571429	0.14078237
1	1157	"tags=18%, list=8%, signal=20%"
GSE4590_LARGE_PRE_BCELL_VS_VPREB_POS_LARGE_PRE_BCELL_UP
GSE4590_LARGE_PRE_BCELL_VS_VPREB_POS_LARGE_PRE_BCELL_UP
135	0.30120987	1.2315105	0.08471075
0.1412941	1	1667	"tags=27%, list=12%, signal=31%"
GSE32423_MEMORY_VS_NAIVE_CD8_TCELL_IL7_IL4_DN
GSE32423_MEMORY_VS_NAIVE_CD8_TCELL_IL7_IL4_DN		127
0.30239806	1.2312675	0.102970295	0.14150247
1	2240	"tags=31%, list=16%, signal=36%"
GSE360_L_DONOVANI_VS_M_TUBERCULOSIS_MAC_UP
GSE360_L_DONOVANI_VS_M_TUBERCULOSIS_MAC_UP		148
0.29424945	1.2310641	0.08997955	0.14168373
1	2015	"tags=24%, list=14%, signal=27%"
GSE18281_CORTICAL_THYMOCYTE_VS_WHOLE_CORTEX_THYMUS_DN
GSE18281_CORTICAL_THYMOCYTE_VS_WHOLE_CORTEX_THYMUS_DN
140	0.2977891	1.2306057	0.1137339
0.14216405	1	2720	"tags=29%, list=19%, signal=36%"
GSE20198_IL12_VS_IL12_IL18_TREATED_ACT_CD4_TCELL_UP
GSE20198_IL12_VS_IL12_IL18_TREATED_ACT_CD4_TCELL_UP
145	0.29783234	1.230193	0.09375	0.1425711	1
1675	"tags=24%, list=12%, signal=27%"
GSE40274_FOXP3_VS_FOXP3_AND_XBP1_TRANSDUCED_ACTIVATED_CD4_TCELL_UP
GSE40274_FOXP3_VS_FOXP3_AND_XBP1_TRANSDUCED_ACTIVATED_CD4_TCELL_UP
	104	0.31337768	1.2301334	0.10433071
0.14254941	1	1552	"tags=15%, list=11%, signal=17%"
GSE1448_ANTI_VALPHA2_VS_VBETA5_DP_THYMOCYTE_DN

GSE1448_ANTI_VALPHA2_VS_VBETA5_DP_THYMOCYTE_DN 158
0.293716 1.2295793 0.10021322 0.1431008 1
2041 "tags=24%, list=14%, signal=28%"
GSE19374_UNINF_VS_LISTERIA_INFECTED_MACROPHAGE_DN
GSE19374_UNINF_VS_LISTERIA_INFECTED_MACROPHAGE_DN 139
0.2992624 1.2295291 0.09836066 0.14308463
1 1915 "tags=22%, list=14%, signal=25%"
GSE21927_BALBC_VS_C57BL6_MONOCYTE_SPLEEN_DN
GSE21927_BALBC_VS_C57BL6_MONOCYTE_SPLEEN_DN 107
0.30939844 1.2290694 0.10838446 0.14357848
1 2325 "tags=29%, list=16%, signal=34%"
GSE18281_SUBCAPSULAR_VS_CENTRAL_CORTICAL_REGION_OF_THYMUS_UP
GSE18281_SUBCAPSULAR_VS_CENTRAL_CORTICAL_REGION_OF_THYMUS_UP
121 0.30214205 1.2289736 0.084632516
0.14362071 1 2474 "tags=33%, list=18%, signal=40%"
GSE21927_UNTREATED_VS_GMCSF_GCSF_TREATED_BONE_MARROW_DN
GSE21927_UNTREATED_VS_GMCSF_GCSF_TREATED_BONE_MARROW_DN
125 0.3057477 1.2289358 0.08418891
0.14358944 1 2194 "tags=29%, list=16%, signal=34%"
GSE27786_LIN_NEG_VS_MONO_MAC_UP GSE27786_LIN_NEG_VS_MONO_MAC_UP
137 0.29718822 1.2284698 0.083333336
0.14404118 1 2792 "tags=34%, list=20%, signal=41%"
GSE22886_IGG_IGA_MEMORY_BCELL_VS_BM_PLASMA_CELL_UP
GSE22886_IGG_IGA_MEMORY_BCELL_VS_BM_PLASMA_CELL_UP 135
0.29953605 1.2278277 0.103792414 0.14475253
1 2126 "tags=30%, list=15%, signal=35%"
GSE21927_SPLEEN_MONOCYTE_VS_GMCSF_GCSF_BONE_MARROW_DN
GSE21927_SPLEEN_MONOCYTE_VS_GMCSF_GCSF_BONE_MARROW_DN
124 0.3023788 1.2275503 0.09832636
0.14500523 1 2146 "tags=27%, list=15%, signal=31%"
GSE22935_UNSTIM_VS_12H_MBOVIS_BCG_STIM_MACROPHAGE_UP
GSE22935_UNSTIM_VS_12H_MBOVIS_BCG_STIM_MACROPHAGE_UP
140 0.29917413 1.2275325 0.090361446
0.14494333 1 1571 "tags=21%, list=11%, signal=23%"
GSE11961_GERMINAL_CENTER_BCELL_DAY7_VS_MEMORY_BCELL_DAY40_UP
GSE11961_GERMINAL_CENTER_BCELL_DAY7_VS_MEMORY_BCELL_DAY40_UP
143 0.2972527 1.227049 0.08723404
0.14543764 1 1248 "tags=18%, list=9%, signal=20%"
GSE1791_CTRL_VS_NEUROMEDINU_IN_T_CELL_LINE_3H_DN
GSE1791_CTRL_VS_NEUROMEDINU_IN_T_CELL_LINE_3H_DN 120
0.30023083 1.2269697 0.093023255 0.14546205
1 1555 "tags=18%, list=11%, signal=20%"
GSE14000_UNSTIM_VS_16H_LPS_DC_DN GSE14000_UNSTIM_VS_16H_LPS_DC_DN
142 0.2988975 1.226774 0.12555066
0.1456174 1 2453 "tags=33%, list=17%, signal=40%"
GSE21063_CTRL_VS_ANTI_IGM_STIM_BCELL_NFATC1_KO_16H_UP
GSE21063_CTRL_VS_ANTI_IGM_STIM_BCELL_NFATC1_KO_16H_UP
151 0.29632136 1.2260842 0.09484536
0.14640653 1 2224 "tags=28%, list=16%, signal=33%"
GSE17974_IL4_AND_ANTI_IL12_VS_UNTREATED_48H_ACT_CD4_TCELL_DN
GSE17974_IL4_AND_ANTI_IL12_VS_UNTREATED_48H_ACT_CD4_TCELL_DN
122 0.30654907 1.2258307 0.11195446
0.14663222 1 1650 "tags=26%, list=12%, signal=29%"
GSE37301_LYMPHOID_PRIMED_MPP_VS_RAG2_KO_NK_CELL_UP

GSE37301_LYMPHOID_PRIMED_MPP_VS_RAG2_KO_NK_CELL_UP 157
0.29318565 1.2258092 0.09594883 0.14657237
1 1950 "tags=28%, list=14%, signal=32%"
GSE23695_CD57_POS_VS_NEG_NK_CELL_UP
GSE23695_CD57_POS_VS_NEG_NK_CELL_UP 134
0.30086407 1.2252625 0.10085837 0.14717568
1 1990 "tags=27%, list=14%, signal=31%"
GSE20715_0H_VS_48H_OZONE_LUNG_UP GSE20715_0H_VS_48H_OZONE_LUNG_UP
167 0.29029202 1.2247277 0.08946322
0.1477853 1 1610 "tags=20%, list=11%, signal=22%"
GSE39820_CTRL_VS_TGFBETA1_IL6_CD4_TCELL_UP
GSE39820_CTRL_VS_TGFBETA1_IL6_CD4_TCELL_UP 149
0.29615662 1.2245454 0.0982906 0.14791423
1 2037 "tags=26%, list=14%, signal=29%"
GSE22886_NAIVE_CD8_TCELL_VS_NKCELL_DN
GSE22886_NAIVE_CD8_TCELL_VS_NKCELL_DN 138
0.2986885 1.2240489 0.09756097 0.14843489
1 1902 "tags=24%, list=13%, signal=27%"
GSE12392_IFNAR_KO_VS_IFNB_KO_CD8_NEG_SPLEEN_DC_DN
GSE12392_IFNAR_KO_VS_IFNB_KO_CD8_NEG_SPLEEN_DC_DN 161
0.2961982 1.2239311 0.10195228 0.14850254
1 1536 "tags=22%, list=11%, signal=24%"
GSE3982_EOSINOPHIL_VS_DC_DN GSE3982_EOSINOPHIL_VS_DC_DN
156 0.2921835 1.223835 0.09978308
0.14853194 1 2283 "tags=28%, list=16%, signal=33%"
GSE17721_POLYIC_VS_PAM3CSK4_6H_BMDC_UP
GSE17721_POLYIC_VS_PAM3CSK4_6H_BMDC_UP 155
0.29512805 1.2233037 0.09033614 0.1490705
1 2552 "tags=26%, list=18%, signal=31%"
GSE17721_POLYIC_VS_PAM3CSK4_8H_BMDC_UP
GSE17721_POLYIC_VS_PAM3CSK4_8H_BMDC_UP 153
0.28839156 1.2230159 0.08865979 0.14933282
1 2249 "tags=27%, list=16%, signal=32%"
GSE16385_IL4_VS_ROSIGLITAZONE_STIM_MACROPHAGE_UP
GSE16385_IL4_VS_ROSIGLITAZONE_STIM_MACROPHAGE_UP 139
0.297518 1.2222455 0.099585064 0.1502371 1
2359 "tags=33%, list=17%, signal=39%"
GSE14000_4H_VS_16H_LPS_DC_DN GSE14000_4H_VS_16H_LPS_DC_DN
120 0.30391234 1.2215146 0.09791667
0.15102297 1 2642 "tags=33%, list=19%, signal=41%"
GSE3203_UNTREATED_VS_IFNB_TREATED_LN_BCELL_UP
GSE3203_UNTREATED_VS_IFNB_TREATED_LN_BCELL_UP 146
0.29488313 1.2214174 0.09414226 0.15106593
1 2431 "tags=27%, list=17%, signal=33%"
GSE39820_CTRL_VS_TGFBETA3_IL6_CD4_TCELL_UP
GSE39820_CTRL_VS_TGFBETA3_IL6_CD4_TCELL_UP 150
0.2920228 1.2211576 0.10560345 0.15131 1
2104 "tags=29%, list=15%, signal=33%"
GSE5679_CTRL_VS_PPARG_LIGAND_ROSIGLITAZONE_AND_RARA_AGONIST_AM580_TREATED_DC_UP
GSE5679_CTRL_VS_PPARG_LIGAND_ROSIGLITAZONE_AND_RARA_AGONIST_AM580_TREATED_DC_UP 147 0.29633987 1.2208892
0.08154506 0.15156195 1 2239 "tags=33%, list=16%, signal=38%"

GSE16522_ANTI_CD3CD28_STIM_VS_UNSTIM_MEMORY_CD8_TCELL_UP
GSE16522_ANTI_CD3CD28_STIM_VS_UNSTIM_MEMORY_CD8_TCELL_UP
149 0.29052627 1.2207538 0.09775967
0.15166931 1 1967 "tags=23%, list=14%, signal=26%"
GSE20715_0H_VS_24H_OZONE_TLR4_KO_LUNG_DN
GSE20715_0H_VS_24H_OZONE_TLR4_KO_LUNG_DN 167
0.28597173 1.2203522 0.08436214 0.1520923
1 2367 "tags=23%, list=17%, signal=28%"
GSE360_CTRL_VS_L_DONOVANI_MAC_DN GSE360_CTRL_VS_L_DONOVANI_MAC_DN
158 0.2909611 1.2196324 0.10666667
0.15286985 1 2414 "tags=27%, list=17%, signal=32%"
GSE17721_CPG_VS_GARDIQUIMOD_24H_BMDC_DN
GSE17721_CPG_VS_GARDIQUIMOD_24H_BMDC_DN 152
0.2933474 1.2193586 0.095744684 0.1531283
1 2011 "tags=25%, list=14%, signal=29%"
GSE6259_CD4_TCELL_VS_CD8_TCELL_UP GSE6259_CD4_TCELL_VS_CD8_TCELL_UP
144 0.2955754 1.2189714 0.09746589
0.15350375 1 393 "tags=8%, list=3%, signal=8%"
GSE12366_NAIVE_VS_MEMORY_BCELL_DN GSE12366_NAIVE_VS_MEMORY_BCELL_DN
139 0.29202473 1.2189511 0.1018711
0.15344776 1 1964 "tags=25%, list=14%, signal=29%"
GSE2770_TGFB_AND_IL4_ACT_VS_ACT_CD4_TCELL_48H_UP
GSE2770_TGFB_AND_IL4_ACT_VS_ACT_CD4_TCELL_48H_UP 142
0.2981145 1.21862 0.09615385 0.15378372 1
2296 "tags=29%, list=16%, signal=34%"
GSE369_PRE_VS_POST_IL6_INJECTION_IFNG_WT_LIVER_DN
GSE369_PRE_VS_POST_IL6_INJECTION_IFNG_WT_LIVER_DN 156
0.29272956 1.2182215 0.08836207 0.15417251
1 2091 "tags=29%, list=15%, signal=33%"
GSE17721_POLYIC_VS_CPG_6H_BMDC_UP GSE17721_POLYIC_VS_CPG_6H_BMDC_UP
159 0.29026625 1.2181824 0.10515873
0.15413608 1 2421 "tags=28%, list=17%, signal=33%"
GSE1740_UNSTIM_VS_IFNA_STIMULATED_MCSF_IFNG_DERIVED_MACROPHAGE_UP
GSE1740_UNSTIM_VS_IFNA_STIMULATED_MCSF_IFNG_DERIVED_MACROPHAGE_UP
102 0.31177607 1.2181145 0.1278826
0.15414117 1 1482 "tags=16%, list=11%, signal=17%"
GSE25088_ROSIGLITAZONE_VS_IL4_AND_ROSIGLITAZONE_STIM_STAT6_KO_MACROP
HAGE_DAY10_UP
GSE25088_ROSIGLITAZONE_VS_IL4_AND_ROSIGLITAZONE_STIM_STAT6_KO_MACROP
HAGE_DAY10_UP 131 0.29775628 1.2179147
0.1 0.15430121 1 1909 "tags=25%, list=14%,
signal=29%"
GSE36888_STAT5_AB_KNOCKIN_VS_WT_TCELL_IL2_TREATED_6H_UP
GSE36888_STAT5_AB_KNOCKIN_VS_WT_TCELL_IL2_TREATED_6H_UP
143 0.29491988 1.2177973 0.12343096
0.1543479 1 1737 "tags=23%, list=12%, signal=26%"
GSE22601_IMMATURE_CD4_SINGLE_POSITIVE_VS_CD8_SINGLE_POSITIVE_THYMOCY
TE_UP
GSE22601_IMMATURE_CD4_SINGLE_POSITIVE_VS_CD8_SINGLE_POSITIVE_THYMOCY
TE_UP 152 0.29429105 1.2171177
0.08884297 0.1551312 1 2347 "tags=33%,
list=17%, signal=39%"
GSE4984_LPS_VS_VEHICLE_CTRL_TREATED_DC_DN
GSE4984_LPS_VS_VEHICLE_CTRL_TREATED_DC_DN 139

0.2936133 1.217104 0.11464968 0.15506308 1
1366 "tags=20%, list=10%, signal=22%"
GSE19401_NAIVE_VS_IMMUNIZED_MOUSE_PLN_FOLLICULAR_DC_DN
GSE19401_NAIVE_VS_IMMUNIZED_MOUSE_PLN_FOLLICULAR_DC_DN
141 0.29511997 1.2170099 0.09915612
0.15509722 1 2239 "tags=25%, list=16%, signal=29%"
GSE10325_LUPUS_BCELL_VS_LUPUS_MYELOID_DN
GSE10325_LUPUS_BCELL_VS_LUPUS_MYELOID_DN 153
0.29164654 1.2169558 0.094412334 0.15508112
1 1917 "tags=23%, list=14%, signal=26%"
GSE32255_WT_UNSTIM_VS_JMJD2D_KNOCKDOWN_4H_LPS_STIM_DC_UP
GSE32255_WT_UNSTIM_VS_JMJD2D_KNOCKDOWN_4H_LPS_STIM_DC_UP
145 0.29309112 1.2168695 0.07628866
0.15509838 1 1936 "tags=25%, list=14%, signal=28%"
GSE5589_LPS_VS_LPS_AND_IL10_STIM_MACROPHAGE_45MIN_DN
GSE5589_LPS_VS_LPS_AND_IL10_STIM_MACROPHAGE_45MIN_DN
138 0.2953835 1.2160267 0.12090164
0.15609968 1 2093 "tags=22%, list=15%, signal=26%"
GSE1432_CTRL_VS_IFNG_1H_MICROGLIA_DN
GSE1432_CTRL_VS_IFNG_1H_MICROGLIA_DN 140
0.29335845 1.2154075 0.09775967 0.15679549
1 2356 "tags=28%, list=17%, signal=33%"
GSE27786_LSK_VS_BCELL_UP GSE27786_LSK_VS_BCELL_UP 140
0.29743996 1.2149075 0.10421286 0.15736018
1 1942 "tags=24%, list=14%, signal=28%"
GSE18281_MEDULLARY_THYMOCYTE_VS_WHOLE_MEDULLA_THYMUS_UP
GSE18281_MEDULLARY_THYMOCYTE_VS_WHOLE_MEDULLA_THYMUS_UP
133 0.29609507 1.2148342 0.11020408
0.15736693 1 1904 "tags=27%, list=13%, signal=31%"
GSE11924_TH1_VS_TH2_CD4_TCELL_UP GSE11924_TH1_VS_TH2_CD4_TCELL_UP
138 0.2970049 1.2142062 0.1090535
0.15808956 1 2010 "tags=23%, list=14%, signal=27%"
GSE17721_LPS_VS_GARDIQUIMOD_8H_BMDC_UP
GSE17721_LPS_VS_GARDIQUIMOD_8H_BMDC_UP 160
0.28884682 1.2133441 0.08067941 0.15910794
1 1087 "tags=14%, list=8%, signal=15%"
GSE26669_CD4_VS_CD8_TCELL_IN_MLR_COSTIM_BLOCK_UP
GSE26669_CD4_VS_CD8_TCELL_IN_MLR_COSTIM_BLOCK_UP 134
0.2925993 1.2132992 0.12314225 0.15908433
1 2149 "tags=26%, list=15%, signal=31%"
GSE41867_DAY6_VS_DAY8_LCMV_CLONE13_EFFECTOR_CD8_TCELL_UP
GSE41867_DAY6_VS_DAY8_LCMV_CLONE13_EFFECTOR_CD8_TCELL_UP
140 0.29553083 1.21303 0.10714286 0.15935506
1 2604 "tags=29%, list=18%, signal=35%"
GSE14308_TH2_VS_NATURAL_TREG_UP GSE14308_TH2_VS_NATURAL_TREG_UP
141 0.29337722 1.2118562 0.10714286
0.160818 1 930 "tags=14%, list=7%, signal=15%"
GSE40274_GATA1_VS_FOXP3_AND_GATA1_TRANSDUCED_ACTIVATED_CD4_TCELL_DN
GSE40274_GATA1_VS_FOXP3_AND_GATA1_TRANSDUCED_ACTIVATED_CD4_TCELL_DN
150 0.2892357 1.210899 0.0985325
0.16197334 1 1023 "tags=17%, list=7%, signal=18%"
GSE3982_EOSINOPHIL_VS_CENT_MEMORY_CD4_TCELL_UP
GSE3982_EOSINOPHIL_VS_CENT_MEMORY_CD4_TCELL_UP 147
0.28865048 1.2108743 0.10337972 0.16192107

1	2159	"tags=28%, list=15%, signal=33%"
GSE15330_LYMPHOID_MULTIPOTENT_VS_GRANULOCYTE_MONOCYTE_PROGENITOR_IKAROS_KO_UP
GSE15330_LYMPHOID_MULTIPOTENT_VS_GRANULOCYTE_MONOCYTE_PROGENITOR_IKAROS_KO_UP		147	0.29254618	1.2103139
0.11594203	0.16258766	1	2150	"tags=26%,
list=15%, signal=30%"
GSE1112_OT1_CD8AB_VS_HY_CD8AA_THYMOCYTE_RTOC_CULTURE_DN
GSE1112_OT1_CD8AB_VS_HY_CD8AA_THYMOCYTE_RTOC_CULTURE_DN
123	0.30322242	1.2096882	0.12970711
0.16332346	1	2012	"tags=24%, list=14%, signal=27%"
GSE17721_PAM3CSK4_VS_GADIQUIMOD_24H_BMDC_UP
GSE17721_PAM3CSK4_VS_GADIQUIMOD_24H_BMDC_UP		149
0.291316	1.2096353	0.1002045	0.16330819	1
2445	"tags=28%, list=17%, signal=33%"
GSE15324_NAIVE_VS_ACTIVATED_ELF4_KO_CD8_TCELL_DN
GSE15324_NAIVE_VS_ACTIVATED_ELF4_KO_CD8_TCELL_DN		148
0.2900031	1.2083282	0.105932206	0.16493313
1	2391	"tags=29%, list=17%, signal=35%"
GSE19198_CTRL_VS_IL21_TREATED_TCELL_24H_DN
GSE19198_CTRL_VS_IL21_TREATED_TCELL_24H_DN	150
0.2895483	1.2081496	0.108932465	0.16509756
1	2361	"tags=29%, list=17%, signal=34%"
GSE5503_PLN_DC_VS_SPLEEN_DC_ACTIVATED_ALLOGENIC_TCELL_UP
GSE5503_PLN_DC_VS_SPLEEN_DC_ACTIVATED_ALLOGENIC_TCELL_UP
145	0.2961895	1.2081221	0.09475806
0.16503738	1	2210	"tags=21%, list=16%, signal=25%"
GSE23502_BM_VS_COLON_TUMOR_MYELOID_DERIVED_SUPPRESSOR_CELL_DN
GSE23502_BM_VS_COLON_TUMOR_MYELOID_DERIVED_SUPPRESSOR_CELL_DN
	138	0.29376963	1.2073607	0.11111111
0.16595988	1	1079	"tags=17%, list=8%, signal=19%"
GSE7764_NKCELL_VS_SPLENOCYTE_DN	GSE7764_NKCELL_VS_SPLENOCYTE_DN
	143	0.29621053	1.2073122	0.12548262
0.16593891	1	1495	"tags=22%, list=11%, signal=24%"
GSE14000_4H_VS_16H_LPS_DC_UP	GSE14000_4H_VS_16H_LPS_DC_UP
	139	0.2930359	1.2071342	0.08668076
0.16605733	1	2617	"tags=37%, list=19%, signal=45%"
GSE5679_CTRL_VS_PPARG_LIGAND_ROSIGLITAZONE_AND_RARA_AGONIST_AM580_TREATED_DC_DN
GSE5679_CTRL_VS_PPARG_LIGAND_ROSIGLITAZONE_AND_RARA_AGONIST_AM580_TREATED_DC_DN		164	0.28649306	1.2068522
0.11045365	0.16633601	1	2040	"tags=25%,
list=14%, signal=29%"
GSE37301_HEMATOPOIETIC_STEM_CELL_VS_RAG2_KO_NK_CELL_DN
GSE37301_HEMATOPOIETIC_STEM_CELL_VS_RAG2_KO_NK_CELL_DN
152	0.28904226	1.2067515	0.10638298
0.16638386	1	1621	"tags=24%, list=11%, signal=26%"
GSE17721_0.5H_VS_8H_CPG_BMDC_DN	GSE17721_0.5H_VS_8H_CPG_BMDC_DN
	159	0.28898275	1.2061057	0.09756097
0.16714373	1	3106	"tags=35%, list=22%, signal=45%"
GSE17721_POLYIC_VS_PAM3CSK4_16H_BMDC_UP
GSE17721_POLYIC_VS_PAM3CSK4_16H_BMDC_UP		153
0.2932661	1.206048	0.10337972	0.16712958	1
1667	"tags=18%, list=12%, signal=21%"

GSE8835_HEALTHY_VS_CLL_CD4_TCELL_DN
GSE8835_HEALTHY_VS_CLL_CD4_TCELL_DN 135
0.29110885 1.2054197 0.11570248 0.1678929
1 1890 "tags=19%, list=13%, signal=22%"
GSE29949_CD8_POS_DC_SPLEEN_VS_DC_BRAIN_UP
GSE29949_CD8_POS_DC_SPLEEN_VS_DC_BRAIN_UP 147
0.29010814 1.2053281 0.11704312 0.16793215
1 1940 "tags=23%, list=14%, signal=27%"
GSE21927_UNTREATED_VS_GMCSF_GCSF_TREATED_BONE_MARROW_UP
GSE21927_UNTREATED_VS_GMCSF_GCSF_TREATED_BONE_MARROW_UP
117 0.30106294 1.2052329 0.12298387
0.16797352 1 1886 "tags=22%, list=13%, signal=25%"
GSE37416_12H_VS_48H_F_TULARENSIS_LVS_NEUTROPHIL_UP
GSE37416_12H_VS_48H_F_TULARENSIS_LVS_NEUTROPHIL_UP 143
0.2951854 1.2048081 0.124223605 0.16845182
1 1891 "tags=26%, list=13%, signal=30%"
GSE27786_LSK_VS_NEUTROPHIL_UP GSE27786_LSK_VS_NEUTROPHIL_UP
137 0.29256898 1.2046636 0.13069306
0.16855639 1 1819 "tags=23%, list=13%, signal=27%"
GSE14000_TRANSLATED_RNA_VS_MRNA_4H_LPS_DC_DN
GSE14000_TRANSLATED_RNA_VS_MRNA_4H_LPS_DC_DN 108
0.29813737 1.2037109 0.14928426 0.16979334
1 2127 "tags=27%, list=15%, signal=31%"
GSE12392_WT_VS_IFNAR_KO_CD8A_NEG_SPLEEN_DC_DN
GSE12392_WT_VS_IFNAR_KO_CD8A_NEG_SPLEEN_DC_DN 123
0.29426634 1.2028552 0.11632653 0.17087911
1 1886 "tags=26%, list=13%, signal=30%"
GSE7460_CD8_TCELL_VS_CD4_TCELL_ACT_UP
GSE7460_CD8_TCELL_VS_CD4_TCELL_ACT_UP 139
0.29675198 1.202852 0.10683761 0.17079459 1
1535 "tags=23%, list=11%, signal=26%"
GSE36078_WT_VS_IL1R_KO_LUNG_DC_UP GSE36078_WT_VS_IL1R_KO_LUNG_DC_UP
150 0.29306504 1.2022178 0.11064718
0.17159128 1 2332 "tags=21%, list=17%, signal=25%"
GSE1448_ANTI_VALPHA2_VS_VBETA5_DP_THYMOCYTE_UP
GSE1448_ANTI_VALPHA2_VS_VBETA5_DP_THYMOCYTE_UP 156
0.29011714 1.2017958 0.114224136 0.17208195
1 2631 "tags=29%, list=19%, signal=35%"
GSE29618_LAIV_VS_TIV_FLU_VACCINE_DAY7_PDC_DN
GSE29618_LAIV_VS_TIV_FLU_VACCINE_DAY7_PDC_DN 139
0.294018 1.2017039 0.13235295 0.17211138 1
2313 "tags=25%, list=16%, signal=30%"
GSE33513_TCF7_KO_VS_HET_EARLY_THYMIC_PROGENITOR_DN
GSE33513_TCF7_KO_VS_HET_EARLY_THYMIC_PROGENITOR_DN 153
0.29186457 1.2010242 0.13987474 0.17299321
1 1633 "tags=22%, list=12%, signal=25%"
GSE17721_POLYIC_VS_CPG_4H_BMDC_UP GSE17721_POLYIC_VS_CPG_4H_BMDC_UP
145 0.29185537 1.2007875 0.11359026
0.17321287 1 2421 "tags=30%, list=17%, signal=35%"
GSE28737_BCL6_HET_VS_BCL6_KO_MARGINAL_ZONE_BCELL_DN
GSE28737_BCL6_HET_VS_BCL6_KO_MARGINAL_ZONE_BCELL_DN
151 0.28771374 1.2007866 0.088421054
0.17312236 1 2258 "tags=25%, list=16%, signal=30%"
GSE39382_IL3_VS_IL3_IL33_TREATED_MAST_CELL_DN

GSE39382_IL3_VS_IL3_IL33_TREATED_MAST_CELL_DN		142
0.29186565	1.2003165	0.13646056	0.17365637
1	2350	"tags=30%, list=17%, signal=36%"
GSE36888_UNTREATED_VS_IL2_TREATED_TCELL_6H_DN
GSE36888_UNTREATED_VS_IL2_TREATED_TCELL_6H_DN		135
0.29207516	1.2000113	0.13305613	0.17397912
1	2391	"tags=30%, list=17%, signal=35%"
GSE17721_LPS_VS_POLYIC_12H_BMDC_DN
GSE17721_LPS_VS_POLYIC_12H_BMDC_DN		150
0.29129615	1.199736 0.109615386	0.17426214	1
2259	"tags=26%, list=16%, signal=31%"
GSE17721_0.5H_VS_24H_GARDIQUIMOD_BMDC_DN
GSE17721_0.5H_VS_24H_GARDIQUIMOD_BMDC_DN		144
0.28873265	1.1994811	0.122171946	0.17450492
1	2365	"tags=27%, list=17%, signal=32%"
GSE16385_MONOCYTE_VS_12H_IL4_TREATED_MACROPHAGE_UP
GSE16385_MONOCYTE_VS_12H_IL4_TREATED_MACROPHAGE_UP		121
0.30046156	1.1993035	0.11504425	0.1746446
1	1837	"tags=22%, list=13%, signal=25%"
GSE36888_UNTREATED_VS_IL2_TREATED_TCELL_2H_UP
GSE36888_UNTREATED_VS_IL2_TREATED_TCELL_2H_UP		138
0.2874899	1.1990483	0.112840466	0.1749046
1	2404	"tags=29%, list=17%, signal=35%"
GSE21033_1H_VS_12H_POLYIC_STIM_DC_UP
GSE21033_1H_VS_12H_POLYIC_STIM_DC_UP		147
0.2912755	1.1987246	0.115226336	0.17527752
1	1311	"tags=15%, list=9%, signal=16%"
GSE1925_3H_VS_24H_IFNG_STIM_MACROPHAGE_UP
GSE1925_3H_VS_24H_IFNG_STIM_MACROPHAGE_UP		120
0.29913652	1.1984223	0.11904762	0.17559393
1	1980	"tags=24%, list=14%, signal=28%"
GSE41867_NAIVE_VS_DAY8_LCMV_CLONE13_EFFECTOR_CD8_TCELL_DN
GSE41867_NAIVE_VS_DAY8_LCMV_CLONE13_EFFECTOR_CD8_TCELL_DN
115	0.30062273	1.1980671	0.14957266
0.17599799	1	2452	"tags=27%, list=17%, signal=32%"
GSE2585_AIRE_KO_VS_WT_CD80_HIGH_MTEC_DN
GSE2585_AIRE_KO_VS_WT_CD80_HIGH_MTEC_DN		146
0.28931293	1.197672 0.13537118	0.17648767	1
2449	"tags=27%, list=17%, signal=32%"
GSE36392_MAC_VS_NEUTROPHIL_IL25_TREATED_LUNG_DN
GSE36392_MAC_VS_NEUTROPHIL_IL25_TREATED_LUNG_DN		146
0.29174832	1.1972426	0.09656652	0.1769687
1	1967	"tags=17%, list=14%, signal=20%"
GSE46606_DAY1_VS_DAY3_CD40L_IL2_IL5_STIMULATED_IRF4MID_BCELL_DN
GSE46606_DAY1_VS_DAY3_CD40L_IL2_IL5_STIMULATED_IRF4MID_BCELL_DN
	150	0.28821406	1.1967771	0.12334802
0.17753047	1	2092	"tags=29%, list=15%, signal=33%"
GSE22886_NAIVE_BCELL_VS_NEUTROPHIL_DN
GSE22886_NAIVE_BCELL_VS_NEUTROPHIL_DN		142
0.29180616	1.1963446	0.13292433	0.1780534
1	1991	"tags=26%, list=14%, signal=30%"
GSE27786_NKCELL_VS_NEUTROPHIL_UP	GSE27786_NKCELL_VS_NEUTROPHIL_UP
	148	0.28846425	1.1961936	0.125
0.17816709	1	2614	"tags=33%, list=19%, signal=40%"

GSE40666_UNTREATED_VS_IFNA_STIM_CD8_TCELL_90MIN_DN
GSE40666_UNTREATED_VS_IFNA_STIM_CD8_TCELL_90MIN_DN 142
0.2917452 1.1961825 0.13107823 0.17808764
1 2720 "tags=37%, list=19%, signal=46%"
GSE22611_UNSTIM_VS_6H_MDP_STIM_NOD2_TRANSDUCED_HEK293T_CELL_UP
GSE22611_UNSTIM_VS_6H_MDP_STIM_NOD2_TRANSDUCED_HEK293T_CELL_UP
139 0.29287133 1.1960921 0.14285715
0.1781224 1 1997 "tags=21%, list=14%, signal=24%"
GSE41867_DAY6_EFFECTOR_VS_DAY30_EXHAUSTED_CD8_TCELL_LCMV_CLONE13_UP
GSE41867_DAY6_EFFECTOR_VS_DAY30_EXHAUSTED_CD8_TCELL_LCMV_CLONE13_UP
133 0.2960548 1.1957113 0.12526096
0.17855144 1 1134 "tags=20%, list=8%, signal=22%"
GSE17721_4_VS_24H_CPG_BMDC_UP GSE17721_4_VS_24H_CPG_BMDC_UP
156 0.28914902 1.195463 0.12738854
0.17879154 1 1181 "tags=12%, list=8%, signal=13%"
GSE360_DC_VS_MAC_L_DONOVANI_UP GSE360_DC_VS_MAC_L_DONOVANI_UP
154 0.28722614 1.1951356 0.102783725
0.17915611 1 2519 "tags=29%, list=18%, signal=35%"
GSE17721_LPS_VS_GARDIQUIMOD_16H_BMDC_UP
GSE17721_LPS_VS_GARDIQUIMOD_16H_BMDC_UP 155
0.28812462 1.1945224 0.135255 0.17994794 1
2378 "tags=26%, list=17%, signal=31%"
GSE5542_UNTREATED_VS_IFNA_TREATED_EPITHELIAL_CELLS_24H_UP
GSE5542_UNTREATED_VS_IFNA_TREATED_EPITHELIAL_CELLS_24H_UP
151 0.28944358 1.1941974 0.12711865
0.18030725 1 2442 "tags=32%, list=17%, signal=38%"
GSE16451_CTRL_VS_WEST_EQUINE_ENC_VIRUS_MATURE_NEURON_CELL_LINE_UP
GSE16451_CTRL_VS_WEST_EQUINE_ENC_VIRUS_MATURE_NEURON_CELL_LINE_UP
145 0.28854644 1.1940202 0.14198783
0.18045995 1 2462 "tags=31%, list=17%, signal=37%"
GSE11864_CSF1_VS_CSF1_PAM3CYS_IN_MAC_DN
GSE11864_CSF1_VS_CSF1_PAM3CYS_IN_MAC_DN 126
0.29559183 1.1939329 0.12631579 0.18047167
1 2524 "tags=35%, list=18%, signal=42%"
GSE43955_TH0_VS_TGFB_IL6_TH17_ACT_CD4_TCELL_1H_UP
GSE43955_TH0_VS_TGFB_IL6_TH17_ACT_CD4_TCELL_1H_UP 159
0.29266363 1.1937642 0.12916666 0.1806228
1 2354 "tags=30%, list=17%, signal=35%"
GSE17721_POLYIC_VS_CPG_2H_BMDC_UP GSE17721_POLYIC_VS_CPG_2H_BMDC_UP
148 0.28558958 1.1936814 0.1199187
0.1806462 1 1919 "tags=20%, list=14%, signal=23%"
GSE12366_PLASMA_CELL_VS_NAIVE_BCELL_UP
GSE12366_PLASMA_CELL_VS_NAIVE_BCELL_UP 124
0.29676518 1.1934061 0.15982722 0.18094824
1 2392 "tags=25%, list=17%, signal=30%"
GSE17721_CTRL_VS_GARDIQUIMOD_6H_BMDC_DN
GSE17721_CTRL_VS_GARDIQUIMOD_6H_BMDC_DN 151
0.28696927 1.1928068 0.13916501 0.18170665
1 2174 "tags=26%, list=15%, signal=30%"
GSE37533_PPARG1_FOXP3_VS_FOXP3_TRANSDUCED_CD4_TCELL_UP
GSE37533_PPARG1_FOXP3_VS_FOXP3_TRANSDUCED_CD4_TCELL_UP
145 0.29033068 1.1926177 0.11691023
0.18186273 1 2271 "tags=26%, list=16%, signal=31%"
GSE3400_UNTREATED_VS_IFNB_TREATED_MEF_DN

GSE3400_UNTREATED_VS_IFNB_TREATED_MEF_DN 84
0.31533208 1.1925919 0.15301724 0.18180451
1 1737 "tags=23%, list=12%, signal=26%"
GSE5099_UNSTIM_VS_MCSF_TREATED_MONOCYTE_DAY7_UP
GSE5099_UNSTIM_VS_MCSF_TREATED_MONOCYTE_DAY7_UP 120
0.29603198 1.192295 0.13900416 0.18211035 1
2421 "tags=32%, list=17%, signal=38%"
GSE25088_CTRL_VS_IL4_AND_ROSIGLITAZONE_STIM_STAT6_KO_MACROPHAGE_UP
GSE25088_CTRL_VS_IL4_AND_ROSIGLITAZONE_STIM_STAT6_KO_MACROPHAGE_UP
135 0.29048976 1.1909609 0.121212125
0.18391088 1 2400 "tags=27%, list=17%, signal=32%"
GSE42021_CD24HI_VS_CD24INT_TCONV_THYMUS_UP
GSE42021_CD24HI_VS_CD24INT_TCONV_THYMUS_UP 153
0.28787187 1.1906987 0.14468086 0.18419239
1 2251 "tags=28%, list=16%, signal=33%"
GSE29949_DC_BRAIN_VS_MONOCYTE_BONE_MARROW_UP
GSE29949_DC_BRAIN_VS_MONOCYTE_BONE_MARROW_UP 151
0.2843581 1.1903993 0.1187905 0.18453582
1 1964 "tags=28%, list=14%, signal=33%"
GSE20484_MCSG_VS_CXCL4_MONOCYTE_DERIVED_MACROPHAGE_UP
GSE20484_MCSG_VS_CXCL4_MONOCYTE_DERIVED_MACROPHAGE_UP
135 0.29277587 1.1899022 0.152809 0.18515094
1 2064 "tags=24%, list=15%, signal=27%"
GSE3982_EOSINOPHIL_VS_EFF_MEMORY_CD4_TCELL_UP
GSE3982_EOSINOPHIL_VS_EFF_MEMORY_CD4_TCELL_UP 144
0.2903756 1.1897984 0.13968958 0.18520173
1 2595 "tags=33%, list=18%, signal=40%"
GSE29949_CD8_NEG_DC_SPLEEN_VS_MONOCYTE_BONE_MARROW_UP
GSE29949_CD8_NEG_DC_SPLEEN_VS_MONOCYTE_BONE_MARROW_UP
138 0.28866944 1.1896132 0.11344538
0.18536593 1 1967 "tags=26%, list=14%, signal=30%"
GSE5589_LPS_VS_LPS_AND_IL10_STIM_MACROPHAGE_180MIN_UP
GSE5589_LPS_VS_LPS_AND_IL10_STIM_MACROPHAGE_180MIN_UP
141 0.29151478 1.189561 0.14285715 0.18533358
1 2311 "tags=30%, list=16%, signal=36%"
GSE25846_IL10_POS_VS_NEG_CD8_TCELL_DAY7_POST_CORONAVIRUS_BRAIN_DN
GSE25846_IL10_POS_VS_NEG_CD8_TCELL_DAY7_POST_CORONAVIRUS_BRAIN_DN
144 0.29033118 1.1894363 0.11683168
0.18542704 1 2812 "tags=33%, list=20%, signal=41%"
GSE10856_CTRL_VS_TNFRSF6B_IN_MACROPHAGE_UP
GSE10856_CTRL_VS_TNFRSF6B_IN_MACROPHAGE_UP 118
0.29599243 1.1893718 0.16780046 0.18541424
1 1847 "tags=21%, list=13%, signal=24%"
GSE5542_UNTREATED_VS_IFNA_AND_IFNG_TREATED_EPITHELIAL_CELLS_6H_DN
GSE5542_UNTREATED_VS_IFNA_AND_IFNG_TREATED_EPITHELIAL_CELLS_6H_DN
141 0.28884253 1.1892958 0.119284295
0.18542093 1 2374 "tags=27%, list=17%, signal=32%"
GSE21360_PRIMARY_VS_QUATERNARY_MEMORY_CD8_TCELL_UP
GSE21360_PRIMARY_VS_QUATERNARY_MEMORY_CD8_TCELL_UP 144
0.2865302 1.1888729 0.12793177 0.18592411
1 2257 "tags=31%, list=16%, signal=37%"
GSE26559_TCF1_KO_VS_WT_LIN_NEG_CELL_UP
GSE26559_TCF1_KO_VS_WT_LIN_NEG_CELL_UP 144
0.28824338 1.1882749 0.11111111 0.18672532

1 2176 "tags=28%, list=15%, signal=33%"
GSE2770_IL4_ACT_VS_ACT_CD4_TCELL_48H_UP
GSE2770_IL4_ACT_VS_ACT_CD4_TCELL_48H_UP 135
0.29159224 1.1879573 0.1440678 0.18708095
1 2113 "tags=22%, list=15%, signal=26%"
GSE29618_BCELL_VS_MONOCYTE_DAY7_FLU_VACCINE_DN
GSE29618_BCELL_VS_MONOCYTE_DAY7_FLU_VACCINE_DN 153
0.28238747 1.1876274 0.1130064 0.1874544
1 2045 "tags=27%, list=14%, signal=32%"
GSE14000_TRANSLATED_RNA_VS_MRNA_DC_DN
GSE14000_TRANSLATED_RNA_VS_MRNA_DC_DN 97
0.30378324 1.1874424 0.13716814 0.18763989
1 1916 "tags=24%, list=14%, signal=27%"
GSE27786_LSK_VS_ERYTHROBLAST_UP GSE27786_LSK_VS_ERYTHROBLAST_UP
145 0.2830825 1.1873937 0.13865547
0.18762298 1 2278 "tags=26%, list=16%, signal=31%"
GSE17721_0.5H_VS_24H_LPS_BMDC_UP GSE17721_0.5H_VS_24H_LPS_BMDC_UP
155 0.2872327 1.186652 0.113168724
0.18859395 1 1957 "tags=23%, list=14%, signal=26%"
GSE2770_IL12_ACT_VS_ACT_CD4_TCELL_2H_DN
GSE2770_IL12_ACT_VS_ACT_CD4_TCELL_2H_DN 142
0.28823557 1.1862788 0.14587738 0.18904783
1 1805 "tags=25%, list=13%, signal=28%"
GSE7568_CTRL_VS_3H_TGFB_TREATED_MACROPHAGES_WITH_IL4_AND_DEXAMETHASONE_UP
GSE7568_CTRL_VS_3H_TGFB_TREATED_MACROPHAGES_WITH_IL4_AND_DEXAMETHASONE_UP 122 0.29437593 1.1861881
0.15401301 0.1890758 1 1761 "tags=11%, list=12%, signal=13%"
GSE37416_0H_VS_24H_F_TULARENSIS_LVS_NEUTROPHIL_DN
GSE37416_0H_VS_24H_F_TULARENSIS_LVS_NEUTROPHIL_DN 145
0.2830287 1.1846905 0.11134454 0.19111508
1 2449 "tags=32%, list=17%, signal=38%"
GSE43863_DAY6_EFF_VS_DAY150_MEM_TFH_CD4_TCELL_UP
GSE43863_DAY6_EFF_VS_DAY150_MEM_TFH_CD4_TCELL_UP 152
0.28246275 1.1840954 0.12295082 0.19191597
1 1790 "tags=22%, list=13%, signal=25%"
GSE37301_GRANULOCYTE_MONOCYTE_PROGENITOR_VS_RAG2_KO_NK_CELL_DN
GSE37301_GRANULOCYTE_MONOCYTE_PROGENITOR_VS_RAG2_KO_NK_CELL_DN
155 0.28468022 1.1829162 0.13913043
0.19352074 1 2904 "tags=32%, list=21%, signal=40%"
GSE38681_WT_VS_LYL1_KO_LYMPHOID_PRIMED_MULTIPOTENT_PROGENITOR_UP
GSE38681_WT_VS_LYL1_KO_LYMPHOID_PRIMED_MULTIPOTENT_PROGENITOR_UP
149 0.2859558 1.1823163 0.13900416
0.19430424 1 1974 "tags=25%, list=14%, signal=29%"
GSE360_L_MAJOR_VS_B_MALAYI_LOW_DOSE_DC_DN
GSE360_L_MAJOR_VS_B_MALAYI_LOW_DOSE_DC_DN 154
0.28384194 1.182285 0.14806867 0.19424406 1
2613 "tags=29%, list=19%, signal=35%"
GSE24574_BCL6_HIGH_TFH_VS_NAIVE_CD4_TCELL_UP
GSE24574_BCL6_HIGH_TFH_VS_NAIVE_CD4_TCELL_UP 149
0.28364918 1.182063 0.12836438 0.19447279 1
1154 "tags=13%, list=8%, signal=14%"
GSE3994_WT_VS_PAC1_KO_ACTIVATED_MAST_CELL_DN

GSE3994_WT_VS_PAC1_KO_ACTIVATED_MAST_CELL_DN 154
0.28235212 1.1820619 0.112426035 0.19437337
1 2414 "tags=27%, list=17%, signal=33%"
GSE17721_POLYIC_VS_GARDIQUIMOD_24H_BMDC_UP
GSE17721_POLYIC_VS_GARDIQUIMOD_24H_BMDC_UP 153
0.2860737 1.1816354 0.13253012 0.1948998
1 1150 "tags=17%, list=8%, signal=18%"
GSE37301_LYMPHOID_PRIMED_MPP_VS_CD4_TCELL_DN
GSE37301_LYMPHOID_PRIMED_MPP_VS_CD4_TCELL_DN 146
0.28375655 1.181338 0.12997903 0.19521782 1
2831 "tags=36%, list=20%, signal=44%"
GSE22611_MUTANT_NOD2_VS_CTRL_TRANSDUCED_HEK293T_CELL_DN
GSE22611_MUTANT_NOD2_VS_CTRL_TRANSDUCED_HEK293T_CELL_DN
137 0.2888107 1.1812428 0.14782609
0.19526652 1 1818 "tags=18%, list=13%, signal=20%"
GSE15330_HSC_VS_LYMPHOID_PRIMED_MULTIPOTENT_PROGENITOR_UP
GSE15330_HSC_VS_LYMPHOID_PRIMED_MULTIPOTENT_PROGENITOR_UP
154 0.28368744 1.1811956 0.12909836
0.1952368 1 2046 "tags=25%, list=14%, signal=29%"
GSE11961_FOLLICULAR_BCELL_VS_GERMINAL_CENTER_BCELL_DAY7_DN
GSE11961_FOLLICULAR_BCELL_VS_GERMINAL_CENTER_BCELL_DAY7_DN
152 0.28579238 1.181151 0.13025211 0.19520283
1 1795 "tags=18%, list=13%, signal=20%"
GSE19888_ADENOSINE_A3R_INH_PRETREAT_AND_ACT_BY_A3R_VS_A3R_INH_AND_TCELL_MEMBRANES_ACT_MAST_CELL_DN
GSE19888_ADENOSINE_A3R_INH_PRETREAT_AND_ACT_BY_A3R_VS_A3R_INH_AND_TCELL_MEMBRANES_ACT_MAST_CELL_DN 147 0.2877685
1.1811403 0.12997903 0.19511895 1
2321 "tags=32%, list=16%, signal=38%"
GSE29949_CD8_POS_DC_SPLEEN_VS_MONOCYTE_BONE_MARROW_DN
GSE29949_CD8_POS_DC_SPLEEN_VS_MONOCYTE_BONE_MARROW_DN
150 0.2841825 1.1809394 0.13664596
0.19530813 1 1809 "tags=20%, list=13%, signal=23%"
GSE32034_LY6C_HIGH_VS_LOW_ROSIGLIZATONE_TREATED_MONOCYTE_UP
GSE32034_LY6C_HIGH_VS_LOW_ROSIGLIZATONE_TREATED_MONOCYTE_UP
156 0.28123826 1.1807876 0.13432837
0.1954518 1 2686 "tags=35%, list=19%, signal=43%"
GSE27786_CD8_TCELL_VS_MONO_MAC_UP GSE27786_CD8_TCELL_VS_MONO_MAC_UP
144 0.28647044 1.180353 0.15184382
0.19602071 1 2048 "tags=23%, list=15%, signal=27%"
GSE11961_FOLLICULAR_BCELL_VS_MEMORY_BCELL_DAY7_UP
GSE11961_FOLLICULAR_BCELL_VS_MEMORY_BCELL_DAY7_UP 145
0.28517196 1.1797075 0.13478261 0.19687666
1 2072 "tags=28%, list=15%, signal=33%"
GSE21546_ELK1_KO_VS_SAP1A_KO_AND_ELK1_KO_ANTI_CD3_STIM_DP_THYMOCYTES_UP
GSE21546_ELK1_KO_VS_SAP1A_KO_AND_ELK1_KO_ANTI_CD3_STIM_DP_THYMOCYTES_UP 131 0.29065132 1.1794437
0.14444445 0.19716801 1 2102 "tags=25%, list=15%, signal=29%"
GSE13484_12H_UNSTIM_VS_YF17D_VACCINE_STIM_PBMC_DN
GSE13484_12H_UNSTIM_VS_YF17D_VACCINE_STIM_PBMC_DN 153
0.28475112 1.1794313 0.13453816 0.19708355
1 2239 "tags=23%, list=16%, signal=27%"

GSE14000_TRANSLATED_RNA_VS_MRNA_4H_LPS_DC_UP
GSE14000_TRANSLATED_RNA_VS_MRNA_4H_LPS_DC_UP 135
0.28971735 1.179069 0.15714286 0.19753139 1
3153 "tags=38%, list=22%, signal=48%"
GSE7852_TREG_VS_TCONV_DN GSE7852_TREG_VS_TCONV_DN 151
0.2827453 1.1788001 0.12681913 0.19783331
1 2103 "tags=28%, list=15%, signal=33%"
GSE26928_NAIVE_VS_CXCR5_POS_CD4_TCELL_DN
GSE26928_NAIVE_VS_CXCR5_POS_CD4_TCELL_DN 137
0.2866255 1.1785423 0.14604463 0.19811165
1 1371 "tags=20%, list=10%, signal=22%"
GSE15330_WT_VS_IKAROS_KO_HSC_UP GSE15330_WT_VS_IKAROS_KO_HSC_UP
151 0.27996647 1.1783347 0.11776447
0.19831944 1 1837 "tags=23%, list=13%, signal=26%"
GSE3720_UNSTIM_VS_PMA_STIM_VD1_GAMMADELTA_TCELL_DN
GSE3720_UNSTIM_VS_PMA_STIM_VD1_GAMMADELTA_TCELL_DN 99
0.30233303 1.1781604 0.16263737 0.19847135
1 1731 "tags=19%, list=12%, signal=22%"
GSE4984_GALECTIN1_VS_LPS_STIM_DC_UP
GSE4984_GALECTIN1_VS_LPS_STIM_DC_UP 120
0.29432726 1.1780231 0.15845825 0.19858938
1 2065 "tags=29%, list=15%, signal=34%"
GSE15767_MED_VS_SCS_MAC_LN_DN GSE15767_MED_VS_SCS_MAC_LN_DN
142 0.28824985 1.1777672 0.12474849
0.19884668 1 1810 "tags=22%, list=13%, signal=25%"
GSE17721_LPS_VS_CPG_0.5H_BMDC_UP GSE17721_LPS_VS_CPG_0.5H_BMDC_UP
152 0.28608015 1.1777169 0.14529915
0.19881538 1 2620 "tags=26%, list=19%, signal=31%"
GSE17301_CTRL_VS_48H_ACD3_ACD28_STIM_CD8_TCELL_UP
GSE17301_CTRL_VS_48H_ACD3_ACD28_STIM_CD8_TCELL_UP 149
0.28326485 1.177661 0.12727273 0.19879313 1
2030 "tags=23%, list=14%, signal=26%"
GSE1460_DP_THYMOCYTE_VS_NAIVE_CD4_TCELL_CORD_BLOOD_DN
GSE1460_DP_THYMOCYTE_VS_NAIVE_CD4_TCELL_CORD_BLOOD_DN
136 0.2875689 1.1776323 0.14042553
0.19873486 1 1450 "tags=15%, list=10%, signal=16%"
GSE17721_CTRL_VS_CPG_8H_BMDC_UP GSE17721_CTRL_VS_CPG_8H_BMDC_UP
137 0.28753456 1.1775233 0.14468086
0.19878052 1 2314 "tags=27%, list=16%, signal=32%"
GSE2128_C57BL6_VS_NOD_THYMOCYTE_DN
GSE2128_C57BL6_VS_NOD_THYMOCYTE_DN 137
0.28607562 1.1774182 0.14 0.19883385 1
1977 "tags=22%, list=14%, signal=25%"
GSE10240_CTRL_VS_IL17_STIM_PRIMARY_BRONCHIAL_EPITHELIAL_CELLS_UP
GSE10240_CTRL_VS_IL17_STIM_PRIMARY_BRONCHIAL_EPITHELIAL_CELLS_UP
147 0.28568605 1.177116 0.14102565
0.19919203 1 1309 "tags=16%, list=9%, signal=17%"
GSE37532_WT_VS_PPARG_KO_VISCERAL_ADIPOSE_TISSUE_TCONV_DN
GSE37532_WT_VS_PPARG_KO_VISCERAL_ADIPOSE_TISSUE_TCONV_DN
150 0.27865526 1.1769962 0.12276786
0.19927542 1 1977 "tags=26%, list=14%, signal=30%"
GSE17721_CTRL_VS_CPG_8H_BMDC_DN GSE17721_CTRL_VS_CPG_8H_BMDC_DN
164 0.2772108 1.1769936 0.1380753
0.19917728 1 2043 "tags=23%, list=14%, signal=27%"

GSE20715_WT_VS_TLR4_KO_6H_OZONE_LUNG_UP
GSE20715_WT_VS_TLR4_KO_6H_OZONE_LUNG_UP 155
0.28264648 1.1769568 0.12017167 0.19912145
1 1707 "tags=19%, list=12%, signal=21%"
GSE37301_HEMATOPOIETIC_STEM_CELL_VS_GRAN_MONO_PROGENITOR_DN
GSE37301_HEMATOPOIETIC_STEM_CELL_VS_GRAN_MONO_PROGENITOR_DN
138 0.2890754 1.1767937 0.1277533
0.19926284 1 2225 "tags=28%, list=16%, signal=33%"
GSE41867_DAY15_EFFECTOR_VS_DAY30_EXHAUSTED_CD8_TCELL_LCMV_CLONE13_UP

GSE41867_DAY15_EFFECTOR_VS_DAY30_EXHAUSTED_CD8_TCELL_LCMV_CLONE13_UP
146 0.28851795 1.1752038
0.14618644 0.2015644 1 1983 "tags=27%,
list=14%, signal=31%"
GSE37301_HEMATOPOIETIC_STEM_CELL_VS_LYMPHOID_PRIMED_MPP_UP
GSE37301_HEMATOPOIETIC_STEM_CELL_VS_LYMPHOID_PRIMED_MPP_UP
130 0.2842069 1.1747282 0.15317287
0.20218998 1 2062 "tags=25%, list=15%, signal=29%"
GSE37301_RAG2_KO_VS_RAG2_AND_ETS1_KO_NK_CELL_UP
GSE37301_RAG2_KO_VS_RAG2_AND_ETS1_KO_NK_CELL_UP 155
0.28224495 1.1747016 0.14660831 0.20212688
1 1912 "tags=23%, list=14%, signal=26%"
GSE25890_CTRL_VS_IL33_IL7_TREATED_NUOCYTES_DN
GSE25890_CTRL_VS_IL33_IL7_TREATED_NUOCYTES_DN 153
0.28277165 1.1743379 0.13441955 0.2026131
1 2001 "tags=26%, list=14%, signal=30%"
GSE26343_UNSTIM_VS_LPS_STIM_NFAT5_KO_MACROPHAGE_UP
GSE26343_UNSTIM_VS_LPS_STIM_NFAT5_KO_MACROPHAGE_UP 154
0.2837499 1.1741091 0.14075631 0.20286357
1 2149 "tags=30%, list=15%, signal=35%"
GSE40274_CTRL_VS_LEF1_TRANSDUCED_ACTIVATED_CD4_TCELL_UP
GSE40274_CTRL_VS_LEF1_TRANSDUCED_ACTIVATED_CD4_TCELL_UP
142 0.28597918 1.1732935 0.13953489
0.20399456 1 2325 "tags=30%, list=16%, signal=35%"
GSE2585_THYMIC_DC_VS_THYMIC_MACROPHAGE_UP
GSE2585_THYMIC_DC_VS_THYMIC_MACROPHAGE_UP 138
0.28715542 1.1727268 0.14592275 0.20478435
1 2188 "tags=22%, list=16%, signal=25%"
GSE17721_CTRL_VS_PAM3CSK4_24H_BMDC_UP
GSE17721_CTRL_VS_PAM3CSK4_24H_BMDC_UP 147
0.2857144 1.172431 0.15151516 0.20513013 1
1684 "tags=22%, list=12%, signal=24%"
GSE17974_CTRL_VS_ACT_IL4_AND_ANTI_IL12_4H_CD4_TCELL_DN
GSE17974_CTRL_VS_ACT_IL4_AND_ANTI_IL12_4H_CD4_TCELL_DN
142 0.2829207 1.1721917 0.12554112
0.20538647 1 1942 "tags=26%, list=14%, signal=30%"
GSE42724_MEMORY_VS_B1_BCELL_UP GSE42724_MEMORY_VS_B1_BCELL_UP
151 0.28362724 1.1719928 0.14128035
0.20558359 1 2479 "tags=28%, list=18%, signal=34%"
GSE5589_LPS_VS_LPS_AND_IL10_STIM_IL10_KO_MACROPHAGE_180MIN_DN
GSE5589_LPS_VS_LPS_AND_IL10_STIM_IL10_KO_MACROPHAGE_180MIN_DN
156 0.2795992 1.171856 0.15959597
0.2056817 1 761 "tags=12%, list=5%, signal=13%"
GSE3982_MAC_VS_NEUTROPHIL_LPS_STIM_DN

GSE3982_MAC_VS_NEUTROPHIL_LPS_STIM_DN 137
0.28474018 1.171561 0.15010571 0.20606029 1
1575 "tags=15%, list=11%, signal=17%"
GSE36888_UNTREATED_VS_IL2_TREATED_STAT5_AB_KNOCKIN_TCELL_6H_DN
GSE36888_UNTREATED_VS_IL2_TREATED_STAT5_AB_KNOCKIN_TCELL_6H_DN
135 0.2860132 1.1713535 0.15505618
0.20626357 1 2824 "tags=34%, list=20%, signal=42%"
GSE7509_UNSTIM_VS_IFNA_STIM_IMMATURE_DC_DN
GSE7509_UNSTIM_VS_IFNA_STIM_IMMATURE_DC_DN 143
0.28214768 1.1710103 0.13507625 0.2067074
1 2332 "tags=30%, list=17%, signal=36%"
GSE22432_MULTIPOTENT_VS_COMMON_DC_PROGENITOR_UNTREATED_DN
GSE22432_MULTIPOTENT_VS_COMMON_DC_PROGENITOR_UNTREATED_DN
142 0.28399524 1.1703608 0.14799154
0.20762171 1 1942 "tags=25%, list=14%, signal=29%"
GSE2826_XID_VS_BTK_KO_BCELL_UP GSE2826_XID_VS_BTK_KO_BCELL_UP
157 0.2794386 1.1702198 0.13374485
0.20772745 1 2056 "tags=22%, list=15%, signal=25%"
GSE22886_IGA_VS_IGM_MEMORY_BCELL_UP
GSE22886_IGA_VS_IGM_MEMORY_BCELL_UP 142
0.2789222 1.1696243 0.13152401 0.2085703
1 1653 "tags=18%, list=12%, signal=21%"
GSE13485_CTRL_VS_DAY21_YF17D_VACCINE_PBMC_UP
GSE13485_CTRL_VS_DAY21_YF17D_VACCINE_PBMC_UP 132
0.28634232 1.1695046 0.15369262 0.20864104
1 2845 "tags=36%, list=20%, signal=44%"
GSE11864_CSF1_VS_CSF1_IFNG_PAM3CYS_IN_MAC_DN
GSE11864_CSF1_VS_CSF1_IFNG_PAM3CYS_IN_MAC_DN 130
0.2878956 1.1694355 0.14845361 0.20864101
1 2444 "tags=28%, list=17%, signal=34%"
GSE37301_LYMPHOID_PRIMED_MPP_VS_PRO_BCELL_DN
GSE37301_LYMPHOID_PRIMED_MPP_VS_PRO_BCELL_DN 130
0.28741014 1.1689883 0.1691023 0.2092418
1 1208 "tags=13%, list=9%, signal=14%"
GSE23398_WT_VS_IL2_KO_CD4_TCELL_SCURFY_MOUSE_DN
GSE23398_WT_VS_IL2_KO_CD4_TCELL_SCURFY_MOUSE_DN 143
0.27836606 1.1686116 0.14822547 0.20969032
1 2756 "tags=37%, list=20%, signal=46%"
GSE39820_TGFBETA3_IL6_VS_TGFBETA3_IL6_IL23A_TREATED_CD4_TCELL_DN
GSE39820_TGFBETA3_IL6_VS_TGFBETA3_IL6_IL23A_TREATED_CD4_TCELL_DN
146 0.2826293 1.1679815 0.14225054
0.21058667 1 1878 "tags=21%, list=13%, signal=23%"
GSE43955_10H_VS_30H_ACT_CD4_TCELL_WITH_TGFB_IL6_UP
GSE43955_10H_VS_30H_ACT_CD4_TCELL_WITH_TGFB_IL6_UP 157
0.28100866 1.167526 0.14648438 0.21117352 1
2284 "tags=22%, list=16%, signal=26%"
GSE5099_DAY3_VS_DAY7_MCSF_TREATED_MACROPHAGE_DN
GSE5099_DAY3_VS_DAY7_MCSF_TREATED_MACROPHAGE_DN 120
0.28878736 1.1675195 0.17327766 0.21107957
1 1561 "tags=13%, list=11%, signal=15%"
GSE3982_BASOPHIL_VS_EFF_MEMORY_CD4_TCELL_DN
GSE3982_BASOPHIL_VS_EFF_MEMORY_CD4_TCELL_DN 132
0.28386325 1.167416 0.13502109 0.21113744 1
2244 "tags=23%, list=16%, signal=27%"

GSE22601_DOUBLE_NEGATIVE_VS_DOUBLE_POSITIVE_THYMOCYTE_DN
GSE22601_DOUBLE_NEGATIVE_VS_DOUBLE_POSITIVE_THYMOCYTE_DN
154 0.28148317 1.1671633 0.13293651
0.2114101 1 1962 "tags=27%, list=14%, signal=31%"
GSE8685_IL2_STARVED_VS_IL2_ACT_IL2_STARVED_CD4_TCELL_UP
GSE8685_IL2_STARVED_VS_IL2_ACT_IL2_STARVED_CD4_TCELL_UP
151 0.27951458 1.166879 0.15463917 0.21172836
1 2108 "tags=28%, list=15%, signal=33%"
GSE40274_LEF1_VS_FOXP3_AND_LEF1_TRANSDUCED_ACTIVATED_CD4_TCELL_UP
GSE40274_LEF1_VS_FOXP3_AND_LEF1_TRANSDUCED_ACTIVATED_CD4_TCELL_UP
146 0.2786478 1.1658314 0.14468086
0.21332274 1 2232 "tags=29%, list=16%, signal=34%"
GSE5589_LPS_VS_LPS_AND_IL6_STIM_MACROPHAGE_45MIN_UP
GSE5589_LPS_VS_LPS_AND_IL6_STIM_MACROPHAGE_45MIN_UP
142 0.284109 1.1657207 0.15800866 0.21340032
1 2079 "tags=30%, list=15%, signal=35%"
GSE17974_CTRL_VS_ACT_IL4_AND_ANTI_IL12_2H_CD4_TCELL_DN
GSE17974_CTRL_VS_ACT_IL4_AND_ANTI_IL12_2H_CD4_TCELL_DN
135 0.2872264 1.164638 0.17586912 0.2150659
1 1702 "tags=21%, list=12%, signal=23%"
GSE5589_WT_VS_IL6_KO_LPS_STIM_MACROPHAGE_45MIN_UP
GSE5589_WT_VS_IL6_KO_LPS_STIM_MACROPHAGE_45MIN_UP 133
0.28945172 1.1640717 0.14439656 0.21586207
1 2964 "tags=32%, list=21%, signal=40%"
GSE37534_UNTREATED_VS_PIOGLITAZONE_TREATED_CD4_TCELL_PPARG1_AND_FOXP3_TRASDUCED_UP
GSE37534_UNTREATED_VS_PIOGLITAZONE_TREATED_CD4_TCELL_PPARG1_AND_FOXP3_TRASDUCED_UP 127 0.28526008 1.1640682
0.1663286 0.21576028 1 1711 "tags=18%, list=12%, signal=20%"
GSE360_L_MAJOR_VS_M_TUBERCULOSIS_DC_UP
GSE360_L_MAJOR_VS_M_TUBERCULOSIS_DC_UP 152
0.27915925 1.1639446 0.15918367 0.21585332
1 2850 "tags=32%, list=20%, signal=39%"
GSE18804_SPLEEN_MACROPHAGE_VS_TUMORAL_MACROPHAGE_DN
GSE18804_SPLEEN_MACROPHAGE_VS_TUMORAL_MACROPHAGE_DN
127 0.28698578 1.1634214 0.16334662
0.21658523 1 2439 "tags=27%, list=17%, signal=32%"
GSE15659_NAIVE_CD4_TCELL_VS_RESTING_TREG_DN
GSE15659_NAIVE_CD4_TCELL_VS_RESTING_TREG_DN 123
0.28892517 1.1630445 0.18454936 0.21707763
1 1783 "tags=17%, list=13%, signal=19%"
GSE3720_LPS_VS_PMA_STIM_VD2_GAMMADELTA_TCELL_DN
GSE3720_LPS_VS_PMA_STIM_VD2_GAMMADELTA_TCELL_DN 122
0.28646746 1.1627725 0.16352202 0.2174212
1 2461 "tags=22%, list=17%, signal=27%"
GSE12392_CD8A_POS_VS_NEG_SPLEEN_DC_UP
GSE12392_CD8A_POS_VS_NEG_SPLEEN_DC_UP 167
0.2754665 1.1625365 0.13865547 0.21768749
1 2085 "tags=25%, list=15%, signal=29%"
GSE36891_UNSTIM_VS_PAM_TLR2_STIM_PERITONEAL_MACROPHAGE_UP
GSE36891_UNSTIM_VS_PAM_TLR2_STIM_PERITONEAL_MACROPHAGE_UP
135 0.28323615 1.161967 0.16563147 0.21848276
1 1713 "tags=21%, list=12%, signal=24%"

GSE37533_UNTREATED_VS_PIOGLIZATONE_TREATED_CD4_TCELL_PPARG1_AND_FOXP3_TRASDUCED_DN
GSE37533_UNTREATED_VS_PIOGLIZATONE_TREATED_CD4_TCELL_PPARG1_AND_FOXP3_TRASDUCED_DN 155 0.2795926 1.1608769 0.16488223 0.2201278 1 1800 "tags=25%, list=13%, signal=28%"
GSE43955_10H_VS_30H_ACT_CD4_TCELL_UP
GSE43955_10H_VS_30H_ACT_CD4_TCELL_UP 159 0.27551547 1.160619 0.14102565 0.22043337 1 1008 "tags=13%, list=7%, signal=13%"
GSE40666_STAT1_KO_VS_STAT4_KO_CD8_TCELL_WITH_IFNA_STIM_90MIN_UP
GSE40666_STAT1_KO_VS_STAT4_KO_CD8_TCELL_WITH_IFNA_STIM_90MIN_UP 125 0.28975087 1.160072 0.16806723 0.22122711 1 2915 "tags=34%, list=21%, signal=43%"
GSE22140_HEALTHY_VS_ARTHRITIC_GERMFREE_MOUSE_CD4_TCELL_DN
GSE22140_HEALTHY_VS_ARTHRITIC_GERMFREE_MOUSE_CD4_TCELL_DN 153 0.28221253 1.1599665 0.17745303 0.22129023 1 1566 "tags=24%, list=11%, signal=26%"
GSE13306_RA_VS_UNTREATED_TREG_DN GSE13306_RA_VS_UNTREATED_TREG_DN 144 0.2818289 1.15955 0.14457831 0.22186764 1 1743 "tags=17%, list=12%, signal=19%"
GSE23925_LIGHT_ZONE_VS_DARK_ZONE_BCELL_UP
GSE23925_LIGHT_ZONE_VS_DARK_ZONE_BCELL_UP 141 0.27985647 1.1594684 0.15448852 0.22189273 1 2071 "tags=23%, list=15%, signal=26%"
GSE11961_FOLLICULAR_BCELL_VS_PLASMA_CELL_DAY7_DN
GSE11961_FOLLICULAR_BCELL_VS_PLASMA_CELL_DAY7_DN 134 0.28432456 1.159352 0.16997792 0.22195785 1 1848 "tags=25%, list=13%, signal=29%"
GSE22611_NOD2_TRANSDUCED_VS_CTRL_HEK293T_STIMULATED_WITH_MDP_2H_UP
GSE22611_NOD2_TRANSDUCED_VS_CTRL_HEK293T_STIMULATED_WITH_MDP_2H_UP 143 0.28368226 1.1590916 0.16969697 0.22226478 1 2828 "tags=36%, list=20%, signal=45%"
GSE360_L_DONOVANI_VS_L_MAJOR_DC_DN
GSE360_L_DONOVANI_VS_L_MAJOR_DC_DN 144 0.28055215 1.1586534 0.17213115 0.22285265 1 2039 "tags=23%, list=14%, signal=27%"
GSE36888_UNTREATED_VS_IL2_TREATED_TCELL_2H_DN
GSE36888_UNTREATED_VS_IL2_TREATED_TCELL_2H_DN 138 0.28262508 1.1585699 0.16803278 0.22286993 1 2311 "tags=22%, list=16%, signal=27%"
GSE27786_LSK_VS_NEUTROPHIL_DN GSE27786_LSK_VS_NEUTROPHIL_DN 143 0.28145248 1.1585118 0.17035398 0.22284359 1 2687 "tags=28%, list=19%, signal=34%"
GSE21546_WT_VS_ELK1_KO_DP_THYMOCYTES_DN
GSE21546_WT_VS_ELK1_KO_DP_THYMOCYTES_DN 142 0.28027466 1.1581848 0.17299578 0.22328061 1 1709 "tags=16%, list=12%, signal=18%"
GSE27291_0H_VS_6H_STIM_GAMMADELTA_TCELL_DN
GSE27291_0H_VS_6H_STIM_GAMMADELTA_TCELL_DN 117 0.29253155 1.1580535 0.17171717 0.22338384 1 2801 "tags=29%, list=20%, signal=36%"
GSE46143_CTRL_VS_LMP2A_TRANSDUCED_CD10_POS_GC_BCELL_DN
GSE46143_CTRL_VS_LMP2A_TRANSDUCED_CD10_POS_GC_BCELL_DN

147 0.277074 1.1574701 0.16593887 0.22423433
1 2093 "tags=27%, list=15%, signal=31%"
GSE27786_LSK_VS_NKTCELL_DN GSE27786_LSK_VS_NKTCELL_DN
132 0.28461894 1.1565912 0.15303983
0.22554454 1 2234 "tags=27%, list=16%, signal=32%"
GSE37301_MULTIPOTENT_PROGENITOR_VS_GRAN_MONO_PROGENITOR_DN
GSE37301_MULTIPOTENT_PROGENITOR_VS_GRAN_MONO_PROGENITOR_DN
155 0.27586654 1.1559021 0.16666667
0.22655237 1 2081 "tags=28%, list=15%, signal=33%"
GSE11864_UNTREATED_VS_CSF1_PAM3CYS_IN_MAC_UP
GSE11864_UNTREATED_VS_CSF1_PAM3CYS_IN_MAC_UP 125
0.2900843 1.1555433 0.17521368 0.22702377
1 2212 "tags=30%, list=16%, signal=35%"
GSE3982_NEUTROPHIL_VS_BASOPHIL_UP GSE3982_NEUTROPHIL_VS_BASOPHIL_UP
142 0.2790322 1.1551957 0.17634408
0.22747152 1 2121 "tags=25%, list=15%, signal=29%"
GSE29164_UNTREATED_VS_CD8_TCELL_TREATED_MELANOMA_DAY3_UP
GSE29164_UNTREATED_VS_CD8_TCELL_TREATED_MELANOMA_DAY3_UP
150 0.27944398 1.1551279 0.16043957
0.22746246 1 2253 "tags=27%, list=16%, signal=32%"
GSE15215_CD2_POS_VS_NEG_PDC_DN GSE15215_CD2_POS_VS_NEG_PDC_DN
145 0.27886054 1.153933 0.14736842
0.22933407 1 3233 "tags=39%, list=23%, signal=50%"
GSE2770_UNTREATED_VS_IL4_TREATED_ACT_CD4_TCELL_48H_UP
GSE2770_UNTREATED_VS_IL4_TREATED_ACT_CD4_TCELL_48H_UP
145 0.27865154 1.1537318 0.18238993
0.22955637 1 2887 "tags=32%, list=20%, signal=40%"
GSE29618_PRE_VS_DAY7_POST_LAIV_FLU_VACCINE_MONOCYTE_DN
GSE29618_PRE_VS_DAY7_POST_LAIV_FLU_VACCINE_MONOCYTE_DN
132 0.2835915 1.1536729 0.19496855
0.22953752 1 1458 "tags=17%, list=10%, signal=18%"
GSE2585_CTEC_VS_THYMIC_DC_DN GSE2585_CTEC_VS_THYMIC_DC_DN
154 0.2796533 1.1529799 0.15789473
0.23058766 1 1865 "tags=16%, list=13%, signal=19%"
GSE21063_CTRL_VS_ANTI_IGM_STIM_BCELL_NFATC1_KO_16H_DN
GSE21063_CTRL_VS_ANTI_IGM_STIM_BCELL_NFATC1_KO_16H_DN
149 0.27295968 1.1526164 0.16255145
0.23108913 1 1898 "tags=22%, list=13%, signal=25%"
GSE6674_UNSTIM_VS_ANTI_IGM_STIM_BCELL_UP
GSE6674_UNSTIM_VS_ANTI_IGM_STIM_BCELL_UP 132
0.28005606 1.1523752 0.19077568 0.23136653
1 2240 "tags=34%, list=16%, signal=40%"
GSE36527_CD69_NEG_VS_POS_TREG_CD62L_LOS_KLRG1_NEG_UP
GSE36527_CD69_NEG_VS_POS_TREG_CD62L_LOS_KLRG1_NEG_UP
156 0.2788363 1.1516235 0.17738791
0.23258194 1 2360 "tags=33%, list=17%, signal=39%"
GSE20715_WT_VS_TLR4_KO_24H_OZONE_LUNG_DN
GSE20715_WT_VS_TLR4_KO_24H_OZONE_LUNG_DN 157
0.2767907 1.1511074 0.19271949 0.23333688
1 2720 "tags=31%, list=19%, signal=38%"
GSE13493_DP_VS_CD4INTCD8POS_THYMOCYTE_DN
GSE13493_DP_VS_CD4INTCD8POS_THYMOCYTE_DN 143
0.27931175 1.1493917 0.17263158 0.23610488
1 2209 "tags=23%, list=16%, signal=27%"

GSE37301_HEMATOPOIETIC_STEM_CELL_VS_PRO_BCELL_UP
GSE37301_HEMATOPOIETIC_STEM_CELL_VS_PRO_BCELL_UP 145
0.27768373 1.1490082 0.15717092 0.23668186
1 2442 "tags=23%, list=17%, signal=27%"
GSE37416_0H_VS_3H_F_TULARENSIS_LVS_NEUTROPHIL_DN
GSE37416_0H_VS_3H_F_TULARENSIS_LVS_NEUTROPHIL_DN 134
0.28222635 1.1485543 0.17355372 0.23732902
1 2457 "tags=33%, list=17%, signal=39%"
GSE360_L_MAJOR_VS_B_MALAYI_LOW_DOSE_MAC_UP
GSE360_L_MAJOR_VS_B_MALAYI_LOW_DOSE_MAC_UP 153
0.27549577 1.1484823 0.17047817 0.23734634
1 2376 "tags=23%, list=17%, signal=27%"
GSE41978_ID2_KO_VS_BIM_KO_KLRG1_LOW_EFFECTOR_CD8_TCELL_DN
GSE41978_ID2_KO_VS_BIM_KO_KLRG1_LOW_EFFECTOR_CD8_TCELL_DN
149 0.2802002 1.1482117 0.16432866
0.23769219 1 2647 "tags=26%, list=19%, signal=32%"
GSE17721_CTRL_VS_POLYIC_8H_BMDC_DN
GSE17721_CTRL_VS_POLYIC_8H_BMDC_DN 162
0.2725463 1.1472214 0.15833333 0.23933002
1 2043 "tags=20%, list=14%, signal=24%"
GSE27786_CD8_TCELL_VS_ERYTHROBLAST_UP
GSE27786_CD8_TCELL_VS_ERYTHROBLAST_UP 149
0.2773325 1.1468469 0.17922607 0.23986039
1 2351 "tags=29%, list=17%, signal=34%"
GSE41867_DAY8_EFFECTOR_VS_DAY30_MEMORY_CD8_TCELL_LCMV_ARMSTRONG_DN
GSE41867_DAY8_EFFECTOR_VS_DAY30_MEMORY_CD8_TCELL_LCMV_ARMSTRONG_DN
145 0.27787164 1.1467701 0.17475729
0.23988487 1 2224 "tags=24%, list=16%, signal=28%"
GSE1740_MCSF_VS_MCSF_AND_IFNG_DAY2_DERIVED_MACROPHAGE_UP
GSE1740_MCSF_VS_MCSF_AND_IFNG_DAY2_DERIVED_MACROPHAGE_UP
101 0.29070634 1.1462668 0.18958333
0.24062821 1 935 "tags=10%, list=7%, signal=11%"
GSE5099_MONOCYTE_VS_CLASSICAL_M1_MACROPHAGE_UP
GSE5099_MONOCYTE_VS_CLASSICAL_M1_MACROPHAGE_UP 138
0.280507 1.1462631 0.17327766 0.24051878 1
2239 "tags=23%, list=16%, signal=27%"
GSE30971_WBP7_HET_VS_KO_MACROPHAGE_2H_LPS_STIM_UP
GSE30971_WBP7_HET_VS_KO_MACROPHAGE_2H_LPS_STIM_UP 137
0.28025615 1.1458259 0.1719457 0.24115424
1 2242 "tags=28%, list=16%, signal=33%"
GSE39820_CTRL_VS_TGFBETA1_IL6_CD4_TCELL_DN
GSE39820_CTRL_VS_TGFBETA1_IL6_CD4_TCELL_DN 151
0.28012407 1.1449865 0.18201284 0.24248971
1 2359 "tags=32%, list=17%, signal=38%"
GSE17721_0.5H_VS_4H_POLYIC_BMDC_DN
GSE17721_0.5H_VS_4H_POLYIC_BMDC_DN 148
0.27470163 1.1449196 0.16450216 0.2424756
1 2494 "tags=30%, list=18%, signal=37%"
GSE41867_LCMV_ARMSTRONG_VS_CLONE13_DAY8_EFFECTOR_CD8_TCELL_UP
GSE41867_LCMV_ARMSTRONG_VS_CLONE13_DAY8_EFFECTOR_CD8_TCELL_UP
151 0.27889788 1.1448308 0.16701461
0.24253227 1 2511 "tags=30%, list=18%, signal=36%"
GSE40685_TREG_VS_FOXP3_KO_TREG_PRECURSOR_UP
GSE40685_TREG_VS_FOXP3_KO_TREG_PRECURSOR_UP 129

0.2838551	1.1442635	0.1970339	0.24341482
1	2358	"tags=29%, list=17%, signal=34%"
GSE34156_UNTREATED_VS_24H_TLR1_TLR2_LIGAND_TREATED_MONOCYTE_UP
GSE34156_UNTREATED_VS_24H_TLR1_TLR2_LIGAND_TREATED_MONOCYTE_UP
116	0.28492504	1.1442448	0.18655097
0.24332768	1	1662	"tags=19%, list=12%, signal=21%"
GSE43955_TH0_VS_TGFB_IL6_IL23_TH17_ACT_CD4_TCELL_52H_DN
GSE43955_TH0_VS_TGFB_IL6_IL23_TH17_ACT_CD4_TCELL_52H_DN
163	0.27416837	1.1441789	0.17735043
0.24332127	1	2726	"tags=32%, list=19%, signal=39%"
GSE9988_ANTI_TREM1_VS_LOW_LPS_MONOCYTE_UP
GSE9988_ANTI_TREM1_VS_LOW_LPS_MONOCYTE_UP	137
0.2787732	1.1437393	0.18987341	0.24396552
1	2842	"tags=31%, list=20%, signal=38%"
GSE28726_NAIVE_VS_ACTIVATED_CD4_TCELL_UP
GSE28726_NAIVE_VS_ACTIVATED_CD4_TCELL_UP	148
0.27887428	1.143737	0.18875502	0.24385318	1
1904	"tags=22%, list=13%, signal=26%"
GSE14000_UNSTIM_VS_16H_LPS_DC_UP	GSE14000_UNSTIM_VS_16H_LPS_DC_UP
131	0.28225905	1.1437228	0.18410042
0.24375804	1	1988	"tags=28%, list=14%, signal=33%"
GSE32986_CURDLAN_HIGHDOSE_VS_GMCSF_AND_CURDLAN_HIGHDOSE_STIM_DC_UP
GSE32986_CURDLAN_HIGHDOSE_VS_GMCSF_AND_CURDLAN_HIGHDOSE_STIM_DC_UP
142	0.27919835	1.1427628	0.1875
0.24531285	1	1005	"tags=15%, list=7%, signal=16%"
GSE11884_WT_VS_FURIN_KO_NAIVE_CD4_TCELL_DN
GSE11884_WT_VS_FURIN_KO_NAIVE_CD4_TCELL_DN	100
0.29067376	1.1425321	0.18867925	0.24561585
1	1516	"tags=17%, list=11%, signal=19%"
GSE5589_IL6_KO_VS_IL10_KO_LPS_AND_IL10_STIM_MACROPHAGE_45MIN_DN
GSE5589_IL6_KO_VS_IL10_KO_LPS_AND_IL10_STIM_MACROPHAGE_45MIN_DN
149	0.27344882	1.1423587	0.17286652
0.24580163	1	1742	"tags=20%, list=12%, signal=23%"
GSE41867_DAY6_EFFECTOR_VS_DAY30_MEMORY_CD8_TCELL_LCMV_ARMSTRONG_UP
GSE41867_DAY6_EFFECTOR_VS_DAY30_MEMORY_CD8_TCELL_LCMV_ARMSTRONG_UP
144	0.2795294	1.1423053	0.20568928
0.245781	1	2489	"tags=33%, list=18%, signal=40%"
GSE24574_BCL6_LOW_TFH_VS_NAIVE_CD4_TCELL_UP
GSE24574_BCL6_LOW_TFH_VS_NAIVE_CD4_TCELL_UP	157
0.27220172	1.1418082	0.15991902	0.24651074
1	1839	"tags=20%, list=13%, signal=23%"
GSE1112_HY_CD8AB_VS_HY_CD8AA_THYMOCYTE_RTOC_CULTURE_UP
GSE1112_HY_CD8AB_VS_HY_CD8AA_THYMOCYTE_RTOC_CULTURE_UP
108	0.28646937	1.1417587	0.20954357
0.2464679	1	1180	"tags=16%, list=8%, signal=17%"
GSE17721_CTRL_VS_PAM3CSK4_6H_BMDC_UP
GSE17721_CTRL_VS_PAM3CSK4_6H_BMDC_UP	147
0.2802259	1.1408608	0.1754386	0.24798194
1	1876	"tags=22%, list=13%, signal=26%"
GSE2770_TGFB_AND_IL4_VS_IL12_TREATED_ACT_CD4_TCELL_2H_UP
GSE2770_TGFB_AND_IL4_VS_IL12_TREATED_ACT_CD4_TCELL_2H_UP
121	0.28298646	1.1408565	0.19789474
0.24787048	1	2689	"tags=31%, list=19%, signal=37%"
GSE20727_ROS_INH_VS_ROS_INH_AND_DNFB_ALLERGEN_TREATED_DC_UP

GSE20727_ROS_INH_VS_ROS_INH_AND_DNFB_ALLERGEN_TREATED_DC_UP
139	0.27446833	1.1407251	0.19139785
0.2479636	1	2350	"tags=25%, list=17%, signal=30%"
GSE1460_INTRATHYMIC_T_PROGENITOR_VS_DP_THYMOCYTE_UP
GSE1460_INTRATHYMIC_T_PROGENITOR_VS_DP_THYMOCYTE_UP
147	0.2759142	1.1404384	0.16907217
0.24832927	1	1614	"tags=19%, list=11%, signal=21%"
GSE37533_PPARG1_FOXP3_VS_FOXP3_TRANSDUCED_CD4_TCELL_PIOGLITAZONE_TRE
ATED_DN
GSE37533_PPARG1_FOXP3_VS_FOXP3_TRANSDUCED_CD4_TCELL_PIOGLITAZONE_TRE
ATED_DN	145	0.27846766	1.1403677
0.21308017	0.24833514	1	2351	"tags=28%,
list=17%, signal=33%"
GSE17721_LPS_VS_GARDIQUIMOD_8H_BMDC_DN
GSE17721_LPS_VS_GARDIQUIMOD_8H_BMDC_DN	151
0.27307433	1.1397983	0.19172113	0.24921486
1	2347	"tags=26%, list=17%, signal=31%"
GSE14000_TRANSLATED_RNA_VS_MRNA_16H_LPS_DC_DN
GSE14000_TRANSLATED_RNA_VS_MRNA_16H_LPS_DC_DN	101
0.2869578	1.1397436	0.17532468	0.24918917
1	2019	"tags=26%, list=14%, signal=30%"
GSE43955_TH0_VS_TGFB_IL6_IL23_TH17_ACT_CD4_TCELL_60H_DN
GSE43955_TH0_VS_TGFB_IL6_IL23_TH17_ACT_CD4_TCELL_60H_DN
152	0.27588025	1.1392145	0.19057377
0.25002486	1	1986	"tags=26%, list=14%, signal=30%"
GSE1112_OT1_VS_HY_CD8AB_THYMOCYTE_RTOC_CULTURE_DN
GSE1112_OT1_VS_HY_CD8AB_THYMOCYTE_RTOC_CULTURE_DN	133
0.2775867	1.1390938	0.19491525	0.2501113
1	2172	"tags=29%, list=15%, signal=33%"
GSE27291_0H_VS_6H_STIM_GAMMADELTA_TCELL_UP
GSE27291_0H_VS_6H_STIM_GAMMADELTA_TCELL_UP	110
0.2883205	1.1386794	0.22546972	0.25073713
1	2735	"tags=29%, list=19%, signal=36%"
GSE8621_UNSTIM_VS_LPS_STIM_MACROPHAGE_UP
GSE8621_UNSTIM_VS_LPS_STIM_MACROPHAGE_UP	140
0.27511817	1.1385461	0.1910828	0.25085545
1	1493	"tags=14%, list=11%, signal=16%"
GSE15659_NAIVE_CD4_TCELL_VS_NONSUPPRESSIVE_TCELL_DN
GSE15659_NAIVE_CD4_TCELL_VS_NONSUPPRESSIVE_TCELL_DN
140	0.27756384	1.1381567	0.19474836
0.25140953	1	1527	"tags=16%, list=11%, signal=18%"
GSE10422_WT_VS_BAFF_TRANSGENIC_LN_BCELL_UP
GSE10422_WT_VS_BAFF_TRANSGENIC_LN_BCELL_UP	132
0.2804258	1.1379803	0.19832985	0.2516137
1	2093	"tags=28%, list=15%, signal=33%"
GSE1740_UNSTIM_VS_IFNA_STIMULATED_MCSF_IFNG_DERIVED_MACROPHAGE_DN
GSE1740_UNSTIM_VS_IFNA_STIMULATED_MCSF_IFNG_DERIVED_MACROPHAGE_DN
	135	0.2723063	1.1374297	0.18526316
0.25249135	1	2106	"tags=22%, list=15%, signal=26%"
GSE21774_CD62L_POS_CD56_DIM_VS_CD62L_NEG_CD56_DIM_NK_CELL_DN
GSE21774_CD62L_POS_CD56_DIM_VS_CD62L_NEG_CD56_DIM_NK_CELL_DN
	145	0.27627552	1.1361454	0.1912088
0.25470966	1	1761	"tags=19%, list=12%, signal=22%"
GSE43955_TH0_VS_TGFB_IL6_IL23_TH17_ACT_CD4_TCELL_60H_UP

GSE43955_TH0_VS_TGFB_IL6_IL23_TH17_ACT_CD4_TCELL_60H_UP
161	0.27236494	1.1346877	0.17634408
0.25714308	1	1438	"tags=17%, list=10%, signal=18%"
GSE29618_LAIV_VS_TIV_FLU_VACCINE_DAY7_PDC_UP
GSE29618_LAIV_VS_TIV_FLU_VACCINE_DAY7_PDC_UP		133
0.2779769	1.1344913	0.18897638	0.25739872
1	1946	"tags=23%, list=14%, signal=26%"
GSE22601_IMMATURE_CD4_SINGLE_POSITIVE_VS_CD4_SINGLE_POSITIVE_THYMOCYTE_DN
GSE22601_IMMATURE_CD4_SINGLE_POSITIVE_VS_CD4_SINGLE_POSITIVE_THYMOCYTE_DN		131	0.27842712	1.1343275
0.22083333	0.25756338	1	2379	"tags=32%, list=17%, signal=38%"
GSE21670_UNTREATED_VS_IL6_TREATED_STAT3_KO_CD4_TCELL_DN
GSE21670_UNTREATED_VS_IL6_TREATED_STAT3_KO_CD4_TCELL_DN
143	0.2750507	1.1341965	0.21975806
0.25766477	1	2422	"tags=28%, list=17%, signal=33%"
GSE37301_HEMATOPOIETIC_STEM_CELL_VS_LYMPHOID_PRIMED_MPP_DN
GSE37301_HEMATOPOIETIC_STEM_CELL_VS_LYMPHOID_PRIMED_MPP_DN
128	0.28051746	1.134046	0.18105263	0.2578064
1	2470	"tags=27%, list=18%, signal=32%"
GSE17721_CPG_VS_GARDIQUIMOD_4H_BMDC_DN
GSE17721_CPG_VS_GARDIQUIMOD_4H_BMDC_DN		152
0.26908535	1.1340419	0.16883117	0.25768614
1	1894	"tags=22%, list=13%, signal=26%"
GSE3982_CTRL_VS_LPS_48H_DC_UP	GSE3982_CTRL_VS_LPS_48H_DC_UP
	131	0.27853954	1.1337614	0.19254659
0.25806805	1	1242	"tags=13%, list=9%, signal=14%"
GSE37301_CD4_TCELL_VS_RAG2_KO_NK_CELL_DN
GSE37301_CD4_TCELL_VS_RAG2_KO_NK_CELL_DN		142
0.27873573	1.1333386	0.20181406	0.25872126
1	2321	"tags=30%, list=16%, signal=35%"
GSE11961_MEMORY_BCELL_DAY7_VS_PLASMA_CELL_DAY7_DN
GSE11961_MEMORY_BCELL_DAY7_VS_PLASMA_CELL_DAY7_DN		147
0.2764768	1.1319634	0.22342733	0.2610807
1	2427	"tags=27%, list=17%, signal=33%"
GSE37532_TREG_VS_TCONV_CD4_TCELL_FROM_LN_DN
GSE37532_TREG_VS_TCONV_CD4_TCELL_FROM_LN_DN		133
0.27743912	1.1319103	0.21042085	0.2610512
1	2004	"tags=26%, list=14%, signal=30%"
GSE43863_LY6C_INT_CXCR5POS_VS_LY6C_LOW_CXCR5NEG_EFFECTOR_CD4_TCELL_UP
GSE43863_LY6C_INT_CXCR5POS_VS_LY6C_LOW_CXCR5NEG_EFFECTOR_CD4_TCELL_UP		155	0.27177107	1.1318285
0.19389978	0.261068	1	1573	"tags=19%, list=11%, signal=21%"
GSE3720_UNSTIM_VS_LPS_STIM_VD2_GAMMADELTA_TCELL_DN
GSE3720_UNSTIM_VS_LPS_STIM_VD2_GAMMADELTA_TCELL_DN		114
0.2913503	1.1317446	0.23770492	0.26109186
1	1964	"tags=21%, list=14%, signal=24%"
GSE26669_CD4_VS_CD8_TCELL_IN_MLR_COSTIM_BLOCK_DN
GSE26669_CD4_VS_CD8_TCELL_IN_MLR_COSTIM_BLOCK_DN		139
0.27472037	1.1312581	0.21311475	0.26183552
1	1686	"tags=20%, list=12%, signal=23%"

GSE26290_WT_VS_PDK1_KO_ANTI_CD3_AND_IL2_STIM_CD8_TCELL_DN
GSE26290_WT_VS_PDK1_KO_ANTI_CD3_AND_IL2_STIM_CD8_TCELL_DN
154 0.27100328 1.1309574 0.18502203
0.26225534 1 2481 "tags=30%, list=18%, signal=36%"
GSE1432_CTRL_VS_IFNG_1H_MICROGLIA_UP
GSE1432_CTRL_VS_IFNG_1H_MICROGLIA_UP 135
0.27429616 1.1305327 0.19088937 0.26294023
1 1718 "tags=21%, list=12%, signal=24%"
GSE6092_UNSTIM_VS_IFNG_STIM_ENDOTHELIAL_CELL_UP
GSE6092_UNSTIM_VS_IFNG_STIM_ENDOTHELIAL_CELL_UP 151
0.27057016 1.1299282 0.2164009 0.26391196
1 2663 "tags=28%, list=19%, signal=35%"
GSE25088_CTRL_VS_ROSIGLITAZONE_STIM_STAT6_KO_MACROPHAGE_UP
GSE25088_CTRL_VS_ROSIGLITAZONE_STIM_STAT6_KO_MACROPHAGE_UP
98 0.28806758 1.1298342 0.24421053
0.26395893 1 1889 "tags=22%, list=13%, signal=26%"
GSE44649_NAIVE_VS_ACTIVATED_CD8_TCELL_MIR155_KO_UP
GSE44649_NAIVE_VS_ACTIVATED_CD8_TCELL_MIR155_KO_UP 158
0.2699613 1.1297195 0.17610063 0.26406428
1 2374 "tags=28%, list=17%, signal=34%"
GSE21927_SPLEEN_VS_C26GM_TUMOR_MONOCYTE_BALBC_DN
GSE21927_SPLEEN_VS_C26GM_TUMOR_MONOCYTE_BALBC_DN 125
0.27862084 1.129561 0.1891348 0.2642335 1
2122 "tags=23%, list=15%, signal=27%"
GSE23308_CTRL_VS_CORTICOSTERONE_TREATED_MACROPHAGE_MINERALCORTICOID_
REC_KO_UP
GSE23308_CTRL_VS_CORTICOSTERONE_TREATED_MACROPHAGE_MINERALCORTICOID_
REC_KO_UP 151 0.27044994 1.1295274
0.171875 0.26416302 1 2317 "tags=30%, list=16%,
signal=36%"
GSE15735_CTRL_VS_HDAC_INHIBITOR_TREATED_CD4_TCELL_12H_DN
GSE15735_CTRL_VS_HDAC_INHIBITOR_TREATED_CD4_TCELL_12H_DN
142 0.2730121 1.1294975 0.2012848 0.264093
1 1621 "tags=25%, list=11%, signal=28%"
GSE22103_UNSTIM_VS_GMCSF_AND_IFNG_STIM_NEUTROPHIL_UP
GSE22103_UNSTIM_VS_GMCSF_AND_IFNG_STIM_NEUTROPHIL_UP
127 0.28013352 1.1291807 0.20250522
0.2645553 1 2644 "tags=33%, list=19%, signal=40%"
GSE28130_ACTIVATED_VS_INDUCEED_TREG_DN
GSE28130_ACTIVATED_VS_INDUCEED_TREG_DN 151
0.2689983 1.1290562 0.18444444 0.2646549
1 807 "tags=11%, list=6%, signal=12%"
GSE2770_TGFB_AND_IL4_VS_IL4_TREATED_ACT_CD4_TCELL_6H_UP
GSE2770_TGFB_AND_IL4_VS_IL4_TREATED_ACT_CD4_TCELL_6H_UP
127 0.2768868 1.1290141 0.2058212
0.2646183 1 2507 "tags=28%, list=18%, signal=34%"
GSE27786_CD8_TCELL_VS_NEUTROPHIL_UP
GSE27786_CD8_TCELL_VS_NEUTROPHIL_UP 140
0.27373245 1.1286917 0.20421052 0.26509267
1 2533 "tags=31%, list=18%, signal=38%"
GSE19888_ADENOSINE_A3R_ACT_VS_TCELL_MEMBRANES_ACT_AND_A3R_INH_PRETRE
AT_IN_MAST_CELL_UP
GSE19888_ADENOSINE_A3R_ACT_VS_TCELL_MEMBRANES_ACT_AND_A3R_INH_PRETRE
AT_IN_MAST_CELL_UP 114 0.28549436 1.128566

0.2178899	0.26520154	1	1768	"tags=20%, list=13%, signal=23%"
GSE43863_NAIVE_VS_LY6C_LOW_CXCR5NEG_CD4_EFF_TCELL_D6_LCMV_DN
GSE43863_NAIVE_VS_LY6C_LOW_CXCR5NEG_CD4_EFF_TCELL_D6_LCMV_DN
	147	0.2702394	1.1280515	0.19690265
0.2660671	1	3342	"tags=33%, list=24%, signal=43%"
GSE37532_WT_VS_PPARG_KO_VISCERAL_ADIPOSE_TISSUE_TCONV_UP
GSE37532_WT_VS_PPARG_KO_VISCERAL_ADIPOSE_TISSUE_TCONV_UP
146	0.27599838	1.1274623	0.20175439
0.2670517	1	2507	"tags=29%, list=18%, signal=35%"
GSE11864_CSF1_PAM3CYS_VS_CSF1_IFNG_PAM3CYS_IN_MAC_DN
GSE11864_CSF1_PAM3CYS_VS_CSF1_IFNG_PAM3CYS_IN_MAC_DN
128	0.27834433	1.1270595	0.19139785
0.26765388	1	1208	"tags=16%, list=9%, signal=18%"
GSE360_L_DONOVANI_VS_T_GONDII_MAC_UP
GSE360_L_DONOVANI_VS_T_GONDII_MAC_UP	147
0.27229452	1.1260954	0.21398304	0.26928186
1	2763	"tags=30%, list=20%, signal=37%"
GSE2197_CPG_DNA_VS_UNTREATED_IN_DC_DN
GSE2197_CPG_DNA_VS_UNTREATED_IN_DC_DN	145
0.27216098	1.1259122	0.22177419	0.2694848
1	1268	"tags=16%, list=9%, signal=17%"
GSE43863_TFH_VS_LY6C_INT_CXCR5POS_EFFECTOR_CD4_TCELL_DN
GSE43863_TFH_VS_LY6C_INT_CXCR5POS_EFFECTOR_CD4_TCELL_DN
151	0.26889944	1.125792	0.18669528	0.2695811
1	1573	"tags=18%, list=11%, signal=20%"
GSE11961_PLASMA_CELL_DAY7_VS_GERMINAL_CENTER_BCELL_DAY40_UP
GSE11961_PLASMA_CELL_DAY7_VS_GERMINAL_CENTER_BCELL_DAY40_UP
141	0.27480373	1.1253563	0.21995927
0.27027488	1	2020	"tags=17%, list=14%, signal=20%"
GSE23321_CD8_STEM_CELL_MEMORY_VS_EFFECTOR_MEMORY_CD8_TCELL_UP
GSE23321_CD8_STEM_CELL_MEMORY_VS_EFFECTOR_MEMORY_CD8_TCELL_UP
	148	0.27223068	1.1240504	0.21666667
0.27261734	1	2403	"tags=29%, list=17%, signal=35%"
GSE2706_R848_VS_LPS_8H_STIM_DC_UP GSE2706_R848_VS_LPS_8H_STIM_DC_UP
	95	0.28692877	1.1240473	0.22981367
0.27249226	1	1800	"tags=17%, list=13%, signal=19%"
GSE27786_CD4_TCELL_VS_ERYTHTROBLAST_UP
GSE27786_CD4_TCELL_VS_ERYTHTROBLAST_UP	147
0.27130875	1.1234443	0.20682302	0.2734967
1	2390	"tags=31%, list=17%, signal=36%"
GSE27786_LIN_NEG_VS_NEUTROPHIL_UP GSE27786_LIN_NEG_VS_NEUTROPHIL_UP
	119	0.28169638	1.1231261	0.21176471
0.2739594	1	2455	"tags=30%, list=17%, signal=36%"
GSE9960_GRAM_POS_VS_GRAM_NEG_AND_POS_SEPSIS_PBMC_DN
GSE9960_GRAM_POS_VS_GRAM_NEG_AND_POS_SEPSIS_PBMC_DN
156	0.26739568	1.1226573	0.1995842
0.2746966	1	2053	"tags=22%, list=15%, signal=25%"
GSE360_T_GONDII_VS_B_MALAYI_HIGH_DOSE_DC_DN
GSE360_T_GONDII_VS_B_MALAYI_HIGH_DOSE_DC_DN	159
0.27001953	1.1223809	0.19427402	0.27507332
1	1869	"tags=22%, list=13%, signal=25%"
GSE17721_POLYIC_VS_PAM3CSK4_2H_BMDC_UP
GSE17721_POLYIC_VS_PAM3CSK4_2H_BMDC_UP	143

0.27108172	1.1222476	0.19795918	0.27519885
1	2041	"tags=24%, list=14%, signal=28%"
GSE29618_MONOCYTE_VS_PDC_DAY7_FLU_VACCINE_DN
GSE29618_MONOCYTE_VS_PDC_DAY7_FLU_VACCINE_DN		150
0.27129865	1.1218994	0.20892495	0.275757 1
1900	"tags=21%, list=13%, signal=24%"
GSE2826_WT_VS_XID_BCELL_DN	GSE2826_WT_VS_XID_BCELL_DN
	164	0.26561394	1.1216809	0.19087137
0.27606857	1	1912	"tags=20%, list=14%, signal=23%"
GSE6674_ANTI_IGM_VS_ANTI_IGM_AND_CPG_STIM_BCELL_DN
GSE6674_ANTI_IGM_VS_ANTI_IGM_AND_CPG_STIM_BCELL_DN	149
0.27030686	1.1214112	0.20159681	0.27646104
1	2388	"tags=30%, list=17%, signal=36%"
GSE22935_WT_VS_MYD88_KO_MACROPHAGE_24H_MBOVIS_BCG_STIM_UP
GSE22935_WT_VS_MYD88_KO_MACROPHAGE_24H_MBOVIS_BCG_STIM_UP
139	0.27465943	1.1210556	0.23312883
0.2770044	1	2559	"tags=29%, list=18%, signal=36%"
GSE4748_CTRL_VS_LPS_STIM_DC_3H_DN GSE4748_CTRL_VS_LPS_STIM_DC_3H_DN
	160	0.2638852	1.1209867	0.21138212
0.2769967	1	2374	"tags=24%, list=17%, signal=29%"
GSE21546_WT_VS_SAP1A_KO_DP_THYMOCYTES_DN
GSE21546_WT_VS_SAP1A_KO_DP_THYMOCYTES_DN	108
0.28067702	1.1208951	0.22559653	0.27702385
1	2094	"tags=23%, list=15%, signal=27%"
GSE23114_PERITONEAL_CAVITY_B1A_BCELL_VS_SPLEEN_BCELL_IN_SLE2C1_MOUSE_UP
GSE23114_PERITONEAL_CAVITY_B1A_BCELL_VS_SPLEEN_BCELL_IN_SLE2C1_MOUSE_UP	153	0.2678941	1.1202372
0.22336066	0.27808997	1	1571	"tags=16%, list=11%, signal=18%"
GSE8835_CD4_VS_CD8_TCELL_UP	GSE8835_CD4_VS_CD8_TCELL_UP
	149	0.27236634	1.1198269	0.21906693
0.27876806	1	2897	"tags=33%, list=21%, signal=41%"
GSE34156_TLR1_TLR2_LIGAND_VS_NOD2_AND_TLR1_TLR2_LIGAND_6H_TREATED_MONOCYTE_UP
GSE34156_TLR1_TLR2_LIGAND_VS_NOD2_AND_TLR1_TLR2_LIGAND_6H_TREATED_MONOCYTE_UP	130	0.2751437	1.1197025
0.20781893	0.27885842	1	2393	"tags=26%, list=17%, signal=31%"
GSE360_DC_VS_MAC_M_TUBERCULOSIS_DN
GSE360_DC_VS_MAC_M_TUBERCULOSIS_DN	147
0.26756567	1.1196516	0.2238193	0.27882844
1	1982	"tags=24%, list=14%, signal=27%"
GSE40274_CTRL_VS_FOXP3_AND_IRF4_TRANSDUCED_ACTIVATED_CD4_TCELL_UP
GSE40274_CTRL_VS_FOXP3_AND_IRF4_TRANSDUCED_ACTIVATED_CD4_TCELL_UP
	120	0.27690738	1.1194528	0.21063395
0.27908072	1	1700	"tags=19%, list=12%, signal=22%"
GSE19198_1H_VS_24H_IL21_TREATED_TCELL_UP
GSE19198_1H_VS_24H_IL21_TREATED_TCELL_UP	153
0.26548585	1.119128 0.22689076	0.27954724	1
1120	"tags=16%, list=8%, signal=18%"
GSE11961_MARGINAL_ZONE_BCELL_VS_MEMORY_BCELL_DAY7_UP
GSE11961_MARGINAL_ZONE_BCELL_VS_MEMORY_BCELL_DAY7_UP
140	0.27069244	1.1174637	0.21802935

0.2825133	1	1498	"tags=17%, list=11%, signal=19%"
GSE3039_CD4_TCELL_VS_B1_BCELL_DN	GSE3039_CD4_TCELL_VS_B1_BCELL_DN
	142	0.27795365	1.1173941	0.24395604
0.2825157	1	1511	"tags=17%, list=11%, signal=19%"
GSE21360_NAIVE_VS_SECONDARY_MEMORY_CD8_TCELL_DN
GSE21360_NAIVE_VS_SECONDARY_MEMORY_CD8_TCELL_DN		137
0.273648	1.1170089	0.2208589	0.28313136	1
1605	"tags=22%, list=11%, signal=24%"
GSE34156_UNTREATED_VS_6H_NOD2_LIGAND_TREATED_MONOCYTE_UP
GSE34156_UNTREATED_VS_6H_NOD2_LIGAND_TREATED_MONOCYTE_UP
141	0.273194	1.1168048	0.22912206	0.28341675
1	2414	"tags=29%, list=17%, signal=35%"
GSE2770_IL12_VS_TGFB_AND_IL12_TREATED_ACT_CD4_TCELL_2H_DN
GSE2770_IL12_VS_TGFB_AND_IL12_TREATED_ACT_CD4_TCELL_2H_DN
124	0.27546877	1.1166613	0.22745901
0.28355592	1	1828	"tags=19%, list=13%, signal=22%"
GSE34156_UNTREATED_VS_24H_NOD2_AND_TLR1_TLR2_LIGAND_TREATED_MONOCYTE_UP
GSE34156_UNTREATED_VS_24H_NOD2_AND_TLR1_TLR2_LIGAND_TREATED_MONOCYTE_UP		119	0.28022242	1.1166569
0.23240939	0.283432	1	1602	"tags=19%, list=11%, signal=22%"
GSE40274_EOS_VS_FOXP3_AND_EOS_TRANSDUCED_ACTIVATED_CD4_TCELL_UP
GSE40274_EOS_VS_FOXP3_AND_EOS_TRANSDUCED_ACTIVATED_CD4_TCELL_UP
	123	0.27765015	1.1165992	0.21888413
0.28342354	1	2560	"tags=29%, list=18%, signal=35%"
GSE22935_WT_VS_MYD88_KO_MACROPHAGE_24H_MBOVIS_BCG_STIM_DN
GSE22935_WT_VS_MYD88_KO_MACROPHAGE_24H_MBOVIS_BCG_STIM_DN
151	0.26787144	1.1163695	0.20876826
0.28373495	1	2003	"tags=25%, list=14%, signal=28%"
GSE7509_DC_VS_MONOCYTE_UP	GSE7509_DC_VS_MONOCYTE_UP		164
0.26571247	1.1153172	0.21978022	0.28568727
1	2293	"tags=25%, list=16%, signal=30%"
GSE19888_ADENOSINE_A3R_ACT_VS_TCELL_MEMBRANES_ACT_IN_MAST_CELL_DN
GSE19888_ADENOSINE_A3R_ACT_VS_TCELL_MEMBRANES_ACT_IN_MAST_CELL_DN
	115	0.28144428	1.1149768	0.24416135
0.28621727	1	1982	"tags=22%, list=14%, signal=25%"
GSE22886_DC_VS_MONOCYTE_UP	GSE22886_DC_VS_MONOCYTE_UP
	145	0.27102882	1.1148081	0.2402464
0.28639874	1	2132	"tags=24%, list=15%, signal=28%"
GSE46606_IRF4HIGH_VS_IRF4MID_CD40L_IL2_IL5_DAY3_STIMULATED_BCELL_UP
GSE46606_IRF4HIGH_VS_IRF4MID_CD40L_IL2_IL5_DAY3_STIMULATED_BCELL_UP
	147	0.26993844	1.1146617	0.22420634
0.28654927	1	1195	"tags=17%, list=8%, signal=18%"
GSE360_L_DONOVANI_VS_M_TUBERCULOSIS_DC_DN
GSE360_L_DONOVANI_VS_M_TUBERCULOSIS_DC_DN	151
0.26968694	1.1145645	0.22083333	0.28661242
1	2424	"tags=29%, list=17%, signal=35%"
GSE3982_CTRL_VS_LPS_4H_MAC_DN	GSE3982_CTRL_VS_LPS_4H_MAC_DN
	160	0.26710144	1.1145213	0.21029083
0.28656512	1	2421	"tags=29%, list=17%, signal=34%"
GSE37605_FOXP3_FUSION_GFP_VS_IRES_GFP_TREG_NOD_DN
GSE37605_FOXP3_FUSION_GFP_VS_IRES_GFP_TREG_NOD_DN		127
0.27858612	1.1144168	0.2275574	0.28662315

1 2361 "tags=30%, list=17%, signal=36%"
GSE22886_CD8_VS_CD4_NAIVE_TCELL_UP
GSE22886_CD8_VS_CD4_NAIVE_TCELL_UP 152
0.26571286 1.1137663 0.23044397 0.28778255
1 2355 "tags=33%, list=17%, signal=39%"
GSE1791_CTRL_VS_NEUROMEDINU_IN_T_CELL_LINE_3H_UP
GSE1791_CTRL_VS_NEUROMEDINU_IN_T_CELL_LINE_3H_UP 102
0.28322008 1.1130936 0.25701943 0.28896812
1 1543 "tags=23%, list=11%, signal=25%"
GSE37605_NOD_VS_C57BL6_IRES_GFP_TREG_DN
GSE37605_NOD_VS_C57BL6_IRES_GFP_TREG_DN 128
0.27421153 1.1125677 0.20726496 0.28983566
1 2422 "tags=25%, list=17%, signal=30%"
GSE13485_DAY7_VS_DAY21_YF17D_VACCINE_PBMC_UP
GSE13485_DAY7_VS_DAY21_YF17D_VACCINE_PBMC_UP 128
0.2706835 1.1121224 0.23516949 0.29053187
1 1876 "tags=20%, list=13%, signal=22%"
GSE11864_UNTREATED_VS_CSF1_IFNG_IN_MAC_UP
GSE11864_UNTREATED_VS_CSF1_IFNG_IN_MAC_UP 136
0.27260992 1.1120397 0.22532189 0.29056504
1 2190 "tags=26%, list=16%, signal=30%"
GSE339_CD4POS_VS_CD8POS_DC_UP GSE339_CD4POS_VS_CD8POS_DC_UP
160 0.26343176 1.1109016 0.23443983
0.2926185 1 2006 "tags=22%, list=14%, signal=25%"
GSE3982_EFF_MEMORY_VS_CENT_MEMORY_CD4_TCELL_UP
GSE3982_EFF_MEMORY_VS_CENT_MEMORY_CD4_TCELL_UP 133
0.27316394 1.1102306 0.22362868 0.29382846
1 1705 "tags=21%, list=12%, signal=24%"
GSE3982_EOSINOPHIL_VS_BASOPHIL_DN GSE3982_EOSINOPHIL_VS_BASOPHIL_DN
130 0.27273476 1.1101494 0.24672489
0.29385108 1 1862 "tags=26%, list=13%, signal=30%"
GSE24726_WT_VS_E2_2_KO_PDC_DN GSE24726_WT_VS_E2_2_KO_PDC_DN
152 0.26555514 1.1099943 0.24678111
0.29403445 1 2648 "tags=34%, list=19%, signal=42%"
GSE22589_HEALTHY_VS_HIV_INFECTED_DC_UP
GSE22589_HEALTHY_VS_HIV_INFECTED_DC_UP 143
0.27471006 1.1093457 0.23780487 0.29517257
1 2551 "tags=31%, list=18%, signal=37%"
GSE26928_CENTR_MEMORY_VS_CXCR5_POS_CD4_TCELL_UP
GSE26928_CENTR_MEMORY_VS_CXCR5_POS_CD4_TCELL_UP 118
0.27868643 1.109101 0.25910065 0.29551247 1
2255 "tags=23%, list=16%, signal=27%"
GSE5589_UNSTIM_VS_45MIN_LPS_AND_IL6_STIM_MACROPHAGE_UP
GSE5589_UNSTIM_VS_45MIN_LPS_AND_IL6_STIM_MACROPHAGE_UP
146 0.26604852 1.1091006 0.2079832
0.2953772 1 2033 "tags=25%, list=14%, signal=29%"
GSE24292_WT_VS_PPARG_KO_MACROPHAGE_UP
GSE24292_WT_VS_PPARG_KO_MACROPHAGE_UP 146
0.26729998 1.1089054 0.265625 0.29564583 1
2710 "tags=33%, list=19%, signal=40%"
GSE21360_PRIMARY_VS_TERTIARY_MEMORY_CD8_TCELL_DN
GSE21360_PRIMARY_VS_TERTIARY_MEMORY_CD8_TCELL_DN 148
0.26894742 1.1087075 0.24404761 0.2958863
1 2469 "tags=32%, list=17%, signal=39%"

GSE28737_FOLLICULAR_VS_MARGINAL_ZONE_BCELL_UP
GSE28737_FOLLICULAR_VS_MARGINAL_ZONE_BCELL_UP 137
0.27065086 1.1086624 0.24042553 0.29582956
1 2130 "tags=23%, list=15%, signal=27%"
GSE6092_IFNG_VS_IFNG_AND_B_BURGDORFERI_INF_ENDOTHELIAL_CELL_DN
GSE6092_IFNG_VS_IFNG_AND_B_BURGDORFERI_INF_ENDOTHELIAL_CELL_DN
142 0.2713753 1.1082748 0.23809524
0.2964233 1 2644 "tags=32%, list=19%, signal=39%"
GSE12198_LOW_IL2_STIM_NK_CELL_VS_HIGH_IL2_STIM_NK_CELL_DN
GSE12198_LOW_IL2_STIM_NK_CELL_VS_HIGH_IL2_STIM_NK_CELL_DN
150 0.26683107 1.10773 0.2238806 0.29738605
1 2648 "tags=25%, list=19%, signal=31%"
GSE32901_TH17_EMRICHED_VS_TH17_NEG_CD4_TCELL_DN
GSE32901_TH17_EMRICHED_VS_TH17_NEG_CD4_TCELL_DN 140
0.27018058 1.107605 0.23044397 0.29749998 1
2191 "tags=25%, list=16%, signal=29%"
GSE13522_CTRL_VS_T_CRUZI_G_STRAIN_INF_SKIN_DN
GSE13522_CTRL_VS_T_CRUZI_G_STRAIN_INF_SKIN_DN 110
0.27549332 1.1075023 0.24197002 0.29756412
1 2295 "tags=25%, list=16%, signal=30%"
GSE1460_DP_THYMOCYTE_VS_THYMIC_STROMAL_CELL_UP
GSE1460_DP_THYMOCYTE_VS_THYMIC_STROMAL_CELL_UP 128
0.27306485 1.1068971 0.26215646 0.29864016
1 2131 "tags=20%, list=15%, signal=24%"
GSE339_EX_VIVO_VS_IN_CULTURE_CD4CD8DN_DC_DN
GSE339_EX_VIVO_VS_IN_CULTURE_CD4CD8DN_DC_DN 158
0.26400188 1.1066422 0.22964509 0.29897973
1 2986 "tags=30%, list=21%, signal=38%"
GSE17974_IL4_AND_ANTI_IL12_VS_UNTREATED_4H_ACT_CD4_TCELL_UP
GSE17974_IL4_AND_ANTI_IL12_VS_UNTREATED_4H_ACT_CD4_TCELL_UP
115 0.27756375 1.1063675 0.25820568
0.29940593 1 1577 "tags=19%, list=11%, signal=21%"
GSE6674_CPG_VS_PL2_3_STIM_BCELL_DN
GSE6674_CPG_VS_PL2_3_STIM_BCELL_DN 140
0.27125233 1.1056926 0.24307036 0.30062133
1 2420 "tags=31%, list=17%, signal=38%"
GSE21927_EL4_VS_MCA203_TUMOR_MONOCYTES_UP
GSE21927_EL4_VS_MCA203_TUMOR_MONOCYTES_UP 117
0.2776316 1.1055009 0.2634855 0.30086267
1 1538 "tags=17%, list=11%, signal=19%"
GSE45365_WT_VS_IFNAR_KO_BCELL_MCMV_INFECTION_UP
GSE45365_WT_VS_IFNAR_KO_BCELL_MCMV_INFECTION_UP 95
0.28396043 1.1049621 0.23777778 0.30177832
1 955 "tags=12%, list=7%, signal=12%"
GSE6269_HEALTHY_VS_FLU_INF_PBMC_UP
GSE6269_HEALTHY_VS_FLU_INF_PBMC_UP 105
0.27604306 1.1047846 0.27196652 0.30201337
1 2699 "tags=36%, list=19%, signal=44%"
GSE15750_WT_VS_TRAF6KO_DAY6_EFF_CD8_TCELL_UP
GSE15750_WT_VS_TRAF6KO_DAY6_EFF_CD8_TCELL_UP 138
0.27052093 1.1047114 0.24364407 0.3020132
1 1977 "tags=22%, list=14%, signal=25%"
GSE3982_DC_VS_CENT_MEMORY_CD4_TCELL_UP
GSE3982_DC_VS_CENT_MEMORY_CD4_TCELL_UP 159

0.26433745	1.1041082	0.23725055	0.30311894
1	2525	"tags=26%, list=18%, signal=31%"
GSE32423_MEMORY_VS_NAIVE_CD8_TCELL_IL7_DN
GSE32423_MEMORY_VS_NAIVE_CD8_TCELL_IL7_DN	144
0.26865998	1.1034893	0.2400881	0.30422643
1	1201	"tags=15%, list=9%, signal=17%"
GSE6566_STRONG_VS_WEAK_DC_STIMULATED_CD4_TCELL_UP
GSE6566_STRONG_VS_WEAK_DC_STIMULATED_CD4_TCELL_UP	114
0.27785954	1.1033297	0.27455357	0.3044088
1	2579	"tags=25%, list=18%, signal=30%"
GSE29617_DAY3_VS_DAY7_TIV_FLU_VACCINE_PBMC_2008_DN
GSE29617_DAY3_VS_DAY7_TIV_FLU_VACCINE_PBMC_2008_DN	101
0.28333142	1.1029091	0.25813448	0.30516496
1	470	"tags=11%, list=3%, signal=11%"
GSE3982_MAC_VS_NKCELL_UP	GSE3982_MAC_VS_NKCELL_UP	151
0.2633421	1.1024238	0.24109015	0.30599555
1	549	"tags=9%, list=4%, signal=9%"
GSE13484_12H_VS_3H_YF17D_VACCINE_STIM_PBMC_DN
GSE13484_12H_VS_3H_YF17D_VACCINE_STIM_PBMC_DN	141
0.2716213	1.1022809	0.26030368	0.30614498
1	1896	"tags=22%, list=13%, signal=25%"
GSE22886_NAIVE_CD4_TCELL_VS_NEUTROPHIL_UP
GSE22886_NAIVE_CD4_TCELL_VS_NEUTROPHIL_UP	143	0.266948
1.1011665	0.23851204	0.30823347	1
2535	"tags=29%, list=18%, signal=35%"
GSE2770_UNTREATED_VS_IL12_TREATED_ACT_CD4_TCELL_48H_UP
GSE2770_UNTREATED_VS_IL12_TREATED_ACT_CD4_TCELL_48H_UP
157	0.26671642	1.1010883	0.24086021
0.3082416	1	1805	"tags=22%, list=13%, signal=25%"
GSE43955_TH0_VS_TGFB_IL6_TH17_ACT_CD4_TCELL_60H_UP
GSE43955_TH0_VS_TGFB_IL6_TH17_ACT_CD4_TCELL_60H_UP	162
0.26657006	1.1010618	0.24307036	0.30816007
1	2034	"tags=20%, list=14%, signal=23%"
GSE17721_LPS_VS_POLYIC_4H_BMDC_UP GSE17721_LPS_VS_POLYIC_4H_BMDC_UP
167	0.25948098	1.1008878	0.23319328
0.30838093	1	2253	"tags=22%, list=16%, signal=25%"
GSE36009_UNSTIM_VS_LPS_STIM_NLRP10_KO_DC_DN
GSE36009_UNSTIM_VS_LPS_STIM_NLRP10_KO_DC_DN	142
0.26443875	1.100808	0.26195425	0.30840054	1
1817	"tags=23%, list=13%, signal=26%"
GSE27786_ERYTHROBLAST_VS_MONO_MAC_DN
GSE27786_ERYTHROBLAST_VS_MONO_MAC_DN	148
0.25982875	1.1002859	0.23168316	0.30927598
1	1825	"tags=22%, list=13%, signal=25%"
GSE1925_CTRL_VS_24H_IFNG_STIM_MACROPHAGE_UP
GSE1925_CTRL_VS_24H_IFNG_STIM_MACROPHAGE_UP	135
0.27266878	1.1002336	0.27108434	0.3092398
1	1686	"tags=19%, list=12%, signal=22%"
GSE31622_WT_VS_KLF3_KO_BCELL_DN	GSE31622_WT_VS_KLF3_KO_BCELL_DN
143	0.26697838	1.1001133	0.23443983
0.30934083	1	2341	"tags=22%, list=17%, signal=27%"
GSE15750_WT_VS_TRAF6KO_DAY10_EFF_CD8_TCELL_UP
GSE15750_WT_VS_TRAF6KO_DAY10_EFF_CD8_TCELL_UP	149
0.2635008	1.09987	0.23188406	0.30968794	1

1301	"tags=19%, list=9%, signal=20%"
GSE14308_TH2_VS_INDUCED_TREG_DN	GSE14308_TH2_VS_INDUCED_TREG_DN
	139	0.26853046	1.0998349	0.25313807
0.30961305	1	2463	"tags=27%, list=17%, signal=32%"
GSE2770_TGFB_AND_IL4_VS_TGFB_AND_IL12_TREATED_ACT_CD4_TCELL_6H_UP
GSE2770_TGFB_AND_IL4_VS_TGFB_AND_IL12_TREATED_ACT_CD4_TCELL_6H_UP
	147	0.26179206	1.0998243	0.2275574
0.3094959	1	1548	"tags=15%, list=11%, signal=17%"
GSE17580_TREG_VS_TEFF_DN	GSE17580_TREG_VS_TEFF_DN	151
0.26918834	1.0996484	0.2473573	0.30970106
1	2175	"tags=25%, list=15%, signal=29%"
GSE20366_CD103_KLRG1_DP_VS_DN_TREG_DN
GSE20366_CD103_KLRG1_DP_VS_DN_TREG_DN	134
0.2671348	1.099545	0.25274727	0.3097691	1
1282	"tags=19%, list=9%, signal=21%"
GSE7460_FOXP3_MUT_VS_WT_ACT_WITH_TGFB_TCONV_UP
GSE7460_FOXP3_MUT_VS_WT_ACT_WITH_TGFB_TCONV_UP	151
0.26359478	1.0993097	0.24481328	0.3100935
1	1795	"tags=22%, list=13%, signal=25%"
GSE29615_CTRL_VS_LAIV_FLU_VACCINE_PBMC_UP
GSE29615_CTRL_VS_LAIV_FLU_VACCINE_PBMC_UP	122
0.2722751	1.0989357	0.25540274	0.31071955
1	1623	"tags=18%, list=12%, signal=20%"
GSE21360_TERTIARY_VS_QUATERNARY_MEMORY_CD8_TCELL_UP
GSE21360_TERTIARY_VS_QUATERNARY_MEMORY_CD8_TCELL_UP
152	0.26343662	1.0978888	0.25523013
0.31269404	1	1054	"tags=14%, list=7%, signal=15%"
GSE27786_NKTCELL_VS_MONO_MAC_UP	GSE27786_NKTCELL_VS_MONO_MAC_UP
	139	0.26921105	1.0974754	0.25462013
0.31343052	1	2792	"tags=37%, list=20%, signal=45%"
GSE22886_CD8_VS_CD4_NAIVE_TCELL_DN
GSE22886_CD8_VS_CD4_NAIVE_TCELL_DN	129
0.26793116	1.0972093	0.26652452	0.31382594
1	2145	"tags=22%, list=15%, signal=25%"
GSE6259_CD4_TCELL_VS_CD8_TCELL_DN	GSE6259_CD4_TCELL_VS_CD8_TCELL_DN
	140	0.2663111	1.0971485	0.24733475
0.31379333	1	2359	"tags=24%, list=17%, signal=29%"
GSE34006_WT_VS_A2AR_KO_TREG_UP	GSE34006_WT_VS_A2AR_KO_TREG_UP
	158	0.2665835	1.0971372	0.234375
0.3136714	1	2338	"tags=25%, list=17%, signal=30%"
GSE5960_TH1_VS_ANERGIC_TH1_UP	GSE5960_TH1_VS_ANERGIC_TH1_UP
	156	0.26202857	1.0963942	0.24603175
0.3150282	1	2975	"tags=35%, list=21%, signal=44%"
GSE26343_UNSTIM_VS_LPS_STIM_MACROPHAGE_DN
GSE26343_UNSTIM_VS_LPS_STIM_MACROPHAGE_DN	151
0.26555672	1.0955803	0.25054467	0.3165892
1	1683	"tags=19%, list=12%, signal=22%"
GSE17721_POLYIC_VS_CPG_24H_BMDC_UP
GSE17721_POLYIC_VS_CPG_24H_BMDC_UP	151
0.26177788	1.0934893	0.2753304	0.32077473
1	1950	"tags=23%, list=14%, signal=26%"
GSE37533_UNTREATED_VS_PIOGLIZATONE_TREATED_CD4_TCELL_FOXP3_TRASDUCED_CD4_TCELL_DN
GSE37533_UNTREATED_VS_PIOGLIZATONE_TREATED_CD4_TCELL_FOXP3_TRASDUCED

_CD4_TCELL_DN		111	0.27627358	1.0933162
0.26694915	0.3209926	1	2327	"tags=23%,
list=16%, signal=28%"
GSE37301_COMMON_LYMPHOID_PROGENITOR_VS_RAG2_KO_NK_CELL_UP
GSE37301_COMMON_LYMPHOID_PROGENITOR_VS_RAG2_KO_NK_CELL_UP
142	0.2623924	1.0924032	0.2290749
0.32275632	1	1662	"tags=16%, list=12%, signal=18%"
GSE17721_LPS_VS_CPG_2H_BMDC_UP	GSE17721_LPS_VS_CPG_2H_BMDC_UP
	156	0.2589079	1.0922083	0.2734694
0.32300943	1	1726	"tags=18%, list=12%, signal=20%"
GSE17721_CTRL_VS_PAM3CSK4_0.5H_BMDC_DN
GSE17721_CTRL_VS_PAM3CSK4_0.5H_BMDC_DN		158
0.25952414	1.0921612	0.24946696	0.3229626
1	1171	"tags=12%, list=8%, signal=13%"
GSE19888_CTRL_VS_A3R_ACT_TREATED_MAST_CELL_PRETREATED_WITH_A3R_INH_D
N
GSE19888_CTRL_VS_A3R_ACT_TREATED_MAST_CELL_PRETREATED_WITH_A3R_INH_D
N		150	0.2634072	1.0916283
0.24944812	0.32386854	1	834	"tags=11%,
list=6%, signal=12%"
GSE3982_MEMORY_CD4_TCELL_VS_BCELL_DN
GSE3982_MEMORY_CD4_TCELL_VS_BCELL_DN		136
0.26462945	1.0915972	0.24554455	0.3237833
1	2232	"tags=25%, list=16%, signal=29%"
GSE41176_WT_VS_TAK1_KO_ANTI_IGM_STIM_BCELL_6H_DN
GSE41176_WT_VS_TAK1_KO_ANTI_IGM_STIM_BCELL_6H_DN		159
0.26073137	1.0915415	0.25475284	0.32374012
1	2442	"tags=29%, list=17%, signal=35%"
GSE21063_3H_VS_16H_ANTI_IGM_STIM_NFATC1_KOBCELL_UP
GSE21063_3H_VS_16H_ANTI_IGM_STIM_NFATC1_KOBCELL_UP		144
0.26331398	1.0911008	0.2699029	0.32451257
1	2003	"tags=22%, list=14%, signal=26%"
GSE22886_NAIVE_CD4_TCELL_VS_12H_ACT_TH1_UP
GSE22886_NAIVE_CD4_TCELL_VS_12H_ACT_TH1_UP	136
0.2679285	1.0910946	0.25894737	0.32438222
1	1788	"tags=19%, list=13%, signal=22%"
GSE15659_CD45RA_NEG_CD4_TCELL_VS_RESTING_TREG_UP
GSE15659_CD45RA_NEG_CD4_TCELL_VS_RESTING_TREG_UP		121
0.2709425	1.0910826	0.29957807	0.32426304
1	2543	"tags=24%, list=18%, signal=29%"
GSE17721_PAM3CSK4_VS_GADIQUIMOD_8H_BMDC_DN
GSE17721_PAM3CSK4_VS_GADIQUIMOD_8H_BMDC_DN	155
0.2647925	1.0910609	0.25979382	0.3241574
1	1251	"tags=15%, list=9%, signal=17%"
GSE37416_12H_VS_48H_F_TULARENSIS_LVS_NEUTROPHIL_DN
GSE37416_12H_VS_48H_F_TULARENSIS_LVS_NEUTROPHIL_DN		106
0.27406508	1.0907872	0.27272728	0.32461	1
1685	"tags=21%, list=12%, signal=23%"
GSE42021_CD24INT_VS_CD24LOW_TREG_THYMUS_UP
GSE42021_CD24INT_VS_CD24LOW_TREG_THYMUS_UP	135
0.27050573	1.0907857	0.28902954	0.32446608
1	1809	"tags=19%, list=13%, signal=21%"
GSE46606_DAY1_VS_DAY3_CD40L_IL2_IL5_STIMULATED_IRF4HIGH_BCELL_DN
GSE46606_DAY1_VS_DAY3_CD40L_IL2_IL5_STIMULATED_IRF4HIGH_BCELL_DN

137	0.26507363	1.0900663	0.27364185
0.32584223	1	2520	"tags=31%, list=18%, signal=37%"
GSE28737_WT_VS_BCL6_HET_FOLLICULAR_BCELL_DN
GSE28737_WT_VS_BCL6_HET_FOLLICULAR_BCELL_DN	140
0.26398033	1.0899172	0.23858921	0.32602412
1	2142	"tags=23%, list=15%, signal=27%"
GSE26488_WT_VS_HDAC7_KO_DOUBLE_POSITIVE_THYMOCYTE_DN
GSE26488_WT_VS_HDAC7_KO_DOUBLE_POSITIVE_THYMOCYTE_DN
126	0.268746	1.0893657	0.26565874	0.3270404
1	2521	"tags=29%, list=18%, signal=35%"
GSE46606_UNSTIM_VS_CD40L_IL2_IL5_3DAY_STIMULATED_IRF4MID_SORTED_BCEL
L_UP
GSE46606_UNSTIM_VS_CD40L_IL2_IL5_3DAY_STIMULATED_IRF4MID_SORTED_BCEL
L_UP	146	0.26271683	1.0889076
0.30470347	0.3278445	1	2655	"tags=34%,
list=19%, signal=41%"
GSE41867_DAY8_EFFECTOR_VS_DAY30_EXHAUSTED_CD8_TCELL_LCMV_CLONE13_DN
GSE41867_DAY8_EFFECTOR_VS_DAY30_EXHAUSTED_CD8_TCELL_LCMV_CLONE13_DN
152	0.26165277	1.0887755	0.26947367
0.32796207	1	2565	"tags=30%, list=18%, signal=37%"
GSE32986_UNSTIM_VS_GMCSF_STIM_DC_DN
GSE32986_UNSTIM_VS_GMCSF_STIM_DC_DN	134
0.26306704	1.0886229	0.27542374	0.3281385
1	2668	"tags=29%, list=19%, signal=36%"
GSE40274_CTRL_VS_LEF1_TRANSDUCED_ACTIVATED_CD4_TCELL_DN
GSE40274_CTRL_VS_LEF1_TRANSDUCED_ACTIVATED_CD4_TCELL_DN
133	0.27083677	1.0885977	0.28658536
0.32805198	1	1612	"tags=19%, list=11%, signal=21%"
GSE10463_CD40L_AND_VA347_VS_CD40L_IN_DC_UP
GSE10463_CD40L_AND_VA347_VS_CD40L_IN_DC_UP	115
0.27107525	1.0885061	0.2811245	0.32810104
1	1913	"tags=18%, list=14%, signal=21%"
GSE32986_UNSTIM_VS_CURDLAN_HIGHDOSE_STIM_DC_DN
GSE32986_UNSTIM_VS_CURDLAN_HIGHDOSE_STIM_DC_DN	126
0.26973274	1.0884197	0.27991885	0.32812324
1	1891	"tags=20%, list=13%, signal=23%"
GSE27786_LIN_NEG_VS_MONO_MAC_DN	GSE27786_LIN_NEG_VS_MONO_MAC_DN
149	0.2637573	1.0882334	0.28542915
0.3283775	1	2837	"tags=30%, list=20%, signal=37%"
GSE360_CTRL_VS_M_TUBERCULOSIS_DC_DN
GSE360_CTRL_VS_M_TUBERCULOSIS_DC_DN	152
0.2597405	1.0879109	0.24740125	0.32890302
1	2010	"tags=24%, list=14%, signal=28%"
GSE16385_MONOCYTE_VS_12H_ROSIGLITAZONE_IL4_TREATED_MACROPHAGE_UP
GSE16385_MONOCYTE_VS_12H_ROSIGLITAZONE_IL4_TREATED_MACROPHAGE_UP
140	0.2645882	1.0878592	0.25431034
0.32886857	1	2468	"tags=28%, list=17%, signal=33%"
GSE22886_NAIVE_CD4_TCELL_VS_NKCELL_UP
GSE22886_NAIVE_CD4_TCELL_VS_NKCELL_UP	140
0.26337084	1.0878422	0.28270042	0.32876045
1	1484	"tags=18%, list=11%, signal=20%"
GSE21927_SPLEEN_VS_TUMOR_MONOCYTE_BALBC_UP
GSE21927_SPLEEN_VS_TUMOR_MONOCYTE_BALBC_UP	101
0.27338788	1.0874687	0.27623126	0.32940516

1	1159	"tags=14%, list=8%, signal=15%"
GSE3982_DC_VS_BASOPHIL_UP	GSE3982_DC_VS_BASOPHIL_UP		148
0.26544318	1.0862849	0.26349893	0.33180073
1	2121	"tags=24%, list=15%, signal=28%"
GSE45365_CD8A_DC_VS_CD11B_DC_DN	GSE45365_CD8A_DC_VS_CD11B_DC_DN
	125	0.26835567	1.0857424	0.28817204
0.33280495	1	2513	"tags=27%, list=18%, signal=33%"
GSE2770_TGFB_AND_IL4_VS_IL4_TREATED_ACT_CD4_TCELL_48H_UP
GSE2770_TGFB_AND_IL4_VS_IL4_TREATED_ACT_CD4_TCELL_48H_UP
143	0.2589523	1.0855951	0.25465837	0.332947
1	2223	"tags=27%, list=16%, signal=32%"
GSE34156_TLR1_TLR2_LIGAND_VS_NOD2_AND_TLR1_TLR2_LIGAND_24H_TREATED_MONOCYTE_UP
GSE34156_TLR1_TLR2_LIGAND_VS_NOD2_AND_TLR1_TLR2_LIGAND_24H_TREATED_MONOCYTE_UP		140	0.26503938	1.0855722
0.2611336	0.33285987	1	1307	"tags=17%, list=9%, signal=19%"
GSE45365_NK_CELL_VS_CD8A_DC_MCMV_INFECTION_DN
GSE45365_NK_CELL_VS_CD8A_DC_MCMV_INFECTION_DN		134
0.26343736	1.0843167	0.25150904	0.33546287
1	1490	"tags=16%, list=11%, signal=18%"
GSE15659_CD45RA_NEG_CD4_TCELL_VS_NONSUPPRESSIVE_TCELL_DN
GSE15659_CD45RA_NEG_CD4_TCELL_VS_NONSUPPRESSIVE_TCELL_DN
139	0.26851383	1.0843165	0.27982646
0.33531275	1	2091	"tags=20%, list=15%, signal=23%"
GSE33424_CD161_INT_VS_NEG_CD8_TCELL_DN
GSE33424_CD161_INT_VS_NEG_CD8_TCELL_DN		148
0.2595392	1.084296	0.24631579	0.33520707	1
2612	"tags=31%, list=19%, signal=38%"
GSE5589_IL6_KO_VS_IL10_KO_LPS_STIM_MACROPHAGE_45MIN_UP
GSE5589_IL6_KO_VS_IL10_KO_LPS_STIM_MACROPHAGE_45MIN_UP
136	0.26417893	1.0841984	0.29723993
0.3352607	1	2119	"tags=21%, list=15%, signal=25%"
GSE16450_IMMATURE_VS_MATURE_NEURON_CELL_LINE_6H_IFNA_STIM_DN
GSE16450_IMMATURE_VS_MATURE_NEURON_CELL_LINE_6H_IFNA_STIM_DN
	148	0.2622647	1.0835521	0.2733051
0.3364635	1	2429	"tags=24%, list=17%, signal=28%"
GSE28737_BCL6_HET_VS_BCL6_KO_FOLLICULAR_BCELL_DN
GSE28737_BCL6_HET_VS_BCL6_KO_FOLLICULAR_BCELL_DN		154
0.25820503	1.0833515	0.26361656	0.33675563
1	1546	"tags=16%, list=11%, signal=18%"
GSE20715_0H_VS_24H_OZONE_LUNG_UP	GSE20715_0H_VS_24H_OZONE_LUNG_UP
	162	0.25816143	1.0832988	0.28309572
0.33671737	1	1825	"tags=22%, list=13%, signal=25%"
GSE10325_LUPUS_CD4_TCELL_VS_LUPUS_MYELOID_UP
GSE10325_LUPUS_CD4_TCELL_VS_LUPUS_MYELOID_UP		146
0.25975505	1.083195	0.23732251	0.33678228	1
2055	"tags=26%, list=15%, signal=30%"
GSE19401_PAM2CSK4_VS_RETINOIC_ACID_AND_PAM2CSK4_STIM_FOLLICULAR_DC_UP
GSE19401_PAM2CSK4_VS_RETINOIC_ACID_AND_PAM2CSK4_STIM_FOLLICULAR_DC_UP		136	0.26598817	1.0830979
0.2829787	0.33683592	1	2053	"tags=20%, list=15%, signal=23%"

GSE9960_HEALTHY_VS_GRAM_NEG_SEPSIS_PBMC_DN
GSE9960_HEALTHY_VS_GRAM_NEG_SEPSIS_PBMC_DN 152
0.2633134 1.0829307 0.28015563 0.33704254
1 2452 "tags=28%, list=17%, signal=34%"
GSE24210_IL35_TREATED_VS_UNTREATED_TCONV_CD4_TCELL_UP
GSE24210_IL35_TREATED_VS_UNTREATED_TCONV_CD4_TCELL_UP
147 0.26216602 1.0826395 0.25760648
0.33751792 1 2373 "tags=28%, list=17%, signal=33%"
GSE21927_SPLENIC_C26GM_TUMOROUS_VS_BONE_MARROW_MONOCYTES_DN
GSE21927_SPLENIC_C26GM_TUMOROUS_VS_BONE_MARROW_MONOCYTES_DN
109 0.2737245 1.0825553 0.28752643
0.33755863 1 2064 "tags=23%, list=15%, signal=27%"
GSE46606_DAY1_VS_DAY3_CD40L_IL2_IL5_STIMULATED_IRF4_KO_BCELL_DN
GSE46606_DAY1_VS_DAY3_CD40L_IL2_IL5_STIMULATED_IRF4_KO_BCELL_DN
138 0.2630813 1.0819741 0.2850679
0.3386657 1 1707 "tags=18%, list=12%, signal=20%"
GSE17721_CPG_VS_GARDIQUIMOD_16H_BMDC_DN
GSE17721_CPG_VS_GARDIQUIMOD_16H_BMDC_DN 149
0.2628957 1.0818584 0.29690722 0.33877894
1 2246 "tags=26%, list=16%, signal=30%"
GSE36476_YOUNG_VS_OLD_DONOR_MEMORY_CD4_TCELL_40H_TSST_ACT_DN
GSE36476_YOUNG_VS_OLD_DONOR_MEMORY_CD4_TCELL_40H_TSST_ACT_DN
140 0.26323926 1.0810772 0.28247422
0.34029266 1 2479 "tags=29%, list=18%, signal=34%"
GSE43955_TH0_VS_TGFB_IL6_TH17_ACT_CD4_TCELL_10H_UP
GSE43955_TH0_VS_TGFB_IL6_TH17_ACT_CD4_TCELL_10H_UP 167
0.25767353 1.0799537 0.27272728 0.3426112
1 2001 "tags=23%, list=14%, signal=26%"
GSE6269_HEALTHY_VS_STAPH_PNEUMO_INF_PBMC_DN
GSE6269_HEALTHY_VS_STAPH_PNEUMO_INF_PBMC_DN 123
0.272062 1.0798521 0.29227558 0.34267038 1
1966 "tags=28%, list=14%, signal=33%"
GSE17721_LPS_VS_PAM3CSK4_4H_BMDC_UP
GSE17721_LPS_VS_PAM3CSK4_4H_BMDC_UP 158
0.25966212 1.0797079 0.26751593 0.3428248
1 2660 "tags=30%, list=19%, signal=36%"
GSE43863_TFH_VS_LY6C_INT_CXCR5POS_MEMORY_CD4_TCELL_DN
GSE43863_TFH_VS_LY6C_INT_CXCR5POS_MEMORY_CD4_TCELL_DN
138 0.26264083 1.0793535 0.28599605
0.34342393 1 2177 "tags=19%, list=15%, signal=22%"
GSE17721_LPS_VS_PAM3CSK4_24H_BMDC_UP
GSE17721_LPS_VS_PAM3CSK4_24H_BMDC_UP 148
0.26130572 1.0792983 0.2780269 0.34338847
1 2543 "tags=32%, list=18%, signal=39%"
GSE17721_PAM3CSK4_VS_CPG_4H_BMDC_UP
GSE17721_PAM3CSK4_VS_CPG_4H_BMDC_UP 127
0.26658693 1.0790445 0.278481 0.34377742 1
1681 "tags=20%, list=12%, signal=23%"
GSE25087_TREG_VS_TCONV_FETUS_UP GSE25087_TREG_VS_TCONV_FETUS_UP
139 0.26046637 1.0789019 0.29310346
0.34392688 1 2742 "tags=32%, list=19%, signal=39%"
GSE7548_NAIVE_VS_DAY7_PCC_IMMUNIZATION_CD4_TCELL_UP
GSE7548_NAIVE_VS_DAY7_PCC_IMMUNIZATION_CD4_TCELL_UP
151 0.26172003 1.078564 0.2647059 0.34450424

1 1985 "tags=24%, list=14%, signal=27%"
GSE3982_DC_VS_MAC_LPS_STIM_DN GSE3982_DC_VS_MAC_LPS_STIM_DN
146 0.26213443 1.0781345 0.26963907
0.34531 1 1674 "tags=19%, list=12%, signal=22%"
GSE23308_WT_VS_MINERALCORTICOID_REC_KO_MACROPHAGE_DN
GSE23308_WT_VS_MINERALCORTICOID_REC_KO_MACROPHAGE_DN
164 0.25617543 1.0772196 0.27004218
0.34713444 1 2249 "tags=22%, list=16%, signal=26%"
GSE9316_CD4_TCELL_BALBC_VS_TH17_ENRI_CD4_TCELL_SKG_PMA_IONO_STIM_FR4NEG_UP
GSE9316_CD4_TCELL_BALBC_VS_TH17_ENRI_CD4_TCELL_SKG_PMA_IONO_STIM_FR4NEG_UP 153 0.2581695 1.0751592
0.29148936 0.35146317 1 2188 "tags=21%, list=16%, signal=24%"
GSE1460_CD4_THYMOCYTE_VS_NAIVE_CD4_TCELL_ADULT_BLOOD_DN
GSE1460_CD4_THYMOCYTE_VS_NAIVE_CD4_TCELL_ADULT_BLOOD_DN
131 0.26862752 1.075116 0.29399142 0.35139722
1 2415 "tags=27%, list=17%, signal=33%"
GSE2826_WT_VS_BTK_KO_BCELL_UP GSE2826_WT_VS_BTK_KO_BCELL_UP
155 0.2608207 1.0750626 0.2742268
0.35136065 1 2118 "tags=26%, list=15%, signal=30%"
GSE6875_WT_VS_FOXP3_KO_TREG_UP GSE6875_WT_VS_FOXP3_KO_TREG_UP
139 0.25990117 1.0743332 0.29130435
0.352765 1 2539 "tags=29%, list=18%, signal=36%"
GSE2585_THYMIC_DC_VS_MTEC_DN GSE2585_THYMIC_DC_VS_MTEC_DN
146 0.2584341 1.073847 0.2915811
0.3536526 1 1733 "tags=19%, list=12%, signal=22%"
GSE40274_CTRL_VS_HELIOS_TRANSDUCED_ACTIVATED_CD4_TCELL_UP
GSE40274_CTRL_VS_HELIOS_TRANSDUCED_ACTIVATED_CD4_TCELL_UP
128 0.26539 1.0737898 0.29805616 0.35362333
1 2361 "tags=28%, list=17%, signal=33%"
GSE32255_WT_VS_JMJD2D_KNOCKDOWN_4H_LPS_STIM_DC_DN
GSE32255_WT_VS_JMJD2D_KNOCKDOWN_4H_LPS_STIM_DC_DN 103
0.2653129 1.0734526 0.31612903 0.3542188
1 1935 "tags=22%, list=14%, signal=26%"
GSE13411_NAIVE_VS_MEMORY_BCELL_UP GSE13411_NAIVE_VS_MEMORY_BCELL_UP
117 0.26931626 1.0730457 0.3014862
0.35492128 1 1280 "tags=14%, list=9%, signal=15%"
GSE36009_WT_VS_NLRP10_KO_DC_DN GSE36009_WT_VS_NLRP10_KO_DC_DN
146 0.25852236 1.0724459 0.2930328
0.35612115 1 2174 "tags=25%, list=15%, signal=29%"
GSE17721_POLYIC_VS_CPG_8H_BMDC_UP GSE17721_POLYIC_VS_CPG_8H_BMDC_UP
148 0.25679436 1.0722065 0.2936345
0.35647798 1 1894 "tags=17%, list=13%, signal=19%"
GSE28737_WT_VS_BCL6_KO_MARGINAL_ZONE_BCELL_DN
GSE28737_WT_VS_BCL6_KO_MARGINAL_ZONE_BCELL_DN 125
0.26561537 1.0717746 0.29885057 0.3573195
1 2632 "tags=30%, list=19%, signal=36%"
GSE17721_CTRL_VS_PAM3CSK4_12H_BMDC_DN
GSE17721_CTRL_VS_PAM3CSK4_12H_BMDC_DN 158
0.25463906 1.0708482 0.2811159 0.3592039
1 1589 "tags=19%, list=11%, signal=21%"
GSE25088_CTRL_VS_IL4_AND_ROSIGLITAZONE_STIM_STAT6_KO_MACROPHAGE_DN
GSE25088_CTRL_VS_IL4_AND_ROSIGLITAZONE_STIM_STAT6_KO_MACROPHAGE_DN

121	0.26527902	1.0706944	0.31673306
0.35937932	1	366	"tags=8%, list=3%, signal=8%"
GSE3982_CTRL_VS_LPS_4H_MAC_UP	GSE3982_CTRL_VS_LPS_4H_MAC_UP
135	0.2615336	1.0692327	0.32489452
0.36253485	1	2037	"tags=21%, list=14%, signal=25%"
GSE42088_UNINF_VS_LEISHMANIA_INF_DC_2H_UP
GSE42088_UNINF_VS_LEISHMANIA_INF_DC_2H_UP	151
0.2562685	1.0689616	0.30620986	0.36295378
1	2424	"tags=30%, list=17%, signal=36%"
GSE29618_LAIV_VS_TIV_FLU_VACCINE_DAY7_BCELL_DN
GSE29618_LAIV_VS_TIV_FLU_VACCINE_DAY7_BCELL_DN	140
0.26000512	1.0689201	0.29795918	0.362882	1
2455	"tags=28%, list=17%, signal=33%"
GSE17721_LPS_VS_CPG_2H_BMDC_DN	GSE17721_LPS_VS_CPG_2H_BMDC_DN
149	0.25597677	1.0676758	0.30590716
0.36550906	1	1363	"tags=14%, list=10%, signal=15%"
GSE36392_TYPE_2_MYELOID_VS_EOSINOPHIL_IL25_TREATED_LUNG_UP
GSE36392_TYPE_2_MYELOID_VS_EOSINOPHIL_IL25_TREATED_LUNG_UP
138	0.26107523	1.0674447	0.2965235
0.36585754	1	2056	"tags=24%, list=15%, signal=28%"
GSE37301_MULTIPOTENT_PROGENITOR_VS_COMMON_LYMPHOID_PROGENITOR_UP
GSE37301_MULTIPOTENT_PROGENITOR_VS_COMMON_LYMPHOID_PROGENITOR_UP
136	0.26104254	1.0664724	0.29273504
0.36791676	1	2373	"tags=31%, list=17%, signal=37%"
GSE15330_HSC_VS_GRANULOCYTE_MONOCYTE_PROGENITOR_UP
GSE15330_HSC_VS_GRANULOCYTE_MONOCYTE_PROGENITOR_UP	146
0.25737077	1.0633792	0.30625	0.3749298	1
2135	"tags=24%, list=15%, signal=28%"
GSE28726_NAIVE_VS_ACTIVATED_NKTCELL_UP
GSE28726_NAIVE_VS_ACTIVATED_NKTCELL_UP	140
0.25828028	1.0633575	0.31453362	0.37481344
1	2205	"tags=21%, list=16%, signal=25%"
GSE22501_PERIPHERAL_BLOOD_VS_CORD_BLOOD_TREG_DN
GSE22501_PERIPHERAL_BLOOD_VS_CORD_BLOOD_TREG_DN	150
0.25789326	1.063301	0.29614604	0.3747793	1
1169	"tags=11%, list=8%, signal=12%"
GSE27786_BCELL_VS_NKTCELL_DN	GSE27786_BCELL_VS_NKTCELL_DN
145	0.25446713	1.0630406	0.30463576
0.37523165	1	1778	"tags=21%, list=13%, signal=24%"
GSE17721_PAM3CSK4_VS_GADIQUIMOD_4H_BMDC_DN
GSE17721_PAM3CSK4_VS_GADIQUIMOD_4H_BMDC_DN	157
0.25538158	1.0629789	0.29012346	0.37520128
1	2831	"tags=33%, list=20%, signal=41%"
GSE5503_LIVER_DC_VS_MLN_DC_ACTIVATED_ALLOGENIC_TCELL_DN
GSE5503_LIVER_DC_VS_MLN_DC_ACTIVATED_ALLOGENIC_TCELL_DN
145	0.2563021	1.0622535	0.30952382
0.37671635	1	2647	"tags=30%, list=19%, signal=37%"
GSE29164_DAY3_VS_DAY7_CD8_TCELL_AND_IL12_TREATED_MELANOMA_DN
GSE29164_DAY3_VS_DAY7_CD8_TCELL_AND_IL12_TREATED_MELANOMA_DN
150	0.2568252	1.0619344	0.3340122
0.3772934	1	1552	"tags=16%, list=11%, signal=18%"
GSE30153_LUPUS_VS_HEALTHY_DONOR_BCELL_DN
GSE30153_LUPUS_VS_HEALTHY_DONOR_BCELL_DN	140
0.2623981	1.0617594	0.3031579	0.3775229

1 1433 "tags=12%, list=10%, signal=13%"
GSE21927_SPLENIC_C26GM_TUMOROUS_VS_4T1_TUMOR_MONOCYTES_DN
GSE21927_SPLENIC_C26GM_TUMOROUS_VS_4T1_TUMOR_MONOCYTES_DN
118 0.26661032 1.0616797 0.32264957
0.37752712 1 1706 "tags=23%, list=12%, signal=26%"
GSE43956_WT_VS_SGK1_KO_TH17_DIFFERENTIATED_CD4_TCELL_UP
GSE43956_WT_VS_SGK1_KO_TH17_DIFFERENTIATED_CD4_TCELL_UP
155 0.25392276 1.0615014 0.30387932
0.37778035 1 2702 "tags=23%, list=19%, signal=28%"
GSE42088_UNINF_VS_LEISHMANIA_INF_DC_24H_DN
GSE42088_UNINF_VS_LEISHMANIA_INF_DC_24H_DN 162
0.25168207 1.0611461 0.32692307 0.37844738
1 2469 "tags=28%, list=17%, signal=33%"
GSE29618_PRE_VS_DAY7_FLU_VACCINE_BCELL_DN
GSE29618_PRE_VS_DAY7_FLU_VACCINE_BCELL_DN 136
0.2553851 1.0602461 0.2905983 0.38030028
1 2205 "tags=25%, list=16%, signal=29%"
GSE10094_LCMV_VS_LISTERIA_IND_EFF_CD4_TCELL_UP
GSE10094_LCMV_VS_LISTERIA_IND_EFF_CD4_TCELL_UP 133
0.26071036 1.0602317 0.3139785 0.38016593
1 1969 "tags=26%, list=14%, signal=29%"
GSE2770_IL4_ACT_VS_ACT_CD4_TCELL_48H_DN
GSE2770_IL4_ACT_VS_ACT_CD4_TCELL_48H_DN 139
0.25719854 1.0602026 0.31625834 0.38007227
1 1583 "tags=14%, list=11%, signal=15%"
GSE20727_CTRL_VS_ROS_INH_AND_DNFB_ALLERGEN_TREATED_DC_UP
GSE20727_CTRL_VS_ROS_INH_AND_DNFB_ALLERGEN_TREATED_DC_UP
137 0.2593841 1.0597967 0.29979035
0.38081494 1 1938 "tags=23%, list=14%, signal=27%"
GSE46025_WT_VS_FOXO1_KO_KLRG1_LOW_CD8_EFFECTOR_TCELL_DN
GSE46025_WT_VS_FOXO1_KO_KLRG1_LOW_CD8_EFFECTOR_TCELL_DN
119 0.2655822 1.0590041 0.32838982
0.38247266 1 2016 "tags=21%, list=14%, signal=24%"
GSE11864_CSF1_PAM3CYS_VS_CSF1_IFNG_PAM3CYS_IN_MAC_UP
GSE11864_CSF1_PAM3CYS_VS_CSF1_IFNG_PAM3CYS_IN_MAC_UP
130 0.25870588 1.0588193 0.33333334
0.38273856 1 2401 "tags=25%, list=17%, signal=29%"
GSE1740_MCSF_VS_MCSF_AND_IFNG_DAY2_DERIVED_MACROPHAGE_WITH_IFNA_STIM
_UP
GSE1740_MCSF_VS_MCSF_AND_IFNG_DAY2_DERIVED_MACROPHAGE_WITH_IFNA_STIM
_UP 133 0.26043138 1.057233 0.31905782
0.38621977 1 2594 "tags=32%, list=18%, signal=39%"
GSE18893_TCONV_VS_TREG_2H_CULTURE_UP
GSE18893_TCONV_VS_TREG_2H_CULTURE_UP 138
0.25496858 1.0570905 0.3067227 0.38639772
1 1666 "tags=18%, list=12%, signal=20%"
GSE37301_HEMATOPOIETIC_STEM_CELL_VS_COMMON_LYMPHOID_PROGENITOR_UP
GSE37301_HEMATOPOIETIC_STEM_CELL_VS_COMMON_LYMPHOID_PROGENITOR_UP
130 0.25404793 1.056241 0.31346577
0.38822407 1 1994 "tags=21%, list=14%, signal=24%"
GSE19772_CTRL_VS_HCMV_INF_MONOCYTES_AND_PI3K_INHIBITION_UP
GSE19772_CTRL_VS_HCMV_INF_MONOCYTES_AND_PI3K_INHIBITION_UP
124 0.26262197 1.0561912 0.33539096
0.3881637 1 2078 "tags=22%, list=15%, signal=25%"

GSE22935_UNSTIM_VS_12H_MBOVIS_BCG_STIM_MACROPHAGE_DN
GSE22935_UNSTIM_VS_12H_MBOVIS_BCG_STIM_MACROPHAGE_DN
151 0.25458238 1.0560949 0.33194155
0.38823414 1 2308 "tags=23%, list=16%, signal=27%"
GSE3920_UNTREATED_VS_IFNA_TREATED_ENDOTHELIAL_CELL_UP
GSE3920_UNTREATED_VS_IFNA_TREATED_ENDOTHELIAL_CELL_UP
147 0.25483954 1.0556899 0.32786885
0.3890297 1 2083 "tags=24%, list=15%, signal=28%"
GSE17721_0.5H_VS_12H_POLYIC_BMDC_DN
GSE17721_0.5H_VS_12H_POLYIC_BMDC_DN 155
0.2536517 1.0554161 0.32007954 0.38950944
1 2042 "tags=24%, list=14%, signal=28%"
GSE43863_TH1_VS_TFH_MEMORY_CD4_TCELL_UP
GSE43863_TH1_VS_TFH_MEMORY_CD4_TCELL_UP 147
0.25449368 1.0550271 0.303719 0.39025292 1
2646 "tags=31%, list=19%, signal=37%"
GSE1460_INTRATHYMIC_T_PROGENITOR_VS_THYMIC_STROMAL_CELL_DN
GSE1460_INTRATHYMIC_T_PROGENITOR_VS_THYMIC_STROMAL_CELL_DN
146 0.25547782 1.0544863 0.32730925
0.39130908 1 1912 "tags=19%, list=14%, signal=22%"
GSE2405_0H_VS_1.5H_A_PHAGOCYTOPHILUM_STIM_NEUTROPHIL_UP
GSE2405_0H_VS_1.5H_A_PHAGOCYTOPHILUM_STIM_NEUTROPHIL_UP
158 0.25067946 1.0544797 0.32489452
0.39115694 1 2718 "tags=25%, list=19%, signal=30%"
GSE21033_CTRL_VS_POLYIC_STIM_DC_1H_UP
GSE21033_CTRL_VS_POLYIC_STIM_DC_1H_UP 132
0.26120096 1.0539966 0.31333333 0.39212126
1 1614 "tags=16%, list=11%, signal=18%"
GSE41176_UNSTIM_VS_ANTI_IGM_STIM_BCELL_1H_DN
GSE41176_UNSTIM_VS_ANTI_IGM_STIM_BCELL_1H_DN 136
0.2573513 1.0539182 0.33620688 0.39212322
1 2083 "tags=27%, list=15%, signal=32%"
GSE21360_SECONDARY_VS_QUATERNARY_MEMORY_CD8_TCELL_DN
GSE21360_SECONDARY_VS_QUATERNARY_MEMORY_CD8_TCELL_DN
136 0.2596063 1.0534761 0.34331337
0.39302173 1 2572 "tags=26%, list=18%, signal=32%"
GSE27859_MACROPHAGE_VS_CD11C_INT_F480_HI_MACROPHAGE_UP
GSE27859_MACROPHAGE_VS_CD11C_INT_F480_HI_MACROPHAGE_UP
136 0.25821877 1.0531707 0.3480084
0.39359328 1 2222 "tags=28%, list=16%, signal=33%"
GSE2770_IL12_VS_TGFB_AND_IL12_TREATED_ACT_CD4_TCELL_2H_UP
GSE2770_IL12_VS_TGFB_AND_IL12_TREATED_ACT_CD4_TCELL_2H_UP
91 0.27405626 1.053066 0.33710408 0.39367878
1 1578 "tags=19%, list=11%, signal=21%"
GSE7460_WT_VS_FOXP3_HET_ACT_WITH_TGFB_TCONV_DN
GSE7460_WT_VS_FOXP3_HET_ACT_WITH_TGFB_TCONV_DN 143
0.2564327 1.0530463 0.31666666 0.39354995
1 1999 "tags=22%, list=14%, signal=25%"
GSE17721_PAM3CSK4_VS_CPG_4H_BMDC_DN
GSE17721_PAM3CSK4_VS_CPG_4H_BMDC_DN 154
0.25174636 1.052983 0.32258064 0.3935374 1
2352 "tags=27%, list=17%, signal=32%"
GSE8621_LPS_STIM_VS_LPS_PRIMED_AND_LPS_STIM_MACROPHAGE_UP
GSE8621_LPS_STIM_VS_LPS_PRIMED_AND_LPS_STIM_MACROPHAGE_UP

154	0.24742395	1.0528336	0.32371134
0.39370018	1	2530	"tags=23%, list=18%, signal=27%"
GSE2770_IL12_ACT_VS_ACT_CD4_TCELL_48H_UP
GSE2770_IL12_ACT_VS_ACT_CD4_TCELL_48H_UP	145
0.25385424	1.0523604	0.32489452	0.39463428
1	3096	"tags=38%, list=22%, signal=48%"
GSE38696_LIGHT_ZONE_VS_DARK_ZONE_BCELL_DN
GSE38696_LIGHT_ZONE_VS_DARK_ZONE_BCELL_DN	105
0.2636316	1.0513619	0.3326316	0.39681107
1	2359	"tags=22%, list=17%, signal=26%"
GSE3982_BCELL_VS_BASOPHIL_UP	GSE3982_BCELL_VS_BASOPHIL_UP
	125	0.25965744	1.0509834	0.3531915
0.3975513	1	2929	"tags=32%, list=21%, signal=40%"
GSE22432_CONVENTIONAL_CDC_VS_PLASMACYTOID_PDC_DN
GSE22432_CONVENTIONAL_CDC_VS_PLASMACYTOID_PDC_DN	153
0.25582474	1.0507989	0.32635984	0.39781135
1	2745	"tags=32%, list=19%, signal=39%"
GSE46606_DAY1_VS_DAY3_CD40L_IL2_IL5_STIMULATED_IRF4HIGH_BCELL_UP
GSE46606_DAY1_VS_DAY3_CD40L_IL2_IL5_STIMULATED_IRF4HIGH_BCELL_UP
	133	0.25524127	1.0505425	0.3204301
0.39823824	1	2018	"tags=23%, list=14%, signal=26%"
GSE5099_DAY3_VS_DAY7_MCSF_TREATED_MACROPHAGE_UP
GSE5099_DAY3_VS_DAY7_MCSF_TREATED_MACROPHAGE_UP	116
0.26171502	1.050464	0.34979424	0.39823943	1
1863	"tags=22%, list=13%, signal=26%"
GSE43863_TFH_VS_LY6C_INT_CXCR5POS_EFFECTOR_CD4_TCELL_UP
GSE43863_TFH_VS_LY6C_INT_CXCR5POS_EFFECTOR_CD4_TCELL_UP
157	0.25024337	1.0503315	0.34745762
0.39837304	1	2482	"tags=24%, list=18%, signal=29%"
GSE21670_UNTREATED_VS_TGFB_TREATED_CD4_TCELL_UP
GSE21670_UNTREATED_VS_TGFB_TREATED_CD4_TCELL_UP	135
0.25781718	1.0501826	0.32832617	0.3985581
1	278	"tags=4%, list=2%, signal=4%"
GSE27786_BCELL_VS_NKCELL_UP	GSE27786_BCELL_VS_NKCELL_UP
	139	0.25560734	1.0500617	0.33119658
0.3986673	1	1925	"tags=18%, list=14%, signal=21%"
GSE21927_BALBC_VS_C57BL6_MONOCYTE_SPLEEN_UP
GSE21927_BALBC_VS_C57BL6_MONOCYTE_SPLEEN_UP	112
0.26432124	1.0499474	0.37313432	0.39875573
1	2033	"tags=26%, list=14%, signal=30%"
GSE11961_PLASMA_CELL_DAY7_VS_GERMINAL_CENTER_BCELL_DAY40_DN
GSE11961_PLASMA_CELL_DAY7_VS_GERMINAL_CENTER_BCELL_DAY40_DN
137	0.25571078	1.0495561	0.33755276
0.39947978	1	1295	"tags=12%, list=9%, signal=14%"
GSE43863_NAIVE_VS_MEMORY_TH1_CD4_TCELL_D150_LCMV_DN
GSE43863_NAIVE_VS_MEMORY_TH1_CD4_TCELL_D150_LCMV_DN
91	0.2701743	1.0491453	0.35176992
0.40027857	1	741	"tags=9%, list=5%, signal=9%"
GSE17721_LPS_VS_GARDIQUIMOD_0.5H_BMDC_UP
GSE17721_LPS_VS_GARDIQUIMOD_0.5H_BMDC_UP	156
0.25134784	1.0488858	0.33541667	0.40075967
1	1991	"tags=24%, list=14%, signal=27%"
GSE3982_DC_VS_BASOPHIL_DN	GSE3982_DC_VS_BASOPHIL_DN	128
0.25861275	1.0486459	0.33194155	0.4011415

1	2776	"tags=33%, list=20%, signal=40%"
GSE13522_CTRL_VS_T_CRUZI_Y_STRAIN_INF_SKIN_IFNG_KO_DN
GSE13522_CTRL_VS_T_CRUZI_Y_STRAIN_INF_SKIN_IFNG_KO_DN
105	0.2653934	1.0483348	0.34632036
0.40166244	1	1871	"tags=19%, list=13%, signal=22%"
GSE17721_LPS_VS_PAM3CSK4_0.5H_BMDC_UP
GSE17721_LPS_VS_PAM3CSK4_0.5H_BMDC_UP	147
0.25539115	1.0482576	0.33264032	0.4016729
1	2479	"tags=23%, list=18%, signal=28%"
GSE17721_LPS_VS_PAM3CSK4_6H_BMDC_UP
GSE17721_LPS_VS_PAM3CSK4_6H_BMDC_UP	160
0.25001404	1.0480868	0.3279678	0.40190843
1	1542	"tags=16%, list=11%, signal=18%"
GSE46606_IRF4HIGH_VS_IRF4MID_CD40L_IL2_IL5_DAY1_STIMULATED_BCELL_DN
GSE46606_IRF4HIGH_VS_IRF4MID_CD40L_IL2_IL5_DAY1_STIMULATED_BCELL_DN
144	0.254018	1.0477607	0.33122364
0.40250006	1	898	"tags=10%, list=6%, signal=11%"
GSE26030_UNSTIM_VS_RESTIM_TH17_DAY5_POST_POLARIZATION_DN
GSE26030_UNSTIM_VS_RESTIM_TH17_DAY5_POST_POLARIZATION_DN
141	0.2548245	1.046811	0.34061137	0.4045423
1	2434	"tags=28%, list=17%, signal=34%"
GSE21033_CTRL_VS_POLYIC_STIM_DC_12H_DN
GSE21033_CTRL_VS_POLYIC_STIM_DC_12H_DN	143
0.25379646	1.0464935	0.32905984	0.40513003
1	2161	"tags=22%, list=15%, signal=25%"
GSE6259_33D1_POS_DC_VS_CD8_TCELL_DN
GSE6259_33D1_POS_DC_VS_CD8_TCELL_DN	99
0.2652023	1.0457486	0.36402568	0.40676278
1	1930	"tags=22%, list=14%, signal=26%"
GSE15930_STIM_VS_STIM_AND_TRICHOSTATINA_72H_CD8_T_CELL_DN
GSE15930_STIM_VS_STIM_AND_TRICHOSTATINA_72H_CD8_T_CELL_DN
158	0.24751125	1.0454569	0.3375	0.40731022
1	2423	"tags=26%, list=17%, signal=31%"
GSE34515_CD16_NEG_MONOCYTE_VS_DC_UP
GSE34515_CD16_NEG_MONOCYTE_VS_DC_UP	147
0.2538714	1.0453198	0.3231441	0.40746832
1	2489	"tags=27%, list=18%, signal=33%"
GSE17721_POLYIC_VS_PAM3CSK4_24H_BMDC_UP
GSE17721_POLYIC_VS_PAM3CSK4_24H_BMDC_UP	153
0.2524682	1.0452322	0.33618844	0.40749168
1	2249	"tags=23%, list=16%, signal=27%"
GSE8621_LPS_PRIMED_UNSTIM_VS_LPS_PRIMED_AND_LPS_STIM_MACROPHAGE_UP
GSE8621_LPS_PRIMED_UNSTIM_VS_LPS_PRIMED_AND_LPS_STIM_MACROPHAGE_UP
152	0.25212657	1.0447445	0.35053763
0.40848902	1	1474	"tags=18%, list=10%, signal=20%"
GSE17721_POLYIC_VS_CPG_1H_BMDC_UP	GSE17721_POLYIC_VS_CPG_1H_BMDC_UP
148	0.25142896	1.0444248	0.34189722
0.40910587	1	2285	"tags=26%, list=16%, signal=31%"
GSE360_DC_VS_MAC_L_MAJOR_DN	GSE360_DC_VS_MAC_L_MAJOR_DN
149	0.25655857	1.0442333	0.36703297
0.40940446	1	1896	"tags=19%, list=13%, signal=21%"
GSE29164_DAY3_VS_DAY7_UNTREATED_MELANOMA_UP
GSE29164_DAY3_VS_DAY7_UNTREATED_MELANOMA_UP	119
0.26175103	1.0436745	0.37744033	0.41059005

1 2239 "tags=24%, list=16%, signal=29%"
GSE45365_CD8A_DC_VS_CD11B_DC_IFNAR_KO_DN
GSE45365_CD8A_DC_VS_CD11B_DC_IFNAR_KO_DN 123
0.2604252 1.0433966 0.36580086 0.41105086
1 2569 "tags=33%, list=18%, signal=40%"
GSE25087_TREG_VS_TCONV_FETUS_DN GSE25087_TREG_VS_TCONV_FETUS_DN
122 0.2582065 1.0433798 0.3531915
0.41090742 1 3020 "tags=39%, list=21%, signal=49%"
GSE360_L_MAJOR_VS_M_TUBERCULOSIS_MAC_UP
GSE360_L_MAJOR_VS_M_TUBERCULOSIS_MAC_UP 142
0.25258452 1.0433036 0.3457557 0.41092813
1 2311 "tags=20%, list=16%, signal=23%"
GSE22432_UNTREATED_VS_TGFB1_TREATED_COMMON_DC_PROGENITOR_UP
GSE22432_UNTREATED_VS_TGFB1_TREATED_COMMON_DC_PROGENITOR_UP
156 0.25121245 1.0429897 0.33808553
0.41152993 1 2417 "tags=26%, list=17%, signal=31%"
GSE22611_NOD2_TRANSD_VS_CTRL_TRANSD_HEK293_MDP_STIM_6H_UP
GSE22611_NOD2_TRANSD_VS_CTRL_TRANSD_HEK293_MDP_STIM_6H_UP
138 0.2538073 1.0422698 0.34745762
0.41307545 1 1400 "tags=14%, list=10%, signal=15%"
GSE3982_CTRL_VS_LPS_1H_NEUTROPHIL_DN
GSE3982_CTRL_VS_LPS_1H_NEUTROPHIL_DN 139
0.25377014 1.0421451 0.33966243 0.4131683
1 1880 "tags=19%, list=13%, signal=21%"
GSE34156_UNTREATED_VS_24H_TLR1_TLR2_LIGAND_TREATED_MONOCYTE_DN
GSE34156_UNTREATED_VS_24H_TLR1_TLR2_LIGAND_TREATED_MONOCYTE_DN
103 0.26842347 1.0420158 0.36784142
0.41331968 1 2388 "tags=26%, list=17%, signal=31%"
GSE46143_CTRL_VS_LMP2A_TRANSDUCED_CD10_POS_GC_BCELL_UP
GSE46143_CTRL_VS_LMP2A_TRANSDUCED_CD10_POS_GC_BCELL_UP
161 0.24840333 1.0419415 0.35112935
0.41333333 1 2151 "tags=19%, list=15%, signal=22%"
GSE18893_TCONV_VS_TREG_2H_TNF_STIM_UP
GSE18893_TCONV_VS_TREG_2H_TNF_STIM_UP 142
0.2536863 1.0419357 0.35331905 0.41317144
1 1337 "tags=14%, list=9%, signal=15%"
GSE37301_MULTIPOTENT_PROGENITOR_VS_CD4_TCELL_UP
GSE37301_MULTIPOTENT_PROGENITOR_VS_CD4_TCELL_UP 141
0.25373992 1.0418038 0.38135594 0.41331896
1 2578 "tags=33%, list=18%, signal=40%"
GSE9960_GRAM_NEG_VS_GRAM_POS_SEPSIS_PBMC_UP
GSE9960_GRAM_NEG_VS_GRAM_POS_SEPSIS_PBMC_UP 134
0.25395885 1.0417324 0.34504133 0.41331196
1 867 "tags=13%, list=6%, signal=13%"
GSE45881_CXCR6HI_VS_CXCR1LO_COLONIC_LAMINA_PROPRIA_DN
GSE45881_CXCR6HI_VS_CXCR1LO_COLONIC_LAMINA_PROPRIA_DN
141 0.2535765 1.0416797 0.36263737
0.4132484 1 2430 "tags=30%, list=17%, signal=36%"
GSE17721_POLYIC_VS_CPG_4H_BMDC_DN GSE17721_POLYIC_VS_CPG_4H_BMDC_DN
144 0.25157475 1.0414551 0.37612614
0.41359925 1 2176 "tags=25%, list=15%, signal=29%"
GSE43955_1H_VS_42H_ACT_CD4_TCELL_WITH_TGFB_IL6_DN
GSE43955_1H_VS_42H_ACT_CD4_TCELL_WITH_TGFB_IL6_DN 159
0.24856013 1.0410537 0.38036808 0.41442475

1	896	"tags=10%, list=6%, signal=11%"
GSE31082_DN_VS_DP_THYMOCYTE_DN	GSE31082_DN_VS_DP_THYMOCYTE_DN
	148	0.2502421	1.0407561	0.36717063
0.4149732	1	2680	"tags=32%, list=19%, signal=39%"
GSE25677_MPL_VS_MPL_AND_R848_STIM_BCELL_UP
GSE25677_MPL_VS_MPL_AND_R848_STIM_BCELL_UP	121
0.25773805	1.0399525	0.37634408	0.41676694
1	2499	"tags=31%, list=18%, signal=37%"
GSE9601_NFKB_INHIBITOR_VS_PI3K_INHIBITOR_TREATED_HCMV_INF_MONOCYTE_UP
GSE9601_NFKB_INHIBITOR_VS_PI3K_INHIBITOR_TREATED_HCMV_INF_MONOCYTE_UP	151	0.25218564	1.0388595
0.36713997	0.41925287	1	2275	"tags=27%, list=16%, signal=32%"
GSE36476_YOUNG_VS_OLD_DONOR_MEMORY_CD4_TCELL_UP
GSE36476_YOUNG_VS_OLD_DONOR_MEMORY_CD4_TCELL_UP	122
0.25818598	1.0385486	0.36637932	0.41983563
1	1106	"tags=13%, list=8%, signal=14%"
GSE17721_LPS_VS_CPG_6H_BMDC_UP	GSE17721_LPS_VS_CPG_6H_BMDC_UP
	166	0.24831499	1.0376905	0.3566879
0.42173386	1	2364	"tags=23%, list=17%, signal=27%"
GSE16385_UNTREATED_VS_12H_IL4_TREATED_MACROPHAGE_UP
GSE16385_UNTREATED_VS_12H_IL4_TREATED_MACROPHAGE_UP
156	0.2494983	1.0376054	0.33950618
0.42176533	1	2221	"tags=22%, list=16%, signal=26%"
GSE18281_CORTEX_VS_MEDULLA_THYMUS_DN
GSE18281_CORTEX_VS_MEDULLA_THYMUS_DN	126
0.25688276	1.0374824	0.35490605	0.4218722
1	1232	"tags=16%, list=9%, signal=17%"
GSE43700_UNTREATED_VS_IL10_TREATED_PBMC_UP
GSE43700_UNTREATED_VS_IL10_TREATED_PBMC_UP	157
0.24736777	1.0370643	0.36034116	0.4226886
1	2360	"tags=22%, list=17%, signal=26%"
GSE17721_PAM3CSK4_VS_GADIQUIMOD_12H_BMDC_DN
GSE17721_PAM3CSK4_VS_GADIQUIMOD_12H_BMDC_DN	157
0.25148827	1.0361794	0.36325678	0.42468533
1	2841	"tags=30%, list=20%, signal=37%"
GSE41867_NAIVE_VS_DAY8_LCMV_ARMSTRONG_EFFECTOR_CD8_TCELL_UP
GSE41867_NAIVE_VS_DAY8_LCMV_ARMSTRONG_EFFECTOR_CD8_TCELL_UP
144	0.2479324	1.0357696	0.38493723
0.42546555	1	2973	"tags=33%, list=21%, signal=41%"
GSE18804_SPLEEN_MACROPHAGE_VS_BRAIN_TUMORAL_MACROPHAGE_DN
GSE18804_SPLEEN_MACROPHAGE_VS_BRAIN_TUMORAL_MACROPHAGE_DN
134	0.25456464	1.0349212	0.3722334
0.4273344	1	2293	"tags=24%, list=16%, signal=28%"
GSE3039_CD4_TCELL_VS_B2_BCELL_DN	GSE3039_CD4_TCELL_VS_B2_BCELL_DN
	146	0.25051454	1.0346745	0.35684648
0.42773074	1	1974	"tags=23%, list=14%, signal=27%"
GSE37301_GRANULOCYTE_MONOCYTE_PROGENITOR_VS_RAG2_KO_NK_CELL_UP
GSE37301_GRANULOCYTE_MONOCYTE_PROGENITOR_VS_RAG2_KO_NK_CELL_UP
	148	0.25202578	1.0346743	0.36477986
0.42755005	1	2024	"tags=24%, list=14%, signal=27%"
GSE17721_LPS_VS_POLYIC_24H_BMDC_DN
GSE17721_LPS_VS_POLYIC_24H_BMDC_DN	150

0.25232923 1.0345911 0.3443299 0.42757145
1 1912 "tags=22%, list=14%, signal=25%"
GSE5589_UNSTIM_VS_45MIN_LPS_AND_IL10_STIM_MACROPHAGE_UP
GSE5589_UNSTIM_VS_45MIN_LPS_AND_IL10_STIM_MACROPHAGE_UP
148 0.24705622 1.0343539 0.375 0.4279781
1 2607 "tags=31%, list=18%, signal=38%"
GSE15930_STIM_VS_STIM_AND_IFNAB_72H_CD8_T_CELL_UP
GSE15930_STIM_VS_STIM_AND_IFNAB_72H_CD8_T_CELL_UP 156
0.24777311 1.03425 0.36514524 0.42804965 1
2290 "tags=22%, list=16%, signal=26%"
GSE17721_LPS_VS_CPG_12H_BMDC_DN GSE17721_LPS_VS_CPG_12H_BMDC_DN
154 0.24398431 1.0331179 0.35639414
0.43066233 1 2393 "tags=21%, list=17%, signal=26%"
GSE7460_FOXP3_MUT_VS_HET_ACT_TCONV_UP
GSE7460_FOXP3_MUT_VS_HET_ACT_TCONV_UP 146 0.249617
1.0330199 0.3838384 0.43072152 1
2502 "tags=28%, list=18%, signal=34%"
GSE8621_UNSTIM_VS_LPS_PRIMED_AND_LPS_STIM_MACROPHAGE_DN
GSE8621_UNSTIM_VS_LPS_PRIMED_AND_LPS_STIM_MACROPHAGE_DN
142 0.24975967 1.033018 0.3716075 0.43054295
1 2018 "tags=23%, list=14%, signal=27%"
GSE23308_WT_VS_MINERALCORTICOID_REC_KO_MACROPHAGE_CORTICOSTERONE_TREATED_UP
GSE23308_WT_VS_MINERALCORTICOID_REC_KO_MACROPHAGE_CORTICOSTERONE_TREATED_UP 145 0.25148076 1.0329621
0.35497835 0.43049568 1 2217 "tags=25%, list=16%, signal=29%"
GSE14769_UNSTIM_VS_120MIN_LPS_BMDM_UP
GSE14769_UNSTIM_VS_120MIN_LPS_BMDM_UP 121
0.25559506 1.0325044 0.37885463 0.4314404
1 1535 "tags=21%, list=11%, signal=23%"
GSE17301_CTRL_VS_48H_ACD3_ACD28_STIM_CD8_TCELL_DN
GSE17301_CTRL_VS_48H_ACD3_ACD28_STIM_CD8_TCELL_DN 159
0.2474238 1.0316238 0.36951983 0.43345907
1 2382 "tags=26%, list=17%, signal=31%"
GSE36891_POLYIC_TLR3_VS_PAM_TLR2_STIM_PERITONEAL_MACROPHAGE_DN
GSE36891_POLYIC_TLR3_VS_PAM_TLR2_STIM_PERITONEAL_MACROPHAGE_DN
130 0.25498727 1.0308609 0.37419355
0.43521303 1 2257 "tags=25%, list=16%, signal=30%"
GSE360_L_DONOVANI_VS_B_MALAYI_HIGH_DOSE_MAC_UP
GSE360_L_DONOVANI_VS_B_MALAYI_HIGH_DOSE_MAC_UP 129
0.25258306 1.0308082 0.36472946 0.43514735
1 1728 "tags=21%, list=12%, signal=24%"
GSE10463_CD40L_AND_VA347_VS_CD40L_IN_DC_DN
GSE10463_CD40L_AND_VA347_VS_CD40L_IN_DC_DN 113
0.25827137 1.0305669 0.37148595 0.43555883
1 1012 "tags=12%, list=7%, signal=12%"
GSE21063_CTRL_VS_ANTI_IGM_STIM_BCELL_3H_DN
GSE21063_CTRL_VS_ANTI_IGM_STIM_BCELL_3H_DN 135
0.2540294 1.0302804 0.39839035 0.4360924
1 2578 "tags=31%, list=18%, signal=38%"
GSE11924_TFH_VS_TH1_CD4_TCELL_UP GSE11924_TFH_VS_TH1_CD4_TCELL_UP
144 0.24712178 1.0302229 0.37708333
0.4360516 1 2454 "tags=29%, list=17%, signal=35%"

GSE6269_HEALTHY_VS_STAPH_AUREUS_INF_PBMC_DN
GSE6269_HEALTHY_VS_STAPH_AUREUS_INF_PBMC_DN 139
0.25155264 1.0301899 0.36460555 0.43595776
1 2530 "tags=23%, list=18%, signal=28%"
GSE13229_IMM_VS_INTMATURE_NKCELL_UP
GSE13229_IMM_VS_INTMATURE_NKCELL_UP 138
0.25177228 1.0294894 0.38266385 0.43749675
1 1668 "tags=17%, list=12%, signal=19%"
GSE2405_0H_VS_24H_A_PHAGOCYTOPHILUM_STIM_NEUTROPHIL_DN
GSE2405_0H_VS_24H_A_PHAGOCYTOPHILUM_STIM_NEUTROPHIL_DN
156 0.24917278 1.0287852 0.38709676
0.43903783 1 1564 "tags=15%, list=11%, signal=16%"
GSE27786_BCELL_VS_NEUTROPHIL_UP GSE27786_BCELL_VS_NEUTROPHIL_UP
143 0.2516243 1.0286422 0.36659878
0.43922952 1 2322 "tags=27%, list=16%, signal=31%"
GSE17301_ACD3_ACD28_VS_ACD3_ACD28_AND_IFNA5_STIM_CD8_TCELL_DN
GSE17301_ACD3_ACD28_VS_ACD3_ACD28_AND_IFNA5_STIM_CD8_TCELL_DN
142 0.24970004 1.0282688 0.3747412
0.4399772 1 2661 "tags=30%, list=19%, signal=37%"
GSE37534_UNTREATED_VS_ROSIGLITAZONE_TREATED_CD4_TCELL_PPARG1_AND_FOX
P3_TRASDUCED_UP
GSE37534_UNTREATED_VS_ROSIGLITAZONE_TREATED_CD4_TCELL_PPARG1_AND_FOX
P3_TRASDUCED_UP 136 0.25125134 1.028143
0.36268345 0.44011042 1 1691 "tags=18%,
list=12%, signal=21%"
GSE17721_CTRL_VS_LPS_24H_BMDC_UP GSE17721_CTRL_VS_LPS_24H_BMDC_UP
150 0.24835075 1.0268308 0.39157894
0.44324538 1 2297 "tags=25%, list=16%, signal=29%"
GSE10325_CD4_TCELL_VS_LUPUS_CD4_TCELL_UP
GSE10325_CD4_TCELL_VS_LUPUS_CD4_TCELL_UP 137
0.25683743 1.0268233 0.39539748 0.44307706
1 1803 "tags=19%, list=13%, signal=22%"
GSE9650_NAIVE_VS_EXHAUSTED_CD8_TCELL_DN
GSE9650_NAIVE_VS_EXHAUSTED_CD8_TCELL_DN 160
0.24196966 1.0267131 0.3910387 0.44315806
1 2018 "tags=24%, list=14%, signal=27%"
GSE6674_UNSTIM_VS_PL2_3_STIM_BCELL_DN
GSE6674_UNSTIM_VS_PL2_3_STIM_BCELL_DN 144
0.2529033 1.0257025 0.4 0.4454426 1
2008 "tags=24%, list=14%, signal=27%"
GSE41867_LCMV_ARMSTRONG_VS_CLONE13_DAY15_EFFECTOR_CD8_TCELL_UP
GSE41867_LCMV_ARMSTRONG_VS_CLONE13_DAY15_EFFECTOR_CD8_TCELL_UP
143 0.24808568 1.0254587 0.38641188
0.44586793 1 2647 "tags=30%, list=19%, signal=37%"
GSE37416_CTRL_VS_3H_F_TULARENSIS_LVS_NEUTROPHIL_DN
GSE37416_CTRL_VS_3H_F_TULARENSIS_LVS_NEUTROPHIL_DN 129
0.25108716 1.0248549 0.39285713 0.44721645
1 3065 "tags=30%, list=22%, signal=38%"
GSE22935_24H_VS_48H_MBOVIS_BCG_STIM_MYD88_KO_MACROPHAGE_UP
GSE22935_24H_VS_48H_MBOVIS_BCG_STIM_MYD88_KO_MACROPHAGE_UP
158 0.24629262 1.0241858 0.35639414
0.44866627 1 1251 "tags=13%, list=9%, signal=14%"
GSE17721_0.5H_VS_24H_POLYIC_BMDC_DN
GSE17721_0.5H_VS_24H_POLYIC_BMDC_DN 149

0.24473006 1.024073 0.39565217 0.44876596 1
2011 "tags=21%, list=14%, signal=25%"
GSE17721_0.5H_VS_12H_GARDIQUIMOD_BMDC_DN
GSE17721_0.5H_VS_12H_GARDIQUIMOD_BMDC_DN 164
0.24275276 1.0240098 0.4054054 0.44873455
1 2058 "tags=19%, list=15%, signal=22%"
GSE30971_2H_VS_4H_LPS_STIM_MACROPHAGE_WBP7_KO_UP
GSE30971_2H_VS_4H_LPS_STIM_MACROPHAGE_WBP7_KO_UP 143
0.24704848 1.0237663 0.41422594 0.4491698
1 2353 "tags=23%, list=17%, signal=27%"
GSE2770_IL12_AND_TGFB_VS_IL4_TREATED_ACT_CD4_TCELL_2H_UP
GSE2770_IL12_AND_TGFB_VS_IL4_TREATED_ACT_CD4_TCELL_2H_UP
141 0.24498451 1.0230376 0.40567952
0.45083427 1 2296 "tags=27%, list=16%, signal=32%"
GSE3982_MAC_VS_BASOPHIL_UP GSE3982_MAC_VS_BASOPHIL_UP
154 0.24434231 1.0229701 0.396728
0.4508012 1 2382 "tags=25%, list=17%, signal=29%"
GSE13229_IMM_VS_INTMATURE_NKCELL_DN
GSE13229_IMM_VS_INTMATURE_NKCELL_DN 143
0.2495325 1.0226665 0.39917696 0.4514 1
2215 "tags=26%, list=16%, signal=30%"
GSE3039_CD4_TCELL_VS_ALPHAALPHA_CD8_TCELL_UP
GSE3039_CD4_TCELL_VS_ALPHAALPHA_CD8_TCELL_UP 157
0.24599104 1.0224842 0.37525773 0.45164943
1 1305 "tags=15%, list=9%, signal=17%"
GSE35685_CD34POS_CD10NEG_CD62LPOS_VS_CD34POS_CD10POS_BONE_MARROW_DN
GSE35685_CD34POS_CD10NEG_CD62LPOS_VS_CD34POS_CD10POS_BONE_MARROW_DN
157 0.24685483 1.0218365 0.3940043
0.45311868 1 2319 "tags=28%, list=16%, signal=33%"
GSE11924_TFH_VS_TH17_CD4_TCELL_UP GSE11924_TFH_VS_TH17_CD4_TCELL_UP
144 0.24524574 1.021311 0.40127388
0.45421144 1 2596 "tags=33%, list=18%, signal=40%"
GSE17721_POLYIC_VS_CPG_12H_BMDC_UP
GSE17721_POLYIC_VS_CPG_12H_BMDC_UP 155
0.24472505 1.0204779 0.410596 0.45610574 1
2419 "tags=28%, list=17%, signal=33%"
GSE20152_SPHK1_KO_VS_HTNFA_OVEREXPRESS_ANKLE_UP
GSE20152_SPHK1_KO_VS_HTNFA_OVEREXPRESS_ANKLE_UP 140
0.24478592 1.0187589 0.3815261 0.46022362
1 1698 "tags=17%, list=12%, signal=19%"
GSE36476_YOUNG_VS_OLD_DONOR_MEMORY_CD4_TCELL_16H_TSST_ACT_UP
GSE36476_YOUNG_VS_OLD_DONOR_MEMORY_CD4_TCELL_16H_TSST_ACT_UP
134 0.24900003 1.0184982 0.40041494
0.4606824 1 2379 "tags=23%, list=17%, signal=28%"
GSE22589_HEALTHY_VS_SIV_INFECTED_DC_UP
GSE22589_HEALTHY_VS_SIV_INFECTED_DC_UP 143
0.2439652 1.0184587 0.3970588 0.46059892
1 2227 "tags=21%, list=16%, signal=25%"
GSE17721_LPS_VS_POLYIC_2H_BMDC_DN GSE17721_LPS_VS_POLYIC_2H_BMDC_DN
137 0.24678577 1.0181859 0.3973799
0.46111426 1 3093 "tags=34%, list=22%, signal=43%"
GSE27786_LIN_NEG_VS_BCELL_DN GSE27786_LIN_NEG_VS_BCELL_DN
130 0.25148013 1.0174775 0.4173387
0.46278352 1 2059 "tags=21%, list=15%, signal=24%"

GSE42021_TREG_PLN_VS_CD24LO_TREG_THYMUS_DN
GSE42021_TREG_PLN_VS_CD24LO_TREG_THYMUS_DN 129
0.25039172 1.0173694 0.4090909 0.46285746
1 1856 "tags=21%, list=13%, signal=24%"
GSE24102_GRANULOCYSTIC_MDSC_VS_NEUTROPHIL_UP
GSE24102_GRANULOCYSTIC_MDSC_VS_NEUTROPHIL_UP 143
0.24530078 1.0172825 0.4057971 0.46288985
1 2682 "tags=27%, list=19%, signal=32%"
GSE25088_CTRL_VS_IL4_STIM_MACROPHAGE_UP
GSE25088_CTRL_VS_IL4_STIM_MACROPHAGE_UP 130
0.25221768 1.0172102 0.43866944 0.46288553
1 875 "tags=12%, list=6%, signal=12%"
GSE9946_IMMATURE_VS_MATURE_STIMULATORY_DC_UP
GSE9946_IMMATURE_VS_MATURE_STIMULATORY_DC_UP 105
0.25654095 1.0170958 0.40248963 0.46297365
1 1795 "tags=20%, list=13%, signal=23%"
GSE5503_MLN_DC_VS_PLN_DC_ACTIVATED_ALLOGENIC_TCELL_DN
GSE5503_MLN_DC_VS_PLN_DC_ACTIVATED_ALLOGENIC_TCELL_DN
142 0.2461193 1.0162505 0.41497976
0.4649037 1 2018 "tags=20%, list=14%, signal=24%"
GSE23505_UNTREATED_VS_4DAY_IL6_IL1_TGFB_TREATED_CD4_TCELL_UP
GSE23505_UNTREATED_VS_4DAY_IL6_IL1_TGFB_TREATED_CD4_TCELL_UP
152 0.24805756 1.0158703 0.410101
0.46569523 1 2228 "tags=24%, list=16%, signal=29%"
GSE21063_WT_VS_NFATC1_KO_BCELL_UP GSE21063_WT_VS_NFATC1_KO_BCELL_UP
152 0.2447484 1.0158589 0.41276595
0.46553454 1 1668 "tags=18%, list=12%, signal=21%"
GSE24142_EARLY_THYMIC_PROGENITOR_VS_DN3_THYMOCYTE_UP
GSE24142_EARLY_THYMIC_PROGENITOR_VS_DN3_THYMOCYTE_UP
163 0.24444029 1.0157714 0.41247484
0.46558288 1 1897 "tags=21%, list=13%, signal=25%"
GSE22342_CD11C_HIGH_VS_LOW_DECIDUAL_MACROPHAGES_UP
GSE22342_CD11C_HIGH_VS_LOW_DECIDUAL_MACROPHAGES_UP 115
0.25176185 1.0156775 0.40733197 0.46562457
1 2712 "tags=34%, list=19%, signal=42%"
GSE3039_CD4_TCELL_VS_NKT_CELL_UP GSE3039_CD4_TCELL_VS_NKT_CELL_UP
155 0.24367675 1.0155658 0.39529914
0.46572074 1 2305 "tags=26%, list=16%, signal=31%"
GSE360_CTRL_VS_B_MALAYI_LOW_DOSE_DC_UP
GSE360_CTRL_VS_B_MALAYI_LOW_DOSE_DC_UP 136
0.24747643 1.0155098 0.42916667 0.46566483
1 1051 "tags=14%, list=7%, signal=15%"
GSE9960_GRAM_NEG_VS_GRAM_NEG_AND_POS_SEPSIS_PBMC_DN
GSE9960_GRAM_NEG_VS_GRAM_NEG_AND_POS_SEPSIS_PBMC_DN
117 0.2534066 1.0152336 0.4047619
0.4661632 1 2702 "tags=32%, list=19%, signal=40%"
GSE2770_IL12_VS_TGFB_AND_IL12_TREATED_ACT_CD4_TCELL_48H_UP
GSE2770_IL12_VS_TGFB_AND_IL12_TREATED_ACT_CD4_TCELL_48H_UP
150 0.242633 1.0149555 0.41649485 0.4666658
1 2856 "tags=33%, list=20%, signal=41%"
GSE17721_CTRL_VS_POLYIC_12H_BMDC_DN
GSE17721_CTRL_VS_POLYIC_12H_BMDC_DN 162
0.23866853 1.0144508 0.42307693 0.46776143
1 1589 "tags=15%, list=11%, signal=17%"

GSE1925_CTRL_VS_24H_IFNG_STIM_IFNG_PRIMED_MACROPHAGE_UP
GSE1925_CTRL_VS_24H_IFNG_STIM_IFNG_PRIMED_MACROPHAGE_UP
158 0.24297947 1.0142666 0.44989774
0.4680173 1 1548 "tags=16%, list=11%, signal=18%"
GSE30971_2H_VS_4H_LPS_STIM_MACROPHAGE_WBP7_KO_DN
GSE30971_2H_VS_4H_LPS_STIM_MACROPHAGE_WBP7_KO_DN 126
0.2479014 1.0139408 0.42793792 0.46862066
1 799 "tags=10%, list=6%, signal=10%"
GSE26030_UNSTIM_VS_RESTIM_TH17_DAY15_POST_POLARIZATION_UP
GSE26030_UNSTIM_VS_RESTIM_TH17_DAY15_POST_POLARIZATION_UP
146 0.24524021 1.0135697 0.39419088
0.4693693 1 2371 "tags=29%, list=17%, signal=35%"
GSE46242_TH1_VS_ANERGIC_TH1_CD4_TCELL_WITH_EGR2_DELETED_UP
GSE46242_TH1_VS_ANERGIC_TH1_CD4_TCELL_WITH_EGR2_DELETED_UP
130 0.25102767 1.0120977 0.42083332
0.47302163 1 2026 "tags=25%, list=14%, signal=28%"
GSE17721_LPS_VS_POLYIC_1H_BMDC_UP GSE17721_LPS_VS_POLYIC_1H_BMDC_UP
156 0.24397948 1.0119963 0.4347826
0.47307587 1 2720 "tags=26%, list=19%, signal=31%"
GSE13411_NAIVE_VS_IGM_MEMORY_BCELL_UP
GSE13411_NAIVE_VS_IGM_MEMORY_BCELL_UP 125
0.25112155 1.0114834 0.4504717 0.47421458
1 1355 "tags=17%, list=10%, signal=18%"
GSE12366_PLASMA_CELL_VS_MEMORY_BCELL_UP
GSE12366_PLASMA_CELL_VS_MEMORY_BCELL_UP 132
0.2520844 1.010754 0.43067226 0.4758546 1
2410 "tags=24%, list=17%, signal=29%"
GSE2585_AIRE_KO_VS_WT_CD80_LOW_MTEC_DN
GSE2585_AIRE_KO_VS_WT_CD80_LOW_MTEC_DN 154
0.24148443 1.0105503 0.42474228 0.47620186
1 1997 "tags=18%, list=14%, signal=21%"
GSE23308_CTRL_VS_CORTICOSTERONE_TREATED_MACROPHAGE_DN
GSE23308_CTRL_VS_CORTICOSTERONE_TREATED_MACROPHAGE_DN
164 0.24042639 1.0100718 0.43157893
0.4772253 1 1668 "tags=17%, list=12%, signal=19%"
GSE25088_WT_VS_STAT6_KO_MACROPHAGE_UP
GSE25088_WT_VS_STAT6_KO_MACROPHAGE_UP 138
0.24737261 1.0099627 0.41983122 0.4773121
1 2037 "tags=24%, list=14%, signal=28%"
GSE9037_CTRL_VS_LPS_1H_STIM_BMDM_UP
GSE9037_CTRL_VS_LPS_1H_STIM_BMDM_UP 122
0.25037616 1.009453 0.41036716 0.47840467 1
1872 "tags=21%, list=13%, signal=24%"
GSE17186_NAIVE_VS_CD21HIGH_TRANSITIONAL_BCELL_CORD_BLOOD_UP
GSE17186_NAIVE_VS_CD21HIGH_TRANSITIONAL_BCELL_CORD_BLOOD_UP
149 0.24301925 1.0094458 0.44676408
0.47822884 1 1832 "tags=23%, list=13%, signal=26%"
GSE46606_IRF4HIGH_VS_WT_CD40L_IL2_IL5_DAY1_STIMULATED_BCELL_DN
GSE46606_IRF4HIGH_VS_WT_CD40L_IL2_IL5_DAY1_STIMULATED_BCELL_DN
145 0.24284878 1.0090365 0.40778688
0.4790687 1 2356 "tags=24%, list=17%, signal=29%"
GSE29164_CD8_TCELL_VS_CD8_TCELL_AND_IL12_TREATED_MELANOMA_DAY3_UP
GSE29164_CD8_TCELL_VS_CD8_TCELL_AND_IL12_TREATED_MELANOMA_DAY3_UP
147 0.24314114 1.0079838 0.42405063

0.4815276	1	3029	"tags=36%, list=21%, signal=45%"
GSE9960_HEALTHY_VS_SEPSIS_PBMC_DN GSE9960_HEALTHY_VS_SEPSIS_PBMC_DN
	97	0.25582147	1.007535 0.39529914
0.4825149	1	1208	"tags=11%, list=9%, signal=12%"
GSE40666_UNTREATED_VS_IFNA_STIM_STAT1_KO_CD8_TCELL_90MIN_UP
GSE40666_UNTREATED_VS_IFNA_STIM_STAT1_KO_CD8_TCELL_90MIN_UP
115	0.25636253	1.0071485	0.4502924
0.48332593	1	2228	"tags=24%, list=16%, signal=29%"
GSE45365_CTRL_VS_MCMV_INFECTION_NK_CELL_DN
GSE45365_CTRL_VS_MCMV_INFECTION_NK_CELL_DN	128
0.24965774	1.0064951	0.41885966	0.4848811
1	1709	"tags=21%, list=12%, signal=24%"
GSE11924_TH1_VS_TH17_CD4_TCELL_UP GSE11924_TH1_VS_TH17_CD4_TCELL_UP
	142	0.24267066	1.0064074	0.42207792
0.48491895	1	1443	"tags=13%, list=10%, signal=14%"
GSE5542_IFNG_VS_IFNA_AND_IFNG_TREATED_EPITHELIAL_CELLS_6H_DN
GSE5542_IFNG_VS_IFNA_AND_IFNG_TREATED_EPITHELIAL_CELLS_6H_DN
	147	0.24621776	1.0061516	0.44067797
0.48536548	1	2735	"tags=31%, list=19%, signal=38%"
GSE36476_YOUNG_VS_OLD_DONOR_MEMORY_CD4_TCELL_72H_TSST_ACT_UP
GSE36476_YOUNG_VS_OLD_DONOR_MEMORY_CD4_TCELL_72H_TSST_ACT_UP
	129	0.24763106	1.005974 0.43951613
0.48560607	1	1273	"tags=12%, list=9%, signal=13%"
GSE3982_CENT_MEMORY_CD4_TCELL_VS_NKCELL_DN
GSE3982_CENT_MEMORY_CD4_TCELL_VS_NKCELL_DN	142
0.24162894	1.0055226	0.42417583	0.48661023
1	2132	"tags=20%, list=15%, signal=23%"
GSE2405_HEAT_KILLED_VS_LIVE_A_PHAGOCYTOPHILUM_STIM_NEUTROPHIL_9H_DN
GSE2405_HEAT_KILLED_VS_LIVE_A_PHAGOCYTOPHILUM_STIM_NEUTROPHIL_9H_DN
	146	0.2397845	1.0052547	0.4248927
0.48708048	1	777	"tags=10%, list=6%, signal=11%"
GSE17721_CTRL_VS_GARDIQUIMOD_12H_BMDC_DN
GSE17721_CTRL_VS_GARDIQUIMOD_12H_BMDC_DN	156
0.24076763	1.0052179	0.42588726	0.48697203
1	1717	"tags=19%, list=12%, signal=21%"
GSE22025_UNTREATED_VS_TGFB1_AND_PROGESTERONE_TREATED_CD4_TCELL_DN
GSE22025_UNTREATED_VS_TGFB1_AND_PROGESTERONE_TREATED_CD4_TCELL_DN
	142	0.24318856	1.0047028	0.46526316
0.48810393	1	1709	"tags=14%, list=12%, signal=16%"
GSE2197_CPG_DNA_VS_UNTREATED_IN_DC_UP
GSE2197_CPG_DNA_VS_UNTREATED_IN_DC_UP	155
0.24006966	1.0043375	0.43067226	0.4888692
1	2707	"tags=31%, list=19%, signal=38%"
GSE360_L_MAJOR_VS_B_MALAYI_HIGH_DOSE_DC_DN
GSE360_L_MAJOR_VS_B_MALAYI_HIGH_DOSE_DC_DN	142
0.24407877	1.0040689	0.41093117	0.4893761
1	2604	"tags=28%, list=18%, signal=34%"
GSE360_DC_VS_MAC_B_MALAYI_LOW_DOSE_DN
GSE360_DC_VS_MAC_B_MALAYI_LOW_DOSE_DN	157
0.24133055	1.0039872	0.4731405	0.48939395
1	1741	"tags=21%, list=12%, signal=24%"
GSE1791_CTRL_VS_NEUROMEDINU_IN_T_CELL_LINE_0.8H_UP
GSE1791_CTRL_VS_NEUROMEDINU_IN_T_CELL_LINE_0.8H_UP	127
0.24798962	1.0036426	0.45396146	0.49008518

1	1177	"tags=13%, list=8%, signal=14%"
GSE360_DC_VS_MAC_UP	GSE360_DC_VS_MAC_UP	135
0.24677931	1.0036378	0.45010182	0.4898991
1	1829	"tags=21%, list=13%, signal=24%"
GSE16522_MEMORY_VS_NAIVE_CD8_TCELL_DN
GSE16522_MEMORY_VS_NAIVE_CD8_TCELL_DN	142
0.2452761	1.0036216	0.4328063	0.4897402
1	1659	"tags=17%, list=12%, signal=19%"
GSE8921_3H_VS_24H_TLR1_2_STIM_MONOCYTE_DN
GSE8921_3H_VS_24H_TLR1_2_STIM_MONOCYTE_DN	143
0.24782121	1.002748 0.4617068	0.4917953	1
2394	"tags=27%, list=17%, signal=33%"
GSE37301_LYMPHOID_PRIMED_MPP_VS_GRAN_MONO_PROGENITOR_UP
GSE37301_LYMPHOID_PRIMED_MPP_VS_GRAN_MONO_PROGENITOR_UP
147	0.2424463	1.0024893	0.4265306
0.49226967	1	2420	"tags=31%, list=17%, signal=37%"
GSE24574_BCL6_HIGH_TFH_VS_TFH_CD4_TCELL_UP
GSE24574_BCL6_HIGH_TFH_VS_TFH_CD4_TCELL_UP	128
0.24609968	1.0024382	0.4453125	0.49220628
1	1692	"tags=17%, list=12%, signal=19%"
GSE16450_CTRL_VS_IFNA_12H_STIM_IMMATURE_NEURON_CELL_LINE_DN
GSE16450_CTRL_VS_IFNA_12H_STIM_IMMATURE_NEURON_CELL_LINE_DN
117	0.25029033	1.0013722	0.4515464
0.4947438	1	1781	"tags=18%, list=13%, signal=20%"
GSE28726_NAIVE_VS_ACTIVATED_VA24NEG_NKTCELL_DN
GSE28726_NAIVE_VS_ACTIVATED_VA24NEG_NKTCELL_DN	141
0.24515302	1.0012052	0.4539749	0.49496064
1	1228	"tags=13%, list=9%, signal=14%"
GSE38696_LIGHT_ZONE_VS_DARK_ZONE_BCELL_UP
GSE38696_LIGHT_ZONE_VS_DARK_ZONE_BCELL_UP	143
0.24253987	1.0011648	0.42765957	0.49486998
1	2458	"tags=26%, list=17%, signal=31%"
GSE22025_UNTREATED_VS_PROGESTERONE_TREATED_CD4_TCELL_UP
GSE22025_UNTREATED_VS_PROGESTERONE_TREATED_CD4_TCELL_UP
143	0.24241033	1.0004976	0.44347826
0.49645683	1	1403	"tags=12%, list=10%, signal=13%"
GSE3982_EOSINOPHIL_VS_NEUTROPHIL_DN
GSE3982_EOSINOPHIL_VS_NEUTROPHIL_DN	133
0.24644299	0.99938625	0.44	0.49913314	1
2043	"tags=20%, list=14%, signal=23%"
GSE27786_LSK_VS_MONO_MAC_UP	GSE27786_LSK_VS_MONO_MAC_UP
130	0.24262333	0.99936604	0.45194274
0.4989776	1	2100	"tags=25%, list=15%, signal=30%"
GSE41978_WT_VS_BIM_KO_KLRG1_LOW_EFFECTOR_CD8_TCELL_DN
GSE41978_WT_VS_BIM_KO_KLRG1_LOW_EFFECTOR_CD8_TCELL_DN
153	0.24108289	0.99924433	0.44698545
0.49908906	1	1749	"tags=19%, list=12%, signal=21%"
GSE30083_SP1_VS_SP4_THYMOCYTE_UP	GSE30083_SP1_VS_SP4_THYMOCYTE_UP
164	0.23655514	0.99858814	0.46296296
0.5006292	1	1769	"tags=18%, list=13%, signal=21%"
GSE14000_4H_VS_16H_LPS_DC_TRANSLATED_RNA_DN
GSE14000_4H_VS_16H_LPS_DC_TRANSLATED_RNA_DN	132
0.24474156	0.9982684	0.4517544	0.5012438
1	1034	"tags=11%, list=7%, signal=12%"

GSE2770_IL12_AND_TGFB_VS_IL4_TREATED_ACT_CD4_TCELL_48H_DN
GSE2770_IL12_AND_TGFB_VS_IL4_TREATED_ACT_CD4_TCELL_48H_DN
102 0.2532137 0.99819815 0.4649123
0.5012408 1 1246 "tags=14%, list=9%, signal=15%"
GSE15330_WT_VS_IKAROS_KO_MEGAKARYOCYTE_ERYTHROID_PROGENITOR_DN
GSE15330_WT_VS_IKAROS_KO_MEGAKARYOCYTE_ERYTHROID_PROGENITOR_DN
153 0.2406653 0.99738973 0.44591612
0.503175 1 2724 "tags=31%, list=19%, signal=38%"
GSE2770_UNTREATED_VS_IL12_TREATED_ACT_CD4_TCELL_48H_DN
GSE2770_UNTREATED_VS_IL12_TREATED_ACT_CD4_TCELL_48H_DN
144 0.23826852 0.9969308 0.45686275
0.5042039 1 2463 "tags=22%, list=17%, signal=27%"
GSE7548_NAIVE_VS_DAY28_PCC_IMMUNIZATION_CD4_TCELL_DN
GSE7548_NAIVE_VS_DAY28_PCC_IMMUNIZATION_CD4_TCELL_DN
141 0.24283776 0.99671483 0.47599164
0.5045788 1 1280 "tags=13%, list=9%, signal=15%"
GSE21927_UNTREATED_VS_GMCSF_IL6_TREATED_BONE_MARROW_DN
GSE21927_UNTREATED_VS_GMCSF_IL6_TREATED_BONE_MARROW_DN
114 0.24675559 0.9965324 0.40963855
0.5048647 1 2380 "tags=25%, list=17%, signal=30%"
GSE3982_BCELL_VS_CENT_MEMORY_CD4_TCELL_DN
GSE3982_BCELL_VS_CENT_MEMORY_CD4_TCELL_DN 125
0.24592471 0.99387693 0.46875 0.5116146 1
1672 "tags=17%, list=12%, signal=19%"
GSE32533_WT_VS_MIR17_OVEREXPRESS_ACT_CD4_TCELL_UP
GSE32533_WT_VS_MIR17_OVEREXPRESS_ACT_CD4_TCELL_UP 138
0.24225567 0.99330467 0.46090534 0.5128859
1 2296 "tags=28%, list=16%, signal=33%"
GSE24634_TEFF_VS_TCONV_DAY10_IN_CULTURE_DN
GSE24634_TEFF_VS_TCONV_DAY10_IN_CULTURE_DN 159
0.2368831 0.99327976 0.45510203 0.5127482
1 1912 "tags=20%, list=14%, signal=23%"
GSE13229_IMM_VS_MATURE_NKCELL_DN GSE13229_IMM_VS_MATURE_NKCELL_DN
142 0.24293752 0.9916652 0.4474273
0.5168482 1 1776 "tags=21%, list=13%, signal=24%"
GSE3982_MAST_CELL_VS_BASOPHIL_UP GSE3982_MAST_CELL_VS_BASOPHIL_UP
139 0.24214976 0.99129856 0.47921225
0.5176507 1 3188 "tags=35%, list=23%, signal=45%"
GSE15930_STIM_VS_STIM_AND_IFNAB_24H_CD8_T_CELL_DN
GSE15930_STIM_VS_STIM_AND_IFNAB_24H_CD8_T_CELL_DN 154
0.23773947 0.9906716 0.49385247 0.51909554
1 2663 "tags=29%, list=19%, signal=35%"
GSE3982_DC_VS_BCELL_UP GSE3982_DC_VS_BCELL_UP 159
0.23876627 0.9905387 0.47895792 0.51922876
1 1279 "tags=11%, list=9%, signal=12%"
GSE29618_MONOCYTE_VS_PDC_DN GSE29618_MONOCYTE_VS_PDC_DN
141 0.24204528 0.98874086 0.47686118
0.52384245 1 1900 "tags=22%, list=13%, signal=25%"
GSE9960_HEALTHY_VS_GRAM_NEG_AND_POS_SEPSIS_PBMC_DN
GSE9960_HEALTHY_VS_GRAM_NEG_AND_POS_SEPSIS_PBMC_DN 114
0.24678873 0.98792577 0.46055436 0.52582157
1 1794 "tags=20%, list=13%, signal=23%"
GSE1460_CD4_THYMOCYTE_VS_THYMIC_STROMAL_CELL_DN
GSE1460_CD4_THYMOCYTE_VS_THYMIC_STROMAL_CELL_DN 146

0.24023817 0.98745316 0.47722772 0.52686256
1 2276 "tags=25%, list=16%, signal=30%"
GSE11924_TFH_VS_TH2_CD4_TCELL_UP GSE11924_TFH_VS_TH2_CD4_TCELL_UP
142 0.23817123 0.987012 0.5031447
0.5278374 1 2379 "tags=27%, list=17%, signal=32%"
GSE42021_TREG_PLN_VS_TREG_PRECURSORS_THYMUS_UP
GSE42021_TREG_PLN_VS_TREG_PRECURSORS_THYMUS_UP 130
0.24292767 0.98686993 0.46169356 0.52800155
1 1585 "tags=14%, list=11%, signal=15%"
GSE41867_DAY8_VS_DAY15_LCMV_ARMSTRONG_EFFECTOR_CD8_TCELL_UP
GSE41867_DAY8_VS_DAY15_LCMV_ARMSTRONG_EFFECTOR_CD8_TCELL_UP
100 0.25209916 0.98630154 0.47619048
0.52931684 1 1048 "tags=8%, list=7%, signal=9%"
GSE22886_IGG_IGA_MEMORY_BCELL_VS_BM_PLASMA_CELL_DN
GSE22886_IGG_IGA_MEMORY_BCELL_VS_BM_PLASMA_CELL_DN 145
0.24016926 0.98626065 0.49790794 0.52921706
1 2148 "tags=24%, list=15%, signal=28%"
GSE18281_CORTEX_VS_MEDULLA_THYMUS_UP
GSE18281_CORTEX_VS_MEDULLA_THYMUS_UP 125
0.24306023 0.9857535 0.4767677 0.5303571
1 1204 "tags=10%, list=9%, signal=11%"
GSE3203_HEALTHY_VS_INFLUENZA_INFECTED_LN_BCELL_UP
GSE3203_HEALTHY_VS_INFLUENZA_INFECTED_LN_BCELL_UP 147
0.2373218 0.98574173 0.49785408 0.53017825
1 1990 "tags=22%, list=14%, signal=25%"
GSE17721_CTRL_VS_LPS_8H_BMDC_DN GSE17721_CTRL_VS_LPS_8H_BMDC_DN
160 0.23376967 0.9855731 0.51521736
0.53042316 1 1553 "tags=16%, list=11%, signal=17%"
GSE1460_NAIVE_CD4_TCELL_ADULT_BLOOD_VS_THYMIC_STROMAL_CELL_UP
GSE1460_NAIVE_CD4_TCELL_ADULT_BLOOD_VS_THYMIC_STROMAL_CELL_UP
131 0.23970224 0.98531 0.5 0.5309098
1 2283 "tags=24%, list=16%, signal=28%"
GSE5099_UNSTIM_VS_MCSF_TREATED_MONOCYTE_DAY7_DN
GSE5099_UNSTIM_VS_MCSF_TREATED_MONOCYTE_DAY7_DN 144
0.24001239 0.98526835 0.48347107 0.5308046
1 1810 "tags=22%, list=13%, signal=24%"
GSE21546_UNSTIM_VS_ANTI_CD3_STIM_ELK1_KO_DP_THYMOCYTES_UP
GSE21546_UNSTIM_VS_ANTI_CD3_STIM_ELK1_KO_DP_THYMOCYTES_UP
107 0.2474836 0.9848181 0.48247424
0.53178436 1 2419 "tags=25%, list=17%, signal=30%"
GSE1925_CTRL_VS_3H_IFNG_STIM_IFNG_PRIMED_MACROPHAGE_UP
GSE1925_CTRL_VS_3H_IFNG_STIM_IFNG_PRIMED_MACROPHAGE_UP
122 0.24269806 0.98445195 0.4893617
0.5325325 1 2423 "tags=26%, list=17%, signal=31%"
GSE24142_ADULT_VS_FETAL_DN2_THYMOCYTE_DN
GSE24142_ADULT_VS_FETAL_DN2_THYMOCYTE_DN 154
0.23690033 0.98426896 0.46638656 0.5327941
1 2359 "tags=23%, list=17%, signal=28%"
GSE29949_CD8_NEG_DC_SPLEEN_VS_CD8_POS_DC_SPLEEN_DN
GSE29949_CD8_NEG_DC_SPLEEN_VS_CD8_POS_DC_SPLEEN_DN 142
0.23872335 0.98352295 0.47975707 0.53457063
1 2149 "tags=22%, list=15%, signal=25%"
GSE22611_UNSTIM_VS_6H_MDP_STIM_MUTANT_NOD2_TRANSDUCED_HEK293T_CELL_D
N

GSE22611_UNSTIM_VS_6H_MDP_STIM_MUTANT_NOD2_TRANSDUCED_HEK293T_CELL_DN 134 0.24187998 0.9831296 0.47510374 0.5353976 1 2607 "tags=24%, list=18%, signal=29%"
GSE6259_FLT3L_INDUCED_DEC205_POS_DC_VS_CD8_TCELL_UP
GSE6259_FLT3L_INDUCED_DEC205_POS_DC_VS_CD8_TCELL_UP 111 0.2498858 0.9828432 0.50429183 0.5359349 1 1904 "tags=22%, list=13%, signal=25%"
GSE29164_DAY3_VS_DAY7_UNTREATED_MELANOMA_DN
GSE29164_DAY3_VS_DAY7_UNTREATED_MELANOMA_DN 153 0.23538028 0.9826007 0.5031315 0.5363882 1 2533 "tags=25%, list=18%, signal=31%"
GSE24671_CTRL_VS_SENDAI_VIRUS_INFECTED_MOUSE_SPLENOCYTES_DN
GSE24671_CTRL_VS_SENDAI_VIRUS_INFECTED_MOUSE_SPLENOCYTES_DN 135 0.23896597 0.98224306 0.4891775 0.5370915 1 2045 "tags=22%, list=14%, signal=26%"
GSE9006_1MONTH_VS_4MONTH_POST_TYPE_1_DIABETES_DX_PBMC_DN
GSE9006_1MONTH_VS_4MONTH_POST_TYPE_1_DIABETES_DX_PBMC_DN 133 0.23859504 0.9816511 0.48806942 0.53843784 1 1811 "tags=15%, list=13%, signal=17%"
GSE1791_CTRL_VS_NEUROMEDINU_IN_T_CELL_LINE_12H_UP
GSE1791_CTRL_VS_NEUROMEDINU_IN_T_CELL_LINE_12H_UP 132 0.24269088 0.9812335 0.4851695 0.53935903 1 2593 "tags=31%, list=18%, signal=38%"
GSE29615_CTRL_VS_DAY7_LAIV_FLU_VACCINE_PBMC_UP
GSE29615_CTRL_VS_DAY7_LAIV_FLU_VACCINE_PBMC_UP 106 0.24978374 0.9808374 0.48187634 0.54022187 1 1866 "tags=14%, list=13%, signal=16%"
GSE27786_ERYTHROBLAST_VS_NEUTROPHIL_DN
GSE27786_ERYTHROBLAST_VS_NEUTROPHIL_DN 147 0.23896083 0.9808338 0.49686846 0.5400133 1 2953 "tags=32%, list=21%, signal=40%"
GSE37605_C57BL6_VS_NOD_FOXP3_FUSION_GFP_TCONV_UP
GSE37605_C57BL6_VS_NOD_FOXP3_FUSION_GFP_TCONV_UP 136 0.241471 0.98079735 0.51914895 0.53989995 1 1516 "tags=19%, list=11%, signal=21%"
GSE3982_CENT_MEMORY_CD4_TCELL_VS_NKCELL_UP
GSE3982_CENT_MEMORY_CD4_TCELL_VS_NKCELL_UP 125 0.24310787 0.98018587 0.46696034 0.54130065 1 1092 "tags=13%, list=8%, signal=14%"
GSE29618_PRE_VS_DAY7_POST_TIV_FLU_VACCINE_PDC_UP
GSE29618_PRE_VS_DAY7_POST_TIV_FLU_VACCINE_PDC_UP 129 0.24344862 0.9785165 0.52663934 0.5455468 1 1927 "tags=16%, list=14%, signal=19%"
GSE21927_SPLEEN_MONOCYTE_VS_GMCSF_GCSF_BONE_MARROW_UP
GSE21927_SPLEEN_MONOCYTE_VS_GMCSF_GCSF_BONE_MARROW_UP 100 0.248018 0.97849005 0.49795082 0.54539096 1 1667 "tags=19%, list=12%, signal=21%"
GSE34205_HEALTHY_VS_FLU_INF_INFANT_PBMC_UP
GSE34205_HEALTHY_VS_FLU_INF_INFANT_PBMC_UP 105 0.24898092 0.9778659 0.4896694 0.546829 1 1891 "tags=20%, list=13%, signal=23%"
GSE43863_DAY6_EFF_VS_DAY150_MEM_TFH_CD4_TCELL_DN
GSE43863_DAY6_EFF_VS_DAY150_MEM_TFH_CD4_TCELL_DN 143

0.23690508 0.9777323 0.50213677 0.54697585
1 1559 "tags=16%, list=11%, signal=18%"
GSE22886_NAIVE_CD4_TCELL_VS_MEMORY_TCELL_UP
GSE22886_NAIVE_CD4_TCELL_VS_MEMORY_TCELL_UP 135
0.23671435 0.97692305 0.47021276 0.54899025
1 1355 "tags=11%, list=10%, signal=12%"
GSE22935_UNSTIM_VS_24H_MBOVIS_BCG_STIM_MACROPHAGE_DN
GSE22935_UNSTIM_VS_24H_MBOVIS_BCG_STIM_MACROPHAGE_DN
157 0.23397577 0.9768046 0.50993377
0.54911363 1 1810 "tags=18%, list=13%, signal=21%"
GSE21546_UNSTIM_VS_ANTI_CD3_STIM_ELK1_KO_DP_THYMOCYTES_DN
GSE21546_UNSTIM_VS_ANTI_CD3_STIM_ELK1_KO_DP_THYMOCYTES_DN
112 0.24384576 0.9758042 0.50931674
0.55164576 1 1665 "tags=15%, list=12%, signal=17%"
GSE9946_MATURE_STIMULATORY_VS_LISTERIA_INF_MATURE_DC_UP
GSE9946_MATURE_STIMULATORY_VS_LISTERIA_INF_MATURE_DC_UP
101 0.25290772 0.97443634 0.5171026
0.5551664 1 2342 "tags=26%, list=17%, signal=31%"
GSE17721_PAM3CSK4_VS_CPG_2H_BMDC_DN
GSE17721_PAM3CSK4_VS_CPG_2H_BMDC_DN 151
0.23747805 0.97316295 0.5338983 0.55837315
1 2079 "tags=21%, list=15%, signal=25%"
GSE2770_IL12_AND_TGFB_ACT_VS_ACT_CD4_TCELL_48H_UP
GSE2770_IL12_AND_TGFB_ACT_VS_ACT_CD4_TCELL_48H_UP 130
0.23903868 0.9730637 0.50718683 0.5584273
1 2286 "tags=24%, list=16%, signal=28%"
GSE11961_PLASMA_CELL_DAY7_VS_MEMORY_BCELL_DAY40_DN
GSE11961_PLASMA_CELL_DAY7_VS_MEMORY_BCELL_DAY40_DN 143
0.23560536 0.9727886 0.49900597 0.55892795
1 741 "tags=7%, list=5%, signal=7%"
GSE26727_WT_VS_KLF2_KO_LPS_STIM_MACROPHAGE_UP
GSE26727_WT_VS_KLF2_KO_LPS_STIM_MACROPHAGE_UP 149
0.2357847 0.9723429 0.52272725 0.55988467
1 2121 "tags=22%, list=15%, signal=26%"
GSE24102_GRANULOCYSTIC_MDSC_VS_NEUTROPHIL_DN
GSE24102_GRANULOCYSTIC_MDSC_VS_NEUTROPHIL_DN 148
0.2351896 0.9722506 0.5346535 0.55991846
1 2018 "tags=22%, list=14%, signal=26%"
GSE360_L_MAJOR_VS_B_MALAYI_HIGH_DOSE_DC_UP
GSE360_L_MAJOR_VS_B_MALAYI_HIGH_DOSE_DC_UP 156
0.23406866 0.9718465 0.52904564 0.56082153
1 2186 "tags=20%, list=15%, signal=23%"
GSE37532_VISCERAL_ADIPOSE_TISSUE_VS_LN_DERIVED_TCONV_CD4_TCELL_UP
GSE37532_VISCERAL_ADIPOSE_TISSUE_VS_LN_DERIVED_TCONV_CD4_TCELL_UP
92 0.25636455 0.9717751 0.5010482
0.56077445 1 1705 "tags=14%, list=12%, signal=16%"
GSE6566_STRONG_VS_WEAK_DC_STIMULATED_CD4_TCELL_DN
GSE6566_STRONG_VS_WEAK_DC_STIMULATED_CD4_TCELL_DN 115
0.24338585 0.9713629 0.5 0.56169885 1
2749 "tags=29%, list=19%, signal=35%"
GSE20715_WT_VS_TLR4_KO_24H_OZONE_LUNG_UP
GSE20715_WT_VS_TLR4_KO_24H_OZONE_LUNG_UP 159
0.23264372 0.97014827 0.51508623 0.5647793
1 1644 "tags=17%, list=12%, signal=19%"

GSE45365_NK_CELL_VS_BCELL_MCMV_INFECTION_UP
GSE45365_NK_CELL_VS_BCELL_MCMV_INFECTION_UP 144
0.23556127 0.9695811 0.52699786 0.56607705
1 2532 "tags=30%, list=18%, signal=36%"
GSE17186_MEMORY_VS_CD21LOW_TRANSITIONAL_BCELL_DN
GSE17186_MEMORY_VS_CD21LOW_TRANSITIONAL_BCELL_DN 145
0.23480295 0.9695108 0.5373737 0.56603885
1 2576 "tags=26%, list=18%, signal=32%"
GSE12507_PDC_CELL_LINE_VS_IMMATUE_T_CELL_LINE_DN
GSE12507_PDC_CELL_LINE_VS_IMMATUE_T_CELL_LINE_DN 109
0.24495046 0.9686525 0.5106383 0.56816185
1 2486 "tags=18%, list=18%, signal=22%"
GSE41867_DAY6_EFFECTOR_VS_DAY30_EXHAUSTED_CD8_TCELL_LCMV_CLONE13_DN
GSE41867_DAY6_EFFECTOR_VS_DAY30_EXHAUSTED_CD8_TCELL_LCMV_CLONE13_DN
144 0.23495315 0.9683591 0.5482546
0.5687345 1 2704 "tags=28%, list=19%, signal=34%"
GSE2770_UNTREATED_VS_TGFB_AND_IL4_TREATED_ACT_CD4_TCELL_4H_DN
GSE2770_UNTREATED_VS_TGFB_AND_IL4_TREATED_ACT_CD4_TCELL_4H_DN
92 0.253711 0.96730804 0.51555556
0.57140636 1 1578 "tags=17%, list=11%, signal=19%"
GSE36888_UNTREATED_VS_IL2_TREATED_STAT5_AB_KNOCKIN_TCELL_17H_UP
GSE36888_UNTREATED_VS_IL2_TREATED_STAT5_AB_KNOCKIN_TCELL_17H_UP
136 0.23487216 0.9671966 0.5540541
0.5714766 1 949 "tags=11%, list=7%, signal=12%"
GSE13485_CTRL_VS_DAY7_YF17D_VACCINE_PBMC_UP
GSE13485_CTRL_VS_DAY7_YF17D_VACCINE_PBMC_UP 105
0.25195214 0.96643925 0.51843816 0.5733815
1 2499 "tags=24%, list=18%, signal=29%"
GSE17721_POLYIC_VS_CPG_2H_BMDC_DN GSE17721_POLYIC_VS_CPG_2H_BMDC_DN
158 0.23381558 0.96587527 0.5482546
0.57472825 1 1594 "tags=14%, list=11%, signal=16%"
GSE3039_NKT_CELL_VS_B1_BCELL_UP GSE3039_NKT_CELL_VS_B1_BCELL_UP
142 0.23315431 0.96509665 0.5433404
0.5766211 1 2204 "tags=21%, list=16%, signal=25%"
GSE22140_GERMFREE_VS_SPF_ARTHRITIC_MOUSE_CD4_TCELL_UP
GSE22140_GERMFREE_VS_SPF_ARTHRITIC_MOUSE_CD4_TCELL_UP
146 0.23656432 0.9634265 0.54471546
0.5810496 1 2420 "tags=29%, list=17%, signal=34%"
GSE13411_IGM_VS_SWITCHED_MEMORY_BCELL_DN
GSE13411_IGM_VS_SWITCHED_MEMORY_BCELL_DN 136
0.23510417 0.9626719 0.55949897 0.5829147
1 2292 "tags=26%, list=16%, signal=30%"
GSE9509_10MIN_VS_30MIN_LPS_AND_IL10_STIM_IL10_KO_MACROPHAGE_DN
GSE9509_10MIN_VS_30MIN_LPS_AND_IL10_STIM_IL10_KO_MACROPHAGE_DN
126 0.23818 0.9624345 0.52953154
0.5832955 1 2047 "tags=18%, list=15%, signal=21%"
GSE3691_IFN_PRODUCING_KILLER_DC_VS_CONVENTIONAL_DC_SPLEEN_DN
GSE3691_IFN_PRODUCING_KILLER_DC_VS_CONVENTIONAL_DC_SPLEEN_DN
153 0.22671153 0.960796 0.5588865
0.58763725 1 1689 "tags=12%, list=12%, signal=14%"
GSE17721_CTRL_VS_GARDIQUIMOD_2H_BMDC_UP
GSE17721_CTRL_VS_GARDIQUIMOD_2H_BMDC_UP 136
0.23556997 0.9601556 0.5673913 0.5891528
1 1876 "tags=21%, list=13%, signal=24%"

GSE3982_BCELL_VS_NKCELL_DN GSE3982_BCELL_VS_NKCELL_DN
143 0.23226334 0.9599672 0.5588235
0.5893988 1 2235 "tags=15%, list=16%, signal=18%"
GSE27786_CD4_TCELL_VS_NEUTROPHIL_UP
GSE27786_CD4_TCELL_VS_NEUTROPHIL_UP 141
0.23348244 0.9595971 0.56962025 0.5901772
1 2345 "tags=26%, list=17%, signal=30%"
GSE21927_EL4_VS_MCA203_TUMOR_MONOCYTES_DN
GSE21927_EL4_VS_MCA203_TUMOR_MONOCYTES_DN 111
0.24304557 0.95629007 0.53218883 0.5990888
1 1122 "tags=12%, list=8%, signal=13%"
GSE5589_LPS_VS_LPS_AND_IL6_STIM_IL10_KO_MACROPHAGE_45MIN_UP
GSE5589_LPS_VS_LPS_AND_IL6_STIM_IL10_KO_MACROPHAGE_45MIN_UP
140 0.23071063 0.95575315 0.5670103
0.6003769 1 1725 "tags=12%, list=12%, signal=14%"
GSE46606_UNSTIM_VS_CD40L_IL2_IL5_3DAY_STIMULATED_IRF4HIGH_SORTED_BCE
LL_UP
GSE46606_UNSTIM_VS_CD40L_IL2_IL5_3DAY_STIMULATED_IRF4HIGH_SORTED_BCE
LL_UP 138 0.23308527 0.95499605
0.5641548 0.6022753 1 1797 "tags=17%,
list=13%, signal=19%"
GSE41867_DAY15_EFFECTOR_VS_DAY30_EXHAUSTED_CD8_TCELL_LCMV_CLONE13_DN

GSE41867_DAY15_EFFECTOR_VS_DAY30_EXHAUSTED_CD8_TCELL_LCMV_CLONE13_DN
129 0.23255241 0.9548511
0.5807128 0.6024259 1 2222 "tags=24%,
list=16%, signal=28%"
GSE18893_CTRL_VS_TNF_TREATED_TCONV_24H_UP
GSE18893_CTRL_VS_TNF_TREATED_TCONV_24H_UP 131
0.23228979 0.9545389 0.58239275 0.60305375
1 2587 "tags=26%, list=18%, signal=31%"
GSE17721_12H_VS_24H_CPG_BMDC_UP GSE17721_12H_VS_24H_CPG_BMDC_UP
156 0.22763973 0.9537538 0.5809129
0.6049802 1 2441 "tags=24%, list=17%, signal=28%"
GSE11864_CSF1_IFNG_VS_CSF1_PAM3CYS_IN_MAC_DN
GSE11864_CSF1_IFNG_VS_CSF1_PAM3CYS_IN_MAC_DN 132
0.2359824 0.95259494 0.57836646 0.60793823
1 2469 "tags=27%, list=17%, signal=32%"
GSE360_L_DONOVANI_VS_L_MAJOR_DC_UP
GSE360_L_DONOVANI_VS_L_MAJOR_DC_UP 150
0.22991413 0.95252436 0.59381443 0.60789007
1 2779 "tags=27%, list=20%, signal=33%"
GSE17721_0.5H_VS_12H_PAM3CSK4_BMDC_UP
GSE17721_0.5H_VS_12H_PAM3CSK4_BMDC_UP 146
0.22952712 0.95167935 0.58913046 0.60998267
1 2595 "tags=27%, list=18%, signal=32%"
GSE34156_NOD2_LIGAND_VS_NOD2_AND_TLR1_TLR2_LIGAND_6H_TREATED_MONOCYT
E_DN
GSE34156_NOD2_LIGAND_VS_NOD2_AND_TLR1_TLR2_LIGAND_6H_TREATED_MONOCYT
E_DN 160 0.22696097 0.9515959
0.58178055 0.60998005 1 1687 "tags=16%,
list=12%, signal=18%"
GSE2405_HEAT_KILLED_VS_LIVE_A_PHAGOCYTOPHILUM_STIM_NEUTROPHIL_24H_DN

GSE2405_HEAT_KILLED_VS_LIVE_A_PHAGOCYTOPHILUM_STIM_NEUTROPHIL_24H_DN
129 0.2356316 0.95156467
0.5835141 0.60982746 1 1169 "tags=12%,
list=8%, signal=13%"
GSE17721_PAM3CSK4_VS_CPG_2H_BMDC_UP
GSE17721_PAM3CSK4_VS_CPG_2H_BMDC_UP 155
0.22754028 0.9514876 0.5585774 0.609798 1
1893 "tags=18%, list=13%, signal=21%"
GSE24972_MARGINAL_ZONE_BCELL_VS_FOLLICULAR_BCELL_DN
GSE24972_MARGINAL_ZONE_BCELL_VS_FOLLICULAR_BCELL_DN
142 0.229405 0.9514305 0.57758623 0.6097271
1 1390 "tags=12%, list=10%, signal=13%"
GSE29615_CTRL_VS_DAY3_LAIV_IFLU_VACCINE_PBMC_DN
GSE29615_CTRL_VS_DAY3_LAIV_IFLU_VACCINE_PBMC_DN 118
0.2343008 0.9508272 0.5609756 0.611201 1
2716 "tags=28%, list=19%, signal=34%"
GSE6269_E_COLI_VS_STREP_PNEUMO_INF_PBMC_UP
GSE6269_E_COLI_VS_STREP_PNEUMO_INF_PBMC_UP 120
0.23859377 0.9503826 0.5619469 0.6121724
1 1703 "tags=17%, list=12%, signal=19%"
GSE3982_MAST_CELL_VS_NEUTROPHIL_DN
GSE3982_MAST_CELL_VS_NEUTROPHIL_DN 143
0.22975361 0.9498848 0.55693585 0.6132716
1 2422 "tags=22%, list=17%, signal=27%"
GSE5099_MONOCYTE_VS_ALTERNATIVE_M2_MACROPHAGE_UP
GSE5099_MONOCYTE_VS_ALTERNATIVE_M2_MACROPHAGE_UP 106
0.23775792 0.94841444 0.5705394 0.61708283
1 1703 "tags=14%, list=12%, signal=16%"
GSE26488_HDAC7_KO_VS_VP16_TRANSGENIC_HDAC7_KO_DOUBLE_POSITIVE_THYMOC
YTE_DN
GSE26488_HDAC7_KO_VS_VP16_TRANSGENIC_HDAC7_KO_DOUBLE_POSITIVE_THYMOC
YTE_DN 160 0.22687034 0.94785786
0.5615551 0.6184387 1 2041 "tags=19%,
list=14%, signal=22%"
GSE5503_LIVER_DC_VS_PLN_DC_ACTIVATED_ALLOGENIC_TCELL_DN
GSE5503_LIVER_DC_VS_PLN_DC_ACTIVATED_ALLOGENIC_TCELL_DN
148 0.2265854 0.94774795 0.5884692
0.61849874 1 2760 "tags=27%, list=20%, signal=33%"
GSE35685_CD34POS_CD38NEG_VS_CD34POS_CD10POS_BONE_MARROW_UP
GSE35685_CD34POS_CD38NEG_VS_CD34POS_CD10POS_BONE_MARROW_UP
160 0.22827028 0.9474502 0.58958334
0.61906683 1 2103 "tags=21%, list=15%, signal=24%"
GSE17721_LPS_VS_POLYIC_1H_BMDC_DN GSE17721_LPS_VS_POLYIC_1H_BMDC_DN
144 0.22895359 0.9471404 0.5960265
0.61966026 1 2317 "tags=24%, list=16%, signal=28%"
GSE19401_UNSTIM_VS_RETINOIC_ACID_STIM_FOLLICULAR_DC_DN
GSE19401_UNSTIM_VS_RETINOIC_ACID_STIM_FOLLICULAR_DC_DN
152 0.2292136 0.947008 0.6197479 0.6197821
1 1997 "tags=20%, list=14%, signal=24%"
GSE6269_STAPH_AUREUS_VS_STREP_PNEUMO_INF_PBMC_DN
GSE6269_STAPH_AUREUS_VS_STREP_PNEUMO_INF_PBMC_DN 133
0.23472981 0.94616723 0.5720339 0.62186325
1 1325 "tags=13%, list=9%, signal=14%"
GSE7460_WT_VS_FOXP3_HET_ACT_TCONV_DN

GSE7460_WT_VS_FOXP3_HET_ACT_TCONV_DN 146
0.23112404 0.94582 0.57419354 0.6225543 1
3028 "tags=32%, list=21%, signal=41%"
GSE3982_DC_VS_NKCELL_UP GSE3982_DC_VS_NKCELL_UP 150
0.22774607 0.94295317 0.5807128 0.63036513
1 1586 "tags=14%, list=11%, signal=16%"
GSE15659_NAIVE_VS_PTPRC_NEG_CD4_TCELL_DN
GSE15659_NAIVE_VS_PTPRC_NEG_CD4_TCELL_DN 137
0.23018286 0.9422692 0.6106557 0.6320043
1 1323 "tags=12%, list=9%, signal=13%"
GSE2706_R848_VS_R848_AND_LPS_8H_STIM_DC_UP
GSE2706_R848_VS_R848_AND_LPS_8H_STIM_DC_UP 118
0.23325352 0.9417106 0.5840517 0.6332637
1 2079 "tags=19%, list=15%, signal=22%"
GSE5589_WT_VS_IL10_KO_LPS_AND_IL6_STIM_MACROPHAGE_45MIN_DN
GSE5589_WT_VS_IL10_KO_LPS_AND_IL6_STIM_MACROPHAGE_45MIN_DN
137 0.23289108 0.94112366 0.58635396
0.63459736 1 1308 "tags=10%, list=9%, signal=11%"
GSE11884_WT_VS_FURIN_KO_NAIVE_CD4_TCELL_UP
GSE11884_WT_VS_FURIN_KO_NAIVE_CD4_TCELL_UP 104
0.23712765 0.9408768 0.5625 0.635034 1
1551 "tags=17%, list=11%, signal=19%"
GSE29618_PRE_VS_DAY7_POST_TIV_FLU_VACCINE_MDC_DN
GSE29618_PRE_VS_DAY7_POST_TIV_FLU_VACCINE_MDC_DN 134
0.23084252 0.9408415 0.5833333 0.63488483
1 1701 "tags=15%, list=12%, signal=17%"
GSE17721_LPS_VS_PAM3CSK4_2H_BMDC_DN
GSE17721_LPS_VS_PAM3CSK4_2H_BMDC_DN 158
0.22426838 0.93774444 0.610998 0.6432004 1
1953 "tags=18%, list=14%, signal=20%"
GSE46606_IRF4_KO_VS_WT_CD40L_IL2_IL5_1DAY_STIMULATED_BCELL_DN
GSE46606_IRF4_KO_VS_WT_CD40L_IL2_IL5_1DAY_STIMULATED_BCELL_DN
128 0.23178503 0.93764 0.57894737
0.64322555 1 2257 "tags=21%, list=16%, signal=25%"
GSE17721_LPS_VS_GARDIQUIMOD_6H_BMDC_UP
GSE17721_LPS_VS_GARDIQUIMOD_6H_BMDC_UP 149
0.22809124 0.93705595 0.5835141 0.64456606
1 2372 "tags=26%, list=17%, signal=31%"
GSE5589_WT_VS_IL6_KO_LPS_STIM_MACROPHAGE_45MIN_DN
GSE5589_WT_VS_IL6_KO_LPS_STIM_MACROPHAGE_45MIN_DN 153
0.22482145 0.9345697 0.6266925 0.65132314
1 2204 "tags=19%, list=16%, signal=22%"
GSE360_CTRL_VS_L_MAJOR_MAC_DN GSE360_CTRL_VS_L_MAJOR_MAC_DN
147 0.22396287 0.933638 0.6476578
0.65364295 1 2081 "tags=20%, list=15%, signal=24%"
GSE35543_IN_VITRO_ITREG_VS_CONVERTED_EX_ITREG_DN
GSE35543_IN_VITRO_ITREG_VS_CONVERTED_EX_ITREG_DN 147
0.22544906 0.93340844 0.6252588 0.654021 1
1702 "tags=16%, list=12%, signal=18%"
GSE45365_HEALTHY_VS_MCMV_INFECTION_CD8_TCELL_IFNAR_KO_UP
GSE45365_HEALTHY_VS_MCMV_INFECTION_CD8_TCELL_IFNAR_KO_UP
104 0.23826143 0.9331539 0.58921164
0.65445983 1 2011 "tags=22%, list=14%, signal=26%"
GSE41176_WT_VS_TAK1_KO_ANTI_IGM_STIM_BCELL_3H_UP

GSE41176_WT_VS_TAK1_KO_ANTI_IGM_STIM_BCELL_3H_UP 146
0.22569524 0.93286145 0.6261062 0.6550323
1 1931 "tags=21%, list=14%, signal=24%"
GSE17974_IL4_AND_ANTI_IL12_VS_UNTREATED_2H_ACT_CD4_TCELL_UP
GSE17974_IL4_AND_ANTI_IL12_VS_UNTREATED_2H_ACT_CD4_TCELL_UP
112 0.2318532 0.9323099 0.6189474
0.65632993 1 1671 "tags=16%, list=12%, signal=18%"
GSE3982_NEUTROPHIL_VS_TH2_UP GSE3982_NEUTROPHIL_VS_TH2_UP
150 0.22225322 0.9315565 0.64344263
0.6581183 1 2121 "tags=19%, list=15%, signal=22%"
GSE15930_STIM_VS_STIM_AND_IL12_24H_CD8_T_CELL_DN
GSE15930_STIM_VS_STIM_AND_IL12_24H_CD8_T_CELL_DN 159
0.22256643 0.9310025 0.66170216 0.6593953
1 2226 "tags=19%, list=16%, signal=22%"
GSE13306_TREG_RA_VS_TCONV_RA_UP GSE13306_TREG_RA_VS_TCONV_RA_UP
143 0.22766268 0.92920935 0.640592 0.664238
1 1860 "tags=17%, list=13%, signal=20%"
GSE2585_THYMIC_DC_VS_MTEC_UP GSE2585_THYMIC_DC_VS_MTEC_UP
130 0.23014174 0.92869264 0.6039823
0.66543174 1 1971 "tags=20%, list=14%, signal=23%"
GSE6259_FLT3L_INDUCED_33D1_POS_DC_VS_BCELL_UP
GSE6259_FLT3L_INDUCED_33D1_POS_DC_VS_BCELL_UP 133
0.22667186 0.9284619 0.62658226 0.66580427
1 2684 "tags=28%, list=19%, signal=34%"
GSE2124_CTRL_VS_LYMPHOTOXIN_BETA_TREATED_MLN_UP
GSE2124_CTRL_VS_LYMPHOTOXIN_BETA_TREATED_MLN_UP 130
0.22988999 0.92811954 0.6163265 0.66649854
1 2434 "tags=17%, list=17%, signal=20%"
GSE17721_LPS_VS_CPG_24H_BMDC_UP GSE17721_LPS_VS_CPG_24H_BMDC_UP
150 0.22349408 0.9275204 0.6325678
0.6679039 1 2209 "tags=22%, list=16%, signal=26%"
GSE3982_DC_VS_NEUTROPHIL_LPS_STIM_DN
GSE3982_DC_VS_NEUTROPHIL_LPS_STIM_DN 147
0.22271566 0.92654985 0.6476578 0.67032033
1 2422 "tags=24%, list=17%, signal=28%"
GSE20715_WT_VS_TLR4_KO_LUNG_DN GSE20715_WT_VS_TLR4_KO_LUNG_DN
142 0.22468579 0.92639816 0.62634987
0.67048645 1 2664 "tags=25%, list=19%, signal=31%"
GSE11961_FOLLICULAR_BCELL_VS_MEMORY_BCELL_DAY40_UP
GSE11961_FOLLICULAR_BCELL_VS_MEMORY_BCELL_DAY40_UP 145
0.22635165 0.9260996 0.62729126 0.671064 1
1446 "tags=13%, list=10%, signal=14%"
GSE4142_GC_BCELL_VS_MEMORY_BCELL_UP
GSE4142_GC_BCELL_VS_MEMORY_BCELL_UP 141
0.22408156 0.9254878 0.6693387 0.67247206
1 1473 "tags=14%, list=10%, signal=16%"
GSE3982_MAST_CELL_VS_EFF_MEMORY_CD4_TCELL_DN
GSE3982_MAST_CELL_VS_EFF_MEMORY_CD4_TCELL_DN 143
0.22291772 0.9242897 0.6528926 0.67554337
1 2403 "tags=20%, list=17%, signal=24%"
GSE16385_MONOCYTE_VS_12H_IFNG_TNF_TREATED_MACROPHAGE_DN
GSE16385_MONOCYTE_VS_12H_IFNG_TNF_TREATED_MACROPHAGE_DN
139 0.22552791 0.92424446 0.65163934
0.6754012 1 2532 "tags=20%, list=18%, signal=24%"

GSE2770_IL12_VS_TGFB_AND_IL12_TREATED_ACT_CD4_TCELL_6H_UP
GSE2770_IL12_VS_TGFB_AND_IL12_TREATED_ACT_CD4_TCELL_6H_UP
133 0.22819287 0.92418593 0.6188341
0.67528737 1 1335 "tags=14%, list=9%, signal=15%"
GSE17721_CTRL_VS_CPG_0.5H_BMDC_DN GSE17721_CTRL_VS_CPG_0.5H_BMDC_DN
150 0.22525118 0.92238355 0.6506024
0.6800202 1 2000 "tags=14%, list=14%, signal=16%"
GSE17721_CTRL_VS_PAM3CSK4_4H_BMDC_DN
GSE17721_CTRL_VS_PAM3CSK4_4H_BMDC_DN 156
0.21865177 0.9194854 0.6958333 0.68766475
1 2110 "tags=17%, list=15%, signal=20%"
GSE40685_NAIVE_CD4_TCELL_VS_TREG_UP
GSE40685_NAIVE_CD4_TCELL_VS_TREG_UP 110
0.23240888 0.9190277 0.62785864 0.68863916
1 2211 "tags=19%, list=16%, signal=22%"
GSE1740_UNSTIM_VS_IFNA_STIMULATED_MCSF_DERIVED_MACROPHAGE_UP
GSE1740_UNSTIM_VS_IFNA_STIMULATED_MCSF_DERIVED_MACROPHAGE_UP
95 0.23911455 0.91900504 0.6367432
0.6884409 1 1500 "tags=14%, list=11%, signal=15%"
GSE18893_CTRL_VS_TNF_TREATED_TREG_2H_DN
GSE18893_CTRL_VS_TNF_TREATED_TREG_2H_DN 139
0.22241446 0.91856384 0.64316237 0.6893505
1 1157 "tags=12%, list=8%, signal=12%"
GSE27786_NEUTROPHIL_VS_MONO_MAC_UP
GSE27786_NEUTROPHIL_VS_MONO_MAC_UP 136
0.22495621 0.9181483 0.6446281 0.69026035
1 2416 "tags=25%, list=17%, signal=30%"
GSE37416_12H_VS_24H_F_TULARENSIS_LVS_NEUTROPHIL_DN
GSE37416_12H_VS_24H_F_TULARENSIS_LVS_NEUTROPHIL_DN 133
0.22664967 0.9173065 0.6398305 0.6922694
1 2326 "tags=24%, list=16%, signal=29%"
GSE3982_EOSINOPHIL_VS_DC_UP GSE3982_EOSINOPHIL_VS_DC_UP
131 0.2285495 0.9163665 0.6557734
0.69460994 1 2421 "tags=25%, list=17%, signal=30%"
GSE18791_CTRL_VS_NEWCASTLE_VIRUS_DC_2H_UP
GSE18791_CTRL_VS_NEWCASTLE_VIRUS_DC_2H_UP 111
0.23146652 0.91552126 0.640592 0.69658947 1
2231 "tags=21%, list=16%, signal=24%"
GSE27859_MACROPHAGE_VS_CD11C_INT_F480_INT_DC_UP
GSE27859_MACROPHAGE_VS_CD11C_INT_F480_INT_DC_UP 137
0.22439392 0.91532665 0.6598778 0.69684434
1 2575 "tags=23%, list=18%, signal=27%"
GSE13306_TREG_VS_TCONV_LAMINA_PROPRIA_DN
GSE13306_TREG_VS_TCONV_LAMINA_PROPRIA_DN 140
0.21927579 0.91526985 0.7006369 0.6967361
1 2103 "tags=21%, list=15%, signal=25%"
GSE5589_UNSTIM_VS_180MIN_LPS_STIM_MACROPHAGE_UP
GSE5589_UNSTIM_VS_180MIN_LPS_STIM_MACROPHAGE_UP 138
0.22307931 0.9146804 0.65217394 0.69809645
1 1513 "tags=14%, list=11%, signal=16%"
GSE19941_IL10_KO_VS_IL10_KO_AND_NFKBP50_KO_UNSTIM_MACROPHAGE_UP
GSE19941_IL10_KO_VS_IL10_KO_AND_NFKBP50_KO_UNSTIM_MACROPHAGE_UP
157 0.2168811 0.91393524 0.70675105
0.6998485 1 2511 "tags=22%, list=18%, signal=26%"

GSE17721_CPG_VS_GARDIQUIMOD_0.5H_BMDC_DN
GSE17721_CPG_VS_GARDIQUIMOD_0.5H_BMDC_DN 153
0.22026332 0.9138727 0.6533613 0.69975084
1 2585 "tags=26%, list=18%, signal=32%"
GSE37301_HEMATOPOIETIC_STEM_CELL_VS_MULTIPOTENT_PROGENITOR_UP
GSE37301_HEMATOPOIETIC_STEM_CELL_VS_MULTIPOTENT_PROGENITOR_UP
157 0.22100034 0.9134717 0.67075664
0.70057565 1 2700 "tags=25%, list=19%, signal=31%"
GSE29615_DAY3_VS_DAY7_LAIV_FLU_VACCINE_PBMC_DN
GSE29615_DAY3_VS_DAY7_LAIV_FLU_VACCINE_PBMC_DN 120
0.2228821 0.91181636 0.70464134 0.70494527
1 2454 "tags=25%, list=17%, signal=30%"
GSE21360_PRIMARY_VS_SECONDARY_MEMORY_CD8_TCELL_DN
GSE21360_PRIMARY_VS_SECONDARY_MEMORY_CD8_TCELL_DN 148
0.2225062 0.9115732 0.6954644 0.70533186
1 1984 "tags=18%, list=14%, signal=21%"
GSE17721_CTRL_VS_LPS_4H_BMDC_DN GSE17721_CTRL_VS_LPS_4H_BMDC_DN
160 0.21839651 0.9111809 0.6789366
0.70610154 1 1508 "tags=13%, list=11%, signal=15%"
GSE5589_LPS_VS_LPS_AND_IL6_STIM_IL10_KO_MACROPHAGE_45MIN_DN
GSE5589_LPS_VS_LPS_AND_IL6_STIM_IL10_KO_MACROPHAGE_45MIN_DN
152 0.21722429 0.91102713 0.6929638
0.7062715 1 1469 "tags=14%, list=10%, signal=15%"
GSE3982_MAC_VS_NEUTROPHIL_DN GSE3982_MAC_VS_NEUTROPHIL_DN
140 0.21834624 0.90968233 0.7008197
0.70966786 1 1847 "tags=16%, list=13%, signal=18%"
GSE45837_WT_VS_GFI1_KO_PDC_UP GSE45837_WT_VS_GFI1_KO_PDC_UP
130 0.22258146 0.9092429 0.69214875
0.71057147 1 1769 "tags=15%, list=13%, signal=17%"
GSE21774_CD56_BRIGHT_VS_DIM_CD62L_POSITIVE_NK_CELL_DN
GSE21774_CD56_BRIGHT_VS_DIM_CD62L_POSITIVE_NK_CELL_DN
137 0.22352502 0.90870345 0.6923077
0.7118026 1 3014 "tags=27%, list=21%, signal=34%"
GSE27670_BLIMP1_VS_LMP1_TRANSDUCED_GC_BCELL_UP
GSE27670_BLIMP1_VS_LMP1_TRANSDUCED_GC_BCELL_UP 158
0.21730143 0.9080656 0.68 0.7132187 1
2445 "tags=22%, list=17%, signal=26%"
GSE17721_PAM3CSK4_VS_GADIQUIMOD_16H_BMDC_UP
GSE17721_PAM3CSK4_VS_GADIQUIMOD_16H_BMDC_UP 151
0.22001758 0.9073984 0.703629 0.7146869 1
2042 "tags=19%, list=14%, signal=22%"
GSE20152_HTNFA_OVERXPRESS_ANKLE_VS_CTRL_SPHK1_KO_ANKLE_UP
GSE20152_HTNFA_OVERXPRESS_ANKLE_VS_CTRL_SPHK1_KO_ANKLE_UP
140 0.21911126 0.9072712 0.7118644
0.7147345 1 3405 "tags=31%, list=24%, signal=40%"
GSE9960_HEALTHY_VS_SEPSIS_PBMC_UP GSE9960_HEALTHY_VS_SEPSIS_PBMC_UP
137 0.2238131 0.90719193 0.6624738
0.71467024 1 1937 "tags=19%, list=14%, signal=22%"
GSE3982_EOSINOPHIL_VS_TH1_UP GSE3982_EOSINOPHIL_VS_TH1_UP
141 0.2203343 0.90709937 0.69690263
0.71462643 1 1855 "tags=19%, list=13%, signal=22%"
GSE29164_UNTREATED_VS_CD8_TCELL_TREATED_MELANOMA_DAY7_DN
GSE29164_UNTREATED_VS_CD8_TCELL_TREATED_MELANOMA_DAY7_DN
137 0.22035863 0.90479827 0.7033195

0.7205516	1	1115	"tags=12%, list=8%, signal=13%"
GSE22886_NAIVE_VS_MEMORY_TCELL_UP GSE22886_NAIVE_VS_MEMORY_TCELL_UP
	139	0.22228111	0.904354 0.6956522
0.7214841	1	2796	"tags=27%, list=20%, signal=34%"
GSE37532_VISCERAL_ADIPOSE_TISSUE_VS_LN_DERIVED_PPARG_KO_TREG_CD4_TCE
LL_DN
GSE37532_VISCERAL_ADIPOSE_TISSUE_VS_LN_DERIVED_PPARG_KO_TREG_CD4_TCE
LL_DN	98	0.23403591	0.90422595
0.65800864	0.7215276	1	1733	"tags=16%,
list=12%, signal=18%"
GSE22601_DOUBLE_NEGATIVE_VS_CD8_SINGLE_POSITIVE_THYMOCYTE_UP
GSE22601_DOUBLE_NEGATIVE_VS_CD8_SINGLE_POSITIVE_THYMOCYTE_UP
	114	0.22657196	0.90390337	0.6631356
0.7221096	1	1098	"tags=10%, list=8%, signal=10%"
GSE3982_MAST_CELL_VS_DC_DN	GSE3982_MAST_CELL_VS_DC_DN
	140	0.21833932	0.90329516	0.706383
0.7234745	1	2350	"tags=22%, list=17%, signal=26%"
GSE46242_TH1_VS_ANERGIC_TH1_CD4_TCELL_DN
GSE46242_TH1_VS_ANERGIC_TH1_CD4_TCELL_DN	110
0.23014237	0.8996554	0.6794055	0.7327765
1	1967	"tags=16%, list=14%, signal=19%"
GSE25123_CTRL_VS_IL4_AND_ROSIGLITAZONE_STIM_MACROPHAGE_DN
GSE25123_CTRL_VS_IL4_AND_ROSIGLITAZONE_STIM_MACROPHAGE_DN
123	0.22551498	0.89831007	0.69365424
0.7360887	1	2355	"tags=25%, list=17%, signal=30%"
GSE27786_LIN_NEG_VS_NEUTROPHIL_DN GSE27786_LIN_NEG_VS_NEUTROPHIL_DN
	145	0.22012554	0.8977322	0.7163561
0.7373684	1	2555	"tags=25%, list=18%, signal=30%"
GSE19772_HCMV_INFL_VS_HCMV_INF_MONOCYTES_AND_PI3K_INHIBITION_UP
GSE19772_HCMV_INFL_VS_HCMV_INF_MONOCYTES_AND_PI3K_INHIBITION_UP
	159	0.21435757	0.897618 0.7133758
0.7373936	1	2660	"tags=23%, list=19%, signal=28%"
GSE13762_CTRL_VS_125_VITAMIND_DAY12_DC_DN
GSE13762_CTRL_VS_125_VITAMIND_DAY12_DC_DN	101
0.2317269	0.8970634	0.6893004	0.7386119
1	2356	"tags=20%, list=17%, signal=24%"
GSE17721_POLYIC_VS_PAM3CSK4_2H_BMDC_DN
GSE17721_POLYIC_VS_PAM3CSK4_2H_BMDC_DN	162
0.21397477	0.89688003	0.71929824	0.73881537
1	1830	"tags=13%, list=13%, signal=15%"
GSE23114_WT_VS_SLE2C1_MOUSE_PERITONEAL_CAVITY_B1A_BCELL_UP
GSE23114_WT_VS_SLE2C1_MOUSE_PERITONEAL_CAVITY_B1A_BCELL_UP
118	0.22140443	0.8963539	0.70264316
0.7399674	1	2218	"tags=19%, list=16%, signal=23%"
GSE36009_UNSTIM_VS_LPS_STIM_DC_DN GSE36009_UNSTIM_VS_LPS_STIM_DC_DN
	137	0.21855377	0.89627343	0.7114968
0.73988944	1	2222	"tags=23%, list=16%, signal=27%"
GSE17721_4H_VS_24H_POLYIC_BMDC_DN GSE17721_4H_VS_24H_POLYIC_BMDC_DN
	157	0.21553357	0.89584035	0.7310195
0.74077433	1	2740	"tags=30%, list=19%, signal=37%"
GSE25123_ROSIGLITAZONE_VS_IL4_AND_ROSIGLITAZONE_STIM_PPARG_KO_MACROP
HAGE_DAY10_DN
GSE25123_ROSIGLITAZONE_VS_IL4_AND_ROSIGLITAZONE_STIM_PPARG_KO_MACROP
HAGE_DAY10_DN	140	0.2168842	0.89409757

0.71079427	0.74512213	1	1247	"tags=14%, list=9%, signal=15%"
GSE17721_CPG_VS_GARDIQUIMOD_12H_BMDC_UP
GSE17721_CPG_VS_GARDIQUIMOD_12H_BMDC_UP	160
0.21550977	0.89384735	0.71487606	0.74548644
1	1676	"tags=13%, list=12%, signal=15%"
GSE2405_HEAT_KILLED_LYSATE_VS_LIVE_A_PHAGOCYTOPHILUM_STIM_NEUTROPHIL_24H_DN
GSE2405_HEAT_KILLED_LYSATE_VS_LIVE_A_PHAGOCYTOPHILUM_STIM_NEUTROPHIL_24H_DN	149	0.2154161	0.89297104
0.7407407	0.7475382	1	1865	"tags=16%, list=13%, signal=18%"
GSE14769_UNSTIM_VS_360MIN_LPS_BMDM_DN
GSE14769_UNSTIM_VS_360MIN_LPS_BMDM_DN	141
0.21536197	0.89121646	0.73617023	0.7518704
1	2249	"tags=18%, list=16%, signal=21%"
GSE21927_SPLEEN_VS_4T1_TUMOR_MONOCYTE_BALBC_UP
GSE21927_SPLEEN_VS_4T1_TUMOR_MONOCYTE_BALBC_UP	129
0.22268243	0.8902156	0.7319149	0.7541914
1	2133	"tags=21%, list=15%, signal=24%"
GSE25502_WT_VS_KLF13_KO_THYMIC_MEMORY_LIKE_CD8_TCELL_UP
GSE25502_WT_VS_KLF13_KO_THYMIC_MEMORY_LIKE_CD8_TCELL_UP
120	0.22075641	0.88877106	0.702407	0.7576308
1	1910	"tags=17%, list=14%, signal=19%"
GSE3982_CTRL_VS_PMA_STIM_EOSINOPHIL_DN
GSE3982_CTRL_VS_PMA_STIM_EOSINOPHIL_DN	131
0.21849185	0.88770527	0.7196653	0.7601036
1	1670	"tags=14%, list=12%, signal=15%"
GSE5099_CLASSICAL_M1_VS_ALTERNATIVE_M2_MACROPHAGE_DN
GSE5099_CLASSICAL_M1_VS_ALTERNATIVE_M2_MACROPHAGE_DN
115	0.22312713	0.8870465	0.6865672
0.7615428	1	928	"tags=8%, list=7%, signal=8%"
GSE16385_MONOCYTE_VS_12H_ROSIGLITAZONE_TREATED_MACROPHAGE_UP
GSE16385_MONOCYTE_VS_12H_ROSIGLITAZONE_TREATED_MACROPHAGE_UP
	144	0.21231903	0.886785	0.76008064
0.7619499	1	1936	"tags=17%, list=14%, signal=19%"
GSE11057_CD4_CENT_MEM_VS_PBMC_DN	GSE11057_CD4_CENT_MEM_VS_PBMC_DN
	138	0.2162281	0.88658494	0.7505241
0.76218945	1	2085	"tags=17%, list=15%, signal=20%"
GSE17721_POLYIC_VS_GARDIQUIMOD_0.5H_BMDC_UP
GSE17721_POLYIC_VS_GARDIQUIMOD_0.5H_BMDC_UP	150
0.21516298	0.886389	0.7240664	0.7623972	1
2027	"tags=16%, list=14%, signal=18%"
GSE22025_UNTREATED_VS_TGFB1_TREATED_CD4_TCELL_DN
GSE22025_UNTREATED_VS_TGFB1_TREATED_CD4_TCELL_DN	142
0.21484458	0.8858482	0.7397541	0.76349455
1	1089	"tags=9%, list=8%, signal=10%"
GSE18281_SUBCAPSULAR_CORTICAL_REGION_VS_WHOLE_CORTEX_THYMUS_UP
GSE18281_SUBCAPSULAR_CORTICAL_REGION_VS_WHOLE_CORTEX_THYMUS_UP
	151	0.21297479	0.8850174	0.76257545
0.76531893	1	2695	"tags=22%, list=19%, signal=27%"
GSE42021_CD24INT_TREG_VS_CD24INT_TCONV_THYMUS_DN
GSE42021_CD24INT_TREG_VS_CD24INT_TCONV_THYMUS_DN	156
0.21200418	0.8848674	0.7531915	0.7654037

1	2343	"tags=21%, list=17%, signal=24%"
GSE17974_IL4_AND_ANTI_IL12_VS_UNTREATED_1H_ACT_CD4_TCELL_DN
GSE17974_IL4_AND_ANTI_IL12_VS_UNTREATED_1H_ACT_CD4_TCELL_DN
95	0.23110986	0.8837014	0.6905263	0.768137
1	516	"tags=5%, list=4%, signal=5%"
GSE15659_NAIVE_CD4_TCELL_VS_RESTING_TREG_UP
GSE15659_NAIVE_CD4_TCELL_VS_RESTING_TREG_UP	116
0.22359726	0.88366395	0.72307694	0.76794064
1	2543	"tags=22%, list=18%, signal=27%"
GSE22432_MULTIPOTENT_PROGENITOR_VS_CDC_UP
GSE22432_MULTIPOTENT_PROGENITOR_VS_CDC_UP	149
0.21169786	0.8827384	0.75365347	0.77002674
1	2321	"tags=23%, list=16%, signal=28%"
GSE27859_MACROPHAGE_VS_DC_DN	GSE27859_MACROPHAGE_VS_DC_DN
130	0.21589729	0.8822212	0.7782178
0.7710932	1	1561	"tags=15%, list=11%, signal=16%"
GSE46606_UNSTIM_VS_CD40L_IL2_IL5_1DAY_STIMULATED_IRF4MID_SORTED_BCEL
L_DN
GSE46606_UNSTIM_VS_CD40L_IL2_IL5_1DAY_STIMULATED_IRF4MID_SORTED_BCEL
L_DN	129	0.21842945	0.8812218
0.7694013	0.77332693	1	1782	"tags=15%,
list=13%, signal=17%"
GSE42021_TCONV_PLN_VS_CD24INT_TCONV_THYMUS_UP
GSE42021_TCONV_PLN_VS_CD24INT_TCONV_THYMUS_UP	148
0.21276627	0.879996 0.7688985	0.77603865	1
2635	"tags=26%, list=19%, signal=32%"
GSE37605_C57BL6_VS_NOD_FOXP3_IRES_GFP_TREG_DN
GSE37605_C57BL6_VS_NOD_FOXP3_IRES_GFP_TREG_DN	145
0.21231224	0.8795385	0.79317695	0.77690053
1	2596	"tags=24%, list=18%, signal=29%"
GSE20152_HTNFA_OVERXPRESS_ANKLE_VS_CTRL_SPHK1_KO_ANKLE_DN
GSE20152_HTNFA_OVERXPRESS_ANKLE_VS_CTRL_SPHK1_KO_ANKLE_DN
129	0.2152326	0.8777787	0.77272725
0.7810168	1	1933	"tags=16%, list=14%, signal=18%"
GSE23321_EFFECTOR_MEMORY_VS_NAIVE_CD8_TCELL_UP
GSE23321_EFFECTOR_MEMORY_VS_NAIVE_CD8_TCELL_UP	133
0.21794371	0.87730587	0.75446427	0.7819247
1	1650	"tags=14%, list=12%, signal=16%"
GSE3982_EOSINOPHIL_VS_MAST_CELL_UP
GSE3982_EOSINOPHIL_VS_MAST_CELL_UP	140
0.21120572	0.8761627	0.77546775	0.78447855
1	2270	"tags=20%, list=16%, signal=24%"
GSE37301_HEMATOPOIETIC_STEM_CELL_VS_MULTIPOTENT_PROGENITOR_DN
GSE37301_HEMATOPOIETIC_STEM_CELL_VS_MULTIPOTENT_PROGENITOR_DN
124	0.21530445	0.8754262	0.75615215
0.7860527	1	1684	"tags=16%, list=12%, signal=18%"
GSE43955_10H_VS_60H_ACT_CD4_TCELL_DN
GSE43955_10H_VS_60H_ACT_CD4_TCELL_DN	157
0.2115536	0.87472796	0.7648305	0.787454 1
1964	"tags=18%, list=14%, signal=20%"
GSE8921_UNSTIM_0H_VS_TLR1_2_STIM_MONOCYTE_24H_UP
GSE8921_UNSTIM_0H_VS_TLR1_2_STIM_MONOCYTE_24H_UP	132
0.21292424	0.8744585	0.76875 0.78781337	1
858	"tags=8%, list=6%, signal=9%"

GSE21927_SPLEEN_VS_C26GM_TUMOR_MONOCYTE_BALBC_UP
GSE21927_SPLEEN_VS_C26GM_TUMOR_MONOCYTE_BALBC_UP 105
0.22180897 0.87213737 0.7108168 0.793155 1
2542 "tags=17%, list=18%, signal=21%"
GSE12003_4D_VS_8D_CULTURE_BM_PROGENITOR_DN
GSE12003_4D_VS_8D_CULTURE_BM_PROGENITOR_DN 90
0.22576398 0.8709399 0.73535794 0.79569155
1 984 "tags=11%, list=7%, signal=12%"
GSE18804_BRAIN_VS_COLON_TUMORAL_MACROPHAGE_DN
GSE18804_BRAIN_VS_COLON_TUMORAL_MACROPHAGE_DN 143
0.20983866 0.8685052 0.78723407 0.8011986
1 1269 "tags=12%, list=9%, signal=13%"
GSE22589_SIV_VS_HIV_AND_SIV_INFECTED_DC_DN
GSE22589_SIV_VS_HIV_AND_SIV_INFECTED_DC_DN 138
0.20934501 0.86414313 0.80353636 0.8112905
1 1856 "tags=18%, list=13%, signal=21%"
GSE22601_DOUBLE_NEGATIVE_VS_IMMATURE_CD4_SP_THYMOCYTE_DN
GSE22601_DOUBLE_NEGATIVE_VS_IMMATURE_CD4_SP_THYMOCYTE_DN
123 0.21558529 0.8636602 0.782 0.8120936
1 2282 "tags=20%, list=16%, signal=24%"
GSE10147_IL3_VS_IL3_AND_HIVP17_STIM_PDC_DN
GSE10147_IL3_VS_IL3_AND_HIVP17_STIM_PDC_DN 111
0.21391259 0.8633082 0.78099173 0.8126344
1 1387 "tags=10%, list=10%, signal=11%"
GSE4748_CTRL_VS_CYANOBACTERIUM_LPSLIKE_STIM_DC_1H_DN
GSE4748_CTRL_VS_CYANOBACTERIUM_LPSLIKE_STIM_DC_1H_DN
151 0.20942807 0.8631815 0.8059072
0.81263953 1 2372 "tags=21%, list=17%, signal=24%"
GSE6269_FLU_VS_STREP_PNEUMO_INF_PBMC_DN
GSE6269_FLU_VS_STREP_PNEUMO_INF_PBMC_DN 122
0.21513493 0.86279804 0.79957354 0.8132045
1 2216 "tags=21%, list=16%, signal=25%"
GSE40685_NAIVE_CD4_TCELL_VS_FOXP3_KO_TREG_PRECURSOR_DN
GSE40685_NAIVE_CD4_TCELL_VS_FOXP3_KO_TREG_PRECURSOR_DN
131 0.212285 0.8623898 0.7877462 0.8138541
1 1478 "tags=12%, list=10%, signal=14%"
GSE24210_IL35_TREATED_VS_RESTING_TREG_UP
GSE24210_IL35_TREATED_VS_RESTING_TREG_UP 147
0.20953032 0.85982454 0.8077754 0.8195368
1 1634 "tags=12%, list=12%, signal=14%"
GSE15659_CD45RA_NEG_CD4_TCELL_VS_RESTING_TREG_DN
GSE15659_CD45RA_NEG_CD4_TCELL_VS_RESTING_TREG_DN 127
0.20987767 0.8575648 0.832 0.82441026 1
2438 "tags=19%, list=17%, signal=23%"
GSE5503_LIVER_DC_VS_SPLEEN_DC_ACTIVATED_ALLOGENIC_TCELL_UP
GSE5503_LIVER_DC_VS_SPLEEN_DC_ACTIVATED_ALLOGENIC_TCELL_UP
147 0.20550476 0.8575612 0.80487806
0.8241071 1 670 "tags=7%, list=5%, signal=7%"
GSE2770_UNTREATED_VS_TGFB_AND_IL12_TREATED_ACT_CD4_TCELL_6H_DN
GSE2770_UNTREATED_VS_TGFB_AND_IL12_TREATED_ACT_CD4_TCELL_6H_DN
139 0.20830995 0.8566968 0.78904665
0.8257735 1 2615 "tags=25%, list=19%, signal=31%"
GSE16450_IMMATURE_VS_MATURE_NEURON_CELL_LINE_12H_IFNA_STIM_DN
GSE16450_IMMATURE_VS_MATURE_NEURON_CELL_LINE_12H_IFNA_STIM_DN

138	0.20653142	0.8564249	0.7946058
0.8260548	1	2472	"tags=22%, list=18%, signal=26%"
GSE35543_IN_VIVO_NTREG_VS_CONVERTED_EX_ITREG_UP
GSE35543_IN_VIVO_NTREG_VS_CONVERTED_EX_ITREG_UP	117
0.2108306	0.8558867	0.79288703	0.8269678
1	1909	"tags=16%, list=14%, signal=19%"
GSE9946_IMMATURE_VS_LISTERIA_INF_MATURE_DC_UP
GSE9946_IMMATURE_VS_LISTERIA_INF_MATURE_DC_UP	102
0.2154872	0.855811 0.7677419	0.82682973	1
3425	"tags=27%, list=24%, signal=36%"
GSE5503_LIVER_DC_VS_PLN_DC_ACTIVATED_ALLOGENIC_TCELL_UP
GSE5503_LIVER_DC_VS_PLN_DC_ACTIVATED_ALLOGENIC_TCELL_UP
155	0.20498613	0.85480416	0.82365143
0.8287809	1	1098	"tags=12%, list=8%, signal=13%"
GSE2585_CD80_HIGH_VS_LOW_MTEC_DN	GSE2585_CD80_HIGH_VS_LOW_MTEC_DN
130	0.20648512	0.8536427	0.81276596
0.83099014	1	2127	"tags=18%, list=15%, signal=22%"
GSE34156_UNTREATED_VS_6H_NOD2_AND_TLR1_TLR2_LIGAND_TREATED_MONOCYTE_
UP
GSE34156_UNTREATED_VS_6H_NOD2_AND_TLR1_TLR2_LIGAND_TREATED_MONOCYTE_
UP	118	0.21182823	0.8529267
0.78688526	0.8322162	1	1869	"tags=17%,
list=13%, signal=19%"
GSE3982_NKCELL_VS_TH1_UP	GSE3982_NKCELL_VS_TH1_UP	114
0.20993385	0.85269296	0.7876289	0.8324356
1	2155	"tags=16%, list=15%, signal=18%"
GSE40493_BCL6_KO_VS_WT_TREG_UP	GSE40493_BCL6_KO_VS_WT_TREG_UP
143	0.20816071	0.8507902	0.80728054
0.83627564	1	1943	"tags=20%, list=14%, signal=23%"
GSE24574_BCL6_LOW_TFH_VS_TCONV_CD4_TCELL_UP
GSE24574_BCL6_LOW_TFH_VS_TCONV_CD4_TCELL_UP	136
0.20920868	0.8490218	0.8308026	0.8398647
1	2195	"tags=17%, list=16%, signal=20%"
GSE360_HIGH_VS_LOW_DOSE_B_MALAYI_DC_UP
GSE360_HIGH_VS_LOW_DOSE_B_MALAYI_DC_UP	150
0.2042455	0.8484371	0.85333335	0.84079736
1	2085	"tags=17%, list=15%, signal=20%"
GSE36476_YOUNG_VS_OLD_DONOR_MEMORY_CD4_TCELL_16H_TSST_ACT_DN
GSE36476_YOUNG_VS_OLD_DONOR_MEMORY_CD4_TCELL_16H_TSST_ACT_DN
127	0.2104014	0.84641933	0.82626265
0.8447811	1	1060	"tags=11%, list=8%, signal=12%"
GSE6090_UNSTIM_VS_DC_SIGN_STIM_DC_DN
GSE6090_UNSTIM_VS_DC_SIGN_STIM_DC_DN	105
0.21338753	0.84590477	0.7995781	0.84556067
1	991	"tags=10%, list=7%, signal=10%"
GSE21927_SPLEEN_VS_TUMOR_MONOCYTE_C57BL6_UP
GSE21927_SPLEEN_VS_TUMOR_MONOCYTE_C57BL6_UP	112
0.2129824	0.8415679	0.80212766	0.85427034
1	1069	"tags=8%, list=8%, signal=9%"
GSE360_DC_VS_MAC_L_MAJOR_UP	GSE360_DC_VS_MAC_L_MAJOR_UP
149	0.20159209	0.8400486	0.8523207
0.85710835	1	2670	"tags=27%, list=19%, signal=33%"
GSE32533_MIR17_KO_VS_MIR17_OVEREXPRESS_ACT_CD4_TCELL_DN
GSE32533_MIR17_KO_VS_MIR17_OVEREXPRESS_ACT_CD4_TCELL_DN

134 0.20539756 0.83978266 0.84934497 0.85734
1 2155 "tags=19%, list=15%, signal=23%"
GSE34205_RSV_VS_FLU_INF_INFANT_PBMC_UP
GSE34205_RSV_VS_FLU_INF_INFANT_PBMC_UP 108
0.21207924 0.839697 0.8262712 0.8572059 1
2669 "tags=21%, list=19%, signal=26%"
GSE22935_WT_VS_MYD88_KO_MACROPHAGE_48H_MBOVIS_BCG_STIM_UP
GSE22935_WT_VS_MYD88_KO_MACROPHAGE_48H_MBOVIS_BCG_STIM_UP
160 0.2023106 0.8393727 0.85714287
0.85753065 1 1220 "tags=11%, list=9%, signal=11%"
GSE12003_MIR223_KO_VS_WT_BM_PROGENITOR_4D_CULTURE_DN
GSE12003_MIR223_KO_VS_WT_BM_PROGENITOR_4D_CULTURE_DN
162 0.19904207 0.83782446 0.8586498
0.86030227 1 845 "tags=7%, list=6%, signal=7%"
GSE12707_AT16L1_HYPOMORPH_VS_WT_THYMUS_DN
GSE12707_AT16L1_HYPOMORPH_VS_WT_THYMUS_DN 95
0.21566091 0.836975 0.80728054 0.86165434 1
1983 "tags=15%, list=14%, signal=17%"
GSE9946_LISTERIA_INF_MATURE_VS_PROSTAGLANDINE2_TREATED_MATURE_DC_UP
GSE9946_LISTERIA_INF_MATURE_VS_PROSTAGLANDINE2_TREATED_MATURE_DC_UP
97 0.21458738 0.83448696 0.83503056
0.86621594 1 3068 "tags=27%, list=22%, signal=34%"
GSE26488_WT_VS_HDAC7_DELTAP_TG_OT2_THYMOCYTE_WITH_PEPTIDE_INJECTION_
UP
GSE26488_WT_VS_HDAC7_DELTAP_TG_OT2_THYMOCYTE_WITH_PEPTIDE_INJECTION_
UP 169 0.1955814 0.8337897 0.892057
0.86728376 1 2222 "tags=18%, list=16%, signal=22%"
GSE17721_CPG_VS_GARDIQUIMOD_0.5H_BMDC_UP
GSE17721_CPG_VS_GARDIQUIMOD_0.5H_BMDC_UP 149
0.20296411 0.83159065 0.8692946 0.871086 1
2620 "tags=19%, list=19%, signal=23%"
GSE27786_NKCELL_VS_ERYTHROBLAST_DN
GSE27786_NKCELL_VS_ERYTHROBLAST_DN 138
0.20166157 0.82672566 0.86070687 0.87979704
1 3049 "tags=23%, list=22%, signal=29%"
GSE19888_CTRL_VS_A3R_ACT_TREATED_MAST_CELL_PRETREATED_WITH_A3R_INH_U
P
GSE19888_CTRL_VS_A3R_ACT_TREATED_MAST_CELL_PRETREATED_WITH_A3R_INH_U
P 129 0.20697185 0.8257288
0.85775864 0.88130134 1 1552 "tags=13%,
list=11%, signal=15%"
GSE16385_IFNG_TNF_VS_ROSIGLITAZONE_STIM_MACROPHAGE_DN
GSE16385_IFNG_TNF_VS_ROSIGLITAZONE_STIM_MACROPHAGE_DN
92 0.21036121 0.82467425 0.8568627
0.8828805 1 1179 "tags=10%, list=8%, signal=11%"
GSE29618_PRE_VS_DAY7_FLU_VACCINE_PDC_UP
GSE29618_PRE_VS_DAY7_FLU_VACCINE_PDC_UP 131
0.20249614 0.8246699 0.85106385 0.88256055
1 1588 "tags=9%, list=11%, signal=10%"
GSE34217_MIR17_92_OVEREXPRESS_VS_WT_ACT_CD8_TCELL_DN
GSE34217_MIR17_92_OVEREXPRESS_VS_WT_ACT_CD8_TCELL_DN
101 0.21013305 0.8229309 0.82071716
0.88533914 1 1384 "tags=13%, list=10%, signal=14%"
GSE13306_RA_VS_UNTREATED_TCONV_DN GSE13306_RA_VS_UNTREATED_TCONV_DN

140	0.20037048	0.8224713	0.9
0.8858131	1	2424	"tags=19%, list=17%, signal=23%"
GSE21063_CTRL_VS_ANTI_IGM_STIM_BCELL_8H_DN
GSE21063_CTRL_VS_ANTI_IGM_STIM_BCELL_8H_DN	129
0.20023245	0.8222917	0.8565217	0.8858038
1	1375	"tags=10%, list=10%, signal=11%"
GSE16385_UNTREATED_VS_12H_ROSIGLITAZONE_IL4_TREATED_MACROPHAGE_DN
GSE16385_UNTREATED_VS_12H_ROSIGLITAZONE_IL4_TREATED_MACROPHAGE_DN
142	0.19852982	0.8139777	0.9154013
0.89969045	1	2593	"tags=23%, list=18%, signal=27%"
GSE22935_UNSTIM_VS_24H_MBOVIS_BCG_STIM_MACROPHAGE_UP
GSE22935_UNSTIM_VS_24H_MBOVIS_BCG_STIM_MACROPHAGE_UP
164	0.19427617	0.80769676	0.9107505
0.9094226	1	2051	"tags=16%, list=15%, signal=18%"
GSE20715_0H_VS_24H_OZONE_LUNG_DN	GSE20715_0H_VS_24H_OZONE_LUNG_DN
162	0.19171421	0.8069058	0.93509126
0.9103444	1	1766	"tags=13%, list=13%, signal=15%"
GSE5589_IL6_KO_VS_IL10_KO_LPS_STIM_MACROPHAGE_180MIN_DN
GSE5589_IL6_KO_VS_IL10_KO_LPS_STIM_MACROPHAGE_180MIN_DN
136	0.19377974	0.8030202	0.9077869
0.91594696	1	2434	"tags=21%, list=17%, signal=25%"
GSE27786_CD8_TCELL_VS_ERYTHROBLAST_DN
GSE27786_CD8_TCELL_VS_ERYTHROBLAST_DN	143
0.19085312	0.7880321	0.9147982	0.93607724
1	1894	"tags=13%, list=13%, signal=15%"
GSE17721_0.5H_VS_24H_LPS_BMDC_DN	GSE17721_0.5H_VS_24H_LPS_BMDC_DN
161	0.1846351	0.78757286	0.94871795
0.936283	1	2350	"tags=22%, list=17%, signal=26%"
GSE13485_CTRL_VS_DAY21_YF17D_VACCINE_PBMC_DN
GSE13485_CTRL_VS_DAY21_YF17D_VACCINE_PBMC_DN	87
0.20585592	0.7830442	0.89837396	0.9414331
1	1091	"tags=7%, list=8%, signal=7%"
GSE29949_MICROGLIA_BRAIN_VS_CD8_NEG_DC_SPLEEN_DN
GSE29949_MICROGLIA_BRAIN_VS_CD8_NEG_DC_SPLEEN_DN	150
0.18959819	0.781142	0.9303279	0.94334394	1
2271	"tags=16%, list=16%, signal=19%"
GSE16385_IFNG_TNF_VS_IL4_STIM_MACROPHAGE_DN
GSE16385_IFNG_TNF_VS_IL4_STIM_MACROPHAGE_DN	117
0.19386706	0.7683445	0.9275053	0.9565512
1	1503	"tags=10%, list=11%, signal=11%"
GSE16385_UNTREATED_VS_12H_IFNG_TNF_TREATED_MACROPHAGE_UP
GSE16385_UNTREATED_VS_12H_IFNG_TNF_TREATED_MACROPHAGE_UP
112	0.19122058	0.76605153	0.92440605
0.9583625	1	2733	"tags=21%, list=19%, signal=25%"
GSE3982_BASOPHIL_VS_NKCELL_DN	GSE3982_BASOPHIL_VS_NKCELL_DN
133	0.18404186	0.7616463	0.94218415
0.9620657	1	2211	"tags=17%, list=16%, signal=19%"
GSE27786_NKTCELL_VS_ERYTHROBLAST_DN
GSE27786_NKTCELL_VS_ERYTHROBLAST_DN	147
0.1839853	0.7543453	0.9734694	0.9677886
1	2331	"tags=16%, list=17%, signal=19%"
GSE29949_MICROGLIA_BRAIN_VS_CD8_POS_DC_SPLEEN_DN
GSE29949_MICROGLIA_BRAIN_VS_CD8_POS_DC_SPLEEN_DN	147
0.18175477	0.74779797	0.96385545	0.9722985

1	574	"tags=5%, list=4%, signal=5%"
GSE29615_DAY3_VS_DAY7_LAIV_FLU_VACCINE_PBMC_UP
GSE29615_DAY3_VS_DAY7_LAIV_FLU_VACCINE_PBMC_UP	105
0.1897453	0.74705607	0.9471459	0.97245723
1	1866	"tags=14%, list=13%, signal=16%"
GSE37301_HEMATOPOIETIC_STEM_CELL_VS_CD4_TCELL_DN
GSE37301_HEMATOPOIETIC_STEM_CELL_VS_CD4_TCELL_DN	103
0.18497925	0.7294479	0.96255505	0.98272717
1	1330	"tags=9%, list=9%, signal=10%"
GSE13411_IGM_VS_SWITCHED_MEMORY_BCELL_UP
GSE13411_IGM_VS_SWITCHED_MEMORY_BCELL_UP	141
0.17626245	0.72719085	0.9801762	0.9835604
1	2346	"tags=16%, list=17%, signal=19%"
GSE42088_UNINF_VS_LEISHMANIA_INF_DC_8H_DN
GSE42088_UNINF_VS_LEISHMANIA_INF_DC_8H_DN	133
0.17737232	0.7246296	0.9812108	0.98443484
1	866	"tags=6%, list=6%, signal=6%"
GSE46606_DAY1_VS_DAY3_CD40L_IL2_IL5_STIMULATED_IRF4_KO_BCELL_UP
GSE46606_DAY1_VS_DAY3_CD40L_IL2_IL5_STIMULATED_IRF4_KO_BCELL_UP
	140	0.17293112	0.7223269	0.9840319
0.9851657	1	2997	"tags=24%, list=21%, signal=30%"
GSE29614_CTRL_VS_DAY3_TIV_FLU_VACCINE_PBMC_DN
GSE29614_CTRL_VS_DAY3_TIV_FLU_VACCINE_PBMC_DN	94
0.18378517	0.7154978	0.9762419	0.9875933
1	520	"tags=5%, list=4%, signal=5%"
GSE14413_UNSTIM_VS_IFNB_STIM_NIH3T3_CELLS_UP
GSE14413_UNSTIM_VS_IFNB_STIM_NIH3T3_CELLS_UP	100
0.17895718	0.7138004	0.9715536	0.9878777
1	2765	"tags=20%, list=20%, signal=25%"
GSE36476_YOUNG_VS_OLD_DONOR_MEMORY_CD4_TCELL_40H_TSST_ACT_UP
GSE36476_YOUNG_VS_OLD_DONOR_MEMORY_CD4_TCELL_40H_TSST_ACT_UP
	135	0.17270543	0.7102981	0.98523206
0.9887368	1	2003	"tags=12%, list=14%, signal=14%"
GSE29949_CD8_NEG_DC_SPLEEN_VS_CD8_POS_DC_SPLEEN_UP
GSE29949_CD8_NEG_DC_SPLEEN_VS_CD8_POS_DC_SPLEEN_UP	151
0.16725545	0.7005419	0.99139786	0.9915586
1	1990	"tags=11%, list=14%, signal=13%"
GSE5589_WT_VS_IL6_KO_LPS_STIM_MACROPHAGE_180MIN_DN
GSE5589_WT_VS_IL6_KO_LPS_STIM_MACROPHAGE_180MIN_DN	98
0.17432027	0.68715227	0.9800885	0.9944106
1	1538	"tags=11%, list=11%, signal=13%"
GSE13522_WT_VS_IFNG_KO_SKIN_DN	GSE13522_WT_VS_IFNG_KO_SKIN_DN
	94	0.17327182	0.6787835	0.9934924
0.9956143	1	2122	"tags=16%, list=15%, signal=19%"
GSE9946_MATURE_STIMULATORY_VS_LISTERIA_INF_MATURE_DC_DN
GSE9946_MATURE_STIMULATORY_VS_LISTERIA_INF_MATURE_DC_DN
95	0.17041332	0.65935177	0.99602383
0.9978767	1	2003	"tags=15%, list=14%, signal=17%"
GSE14413_UNSTIM_VS_IFNB_STIM_L929_CELLS_DN
GSE14413_UNSTIM_VS_IFNB_STIM_L929_CELLS_DN	89
0.1685827	0.63843125	0.9918033	0.9990974
1	639	"tags=6%, list=5%, signal=6%"
GSE14026_TH1_VS_TH17_UP	GSE14026_TH1_VS_TH17_UP	135
0.1506534	0.6205012	1	0.9994897	1

2920 "tags=22%, list=21%, signal=28%"
GSE11367_CTRL_VS_IL17_TREATED_SMOOTH_MUSCLE_CELL_UP
GSE11367_CTRL_VS_IL17_TREATED_SMOOTH_MUSCLE_CELL_UP
101 0.117878236 0.4598367 1 0.99999917
1 2644 "tags=14%, list=19%, signal=17%"

Table S5B
NAME GS
 follow link to MSigDB GS DETAILS SIZE
ES NES NOM p-val FDR q-val FWER p-val
RANK AT MAX LEADING EDGE
GSE14415_NATURAL_TREG_VS_FOXP3_KO_NATURAL_TREG_DN
GSE14415_NATURAL_TREG_VS_FOXP3_KO_NATURAL_TREG_DN Details ...
116 -0.7920045 -3.1605818 0 0 0
1856 "tags=73%, list=13%, signal=84%"
GSE14415_INDUCED_VS_NATURAL_TREG_UP
GSE14415_INDUCED_VS_NATURAL_TREG_UP Details ...
115 -0.7845523 -3.1368103 0 0 0
1883 "tags=70%, list=13%, signal=81%"
GSE13547_WT_VS_ZFX_KO_BCELL_UP GSE13547_WT_VS_ZFX_KO_BCELL_UP
Details ... 122 -0.73388404 -2.9528754 0
0 0 1258 "tags=50%, list=9%, signal=54%"
GSE15330_HSC_VS_MEGAKARYOCYTE_ERYTHROID_PROGENITOR_UP
GSE15330_HSC_VS_MEGAKARYOCYTE_ERYTHROID_PROGENITOR_UP
Details ... 125 -0.6965992 -2.807849 0
0 0 1437 "tags=49%, list=10%, signal=54%"
GSE13547_CTRL_VS_ANTI_IGM_STIM_ZFX_KO_BCELL_12H_UP
GSE13547_CTRL_VS_ANTI_IGM_STIM_ZFX_KO_BCELL_12H_UP Details ...
107 -0.70223194 -2.8053808 0 0 0
2469 "tags=63%, list=17%, signal=75%"
GSE14699_NAIVE_VS_DELETIONAL_TOLERANCE_CD8_TCELL_UP
GSE14699_NAIVE_VS_DELETIONAL_TOLERANCE_CD8_TCELL_UP
Details ... 113 -0.7080644 -2.7982972 0
0 0 1414 "tags=50%, list=10%, signal=55%"
GSE13547_CTRL_VS_ANTI_IGM_STIM_ZFX_KO_BCELL_12H_DN
GSE13547_CTRL_VS_ANTI_IGM_STIM_ZFX_KO_BCELL_12H_DN Details ...
118 -0.6795874 -2.71107 0 0 0
1437 "tags=45%, list=10%, signal=50%"
GSE14415_FOXP3_KO_NATURAL_TREG_VS_TCONV_UP
GSE14415_FOXP3_KO_NATURAL_TREG_VS_TCONV_UP Details ...
130 -0.65910107 -2.702069 0 0 0
1535 "tags=46%, list=11%, signal=51%"
GOLDRATH_EFF_VS_MEMORY_CD8_TCELL_DN
GOLDRATH_EFF_VS_MEMORY_CD8_TCELL_DN Details ...
150 -0.64228404 -2.693487 0 0 0
1500 "tags=40%, list=11%, signal=44%"
GSE13547_2H_VS_12_H_ANTI_IGM_STIM_ZFX_KO_BCELL_UP
GSE13547_2H_VS_12_H_ANTI_IGM_STIM_ZFX_KO_BCELL_UP Details ...
118 -0.6657932 -2.6603973 0 0 0
1591 "tags=47%, list=11%, signal=52%"
GSE9650_NAIVE_VS_EFF_CD8_TCELL_UP GSE9650_NAIVE_VS_EFF_CD8_TCELL_UP
Details ... 156 -0.631967 -2.6433086 0
0 0 1424 "tags=39%, list=10%, signal=43%"
GSE40068_BCL6_POS_VS_NEG_CXCR5_POS_TFH_DN
GSE40068_BCL6_POS_VS_NEG_CXCR5_POS_TFH_DN Details ...
132 -0.64637655 -2.6399653 0 0 0
1610 "tags=47%, list=11%, signal=53%"
GSE14699_NAIVE_VS_ACT_CD8_TCELL_DN
GSE14699_NAIVE_VS_ACT_CD8_TCELL_DN Details ...
136 -0.6289371 -2.583616 0 0 0
1435 "tags=44%, list=10%, signal=49%"

GSE14415_INDUCED_TREG_VS_TCONV_DN GSE14415_INDUCED_TREG_VS_TCONV_DN
Details ... 122 -0.6482624 -2.5814164 0
0 0 1704 "tags=50%, list=12%, signal=56%"
KAECH_NAIVE_VS_DAY8_EFF_CD8_TCELL_UP
KAECH_NAIVE_VS_DAY8_EFF_CD8_TCELL_UP Details ...
158 -0.620211 -2.5668218 0 0 0
1435 "tags=39%, list=10%, signal=43%"
GSE33162_UNTREATED_VS_4H_LPS_STIM_HDAC3_HET_MACROPHAGE_UP
GSE33162_UNTREATED_VS_4H_LPS_STIM_HDAC3_HET_MACROPHAGE_UP
Details ... 154 -0.6143706 -2.5665185 0
0 0 1509 "tags=36%, list=11%, signal=40%"
GSE3920_IFNA_VS_IFNG_TREATED_ENDOTHELIAL_CELL_UP
GSE3920_IFNA_VS_IFNG_TREATED_ENDOTHELIAL_CELL_UP Details ...
127 -0.63924104 -2.56532 0 0 0
3190 "tags=61%, list=23%, signal=78%"
GSE15750_DAY6_VS_DAY10_EFF_CD8_TCELL_DN
GSE15750_DAY6_VS_DAY10_EFF_CD8_TCELL_DN Details ...
133 -0.62883496 -2.5613303 0 0 0
2101 "tags=45%, list=15%, signal=53%"
GSE14415_ACT_VS_CTRL_NATURAL_TREG_DN
GSE14415_ACT_VS_CTRL_NATURAL_TREG_DN Details ...
128 -0.63416433 -2.546876 0 0 0
1679 "tags=41%, list=12%, signal=46%"
GSE20727_H2O2_VS_ROS_INHIBITOR_TREATED_DC_DN
GSE20727_H2O2_VS_ROS_INHIBITOR_TREATED_DC_DN Details ...
142 -0.6074251 -2.5127246 0 0 0
1779 "tags=39%, list=13%, signal=45%"
GSE15750_DAY6_VS_DAY10_TRAF6KO_EFF_CD8_TCELL_DN
GSE15750_DAY6_VS_DAY10_TRAF6KO_EFF_CD8_TCELL_DN
141 -0.6086538 -2.5051148 0 0 0
2312 "tags=49%, list=16%, signal=58%"
GOLDRATH_NAIVE_VS_EFF_CD8_TCELL_UP
GOLDRATH_NAIVE_VS_EFF_CD8_TCELL_UP
151 -0.60138685 -2.4974768 0 0 0
1740 "tags=43%, list=12%, signal=49%"
GSE33162_UNTREATED_VS_4H_LPS_STIM_HDAC3_HET_MACROPHAGE_DN
GSE33162_UNTREATED_VS_4H_LPS_STIM_HDAC3_HET_MACROPHAGE_DN
152 -0.5962253 -2.4951632 0 0 0
1625 "tags=36%, list=12%, signal=40%"
GSE13547_WT_VS_ZFX_KO_BCELL_ANTI_IGM_STIM_2H_DN
GSE13547_WT_VS_ZFX_KO_BCELL_ANTI_IGM_STIM_2H_DN
119 -0.62349457 -2.4949677 0 0 0
2943 "tags=54%, list=21%, signal=67%"
GSE9650_EFFECTOR_VS_MEMORY_CD8_TCELL_DN
GSE9650_EFFECTOR_VS_MEMORY_CD8_TCELL_DN
156 -0.59121096 -2.4929132 0 0 0
1585 "tags=40%, list=11%, signal=44%"
GSE19888_CTRL_VS_A3R_INHIBITOR_TREATED_MAST_CELL_DN
GSE19888_CTRL_VS_A3R_INHIBITOR_TREATED_MAST_CELL_DN
129 -0.6117933 -2.4816866 0 0 0
2419 "tags=46%, list=17%, signal=55%"
GSE15330_LYMPHOID_MULTIPOTENT_VS_MEGAKARYOCYTE_ERYTHROID_PROGENITOR_
IKAROS_KO_UP
GSE15330_LYMPHOID_MULTIPOTENT_VS_MEGAKARYOCYTE_ERYTHROID_PROGENITOR_

IKAROS_KO_UP 132 -0.60986847 -2.4799666
0 0 0 1437 "tags=42%, list=10%, signal=46%"
GSE40068_CXCR5POS_BCL6POS_TFH_VS_CXCR5NEG_BCL6NEG_CD4_TCELL_UP
GSE40068_CXCR5POS_BCL6POS_TFH_VS_CXCR5NEG_BCL6NEG_CD4_TCELL_UP
146 -0.5957934 -2.4718382 0 0
0 1779 "tags=42%, list=13%, signal=47%"
GSE15330_HSC_VS_MEGAKARYOCYTE_ERYTHROID_PROGENITOR_DN
GSE15330_HSC_VS_MEGAKARYOCYTE_ERYTHROID_PROGENITOR_DN
125 -0.61609095 -2.4651613 0 0 0
1437 "tags=42%, list=10%, signal=46%"
KAECH_DAY8_EFF_VS_MEMORY_CD8_TCELL_DN
KAECH_DAY8_EFF_VS_MEMORY_CD8_TCELL_DN
150 -0.5915394 -2.4542527 0 0 0
1509 "tags=39%, list=11%, signal=44%"
GSE40274_CTRL_VS_EOS_TRANSDUCED_ACTIVATED_CD4_TCELL_DN
GSE40274_CTRL_VS_EOS_TRANSDUCED_ACTIVATED_CD4_TCELL_DN
115 -0.6144799 -2.4520206 0 0 0
1833 "tags=40%, list=13%, signal=46%"
GSE40068_CXCR5POS_BCL6POS_TFH_VS_CXCR5NEG_BCL6NEG_CD4_TCELL_DN
GSE40068_CXCR5POS_BCL6POS_TFH_VS_CXCR5NEG_BCL6NEG_CD4_TCELL_DN
157 -0.58192754 -2.449242 0 0
0 2174 "tags=43%, list=15%, signal=51%"
GSE39110_UNTREATED_VS_IL2_TREATED_CD8_TCELL_DAY6_POST_IMMUNIZATION_D
N
GSE39110_UNTREATED_VS_IL2_TREATED_CD8_TCELL_DAY6_POST_IMMUNIZATION_D
N 139 -0.60133344 -2.435259 0
0 0 1288 "tags=35%, list=9%, signal=38%"
GSE13946_CTRL_VS_DSS_COLITIS_GD_TCELL_FROM_COLON_DN
GSE13946_CTRL_VS_DSS_COLITIS_GD_TCELL_FROM_COLON_DN
134 -0.5928245 -2.424504 0 0 0
1435 "tags=40%, list=10%, signal=44%"
GSE39110_DAY3_VS_DAY6_POST_IMMUNIZATION_CD8_TCELL_UP
GSE39110_DAY3_VS_DAY6_POST_IMMUNIZATION_CD8_TCELL_UP
142 -0.58643067 -2.4179509 0 0 0
1726 "tags=37%, list=12%, signal=42%"
GSE14415_INDUCED_TREG_VS_FOXP3_KO_INDUCED_TREG_UP
GSE14415_INDUCED_TREG_VS_FOXP3_KO_INDUCED_TREG_UP
138 -0.5784511 -2.389455 0 0 0
1397 "tags=38%, list=10%, signal=42%"
GSE2770_UNTREATED_VS_ACT_CD4_TCELL_6H_DN
GSE2770_UNTREATED_VS_ACT_CD4_TCELL_6H_DN
133 -0.58293545 -2.3849335 0 0 0
1591 "tags=34%, list=11%, signal=38%"
GSE10239_NAIVE_VS_DAY4.5_EFF_CD8_TCELL_UP
GSE10239_NAIVE_VS_DAY4.5_EFF_CD8_TCELL_UP
135 -0.5845982 -2.3520055 0 0 0
1277 "tags=35%, list=9%, signal=38%"
GSE19888_ADENOSINE_A3R_INH_PRETREAT_AND_ACT_BY_A3R_VS_A3R_INH_AND_TC
ELL_MEMBRANES_ACT_MAST_CELL_UP
GSE19888_ADENOSINE_A3R_INH_PRETREAT_AND_ACT_BY_A3R_VS_A3R_INH_AND_TC
ELL_MEMBRANES_ACT_MAST_CELL_UP
133 -0.5728688 -2.3414397 0 0 0
1796 "tags=41%, list=13%, signal=46%"
GSE39110_UNTREATED_VS_IL2_TREATED_CD8_TCELL_DAY3_POST_IMMUNIZATION_U

P
GSE39110_UNTREATED_VS_IL2_TREATED_CD8_TCELL_DAY3_POST_IMMUNIZATION_UP	136	-0.56684196	-2.3389866	0
0	0	1830	"tags=36%, list=13%, signal=41%"
GSE11386_NAIVE_VS_MEMORY_BCELL_DN GSE11386_NAIVE_VS_MEMORY_BCELL_DN
	122	-0.5750599	-2.335291	0	0
0	1445	"tags=38%, list=10%, signal=42%"
KAECH_NAIVE_VS_DAY15_EFF_CD8_TCELL_UP
KAECH_NAIVE_VS_DAY15_EFF_CD8_TCELL_UP
159	-0.56162095	-2.3331335	0	0	0
1435	"tags=35%, list=10%, signal=38%"
GSE40274_FOXP3_VS_FOXP3_AND_PBX1_TRANSDUCED_ACTIVATED_CD4_TCELL_DN
GSE40274_FOXP3_VS_FOXP3_AND_PBX1_TRANSDUCED_ACTIVATED_CD4_TCELL_DN
	150	-0.5631535	-2.3232806	0	0
0	2397	"tags=45%, list=17%, signal=54%"
GSE13547_WT_VS_ZFX_KO_BCELL_ANTI_IGM_STIM_12H_DN
GSE13547_WT_VS_ZFX_KO_BCELL_ANTI_IGM_STIM_12H_DN
119	-0.5782173	-2.3226357	0	0	0
1135	"tags=32%, list=8%, signal=34%"
GSE36078_WT_VS_IL1R_KO_LUNG_DC_AFTER_AD5_INF_DN
GSE36078_WT_VS_IL1R_KO_LUNG_DC_AFTER_AD5_INF_DN
151	-0.55560154	-2.3158097	0	0	0
2169	"tags=44%, list=15%, signal=51%"
GSE19941_LPS_VS_LPS_AND_IL10_STIM_IL10_KO_MACROPHAGE_DN
GSE19941_LPS_VS_LPS_AND_IL10_STIM_IL10_KO_MACROPHAGE_DN
109	-0.5809035	-2.2920947	0	0	0
1895	"tags=44%, list=13%, signal=50%"
GSE14769_UNSTIM_VS_40MIN_LPS_BMDM_DN
GSE14769_UNSTIM_VS_40MIN_LPS_BMDM_DN
152	-0.5485254	-2.2885516	0	0	0
1776	"tags=39%, list=13%, signal=45%"
KAECH_DAY8_EFF_VS_DAY15_EFF_CD8_TCELL_DN
KAECH_DAY8_EFF_VS_DAY15_EFF_CD8_TCELL_DN
150	-0.55680007	-2.2883444	0	0	0
1509	"tags=37%, list=11%, signal=41%"
GSE14415_INDUCED_TREG_VS_FOXP3_KO_INDUCED_TREG_IL2_CULTURE_UP
GSE14415_INDUCED_TREG_VS_FOXP3_KO_INDUCED_TREG_IL2_CULTURE_UP
	142	-0.5457511	-2.2837431	0	0
0	903	"tags=31%, list=6%, signal=33%"
GSE15930_NAIVE_VS_48H_IN_VITRO_STIM_CD8_TCELL_UP
GSE15930_NAIVE_VS_48H_IN_VITRO_STIM_CD8_TCELL_UP
148	-0.55392563	-2.2784965	0	0	0
1980	"tags=37%, list=14%, signal=43%"
GSE9650_EFFECTOR_VS_EXHAUSTED_CD8_TCELL_DN
GSE9650_EFFECTOR_VS_EXHAUSTED_CD8_TCELL_DN
157	-0.5415937	-2.2548301	0	0	0
2297	"tags=37%, list=16%, signal=44%"
GSE40274_FOXP3_VS_FOXP3_AND_GATA1_TRANSDUCED_ACTIVATED_CD4_TCELL_DN
GSE40274_FOXP3_VS_FOXP3_AND_GATA1_TRANSDUCED_ACTIVATED_CD4_TCELL_DN
	150	-0.5453015	-2.242668	0	0
0	2468	"tags=44%, list=17%, signal=53%"
GSE36891_POLYIC_TLR3_VS_PAM_TLR2_STIM_PERITONEAL_MACROPHAGE_UP
GSE36891_POLYIC_TLR3_VS_PAM_TLR2_STIM_PERITONEAL_MACROPHAGE_UP
	121	-0.557561	-2.227713	0	0

0	2671	"tags=43%, list=19%, signal=53%"
GSE40274_FOXP3_VS_FOXP3_AND_HELIOS_TRANSDUCED_ACTIVATED_CD4_TCELL_DN

GSE40274_FOXP3_VS_FOXP3_AND_HELIOS_TRANSDUCED_ACTIVATED_CD4_TCELL_DN
		95	-0.56621265	-2.2239773	0
0	0	1592	"tags=38%, list=11%, signal=42%"
GSE2935_UV_INACTIVATED_VS_LIVE_SENDAI_VIRUS_INF_MACROPHAGE_UP
GSE2935_UV_INACTIVATED_VS_LIVE_SENDAI_VIRUS_INF_MACROPHAGE_UP
	145	-0.53928113	-2.2210987	0	0
0	1775	"tags=38%, list=13%, signal=43%"
GSE21670_TGFB_VS_IL6_TREATED_STAT3_KO_CD4_TCELL_UP
GSE21670_TGFB_VS_IL6_TREATED_STAT3_KO_CD4_TCELL_UP
126	-0.54753584	-2.2203841	0	0	0
2035	"tags=34%, list=14%, signal=40%"
GSE14415_ACT_TCONV_VS_ACT_NATURAL_TREG_UP
GSE14415_ACT_TCONV_VS_ACT_NATURAL_TREG_UP
139	-0.5312784	-2.1947093	0	0	0
1435	"tags=32%, list=10%, signal=36%"
GSE37301_PRO_BCELL_VS_CD4_TCELL_DN
GSE37301_PRO_BCELL_VS_CD4_TCELL_DN
125	-0.5380688	-2.1930177	0	0	0
1546	"tags=28%, list=11%, signal=31%"
GSE39110_DAY3_VS_DAY6_POST_IMMUNIZATION_CD8_TCELL_WITH_IL2_TREATMENT_DN
GSE39110_DAY3_VS_DAY6_POST_IMMUNIZATION_CD8_TCELL_WITH_IL2_TREATMENT_DN		146	-0.52773595	-2.187285	0
0	0	1580	"tags=34%, list=11%, signal=38%"
GSE17974_0H_VS_24H_IN_VITRO_ACT_CD4_TCELL_UP
GSE17974_0H_VS_24H_IN_VITRO_ACT_CD4_TCELL_UP
109	-0.54748213	-2.1613684	0	0	0
1856	"tags=35%, list=13%, signal=40%"
GSE38304_MYC_NEG_VS_POS_GC_BCELL_UP
GSE38304_MYC_NEG_VS_POS_GC_BCELL_UP
161	-0.5194925	-2.1546118	0	0	0
1459	"tags=33%, list=10%, signal=36%"
GSE17974_1.5H_VS_72H_IL4_AND_ANTI_IL12_ACT_CD4_TCELL_UP
GSE17974_1.5H_VS_72H_IL4_AND_ANTI_IL12_ACT_CD4_TCELL_UP
126	-0.5331048	-2.1544347	0	0	0
2352	"tags=43%, list=17%, signal=51%"
GSE3920_UNTREATED_VS_IFNG_TREATED_FIBROBLAST_DN
GSE3920_UNTREATED_VS_IFNG_TREATED_FIBROBLAST_DN
135	-0.52955097	-2.1427555	0	0	0
2476	"tags=41%, list=18%, signal=49%"
GSE5142_HTERT_TRANSDUCED_VS_CTRL_CD8_TCELL_LATE_PASSAGE_CLONE_DN
GSE5142_HTERT_TRANSDUCED_VS_CTRL_CD8_TCELL_LATE_PASSAGE_CLONE_DN
	140	-0.5166635	-2.1351824	0	0
0	2139	"tags=38%, list=15%, signal=44%"
GSE16450_IMMATURE_VS_MATURE_NEURON_CELL_LINE_6H_IFNA_STIM_UP
GSE16450_IMMATURE_VS_MATURE_NEURON_CELL_LINE_6H_IFNA_STIM_UP
	142	-0.51842004	-2.1302893	0	0
0	1697	"tags=25%, list=12%, signal=29%"
GSE21670_UNTREATED_VS_TGFB_TREATED_CD4_TCELL_DN
GSE21670_UNTREATED_VS_TGFB_TREATED_CD4_TCELL_DN
148	-0.51635283	-2.1223447	0	0	0

1663 "tags=28%, list=12%, signal=32%"
GSE17974_CTRL_VS_ACT_IL4_AND_ANTI_IL12_12H_CD4_TCELL_UP
GSE17974_CTRL_VS_ACT_IL4_AND_ANTI_IL12_12H_CD4_TCELL_UP
116 -0.52484226 -2.1101696 0 0 0
1856 "tags=35%, list=13%, signal=40%"
GSE7460_TREG_VS_TCONV_ACT_WITH_TGFB_UP
GSE7460_TREG_VS_TCONV_ACT_WITH_TGFB_UP
151 -0.50799066 -2.1040955 0 0 0
2085 "tags=35%, list=15%, signal=41%"
GSE36078_UNTREATED_VS_AD5_INF_IL1R_KO_MOUSE_LUNG_DC_UP
GSE36078_UNTREATED_VS_AD5_INF_IL1R_KO_MOUSE_LUNG_DC_UP
139 -0.5048025 -2.0860074 0 0 0
2647 "tags=40%, list=19%, signal=49%"
GSE9650_NAIVE_VS_MEMORY_CD8_TCELL_UP
GSE9650_NAIVE_VS_MEMORY_CD8_TCELL_UP
151 -0.50124764 -2.0846174 0 0 0
1780 "tags=34%, list=13%, signal=39%"
GSE40277_GATA1_AND_SATB1_TRANSDUCED_VS_CTRL_CD4_TCELL_DN
GSE40277_GATA1_AND_SATB1_TRANSDUCED_VS_CTRL_CD4_TCELL_DN
142 -0.51090306 -2.0796504 0 0 0
2528 "tags=40%, list=18%, signal=48%"
GSE3720_VD1_VS_VD2_GAMMADELTA_TCELL_WITH_LPS_STIM_UP
GSE3720_VD1_VS_VD2_GAMMADELTA_TCELL_WITH_LPS_STIM_UP
142 -0.4980894 -2.0715783 0 0 0
1784 "tags=36%, list=13%, signal=41%"
GSE5679_CTRL_VS_RARA_AGONIST_AM580_TREATED_DC_DN
GSE5679_CTRL_VS_RARA_AGONIST_AM580_TREATED_DC_DN
145 -0.50175625 -2.071555 0 0 0
2312 "tags=39%, list=16%, signal=46%"
GSE7460_FOXP3_MUT_VS_WT_ACT_WITH_TGFB_TCONV_DN
GSE7460_FOXP3_MUT_VS_WT_ACT_WITH_TGFB_TCONV_DN
144 -0.49893 -2.0677054 0 0 0
1459 "tags=31%, list=10%, signal=34%"
GSE27241_CTRL_VS_DIGOXIN_TREATED_CD4_TCELL_IN_TH17_POLARIZING_CONDIT
IONS_UP
GSE27241_CTRL_VS_DIGOXIN_TREATED_CD4_TCELL_IN_TH17_POLARIZING_CONDIT
IONS_UP 124 -0.5134458 -2.062391 0
0 0 1979 "tags=32%, list=14%, signal=37%"
GSE43863_TH1_VS_LY6C_INT_CXCR5POS_EFFECTOR_CD4_TCELL_DN
GSE43863_TH1_VS_LY6C_INT_CXCR5POS_EFFECTOR_CD4_TCELL_DN
144 -0.50202614 -2.0606759 0 0 0
1460 "tags=32%, list=10%, signal=35%"
GSE18893_TCONV_VS_TREG_2H_TNF_STIM_DN
GSE18893_TCONV_VS_TREG_2H_TNF_STIM_DN
110 -0.52309555 -2.0604196 0 0 0
1803 "tags=33%, list=13%, signal=37%"
GSE2770_UNTREATED_VS_ACT_CD4_TCELL_2H_UP
GSE2770_UNTREATED_VS_ACT_CD4_TCELL_2H_UP
135 -0.50621295 -2.0550065 0 1.07E-05 0.001
2272 "tags=39%, list=16%, signal=45%"
GSE18791_CTRL_VS_NEWCASTLE_VIRUS_DC_2H_DN
GSE18791_CTRL_VS_NEWCASTLE_VIRUS_DC_2H_DN
104 -0.5224027 -2.0547254 0 1.06E-05 0.001
2088 "tags=40%, list=15%, signal=47%"

GSE36078_UNTREATED_VS_AD5_T425A_HEXON_INF_MOUSE_LUNG_DC_UP
GSE36078_UNTREATED_VS_AD5_T425A_HEXON_INF_MOUSE_LUNG_DC_UP
143 -0.4977823 -2.0531762 0 1.04E-05 0.001
2532 "tags=37%, list=18%, signal=45%"
KAECH_NAIVE_VS_MEMORY_CD8_TCELL_UP
KAECH_NAIVE_VS_MEMORY_CD8_TCELL_UP
150 -0.4904864 -2.0515137 0 1.03E-05 0.001
1780 "tags=34%, list=13%, signal=38%"
GSE17974_CTRL_VS_ACT_IL4_AND_ANTI_IL12_24H_CD4_TCELL_UP
GSE17974_CTRL_VS_ACT_IL4_AND_ANTI_IL12_24H_CD4_TCELL_UP
119 -0.5101287 -2.0445297 0 1.02E-05 0.001
1796 "tags=31%, list=13%, signal=35%"
GSE24142_ADULT_VS_FETAL_DN3_THYMOCYTE_UP
GSE24142_ADULT_VS_FETAL_DN3_THYMOCYTE_UP
149 -0.4868879 -2.0444221 0 1.01E-05 0.001
1769 "tags=32%, list=13%, signal=36%"
GSE20727_H2O2_VS_ROS_INHIBITOR_TREATED_DC_UP
GSE20727_H2O2_VS_ROS_INHIBITOR_TREATED_DC_UP
144 -0.49649495 -2.0414984 0 9.94E-06 0.001
1790 "tags=34%, list=13%, signal=39%"
GSE4590_SMALL_VS_VPREB_POS_LARGE_PRE_BCELL_UP
GSE4590_SMALL_VS_VPREB_POS_LARGE_PRE_BCELL_UP
124 -0.5083874 -2.0400062 0 9.83E-06 0.001
1941 "tags=34%, list=14%, signal=39%"
GSE7568_IL4_VS_IL4_AND_DEXAMETHASONE_TREATED_MACROPHAGE_DN
GSE7568_IL4_VS_IL4_AND_DEXAMETHASONE_TREATED_MACROPHAGE_DN
117 -0.50572276 -2.0266025 0 9.71E-06 0.001
2081 "tags=40%, list=15%, signal=47%"
GSE10239_NAIVE_VS_KLRG1HIGH_EFF_CD8_TCELL_UP
GSE10239_NAIVE_VS_KLRG1HIGH_EFF_CD8_TCELL_UP
138 -0.4971928 -2.0223482 0 9.60E-06 0.001
1509 "tags=30%, list=11%, signal=34%"
GSE19941_UNSTIM_VS_LPS_AND_IL10_STIM_IL10_KO_NFKBP50_KO_MACROPHAGE_UP
GSE19941_UNSTIM_VS_LPS_AND_IL10_STIM_IL10_KO_NFKBP50_KO_MACROPHAGE_UP 129 -0.49560422 -2.0143943 0
9.49E-06 0.001 1737 "tags=28%, list=12%, signal=32%"
GSE34179_THPOK_KO_VS_WT_VA14I_NKTCELL_UP
GSE34179_THPOK_KO_VS_WT_VA14I_NKTCELL_UP
132 -0.49024674 -2.0110896 0 9.39E-06 0.001
1980 "tags=28%, list=14%, signal=32%"
GSE14769_UNSTIM_VS_80MIN_LPS_BMDM_DN
GSE14769_UNSTIM_VS_80MIN_LPS_BMDM_DN
150 -0.48262945 -2.005419 0 9.28E-06 0.001
3811 "tags=49%, list=27%, signal=67%"
GSE7852_LN_VS_THYMUS_TCONV_UP GSE7852_LN_VS_THYMUS_TCONV_UP
138 -0.49276036 -2.004537 0 9.18E-06
0.001 1691 "tags=33%, list=12%, signal=38%"
GSE24142_ADULT_VS_FETAL_EARLY_THYMIC_PROGENITOR_UP
GSE24142_ADULT_VS_FETAL_EARLY_THYMIC_PROGENITOR_UP
154 -0.48719388 -2.0014734 0 9.08E-06 0.001
1848 "tags=30%, list=13%, signal=34%"
GSE14769_UNSTIM_VS_60MIN_LPS_BMDM_DN
GSE14769_UNSTIM_VS_60MIN_LPS_BMDM_DN

154	-0.47639263	-1.9983352	0	8.98E-06 0.001
2193	"tags=37%, list=16%, signal=43%"
GSE37532_WT_VS_PPARG_KO_LN_TCONV_UP
GSE37532_WT_VS_PPARG_KO_LN_TCONV_UP
136	-0.4837034	-1.9957852	0	8.89E-06 0.001
1371	"tags=31%, list=10%, signal=34%"
GSE21063_CTRL_VS_ANTI_IGM_STIM_BCELL_NFATC1_KO_3H_DN
GSE21063_CTRL_VS_ANTI_IGM_STIM_BCELL_NFATC1_KO_3H_DN
116	-0.49512774	-1.9882954	0	1.76E-05 0.002
1928	"tags=33%, list=14%, signal=38%"
GSE5455_HEALTHY_VS_TUMOR_BEARING_MOUSE_SPLEEN_MONOCYTE_24H_INCUBATIO
N_UP
GSE5455_HEALTHY_VS_TUMOR_BEARING_MOUSE_SPLEEN_MONOCYTE_24H_INCUBATIO
N_UP	147	-0.48224577	-1.9864553	0
1.75E-05 0.002	2267	"tags=37%, list=16%, signal=44%"
GSE14415_NATURAL_TREG_VS_FOXP3_KO_NATURAL_TREG_UP
GSE14415_NATURAL_TREG_VS_FOXP3_KO_NATURAL_TREG_UP
113	-0.49847847	-1.9846256	0	1.73E-05 0.002
1823	"tags=32%, list=13%, signal=36%"
GSE17974_0H_VS_48H_IN_VITRO_ACT_CD4_TCELL_UP
GSE17974_0H_VS_48H_IN_VITRO_ACT_CD4_TCELL_UP
109	-0.49980432	-1.9804904	0.001845019	2.55E-05
0.003	1796	"tags=33%, list=13%, signal=38%"
GSE25146_UNSTIM_VS_HELIOBACTER_PYLORI_LPS_STIM_AGS_CELL_DN
GSE25146_UNSTIM_VS_HELIOBACTER_PYLORI_LPS_STIM_AGS_CELL_DN
125	-0.49395218	-1.9797726	0	2.52E-05 0.003
1726	"tags=34%, list=12%, signal=38%"
GSE37533_UNTREATED_VS_PIOGLIZATONE_TREATED_CD4_TCELL_PPARG2_AND_FOXP
3_TRASDUCED_UP
GSE37533_UNTREATED_VS_PIOGLIZATONE_TREATED_CD4_TCELL_PPARG2_AND_FOXP
3_TRASDUCED_UP	111	-0.5002202	-1.9791722
0	2.50E-05 0.003	3452	"tags=42%, list=24%, signal=56%"
GSE24671_CTRL_VS_BAKIMULC_INFECTED_MOUSE_SPLENOCYTES_DN
GSE24671_CTRL_VS_BAKIMULC_INFECTED_MOUSE_SPLENOCYTES_DN
132	-0.4854279	-1.977853	0	2.47E-05 0.003
1937	"tags=34%, list=14%, signal=39%"
GSE7460_CTRL_VS_TGFB_TREATED_ACT_CD8_TCELL_DN
GSE7460_CTRL_VS_TGFB_TREATED_ACT_CD8_TCELL_DN
135	-0.48070166	-1.9777474	0	2.45E-05 0.003
1141	"tags=23%, list=8%, signal=25%"
GSE11961_GERMINAL_CENTER_BCELL_DAY7_VS_GERMINAL_CENTER_BCELL_DAY40_D
N
GSE11961_GERMINAL_CENTER_BCELL_DAY7_VS_GERMINAL_CENTER_BCELL_DAY40_D
N	138	-0.4767416	-1.9774413	0
2.43E-05 0.003	3190	"tags=46%, list=23%, signal=58%"
GSE20366_EX_VIVO_VS_DEC205_CONVERSION_DN
GSE20366_EX_VIVO_VS_DEC205_CONVERSION_DN
135	-0.4902983	-1.9732864	0	2.40E-05 0.003
1638	"tags=33%, list=12%, signal=37%"
GSE14350_TREG_VS_TEFF_UP	GSE14350_TREG_VS_TEFF_UP
130	-0.48523003	-1.9720023	0	2.38E-05 0.003
1532	"tags=28%, list=11%, signal=32%"
GSE23505_IL6_IL1_VS_IL6_IL1_TGFB_TREATED_CD4_TCELL_UP
GSE23505_IL6_IL1_VS_IL6_IL1_TGFB_TREATED_CD4_TCELL_UP

133 -0.47794363 -1.9685946 0 3.16E-05 0.004
2043 "tags=28%, list=14%, signal=32%"
GSE20366_TREG_VS_NAIVE_CD4_TCELL_HOMEOSTATIC_CONVERSION_DN
GSE20366_TREG_VS_NAIVE_CD4_TCELL_HOMEOSTATIC_CONVERSION_DN
151 -0.4762983 -1.9632642 0 3.13E-05 0.004
1414 "tags=28%, list=10%, signal=31%"
GSE10239_NAIVE_VS_KLRG1INT_EFF_CD8_TCELL_UP
GSE10239_NAIVE_VS_KLRG1INT_EFF_CD8_TCELL_UP
131 -0.4812607 -1.9620762 0 3.10E-05 0.004
1533 "tags=29%, list=11%, signal=32%"
GSE17974_CTRL_VS_ACT_IL4_AND_ANTI_IL12_6H_CD4_TCELL_UP
GSE17974_CTRL_VS_ACT_IL4_AND_ANTI_IL12_6H_CD4_TCELL_UP
116 -0.48734453 -1.9593487 0 3.07E-05 0.004
1277 "tags=29%, list=9%, signal=32%"
GSE8678_IL7R_LOW_VS_HIGH_EFF_CD8_TCELL_DN
GSE8678_IL7R_LOW_VS_HIGH_EFF_CD8_TCELL_DN
143 -0.47840375 -1.9588841 0 3.81E-05 0.005
1392 "tags=27%, list=10%, signal=30%"
GSE15324_ELF4_KO_VS_WT_ACTIVATED_CD8_TCELL_UP
GSE15324_ELF4_KO_VS_WT_ACTIVATED_CD8_TCELL_UP
146 -0.47886178 -1.9566467 0 3.77E-05 0.005
1872 "tags=30%, list=13%, signal=34%"
GSE14386_UNTREATED_VS_IFNA_TREATED_ACT_PBMC_MS_PATIENT_DN
GSE14386_UNTREATED_VS_IFNA_TREATED_ACT_PBMC_MS_PATIENT_DN
125 -0.4803927 -1.9551392 0 3.74E-05 0.005
1498 "tags=30%, list=11%, signal=34%"
GSE17974_CTRL_VS_ACT_IL4_AND_ANTI_IL12_72H_CD4_TCELL_UP
GSE17974_CTRL_VS_ACT_IL4_AND_ANTI_IL12_72H_CD4_TCELL_UP
124 -0.4821644 -1.949261 0 4.46E-05 0.006
1856 "tags=35%, list=13%, signal=40%"
GSE11961_UNSTIM_VS_ANTI_IGM_AND_CD40_STIM_6H_FOLLICULAR_BCELL_DN
GSE11961_UNSTIM_VS_ANTI_IGM_AND_CD40_STIM_6H_FOLLICULAR_BCELL_DN
147 -0.4772322 -1.9465133 0 4.42E-05
0.006 1397 "tags=29%, list=10%, signal=32%"
GSE2706_R848_VS_R848_AND_LPS_2H_STIM_DC_DN
GSE2706_R848_VS_R848_AND_LPS_2H_STIM_DC_DN
124 -0.48629445 -1.9454728 0 4.38E-05 0.006
1415 "tags=26%, list=10%, signal=28%"
GSE14350_IL2RB_KO_VS_WT_TEFF_UP GSE14350_IL2RB_KO_VS_WT_TEFF_UP
156 -0.4641211 -1.9451172 0 4.34E-05
0.006 1379 "tags=29%, list=10%, signal=32%"
GSE17974_CTRL_VS_ACT_IL4_AND_ANTI_IL12_48H_CD4_TCELL_UP
GSE17974_CTRL_VS_ACT_IL4_AND_ANTI_IL12_48H_CD4_TCELL_UP
105 -0.48746765 -1.944893 0 4.30E-05 0.006
1982 "tags=32%, list=14%, signal=37%"
GSE21670_TGFB_VS_TGFB_AND_IL6_TREATED_STAT3_KO_CD4_TCELL_UP
GSE21670_TGFB_VS_TGFB_AND_IL6_TREATED_STAT3_KO_CD4_TCELL_UP
131 -0.46941835 -1.9338766 0 5.68E-05 0.008
1726 "tags=29%, list=12%, signal=33%"
GSE7460_FOXP3_MUT_VS_WT_ACT_TCONV_DN
GSE7460_FOXP3_MUT_VS_WT_ACT_TCONV_DN
148 -0.47044086 -1.9325032 0 6.35E-05 0.008
1459 "tags=30%, list=10%, signal=34%"
GSE42021_TREG_PLN_VS_CD24HI_TREG_THYMUS_UP

GSE42021_TREG_PLN_VS_CD24HI_TREG_THYMUS_UP
165 -0.4592268 -1.9314227 0 6.29E-05 0.008
2159 "tags=32%, list=15%, signal=37%"
GSE25123_CTRL_VS_IL4_AND_ROSIGLITAZONE_STIM_PPARG_KO_MACROPHAGE_DN
GSE25123_CTRL_VS_IL4_AND_ROSIGLITAZONE_STIM_PPARG_KO_MACROPHAGE_DN
137 -0.4721956 -1.927797 0 6.24E-05
0.008 2324 "tags=39%, list=16%, signal=47%"
GSE37301_CD4_TCELL_VS_GRANULOCYTE_MONOCYTE_PROGENITOR_DN
GSE37301_CD4_TCELL_VS_GRANULOCYTE_MONOCYTE_PROGENITOR_DN
156 -0.4608469 -1.927765 0 6.19E-05 0.008
1362 "tags=26%, list=10%, signal=29%"
GSE22229_UNTREATED_VS_IMMUNOSUPP_THERAPY_RENAL_TRANSPLANT_PATIENT_PB
MC_DN
GSE22229_UNTREATED_VS_IMMUNOSUPP_THERAPY_RENAL_TRANSPLANT_PATIENT_PB
MC_DN 148 -0.46660966 -1.9268565 0
6.82E-05 0.008 2113 "tags=28%, list=15%, signal=32%"
GSE39916_B_CELL_SPLEEN_VS_PLASMA_CELL_BONE_MARROW_UP
GSE39916_B_CELL_SPLEEN_VS_PLASMA_CELL_BONE_MARROW_UP
151 -0.4677802 -1.9239699 0 6.77E-05 0.008
1492 "tags=27%, list=11%, signal=30%"
GSE8921_UNSTIM_VS_TLR1_2_STIM_MONOCYTE_24H_UP
GSE8921_UNSTIM_VS_TLR1_2_STIM_MONOCYTE_24H_UP
140 -0.4685067 -1.9223025 0 7.39E-05 0.009
2130 "tags=35%, list=15%, signal=41%"
GSE7460_TREG_VS_TCONV_ACT_UP GSE7460_TREG_VS_TCONV_ACT_UP
140 -0.47009012 -1.9156157 0 7.34E-05
0.009 2386 "tags=37%, list=17%, signal=44%"
GSE411_UNSTIM_VS_400MIN_IL6_STIM_MACROPHAGE_UP
GSE411_UNSTIM_VS_400MIN_IL6_STIM_MACROPHAGE_UP
129 -0.47205856 -1.914355 0 7.28E-05 0.009
2485 "tags=33%, list=18%, signal=40%"
GSE20366_EX_VIVO_VS_HOMEOSTATIC_CONVERSION_TREG_UP
GSE20366_EX_VIVO_VS_HOMEOSTATIC_CONVERSION_TREG_UP
137 -0.4636352 -1.9112283 0 8.54E-05 0.011
2152 "tags=34%, list=15%, signal=39%"
GSE20366_CD103_POS_VS_CD103_KLRG1_DP_TREG_UP
GSE20366_CD103_POS_VS_CD103_KLRG1_DP_TREG_UP
148 -0.4642418 -1.9102267 0 9.12E-05 0.012
2044 "tags=34%, list=14%, signal=39%"
GSE14308_INDUCED_VS_NATURAL_TREG_DN
GSE14308_INDUCED_VS_NATURAL_TREG_DN
137 -0.46615615 -1.9100958 0 9.05E-05 0.012
1999 "tags=30%, list=14%, signal=35%"
GSE9988_LPS_VS_CTRL_TREATED_MONOCYTE_UP
GSE9988_LPS_VS_CTRL_TREATED_MONOCYTE_UP
132 -0.47195947 -1.9050567 0 8.98E-05 0.012
2610 "tags=39%, list=18%, signal=47%"
GSE3565_CTRL_VS_LPS_INJECTED_DUSP1_KO_SPLENOCYTES_DN
GSE3565_CTRL_VS_LPS_INJECTED_DUSP1_KO_SPLENOCYTES_DN
140 -0.46406335 -1.9034745 0 8.91E-05 0.012
1703 "tags=36%, list=12%, signal=40%"
GSE17580_UNINFECTED_VS_S_MANSONI_INF_TREG_UP
GSE17580_UNINFECTED_VS_S_MANSONI_INF_TREG_UP
147 -0.46115103 -1.9019974 0 9.47E-05 0.013

1883 "tags=31%, list=13%, signal=35%"
GSE5679_CTRL_VS_PPARG_LIGAND_ROSIGLITAZONE_TREATED_DC_DN
GSE5679_CTRL_VS_PPARG_LIGAND_ROSIGLITAZONE_TREATED_DC_DN
141 -0.46007806 -1.8971163 0 1.07E-04 0.015
2307 "tags=38%, list=16%, signal=45%"
GSE2706_UNSTIM_VS_2H_LPS_AND_R848_DC_DN
GSE2706_UNSTIM_VS_2H_LPS_AND_R848_DC_DN
135 -0.46122292 -1.8947419 0 1.06E-04 0.015
2508 "tags=37%, list=18%, signal=45%"
GSE7852_LN_VS_THYMUS_TREG_UP GSE7852_LN_VS_THYMUS_TREG_UP
136 -0.46660927 -1.8940452 0 1.05E-04
0.015 1724 "tags=32%, list=12%, signal=36%"
GSE36891_UNSTIM_VS_POLYIC_TLR3_STIM_PERITONEAL_MACROPHAGE_UP
GSE36891_UNSTIM_VS_POLYIC_TLR3_STIM_PERITONEAL_MACROPHAGE_UP
124 -0.46061397 -1.8906249 0 1.23E-04
0.018 1961 "tags=27%, list=14%, signal=31%"
GSE29618_BCELL_VS_PDC_UP GSE29618_BCELL_VS_PDC_UP
135 -0.4663299 -1.8899939 0 1.22E-04 0.018
1749 "tags=33%, list=12%, signal=38%"
GSE40685_NAIVE_CD4_TCELL_VS_TREG_DN
GSE40685_NAIVE_CD4_TCELL_VS_TREG_DN
139 -0.46134606 -1.889884 0 1.21E-04 0.018
1766 "tags=32%, list=13%, signal=36%"
GSE7219_UNSTIM_VS_LPS_AND_ANTI_CD40_STIM_DC_DN
GSE7219_UNSTIM_VS_LPS_AND_ANTI_CD40_STIM_DC_DN
143 -0.45175028 -1.8890176 0 1.20E-04 0.018
1498 "tags=30%, list=11%, signal=33%"
GSE37605_FOXP3_FUSION_GFP_VS_IRES_GFP_TREG_C57BL6_UP
GSE37605_FOXP3_FUSION_GFP_VS_IRES_GFP_TREG_C57BL6_UP
162 -0.44733638 -1.8881068 0 1.19E-04 0.018
1591 "tags=26%, list=11%, signal=29%"
GSE17721_CTRL_VS_CPG_1H_BMDC_DN GSE17721_CTRL_VS_CPG_1H_BMDC_DN
157 -0.44976962 -1.8853647 0 1.18E-04
0.018 2188 "tags=25%, list=16%, signal=29%"
GSE17974_1H_VS_72H_UNTREATED_IN_VITRO_CD4_TCELL_UP
GSE17974_1H_VS_72H_UNTREATED_IN_VITRO_CD4_TCELL_UP
126 -0.4623502 -1.8845193 0 1.17E-04 0.018
1979 "tags=33%, list=14%, signal=38%"
GSE20366_EX_VIVO_VS_HOMEOSTATIC_CONVERSION_NAIVE_CD4_TCELL_UP
GSE20366_EX_VIVO_VS_HOMEOSTATIC_CONVERSION_NAIVE_CD4_TCELL_UP
127 -0.46469375 -1.8837684 0 1.17E-04
0.018 1848 "tags=35%, list=13%, signal=40%"
GSE25123_WT_VS_PPARG_KO_MACROPHAGE_IL4_STIM_UP
GSE25123_WT_VS_PPARG_KO_MACROPHAGE_IL4_STIM_UP
139 -0.4575653 -1.8821361 0 1.22E-04 0.019
2267 "tags=35%, list=16%, signal=41%"
GSE40274_FOXP3_VS_FOXP3_AND_EOS_TRANSDUCED_ACTIVATED_CD4_TCELL_UP
GSE40274_FOXP3_VS_FOXP3_AND_EOS_TRANSDUCED_ACTIVATED_CD4_TCELL_UP
135 -0.4567287 -1.8816426 0 1.21E-04
0.019 1726 "tags=33%, list=12%, signal=37%"
GSE26343_UNSTIM_VS_LPS_STIM_NFAT5_KO_MACROPHAGE_DN
GSE26343_UNSTIM_VS_LPS_STIM_NFAT5_KO_MACROPHAGE_DN
167 -0.44697267 -1.8793068 0 1.26E-04 0.02
2610 "tags=37%, list=18%, signal=44%"

GSE32986_CURDLAN_HIGHDOSE_VS_GMCSF_AND_CURDLAN_HIGHDOSE_STIM_DC_DN
GSE32986_CURDLAN_HIGHDOSE_VS_GMCSF_AND_CURDLAN_HIGHDOSE_STIM_DC_DN
155 -0.45083338 -1.8775165 0 1.31E-04
0.021 2610 "tags=39%, list=18%, signal=48%"
GSE2770_UNTREATED_VS_ACT_CD4_TCELL_48H_DN
GSE2770_UNTREATED_VS_ACT_CD4_TCELL_48H_DN
102 -0.47754577 -1.875178 0 1.47E-04 0.024
1823 "tags=31%, list=13%, signal=36%"
GSE40277_EOS_AND_LEF1_TRANSDUCED_VS_GATA1_AND_SATB1_TRANSDUCED_CD4_T
CELL_UP
GSE40277_EOS_AND_LEF1_TRANSDUCED_VS_GATA1_AND_SATB1_TRANSDUCED_CD4_T
CELL_UP 152 -0.45159155 -1.8748463 0
1.51E-04 0.025 1853 "tags=33%, list=13%, signal=37%"
GSE20366_EX_VIVO_VS_DEC205_CONVERSION_NAIVE_CD4_TCELL_DN
GSE20366_EX_VIVO_VS_DEC205_CONVERSION_NAIVE_CD4_TCELL_DN
147 -0.44703776 -1.8647822 0 1.83E-04 0.031
3051 "tags=39%, list=22%, signal=50%"
GSE22886_NAIVE_BCELL_VS_BLOOD_PLASMA_CELL_UP
GSE22886_NAIVE_BCELL_VS_BLOOD_PLASMA_CELL_UP
130 -0.458609 -1.8641526 0 1.82E-04 0.031
2972 "tags=38%, list=21%, signal=47%"
GSE33424_CD161_HIGH_VS_NEG_CD8_TCELL_UP
GSE33424_CD161_HIGH_VS_NEG_CD8_TCELL_UP
156 -0.44705403 -1.8633822 0 1.81E-04 0.031
2288 "tags=35%, list=16%, signal=41%"
GSE41176_WT_VS_TAK1_KO_ANTI_IGM_STIM_BCELL_6H_UP
GSE41176_WT_VS_TAK1_KO_ANTI_IGM_STIM_BCELL_6H_UP
150 -0.44440928 -1.8622257 0 1.80E-04 0.031
2032 "tags=28%, list=14%, signal=32%"
GSE41867_DAY8_VS_DAY15_LCMV_CLONE13_EFFECTOR_CD8_TCELL_UP
GSE41867_DAY8_VS_DAY15_LCMV_CLONE13_EFFECTOR_CD8_TCELL_UP
145 -0.4533047 -1.8606536 0 1.84E-04 0.032
1452 "tags=26%, list=10%, signal=29%"
GSE21360_SECONDARY_VS_QUATERNARY_MEMORY_CD8_TCELL_UP
GSE21360_SECONDARY_VS_QUATERNARY_MEMORY_CD8_TCELL_UP
151 -0.44985697 -1.8601979 0 1.83E-04 0.032
1254 "tags=24%, list=9%, signal=26%"
GSE36078_UNTREATED_VS_AD5_T425A_HEXON_INF_IL1R_KO_MOUSE_LUNG_DC_DN
GSE36078_UNTREATED_VS_AD5_T425A_HEXON_INF_IL1R_KO_MOUSE_LUNG_DC_DN
132 -0.4601158 -1.8588428 0 1.82E-04
0.032 2618 "tags=30%, list=19%, signal=36%"
GSE37301_CD4_TCELL_VS_GRANULOCYTE_MONOCYTE_PROGENITOR_UP
GSE37301_CD4_TCELL_VS_GRANULOCYTE_MONOCYTE_PROGENITOR_UP
134 -0.45664847 -1.8565542 0 1.97E-04 0.035
1665 "tags=27%, list=12%, signal=30%"
GSE4590_PRE_BCELL_VS_LARGE_PRE_BCELL_UP
GSE4590_PRE_BCELL_VS_LARGE_PRE_BCELL_UP
121 -0.46261832 -1.8487403 0 2.27E-04 0.041
2180 "tags=31%, list=15%, signal=36%"
GSE32255_WT_VS_JMJD2D_KNOCKDOWN_4H_LPS_STIM_DC_UP
GSE32255_WT_VS_JMJD2D_KNOCKDOWN_4H_LPS_STIM_DC_UP
108 -0.46539435 -1.8447201 0 2.42E-04 0.044
1329 "tags=26%, list=9%, signal=28%"
GSE14415_FOXP3_KO_NATURAL_TREG_VS_TCONV_DN

GSE14415_FOXP3_KO_NATURAL_TREG_VS_TCONV_DN
142 -0.45255578 -1.8433526 0 2.40E-04 0.044
1397 "tags=29%, list=10%, signal=32%"
GSE28408_LY6G_POS_VS_NEG_DC_UP GSE28408_LY6G_POS_VS_NEG_DC_UP
139 -0.44717345 -1.8406501 0 2.54E-04
0.047 2526 "tags=32%, list=18%, signal=39%"
GSE21670_IL6_VS_TGFB_AND_IL6_TREATED_STAT3_KO_CD4_TCELL_DN
GSE21670_IL6_VS_TGFB_AND_IL6_TREATED_STAT3_KO_CD4_TCELL_DN
141 -0.44594896 -1.8290899 0 3.25E-04 0.061
2238 "tags=33%, list=16%, signal=38%"
GSE41867_NAIVE_VS_DAY15_LCMV_EFFECTOR_CD8_TCELL_DN
GSE41867_NAIVE_VS_DAY15_LCMV_EFFECTOR_CD8_TCELL_DN
143 -0.44458497 -1.828187 0 3.28E-04 0.062
1367 "tags=26%, list=10%, signal=28%"
GSE8921_UNSTIM_VS_TLR1_2_STIM_MONOCYTE_6H_UP
GSE8921_UNSTIM_VS_TLR1_2_STIM_MONOCYTE_6H_UP
138 -0.4408534 -1.8234066 0 3.67E-04 0.07
3126 "tags=43%, list=22%, signal=54%"
GSE17974_0H_VS_6H_IN_VITRO_ACT_CD4_TCELL_UP
GSE17974_0H_VS_6H_IN_VITRO_ACT_CD4_TCELL_UP
125 -0.44687608 -1.8222996 0 3.70E-04 0.071
1461 "tags=25%, list=10%, signal=27%"
GSE9988_LPS_VS_VEHICLE_TREATED_MONOCYTE_UP
GSE9988_LPS_VS_VEHICLE_TREATED_MONOCYTE_UP
129 -0.44554713 -1.820809 0 3.78E-04 0.073
2159 "tags=32%, list=15%, signal=37%"
GSE19825_NAIVE_VS_IL2RAHIGH_DAY3_EFF_CD8_TCELL_UP
GSE19825_NAIVE_VS_IL2RAHIGH_DAY3_EFF_CD8_TCELL_UP
133 -0.4467233 -1.8161142 0 3.96E-04 0.076
4635 "tags=46%, list=33%, signal=68%"
GSE3565_DUSP1_VS_WT_SPLENOCYTES_POST_LPS_INJECTION_UP
GSE3565_DUSP1_VS_WT_SPLENOCYTES_POST_LPS_INJECTION_UP
120 -0.4536339 -1.8159174 0 3.98E-04 0.077
1371 "tags=28%, list=10%, signal=31%"
GSE23505_UNTREATED_VS_4DAY_IL6_IL1_TGFB_TREATED_CD4_TCELL_DN
GSE23505_UNTREATED_VS_4DAY_IL6_IL1_TGFB_TREATED_CD4_TCELL_DN
139 -0.44955176 -1.8129193 0 4.16E-04
0.081 1555 "tags=26%, list=11%, signal=29%"
GSE7219_UNSTIM_VS_LPS_AND_ANTI_CD40_STIM_NIK_NFKB2_KO_DC_DN
GSE7219_UNSTIM_VS_LPS_AND_ANTI_CD40_STIM_NIK_NFKB2_KO_DC_DN
163 -0.4294952 -1.8105186 0 4.18E-04 0.082
1397 "tags=31%, list=10%, signal=34%"
GSE46606_UNSTIM_VS_CD40L_IL2_IL5_1DAY_STIMULATED_IRF4HIGH_SORTED_BCE
LL_DN
GSE46606_UNSTIM_VS_CD40L_IL2_IL5_1DAY_STIMULATED_IRF4HIGH_SORTED_BCE
LL_DN 153 -0.44271144 -1.8101344 0
4.30E-04 0.085 1776 "tags=29%, list=13%, signal=33%"
GSE24142_EARLY_THYMIC_PROGENITOR_VS_DN2_THYMOCYTE_ADULT_UP
GSE24142_EARLY_THYMIC_PROGENITOR_VS_DN2_THYMOCYTE_ADULT_UP
156 -0.43368486 -1.8056655 0 4.72E-04 0.094
1166 "tags=22%, list=8%, signal=23%"
GSE21546_WT_VS_SAP1A_KO_AND_ELK1_KO_ANTI_CD3_STIM_DP_THYMOCYTES_UP
GSE21546_WT_VS_SAP1A_KO_AND_ELK1_KO_ANTI_CD3_STIM_DP_THYMOCYTES_UP
136 -0.4424958 -1.8044275 0 4.79E-04

0.096	2646	"tags=35%, list=19%, signal=43%"
GSE23925_DARK_ZONE_VS_NAIVE_BCELL_DN
GSE23925_DARK_ZONE_VS_NAIVE_BCELL_DN
149	-0.4325648	-1.80409	0	4.81E-04	0.097
1844	"tags=29%, list=13%, signal=33%"
GSE17812_WT_VS_THPOK_KO_MEMORY_CD8_TCELL_UP
GSE17812_WT_VS_THPOK_KO_MEMORY_CD8_TCELL_UP
155	-0.4351937	-1.8023919	0	4.88E-04	0.099
1462	"tags=28%, list=10%, signal=31%"
GSE21546_UNSTIM_VS_ANTI_CD3_STIM_SAP1A_KO_DP_THYMOCYTES_UP
GSE21546_UNSTIM_VS_ANTI_CD3_STIM_SAP1A_KO_DP_THYMOCYTES_UP
144	-0.43539137	-1.7997974	0	5.28E-04	0.108
2619	"tags=33%, list=19%, signal=41%"
GSE3039_B2_VS_B1_BCELL_UP	GSE3039_B2_VS_B1_BCELL_UP
141	-0.4346198	-1.7988439	0	5.53E-04	0.114
2215	"tags=35%, list=16%, signal=42%"
GSE7852_TREG_VS_TCONV_UP	GSE7852_TREG_VS_TCONV_UP
159	-0.43105733	-1.7986937	0	5.55E-04	0.115
2636	"tags=38%, list=19%, signal=46%"
GSE2770_TGFB_AND_IL4_VS_IL12_TREATED_ACT_CD4_TCELL_2H_DN
GSE2770_TGFB_AND_IL4_VS_IL12_TREATED_ACT_CD4_TCELL_2H_DN
137	-0.4415902	-1.7984253	0	5.57E-04	0.116
2159	"tags=32%, list=15%, signal=38%"
GSE17974_0H_VS_72H_IN_VITRO_ACT_CD4_TCELL_UP
GSE17974_0H_VS_72H_IN_VITRO_ACT_CD4_TCELL_UP
107	-0.45646754	-1.7982774	0	5.54E-04	0.116
2064	"tags=31%, list=15%, signal=36%"
GSE2770_IL12_AND_TGFB_VS_IL4_TREATED_ACT_CD4_TCELL_48H_UP
GSE2770_IL12_AND_TGFB_VS_IL4_TREATED_ACT_CD4_TCELL_48H_UP
137	-0.43821752	-1.7979449	0.001904762	5.51E-04
0.116	1531	"tags=31%, list=11%, signal=35%"
GSE7348_UNSTIM_VS_LPS_STIM_MACROPHAGE_UP
GSE7348_UNSTIM_VS_LPS_STIM_MACROPHAGE_UP
117	-0.4473757	-1.7977605	0	5.48E-04	0.116
2162	"tags=34%, list=15%, signal=40%"
GSE12003_MIR223_KO_VS_WT_BM_PROGENITOR_4D_CULTURE_UP
GSE12003_MIR223_KO_VS_WT_BM_PROGENITOR_4D_CULTURE_UP
146	-0.43254387	-1.797382	0	5.45E-04	0.116
1712	"tags=30%, list=12%, signal=34%"
GSE3920_IFNA_VS_IFNB_TREATED_ENDOTHELIAL_CELL_UP
GSE3920_IFNA_VS_IFNB_TREATED_ENDOTHELIAL_CELL_UP
136	-0.44366974	-1.7970116	0	5.46E-04	0.117
2704	"tags=40%, list=19%, signal=49%"
GSE32986_GMCSF_VS_GMCSF_AND_CURDLAN_LOWDOSE_STIM_DC_DN
GSE32986_GMCSF_VS_GMCSF_AND_CURDLAN_LOWDOSE_STIM_DC_DN
106	-0.45065296	-1.7956415	0	5.43E-04	0.117
1470	"tags=25%, list=10%, signal=28%"
GSE7768_OVA_ALONE_VS_OVA_WITH_LPS_IMMUNIZED_MOUSE_WHOLE_SPLEEN_6H_DN

GSE7768_OVA_ALONE_VS_OVA_WITH_LPS_IMMUNIZED_MOUSE_WHOLE_SPLEEN_6H_DN
142	-0.4348279	-1.7915626	0
5.95E-04	0.128	4028	"tags=48%, list=29%, signal=66%"
GSE3039_NKT_CELL_VS_B2_BCELL_DN	GSE3039_NKT_CELL_VS_B2_BCELL_DN
154	-0.42342633	-1.7893492	0	6.09E-04

0.132 2234 "tags=34%, list=16%, signal=40%"
GSE411_UNSTIM_VS_400MIN_IL6_STIM_SOCS3_KO_MACROPHAGE_UP
GSE411_UNSTIM_VS_400MIN_IL6_STIM_SOCS3_KO_MACROPHAGE_UP
134 -0.43904307 -1.7893274 0 6.06E-04 0.132
2129 "tags=35%, list=15%, signal=41%"
GSE30083_SP1_VS_SP2_THYMOCYTE_DN GSE30083_SP1_VS_SP2_THYMOCYTE_DN
139 -0.43508726 -1.7891263 0 6.07E-04
0.132 1141 "tags=18%, list=8%, signal=19%"
GSE19825_NAIVE_VS_DAY3_EFF_CD8_TCELL_UP
GSE19825_NAIVE_VS_DAY3_EFF_CD8_TCELL_UP
144 -0.43258572 -1.7890601 0 6.04E-04 0.132
5310 "tags=49%, list=38%, signal=78%"
GSE30971_CTRL_VS_LPS_STIM_MACROPHAGE_WBP7_HET_2H_UP
GSE30971_CTRL_VS_LPS_STIM_MACROPHAGE_WBP7_HET_2H_UP
144 -0.43443522 -1.7879193 0 6.10E-04 0.134
2159 "tags=31%, list=15%, signal=37%"
GSE17301_IFNA2_VS_IFNA5_STIM_ACD3_ACD28_ACT_CD8_TCELL_UP
GSE17301_IFNA2_VS_IFNA5_STIM_ACD3_ACD28_ACT_CD8_TCELL_UP
144 -0.43823898 -1.7876006 0 6.11E-04 0.135
1148 "tags=25%, list=8%, signal=27%"
GSE45365_CD8A_DC_VS_CD11B_DC_IFNAR_KO_MCMV_INFECTION_DN
GSE45365_CD8A_DC_VS_CD11B_DC_IFNAR_KO_MCMV_INFECTION_DN
108 -0.45199355 -1.7863525 0 6.30E-04 0.14
2964 "tags=37%, list=21%, signal=47%"
GSE13522_CTRL_VS_T_CRUZI_Y_STRAIN_INF_SKIN_IFNAR_KO_UP
GSE13522_CTRL_VS_T_CRUZI_Y_STRAIN_INF_SKIN_IFNAR_KO_UP
152 -0.42371684 -1.7854992 0 6.44E-04 0.143
1315 "tags=27%, list=9%, signal=29%"
GSE36476_CTRL_VS_TSST_ACT_72H_MEMORY_CD4_TCELL_OLD_UP
GSE36476_CTRL_VS_TSST_ACT_72H_MEMORY_CD4_TCELL_OLD_UP
132 -0.43953934 -1.7844619 0 6.49E-04 0.144
1713 "tags=27%, list=12%, signal=31%"
GSE27786_CD8_TCELL_VS_NKCELL_UP GSE27786_CD8_TCELL_VS_NKCELL_UP
142 -0.43031996 -1.7834033 0 6.50E-04
0.145 2306 "tags=27%, list=16%, signal=32%"
GSE4984_UNTREATED_VS_LPS_TREATED_DC_UP
GSE4984_UNTREATED_VS_LPS_TREATED_DC_UP
110 -0.4554997 -1.7815875 0 6.77E-04 0.152
3058 "tags=33%, list=22%, signal=41%"
GSE21670_STAT3_KO_VS_WT_CD4_TCELL_IL6_TREATED_DN
GSE21670_STAT3_KO_VS_WT_CD4_TCELL_IL6_TREATED_DN
158 -0.43046772 -1.7807931 0 6.77E-04 0.152
2095 "tags=29%, list=15%, signal=34%"
GSE28237_FOLLICULAR_VS_LATE_GC_BCELL_UP
GSE28237_FOLLICULAR_VS_LATE_GC_BCELL_UP
141 -0.43258014 -1.779987 0.001886793 6.83E-04
0.153 3231 "tags=32%, list=23%, signal=41%"
GSE3982_MEMORY_CD4_TCELL_VS_TH1_UP
GSE3982_MEMORY_CD4_TCELL_VS_TH1_UP
114 -0.4465574 -1.7791722 0 6.92E-04 0.156
1625 "tags=28%, list=12%, signal=31%"
GSE40274_CTRL_VS_FOXP3_TRANSDUCED_ACTIVATED_CD4_TCELL_DN
GSE40274_CTRL_VS_FOXP3_TRANSDUCED_ACTIVATED_CD4_TCELL_DN
138 -0.43532634 -1.7776823 0 7.05E-04 0.16

1715	"tags=33%, list=12%, signal=37%"
GSE20366_TREG_VS_NAIVE_CD4_TCELL_DN
GSE20366_TREG_VS_NAIVE_CD4_TCELL_DN
153	-0.42875808	-1.7776456	0	7.01E-04	0.16
2170	"tags=32%, list=15%, signal=37%"
GSE7460_CTRL_VS_TGFB_TREATED_ACT_FOXP3_HET_TCONV_DN
GSE7460_CTRL_VS_TGFB_TREATED_ACT_FOXP3_HET_TCONV_DN
144	-0.4300766	-1.7771516	0	7.06E-04	0.162
790	"tags=19%, list=6%, signal=20%"
GSE24492_LYVE_NEG_VS_POS_MACROPHAGE_UP
GSE24492_LYVE_NEG_VS_POS_MACROPHAGE_UP
129	-0.4347614	-1.7764466	0	7.15E-04	0.163
1431	"tags=24%, list=10%, signal=26%"
GSE7852_TREG_VS_TCONV_THYMUS_UP	GSE7852_TREG_VS_TCONV_THYMUS_UP
157	-0.42323825	-1.7741575	0	7.32E-04
0.168	1208	"tags=27%, list=9%, signal=30%"
GSE7460_CTRL_VS_TGFB_TREATED_ACT_TREG_DN
GSE7460_CTRL_VS_TGFB_TREATED_ACT_TREG_DN
140	-0.43314743	-1.7740017	0	7.28E-04	0.168
1500	"tags=22%, list=11%, signal=25%"
GSE3920_UNTREATED_VS_IFNG_TREATED_ENDOTHELIAL_CELL_UP
GSE3920_UNTREATED_VS_IFNG_TREATED_ENDOTHELIAL_CELL_UP
121	-0.44257233	-1.7726724	0	7.37E-04	0.17
2249	"tags=30%, list=16%, signal=35%"
GSE2706_UNSTIM_VS_2H_LPS_DC_DN	GSE2706_UNSTIM_VS_2H_LPS_DC_DN
133	-0.43729165	-1.7708048	0	7.41E-04
0.171	2610	"tags=37%, list=18%, signal=45%"
GSE30083_SP2_VS_SP3_THYMOCYTE_UP	GSE30083_SP2_VS_SP3_THYMOCYTE_UP
151	-0.4257126	-1.763643	0	8.14E-04
0.186	2254	"tags=25%, list=16%, signal=29%"
GSE7509_UNSTIM_VS_FCGRIIB_STIM_MONOCYTE_DN
GSE7509_UNSTIM_VS_FCGRIIB_STIM_MONOCYTE_DN
119	-0.4364312	-1.7591431	0	8.94E-04	0.2
3397	"tags=45%, list=24%, signal=58%"
GSE41176_UNSTIM_VS_ANTI_IGM_STIM_BCELL_1H_UP
GSE41176_UNSTIM_VS_ANTI_IGM_STIM_BCELL_1H_UP
154	-0.4240426	-1.7585852	0	8.94E-04	0.201
1704	"tags=26%, list=12%, signal=29%"
GSE21927_SPLENIC_VS_TUMOR_MONOCYTES_FROM_C26GM_TUMOROUS_MICE_BALBC_DN
GSE21927_SPLENIC_VS_TUMOR_MONOCYTES_FROM_C26GM_TUMOROUS_MICE_BALBC_DN	138	-0.431465	-1.7583712	0
8.90E-04	0.201	1784	"tags=28%, list=13%, signal=31%"
GSE9988_LOW_LPS_VS_CTRL_TREATED_MONOCYTE_UP
GSE9988_LOW_LPS_VS_CTRL_TREATED_MONOCYTE_UP
139	-0.4373939	-1.7575064	0	9.09E-04	0.207
2159	"tags=32%, list=15%, signal=38%"
GSE36476_YOUNG_VS_OLD_DONOR_MEMORY_CD4_TCELL_72H_TSST_ACT_DN
GSE36476_YOUNG_VS_OLD_DONOR_MEMORY_CD4_TCELL_72H_TSST_ACT_DN
130	-0.43232122	-1.7574618	0.001960784
9.09E-04	0.208	2536	"tags=33%, list=18%, signal=40%"
GSE24142_ADULT_VS_FETAL_DN2_THYMOCYTE_UP
GSE24142_ADULT_VS_FETAL_DN2_THYMOCYTE_UP
163	-0.42160857	-1.7552264	0	9.51E-04	0.215

1414 "tags=28%, list=10%, signal=30%"
GSE45365_NK_CELL_VS_CD8A_DC_MCMV_INFECTION_UP
GSE45365_NK_CELL_VS_CD8A_DC_MCMV_INFECTION_UP
115 -0.43761927 -1.753521 0 9.82E-04 0.222
3222 "tags=37%, list=23%, signal=48%"
GSE3039_NKT_CELL_VS_ALPHABETA_CD8_TCELL_DN
GSE3039_NKT_CELL_VS_ALPHABETA_CD8_TCELL_DN
139 -0.43185902 -1.7534653 0 9.81E-04 0.223
2304 "tags=35%, list=16%, signal=41%"
GSE27241_WT_VS_RORGT_KO_TH17_POLARIZED_CD4_TCELL_TREATED_WITH_DIGOXIN_DN
GSE27241_WT_VS_RORGT_KO_TH17_POLARIZED_CD4_TCELL_TREATED_WITH_DIGOXIN_DN 148 -0.42632693 -1.7508172 0
0.001011349 0.231 1695 "tags=31%, list=12%, signal=35%"
GSE7348_UNSTIM_VS_LPS_STIM_MACROPHAGE_DN
GSE7348_UNSTIM_VS_LPS_STIM_MACROPHAGE_DN
144 -0.42650792 -1.7501694 0 0.001018158
0.233 2215 "tags=31%, list=16%, signal=37%"
GSE30083_SP3_VS_SP4_THYMOCYTE_DN GSE30083_SP3_VS_SP4_THYMOCYTE_DN
146 -0.4204882 -1.7498161 0
0.00101739 0.234 1621 "tags=30%, list=11%, signal=34%"
GSE30962_PRIMARY_VS_SECONDARY_ACUTE_LCMV_INF_CD8_TCELL_DN
GSE30962_PRIMARY_VS_SECONDARY_ACUTE_LCMV_INF_CD8_TCELL_DN
147 -0.41950104 -1.7494553 0 0.001012807
0.234 1184 "tags=22%, list=8%, signal=24%"
GSE7852_THYMUS_VS_FAT_TCONV_DN GSE7852_THYMUS_VS_FAT_TCONV_DN
148 -0.42083126 -1.7474222 0
0.001045847 0.241 1830 "tags=31%, list=13%, signal=35%"
GSE23984_CTRL_VS_HYPOCALEMIC_VITAMIND_ANALOG_TCELL_DN
GSE23984_CTRL_VS_HYPOCALEMIC_VITAMIND_ANALOG_TCELL_DN
150 -0.42222592 -1.7452188 0 0.001101285
0.25 2187 "tags=31%, list=15%, signal=37%"
GSE42021_TCONV_PLN_VS_TREG_PRECURSORS_THYMUS_DN
GSE42021_TCONV_PLN_VS_TREG_PRECURSORS_THYMUS_DN
165 -0.4120094 -1.7450843 0 0.001096391
0.25 2215 "tags=30%, list=16%, signal=35%"
GSE15930_NAIVE_VS_48H_IN_VITRO_STIM_IFNAB_CD8_TCELL_UP
GSE15930_NAIVE_VS_48H_IN_VITRO_STIM_IFNAB_CD8_TCELL_UP
152 -0.4187882 -1.7449926 0 0.00109525
0.251 3035 "tags=38%, list=22%, signal=47%"
GSE33425_CD8_ALPHAALPHA_VS_ALPHABETA_CD161_HIGH_TCELL_UP
GSE33425_CD8_ALPHAALPHA_VS_ALPHABETA_CD161_HIGH_TCELL_UP
160 -0.41971055 -1.7449284 0 0.00109787
0.251 2842 "tags=38%, list=20%, signal=46%"
GSE6092_CTRL_VS_BORRELIA_BIRGDOFERI_INF_ENDOTHELIAL_CELL_DN
GSE6092_CTRL_VS_BORRELIA_BIRGDOFERI_INF_ENDOTHELIAL_CELL_DN
118 -0.43859532 -1.7446386 0 0.001096782
0.251 3811 "tags=46%, list=27%, signal=62%"
GSE3982_EFF_MEMORY_VS_CENT_MEMORY_CD4_TCELL_DN
GSE3982_EFF_MEMORY_VS_CENT_MEMORY_CD4_TCELL_DN
130 -0.42939907 -1.7427028 0 0.001117696
0.256 1990 "tags=26%, list=14%, signal=30%"
GSE26495_NAIVE_VS_PD1HIGH_CD8_TCELL_UP
GSE26495_NAIVE_VS_PD1HIGH_CD8_TCELL_UP

113	-0.4412894	-1.7381017	0	0.00119317
0.27	2156	"tags=28%, list=15%, signal=33%"
GSE32986_GMCSF_VS_GMCSF_AND_CURDLAN_HIGHDOSE_STIM_DC_DN
GSE32986_GMCSF_VS_GMCSF_AND_CURDLAN_HIGHDOSE_STIM_DC_DN
129	-0.428331	-1.7367469	0	0.001217012
0.274	1857	"tags=26%, list=13%, signal=30%"
GSE7764_IL15_TREATED_VS_CTRL_NK_CELL_24H_DN
GSE7764_IL15_TREATED_VS_CTRL_NK_CELL_24H_DN
147	-0.41486752	-1.7349398	0	0.001233721
0.278	2527	"tags=37%, list=18%, signal=45%"
GSE41867_NAIVE_VS_DAY15_LCMV_ARMSTRONG_EFFECTOR_CD8_TCELL_DN
GSE41867_NAIVE_VS_DAY15_LCMV_ARMSTRONG_EFFECTOR_CD8_TCELL_DN
	144	-0.4238393	-1.7341692	0
0.001239448	0.279	2251	"tags=31%, list=16%, signal=36%"
GSE15659_RESTING_TREG_VS_NONSUPPRESSIVE_TCELL_UP
GSE15659_RESTING_TREG_VS_NONSUPPRESSIVE_TCELL_UP
105	-0.43776393	-1.729139	0	0.00134586
0.306	2241	"tags=28%, list=16%, signal=33%"
GSE7219_WT_VS_NIK_NFKB2_KO_DC_UP	GSE7219_WT_VS_NIK_NFKB2_KO_DC_UP
	159	-0.4179214	-1.726142	0
0.001408239	0.32	1767	"tags=35%, list=13%, signal=40%"
GSE24142_EARLY_THYMIC_PROGENITOR_VS_DN2_THYMOCYTE_FETAL_UP
GSE24142_EARLY_THYMIC_PROGENITOR_VS_DN2_THYMOCYTE_FETAL_UP
170	-0.40678644	-1.7241437	0	0.001441633
0.327	1209	"tags=23%, list=9%, signal=25%"
GSE3920_UNTREATED_VS_IFNA_TREATED_FIBROBLAST_UP
GSE3920_UNTREATED_VS_IFNA_TREATED_FIBROBLAST_UP
139	-0.41850185	-1.7232732	0	0.001449754
0.329	3811	"tags=46%, list=27%, signal=62%"
GSE14350_TREG_VS_TEFF_IN_IL2RB_KO_UP
GSE14350_TREG_VS_TEFF_IN_IL2RB_KO_UP
138	-0.41858697	-1.7227509	0	0.001450695
0.331	2753	"tags=36%, list=20%, signal=45%"
GSE17974_CTRL_VS_ACT_IL4_AND_ANTI_IL12_4H_CD4_TCELL_UP
GSE17974_CTRL_VS_ACT_IL4_AND_ANTI_IL12_4H_CD4_TCELL_UP
122	-0.42628375	-1.7209926	0	0.001483399
0.339	1790	"tags=28%, list=13%, signal=32%"
GSE37301_MULTIPOTENT_PROGENITOR_VS_LYMPHOID_PRIMED_MPP_UP
GSE37301_MULTIPOTENT_PROGENITOR_VS_LYMPHOID_PRIMED_MPP_UP
115	-0.42850593	-1.718914	0	0.001547468
0.35	1780	"tags=30%, list=13%, signal=34%"
GSE11961_MEMORY_BCELL_DAY7_VS_GERMINAL_CENTER_BCELL_DAY7_DN
GSE11961_MEMORY_BCELL_DAY7_VS_GERMINAL_CENTER_BCELL_DAY7_DN
145	-0.4165254	-1.7178406	0	0.00155498
0.353	1725	"tags=29%, list=12%, signal=33%"
GSE9988_LOW_LPS_VS_VEHICLE_TREATED_MONOCYTE_UP
GSE9988_LOW_LPS_VS_VEHICLE_TREATED_MONOCYTE_UP
135	-0.42258197	-1.7163696	0	0.001580156
0.358	2159	"tags=31%, list=15%, signal=36%"
GSE40273_GATA1_KO_VS_WT_TREG_DN	GSE40273_GATA1_KO_VS_WT_TREG_DN
	133	-0.41719845	-1.715808	0
0.001580624	0.359	1980	"tags=33%, list=14%, signal=38%"
GSE27434_WT_VS_DNMT1_KO_TREG_DN	GSE27434_WT_VS_DNMT1_KO_TREG_DN
	168	-0.40659755	-1.7140023	0

0.001646512	0.371	1864	"tags=26%, list=13%, signal=30%"
GSE41867_LCMV_ARMSTRONG_VS_CLONE13_DAY15_EFFECTOR_CD8_TCELL_DN
GSE41867_LCMV_ARMSTRONG_VS_CLONE13_DAY15_EFFECTOR_CD8_TCELL_DN
	92	-0.45006344	-1.7137609	0
0.001646591	0.372	3938	"tags=43%, list=28%, signal=60%"
GSE15324_NAIVE_VS_ACTIVATED_CD8_TCELL_UP
GSE15324_NAIVE_VS_ACTIVATED_CD8_TCELL_UP
132	-0.42250827	-1.7137288	0	0.001639898
0.372	2279	"tags=31%, list=16%, signal=37%"
GSE17974_0H_VS_12H_IN_VITRO_ACT_CD4_TCELL_UP
GSE17974_0H_VS_12H_IN_VITRO_ACT_CD4_TCELL_UP
111	-0.43642333	-1.7134228	0.001838235
0.001639988	0.374	1856	"tags=29%, list=13%, signal=33%"
GSE23925_LIGHT_ZONE_VS_NAIVE_BCELL_UP
GSE23925_LIGHT_ZONE_VS_NAIVE_BCELL_UP
147	-0.41501272	-1.7130873	0	0.001647034
0.377	1594	"tags=27%, list=11%, signal=30%"
GSE14769_UNSTIM_VS_120MIN_LPS_BMDM_DN
GSE14769_UNSTIM_VS_120MIN_LPS_BMDM_DN
156	-0.40956306	-1.7123936	0	0.001660672
0.381	2377	"tags=31%, list=17%, signal=37%"
GSE42021_CD24HI_TREG_VS_CD24HI_TCONV_THYMUS_DN
GSE42021_CD24HI_TREG_VS_CD24HI_TCONV_THYMUS_DN
168	-0.40779024	-1.7108119	0	0.001697779
0.386	1864	"tags=24%, list=13%, signal=28%"
GSE23502_BM_VS_COLON_TUMOR_MYELOID_DERIVED_SUPPRESSOR_CELL_UP
GSE23502_BM_VS_COLON_TUMOR_MYELOID_DERIVED_SUPPRESSOR_CELL_UP
	154	-0.41135037	-1.7092764	0
0.001731369	0.395	2000	"tags=29%, list=14%, signal=34%"
GSE22886_NAIVE_CD4_TCELL_VS_MONOCYTE_UP
GSE22886_NAIVE_CD4_TCELL_VS_MONOCYTE_UP
140	-0.41370577	-1.7091104	0	0.001724498
0.395	1551	"tags=24%, list=11%, signal=27%"
GSE25085_FETAL_LIVER_VS_FETAL_BM_SP4_THYMIC_IMPLANT_DN
GSE25085_FETAL_LIVER_VS_FETAL_BM_SP4_THYMIC_IMPLANT_DN
146	-0.40755415	-1.706357	0	0.001820462
0.415	2113	"tags=28%, list=15%, signal=33%"
GSE9601_UNTREATED_VS_PI3K_INHIBITOR_TREATED_HCMV_INF_MONOCYTE_DN
GSE9601_UNTREATED_VS_PI3K_INHIBITOR_TREATED_HCMV_INF_MONOCYTE_DN
	110	-0.4317575	-1.7055491	0.001869159
0.001829827	0.418	1829	"tags=29%, list=13%, signal=33%"
GSE37301_CD4_TCELL_VS_RAG2_KO_NK_CELL_UP
GSE37301_CD4_TCELL_VS_RAG2_KO_NK_CELL_UP
119	-0.42487282	-1.705049	0	0.001829298
0.42	2047	"tags=26%, list=15%, signal=30%"
GSE18791_UNSTIM_VS_NEWCATSLE_VIRUS_DC_10H_DN
GSE18791_UNSTIM_VS_NEWCATSLE_VIRUS_DC_10H_DN
126	-0.41956997	-1.704802	0	0.001825447
0.421	2035	"tags=37%, list=14%, signal=42%"
GSE7460_TCONV_VS_TREG_THYMUS_DN	GSE7460_TCONV_VS_TREG_THYMUS_DN
	157	-0.4090257	-1.7047523	0.001941748
0.001821588	0.422	1128	"tags=24%, list=8%, signal=25%"
GSE16266_LPS_VS_HEATSHOCK_AND_LPS_STIM_MEF_UP
GSE16266_LPS_VS_HEATSHOCK_AND_LPS_STIM_MEF_UP

155 -0.40561864 -1.7041749 0 0.00184365
0.428 2468 "tags=34%, list=17%, signal=40%"
GSE40274_CTRL_VS_FOXP3_AND_XBP1_TRANSDUCED_ACTIVATED_CD4_TCELL_DN
GSE40274_CTRL_VS_FOXP3_AND_XBP1_TRANSDUCED_ACTIVATED_CD4_TCELL_DN
128 -0.41436398 -1.7037076 0
0.001842958 0.43 1621 "tags=27%, list=11%, signal=30%"
GSE2128_C57BL6_VS_NOD_CD4CD8_DP_THYMOCYTE_DN
GSE2128_C57BL6_VS_NOD_CD4CD8_DP_THYMOCYTE_DN
131 -0.41643918 -1.7030046 0 0.001865048
0.436 2732 "tags=37%, list=19%, signal=45%"
GSE40666_WT_VS_STAT1_KO_CD8_TCELL_DN
GSE40666_WT_VS_STAT1_KO_CD8_TCELL_DN
151 -0.410175 -1.7027069 0 0.001867568
0.438 2501 "tags=34%, list=18%, signal=41%"
GSE34392_ST2_KO_VS_WT_DAY8_LCMV_EFFECTOR_CD8_TCELL_DN
GSE34392_ST2_KO_VS_WT_DAY8_LCMV_EFFECTOR_CD8_TCELL_DN
166 -0.40624893 -1.6976887 0 0.002046457
0.471 1776 "tags=24%, list=13%, signal=27%"
GSE14000_UNSTIM_VS_4H_LPS_DC_TRANSLATED_RNA_DN
GSE14000_UNSTIM_VS_4H_LPS_DC_TRANSLATED_RNA_DN
124 -0.4271932 -1.6975696 0 0.002038676
0.471 2163 "tags=35%, list=15%, signal=42%"
GSE30083_SP1_VS_SP4_THYMOCYTE_DN GSE30083_SP1_VS_SP4_THYMOCYTE_DN
138 -0.41596007 -1.6966252 0
0.002065866 0.477 1236 "tags=25%, list=9%, signal=28%"
GSE18791_CTRL_VS_NEWCASTLE_VIRUS_DC_10H_DN
GSE18791_CTRL_VS_NEWCASTLE_VIRUS_DC_10H_DN
122 -0.41945544 -1.6963807 0 0.002073929
0.478 2544 "tags=39%, list=18%, signal=47%"
GSE9988_ANTI_TREM1_AND_LPS_VS_CTRL_TREATED_MONOCYTES_UP
GSE9988_ANTI_TREM1_AND_LPS_VS_CTRL_TREATED_MONOCYTES_UP
140 -0.412152 -1.6959745 0 0.002075643
0.479 3155 "tags=42%, list=22%, signal=54%"
GSE13522_CTRL_VS_T_CRUZI_Y_STRAIN_INF_SKIN_129_MOUSE_DN
GSE13522_CTRL_VS_T_CRUZI_Y_STRAIN_INF_SKIN_129_MOUSE_DN
140 -0.41822404 -1.6956306 0 0.002086794
0.482 1690 "tags=24%, list=12%, signal=27%"
GSE2770_IL12_VS_TGFB_AND_IL12_TREATED_ACT_CD4_TCELL_48H_DN
GSE2770_IL12_VS_TGFB_AND_IL12_TREATED_ACT_CD4_TCELL_48H_DN
134 -0.4146144 -1.6951514 0 0.002107235
0.483 2369 "tags=34%, list=17%, signal=40%"
GSE19198_CTRL_VS_IL21_TREATED_TCELL_24H_UP
GSE19198_CTRL_VS_IL21_TREATED_TCELL_24H_UP
167 -0.40100425 -1.6948799 0 0.002108768
0.484 2215 "tags=30%, list=16%, signal=35%"
GSE3691_IFN_PRODUCING_KILLER_DC_VS_CONVENTIONAL_DC_SPLEEN_UP
GSE3691_IFN_PRODUCING_KILLER_DC_VS_CONVENTIONAL_DC_SPLEEN_UP
139 -0.41719437 -1.6931294 0
0.002157296 0.494 1590 "tags=30%, list=11%, signal=34%"
GSE37301_HEMATOPOIETIC_STEM_CELL_VS_GRAN_MONO_PROGENITOR_UP
GSE37301_HEMATOPOIETIC_STEM_CELL_VS_GRAN_MONO_PROGENITOR_UP
127 -0.41651756 -1.6930326 0 0.002152406
0.495 2079 "tags=30%, list=15%, signal=35%"
GSE5542_IFNG_VS_IFNA_TREATED_EPITHELIAL_CELLS_24H_DN

GSE5542_IFNG_VS_IFNA_TREATED_EPITHELIAL_CELLS_24H_DN
137	-0.4108148	-1.6878282	0	0.002330172
0.524	3575	"tags=38%, list=25%, signal=50%"
GSE10500_ARTHRITIC_SYNOVIAL_FLUID_VS_HEALTHY_MACROPHAGE_DN
GSE10500_ARTHRITIC_SYNOVIAL_FLUID_VS_HEALTHY_MACROPHAGE_DN
102	-0.43378052	-1.6872098	0	0.002361488
0.531	3211	"tags=38%, list=23%, signal=49%"
GSE40666_WT_VS_STAT4_KO_CD8_TCELL_WITH_IFNA_STIM_90MIN_UP
GSE40666_WT_VS_STAT4_KO_CD8_TCELL_WITH_IFNA_STIM_90MIN_UP
140	-0.40956974	-1.6866432	0	0.002377344
0.535	2287	"tags=35%, list=16%, signal=41%"
GSE39556_CD8A_DC_VS_NK_CELL_MOUSE_3H_POST_POLYIC_INJ_DN
GSE39556_CD8A_DC_VS_NK_CELL_MOUSE_3H_POST_POLYIC_INJ_DN
138	-0.41577122	-1.6859304	0	0.002390242
0.537	1665	"tags=25%, list=12%, signal=28%"
GSE32423_MEMORY_VS_NAIVE_CD8_TCELL_UP
GSE32423_MEMORY_VS_NAIVE_CD8_TCELL_UP
139	-0.41051954	-1.6845796	0	0.002445511
0.546	791	"tags=18%, list=6%, signal=19%"
GSE22886_UNSTIM_VS_IL2_STIM_NKCELL_UP
GSE22886_UNSTIM_VS_IL2_STIM_NKCELL_UP
131	-0.41428918	-1.682951	0	0.002491413
0.556	2185	"tags=27%, list=15%, signal=32%"
GSE39556_CD8A_DC_VS_NK_CELL_DN	GSE39556_CD8A_DC_VS_NK_CELL_DN
	155	-0.4077389	-1.6827667	0
0.002488461	0.558	2278	"tags=25%, list=16%, signal=29%"
GSE43863_LY6C_INT_CXCR5POS_VS_LY6C_LOW_CXCR5NEG_EFFECTOR_CD4_TCELL_D
N
GSE43863_LY6C_INT_CXCR5POS_VS_LY6C_LOW_CXCR5NEG_EFFECTOR_CD4_TCELL_D
N		162	-0.4056476	-1.6822586	0
0.002503772	0.562	2199	"tags=28%, list=16%, signal=33%"
GSE2706_UNSTIM_VS_8H_LPS_AND_R848_DC_DN
GSE2706_UNSTIM_VS_8H_LPS_AND_R848_DC_DN
136	-0.41614187	-1.6819981	0	0.002503834
0.562	1703	"tags=27%, list=12%, signal=31%"
KAECH_DAY15_EFF_VS_MEMORY_CD8_TCELL_DN
KAECH_DAY15_EFF_VS_MEMORY_CD8_TCELL_DN
156	-0.40125436	-1.681171	0	0.002528023
0.565	1371	"tags=25%, list=10%, signal=27%"
GSE45739_UNSTIM_VS_ACD3_ACD28_STIM_WT_CD4_TCELL_UP
GSE45739_UNSTIM_VS_ACD3_ACD28_STIM_WT_CD4_TCELL_UP
144	-0.40830827	-1.6810687	0	0.002531049
0.568	2496	"tags=34%, list=18%, signal=41%"
GSE22611_NOD2_TRANSD_VS_CTRL_TRANSD_HEK293_MDP_STIM_2H_DN
GSE22611_NOD2_TRANSD_VS_CTRL_TRANSD_HEK293_MDP_STIM_2H_DN
103	-0.43041345	-1.6796762	0	0.002581268
0.575	6024	"tags=59%, list=43%, signal=103%"
GSE2770_UNTREATED_VS_TGFB_AND_IL4_TREATED_ACT_CD4_TCELL_4H_UP
GSE2770_UNTREATED_VS_TGFB_AND_IL4_TREATED_ACT_CD4_TCELL_4H_UP
	133	-0.4083193	-1.6773455	0
0.002675503	0.59	1916	"tags=27%, list=14%, signal=31%"
GSE16385_UNTREATED_VS_12H_ROSIGLITAZONE_IFNG_TNF_TREATED_MACROPHAGE_
UP
GSE16385_UNTREATED_VS_12H_ROSIGLITAZONE_IFNG_TNF_TREATED_MACROPHAGE_

UP 152 -0.40473694 -1.676427 0
0.00271062 0.595 2117 "tags=23%, list=15%, signal=27%"
GSE12845_PRE_GC_VS_DARKZONE_GC_TONSIL_BCELL_UP
GSE12845_PRE_GC_VS_DARKZONE_GC_TONSIL_BCELL_UP
142 -0.40907437 -1.6758696 0.001869159
0.002736464 0.601 1747 "tags=27%, list=12%, signal=31%"
GSE17580_UNINFECTED_VS_S_MANSONI_INF_TEFF_UP
GSE17580_UNINFECTED_VS_S_MANSONI_INF_TEFF_UP
152 -0.40723372 -1.6753327 0 0.002747434
0.606 2129 "tags=30%, list=15%, signal=34%"
GSE3982_MEMORY_CD4_TCELL_VS_TH2_UP
GSE3982_MEMORY_CD4_TCELL_VS_TH2_UP
121 -0.41771284 -1.6751261 0 0.002752425
0.609 2155 "tags=25%, list=15%, signal=29%"
GSE3982_MAC_VS_EFF_MEMORY_CD4_TCELL_DN
GSE3982_MAC_VS_EFF_MEMORY_CD4_TCELL_DN
129 -0.4136786 -1.6744858 0.001941748
0.002769218 0.612 1610 "tags=22%, list=11%, signal=24%"
GSE6259_33D1_POS_VS_DEC205_POS_FLT3L_INDUCED_SPLENIC_DC_UP
GSE6259_33D1_POS_VS_DEC205_POS_FLT3L_INDUCED_SPLENIC_DC_UP
146 -0.41005686 -1.6742923 0 0.002765406
0.612 2183 "tags=25%, list=15%, signal=30%"
GSE2706_UNSTIM_VS_2H_R848_DC_DN GSE2706_UNSTIM_VS_2H_R848_DC_DN
137 -0.4067678 -1.6739638 0
0.002779004 0.613 2627 "tags=34%, list=19%, signal=41%"
GSE2405_0H_VS_12H_A_PHAGOCYTOPHILUM_STIM_NEUTROPHIL_UP
GSE2405_0H_VS_12H_A_PHAGOCYTOPHILUM_STIM_NEUTROPHIL_UP
149 -0.40432888 -1.6736842 0 0.002783784
0.615 1790 "tags=25%, list=13%, signal=28%"
GSE7852_THYMUS_VS_FAT_TREG_DN GSE7852_THYMUS_VS_FAT_TREG_DN
141 -0.4037398 -1.6722692 0
0.002872137 0.629 1271 "tags=21%, list=9%, signal=23%"
GSE16385_ROSIGLITAZONE_IL4_VS_IFNG_TNF_STIM_MACROPHAGE_UP
GSE16385_ROSIGLITAZONE_IL4_VS_IFNG_TNF_STIM_MACROPHAGE_UP
147 -0.40205547 -1.6719382 0 0.002879381
0.632 1856 "tags=27%, list=13%, signal=31%"
GSE7852_TREG_VS_TCONV_LN_DN GSE7852_TREG_VS_TCONV_LN_DN
155 -0.40116882 -1.6715355 0
0.00289822 0.635 2280 "tags=33%, list=16%, signal=39%"
GSE25123_WT_VS_PPARG_KO_MACROPHAGE_ROSIGLITAZONE_STIM_UP
GSE25123_WT_VS_PPARG_KO_MACROPHAGE_ROSIGLITAZONE_STIM_UP
131 -0.41535485 -1.6712002 0 0.002896967
0.636 3344 "tags=40%, list=24%, signal=53%"
GSE41867_DAY6_VS_DAY15_LCMV_CLONE13_EFFECTOR_CD8_TCELL_UP
GSE41867_DAY6_VS_DAY15_LCMV_CLONE13_EFFECTOR_CD8_TCELL_UP
105 -0.4267985 -1.6698467 0 0.002972104
0.646 4708 "tags=45%, list=33%, signal=67%"
GSE44649_WT_VS_MIR155_KO_NAIVE_CD8_TCELL_UP
GSE44649_WT_VS_MIR155_KO_NAIVE_CD8_TCELL_UP
148 -0.4044302 -1.6694118 0 0.002990403
0.648 2264 "tags=30%, list=16%, signal=35%"
GSE37301_PRO_BCELL_VS_GRANULOCYTE_MONOCYTE_PROGENITOR_UP
GSE37301_PRO_BCELL_VS_GRANULOCYTE_MONOCYTE_PROGENITOR_UP
115 -0.41971475 -1.6652846 0.001883239

0.003183652	0.672	1996	"tags=34%, list=14%, signal=39%"
GSE22886_NAIVE_CD4_TCELL_VS_48H_ACT_TH2_UP
GSE22886_NAIVE_CD4_TCELL_VS_48H_ACT_TH2_UP
132	-0.40502128	-1.6648492	0	0.003200981
0.675	1327	"tags=22%, list=9%, signal=24%"
GSE37605_TREG_VS_TCONV_NOD_FOXP3_FUSION_GFP_UP
GSE37605_TREG_VS_TCONV_NOD_FOXP3_FUSION_GFP_UP
104	-0.42222226	-1.6647601	0.001937985
0.003198677	0.675	2945	"tags=31%, list=21%, signal=39%"
GSE7852_TREG_VS_TCONV_THYMUS_DN	GSE7852_TREG_VS_TCONV_THYMUS_DN
132	-0.40441057	-1.6613408	0
0.003386346	0.698	1844	"tags=27%, list=13%, signal=31%"
GSE9988_ANTI_TREM1_VS_LPS_MONOCYTE_DN
GSE9988_ANTI_TREM1_VS_LPS_MONOCYTE_DN
145	-0.40049943	-1.6610361	0	0.003391854
0.701	2366	"tags=32%, list=17%, signal=39%"
GSE3982_MAC_VS_TH2_UP	GSE3982_MAC_VS_TH2_UP
156	-0.39934215	-1.6589198	0	0.003474759
0.71	1643	"tags=23%, list=12%, signal=26%"
GSE37301_MULTIPOTENT_PROGENITOR_VS_LYMPHOID_PRIMED_MPP_DN
GSE37301_MULTIPOTENT_PROGENITOR_VS_LYMPHOID_PRIMED_MPP_DN
153	-0.4017089	-1.6582713	0	0.003510292
0.715	1968	"tags=29%, list=14%, signal=34%"
GSE19772_CTRL_VS_HCMV_INF_MONOCYTES_UP
GSE19772_CTRL_VS_HCMV_INF_MONOCYTES_UP
152	-0.40011454	-1.6581514	0	0.003501556
0.715	1918	"tags=30%, list=14%, signal=34%"
GSE40666_NAIVE_VS_EFFECTOR_CD8_TCELL_UP
GSE40666_NAIVE_VS_EFFECTOR_CD8_TCELL_UP
146	-0.40344888	-1.6574683	0	0.003537078
0.718	1948	"tags=30%, list=14%, signal=35%"
GSE11961_MEMORY_BCELL_DAY7_VS_PLASMA_CELL_DAY7_UP
GSE11961_MEMORY_BCELL_DAY7_VS_PLASMA_CELL_DAY7_UP
149	-0.39753613	-1.6571681	0	0.003542164
0.72	2318	"tags=30%, list=16%, signal=36%"
GSE19923_WT_VS_HEB_AND_E2A_KO_DP_THYMOCYTE_DN
GSE19923_WT_VS_HEB_AND_E2A_KO_DP_THYMOCYTE_DN
166	-0.39069232	-1.6543739	0	0.003661465
0.729	1827	"tags=28%, list=13%, signal=31%"
GSE41087_WT_VS_FOXP3_MUT_ANTI_CD3_CD28_STIM_CD4_TCELL_UP
GSE41087_WT_VS_FOXP3_MUT_ANTI_CD3_CD28_STIM_CD4_TCELL_UP
139	-0.40468597	-1.6543543	0	0.003652333
0.729	2648	"tags=35%, list=19%, signal=42%"
GSE3982_MAST_CELL_VS_BASOPHIL_DN	GSE3982_MAST_CELL_VS_BASOPHIL_DN
139	-0.40410802	-1.6533144	0
0.003708327	0.735	1871	"tags=27%, list=13%, signal=30%"
GSE40274_HELIOS_VS_FOXP3_AND_HELIOS_TRANSDUCED_ACTIVATED_CD4_TCELL_U
P
GSE40274_HELIOS_VS_FOXP3_AND_HELIOS_TRANSDUCED_ACTIVATED_CD4_TCELL_U
P	149	-0.4046524	-1.6509312	0
0.00385301	0.752	1942	"tags=30%, list=14%, signal=34%"
GSE5455_HEALTHY_VS_TUMOR_BEARING_MOUSE_SPLEEN_MONOCYTE_UP
GSE5455_HEALTHY_VS_TUMOR_BEARING_MOUSE_SPLEEN_MONOCYTE_UP
151	-0.40046245	-1.6505505	0	0.003859492

0.754 2441 "tags=29%, list=17%, signal=35%"
GSE41867_NAIVE_VS_DAY30_LCMV_CLONE13_EXHAUSTED_CD8_TCELL_UP
GSE41867_NAIVE_VS_DAY30_LCMV_CLONE13_EXHAUSTED_CD8_TCELL_UP
132 -0.4006375 -1.6505108 0.001872659
0.003849916 0.755 1254 "tags=22%, list=9%, signal=24%"
GSE4590_PRE_BCELL_VS_SMALL_PRE_BCELL_UP
GSE4590_PRE_BCELL_VS_SMALL_PRE_BCELL_UP
125 -0.41432658 -1.6487678 0 0.003955693
0.762 2073 "tags=26%, list=15%, signal=30%"
GSE15324_NAIVE_VS_ACTIVATED_ELF4_KO_CD8_TCELL_UP
GSE15324_NAIVE_VS_ACTIVATED_ELF4_KO_CD8_TCELL_UP
140 -0.40382847 -1.6473854 0.003780718
0.004020309 0.767 1913 "tags=27%, list=14%, signal=31%"
GSE28237_FOLLICULAR_VS_EARLY_GC_BCELL_UP
GSE28237_FOLLICULAR_VS_EARLY_GC_BCELL_UP
120 -0.40780398 -1.6473048 0 0.004018193
0.767 2648 "tags=33%, list=19%, signal=41%"
GSE21774_CD62L_POS_CD56_BRIGHT_VS_CD62L_NEG_CD56_DIM_NK_CELL_DN
GSE21774_CD62L_POS_CD56_BRIGHT_VS_CD62L_NEG_CD56_DIM_NK_CELL_DN
137 -0.40557793 -1.6470437 0.001912046
0.004021317 0.77 2392 "tags=28%, list=17%, signal=33%"
GSE40666_UNTREATED_VS_IFNA_STIM_STAT1_KO_CD8_TCELL_90MIN_DN
GSE40666_UNTREATED_VS_IFNA_STIM_STAT1_KO_CD8_TCELL_90MIN_DN
132 -0.4092058 -1.646763 0 0.004021945
0.772 2215 "tags=28%, list=16%, signal=33%"
GSE36078_UNTREATED_VS_AD5_INF_MOUSE_LUNG_DC_UP
GSE36078_UNTREATED_VS_AD5_INF_MOUSE_LUNG_DC_UP
148 -0.3984089 -1.6461449 0 0.004035769
0.774 3762 "tags=31%, list=27%, signal=42%"
GSE18791_CTRL_VS_NEWCASTLE_VIRUS_DC_4H_DN
GSE18791_CTRL_VS_NEWCASTLE_VIRUS_DC_4H_DN
119 -0.40910628 -1.6439706 0 0.004157482
0.788 1766 "tags=28%, list=13%, signal=31%"
GSE45365_HEALTHY_VS_MCMV_INFECTION_CD11B_DC_IFNAR_KO_UP
GSE45365_HEALTHY_VS_MCMV_INFECTION_CD11B_DC_IFNAR_KO_UP
118 -0.41210642 -1.6405944 0.001904762
0.004374199 0.81 2212 "tags=31%, list=16%, signal=37%"
GSE1460_INTRATHYMIC_T_PROGENITOR_VS_CD4_THYMOCYTE_DN
GSE1460_INTRATHYMIC_T_PROGENITOR_VS_CD4_THYMOCYTE_DN
154 -0.39080632 -1.6405138 0 0.004371047
0.811 1790 "tags=23%, list=13%, signal=26%"
GSE20727_CTRL_VS_H2O2_TREATED_DC_DN
GSE20727_CTRL_VS_H2O2_TREATED_DC_DN
134 -0.40041155 -1.6381507 0 0.004570018
0.823 1909 "tags=25%, list=14%, signal=28%"
GSE22935_UNSTIM_VS_48H_MBOVIS_BCG_STIM_MYD88_KO_MACROPHAGE_UP
GSE22935_UNSTIM_VS_48H_MBOVIS_BCG_STIM_MYD88_KO_MACROPHAGE_UP
135 -0.40182483 -1.6375403 0.002
0.004600104 0.824 2738 "tags=35%, list=19%, signal=43%"
GSE11057_NAIVE_VS_EFF_MEMORY_CD4_TCELL_UP
GSE11057_NAIVE_VS_EFF_MEMORY_CD4_TCELL_UP
115 -0.40773228 -1.6374357 0.001926782
0.004591039 0.825 1837 "tags=27%, list=13%, signal=31%"
GSE25123_IL4_VS_IL4_AND_ROSIGLITAZONE_STIM_MACROPHAGE_DAY10_DN

GSE25123_IL4_VS_IL4_AND_ROSIGLITAZONE_STIM_MACROPHAGE_DAY10_DN
124 -0.4075708 -1.6363295 0
0.004643772 0.829 1307 "tags=20%, list=9%, signal=22%"
GSE3720_VD1_VS_VD2_GAMMADELTA_TCELL_WITH_PMA_STIM_UP
GSE3720_VD1_VS_VD2_GAMMADELTA_TCELL_WITH_PMA_STIM_UP
136 -0.39855462 -1.635717 0 0.004680765
0.83 2694 "tags=33%, list=19%, signal=41%"
GSE21379_TFH_VS_NON_TFH_CD4_TCELL_DN
GSE21379_TFH_VS_NON_TFH_CD4_TCELL_DN
136 -0.39534524 -1.6353234 0.001980198
0.004694788 0.831 5045 "tags=49%, list=36%, signal=75%"
GSE35825_IFNA_VS_IFNG_STIM_MACROPHAGE_UP
GSE35825_IFNA_VS_IFNG_STIM_MACROPHAGE_UP
133 -0.4040404 -1.6352285 0 0.004688252
0.832 2377 "tags=30%, list=17%, signal=36%"
GSE411_UNSTIM_VS_100MIN_IL6_STIM_SOCS3_KO_MACROPHAGE_UP
GSE411_UNSTIM_VS_100MIN_IL6_STIM_SOCS3_KO_MACROPHAGE_UP
141 -0.39980486 -1.6322647 0.001904762
0.00488818 0.847 2169 "tags=34%, list=15%, signal=40%"
GSE20366_TREG_VS_NAIVE_CD4_TCELL_DEC205_CONVERSION_DN
GSE20366_TREG_VS_NAIVE_CD4_TCELL_DEC205_CONVERSION_DN
130 -0.40166467 -1.6289277 0 0.005119196
0.862 2313 "tags=28%, list=16%, signal=33%"
GSE9988_ANTI_TREM1_VS_LOW_LPS_MONOCYTE_DN
GSE9988_ANTI_TREM1_VS_LOW_LPS_MONOCYTE_DN
146 -0.39358625 -1.6288894 0 0.00510888
0.862 2155 "tags=30%, list=15%, signal=35%"
GSE2706_R848_VS_LPS_2H_STIM_DC_DN GSE2706_R848_VS_LPS_2H_STIM_DC_DN
110 -0.41399896 -1.6286458 0.001976285
0.005121483 0.864 2115 "tags=27%, list=15%, signal=32%"
GSE9509_LPS_VS_LPS_AND_IL10_STIM_IL10_KO_MACROPHAGE_10MIN_UP
GSE9509_LPS_VS_LPS_AND_IL10_STIM_IL10_KO_MACROPHAGE_10MIN_UP
158 -0.3894055 -1.6254147 0
0.005360055 0.882 2677 "tags=32%, list=19%, signal=39%"
GSE20366_TREG_VS_NAIVE_CD4_TCELL_DEC205_CONVERSION_UP
GSE20366_TREG_VS_NAIVE_CD4_TCELL_DEC205_CONVERSION_UP
151 -0.39212754 -1.625332 0.001923077
0.005354077 0.883 1779 "tags=26%, list=13%, signal=29%"
GSE7460_WT_VS_FOXP3_HET_ACT_TCONV_UP
GSE7460_WT_VS_FOXP3_HET_ACT_TCONV_UP
142 -0.39394847 -1.6235074 0.001964637
0.005480834 0.89 1459 "tags=23%, list=10%, signal=26%"
GSE30971_CTRL_VS_LPS_STIM_MACROPHAGE_WBP7_KO_4H_DN
GSE30971_CTRL_VS_LPS_STIM_MACROPHAGE_WBP7_KO_4H_DN
132 -0.3960121 -1.6232682 0 0.005477131
0.891 2344 "tags=34%, list=17%, signal=40%"
GSE36888_UNTREATED_VS_IL2_TREATED_STAT5_AB_KNOCKIN_TCELL_2H_UP
GSE36888_UNTREATED_VS_IL2_TREATED_STAT5_AB_KNOCKIN_TCELL_2H_UP
141 -0.3949722 -1.6231383 0
0.005470975 0.892 2610 "tags=34%, list=18%, signal=41%"
GSE15735_2H_VS_12H_HDAC_INHIBITOR_TREATED_CD4_TCELL_UP
GSE15735_2H_VS_12H_HDAC_INHIBITOR_TREATED_CD4_TCELL_UP
150 -0.39382732 -1.6220725 0.001851852
0.005556309 0.894 2183 "tags=33%, list=15%, signal=38%"

GSE36009_WT_VS_NLRP10_KO_DC_UP GSE36009_WT_VS_NLRP10_KO_DC_UP
142 -0.38960186 -1.622033 0
0.005540014 0.894 2125 "tags=27%, list=15%, signal=31%"
GSE40666_UNTREATED_VS_IFNA_STIM_STAT4_KO_EFFECTOR_CD8_TCELL_90MIN_DN

GSE40666_UNTREATED_VS_IFNA_STIM_STAT4_KO_EFFECTOR_CD8_TCELL_90MIN_DN
127 -0.40544745 -1.6214943
0.003891051 0.005575488 0.9 1817 "tags=28%,
list=13%, signal=31%"
GSE39556_UNTREATED_VS_3H_POLYIC_INJ_MOUSE_CD8A_DC_DN
GSE39556_UNTREATED_VS_3H_POLYIC_INJ_MOUSE_CD8A_DC_DN
151 -0.38719705 -1.6202565 0.001798561
0.005694254 0.904 1817 "tags=23%, list=13%, signal=26%"
GSE7219_UNSTIM_VS_LPS_AND_ANTI_CD40_STIM_DC_UP
GSE7219_UNSTIM_VS_LPS_AND_ANTI_CD40_STIM_DC_UP
140 -0.39107957 -1.6169652 0.003831418
0.005976492 0.917 1257 "tags=26%, list=9%, signal=28%"
GSE4590_PRE_BCELL_VS_LARGE_PRE_BCELL_DN
GSE4590_PRE_BCELL_VS_LARGE_PRE_BCELL_DN
122 -0.3981517 -1.615896 0 0.006073548
0.919 2476 "tags=30%, list=18%, signal=35%"
GSE17974_IL4_AND_ANTI_IL12_VS_UNTREATED_24H_ACT_CD4_TCELL_DN
GSE17974_IL4_AND_ANTI_IL12_VS_UNTREATED_24H_ACT_CD4_TCELL_DN
112 -0.41148978 -1.6151416 0
0.006114328 0.921 830 "tags=22%, list=6%, signal=24%"
GSE21546_UNSTIM_VS_ANTI_CD3_STIM_DP_THYMOCYTES_UP
GSE21546_UNSTIM_VS_ANTI_CD3_STIM_DP_THYMOCYTES_UP
134 -0.3981407 -1.614012 0 0.006205672
0.923 3203 "tags=37%, list=23%, signal=48%"
GSE17974_0.5H_VS_72H_IL4_AND_ANTI_IL12_ACT_CD4_TCELL_UP
GSE17974_0.5H_VS_72H_IL4_AND_ANTI_IL12_ACT_CD4_TCELL_UP
129 -0.3945458 -1.6128851 0 0.006289237
0.927 2419 "tags=31%, list=17%, signal=37%"
GSE9988_ANTI_TREM1_VS_ANTI_TREM1_AND_LPS_MONOCYTE_DN
GSE9988_ANTI_TREM1_VS_ANTI_TREM1_AND_LPS_MONOCYTE_DN
134 -0.39877194 -1.6123699 0.001953125
0.006312195 0.929 2649 "tags=34%, list=19%, signal=41%"
GSE29618_BCELL_VS_MONOCYTE_UP GSE29618_BCELL_VS_MONOCYTE_UP
120 -0.4012917 -1.6123375 0
0.006296559 0.929 621 "tags=15%, list=4%, signal=16%"
GSE3691_IFN_PRODUCING_KILLER_DC_VS_PLASMACYTOID_DC_SPLEEN_UP
GSE3691_IFN_PRODUCING_KILLER_DC_VS_PLASMACYTOID_DC_SPLEEN_UP
139 -0.38944462 -1.6108602 0.002
0.006407614 0.934 1885 "tags=28%, list=13%, signal=32%"
GSE2706_UNSTIM_VS_8H_R848_DC_DN GSE2706_UNSTIM_VS_8H_R848_DC_DN
135 -0.3910702 -1.6093477 0.001872659
0.006528288 0.937 2433 "tags=36%, list=17%, signal=43%"
GSE40277_EOS_AND_LEF1_TRANSDUCED_VS_CTRL_CD4_TCELL_UP
GSE40277_EOS_AND_LEF1_TRANSDUCED_VS_CTRL_CD4_TCELL_UP
144 -0.38867775 -1.609068 0 0.006540928
0.937 2193 "tags=33%, list=16%, signal=39%"
GSE41176_WT_VS_TAK1_KO_ANTI_IGM_STIM_BCELL_1H_UP
GSE41176_WT_VS_TAK1_KO_ANTI_IGM_STIM_BCELL_1H_UP
141 -0.3887196 -1.6076639 0.001851852

0.00664602 0.941 4475 "tags=43%, list=32%, signal=62%"
GSE17721_PAM3CSK4_VS_GADIQUIMOD_1H_BMDC_UP
GSE17721_PAM3CSK4_VS_GADIQUIMOD_1H_BMDC_UP
169 -0.3826013 -1.6056609 0.001930502
0.006805286 0.943 2524 "tags=27%, list=18%, signal=33%"
GSE24574_BCL6_HIGH_VS_LOW_TFH_CD4_TCELL_UP
GSE24574_BCL6_HIGH_VS_LOW_TFH_CD4_TCELL_UP
140 -0.39106673 -1.6049176 0.001838235
0.006873612 0.943 2344 "tags=30%, list=17%, signal=36%"
GSE29618_BCELL_VS_MDC_UP GSE29618_BCELL_VS_MDC_UP
126 -0.39352882 -1.6046565 0.001937985
0.006894464 0.945 2084 "tags=28%, list=15%, signal=32%"
GSE41867_NAIVE_VS_DAY6_LCMV_ARMSTRONG_EFFECTOR_CD8_TCELL_UP
GSE41867_NAIVE_VS_DAY6_LCMV_ARMSTRONG_EFFECTOR_CD8_TCELL_UP
144 -0.3851613 -1.6032231 0 0.00703307
0.949 1981 "tags=25%, list=14%, signal=29%"
GSE14000_UNSTIM_VS_16H_LPS_DC_TRANSLATED_RNA_DN
GSE14000_UNSTIM_VS_16H_LPS_DC_TRANSLATED_RNA_DN
124 -0.39434585 -1.602858 0.001960784
0.007043854 0.949 2248 "tags=35%, list=16%, signal=41%"
GSE23502_WT_VS_HDC_KO_MYELOID_DERIVED_SUPPRESSOR_CELL_BM_UP
GSE23502_WT_VS_HDC_KO_MYELOID_DERIVED_SUPPRESSOR_CELL_BM_UP
136 -0.3875138 -1.6025996 0 0.007042928
0.95 2322 "tags=32%, list=16%, signal=37%"
GSE29164_CD8_TCELL_VS_CD8_TCELL_AND_IL12_TREATED_MELANOMA_DAY3_DN
GSE29164_CD8_TCELL_VS_CD8_TCELL_AND_IL12_TREATED_MELANOMA_DAY3_DN
153 -0.38712052 -1.6014016 0.003745318
0.007158344 0.955 1459 "tags=26%, list=10%, signal=29%"
GSE26928_EFF_MEMORY_VS_CXCR5_POS_CD4_TCELL_DN
GSE26928_EFF_MEMORY_VS_CXCR5_POS_CD4_TCELL_DN
102 -0.40692365 -1.6012783 0 0.00714545
0.955 4356 "tags=54%, list=31%, signal=77%"
GSE45365_BCELL_VS_CD8_TCELL_UP GSE45365_BCELL_VS_CD8_TCELL_UP
98 -0.4156733 -1.6010911 0.002008032
0.007144159 0.956 4346 "tags=41%, list=31%, signal=59%"
GSE40184_HEALTHY_VS_HCV_INFECTED_DONOR_PBMC_UP
GSE40184_HEALTHY_VS_HCV_INFECTED_DONOR_PBMC_UP
142 -0.39269647 -1.5997213 0 0.007251534
0.959 3799 "tags=44%, list=27%, signal=60%"
GSE20366_TREG_VS_TCONV_UP GSE20366_TREG_VS_TCONV_UP
140 -0.38772774 -1.599565 0.001937985
0.007254666 0.959 2199 "tags=27%, list=16%, signal=32%"
GSE41867_NAIVE_VS_DAY6_LCMV_ARMSTRONG_EFFECTOR_CD8_TCELL_DN
GSE41867_NAIVE_VS_DAY6_LCMV_ARMSTRONG_EFFECTOR_CD8_TCELL_DN
146 -0.38654312 -1.5985401 0 0.007346891
0.96 2069 "tags=29%, list=15%, signal=34%"
GSE37605_FOXP3_FUSION_GFP_VS_IRES_GFP_TREG_C57BL6_DN
GSE37605_FOXP3_FUSION_GFP_VS_IRES_GFP_TREG_C57BL6_DN
142 -0.38845545 -1.5981811 0 0.00735909
0.962 2631 "tags=30%, list=19%, signal=37%"
GSE23568_ID3_TRANSDUCED_VS_ID3_KO_CD8_TCELL_DN
GSE23568_ID3_TRANSDUCED_VS_ID3_KO_CD8_TCELL_DN
146 -0.3850438 -1.5972935 0 0.007444131
0.964 1771 "tags=29%, list=13%, signal=33%"

GSE21063_CTRL_VS_ANTI_IGM_STIM_BCELL_NFATC1_KO_8H_UP
GSE21063_CTRL_VS_ANTI_IGM_STIM_BCELL_NFATC1_KO_8H_UP
135 -0.3900473 -1.5964215 0.001908397
0.007528677 0.964 2119 "tags=28%, list=15%, signal=33%"
GSE411_WT_VS_SOCS3_KO_MACROPHAGE_IL6_STIM_400MIN_DN
GSE411_WT_VS_SOCS3_KO_MACROPHAGE_IL6_STIM_400MIN_DN
137 -0.392869 -1.5951787 0 0.007637747
0.966 2638 "tags=35%, list=19%, signal=43%"
GSE21033_CTRL_VS_POLYIC_STIM_DC_6H_UP
GSE21033_CTRL_VS_POLYIC_STIM_DC_6H_UP
143 -0.38670015 -1.5951362 0.003861004
0.007626167 0.966 2440 "tags=26%, list=17%, signal=31%"
GSE25123_WT_VS_PPARG_KO_MACROPHAGE_IL4_AND_ROSIGLITAZONE_STIM_DN
GSE25123_WT_VS_PPARG_KO_MACROPHAGE_IL4_AND_ROSIGLITAZONE_STIM_DN
141 -0.38988605 -1.5944293 0.001901141
0.007684799 0.967 1855 "tags=22%, list=13%, signal=25%"
GSE2770_TGFB_AND_IL4_VS_TGFB_AND_IL12_TREATED_ACT_CD4_TCELL_2H_UP
GSE2770_TGFB_AND_IL4_VS_TGFB_AND_IL12_TREATED_ACT_CD4_TCELL_2H_UP
102 -0.40368718 -1.5943532 0.003663004
0.007682345 0.968 2266 "tags=25%, list=16%, signal=29%"
GSE45365_HEALTHY_VS_MCMV_INFECTION_BCELL_IFNAR_KO_DN
GSE45365_HEALTHY_VS_MCMV_INFECTION_BCELL_IFNAR_KO_DN
135 -0.38769436 -1.592725 0 0.007860198
0.969 2192 "tags=28%, list=16%, signal=33%"
GSE42021_CD24HI_VS_CD24LOW_TREG_THYMUS_DN
GSE42021_CD24HI_VS_CD24LOW_TREG_THYMUS_DN
132 -0.39510512 -1.5922682 0.003929273
0.007877513 0.969 1841 "tags=25%, list=13%, signal=28%"
GSE4748_CTRL_VS_LPS_AND_CYANOBACTERIUM_LPSLIKE_STIM_DC_3H_UP
GSE4748_CTRL_VS_LPS_AND_CYANOBACTERIUM_LPSLIKE_STIM_DC_3H_UP
150 -0.3790808 -1.5915681 0
0.007953077 0.971 1610 "tags=20%, list=11%, signal=22%"
GSE39556_UNTREATED_VS_3H_POLYIC_INJ_MOUSE_NK_CELL_DN
GSE39556_UNTREATED_VS_3H_POLYIC_INJ_MOUSE_NK_CELL_DN
150 -0.38327104 -1.5912834 0.001915709
0.007963408 0.971 1817 "tags=23%, list=13%, signal=26%"
GSE21379_TFH_VS_NON_TFH_SAP_KO_CD4_TCELL_DN
GSE21379_TFH_VS_NON_TFH_SAP_KO_CD4_TCELL_DN
147 -0.38695747 -1.5893068 0.004008016
0.008171191 0.975 3574 "tags=39%, list=25%, signal=51%"
GSE42021_CD24HI_VS_CD24INT_TCONV_THYMUS_DN
GSE42021_CD24HI_VS_CD24INT_TCONV_THYMUS_DN
145 -0.38188788 -1.5892704 0.001897533
0.008158566 0.975 2403 "tags=31%, list=17%, signal=37%"
GSE17301_ACD3_ACD28_VS_ACD3_ACD28_AND_IFNA2_STIM_CD8_TCELL_DN
GSE17301_ACD3_ACD28_VS_ACD3_ACD28_AND_IFNA2_STIM_CD8_TCELL_DN
145 -0.38286 -1.5888107 0.005928854
0.00818354 0.976 1237 "tags=22%, list=9%, signal=24%"
GSE40274_GATA1_VS_FOXP3_AND_GATA1_TRANSDUCED_ACTIVATED_CD4_TCELL_UP
GSE40274_GATA1_VS_FOXP3_AND_GATA1_TRANSDUCED_ACTIVATED_CD4_TCELL_UP
155 -0.38191912 -1.5886055 0
0.008181967 0.976 2145 "tags=28%, list=15%, signal=33%"
GSE10147_IL3_VS_IL3_AND_HIVP17_STIM_PDC_UP
GSE10147_IL3_VS_IL3_AND_HIVP17_STIM_PDC_UP

126	-0.39307868	-1.5881659	0	0.008213279
0.978	2847	"tags=40%, list=20%, signal=49%"
GSE360_T_GONDII_VS_B_MALAYI_LOW_DOSE_DC_UP
GSE360_T_GONDII_VS_B_MALAYI_LOW_DOSE_DC_UP
136	-0.38702202	-1.5869563	0	0.008336738
0.981	1766	"tags=24%, list=13%, signal=27%"
GSE30971_WBP7_HET_VS_KO_MACROPHAGE_DN
GSE30971_WBP7_HET_VS_KO_MACROPHAGE_DN
142	-0.3869305	-1.586428	0	0.008387533
0.981	1428	"tags=21%, list=10%, signal=23%"
GSE24142_EARLY_THYMIC_PROGENITOR_VS_DN2_THYMOCYTE_UP
GSE24142_EARLY_THYMIC_PROGENITOR_VS_DN2_THYMOCYTE_UP
163	-0.3767616	-1.5849375	0	0.008525218
0.981	1051	"tags=22%, list=7%, signal=24%"
GSE7460_WT_VS_FOXP3_HET_ACT_WITH_TGFB_TCONV_UP
GSE7460_WT_VS_FOXP3_HET_ACT_WITH_TGFB_TCONV_UP
134	-0.3922477	-1.5832243	0	0.008697243
0.984	1459	"tags=22%, list=10%, signal=25%"
GSE14308_TH2_VS_TH17_DN	GSE14308_TH2_VS_TH17_DN
137	-0.388811	-1.5831475	0	0.008676926
0.984	1948	"tags=26%, list=14%, signal=30%"
GSE9988_ANTI_TREM1_AND_LPS_VS_VEHICLE_TREATED_MONOCYTES_UP
GSE9988_ANTI_TREM1_AND_LPS_VS_VEHICLE_TREATED_MONOCYTES_UP
125	-0.38897938	-1.5814885	0.007751938
0.008886628	0.988	2911	"tags=38%, list=21%, signal=48%"
GSE13522_WT_VS_IFNAR_KO_SKING_T_CRUZI_Y_STRAIN_INF_DN
GSE13522_WT_VS_IFNAR_KO_SKING_T_CRUZI_Y_STRAIN_INF_DN
143	-0.38091114	-1.5814135	0.001897533
0.008870332	0.988	2980	"tags=29%, list=21%, signal=37%"
GSE7460_FOXP3_MUT_VS_WT_ACT_TCONV_UP
GSE7460_FOXP3_MUT_VS_WT_ACT_TCONV_UP
153	-0.38261318	-1.5806271	0.001838235
0.008977253	0.988	1481	"tags=24%, list=10%, signal=26%"
GSE22886_IGG_IGA_MEMORY_BCELL_VS_BLOOD_PLASMA_CELL_UP
GSE22886_IGG_IGA_MEMORY_BCELL_VS_BLOOD_PLASMA_CELL_UP
138	-0.38305852	-1.5805715	0	0.00896287
0.988	3293	"tags=37%, list=23%, signal=48%"
GSE33292_DN3_THYMOCYTE_VS_TCF1_KO_TCELL_LYMPHOMA_UP
GSE33292_DN3_THYMOCYTE_VS_TCF1_KO_TCELL_LYMPHOMA_UP
160	-0.37645853	-1.580569	0	0.008940006
0.988	1998	"tags=24%, list=14%, signal=27%"
GSE20754_WT_VS_TCF1_KO_MEMORY_CD8_TCELL_UP
GSE20754_WT_VS_TCF1_KO_MEMORY_CD8_TCELL_UP
143	-0.38400957	-1.5804969	0.005725191
0.008936618	0.989	1867	"tags=28%, list=13%, signal=32%"
GSE36392_TYPE_2_MYELOID_VS_EOSINOPHIL_IL25_TREATED_LUNG_DN
GSE36392_TYPE_2_MYELOID_VS_EOSINOPHIL_IL25_TREATED_LUNG_DN
134	-0.3934251	-1.5799325	0	0.008979996
0.99	1766	"tags=25%, list=13%, signal=28%"
GSE360_HIGH_DOSE_B_MALAYI_VS_M_TUBERCULOSIS_MAC_DN
GSE360_HIGH_DOSE_B_MALAYI_VS_M_TUBERCULOSIS_MAC_DN
147	-0.384121	-1.578668	0	0.009110952
0.991	1194	"tags=19%, list=8%, signal=21%"
GSE41867_NAIVE_VS_DAY6_LCMV_EFFECTOR_CD8_TCELL_DN

GSE41867_NAIVE_VS_DAY6_LCMV_EFFECTOR_CD8_TCELL_DN
142	-0.3829728	-1.5779043	0	0.00919175
0.991	2315	"tags=35%, list=16%, signal=42%"
GSE24634_TREG_VS_TCONV_POST_DAY7_IL4_CONVERSION_DN
GSE24634_TREG_VS_TCONV_POST_DAY7_IL4_CONVERSION_DN
152	-0.37667254	-1.5761434	0	0.009391294
0.992	3217	"tags=36%, list=23%, signal=46%"
GSE14308_TH2_VS_NATURAL_TREG_DN	GSE14308_TH2_VS_NATURAL_TREG_DN
	143	-0.3813774	-1.575658	0.005607477
0.009452529	0.992	1865	"tags=25%, list=13%, signal=29%"
GSE3920_IFNA_VS_IFNG_TREATED_FIBROBLAST_UP
GSE3920_IFNA_VS_IFNG_TREATED_FIBROBLAST_UP
130	-0.389403	-1.5749099	0.001851852
0.00954272	0.993	2130	"tags=28%, list=15%, signal=33%"
GSE39022_LN_VS_SPLEEN_DC_DN	GSE39022_LN_VS_SPLEEN_DC_DN
	128	-0.38862827	-1.5748267	0.003745318
0.009531415	0.993	1089	"tags=20%, list=8%, signal=22%"
GSE33425_CD161_HIGH_VS_INT_CD8_TCELL_UP
GSE33425_CD161_HIGH_VS_INT_CD8_TCELL_UP
158	-0.37713292	-1.574707	0	0.009522329
0.993	2517	"tags=35%, list=18%, signal=42%"
GSE21360_PRIMARY_VS_QUATERNARY_MEMORY_CD8_TCELL_DN
GSE21360_PRIMARY_VS_QUATERNARY_MEMORY_CD8_TCELL_DN
133	-0.38125676	-1.5740275	0	0.009601087
0.993	1621	"tags=26%, list=11%, signal=29%"
GSE18791_CTRL_VS_NEWCASTLE_VIRUS_DC_1H_DN
GSE18791_CTRL_VS_NEWCASTLE_VIRUS_DC_1H_DN
102	-0.40658554	-1.5740261	0.005859375
0.009577264	0.993	2140	"tags=29%, list=15%, signal=34%"
GSE21063_CTRL_VS_ANTI_IGM_STIM_BCELL_8H_UP
GSE21063_CTRL_VS_ANTI_IGM_STIM_BCELL_8H_UP
123	-0.3939288	-1.5734949	0	0.009649226
0.993	3761	"tags=38%, list=27%, signal=52%"
GSE40441_NRP1_POS_INDUCED_TREG_VS_NRP1_NEG_NATURAL_TREG_UP
GSE40441_NRP1_POS_INDUCED_TREG_VS_NRP1_NEG_NATURAL_TREG_UP
153	-0.3800746	-1.5731547	0	0.009666836
0.994	2452	"tags=29%, list=17%, signal=34%"
GSE37301_MULTIPOTENT_PROGENITOR_VS_PRO_BCELL_DN
GSE37301_MULTIPOTENT_PROGENITOR_VS_PRO_BCELL_DN
144	-0.3832089	-1.5711347	0	0.009920492
0.996	1860	"tags=24%, list=13%, signal=28%"
GSE16385_ROSIGLITAZONE_IFNG_TNF_VS_IL4_STIM_MACROPHAGE_DN
GSE16385_ROSIGLITAZONE_IFNG_TNF_VS_IL4_STIM_MACROPHAGE_DN
137	-0.38359874	-1.5708115	0	0.009933322
0.996	1413	"tags=21%, list=10%, signal=23%"
GSE3982_BCELL_VS_TH2_UP	GSE3982_BCELL_VS_TH2_UP
127	-0.38279164	-1.5706658	0.002053388
0.009931757	0.996	2553	"tags=27%, list=18%, signal=32%"
GSE9601_UNTREATED_VS_NFKB_INHIBITOR_TREATED_HCMV_INF_MONOCYTE_DN
GSE9601_UNTREATED_VS_NFKB_INHIBITOR_TREATED_HCMV_INF_MONOCYTE_DN
	148	-0.37926155	-1.5694505	0
0.010113304	0.997	2327	"tags=31%, list=16%, signal=37%"
GSE43863_DAY6_EFF_VS_DAY150_MEM_LY6C_INT_CXCR5POS_CD4_TCELL_UP
GSE43863_DAY6_EFF_VS_DAY150_MEM_LY6C_INT_CXCR5POS_CD4_TCELL_UP

148	-0.375459	-1.569418	0
0.010090707	0.997	2162	"tags=28%, list=15%, signal=33%"
GSE9509_LPS_VS_LPS_AND_IL10_STIM_IL10_KO_MACROPHAGE_20MIN_UP
GSE9509_LPS_VS_LPS_AND_IL10_STIM_IL10_KO_MACROPHAGE_20MIN_UP
133	-0.38634774	-1.568417	0
0.010213438	0.997	4123	"tags=38%, list=29%, signal=54%"
GSE30083_SP2_VS_SP4_THYMOCYTE_DN	GSE30083_SP2_VS_SP4_THYMOCYTE_DN
147	-0.37914845	-1.568044	0
0.010231699	0.997	1345	"tags=28%, list=10%, signal=31%"
GSE33292_WT_VS_TCF1_KO_DN3_THYMOCYTE_UP
GSE33292_WT_VS_TCF1_KO_DN3_THYMOCYTE_UP
141	-0.38339424	-1.5678911	0	0.010235659
0.997	1666	"tags=23%, list=12%, signal=26%"
GSE43955_TGFB_IL6_VS_TGFB_IL6_IL23_TH17_ACT_CD4_TCELL_52H_DN
GSE43955_TGFB_IL6_VS_TGFB_IL6_IL23_TH17_ACT_CD4_TCELL_52H_DN
166	-0.37320387	-1.5676335	0
0.010261814	0.997	2193	"tags=27%, list=16%, signal=32%"
GSE17974_2H_VS_72H_UNTREATED_IN_VITRO_CD4_TCELL_UP
GSE17974_2H_VS_72H_UNTREATED_IN_VITRO_CD4_TCELL_UP
121	-0.39326876	-1.5667521	0.004081633
0.010395561	0.997	1070	"tags=21%, list=8%, signal=23%"
GSE12845_IGD_NEG_BLOOD_VS_NAIVE_TONSIL_BCELL_DN
GSE12845_IGD_NEG_BLOOD_VS_NAIVE_TONSIL_BCELL_DN
129	-0.38530824	-1.5665599	0.001824818
0.010398964	0.998	2810	"tags=33%, list=20%, signal=40%"
GSE15930_NAIVE_VS_72H_IN_VITRO_STIM_CD8_TCELL_UP
GSE15930_NAIVE_VS_72H_IN_VITRO_STIM_CD8_TCELL_UP
159	-0.37415916	-1.5650027	0.002016129
0.010643037	0.998	3210	"tags=35%, list=23%, signal=45%"
GSE24634_NAIVE_CD4_TCELL_VS_DAY5_IL4_CONV_TREG_UP
GSE24634_NAIVE_CD4_TCELL_VS_DAY5_IL4_CONV_TREG_UP
128	-0.38941243	-1.5629247	0.003738318
0.010964051	0.998	2038	"tags=23%, list=14%, signal=27%"
GSE3565_CTRL_VS_LPS_INJECTED_SPLENOCYTES_DN
GSE3565_CTRL_VS_LPS_INJECTED_SPLENOCYTES_DN
138	-0.3776588	-1.5627165	0	0.010964064
0.998	1507	"tags=27%, list=11%, signal=30%"
GSE28726_ACT_CD4_TCELL_VS_ACT_VA24NEG_NKTCELL_DN
GSE28726_ACT_CD4_TCELL_VS_ACT_VA24NEG_NKTCELL_DN
146	-0.3731603	-1.5622858	0.003868472
0.011006103	0.998	1703	"tags=26%, list=12%, signal=29%"
GSE2124_CTRL_VS_LYMPHOTOXIN_BETA_TREATED_MLN_DN
GSE2124_CTRL_VS_LYMPHOTOXIN_BETA_TREATED_MLN_DN
144	-0.37921768	-1.5616679	0	0.01106583
0.998	1898	"tags=26%, list=13%, signal=30%"
GSE14415_NATURAL_TREG_VS_TCONV_UP GSE14415_NATURAL_TREG_VS_TCONV_UP
118	-0.391216	-1.561366	0.005607477
0.011081518	0.998	1852	"tags=27%, list=13%, signal=31%"
GSE9650_EXHAUSTED_VS_MEMORY_CD8_TCELL_UP
GSE9650_EXHAUSTED_VS_MEMORY_CD8_TCELL_UP
150	-0.37265775	-1.5611756	0.001953125
0.011088993	0.999	1551	"tags=24%, list=11%, signal=27%"
GSE37534_UNTREATED_VS_PIOGLITAZONE_TREATED_CD4_TCELL_PPARG1_AND_FOXP
3_TRASDUCED_DN

GSE37534_UNTREATED_VS_PIOGLITAZONE_TREATED_CD4_TCELL_PPARG1_AND_FOXP3_TRASDUCED_DN	154	-0.37062842	-1.5595386	0.001915709	0.011313331	0.999	1138	"tags=21%, list=8%, signal=23%"
GSE11864_CSF1_VS_CSF1_IFNG_IN_MAC_UP	GSE11864_CSF1_VS_CSF1_IFNG_IN_MAC_UP	129	-0.38420635	-1.5594331	0.00204918	0.011304654	0.999	1441	"tags=22%, list=10%, signal=24%"
GSE36078_WT_VS_IL1R_KO_LUNG_DC_AFTER_AD5_T425A_HEXON_INF_DN	GSE36078_WT_VS_IL1R_KO_LUNG_DC_AFTER_AD5_T425A_HEXON_INF_DN	147	-0.37662497	-1.5593892	0	0.011287887	0.999	1282	"tags=20%, list=9%, signal=22%"
GSE13306_TREG_VS_TCONV_UP	GSE13306_TREG_VS_TCONV_UP	150	-0.37699074	-1.5574917	0.008281574	0.011592092	0.999	3394	"tags=34%, list=24%, signal=44%"
GSE46606_IRF4_KO_VS_WT_UNSTIM_BCELL_DN	GSE46606_IRF4_KO_VS_WT_UNSTIM_BCELL_DN	147	-0.3785874	-1.5568209	0	0.011667149	1	1978	"tags=26%, list=14%, signal=30%"
GSE411_UNSTIM_VS_100MIN_IL6_STIM_MACROPHAGE_DN	GSE411_UNSTIM_VS_100MIN_IL6_STIM_MACROPHAGE_DN	146	-0.37131122	-1.5565313	0	0.011694912	1	1174	"tags=18%, list=8%, signal=20%"
GSE1460_DP_VS_CD4_THYMOCYTE_DN	GSE1460_DP_VS_CD4_THYMOCYTE_DN	145	-0.37388277	-1.5560324	0.003960396	0.011757704	1	2000	"tags=25%, list=14%, signal=29%"
GSE3565_DUSP1_VS_WT_SPLENOCYTES_DN	GSE3565_DUSP1_VS_WT_SPLENOCYTES_DN	147	-0.3753375	-1.5557033	0.001960784	0.011792945	1	1229	"tags=24%, list=9%, signal=27%"
GSE36888_UNTREATED_VS_IL2_TREATED_TCELL_17H_DN	GSE36888_UNTREATED_VS_IL2_TREATED_TCELL_17H_DN	132	-0.37979093	-1.5548837	0	0.011906049	1	1534	"tags=24%, list=11%, signal=27%"
GSE15659_RESTING_VS_ACTIVATED_TREG_UP	GSE15659_RESTING_VS_ACTIVATED_TREG_UP	102	-0.40075338	-1.55383	0.005703422	0.012059356	1	2876	"tags=28%, list=20%, signal=35%"
GSE31082_CD4_VS_CD8_SP_THYMOCYTE_UP	GSE31082_CD4_VS_CD8_SP_THYMOCYTE_UP	158	-0.3706068	-1.553788	0.001834862	0.012035477	1	2694	"tags=32%, list=19%, signal=39%"
GSE7460_CTRL_VS_TGFB_TREATED_ACT_TCONV_DN	GSE7460_CTRL_VS_TGFB_TREATED_ACT_TCONV_DN	147	-0.37358937	-1.5533413	0.001968504	0.012096528	1	2410	"tags=31%, list=17%, signal=37%"
GSE34515_CD16_POS_MONOCYTE_VS_DC_DN	GSE34515_CD16_POS_MONOCYTE_VS_DC_DN	142	-0.37307575	-1.5522475	0	0.012252037	1	1597	"tags=25%, list=11%, signal=28%"
GSE2770_IL12_ACT_VS_ACT_CD4_TCELL_2H_UP	GSE2770_IL12_ACT_VS_ACT_CD4_TCELL_2H_UP	123	-0.38340434	-1.5518851	0.003921569	0.012297231	1	1663	"tags=25%, list=12%, signal=28%"

GSE39916_B_CELL_SPLEEN_VS_PLASMA_CELL_BONE_MARROW_DN
GSE39916_B_CELL_SPLEEN_VS_PLASMA_CELL_BONE_MARROW_DN
152 -0.37475324 -1.5515947 0.003642987
0.012313554 1 1279 "tags=26%, list=9%, signal=28%"
GSE39820_IL1B_IL6_VS_IL1B_IL6_IL23A_TREATED_CD4_TCELL_DN
GSE39820_IL1B_IL6_VS_IL1B_IL6_IL23A_TREATED_CD4_TCELL_DN
155 -0.36904684 -1.5513971 0.001960784
0.012318108 1 1841 "tags=21%, list=13%, signal=24%"
GSE14308_TH17_VS_NATURAL_TREG_DN GSE14308_TH17_VS_NATURAL_TREG_DN
146 -0.37371016 -1.5490752 0.003968254
0.012660816 1 2004 "tags=25%, list=14%, signal=28%"
GSE6875_TCONV_VS_FOXP3_KO_TREG_DN GSE6875_TCONV_VS_FOXP3_KO_TREG_DN
151 -0.3707084 -1.5483683 0.003968254
0.012750438 1 2524 "tags=30%, list=18%, signal=37%"
GSE26030_UNSTIM_VS_RESTIM_TH1_DAY5_POST_POLARIZATION_UP
GSE26030_UNSTIM_VS_RESTIM_TH1_DAY5_POST_POLARIZATION_UP
146 -0.3759942 -1.5479959 0.003738318
0.012788188 1 2481 "tags=35%, list=18%, signal=42%"
GSE7460_TCONV_VS_TREG_LN_UP GSE7460_TCONV_VS_TREG_LN_UP
151 -0.37171885 -1.5478672 0
0.012780373 1 1790 "tags=25%, list=13%, signal=29%"
GSE21670_TGFB_VS_IL6_TREATED_STAT3_KO_CD4_TCELL_DN
GSE21670_TGFB_VS_IL6_TREATED_STAT3_KO_CD4_TCELL_DN
132 -0.38174292 -1.5472623 0.003738318
0.012872488 1 2174 "tags=29%, list=15%, signal=34%"
GSE33513_TCF7_KO_VS_HET_EARLY_THYMIC_PROGENITOR_UP
GSE33513_TCF7_KO_VS_HET_EARLY_THYMIC_PROGENITOR_UP
140 -0.3705536 -1.5461624 0 0.013053688
1 2525 "tags=26%, list=18%, signal=32%"
GSE11057_NAIVE_VS_CENT_MEMORY_CD4_TCELL_UP
GSE11057_NAIVE_VS_CENT_MEMORY_CD4_TCELL_UP
113 -0.38793334 -1.543202 0.005703422
0.013585694 1 1604 "tags=21%, list=11%, signal=24%"
GSE12392_IFNAR_KO_VS_IFNB_KO_CD8_NEG_SPLEEN_DC_UP
GSE12392_IFNAR_KO_VS_IFNB_KO_CD8_NEG_SPLEEN_DC_UP
140 -0.3714002 -1.5428867 0.001960784
0.01361375 1 2538 "tags=31%, list=18%, signal=37%"
GSE3920_IFNA_VS_IFNG_TREATED_FIBROBLAST_DN
GSE3920_IFNA_VS_IFNG_TREATED_FIBROBLAST_DN
149 -0.37206423 -1.5420909 0 0.013742727
1 1435 "tags=25%, list=10%, signal=27%"
GSE36476_CTRL_VS_TSST_ACT_72H_MEMORY_CD4_TCELL_YOUNG_UP
GSE36476_CTRL_VS_TSST_ACT_72H_MEMORY_CD4_TCELL_YOUNG_UP
137 -0.37433842 -1.5420091 0.001883239
0.01372524 1 3215 "tags=38%, list=23%, signal=49%"
GSE43863_DAY6_EFF_VS_DAY150_MEM_TH1_CD4_TCELL_UP
GSE43863_DAY6_EFF_VS_DAY150_MEM_TH1_CD4_TCELL_UP
164 -0.3715226 -1.5416261 0 0.013779253
1 1962 "tags=28%, list=14%, signal=32%"
GSE37532_TREG_VS_TCONV_PPARG_KO_CD4_TCELL_FROM_VISCERAL_ADIPOSE_TISS
UE_DN
GSE37532_TREG_VS_TCONV_PPARG_KO_CD4_TCELL_FROM_VISCERAL_ADIPOSE_TISS
UE_DN 143 -0.37622952 -1.5415205 0
0.013774742 1 2261 "tags=29%, list=16%, signal=35%"

GSE18791_CTRL_VS_NEWCASTLE_VIRUS_DC_16H_DN
GSE18791_CTRL_VS_NEWCASTLE_VIRUS_DC_16H_DN
124	-0.3859528	-1.5401498	0.001937985
0.013994218	1	2433	"tags=32%, list=17%, signal=39%"
GSE26669_CTRL_VS_COSTIM_BLOCK_MLR_CD8_TCELL_DN
GSE26669_CTRL_VS_COSTIM_BLOCK_MLR_CD8_TCELL_DN
144	-0.3757066	-1.5394946	0.001964637
0.014063743	1	2193	"tags=30%, list=16%, signal=35%"
GSE23568_CTRL_VS_ID3_TRANSDUCED_CD8_TCELL_UP
GSE23568_CTRL_VS_ID3_TRANSDUCED_CD8_TCELL_UP
155	-0.37122428	-1.5394722	0	0.014036471
1	1729	"tags=27%, list=12%, signal=31%"
GSE32423_MEMORY_VS_NAIVE_CD8_TCELL_DN
GSE32423_MEMORY_VS_NAIVE_CD8_TCELL_DN
145	-0.37254113	-1.5391439	0	0.014068268
1	2159	"tags=27%, list=15%, signal=31%"
GSE33424_CD161_INT_VS_NEG_CD8_TCELL_UP
GSE33424_CD161_INT_VS_NEG_CD8_TCELL_UP
141	-0.37332758	-1.5378532	0	0.014310532
1	1487	"tags=27%, list=11%, signal=30%"
GSE14000_UNSTIM_VS_4H_LPS_DC_DN	GSE14000_UNSTIM_VS_4H_LPS_DC_DN
	135	-0.373428	-1.5376171	0.001855288
0.014334561	1	2409	"tags=33%, list=17%, signal=40%"
GSE10273_HIGH_VS_LOW_IL7_TREATED_IRF4_8_NULL_PRE_BCELL_UP
GSE10273_HIGH_VS_LOW_IL7_TREATED_IRF4_8_NULL_PRE_BCELL_UP
133	-0.37508094	-1.537358	0.003875969
0.014336224	1	1473	"tags=23%, list=10%, signal=26%"
GSE21670_UNTREATED_VS_IL6_TREATED_CD4_TCELL_DN
GSE21670_UNTREATED_VS_IL6_TREATED_CD4_TCELL_DN
144	-0.37373534	-1.5372033	0.003831418
0.014330746	1	2123	"tags=31%, list=15%, signal=36%"
GSE3982_BASOPHIL_VS_EFF_MEMORY_CD4_TCELL_UP
GSE3982_BASOPHIL_VS_EFF_MEMORY_CD4_TCELL_UP
154	-0.37476215	-1.536357	0	0.014468149
1	1922	"tags=28%, list=14%, signal=32%"
GSE29949_MICROGLIA_BRAIN_VS_CD8_POS_DC_SPLEEN_UP
GSE29949_MICROGLIA_BRAIN_VS_CD8_POS_DC_SPLEEN_UP
146	-0.37522098	-1.5348407	0.001886793
0.014753754	1	791	"tags=18%, list=6%, signal=19%"
GSE26156_DOUBLE_POSITIVE_VS_CD4_SINGLE_POSITIVE_THYMOCYTE_UP
GSE26156_DOUBLE_POSITIVE_VS_CD4_SINGLE_POSITIVE_THYMOCYTE_UP
	138	-0.38006675	-1.5330132	0.003868472
0.0150971	1	1851	"tags=27%, list=13%, signal=31%"
GSE11961_MARGINAL_ZONE_BCELL_VS_PLASMA_CELL_DAY7_UP
GSE11961_MARGINAL_ZONE_BCELL_VS_PLASMA_CELL_DAY7_UP
142	-0.3753825	-1.5320857	0.001964637
0.015228242	1	1983	"tags=27%, list=14%, signal=31%"
GSE7460_CTRL_VS_TGFB_TREATED_ACT_FOXP3_MUT_TCONV_DN
GSE7460_CTRL_VS_TGFB_TREATED_ACT_FOXP3_MUT_TCONV_DN
145	-0.37357217	-1.5316029	0	0.015293329
1	890	"tags=16%, list=6%, signal=17%"
GSE15659_NAIVE_CD4_TCELL_VS_NONSUPPRESSIVE_TCELL_UP
GSE15659_NAIVE_CD4_TCELL_VS_NONSUPPRESSIVE_TCELL_UP
107	-0.3907602	-1.5314478	0.003717472

0.015287688	1	1826	"tags=22%, list=13%, signal=26%"
GSE12003_MIR223_KO_VS_WT_BM_PROGENITOR_8D_CULTURE_UP
GSE12003_MIR223_KO_VS_WT_BM_PROGENITOR_8D_CULTURE_UP
92	-0.399274	-1.5306332	0.009433962
0.015433541	1	1506	"tags=20%, list=11%, signal=22%"
GSE29614_CTRL_VS_TIV_FLU_VACCINE_PBMC_2007_UP
GSE29614_CTRL_VS_TIV_FLU_VACCINE_PBMC_2007_UP
94	-0.39685437	-1.5296669	0.003861004
0.01557901	1	5030	"tags=40%, list=36%, signal=62%"
GSE22886_IL2_VS_IL15_STIM_NKCELL_UP
GSE22886_IL2_VS_IL15_STIM_NKCELL_UP
137	-0.37364414	-1.5290498	0.001811594
0.015687708	1	4087	"tags=36%, list=29%, signal=51%"
GSE25123_WT_VS_PPARG_KO_MACROPHAGE_IL4_STIM_DN
GSE25123_WT_VS_PPARG_KO_MACROPHAGE_IL4_STIM_DN
158	-0.3635413	-1.5270051	0.003921569
0.016145546	1	1498	"tags=25%, list=11%, signal=28%"
GSE14308_TH17_VS_NAIVE_CD4_TCELL_DN
GSE14308_TH17_VS_NAIVE_CD4_TCELL_DN
145	-0.36853176	-1.5266541	0.003868472
0.01620421	1	1305	"tags=19%, list=9%, signal=20%"
GSE42021_TREG_PLN_VS_CD24INT_TREG_THYMUS_UP
GSE42021_TREG_PLN_VS_CD24INT_TREG_THYMUS_UP
166	-0.36246103	-1.5263904	0	0.01621985
1	1864	"tags=25%, list=13%, signal=28%"
GSE3920_UNTREATED_VS_IFNA_TREATED_FIBROBLAST_DN
GSE3920_UNTREATED_VS_IFNA_TREATED_FIBROBLAST_DN
139	-0.37284708	-1.526146	0.001769912
0.016242694	1	2395	"tags=36%, list=17%, signal=43%"
GSE21379_WT_VS_SAP_KO_TFH_CD4_TCELL_DN
GSE21379_WT_VS_SAP_KO_TFH_CD4_TCELL_DN
130	-0.37805614	-1.525722	0.007246377
0.016297333	1	1394	"tags=22%, list=10%, signal=24%"
GSE9601_NFKB_INHIBITOR_VS_PI3K_INHIBITOR_TREATED_HCMV_INF_MONOCYTE_D
N
GSE9601_NFKB_INHIBITOR_VS_PI3K_INHIBITOR_TREATED_HCMV_INF_MONOCYTE_D
N		164	-0.3611752	-1.5250634
0.001862197	0.016413728	1	2649	"tags=32%,
list=19%, signal=39%"
GSE411_WT_VS_SOCS3_KO_MACROPHAGE_IL6_STIM_400MIN_UP
GSE411_WT_VS_SOCS3_KO_MACROPHAGE_IL6_STIM_400MIN_UP
147	-0.36771086	-1.5247216	0.003780718
0.016443007	1	2178	"tags=33%, list=15%, signal=38%"
GSE13738_RESTING_VS_TCR_ACTIVATED_CD4_TCELL_UP
GSE13738_RESTING_VS_TCR_ACTIVATED_CD4_TCELL_UP
105	-0.38600603	-1.5235456	0.009328358
0.016647317	1	1796	"tags=24%, list=13%, signal=27%"
GSE40274_CTRL_VS_FOXP3_AND_HELIOS_TRANSDUCED_ACTIVATED_CD4_TCELL_UP
GSE40274_CTRL_VS_FOXP3_AND_HELIOS_TRANSDUCED_ACTIVATED_CD4_TCELL_UP
	140	-0.36952636	-1.5231675	0.009823183
0.01668325	1	1516	"tags=26%, list=11%, signal=29%"
GSE32533_WT_VS_MIR17_KO_ACT_CD4_TCELL_UP
GSE32533_WT_VS_MIR17_KO_ACT_CD4_TCELL_UP
143	-0.37119168	-1.5213677	0.001890359

0.017038055	1	3819	"tags=38%, list=27%, signal=52%"
GSE22025_PROGESTERONE_VS_TGFB1_AND_PROGESTERONE_TREATED_CD4_TCELL_UP

GSE22025_PROGESTERONE_VS_TGFB1_AND_PROGESTERONE_TREATED_CD4_TCELL_UP
	136	-0.36904004	-1.51937 0.005576208
0.017478965	1	2199	"tags=30%, list=16%, signal=35%"
GSE24972_MARGINAL_ZONE_BCELL_VS_FOLLICULAR_BCELL_IRF8_KO_DN
GSE24972_MARGINAL_ZONE_BCELL_VS_FOLLICULAR_BCELL_IRF8_KO_DN
127	-0.37519255	-1.5193173	0.001923077
0.017451322	1	2263	"tags=24%, list=16%, signal=29%"
GSE29618_BCELL_VS_PDC_DAY7_FLU_VACCINE_UP
GSE29618_BCELL_VS_PDC_DAY7_FLU_VACCINE_UP
130	-0.37281516	-1.5190499	0.005597015
0.017479705	1	2352	"tags=32%, list=17%, signal=38%"
GSE18791_CTRL_VS_NEWCASTLE_VIRUS_DC_12H_DN
GSE18791_CTRL_VS_NEWCASTLE_VIRUS_DC_12H_DN
119	-0.3811071	-1.5180405	0.001912046
0.017665239	1	1766	"tags=30%, list=13%, signal=34%"
GSE6674_ANTI_IGM_VS_CPG_STIM_BCELL_DN
GSE6674_ANTI_IGM_VS_CPG_STIM_BCELL_DN
142	-0.3655789	-1.5175864	0.005802708
0.017734513	1	2654	"tags=35%, list=19%, signal=42%"
GSE22886_NAIVE_CD4_TCELL_VS_48H_ACT_TH1_UP
GSE22886_NAIVE_CD4_TCELL_VS_48H_ACT_TH1_UP
123	-0.37457746	-1.517539	0.008896797
0.017704742	1	1519	"tags=21%, list=11%, signal=23%"
GSE37534_UNTREATED_VS_ROSIGLITAZONE_TREATED_CD4_TCELL_PPARG1_AND_FOXP3_TRASDUCED_DN
GSE37534_UNTREATED_VS_ROSIGLITAZONE_TREATED_CD4_TCELL_PPARG1_AND_FOXP3_TRASDUCED_DN	141	-0.3712036	-1.5174737
0.005639098	0.017680367	1	1108	"tags=19%, list=8%, signal=21%"
GSE7852_TREG_VS_TCONV_FAT_UP	GSE7852_TREG_VS_TCONV_FAT_UP
	153	-0.364358	-1.5165142	0.001886793
0.017891651	1	1830	"tags=25%, list=13%, signal=28%"
GSE30962_PRIMARY_VS_SECONDARY_CHRONIC_LCMV_INF_CD8_TCELL_DN
GSE30962_PRIMARY_VS_SECONDARY_CHRONIC_LCMV_INF_CD8_TCELL_DN
146	-0.36700144	-1.5154579	0	0.018093174
1	1038	"tags=20%, list=7%, signal=21%"
GSE11961_GERMINAL_CENTER_BCELL_DAY7_VS_MEMORY_BCELL_DAY40_DN
GSE11961_GERMINAL_CENTER_BCELL_DAY7_VS_MEMORY_BCELL_DAY40_DN
	144	-0.370135	-1.5151734	0.007920792
0.018123206	1	1998	"tags=24%, list=14%, signal=28%"
GSE32986_GMCSF_VS_GMCSF_AND_CURDLAN_HIGHDOSE_STIM_DC_UP
GSE32986_GMCSF_VS_GMCSF_AND_CURDLAN_HIGHDOSE_STIM_DC_UP
149	-0.37023675	-1.5148554	0.004024145
0.018168868	1	1196	"tags=20%, list=8%, signal=22%"
GSE21774_CD62L_POS_CD56_DIM_VS_CD62L_NEG_CD56_DIM_NK_CELL_UP
GSE21774_CD62L_POS_CD56_DIM_VS_CD62L_NEG_CD56_DIM_NK_CELL_UP
	155	-0.3597564	-1.5132734	0.001869159
0.0185067	1	2095	"tags=28%, list=15%, signal=33%"
GSE4590_PRE_BCELL_VS_VPREB_POS_LARGE_PRE_BCELL_DN
GSE4590_PRE_BCELL_VS_VPREB_POS_LARGE_PRE_BCELL_DN
116	-0.3841882	-1.5132581	0.005660377

0.018472442	1	2664	"tags=35%, list=19%, signal=43%"
GSE26928_NAIVE_VS_CENT_MEMORY_CD4_TCELL_UP
GSE26928_NAIVE_VS_CENT_MEMORY_CD4_TCELL_UP
110	-0.38489863	-1.513071	0.005825243
0.018472485	1	2398	"tags=27%, list=17%, signal=33%"
GSE32986_UNSTIM_VS_GMCSF_STIM_DC_UP
GSE32986_UNSTIM_VS_GMCSF_STIM_DC_UP
107	-0.37879616	-1.5126462	0.003846154
0.01856103	1	1509	"tags=21%, list=11%, signal=23%"
GSE557_CIITA_KO_VS_I_AB_KO_DC_UP	GSE557_CIITA_KO_VS_I_AB_KO_DC_UP
	136	-0.3672955	-1.5120337	0.005791506
0.018693807	1	2584	"tags=33%, list=18%, signal=40%"
GSE37301_HEMATOPOIETIC_STEM_CELL_VS_RAG2_KO_NK_CELL_UP
GSE37301_HEMATOPOIETIC_STEM_CELL_VS_RAG2_KO_NK_CELL_UP
130	-0.37260413	-1.5104588	0.009881423
0.019041367	1	2006	"tags=30%, list=14%, signal=35%"
GSE40274_FOXP3_VS_FOXP3_AND_XBP1_TRANSDUCED_ACTIVATED_CD4_TCELL_DN
GSE40274_FOXP3_VS_FOXP3_AND_XBP1_TRANSDUCED_ACTIVATED_CD4_TCELL_DN
	113	-0.37581694	-1.5103732	0.005628518
0.019023368	1	1139	"tags=23%, list=8%, signal=25%"
GSE16450_CTRL_VS_IFNA_6H_STIM_MATURE_NEURON_CELL_LINE_UP
GSE16450_CTRL_VS_IFNA_6H_STIM_MATURE_NEURON_CELL_LINE_UP
139	-0.36727422	-1.5102942	0.001855288
0.019010516	1	2053	"tags=27%, list=15%, signal=31%"
GSE22886_UNSTIM_VS_IL15_STIM_NKCELL_UP
GSE22886_UNSTIM_VS_IL15_STIM_NKCELL_UP
128	-0.37532687	-1.5092032	0.006085193
0.019251488	1	4049	"tags=36%, list=29%, signal=50%"
GSE28737_BCL6_HET_VS_BCL6_KO_MARGINAL_ZONE_BCELL_UP
GSE28737_BCL6_HET_VS_BCL6_KO_MARGINAL_ZONE_BCELL_UP
138	-0.36466327	-1.5087384	0.003663004
0.019336183	1	2663	"tags=23%, list=19%, signal=28%"
GSE3720_VD1_VS_VD2_GAMMADELTA_TCELL_WITH_PMA_STIM_DN
GSE3720_VD1_VS_VD2_GAMMADELTA_TCELL_WITH_PMA_STIM_DN
147	-0.36413127	-1.5038754	0.009578544
0.02054777	1	2494	"tags=31%, list=18%, signal=38%"
GSE7460_CTRL_VS_FOXP3_OVEREXPR_TCONV_DN
GSE7460_CTRL_VS_FOXP3_OVEREXPR_TCONV_DN
136	-0.3695049	-1.5037607	0.001828154
0.02054387	1	1746	"tags=24%, list=12%, signal=27%"
GSE36392_MAC_VS_NEUTROPHIL_IL25_TREATED_LUNG_UP
GSE36392_MAC_VS_NEUTROPHIL_IL25_TREATED_LUNG_UP
127	-0.37187994	-1.5030686	0.003976143
0.02069585	1	1506	"tags=24%, list=11%, signal=26%"
GSE24574_BCL6_HIGH_TFH_VS_TFH_CD4_TCELL_DN
GSE24574_BCL6_HIGH_TFH_VS_TFH_CD4_TCELL_DN
139	-0.36362368	-1.5030416	0.003642987
0.020662997	1	1784	"tags=26%, list=13%, signal=29%"
GSE2706_R848_VS_LPS_2H_STIM_DC_UP	GSE2706_R848_VS_LPS_2H_STIM_DC_UP
	98	-0.38970792	-1.5026691	0.00996016
0.0207255	1	1979	"tags=22%, list=14%, signal=26%"
GSE15733_BM_VS_SPLEEN_MEMORY_CD4_TCELL_DN
GSE15733_BM_VS_SPLEEN_MEMORY_CD4_TCELL_DN
141	-0.36668637	-1.5018934	0.009615385

0.020884395	1	2397	"tags=32%, list=17%, signal=38%"
GSE32034_UNTREATED_VS_ROSIGLIZATONE_TREATED_LY6C_LOW_MONOCYTE_UP
GSE32034_UNTREATED_VS_ROSIGLIZATONE_TREATED_LY6C_LOW_MONOCYTE_UP
	97	-0.38927907	-1.5013443	0.008
0.020975912	1	2307	"tags=22%, list=16%, signal=26%"
GSE45365_WT_VS_IFNAR_KO_CD11B_DC_UP
GSE45365_WT_VS_IFNAR_KO_CD11B_DC_UP
118	-0.3785824	-1.501174	0.007662835
0.02098594	1	3127	"tags=34%, list=22%, signal=43%"
GSE2770_IL12_AND_TGFB_ACT_VS_ACT_CD4_TCELL_6H_UP
GSE2770_IL12_AND_TGFB_ACT_VS_ACT_CD4_TCELL_6H_UP
127	-0.3647545	-1.4991646	0.011428571
0.021526469	1	1566	"tags=23%, list=11%, signal=25%"
GSE30971_WBP7_HET_VS_KO_MACROPHAGE_2H_LPS_STIM_DN
GSE30971_WBP7_HET_VS_KO_MACROPHAGE_2H_LPS_STIM_DN
148	-0.36410996	-1.4984576	0.003816794
0.021689579	1	1428	"tags=19%, list=10%, signal=21%"
GSE15930_NAIVE_VS_72H_IN_VITRO_STIM_IL12_CD8_TCELL_UP
GSE15930_NAIVE_VS_72H_IN_VITRO_STIM_IL12_CD8_TCELL_UP
154	-0.36178815	-1.4980485	0.005649718
0.021765778	1	2880	"tags=31%, list=20%, signal=38%"
GSE37605_C57BL6_VS_NOD_FOXP3_IRES_GFP_TCONV_UP
GSE37605_C57BL6_VS_NOD_FOXP3_IRES_GFP_TCONV_UP
116	-0.37768224	-1.4975294	0	0.021879816
1	2720	"tags=26%, list=19%, signal=32%"
GSE11961_FOLLICULAR_BCELL_VS_GERMINAL_CENTER_BCELL_DAY40_UP
GSE11961_FOLLICULAR_BCELL_VS_GERMINAL_CENTER_BCELL_DAY40_UP
151	-0.36406106	-1.4974225	0.003780718
0.021868287	1	2423	"tags=31%, list=17%, signal=37%"
GSE18791_CTRL_VS_NEWCASTLE_VIRUS_DC_6H_DN
GSE18791_CTRL_VS_NEWCASTLE_VIRUS_DC_6H_DN
134	-0.36752978	-1.4973265	0.005524862
0.02185338	1	1766	"tags=27%, list=13%, signal=30%"
GSE5542_UNTREATED_VS_IFNA_TREATED_EPITHELIAL_CELLS_24H_DN
GSE5542_UNTREATED_VS_IFNA_TREATED_EPITHELIAL_CELLS_24H_DN
117	-0.37506157	-1.4969196	0	0.021931684
1	1409	"tags=21%, list=10%, signal=24%"
GSE11057_EFF_MEM_VS_CENT_MEM_CD4_TCELL_DN
GSE11057_EFF_MEM_VS_CENT_MEM_CD4_TCELL_DN
113	-0.37705088	-1.4967785	0.011976048
0.021916803	1	1557	"tags=27%, list=11%, signal=31%"
GSE40493_BCL6_KO_VS_WT_TREG_DN	GSE40493_BCL6_KO_VS_WT_TREG_DN
	77	-0.39989245	-1.4955338	0.011173184
0.022262696	1	4496	"tags=42%, list=32%, signal=61%"
GSE22886_NAIVE_TCELL_VS_MONOCYTE_UP
GSE22886_NAIVE_TCELL_VS_MONOCYTE_UP
142	-0.35974187	-1.4952843	0.010889292
0.022301024	1	1236	"tags=20%, list=9%, signal=21%"
GSE369_PRE_VS_POST_IL6_INJECTION_IFNG_KO_LIVER_DN
GSE369_PRE_VS_POST_IL6_INJECTION_IFNG_KO_LIVER_DN
143	-0.36456856	-1.4946275	0.003929273
0.022434527	1	2381	"tags=32%, list=17%, signal=38%"
GSE21670_STAT3_KO_VS_WT_CD4_TCELL_TGFB_IL6_TREATED_DN
GSE21670_STAT3_KO_VS_WT_CD4_TCELL_TGFB_IL6_TREATED_DN

150	-0.36227483	-1.4941268	0.003824092
0.0225533	1	1840	"tags=25%, list=13%, signal=29%"
GSE13306_TREG_VS_TCONV_LAMINA_PROPRIA_UP
GSE13306_TREG_VS_TCONV_LAMINA_PROPRIA_UP
152	-0.36306536	-1.4932859	0.001930502
0.022746151	1	2619	"tags=32%, list=19%, signal=38%"
GSE34156_NOD2_LIGAND_VS_NOD2_AND_TLR1_TLR2_LIGAND_24H_TREATED_MONOCY
TE_DN
GSE34156_NOD2_LIGAND_VS_NOD2_AND_TLR1_TLR2_LIGAND_24H_TREATED_MONOCY
TE_DN	99	-0.38165697	-1.4928035
0.007476636	0.02286416	1	1376	"tags=21%,
list=10%, signal=23%"
GSE14350_IL2RB_KO_VS_WT_TREG_DN	GSE14350_IL2RB_KO_VS_WT_TREG_DN
	143	-0.36285883	-1.4921446	0
0.023028769	1	1962	"tags=26%, list=14%, signal=30%"
GSE24671_CTRL_VS_SENDAI_VIRUS_INFECTED_MOUSE_SPLENOCYTES_UP
GSE24671_CTRL_VS_SENDAI_VIRUS_INFECTED_MOUSE_SPLENOCYTES_UP
126	-0.36812678	-1.491143	0.009940358
0.023273755	1	1362	"tags=21%, list=10%, signal=24%"
GSE25677_MPL_VS_R848_STIM_BCELL_UP
GSE25677_MPL_VS_R848_STIM_BCELL_UP
147	-0.363101	-1.4909011	0.001949318
0.02329682	1	1702	"tags=24%, list=12%, signal=28%"
GSE15930_NAIVE_VS_24H_IN_VITRO_STIM_IL12_CD8_TCELL_UP
GSE15930_NAIVE_VS_24H_IN_VITRO_STIM_IL12_CD8_TCELL_UP
156	-0.35393035	-1.4905528	0.001841621
0.023364244	1	1462	"tags=21%, list=10%, signal=23%"
GSE13738_TCR_VS_BYSTANDER_ACTIVATED_CD4_TCELL_UP
GSE13738_TCR_VS_BYSTANDER_ACTIVATED_CD4_TCELL_UP
135	-0.36289656	-1.4883354	0.007736944
0.024018528	1	1463	"tags=21%, list=10%, signal=24%"
GSE23505_UNTREATED_VS_4DAY_IL6_IL1_TREATED_CD4_TCELL_DN
GSE23505_UNTREATED_VS_4DAY_IL6_IL1_TREATED_CD4_TCELL_DN
154	-0.35585704	-1.487304	0.005617978
0.024298344	1	2433	"tags=22%, list=17%, signal=26%"
GSE3982_MAST_CELL_VS_TH2_UP	GSE3982_MAST_CELL_VS_TH2_UP
	145	-0.36104715	-1.4872203	0.003853565
0.024284111	1	2242	"tags=26%, list=16%, signal=30%"
GSE1112_HY_CD8AB_VS_HY_CD8AA_THYMOCYTE_RTOC_CULTURE_DN
GSE1112_HY_CD8AB_VS_HY_CD8AA_THYMOCYTE_RTOC_CULTURE_DN
129	-0.36524975	-1.4872161	0	0.02424138
1	1881	"tags=26%, list=13%, signal=29%"
GSE1460_DP_THYMOCYTE_VS_NAIVE_CD4_TCELL_ADULT_BLOOD_DN
GSE1460_DP_THYMOCYTE_VS_NAIVE_CD4_TCELL_ADULT_BLOOD_DN
137	-0.36388308	-1.4866486	0	0.02437649
1	1681	"tags=24%, list=12%, signal=27%"
GSE41176_UNSTIM_VS_ANTI_IGM_STIM_BCELL_3H_UP
GSE41176_UNSTIM_VS_ANTI_IGM_STIM_BCELL_3H_UP
147	-0.35940894	-1.485948	0.001930502
0.02455374	1	2654	"tags=31%, list=19%, signal=37%"
GSE36888_STAT5_AB_KNOCKIN_VS_WT_TCELL_IL2_TREATED_17H_UP
GSE36888_STAT5_AB_KNOCKIN_VS_WT_TCELL_IL2_TREATED_17H_UP
157	-0.35972375	-1.4848571	0.005484461
0.02484616	1	1371	"tags=22%, list=10%, signal=24%"

GSE8835_CD4_VS_CD8_TCELL_CLL_PATIENT_DN
GSE8835_CD4_VS_CD8_TCELL_CLL_PATIENT_DN
149 -0.36345094 -1.4842242 0.003868472
0.025011124 1 2344 "tags=32%, list=17%, signal=38%"
GSE24142_DN2_VS_DN3_THYMOCYTE_FETAL_DN
GSE24142_DN2_VS_DN3_THYMOCYTE_FETAL_DN
162 -0.3563903 -1.4842122 0.010989011
0.024967432 1 1996 "tags=27%, list=14%, signal=31%"
GSE32986_GMCSF_AND_CURDLAN_LOWDOSE_VS_GMCSF_AND_CURDLAN_HIGHDOSE_STIM_DC_DN
GSE32986_GMCSF_AND_CURDLAN_LOWDOSE_VS_GMCSF_AND_CURDLAN_HIGHDOSE_STIM_DC_DN 118 -0.3764554 -1.4840193
0.013011153 0.024971196 1 2089 "tags=23%, list=15%, signal=27%"
GSE23505_UNTREATED_VS_4DAY_IL6_IL1_IL23_TREATED_CD4_TCELL_UP
GSE23505_UNTREATED_VS_4DAY_IL6_IL1_IL23_TREATED_CD4_TCELL_UP
136 -0.36427218 -1.4823498 0.003780718
0.025464 1 3804 "tags=43%, list=27%, signal=58%"
GSE16385_IFNG_TNF_VS_UNSTIM_MACROPHAGE_ROSIGLITAZONE_TREATED_DN
GSE16385_IFNG_TNF_VS_UNSTIM_MACROPHAGE_ROSIGLITAZONE_TREATED_DN
157 -0.35791266 -1.4822235 0.003937008
0.02545575 1 2078 "tags=27%, list=15%, signal=31%"
GSE30083_SP1_VS_SP3_THYMOCYTE_DN GSE30083_SP1_VS_SP3_THYMOCYTE_DN
134 -0.36318672 -1.4819132 0.014
0.025505606 1 1780 "tags=27%, list=13%, signal=30%"
GSE9988_ANTI_TREM1_VS_CTRL_TREATED_MONOCYTES_UP
GSE9988_ANTI_TREM1_VS_CTRL_TREATED_MONOCYTES_UP
148 -0.36064413 -1.4811655 0.003710575
0.025656462 1 1926 "tags=26%, list=14%, signal=30%"
GSE43863_TH1_VS_TFH_EFFECTOR_CD4_TCELL_DN
GSE43863_TH1_VS_TFH_EFFECTOR_CD4_TCELL_DN
132 -0.36026138 -1.4810024 0.001848429
0.025654135 1 2340 "tags=33%, list=17%, signal=39%"
GSE12392_WT_VS_IFNB_KO_CD8A_NEG_SPLEEN_DC_DN
GSE12392_WT_VS_IFNB_KO_CD8A_NEG_SPLEEN_DC_DN
147 -0.35843778 -1.479397 0.005791506
0.026140004 1 1928 "tags=27%, list=14%, signal=31%"
GSE7764_NKCELL_VS_SPLENOCYTE_UP GSE7764_NKCELL_VS_SPLENOCYTE_UP
145 -0.36274195 -1.4778345 0.006097561
0.02658226 1 1362 "tags=24%, list=10%, signal=26%"
GSE36888_UNTREATED_VS_IL2_TREATED_STAT5_AB_KNOCKIN_TCELL_6H_UP
GSE36888_UNTREATED_VS_IL2_TREATED_STAT5_AB_KNOCKIN_TCELL_6H_UP
132 -0.36613113 -1.477708 0.009803922
0.026598373 1 2097 "tags=29%, list=15%, signal=33%"
GSE5589_LPS_VS_LPS_AND_IL10_STIM_IL10_KO_MACROPHAGE_45MIN_UP
GSE5589_LPS_VS_LPS_AND_IL10_STIM_IL10_KO_MACROPHAGE_45MIN_UP
143 -0.36145604 -1.4776706 0.001956947
0.026557256 1 3190 "tags=34%, list=23%, signal=43%"
GSE35825_UNTREATED_VS_IFNA_STIM_MACROPHAGE_DN
GSE35825_UNTREATED_VS_IFNA_STIM_MACROPHAGE_DN
136 -0.362575 -1.477426 0.005769231
0.026587533 1 2006 "tags=23%, list=14%, signal=26%"
GSE6259_BCELL_VS_CD8_TCELL_DN GSE6259_BCELL_VS_CD8_TCELL_DN
129 -0.36597022 -1.4773626 0.005628518

0.026555805	1	1979	"tags=29%, list=14%, signal=33%"
GSE17721_0.5H_VS_4H_GARDIQUIMOD_BMDC_DN
GSE17721_0.5H_VS_4H_GARDIQUIMOD_BMDC_DN
153	-0.35132587	-1.4766766	0.005703422
0.02676632	1	2194	"tags=25%, list=16%, signal=30%"
GSE13887_HEALTHY_VS_LUPUS_RESTING_CD4_TCELL_UP
GSE13887_HEALTHY_VS_LUPUS_RESTING_CD4_TCELL_UP
90	-0.38570908	-1.4755622	0.011299435
0.027116647	1	1958	"tags=26%, list=14%, signal=29%"
GSE11961_MARGINAL_ZONE_BCELL_VS_MEMORY_BCELL_DAY40_UP
GSE11961_MARGINAL_ZONE_BCELL_VS_MEMORY_BCELL_DAY40_UP
140	-0.35358426	-1.4735304	0.007233273
0.027769795	1	2551	"tags=29%, list=18%, signal=35%"
GSE29164_UNTREATED_VS_CD8_TCELL_AND_IL12_TREATED_MELANOMA_DAY3_UP
GSE29164_UNTREATED_VS_CD8_TCELL_AND_IL12_TREATED_MELANOMA_DAY3_UP
	143	-0.3559954	-1.4735003	0.007590133
0.027726972	1	1537	"tags=20%, list=11%, signal=22%"
GSE11961_MARGINAL_ZONE_BCELL_VS_GERMINAL_CENTER_BCELL_DAY7_UP
GSE11961_MARGINAL_ZONE_BCELL_VS_GERMINAL_CENTER_BCELL_DAY7_UP
	131	-0.361546	-1.4729723	0.007648184
0.02785382	1	2058	"tags=27%, list=15%, signal=32%"
GSE17721_CTRL_VS_PAM3CSK4_1H_BMDC_DN
GSE17721_CTRL_VS_PAM3CSK4_1H_BMDC_DN
162	-0.3525055	-1.4728607	0.001897533
0.027845956	1	3339	"tags=32%, list=24%, signal=42%"
GSE43863_NAIVE_VS_MEMORY_TH1_CD4_TCELL_D150_LCMV_UP
GSE43863_NAIVE_VS_MEMORY_TH1_CD4_TCELL_D150_LCMV_UP
95	-0.37673432	-1.4723111	0.005836576
0.027952805	1	3045	"tags=32%, list=22%, signal=40%"
GSE14908_RESTING_VS_HDM_STIM_CD4_TCELL_ATOPIC_PATIENT_DN
GSE14908_RESTING_VS_HDM_STIM_CD4_TCELL_ATOPIC_PATIENT_DN
146	-0.35597453	-1.4720733	0.0078125
0.027982987	1	1342	"tags=21%, list=10%, signal=22%"
GSE14769_UNSTIM_VS_20MIN_LPS_BMDM_DN
GSE14769_UNSTIM_VS_20MIN_LPS_BMDM_DN
147	-0.355604	-1.4717151	0.012867647
0.02805105	1	2344	"tags=31%, list=17%, signal=37%"
GSE13522_WT_VS_IFNAR_KO_SKING_T_CRUZI_Y_STRAIN_INF_UP
GSE13522_WT_VS_IFNAR_KO_SKING_T_CRUZI_Y_STRAIN_INF_UP
142	-0.36218297	-1.4716384	0.013384321
0.028027814	1	1254	"tags=23%, list=9%, signal=25%"
GSE24634_TREG_VS_TCONV_POST_DAY5_IL4_CONVERSION_DN
GSE24634_TREG_VS_TCONV_POST_DAY5_IL4_CONVERSION_DN
131	-0.3653526	-1.4716151	0.005928854
0.027979026	1	2925	"tags=34%, list=21%, signal=42%"
GSE5589_IL6_KO_VS_IL10_KO_LPS_AND_IL6_STIM_MACROPHAGE_45MIN_DN
GSE5589_IL6_KO_VS_IL10_KO_LPS_AND_IL6_STIM_MACROPHAGE_45MIN_DN
	144	-0.3608059	-1.4707578	0.003898636
0.028192926	1	2324	"tags=26%, list=16%, signal=31%"
GSE3039_NKT_CELL_VS_ALPHAALPHA_CD8_TCELL_UP
GSE3039_NKT_CELL_VS_ALPHAALPHA_CD8_TCELL_UP
149	-0.3515904	-1.4701533	0.01119403
0.02833866	1	2180	"tags=29%, list=15%, signal=34%"
GSE10240_CTRL_VS_IL22_STIM_PRIMARY_BRONCHIAL_EPITHELIAL_CELLS_UP

GSE10240_CTRL_VS_IL22_STIM_PRIMARY_BRONCHIAL_EPITHELIAL_CELLS_UP
146 -0.35707977 -1.469526 0.003676471
0.028500061 1 2366 "tags=26%, list=17%, signal=31%"
GSE24210_IL35_TREATED_VS_RESTING_TREG_DN
GSE24210_IL35_TREATED_VS_RESTING_TREG_DN
144 -0.35632658 -1.4692506 0.005725191
0.028552387 1 3241 "tags=28%, list=23%, signal=36%"
GSE29617_CTRL_VS_DAY7_TIV_FLU_VACCINE_PBMC_2008_UP
GSE29617_CTRL_VS_DAY7_TIV_FLU_VACCINE_PBMC_2008_UP
133 -0.35998914 -1.4690957 0.004040404
0.02855417 1 1975 "tags=27%, list=14%, signal=31%"
GSE10239_MEMORY_VS_DAY4.5_EFF_CD8_TCELL_UP
GSE10239_MEMORY_VS_DAY4.5_EFF_CD8_TCELL_UP
139 -0.35690632 -1.4689927 0.00990099
0.028534884 1 1704 "tags=24%, list=12%, signal=27%"
GSE46606_DAY1_VS_DAY3_CD40L_IL2_IL5_STIMULATED_BCELL_UP
GSE46606_DAY1_VS_DAY3_CD40L_IL2_IL5_STIMULATED_BCELL_UP
110 -0.36948156 -1.468469 0.015267176
0.028672354 1 2290 "tags=26%, list=16%, signal=31%"
GSE13485_DAY3_VS_DAY21_YF17D_VACCINE_PBMC_DN
GSE13485_DAY3_VS_DAY21_YF17D_VACCINE_PBMC_DN
98 -0.37662417 -1.4683855 0.015384615
0.028647155 1 5261 "tags=50%, list=37%, signal=79%"
GSE21033_CTRL_VS_POLYIC_STIM_DC_24H_UP
GSE21033_CTRL_VS_POLYIC_STIM_DC_24H_UP
111 -0.37217104 -1.4662181 0.012121212
0.029348455 1 2263 "tags=29%, list=16%, signal=34%"
GSE38697_LIGHT_ZONE_VS_DARK_ZONE_BCELL_DN
GSE38697_LIGHT_ZONE_VS_DARK_ZONE_BCELL_DN
84 -0.39096275 -1.465943 0.01778656
0.02936807 1 6627 "tags=63%, list=47%, signal=118%"
GSE16266_LPS_VS_HEATSHOCK_AND_LPS_STIM_MEF_DN
GSE16266_LPS_VS_HEATSHOCK_AND_LPS_STIM_MEF_DN
144 -0.35621834 -1.4657763 0.00591716
0.029366583 1 1775 "tags=23%, list=13%, signal=26%"
GSE37301_PRO_BCELL_VS_CD4_TCELL_UP
GSE37301_PRO_BCELL_VS_CD4_TCELL_UP
127 -0.3625197 -1.4656931 0.005825243
0.029344555 1 1371 "tags=25%, list=10%, signal=28%"
GSE5099_CLASSICAL_M1_VS_ALTERNATIVE_M2_MACROPHAGE_UP
GSE5099_CLASSICAL_M1_VS_ALTERNATIVE_M2_MACROPHAGE_UP
128 -0.36252552 -1.465542 0.016885553
0.029344842 1 1602 "tags=25%, list=11%, signal=28%"
GSE21546_ELK1_KO_VS_SAP1A_KO_AND_ELK1_KO_ANTI_CD3_STIM_DP_THYMOCYTES_DN
GSE21546_ELK1_KO_VS_SAP1A_KO_AND_ELK1_KO_ANTI_CD3_STIM_DP_THYMOCYTES_DN 139 -0.35563412 -1.4648008
0.016853932 0.029575704 1 1457 "tags=20%, list=10%, signal=22%"
GSE22886_CD8_TCELL_VS_BCELL_NAIVE_DN
GSE22886_CD8_TCELL_VS_BCELL_NAIVE_DN
129 -0.35799077 -1.4641645 0.01369863
0.029754983 1 2515 "tags=27%, list=18%, signal=33%"
GSE17721_LPS_VS_GARDIQUIMOD_2H_BMDC_UP

GSE17721_LPS_VS_GARDIQUIMOD_2H_BMDC_UP
160 -0.35170442 -1.4641201 0.007633588
0.02972215 1 1821 "tags=21%, list=13%, signal=24%"
GSE2405_S_AUREUS_VS_A_PHAGOCYTOPHILUM_NEUTROPHIL_UP
GSE2405_S_AUREUS_VS_A_PHAGOCYTOPHILUM_NEUTROPHIL_UP
144 -0.35381833 -1.4626156 0.013592233
0.030239359 1 1783 "tags=22%, list=13%, signal=24%"
GSE4142_NAIVE_BCELL_VS_PLASMA_CELL_UP
GSE4142_NAIVE_BCELL_VS_PLASMA_CELL_UP
146 -0.35350764 -1.4603666 0.003759399
0.03105087 1 2123 "tags=27%, list=15%, signal=32%"
GSE18791_CTRL_VS_NEWCASTLE_VIRUS_DC_8H_DN
GSE18791_CTRL_VS_NEWCASTLE_VIRUS_DC_8H_DN
125 -0.36069506 -1.4597828 0.007575758
0.031233922 1 2377 "tags=31%, list=17%, signal=37%"
GSE13306_TREG_VS_TCONV_SPLEEN_UP GSE13306_TREG_VS_TCONV_SPLEEN_UP
133 -0.3585731 -1.459054 0.0056926
0.031462796 1 1783 "tags=24%, list=13%, signal=27%"
GSE2128_C57BL6_VS_NOD_CD4CD8_DP_THYMOCYTE_UP
GSE2128_C57BL6_VS_NOD_CD4CD8_DP_THYMOCYTE_UP
139 -0.35383862 -1.4579588 0.00952381
0.031836174 1 1323 "tags=21%, list=9%, signal=23%"
GSE21546_WT_VS_SAP1A_KO_AND_ELK1_KO_DP_THYMOCYTES_DN
GSE21546_WT_VS_SAP1A_KO_AND_ELK1_KO_DP_THYMOCYTES_DN
151 -0.35410497 -1.4577767 0.012448133
0.031837486 1 2291 "tags=28%, list=16%, signal=34%"
GSE34006_WT_VS_A2AR_KO_TREG_DN GSE34006_WT_VS_A2AR_KO_TREG_DN
144 -0.35297114 -1.4568961 0.005639098
0.03213216 1 1766 "tags=22%, list=13%, signal=24%"
GSE3039_ALPHAALPHA_CD8_TCELL_VS_B2_BCELL_DN
GSE3039_ALPHAALPHA_CD8_TCELL_VS_B2_BCELL_DN
152 -0.34905532 -1.4552296 0 0.032788843
1 2169 "tags=28%, list=15%, signal=33%"
GSE41978_ID2_KO_VS_BIM_KO_KLRG1_LOW_EFFECTOR_CD8_TCELL_UP
GSE41978_ID2_KO_VS_BIM_KO_KLRG1_LOW_EFFECTOR_CD8_TCELL_UP
134 -0.35137716 -1.4551022 0 0.032788847
1 1766 "tags=25%, list=13%, signal=28%"
GSE37533_PPARG2_FOXP3_VS_FOXP3_TRANSDUCED_CD4_TCELL_DN
GSE37533_PPARG2_FOXP3_VS_FOXP3_TRANSDUCED_CD4_TCELL_DN
131 -0.35611147 -1.4548236 0.023255814
0.032824796 1 1817 "tags=24%, list=13%, signal=28%"
GSE41867_DAY6_VS_DAY15_LCMV_ARMSTRONG_EFFECTOR_CD8_TCELL_UP
GSE41867_DAY6_VS_DAY15_LCMV_ARMSTRONG_EFFECTOR_CD8_TCELL_UP
129 -0.35681468 -1.4548157 0.01084991
0.032772973 1 2610 "tags=27%, list=18%, signal=33%"
GSE41867_DAY6_EFFECTOR_VS_DAY30_MEMORY_CD8_TCELL_LCMV_ARMSTRONG_DN
GSE41867_DAY6_EFFECTOR_VS_DAY30_MEMORY_CD8_TCELL_LCMV_ARMSTRONG_DN
143 -0.35609585 -1.4547808 0.007692308
0.032734297 1 2494 "tags=33%, list=18%, signal=40%"
GSE30971_CTRL_VS_LPS_STIM_MACROPHAGE_WBP7_KO_2H_UP
GSE30971_CTRL_VS_LPS_STIM_MACROPHAGE_WBP7_KO_2H_UP
140 -0.35409912 -1.4543502 0.006085193
0.032803327 1 1387 "tags=19%, list=10%, signal=20%"
GSE13547_CTRL_VS_ANTI_IGM_STIM_ZFX_KO_BCELL_2H_DN

GSE13547_CTRL_VS_ANTI_IGM_STIM_ZFX_KO_BCELL_2H_DN
141	-0.35874537	-1.4542654	0.011627907
0.03278618	1	1277	"tags=21%, list=9%, signal=22%"
GSE20366_CD103_POS_VS_NEG_TREG_KLRG1NEG_DN
GSE20366_CD103_POS_VS_NEG_TREG_KLRG1NEG_DN
158	-0.35023582	-1.4524345	0.005725191
0.033469006	1	2452	"tags=27%, list=17%, signal=33%"
GSE5589_LPS_AND_IL10_VS_LPS_AND_IL6_STIM_MACROPHAGE_45MIN_UP
GSE5589_LPS_AND_IL10_VS_LPS_AND_IL6_STIM_MACROPHAGE_45MIN_UP
	144	-0.35661206	-1.4516827	0.011406845
0.033733796	1	2311	"tags=28%, list=16%, signal=33%"
GSE15930_NAIVE_VS_48H_IN_VITRO_STIM_IL12_CD8_TCELL_UP
GSE15930_NAIVE_VS_48H_IN_VITRO_STIM_IL12_CD8_TCELL_UP
151	-0.34858328	-1.4498498	0.00589391
0.03445326	1	3024	"tags=30%, list=21%, signal=38%"
GSE27896_HDAC6_KO_VS_WT_TREG_UP	GSE27896_HDAC6_KO_VS_WT_TREG_UP
	126	-0.3603903	-1.4498198	0.011976048
0.034416292	1	1656	"tags=25%, list=12%, signal=28%"
GSE19888_ADENOSINE_A3R_INH_VS_INH_PRETREAT_AND_ACT_WITH_TCELL_MEMBRANES_MAST_CELL_UP
GSE19888_ADENOSINE_A3R_INH_VS_INH_PRETREAT_AND_ACT_WITH_TCELL_MEMBRANES_MAST_CELL_UP	137	-0.35286137	-1.4497313
0.003937008	0.03439944	1	2187	"tags=28%, list=15%, signal=33%"
GSE19198_CTRL_VS_IL21_TREATED_TCELL_1H_UP
GSE19198_CTRL_VS_IL21_TREATED_TCELL_1H_UP
106	-0.3643069	-1.4489841	0.018691588
0.034665376	1	2570	"tags=28%, list=18%, signal=34%"
GSE17301_IFNA2_VS_IFNA2_AND_ACD3_ACD28_STIM_CD8_TCELL_UP
GSE17301_IFNA2_VS_IFNA2_AND_ACD3_ACD28_STIM_CD8_TCELL_UP
137	-0.3554477	-1.4480821	0.011320755
0.034996334	1	1198	"tags=19%, list=8%, signal=21%"
GSE40274_CTRL_VS_FOXP3_AND_LEF1_TRANSDUCED_ACTIVATED_CD4_TCELL_UP
GSE40274_CTRL_VS_FOXP3_AND_LEF1_TRANSDUCED_ACTIVATED_CD4_TCELL_UP
	132	-0.35729647	-1.4471723	0.009276438
0.03535444	1	1776	"tags=24%, list=13%, signal=27%"
GSE10239_NAIVE_VS_MEMORY_CD8_TCELL_UP
GSE10239_NAIVE_VS_MEMORY_CD8_TCELL_UP
143	-0.35092422	-1.4466828	0.007766991
0.035502356	1	2484	"tags=28%, list=18%, signal=34%"
GSE6259_33D1_POS_VS_DEC205_POS_SPLENIC_DC_DN
GSE6259_33D1_POS_VS_DEC205_POS_SPLENIC_DC_DN
141	-0.34812766	-1.4463518	0.005681818
0.035583932	1	1544	"tags=19%, list=11%, signal=21%"
GSE20727_DNFB_ALLERGEN_VS_ROS_INH_AND_DNFB_ALLERGEN_TREATED_DC_UP
GSE20727_DNFB_ALLERGEN_VS_ROS_INH_AND_DNFB_ALLERGEN_TREATED_DC_UP
	111	-0.36627916	-1.4449832	0.018
0.0361041	1	1934	"tags=22%, list=14%, signal=25%"
GSE22886_TCELL_VS_BCELL_NAIVE_DN	GSE22886_TCELL_VS_BCELL_NAIVE_DN
	130	-0.358399	-1.4448448	0.005681818
0.036101505	1	2254	"tags=27%, list=16%, signal=32%"
GSE18804_BRAIN_VS_COLON_TUMORAL_MACROPHAGE_UP
GSE18804_BRAIN_VS_COLON_TUMORAL_MACROPHAGE_UP
123	-0.35824454	-1.4434048	0.013307985

0.036699265	1	2910	"tags=31%, list=21%, signal=39%"
GSE25088_CTRL_VS_IL4_STIM_STAT6_KO_MACROPHAGE_UP
GSE25088_CTRL_VS_IL4_STIM_STAT6_KO_MACROPHAGE_UP
121	-0.3556689	-1.4432187	0.00998004
0.03671663	1	2135	"tags=28%, list=15%, signal=33%"
GSE15330_MEGAKARYOCYTE_ERYTHROID_VS_GRANULOCYTE_MONOCYTE_PROGENITOR_DN
GSE15330_MEGAKARYOCYTE_ERYTHROID_VS_GRANULOCYTE_MONOCYTE_PROGENITOR_DN	162	-0.3448642	-1.4428735
0.009803922	0.036800794	1	1240	"tags=19%, list=9%, signal=20%"
GSE2770_IL12_VS_IL4_TREATED_ACT_CD4_TCELL_48H_DN
GSE2770_IL12_VS_IL4_TREATED_ACT_CD4_TCELL_48H_DN
136	-0.35081005	-1.4422699	0.003780718
0.03699376	1	1478	"tags=23%, list=10%, signal=25%"
GSE16450_IMMATURE_VS_MATURE_NEURON_CELL_LINE_UP
GSE16450_IMMATURE_VS_MATURE_NEURON_CELL_LINE_UP
122	-0.35828334	-1.4420614	0.018	0.03701054
1	2028	"tags=24%, list=14%, signal=28%"
GSE19825_NAIVE_VS_IL2RALOW_DAY3_EFF_CD8_TCELL_UP
GSE19825_NAIVE_VS_IL2RALOW_DAY3_EFF_CD8_TCELL_UP
145	-0.35021374	-1.4417961	0.011111111
0.037056677	1	4548	"tags=39%, list=32%, signal=56%"
GSE7568_CTRL_VS_3H_TGFB_TREATED_MACROPHAGES_WITH_IL4_AND_DEXAMETHASONE_DN
GSE7568_CTRL_VS_3H_TGFB_TREATED_MACROPHAGES_WITH_IL4_AND_DEXAMETHASONE_DN	123	-0.35228914	-1.4415959
0.019120459	0.037081636	1	2530	"tags=33%, list=18%, signal=39%"
GSE24634_NAIVE_CD4_TCELL_VS_DAY7_IL4_CONV_TREG_UP
GSE24634_NAIVE_CD4_TCELL_VS_DAY7_IL4_CONV_TREG_UP
125	-0.35685515	-1.4413378	0.013157895
0.037167456	1	1856	"tags=23%, list=13%, signal=26%"
GSE7852_LN_VS_FAT_TCONV_UP	GSE7852_LN_VS_FAT_TCONV_UP
	125	-0.35446483	-1.4407492	0.00967118
0.037386302	1	2355	"tags=28%, list=17%, signal=33%"
GSE34515_CD16_POS_MONOCYTE_VS_DC_UP
GSE34515_CD16_POS_MONOCYTE_VS_DC_UP
148	-0.3427232	-1.4403746	0.015151516
0.037517034	1	2137	"tags=30%, list=15%, signal=35%"
GSE13547_CTRL_VS_ANTI_IGM_STIM_BCELL_2H_DN
GSE13547_CTRL_VS_ANTI_IGM_STIM_BCELL_2H_DN
128	-0.35663506	-1.4397349	0.015717093
0.03773164	1	1779	"tags=23%, list=13%, signal=26%"
GSE8685_IL2_STARVED_VS_IL21_ACT_IL2_STARVED_CD4_TCELL_UP
GSE8685_IL2_STARVED_VS_IL21_ACT_IL2_STARVED_CD4_TCELL_UP
146	-0.3470194	-1.4396483	0.009633912
0.037708428	1	2331	"tags=29%, list=17%, signal=34%"
GSE46606_IRF4MID_VS_WT_CD40L_IL2_IL5_DAY1_STIMULATED_BCELL_DN
GSE46606_IRF4MID_VS_WT_CD40L_IL2_IL5_DAY1_STIMULATED_BCELL_DN
	151	-0.34498194	-1.4390501	0.01171875
0.03794087	1	3198	"tags=34%, list=23%, signal=44%"
GSE9239_CTRL_VS_TNF_INHIBITOR_TREATED_DC_UP
GSE9239_CTRL_VS_TNF_INHIBITOR_TREATED_DC_UP

145	-0.3506185	-1.438765	0.005964215
0.03802984	1	2156	"tags=27%, list=15%, signal=31%"
GSE7460_TCONV_VS_TREG_LN_DN	GSE7460_TCONV_VS_TREG_LN_DN
	156	-0.35018542	-1.4385186	0.017716536
0.038078822	1	1963	"tags=28%, list=14%, signal=32%"
GSE7509_UNSTIM_VS_TNFA_IL1B_IL6_PGE_STIM_DC_DN
GSE7509_UNSTIM_VS_TNFA_IL1B_IL6_PGE_STIM_DC_DN
61	-0.40309063	-1.4381119	0.03914591
0.038212795	1	4459	"tags=41%, list=32%, signal=60%"
GSE14308_TH2_VS_INDUCED_TREG_UP	GSE14308_TH2_VS_INDUCED_TREG_UP
	142	-0.3471866	-1.436049	0.011152417
0.03908254	1	2145	"tags=30%, list=15%, signal=35%"
GSE19941_LPS_VS_LPS_AND_IL10_STIM_IL10_KO_NFKBP50_KO_MACROPHAGE_DN
GSE19941_LPS_VS_LPS_AND_IL10_STIM_IL10_KO_NFKBP50_KO_MACROPHAGE_DN
	152	-0.3469133	-1.4353691	0.005847953
0.03935055	1	1055	"tags=18%, list=7%, signal=19%"
GSE36888_STAT5_AB_KNOCKIN_VS_WT_TCELL_IL2_TREATED_2H_UP
GSE36888_STAT5_AB_KNOCKIN_VS_WT_TCELL_IL2_TREATED_2H_UP
136	-0.35337403	-1.4351662	0.006085193
0.03939486	1	1840	"tags=22%, list=13%, signal=25%"
GSE30971_CTRL_VS_LPS_STIM_MACROPHAGE_WBP7_KO_4H_UP
GSE30971_CTRL_VS_LPS_STIM_MACROPHAGE_WBP7_KO_4H_UP
141	-0.34706974	-1.4324799	0.004024145
0.040604547	1	1387	"tags=18%, list=10%, signal=20%"
GSE41867_DAY15_EFFECTOR_VS_DAY30_MEMORY_CD8_TCELL_LCMV_ARMSTRONG_UP
GSE41867_DAY15_EFFECTOR_VS_DAY30_MEMORY_CD8_TCELL_LCMV_ARMSTRONG_UP
	142	-0.34724078	-1.4319762	0.009363296
0.040779322	1	1455	"tags=21%, list=10%, signal=23%"
GSE11961_MARGINAL_ZONE_BCELL_VS_GERMINAL_CENTER_BCELL_DAY40_DN
GSE11961_MARGINAL_ZONE_BCELL_VS_GERMINAL_CENTER_BCELL_DAY40_DN
	152	-0.34300148	-1.43184	0.011363637
0.040781505	1	1925	"tags=28%, list=14%, signal=32%"
GSE19401_RETINOIC_ACID_VS_RETINOIC_ACID_AND_PAM2CSK4_STIM_FOLLICULAR_DC_UP
GSE19401_RETINOIC_ACID_VS_RETINOIC_ACID_AND_PAM2CSK4_STIM_FOLLICULAR_DC_UP	148	-0.34972593	-1.4311016
0.008048289	0.04109727	1	2630	"tags=26%, list=19%, signal=32%"
GSE17721_PAM3CSK4_VS_CPG_16H_BMDC_UP
GSE17721_PAM3CSK4_VS_CPG_16H_BMDC_UP
137	-0.35156447	-1.430512	0.007648184
0.041290846	1	798	"tags=15%, list=6%, signal=15%"
GSE14308_TH1_VS_NAIVE_CD4_TCELL_DN
GSE14308_TH1_VS_NAIVE_CD4_TCELL_DN
129	-0.35422125	-1.4291815	0.019011406
0.041912172	1	3177	"tags=33%, list=23%, signal=43%"
GSE4748_LPS_VS_LPS_AND_CYANOBACTERIUM_LPSLIKE_STIM_DC_3H_UP
GSE4748_LPS_VS_LPS_AND_CYANOBACTERIUM_LPSLIKE_STIM_DC_3H_UP
129	-0.34994993	-1.4274215	0.017142856
0.0427548	1	1594	"tags=27%, list=11%, signal=30%"
GSE32423_MEMORY_VS_NAIVE_CD8_TCELL_IL7_UP
GSE32423_MEMORY_VS_NAIVE_CD8_TCELL_IL7_UP
128	-0.35183257	-1.4272683	0.007677543
0.042764705	1	1800	"tags=26%, list=13%, signal=29%"

GSE17974_IL4_AND_ANTI_IL12_VS_UNTREATED_12H_ACT_CD4_TCELL_DN
GSE17974_IL4_AND_ANTI_IL12_VS_UNTREATED_12H_ACT_CD4_TCELL_DN
120 -0.35169765 -1.4271461 0.013435701
0.04276635 1 546 "tags=12%, list=4%, signal=12%"
GSE12366_GC_VS_MEMORY_BCELL_DN GSE12366_GC_VS_MEMORY_BCELL_DN
133 -0.34798455 -1.4253794 0.012915129
0.043556444 1 1245 "tags=21%, list=9%, signal=23%"
GSE17974_CTRL_VS_ACT_IL4_AND_ANTI_IL12_6H_CD4_TCELL_DN
GSE17974_CTRL_VS_ACT_IL4_AND_ANTI_IL12_6H_CD4_TCELL_DN
132 -0.3486515 -1.4250276 0.005769231
0.043667547 1 3176 "tags=34%, list=23%, signal=44%"
GSE5542_UNTREATED_VS_IFNA_TREATED_EPITHELIAL_CELLS_6H_DN
GSE5542_UNTREATED_VS_IFNA_TREATED_EPITHELIAL_CELLS_6H_DN
127 -0.3553401 -1.4245911 0.011538462
0.043825354 1 1399 "tags=20%, list=10%, signal=23%"
GSE14308_TH1_VS_NATURAL_TREG_DN GSE14308_TH1_VS_NATURAL_TREG_DN
135 -0.3516589 -1.424096 0.015296367
0.044001088 1 1873 "tags=25%, list=13%, signal=29%"
GSE3982_CENT_MEMORY_CD4_TCELL_VS_TH2_UP
GSE3982_CENT_MEMORY_CD4_TCELL_VS_TH2_UP
125 -0.35021257 -1.4232143 0.009560229
0.04437639 1 2638 "tags=26%, list=19%, signal=32%"
GSE411_WT_VS_SOCS3_KO_MACROPHAGE_DN
GSE411_WT_VS_SOCS3_KO_MACROPHAGE_DN
140 -0.3482872 -1.4229469 0.012216405
0.04444966 1 2071 "tags=29%, list=15%, signal=34%"
GSE11057_EFF_MEM_VS_CENT_MEM_CD4_TCELL_UP
GSE11057_EFF_MEM_VS_CENT_MEM_CD4_TCELL_UP
137 -0.34853953 -1.422815 0.014814815
0.04443914 1 1713 "tags=22%, list=12%, signal=25%"
GSE17721_CTRL_VS_CPG_4H_BMDC_DN GSE17721_CTRL_VS_CPG_4H_BMDC_DN
154 -0.33822453 -1.4220054 0.015810277
0.04479162 1 2106 "tags=23%, list=15%, signal=27%"
GSE1925_CTRL_VS_3H_IFNG_STIM_MACROPHAGE_UP
GSE1925_CTRL_VS_3H_IFNG_STIM_MACROPHAGE_UP
150 -0.34501168 -1.421397 0.011090573
0.04500336 1 1703 "tags=21%, list=12%, signal=23%"
GSE21670_STAT3_KO_VS_WT_CD4_TCELL_DN
GSE21670_STAT3_KO_VS_WT_CD4_TCELL_DN
149 -0.3422315 -1.4207034 0.013257576
0.04528015 1 1864 "tags=27%, list=13%, signal=31%"
GSE411_100MIN_VS_400MIN_IL6_STIM_SOCS3_KO_MACROPHAGE_DN
GSE411_100MIN_VS_400MIN_IL6_STIM_SOCS3_KO_MACROPHAGE_DN
140 -0.34540612 -1.4191668 0.025540275
0.04607808 1 2821 "tags=30%, list=20%, signal=37%"
GSE30962_ACUTE_VS_CHRONIC_LCMV_SECONDARY_INF_CD8_TCELL_UP
GSE30962_ACUTE_VS_CHRONIC_LCMV_SECONDARY_INF_CD8_TCELL_UP
144 -0.34655204 -1.4189858 0.011472276
0.04610455 1 1379 "tags=21%, list=10%, signal=23%"
GSE25085_FETAL_BM_VS_ADULT_BM_SP4_THYMIC_IMPLANT_UP
GSE25085_FETAL_BM_VS_ADULT_BM_SP4_THYMIC_IMPLANT_UP
143 -0.3455736 -1.4189442 0.0227704
0.04605495 1 1797 "tags=29%, list=13%, signal=33%"
GSE11961_MEMORY_BCELL_DAY7_VS_GERMINAL_CENTER_BCELL_DAY7_UP

GSE11961_MEMORY_BCELL_DAY7_VS_GERMINAL_CENTER_BCELL_DAY7_UP
151 -0.3395474 -1.4187548 0.005780347
0.046077576 1 1135 "tags=17%, list=8%, signal=18%"
GSE25087_FETAL_VS_ADULT_TCONV_DN GSE25087_FETAL_VS_ADULT_TCONV_DN
119 -0.35847417 -1.4187078 0.021442495
0.046036016 1 2252 "tags=29%, list=16%, signal=34%"
GSE8921_UNSTIM_VS_TLR1_2_STIM_MONOCYTE_3H_DN
GSE8921_UNSTIM_VS_TLR1_2_STIM_MONOCYTE_3H_DN
145 -0.34350112 -1.4185735 0.009560229
0.046029966 1 2322 "tags=28%, list=16%, signal=33%"
GSE17974_IL4_AND_ANTI_IL12_VS_UNTREATED_6H_ACT_CD4_TCELL_DN
GSE17974_IL4_AND_ANTI_IL12_VS_UNTREATED_6H_ACT_CD4_TCELL_DN
134 -0.34727436 -1.4184641 0.019120459
0.046004243 1 2943 "tags=33%, list=21%, signal=41%"
GSE21927_SPLENIC_C26GM_TUMOROUS_VS_BONE_MARROW_MONOCYTES_UP
GSE21927_SPLENIC_C26GM_TUMOROUS_VS_BONE_MARROW_MONOCYTES_UP
106 -0.36168784 -1.41809 0.01734104 0.046111736
1 2480 "tags=22%, list=18%, signal=26%"
GSE20366_CD103_KLRG1_DP_VS_DN_TREG_UP
GSE20366_CD103_KLRG1_DP_VS_DN_TREG_UP
140 -0.34112644 -1.4158478 0.015779093
0.047259927 1 2139 "tags=26%, list=15%, signal=30%"
GSE4984_LPS_VS_VEHICLE_CTRL_TREATED_DC_UP
GSE4984_LPS_VS_VEHICLE_CTRL_TREATED_DC_UP
122 -0.35435832 -1.4157892 0.015296367
0.0472232 1 1833 "tags=20%, list=13%, signal=23%"
GSE6674_ANTI_IGM_VS_CPG_STIM_BCELL_UP
GSE6674_ANTI_IGM_VS_CPG_STIM_BCELL_UP
136 -0.34985718 -1.4151995 0.012891345
0.047485404 1 1934 "tags=28%, list=14%, signal=32%"
GSE41867_DAY15_EFFECTOR_VS_DAY30_MEMORY_CD8_TCELL_LCMV_ARMSTRONG_DN
GSE41867_DAY15_EFFECTOR_VS_DAY30_MEMORY_CD8_TCELL_LCMV_ARMSTRONG_DN
104 -0.3621508 -1.4149308 0.025925925
0.047556482 1 2585 "tags=32%, list=18%, signal=39%"
GSE5679_PPARG_LIGAND_ROSIGLITAZONE_VS_ROSIGLITAZONE_AND_RARA_AGONIST_AM580_TREATED_DC_DN
GSE5679_PPARG_LIGAND_ROSIGLITAZONE_VS_ROSIGLITAZONE_AND_RARA_AGONIST_AM580_TREATED_DC_DN
137 -0.33958596 -1.4146036 0.022857143
0.04767885 1 3298 "tags=39%, list=23%, signal=51%"
GSE25087_FETAL_VS_ADULT_TREG_DN GSE25087_FETAL_VS_ADULT_TREG_DN
125 -0.35106227 -1.4144375 0.017110266
0.047697544 1 2402 "tags=30%, list=17%, signal=36%"
GSE46606_UNSTIM_VS_CD40L_IL2_IL5_1DAY_STIMULATED_IRF4MID_SORTED_BCELL_UP
GSE46606_UNSTIM_VS_CD40L_IL2_IL5_1DAY_STIMULATED_IRF4MID_SORTED_BCELL_UP 149 -0.34136638 -1.4143286
0.012244898 0.0477081 1 2080 "tags=28%, list=15%, signal=33%"
GSE37301_HEMATOPOIETIC_STEM_CELL_VS_COMMON_LYMPHOID_PROGENITOR_DN
GSE37301_HEMATOPOIETIC_STEM_CELL_VS_COMMON_LYMPHOID_PROGENITOR_DN
149 -0.3416174 -1.4142467 0.012750455
0.047680248 1 1759 "tags=23%, list=12%, signal=26%"
GSE11961_FOLLICULAR_BCELL_VS_MEMORY_BCELL_DAY40_DN

GSE11961_FOLLICULAR_BCELL_VS_MEMORY_BCELL_DAY40_DN
146 -0.3417169 -1.4139801 0.01509434
0.04775929 1 2762 "tags=27%, list=20%, signal=33%"
GSE21546_WT_VS_ELK1_KO_DP_THYMOCYTES_UP
GSE21546_WT_VS_ELK1_KO_DP_THYMOCYTES_UP
149 -0.34272572 -1.413251 0.016759777
0.048128504 1 2347 "tags=23%, list=17%, signal=28%"
GSE17721_CTRL_VS_LPS_2H_BMDC_DN GSE17721_CTRL_VS_LPS_2H_BMDC_DN
153 -0.33523554 -1.4129443 0.009596929
0.048228268 1 3308 "tags=31%, list=23%, signal=41%"
GSE2197_IMMUNOSUPPRESSIVE_DNA_VS_UNTREATED_IN_DC_UP
GSE2197_IMMUNOSUPPRESSIVE_DNA_VS_UNTREATED_IN_DC_UP
144 -0.3429833 -1.4125841 0.00990099
0.048355985 1 1723 "tags=23%, list=12%, signal=26%"
GSE17186_BLOOD_VS_CORD_BLOOD_CD21HIGH_TRANSITIONAL_BCELL_DN
GSE17186_BLOOD_VS_CORD_BLOOD_CD21HIGH_TRANSITIONAL_BCELL_DN
141 -0.34862748 -1.4120777 0.025490196
0.04855569 1 1826 "tags=27%, list=13%, signal=31%"
GSE22935_UNSTIM_VS_48H_MBOVIS_BCG_STIM_MACROPHAGE_DN
GSE22935_UNSTIM_VS_48H_MBOVIS_BCG_STIM_MACROPHAGE_DN
145 -0.34158736 -1.411849 0.015968064
0.04859328 1 2553 "tags=27%, list=18%, signal=32%"
GSE34156_TLR1_TLR2_LIGAND_VS_NOD2_AND_TLR1_TLR2_LIGAND_24H_TREATED_M
ONOCYTE_DN
GSE34156_TLR1_TLR2_LIGAND_VS_NOD2_AND_TLR1_TLR2_LIGAND_24H_TREATED_M
ONOCYTE_DN 143 -0.33926222 -1.411464
0.017307693 0.048769463 1 2493 "tags=26%,
list=18%, signal=31%"
GSE13229_IMM_VS_MATURE_NKCELL_UP GSE13229_IMM_VS_MATURE_NKCELL_UP
148 -0.33906743 -1.4114366 0.007797271
0.048705827 1 2912 "tags=28%, list=21%, signal=35%"
GSE37301_COMMON_LYMPHOID_PROGENITOR_VS_GRAN_MONO_PROGENITOR_DN
GSE37301_COMMON_LYMPHOID_PROGENITOR_VS_GRAN_MONO_PROGENITOR_DN
139 -0.34593707 -1.411269 0.007722008
0.04874572 1 1463 "tags=18%, list=10%, signal=20%"
GSE13411_IGM_MEMORY_BCELL_VS_PLASMA_CELL_UP
GSE13411_IGM_MEMORY_BCELL_VS_PLASMA_CELL_UP
132 -0.34695137 -1.4109342 0.015065913
0.04884855 1 1800 "tags=22%, list=13%, signal=25%"
GSE17721_CTRL_VS_POLYIC_1H_BMDC_DN
GSE17721_CTRL_VS_POLYIC_1H_BMDC_DN
157 -0.3355344 -1.4106501 0.007532957
0.048930828 1 2424 "tags=24%, list=17%, signal=29%"
GSE7348_LPS_VS_TOLERIZED_AND_LPS_STIM_MACROPHAGE_DN
GSE7348_LPS_VS_TOLERIZED_AND_LPS_STIM_MACROPHAGE_DN
147 -0.33640367 -1.4105351 0.009746589
0.048912827 1 2125 "tags=25%, list=15%, signal=29%"
GSE7568_IL4_VS_IL4_AND_TGFB_TREATED_MACROPHAGE_24H_UP
GSE7568_IL4_VS_IL4_AND_TGFB_TREATED_MACROPHAGE_24H_UP
132 -0.3433238 -1.4097204 0.017208412
0.04935255 1 1656 "tags=22%, list=12%, signal=25%"
GSE13738_RESTING_VS_BYSTANDER_ACTIVATED_CD4_TCELL_UP
GSE13738_RESTING_VS_BYSTANDER_ACTIVATED_CD4_TCELL_UP
98 -0.36004117 -1.4083543 0.03206413

0.050028063	1	1729	"tags=17%, list=12%, signal=20%"
GSE6092_UNSTIM_VS_IFNG_STIM_AND_B_BURGDORFERI_INF_ENDOTHELIAL_CELL_DN
GSE6092_UNSTIM_VS_IFNG_STIM_AND_B_BURGDORFERI_INF_ENDOTHELIAL_CELL_DN	143	-0.33892685	-1.4079615
0.025242718	0.05018612	1	4617	"tags=36%, list=33%, signal=53%"
GSE12366_GC_VS_NAIVE_BCELL_DN	GSE12366_GC_VS_NAIVE_BCELL_DN
	119	-0.34970358	-1.4067029	0.015873017
0.05082219	1	1509	"tags=22%, list=11%, signal=24%"
GSE22601_IMMATURE_CD4_SINGLE_POSITIVE_VS_CD8_SINGLE_POSITIVE_THYMOCYTE_DN
GSE22601_IMMATURE_CD4_SINGLE_POSITIVE_VS_CD8_SINGLE_POSITIVE_THYMOCYTE_DN	146	-0.3425494	-1.4062995
0.025145067	0.05097737	1	1345	"tags=25%, list=10%, signal=28%"
GSE46606_IRF4_KO_VS_WT_UNSTIM_BCELL_UP
GSE46606_IRF4_KO_VS_WT_UNSTIM_BCELL_UP
130	-0.34704062	-1.4061182	0.015625	0.0510042
1	2213	"tags=32%, list=16%, signal=37%"
GSE31082_DP_VS_CD4_SP_THYMOCYTE_DN
GSE31082_DP_VS_CD4_SP_THYMOCYTE_DN
141	-0.34292668	-1.4059819	0.019607844
0.050993282	1	1634	"tags=23%, list=12%, signal=26%"
GSE15735_CTRL_VS_HDAC_INHIBITOR_TREATED_CD4_TCELL_2H_UP
GSE15735_CTRL_VS_HDAC_INHIBITOR_TREATED_CD4_TCELL_2H_UP
155	-0.3355691	-1.4051466	0.007476636
0.05138359	1	1207	"tags=20%, list=9%, signal=22%"
GSE3039_ALPHAALPHA_CD8_TCELL_VS_B1_BCELL_UP
GSE3039_ALPHAALPHA_CD8_TCELL_VS_B1_BCELL_UP
152	-0.33942243	-1.4047511	0.017408123
0.051560737	1	1919	"tags=26%, list=14%, signal=29%"
GSE37301_PRO_BCELL_VS_GRANULOCYTE_MONOCYTE_PROGENITOR_DN
GSE37301_PRO_BCELL_VS_GRANULOCYTE_MONOCYTE_PROGENITOR_DN
139	-0.34064823	-1.4045113	0.022181146
0.051646262	1	1270	"tags=23%, list=9%, signal=25%"
GSE15930_NAIVE_VS_72H_IN_VITRO_STIM_IFNAB_CD8_TCELL_UP
GSE15930_NAIVE_VS_72H_IN_VITRO_STIM_IFNAB_CD8_TCELL_UP
156	-0.3378822	-1.4041125	0.018181818
0.051791582	1	3210	"tags=31%, list=23%, signal=39%"
GSE17186_NAIVE_VS_CD21HIGH_TRANSITIONAL_BCELL_CORD_BLOOD_DN
GSE17186_NAIVE_VS_CD21HIGH_TRANSITIONAL_BCELL_CORD_BLOOD_DN
133	-0.34729347	-1.4039086	0.021194605
0.051834494	1	2170	"tags=29%, list=15%, signal=33%"
GSE40666_WT_VS_STAT1_KO_CD8_TCELL_UP
GSE40666_WT_VS_STAT1_KO_CD8_TCELL_UP
143	-0.3439811	-1.4038177	0.022514071
0.05180521	1	2152	"tags=31%, list=15%, signal=37%"
GSE7460_TCONV_VS_TREG_THYMUS_UP	GSE7460_TCONV_VS_TREG_THYMUS_UP
	148	-0.34373936	-1.4032303	0.013384321
0.05205345	1	1858	"tags=26%, list=13%, signal=30%"
GSE17301_IFNA2_VS_IFNA2_AND_ACD3_ACD28_STIM_CD8_TCELL_DN
GSE17301_IFNA2_VS_IFNA2_AND_ACD3_ACD28_STIM_CD8_TCELL_DN
143	-0.34218448	-1.4030936	0.014141414

0.052057277	1	1814	"tags=25%, list=13%, signal=29%"
GSE37416_CTRL_VS_0H_F_TULARENSIS_LVS_NEUTROPHIL_DN
GSE37416_CTRL_VS_0H_F_TULARENSIS_LVS_NEUTROPHIL_DN
91	-0.3659198	-1.4029154	0.03364486
0.05206383	1	1535	"tags=21%, list=11%, signal=23%"
GSE29614_DAY3_VS_DAY7_TIV_FLU_VACCINE_PBMC_UP
GSE29614_DAY3_VS_DAY7_TIV_FLU_VACCINE_PBMC_UP
108	-0.3552803	-1.4027113	0.018050542
0.052113228	1	5271	"tags=51%, list=37%, signal=81%"
GSE22229_UNTREATED_VS_IMMUNOSUPP_THERAPY_RENAL_TRANSPLANT_PATIENT_PBMC_UP
GSE22229_UNTREATED_VS_IMMUNOSUPP_THERAPY_RENAL_TRANSPLANT_PATIENT_PBMC_UP	147	-0.34030217	-1.4022366
0.009823183	0.052317675	1	3574	"tags=36%, list=25%, signal=48%"
GSE16266_CTRL_VS_LPS_STIM_MEF_UP	GSE16266_CTRL_VS_LPS_STIM_MEF_UP
139	-0.34326664	-1.4011056	0.017578125
0.052906796	1	1885	"tags=27%, list=13%, signal=30%"
GSE23505_UNTREATED_VS_4DAY_IL6_IL1_IL23_TREATED_CD4_TCELL_DN
GSE23505_UNTREATED_VS_4DAY_IL6_IL1_IL23_TREATED_CD4_TCELL_DN
139	-0.33958516	-1.4010316	0.011131725
0.05288845	1	1919	"tags=25%, list=14%, signal=29%"
GSE22601_DOUBLE_POSITIVE_VS_CD4_SINGLE_POSITIVE_THYMOCYTE_UP
GSE22601_DOUBLE_POSITIVE_VS_CD4_SINGLE_POSITIVE_THYMOCYTE_UP
125	-0.34502026	-1.4005075	0.026923077
0.053110678	1	2530	"tags=25%, list=18%, signal=30%"
GSE7460_CTRL_VS_FOXP3_OVEREXPR_TCONV_1_DN
GSE7460_CTRL_VS_FOXP3_OVEREXPR_TCONV_1_DN
143	-0.33853567	-1.4004107	0.011257036
0.053106874	1	3044	"tags=31%, list=22%, signal=39%"
GSE37532_VISCERAL_ADIPOSE_TISSUE_VS_LN_DERIVED_TCONV_CD4_TCELL_DN
GSE37532_VISCERAL_ADIPOSE_TISSUE_VS_LN_DERIVED_TCONV_CD4_TCELL_DN
79	-0.37225145	-1.3997873	0.023121387
0.053422213	1	6666	"tags=65%, list=47%, signal=122%"
GSE20727_CTRL_VS_H2O2_TREATED_DC_UP
GSE20727_CTRL_VS_H2O2_TREATED_DC_UP
131	-0.34704214	-1.3986177	0.015564202
0.05406328	1	1625	"tags=23%, list=12%, signal=26%"
GSE13484_UNSTIM_VS_YF17D_VACCINE_STIM_PBMC_DN
GSE13484_UNSTIM_VS_YF17D_VACCINE_STIM_PBMC_DN
139	-0.3382477	-1.3975886	0.020560747
0.05462396	1	1912	"tags=27%, list=14%, signal=30%"
GSE15930_NAIVE_VS_72H_IN_VITRO_STIM_TRICHOSTATINA_CD8_TCELL_UP
GSE15930_NAIVE_VS_72H_IN_VITRO_STIM_TRICHOSTATINA_CD8_TCELL_UP
151	-0.33722562	-1.3965578	0.009276438
0.05519145	1	3035	"tags=31%, list=22%, signal=39%"
GSE21927_SPLEEN_C57BL6_VS_EL4_TUMOR_BALBC_MONOCYTES_UP
GSE21927_SPLEEN_C57BL6_VS_EL4_TUMOR_BALBC_MONOCYTES_UP
131	-0.34394348	-1.3965154	0.011904762
0.055149373	1	1251	"tags=19%, list=9%, signal=21%"
GSE24634_TEFF_VS_TCONV_DAY3_IN_CULTURE_DN
GSE24634_TEFF_VS_TCONV_DAY3_IN_CULTURE_DN
140	-0.33968723	-1.3962908	0.020599252
0.05520458	1	2403	"tags=33%, list=17%, signal=39%"

GSE557_WT_VS_I_AB_KO_DC_UP   GSE557_WT_VS_I_AB_KO_DC_UP
        137   -0.34084037   -1.3956683   0.014198783
0.05554931   1   2498   "tags=31%, list=18%, signal=37%"
GSE6674_CPG_VS_CPG_AND_ANTI_IGM_STIM_BCELL_DN
GSE6674_CPG_VS_CPG_AND_ANTI_IGM_STIM_BCELL_DN
135   -0.3386008   -1.3950258   0.014760148
0.055886507   1   1887   "tags=24%, list=13%, signal=27%"
GSE30971_WBP7_HET_VS_KO_MACROPHAGE_4H_LPS_STIM_DN
GSE30971_WBP7_HET_VS_KO_MACROPHAGE_4H_LPS_STIM_DN
139   -0.34005034   -1.3947867   0.022944551
0.055943966   1   2152   "tags=25%, list=15%, signal=29%"
GSE22886_DAY1_VS_DAY7_MONOCYTE_IN_CULTURE_UP
GSE22886_DAY1_VS_DAY7_MONOCYTE_IN_CULTURE_UP
151   -0.3379749   -1.3946328   0.015267176
0.05596626   1   2648   "tags=29%, list=19%, signal=35%"
GSE29164_DAY3_VS_DAY7_CD8_TCELL_AND_IL12_TREATED_MELANOMA_UP
GSE29164_DAY3_VS_DAY7_CD8_TCELL_AND_IL12_TREATED_MELANOMA_UP
        148   -0.33616933   -1.3929447   0.015009381
0.056966327   1   2079   "tags=29%, list=15%, signal=34%"
GSE16266_CTRL_VS_LPS_STIM_MEF_DN GSE16266_CTRL_VS_LPS_STIM_MEF_DN
        147   -0.33332872   -1.3925725   0.02504817
0.057127073   1   2468   "tags=23%, list=17%, signal=28%"
GSE12392_WT_VS_IFNB_KO_CD8A_POS_SPLEEN_DC_DN
GSE12392_WT_VS_IFNB_KO_CD8A_POS_SPLEEN_DC_DN
137   -0.34280774   -1.3923391   0.015717093
0.05719202   1   2254   "tags=25%, list=16%, signal=29%"
GSE15271_CXCR4_POS_VS_NEG_GC_BCELL_DN
GSE15271_CXCR4_POS_VS_NEG_GC_BCELL_DN
130   -0.34052005   -1.3921616   0.016917294
0.057195615   1   1918   "tags=27%, list=14%, signal=31%"
GSE40274_CTRL_VS_FOXP3_AND_GATA1_TRANSDUCED_ACTIVATED_CD4_TCELL_UP
GSE40274_CTRL_VS_FOXP3_AND_GATA1_TRANSDUCED_ACTIVATED_CD4_TCELL_UP
        117   -0.35359767   -1.3916835   0.024253732
0.057405848   1   1128   "tags=18%, list=8%, signal=19%"
GSE22886_NAIVE_VS_IGM_MEMORY_BCELL_UP
GSE22886_NAIVE_VS_IGM_MEMORY_BCELL_UP
146   -0.33566606   -1.3912783   0.020522388
0.057606775   1   2394   "tags=27%, list=17%, signal=32%"
GSE3982_BASOPHIL_VS_NKCELL_UP   GSE3982_BASOPHIL_VS_NKCELL_UP
        139   -0.33928975   -1.3909726   0.022813689
0.05775083   1   2475   "tags=27%, list=18%, signal=33%"
GSE22229_RENAL_TRANSPLANT_VS_HEALTHY_PBMC_DN
GSE22229_RENAL_TRANSPLANT_VS_HEALTHY_PBMC_DN
143   -0.33376166   -1.390558   0.011695907
0.057933204   1   2844   "tags=29%, list=20%, signal=36%"
GSE33425_CD161_INT_VS_NEG_CD8_TCELL_DN
GSE33425_CD161_INT_VS_NEG_CD8_TCELL_DN
158   -0.33839634   -1.390461   0.015414258
0.057920408   1   2288   "tags=30%, list=16%, signal=36%"
GSE42021_TCONV_PLN_VS_TREG_PRECURSORS_THYMUS_UP
GSE42021_TCONV_PLN_VS_TREG_PRECURSORS_THYMUS_UP
146   -0.33617404   -1.3904556   0.030357143
0.057842072   1   2507   "tags=29%, list=18%, signal=35%"
GSE1791_CTRL_VS_NEUROMEDINU_IN_T_CELL_LINE_6H_UP

GSE1791_CTRL_VS_NEUROMEDINU_IN_T_CELL_LINE_6H_UP
141	-0.3373292	-1.3904444	0.01734104
0.057767518	1	1426	"tags=21%, list=10%, signal=23%"
GSE40273_EOS_KO_VS_WT_TREG_DN	GSE40273_EOS_KO_VS_WT_TREG_DN
	139	-0.33892757	-1.3901637	0.020872865
0.05787614	1	2006	"tags=31%, list=14%, signal=36%"
GSE4984_UNTREATED_VS_VEHICLE_CTRL_TREATED_DC_UP
GSE4984_UNTREATED_VS_VEHICLE_CTRL_TREATED_DC_UP
123	-0.345767	-1.3901173	0.02946593
0.05782557	1	2649	"tags=32%, list=19%, signal=39%"
GSE20366_TREG_VS_TCONV_DN GSE20366_TREG_VS_TCONV_DN
152	-0.33547205	-1.3896526	0.023941068
0.0580484	1	1363	"tags=21%, list=10%, signal=23%"
GSE369_SOCS3_KO_VS_WT_LIVER_POST_IL6_INJECTION_UP
GSE369_SOCS3_KO_VS_WT_LIVER_POST_IL6_INJECTION_UP
138	-0.34610817	-1.3896475	0.02734375
0.05796924	1	1356	"tags=18%, list=10%, signal=20%"
GSE6092_IFNG_VS_IFNG_AND_B_BURGDORFERI_INF_ENDOTHELIAL_CELL_UP
GSE6092_IFNG_VS_IFNG_AND_B_BURGDORFERI_INF_ENDOTHELIAL_CELL_UP
	140	-0.3381321	-1.3893822	0.017274473
0.058056433	1	2030	"tags=27%, list=14%, signal=31%"
GSE1925_CTRL_VS_IFNG_PRIMED_MACROPHAGE_3H_IFNG_STIM_UP
GSE1925_CTRL_VS_IFNG_PRIMED_MACROPHAGE_3H_IFNG_STIM_UP
135	-0.3424309	-1.389325	0.021696253
0.0580176	1	2518	"tags=30%, list=18%, signal=37%"
GSE32986_CURDLAN_LOWDOSE_VS_CURDLAN_HIGHDOSE_STIM_DC_DN
GSE32986_CURDLAN_LOWDOSE_VS_CURDLAN_HIGHDOSE_STIM_DC_DN
161	-0.33641416	-1.3887707	0.015355086
0.058322694	1	2962	"tags=33%, list=21%, signal=41%"
GSE29618_PRE_VS_DAY7_FLU_VACCINE_MDC_UP
GSE29618_PRE_VS_DAY7_FLU_VACCINE_MDC_UP
134	-0.34129044	-1.3887086	0.014141414
0.058278758	1	2410	"tags=22%, list=17%, signal=27%"
GSE45365_CTRL_VS_MCMV_INFECTION_NK_CELL_UP
GSE45365_CTRL_VS_MCMV_INFECTION_NK_CELL_UP
143	-0.33911988	-1.3882154	0.007476636
0.058495656	1	2385	"tags=26%, list=17%, signal=31%"
GSE2706_R848_VS_LPS_8H_STIM_DC_DN GSE2706_R848_VS_LPS_8H_STIM_DC_DN
	122	-0.34439886	-1.3877944	0.013059702
0.05869557	1	2081	"tags=28%, list=15%, signal=32%"
GSE14699_NAIVE_VS_ACT_CD8_TCELL_UP
GSE14699_NAIVE_VS_ACT_CD8_TCELL_UP
148	-0.33594114	-1.3873967	0.015267176
0.058886655	1	2080	"tags=28%, list=15%, signal=33%"
GSE26351_UNSTIM_VS_WNT_PATHWAY_STIM_HEMATOPOIETIC_PROGENITORS_UP
GSE26351_UNSTIM_VS_WNT_PATHWAY_STIM_HEMATOPOIETIC_PROGENITORS_UP
	129	-0.3420762	-1.3872902	0.026070764
0.058868967	1	2931	"tags=29%, list=21%, signal=37%"
GSE15330_HSC_VS_GRANULOCYTE_MONOCYTE_PROGENITOR_IKAROS_KO_UP
GSE15330_HSC_VS_GRANULOCYTE_MONOCYTE_PROGENITOR_IKAROS_KO_UP
	155	-0.334699	-1.3862212	0.020446097
0.05947288	1	1494	"tags=19%, list=11%, signal=21%"
GSE13411_NAIVE_BCELL_VS_PLASMA_CELL_UP
GSE13411_NAIVE_BCELL_VS_PLASMA_CELL_UP

130	-0.33939525	-1.3857026	0.026666667
0.05974706	1	1578	"tags=20%, list=11%, signal=22%"
GSE7768_OVA_ALONE_VS_OVA_WITH_MPL_IMMUNIZED_MOUSE_WHOLE_SPLEEN_6H_DN

GSE7768_OVA_ALONE_VS_OVA_WITH_MPL_IMMUNIZED_MOUSE_WHOLE_SPLEEN_6H_DN
		128	-0.3390372	-1.3849725
0.016161617	0.06014785	1	1367	"tags=20%,
list=10%, signal=21%"
GSE1460_INTRATHYMIC_T_PROGENITOR_VS_NAIVE_CD4_TCELL_CORD_BLOOD_DN
GSE1460_INTRATHYMIC_T_PROGENITOR_VS_NAIVE_CD4_TCELL_CORD_BLOOD_DN
	145	-0.33543682	-1.384629	0.021072797
0.06031033	1	1660	"tags=20%, list=12%, signal=22%"
GSE19198_1H_VS_6H_IL21_TREATED_TCELL_UP
GSE19198_1H_VS_6H_IL21_TREATED_TCELL_UP
98	-0.35739478	-1.3839123	0.014787431
0.06070108	1	3276	"tags=27%, list=23%, signal=34%"
GSE7852_THYMUS_VS_FAT_TREG_UP	GSE7852_THYMUS_VS_FAT_TREG_UP
	137	-0.34098658	-1.3830891	0.025242718
0.06121087	1	1593	"tags=20%, list=11%, signal=22%"
GSE13306_LAMINA_PROPRIA_VS_SPLEEN_TREG_UP
GSE13306_LAMINA_PROPRIA_VS_SPLEEN_TREG_UP
141	-0.33853906	-1.3830768	0.022304833
0.06113245	1	1429	"tags=23%, list=10%, signal=25%"
GSE8515_IL1_VS_IL6_4H_STIM_MAC_UP GSE8515_IL1_VS_IL6_4H_STIM_MAC_UP
	151	-0.33266577	-1.3820671	0.018691588
0.061739862	1	1724	"tags=22%, list=12%, signal=25%"
GSE40274_IRF4_VS_FOXP3_AND_IRF4_TRANSDUCED_ACTIVATED_CD4_TCELL_DN
GSE40274_IRF4_VS_FOXP3_AND_IRF4_TRANSDUCED_ACTIVATED_CD4_TCELL_DN
	89	-0.36289027	-1.3817348	0.029906543
0.061864045	1	3489	"tags=35%, list=25%, signal=46%"
GSE45739_UNSTIM_VS_ACD3_ACD28_STIM_NRAS_KO_CD4_TCELL_UP
GSE45739_UNSTIM_VS_ACD3_ACD28_STIM_NRAS_KO_CD4_TCELL_UP
147	-0.33307126	-1.3817189	0.016161617
0.06179201	1	1837	"tags=27%, list=13%, signal=30%"
GSE40666_STAT1_KO_VS_STAT4_KO_CD8_TCELL_WITH_IFNA_STIM_90MIN_DN
GSE40666_STAT1_KO_VS_STAT4_KO_CD8_TCELL_WITH_IFNA_STIM_90MIN_DN
	106	-0.3550012	-1.3813579	0.036608864
0.061950594	1	3693	"tags=36%, list=26%, signal=48%"
GSE360_T_GONDII_VS_B_MALAYI_HIGH_DOSE_DC_UP
GSE360_T_GONDII_VS_B_MALAYI_HIGH_DOSE_DC_UP
139	-0.33793122	-1.3812283	0.013157895
0.061953258	1	1910	"tags=22%, list=14%, signal=26%"
GSE3337_CTRL_VS_4H_IFNG_IN_CD8POS_DC_UP
GSE3337_CTRL_VS_4H_IFNG_IN_CD8POS_DC_UP
146	-0.33406538	-1.37989 0.014	0.06280355	1
1413	"tags=24%, list=10%, signal=26%"
GSE45739_NRAS_KO_VS_WT_UNSTIM_CD4_TCELL_DN
GSE45739_NRAS_KO_VS_WT_UNSTIM_CD4_TCELL_DN
131	-0.34460053	-1.3798337	0.02919708
0.06275935	1	2373	"tags=28%, list=17%, signal=34%"
GSE17974_0H_VS_0.5H_IN_VITRO_ACT_CD4_TCELL_DN
GSE17974_0H_VS_0.5H_IN_VITRO_ACT_CD4_TCELL_DN
115	-0.34907237	-1.3796731	0.04032258
0.06280686	1	2177	"tags=29%, list=15%, signal=34%"

GSE15659_NAIVE_VS_PTPRC_NEG_CD4_TCELL_UP
GSE15659_NAIVE_VS_PTPRC_NEG_CD4_TCELL_UP
107 -0.3475855 -1.3795513 0.028355388
0.06282655 1 2067 "tags=21%, list=15%, signal=25%"
GSE12845_IGD_POS_BLOOD_VS_NAIVE_TONSIL_BCELL_UP
GSE12845_IGD_POS_BLOOD_VS_NAIVE_TONSIL_BCELL_UP
121 -0.347001 -1.3794764 0.02264151
0.06280207 1 827 "tags=16%, list=6%, signal=17%"
GSE26495_NAIVE_VS_PD1LOW_CD8_TCELL_UP
GSE26495_NAIVE_VS_PD1LOW_CD8_TCELL_UP
111 -0.34837586 -1.3793185 0.028790787
0.06282073 1 2156 "tags=23%, list=15%, signal=26%"
GSE10147_IL3_VS_IL3_AND_CPG_STIM_PDC_DN
GSE10147_IL3_VS_IL3_AND_CPG_STIM_PDC_DN
124 -0.34225163 -1.3793004 0.026515152
0.06274936 1 2592 "tags=27%, list=18%, signal=33%"
GSE29949_CD8_NEG_DC_SPLEEN_VS_DC_BRAIN_DN
GSE29949_CD8_NEG_DC_SPLEEN_VS_DC_BRAIN_DN
145 -0.32955557 -1.379173 0.02534113
0.06274874 1 1797 "tags=23%, list=13%, signal=26%"
GSE44649_WT_VS_MIR155_KO_NAIVE_CD8_TCELL_DN
GSE44649_WT_VS_MIR155_KO_NAIVE_CD8_TCELL_DN
137 -0.33628103 -1.3790838 0.029906543
0.06272996 1 2035 "tags=25%, list=14%, signal=29%"
GSE27786_LSK_VS_LIN_NEG_CELL_UP GSE27786_LSK_VS_LIN_NEG_CELL_UP
148 -0.33393064 -1.3790776 0.013539651
0.06265 1 1555 "tags=24%, list=11%, signal=26%"
GSE6875_TCONV_VS_TREG_UP GSE6875_TCONV_VS_TREG_UP
147 -0.33428124 -1.3790543 0.018656716
0.062588334 1 2826 "tags=31%, list=20%, signal=39%"
GSE369_IFNG_KO_VS_WT_LIVER_UP GSE369_IFNG_KO_VS_WT_LIVER_UP
139 -0.3338506 -1.378791 0.020872865
0.06266712 1 1616 "tags=24%, list=11%, signal=27%"
GSE26890_CXCR1_NEG_VS_POS_EFFECTOR_CD8_TCELL_UP
GSE26890_CXCR1_NEG_VS_POS_EFFECTOR_CD8_TCELL_UP
131 -0.33742723 -1.3783417 0.026768642
0.0628891 1 2163 "tags=30%, list=15%, signal=35%"
GSE13173_UNTREATED_VS_IL12_TREATED_ACT_CD8_TCELL_DN
GSE13173_UNTREATED_VS_IL12_TREATED_ACT_CD8_TCELL_DN
107 -0.35362226 -1.3767152 0.03305785
0.06387838 1 2856 "tags=35%, list=20%, signal=43%"
GSE14308_NAIVE_CD4_TCELL_VS_NATURAL_TREG_DN
GSE14308_NAIVE_CD4_TCELL_VS_NATURAL_TREG_DN
134 -0.33788857 -1.3762252 0.017110266
0.06413805 1 1759 "tags=25%, list=12%, signal=28%"
GSE18791_CTRL_VS_NEWCASTLE_VIRUS_DC_14H_DN
GSE18791_CTRL_VS_NEWCASTLE_VIRUS_DC_14H_DN
117 -0.34342086 -1.375952 0.0227704
0.06425589 1 2377 "tags=24%, list=17%, signal=29%"
GSE3982_EFF_MEMORY_CD4_TCELL_VS_NKCELL_DN
GSE3982_EFF_MEMORY_CD4_TCELL_VS_NKCELL_DN
135 -0.3378896 -1.3738575 0.02504817
0.065689854 1 1938 "tags=21%, list=14%, signal=25%"
GSE4748_CTRL_VS_CYANOBACTERIUM_LPSLIKE_STIM_DC_3H_UP

GSE4748_CTRL_VS_CYANOBACTERIUM_LPSLIKE_STIM_DC_3H_UP
133	-0.34132734	-1.373805	0.017475728
0.0656482	1	2122	"tags=28%, list=15%, signal=32%"
GSE3982_BASOPHIL_VS_TH2_UP	GSE3982_BASOPHIL_VS_TH2_UP
	130	-0.3332055	-1.3736064	0.02385686
0.06570428	1	2481	"tags=27%, list=18%, signal=32%"
GSE20715_0H_VS_48H_OZONE_TLR4_KO_LUNG_UP
GSE20715_0H_VS_48H_OZONE_TLR4_KO_LUNG_UP
166	-0.33113247	-1.3736047	0.014760148
0.065618046	1	2583	"tags=27%, list=18%, signal=32%"
GSE6092_B_BURGDOFERI_VS_B_BURGDORFERI_AND_IFNG_STIM_ENDOTHELIAL_CELL_UP
GSE6092_B_BURGDOFERI_VS_B_BURGDORFERI_AND_IFNG_STIM_ENDOTHELIAL_CELL_UP	137	-0.33695415	-1.3732721
0.027726432	0.06579477	1	2087	"tags=27%, list=15%, signal=31%"
GSE23321_CENTRAL_MEMORY_VS_NAIVE_CD8_TCELL_UP
GSE23321_CENTRAL_MEMORY_VS_NAIVE_CD8_TCELL_UP
160	-0.32708675	-1.3729998	0.012448133
0.065895244	1	2214	"tags=28%, list=16%, signal=33%"
GSE16697_CD4_TCELL_VS_TFH_CD4_TCELL_DN
GSE16697_CD4_TCELL_VS_TFH_CD4_TCELL_DN
150	-0.33210006	-1.3729975	0.017110266
0.06580796	1	1052	"tags=20%, list=7%, signal=21%"
GSE25123_CTRL_VS_IL4_STIM_MACROPHAGE_DN
GSE25123_CTRL_VS_IL4_STIM_MACROPHAGE_DN
132	-0.33714667	-1.3726869	0.028846154
0.06593131	1	1769	"tags=22%, list=13%, signal=25%"
GSE3994_WT_VS_PAC1_KO_ACTIVATED_MAST_CELL_UP
GSE3994_WT_VS_PAC1_KO_ACTIVATED_MAST_CELL_UP
153	-0.3273305	-1.3724318	0.020599252
0.066022106	1	2693	"tags=23%, list=19%, signal=28%"
GSE17721_CTRL_VS_LPS_1H_BMDC_DN	GSE17721_CTRL_VS_LPS_1H_BMDC_DN
	157	-0.32988983	-1.3724083	0.016513761
0.06595611	1	1910	"tags=20%, list=14%, signal=23%"
GSE36826_WT_VS_IL1R_KO_SKIN_UP	GSE36826_WT_VS_IL1R_KO_SKIN_UP
	137	-0.33745074	-1.3723115	0.021484375
0.065931186	1	2120	"tags=26%, list=15%, signal=31%"
GSE13306_RA_VS_UNTREATED_MEM_CD4_TCELL_UP
GSE13306_RA_VS_UNTREATED_MEM_CD4_TCELL_UP
142	-0.33594278	-1.3719822	0.018484289
0.066064335	1	2163	"tags=27%, list=15%, signal=32%"
GSE24210_CTRL_VS_IL35_TREATED_TCONV_CD4_TCELL_UP
GSE24210_CTRL_VS_IL35_TREATED_TCONV_CD4_TCELL_UP
146	-0.33024165	-1.371661	0.024809161
0.066217385	1	1139	"tags=20%, list=8%, signal=21%"
GSE9006_HEALTHY_VS_TYPE_1_DIABETES_PBMC_1MONTH_POST_DX_DN
GSE9006_HEALTHY_VS_TYPE_1_DIABETES_PBMC_1MONTH_POST_DX_DN
129	-0.33784512	-1.3714085	0.026369167
0.06631269	1	1737	"tags=20%, list=12%, signal=23%"
GSE9946_IMMATURE_VS_LISTERIA_INF_MATURE_DC_DN
GSE9946_IMMATURE_VS_LISTERIA_INF_MATURE_DC_DN
101	-0.352971	-1.3709166	0.048449613
0.0666118	1	2607	"tags=29%, list=18%, signal=35%"

GSE37301_HEMATOPOIETIC_STEM_CELL_VS_PRO_BCELL_DN
GSE37301_HEMATOPOIETIC_STEM_CELL_VS_PRO_BCELL_DN
124 -0.33937064 -1.3708513 0.026515152 0.066583
1 1099 "tags=18%, list=8%, signal=19%"
GSE3982_EOSINOPHIL_VS_TH2_UP GSE3982_EOSINOPHIL_VS_TH2_UP
137 -0.33682886 -1.3707452 0.021113243
0.06658183 1 2491 "tags=24%, list=18%, signal=29%"
GSE22935_UNSTIM_VS_24H_MBOVIS_BCG_STIM_MYD88_KO_MACROPHAGE_UP
GSE22935_UNSTIM_VS_24H_MBOVIS_BCG_STIM_MYD88_KO_MACROPHAGE_UP
149 -0.3322963 -1.3696493 0.017208412
0.06729505 1 1299 "tags=22%, list=9%, signal=24%"
GSE43955_TH0_VS_TGFB_IL6_TH17_ACT_CD4_TCELL_52H_UP
GSE43955_TH0_VS_TGFB_IL6_TH17_ACT_CD4_TCELL_52H_UP
169 -0.32364118 -1.369157 0.017045455
0.06758335 1 1852 "tags=22%, list=13%, signal=26%"
GSE43955_1H_VS_60H_ACT_CD4_TCELL_WITH_TGFB_IL6_DN
GSE43955_1H_VS_60H_ACT_CD4_TCELL_WITH_TGFB_IL6_DN
155 -0.32922453 -1.3687468 0.03076923
0.06778475 1 1851 "tags=19%, list=13%, signal=21%"
GSE16450_IMMATURE_VS_MATURE_NEURON_CELL_LINE_12H_IFNA_STIM_UP
GSE16450_IMMATURE_VS_MATURE_NEURON_CELL_LINE_12H_IFNA_STIM_UP
153 -0.3282449 -1.3673035 0.023210831
0.06874019 1 2629 "tags=35%, list=19%, signal=43%"
GSE37301_COMMON_LYMPHOID_PROGENITOR_VS_RAG2_KO_NK_CELL_DN
GSE37301_COMMON_LYMPHOID_PROGENITOR_VS_RAG2_KO_NK_CELL_DN
137 -0.33365992 -1.366893 0.03448276
0.06894894 1 2506 "tags=28%, list=18%, signal=33%"
GSE27241_CTRL_VS_DIGOXIN_TREATED_RORGT_KO_CD4_TCELL_IN_TH17_POLARIZI
NG_CONDITIONS_DN
GSE27241_CTRL_VS_DIGOXIN_TREATED_RORGT_KO_CD4_TCELL_IN_TH17_POLARIZI
NG_CONDITIONS_DN 139 -0.33370885 -1.3665588
0.02020202 0.0691239 1 2167 "tags=27%,
list=15%, signal=31%"
GSE5589_WT_VS_IL6_KO_LPS_AND_IL10_STIM_MACROPHAGE_180MIN_UP
GSE5589_WT_VS_IL6_KO_LPS_AND_IL10_STIM_MACROPHAGE_180MIN_UP
76 -0.36455992 -1.3660924 0.053333335
0.069374114 1 1647 "tags=17%, list=12%, signal=19%"
GSE13485_DAY1_VS_DAY7_YF17D_VACCINE_PBMC_UP
GSE13485_DAY1_VS_DAY7_YF17D_VACCINE_PBMC_UP
108 -0.34121013 -1.3657607 0.04288499
0.06955868 1 4870 "tags=41%, list=35%, signal=62%"
GOLDRATH_NAIVE_VS_MEMORY_CD8_TCELL_UP
GOLDRATH_NAIVE_VS_MEMORY_CD8_TCELL_UP
156 -0.32526916 -1.3657315 0.014953272
0.06948847 1 1776 "tags=27%, list=13%, signal=30%"
GSE29617_CTRL_VS_TIV_FLU_VACCINE_PBMC_2008_UP
GSE29617_CTRL_VS_TIV_FLU_VACCINE_PBMC_2008_UP
115 -0.34414232 -1.3644235 0.02443609
0.07036314 1 3866 "tags=43%, list=27%, signal=58%"
GSE23505_IL6_IL1_VS_IL6_IL1_IL23_TREATED_CD4_TCELL_DN
GSE23505_IL6_IL1_VS_IL6_IL1_IL23_TREATED_CD4_TCELL_DN
149 -0.32410327 -1.3637096 0.015779093
0.07083572 1 2368 "tags=29%, list=17%, signal=34%"
GSE41867_NAIVE_VS_DAY8_LCMV_EFFECTOR_CD8_TCELL_DN

GSE41867_NAIVE_VS_DAY8_LCMV_EFFECTOR_CD8_TCELL_DN
136	-0.3307504	-1.361953	0.02734375
0.072009355	1	1660	"tags=24%, list=12%, signal=27%"
GSE5542_IFNG_VS_IFNA_TREATED_EPITHELIAL_CELLS_6H_DN
GSE5542_IFNG_VS_IFNA_TREATED_EPITHELIAL_CELLS_6H_DN
137	-0.33203843	-1.3607789	0.026119404
0.0727845	1	823	"tags=14%, list=6%, signal=15%"
GSE11961_MEMORY_BCELL_DAY40_VS_GERMINAL_CENTER_BCELL_DAY40_DN
GSE11961_MEMORY_BCELL_DAY40_VS_GERMINAL_CENTER_BCELL_DAY40_DN
	137	-0.33226076	-1.3605698	0.035916824
0.0728519	1	2359	"tags=24%, list=17%, signal=29%"
GSE32986_UNSTIM_VS_GMCSF_AND_CURDLAN_HIGHDOSE_STIM_DC_DN
GSE32986_UNSTIM_VS_GMCSF_AND_CURDLAN_HIGHDOSE_STIM_DC_DN
116	-0.3395405	-1.3602078	0.03499079
0.07303683	1	1216	"tags=18%, list=9%, signal=20%"
GSE42724_MEMORY_BCELL_VS_PLASMABLAST_UP
GSE42724_MEMORY_BCELL_VS_PLASMABLAST_UP
158	-0.32756126	-1.3601718	0.0295858
0.07297886	1	1856	"tags=23%, list=13%, signal=26%"
GSE29618_PRE_VS_DAY7_POST_LAIV_FLU_VACCINE_MDC_DN
GSE29618_PRE_VS_DAY7_POST_LAIV_FLU_VACCINE_MDC_DN
134	-0.33304316	-1.3599685	0.032608695
0.0730674	1	2664	"tags=31%, list=19%, signal=38%"
GSE3691_CONVENTIONAL_VS_PLASMACYTOID_DC_SPLEEN_UP
GSE3691_CONVENTIONAL_VS_PLASMACYTOID_DC_SPLEEN_UP
146	-0.32977518	-1.3598171	0.013539651
0.0730793	1	1187	"tags=16%, list=8%, signal=17%"
GSE34006_A2AR_KO_VS_A2AR_AGONIST_TREATED_TREG_UP
GSE34006_A2AR_KO_VS_A2AR_AGONIST_TREATED_TREG_UP
146	-0.32229784	-1.3596056	0.02457467
0.07315706	1	2790	"tags=34%, list=20%, signal=41%"
GSE9878_CTRL_VS_EBF_TRANSDUCED_PAX5_KO_PRO_BCELL_UP
GSE9878_CTRL_VS_EBF_TRANSDUCED_PAX5_KO_PRO_BCELL_UP
149	-0.326276	-1.3585081	0.040229887
0.073911056	1	1413	"tags=21%, list=10%, signal=23%"
GSE13493_DP_VS_CD4INTCD8POS_THYMOCYTE_UP
GSE13493_DP_VS_CD4INTCD8POS_THYMOCYTE_UP
138	-0.329724	-1.3581988	0.026515152
0.07409296	1	1694	"tags=22%, list=12%, signal=24%"
GSE3982_CTRL_VS_LPS_48H_DC_DN	GSE3982_CTRL_VS_LPS_48H_DC_DN
	146	-0.32707295	-1.358112	0.02
0.07407449	1	1457	"tags=22%, list=10%, signal=24%"
GSE2405_S_AUREUS_VS_A_PHAGOCYTOPHILUM_NEUTROPHIL_DN
GSE2405_S_AUREUS_VS_A_PHAGOCYTOPHILUM_NEUTROPHIL_DN
140	-0.3333784	-1.3576816	0.023166023
0.074332796	1	3035	"tags=33%, list=22%, signal=41%"
GSE36888_UNTREATED_VS_IL2_TREATED_TCELL_6H_UP
GSE36888_UNTREATED_VS_IL2_TREATED_TCELL_6H_UP
129	-0.33733347	-1.3563557	0.032319393
0.0752962	1	2097	"tags=30%, list=15%, signal=35%"
GSE22611_NOD2_TRANSD_VS_CTRL_TRANSD_HEK293_MDP_STIM_2H_UP
GSE22611_NOD2_TRANSD_VS_CTRL_TRANSD_HEK293_MDP_STIM_2H_UP
129	-0.3368325	-1.3559738	0.029850746
0.075486824	1	2264	"tags=21%, list=16%, signal=25%"

GSE41176_UNSTIM_VS_ANTI_IGM_STIM_BCELL_6H_DN
GSE41176_UNSTIM_VS_ANTI_IGM_STIM_BCELL_6H_DN
164 -0.31961262 -1.3558834 0.017578125
0.07547222 1 1452 "tags=19%, list=10%, signal=21%"
GSE25088_CTRL_VS_IL4_AND_ROSIGLITAZONE_STIM_MACROPHAGE_UP
GSE25088_CTRL_VS_IL4_AND_ROSIGLITAZONE_STIM_MACROPHAGE_UP
141 -0.33033034 -1.3554124 0.02745098
0.0757699 1 1507 "tags=21%, list=11%, signal=24%"
GSE16385_ROSIGLITAZONE_IL4_VS_IL4_ALONE_STIM_MACROPHAGE_12H_DN
GSE16385_ROSIGLITAZONE_IL4_VS_IL4_ALONE_STIM_MACROPHAGE_12H_DN
149 -0.32873422 -1.3553934 0.02471483
0.07568915 1 1977 "tags=23%, list=14%, signal=27%"
GSE40274_FOXP3_VS_FOXP3_AND_IRF4_TRANSDUCED_ACTIVATED_CD4_TCELL_UP
GSE40274_FOXP3_VS_FOXP3_AND_IRF4_TRANSDUCED_ACTIVATED_CD4_TCELL_UP
144 -0.33010486 -1.3553576 0.020912547
0.07562347 1 1274 "tags=23%, list=9%, signal=25%"
GSE12003_4D_VS_8D_CULTURE_BM_PROGENITOR_UP
GSE12003_4D_VS_8D_CULTURE_BM_PROGENITOR_UP
126 -0.3363361 -1.3545009 0.024242423
0.07620021 1 1827 "tags=24%, list=13%, signal=27%"
GSE411_100MIN_VS_400MIN_IL6_STIM_MACROPHAGE_UP
GSE411_100MIN_VS_400MIN_IL6_STIM_MACROPHAGE_UP
142 -0.32871926 -1.3539578 0.034883723
0.076575555 1 1600 "tags=24%, list=11%, signal=27%"
GSE29949_MICROGLIA_VS_DC_BRAIN_DN GSE29949_MICROGLIA_VS_DC_BRAIN_DN
148 -0.32842246 -1.353636 0.029354207
0.07675834 1 3381 "tags=30%, list=24%, signal=39%"
GSE22935_24H_VS_48H_MBOVIS_BCG_STIM_MACROPHAGE_UP
GSE22935_24H_VS_48H_MBOVIS_BCG_STIM_MACROPHAGE_UP
131 -0.3330339 -1.3529749 0.03358209
0.07721063 1 2491 "tags=24%, list=18%, signal=29%"
GSE10325_MYELOID_VS_LUPUS_MYELOID_UP
GSE10325_MYELOID_VS_LUPUS_MYELOID_UP
138 -0.3258486 -1.3521364 0.027881041
0.07778831 1 4668 "tags=39%, list=33%, signal=58%"
GSE9006_HEALTHY_VS_TYPE_2_DIABETES_PBMC_AT_DX_DN
GSE9006_HEALTHY_VS_TYPE_2_DIABETES_PBMC_AT_DX_DN
144 -0.32821205 -1.3520273 0.01775148
0.07776141 1 1741 "tags=21%, list=12%, signal=24%"
GSE26030_UNSTIM_VS_RESTIM_TH17_DAY5_POST_POLARIZATION_UP
GSE26030_UNSTIM_VS_RESTIM_TH17_DAY5_POST_POLARIZATION_UP
145 -0.3302312 -1.3518531 0.013513514
0.07780653 1 2419 "tags=32%, list=17%, signal=39%"
GSE3203_INFLUENZA_INF_VS_IFNB_TREATED_LN_BCELL_DN
GSE3203_INFLUENZA_INF_VS_IFNB_TREATED_LN_BCELL_DN
144 -0.32832295 -1.3516482 0.0332681
0.0778724 1 2067 "tags=26%, list=15%, signal=31%"
GSE22025_UNTREATED_VS_TGFB1_TREATED_CD4_TCELL_UP
GSE22025_UNTREATED_VS_TGFB1_TREATED_CD4_TCELL_UP
144 -0.33031833 -1.3513831 0.030241935
0.07799462 1 1895 "tags=22%, list=13%, signal=25%"
GSE37532_TREG_VS_TCONV_CD4_TCELL_FROM_VISCERAL_ADIPOSE_TISSUE_UP
GSE37532_TREG_VS_TCONV_CD4_TCELL_FROM_VISCERAL_ADIPOSE_TISSUE_UP
98 -0.34272265 -1.3503857 0.044660196

0.07867402 1 4091 "tags=30%, list=29%, signal=41%"
GSE27241_WT_CTRL_VS_DIGOXIN_TREATED_RORGT_KO_CD4_TCELL_IN_TH17_POLAR
IZING_CONDITIONS_UP
GSE27241_WT_CTRL_VS_DIGOXIN_TREATED_RORGT_KO_CD4_TCELL_IN_TH17_POLAR
IZING_CONDITIONS_UP
136 -0.32704455 -1.3501897 0.039138943
0.07872808 1 2588 "tags=26%, list=18%, signal=31%"
GSE22886_NAIVE_BCELL_VS_DC_UP GSE22886_NAIVE_BCELL_VS_DC_UP
115 -0.33680034 -1.3497995 0.037037037
0.07894052 1 2264 "tags=24%, list=16%, signal=29%"
GSE42724_MEMORY_VS_B1_BCELL_DN GSE42724_MEMORY_VS_B1_BCELL_DN
132 -0.33422044 -1.3497225 0.035714287
0.07890957 1 2254 "tags=28%, list=16%, signal=33%"
GSE7831_CPG_VS_INFLUENZA_STIM_PDC_1H_DN
GSE7831_CPG_VS_INFLUENZA_STIM_PDC_1H_DN
162 -0.32135686 -1.3492802 0.023255814
0.07917002 1 2180 "tags=23%, list=15%, signal=27%"
GSE37416_0H_VS_12H_F_TULARENSIS_LVS_NEUTROPHIL_DN
GSE37416_0H_VS_12H_F_TULARENSIS_LVS_NEUTROPHIL_DN
150 -0.3241756 -1.3489507 0.035650622
0.07934074 1 1283 "tags=21%, list=9%, signal=23%"
GSE360_HIGH_DOSE_B_MALAYI_VS_M_TUBERCULOSIS_DC_DN
GSE360_HIGH_DOSE_B_MALAYI_VS_M_TUBERCULOSIS_DC_DN
146 -0.32449096 -1.348889 0.029411765
0.07928733 1 1116 "tags=18%, list=8%, signal=19%"
GSE7400_CTRL_VS_CSF3_IN_VIVO_TREATED_PBMC_UP
GSE7400_CTRL_VS_CSF3_IN_VIVO_TREATED_PBMC_UP
132 -0.32688603 -1.3475524 0.020833334
0.08026877 1 2596 "tags=24%, list=18%, signal=29%"
GSE31082_DN_VS_CD4_SP_THYMOCYTE_DN
GSE31082_DN_VS_CD4_SP_THYMOCYTE_DN
138 -0.33511484 -1.3474523 0.017441861
0.08025277 1 1546 "tags=26%, list=11%, signal=29%"
GSE44649_WT_VS_MIR155_KO_ACTIVATED_CD8_TCELL_DN
GSE44649_WT_VS_MIR155_KO_ACTIVATED_CD8_TCELL_DN
138 -0.3317793 -1.347445 0.027290449
0.080161296 1 2213 "tags=31%, list=16%, signal=37%"
GSE9037_CTRL_VS_LPS_4H_STIM_BMDM_DN
GSE9037_CTRL_VS_LPS_4H_STIM_BMDM_DN
133 -0.32698584 -1.3465677 0.027131783
0.08074959 1 1347 "tags=17%, list=10%, signal=19%"
GSE37301_COMMON_LYMPHOID_PROGENITOR_VS_PRO_BCELL_UP
GSE37301_COMMON_LYMPHOID_PROGENITOR_VS_PRO_BCELL_UP
142 -0.3219318 -1.3464531 0.0332681
0.08073303 1 1460 "tags=18%, list=10%, signal=19%"
GSE22025_UNTREATED_VS_PROGESTERONE_TREATED_CD4_TCELL_DN
GSE22025_UNTREATED_VS_PROGESTERONE_TREATED_CD4_TCELL_DN
141 -0.3303309 -1.3462765 0.01981982
0.08077764 1 1583 "tags=20%, list=11%, signal=22%"
GSE15624_3H_VS_6H_HALOFUGINONE_TREATED_CD4_TCELL_UP
GSE15624_3H_VS_6H_HALOFUGINONE_TREATED_CD4_TCELL_UP
132 -0.33269995 -1.3460944 0.020952381
0.08083351 1 1063 "tags=20%, list=8%, signal=22%"
GSE22886_NAIVE_CD4_TCELL_VS_DC_UP GSE22886_NAIVE_CD4_TCELL_VS_DC_UP

138	-0.3325266	-1.345823	0.03358209
0.08098286	1	657	"tags=15%, list=5%, signal=16%"
GSE23925_LIGHT_ZONE_VS_NAIVE_BCELL_DN
GSE23925_LIGHT_ZONE_VS_NAIVE_BCELL_DN
143	-0.3300071	-1.3456762	0.03358209
0.0810061	1	1437	"tags=21%, list=10%, signal=23%"
GSE7831_1H_VS_4H_INFLUENZA_STIM_PDC_UP
GSE7831_1H_VS_4H_INFLUENZA_STIM_PDC_UP
159	-0.32187718	-1.3455491	0.026070764
0.0810173	1	2118	"tags=26%, list=15%, signal=30%"
GSE9988_LPS_VS_LPS_AND_ANTI_TREM1_MONOCYTE_UP
GSE9988_LPS_VS_LPS_AND_ANTI_TREM1_MONOCYTE_UP
150	-0.31977898	-1.3447889	0.02357564
0.08154727	1	2508	"tags=29%, list=18%, signal=34%"
GSE29164_UNTREATED_VS_CD8_TCELL_TREATED_MELANOMA_DAY3_DN
GSE29164_UNTREATED_VS_CD8_TCELL_TREATED_MELANOMA_DAY3_DN
147	-0.32426757	-1.3446641	0.03846154
0.08154458	1	1409	"tags=20%, list=10%, signal=22%"
GSE40274_FOXP3_VS_FOXP3_AND_LEF1_TRANSDUCED_ACTIVATED_CD4_TCELL_UP
GSE40274_FOXP3_VS_FOXP3_AND_LEF1_TRANSDUCED_ACTIVATED_CD4_TCELL_UP
146	-0.32612464	-1.3440647	0.024299065
0.08194377	1	1704	"tags=27%, list=12%, signal=31%"
GSE15330_HSC_VS_MEGAKARYOCYTE_ERYTHROID_PROGENITOR_IKAROS_KO_DN
GSE15330_HSC_VS_MEGAKARYOCYTE_ERYTHROID_PROGENITOR_IKAROS_KO_DN
150	-0.3282676	-1.3438857	0.029684601
0.08200055	1	977	"tags=13%, list=7%, signal=13%"
GSE11818_WT_VS_DICER_KO_TREG_DN	GSE11818_WT_VS_DICER_KO_TREG_DN
120	-0.33319998	-1.3429075	0.05263158
0.082742475	1	1749	"tags=23%, list=12%, signal=25%"
GSE9006_1MONTH_VS_4MONTH_POST_TYPE_1_DIABETES_DX_PBMC_UP
GSE9006_1MONTH_VS_4MONTH_POST_TYPE_1_DIABETES_DX_PBMC_UP
134	-0.3252818	-1.342808	0.034816246
0.082727894	1	2619	"tags=29%, list=19%, signal=35%"
GSE10325_BCELL_VS_LUPUS_BCELL_UP	GSE10325_BCELL_VS_LUPUS_BCELL_UP
133	-0.3316119	-1.342476	0.029469548
0.082949646	1	5132	"tags=47%, list=36%, signal=73%"
GSE37319_WT_VS_RC3H1_KO_CD44LOW_CD8_TCELL_DN
GSE37319_WT_VS_RC3H1_KO_CD44LOW_CD8_TCELL_DN
105	-0.3436834	-1.3419846	0.031894933
0.08325389	1	2095	"tags=26%, list=15%, signal=30%"
GSE13522_WT_VS_IFNAR_KO_SKIN_UP	GSE13522_WT_VS_IFNAR_KO_SKIN_UP
142	-0.32260528	-1.3419467	0.032818533
0.08318792	1	1523	"tags=25%, list=11%, signal=27%"
GSE18893_CTRL_VS_TNF_TREATED_TREG_2H_UP
GSE18893_CTRL_VS_TNF_TREATED_TREG_2H_UP
140	-0.32702368	-1.3414968	0.03448276
0.0834802	1	1534	"tags=22%, list=11%, signal=25%"
GSE17721_CTRL_VS_POLYIC_2H_BMDC_DN
GSE17721_CTRL_VS_POLYIC_2H_BMDC_DN
157	-0.32038945	-1.3400034	0.031128405
0.08470573	1	1718	"tags=20%, list=12%, signal=23%"
GSE26669_CTRL_VS_COSTIM_BLOCK_MLR_CD4_TCELL_DN
GSE26669_CTRL_VS_COSTIM_BLOCK_MLR_CD4_TCELL_DN
136	-0.32900903	-1.3399769	0.029962547

0.08463118	1	1612	"tags=22%, list=11%, signal=25%"
GSE29949_DC_BRAIN_VS_MONOCYTE_BONE_MARROW_DN
GSE29949_DC_BRAIN_VS_MONOCYTE_BONE_MARROW_DN
152	-0.3210419	-1.3394761	0.034552846
0.08499774	1	3786	"tags=36%, list=27%, signal=49%"
GSE15735_CTRL_VS_HDAC_INHIBITOR_TREATED_CD4_TCELL_2H_DN
GSE15735_CTRL_VS_HDAC_INHIBITOR_TREATED_CD4_TCELL_2H_DN
133	-0.3281911	-1.3388041	0.032323234
0.08550763	1	1139	"tags=20%, list=8%, signal=21%"
GSE19941_LPS_VS_LPS_AND_IL10_STIM_IL10_KO_NFKBP50_KO_MACROPHAGE_UP
GSE19941_LPS_VS_LPS_AND_IL10_STIM_IL10_KO_NFKBP50_KO_MACROPHAGE_UP
	138	-0.32769185	-1.338725	0.038674034
0.08547884	1	2521	"tags=30%, list=18%, signal=37%"
GSE15659_NONSUPPRESSIVE_TCELL_VS_ACTIVATED_TREG_UP
GSE15659_NONSUPPRESSIVE_TCELL_VS_ACTIVATED_TREG_UP
111	-0.33737352	-1.3385133	0.050880626
0.08554154	1	4646	"tags=43%, list=33%, signal=64%"
GSE22886_TH1_VS_TH2_12H_ACT_UP	GSE22886_TH1_VS_TH2_12H_ACT_UP
	152	-0.318778	-1.3380332	0.038387716
0.08586899	1	2300	"tags=24%, list=16%, signal=28%"
GSE23502_WT_VS_HDC_KO_MYELOID_DERIVED_SUPPRESSOR_CELL_COLON_TUMOR_DN

GSE23502_WT_VS_HDC_KO_MYELOID_DERIVED_SUPPRESSOR_CELL_COLON_TUMOR_DN
		146	-0.3255575	-1.3375412
0.029739777	0.0861789	1	6728	"tags=62%,
list=48%, signal=118%"
GSE23925_LIGHT_ZONE_VS_DARK_ZONE_BCELL_DN
GSE23925_LIGHT_ZONE_VS_DARK_ZONE_BCELL_DN
147	-0.32610723	-1.337534	0.031128405
0.08608218	1	1192	"tags=18%, list=8%, signal=20%"
GSE43955_10H_VS_30H_ACT_CD4_TCELL_WITH_TGFB_IL6_DN
GSE43955_10H_VS_30H_ACT_CD4_TCELL_WITH_TGFB_IL6_DN
161	-0.31785336	-1.3369812	0.035250463
0.086504646	1	2475	"tags=25%, list=18%, signal=30%"
GSE19401_NAIVE_VS_IMMUNIZED_MOUSE_PLN_FOLLICULAR_DC_UP
GSE19401_NAIVE_VS_IMMUNIZED_MOUSE_PLN_FOLLICULAR_DC_UP
153	-0.32002413	-1.336665	0.041501977
0.08667413	1	816	"tags=19%, list=6%, signal=20%"
GSE17974_IL4_AND_ANTI_IL12_VS_UNTREATED_24H_ACT_CD4_TCELL_UP
GSE17974_IL4_AND_ANTI_IL12_VS_UNTREATED_24H_ACT_CD4_TCELL_UP
	106	-0.3419156	-1.3365624	0.053407
0.08667988	1	3753	"tags=36%, list=27%, signal=48%"
GSE17974_0H_VS_2H_IN_VITRO_ACT_CD4_TCELL_UP
GSE17974_0H_VS_2H_IN_VITRO_ACT_CD4_TCELL_UP
126	-0.33335316	-1.3363044	0.032882012
0.08681096	1	2635	"tags=29%, list=19%, signal=36%"
GSE41176_UNSTIM_VS_ANTI_IGM_STIM_TAK1_KO_BCELL_6H_UP
GSE41176_UNSTIM_VS_ANTI_IGM_STIM_TAK1_KO_BCELL_6H_UP
139	-0.32486045	-1.3360432	0.036217302
0.0869206	1	3117	"tags=31%, list=22%, signal=39%"
GSE24634_TEFF_VS_TCONV_DAY7_IN_CULTURE_DN
GSE24634_TEFF_VS_TCONV_DAY7_IN_CULTURE_DN
146	-0.32647306	-1.3352154	0.030075189
0.08755767	1	1128	"tags=18%, list=8%, signal=20%"

GSE37416_0H_VS_48H_F_TULARENSIS_LVS_NEUTROPHIL_DN
GSE37416_0H_VS_48H_F_TULARENSIS_LVS_NEUTROPHIL_DN
147	-0.32404113	-1.3339194	0.036764707
0.08862142	1	1271	"tags=21%, list=9%, signal=23%"
GSE25123_CTRL_VS_IL4_STIM_PPARG_KO_MACROPHAGE_DN
GSE25123_CTRL_VS_IL4_STIM_PPARG_KO_MACROPHAGE_DN
143	-0.32337755	-1.3338804	0.042226486
0.08855653	1	2252	"tags=22%, list=16%, signal=26%"
GSE13522_WT_VS_IFNG_KO_SKING_T_CRUZI_Y_STRAIN_INF_DN
GSE13522_WT_VS_IFNG_KO_SKING_T_CRUZI_Y_STRAIN_INF_DN
95	-0.3496398	-1.3323616	0.049360145
0.08986935	1	2355	"tags=25%, list=17%, signal=30%"
GSE21927_SPLEEN_VS_TUMOR_MONOCYTE_C57BL6_DN
GSE21927_SPLEEN_VS_TUMOR_MONOCYTE_C57BL6_DN
108	-0.33824134	-1.3322666	0.04423077
0.08984382	1	2820	"tags=30%, list=20%, signal=37%"
GSE7768_OVA_WITH_LPS_VS_OVA_WITH_MPL_IMMUNIZED_MOUSE_WHOLE_SPLEEN_6H_DN
GSE7768_OVA_WITH_LPS_VS_OVA_WITH_MPL_IMMUNIZED_MOUSE_WHOLE_SPLEEN_6H_DN	129	-0.33139968	-1.3315215
0.02918288	0.090410545	1	2491	"tags=27%, list=18%, signal=33%"
GSE40655_FOXO1_KO_VS_WT_NTREG_UP	GSE40655_FOXO1_KO_VS_WT_NTREG_UP
133	-0.3262334	-1.3314921	0.04725898
0.09033099	1	2972	"tags=34%, list=21%, signal=42%"
GSE26351_WNT_VS_BMP_PATHWAY_STIM_HEMATOPOIETIC_PROGENITORS_DN
GSE26351_WNT_VS_BMP_PATHWAY_STIM_HEMATOPOIETIC_PROGENITORS_DN
135	-0.32818794	-1.3313221	0.05078125
0.0903585	1	1280	"tags=19%, list=9%, signal=20%"
GSE2585_CD80_HIGH_VS_LOW_AIRE_KO_MTEC_UP
GSE2585_CD80_HIGH_VS_LOW_AIRE_KO_MTEC_UP
151	-0.31697854	-1.3310415	0.043809526
0.09051692	1	2180	"tags=21%, list=15%, signal=24%"
GSE11924_TH2_VS_TH17_CD4_TCELL_UP	GSE11924_TH2_VS_TH17_CD4_TCELL_UP
137	-0.32520145	-1.3310032	0.027777778
0.09044539	1	2186	"tags=23%, list=15%, signal=27%"
GSE13493_CD4INTCD8POS_VS_CD8POS_THYMOCYTE_UP
GSE13493_CD4INTCD8POS_VS_CD8POS_THYMOCYTE_UP
145	-0.32171118	-1.3308933	0.041431263
0.09044789	1	1948	"tags=25%, list=14%, signal=29%"
GSE4142_NAIVE_VS_GC_BCELL_UP	GSE4142_NAIVE_VS_GC_BCELL_UP
135	-0.32733276	-1.3302361	0.032015067
0.09096576	1	2013	"tags=20%, list=14%, signal=23%"
GSE41176_UNSTIM_VS_ANTI_IGM_STIM_BCELL_24H_UP
GSE41176_UNSTIM_VS_ANTI_IGM_STIM_BCELL_24H_UP
134	-0.32341996	-1.3301837	0.02793296
0.090906754	1	3960	"tags=31%, list=28%, signal=42%"
GSE2706_2H_VS_8H_R848_STIM_DC_UP	GSE2706_2H_VS_8H_R848_STIM_DC_UP
137	-0.3219993	-1.3297628	0.03314917
0.09118659	1	1415	"tags=19%, list=10%, signal=21%"
GSE7348_UNSTIM_VS_TOLERIZED_AND_LPS_STIM_MACROPHAGE_DN
GSE7348_UNSTIM_VS_TOLERIZED_AND_LPS_STIM_MACROPHAGE_DN
135	-0.32479188	-1.3295453	0.044401545
0.09127137	1	2720	"tags=30%, list=19%, signal=36%"

GSE14413_UNSTIM_VS_IFNB_STIM_NIH3T3_CELLS_DN
GSE14413_UNSTIM_VS_IFNB_STIM_NIH3T3_CELLS_DN
98	-0.34149268	-1.3295346	0.05511811
0.091175064	1	1740	"tags=22%, list=12%, signal=25%"
GSE7509_FCGRIIB_VS_TNFA_IL1B_IL6_PGE_STIM_DC_DN
GSE7509_FCGRIIB_VS_TNFA_IL1B_IL6_PGE_STIM_DC_DN
102	-0.34068927	-1.3290151	0.062381852
0.09154175	1	3000	"tags=30%, list=21%, signal=38%"
GSE34156_NOD2_LIGAND_VS_TLR1_TLR2_LIGAND_24H_TREATED_MONOCYTE_DN
GSE34156_NOD2_LIGAND_VS_TLR1_TLR2_LIGAND_24H_TREATED_MONOCYTE_DN
	111	-0.33477736	-1.3288411	0.057692308
0.091605514	1	2368	"tags=26%, list=17%, signal=31%"
GSE10273_HIGH_VS_LOW_IL7_TREATED_IRF4_8_NULL_PRE_BCELL_DN
GSE10273_HIGH_VS_LOW_IL7_TREATED_IRF4_8_NULL_PRE_BCELL_DN
136	-0.32158253	-1.3288312	0.04263566
0.091509126	1	1146	"tags=21%, list=8%, signal=23%"
GSE16385_ROSIGLITAZONE_IFNG_TNF_VS_IL4_STIM_MACROPHAGE_UP
GSE16385_ROSIGLITAZONE_IFNG_TNF_VS_IL4_STIM_MACROPHAGE_UP
160	-0.31753004	-1.328658	0.023809524
0.09156035	1	2413	"tags=25%, list=17%, signal=30%"
GSE3920_IFNA_VS_IFNB_TREATED_ENDOTHELIAL_CELL_DN
GSE3920_IFNA_VS_IFNB_TREATED_ENDOTHELIAL_CELL_DN
127	-0.32944143	-1.3286417	0.042201836
0.09146614	1	1079	"tags=18%, list=8%, signal=19%"
GSE26928_EFF_MEM_VS_CENTR_MEM_CD4_TCELL_DN
GSE26928_EFF_MEM_VS_CENTR_MEM_CD4_TCELL_DN
93	-0.34825438	-1.328455	0.04606526
0.09154125	1	2242	"tags=23%, list=16%, signal=27%"
GSE14000_UNSTIM_VS_16H_LPS_DC_TRANSLATED_RNA_UP
GSE14000_UNSTIM_VS_16H_LPS_DC_TRANSLATED_RNA_UP
143	-0.32531595	-1.3277316	0.041904762
0.09210377	1	1500	"tags=23%, list=11%, signal=26%"
GSE14699_NAIVE_VS_DELETIONAL_TOLERANCE_CD8_TCELL_DN
GSE14699_NAIVE_VS_DELETIONAL_TOLERANCE_CD8_TCELL_DN
146	-0.32221973	-1.327058	0.0331384
0.09262715	1	2079	"tags=25%, list=15%, signal=29%"
GSE2405_0H_VS_3H_A_PHAGOCYTOPHILUM_STIM_NEUTROPHIL_DN
GSE2405_0H_VS_3H_A_PHAGOCYTOPHILUM_STIM_NEUTROPHIL_DN
115	-0.33350715	-1.3265734	0.047709923
0.092953496	1	1876	"tags=17%, list=13%, signal=20%"
GSE4142_GC_BCELL_VS_MEMORY_BCELL_DN
GSE4142_GC_BCELL_VS_MEMORY_BCELL_DN
137	-0.3248844	-1.3249245	0.031067962
0.09438056	1	1383	"tags=18%, list=10%, signal=19%"
GSE9960_GRAM_NEG_VS_GRAM_NEG_AND_POS_SEPSIS_PBMC_UP
GSE9960_GRAM_NEG_VS_GRAM_NEG_AND_POS_SEPSIS_PBMC_UP
119	-0.3323465	-1.3243321	0.056751467
0.09485608	1	2391	"tags=26%, list=17%, signal=31%"
GSE39820_CTRL_VS_TGFBETA1_IL6_IL23A_CD4_TCELL_UP
GSE39820_CTRL_VS_TGFBETA1_IL6_IL23A_CD4_TCELL_UP
134	-0.32275853	-1.3237014	0.053639848
0.095356375	1	2638	"tags=27%, list=19%, signal=33%"
GSE43955_TH0_VS_TGFB_IL6_TH17_ACT_CD4_TCELL_4H_UP
GSE43955_TH0_VS_TGFB_IL6_TH17_ACT_CD4_TCELL_4H_UP

152 -0.3207426 -1.3232032 0.028 0.09572591
1 1021 "tags=16%, list=7%, signal=17%"
GSE28783_CTRL_ANTI_MIR_VS_UNTREATED_ATHEROSCLEROSIS_MACROPHAGE_DN
GSE28783_CTRL_ANTI_MIR_VS_UNTREATED_ATHEROSCLEROSIS_MACROPHAGE_DN
144 -0.32212445 -1.3230287 0.037593983
0.09577991 1 3194 "tags=33%, list=23%, signal=43%"
GSE3337_CTRL_VS_16H_IFNG_IN_CD8POS_DC_UP
GSE3337_CTRL_VS_16H_IFNG_IN_CD8POS_DC_UP
166 -0.31304243 -1.3225836 0.035849057
0.096123114 1 2426 "tags=26%, list=17%, signal=31%"
GSE37533_PPARG1_FOXP3_VS_FOXP3_TRANSDUCED_CD4_TCELL_PIOGLITAZONE_TRE
ATED_UP
GSE37533_PPARG1_FOXP3_VS_FOXP3_TRANSDUCED_CD4_TCELL_PIOGLITAZONE_TRE
ATED_UP 139 -0.32259455 -1.322379
0.041044775 0.096229 1 1138 "tags=17%, list=8%,
signal=19%"
GSE7831_CPG_VS_INFLUENZA_STIM_PDC_4H_UP
GSE7831_CPG_VS_INFLUENZA_STIM_PDC_4H_UP
138 -0.32809475 -1.3220218 0.0407767
0.09645309 1 1480 "tags=19%, list=10%, signal=21%"
GSE3982_MAC_VS_TH1_UP GSE3982_MAC_VS_TH1_UP
141 -0.326903 -1.3219024 0.052532833
0.09645908 1 1277 "tags=20%, list=9%, signal=22%"
GSE3982_MAC_VS_BASOPHIL_DN GSE3982_MAC_VS_BASOPHIL_DN
130 -0.326356 -1.3216232 0.036659878
0.09662763 1 2749 "tags=25%, list=19%, signal=31%"
GSE45739_UNSTIM_VS_ACD3_ACD28_STIM_NRAS_KO_CD4_TCELL_DN
GSE45739_UNSTIM_VS_ACD3_ACD28_STIM_NRAS_KO_CD4_TCELL_DN
144 -0.32330823 -1.3215073 0.03815261
0.09663879 1 1145 "tags=15%, list=8%, signal=16%"
GSE46606_UNSTIM_VS_CD40L_IL2_IL5_DAY1_STIMULATED_BCELL_UP
GSE46606_UNSTIM_VS_CD40L_IL2_IL5_DAY1_STIMULATED_BCELL_UP
129 -0.32419643 -1.3215054 0.046558704
0.096529104 1 1585 "tags=24%, list=11%, signal=27%"
GSE2770_IL4_ACT_VS_ACT_CD4_TCELL_2H_DN
GSE2770_IL4_ACT_VS_ACT_CD4_TCELL_2H_DN
139 -0.32224816 -1.3214077 0.047984645
0.09651149 1 1704 "tags=24%, list=12%, signal=27%"
GSE40274_CTRL_VS_HELIOS_TRANSDUCED_ACTIVATED_CD4_TCELL_DN
GSE40274_CTRL_VS_HELIOS_TRANSDUCED_ACTIVATED_CD4_TCELL_DN
145 -0.3196422 -1.321401 0.038535647
0.09641071 1 2362 "tags=27%, list=17%, signal=32%"
GSE43863_NAIVE_VS_TFH_CD4_EFF_TCELL_D6_LCMV_DN
GSE43863_NAIVE_VS_TFH_CD4_EFF_TCELL_D6_LCMV_DN
101 -0.3338456 -1.3211623 0.04743083
0.09651757 1 2394 "tags=25%, list=17%, signal=30%"
GSE27241_CTRL_VS_DIGOXIN_TREATED_CD4_TCELL_IN_TH17_POLARIZING_CONDIT
IONS_DN
GSE27241_CTRL_VS_DIGOXIN_TREATED_CD4_TCELL_IN_TH17_POLARIZING_CONDIT
IONS_DN 144 -0.32493007 -1.3211592
0.029013539 0.09641329 1 1766 "tags=22%,
list=13%, signal=24%"
GSE21546_WT_VS_SAP1A_KO_AND_ELK1_KO_DP_THYMOCYTES_UP
GSE21546_WT_VS_SAP1A_KO_AND_ELK1_KO_DP_THYMOCYTES_UP

150	-0.31934643	-1.3210443	0.036764707
0.09642986	1	2193	"tags=22%, list=16%, signal=26%"
GSE9037_WT_VS_IRAK4_KO_BMDM_UP	GSE9037_WT_VS_IRAK4_KO_BMDM_UP
	141	-0.3224034	-1.3207827	0.042307694
0.09655534	1	1767	"tags=23%, list=13%, signal=26%"
GSE19923_HEB_KO_VS_HEB_AND_E2A_KO_DP_THYMOCYTE_DN
GSE19923_HEB_KO_VS_HEB_AND_E2A_KO_DP_THYMOCYTE_DN
159	-0.31643692	-1.3188634	0.0390625
0.09828374	1	1356	"tags=18%, list=10%, signal=19%"
GSE42021_CD24INT_VS_CD24LOW_TREG_THYMUS_DN
GSE42021_CD24INT_VS_CD24LOW_TREG_THYMUS_DN
145	-0.32162872	-1.3187048	0.031307552
0.09832748	1	2579	"tags=28%, list=18%, signal=34%"
GSE6259_BCELL_VS_CD8_TCELL_UP	GSE6259_BCELL_VS_CD8_TCELL_UP
	127	-0.3255602	-1.318685	0.044315994
0.098247245	1	1349	"tags=17%, list=10%, signal=18%"
GSE36826_NORMAL_VS_STAPH_AUREUS_INF_SKIN_DN
GSE36826_NORMAL_VS_STAPH_AUREUS_INF_SKIN_DN
150	-0.3225478	-1.3186682	0.023076924
0.09814742	1	1953	"tags=24%, list=14%, signal=28%"
GSE11961_MEMORY_BCELL_DAY7_VS_GERMINAL_CENTER_BCELL_DAY40_DN
GSE11961_MEMORY_BCELL_DAY7_VS_GERMINAL_CENTER_BCELL_DAY40_DN
	142	-0.31931952	-1.318309	0.038986355
0.09836568	1	2551	"tags=28%, list=18%, signal=34%"
GSE46606_UNSTIM_VS_CD40L_IL2_IL5_DAY3_STIMULATED_BCELL_UP
GSE46606_UNSTIM_VS_CD40L_IL2_IL5_DAY3_STIMULATED_BCELL_UP
128	-0.32636243	-1.3177655	0.06066536
0.09881297	1	1544	"tags=22%, list=11%, signal=24%"
GSE8835_CD4_VS_CD8_TCELL_CLL_PATIENT_UP
GSE8835_CD4_VS_CD8_TCELL_CLL_PATIENT_UP
147	-0.31884417	-1.3176124	0.038535647
0.09886736	1	2297	"tags=26%, list=16%, signal=31%"
GSE22886_NAIVE_BCELL_VS_MONOCYTE_UP
GSE22886_NAIVE_BCELL_VS_MONOCYTE_UP
118	-0.3254724	-1.3168362	0.04761905
0.09956004	1	2212	"tags=27%, list=16%, signal=32%"
GSE26030_TH1_VS_TH17_DAY5_POST_POLARIZATION_UP
GSE26030_TH1_VS_TH17_DAY5_POST_POLARIZATION_UP
143	-0.31714615	-1.316767	0.030245747
0.099505305	1	1665	"tags=24%, list=12%, signal=27%"
GSE9006_TYPE_1_DIABETES_AT_DX_VS_4MONTH_POST_DX_PBMC_DN
GSE9006_TYPE_1_DIABETES_AT_DX_VS_4MONTH_POST_DX_PBMC_DN
124	-0.32537493	-1.3158045	0.05380334
0.10037085	1	1462	"tags=17%, list=10%, signal=19%"
GSE43863_TFH_VS_LY6C_LOW_CXCR5NEG_EFFECTOR_CD4_TCELL_DN
GSE43863_TFH_VS_LY6C_LOW_CXCR5NEG_EFFECTOR_CD4_TCELL_DN
167	-0.3156411	-1.3157628	0.03187251
0.10028984	1	1712	"tags=18%, list=12%, signal=20%"
GSE17186_NAIVE_VS_CD21LOW_TRANSITIONAL_BCELL_CORD_BLOOD_UP
GSE17186_NAIVE_VS_CD21LOW_TRANSITIONAL_BCELL_CORD_BLOOD_UP
152	-0.31533593	-1.3143059	0.034026466
0.10168451	1	1377	"tags=19%, list=10%, signal=21%"
GSE18791_UNSTIM_VS_NEWCATSLE_VIRUS_DC_18H_DN
GSE18791_UNSTIM_VS_NEWCATSLE_VIRUS_DC_18H_DN

109	-0.3366663	-1.3141992	0.059160307
0.10167539	1	2406	"tags=27%, list=17%, signal=32%"
GSE15659_CD45RA_NEG_CD4_TCELL_VS_ACTIVATED_TREG_UP
GSE15659_CD45RA_NEG_CD4_TCELL_VS_ACTIVATED_TREG_UP
110	-0.32962027	-1.314008	0.056925997
0.10175215	1	4381	"tags=42%, list=31%, signal=60%"
GSE10240_IL22_VS_IL22_AND_IL17_STIM_PRIMARY_BRONCHIAL_EPITHELIAL_CELLS_UP
GSE10240_IL22_VS_IL22_AND_IL17_STIM_PRIMARY_BRONCHIAL_EPITHELIAL_CELLS_UP	147	-0.31823874	-1.3137344
0.045081966	0.10192301	1	1856	"tags=27%, list=13%, signal=31%"
GSE1448_CTRL_VS_ANTI_VALPHA2_DP_THYMOCYTE_DN
GSE1448_CTRL_VS_ANTI_VALPHA2_DP_THYMOCYTE_DN
160	-0.31657678	-1.3132914	0.042435423
0.10225179	1	2178	"tags=21%, list=15%, signal=24%"
GSE7852_LN_VS_FAT_TCONV_DN	GSE7852_LN_VS_FAT_TCONV_DN
151	-0.31961796	-1.3123419	0.030828517
0.10313759	1	1594	"tags=23%, list=11%, signal=26%"
GSE8921_UNSTIM_0H_VS_TLR1_2_STIM_MONOCYTE_6H_DN
GSE8921_UNSTIM_0H_VS_TLR1_2_STIM_MONOCYTE_6H_DN
130	-0.32069576	-1.3122443	0.046846848
0.10311317	1	1927	"tags=23%, list=14%, signal=26%"
GSE22045_TREG_VS_TCONV_DN GSE22045_TREG_VS_TCONV_DN
102	-0.33350414	-1.3119526	0.06422018
0.10332707	1	2292	"tags=26%, list=16%, signal=31%"
GSE3039_CD4_TCELL_VS_ALPHABETA_CD8_TCELL_UP
GSE3039_CD4_TCELL_VS_ALPHABETA_CD8_TCELL_UP
144	-0.3193564	-1.311828	0.031936128
0.10332932	1	2199	"tags=25%, list=16%, signal=29%"
GSE22601_DOUBLE_NEGATIVE_VS_CD4_SINGLE_POSITIVE_THYMOCYTE_UP
GSE22601_DOUBLE_NEGATIVE_VS_CD4_SINGLE_POSITIVE_THYMOCYTE_UP
147	-0.31317496	-1.3114042	0.031894933
0.103626445	1	1349	"tags=22%, list=10%, signal=25%"
GSE28737_FOLLICULAR_VS_MARGINAL_ZONE_BCELL_BCL6_HET_UP
GSE28737_FOLLICULAR_VS_MARGINAL_ZONE_BCELL_BCL6_HET_UP
130	-0.3241411	-1.3114016	0.05162524
0.10351431	1	2211	"tags=28%, list=16%, signal=33%"
GSE27786_BCELL_VS_CD4_TCELL_DN	GSE27786_BCELL_VS_CD4_TCELL_DN
138	-0.32312733	-1.3112867	0.033771105
0.10352446	1	2108	"tags=23%, list=15%, signal=27%"
GSE31082_DP_VS_CD8_SP_THYMOCYTE_DN
GSE31082_DP_VS_CD8_SP_THYMOCYTE_DN
147	-0.31758675	-1.3110763	0.035781544
0.10360309	1	1845	"tags=22%, list=13%, signal=26%"
GSE27786_LIN_NEG_VS_CD4_TCELL_DN	GSE27786_LIN_NEG_VS_CD4_TCELL_DN
142	-0.31741565	-1.310868	0.037664782
0.10369257	1	1621	"tags=22%, list=11%, signal=24%"
GSE19941_UNSTIM_VS_LPS_STIM_IL10_KO_NFKBP50_KO_MACROPHAGE_DN
GSE19941_UNSTIM_VS_LPS_STIM_IL10_KO_NFKBP50_KO_MACROPHAGE_DN
134	-0.32595766	-1.3096942	0.042201836
0.104776345	1	2594	"tags=25%, list=18%, signal=31%"
GSE41176_UNSTIM_VS_ANTI_IGM_STIM_TAK1_KO_BCELL_24H_DN
GSE41176_UNSTIM_VS_ANTI_IGM_STIM_TAK1_KO_BCELL_24H_DN

143	-0.3176259	-1.3094466	0.041431263
0.10492218	1	1871	"tags=24%, list=13%, signal=27%"
GSE14350_IL2RB_KO_VS_WT_TREG_UP	GSE14350_IL2RB_KO_VS_WT_TREG_UP
	154	-0.31205568	-1.3092524	0.030534351
0.10499615	1	2317	"tags=27%, list=16%, signal=32%"
GSE3039_ALPHABETA_CD8_TCELL_VS_B1_BCELL_UP
GSE3039_ALPHABETA_CD8_TCELL_VS_B1_BCELL_UP
127	-0.32188505	-1.3091393	0.047358833
0.104995154	1	2145	"tags=28%, list=15%, signal=32%"
GSE37605_C57BL6_VS_NOD_FOXP3_FUSION_GFP_TREG_DN
GSE37605_C57BL6_VS_NOD_FOXP3_FUSION_GFP_TREG_DN
153	-0.31998837	-1.3089956	0.033663366
0.10503101	1	1591	"tags=24%, list=11%, signal=27%"
GSE13522_WT_VS_IFNG_KO_SKIN_UP	GSE13522_WT_VS_IFNG_KO_SKIN_UP
	97	-0.33803377	-1.3087052	0.054924242
0.10522121	1	6143	"tags=54%, list=44%, signal=94%"
GSE29949_MICROGLIA_VS_DC_BRAIN_UP GSE29949_MICROGLIA_VS_DC_BRAIN_UP
	151	-0.31386703	-1.3072543	0.039138943
0.106637895	1	1815	"tags=19%, list=13%, signal=22%"
GSE22589_HIV_VS_HIV_AND_SIV_INFECTED_DC_UP
GSE22589_HIV_VS_HIV_AND_SIV_INFECTED_DC_UP
144	-0.31535715	-1.3070134	0.046554934
0.106778845	1	2822	"tags=28%, list=20%, signal=34%"
GSE25677_MPL_VS_MPL_AND_R848_STIM_BCELL_DN
GSE25677_MPL_VS_MPL_AND_R848_STIM_BCELL_DN
98	-0.3335782	-1.3069999	0.066287875
0.10668308	1	2867	"tags=40%, list=20%, signal=50%"
GSE17721_PAM3CSK4_VS_GADIQUIMOD_0.5H_BMDC_UP
GSE17721_PAM3CSK4_VS_GADIQUIMOD_0.5H_BMDC_UP
156	-0.31388032	-1.3063778	0.037328094
0.10725143	1	2562	"tags=27%, list=18%, signal=33%"
GSE30971_WBP7_HET_VS_KO_MACROPHAGE_UP
GSE30971_WBP7_HET_VS_KO_MACROPHAGE_UP
147	-0.31305695	-1.3061789	0.043726236
0.10736701	1	1259	"tags=17%, list=9%, signal=18%"
GSE37532_WT_VS_PPARG_KO_VISCERAL_ADIPOSE_TISSUE_TREG_DN
GSE37532_WT_VS_PPARG_KO_VISCERAL_ADIPOSE_TISSUE_TREG_DN
159	-0.31252196	-1.3058137	0.054003723
0.1075987	1	1769	"tags=23%, list=13%, signal=26%"
GSE17974_IL4_AND_ANTI_IL12_VS_UNTREATED_1H_ACT_CD4_TCELL_UP
GSE17974_IL4_AND_ANTI_IL12_VS_UNTREATED_1H_ACT_CD4_TCELL_UP
112	-0.32384002	-1.3052412	0.06273764
0.108042166	1	2170	"tags=20%, list=15%, signal=23%"
GSE5679_RARA_AGONIST_AM580_VS_AM580_AND_ROSIGLITAZONE_TREATED_DC_DN
GSE5679_RARA_AGONIST_AM580_VS_AM580_AND_ROSIGLITAZONE_TREATED_DC_DN
	152	-0.31558755	-1.3049016	0.042944785
0.10827009	1	1573	"tags=21%, list=11%, signal=23%"
GSE4590_PRE_BCELL_VS_SMALL_PRE_BCELL_DN
GSE4590_PRE_BCELL_VS_SMALL_PRE_BCELL_DN
92	-0.34020463	-1.3041568	0.06616257
0.108901896	1	2590	"tags=30%, list=18%, signal=37%"
GSE22935_UNSTIM_VS_12H_MBOVIS_BCG_STIM_MYD88_KO_MACROPHAGE_UP
GSE22935_UNSTIM_VS_12H_MBOVIS_BCG_STIM_MYD88_KO_MACROPHAGE_UP
	151	-0.3153504	-1.3037966	0.03846154

0.1091944	1	3705	"tags=34%, list=26%, signal=46%"
GSE21380_NON_TFH_VS_TFH_CD4_TCELL_UP
GSE21380_NON_TFH_VS_TFH_CD4_TCELL_UP
143	-0.31932288	-1.3028718	0.041493777
0.11009271	1	2377	"tags=29%, list=17%, signal=35%"
GSE23568_ID3_KO_VS_WT_CD8_TCELL_DN
GSE23568_ID3_KO_VS_WT_CD8_TCELL_DN
161	-0.30778503	-1.3021698	0.035051547
0.11070151	1	1883	"tags=29%, list=13%, signal=33%"
GSE29618_PRE_VS_DAY7_FLU_VACCINE_MDC_DN
GSE29618_PRE_VS_DAY7_FLU_VACCINE_MDC_DN
132	-0.31659228	-1.3020039	0.037243947
0.11075958	1	2237	"tags=24%, list=16%, signal=29%"
GSE42021_TREG_VS_TCONV_PLN_UP	GSE42021_TREG_VS_TCONV_PLN_UP
138	-0.319337	-1.3013281	0.05464481
0.111392595	1	2184	"tags=28%, list=15%, signal=32%"
GSE8921_3H_VS_24H_TLR1_2_STIM_MONOCYTE_UP
GSE8921_3H_VS_24H_TLR1_2_STIM_MONOCYTE_UP
135	-0.32037085	-1.3008181	0.05697446
0.111835815	1	823	"tags=16%, list=6%, signal=17%"
GSE11057_NAIVE_VS_MEMORY_CD4_TCELL_UP
GSE11057_NAIVE_VS_MEMORY_CD4_TCELL_UP
107	-0.3329441	-1.2998577	0.07156673
0.112744115	1	2352	"tags=22%, list=17%, signal=27%"
GSE37533_PPARG1_FOXP3_VS_PPARG2_FOXP3_TRANSDUCED_CD4_TCELL_PIOGLITAZONE_TREATED_DN
GSE37533_PPARG1_FOXP3_VS_PPARG2_FOXP3_TRANSDUCED_CD4_TCELL_PIOGLITAZONE_TREATED_DN	140	-0.31335512	-1.2992357
0.04699248	0.11332086	1	1506	"tags=21%, list=11%, signal=23%"
GSE17721_LPS_VS_GARDIQUIMOD_1H_BMDC_UP
GSE17721_LPS_VS_GARDIQUIMOD_1H_BMDC_UP
160	-0.3073048	-1.298962	0.04109589
0.113482244	1	2025	"tags=22%, list=14%, signal=25%"
GSE14308_TH2_VS_NAIVE_CD4_TCELL_DN
GSE14308_TH2_VS_NAIVE_CD4_TCELL_DN
136	-0.3171336	-1.2986308	0.0498155
0.1137023	1	2075	"tags=21%, list=15%, signal=24%"
GSE7852_TREG_VS_TCONV_LN_UP	GSE7852_TREG_VS_TCONV_LN_UP
148	-0.31243855	-1.2984403	0.054844607
0.11382756	1	1779	"tags=26%, list=13%, signal=29%"
GSE29618_MONOCYTE_VS_MDC_UP	GSE29618_MONOCYTE_VS_MDC_UP
149	-0.31525698	-1.2978473	0.03468208
0.11440237	1	2081	"tags=26%, list=15%, signal=30%"
GSE5503_MLN_DC_VS_SPLEEN_DC_ACTIVATED_ALLOGENIC_TCELL_UP
GSE5503_MLN_DC_VS_SPLEEN_DC_ACTIVATED_ALLOGENIC_TCELL_UP
156	-0.30933383	-1.2973918	0.040384617
0.11477885	1	1277	"tags=18%, list=9%, signal=20%"
GSE17721_CPG_VS_GARDIQUIMOD_12H_BMDC_DN
GSE17721_CPG_VS_GARDIQUIMOD_12H_BMDC_DN
147	-0.3122301	-1.2972476	0.037037037
0.1148305	1	1837	"tags=24%, list=13%, signal=27%"
GSE6259_33D1_POS_DC_VS_CD4_TCELL_DN
GSE6259_33D1_POS_DC_VS_CD4_TCELL_DN

142	-0.3153454	-1.2969589	0.04477612
0.115007885	1	2388	"tags=26%, list=17%, signal=31%"
GSE42021_CD24INT_VS_CD24LOW_TCONV_THYMUS_DN
GSE42021_CD24INT_VS_CD24LOW_TCONV_THYMUS_DN
136	-0.31721604	-1.296714	0.048076924
0.11515027	1	1766	"tags=19%, list=13%, signal=22%"
GSE17580_TREG_VS_TEFF_UP	GSE17580_TREG_VS_TEFF_UP
152	-0.31279215	-1.2952721	0.046468403
0.11661819	1	2188	"tags=27%, list=16%, signal=32%"
GSE22601_IMMATURE_CD4_SINGLE_POSITIVE_VS_DOUBLE_POSITIVE_THYMOCYTE_DN
GSE22601_IMMATURE_CD4_SINGLE_POSITIVE_VS_DOUBLE_POSITIVE_THYMOCYTE_DN	145	-0.31586003	-1.2952498
0.042226486	0.11650902	1	1918	"tags=24%, list=14%, signal=28%"
GSE23502_BM_VS_COLON_TUMOR_HDC_KO_MYELOID_DERIVED_SUPPRESSOR_CELL_DN

GSE23502_BM_VS_COLON_TUMOR_HDC_KO_MYELOID_DERIVED_SUPPRESSOR_CELL_DN
	140	-0.3150156	-1.2951254
0.03984064	0.11652983	1	1463	"tags=21%, list=10%, signal=23%"
GSE3982_BASOPHIL_VS_TH1_UP	GSE3982_BASOPHIL_VS_TH1_UP
	136	-0.31415612	-1.2947803	0.05544933
0.11680029	1	2214	"tags=25%, list=16%, signal=29%"
GSE26488_WT_VS_HDAC7_DELTAP_TG_OT2_THYMOCYTE_WITH_PEPTIDE_INJECTION_DN
GSE26488_WT_VS_HDAC7_DELTAP_TG_OT2_THYMOCYTE_WITH_PEPTIDE_INJECTION_DN	167	-0.30823722	-1.2941617
0.04537205	0.11737901	1	2327	"tags=24%, list=16%, signal=28%"
GSE21380_NON_TFH_VS_TFH_CD4_TCELL_DN
GSE21380_NON_TFH_VS_TFH_CD4_TCELL_DN
152	-0.31434393	-1.2940624	0.054924242
0.11736517	1	1979	"tags=26%, list=14%, signal=30%"
GSE26290_WT_VS_PDK1_KO_ANTI_CD3_AND_IL2_STIM_CD8_TCELL_UP
GSE26290_WT_VS_PDK1_KO_ANTI_CD3_AND_IL2_STIM_CD8_TCELL_UP
133	-0.3187794	-1.293571	0.055445544
0.11775576	1	2409	"tags=26%, list=17%, signal=31%"
GSE27786_CD8_TCELL_VS_NKTCELL_UP	GSE27786_CD8_TCELL_VS_NKTCELL_UP
	150	-0.3098136	-1.2931077	0.03984064
0.118154354	1	993	"tags=16%, list=7%, signal=17%"
GSE40274_XBP1_VS_FOXP3_AND_XBP1_TRANSDUCED_ACTIVATED_CD4_TCELL_UP
GSE40274_XBP1_VS_FOXP3_AND_XBP1_TRANSDUCED_ACTIVATED_CD4_TCELL_UP
	149	-0.3114174	-1.2929902	0.051376145
0.11816991	1	1480	"tags=23%, list=10%, signal=25%"
GSE25088_IL4_VS_IL4_AND_ROSIGLITAZONE_STIM_STAT6_KO_MACROPHAGE_DAY10_DN
GSE25088_IL4_VS_IL4_AND_ROSIGLITAZONE_STIM_STAT6_KO_MACROPHAGE_DAY10_DN	137	-0.31829554	-1.292985
0.049212597	0.11805028	1	1895	"tags=24%, list=13%, signal=28%"
GSE24574_BCL6_HIGH_TFH_VS_TCONV_CD4_TCELL_UP
GSE24574_BCL6_HIGH_TFH_VS_TCONV_CD4_TCELL_UP
142	-0.31159946	-1.2926773	0.056603774

0.11825312	1	1164	"tags=16%, list=8%, signal=17%"
GSE36826_NORMAL_VS_STAPH_AUREUS_INF_IL1R_KO_SKIN_UP
GSE36826_NORMAL_VS_STAPH_AUREUS_INF_IL1R_KO_SKIN_UP
155	-0.30896795	-1.2921555	0.040152963
0.118700385	1	3139	"tags=33%, list=22%, signal=42%"
GSE12366_NAIVE_VS_MEMORY_BCELL_UP GSE12366_NAIVE_VS_MEMORY_BCELL_UP
	122	-0.3235222	-1.2913907	0.056338027
0.119436815	1	2377	"tags=24%, list=17%, signal=28%"
GSE27291_0H_VS_7D_STIM_GAMMADELTA_TCELL_UP
GSE27291_0H_VS_7D_STIM_GAMMADELTA_TCELL_UP
121	-0.31766537	-1.2913512	0.05451128
0.1193507	1	3050	"tags=36%, list=22%, signal=46%"
GSE3982_DC_VS_TH2_UP	GSE3982_DC_VS_TH2_UP
144	-0.31590566	-1.2912791	0.04174573
0.119317584	1	3360	"tags=30%, list=24%, signal=39%"
GSE42724_NAIVE_BCELL_VS_PLASMABLAST_UP
GSE42724_NAIVE_BCELL_VS_PLASMABLAST_UP
135	-0.3167117	-1.2911615	0.062030077
0.11932564	1	1856	"tags=24%, list=13%, signal=27%"
GSE41176_WT_VS_TAK1_KO_UNSTIM_BCELL_DN
GSE41176_WT_VS_TAK1_KO_UNSTIM_BCELL_DN
131	-0.3174781	-1.2904288	0.06126482
0.119990334	1	4801	"tags=41%, list=34%, signal=62%"
GSE4590_SMALL_VS_LARGE_PRE_BCELL_UP
GSE4590_SMALL_VS_LARGE_PRE_BCELL_UP
149	-0.30970493	-1.2904173	0.05871212
0.119878665	1	1487	"tags=21%, list=11%, signal=23%"
GSE13522_CTRL_VS_T_CRUZI_Y_STRAIN_INF_SKIN_BALBC_MOUSE_DN
GSE13522_CTRL_VS_T_CRUZI_Y_STRAIN_INF_SKIN_BALBC_MOUSE_DN
94	-0.33534712	-1.290278	0.07293666
0.119914964	1	2397	"tags=29%, list=17%, signal=34%"
GSE9316_IL6_KO_VS_IFNG_KO_INVIVO_EXPANDED_CD4_TCELL_DN
GSE9316_IL6_KO_VS_IFNG_KO_INVIVO_EXPANDED_CD4_TCELL_DN
167	-0.3067865	-1.2896898	0.049056605
0.12046123	1	1776	"tags=23%, list=13%, signal=26%"
GSE29949_MICROGLIA_BRAIN_VS_MONOCYTE_BONE_MARROW_DN
GSE29949_MICROGLIA_BRAIN_VS_MONOCYTE_BONE_MARROW_DN
146	-0.31471592	-1.2895011	0.05950096
0.12057587	1	1001	"tags=17%, list=7%, signal=18%"
GSE8515_CTRL_VS_IL1_4H_STIM_MAC_DN
GSE8515_CTRL_VS_IL1_4H_STIM_MAC_DN
152	-0.31336576	-1.2884951	0.051526718
0.12156887	1	2593	"tags=29%, list=18%, signal=35%"
GSE40273_XBP1_KO_VS_WT_TREG_DN	GSE40273_XBP1_KO_VS_WT_TREG_DN
	146	-0.31188282	-1.2881753	0.045454547
0.12181306	1	1751	"tags=24%, list=12%, signal=27%"
GSE40666_STAT1_KO_VS_STAT4_KO_CD8_TCELL_DN
GSE40666_STAT1_KO_VS_STAT4_KO_CD8_TCELL_DN
100	-0.32846916	-1.2873518	0.061657034
0.12266773	1	3822	"tags=32%, list=27%, signal=44%"
GSE17186_MEMORY_VS_NAIVE_BCELL_UP GSE17186_MEMORY_VS_NAIVE_BCELL_UP
	143	-0.30938908	-1.2860919	0.05490196
0.124001555	1	1574	"tags=22%, list=11%, signal=25%"
GSE29617_CTRL_VS_DAY3_TIV_FLU_VACCINE_PBMC_2008_UP

GSE29617_CTRL_VS_DAY3_TIV_FLU_VACCINE_PBMC_2008_UP
117	-0.32331094	-1.2857876	0.065737054
0.124243565	1	3196	"tags=34%, list=23%, signal=44%"
GSE32255_WT_UNSTIM_VS_JMJD2D_KNOCKDOWN_4H_LPS_STIM_DC_DN
GSE32255_WT_UNSTIM_VS_JMJD2D_KNOCKDOWN_4H_LPS_STIM_DC_DN
139	-0.31348076	-1.2852949	0.05533597
0.12468035	1	2085	"tags=24%, list=15%, signal=28%"
GSE7348_LPS_VS_TOLERIZED_AND_LPS_STIM_MACROPHAGE_UP
GSE7348_LPS_VS_TOLERIZED_AND_LPS_STIM_MACROPHAGE_UP
141	-0.31021613	-1.2852092	0.04789272
0.1246499	1	2098	"tags=23%, list=15%, signal=27%"
GSE3982_DC_VS_TH1_UP	GSE3982_DC_VS_TH1_UP
142	-0.3076197	-1.2848339	0.053016454
0.12494725	1	2648	"tags=27%, list=19%, signal=33%"
GSE19198_1H_VS_6H_IL21_TREATED_TCELL_DN
GSE19198_1H_VS_6H_IL21_TREATED_TCELL_DN
167	-0.30101338	-1.2837803	0.05882353
0.12604237	1	1790	"tags=21%, list=13%, signal=24%"
GSE25123_WT_VS_PPARG_KO_MACROPHAGE_UP
GSE25123_WT_VS_PPARG_KO_MACROPHAGE_UP
138	-0.31671935	-1.283554	0.06902985	0.12615
1	2037	"tags=25%, list=14%, signal=29%"
GSE19825_CD24LOW_VS_IL2RA_HIGH_DAY3_EFF_CD8_TCELL_UP
GSE19825_CD24LOW_VS_IL2RA_HIGH_DAY3_EFF_CD8_TCELL_UP
142	-0.31502005	-1.2832822	0.053949904
0.12633683	1	1752	"tags=25%, list=12%, signal=28%"
GSE9650_GP33_VS_GP276_LCMV_SPECIFIC_EXHAUSTED_CD8_TCELL_UP
GSE9650_GP33_VS_GP276_LCMV_SPECIFIC_EXHAUSTED_CD8_TCELL_UP
177	-0.3004145	-1.283168	0.04323308
0.12634265	1	4056	"tags=35%, list=29%, signal=49%"
GSE23568_CTRL_TRANSDUCED_VS_WT_CD8_TCELL_UP
GSE23568_CTRL_TRANSDUCED_VS_WT_CD8_TCELL_UP
161	-0.30603424	-1.2823282	0.048732944
0.12723951	1	2288	"tags=29%, list=16%, signal=34%"
GSE24634_TREG_VS_TCONV_POST_DAY3_IL4_CONVERSION_DN
GSE24634_TREG_VS_TCONV_POST_DAY3_IL4_CONVERSION_DN
150	-0.31376666	-1.2819613	0.053435113
0.1275526	1	3238	"tags=32%, list=23%, signal=41%"
GSE4748_CTRL_VS_CYANOBACTERIUM_LPSLIKE_STIM_DC_1H_UP
GSE4748_CTRL_VS_CYANOBACTERIUM_LPSLIKE_STIM_DC_1H_UP
147	-0.30893272	-1.28072	0.054820415	0.12890549
1	2210	"tags=25%, list=16%, signal=30%"
GSE22919_RESTING_VS_IL2_IL12_IL15_STIM_NK_CELL_DN
GSE22919_RESTING_VS_IL2_IL12_IL15_STIM_NK_CELL_DN
137	-0.3098196	-1.280699	0.06367041
0.12880406	1	2055	"tags=27%, list=15%, signal=31%"
GSE360_CTRL_VS_T_GONDII_DC_DN	GSE360_CTRL_VS_T_GONDII_DC_DN
	137	-0.31208947	-1.2806373	0.061068702
0.12874646	1	1607	"tags=20%, list=11%, signal=23%"
GSE7509_DC_VS_MONOCYTE_WITH_FCGRIIB_STIM_DN
GSE7509_DC_VS_MONOCYTE_WITH_FCGRIIB_STIM_DN
147	-0.3090092	-1.2799808	0.072407044
0.12939063	1	1766	"tags=24%, list=13%, signal=27%"
GSE41978_ID2_KO_VS_ID2_KO_AND_BIM_KO_KLRG1_LOW_EFFECTOR_CD8_TCELL_UP

GSE41978_ID2_KO_VS_ID2_KO_AND_BIM_KO_KLRG1_LOW_EFFECTOR_CD8_TCELL_UP
161 -0.3068113 -1.2798074
0.044198897 0.12947193 1 3244 "tags=26%,
list=23%, signal=33%"
GSE46606_IRF4HIGH_VS_IRF4MID_CD40L_IL2_IL5_DAY3_STIMULATED_BCELL_DN
GSE46606_IRF4HIGH_VS_IRF4MID_CD40L_IL2_IL5_DAY3_STIMULATED_BCELL_DN
132 -0.3135032 -1.2793934 0.06212425
0.12983268 1 1098 "tags=17%, list=8%, signal=18%"
GSE22886_NAIVE_BCELL_VS_NEUTROPHIL_UP
GSE22886_NAIVE_BCELL_VS_NEUTROPHIL_UP
118 -0.31560564 -1.2790552 0.059479553
0.13011782 1 1145 "tags=17%, list=8%, signal=18%"
GSE41867_DAY8_VS_DAY15_LCMV_ARMSTRONG_EFFECTOR_CD8_TCELL_DN
GSE41867_DAY8_VS_DAY15_LCMV_ARMSTRONG_EFFECTOR_CD8_TCELL_DN
93 -0.32953006 -1.2787627 0.07212476
0.13034019 1 2295 "tags=19%, list=16%, signal=23%"
GSE29618_PRE_VS_DAY7_FLU_VACCINE_MONOCYTE_DN
GSE29618_PRE_VS_DAY7_FLU_VACCINE_MONOCYTE_DN
125 -0.3195306 -1.2786528 0.057692308
0.13034779 1 1724 "tags=21%, list=12%, signal=23%"
GSE36476_YOUNG_VS_OLD_DONOR_MEMORY_CD4_TCELL_DN
GSE36476_YOUNG_VS_OLD_DONOR_MEMORY_CD4_TCELL_DN
134 -0.3120255 -1.2786413 0.058479533
0.13022612 1 5234 "tags=50%, list=37%, signal=79%"
GSE1460_INTRATHYMIC_T_PROGENITOR_VS_DP_THYMOCYTE_DN
GSE1460_INTRATHYMIC_T_PROGENITOR_VS_DP_THYMOCYTE_DN
149 -0.30649808 -1.2784046 0.049523808
0.13038881 1 1807 "tags=17%, list=13%, signal=20%"
GSE40666_NAIVE_VS_EFFECTOR_CD8_TCELL_DN
GSE40666_NAIVE_VS_EFFECTOR_CD8_TCELL_DN
143 -0.30873325 -1.2783146 0.04779412
0.13035557 1 1498 "tags=20%, list=11%, signal=22%"
GSE45365_NK_CELL_VS_CD11B_DC_DN GSE45365_NK_CELL_VS_CD11B_DC_DN
156 -0.3047055 -1.2761308 0.045009784
0.13285969 1 1349 "tags=21%, list=10%, signal=22%"
GSE8921_UNSTIM_0H_VS_TLR1_2_STIM_MONOCYTE_12H_DN
GSE8921_UNSTIM_0H_VS_TLR1_2_STIM_MONOCYTE_12H_DN
141 -0.309427 -1.2759668 0.060037524
0.13293174 1 2212 "tags=24%, list=16%, signal=28%"
GSE17301_CTRL_VS_48H_IFNA2_STIM_CD8_TCELL_UP
GSE17301_CTRL_VS_48H_IFNA2_STIM_CD8_TCELL_UP
148 -0.30911875 -1.2758261 0.058708414
0.13297671 1 1856 "tags=24%, list=13%, signal=27%"
GSE360_L_DONOVANI_VS_M_TUBERCULOSIS_MAC_DN
GSE360_L_DONOVANI_VS_M_TUBERCULOSIS_MAC_DN
150 -0.3103373 -1.275475 0.054924242
0.13329865 1 943 "tags=15%, list=7%, signal=16%"
GSE9037_CTRL_VS_LPS_1H_STIM_IRAK4_KO_BMDM_UP
GSE9037_CTRL_VS_LPS_1H_STIM_IRAK4_KO_BMDM_UP
142 -0.31462774 -1.2754025 0.08256881
0.13325174 1 3621 "tags=38%, list=26%, signal=51%"
GSE15930_NAIVE_VS_24H_IN_VITRO_STIM_CD8_TCELL_UP
GSE15930_NAIVE_VS_24H_IN_VITRO_STIM_CD8_TCELL_UP

159	-0.30683926	-1.2743063	0.06457564
0.13446787	1	1462	"tags=18%, list=10%, signal=19%"
GSE17974_CTRL_VS_ACT_IL4_AND_ANTI_IL12_0.5H_CD4_TCELL_DN
GSE17974_CTRL_VS_ACT_IL4_AND_ANTI_IL12_0.5H_CD4_TCELL_DN
114	-0.3203795	-1.2742707	0.07171315
0.13437937	1	2177	"tags=25%, list=15%, signal=30%"
GSE22443_NAIVE_VS_ACT_AND_IL2_TREATED_CD8_TCELL_DN
GSE22443_NAIVE_VS_ACT_AND_IL2_TREATED_CD8_TCELL_DN
157	-0.3048294	-1.2741368	0.050290134
0.1344336	1	4250	"tags=31%, list=30%, signal=44%"
GSE17974_CTRL_VS_ACT_IL4_AND_ANTI_IL12_1H_CD4_TCELL_DN
GSE17974_CTRL_VS_ACT_IL4_AND_ANTI_IL12_1H_CD4_TCELL_DN
123	-0.31584957	-1.2738124	0.06410257	0.134747
1	3149	"tags=31%, list=22%, signal=39%"
GSE32423_CTRL_VS_IL7_IL4_MEMORY_CD8_TCELL_UP
GSE32423_CTRL_VS_IL7_IL4_MEMORY_CD8_TCELL_UP
148	-0.30688134	-1.2736496	0.064638786
0.13481912	1	1585	"tags=20%, list=11%, signal=23%"
GSE29614_CTRL_VS_DAY7_TIV_FLU_VACCINE_PBMC_UP
GSE29614_CTRL_VS_DAY7_TIV_FLU_VACCINE_PBMC_UP
100	-0.32845134	-1.2734251	0.08943089
0.13494484	1	4638	"tags=43%, list=33%, signal=64%"
GSE46606_UNSTIM_VS_CD40L_IL2_IL5_1DAY_STIMULATED_IRF4_KO_BCELL_DN
GSE46606_UNSTIM_VS_CD40L_IL2_IL5_1DAY_STIMULATED_IRF4_KO_BCELL_DN
	135	-0.31017658	-1.2730248	0.075925924
0.13533841	1	900	"tags=17%, list=6%, signal=18%"
GSE40274_CTRL_VS_FOXP3_AND_IRF4_TRANSDUCED_ACTIVATED_CD4_TCELL_DN
GSE40274_CTRL_VS_FOXP3_AND_IRF4_TRANSDUCED_ACTIVATED_CD4_TCELL_DN
	119	-0.31434646	-1.2727728	0.083984375
0.13549381	1	2735	"tags=32%, list=19%, signal=39%"
GSE13411_SWITCHED_MEMORY_BCELL_VS_PLASMA_CELL_UP
GSE13411_SWITCHED_MEMORY_BCELL_VS_PLASMA_CELL_UP
136	-0.31291562	-1.2724725	0.06563707
0.13573918	1	1483	"tags=20%, list=11%, signal=22%"
GSE40685_TREG_VS_FOXP3_KO_TREG_PRECURSOR_DN
GSE40685_TREG_VS_FOXP3_KO_TREG_PRECURSOR_DN
129	-0.31225106	-1.272339	0.06187625	0.135764
1	1829	"tags=22%, list=13%, signal=26%"
GSE29164_UNTREATED_VS_CD8_TCELL_AND_IL12_TREATED_MELANOMA_DAY7_DN
GSE29164_UNTREATED_VS_CD8_TCELL_AND_IL12_TREATED_MELANOMA_DAY7_DN
	145	-0.30841437	-1.271188	0.059160307
0.13703036	1	1813	"tags=19%, list=13%, signal=22%"
GSE19198_6H_VS_24H_IL21_TREATED_TCELL_DN
GSE19198_6H_VS_24H_IL21_TREATED_TCELL_DN
139	-0.30961862	-1.2697952	0.0625	0.13867834
1	2259	"tags=24%, list=16%, signal=28%"
GSE23321_CENTRAL_VS_EFFECTOR_MEMORY_CD8_TCELL_UP
GSE23321_CENTRAL_VS_EFFECTOR_MEMORY_CD8_TCELL_UP
156	-0.30360964	-1.2696015	0.056092843
0.13880548	1	1588	"tags=20%, list=11%, signal=22%"
GSE1925_CTRL_VS_IFNG_PRIMED_MACROPHAGE_24H_IFNG_STIM_UP
GSE1925_CTRL_VS_IFNG_PRIMED_MACROPHAGE_24H_IFNG_STIM_UP
154	-0.30674177	-1.2695086	0.065476194
0.13879974	1	1270	"tags=17%, list=9%, signal=18%"

GSE26488_CTRL_VS_PEPTIDE_INJECTION_HDAC7_DELTAP_TG_OT2_THYMOCYTE_DN
GSE26488_CTRL_VS_PEPTIDE_INJECTION_HDAC7_DELTAP_TG_OT2_THYMOCYTE_DN
161 -0.30021644 -1.269358 0.042307694
0.13887548 1 2327 "tags=29%, list=16%, signal=35%"
GSE13522_CTRL_VS_T_CRUZI_Y_STRAIN_INF_SKIN_129_MOUSE_UP
GSE13522_CTRL_VS_T_CRUZI_Y_STRAIN_INF_SKIN_129_MOUSE_UP
96 -0.32827666 -1.2693546 0.073033705
0.13874231 1 4211 "tags=34%, list=30%, signal=49%"
GSE28737_WT_VS_BCL6_HET_MARGINAL_ZONE_BCELL_DN
GSE28737_WT_VS_BCL6_HET_MARGINAL_ZONE_BCELL_DN
132 -0.31308562 -1.2691503 0.06792453
0.13889554 1 2433 "tags=29%, list=17%, signal=34%"
GSE22886_NAIVE_CD8_TCELL_VS_MONOCYTE_UP
GSE22886_NAIVE_CD8_TCELL_VS_MONOCYTE_UP
148 -0.3112072 -1.2690803 0.063432835
0.13885559 1 1837 "tags=20%, list=13%, signal=23%"
GSE2770_TGFB_AND_IL4_ACT_VS_ACT_CD4_TCELL_2H_DN
GSE2770_TGFB_AND_IL4_ACT_VS_ACT_CD4_TCELL_2H_DN
135 -0.3124023 -1.26904 0.060661763 0.13877182
1 1704 "tags=22%, list=12%, signal=25%"
GSE37534_GW1929_VS_ROSIGLITAZONE_TREATED_CD4_TCELL_PPARG1_FOXP3_TRAN
SDUCED_DN
GSE37534_GW1929_VS_ROSIGLITAZONE_TREATED_CD4_TCELL_PPARG1_FOXP3_TRAN
SDUCED_DN 117 -0.3135131 -1.2689575
0.061594203 0.13872877 1 1766 "tags=25%,
list=13%, signal=28%"
GSE18804_SPLEEN_MACROPHAGE_VS_BRAIN_TUMORAL_MACROPHAGE_UP
GSE18804_SPLEEN_MACROPHAGE_VS_BRAIN_TUMORAL_MACROPHAGE_UP
132 -0.3114846 -1.268467 0.07635009
0.1392013 1 2685 "tags=30%, list=19%, signal=36%"
GSE18893_TCONV_VS_TREG_24H_CULTURE_UP
GSE18893_TCONV_VS_TREG_24H_CULTURE_UP
158 -0.3018386 -1.2684549 0.053892214
0.13908422 1 2279 "tags=28%, list=16%, signal=34%"
GSE9960_HEALTHY_VS_GRAM_POS_SEPSIS_PBMC_DN
GSE9960_HEALTHY_VS_GRAM_POS_SEPSIS_PBMC_DN
128 -0.31135184 -1.2683945 0.07495429
0.13902122 1 2276 "tags=26%, list=16%, signal=30%"
GSE19198_6H_VS_24H_IL21_TREATED_TCELL_UP
GSE19198_6H_VS_24H_IL21_TREATED_TCELL_UP
137 -0.308844 -1.2683126 0.0726257
0.13899148 1 2215 "tags=22%, list=16%, signal=26%"
GSE17721_POLYIC_VS_CPG_1H_BMDC_DN GSE17721_POLYIC_VS_CPG_1H_BMDC_DN
164 -0.30158034 -1.2681521 0.06569343
0.13907379 1 1814 "tags=20%, list=13%, signal=23%"
GSE43863_TH1_VS_TFH_MEMORY_CD4_TCELL_DN
GSE43863_TH1_VS_TFH_MEMORY_CD4_TCELL_DN
160 -0.30361947 -1.2679529 0.07037037
0.13919206 1 1295 "tags=22%, list=9%, signal=24%"
GSE3982_DC_VS_EFF_MEMORY_CD4_TCELL_DN
GSE3982_DC_VS_EFF_MEMORY_CD4_TCELL_DN
121 -0.31782898 -1.2677292 0.071969695
0.13932514 1 331 "tags=9%, list=2%, signal=9%"
GSE17721_LPS_VS_PAM3CSK4_1H_BMDC_DN

GSE17721_LPS_VS_PAM3CSK4_1H_BMDC_DN
160	-0.3032803	-1.2675611	0.061797753
0.13940375	1	2720	"tags=25%, list=19%, signal=31%"
GSE43955_TH0_VS_TGFB_IL6_TH17_ACT_CD4_TCELL_20H_DN
GSE43955_TH0_VS_TGFB_IL6_TH17_ACT_CD4_TCELL_20H_DN
161	-0.302291	-1.2674441	0.053016454
0.1394072	1	1852	"tags=20%, list=13%, signal=23%"
GSE37301_PRO_BCELL_VS_RAG2_KO_NK_CELL_DN
GSE37301_PRO_BCELL_VS_RAG2_KO_NK_CELL_DN
149	-0.30488768	-1.2670354	0.06936416
0.13977455	1	1779	"tags=27%, list=13%, signal=30%"
GSE17721_CTRL_VS_POLYIC_2H_BMDC_UP
GSE17721_CTRL_VS_POLYIC_2H_BMDC_UP
147	-0.30653176	-1.2669696	0.060546875
0.13972118	1	1317	"tags=17%, list=9%, signal=19%"
GSE20715_0H_VS_24H_OZONE_TLR4_KO_LUNG_UP
GSE20715_0H_VS_24H_OZONE_TLR4_KO_LUNG_UP
161	-0.30441982	-1.266534	0.05586592
0.14016952	1	1736	"tags=23%, list=12%, signal=26%"
GSE19512_NAUTRAL_VS_INDUCED_TREG_UP
GSE19512_NAUTRAL_VS_INDUCED_TREG_UP
151	-0.30674654	-1.2663212	0.07168459
0.14031713	1	3735	"tags=35%, list=26%, signal=47%"
GSE24634_IL4_VS_CTRL_TREATED_NAIVE_CD4_TCELL_DAY3_DN
GSE24634_IL4_VS_CTRL_TREATED_NAIVE_CD4_TCELL_DAY3_DN
143	-0.30882522	-1.2660785	0.064327486
0.14049752	1	1347	"tags=20%, list=10%, signal=22%"
GSE360_L_DONOVANI_VS_L_MAJOR_MAC_DN
GSE360_L_DONOVANI_VS_L_MAJOR_MAC_DN
148	-0.30992928	-1.2657213	0.061657034
0.14085236	1	1130	"tags=18%, list=8%, signal=20%"
GSE32423_MEMORY_VS_NAIVE_CD8_TCELL_IL7_IL4_UP
GSE32423_MEMORY_VS_NAIVE_CD8_TCELL_IL7_IL4_UP
142	-0.30612996	-1.2652612	0.06428572
0.14129588	1	3645	"tags=30%, list=26%, signal=40%"
GSE40441_NRP1_POS_INDUCED_TREG_VS_NRP1_NEG_NATURAL_TREG_DN
GSE40441_NRP1_POS_INDUCED_TREG_VS_NRP1_NEG_NATURAL_TREG_DN
158	-0.29999483	-1.2649543	0.05950096
0.14152552	1	1694	"tags=18%, list=12%, signal=21%"
GSE27786_LIN_NEG_VS_CD8_TCELL_DN	GSE27786_LIN_NEG_VS_CD8_TCELL_DN
	149	-0.30368152	-1.2647946	0.073076926
0.14160122	1	1480	"tags=18%, list=10%, signal=20%"
GSE19198_CTRL_VS_IL21_TREATED_TCELL_1H_DN
GSE19198_CTRL_VS_IL21_TREATED_TCELL_1H_DN
143	-0.3064522	-1.2646135	0.07102804
0.14170611	1	3815	"tags=40%, list=27%, signal=54%"
GSE13485_DAY7_VS_DAY21_YF17D_VACCINE_PBMC_DN
GSE13485_DAY7_VS_DAY21_YF17D_VACCINE_PBMC_DN
97	-0.32626063	-1.2642993	0.09126984
0.14199854	1	5692	"tags=51%, list=40%, signal=84%"
GSE20715_0H_VS_6H_OZONE_LUNG_DN	GSE20715_0H_VS_6H_OZONE_LUNG_DN
	160	-0.30299518	-1.2637998	0.06584362
0.14250632	1	2310	"tags=20%, list=16%, signal=24%"
GSE32901_NAIVE_VS_TH1_CD4_TCELL_UP

GSE32901_NAIVE_VS_TH1_CD4_TCELL_UP
96	-0.32867342	-1.2637093	0.09248555
0.14249532	1	1865	"tags=23%, list=13%, signal=26%"
GSE21546_WT_VS_SAP1A_KO_ANTI_CD3_STIM_DP_THYMOCYTES_DN
GSE21546_WT_VS_SAP1A_KO_ANTI_CD3_STIM_DP_THYMOCYTES_DN
138	-0.30822095	-1.2635617	0.05667276
0.14254676	1	1366	"tags=17%, list=10%, signal=19%"
GSE17186_MEMORY_VS_CD21HIGH_TRANSITIONAL_BCELL_UP
GSE17186_MEMORY_VS_CD21HIGH_TRANSITIONAL_BCELL_UP
146	-0.3052136	-1.263413	0.068571426
0.14260909	1	2207	"tags=24%, list=16%, signal=28%"
GSE36078_UNTREATED_VS_AD5_T425A_HEXON_INF_IL1R_KO_MOUSE_LUNG_DC_UP
GSE36078_UNTREATED_VS_AD5_T425A_HEXON_INF_IL1R_KO_MOUSE_LUNG_DC_UP
	135	-0.3128045	-1.2628012	0.0655106
0.14325823	1	5149	"tags=47%, list=36%, signal=73%"
GSE17186_NAIVE_VS_CD21LOW_TRANSITIONAL_BCELL_DN
GSE17186_NAIVE_VS_CD21LOW_TRANSITIONAL_BCELL_DN
124	-0.3133104	-1.2622676	0.08143939
0.14384478	1	1737	"tags=24%, list=12%, signal=27%"
GSE12839_CTRL_VS_IL12_TREATED_PBMC_DN
GSE12839_CTRL_VS_IL12_TREATED_PBMC_DN
86	-0.32778546	-1.2622622	0.09191176
0.1437108	1	4143	"tags=36%, list=29%, signal=51%"
GSE3039_ALPHABETA_CD8_TCELL_VS_B2_BCELL_UP
GSE3039_ALPHABETA_CD8_TCELL_VS_B2_BCELL_UP
145	-0.3059947	-1.2622076	0.07517482
0.14364807	1	2496	"tags=26%, list=18%, signal=32%"
GSE11818_WT_VS_DICER_KO_TREG_UP	GSE11818_WT_VS_DICER_KO_TREG_UP
	116	-0.31267577	-1.2619511	0.06095238
0.14384285	1	3351	"tags=26%, list=24%, signal=34%"
GSE40274_CTRL_VS_XBP1_TRANSDUCED_ACTIVATED_CD4_TCELL_UP
GSE40274_CTRL_VS_XBP1_TRANSDUCED_ACTIVATED_CD4_TCELL_UP
136	-0.30839637	-1.2617928	0.06849315
0.14391002	1	1829	"tags=22%, list=13%, signal=25%"
GSE24634_TEFF_VS_TCONV_DAY5_IN_CULTURE_DN
GSE24634_TEFF_VS_TCONV_DAY5_IN_CULTURE_DN
125	-0.31369767	-1.261789	0.08384458
0.14377402	1	1766	"tags=22%, list=13%, signal=24%"
GSE25088_CTRL_VS_ROSIGLITAZONE_STIM_MACROPHAGE_UP
GSE25088_CTRL_VS_ROSIGLITAZONE_STIM_MACROPHAGE_UP
138	-0.30458635	-1.2617469	0.05839416
0.14368811	1	1946	"tags=25%, list=14%, signal=29%"
GSE3982_MAC_VS_BCELL_UP	GSE3982_MAC_VS_BCELL_UP
161	-0.3016513	-1.2612003	0.054104477
0.14425786	1	720	"tags=15%, list=5%, signal=16%"
GSE40443_INDUCED_VS_TOTAL_TREG_DN	GSE40443_INDUCED_VS_TOTAL_TREG_DN
	136	-0.3074402	-1.2607272	0.09193246
0.14473122	1	1067	"tags=18%, list=8%, signal=20%"
GSE11961_MEMORY_BCELL_DAY7_VS_MEMORY_BCELL_DAY40_DN
GSE11961_MEMORY_BCELL_DAY7_VS_MEMORY_BCELL_DAY40_DN
147	-0.3083153	-1.2607205	0.06743738
0.14460695	1	2547	"tags=30%, list=18%, signal=36%"
GSE8921_UNSTIM_0H_VS_TLR1_2_STIM_MONOCYTE_24H_DN
GSE8921_UNSTIM_0H_VS_TLR1_2_STIM_MONOCYTE_24H_DN

151	-0.30196738	-1.260643	0.06581353
0.14457493	1	1922	"tags=24%, list=14%, signal=27%"
GSE2585_CTEC_VS_MTEC_THYMUS_DN	GSE2585_CTEC_VS_MTEC_THYMUS_DN
	139	-0.30542955	-1.2603799	0.04819277
0.1447843	1	1871	"tags=22%, list=13%, signal=25%"
GSE43863_TFH_VS_LY6C_LOW_CXCR5NEG_EFFECTOR_CD4_TCELL_UP
GSE43863_TFH_VS_LY6C_LOW_CXCR5NEG_EFFECTOR_CD4_TCELL_UP
148	-0.30563176	-1.2603782	0.053030305
0.14464761	1	1445	"tags=21%, list=10%, signal=23%"
GSE4984_UNTREATED_VS_GALECTIN1_TREATED_DC_DN
GSE4984_UNTREATED_VS_GALECTIN1_TREATED_DC_DN
121	-0.3151856	-1.2591449	0.08560311
0.1461017	1	1409	"tags=20%, list=10%, signal=22%"
GSE17721_CTRL_VS_GARDIQUIMOD_1H_BMDC_DN
GSE17721_CTRL_VS_GARDIQUIMOD_1H_BMDC_DN
156	-0.2989484	-1.2574184	0.06138614
0.1482526	1	1727	"tags=20%, list=12%, signal=22%"
GSE9988_ANTI_TREM1_VS_VEHICLE_TREATED_MONOCYTES_UP
GSE9988_ANTI_TREM1_VS_VEHICLE_TREATED_MONOCYTES_UP
135	-0.31097436	-1.2573432	0.07438017
0.14820392	1	1926	"tags=22%, list=14%, signal=25%"
GSE1791_CTRL_VS_NEUROMEDINU_IN_T_CELL_LINE_0.8H_DN
GSE1791_CTRL_VS_NEUROMEDINU_IN_T_CELL_LINE_0.8H_DN
115	-0.31490934	-1.2572455	0.103846155
0.14819887	1	1770	"tags=21%, list=13%, signal=24%"
GSE12845_NAIVE_VS_DARKZONE_GC_TONSIL_BCELL_UP
GSE12845_NAIVE_VS_DARKZONE_GC_TONSIL_BCELL_UP
133	-0.30715427	-1.2568986	0.06614786
0.1485246	1	3663	"tags=37%, list=26%, signal=49%"
GSE5679_PPARG_LIGAND_ROSIGLITAZONE_VS_RARA_AGONIST_AM580_TREATED_DC_
DN
GSE5679_PPARG_LIGAND_ROSIGLITAZONE_VS_RARA_AGONIST_AM580_TREATED_DC_
DN		146	-0.30425364	-1.2565835
0.071969695	0.14881323	1	2236	"tags=27%,
list=16%, signal=31%"
GSE43863_NAIVE_VS_MEMORY_LY6C_INT_CXCR5POS_CD4_TCELL_D150_LCMV_UP
GSE43863_NAIVE_VS_MEMORY_LY6C_INT_CXCR5POS_CD4_TCELL_D150_LCMV_UP
	143	-0.30844343	-1.2565106	0.088
0.14877929	1	1698	"tags=25%, list=12%, signal=28%"
GSE2706_2H_VS_8H_LPS_STIM_DC_UP	GSE2706_2H_VS_8H_LPS_STIM_DC_UP
	146	-0.30534402	-1.2564396	0.082
0.14873588	1	2113	"tags=24%, list=15%, signal=28%"
GSE11961_GERMINAL_CENTER_BCELL_DAY7_VS_PLASMA_CELL_DAY7_DN
GSE11961_GERMINAL_CENTER_BCELL_DAY7_VS_PLASMA_CELL_DAY7_DN
143	-0.30552873	-1.2561074	0.07279693
0.14904222	1	1800	"tags=27%, list=13%, signal=30%"
GSE36476_CTRL_VS_TSST_ACT_40H_MEMORY_CD4_TCELL_OLD_UP
GSE36476_CTRL_VS_TSST_ACT_40H_MEMORY_CD4_TCELL_OLD_UP
126	-0.31412303	-1.2557474	0.098148145
0.14941436	1	1996	"tags=21%, list=14%, signal=25%"
GSE3982_EOSINOPHIL_VS_NKCELL_UP	GSE3982_EOSINOPHIL_VS_NKCELL_UP
	152	-0.3028532	-1.2554344	0.072407044
0.14969142	1	2648	"tags=25%, list=19%, signal=30%"
GSE13522_CTRL_VS_T_CRUZI_Y_STRAIN_INF_SKIN_IFNAR_KO_DN

GSE13522_CTRL_VS_T_CRUZI_Y_STRAIN_INF_SKIN_IFNAR_KO_DN
149 -0.29948694 -1.254991 0.07114624
0.15015528 1 1498 "tags=19%, list=11%, signal=22%"
GSE22611_MUTANT_NOD2_TRANSDUCED_VS_CTRL_HEK293T_STIMULATED_WITH_MDP_
6H_UP
GSE22611_MUTANT_NOD2_TRANSDUCED_VS_CTRL_HEK293T_STIMULATED_WITH_MDP_
6H_UP 137 -0.30486402 -1.2548347
0.060661763 0.15020652 1 1770 "tags=18%,
list=13%, signal=21%"
GSE20484_MCSG_VS_CXCL4_MONOCYTE_DERIVED_MACROPHAGE_DN
GSE20484_MCSG_VS_CXCL4_MONOCYTE_DERIVED_MACROPHAGE_DN
122 -0.3167839 -1.2530364 0.08851224
0.15252909 1 1371 "tags=17%, list=10%, signal=19%"
GSE22886_NAIVE_VS_IGG_IGA_MEMORY_BCELL_UP
GSE22886_NAIVE_VS_IGG_IGA_MEMORY_BCELL_UP
138 -0.3048553 -1.2524573 0.07285974
0.15314402 1 3350 "tags=32%, list=24%, signal=41%"
GSE10325_CD4_TCELL_VS_BCELL_DN GSE10325_CD4_TCELL_VS_BCELL_DN
130 -0.31016397 -1.2517688 0.07509158
0.15389352 1 2288 "tags=27%, list=16%, signal=32%"
GSE35825_UNTREATED_VS_IFNG_STIM_MACROPHAGE_UP
GSE35825_UNTREATED_VS_IFNG_STIM_MACROPHAGE_UP
157 -0.30312276 -1.2514782 0.07115385
0.15416351 1 1766 "tags=22%, list=13%, signal=24%"
GSE29618_MONOCYTE_VS_PDC_DAY7_FLU_VACCINE_UP
GSE29618_MONOCYTE_VS_PDC_DAY7_FLU_VACCINE_UP
151 -0.30299535 -1.2508626 0.067961164
0.15487604 1 1277 "tags=19%, list=9%, signal=21%"
GSE360_L_MAJOR_VS_B_MALAYI_LOW_DOSE_DC_UP
GSE360_L_MAJOR_VS_B_MALAYI_LOW_DOSE_DC_UP
154 -0.30077085 -1.2506328 0.07948244
0.15501647 1 1498 "tags=19%, list=11%, signal=21%"
GSE21063_CTRL_VS_ANTI_IGM_STIM_BCELL_3H_UP
GSE21063_CTRL_VS_ANTI_IGM_STIM_BCELL_3H_UP
119 -0.31174642 -1.2504581 0.08829175
0.1551145 1 2174 "tags=25%, list=15%, signal=30%"
GSE37534_UNTREATED_VS_GW1929_TREATED_CD4_TCELL_PPARG1_AND_FOXP3_TRAS
DUCED_UP
GSE37534_UNTREATED_VS_GW1929_TREATED_CD4_TCELL_PPARG1_AND_FOXP3_TRAS
DUCED_UP 150 -0.30410329 -1.2503356
0.07238095 0.1551393 1 1867 "tags=24%,
list=13%, signal=27%"
GSE37336_LY6C_POS_VS_NEG_NAIVE_CD4_TCELL_UP
GSE37336_LY6C_POS_VS_NEG_NAIVE_CD4_TCELL_UP
110 -0.31669837 -1.250283 0.07927928
0.15506484 1 3664 "tags=32%, list=26%, signal=43%"
GSE4590_LARGE_PRE_BCELL_VS_VPREB_POS_LARGE_PRE_BCELL_DN
GSE4590_LARGE_PRE_BCELL_VS_VPREB_POS_LARGE_PRE_BCELL_DN
142 -0.30570525 -1.250025 0.073033705
0.15528305 1 2170 "tags=25%, list=15%, signal=29%"
GSE22601_DOUBLE_POSITIVE_VS_CD8_SINGLE_POSITIVE_THYMOCYTE_UP
GSE22601_DOUBLE_POSITIVE_VS_CD8_SINGLE_POSITIVE_THYMOCYTE_UP
140 -0.30748403 -1.249832 0.087649405
0.1553982 1 1719 "tags=21%, list=12%, signal=23%"

GSE8921_UNSTIM_VS_TLR1_2_STIM_MONOCYTE_12H_UP
GSE8921_UNSTIM_VS_TLR1_2_STIM_MONOCYTE_12H_UP
150 -0.30288365 -1.2493008 0.06403013
0.15600306 1 1744 "tags=21%, list=12%, signal=23%"
GSE339_CD8POS_VS_CD4CD8DN_DC_DN GSE339_CD8POS_VS_CD4CD8DN_DC_DN
160 -0.2999063 -1.24865 0.073126145
0.15676595 1 1736 "tags=21%, list=12%, signal=24%"
GSE18281_CORTICAL_VS_MEDULLARY_THYMOCYTE_DN
GSE18281_CORTICAL_VS_MEDULLARY_THYMOCYTE_DN
139 -0.30776468 -1.2484633 0.09222423
0.15685679 1 1842 "tags=17%, list=13%, signal=19%"
GSE19888_ADENOSINE_A3R_INH_VS_TCELL_MEMBRANES_ACT_MAST_CELL_UP
GSE19888_ADENOSINE_A3R_INH_VS_TCELL_MEMBRANES_ACT_MAST_CELL_UP
136 -0.30563617 -1.2480031 0.077634014
0.15736148 1 1766 "tags=23%, list=13%, signal=26%"
GSE36476_CTRL_VS_TSST_ACT_40H_MEMORY_CD4_TCELL_YOUNG_UP
GSE36476_CTRL_VS_TSST_ACT_40H_MEMORY_CD4_TCELL_YOUNG_UP
134 -0.30659455 -1.2478414 0.07372401
0.15743329 1 1719 "tags=24%, list=12%, signal=27%"
GSE18203_CTRL_VS_INTRATUMORAL_CPG_INJ_MC38_TUMOR_UP
GSE18203_CTRL_VS_INTRATUMORAL_CPG_INJ_MC38_TUMOR_UP
147 -0.29905704 -1.2477186 0.061913695
0.15746933 1 1279 "tags=17%, list=9%, signal=19%"
GSE6259_FLT3L_INDUCED_DEC205_POS_DC_VS_BCELL_UP
GSE6259_FLT3L_INDUCED_DEC205_POS_DC_VS_BCELL_UP
119 -0.3042479 -1.247619 0.09881423
0.15746534 1 2385 "tags=24%, list=17%, signal=29%"
GSE42021_CD24HI_VS_CD24LOW_TCONV_THYMUS_DN
GSE42021_CD24HI_VS_CD24LOW_TCONV_THYMUS_DN
141 -0.30616754 -1.247485 0.08997955
0.15750702 1 2215 "tags=28%, list=16%, signal=33%"
GSE7348_UNSTIM_VS_TOLERIZED_AND_LPS_STIM_MACROPHAGE_UP
GSE7348_UNSTIM_VS_TOLERIZED_AND_LPS_STIM_MACROPHAGE_UP
118 -0.31390458 -1.2471135 0.09039548
0.15784734 1 2918 "tags=31%, list=21%, signal=38%"
GSE6681_DELETED_FOXP3_VS_WT_TREG_UP
GSE6681_DELETED_FOXP3_VS_WT_TREG_UP
129 -0.30782005 -1.2470199 0.079303674
0.15783529 1 2648 "tags=18%, list=19%, signal=22%"
GSE19888_CTRL_VS_T_CELL_MEMBRANES_ACT_MAST_CELL_DN
GSE19888_CTRL_VS_T_CELL_MEMBRANES_ACT_MAST_CELL_DN
148 -0.30050495 -1.2467533 0.060037524
0.15802519 1 2372 "tags=23%, list=17%, signal=27%"
GSE19923_E2A_KO_VS_HEB_AND_E2A_KO_DP_THYMOCYTE_DN
GSE19923_E2A_KO_VS_HEB_AND_E2A_KO_DP_THYMOCYTE_DN
114 -0.3112518 -1.2466048 0.09608541
0.15808961 1 4525 "tags=39%, list=32%, signal=56%"
GSE14026_TH1_VS_TH17_DN GSE14026_TH1_VS_TH17_DN
143 -0.30194935 -1.2465669 0.09266409
0.15800482 1 2732 "tags=24%, list=19%, signal=30%"
GSE3982_BCELL_VS_EFF_MEMORY_CD4_TCELL_UP
GSE3982_BCELL_VS_EFF_MEMORY_CD4_TCELL_UP
140 -0.30275428 -1.2463081 0.07581227
0.15819588 1 2133 "tags=21%, list=15%, signal=24%"

GSE9960_GRAM_NEG_VS_GRAM_POS_SEPSIS_PBMC_DN
GSE9960_GRAM_NEG_VS_GRAM_POS_SEPSIS_PBMC_DN
138 -0.30239713 -1.2458395 0.0626186
0.1587343 1 1196 "tags=17%, list=8%, signal=19%"
GSE45365_NK_CELL_VS_BCELL_UP GSE45365_NK_CELL_VS_BCELL_UP
148 -0.30068925 -1.2456917 0.09193246
0.15880005 1 2140 "tags=26%, list=15%, signal=30%"
GSE7460_CTRL_VS_TGFB_TREATED_ACT_FOXP3_MUT_TCONV_UP
GSE7460_CTRL_VS_TGFB_TREATED_ACT_FOXP3_MUT_TCONV_UP
139 -0.30538046 -1.2445728 0.08429119
0.16022137 1 1527 "tags=17%, list=11%, signal=19%"
GSE13229_MATURE_VS_INTMATURE_NKCELL_UP
GSE13229_MATURE_VS_INTMATURE_NKCELL_UP
124 -0.30694413 -1.2442236 0.09001957
0.1605785 1 2471 "tags=21%, list=18%, signal=25%"
GSE42021_TREG_PLN_VS_TREG_PRECURSORS_THYMUS_DN
GSE42021_TREG_PLN_VS_TREG_PRECURSORS_THYMUS_DN
137 -0.3022377 -1.2439574 0.084870845
0.16078626 1 1841 "tags=23%, list=13%, signal=26%"
GSE25088_WT_VS_STAT6_KO_MACROPHAGE_ROSIGLITAZONE_STIM_DN
GSE25088_WT_VS_STAT6_KO_MACROPHAGE_ROSIGLITAZONE_STIM_DN
134 -0.30631045 -1.2437898 0.09126984
0.16086242 1 1480 "tags=17%, list=10%, signal=19%"
GSE12001_MIR223_KO_VS_WT_NEUTROPHIL_DN
GSE12001_MIR223_KO_VS_WT_NEUTROPHIL_DN
138 -0.30337164 -1.2421998 0.088176355
0.16292772 1 1954 "tags=24%, list=14%, signal=27%"
GSE5542_IFNA_VS_IFNA_AND_IFNG_TREATED_EPITHELIAL_CELLS_24H_UP
GSE5542_IFNA_VS_IFNA_AND_IFNG_TREATED_EPITHELIAL_CELLS_24H_UP
135 -0.30587474 -1.2417873 0.09423077
0.163387 1 2901 "tags=29%, list=21%, signal=36%"
GSE40274_SATB1_VS_FOXP3_AND_SATB1_TRANSDUCED_ACTIVATED_CD4_TCELL_DN
GSE40274_SATB1_VS_FOXP3_AND_SATB1_TRANSDUCED_ACTIVATED_CD4_TCELL_DN
86 -0.32188147 -1.2413825 0.115530305
0.16385604 1 2885 "tags=31%, list=20%, signal=39%"
GSE27241_WT_CTRL_VS_DIGOXIN_TREATED_RORGT_KO_CD4_TCELL_IN_TH17_POLARIZING_CONDITIONS_DN
GSE27241_WT_CTRL_VS_DIGOXIN_TREATED_RORGT_KO_CD4_TCELL_IN_TH17_POLARIZING_CONDITIONS_DN
132 -0.3044781 -1.2411473 0.08990826
0.16403149 1 1855 "tags=21%, list=13%, signal=24%"
GSE26488_WT_VS_VP16_TRANSGENIC_HDAC7_KO_DOUBLE_POSITIVE_THYMOCYTE_UP

GSE26488_WT_VS_VP16_TRANSGENIC_HDAC7_KO_DOUBLE_POSITIVE_THYMOCYTE_UP
158 -0.30097985 -1.2411416
0.07456979 0.1638941 1 3338 "tags=32%, list=24%, signal=42%"
GSE32901_TH17_EMRICHED_VS_TH17_NEG_CD4_TCELL_UP
GSE32901_TH17_EMRICHED_VS_TH17_NEG_CD4_TCELL_UP
94 -0.32611218 -1.240977 0.107344635
0.1640119 1 2362 "tags=20%, list=17%, signal=24%"
GSE22886_TH1_VS_TH2_48H_ACT_UP GSE22886_TH1_VS_TH2_48H_ACT_UP
156 -0.2931402 -1.2408177 0.062381852
0.16409269 1 3384 "tags=28%, list=24%, signal=36%"

GSE5542_UNTREATED_VS_IFNA_AND_IFNG_TREATED_EPITHELIAL_CELLS_24H_DN
GSE5542_UNTREATED_VS_IFNA_AND_IFNG_TREATED_EPITHELIAL_CELLS_24H_DN
148 -0.299834 -1.2406403 0.08023483
0.16420412 1 2404 "tags=25%, list=17%, signal=30%"
GSE39820_CTRL_VS_TGFBETA3_IL6_CD4_TCELL_DN
GSE39820_CTRL_VS_TGFBETA3_IL6_CD4_TCELL_DN
147 -0.29887515 -1.2398491 0.08015267
0.16521569 1 1882 "tags=21%, list=13%, signal=24%"
GSE5099_UNSTIM_VS_MCSF_TREATED_MONOCYTE_DAY3_UP
GSE5099_UNSTIM_VS_MCSF_TREATED_MONOCYTE_DAY3_UP
136 -0.30536214 -1.2396958 0.09315589 0.165278
1 1719 "tags=21%, list=12%, signal=24%"
GSE29618_MONOCYTE_VS_MDC_DAY7_FLU_VACCINE_UP
GSE29618_MONOCYTE_VS_MDC_DAY7_FLU_VACCINE_UP
156 -0.29420984 -1.2395196 0.09056604
0.1653649 1 2084 "tags=22%, list=15%, signal=26%"
GSE22886_TH1_VS_TH2_48H_ACT_DN GSE22886_TH1_VS_TH2_48H_ACT_DN
142 -0.30268672 -1.2393891 0.09803922
0.16539943 1 1299 "tags=18%, list=9%, signal=20%"
GSE19198_CTRL_VS_IL21_TREATED_TCELL_6H_DN
GSE19198_CTRL_VS_IL21_TREATED_TCELL_6H_DN
137 -0.30182976 -1.2390233 0.082 0.16580155
1 2091 "tags=24%, list=15%, signal=28%"
GSE36392_EOSINOPHIL_VS_NEUTROPHIL_IL25_TREATED_LUNG_UP
GSE36392_EOSINOPHIL_VS_NEUTROPHIL_IL25_TREATED_LUNG_UP
160 -0.29764673 -1.2389052 0.08007449
0.16582507 1 2261 "tags=26%, list=16%, signal=31%"
GSE12198_LOW_IL2_STIM_NK_CELL_VS_HIGH_IL2_STIM_NK_CELL_UP
GSE12198_LOW_IL2_STIM_NK_CELL_VS_HIGH_IL2_STIM_NK_CELL_UP
143 -0.29868063 -1.2386259 0.086629 0.16609314
1 3128 "tags=32%, list=22%, signal=41%"
GSE30153_LUPUS_VS_HEALTHY_DONOR_BCELL_UP
GSE30153_LUPUS_VS_HEALTHY_DONOR_BCELL_UP
143 -0.3021259 -1.2382553 0.07622504
0.1665074 1 832 "tags=14%, list=6%, signal=15%"
GSE19198_1H_VS_24H_IL21_TREATED_TCELL_DN
GSE19198_1H_VS_24H_IL21_TREATED_TCELL_DN
139 -0.30339342 -1.2380362 0.08734403
0.16667803 1 2278 "tags=22%, list=16%, signal=26%"
GSE17721_PAM3CSK4_VS_GADIQUIMOD_2H_BMDC_UP
GSE17721_PAM3CSK4_VS_GADIQUIMOD_2H_BMDC_UP
167 -0.2934609 -1.2380008 0.07224335
0.16656846 1 2032 "tags=21%, list=14%, signal=24%"
GSE18791_CTRL_VS_NEWCASTLE_VIRUS_DC_18H_DN
GSE18791_CTRL_VS_NEWCASTLE_VIRUS_DC_18H_DN
113 -0.31135216 -1.2373996 0.10344828
0.16725446 1 2377 "tags=27%, list=17%, signal=33%"
GSE15330_GRANULOCYTE_MONOCYTE_PROGENITOR_VS_PRO_BCELL_DN
GSE15330_GRANULOCYTE_MONOCYTE_PROGENITOR_VS_PRO_BCELL_DN
152 -0.29749817 -1.2369593 0.09019608
0.16773961 1 2059 "tags=21%, list=15%, signal=24%"
GSE21927_SPLEEN_VS_BONE_MARROW_MONOCYTE_BALBC_UP
GSE21927_SPLEEN_VS_BONE_MARROW_MONOCYTE_BALBC_UP
129 -0.3049291 -1.2367914 0.08382066

0.1678119	1	3110	"tags=27%, list=22%, signal=34%"
GSE25502_WT_VS_KLF13_KO_THYMIC_MEMORY_LIKE_CD8_TCELL_DN
GSE25502_WT_VS_KLF13_KO_THYMIC_MEMORY_LIKE_CD8_TCELL_DN
111	-0.31377748	-1.2366945	0.10153256
0.16781063	1	5032	"tags=41%, list=36%, signal=63%"
GSE22886_IGM_MEMORY_BCELL_VS_BLOOD_PLASMA_CELL_UP
GSE22886_IGM_MEMORY_BCELL_VS_BLOOD_PLASMA_CELL_UP
122	-0.30549622	-1.2366596	0.09881423
0.16771032	1	4475	"tags=43%, list=32%, signal=63%"
GSE9946_MATURE_STIMULATORY_VS_PROSTAGLANDINE2_TREATED_MATURE_DC_DN
GSE9946_MATURE_STIMULATORY_VS_PROSTAGLANDINE2_TREATED_MATURE_DC_DN
	98	-0.3191417	-1.2365967	0.09497207
0.16764636	1	5025	"tags=41%, list=36%, signal=63%"
GSE2706_2H_VS_8H_R848_STIM_DC_DN	GSE2706_2H_VS_8H_R848_STIM_DC_DN
	122	-0.3056386	-1.2364935	0.08969466
0.16764112	1	781	"tags=13%, list=6%, signal=14%"
GSE33424_CD161_HIGH_VS_INT_CD8_TCELL_UP
GSE33424_CD161_HIGH_VS_INT_CD8_TCELL_UP
155	-0.29911095	-1.2361745	0.08416834
0.16797867	1	1759	"tags=25%, list=12%, signal=28%"
GSE29949_CD8_POS_DC_SPLEEN_VS_MONOCYTE_BONE_MARROW_UP
GSE29949_CD8_POS_DC_SPLEEN_VS_MONOCYTE_BONE_MARROW_UP
150	-0.29529753	-1.2356497	0.08608059
0.16854541	1	938	"tags=11%, list=7%, signal=12%"
GSE9988_LPS_VS_LPS_AND_ANTI_TREM1_MONOCYTE_DN
GSE9988_LPS_VS_LPS_AND_ANTI_TREM1_MONOCYTE_DN
132	-0.30418432	-1.2352034	0.08365019
0.16907081	1	1367	"tags=19%, list=10%, signal=21%"
GSE17974_IL4_AND_ANTI_IL12_VS_UNTREATED_2H_ACT_CD4_TCELL_DN
GSE17974_IL4_AND_ANTI_IL12_VS_UNTREATED_2H_ACT_CD4_TCELL_DN
114	-0.30964804	-1.2351508	0.108411215
0.16899608	1	2807	"tags=27%, list=20%, signal=34%"
GSE15659_TREG_VS_TCONV_DN	GSE15659_TREG_VS_TCONV_DN
112	-0.31130934	-1.2349855	0.10344828
0.16906562	1	2641	"tags=26%, list=19%, signal=32%"
GSE17974_IL4_AND_ANTI_IL12_VS_UNTREATED_12H_ACT_CD4_TCELL_UP
GSE17974_IL4_AND_ANTI_IL12_VS_UNTREATED_12H_ACT_CD4_TCELL_UP
	125	-0.3053889	-1.2345158	0.087037034
0.16962047	1	2087	"tags=22%, list=15%, signal=26%"
GSE41867_NAIVE_VS_DAY8_LCMV_ARMSTRONG_EFFECTOR_CD8_TCELL_DN
GSE41867_NAIVE_VS_DAY8_LCMV_ARMSTRONG_EFFECTOR_CD8_TCELL_DN
147	-0.30000383	-1.23431	0.09039548	0.16976677
1	2876	"tags=30%, list=20%, signal=37%"
GSE26343_WT_VS_NFAT5_KO_MACROPHAGE_DN
GSE26343_WT_VS_NFAT5_KO_MACROPHAGE_DN
156	-0.29494694	-1.2340273	0.077067666
0.17003508	1	2501	"tags=31%, list=18%, signal=38%"
GSE2770_TGFB_AND_IL4_VS_TGFB_AND_IL12_TREATED_ACT_CD4_TCELL_6H_DN
GSE2770_TGFB_AND_IL4_VS_TGFB_AND_IL12_TREATED_ACT_CD4_TCELL_6H_DN
	141	-0.2992035	-1.2334027	0.08576998
0.17083445	1	1278	"tags=16%, list=9%, signal=18%"
GSE17721_0.5H_VS_4H_CPG_BMDC_DN	GSE17721_0.5H_VS_4H_CPG_BMDC_DN
	159	-0.29467228	-1.233093	0.08527132
0.17110543	1	1467	"tags=18%, list=10%, signal=19%"

GSE7852_LN_VS_FAT_TREG_DN GSE7852_LN_VS_FAT_TREG_DN
155 -0.29762566 -1.2329758 0.07089552
0.1711408 1 2496 "tags=27%, list=18%, signal=33%"
GSE17721_LPS_VS_GARDIQUIMOD_24H_BMDC_UP
GSE17721_LPS_VS_GARDIQUIMOD_24H_BMDC_UP
149 -0.29767317 -1.2322341 0.08285164
0.17208706 1 1829 "tags=20%, list=13%, signal=23%"
GSE11057_CD4_CENT_MEM_VS_PBMC_UP GSE11057_CD4_CENT_MEM_VS_PBMC_UP
136 -0.30295482 -1.232059 0.10326087
0.1722052 1 1250 "tags=18%, list=9%, signal=19%"
GSE27786_CD4_TCELL_VS_NKCELL_UP GSE27786_CD4_TCELL_VS_NKCELL_UP
123 -0.3101608 -1.2319362 0.11389961
0.17222755 1 1803 "tags=24%, list=13%, signal=27%"
GSE21360_NAIVE_VS_QUATERNARY_MEMORY_CD8_TCELL_UP
GSE21360_NAIVE_VS_QUATERNARY_MEMORY_CD8_TCELL_UP
148 -0.2962889 -1.231856 0.074 0.17220274
1 2278 "tags=25%, list=16%, signal=29%"
GSE45881_CXCR6HI_VS_CXCR1LO_COLONIC_LAMINA_PROPRIA_UP
GSE45881_CXCR6HI_VS_CXCR1LO_COLONIC_LAMINA_PROPRIA_UP
122 -0.30624062 -1.2317897 0.096834265
0.17215274 1 2946 "tags=30%, list=21%, signal=37%"
GSE10325_CD4_TCELL_VS_MYELOID_UP GSE10325_CD4_TCELL_VS_MYELOID_UP
152 -0.29603916 -1.2315809 0.09055118
0.17231351 1 1837 "tags=21%, list=13%, signal=24%"
GSE21678_WT_VS_FOXO1_FOXO3_KO_TREG_DN
GSE21678_WT_VS_FOXO1_FOXO3_KO_TREG_DN
112 -0.3100942 -1.2312095 0.09869646
0.17271239 1 2030 "tags=26%, list=14%, signal=30%"
GSE18791_CTRL_VS_NEWCASTLE_VIRUS_DC_1H_UP
GSE18791_CTRL_VS_NEWCASTLE_VIRUS_DC_1H_UP
103 -0.3124181 -1.2303623 0.10536044
0.17384893 1 5424 "tags=49%, list=38%, signal=78%"
GSE37533_PPARG1_FOXP3_VS_FOXP3_TRANSDUCED_CD4_TCELL_DN
GSE37533_PPARG1_FOXP3_VS_FOXP3_TRANSDUCED_CD4_TCELL_DN
137 -0.2981355 -1.2303131 0.09210526
0.17375779 1 1817 "tags=23%, list=13%, signal=27%"
GSE32423_CTRL_VS_IL4_MEMORY_CD8_TCELL_UP
GSE32423_CTRL_VS_IL4_MEMORY_CD8_TCELL_UP
139 -0.30208826 -1.2301512 0.09775967
0.17383792 1 2022 "tags=23%, list=14%, signal=27%"
GSE43955_1H_VS_20H_ACT_CD4_TCELL_WITH_TGFB_IL6_DN
GSE43955_1H_VS_20H_ACT_CD4_TCELL_WITH_TGFB_IL6_DN
169 -0.2920538 -1.2299428 0.07879925
0.17398995 1 3136 "tags=31%, list=22%, signal=40%"
GSE2706_UNSTIM_VS_8H_LPS_DC_DN GSE2706_UNSTIM_VS_8H_LPS_DC_DN
131 -0.30071437 -1.22907 0.096339114
0.17516649 1 2081 "tags=24%, list=15%, signal=27%"
GSE27291_6H_VS_7D_STIM_GAMMADELTA_TCELL_DN
GSE27291_6H_VS_7D_STIM_GAMMADELTA_TCELL_DN
114 -0.30672193 -1.2289894 0.1003937
0.1751328 1 2933 "tags=30%, list=21%, signal=37%"
GSE21360_TERTIARY_VS_QUATERNARY_MEMORY_CD8_TCELL_DN
GSE21360_TERTIARY_VS_QUATERNARY_MEMORY_CD8_TCELL_DN
147 -0.29761064 -1.2286974 0.106589146

0.17540501	1	2433	"tags=27%, list=17%, signal=32%"
GSE17721_LPS_VS_CPG_4H_BMDC_UP	GSE17721_LPS_VS_CPG_4H_BMDC_UP
	157	-0.29289433	-1.2281048	0.08102767
0.17613779	1	2038	"tags=24%, list=14%, signal=27%"
GSE32901_TH1_VS_TH17_ENRICHED_CD4_TCELL_UP
GSE32901_TH1_VS_TH17_ENRICHED_CD4_TCELL_UP
137	-0.30053493	-1.2276468	0.08856089
0.1766536	1	2097	"tags=25%, list=15%, signal=29%"
GSE40274_CTRL_VS_GATA1_TRANSDUCED_ACTIVATED_CD4_TCELL_UP
GSE40274_CTRL_VS_GATA1_TRANSDUCED_ACTIVATED_CD4_TCELL_UP
106	-0.30977634	-1.2273424	0.12922466
0.17697795	1	1357	"tags=17%, list=10%, signal=19%"
GSE43956_WT_VS_SGK1_KO_TH17_DIFFERENTIATED_CD4_TCELL_DN
GSE43956_WT_VS_SGK1_KO_TH17_DIFFERENTIATED_CD4_TCELL_DN
151	-0.2954825	-1.227149	0.09158879
0.17710726	1	1328	"tags=17%, list=9%, signal=18%"
GSE21546_WT_VS_SAP1A_KO_DP_THYMOCYTES_UP
GSE21546_WT_VS_SAP1A_KO_DP_THYMOCYTES_UP
124	-0.30496922	-1.2271148	0.119544595
0.17700896	1	1775	"tags=22%, list=13%, signal=25%"
GSE41978_ID2_KO_AND_BIM_KO_VS_BIM_KO_KLRG1_LOW_EFFECTOR_CD8_TCELL_DN

GSE41978_ID2_KO_AND_BIM_KO_VS_BIM_KO_KLRG1_LOW_EFFECTOR_CD8_TCELL_DN
		142	-0.3010641	-1.2268234
0.087272726	0.17730781	1	2507	"tags=27%,
list=18%, signal=33%"
GSE22886_DC_VS_MONOCYTE_DN	GSE22886_DC_VS_MONOCYTE_DN
	133	-0.30208862	-1.226051	0.11088296
0.1782823	1	2536	"tags=28%, list=18%, signal=34%"
GSE19401_UNSTIM_VS_RETINOIC_ACID_AND_PAM2CSK4_STIM_FOLLICULAR_DC_DN
GSE19401_UNSTIM_VS_RETINOIC_ACID_AND_PAM2CSK4_STIM_FOLLICULAR_DC_DN
	165	-0.29128382	-1.2257432	0.085192695
0.17858887	1	1428	"tags=17%, list=10%, signal=19%"
GSE19888_CTRL_VS_TCELL_MEMBRANES_ACT_MAST_CELL_PRETREAT_A3R_INH_DN
GSE19888_CTRL_VS_TCELL_MEMBRANES_ACT_MAST_CELL_PRETREAT_A3R_INH_DN
	143	-0.30023965	-1.2256824	0.0961183
0.17852762	1	1770	"tags=22%, list=13%, signal=25%"
GSE20366_TREG_VS_NAIVE_CD4_TCELL_UP
GSE20366_TREG_VS_NAIVE_CD4_TCELL_UP
141	-0.29931247	-1.2255603	0.10721248
0.17856862	1	2085	"tags=28%, list=15%, signal=32%"
GSE3203_UNTREATED_VS_IFNB_TREATED_LN_BCELL_DN
GSE3203_UNTREATED_VS_IFNB_TREATED_LN_BCELL_DN
132	-0.29988226	-1.225453	0.09193246
0.17856617	1	2297	"tags=23%, list=16%, signal=28%"
GSE27786_BCELL_VS_NKCELL_DN	GSE27786_BCELL_VS_NKCELL_DN
	154	-0.29507127	-1.2250487	0.09195402
0.17905198	1	1259	"tags=18%, list=9%, signal=19%"
GSE13485_CTRL_VS_DAY3_YF17D_VACCINE_PBMC_DN
GSE13485_CTRL_VS_DAY3_YF17D_VACCINE_PBMC_DN
129	-0.29995486	-1.2246616	0.10444874
0.17946462	1	1766	"tags=24%, list=13%, signal=27%"
GSE40225_WT_VS_RIP_B7X_DIABETIC_MOUSE_PANCREATIC_CD8_TCELL_UP
GSE40225_WT_VS_RIP_B7X_DIABETIC_MOUSE_PANCREATIC_CD8_TCELL_UP

119	-0.30640516	-1.2245208	0.108778626
0.17950669	1	1612	"tags=19%, list=11%, signal=22%"
GSE21033_CTRL_VS_POLYIC_STIM_DC_6H_DN
GSE21033_CTRL_VS_POLYIC_STIM_DC_6H_DN
107	-0.30990204	-1.2244854	0.11324376
0.17940423	1	1510	"tags=16%, list=11%, signal=18%"
GSE12198_NK_VS_NK_ACT_EXPANSION_SYSTEM_DERIVED_NK_CELL_UP
GSE12198_NK_VS_NK_ACT_EXPANSION_SYSTEM_DERIVED_NK_CELL_UP
146	-0.29803926	-1.2244545	0.10037175
0.17929779	1	1712	"tags=21%, list=12%, signal=23%"
GSE29949_MICROGLIA_BRAIN_VS_CD8_NEG_DC_SPLEEN_UP
GSE29949_MICROGLIA_BRAIN_VS_CD8_NEG_DC_SPLEEN_UP
147	-0.295315	-1.2243435	0.09090909
0.17932141	1	1391	"tags=16%, list=10%, signal=17%"
GSE3982_NKCELL_VS_TH2_UP	GSE3982_NKCELL_VS_TH2_UP
141	-0.29804215	-1.2243183	0.09586466
0.17921585	1	4898	"tags=47%, list=35%, signal=71%"
GSE21546_SAP1A_KO_VS_SAP1A_KO_AND_ELK1_KO_ANTI_CD3_STIM_DP_THYMOCYTE
S_DN
GSE21546_SAP1A_KO_VS_SAP1A_KO_AND_ELK1_KO_ANTI_CD3_STIM_DP_THYMOCYTE
S_DN	157	-0.2941895	-1.2236695
0.09861933	0.18005006	1	2287	"tags=27%,
list=16%, signal=32%"
GSE5589_LPS_VS_LPS_AND_IL10_STIM_IL6_KO_MACROPHAGE_45MIN_UP
GSE5589_LPS_VS_LPS_AND_IL10_STIM_IL6_KO_MACROPHAGE_45MIN_UP
139	-0.29805937	-1.2234976	0.0952381
0.18016763	1	2087	"tags=22%, list=15%, signal=26%"
GSE17721_LPS_VS_CPG_1H_BMDC_DN	GSE17721_LPS_VS_CPG_1H_BMDC_DN
167	-0.2917161	-1.2231857	0.08366534
0.18051933	1	1800	"tags=21%, list=13%, signal=24%"
GSE22282_HYPOXIA_VS_NORMOXIA_MYELOID_DC_DN
GSE22282_HYPOXIA_VS_NORMOXIA_MYELOID_DC_DN
134	-0.2965684	-1.2216551	0.10700389
0.18267424	1	1309	"tags=15%, list=9%, signal=16%"
GSE25123_ROSIGLITAZONE_VS_IL4_AND_ROSIGLITAZONE_STIM_MACROPHAGE_DAY1
0_DN
GSE25123_ROSIGLITAZONE_VS_IL4_AND_ROSIGLITAZONE_STIM_MACROPHAGE_DAY1
0_DN	86	-0.32178506	-1.221208
0.14392523	0.18320648	1	2950	"tags=30%,
list=21%, signal=38%"
GSE21360_NAIVE_VS_QUATERNARY_MEMORY_CD8_TCELL_DN
GSE21360_NAIVE_VS_QUATERNARY_MEMORY_CD8_TCELL_DN
145	-0.29445603	-1.2208871	0.08704062
0.18353215	1	1912	"tags=23%, list=14%, signal=27%"
GSE42021_TREG_PLN_VS_CD24INT_TREG_THYMUS_DN
GSE42021_TREG_PLN_VS_CD24INT_TREG_THYMUS_DN
134	-0.29847062	-1.2208867	0.10694184
0.18337624	1	1108	"tags=18%, list=8%, signal=19%"
GSE22886_NAIVE_BCELL_VS_BM_PLASMA_CELL_DN
GSE22886_NAIVE_BCELL_VS_BM_PLASMA_CELL_DN
153	-0.29051885	-1.2208141	0.10625	0.18334615
1	1038	"tags=17%, list=7%, signal=18%"
GSE16385_ROSIGLITAZONE_IL4_VS_IFNG_TNF_STIM_MACROPHAGE_DN
GSE16385_ROSIGLITAZONE_IL4_VS_IFNG_TNF_STIM_MACROPHAGE_DN

143 -0.29628575 -1.2202307 0.096525095
0.18409523 1 5750 "tags=55%, list=41%, signal=92%"
GSE339_EX_VIVO_VS_IN_CULTURE_CD4POS_DC_DN
GSE339_EX_VIVO_VS_IN_CULTURE_CD4POS_DC_DN
149 -0.29183766 -1.2190973 0.09784736
0.18571015 1 2241 "tags=24%, list=16%, signal=28%"
GSE16451_CTRL_VS_WEST_EQUINE_ENC_VIRUS_IMMATURE_NEURON_CELL_LINE_UP
GSE16451_CTRL_VS_WEST_EQUINE_ENC_VIRUS_IMMATURE_NEURON_CELL_LINE_UP
154 -0.29178905 -1.2188597 0.08039216
0.18593152 1 2283 "tags=24%, list=16%, signal=28%"
GSE23321_CD8_STEM_CELL_MEMORY_VS_CENTRAL_MEMORY_CD8_TCELL_UP
GSE23321_CD8_STEM_CELL_MEMORY_VS_CENTRAL_MEMORY_CD8_TCELL_UP
145 -0.2971457 -1.2186779 0.09416196
0.18605585 1 3964 "tags=34%, list=28%, signal=47%"
GSE22886_CTRL_VS_LPS_24H_DC_DN GSE22886_CTRL_VS_LPS_24H_DC_DN
141 -0.29661605 -1.2184187 0.11111111
0.1862725 1 1158 "tags=21%, list=8%, signal=22%"
GSE22886_DAY0_VS_DAY7_MONOCYTE_IN_CULTURE_UP
GSE22886_DAY0_VS_DAY7_MONOCYTE_IN_CULTURE_UP
135 -0.3031358 -1.2183642 0.10377359
0.1862146 1 2501 "tags=31%, list=18%, signal=37%"
GSE28726_ACT_CD4_TCELL_VS_ACT_NKTCELL_DN
GSE28726_ACT_CD4_TCELL_VS_ACT_NKTCELL_DN
142 -0.2958838 -1.2183388 0.09293681
0.18610546 1 2457 "tags=26%, list=17%, signal=31%"
GSE6269_HEALTHY_VS_E_COLI_INF_PBMC_UP
GSE6269_HEALTHY_VS_E_COLI_INF_PBMC_UP
110 -0.30920735 -1.2182595 0.10748561
0.18606132 1 2685 "tags=27%, list=19%, signal=33%"
GSE43863_NAIVE_VS_TH1_EFF_CD4_TCELL_D6_LCMV_DN
GSE43863_NAIVE_VS_TH1_EFF_CD4_TCELL_D6_LCMV_DN
98 -0.31768626 -1.2180753 0.12523021
0.18619914 1 2457 "tags=22%, list=17%, signal=27%"
GSE1740_MCSF_VS_MCSF_AND_IFNG_DAY2_DERIVED_MACROPHAGE_DN
GSE1740_MCSF_VS_MCSF_AND_IFNG_DAY2_DERIVED_MACROPHAGE_DN
124 -0.3022222 -1.2179756 0.12915128
0.1861904 1 2339 "tags=26%, list=17%, signal=31%"
GSE17580_TREG_VS_TEFF_S_MANSONI_INF_DN
GSE17580_TREG_VS_TEFF_S_MANSONI_INF_DN
151 -0.295851 -1.217721 0.08364312
0.18644662 1 2237 "tags=26%, list=16%, signal=31%"
GSE22025_TGFB1_VS_TGFB1_AND_PROGESTERONE_TREATED_CD4_TCELL_UP
GSE22025_TGFB1_VS_TGFB1_AND_PROGESTERONE_TREATED_CD4_TCELL_UP
147 -0.29354265 -1.2176249 0.08514493
0.1864482 1 1055 "tags=14%, list=7%, signal=15%"
GSE2405_0H_VS_3H_A_PHAGOCYTOPHILUM_STIM_NEUTROPHIL_UP
GSE2405_0H_VS_3H_A_PHAGOCYTOPHILUM_STIM_NEUTROPHIL_UP
161 -0.29255617 -1.2175921 0.07954545
0.18635195 1 3910 "tags=35%, list=28%, signal=48%"
GSE17721_CTRL_VS_PAM3CSK4_8H_BMDC_DN
GSE17721_CTRL_VS_PAM3CSK4_8H_BMDC_DN
160 -0.28921852 -1.2174057 0.10547667
0.18648967 1 2081 "tags=24%, list=15%, signal=28%"
GSE14908_RESTING_VS_HDM_STIM_CD4_TCELL_NONATOPIC_PATIENT_UP

GSE14908_RESTING_VS_HDM_STIM_CD4_TCELL_NONATOPIC_PATIENT_UP
138 -0.29651758 -1.2167991 0.10707804
0.18728542 1 2182 "tags=24%, list=15%, signal=28%"
GSE22886_DAY0_VS_DAY1_MONOCYTE_IN_CULTURE_DN
GSE22886_DAY0_VS_DAY1_MONOCYTE_IN_CULTURE_DN
161 -0.28619716 -1.2165294 0.10018553
0.18754542 1 1712 "tags=20%, list=12%, signal=23%"
GSE21546_UNSTIM_VS_ANTI_CD3_STIM_SAP1A_KO_AND_ELK1_KO_DP_THYMOCYTES_UP
GSE21546_UNSTIM_VS_ANTI_CD3_STIM_SAP1A_KO_AND_ELK1_KO_DP_THYMOCYTES_UP 106 -0.3066124 -1.2161599
0.12859885 0.18795972 1 2097 "tags=24%, list=15%, signal=27%"
GSE17721_0.5H_VS_4H_GARDIQUIMOD_BMDC_UP
GSE17721_0.5H_VS_4H_GARDIQUIMOD_BMDC_UP
143 -0.29470196 -1.2157917 0.104166664
0.18838009 1 1679 "tags=21%, list=12%, signal=24%"
GSE40443_INDUCED_VS_TOTAL_TREG_UP GSE40443_INDUCED_VS_TOTAL_TREG_UP
157 -0.28647634 -1.2153515 0.08416834
0.18891478 1 4026 "tags=34%, list=29%, signal=48%"
GSE46606_UNSTIM_VS_CD40L_IL2_IL5_3DAY_STIMULATED_IRF4HIGH_SORTED_BCELL_DN
GSE46606_UNSTIM_VS_CD40L_IL2_IL5_3DAY_STIMULATED_IRF4HIGH_SORTED_BCELL_DN 136 -0.29523474 -1.2149523
0.11516315 0.18942001 1 3749 "tags=30%, list=27%, signal=41%"
GSE14308_TH1_VS_TH17_DN GSE14308_TH1_VS_TH17_DN
139 -0.29252893 -1.2145586 0.11700183
0.18989521 1 2307 "tags=25%, list=16%, signal=30%"
GSE37533_PPARG2_FOXP3_VS_FOXP3_TRANSDUCED_CD4_TCELL_UP
GSE37533_PPARG2_FOXP3_VS_FOXP3_TRANSDUCED_CD4_TCELL_UP
142 -0.2967353 -1.2141191 0.11153119 0.19038
1 2232 "tags=26%, list=16%, signal=31%"
GSE24142_EARLY_THYMIC_PROGENITOR_VS_DN3_THYMOCYTE_FETAL_DN
GSE24142_EARLY_THYMIC_PROGENITOR_VS_DN3_THYMOCYTE_FETAL_DN
156 -0.29495555 -1.2140986 0.09487666
0.19025102 1 2085 "tags=24%, list=15%, signal=28%"
GSE29617_DAY3_VS_DAY7_TIV_FLU_VACCINE_PBMC_2008_UP
GSE29617_DAY3_VS_DAY7_TIV_FLU_VACCINE_PBMC_2008_UP
124 -0.3040122 -1.2138627 0.119140625
0.19046575 1 1105 "tags=14%, list=8%, signal=15%"
GSE22033_UNTREATED_VS_MRL24_TREATED_MEF_DN
GSE22033_UNTREATED_VS_MRL24_TREATED_MEF_DN
149 -0.29148492 -1.2135253 0.08695652
0.19082129 1 2071 "tags=19%, list=15%, signal=22%"
GSE11864_CSF1_VS_CSF1_IFNG_PAM3CYS_IN_MAC_UP
GSE11864_CSF1_VS_CSF1_IFNG_PAM3CYS_IN_MAC_UP
124 -0.29975355 -1.2133338 0.105065666
0.19096233 1 2934 "tags=27%, list=21%, signal=33%"
GSE30971_2H_VS_4H_LPS_STIM_MACROPHAGE_WBP7_HET_DN
GSE30971_2H_VS_4H_LPS_STIM_MACROPHAGE_WBP7_HET_DN
105 -0.30346462 -1.2130183 0.106814 0.19130847
1 2504 "tags=24%, list=18%, signal=29%"
GSE5542_IFNG_VS_IFNA_AND_IFNG_TREATED_EPITHELIAL_CELLS_24H_DN

GSE5542_IFNG_VS_IFNA_AND_IFNG_TREATED_EPITHELIAL_CELLS_24H_DN
154 -0.2873211 -1.2125835 0.0967118
0.1918426 1 2974 "tags=27%, list=21%, signal=34%"
GSE22886_NAIVE_CD4_TCELL_VS_NEUTROPHIL_DN
GSE22886_NAIVE_CD4_TCELL_VS_NEUTROPHIL_DN
141 -0.2934104 -1.2115326 0.11516315
0.19337513 1 3714 "tags=34%, list=26%, signal=46%"
GSE8921_UNSTIM_0H_VS_TLR1_2_STIM_MONOCYTE_3H_UP
GSE8921_UNSTIM_0H_VS_TLR1_2_STIM_MONOCYTE_3H_UP
135 -0.29672223 -1.2109268 0.11657559
0.19420582 1 1803 "tags=19%, list=13%, signal=22%"
GSE32901_NAIVE_VS_TH17_ENRICHED_CD4_TCELL_UP
GSE32901_NAIVE_VS_TH17_ENRICHED_CD4_TCELL_UP
118 -0.30126566 -1.2105725 0.11434109
0.19460481 1 2241 "tags=25%, list=16%, signal=29%"
GSE6259_FLT3L_INDUCED_DEC205_POS_DC_VS_CD4_TCELL_DN
GSE6259_FLT3L_INDUCED_DEC205_POS_DC_VS_CD4_TCELL_DN
91 -0.31914666 -1.2103479 0.12692308
0.19478913 1 6367 "tags=49%, list=45%, signal=90%"
GSE7548_NAIVE_VS_DAY7_PCC_IMMUNIZATION_CD4_TCELL_DN
GSE7548_NAIVE_VS_DAY7_PCC_IMMUNIZATION_CD4_TCELL_DN
141 -0.29186815 -1.2099253 0.10172745
0.1952969 1 1117 "tags=16%, list=8%, signal=18%"
GSE3720_UNSTIM_VS_LPS_STIM_VD1_GAMMADELTA_TCELL_DN
GSE3720_UNSTIM_VS_LPS_STIM_VD1_GAMMADELTA_TCELL_DN
119 -0.3036739 -1.2092245 0.10071942
0.19625285 1 962 "tags=14%, list=7%, signal=15%"
GSE29164_CD8_TCELL_VS_CD8_TCELL_AND_IL12_TREATED_MELANOMA_DAY7_UP
GSE29164_CD8_TCELL_VS_CD8_TCELL_AND_IL12_TREATED_MELANOMA_DAY7_UP
125 -0.30219215 -1.2090056 0.13207547
0.19645873 1 1585 "tags=21%, list=11%, signal=23%"
GSE2706_R848_VS_R848_AND_LPS_8H_STIM_DC_DN
GSE2706_R848_VS_R848_AND_LPS_8H_STIM_DC_DN
125 -0.2976225 -1.2082728 0.12737642
0.19748968 1 2248 "tags=24%, list=16%, signal=28%"
GSE22886_CD4_TCELL_VS_BCELL_NAIVE_DN
GSE22886_CD4_TCELL_VS_BCELL_NAIVE_DN
127 -0.29531196 -1.2074374 0.10940499
0.19866404 1 2694 "tags=27%, list=19%, signal=33%"
GSE42021_TREG_PLN_VS_CD24LO_TREG_THYMUS_UP
GSE42021_TREG_PLN_VS_CD24LO_TREG_THYMUS_UP
135 -0.29710674 -1.2072436 0.10881801
0.19883226 1 2028 "tags=23%, list=14%, signal=27%"
GSE36009_UNSTIM_VS_LPS_STIM_DC_UP GSE36009_UNSTIM_VS_LPS_STIM_DC_UP
142 -0.29206234 -1.2072366 0.12222222
0.19868207 1 2377 "tags=27%, list=17%, signal=32%"
GSE43863_NAIVE_VS_LY6C_INT_CXCR5POS_CD4_EFF_TCELL_D6_LCMV_DN
GSE43863_NAIVE_VS_LY6C_INT_CXCR5POS_CD4_EFF_TCELL_D6_LCMV_DN
100 -0.31062764 -1.2061384 0.14181818
0.2002521 1 4588 "tags=35%, list=33%, signal=51%"
GSE2770_UNTREATED_VS_TGFB_AND_IL12_TREATED_ACT_CD4_TCELL_48H_DN
GSE2770_UNTREATED_VS_TGFB_AND_IL12_TREATED_ACT_CD4_TCELL_48H_DN
137 -0.29424205 -1.2060035 0.116504855
0.20030767 1 1848 "tags=21%, list=13%, signal=24%"

GSE7831_UNSTIM_VS_CPG_STIM_PDC_1H_DN
GSE7831_UNSTIM_VS_CPG_STIM_PDC_1H_DN
150 -0.2907642 -1.205473 0.10646388
0.20098877 1 1593 "tags=23%, list=11%, signal=25%"
GSE360_CTRL_VS_M_TUBERCULOSIS_MAC_DN
GSE360_CTRL_VS_M_TUBERCULOSIS_MAC_DN
155 -0.2880606 -1.2051789 0.10638298
0.20130384 1 928 "tags=13%, list=7%, signal=14%"
GSE13493_CD4INTCD8POS_VS_CD8POS_THYMOCYTE_DN
GSE13493_CD4INTCD8POS_VS_CD8POS_THYMOCYTE_DN
133 -0.29805064 -1.2051543 0.118320614
0.20117868 1 2375 "tags=22%, list=17%, signal=26%"
GSE17721_CTRL_VS_GARDIQUIMOD_4H_BMDC_DN
GSE17721_CTRL_VS_GARDIQUIMOD_4H_BMDC_DN
153 -0.29236597 -1.2051092 0.09266409
0.20107982 1 2956 "tags=24%, list=21%, signal=30%"
GSE37605_C57BL6_VS_NOD_FOXP3_IRES_GFP_TREG_UP
GSE37605_C57BL6_VS_NOD_FOXP3_IRES_GFP_TREG_UP
84 -0.3211023 -1.2049253 0.1438849
0.20122494 1 3754 "tags=29%, list=27%, signal=39%"
GSE3982_MAST_CELL_VS_MAC_UP GSE3982_MAST_CELL_VS_MAC_UP
129 -0.29701862 -1.2047279 0.11786372
0.20137995 1 1521 "tags=19%, list=11%, signal=21%"
GSE43863_TFH_VS_LY6C_INT_CXCR5POS_MEMORY_CD4_TCELL_UP
GSE43863_TFH_VS_LY6C_INT_CXCR5POS_MEMORY_CD4_TCELL_UP
140 -0.29148692 -1.204222 0.118644066
0.20200992 1 2377 "tags=24%, list=17%, signal=28%"
GSE5589_UNSTIM_VS_180MIN_LPS_AND_IL10_STIM_MACROPHAGE_UP
GSE5589_UNSTIM_VS_180MIN_LPS_AND_IL10_STIM_MACROPHAGE_UP
150 -0.29266357 -1.2038248 0.11111111
0.20251457 1 2204 "tags=25%, list=16%, signal=29%"
GSE19401_UNSTIM_VS_PAM2CSK4_STIM_FOLLICULAR_DC_UP
GSE19401_UNSTIM_VS_PAM2CSK4_STIM_FOLLICULAR_DC_UP
153 -0.2897857 -1.2028613 0.10948905
0.20391814 1 2192 "tags=27%, list=16%, signal=31%"
GSE27670_CTRL_VS_LMP1_TRANSDUCED_GC_BCELL_DN
GSE27670_CTRL_VS_LMP1_TRANSDUCED_GC_BCELL_DN
158 -0.2914261 -1.202747 0.11376147
0.20395847 1 2964 "tags=23%, list=21%, signal=29%"
GSE2770_TGFB_AND_IL4_VS_IL4_TREATED_ACT_CD4_TCELL_2H_UP
GSE2770_TGFB_AND_IL4_VS_IL4_TREATED_ACT_CD4_TCELL_2H_UP
144 -0.29132718 -1.2026865 0.10873786
0.20388064 1 1438 "tags=14%, list=10%, signal=15%"
GSE45365_WT_VS_IFNAR_KO_CD11B_DC_MCMV_INFECTION_UP
GSE45365_WT_VS_IFNAR_KO_CD11B_DC_MCMV_INFECTION_UP
118 -0.30333644 -1.2020396 0.13211009
0.20483746 1 2212 "tags=25%, list=16%, signal=30%"
GSE3982_MAST_CELL_VS_TH1_UP GSE3982_MAST_CELL_VS_TH1_UP
135 -0.29224786 -1.2019689 0.10526316
0.20478505 1 2113 "tags=21%, list=15%, signal=25%"
GSE13306_RA_VS_UNTREATED_TCONV_UP GSE13306_RA_VS_UNTREATED_TCONV_UP
143 -0.2883892 -1.2011833 0.102713175
0.20593962 1 2553 "tags=26%, list=18%, signal=31%"
GSE2770_UNTREATED_VS_TGFB_AND_IL12_TREATED_ACT_CD4_TCELL_6H_UP

GSE2770_UNTREATED_VS_TGFB_AND_IL12_TREATED_ACT_CD4_TCELL_6H_UP
132 -0.2940863 -1.2007546 0.10980392
0.20650128 1 1848 "tags=21%, list=13%, signal=24%"
GSE360_L_DONOVANI_VS_B_MALAYI_LOW_DOSE_DC_UP
GSE360_L_DONOVANI_VS_B_MALAYI_LOW_DOSE_DC_UP
157 -0.28900394 -1.2006905 0.108986616
0.2064429 1 3037 "tags=27%, list=22%, signal=34%"
GSE4590_PRE_BCELL_VS_VPREB_POS_LARGE_PRE_BCELL_UP
GSE4590_PRE_BCELL_VS_VPREB_POS_LARGE_PRE_BCELL_UP
123 -0.29879346 -1.2006176 0.13267326
0.20639013 1 2199 "tags=20%, list=16%, signal=24%"
GSE27786_CD4_VS_CD8_TCELL_UP GSE27786_CD4_VS_CD8_TCELL_UP
135 -0.2937344 -1.2002162 0.11376147
0.20687449 1 1797 "tags=21%, list=13%, signal=24%"
GSE22601_DOUBLE_NEGATIVE_VS_CD8_SINGLE_POSITIVE_THYMOCYTE_DN
GSE22601_DOUBLE_NEGATIVE_VS_CD8_SINGLE_POSITIVE_THYMOCYTE_DN
119 -0.29710227 -1.1999516 0.12720157
0.20715868 1 2640 "tags=25%, list=19%, signal=31%"
GSE14386_UNTREATED_VS_IFNA_TREATED_ACT_PBMC_MS_PATIENT_UP
GSE14386_UNTREATED_VS_IFNA_TREATED_ACT_PBMC_MS_PATIENT_UP
97 -0.30907488 -1.1998351 0.14478764
0.20718865 1 4960 "tags=47%, list=35%, signal=73%"
GSE5542_UNTREATED_VS_IFNG_TREATED_EPITHELIAL_CELLS_24H_DN
GSE5542_UNTREATED_VS_IFNG_TREATED_EPITHELIAL_CELLS_24H_DN
126 -0.29815537 -1.1994684 0.12523365
0.20762677 1 1482 "tags=18%, list=11%, signal=20%"
GSE39152_CD103_NEG_VS_POS_MEMORY_CD8_TCELL_UP
GSE39152_CD103_NEG_VS_POS_MEMORY_CD8_TCELL_UP
163 -0.282578 -1.1992532 0.11811024
0.20779845 1 1575 "tags=17%, list=11%, signal=19%"
GSE10240_IL22_VS_IL22_AND_IL17_STIM_PRIMARY_BRONCHIAL_EPITHELIAL_CELLS_DN
GSE10240_IL22_VS_IL22_AND_IL17_STIM_PRIMARY_BRONCHIAL_EPITHELIAL_CELLS_DN 144 -0.29277813 -1.1990736
0.12138728 0.20793292 1 1885 "tags=20%, list=13%, signal=23%"
GSE22935_WT_VS_MYD88_KO_MACROPHAGE_DN
GSE22935_WT_VS_MYD88_KO_MACROPHAGE_DN
145 -0.2914068 -1.1988407 0.112840466
0.20813128 1 3460 "tags=28%, list=25%, signal=36%"
GSE17721_CTRL_VS_PAM3CSK4_2H_BMDC_DN
GSE17721_CTRL_VS_PAM3CSK4_2H_BMDC_DN
171 -0.28504708 -1.1987381 0.115384616
0.20812637 1 3187 "tags=30%, list=23%, signal=38%"
GSE27786_NKTCELL_VS_ERYTHROBLAST_UP
GSE27786_NKTCELL_VS_ERYTHROBLAST_UP
152 -0.28783745 -1.1985583 0.10357816
0.20826328 1 1407 "tags=20%, list=10%, signal=22%"
GSE10325_LUPUS_CD4_TCELL_VS_LUPUS_BCELL_DN
GSE10325_LUPUS_CD4_TCELL_VS_LUPUS_BCELL_DN
125 -0.29558918 -1.1984807 0.12859885
0.20823026 1 2325 "tags=25%, list=16%, signal=29%"
GSE24142_ADULT_VS_FETAL_DN3_THYMOCYTE_DN
GSE24142_ADULT_VS_FETAL_DN3_THYMOCYTE_DN

156	-0.28789052	-1.1984469	0.120229006
0.20812237	1	1946	"tags=19%, list=14%, signal=22%"
GSE16450_CTRL_VS_IFNA_12H_STIM_IMMATURE_NEURON_CELL_LINE_UP
GSE16450_CTRL_VS_IFNA_12H_STIM_IMMATURE_NEURON_CELL_LINE_UP
129	-0.2936375	-1.1975152	0.13060428
0.20950781	1	2317	"tags=27%, list=16%, signal=32%"
GSE24634_NAIVE_CD4_TCELL_VS_DAY3_IL4_CONV_TREG_UP
GSE24634_NAIVE_CD4_TCELL_VS_DAY3_IL4_CONV_TREG_UP
138	-0.29166982	-1.1971396	0.10176125
0.21000852	1	3948	"tags=33%, list=28%, signal=46%"
GSE24081_CONTROLLER_VS_PROGRESSOR_HIV_SPECIFIC_CD8_TCELL_UP
GSE24081_CONTROLLER_VS_PROGRESSOR_HIV_SPECIFIC_CD8_TCELL_UP
134	-0.29010418	-1.1968573	0.121212125
0.21031821	1	1523	"tags=19%, list=11%, signal=22%"
GSE360_DC_VS_MAC_M_TUBERCULOSIS_UP
GSE360_DC_VS_MAC_M_TUBERCULOSIS_UP
136	-0.2926198	-1.1967844	0.124282986
0.21025886	1	1817	"tags=21%, list=13%, signal=24%"
GSE17974_2.5H_VS_72H_IL4_AND_ANTI_IL12_ACT_CD4_TCELL_UP
GSE17974_2.5H_VS_72H_IL4_AND_ANTI_IL12_ACT_CD4_TCELL_UP
132	-0.29689285	-1.1964945	0.13358779
0.2105949	1	1979	"tags=25%, list=14%, signal=29%"
GSE10325_BCELL_VS_MYELOID_UP	GSE10325_BCELL_VS_MYELOID_UP
	128	-0.29245284	-1.1964788	0.12734865
0.21045516	1	1145	"tags=16%, list=8%, signal=17%"
GSE18281_SUBCAPSULAR_VS_PERIMEDULLARY_CORTICAL_REGION_OF_THYMUS_UP
GSE18281_SUBCAPSULAR_VS_PERIMEDULLARY_CORTICAL_REGION_OF_THYMUS_UP
	155	-0.28377458	-1.1962042	0.116071425
0.21075991	1	1295	"tags=17%, list=9%, signal=18%"
GSE16450_CTRL_VS_IFNA_6H_STIM_IMMATURE_NEURON_CELL_LINE_UP
GSE16450_CTRL_VS_IFNA_6H_STIM_IMMATURE_NEURON_CELL_LINE_UP
142	-0.29305542	-1.1956543	0.12475248
0.21157178	1	2405	"tags=23%, list=17%, signal=28%"
GSE32255_UNSTIM_VS_4H_LPS_STIM_DC_UP
GSE32255_UNSTIM_VS_4H_LPS_STIM_DC_UP
150	-0.28802782	-1.1950558	0.11619048
0.21241905	1	1979	"tags=28%, list=14%, signal=32%"
GSE11924_TH1_VS_TH17_CD4_TCELL_DN GSE11924_TH1_VS_TH17_CD4_TCELL_DN
	147	-0.29009482	-1.1949751	0.10789981
0.2123886	1	1865	"tags=25%, list=13%, signal=29%"
GSE30971_CTRL_VS_LPS_STIM_MACROPHAGE_WBP7_HET_4H_UP
GSE30971_CTRL_VS_LPS_STIM_MACROPHAGE_WBP7_HET_4H_UP
157	-0.28591	-1.1945177	0.11382114	0.21300901
1	1091	"tags=16%, list=8%, signal=17%"
GSE12484_HEALTHY_VS_PERIDONTITIS_NEUTROPHILS_UP
GSE12484_HEALTHY_VS_PERIDONTITIS_NEUTROPHILS_UP
108	-0.30023012	-1.1941648	0.12614259
0.21343102	1	5470	"tags=50%, list=39%, signal=81%"
GSE37534_PIOGLITAZONE_VS_ROSIGLITAZONE_TREATED_CD4_TCELL_PPARG1_FOXP
3_TRANSDUCED_DN
GSE37534_PIOGLITAZONE_VS_ROSIGLITAZONE_TREATED_CD4_TCELL_PPARG1_FOXP
3_TRANSDUCED_DN	129	-0.29469755	-1.193925
0.1254902	0.21369094	1	3228	"tags=28%,
list=23%, signal=36%"

GSE16385_ROSIGLITAZONE_IL4_VS_ROSIGLITAZONE_ALONE_STIM_MACROPHAGE_UP

GSE16385_ROSIGLITAZONE_IL4_VS_ROSIGLITAZONE_ALONE_STIM_MACROPHAGE_UP
140 -0.2915581 -1.1934503 0.125
0.21431366 1 2085 "tags=18%, list=15%, signal=21%"
GSE22611_UNSTIM_VS_6H_MDP_STIM_NOD2_TRANSDUCED_HEK293T_CELL_DN
GSE22611_UNSTIM_VS_6H_MDP_STIM_NOD2_TRANSDUCED_HEK293T_CELL_DN
135 -0.29247987 -1.1931098 0.14147286
0.2147079 1 1507 "tags=17%, list=11%, signal=19%"
GSE17721_4_VS_24H_CPG_BMDC_DN GSE17721_4_VS_24H_CPG_BMDC_DN
144 -0.29134887 -1.193027 0.10526316
0.21467665 1 1679 "tags=20%, list=12%, signal=23%"
GSE5589_IL6_KO_VS_IL10_KO_LPS_STIM_MACROPHAGE_45MIN_DN
GSE5589_IL6_KO_VS_IL10_KO_LPS_STIM_MACROPHAGE_45MIN_DN
149 -0.29155263 -1.1928312 0.1309298
0.2148365 1 1844 "tags=23%, list=13%, signal=27%"
GSE24210_TCONV_VS_TREG_UP GSE24210_TCONV_VS_TREG_UP
140 -0.2917585 -1.1923085 0.12055336
0.21554166 1 2342 "tags=27%, list=17%, signal=32%"
GSE5142_CTRL_VS_HTERT_TRANSDUCED_CD8_TCELL_EARLY_PASSAGE_CLONE_UP
GSE5142_CTRL_VS_HTERT_TRANSDUCED_CD8_TCELL_EARLY_PASSAGE_CLONE_UP
147 -0.28578535 -1.1920404 0.11153119
0.21583247 1 2344 "tags=27%, list=17%, signal=32%"
GSE11961_FOLLICULAR_BCELL_VS_GERMINAL_CENTER_BCELL_DAY7_UP
GSE11961_FOLLICULAR_BCELL_VS_GERMINAL_CENTER_BCELL_DAY7_UP
141 -0.28846627 -1.1918993 0.123595506
0.21590005 1 2132 "tags=25%, list=15%, signal=29%"
GSE17974_0H_VS_4H_IN_VITRO_ACT_CD4_TCELL_UP
GSE17974_0H_VS_4H_IN_VITRO_ACT_CD4_TCELL_UP
132 -0.29447752 -1.1909658 0.14203455
0.21730281 1 1625 "tags=17%, list=12%, signal=20%"
GSE4748_CTRL_VS_LPS_STIM_DC_3H_UP GSE4748_CTRL_VS_LPS_STIM_DC_3H_UP
161 -0.2882923 -1.1906574 0.13929313
0.21768858 1 1943 "tags=19%, list=14%, signal=21%"
GSE9946_IMMATURE_VS_MATURE_STIMULATORY_DC_DN
GSE9946_IMMATURE_VS_MATURE_STIMULATORY_DC_DN
98 -0.30765063 -1.1904298 0.14393939
0.21789274 1 4849 "tags=39%, list=34%, signal=59%"
GSE17974_IL4_AND_ANTI_IL12_VS_UNTREATED_72H_ACT_CD4_TCELL_DN
GSE17974_IL4_AND_ANTI_IL12_VS_UNTREATED_72H_ACT_CD4_TCELL_DN
133 -0.2938712 -1.1902746 0.13729128
0.21795627 1 2193 "tags=26%, list=16%, signal=31%"
GSE22886_NAIVE_TCELL_VS_MONOCYTE_DN
GSE22886_NAIVE_TCELL_VS_MONOCYTE_DN
156 -0.28513825 -1.1894251 0.13396226
0.21927555 1 1703 "tags=21%, list=12%, signal=24%"
GSE19888_ADENOSINE_A3R_INH_PRETREAT_AND_ACT_BY_A3R_VS_TCELL_MEMBRANES_ACT_MAST_CELL_UP
GSE19888_ADENOSINE_A3R_INH_PRETREAT_AND_ACT_BY_A3R_VS_TCELL_MEMBRANES_ACT_MAST_CELL_UP
144 -0.29015675 -1.1892704 0.1342155
0.21933551 1 1770 "tags=24%, list=13%, signal=27%"
GSE40274_CTRL_VS_GATA1_TRANSDUCED_ACTIVATED_CD4_TCELL_DN
GSE40274_CTRL_VS_GATA1_TRANSDUCED_ACTIVATED_CD4_TCELL_DN

133	-0.29494992	-1.1892352	0.13267326
0.21922423	1	1538	"tags=18%, list=11%, signal=20%"
GSE21546_UNSTIM_VS_ANTI_CD3_STIM_SAP1A_KO_DP_THYMOCYTES_DN
GSE21546_UNSTIM_VS_ANTI_CD3_STIM_SAP1A_KO_DP_THYMOCYTES_DN
90	-0.31148556	-1.1886593	0.14545454
0.22003676	1	4609	"tags=43%, list=33%, signal=64%"
GSE22025_TGFB1_VS_TGFB1_AND_PROGESTERONE_TREATED_CD4_TCELL_DN
GSE22025_TGFB1_VS_TGFB1_AND_PROGESTERONE_TREATED_CD4_TCELL_DN
150	-0.28782344	-1.1880159	0.1299639
0.22101106	1	925	"tags=11%, list=7%, signal=12%"
GSE3982_EOSINOPHIL_VS_MAC_UP	GSE3982_EOSINOPHIL_VS_MAC_UP
135	-0.29223967	-1.1878786	0.14312617
0.22105815	1	2619	"tags=26%, list=19%, signal=32%"
GSE15659_CD45RA_NEG_CD4_TCELL_VS_NONSUPPRESSIVE_TCELL_UP
GSE15659_CD45RA_NEG_CD4_TCELL_VS_NONSUPPRESSIVE_TCELL_UP
109	-0.3033467	-1.1875472	0.14700194
0.22149612	1	2036	"tags=18%, list=14%, signal=21%"
GSE9509_10MIN_VS_30MIN_LPS_STIM_IL10_KO_MACROPHAGE_DN
GSE9509_10MIN_VS_30MIN_LPS_STIM_IL10_KO_MACROPHAGE_DN
132	-0.29306665	-1.1874245	0.107421875
0.22155286	1	4123	"tags=39%, list=29%, signal=54%"
GSE17721_LPS_VS_GARDIQUIMOD_4H_BMDC_UP
GSE17721_LPS_VS_GARDIQUIMOD_4H_BMDC_UP
152	-0.28350258	-1.1872005	0.12	0.22174986
1	2344	"tags=22%, list=17%, signal=27%"
GSE22886_IGA_VS_IGM_MEMORY_BCELL_DN
GSE22886_IGA_VS_IGM_MEMORY_BCELL_DN
127	-0.29036844	-1.1869353	0.1311787
0.22203366	1	2631	"tags=25%, list=19%, signal=31%"
GSE24634_IL4_VS_CTRL_TREATED_NAIVE_CD4_TCELL_DAY5_DN
GSE24634_IL4_VS_CTRL_TREATED_NAIVE_CD4_TCELL_DAY5_DN
128	-0.29627487	-1.1865137	0.13320826
0.22263637	1	1766	"tags=20%, list=13%, signal=23%"
GSE40274_XBP1_VS_FOXP3_AND_XBP1_TRANSDUCED_ACTIVATED_CD4_TCELL_DN
GSE40274_XBP1_VS_FOXP3_AND_XBP1_TRANSDUCED_ACTIVATED_CD4_TCELL_DN
145	-0.28687128	-1.1863049	0.115830116
0.22281821	1	2961	"tags=30%, list=21%, signal=37%"
GSE9037_CTRL_VS_LPS_1H_STIM_IRAK4_KO_BMDM_DN
GSE9037_CTRL_VS_LPS_1H_STIM_IRAK4_KO_BMDM_DN
145	-0.28949744	-1.1861956	0.12984496
0.22282124	1	1962	"tags=24%, list=14%, signal=28%"
GSE19888_ADENOSINE_A3R_ACT_VS_TCELL_MEMBRANES_ACT_AND_A3R_INH_PRETREAT_IN_MAST_CELL_DN
GSE19888_ADENOSINE_A3R_ACT_VS_TCELL_MEMBRANES_ACT_AND_A3R_INH_PRETREAT_IN_MAST_CELL_DN
138	-0.28788775	-1.1860596	0.14750957
0.22288999	1	3041	"tags=28%, list=22%, signal=36%"
GSE7548_DAY7_VS_DAY28_PCC_IMMUNIZATION_CD4_TCELL_UP
GSE7548_DAY7_VS_DAY28_PCC_IMMUNIZATION_CD4_TCELL_UP
155	-0.28487474	-1.1860209	0.121157326
0.22278562	1	1498	"tags=15%, list=11%, signal=17%"
GSE22886_NAIVE_TCELL_VS_NEUTROPHIL_DN
GSE22886_NAIVE_TCELL_VS_NEUTROPHIL_DN
136	-0.2903161	-1.1857685	0.14736842

0.22305275	1	3051	"tags=29%, list=22%, signal=37%"
GSE43863_NAIVE_VS_LY6C_INT_CXCR5POS_CD4_EFF_TCELL_D6_LCMV_UP
GSE43863_NAIVE_VS_LY6C_INT_CXCR5POS_CD4_EFF_TCELL_D6_LCMV_UP
	124	-0.29320002	-1.1855179	0.14509805
0.22334638	1	2381	"tags=26%, list=17%, signal=31%"
GSE19888_ADENOSINE_A3R_INH_VS_ACT_WITH_INHIBITOR_PRETREATMENT_IN_MAST_CELL_UP
GSE19888_ADENOSINE_A3R_INH_VS_ACT_WITH_INHIBITOR_PRETREATMENT_IN_MAST_CELL_UP	135	-0.28765228	-1.1854378
0.13670412	0.22332111	1	1770	"tags=19%, list=13%, signal=21%"
GSE15735_CTRL_VS_HDAC_INHIBITOR_TREATED_CD4_TCELL_12H_UP
GSE15735_CTRL_VS_HDAC_INHIBITOR_TREATED_CD4_TCELL_12H_UP
145	-0.2891555	-1.1853534	0.12432432
0.2232854	1	2283	"tags=26%, list=16%, signal=31%"
GSE360_CTRL_VS_L_MAJOR_DC_DN	GSE360_CTRL_VS_L_MAJOR_DC_DN
	148	-0.28845057	-1.1850501	0.14176245
0.22362082	1	3083	"tags=30%, list=22%, signal=38%"
GSE19888_ADENOSINE_A3R_INH_PRETREAT_AND_ACT_BY_A3R_VS_TCELL_MEMBRANES_ACT_MAST_CELL_DN
GSE19888_ADENOSINE_A3R_INH_PRETREAT_AND_ACT_BY_A3R_VS_TCELL_MEMBRANES_ACT_MAST_CELL_DN
137	-0.29130942	-1.1843547	0.12653062
0.22465476	1	2372	"tags=25%, list=17%, signal=30%"
GSE4590_SMALL_VS_LARGE_PRE_BCELL_DN
GSE4590_SMALL_VS_LARGE_PRE_BCELL_DN
125	-0.29336783	-1.183773	0.14611006
0.22555466	1	2133	"tags=24%, list=15%, signal=28%"
GSE46606_UNSTIM_VS_CD40L_IL2_IL5_3DAY_STIMULATED_IRF4MID_SORTED_BCELL_DN
GSE46606_UNSTIM_VS_CD40L_IL2_IL5_3DAY_STIMULATED_IRF4MID_SORTED_BCELL_DN	151	-0.28437006	-1.1837443
0.12172285	0.22543113	1	2238	"tags=25%, list=16%, signal=30%"
GSE15930_NAIVE_VS_24H_IN_VITRO_STIM_INFAB_CD8_TCELL_UP
GSE15930_NAIVE_VS_24H_IN_VITRO_STIM_INFAB_CD8_TCELL_UP
162	-0.2842196	-1.1836275	0.1374502
0.22546315	1	1462	"tags=17%, list=10%, signal=18%"
GSE16450_CTRL_VS_IFNA_6H_STIM_IMMATURE_NEURON_CELL_LINE_DN
GSE16450_CTRL_VS_IFNA_6H_STIM_IMMATURE_NEURON_CELL_LINE_DN
128	-0.2885116	-1.1835147	0.12256809
0.22549349	1	1531	"tags=21%, list=11%, signal=23%"
GSE32034_UNTREATED_VS_ROSIGLIZATONE_TREATED_LY6C_HIGH_MONOCYTE_DN
GSE32034_UNTREATED_VS_ROSIGLIZATONE_TREATED_LY6C_HIGH_MONOCYTE_DN
	139	-0.28920478	-1.1834246	0.14478764
0.22547153	1	1597	"tags=20%, list=11%, signal=22%"
GSE5542_IFNA_VS_IFNA_AND_IFNG_TREATED_EPITHELIAL_CELLS_6H_DN
GSE5542_IFNA_VS_IFNA_AND_IFNG_TREATED_EPITHELIAL_CELLS_6H_DN
	154	-0.28831542	-1.1826733	0.13017751
0.2266355	1	2089	"tags=23%, list=15%, signal=26%"
GSE37301_MULTIPOTENT_PROGENITOR_VS_GRAN_MONO_PROGENITOR_UP
GSE37301_MULTIPOTENT_PROGENITOR_VS_GRAN_MONO_PROGENITOR_UP
158	-0.28465128	-1.1815263	0.12301587
0.22849217	1	770	"tags=12%, list=5%, signal=13%"

GSE7460_FOXP3_MUT_VS_HET_ACT_TCONV_DN
GSE7460_FOXP3_MUT_VS_HET_ACT_TCONV_DN
146	-0.28133082	-1.1814779	0.11710037
0.22841313	1	1417	"tags=14%, list=10%, signal=15%"
GSE26912_TUMORICIDAL_VS_CTRL_MACROPHAGE_UP
GSE26912_TUMORICIDAL_VS_CTRL_MACROPHAGE_UP
154	-0.28633133	-1.1810048	0.118236475
0.22908442	1	2138	"tags=17%, list=15%, signal=20%"
GSE17721_CPG_VS_GARDIQUIMOD_1H_BMDC_UP
GSE17721_CPG_VS_GARDIQUIMOD_1H_BMDC_UP
166	-0.2844629	-1.1806247	0.13011153
0.2295911	1	1723	"tags=19%, list=12%, signal=21%"
GSE11057_CD4_EFF_MEM_VS_PBMC_UP	GSE11057_CD4_EFF_MEM_VS_PBMC_UP
	140	-0.28682113	-1.1804824	0.12741312
0.22966191	1	1125	"tags=16%, list=8%, signal=17%"
GSE37532_VISCERAL_ADIPOSE_TISSUE_VS_LN_DERIVED_TREG_CD4_TCELL_DN
GSE37532_VISCERAL_ADIPOSE_TISSUE_VS_LN_DERIVED_TREG_CD4_TCELL_DN
	115	-0.29474074	-1.1802554	0.16730037
0.2298445	1	3041	"tags=28%, list=22%, signal=35%"
GSE7568_CTRL_VS_24H_TGFB_TREATED_MACROPHAGES_WITH_IL4_AND_DEXAMETHAS
ONE_UP
GSE7568_CTRL_VS_24H_TGFB_TREATED_MACROPHAGES_WITH_IL4_AND_DEXAMETHAS
ONE_UP		138	-0.2846679	-1.1802185
0.118473895	0.2297285	1	1483	"tags=17%,
list=11%, signal=19%"
GSE5589_IL6_KO_VS_IL10_KO_LPS_AND_IL10_STIM_MACROPHAGE_180MIN_DN
GSE5589_IL6_KO_VS_IL10_KO_LPS_AND_IL10_STIM_MACROPHAGE_180MIN_DN
	151	-0.28114557	-1.1796681	0.10838446
0.23052101	1	2080	"tags=24%, list=15%, signal=28%"
GSE5589_LPS_VS_LPS_AND_IL10_STIM_IL10_KO_MACROPHAGE_180MIN_UP
GSE5589_LPS_VS_LPS_AND_IL10_STIM_IL10_KO_MACROPHAGE_180MIN_UP
	137	-0.2884843	-1.1791736	0.14694656
0.23122084	1	4002	"tags=36%, list=28%, signal=50%"
GSE11961_FOLLICULAR_BCELL_VS_PLASMA_CELL_DAY7_UP
GSE11961_FOLLICULAR_BCELL_VS_PLASMA_CELL_DAY7_UP
148	-0.28383327	-1.1789486	0.12969925
0.23146434	1	1975	"tags=22%, list=14%, signal=25%"
GSE2770_IL12_ACT_VS_ACT_CD4_TCELL_6H_DN
GSE2770_IL12_ACT_VS_ACT_CD4_TCELL_6H_DN
114	-0.29416072	-1.1788296	0.17216118
0.23151174	1	1934	"tags=21%, list=14%, signal=24%"
GSE38697_LIGHT_ZONE_VS_DARK_ZONE_BCELL_UP
GSE38697_LIGHT_ZONE_VS_DARK_ZONE_BCELL_UP
127	-0.28897503	-1.1788081	0.1575985
0.23137552	1	2917	"tags=30%, list=21%, signal=37%"
GSE22935_UNSTIM_VS_48H_MBOVIS_BCG_STIM_MYD88_KO_MACROPHAGE_DN
GSE22935_UNSTIM_VS_48H_MBOVIS_BCG_STIM_MYD88_KO_MACROPHAGE_DN
	140	-0.28774017	-1.1787847	0.13127413
0.23124345	1	2504	"tags=20%, list=18%, signal=24%"
GSE7460_CD8_TCELL_VS_CD4_TCELL_ACT_DN
GSE7460_CD8_TCELL_VS_CD4_TCELL_ACT_DN
143	-0.29028302	-1.1786495	0.14754099
0.23130916	1	1837	"tags=24%, list=13%, signal=28%"
GSE21033_1H_VS_12H_POLYIC_STIM_DC_DN

GSE21033_1H_VS_12H_POLYIC_STIM_DC_DN
104	-0.29951036	-1.1782773	0.1541502
0.23179874	1	2796	"tags=31%, list=20%, signal=38%"
GSE36888_UNTREATED_VS_IL2_TREATED_TCELL_17H_UP
GSE36888_UNTREATED_VS_IL2_TREATED_TCELL_17H_UP
139	-0.28640008	-1.1781099	0.14738806
0.23192433	1	764	"tags=14%, list=5%, signal=14%"
GSE22313_HEALTHY_VS_SLE_MOUSE_CD4_TCELL_UP
GSE22313_HEALTHY_VS_SLE_MOUSE_CD4_TCELL_UP
125	-0.2891513	-1.1780385	0.14478764
0.23187882	1	2427	"tags=22%, list=17%, signal=27%"
GSE14308_TH17_VS_INDUCED_TREG_UP	GSE14308_TH17_VS_INDUCED_TREG_UP
	145	-0.2841142	-1.1778785	0.13111547
0.23199019	1	1471	"tags=18%, list=10%, signal=20%"
GSE16385_UNTREATED_VS_12H_ROSIGLITAZONE_TREATED_MACROPHAGE_DN
GSE16385_UNTREATED_VS_12H_ROSIGLITAZONE_TREATED_MACROPHAGE_DN
	110	-0.29660633	-1.1777498	0.15719697
0.2320348	1	2000	"tags=19%, list=14%, signal=22%"
GSE9988_LOW_LPS_VS_ANTI_TREM1_AND_LPS_MONOCYTE_DN
GSE9988_LOW_LPS_VS_ANTI_TREM1_AND_LPS_MONOCYTE_DN
129	-0.29126924	-1.1777313	0.1444867
0.23189144	1	1367	"tags=19%, list=10%, signal=20%"
GSE3982_DC_VS_BCELL_DN	GSE3982_DC_VS_BCELL_DN
121	-0.29668844	-1.1776468	0.1693989	0.231852
1	1715	"tags=22%, list=12%, signal=25%"
GSE39556_UNTREATED_VS_3H_POLYIC_INJ_MOUSE_CD8A_DC_UP
GSE39556_UNTREATED_VS_3H_POLYIC_INJ_MOUSE_CD8A_DC_UP
153	-0.28654608	-1.1766998	0.13528337
0.23344055	1	1536	"tags=18%, list=11%, signal=20%"
GSE13484_3H_UNSTIM_VS_YF17D_VACCINE_STIM_PBMC_DN
GSE13484_3H_UNSTIM_VS_YF17D_VACCINE_STIM_PBMC_DN
147	-0.28274167	-1.17653	0.1344697	0.2335725
1	1665	"tags=21%, list=12%, signal=24%"
GSE16755_CTRL_VS_IFNA_TREATED_MAC_UP
GSE16755_CTRL_VS_IFNA_TREATED_MAC_UP
134	-0.2929363	-1.1764781	0.16856061
0.23350562	1	2467	"tags=26%, list=17%, signal=31%"
GSE4984_GALECTIN1_VS_VEHICLE_CTRL_TREATED_DC_UP
GSE4984_GALECTIN1_VS_VEHICLE_CTRL_TREATED_DC_UP
122	-0.29362142	-1.1763954	0.14925373
0.23347999	1	1604	"tags=20%, list=11%, signal=23%"
GSE369_PRE_VS_POST_IL6_INJECTION_IFNG_WT_LIVER_UP
GSE369_PRE_VS_POST_IL6_INJECTION_IFNG_WT_LIVER_UP
138	-0.28978318	-1.1763543	0.1597633
0.23337506	1	1591	"tags=14%, list=11%, signal=15%"
GSE28449_WT_VS_LRF_KO_GERMINAL_CENTER_BCELL_UP
GSE28449_WT_VS_LRF_KO_GERMINAL_CENTER_BCELL_UP
142	-0.28789324	-1.1762958	0.15879017
0.23329704	1	3383	"tags=31%, list=24%, signal=40%"
GSE8685_IL2_ACT_IL2_STARVED_VS_IL21_ACT_IL2_STARVED_CD4_TCELL_DN
GSE8685_IL2_ACT_IL2_STARVED_VS_IL21_ACT_IL2_STARVED_CD4_TCELL_DN
	104	-0.29662427	-1.176162	0.15869981
0.23334397	1	3927	"tags=38%, list=28%, signal=52%"
GSE2585_CTEC_VS_MTEC_THYMUS_UP	GSE2585_CTEC_VS_MTEC_THYMUS_UP

148 -0.2845212 -1.1749004 0.14897579
0.23548411 1 2075 "tags=20%, list=15%, signal=24%"
GSE9037_CTRL_VS_LPS_4H_STIM_IRAK4_KO_BMDM_DN
GSE9037_CTRL_VS_LPS_4H_STIM_IRAK4_KO_BMDM_DN
153 -0.28299674 -1.1744806 0.12820514
0.23606123 1 2287 "tags=22%, list=16%, signal=25%"
GSE369_SOCS3_KO_VS_IFNG_KO_LIVER_UP
GSE369_SOCS3_KO_VS_IFNG_KO_LIVER_UP
127 -0.29200482 -1.1744562 0.16666667
0.2359255 1 3052 "tags=26%, list=22%, signal=33%"
GSE37605_TREG_VS_TCONV_NOD_FOXP3_FUSION_GFP_DN
GSE37605_TREG_VS_TCONV_NOD_FOXP3_FUSION_GFP_DN
134 -0.28689983 -1.1740195 0.16111112
0.23653586 1 2631 "tags=25%, list=19%, signal=31%"
GSE19941_UNSTIM_VS_LPS_AND_IL10_STIM_IL10_KO_MACROPHAGE_UP
GSE19941_UNSTIM_VS_LPS_AND_IL10_STIM_IL10_KO_MACROPHAGE_UP
152 -0.28191292 -1.1738335 0.1344383
0.2367089 1 1731 "tags=19%, list=12%, signal=22%"
GSE2770_UNTREATED_VS_IL12_TREATED_ACT_CD4_TCELL_6H_UP
GSE2770_UNTREATED_VS_IL12_TREATED_ACT_CD4_TCELL_6H_UP
144 -0.28249267 -1.1734979 0.12569316
0.23714498 1 2052 "tags=23%, list=15%, signal=27%"
GSE37533_PPARG1_FOXP3_VS_PPARG2_FOXP3_TRANSDUCED_CD4_TCELL_DN
GSE37533_PPARG1_FOXP3_VS_PPARG2_FOXP3_TRANSDUCED_CD4_TCELL_DN
119 -0.29590017 -1.1733313 0.17448406
0.2372762 1 2028 "tags=24%, list=14%, signal=28%"
GSE22589_SIV_VS_HIV_AND_SIV_INFECTED_DC_UP
GSE22589_SIV_VS_HIV_AND_SIV_INFECTED_DC_UP
141 -0.28554055 -1.1732562 0.15779468
0.23722237 1 1463 "tags=15%, list=10%, signal=16%"
GSE16755_CTRL_VS_IFNA_TREATED_MAC_DN
GSE16755_CTRL_VS_IFNA_TREATED_MAC_DN
144 -0.28390768 -1.1727191 0.1409002
0.23807219 1 1546 "tags=19%, list=11%, signal=22%"
GSE36392_EOSINOPHIL_VS_MAC_IL25_TREATED_LUNG_UP
GSE36392_EOSINOPHIL_VS_MAC_IL25_TREATED_LUNG_UP
155 -0.28024167 -1.1726664 0.13718411
0.23799133 1 3469 "tags=25%, list=25%, signal=33%"
GSE6681_DELETED_FOXP3_VS_WT_TREG_DN
GSE6681_DELETED_FOXP3_VS_WT_TREG_DN
153 -0.28079212 -1.1725512 0.15607986
0.23803256 1 1817 "tags=22%, list=13%, signal=25%"
GSE11057_PBMC_VS_MEM_CD4_TCELL_UP GSE11057_PBMC_VS_MEM_CD4_TCELL_UP
136 -0.289231 -1.172398 0.17095588
0.23812689 1 2693 "tags=24%, list=19%, signal=29%"
GSE360_L_MAJOR_VS_T_GONDII_DC_DN GSE360_L_MAJOR_VS_T_GONDII_DC_DN
137 -0.2819211 -1.1723063 0.13188976
0.23812123 1 3024 "tags=26%, list=21%, signal=32%"
GSE17974_CTRL_VS_ACT_IL4_AND_ANTI_IL12_0.5H_CD4_TCELL_UP
GSE17974_CTRL_VS_ACT_IL4_AND_ANTI_IL12_0.5H_CD4_TCELL_UP
102 -0.30239558 -1.1718221 0.16541353
0.23886053 1 5026 "tags=41%, list=36%, signal=63%"
GSE41867_DAY6_VS_DAY8_LCMV_CLONE13_EFFECTOR_CD8_TCELL_DN
GSE41867_DAY6_VS_DAY8_LCMV_CLONE13_EFFECTOR_CD8_TCELL_DN

138 -0.28641742 -1.1715474 0.14734773
0.23918098 1 1715 "tags=20%, list=12%, signal=22%"
GSE35685_CD34POS_CD38NEG_VS_CD34POS_CD10POS_BONE_MARROW_DN
GSE35685_CD34POS_CD38NEG_VS_CD34POS_CD10POS_BONE_MARROW_DN
148 -0.28280815 -1.1710929 0.14391144
0.23983315 1 770 "tags=15%, list=5%, signal=16%"
GSE35825_UNTREATED_VS_IFNG_STIM_MACROPHAGE_DN
GSE35825_UNTREATED_VS_IFNG_STIM_MACROPHAGE_DN
144 -0.28514174 -1.171004 0.16159695
0.23981106 1 1968 "tags=24%, list=14%, signal=27%"
GSE5589_WT_VS_IL10_KO_LPS_STIM_MACROPHAGE_45MIN_DN
GSE5589_WT_VS_IL10_KO_LPS_STIM_MACROPHAGE_45MIN_DN
144 -0.28248936 -1.1708789 0.15444015
0.2398469 1 3481 "tags=31%, list=25%, signal=41%"
GSE32423_CTRL_VS_IL7_MEMORY_CD8_TCELL_DN
GSE32423_CTRL_VS_IL7_MEMORY_CD8_TCELL_DN
141 -0.28647557 -1.1708021 0.17021276
0.23981734 1 2754 "tags=24%, list=20%, signal=30%"
GSE23502_WT_VS_HDC_KO_MYELOID_DERIVED_SUPPRESSOR_CELL_BM_DN
GSE23502_WT_VS_HDC_KO_MYELOID_DERIVED_SUPPRESSOR_CELL_BM_DN
145 -0.28353468 -1.1705264 0.13384321
0.24012566 1 1126 "tags=19%, list=8%, signal=20%"
GSE32034_LY6C_HIGH_VS_LOW_MONOCYTE_DN
GSE32034_LY6C_HIGH_VS_LOW_MONOCYTE_DN
146 -0.2855731 -1.1699523 0.13711151
0.24102151 1 1870 "tags=24%, list=13%, signal=27%"
GSE3982_DC_VS_NKCELL_DN GSE3982_DC_VS_NKCELL_DN
124 -0.2882664 -1.1698408 0.14068441
0.24103366 1 2306 "tags=27%, list=16%, signal=32%"
GSE3982_EFF_MEMORY_CD4_TCELL_VS_NKCELL_UP
GSE3982_EFF_MEMORY_CD4_TCELL_VS_NKCELL_UP
127 -0.2894528 -1.169641 0.16377649
0.24121536 1 2562 "tags=24%, list=18%, signal=30%"
GSE10325_LUPUS_CD4_TCELL_VS_LUPUS_BCELL_UP
GSE10325_LUPUS_CD4_TCELL_VS_LUPUS_BCELL_UP
157 -0.28029177 -1.1693854 0.12749004
0.24154735 1 1231 "tags=15%, list=9%, signal=17%"
GSE27670_CTRL_VS_BLIMP1_TRANSDUCED_GC_BCELL_DN
GSE27670_CTRL_VS_BLIMP1_TRANSDUCED_GC_BCELL_DN
154 -0.28189266 -1.169242 0.15637065
0.24163625 1 2122 "tags=19%, list=15%, signal=23%"
GSE29618_PRE_VS_DAY7_POST_LAIV_FLU_VACCINE_BCELL_DN
GSE29618_PRE_VS_DAY7_POST_LAIV_FLU_VACCINE_BCELL_DN
135 -0.2829192 -1.1686702 0.15799257
0.24250557 1 1582 "tags=19%, list=11%, signal=21%"
GSE25085_FETAL_BM_VS_ADULT_BM_SP4_THYMIC_IMPLANT_DN
GSE25085_FETAL_BM_VS_ADULT_BM_SP4_THYMIC_IMPLANT_DN
152 -0.27919018 -1.1681294 0.16165413
0.24332057 1 1770 "tags=22%, list=13%, signal=25%"
GSE13485_DAY1_VS_DAY3_YF17D_VACCINE_PBMC_DN
GSE13485_DAY1_VS_DAY3_YF17D_VACCINE_PBMC_DN
125 -0.2888945 -1.1676648 0.17358491
0.24398856 1 1295 "tags=19%, list=9%, signal=21%"
GSE9037_CTRL_VS_LPS_1H_STIM_BMDM_DN

GSE9037_CTRL_VS_LPS_1H_STIM_BMDM_DN
151	-0.27941072	-1.1674589	0.13539653
0.24417876	1	1674	"tags=17%, list=12%, signal=19%"
GSE40274_FOXP3_VS_FOXP3_AND_LEF1_TRANSDUCED_ACTIVATED_CD4_TCELL_DN
GSE40274_FOXP3_VS_FOXP3_AND_LEF1_TRANSDUCED_ACTIVATED_CD4_TCELL_DN
148	-0.28368506	-1.1670552	0.14661655
0.24475679	1	1942	"tags=20%, list=14%, signal=22%"
GSE5455_EX_VIVO_VS_POST_24H_INCUBATION_MONOCYTES_FROM_TUMOR_BEARING_MOUSE_DN
GSE5455_EX_VIVO_VS_POST_24H_INCUBATION_MONOCYTES_FROM_TUMOR_BEARING_MOUSE_DN	141	-0.28056565	-1.1654868
0.13450292	0.24756776	1	2236	"tags=24%, list=16%, signal=28%"
GSE45739_NRAS_KO_VS_WT_ACD3_ACD28_STIM_CD4_TCELL_DN
GSE45739_NRAS_KO_VS_WT_ACD3_ACD28_STIM_CD4_TCELL_DN
132	-0.28589478	-1.1653135	0.15238096
0.24773277	1	2287	"tags=24%, list=16%, signal=29%"
GSE20727_ROS_INH_VS_ROS_INH_AND_DNFB_ALLERGEN_TREATED_DC_DN
GSE20727_ROS_INH_VS_ROS_INH_AND_DNFB_ALLERGEN_TREATED_DC_DN
110	-0.29515073	-1.1652842	0.18014705
0.24760383	1	3746	"tags=27%, list=27%, signal=37%"
GSE14769_UNSTIM_VS_20MIN_LPS_BMDM_UP
GSE14769_UNSTIM_VS_20MIN_LPS_BMDM_UP
141	-0.2870881	-1.164337	0.1539924
0.24918231	1	2857	"tags=21%, list=20%, signal=26%"
GSE6269_E_COLI_VS_STAPH_AUREUS_INF_PBMC_UP
GSE6269_E_COLI_VS_STAPH_AUREUS_INF_PBMC_UP
115	-0.2895306	-1.1643103	0.17373738
0.24904637	1	2137	"tags=23%, list=15%, signal=27%"
GSE30083_SP2_VS_SP4_THYMOCYTE_UP	GSE30083_SP2_VS_SP4_THYMOCYTE_UP
148	-0.282206	-1.1635017	0.14862385
0.2503427	1	4165	"tags=33%, list=30%, signal=46%"
GSE11057_NAIVE_CD4_VS_PBMC_CD4_TCELL_UP
GSE11057_NAIVE_CD4_VS_PBMC_CD4_TCELL_UP
117	-0.29324862	-1.1634098	0.18430656
0.2503577	1	1837	"tags=20%, list=13%, signal=22%"
GSE24142_DN2_VS_DN3_THYMOCYTE_ADULT_DN
GSE24142_DN2_VS_DN3_THYMOCYTE_ADULT_DN
140	-0.28268954	-1.1634037	0.17260788
0.2501846	1	1264	"tags=15%, list=9%, signal=16%"
GSE5589_WT_VS_IL6_KO_LPS_STIM_MACROPHAGE_180MIN_UP
GSE5589_WT_VS_IL6_KO_LPS_STIM_MACROPHAGE_180MIN_UP
101	-0.29667568	-1.1632061	0.20817845
0.2503657	1	3132	"tags=26%, list=22%, signal=33%"
GSE2706_2H_VS_8H_R848_AND_LPS_STIM_DC_DN
GSE2706_2H_VS_8H_R848_AND_LPS_STIM_DC_DN
127	-0.28615072	-1.1628041	0.17260788
0.25095809	1	2047	"tags=20%, list=15%, signal=24%"
GSE13522_CTRL_VS_T_CRUZI_G_STRAIN_INF_SKIN_UP
GSE13522_CTRL_VS_T_CRUZI_G_STRAIN_INF_SKIN_UP
144	-0.28211963	-1.1626085	0.17588933
0.25114995	1	1851	"tags=23%, list=13%, signal=26%"
GSE24142_EARLY_THYMIC_PROGENITOR_VS_DN3_THYMOCYTE_DN
GSE24142_EARLY_THYMIC_PROGENITOR_VS_DN3_THYMOCYTE_DN

162	-0.27899367	-1.161993	0.1391129
0.2521311	1	2028	"tags=22%, list=14%, signal=26%"
GSE12963_UNINF_VS_ENV_AND_NEF_DEFICIENT_HIV1_INF_CD4_TCELL_UP
GSE12963_UNINF_VS_ENV_AND_NEF_DEFICIENT_HIV1_INF_CD4_TCELL_UP
	106	-0.292956	-1.1617459	0.17137477
0.2524303	1	1996	"tags=22%, list=14%, signal=25%"
GSE37532_WT_VS_PPARG_KO_LN_TREG_DN
GSE37532_WT_VS_PPARG_KO_LN_TREG_DN
88	-0.3043599	-1.1614915	0.17765568
0.2527356	1	5928	"tags=47%, list=42%, signal=80%"
GSE14000_4H_VS_16H_LPS_DC_TRANSLATED_RNA_UP
GSE14000_4H_VS_16H_LPS_DC_TRANSLATED_RNA_UP
141	-0.2793776	-1.1612916	0.13831776
0.25292617	1	1823	"tags=20%, list=13%, signal=23%"
GSE7831_UNSTIM_VS_INFLUENZA_STIM_PDC_1H_DN
GSE7831_UNSTIM_VS_INFLUENZA_STIM_PDC_1H_DN
139	-0.2856523	-1.1611848	0.16061185
0.2529388	1	1827	"tags=22%, list=13%, signal=25%"
GSE43955_TH0_VS_TGFB_IL6_TH17_ACT_CD4_TCELL_20H_UP
GSE43955_TH0_VS_TGFB_IL6_TH17_ACT_CD4_TCELL_20H_UP
152	-0.2802278	-1.1611212	0.14531548
0.25288123	1	2615	"tags=28%, list=19%, signal=34%"
GSE17721_CTRL_VS_CPG_2H_BMDC_DN	GSE17721_CTRL_VS_CPG_2H_BMDC_DN
	157	-0.28061008	-1.1610469	0.16334662
0.2528501	1	2000	"tags=20%, list=14%, signal=23%"
GSE39820_CTRL_VS_IL1B_IL6_CD4_TCELL_DN
GSE39820_CTRL_VS_IL1B_IL6_CD4_TCELL_DN
146	-0.2794856	-1.1608456	0.1539961
0.2530739	1	1890	"tags=20%, list=13%, signal=23%"
GSE24142_EARLY_THYMIC_PROGENITOR_VS_DN2_THYMOCYTE_FETAL_DN
GSE24142_EARLY_THYMIC_PROGENITOR_VS_DN2_THYMOCYTE_FETAL_DN
156	-0.2781436	-1.1606694	0.15605749
0.2532402	1	2096	"tags=21%, list=15%, signal=25%"
GSE41176_UNSTIM_VS_ANTI_IGM_STIM_BCELL_24H_DN
GSE41176_UNSTIM_VS_ANTI_IGM_STIM_BCELL_24H_DN
144	-0.2820912	-1.1605579	0.14931238
0.2532385	1	1329	"tags=18%, list=9%, signal=20%"
GSE24142_DN2_VS_DN3_THYMOCYTE_FETAL_UP
GSE24142_DN2_VS_DN3_THYMOCYTE_FETAL_UP
156	-0.27828848	-1.160468	0.14821763
0.25321704	1	1455	"tags=17%, list=10%, signal=18%"
GSE21360_NAIVE_VS_PRIMARY_MEMORY_CD8_TCELL_DN
GSE21360_NAIVE_VS_PRIMARY_MEMORY_CD8_TCELL_DN
147	-0.28047407	-1.1601803	0.14559387
0.25357834	1	2344	"tags=25%, list=17%, signal=30%"
GSE39820_TGFBETA3_IL6_VS_TGFBETA3_IL6_IL23A_TREATED_CD4_TCELL_UP
GSE39820_TGFBETA3_IL6_VS_TGFBETA3_IL6_IL23A_TREATED_CD4_TCELL_UP
	146	-0.27894124	-1.1600342	0.14990138
0.2536583	1	2992	"tags=27%, list=21%, signal=34%"
GSE13306_RA_VS_UNTREATED_TREG_UP	GSE13306_RA_VS_UNTREATED_TREG_UP
	142	-0.28036672	-1.1591849	0.16853933
0.25510913	1	1953	"tags=21%, list=14%, signal=24%"
GSE2405_0H_VS_6H_A_PHAGOCYTOPHILUM_STIM_NEUTROPHIL_UP
GSE2405_0H_VS_6H_A_PHAGOCYTOPHILUM_STIM_NEUTROPHIL_UP

131	-0.28686497	-1.1591334	0.15151516
0.25502166	1	1110	"tags=17%, list=8%, signal=18%"
GSE1460_CD4_THYMOCYTE_VS_NAIVE_CD4_TCELL_CORD_BLOOD_DN
GSE1460_CD4_THYMOCYTE_VS_NAIVE_CD4_TCELL_CORD_BLOOD_DN
129	-0.28675565	-1.1586211	0.18252428
0.25581354	1	717	"tags=10%, list=5%, signal=11%"
GSE6674_PL2_3_VS_ANTI_IGM_AND_CPG_STIM_BCELL_DN
GSE6674_PL2_3_VS_ANTI_IGM_AND_CPG_STIM_BCELL_DN
147	-0.2801512	-1.158526	0.16058394
0.25582185	1	661	"tags=12%, list=5%, signal=13%"
GSE37416_0H_VS_6H_F_TULARENSIS_LVS_NEUTROPHIL_DN
GSE37416_0H_VS_6H_F_TULARENSIS_LVS_NEUTROPHIL_DN
142	-0.28320074	-1.1581	0.17142858	0.2564421
1	1285	"tags=19%, list=9%, signal=21%"
GSE2706_2H_VS_8H_LPS_STIM_DC_DN	GSE2706_2H_VS_8H_LPS_STIM_DC_DN
	114	-0.29342207	-1.1575094	0.20281124
0.2573914	1	2486	"tags=29%, list=18%, signal=35%"
GSE37532_VISCERAL_ADIPOSE_TISSUE_VS_LN_DERIVED_PPARG_KO_TCONV_CD4_TC
ELL_DN
GSE37532_VISCERAL_ADIPOSE_TISSUE_VS_LN_DERIVED_PPARG_KO_TCONV_CD4_TC
ELL_DN	72	-0.31559896	-1.156401
0.20437956	0.25940353	1	3819	"tags=33%,
list=27%, signal=45%"
GSE9946_MATURE_STIMULATORY_VS_PROSTAGLANDINE2_TREATED_MATURE_DC_UP
GSE9946_MATURE_STIMULATORY_VS_PROSTAGLANDINE2_TREATED_MATURE_DC_UP
	89	-0.2995565	-1.1560391	0.1747212
0.25991586	1	1811	"tags=20%, list=13%, signal=23%"
GSE8685_IL15_ACT_IL2_STARVED_VS_IL21_ACT_IL2_STARVED_CD4_TCELL_DN
GSE8685_IL15_ACT_IL2_STARVED_VS_IL21_ACT_IL2_STARVED_CD4_TCELL_DN
	132	-0.28458124	-1.1558971	0.17988394
0.26002952	1	1712	"tags=20%, list=12%, signal=22%"
GSE24726_WT_VS_E2_2_KO_PDC_DAY6_POST_DELETION_DN
GSE24726_WT_VS_E2_2_KO_PDC_DAY6_POST_DELETION_DN
153	-0.27745277	-1.155412	0.16360295
0.26079816	1	2280	"tags=25%, list=16%, signal=30%"
GSE17974_CTRL_VS_ACT_IL4_AND_ANTI_IL12_1H_CD4_TCELL_UP
GSE17974_CTRL_VS_ACT_IL4_AND_ANTI_IL12_1H_CD4_TCELL_UP
127	-0.28516662	-1.1548287	0.17919075
0.26171753	1	2487	"tags=25%, list=18%, signal=30%"
GSE10325_CD4_TCELL_VS_LUPUS_CD4_TCELL_DN
GSE10325_CD4_TCELL_VS_LUPUS_CD4_TCELL_DN
134	-0.28275478	-1.1538925	0.18669131
0.26330984	1	1912	"tags=24%, list=14%, signal=27%"
GSE30971_WBP7_HET_VS_KO_MACROPHAGE_4H_LPS_STIM_UP
GSE30971_WBP7_HET_VS_KO_MACROPHAGE_4H_LPS_STIM_UP
137	-0.2847386	-1.1537621	0.17680608
0.2633867	1	1680	"tags=21%, list=12%, signal=24%"
GSE18281_SUBCAPSULAR_CORTICAL_REGION_VS_WHOLE_MEDULLA_THYMUS_DN
GSE18281_SUBCAPSULAR_CORTICAL_REGION_VS_WHOLE_MEDULLA_THYMUS_DN
	141	-0.27614772	-1.1534857	0.14285715
0.26373377	1	2636	"tags=18%, list=19%, signal=22%"
GSE14769_UNSTIM_VS_40MIN_LPS_BMDM_UP
GSE14769_UNSTIM_VS_40MIN_LPS_BMDM_UP
141	-0.277864	-1.1531981	0.16788322

0.26409924	1	4283	"tags=37%, list=30%, signal=52%"
GSE9988_ANTI_TREM1_VS_LPS_MONOCYTE_UP
GSE9988_ANTI_TREM1_VS_LPS_MONOCYTE_UP
147	-0.28258258	-1.1528947	0.17210145
0.26448727	1	1712	"tags=22%, list=12%, signal=25%"
GSE34156_TLR1_TLR2_LIGAND_VS_NOD2_AND_TLR1_TLR2_LIGAND_6H_TREATED_MONOCYTE_DN
GSE34156_TLR1_TLR2_LIGAND_VS_NOD2_AND_TLR1_TLR2_LIGAND_6H_TREATED_MONOCYTE_DN	101	-0.2931948	-1.152874
0.20488721	0.2643408	1	2519	"tags=25%, list=18%, signal=30%"
GSE40274_HELIOS_VS_FOXP3_AND_HELIOS_TRANSDUCED_ACTIVATED_CD4_TCELL_DN
GSE40274_HELIOS_VS_FOXP3_AND_HELIOS_TRANSDUCED_ACTIVATED_CD4_TCELL_DN	143	-0.28008422	-1.1524769
0.1743295	0.26490697	1	1399	"tags=19%, list=10%, signal=21%"
GSE18281_CORTICAL_THYMOCYTE_VS_WHOLE_CORTEX_THYMUS_UP
GSE18281_CORTICAL_THYMOCYTE_VS_WHOLE_CORTEX_THYMUS_UP
149	-0.27775088	-1.15234 0.162	0.264976 1
5487	"tags=45%, list=39%, signal=73%"
GSE13738_TCR_VS_BYSTANDER_ACTIVATED_CD4_TCELL_DN
GSE13738_TCR_VS_BYSTANDER_ACTIVATED_CD4_TCELL_DN
133	-0.27787286	-1.1520265	0.18181819
0.2653821	1	2139	"tags=25%, list=15%, signal=29%"
GSE22886_NAIVE_CD8_TCELL_VS_MONOCYTE_DN
GSE22886_NAIVE_CD8_TCELL_VS_MONOCYTE_DN
154	-0.2771664	-1.151649	0.16171004	0.265902
1	1703	"tags=19%, list=12%, signal=22%"
GSE17974_0H_VS_1H_IN_VITRO_ACT_CD4_TCELL_DN
GSE17974_0H_VS_1H_IN_VITRO_ACT_CD4_TCELL_DN
130	-0.2788057	-1.1508341	0.16171004
0.2673389	1	2402	"tags=29%, list=17%, signal=35%"
GSE2405_HEAT_KILLED_LYSATE_VS_LIVE_A_PHAGOCYTOPHILUM_STIM_NEUTROPHIL_24H_UP
GSE2405_HEAT_KILLED_LYSATE_VS_LIVE_A_PHAGOCYTOPHILUM_STIM_NEUTROPHIL_24H_UP	162	-0.2746856	-1.1504494
0.18076923	0.26791298	1	2828	"tags=31%, list=20%, signal=39%"
GSE43955_TH0_VS_TGFB_IL6_TH17_ACT_CD4_TCELL_30H_UP
GSE43955_TH0_VS_TGFB_IL6_TH17_ACT_CD4_TCELL_30H_UP
162	-0.27292773	-1.1495215	0.19135803
0.26954627	1	2450	"tags=23%, list=17%, signal=27%"
GSE19888_NO_PRETREAT_VS_ADENOSINE_A3R_INHIBITOR_PRETREATED_MAST_CELL_TCELL_MEMBRANES_ACT_DN
GSE19888_NO_PRETREAT_VS_ADENOSINE_A3R_INHIBITOR_PRETREATED_MAST_CELL_TCELL_MEMBRANES_ACT_DN
143	-0.27605847	-1.1488594	0.17196262
0.27063555	1	1467	"tags=20%, list=10%, signal=22%"
GSE14908_RESTING_VS_HDM_STIM_CD4_TCELL_NONATOPIC_PATIENT_DN
GSE14908_RESTING_VS_HDM_STIM_CD4_TCELL_NONATOPIC_PATIENT_DN
147	-0.277843	-1.1488165	0.1641791
0.27053544	1	2288	"tags=26%, list=16%, signal=31%"
GSE3982_MAST_CELL_VS_NKCELL_UP	GSE3982_MAST_CELL_VS_NKCELL_UP

146	-0.28023407	-1.14873	0.18878505
0.27049333	1	1507	"tags=19%, list=11%, signal=21%"
GSE39820_CTRL_VS_IL1B_IL6_CD4_TCELL_UP
GSE39820_CTRL_VS_IL1B_IL6_CD4_TCELL_UP
143	-0.2814285	-1.148403	0.18738404
0.27094254	1	1909	"tags=26%, list=14%, signal=30%"
GSE3982_BCELL_VS_CENT_MEMORY_CD4_TCELL_UP
GSE3982_BCELL_VS_CENT_MEMORY_CD4_TCELL_UP
137	-0.27925816	-1.1478415	0.17311233
0.27189088	1	2162	"tags=22%, list=15%, signal=26%"
GSE44649_WT_VS_MIR155_KO_ACTIVATED_CD8_TCELL_UP
GSE44649_WT_VS_MIR155_KO_ACTIVATED_CD8_TCELL_UP
143	-0.27473253	-1.1477696	0.17115384
0.27184668	1	2491	"tags=25%, list=18%, signal=30%"
GSE36476_CTRL_VS_TSST_ACT_16H_MEMORY_CD4_TCELL_OLD_UP
GSE36476_CTRL_VS_TSST_ACT_16H_MEMORY_CD4_TCELL_OLD_UP
135	-0.282328	-1.1471128	0.15327103
0.27293962	1	1625	"tags=21%, list=12%, signal=23%"
GSE17721_0.5H_VS_12H_CPG_BMDC_DN	GSE17721_0.5H_VS_12H_CPG_BMDC_DN
156	-0.2739415	-1.1469383	0.15254237
0.2730839	1	2473	"tags=23%, list=18%, signal=28%"
GSE16385_MONOCYTE_VS_12H_ROSIGLITAZONE_IFNG_TNF_TREATED_MACROPHAGE_UP
GSE16385_MONOCYTE_VS_12H_ROSIGLITAZONE_IFNG_TNF_TREATED_MACROPHAGE_UP	148	-0.27618566	-1.1465629
0.18359375	0.27365658	1	1823	"tags=19%, list=13%, signal=21%"
GSE4535_BM_DERIVED_DC_VS_FOLLICULAR_DC_DN
GSE4535_BM_DERIVED_DC_VS_FOLLICULAR_DC_DN
140	-0.28125617	-1.1463345	0.19581749
0.27393943	1	2589	"tags=29%, list=18%, signal=35%"
GSE3039_CD4_TCELL_VS_B2_BCELL_UP	GSE3039_CD4_TCELL_VS_B2_BCELL_UP
144	-0.2735605	-1.1462735	0.15601504
0.27385548	1	2199	"tags=24%, list=16%, signal=28%"
GSE40274_FOXP3_VS_FOXP3_AND_SATB1_TRANSDUCED_ACTIVATED_CD4_TCELL_DN
GSE40274_FOXP3_VS_FOXP3_AND_SATB1_TRANSDUCED_ACTIVATED_CD4_TCELL_DN
141	-0.27869585	-1.1462098	0.1796875
0.2737807	1	1455	"tags=22%, list=10%, signal=24%"
GSE18791_UNSTIM_VS_NEWCATSLE_VIRUS_DC_2H_DN
GSE18791_UNSTIM_VS_NEWCATSLE_VIRUS_DC_2H_DN
100	-0.29528967	-1.1459186	0.20527308
0.27419066	1	3797	"tags=35%, list=27%, signal=48%"
GSE25123_CTRL_VS_IL4_AND_ROSIGLITAZONE_STIM_PPARG_KO_MACROPHAGE_UP
GSE25123_CTRL_VS_IL4_AND_ROSIGLITAZONE_STIM_PPARG_KO_MACROPHAGE_UP
142	-0.27894723	-1.1458749	0.16697589
0.27408758	1	3270	"tags=27%, list=23%, signal=35%"
GSE1925_CTRL_VS_24H_IFNG_STIM_MACROPHAGE_DN
GSE1925_CTRL_VS_24H_IFNG_STIM_MACROPHAGE_DN
133	-0.27673274	-1.1453055	0.1880998
0.2750525	1	1396	"tags=20%, list=10%, signal=21%"
GSE43863_NAIVE_VS_MEMORY_TFH_CD4_TCELL_D150_LCMV_UP
GSE43863_NAIVE_VS_MEMORY_TFH_CD4_TCELL_D150_LCMV_UP
148	-0.27505565	-1.1451548	0.17836812
0.2751547	1	1865	"tags=22%, list=13%, signal=25%"

GSE22432_MULTIPOTENT_VS_COMMON_DC_PROGENITOR_DN
GSE22432_MULTIPOTENT_VS_COMMON_DC_PROGENITOR_DN
155 -0.27457723 -1.1450807 0.18164794
0.27510232 1 1295 "tags=16%, list=9%, signal=18%"
GSE24634_IL4_VS_CTRL_TREATED_NAIVE_CD4_TCELL_DAY7_DN
GSE24634_IL4_VS_CTRL_TREATED_NAIVE_CD4_TCELL_DAY7_DN
142 -0.28056616 -1.1446627 0.17877094
0.27573094 1 1341 "tags=17%, list=10%, signal=18%"
GSE369_SOCS3_KO_VS_WT_LIVER_POST_IL6_INJECTION_DN
GSE369_SOCS3_KO_VS_WT_LIVER_POST_IL6_INJECTION_DN
155 -0.27481467 -1.1440346 0.16446126
0.27678505 1 1934 "tags=23%, list=14%, signal=27%"
GSE14000_UNSTIM_VS_4H_LPS_DC_TRANSLATED_RNA_UP
GSE14000_UNSTIM_VS_4H_LPS_DC_TRANSLATED_RNA_UP
125 -0.2829895 -1.1439867 0.17293233
0.27668554 1 1354 "tags=19%, list=10%, signal=21%"
GSE36826_WT_VS_IL1R_KO_SKIN_STAPH_AUREUS_INF_DN
GSE36826_WT_VS_IL1R_KO_SKIN_STAPH_AUREUS_INF_DN
149 -0.27440616 -1.1435486 0.16981132
0.27735502 1 1783 "tags=21%, list=13%, signal=24%"
GSE18804_SPLEEN_MACROPHAGE_VS_TUMORAL_MACROPHAGE_UP
GSE18804_SPLEEN_MACROPHAGE_VS_TUMORAL_MACROPHAGE_UP
123 -0.2830653 -1.1434642 0.18074656
0.27733937 1 1222 "tags=15%, list=9%, signal=17%"
GSE2128_CTRL_VS_MIMETOPE_NEGATIVE_SELECTION_DP_THYMOCYTE_NOD_UP
GSE2128_CTRL_VS_MIMETOPE_NEGATIVE_SELECTION_DP_THYMOCYTE_NOD_UP
148 -0.27750954 -1.1433216 0.1906542
0.27740213 1 1424 "tags=19%, list=10%, signal=21%"
GSE11864_CSF1_VS_CSF1_PAM3CYS_IN_MAC_UP
GSE11864_CSF1_VS_CSF1_PAM3CYS_IN_MAC_UP
133 -0.28083602 -1.1433052 0.18395303
0.2772344 1 1461 "tags=20%, list=10%, signal=22%"
GSE36527_CD62L_HIGH_CD69_NEG_VS_CD62L_LOW_CD69_POS_TREG_KLRG1_NEG_UP

GSE36527_CD62L_HIGH_CD69_NEG_VS_CD62L_LOW_CD69_POS_TREG_KLRG1_NEG_UP
157 -0.2700611 -1.1427884
0.17557251 0.27806726 1 2142 "tags=22%,
list=15%, signal=25%"
GSE3920_IFNB_VS_IFNG_TREATED_ENDOTHELIAL_CELL_UP
GSE3920_IFNB_VS_IFNG_TREATED_ENDOTHELIAL_CELL_UP
127 -0.2850801 -1.142217 0.19960861
0.2790176 1 1462 "tags=20%, list=10%, signal=23%"
GSE3982_DC_VS_NEUTROPHIL_DN GSE3982_DC_VS_NEUTROPHIL_DN
134 -0.28182036 -1.1420531 0.19165085
0.2791832 1 1621 "tags=19%, list=11%, signal=21%"
GSE3982_BASOPHIL_VS_CENT_MEMORY_CD4_TCELL_UP
GSE3982_BASOPHIL_VS_CENT_MEMORY_CD4_TCELL_UP
139 -0.2792757 -1.1415836 0.18959108
0.27994546 1 1445 "tags=17%, list=10%, signal=19%"
GSE43955_10H_VS_60H_ACT_CD4_TCELL_WITH_TGFB_IL6_DN
GSE43955_10H_VS_60H_ACT_CD4_TCELL_WITH_TGFB_IL6_DN
165 -0.27086365 -1.1408209 0.19585687
0.28133726 1 2553 "tags=25%, list=18%, signal=30%"
GSE26488_CTRL_VS_PEPTIDE_INJECTION_OT2_THYMOCYTE_DN

GSE26488_CTRL_VS_PEPTIDE_INJECTION_OT2_THYMOCYTE_DN
140	-0.27725527	-1.1405559	0.20229007
0.28167754	1	1585	"tags=21%, list=11%, signal=23%"
GSE34156_UNTREATED_VS_6H_TLR1_TLR2_LIGAND_TREATED_MONOCYTE_DN
GSE34156_UNTREATED_VS_6H_TLR1_TLR2_LIGAND_TREATED_MONOCYTE_DN
	130	-0.2807025	-1.1404277	0.19850187
0.2817416	1	1317	"tags=20%, list=9%, signal=22%"
GSE22589_HEALTHY_VS_HIV_INFECTED_DC_DN
GSE22589_HEALTHY_VS_HIV_INFECTED_DC_DN
153	-0.27280375	-1.1403669	0.2	0.28167006
1	2535	"tags=25%, list=18%, signal=30%"
GSE25123_CTRL_VS_ROSIGLITAZONE_STIM_PPARG_KO_MACROPHAGE_DN
GSE25123_CTRL_VS_ROSIGLITAZONE_STIM_PPARG_KO_MACROPHAGE_DN
143	-0.27672756	-1.1402103	0.19075145
0.28179437	1	1600	"tags=15%, list=11%, signal=17%"
GSE25846_IL10_POS_VS_NEG_CD8_TCELL_DAY7_POST_CORONAVIRUS_BRAIN_UP
GSE25846_IL10_POS_VS_NEG_CD8_TCELL_DAY7_POST_CORONAVIRUS_BRAIN_UP
	130	-0.27796432	-1.1389753	0.20113853
0.28409708	1	3745	"tags=38%, list=27%, signal=51%"
GSE557_WT_VS_CIITA_KO_DC_UP	GSE557_WT_VS_CIITA_KO_DC_UP
	136	-0.27890432	-1.1389581	0.20073664
0.2839313	1	2492	"tags=28%, list=18%, signal=34%"
GSE17721_LPS_VS_POLYIC_2H_BMDC_UP GSE17721_LPS_VS_POLYIC_2H_BMDC_UP
	167	-0.27081063	-1.1389424	0.18045112
0.28376275	1	2155	"tags=20%, list=15%, signal=23%"
GSE3982_MAST_CELL_VS_BCELL_DN	GSE3982_MAST_CELL_VS_BCELL_DN
	130	-0.28399396	-1.1389337	0.21031746
0.28358096	1	3714	"tags=36%, list=26%, signal=49%"
GSE39382_IL3_VS_IL3_IL33_TREATED_MAST_CELL_UP
GSE39382_IL3_VS_IL3_IL33_TREATED_MAST_CELL_UP
151	-0.2746229	-1.1385067	0.20306513
0.28425342	1	1941	"tags=21%, list=14%, signal=24%"
GSE22611_MUTANT_NOD2_TRANSDUCED_VS_CTRL_HEK293T_STIMULATED_WITH_MDP_6H_DN
GSE22611_MUTANT_NOD2_TRANSDUCED_VS_CTRL_HEK293T_STIMULATED_WITH_MDP_6H_DN	144	-0.27966326	-1.1382349
0.18568665	0.28460637	1	2651	"tags=24%, list=19%, signal=29%"
GSE35543_IN_VIVO_NTREG_VS_IN_VITRO_ITREG_DN
GSE35543_IN_VIVO_NTREG_VS_IN_VITRO_ITREG_DN
103	-0.2954092	-1.1372266	0.21290323
0.28643593	1	2457	"tags=30%, list=17%, signal=36%"
GSE18893_TCONV_VS_TREG_24H_TNF_STIM_DN
GSE18893_TCONV_VS_TREG_24H_TNF_STIM_DN
135	-0.27844775	-1.1370637	0.18582375
0.2865554	1	2882	"tags=27%, list=20%, signal=34%"
GSE29615_CTRL_VS_DAY7_LAIV_FLU_VACCINE_PBMC_DN
GSE29615_CTRL_VS_DAY7_LAIV_FLU_VACCINE_PBMC_DN
87	-0.29853258	-1.1370147	0.21441442
0.28646666	1	3107	"tags=26%, list=22%, signal=34%"
GSE28130_ACTIVATED_VS_INDUCEED_TREG_UP
GSE28130_ACTIVATED_VS_INDUCEED_TREG_UP
148	-0.27094242	-1.1366795	0.18106996
0.2869342	1	1696	"tags=16%, list=12%, signal=17%"

GSE21063_3H_VS_16H_ANTI_IGM_STIM_BCELL_UP
GSE21063_3H_VS_16H_ANTI_IGM_STIM_BCELL_UP
147 -0.27515018 -1.1366417 0.19066148
0.28680196 1 1285 "tags=16%, list=9%, signal=17%"
GSE12366_GC_BCELL_VS_PLASMA_CELL_DN
GSE12366_GC_BCELL_VS_PLASMA_CELL_DN
129 -0.28122136 -1.1362233 0.22118959
0.28745052 1 2443 "tags=24%, list=17%, signal=29%"
GSE22140_GERMFREE_VS_SPF_MOUSE_CD4_TCELL_UP
GSE22140_GERMFREE_VS_SPF_MOUSE_CD4_TCELL_UP
148 -0.27469248 -1.1356239 0.19622642
0.28846037 1 1766 "tags=20%, list=13%, signal=22%"
GSE3982_MAC_VS_BCELL_DN GSE3982_MAC_VS_BCELL_DN
124 -0.28607804 -1.1353081 0.19662921
0.28895137 1 3611 "tags=31%, list=26%, signal=42%"
GSE32034_LY6C_HIGH_VS_LOW_ROSIGLIZATONE_TREATED_MONOCYTE_DN
GSE32034_LY6C_HIGH_VS_LOW_ROSIGLIZATONE_TREATED_MONOCYTE_DN
159 -0.2675929 -1.1350352 0.18290259
0.28935096 1 906 "tags=13%, list=6%, signal=14%"
GSE37533_UNTREATED_VS_PIOGLIZATONE_TREATED_CD4_TCELL_PPARG1_AND_FOXP
3_TRASDUCED_UP
GSE37533_UNTREATED_VS_PIOGLIZATONE_TREATED_CD4_TCELL_PPARG1_AND_FOXP
3_TRASDUCED_UP 151 -0.27508304 -1.1347321
0.19886364 0.28977945 1 1770 "tags=26%,
list=13%, signal=29%"
GSE13762_CTRL_VS_125_VITAMIND_DAY5_DC_DN
GSE13762_CTRL_VS_125_VITAMIND_DAY5_DC_DN
106 -0.28589788 -1.1342335 0.20670392
0.2906193 1 4981 "tags=41%, list=35%, signal=62%"
GSE17721_LPS_VS_PAM3CSK4_0.5H_BMDC_DN
GSE17721_LPS_VS_PAM3CSK4_0.5H_BMDC_DN
149 -0.27356625 -1.1338805 0.20992367
0.29114622 1 2050 "tags=20%, list=15%, signal=23%"
GSE2706_UNSTIM_VS_2H_LPS_DC_UP GSE2706_UNSTIM_VS_2H_LPS_DC_UP
79 -0.30447373 -1.1338497 0.23818526
0.29101452 1 3639 "tags=43%, list=26%, signal=58%"
GSE28737_BCL6_HET_VS_BCL6_KO_FOLLICULAR_BCELL_UP
GSE28737_BCL6_HET_VS_BCL6_KO_FOLLICULAR_BCELL_UP
140 -0.27644113 -1.1337547 0.21839081
0.29101342 1 1101 "tags=15%, list=8%, signal=16%"
GSE20198_UNTREATED_VS_IL12_IL18_TREATED_ACT_CD4_TCELL_DN
GSE20198_UNTREATED_VS_IL12_IL18_TREATED_ACT_CD4_TCELL_DN
141 -0.2773349 -1.1337475 0.19924812
0.29082772 1 1747 "tags=21%, list=12%, signal=23%"
GSE19888_ADENOSINE_A3R_INH_VS_INH_PRETREAT_AND_ACT_WITH_TCELL_MEMBRA
NES_MAST_CELL_DN
GSE19888_ADENOSINE_A3R_INH_VS_INH_PRETREAT_AND_ACT_WITH_TCELL_MEMBRA
NES_MAST_CELL_DN 137 -0.27697065 -1.1332389
0.21637426 0.29163674 1 1571 "tags=14%,
list=11%, signal=15%"
GSE42088_UNINF_VS_LEISHMANIA_INF_DC_8H_UP
GSE42088_UNINF_VS_LEISHMANIA_INF_DC_8H_UP
139 -0.27412355 -1.1330603 0.1821632
0.29179227 1 2087 "tags=24%, list=15%, signal=28%"

GSE32164_RESTING_DIFFERENTIATED_VS_CMYC_INHIBITED_MACROPHAGE_UP
GSE32164_RESTING_DIFFERENTIATED_VS_CMYC_INHIBITED_MACROPHAGE_UP
163 -0.27252045 -1.1328096 0.20703125
0.29210493 1 2570 "tags=25%, list=18%, signal=30%"
GSE21670_UNTREATED_VS_TGFB_TREATED_STAT3_KO_CD4_TCELL_DN
GSE21670_UNTREATED_VS_TGFB_TREATED_STAT3_KO_CD4_TCELL_DN
156 -0.27111575 -1.1327477 0.19460501
0.29202285 1 1841 "tags=22%, list=13%, signal=26%"
GSE22935_24H_VS_48H_MBOVIS_BCG_STIM_MYD88_KO_MACROPHAGE_DN
GSE22935_24H_VS_48H_MBOVIS_BCG_STIM_MYD88_KO_MACROPHAGE_DN
137 -0.27897948 -1.1321907 0.1852552
0.2930051 1 3604 "tags=31%, list=26%, signal=42%"
GSE3720_LPS_VS_PMA_STIM_VD2_GAMMADELTA_TCELL_UP
GSE3720_LPS_VS_PMA_STIM_VD2_GAMMADELTA_TCELL_UP
102 -0.28602663 -1.1321863 0.20883535
0.29281408 1 2848 "tags=31%, list=20%, signal=39%"
GSE7509_DC_VS_MONOCYTE_WITH_FCGRIIB_STIM_UP
GSE7509_DC_VS_MONOCYTE_WITH_FCGRIIB_STIM_UP
152 -0.27017337 -1.131312 0.188 0.29446313
1 1507 "tags=19%, list=11%, signal=21%"
GSE26290_CTRL_VS_AKT_INHIBITOR_TREATED_ANTI_CD3_AND_IL2_STIM_CD8_TCELL_DN
GSE26290_CTRL_VS_AKT_INHIBITOR_TREATED_ANTI_CD3_AND_IL2_STIM_CD8_TCELL_DN 149 -0.27180332 -1.131112
0.2043222 0.29467395 1 2217 "tags=23%,
list=16%, signal=28%"
GSE29949_MICROGLIA_BRAIN_VS_MONOCYTE_BONE_MARROW_UP
GSE29949_MICROGLIA_BRAIN_VS_MONOCYTE_BONE_MARROW_UP
148 -0.27379102 -1.1309351 0.18450184
0.29486004 1 2290 "tags=19%, list=16%, signal=22%"
GSE8835_HEALTHY_VS_CLL_CD8_TCELL_DN
GSE8835_HEALTHY_VS_CLL_CD8_TCELL_DN
146 -0.27780282 -1.1307638 0.19805825
0.29500312 1 2073 "tags=20%, list=15%, signal=23%"
GSE46606_IRF4HIGH_VS_WT_CD40L_IL2_IL5_DAY1_STIMULATED_BCELL_UP
GSE46606_IRF4HIGH_VS_WT_CD40L_IL2_IL5_DAY1_STIMULATED_BCELL_UP
138 -0.28022823 -1.1293381 0.20696326
0.2977367 1 1471 "tags=20%, list=10%, signal=22%"
GSE5589_LPS_VS_LPS_AND_IL6_STIM_IL6_KO_MACROPHAGE_45MIN_UP
GSE5589_LPS_VS_LPS_AND_IL6_STIM_IL6_KO_MACROPHAGE_45MIN_UP
156 -0.27258804 -1.1285337 0.18538325
0.29919347 1 1540 "tags=19%, list=11%, signal=21%"
GSE36888_UNTREATED_VS_IL2_TREATED_STAT5_AB_KNOCKIN_TCELL_2H_DN
GSE36888_UNTREATED_VS_IL2_TREATED_STAT5_AB_KNOCKIN_TCELL_2H_DN
136 -0.2774205 -1.1281893 0.20245399
0.29968432 1 1927 "tags=22%, list=14%, signal=25%"
GSE29618_BCELL_VS_MDC_DAY7_FLU_VACCINE_UP
GSE29618_BCELL_VS_MDC_DAY7_FLU_VACCINE_UP
125 -0.28024346 -1.1281393 0.212 0.29959968
1 2081 "tags=20%, list=15%, signal=23%"
GSE1432_1H_VS_24H_IFNG_MICROGLIA_UP
GSE1432_1H_VS_24H_IFNG_MICROGLIA_UP
162 -0.27220473 -1.1263716 0.20736434
0.30309737 1 1523 "tags=17%, list=11%, signal=19%"

GSE17721_12H_VS_24H_LPS_BMDC_UP	GSE17721_12H_VS_24H_LPS_BMDC_UP
	161	-0.26792917	-1.1259265	0.1961165
0.30384806	1	2108	"tags=22%, list=15%, signal=25%"
GSE29618_PRE_VS_DAY7_POST_TIV_FLU_VACCINE_MONOCYTE_DN
GSE29618_PRE_VS_DAY7_POST_TIV_FLU_VACCINE_MONOCYTE_DN
136	-0.27435806	-1.1258281	0.22072937
0.30385256	1	1961	"tags=20%, list=14%, signal=23%"
GSE37533_UNTREATED_VS_PIOGLIZATONE_TREATED_CD4_TCELL_PPARG2_AND_FOXP3_TRASDUCED_DN
GSE37533_UNTREATED_VS_PIOGLIZATONE_TREATED_CD4_TCELL_PPARG2_AND_FOXP3_TRASDUCED_DN	123	-0.27839023	-1.1258273
0.22868218	0.30364832	1	1331	"tags=20%, list=9%, signal=21%"
GSE8515_CTRL_VS_IL6_4H_STIM_MAC_UP
GSE8515_CTRL_VS_IL6_4H_STIM_MAC_UP
129	-0.27537996	-1.1256602	0.19736843
0.3038002	1	1139	"tags=16%, list=8%, signal=17%"
GSE34156_NOD2_LIGAND_VS_NOD2_AND_TLR1_TLR2_LIGAND_6H_TREATED_MONOCYTE_UP
GSE34156_NOD2_LIGAND_VS_NOD2_AND_TLR1_TLR2_LIGAND_6H_TREATED_MONOCYTE_UP	113	-0.28299528	-1.1250248
0.2259887	0.3049536	1	2368	"tags=25%, list=17%, signal=30%"
GSE28783_ANTI_MIR33_VS_UNTREATED_ATHEROSCLEROSIS_MACROPHAGE_DN
GSE28783_ANTI_MIR33_VS_UNTREATED_ATHEROSCLEROSIS_MACROPHAGE_DN
	135	-0.27390066	-1.1243539	0.20272905
0.30615094	1	1878	"tags=16%, list=13%, signal=19%"
GSE14308_TH1_VS_INDUCED_TREG_UP	GSE14308_TH1_VS_INDUCED_TREG_UP
	139	-0.27488866	-1.123908	0.22846442
0.3069617	1	1251	"tags=17%, list=9%, signal=18%"
GSE28237_EARLY_VS_LATE_GC_BCELL_UP
GSE28237_EARLY_VS_LATE_GC_BCELL_UP
138	-0.2739144	-1.1234972	0.20643939
0.30763164	1	3736	"tags=26%, list=26%, signal=35%"
GSE18281_SUBCAPSULAR_VS_CENTRAL_CORTICAL_REGION_OF_THYMUS_DN
GSE18281_SUBCAPSULAR_VS_CENTRAL_CORTICAL_REGION_OF_THYMUS_DN
	157	-0.2699049	-1.1231811	0.21553399
0.30810127	1	1776	"tags=24%, list=13%, signal=27%"
GSE41176_UNSTIM_VS_ANTI_IGM_STIM_BCELL_3H_DN
GSE41176_UNSTIM_VS_ANTI_IGM_STIM_BCELL_3H_DN
134	-0.27517033	-1.1231302	0.23300971
0.30800498	1	1616	"tags=20%, list=11%, signal=23%"
GSE34205_HEALTHY_VS_RSV_INF_INFANT_PBMC_UP
GSE34205_HEALTHY_VS_RSV_INF_INFANT_PBMC_UP
111	-0.28144562	-1.1226968	0.23175183
0.3087753	1	2033	"tags=20%, list=14%, signal=23%"
GSE22611_NOD2_TRANSD_VS_CTRL_TRANSD_HEK293_MDP_STIM_6H_DN
GSE22611_NOD2_TRANSD_VS_CTRL_TRANSD_HEK293_MDP_STIM_6H_DN
134	-0.27696702	-1.1224673	0.21904762
0.30904344	1	2132	"tags=19%, list=15%, signal=23%"
GSE32034_UNTREATED_VS_ROSIGLIZATONE_TREATED_LY6C_HIGH_MONOCYTE_UP
GSE32034_UNTREATED_VS_ROSIGLIZATONE_TREATED_LY6C_HIGH_MONOCYTE_UP
	156	-0.26926583	-1.1217287	0.21001926
0.31039777	1	1840	"tags=21%, list=13%, signal=23%"

GSE17974_IL4_AND_ANTI_IL12_VS_UNTREATED_4H_ACT_CD4_TCELL_DN
GSE17974_IL4_AND_ANTI_IL12_VS_UNTREATED_4H_ACT_CD4_TCELL_DN
101 -0.29000595 -1.1216261 0.23333333
0.31041634 1 4495 "tags=40%, list=32%, signal=58%"
GSE17721_LPS_VS_POLYIC_6H_BMDC_UP GSE17721_LPS_VS_POLYIC_6H_BMDC_UP
146 -0.2716769 -1.1211935 0.20641282
0.31112674 1 898 "tags=14%, list=6%, signal=14%"
GSE23925_DARK_ZONE_VS_NAIVE_BCELL_UP
GSE23925_DARK_ZONE_VS_NAIVE_BCELL_UP
145 -0.27152482 -1.1211326 0.22789784
0.31105602 1 1207 "tags=17%, list=9%, signal=18%"
GSE2128_CTRL_VS_MIMETOPE_NEGATIVE_SELECTION_DP_THYMOCYTE_C57BL6_UP
GSE2128_CTRL_VS_MIMETOPE_NEGATIVE_SELECTION_DP_THYMOCYTE_C57BL6_UP
125 -0.27757394 -1.120437 0.23062016
0.31232145 1 1895 "tags=24%, list=13%, signal=27%"
GSE360_L_MAJOR_VS_B_MALAYI_HIGH_DOSE_MAC_UP
GSE360_L_MAJOR_VS_B_MALAYI_HIGH_DOSE_MAC_UP
144 -0.2715149 -1.119924 0.21346153
0.3132651 1 2554 "tags=23%, list=18%, signal=28%"
GSE33162_HDAC3_KO_VS_HDAC3_KO_MACROPHAGE_UP
GSE33162_HDAC3_KO_VS_HDAC3_KO_MACROPHAGE_UP
142 -0.272211 -1.1198344 0.21541502
0.3132425 1 2407 "tags=25%, list=17%, signal=29%"
GSE41978_WT_VS_ID2_KO_KLRG1_LOW_EFFECTOR_CD8_TCELL_DN
GSE41978_WT_VS_ID2_KO_KLRG1_LOW_EFFECTOR_CD8_TCELL_DN
164 -0.26539674 -1.1196116 0.1920792
0.31352285 1 4011 "tags=33%, list=28%, signal=45%"
GSE17721_LPS_VS_CPG_12H_BMDC_UP GSE17721_LPS_VS_CPG_12H_BMDC_UP
161 -0.2659761 -1.1189872 0.20864661
0.31466565 1 1766 "tags=20%, list=13%, signal=22%"
GSE19888_ADENOSINE_A3R_ACT_VS_A3R_ACT_WITH_A3R_INH_PRETREATMENT_IN_M
AST_CELL_DN
GSE19888_ADENOSINE_A3R_ACT_VS_A3R_ACT_WITH_A3R_INH_PRETREATMENT_IN_M
AST_CELL_DN 141 -0.27233738 -1.1187764
0.23846154 0.31492275 1 1879 "tags=19%,
list=13%, signal=22%"
GSE16385_ROSIGLITAZONE_IL4_VS_IL4_ALONE_STIM_MACROPHAGE_12H_UP
GSE16385_ROSIGLITAZONE_IL4_VS_IL4_ALONE_STIM_MACROPHAGE_12H_UP
146 -0.26812208 -1.118761 0.21121496
0.31474203 1 1100 "tags=15%, list=8%, signal=16%"
GSE3720_LPS_VS_PMA_STIM_VD1_GAMMADELTA_TCELL_UP
GSE3720_LPS_VS_PMA_STIM_VD1_GAMMADELTA_TCELL_UP
95 -0.29120645 -1.1185707 0.22990654
0.3149557 1 1527 "tags=19%, list=11%, signal=21%"
GSE6269_FLU_VS_STAPH_AUREUS_INF_PBMC_UP
GSE6269_FLU_VS_STAPH_AUREUS_INF_PBMC_UP
122 -0.2753497 -1.1182641 0.22490707
0.3154091 1 1174 "tags=16%, list=8%, signal=17%"
GSE46606_IRF4MID_VS_WT_CD40L_IL2_IL5_DAY1_STIMULATED_BCELL_UP
GSE46606_IRF4MID_VS_WT_CD40L_IL2_IL5_DAY1_STIMULATED_BCELL_UP
141 -0.26788214 -1.1180276 0.21252371
0.31571254 1 2304 "tags=26%, list=16%, signal=31%"
GSE3982_EFF_MEMORY_CD4_TCELL_VS_TH2_UP
GSE3982_EFF_MEMORY_CD4_TCELL_VS_TH2_UP

129 -0.27671295 -1.1174567 0.22686026
0.3167093 1 1010 "tags=9%, list=7%, signal=10%"
GSE2706_LPS_VS_R848_AND_LPS_8H_STIM_DC_DN
GSE2706_LPS_VS_R848_AND_LPS_8H_STIM_DC_DN
124 -0.27824834 -1.1174383 0.2193676
0.3165386 1 2019 "tags=19%, list=14%, signal=22%"
GSE21380_NON_TFH_VS_GERMINAL_CENTER_TFH_CD4_TCELL_DN
GSE21380_NON_TFH_VS_GERMINAL_CENTER_TFH_CD4_TCELL_DN
142 -0.27044106 -1.1168938 0.21194606
0.31750506 1 1483 "tags=19%, list=11%, signal=21%"
GSE19401_PAM2CSK4_VS_RETINOIC_ACID_STIM_FOLLICULAR_DC_DN
GSE19401_PAM2CSK4_VS_RETINOIC_ACID_STIM_FOLLICULAR_DC_DN
142 -0.27207378 -1.116542 0.21663442
0.31805798 1 2534 "tags=22%, list=18%, signal=26%"
GSE19941_IL10_KO_VS_IL10_KO_AND_NFKBP50_KO_UNSTIM_MACROPHAGE_DN
GSE19941_IL10_KO_VS_IL10_KO_AND_NFKBP50_KO_UNSTIM_MACROPHAGE_DN
155 -0.26738846 -1.1163875 0.2077206
0.31817505 1 2366 "tags=23%, list=17%, signal=27%"
GSE9509_LPS_VS_LPS_AND_IL10_STIM_IL10_KO_MACROPHAGE_30MIN_UP
GSE9509_LPS_VS_LPS_AND_IL10_STIM_IL10_KO_MACROPHAGE_30MIN_UP
136 -0.27452278 -1.1159198 0.22612086
0.318998 1 1357 "tags=18%, list=10%, signal=19%"
GSE39820_CTRL_VS_IL1B_IL6_IL23A_CD4_TCELL_DN
GSE39820_CTRL_VS_IL1B_IL6_IL23A_CD4_TCELL_DN
153 -0.26946256 -1.1150453 0.22814499
0.32075307 1 1783 "tags=18%, list=13%, signal=21%"
GSE21774_CD62L_POS_CD56_BRIGHT_VS_CD62L_NEG_CD56_DIM_NK_CELL_UP
GSE21774_CD62L_POS_CD56_BRIGHT_VS_CD62L_NEG_CD56_DIM_NK_CELL_UP
148 -0.27603337 -1.1148205 0.21387284
0.3210587 1 1885 "tags=21%, list=13%, signal=24%"
GSE3982_EOSINOPHIL_VS_BASOPHIL_UP GSE3982_EOSINOPHIL_VS_BASOPHIL_UP
140 -0.2739172 -1.1145871 0.23020259
0.32133222 1 2625 "tags=27%, list=19%, signal=33%"
GSE18791_UNSTIM_VS_NEWCATSLE_VIRUS_DC_6H_DN
GSE18791_UNSTIM_VS_NEWCATSLE_VIRUS_DC_6H_DN
116 -0.2785619 -1.1140336 0.21375465
0.3223613 1 1766 "tags=22%, list=13%, signal=24%"
GSE41867_MEMORY_VS_EXHAUSTED_CD8_TCELL_DAY30_LCMV_UP
GSE41867_MEMORY_VS_EXHAUSTED_CD8_TCELL_DAY30_LCMV_UP
144 -0.27229843 -1.1139839 0.22287968
0.32226676 1 1934 "tags=22%, list=14%, signal=25%"
GSE11961_MARGINAL_ZONE_BCELL_VS_MEMORY_BCELL_DAY7_DN
GSE11961_MARGINAL_ZONE_BCELL_VS_MEMORY_BCELL_DAY7_DN
142 -0.27050674 -1.1134232 0.2394636
0.32332125 1 5825 "tags=48%, list=41%, signal=81%"
GSE22501_PERIPHERAL_BLOOD_VS_CORD_BLOOD_TREG_UP
GSE22501_PERIPHERAL_BLOOD_VS_CORD_BLOOD_TREG_UP
151 -0.27033144 -1.1133375 0.22884615
0.32330894 1 2758 "tags=20%, list=20%, signal=24%"
GSE21678_WT_VS_FOXO1_FOXO3_KO_TREG_UP
GSE21678_WT_VS_FOXO1_FOXO3_KO_TREG_UP
126 -0.27851486 -1.1130089 0.22332016
0.32386455 1 1484 "tags=21%, list=11%, signal=23%"
GSE15750_WT_VS_TRAF6KO_DAY6_EFF_CD8_TCELL_DN

GSE15750_WT_VS_TRAF6KO_DAY6_EFF_CD8_TCELL_DN
135 -0.27109402 -1.1129925 0.20729366
0.3236878 1 3174 "tags=23%, list=22%, signal=29%"
GSE21670_STAT3_KO_VS_WT_CD4_TCELL_UP
GSE21670_STAT3_KO_VS_WT_CD4_TCELL_UP
137 -0.26823142 -1.1115326 0.20898438
0.32664534 1 2377 "tags=22%, list=17%, signal=26%"
GSE21546_ELK1_KO_VS_SAP1A_KO_AND_ELK1_KO_DP_THYMOCYTES_UP
GSE21546_ELK1_KO_VS_SAP1A_KO_AND_ELK1_KO_DP_THYMOCYTES_UP
108 -0.2808647 -1.111479 0.24723247
0.32654878 1 1766 "tags=21%, list=13%, signal=24%"
GSE32533_WT_VS_MIR17_OVEREXPRESS_ACT_CD4_TCELL_DN
GSE32533_WT_VS_MIR17_OVEREXPRESS_ACT_CD4_TCELL_DN
149 -0.26866257 -1.1113318 0.2401501
0.32664743 1 3523 "tags=28%, list=25%, signal=37%"
GSE26030_TH1_VS_TH17_DAY15_POST_POLARIZATION_UP
GSE26030_TH1_VS_TH17_DAY15_POST_POLARIZATION_UP
139 -0.27128163 -1.1112967 0.22393823
0.32650492 1 1965 "tags=21%, list=14%, signal=24%"
GSE14769_20MIN_VS_360MIN_LPS_BMDM_DN
GSE14769_20MIN_VS_360MIN_LPS_BMDM_DN
145 -0.26917994 -1.1112202 0.2278481
0.3264537 1 2021 "tags=21%, list=14%, signal=24%"
GSE16385_UNTREATED_VS_12H_ROSIGLITAZONE_IFNG_TNF_TREATED_MACROPHAGE_
DN
GSE16385_UNTREATED_VS_12H_ROSIGLITAZONE_IFNG_TNF_TREATED_MACROPHAGE_
DN 129 -0.27439815 -1.1111685
0.22972973 0.32635334 1 1842 "tags=20%,
list=13%, signal=23%"
GSE32423_IL7_VS_IL7_IL4_NAIVE_CD8_TCELL_UP
GSE32423_IL7_VS_IL7_IL4_NAIVE_CD8_TCELL_UP
146 -0.26665086 -1.1110975 0.22033899
0.3262967 1 3420 "tags=33%, list=24%, signal=43%"
GSE42724_NAIVE_VS_B1_BCELL_UP GSE42724_NAIVE_VS_B1_BCELL_UP
144 -0.27339932 -1.1108856 0.22678572
0.3265334 1 2282 "tags=17%, list=16%, signal=20%"
GSE36476_CTRL_VS_TSST_ACT_16H_MEMORY_CD4_TCELL_YOUNG_UP
GSE36476_CTRL_VS_TSST_ACT_16H_MEMORY_CD4_TCELL_YOUNG_UP
138 -0.26883045 -1.1102095 0.23747681
0.3277964 1 1736 "tags=21%, list=12%, signal=24%"
GSE8921_UNSTIM_0H_VS_TLR1_2_STIM_MONOCYTE_3H_DN
GSE8921_UNSTIM_0H_VS_TLR1_2_STIM_MONOCYTE_3H_DN
138 -0.26897505 -1.110155 0.22574627
0.32771155 1 4918 "tags=36%, list=35%, signal=55%"
GSE15324_NAIVE_VS_ACTIVATED_CD8_TCELL_DN
GSE15324_NAIVE_VS_ACTIVATED_CD8_TCELL_DN
159 -0.2676518 -1.1099489 0.2112676
0.32795683 1 686 "tags=10%, list=5%, signal=10%"
GSE2585_CTEC_VS_THYMIC_MACROPHAGE_UP
GSE2585_CTEC_VS_THYMIC_MACROPHAGE_UP
142 -0.2697774 -1.1099249 0.23339659
0.32779485 1 2073 "tags=20%, list=15%, signal=23%"
GSE13485_DAY3_VS_DAY21_YF17D_VACCINE_PBMC_UP
GSE13485_DAY3_VS_DAY21_YF17D_VACCINE_PBMC_UP

126	-0.27556226	-1.1094333	0.23731884
0.32865328	1	1872	"tags=22%, list=13%, signal=25%"
GSE43955_1H_VS_10H_ACT_CD4_TCELL_WITH_TGFB_IL6_DN
GSE43955_1H_VS_10H_ACT_CD4_TCELL_WITH_TGFB_IL6_DN
156	-0.26769924	-1.108621	0.22348484
0.33019468	1	2151	"tags=23%, list=15%, signal=27%"
GSE1460_INTRATHYMIC_T_PROGENITOR_VS_NAIVE_CD4_TCELL_ADULT_BLOOD_DN
GSE1460_INTRATHYMIC_T_PROGENITOR_VS_NAIVE_CD4_TCELL_ADULT_BLOOD_DN
140	-0.27334023	-1.108317	0.23863636
0.33066133	1	2156	"tags=21%, list=15%, signal=25%"
GSE20715_0H_VS_6H_OZONE_TLR4_KO_LUNG_DN
GSE20715_0H_VS_6H_OZONE_TLR4_KO_LUNG_DN
154	-0.26364225	-1.1075909	0.23505977
0.3320371	1	1839	"tags=17%, list=13%, signal=19%"
GSE5589_WT_VS_IL6_KO_LPS_AND_IL6_STIM_MACROPHAGE_45MIN_DN
GSE5589_WT_VS_IL6_KO_LPS_AND_IL6_STIM_MACROPHAGE_45MIN_DN
154	-0.26613355	-1.1074216	0.23031496
0.33222055	1	3523	"tags=29%, list=25%, signal=38%"
GSE360_DC_VS_MAC_T_GONDII_DN	GSE360_DC_VS_MAC_T_GONDII_DN
151	-0.26710725	-1.1072564	0.22200392
0.3323599	1	1929	"tags=18%, list=14%, signal=20%"
GSE15624_3H_VS_6H_HALOFUGINONE_TREATED_CD4_TCELL_DN
GSE15624_3H_VS_6H_HALOFUGINONE_TREATED_CD4_TCELL_DN
116	-0.27405307	-1.1069657	0.243083	0.33277223
1	1740	"tags=22%, list=12%, signal=24%"
GSE13411_NAIVE_VS_SWITCHED_MEMORY_BCELL_UP
GSE13411_NAIVE_VS_SWITCHED_MEMORY_BCELL_UP
140	-0.2717879	-1.1063371	0.2647619
0.3339608	1	4827	"tags=41%, list=34%, signal=62%"
GSE45382_UNTREATED_VS_TGFB_TREATED_MACROPHAGES_DN
GSE45382_UNTREATED_VS_TGFB_TREATED_MACROPHAGES_DN
130	-0.27236912	-1.1063087	0.25045705
0.33380628	1	2248	"tags=27%, list=16%, signal=32%"
GSE22025_PROGESTERONE_VS_TGFB1_AND_PROGESTERONE_TREATED_CD4_TCELL_DN

GSE22025_PROGESTERONE_VS_TGFB1_AND_PROGESTERONE_TREATED_CD4_TCELL_DN
142	-0.269834	-1.1058898	0.251938
0.33457005	1	1913	"tags=20%, list=14%, signal=23%"
GSE26030_UNSTIM_VS_RESTIM_TH1_DAY15_POST_POLARIZATION_UP
GSE26030_UNSTIM_VS_RESTIM_TH1_DAY15_POST_POLARIZATION_UP
157	-0.26261476	-1.1056509	0.20512821
0.3349135	1	1089	"tags=14%, list=8%, signal=15%"
GSE41978_KLRG1_HIGH_VS_LOW_EFFECTOR_CD8_TCELL_UP
GSE41978_KLRG1_HIGH_VS_LOW_EFFECTOR_CD8_TCELL_UP
165	-0.26372093	-1.1056461	0.23647295
0.33470437	1	4239	"tags=35%, list=30%, signal=50%"
GSE29949_CD8_NEG_DC_SPLEEN_VS_MONOCYTE_BONE_MARROW_DN
GSE29949_CD8_NEG_DC_SPLEEN_VS_MONOCYTE_BONE_MARROW_DN
148	-0.27061883	-1.1054627	0.2523719
0.33490875	1	2424	"tags=21%, list=17%, signal=25%"
GSE20198_IL12_VS_IL12_IL18_TREATED_ACT_CD4_TCELL_DN
GSE20198_IL12_VS_IL12_IL18_TREATED_ACT_CD4_TCELL_DN
150	-0.26767257	-1.105291	0.23722628
0.3350606	1	1487	"tags=20%, list=11%, signal=22%"

GSE2706_UNSTIM_VS_2H_LPS_AND_R848_DC_UP
GSE2706_UNSTIM_VS_2H_LPS_AND_R848_DC_UP
95 -0.28147414 -1.1049243 0.26901668
0.3356463 1 1721 "tags=23%, list=12%, signal=26%"
GSE27786_NKCELL_VS_ERYTHROBLAST_UP
GSE27786_NKCELL_VS_ERYTHROBLAST_UP
140 -0.271167 -1.1047777 0.26325756
0.3357682 1 1821 "tags=18%, list=13%, signal=20%"
GSE28783_ANTI_MIR33_VS_CTRL_ATHEROSCLEROSIS_MACROPHAGE_UP
GSE28783_ANTI_MIR33_VS_CTRL_ATHEROSCLEROSIS_MACROPHAGE_UP
147 -0.266348 -1.1039455 0.23781677
0.33742213 1 3175 "tags=28%, list=22%, signal=36%"
GSE24634_TREG_VS_TCONV_POST_DAY10_IL4_CONVERSION_DN
GSE24634_TREG_VS_TCONV_POST_DAY10_IL4_CONVERSION_DN
152 -0.2664854 -1.1039104 0.22463769
0.33727887 1 2111 "tags=21%, list=15%, signal=24%"
GSE17721_POLYIC_VS_PAM3CSK4_0.5H_BMDC_DN
GSE17721_POLYIC_VS_PAM3CSK4_0.5H_BMDC_DN
144 -0.2725854 -1.1038173 0.22900763
0.33725387 1 1665 "tags=22%, list=12%, signal=24%"
GSE27786_NKCELL_VS_NEUTROPHIL_DN GSE27786_NKCELL_VS_NEUTROPHIL_DN
131 -0.27283162 -1.1031041 0.23756906
0.338638 1 1009 "tags=14%, list=7%, signal=15%"
GSE22432_MULTIPOTENT_PROGENITOR_VS_PDC_UP
GSE22432_MULTIPOTENT_PROGENITOR_VS_PDC_UP
147 -0.26707464 -1.1027402 0.2513863
0.33928043 1 1268 "tags=14%, list=9%, signal=15%"
GSE12963_ENV_NEF_VS_ENV_NEF_AND_VPR_DEFICIENT_HIV1_INF_CD4_TCELL_UP
GSE12963_ENV_NEF_VS_ENV_NEF_AND_VPR_DEFICIENT_HIV1_INF_CD4_TCELL_UP
96 -0.28349155 -1.1020837 0.26785713
0.34057087 1 1638 "tags=18%, list=12%, signal=20%"
GSE22935_UNSTIM_VS_48H_MBOVIS_BCG_STIM_MACROPHAGE_UP
GSE22935_UNSTIM_VS_48H_MBOVIS_BCG_STIM_MACROPHAGE_UP
144 -0.269036 -1.1017584 0.25384617
0.34111562 1 1749 "tags=15%, list=12%, signal=17%"
GSE11961_FOLLICULAR_BCELL_VS_MARGINAL_ZONE_BCELL_UP
GSE11961_FOLLICULAR_BCELL_VS_MARGINAL_ZONE_BCELL_UP
132 -0.27035737 -1.101234 0.24288425
0.34208944 1 2074 "tags=19%, list=15%, signal=22%"
GSE45365_HEALTHY_VS_MCMV_INFECTION_CD8A_DC_DN
GSE45365_HEALTHY_VS_MCMV_INFECTION_CD8A_DC_DN
153 -0.2668669 -1.1001921 0.24801587
0.34419823 1 2412 "tags=22%, list=17%, signal=26%"
GSE7764_IL15_NK_CELL_24H_VS_SPLENOCYTE_DN
GSE7764_IL15_NK_CELL_24H_VS_SPLENOCYTE_DN
141 -0.26834568 -1.1001283 0.2560297
0.34412277 1 1491 "tags=17%, list=11%, signal=19%"
GSE21063_WT_VS_NFATC1_KO_8H_ANTI_IGM_STIM_BCELL_DN
GSE21063_WT_VS_NFATC1_KO_8H_ANTI_IGM_STIM_BCELL_DN
124 -0.2755903 -1.1000254 0.28248587
0.3441365 1 3531 "tags=38%, list=25%, signal=50%"
GSE37336_LY6C_POS_VS_NEG_NAIVE_CD4_TCELL_DN
GSE37336_LY6C_POS_VS_NEG_NAIVE_CD4_TCELL_DN
100 -0.2799221 -1.0995101 0.27011493

0.34510386	1	4889	"tags=41%, list=35%, signal=62%"
GSE7831_UNSTIM_VS_CPG_STIM_PDC_4H_DN
GSE7831_UNSTIM_VS_CPG_STIM_PDC_4H_DN
157	-0.2636708	-1.0992264	0.23608445
0.3455183	1	1303	"tags=15%, list=9%, signal=17%"
GSE42021_CD24INT_VS_CD24LOW_TCONV_THYMUS_UP
GSE42021_CD24INT_VS_CD24LOW_TCONV_THYMUS_UP
131	-0.27013573	-1.098581	0.26268116
0.34680527	1	1467	"tags=17%, list=10%, signal=19%"
GSE6259_33D1_POS_VS_DEC205_POS_SPLENIC_DC_UP
GSE6259_33D1_POS_VS_DEC205_POS_SPLENIC_DC_UP
125	-0.2726393	-1.0985395	0.23449612
0.3466777	1	1817	"tags=20%, list=13%, signal=23%"
GSE11864_UNTREATED_VS_CSF1_IFNG_PAM3CYS_IN_MAC_UP
GSE11864_UNTREATED_VS_CSF1_IFNG_PAM3CYS_IN_MAC_UP
129	-0.27637762	-1.0982542	0.28290766
0.34710935	1	1771	"tags=21%, list=13%, signal=24%"
GSE26351_UNSTIM_VS_WNT_PATHWAY_STIM_HEMATOPOIETIC_PROGENITORS_DN
GSE26351_UNSTIM_VS_WNT_PATHWAY_STIM_HEMATOPOIETIC_PROGENITORS_DN
	145	-0.26601824	-1.097191	0.23076923
0.3493245	1	1582	"tags=19%, list=11%, signal=22%"
GSE7219_WT_VS_NIK_NFKB2_KO_LPS_AND_ANTI_CD40_STIM_DC_DN
GSE7219_WT_VS_NIK_NFKB2_KO_LPS_AND_ANTI_CD40_STIM_DC_DN
148	-0.26356116	-1.0969844	0.26434427
0.34959108	1	1654	"tags=20%, list=12%, signal=22%"
GSE1740_MCSF_VS_MCSF_AND_IFNG_DAY2_DERIVED_MACROPHAGE_WITH_IFNA_STIM
_DN
GSE1740_MCSF_VS_MCSF_AND_IFNG_DAY2_DERIVED_MACROPHAGE_WITH_IFNA_STIM
_DN	108	-0.28011236	-1.0967375
0.25765765	0.34990528	1	2756	"tags=27%,
list=20%, signal=33%"
GSE360_DC_VS_MAC_T_GONDII_UP	GSE360_DC_VS_MAC_T_GONDII_UP
	138	-0.26953262	-1.0967294	0.26418787
0.34970057	1	3381	"tags=30%, list=24%, signal=40%"
GSE35435_RESTING_VS_IL4_TREATED_MACROPHAGE_UP
GSE35435_RESTING_VS_IL4_TREATED_MACROPHAGE_UP
127	-0.27233157	-1.0964214	0.2576336
0.3501795	1	1270	"tags=18%, list=9%, signal=20%"
GSE25088_CTRL_VS_IL4_STIM_STAT6_KO_MACROPHAGE_DN
GSE25088_CTRL_VS_IL4_STIM_STAT6_KO_MACROPHAGE_DN
117	-0.27259132	-1.0959269	0.25	0.35109526
1	2861	"tags=25%, list=20%, signal=31%"
GSE3982_MAST_CELL_VS_NKCELL_DN	GSE3982_MAST_CELL_VS_NKCELL_DN
	143	-0.2670652	-1.0958445	0.27643785
0.3510704	1	1161	"tags=12%, list=8%, signal=13%"
GSE1925_3H_VS_24H_IFNG_STIM_MACROPHAGE_DN
GSE1925_3H_VS_24H_IFNG_STIM_MACROPHAGE_DN
128	-0.2720639	-1.0955932	0.23962264
0.3514481	1	1766	"tags=20%, list=13%, signal=23%"
GSE21927_GMCSF_IL6_VS_GMCSF_GCSF_TREATED_BONE_MARROW_UP
GSE21927_GMCSF_IL6_VS_GMCSF_GCSF_TREATED_BONE_MARROW_UP
104	-0.27842793	-1.0952816	0.24859813
0.35191846	1	3465	"tags=34%, list=25%, signal=44%"
GSE5589_LPS_VS_LPS_AND_IL10_STIM_IL6_KO_MACROPHAGE_180MIN_UP

GSE5589_LPS_VS_LPS_AND_IL10_STIM_IL6_KO_MACROPHAGE_180MIN_UP
140 -0.2648775 -1.0949455 0.23703703
0.3524955 1 1799 "tags=21%, list=13%, signal=24%"
GSE5589_IL6_KO_VS_IL10_KO_LPS_AND_IL10_STIM_MACROPHAGE_45MIN_UP
GSE5589_IL6_KO_VS_IL10_KO_LPS_AND_IL10_STIM_MACROPHAGE_45MIN_UP
149 -0.26271322 -1.0948589 0.25788498
0.352476 1 939 "tags=11%, list=7%, signal=11%"
GSE2770_UNTREATED_VS_IL4_TREATED_ACT_CD4_TCELL_6H_UP
GSE2770_UNTREATED_VS_IL4_TREATED_ACT_CD4_TCELL_6H_UP
155 -0.2663982 -1.0943407 0.238921 0.3534194
1 1831 "tags=21%, list=13%, signal=24%"
GSE12845_IGD_POS_VS_NEG_BLOOD_BCELL_UP
GSE12845_IGD_POS_VS_NEG_BLOOD_BCELL_UP
128 -0.27186972 -1.0943363 0.26732674
0.35320395 1 1692 "tags=19%, list=12%, signal=21%"
GSE16385_UNTREATED_VS_12H_ROSIGLITAZONE_TREATED_MACROPHAGE_UP
GSE16385_UNTREATED_VS_12H_ROSIGLITAZONE_TREATED_MACROPHAGE_UP
107 -0.27445704 -1.0931284 0.25868726
0.35583025 1 4210 "tags=35%, list=30%, signal=49%"
GSE43957_UNTREATED_VS_NACL_TREATED_ANTI_CD3_CD28_STIM_CD4_TCELL_UP
GSE43957_UNTREATED_VS_NACL_TREATED_ANTI_CD3_CD28_STIM_CD4_TCELL_UP
165 -0.26037693 -1.0925386 0.2541744
0.3569075 1 3420 "tags=30%, list=24%, signal=40%"
GSE1925_CTRL_VS_3H_IFNG_STIM_MACROPHAGE_DN
GSE1925_CTRL_VS_3H_IFNG_STIM_MACROPHAGE_DN
138 -0.26145175 -1.091415 0.2632613
0.35929537 1 3108 "tags=26%, list=22%, signal=33%"
GSE4142_PLASMA_CELL_VS_MEMORY_BCELL_UP
GSE4142_PLASMA_CELL_VS_MEMORY_BCELL_UP
132 -0.27063665 -1.0906646 0.27734375
0.36085925 1 4649 "tags=33%, list=33%, signal=48%"
GSE5679_RARA_AGONIST_AM580_VS_AM580_AND_ROSIGLITAZONE_TREATED_DC_UP
GSE5679_RARA_AGONIST_AM580_VS_AM580_AND_ROSIGLITAZONE_TREATED_DC_UP
155 -0.2634685 -1.0904083 0.26499033
0.36126077 1 2441 "tags=21%, list=17%, signal=25%"
GSE5589_WT_VS_IL10_KO_LPS_AND_IL10_STIM_MACROPHAGE_180MIN_DN
GSE5589_WT_VS_IL10_KO_LPS_AND_IL10_STIM_MACROPHAGE_180MIN_DN
93 -0.2800408 -1.0903487 0.2951128
0.36117327 1 5116 "tags=43%, list=36%, signal=67%"
GSE14908_ATOPIC_VS_NONATOPIC_PATIENT_RESTING_CD4_TCELL_UP
GSE14908_ATOPIC_VS_NONATOPIC_PATIENT_RESTING_CD4_TCELL_UP
147 -0.26049227 -1.0902797 0.26459143
0.36111832 1 1491 "tags=15%, list=11%, signal=17%"
GSE17721_CTRL_VS_LPS_0.5H_BMDC_DN GSE17721_CTRL_VS_LPS_0.5H_BMDC_DN
157 -0.2627687 -1.0902236 0.29300568
0.36101907 1 3596 "tags=32%, list=25%, signal=43%"
GSE13306_RA_VS_UNTREATED_MEM_CD4_TCELL_DN
GSE13306_RA_VS_UNTREATED_MEM_CD4_TCELL_DN
147 -0.26336253 -1.0900201 0.26508227
0.36128676 1 1138 "tags=12%, list=8%, signal=12%"
GSE45365_NK_CELL_VS_BCELL_DN GSE45365_NK_CELL_VS_BCELL_DN
104 -0.27689847 -1.0897363 0.2992126
0.36171013 1 2560 "tags=21%, list=18%, signal=26%"
GSE3982_MAST_CELL_VS_EFF_MEMORY_CD4_TCELL_UP

GSE3982_MAST_CELL_VS_EFF_MEMORY_CD4_TCELL_UP
146	-0.26259616	-1.0897263	0.24157304
0.36149886	1	1635	"tags=16%, list=12%, signal=18%"
GSE32423_IL7_VS_IL7_IL4_NAIVE_CD8_TCELL_DN
GSE32423_IL7_VS_IL7_IL4_NAIVE_CD8_TCELL_DN
149	-0.26312694	-1.0891317	0.25656566
0.36264697	1	2593	"tags=27%, list=18%, signal=33%"
GSE557_WT_VS_CIITA_KO_DC_DN	GSE557_WT_VS_CIITA_KO_DC_DN
	144	-0.26391616	-1.0890038	0.2781955
0.36270154	1	1680	"tags=19%, list=12%, signal=21%"
GSE360_L_MAJOR_VS_M_TUBERCULOSIS_DC_DN
GSE360_L_MAJOR_VS_M_TUBERCULOSIS_DC_DN
151	-0.2628485	-1.0882691	0.27321428
0.36423844	1	1223	"tags=14%, list=9%, signal=15%"
GSE15659_TREG_VS_TCONV_UP GSE15659_TREG_VS_TCONV_UP
108	-0.27463877	-1.0882374	0.2851711
0.36409336	1	2640	"tags=23%, list=19%, signal=28%"
GSE40666_WT_VS_STAT1_KO_CD8_TCELL_WITH_IFNA_STIM_90MIN_UP
GSE40666_WT_VS_STAT1_KO_CD8_TCELL_WITH_IFNA_STIM_90MIN_UP
143	-0.2640872	-1.0879138	0.26386234
0.36461473	1	1873	"tags=21%, list=13%, signal=24%"
GSE37533_PPARG1_FOXP3_VS_PPARG2_FOXP3_TRANSDUCED_CD4_TCELL_PIOGLITAZ
ONE_TREATED_UP
GSE37533_PPARG1_FOXP3_VS_PPARG2_FOXP3_TRANSDUCED_CD4_TCELL_PIOGLITAZ
ONE_TREATED_UP		142	-0.26283446	-1.0874026
0.25291827	0.3656209	1	2315	"tags=24%,
list=16%, signal=28%"
GSE5589_UNSTIM_VS_45MIN_LPS_AND_IL10_STIM_MACROPHAGE_DN
GSE5589_UNSTIM_VS_45MIN_LPS_AND_IL10_STIM_MACROPHAGE_DN
144	-0.2630309	-1.0872711	0.26482213
0.36569118	1	2331	"tags=23%, list=17%, signal=27%"
GSE15659_NAIVE_CD4_TCELL_VS_ACTIVATED_TREG_UP
GSE15659_NAIVE_CD4_TCELL_VS_ACTIVATED_TREG_UP
117	-0.26872188	-1.0872556	0.29571983
0.36549446	1	3249	"tags=28%, list=23%, signal=36%"
GSE17721_POLYIC_VS_GARDIQUIMOD_8H_BMDC_UP
GSE17721_POLYIC_VS_GARDIQUIMOD_8H_BMDC_UP
160	-0.25949377	-1.0871359	0.24821429
0.36552718	1	1675	"tags=18%, list=12%, signal=20%"
GSE40274_CTRL_VS_FOXP3_AND_PBX1_TRANSDUCED_ACTIVATED_CD4_TCELL_UP
GSE40274_CTRL_VS_FOXP3_AND_PBX1_TRANSDUCED_ACTIVATED_CD4_TCELL_UP
	122	-0.27090278	-1.087135	0.2651515
0.36529896	1	2159	"tags=20%, list=15%, signal=24%"
GSE41176_UNSTIM_VS_ANTI_IGM_STIM_TAK1_KO_BCELL_3H_DN
GSE41176_UNSTIM_VS_ANTI_IGM_STIM_TAK1_KO_BCELL_3H_DN
125	-0.27090913	-1.0866948	0.27634194
0.36609948	1	5299	"tags=40%, list=38%, signal=63%"
GSE15930_STIM_VS_STIM_AND_IFNAB_48H_CD8_T_CELL_DN
GSE15930_STIM_VS_STIM_AND_IFNAB_48H_CD8_T_CELL_DN
160	-0.2619668	-1.0866667	0.26459143
0.3659377	1	2067	"tags=23%, list=15%, signal=27%"
GSE2770_TGFB_AND_IL4_ACT_VS_ACT_CD4_TCELL_6H_DN
GSE2770_TGFB_AND_IL4_ACT_VS_ACT_CD4_TCELL_6H_DN
135	-0.26909295	-1.0864981	0.2835249

0.36610362	1	2233	"tags=24%, list=16%, signal=28%"
GSE17721_PAM3CSK4_VS_CPG_1H_BMDC_DN
GSE17721_PAM3CSK4_VS_CPG_1H_BMDC_DN
162	-0.259841	-1.0862994	0.28	0.36636043
1	1442	"tags=17%, list=10%, signal=19%"
GSE7548_NAIVE_VS_DAY28_PCC_IMMUNIZATION_CD4_TCELL_UP
GSE7548_NAIVE_VS_DAY28_PCC_IMMUNIZATION_CD4_TCELL_UP
152	-0.2615477	-1.085921	0.259542	0.36704817
1	2476	"tags=23%, list=18%, signal=28%"
GSE27786_BCELL_VS_NKTCELL_UP	GSE27786_BCELL_VS_NKTCELL_UP
	142	-0.2631649	-1.0857766	0.2725528
0.36714488	1	1316	"tags=18%, list=9%, signal=19%"
GSE24972_MARGINAL_ZONE_BCELL_VS_FOLLICULAR_BCELL_UP
GSE24972_MARGINAL_ZONE_BCELL_VS_FOLLICULAR_BCELL_UP
134	-0.2667057	-1.0853218	0.27186313
0.3679917	1	1261	"tags=13%, list=9%, signal=15%"
GSE3982_DC_VS_MAC_UP	GSE3982_DC_VS_MAC_UP
130	-0.26831424	-1.0852976	0.27985075
0.36782166	1	1699	"tags=19%, list=12%, signal=22%"
GSE21033_CTRL_VS_POLYIC_STIM_DC_1H_DN
GSE21033_CTRL_VS_POLYIC_STIM_DC_1H_DN
133	-0.26483166	-1.0852833	0.27220076
0.36762658	1	2405	"tags=23%, list=17%, signal=28%"
GSE36527_CD62L_HIGH_CD69_NEG_VS_CD62L_LOW_CD69_POS_TREG_KLRG1_NEG_DN

GSE36527_CD62L_HIGH_CD69_NEG_VS_CD62L_LOW_CD69_POS_TREG_KLRG1_NEG_DN
		151	-0.26110664	-1.0846162
0.28957528	0.3689881	1	2427	"tags=24%,
list=17%, signal=28%"
GSE17974_CTRL_VS_ACT_IL4_AND_ANTI_IL12_2H_CD4_TCELL_UP
GSE17974_CTRL_VS_ACT_IL4_AND_ANTI_IL12_2H_CD4_TCELL_UP
125	-0.26694	-1.0843885	0.31296295	0.36930367
1	2511	"tags=26%, list=18%, signal=32%"
GSE41978_WT_VS_BIM_KO_KLRG1_LOW_EFFECTOR_CD8_TCELL_UP
GSE41978_WT_VS_BIM_KO_KLRG1_LOW_EFFECTOR_CD8_TCELL_UP
136	-0.26438498	-1.0840714	0.26213592
0.36984456	1	3677	"tags=32%, list=26%, signal=43%"
GSE27786_LSK_VS_NKCELL_UP	GSE27786_LSK_VS_NKCELL_UP
141	-0.26561382	-1.0839574	0.27671754
0.36986914	1	1870	"tags=24%, list=13%, signal=28%"
GSE21670_IL6_VS_TGFB_AND_IL6_TREATED_CD4_TCELL_UP
GSE21670_IL6_VS_TGFB_AND_IL6_TREATED_CD4_TCELL_UP
123	-0.2669255	-1.0838944	0.28707224
0.3697851	1	1631	"tags=18%, list=12%, signal=20%"
GSE17721_CPG_VS_GARDIQUIMOD_6H_BMDC_UP
GSE17721_CPG_VS_GARDIQUIMOD_6H_BMDC_UP
160	-0.25925046	-1.0836765	0.2642857
0.3700807	1	1035	"tags=13%, list=7%, signal=13%"
GSE3982_EFF_MEMORY_CD4_TCELL_VS_TH1_UP
GSE3982_EFF_MEMORY_CD4_TCELL_VS_TH1_UP
128	-0.26995754	-1.0832856	0.28235295
0.37073752	1	1889	"tags=16%, list=13%, signal=19%"
GSE32423_IL7_VS_IL7_IL4_MEMORY_CD8_TCELL_UP
GSE32423_IL7_VS_IL7_IL4_MEMORY_CD8_TCELL_UP

127 -0.26663676 -1.0832148 0.2848837
0.37067902 1 1328 "tags=18%, list=9%, signal=20%"
GSE42724_NAIVE_VS_MEMORY_BCELL_UP GSE42724_NAIVE_VS_MEMORY_BCELL_UP
140 -0.26553094 -1.0829535 0.28988326
0.3710489 1 2168 "tags=20%, list=15%, signal=23%"
GSE20500_CTRL_VS_RETINOIC_ACID_TREATED_CD4_TCELL_DN
GSE20500_CTRL_VS_RETINOIC_ACID_TREATED_CD4_TCELL_DN
142 -0.26146704 -1.0825182 0.2556818
0.37189633 1 3860 "tags=32%, list=27%, signal=44%"
GSE33162_HDAC3_KO_VS_HDAC3_KO_4H_LPS_STIM_MACROPHAGE_UP
GSE33162_HDAC3_KO_VS_HDAC3_KO_4H_LPS_STIM_MACROPHAGE_UP
151 -0.26294637 -1.0822942 0.26181102
0.37219593 1 1879 "tags=19%, list=13%, signal=22%"
GSE22886_NAIVE_TCELL_VS_DC_UP GSE22886_NAIVE_TCELL_VS_DC_UP
124 -0.26984018 -1.0822815 0.29433963
0.37199792 1 1172 "tags=16%, list=8%, signal=17%"
GSE17186_NAIVE_VS_CD21HIGH_TRANSITIONAL_BCELL_UP
GSE17186_NAIVE_VS_CD21HIGH_TRANSITIONAL_BCELL_UP
141 -0.25989676 -1.0815765 0.26824456
0.37344772 1 1984 "tags=20%, list=14%, signal=23%"
GSE8685_IL2_STARVED_VS_IL15_ACT_IL2_STARVED_CD4_TCELL_UP
GSE8685_IL2_STARVED_VS_IL15_ACT_IL2_STARVED_CD4_TCELL_UP
132 -0.2652332 -1.0812609 0.2952381
0.37398314 1 1779 "tags=18%, list=13%, signal=21%"
GSE17721_POLYIC_VS_GARDIQUIMOD_16H_BMDC_UP
GSE17721_POLYIC_VS_GARDIQUIMOD_16H_BMDC_UP
145 -0.2581415 -1.0807074 0.29445508
0.3750461 1 1591 "tags=17%, list=11%, signal=18%"
GSE14308_TH2_VS_TH1_DN GSE14308_TH2_VS_TH1_DN
149 -0.26099655 -1.080373 0.28761905
0.3756209 1 2149 "tags=23%, list=15%, signal=27%"
GSE11961_MARGINAL_ZONE_BCELL_VS_GERMINAL_CENTER_BCELL_DAY40_UP
GSE11961_MARGINAL_ZONE_BCELL_VS_GERMINAL_CENTER_BCELL_DAY40_UP
140 -0.26331192 -1.0803692 0.27364185
0.37539375 1 1631 "tags=18%, list=12%, signal=20%"
GSE39152_SPLEEN_CD103_NEG_VS_BRAIN_CD103_POS_MEMORY_CD8_TCELL_DN
GSE39152_SPLEEN_CD103_NEG_VS_BRAIN_CD103_POS_MEMORY_CD8_TCELL_DN
170 -0.25971475 -1.0803219 0.28820115
0.37527958 1 2738 "tags=24%, list=19%, signal=30%"
GSE29615_CTRL_VS_DAY3_LAIV_IFLU_VACCINE_PBMC_UP
GSE29615_CTRL_VS_DAY3_LAIV_IFLU_VACCINE_PBMC_UP
136 -0.26300058 -1.0798881 0.28599605
0.3760451 1 2031 "tags=21%, list=14%, signal=24%"
GSE27859_DC_VS_CD11C_INT_F480_INT_DC_UP
GSE27859_DC_VS_CD11C_INT_F480_INT_DC_UP
148 -0.26392856 -1.0788307 0.28465804
0.37838125 1 1953 "tags=20%, list=14%, signal=23%"
GSE6674_UNSTIM_VS_CPG_STIM_BCELL_DN
GSE6674_UNSTIM_VS_CPG_STIM_BCELL_DN
132 -0.26491046 -1.0787892 0.29504952
0.3782473 1 2494 "tags=24%, list=18%, signal=29%"
GSE22432_PDC_VS_TGFB1_TREATEDCOMMON_DC_PROGENITOR_DN
GSE22432_PDC_VS_TGFB1_TREATEDCOMMON_DC_PROGENITOR_DN
149 -0.26256907 -1.0784473 0.30812854

0.3788318	1	1665	"tags=23%, list=12%, signal=26%"
GSE5455_HEALTHY_VS_TUMOR_BEARING_MOUSE_SPLEEN_MONOCYTE_DN
GSE5455_HEALTHY_VS_TUMOR_BEARING_MOUSE_SPLEEN_MONOCYTE_DN
156	-0.2580062	-1.078079	0.24950884
0.37949494	1	1135	"tags=13%, list=8%, signal=14%"
GSE11864_UNTREATED_VS_CSF1_IFNG_PAM3CYS_IN_MAC_DN
GSE11864_UNTREATED_VS_CSF1_IFNG_PAM3CYS_IN_MAC_DN
137	-0.2602288	-1.0780315	0.3003663
0.37937632	1	1536	"tags=18%, list=11%, signal=20%"
GSE9960_GRAM_POS_VS_GRAM_NEG_AND_POS_SEPSIS_PBMC_UP
GSE9960_GRAM_POS_VS_GRAM_NEG_AND_POS_SEPSIS_PBMC_UP
150	-0.25970453	-1.0776248	0.28626692
0.38008246	1	1399	"tags=17%, list=10%, signal=19%"
GSE9960_HEALTHY_VS_GRAM_NEG_SEPSIS_PBMC_UP
GSE9960_HEALTHY_VS_GRAM_NEG_SEPSIS_PBMC_UP
145	-0.2621516	-1.0775098	0.26857144
0.3801345	1	1378	"tags=18%, list=10%, signal=20%"
GSE24972_WT_VS_IRF8_KO_MARGINAL_ZONE_SPLEEN_BCELL_UP
GSE24972_WT_VS_IRF8_KO_MARGINAL_ZONE_SPLEEN_BCELL_UP
147	-0.26074126	-1.0774809	0.29651162
0.37997663	1	1074	"tags=16%, list=8%, signal=17%"
GSE360_T_GONDII_VS_M_TUBERCULOSIS_DC_DN
GSE360_T_GONDII_VS_M_TUBERCULOSIS_DC_DN
150	-0.2572736	-1.0774401	0.28703704
0.37983254	1	1516	"tags=18%, list=11%, signal=20%"
GSE17974_0H_VS_1H_IN_VITRO_ACT_CD4_TCELL_UP
GSE17974_0H_VS_1H_IN_VITRO_ACT_CD4_TCELL_UP
112	-0.27434194	-1.0773841	0.28962818
0.37971935	1	2615	"tags=27%, list=19%, signal=33%"
GSE40274_FOXP3_VS_FOXP3_AND_GATA1_TRANSDUCED_ACTIVATED_CD4_TCELL_UP
GSE40274_FOXP3_VS_FOXP3_AND_GATA1_TRANSDUCED_ACTIVATED_CD4_TCELL_UP
	140	-0.26516572	-1.0773001	0.29389313
0.3796992	1	2262	"tags=19%, list=16%, signal=22%"
GSE13887_RESTING_VS_NO_TREATED_CD4_TCELL_UP
GSE13887_RESTING_VS_NO_TREATED_CD4_TCELL_UP
100	-0.27650306	-1.0771366	0.3007663
0.37984437	1	6817	"tags=56%, list=48%, signal=108%"
GSE369_PRE_VS_POST_IL6_INJECTION_SOCS3_KO_LIVER_UP
GSE369_PRE_VS_POST_IL6_INJECTION_SOCS3_KO_LIVER_UP
141	-0.2615482	-1.0771271	0.29205176
0.3796342	1	1926	"tags=21%, list=14%, signal=24%"
GSE16385_MONOCYTE_VS_MACROPHAGE_DN
GSE16385_MONOCYTE_VS_MACROPHAGE_DN
136	-0.26135907	-1.0769669	0.28219697
0.37979963	1	2607	"tags=21%, list=18%, signal=25%"
GSE2770_TGFB_AND_IL4_VS_IL12_TREATED_ACT_CD4_TCELL_6H_UP
GSE2770_TGFB_AND_IL4_VS_IL12_TREATED_ACT_CD4_TCELL_6H_UP
149	-0.26031289	-1.0768974	0.2932039
0.37973747	1	1962	"tags=21%, list=14%, signal=24%"
GSE22589_HEALTHY_VS_SIV_INFECTED_DC_DN
GSE22589_HEALTHY_VS_SIV_INFECTED_DC_DN
155	-0.25716385	-1.0765767	0.2962264
0.38026145	1	1779	"tags=16%, list=13%, signal=18%"
GSE24210_RESTING_TREG_VS_TCONV_UP GSE24210_RESTING_TREG_VS_TCONV_UP

145	-0.26376837	-1.0758293	0.28917912
0.3818467	1	1998	"tags=23%, list=14%, signal=27%"
GSE31082_DN_VS_CD8_SP_THYMOCYTE_DN
GSE31082_DN_VS_CD8_SP_THYMOCYTE_DN
155	-0.25891593	-1.0756813	0.2840691
0.3819717	1	1890	"tags=20%, list=13%, signal=23%"
GSE27786_NKTCELL_VS_NEUTROPHIL_UP GSE27786_NKTCELL_VS_NEUTROPHIL_UP
136	-0.2648174	-1.0756615	0.28375733
0.38178697	1	805	"tags=15%, list=6%, signal=16%"
GSE26669_CD4_VS_CD8_TCELL_IN_MLR_UP
GSE26669_CD4_VS_CD8_TCELL_IN_MLR_UP
148	-0.2580397	-1.0756592	0.27992633
0.38155603	1	2426	"tags=25%, list=17%, signal=30%"
GSE2585_CD80_HIGH_VS_LOW_MTEC_UP GSE2585_CD80_HIGH_VS_LOW_MTEC_UP
129	-0.26175973	-1.0756179	0.28764477
0.38142982	1	6206	"tags=53%, list=44%, signal=93%"
GSE37416_CTRL_VS_24H_F_TULARENSIS_LVS_NEUTROPHIL_DN
GSE37416_CTRL_VS_24H_F_TULARENSIS_LVS_NEUTROPHIL_DN
143	-0.2607269	-1.0755292	0.27431905
0.38141993	1	1638	"tags=20%, list=12%, signal=23%"
GSE32986_CURDLAN_LOWDOSE_VS_GMCSF_AND_CURDLAN_LOWDOSE_STIM_DC_DN
GSE32986_CURDLAN_LOWDOSE_VS_GMCSF_AND_CURDLAN_LOWDOSE_STIM_DC_DN
140	-0.26509026	-1.0752397	0.2848837
0.3818638	1	1804	"tags=22%, list=13%, signal=25%"
GSE8384_CTRL_VS_B_ABORTUS_4H_MAC_CELL_LINE_DN
GSE8384_CTRL_VS_B_ABORTUS_4H_MAC_CELL_LINE_DN
161	-0.25960928	-1.0751237	0.28355387
0.38190395	1	1776	"tags=20%, list=13%, signal=22%"
GSE13522_CTRL_VS_T_CRUZI_BRAZIL_STRAIN_INF_SKIN_DN
GSE13522_CTRL_VS_T_CRUZI_BRAZIL_STRAIN_INF_SKIN_DN
147	-0.26055753	-1.0750513	0.270073	0.3818382
1	1991	"tags=18%, list=14%, signal=21%"
GSE24142_EARLY_THYMIC_PROGENITOR_VS_DN3_THYMOCYTE_FETAL_UP
GSE24142_EARLY_THYMIC_PROGENITOR_VS_DN3_THYMOCYTE_FETAL_UP
151	-0.257808	-1.0742807	0.28675136
0.3834974	1	1262	"tags=17%, list=9%, signal=18%"
GSE14000_TRANSLATED_RNA_VS_MRNA_16H_LPS_DC_UP
GSE14000_TRANSLATED_RNA_VS_MRNA_16H_LPS_DC_UP
106	-0.2744114	-1.0737712	0.28651685
0.3845027	1	2675	"tags=23%, list=19%, signal=28%"
GSE43955_10H_VS_30H_ACT_CD4_TCELL_DN
GSE43955_10H_VS_30H_ACT_CD4_TCELL_DN
170	-0.25154147	-1.0729269	0.27	0.3863364
1	2191	"tags=18%, list=16%, signal=21%"
GSE17974_0H_VS_2H_IN_VITRO_ACT_CD4_TCELL_DN
GSE17974_0H_VS_2H_IN_VITRO_ACT_CD4_TCELL_DN
128	-0.26709023	-1.0728606	0.3040153
0.38626322	1	821	"tags=13%, list=6%, signal=14%"
GSE24142_DN2_VS_DN3_THYMOCYTE_DN GSE24142_DN2_VS_DN3_THYMOCYTE_DN
162	-0.25901765	-1.0725305	0.31384015
0.38680792	1	2138	"tags=20%, list=15%, signal=23%"
GSE5589_WT_VS_IL10_KO_LPS_STIM_MACROPHAGE_180MIN_DN
GSE5589_WT_VS_IL10_KO_LPS_STIM_MACROPHAGE_180MIN_DN
129	-0.26467168	-1.0724916	0.29718876

0.38667828	1	1670	"tags=16%, list=12%, signal=18%"
GSE37533_PPARG2_FOXP3_VS_FOXP3_TRANSDUCED_CD4_TCELL_PIOGLITAZONE_TREATED_UP
GSE37533_PPARG2_FOXP3_VS_FOXP3_TRANSDUCED_CD4_TCELL_PIOGLITAZONE_TREATED_UP	147	-0.25657701	-1.0719016
0.2889306	0.38787422	1	2102	"tags=19%, list=15%, signal=22%"
GSE36095_WT_VS_HDAC9_KO_TREG_DN	GSE36095_WT_VS_HDAC9_KO_TREG_DN
	95	-0.27654833	-1.0716459	0.32518798
0.38826847	1	3176	"tags=23%, list=23%, signal=30%"
GSE16697_CD4_TCELL_VS_TFH_CD4_TCELL_UP
GSE16697_CD4_TCELL_VS_TFH_CD4_TCELL_UP
149	-0.26056594	-1.0716121	0.29259259	0.388109
1	1858	"tags=21%, list=13%, signal=24%"
GSE1460_NAIVE_CD4_TCELL_CORD_BLOOD_VS_THYMIC_STROMAL_CELL_UP
GSE1460_NAIVE_CD4_TCELL_CORD_BLOOD_VS_THYMIC_STROMAL_CELL_UP
	127	-0.2657812	-1.0714707	0.29961833
0.3882176	1	2369	"tags=19%, list=17%, signal=23%"
GSE11961_MEMORY_BCELL_DAY7_VS_GERMINAL_CENTER_BCELL_DAY40_UP
GSE11961_MEMORY_BCELL_DAY7_VS_GERMINAL_CENTER_BCELL_DAY40_UP
	136	-0.26385036	-1.0713938	0.29174665
0.38817316	1	2163	"tags=21%, list=15%, signal=25%"
GSE10211_UV_INACT_SENDAI_VS_LIVE_SENDAI_VIRUS_TRACHEAL_EPITHELIAL_CELLS_UP
GSE10211_UV_INACT_SENDAI_VS_LIVE_SENDAI_VIRUS_TRACHEAL_EPITHELIAL_CELLS_UP	118	-0.26452982	-1.071188
0.3364486	0.3884449	1	2373	"tags=22%, list=17%, signal=26%"
GSE12198_CTRL_VS_HIGH_IL2_STIM_NK_CELL_DN
GSE12198_CTRL_VS_HIGH_IL2_STIM_NK_CELL_DN
160	-0.2557372	-1.0705712	0.30938125
0.3897217	1	1397	"tags=16%, list=10%, signal=18%"
GSE18281_SUBCAPSULAR_VS_PERIMEDULLARY_CORTICAL_REGION_OF_THYMUS_DN
GSE18281_SUBCAPSULAR_VS_PERIMEDULLARY_CORTICAL_REGION_OF_THYMUS_DN
	140	-0.2609371	-1.0703744	0.2818533
0.38996503	1	2670	"tags=22%, list=19%, signal=27%"
GSE36888_UNTREATED_VS_IL2_TREATED_STAT5_AB_KNOCKIN_TCELL_17H_DN
GSE36888_UNTREATED_VS_IL2_TREATED_STAT5_AB_KNOCKIN_TCELL_17H_DN
	137	-0.2612603	-1.0700161	0.3045977
0.3906049	1	2283	"tags=23%, list=16%, signal=28%"
GSE41867_DAY6_VS_DAY15_LCMV_ARMSTRONG_EFFECTOR_CD8_TCELL_DN
GSE41867_DAY6_VS_DAY15_LCMV_ARMSTRONG_EFFECTOR_CD8_TCELL_DN
130	-0.26650485	-1.069357	0.31688803
0.39198545	1	627	"tags=11%, list=4%, signal=11%"
GSE21927_HEALTHY_VS_TUMOROUS_BALBC_MOUSE_MONOCYTE_UP
GSE21927_HEALTHY_VS_TUMOROUS_BALBC_MOUSE_MONOCYTE_UP
121	-0.26816407	-1.0692475	0.33665338
0.39203992	1	2843	"tags=26%, list=20%, signal=33%"
GSE2770_IL12_AND_TGFB_VS_IL4_TREATED_ACT_CD4_TCELL_6H_DN
GSE2770_IL12_AND_TGFB_VS_IL4_TREATED_ACT_CD4_TCELL_6H_DN
146	-0.25958392	-1.0688614	0.29766536
0.39275387	1	5484	"tags=51%, list=39%, signal=82%"
GSE35435_RESTING_VS_IL4_TREATED_MACROPHAGE_DN
GSE35435_RESTING_VS_IL4_TREATED_MACROPHAGE_DN

126	-0.2637974	-1.0685339	0.31407943
0.39329717	1	1869	"tags=21%, list=13%, signal=24%"
GSE20727_CTRL_VS_DNFB_ALLERGEN_TREATED_DC_DN
GSE20727_CTRL_VS_DNFB_ALLERGEN_TREATED_DC_DN
113	-0.2680647	-1.0675602	0.28977272
0.3954916	1	2084	"tags=18%, list=15%, signal=21%"
GSE1791_CTRL_VS_NEUROMEDINU_IN_T_CELL_LINE_6H_DN
GSE1791_CTRL_VS_NEUROMEDINU_IN_T_CELL_LINE_6H_DN
136	-0.26339963	-1.0674939	0.2932039
0.39541367	1	2481	"tags=28%, list=18%, signal=34%"
GSE24142_EARLY_THYMIC_PROGENITOR_VS_DN3_THYMOCYTE_ADULT_UP
GSE24142_EARLY_THYMIC_PROGENITOR_VS_DN3_THYMOCYTE_ADULT_UP
159	-0.25592655	-1.0673232	0.2768031
0.39560294	1	1159	"tags=14%, list=8%, signal=15%"
GSE17721_CPG_VS_GARDIQUIMOD_4H_BMDC_UP
GSE17721_CPG_VS_GARDIQUIMOD_4H_BMDC_UP
156	-0.25676388	-1.0673181	0.29963234
0.39537677	1	1662	"tags=19%, list=12%, signal=21%"
GSE42021_TCONV_PLN_VS_CD24HI_TCONV_THYMUS_UP
GSE42021_TCONV_PLN_VS_CD24HI_TCONV_THYMUS_UP
144	-0.26172835	-1.0658702	0.30508474
0.39875925	1	1752	"tags=21%, list=12%, signal=24%"
GSE27786_LIN_NEG_VS_NKCELL_DN	GSE27786_LIN_NEG_VS_NKCELL_DN
	154	-0.25285593	-1.0657614	0.29928315
0.3988147	1	1347	"tags=18%, list=10%, signal=19%"
GSE3982_DC_VS_CENT_MEMORY_CD4_TCELL_DN
GSE3982_DC_VS_CENT_MEMORY_CD4_TCELL_DN
118	-0.267887	-1.0654465	0.31214952
0.39936033	1	1845	"tags=18%, list=13%, signal=20%"
GSE27786_CD4_VS_CD8_TCELL_DN	GSE27786_CD4_VS_CD8_TCELL_DN
	136	-0.26138198	-1.0651383	0.3135436
0.39985886	1	1602	"tags=16%, list=11%, signal=18%"
GSE1740_UNSTIM_VS_IFNA_STIMULATED_MCSF_DERIVED_MACROPHAGE_DN
GSE1740_UNSTIM_VS_IFNA_STIMULATED_MCSF_DERIVED_MACROPHAGE_DN
	109	-0.26904705	-1.0650963	0.32495165
0.39972985	1	2159	"tags=28%, list=15%, signal=32%"
GSE20152_SPHK1_KO_VS_WT_HTNFA_OVERXPRESS_ANKLE_UP
GSE20152_SPHK1_KO_VS_WT_HTNFA_OVERXPRESS_ANKLE_UP
136	-0.25768572	-1.0650398	0.3229572
0.39963126	1	2440	"tags=21%, list=17%, signal=25%"
GSE1925_3H_VS_24H_IFNG_STIM_IFNG_PRIMED_MACROPHAGE_UP
GSE1925_3H_VS_24H_IFNG_STIM_IFNG_PRIMED_MACROPHAGE_UP
161	-0.25515386	-1.0647917	0.3031496
0.40002593	1	1878	"tags=22%, list=13%, signal=25%"
GSE13887_RESTING_VS_NO_TREATED_CD4_TCELL_DN
GSE13887_RESTING_VS_NO_TREATED_CD4_TCELL_DN
82	-0.28254035	-1.0647548	0.32780847
0.3998766	1	2987	"tags=26%, list=21%, signal=32%"
GSE16386_IL4_VS_IL4_AND_ROSIGLITAZONE_STIM_MACROPHAGE_6H_DN
GSE16386_IL4_VS_IL4_AND_ROSIGLITAZONE_STIM_MACROPHAGE_6H_DN
136	-0.2596495	-1.0641798	0.3095685
0.40106928	1	2186	"tags=19%, list=15%, signal=22%"
GSE6269_STAPH_AUREUS_VS_STREP_PNEUMO_INF_PBMC_UP
GSE6269_STAPH_AUREUS_VS_STREP_PNEUMO_INF_PBMC_UP

137	-0.25786978	-1.0637418	0.31496063
0.40190658	1	2158	"tags=22%, list=15%, signal=26%"
GSE29618_BCELL_VS_MONOCYTE_DAY7_FLU_VACCINE_UP
GSE29618_BCELL_VS_MONOCYTE_DAY7_FLU_VACCINE_UP
126	-0.26501125	-1.063599	0.3326886
0.4020283	1	2410	"tags=21%, list=17%, signal=26%"
GSE34156_UNTREATED_VS_6H_NOD2_LIGAND_TREATED_MONOCYTE_DN
GSE34156_UNTREATED_VS_6H_NOD2_LIGAND_TREATED_MONOCYTE_DN
136	-0.2602814	-1.0633779	0.3139535
0.40233797	1	2491	"tags=26%, list=18%, signal=31%"
GSE46606_DAY1_VS_DAY3_CD40L_IL2_IL5_STIMULATED_BCELL_DN
GSE46606_DAY1_VS_DAY3_CD40L_IL2_IL5_STIMULATED_BCELL_DN
138	-0.25962216	-1.062843	0.2981132
0.40341735	1	2475	"tags=22%, list=18%, signal=27%"
GSE26559_TCF1_KO_VS_WT_LIN_NEG_CELL_DN
GSE26559_TCF1_KO_VS_WT_LIN_NEG_CELL_DN
143	-0.25471377	-1.0625126	0.29821074
0.4039654	1	4748	"tags=38%, list=34%, signal=57%"
GSE24726_WT_VS_E2_2_KO_PDC_DAY4_POST_DELETION_UP
GSE24726_WT_VS_E2_2_KO_PDC_DAY4_POST_DELETION_UP
147	-0.26035476	-1.061993	0.30268198
0.4050532	1	1622	"tags=19%, list=11%, signal=21%"
GSE1460_DP_THYMOCYTE_VS_THYMIC_STROMAL_CELL_DN
GSE1460_DP_THYMOCYTE_VS_THYMIC_STROMAL_CELL_DN
145	-0.2564782	-1.0619811	0.33973128
0.40483603	1	1146	"tags=15%, list=8%, signal=16%"
GSE35543_IN_VIVO_NTREG_VS_CONVERTED_EX_ITREG_DN
GSE35543_IN_VIVO_NTREG_VS_CONVERTED_EX_ITREG_DN
136	-0.25853756	-1.0609125	0.3108911
0.4073124	1	6067	"tags=49%, list=43%, signal=86%"
GSE26928_NAIVE_VS_EFF_MEMORY_CD4_TCELL_UP
GSE26928_NAIVE_VS_EFF_MEMORY_CD4_TCELL_UP
105	-0.26857185	-1.0593253	0.33826247
0.41099578	1	2085	"tags=22%, list=15%, signal=26%"
GSE40274_CTRL_VS_FOXP3_AND_EOS_TRANSDUCED_ACTIVATED_CD4_TCELL_DN
GSE40274_CTRL_VS_FOXP3_AND_EOS_TRANSDUCED_ACTIVATED_CD4_TCELL_DN
	128	-0.26062548	-1.0591547	0.3255814
0.4111904	1	2840	"tags=24%, list=20%, signal=30%"
GSE40274_LEF1_VS_FOXP3_AND_LEF1_TRANSDUCED_ACTIVATED_CD4_TCELL_DN
GSE40274_LEF1_VS_FOXP3_AND_LEF1_TRANSDUCED_ACTIVATED_CD4_TCELL_DN
	143	-0.25997776	-1.0590807	0.32089552
0.4111225	1	2439	"tags=23%, list=17%, signal=28%"
GSE26488_WT_VS_VP16_TRANSGENIC_HDAC7_KO_DOUBLE_POSITIVE_THYMOCYTE_DN

GSE26488_WT_VS_VP16_TRANSGENIC_HDAC7_KO_DOUBLE_POSITIVE_THYMOCYTE_DN
		135	-0.26159242	-1.0587027
0.32777777	0.41178128	1	1702	"tags=20%,
list=12%, signal=23%"
GSE10325_CD4_TCELL_VS_MYELOID_DN	GSE10325_CD4_TCELL_VS_MYELOID_DN
	151	-0.2541007	-1.0585505	0.33078393
0.41192898	1	2593	"tags=26%, list=18%, signal=32%"
GSE6269_HEALTHY_VS_E_COLI_INF_PBMC_DN
GSE6269_HEALTHY_VS_E_COLI_INF_PBMC_DN
119	-0.26535845	-1.058163	0.32827324

0.4126521	1	1461	"tags=16%, list=10%, signal=18%"
GSE6259_33D1_POS_DC_VS_BCELL_UP	GSE6259_33D1_POS_DC_VS_BCELL_UP
	118	-0.26669416	-1.0578796	0.3276515
0.41310883	1	4083	"tags=39%, list=29%, signal=54%"
GSE11057_PBMC_VS_MEM_CD4_TCELL_DN GSE11057_PBMC_VS_MEM_CD4_TCELL_DN
	131	-0.26170304	-1.057173	0.31176472
0.41466326	1	1310	"tags=16%, list=9%, signal=18%"
GSE37534_GW1929_VS_PIOGLITAZONE_TREATED_CD4_TCELL_PPARG1_FOXP3_TRANSDUCED_UP
GSE37534_GW1929_VS_PIOGLITAZONE_TREATED_CD4_TCELL_PPARG1_FOXP3_TRANSDUCED_UP	130	-0.2611438	-1.0568303
0.34108528	0.41527426	1	1557	"tags=18%, list=11%, signal=21%"
GSE45365_NK_CELL_VS_CD8_TCELL_MCMV_INFECTION_DN
GSE45365_NK_CELL_VS_CD8_TCELL_MCMV_INFECTION_DN
145	-0.25606993	-1.0566474	0.3273453
0.4154983	1	2867	"tags=30%, list=20%, signal=38%"
GSE26488_HDAC7_KO_VS_VP16_TRANSGENIC_HDAC7_KO_DOUBLE_POSITIVE_THYMOCYTE_UP
GSE26488_HDAC7_KO_VS_VP16_TRANSGENIC_HDAC7_KO_DOUBLE_POSITIVE_THYMOCYTE_UP	110	-0.26793072	-1.0565516
0.33462283	0.41548285	1	2124	"tags=18%, list=15%, signal=21%"
GSE24634_IL4_VS_CTRL_TREATED_NAIVE_CD4_TCELL_DAY5_UP
GSE24634_IL4_VS_CTRL_TREATED_NAIVE_CD4_TCELL_DAY5_UP
152	-0.25398296	-1.0554205	0.32588455
0.41806054	1	2180	"tags=24%, list=15%, signal=28%"
GSE17721_POLYIC_VS_GARDIQUIMOD_2H_BMDC_UP
GSE17721_POLYIC_VS_GARDIQUIMOD_2H_BMDC_UP
147	-0.25438145	-1.0538349	0.31261426
0.42181653	1	2629	"tags=24%, list=19%, signal=30%"
GSE12963_UNINF_VS_ENV_AND_NEF_AND_VPR_DEFICIENT_HIV1_INF_CD4_TCELL_UP
GSE12963_UNINF_VS_ENV_AND_NEF_AND_VPR_DEFICIENT_HIV1_INF_CD4_TCELL_UP	102	-0.27184895	-1.0531713
0.35977858	0.4232116	1	3425	"tags=29%, list=24%, signal=39%"
GSE23321_CENTRAL_VS_EFFECTOR_MEMORY_CD8_TCELL_DN
GSE23321_CENTRAL_VS_EFFECTOR_MEMORY_CD8_TCELL_DN
143	-0.2577572	-1.0530596	0.33638024
0.42325044	1	1142	"tags=15%, list=8%, signal=17%"
GSE24292_WT_VS_PPARG_KO_MACROPHAGE_DN
GSE24292_WT_VS_PPARG_KO_MACROPHAGE_DN
147	-0.25288913	-1.0530463	0.33205375
0.4230298	1	1998	"tags=20%, list=14%, signal=24%"
GSE40274_CTRL_VS_SATB1_TRANSDUCED_ACTIVATED_CD4_TCELL_DN
GSE40274_CTRL_VS_SATB1_TRANSDUCED_ACTIVATED_CD4_TCELL_DN
135	-0.25629053	-1.052727	0.34808853
0.42359224	1	2292	"tags=24%, list=16%, signal=29%"
GSE13493_DP_VS_CD8POS_THYMOCYTE_UP
GSE13493_DP_VS_CD8POS_THYMOCYTE_UP
137	-0.25593445	-1.0526417	0.33723196
0.42357454	1	1537	"tags=16%, list=11%, signal=18%"
GSE9006_HEALTHY_VS_TYPE_1_DIABETES_PBMC_4MONTH_POST_DX_DN

GSE9006_HEALTHY_VS_TYPE_1_DIABETES_PBMC_4MONTH_POST_DX_DN
127	-0.26063025	-1.0524876	0.33707866
0.4237143	1	1536	"tags=14%, list=11%, signal=16%"
GSE28737_WT_VS_BCL6_HET_MARGINAL_ZONE_BCELL_UP
GSE28737_WT_VS_BCL6_HET_MARGINAL_ZONE_BCELL_UP
134	-0.25551817	-1.051784	0.33333334	0.425258
1	2591	"tags=22%, list=18%, signal=26%"
GSE9037_WT_VS_IRAK4_KO_LPS_4H_STIM_BMDM_UP
GSE9037_WT_VS_IRAK4_KO_LPS_4H_STIM_BMDM_UP
148	-0.25427723	-1.0517602	0.32854208
0.42507228	1	1641	"tags=16%, list=12%, signal=17%"
GSE12198_CTRL_VS_HIGH_IL2_STIM_NK_CELL_UP
GSE12198_CTRL_VS_HIGH_IL2_STIM_NK_CELL_UP
157	-0.25133577	-1.0516806	0.33918127
0.42503628	1	885	"tags=13%, list=6%, signal=14%"
GSE22611_MUTANT_NOD2_VS_CTRL_TRANSDUCED_HEK293T_CELL_UP
GSE22611_MUTANT_NOD2_VS_CTRL_TRANSDUCED_HEK293T_CELL_UP
130	-0.26025027	-1.0513572	0.33776093
0.42561984	1	2117	"tags=21%, list=15%, signal=24%"
GSE45365_CD8A_DC_VS_CD11B_DC_IFNAR_KO_UP
GSE45365_CD8A_DC_VS_CD11B_DC_IFNAR_KO_UP
123	-0.2597329	-1.0509331	0.34008098
0.4264395	1	2915	"tags=24%, list=21%, signal=30%"
GSE4748_CYANOBACTERIUM_LPSLIKE_VS_LPS_AND_CYANOBACTERIUM_LPSLIKE_STIM_DC_3H_UP
GSE4748_CYANOBACTERIUM_LPSLIKE_VS_LPS_AND_CYANOBACTERIUM_LPSLIKE_STIM_DC_3H_UP	121	-0.26387328	-1.0508406
0.36363637	0.4264035	1	2439	"tags=24%, list=17%, signal=29%"
GSE360_CTRL_VS_B_MALAYI_HIGH_DOSE_DC_DN
GSE360_CTRL_VS_B_MALAYI_HIGH_DOSE_DC_DN
148	-0.25538585	-1.0504636	0.34022558
0.42711523	1	2237	"tags=21%, list=16%, signal=25%"
GSE37301_LYMPHOID_PRIMED_MPP_VS_CD4_TCELL_UP
GSE37301_LYMPHOID_PRIMED_MPP_VS_CD4_TCELL_UP
129	-0.25949338	-1.0503228	0.33898306
0.42721486	1	2614	"tags=22%, list=19%, signal=26%"
GSE13229_MATURE_VS_INTMATURE_NKCELL_DN
GSE13229_MATURE_VS_INTMATURE_NKCELL_DN
139	-0.25418612	-1.0494707	0.3425926
0.4291971	1	2493	"tags=24%, list=18%, signal=29%"
GSE360_CTRL_VS_L_DONOVANI_DC_DN	GSE360_CTRL_VS_L_DONOVANI_DC_DN
	154	-0.2501317	-1.0491263	0.33461538
0.42984942	1	3051	"tags=28%, list=22%, signal=35%"
GSE23505_IL6_IL1_IL23_VS_IL6_IL1_TGFB_TREATED_CD4_TCELL_UP
GSE23505_IL6_IL1_IL23_VS_IL6_IL1_TGFB_TREATED_CD4_TCELL_UP
159	-0.25350893	-1.0485314	0.33333334
0.4310741	1	2230	"tags=21%, list=16%, signal=25%"
GSE37319_WT_VS_RC3H1_KO_CD44LOW_CD8_TCELL_UP
GSE37319_WT_VS_RC3H1_KO_CD44LOW_CD8_TCELL_UP
104	-0.26666027	-1.0481877	0.3529412
0.43171126	1	2378	"tags=24%, list=17%, signal=29%"
GSE12198_CTRL_VS_LOW_IL2_STIM_NK_CELL_UP
GSE12198_CTRL_VS_LOW_IL2_STIM_NK_CELL_UP

141	-0.25541282	-1.0480876	0.3452381
0.43171817	1	2537	"tags=26%, list=18%, signal=32%"
GSE12845_NAIVE_VS_PRE_GC_TONSIL_BCELL_UP
GSE12845_NAIVE_VS_PRE_GC_TONSIL_BCELL_UP
124	-0.25818872	-1.0479901	0.35963303
0.43169743	1	2971	"tags=27%, list=21%, signal=34%"
GSE8835_HEALTHY_VS_CLL_CD4_TCELL_UP
GSE8835_HEALTHY_VS_CLL_CD4_TCELL_UP
142	-0.25592563	-1.0477298	0.34264433
0.43216535	1	2292	"tags=20%, list=16%, signal=24%"
GSE21360_NAIVE_VS_PRIMARY_MEMORY_CD8_TCELL_UP
GSE21360_NAIVE_VS_PRIMARY_MEMORY_CD8_TCELL_UP
142	-0.25762415	-1.0476775	0.35188866
0.43203554	1	2838	"tags=30%, list=20%, signal=37%"
GSE1566_WT_VS_EZH2_KO_LN_TCELL_DN GSE1566_WT_VS_EZH2_KO_LN_TCELL_DN
116	-0.26278308	-1.047154	0.3444882
0.4331032	1	2414	"tags=21%, list=17%, signal=25%"
GSE19888_CTRL_VS_TCELL_MEMBRANES_ACT_MAST_CELL_PRETREAT_A3R_INH_UP
GSE19888_CTRL_VS_TCELL_MEMBRANES_ACT_MAST_CELL_PRETREAT_A3R_INH_UP
152	-0.25274995	-1.0469124	0.34637964
0.43348274	1	4129	"tags=34%, list=29%, signal=48%"
GSE43955_TH0_VS_TGFB_IL6_TH17_ACT_CD4_TCELL_1H_DN
GSE43955_TH0_VS_TGFB_IL6_TH17_ACT_CD4_TCELL_1H_DN
164	-0.2459965	-1.0462326	0.3705104
0.43496805	1	2713	"tags=20%, list=19%, signal=25%"
GSE19888_ADENOSINE_A3R_INH_VS_ACT_IN_MAST_CELL_DN
GSE19888_ADENOSINE_A3R_INH_VS_ACT_IN_MAST_CELL_DN
127	-0.25739115	-1.0457736	0.3358349
0.43586284	1	2398	"tags=24%, list=17%, signal=28%"
GSE8868_SPLEEN_VS_INTESTINE_CD11B_POS_CD11C_NEG_DC_DN
GSE8868_SPLEEN_VS_INTESTINE_CD11B_POS_CD11C_NEG_DC_DN
145	-0.25274923	-1.0454988	0.3403846
0.43630612	1	1986	"tags=20%, list=14%, signal=23%"
GSE34156_NOD2_LIGAND_VS_TLR1_TLR2_LIGAND_6H_TREATED_MONOCYTE_UP
GSE34156_NOD2_LIGAND_VS_TLR1_TLR2_LIGAND_6H_TREATED_MONOCYTE_UP
164	-0.24947548	-1.0449855	0.3612663
0.43743286	1	2487	"tags=24%, list=18%, signal=29%"
GSE12845_IGD_POS_BLOOD_VS_PRE_GC_TONSIL_BCELL_UP
GSE12845_IGD_POS_BLOOD_VS_PRE_GC_TONSIL_BCELL_UP
132	-0.25506628	-1.0445565	0.36960602
0.43831685	1	2138	"tags=19%, list=15%, signal=22%"
GSE28737_WT_VS_BCL6_KO_FOLLICULAR_BCELL_UP
GSE28737_WT_VS_BCL6_KO_FOLLICULAR_BCELL_UP
143	-0.250565	-1.04451	0.3576779	0.438184	1
3305	"tags=30%, list=23%, signal=39%"
GSE21380_TFH_VS_GERMINAL_CENTER_TFH_CD4_TCELL_UP
GSE21380_TFH_VS_GERMINAL_CENTER_TFH_CD4_TCELL_UP
150	-0.25025478	-1.0442197	0.31976745
0.43865925	1	4463	"tags=34%, list=32%, signal=49%"
GSE20500_RETINOIC_ACID_VS_RARA_ANTAGONIST_TREATED_CD4_TCELL_DN
GSE20500_RETINOIC_ACID_VS_RARA_ANTAGONIST_TREATED_CD4_TCELL_DN
144	-0.25395137	-1.0434983	0.33458647
0.4402664	1	2480	"tags=24%, list=18%, signal=29%"
GSE11961_MEMORY_BCELL_DAY40_VS_GERMINAL_CENTER_BCELL_DAY40_UP

GSE11961_MEMORY_BCELL_DAY40_VS_GERMINAL_CENTER_BCELL_DAY40_UP
137 -0.25456464 -1.0434031 0.34387353
0.44025344 1 2299 "tags=21%, list=16%, signal=25%"
GSE15624_CTRL_VS_3H_HALOFUGINONE_TREATED_CD4_TCELL_UP
GSE15624_CTRL_VS_3H_HALOFUGINONE_TREATED_CD4_TCELL_UP
130 -0.2583256 -1.0428182 0.3653846
0.44153783 1 2377 "tags=25%, list=17%, signal=30%"
GSE29615_CTRL_VS_LAIV_FLU_VACCINE_PBMC_DN
GSE29615_CTRL_VS_LAIV_FLU_VACCINE_PBMC_DN
90 -0.27393547 -1.0423194 0.37449393
0.4425211 1 4259 "tags=36%, list=30%, signal=51%"
GSE9006_TYPE_1_VS_TYPE_2_DIABETES_PBMC_AT_DX_DN
GSE9006_TYPE_1_VS_TYPE_2_DIABETES_PBMC_AT_DX_DN
143 -0.25251183 -1.0421293 0.39034206
0.4427357 1 2169 "tags=22%, list=15%, signal=26%"
GSE16522_ANTI_CD3CD28_STIM_VS_UNSTIM_NAIVE_CD8_TCELL_UP
GSE16522_ANTI_CD3CD28_STIM_VS_UNSTIM_NAIVE_CD8_TCELL_UP
145 -0.25622502 -1.042039 0.34095237
0.4426986 1 1999 "tags=17%, list=14%, signal=20%"
GSE2706_UNSTIM_VS_2H_R848_DC_UP GSE2706_UNSTIM_VS_2H_R848_DC_UP
113 -0.2669008 -1.0414833 0.35882354
0.44388828 1 3639 "tags=38%, list=26%, signal=51%"
GSE17721_CTRL_VS_CPG_24H_BMDC_UP GSE17721_CTRL_VS_CPG_24H_BMDC_UP
154 -0.24794734 -1.0414485 0.33901516
0.44372904 1 2886 "tags=27%, list=20%, signal=33%"
GSE45365_WT_VS_IFNAR_KO_CD8A_DC_DN
GSE45365_WT_VS_IFNAR_KO_CD8A_DC_DN
146 -0.24879545 -1.0411959 0.3618182
0.44411346 1 2081 "tags=21%, list=15%, signal=25%"
GSE25088_ROSIGLITAZONE_VS_IL4_AND_ROSIGLITAZONE_STIM_MACROPHAGE_DAY1
0_UP
GSE25088_ROSIGLITAZONE_VS_IL4_AND_ROSIGLITAZONE_STIM_MACROPHAGE_DAY1
0_UP 133 -0.25683218 -1.0407429
0.3544776 0.4450318 1 1725 "tags=20%,
list=12%, signal=22%"
GSE27859_CD11C_INT_F480_HI_MACROPHAGE_VS_CD11C_ING_F480_INT_DC_DN
GSE27859_CD11C_INT_F480_HI_MACROPHAGE_VS_CD11C_ING_F480_INT_DC_DN
162 -0.247793 -1.0401951 0.3507014
0.44620296 1 1980 "tags=21%, list=14%, signal=24%"
GSE21774_CD56_BRIGHT_VS_DIM_CD62L_POSITIVE_NK_CELL_UP
GSE21774_CD56_BRIGHT_VS_DIM_CD62L_POSITIVE_NK_CELL_UP
154 -0.25219244 -1.0399923 0.35700196
0.44645983 1 2121 "tags=18%, list=15%, signal=20%"
GSE360_T_GONDII_VS_M_TUBERCULOSIS_DC_UP
GSE360_T_GONDII_VS_M_TUBERCULOSIS_DC_UP
143 -0.25355864 -1.0399703 0.3761468
0.4462546 1 3351 "tags=29%, list=24%, signal=38%"
GSE13762_CTRL_VS_125_VITAMIND_DAY12_DC_UP
GSE13762_CTRL_VS_125_VITAMIND_DAY12_DC_UP
107 -0.26367235 -1.0398718 0.3572744
0.44624123 1 1874 "tags=17%, list=13%, signal=19%"
GSE3982_CTRL_VS_LPS_1H_NEUTROPHIL_UP
GSE3982_CTRL_VS_LPS_1H_NEUTROPHIL_UP
132 -0.2543679 -1.038705 0.33951762

0.44901633	1	2503	"tags=27%, list=18%, signal=33%"
GSE37532_VISCERAL_ADIPOSE_TISSUE_VS_LN_DERIVED_TREG_CD4_TCELL_UP
GSE37532_VISCERAL_ADIPOSE_TISSUE_VS_LN_DERIVED_TREG_CD4_TCELL_UP
	129	-0.2620995	-1.0381607	0.3693182
0.45018473	1	1906	"tags=21%, list=14%, signal=24%"
GSE22886_NAIVE_CD4_TCELL_VS_12H_ACT_TH2_UP
GSE22886_NAIVE_CD4_TCELL_VS_12H_ACT_TH2_UP
129	-0.25765583	-1.0380374	0.37148216
0.45022398	1	1719	"tags=16%, list=12%, signal=17%"
GSE6259_FLT3L_INDUCED_VS_WT_SPLENIC_DC_33D1_POS_UP
GSE6259_FLT3L_INDUCED_VS_WT_SPLENIC_DC_33D1_POS_UP
136	-0.25618798	-1.0377066	0.3457249
0.45084843	1	2163	"tags=23%, list=15%, signal=27%"
GSE13887_ACT_CD4_VS_NO_TREATED_CD4_TCELL_UP
GSE13887_ACT_CD4_VS_NO_TREATED_CD4_TCELL_UP
89	-0.26771572	-1.0375776	0.3824092
0.45091575	1	7398	"tags=57%, list=52%, signal=120%"
GSE36009_WT_VS_NLRP10_KO_DC_LPS_STIM_DN
GSE36009_WT_VS_NLRP10_KO_DC_LPS_STIM_DN
136	-0.2549014	-1.0373535	0.36213234
0.45122644	1	2005	"tags=16%, list=14%, signal=19%"
GSE25088_WT_VS_STAT6_KO_MACROPHAGE_ROSIGLITAZONE_AND_IL4_STIM_DN
GSE25088_WT_VS_STAT6_KO_MACROPHAGE_ROSIGLITAZONE_AND_IL4_STIM_DN
	135	-0.2568044	-1.0366402	0.38
0.45288804	1	1074	"tags=13%, list=8%, signal=13%"
GSE10239_MEMORY_VS_KLRG1HIGH_EFF_CD8_TCELL_UP
GSE10239_MEMORY_VS_KLRG1HIGH_EFF_CD8_TCELL_UP
138	-0.2571423	-1.0364915	0.3548387
0.45301595	1	3181	"tags=25%, list=23%, signal=32%"
GSE20198_UNTREATED_VS_IFNA_TREATED_ACT_CD4_TCELL_UP
GSE20198_UNTREATED_VS_IFNA_TREATED_ACT_CD4_TCELL_UP
141	-0.25269628	-1.0357755	0.378119 0.45465738
1	2856	"tags=25%, list=20%, signal=31%"
GSE37532_WT_VS_PPARG_KO_LN_TREG_UP
GSE37532_WT_VS_PPARG_KO_LN_TREG_UP
141	-0.2540541	-1.035354	0.35440612
0.45550114	1	2022	"tags=21%, list=14%, signal=25%"
GSE9316_IL6_KO_VS_IFNG_KO_INVIVO_EXPANDED_CD4_TCELL_UP
GSE9316_IL6_KO_VS_IFNG_KO_INVIVO_EXPANDED_CD4_TCELL_UP
160	-0.25063795	-1.0352305	0.3824092
0.45557177	1	1147	"tags=13%, list=8%, signal=14%"
GSE17721_POLYIC_VS_GARDIQUIMOD_2H_BMDC_DN
GSE17721_POLYIC_VS_GARDIQUIMOD_2H_BMDC_DN
158	-0.24622785	-1.0351475	0.36501902
0.45551768	1	1426	"tags=15%, list=10%, signal=17%"
GSE46242_CTRL_VS_EGR2_DELETED_ANERGIC_TH1_CD4_TCELL_DN
GSE46242_CTRL_VS_EGR2_DELETED_ANERGIC_TH1_CD4_TCELL_DN
144	-0.25215006	-1.0347927	0.37159532
0.4561646	1	2911	"tags=31%, list=21%, signal=38%"
GSE26928_NAIVE_VS_EFF_MEMORY_CD4_TCELL_DN
GSE26928_NAIVE_VS_EFF_MEMORY_CD4_TCELL_DN
137	-0.25355604	-1.0347359	0.37310606
0.4560628	1	1806	"tags=21%, list=13%, signal=24%"
GSE19401_PLN_VS_PEYERS_PATCH_FOLLICULAR_DC_UP

GSE19401_PLN_VS_PEYERS_PATCH_FOLLICULAR_DC_UP
136	-0.25141746	-1.034607	0.36363637	0.456132
1	2335	"tags=21%, list=17%, signal=25%"
GSE43955_TH0_VS_TGFB_IL6_TH17_ACT_CD4_TCELL_52H_DN
GSE43955_TH0_VS_TGFB_IL6_TH17_ACT_CD4_TCELL_52H_DN
158	-0.24808842	-1.0341362	0.36007828
0.45712987	1	2106	"tags=16%, list=15%, signal=18%"
GSE12707_AT16L1_HYPOMORPH_VS_WT_THYMUS_UP
GSE12707_AT16L1_HYPOMORPH_VS_WT_THYMUS_UP
100	-0.26391032	-1.0338868	0.39499035
0.4575346	1	2861	"tags=27%, list=20%, signal=34%"
GSE25123_CTRL_VS_ROSIGLITAZONE_STIM_PPARG_KO_MACROPHAGE_UP
GSE25123_CTRL_VS_ROSIGLITAZONE_STIM_PPARG_KO_MACROPHAGE_UP
134	-0.2549452	-1.0334513	0.36783734
0.45843497	1	1744	"tags=20%, list=12%, signal=23%"
GSE22886_DAY0_VS_DAY1_MONOCYTE_IN_CULTURE_UP
GSE22886_DAY0_VS_DAY1_MONOCYTE_IN_CULTURE_UP
136	-0.25181744	-1.0333451	0.3976143
0.45844558	1	2570	"tags=23%, list=18%, signal=28%"
GSE21379_WT_VS_SAP_KO_TFH_CD4_TCELL_UP
GSE21379_WT_VS_SAP_KO_TFH_CD4_TCELL_UP
161	-0.24453369	-1.0329651	0.36278194
0.45923096	1	3159	"tags=22%, list=22%, signal=28%"
GSE23114_PERITONEAL_CAVITY_B1A_BCELL_VS_SPLEEN_BCELL_UP
GSE23114_PERITONEAL_CAVITY_B1A_BCELL_VS_SPLEEN_BCELL_UP
119	-0.25887844	-1.0329262	0.38619402
0.45907044	1	1279	"tags=14%, list=9%, signal=16%"
GSE21033_3H_VS_24H_POLYIC_STIM_DC_UP
GSE21033_3H_VS_24H_POLYIC_STIM_DC_UP
121	-0.25905877	-1.0326369	0.3778626
0.45956802	1	2859	"tags=27%, list=20%, signal=34%"
GSE29618_PRE_VS_DAY7_FLU_VACCINE_MONOCYTE_UP
GSE29618_PRE_VS_DAY7_FLU_VACCINE_MONOCYTE_UP
122	-0.25743723	-1.0324895	0.38919926
0.45969653	1	3490	"tags=30%, list=25%, signal=39%"
GSE5542_UNTREATED_VS_IFNA_AND_IFNG_TREATED_EPITHELIAL_CELLS_6H_UP
GSE5542_UNTREATED_VS_IFNA_AND_IFNG_TREATED_EPITHELIAL_CELLS_6H_UP
	122	-0.25638086	-1.0321497	0.35823753
0.4603243	1	3210	"tags=34%, list=23%, signal=43%"
GSE22443_NAIVE_VS_ACT_AND_IL2_TREATED_CD8_TCELL_UP
GSE22443_NAIVE_VS_ACT_AND_IL2_TREATED_CD8_TCELL_UP
164	-0.24577738	-1.0320916	0.3605948
0.46022302	1	4638	"tags=37%, list=33%, signal=55%"
GSE15930_STIM_VS_STIM_AND_TRICHOSTATINA_48H_CD8_T_CELL_DN
GSE15930_STIM_VS_STIM_AND_TRICHOSTATINA_48H_CD8_T_CELL_DN
151	-0.2466399	-1.0320461	0.37236086
0.4600852	1	1724	"tags=17%, list=12%, signal=19%"
GSE9960_HEALTHY_VS_GRAM_NEG_AND_POS_SEPSIS_PBMC_UP
GSE9960_HEALTHY_VS_GRAM_NEG_AND_POS_SEPSIS_PBMC_UP
115	-0.25753102	-1.0308005	0.36960602
0.46313286	1	2187	"tags=18%, list=15%, signal=21%"
GSE36392_TYPE_2_MYELOID_VS_NEUTROPHIL_IL25_TREATED_LUNG_UP
GSE36392_TYPE_2_MYELOID_VS_NEUTROPHIL_IL25_TREATED_LUNG_UP
151	-0.24757347	-1.0307443	0.36923078

0.4630061	1	2475	"tags=26%, list=18%, signal=32%"
GSE24634_IL4_VS_CTRL_TREATED_NAIVE_CD4_TCELL_DAY3_UP
GSE24634_IL4_VS_CTRL_TREATED_NAIVE_CD4_TCELL_DAY3_UP
152	-0.24813089	-1.0299789	0.36734694
0.4647309	1	742	"tags=13%, list=5%, signal=13%"
GSE20366_CD103_POS_VS_CD103_KLRG1_DP_TREG_DN
GSE20366_CD103_POS_VS_CD103_KLRG1_DP_TREG_DN
152	-0.24695003	-1.0299287	0.37200737
0.4645918	1	1561	"tags=17%, list=11%, signal=19%"
GSE22443_IL2_VS_IL12_TREATED_ACT_CD8_TCELL_UP
GSE22443_IL2_VS_IL12_TREATED_ACT_CD8_TCELL_UP
148	-0.24861062	-1.0292966	0.37545788
0.46601912	1	4162	"tags=34%, list=29%, signal=47%"
GSE43955_TH0_VS_TGFB_IL6_TH17_ACT_CD4_TCELL_30H_DN
GSE43955_TH0_VS_TGFB_IL6_TH17_ACT_CD4_TCELL_30H_DN
151	-0.24653152	-1.0290852	0.3752345
0.4662674	1	1867	"tags=19%, list=13%, signal=21%"
GSE45365_HEALTHY_VS_MCMV_INFECTION_CD8A_DC_IFNAR_KO_DN
GSE45365_HEALTHY_VS_MCMV_INFECTION_CD8A_DC_IFNAR_KO_DN
148	-0.24840729	-1.02857	0.3775322	0.46736202
1	1724	"tags=18%, list=12%, signal=20%"
GSE21927_C26GM_VS_4T1_TUMOR_MONOCYTE_BALBC_DN
GSE21927_C26GM_VS_4T1_TUMOR_MONOCYTE_BALBC_DN
130	-0.2530298	-1.0283891	0.4173077
0.46756274	1	1471	"tags=17%, list=10%, signal=19%"
GSE16385_IFNG_TNF_VS_IL4_STIM_MACROPHAGE_ROSIGLITAZONE_TREATED_UP
GSE16385_IFNG_TNF_VS_IL4_STIM_MACROPHAGE_ROSIGLITAZONE_TREATED_UP
118	-0.25612217	-1.0282938	0.3776091
0.46756393	1	1299	"tags=14%, list=9%, signal=16%"
GSE17974_IL4_AND_ANTI_IL12_VS_UNTREATED_6H_ACT_CD4_TCELL_UP
GSE17974_IL4_AND_ANTI_IL12_VS_UNTREATED_6H_ACT_CD4_TCELL_UP
115	-0.2584172	-1.0281653	0.38519925
0.46763387	1	3180	"tags=25%, list=23%, signal=32%"
GSE3982_CENT_MEMORY_CD4_TCELL_VS_TH1_UP
GSE3982_CENT_MEMORY_CD4_TCELL_VS_TH1_UP
129	-0.24907605	-1.0279324	0.4163424
0.46799403	1	1179	"tags=15%, list=8%, signal=16%"
GSE24574_BCL6_LOW_TFH_VS_NAIVE_CD4_TCELL_DN
GSE24574_BCL6_LOW_TFH_VS_NAIVE_CD4_TCELL_DN
139	-0.25121486	-1.0278374	0.3901515
0.46797925	1	1875	"tags=19%, list=13%, signal=22%"
GSE21379_TFH_VS_NON_TFH_CD4_TCELL_UP
GSE21379_TFH_VS_NON_TFH_CD4_TCELL_UP
140	-0.25103056	-1.0268734	0.3877551
0.4702673	1	2167	"tags=19%, list=15%, signal=23%"
GSE27786_BCELL_VS_MONO_MAC_UP	GSE27786_BCELL_VS_MONO_MAC_UP
124	-0.25389868	-1.0267543	0.36673346
0.47033018	1	2426	"tags=24%, list=17%, signal=29%"
GSE2770_TGFB_AND_IL4_VS_TGFB_AND_IL12_TREATED_ACT_CD4_TCELL_48H_UP
GSE2770_TGFB_AND_IL4_VS_TGFB_AND_IL12_TREATED_ACT_CD4_TCELL_48H_UP
119	-0.25693578	-1.0253863	0.40373832
0.47371158	1	1211	"tags=17%, list=9%, signal=18%"
GSE30083_SP1_VS_SP2_THYMOCYTE_UP	GSE30083_SP1_VS_SP2_THYMOCYTE_UP
155	-0.24544336	-1.0249215	0.4043716

0.47469234	1	2261	"tags=23%, list=16%, signal=27%"
GSE12845_IGD_POS_BLOOD_VS_DARKZONE_GC_TONSIL_BCELL_UP
GSE12845_IGD_POS_BLOOD_VS_DARKZONE_GC_TONSIL_BCELL_UP
123	-0.25675333	-1.0245559	0.38333333
0.47540238	1	1279	"tags=15%, list=9%, signal=17%"
GSE29618_LAIV_VS_TIV_FLU_VACCINE_DAY7_BCELL_UP
GSE29618_LAIV_VS_TIV_FLU_VACCINE_DAY7_BCELL_UP
130	-0.25011298	-1.0243623	0.4060721
0.4756433	1	3064	"tags=24%, list=22%, signal=30%"
GSE9988_LOW_LPS_VS_ANTI_TREM1_AND_LPS_MONOCYTE_UP
GSE9988_LOW_LPS_VS_ANTI_TREM1_AND_LPS_MONOCYTE_UP
144	-0.24861237	-1.024134	0.41902834
0.4759633	1	2836	"tags=29%, list=20%, signal=36%"
GSE25088_CTRL_VS_IL4_STIM_MACROPHAGE_DN
GSE25088_CTRL_VS_IL4_STIM_MACROPHAGE_DN
136	-0.251504	-1.0240623	0.41142857
0.4758976	1	1703	"tags=15%, list=12%, signal=17%"
GSE36527_CD62L_HIGH_VS_CD62L_LOW_TREG_CD69_NEG_KLRG1_NEG_DN
GSE36527_CD62L_HIGH_VS_CD62L_LOW_TREG_CD69_NEG_KLRG1_NEG_DN
156	-0.24489431	-1.0232649	0.40618956
0.47770604	1	2980	"tags=27%, list=21%, signal=34%"
GSE19772_CTRL_VS_HCMV_INF_MONOCYTES_AND_PI3K_INHIBITION_DN
GSE19772_CTRL_VS_HCMV_INF_MONOCYTES_AND_PI3K_INHIBITION_DN
157	-0.24273176	-1.0228053	0.37284896
0.47867343	1	2371	"tags=20%, list=17%, signal=23%"
GSE21033_1H_VS_24H_POLYIC_STIM_DC_UP
GSE21033_1H_VS_24H_POLYIC_STIM_DC_UP
126	-0.25158215	-1.0227942	0.38666666
0.47844455	1	2307	"tags=26%, list=16%, signal=31%"
GSE7596_AKT_TRANSD_VS_CTRL_CD4_TCONV_WITH_TGFB_DN
GSE7596_AKT_TRANSD_VS_CTRL_CD4_TCONV_WITH_TGFB_DN
89	-0.26752034	-1.022331	0.3815261	0.479418
1	2452	"tags=19%, list=17%, signal=23%"
GSE1112_OT1_CD8AB_VS_HY_CD8AA_THYMOCYTE_RTOC_CULTURE_UP
GSE1112_OT1_CD8AB_VS_HY_CD8AA_THYMOCYTE_RTOC_CULTURE_UP
95	-0.26360223	-1.0222957	0.39130434	0.479249
1	816	"tags=12%, list=6%, signal=12%"
GSE15330_LYMPHOID_MULTIPOTENT_VS_MEGAKARYOCYTE_ERYTHROID_PROGENITOR_
DN
GSE15330_LYMPHOID_MULTIPOTENT_VS_MEGAKARYOCYTE_ERYTHROID_PROGENITOR_
DN	157	-0.24622712	-1.0222776
0.38963532	0.4790312	1	2454	"tags=21%,
list=17%, signal=25%"
GSE36891_UNSTIM_VS_POLYIC_TLR3_STIM_PERITONEAL_MACROPHAGE_DN
GSE36891_UNSTIM_VS_POLYIC_TLR3_STIM_PERITONEAL_MACROPHAGE_DN
	148	-0.24537201	-1.0220852	0.39387757
0.4792727	1	1797	"tags=20%, list=13%, signal=22%"
GSE17721_LPS_VS_POLYIC_0.5H_BMDC_DN
GSE17721_LPS_VS_POLYIC_0.5H_BMDC_DN
163	-0.24270461	-1.0216278	0.38973385
0.4801727	1	3606	"tags=34%, list=26%, signal=45%"
GSE39864_WT_VS_GATA3_KO_TREG_UP	GSE39864_WT_VS_GATA3_KO_TREG_UP
	157	-0.24547218	-1.0215715	0.39661655
0.48006114	1	1717	"tags=15%, list=12%, signal=17%"

GSE14308_NAIVE_CD4_TCELL_VS_NATURAL_TREG_UP
GSE14308_NAIVE_CD4_TCELL_VS_NATURAL_TREG_UP
127 -0.2536942 -1.02153 0.43141153 0.4798905
1 2596 "tags=20%, list=18%, signal=25%"
GSE37301_COMMON_LYMPHOID_PROGENITOR_VS_CD4_TCELL_UP
GSE37301_COMMON_LYMPHOID_PROGENITOR_VS_CD4_TCELL_UP
142 -0.2480673 -1.0207024 0.38963532
0.48183402 1 2179 "tags=23%, list=15%, signal=26%"
GSE5589_WT_VS_IL10_KO_LPS_AND_IL10_STIM_MACROPHAGE_45MIN_DN
GSE5589_WT_VS_IL10_KO_LPS_AND_IL10_STIM_MACROPHAGE_45MIN_DN
141 -0.24695192 -1.0200281 0.41304347
0.4833684 1 2479 "tags=23%, list=18%, signal=28%"
GSE37533_PPARG1_FOXP3_VS_PPARG2_FOXP3_TRANSDUCED_CD4_TCELL_UP
GSE37533_PPARG1_FOXP3_VS_PPARG2_FOXP3_TRANSDUCED_CD4_TCELL_UP
131 -0.25076857 -1.0198424 0.39655173
0.48362187 1 998 "tags=15%, list=7%, signal=15%"
GSE39820_TGFBETA1_VS_TGFBETA3_IN_IL6_TREATED_CD4_TCELL_UP
GSE39820_TGFBETA1_VS_TGFBETA3_IN_IL6_TREATED_CD4_TCELL_UP
158 -0.24677101 -1.0193741 0.4266145
0.4846312 1 2382 "tags=23%, list=17%, signal=27%"
GSE7831_1H_VS_4H_CPG_STIM_PDC_UP GSE7831_1H_VS_4H_CPG_STIM_PDC_UP
152 -0.24517359 -1.0190983 0.4050633
0.48510218 1 2283 "tags=20%, list=16%, signal=23%"
GSE5589_LPS_VS_LPS_AND_IL10_STIM_MACROPHAGE_180MIN_DN
GSE5589_LPS_VS_LPS_AND_IL10_STIM_MACROPHAGE_180MIN_DN
144 -0.24804413 -1.0181195 0.3938224
0.48740906 1 756 "tags=12%, list=5%, signal=12%"
GSE27859_MACROPHAGE_VS_DC_UP GSE27859_MACROPHAGE_VS_DC_UP
137 -0.24721941 -1.017594 0.40038314
0.48855126 1 1316 "tags=18%, list=9%, signal=19%"
GSE27786_CD4_TCELL_VS_MONO_MAC_UP GSE27786_CD4_TCELL_VS_MONO_MAC_UP
130 -0.24799544 -1.0170069 0.404
0.48985305 1 2377 "tags=22%, list=17%, signal=27%"
GSE10325_LUPUS_CD4_TCELL_VS_LUPUS_MYELOID_DN
GSE10325_LUPUS_CD4_TCELL_VS_LUPUS_MYELOID_DN
152 -0.24297826 -1.0159748 0.41291586
0.49237016 1 1537 "tags=17%, list=11%, signal=19%"
GSE22886_NEUTROPHIL_VS_DC_UP GSE22886_NEUTROPHIL_VS_DC_UP
136 -0.2494004 -1.0157967 0.41523808
0.49257988 1 1139 "tags=12%, list=8%, signal=13%"
GSE45365_NK_CELL_VS_CD8_TCELL_UP GSE45365_NK_CELL_VS_CD8_TCELL_UP
116 -0.25573096 -1.015577 0.42256215
0.49294007 1 2280 "tags=23%, list=16%, signal=28%"
GSE14769_UNSTIM_VS_240MIN_LPS_BMDM_DN
GSE14769_UNSTIM_VS_240MIN_LPS_BMDM_DN
154 -0.24611935 -1.015381 0.41894352
0.49318787 1 1591 "tags=18%, list=11%, signal=20%"
GSE25123_IL4_VS_IL4_AND_ROSIGLITAZONE_STIM_PPARG_KO_MACROPHAGE_DAY10_DN
GSE25123_IL4_VS_IL4_AND_ROSIGLITAZONE_STIM_PPARG_KO_MACROPHAGE_DAY10_DN 137 -0.24830006 -1.015301
0.41828793 0.49311697 1 2409 "tags=23%, list=17%, signal=28%"
GSE5099_MONOCYTE_VS_ALTERNATIVE_M2_MACROPHAGE_DN

GSE5099_MONOCYTE_VS_ALTERNATIVE_M2_MACROPHAGE_DN
117 -0.25213075 -1.0148672 0.42264152
0.49396926 1 1332 "tags=14%, list=9%, signal=15%"
GSE7460_TREG_VS_TCONV_ACT_DN GSE7460_TREG_VS_TCONV_ACT_DN
139 -0.24672511 -1.0147482 0.40968344
0.49398398 1 1560 "tags=15%, list=11%, signal=17%"
GSE3982_BCELL_VS_BASOPHIL_DN GSE3982_BCELL_VS_BASOPHIL_DN
154 -0.24453612 -1.0144516 0.40576923
0.4945157 1 2501 "tags=23%, list=18%, signal=28%"
GSE3400_UNTREATED_VS_IFNB_TREATED_MEF_UP
GSE3400_UNTREATED_VS_IFNB_TREATED_MEF_UP
124 -0.25222045 -1.0143533 0.4124294
0.49451306 1 2354 "tags=27%, list=17%, signal=33%"
GSE17974_IL4_AND_ANTI_IL12_VS_UNTREATED_0.5H_ACT_CD4_TCELL_DN
GSE17974_IL4_AND_ANTI_IL12_VS_UNTREATED_0.5H_ACT_CD4_TCELL_DN
100 -0.26145518 -1.013781 0.42424244
0.495794 1 4768 "tags=41%, list=34%, signal=61%"
GSE43863_TH1_VS_LY6C_INT_CXCR5POS_MEMORY_CD4_TCELL_DN
GSE43863_TH1_VS_LY6C_INT_CXCR5POS_MEMORY_CD4_TCELL_DN
146 -0.24417503 -1.0132405 0.39245284
0.4969978 1 4587 "tags=36%, list=33%, signal=52%"
GSE14769_40MIN_VS_360MIN_LPS_BMDM_DN
GSE14769_40MIN_VS_360MIN_LPS_BMDM_DN
142 -0.24512 -1.0131773 0.42322835 0.4968952
1 2249 "tags=21%, list=16%, signal=25%"
GSE2706_UNSTIM_VS_8H_R848_DC_UP GSE2706_UNSTIM_VS_8H_R848_DC_UP
128 -0.25042367 -1.013109 0.41505793
0.49679086 1 3241 "tags=27%, list=23%, signal=35%"
GSE19374_UNINF_VS_LISTERIA_INFECTED_MACROPHAGE_UP
GSE19374_UNINF_VS_LISTERIA_INFECTED_MACROPHAGE_UP
135 -0.25000048 -1.0129656 0.41747573
0.49689612 1 2614 "tags=21%, list=19%, signal=25%"
GSE14413_UNSTIM_VS_IFNB_STIM_RAW264_CELLS_DN
GSE14413_UNSTIM_VS_IFNB_STIM_RAW264_CELLS_DN
81 -0.26885635 -1.0128627 0.4512195
0.49689204 1 7495 "tags=63%, list=53%, signal=133%"
GSE2770_UNTREATED_VS_TGFB_AND_IL12_TREATED_ACT_CD4_TCELL_2H_UP
GSE2770_UNTREATED_VS_TGFB_AND_IL12_TREATED_ACT_CD4_TCELL_2H_UP
144 -0.24676321 -1.011692 0.4228457
0.49979177 1 3698 "tags=28%, list=26%, signal=37%"
GSE43863_TH1_VS_TFH_EFFECTOR_CD4_TCELL_UP
GSE43863_TH1_VS_TFH_EFFECTOR_CD4_TCELL_UP
145 -0.2427094 -1.0110345 0.4183486
0.5012571 1 3850 "tags=32%, list=27%, signal=44%"
GSE26928_CENTR_MEMORY_VS_CXCR5_POS_CD4_TCELL_DN
GSE26928_CENTR_MEMORY_VS_CXCR5_POS_CD4_TCELL_DN
113 -0.25323433 -1.0105312 0.4187867
0.5023506 1 3064 "tags=27%, list=22%, signal=34%"
GSE29618_PRE_VS_DAY7_POST_LAIV_FLU_VACCINE_BCELL_UP
GSE29618_PRE_VS_DAY7_POST_LAIV_FLU_VACCINE_BCELL_UP
150 -0.24194618 -1.0104624 0.41992188
0.5022495 1 3154 "tags=27%, list=22%, signal=35%"
GSE30083_SP1_VS_SP3_THYMOCYTE_UP GSE30083_SP1_VS_SP3_THYMOCYTE_UP
152 -0.24243928 -1.0094026 0.40530303

0.504917 1 1864 "tags=17%, list=13%, signal=19%"
GSE21063_3H_VS_16H_ANTI_IGM_STIM_BCELL_DN
GSE21063_3H_VS_16H_ANTI_IGM_STIM_BCELL_DN
126 -0.2501942 -1.0092192 0.43106797
0.50512654 1 2043 "tags=21%, list=14%, signal=24%"
GSE3982_MAST_CELL_VS_CENT_MEMORY_CD4_TCELL_DN
GSE3982_MAST_CELL_VS_CENT_MEMORY_CD4_TCELL_DN
135 -0.24790427 -1.0071231 0.43486974
0.5105927 1 2740 "tags=24%, list=19%, signal=30%"
GSE15330_WT_VS_IKAROS_KO_GRANULOCYTE_MONOCYTE_PROGENITOR_UP
GSE15330_WT_VS_IKAROS_KO_GRANULOCYTE_MONOCYTE_PROGENITOR_UP
152 -0.24204347 -1.0058002 0.4502924
0.5139162 1 965 "tags=12%, list=7%, signal=13%"
GSE25087_TREG_VS_TCONV_ADULT_DN GSE25087_TREG_VS_TCONV_ADULT_DN
128 -0.25085792 -1.0037943 0.46139705
0.51919854 1 2507 "tags=23%, list=18%, signal=27%"
GSE17721_CPG_VS_GARDIQUIMOD_16H_BMDC_UP
GSE17721_CPG_VS_GARDIQUIMOD_16H_BMDC_UP
150 -0.24280442 -1.0031472 0.47504622
0.5207165 1 2606 "tags=23%, list=18%, signal=28%"
GSE18804_SPLEEN_MACROPHAGE_VS_COLON_TUMORAL_MACROPHAGE_DN
GSE18804_SPLEEN_MACROPHAGE_VS_COLON_TUMORAL_MACROPHAGE_DN
148 -0.24367598 -1.0029712 0.48015872
0.5209378 1 1817 "tags=18%, list=13%, signal=21%"
GSE41867_NAIVE_VS_DAY15_LCMV_CONE13_EFFECTOR_CD8_TCELL_DN
GSE41867_NAIVE_VS_DAY15_LCMV_CONE13_EFFECTOR_CD8_TCELL_DN
124 -0.24675809 -1.0028622 0.46022728
0.5209618 1 1800 "tags=18%, list=13%, signal=20%"
GSE29618_PRE_VS_DAY7_POST_LAIV_FLU_VACCINE_MDC_UP
GSE29618_PRE_VS_DAY7_POST_LAIV_FLU_VACCINE_MDC_UP
134 -0.2459861 -1.0027094 0.46626985
0.52108085 1 1841 "tags=15%, list=13%, signal=17%"
GSE46606_IRF4HIGH_VS_IRF4MID_CD40L_IL2_IL5_DAY1_STIMULATED_BCELL_UP
GSE46606_IRF4HIGH_VS_IRF4MID_CD40L_IL2_IL5_DAY1_STIMULATED_BCELL_UP
153 -0.24214627 -1.0026991 0.4402985
0.5208217 1 2518 "tags=26%, list=18%, signal=31%"
GSE28737_WT_VS_BCL6_HET_FOLLICULAR_BCELL_UP
GSE28737_WT_VS_BCL6_HET_FOLLICULAR_BCELL_UP
143 -0.24236369 -1.0025951 0.43454546
0.52082586 1 2648 "tags=24%, list=19%, signal=30%"
GSE20198_UNTREATED_VS_IL12_TREATED_ACT_CD4_TCELL_DN
GSE20198_UNTREATED_VS_IL12_TREATED_ACT_CD4_TCELL_DN
145 -0.24217725 -1.0014327 0.44356436
0.52378124 1 1596 "tags=18%, list=11%, signal=20%"
GSE17721_CTRL_VS_POLYIC_4H_BMDC_DN
GSE17721_CTRL_VS_POLYIC_4H_BMDC_DN
145 -0.24501827 -1.0003647 0.4493927
0.5264008 1 1855 "tags=17%, list=13%, signal=19%"
GSE28783_ANTI_MIR33_VS_CTRL_ATHEROSCLEROSIS_MACROPHAGE_DN
GSE28783_ANTI_MIR33_VS_CTRL_ATHEROSCLEROSIS_MACROPHAGE_DN
131 -0.24638808 -0.99959224 0.44962686
0.52825475 1 1651 "tags=16%, list=12%, signal=18%"
GSE17721_POLYIC_VS_PAM3CSK4_1H_BMDC_DN
GSE17721_POLYIC_VS_PAM3CSK4_1H_BMDC_DN

168	-0.23758514	-0.9995351	0.43103448
0.52813596	1	2498	"tags=23%, list=18%, signal=28%"
GSE21063_WT_VS_NFATC1_KO_16H_ANTI_IGM_STIM_BCELL_UP
GSE21063_WT_VS_NFATC1_KO_16H_ANTI_IGM_STIM_BCELL_UP
141	-0.24193181	-0.9992378	0.45057034
0.52870244	1	2493	"tags=26%, list=18%, signal=32%"
GSE13485_CTRL_VS_DAY1_YF17D_VACCINE_PBMC_DN
GSE13485_CTRL_VS_DAY1_YF17D_VACCINE_PBMC_DN
91	-0.2600392	-0.9992097	0.4521576
0.52849364	1	5316	"tags=44%, list=38%, signal=70%"
GSE21546_SAP1A_KO_VS_SAP1A_KO_AND_ELK1_KO_DP_THYMOCYTES_UP
GSE21546_SAP1A_KO_VS_SAP1A_KO_AND_ELK1_KO_DP_THYMOCYTES_UP
153	-0.2411399	-0.99912745	0.47878787
0.52843976	1	1392	"tags=16%, list=10%, signal=18%"
GSE5589_WT_VS_IL6_KO_LPS_AND_IL10_STIM_MACROPHAGE_180MIN_DN
GSE5589_WT_VS_IL6_KO_LPS_AND_IL10_STIM_MACROPHAGE_180MIN_DN
109	-0.25602043	-0.99756235	0.47591522
0.53242844	1	2076	"tags=22%, list=15%, signal=26%"
GSE37533_UNTREATED_VS_PIOGLIZATONE_TREATED_CD4_TCELL_FOXP3_TRASDUCED_CD4_TCELL_UP
GSE37533_UNTREATED_VS_PIOGLIZATONE_TREATED_CD4_TCELL_FOXP3_TRASDUCED_CD4_TCELL_UP		119	-0.24757294	-0.9972763
0.45708954	0.5329337	1	1482	"tags=13%, list=11%, signal=14%"
GSE39820_TGFBETA1_VS_TGFBETA3_IN_IL6_IL23A_TREATED_CD4_TCELL_DN
GSE39820_TGFBETA1_VS_TGFBETA3_IN_IL6_IL23A_TREATED_CD4_TCELL_DN
	139	-0.24519502	-0.9966675	0.47448015
0.53435314	1	1841	"tags=17%, list=13%, signal=20%"
GSE42088_UNINF_VS_LEISHMANIA_INF_DC_4H_UP
GSE42088_UNINF_VS_LEISHMANIA_INF_DC_4H_UP
159	-0.23913495	-0.9965456	0.45276293	0.534391
1	2025	"tags=22%, list=14%, signal=25%"
GSE15624_CTRL_VS_6H_HALOFUGINONE_TREATED_CD4_TCELL_UP
GSE15624_CTRL_VS_6H_HALOFUGINONE_TREATED_CD4_TCELL_UP
100	-0.2561481	-0.99646986	0.45746693
0.53431046	1	3712	"tags=38%, list=26%, signal=51%"
GSE46606_UNSTIM_VS_CD40L_IL2_IL5_3DAY_STIMULATED_IRF4_KO_BCELL_DN
GSE46606_UNSTIM_VS_CD40L_IL2_IL5_3DAY_STIMULATED_IRF4_KO_BCELL_DN
	106	-0.25428027	-0.9961256	0.44660193
0.5350057	1	2022	"tags=22%, list=14%, signal=25%"
GSE10325_BCELL_VS_MYELOID_DN	GSE10325_BCELL_VS_MYELOID_DN
	147	-0.2396843	-0.995204	0.46792454
0.5372767	1	2570	"tags=26%, list=18%, signal=31%"
GSE13411_PLASMA_CELL_VS_MEMORY_BCELL_DN
GSE13411_PLASMA_CELL_VS_MEMORY_BCELL_DN
134	-0.24079083	-0.9951198	0.44742268
0.5372309	1	1578	"tags=16%, list=11%, signal=17%"
GSE34515_CD16_NEG_VS_POS_MONOCYTE_DN
GSE34515_CD16_NEG_VS_POS_MONOCYTE_DN
128	-0.24420807	-0.99493086	0.46837944	0.53745
1	1174	"tags=15%, list=8%, signal=16%"
GSE23321_CENTRAL_MEMORY_VS_NAIVE_CD8_TCELL_DN
GSE23321_CENTRAL_MEMORY_VS_NAIVE_CD8_TCELL_DN
155	-0.23827906	-0.99452037	0.4910072

0.53831005	1	4642	"tags=34%, list=33%, signal=50%"
GSE37563_WT_VS_CTLA4_KO_CD4_TCELL_D4_POST_IMMUNIZATION_UP
GSE37563_WT_VS_CTLA4_KO_CD4_TCELL_D4_POST_IMMUNIZATION_UP
125	-0.25056764	-0.99415106	0.48449612
0.5390804	1	2899	"tags=28%, list=21%, signal=35%"
GSE22886_NAIVE_CD8_TCELL_VS_NEUTROPHIL_DN
GSE22886_NAIVE_CD8_TCELL_VS_NEUTROPHIL_DN
140	-0.24009484	-0.9937694	0.47227535
0.5397885	1	3036	"tags=26%, list=22%, signal=33%"
GSE6259_33D1_POS_VS_DEC205_POS_FLT3L_INDUCED_SPLENIC_DC_DN
GSE6259_33D1_POS_VS_DEC205_POS_FLT3L_INDUCED_SPLENIC_DC_DN
143	-0.23882394	-0.9937317	0.46927375
0.5396059	1	2751	"tags=25%, list=19%, signal=31%"
GSE41978_WT_VS_ID2_KO_KLRG1_LOW_EFFECTOR_CD8_TCELL_UP
GSE41978_WT_VS_ID2_KO_KLRG1_LOW_EFFECTOR_CD8_TCELL_UP
155	-0.23941967	-0.99325883	0.46564886
0.54064703	1	4239	"tags=32%, list=30%, signal=46%"
GSE34156_UNTREATED_VS_6H_TLR1_TLR2_LIGAND_TREATED_MONOCYTE_UP
GSE34156_UNTREATED_VS_6H_TLR1_TLR2_LIGAND_TREATED_MONOCYTE_UP
141	-0.2402184	-0.9917872	0.4763705
0.5444303	1	2254	"tags=21%, list=16%, signal=25%"
GSE17721_LPS_VS_PAM3CSK4_2H_BMDC_UP
GSE17721_LPS_VS_PAM3CSK4_2H_BMDC_UP
164	-0.23668723	-0.9915324	0.4686347
0.54484785	1	2424	"tags=25%, list=17%, signal=30%"
GSE13485_CTRL_VS_DAY3_YF17D_VACCINE_PBMC_UP
GSE13485_CTRL_VS_DAY3_YF17D_VACCINE_PBMC_UP
108	-0.25393352	-0.9913206	0.46931407
0.5451639	1	1631	"tags=18%, list=12%, signal=20%"
GSE26669_CD4_VS_CD8_TCELL_IN_MLR_DN
GSE26669_CD4_VS_CD8_TCELL_IN_MLR_DN
141	-0.24342237	-0.9908216	0.48091602
0.5463389	1	1946	"tags=18%, list=14%, signal=20%"
GSE5589_LPS_AND_IL10_VS_LPS_AND_IL6_STIM_IL10_KO_MACROPHAGE_45MIN_UP

GSE5589_LPS_AND_IL10_VS_LPS_AND_IL6_STIM_IL10_KO_MACROPHAGE_45MIN_UP
149	-0.23890667	-0.99050045
0.49712092	0.54691607	1	2248	"tags=21%, list=16%, signal=24%"
GSE1925_CTRL_VS_3H_IFNG_STIM_IFNG_PRIMED_MACROPHAGE_DN
GSE1925_CTRL_VS_3H_IFNG_STIM_IFNG_PRIMED_MACROPHAGE_DN
163	-0.2367291	-0.99047947	0.47126436
0.54668117	1	2246	"tags=21%, list=16%, signal=25%"
GSE2585_CTEC_VS_THYMIC_DC_UP	GSE2585_CTEC_VS_THYMIC_DC_UP
140	-0.23948796	-0.99017245	0.47777778
0.5472375	1	2257	"tags=19%, list=16%, signal=22%"
GSE17974_0.5H_VS_72H_UNTREATED_IN_VITRO_CD4_TCELL_UP
GSE17974_0.5H_VS_72H_UNTREATED_IN_VITRO_CD4_TCELL_UP
115	-0.25225174	-0.9898684	0.48518518
0.54781514	1	1638	"tags=17%, list=12%, signal=20%"
GSE32986_UNSTIM_VS_GMCSF_AND_CURDLAN_LOWDOSE_STIM_DC_DN
GSE32986_UNSTIM_VS_GMCSF_AND_CURDLAN_LOWDOSE_STIM_DC_DN
131	-0.24506128	-0.98976505	0.47058824
0.5478116	1	1597	"tags=17%, list=11%, signal=19%"

GSE18281_SUBCAPSULAR_CORTICAL_REGION_VS_WHOLE_CORTEX_THYMUS_DN
GSE18281_SUBCAPSULAR_CORTICAL_REGION_VS_WHOLE_CORTEX_THYMUS_DN
138 -0.24108349 -0.98960435 0.46395564
0.54797953 1 2422 "tags=20%, list=17%, signal=24%"
GSE6875_TCONV_VS_FOXP3_KO_TREG_UP GSE6875_TCONV_VS_FOXP3_KO_TREG_UP
136 -0.24187772 -0.9892173 0.4797048
0.5488005 1 2278 "tags=19%, list=16%, signal=23%"
GSE6674_UNSTIM_VS_ANTI_IGM_AND_CPG_STIM_BCELL_DN
GSE6674_UNSTIM_VS_ANTI_IGM_AND_CPG_STIM_BCELL_DN
142 -0.23891267 -0.98851156 0.47338402
0.55049086 1 1726 "tags=22%, list=12%, signal=25%"
GSE6259_FLT3L_INDUCED_33D1_POS_DC_VS_CD4_TCELL_UP
GSE6259_FLT3L_INDUCED_33D1_POS_DC_VS_CD4_TCELL_UP
146 -0.24024853 -0.9880631 0.46034816
0.5514256 1 2539 "tags=25%, list=18%, signal=30%"
GSE8685_IL2_ACT_IL2_STARVED_VS_IL15_ACT_IL2_STARVED_CD4_TCELL_UP
GSE8685_IL2_ACT_IL2_STARVED_VS_IL15_ACT_IL2_STARVED_CD4_TCELL_UP
142 -0.24088992 -0.98801786 0.4892368
0.55125487 1 1072 "tags=13%, list=8%, signal=14%"
GSE27786_BCELL_VS_CD8_TCELL_DN GSE27786_BCELL_VS_CD8_TCELL_DN
139 -0.24197663 -0.9880122 0.45773196
0.550976 1 2666 "tags=24%, list=19%, signal=29%"
GSE21063_CTRL_VS_ANTI_IGM_STIM_BCELL_16H_DN
GSE21063_CTRL_VS_ANTI_IGM_STIM_BCELL_16H_DN
138 -0.23992765 -0.98705304 0.48228347
0.55334866 1 2404 "tags=20%, list=17%, signal=23%"
GSE24574_BCL6_HIGH_TFH_VS_TCONV_CD4_TCELL_DN
GSE24574_BCL6_HIGH_TFH_VS_TCONV_CD4_TCELL_DN
138 -0.24336416 -0.9868586 0.47916666
0.55359524 1 3298 "tags=26%, list=23%, signal=34%"
GSE15324_ELF4_KO_VS_WT_NAIVE_CD8_TCELL_DN
GSE15324_ELF4_KO_VS_WT_NAIVE_CD8_TCELL_DN
148 -0.23773846 -0.98640984 0.50769234
0.5545555 1 3490 "tags=30%, list=25%, signal=40%"
GSE22611_NOD2_VS_MUTANT_NOD2_TRANSDUCED_HEK293T_CELL_DN
GSE22611_NOD2_VS_MUTANT_NOD2_TRANSDUCED_HEK293T_CELL_DN
134 -0.23717979 -0.985595 0.46868688
0.5565442 1 2437 "tags=21%, list=17%, signal=25%"
GSE6674_UNSTIM_VS_ANTI_IGM_STIM_BCELL_DN
GSE6674_UNSTIM_VS_ANTI_IGM_STIM_BCELL_DN
95 -0.25820833 -0.9855465 0.44204322
0.5563794 1 5188 "tags=37%, list=37%, signal=58%"
GSE13522_WT_VS_IFNG_KO_SKING_T_CRUZI_Y_STRAIN_INF_UP
GSE13522_WT_VS_IFNG_KO_SKING_T_CRUZI_Y_STRAIN_INF_UP
90 -0.2608125 -0.98532844 0.4895238
0.5566931 1 5718 "tags=43%, list=41%, signal=72%"
GSE360_CTRL_VS_B_MALAYI_LOW_DOSE_DC_DN
GSE360_CTRL_VS_B_MALAYI_LOW_DOSE_DC_DN
148 -0.2403312 -0.9846408 0.4770115
0.55838376 1 2553 "tags=23%, list=18%, signal=28%"
GSE4811_CLASSSICALY_ACTIVATED_VS_TYPE_2_ACTIVATED_MACROPHAGE_DN
GSE4811_CLASSSICALY_ACTIVATED_VS_TYPE_2_ACTIVATED_MACROPHAGE_DN
143 -0.23925462 -0.9837222 0.49510762
0.56065255 1 5273 "tags=38%, list=37%, signal=60%"

GSE31622_WT_VS_KLF3_KO_BCELL_UP	GSE31622_WT_VS_KLF3_KO_BCELL_UP
	149	-0.23777737	-0.98342	0.47211897
0.5612257	1	3344	"tags=27%, list=24%, signal=35%"
GSE360_L_MAJOR_VS_T_GONDII_MAC_UP	GSE360_L_MAJOR_VS_T_GONDII_MAC_UP
	153	-0.23990281	-0.98274	0.52327746	0.562839
1	3101	"tags=24%, list=22%, signal=31%"
GSE43955_TH0_VS_TGFB_IL6_TH17_ACT_CD4_TCELL_42H_DN
GSE43955_TH0_VS_TGFB_IL6_TH17_ACT_CD4_TCELL_42H_DN
165	-0.23464088	-0.9821416	0.5095785
0.5642064	1	2279	"tags=18%, list=16%, signal=21%"
GSE18893_CTRL_VS_TNF_TREATED_TCONV_24H_DN
GSE18893_CTRL_VS_TNF_TREATED_TCONV_24H_DN
143	-0.23827653	-0.98172575	0.48761904	0.565085
1	1852	"tags=19%, list=13%, signal=22%"
GSE32901_TH1_VS_TH17_NEG_CD4_TCELL_UP
GSE32901_TH1_VS_TH17_NEG_CD4_TCELL_UP
95	-0.2531898	-0.9817203	0.49438202
0.5648034	1	2639	"tags=19%, list=19%, signal=23%"
GSE4142_NAIVE_BCELL_VS_PLASMA_CELL_DN
GSE4142_NAIVE_BCELL_VS_PLASMA_CELL_DN
146	-0.23899685	-0.9814038	0.5	0.5653765
1	2213	"tags=20%, list=16%, signal=23%"
GSE11924_TH1_VS_TH2_CD4_TCELL_DN	GSE11924_TH1_VS_TH2_CD4_TCELL_DN
	146	-0.23609993	-0.98033893	0.48906562
0.5680914	1	2508	"tags=24%, list=18%, signal=29%"
GSE19512_NAUTRAL_VS_INDUCED_TREG_DN
GSE19512_NAUTRAL_VS_INDUCED_TREG_DN
145	-0.23844709	-0.9788065	0.49499	0.5721818
1	2078	"tags=17%, list=15%, signal=19%"
GSE21546_WT_VS_ELK1_KO_ANTI_CD3_STIM_DP_THYMOCYTES_UP
GSE21546_WT_VS_ELK1_KO_ANTI_CD3_STIM_DP_THYMOCYTES_UP
143	-0.23562385	-0.9786957	0.5122873
0.57220346	1	3233	"tags=27%, list=23%, signal=35%"
GSE17721_PAM3CSK4_VS_CPG_0.5H_BMDC_UP
GSE17721_PAM3CSK4_VS_CPG_0.5H_BMDC_UP
151	-0.2372458	-0.97787505	0.49256507
0.5742242	1	3926	"tags=34%, list=28%, signal=46%"
GSE14769_UNSTIM_VS_80MIN_LPS_BMDM_UP
GSE14769_UNSTIM_VS_80MIN_LPS_BMDM_UP
127	-0.24159575	-0.9774133	0.4970874
0.57526124	1	2357	"tags=23%, list=17%, signal=27%"
GSE3982_BCELL_VS_TH1_UP	GSE3982_BCELL_VS_TH1_UP
126	-0.24055098	-0.97736895	0.48979592
0.57508826	1	3367	"tags=34%, list=24%, signal=44%"
GSE17721_CPG_VS_GARDIQUIMOD_2H_BMDC_DN
GSE17721_CPG_VS_GARDIQUIMOD_2H_BMDC_DN
157	-0.22933967	-0.97662616	0.5118577
0.57691246	1	1587	"tags=16%, list=11%, signal=18%"
GSE3982_MAST_CELL_VS_BCELL_UP	GSE3982_MAST_CELL_VS_BCELL_UP
	145	-0.23431508	-0.9751779	0.5041494
0.5807008	1	1770	"tags=16%, list=13%, signal=18%"
GSE26343_WT_VS_NFAT5_KO_MACROPHAGE_LPS_STIM_UP
GSE26343_WT_VS_NFAT5_KO_MACROPHAGE_LPS_STIM_UP
147	-0.23487978	-0.9742986	0.5304183

0.5828916	1	3756	"tags=35%, list=27%, signal=47%"
GSE18148_CBFB_KO_VS_WT_TREG_UP	GSE18148_CBFB_KO_VS_WT_TREG_UP
	135	-0.24186772	-0.97374284	0.5156863
0.58415854	1	4529	"tags=36%, list=32%, signal=52%"
GSE42724_B1_BCELL_VS_PLASMABLAST_DN
GSE42724_B1_BCELL_VS_PLASMABLAST_DN
134	-0.23794375	-0.9730572	0.49380532
0.58577275	1	2777	"tags=24%, list=20%, signal=29%"
GSE35825_UNTREATED_VS_IFNA_STIM_MACROPHAGE_UP
GSE35825_UNTREATED_VS_IFNA_STIM_MACROPHAGE_UP
135	-0.23944159	-0.9724443	0.5084112
0.58718467	1	1920	"tags=21%, list=14%, signal=25%"
GSE37301_LYMPHOID_PRIMED_MPP_VS_RAG2_KO_NK_CELL_DN
GSE37301_LYMPHOID_PRIMED_MPP_VS_RAG2_KO_NK_CELL_DN
126	-0.24226761	-0.9723406	0.5064935
0.5871702	1	2708	"tags=22%, list=19%, signal=27%"
GSE17721_PAM3CSK4_VS_GADIQUIMOD_6H_BMDC_DN
GSE17721_PAM3CSK4_VS_GADIQUIMOD_6H_BMDC_DN
159	-0.23217535	-0.97078556	0.519084 0.5913302
1	2388	"tags=23%, list=17%, signal=27%"
GSE39820_IL1B_IL6_VS_IL1B_IL6_IL23A_TREATED_CD4_TCELL_UP
GSE39820_IL1B_IL6_VS_IL1B_IL6_IL23A_TREATED_CD4_TCELL_UP
137	-0.23372355	-0.9706463	0.5225933
0.5914304	1	2130	"tags=16%, list=15%, signal=19%"
GSE27786_NKCELL_VS_NKTCELL_DN	GSE27786_NKCELL_VS_NKTCELL_DN
	123	-0.24220805	-0.9702602	0.5219885
0.59224427	1	2024	"tags=20%, list=14%, signal=23%"
GSE25677_R848_VS_MPL_AND_R848_STIM_BCELL_UP
GSE25677_R848_VS_MPL_AND_R848_STIM_BCELL_UP
104	-0.24942584	-0.96995896	0.5120594
0.5927992	1	6180	"tags=55%, list=44%, signal=97%"
GSE8515_CTRL_VS_IL1_4H_STIM_MAC_UP
GSE8515_CTRL_VS_IL1_4H_STIM_MAC_UP
135	-0.23840374	-0.96880084	0.5175097
0.5957355	1	2313	"tags=21%, list=16%, signal=25%"
GSE18281_PERIMEDULLARY_CORTICAL_REGION_VS_WHOLE_MEDULLA_THYMUS_DN
GSE18281_PERIMEDULLARY_CORTICAL_REGION_VS_WHOLE_MEDULLA_THYMUS_DN
	106	-0.24614905	-0.96803427	0.55263156
0.5975953	1	2960	"tags=25%, list=21%, signal=31%"
GSE42724_NAIVE_VS_MEMORY_BCELL_DN GSE42724_NAIVE_VS_MEMORY_BCELL_DN
	136	-0.23567253	-0.96759266	0.5443038
0.5985367	1	670	"tags=10%, list=5%, signal=11%"
GSE360_CTRL_VS_T_GONDII_DC_UP	GSE360_CTRL_VS_T_GONDII_DC_UP
	156	-0.23294196	-0.9670531	0.5404896
0.59975064	1	2164	"tags=19%, list=15%, signal=22%"
GSE29618_PRE_VS_DAY7_POST_TIV_FLU_VACCINE_MDC_UP
GSE29618_PRE_VS_DAY7_POST_TIV_FLU_VACCINE_MDC_UP
132	-0.23887484	-0.9669543	0.5243665
0.59970623	1	2420	"tags=20%, list=17%, signal=24%"
GSE15215_CD2_POS_VS_NEG_PDC_UP	GSE15215_CD2_POS_VS_NEG_PDC_UP
	147	-0.23287214	-0.9668037	0.5584906
0.59984326	1	1404	"tags=13%, list=10%, signal=14%"
GSE20715_WT_VS_TLR4_KO_6H_OZONE_LUNG_DN
GSE20715_WT_VS_TLR4_KO_6H_OZONE_LUNG_DN

143 -0.23688275 -0.9662926 0.53763443
0.6009961 1 2664 "tags=22%, list=19%, signal=26%"
GSE15330_LYMPHOID_MULTIPOTENT_VS_PRO_BCELL_UP
GSE15330_LYMPHOID_MULTIPOTENT_VS_PRO_BCELL_UP
162 -0.22702836 -0.96620876 0.5425926
0.60090464 1 2152 "tags=18%, list=15%, signal=21%"
GSE21063_WT_VS_NFATC1_KO_3H_ANTI_IGM_STIM_BCELL_UP
GSE21063_WT_VS_NFATC1_KO_3H_ANTI_IGM_STIM_BCELL_UP
147 -0.23235852 -0.9653367 0.535316 0.6031129
1 1254 "tags=13%, list=9%, signal=14%"
GSE14308_NAIVE_CD4_TCELL_VS_INDUCED_TREG_UP
GSE14308_NAIVE_CD4_TCELL_VS_INDUCED_TREG_UP
127 -0.23856117 -0.9648113 0.5222222
0.6043354 1 3602 "tags=30%, list=26%, signal=40%"
GSE41867_DAY6_VS_DAY15_LCMV_CLONE13_EFFECTOR_CD8_TCELL_DN
GSE41867_DAY6_VS_DAY15_LCMV_CLONE13_EFFECTOR_CD8_TCELL_DN
98 -0.24692184 -0.9640945 0.5310219 0.606133
1 3577 "tags=29%, list=25%, signal=38%"
GSE15930_STIM_VS_STIM_AND_TRICHOSTATINA_24H_CD8_T_CELL_UP
GSE15930_STIM_VS_STIM_AND_TRICHOSTATINA_24H_CD8_T_CELL_UP
164 -0.23169664 -0.96403223 0.5654649
0.60598886 1 2151 "tags=18%, list=15%, signal=21%"
GSE2405_HEAT_KILLED_VS_LIVE_A_PHAGOCYTOPHILUM_STIM_NEUTROPHIL_24H_UP

GSE2405_HEAT_KILLED_VS_LIVE_A_PHAGOCYTOPHILUM_STIM_NEUTROPHIL_24H_UP
118 -0.24371818 -0.9638265
0.51498127 0.6062425 1 2437 "tags=20%,
list=17%, signal=24%"
GSE3982_EOSINOPHIL_VS_BCELL_UP GSE3982_EOSINOPHIL_VS_BCELL_UP
159 -0.23320945 -0.9634766 0.5487572
0.60693336 1 2211 "tags=19%, list=16%, signal=22%"
GSE17721_POLYIC_VS_CPG_16H_BMDC_UP
GSE17721_POLYIC_VS_CPG_16H_BMDC_UP
156 -0.23277056 -0.96320236 0.5571688
0.6073996 1 2931 "tags=26%, list=21%, signal=33%"
GSE25147_UNSTIM_VS_HELIOBACTER_PYLORI_LPS_STIM_MKN45_CELL_DN
GSE25147_UNSTIM_VS_HELIOBACTER_PYLORI_LPS_STIM_MKN45_CELL_DN
124 -0.23993516 -0.9624382 0.5247706
0.6093039 1 2318 "tags=18%, list=16%, signal=21%"
GSE17721_0.5H_VS_24H_CPG_BMDC_DN GSE17721_0.5H_VS_24H_CPG_BMDC_DN
157 -0.23100019 -0.961497 0.5588785
0.61173135 1 2501 "tags=20%, list=18%, signal=24%"
GSE1432_CTRL_VS_IFNG_24H_MICROGLIA_UP
GSE1432_CTRL_VS_IFNG_24H_MICROGLIA_UP
154 -0.230387 -0.96144164 0.5447316
0.61156803 1 2101 "tags=20%, list=15%, signal=23%"
GSE39820_TGFBETA1_IL6_VS_TGFBETA1_IL6_IL23A_TREATED_CD4_TCELL_UP
GSE39820_TGFBETA1_IL6_VS_TGFBETA1_IL6_IL23A_TREATED_CD4_TCELL_UP
159 -0.22887135 -0.9606662 0.52845526
0.61348426 1 2118 "tags=19%, list=15%, signal=23%"
GSE17186_CD21LOW_VS_CD21HIGH_TRANSITIONAL_BCELL_CORD_BLOOD_DN
GSE17186_CD21LOW_VS_CD21HIGH_TRANSITIONAL_BCELL_CORD_BLOOD_DN
147 -0.2331259 -0.9605839 0.53394496
0.613405 1 2311 "tags=22%, list=16%, signal=26%"

GSE41176_WT_VS_TAK1_KO_ANTI_IGM_STIM_BCELL_1H_DN
GSE41176_WT_VS_TAK1_KO_ANTI_IGM_STIM_BCELL_1H_DN
144 -0.23257507 -0.9605452 0.5510204
0.6131889 1 5662 "tags=46%, list=40%, signal=76%"
GSE360_L_DONOVANI_VS_B_MALAYI_HIGH_DOSE_DC_UP
GSE360_L_DONOVANI_VS_B_MALAYI_HIGH_DOSE_DC_UP
150 -0.23158097 -0.9596438 0.5611111
0.6154961 1 3164 "tags=30%, list=22%, signal=38%"
GSE17721_CTRL_VS_POLYIC_24H_BMDC_DN
GSE17721_CTRL_VS_POLYIC_24H_BMDC_DN
155 -0.22767687 -0.95880616 0.53937006
0.61758566 1 1770 "tags=14%, list=13%, signal=16%"
GSE19923_WT_VS_HEB_KO_DP_THYMOCYTE_UP
GSE19923_WT_VS_HEB_KO_DP_THYMOCYTE_UP
159 -0.23159578 -0.95872957 0.5546875
0.61749256 1 2995 "tags=28%, list=21%, signal=35%"
GSE2128_C57BL6_VS_NOD_THYMOCYTE_MIMETOPE_NEGATIVE_SELECTION_UP
GSE2128_C57BL6_VS_NOD_THYMOCYTE_MIMETOPE_NEGATIVE_SELECTION_UP
145 -0.23048449 -0.95869863 0.5658153
0.6172527 1 3582 "tags=26%, list=25%, signal=34%"
GSE23114_WT_VS_SLE2C1_MOUSE_PERITONEAL_CAVITY_B1A_BCELL_DN
GSE23114_WT_VS_SLE2C1_MOUSE_PERITONEAL_CAVITY_B1A_BCELL_DN
121 -0.23856336 -0.95799917 0.5394737
0.6189161 1 1704 "tags=12%, list=12%, signal=14%"
GSE14415_INDUCED_TREG_VS_FOXP3_KO_INDUCED_TREG_IL2_CULTURE_DN
GSE14415_INDUCED_TREG_VS_FOXP3_KO_INDUCED_TREG_IL2_CULTURE_DN
94 -0.250244 -0.95671916 0.5277778
0.62224305 1 1414 "tags=14%, list=10%, signal=15%"
GSE339_CD4POS_VS_CD8POS_DC_IN_CULTURE_UP
GSE339_CD4POS_VS_CD8POS_DC_IN_CULTURE_UP
157 -0.23143084 -0.9565451 0.5685185 0.622442
1 3615 "tags=32%, list=26%, signal=42%"
GSE8515_CTRL_VS_IL6_4H_STIM_MAC_DN
GSE8515_CTRL_VS_IL6_4H_STIM_MAC_DN
139 -0.23510446 -0.95611423 0.5486381
0.62334514 1 4453 "tags=36%, list=32%, signal=52%"
GSE45365_WT_VS_IFNAR_KO_CD8A_DC_MCMV_INFECTION_DN
GSE45365_WT_VS_IFNAR_KO_CD8A_DC_MCMV_INFECTION_DN
123 -0.23850058 -0.956078 0.53231937 0.623134
1 1289 "tags=11%, list=9%, signal=12%"
GSE25890_CTRL_VS_IL33_IL7_TREATED_NUOCYTES_UP
GSE25890_CTRL_VS_IL33_IL7_TREATED_NUOCYTES_UP
136 -0.23649885 -0.95481044 0.5689655
0.62646836 1 1718 "tags=16%, list=12%, signal=18%"
GSE40274_CTRL_VS_FOXP3_AND_SATB1_TRANSDUCED_ACTIVATED_CD4_TCELL_DN
GSE40274_CTRL_VS_FOXP3_AND_SATB1_TRANSDUCED_ACTIVATED_CD4_TCELL_DN
115 -0.24080369 -0.95394677 0.5509804
0.6286248 1 2515 "tags=23%, list=18%, signal=27%"
GSE11924_TH2_VS_TH17_CD4_TCELL_DN GSE11924_TH2_VS_TH17_CD4_TCELL_DN
143 -0.23110776 -0.9536745 0.57247704
0.6290472 1 2248 "tags=20%, list=16%, signal=24%"
GSE9037_WT_VS_IRAK4_KO_LPS_1H_STIM_BMDM_UP
GSE9037_WT_VS_IRAK4_KO_LPS_1H_STIM_BMDM_UP
140 -0.22898081 -0.95319813 0.5694444

0.6300813	1	2508	"tags=21%, list=18%, signal=25%"
GSE22935_UNSTIM_VS_24H_MBOVIS_BCG_STIM_MYD88_KO_MACROPHAGE_DN
GSE22935_UNSTIM_VS_24H_MBOVIS_BCG_STIM_MYD88_KO_MACROPHAGE_DN
	138	-0.23336828	-0.9531478	0.5673624
0.62990165	1	3282	"tags=25%, list=23%, signal=32%"
GSE2405_0H_VS_6H_A_PHAGOCYTOPHILUM_STIM_NEUTROPHIL_DN
GSE2405_0H_VS_6H_A_PHAGOCYTOPHILUM_STIM_NEUTROPHIL_DN
119	-0.23738201	-0.9529868	0.5536723
0.6300389	1	3066	"tags=19%, list=22%, signal=24%"
GSE29949_CD8_POS_DC_SPLEEN_VS_DC_BRAIN_DN
GSE29949_CD8_POS_DC_SPLEEN_VS_DC_BRAIN_DN
139	-0.2319687	-0.95292014	0.567619 0.6298995
1	3693	"tags=28%, list=26%, signal=38%"
GSE37416_CTRL_VS_0H_F_TULARENSIS_LVS_NEUTROPHIL_UP
GSE37416_CTRL_VS_0H_F_TULARENSIS_LVS_NEUTROPHIL_UP
107	-0.24352038	-0.9528439	0.5533981
0.6297895	1	3019	"tags=29%, list=21%, signal=37%"
GSE30083_SP3_VS_SP4_THYMOCYTE_UP	GSE30083_SP3_VS_SP4_THYMOCYTE_UP
	150	-0.22872564	-0.9527472	0.55839413
0.6297389	1	3452	"tags=23%, list=24%, signal=31%"
GSE27896_HDAC6_KO_VS_WT_TREG_DN	GSE27896_HDAC6_KO_VS_WT_TREG_DN
	130	-0.23149027	-0.9520021	0.55841583
0.6315819	1	3029	"tags=28%, list=21%, signal=35%"
GSE3982_BCELL_VS_NKCELL_UP	GSE3982_BCELL_VS_NKCELL_UP
	137	-0.23407334	-0.95114714	0.5731225
0.63374364	1	2468	"tags=24%, list=17%, signal=29%"
GSE19923_WT_VS_E2A_KO_DP_THYMOCYTE_DN
GSE19923_WT_VS_E2A_KO_DP_THYMOCYTE_DN
140	-0.23041031	-0.9498807	0.5743119
0.6371277	1	1148	"tags=11%, list=8%, signal=12%"
GSE29618_PDC_VS_MDC_UP	GSE29618_PDC_VS_MDC_UP
153	-0.22835313	-0.94891524	0.5973025
0.6394967	1	2278	"tags=22%, list=16%, signal=25%"
GSE20366_CD103_POS_VS_NEG_TREG_KLRG1NEG_UP
GSE20366_CD103_POS_VS_NEG_TREG_KLRG1NEG_UP
136	-0.22987579	-0.9487918	0.5808967
0.63952255	1	2379	"tags=20%, list=17%, signal=24%"
GSE4142_NAIVE_VS_MEMORY_BCELL_UP	GSE4142_NAIVE_VS_MEMORY_BCELL_UP
	144	-0.22979769	-0.9477369	0.58733207
0.642234 1	4178	"tags=32%, list=30%, signal=45%"
GSE20151_CTRL_VS_FUSOBACT_NUCLEATUM_NEUTROPHIL_UP
GSE20151_CTRL_VS_FUSOBACT_NUCLEATUM_NEUTROPHIL_UP
140	-0.22832958	-0.9474358	0.582846 0.6427568
1	2114	"tags=17%, list=15%, signal=20%"
GSE46242_CTRL_VS_EGR2_DELETED_TH1_CD4_TCELL_DN
GSE46242_CTRL_VS_EGR2_DELETED_TH1_CD4_TCELL_DN
125	-0.2412636	-0.94737077	0.589942 0.6426111
1	1771	"tags=17%, list=13%, signal=19%"
GSE19772_CTRL_VS_HCMV_INF_MONOCYTES_DN
GSE19772_CTRL_VS_HCMV_INF_MONOCYTES_DN
112	-0.23601095	-0.94553095	0.57042253
0.6474906	1	558	"tags=8%, list=4%, signal=8%"
GSE5589_IL6_KO_VS_IL10_KO_LPS_STIM_MACROPHAGE_180MIN_UP
GSE5589_IL6_KO_VS_IL10_KO_LPS_STIM_MACROPHAGE_180MIN_UP

137 -0.23183385 -0.9447099 0.5764023
0.64946514 1 1849 "tags=16%, list=13%, signal=18%"
GSE5589_WT_VS_IL6_KO_LPS_AND_IL10_STIM_MACROPHAGE_45MIN_DN
GSE5589_WT_VS_IL6_KO_LPS_AND_IL10_STIM_MACROPHAGE_45MIN_DN
154 -0.2286798 -0.94411737 0.5900383
0.6508016 1 1387 "tags=16%, list=10%, signal=17%"
GSE16385_IL4_VS_ROSIGLITAZONE_STIM_MACROPHAGE_DN
GSE16385_IL4_VS_ROSIGLITAZONE_STIM_MACROPHAGE_DN
120 -0.23832864 -0.9435993 0.5681382
0.65194845 1 2867 "tags=28%, list=20%, signal=35%"
GSE22103_LPS_VS_GMCSF_AND_IFNG_STIM_NEUTROPHIL_DN
GSE22103_LPS_VS_GMCSF_AND_IFNG_STIM_NEUTROPHIL_DN
145 -0.22460316 -0.9433045 0.6289425
0.6524391 1 3817 "tags=35%, list=27%, signal=48%"
GSE7218_UNSTIM_VS_ANTIGEN_STIM_THROUGH_IGM_BCELL_UP
GSE7218_UNSTIM_VS_ANTIGEN_STIM_THROUGH_IGM_BCELL_UP
106 -0.23913185 -0.9427287 0.58431375
0.65375704 1 3400 "tags=30%, list=24%, signal=39%"
GSE43955_1H_VS_42H_ACT_CD4_TCELL_DN
GSE43955_1H_VS_42H_ACT_CD4_TCELL_DN
160 -0.22625476 -0.9421786 0.594041 0.65499705
1 2571 "tags=24%, list=18%, signal=29%"
GSE3982_BASOPHIL_VS_CENT_MEMORY_CD4_TCELL_DN
GSE3982_BASOPHIL_VS_CENT_MEMORY_CD4_TCELL_DN
130 -0.23144196 -0.94166577 0.6007984
0.65613145 1 2693 "tags=22%, list=19%, signal=26%"
GSE18893_CTRL_VS_TNF_TREATED_TREG_24H_UP
GSE18893_CTRL_VS_TNF_TREATED_TREG_24H_UP
143 -0.22669141 -0.9415122 0.5946463
0.65622723 1 2238 "tags=19%, list=16%, signal=22%"
GSE21670_TGFB_VS_IL6_TREATED_CD4_TCELL_DN
GSE21670_TGFB_VS_IL6_TREATED_CD4_TCELL_DN
145 -0.23020722 -0.9410995 0.6007752
0.6570879 1 3921 "tags=32%, list=28%, signal=44%"
GSE20500_RETINOIC_ACID_VS_RARA_ANTAGONIST_TREATED_CD4_TCELL_UP
GSE20500_RETINOIC_ACID_VS_RARA_ANTAGONIST_TREATED_CD4_TCELL_UP
141 -0.23036562 -0.9410225 0.6007752
0.6569691 1 2212 "tags=20%, list=16%, signal=23%"
GSE17721_CTRL_VS_GARDIQUIMOD_8H_BMDC_DN
GSE17721_CTRL_VS_GARDIQUIMOD_8H_BMDC_DN
160 -0.22623698 -0.9406375 0.57481754
0.6577053 1 2179 "tags=19%, list=15%, signal=23%"
GSE16385_IFNG_TNF_VS_UNSTIM_MACROPHAGE_ROSIGLITAZONE_TREATED_UP
GSE16385_IFNG_TNF_VS_UNSTIM_MACROPHAGE_ROSIGLITAZONE_TREATED_UP
108 -0.23858316 -0.93954736 0.6
0.6605146 1 1546 "tags=15%, list=11%, signal=17%"
GSE18791_UNSTIM_VS_NEWCATSLE_VIRUS_DC_1H_UP
GSE18791_UNSTIM_VS_NEWCATSLE_VIRUS_DC_1H_UP
103 -0.2418953 -0.93849206 0.5725047
0.66327554 1 4567 "tags=40%, list=32%, signal=58%"
GSE14413_UNSTIM_VS_IFNB_STIM_L929_CELLS_UP
GSE14413_UNSTIM_VS_IFNB_STIM_L929_CELLS_UP
70 -0.2562036 -0.93754405 0.5574074 0.665605
1 2628 "tags=23%, list=19%, signal=28%"

GSE28737_FOLLICULAR_VS_MARGINAL_ZONE_BCELL_BCL6_HET_DN
GSE28737_FOLLICULAR_VS_MARGINAL_ZONE_BCELL_BCL6_HET_DN
146 -0.22524585 -0.93677455 0.60820895
0.66755813 1 2076 "tags=21%, list=15%, signal=25%"
GSE46606_IRF4_KO_VS_WT_CD40L_IL2_IL5_1DAY_STIMULATED_BCELL_UP
GSE46606_IRF4_KO_VS_WT_CD40L_IL2_IL5_1DAY_STIMULATED_BCELL_UP
123 -0.23387714 -0.93658555 0.61
0.66776884 1 2030 "tags=20%, list=14%, signal=24%"
GSE3982_MAC_VS_CENT_MEMORY_CD4_TCELL_DN
GSE3982_MAC_VS_CENT_MEMORY_CD4_TCELL_DN
123 -0.23486543 -0.935607 0.60828626
0.67022926 1 2666 "tags=24%, list=19%, signal=29%"
GSE26290_CTRL_VS_AKT_INHIBITOR_TREATED_ANTI_CD3_AND_IL2_STIM_CD8_TCELL_UP
GSE26290_CTRL_VS_AKT_INHIBITOR_TREATED_ANTI_CD3_AND_IL2_STIM_CD8_TCELL_UP 140 -0.23099962 -0.9353174
0.57699805 0.6707392 1 2610 "tags=23%, list=18%, signal=28%"
GSE28737_FOLLICULAR_VS_MARGINAL_ZONE_BCELL_DN
GSE28737_FOLLICULAR_VS_MARGINAL_ZONE_BCELL_DN
143 -0.22669536 -0.9352827 0.606679 0.6705112
1 1224 "tags=10%, list=9%, signal=11%"
GSE13173_UNTREATED_VS_IL12_TREATED_ACT_CD8_TCELL_UP
GSE13173_UNTREATED_VS_IL12_TREATED_ACT_CD8_TCELL_UP
92 -0.24137741 -0.93337595 0.59266055
0.6756019 1 5217 "tags=40%, list=37%, signal=63%"
GSE10422_WT_VS_BAFF_TRANSGENIC_LN_BCELL_DN
GSE10422_WT_VS_BAFF_TRANSGENIC_LN_BCELL_DN
96 -0.24326345 -0.9326266 0.58878505
0.6773868 1 1600 "tags=16%, list=11%, signal=18%"
GSE5589_WT_VS_IL6_KO_LPS_AND_IL6_STIM_MACROPHAGE_45MIN_UP
GSE5589_WT_VS_IL6_KO_LPS_AND_IL6_STIM_MACROPHAGE_45MIN_UP
140 -0.22481112 -0.9312113 0.64547205
0.6811117 1 3483 "tags=29%, list=25%, signal=38%"
GSE22282_HYPOXIA_VS_NORMOXIA_MYELOID_DC_UP
GSE22282_HYPOXIA_VS_NORMOXIA_MYELOID_DC_UP
158 -0.22279872 -0.93112826 0.6316832
0.68100774 1 2138 "tags=20%, list=15%, signal=23%"
GSE46025_WT_VS_FOXO1_KO_KLRG1_LOW_CD8_EFFECTOR_TCELL_UP
GSE46025_WT_VS_FOXO1_KO_KLRG1_LOW_CD8_EFFECTOR_TCELL_UP
133 -0.2313305 -0.9310905 0.6191406
0.6807667 1 1851 "tags=16%, list=13%, signal=18%"
GSE3982_DC_VS_EFF_MEMORY_CD4_TCELL_UP
GSE3982_DC_VS_EFF_MEMORY_CD4_TCELL_UP
152 -0.2246481 -0.93103296 0.6545802
0.6805932 1 3363 "tags=29%, list=24%, signal=38%"
GSE23114_PERITONEAL_CAVITY_B1A_BCELL_VS_SPLEEN_BCELL_IN_SLE2C1_MOUSE_DN
GSE23114_PERITONEAL_CAVITY_B1A_BCELL_VS_SPLEEN_BCELL_IN_SLE2C1_MOUSE_DN 133 -0.22802393 -0.93059653
0.60714287 0.68147343 1 1986 "tags=19%, list=14%, signal=22%"
GSE41978_ID2_KO_AND_BIM_KO_VS_BIM_KO_KLRG1_LOW_EFFECTOR_CD8_TCELL_UP

GSE41978_ID2_KO_AND_BIM_KO_VS_BIM_KO_KLRG1_LOW_EFFECTOR_CD8_TCELL_UP
148 -0.22389895 -0.93029237
0.63915545 0.6819905 1 2652 "tags=20%,
list=19%, signal=25%"
GSE22342_CD11C_HIGH_VS_LOW_DECIDUAL_MACROPHAGES_DN
GSE22342_CD11C_HIGH_VS_LOW_DECIDUAL_MACROPHAGES_DN
115 -0.23447478 -0.9299086 0.6078067
0.68274224 1 2022 "tags=18%, list=14%, signal=21%"
GSE15330_HSC_VS_GRANULOCYTE_MONOCYTE_PROGENITOR_DN
GSE15330_HSC_VS_GRANULOCYTE_MONOCYTE_PROGENITOR_DN
144 -0.22687787 -0.9298312 0.64510167
0.6826134 1 1764 "tags=16%, list=12%, signal=18%"
GSE9037_CTRL_VS_LPS_4H_STIM_IRAK4_KO_BMDM_UP
GSE9037_CTRL_VS_LPS_4H_STIM_IRAK4_KO_BMDM_UP
134 -0.22565287 -0.92878073 0.6405959 0.685329
1 2051 "tags=19%, list=15%, signal=22%"
GSE9601_UNTREATED_VS_NFKB_INHIBITOR_TREATED_HCMV_INF_MONOCYTE_UP
GSE9601_UNTREATED_VS_NFKB_INHIBITOR_TREATED_HCMV_INF_MONOCYTE_UP
121 -0.2321548 -0.9270581 0.6183206
0.68992496 1 2297 "tags=22%, list=16%, signal=26%"
GSE19941_UNSTIM_VS_LPS_STIM_IL10_KO_MACROPHAGE_UP
GSE19941_UNSTIM_VS_LPS_STIM_IL10_KO_MACROPHAGE_UP
141 -0.22503583 -0.9255568 0.6431373
0.6938627 1 3205 "tags=28%, list=23%, signal=36%"
GSE40274_FOXP3_VS_FOXP3_AND_HELIOS_TRANSDUCED_ACTIVATED_CD4_TCELL_UP

GSE40274_FOXP3_VS_FOXP3_AND_HELIOS_TRANSDUCED_ACTIVATED_CD4_TCELL_UP
106 -0.23449983 -0.92352164
0.6380597 0.6992883 1 3499 "tags=25%,
list=25%, signal=34%"
GSE45365_HEALTHY_VS_MCMV_INFECTION_CD8_TCELL_IFNAR_KO_DN
GSE45365_HEALTHY_VS_MCMV_INFECTION_CD8_TCELL_IFNAR_KO_DN
109 -0.23243187 -0.92158836 0.63582677
0.70438784 1 2824 "tags=23%, list=20%, signal=28%"
GSE10240_IL17_VS_IL17_AND_IL22_STIM_PRIMARY_BRONCHIAL_EPITHELIAL_CEL
LS_DN
GSE10240_IL17_VS_IL17_AND_IL22_STIM_PRIMARY_BRONCHIAL_EPITHELIAL_CEL
LS_DN 150 -0.22394736 -0.92135084
0.67248064 0.70470625 1 4783 "tags=34%,
list=34%, signal=51%"
GSE19941_IL10_KO_VS_IL10_KO_AND_NFKBP50_KO_LPS_AND_IL10_STIM_MACROPH
AGE_UP
GSE19941_IL10_KO_VS_IL10_KO_AND_NFKBP50_KO_LPS_AND_IL10_STIM_MACROPH
AGE_UP 143 -0.2229135 -0.9202552
0.63147795 0.7075091 1 2313 "tags=21%,
list=16%, signal=25%"
GSE13485_DAY1_VS_DAY21_YF17D_VACCINE_PBMC_UP
GSE13485_DAY1_VS_DAY21_YF17D_VACCINE_PBMC_UP
121 -0.22902147 -0.9192781 0.6401591
0.7099013 1 1308 "tags=15%, list=9%, signal=16%"
GSE21670_UNTREATED_VS_TGFB_TREATED_STAT3_KO_CD4_TCELL_UP
GSE21670_UNTREATED_VS_TGFB_TREATED_STAT3_KO_CD4_TCELL_UP
142 -0.22536819 -0.91828334 0.67241377
0.71236503 1 2342 "tags=20%, list=17%, signal=23%"

GSE17721_CPG_VS_GARDIQUIMOD_8H_BMDC_DN
GSE17721_CPG_VS_GARDIQUIMOD_8H_BMDC_DN
145 -0.22024935 -0.91758627 0.6632653
0.71397126 1 1749 "tags=15%, list=12%, signal=17%"
GSE37301_MULTIPOTENT_PROGENITOR_VS_PRO_BCELL_UP
GSE37301_MULTIPOTENT_PROGENITOR_VS_PRO_BCELL_UP
148 -0.2187401 -0.91684943 0.6528302
0.7156835 1 3146 "tags=27%, list=22%, signal=34%"
GSE18791_UNSTIM_VS_NEWCATSLE_VIRUS_DC_6H_UP
GSE18791_UNSTIM_VS_NEWCATSLE_VIRUS_DC_6H_UP
127 -0.22975156 -0.9140689 0.6531008
0.7231006 1 2008 "tags=20%, list=14%, signal=23%"
GSE34217_MIR17_92_OVEREXPRESS_VS_WT_ACT_CD8_TCELL_UP
GSE34217_MIR17_92_OVEREXPRESS_VS_WT_ACT_CD8_TCELL_UP
102 -0.23603222 -0.913764 0.609127 0.7235855
1 3379 "tags=26%, list=24%, signal=35%"
GSE41176_UNSTIM_VS_ANTI_IGM_STIM_TAK1_KO_BCELL_6H_DN
GSE41176_UNSTIM_VS_ANTI_IGM_STIM_TAK1_KO_BCELL_6H_DN
129 -0.22804968 -0.91362315 0.6343874
0.72361815 1 6163 "tags=44%, list=44%, signal=78%"
GSE29618_PRE_VS_DAY7_POST_LAIV_FLU_VACCINE_PDC_UP
GSE29618_PRE_VS_DAY7_POST_LAIV_FLU_VACCINE_PDC_UP
140 -0.22236039 -0.91243756 0.6640777
0.72646874 1 3880 "tags=34%, list=27%, signal=46%"
GSE29949_CD8_NEG_DC_SPLEEN_VS_DC_BRAIN_UP
GSE29949_CD8_NEG_DC_SPLEEN_VS_DC_BRAIN_UP
144 -0.22145893 -0.91186714 0.66368514
0.7277006 1 4067 "tags=31%, list=29%, signal=42%"
GSE37605_C57BL6_VS_NOD_FOXP3_FUSION_GFP_TREG_UP
GSE37605_C57BL6_VS_NOD_FOXP3_FUSION_GFP_TREG_UP
82 -0.23875491 -0.911764 0.6451613
0.7276107 1 6543 "tags=50%, list=46%, signal=93%"
GSE10239_MEMORY_VS_KLRG1INT_EFF_CD8_TCELL_UP
GSE10239_MEMORY_VS_KLRG1INT_EFF_CD8_TCELL_UP
132 -0.22607748 -0.91086555 0.69285715 0.729706
1 3181 "tags=26%, list=23%, signal=33%"
GSE17721_CTRL_VS_POLYIC_6H_BMDC_DN
GSE17721_CTRL_VS_POLYIC_6H_BMDC_DN
159 -0.21892008 -0.9107478 0.69507575
0.7296628 1 2048 "tags=17%, list=15%, signal=20%"
GSE15330_WT_VS_IKAROS_KO_LYMPHOID_MULTIPOTENT_PROGENITOR_DN
GSE15330_WT_VS_IKAROS_KO_LYMPHOID_MULTIPOTENT_PROGENITOR_DN
138 -0.2227127 -0.9085108 0.66988415
0.73543817 1 1361 "tags=13%, list=10%, signal=14%"
GSE24492_LYVE_NEG_VS_POS_MACROPHAGE_DN
GSE24492_LYVE_NEG_VS_POS_MACROPHAGE_DN
139 -0.22353783 -0.9071511 0.70321363
0.73883057 1 2446 "tags=21%, list=17%, signal=25%"
GSE8621_UNSTIM_VS_LPS_PRIMED_UNSTIM_MACROPHAGE_UP
GSE8621_UNSTIM_VS_LPS_PRIMED_UNSTIM_MACROPHAGE_UP
149 -0.22021124 -0.90627 0.6959847 0.7408996
1 3036 "tags=26%, list=22%, signal=32%"
GSE360_T_GONDII_VS_M_TUBERCULOSIS_MAC_DN
GSE360_T_GONDII_VS_M_TUBERCULOSIS_MAC_DN

147	-0.22121516	-0.9060813	0.7048872
0.74101967	1	1702	"tags=15%, list=12%, signal=17%"
GSE3203_WT_VS_IFNAR1_KO_INFLUENZA_INFECTED_LN_BCELL_UP
GSE3203_WT_VS_IFNAR1_KO_INFLUENZA_INFECTED_LN_BCELL_UP
135	-0.22158407	-0.90484136	0.6928839
0.7441031	1	2379	"tags=16%, list=17%, signal=19%"
GSE43863_NAIVE_VS_LY6C_LOW_CXCR5NEG_CD4_EFF_TCELL_D6_LCMV_UP
GSE43863_NAIVE_VS_LY6C_LOW_CXCR5NEG_CD4_EFF_TCELL_D6_LCMV_UP
	91	-0.2322021	-0.90412575	0.6582734
0.7456494	1	3210	"tags=27%, list=23%, signal=35%"
GSE22033_WT_VS_PPARG_KO_MEF_UP	GSE22033_WT_VS_PPARG_KO_MEF_UP
	121	-0.223844	-0.9036483	0.68233085
0.7466075	1	3832	"tags=31%, list=27%, signal=42%"
GSE37605_NOD_VS_C57BL6_IRES_GFP_TREG_UP
GSE37605_NOD_VS_C57BL6_IRES_GFP_TREG_UP
118	-0.2251596	-0.90337634	0.6978417	0.746998
1	2335	"tags=19%, list=17%, signal=23%"
GSE21360_NAIVE_VS_SECONDARY_MEMORY_CD8_TCELL_UP
GSE21360_NAIVE_VS_SECONDARY_MEMORY_CD8_TCELL_UP
149	-0.21750316	-0.90206873	0.69274807	0.75014
1	2215	"tags=17%, list=16%, signal=20%"
GSE9316_CD4_TCELL_BALBC_VS_TH17_ENRI_CD4_TCELL_SKG_PMA_IONO_STIM_FR4
NEG_DN
GSE9316_CD4_TCELL_BALBC_VS_TH17_ENRI_CD4_TCELL_SKG_PMA_IONO_STIM_FR4
NEG_DN	142	-0.22010769	-0.9009359	0.734375
0.7528951	1	1976	"tags=19%, list=14%, signal=22%"
GSE26928_NAIVE_VS_CXCR5_POS_CD4_TCELL_UP
GSE26928_NAIVE_VS_CXCR5_POS_CD4_TCELL_UP
120	-0.22688381	-0.90092444	0.7029703
0.7525443	1	2256	"tags=18%, list=16%, signal=22%"
GSE46606_IRF4MID_VS_WT_CD40L_IL2_IL5_DAY3_STIMULATED_BCELL_UP
GSE46606_IRF4MID_VS_WT_CD40L_IL2_IL5_DAY3_STIMULATED_BCELL_UP
	142	-0.22002432	-0.900271	0.68725866
0.75398606	1	1573	"tags=15%, list=11%, signal=17%"
GSE36888_STAT5_AB_KNOCKIN_VS_WT_TCELL_IL2_TREATED_17H_DN
GSE36888_STAT5_AB_KNOCKIN_VS_WT_TCELL_IL2_TREATED_17H_DN
145	-0.21795142	-0.90017873	0.69942194
0.75386906	1	1545	"tags=13%, list=11%, signal=15%"
GSE32034_UNTREATED_VS_ROSIGLIZATONE_TREATED_LY6C_LOW_MONOCYTE_DN
GSE32034_UNTREATED_VS_ROSIGLIZATONE_TREATED_LY6C_LOW_MONOCYTE_DN
	117	-0.22614902	-0.9001012	0.6826004
0.7537041	1	4270	"tags=33%, list=30%, signal=47%"
GSE17721_POLYIC_VS_GARDIQUIMOD_12H_BMDC_UP
GSE17721_POLYIC_VS_GARDIQUIMOD_12H_BMDC_UP
147	-0.2166872	-0.89896333	0.70233464
0.75648177	1	1797	"tags=14%, list=13%, signal=15%"
GSE22601_IMMATURE_CD4_SINGLE_POSITIVE_VS_DOUBLE_POSITIVE_THYMOCYTE_U
P
GSE22601_IMMATURE_CD4_SINGLE_POSITIVE_VS_DOUBLE_POSITIVE_THYMOCYTE_U
P	105	-0.23015158	-0.89827853
0.6969112	0.75798416	1	5914	"tags=47%,
list=42%, signal=80%"
GSE27670_CTRL_VS_BLIMP1_TRANSDUCED_GC_BCELL_UP
GSE27670_CTRL_VS_BLIMP1_TRANSDUCED_GC_BCELL_UP

162	-0.21216647	-0.89786446	0.743295 0.7587318
1	2257	"tags=17%, list=16%, signal=20%"
GSE13887_ACT_CD4_VS_NO_TREATED_CD4_TCELL_DN
GSE13887_ACT_CD4_VS_NO_TREATED_CD4_TCELL_DN
96	-0.23324333	-0.8976279	0.68421054
0.7589838	1	2309	"tags=16%, list=16%, signal=19%"
GSE25088_ROSIGLITAZONE_VS_IL4_AND_ROSIGLITAZONE_STIM_MACROPHAGE_DAY1
0_DN
GSE25088_ROSIGLITAZONE_VS_IL4_AND_ROSIGLITAZONE_STIM_MACROPHAGE_DAY1
0_DN	144	-0.2179811	-0.8975347
0.7101728	0.7588612	1	1282	"tags=13%,
list=9%, signal=14%"
GSE8835_CD4_VS_CD8_TCELL_DN	GSE8835_CD4_VS_CD8_TCELL_DN
	139	-0.21698809	-0.89723974	0.7337278
0.7593051	1	4494	"tags=35%, list=32%, signal=51%"
GSE24634_NAIVE_CD4_TCELL_VS_DAY10_IL4_CONV_TREG_UP
GSE24634_NAIVE_CD4_TCELL_VS_DAY10_IL4_CONV_TREG_UP
131	-0.2181194	-0.8969407	0.70857143
0.75971043	1	1528	"tags=15%, list=11%, signal=16%"
GSE2706_LPS_VS_R848_AND_LPS_2H_STIM_DC_DN
GSE2706_LPS_VS_R848_AND_LPS_2H_STIM_DC_DN
98	-0.22942641	-0.8962232	0.6891386
0.7612845	1	4433	"tags=36%, list=31%, signal=52%"
GSE22886_TH1_VS_TH2_12H_ACT_DN	GSE22886_TH1_VS_TH2_12H_ACT_DN
	132	-0.21982248	-0.8946201	0.7191919
0.76525646	1	2182	"tags=15%, list=15%, signal=18%"
GSE19923_WT_VS_E2A_KO_DP_THYMOCYTE_UP
GSE19923_WT_VS_E2A_KO_DP_THYMOCYTE_UP
170	-0.2131952	-0.8939974	0.765873 0.76649815
1	1236	"tags=13%, list=9%, signal=14%"
GSE37605_TREG_VS_TCONV_C57BL6_FOXP3_FUSION_GFP_UP
GSE37605_TREG_VS_TCONV_C57BL6_FOXP3_FUSION_GFP_UP
101	-0.22922903	-0.89366007	0.6802218
0.76704854	1	2247	"tags=16%, list=16%, signal=19%"
GSE25123_WT_VS_PPARG_KO_MACROPHAGE_ROSIGLITAZONE_STIM_DN
GSE25123_WT_VS_PPARG_KO_MACROPHAGE_ROSIGLITAZONE_STIM_DN
113	-0.22179165	-0.89296925	0.71789885
0.76851153	1	2193	"tags=18%, list=16%, signal=21%"
GSE1925_CTRL_VS_IFNG_PRIMED_MACROPHAGE_3H_IFNG_STIM_DN
GSE1925_CTRL_VS_IFNG_PRIMED_MACROPHAGE_3H_IFNG_STIM_DN
94	-0.2306852	-0.8923137	0.6859345
0.76983285	1	981	"tags=9%, list=7%, signal=9%"
GSE1566_WT_VS_EZH2_KO_LN_TCELL_UP GSE1566_WT_VS_EZH2_KO_LN_TCELL_UP
	117	-0.22414869	-0.8919772	0.6829746
0.77032447	1	598	"tags=7%, list=4%, signal=7%"
GSE339_CD4POS_VS_CD4CD8DN_DC_IN_CULTURE_UP
GSE339_CD4POS_VS_CD4CD8DN_DC_IN_CULTURE_UP
156	-0.21575181	-0.89112157	0.7538168
0.7721872	1	3761	"tags=28%, list=27%, signal=38%"
GSE36888_STAT5_AB_KNOCKIN_VS_WT_TCELL_IL2_TREATED_2H_DN
GSE36888_STAT5_AB_KNOCKIN_VS_WT_TCELL_IL2_TREATED_2H_DN
132	-0.21910106	-0.8908802	0.71794873
0.7724295	1	1250	"tags=14%, list=9%, signal=15%"
GSE6259_DEC205_POS_DC_VS_CD8_TCELL_DN

GSE6259_DEC205_POS_DC_VS_CD8_TCELL_DN
118	-0.22199853	-0.89050883	0.7243948
0.7730576	1	2803	"tags=21%, list=20%, signal=26%"
GSE24210_IL35_TREATED_VS_UNTREATED_TCONV_CD4_TCELL_DN
GSE24210_IL35_TREATED_VS_UNTREATED_TCONV_CD4_TCELL_DN
150	-0.21275161	-0.88841814	0.7642726
0.77827007	1	2910	"tags=23%, list=21%, signal=29%"
GSE11367_CTRL_VS_IL17_TREATED_SMOOTH_MUSCLE_CELL_DN
GSE11367_CTRL_VS_IL17_TREATED_SMOOTH_MUSCLE_CELL_DN
86	-0.23259725	-0.88751984	0.7100372
0.7802435	1	733	"tags=10%, list=5%, signal=11%"
GSE13485_DAY1_VS_DAY3_YF17D_VACCINE_PBMC_UP
GSE13485_DAY1_VS_DAY3_YF17D_VACCINE_PBMC_UP
101	-0.22638428	-0.88664246	0.72088355
0.7821078	1	5352	"tags=43%, list=38%, signal=68%"
GSE22589_HIV_VS_HIV_AND_SIV_INFECTED_DC_DN
GSE22589_HIV_VS_HIV_AND_SIV_INFECTED_DC_DN
128	-0.21969563	-0.8860965	0.7308448
0.7831167	1	4389	"tags=36%, list=31%, signal=52%"
GSE2706_R848_VS_R848_AND_LPS_2H_STIM_DC_UP
GSE2706_R848_VS_R848_AND_LPS_2H_STIM_DC_UP
103	-0.22325458	-0.8857534	0.6780952
0.7836271	1	3058	"tags=28%, list=22%, signal=36%"
GSE26488_CTRL_VS_PEPTIDE_INJECTION_OT2_THYMOCYTE_UP
GSE26488_CTRL_VS_PEPTIDE_INJECTION_OT2_THYMOCYTE_UP
102	-0.2241666	-0.88496226	0.72504705
0.78526014	1	1948	"tags=16%, list=14%, signal=18%"
GSE40666_UNTREATED_VS_IFNA_STIM_CD8_TCELL_90MIN_UP
GSE40666_UNTREATED_VS_IFNA_STIM_CD8_TCELL_90MIN_UP
97	-0.22399743	-0.8844269	0.72583824
0.7863094	1	4184	"tags=31%, list=30%, signal=44%"
GSE26023_PHD3_KO_VS_WT_NEUTROPHIL_HYPOXIA_UP
GSE26023_PHD3_KO_VS_WT_NEUTROPHIL_HYPOXIA_UP
155	-0.2138221	-0.8831538	0.74563104
0.7892225	1	2598	"tags=21%, list=18%, signal=25%"
GSE17721_CTRL_VS_POLYIC_0.5H_BMDC_DN
GSE17721_CTRL_VS_POLYIC_0.5H_BMDC_DN
168	-0.20935647	-0.8829324	0.7653061
0.7893885	1	3599	"tags=27%, list=26%, signal=36%"
GSE13485_DAY3_VS_DAY7_YF17D_VACCINE_PBMC_UP
GSE13485_DAY3_VS_DAY7_YF17D_VACCINE_PBMC_UP
106	-0.22715975	-0.88245255	0.7327273
0.7902178	1	3025	"tags=25%, list=21%, signal=32%"
GSE20715_WT_VS_TLR4_KO_48H_OZONE_LUNG_UP
GSE20715_WT_VS_TLR4_KO_48H_OZONE_LUNG_UP
158	-0.21117684	-0.88188875	0.78019804
0.79131097	1	1990	"tags=17%, list=14%, signal=20%"
GSE13547_WT_VS_ZFX_KO_BCELL_ANTI_IGM_STIM_12H_UP
GSE13547_WT_VS_ZFX_KO_BCELL_ANTI_IGM_STIM_12H_UP
113	-0.22258025	-0.8817759	0.7208413
0.79120576	1	5811	"tags=43%, list=41%, signal=73%"
GSE17721_CTRL_VS_CPG_12H_BMDC_DN	GSE17721_CTRL_VS_CPG_12H_BMDC_DN
	158	-0.20962077	-0.87912726	0.7758945
0.7976183	1	1022	"tags=10%, list=7%, signal=11%"

GSE17721_CTRL_VS_GARDIQUIMOD_2H_BMDC_DN
GSE17721_CTRL_VS_GARDIQUIMOD_2H_BMDC_DN
156 -0.21222106 -0.8787478 0.7590822
0.79822975 1 3321 "tags=29%, list=24%, signal=38%"
GSE2770_IL12_VS_IL4_TREATED_ACT_CD4_TCELL_2H_DN
GSE2770_IL12_VS_IL4_TREATED_ACT_CD4_TCELL_2H_DN
124 -0.21777 -0.8780447 0.7803468 0.7996157
1 1542 "tags=13%, list=11%, signal=14%"
GSE24972_WT_VS_IRF8_KO_MARGINAL_ZONE_SPLEEN_BCELL_DN
GSE24972_WT_VS_IRF8_KO_MARGINAL_ZONE_SPLEEN_BCELL_DN
127 -0.21595626 -0.87798905 0.77559054
0.7993722 1 5368 "tags=41%, list=38%, signal=65%"
GSE22935_WT_VS_MYD88_KO_MACROPHAGE_12H_MBOVIS_BCG_STIM_UP
GSE22935_WT_VS_MYD88_KO_MACROPHAGE_12H_MBOVIS_BCG_STIM_UP
147 -0.21378715 -0.8762883 0.734252 0.8032285
1 1680 "tags=14%, list=12%, signal=15%"
GSE9946_IMMATURE_VS_PROSTAGLANDINE2_TREATED_MATURE_DC_UP
GSE9946_IMMATURE_VS_PROSTAGLANDINE2_TREATED_MATURE_DC_UP
107 -0.22394171 -0.87549305 0.7348485
0.80486727 1 2842 "tags=21%, list=20%, signal=27%"
GSE33162_HDAC3_KO_VS_HDAC3_KO_4H_LPS_STIM_MACROPHAGE_DN
GSE33162_HDAC3_KO_VS_HDAC3_KO_4H_LPS_STIM_MACROPHAGE_DN
165 -0.20894434 -0.8741199 0.7556326
0.8079242 1 3558 "tags=27%, list=25%, signal=35%"
GSE3039_ALPHAALPHA_VS_ALPHABETA_CD8_TCELL_DN
GSE3039_ALPHAALPHA_VS_ALPHABETA_CD8_TCELL_DN
153 -0.2091883 -0.873218 0.7918149
0.8097717 1 2174 "tags=18%, list=15%, signal=21%"
GSE41867_NAIVE_VS_DAY15_LCMV_CONE13_EFFECTOR_CD8_TCELL_UP
GSE41867_NAIVE_VS_DAY15_LCMV_CONE13_EFFECTOR_CD8_TCELL_UP
100 -0.22774315 -0.8719187 0.74038464
0.81254625 1 5053 "tags=43%, list=36%, signal=67%"
GSE20198_UNTREATED_VS_IL12_IL18_TREATED_ACT_CD4_TCELL_UP
GSE20198_UNTREATED_VS_IL12_IL18_TREATED_ACT_CD4_TCELL_UP
136 -0.2129868 -0.8710891 0.7765151
0.8141965 1 2584 "tags=21%, list=18%, signal=26%"
GSE21927_HEALTHY_VS_TUMOROUS_BALBC_MOUSE_MONOCYTE_DN
GSE21927_HEALTHY_VS_TUMOROUS_BALBC_MOUSE_MONOCYTE_DN
90 -0.22943242 -0.8694345 0.76208895
0.81785756 1 1223 "tags=8%, list=9%, signal=8%"
GSE29618_PRE_VS_DAY7_POST_TIV_FLU_VACCINE_BCELL_UP
GSE29618_PRE_VS_DAY7_POST_TIV_FLU_VACCINE_BCELL_UP
152 -0.20708042 -0.8664717 0.8152381
0.8245834 1 2827 "tags=19%, list=20%, signal=24%"
GSE3982_MAC_VS_NKCELL_DN GSE3982_MAC_VS_NKCELL_DN
134 -0.2101469 -0.86632794 0.7973231
0.8245099 1 1873 "tags=17%, list=13%, signal=20%"
GSE11961_FOLLICULAR_BCELL_VS_MARGINAL_ZONE_BCELL_DN
GSE11961_FOLLICULAR_BCELL_VS_MARGINAL_ZONE_BCELL_DN
150 -0.21035042 -0.86596775 0.80237156
0.8250047 1 4503 "tags=34%, list=32%, signal=49%"
GSE360_L_MAJOR_VS_T_GONDII_DC_UP GSE360_L_MAJOR_VS_T_GONDII_DC_UP
134 -0.21293506 -0.86539894 0.7679558
0.8259769 1 2133 "tags=14%, list=15%, signal=17%"

GSE18148_CBFB_KO_VS_WT_TREG_DN	GSE18148_CBFB_KO_VS_WT_TREG_DN
	145	-0.20909567	-0.86508393	0.80265653
0.826302	1	5150	"tags=39%, list=36%, signal=61%"
GSE21927_SPLENIC_VS_TUMOR_MONOCYTES_FROM_C26GM_TUMOROUS_MICE_BALBC_UP
GSE21927_SPLENIC_VS_TUMOR_MONOCYTES_FROM_C26GM_TUMOROUS_MICE_BALBC_UP		118	-0.21443643	-0.86350995
0.7906067	0.8296534	1	2402	"tags=20%, list=17%, signal=24%"
GSE21033_3H_VS_12H_POLYIC_STIM_DC_DN
GSE21033_3H_VS_12H_POLYIC_STIM_DC_DN
110	-0.21809532	-0.8633202	0.7973485
0.8296863	1	3598	"tags=27%, list=25%, signal=36%"
GSE21670_TGFB_VS_IL6_TREATED_CD4_TCELL_UP
GSE21670_TGFB_VS_IL6_TREATED_CD4_TCELL_UP
131	-0.21207316	-0.86274445	0.7895717
0.8306899	1	3977	"tags=27%, list=28%, signal=37%"
GSE17974_IL4_AND_ANTI_IL12_VS_UNTREATED_72H_ACT_CD4_TCELL_UP
GSE17974_IL4_AND_ANTI_IL12_VS_UNTREATED_72H_ACT_CD4_TCELL_UP
	109	-0.2189418	-0.86151266	0.7533719
0.83324057	1	3601	"tags=32%, list=26%, signal=43%"
GSE21063_WT_VS_NFATC1_KO_16H_ANTI_IGM_STIM_BCELL_DN
GSE21063_WT_VS_NFATC1_KO_16H_ANTI_IGM_STIM_BCELL_DN
115	-0.2173244	-0.8611209	0.77024484
0.8337481	1	1766	"tags=15%, list=13%, signal=17%"
GSE2770_IL12_AND_TGFB_VS_IL4_TREATED_ACT_CD4_TCELL_2H_DN
GSE2770_IL12_AND_TGFB_VS_IL4_TREATED_ACT_CD4_TCELL_2H_DN
125	-0.21146618	-0.860494	0.8	0.8348169
1	2505	"tags=20%, list=18%, signal=24%"
GSE18791_UNSTIM_VS_NEWCATSLE_VIRUS_DC_1H_DN
GSE18791_UNSTIM_VS_NEWCATSLE_VIRUS_DC_1H_DN
111	-0.21685319	-0.8587831	0.7924528
0.8383397	1	1590	"tags=15%, list=11%, signal=17%"
GSE22886_NEUTROPHIL_VS_MONOCYTE_UP
GSE22886_NEUTROPHIL_VS_MONOCYTE_UP
129	-0.21175924	-0.85856485	0.7918288
0.8384667	1	4555	"tags=36%, list=32%, signal=53%"
GSE28726_NAIVE_CD4_TCELL_VS_NAIVE_VA24NEG_NKTCELL_DN
GSE28726_NAIVE_CD4_TCELL_VS_NAIVE_VA24NEG_NKTCELL_DN
146	-0.21073566	-0.8567862	0.81409	0.8421002
1	3155	"tags=23%, list=22%, signal=29%"
GSE8515_IL1_VS_IL6_4H_STIM_MAC_DN	GSE8515_IL1_VS_IL6_4H_STIM_MAC_DN
	132	-0.20832777	-0.85445195	0.8241966
0.8470784	1	2954	"tags=21%, list=21%, signal=27%"
GSE45365_NK_CELL_VS_CD11B_DC_UP	GSE45365_NK_CELL_VS_CD11B_DC_UP
	136	-0.21063752	-0.8539066	0.822314
0.84790075	1	3578	"tags=23%, list=25%, signal=30%"
GSE17721_CPG_VS_GARDIQUIMOD_2H_BMDC_UP
GSE17721_CPG_VS_GARDIQUIMOD_2H_BMDC_UP
157	-0.20482144	-0.8537648	0.8352941
0.8477952	1	1938	"tags=15%, list=14%, signal=18%"
GSE2128_CTRL_VS_MIMETOPE_NEGATIVE_SELECTION_DP_THYMOCYTE_NOD_DN
GSE2128_CTRL_VS_MIMETOPE_NEGATIVE_SELECTION_DP_THYMOCYTE_NOD_DN
	115	-0.21577698	-0.8533772	0.8125

0.8482239 1 3218 "tags=25%, list=23%, signal=32%"
GSE17974_0H_VS_0.5H_IN_VITRO_ACT_CD4_TCELL_UP
GSE17974_0H_VS_0.5H_IN_VITRO_ACT_CD4_TCELL_UP
110 -0.21466307 -0.8532867 0.77203065
0.84800845 1 1533 "tags=11%, list=11%, signal=12%"
GSE22527_ANTI_CD3_INVIVO_VS_UNTREATED_MOUSE_TREG_UP
GSE22527_ANTI_CD3_INVIVO_VS_UNTREATED_MOUSE_TREG_UP
150 -0.20377257 -0.8515139 0.8431772 0.851531
1 1051 "tags=10%, list=7%, signal=11%"
GSE45365_HEALTHY_VS_MCMV_INFECTION_CD11B_DC_UP
GSE45365_HEALTHY_VS_MCMV_INFECTION_CD11B_DC_UP
113 -0.21304443 -0.84865165 0.82608694
0.85740876 1 3267 "tags=23%, list=23%, signal=30%"
GSE29614_CTRL_VS_DAY3_TIV_FLU_VACCINE_PBMC_UP
GSE29614_CTRL_VS_DAY3_TIV_FLU_VACCINE_PBMC_UP
100 -0.2192383 -0.8434375 0.8064516
0.86805284 1 5174 "tags=41%, list=37%, signal=64%"
GSE9946_LISTERIA_INF_MATURE_VS_PROSTAGLANDINE2_TREATED_MATURE_DC_DN
GSE9946_LISTERIA_INF_MATURE_VS_PROSTAGLANDINE2_TREATED_MATURE_DC_DN
85 -0.22426693 -0.8434107 0.765748
0.86768913 1 3871 "tags=29%, list=27%, signal=40%"
GSE17721_POLYIC_VS_CPG_0.5H_BMDC_UP
GSE17721_POLYIC_VS_CPG_0.5H_BMDC_UP
144 -0.20349865 -0.841997 0.8574007
0.8702774 1 4794 "tags=40%, list=34%, signal=59%"
GSE32423_CTRL_VS_IL7_MEMORY_CD8_TCELL_UP
GSE32423_CTRL_VS_IL7_MEMORY_CD8_TCELL_UP
142 -0.20445642 -0.8401311 0.8407767
0.8737517 1 4326 "tags=33%, list=31%, signal=47%"
GSE29618_LAIV_VS_TIV_FLU_VACCINE_DAY7_MDC_DN
GSE29618_LAIV_VS_TIV_FLU_VACCINE_DAY7_MDC_DN
141 -0.20662323 -0.8386887 0.86050725 0.876237
1 2760 "tags=23%, list=20%, signal=28%"
GSE3203_INFLUENZA_INF_VS_IFNB_TREATED_LN_BCELL_UP
GSE3203_INFLUENZA_INF_VS_IFNB_TREATED_LN_BCELL_UP
152 -0.20123757 -0.8385363 0.8693069
0.8761231 1 2559 "tags=16%, list=18%, signal=20%"
GSE16385_UNTREATED_VS_12H_IL4_TREATED_MACROPHAGE_DN
GSE16385_UNTREATED_VS_12H_IL4_TREATED_MACROPHAGE_DN
135 -0.20593795 -0.83851546 0.8596491
0.8757405 1 2959 "tags=23%, list=21%, signal=29%"
GSE36392_TYPE_2_MYELOID_VS_MAC_IL25_TREATED_LUNG_UP
GSE36392_TYPE_2_MYELOID_VS_MAC_IL25_TREATED_LUNG_UP
149 -0.19987425 -0.8381888 0.87666035
0.8759828 1 3926 "tags=29%, list=28%, signal=40%"
GSE17974_IL4_AND_ANTI_IL12_VS_UNTREATED_48H_ACT_CD4_TCELL_UP
GSE17974_IL4_AND_ANTI_IL12_VS_UNTREATED_48H_ACT_CD4_TCELL_UP
119 -0.2095944 -0.83795345 0.82472324
0.87601805 1 1667 "tags=16%, list=12%, signal=18%"
GSE41176_WT_VS_TAK1_KO_UNSTIM_BCELL_UP
GSE41176_WT_VS_TAK1_KO_UNSTIM_BCELL_UP
134 -0.20633851 -0.83723855 0.85265225
0.87703395 1 1349 "tags=11%, list=10%, signal=12%"
GSE22601_DOUBLE_NEGATIVE_VS_DOUBLE_POSITIVE_THYMOCYTE_UP

GSE22601_DOUBLE_NEGATIVE_VS_DOUBLE_POSITIVE_THYMOCYTE_UP
120 -0.20558639 -0.83563197 0.84139264
0.87984425 1 1516 "tags=13%, list=11%, signal=14%"
GSE21546_UNSTIM_VS_ANTI_CD3_STIM_SAP1A_KO_AND_ELK1_KO_DP_THYMOCYTES_DN
GSE21546_UNSTIM_VS_ANTI_CD3_STIM_SAP1A_KO_AND_ELK1_KO_DP_THYMOCYTES_DN 87 -0.21978338 -0.83500767
0.8089669 0.8806743 1 6060 "tags=52%, list=43%, signal=90%"
GSE7460_CTRL_VS_FOXP3_OVEREXPR_TCONV_UP
GSE7460_CTRL_VS_FOXP3_OVEREXPR_TCONV_UP
136 -0.20633942 -0.8349377 0.8593156
0.8803959 1 655 "tags=6%, list=5%, signal=6%"
GSE34156_NOD2_LIGAND_VS_NOD2_AND_TLR1_TLR2_LIGAND_24H_TREATED_MONOCYTE_UP
GSE34156_NOD2_LIGAND_VS_NOD2_AND_TLR1_TLR2_LIGAND_24H_TREATED_MONOCYTE_UP 121 -0.2094237 -0.8316517
0.8797595 0.88645035 1 1304 "tags=10%, list=9%, signal=11%"
GSE16450_CTRL_VS_IFNA_12H_STIM_MATURE_NEURON_CELL_LINE_UP
GSE16450_CTRL_VS_IFNA_12H_STIM_MATURE_NEURON_CELL_LINE_UP
135 -0.20309998 -0.82971936 0.87202924
0.8897577 1 4347 "tags=32%, list=31%, signal=46%"
GSE6259_DEC205_POS_DC_VS_BCELL_UP GSE6259_DEC205_POS_DC_VS_BCELL_UP
128 -0.20397303 -0.8289255 0.8731618
0.8908646 1 4141 "tags=26%, list=29%, signal=36%"
GSE22033_UNTREATED_VS_ROSIGLITAZONE_TREATED_MEF_DN
GSE22033_UNTREATED_VS_ROSIGLITAZONE_TREATED_MEF_DN
134 -0.20342676 -0.82859194 0.8763441
0.89107263 1 1796 "tags=15%, list=13%, signal=17%"
GSE7459_UNTREATED_VS_IL6_TREATED_ACT_CD4_TCELL_UP
GSE7459_UNTREATED_VS_IL6_TREATED_ACT_CD4_TCELL_UP
108 -0.20708695 -0.82859105 0.8451243
0.89064664 1 4899 "tags=41%, list=35%, signal=62%"
GSE46606_DAY1_VS_DAY3_CD40L_IL2_IL5_STIMULATED_IRF4MID_BCELL_UP
GSE46606_DAY1_VS_DAY3_CD40L_IL2_IL5_STIMULATED_IRF4MID_BCELL_UP
152 -0.19679981 -0.82661635 0.8844697
0.89387625 1 2901 "tags=20%, list=21%, signal=25%"
GSE23308_CTRL_VS_CORTICOSTERONE_TREATED_MACROPHAGE_UP
GSE23308_CTRL_VS_CORTICOSTERONE_TREATED_MACROPHAGE_UP
155 -0.19657715 -0.82066023 0.8857678
0.9041597 1 1138 "tags=9%, list=8%, signal=10%"
GSE13887_HEALTHY_VS_LUPUS_RESTING_CD4_TCELL_DN
GSE13887_HEALTHY_VS_LUPUS_RESTING_CD4_TCELL_DN
85 -0.21401925 -0.81650424 0.8464351
0.9108834 1 2974 "tags=26%, list=21%, signal=33%"
GSE29618_PRE_VS_DAY7_POST_TIV_FLU_VACCINE_MONOCYTE_UP
GSE29618_PRE_VS_DAY7_POST_TIV_FLU_VACCINE_MONOCYTE_UP
127 -0.19850858 -0.8153041 0.9019608
0.91252583 1 3237 "tags=23%, list=23%, signal=29%"
GSE34156_UNTREATED_VS_24H_NOD2_AND_TLR1_TLR2_LIGAND_TREATED_MONOCYTE_DN
GSE34156_UNTREATED_VS_24H_NOD2_AND_TLR1_TLR2_LIGAND_TREATED_MONOCYTE_DN 96 -0.20850329 -0.81525433

0.86460346	0.91217977	1	2934	"tags=22%, list=21%, signal=27%"
GSE40274_EOS_VS_FOXP3_AND_EOS_TRANSDUCED_ACTIVATED_CD4_TCELL_DN
GSE40274_EOS_VS_FOXP3_AND_EOS_TRANSDUCED_ACTIVATED_CD4_TCELL_DN
	93	-0.20919271	-0.81269 0.8613281
0.9159439	1	3092	"tags=25%, list=22%, signal=31%"
GSE11961_MEMORY_BCELL_DAY7_VS_MEMORY_BCELL_DAY40_UP
GSE11961_MEMORY_BCELL_DAY7_VS_MEMORY_BCELL_DAY40_UP
142	-0.19864307	-0.8087066	0.8878676
0.92190015	1	2091	"tags=18%, list=15%, signal=20%"
GSE15767_MED_VS_SCS_MAC_LN_UP	GSE15767_MED_VS_SCS_MAC_LN_UP
	155	-0.19437589	-0.8078987	0.91783565
0.92274034	1	3952	"tags=29%, list=28%, signal=40%"
GSE13485_PRE_VS_POST_YF17D_VACCINATION_PBMC_UP
GSE13485_PRE_VS_POST_YF17D_VACCINATION_PBMC_UP
119	-0.20120087	-0.80668545	0.9055118
0.92424685	1	2184	"tags=17%, list=15%, signal=20%"
GSE45365_BCELL_VS_CD8_TCELL_DN	GSE45365_BCELL_VS_CD8_TCELL_DN
	107	-0.20431525	-0.80628914	0.86278194
0.92443454	1	4648	"tags=37%, list=33%, signal=55%"
GSE5589_LPS_AND_IL10_VS_LPS_AND_IL6_STIM_IL10_KO_MACROPHAGE_45MIN_DN

GSE5589_LPS_AND_IL10_VS_LPS_AND_IL6_STIM_IL10_KO_MACROPHAGE_45MIN_DN
		148	-0.1961528	-0.80592954
0.9138577	0.9245499	1	3979	"tags=30%, list=28%, signal=41%"
GSE18281_PERIMEDULLARY_CORTICAL_REGION_VS_WHOLE_CORTEX_THYMUS_DN
GSE18281_PERIMEDULLARY_CORTICAL_REGION_VS_WHOLE_CORTEX_THYMUS_DN
	143	-0.19606467	-0.80549496	0.90909094
0.92478335	1	2936	"tags=22%, list=21%, signal=28%"
GSE9006_TYPE_1_DIABETES_AT_DX_VS_1MONTH_POST_DX_PBMC_DN
GSE9006_TYPE_1_DIABETES_AT_DX_VS_1MONTH_POST_DX_PBMC_DN
135	-0.19456692	-0.79890585	0.9298246
0.93405104	1	3693	"tags=29%, list=26%, signal=39%"
GSE7218_UNSTIM_VS_ANTIGEN_STIM_THROUGH_IGM_BCELL_DN
GSE7218_UNSTIM_VS_ANTIGEN_STIM_THROUGH_IGM_BCELL_DN
98	-0.20341612	-0.7988638	0.8740458
0.93366814	1	2409	"tags=19%, list=17%, signal=23%"
GSE4142_PLASMA_CELL_VS_GC_BCELL_UP
GSE4142_PLASMA_CELL_VS_GC_BCELL_UP
134	-0.19492729	-0.7933548	0.922	0.9407581
1	3697	"tags=30%, list=26%, signal=40%"
GSE33374_CD8_ALPHAALPHA_VS_ALPHABETA_CD161_HIGH_TCELL_UP
GSE33374_CD8_ALPHAALPHA_VS_ALPHABETA_CD161_HIGH_TCELL_UP
166	-0.18706845	-0.7888824	0.95194083	0.94614
1	3139	"tags=21%, list=22%, signal=27%"
GSE16385_ROSIGLITAZONE_VS_UNTREATED_IFNG_TNF_STIM_MACROPHAGE_UP
GSE16385_ROSIGLITAZONE_VS_UNTREATED_IFNG_TNF_STIM_MACROPHAGE_UP
	147	-0.19023392	-0.78824604	0.9161905
0.9464893	1	3296	"tags=25%, list=23%, signal=32%"
GSE7459_UNTREATED_VS_IL6_TREATED_ACT_CD4_TCELL_DN
GSE7459_UNTREATED_VS_IL6_TREATED_ACT_CD4_TCELL_DN
112	-0.19651039	-0.786832	0.9082031
0.9478095	1	1961	"tags=19%, list=14%, signal=22%"

GSE12505_WT_VS_E2_2_HET_PDC_UP	GSE12505_WT_VS_E2_2_HET_PDC_UP
	102	-0.20162448	-0.78563887	0.9042357
0.9488328	1	4347	"tags=30%, list=31%, signal=44%"
GSE18281_PERIMEDULLARY_CORTICAL_REGION_VS_WHOLE_MEDULLA_THYMUS_UP
GSE18281_PERIMEDULLARY_CORTICAL_REGION_VS_WHOLE_MEDULLA_THYMUS_UP
	146	-0.1905889	-0.7838161	0.9510358
0.95061165	1	1569	"tags=11%, list=11%, signal=12%"
GSE46606_UNSTIM_VS_CD40L_IL2_IL5_3DAY_STIMULATED_IRF4_KO_BCELL_UP
GSE46606_UNSTIM_VS_CD40L_IL2_IL5_3DAY_STIMULATED_IRF4_KO_BCELL_UP
	94	-0.20253836	-0.7829601	0.8820225
0.95115376	1	4684	"tags=32%, list=33%, signal=47%"
GSE3203_HEALTHY_VS_INFLUENZA_INFECTED_LN_BCELL_DN
GSE3203_HEALTHY_VS_INFLUENZA_INFECTED_LN_BCELL_DN
136	-0.19077645	-0.78039247	0.9203036
0.95369095	1	4065	"tags=29%, list=29%, signal=40%"
GSE12003_4D_VS_8D_CULTURE_MIR223_KO_BM_PROGENITOR_UP
GSE12003_4D_VS_8D_CULTURE_MIR223_KO_BM_PROGENITOR_UP
115	-0.1933463	-0.7786941	0.9175824
0.95515347	1	2702	"tags=20%, list=19%, signal=25%"
GSE37301_RAG2_KO_VS_RAG2_AND_ETS1_KO_NK_CELL_DN
GSE37301_RAG2_KO_VS_RAG2_AND_ETS1_KO_NK_CELL_DN
152	-0.18845354	-0.7782241	0.9539347
0.9552481	1	2243	"tags=18%, list=16%, signal=21%"
GSE45365_NK_CELL_VS_CD8A_DC_DN	GSE45365_NK_CELL_VS_CD8A_DC_DN
	142	-0.19102463	-0.77626383	0.9365942
0.95694035	1	4792	"tags=35%, list=34%, signal=53%"
GSE27092_WT_VS_HDAC7_PHOSPHO_DEFICIENT_CD8_TCELL_UP
GSE27092_WT_VS_HDAC7_PHOSPHO_DEFICIENT_CD8_TCELL_UP
169	-0.18606974	-0.7759046	0.96374047
0.9568615	1	2950	"tags=20%, list=21%, signal=25%"
GSE23502_BM_VS_COLON_TUMOR_HDC_KO_MYELOID_DERIVED_SUPPRESSOR_CELL_UP

GSE23502_BM_VS_COLON_TUMOR_HDC_KO_MYELOID_DERIVED_SUPPRESSOR_CELL_UP
		132	-0.19129671	-0.7747708
0.92460316	0.95761675	1	4005	"tags=30%,
list=28%, signal=41%"
GSE13522_CTRL_VS_T_CRUZI_BRAZIL_STRAIN_INF_SKIN_UP
GSE13522_CTRL_VS_T_CRUZI_BRAZIL_STRAIN_INF_SKIN_UP
106	-0.19869724	-0.7746795	0.91369605
0.95725936	1	2517	"tags=18%, list=18%, signal=22%"
GSE41176_UNSTIM_VS_ANTI_IGM_STIM_TAK1_KO_BCELL_1H_DN
GSE41176_UNSTIM_VS_ANTI_IGM_STIM_TAK1_KO_BCELL_1H_DN
140	-0.18405415	-0.7593534	0.9530075
0.9713231	1	4553	"tags=34%, list=32%, signal=50%"
GSE35543_IN_VIVO_NTREG_VS_IN_VITRO_ITREG_UP
GSE35543_IN_VIVO_NTREG_VS_IN_VITRO_ITREG_UP
140	-0.18360125	-0.7577559	0.95849055
0.97224206	1	4966	"tags=38%, list=35%, signal=58%"
GSE22025_UNTREATED_VS_TGFB1_AND_PROGESTERONE_TREATED_CD4_TCELL_UP
GSE22025_UNTREATED_VS_TGFB1_AND_PROGESTERONE_TREATED_CD4_TCELL_UP
	159	-0.17952253	-0.75465494	0.9587426
0.97424793	1	938	"tags=8%, list=7%, signal=8%"
GSE4142_NAIVE_VS_MEMORY_BCELL_DN	GSE4142_NAIVE_VS_MEMORY_BCELL_DN
	139	-0.1852499	-0.75445044	0.9559387

0.97395647	1	3004	"tags=19%, list=21%, signal=24%"
GSE13887_RESTING_VS_ACT_CD4_TCELL_UP
GSE13887_RESTING_VS_ACT_CD4_TCELL_UP
69	-0.20599985	-0.7488752	0.9166667
0.97765076	1	5525	"tags=41%, list=39%, signal=66%"
GSE46606_UNSTIM_VS_CD40L_IL2_IL5_1DAY_STIMULATED_IRF4HIGH_SORTED_BCELL_UP
GSE46606_UNSTIM_VS_CD40L_IL2_IL5_1DAY_STIMULATED_IRF4HIGH_SORTED_BCELL_UP	146	-0.17987539	-0.7482301
0.9756098	0.9776409	1	4220	"tags=28%, list=30%, signal=40%"
GSE21380_NON_TFH_VS_GERMINAL_CENTER_TFH_CD4_TCELL_UP
GSE21380_NON_TFH_VS_GERMINAL_CENTER_TFH_CD4_TCELL_UP
148	-0.18136021	-0.74767286	0.9613153
0.97757095	1	3871	"tags=27%, list=27%, signal=37%"
GSE19888_ADENOSINE_A3R_INH_VS_ACT_WITH_INHIBITOR_PRETREATMENT_IN_MAST_CELL_DN
GSE19888_ADENOSINE_A3R_INH_VS_ACT_WITH_INHIBITOR_PRETREATMENT_IN_MAST_CELL_DN	146	-0.18038781	-0.74654067
0.980916	0.9779101	1	1582	"tags=11%, list=11%, signal=12%"
GSE24814_STAT5_KO_VS_WT_PRE_BCELL_UP
GSE24814_STAT5_KO_VS_WT_PRE_BCELL_UP
145	-0.17903565	-0.7441626	0.98839456
0.97905207	1	5541	"tags=43%, list=39%, signal=71%"
GSE15930_STIM_VS_STIM_AND_IL12_72H_CD8_T_CELL_DN
GSE15930_STIM_VS_STIM_AND_IL12_72H_CD8_T_CELL_DN
164	-0.1742915	-0.7379195	0.9868668
0.9823974	1	4580	"tags=32%, list=32%, signal=47%"
GSE10094_LCMV_VS_LISTERIA_IND_EFF_CD4_TCELL_DN
GSE10094_LCMV_VS_LISTERIA_IND_EFF_CD4_TCELL_DN
142	-0.17891344	-0.737574	0.972167	0.9821277
1	1159	"tags=8%, list=8%, signal=8%"
GSE40666_WT_VS_STAT4_KO_CD8_TCELL_UP
GSE40666_WT_VS_STAT4_KO_CD8_TCELL_UP
112	-0.18340245	-0.73295027	0.95619047
0.98428196	1	3213	"tags=21%, list=23%, signal=28%"
GSE5589_WT_VS_IL10_KO_LPS_AND_IL6_STIM_MACROPHAGE_45MIN_UP
GSE5589_WT_VS_IL10_KO_LPS_AND_IL6_STIM_MACROPHAGE_45MIN_UP
139	-0.18118496	-0.73231435	0.9830508
0.9841556	1	4287	"tags=31%, list=30%, signal=44%"
GSE6259_33D1_POS_DC_VS_CD8_TCELL_UP
GSE6259_33D1_POS_DC_VS_CD8_TCELL_UP
102	-0.18604335	-0.73208714	0.957935	0.9838159
1	5382	"tags=47%, list=38%, signal=76%"
GSE28737_WT_VS_BCL6_KO_MARGINAL_ZONE_BCELL_UP
GSE28737_WT_VS_BCL6_KO_MARGINAL_ZONE_BCELL_UP
134	-0.17967814	-0.729679	0.97551787
0.98462456	1	3812	"tags=27%, list=27%, signal=36%"
GSE14413_UNSTIM_VS_IFNB_STIM_RAW264_CELLS_UP
GSE14413_UNSTIM_VS_IFNB_STIM_RAW264_CELLS_UP
93	-0.18374951	-0.7090829	0.97307694
0.9928444	1	3223	"tags=23%, list=23%, signal=29%"
GSE13306_TREG_RA_VS_TCONV_RA_DN	GSE13306_TREG_RA_VS_TCONV_RA_DN

151	-0.17076957	-0.70867383	0.98703706
0.99250424	1	5101	"tags=36%, list=36%, signal=56%"
GSE360_L_DONOVANI_VS_T_GONDII_DC_UP
GSE360_L_DONOVANI_VS_T_GONDII_DC_UP
142	-0.17049739	-0.700875	0.98846155
0.99438924	1	2028	"tags=13%, list=14%, signal=15%"
GSE10147_IL3_AND_HIVP17_VS_IL3_AND_CPG_STIM_PDC_UP
GSE10147_IL3_AND_HIVP17_VS_IL3_AND_CPG_STIM_PDC_UP
119	-0.17258148	-0.6980499	0.9846449
0.9947107	1	5578	"tags=45%, list=40%, signal=73%"
GSE19888_CTRL_VS_A3R_ACTIVATION_MAST_CELL_DN
GSE19888_CTRL_VS_A3R_ACTIVATION_MAST_CELL_DN
130	-0.17205955	-0.69525015	0.9961165
0.9949672	1	6035	"tags=49%, list=43%, signal=85%"
GSE17721_0.5H_VS_4H_LPS_BMDC_DN	GSE17721_0.5H_VS_4H_LPS_BMDC_DN
157	-0.16709991	-0.69454396	0.99614644
0.9946798	1	2377	"tags=15%, list=17%, signal=18%"
GSE13762_CTRL_VS_125_VITAMIND_DAY5_DC_UP
GSE13762_CTRL_VS_125_VITAMIND_DAY5_DC_UP
97	-0.17522098	-0.68107176	0.98526704
0.9970947	1	3570	"tags=22%, list=25%, signal=29%"
GSE15750_WT_VS_TRAF6KO_DAY10_EFF_CD8_TCELL_DN
GSE15750_WT_VS_TRAF6KO_DAY10_EFF_CD8_TCELL_DN
143	-0.16448598	-0.67628247	0.9906015
0.9974378	1	5018	"tags=36%, list=36%, signal=55%"
GSE7568_CTRL_VS_24H_TGFB_TREATED_MACROPHAGES_WITH_IL4_AND_DEXAMETHASONE_DN
GSE7568_CTRL_VS_24H_TGFB_TREATED_MACROPHAGES_WITH_IL4_AND_DEXAMETHASONE_DN	144	-0.15906925	-0.65975505
0.99809885	0.99911815	1	368	"tags=3%, list=3%, signal=4%"
GSE2585_AIRE_KO_VS_WT_CD80_LOW_MTEC_UP
GSE2585_AIRE_KO_VS_WT_CD80_LOW_MTEC_UP
129	-0.15680331	-0.6382708	1	1	1
2875	"tags=16%, list=20%, signal=19%"
GSE41176_UNSTIM_VS_ANTI_IGM_STIM_TAK1_KO_BCELL_3H_UP
GSE41176_UNSTIM_VS_ANTI_IGM_STIM_TAK1_KO_BCELL_3H_UP
130	-0.14871539	-0.60909206	1	1	1
3974	"tags=25%, list=28%, signal=35%"
GSE27786_BCELL_VS_ERYTHROBLAST_DN GSE27786_BCELL_VS_ERYTHROBLAST_DN
151	-0.14667778	-0.6060053	1	1
1	4005	"tags=25%, list=28%, signal=34%"
GSE37605_TREG_VS_TCONV_C57BL6_FOXP3_IRES_GFP_UP
GSE37605_TREG_VS_TCONV_C57BL6_FOXP3_IRES_GFP_UP
96	-0.14757884	-0.5692266	0.9980916	1
1	6046	"tags=45%, list=43%, signal=78%"
GSE18791_UNSTIM_VS_NEWCATSLE_VIRUS_DC_2H_UP
GSE18791_UNSTIM_VS_NEWCATSLE_VIRUS_DC_2H_UP
107	-0.12452007	-0.48570925	1	0.9999984
1	5168	"tags=32%, list=37%, signal=50%"
