# Supplementary material for: Inflation vs. Exhaustion of Antiviral CD8+ T-Cell Populations in Persistent Infections: Two Sides of the Same Coin?
Source: Front Immunol. 2019 Mar 6;10:197. doi: 10.3389/fimmu.2019.00197 (PMC6414785; doi:10.3389/fimmu.2019.00197)
Supplement: Table S2 — (A–C) TOM matrices of blue and turquoise modules. Table matrices with topological overlay measures (TOM) of blue modules detected including all samples (A) or excluding possible outliers (B); TOM matrix of turquoise module including all inflating samples (C). [file Table_2.pdf]

Table S2A

NAMES 1110067D22RIK 1700025G04RIK 1700034H14RIK
2010012O05RIK 2010305A19RIK 2310004I24RIK 2310044G17RIK
2610524H06RIK 2610528E23RIK 2810408M09RIK 2900062L11RIK
3110057O12RIK 4632434I11RIK 4930503L19RIK 5730469M10RIK
6330403K07RIK 6330442E10RIK ABHD5 ACPL2 ACSBG1 ACSS2
ACTN1 ADA ADAM9 ADD1 ADK ADSL AHNAK AI451617
AI747699 AIM1 AKR1E1 AKT1S1 AMIGO2 ANXA1 ANXA2 ANXA4
ANXA5 AP1S2 APAF1 APEX1 APLP2 ARHGAP26 ARL8A ART4
AS3MT ASAH1 ASH1L ASS1 ATG5 ATN1 ATP1B1 ATP2A2
ATP2A3 ATP2B1 ATP6V1B2 ATP6V1E1 ATPAF2 B3GALNT1 B3GNT5 BAZ1A
BBS9 BC016495 BC057170 BCAP31 BCL2L11 BEX2 BOLA2 BSCL2
BSDC1 BTBD10 BTG3 BTLA BZW2 C1QBP CAMK2N1 CAMP
CAPG CAPN2 CAR5B CARHSP1 CASP1 CASP4 CBLB CCDC109B
CCDC125 CCDC28B CCDC86 CCDC88C CCL2 CCL3 CCL4 CCL5
CCL6 CCL9 CCR5 CCR7 CCR9 CCRL2 CCT3 CD244
CD3EAP CD44 CD48 CD74 CD80 CD82 CD9 CDC37
CDC42EP3 CEP78 CERCAM CHCHD10 CHD4 CHPT1 CHST12 CHSY1
CLDN10 CLDND2 CLEC2I CLEC4D CLEC4N CLGN CLYBL CMAH
CNR2 COBLL1 COMMD6 COPS4 COQ10B CORO2A CPEB2 CREM
CRIP2 CRMP1 CRYBG3 CTNNBIP1 CTPS CTSD CTSE CX3CR1
CXCL2 CXCR6 CYB5 CYFIP1 CYP17A1 CYSLTR2 D10WSU52E
D16ERTD472E D1ERTD622E DAPK2 DAPL1 DBNDD2 DEK
DENND5A DGAT1 DGKA DHRS7 DKKL1 DNAJB13 DPH5 DPP4
DPYSL2 DTD1 DUS2L DUSP1 DUSP16 DUSP19 DUSP6 E2F2
EBPL ECE1 ECE2 EFHA1 EGR2 EIF2S2 EIF4E3 ELOVL1
EMP1 EMP3 ENDOD1 ENPP1 ENTPD1 ENTPD5 EOMES ERMN
ERRFI1 ESM1 ETAA1 ETS2 EVL EXOSC2 EXT1 F10
F2RL1 F2RL3 FAM134B FASL FCGR2B FCHSD2 FDPS FGFR1OP
FGL2 FOXP1 FUCA2 FYN FZD5 G0S2 GAB3
GABARAPL1 GABARAPL2 GALK1 GALNT3 GARNL4 GART
GAS5 GBA GBP4 GCNT1 GEM GGT1 GIMAP7 GM885
GMDS GNA15 GNPTAB GOLGA7 GOLIM4 GOLM1 GPR141 GPX1
GPX8 GRK5 GRWD1 GSTO1 GSTT2 GZMK H2-AA H2-AB1
H2-EB1 H2-Q10 HACE1 HBA-A1 HBB-B1 HBS1L HDDC2 HDGFRP3
HERPUD2 HIST1H1C HIST1H2AC HIST1H2BG HIST1H4C HIST1H4D
HIST1H4M HIST2H2BE HIST3H2A HIST4H4 HPCAL1 HPSE HSD17B12
HSPA1A HVCN1 ICA1 IDE IDH1 IER3 IFI204 IFNG
IFNGR1 IFT57 IFT80 IIGP1 IL10RA IL10RB IL15 IL18
IL18R1 IL18RAP IL1R2 IL2RB IL6ST ILDR1 IMPDH2 INSL6
IRAK2 ISG20 ITGB2 JAZF1 KCNJ8 KCNK5 KDELC1 KIF5C
KLF7 KLHDC10 KLRA10 KLRB1C KLRB1F KLRD1 KLRE1 KLRG1
KLRK1 LAIR1 LAT2 LATS2 LCN2 LCN4 LEF1 LGALS1
LGALS3 LMNA LOC547323 LPIN1 LRPPRC LRRC40 LRRK1
LTB LXN LY6E LY96 LYPD6B LYSMD2 LYZ2 MAN2A1
MAN2A2 MAP2K6 MAPKAPK3 MAPRE2 METTL1 MIPEP MKI67IP MLF1
MOSPD1 MPND MRPL23 MRPL48 MRPS27 MRPS28 MRRF MS4A4C
MS4A6C MS4A6D MSC MTHFD1 MX1 MXI1 MYADM MYB
MYC MYL4 MYO1F MYO1G MYO5A N4BP2 N6AMT1 NAIP2
NAIP5 NCALD NDFIP2 NECAP2 NEDD4L NFYB NGP NME4
NOD1 NQO2 NR4A1 NR4A2 NRN1 NRP1 NSG2 NSMAF
NUP50 OASL2 OCIAD2 OSBPL3 PACSIN1 PARVG PCYT1A PDE4B
PDE4D PDK1 PDLIM1 PECAM1 PELI1 PHF11 PHF21A PHPT1
PHTF2 PLEK PLEKHB2 PLEKHF1 PLSCR1 PLXDC1 PMAIP1 PMS2

PNO1 POLDIP2 POLK PPP1R9B PPP3CA PPT1 PQLC3 PRDM1
PRDX4 PRKCZ PRKX PRPS2 PRR13 PRUNE PSAP PTGR1
PTGS2 PTPLAD1 PTPN11 PTPN6 PUS7L PVR QPRT RAB18
RABGAP1L RAI12 RAMP1 RAP2A RAPGEF6 RBM38 RCN3 REEP5
REXO2 RFC2 RGS1 RGS10 RGS16 RHOQ ROM1 RORA
RPL12 RRAS2 RREB1 RRN3 RSL1 RTP4 RUVBL2 RYK
S100A4 S100A8 S100A9 SAA3 SAMD3 SAMSN1 SAP30BP SATB1
SCMH1 SDCBP2 SEC24D SELL SEPHS1 SEPP1 SERPINA3F
SERPINB1B SERPINB6A SERPINI1 SESN1 SF3B3 SFMBT1
SGCB SHMT2 SIDT1 SIT1 SLAMF6 SLAMF7 SLC11A2 SLC15A2
SLC20A1 SLC25A24 SLC27A4 SLC2A3 SLC30A4 SLC6A19 SLC9A7 SLC9A9
SLFN1 SMAD3 SMARCC1 SMPD1 SMPDL3B SND1 SNX10 SNX18
SOCS3 SPRED1 SRM SSR4 ST3GAL4 ST3GAL6 ST7L ST8SIA4
STARD10 STARD3NL STARD7 STT3B SUHW4 SUOX SURF4 SWAP70
SYAP1 SYT11 SYTL2 SYTL3 TAF9B TANK TBL1X TBX21
TCF7 TCTE3 TEX2 TEX9 TFRC TGTP THADA TIMM10
TLE1 TLR1 TLR2 TLR7 TM6SF1 TMEM108 TMEM111 TMEM184C
TMEM2 TMEM50B TMEM51 TMEM65 TNFAIP3 TNFRSF18 TNFRSF26 TNFSF10
TNFSF14 TNFSF8 TNFSF9 TPCN1 TPPP3 TRAF1 TRAPPC2 TRDMT1
TRIB2 TRIM17 TRIM36 TRIM56 TRMT1 TRP53 TRPC4AP TRPM7
TRPS1 TSEN15 TSPAN13 TSPAN3 TSPAN31 TSR1 TUBB3 TUSC4
TXNDC5 UAP1 UAP1L1 UBA6 UBASH3B UNC84A URB2 USP18
USP28 UTP15 UVRAG VAMP8 VAV3 VIM VPREB1 VPS54
WBP5 WDR74 XKRX ZBTB2 ZEB2 ZFP238 ZFP281 ZFP455
ZFP52 ZFP715 ZHX2 ZMYM2
1110067D22RIK 1 0.488957704 0.362983089
0.312065077 0.346955893 0.281260226 0.164785528
0.188802012 0.068871962 0.001905681 0.012292253
0.368714792 0.135606394 0.196748461 0.252849273
0.346196641 0.112802147 0.385510734 0.208633929
0.344633654 0.071964497 0.315039116 0.083206089
0.28713807 0.08346722 0.053910156 0.212353723
0.390063076 0.065509251 0.230573345 0.314530303
0.128509163 0.393689306 0.499797419 0.323804815
0.49327416 0.352694434 0.025949797 0.201491128
0.412959209 0.336503871 0.334233573 0.256837543
0.032967514 0.469603483 0.187173972 0.361061177
0.210471796 0.021916391 0.19778617 0.436462727
0.434699706 0.309685763 0.2062022 0.121311176
0.118992607 0.188534461 0.188918742 0.266032055
0.179110128 0.125177275 0.450197757 0.064392507
0.130837307 0.253587224 0.167366227 0.227382586
0.306731805 0.326664391 0.026538455 0.357038076
0.001014614 0.336582303 0.355810789 0.241131696
0.041519583 0.011087861 0.188891003 0.25456243
0.419021779 0.475116707 0.431809491 0.445797838
0.142719708 0.297869238 0.344453369 0.135080285
0.412897505 0.125372973 0.108470552 0.34043023
0.44222681 0.48708056 0.178414779 0.251179662
0.327084072 0.454471613 0.3216296 0.324286475
0.23741214 0.072223535 0.405955904 0.46890506
0.379209626 0.27848022 0.134717607 0.345027722
0.063831668 0.318618135 0.279683169 0.172194624
0.428999514 0.015140912 0.196457912 0.267709695

0.269646717	0.379095395	0.484958306	0.156284269
0.373173433	0.018759806	0.099457533	0.04771609
0.265199435	0.147869163	0.463076906	0.387083932
0.211920103	0.301305943	0.237539142	0.003447291
0.028110169	0.23918091	0.385100637	0.381611135
0.369237892	0.398666924	0.159515259	0.329354289
0.333557189	0.427600451	0.038315719	0.096715337
0.330279166	0.237328747	0.210428244	0.345807139
0.239692835	0.117570211	0.115102471	0.476726691
0.411574929	0.073686541	0.347571979	0.343459714
0.220582514	0.297114397	0.107109103	0.212007712
0.04039093	0.462662277	0.033708082	0.332825498
0.187045067	0.128425796	0.026120333	0.230549514
0.205562419	0.353031988	0.466868652	0.114900594
0.247599679	0.3218867	0.294781332	0.289506414
0.003946873	0.059828265	0.273924057	0.458501588
0.222219643	0.482614218	0.279636824	0.299076199
0.249344534	0.387844443	0.007986195	0.474056356
0.494197426	0.268717089	0.282694162	0.381322483
0.285390108	0.374286537	0.056729764	0.345733865
0.316804743	0.089507333	0.460178823	0.130887693
0.453178754	0.033721615	0.263107817	0.379521733
0.324178902	0.009364512	0.171712889	0.308745772
0.036498885	0.077501828	0.371348213	0.320401386
0.28038409	0.384921338	0.434274392	0.265615874
0.332557039	0.230518816	0.018784849	0.299993187
0.39594846	0.300351792	0.416875103	0.206755134
0.299382957	0.437232889	0.37427512	0.14194643
0.035680602	0.267073113	0.016285684	0.260457949
0.390759001	0.075228162	0.309688671	0.208800367
0.069115939	0.479461869	0.177271681	0.244832957
0.130246323	0.275297602	0.243898897	0.139039883
0.12584815	0.148276142	0.281162426	0.290884264
0.029465729	0.075537171	0.033161014	0.266182136
0.076167243	0.219337391	0.215174954	0.326136735
0.261745717	0.073745951	0.284378297	0.287553008
0.108820485	0.01606097	0.474082639	0.353971459
0.280712766	0.139307051	0.048692426	0.231410214
0.399565145	0.219410892	0.252452199	0.399044869
0.041075483	0.451123998	0.162217892	0.101463119
0.277877747	0.202463071	0.409855154	0.043875518
0.356617241	0.430643812	0.318478819	0.143505945
0.360539091	0.339530905	0.025283134	0.35450398
0.336531927	0.028474273	0.490665133	0.122030145
0.024850305	0.361905724	0.31379928	0.074680964
0.078663922	0.219089152	0.04035813	0.249408455
0.471181973	0.319928967	0.323195076	0.239311612
0.029475313	0.004252509	0.222303384	0.376209355
0.489892443	0.476730561	0.254782737	0.260317841
0.2121861	0.244734587	0.141710989	0.459521336
0.296853099	0.508718448	0.009015989	0.358377943
0.483055485	0.240565736	0.063690267	0.234792281
0.339799391	0.042143943	0.243473873	0.46378464
0.400178784	0.264384399	0.256322846	0.384055182

0.012792785	0.038377691	0.329931436	0.190546835
0.281912908	0.192306217	0.063653902	0.305189616
0.028955516	0.408901357	0.407872878	0.046050213
0.220445588	0.304721489	0.334973268	0.035685136
0.405904329	0.491735745	0.494945702	0.185958345
0.37347214	0.438310513	0.301396513	0.095161901
0.020444719	0.494141843	0.346367155	0.014297421
0.423921044	0.192881338	0.005039617	0.468520442
0.173602017	0.444128667	0.003324549	0.144876581
0.062342607	0.413674118	0.449823259	0.326170759
0.161919903	0.35446669	0.016815813	0.500961498
0.447672606	0.01546396	0.148598995	0.353578234
0.323289804	0.507925971	0.256985723	0.384000203
0.320902604	0.46537115	0.173144653	0.227598023
0.367650451	0.467172286	0.347989823	0.446094079
0.395686891	0.360149067	0.2272493	0.011374812
0.120909847	0.184752578	0.190601012	0.051261391
0.28034508	0.111958042	0.46870552	0.421562819
0.432997047	0.006635153	0.133309974	0.366787285
0.399956686	0.439372555	0.045016799	0.009211745
0.013507237	0.012365018	0.311479271	0.246485602
0.454671415	0.145316706	0.004817473	0.043423753
0.24574366	0.319892979	0.464379256	0.321211962
0.389560173	0.087396742	0.166712409	0.346155657
0.057025491	0.29892488	0.431568162	0.439534873
0.063262576	0.249273731	0.381539391	0.43860143
0.070826446	0.287508405	0.368905609	0.216009738
0.031947957	0.081767709	0.060645797	0.380048676
0.475952445	0.046537502	0.020488202	0.125901963
0.078827564	0.43681998	0.122983956	0.473191617
0.378761939	0.370701869	0.086075421	0.457888326
0.029404139	0.456057714	0.276837002	0.04289528
0.329186371	0.019018454	0.460230952	0.087753788
0.270079552	0.358439099	0.173332457	0.023306729
0.194156739	0.052466196	0.415631152	0.407950582
0.053508679	0.234823183	0.295293668	0.378868227
0.234648312	0.318275751	0.47744524	0.289128412
0.065979863	0.192547696	0.345730461	0.342358237
0.218609705	0.447827596	0.202279206	0.502079353
0.352478954	0.459048265	0.440066525	0.275810542
0.2052446	0.339972293	0.25993478	0.011483441
0.315924012	0.381337233	0.356576019	0.110217048
0.191720288	0.331309329	0.071766413	0.237685728
0.285460046	0.233318418	0.110460848	0.389654348
0.363309218	0.339405283	0.059554772	0.05325617
0.513670179	0.43384203	0.127414464	0.320320932
0.186136673	0.269599502	0.389039426	0.377723434
0.342952801	0.37664717	0.363010594	0.271919658
0.389886601	0.229509266	0.495504162	0.101775948
0.078223378	0.368727552	0.060396329	0.100514623
0.120976241	0.03154098	0.248601669	0.363643826
0.308451166	0.294226205	0.297597127	0.085452436
0.308086972	0.387240779	0.313783811	0.147889326
0.054564051	0.457299742	0.009700417	0.062529991

0.321258061	0.360295272	0.281715576	0.229270542
0.247373071	0.173596768	0.246351453	0.244358215
0.194213983	0.343941669	0.361094082	0.037460058
0.017962147	0.452613713	0.344337144	0.038888834
0.109364562	0.346932353	0.348591207	0.165197465
0.030077984	0.441270066	0.226771729	0.357293194
0.295234705	0.077929004	0.361948268	0.113044343
0.087600943	0.440473477	0.353395394	0.484450142
0.36630393	0.320295222	0.447154077	0.375514903
0.178413655	0.366835285	0.02436364	0.407198712
0.29151007			
1700025G04RIK	0.488957704	1 0.453833524	
0.493032105	0.490469994	0.464089917	0.259871069
0.233009614	0.106012306	0.007031388	0.029799455
0.499778158	0.251149922	0.334635282	0.407635985
0.478861242	0.15370001	0.402450498	0.284164827
0.478476854	0.122623092	0.459773202	0.112207106
0.323646563	0.119618098	0.082566286	0.310002995
0.507240431	0.094238633	0.420904714	0.464175968
0.109205969	0.509212901	0.526895921	0.503040263
0.521054646	0.470277452	0.012663121	0.376214216
0.490216495	0.512435961	0.479630269	0.388937149
0.052709353	0.501063231	0.298638898	0.451708699
0.284619101	0.036884728	0.311493423	0.461623015
0.491543633	0.518042203	0.283039996	0.201189066
0.259371445	0.29838846	0.318935422	0.406792943
0.274787934	0.214156056	0.492364837	0.115674039
0.189406286	0.304666086	0.166651848	0.318016199
0.395880609	0.468292546	0.021699732	0.466831624
0.00405253	0.452219378	0.422529435	0.365687661
0.047369624	0.025947053	0.195996929	0.410590724
0.518136419	0.477478907	0.528717285	0.526837607
0.208825023	0.32820923	0.446091995	0.204339288
0.510094267	0.224085684	0.128032823	0.47970244
0.529925342	0.520406382	0.231973024	0.293624727
0.511308707	0.508427842	0.499460216	0.501554157
0.410726938	0.054240164	0.493457963	0.529741625
0.474542928	0.357078871	0.173586218	0.489386727
0.067397988	0.429602903	0.415550317	0.177289631
0.520264783	0.023571383	0.237409451	0.435776595
0.242077944	0.513945474	0.511186162	0.227914523
0.461177417	0.022968655	0.160925483	0.036060903
0.387046062	0.160201341	0.504771931	0.525801527
0.290399258	0.445013696	0.322136094	0.001837435
0.029169345	0.201227686	0.511029278	0.421783596
0.501084084	0.44759767	0.281695749	0.477019203
0.512089245	0.528022088	0.082351034	0.117730302
0.497243222	0.201949377	0.27752432	0.521947502
0.357226185	0.141809298	0.151494479	0.529537178
0.470814808	0.102023716	0.400088153	0.473985344
0.41335997	0.403086989	0.127109904	0.333238996
0.064486187	0.487980553	0.029715273	0.398062018
0.33019116	0.167334242	0.012091014	0.325478272
0.356087829	0.516310726	0.488622668	0.122827008

0.382099831	0.469096158	0.398889349	0.514017851
0.014639639	0.052662037	0.352662498	0.529818468
0.372447756	0.520724709	0.348561461	0.342635185
0.321636345	0.513080216	0.003434735	0.50920969
0.522427325	0.376004837	0.472033586	0.474492796
0.443767584	0.520994639	0.050927368	0.478807566
0.438009407	0.0912113	0.529682758	0.185649523
0.490360542	0.04883361	0.396094695	0.503575716
0.507065019	0.010188635	0.295119523	0.464906967
0.027908876	0.132083607	0.498540391	0.480457703
0.457199368	0.507762633	0.522612341	0.424073466
0.497273096	0.280215024	0.013266005	0.357086098
0.505497351	0.453648717	0.482212033	0.293369096
0.41836834	0.511395202	0.497531871	0.267585879
0.053837856	0.384507813	0.026809542	0.391574584
0.470921796	0.152972391	0.468429795	0.158747979
0.078488409	0.527628997	0.194380012	0.292711957
0.139462223	0.48837042	0.298926513	0.303785278
0.262909073	0.193547449	0.414755873	0.45003621
0.05065862	0.111035791	0.013591984	0.449516585
0.1776603	0.419743498	0.422295723	0.49508281
0.395316488	0.135035857	0.450201075	0.322668571
0.127032155	0.016394757	0.518668958	0.467129935
0.421557348	0.187278891	0.044951167	0.419562308
0.529375599	0.41241728	0.341942681	0.516783635
0.030652104	0.52589732	0.305698033	0.141814739
0.441672436	0.312179461	0.515946496	0.078466481
0.467982967	0.50185546	0.353240043	0.217855063
0.469663968	0.491863403	0.038366754	0.485377996
0.485012136	0.036356149	0.518930537	0.139306582
0.022401028	0.499870041	0.519662633	0.085193922
0.086790243	0.412853061	0.063079365	0.332607282
0.526426963	0.473440792	0.519110304	0.23757706
0.050311373	0.013118694	0.228200514	0.452207511
0.522412896	0.516558228	0.298404339	0.429313286
0.284636796	0.397387255	0.190443503	0.529180163
0.20667614	0.515997587	0.007205882	0.509831602
0.521423074	0.370010721	0.092611884	0.386955948
0.479865942	0.056539317	0.238069462	0.500459948
0.512088443	0.393581771	0.421418902	0.490595093
0.021737781	0.05559524	0.45073384	0.203283409
0.466422787	0.252998502	0.055278074	0.402245031
0.053426512	0.502017657	0.515712132	0.072328141
0.434795112	0.380026178	0.429972108	0.043835771
0.50845355	0.494698768	0.528597826	0.197414052
0.515541046	0.51659032	0.473151704	0.086836152
0.018023595	0.517614771	0.479131295	0.027857065
0.526509466	0.312171572	0.016882065	0.512100337
0.221506401	0.465572897	0.005274608	0.088071934
0.041457334	0.513542895	0.492999844	0.461016053
0.33179597	0.426788022	0.005458611	0.517537419
0.506455105	0.014991219	0.155820723	0.521260591
0.500316977	0.524711932	0.37061899	0.520118314
0.499111812	0.507998209	0.299878728	0.311590734

0.522529996	0.490845906	0.49999741	0.49432551
0.527462011	0.48356311	0.335597568	0.020288434
0.159209631	0.27658558	0.392773728	0.105696308
0.40775897	0.164061131	0.510097167	0.525117305
0.525990963	0.005047048	0.192532291	0.518895664
0.50829755	0.520478364	0.021096261	0.00690037
0.007927597	0.022006952	0.436821088	0.272390296
0.522271876	0.319765381	0.005153679	0.058326223
0.334088989	0.456764943	0.509020385	0.442392804
0.50701374	0.092666388	0.365457071	0.463313651
0.07847184	0.408984944	0.530516495	0.495249814
0.043250964	0.281198213	0.435485666	0.528046827
0.079995656	0.362881127	0.505809359	0.390367484
0.013198183	0.090349891	0.076043431	0.500407904
0.510583238	0.09544771	0.039772232	0.194353146
0.152476366	0.524136549	0.199175769	0.53176023
0.517723455	0.50309816	0.08079827	0.493454904
0.049107557	0.496563668	0.387996158	0.076753829
0.320919702	0.013201206	0.491197949	0.196229724
0.405678289	0.474873611	0.234691478	0.016993604
0.202373561	0.059125039	0.529322854	0.518807535
0.033516873	0.287910202	0.338615169	0.466764917
0.361755372	0.510778813	0.515707212	0.489724984
0.110388116	0.149398167	0.482160912	0.478216622
0.300045154	0.527704434	0.365422267	0.519569002
0.489621976	0.506147251	0.518178833	0.422743305
0.267463136	0.481368663	0.464662284	0.026653608
0.413750031	0.527989523	0.424568072	0.182803021
0.305368879	0.44671688	0.056072042	0.211974263
0.378968689	0.394593244	0.177021791	0.499572932
0.464621006	0.483610022	0.048834429	0.07216551
0.532606843	0.488046684	0.182511676	0.509897079
0.192378787	0.408139088	0.51502586	0.510382657
0.437153949	0.526390116	0.508016246	0.481023103
0.482515129	0.313583115	0.530193239	0.116788863
0.099565857	0.487760131	0.122053882	0.101722476
0.170833817	0.029130114	0.348304669	0.486677603
0.437396036	0.450592084	0.392777122	0.09381075
0.367609281	0.42839981	0.412532253	0.2560896
0.097065275	0.508517001	0.010110414	0.062300532
0.444679487	0.521276549	0.448820076	0.27413995
0.342502338	0.151308428	0.352645296	0.377954638
0.411119905	0.422087515	0.520049731	0.041341529
0.024683372	0.52887343	0.448021626	0.020362788
0.09808206	0.459942282	0.334203915	0.297103365
0.022051783	0.508774355	0.428987879	0.490277759
0.418756539	0.074568377	0.427156336	0.106893419
0.068413954	0.478276631	0.453088664	0.512167936
0.390699008	0.488935561	0.464801659	0.518946227
0.133347726	0.440293199	0.034684631	0.514451843
0.481754772			
1700034H14RIK	0.362983089	0.453833524	1
0.315204273	0.219644724	0.186156763	0.09296202
0.08538874	0.074796324	0.001423338	0.014373516

0.295388019	0.083255562	0.093438968	0.156374517
0.366416234	0.085222188	0.209358238	0.127671477
0.290047358	0.034557691	0.298855926	0.05911745
0.223426971	0.083951923	0.04868205	0.182183831
0.382303234	0.058694876	0.101308746	0.252334791
0.103623879	0.360233407	0.45571574	0.293976576
0.446050262	0.339223978	0.011486278	0.104498609
0.378706635	0.371586547	0.288403313	0.177726443
0.026080308	0.400431593	0.112483082	0.290527242
0.109070221	0.023585773	0.084027412	0.369128917
0.385792969	0.227919161	0.10255593	0.072792164
0.068601702	0.068657572	0.135499145	0.177991755
0.103186645	0.046932596	0.408741384	0.050138738
0.064674656	0.119186116	0.125149066	0.110762723
0.214376053	0.285595749	0.016861428	0.28584945
4.05E-05	0.281675914	0.268977808	0.193194645
0.039559735	0.008791064	0.139052074	0.169009403
0.407158297	0.438698751	0.448418865	0.460062015
0.09661775	0.197934314	0.23104086	0.09005581
0.390694274	0.073440332	0.080959064	0.365756263
0.45694518	0.451250242	0.141409883	0.192820913
0.314061491	0.408718536	0.187423212	0.187370532
0.175684253	0.08463734	0.36091141	0.458371294
0.324071802	0.20669808	0.111972923	0.301721597
0.052279259	0.169387235	0.22335384	0.09238556
0.421720164	0.015943913	0.155583747	0.149503269
0.176138804	0.370238493	0.46404963	0.096690146
0.220123508	0.016217861	0.1083067	0.043697693
0.146549831	0.138895619	0.398629371	0.372915093
0.088467919	0.172778731	0.12975214	0.005309243
0.030324762	0.177915757	0.350447077	0.198024118
0.354487412	0.383007202	0.118288757	0.276591929
0.30418036	0.442409344	0.020440325	0.063146722
0.214006847	0.170798012	0.142488647	0.238447543
0.175333311	0.066798063	0.073809362	0.47692453
0.34690407	0.051355123	0.157466852	0.343878781
0.118150643	0.221095669	0.068304399	0.109972235
0.023307524	0.432740889	0.038386156	0.15917483
0.159185751	0.088928429	0.019228112	0.102619533
0.114939882	0.334147132	0.40175131	0.081345235
0.128147904	0.281763507	0.124096495	0.268088736
0.00505305	0.048923503	0.108740293	0.43533992
0.1313843	0.48242111	0.167080078	0.222088175
0.178327854	0.406947076	0.009771349	0.421270113
0.467969803	0.111636551	0.195023217	0.294809455
0.197456077	0.376149866	0.028657426	0.316339061
0.240186286	0.067224358	0.469690636	0.100544915
0.409011826	0.032270369	0.114604121	0.412723653
0.316160501	0.007667361	0.075249295	0.253210501
0.043407135	0.035461153	0.345947466	0.278056789
0.211162456	0.413651358	0.446992126	0.193205354
0.206147268	0.121997946	0.020270897	0.217880155
0.404249151	0.160221059	0.36534986	0.117849574
0.16923469	0.407956126	0.36165838	0.080411433

0.028729745	0.126024622	0.011661169	0.186975454
0.379195689	0.041172928	0.196184661	0.136321177
0.064329091	0.465494109	0.166679207	0.199994416
0.135682239	0.167122108	0.140188714	0.070068692
0.077811639	0.114980108	0.204549272	0.186513394
0.015817908	0.05458369	0.042667399	0.13066544
0.04188282	0.109632766	0.100162139	0.261610432
0.116501145	0.043350587	0.176331146	0.237224702
0.110181501	0.017535803	0.443627773	0.318355665
0.218932404	0.09654765	0.043507413	0.1103093
0.380704019	0.118238913	0.10605668	0.360926095
0.03795836	0.457584605	0.094686924	0.083999193
0.162504062	0.121378272	0.418033034	0.036495162
0.208121944	0.37611855	0.211488637	0.12013592
0.295447885	0.267646196	0.018730966	0.322069236
0.286669904	0.025351404	0.467838089	0.09798554
0.0267043	0.364043128	0.32598148	0.06848609
0.078934297	0.122413255	0.019984338	0.202173081
0.455351757	0.326010795	0.305297853	0.213218559
0.023083755	0.002795096	0.177205226	0.262806271
0.427608752	0.46593584	0.161521333	0.168026409
0.134161751	0.180986483	0.053242655	0.449618016
0.2229276	0.464791419	0.009869707	0.387003384
0.436195733	0.094889611	0.064131779	0.117768283
0.2866969	0.036393205	0.135769155	0.422541301
0.384924136	0.190159337	0.183488445	0.316962071
0.008854066	0.021361764	0.265501719	0.18547234
0.161113191	0.146569179	0.078308006	0.14508602
0.023371685	0.359185174	0.410720593	0.055022358
0.129465479	0.148834622	0.284204092	0.030790351
0.430688305	0.470318649	0.446085362	0.099454941
0.352369885	0.432578824	0.239538646	0.085456713
0.025936929	0.462593634	0.286387984	0.012412202
0.418069153	0.074644414	0.00387292	0.427348926
0.108838158	0.365567792	0.0055843	0.129924252
0.07166423	0.440830466	0.409157713	0.250991774
0.069809191	0.247529509	0.015654013	0.489107626
0.405307593	0.022624459	0.137647356	0.336724665
0.346376456	0.461650827	0.203363765	0.401773424
0.258700257	0.425295457	0.090199765	0.167153091
0.353551304	0.452146969	0.332336377	0.406524287
0.379726255	0.342683369	0.156075272	0.010115863
0.154629063	0.077928306	0.116273112	0.04816175
0.174826886	0.102845985	0.444995901	0.426745065
0.433704755	0.008713251	0.064546861	0.340635798
0.362900717	0.426979421	0.046904639	0.01403475
0.017976145	0.020815619	0.183057193	0.193427476
0.448314159	0.064380657	0.005393896	0.021145417
0.154298484	0.292789135	0.426076676	0.294125176
0.409082153	0.065953555	0.088378477	0.32082106
0.066658079	0.226272628	0.433403258	0.386800511
0.058921885	0.195221803	0.189535088	0.429946969
0.082178473	0.201135591	0.299162727	0.124039833
0.028837513	0.083547262	0.068316035	0.400807882

0.429020416	0.03371648	0.018939249	0.046441622
0.048757346	0.429545486	0.082286782	0.472162245
0.360643472	0.380146147	0.079068001	0.422988901
0.028211609	0.40406162	0.199926235	0.027556403
0.209354718	0.01942398	0.425629712	0.083766894
0.113783773	0.283279467	0.186620461	0.028101242
0.201426341	0.048052651	0.424603877	0.438412982
0.060649163	0.134720377	0.204987827	0.307011265
0.109438773	0.321808561	0.424340115	0.168452175
0.038897509	0.147100812	0.302582468	0.265087036
0.090466087	0.461472669	0.140680614	0.491413765
0.323725596	0.435827316	0.420502321	0.183959031
0.142207436	0.222456285	0.203180003	0.008285833
0.204960182	0.378934889	0.180749002	0.105908096
0.133631429	0.241270012	0.045726307	0.114741108
0.202111373	0.134323222	0.063294509	0.406830986
0.25252422	0.281476645	0.027394718	0.061937381
0.479378587	0.396717424	0.080113834	0.282274371
0.138229332	0.190671765	0.406952757	0.402190443
0.20783954	0.34902703	0.329655936	0.213125642
0.329246689	0.140531585	0.45374966	0.039896612
0.092324929	0.269441883	0.041809271	0.090256861
0.08132476	0.039373408	0.187775042	0.350213652
0.18789333	0.227868516	0.122348266	0.075568011
0.205750509	0.200021767	0.221603877	0.076538663
0.041141493	0.413076887	0.011656223	0.052888402
0.180310018	0.373354561	0.188709251	0.174223758
0.20055478	0.142845559	0.152184884	0.103373249
0.090020815	0.19494919	0.370494373	0.031001135
0.010766464	0.455849031	0.17101448	0.045852513
0.084653819	0.251906055	0.18657859	0.115356218
0.038387848	0.424936998	0.109761222	0.252807319
0.168595236	0.059190186	0.288717851	0.07154159
0.067401284	0.378329756	0.271304306	0.451389904
0.245107994	0.330894406	0.375782653	0.338474893
0.139984713	0.236656864	0.020729414	0.390383265
0.176760181			
2010012005RIK	0.312065077	0.493032105	0.315204273
1	0.384160828	0.337073903	0.339610073
0.262123314	0.182659608	0.033642713	0.065002065
0.338630072	0.260002822	0.340387701	0.319939142
0.398851971	0.199872084	0.31505347	0.3541312
0.396269461	0.212674942	0.401697443	0.219833394
0.263700481	0.164560377	0.164084931	0.285657115
0.447989331	0.179367868	0.313315736	0.366952971
0.151350246	0.372353808	0.460366701	0.372171541
0.439719245	0.28234292	0.020743879	0.343815908
0.381166007	0.369356555	0.396513189	0.402704045
0.139144254	0.43175691	0.365630284	0.347453301
0.2655557	0.091743552	0.268213342	0.403402876
0.420328932	0.352201923	0.316874022	0.299089888
0.221712679	0.254642225	0.21567117	0.2912956
0.348564089	0.217950762	0.430217199	0.209706211
0.18951772	0.321878719	0.189935204	0.349222666

0.274577085	0.330145173	0.065010803	0.275382524
0.014664468	0.396902152	0.346624293	0.243635023
0.108836362	0.075520976	0.221906162	0.400803629
0.454472849	0.397107164	0.464182811	0.454021068
0.242108423	0.325010493	0.299091948	0.289437163
0.39593929	0.312486258	0.187161145	0.395088326
0.469311545	0.469199847	0.268505233	0.30009109
0.397368438	0.454466768	0.366153657	0.389274203
0.306083566	0.081604876	0.40554726	0.476945554
0.356664027	0.247966095	0.23388573	0.386983492
0.137615384	0.314250608	0.409663481	0.171258538
0.470944697	0.080896767	0.257849456	0.39512638
0.271084153	0.37711904	0.456371084	0.322917183
0.34726621	0.0625112	0.150692107	0.077659605
0.33741288	0.118065355	0.409593953	0.459123462
0.29214202	0.351214778	0.365726661	0.012822875
0.069816329	0.205025371	0.356129964	0.335705625
0.366410301	0.304805426	0.321705087	0.311598668
0.324469953	0.454080304	0.07178806	0.220868796
0.390435694	0.208962518	0.339681287	0.359724234
0.347118354	0.238491988	0.24661469	0.498225589
0.407628588	0.201444951	0.291042039	0.399295793
0.353485885	0.333998013	0.220352168	0.349856296
0.153870473	0.41079629	0.061518437	0.328310544
0.246329352	0.170034172	0.033174488	0.320483924
0.347153857	0.400815231	0.385173047	0.208207864
0.377131744	0.394225675	0.297291064	0.358158409
0.050126791	0.112768167	0.23715057	0.480790721
0.258731975	0.455274854	0.367621033	0.32397585
0.338009925	0.423151461	0.004595882	0.441426484
0.466356059	0.248283411	0.391609306	0.351432775
0.300917661	0.450505449	0.033984253	0.397015982
0.268394442	0.175064606	0.48961928	0.252899212
0.407691968	0.116753045	0.341771621	0.359436007
0.30931204	0.023769797	0.284597297	0.39541136
0.055238268	0.211060506	0.3558339	0.409172843
0.39508559	0.383138264	0.479251033	0.28686888
0.393626153	0.331002729	0.039824507	0.318260577
0.354858727	0.280886049	0.422813498	0.35127119
0.257825844	0.454560092	0.33328372	0.211478729
0.139369751	0.363941223	0.06833315	0.373194471
0.326841498	0.242331296	0.311893768	0.17249973
0.132546383	0.497736907	0.167726419	0.196012251
0.109183116	0.364559483	0.353505679	0.298960578
0.244462685	0.267996357	0.323210673	0.369102695
0.132399827	0.193063811	0.024387573	0.272271286
0.19295636	0.306805495	0.296021241	0.312736149
0.331560265	0.195581513	0.38713625	0.260147893
0.181425077	0.052701105	0.478142547	0.396858474
0.274661106	0.287373607	0.097913908	0.261273152
0.462264913	0.280346566	0.232642931	0.450354745
0.070463951	0.455665142	0.325680517	0.212954778
0.32269337	0.361468205	0.468404994	0.102457547
0.332276435	0.446902616	0.341962212	0.278469562

0.370151313	0.399562446	0.109667038	0.325595505
0.314237157	0.095317027	0.478866792	0.17518318
0.060736913	0.353320202	0.339024352	0.155798342
0.150218433	0.378640703	0.143813133	0.348061464
0.495970075	0.403874559	0.368278256	0.212250819
0.131324156	0.035827787	0.225618978	0.300430357
0.446068072	0.4554911	0.331516392	0.347947423
0.352528513	0.322081407	0.182474651	0.478032602
0.174198182	0.459035441	0.029696768	0.348308905
0.437044675	0.278886031	0.153115473	0.369929743
0.363131962	0.142375532	0.283454512	0.453982707
0.416359702	0.358590484	0.351113108	0.357903907
0.079929088	0.13701372	0.358374999	0.160193024
0.36831363	0.316327455	0.040900647	0.329237173
0.133376329	0.43459571	0.417537186	0.145307377
0.31062464	0.306883228	0.365714037	0.115604091
0.388532264	0.431245815	0.468062802	0.270148586
0.344627416	0.444201425	0.288064391	0.142794172
0.048451037	0.448946821	0.336079469	0.074957688
0.461589936	0.2349079	0.054646771	0.478026085
0.312972283	0.374755327	0.019266897	0.058789861
0.067699908	0.414400359	0.433507272	0.369974421
0.249688392	0.377799876	0.006949766	0.469536877
0.459218622	0.041060231	0.173128007	0.353482842
0.312903868	0.491897243	0.271779915	0.396996714
0.321745638	0.437308284	0.329065556	0.344859879
0.421755938	0.417856714	0.369860525	0.436537571
0.449382795	0.371554162	0.38299075	0.073714742
0.169227576	0.284151166	0.278825066	0.159625229
0.287508091	0.225663359	0.440783103	0.447323854
0.438873528	0.022404548	0.270147287	0.357773899
0.44552005	0.476447423	0.040499669	0.012652821
0.010791348	0.046561596	0.379330188	0.28839289
0.455325181	0.240128829	0.02680685	0.133759642
0.359061392	0.335056489	0.463080162	0.407498347
0.353373441	0.16357653	0.306645403	0.400961369
0.12883998	0.239692933	0.458559944	0.452191931
0.080244676	0.296472614	0.314696732	0.490887259
0.052081544	0.352780865	0.338564495	0.370098641
0.02555248	0.145431254	0.136545613	0.39050848
0.422935465	0.152732525	0.089900471	0.145272571
0.249082937	0.431544908	0.281720915	0.494546354
0.429181122	0.440532277	0.10991303	0.44931248
0.102921025	0.433171722	0.306182192	0.096854142
0.297903188	0.042986409	0.399417794	0.179088223
0.329625981	0.345208678	0.218985027	0.03948265
0.145124305	0.117205678	0.472092038	0.415377509
0.057394098	0.340753279	0.33975398	0.373200164
0.332165539	0.401756188	0.453204228	0.308628866
0.214027864	0.102636945	0.38601261	0.311803274
0.297223239	0.468454383	0.330959357	0.481890461
0.330060819	0.428790596	0.425100388	0.399961205
0.223599847	0.340946307	0.363198581	0.084611487
0.395737664	0.442002198	0.345881238	0.222042145

0.331156592	0.281593178	0.107734	0.150395533
0.230879676	0.312152882	0.26151137	0.432933672
0.333993317	0.313610668	0.094665841	0.135646959
0.49959966	0.408621253	0.254709496	0.385972651
0.249922403	0.384051931	0.372828759	0.360981807
0.302174755	0.403965187	0.387457337	0.350462781
0.415091989	0.369611107	0.473682137	0.148790226
0.128002782	0.2982634	0.206810251	0.157872042
0.264434649	0.07193693	0.212451044	0.385550776
0.256105307	0.404328462	0.274477995	0.162347332
0.333533781	0.278188134	0.386176604	0.323395402
0.186128686	0.454952055	0.031834266	0.120965483
0.272004726	0.376822309	0.320208314	0.310680726
0.248435717	0.163173583	0.374127231	0.299711443
0.296338167	0.278451877	0.382358248	0.101861167
0.080900493	0.477501188	0.319576998	0.0409303
0.11452713	0.293887927	0.290838621	0.325763784
0.046179254	0.420561932	0.316339007	0.361927763
0.389320388	0.146265763	0.319740766	0.134322218
0.130759321	0.40964802	0.312522272	0.448893834
0.298145707	0.409845533	0.390510399	0.370476777
0.152969071	0.262285326	0.108403036	0.419560445
0.296662283			
2010305A19RIK	0.346955893	0.490469994	0.219644724
0.384160828	1	0.205778576	0.160956211
0.117264981	0.079852637	0.008463354	0.039907106
0.299002266	0.153981837	0.166864581	0.154332832
0.418109844	0.159671169	0.209080354	0.201821769
0.355017592	0.077626791	0.384127003	0.106061051
0.238123504	0.123426177	0.099908409	0.203352341
0.44132487	0.107965999	0.151114143	0.307900119
0.146261985	0.3957702	0.456701716	0.361809811
0.450467556	0.299688086	0.026348821	0.156989336
0.377024762	0.379967649	0.356483809	0.244047224
0.062387061	0.408995699	0.187186097	0.2912349
0.128155724	0.056424094	0.108200403	0.378167695
0.404533564	0.263174598	0.140539716	0.135283888
0.133159994	0.123773156	0.173366646	0.209642111
0.19005404	0.078702572	0.431532683	0.079873296
0.112241916	0.173001482	0.189247342	0.177540719
0.238564193	0.296653231	0.033279637	0.264998276
0.004408819	0.328105673	0.286617187	0.178999443
0.078540985	0.038313459	0.203658426	0.244631191
0.463996685	0.414524539	0.502049365	0.495360008
0.170559098	0.254875157	0.245806509	0.17673521
0.397577424	0.144079332	0.093499009	0.434628057
0.505445449	0.47956012	0.193393602	0.237773118
0.363840955	0.444116791	0.208194197	0.248453215
0.178571706	0.093218799	0.390289805	0.505269505
0.339686761	0.217503513	0.172745546	0.364664516
0.098577649	0.171330923	0.303252741	0.151864813
0.473253993	0.030138221	0.216263334	0.222711859
0.193765629	0.377880501	0.475972805	0.169181358
0.263386077	0.039616799	0.104149585	0.072407731

0.196845404	0.128876523	0.396296179	0.44764928
0.153641015	0.257440542	0.194396156	0.011937012
0.060890433	0.19518034	0.393963431	0.231350901
0.379829525	0.329619507	0.184521704	0.300632253
0.317336365	0.500300879	0.018860295	0.110043605
0.228755378	0.203680372	0.219441395	0.283882421
0.231399103	0.108966763	0.149062063	0.511136791
0.369678183	0.110954427	0.178057129	0.39734372
0.196010566	0.257279248	0.131235146	0.209889442
0.070186017	0.422855409	0.064590498	0.182054868
0.138707427	0.166866977	0.024967587	0.154251132
0.159160146	0.388259825	0.389418715	0.136955254
0.229647466	0.343956383	0.15925802	0.319079483
0.019241348	0.072330587	0.125722474	0.495160016
0.164060988	0.498352476	0.22868995	0.246851375
0.243887443	0.43754953	0.004012004	0.422910268
0.478806198	0.124191869	0.265686133	0.309459792
0.206182605	0.435084661	0.022654939	0.395235117
0.246203399	0.111245471	0.521446322	0.175080306
0.414582165	0.073502839	0.187664317	0.407206786
0.345406119	0.015039439	0.112288316	0.316170659
0.06427962	0.074478047	0.36300678	0.331708953
0.246253392	0.427549313	0.509025088	0.192794168
0.25575699	0.180226524	0.032471675	0.250839697
0.398590271	0.206547004	0.385997531	0.175625305
0.170398569	0.433236271	0.352223904	0.115893299
0.067655456	0.197095812	0.022198814	0.227800579
0.33999044	0.115757971	0.199510547	0.174347358
0.11212157	0.50869121	0.167561042	0.18226755
0.119376387	0.239878602	0.20141269	0.147001187
0.149852632	0.172417425	0.219276351	0.225734064
0.047396951	0.125253958	0.037463294	0.194503317
0.110067276	0.182733818	0.169199863	0.306137693
0.208257858	0.109321479	0.244251027	0.212589056
0.135680309	0.040732079	0.477582377	0.354612558
0.239399886	0.149704853	0.08018928	0.159748136
0.459899314	0.131552035	0.114153332	0.42744466
0.043542317	0.50243909	0.188480425	0.14870285
0.238681627	0.213234937	0.468186797	0.040402754
0.234083744	0.425741184	0.249501169	0.178741196
0.335929874	0.315558551	0.04726179	0.3211225
0.311116435	0.064227136	0.498233468	0.16116159
0.052596845	0.382474994	0.3552489	0.114252793
0.112937418	0.202865655	0.046157915	0.253157404
0.495940678	0.407868522	0.360758973	0.198949458
0.065533208	0.021969508	0.21678246	0.227218516
0.439561659	0.489804183	0.237586721	0.234833433
0.210028754	0.20712738	0.07787972	0.495528886
0.164852835	0.469254015	0.023030594	0.405196702
0.439370714	0.137590819	0.10820617	0.180358823
0.326298084	0.070099652	0.182419836	0.443114503
0.405875295	0.219998728	0.196289863	0.350828234
0.025549967	0.060389859	0.254739621	0.158214286
0.222333405	0.200531376	0.055384039	0.180092014

0.067149618	0.408718568	0.46484567	0.078810415
0.170416824	0.15859656	0.334582381	0.06734626
0.431343561	0.463260606	0.46119696	0.154862185
0.392131511	0.457205826	0.251528507	0.122437228
0.045940418	0.462014533	0.293657852	0.033967538
0.49096965	0.09679906	0.024533438	0.461377859
0.175158979	0.364674473	0.002435678	0.064136673
0.078763728	0.475487579	0.411541268	0.300208663
0.097765753	0.279642483	0.019192076	0.507452197
0.442774624	0.036909686	0.169985299	0.380263342
0.35123185	0.487609562	0.204380704	0.427404591
0.279773345	0.442728219	0.162001387	0.234010307
0.396630721	0.45052305	0.353827429	0.413549947
0.462652247	0.385956612	0.21952823	0.031041367
0.109194065	0.124729342	0.132286831	0.08564199
0.18358225	0.148833173	0.449542141	0.488918214
0.47007894	0.019645952	0.118985538	0.372356182
0.416669006	0.47642643	0.040655744	0.006128004
0.00835542	0.033148095	0.236086802	0.226123566
0.469354461	0.09618163	0.017188132	0.048965676
0.188687891	0.307723247	0.463928398	0.363408557
0.409773143	0.12496678	0.139585932	0.375773113
0.095428893	0.214379368	0.478158298	0.422575633
0.08403838	0.228616777	0.208100706	0.492397724
0.063311296	0.262798345	0.327168902	0.204518392
0.037769936	0.119847964	0.09661283	0.444307386
0.434198767	0.092329826	0.056077067	0.06496964
0.116378269	0.471448537	0.155980658	0.522651677
0.410963109	0.460596734	0.126744016	0.428185257
0.062554073	0.427503981	0.238671489	0.029385497
0.235001879	0.035503237	0.435255155	0.099645501
0.173199847	0.302088004	0.139377248	0.047940033
0.133609765	0.091632709	0.490330142	0.470535152
0.072458843	0.189372903	0.24444729	0.327142429
0.151456862	0.395973031	0.439141832	0.17057267
0.093532652	0.137114	0.350223132	0.272714826
0.156758381	0.513964654	0.204667032	0.505843244
0.318242423	0.438289782	0.44454784	0.216926195
0.22033859	0.220622616	0.267510987	0.024980088
0.238851998	0.464991392	0.209657072	0.140933746
0.218418266	0.224588591	0.073372069	0.097867497
0.193396734	0.168146895	0.153217921	0.464641843
0.269769359	0.287192995	0.060121353	0.087941652
0.506936895	0.393494452	0.095044183	0.323871957
0.192576486	0.243489712	0.419360617	0.428705121
0.208754665	0.418411931	0.380295587	0.282557196
0.375473102	0.206874284	0.468610339	0.052549523
0.086910685	0.281720654	0.107808259	0.13612758
0.158498531	0.056679259	0.171563136	0.409611999
0.199422575	0.320220322	0.171792941	0.104496146
0.267611966	0.190852499	0.271372156	0.1405945
0.092138438	0.439483382	0.024003467	0.100589938
0.224214996	0.381827992	0.228016913	0.21660963
0.202790906	0.179962038	0.247176077	0.138416771

0.144847743	0.195390004	0.377697248	0.040615576
0.036170587	0.514744526	0.178557026	0.050067412
0.128142964	0.244099221	0.223462739	0.176805573
0.054176347	0.452114953	0.150829942	0.266051152
0.209876411	0.105122763	0.294593573	0.07454437
0.081616826	0.394349611	0.241088221	0.465092795
0.245774477	0.420431702	0.368947695	0.390563888
0.110309036	0.194331112	0.04374879	0.434461371
0.184190451			
2310004I24RIK	0.281260226	0.464089917	0.186156763
0.337073903	0.205778576	1	0.18965277
0.189215913	0.08851094	0.012425173	0.018757036
0.201190166	0.111272986	0.200718969	0.234222868
0.293027443	0.063619826	0.204676179	0.17123974
0.283735166	0.132366489	0.24146853	0.095245316
0.11967116	0.051332947	0.055524406	0.128971469
0.378254052	0.062221564	0.246299572	0.31749156
0.049872557	0.329951439	0.458321553	0.230222239
0.432613159	0.272177681	0.006492438	0.243097024
0.368681904	0.299548282	0.300170787	0.210025608
0.053855534	0.45735565	0.19201954	0.17974016
0.192314099	0.023830332	0.235265871	0.435161058
0.428094999	0.223862623	0.22185142	0.162115762
0.108360788	0.203653289	0.099153202	0.281981637
0.17328747	0.195490636	0.422229198	0.118502575
0.116371512	0.212552152	0.064064617	0.216413363
0.139029904	0.343870333	0.025989163	0.180510036
0.00749069	0.268783887	0.171402701	0.134230271
0.029861704	0.019451725	0.079153749	0.236505317
0.387302283	0.389241152	0.372698216	0.37788327
0.087007707	0.148359178	0.15581176	0.117027485
0.370112501	0.164437568	0.084958679	0.276837016
0.382792297	0.442302992	0.104481705	0.126807873
0.369579748	0.442988046	0.232129792	0.219091533
0.179544346	0.0223671	0.408669118	0.410676432
0.364363273	0.115018134	0.082325933	0.357970059
0.044634095	0.227205869	0.219429706	0.068133261
0.401023092	0.033669797	0.096291699	0.236037235
0.134656017	0.341071667	0.42425789	0.155746978
0.182098254	0.014716404	0.060478959	0.021391028
0.198581049	0.043420484	0.44061814	0.392836255
0.185122522	0.183415344	0.209395633	0.001980498
0.015345202	0.087187963	0.313423202	0.203311977
0.308594777	0.300799771	0.1514299	0.31810814
0.228585081	0.363084387	0.080372516	0.105756356
0.236184213	0.086638449	0.150551282	0.21882244
0.155136022	0.128143271	0.099202578	0.434954583
0.434325854	0.076803501	0.216364351	0.264665106
0.220967488	0.151751667	0.088520265	0.170490146
0.065864998	0.402721638	0.01299669	0.228168626
0.145468639	0.054582298	0.012596499	0.2317211
0.216032755	0.367844218	0.410539491	0.080823863
0.191829794	0.213779272	0.231297299	0.302153384
0.017899066	0.039611743	0.210465401	0.421730008

0.252412005	0.389373104	0.183877869	0.152523558
0.145883019	0.347989723	0.002209446	0.454099077
0.439569015	0.21493822	0.207735676	0.190774256
0.175893551	0.382889033	0.022351403	0.258449005
0.265373422	0.066996903	0.403289726	0.091455975
0.400313427	0.034302679	0.23138954	0.300158687
0.262758411	0.008250372	0.235171739	0.298241354
0.012167881	0.148887972	0.30410193	0.277310004
0.231695144	0.309421829	0.385027859	0.167246267
0.213760224	0.186854313	0.009823297	0.142975317
0.303118216	0.170900203	0.440468254	0.198408125
0.208506945	0.440410009	0.311380117	0.162423951
0.052370194	0.240055822	0.028300751	0.190759995
0.325569471	0.111085117	0.199778234	0.067689077
0.036806017	0.444118838	0.061999608	0.090160686
0.039581391	0.223276286	0.188466292	0.163439858
0.104998145	0.109533896	0.173117107	0.218849299
0.06769673	0.06318772	0.005692311	0.153268238
0.076228847	0.169182248	0.177470357	0.289185683
0.185911858	0.069554841	0.193701296	0.128708888
0.0616153	0.011787679	0.445900494	0.253859646
0.311745125	0.138206017	0.026986548	0.167676194
0.398645234	0.234028471	0.215570304	0.42345842
0.024342499	0.378386701	0.156224908	0.071620242
0.156814138	0.167787927	0.395299531	0.070427572
0.229250688	0.442242614	0.167193597	0.113559041
0.386345326	0.263117724	0.043399462	0.32464489
0.305740935	0.02503861	0.432433614	0.053890945
0.013997353	0.244894606	0.287781956	0.049537288
0.047714769	0.216756152	0.091952701	0.152620785
0.448353474	0.266302022	0.336383693	0.087394908
0.044931096	0.006904059	0.084891892	0.172748221
0.455469809	0.401424818	0.147823381	0.166682576
0.167247669	0.171516234	0.177282804	0.416991355
0.086876284	0.444532939	0.006598865	0.281137298
0.437874605	0.225009965	0.046263305	0.247352399
0.372701962	0.054481292	0.15154774	0.443579463
0.393607736	0.195082791	0.202962244	0.232791064
0.03644355	0.064881399	0.198215184	0.065326381
0.231356083	0.146219493	0.01217147	0.235337348
0.044807316	0.430178515	0.331207887	0.054637097
0.237290901	0.232448041	0.176919656	0.037614222
0.328087379	0.400580531	0.462978211	0.142732454
0.291671428	0.400684942	0.162576898	0.045339496
0.00989736	0.430920791	0.296987942	0.027086551
0.378954994	0.218536803	0.014466262	0.459550142
0.151112747	0.410429392	0.016808203	0.030327002
0.017628598	0.321979475	0.446349889	0.295512156
0.223702798	0.216961225	0.001509388	0.415266807
0.443613991	0.007806634	0.057942632	0.308226893
0.255395849	0.475204376	0.132954197	0.330220453
0.187887482	0.427747015	0.176321428	0.151714569
0.382147523	0.386526366	0.337779531	0.439210598
0.373365675	0.232487536	0.194313972	0.025916083

0.08536496	0.222969489	0.202195325	0.077450772
0.251462394	0.084671828	0.409440563	0.360936818
0.369994485	0.004130583	0.174877198	0.325550736
0.414619998	0.419037345	0.013442606	0.007466806
0.004358312	0.010041731	0.244047315	0.121810093
0.411476476	0.201099174	0.005878679	0.075336853
0.203604691	0.171709499	0.441320454	0.257993647
0.292457299	0.055280806	0.200058617	0.282846651
0.038637613	0.2564992	0.388283583	0.458384238
0.022983382	0.130233401	0.202980847	0.424746029
0.018585715	0.158048954	0.216918454	0.198236045
0.006683983	0.044543115	0.045388029	0.302694521
0.419987952	0.046989186	0.021822085	0.125282144
0.11143144	0.364668618	0.115164646	0.419589743
0.382184598	0.342687505	0.031109335	0.454051873
0.033357338	0.427384833	0.139348792	0.067734516
0.143877567	0.011089505	0.370584498	0.092815624
0.226549849	0.286070444	0.115753104	0.007822149
0.066561225	0.031658247	0.387542186	0.328095985
0.013832595	0.197544643	0.158670827	0.232763396
0.245035318	0.317384487	0.460337682	0.221009237
0.106679825	0.039945043	0.311100103	0.270873113
0.196069694	0.377337278	0.167936439	0.432481793
0.309777253	0.4038075	0.385387316	0.239603827
0.088027122	0.243668772	0.303041835	0.037326691
0.216113854	0.35597295	0.231114345	0.082089625
0.142602276	0.154452186	0.050925088	0.107914547
0.120118541	0.251535929	0.109537015	0.339506314
0.246027302	0.288181444	0.047089907	0.045378892
0.469146921	0.41560081	0.174751556	0.304289886
0.098649172	0.196985591	0.304388474	0.280656272
0.191686121	0.354068455	0.348193446	0.303193007
0.431334976	0.196275501	0.465406793	0.126440905
0.045995047	0.20518331	0.07937267	0.049435324
0.103567283	0.018521473	0.111779013	0.263200053
0.143983704	0.210906366	0.199342819	0.063535938
0.15206854	0.191799522	0.19367802	0.193467755
0.069450859	0.451991255	0.007332015	0.034405259
0.14230119	0.32908071	0.162347137	0.132582262
0.117371914	0.057507311	0.173658841	0.241060205
0.202089099	0.171177333	0.343119529	0.047897195
0.030842429	0.390746714	0.22755791	0.009684402
0.046778613	0.24134992	0.157140754	0.176126825
0.009559823	0.377621486	0.2473339	0.200869825
0.247530683	0.050876417	0.361720644	0.076214581
0.057264026	0.417585518	0.171122861	0.424638531
0.152543834	0.300986134	0.430988204	0.332072834
0.073755085	0.169521149	0.04314156	0.368814847
0.186419945			
2310044G17RIK	0.164785528	0.259871069	0.09296202
0.339610073	0.160956211	0.18965277	1
0.194008831	0.174803489	0.091231567	0.070642098
0.133039994	0.125767866	0.186154145	0.115970758
0.303771214	0.115146784	0.102996235	0.271965346

0.290869459	0.273218002	0.28482792	0.263967751
0.075522027	0.09473573	0.17469013	0.098643816
0.332080355	0.18017296	0.154760959	0.292170025
0.093743543	0.221908421	0.213824501	0.19287291
0.189845284	0.138782391	0.023768534	0.176666826
0.223029084	0.221993114	0.310964414	0.296824518
0.219583522	0.205942752	0.304447999	0.146986413
0.13662406	0.111684177	0.141897859	0.210120586
0.189931003	0.153703437	0.225737338	0.283680515
0.08915333	0.13536564	0.043845125	0.207429487
0.264049121	0.162688289	0.198037297	0.248963496
0.12283769	0.201908843	0.089100283	0.223114125
0.063799771	0.221361955	0.130202294	0.092531415
0.034091655	0.307379001	0.154830097	0.052703059
0.12748127	0.108544974	0.103907275	0.318971734
0.306367049	0.185498707	0.266754732	0.239263674
0.11957223	0.137235967	0.085138157	0.215602223
0.227065223	0.267660629	0.138615373	0.285677696
0.2624122	0.222318593	0.141280191	0.143073905
0.255552226	0.254700394	0.120498856	0.134897977
0.082085498	0.049326635	0.209355789	0.249632933
0.204891799	0.053191952	0.14107371	0.289696651
0.156581399	0.089675263	0.327213252	0.070084054
0.316035245	0.174234419	0.112359344	0.255228971
0.140264793	0.218081276	0.2075232	0.26997556
0.114641084	0.078152813	0.048724755	0.089763874
0.124912681	0.024918753	0.177670409	0.301656325
0.17524632	0.110402349	0.237724476	0.029013943
0.077255272	0.082398716	0.1769308	0.121318837
0.238740157	0.128056437	0.176055601	0.183181571
0.134620534	0.260735062	0.037064732	0.262727693
0.175801615	0.090770257	0.267047506	0.151187179
0.146006678	0.265810328	0.230217208	0.277812817
0.239372738	0.234525478	0.094247266	0.280406117
0.149792146	0.130862827	0.229525673	0.183558328
0.248297205	0.175961965	0.055503094	0.139574665
0.068733448	0.067830339	0.058791455	0.195057019
0.163571405	0.219338694	0.159411725	0.19883972
0.180633097	0.229748943	0.135156309	0.2114888
0.092872348	0.136205782	0.084013 0.258633886	
0.176996516	0.232167078	0.179683945	0.123828403
0.185136024	0.282532906	0.002450999	0.206717723
0.215402289	0.078978979	0.201220631	0.154465365
0.077460994	0.30886466	0.010673995	0.271431585
0.143340283	0.194578164	0.289050482	0.16285761
0.193069899	0.147123193	0.160767065	0.16901571
0.137676715	0.034431949	0.184550398	0.323923219
0.048450296	0.268598279	0.224844898	0.292014137
0.231801926	0.231139417	0.309502064	0.06639476
0.159802813	0.223305507	0.066504233	0.122743532
0.190544359	0.066264523	0.234746919	0.250325741
0.109543845	0.249514285	0.152342079	0.089480021
0.217706712	0.213322712	0.110390243	0.170048195
0.155197287	0.240152068	0.094923543	0.08274871

0.102677289	0.264424847	0.046463186	0.038085969
0.025186565	0.177821981	0.230501841	0.153312214
0.09393243	0.195923291	0.095321109	0.125862406
0.250970676	0.178449096	0.019099739	0.054043279
0.098813483	0.086007088	0.081874083	0.174024745
0.133999581	0.143339827	0.147075112	0.072617002
0.108579708	0.084350877	0.253051525	0.267901074
0.175962617	0.263578935	0.111969338	0.056822317
0.290571905	0.132867601	0.082973437	0.275427601
0.086475135	0.260479682	0.16677764	0.153018606
0.079718306	0.237543218	0.328071791	0.084652511
0.160927121	0.24641238	0.167687884	0.164599091
0.200664156	0.262155362	0.195703232	0.173748423
0.167425347	0.130638116	0.23390921	0.090462124
0.081962845	0.179087294	0.178062622	0.14342839
0.120185348	0.187425673	0.255247013	0.221705017
0.267488376	0.297104384	0.217546207	0.064719113
0.198498082	0.0486002	0.08260797	0.091567989
0.202952476	0.213689692	0.199146692	0.112475334
0.293293011	0.103353125	0.127446325	0.249761663
0.05285411	0.215819644	0.062399082	0.176321269
0.189498569	0.132988256	0.114354622	0.256984749
0.217307219	0.214408278	0.187400311	0.219924377
0.25140378	0.144228507	0.11707711	0.18528385
0.195059983	0.24230119	0.156847138	0.039552048
0.127117497	0.197843161	0.007875242	0.142639179
0.178175791	0.241913288	0.258500247	0.149818457
0.17073869	0.129160082	0.19849323	0.175228879
0.179760178	0.191885614	0.220729496	0.226308476
0.178024668	0.214401524	0.089150854	0.113562436
0.058921188	0.197892514	0.206573464	0.098562749
0.295230962	0.12232848	0.089933216	0.264530134
0.253545168	0.164360129	0.039778569	0.012645623
0.046765775	0.239180038	0.208435554	0.282540658
0.10569759	0.235371885	0.004189832	0.227434194
0.233657373	0.047831143	0.07532012	0.174905231
0.138597142	0.254507094	0.06400214	0.196335333
0.115513775	0.194892993	0.208689814	0.219566848
0.261716098	0.177519082	0.255232568	0.240260676
0.29003977	0.205544951	0.318763686	0.165276951
0.07499018	0.196388314	0.082726009	0.120288079
0.174559661	0.138503119	0.222546511	0.276393411
0.218980874	0.045355379	0.25883728	0.186954033
0.301930892	0.313287848	0.041791457	0.010993962
0.00502208	0.036902638	0.252128869	0.123528979
0.237661477	0.089623262	0.065735349	0.233543862
0.180621572	0.140049941	0.229275885	0.331456367
0.183578608	0.159356826	0.128345966	0.313484493
0.093762994	0.125593685	0.243645792	0.247138423
0.07671603	0.130886992	0.098586799	0.31356154
0.009005361	0.192817118	0.14512584	0.171017379
0.024759359	0.104720849	0.120604549	0.258926775
0.17817586	0.119713512	0.093064378	0.065667952
0.247232864	0.249199683	0.203962329	0.278252448

0.288967503	0.330067415	0.065267017	0.229862596
0.125634265	0.200059456	0.090917315	0.063987671
0.109124841	0.074639199	0.174896364	0.069839509
0.136658398	0.215721241	0.082615608	0.043530635
0.030933931	0.114693839	0.295142796	0.22990618
0.04264938	0.232793719	0.157596144	0.216144416
0.221961491	0.290027663	0.215853172	0.093911623
0.285870739	0.021244627	0.287691066	0.161444926
0.179816179	0.269298728	0.133378055	0.24061907
0.201223322	0.237023975	0.2089577	0.260035731
0.067417954	0.162103217	0.276494357	0.154959156
0.232150538	0.281444105	0.15515884	0.109850354
0.159740597	0.078097607	0.138264067	0.043355427
0.046867786	0.219192535	0.227029086	0.315892543
0.184449955	0.17298661	0.138694052	0.124929009
0.252552259	0.224852465	0.227529046	0.244199457
0.151915436	0.209027006	0.199735468	0.190818061
0.083624578	0.2198147	0.262683165	0.238156898
0.243360842	0.263812364	0.226080908	0.147955829
0.07249267	0.116395181	0.196211261	0.115073732
0.225158577	0.084483291	0.038103797	0.251057576
0.045308538	0.268974178	0.095084106	0.13845917
0.144441056	0.076361996	0.219712657	0.256476768
0.193254891	0.221120274	0.04718498	0.121954357
0.051582118	0.211539088	0.0943055	0.14889564
0.056891816	0.070388155	0.224427897	0.150412958
0.093948443	0.063607845	0.212067898	0.159943843
0.175861651	0.282993859	0.109349541	0.037679534
0.069126955	0.143511299	0.109291184	0.18882923
0.042080334	0.190943887	0.117851279	0.139653396
0.253536796	0.16222525	0.175345457	0.095811475
0.151403861	0.206685212	0.103005263	0.201460586
0.080795152	0.322485968	0.186019361	0.198017187
0.072624998	0.071237055	0.209010164	0.273651694
0.058459722			
2610524H06RIK	0.188802012	0.233009614	0.08538874
0.262123314	0.117264981	0.189215913	0.194008831
1	0.041890179	0.017554993	0.005009484
0.120254539	0.026589022	0.095841774	0.051017797
0.237241723	0.024643711	0.127430978	0.198194255
0.273075358	0.124568568	0.185220907	0.086181526
0.025306167	0.017730282	0.039438857	0.031224961
0.307422138	0.049916994	0.145172134	0.324570495
0.035978457	0.239906915	0.209787289	0.121226499
0.182400112	0.169334802	0.024752796	0.073220631
0.273109739	0.163686322	0.281020688	0.202543419
0.052740296	0.230037922	0.186768743	0.115902506
0.110191955	0.015749264	0.163316106	0.245658814
0.173524171	0.101191804	0.192008851	0.122039548
0.013432199	0.128442833	0.009124232	0.298879803
0.160502803	0.167878032	0.177514704	0.063298417
0.043151899	0.211271794	0.028844172	0.225358406
0.027012534	0.279988408	0.077679999	0.09190698
0.013257481	0.296259126	0.130805508	0.019187333

0.044750843	0.016164725	0.044490453	0.227787052
0.283856687	0.213143683	0.201337701	0.182001883
0.031814734	0.112102716	0.060697287	0.071447287
0.234670373	0.128403964	0.091263225	0.203144307
0.200042331	0.195194024	0.035786663	0.087607227
0.191357485	0.256201649	0.102259472	0.085988437
0.02593095	0.014069698	0.193334995	0.203303639
0.257778557	0.031380841	0.05700774	0.314108174
0.057366532	0.067316165	0.22559674	0.039759973
0.288354782	0.041674428	0.055395744	0.188476849
0.083080059	0.223811596	0.178514574	0.177506615
0.101754583	0.021469497	0.007895571	0.031452621
0.062265021	0.006872623	0.185452421	0.241094078
0.124354561	0.033173471	0.176186462	0.006814333
0.015922736	0.045487435	0.169416459	0.164464648
0.239290826	0.146835456	0.047730496	0.223442717
0.098137305	0.194995017	0.025575405	0.105444274
0.15619036	0.045466302	0.168581707	0.119689426
0.072964512	0.154576996	0.099781715	0.231819142
0.280830485	0.102970313	0.13466015	0.220494662
0.038526487	0.076927586	0.108291529	0.087305305
0.072685603	0.160752453	0.013315797	0.217154166
0.015718366	0.014737146	0.050589068	0.254912415
0.072900761	0.160441617	0.173404073	0.111124981
0.119930073	0.147601475	0.204654186	0.150753774
0.007396658	0.069939	0.162796489	0.213573103
0.250388219	0.208379896	0.099593751	0.061618078
0.111805566	0.242317753	0.002889186	0.212137932
0.189361897	0.149485187	0.121090927	0.143734639
0.042749021	0.245196627	0.022646846	0.218821673
0.220976708	0.096887517	0.232261298	0.065253771
0.20242683	0.034672734	0.133364356	0.125930461
0.106280408	0.012851219	0.210854461	0.287623829
0.011127187	0.114369319	0.238923142	0.232450547
0.141085733	0.201711311	0.250427976	0.033551032
0.112059737	0.201608422	0.027041627	0.084694059
0.186703853	0.024290819	0.248813293	0.116167358
0.182887356	0.231522574	0.144786523	0.023389372
0.067399082	0.212711954	0.062628896	0.094953322
0.166023436	0.053082697	0.06567535	0.052958168
0.026810178	0.217026132	0.015755051	0.018359444
0.006461125	0.123930786	0.219130273	0.038760941
0.015839908	0.050571804	0.037342643	0.058127061
0.086551514	0.050343157	0.010220928	0.027235121
0.016441608	0.036065062	0.041980174	0.189945125
0.065907997	0.037350545	0.088697927	0.02646713
0.02666335	0.025102474	0.227958433	0.235900368
0.256443185	0.094995802	0.039856751	0.020185099
0.226744212	0.13679862	0.129273864	0.235214446
0.054150187	0.216073652	0.036603721	0.051795278
0.037464716	0.138324635	0.277976755	0.027840343
0.202479255	0.236254245	0.147312851	0.048483898
0.220079458	0.23231218	0.054819102	0.205672369
0.202157008	0.035688441	0.203630572	0.022549818

0.026658521	0.142753516	0.118464712	0.057998235
0.038010768	0.094221246	0.123431097	0.127533527
0.223325224	0.197286597	0.159365345	0.029134299
0.044292144	0.005403318	0.040077757	0.09392414
0.209076701	0.178665831	0.153280757	0.055333207
0.201340813	0.02951298	0.106318285	0.199646374
0.045027512	0.21771954	0.019687178	0.127430856
0.178722274	0.197809862	0.024052835	0.205080378
0.241866028	0.066027685	0.141663731	0.209208932
0.227402419	0.064408619	0.047042809	0.18862736
0.064750914	0.078938492	0.110663066	0.011154161
0.056843101	0.072481089	0.002083111	0.097842569
0.032903098	0.21941779	0.230470525	0.024254807
0.121504242	0.117325102	0.141569098	0.057294009
0.133761586	0.182083831	0.213834345	0.148097999
0.166969787	0.167867234	0.054213351	0.04858621
0.016009821	0.184526032	0.254025166	0.023938559
0.245438037	0.20699188	0.010267831	0.255250147
0.189166124	0.195573767	0.026517816	0.013254082
0.015407716	0.194808049	0.19886373	0.312899863
0.076549804	0.249931694	0.003458885	0.201128971
0.211861508	0.008120374	0.018191701	0.140178208
0.098544225	0.253151935	0.030747745	0.139499225
0.07966777	0.172386119	0.067129242	0.119515992
0.221698677	0.150496565	0.278678862	0.260282387
0.238758235	0.158272381	0.202841629	0.031925046
0.015946806	0.209278505	0.026836248	0.014121021
0.264165591	0.026507446	0.227211701	0.223440159
0.172233721	0.014074191	0.142654747	0.172862061
0.304120061	0.28972591	0.030886345	0.006210221
0.007707564	0.002864003	0.256347516	0.082094088
0.20393748	0.037894725	0.016392257	0.139057575
0.085368815	0.085653175	0.202757026	0.266544986
0.166075674	0.056963084	0.031753451	0.286103467
0.01384605	0.207638216	0.187353035	0.239066645
0.030014037	0.075695116	0.151649439	0.261059569
0.001674927	0.129212886	0.132455969	0.045118091
0.016714452	0.025932993	0.021689261	0.223132877
0.165324436	0.01684381	0.011845039	0.064063712
0.060423041	0.222137626	0.054265699	0.228397575
0.264502561	0.262974714	0.014643932	0.22146328
0.016578624	0.192058638	0.048268823	0.042470657
0.08650103	0.028075416	0.169373895	0.007520345
0.138068385	0.277483431	0.020093963	0.009291176
0.010154984	0.030659392	0.218544617	0.174170262
0.014609457	0.123612027	0.126309332	0.228891511
0.27288244	0.205595296	0.215049797	0.084079596
0.078124025	0.012419514	0.306575638	0.216348603
0.135227192	0.212081379	0.026613049	0.214482035
0.242461892	0.247662136	0.180389381	0.191053915
0.015627915	0.201630519	0.221806628	0.045928221
0.212753921	0.206489326	0.211220179	0.019231291
0.065076955	0.05561052	0.070843979	0.069181292
0.030421489	0.252884699	0.057885909	0.269038244

0.252296842	0.224479951	0.100138429	0.020791637
0.233530324	0.246065423	0.124070939	0.202257145
0.093733792	0.11364305	0.177767491	0.152472767
0.037984552	0.174766645	0.275584403	0.205913539
0.24995581	0.190068027	0.212031031	0.19580757
0.015731031	0.126127113	0.031764334	0.049378853
0.102653535	0.019492875	0.012353579	0.210410305
0.024013835	0.173821654	0.074024153	0.04868768
0.10019291	0.115593937	0.159429761	0.151249276
0.039377312	0.199348432	0.011642516	0.040948933
0.032156256	0.17113178	0.048900637	0.072238785
0.014250632	0.025090542	0.130791715	0.21688194
0.0567928	0.037786131	0.17455277	0.11145811
0.064610959	0.232966933	0.165571065	0.016569193
0.021521994	0.185026614	0.082438241	0.038069059
0.010165031	0.150947892	0.086142767	0.106132414
0.247609507	0.064558152	0.251944019	0.056172746
0.089067383	0.239865354	0.083958129	0.180685211
0.079237877	0.222204789	0.225834072	0.181196025
0.06463758	0.080060306	0.062836158	0.270011859
0.035886168			
2610528E23RIK	0.068871962	0.106012306	0.074796324
0.182659608	0.079852637	0.08851094	0.174803489
0.041890179	1	0.014785549	0.022307705
0.064170427	0.013572855	0.04071807	0.066775875
0.185447549	0.050159862	0.041466345	0.179983028
0.131113747	0.075796087	0.167596744	0.124076682
0.041531892	0.109059957	0.162840105	0.125527131
0.154278646	0.186660688	0.052759041	0.110355611
0.039393416	0.081024557	0.083673102	0.099301477
0.068734126	0.060888168	0.001125178	0.072786095
0.087660018	0.15463854	0.143067332	0.181340269
0.102428167	0.0678419	0.212575525	0.100355734
0.011854877	0.075218561	0.046800118	0.070805242
0.068521606	0.084388331	0.092176522	0.159206526
0.009849067	0.020823969	0.016472762	0.061299971
0.160662249	0.039779278	0.074095168	0.075992898
0.011166298	0.066361348	0.034761222	0.090044149
0.038452904	0.084571298	0.020597515	0.041171548
0.002143981	0.160738456	0.095450131	0.066853213
0.121059295	0.019283111	0.053071489	0.162744706
0.135710784	0.070496888	0.134705457	0.120988273
0.023351037	0.094357268	0.053577303	0.098431739
0.100473897	0.153709296	0.021479848	0.169830614
0.131694791	0.086899218	0.065955041	0.147419267
0.12618098	0.095417662	0.079167365	0.079705996
0.083069837	0.038930599	0.079305614	0.108142534
0.067730132	0.04723264	0.14796305	0.102682796
0.103550612	0.01965703	0.190333821	0.004206203
0.146519804	0.138777103	0.113621309	0.109604506
0.031285522	0.09729866	0.087589375	0.1920905
0.058548538	0.010542159	0.022022071	0.04871698
0.009895993	0.012629858	0.060257338	0.132542729
0.019551703	0.02396995	0.123663538	0.023808143

0.052213326	0.013637988	0.05871614	0.048668282
0.110803626	0.05446353	0.154494876	0.0589917
0.071799788	0.129685922	0.008824206	0.118505333
0.104325744	0.020660845	0.196252896	0.061673532
0.150401085	0.097286755	0.105240431	0.13484649
0.082146458	0.144712553	0.025380341	0.16456083
0.03741653	0.100439658	0.096737191	0.049288517
0.066964038	0.0727282	0.053711986	0.056657311
0.046929986	0.009692554	0.009432875	0.073018162
0.025468285	0.095658244	0.052310786	0.118456243
0.067048057	0.149523404	0.040758264	0.099075519
0.026603294	0.026903398	0.02163015	0.104579717
0.047170119	0.104397697	0.064293736	0.079017295
0.159207729	0.155853835	0.001557736	0.076970602
0.090544916	0.022895544	0.101393146	0.079369599
0.090076625	0.156031456	0.000852537	0.128033277
0.040193767	0.121134899	0.142239368	0.154329759
0.068945518	0.143567983	0.041749576	0.098994581
0.054810089	0.005256008	0.080138115	0.15648896
0.043489214	0.069989994	0.100205223	0.166115494
0.188742285	0.126473808	0.1543362	0.088485572
0.092593785	0.088872047	0.016885336	0.107336085
0.090021157	0.007416124	0.087952626	0.087907905
0.03059656	0.105581951	0.065453122	0.011542138
0.166539322	0.086742329	0.015130417	0.193127385
0.074134034	0.056993299	0.093035943	0.012681253
0.078711035	0.117952353	0.046264294	0.033831941
0.024989172	0.062077972	0.139789412	0.035302446
0.018806514	0.114483273	0.089931673	0.09119759
0.073694262	0.090624821	0.007774509	0.012240145
0.010318351	0.02178726	0.019137887	0.052513382
0.015898044	0.013697056	0.096606059	0.047353857
0.109577355	0.08174286	0.10505188	0.153892727
0.049176294	0.142753997	0.07081262	0.003970514
0.123674708	0.057287001	0.021639145	0.107060112
0.021956807	0.12409474	0.037959897	0.151253916
0.034581535	0.140251376	0.172094895	0.022557719
0.054570709	0.085692919	0.12280349	0.177556422
0.059332987	0.131841519	0.034804841	0.064315226
0.050961977	0.084139966	0.095907502	0.037022394
0.078123961	0.097245945	0.099957485	0.168848459
0.134920645	0.109204926	0.065676656	0.195344243
0.119312962	0.175732853	0.096868128	0.057315089
0.10959457	0.003893667	0.060603797	0.054592301
0.068599364	0.090898978	0.1241941	0.079975086
0.203067274	0.090444136	0.007120647	0.11053942
0.010215607	0.081318491	0.04843786	0.099854583
0.070776436	0.034781036	0.136579032	0.128632571
0.069685709	0.172093096	0.060190613	0.083311561
0.122370694	0.134603917	0.113077557	0.076889762
0.065735512	0.040145522	0.152186503	0.035385885
0.062131851	0.147742716	0.004092048	0.034739951
0.052536397	0.088157094	0.11406336	0.16634486
0.072295979	0.040710882	0.122269121	0.130547842

0.099128054	0.078201955	0.082181778	0.068478567
0.065079765	0.099657318	0.053386666	0.094522674
0.079108892	0.078736764	0.085747289	0.014754462
0.131366955	0.037416962	0.01069305	0.104251658
0.178023847	0.048339501	0.058621921	0.00289099
0.035246894	0.123578098	0.086070061	0.113574285
0.031763031	0.117987012	0.000844156	0.097009768
0.087875816	0.06194918	0.039124465	0.069467396
0.073944402	0.096240538	0.08541463	0.105034599
0.058414167	0.074023545	0.021853662	0.206403272
0.120691372	0.075851966	0.114349452	0.096942322
0.118939749	0.113930668	0.226215154	0.095587153
0.056989305	0.06299778	0.045877482	0.036425642
0.060658722	0.124915457	0.090379306	0.130063402
0.099310781	0.053410984	0.048336969	0.072600408
0.119017856	0.142526069	0.013610959	0.04083131
0.002550964	0.033509694	0.104927072	0.126879657
0.111351891	0.009704141	0.04540353	0.037632649
0.046114079	0.107481123	0.087251518	0.188314076
0.090591022	0.082456676	0.037002423	0.163142565
0.070838817	0.036790725	0.115084868	0.09194281
0.033474208	0.131652793	0.035710407	0.144727712
0.008135683	0.1276977	0.058374332	0.044357182
0.004606353	0.109221568	0.153468947	0.133421696
0.064445401	0.030156661	0.032295016	0.00295525
0.098290232	0.109439535	0.063514305	0.128708677
0.126431721	0.163061218	0.026221238	0.09819777
0.031426303	0.069648355	0.081594867	0.009345686
0.044695574	0.031945832	0.062372809	0.031212933
0.033026607	0.081633899	0.05448139	0.040083581
0.025595831	0.079896239	0.150068236	0.124918983
0.025650067	0.09595753	0.127346658	0.105910148
0.08972146	0.149899635	0.078484181	0.045927399
0.107019341	0.002606238	0.123182098	0.054368989
0.030935224	0.131576168	0.073849324	0.106652297
0.087056439	0.101882943	0.087495169	0.194831982
0.01127687	0.072257773	0.116951569	0.021932145
0.141245866	0.126829198	0.067119822	0.064730771
0.142538141	0.053961253	0.018978846	0.002656233
0.038451047	0.081178274	0.040888858	0.164306084
0.064464575	0.061190772	0.009089132	0.16908152
0.100851892	0.0940919	0.063416836	0.124200106
0.109285993	0.19237568	0.097638685	0.095362995
0.031973661	0.081876986	0.099452875	0.082138444
0.081090035	0.196700266	0.088818121	0.014895006
0.045590733	0.039865579	0.066934403	0.117023265
0.140268717	0.067879729	0.024941082	0.124622132
0.020264656	0.135502102	0.008111828	0.031519656
0.101233465	0.031276768	0.141517764	0.108940855
0.058047783	0.085214376	0.013025359	0.071651442
0.018091733	0.122985346	0.077285828	0.11058616
0.033478954	0.025613747	0.140956132	0.049820565
0.029836475	0.005989522	0.118465017	0.036694976
0.036681518	0.130537937	0.045646747	0.015614583

0.011407314	0.055699586	0.023136802	0.083968599
0.05127157	0.080370501	0.057464941	0.086161269
0.125000207	0.072644749	0.049851871	0.017255892
0.040160752	0.067266264	0.079530671	0.078609002
0.054871885	0.175055456	0.060726108	0.068135861
0.012158802	0.038306171	0.106236337	0.115946442
0.037149651			
2810408M09RIK	0.001905681	0.007031388	0.001423338
0.033642713	0.008463354	0.012425173	0.091231567
0.017554993	0.014785549	1	0.01931244
0.00401805	0.007487396	0.051734733	0.004528159
0.017409032	0.041702817	0.001727934	0.067159042
0.017374791	0.105793097	0.030134158	0.070477784
0.001368513	0.031551227	0.082599257	0.008138289
0.019588026	0.087304756	0.017171728	0.019257551
0.011545247	0.005469217	0.00340409	0.015437645
0.002610907	0.001116352	0.001268496	0.025817639
0.004419586	0.009037394	0.019500583	0.065188508
0.119138175	0.003606012	0.095537848	0.00493837
0.001075002	0.071657187	0.015991809	0.003534353
0.004439045	0.006804624	0.027272503	0.099319149
0.00526931	0.012488904	0.002766355	0.011613581
0.08301777	0.032765391	0.00400533	0.036256499
0.001183878	0.018190889	0.010802474	0.042823956
0.00083463	0.006700536	0.019340723	0.000678414
0.019831984	0.020240447	0.00698353	0.000945962
0.085160369	0.106874164	0.015465762	0.057595391
0.013611344	0.001753092	0.011298331	0.006944867
0.033342626	0.011321518	0.001601509	0.070108774
0.00477489	0.124954741	0.011494448	0.019038094
0.010089463	0.004775058	0.011506485	0.014822052
0.017013584	0.00658305	0.007001459	0.009694668
0.004581492	0.004410678	0.005040832	0.007694477
0.004696175	0.001847248	0.049263622	0.016403633
0.055239115	0.001885706	0.053118004	0.003440009
0.014452103	0.083410419	0.021134346	0.037946608
0.00359112	0.006586077	0.00383231	0.095155577
0.004136686	0.020256518	0.000745845	0.021176446
0.00230812	0.000807304	0.002070932	0.020746337
0.016899069	0.003421763	0.03314654	0.027981942
0.046354683	0.001046344	0.004191329	0.003756888
0.00665179	0.000314017	0.046111718	0.003910597
0.003234484	0.010087939	0.001527023	0.058787708
0.011965052	0.001891635	0.063480297	0.005880537
0.025915143	0.054628566	0.079109941	0.008983447
0.00627909	0.127967887	0.001845396	0.02023356
0.010789899	0.012099899	0.064920005	0.051075182
0.109724843	0.002119655	0.029196419	0.006625412
0.001407343	0.008555505	0.003535964	0.027797036
0.018697177	0.011498244	0.000894978	0.05572427
0.044859289	0.018794565	0.005722027	0.019548652
0.042186223	0.015548943	0.001962496	0.009688815
0.012945828	0.004965515	0.019629699	0.004537855
0.027916836	0.012074204	1.27E-05	0.003681491

0.003965697	0.002554276	0.038201483	0.004464694
0.00209331	0.021025302	8.81E-05	0.021062245
0.002684179	0.051587433	0.010966968	0.075283739
0.002825614	0.101423491	0.013387007	0.003161122
0.003713672	0.002870759	0.042479066	0.027423913
0.01282799	0.061663381	0.005370145	0.025934314
0.025014716	0.006445283	0.014809821	0.002054934
0.012705627	0.032597861	0.008596257	0.009258767
0.003130458	0.000304312	0.007018114	0.027007156
0.002459201	0.007705211	0.002115067	0.000880928
0.143388434	0.023391019	0.05605025	0.020339786
0.001058131	0.098263659	0.002051485	0.002478231
0.041829354	0.008371491	0.002832034	0.000672817
0.00101997	0.025639773	0.036756549	0.057665309
0.029387909	0.023203126	0.003348282	0.006759176
0.132431654	0.103792091	0.000723632	0.004483925
0.04234069	0.020978278	0.018588918	0.004240387
0.010200407	0.056914495	0.025652756	0.000967322
0.021097443	0.079372595	0.006899853	0.016192304
0.005499403	0.04363769	0.04652881	0.001769638
0.016934985	0.01407858	0.001763695	0.01355089
0.004658798	0.00726514	0.039416145	0.07430052
0.01081115	0.087284367	0.016485079	0.002317912
0.00402814	0.008114657	0.010567853	0.040204969
0.007871939	0.016904742	0.045198511	0.003331725
0.004897673	0.108117957	0.005724585	0.020849969
0.051911205	0.005711722	0.00685496	0.062448814
0.038717652	0.046117795	0.083220002	0.036411906
0.009112668	0.029791452	0.01038647	0.002090653
0.158276025	0.041168163	0.006763642	0.001205143
0.003307922	0.004741827	0.031784223	0.02191493
0.06920836	0.0051194	0.001575136	0.008446098
0.000180354	0.003238396	0.049146603	0.003815394
0.002700706	0.010481747	0.056297311	0.042695145
0.009715601	0.093175747	0.01341373	0.00549743
0.007238334	0.008779166	0.008033087	0.005743717
0.13869429	0.047180949	0.006291985	0.000879384
0.006945094	0.021463642	0.000148417	0.003230728
0.093498833	0.008967072	0.010244929	0.037257952
0.022643183	0.003030439	0.013401319	0.098198721
0.003677054	0.002345264	0.004444594	0.027942313
0.003994527	0.005943977	0.002402405	0.025785687
0.040597258	0.003019077	0.005572325	0.024666804
0.013221605	0.012357084	0.084608555	0.008005005
0.067343186	0.001521327	0.007586391	0.000148323
0.005277287	0.007647497	0.004201362	0.015753315
0.017432616	0.012408917	0.000136069	0.004370957
0.007667402	0.027346072	0.004636459	0.005334054
0.003858584	0.005761237	0.002948399	0.006637938
0.005388914	0.003529818	0.015461905	0.038465657
0.01450987	0.002320688	0.010876524	0.005656696
0.016990399	0.00905868	0.070064777	0.124977168
0.001113151	0.028792073	0.008092306	0.006345659
0.006686339	0.033319174	0.004755478	0.010725343

0.007299136	0.05092222	0.042395538	0.004598532
0.015367647	0.012774567	0.001054881	0.000611393
0.000347029	0.014758286	0.018459645	0.012435547
0.005969844	0.011807733	0.090585534	0.069136689
0.012960101	0.006672593	0.005978669	0.03271462
0.003099795	0.041706162	0.023873429	0.020638039
0.014266574	0.001373175	0.009721401	0.007471371
0.009242959	0.010218411	0.002576555	0.014404574
5.92E-05	0.024060845	0.004571663	0.01873112
0.000664654	0.025165043	0.025900009	0.009241945
0.002292007	0.078983822	0.091365556	0.000717096
0.128592803	0.006601991	0.072982906	0.00951264
0.015534588	0.023563774	0.006585204	0.005027418
0.009802678	0.004438741	0.008112507	0.006761908
0.004500191	0.020989737	0.002324653	0.004313395
0.013714722	0.006405061	0.000931374	0.015600952
0.000159669	0.059196777	0.015045337	0.007209085
0.005021462	0.017881396	0.014894342	0.008086301
0.035191543	0.027661491	0.004299055	0.004050611
0.098766032	6.45E-05	0.016656412	0.004421911
0.018470653	0.009856538	0.012381624	0.005028063
0.00419787	0.004135648	0.004733128	0.031681764
0.001995116	0.005536926	0.036143578	0.105831614
0.022699057	0.017867123	0.005434774	0.01543562
0.051061035	0.001122003	0.003887163	0.00015326
0.000643627	0.025219158	0.049364416	0.015837617
0.004911091	0.004204906	0.007097438	0.032808773
0.005663874	0.004013595	0.020617821	0.016565701
0.025367257	0.02361761	0.004923066	0.004838156
0.000527341	0.009856621	0.01162401	0.026936627
0.009336219	0.045049244	0.004562839	0.012807717
0.001787128	0.001462092	0.092254046	0.032864054
0.100686231	0.020935355	0.000350382	0.01211399
0.000509577	0.042427089	0.00095075	0.007332193
0.010166112	0.000580052	0.020570289	0.093505999
0.112384848	0.005694921	0.007190513	0.044734507
0.001416389	0.006339746	0.007296352	0.01982807
0.001132815	0.002721504	0.045495132	0.014404549
0.025217228	0.000247132	0.006703687	0.007999644
0.088639031	0.011044865	0.003809895	0.003205939
0.000548842	0.001677504	0.002561728	0.018429953
0.014590248	0.003909843	0.012960096	0.004201169
0.026782394	0.037398507	0.002829671	0.000888323
0.008396423	0.004287005	0.001923126	0.003475686
0.001665317	0.031864859	0.002181666	0.005953694
0.000679831	0.000186272	0.080983359	0.008818065
0.001666122			
2900062L11RIK	0.012292253	0.029799455	0.014373516
0.065002065	0.039907106	0.018757036	0.070642098
0.005009484	0.022307705	0.01931244	1
0.020530463	0.022128369	0.025860239	0.009869412
0.062037433	0.109939235	0.004625364	0.080951579
0.038402776	0.029698569	0.095000041	0.051896828
0.019669647	0.164192181	0.196716291	0.079923147

0.04271609	0.173648354	0.019796046	0.024975198
0.029942102	0.018732805	0.01911008	0.067044466
0.017563374	0.008795035	0.00121314	0.030164873
0.013702131	0.047624256	0.042032019	0.090371091
0.073416942	0.013653867	0.10660647	0.033886833
0.001191064	0.157146372	0.006104594	0.012890955
0.019519728	0.039027438	0.014157317	0.079192187
0.035674792	0.006682792	0.032347974	0.010047267
0.10238091	0.008315631	0.021605085	0.024017783
0.005058148	0.012328705	0.041107483	0.025925605
0.020620519	0.01245138	0.006088737	0.010505211
0.007166549	0.039305789	0.035083405	0.030257556
0.141468188	0.07594596	0.051614866	0.07531404
0.038056915	0.012277696	0.052706807	0.043855522
0.041399229	0.039289304	0.024542366	0.103451727
0.020208073	0.100353321	0.004439594	0.07573144
0.049651492	0.026080555	0.05214317	0.079890424
0.04649384	0.02311804	0.017992485	0.039042309
0.033858796	0.030405702	0.021517313	0.037793756
0.01138186	0.032911771	0.150928736	0.02555636
0.087184604	0.002204487	0.092620632	0.004636063
0.040502432	0.047691963	0.109816866	0.056381241
0.006198922	0.020355122	0.025812148	0.095460783
0.021862702	0.016425845	0.016596667	0.038680488
0.003819722	0.017279666	0.013034992	0.051294295
0.006743099	0.01978352	0.036918044	0.032085128
0.092812598	0.004456989	0.019315252	0.008749639
0.024746846	0.008620796	0.139303033	0.011075436
0.028821507	0.052368886	0.000625385	0.040621939
0.027279865	0.008281271	0.117285881	0.026507915
0.108854492	0.022365094	0.084886097	0.040403262
0.015206242	0.116966935	0.002943108	0.061374952
0.030901538	0.072781389	0.058360361	0.050466427
0.059965306	0.018546626	0.097018815	0.006264236
0.01654497	0.037210282	0.00160336	0.014833712
0.008742747	0.041262071	0.010750976	0.063841107
0.054762607	0.0789653	0.005909896	0.04587678
0.077719438	0.011074358	0.001966666	0.038018344
0.00922676	0.029093776	0.022637405	0.025797764
0.098307865	0.04187962	7.48E-05	0.016605313
0.025185691	0.00207083	0.081197747	0.024144171
0.037915546	0.052957553	2.14E-05	0.05634466
0.005530342	0.056190616	0.048303571	0.188391329
0.015042431	0.201781054	0.015045653	0.033212713
0.024762134	0.005090794	0.016811062	0.04579474
0.060273845	0.020956673	0.019800017	0.053397823
0.075422923	0.034183716	0.053415908	0.037858913
0.043689463	0.022516599	0.009799205	0.052239195
0.020271332	0.004008768	0.018079479	0.024759023
0.003602006	0.026559203	0.015425811	0.005631378
0.12419734	0.025055405	0.005819256	0.081784902
0.012385616	0.087174948	0.025911286	0.005817908
0.120029739	0.038441146	0.043921069	0.020476078
0.029143716	0.046432889	0.038260803	0.060730991

0.064856823	0.072422958	0.038411989	0.036311946
0.040499997	0.149577284	0.003150735	0.015195745
0.056193845	0.035024901	0.026857378	0.013686722
0.011642327	0.034782087	0.066374418	0.013697108
0.076981628	0.116286974	0.028924975	0.044082513
0.007439439	0.051298842	0.070086802	0.004195836
0.050021622	0.013264406	0.001591876	0.036743505
0.003798694	0.040202592	0.060331105	0.194636936
0.046295766	0.116487075	0.049392265	0.004898557
0.010361247	0.024395666	0.036323014	0.14706486
0.015546712	0.037652454	0.024650628	0.011110487
0.011273154	0.13474873	0.029418918	0.063509464
0.097759456	0.035418058	0.043768598	0.150281537
0.104584127	0.089039144	0.01627125	0.123867249
0.037168863	0.092512831	0.040244483	0.024266122
0.141116816	0.057571014	0.046125045	0.011635238
0.015984571	0.029810986	0.064454852	0.084660655
0.099248563	0.052400171	0.000795837	0.039218004
0.001288144	0.018500398	0.049289477	0.038578208
0.018100563	0.007251398	0.199400019	0.044389066
0.016622728	0.086090007	0.012869666	0.021503958
0.029126272	0.049078801	0.037318892	0.022252418
0.027995847	0.025761663	0.044373063	0.021410445
0.032202715	0.06459211	0.007214238	0.005376222
0.080055692	0.027202171	0.037604155	0.099795818
0.034742668	0.00358884	0.061632338	0.10275458
0.034699862	0.019191328	0.020342935	0.018154257
0.021326814	0.034152998	0.033341294	0.067669853
0.113248393	0.020058028	0.012938359	0.016283409
0.047284671	0.004801213	0.064969211	0.025844801
0.069018805	0.009120625	0.001247431	0.000174803
0.025183024	0.042528673	0.019731974	0.023946696
0.008796094	0.026594746	0.000609059	0.028040212
0.026077261	0.098305516	0.040767422	0.028407919
0.031380611	0.022945281	0.047592684	0.042999886
0.031978107	0.021686807	0.016432594	0.13038754
0.037566873	0.022642688	0.022049954	0.018252156
0.047113332	0.046907458	0.110193037	0.08604388
0.011817241	0.012943014	0.019042927	0.041921793
0.007069152	0.110767575	0.020109896	0.046200784
0.03740406	0.068639418	0.014326223	0.021758362
0.029566553	0.037820024	0.002161454	0.001176651
0.000217522	0.089387423	0.026923632	0.057202151
0.031470506	0.004886912	0.061623849	0.01175273
0.011013633	0.059167273	0.026886442	0.068722943
0.024282505	0.076383863	0.033505511	0.046016328
0.088561969	0.003761422	0.043553963	0.02364365
0.022112522	0.054417362	0.005308945	0.046561735
0.017334741	0.078994732	0.021333294	0.039272377
0.001944536	0.100947096	0.123542846	0.041512104
0.017459394	0.124453171	0.1278984	0.000582497
0.116419197	0.032424129	0.067442557	0.043035404
0.034671763	0.062620623	0.045889389	0.021746727
0.058262577	0.019204597	0.065324707	0.000879054

0.011785643	0.021316297	0.017081914	0.047101801
0.009563805	0.013310242	0.011866287	0.067046631
0.007826349	0.112366807	0.059089374	0.046418506
0.021511665	0.023247879	0.045790607	0.023519225
0.017978813	0.069868877	0.018680901	0.011444334
0.06989628	0.001641426	0.028689618	0.008997075
0.009870686	0.04811203	0.075659445	0.028701668
0.012971203	0.01985275	0.026739875	0.057514975
0.023400776	0.010420109	0.046186928	0.015161281
0.038600102	0.059669154	0.009411833	0.071736644
0.163002032	0.021153198	0.005568924	4.76E-05
0.019690136	0.017370261	0.054870449	0.051441138
0.010364395	0.009926552	0.003538446	0.127828425
0.026842076	0.016627833	0.006881204	0.038392865
0.05767079	0.101696456	0.027701083	0.035432997
0.006485579	0.035788356	0.025182777	0.035238441
0.02105471	0.068847843	0.022435638	0.000992404
0.018346345	0.01032674	0.136608839	0.107390647
0.127648102	0.051442295	0.012466574	0.045967591
0.009877421	0.091790408	0.001634565	0.013321587
0.054047112	0.003534196	0.063817306	0.050097534
0.072040149	0.024128487	0.012836661	0.083694051
0.013437492	0.034545418	0.060289035	0.052926704
0.023798229	0.019806106	0.106393287	0.008656235
0.027400351	0.001336771	0.031959238	0.004140597
0.030434895	0.045482411	0.005143132	0.008929433
0.008769227	0.007720743	0.004951198	0.064598296
0.068735034	0.029261561	0.023233672	0.0297766
0.029527041	0.045353511	0.007025565	0.002321593
0.007289368	0.013404469	0.021909462	0.022539582
0.013950223	0.086648826	0.010171418	0.02323798
0.001166498	0.006523363	0.041405443	0.029212008
0.014128702			
3110057O12RIK	0.368714792	0.499778158	0.295388019
0.338630072	0.299002266	0.201190166	0.133039994
0.120254539	0.064170427	0.00401805	0.020530463
1	0.1546251	0.171653548	0.179287029
0.368893143	0.146623502	0.26591263	0.184333119
0.305694544	0.054366208	0.32478134	0.076208252
0.26396395	0.111832969	0.063173497	0.214496119
0.412710934	0.074578438	0.164006731	0.276419311
0.139020868	0.394725415	0.493357815	0.341084073
0.496892417	0.331822386	0.025849077	0.151774363
0.394085262	0.367973308	0.303943289	0.230915963
0.035319072	0.447257964	0.163463238	0.317993884
0.136984725	0.035491862	0.121872027	0.398768268
0.438974875	0.253021856	0.134908809	0.101795786
0.131492384	0.121438404	0.216904963	0.200102735
0.157034179	0.075906609	0.455731287	0.053127264
0.084184439	0.1682616	0.187559029	0.168287547
0.2713633	0.296801692	0.020815194	0.294437091
0.001861694	0.285174911	0.317840324	0.214109197
0.053770802	0.022119744	0.20566625	0.207465314
0.44054073	0.459043458	0.490241638	0.493861659

0.178239978	0.266671484	0.30641098	0.141362842
0.405066145	0.114265204	0.108412043	0.384368586
0.497317714	0.503191527	0.188261897	0.244543115
0.343837156	0.448401612	0.263564579	0.280977331
0.213679389	0.085483866	0.396529495	0.510458808
0.353634324	0.281165932	0.155285656	0.341217571
0.06865831	0.241876823	0.244892782	0.184492453
0.454521762	0.01444348	0.214883828	0.215539317
0.213995339	0.391737434	0.508113184	0.137392608
0.330064173	0.02949844	0.118839835	0.053532109
0.24549462	0.18158501	0.438868272	0.42530866
0.153254948	0.265623189	0.179615635	0.006363706
0.04341208	0.212313664	0.409970078	0.284232788
0.35942047	0.381138904	0.162018614	0.301864326
0.322053756	0.482108149	0.028761988	0.077422975
0.275462512	0.208413564	0.197267869	0.290965181
0.248572625	0.088130351	0.113405998	0.510062974
0.378098127	0.075513161	0.226650392	0.360567461
0.185016326	0.302820838	0.099117713	0.204130472
0.036726737	0.466205153	0.051501898	0.223527893
0.163101088	0.170321926	0.019859368	0.155396147
0.183261386	0.382069335	0.437916905	0.108854717
0.227323853	0.29492666	0.189831833	0.313038799
0.006935747	0.061624766	0.161425398	0.497597615
0.153764968	0.517806104	0.241967496	0.26124795
0.246570573	0.416286725	0.007168281	0.462110729
0.509886001	0.172847398	0.271084539	0.323011536
0.238618201	0.409660373	0.043665668	0.352684202
0.269194382	0.083001553	0.506309556	0.150496922
0.449903415	0.043630407	0.187933176	0.421276557
0.370109784	0.012159623	0.112239824	0.26607784
0.049162286	0.048059301	0.348977843	0.287253451
0.234091468	0.414896569	0.481936569	0.23056188
0.296263237	0.17790199	0.021561981	0.293408472
0.410183184	0.22612353	0.396975565	0.162661051
0.200103264	0.442432244	0.386876836	0.095052354
0.03694252	0.186773216	0.019518641	0.237226283
0.365583633	0.078312869	0.236888711	0.192340392
0.092289104	0.511229999	0.202935659	0.248100547
0.156860421	0.241092713	0.191748552	0.14424841
0.16252633	0.140892486	0.246500965	0.237017946
0.023770619	0.092250387	0.035345449	0.256638765
0.109160723	0.22427702	0.207733659	0.300151157
0.221677609	0.099313237	0.281059414	0.247872109
0.128202539	0.02365629	0.485744054	0.323682712
0.245055799	0.117706205	0.058844671	0.218401068
0.434228931	0.156381538	0.143688239	0.411442627
0.041439198	0.48509188	0.172528527	0.121952347
0.290106972	0.19896642	0.435920783	0.036669095
0.246732183	0.429519508	0.271838666	0.152814977
0.354508594	0.281296826	0.027253488	0.343514764
0.334417382	0.039603782	0.521617104	0.155341843
0.034197287	0.382056237	0.351179857	0.085570806
0.093193295	0.198205832	0.026082169	0.245946354

0.500035277	0.354129397	0.340396154	0.236560115
0.03596599	0.013000889	0.233735167	0.312719693
0.48046598	0.516819842	0.234800691	0.275991815
0.188043253	0.218263449	0.074474689	0.501535818
0.233133496	0.507665602	0.012306166	0.399886027
0.481685831	0.158886252	0.085793584	0.171604161
0.33355001	0.04109492	0.181002322	0.473204341
0.392245808	0.221513524	0.221444481	0.344008177
0.011942256	0.028755207	0.280013352	0.202816078
0.226171462	0.170148707	0.091624829	0.199255555
0.038598053	0.409937734	0.451999788	0.050633983
0.166489349	0.186443045	0.308570415	0.038903799
0.450888209	0.50466207	0.496622269	0.136343706
0.402211452	0.473270844	0.27517124	0.10428536
0.03115624	0.505825547	0.2875485	0.019985286
0.462421648	0.117516621	0.012193076	0.469737559
0.145894112	0.410915484	0.003399041	0.109552086
0.074493848	0.466675769	0.438785786	0.264213565
0.112485657	0.272601644	0.014121876	0.531303178
0.463301765	0.026526531	0.169376582	0.387048067
0.379953294	0.507396276	0.254398427	0.438467423
0.299681202	0.474271949	0.154364228	0.221205205
0.392473906	0.490287599	0.346707606	0.430003954
0.436026256	0.360562555	0.199331982	0.012821933
0.102010613	0.114924852	0.160195072	0.052382811
0.180853166	0.12797305	0.483667146	0.461774195
0.486531894	0.010589839	0.094270954	0.380950049
0.402762233	0.454530697	0.038789169	0.010183821
0.015099421	0.026022257	0.222985944	0.238692188
0.475676024	0.118153397	0.007691522	0.030551326
0.209552154	0.304312196	0.478704058	0.307125307
0.416552277	0.091367843	0.154319581	0.330553892
0.074331261	0.236619425	0.482265641	0.429469222
0.066754558	0.227523574	0.282224333	0.466614918
0.09296691	0.27469281	0.342372184	0.202624669
0.031308604	0.098485453	0.067989094	0.414414467
0.479285276	0.073414123	0.039099334	0.08300356
0.084139573	0.460200338	0.136866443	0.514381228
0.394248688	0.416431056	0.104589682	0.447340494
0.034492714	0.462355384	0.289306925	0.036666824
0.283613112	0.022637694	0.478019134	0.092936371
0.195215754	0.297877595	0.142121048	0.032974114
0.188003127	0.070385662	0.462069071	0.462663003
0.065383415	0.165533771	0.269466277	0.309994489
0.164164266	0.361854615	0.469980355	0.232792755
0.054163991	0.198728368	0.326174471	0.296462782
0.151580661	0.500929948	0.192081177	0.527987783
0.325849011	0.446347062	0.466145616	0.218678959
0.225659415	0.248990013	0.233817153	0.016057974
0.264385767	0.432213751	0.247075203	0.138637374
0.204112908	0.291847948	0.047997542	0.168679394
0.273189963	0.158830058	0.110575561	0.424613681
0.269209268	0.309273544	0.0399753	0.061938298
0.525942085	0.405047947	0.093967032	0.300667854

0.180624433	0.240239481	0.428539709	0.42994818
0.242720748	0.410889961	0.370445358	0.260531097
0.376673085	0.184447597	0.497117402	0.05734339
0.090645404	0.299019033	0.075197021	0.114163099
0.125815128	0.040371152	0.240358202	0.373535619
0.283924413	0.270598998	0.193513736	0.085068118
0.28684636	0.263278613	0.27803493	0.122399262
0.068387883	0.460572619	0.013445491	0.074547286
0.304643763	0.381554814	0.280860771	0.228755435
0.251583999	0.176056327	0.233825938	0.166014718
0.173796213	0.278414598	0.381910155	0.031933279
0.017316167	0.500423122	0.248099831	0.044918084
0.088869252	0.243965579	0.270699093	0.146427842
0.042392654	0.473287652	0.165765187	0.303206614
0.217130867	0.077855785	0.317041677	0.086827078
0.067199631	0.425508985	0.304941563	0.501150185
0.311726224	0.364615121	0.405311377	0.394058797
0.155361188	0.277541343	0.023994257	0.416301946
0.259557949			
4632434I11RIK	0.135606394	0.251149922	0.083255562
0.260002822	0.153981837	0.111272986	0.125767866
0.026589022	0.013572855	0.007487396	0.022128369
0.1546251	1	0.087034307	0.033813847
0.238960456	0.107326883	0.041371676	0.156193556
0.189147199	0.047591999	0.272667212	0.053477734
0.044362815	0.102223515	0.096193102	0.097749451
0.252315514	0.101897342	0.097747857	0.168161685
0.035573006	0.181981272	0.211694272	0.260222137
0.208868569	0.109459923	0.001648338	0.07078995
0.159556933	0.214667178	0.200194996	0.222633411
0.047856966	0.186388877	0.175707876	0.148165027
0.004260752	0.067299754	0.037612587	0.164863809
0.205828437	0.152880869	0.048292429	0.094049574
0.033252868	0.041028014	0.05529898	0.103777395
0.158401686	0.034086921	0.209375027	0.022020558
0.005949718	0.053392068	0.057509363	0.089604625
0.055482702	0.130216609	0.006190556	0.1047755
0.002210421	0.181924338	0.148417379	0.057803416
0.082594402	0.045169325	0.078917466	0.200018164
0.248648858	0.165095663	0.291190828	0.265193273
0.071954667	0.102817099	0.109181473	0.118142941
0.184135003	0.1371715	0.008213539	0.26872108
0.28654204	0.24083141	0.0690298	0.13379308
0.230827952	0.213307919	0.104529962	0.131349442
0.06393442	0.017619854	0.19131061	0.272310169
0.147273618	0.093819276	0.138675658	0.200074918
0.06079943	0.023287605	0.238823863	0.011340885
0.259903608	0.019167031	0.137625906	0.175403102
0.019229797	0.177636688	0.233829191	0.142089276
0.119677807	0.00922936	0.021476586	0.026590362
0.015154868	0.026993066	0.174397746	0.288933523
0.035608848	0.048397214	0.091275429	0.008579662
0.046251925	0.011156645	0.191189955	0.071174188
0.170094024	0.121662048	0.128662633	0.133498767

0.165593959	0.282755891	0.00142888	0.044486281
0.149297223	0.01945956	0.184678316	0.158628024
0.184754989	0.042637114	0.098708317	0.271325338
0.171900202	0.105972435	0.036426794	0.248320869
0.064882565	0.187415925	0.076351539	0.126916174
0.046756674	0.194796894	0.047478657	0.061030453
0.023707497	0.043698185	0.001334046	0.071317713
0.034260018	0.243270331	0.15963395	0.0755128
0.155506951	0.228126359	0.061662162	0.237094388
0.015415274	0.012210204	0.033531465	0.279881779
0.089423636	0.241150166	0.069865708	0.061388316
0.17546791	0.231901935	0.000120189	0.198862691
0.234488299	0.039509764	0.25610465	0.149758101
0.089898378	0.271242933	0.000856655	0.243348883
0.09789753	0.055160555	0.286772397	0.173373808
0.181705025	0.088619298	0.069560232	0.202324108
0.194155672	0.004291602	0.063141289	0.193684864
0.024549759	0.030257809	0.156399467	0.206467167
0.17814989	0.200800787	0.288198799	0.082920658
0.175754625	0.075310922	0.005779356	0.13480989
0.169259928	0.017171253	0.18497381	0.062863998
0.068648242	0.219498639	0.161554232	0.005187057
0.069013389	0.095539448	0.006455423	0.150799203
0.130339656	0.090268946	0.087098693	0.015549642
0.083787617	0.273432943	0.058188622	0.055800266
0.032647322	0.205209977	0.103359233	0.118260358
0.085405227	0.070656159	0.076044184	0.075859699
0.034666183	0.125249517	0.001653796	0.087395962
0.074818292	0.131291253	0.124134395	0.145539362
0.059046691	0.055546255	0.194563519	0.030842106
0.061215133	0.048040118	0.244961143	0.196080267
0.104769112	0.060047736	0.049554533	0.024257134
0.284249649	0.087922741	0.023553638	0.24836656
0.002769859	0.252252458	0.089637485	0.151240865
0.139073373	0.20781257	0.259057591	0.003632255
0.096662039	0.221103271	0.111981999	0.116474728
0.183477905	0.187900585	0.015075021	0.132767313
0.146781861	0.076304324	0.256971917	0.067388925
0.045312498	0.197221202	0.212920611	0.096574102
0.068692397	0.17668115	0.020783596	0.210692496
0.268358512	0.292288285	0.21264687	0.047686667
0.077669314	0.019336451	0.075950656	0.092621357
0.202825119	0.2497141	0.142451468	0.197060458
0.174535094	0.077629239	0.003372679	0.275448149
0.006845567	0.213167199	0.019576446	0.207295154
0.206365324	0.067720707	0.109489024	0.126384078
0.173347429	0.051162256	0.04150553	0.222070804
0.195723911	0.083612507	0.079746079	0.164149051
0.01819586	0.018524555	0.138303456	0.033893001
0.076555131	0.076638419	0.007431572	0.034288444
0.046607192	0.222915104	0.245177148	0.038700836
0.130698316	0.029785203	0.201031636	0.057132825
0.222252324	0.205915255	0.22348796	0.044483347
0.191564191	0.245189892	0.149278897	0.057883642

0.042617154	0.216931472	0.123141389	0.007317772
0.27672437	0.046550013	0.028209397	0.231670696
0.11048423	0.15201728	0.000483072	0.000885039
0.016612605	0.248639232	0.19712425	0.153342467
0.041528755	0.134269295	0.000213426	0.244424902
0.239673687	0.034686631	0.041732448	0.210331756
0.201259067	0.232844789	0.100506108	0.250405516
0.185290936	0.215889687	0.033719198	0.188840895
0.241255565	0.212482655	0.174162912	0.189390568
0.279481068	0.209228844	0.203135855	0.032567556
0.008272754	0.051297262	0.046833666	0.010171058
0.078306731	0.075676488	0.211022677	0.261776222
0.265350826	0.021392626	0.031434679	0.191548769
0.229700246	0.24724563	0.001338678	0.000378082
0.000491164	0.025839854	0.130386169	0.101854357
0.23255647	0.021906431	0.021433945	0.016541808
0.031089016	0.192082419	0.236431881	0.236924831
0.181230461	0.063758582	0.072432136	0.207969063
0.043297953	0.075379156	0.277813644	0.21487225
0.016833004	0.088628654	0.063199279	0.272600012
0.015522118	0.187470727	0.168507186	0.094522945
0.001118065	0.059058588	0.052539044	0.225258954
0.201870847	0.091584874	0.066897308	0.00368303
0.111085287	0.229254653	0.08861318	0.28100664
0.229119819	0.286121713	0.028156432	0.201806514
0.015095768	0.215772434	0.149269594	0.004899192
0.051905551	0.011091202	0.19681308	0.02490189
0.070440791	0.131406905	0.012992978	0.023899512
0.016040542	0.075033029	0.292059001	0.253297135
0.01335891	0.049552616	0.118823899	0.150084047
0.093613038	0.280916729	0.208396358	0.090000376
0.058233107	0.005261095	0.187373232	0.122424871
0.048259271	0.282527354	0.08463662	0.245853145
0.13578257	0.187637826	0.226031599	0.152071366
0.034136907	0.104961161	0.20106641	0.011503967
0.146079033	0.293937543	0.074054369	0.062229749
0.187333257	0.108189149	0.003553587	0.001837112
0.083827927	0.104548495	0.064450102	0.253475619
0.11180611	0.131159536	0.004200301	0.051593744
0.246752867	0.168293645	0.014885024	0.188001084
0.084573767	0.174144276	0.204084512	0.218719492
0.02991271	0.247001719	0.204692106	0.20569317
0.197126117	0.127156946	0.225871462	0.006654902
0.011156578	0.115367941	0.095298604	0.084421286
0.142518024	0.022721012	0.044032496	0.222789643
0.063102623	0.26808005	0.019197803	0.009332452
0.138176692	0.046920724	0.172701191	0.101350132
0.05903996	0.224286011	0.003292904	0.061440608
0.082404259	0.206986584	0.167511569	0.090220861
0.042930546	0.025898	0.188787911	0.071346607
0.116417834	0.010903835	0.203785827	0.003576413
0.022218984	0.281988408	0.065356102	0.005627854
0.004408828	0.086548478	0.032814391	0.0560495
0.02663222	0.232726488	0.074611536	0.13190122

0.122574275	0.043316338	0.116817587	0.003829659
0.008512865	0.181397807	0.116491026	0.226363363
0.07245368	0.297704493	0.152384973	0.202717118
0.003076689	0.065540582	0.025299622	0.208690826
0.074060691			
4930503L19RIK	0.196748461	0.334635282	0.093438968
0.340387701	0.166864581	0.200718969	0.186154145
0.095841774	0.04071807	0.051734733	0.025860239
0.171653548	0.087034307	1	0.053813115
0.280372159	0.070902892	0.081607854	0.201681932
0.283149258	0.114131852	0.281120844	0.084815085
0.035868217	0.040840014	0.062719181	0.053100063
0.356701339	0.071224795	0.170810207	0.310512583
0.040749177	0.270487383	0.292896725	0.238242519
0.277773131	0.170878902	0.019078151	0.093400517
0.268352391	0.231480915	0.294903491	0.265012724
0.070836778	0.298158648	0.222383759	0.146227591
0.068103391	0.043085188	0.106975931	0.27847592
0.282809363	0.169932004	0.113258682	0.127632555
0.070219007	0.109801184	0.036468476	0.250450707
0.193251567	0.116540168	0.280171865	0.065198799
0.044739017	0.126254363	0.046860552	0.185566644
0.041195604	0.254600679	0.03640558	0.121431036
0.031038894	0.272898866	0.152779472	0.034221922
0.058315723	0.081872519	0.06103817	0.281217086
0.342642117	0.248026598	0.31777766	0.289294277
0.102886116	0.112364843	0.093788326	0.117882905
0.262467358	0.172269216	0.067491849	0.282266386
0.315738651	0.305623048	0.056469922	0.106204836
0.296300708	0.320955144	0.133125399	0.140589675
0.048000962	0.015290331	0.274555536	0.321404599
0.26874858	0.058167101	0.093859734	0.339485645
0.054770567	0.067373028	0.292360508	0.088129991
0.352265999	0.033576683	0.087819431	0.247001402
0.059930646	0.266377188	0.284540994	0.178756162
0.127611139	0.065274575	0.019429473	0.026842311
0.08468195	0.015980286	0.262250983	0.364983935
0.121342392	0.065590679	0.142856189	0.007231735
0.031551678	0.038373459	0.251665977	0.127799364
0.249827899	0.171765762	0.093255267	0.230324384
0.167084499	0.306348861	0.024327479	0.079762105
0.184028512	0.038773448	0.198255638	0.186501165
0.127284036	0.09579139	0.118045107	0.332718692
0.30407358	0.116979691	0.09169135	0.279367679
0.079385581	0.136808439	0.094683306	0.158468207
0.090407793	0.245901518	0.021239086	0.138162555
0.029428541	0.0537342	0.015198756	0.175146662
0.107149572	0.289317609	0.236667326	0.090633377
0.198715478	0.223134056	0.152527281	0.277017763
0.030329356	0.047969486	0.099802643	0.341771128
0.227207352	0.291958272	0.097673966	0.060295074
0.149804215	0.296835525	0.001241414	0.2907915
0.292305959	0.102433708	0.249235356	0.166938463
0.069015323	0.346725342	0.008911075	0.29281463

0.20156289	0.068360285	0.335770372	0.121204383
0.267727818	0.056462837	0.132754036	0.20506727
0.202008155	0.011006188	0.162224679	0.294784154
0.015024609	0.090969588	0.237239646	0.2788212
0.192426004	0.24808675	0.345564276	0.059426194
0.171443054	0.140147069	0.016485888	0.111455931
0.222043132	0.041915166	0.306340906	0.100512802
0.154991621	0.314745732	0.20339378	0.037226271
0.071401873	0.179700186	0.065566059	0.127399446
0.184984626	0.132264813	0.085306679	0.042795643
0.044834942	0.334456905	0.026470918	0.031409938
0.013160635	0.249767874	0.165761756	0.133259155
0.099296228	0.063712445	0.054829305	0.079375138
0.084166686	0.100533607	0.004961589	0.079526913
0.127994901	0.119830366	0.128400123	0.226694669
0.112616215	0.153822601	0.164242987	0.033161349
0.039572429	0.031226091	0.322901111	0.254929261
0.237425422	0.082246245	0.039235277	0.064822721
0.354724622	0.161444169	0.069114375	0.345481323
0.028183477	0.298914491	0.118504702	0.09582067
0.102332251	0.222914636	0.342311665	0.02305005
0.173425485	0.330744844	0.1313682	0.081129639
0.302729215	0.254196178	0.063152234	0.228525122
0.246734786	0.056658035	0.315925762	0.051546673
0.029255156	0.208960837	0.216244613	0.068405084
0.0448621	0.184156859	0.081547285	0.179526233
0.33884501	0.30005407	0.26354177	0.032131333
0.072928343	0.058779994	0.052123308	0.105113961
0.297561245	0.29056479	0.163363103	0.139131252
0.210768387	0.04914273	0.062777476	0.323971165
0.027239576	0.294972593	0.015704826	0.204209249
0.274181282	0.161919055	0.051453998	0.208902374
0.301703881	0.05534053	0.079966758	0.310589169
0.276085332	0.074979765	0.06722806	0.21558469
0.057660424	0.071863425	0.135028364	0.018996211
0.084219255	0.070712654	0.004778409	0.070649246
0.085975108	0.320924492	0.304247722	0.035099467
0.193608948	0.066071894	0.194179655	0.055022673
0.226700729	0.262009413	0.311198813	0.091913323
0.23578473	0.28405086	0.116281304	0.044724763
0.020625544	0.279868892	0.225386255	0.070754214
0.338891925	0.143461671	0.069175742	0.338384494
0.157462374	0.251286454	0.011033716	0.007026639
0.01423694	0.272912354	0.279583597	0.277377551
0.097190338	0.210362131	0.00108064	0.297264052
0.327392744	0.014462575	0.030357183	0.240039476
0.189786346	0.33618384	0.057474619	0.251129141
0.162447346	0.278896093	0.124355065	0.161497491
0.318266244	0.247426317	0.284347539	0.301495857
0.354709162	0.219373024	0.234836586	0.0406032
0.016447166	0.13482852	0.052402596	0.038658534
0.193621911	0.053190318	0.296083528	0.312995168
0.292228168	0.012931877	0.113915394	0.243513115
0.358221579	0.349477232	0.010323069	0.002758148

0.002744773	0.013993658	0.227707467	0.087027274
0.293536753	0.089525571	0.01939563	0.086790305
0.097574925	0.151314823	0.313769596	0.299211827
0.214867698	0.058195593	0.092610387	0.293651339
0.027950731	0.166357725	0.312813057	0.322683702
0.022573544	0.076981518	0.120429877	0.358737879
0.005041964	0.173176774	0.194200752	0.094637778
0.007572409	0.035499736	0.029704776	0.26882978
0.263751003	0.096344441	0.066914163	0.056134251
0.119846176	0.29109347	0.126666922	0.336473547
0.335557108	0.351635364	0.024688549	0.293446148
0.034100513	0.294978228	0.097190903	0.038434763
0.070055691	0.01930244	0.253208207	0.027240264
0.141533897	0.239636259	0.022284447	0.012073152
0.014165312	0.046591456	0.335305228	0.264665099
0.013234791	0.08305298	0.129555779	0.218408775
0.216675604	0.323377697	0.303359645	0.128992096
0.090099087	0.015060256	0.303925902	0.219522961
0.112048566	0.317654019	0.06197016	0.307496589
0.225538056	0.278819771	0.276443482	0.198099486
0.041102017	0.186631253	0.304737863	0.092419132
0.20402647	0.337932248	0.144479815	0.049275054
0.13695831	0.089767507	0.034299641	0.038088249
0.056826415	0.235570013	0.116979995	0.319903104
0.21165955	0.225446231	0.053415918	0.031462101
0.332099324	0.267715683	0.081588916	0.266006885
0.088106503	0.159807906	0.240354927	0.227263536
0.04131464	0.300859534	0.311733295	0.315563714
0.324918891	0.171447631	0.309115318	0.077137026
0.020970159	0.154962184	0.102521899	0.057046042
0.144442076	0.021703006	0.025237936	0.260922127
0.04558865	0.286103112	0.063994844	0.051564747
0.125584707	0.085115315	0.19336423	0.176254193
0.09924674	0.306382543	0.017337861	0.048735723
0.064662475	0.23742191	0.110407919	0.083990478
0.032089161	0.029419834	0.195821916	0.171796658
0.148388806	0.048032436	0.246011687	0.045485944
0.06101472	0.335785346	0.133683083	0.010700955
0.019798935	0.156782641	0.066565217	0.069803117
0.012494182	0.262141218	0.114617884	0.141261485
0.220081821	0.055021938	0.242713398	0.033544937
0.040069204	0.294037485	0.106979328	0.283667488
0.078137269	0.330718186	0.261230213	0.270117358
0.035566431	0.083553942	0.053296258	0.305427381
0.067410868			
5730469M10RIK	0.252849273	0.407635985	0.156374517
0.319939142	0.154332832	0.234222868	0.115970758
0.051017797	0.066775875	0.004528159	0.009869412
0.179287029	0.033813847	0.053813115	1
0.29195986	0.018991047	0.094467718	0.116025732
0.253101752	0.03651327	0.228935218	0.035689198
0.04587135	0.028145684	0.030758504	0.088933089
0.339362886	0.038599446	0.140236478	0.262941527
0.016015757	0.277339074	0.406133303	0.205424511

0.370841326	0.255871912	0.000709908	0.098215072
0.325367163	0.321694891	0.269723785	0.177559019
0.022049317	0.378373106	0.144673892	0.170922357
0.070444771	0.010511057	0.094207215	0.365906572
0.361417008	0.210588787	0.091070323	0.076943273
0.031082235	0.049841901	0.033003389	0.212662677
0.101049364	0.059484962	0.362941627	0.065949302
0.036779168	0.081979726	0.018824507	0.103139103
0.065102221	0.296209872	0.008334065	0.165396657
0.001219552	0.252490616	0.147710445	0.096213474
0.017419375	0.005435617	0.030068908	0.200547452
0.346006311	0.344384893	0.345520888	0.353383639
0.02428801	0.091265597	0.121344904	0.04516722
0.338010653	0.086023587	0.032612894	0.268149795
0.356387455	0.384376508	0.041856122	0.103984722
0.344823894	0.375130449	0.180193646	0.142741824
0.106811951	0.011218044	0.35039496	0.363700852
0.301798937	0.077516827	0.052690849	0.290335722
0.019772833	0.066963227	0.20934741	0.014925858
0.361086918	0.021136574	0.063097782	0.163552498
0.043268462	0.305928853	0.380004861	0.098521626
0.118374778	0.004528958	0.042208913	0.007917936
0.048563885	0.016644847	0.372744519	0.343201235
0.040544525	0.053235725	0.103233949	0.001039981
0.006249808	0.028959105	0.249277023	0.111216153
0.284759551	0.28390968	0.086304769	0.263139123
0.220223771	0.334677498	0.026622084	0.036869434
0.199642636	0.028317471	0.119007082	0.175268824
0.118133327	0.042343529	0.038801284	0.405953844
0.360879875	0.038014771	0.084380095	0.257860595
0.063369558	0.124275123	0.03318067	0.052439579
0.01977981	0.361553714	0.006221386	0.126038072
0.093746658	0.014039519	0.004279669	0.109830588
0.063570482	0.324985349	0.347483183	0.038178452
0.082582029	0.215126721	0.124182841	0.26886785
0.009682571	0.014809985	0.094109114	0.36160203
0.179622569	0.354347371	0.063322024	0.074926256
0.117851627	0.341634887	0.00234213	0.393352782
0.395541969	0.099632841	0.164345981	0.174421653
0.149516752	0.356539739	0.00674124	0.22575464
0.20476828	0.030583519	0.373839011	0.057328407
0.333634983	0.019644505	0.079583274	0.302362129
0.220135816	0.002340062	0.123270659	0.270694585
0.005863314	0.043976754	0.278899475	0.276028529
0.243573273	0.302167747	0.355580631	0.138510004
0.165575515	0.077240906	0.0048927	0.110758821
0.283136713	0.054599976	0.383338326	0.066707141
0.157428498	0.388553876	0.279577277	0.062101295
0.027555995	0.130318971	0.010148109	0.169533966
0.31739235	0.029400192	0.183788127	0.018416613
0.016152291	0.400948973	0.033202768	0.05298758
0.018787737	0.157096907	0.111497073	0.040190495
0.027057671	0.045679325	0.102912415	0.119596282
0.020886823	0.024613102	0.003347819	0.049580064

0.016056571	0.052816109	0.057397547	0.226719019
0.039268837	0.016437094	0.128430038	0.058343465
0.030377337	0.006674575	0.397233165	0.243945505
0.241436501	0.054826411	0.011148822	0.035936872
0.344690103	0.184977076	0.085260044	0.35641835
0.010282844	0.349908579	0.041990416	0.044008736
0.06759778	0.102501825	0.375885979	0.031485195
0.172591626	0.359244337	0.126134835	0.070913361
0.295290931	0.245095557	0.014797805	0.275781606
0.239508461	0.010819424	0.379802614	0.016682158
0.007683318	0.233321993	0.279728169	0.034060059
0.027883628	0.134940127	0.029801526	0.143667272
0.404233541	0.255861295	0.296788597	0.046394481
0.017692302	0.001117473	0.042171499	0.145304662
0.380043974	0.35668823	0.095772992	0.10789376
0.128592552	0.093305098	0.03715074	0.372600069
0.030429589	0.383962533	0.003042781	0.276005368
0.381826168	0.106174993	0.027983461	0.170247828
0.293118103	0.028622855	0.048165433	0.38286204
0.374931523	0.125745254	0.128378905	0.199855293
0.013858079	0.01965261	0.212645491	0.030779026
0.105334944	0.075712754	0.005665461	0.065437262
0.015845583	0.3550806	0.290470223	0.037686342
0.187783726	0.084528946	0.169659699	0.018813289
0.322225515	0.359351375	0.398286825	0.043209113
0.23950937	0.369914346	0.157378252	0.023016667
0.006519877	0.383554976	0.273634846	0.010446334
0.334402819	0.113034352	0.003816457	0.397644305
0.092380835	0.327928767	0.018881424	0.013511442
0.008432201	0.302052833	0.403617545	0.2523024
0.086263472	0.187634664	0.000621456	0.371927259
0.375316611	0.004630116	0.023425624	0.264269299
0.243189588	0.409723782	0.103802754	0.314788002
0.171131608	0.369848353	0.046710109	0.133497446
0.3452901	0.349238478	0.311443481	0.39404019
0.317026152	0.218924984	0.168758999	0.012084769
0.090739688	0.087541831	0.094440768	0.03891729
0.205872526	0.040787686	0.360276165	0.325871177
0.332678709	0.002615172	0.047242669	0.280075373
0.354625662	0.380568079	0.006735682	0.00904463
0.002545404	0.007190281	0.188051368	0.084655602
0.384533359	0.047010627	0.002547341	0.0213081
0.071824484	0.183440542	0.37806428	0.246474702
0.278990587	0.020973469	0.057280635	0.263048441
0.022810886	0.208692023	0.354681475	0.388584862
0.008838347	0.089014394	0.108151528	0.383254244
0.009959053	0.123368193	0.180942338	0.061243575
0.002621088	0.022884199	0.027469792	0.285713602
0.358475835	0.012960886	0.00696862	0.027108861
0.037592939	0.325937126	0.035749415	0.381895694
0.339880231	0.3105153	0.011116034	0.412954803
0.01544169	0.357510843	0.102758707	0.031102395
0.05556832	0.004330984	0.310961102	0.060544896
0.079618666	0.241816743	0.094081482	0.003724603

0.038951678	0.013170721	0.360127747	0.314386445
0.006209824	0.072751318	0.115694516	0.215830409
0.15298354	0.287604651	0.394789752	0.170450982
0.035181951	0.011821399	0.267806916	0.220250699
0.046723449	0.347128126	0.06676446	0.39225834
0.285569518	0.369623377	0.334969527	0.225086016
0.025979739	0.208906893	0.258267607	0.012917802
0.172316515	0.31206865	0.145494996	0.041724463
0.089324768	0.137657814	0.016083261	0.025577114
0.091584605	0.197763654	0.030269418	0.318225211
0.200630609	0.239727555	0.010065426	0.028787495
0.410039466	0.379346589	0.074880732	0.284680926
0.052716008	0.181138166	0.285201424	0.258270417
0.069087364	0.294972972	0.28668608	0.236821324
0.345201518	0.140604143	0.410289971	0.031927482
0.035158925	0.166513436	0.024342063	0.030062838
0.052501145	0.010794368	0.049140876	0.238885156
0.062267471	0.186323424	0.044377911	0.030837549
0.109447109	0.112607408	0.160830186	0.083792057
0.024183788	0.392443538	0.003898091	0.013908854
0.059703565	0.332295322	0.131743099	0.067226456
0.046110939	0.019879903	0.116359618	0.12568513
0.082711721	0.050244543	0.343671691	0.025321085
0.008670947	0.350600373	0.143693267	0.00491298
0.017594427	0.217697705	0.040275441	0.072584259
0.005265368	0.334272479	0.127518324	0.171751139
0.188754078	0.017262621	0.282095103	0.025343646
0.023501321	0.343946836	0.164142493	0.369676887
0.096198975	0.279942565	0.362335926	0.267996804
0.02625431	0.137444809	0.020310908	0.320538239
0.099636198			
6330403K07RIK	0.346196641	0.478861242	0.366416234
0.398851971	0.418109844	0.293027443	0.303771214
0.237241723	0.185447549	0.017409032	0.062037433
0.368893143	0.238960456	0.280372159	0.29195986
1	0.238759659	0.369454637	0.365765182
0.406110211	0.159555932	0.426106494	0.219898017
0.367482319	0.202195963	0.17868518	0.338274154
0.455204025	0.198835066	0.250796281	0.342837335
0.235861664	0.390698396	0.443494387	0.384751147
0.426278288	0.302932484	0.035172046	0.279506085
0.388134144	0.384259133	0.39744551	0.394940281
0.127398003	0.399900604	0.33956451	0.414870303
0.24133174	0.098263455	0.212371942	0.377159127
0.385633831	0.340140961	0.287070233	0.261542131
0.191377082	0.207196855	0.2345167	0.25076329
0.33544401	0.154722926	0.408378554	0.174690859
0.195387931	0.324212336	0.280143478	0.323440522
0.350371472	0.314054216	0.078411928	0.309581399
0.006710322	0.411655668	0.424585963	0.283298289
0.138938614	0.060144181	0.312095099	0.368331803
0.468096676	0.407007649	0.485433078	0.475193139
0.258912523	0.415825034	0.364067407	0.294449234
0.404013729	0.262452501	0.189491958	0.429948103

0.48780637	0.45632697	0.33040388	0.390384802
0.363998289	0.439410105	0.351574756	0.398179486
0.30269106	0.152220883	0.379487331	0.483589443
0.343438326	0.318801927	0.286564111	0.373266361
0.17845958	0.302297873	0.411034597	0.210694023
0.480983865	0.07500324	0.338413507	0.362578715
0.361064719	0.384093631	0.452190398	0.315394272
0.409507682	0.067159688	0.178085626	0.126951017
0.328891108	0.183493994	0.379766261	0.443022
0.264260742	0.385784059	0.367106188	0.022060359
0.099197268	0.314149096	0.366900549	0.387109461
0.386761526	0.322330287	0.312723972	0.300180546
0.335231775	0.479816862	0.041600765	0.230181751
0.380850823	0.324801345	0.377234667	0.366359298
0.381309999	0.232775035	0.263114556	0.503032308
0.376650923	0.206680775	0.306598052	0.429506356
0.310375867	0.39427055	0.244208898	0.328940316
0.134401702	0.403655286	0.101580951	0.335279649
0.247915553	0.219112177	0.055762533	0.285520189
0.285176126	0.371603186	0.368940081	0.252718175
0.362564095	0.423966161	0.279753585	0.317404238
0.034683173	0.146273193	0.222525897	0.474131611
0.205851116	0.482039231	0.399500157	0.413803808
0.400774384	0.447595819	0.00918417	0.413727961
0.458644394	0.220300147	0.373595943	0.408625009
0.328413446	0.440584738	0.03919677	0.423762038
0.255905347	0.216316946	0.510453432	0.286989537
0.401316422	0.13163954	0.314209224	0.387583535
0.315795872	0.028879723	0.215349591	0.377434276
0.101663551	0.158699887	0.372766172	0.40273976
0.381574693	0.425441847	0.503891415	0.310598754
0.408207181	0.339938644	0.063479247	0.40386871
0.393758559	0.30054803	0.387934169	0.337662229
0.237687839	0.431274257	0.341484389	0.186788961
0.1363039	0.344027516	0.04975047	0.39617275
0.339596608	0.190834346	0.330256186	0.272310121
0.180185641	0.492177866	0.252523844	0.269588089
0.174607032	0.322255505	0.376790808	0.224539691
0.211151166	0.316942609	0.36568433	0.380843792
0.101285961	0.205638432	0.062905225	0.262516071
0.148426649	0.263059509	0.242942155	0.297967793
0.313998598	0.165552302	0.38880488	0.352496785
0.239434553	0.072583453	0.466355628	0.430048891
0.246338985	0.297966481	0.139840806	0.232259406
0.447726202	0.215496202	0.208592691	0.419972097
0.094373735	0.485849473	0.287999087	0.247304413
0.338828534	0.35589774	0.479203453	0.087611313
0.34191602	0.414963708	0.426667165	0.306789895
0.328745036	0.399857133	0.100297199	0.32271079
0.303765239	0.111623791	0.476238989	0.237582109
0.091502573	0.389392691	0.341185217	0.202444326
0.20185691	0.327493374	0.107740029	0.403704675
0.483840426	0.42253824	0.3490617	0.321070045
0.121492566	0.025972845	0.332383874	0.356557954

0.421430404	0.462759673	0.396593079	0.354975496
0.375063308	0.34397793	0.157760338	0.4753006
0.286340756	0.454475187	0.044488483	0.382360055
0.41760316	0.236263981	0.182054774	0.322575038
0.325820274	0.148885374	0.339718124	0.429162819
0.411757417	0.386710448	0.347493232	0.397565235
0.060436257	0.124802101	0.4033424	0.247274244
0.358818067	0.363876734	0.079270217	0.330443238
0.118121215	0.399713462	0.453563424	0.15952851
0.243655769	0.306710976	0.431205722	0.13153189
0.40914635	0.44147196	0.44590538	0.299595853
0.368223059	0.436030098	0.306251311	0.212914163
0.077896938	0.440487142	0.330591373	0.058116546
0.479908269	0.180726005	0.036001152	0.457668592
0.330211625	0.350179341	0.012249785	0.110926495
0.133112833	0.460631749	0.404638047	0.358386062
0.171735984	0.421101177	0.024187423	0.48478841
0.429179327	0.064713593	0.264733829	0.354499067
0.328322798	0.477571222	0.322330835	0.406458988
0.331839261	0.417970718	0.285292383	0.39303184
0.400209677	0.42254661	0.370542005	0.418396231
0.449110183	0.418844428	0.384952547	0.065764471
0.223037315	0.238002464	0.221931913	0.148909007
0.255883897	0.257233883	0.442956578	0.475740586
0.44631299	0.036072623	0.229935504	0.358456902
0.425615525	0.48220012	0.080750927	0.017685858
0.016192909	0.055732128	0.372441595	0.38139274
0.457349536	0.158887926	0.032920611	0.109623066
0.356944829	0.380401119	0.442660772	0.42874867
0.394890915	0.214673254	0.228932667	0.423782522
0.166345281	0.227562258	0.459490222	0.417341689
0.142335111	0.389871674	0.346998191	0.489757288
0.087627614	0.419848834	0.365294063	0.331372892
0.057325465	0.203328728	0.180455779	0.44013939
0.408001919	0.135709777	0.085950457	0.118136915
0.204344898	0.45923199	0.265632002	0.506835514
0.418626164	0.461952728	0.183683814	0.426086394
0.11553258	0.407256977	0.361940843	0.064296615
0.391322556	0.068182575	0.407168835	0.163401281
0.29271853	0.348785984	0.273926183	0.075616424
0.22114806	0.15424355	0.480998957	0.452854056
0.116830925	0.365242356	0.417957297	0.415718611
0.280483238	0.395185774	0.425102831	0.275714276
0.184812682	0.186971497	0.383952831	0.302313815
0.272244217	0.496631527	0.324700553	0.490893948
0.332975497	0.439902846	0.427822015	0.385442754
0.29638639	0.327769877	0.304409869	0.052189135
0.419482853	0.446804449	0.370443043	0.238610637
0.348348244	0.332679218	0.151293968	0.171704818
0.284075358	0.251872448	0.24926242	0.470487975
0.343818683	0.302143326	0.115123907	0.168291503
0.491748092	0.399985551	0.218365121	0.362639289
0.330266985	0.407432352	0.406693494	0.40540081
0.353845489	0.392924092	0.385775924	0.296348054

0.37281706	0.383320836	0.453386993	0.122018538
0.172747618	0.323368126	0.176765565	0.227314358
0.27699929	0.107173654	0.259553954	0.424750556
0.293479895	0.407800887	0.273231768	0.198979291
0.421055958	0.320816253	0.435736349	0.261817357
0.165876392	0.423550304	0.040449819	0.168444141
0.312141617	0.38266011	0.341691261	0.377234122
0.309255754	0.27253707	0.401348166	0.251508997
0.222254672	0.317724833	0.379400336	0.099765156
0.074092821	0.500742705	0.314422918	0.08648789
0.192573583	0.30139599	0.368309576	0.310196892
0.087495765	0.42192768	0.256317727	0.404084595
0.374435172	0.192100116	0.297990023	0.167396351
0.17732186	0.386828182	0.369197061	0.440353623
0.393232001	0.418760305	0.364729083	0.370847556
0.214474755	0.30821278	0.102019312	0.436748187
0.290004234			
6330442E10RIK	0.112802147	0.15370001	0.085222188
0.199872084	0.159671169	0.063619826	0.115146784
0.024643711	0.050159862	0.041702817	0.109939235
0.146623502	0.107326883	0.070902892	0.018991047
0.238759659	1	0.049260311	0.202960977
0.173602858	0.042383587	0.291555658	0.09133857
0.074255654	0.179994519	0.173218205	0.112211906
0.202012689	0.182154125	0.047727033	0.122178536
0.150348279	0.141178656	0.119755868	0.234480421
0.120021016	0.081844084	0.042399743	0.034694703
0.112094753	0.1662171	0.169934038	0.224174576
0.092091311	0.098263907	0.174650511	0.178139118
0.017893148	0.159320239	0.019119419	0.087904888
0.106878891	0.106099602	0.039238191	0.090905195
0.074438611	0.025722181	0.067363384	0.06006633
0.175847789	0.018595188	0.118435584	0.038925651
0.025905796	0.055894354	0.167235565	0.085828981
0.068878404	0.081701926	0.052008102	0.095359073
0.015851309	0.174253757	0.205005219	0.048364792
0.230262002	0.161477638	0.201071799	0.164961816
0.196065907	0.109693919	0.231861961	0.200738208
0.181382535	0.185308022	0.127432492	0.179490428
0.123913365	0.125639549	0.061204402	0.280314427
0.221348077	0.14422829	0.125502602	0.234557015
0.131990779	0.133061285	0.073162565	0.115815613
0.043351935	0.113239157	0.105491329	0.192310545
0.091098558	0.123604732	0.271412098	0.139910014
0.170249182	0.022898831	0.248319255	0.121784968
0.201707745	0.042190361	0.267299604	0.138851834
0.071381248	0.134368078	0.145247364	0.170161873
0.146318067	0.132898645	0.060910359	0.126905932
0.056805385	0.073213377	0.088397599	0.199196607
0.039998983	0.064139019	0.093981196	0.061368697
0.171477714	0.084070168	0.14267521	0.087137965
0.148879208	0.081660121	0.130067398	0.078831064
0.128954937	0.22915501	0.00248536	0.075081439
0.112418234	0.093853619	0.280614515	0.131369838

0.211800088	0.058695491	0.180992973	0.190193292
0.093753868	0.188784242	0.034559105	0.257133888
0.043398954	0.24034578	0.143374164	0.135873198
0.08393115	0.112596586	0.185651172	0.052748743
0.025863347	0.19333554	0.035847869	0.056855551
0.04540137	0.139046462	0.085008493	0.174120741
0.162191915	0.236997618	0.043833353	0.141700091
0.06514006	0.101715365	0.021539572	0.187467461
0.043534222	0.184808638	0.093625066	0.09562694
0.269149969	0.195121319	0.002819692	0.106095685
0.141529709	0.022246915	0.213849513	0.166235199
0.068670569	0.19606193	0.004099216	0.259494023
0.063684637	0.138542646	0.224971308	0.297472695
0.11483434	0.190336319	0.053411369	0.155220769
0.136737315	0.037252377	0.033670737	0.148333286
0.131334107	0.024962336	0.131822836	0.175431503
0.135209949	0.186975477	0.242550711	0.063008061
0.148261937	0.092616688	0.076206578	0.211175293
0.147180372	0.027329576	0.100367221	0.057863116
0.041512825	0.132009673	0.106808312	0.01431071
0.12121032	0.079578644	0.038789653	0.145959492
0.084564016	0.108889985	0.060744999	0.108581095
0.222238262	0.179526043	0.116706629	0.079616002
0.068226484	0.138094223	0.131840275	0.081300197
0.127151611	0.111827792	0.066693236	0.063872975
0.048697588	0.227952252	0.055170952	0.064362175
0.144257646	0.0863342	0.072255125	0.092826175
0.06034152	0.170767288	0.176005624	0.052829735
0.133822578	0.170523241	0.150675601	0.207848801
0.05696702	0.081318433	0.172300919	0.035147699
0.193120473	0.036412971	0.013309167	0.149010176
0.058875273	0.203176044	0.111692513	0.276761868
0.135007348	0.246657749	0.205173082	0.014004235
0.078148198	0.126925664	0.177820259	0.17115496
0.095041198	0.151313554	0.083554293	0.090284345
0.095122134	0.197975779	0.166901013	0.198084258
0.178519124	0.196868198	0.144139124	0.242756686
0.176785503	0.124474716	0.0261628	0.305208887
0.172761343	0.291964636	0.129981217	0.099484151
0.134433535	0.153966279	0.179895589	0.100742244
0.111611722	0.170444181	0.24949096	0.186964962
0.235038208	0.058778181	0.010727978	0.185591702
0.046171114	0.131075679	0.085972307	0.168745166
0.112831856	0.038569215	0.208493366	0.08498118
0.097432534	0.093057755	0.074860173	0.127853127
0.126170454	0.076757619	0.05560617	0.171211124
0.040483819	0.059662789	0.137555 0.066674566	
0.05582004	0.106682432	0.048184005	0.026210646
0.13732019	0.123323986	0.22429571	0.086522715
0.059225757	0.021997615	0.265996557	0.137316979
0.16086206	0.136693708	0.126586606	0.071981049
0.153735768	0.154223015	0.117683416	0.186826919
0.174506846	0.12952741	0.08831559	0.073496032
0.221755126	0.022015521	0.130286046	0.140859756

0.160687334	0.079460784	0.002293739	0.014654642
0.112100476	0.228507919	0.103630885	0.117838744
0.0163967	0.156521063	0.016983546	0.172834327
0.136677717	0.128744255	0.134633689	0.141024924
0.148593229	0.142689629	0.101779224	0.175895565
0.146007506	0.121790495	0.073624573	0.268189263
0.152235962	0.134861024	0.128486152	0.112858011
0.212885695	0.230663432	0.219903866	0.064872508
0.034236661	0.034388319	0.021264045	0.063723419
0.04712298	0.127719638	0.143200135	0.221652887
0.187795715	0.103564708	0.040793239	0.124572655
0.149849664	0.184988822	0.042378526	0.006852247
0.005673803	0.118246771	0.108498021	0.19101735
0.156453981	0.017055088	0.077777312	0.03657709
0.059060123	0.201849774	0.140554348	0.250125773
0.159118602	0.173574454	0.039385795	0.213051117
0.136286717	0.045350381	0.19353148	0.117182207
0.116194721	0.155038415	0.065761286	0.199675072
0.038039201	0.276382362	0.158230658	0.067095705
0.049873499	0.157478409	0.112649365	0.219943685
0.113665271	0.194255475	0.197352916	0.009900735
0.121761311	0.184420637	0.144397439	0.207739141
0.162467104	0.257677654	0.159401416	0.113840472
0.108198034	0.11868153	0.183853569	0.007762311
0.099359272	0.090535483	0.132081485	0.039820942
0.047422398	0.105001432	0.034267108	0.123203855
0.039112248	0.205221476	0.22264335	0.216705691
0.116783656	0.059041985	0.193787367	0.16295626
0.062824384	0.22185503	0.113890556	0.053520467
0.067163798	0.064898099	0.159349246	0.085410903
0.03992377	0.231191353	0.066857032	0.169263721
0.096819477	0.131374701	0.150092808	0.122006173
0.119644618	0.07361892	0.11410356	0.059463745
0.165248162	0.228557608	0.071365487	0.127432562
0.234240732	0.107518583	0.054818204	0.01436715
0.091679266	0.056318624	0.127040252	0.23949622
0.095203243	0.088383288	0.053922223	0.109626493
0.156664885	0.101774904	0.028095436	0.136785638
0.202523891	0.178536257	0.175276842	0.200762166
0.029214806	0.162112036	0.148540737	0.119660401
0.105780959	0.146335143	0.129153348	0.010994949
0.073382169	0.100770773	0.14759892	0.242104572
0.232310213	0.111173764	0.040180196	0.237248763
0.053928216	0.264988034	0.020738339	0.092586866
0.221894684	0.046426561	0.221888798	0.086321511
0.139605931	0.121449417	0.062469485	0.196364912
0.080541403	0.138709514	0.144390095	0.155531934
0.063248932	0.120982913	0.252726465	0.041707509
0.058308483	0.031948552	0.132432709	0.033412076
0.070382339	0.228324196	0.049832742	0.085687754
0.071114858	0.063245981	0.058503199	0.062438529
0.12934915	0.149291808	0.036726088	0.137175875
0.105290867	0.132893347	0.064977265	0.032239948
0.058875368	0.103474782	0.119221676	0.136306359

0.111026172	0.270858889	0.079469375	0.140402222
0.046247621	0.064028641	0.063347799	0.16881312
0.049133718
ABHD5	0.385510734	0.402450498	0.209358238
0.31505347	0.209080354	0.204676179	0.102996235
0.127430978	0.041466345	0.001727934	0.004625364
0.26591263	0.041371676	0.081607854	0.094467718
0.369454637	0.049260311	1	0.151953991
0.349504146	0.049317402	0.280835504	0.061613561
0.118367038	0.035337108	0.031162033	0.07476437
0.410889027	0.039192867	0.117933272	0.330305633
0.094367756	0.394259486	0.393040609	0.232993662
0.371287761	0.349339148	0.031478414	0.071791831
0.426935881	0.303145441	0.340359582	0.194058796
0.026383654	0.37721991	0.127000685	0.262915495
0.113047777	0.012708734	0.093669486	0.373998361
0.314200119	0.178779701	0.121065945	0.065271984
0.026869269	0.086519806	0.044347829	0.259008312
0.107771648	0.065542241	0.337605825	0.039065279
0.058186336	0.157868555	0.091171124	0.140606105
0.101948374	0.345669895	0.040738139	0.251627903
0.001088262	0.34928579	0.272125532	0.067573749
0.032855967	0.006271095	0.101474808	0.195716886
0.424990551	0.424608205	0.378754084	0.373537109
0.058077009	0.192151828	0.178332029	0.066377999
0.392018781	0.06175738	0.077444845	0.342902697
0.381787782	0.375289481	0.078936909	0.166286911
0.267380288	0.40187968	0.158999845	0.14610882
0.067221051	0.055218587	0.31925276	0.387023791
0.368325853	0.108295926	0.072461542	0.362553763
0.052190165	0.139063454	0.242304009	0.094681084
0.427415919	0.016288515	0.104593324	0.171286909
0.194385192	0.375217928	0.371844088	0.1047723
0.217538222	0.016667193	0.029962679	0.048714335
0.107510976	0.05088869	0.343594397	0.34326701
0.113094173	0.099777851	0.132104181	0.00661216
0.02274104	0.161408004	0.330176813	0.254919506
0.392243406	0.345977195	0.05762504	0.312708125
0.228453692	0.374733062	0.015619106	0.079647114
0.205955003	0.151830631	0.167631673	0.218033085
0.11388655	0.095244645	0.072657926	0.414580374
0.373642729	0.051229324	0.181566105	0.342946816
0.067515134	0.171131194	0.078289898	0.086913865
0.03197215	0.344389784	0.026242282	0.222691663
0.051633541	0.047186288	0.050642172	0.152869356
0.081993825	0.265331035	0.350179937	0.088460986
0.111136804	0.250273708	0.194703205	0.217529534
0.002009277	0.062924812	0.164038246	0.376249995
0.188479384	0.427485352	0.145503757	0.144846237
0.167192357	0.39678685	0.01138379	0.37105833
0.380501139	0.142268786	0.164872143	0.298125486
0.106099729	0.347670714	0.033453443	0.333066559
0.311092289	0.078225514	0.417519104	0.069569095
0.379884704	0.022725084	0.111764592	0.307913821

0.239383403	0.008243762	0.099210923	0.299947288
0.031010657	0.051122806	0.39321364	0.290878888
0.181585362	0.394952497	0.419695628	0.087471822
0.183799675	0.143225634	0.025901481	0.186272175
0.391095467	0.085569681	0.359692288	0.110795158
0.231450017	0.369701742	0.310675806	0.045313338
0.028966607	0.169999313	0.014401774	0.136650159
0.346704291	0.033280064	0.142093825	0.151960951
0.04192569	0.393544326	0.070429936	0.080281653
0.047530688	0.153115771	0.176388581	0.040394331
0.031548881	0.078332126	0.092293202	0.102541262
0.026381838	0.04079293	0.043913831	0.064892419
0.019806711	0.055881731	0.054386403	0.2850565
0.098350668	0.03080978	0.130564312	0.125939096
0.06207693	0.016897522	0.395376478	0.352107507
0.282554189	0.088321573	0.044035674	0.05641395
0.342634194	0.118771203	0.118510671	0.335075574
0.051789845	0.414042378	0.053639117	0.051813234
0.081156462	0.118577299	0.41238962	0.022651867
0.279877417	0.355070959	0.239977838	0.062712556
0.294823545	0.293951515	0.023209654	0.343365482
0.309836804	0.022873699	0.393580635	0.061868285
0.024933442	0.314300674	0.238534179	0.053359328
0.053630211	0.09167243	0.038232586	0.188461509
0.388944428	0.299560247	0.25519623	0.116199429
0.020745376	0.001810778	0.108830385	0.2428111
0.380161814	0.376942719	0.190279639	0.10233667
0.171486646	0.074421762	0.085728683	0.371691943
0.208830141	0.418202589	0.013566545	0.298222248
0.355742697	0.140892755	0.031232429	0.149630157
0.30229212	0.036825664	0.176453523	0.366414153
0.364776573	0.101921463	0.084946019	0.35014449
0.015171687	0.035399605	0.222038439	0.074648339
0.092632048	0.101742688	0.023817408	0.14313511
0.01831458	0.32687271	0.409134727	0.027784181
0.118868458	0.154321031	0.272151252	0.032875642
0.316770547	0.396889448	0.388283786	0.140783262
0.332985154	0.334987645	0.152431844	0.072262428
0.019414046	0.379799131	0.352104915	0.010440601
0.401224552	0.11763414	0.002531247	0.404884549
0.127746598	0.34737087	0.005239901	0.101497172
0.054205702	0.395525385	0.347629786	0.335933586
0.052773211	0.333921969	0.020569685	0.416826657
0.351292971	0.013068456	0.070929963	0.274951244
0.242268637	0.431715796	0.089203992	0.298187195
0.185786274	0.342284842	0.06786439	0.156424321
0.319042779	0.347515123	0.376872049	0.404158147
0.362280659	0.319578944	0.177371258	0.010915646
0.057192304	0.1027833	0.049210886	0.020159815
0.251709099	0.051819317	0.416524398	0.398858627
0.347569566	0.009894147	0.090562942	0.306972568
0.388719461	0.434539757	0.067614704	0.009875905
0.017617125	0.00536546	0.25343791	0.148441859
0.384761998	0.042651566	0.007518593	0.044941565

0.126099032	0.210194852	0.363096431	0.330928911
0.36749471	0.06430165	0.044264859	0.378912873
0.028472526	0.298638653	0.351681706	0.359615509
0.062783462	0.136896318	0.238597589	0.403555886
0.019455099	0.205551856	0.280496586	0.070249684
0.045498789	0.051469797	0.037223311	0.403581692
0.34650643	0.016782132	0.009739701	0.054516539
0.036137056	0.409883864	0.057471804	0.415430643
0.363784052	0.381449152	0.049846211	0.375709818
0.015264114	0.344829829	0.123070948	0.023059332
0.189500118	0.027820521	0.367025009	0.020535716
0.112147558	0.373953084	0.072899151	0.021401378
0.077696612	0.0364963	0.368734225	0.365098402
0.049319456	0.142666082	0.206029337	0.371694474
0.168708495	0.290459179	0.37463961	0.137470198
0.043509793	0.093733307	0.373664522	0.318131052
0.115396348	0.402967507	0.060127006	0.423496362
0.364096139	0.436081604	0.355332001	0.193878619
0.068178894	0.262641447	0.20907973	0.009560674
0.246469707	0.336046956	0.260951429	0.040173062
0.083351447	0.170936983	0.08422052	0.1533676
0.114930378	0.181077347	0.053937002	0.420704685
0.346864554	0.321779469	0.072920965	0.032047445
0.425432463	0.399410137	0.084510677	0.27490108
0.119987791	0.16209815	0.367613646	0.342053527
0.130968462	0.300704269	0.36803538	0.217617457
0.328403914	0.16155818	0.389220426	0.086982345
0.036894302	0.276700058	0.0251603	0.066195862
0.068297562	0.029755257	0.067163035	0.360949584
0.082809263	0.222774576	0.125772174	0.065659747
0.197325046	0.232206425	0.23390728	0.071429014
0.028976923	0.346858015	0.011214232	0.048629884
0.092466687	0.309167477	0.117636929	0.118812746
0.0748127	0.101666665	0.152449022	0.14890838
0.05524487	0.133072	0.305010088	0.04366301
0.021079894	0.417775225	0.214295365	0.046474042
0.067157874	0.306808814	0.205056615	0.059694871
0.026584844	0.327809592	0.082672023	0.219656514
0.229239872	0.070038816	0.346203455	0.085831292
0.098829957	0.37903683	0.217343803	0.367574396
0.203487227	0.312443544	0.370204489	0.316239358
0.155647201	0.224182039	0.025186783	0.419322516
0.08178934			
ACPL2	0.208633929	0.284164827	0.127671477
0.3541312	0.201821769	0.17123974	0.271965346
0.198194255	0.179983028	0.067159042	0.080951579
0.184333119	0.156193556	0.201681932	0.116025732
0.365765182	0.202960977	0.151953991	1
0.324247959	0.22707292	0.348580843	0.30158174
0.137723015	0.158127318	0.222460768	0.146407315
0.371012861	0.23537559	0.12421339	0.292638883
0.198753744	0.262304875	0.234525172	0.245676927
0.214766219	0.169845085	0.052739412	0.149016533
0.25248754	0.259415888	0.333642129	0.320036967

0.230125762	0.215621345	0.286138461	0.210260303
0.136523706	0.15708192	0.122602437	0.213956964
0.200051012	0.166005747	0.221161253	0.26704052
0.098030619	0.126262463	0.07191404	0.188022904
0.286190904	0.125626893	0.214815083	0.199706384
0.125576013	0.22823903	0.187726517	0.240507663
0.1068653	0.223048007	0.17847805	0.129539485
0.026563596	0.343591378	0.22811963	0.076131202
0.206982039	0.137472393	0.206867191	0.301373286
0.350254	0.219075146	0.323843709	0.289479007
0.198776022	0.221782348	0.126834206	0.281313631
0.255464333	0.25741313	0.189957831	0.359127627
0.316250186	0.248837447	0.219386313	0.211693336
0.249676653	0.27299607	0.133256685	0.157564656
0.09936065	0.118242819	0.215496357	0.29407506
0.217837255	0.097479651	0.230537279	0.301251717
0.243320117	0.116967439	0.359683287	0.139343297
0.360067002	0.160195135	0.200975851	0.245251374
0.2311118	0.254588309	0.239553212	0.302940002
0.166640392	0.135745015	0.072870622	0.17751145
0.166817925	0.060120832	0.186394107	0.327027228
0.191227955	0.152104085	0.268575067	0.059289276
0.150729706	0.167248887	0.216397957	0.166819614
0.281622815	0.157247201	0.201062936	0.190401287
0.169364876	0.317734157	0.0245551	0.297672905
0.198241008	0.179359213	0.321623338	0.183165012
0.198783076	0.291963786	0.305657195	0.3187296
0.23747047	0.290412684	0.12398473	0.352076893
0.145711123	0.193792217	0.305942131	0.234996735
0.246203722	0.200042387	0.12695533	0.162913256
0.078474411	0.136712004	0.105893235	0.192269475
0.172836097	0.227498511	0.174321558	0.290773325
0.231064586	0.284837217	0.133524547	0.219006904
0.072971344	0.22148073	0.089129317	0.295153873
0.145070439	0.287866173	0.251342974	0.198940876
0.253043937	0.33236866	0.006343631	0.219989922
0.244511224	0.084721572	0.231276175	0.216185501
0.096661076	0.334730137	0.017690253	0.340800616
0.15832943	0.279062221	0.344259861	0.244346634
0.219721494	0.200299979	0.172402266	0.212688371
0.171628046	0.052674508	0.145065939	0.321747338
0.113619406	0.199425081	0.263695252	0.31353322
0.231513431	0.291395195	0.369376271	0.089518727
0.193699779	0.273736418	0.122825299	0.189661446
0.243151782	0.087060328	0.234752883	0.274640604
0.11599423	0.263599208	0.182306639	0.072503162
0.23712109	0.204532857	0.113854648	0.194432899
0.178201273	0.235661276	0.10604413	0.179276868
0.191210671	0.29808714	0.100464598	0.075911735
0.058840699	0.183677488	0.285039007	0.153901774
0.125354806	0.264715948	0.132141827	0.15147792
0.210121198	0.254799088	0.058933905	0.079365492
0.125149175	0.110066037	0.097880986	0.187818654
0.167958092	0.196837196	0.17954842	0.124837639

0.186987114	0.144839346	0.277967024	0.330470423
0.167394658	0.301223379	0.202934422	0.073468338
0.314748264	0.10587895	0.084319876	0.281639165
0.146976919	0.314712342	0.195865804	0.232962244
0.125019675	0.268063346	0.367132278	0.07418839
0.17908937	0.254223191	0.233070753	0.22231029
0.201323586	0.28665388	0.213236042	0.195337835
0.188630421	0.200736506	0.271539898	0.182745029
0.15425176	0.243035418	0.204047437	0.22788671
0.202980254	0.176473694	0.196716192	0.294364217
0.296771866	0.360707072	0.224087152	0.132735744
0.222914429	0.068168367	0.16990619	0.134824894
0.220281402	0.256683306	0.269893863	0.148330958
0.328088029	0.129111374	0.112046169	0.287941159
0.118209714	0.246150971	0.10943277	0.22281227
0.208711787	0.117338333	0.178505413	0.215277715
0.216567758	0.243043324	0.263918879	0.239588359
0.265351761	0.179158911	0.137464	0.246966733
0.164338678	0.231027189	0.196761854	0.084400031
0.142854378	0.256056107	0.024982983	0.163092695
0.207377939	0.245192431	0.326453679	0.173448805
0.133445675	0.14601261	0.278927368	0.229209766
0.222420144	0.230351378	0.24133095	0.286512099
0.224347447	0.243614657	0.117693685	0.212438047
0.120594645	0.226858139	0.222115874	0.11155972
0.346292599	0.094919515	0.09687434	0.282551855
0.308484045	0.175019588	0.030354198	0.033238014
0.11750029	0.310124871	0.216520788	0.287881203
0.080274624	0.286622531	0.015897326	0.274552181
0.250152226	0.098372269	0.153014349	0.205365181
0.177891846	0.279521019	0.102931595	0.236710349
0.153175888	0.214518991	0.227202423	0.275068569
0.277515829	0.211183817	0.279550226	0.254139484
0.330074294	0.27734165	0.332188734	0.158026202
0.096766366	0.174966977	0.075700047	0.116070967
0.161860487	0.197490534	0.260908581	0.3331182
0.264631947	0.0867563	0.238772978	0.21402667
0.314680659	0.34871124	0.091924703	0.018530187
0.011446043	0.063255972	0.253466407	0.213381156
0.271151395	0.0728247	0.097951402	0.210843559
0.227219063	0.195503876	0.250099893	0.387019566
0.238124486	0.254879212	0.116043924	0.368261921
0.147618288	0.133123347	0.284988609	0.250084757
0.163176549	0.211522207	0.134091314	0.347242648
0.022104494	0.268232555	0.197596541	0.184610651
0.064607666	0.186354885	0.176187124	0.327863971
0.200194399	0.164089605	0.140205144	0.066106491
0.248640726	0.300270794	0.263702334	0.32645298
0.311083305	0.387320465	0.143072524	0.240981914
0.154215911	0.217884401	0.136124626	0.051930561
0.196021813	0.139640471	0.21221777	0.066082525
0.151292056	0.243335096	0.107103915	0.101562127
0.062941453	0.202888567	0.338199381	0.291547268
0.113006959	0.266987456	0.2255053	0.276268383

0.190521888	0.322469224	0.229492296	0.099970268
0.259290715	0.062328967	0.320626598	0.18585401
0.19229601	0.330242322	0.152736853	0.283624418
0.225383238	0.269648688	0.243876044	0.252875592
0.125204549	0.177920274	0.242803707	0.133186445
0.275015993	0.324081808	0.176449095	0.159100281
0.218880795	0.113099181	0.194194828	0.071738636
0.074062803	0.177925025	0.266085222	0.380396166
0.214397184	0.195790455	0.18564202	0.173220372
0.283933625	0.238239144	0.210577087	0.255751905
0.256659533	0.2340951	0.256373106	0.253867221
0.115865008	0.246453164	0.290467582	0.22470788
0.237438236	0.276712852	0.246110175	0.136416145
0.117695688	0.153461755	0.217901227	0.211434964
0.297926381	0.147144855	0.065135735	0.323383413
0.075609555	0.313544912	0.111890063	0.203572767
0.212567605	0.103958764	0.280081789	0.240752983
0.224373997	0.232233673	0.074033255	0.219091387
0.089064238	0.239777743	0.124339147	0.240418923
0.097923195	0.155206259	0.270573798	0.132597355
0.093872313	0.102303362	0.235626323	0.17841949
0.190068485	0.341756282	0.125382096	0.097985185
0.118183746	0.15950153	0.182964325	0.189395313
0.101878006	0.22390335	0.105665442	0.182475535
0.249585117	0.258104924	0.177809129	0.140999392
0.221602978	0.222361741	0.146754316	0.230457704
0.143033145	0.371618266	0.192555465	0.228984347
0.13987399	0.100623451	0.216658807	0.314912306
0.080833984			
ACSBG1	0.344633654	0.478476854	0.290047358
0.396269461	0.355017592	0.283735166	0.290869459
0.273075358	0.131113747	0.017374791	0.038402776
0.305694544	0.189147199	0.283149258	0.253101752
0.406110211	0.173602858	0.349504146	0.324247959
1	0.180558197	0.354959838	0.193115158
0.260917563	0.127075367	0.131448797	0.228672358
0.459559598	0.14706902	0.259277461	0.360786578
0.179369228	0.401858403	0.444820774	0.313362866
0.424105922	0.303983169	0.040288426	0.265644774
0.405894711	0.352195011	0.382776593	0.345314982
0.110467583	0.420487576	0.30445865	0.327056364
0.253862144	0.0657365	0.239255851	0.403695521
0.389232406	0.288355585	0.302028405	0.245337212
0.15115709	0.253634649	0.162494021	0.270679531
0.305966881	0.195759222	0.40839209	0.152114768
0.18367625	0.349624115	0.208165084	0.337482041
0.25839718	0.344700194	0.07990703	0.258989795
0.010240772	0.376722498	0.342744385	0.192116378
0.102137984	0.049488444	0.235351417	0.344977586
0.470026827	0.411390039	0.455482164	0.444102483
0.201575391	0.340540951	0.279234328	0.247415924
0.407576523	0.243667552	0.162821711	0.397911684
0.458568522	0.450057897	0.236160199	0.289975998
0.362641817	0.454225538	0.300384664	0.329788271

0.220598424	0.095676757	0.388386515	0.465333294
0.373047792	0.222762103	0.206626005	0.404606829
0.142236314	0.289085936	0.35997674	0.19168135
0.478868233	0.06136591	0.242207761	0.345020752
0.314622872	0.384650831	0.437157367	0.285072687
0.338264486	0.054072548	0.101635104	0.096334749
0.293716235	0.111188496	0.39163698	0.439936119
0.299888876	0.31918836	0.349110884	0.014926413
0.067192324	0.254458258	0.36694401	0.366961022
0.389104432	0.316110042	0.237937578	0.327592135
0.27352116	0.450281521	0.044958072	0.217243126
0.329053445	0.257598717	0.31635681	0.318226374
0.282200265	0.238087176	0.232763084	0.485549473
0.409220452	0.180710397	0.32118462	0.378398993
0.276574471	0.294912944	0.22188509	0.306432976
0.136959242	0.393386642	0.064286546	0.341198202
0.166963732	0.158193177	0.055373861	0.314848259
0.263972879	0.364917158	0.378824008	0.217846582
0.335088805	0.346421668	0.301710997	0.306105991
0.024820138	0.124692457	0.253480694	0.464825973
0.230644238	0.461357344	0.35577028	0.31839677
0.310012994	0.431375988	0.006414827	0.422321284
0.44667942	0.238675903	0.315217324	0.341509284
0.2442676	0.432297724	0.038546979	0.37636422
0.280200907	0.185861426	0.487532236	0.214874505
0.407247564	0.095907622	0.331089187	0.350004155
0.296355235	0.022720489	0.241839942	0.355448256
0.064305489	0.182948719	0.37706019	0.357501782
0.319098225	0.402502072	0.484146359	0.223874543
0.340223671	0.338342603	0.04787191	0.302830988
0.378811049	0.25268697	0.410197893	0.315979002
0.245651954	0.435710174	0.330632361	0.169452645
0.116446361	0.353831218	0.048185725	0.305559511
0.335143208	0.182048251	0.262328406	0.231116752
0.123998684	0.478679415	0.158885993	0.179194137
0.101734216	0.295885087	0.353599574	0.207575455
0.163424885	0.241951295	0.268482872	0.309167599
0.111829558	0.164738874	0.040820175	0.223936951
0.125306065	0.229208686	0.222245393	0.316236839
0.312685057	0.146909932	0.31286074	0.251404126
0.15975814	0.052121083	0.464777624	0.371877843
0.288310613	0.259031349	0.106737021	0.204618913
0.44429836	0.213592176	0.23354132	0.430561004
0.079816383	0.465802065	0.246294696	0.179518185
0.266719074	0.307010286	0.470735098	0.071500546
0.335998608	0.432594851	0.351391703	0.222062234
0.355556814	0.361371472	0.090601938	0.337887355
0.323852963	0.085750374	0.463861579	0.169510405
0.064695488	0.334270072	0.314037022	0.147568623
0.140102028	0.286315093	0.127989596	0.312899449
0.473593479	0.375334438	0.345242712	0.21788585
0.102364944	0.022103717	0.237149791	0.288530721
0.432406081	0.441949079	0.33540487	0.271068901
0.327802156	0.249326032	0.198452955	0.457547923

0.229894838	0.455540326	0.033858453	0.34673739
0.41600967	0.265590853	0.119284263	0.319249089
0.353743607	0.123751108	0.332491333	0.432475677
0.412846831	0.299541592	0.264795021	0.350303727
0.062459272	0.132497344	0.315275838	0.152504318
0.30922378	0.286759289	0.040197354	0.331959886
0.097720014	0.413308852	0.439725679	0.105597017
0.235709397	0.308076115	0.346663021	0.107872735
0.372028336	0.428558605	0.449825844	0.30546431
0.361714455	0.41588048	0.236488954	0.156141047
0.049664308	0.431949175	0.331745249	0.052200919
0.470197201	0.210383864	0.030800698	0.467666593
0.300748615	0.371814714	0.010453344	0.081490907
0.084650839	0.428857869	0.408753945	0.352788993
0.184954079	0.385946616	0.018020505	0.466847042
0.433234472	0.037149029	0.170512817	0.343375708
0.29338052	0.486088319	0.2198813	0.372804748
0.265360513	0.413781026	0.263840185	0.305510261
0.398207929	0.401976408	0.384773905	0.43417655
0.446322312	0.344842922	0.334724612	0.056306441
0.132117443	0.275738002	0.185406691	0.105878099
0.270176155	0.178424674	0.441313859	0.457861018
0.421207002	0.025041266	0.260073634	0.357267272
0.446351965	0.485564741	0.063746932	0.010566651
0.011803316	0.027258572	0.367649592	0.285133852
0.444160044	0.165743441	0.026119365	0.131432208
0.318096471	0.287556649	0.442828776	0.375378843
0.371825507	0.17370035	0.203153328	0.393341815
0.099508909	0.252551345	0.436239737	0.433694797
0.105906264	0.292421246	0.333420645	0.48444214
0.044790496	0.337808749	0.311151296	0.283073068
0.046404534	0.136754955	0.116805326	0.420286588
0.404420535	0.1030489	0.060312943	0.141910517
0.1820269	0.448873105	0.222419402	0.488896823
0.425208681	0.449795038	0.122229436	0.430993342
0.078276694	0.412290319	0.260063533	0.062754999
0.332642144	0.053884257	0.400502115	0.102927277
0.309787872	0.342763029	0.171272967	0.04716912
0.13333206	0.110370291	0.459169174	0.416494024
0.075738412	0.347345853	0.334785469	0.37047802
0.298498774	0.380748125	0.434934628	0.243354737
0.181219342	0.131816015	0.38569486	0.299023364
0.308504454	0.46924912	0.245652779	0.474879276
0.34547831	0.444135594	0.415404531	0.339398141
0.211961104	0.308911759	0.29541835	0.052716302
0.369051166	0.43216789	0.362928887	0.15404095
0.267069897	0.251809462	0.149018467	0.179125446
0.201621037	0.267712449	0.228085299	0.45629297
0.336491479	0.31274565	0.139833187	0.108190484
0.489534409	0.414111707	0.212320687	0.332490385
0.264382757	0.324360412	0.383996962	0.372838416
0.291040487	0.389526096	0.403153728	0.301435114
0.400626639	0.342322242	0.454345029	0.161711071
0.101328378	0.288068485	0.144201928	0.162535044

0.233117085	0.069656732	0.17695198	0.383966034
0.22237847	0.351026802	0.293335477	0.158135911
0.330771003	0.299109428	0.363973914	0.262276369
0.135350336	0.427067035	0.030163865	0.128230102
0.247805015	0.361707864	0.254111232	0.288112761
0.206082585	0.195558477	0.335815879	0.273423942
0.210115432	0.274369528	0.362027324	0.094941719
0.077948917	0.481808685	0.304925572	0.058098729
0.145167161	0.288850444	0.347024292	0.248130334
0.053407757	0.401943063	0.245188108	0.327933858
0.362844325	0.16271466	0.337277754	0.14517461
0.160438435	0.408569912	0.284380302	0.431179462
0.311397817	0.399493082	0.389291678	0.373795708
0.181687102	0.257473551	0.089102474	0.443913887
0.227510693			
ACSS2	0.071964497	0.122623092	0.034557691
0.212674942	0.077626791	0.132366489	0.273218002
0.124568568	0.075796087	0.105793097	0.029698569
0.054366208	0.047591999	0.114131852	0.03651327
0.159555932	0.042383587	0.049317402	0.22707292
0.180558197	1	0.155774089	0.151318862
0.013942883	0.03139326	0.099186929	0.028964549
0.194978275	0.108334125	0.130962798	0.212690004
0.032747075	0.113146107	0.093728679	0.087805515
0.078662418	0.058874948	0.020951922	0.09927264
0.117830997	0.097307818	0.197244091	0.219458855
0.145481338	0.102448795	0.266811002	0.059583098
0.064108733	0.053836991	0.124076753	0.111343751
0.086737727	0.071350891	0.168267279	0.221233336
0.030957227	0.106962546	0.00772227	0.175938299
0.23609664	0.169009957	0.086930203	0.141867516
0.050171459	0.155837757	0.026802561	0.211028262
0.01375925	0.140107296	0.102085755	0.032026201
0.046632973	0.199752328	0.068919698	0.011056073
0.088164038	0.076283441	0.039887385	0.252041622
0.165765976	0.079569314	0.118571869	0.098909811
0.045968904	0.074161576	0.028000097	0.120767093
0.110948821	0.243624496	0.074743751	0.142288318
0.114649384	0.096282842	0.042177816	0.068834567
0.141967918	0.131791296	0.060066823	0.067034183
0.021445985	0.012727807	0.102487121	0.109369439
0.116157183	0.015658809	0.079789659	0.193499728
0.093716514	0.029702213	0.225565734	0.028857018
0.169872162	0.118308739	0.055441074	0.195241166
0.051033246	0.108682154	0.083798451	0.252669751
0.050211681	0.047457903	0.008492047	0.041245111
0.050193537	0.003455956	0.079930487	0.167079092
0.10803653	0.028526828	0.177188381	0.018041871
0.034321466	0.02166266	0.079667428	0.072057145
0.122757964	0.047847196	0.086059255	0.103850974
0.049767498	0.11445515	0.027165899	0.153314397
0.098141552	0.025554468	0.195229587	0.072706676
0.074424834	0.172415379	0.159586871	0.126616142
0.141213645	0.19497024	0.055723091	0.147651057

0.055223029	0.054251383	0.149140361	0.109373814
0.181123571	0.069384661	0.022787108	0.106796256
0.013006248	0.020478382	0.043085645	0.205875711
0.080474391	0.10826197	0.067208067	0.137686263
0.134378768	0.113450283	0.110512593	0.116464573
0.060710333	0.07366182	0.071042382	0.121257813
0.164558727	0.096362861	0.078875092	0.038365709
0.102650474	0.140362767	0.000606533	0.09601768
0.089018142	0.065994467	0.119829346	0.067041314
0.026273297	0.170290128	0.004602999	0.150683467
0.086729461	0.124964544	0.132819172	0.108360488
0.087485554	0.094914786	0.112166958	0.060350054
0.053522578	0.024836986	0.21283301	0.226724077
0.015794958	0.198405673	0.115797137	0.178614522
0.13417566	0.10187328	0.152275185	0.021334267
0.079211645	0.170444868	0.034241967	0.052676246
0.079834037	0.013619986	0.128326278	0.121131342
0.072517384	0.125965995	0.06186243	0.03086541
0.170298075	0.18358244	0.105527494	0.08924386
0.063367835	0.146294224	0.036122162	0.028439245
0.045280147	0.119660065	0.00958284	0.007788673
0.003396522	0.115751174	0.192824501	0.07986499
0.035467752	0.070978847	0.026294776	0.049624741
0.212369026	0.109732996	0.005765308	0.018510751
0.050172113	0.040371074	0.044788244	0.09578002
0.059176773	0.082096523	0.079427811	0.012993947
0.032722303	0.060126256	0.118897734	0.148773846
0.124449088	0.129309159	0.061245241	0.016730337
0.154079565	0.103502864	0.057389413	0.15293981
0.048903771	0.114801302	0.070966056	0.093524354
0.031083532	0.191490214	0.175643973	0.042942171
0.098479944	0.132201325	0.091528487	0.076034087
0.119119731	0.157812523	0.143702524	0.088659353
0.090629765	0.088276006	0.100509435	0.029019614
0.050680728	0.071905796	0.074075274	0.094813651
0.054514558	0.136620653	0.224697028	0.123793197
0.124815699	0.159507726	0.110415382	0.014420341
0.137996859	0.038122368	0.027338183	0.033044696
0.09276642	0.085985495	0.134814675	0.053203858
0.232692877	0.02734342	0.08804952	0.110694474
0.012199635	0.094419645	0.04093926	0.065587222
0.079175691	0.130600694	0.050731208	0.222870354
0.136213903	0.13505413	0.099734346	0.102080885
0.125919612	0.055868108	0.041062979	0.089368203
0.182653091	0.181724638	0.066034722	0.005647591
0.052641862	0.083122061	0.000660446	0.058561872
0.110350315	0.129836106	0.12466232	0.060178358
0.127685044	0.060016865	0.093687859	0.121298257
0.065136109	0.075023962	0.100128515	0.132830582
0.078257969	0.089178464	0.02872013	0.055516695
0.031532544	0.080336251	0.11985662	0.06813344
0.148675832	0.134696935	0.07109797	0.134933153
0.22477417	0.078255324	0.03465797	0.002025147
0.013930616	0.101708582	0.096409406	0.199061909

0.078926976	0.146539512	0.000936221	0.092986067
0.114075565	0.018085479	0.017655044	0.074726278
0.049246738	0.122273195	0.01891544	0.077507747
0.043634353	0.082495333	0.101710347	0.130209385
0.141480416	0.066150917	0.148476532	0.123868144
0.155069438	0.091161537	0.243629694	0.118416688
0.0140044	0.192753982	0.029319481	0.047703162
0.138170056	0.044917734	0.10112271	0.130625845
0.091133698	0.032189113	0.178472065	0.086088923
0.182921881	0.167363433	0.017598874	0.00484995
0.00201833	0.011182965	0.186082395	0.059810605
0.103642253	0.048647998	0.050807291	0.21602221
0.073220843	0.050990108	0.10545085	0.207670537
0.072194463	0.084789916	0.056177417	0.189996489
0.028274503	0.075129154	0.107089913	0.130714423
0.031435519	0.057888037	0.057237714	0.162495024
0.00067044	0.106285008	0.062135625	0.064810728
0.010944476	0.036587304	0.040195967	0.122420726
0.072551089	0.060273141	0.050023143	0.038992392
0.158497213	0.113897912	0.098541288	0.126599345
0.164632534	0.183355606	0.019203445	0.109595853
0.054685399	0.091281906	0.033625127	0.046653212
0.041053571	0.043880836	0.070246419	0.017813583
0.100385054	0.132906753	0.015718015	0.016267906
0.003839443	0.057734702	0.143105469	0.094276696
0.013566103	0.116335861	0.085620253	0.115735074
0.223413178	0.163611681	0.101052676	0.045554588
0.182676667	0.003107113	0.186126989	0.091753288
0.114191	0.119263752	0.037806007	0.102182012
0.107153867	0.111316987	0.090042111	0.177604596
0.014408264	0.098039933	0.208093291	0.150430514
0.152627368	0.142558929	0.105484749	0.03131755
0.097065117	0.023977778	0.076487168	0.017842618
0.012609557	0.201247172	0.121220405	0.166080058
0.113719116	0.096740849	0.109545597	0.044272547
0.114594408	0.112825096	0.137191239	0.141938337
0.085717013	0.113419435	0.083168475	0.075243153
0.01832374	0.106151054	0.15633852	0.169502512
0.145050909	0.206009545	0.101204538	0.134399844
0.017936658	0.049431068	0.104934419	0.062417589
0.174140465	0.035085678	0.00554569	0.126120614
0.010451601	0.167637127	0.038651719	0.056536341
0.068940349	0.036637622	0.124307274	0.231540755
0.112091946	0.103303927	0.024473746	0.064651114
0.014904059	0.092655152	0.034376435	0.064151204
0.009111378	0.017379958	0.150942402	0.146422521
0.068618533	0.013785826	0.095418608	0.106416478
0.154055039	0.132389468	0.067815833	0.013486589
0.021132308	0.074791744	0.037128259	0.068366874
0.014280355	0.075241836	0.088278201	0.058304904
0.196443657	0.085142523	0.106293657	0.042136214
0.077489593	0.106073034	0.035566097	0.083039312
0.031211725	0.182855423	0.093015698	0.095457613
0.026618857	0.024926877	0.144250445	0.145558411

0.019335035			
ACTN1	0.315039116	0.459773202	0.298855926
0.401697443	0.384127003	0.24146853	0.28482792
0.185220907	0.167596744	0.030134158	0.095000041
0.32478134	0.272667212	0.281120844	0.228935218
0.426106494	0.291555658	0.280835504	0.348580843
0.354959838	0.155774089	1	0.219413588
0.327699861	0.236368691	0.211058855	0.321121134
0.444130387	0.225382216	0.217133663	0.307951904
0.242532688	0.368942238	0.41396377	0.368278553
0.403410634	0.268051147	0.039375976	0.249580679
0.350679326	0.376018515	0.359735808	0.377244751
0.147995155	0.368623612	0.32848999	0.36449545
0.186957676	0.13787646	0.163658935	0.340522276
0.369401189	0.303659208	0.228419198	0.255658966
0.225989488	0.178051621	0.250110788	0.201910522
0.332873525	0.127327407	0.391513518	0.163064491
0.169211836	0.262476948	0.297429131	0.285614574
0.3133053	0.280168743	0.073956366	0.260707494
0.0130961	0.350260888	0.378867031	0.246100349
0.168524668	0.106425834	0.323962437	0.342036977
0.455399403	0.369516897	0.490054075	0.472081934
0.307131001	0.37877025	0.325294237	0.327046069
0.372288998	0.271310447	0.172894732	0.443221737
0.489491674	0.440385605	0.328900104	0.367920933
0.3601565	0.414242383	0.300757465	0.366463754
0.269577779	0.154819383	0.359040908	0.47916694
0.312197995	0.2987247	0.312527274	0.357454145
0.195651846	0.242150574	0.387142711	0.228907239
0.467404707	0.076820661	0.348452603	0.337573952
0.298571795	0.362715216	0.435996518	0.307817089
0.364718358	0.09569231	0.175451893	0.137135619
0.305020021	0.183847435	0.349970152	0.447502658
0.234565504	0.368499645	0.319832076	0.030116179
0.131670818	0.274603993	0.361087573	0.315701221
0.364086481	0.287095662	0.325949801	0.272548089
0.297288505	0.484223103	0.032929767	0.214706926
0.329240197	0.287908286	0.371484443	0.33061639
0.378334998	0.205274353	0.283510643	0.490060852
0.342251885	0.229288491	0.234577798	0.409974605
0.300117961	0.382996473	0.249234236	0.349135623
0.154045959	0.379673896	0.128868152	0.257480725
0.208323286	0.269694816	0.047304682	0.235911589
0.266030797	0.374260492	0.336729458	0.253055524
0.368493707	0.396109783	0.219365385	0.323196272
0.056295777	0.14625932	0.162700495	0.472222366
0.166157007	0.466090949	0.363130776	0.36122636
0.390387282	0.43438456	0.006668877	0.384007239
0.438886718	0.165649711	0.370828827	0.354752211
0.283537309	0.440111104	0.028296136	0.40229582
0.22079498	0.213288564	0.5063812	0.322586522
0.375041923	0.165972423	0.275863477	0.379941111
0.320463251	0.03265095	0.177821509	0.326440198
0.118424006	0.139900096	0.343138594	0.356483852

0.341771912	0.410850633	0.503286414	0.270929006
0.375807562	0.296147842	0.067980699	0.374854615
0.370649356	0.260324745	0.359358097	0.297757813
0.183746206	0.410149919	0.322174608	0.164615616
0.154591872	0.294170578	0.056427647	0.362326653
0.301551372	0.234252938	0.271484371	0.250418492
0.216212301	0.48258612	0.247190692	0.245689848
0.17105771	0.312453203	0.329973901	0.259660454
0.268246565	0.309489807	0.320898749	0.335151932
0.108977101	0.254844972	0.058684787	0.263940378
0.216801925	0.281028544	0.253886142	0.278915609
0.304667369	0.226573567	0.38071629	0.29589953
0.249063585	0.095430514	0.447291457	0.374635895
0.219539419	0.275365004	0.160674168	0.231573863
0.451844194	0.178669665	0.1475994	0.412979501
0.087717127	0.476311353	0.33089459	0.285036159
0.349778002	0.370582977	0.467343701	0.07296342
0.275425577	0.398271208	0.371627292	0.322169982
0.313844931	0.344617274	0.116473936	0.295937901
0.286470895	0.147507071	0.463809441	0.275721723
0.113148903	0.368511153	0.344575632	0.223140664
0.218297143	0.326572406	0.098360909	0.394502789
0.47285152	0.427924737	0.348602181	0.286139724
0.156312701	0.061402975	0.324380803	0.295846139
0.395084256	0.454163679	0.378464537	0.364302558
0.358731587	0.310557782	0.11770931	0.47178333
0.216312911	0.425471712	0.055162612	0.379514588
0.393920037	0.192479152	0.220316452	0.282869594
0.309619207	0.155698451	0.283946016	0.410248683
0.38520598	0.336802036	0.303325479	0.344468216
0.067504181	0.129390221	0.346491373	0.225719957
0.324573099	0.336185615	0.080397629	0.25572059
0.162496791	0.385741051	0.451059454	0.170217589
0.217703053	0.223681999	0.40086439	0.149443199
0.402645221	0.417444443	0.421973831	0.25893058
0.36175326	0.42890915	0.282599137	0.22026392
0.09921299	0.418120579	0.276722682	0.084293902
0.478960845	0.138654646	0.073636672	0.434437198
0.308885541	0.318521624	0.009055933	0.076942422
0.135036073	0.457919585	0.376871652	0.297652143
0.147388981	0.354161909	0.021626764	0.468650541
0.416253526	0.085162253	0.269163212	0.355128079
0.332525187	0.450997655	0.296128264	0.409375197
0.308558487	0.401053078	0.286842745	0.38494003
0.393221044	0.406555053	0.345667538	0.384711041
0.455143823	0.379487758	0.365417795	0.080950285
0.176091602	0.193496526	0.198082572	0.17075055
0.195799749	0.278857628	0.418712045	0.473917023
0.447010299	0.047627454	0.206388108	0.346300252
0.407314128	0.464500683	0.066713966	0.013655974
0.012665072	0.081844455	0.314346431	0.353162759
0.440421358	0.15097356	0.046086295	0.103143435
0.317332589	0.353168901	0.428868089	0.386002711
0.378777661	0.232487197	0.231370206	0.385706293

0.188872388	0.187542367	0.460748463	0.394123386
0.144711627	0.352320532	0.277433521	0.482386046
0.087778886	0.404215435	0.325273044	0.331683231
0.054880792	0.222366565	0.190875179	0.431989828
0.387562615	0.203454142	0.140755298	0.095419021
0.241740026	0.445539628	0.304073364	0.500634818
0.407210046	0.469664047	0.205892311	0.396327319
0.138139471	0.389535948	0.354096165	0.054639604
0.337247189	0.07661266	0.390157071	0.168303808
0.252835406	0.28773897	0.214736092	0.091990522
0.182462114	0.188820568	0.484848647	0.45260689
0.122679751	0.304519441	0.377561117	0.349986136
0.23337981	0.410278535	0.399233255	0.236563243
0.190631542	0.172055706	0.351917645	0.255147527
0.236732657	0.496935257	0.321746449	0.47149558
0.298154479	0.404405415	0.415490457	0.335065247
0.314800594	0.262668838	0.277654013	0.07209837
0.367997221	0.460890678	0.291103948	0.263749834
0.373291283	0.292303845	0.128459644	0.124348696
0.256039249	0.208302376	0.287399517	0.46436834
0.276212649	0.2685416	0.105400931	0.182638165
0.470315445	0.360841313	0.177399291	0.32722722
0.319505491	0.379222809	0.396505047	0.404556572
0.27861049	0.398442446	0.369842875	0.285722156
0.35336075	0.344107094	0.429256223	0.087777506
0.162198322	0.272770449	0.231386612	0.242925743
0.303604344	0.120052854	0.232605012	0.404668889
0.264415007	0.397531452	0.217959721	0.198971772
0.392933169	0.242058956	0.402156226	0.255565253
0.208768356	0.405033092	0.05163402	0.195006542
0.294223237	0.366910818	0.329946017	0.360518884
0.288282395	0.263629378	0.402117645	0.203363847
0.222625488	0.264506422	0.364313591	0.086872152
0.087472967	0.499249013	0.246284607	0.086814349
0.177427719	0.232301335	0.306073903	0.302393863
0.102737841	0.415287706	0.221823884	0.35501896
0.317710613	0.200227768	0.262519055	0.131345587
0.147835033	0.358211587	0.313837171	0.422620688
0.331938763	0.438914401	0.326339785	0.365383763
0.168670645	0.2447377	0.107659252	0.417294409
0.255619957			

ADA 0.083206089 0.112207106 0.05911745

0.219833394	0.106061051	0.095245316	0.263967751
0.086181526	0.124076682	0.070477784	0.051896828
0.076208252	0.053477734	0.084815085	0.035689198
0.219898017	0.09133857	0.061613561	0.30158174
0.193115158	0.151318862	0.219413588	1
0.036850839	0.088978175	0.18675151	0.069593269
0.203697155	0.209557468	0.074383132	0.172804125
0.0858626	0.112859197	0.081792829	0.118700265
0.069704763	0.06346654	0.027047402	0.070531957
0.108822016	0.127460855	0.205237898	0.263126837
0.176666869	0.074906018	0.293146917	0.108242911
0.044970239	0.102996781	0.066782627	0.080339604

0.067565385	0.077434114	0.138788765	0.21630551
0.028375453	0.051213492	0.013778243	0.101449438
0.268182645	0.076669416	0.074110985	0.138291579
0.050570212	0.135785333	0.070077096	0.177204002
0.029190397	0.107613224	0.142845768	0.044281901
0.022678419	0.229655847	0.125109922	0.024479756
0.205855391	0.09033493	0.100765211	0.236905573
0.175227662	0.076776874	0.145614163	0.120028731
0.069918534	0.143137241	0.052622483	0.174382049
0.109196744	0.228724805	0.093613758	0.206794329
0.138297261	0.089041503	0.085451561	0.161486038
0.121268916	0.111931518	0.068752398	0.087750653
0.032480736	0.054544401	0.082528353	0.118072656
0.088866432	0.038385647	0.18486906	0.158092408
0.196699996	0.024887684	0.276744543	0.037343414
0.181137331	0.159960236	0.140650299	0.174834495
0.088407292	0.112993688	0.083570944	0.306039169
0.080136687	0.081945564	0.024844834	0.109577683
0.046127602	0.012888889	0.059782781	0.161300496
0.064337151	0.03532094	0.193834804	0.056292655
0.092863699	0.050040539	0.079329821	0.086471835
0.140146932	0.051266131	0.123268206	0.077757034
0.065496626	0.141949518	0.009766926	0.200317215
0.112676853	0.058873808	0.30817437	0.081596102
0.139084063	0.186243721	0.23250725	0.137210836
0.099615186	0.280269838	0.046907856	0.209674077
0.043254407	0.109195232	0.2196238	0.111295139
0.168385576	0.065209725	0.087297163	0.09532073
0.020529637	0.042714414	0.082644217	0.143365134
0.060104277	0.094392177	0.053075727	0.242226813
0.141246814	0.168762528	0.074448588	0.10377932
0.065646185	0.134825128	0.039769485	0.119989155
0.07815534	0.113386095	0.106236682	0.082112307
0.200199067	0.172267923	0.003173253	0.076660421
0.086323911	0.036962603	0.136650516	0.102778762
0.043558863	0.175634542	0.003738792	0.19572029
0.06192501	0.229072423	0.156881921	0.211073014
0.078033106	0.191642027	0.080513913	0.081559766
0.059034273	0.039741551	0.116263126	0.213220448
0.063273984	0.12006276	0.128865039	0.199714921
0.158065106	0.138338598	0.183238396	0.037528622
0.1043132	0.181573567	0.091417391	0.118079599
0.101143501	0.015425785	0.095686428	0.133648705
0.047307115	0.109425935	0.06479335	0.022785094
0.242551859	0.153122454	0.09013367	0.145119298
0.065464474	0.121686519	0.050397379	0.060965797
0.124163292	0.121099469	0.036361585	0.022686959
0.015738047	0.095020395	0.240469312	0.058591453
0.037633937	0.127353399	0.047775714	0.066512116
0.159416904	0.182568903	0.031737708	0.018264443
0.043226349	0.034023955	0.031504459	0.075668616
0.042794097	0.082656719	0.109133497	0.031038221
0.092553108	0.155368493	0.110938657	0.202295327
0.072620594	0.18285953	0.152376186	0.011749269

0.148099319	0.057425796	0.032160923	0.126576987
0.098429317	0.137564284	0.06962304	0.19717104
0.044287991	0.242330634	0.195777736	0.044658093
0.0875555	0.101872296	0.167450641	0.150091661
0.077483598	0.169930519	0.165075003	0.077230138
0.071551963	0.177302559	0.100916008	0.07155606
0.14744474	0.108344193	0.085602737	0.227874464
0.148817415	0.12668391	0.148970746	0.233536816
0.122638787	0.219955381	0.09972652	0.048142052
0.187522214	0.041469801	0.080523419	0.055307011
0.073890504	0.092966889	0.228238267	0.081665076
0.324943061	0.045268938	0.044259275	0.11590446
0.030201241	0.086740807	0.106663681	0.089969568
0.067904047	0.06821844	0.128501333	0.174984089
0.093221228	0.218736385	0.132267682	0.087116341
0.121659483	0.089490363	0.058872442	0.114611493
0.157713003	0.16877749	0.112691084	0.021229122
0.055904865	0.149121172	0.005799662	0.051720059
0.139289964	0.099535166	0.15357238	0.119452941
0.075449281	0.054399972	0.163898067	0.21832621
0.082951919	0.078610538	0.08506337	0.156291424
0.085740053	0.091613385	0.042348227	0.154013943
0.113892635	0.076201304	0.108025556	0.073578533
0.165562197	0.062064929	0.068314738	0.116236772
0.300079517	0.055512759	0.038552506	0.006869939
0.059221639	0.140078595	0.078422225	0.173534928
0.034938513	0.18602042	0.006383157	0.101970925
0.094946266	0.067804717	0.056243413	0.075488904
0.064660362	0.107378786	0.043795561	0.09343381
0.058517085	0.072226168	0.087954799	0.238239509
0.130287173	0.069602007	0.141809973	0.10361568
0.15901258	0.139901728	0.309293273	0.143415443
0.04220014	0.117968018	0.022283992	0.066928747
0.087493795	0.096156173	0.099756539	0.157273563
0.102817	0.100671434	0.120119044	0.08153951
0.154936475	0.170224193	0.055681002	0.014180313
0.005129617	0.038746733	0.170017683	0.142635368
0.108573764	0.019293259	0.104913257	0.152367806
0.082475066	0.093209839	0.092389419	0.267536072
0.096191251	0.175063975	0.036977264	0.234653863
0.080503178	0.051686127	0.117859169	0.100187998
0.092164128	0.134170625	0.060549863	0.167608158
0.004541269	0.189656336	0.079769575	0.05953178
0.037537538	0.111222017	0.11190537	0.165655661
0.063583202	0.078450075	0.081035005	0.016094115
0.161176423	0.12989196	0.119927237	0.14063538
0.156717043	0.218384879	0.06258962	0.093154789
0.098613735	0.074210968	0.073064169	0.028535492
0.076524466	0.10793986	0.069770796	0.023905211
0.066901662	0.121721785	0.040198205	0.065119759
0.014061136	0.142549714	0.163382963	0.128064612
0.058007473	0.140641293	0.166597021	0.15199984
0.148247221	0.179375253	0.080746424	0.040459974
0.172816228	0.011576586	0.184906904	0.077123466

0.07671788	0.147289552	0.052163298	0.109435434
0.103605944	0.112119391	0.091189377	0.19810361
0.031544618	0.088740602	0.14797079	0.110602426
0.202306463	0.156174649	0.105834032	0.065553741
0.159552898	0.043603627	0.121434641	0.015685388
0.027274047	0.116664753	0.129677813	0.209852748
0.104309146	0.082346727	0.111176818	0.109494695
0.108127955	0.097581344	0.119727945	0.13783474
0.184242782	0.172368945	0.108156706	0.105484221
0.026272254	0.099242447	0.141250323	0.117673787
0.099552279	0.264364838	0.088304254	0.066755291
0.052212242	0.055932576	0.116602233	0.173980886
0.261998349	0.10484818	0.012468786	0.173974043
0.017212115	0.206078134	0.023650624	0.10955837
0.137365837	0.042798546	0.192742259	0.184929417
0.13195783	0.084203267	0.054706321	0.157636325
0.022641327	0.105494795	0.057463411	0.13488662
0.021148637	0.052028383	0.222745852	0.084170331
0.040328356	0.01710871	0.102502196	0.135383104
0.152273671	0.155288375	0.060804879	0.056857552
0.04895171	0.069751901	0.050266029	0.07505322
0.067001671	0.078309088	0.056561181	0.090883906
0.186373885	0.169653944	0.067799672	0.069969321
0.136600564	0.081315889	0.064783635	0.078305932
0.064080529	0.225142627	0.066251753	0.089317776
0.051148253	0.036790373	0.188426905	0.150751748
0.024515686			
ADAM9	0.28713807	0.323646563	0.223426971
0.263700481	0.238123504	0.11967116	0.075522027
0.025306167	0.041531892	0.001368513	0.019669647
0.26396395	0.044362815	0.035868217	0.04587135
0.367482319	0.074255654	0.118367038	0.137723015
0.260917563	0.013942883	0.327699861	0.036850839
1	0.08621862	0.058760537	0.154360207
0.311687832	0.069591081	0.066784046	0.18617769
0.094478387	0.283382622	0.29948192	0.295779905
0.296855781	0.239122205	0.013367828	0.043462193
0.262565567	0.319685688	0.255489794	0.182088078
0.020803555	0.241831795	0.116472611	0.327223719
0.031070992	0.028211113	0.029828491	0.226768772
0.243192258	0.194263678	0.045193622	0.048613159
0.035342043	0.025576261	0.073802402	0.107122209
0.10262303	0.01573678	0.274731759	0.025316315
0.039670053	0.065666732	0.11285152	0.072234647
0.159623985	0.181453122	0.014293359	0.246683455
4.73E-05	0.255978701	0.319354651	0.135773089
0.064393376	0.010000536	0.142768611	0.155592405
0.333886806	0.298341073	0.391792411	0.391609942
0.059752529	0.2259443	0.273577413	0.070878084
0.285886704	0.055693607	0.033434283	0.388198571
0.390618599	0.319574871	0.101552663	0.279480525
0.225978107	0.283543587	0.134937068	0.168642048
0.084662894	0.097112241	0.24105365	0.364744821
0.20705641	0.232188069	0.147668653	0.221774001

0.065725298	0.044370681	0.231711083	0.054093847
0.339720728	0.013278656	0.22170873	0.13599765
0.094533087	0.270857312	0.333572166	0.095341788
0.235348697	0.015900948	0.068773092	0.055437812
0.054111843	0.111985437	0.240361734	0.292012959
0.030414934	0.088410164	0.091455374	0.011977845
0.041357976	0.115210287	0.275011374	0.157908762
0.300624456	0.261240315	0.090212152	0.182065635
0.266731914	0.396837089	0.002776612	0.038075825
0.170349459	0.134093711	0.196871199	0.212933612
0.193471429	0.031727209	0.067722617	0.372052279
0.215006612	0.056297045	0.077049221	0.346389042
0.048149312	0.286646142	0.057400217	0.064953698
0.016871995	0.293448191	0.062914357	0.098605628
0.064125469	0.082476737	0.018821363	0.062391502
0.034440279	0.242690921	0.248071689	0.085697628
0.10072149	0.308505351	0.080683163	0.196328237
0.006575097	0.037205309	0.053182006	0.334608263
0.073281693	0.389970192	0.085986899	0.15970762
0.248442403	0.348370058	0.00705056	0.262286683
0.330666736	0.050418013	0.18579171	0.304764306
0.175568773	0.29822929	0.008393602	0.320892325
0.149887884	0.066983069	0.399655141	0.134570419
0.275884548	0.048550241	0.060451233	0.356243381
0.25434198	0.010668502	0.037652043	0.211255491
0.065231696	0.013397131	0.285509299	0.24410352
0.195476683	0.373855359	0.393341959	0.156402466
0.201300087	0.076237725	0.027447311	0.281869917
0.340463141	0.070429161	0.22692724	0.050406047
0.09831913	0.277310602	0.262697951	0.032725447
0.031732592	0.097367359	0.004977631	0.188736262
0.265224985	0.02326508	0.173308592	0.100526411
0.081138853	0.35307628	0.169955361	0.18235596
0.11862646	0.136599811	0.121946324	0.02769526
0.038647526	0.083167732	0.134332572	0.117609319
0.009296843	0.058473892	0.058125961	0.070912371
0.022157047	0.053862687	0.049060172	0.189637736
0.046323456	0.024677291	0.162776828	0.132861459
0.08678466	0.036160184	0.318979284	0.308020062
0.125650713	0.055818106	0.056825527	0.035520717
0.301150067	0.063734899	0.037389101	0.258895698
0.025577695	0.398227856	0.045993556	0.113058949
0.128183651	0.131207062	0.346713169	0.015345674
0.165320796	0.251882176	0.255676253	0.112850417
0.179609093	0.230453887	0.014968131	0.212486937
0.188949678	0.037645953	0.345661173	0.088295881
0.050573368	0.352500611	0.271464683	0.108386957
0.090382194	0.100813356	0.008675962	0.27902491
0.336270917	0.34085443	0.233279721	0.183099706
0.023636795	0.004820472	0.202586377	0.246568428
0.271204894	0.357646804	0.207590981	0.165781492
0.168887255	0.106300368	0.015612633	0.348019034
0.117564339	0.318428791	0.018579472	0.359910513
0.284559526	0.055801564	0.077341289	0.095871475

0.178665542	0.033371626	0.076743393	0.277861777
0.284522819	0.130281529	0.099190829	0.298740334
0.006104759	0.015275633	0.276125122	0.136864715
0.092000869	0.110471092	0.07896692	0.049568427
0.020609303	0.241108097	0.369828524	0.0457515
0.085380383	0.052355455	0.351222423	0.037991358
0.358452461	0.340990081	0.293028542	0.053499931
0.295642916	0.321280953	0.235041679	0.108976302
0.053127212	0.315535009	0.201016548	0.008710624
0.362280277	0.035733799	0.004983978	0.293363771
0.107727738	0.216581606	0.000789079	0.06235975
0.087665129	0.413499511	0.259048121	0.194885121
0.021394003	0.242297952	0.030446434	0.378790231
0.271372992	0.036347299	0.138578839	0.263770025
0.29203361	0.316667772	0.193629859	0.332553792
0.232686692	0.287452934	0.036292965	0.236492908
0.255403053	0.33175493	0.241623329	0.264209964
0.314647477	0.364762509	0.177558346	0.011104803
0.094729142	0.037823732	0.03627428	0.035011266
0.103323295	0.073940952	0.313413157	0.382607429
0.346608407	0.0217975	0.024420708	0.254083509
0.258395915	0.334792071	0.043482848	0.007385275
0.007267195	0.027675267	0.156578609	0.226522474
0.337328661	0.01410834	0.010151184	0.009903382
0.057222373	0.330275008	0.296856165	0.297486073
0.359669639	0.075873747	0.027457421	0.30339304
0.069418435	0.133109472	0.344499115	0.252015032
0.073681492	0.215128217	0.131160217	0.342733243
0.078633903	0.274507442	0.271161019	0.052448272
0.04154486	0.088833873	0.06890055	0.388830172
0.28509115	0.027428646	0.021304113	0.010163107
0.030499331	0.363213027	0.045072234	0.385020367
0.271099506	0.34956749	0.107160683	0.276435253
0.036083159	0.263008526	0.239618793	0.005879661
0.138230291	0.027379964	0.308820665	0.044625204
0.053873559	0.209987795	0.10250357	0.052948089
0.124407831	0.060123955	0.364210251	0.404657266
0.080348132	0.071691968	0.235677116	0.286679538
0.072546707	0.279274922	0.270209955	0.106877836
0.023265939	0.111726219	0.243162966	0.175733986
0.034850156	0.406700565	0.072304104	0.371525878
0.228632931	0.318420536	0.315797273	0.166127719
0.122464717	0.148412384	0.143917401	0.004657424
0.199185695	0.33031331	0.141727489	0.068781848
0.14788752	0.259969958	0.042529985	0.032179337
0.21822513	0.081354983	0.041458799	0.379449771
0.189880947	0.186559238	0.020562559	0.058815511
0.342094703	0.263781648	0.025703781	0.217179089
0.153613894	0.222381971	0.350647617	0.382345005
0.088272789	0.271094864	0.248513381	0.148138044
0.208287748	0.142256978	0.30312426	0.009545558
0.067790983	0.227823617	0.029468555	0.138780575
0.087837879	0.049521913	0.100661532	0.359750025
0.117117974	0.241152614	0.040534144	0.054397259

0.28654728	0.138759273	0.259081519	0.043700502
0.027646495	0.270917853	0.014002544	0.069843746
0.132680258	0.292763359	0.205694341	0.141567963
0.117493503	0.136849914	0.190278092	0.058277477
0.044023012	0.06838125	0.276757209	0.015282236
0.00954716	0.392142607	0.104146014	0.059731113
0.114060223	0.181180357	0.085249402	0.058684147
0.062595624	0.325638	0.059382717	0.259505236
0.140992406	0.055385563	0.167867702	0.03646015
0.046154854	0.237407952	0.278418323	0.31613357
0.237422672	0.322600658	0.22146671	0.260592278
0.061667044	0.202387718	0.016201556	0.314548356
0.099865445			
ADD1	0.08346722	0.119618098	0.083951923
0.164560377	0.123426177	0.051332947	0.09473573
0.017730282	0.109059957	0.031551227	0.164192181
0.111832969	0.102223515	0.040840014	0.028145684
0.202195963	0.179994519	0.035337108	0.158127318
0.127075367	0.03139326	0.236368691	0.088978175
0.08621862	1	0.186742619	0.15608458
0.152915891	0.199348606	0.033495791	0.083524821
0.120796478	0.09727935	0.094018291	0.186045571
0.091077876	0.063943168	0.014207623	0.033635286
0.081754838	0.161127996	0.127260675	0.181069479
0.094916855	0.071115024	0.150777221	0.155748195
0.016896439	0.189857701	0.013802195	0.064266509
0.081620006	0.094443246	0.030432974	0.080784429
0.073232956	0.012753191	0.083439334	0.037808178
0.135404763	0.011039599	0.091387245	0.061747867
0.025800933	0.035692858	0.130492399	0.055611035
0.069537524	0.059668676	0.034060633	0.073760568
0.007245718	0.134244373	0.162514574	0.074844354
0.197913131	0.121679596	0.142811539	0.127350993
0.14755051	0.082031394	0.189877773	0.168040549
0.141030287	0.143961688	0.111975619	0.138113054
0.097520853	0.102790582	0.061239875	0.232450558
0.182624446	0.111845631	0.151205302	0.226744689
0.112916904	0.098455697	0.068884308	0.099223007
0.065033622	0.135232121	0.081644404	0.150202493
0.062636231	0.127794777	0.263113791	0.09387517
0.134084315	0.019793662	0.196847385	0.059044041
0.155350931	0.066032612	0.245489422	0.098043715
0.061337352	0.102406716	0.116968645	0.139954671
0.107342273	0.093735445	0.131850914	0.104858109
0.045195722	0.097774184	0.066774886	0.152206441
0.023393972	0.059661008	0.072865098	0.058555274
0.166634584	0.07239656	0.095453214	0.055641643
0.11355515	0.065567862	0.154912032	0.054821887
0.113189514	0.186305287	0.003096764	0.068755338
0.098302241	0.077713276	0.229399444	0.093669614
0.224108591	0.046296744	0.123730124	0.157145269
0.0675908	0.143155917	0.020574616	0.210813649
0.044394536	0.216929094	0.097571854	0.079010374
0.063659494	0.091383189	0.167885849	0.035327492

0.059735518	0.144040057	0.02382672	0.035856662
0.041452695	0.112708572	0.063150022	0.128329088
0.098828665	0.208375512	0.026368655	0.114342418
0.092467149	0.085949525	0.012547964	0.141669224
0.026760453	0.14545213	0.067989611	0.094467872
0.243632346	0.163480152	0.004526033	0.081715328
0.114093591	0.013798423	0.162163942	0.127637625
0.089477735	0.159928591	0.002969484	0.185801819
0.039338585	0.107478891	0.182236872	0.2701413
0.083461024	0.189928315	0.031710947	0.142205075
0.10300664	0.029905204	0.0248208	0.114948337
0.143424065	0.021766134	0.099553172	0.147154789
0.143090681	0.155098664	0.193030087	0.088125164
0.126918416	0.061183518	0.070635032	0.193206597
0.115349453	0.028102386	0.076217657	0.055101284
0.025883453	0.104101917	0.084548475	0.020417575
0.112559992	0.054385724	0.029526125	0.172778015
0.071727032	0.08158554	0.073461552	0.068356596
0.192273308	0.144497321	0.136715526	0.093699559
0.105401412	0.089549736	0.099499406	0.055906591
0.107692308	0.135457756	0.086250911	0.070985708
0.037345373	0.1702966	0.058250358	0.038870613
0.094946254	0.052472567	0.040875796	0.061207158
0.035312824	0.106353274	0.153280366	0.073020965
0.183919	0.142380882	0.118411401	0.165656152
0.036735725	0.087105696	0.12901329	0.023730031
0.147230543	0.029698457	0.009104649	0.111570715
0.050373078	0.162679898	0.093949509	0.265379418
0.099912138	0.182983961	0.168927191	0.025792664
0.051364943	0.091441269	0.147361543	0.204706796
0.063157706	0.119092596	0.074370186	0.064606138
0.061532677	0.151654589	0.129785155	0.170521716
0.147740669	0.161527405	0.12985423	0.233930003
0.190251336	0.107614076	0.021600923	0.283082156
0.139027263	0.241285623	0.105012684	0.108934967
0.115810481	0.090688478	0.153020536	0.085454459
0.081808159	0.134574962	0.17875153	0.169532008
0.189348356	0.082947092	0.00709446	0.148082417
0.042337917	0.098854387	0.070652531	0.149404376
0.087950034	0.021948709	0.24301323	0.067554596
0.066141185	0.100103259	0.04812616	0.097362532
0.106100812	0.097166417	0.077569387	0.117721772
0.035266318	0.045247356	0.149431553	0.099985483
0.053337767	0.118677944	0.079571	0.021240272
0.127088186	0.091378615	0.164203677	0.167185227
0.047621718	0.017327786	0.212345322	0.11871498
0.144530405	0.107169954	0.096329033	0.048002298
0.106252964	0.129392186	0.110900934	0.157403277
0.168561247	0.102250688	0.066172569	0.063407934
0.168101789	0.013978801	0.093486482	0.106843354
0.128113442	0.055185573	0.006487793	0.01700753
0.121112717	0.184450839	0.084383781	0.08296283
0.01230822	0.113520674	0.012225465	0.135540539
0.10289816	0.166090948	0.156894133	0.10682491

0.130684238	0.106919862	0.133014723	0.154350724
0.120928854	0.0948249	0.058413624	0.260441944
0.121789053	0.109946879	0.098092536	0.087270651
0.15494493	0.183335191	0.191151687	0.073872183
0.097931335	0.022595154	0.027617812	0.112426605
0.032308924	0.187207884	0.10839094	0.172076072
0.150478286	0.087948701	0.03095825	0.093050931
0.109110823	0.143462961	0.042423753	0.016639087
0.009475657	0.22091835	0.076233039	0.184324524
0.129609828	0.012394188	0.06352915	0.025406253
0.058831241	0.196099464	0.107264797	0.197016647
0.129009201	0.12622746	0.037421793	0.162286011
0.216884462	0.029784673	0.158053819	0.0888391
0.097840486	0.158943838	0.040795297	0.157202885
0.076557342	0.217600227	0.109636247	0.069312564
0.036915269	0.184653294	0.181431671	0.173671155
0.086816068	0.164863918	0.182060549	0.00605489
0.097025471	0.140477471	0.124609208	0.165744842
0.123029552	0.197663749	0.150003664	0.092507823
0.145985617	0.087789088	0.185008388	0.009250294
0.069156153	0.073551481	0.095869258	0.093725555
0.026876452	0.071929567	0.085012522	0.126660826
0.071495333	0.170067575	0.183313775	0.183382285
0.113716536	0.049675143	0.167764584	0.120188706
0.04259714	0.177753021	0.086527725	0.045051522
0.063127177	0.052581418	0.113061747	0.056526012
0.022366799	0.185510313	0.093950777	0.135065508
0.073905056	0.102550965	0.116101827	0.120066949
0.11105645	0.055215011	0.086147313	0.046035389
0.131837045	0.174787329	0.049894948	0.200520227
0.224213125	0.10392923	0.043280296	0.008388661
0.097520453	0.039464658	0.09796792	0.18867088
0.061598141	0.060668917	0.027480097	0.187924441
0.120378093	0.080525005	0.033744798	0.111939788
0.160119363	0.188451276	0.140222045	0.160428763
0.033393472	0.117978712	0.103033059	0.080162168
0.074955155	0.129649451	0.101150013	0.006954049
0.140383806	0.066441019	0.136877315	0.214660199
0.176667585	0.145817795	0.05518899	0.17804334
0.048659374	0.196451733	0.013222876	0.111041293
0.179007171	0.032325873	0.179968341	0.060666775
0.134169878	0.094948738	0.067832486	0.143851527
0.05782914	0.127073115	0.149466769	0.147546132
0.088578811	0.107160855	0.196459615	0.026245619
0.036622816	0.028739436	0.119105892	0.035964887
0.045626392	0.177037575	0.035498962	0.084416693
0.064607496	0.046546401	0.038092855	0.090124588
0.141820658	0.119527636	0.031828364	0.118873846
0.079556905	0.102121298	0.042738671	0.03220366
0.056096833	0.072835184	0.117011717	0.105942733
0.092315672	0.21778848	0.057911095	0.096562916
0.041444325	0.053312137	0.069430244	0.124124353
0.049904244			

ADK 0.053910156 0.082566286 0.04868205

0.164084931	0.099908409	0.055524406	0.17469013
0.039438857	0.162840105	0.082599257	0.196716291
0.063173497	0.096193102	0.062719181	0.030758504
0.17868518	0.173218205	0.031162033	0.222460768
0.131448797	0.099186929	0.211058855	0.18675151
0.058760537	0.186742619	1 0.108899866	
0.137955493	0.263886987	0.044777475	0.095410422
0.1296961	0.073034317	0.057295275	0.126195284
0.05117087	0.039163832	0.02493021	0.052173733
0.061438354	0.112463518	0.137723248	0.21200027
0.212189452	0.046156572	0.228711527	0.100495395
0.026370898	0.239308752	0.024807423	0.046716323
0.049639072	0.073877025	0.061321426	0.160048033
0.071481173	0.023240668	0.038205123	0.044419677
0.216572933	0.028441674	0.056352537	0.142542124
0.04957204	0.062858791	0.118035354	0.095446821
0.039020626	0.054706036	0.09987114	0.036916943
0.025577232	0.145832683	0.113773444	0.038461458
0.253726508	0.184070691	0.121246316	0.175215567
0.123041282	0.049038104	0.128662475	0.107036418
0.125412168	0.130055771	0.059046756	0.185092219
0.070886986	0.181016483	0.07078708	0.189916165
0.121455461	0.068884624	0.146600789	0.186029415
0.095622276	0.074081743	0.050861205	0.08276462
0.043557126	0.116953237	0.058550626	0.097512755
0.04855845	0.055297755	0.249406126	0.092546143
0.205083618	0.017051843	0.225553003	0.050556923
0.127281319	0.173531268	0.1943027	0.129662194
0.07471703	0.074089839	0.067345248	0.23042806
0.070179447	0.123617433	0.07248842	0.15350256
0.044613429	0.034285491	0.039948863	0.124523567
0.04248063	0.055363084	0.119169613	0.096371668
0.192640007	0.063844117	0.060346037	0.049442867
0.094942054	0.034235353	0.165628061	0.043807476
0.064135422	0.127286873	0.005114682	0.162745838
0.078783258	0.074718419	0.281379569	0.069464292
0.174019796	0.113888401	0.198694814	0.108387606
0.055428609	0.247308798	0.020488547	0.174401303
0.060818371	0.135942634	0.174494258	0.096420574
0.181272994	0.050215284	0.164346129	0.040137563
0.039978368	0.105846554	0.05612277	0.064080631
0.051572336	0.080914567	0.035557029	0.195084044
0.118178658	0.170800069	0.031946785	0.087004614
0.169513928	0.136424382	0.014011015	0.096129871
0.033966198	0.089163807	0.082985637	0.078054796
0.217358094	0.1297241	0.002863683	0.050777184
0.067346139	0.013072039	0.136174289	0.084298755
0.056110294	0.134666973	0.001521016	0.161536461
0.02949265	0.178127239	0.129461341	0.279700689
0.052118252	0.265143604	0.047144817	0.078417548
0.054783297	0.043957674	0.051444301	0.137359613
0.144469862	0.081089048	0.082132223	0.14884185
0.143439316	0.110265101	0.148176936	0.052061645
0.096831602	0.09717792	0.114252716	0.13068454

0.074704728	0.021203225	0.0585584	0.097661848
0.020289201	0.076000713	0.048293376	0.033313088
0.226470872	0.087040603	0.057136146	0.154228808
0.044176013	0.163495526	0.050413339	0.070069845
0.198030813	0.09734403	0.063624812	0.034248474
0.04026261	0.082742891	0.147311583	0.074666333
0.089586148	0.185304768	0.059781512	0.067200563
0.134853668	0.22086044	0.053516685	0.020545035
0.095786439	0.03747708	0.031103183	0.048183419
0.042557417	0.12796178	0.114911668	0.049237148
0.170680813	0.200504771	0.081303711	0.148580676
0.033577404	0.162947021	0.184450554	0.016609956
0.118111444	0.031125876	0.010786129	0.092108054
0.079233795	0.113675498	0.117552809	0.276177209
0.0591886	0.224250504	0.144596518	0.042328699
0.048339768	0.069190619	0.134621327	0.208471319
0.046923492	0.116376342	0.179413375	0.045863599
0.042630464	0.222390843	0.079375092	0.14718749
0.189062189	0.099866418	0.083865281	0.281833374
0.199417424	0.125015072	0.091539775	0.257273717
0.095630364	0.207093993	0.083953764	0.063644067
0.231739424	0.118327682	0.105872102	0.044672981
0.049937068	0.077659158	0.195536121	0.109768651
0.259727021	0.065785133	0.02038685	0.096264183
0.028406107	0.060100902	0.132742741	0.089037663
0.050201942	0.029247382	0.225000456	0.109918718
0.054378067	0.214417184	0.079362225	0.061841204
0.085292492	0.091834735	0.061734767	0.084870956
0.127979178	0.157506658	0.1088656	0.046673609
0.055678844	0.151243781	0.018990417	0.030504556
0.225427268	0.06991791	0.119762729	0.232738776
0.056173846	0.023929665	0.162274129	0.220480195
0.078524216	0.059753084	0.059796592	0.098409097
0.067137486	0.078539134	0.052401937	0.172993827
0.179489587	0.057434599	0.058797613	0.113335619
0.131112985	0.021244494	0.154582616	0.078382545
0.209849894	0.033510105	0.01808983	0.007099282
0.111355	0.121348221	0.054769298	0.096114039
0.018238224	0.117247761	0.012299762	0.081135836
0.068870625	0.168884094	0.120367716	0.065771196
0.064736692	0.072979565	0.066337004	0.089364042
0.061483554	0.056037258	0.092419331	0.26081559
0.09515046	0.058463706	0.084705231	0.063967359
0.124263495	0.130191482	0.25362306	0.196947453
0.089132669	0.050537216	0.024796007	0.164348718
0.036267168	0.19474701	0.06837658	0.129113572
0.089458544	0.129101339	0.082430682	0.061333424
0.096479042	0.119091563	0.056024528	0.01431009
0.004705044	0.161116551	0.099469896	0.152162621
0.085561469	0.016590004	0.13645794	0.093749781
0.074457989	0.112076594	0.070302401	0.20296725
0.079061612	0.195131586	0.045534339	0.1620247
0.207241995	0.022624812	0.101546348	0.066829939
0.129472456	0.142269039	0.031456505	0.127357768

0.018113147	0.191996821	0.065818138	0.081829624
0.046373175	0.194126776	0.225398966	0.13272995
0.048204665	0.184034593	0.204954655	0.008450224
0.178902889	0.100181399	0.166302586	0.114674686
0.106075511	0.173131557	0.1432563	0.063811314
0.229677624	0.053062768	0.102610742	0.015098409
0.056151494	0.132222044	0.053045843	0.076148507
0.034123853	0.064858482	0.07216746	0.142849272
0.028720641	0.208680502	0.1398175	0.117295286
0.108041417	0.094059232	0.146520695	0.101098802
0.06770921	0.152751298	0.055289058	0.029552286
0.172808975	0.022192561	0.111379995	0.041320523
0.043448902	0.126619743	0.094691623	0.084182327
0.057974211	0.073382537	0.072082616	0.144083545
0.075905832	0.04628778	0.100572907	0.107919721
0.13712277	0.136174833	0.052966832	0.157119588
0.2160108	0.048618492	0.106893156	0.006716979
0.036092319	0.056451325	0.177465716	0.160372764
0.054385043	0.04435889	0.081673123	0.233228793
0.079158458	0.059806645	0.073752528	0.101839629
0.174248709	0.184152715	0.087591449	0.096478174
0.028398455	0.082838143	0.088177639	0.080676868
0.061972543	0.191780351	0.063798284	0.021330324
0.113178602	0.040396092	0.208224145	0.221441604
0.250160433	0.18490416	0.022078923	0.142810685
0.02011317	0.191366376	0.015596979	0.15393382
0.143493314	0.022534088	0.171919593	0.115859191
0.205032106	0.061899219	0.097924552	0.193644976
0.026078853	0.085364752	0.07980384	0.133003168
0.047238951	0.092632444	0.219296124	0.035325552
0.034257813	0.01783968	0.081036294	0.083799408
0.143569856	0.126584369	0.028406954	0.088512236
0.085774328	0.038083469	0.038678542	0.127069087
0.138022208	0.06845214	0.040407977	0.083570612
0.10767089	0.175710677	0.033042832	0.048090727
0.10934526	0.048801541	0.061963097	0.060787905
0.056305505	0.197688024	0.038246842	0.067134986
0.037957878	0.027695453	0.179224646	0.102584785
0.024630259			
ADSL	0.212353723	0.310002995	0.182183831
0.285657115	0.203352341	0.128971469	0.098643816
0.031224961	0.125527131	0.008138289	0.079923147
0.214496119	0.097749451	0.053100063	0.088933089
0.338274154	0.112211906	0.07476437	0.146407315
0.228672358	0.028964549	0.321121134	0.069593269
0.154360207	0.15608458	0.108899866	1
0.2919785	0.113604405	0.074179719	0.170760221
0.088525666	0.226506465	0.277984411	0.290716969
0.268616396	0.183344468	0.005306095	0.071251916
0.214773836	0.333994337	0.233762251	0.206841883
0.054198017	0.220538089	0.154329749	0.260337135
0.04214633	0.081430696	0.031249375	0.201822358
0.24054244	0.208638245	0.048936852	0.073783546
0.084993818	0.02559375	0.124769794	0.095208254

0.123603612	0.018419484	0.262575057	0.075359188
0.047739298	0.052613448	0.107345426	0.071343478
0.141273797	0.161193822	0.015895488	0.180477033
0.00114273	0.227166308	0.2390219	0.16209295
0.077470114	0.034615047	0.114357381	0.178973263
0.300869011	0.240651747	0.374944338	0.370338997
0.098346629	0.167125619	0.20904837	0.10198321
0.251799802	0.086351548	0.054293201	0.358088778
0.37504115	0.301474389	0.155505457	0.240395324
0.258766096	0.258716182	0.148240181	0.171704249
0.148234902	0.09944386	0.235151338	0.341884568
0.175963177	0.19273517	0.166797297	0.200154338
0.072682691	0.05503435	0.246400953	0.048166165
0.315597525	0.042055329	0.199714484	0.146052961
0.087112868	0.240428691	0.314370391	0.118556089
0.173159731	0.029411475	0.188796099	0.054389882
0.072132394	0.124086704	0.221761889	0.299535241
0.037505732	0.117572239	0.093093444	0.01703899
0.065994975	0.097425495	0.225898061	0.102120348
0.248052617	0.205896174	0.153901158	0.154764949
0.247016563	0.371218621	0.007858022	0.061358887
0.176393715	0.100495562	0.201581449	0.187580797
0.23256398	0.045541728	0.076690652	0.359512716
0.196587255	0.073215615	0.049470898	0.318294373
0.091300088	0.262558993	0.066337189	0.075198779
0.035918762	0.271319914	0.07338678	0.073378444
0.157472141	0.099032803	0.012186165	0.05775835
0.067231702	0.26528852	0.213984914	0.077187125
0.101977507	0.304507493	0.062407526	0.227402168
0.041028483	0.047421956	0.036814774	0.320625737
0.070396488	0.338131149	0.094924349	0.143537513
0.238912936	0.32705379	0.005081135	0.247237266
0.312980261	0.039541188	0.210354901	0.225660272
0.203775877	0.313015181	0.005796097	0.278053958
0.112163973	0.069321439	0.37571344	0.16727597
0.237681005	0.08426723	0.054662778	0.335497666
0.232871769	0.013255432	0.045614564	0.210629776
0.073041409	0.027584156	0.228522864	0.252796573
0.248340946	0.318975058	0.366915841	0.194637913
0.201640362	0.067298693	0.030136031	0.233907037
0.270144095	0.085386406	0.220412313	0.077573128
0.07512892	0.27314107	0.229937891	0.068163268
0.059272893	0.09041353	0.013269626	0.240369124
0.223519672	0.056568436	0.191124684	0.068760635
0.103884407	0.345019513	0.154770542	0.14651852
0.134754774	0.148047513	0.112934165	0.059528925
0.089787156	0.138215371	0.16187898	0.139059352
0.022403209	0.085381699	0.038230606	0.066163059
0.055443401	0.066499573	0.057687166	0.159012843
0.056967819	0.052843533	0.187104997	0.155833516
0.148188583	0.045592466	0.304238377	0.269033169
0.108172809	0.094504942	0.062393051	0.053228912
0.305073247	0.077803874	0.028186705	0.262950463
0.029125641	0.353572676	0.095612962	0.147956161

0.127424025	0.154127397	0.33763309	0.03964038
0.120498645	0.242047323	0.193673146	0.164660011
0.171744292	0.220143915	0.034706076	0.17887908
0.158082853	0.057053306	0.322713666	0.111974776
0.054344751	0.299244142	0.288286611	0.122829156
0.118527464	0.141921188	0.01886647	0.283055673
0.33369938	0.341493186	0.248357089	0.15313496
0.055910918	0.016416492	0.142580883	0.178730788
0.246909291	0.329431669	0.168656742	0.197035586
0.17774666	0.171543546	0.017639479	0.339494195
0.078192434	0.279952446	0.023693566	0.330216259
0.266181718	0.048762938	0.136285198	0.113202001
0.170853121	0.065922068	0.0639403	0.26755108
0.275515713	0.160597835	0.149186419	0.220415757
0.016963472	0.029532094	0.27512232	0.156425432
0.106502954	0.147950117	0.082757062	0.056200111
0.059170667	0.240396679	0.310190495	0.136513666
0.108890491	0.050568073	0.287170565	0.058902983
0.342893184	0.296148747	0.275120959	0.054911864
0.237967281	0.322338722	0.227781389	0.095928738
0.061938843	0.290626165	0.168714798	0.028714792
0.330177554	0.034747836	0.022718386	0.27872379
0.112876351	0.185052544	0.006546605	0.041284287
0.081506497	0.356122949	0.256269115	0.171494116
0.029440743	0.186279447	0.010984471	0.335553319
0.268286614	0.066924035	0.149667101	0.247954439
0.283812021	0.288990002	0.21327695	0.336754713
0.22452498	0.274343653	0.067677217	0.255827333
0.266940827	0.304064993	0.218592613	0.244565884
0.298712256	0.304674267	0.207833672	0.03424403
0.198613398	0.037772155	0.077563999	0.122855184
0.088267356	0.161344044	0.276706269	0.343108181
0.331542906	0.025578761	0.03929606	0.22928215
0.246614453	0.311496223	0.0318187	0.014348467
0.008716439	0.100059143	0.139389981	0.188411143
0.32193335	0.029040561	0.01833516	0.017670351
0.08963463	0.31396393	0.284486462	0.279401193
0.291674761	0.079725834	0.063311507	0.265312008
0.155188392	0.097145075	0.342395289	0.246968494
0.060312157	0.184154019	0.085160932	0.334265231
0.099845961	0.233700372	0.212161364	0.102047799
0.021453203	0.126838271	0.140041392	0.323352172
0.261627306	0.075343461	0.064200984	0.014219691
0.069226537	0.311930433	0.091485603	0.361928066
0.263428941	0.330434 0.103642654	0.269201603	
0.089945231	0.247746587	0.232765359	0.01948414
0.095011148	0.029359415	0.259440133	0.156552685
0.048736965	0.165005601	0.205209245	0.056895458
0.146714962	0.082703697	0.363381497	0.366556096
0.067858901	0.08356745	0.194529985	0.218903737
0.075404673	0.297643482	0.255440423	0.114944325
0.052863592	0.076970994	0.213185849	0.141722768
0.038146978	0.373432266	0.15413373	0.33794129
0.190205866	0.272880607	0.291137437	0.19745913

0.13359526	0.131347484	0.16903495	0.017936331
0.183159801	0.325245465	0.103036864	0.174791926
0.192647032	0.216271217	0.036373113	0.021513297
0.183257467	0.088427302	0.073916803	0.336225646
0.13929244	0.153753249	0.01675365	0.140172952
0.31604965	0.236016898	0.052324851	0.228039252
0.123602083	0.262967729	0.298975516	0.319837727
0.104543268	0.262311043	0.219757495	0.160990376
0.203640883	0.154894107	0.288580563	0.011186536
0.136889263	0.160159195	0.08177866	0.122184582
0.105270599	0.079780331	0.132067169	0.293851241
0.100232044	0.246829004	0.038806195	0.088258165
0.219479424	0.084150794	0.231539255	0.06000805
0.07673206	0.268975827	0.028981862	0.07280639
0.101372357	0.296894361	0.230744723	0.13740426
0.162847305	0.114396313	0.192968752	0.05585577
0.059266292	0.074498146	0.285395847	0.027649321
0.017968529	0.357108634	0.083798359	0.047573407
0.082303869	0.135660532	0.067558089	0.140168759
0.069479824	0.306640362	0.074459365	0.22195861
0.136803467	0.062608694	0.136554476	0.041269498
0.049416628	0.209360078	0.230837636	0.292122698
0.155727062	0.328644703	0.193000343	0.227731546
0.054031539	0.13831099	0.039085658	0.27040651
0.108292028			
AHNAK	0.390063076	0.507240431	0.382303234
0.447989331	0.44132487	0.378254052	0.332080355
0.307422138	0.154278646	0.019588026	0.04271609
0.412710934	0.252315514	0.356701339	0.339362886
0.455204025	0.202012689	0.410889027	0.371012861
0.459559598	0.194978275	0.444130387	0.203697155
0.311687832	0.152915891	0.137955493	0.2919785
1	0.156623131	0.341028209	0.427478939
0.179102931	0.418732675	0.483189078	0.419484504
0.464744418	0.35542372	0.032355918	0.338144494
0.412165883	0.417025791	0.454021007	0.424320741
0.111562219	0.457389663	0.35869147	0.415078634
0.302669678	0.07154376	0.302831162	0.428392818
0.432606628	0.390242214	0.347692987	0.274299731
0.206420033	0.295182055	0.230321109	0.348491311
0.346641885	0.234265563	0.444544226	0.169323481
0.197572166	0.380298991	0.223658666	0.383680534
0.322317751	0.380836547	0.0688466	0.347239543
0.010043248	0.459371093	0.419465034	0.262051463
0.100648698	0.054747067	0.258208764	0.419372548
0.467603714	0.433672626	0.470065227	0.463336907
0.251690887	0.384234179	0.360948205	0.278356984
0.418606006	0.284910756	0.201709255	0.448785129
0.475914489	0.485821236	0.281302289	0.336447474
0.387458897	0.47323571	0.411163483	0.430100876
0.311596631	0.09286013	0.415682511	0.489720341
0.383375085	0.292977739	0.235145092	0.426017841
0.136284166	0.384067164	0.439022837	0.218788214
0.482464153	0.060117358	0.28081362	0.422944386

0.336197444	0.417613708	0.47389257	0.317692259
0.427041836	0.056487258	0.141554771	0.085084534
0.387850864	0.143248783	0.432458699	0.455708053
0.342455958	0.391629714	0.39594357	0.011476575
0.065331773	0.264109259	0.383050445	0.432387233
0.43861177	0.337110633	0.295443312	0.362548102
0.377337272	0.461098228	0.070961162	0.216164704
0.443914981	0.266726482	0.356685439	0.421042187
0.362680323	0.248527968	0.244256066	0.507628874
0.428129477	0.185960885	0.381055125	0.457238131
0.351258416	0.37906369	0.225359278	0.370969347
0.131341761	0.432127232	0.062159241	0.412813348
0.241962559	0.1879467	0.042517801	0.365605157
0.357909986	0.393913737	0.415196426	0.218795969
0.406589533	0.436975767	0.36956084	0.393003137
0.026829285	0.121921015	0.31006605	0.49082659
0.302273896	0.47966584	0.418367756	0.372957258
0.367914916	0.446062526	0.006514993	0.462933801
0.484278436	0.316339542	0.421595319	0.431597244
0.328790776	0.443985689	0.052589604	0.459588467
0.343212421	0.180349203	0.498850788	0.246731402
0.435055804	0.096546619	0.391379893	0.36948577
0.356668249	0.02393193	0.298890638	0.442154001
0.05828732	0.193319331	0.430565191	0.452325057
0.412265256	0.433123633	0.486285497	0.308162125
0.443312205	0.377384032	0.041170894	0.371035752
0.407006988	0.325390443	0.435780413	0.368559456
0.329367321	0.463938245	0.354010541	0.200689708
0.114413476	0.406451416	0.059775498	0.387163011
0.351731563	0.206011157	0.350547925	0.234225073
0.131377303	0.506332334	0.194496095	0.235734553
0.127950445	0.398011563	0.393261124	0.280324867
0.231282857	0.266298686	0.350880433	0.391455155
0.109060906	0.178440954	0.033037204	0.316471408
0.173414613	0.328380364	0.31894196	0.378364287
0.385129028	0.182984519	0.418231149	0.303903178
0.182010528	0.046587357	0.491817804	0.462422205
0.323756063	0.281267218	0.100181865	0.296842708
0.459828136	0.306389696	0.294113382	0.4507004
0.079107862	0.470653919	0.309826286	0.202174452
0.361671615	0.368069927	0.47048806	0.09166205
0.413387163	0.458746088	0.405623162	0.261822141
0.384561599	0.458161085	0.091222285	0.370276208
0.367865951	0.08357062	0.496596941	0.189778075
0.058047759	0.41881702	0.380092761	0.150454511
0.147338998	0.372914896	0.126605194	0.374110636
0.502925763	0.44748628	0.383187492	0.255573503
0.103848683	0.025242223	0.267987342	0.378688661
0.471268219	0.473449348	0.376687559	0.370845233
0.369431714	0.329981264	0.216495978	0.486194244
0.251638456	0.487984078	0.027571378	0.385683843
0.45826783	0.334772617	0.135811324	0.387093657
0.374441647	0.121757171	0.346118501	0.470060967
0.423716777	0.37451226	0.359177468	0.441681342

0.060611852	0.119880152	0.404556627	0.189476304
0.389792528	0.319630956	0.052311885	0.399067971
0.103098761	0.441305828	0.446187001	0.114633071
0.324418845	0.375579868	0.425232451	0.101736506
0.398597394	0.459139537	0.488881229	0.31061955
0.400862795	0.450311255	0.331399873	0.155109678
0.045384316	0.471559814	0.407158599	0.055765391
0.471594601	0.276036748	0.034666218	0.493026935
0.323538478	0.405760571	0.014946359	0.091147956
0.079971671	0.431142 0.446904268	0.434351532	
0.256596732	0.454312228	0.011606105	0.492860717
0.470070368	0.036451922	0.192835872	0.377494428
0.356336108	0.514469006	0.298977168	0.400359005
0.374258427	0.453155777	0.330616387	0.35976336
0.421078412	0.437934986	0.433824327	0.456467123
0.453663471	0.436918229	0.39349141	0.052920274
0.158459567	0.313302652	0.274479683	0.118518527
0.348802077	0.208988029	0.466920305	0.45799279
0.44975394	0.020211624	0.274922707	0.385682999
0.455396289	0.487713863	0.053287276	0.01341256
0.014103473	0.033974811	0.436785557	0.328363227
0.467208329	0.240859202	0.021636615	0.128251586
0.389513076	0.381955724	0.475933172	0.458887687
0.401756213	0.168561902	0.292446522	0.464707894
0.112768439	0.308550493	0.463419716	0.463170128
0.093659117	0.331254259	0.413856149	0.494082045
0.060547872	0.40022682	0.414614984	0.369308487
0.035654989	0.143839618	0.121619439	0.448464511
0.445278024	0.127399111	0.069881239	0.181821139
0.211574389	0.451768679	0.266456977	0.505425532
0.433785442	0.460258213	0.121417494	0.463027592
0.081488934	0.452269914	0.342788403	0.089436073
0.380346842	0.044321625	0.429023264	0.145740432
0.387159178	0.42625986	0.215698333	0.040721322
0.17337316	0.113221493	0.470259944	0.426036317
0.06897851	0.372504014	0.393142964	0.454723057
0.371186352	0.444096409	0.472674584	0.35119676
0.183982708	0.149301436	0.455244728	0.389205675
0.348033157	0.479462828	0.318545505	0.502336116
0.403181412	0.454626207	0.442537654	0.422510211
0.253291836	0.412684849	0.38967247	0.060713457
0.450708648	0.440046908	0.43108915	0.203065699
0.32762691	0.337952714	0.122837261	0.223431096
0.277550432	0.346839432	0.246292744	0.459814938
0.420994025	0.388782122	0.113753979	0.115760923
0.519532875	0.430622962	0.253855464	0.431126238
0.280907142	0.400537385	0.416030269	0.411142122
0.361461981	0.409250572	0.433486076	0.387406939
0.424624021	0.386685227	0.491638477	0.18176878
0.126579624	0.378402231	0.168687558	0.166447242
0.256543944	0.06671383	0.246996458	0.454757369
0.310857355	0.440322074	0.351244367	0.161680086
0.389563625	0.371306936	0.436463487	0.311902435
0.155772243	0.465890048	0.027095903	0.123607086

0.335355452	0.399191189	0.351729209	0.340393125
0.278026933	0.200552861	0.396222054	0.352591077
0.305672447	0.355707631	0.392926601	0.099390009
0.070167207	0.489884312	0.400831125	0.050146207
0.13186452	0.367365679	0.388829342	0.299158526
0.048446588	0.434037393	0.331791724	0.422463023
0.43502778	0.153807692	0.350062991	0.162239314
0.148361862	0.436744372	0.376707204	0.46950975
0.377976103	0.452737825	0.419756087	0.39077779
0.203431973	0.337852227	0.088469079	0.4388481
0.337052108			
AI451617	0.065509251	0.094238633	0.058694876
0.179367868	0.107965999	0.062221564	0.18017296
0.049916994	0.186660688	0.087304756	0.173648354
0.074578438	0.101897342	0.071224795	0.038599446
0.198835066	0.182154125	0.039192867	0.23537559
0.14706902	0.108334125	0.225382216	0.209557468
0.069591081	0.199348606	0.263886987	0.113604405
0.156623131	1	0.046043544	0.107976091
0.153727748	0.085650686	0.067142233	0.135019168
0.059910207	0.048483971	0.027536537	0.0579123
0.074579316	0.127286128	0.152332905	0.225419216
0.231292239	0.054477777	0.234696245	0.11588149
0.035128371	0.238617857	0.0286179	0.054867024
0.056731504	0.077416362	0.067752731	0.159861751
0.06647201	0.02645585	0.041674329	0.051537297
0.217065431	0.030352081	0.064258646	0.159358501
0.053453128	0.070667095	0.134566479	0.104691835
0.043971349	0.064745672	0.115928889	0.044505059
0.024571129	0.164474888	0.130882736	0.041244152
0.257490979	0.181045259	0.139306667	0.182372813
0.140811526	0.059558722	0.144116116	0.120420489
0.13889333	0.146158994	0.066202069	0.19782848
0.083481911	0.178993089	0.090372653	0.20814645
0.136444142	0.078791532	0.162042902	0.203151826
0.103515268	0.084732696	0.060406259	0.089080471
0.051005057	0.138928009	0.06575102	0.110182075
0.058376718	0.061888612	0.258549089	0.105446636
0.226646817	0.023559879	0.239233701	0.059775346
0.144924994	0.189095022	0.203196062	0.133215983
0.097590016	0.088120132	0.077508791	0.237445771
0.08015057	0.137545891	0.07959184	0.175780711
0.052263735	0.041731454	0.046725212	0.13720683
0.051083	0.062614472	0.127234249	0.106266738
0.21412046	0.081945225	0.069211765	0.059883714
0.110461467	0.042405229	0.162416484	0.051385545
0.071615887	0.141614606	0.00574237	0.18439797
0.090087963	0.092671534	0.295778291	0.075928745
0.182653266	0.133974276	0.204784917	0.123358081
0.064576663	0.247754694	0.02540098	0.19579959
0.063359959	0.147358292	0.195106395	0.102945557
0.187589162	0.058432461	0.182833546	0.049556007
0.045467071	0.109390994	0.07171358	0.070715597
0.06139617	0.087904881	0.042101156	0.20791872

0.120981848	0.184201609	0.037603529	0.094301773
0.154973673	0.163460473	0.017200847	0.107942254
0.038218566	0.104159383	0.101136829	0.094422481
0.23337749	0.149335476	0.005852515	0.059625048
0.077747498	0.016325839	0.141602831	0.098395249
0.059194756	0.149746613	0.002337293	0.178029682
0.036449778	0.204933087	0.146155259	0.280107502
0.061185821	0.262974346	0.049934274	0.089831082
0.061091966	0.044363248	0.055358095	0.149846253
0.165346643	0.086684516	0.096681867	0.165835602
0.152125664	0.127923639	0.166797289	0.055214975
0.107739065	0.108487601	0.135983745	0.147442151
0.088693225	0.024620136	0.068286827	0.113565956
0.025292256	0.086679199	0.057425733	0.034296125
0.237037636	0.093202521	0.067677269	0.165137555
0.052716701	0.16190584	0.055381309	0.086175541
0.215287866	0.10994737	0.075761295	0.039710188
0.048658377	0.08518033	0.163184141	0.074427308
0.086847417	0.20267933	0.063634584	0.071109213
0.140441794	0.231570657	0.070952081	0.021498751
0.090002131	0.037103028	0.031045337	0.05509362
0.048041028	0.134440531	0.122543948	0.062434553
0.200900187	0.204976004	0.093433726	0.169537245
0.04001072	0.181588087	0.207677709	0.018658925
0.129167807	0.034516602	0.013051249	0.101288655
0.099876921	0.128791943	0.121322416	0.275760945
0.060805144	0.228266401	0.162864917	0.041360754
0.056301481	0.077732352	0.154017132	0.210397418
0.053756951	0.130378603	0.184524915	0.055303285
0.050600734	0.234626751	0.091096766	0.163537879
0.20244426	0.114416665	0.091757758	0.290225533
0.229911734	0.122404249	0.102468657	0.274439035
0.10815197	0.223262237	0.090101189	0.080038935
0.237869364	0.102785349	0.115847379	0.054859974
0.058532664	0.089127147	0.211683362	0.113450512
0.27415231	0.071975964	0.02421574	0.108316195
0.040852616	0.070886098	0.145023606	0.099382544
0.058384989	0.032775968	0.236799645	0.11457593
0.062321031	0.235994181	0.097747647	0.071674433
0.097078025	0.096198778	0.067292213	0.098926404
0.138322898	0.162114517	0.123328548	0.057300575
0.057043877	0.170729821	0.023627098	0.03693744
0.23413651	0.077740764	0.137012425	0.252355948
0.056770413	0.031128811	0.178888436	0.238519603
0.089568099	0.070481247	0.069744494	0.117606983
0.076919793	0.088669685	0.057690868	0.193923966
0.193958252	0.067343284	0.070948016	0.123833996
0.146422599	0.024477986	0.140384877	0.090411591
0.223647605	0.039874811	0.025119317	0.011549255
0.134903641	0.138620537	0.063396338	0.109055806
0.019562667	0.135654044	0.01473175	0.093925095
0.078358089	0.182976651	0.13494542	0.073383272
0.073896263	0.08497761	0.072343229	0.100265489
0.069212636	0.06415136	0.09792484	0.273065917

0.106853342	0.067540192	0.100417362	0.075644687
0.137209425	0.146505512	0.264892072	0.199654757
0.106300443	0.054426902	0.028134313	0.164421538
0.043054434	0.219503758	0.081322257	0.144772367
0.101635179	0.140446997	0.094829811	0.070053741
0.110500685	0.135949762	0.076404023	0.020636131
0.007505861	0.158729038	0.10984315	0.170647976
0.097959756	0.01887333	0.14413307	0.106345754
0.094920972	0.123513674	0.079504913	0.223585908
0.092330905	0.213502582	0.04674602	0.182824342
0.214701521	0.028436125	0.114233745	0.075643552
0.152787846	0.156981608	0.039287886	0.142008652
0.021279036	0.207417649	0.076018171	0.088470429
0.060107678	0.220316311	0.243532239	0.151526752
0.055985789	0.17761891	0.203377109	0.010484578
0.181553594	0.114482968	0.181607023	0.129739799
0.120615436	0.19152142	0.153317192	0.073789034
0.208027711	0.061322611	0.111596837	0.020950557
0.071332844	0.150545463	0.062127817	0.075109578
0.038457456	0.077674776	0.083320908	0.156194049
0.037645094	0.227458783	0.15423017	0.13251224
0.128957446	0.109522588	0.167950119	0.119837331
0.075561662	0.164786921	0.064184177	0.034251824
0.178358214	0.028107601	0.12793244	0.050726582
0.051018408	0.142709252	0.096850428	0.097692787
0.069798214	0.086445627	0.08248351	0.155859596
0.07975529	0.055866427	0.106217875	0.121564556
0.155176677	0.148272406	0.063550235	0.169696122
0.214401686	0.05625968	0.123588757	0.010172145
0.041826427	0.062339224	0.187337282	0.180864514
0.065241817	0.053629034	0.091624978	0.253257354
0.091203554	0.070509042	0.091510138	0.113365619
0.190950045	0.190730063	0.102646886	0.109493656
0.034639258	0.091413634	0.100902534	0.086636774
0.069577611	0.203469881	0.07397279	0.02837795
0.127639293	0.047669103	0.202407412	0.232483078
0.251397164	0.217002504	0.027202602	0.160008786
0.022842595	0.200667724	0.01912148	0.190938997
0.156009428	0.028606664	0.187486584	0.118777866
0.220480369	0.070417046	0.110738926	0.213806558
0.029078609	0.09683376	0.08573769	0.149302166
0.052969591	0.108671393	0.224467398	0.040212565
0.034183157	0.0238492	0.092290847	0.104848287
0.149508693	0.143154572	0.036158349	0.112374015
0.090586099	0.045895478	0.049994687	0.130943534
0.158165906	0.077291899	0.040343362	0.092758073
0.120699779	0.199065581	0.039880278	0.059191519
0.13686975	0.05800148	0.073121772	0.070116116
0.067445316	0.212136121	0.045605107	0.075802632
0.053611785	0.034181804	0.198136645	0.117119472
0.027268936			
AI747699 0.230573345	0.420904714	0.101308746	
0.313315736	0.151114143	0.246299572	0.154760959
0.145172134	0.052759041	0.017171728	0.019796046

0.164006731	0.097747857	0.170810207	0.140236478
0.250796281	0.047727033	0.117933272	0.12421339
0.259277461	0.130962798	0.217133663	0.074383132
0.066784046	0.033495791	0.044777475	0.074179719
0.341028209	0.046043544	1	0.309674904
0.028925562	0.29555117	0.406430429	0.207698645
0.384225972	0.2166188	0.007034122	0.197261442
0.316645305	0.247586168	0.281225118	0.174561475
0.047498279	0.421284324	0.148305964	0.129329712
0.123743516	0.021667682	0.186021275	0.404764487
0.395640129	0.203302054	0.168293938	0.137315769
0.100862783	0.197646122	0.063718654	0.290793362
0.142148192	0.18551672	0.384155824	0.09376848
0.097532723	0.160865962	0.040433993	0.167735579
0.081439385	0.314720174	0.02078261	0.141035044
0.009666507	0.240124559	0.119810457	0.072656942
0.022174756	0.023671054	0.049534535	0.226017938
0.344641159	0.327507142	0.324677119	0.325138523
0.065136202	0.089646353	0.095584939	0.092600118
0.323476205	0.144765468	0.054253568	0.239655251
0.332841808	0.39906707	0.0696513	0.074432713
0.352488818	0.408368495	0.141539664	0.144962724
0.104483562	0.010859749	0.383296011	0.364710538
0.330544326	0.065152462	0.053557595	0.347550362
0.032656723	0.130744303	0.207563653	0.049201247
0.357713123	0.028200652	0.059882779	0.209857504
0.073925079	0.287042049	0.371020278	0.122025325
0.113531896	0.01258426	0.033305278	0.013763451
0.141276555	0.022449521	0.401076912	0.369858432
0.165301149	0.126849115	0.155290334	0.00137082
0.011014597	0.046352462	0.283689897	0.125457147
0.260608012	0.239695119	0.110185899	0.302177595
0.193711265	0.318169953	0.058513877	0.080253836
0.173095681	0.048785744	0.105829074	0.194207919
0.099383941	0.099373482	0.079613693	0.379648062
0.413223723	0.064508087	0.139568106	0.226260595
0.183708571	0.100146681	0.07072725	0.143134071
0.068617472	0.350787155	0.008153895	0.150949013
0.083068105	0.041760371	0.007363299	0.187644057
0.158419047	0.349380827	0.36633705	0.057188034
0.15448614	0.186399956	0.1720823	0.289459198
0.022509921	0.025866628	0.155165255	0.386285773
0.282707465	0.329450174	0.119860602	0.087477248
0.091780455	0.292657876	0.000157676	0.410483027
0.386293437	0.155058442	0.184063426	0.152832255
0.104568253	0.346367767	0.01400483	0.231771319
0.243233803	0.046786695	0.35149905	0.065690096
0.355014565	0.028533887	0.194437547	0.243566448
0.23528607	0.008194459	0.199545995	0.289069167
0.006885025	0.148082342	0.258887492	0.245533846
0.187230787	0.25160494	0.336417436	0.096262437
0.148651378	0.13569263	0.006579908	0.082097008
0.245386992	0.116436179	0.406272338	0.150123996
0.191291463	0.398751351	0.262020498	0.117796116

0.046711114	0.190704437	0.026909702	0.118468269
0.267971018	0.119323263	0.119383692	0.039957036
0.025246133	0.396664196	0.032813768	0.047175391
0.019663087	0.219924924	0.130264977	0.160956728
0.091873321	0.075481032	0.103113475	0.137320127
0.073653842	0.054229994	0.0019891	0.11663465
0.083026248	0.142216618	0.154926706	0.280971855
0.166317784	0.068507397	0.132618834	0.063852031
0.034785581	0.009334675	0.4020614	0.213272994
0.305513547	0.102319214	0.018916647	0.127252116
0.378116067	0.207793601	0.157606746	0.410337295
0.013576731	0.327080327	0.13115575	0.051610566
0.110524946	0.128847051	0.347177316	0.057785891
0.196740415	0.425335269	0.098299797	0.074876457
0.384449075	0.241775064	0.042100088	0.277089511
0.282556089	0.021833502	0.383948933	0.036207919
0.009911886	0.19868543	0.251707951	0.03333987
0.028651279	0.176369425	0.090006465	0.109645358
0.401917136	0.238081396	0.320254677	0.043904036
0.044180418	0.012544024	0.048641846	0.106581794
0.41398786	0.349062017	0.097699974	0.113347442
0.119345849	0.108406309	0.147259043	0.372893513
0.038441692	0.391691898	0.005216021	0.235302268
0.391047506	0.194086017	0.032063696	0.199316197
0.364029115	0.043122274	0.101213599	0.39931761
0.348841319	0.121430853	0.122313975	0.19846915
0.037905126	0.06568332	0.13213939	0.031905398
0.160066943	0.094697987	0.005445627	0.172628734
0.04226351	0.417069295	0.287220708	0.035827794
0.246263832	0.153412555	0.147404457	0.030955682
0.272786667	0.339898226	0.416904972	0.104841846
0.260320074	0.352224739	0.132142066	0.027259154
0.006372481	0.374729236	0.256168482	0.022523195
0.342986348	0.183310355	0.022035767	0.419687849
0.109105773	0.377941206	0.0062371	0.010728569
0.008460341	0.270420062	0.400545485	0.283011596
0.186443639	0.172482343	0.000173376	0.357653266
0.4114765	0.004952427	0.033745286	0.280401552
0.213041384	0.427375645	0.074868524	0.284966819
0.160655346	0.38519049	0.155119437	0.099461085
0.351036046	0.331450649	0.29362557	0.388309242
0.349404678	0.193077439	0.155170107	0.027832498
0.037023174	0.194086021	0.137928001	0.056348774
0.241198521	0.052997933	0.350710025	0.317694544
0.324059228	0.003097676	0.149580456	0.295512263
0.389687046	0.371626433	0.005923764	0.001847473
0.00149405	0.007033911	0.214979053	0.069103608
0.356523766	0.171308055	0.005566686	0.07435111
0.119536169	0.138525339	0.404850209	0.231181702
0.237807477	0.040884696	0.175133725	0.247847397
0.025214704	0.230048131	0.343595105	0.435144063
0.013329519	0.074095102	0.126040949	0.383750812
0.008989573	0.109494821	0.186269284	0.162874241
0.003316492	0.027007263	0.02825019	0.256278955

0.374150216	0.04825118	0.02179787	0.10888124
0.108708061	0.317915101	0.090068091	0.368997194
0.347139284	0.308241274	0.019737185	0.40396504
0.032208139	0.394432311	0.083181065	0.045035083
0.08132952	0.007420327	0.322711223	0.064821576
0.187428863	0.253173487	0.055620102	0.004849541
0.029623307	0.023199299	0.346449131	0.276187423
0.006871263	0.137958559	0.09157062	0.191256161
0.193934291	0.294482835	0.419160342	0.151146276
0.104226066	0.020072085	0.28220177	0.238797168
0.176410156	0.327068886	0.124272607	0.372058199
0.265826057	0.346927694	0.339983326	0.180166039
0.060460855	0.203897163	0.320715865	0.038236682
0.149120422	0.329303846	0.148993235	0.05329342
0.101826162	0.098144765	0.035186587	0.061893544
0.066252215	0.260477491	0.100917936	0.294297782
0.219080252	0.257963246	0.043860514	0.02856761
0.417694088	0.365771887	0.1166437	0.272047966
0.060421053	0.135085434	0.250525355	0.236108088
0.11169765	0.334204121	0.32711651	0.319993004
0.425928498	0.13748321	0.416103247	0.104093176
0.026357857	0.176779631	0.079848244	0.031012889
0.082134794	0.010902664	0.060767554	0.228371543
0.087239823	0.204090726	0.163713062	0.035148429
0.093891424	0.111619937	0.139269367	0.166622449
0.059744789	0.416981937	0.005359779	0.02474871
0.093508116	0.283832664	0.1026259	0.077417128
0.067430363	0.032239426	0.124430226	0.192034602
0.176787072	0.096432811	0.294079038	0.031552242
0.033984818	0.34256885	0.140490409	0.00441615
0.029622212	0.205702809	0.096947568	0.132595015
0.005206842	0.334736116	0.199650351	0.139809866
0.189437043	0.035955394	0.339879498	0.044514412
0.033897255	0.384065794	0.110252717	0.377215161
0.083348332	0.277634992	0.392899653	0.308587387
0.034186611	0.1014801	0.037155781	0.327305502
0.118105933			
AIM1	0.314530303	0.464175968	0.252334791
0.366952971	0.307900119	0.31749156	0.292170025
0.324570495	0.110355611	0.019257551	0.024975198
0.276419311	0.168161685	0.310512583	0.262941527
0.342837335	0.122178536	0.330305633	0.292638883
0.360786578	0.212690004	0.307951904	0.172804125
0.18617769	0.083524821	0.095410422	0.170760221
0.427478939	0.107976091	0.309674904	1
0.114525481	0.378178634	0.442335694	0.277427128
0.417563954	0.286535518	0.030897092	0.287407637
0.398888795	0.307323858	0.360891027	0.319239732
0.094891752	0.441795582	0.288431379	0.270973908
0.278569732	0.044819855	0.313703164	0.427054641
0.397400905	0.277906127	0.342440223	0.240442564
0.138120108	0.322060289	0.126619307	0.328595845
0.283810906	0.278680043	0.403122703	0.147863674
0.181570045	0.369016429	0.135535267	0.356123503

0.196204354	0.362306261	0.071109204	0.232587967
0.013122722	0.362162115	0.278762143	0.151924202
0.067560229	0.040503497	0.160440178	0.342154224
0.432534089	0.39874747	0.399437293	0.391942845
0.159356975	0.264344165	0.224953799	0.205039045
0.391002658	0.241038639	0.154580064	0.326387848
0.405028036	0.434387793	0.17702543	0.212250259
0.356420955	0.45137237	0.293975267	0.29903076
0.194976379	0.049475268	0.393804704	0.426532586
0.38569343	0.165565616	0.145893468	0.406125682
0.100079523	0.304668993	0.329329995	0.155176465
0.443963282	0.053766409	0.168316038	0.348162566
0.256711907	0.362541353	0.41355962	0.262087015
0.287358902	0.040312208	0.071744057	0.058572008
0.286454432	0.069833023	0.408138633	0.415699269
0.339334109	0.26750093	0.3419755	0.007280259
0.038390033	0.177164606	0.340919304	0.342685396
0.350215496	0.299988684	0.197920125	0.339601657
0.250089629	0.39197462	0.069102166	0.196287487
0.32458829	0.178564471	0.260658238	0.300459589
0.227101587	0.242771918	0.194870967	0.447262748
0.437157152	0.151303186	0.339120662	0.320785915
0.269893161	0.231042966	0.188741524	0.29027674
0.129310513	0.381705331	0.033211039	0.366283739
0.146633245	0.106781708	0.041031642	0.362922126
0.284767546	0.353013234	0.387779559	0.171500242
0.316857675	0.296056591	0.349742525	0.296398542
0.020308819	0.09723421	0.308823356	0.436121071
0.299095283	0.414658858	0.31693503	0.246990414
0.240692692	0.383057959	0.004105893	0.433117633
0.427831752	0.296204599	0.292838613	0.295626668
0.20836706	0.402531356	0.043070311	0.330083344
0.296627454	0.14499618	0.434021841	0.159430411
0.401801041	0.066389504	0.361840178	0.30051579
0.268237936	0.018469588	0.309522023	0.360910557
0.0313675	0.223120225	0.345375053	0.340713316
0.297949924	0.344246321	0.426871099	0.18947548
0.305031436	0.335446065	0.030060642	0.230334081
0.333804351	0.230136499	0.428359623	0.31681815
0.288336672	0.431761765	0.309224518	0.165881514
0.09643828	0.372004479	0.056295194	0.258969182
0.320450714	0.177704945	0.239122945	0.16354537
0.077844427	0.447982735	0.100732753	0.127412531
0.061146413	0.302045949	0.330105397	0.218940265
0.144513149	0.188440898	0.221982641	0.274182514
0.121102595	0.126264851	0.01806596	0.211717014
0.117676105	0.230000515	0.235801218	0.315630788
0.320635486	0.13538262	0.277022913	0.184716707
0.105642677	0.031263478	0.448149487	0.337438442
0.326082609	0.231731322	0.068647064	0.207674364
0.418579205	0.266148352	0.292771531	0.427735386
0.061140327	0.410118347	0.22176598	0.128439515
0.22583827	0.272702114	0.42898185	0.075849942
0.345115882	0.441009075	0.285119686	0.167595418

0.384532936	0.352901634	0.082193658	0.334485358
0.329674906	0.059276116	0.437109691	0.109888721
0.037400546	0.27800727	0.280947307	0.09931605
0.09105469	0.280128907	0.15331289	0.245808504
0.449226077	0.311830354	0.33092442	0.145326285
0.083603255	0.017765229	0.157695499	0.249739299
0.440092699	0.406012854	0.271518272	0.229780637
0.285909669	0.208716738	0.2605807	0.42410816
0.168837496	0.444650324	0.020512975	0.292972326
0.414108279	0.331390905	0.078877396	0.343709887
0.377587398	0.101387918	0.301014101	0.431172046
0.396664801	0.257409046	0.239029014	0.318288441
0.065302045	0.128386851	0.267863601	0.098052934
0.286830821	0.228956279	0.021398179	0.36034884
0.079839484	0.422192203	0.384070704	0.074853945
0.271843715	0.342894735	0.281950998	0.081083324
0.326350829	0.401849531	0.449149907	0.288652511
0.326019774	0.388295991	0.205094985	0.100576531
0.025666482	0.417406548	0.332275152	0.045530251
0.421954021	0.293342647	0.026596254	0.463556591
0.268231818	0.394993239	0.014362186	0.055518609
0.043689858	0.363068528	0.415504014	0.370082528
0.247382167	0.355314135	0.007140164	0.428080045
0.433218013	0.018902195	0.106221641	0.316525385
0.255121105	0.478837396	0.170699671	0.33071524
0.239832338	0.405998042	0.275598873	0.241311469
0.386791336	0.374470917	0.371227371	0.437636037
0.411506491	0.294175802	0.301921211	0.047332726
0.090394235	0.335972228	0.196231033	0.079522087
0.323176596	0.127053812	0.421617534	0.401916273
0.381142607	0.013292004	0.286531019	0.339159615
0.444622579	0.453352875	0.038514868	0.00830174
0.007929778	0.014768846	0.376592381	0.208972409
0.413160481	0.214696745	0.016928385	0.151078278
0.300364923	0.235552458	0.4324781	0.341624742
0.319665979	0.123460982	0.223937297	0.353217945
0.062321207	0.273624156	0.397698683	0.446431502
0.061696763	0.215916268	0.329304799	0.450203662
0.025801263	0.268634926	0.284769324	0.268098631
0.023768933	0.084640362	0.074760261	0.355737013
0.398494709	0.082164119	0.04274857	0.193933137
0.172028026	0.402529424	0.19440026	0.442465519
0.408741391	0.395808557	0.067865482	0.432388447
0.057314788	0.41528133	0.200370623	0.078889813
0.26777017	0.03304586	0.377454348	0.083536876
0.356433321	0.349437469	0.128842138	0.022190223
0.089115162	0.071493587	0.410783237	0.35396899
0.037497555	0.326350804	0.266560033	0.338803101
0.353383783	0.343089177	0.44172672	0.255779383
0.176008063	0.081811979	0.365492048	0.311578133
0.349992711	0.410376379	0.20987814	0.440635413
0.338715727	0.423836109	0.3890102	0.32768767
0.146792476	0.332176583	0.322357508	0.05863848
0.340048725	0.386817379	0.362671544	0.114338023

0.212920213	0.208261188	0.118006561	0.191120041
0.158528679	0.327910527	0.198926248	0.396265709
0.347610861	0.318209097	0.130055944	0.070820198
0.471184853	0.414947644	0.237288193	0.325058989
0.194437462	0.275458445	0.333215744	0.312904323
0.261103386	0.366489805	0.392694888	0.315917672
0.425983263	0.311695633	0.449139233	0.22794074
0.069362888	0.273905122	0.123991626	0.103874933
0.192796038	0.040493468	0.138670053	0.321930495
0.189858853	0.31585657	0.324312937	0.116312524
0.25496225	0.289889767	0.30760478	0.285289824
0.114681053	0.429428106	0.018124067	0.083586799
0.209462091	0.332812599	0.212138908	0.222134889
0.154609552	0.120344176	0.282280647	0.340088203
0.241844663	0.249890747	0.338614679	0.095218764
0.073451021	0.427777088	0.327487854	0.029164494
0.091824688	0.299516372	0.305406512	0.217122229
0.025198578	0.372089758	0.280370929	0.284957691
0.369313304	0.119479388	0.370416457	0.137300814
0.129383918	0.422680208	0.239640084	0.414917108
0.246708997	0.34496099	0.414847186	0.355165267
0.146971651	0.230710701	0.080527363	0.411471002
0.217809202			
AKR1E1	0.128509163	0.109205969	0.103623879
0.151350246	0.146261985	0.049872557	0.093743543
0.035978457	0.039393416	0.011545247	0.029942102
0.139020868	0.035573006	0.040749177	0.016015757
0.235861664	0.150348279	0.094367756	0.198753744
0.179369228	0.032747075	0.242532688	0.0858626
0.094478387	0.120796478	0.1296961	0.088525666
0.179102931	0.153727748	0.028925562	0.114525481
1	0.138128289	0.085378709	0.158365963
0.082608035	0.092126319	0.09547588	0.020924934
0.116106861	0.132740597	0.162198633	0.17731795
0.066558432	0.070386217	0.131235027	0.202400127
0.021656576	0.078571177	0.018852827	0.068862352
0.06385543	0.068076839	0.046986796	0.068770697
0.016545945	0.022194671	0.027105609	0.056596161
0.13730689	0.015807702	0.076691415	0.026695467
0.018643201	0.082826164	0.219034352	0.090263883
0.074142268	0.079019303	0.095164779	0.102932929
0.004545761	0.190670811	0.26036861	0.036617996
0.253852426	0.053116891	0.268677536	0.122531663
0.175190338	0.10194119	0.175749047	0.151147015
0.098779206	0.274380248	0.131082985	0.133727622
0.111689018	0.076344823	0.064029	0.255207068
0.166030325	0.09707932	0.095858558	0.293174873
0.07906531	0.103362631	0.06002066	0.085420421
0.025369522	0.19729984	0.070301183	0.138417554
0.084277984	0.119351755	0.253641639	0.121499314
0.231366644	0.024854989	0.206779624	0.130428374
0.173360928	0.037991048	0.280485981	0.097368661
0.133509104	0.124804737	0.099932706	0.152055125
0.166199716	0.095986398	0.028474614	0.222182739

0.039786568	0.071128556	0.059168247	0.13420502
0.035103461	0.04458329	0.101301197	0.088101368
0.170597857	0.173173056	0.114942764	0.14244198
0.162393547	0.07962023	0.07215414	0.069700957
0.094587986	0.176459508	0.001049348	0.100121962
0.09106712	0.174137964	0.288159296	0.102153143
0.15346876	0.080717659	0.178779443	0.144157729
0.07437741	0.176503812	0.058563557	0.246736849
0.01967598	0.209758085	0.171714995	0.087195502
0.060320117	0.077180808	0.234835368	0.079723584
0.011876448	0.120354834	0.117957619	0.062847036
0.024751551	0.078030386	0.061621422	0.247993034
0.112769717	0.201586527	0.052347444	0.075980861
0.016003441	0.154437733	0.031737305	0.127880559
0.034235826	0.157695204	0.112435054	0.13260653
0.277486854	0.181351578	0.020261151	0.073075061
0.097578124	0.026174165	0.120763177	0.193769196
0.050842815	0.142477576	0.008351173	0.24414619
0.069587374	0.216740676	0.180024737	0.231428934
0.090654	0.154243711 0.046591	0.121870586	0.087496419
0.031136364	0.027306246	0.130076682	0.197102683
0.018154933	0.146583263	0.153844779	0.097268047
0.191030109	0.204371037	0.043426012	0.116338449
0.121793093	0.137240379	0.254464657	0.153812008
0.018359347	0.073953636	0.055965586	0.044994686
0.094032481	0.086242989	0.007767624	0.109983405
0.079760113	0.020681057	0.120275157	0.078751181
0.04166065	0.050728078	0.211097748	0.22376995
0.12557291	0.138402278	0.087992944	0.079360695
0.071665627	0.171393831	0.024415214	0.029663517
0.103043562	0.054216774	0.052085157	0.034299913
0.165714362	0.187099023	0.030894123	0.028395914
0.030002445	0.024798293	0.077604031	0.036300609
0.061408583	0.111934339	0.070326136	0.147722334
0.206239818	0.109091909	0.227026097	0.051680067
0.087036825	0.249198736	0.012844365	0.128319732
0.02074892	0.017985989	0.097115609	0.114301663
0.174703254	0.042874505	0.218075035	0.076318232
0.174386999	0.176311886	0.007483593	0.091883219
0.08562308	0.266224324	0.128452714	0.061156659
0.137805307	0.062702099	0.086156805	0.079382539
0.183138322	0.117857237	0.171007371	0.249731435
0.187182498	0.090542677	0.286583096	0.219729492
0.060399879	0.024273702	0.29595868	0.119494398
0.235056624	0.079792748	0.161988254	0.092482725
0.03276261	0.258781169	0.129485365	0.077759689
0.121753061	0.313881595	0.101115515	0.239956256
0.037423055	0.013602991	0.126582701	0.12690836
0.101194523	0.138580413	0.135570293	0.074534496
0.03445909	0.147668698	0.064668362	0.06901341
0.102936013	0.143106398	0.086649077	0.101941865
0.066691896	0.038391229	0.195776751	0.032008503
0.054334068	0.141784632	0.085191325	0.039879961
0.117343261	0.052887805	0.031676489	0.069391857

0.078318589	0.210075697	0.061436678	0.029008678
0.031266343	0.283124347	0.158659866	0.116111174
0.108206359	0.087363667	0.120152768	0.133005268
0.099879289	0.075853773	0.301594779	0.234093908
0.091289492	0.09886046	0.037440212	0.181268343
0.019690817	0.028687453	0.104358607	0.189673046
0.058712886	0.003616127	0.057506589	0.211321245
0.210063571	0.069439582	0.122027258	0.008687204
0.214491976	0.082304382	0.135343911	0.087344192
0.130469051	0.138146426	0.093981327	0.098598962
0.108639367	0.078596625	0.115752273	0.095423439
0.078298146	0.03357581	0.251092728	0.102618446
0.093898137	0.126009198	0.092807484	0.156843247
0.233298723	0.188735857	0.044466902	0.038464295
0.036333065	0.009223861	0.026697129	0.050744824
0.097592702	0.11939309	0.188807945	0.12958808
0.158736178	0.038680756	0.093268588	0.119480373
0.160631672	0.151962367	0.016150279	0.016397737
0.044596449	0.112371658	0.267057885	0.118548077
0.005562915	0.094458163	0.038501616	0.057976478
0.166975944	0.09313651	0.247101668	0.157804585
0.233107968	0.012352138	0.230525421	0.086006709
0.053074169	0.13102963	0.079152039	0.225102361
0.21731314	0.102090751	0.153443642	0.029123954
0.295805818	0.145671244	0.02934479	0.156714607
0.191731458	0.115248373	0.227267283	0.075168854
0.058299997	0.072461066	0.007639439	0.058472307
0.16448042	0.076840286	0.160236378	0.128383274
0.219281116	0.188162084	0.082046435	0.052218728
0.076395825	0.147759492	0.005318448	0.181369577
0.158141057	0.102288459	0.01040879	0.039552614
0.124390445	0.031342097	0.186772498	0.048796316
0.213268192	0.162825631	0.180240729	0.224663949
0.081556461	0.266711122	0.211499643	0.057179817
0.15184014	0.078034162	0.035089721	0.050331245
0.125404407	0.162502507	0.087336459	0.041530915
0.186693382	0.032848864	0.131993106	0.101744643
0.122123281	0.108600764	0.104944974	0.086358527
0.074355627	0.06808959	0.024570449	0.18582742
0.160241365	0.107929004	0.058171918	0.148867143
0.102806576	0.125825382	0.03624069	0.083909265
0.043427329	0.074148953	0.233292628	0.119833414
0.085213794	0.10535245	0.094770654	0.115992567
0.088382202	0.029164288	0.106096925	0.308603496
0.14366883	0.164586371	0.17905715	0.032918993
0.103941696	0.128954683	0.069344786	0.0709767
0.155476706	0.089472595	0.018322661	0.058575734
0.105279093	0.052553536	0.331508803	0.194559477
0.141950117	0.033543106	0.242318885	0.042588517
0.193100502	0.021664945	0.126334307	0.269974142
0.077243422	0.236329004	0.056635209	0.067921863
0.076645604	0.065196529	0.260949339	0.064054484
0.103285009	0.087293572	0.185318056	0.042575863
0.198259112	0.212954184	0.037549974	0.019278338

0.036204919	0.096014371	0.050044773	0.064206046
0.187261124	0.062924793	0.206659058	0.124553542
0.076351108	0.112078171	0.031904887	0.217925665
0.098225917	0.02051507	0.134429072	0.109540497
0.210786003	0.060381061	0.05396571	0.13455296
0.080397465	0.130149264	0.092643495	0.176644334
0.20689782	0.061956196	0.103991666	0.114819267
0.084665789	0.058046504	0.161057663	0.028184604
AKT1S1	0.393689306	0.509212901	0.360233407
0.372353808	0.3957702	0.329951439	0.221908421
0.239906915	0.081024557	0.005469217	0.018732805
0.394725415	0.181981272	0.270487383	0.277339074
0.390698396	0.141178656	0.394259486	0.262304875
0.401858403	0.113146107	0.368942238	0.112859197
0.283382622	0.09727935	0.073034317	0.226506465
0.418732675	0.085650686	0.29555117	0.378178634
0.138128289	1	0.506720974	0.37290347
0.497491962	0.36281814	0.034246615	0.257168584
0.418660131	0.370099814	0.393888301	0.318251727
0.050711597	0.484635772	0.244416388	0.379418871
0.250910518	0.032271475	0.250438457	0.45078398
0.450594791	0.353187985	0.260842617	0.168411514
0.159269618	0.262480594	0.206719484	0.323406053
0.239330243	0.180479093	0.463537398	0.087254432
0.161176963	0.310573048	0.184518675	0.29456058
0.309932883	0.359464442	0.037128802	0.361160509
0.003793509	0.389490684	0.380019704	0.236685005
0.054184259	0.022387427	0.209310758	0.324398803
0.442503703	0.465892856	0.449035745	0.455181812
0.18297789	0.325034091	0.353984265	0.181141698
0.420855706	0.177064111	0.130754482	0.386541196
0.458229356	0.501232816	0.204671838	0.269880542
0.356127662	0.47527575	0.358692487	0.372023996
0.254755179	0.068360053	0.425266099	0.486661575
0.395952331	0.278603095	0.158720924	0.388138157
0.081014632	0.345660995	0.342108431	0.204585792
0.454159908	0.022887036	0.217994212	0.344153064
0.278458743	0.391070773	0.490243257	0.207364779
0.40127251	0.028790551	0.102333237	0.054640668
0.314028651	0.136685965	0.468725377	0.422715119
0.2889035	0.347874057	0.297477299	0.004610831
0.035537805	0.23834116	0.39816245	0.412138151
0.406934238	0.374037492	0.198469988	0.360875453
0.359727571	0.44395739	0.048722601	0.128400233
0.378921761	0.24016017	0.257141723	0.393062203
0.275845956	0.157210183	0.155014916	0.492797446
0.435686109	0.104686596	0.377862367	0.389919661
0.278741859	0.325737297	0.143635241	0.284805358
0.067910908	0.459887289	0.038035664	0.376831563
0.193128285	0.155483737	0.028177827	0.295269183
0.262101962	0.379743289	0.463237346	0.142553578
0.322586906	0.367832455	0.350179417	0.344389982
0.008101617	0.072510389	0.311866475	0.484183598
0.280880065	0.485390118	0.332551803	0.312584622

0.284463775	0.400563058	0.005387632	0.485074275
0.501179041	0.307981891	0.347010231	0.407868449
0.297043918	0.403836893	0.056805353	0.401033383
0.353576306	0.111041301	0.478198888	0.163380719
0.458973344	0.047395052	0.340749109	0.371566903
0.352866727	0.012981898	0.22895017	0.371509831
0.038143119	0.118852004	0.406425855	0.375203022
0.32854186	0.400003882	0.455546802	0.272885572
0.381706792	0.285864697	0.022548234	0.316434996
0.396877288	0.32828328	0.438453377	0.260687762
0.335255734	0.457425535	0.368863073	0.166105849
0.053785732	0.335712503	0.027837007	0.295424415
0.374853692	0.12070034	0.322720806	0.218425722
0.083023953	0.499065558	0.170367856	0.22970967
0.117129375	0.346958163	0.295457457	0.201772574
0.169720031	0.177896437	0.297742058	0.324165322
0.051992311	0.105908206	0.027324933	0.315484728
0.118194699	0.283474765	0.281764176	0.375996384
0.346457572	0.115279665	0.338292409	0.273200369
0.119178677	0.021958672	0.492561497	0.397339426
0.319132484	0.176517157	0.060126467	0.278605449
0.434711934	0.268371606	0.284797267	0.434209682
0.048493322	0.46201773	0.21928397	0.12714942
0.324852664	0.262319262	0.432489673	0.053685334
0.401768418	0.461118563	0.34247578	0.171651894
0.392126025	0.398200316	0.040044098	0.363625605
0.366815642	0.041442119	0.504534799	0.141867245
0.030972864	0.389949648	0.350161037	0.090357567
0.089859191	0.282939344	0.065560628	0.286760475
0.492626575	0.373749195	0.352763346	0.225656542
0.047549192	0.011147423	0.229402614	0.375073458
0.501499036	0.484193808	0.297041399	0.305210789
0.263696084	0.268953392	0.181890588	0.47902983
0.255769565	0.513632298	0.012497107	0.368427033
0.488541664	0.307965608	0.078612503	0.30265313
0.370504144	0.058843906	0.279482286	0.480662774
0.413502394	0.293285931	0.283847085	0.421546981
0.023297719	0.063029339	0.348059185	0.172320736
0.327252595	0.222725043	0.051432393	0.358197553
0.0467024	0.439764186	0.422825616	0.056467976
0.278240319	0.339188911	0.37113377	0.050364037
0.402396001	0.487072277	0.50809306	0.231605186
0.397491168	0.449751112	0.321998863	0.105606437
0.024009104	0.496361439	0.384407209	0.023890798
0.449050023	0.245695339	0.012429488	0.491674818
0.219314511	0.449972757	0.004237987	0.106819852
0.058689048	0.419005957	0.460164859	0.386176159
0.211894657	0.398068304	0.01397722	0.505519244
0.472189351	0.017860052	0.152217489	0.368837852
0.343081568	0.525076958	0.261880872	0.392170296
0.353024078	0.475501829	0.238752297	0.265958848
0.394740867	0.463620244	0.39357092	0.45914997
0.428905486	0.395497865	0.283607945	0.01988959
0.10896954	0.246045897	0.221701201	0.065152121

0.325629171	0.132329753	0.474869983	0.439873982
0.446498551	0.008895741	0.184868055	0.381354738
0.43369439	0.462824336	0.040519313	0.007329349
0.010982506	0.015166961	0.377334904	0.260782287
0.46390628	0.194013208	0.008095857	0.07090649
0.287275596	0.343052175	0.484152845	0.376889881
0.390829653	0.109178108	0.219810215	0.397337819
0.065447288	0.323320617	0.450240175	0.465832914
0.066871951	0.263431991	0.409761978	0.466694192
0.059534089	0.328900922	0.404594385	0.277515931
0.030957093	0.091349072	0.06924081	0.409638626
0.479203254	0.071962605	0.032894063	0.166376576
0.119694075	0.449038455	0.167185601	0.491572938
0.407279583	0.399183964	0.092457413	0.468976893
0.041209507	0.473577412	0.296837935	0.055083609
0.343740097	0.023390991	0.460622126	0.099708795
0.346827159	0.401468768	0.161846434	0.025208443
0.162698328	0.066207313	0.44001949	0.412762973
0.051334219	0.280785075	0.319657272	0.414515976
0.300033355	0.377105267	0.492164996	0.32307326
0.101402662	0.169213395	0.401072722	0.381529173
0.295112377	0.463012889	0.242208692	0.508488912
0.387207218	0.462868355	0.45009942	0.327576857
0.226613831	0.379600748	0.326987224	0.022445738
0.364763549	0.411832329	0.394149786	0.130443866
0.235701118	0.334670234	0.084501441	0.239153313
0.277242754	0.295647467	0.16021515	0.408106936
0.40568852	0.380444983	0.08211706	0.062310979
0.528427503	0.439581967	0.157381282	0.371465349
0.209371735	0.312754071	0.39524626	0.39284659
0.347657261	0.403957393	0.400206663	0.34020338
0.423564179	0.280445005	0.507640743	0.135986879
0.081187756	0.393974201	0.092148227	0.113483773
0.161966556	0.035172892	0.239409423	0.408468028
0.31488963	0.359746182	0.358128946	0.099419479
0.336139831	0.387644517	0.358985472	0.207541589
0.08106182	0.476688623	0.013280331	0.077332816
0.344606358	0.36922666	0.310753182	0.253548919
0.248678055	0.175204955	0.3016606	0.307001468
0.257115761	0.351025131	0.370847353	0.050461027
0.032467696	0.471466011	0.377600518	0.036260563
0.113552945	0.369813885	0.374780604	0.205308789
0.030858865	0.449025407	0.279547874	0.386449391
0.356274768	0.096619382	0.375109108	0.118637607
0.097774915	0.457351734	0.357933294	0.49216131
0.3630211	0.379172371	0.453659809	0.396172923
0.171009342	0.353163539	0.037157377	0.424888159
0.312416836			

AMIGO2 0.499797419 0.526895921 0.45571574

0.460366701	0.456701716	0.458321553	0.213824501
0.209787289	0.083673102	0.00340409	0.01911008
0.493357815	0.211694272	0.292896725	0.406133303
0.443494387	0.119755868	0.393040609	0.234525172
0.444820774	0.093728679	0.41396377	0.081792829

0.29948192	0.094018291	0.057295275	0.277984411
0.483189078	0.067142233	0.406430429	0.442335694
0.085378709	0.506720974	1	0.473803555
0.512419697	0.495588955	0.009444954	0.343407663
0.500227039	0.501214398	0.446008842	0.337311923
0.034097809	0.4934337	0.248181941	0.428088965
0.275424257	0.023566884	0.301667452	0.488800975
0.50290134	0.506465436	0.251567366	0.158257679
0.225369932	0.276122186	0.306712184	0.405383759
0.222381133	0.195691338	0.515468941	0.088183719
0.16111732	0.268323851	0.135629339	0.275903058
0.3820008	0.475597945	0.013890906	0.485106128
0.002081001	0.416428677	0.391193565	0.365705914
0.031625006	0.014924812	0.16083037	0.362347593
0.499928133	0.506148037	0.514696988	0.523318116
0.171020692	0.286498213	0.436787862	0.158887117
0.517144735	0.178710546	0.109056055	0.441775078
0.51990826	0.525295215	0.191952307	0.25343198
0.505263865	0.517222376	0.493152576	0.468431344
0.401123376	0.042065188	0.512038423	0.530047945
0.490315983	0.344639439	0.136934544	0.473929691
0.046781416	0.433050818	0.364660683	0.154077835
0.503205415	0.01445892	0.198385115	0.390419404
0.209027078	0.514445229	0.524767104	0.181249653
0.436692739	0.01463926	0.146055062	0.02475139
0.354599251	0.15084677	0.506412915	0.508238357
0.256844193	0.404790322	0.274636839	0.000381384
0.019056846	0.176990299	0.517281443	0.400891003
0.486905654	0.486969095	0.235766101	0.491630993
0.51503932	0.513662435	0.084801663	0.086878015
0.47734047	0.173850078	0.227142614	0.50408788
0.313676416	0.11090444	0.112938975	0.527955811
0.487574389	0.072088494	0.39689351	0.436948057
0.372907306	0.367558058	0.094028126	0.281717705
0.042190136	0.510475281	0.020304806	0.38357214
0.329366339	0.137043168	0.008171131	0.294510683
0.325736715	0.518178592	0.495054119	0.091907616
0.329454278	0.428758331	0.390364211	0.503049451
0.007989287	0.037955098	0.361319451	0.528939895
0.37457819	0.524811691	0.304766903	0.308870267
0.275635979	0.494100025	0.003175642	0.505740842
0.524447785	0.390783539	0.43073966	0.455138693
0.439489225	0.501223814	0.055994114	0.439392575
0.45820146	0.066167946	0.516992249	0.145085023
0.514327455	0.031689356	0.360768078	0.511092939
0.51708687	0.007050852	0.273697198	0.431317034
0.0194016	0.10413741	0.490024281	0.445526098
0.422716757	0.494701466	0.501046549	0.420864833
0.463512085	0.238198317	0.00843621	0.321946371
0.50757015	0.454677709	0.500457677	0.24989934
0.439847318	0.521738301	0.518941547	0.252616822
0.034816644	0.344883047	0.019426173	0.351402353
0.501909185	0.112980528	0.471235601	0.135223765
0.057339053	0.530916929	0.175436063	0.288653

0.130086116	0.459356567	0.252902377	0.261546466
0.226725323	0.152078472	0.392151099	0.419929483
0.033095358	0.079559012	0.010347465	0.444874715
0.143335835	0.393134342	0.402182133	0.5006322
0.357505989	0.102811759	0.410349923	0.307441528
0.101354138	0.009568356	0.528315794	0.431708961
0.438353697	0.147328285	0.030724864	0.412604481
0.515170484	0.421365088	0.357233535	0.513228011
0.023083876	0.517085013	0.254824425	0.107588005
0.411104695	0.258309663	0.495152768	0.068340861
0.459927344	0.512211211	0.314486742	0.176768424
0.485972908	0.461908726	0.024184265	0.502020421
0.499189974	0.022781623	0.53063224	0.110571219
0.01407985	0.485285602	0.516743685	0.061412376
0.064523371	0.367934324	0.045086256	0.284768524
0.530139616	0.430849556	0.514497728	0.216708152
0.031815427	0.007095118	0.196215524	0.457243072
0.513123938	0.528662704	0.250792226	0.393498902
0.2335141	0.371787546	0.174159745	0.530796978
0.200623831	0.518626411	0.003593143	0.507406793
0.514416478	0.360049876	0.068704234	0.34789371
0.488762864	0.037640479	0.201645512	0.518858598
0.515084039	0.359347327	0.400336166	0.472370453
0.01297602	0.036078875	0.428375617	0.190658505
0.434533339	0.208461382	0.052605605	0.382348262
0.034116535	0.511338261	0.496249095	0.052247843
0.425208718	0.373096336	0.389893255	0.028133493
0.518528671	0.519341477	0.515892476	0.159843084
0.515390643	0.528589288	0.477570963	0.064952895
0.011379409	0.517499368	0.48032008	0.018362084
0.506757414	0.313172213	0.009057397	0.521455655
0.177022331	0.469312259	0.004006515	0.096360936
0.031253916	0.498149632	0.507695312	0.437694103
0.329530864	0.391767937	0.004320805	0.528718128
0.520414543	0.009375255	0.128898653	0.5258829
0.508544894	0.521909185	0.356199522	0.521185936
0.494300449	0.513770083	0.253945498	0.262905578
0.515769735	0.516723561	0.491457913	0.510927279
0.507858388	0.454523538	0.282809536	0.011402941
0.144702626	0.24811594	0.395614642	0.079755443
0.413321189	0.130895715	0.523835969	0.50674261
0.528060299	0.002186675	0.157287113	0.526299369
0.500082397	0.507641142	0.016380709	0.005856443
0.008001381	0.015291645	0.402434422	0.235064638
0.529543185	0.313493832	0.00186226	0.041651587
0.296165165	0.435663709	0.524523773	0.397692816
0.508410936	0.066534131	0.337883854	0.426152431
0.058817375	0.439093473	0.527993035	0.510203594
0.03137818	0.242670945	0.430901638	0.514405448
0.078184021	0.314917063	0.497638727	0.344706645
0.009860032	0.068004473	0.055297986	0.47629506
0.504100763	0.068930338	0.025613341	0.189040241
0.113828285	0.516699692	0.156871939	0.525478299
0.504846271	0.469257106	0.06133798	0.514727052

0.032387358	0.512445834	0.359208101	0.071812053
0.291171696	0.008005811	0.515075212	0.179775625
0.381374178	0.466570033	0.220519589	0.011018496
0.203490325	0.041167647	0.511110957	0.507162907
0.024957281	0.243646033	0.298050812	0.441854941
0.336825733	0.479259219	0.509717632	0.505281398
0.078331893	0.145153579	0.456431515	0.487072291
0.265537861	0.514414157	0.326582361	0.530129515
0.497108202	0.517103002	0.528364948	0.384390775
0.233315087	0.480698433	0.442046108	0.016708225
0.373473269	0.505137329	0.405710564	0.15342284
0.255176808	0.448914428	0.040197072	0.223822032
0.385081583	0.382108667	0.132706338	0.470207585
0.457718687	0.494337239	0.034707222	0.052292842
0.527474057	0.507183492	0.156797291	0.490433153
0.155337246	0.362801892	0.511335811	0.501427383
0.433304962	0.522915893	0.495447668	0.464382282
0.493420965	0.263828074	0.518843179	0.105176164
0.082104299	0.496932495	0.088374482	0.076751635
0.1294759	0.019523806	0.352862572	0.454409437
0.444400107	0.400980822	0.387435618	0.071322121
0.325776473	0.441885416	0.365308749	0.212918491
0.069148567	0.515479896	0.00636029	0.043523492
0.441306793	0.522587859	0.430066881	0.232997231
0.328415382	0.125897301	0.297808521	0.36558504
0.395208426	0.422384932	0.524180456	0.031063889
0.014628392	0.514267695	0.453008377	0.014793463
0.077859766	0.474306593	0.308402006	0.250756207
0.01514236	0.524682826	0.416796549	0.470941116
0.382499894	0.053138964	0.454304856	0.088873674
0.05191735	0.502347646	0.449749751	0.521601615
0.374925719	0.44829957	0.477136063	0.520519574
0.122084084	0.46264303	0.021794934	0.501581679
0.493999774			
ANXA1	0.323804815	0.503040263	0.293976576
0.372171541	0.361809811	0.230222239	0.19287291
0.121226499	0.099301477	0.015437645	0.067044466
0.341084073	0.260222137	0.238242519	0.205424511
0.384751147	0.234480421	0.232993662	0.245676927
0.313362866	0.087805515	0.368278553	0.118700265
0.295779905	0.186045571	0.126195284	0.290716969
0.419484504	0.135019168	0.207698645	0.277427128
0.158365963	0.37290347	0.473803555	1
0.476479995	0.28271097	0.022133815	0.212778461
0.353831079	0.380906773	0.318521172	0.306771263
0.072935587	0.421798593	0.238888091	0.345513376
0.141026764	0.084811122	0.131693082	0.374076188
0.438622567	0.317371973	0.154115514	0.162918411
0.247892035	0.146353784	0.305290655	0.188916254
0.235838191	0.092373188	0.454941321	0.091892144
0.124176669	0.180429801	0.229361462	0.205467701
0.31575153	0.274107724	0.026886255	0.286449206
0.007230044	0.293778441	0.342559952	0.26382219
0.092451526	0.069803601	0.249973243	0.28088445

0.442887171	0.408450426	0.512231677	0.508324249
0.269869243	0.297772525	0.339231888	0.230753765
0.383387202	0.189093749	0.116014358	0.412769828
0.517057477	0.503286231	0.258351776	0.294971838
0.378025058	0.440439786	0.297894098	0.355168116
0.277839424	0.097937127	0.401160348	0.522721595
0.324358862	0.320459074	0.22810721	0.341577971
0.104820339	0.22747718	0.320092938	0.201712533
0.458113616	0.03055399	0.283043404	0.290544056
0.201900537	0.369708061	0.50270495	0.201870245
0.351282235	0.055387293	0.175265625	0.068945452
0.282046443	0.201121818	0.416528535	0.45771467
0.178479067	0.350424696	0.225464933	0.01137166
0.074919683	0.199893183	0.399141546	0.269694859
0.344667288	0.327079079	0.265416604	0.283118186
0.329297856	0.505118465	0.030952306	0.110328136
0.313449537	0.209085906	0.267882181	0.341549985
0.336911057	0.110412457	0.175804277	0.517963539
0.360923311	0.129573801	0.203405574	0.374995802
0.277102824	0.370480452	0.142928753	0.29504588
0.076184654	0.448436	0.078166997	0.210360027
0.213765282	0.256536642	0.016184702	0.176036975
0.233431022	0.417662794	0.403530952	0.144866779
0.314873544	0.365017978	0.191969	0.349613772
0.031950994	0.073079989	0.145414064	0.515394948
0.158490557	0.501117157	0.279278634	0.291598313
0.314950828	0.418736336	0.003829763	0.441954435
0.503577174	0.158790706	0.366017438	0.339597292
0.294437757	0.437410327	0.027509149	0.371615973
0.228778552	0.111247632	0.520835479	0.238463333
0.421846189	0.092084461	0.231598144	0.422006392
0.377904974	0.018206622	0.137595237	0.285090183
0.067687114	0.076281257	0.326818969	0.315811322
0.306323833	0.403677644	0.498361528	0.28732005
0.361623974	0.202182321	0.028624888	0.331812905
0.380462702	0.274991872	0.387362809	0.207830286
0.18686999	0.442677498	0.36319902	0.144597289
0.075251953	0.228239962	0.031910584	0.309862075
0.329218202	0.164343322	0.2747983	0.177326951
0.14440741	0.525351505	0.235361956	0.267173975
0.177363828	0.322535835	0.225882299	0.253541096
0.296234962	0.207345939	0.307102496	0.303607719
0.04933159	0.168880928	0.029561161	0.326461475
0.234256546	0.324891255	0.295982457	0.29298712
0.283049938	0.19048643	0.367801576	0.262552887
0.173939355	0.045115827	0.486702077	0.328303808
0.223894486	0.166412421	0.085502638	0.282302904
0.46975052	0.184085913	0.130903078	0.436937603
0.040734389	0.490209827	0.294482295	0.201246833
0.38647083	0.29179105	0.449203715	0.051369724
0.253508974	0.435718287	0.292573011	0.240925325
0.351523831	0.310480052	0.055116451	0.311380561
0.310210507	0.079290768	0.520468037	0.216966239
0.056098156	0.378271977	0.380675355	0.132216054

0.134102737	0.299098361	0.042965666	0.320639447
0.513270301	0.397442758	0.374512425	0.245640739
0.083248541	0.051085013	0.268008576	0.300644298
0.459311078	0.515163849	0.283524555	0.378717317
0.252722427	0.297610111	0.079527215	0.518519823
0.167169036	0.480085797	0.021574112	0.410330033
0.467043441	0.173444091	0.154523698	0.227019742
0.329908433	0.073619155	0.182581925	0.466322906
0.393926379	0.28576596	0.283722131	0.330783048
0.026353634	0.056943925	0.31969174	0.220018911
0.301804025	0.232574615	0.09199444	0.210422916
0.093489757	0.424193545	0.448614103	0.097614742
0.221906885	0.180028478	0.362101982	0.071163093
0.455524965	0.477028684	0.48313214	0.153534839
0.390281519	0.486838434	0.329513793	0.132167314
0.051572506	0.48699026	0.261938256	0.047886021
0.479629868	0.122753592	0.051128696	0.465673053
0.194579695	0.375745364	0.00330125	0.066062392
0.080854225	0.465679066	0.430098662	0.261395661
0.14295672	0.289166533	0.011337946	0.519299261
0.469508624	0.047385594	0.215497014	0.40074312
0.38781222	0.493043382	0.310520373	0.458931216
0.35130128	0.471670562	0.233427319	0.300793489
0.410343174	0.47929566	0.330171544	0.408001784
0.462318699	0.366871438	0.274377378	0.034200524
0.131004814	0.136945511	0.213393209	0.118335044
0.179437347	0.203412684	0.455148547	0.476010461
0.498171223	0.019277332	0.124994465	0.378576959
0.405144703	0.457108871	0.02987731	0.007576936
0.008262128	0.062172324	0.259498168	0.273625218
0.476152828	0.159875385	0.017820585	0.045555315
0.241430578	0.363198743	0.484292919	0.327955007
0.395587618	0.134540519	0.23763831	0.334101342
0.131622055	0.193654002	0.503125088	0.432050807
0.07633174	0.268499014	0.254602241	0.48719906
0.108388589	0.338396092	0.342736064	0.300848416
0.025743826	0.14320191	0.114539598	0.411873106
0.464205697	0.170580741	0.101943395	0.086752176
0.166188322	0.456637952	0.224795902	0.526448081
0.404116618	0.442796758	0.145203193	0.437631572
0.082973257	0.454698859	0.359446585	0.041296974
0.269211717	0.031018242	0.451881695	0.170090369
0.224191662	0.267773353	0.175972499	0.049417317
0.182638317	0.114809962	0.494895474	0.472018091
0.071281277	0.199315715	0.299217799	0.309928322
0.188449394	0.403028084	0.456754046	0.261288653
0.102672288	0.17108498	0.318932593	0.261341265
0.178041612	0.515154063	0.298546706	0.516663432
0.294183075	0.419208872	0.463888353	0.275027793
0.308289791	0.252223301	0.267864759	0.038755657
0.299447699	0.471894396	0.241055255	0.225506787
0.314911548	0.319307719	0.055495175	0.109401401
0.295382224	0.186110762	0.197004854	0.432337109
0.254917918	0.276806752	0.045623592	0.109289406

0.518770128	0.379518071	0.109162011	0.317868556
0.215217329	0.323923226	0.412876517	0.426630782
0.260189554	0.437802088	0.364477371	0.29142623
0.381417233	0.241399874	0.488007416	0.049699677
0.123018785	0.290195736	0.166901306	0.155536368
0.200513645	0.060873381	0.271469491	0.378668199
0.312506941	0.35864678	0.198946325	0.112981902
0.33634902	0.228255521	0.336928825	0.182633671
0.136873799	0.464327844	0.024526533	0.111753224
0.341885825	0.38756879	0.360777237	0.274896355
0.305503662	0.196535466	0.323512136	0.179456903
0.249444855	0.267769318	0.38825967	0.038375211
0.036234715	0.511763275	0.230819554	0.04403968
0.116399989	0.224903154	0.245064771	0.239012555
0.057989649	0.481796514	0.216341809	0.345022893
0.254022881	0.1054379	0.280164952	0.082945401
0.068162995	0.397552037	0.318640393	0.491799511
0.302880025	0.410157894	0.370328083	0.397901622
0.112874281	0.256393038	0.045786238	0.407695669
0.298155712			
ANXA2	0.49327416	0.521054646	0.446050262
0.439719245	0.450467556	0.432613159	0.189845284
0.182400112	0.068734126	0.002610907	0.017563374
0.496892417	0.208868569	0.277773131	0.370841326
0.426278288	0.120021016	0.371287761	0.214766219
0.424105922	0.078662418	0.403410634	0.069704763
0.296855781	0.091077876	0.05117087	0.268616396
0.464744418	0.059910207	0.384225972	0.417563954
0.082608035	0.497491962	0.512419697	0.476479995
1	0.487774438	0.009620442	0.315710301
0.485570724	0.488713062	0.423481343	0.317766713
0.028301356	0.489748438	0.225996201	0.422453813
0.246476155	0.021433433	0.269084494	0.479179032
0.505206518	0.498583656	0.218195733	0.138194685
0.22940806	0.256232243	0.325787509	0.38159511
0.203340589	0.171165754	0.51739593	0.072102377
0.146800792	0.239819818	0.136216476	0.250143721
0.384421558	0.453290237	0.010660952	0.495018113
0.001807958	0.391287942	0.385236717	0.362169169
0.028606818	0.014253931	0.159735674	0.338758343
0.485399362	0.50367289	0.510727061	0.521814928
0.171349684	0.275727583	0.443807939	0.148972914
0.50317576	0.160760112	0.097011521	0.430992797
0.516331119	0.526394276	0.18185948	0.244401201
0.491208939	0.50728584	0.476968869	0.459562628
0.388043087	0.040477235	0.506068743	0.529041081
0.477983087	0.355451997	0.131094051	0.455377877
0.041779184	0.41412869	0.345659758	0.160854899
0.48783863	0.010517122	0.195180378	0.3698942
0.193462955	0.503482181	0.527546264	0.16204504
0.435338276	0.013585341	0.142286481	0.022447904
0.343714432	0.161306497	0.504705682	0.497322798
0.237113554	0.401298167	0.246792867	0.000909874
0.018010171	0.170634508	0.523298411	0.385961781

0.471017837	0.488339497	0.221763052	0.477711781
0.518696727	0.510248924	0.074090699	0.072932657
0.456760461	0.168092031	0.212115058	0.503624337
0.304195568	0.093186968	0.103070647	0.520139632
0.472524508	0.063521733	0.378664781	0.423254975
0.357928183	0.368709363	0.083441126	0.273498772
0.03519423	0.513593602	0.019767035	0.354481204
0.310579424	0.144906999	0.006475553	0.265550623
0.305655589	0.514969627	0.496261544	0.082345264
0.319518155	0.418757447	0.365880362	0.496012373
0.006390849	0.032837683	0.338284908	0.527115597
0.352673369	0.523304031	0.285661791	0.294196725
0.265224586	0.47873265	0.002934494	0.502619605
0.525232986	0.370102576	0.425794179	0.45003833
0.430390039	0.486465112	0.054335882	0.429739986
0.448970867	0.057563688	0.509641457	0.138669939
0.513933957	0.028127935	0.338627602	0.513684909
0.529559314	0.006207566	0.24293499	0.404723661
0.018607904	0.085011803	0.473692489	0.423940079
0.399439676	0.485073472	0.490866954	0.413575371
0.453321923	0.214793322	0.007040765	0.317054128
0.502020946	0.45332974	0.488517842	0.222985386
0.426956303	0.512221822	0.520551851	0.237394549
0.028762817	0.316972292	0.016087629	0.331345357
0.494209503	0.104176028	0.454809485	0.132587215
0.055193356	0.526695156	0.179441235	0.298964529
0.136313332	0.456598573	0.228342688	0.257437199
0.235704291	0.137819454	0.377146757	0.40057043
0.026005037	0.074983734	0.0097372	0.473242355
0.151548042	0.405177822	0.413707977	0.493581874
0.351684333	0.102036658	0.402857332	0.298357823
0.095014453	0.008402561	0.522205373	0.4135368
0.421911454	0.127944074	0.028001993	0.430554245
0.506692625	0.398445015	0.32795922	0.502985063
0.019408652	0.511042333	0.250235984	0.10201648
0.426088383	0.245355126	0.477645098	0.058153727
0.441410223	0.505333082	0.298597606	0.165569083
0.480764514	0.439287614	0.019690009	0.493780073
0.495594905	0.020575646	0.531065895	0.111300717
0.012692064	0.485280037	0.515435592	0.055988016
0.059365124	0.352701579	0.034638011	0.273566825
0.524448709	0.41976362	0.507340845	0.214005358
0.02744471	0.007972092	0.195816955	0.456781961
0.511019861	0.532667072	0.238618712	0.397074412
0.214591052	0.356480621	0.152253668	0.529218198
0.194646055	0.517737236	0.002749385	0.508398859
0.514415541	0.337115291	0.064916872	0.318056586
0.478088988	0.030661735	0.182147423	0.517158956
0.499153844	0.334598661	0.378501296	0.467326879
0.009563521	0.028674641	0.412181577	0.193212372
0.417723603	0.189512556	0.058248849	0.349793192
0.03041734	0.503770384	0.490270925	0.044134608
0.407747816	0.337507914	0.384379948	0.023696732
0.521996731	0.522706066	0.5149319	0.14058178

0.518552041	0.529258289	0.491580307	0.060314558
0.010472069	0.520581111	0.457892193	0.016227298
0.497459043	0.285604426	0.008784158	0.512205107
0.157759043	0.471261888	0.002601968	0.096022117
0.029885045	0.494156935	0.502410502	0.411183863
0.306640096	0.369914115	0.00426668	0.529564184
0.517551064	0.008639082	0.127786545	0.529408097
0.518428858	0.516198169	0.355735939	0.523296473
0.505512807	0.517546674	0.236475376	0.249423694
0.502801591	0.52366679	0.470642132	0.498282648
0.500062641	0.450343086	0.261159607	0.008692471
0.126422338	0.218608188	0.379917584	0.072069956
0.386021765	0.12265427	0.519734137	0.499421987
0.530467663	0.001591067	0.136147756	0.52251171
0.482908033	0.491906199	0.014219133	0.004882271
0.007428964	0.014733156	0.375181835	0.224803963
0.52380123	0.303413943	0.001121078	0.032827164
0.272612746	0.437389597	0.52348286	0.377791159
0.505992982	0.060959828	0.326009097	0.405593232
0.054496082	0.426381952	0.526295595	0.500888536
0.029000661	0.229621255	0.41912155	0.502130263
0.084978077	0.306407527	0.503678102	0.329567967
0.009314338	0.063758007	0.048943632	0.465412678
0.510208426	0.069311783	0.02531453	0.172665402
0.103617437	0.509718826	0.147870644	0.519919942
0.489227069	0.455723428	0.061097172	0.506103257
0.028168316	0.514176041	0.364178696	0.063004464
0.281964601	0.006742099	0.52276118	0.172590009
0.361307066	0.446316072	0.198684933	0.010465361
0.202782727	0.038632168	0.502299985	0.50455103
0.024068582	0.215504228	0.284753273	0.425919416
0.306576753	0.46711957	0.507230009	0.499022011
0.065502601	0.157908383	0.435263287	0.476500165
0.243601476	0.510130501	0.314520577	0.527844945
0.477919365	0.505470722	0.52847645	0.354730095
0.242288289	0.458399855	0.420857122	0.013875154
0.351161252	0.499366245	0.379398735	0.147002789
0.247470571	0.456835559	0.033175493	0.219224979
0.400263087	0.353885235	0.123958813	0.454925625
0.439947229	0.481351894	0.029191422	0.045901165
0.524064599	0.493083543	0.131246196	0.472746242
0.145747876	0.344520444	0.508983499	0.503022092
0.415267247	0.523390248	0.47994972	0.451577791
0.482415074	0.238461366	0.516759334	0.086687137
0.073512689	0.502786444	0.08278707	0.072410023
0.119385113	0.017276699	0.362215478	0.444898413
0.460998633	0.390816027	0.370846811	0.063387736
0.319553316	0.42990548	0.350670671	0.191532408
0.062976157	0.513125216	0.005467782	0.040512618
0.4641662	0.510944212	0.435908303	0.221427434
0.332155237	0.124489102	0.285697911	0.337909283
0.393726141	0.418309676	0.513001244	0.024155806
0.011576532	0.508414182	0.43187209	0.013793696
0.073407895	0.457184288	0.298039263	0.229869472

0.014472131	0.530674434	0.393629523	0.463848921
0.353572642	0.047418474	0.44367534	0.077903175
0.043052445	0.497330811	0.446118176	0.525205771
0.370717402	0.435128646	0.471544927	0.520504593
0.112922784	0.463385342	0.016838444	0.4872983
0.496827457			
ANXA4	0.352694434	0.470277452	0.339223978
0.28234292	0.299688086	0.272177681	0.138782391
0.169334802	0.060888168	0.001116352	0.008795035
0.331822386	0.109459923	0.170878902	0.255871912
0.302932484	0.081844084	0.349339148	0.169845085
0.303983169	0.058874948	0.268051147	0.06346654
0.239122205	0.063943168	0.039163832	0.183344468
0.35542372	0.048483971	0.2166188	0.286535518
0.092126319	0.36281814	0.495588955	0.28271097
0.487774438	1	0.015351499	0.185718032
0.395989454	0.313005573	0.295186639	0.218087312
0.023735425	0.470057055	0.157556764	0.313226615
0.203216799	0.014923969	0.190338188	0.43458256
0.434273055	0.287866767	0.184466151	0.100049781
0.100290934	0.166376518	0.166723127	0.253152044
0.144224299	0.116201192	0.444072043	0.05561881
0.10479339	0.217125661	0.120465598	0.195574045
0.26849541	0.318230568	0.017719172	0.333366098
0.000579529	0.295580025	0.300600182	0.229065517
0.028096958	0.007040202	0.139563405	0.221260403
0.38355976	0.466852945	0.39598514	0.415044709
0.110577633	0.24081455	0.302629758	0.104775696
0.393402427	0.104197004	0.094076257	0.294048716
0.40885654	0.472155464	0.139922798	0.202609043
0.314293874	0.437504661	0.306269819	0.285443022
0.229910082	0.052092617	0.398208246	0.439341279
0.368928482	0.240476385	0.102820658	0.317758713
0.043890565	0.315297748	0.237567621	0.1316558
0.394749515	0.011228338	0.151646253	0.231678455
0.218060418	0.360155042	0.472461201	0.125950141
0.319615666	0.012726164	0.08796518	0.031075779
0.230708794	0.120372678	0.470463118	0.358160439
0.178140883	0.252683922	0.198350612	0.001391893
0.018102685	0.192831625	0.357282305	0.333152275
0.335592632	0.399478947	0.135550896	0.318430299
0.311779283	0.390202273	0.04549152	0.07415086
0.304878915	0.180763135	0.16746169	0.307221092
0.202382164	0.094951719	0.085322563	0.450945133
0.404361103	0.053838268	0.31913395	0.298592256
0.194390464	0.249274956	0.078633889	0.172547984
0.028612839	0.460167932	0.021590356	0.302470409
0.189169188	0.091790731	0.017377322	0.20487472
0.189516431	0.338941021	0.473566653	0.084779263
0.203609744	0.278705272	0.270680784	0.271863396
0.002729306	0.0442506	0.262370998	0.430212151
0.214911091	0.452575972	0.234257668	0.25391693
0.20265357	0.356414622	0.006947824	0.474363834
0.482651415	0.261943547	0.245576886	0.332319291

0.269231839	0.347580127	0.055087409	0.295782257
0.30365801	0.065282107	0.424012953	0.099737289
0.441972251	0.02306562	0.227555685	0.360161756
0.304089024	0.005947891	0.163068071	0.27613783
0.023781893	0.065169007	0.339329823	0.287636027
0.255171203	0.353098961	0.394417186	0.253612621
0.293020384	0.19132137	0.012159723	0.249628401
0.369041321	0.272234118	0.413908213	0.175830625
0.291467567	0.425395013	0.36202349	0.134961437
0.025479462	0.23170129	0.01330902	0.229680271
0.389039282	0.058011551	0.297419119	0.154966637
0.047989819	0.456083697	0.142864179	0.214969348
0.107025264	0.24531391	0.202278654	0.119563761
0.105452441	0.114796487	0.25556851	0.263631098
0.021800884	0.053951174	0.022953111	0.235395998
0.06003889	0.193097874	0.192375868	0.303065736
0.216091145	0.056183275	0.247681211	0.255870972
0.084989951	0.009768517	0.455216412	0.309072243
0.277791418	0.112650254	0.03237258	0.208513298
0.370003103	0.221900589	0.251174634	0.3785042
0.031469131	0.415548683	0.134188874	0.075769546
0.235675568	0.163960093	0.377978174	0.041466095
0.322855494	0.41602268	0.264697789	0.117269664
0.35260157	0.302223832	0.017885452	0.346212394
0.323363572	0.018411023	0.46967194	0.088019466
0.015616595	0.324343298	0.294967227	0.053517696
0.058141816	0.193895938	0.032303 0.203664981	
0.450369918	0.275586564	0.305744415	0.198369027
0.020413694	0.002148854	0.171399411	0.345575573
0.486748258	0.454539085	0.201251853	0.225263953
0.170937743	0.22192575	0.124946468	0.434373617
0.25593372	0.496040116	0.005049987	0.331910298
0.481832253	0.221182238	0.047519839	0.209473664
0.330175982	0.030826524	0.195855375	0.456332455
0.384199809	0.238784316	0.240802554	0.336847976
0.009212701	0.027004186	0.298771739	0.161181424
0.25542233	0.157140379	0.050419409	0.273123899
0.020636747	0.395490633	0.365817683	0.036972449
0.212009605	0.28370751	0.279245872	0.024500624
0.386948402	0.478337984	0.487698385	0.147009182
0.341572207	0.423979183	0.277734438	0.067628099
0.012872802	0.487599289	0.328824593	0.011438894
0.384610382	0.189054211	0.003019654	0.452025853
0.138972129	0.448216787	0.004146052	0.136689565
0.042945897	0.373825353	0.451532879	0.294457348
0.165290067	0.305803696	0.01093497	0.476297598
0.435776744	0.009810924	0.109758914	0.333760651
0.305622991	0.492210852	0.231126472	0.362283076
0.292121525	0.459062053	0.14563072	0.185516725
0.349613003	0.456194895	0.329027175	0.439484905
0.360415307	0.31194098	0.189151779	0.007565261
0.116040861	0.164732547	0.196871199	0.04249344
0.271994723	0.089961875	0.452240106	0.381428384
0.406972385	0.003575851	0.113289341	0.348023196

0.375996141	0.409433422	0.033727029	0.008761141
0.013261159	0.009094802	0.27381114	0.200193494
0.437482333	0.145883032	0.002419055	0.03332636
0.220842289	0.282691707	0.451086065	0.275528143
0.362661708	0.060570166	0.15646147	0.301823863
0.042614839	0.295028	0.405873627	0.430505993
0.042154415	0.202859973	0.344865644	0.407872748
0.059385718	0.231653991	0.327299898	0.187104433
0.020687646	0.060514574	0.045295697	0.338452887
0.472209886	0.033943627	0.013792513	0.122821578
0.061866215	0.40214068	0.098663457	0.441086168
0.35373004	0.329371494	0.057682953	0.454763234
0.020207942	0.449175761	0.235659607	0.043153284
0.272000114	0.011774007	0.443482141	0.084155487
0.238770449	0.329685478	0.165221701	0.013938625
0.178317993	0.035585459	0.381642974	0.373072825
0.036011304	0.195388066	0.245222081	0.333271164
0.21639493	0.285222059	0.475053957	0.288806838
0.050302454	0.154382024	0.310683243	0.324203142
0.183342441	0.409527729	0.178450127	0.479345333
0.334657386	0.441000481	0.418663875	0.248759942
0.156531647	0.323232795	0.241770013	0.008981636
0.274273817	0.345727008	0.319994344	0.092490975
0.156021227	0.300559217	0.050562052	0.221404915
0.260070584	0.222779917	0.082167884	0.347667454
0.331851112	0.322351638	0.041411622	0.040623528
0.495196495	0.427212541	0.11855185	0.298631088
0.142968816	0.233297291	0.359684871	0.341993508
0.313178864	0.352050443	0.334475418	0.252339067
0.380191722	0.191498267	0.488418762	0.092107298
0.065794955	0.334210354	0.045803892	0.071878127
0.091361483	0.021426525	0.230546783	0.313869401
0.279151373	0.248247653	0.262367324	0.067661149
0.249584319	0.360824576	0.263301931	0.126840576
0.041489208	0.452008896	0.006889342	0.042014527
0.279027372	0.344219704	0.251529809	0.186821264
0.217481787	0.127424345	0.199360635	0.229409438
0.18252434	0.325385648	0.348830344	0.031499452
0.01169409	0.413557759	0.331197581	0.026231734
0.076081964	0.330893339	0.288374238	0.144315474
0.019442056	0.422730991	0.21777395	0.318249119
0.262395494	0.054552363	0.360590411	0.091189381
0.06729205	0.43364976	0.321632359	0.472849029
0.317274294	0.279251552	0.453150605	0.349432138
0.154911046	0.349124972	0.017578368	0.375559283
0.281294872			
ANXA5	0.025949797	0.012663121	0.011486278
0.020743879	0.026348821	0.006492438	0.023768534
0.024752796	0.001125178	0.001268496	0.00121314
0.025849077	0.001648338	0.019078151	0.000709908
0.035172046	0.042399743	0.031478414	0.052739412
0.040288426	0.020951922	0.039375976	0.027047402
0.013367828	0.014207623	0.02493021	0.005306095
0.032355918	0.027536537	0.007034122	0.030897092

0.09547588	0.034246615	0.009444954	0.022133815
0.009620442	0.015351499	1	0.002565626
0.023619934	0.010743861	0.033173853	0.0325376
0.019293378	0.01031151	0.024886386	0.032304839
0.001267526	0.01474895	0.00797597	0.010830998
0.006416208	0.005302876	0.016816944	0.016874167
0.000602094	0.026316043	0.002133126	0.021878237
0.038121692	0.013916546	0.008088333	0.00146532
0.000336852	0.046806679	0.092335189	0.039023201
0.008062424	0.015628738	0.049562946	0.018606478
0.00110717	0.04175373	0.062200321	0.000417548
0.076208137	0.010736019	0.098711685	0.024565868
0.030234046	0.016451703	0.020809758	0.015519429
0.025382331	0.075492219	0.019372458	0.035200829
0.01642425	0.017471598	0.003134926	0.038973806
0.018714752	0.010685993	0.012993008	0.049320887
0.007072717	0.01488171	0.005324443	0.009394683
0.000281051	0.034686716	0.007744921	0.01683372
0.019447485	0.014361029	0.045094628	0.032500409
0.087938032	0.005313051	0.038854979	0.051995331
0.027826154	0.00798578	0.054086888	0.024424968
0.030683627	0.01934137	0.00967886	0.040329726
0.041695962	0.005966353	0.000124094	0.10514573
0.002750239	0.010338291	0.006646966	0.018960457
0.035469384	0.005846418	0.030900883	0.045334715
0.050809029	0.027455764	0.024439102	0.066583657
0.029863559	0.010750853	0.005840672	0.01591121
0.008249767	0.021550629	1.47E-05	0.040542355
0.011625552	0.050795519	0.068037851	0.01652733
0.015681883	0.04128854	0.071902607	0.015413789
0.01332354	0.064883213	0.02949155	0.041234185
0.002169322	0.031670441	0.084604525	0.039561207
0.037413822	0.007153303	0.066478141	0.034401769
0.000642025	0.023915349	0.039164895	0.031984851
0.002562024	0.007067196	0.007847473	0.098868653
0.042385176	0.025494733	0.026781223	0.007660685
0.000686666	0.028742895	0.019102785	0.016567781
0.015039026	0.021387714	0.03727805	0.021243306
0.050310952	0.023552824	0.000300195	0.00846847
0.009596052	0.011531858	0.020011124	0.045265161
0.00183163	0.017142321	0.001222249	0.057450538
0.026921083	0.086608121	0.022383542	0.044532076
0.01331546	0.037273778	0.024715078	0.00973958
0.011104651	0.001952182	0.009419449	0.025338443
0.051476868	0.009907695	0.027759848	0.021444736
0.00758849	0.026681642	0.028948685	0.000896802
0.014998765	0.057124527	0.025148272	0.04372881
0.025311424	0.000916387	0.0097812	0.013733702
0.016462671	0.010467354	0.009615779	2.94E-05
0.034885082	0.027871417	0.001198171	0.009178333
0.008644246	0.015797944	0.00248463	0.113810421
0.057780511	0.013240978	0.018608431	0.009018852
0.008693793	0.015610279	0.054560936	0.007135716
0.003921275	0.018712832	0.002920726	0.00377095

0.024978598	0.05255356	0.022099558	0.009013772
0.00564473	0.008412122	0.008354159	0.018591687
0.029536701	0.016508846	0.015434448	0.006995928
0.020555071	0.078363812	0.01270779	0.042077686
0.0158107	0.01995613	0.102602273	0.001266126
0.017389503	0.002361547	0.007125227	0.012877446
0.008320153	0.022960992	0.007230366	0.040550169
0.014412567	0.042081879	0.022812374	3.98E-05
0.031308357	0.012535462	0.065247247	0.013204345
0.012180219	0.026261293	0.01075148	0.015551218
0.021480264	0.070100244	0.013744347	0.051679948
0.090458168	0.028335429	0.006468633	0.066962337
0.040672095	0.00712668	0.015919234	0.047395013
0.012518024	0.03541841	0.007874482	0.024587936
0.031823454	0.005864497	0.065154294	0.024527283
0.010239258	0.013611163	0.10331517	0.011498586
0.059841399	0.001506891	0.003124288	0.013650066
0.027067869	0.013402834	0.083537664	0.012837645
0.007667144	0.02540745	0.019932519	0.014620018
0.013581164	0.028587318	0.084988038	0.010053632
0.010900771	0.004460054	0.001472285	0.057453624
0.015268142	0.026494337	0.011788023	0.008352876
0.003351752	0.016508954	0.004039205	0.010968196
0.012231686	0.010373224	0.042127117	0.00473925
0.003495491	0.00881433	0.05760511	0.056199599
0.009339291	0.013076787	0.010225977	0.078195437
0.027724344	0.008410789	0.006025795	0.091917914
0.063142469	0.009636116	0.020061832	0.000879284
0.028789425	0.010315388	0.004685959	0.014081824
0.057218197	0.009716874	1.73E-05	0.001743562
0.048818064	0.03098699	0.006367054	0.033046989
0.001312721	0.063686427	0.004903466	0.016682119
0.010284116	0.027828618	0.020498232	0.011819372
0.008413071	0.014878113	0.004037239	0.009561576
0.010988236	0.007815707	0.004628386	0.037516154
0.011861329	0.008802065	0.022971585	0.01243591
0.026701284	0.040860773	0.035076768	0.015946196
5.46E-05	0.020793232	0.000592081	0.000259044
0.015245477	0.010113702	0.017154214	0.02810745
0.014652808	0.073331469	0.017901004	0.013852122
0.022315181	0.024133365	0.016503182	0.000121233
0.000233974	0.001577013	0.033740402	0.049137518
0.01174598	0.000754161	0.059008685	0.027743154
0.007001392	0.016784294	0.010585596	0.049898392
0.023592872	0.095937526	0.001107387	0.048663954
0.006682315	0.016412189	0.01379399	0.009933739
0.085365827	0.034430545	0.052445263	0.019943051
0.001399401	0.07090223	0.036632366	0.00411816
0.011000063	0.032160471	0.01542106	0.039554275
0.008005826	0.010887483	0.012090767	0.000559069
0.015974871	0.026203171	0.018098832	0.019554329
0.02013764	0.037705859	0.036568511	0.00808249
0.002975544	0.009882821	0.016666187	6.33E-05
0.082411625	0.067804684	0.015470058	0.001311494

0.02567646	0.03429929	0.000166306	0.059098547
0.001963123	0.064370039	0.01853031	0.020737922
0.058513158	0.023991866	0.055471822	0.053700822
0.021279958	0.019981556	0.009180809	0.002904303
0.020452543	0.023067411	0.038264541	0.024056572
0.045640197	0.02437303	0.001745201	0.015218025
0.019960806	0.017640465	0.012664855	0.012161643
0.008658626	0.013775709	0.011523133	0.002573236
0.040223461	0.022736764	0.037172096	0.002981157
0.01981477	0.010732123	0.023198942	0.007610654
0.007601168	0.011705509	0.021900418	0.040330817
0.043202264	0.022132533	0.072049265	0.011167961
0.014014764	0.011542748	0.002961917	0.012251096
0.092442803	0.013893552	0.02447663	0.026811179
0.003547427	0.014594517	0.029751836	0.014668007
0.011535671	0.032079007	0.009533691	0.017210107
0.000699996	0.029339498	0.011493489	0.086303673
0.058245767	0.024303276	0.001589125	0.051804046
0.004341071	0.039082194	0.01412117	0.006608501
0.061639654	0.026479125	0.047330013	0.016974716
0.01043961	0.007873201	0.001222688	0.104041608
0.015686389	0.008860788	0.006671859	0.032878085
0.002816401	0.053638895	0.046071815	0.016530485
0.004075943	0.003025726	0.00799511	0.003028311
0.053508831	0.026754164	0.017886586	0.03960223
0.006006403	0.016171215	0.094351026	0.002051961
0.054517718	0.009639185	0.00200218	0.01859451
0.02613448	0.103822276	0.015602454	0.001240467
0.029823951	0.014484685	0.01512749	0.010205118
0.045143804	0.031074166	0.009529493	0.019438448
0.006896453	0.014209335	0.017260622	0.029441602
0.001462804			
AP1S2	0.201491128	0.376214216	0.104498609
0.343815908	0.156989336	0.243097024	0.176666826
0.073220631	0.072786095	0.025817639	0.030164873
0.151774363	0.07078995	0.093400517	0.098215072
0.279506085	0.034694703	0.071791831	0.149016533
0.265644774	0.09927264	0.249580679	0.070531957
0.043462193	0.033635286	0.052173733	0.071251916
0.338144494	0.0579123	0.197261442	0.287407637
0.020924934	0.257168584	0.343407663	0.212778461
0.315710301	0.185718032	0.002565626	1
0.27340714	0.269429362	0.290821378	0.218990711
0.058948228	0.33441832	0.207703498	0.139924599
0.072721027	0.026241438	0.111897868	0.325014142
0.327945953	0.207585312	0.109662859	0.135739267
0.072745346	0.082483513	0.038050352	0.233463828
0.162971985	0.099391289	0.32613384	0.117659129
0.066704171	0.100154993	0.026432643	0.1385472
0.050594091	0.26913028	0.017527532	0.124198475
0.008372382	0.254476843	0.127653173	0.060623489
0.02715347	0.024536685	0.032639876	0.273982745
0.334410784	0.27014616	0.321152205	0.315799535
0.041813641	0.086809451	0.097077439	0.072286487

0.293143187	0.156055081	0.036168404	0.264801132
0.326143648	0.345942397	0.055172029	0.093035066
0.354411731	0.351365228	0.145317809	0.147850861
0.080571059	0.010330508	0.328645202	0.335737776
0.271107095	0.058354504	0.061080969	0.311419963
0.031522392	0.061851019	0.252157107	0.022124228
0.345476014	0.043201708	0.064371478	0.229491283
0.047554532	0.264702607	0.325068108	0.145997083
0.106248513	0.011046146	0.03502415	0.012859344
0.070603443	0.01412873	0.317875545	0.358577791
0.078323899	0.082246576	0.135195769	0.00245743
0.012143893	0.028228719	0.236533707	0.097432932
0.254541662	0.199551708	0.112704354	0.244165156
0.183769487	0.314081857	0.034098005	0.072320854
0.176581013	0.030939615	0.148804623	0.181722485
0.115067501	0.071784484	0.065870876	0.362474636
0.340108836	0.069603444	0.075274772	0.24604107
0.128470723	0.114290097	0.054889139	0.082433312
0.066078676	0.296627858	0.008524849	0.113783014
0.070847197	0.025501731	0.006156639	0.144232173
0.094835131	0.330212518	0.284457338	0.051837495
0.120297158	0.215433896	0.132073748	0.284410235
0.040052068	0.020787263	0.091012416	0.350089993
0.220112559	0.301753488	0.074623569	0.057476793
0.118139378	0.304633163	0.000485738	0.338349935
0.338156986	0.091392994	0.198214848	0.148539219
0.110145537	0.354379607	0.004246414	0.237954719
0.181052407	0.04125321	0.344816021	0.078702716
0.290071303	0.036697441	0.114484663	0.240931095
0.202471395	0.006010929	0.173055945	0.308155543
0.007510292	0.115157035	0.24492997	0.280879784
0.246872332	0.254782363	0.338573516	0.097342703
0.163452475	0.094349245	0.007062869	0.092938346
0.228996143	0.06205025	0.349986126	0.100224156
0.138713533	0.35816978	0.230670141	0.103105235
0.055897513	0.173564014	0.023174608	0.153224154
0.234131591	0.097006055	0.134330884	0.021629772
0.022923651	0.368721921	0.023542761	0.034089247
0.014310373	0.209528211	0.128825816	0.098967677
0.05672624	0.06858204	0.07872232	0.116959761
0.070604646	0.046564335	0.002079883	0.056904263
0.04947348	0.073907062	0.083716363	0.229714764
0.075025037	0.043419372	0.135006311	0.046460949
0.034444018	0.012245284	0.362900209	0.230660022
0.233291488	0.086576783	0.017921502	0.058867193
0.362497255	0.19254821	0.07359726	0.374427403
0.011953261	0.316569574	0.092947731	0.060848007
0.076530625	0.149532766	0.356533563	0.043115115
0.16735052	0.360283471	0.110888076	0.080031883
0.30235163	0.252619606	0.042782979	0.234392826
0.221673475	0.022776756	0.338471802	0.025843056
0.011557856	0.197000102	0.255149772	0.044220232
0.030005274	0.194727821	0.079860686	0.148795891
0.372509117	0.269559908	0.310678923	0.030627344

0.050802982	0.009810352	0.037061969	0.100958971
0.333909641	0.311192246	0.10883888	0.113958648
0.166386915	0.084359029	0.069197818	0.344253536
0.020666056	0.328714434	0.006542287	0.234208759
0.323756337	0.136702788	0.035672084	0.238459548
0.298103536	0.051330892	0.058489007	0.33953864
0.336938813	0.104717475	0.103169904	0.18232743
0.045798931	0.062388394	0.163537143	0.023184412
0.119857648	0.08209826	0.003706254	0.082482331
0.047456681	0.361152602	0.273305365	0.054574613
0.254660261	0.076475215	0.163703878	0.034530049
0.262307259	0.291370496	0.350844604	0.066799258
0.223647493	0.324783696	0.132124938	0.025012781
0.008437974	0.318149887	0.23410585	0.024630109
0.331520333	0.131621582	0.023740777	0.372360521
0.122705532	0.283309966	0.011060289	0.005826643
0.008136109	0.266707071	0.345853836	0.270070052
0.111191487	0.179662977	0.000278856	0.318899744
0.353884227	0.006880359	0.026515807	0.248512309
0.20183782	0.368147077	0.073600734	0.27668248
0.152488402	0.326946437	0.096846659	0.143498105
0.335269932	0.288176441	0.27892264	0.339073983
0.33005289	0.202588096	0.217425955	0.039079461
0.0518828	0.129395723	0.093627259	0.085980852
0.197184312	0.057129902	0.304842829	0.313418097
0.299493337	0.004498734	0.099704508	0.257210388
0.358033822	0.359690912	0.005324737	0.003022303
0.001015982	0.011041631	0.217736487	0.070970391
0.331625207	0.089835334	0.007670901	0.057813971
0.083531645	0.153259848	0.350018464	0.261985188
0.223669613	0.034745664	0.111229557	0.263340509
0.030571187	0.170495452	0.326179068	0.373059111
0.011765731	0.075567775	0.089483876	0.373849905
0.006745188	0.13139447	0.165269639	0.112651504
0.00255322	0.02726025	0.036917929	0.260448245
0.307730509	0.039601548	0.022369713	0.039371817
0.096987035	0.298267738	0.06888602	0.35185334
0.334530444	0.319813418	0.016666758	0.356338968
0.038530723	0.323215535	0.089031603	0.035137202
0.045812033	0.007957661	0.265762102	0.076977634
0.103973758	0.221934511	0.060727606	0.005772024
0.022483124	0.022229172	0.352252293	0.276008001
0.00626596	0.09808796	0.101673146	0.191209573
0.189598064	0.310740216	0.348106559	0.13947902
0.100836216	0.009520851	0.26939989	0.193693038
0.082941248	0.319737791	0.096782239	0.336902646
0.2348429	0.309549907	0.303386862	0.233623444
0.037976361	0.180432001	0.32930339	0.04319501
0.166877725	0.326520602	0.13178497	0.050656479
0.116560839	0.101638322	0.030677916	0.0214168
0.062817353	0.243201488	0.083078355	0.304753303
0.182918567	0.208090081	0.025137382	0.039958022
0.366223619	0.319864246	0.095619268	0.282875164
0.055266085	0.187016412	0.238511289	0.225293274

0.064703376	0.301407835	0.290107967	0.295368196
0.355153511	0.171632	0.358281403	0.048390713
0.02812027	0.146182011	0.073822474	0.03388431
0.085209669	0.01481479	0.036495237	0.231525871
0.048857593	0.229177461	0.062820474	0.036376101
0.105307888	0.08147289	0.165296931	0.142938445
0.063116971	0.358337135	0.006993172	0.021838764
0.05441962	0.27936349	0.116775973	0.061914147
0.04359591	0.022519949	0.149974955	0.148275014
0.121129176	0.046469532	0.287510277	0.028925494
0.031625531	0.330887405	0.112155289	0.004306761
0.026184394	0.17724396	0.045642536	0.124068814
0.006109936	0.297953273	0.165514596	0.145095164
0.212138675	0.029990054	0.254462653	0.028101373
0.028850473	0.305769569	0.115382041	0.320416393
0.069311722	0.305596204	0.305435171	0.264344496
0.019560081	0.09110026	0.044748829	0.303590131
0.079311747
APAF1	0.412959209	0.490216495	0.378706635
0.381166007	0.377024762	0.368681904	0.223029084
0.273109739	0.087660018	0.004419586	0.013702131
0.394085262	0.159556933	0.268352391	0.325367163
0.388134144	0.112094753	0.426935881	0.25248754
0.405894711	0.117830997	0.350679326	0.108822016
0.262565567	0.081754838	0.061438354	0.214773836
0.412165883	0.074579316	0.316645305	0.398888795
0.116106861	0.418660131	0.500227039	0.353831079
0.485570724	0.395989454	0.023619934	0.27340714
1	0.381961697	0.399139608	0.311505877
0.046151855	0.487069235	0.24013393	0.368405809
0.29480581	0.024979196	0.297363476	0.456253255
0.444401285	0.356653216	0.289405696	0.166277315
0.136411052	0.272105743	0.186524057	0.360948049
0.223577391	0.207516784	0.450688925	0.094694733
0.154534757	0.327428922	0.14953614	0.303947957
0.288532206	0.399847797	0.034950875	0.369423888
0.003111707	0.398956786	0.361670556	0.239283833
0.043485869	0.015510673	0.175052333	0.322842483
0.416308153	0.461617635	0.411400706	0.421229609
0.153794284	0.302401936	0.334793125	0.159713794
0.409331728	0.17220864	0.14118618	0.373370395
0.42235176	0.480965173	0.18126548	0.247249218
0.361515639	0.463561397	0.386385145	0.363465482
0.268145414	0.056438715	0.416227689	0.45317488
0.395577673	0.256164004	0.137973727	0.405187886
0.067404266	0.400211871	0.332157143	0.172132935
0.427853475	0.022968366	0.188006341	0.335761146
0.281614624	0.415681443	0.470713746	0.198817937
0.383398782	0.0215736	0.097957991	0.043290472
0.322752285	0.117039417	0.472181649	0.394732055
0.285645466	0.317756281	0.297486518	0.002926451
0.026370153	0.220180631	0.386694335	0.422488263
0.421911638	0.374572895	0.187170549	0.38836355
0.362557328	0.403869268	0.068854261	0.125839942

0.396730241	0.21630261	0.237451506	0.387326014
0.258690366	0.163553175	0.13672101	0.467897722
0.439349731	0.092005055	0.414370018	0.383672119
0.27366452	0.299084748	0.128138808	0.258255924
0.058594809	0.449799726	0.027686457	0.41305434
0.209788257	0.116179578	0.027130079	0.320175275
0.284668715	0.360318048	0.463941279	0.128830281
0.297654307	0.353550576	0.380754532	0.355158658
0.006345879	0.06806969	0.357236657	0.452432131
0.315722445	0.455685054	0.323677216	0.302614815
0.262352517	0.408967258	0.006779455	0.484661703
0.483884876	0.361026049	0.33051316	0.398939992
0.300650756	0.392100261	0.066512205	0.386102611
0.387673129	0.101362411	0.442925275	0.140058709
0.449124404	0.037965674	0.339325217	0.360869874
0.35153866	0.011160725	0.263590726	0.383497112
0.028246141	0.126526185	0.425481377	0.385249901
0.335954911	0.40769608	0.419704012	0.279512241
0.373160815	0.291830015	0.018326053	0.295865444
0.409381998	0.318637448	0.442205784	0.270302482
0.378009235	0.449399746	0.373839947	0.177092471
0.047588496	0.344005101	0.028643357	0.294577028
0.376369003	0.10405274	0.3396389	0.190164035
0.06563074	0.473097767	0.148280506	0.214611305
0.101623332	0.33776712	0.296244133	0.18634061
0.145577816	0.160409268	0.294468926	0.32969561
0.049680365	0.085528554	0.022500323	0.289088984
0.093810175	0.262698104	0.267122064	0.389490172
0.315621729	0.093334819	0.323925996	0.270874944
0.108008842	0.016204126	0.47490166	0.398937515
0.359244032	0.174111211	0.047760529	0.271564795
0.403394833	0.309098824	0.344774914	0.414538739
0.046202723	0.42722999	0.198351747	0.107315035
0.290608217	0.242147799	0.40919818	0.063792129
0.419470073	0.447285119	0.332225829	0.15788661
0.387321686	0.403113807	0.036765723	0.397455432
0.389347253	0.031654458	0.479041765	0.11287752
0.023251808	0.38534693	0.355477568	0.07639166
0.07877952	0.275752057	0.07175245	0.265069368
0.470650931	0.359134062	0.361685945	0.209814138
0.039238678	0.005698767	0.197465118	0.383850699
0.495678065	0.4556163	0.272736536	0.283760428
0.249984439	0.26446193	0.213023915	0.449595647
0.26377838	0.500894389	0.009209211	0.366799731
0.480180635	0.332802702	0.06563035	0.313452191
0.371871832	0.054810168	0.279623979	0.471249315
0.404048455	0.297281878	0.299550988	0.416591486
0.02313589	0.056382881	0.354910798	0.157290689
0.330688867	0.214656715	0.042124349	0.381079599
0.038916698	0.425868981	0.410883952	0.053526613
0.296496708	0.387756688	0.34648098	0.042819867
0.376302747	0.469143451	0.498802476	0.228033702
0.395799963	0.429063616	0.312811829	0.089256914
0.017723802	0.483964047	0.420709415	0.022101473

0.411836083	0.291050116	0.007966557	0.479223746
0.212997044	0.453654998	0.008735205	0.114209054
0.047816299	0.39686646	0.461335663	0.405348038
0.246677161	0.402212947	0.009679406	0.477509561
0.458286469	0.013291252	0.127261502	0.368509099
0.345281932	0.512376215	0.252245973	0.363663627
0.345254191	0.461931838	0.224971981	0.246735999
0.387026993	0.443184377	0.422320815	0.461474013
0.396727981	0.381922386	0.274497708	0.016933591
0.120186709	0.27179877	0.249349206	0.061035736
0.374562691	0.120565774	0.462454879	0.401748939
0.414522502	0.006127362	0.197024539	0.386860491
0.419684179	0.441169878	0.038457058	0.009533262
0.013427649	0.011887174	0.385458339	0.244005611
0.44532516	0.218477827	0.005767887	0.072989271
0.308802836	0.327692147	0.466346045	0.371172315
0.397735783	0.089896709	0.224647256	0.400148943
0.055070838	0.368865233	0.419857372	0.459158512
0.054324838	0.24775322	0.431179351	0.439137842
0.050229414	0.299172816	0.395859024	0.267134181
0.025223011	0.077661192	0.060378317	0.406474303
0.467213787	0.054876272	0.02367596	0.182317077
0.107140715	0.415728972	0.152373703	0.458969332
0.404582027	0.395239128	0.069251675	0.468723336
0.031824839	0.461227999	0.273954281	0.073126182
0.33349146	0.018864118	0.43849121	0.099597698
0.354662066	0.429296963	0.175721737	0.017377423
0.162927116	0.051993758	0.405838524	0.386297052
0.040202565	0.285926149	0.309077772	0.421214544
0.331327336	0.376415199	0.487726997	0.356046443
0.093737687	0.1442392	0.417427803	0.412157676
0.29351947	0.424065712	0.229202869	0.485807504
0.419986739	0.452234602	0.426175936	0.342273722
0.184127674	0.418097817	0.343057552	0.022287495
0.370430677	0.38457636	0.423098997	0.120230404
0.211423132	0.326557319	0.079135395	0.277057067
0.267634766	0.330445154	0.135379172	0.406347106
0.429717248	0.410147486	0.074283985	0.055422104
0.509521943	0.442419305	0.191385188	0.39148729
0.189450299	0.302254249	0.402256319	0.386035789
0.361010997	0.373504228	0.414017734	0.352496239
0.418588794	0.277969186	0.498044376	0.168348056
0.07547436	0.396011956	0.077018775	0.09408885
0.142365199	0.029999909	0.23374882	0.397078099
0.304123653	0.338362055	0.365767042	0.096520971
0.306623961	0.419098503	0.342710295	0.21178762
0.071079737	0.467015689	0.010359783	0.060777709
0.315567121	0.385009259	0.295167309	0.242060763
0.232521234	0.144766419	0.27595074	0.343201169
0.260864195	0.361318839	0.382725199	0.058278765
0.027630079	0.434251309	0.423613015	0.029836734
0.092032735	0.405024581	0.366989339	0.198302417
0.022696027	0.421339656	0.303355603	0.381032802
0.373687004	0.083693311	0.386024161	0.12751366

0.098379796	0.455248756	0.359069678	0.473505873
0.355288192	0.369595719	0.463810064	0.386466486
0.184404835	0.37431473	0.036263339	0.418643005
0.325396603			
APEX1	0.336503871	0.512435961	0.371586547
0.369356555	0.379967649	0.299548282	0.221993114
0.163686322	0.15463854	0.009037394	0.047624256
0.367973308	0.214667178	0.231480915	0.321694891
0.384259133	0.1662171	0.303145441	0.259415888
0.352195011	0.097307818	0.376018515	0.127460855
0.319685688	0.161127996	0.112463518	0.333994337
0.417025791	0.127286128	0.247586168	0.307323858
0.132740597	0.370099814	0.501214398	0.380906773
0.488713062	0.313005573	0.010743861	0.269429362
0.381961697	1	0.348348676	0.325451294
0.068935291	0.44991351	0.262821665	0.38061086
0.211197139	0.058888072	0.188443823	0.410210808
0.451154965	0.363620431	0.211979424	0.184693141
0.195953704	0.163143058	0.273519417	0.235678445
0.23961446	0.120492198	0.466077867	0.132049364
0.141657125	0.221916898	0.180015391	0.236561579
0.344681484	0.30789564	0.026983065	0.326644792
0.002913579	0.346753904	0.361196535	0.340689695
0.071508658	0.032154173	0.20723475	0.311559789
0.438223613	0.436562874	0.48639699	0.493773672
0.199859071	0.308423354	0.35977506	0.202911606
0.407663719	0.195919772	0.137488639	0.387497262
0.496437132	0.506948772	0.254041065	0.305322637
0.388150142	0.455476235	0.375514414	0.385617215
0.36657055	0.089547645	0.419536371	0.505170345
0.348098127	0.322488856	0.203486975	0.34232098
0.089525283	0.29856868	0.341815128	0.141993628
0.456206277	0.039499024	0.256561571	0.311305948
0.235695388	0.383111593	0.508165966	0.215589461
0.363216167	0.032393384	0.208212784	0.055119235
0.289285884	0.175550419	0.447824138	0.439622951
0.189948352	0.350560658	0.265467827	0.006828597
0.04959006	0.210713259	0.372573096	0.310241358
0.360076093	0.360328888	0.282932377	0.302686196
0.367951112	0.477434501	0.051547425	0.126895579
0.374242249	0.209403552	0.268727877	0.359864502
0.344771035	0.132680843	0.154174429	0.522025704
0.393466444	0.115295634	0.256354703	0.383357038
0.300255441	0.360471606	0.1331163	0.254252116
0.066361036	0.467676575	0.051968482	0.274371791
0.323874489	0.161999524	0.017424941	0.216899691
0.270482182	0.409878371	0.432727764	0.139026344
0.288166645	0.388419514	0.239614053	0.346910766
0.024605307	0.070147863	0.195754786	0.495074916
0.201093993	0.494783964	0.303849395	0.337785607
0.316678385	0.424911149	0.005701416	0.471918519
0.515320563	0.212055805	0.357019165	0.369969173
0.372423377	0.434019073	0.03376559	0.36913971
0.255908774	0.111635435	0.50623513	0.205296875

0.435492206	0.073261629	0.253746296	0.421710412
0.345275704	0.013177207	0.186057664	0.334597306
0.05082591	0.099899501	0.350998754	0.370352992
0.374394608	0.405236739	0.479568739	0.365929937
0.388336232	0.22880484	0.024868395	0.341672788
0.385879048	0.316170269	0.42110444	0.248281403
0.238789467	0.46371351	0.373126156	0.198168176
0.071694723	0.269191121	0.029762476	0.371036283
0.372087065	0.129806489	0.36706365	0.157885274
0.10805262	0.523574337	0.223467168	0.280545192
0.168090299	0.319785467	0.260939806	0.209870451
0.210839325	0.218988097	0.376974641	0.377485982
0.049415905	0.124171141	0.028101927	0.284937696
0.13239879	0.274904799	0.258141065	0.301194897
0.25807677	0.117529545	0.371158293	0.330600457
0.17569772	0.03043692	0.498200005	0.373133545
0.247286219	0.196291238	0.065881377	0.25515176
0.450198231	0.244133952	0.195056621	0.434515015
0.046254556	0.479748568	0.248783407	0.170137045
0.336941402	0.272405784	0.455273758	0.078387644
0.304749009	0.441970286	0.327225695	0.249152664
0.354867841	0.362919135	0.047691874	0.332436424
0.310001913	0.053142677	0.515204602	0.160013866
0.039991568	0.384209348	0.371176166	0.118364092
0.126490263	0.313208082	0.055943876	0.324462064
0.516363805	0.382696797	0.369799922	0.266701595
0.064772822	0.013302433	0.245120182	0.35314861
0.477668541	0.503277923	0.277688912	0.357655083
0.26520554	0.368439134	0.108262816	0.505411552
0.207174709	0.496982275	0.014891366	0.397616225
0.485936989	0.208130052	0.130890264	0.275477069
0.339468408	0.079299017	0.207914869	0.479142538
0.424572083	0.36733572	0.380521568	0.358414494
0.027208324	0.056929633	0.394068475	0.232735501
0.352934504	0.273084305	0.075714675	0.271491242
0.067673691	0.430170623	0.420097393	0.119602299
0.265299533	0.261250326	0.367509063	0.063271964
0.451888765	0.487405084	0.5001201	0.173132681
0.362984222	0.487470448	0.350963838	0.118318651
0.036664281	0.501784838	0.319425415	0.036042587
0.456147973	0.168324469	0.019821521	0.482960356
0.214713724	0.400650506	0.010279148	0.094072413
0.070795927	0.441737694	0.465335295	0.313738265
0.188373332	0.336280102	0.009033493	0.515189068
0.472650769	0.033350905	0.191799753	0.378012784
0.36652677	0.509958424	0.351751998	0.44255833
0.361282563	0.481334881	0.227105852	0.311120182
0.413332379	0.482760802	0.351432265	0.444339151
0.433057808	0.384659421	0.298978184	0.030833603
0.236902941	0.177161773	0.288359806	0.134580146
0.243999554	0.206780143	0.466784307	0.454489251
0.475811958	0.012497048	0.149609221	0.373536087
0.414855312	0.464417887	0.03594794	0.013554625
0.012984233	0.046094588	0.307746796	0.290039045

0.488048893	0.181478455	0.010990167	0.052319499
0.294822124	0.38455767	0.487699192	0.358944447
0.393456562	0.111882334	0.249350668	0.360543674
0.125067599	0.234158229	0.486105819	0.453246005
0.065297831	0.300391333	0.299268645	0.483598438
0.098832421	0.329038072	0.357445353	0.309038437
0.021926358	0.130901509	0.12134534	0.396583487
0.474583173	0.099101124	0.053786924	0.101650218
0.143066232	0.447054734	0.193917495	0.514978326
0.408229247	0.415316403	0.10747578	0.473892414
0.070150952	0.461385538	0.353561067	0.06832887
0.288331399	0.024305188	0.444788368	0.217638892
0.249799966	0.317321843	0.298283648	0.033305567
0.23557952	0.083874391	0.477341773	0.451550027
0.058001009	0.25180842	0.325152853	0.358752857
0.238132668	0.377050095	0.480309375	0.322839337
0.108340436	0.144574161	0.341629826	0.300242383
0.193696436	0.490891106	0.329247139	0.522600228
0.327123944	0.448195577	0.457918883	0.346799786
0.246700718	0.320481584	0.303203368	0.030210486
0.34491141	0.435449789	0.30493056	0.225159481
0.293195971	0.354426467	0.063544894	0.139328768
0.316077709	0.239061013	0.160963728	0.419664476
0.306459514	0.304355286	0.042573215	0.118054817
0.52795578	0.42238508	0.167024442	0.362954693
0.210860929	0.36785399	0.404382663	0.398146218
0.341245601	0.409490295	0.360912975	0.300464251
0.39557336	0.283518671	0.508862738	0.074502063
0.146695756	0.319933403	0.124991572	0.135135672
0.176498462	0.056398672	0.303082437	0.37667898
0.32291461	0.354606281	0.23132376	0.12403936
0.335603635	0.293251995	0.355296889	0.1984887
0.111140869	0.480954764	0.020271654	0.08418151
0.318083853	0.404975855	0.371782977	0.287623271
0.327682998	0.174762873	0.311367401	0.224819412
0.246580774	0.307311616	0.408065078	0.054364878
0.028454704	0.48845823	0.299971055	0.038872285
0.11231745	0.295904478	0.26185406	0.280638174
0.043398226	0.470498182	0.270230622	0.388959236
0.312279702	0.093126186	0.310097879	0.101415136
0.086734577	0.417571981	0.374320985	0.496592981
0.33977853	0.379264798	0.410108041	0.376363492
0.144958305	0.318264495	0.049342396	0.408615992
0.347572669			

APLP2 0.334233573 0.479630269 0.288403313

0.396513189	0.356483809	0.300170787	0.310964414
0.281020688	0.143067332	0.019500583	0.042032019
0.303943289	0.200194996	0.294903491	0.269723785
0.39744551	0.169934038	0.340359582	0.333642129
0.382776593	0.197244091	0.359735808	0.205237898
0.255489794	0.127260675	0.137723248	0.233762251
0.454021007	0.152332905	0.281225118	0.360891027
0.162198633	0.393888301	0.446008842	0.318521172
0.423481343	0.295186639	0.033173853	0.290821378

0.399139608	0.348348676	1	0.355957181
0.118315284	0.423234369	0.32205684	0.324282171
0.257363916	0.068451502	0.258507077	0.40813883
0.393620897	0.303100218	0.321798585	0.26568505
0.16508979	0.268642263	0.163110255	0.284225459
0.322810397	0.214418065	0.409821494	0.170362614
0.21130434	0.356958645	0.192712763	0.348099868
0.256076743	0.345056058	0.080159853	0.255578029
0.010323304	0.384969115	0.334132148	0.198556871
0.099728311	0.051541694	0.220708089	0.362514293
0.462775207	0.40432801	0.44812451	0.438435097
0.197166547	0.328399432	0.27645343	0.253144252
0.404974375	0.262658478	0.160055736	0.387174917
0.451756847	0.450357338	0.240539485	0.283616285
0.36961329	0.454638958	0.308542511	0.341983659
0.232149574	0.084714758	0.394452686	0.461297487
0.370029432	0.217877654	0.203670918	0.403305794
0.139938078	0.285241428	0.370101975	0.172792775
0.473348878	0.068814867	0.235475304	0.363443809
0.300894143	0.376537393	0.435170636	0.29921023
0.331689707	0.05108722	0.105798537	0.088519721
0.296006566	0.104040802	0.395827692	0.439843611
0.306465788	0.325215294	0.367008818	0.013329672
0.062169926	0.22807303	0.359054071	0.357170445
0.379646245	0.309459143	0.251495762	0.327708417
0.278535457	0.442993851	0.048840453	0.226695337
0.33916834	0.239353595	0.319118363	0.325958866
0.287039549	0.247720369	0.237006169	0.481718245
0.414665611	0.187015629	0.317632863	0.366968385
0.298861016	0.29138134	0.225240181	0.31212792
0.147006941	0.393754141	0.057712347	0.345285521
0.180196643	0.150543894	0.049325414	0.328891653
0.275130833	0.369220787	0.379915403	0.214802663
0.342939135	0.349793479	0.312972077	0.309626077
0.029060626	0.118455813	0.259879708	0.462544162
0.246777003	0.451620877	0.352040458	0.313635327
0.30714283	0.423666576	0.00515604	0.425405989
0.446419769	0.246608392	0.324765009	0.336894796
0.254214934	0.431328821	0.036018771	0.36930691
0.274783443	0.184314055	0.480305503	0.215901439
0.404434756	0.098921618	0.347957178	0.343472817
0.292376385	0.023566901	0.262841071	0.371437541
0.056756027	0.207264666	0.368972786	0.367384095
0.335521218	0.389857518	0.47505631	0.233595717
0.345800157	0.342676367	0.044722909	0.294013138
0.367524349	0.265093503	0.414087729	0.33514483
0.251668802	0.437711957	0.326149589	0.18777006
0.124385642	0.371020315	0.051454509	0.315446464
0.332805662	0.197647769	0.273988416	0.205944886
0.118434635	0.477122405	0.150693655	0.173181242
0.095591746	0.309162352	0.359496365	0.223207675
0.169690338	0.252697557	0.277779908	0.322889371
0.123706348	0.1671517	0.032517009	0.227272623
0.130784	0.238344172	0.232764443	0.31485477

0.320352517	0.148514701	0.318236092	0.245122456
0.154207253	0.049923287	0.464787643	0.374470109
0.289925676	0.273773518	0.100187953	0.211811624
0.445138098	0.232169772	0.244154792	0.435737604
0.072952591	0.456901482	0.256713198	0.1803041
0.268097233	0.315640726	0.466108548	0.081232222
0.342389175	0.436695678	0.343897439	0.22799786
0.360157809	0.37290503	0.097380212	0.331161717
0.319008656	0.085375715	0.460600916	0.158290315
0.060380402	0.321071249	0.314943428	0.145123811
0.134976915	0.306172392	0.141727601	0.312352632
0.473150635	0.367597826	0.350534882	0.205527376
0.108910977	0.022442088	0.223040644	0.281757981
0.433460439	0.436605933	0.329160453	0.275496512
0.335516344	0.263501478	0.216434693	0.455207091
0.204630295	0.453066694	0.032152904	0.340272721
0.417846951	0.280727724	0.119790584	0.343311545
0.356400417	0.131529779	0.325035125	0.43295125
0.415263819	0.313466473	0.279534015	0.34652665
0.068813857	0.144726598	0.31917326	0.145953804
0.325294563	0.295169958	0.036088383	0.349996697
0.102519544	0.419971793	0.426321216	0.112383516
0.256991165	0.324255821	0.342733323	0.110395616
0.367233899	0.422898417	0.45085227	0.306061564
0.352455762	0.415071435	0.240175113	0.14689769
0.045325782	0.4298252	0.32643551	0.051891736
0.462974213	0.227854869	0.033051773	0.468490776
0.308465974	0.374025352	0.011342314	0.069324104
0.074373504	0.415788593	0.413625409	0.365156144
0.203019769	0.384899943	0.013855081	0.460033352
0.435862806	0.034943183	0.164978632	0.340004261
0.288528412	0.484870675	0.222435248	0.369412708
0.268856344	0.415273056	0.286661913	0.308803201
0.400262686	0.399252814	0.37835095	0.433346082
0.441766483	0.342958539	0.348232506	0.062502062
0.138462143	0.295581497	0.199646976	0.11590176
0.282821319	0.179715681	0.433916235	0.449418299
0.415721793	0.022990941	0.270528587	0.354521841
0.446416087	0.479972051	0.053867033	0.009862426
0.008818409	0.027481283	0.381615729	0.275659483
0.440575318	0.178205943	0.02560788	0.138869498
0.319390748	0.287802046	0.443484733	0.381113615
0.359598221	0.170159025	0.223323422	0.383862442
0.102044376	0.249548308	0.432619085	0.440027785
0.095114551	0.28803435	0.324904304	0.482134155
0.042991656	0.33342082	0.310314118	0.300420778
0.036661137	0.130649706	0.119710807	0.407067865
0.405068842	0.106574477	0.060972114	0.14822643
0.197415032	0.440399697	0.22809362	0.482367941
0.424138796	0.441774113	0.113436624	0.434415686
0.089009949	0.413810564	0.258060974	0.066149469
0.312152372	0.049269575	0.393770758	0.114176405
0.324061331	0.342222345	0.182054737	0.04228393
0.128173508	0.1063054	0.456062745	0.406780131

0.064854465	0.36178588	0.326024973	0.367467044
0.315098424	0.378801415	0.436904892	0.252016843
0.19984102	0.11457441	0.378516857	0.294673215
0.319923317	0.460223322	0.262357484	0.469739223
0.341461642	0.439581314	0.412813462	0.356412717
0.20778819	0.317851837	0.312061943	0.056437006
0.37170178	0.427801803	0.363606068	0.158549152
0.273559226	0.251730818	0.147013569	0.165061416
0.198838385	0.286844441	0.237821576	0.445487927
0.338514467	0.308570787	0.13430329	0.111548861
0.487812164	0.414670328	0.226501211	0.336417727
0.25293155	0.335731552	0.371498212	0.360923019
0.30247791	0.388116173	0.399448902	0.306544533
0.407695793	0.356586901	0.455520708	0.168044892
0.104418202	0.288438531	0.155109259	0.154667224
0.239191913	0.06665962	0.177602293	0.370475241
0.224131263	0.358536976	0.302890091	0.152195254
0.324134469	0.294721506	0.364583456	0.282160659
0.141098859	0.431261361	0.027307854	0.122037333
0.244897991	0.36164731	0.258008284	0.282391084
0.207296906	0.17971099	0.341270716	0.289165911
0.225947072	0.266303573	0.36231969	0.097393054
0.081955195	0.472650341	0.305346138	0.048860467
0.143635792	0.295550762	0.32839665	0.268743117
0.046850147	0.40064198	0.269120863	0.330923833
0.375795577	0.157196534	0.337435652	0.150737227
0.154939227	0.407539099	0.282859568	0.430058686
0.298713231	0.394669748	0.392464681	0.371128766
0.161503251	0.253342429	0.097613295	0.43592621
0.236474105			
ARHGAP26 0.256837543	0.388937149	0.177726443	
0.402704045	0.244047224	0.210025608	0.296824518
0.202543419	0.181340269	0.065188508	0.090371091
0.230915963	0.222633411	0.265012724	0.177559019
0.394940281	0.224174576	0.194058796	0.320036967
0.345314982	0.219458855	0.377244751	0.263126837
0.182088078	0.181069479	0.21200027	0.206841883
0.424320741	0.225419216	0.174561475	0.319239732
0.17731795	0.318251727	0.337311923	0.306771263
0.317766713	0.218087312	0.0325376	0.218990711
0.311505877	0.325451294	0.355957181	1
0.19946226	0.310372308	0.312260096	0.23369382
0.167521877	0.146379123	0.160010594	0.294468723
0.302219313	0.217817659	0.236681757	0.281969254
0.161855486	0.162757939	0.132387074	0.215121443
0.314658992	0.148660415	0.315899099	0.209400529
0.139787511	0.244457943	0.193646505	0.273548831
0.163629774	0.269320353	0.107101953	0.171911632
0.025892813	0.34534599	0.238207316	0.132048566
0.169650884	0.136728791	0.218567065	0.305837195
0.415453062	0.2976373	0.413242231	0.383558172
0.242629685	0.258452502	0.173639104	0.302418474
0.322770029	0.291475692	0.188562657	0.397339255
0.409599357	0.356433159	0.247253458	0.246969085

0.332459072	0.361183403	0.206104312	0.2405798
0.173116852	0.102410999	0.303692195	0.394812325
0.279422255	0.153794661	0.248575923	0.349640816
0.193495621	0.177859599	0.357736118	0.160264762
0.428224106	0.1209233	0.234417135	0.270302221
0.233992088	0.31963815	0.345765336	0.312060509
0.221066352	0.112515979	0.113580849	0.123470259
0.235198796	0.086165018	0.281617694	0.411716305
0.221232544	0.228288212	0.292403639	0.03439328
0.122448366	0.174514895	0.290009493	0.215683343
0.323839048	0.21955145	0.26663826	0.243198219
0.23092327	0.403657096	0.039610028	0.251344428
0.236945169	0.181618371	0.311223615	0.2305879
0.268820093	0.251836018	0.2854942	0.417740377
0.311944471	0.257471824	0.167376804	0.381448584
0.227143973	0.234900997	0.263483016	0.295840601
0.208021563	0.296089046	0.103295836	0.20481072
0.134489299	0.1675868	0.056910535	0.225697516
0.245976486	0.322725713	0.26219798	0.248875601
0.297842117	0.318788937	0.170333049	0.296978814
0.077002587	0.163163365	0.124721562	0.398422762
0.17290729	0.377708175	0.291674625	0.24052965
0.28174391	0.391601622	0.005390689	0.319121433
0.351409984	0.127865147	0.274312243	0.24322777
0.163114774	0.411185878	0.021254213	0.370027982
0.1977378	0.222951592	0.4330872	0.268005047
0.306040917	0.175913314	0.223647645	0.292299058
0.24619617	0.038444396	0.185479488	0.34124334
0.088368412	0.191904227	0.304717488	0.345736791
0.262208437	0.347552302	0.444750905	0.15540585
0.25132075	0.283478651	0.074161734	0.236160424
0.300864567	0.146335087	0.321308523	0.295217742
0.144418989	0.358040871	0.25608747	0.117910989
0.197603194	0.251074519	0.097116286	0.265731531
0.240921849	0.26303625	0.163729846	0.165339209
0.178181979	0.40507294	0.131875557	0.119570871
0.081624587	0.234041396	0.300281707	0.232481426
0.20080288	0.267177226	0.199108363	0.226687278
0.174310709	0.248839618	0.037525734	0.149627406
0.188387153	0.193910948	0.177574003	0.241995924
0.229591489	0.231116977	0.267374468	0.169081986
0.196784113	0.103664973	0.379258233	0.346503105
0.21158609	0.286815622	0.15351872	0.142915066
0.404313802	0.141043891	0.118276028	0.372739687
0.09801644	0.396213907	0.275376474	0.244498265
0.207779936	0.320524968	0.432930846	0.082261247
0.19847156	0.348315222	0.259798063	0.261788305
0.281824478	0.312713694	0.167809408	0.255885824
0.249029399	0.161912003	0.378120635	0.194535253
0.110873107	0.297503813	0.281618138	0.202847664
0.189262805	0.261634714	0.164824472	0.287006354
0.402873401	0.398817813	0.30306724	0.162836867
0.196451795	0.072987176	0.195275115	0.169053367
0.322102801	0.360157462	0.290332373	0.236472425

0.317890584	0.199554234	0.121364377	0.392589271
0.127663729	0.345148444	0.065688495	0.294316457
0.312005443	0.159891222	0.188872451	0.250390014
0.288367283	0.196486357	0.250722617	0.342922926
0.339708437	0.241494463	0.214242125	0.281264309
0.124930409	0.180161242	0.223001852	0.114240204
0.222242015	0.279595522	0.033294887	0.202194768
0.199573467	0.338482642	0.38825294	0.171625579
0.17778934	0.181672205	0.293300855	0.177580473
0.310890561	0.325302573	0.347757946	0.259242039
0.290142344	0.347917231	0.168998013	0.180504807
0.090424359	0.331158509	0.259606557	0.115281688
0.421147697	0.128287252	0.101635 0.379125127	
0.303514162	0.2580154	0.02468764	0.037312709
0.093317077	0.378243214	0.314939452	0.305097896
0.133507732	0.277166494	0.009124371	0.374428225
0.354760211	0.077729526	0.167294695	0.28566984
0.254038352	0.381810044	0.1664295	0.328680345
0.211651841	0.31903945	0.271551379	0.292462545
0.359992118	0.310061095	0.328636278	0.339018633
0.408370005	0.317693837	0.316825933	0.122567135
0.122201622	0.200303225	0.144146639	0.15136803
0.183064748	0.228625438	0.350759082	0.406012117
0.362915792	0.053043285	0.244126763	0.287456086
0.385965194	0.422392245	0.056857963	0.015677153
0.011695194	0.070507317	0.253265913	0.24418854
0.366698939	0.133842469	0.062775933	0.166022783
0.278308889	0.239737075	0.355269587	0.389144374
0.300194421	0.212467567	0.193777181	0.378999123
0.153729254	0.167965711	0.385044845	0.345531871
0.116042898	0.242777641	0.186139942	0.432195611
0.034960828	0.279376329	0.247022522	0.267765663
0.040300039	0.178587635	0.166106879	0.373566511
0.301575563	0.195326631	0.147148852	0.087723712
0.271866655	0.37311121	0.297581888	0.423371326
0.379783724	0.438524751	0.132582826	0.337355631
0.138594547	0.318852051	0.207357965	0.07774912
0.227217163	0.084127232	0.301887931	0.116971803
0.207138124	0.272914692	0.142981706	0.071936069
0.090662326	0.174339396	0.424461835	0.369224832
0.084333894	0.275766473	0.26884646	0.292475745
0.21755377	0.386208399	0.331937242	0.153382217
0.239818093	0.075758819	0.348956658	0.230790781
0.219740989	0.416499555	0.227921222	0.384868277
0.269238551	0.343889558	0.33687036	0.272000165
0.173150395	0.20888366	0.293463176	0.131843942
0.28653155	0.405649032	0.215820298	0.206034366
0.283531382	0.153714389	0.133107606	0.089207708
0.126687664	0.200094354	0.283123875	0.424216072
0.23684158	0.242312094	0.126095219	0.166974688
0.39055286	0.313516989	0.218137218	0.312148882
0.252008701	0.273056958	0.32429576	0.319549681
0.167639231	0.336205984	0.344994753	0.285605061
0.321691182	0.305560963	0.353359382	0.123058356

0.126375291 0.194786229 0.243460113 0.192151575
0.296143383 0.116109875 0.116160772 0.356261873
0.136175953 0.337601889 0.153593992 0.190274384
0.250262435 0.149461984 0.284931122 0.277981106
0.237033201 0.337710817 0.056538891 0.17653143
0.153629497 0.312841734 0.192502709 0.274864218
0.154144712 0.15521286 0.310465514 0.176823682
0.170741523 0.158028711 0.312958347 0.129433332
0.135087843 0.426967753 0.17802052 0.065974082
0.107674508 0.190199183 0.20974068 0.249322891
0.07753048 0.323356045 0.171485526 0.216348578
0.262664367 0.199725233 0.23485565 0.128630706
0.15935574 0.307379383 0.179800498 0.334681052
0.192450625 0.417105374 0.273982838 0.303755525
0.13560683 0.134617231 0.163243972 0.373498429
0.150816625
ARL8A 0.032967514 0.052709353 0.026080308
0.139144254 0.062387061 0.053855534 0.219583522
0.052740296 0.102428167 0.119138175 0.073416942
0.035319072 0.047856966 0.070836778 0.022049317
0.127398003 0.092091311 0.026383654 0.230125762
0.110467583 0.145481338 0.147995155 0.176666869
0.020803555 0.094916855 0.212189452 0.054198017
0.111562219 0.231292239 0.047498279 0.094891752
0.066558432 0.050711597 0.034097809 0.072935587
0.028301356 0.023735425 0.019293378 0.058948228
0.046151855 0.068935291 0.118315284 0.19946226
1 0.030618638 0.246655625 0.055986368
0.024812545 0.118333106 0.040867076 0.033167133
0.029814407 0.043361762 0.088634164 0.201226913
0.027077457 0.033615297 0.009653144 0.051082286
0.226593052 0.056133403 0.032786151 0.130280636
0.030096982 0.082178092 0.056541559 0.127004586
0.015298719 0.048717791 0.107066936 0.017538575
0.039319911 0.130055247 0.068260927 0.014430592
0.22537651 0.130790368 0.079087838 0.17738859
0.092021996 0.02921003 0.077306894 0.059823652
0.061762421 0.091657062 0.025241175 0.168474184
0.048030713 0.219881622 0.060059573 0.1249625
0.071966418 0.039886629 0.05955753 0.114091022
0.070848686 0.051155734 0.03876879 0.054733543
0.022978876 0.043256986 0.03788569 0.057499078
0.038296981 0.02125696 0.175943736 0.083843472
0.190052969 0.010725476 0.199781511 0.026087449
0.093906768 0.161014199 0.114205732 0.124108568
0.047656269 0.052809487 0.036567614 0.259495092
0.03992017 0.08601141 0.019347863 0.099607147
0.025726903 0.00762711 0.023704174 0.091976228
0.040272631 0.022059096 0.13282185 0.066181333
0.110677883 0.026918822 0.035461657 0.040735131
0.068810407 0.018139136 0.121171472 0.033341108
0.031348654 0.074788402 0.006290384 0.17275886
0.060989228 0.032702398 0.248505232 0.042859135
0.107354623 0.14271194 0.213834975 0.067917764

0.044124492	0.280550359	0.020116757	0.12434515
0.033412397	0.071336862	0.190086328	0.094276988
0.169552622	0.026285056	0.097529776	0.044925108
0.011931429	0.036478533	0.056849864	0.090678811
0.04244314	0.052304157	0.019674933	0.206252224
0.11091892	0.109864092	0.035245721	0.062894852
0.108769078	0.099854849	0.015858252	0.06002513
0.041826597	0.051138297	0.072209287	0.048607748
0.149895911	0.091819628	0.00239788	0.031751223
0.037667237	0.015068845	0.099199287	0.049610319
0.024338551	0.101776714	0.000977734	0.117479359
0.024050038	0.196828609	0.081239501	0.207817105
0.031590718	0.231642434	0.050968839	0.038580614
0.02707154	0.030019902	0.084412227	0.129046987
0.06543595	0.100889678	0.060167822	0.127094629
0.109740038	0.068714851	0.099619499	0.022039084
0.064403916	0.121854289	0.076279403	0.074268454
0.044155367	0.006937365	0.044638763	0.093542032
0.018163369	0.05222164	0.027067084	0.018043983
0.272580203	0.098626985	0.0803426	0.105222
0.025111144	0.129061128	0.025562981	0.034069154
0.128853184	0.059704747	0.025808678	0.012026804
0.011052611	0.065732864	0.163527429	0.060463684
0.037355365	0.105825611	0.028764793	0.044310827
0.163088516	0.198224004	0.023017462	0.010606106
0.050165252	0.026416281	0.023890287	0.03443055
0.025971404	0.085931067	0.077145655	0.016910513
0.089391191	0.187641617	0.052431849	0.113919424
0.03229375	0.145508789	0.15079149	0.007012978
0.082872653	0.0340673	0.013019538	0.067517704
0.064408521	0.06818937	0.066613791	0.203911429
0.030523304	0.209117359	0.10715367	0.028210629
0.0393937	0.048536818	0.099194974	0.144656373
0.035975661	0.095697516	0.15595519	0.032873084
0.03061433	0.208056237	0.045943314	0.062040695
0.16267805	0.055069153	0.04629523	0.229217413
0.151613796	0.106815273	0.131072617	0.178166064
0.060759577	0.144484856	0.055564549	0.029550049
0.23100013	0.072502493	0.056223605	0.022950812
0.030402691	0.042112767	0.162605674	0.061822539
0.254346999	0.031333023	0.023963468	0.057397877
0.011752877	0.035592997	0.120413553	0.044066461
0.027949397	0.036105666	0.147874119	0.12529649
0.045499843	0.225941552	0.081713048	0.039028126
0.059632397	0.05835036	0.040723299	0.055151184
0.166917996	0.157782101	0.063996332	0.013080062
0.038893538	0.11752516	0.003458963	0.024750101
0.167462821	0.048881151	0.078500845	0.121177556
0.053298802	0.025067099	0.099933342	0.235624729
0.038669223	0.032014028	0.036652364	0.10952838
0.038377407	0.044113368	0.021829814	0.135034685
0.134901643	0.031725527	0.049932056	0.107304151
0.088126592	0.032775022	0.111513146	0.054752485
0.238673974	0.020724473	0.032432477	0.002305784

0.046051264	0.070336971	0.034495259	0.093087415
0.026642465	0.101213991	0.003507865	0.045097984
0.044829646	0.082534578	0.039709723	0.036327516
0.030968359	0.047854907	0.026933234	0.047239667
0.030365915	0.031491943	0.061374606	0.188335408
0.069829367	0.029150426	0.070909034	0.046275205
0.08807993	0.077697686	0.242055042	0.1670863
0.03058384	0.079994715	0.017283281	0.08283822
0.040109724	0.104170937	0.043172695	0.082670801
0.050166408	0.123298733	0.091920097	0.037063512
0.080600273	0.088007524	0.035069373	0.008465596
0.002881641	0.049346018	0.099095237	0.098758612
0.051286316	0.015439576	0.141396326	0.130085897
0.054063543	0.053803706	0.04281343	0.169091019
0.043066656	0.160924712	0.032501761	0.134678062
0.075720995	0.018726273	0.060343683	0.046993495
0.06976697	0.092592801	0.025346719	0.089710058
0.0027189	0.131807068	0.037277964	0.045099217
0.028564565	0.112245215	0.117437989	0.085998504
0.025938584	0.093672849	0.108425615	0.007546136
0.176345326	0.062112836	0.110826333	0.070998352
0.084283975	0.128983697	0.051358649	0.041776868
0.096565388	0.031888231	0.049254575	0.026488985
0.042193289	0.094383396	0.028224092	0.023902055
0.039823728	0.055704135	0.023225382	0.070359264
0.006626194	0.150885784	0.091226916	0.065229338
0.045136319	0.094032828	0.107951851	0.076893118
0.094194801	0.112517471	0.034685303	0.020394932
0.177746778	0.005004357	0.098944604	0.032555989
0.051140174	0.076051611	0.043473266	0.049249524
0.044194948	0.048333616	0.041330952	0.13374087
0.021160148	0.039666765	0.099334904	0.147237818
0.123464468	0.090664389	0.050873625	0.058405185
0.148020693	0.020248799	0.084280519	0.00494102
0.012877877	0.071917361	0.125630293	0.11525726
0.045809622	0.033929781	0.081973638	0.120523996
0.04873818	0.041817559	0.081755376	0.081429524
0.141590817	0.124383747	0.051321659	0.050619177
0.011808141	0.052263808	0.069909166	0.074464589
0.048327078	0.193624731	0.03883619	0.03700199
0.037885487	0.022465938	0.123966677	0.159165407
0.246343714	0.104657753	0.006344166	0.095521832
0.007627481	0.148664174	0.009529924	0.101144341
0.086393039	0.01549093	0.125725654	0.162856468
0.149178995	0.038538152	0.063800064	0.153799473
0.010729672	0.051189795	0.037192238	0.101929408
0.011169215	0.032776015	0.174311571	0.04569356
0.031991072	0.00655256	0.050584601	0.09573588
0.152357556	0.080477209	0.026180402	0.04454561
0.032423044	0.028217088	0.025099784	0.061242831
0.072032222	0.035588059	0.037566656	0.046965365
0.117974984	0.149406436	0.026594147	0.033068263
0.08833035	0.033804477	0.029797703	0.0333119
0.030169751	0.145686354	0.02550536	0.042125427

0.024116235 0.013505375 0.173553402 0.073765577
0.011486743
ART4 0.469603483 0.501063231 0.400431593
0.43175691 0.408995699 0.45735565 0.205942752
0.230037922 0.0678419 0.003606012 0.013653867
0.447257964 0.186388877 0.298158648 0.378373106
0.399900604 0.098263907 0.37721991 0.215621345
0.420487576 0.102448795 0.368623612 0.074906018
0.241831795 0.071115024 0.046156572 0.220538089
0.457389663 0.054477777 0.421284324 0.441795582
0.070386217 0.484635772 0.4934337 0.421798593
0.489748438 0.470057055 0.01031151 0.33441832
0.487069235 0.44991351 0.423234369 0.310372308
0.030618638 1 0.230283216 0.371229981
0.282734587 0.018232922 0.332860878 0.486882075
0.487508542 0.462586968 0.260645606 0.149877455
0.19717239 0.312871266 0.250379881 0.43212265
0.206300635 0.232817423 0.493307032 0.080217376
0.15103061 0.276231875 0.11082462 0.280175115
0.31422573 0.478317559 0.014141608 0.439863926
0.003115323 0.392762377 0.341584945 0.293761801
0.025384433 0.013451947 0.133141888 0.34550843
0.470829499 0.485383016 0.467878659 0.475446516
0.147351356 0.248701031 0.372112886 0.140595934
0.491544043 0.169840777 0.103234961 0.39296715
0.473561992 0.497467369 0.155846811 0.208933288
0.479989597 0.501155941 0.456535247 0.419464796
0.339119601 0.030383001 0.496072039 0.491508877
0.490094781 0.279660591 0.111189401 0.469384271
0.039110121 0.430079014 0.334460431 0.144652173
0.473302376 0.012529813 0.160252076 0.375953726
0.190217394 0.489137087 0.49107445 0.167511955
0.389027762 0.012917533 0.107656755 0.019845047
0.33863001 0.113099157 0.496503942 0.479811177
0.273764934 0.352906112 0.262440477 0.001305419
0.014481544 0.150890886 0.490863155 0.384526441
0.456361607 0.45958329 0.199478353 0.489960693
0.460874204 0.465754093 0.095697727 0.081938176
0.444442506 0.146555765 0.200180086 0.466942342
0.263361016 0.112095573 0.10247029 0.489878391
0.489434571 0.064743893 0.408082431 0.393443868
0.346877766 0.309515274 0.086262063 0.268094981
0.041345114 0.481609214 0.014715941 0.38889249
0.269155713 0.11141858 0.008002474 0.313054311
0.321485631 0.489163367 0.482631455 0.081576353
0.312103731 0.377285271 0.411405685 0.475081986
0.006315224 0.034211526 0.393592689 0.496772673
0.41107938 0.483998398 0.281143401 0.261114957
0.234009873 0.45438724 0.002704254 0.492184151
0.493210782 0.422315802 0.392672061 0.407646706
0.370607383 0.469339857 0.058237313 0.401741981
0.469758039 0.058474747 0.47422614 0.120253769
0.496764477 0.025237847 0.368478313 0.455620023
0.476051404 0.006477886 0.298929491 0.414794237

0.013702296	0.114460735	0.463120193	0.414184044
0.380148196	0.448679898	0.459938303	0.35094604
0.414620804	0.23549558	0.006613952	0.27007812
0.466861864	0.403836837	0.494916678	0.239915372
0.459930524	0.501561987	0.486160823	0.234362935
0.030590247	0.34580751	0.020330807	0.301041309
0.473015174	0.108581304	0.412540458	0.119259082
0.04489971	0.495932615	0.132521881	0.228178836
0.094485943	0.441810655	0.239482345	0.254657657
0.199651445	0.126014827	0.32613698	0.369149799
0.035205161	0.068074733	0.007215802	0.412737787
0.133145283	0.371946332	0.393010921	0.492499661
0.353968344	0.096395364	0.363593439	0.248657663
0.079075502	0.007348351	0.501429785	0.394597308
0.460918115	0.133449982	0.025029541	0.396027675
0.485348655	0.43527854	0.38787446	0.495356888
0.020772524	0.473310515	0.231761252	0.087344867
0.35911148	0.234742376	0.460959577	0.064529603
0.452121407	0.50142213	0.276107207	0.144301164
0.491089545	0.433604167	0.022875256	0.491477765
0.495750677	0.018812445	0.497742913	0.088943842
0.010757459	0.432017565	0.467279218	0.049156163
0.050800355	0.340942324	0.051069725	0.241927729
0.498222169	0.384815849	0.484912262	0.170399989
0.027933477	0.006714006	0.157418542	0.406380693
0.499850778	0.489843112	0.222536699	0.339302014
0.211190657	0.308532351	0.205229469	0.493475368
0.17442245	0.495998901	0.002603268	0.450580016
0.492306286	0.391995677	0.052736188	0.344153745
0.493452144	0.032625435	0.194723307	0.499874374
0.487631075	0.30738133	0.346182091	0.43686722
0.013549823	0.036333471	0.369900096	0.143053358
0.393793637	0.177203701	0.035847659	0.382420067
0.030113476	0.49849054	0.457586185	0.039603777
0.425383677	0.377506173	0.339176992	0.023969962
0.465652807	0.486536021	0.497006932	0.157489705
0.481253536	0.490306373	0.413836796	0.052114549
0.008090426	0.489860587	0.470500092	0.017410939
0.469891721	0.356201746	0.008465326	0.503105458
0.163621795	0.468902391	0.00434454	0.079955921
0.022673977	0.448329676	0.490139079	0.432286876
0.35699163	0.366999967	0.002999938	0.490779368
0.50170267	0.006274471	0.098175443	0.488654271
0.454657998	0.502532266	0.287079799	0.470804674
0.44014167	0.489517104	0.244162173	0.223236372
0.490151243	0.479371621	0.474162798	0.497645123
0.477461644	0.402902777	0.25666032	0.009946221
0.104420602	0.27198674	0.366591081	0.063459291
0.435080538	0.104045277	0.498262805	0.464705223
0.485313139	0.00115994	0.167211513	0.497200677
0.487852621	0.479564215	0.013481103	0.004841955
0.007050329	0.010012889	0.394257352	0.195208916
0.493111984	0.337196999	0.001191158	0.047171344
0.277845793	0.370107619	0.500600107	0.364481008

0.460935569	0.056193663	0.324930936	0.396491022
0.042648777	0.453087849	0.486424567	0.499070632
0.024856651	0.200465127	0.42262319	0.482454012
0.053507918	0.272236954	0.456824961	0.314110012
0.008112516	0.052177817	0.040904067	0.431699066
0.481168092	0.059048098	0.020876711	0.212840138
0.1057164	0.478316322	0.141163439	0.485732032
0.482806103	0.432716696	0.046018681	0.495539573
0.024752826	0.495660137	0.296668803	0.076655831
0.261742227	0.006519704	0.486776424	0.140410816
0.397794542	0.459001402	0.168477014	0.007518379
0.153020573	0.032792191	0.468762924	0.454366088
0.01812116	0.230384145	0.258926223	0.410187611
0.354921397	0.442600402	0.495187104	0.482834848
0.075052742	0.116597296	0.441114965	0.484258986
0.283071089	0.468500094	0.276521954	0.49435186
0.481305301	0.49172349	0.494334253	0.355527661
0.187867331	0.472869991	0.437630706	0.017793894
0.346079617	0.466080753	0.397488152	0.12072097
0.215963087	0.38252578	0.03761437	0.241682442
0.318759944	0.404601914	0.121455179	0.43125544
0.452886325	0.489192642	0.037908776	0.038799668
0.500915289	0.491773668	0.158376876	0.463924269
0.132756716	0.313647889	0.466491018	0.44894335
0.383753828	0.492948685	0.480573955	0.46301945
0.493354968	0.241820536	0.496790439	0.129472906
0.059696566	0.467144642	0.078014936	0.060713511
0.114468891	0.014472396	0.285510825	0.411681406
0.383409146	0.36449165	0.400951855	0.060622735
0.277700152	0.423420554	0.323645004	0.214610162
0.06105105	0.496363256	0.004954145	0.035460916
0.387321313	0.47942641	0.363988777	0.198045967
0.26225535	0.099262364	0.263774056	0.393268595
0.396498481	0.384435174	0.485300211	0.030745305
0.01512512	0.473505156	0.452455658	0.01090102
0.061172193	0.462912025	0.293666608	0.215709795
0.010387274	0.48578488	0.414303175	0.416373797
0.373251886	0.04646126	0.46846378	0.08411959
0.047564565	0.497441071	0.387727083	0.492834539
0.324184063	0.407829488	0.476253839	0.495643738
0.113082254	0.417797833	0.019996352	0.475385293
0.442113145			
AS3MT	0.187173972	0.298638898	0.112483082
0.365630284	0.187186097	0.19201954	0.304447999
0.186768743	0.212575525	0.095537848	0.10660647
0.163463238	0.175707876	0.222383759	0.144673892
0.33956451	0.174650511	0.127000685	0.286138461
0.30445865	0.266811002	0.32848999	0.293146917
0.116472611	0.150777221	0.228711527	0.154329749
0.35869147	0.234696245	0.148305964	0.288431379
0.131235027	0.244416388	0.248181941	0.238888091
0.225996201	0.157556764	0.024886386	0.207703498
0.24013393	0.262821665	0.32205684	0.312260096
0.246655625	0.230283216	1	0.178544989

0.144574573	0.155502544	0.143545258	0.226450391
0.223025511	0.181077656	0.231121749	0.315184002
0.133299475	0.138115547	0.078271794	0.192514023
0.302583671	0.15324663	0.233385208	0.266912093
0.137933576	0.211647448	0.132877132	0.244312314
0.100776675	0.22432926	0.125409539	0.115265668
0.034900476	0.314804327	0.183840584	0.086733973
0.167140199	0.147197806	0.152571789	0.319745165
0.338204163	0.212972472	0.319388902	0.290054546
0.17804551	0.18187916	0.110084727	0.279263101
0.251169023	0.308331862	0.158111437	0.331707818
0.314692085	0.263088076	0.197750971	0.178298409
0.285011076	0.281884405	0.138839964	0.167975147
0.125737558	0.076406413	0.236499552	0.297587662
0.217089343	0.087622805	0.20439961	0.297681199
0.192619486	0.114860612	0.348182926	0.099185357
0.349777743	0.180282625	0.169962764	0.257889673
0.173215643	0.246529286	0.250660135	0.310433457
0.136950984	0.105401725	0.082775033	0.112772613
0.162012752	0.045889095	0.204588809	0.339773964
0.183466409	0.158708032	0.26929357	0.037036053
0.110981299	0.11395268	0.208766103	0.13993572
0.261938393	0.151579399	0.244343092	0.191428989
0.168589563	0.312107817	0.038948971	0.280963106
0.193150861	0.123153222	0.282586498	0.178192918
0.199079038	0.268811215	0.27703632	0.324282634
0.250591848	0.274137664	0.110576222	0.318926699
0.194240158	0.167808275	0.261406278	0.235877535
0.260844669	0.211572671	0.084974847	0.147850612
0.104115667	0.110240187	0.057258504	0.197868906
0.20009691	0.256777891	0.185147124	0.235043071
0.232954196	0.270429982	0.124552845	0.244553156
0.116601496	0.154321685	0.085969216	0.303798075
0.160573302	0.277002265	0.22581908	0.172376277
0.215556096	0.320009857	0.00347346	0.236300866
0.25725059	0.084963916	0.235837331	0.181298277
0.110553072	0.346553062	0.011394303	0.306918981
0.147665699	0.223681934	0.338684604	0.230116907
0.222706273	0.192245866	0.18268832	0.212689191
0.173104369	0.038613289	0.182745914	0.325686435
0.073134381	0.253032491	0.243973987	0.314306027
0.254673817	0.270090348	0.356744479	0.104753114
0.186531289	0.24591846	0.076439421	0.153628338
0.223218497	0.094770754	0.255116071	0.277974327
0.109337333	0.281597443	0.182388958	0.114034789
0.24485446	0.215631093	0.110799978	0.208792412
0.17792745	0.286062947	0.112935903	0.108767257
0.150109364	0.31084951	0.079871527	0.065769295
0.046234142	0.200066873	0.261553668	0.201877006
0.150003062	0.251237732	0.141771061	0.175055475
0.241587834	0.236207432	0.0271271	0.084945346
0.150421673	0.127530564	0.117341362	0.187454158
0.165155518	0.196040074	0.185919531	0.111180734
0.16078518	0.108859492	0.290678925	0.29465376

0.173001646	0.299133229	0.1429836	0.086009778
0.330289655	0.131424362	0.086343263	0.305502129
0.095735209	0.305415888	0.232409758	0.216715634
0.127149006	0.272218456	0.363948505	0.093447676
0.162592605	0.273637092	0.185486561	0.234177763
0.217982637	0.276777644	0.210638565	0.192608798
0.184542684	0.167658338	0.278476939	0.139720719
0.108239253	0.22048836	0.218754622	0.191340547
0.168202095	0.222512037	0.229658919	0.258246227
0.311903081	0.343274724	0.249698314	0.101952813
0.23513343	0.072494062	0.127343467	0.112127584
0.234210805	0.260434012	0.2231594	0.157072715
0.2959816	0.152957053	0.11874305	0.297673978
0.072928436	0.250737822	0.073226455	0.218980665
0.224676161	0.121907139	0.171918564	0.241203194
0.231160394	0.239024596	0.207592694	0.255505613
0.278320627	0.195739974	0.165708745	0.21143951
0.185666026	0.240810027	0.186317836	0.068104905
0.172414039	0.253252616	0.015954846	0.159201874
0.225578277	0.26947551	0.299129477	0.198482402
0.175069636	0.141832145	0.236694933	0.203431164
0.225762706	0.230907266	0.256293593	0.240532719
0.209961642	0.259835009	0.118950828	0.152498814
0.084521467	0.237057763	0.213393986	0.128528745
0.336469322	0.105098	0.120276969	0.296127907
0.291652011	0.184974128	0.03860276	0.018894591
0.069448487	0.286324712	0.238903769	0.27762803
0.118913942	0.242783688	0.005791886	0.272732586
0.269637374	0.072368627	0.118201704	0.21087656
0.17919434	0.288920704	0.105382042	0.244159016
0.149546995	0.232484745	0.251596369	0.249453035
0.294016399	0.219126478	0.271048625	0.263917945
0.329255962	0.246466278	0.325357561	0.17643423
0.110709284	0.194434361	0.113947053	0.172854769
0.162298441	0.20337125	0.257908978	0.320789221
0.267341063	0.057170976	0.258315254	0.215793964
0.321207517	0.345592711	0.048134432	0.014790037
0.006619362	0.066107941	0.243901028	0.175845235
0.279228746	0.109894872	0.076539146	0.210792991
0.226084475	0.18113103	0.267470652	0.354561448
0.22015223	0.199532764	0.167797586	0.333263468
0.142825152	0.126406597	0.29289948	0.27315895
0.098636199	0.183150735	0.113780295	0.353751779
0.018252398	0.222925058	0.175837876	0.222473827
0.030624911	0.153158636	0.168325631	0.296542491
0.213989842	0.17840528	0.141547342	0.067220102
0.297106747	0.28747913	0.268660456	0.326599067
0.316366497	0.369747361	0.100282021	0.260485171
0.164335635	0.233204475	0.129419721	0.068426308
0.146593911	0.087199499	0.210764298	0.110117298
0.156298288	0.221050884	0.121216345	0.063347274
0.052823795	0.157850536	0.343801694	0.279459044
0.062818055	0.260206367	0.191169004	0.235698079
0.197386197	0.32986291	0.247246836	0.110257304

0.30213742	0.036039308	0.29856598	0.1729933
0.187420039	0.320362389	0.191300136	0.285229025
0.213777246	0.264844126	0.248835049	0.265222966
0.109468644	0.169166931	0.281804879	0.166630455
0.243041097	0.327692254	0.152743943	0.170907377
0.231074043	0.102813067	0.143292675	0.050186454
0.068572841	0.201428452	0.278184922	0.352639235
0.188320095	0.184572034	0.130638052	0.174535331
0.293278367	0.245234174	0.233332752	0.268854524
0.200377147	0.2374125	0.239245691	0.233902472
0.114437051	0.258195584	0.280258994	0.251486177
0.260766321	0.280421626	0.263018544	0.122485816
0.106023801	0.13628825	0.257469815	0.160749924
0.281546955	0.114059635	0.065217285	0.286605013
0.073893444	0.302717456	0.106407302	0.178326401
0.171495499	0.089612949	0.246214909	0.284180115
0.247611431	0.255866417	0.060969565	0.160382447
0.082625143	0.246583829	0.125219188	0.207476546
0.095725393	0.10484111	0.248874067	0.138202062
0.126238476	0.091610076	0.247090802	0.154072099
0.174706716	0.331123832	0.114524565	0.051540689
0.091225267	0.150042704	0.135877882	0.246643802
0.064177209	0.234328042	0.143842937	0.167332612
0.245655132	0.192315288	0.180243532	0.107798086
0.160471575	0.228638445	0.128517253	0.241351305
0.115330481	0.36431263	0.203823289	0.228829627
0.090470352	0.086123959	0.217158798	0.300350747

0.09043562

ASAH1 0.361061177 0.451708699 0.290527242

0.347453301	0.2912349	0.17974016	0.146986413
0.115902506	0.100355734	0.00493837	0.033886833
0.317993884	0.148165027	0.146227591	0.170922357
0.414870303	0.178139118	0.262915495	0.210260303
0.327056364	0.059583098	0.36449545	0.108242911
0.327223719	0.155748195	0.100495395	0.260337135
0.415078634	0.11588149	0.129329712	0.270973908
0.202400127	0.379418871	0.428088965	0.345513376
0.422453813	0.313226615	0.032304839	0.139924599
0.368405809	0.38061086	0.324282171	0.23369382
0.055986368	0.371229981	0.178544989	1
0.130819703	0.05745258	0.101527997	0.34178404
0.363136952	0.238624042	0.139096571	0.120261618
0.117609293	0.098714912	0.201750732	0.176722729
0.177014327	0.062652098	0.3914455	0.073744635
0.105186141	0.172823174	0.245689575	0.166804262
0.298983674	0.272702604	0.037956274	0.284770562
0.001333757	0.315779691	0.3337161	0.232775653
0.090887229	0.027818524	0.26203111	0.202445718
0.437909234	0.410812385	0.484815056	0.481565412
0.184556372	0.314696776	0.306674976	0.169080802
0.386143619	0.123470663	0.118027383	0.428658165
0.487101998	0.443616508	0.242251125	0.307004332
0.318140687	0.403754852	0.231603037	0.267503594
0.210920814	0.154359288	0.347482337	0.477922367

0.3129552	0.298009404	0.206588797	0.323106616
0.113757906	0.19537776	0.274840876	0.173776537
0.44984389	0.029664191	0.272971918	0.201542699
0.266399218	0.370771784	0.451520533	0.164276377
0.318835268	0.04227815	0.149353843	0.099665763
0.208895324	0.220584986	0.360254968	0.402397433
0.138229541	0.269253622	0.198754208	0.01658702
0.075552037	0.278289371	0.369654882	0.272641273
0.372451525	0.343971047	0.185379613	0.269931183
0.31273989	0.481474666	0.017816607	0.11204777
0.246244144	0.284405638	0.236294863	0.265852802
0.271092061	0.110200172	0.146660476	0.4868262
0.329865203	0.106400268	0.19760596	0.398016689
0.171331491	0.314503752	0.135477517	0.191111119
0.05336239	0.404415322	0.091654174	0.205413401
0.17446822	0.189743404	0.03888583	0.142703948
0.155190223	0.340385623	0.362085778	0.155724744
0.214567256	0.332328315	0.160190417	0.281577763
0.012915874	0.09449222	0.127958209	0.455422482
0.128907716	0.495425511	0.258377693	0.319476109
0.288558119	0.432043983	0.012094736	0.390650796
0.452877177	0.128348538	0.240320381	0.330933557
0.242765466	0.40039779	0.032960033	0.378931505
0.231707976	0.129507569	0.501618936	0.191041066
0.392007825	0.074873845	0.169321855	0.414940849
0.329927112	0.018056459	0.096173817	0.279871847
0.0970151	0.056421752	0.357851895	0.307356529
0.232380731	0.436067541	0.48907718	0.233246071
0.278226957	0.189464714	0.046715244	0.330875675
0.412151515	0.215693028	0.344755685	0.177315904
0.15909685	0.39658661	0.348807105	0.098881345
0.061811106	0.182700232	0.021317265	0.261532712
0.342453231	0.084204558	0.231963777	0.242848343
0.138938792	0.473371976	0.264088537	0.276380477
0.208363783	0.195052528	0.21774187	0.116419747
0.136970319	0.20045675	0.268739151	0.246102202
0.033772287	0.121498844	0.077811794	0.192810934
0.085900055	0.168050767	0.148926688	0.270363644
0.189846787	0.092700243	0.263072606	0.310035081
0.186822647	0.047697887	0.441723574	0.360708282
0.206652154	0.156823619	0.100378014	0.154813975
0.409921724	0.115810607	0.115137964	0.372311218
0.065918183	0.485425553	0.163403156	0.165434071
0.255830475	0.208812479	0.443877837	0.04126995
0.218769534	0.373606527	0.315573009	0.198265962
0.289459166	0.296928491	0.042017787	0.306557991
0.286380508	0.066005144	0.469967249	0.199426886
0.067060531	0.397320063	0.337148491	0.137893188
0.148086591	0.182242701	0.034987698	0.284854037
0.459468158	0.389610072	0.316505373	0.312356609
0.055482993	0.012964262	0.302763035	0.29465948
0.402488566	0.470491329	0.273325251	0.255987112
0.220675065	0.239123815	0.069367342	0.463469271
0.286525533	0.446856776	0.028700966	0.405843498

0.407960857	0.124519757	0.126627477	0.164028105
0.282354694	0.071586459	0.213526733	0.406566002
0.378807931	0.251744356	0.227342488	0.355126364
0.019749221	0.046635476	0.293792655	0.267183473
0.21788215	0.227554293	0.125378183	0.189817202
0.055289215	0.357276221	0.45617435	0.090452241
0.131673252	0.179233539	0.349809322	0.06934048
0.432763864	0.454005309	0.426160007	0.166124174
0.378185833	0.428574467	0.25459736	0.167680152
0.063278332	0.439497009	0.280325232	0.025936661
0.459619024	0.088191924	0.014145507	0.422371865
0.182066209	0.332797035	0.004180862	0.141572976
0.138009862	0.481764149	0.377460754	0.270056249
0.081855438	0.282442284	0.033402573	0.492773805
0.399055672	0.054065515	0.246898977	0.350812816
0.356126613	0.451340418	0.273266806	0.41560177
0.282089061	0.406327029	0.148844114	0.264832313
0.362290133	0.436513004	0.337162914	0.384394392
0.419159754	0.395197576	0.220895131	0.025295914
0.170304083	0.107409238	0.130145849	0.077203404
0.164170643	0.174473266	0.43607073	0.468498274
0.451539465	0.025766552	0.100277462	0.34678403
0.375651911	0.446095931	0.076520962	0.015664551
0.018090498	0.043331995	0.213922414	0.3024352
0.445006034	0.078930353	0.01748089	0.039899843
0.21479028	0.330115653	0.419392814	0.34907902
0.420569017	0.142008822	0.122291485	0.365868402
0.122981145	0.204164391	0.452032765	0.374058374
0.123365216	0.299886178	0.247085445	0.454029645
0.124718562	0.305465297	0.331956554	0.19022173
0.059572119	0.157387174	0.12311746	0.44613595
0.404719876	0.079012018	0.051077498	0.05932858
0.094823373	0.455953509	0.150517855	0.495319115
0.378140173	0.434137478	0.170528502	0.395645565
0.064086228	0.38879985	0.300786507	0.030378158
0.313305156	0.047660192	0.418962394	0.10605122
0.162796465	0.291097101	0.206812072	0.07096664
0.244684701	0.110084578	0.459309093	0.47079905
0.122485836	0.201020258	0.311309863	0.338824396
0.142224889	0.361507045	0.399161883	0.178883034
0.074725948	0.232660482	0.329266116	0.263409273
0.140053402	0.498975589	0.204438251	0.490011131
0.314357586	0.425047534	0.423926901	0.222714326
0.259433981	0.217765538	0.217197831	0.018108358
0.269529921	0.423414508	0.235775805	0.169561782
0.227162174	0.282129124	0.087581572	0.134731963
0.26569517	0.135266295	0.13008873	0.454351105
0.262044868	0.277295503	0.058517719	0.109893441
0.473473048	0.370885566	0.102106272	0.293544188
0.242093686	0.26360561	0.425468157	0.436953976
0.257798629	0.37186285	0.350853598	0.229886284
0.322321376	0.216176068	0.43293654	0.052298134
0.138034875	0.283395647	0.088096082	0.178159501
0.158384343	0.080778662	0.241344159	0.406540036

0.25059837	0.28695797	0.165405548	0.129861884
0.32709296	0.239393934	0.29761078	0.121139562
0.084763427	0.395201813	0.026379605	0.121023228
0.263284465	0.372580011	0.264039834	0.27660147
0.275606488	0.260302755	0.255423177	0.131505946
0.123661595	0.247336633	0.363788701	0.047152498
0.028513178	0.493646342	0.204987723	0.090390174
0.161992733	0.238978831	0.27809423	0.170140182
0.085932555	0.42676818	0.135928835	0.297854566
0.21194273	0.123156836	0.265735496	0.108072168
0.111809899	0.360483641	0.302420118	0.437404166
0.336538523	0.388460473	0.338079169	0.35779796
0.18347167	0.251603096	0.042346565	0.411252696
0.212772112			
ASH1L 0.210471796	0.284619101	0.109070221	
0.2655557	0.128155724	0.192314099	0.13662406
0.110191955	0.011854877	0.001075002	0.001191064
0.136984725	0.004260752	0.068103391	0.070444771
0.24133174	0.017893148	0.113047777	0.136523706
0.253862144	0.064108733	0.186957676	0.044970239
0.031070992	0.016896439	0.026370898	0.04214633
0.302669678	0.035128371	0.123743516	0.278569732
0.021656576	0.250910518	0.275424257	0.141026764
0.246476155	0.203216799	0.001267526	0.072721027
0.29480581	0.211197139	0.257363916	0.167521877
0.024812545	0.282734587	0.144574573	0.130819703
1 0.007299282	0.122795001	0.285829244	
0.233298462	0.126501934	0.1283787	0.083883007
0.003159251	0.08291709	0.01281828	0.250457068
0.109367059	0.100161934	0.240546862	0.021537331
0.005473978	0.131307217	0.022425441	0.160043056
0.037414912	0.27959197	0.009988638	0.122030165
0.001010554	0.255378512	0.133280018	0.035639495
0.024575743	0.00334213	0.034577486	0.195033142
0.296070948	0.264448739	0.242463192	0.233820262
0.014792986	0.099526106	0.080429947	0.051921826
0.262770094	0.08940752	0.010261599	0.212635631
0.246395088	0.255869632	0.021249519	0.085745579
0.230429237	0.286121208	0.136930693	0.099624412
0.044301664	0.008974701	0.240479105	0.252683111
0.284265184	0.044932482	0.046947204	0.291129518
0.030313799	0.06158217	0.198571472	0.004418092
0.298734818	0.016491445	0.051940553	0.160203388
0.035234248	0.254942818	0.241345136	0.118432819
0.100333015	0.001766495	0.002156472	0.014449313
0.011444756	0.007571021	0.249920999	0.26240393
0.055047527	0.027107893	0.1220067	0.002155772
0.00869045	0.015862346	0.197280593	0.140330964
0.257167744	0.199274975	0.050685343	0.235369367
0.132430992	0.234800923	0.003372535	0.060282551
0.157803239	0.022989017	0.129494147	0.13106177
0.0765774	0.085026626	0.056402082	0.283636924
0.301152976	0.056260607	0.113984841	0.21902666
0.035970662	0.086621517	0.056217582	0.061022499

0.030016762	0.221279513	0.008232743	0.168862026
0.015391745	0.005255634	0.004369196	0.165052052
0.034749462	0.206364512	0.235983658	0.062890227
0.090526526	0.160461336	0.159671748	0.178009887
0.001465774	0.012400107	0.121824121	0.255921039
0.208260042	0.25614138	0.085025316	0.063619162
0.105349396	0.273238047	0.000887923	0.270950678
0.254779798	0.119903488	0.123076319	0.150133746
0.066684408	0.274464428	0.002144579	0.208629519
0.217434402	0.052510312	0.272758987	0.050484527
0.24748852	0.0203957	0.09936195	0.181215697
0.140116504	0.000672439	0.15535667	0.25515925
0.007258494	0.058586878	0.251961747	0.244796939
0.163862889	0.23840665	0.277747064	0.055915401
0.12022081	0.123201811	0.004902469	0.08862841
0.224189916	0.014632377	0.297133433	0.077034634
0.164327782	0.280708149	0.188389598	0.007177678
0.036088037	0.159779084	0.004352524	0.107184095
0.218241331	0.028153197	0.093938611	0.018057163
0.017676689	0.272938336	0.020427209	0.028638609
0.009794003	0.125276388	0.149316317	0.032133199
0.010142325	0.034841391	0.048758055	0.07408191
0.034874893	0.028920638	0.002459007	0.033358667
0.005625914	0.036492455	0.042370472	0.198175093
0.037819161	0.008600984	0.093123694	0.0342283
0.025512973	0.011323534	0.281095606	0.222161883
0.255435394	0.059369157	0.020064202	0.010347287
0.255703739	0.145711491	0.10711679	0.262698856
0.006184824	0.258397395	0.026851546	0.038402862
0.043913818	0.103105684	0.299748732	0.003191567
0.182621322	0.269946502	0.131853063	0.048069546
0.236927859	0.220516594	0.007317766	0.239761451
0.213734963	0.016517698	0.258028149	0.015498356
0.012984799	0.174079382	0.16304641	0.037908773
0.029258873	0.094584451	0.048535636	0.120059392
0.278273166	0.203252527	0.197075294	0.034643102
0.022841989	0.000357137	0.038449539	0.114430677
0.268324198	0.235910753	0.114288573	0.066158454
0.144570441	0.040937136	0.015609593	0.250678488
0.017713071	0.272623415	0.007232369	0.171995792
0.246009133	0.138968568	0.020169434	0.16948519
0.255283043	0.037464048	0.085511976	0.267987129
0.272553963	0.073007413	0.067190266	0.189360206
0.018891074	0.018958207	0.138389039	0.015805439
0.069200457	0.062039287	0.001286513	0.072496237
0.012009734	0.253911509	0.245407082	0.016490771
0.132631291	0.098489944	0.143928517	0.028528813
0.191201778	0.244842461	0.273663977	0.082743808
0.187703525	0.230216758	0.084747697	0.030841161
0.0093418	0.251870921	0.26794314	0.004035183
0.263123358	0.145870948	0.001405675	0.300103471
0.127918132	0.243975197	0.005437424	0.002912232
0.008274653	0.226056189	0.26446237	0.270164415
0.077220079	0.215037798	0.000488438	0.257246357

0.259632577	0.004598231	0.012498825	0.17620873
0.141696204	0.306227868	0.046334113	0.188231725
0.104899807	0.238312097	0.017064879	0.110512866
0.250408114	0.214211523	0.290361835	0.308640009
0.25520421	0.176518352	0.161741937	0.012394176
0.008264076	0.133526704	0.045059557	0.003082937
0.233589602	0.028397917	0.273217627	0.252816217
0.221328372	0.00563628	0.06856192	0.203790337
0.312641269	0.314628087	0.004019764	0.000594466
0.002087528	0.00129227	0.212225031	0.076814918
0.259333708	0.022793787	0.006065242	0.034950112
0.054184271	0.112466271	0.258594861	0.23632067
0.209112226	0.030551273	0.032804505	0.258348922
0.007202116	0.211643233	0.236970706	0.280248311
0.012746467	0.074938467	0.131358115	0.295056222
0.002548949	0.114923423	0.147083669	0.034946541
0.003136508	0.020516711	0.017486361	0.237896988
0.229425501	0.006970308	0.004296025	0.005954849
0.037796049	0.254302453	0.034322684	0.275273971
0.288382834	0.264603233	0.006861982	0.282321853
0.001856912	0.246867548	0.062952509	0.006955131
0.073908703	0.00789588	0.219839889	0.005931525
0.100038637	0.250489425	0.011407342	0.004977353
0.011043522	0.016570062	0.256520049	0.215578523
0.006647766	0.089649032	0.116501208	0.221145861
0.200181279	0.217277204	0.275303881	0.11738117
0.043553957	0.0034979	0.268920543	0.210451604
0.080230045	0.252151864	0.032399359	0.273492635
0.26032549	0.290695234	0.226062903	0.186491304
0.007501751	0.186054476	0.215112233	0.005391014
0.183281685	0.23322349	0.179091966	0.013804302
0.062813246	0.082529616	0.007273551	0.008824822
0.048333063	0.214619281	0.023678757	0.274433062
0.220507176	0.222293957	0.008275636	0.016568051
0.290134119	0.29028404	0.038884885	0.22548121
0.067886831	0.121838672	0.219188232	0.1847535
0.044401826	0.209599618	0.259089741	0.202666064
0.272927125	0.147840277	0.27749438	0.044864832
0.00214211	0.141240397	0.017145665	0.034161809
0.062783237	0.0094067	0.020821378	0.210589532
0.033653355	0.161624225	0.041073065	0.014260158
0.096782181	0.11641558	0.148311952	0.103143474
0.015590316	0.260539741	0.002002925	0.021671094
0.039740792	0.213320249	0.069990999	0.064744614
0.017475286	0.016106289	0.108544051	0.15263603
0.059693477	0.01397243	0.226526669	0.023874723
0.015804651	0.26591212	0.157709848	0.005043446
0.003140943	0.20239635	0.062551258	0.03016918
0.00693565	0.20748708	0.088647243	0.128404323
0.218926543	0.033414469	0.265012003	0.004127446
0.022601121	0.27766241	0.10924706	0.242616029
0.087351412	0.223920547	0.275362409	0.206662931
0.008419301	0.10160304	0.022392415	0.276863407
0.04966694			

ASS1 0.021916391 0.036884728 0.023585773
0.091743552 0.056424094 0.023830332 0.111684177
0.015749264 0.075218561 0.071657187 0.157146372
0.035491862 0.067299754 0.043085188 0.010511057
0.098263455 0.159320239 0.012708734 0.15708192
0.0657365 0.053836991 0.13787646 0.102996781
0.028211113 0.189857701 0.239308752 0.081430696
0.07154376 0.238617857 0.021667682 0.044819855
0.078571177 0.032271475 0.023566884 0.084811122
0.021433433 0.014923969 0.01474895 0.026241438
0.024979196 0.058888072 0.068451502 0.146379123
0.118333106 0.018232922 0.155502544 0.05745258
0.007299282 1 0.010686198 0.017504247
0.02154033 0.037203292 0.030218528 0.107953743
0.045903335 0.011352894 0.023814647 0.018404779
0.152986517 0.01440897 0.023965689 0.059014033
0.015893521 0.031262349 0.082675504 0.054616754
0.020961615 0.021523365 0.050986469 0.016444421
0.021961683 0.075621472 0.068836695 0.021355466
0.262992918 0.170575492 0.107282304 0.103618156
0.061080334 0.019127304 0.070525158 0.054137442
0.094683501 0.083265193 0.032196848 0.159957485
0.029896204 0.135008549 0.03964259 0.114484532
0.064933038 0.030648278 0.082553871 0.133926248
0.047727563 0.031774979 0.026057428 0.047300512
0.025186869 0.06685778 0.024097346 0.048599036
0.019003935 0.038631104 0.236278838 0.043934756
0.176498254 0.00526384 0.145934381 0.033950983
0.064472625 0.088573518 0.170177164 0.075731322
0.027322503 0.033854927 0.030327363 0.164079173
0.041338837 0.099866781 0.04548839 0.103393926
0.02166811 0.021085209 0.015157193 0.068564589
0.015999061 0.020218883 0.067352332 0.074095191
0.165363549 0.025263458 0.028345153 0.023225784
0.042228844 0.012748575 0.130494181 0.017018644
0.033860458 0.068374259 0.00237284 0.086173741
0.04599421 0.030020244 0.207510135 0.036511914
0.144954221 0.060750496 0.165364641 0.05295467
0.021769626 0.213577155 0.007699021 0.106484293
0.023447462 0.105840554 0.133877675 0.081645849
0.095148871 0.021065947 0.174665168 0.017371388
0.015550925 0.081953105 0.026683659 0.032452537
0.023371828 0.041275832 0.013028349 0.154645866
0.089907439 0.107649131 0.012878112 0.051604451
0.121274245 0.080966671 0.00470035 0.048622354
0.014071727 0.042404979 0.049737591 0.046925699
0.166712389 0.06524152 0.001301741 0.020802
0.029698863 0.005132663 0.103500698 0.045821118
0.028543349 0.072612125 0.000944925 0.098160433
0.01123768 0.138036132 0.067018726 0.26817221
0.021351697 0.26902371 0.02372008 0.038261231
0.029654923 0.034924482 0.025196274 0.069045488
0.10596191 0.031107158 0.034969856 0.080429025
0.07986573 0.053726365 0.079545428 0.028340155

0.060583367	0.060115405	0.070845106	0.093141198
0.033120965	0.005797845	0.023657578	0.04911065
0.007747259	0.033656403	0.020903908	0.007690959
0.192267528	0.042623971	0.040266687	0.10042614
0.016288074	0.101440093	0.022035694	0.032579405
0.20349019	0.046822953	0.055645002	0.023342049
0.02990367	0.053880082	0.088604099	0.059221722
0.072563672	0.104812737	0.035718734	0.033397264
0.069520996	0.228982057	0.027989127	0.015296387
0.088292015	0.035845242	0.027243654	0.019961784
0.018639584	0.10687686	0.09116783	0.018496485
0.124429956	0.221539236	0.036443338	0.082205035
0.01272752	0.089891548	0.164680895	0.007598196
0.062217877	0.014784204	0.003695786	0.044177156
0.043663785	0.055017857	0.067982484	0.275704513
0.049869638	0.183081687	0.073091355	0.020241747
0.019588179	0.030454778	0.080009638	0.174392015
0.021201696	0.059873546	0.112316127	0.01833205
0.018991235	0.238677182	0.037632075	0.10749703
0.201336605	0.055790491	0.044228036	0.258608991
0.185181827	0.086420954	0.035960858	0.195156182
0.045990063	0.134613511	0.040224562	0.042708751
0.19607534	0.147002984	0.083888795	0.0221591
0.020293354	0.037399103	0.140440389	0.09629584
0.180604591	0.038644407	0.005099066	0.048601123
0.009326533	0.025001982	0.119854576	0.044607006
0.020809398	0.013380241	0.245617276	0.056512383
0.024605596	0.150238989	0.041712499	0.027453305
0.035723292	0.052899737	0.035370613	0.043461918
0.066296702	0.069156198	0.058865359	0.026937489
0.027603176	0.095493121	0.014370398	0.010565793
0.148850217	0.031385878	0.063767812	0.123025178
0.02797979	0.008607928	0.105069566	0.189277806
0.039080062	0.025466149	0.025480611	0.051497802
0.032427675	0.038066002	0.030637027	0.15283662
0.204091839	0.024801587	0.02326168	0.075465875
0.068713248	0.00919797	0.154269417	0.034217996
0.140366054	0.012263969	0.01077617	0.001922016
0.065326767	0.064837773	0.022329244	0.04311663
0.00884118	0.059489769	0.00440266	0.037504566
0.032140135	0.162069417	0.070190611	0.03330779
0.036454117	0.031172882	0.046907866	0.048494452
0.039746884	0.023961689	0.046124482	0.194468415
0.049297071	0.025796443	0.038926883	0.026176128
0.067761844	0.074792303	0.16936485	0.117378906
0.034092293	0.024097054	0.012292111	0.076074539
0.013847429	0.131812528	0.030764478	0.066043311
0.047429798	0.147680013	0.030836035	0.028677202
0.045523209	0.056738214	0.025743874	0.009386926
0.003232991	0.15801445	0.047150068	0.110573269
0.039287482	0.006887762	0.127384935	0.041141501
0.029957595	0.073734373	0.031684532	0.121510951
0.037047851	0.155163548	0.027012859	0.087135923
0.137705432	0.007569412	0.05401418	0.028409038

0.069418914 0.092336846 0.014246464 0.063427578
0.012509792 0.139506875 0.036088448 0.039173896
0.023553092 0.158875878 0.155800078 0.068525707
0.019946697 0.160742271 0.190239521 0.003222664
0.141750553 0.047032941 0.108732762 0.057490509
0.052178467 0.099385742 0.08671847 0.026145963
0.144992265 0.023450703 0.081331884 0.006275352
0.034614128 0.081292011 0.023161344 0.03874953
0.017928598 0.027647724 0.025104711 0.115113761
0.011906607 0.20604014 0.074913201 0.062812661
0.065291829 0.04310838 0.097346508 0.050087526
0.034558738 0.09274106 0.02292586 0.014604182
0.094471099 0.009569871 0.056071564 0.017928281
0.02072096 0.067001553 0.053882854 0.038515014
0.023727587 0.029770283 0.033466755 0.078021833
0.041921419 0.020704388 0.053565109 0.078355351
0.082040902 0.076961438 0.022689979 0.108140551
0.186728944 0.025567494 0.041089453 0.00196493
0.022136339 0.025705051 0.094621683 0.08544399
0.023253117 0.019395103 0.035957754 0.155063887
0.034927377 0.022945717 0.027380966 0.051790841
0.133653233 0.115688072 0.043865722 0.051407528
0.008276881 0.042681896 0.042981199 0.044361756
0.026734605 0.113882488 0.027004586 0.007105615
0.078200668 0.017405353 0.142939516 0.202192968
0.219196087 0.111974044 0.012011715 0.080365933
0.011879384 0.1330914 0.003976871 0.07649891
0.095111687 0.008272154 0.109173921 0.079087026
0.135264052 0.026891229 0.062057067 0.17717785
0.016840796 0.041809066 0.058848471 0.103680347
0.023193054 0.043376873 0.162796037 0.016984502
0.026199886 0.005598105 0.039371435 0.039976693
0.081266751 0.066118258 0.012553802 0.052185422
0.031792023 0.013171106 0.016176061 0.053040053
0.123000346 0.032015458 0.019153034 0.044482922
0.053119437 0.119815883 0.011706016 0.020637243
0.045266559 0.020123335 0.033617126 0.026929896
0.029998907 0.122918018 0.013876405 0.031826856
0.016061358 0.011305793 0.094675506 0.047537401
0.015130586
ATG5 0.19778617 0.311493423 0.084027412
0.268213342 0.108200403 0.235265871 0.141897859
0.163316106 0.046800118 0.015991809 0.006104594
0.121872027 0.037612587 0.106975931 0.094207215
0.212371942 0.019119419 0.093669486 0.122602437
0.239255851 0.124076753 0.163658935 0.066782627
0.029828491 0.013802195 0.024807423 0.031249375
0.302831162 0.0286179 0.186021275 0.313703164
0.018852827 0.250438457 0.301667452 0.131693082
0.269084494 0.190338188 0.00797597 0.111897868
0.297363476 0.188443823 0.258507077 0.160010594
0.040867076 0.332860878 0.143545258 0.101527997
0.122795001 0.010686198 1 0.340001284
0.275098412 0.13357731 0.159736066 0.098731021

0.029050793	0.141132548	0.017893636	0.324819026
0.113531179	0.173132849	0.266055383	0.077214
0.060509976	0.139114008	0.019425004	0.158183462
0.030921004	0.315568836	0.034009088	0.100913952
0.010171445	0.243865866	0.098247208	0.02711225
0.015167476	0.011398399	0.024875108	0.206974198
0.291613899	0.265396726	0.229252397	0.223232402
0.02856595	0.067461106	0.061153441	0.050130487
0.274193971	0.100171837	0.058263734	0.183685025
0.233840668	0.277266463	0.034810058	0.05626286
0.262132462	0.323889786	0.115816712	0.094639838
0.045257315	0.00644998	0.281131475	0.254585103
0.304617331	0.030683844	0.030582106	0.324148603
0.025122597	0.092238583	0.183805252	0.026007476
0.300298295	0.031266741	0.033294455	0.179353898
0.064519485	0.2471233	0.252505519	0.109876293
0.08465077	0.00936275	0.012258404	0.012103581
0.079538058	0.007722209	0.29387088	0.281211884
0.133412593	0.052937313	0.127926583	0.001627166
0.006627503	0.030904624	0.201269128	0.116828624
0.232805433	0.189210709	0.049633813	0.266222564
0.123333513	0.221653269	0.052839961	0.078619969
0.153708627	0.032408639	0.102053773	0.135314108
0.060736923	0.111445903	0.054587749	0.282236666
0.367267175	0.048043181	0.111969903	0.18826362
0.077147791	0.065725864	0.057573178	0.073450337
0.056729138	0.241424882	0.004439757	0.170495918
0.036434321	0.013311913	0.016245906	0.199612224
0.101549172	0.233910549	0.266815653	0.04716835
0.092526142	0.135693205	0.195630741	0.2033433
0.010956582	0.029831396	0.159661235	0.271352899
0.305081778	0.242800086	0.083142536	0.049136394
0.073424546	0.246434693	0.001283209	0.312037223
0.269260706	0.155133593	0.125664866	0.122888717
0.056008282	0.276019676	0.014525869	0.186469513
0.241003188	0.043209447	0.260171945	0.037919604
0.269495499	0.017012948	0.12383345	0.161520508
0.143622664	0.006951338	0.211999582	0.276830925
0.00403621	0.139694873	0.236270091	0.221260891
0.153359896	0.202475674	0.260220484	0.046329665
0.110886009	0.116892364	0.008136391	0.060638884
0.198069734	0.047130388	0.340603336	0.116581737
0.201270586	0.310102481	0.192123152	0.056839018
0.037605815	0.179610855	0.040411375	0.086572909
0.215919165	0.0611995	0.084387026	0.02903729
0.012879691	0.282313374	0.012785075	0.017618653
0.006935429	0.149166997	0.131331692	0.064503325
0.026887448	0.045890757	0.044982999	0.067222763
0.073351054	0.028410837	0.002389836	0.037518401
0.023890997	0.049677796	0.05676185	0.224370695
0.08668895	0.032354406	0.083983342	0.033481741
0.020194082	0.007093177	0.300720157	0.196936214
0.31022794	0.07999869	0.014808556	0.046761905
0.275509356	0.197118932	0.131566711	0.307884869

0.022276467	0.240812097	0.052468881	0.027531419
0.041115966	0.098195922	0.290264239	0.036539459
0.192050513	0.322603775	0.094025743	0.036645921
0.307207156	0.220496794	0.037743063	0.243478858
0.237771353	0.014242947	0.268918126	0.015629563
0.00743581	0.142450968	0.163105272	0.023449539
0.018543685	0.107235327	0.110029881	0.088916852
0.291686967	0.180065353	0.221198998	0.022061264
0.029952542	0.00320922	0.022803046	0.086272802
0.305859863	0.236068942	0.087097299	0.058780288
0.125691909	0.044825664	0.151677671	0.257781883
0.032507171	0.293710009	0.005629266	0.153867416
0.272928177	0.202106084	0.01475842	0.204172604
0.31053233	0.03948547	0.092580492	0.294009797
0.284890523	0.06251153	0.061522393	0.164426966
0.046890593	0.061461417	0.107808849	0.012990717
0.072599693	0.05816034	0.00172248	0.124807154
0.026091879	0.310711471	0.224988906	0.023498062
0.187579309	0.134692707	0.111971585	0.025616702
0.178302641	0.242142631	0.30647839	0.101424031
0.185848482	0.236412553	0.073948278	0.01936409
0.004254193	0.263800938	0.260156834	0.016840929
0.26066418	0.229021058	0.008964776	0.332600506
0.105694275	0.29291029	0.022984716	0.009591143
0.006155052	0.19914756	0.300259615	0.292395686
0.116734384	0.18287837	0.000392943	0.253339844
0.298510453	0.003004469	0.014977602	0.183274701
0.132425766	0.331733275	0.034468632	0.184526011
0.098218189	0.262162698	0.088186448	0.082519611
0.27527245	0.217915328	0.282774423	0.331203204
0.261764949	0.14408584	0.150772431	0.023094618
0.022999715	0.17701149	0.062395559	0.025217539
0.285414271	0.027380078	0.276520125	0.238156534
0.218663269	0.002990997	0.137875881	0.216007216
0.343990695	0.31249406	0.009653847	0.005307295
0.002186384	0.002273031	0.222969002	0.047899905
0.26019435	0.091708005	0.005494645	0.100221652
0.093756064	0.088470303	0.28499975	0.213033828
0.177952415	0.027746911	0.073322871	0.23559684
0.011299898	0.239799224	0.236816794	0.336410622
0.011680336	0.04799451	0.117608683	0.298382765
0.002434088	0.088466452	0.134522281	0.073058141
0.004136695	0.014839194	0.016463551	0.207315246
0.254148274	0.01642651	0.008498281	0.085126923
0.060855616	0.243166151	0.050760966	0.268700543
0.296355822	0.249603907	0.008663924	0.313656036
0.014802047	0.281550321	0.045995547	0.045307547
0.055206282	0.00897679	0.224384882	0.021398973
0.13048074	0.258154182	0.031046691	0.00296752
0.013518821	0.013867111	0.252342165	0.193816646
0.004951971	0.111413303	0.079654391	0.185004704
0.246560668	0.221925352	0.312289024	0.116642346
0.078177736	0.008667069	0.27501408	0.226809941
0.138446384	0.235246973	0.04642814	0.272214757

0.256812579	0.28998633	0.239429182	0.177448027
0.01909819	0.209032231	0.271612832	0.038362936
0.15472959	0.23290345	0.166158336	0.022596478
0.053304189	0.06304067	0.041022186	0.061758956
0.035124136	0.286405938	0.056275783	0.24850507
0.22474743	0.241710326	0.050266324	0.017209406
0.308284084	0.314286068	0.136368187	0.226644278
0.042511504	0.104291577	0.189162576	0.162951534
0.060355856	0.226115011	0.286949891	0.255959218
0.344061616	0.134221571	0.303998464	0.172524785
0.014065147	0.134487592	0.035194381	0.019153624
0.054632992	0.008968021	0.021336395	0.183170613
0.031051381	0.153421709	0.102806662	0.030950325
0.070323556	0.102349156	0.120312214	0.122713732
0.036804122	0.298170154	0.00442271	0.01634453
0.034895245	0.212210561	0.058995017	0.046916716
0.023801155	0.017020208	0.094868349	0.217106421
0.085413173	0.051636107	0.222718706	0.055440892
0.036743057	0.25438237	0.150721851	0.004513408
0.017519142	0.200129191	0.070586817	0.062583291
0.003190675	0.215806584	0.126342687	0.103452368
0.212168032	0.031166213	0.321591485	0.045885849
0.04559909	0.314554864	0.082624676	0.25908181
0.057942292	0.213072425	0.325579503	0.223367791
0.037260097	0.082306469	0.041561946	0.276718842
0.051981052			

ATN1 0.436462727 0.461623015 0.369128917

0.403402876	0.378167695	0.435161058	0.210120586
0.245658814	0.070805242	0.003534353	0.012890955
0.398768268	0.164863809	0.27847592	0.365906572
0.377159127	0.087904888	0.373998361	0.213956964
0.403695521	0.111343751	0.340522276	0.080339604
0.226768772	0.064266509	0.046716323	0.201822358
0.428392818	0.054867024	0.404764487	0.427054641
0.068862352	0.45078398	0.488800975	0.374076188
0.479179032	0.43458256	0.010830998	0.325014142
0.456253255	0.410210808	0.40813883	0.294468723
0.033167133	0.486882075	0.226450391	0.34178404
0.285829244	0.017504247	0.340001284	1
0.454989798	0.42130874	0.276774206	0.154877278
0.168987446	0.317000042	0.206047889	0.42071825
0.205772242	0.243867373	0.448342313	0.08677841
0.163641999	0.292865391	0.103448493	0.2839358
0.287120598	0.456003378	0.01798816	0.392801354
0.00284043	0.382306214	0.317165717	0.264183634
0.025728106	0.011787099	0.124485901	0.333528538
0.438428755	0.442225916	0.421587492	0.427383603
0.128700672	0.239583963	0.334368916	0.134745513
0.457920124	0.167938002	0.100177945	0.363752055
0.426162189	0.464007945	0.148498459	0.198078495
0.43805959	0.46192656	0.415784934	0.384005785
0.303200518	0.029877361	0.451933411	0.441230696
0.456046306	0.244368055	0.104336231	0.443918091
0.041402865	0.398230847	0.317535177	0.127810599

0.439312413	0.015364567	0.148252965	0.360284919
0.194949906	0.448278427	0.449733245	0.17019371
0.356640825	0.011907091	0.094237335	0.021338856
0.31338652	0.095432848	0.490135932	0.437017991
0.275816179	0.326309535	0.269812542	0.00064936
0.014001154	0.147596668	0.439849923	0.373034039
0.431476386	0.415644825	0.185710163	0.460634076
0.409660077	0.420935115	0.088729288	0.091462515
0.411621133	0.146977418	0.19540488	0.424079253
0.239270026	0.123786752	0.103867702	0.444782307
0.458002587	0.067285748	0.40213656	0.365577188
0.326057722	0.276146244	0.091092391	0.248183805
0.046254028	0.444116625	0.014023146	0.389473089
0.245365949	0.095992072	0.010371379	0.322532294
0.296335726	0.436740135	0.476327973	0.085524541
0.29098692	0.347291746	0.409315363	0.423359943
0.006515375	0.0364806	0.395915969	0.446093465
0.398932184	0.437011201	0.270580942	0.251669034
0.219515744	0.421551021	0.002636774	0.482121448
0.464871695	0.409650847	0.350327815	0.377248721
0.338876637	0.430217831	0.054727389	0.372472215
0.439518291	0.063565097	0.431633359	0.112555404
0.448760386	0.02582496	0.365489209	0.405866228
0.415409107	0.007136686	0.30461172	0.402449466
0.013662653	0.130411105	0.43994991	0.390574705
0.35721522	0.413866243	0.421818559	0.316717071
0.379247777	0.242025763	0.007658145	0.247996976
0.429174116	0.375997267	0.455179152	0.246143293
0.433847087	0.456981708	0.436571208	0.230763297
0.033864266	0.351554205	0.02037126	0.282672318
0.437024805	0.10501023	0.385410583	0.117771598
0.042443739	0.447023018	0.11749905	0.1990198
0.081550214	0.395929893	0.24501386	0.223464122
0.163270121	0.127717189	0.30318915	0.347981472
0.041224686	0.065166739	0.007730434	0.346059458
0.106385078	0.314297121	0.334381013	0.45774827
0.331257601	0.083158567	0.324974521	0.233844684
0.074813597	0.007846	0.455483591	0.373593654
0.436815311	0.140929158	0.025836801	0.335595568
0.441337502	0.405631393	0.395880857	0.453305015
0.021975376	0.43296887	0.207351752	0.082438178
0.307625783	0.220978121	0.429339907	0.067897313
0.440223392	0.457129566	0.268609229	0.136238243
0.445409786	0.412719036	0.025579337	0.451364378
0.450747144	0.019083293	0.445196119	0.080309569
0.011276552	0.388605589	0.416692746	0.049575803
0.049986579	0.31358268	0.061538195	0.226641552
0.449473205	0.353945484	0.44016592	0.158652956
0.029092982	0.00481486	0.146281936	0.368993503
0.493223353	0.435078481	0.21589478	0.293906802
0.209906207	0.28548608	0.229445131	0.441808149
0.17195987	0.474128987	0.003368654	0.405270892
0.482498195	0.387549153	0.049098016	0.342101397
0.452860583	0.036799213	0.206227084	0.456175466

0.454601219	0.295735026	0.319879969	0.404630529
0.016280376	0.04394851	0.3452099	0.128357324
0.371204072	0.17860226	0.029198317	0.392131082
0.029603517	0.454083526	0.419197697	0.040972594
0.394224491	0.39022949	0.313501324	0.026516819
0.412849264	0.436914064	0.486905084	0.170483867
0.434908385	0.435759158	0.358556513	0.052672385
0.00820083	0.467500328	0.450497321	0.016222602
0.432154347	0.356578265	0.007089677	0.459905229
0.169381356	0.464807703	0.004694054	0.075582704
0.022747712	0.407740415	0.461971618	0.423147913
0.335582765	0.359507788	0.003557345	0.440650116
0.450002187	0.006192554	0.092053705	0.434742432
0.395299272	0.480704626	0.254106652	0.416438144
0.381270631	0.460619555	0.23284811	0.212210129
0.446680131	0.425884097	0.445924251	0.459048607
0.435352575	0.368877019	0.25099143	0.011867853
0.10607325	0.28944268	0.325047615	0.06225669
0.427674977	0.097529334	0.450377563	0.426024831
0.430300372	0.00167544	0.179284948	0.451248161
0.454615239	0.446019494	0.015358422	0.004755843
0.006363276	0.008531642	0.391533092	0.18641882
0.446534701	0.298408306	0.001831817	0.055688979
0.265692288	0.328837537	0.450953678	0.347650566
0.419797384	0.058472269	0.288673747	0.379710013
0.040829162	0.430874571	0.434004348	0.456918496
0.026580862	0.195332084	0.400898001	0.443401005
0.044330933	0.255373213	0.408524099	0.290515814
0.008999243	0.050698647	0.042110974	0.402401702
0.469162467	0.050089391	0.017841383	0.210260418
0.102092667	0.439529664	0.130624519	0.440275039
0.446494203	0.40108745	0.044312086	0.454924756
0.026212073	0.457376477	0.260202753	0.072087125
0.250889965	0.007611426	0.432678494	0.123416344
0.384879271	0.4408152	0.166638749	0.007769918
0.138405757	0.031857276	0.426760289	0.410498
0.01820108	0.245713834	0.246660863	0.390164723
0.353178311	0.403691172	0.483188892	0.428217491
0.083119167	0.102638992	0.420517252	0.448349167
0.290651348	0.424333792	0.253754604	0.445718464
0.455103858	0.456491198	0.443745377	0.345418316
0.16796166	0.446815831	0.409683438	0.017306535
0.332277949	0.422060502	0.396425403	0.107258843
0.197574146	0.339974256	0.047133272	0.228738735
0.275251715	0.394317455	0.119431863	0.40381537
0.435026495	0.454633606	0.046665304	0.039421273
0.476216081	0.458739752	0.167400734	0.429209634
0.131240494	0.296113649	0.421895659	0.404371655
0.373920394	0.441186432	0.4494277	0.424513634
0.45427338	0.245373545	0.485024543	0.145927174
0.057740823	0.425609416	0.072708307	0.059877473
0.112535399	0.015191502	0.248020578	0.383515285
0.333769311	0.335863825	0.400257919	0.061548716
0.261047965	0.407543309	0.306754765	0.212850626

0.05768294	0.465764735	0.005095122	0.035869756
0.334681521	0.437741614	0.316131345	0.18731375
0.231350958	0.096267616	0.249474402	0.387057738
0.344595595	0.349184802	0.44090084	0.035006821
0.018003547	0.430830719	0.426908586	0.011477545
0.066718802	0.445932367	0.288159221	0.209280982
0.010331131	0.430571216	0.393523365	0.382742013
0.368756591	0.0497721	0.438820063	0.090555096
0.05516611	0.452845174	0.352376907	0.45815672
0.302964808	0.376617572	0.466934733	0.448460644
0.110557444	0.383544393	0.023551145	0.444813017
0.385893158			
ATP1B1 0.434699706	0.491543633	0.385792969	
0.420328932	0.404533564	0.428094999	0.189931003
0.173524171	0.068521606	0.004439045	0.019519728
0.438974875	0.205828437	0.282809363	0.361417008
0.385633831	0.106878891	0.314200119	0.200051012
0.389232406	0.086737727	0.369401189	0.067565385
0.243192258	0.081620006	0.049639072	0.24054244
0.432606628	0.056731504	0.395640129	0.397400905
0.06385543	0.450594791	0.50290134	0.438622567
0.505206518	0.434273055	0.006416208	0.327945953
0.444401285	0.451154965	0.393620897	0.302219313
0.029814407	0.487508542	0.223025511	0.363136952
0.233298462	0.02154033	0.275098412	0.454989798
1 0.475409688	0.212652786	0.142197162	
0.238796846	0.259879109	0.297685288	0.373247865
0.197562916	0.186696773	0.49058862	0.077969327
0.140832489	0.220706458	0.108242804	0.240850077
0.325601199	0.429389145	0.009286843	0.427950428
0.00327555	0.357740696	0.326615399	0.32318856
0.025194691	0.017076406	0.129598975	0.331750142
0.448382173	0.457503831	0.470333034	0.479596496
0.157009482	0.231021781	0.378482273	0.142455769
0.462490318	0.167961112	0.087808997	0.388907584
0.476229898	0.494544732	0.158864062	0.206664616
0.481364818	0.479047531	0.448526608	0.428326336
0.370981131	0.029295531	0.485139055	0.491174537
0.44721428	0.298378676	0.115768468	0.431066461
0.03595422	0.384118618	0.325006892	0.132628577
0.452473219	0.011590908	0.165486903	0.360961544
0.157104047	0.464304242	0.491711859	0.15616753
0.373433746	0.012872358	0.128344539	0.016986762
0.323410488	0.125534374	0.504850665	0.474712345
0.225232971	0.362015169	0.231839005	0.00123222
0.015083608	0.130664277	0.478197129	0.330091501
0.425439904	0.435996023	0.218690407	0.447090422
0.473347252	0.467958841	0.087746672	0.068904946
0.42734079	0.12808371	0.192214684	0.464594965
0.278161792	0.088338631	0.096074225	0.484486268
0.452793557	0.06104521	0.335824733	0.381747459
0.361635178	0.322330383	0.075332842	0.263740235
0.037303536	0.478506741	0.01526235	0.31813276
0.297542875	0.123157931	0.004196609	0.259269472

0.310322254	0.50004144	0.48718658	0.071401336
0.304925605	0.38111406	0.343995087	0.486841105
0.009401268	0.027337297	0.314859314	0.495295519
0.3578807	0.474429654	0.254786021	0.249086176
0.23326658	0.438668865	0.001912126	0.492785646
0.492216367	0.351081918	0.407996383	0.387104615
0.391980339	0.462731867	0.04307506	0.388252961
0.412143678	0.04977641	0.469326592	0.12682153
0.477601161	0.027058999	0.327514816	0.465458362
0.488771394	0.005509314	0.257081809	0.385345573
0.013583869	0.094310741	0.426625508	0.398791001
0.383472049	0.434130634	0.451192476	0.380011253
0.41548245	0.196562866	0.005219762	0.268211869
0.446035287	0.407359924	0.470382324	0.213766117
0.397207993	0.488670663	0.476468074	0.243664988
0.029437113	0.303915133	0.017585661	0.307291881
0.448809939	0.114693652	0.41567586	0.098147652
0.04673982	0.494567075	0.142724734	0.24367335
0.105794341	0.450270504	0.207127624	0.280183736
0.242908652	0.124053284	0.339873389	0.375589612
0.029852054	0.071093165	0.005490403	0.442805899
0.165839217	0.406019059	0.420818725	0.460198467
0.330575823	0.105331056	0.376744501	0.246654298
0.081158854	0.007287881	0.491010841	0.371184778
0.407155306	0.121235283	0.022833897	0.422422707
0.482823354	0.413199717	0.311028315	0.486390874
0.015316126	0.465140291	0.254568818	0.09297541
0.39046124	0.23481902	0.445089587	0.06266436
0.39830627	0.485925159	0.252487128	0.155229815
0.468629152	0.406595769	0.021101713	0.457806463
0.462310833	0.019191267	0.494705129	0.092578691
0.010313677	0.430712479	0.48545919	0.048548817
0.05066634	0.358462989	0.03896674	0.243385541
0.495392368	0.385838609	0.489933807	0.168347808
0.029108289	0.010161324	0.156117329	0.387034809
0.507762541	0.491873115	0.206597399	0.371530326
0.197712005	0.331283268	0.154834922	0.49596943
0.14026329	0.483081224	0.002148388	0.458641143
0.509535022	0.331941967	0.059578862	0.31862626
0.465176817	0.030030317	0.154029069	0.491244555
0.46955145	0.309948348	0.360253492	0.408901528
0.011470279	0.030145148	0.367497378	0.152513904
0.401786518	0.171856211	0.040892177	0.322747407
0.033225519	0.487809972	0.441754755	0.04353666
0.425293171	0.309172808	0.334311352	0.022251325
0.477116411	0.477876233	0.504858999	0.123840278
0.467182655	0.498335181	0.443766938	0.048085589
0.008264584	0.494612204	0.421502743	0.018904755
0.458734363	0.29342157	0.012234885	0.485241006
0.145798245	0.464013825	0.003369245	0.064335808
0.020699588	0.441873942	0.480024145	0.384920005
0.33720148	0.32544591	0.001891244	0.485097481
0.495977835	0.006969011	0.102116095	0.493248802
0.474599792	0.484584637	0.312777998	0.485696062

0.461258859	0.492436529	0.240247151	0.225061995
0.482691041	0.483066039	0.437258329	0.470166553
0.469124799	0.397247681	0.247506605	0.009962629
0.11190959	0.221410675	0.396515156	0.077263735
0.371464941	0.112973313	0.479753583	0.456191107
0.491321562	0.001078125	0.137865577	0.484898678
0.459223811	0.458085428	0.009045631	0.004028476
0.005222974	0.014244211	0.350855154	0.187986458
0.487946165	0.340392856	0.001160078	0.034170904
0.256415189	0.387497242	0.496533897	0.344916102
0.448951769	0.051903073	0.354828846	0.367866068
0.048708058	0.391505529	0.492187949	0.482709551
0.020900792	0.193437461	0.361640066	0.472200482
0.065031795	0.266501865	0.446156731	0.326811425
0.005442839	0.053627184	0.043493041	0.414172776
0.500498586	0.073063078	0.026762453	0.172414404
0.1116573	0.463017391	0.145999757	0.481219058
0.463229984	0.420927222	0.047149755	0.481452123
0.02834382	0.487787064	0.31923201	0.06755378
0.228673742	0.005140522	0.477943603	0.178969016
0.348727284	0.405954971	0.176927744	0.007468961
0.15912952	0.033225469	0.469514794	0.456214
0.01626415	0.197135335	0.243484854	0.373377949
0.305761292	0.4438826	0.496690106	0.483888409
0.069524066	0.11068085	0.402766369	0.437588033
0.232322689	0.466274285	0.306318936	0.486814796
0.436656675	0.463662655	0.49073483	0.339658376
0.204138553	0.427318018	0.425003376	0.018229274
0.318479453	0.468178852	0.33660746	0.138793941
0.233206063	0.393549019	0.026846734	0.180449545
0.339522953	0.361000482	0.12180302	0.412538014
0.395426107	0.443737729	0.024617897	0.042111434
0.490403209	0.461065184	0.133210957	0.452962355
0.121554102	0.32026538	0.456214391	0.44712321
0.362996643	0.495688137	0.447558241	0.451497889
0.470883489	0.222820135	0.503795115	0.084538946
0.06334614	0.440998267	0.089166322	0.059132768
0.112393262	0.0141104	0.311474551	0.395483862
0.401565464	0.365478217	0.335926052	0.055861466
0.271261431	0.364322289	0.312821876	0.200860174
0.065654981	0.491623451	0.00479499	0.033617073
0.400700928	0.475224832	0.397298986	0.192443871
0.287669034	0.093945443	0.263363961	0.335948658
0.419307082	0.361319663	0.482239858	0.022936137
0.012229496	0.466523079	0.392945768	0.008835643
0.056810621	0.412357749	0.242625098	0.230641848
0.010347588	0.49350056	0.407887596	0.412514097
0.334573457	0.039904684	0.41931579	0.066929587
0.034646915	0.468635242	0.383109925	0.490201088
0.302810901	0.40613145	0.453394804	0.48436762
0.087196575	0.393293407	0.017701358	0.447678867
0.466237137			

ATP2A2 0.309685763 0.518042203 0.227919161

0.352201923	0.263174598	0.223862623	0.153703437

0.101191804	0.084388331	0.006804624	0.039027438
0.253021856	0.152880869	0.169932004	0.210588787
0.340140961	0.106099602	0.178779701	0.166005747
0.288355585	0.071350891	0.303659208	0.077434114
0.194263678	0.094443246	0.073877025	0.208638245
0.390242214	0.077416362	0.203302054	0.277906127
0.068076839	0.353187985	0.506465436	0.317371973
0.498583656	0.287866767	0.005302876	0.207585312
0.356653216	0.363620431	0.303100218	0.217817659
0.043361762	0.462586968	0.181077656	0.238624042
0.126501934	0.037203292	0.13357731	0.42130874
0.475409688	1	0.139838771	0.130797771
0.177516965	0.131502557	0.192362669	0.208152356
0.169877823	0.094590879	0.483176369	0.084985057
0.125913696	0.145160308	0.102961589	0.160077469
0.232697598	0.297803499	0.013919564	0.233126071
0.003028589	0.262036673	0.221549747	0.227365709
0.039133468	0.025023554	0.119303537	0.226395017
0.414423715	0.419922253	0.465852647	0.479805745
0.126122034	0.182224225	0.240123472	0.137802455
0.389247873	0.142958285	0.064855359	0.347928236
0.477477113	0.516702255	0.153496664	0.179860526
0.399790602	0.460611623	0.246524226	0.277338366
0.24425578	0.040996654	0.442460648	0.500341355
0.340212172	0.203230613	0.120224868	0.334468966
0.050642773	0.186998405	0.238503702	0.086594723
0.43149006	0.022739657	0.153801946	0.234981482
0.123657033	0.356480358	0.511431336	0.141728483
0.232835399	0.017866905	0.118228791	0.026698845
0.190984343	0.097747255	0.470572809	0.435536215
0.129831747	0.254324787	0.177565621	0.002694328
0.024890898	0.108165458	0.371351689	0.192754613
0.327632274	0.341205575	0.197545087	0.303039704
0.309207973	0.4605861	0.036770503	0.076890281
0.247384459	0.11209607	0.169884209	0.265185184
0.219298335	0.078750227	0.100394965	0.506053204
0.401543159	0.073413653	0.168092894	0.312446506
0.245787997	0.229875592	0.080977834	0.183920685
0.048698583	0.476511461	0.024940381	0.171095217
0.210054258	0.106976853	0.006393954	0.151841904
0.178524274	0.428352106	0.450019338	0.078419318
0.209211911	0.277079678	0.171741544	0.341474596
0.021260635	0.032856377	0.138934194	0.496762639
0.177842721	0.470193811	0.182023361	0.199623882
0.193905345	0.389951902	0.001535241	0.481158258
0.519776172	0.146626946	0.256865873	0.235610573
0.26155374	0.419085007	0.016689731	0.305238526
0.233303531	0.061102693	0.484309823	0.129803305
0.43590425	0.046003141	0.194608465	0.402300559
0.349449836	0.00713469	0.139517309	0.28182361
0.023695272	0.078654872	0.315369332	0.288932475
0.260354053	0.367342882	0.4513614	0.254522233
0.262540455	0.141407817	0.010633799	0.201846047
0.356241904	0.242838782	0.427736141	0.162066025

0.167564398	0.465496768	0.360769215	0.176542166
0.046214083	0.195662731	0.015760004	0.236572859
0.35891932	0.109071876	0.256622653	0.081227184
0.059780472	0.521545768	0.124097981	0.167265033
0.090884327	0.250702489	0.160926954	0.177520623
0.167414573	0.138633882	0.251911986	0.264593618
0.036878078	0.082444327	0.009354411	0.223329592
0.114518171	0.218595553	0.210885785	0.297336266
0.191670919	0.084408559	0.247693762	0.189627033
0.089244381	0.015865199	0.496331359	0.277257177
0.251052148	0.123030022	0.033531482	0.204669122
0.454509868	0.188360185	0.135673696	0.451858414
0.01780722	0.459536175	0.193936562	0.104845612
0.243774416	0.186726056	0.429224364	0.054510907
0.212832724	0.461181017	0.191514099	0.159088073
0.374021611	0.272558828	0.031481897	0.31896317
0.306319298	0.031848633	0.513051588	0.091299613
0.019958555	0.32215553	0.374555015	0.06590666
0.065052106	0.243883484	0.038711801	0.201853784
0.514405096	0.332116075	0.390620385	0.141869162
0.044664642	0.013142749	0.139278836	0.21657129
0.491914683	0.497921658	0.169484488	0.2468493
0.169777284	0.2537792	0.085711125	0.503377992
0.094703276	0.494506107	0.007096008	0.384005754
0.501519467	0.157304346	0.077177421	0.206414679
0.347890002	0.046700243	0.118882524	0.486688219
0.419442019	0.244678882	0.246082795	0.269950709
0.01745806	0.042494729	0.249127263	0.129543905
0.2703417	0.169344805	0.035347221	0.189838303
0.047905411	0.454147398	0.390416099	0.069842732
0.220209895	0.168999666	0.23733049	0.037092918
0.43784482	0.479344238	0.508917773	0.103086687
0.354010198	0.48982315	0.250149698	0.060385057
0.017072437	0.502988079	0.270356457	0.023751564
0.4401923	0.126880697	0.018618725	0.482408026
0.132957182	0.419409686	0.003078163	0.038495378
0.030009347	0.407426349	0.478009312	0.261189779
0.155235518	0.211935624	0.004134169	0.503637493
0.486558036	0.015523303	0.108306805	0.385258744
0.353848172	0.509399895	0.223048401	0.432942923
0.265098552	0.498628479	0.175643075	0.1970154
0.409659534	0.486425536	0.32294728	0.43555627
0.42614383	0.301521815	0.199670549	0.021025233
0.129363395	0.13087213	0.213209197	0.103999917
0.183595671	0.120967349	0.44751514	0.43433837
0.465027231	0.005587488	0.101603353	0.373659561
0.40461924	0.443949746	0.012182528	0.004173279
0.003211232	0.025064238	0.216920999	0.162433225
0.479486814	0.15014155	0.005914238	0.032621972
0.175447575	0.259561116	0.501437024	0.278465158
0.364702393	0.0665907	0.207372629	0.293901669
0.072312758	0.213501306	0.477001102	0.471676291
0.030208704	0.174082229	0.186378521	0.474201829
0.055430463	0.207270609	0.274251441	0.224770829

0.007592672	0.069638133	0.070224771	0.360959035
0.491872073	0.075264569	0.037023897	0.07838441
0.109304121	0.429835117	0.128748077	0.49906851
0.395676801	0.39009405	0.061616996	0.479721609
0.055837794	0.475640746	0.225958077	0.034067302
0.157725366	0.010753085	0.443592156	0.15809486
0.18198721	0.25917136	0.173185992	0.015948159
0.121109898	0.045874862	0.465261543	0.424350039
0.024360187	0.162620026	0.187109094	0.245492292
0.167111031	0.365268543	0.493067113	0.233552487
0.081498532	0.077310426	0.295557126	0.253436635
0.137773101	0.468269187	0.246873801	0.50824942
0.299771837	0.430818748	0.457520908	0.232855667
0.170654534	0.216696398	0.282015888	0.019719413
0.214472485	0.430559795	0.193031197	0.133363339
0.201770622	0.235909316	0.035695636	0.073363485
0.203696087	0.181355076	0.118482542	0.384363641
0.223927716	0.274854521	0.025691165	0.066989765
0.52895493	0.414892818	0.096257373	0.314554692
0.116879644	0.25058274	0.370430974	0.372091562
0.235482172	0.422061605	0.34809339	0.293641302
0.418059869	0.185607056	0.517198539	0.042682811
0.077660869	0.244228207	0.098265191	0.073024857
0.114717245	0.025530144	0.198109386	0.317115734
0.225846712	0.25537994	0.168858067	0.063793354
0.210798681	0.184618468	0.231027233	0.145547929
0.072379722	0.499633104	0.009531302	0.046800715
0.223575637	0.389210109	0.253080549	0.161568335
0.211888823	0.096518455	0.212805201	0.16390233
0.206972297	0.199355139	0.39533585	0.024840087
0.019025966	0.466553051	0.189772904	0.014389065
0.075220498	0.22950577	0.152664231	0.214286788
0.019204663	0.479909436	0.224167058	0.262403
0.210318035	0.051012995	0.313637671	0.057713981
0.040670708	0.417948876	0.23348979	0.503412404
0.199380924	0.357185951	0.42180949	0.384400527
0.060185412	0.204800096	0.029002941	0.390567042
0.246372129
ATP2A3	0.2062022	0.283039996	0.10255593
0.316874022	0.140539716	0.22185142	0.225737338
0.192008851	0.092176522	0.027272503	0.014157317
0.134908809	0.048292429	0.113258682	0.091070323
0.287070233	0.039238191	0.121065945	0.221161253
0.302028405	0.168267279	0.228419198	0.138788765
0.045193622	0.030432974	0.061321426	0.048936852
0.347692987	0.067752731	0.168293938	0.342440223
0.046986796	0.260842617	0.251567366	0.154115514
0.218195733	0.184466151	0.016816944	0.109662859
0.289405696	0.211979424	0.321798585	0.236681757
0.088634164	0.260645606	0.231121749	0.139096571
0.1283787	0.030218528	0.159736066	0.276774206
0.212652786	0.139838771	1	0.156314801
0.031212083	0.123327619	0.018544033	0.290782481
0.196969187	0.154861398	0.218393617	0.122255641

0.081632167	0.196467013	0.040940904	0.21670339
0.039127819	0.298522079	0.088232586	0.102435176
0.013038239	0.327934781	0.145095512	0.031699844
0.049456015	0.025274999	0.050103274	0.273663227
0.321792563	0.236552559	0.24934173	0.230889166
0.046621202	0.121131047	0.076981809	0.092538654
0.272847257	0.159079061	0.098030923	0.248501605
0.248624509	0.239751088	0.067270793	0.107897857
0.247962505	0.296811788	0.120995501	0.114105708
0.046019015	0.021310017	0.238207131	0.249787206
0.270108767	0.041264533	0.069938974	0.333304253
0.070314643	0.084973381	0.269347864	0.037855353
0.333525068	0.078922105	0.068808237	0.225403667
0.110322351	0.249771703	0.221701511	0.205662648
0.112637644	0.026262663	0.019106662	0.042309551
0.086784979	0.011777164	0.220302405	0.290096743
0.143613017	0.062394071	0.204737052	0.009199824
0.022850732	0.059043554	0.190253604	0.152000273
0.267879349	0.166481801	0.074828914	0.240227174
0.126053909	0.243472422	0.039969057	0.158888422
0.179726563	0.061479009	0.196552681	0.144999493
0.097962884	0.189573318	0.110784858	0.284338689
0.312375742	0.115069084	0.120653621	0.254962161
0.078677421	0.096326971	0.123556625	0.094803202
0.112555009	0.200480997	0.016796353	0.200251746
0.039623048	0.02340891	0.049159943	0.239194119
0.105463419	0.207847707	0.20270158	0.111838894
0.1283049	0.18544231	0.195806389	0.191177971
0.021149947	0.080546938	0.145946095	0.259107851
0.247392484	0.246014911	0.121813283	0.079741779
0.133965698	0.286704499	0.002230729	0.250700028
0.234521308	0.131700439	0.15070504	0.1577506
0.066710616	0.297432717	0.014539472	0.246337231
0.20977184	0.107490421	0.282859377	0.083104813
0.233099274	0.047697357	0.141179477	0.163424208
0.13300815	0.017566438	0.216852628	0.335748605
0.01629452	0.183025029	0.264529939	0.273749499
0.195346287	0.237270185	0.298520316	0.053875189
0.136268294	0.186698477	0.032779724	0.099654742
0.214012661	0.045322074	0.284945338	0.169124143
0.16927491	0.279210447	0.171868306	0.052307869
0.090510947	0.229810301	0.063681651	0.131790369
0.198545702	0.093195386	0.095159452	0.058489541
0.035645372	0.268956336	0.021844518	0.024097483
0.011535568	0.148084754	0.225817268	0.064700597
0.030330483	0.097446166	0.057068629	0.085511275
0.128714707	0.063230065	0.011301669	0.03260715
0.027458746	0.043642697	0.048848451	0.207509216
0.089565753	0.04959972	0.11031907	0.048380254
0.045699012	0.027357058	0.274544073	0.267724098
0.252703981	0.15400318	0.047158273	0.034351365
0.278954836	0.156860583	0.118767996	0.285626671
0.064377203	0.261801176	0.065430781	0.066270951
0.048323883	0.161427392	0.332058577	0.057198481

0.208685268	0.278889031	0.161913503	0.070155941
0.241823111	0.272356929	0.087185938	0.220234891
0.20800865	0.043305916	0.246620793	0.033927208
0.029021462	0.168257616	0.160622528	0.069795367
0.04867543	0.130437077	0.165946893	0.15830205
0.275289327	0.243217499	0.20925871	0.038193606
0.0696746	0.007555344	0.045461632	0.102474187
0.243070832	0.219188441	0.163511862	0.073941741
0.232189437	0.055971582	0.141093729	0.247627116
0.052115777	0.25475242	0.02433906	0.166195466
0.21774796	0.185469947	0.035556663	0.249839009
0.261250135	0.098846824	0.153752869	0.247166496
0.280559993	0.093536905	0.070549749	0.198126098
0.093338533	0.127901401	0.145325212	0.021120087
0.07994197	0.108380311	0.003518454	0.142334053
0.055480125	0.266890992	0.259407049	0.056801406
0.160714302	0.1411136	0.166671748	0.073305093
0.174588931	0.216850083	0.253887988	0.171963015
0.189802318	0.2139613	0.07437666	0.054079879
0.018032298	0.22232199	0.266865001	0.030678864
0.290340839	0.191921483	0.018155741	0.300465133
0.206356318	0.21824164	0.035551788	0.014976458
0.02141491	0.232331381	0.243622756	0.336236074
0.086548369	0.260510622	0.003211553	0.242047242
0.253995517	0.013082152	0.032517783	0.172845173
0.127554421	0.293937824	0.046142385	0.182014363
0.100009682	0.213611397	0.116568029	0.153463609
0.267988845	0.189516548	0.2991145	0.294384452
0.279703027	0.186576806	0.250376202	0.059728772
0.04123743	0.203940699	0.048705987	0.038763135
0.262221969	0.052278722	0.2566478	0.269606508
0.212738439	0.015039079	0.182324782	0.204280626
0.339857727	0.335176038	0.035209096	0.010445342
0.004720358	0.006976719	0.280143017	0.094434824
0.25014094	0.059710551	0.021448903	0.158240543
0.121777203	0.112960391	0.246638436	0.305712271
0.194222221	0.073315363	0.063925967	0.318451641
0.030935749	0.200333077	0.23380232	0.28593945
0.039262989	0.094581193	0.134754879	0.315858815
0.003788627	0.149790673	0.1466744	0.084035735
0.014924084	0.039845951	0.046190317	0.258733381
0.202095657	0.029899848	0.020072768	0.073181428
0.098086404	0.262919819	0.084286063	0.277279151
0.305793453	0.307555863	0.024404367	0.268712078
0.041965004	0.227600517	0.06376453	0.044441175
0.083373068	0.035760273	0.196797877	0.023716545
0.132287969	0.279767088	0.051048841	0.013978301
0.019751894	0.04059173	0.275068351	0.2165791
0.019010794	0.174895574	0.138056285	0.239870748
0.268562191	0.255205434	0.254061194	0.09984444
0.141508451	0.014418141	0.323050522	0.213027984
0.151104311	0.258391203	0.059893033	0.258944847
0.262767918	0.285661718	0.222107354	0.240670893
0.028724984	0.212799108	0.267913255	0.060161764

0.228339889	0.253869912	0.20623458	0.039759182
0.090419333	0.07226626	0.103149414	0.058954587
0.04023033	0.268034498	0.093797484	0.312920471
0.246015445	0.229958075	0.10373017	0.046024492
0.279292535	0.284601352	0.176979179	0.241216539
0.098044705	0.160433818	0.204829246	0.184630586
0.07052014	0.211820928	0.300957967	0.235136697
0.287835818	0.231989934	0.255957053	0.18278616
0.0334508	0.135870087	0.063034231	0.056229568
0.118788547	0.03109916	0.022512016	0.239704669
0.031210931	0.208395244	0.097201927	0.067950346
0.118872437	0.113302705	0.187793287	0.168164613
0.06793855	0.245117332	0.015904474	0.049138786
0.037877695	0.218826969	0.068750007	0.08145783
0.030485437	0.037947816	0.160253289	0.207815319
0.069290613	0.053979805	0.22009174	0.119429199
0.083911236	0.277225811	0.153817992	0.018841408
0.041287662	0.199585012	0.09697018	0.088995531
0.01391565	0.19282145	0.117140856	0.132492761
0.270210146	0.083920236	0.257391703	0.081801218
0.114348639	0.260512464	0.103097101	0.221250253
0.08485044	0.273325977	0.2525686	0.210564012
0.068136819	0.088493077	0.100270467	0.304890113
0.049627061			
ATP2B1 0.121311176	0.201189066	0.072792164	
0.299089888	0.135283888	0.162115762	0.283680515
0.122039548	0.159206526	0.099319149	0.079192187
0.101795786	0.094049574	0.127632555	0.076943273
0.261542131	0.090905195	0.065271984	0.26704052
0.245337212	0.221233336	0.255658966	0.21630551
0.048613159	0.080784429	0.160048033	0.073783546
0.274299731	0.159861751	0.137315769	0.240442564
0.068770697	0.168411514	0.158257679	0.162918411
0.138194685	0.100049781	0.016874167	0.135739267
0.166277315	0.184693141	0.26568505	0.281969254
0.201226913	0.149877455	0.315184002	0.120261618
0.083883007	0.107953743	0.098731021	0.154877278
0.142197162	0.130797771	0.156314801	1
0.072850213	0.085212465	0.02884632	0.162471924
0.275736479	0.112399797	0.148945833	0.215475476
0.088199762	0.140057897	0.063735377	0.193081223
0.039925248	0.169713893	0.104647388	0.067141512
0.033679218	0.263236069	0.127041334	0.035744744
0.115562728	0.104917593	0.073637613	0.305347322
0.247562899	0.13297928	0.215578478	0.190273412
0.087143781	0.121318446	0.067580249	0.169388247
0.174818528	0.256481899	0.091219826	0.246515665
0.210446912	0.168145422	0.105992709	0.134049386
0.2172633	0.195816986	0.097748346	0.119007466
0.058130129	0.039020558	0.160707459	0.194946566
0.151282391	0.043794839	0.133895021	0.232263105
0.13268455	0.049513745	0.305884891	0.042716084
0.255850422	0.169408967	0.108389094	0.237186658
0.090867406	0.168441382	0.156065051	0.281887537

0.091253504	0.064286766	0.035972738	0.073819955
0.08092156	0.015772404	0.128302096	0.249984175
0.112632749	0.076061817	0.201684467	0.028741752
0.069277934	0.051548323	0.131965127	0.093000934
0.190163309	0.089768122	0.146105986	0.135558014
0.105077683	0.211017786	0.028449466	0.21309347
0.144387902	0.059539348	0.265912082	0.122739358
0.13998389	0.196359813	0.182878484	0.220030203
0.181615519	0.207696436	0.059408402	0.237631071
0.114023669	0.114132357	0.177859825	0.128776991
0.221247519	0.129848442	0.048473017	0.108535291
0.049703191	0.050477706	0.044763693	0.171281052
0.110834146	0.179127791	0.112561002	0.155128211
0.155350747	0.202799603	0.105447213	0.17817287
0.107036835	0.101415904	0.061140213	0.202812026
0.140914454	0.176751811	0.120521186	0.080189868
0.174429018	0.230976373	0.0013844	0.152350483
0.161945549	0.056632575	0.181245527	0.12071982
0.068438284	0.259286989	0.00455653	0.229451337
0.100767413	0.154117271	0.232332218	0.168070394
0.141565815	0.137944394	0.114285952	0.132021462
0.104504806	0.029997695	0.1740405	0.286861374
0.041812876	0.220358078	0.175541186	0.256103929
0.219431259	0.184447323	0.253410873	0.058841876
0.136392521	0.16061394	0.054043458	0.106900391
0.145270668	0.038877604	0.179184141	0.176366546
0.07635041	0.195477625	0.113438188	0.071033425
0.19958321	0.192736592	0.088987387	0.163232611
0.114073545	0.206052059	0.080852715	0.053248352
0.085482137	0.207931436	0.031397691	0.024473615
0.01689635	0.154063585	0.213987904	0.118518722
0.07371079	0.155452305	0.065549789	0.09512107
0.223791747	0.150287076	0.014836759	0.031910469
0.080401669	0.055772588	0.055681123	0.130650965
0.085036379	0.110566375	0.131679586	0.047087103
0.087139968	0.078421087	0.195436404	0.226897905
0.129482472	0.207061849	0.093691072	0.035618388
0.239246857	0.112032625	0.048950642	0.223032247
0.066661455	0.206721138	0.129639573	0.146916939
0.060531092	0.238197013	0.272663103	0.069328507
0.124119672	0.190926951	0.146319306	0.136047042
0.150657848	0.223387616	0.181878236	0.129017944
0.121809147	0.117300159	0.17760913	0.068683198
0.073041431	0.141513581	0.146683337	0.14145558
0.099909506	0.187749751	0.212828863	0.212301379
0.210584065	0.262894174	0.18121437	0.041766564
0.187119399	0.04908908	0.058093183	0.067868057
0.147847118	0.162065759	0.185613485	0.102163082
0.291698819	0.07563474	0.086333725	0.195667309
0.029477527	0.158955629	0.057612565	0.140251528
0.138833474	0.106822036	0.100834197	0.249112175
0.166480142	0.189060604	0.124448605	0.164743175
0.201988218	0.106373784	0.082358048	0.143280844
0.18418193	0.214710534	0.136012511	0.026086393

0.090866154	0.1502078	0.005155338	0.088904137
0.163967519	0.190834367	0.204873438	0.143987723
0.158980984	0.076975587	0.170469357	0.155575322
0.139637331	0.140435009	0.164145865	0.159614372
0.134372712	0.166635395	0.070902919	0.089363911
0.05415803	0.145402887	0.158565958	0.083519075
0.239872885	0.097960628	0.09543072	0.205048019
0.250362419	0.115565794	0.038068383	0.006433662
0.037021956	0.190106581	0.15758031	0.234471457
0.072360517	0.196425505	0.002886855	0.171891838
0.179062537	0.044880934	0.055698871	0.135366141
0.107067901	0.192276746	0.054367501	0.155867525
0.090789459	0.145752425	0.155298796	0.21676239
0.213511216	0.131829577	0.201662765	0.182081371
0.235713851	0.169686414	0.318271865	0.165487053
0.060644627	0.152953638	0.058749374	0.121292428
0.135390949	0.117838039	0.166168241	0.224129655
0.170065154	0.043442277	0.19094976	0.143203325
0.241989405	0.253107153	0.030674314	0.011124363
0.002740888	0.038851587	0.217830098	0.110203084
0.184573864	0.062308363	0.064485531	0.188635571
0.122279062	0.117513162	0.175326533	0.293960333
0.140124279	0.129829584	0.098188139	0.266548486
0.083827813	0.087139332	0.193131969	0.191350432
0.061111395	0.110140657	0.071559433	0.256253333
0.005962105	0.175893923	0.110614377	0.127221776
0.018087014	0.087577558	0.109612881	0.20997314
0.130173559	0.107072922	0.089309301	0.037493282
0.215252801	0.195921579	0.157564594	0.220783475
0.23531072	0.279253159	0.053522252	0.176073728
0.123123285	0.148026714	0.079914755	0.04112286
0.065691872	0.065441453	0.127253297	0.064201669
0.092092272	0.165376243	0.062288324	0.039569799
0.019531409	0.09854343	0.245513818	0.184421835
0.034440024	0.169886203	0.139571643	0.17251168
0.194948951	0.25246549	0.160340974	0.073157811
0.251823497	0.011928651	0.234354037	0.117389947
0.117321716	0.215647867	0.103035504	0.183559911
0.151860777	0.179471382	0.161063215	0.244581439
0.046365378	0.124659234	0.248580596	0.143666183
0.201182002	0.23426048	0.12346373	0.088873575
0.165043902	0.06097927	0.114214881	0.020922568
0.036520446	0.189243879	0.188013516	0.262986531
0.138582	0.126367145	0.103408356	0.117467585
0.192094617	0.170518977	0.169537846	0.20730736
0.123390303	0.203043679	0.15350561	0.149424371
0.050855459	0.173826084	0.207873454	0.201443304
0.189680298	0.262301684	0.169799199	0.091989668
0.059889418	0.08499514	0.177884543	0.101942636
0.207297588	0.074821724	0.022734902	0.207140817
0.026164849	0.244609366	0.052787207	0.106672576
0.128136302	0.054864127	0.198534244	0.213539624
0.174332636	0.168544418	0.041746415	0.100539862
0.033098111	0.168898674	0.08156989	0.104751036

0.037992303	0.049384192	0.219074787	0.123518689
0.078027404	0.035359399	0.16910204	0.117341098
0.157482842	0.226413278	0.07993214	0.029054214
0.05503446	0.106786454	0.062766322	0.160027024
0.03700006	0.145737546	0.112166096	0.114159866
0.22546329	0.128711392	0.125704224	0.063968126
0.112624174	0.149925969	0.080403021	0.149915547
0.063230984	0.284661408	0.133458447	0.152245509
0.042491147	0.051605003	0.187096212	0.217562192
0.041653234			
ATP6V1B2 0.118992607	0.259371445	0.068601702	
0.221712679	0.133159994	0.108360788	0.08915333
0.013432199	0.009849067	0.00526931	0.035674792
0.131492384	0.033252868	0.070219007	0.031082235
0.191377082	0.074438611	0.026869269	0.098030619
0.15115709	0.030957227	0.225989488	0.028375453
0.035342043	0.073232956	0.071481173	0.084993818
0.206420033	0.06647201	0.100862783	0.138120108
0.016545945	0.159269618	0.225369932	0.247892035
0.22940806	0.100290934	0.000602094	0.072745346
0.136411052	0.195953704	0.16508979	0.161855486
0.027077457	0.19717239	0.133299475	0.117609293
0.003159251	0.045903335	0.029050793	0.168987446
0.238796846	0.177516965	0.031212083	0.072850213
1 0.03402861	0.066604871	0.088576519	
0.119722499	0.027753998	0.23604074	0.015005408
0.008965865	0.032086283	0.032894986	0.05939036
0.056809255	0.113856758	0.001932796	0.097946173
0.00213518	0.132910918	0.106400261	0.066499637
0.040760952	0.033886588	0.045998722	0.170656423
0.212749193	0.159913841	0.273368327	0.264096731
0.047313518	0.065155256	0.103991478	0.083173844
0.167140973	0.108228691	0.003294597	0.221270966
0.273503893	0.259231205	0.044145701	0.090201335
0.245517249	0.211858754	0.088749637	0.133317708
0.071066252	0.008421468	0.213308967	0.274530425
0.135549918	0.087426605	0.08787986	0.168777318
0.030158638	0.017212657	0.183825473	0.007404224
0.223106101	0.01018994	0.092316068	0.15809261
0.009420494	0.156898599	0.252722638	0.090794784
0.092037368	0.005304407	0.011363595	0.010222932
0.010075352	0.023038575	0.200248764	0.271340071
0.02160163	0.052381534	0.063588418	0.002837494
0.022064213	0.005921026	0.18914385	0.048118666
0.144593038	0.121702639	0.124267811	0.124004015
0.169721987	0.269040586	0.001727792	0.023074217
0.115147718	0.010453414	0.119972694	0.156016273
0.140720799	0.019677218	0.058892815	0.261350699
0.168936997	0.060326216	0.026687589	0.187453946
0.069486015	0.149217272	0.037958776	0.099427973
0.030459299	0.218902875	0.022093251	0.03849495
0.027095055	0.034081949	0.00017213	0.048316442
0.023111128	0.270868616	0.181423583	0.035397337
0.124037692	0.191886248	0.049504993	0.245030543

0.021284676	0.004138084	0.026160372	0.281686971
0.084487242	0.2276685	0.042263632	0.042051872
0.116615385	0.197490717	1.64E-05	0.212327072
0.252894745	0.029344793	0.23427187	0.113750263
0.102498953	0.246898795	0.000426715	0.192705133
0.083585796	0.024539978	0.265458589	0.120504307
0.185149933	0.058581715	0.061320972	0.208695714
0.209493084	0.001441474	0.053588289	0.160897845
0.011801883	0.024433345	0.130629817	0.169936904
0.167274179	0.17328395	0.252229475	0.096885151
0.146305712	0.040302205	0.001783805	0.093993211
0.153602092	0.020718075	0.189418037	0.037825494
0.057022478	0.222522575	0.159728652	0.009611246
0.04174541	0.075305821	0.003170738	0.119982246
0.131105198	0.083841178	0.094869626	0.006979806
0.044550009	0.278322723	0.044337485	0.051512949
0.027819458	0.213384875	0.059712035	0.132185014
0.103404234	0.050299491	0.076896508	0.080103847
0.021986456	0.083277376	0.000477987	0.105113224
0.085695186	0.147595187	0.140749469	0.138487425
0.046403561	0.04084808	0.154299762	0.026049947
0.032678047	0.021680635	0.244717684	0.146672774
0.097273457	0.036973998	0.020594676	0.029601301
0.280662614	0.092649585	0.017745791	0.257316164
0.00093271	0.236015343	0.088374744	0.100531855
0.14535214	0.153221167	0.225174177	0.003109723
0.079225254	0.23262697	0.070167566	0.093366362
0.191032374	0.150751483	0.008697914	0.12412611
0.138459002	0.040568023	0.262959012	0.039240799
0.019439054	0.173286342	0.23642842	0.052469912
0.034957337	0.184490486	0.011184975	0.147955129
0.27187096	0.241824414	0.240459066	0.031853552
0.052903629	0.019112602	0.049189687	0.073700142
0.220372022	0.25912275	0.086427375	0.184408486
0.112706373	0.082564001	0.002594408	0.28039342
0.003388657	0.217219522	0.006828815	0.210149455
0.232673499	0.054404226	0.074636791	0.115102719
0.168422079	0.026853978	0.019998846	0.235453238
0.194675667	0.07702128	0.072442562	0.129009304
0.009087396	0.011852135	0.116381057	0.028275269
0.087001197	0.052266918	0.005935631	0.025751347
0.033814872	0.242622622	0.207190333	0.029785091
0.159791599	0.020499794	0.153354599	0.028915867
0.234886531	0.213770859	0.238313658	0.021896167
0.18003036	0.264662687	0.167057577	0.026670087
0.019006338	0.232730066	0.102618362	0.005590637
0.248117363	0.03840773	0.027041669	0.230331606
0.063658973	0.167832784	0.000194853	0.000585258
0.006739344	0.217691817	0.216733036	0.122388578
0.049746719	0.09151512	7.63E-05	0.245164089
0.255521012	0.01624075	0.02601125	0.22539236
0.213579058	0.233294388	0.096075876	0.259800538
0.186963828	0.244869288	0.035475127	0.134722397
0.233274426	0.237049905	0.142164515	0.182113713

0.256215302	0.173397762	0.147382728	0.02052877
0.007785781	0.037293035	0.058807244	0.015102133
0.065000678	0.054888943	0.199334446	0.238568151
0.266297963	0.007883535	0.019113057	0.190463259
0.20341921	0.220733681	0.000299554	0.000171585
5.88E-05	0.01923126	0.103462955	0.061708579
0.235194847	0.027044052	0.009023183	0.007175801
0.020096202	0.175914056	0.254864581	0.173124967
0.166215408	0.033730296	0.094559835	0.154455531
0.029071498	0.065289128	0.278012292	0.228048958
0.006263014	0.05912835	0.043703339	0.258122044
0.016742534	0.130411095	0.148340054	0.07238055
0.000249444	0.03371439	0.036745456	0.185337802
0.231412342	0.084678608	0.05326269	0.003914631
0.096367841	0.214605668	0.062555815	0.267550498
0.208714575	0.237802219	0.017865774	0.21359768
0.019542723	0.234646393	0.130901685	0.001486838
0.028688875	0.003408758	0.205624431	0.036870106
0.055002255	0.101867258	0.012229712	0.011312705
0.013341304	0.038058108	0.279508144	0.234535548
0.005062965	0.033646839	0.073362376	0.10832096
0.069341701	0.254084608	0.224570669	0.095907768
0.044660744	0.003775185	0.143292364	0.102581838
0.031587448	0.261821827	0.096031056	0.242739887
0.113519993	0.17655391	0.234431969	0.122279197
0.030811113	0.085265141	0.199626309	0.006352083
0.094714172	0.281764932	0.05002058	0.04374681
0.164329296	0.108625222	0.001790059	0.001070756
0.08527605	0.095308912	0.048624491	0.205498413
0.085806716	0.111178091	0.001567425	0.036842585
0.252994855	0.163682266	0.007938953	0.17391582
0.044348148	0.156624133	0.178357064	0.199259754
0.029570482	0.261025206	0.173406576	0.206965839
0.207515929	0.090198837	0.242402036	0.002472631
0.007882619	0.106111133	0.090629763	0.044310215
0.090897136	0.009642777	0.045830145	0.175964389
0.064411981	0.222485135	0.014214062	0.004670426
0.100524924	0.036324489	0.131218786	0.080024062
0.040913912	0.24959976	0.001747046	0.02989756
0.081955791	0.199791759	0.166836767	0.052970349
0.042334667	0.015945734	0.14697837	0.056368407
0.142450799	0.009933955	0.200941688	0.001253195
0.010683453	0.25645803	0.047423237	0.00169401
0.004594213	0.077673438	0.018715661	0.069042466
0.012181696	0.259625758	0.091587448	0.117212066
0.091842427	0.018512274	0.112392956	0.002270224
0.002985476	0.177453915	0.096862239	0.244926176
0.051901396	0.253325886	0.161799283	0.208580958
0.001194776	0.058845464	0.012083284	0.183083027
0.078137735			
ATP6V1E1	0.188534461	0.29838846	0.068657572
0.254642225	0.123773156	0.203653289	0.13536564
0.128442833	0.020823969	0.012488904	0.006682792
0.121438404	0.041028014	0.109801184	0.049841901

0.207196855	0.025722181	0.086519806	0.126262463
0.253634649	0.106962546	0.178051621	0.051213492
0.025576261	0.012753191	0.023240668	0.02559375
0.295182055	0.02645585	0.197646122	0.322060289
0.022194671	0.262480594	0.276122186	0.146353784
0.256232243	0.166376518	0.026316043	0.082483513
0.272105743	0.163143058	0.268642263	0.162757939
0.033615297	0.312871266	0.138115547	0.098714912
0.08291709	0.011352894	0.141132548	0.317000042
0.259879109	0.131502557	0.123327619	0.085212465
0.03402861	1	0.017777288	0.330926859
0.127816309	0.163723182	0.256502082	0.042669782
0.056834122	0.151484675	0.025116163	0.172539867
0.028422933	0.285356736	0.03158994	0.100269487
0.015385508	0.236753639	0.104213511	0.020448669
0.019085058	0.01792596	0.029471283	0.218295519
0.288945333	0.243447682	0.22774387	0.21750285
0.036433637	0.075733523	0.064781969	0.053956913
0.251383328	0.099155516	0.037052728	0.190704435
0.229836766	0.268631885	0.030193228	0.057997642
0.241547196	0.313692657	0.094412794	0.095164434
0.027450629	0.006573517	0.270766879	0.25402659
0.295775895	0.032122131	0.034827474	0.340650431
0.026847874	0.064007427	0.193059034	0.044697206
0.29071885	0.017335381	0.039440371	0.208825169
0.053302425	0.226429548	0.238681483	0.111548765
0.092848025	0.013233445	0.007974112	0.014279941
0.06899752	0.007616155	0.272751246	0.28170913
0.158052199	0.05442659	0.126843913	0.002163848
0.008850439	0.030781325	0.223210704	0.126687632
0.22746831	0.164641504	0.042578244	0.267777194
0.118794268	0.224814763	0.023374748	0.064639924
0.131920094	0.033278096	0.113281222	0.149885705
0.058525722	0.087733013	0.062520839	0.26399364
0.345165205	0.056251294	0.119347272	0.187843724
0.070600483	0.071166992	0.060667618	0.091680784
0.062293757	0.220512671	0.006004687	0.157005778
0.018423121	0.022445475	0.016322066	0.210038376
0.075100058	0.229037659	0.251473058	0.052210085
0.126323993	0.141054672	0.210671179	0.194795155
0.007975325	0.026194728	0.167050137	0.275381269
0.323295204	0.234926604	0.073600233	0.039140762
0.077904023	0.228003355	0.000346871	0.283306297
0.251800054	0.142052069	0.141137559	0.127623347
0.047213334	0.260567024	0.010134656	0.209819917
0.245485078	0.040009568	0.255334509	0.045912212
0.259504476	0.018283421	0.159927897	0.149286792
0.151969888	0.007705135	0.187401916	0.272849645
0.005138919	0.109806467	0.227240276	0.208303216
0.131731569	0.191814475	0.258605705	0.037033884
0.111768331	0.12301084	0.007755603	0.061750724
0.188339601	0.043232312	0.311055801	0.087975378
0.193553969	0.284818122	0.176211742	0.045692277
0.032593026	0.201325184	0.025984235	0.072728642

0.184227692	0.070463372	0.068023343	0.036272644
0.015200718	0.271508709	0.011665925	0.017203086
0.006167152	0.182751822	0.132139915	0.071890004
0.033679357	0.038500029	0.034288797	0.057932574
0.065798421	0.03596027	0.002654494	0.050983315
0.039993327	0.062111668	0.077811523	0.250531645
0.11896704	0.049528276	0.08618555	0.023885421
0.015937501	0.009289006	0.285361156	0.190549384
0.30803489	0.059970381	0.017487135	0.050534698
0.283455917	0.161036819	0.113188954	0.309558354
0.015204287	0.240814452	0.058974615	0.03321463
0.053255382	0.115661454	0.272194534	0.019427847
0.20772228	0.324193517	0.093478004	0.032977644
0.31282485	0.217368934	0.032458953	0.227329094
0.245179799	0.018209257	0.261315751	0.020377887
0.009345341	0.143356681	0.153778398	0.026729359
0.016337297	0.11194964	0.084218055	0.092930428
0.27641659	0.189467948	0.222425423	0.018103361
0.030326673	0.010994565	0.026835399	0.079241348
0.292963569	0.230858936	0.103077476	0.064121146
0.133370844	0.031709196	0.139198466	0.252348645
0.026060902	0.277574947	0.006757356	0.151564526
0.255127418	0.235239302	0.014212252	0.200619031
0.308039793	0.029796532	0.087289443	0.276880324
0.256742472	0.04932021	0.040829402	0.180195451
0.034817223	0.064310994	0.090408159	0.010443795
0.067368595	0.04543054	0.001630401	0.104203101
0.027739253	0.307874963	0.236116885	0.014194314
0.183117991	0.090197932	0.123995226	0.024993851
0.165390942	0.229014212	0.287990277	0.09918064
0.204679597	0.221490179	0.074835325	0.020036756
0.005254865	0.245031475	0.236880294	0.017128459
0.270752572	0.209816125	0.01439101	0.314999253
0.107512625	0.283793092	0.004019321	0.006720876
0.006317127	0.200243099	0.266128826	0.298415258
0.097001464	0.187593262	0.00157142	0.245898158
0.287384371	0.003257347	0.014727813	0.191820099
0.1263224	0.316128643	0.030056417	0.17557563
0.100770654	0.251296289	0.084340301	0.084591569
0.254182606	0.206620185	0.256551241	0.29559032
0.278708157	0.152680233	0.150298408	0.0192299
0.009671447	0.183573255	0.037006656	0.017991558
0.257173794	0.021527781	0.257066647	0.245416993
0.215401285	0.003817946	0.125727716	0.217493453
0.334432917	0.300328096	0.007915869	0.000862
0.001039616	0.002052104	0.241195882	0.047347367
0.241488748	0.075495682	0.00700169	0.0909841
0.065504603	0.087894117	0.278431583	0.216998037
0.172406079	0.032835318	0.057933209	0.236246573
0.009484806	0.222885135	0.22992909	0.319769415
0.01331979	0.046099847	0.121519167	0.292119048
0.002029735	0.102652078	0.147757328	0.064266004
0.006042297	0.013919838	0.012804244	0.210860285
0.243762645	0.024991245	0.013102474	0.084316116

0.057880359	0.247476401	0.050928681	0.264049516
0.283821957	0.258209775	0.011522872	0.277865148
0.01502142	0.272942341	0.047721446	0.024662199
0.051335743	0.009893343	0.225859468	0.012628708
0.157461691	0.254292149	0.014931774	0.004171455
0.008499045	0.016295706	0.248561312	0.189752121
0.005816706	0.090865322	0.078007248	0.184601822
0.232477023	0.222548616	0.292402443	0.098162756
0.069953698	0.011155155	0.274337385	0.220488366
0.160457186	0.236279056	0.038143812	0.256246847
0.227759193	0.267509678	0.233237362	0.149388685
0.024329565	0.178578843	0.268972418	0.029754495
0.145906401	0.247779078	0.161033317	0.016985446
0.060157735	0.061003551	0.042945849	0.048524166
0.033967195	0.272592081	0.071586553	0.248197959
0.231947324	0.229129385	0.078993986	0.012288238
0.295353828	0.277819904	0.069390951	0.209454794
0.048595174	0.098890397	0.183505076	0.167807156
0.044699251	0.242886413	0.294294609	0.276564541
0.341255875	0.127692344	0.282962176	0.122672566
0.00864335	0.148592072	0.038329343	0.023563662
0.06632975	0.00757907	0.016294535	0.197198946
0.030735043	0.179780928	0.117921227	0.024923794
0.0800851	0.095564292	0.127625908	0.120989267
0.034457407	0.280810795	0.004802703	0.020602241
0.043651093	0.186551787	0.058703921	0.040712415
0.019078116	0.019373279	0.112205311	0.212972675
0.096938383	0.042198397	0.1928073	0.03060222
0.043053329	0.257470172	0.128420158	0.00490875
0.020636228	0.187303466	0.072764818	0.047260434
0.003913342	0.213457732	0.115030884	0.101974171
0.204672239	0.033913068	0.313090178	0.030560038
0.034489915	0.301791409	0.073618095	0.248435425
0.057752322	0.222911481	0.298114001	0.242679863
0.026444075	0.073812468	0.027762366	0.278254601
0.043921111			
ATPAF2 0.188918742	0.318935422	0.135499145	
0.21567117	0.173366646	0.099153202	0.043845125
0.009124232	0.016472762	0.002766355	0.032347974
0.216904963	0.05529898	0.036468476	0.033003389
0.2345167	0.067363384	0.044347829	0.07191404
0.162494021	0.00772227	0.250110788	0.013778243
0.073802402	0.083439334	0.038205123	0.124769794
0.230321109	0.041674329	0.063718654	0.126619307
0.027105609	0.206719484	0.306712184	0.305290655
0.325787509	0.166723127	0.002133126	0.038050352
0.186524057	0.273519417	0.163110255	0.132387074
0.009653144	0.250379881	0.078271794	0.201750732
0.01281828	0.023814647	0.017893636	0.206047889
0.297685288	0.192362669	0.018544033	0.02884632
0.066604871	0.017777288	1	0.077312804
0.06355986	0.00986142	0.310413081	0.011007334
0.013242631	0.024341772	0.051573142	0.038583271
0.109253878	0.131111991	0.001384267	0.195783793

0.001074942	0.141120818	0.179007684	0.128934171
0.030556633	0.018368858	0.068770479	0.116472454
0.253057244	0.248816502	0.362796507	0.369696937
0.057462265	0.098332473	0.201550958	0.052819373
0.218290339	0.044687949	0.0137845	0.277197944
0.368154355	0.342923879	0.047056553	0.138758173
0.241844171	0.253450077	0.123250977	0.145207774
0.096069631	0.024915795	0.249434321	0.360319073
0.168291164	0.205535573	0.095047037	0.168833401
0.021461393	0.025798988	0.161799965	0.031666892
0.263815454	0.003750483	0.133644103	0.107652711
0.020766894	0.217081941	0.359109751	0.05103457
0.1479352	0.010559032	0.076571608	0.011262606
0.034887482	0.091141666	0.268116751	0.284587807
0.014261275	0.058588294	0.041503726	0.002534936
0.021359914	0.028901504	0.259071813	0.070757184
0.192332787	0.217172884	0.089335791	0.145758744
0.264188547	0.357430182	0.003197824	0.011545896
0.135235559	0.029625904	0.104920556	0.187442138
0.165248624	0.009784572	0.033753048	0.342653138
0.186755097	0.0286206	0.035725432	0.237640534
0.042555953	0.235080684	0.021604037	0.060802918
0.007170019	0.314611996	0.031211492	0.043512336
0.052839824	0.070693806	0.000840975	0.032062874
0.029145677	0.294582977	0.261919764	0.028022274
0.086148876	0.238050795	0.045229998	0.242297147
0.01247306	0.008069559	0.027382588	0.344494993
0.0618432	0.339231882	0.044882052	0.069389856
0.14807244	0.258295768	0.000980169	0.27691335
0.350380618	0.033017248	0.210702551	0.186246515
0.15143391	0.271558344	0.002764889	0.226802635
0.109563655	0.018228934	0.347020779	0.100932021
0.260748606	0.030549134	0.039683943	0.34469051
0.297080537	0.002606184	0.025601556	0.13995941
0.020634729	0.004920109	0.177389048	0.178468212
0.158655086	0.263670558	0.319101762	0.150009951
0.170909362	0.031409125	0.004080734	0.166791911
0.242355445	0.047480129	0.220627517	0.02434405
0.071432638	0.270601481	0.246738859	0.024402754
0.015084143	0.052933661	0.003603817	0.135924573
0.206518955	0.024079972	0.131324008	0.021908738
0.047775104	0.35515517	0.124371931	0.156093669
0.10603632	0.168576445	0.049653699	0.054719966
0.085931771	0.03482549	0.097478054	0.086590828
0.003636075	0.045095923	0.006587451	0.116726547
0.063401153	0.104341711	0.095038582	0.16050658
0.032945068	0.036071714	0.168269502	0.059904358
0.0480609	0.014733988	0.310274957	0.182896038
0.102896299	0.022050915	0.01881507	0.050423669
0.298991766	0.071260438	0.019192802	0.262356716
0.004017899	0.325674321	0.054697028	0.082692108
0.170187974	0.104716661	0.266192285	0.006799096
0.093713908	0.257380637	0.105166723	0.085151503
0.198378071	0.155743131	0.005902253	0.174497499

0.172407188	0.021663789	0.356532263	0.05264328
0.017463812	0.28293164	0.2979011	0.046922441
0.041360293	0.115887565	0.002207213	0.175814358
0.340778823	0.271282027	0.249285489	0.086116241
0.017467356	0.018894391	0.098206638	0.156783282
0.292147979	0.373720664	0.096148703	0.217220953
0.086018183	0.090218772	0.004595849	0.361889292
0.022805833	0.304817258	0.004251413	0.323478977
0.320356999	0.039139066	0.068568694	0.065718314
0.178178433	0.012163321	0.020867812	0.303742888
0.233959755	0.080234947	0.087392582	0.191866369
0.002012951	0.004135775	0.178431459	0.087774959
0.077435343	0.049190491	0.065373336	0.022782849
0.017736505	0.254852166	0.280852436	0.022526858
0.102498432	0.021352125	0.221645248	0.014248973
0.368322122	0.331236134	0.312276903	0.015231846
0.25284953	0.359398784	0.284645022	0.034252224
0.02218572	0.339482067	0.129504994	0.010737431
0.297554729	0.02524522	0.015846711	0.277069533
0.04431768	0.224763531	0.000237946	0.014357444
0.018638101	0.323283044	0.276437446	0.117655256
0.026462771	0.112996279	0.002078912	0.357855573
0.302403056	0.018911639	0.053866741	0.286366211
0.341386649	0.300159655	0.182670104	0.365341002
0.276423789	0.330172122	0.027108578	0.143470024
0.252006023	0.360623718	0.173235218	0.232829661
0.287380848	0.258485792	0.108478417	0.004650795
0.034383799	0.018587435	0.056911857	0.034779829
0.065581894	0.057069728	0.284482085	0.308138192
0.368083129	0.006181662	0.009359258	0.239346115
0.216821126	0.262695703	0.003470747	0.001381083
0.001660455	0.042817676	0.088401975	0.100867996
0.31995386	0.021108174	0.003821528	0.002343307
0.028009077	0.295939045	0.320577616	0.182345711
0.273179417	0.025820086	0.045625611	0.176976747
0.040643657	0.089962275	0.362004666	0.254777692
0.011318857	0.089946898	0.067246889	0.299060109
0.085423153	0.160696199	0.225054565	0.046238495
0.003807116	0.043047751	0.031115831	0.257220676
0.325750694	0.048866422	0.034115668	0.006741026
0.032209005	0.286345378	0.037308467	0.351805175
0.230289418	0.268035651	0.035242575	0.271250704
0.017978765	0.298778148	0.230191409	0.005179259
0.052946713	0.004037053	0.310280846	0.070348006
0.039281608	0.126197916	0.040960896	0.015407508
0.062518486	0.032086882	0.332086182	0.345312317
0.015890856	0.024597066	0.111328844	0.155238061
0.046827063	0.260922469	0.289042434	0.125685461
0.011449496	0.049549075	0.157881531	0.134014187
0.018192545	0.355186597	0.076121307	0.347237958
0.152671725	0.244367893	0.312743161	0.107235099
0.080170151	0.102644466	0.143623863	0.00552376
0.109667902	0.316848158	0.062770907	0.068752712
0.139347421	0.220585768	0.003958266	0.009299056

0.215320544	0.065364296	0.026246551	0.255879626
0.109156343	0.142260272	0.00243237	0.029757855
0.332988925	0.213164587	0.008899469	0.189017091
0.056812474	0.157099491	0.285535188	0.312902836
0.042529667	0.297372279	0.191178217	0.163463584
0.21083851	0.066826647	0.319696036	0.001951793
0.028433788	0.167847248	0.033245877	0.054570831
0.053345006	0.013682521	0.107645098	0.234333207
0.12000284	0.204686576	0.018185395	0.017300406
0.150073215	0.063753096	0.151104631	0.033229792
0.024892909	0.305483985	0.006234929	0.026105371
0.137325572	0.262170198	0.25340069	0.073036205
0.082355387	0.038125608	0.127912636	0.040941916
0.0843646	0.038850421	0.262079969	0.003370119
0.002859944	0.335715179	0.065294413	0.00868873
0.01919838	0.10794263	0.029635553	0.038926291
0.02247733	0.360572491	0.058693116	0.180062838
0.080374241	0.01534025	0.137420533	0.0070413
0.005551775	0.228532667	0.190940702	0.342541086
0.11262126	0.263689105	0.210245577	0.248879397
0.012112103	0.124564235	0.0046983	0.226558329
0.116856737			
B3GALNT1 0.266032055	0.406792943	0.177991755	
0.2912956	0.209642111	0.281981637	0.207429487
0.298879803	0.061299971	0.011613581	0.010047267
0.200102735	0.103777395	0.250450707	0.212662677
0.25076329	0.06006633	0.259008312	0.188022904
0.270679531	0.175938299	0.201910522	0.101449438
0.107122209	0.037808178	0.044419677	0.095208254
0.348491311	0.051537297	0.290793362	0.328595845
0.056596161	0.323406053	0.405383759	0.188916254
0.38159511	0.253152044	0.021878237	0.233463828
0.360948049	0.235678445	0.284225459	0.215121443
0.051082286	0.43212265	0.192514023	0.176722729
0.250457068	0.018404779	0.324819026	0.42071825
0.373247865	0.208152356	0.290782481	0.162471924
0.088576519	0.330926859	0.077312804	1
0.184737126	0.305650295	0.366469396	0.095461879
0.131810754	0.303860844	0.068448923	0.284303246
0.121087913	0.347862824	0.043365084	0.175761816
0.011768127	0.268057954	0.178905852	0.096036631
0.029206353	0.018975208	0.08351222	0.251899854
0.35358385	0.361917969	0.309092218	0.309028735
0.088937978	0.15959757	0.146712358	0.116496957
0.339708364	0.16365552	0.105316237	0.231413284
0.31682627	0.382641918	0.093745129	0.117746777
0.307255021	0.408266754	0.224985355	0.206482525
0.131684148	0.019890469	0.363762734	0.350049352
0.369832931	0.098935037	0.07267902	0.364162726
0.048028289	0.262346084	0.222491941	0.100884427
0.362532331	0.028205587	0.087073982	0.263594991
0.165083066	0.309225954	0.357288258	0.166941502
0.194368086	0.018426061	0.037076462	0.025533308
0.219301159	0.03591999	0.399148304	0.343349921

0.298777495	0.174173922	0.244706889	0.002021728
0.014734141	0.102452369	0.291176864	0.262120381
0.284974366	0.269730728	0.115687672	0.324648962
0.182958959	0.301903997	0.079908292	0.122807135
0.244122491	0.102200046	0.154409682	0.224788961
0.132824166	0.175711887	0.112345159	0.367598546
0.429034436	0.083288733	0.302978585	0.228741375
0.199549645	0.13868187	0.110442545	0.200929421
0.079830703	0.341977845	0.011987349	0.313962389
0.097892951	0.054284	0.023806406	0.326381774
0.228405313	0.303459109	0.376427118	0.09555336
0.221390365	0.193304027	0.330110167	0.249178585
0.009351094	0.052817724	0.317925083	0.365747278
0.31594422	0.3374534	0.211969437	0.148232742
0.13853274	0.299597488	0.002501848	0.409677454
0.373969016	0.306634794	0.202650506	0.204865625
0.140156989	0.326540363	0.042009677	0.234356461
0.296801273	0.07867046	0.342057961	0.081691442
0.367530173	0.028776311	0.310192204	0.233984994
0.219753059	0.010784948	0.30398929	0.281635811
0.011161874	0.192655845	0.287658808	0.248792338
0.208210996	0.264562728	0.331198495	0.125991381
0.209080732	0.249501145	0.012669562	0.135556478
0.269072535	0.16853895	0.409886723	0.229081857
0.282577364	0.386986564	0.267576676	0.133044072
0.049993167	0.302838121	0.041465075	0.161935618
0.286381237	0.113993632	0.173027823	0.096483727
0.03383455	0.375828631	0.050711885	0.074651338
0.030071793	0.237164636	0.227244507	0.163058151
0.089616121	0.099927939	0.140324262	0.187202708
0.085849308	0.063397214	0.006895404	0.15935582
0.074138098	0.172961825	0.191253767	0.289861842
0.255279565	0.081588733	0.18219514	0.110010072
0.050381937	0.011871967	0.391048212	0.236009989
0.345719902	0.142372627	0.030476993	0.169303955
0.346598406	0.260638788	0.309735206	0.378145349
0.033220459	0.323988246	0.142163932	0.062317255
0.146709375	0.171846859	0.342985125	0.057906028
0.290295552	0.408143851	0.179288843	0.088493082
0.38059683	0.264178872	0.046833634	0.308990991
0.309826468	0.025688864	0.372832748	0.052753224
0.014331506	0.203451786	0.222232972	0.045547409
0.041529643	0.199093356	0.127043191	0.142105398
0.381342363	0.220420997	0.280917851	0.078234595
0.041962786	0.008027027	0.082227819	0.177972221
0.416252154	0.33859707	0.164598164	0.145345283
0.178364844	0.131571257	0.283746631	0.351078554
0.109727434	0.400522038	0.00788386	0.222773109
0.381792409	0.334193591	0.034851197	0.277190067
0.367803951	0.052523653	0.212030376	0.392541398
0.34298495	0.164938175	0.162057785	0.233098957
0.042777651	0.082111258	0.17734656	0.051082323
0.206834529	0.131800454	0.009492235	0.305411051
0.040124827	0.388852173	0.2990884	0.034577729

0.243656318	0.303666108	0.177381504	0.038775847
0.258361932	0.348116914	0.411884721	0.203866431
0.267953082	0.32688332	0.142914435	0.046822574
0.008855273	0.371958838	0.294985911	0.025511396
0.33483105	0.326051683	0.012748013	0.416268778
0.170622952	0.397683893	0.012128057	0.036169855
0.017402982	0.273801472	0.387962761	0.305777874
0.256250376	0.256482202	0.002946223	0.357318017
0.391687413	0.006086769	0.050114496	0.262711502
0.19917133	0.431661065	0.102955795	0.260786846
0.170724718	0.367210204	0.196605111	0.138380805
0.330580674	0.32109957	0.321086833	0.404720279
0.334116129	0.197744567	0.19525386	0.023270645
0.047359393	0.319496536	0.1595753	0.042139197
0.317005396	0.063770329	0.368341104	0.312807736
0.308389578	0.004378691	0.237293826	0.291678893
0.394978485	0.376900958	0.01905644	0.004768378
0.005195075	0.004996431	0.299290281	0.116843426
0.346852847	0.216812294	0.006605748	0.123385498
0.211928762	0.150449912	0.384850647	0.234964921
0.251290036	0.061301226	0.177988519	0.263632971
0.026042955	0.28473761	0.321758595	0.417399824
0.027551047	0.121137379	0.268962885	0.369549284
0.012371839	0.161090279	0.209593778	0.185125778
0.011260933	0.037337678	0.032198294	0.267523884
0.365037612	0.04142294	0.018355217	0.203934325
0.105461191	0.324257256	0.114240017	0.357510284
0.347458672	0.301943634	0.028729726	0.396770944
0.02480462	0.384353749	0.11902443	0.072648767
0.174381152	0.014144598	0.330706738	0.049862262
0.320247111	0.296924682	0.073892647	0.007288472
0.049825978	0.030965124	0.322146932	0.26817685
0.014574754	0.228672355	0.162418102	0.24662116
0.322908333	0.265583113	0.413717271	0.215558712
0.112574952	0.04598166	0.306096446	0.280300625
0.310813935	0.318484993	0.130405785	0.371274403
0.302318698	0.369687128	0.330179196	0.23603465
0.079491133	0.283226864	0.276313901	0.039601926
0.237335362	0.303075584	0.294111595	0.058782894
0.121072681	0.139801026	0.070839233	0.184935688
0.102382528	0.315802395	0.119596902	0.301634517
0.292481885	0.295178242	0.095009229	0.030793299
0.412803798	0.383261046	0.192208083	0.259251859
0.106525438	0.172898647	0.261980024	0.236271716
0.188013723	0.308501463	0.340848954	0.287271298
0.411919255	0.205504922	0.407152179	0.24671441
0.032632698	0.216608132	0.067555753	0.047649229
0.10768278	0.015902	0.087877021	0.236259772
0.131277138	0.212244234	0.300026743	0.061443298
0.152153971	0.238373647	0.196914389	0.220228601
0.062113755	0.39400679	0.007253823	0.037525062
0.143806071	0.270815803	0.136620669	0.126960124
0.091282028	0.059634746	0.173082198	0.333679748
0.210580117	0.184561708	0.281552046	0.065083675

0.045281237	0.337453185	0.284563735	0.011530241
0.047175181	0.259191116	0.221744228	0.1348889
0.008515866	0.313286236	0.248888352	0.194991722
0.289168469	0.06201453	0.386016261	0.092064368
0.076182498	0.405332598	0.16183936	0.366531873
0.160392865	0.253998562	0.416203479	0.305786012
0.097046173	0.175525937	0.044472872	0.342535697
0.163667146			
B3GNT5	0.179110128	0.274787934	0.103186645
0.348564089	0.19005404	0.17328747	0.264049121
0.160502803	0.160662249	0.08301777	0.10238091
0.157034179	0.158401686	0.193251567	0.101049364
0.33544401	0.175847789	0.107771648	0.286190904
0.305966881	0.23609664	0.332873525	0.268182645
0.10262303	0.135404763	0.216572933	0.123603612
0.346641885	0.217065431	0.142148192	0.283810906
0.13730689	0.239330243	0.222381133	0.235838191
0.203340589	0.144224299	0.038121692	0.162971985
0.223577391	0.23961446	0.322810397	0.314658992
0.226593052	0.206300635	0.302583671	0.177014327
0.109367059	0.152986517	0.113531179	0.205772242
0.197562916	0.169877823	0.196969187	0.275736479
0.119722499	0.127816309	0.06355986	0.184737126
1	0.128294491	0.210075587	0.204365357
0.131441382	0.193608537	0.138864726	0.21544666
0.086338802	0.207282396	0.137082287	0.108937435
0.034002164	0.315386314	0.18878841	0.064353576
0.171733583	0.149739791	0.154351988	0.320666448
0.325982397	0.194261863	0.303055468	0.271726127
0.169273173	0.167064864	0.109753981	0.261655613
0.235309441	0.266918568	0.129014393	0.331529244
0.296303352	0.240068376	0.183877982	0.179106277
0.258425545	0.262215065	0.120138957	0.154820387
0.089374689	0.075951604	0.215217734	0.27904957
0.201418745	0.073963007	0.189263194	0.290766299
0.202209162	0.084809755	0.353596195	0.103043463
0.335109729	0.153100469	0.158475227	0.265127422
0.157897599	0.227223335	0.226850501	0.280499465
0.140956399	0.108949609	0.060491103	0.128387015
0.138034596	0.040712104	0.180639938	0.321960048
0.175583894	0.142629314	0.243529608	0.043507346
0.118201841	0.107916192	0.201632227	0.130103872
0.253408188	0.135608044	0.201768793	0.181456102
0.156497678	0.299117831	0.023829752	0.264069387
0.179249591	0.122649948	0.304522842	0.176062554
0.175194014	0.246132084	0.274936895	0.299612853
0.230620851	0.268571072	0.094598776	0.315351037
0.161404294	0.169952955	0.263515381	0.222056569
0.263667116	0.18934065	0.092448899	0.129536261
0.070945117	0.117599515	0.065820636	0.172669363
0.15244328	0.233599166	0.165370476	0.233850392
0.215257815	0.267643849	0.126393884	0.223044472
0.101225952	0.1543665	0.074354094	0.285078592
0.152778629	0.259804433	0.200982019	0.147890488

0.223958354	0.302014025	0.002114485	0.210128607
0.232351357	0.067669905	0.234411471	0.182177672
0.0881346	0.323469264	0.009326293	0.314049834
0.140214878	0.222689765	0.321290183	0.216288869
0.203939586	0.190387303	0.168149374	0.194242055
0.162793088	0.044402631	0.144973541	0.321963577
0.079043975	0.221054609	0.235622466	0.299675138
0.236228694	0.257002668	0.342987328	0.078240288
0.182141895	0.224236292	0.084356874	0.155032996
0.211951368	0.079743013	0.228001961	0.240273525
0.102219457	0.254639293	0.165629852	0.085734266
0.233751384	0.205285056	0.095412387	0.182862423
0.160143021	0.269902044	0.101336907	0.116857526
0.15300747	0.286514064	0.070661246	0.054388379
0.040053825	0.198227368	0.235542496	0.174933147
0.133582646	0.234358255	0.113690211	0.139483465
0.229098846	0.238928387	0.030632064	0.073491569
0.144350533	0.109716277	0.099875886	0.182764836
0.161015611	0.191097784	0.17114289	0.090002662
0.140028303	0.119497902	0.266666184	0.292829747
0.160251701	0.26775395	0.15606777	0.071614077
0.314085624	0.11208755	0.069136185	0.285643029
0.09328799	0.29210856	0.204307028	0.205165404
0.111758021	0.27028256	0.344634042	0.070047915
0.165283727	0.253509333	0.191951761	0.199993489
0.200219044	0.273696875	0.207510818	0.175383274
0.173578764	0.178774986	0.256337081	0.141717024
0.119469976	0.211845847	0.20044357	0.187300958
0.156046011	0.195803897	0.200634606	0.267071374
0.285511232	0.34252323	0.232487879	0.088548641
0.231464615	0.085248283	0.121981707	0.106116739
0.210666735	0.240003141	0.237688491	0.141707812
0.316634965	0.120455795	0.107478877	0.27557077
0.065090508	0.229136298	0.085330909	0.206752655
0.20031593	0.120582319	0.157087203	0.238796844
0.211982218	0.220317523	0.199653782	0.228925723
0.256098632	0.156917797	0.121630888	0.213372858
0.168405977	0.246777864	0.173313755	0.057858912
0.139996989	0.21945644	0.014895129	0.141302248
0.211306828	0.24888751	0.293936128	0.1631587
0.159136896	0.117088329	0.245241761	0.204592546
0.205916117	0.21036481	0.230615093	0.234817098
0.205852294	0.234210946	0.110979125	0.155706918
0.090291645	0.212231327	0.199683487	0.103323183
0.327974163	0.095598346	0.124044212	0.272005691
0.266910495	0.166507527	0.019552566	0.015815671
0.073468514	0.277257222	0.21032039	0.276874333
0.085058148	0.249453939	0.008844507	0.253215133
0.244473978	0.074290878	0.114225698	0.198833802
0.163522983	0.264825109	0.082887753	0.224187355
0.140927902	0.208655885	0.230648394	0.257194059
0.270158834	0.198238406	0.252975154	0.237439114
0.319160111	0.245931562	0.334254308	0.170708624
0.076915335	0.169410795	0.076062465	0.135872527

0.15182413	0.171962928	0.235649263	0.311873108
0.248228498	0.066334902	0.226463055	0.203674703
0.303082648	0.327524354	0.050343805	0.00906768
0.004064143	0.057581173	0.250836741	0.156014868
0.255049572	0.078453747	0.085675131	0.200285652
0.178568863	0.174250998	0.244087105	0.35954927
0.208668224	0.212362878	0.129965836	0.33419783
0.129146287	0.117089531	0.271173252	0.248861624
0.110882255	0.161387751	0.103097963	0.334268192
0.015426914	0.23973643	0.173950981	0.187548496
0.035821387	0.143873266	0.152976831	0.293308204
0.192059877	0.175651643	0.141583258	0.058813045
0.270983258	0.27803289	0.241052986	0.307115656
0.297361782	0.364519856	0.110555684	0.231737056
0.173229278	0.21081544	0.119805304	0.040885165
0.132826029	0.09965345	0.195326757	0.077158636
0.138790166	0.215493337	0.086848178	0.074236347
0.040854909	0.165832823	0.326434134	0.266023717
0.070271158	0.236039228	0.182849292	0.234669218
0.190812374	0.317401131	0.221270842	0.093634811
0.283919336	0.037108885	0.293146667	0.162735278
0.180280441	0.306247071	0.155981201	0.262219986
0.199488227	0.245989215	0.231180732	0.250916254
0.107507552	0.156837848	0.264788183	0.136927681
0.241186512	0.317473264	0.151810711	0.13803251
0.204350592	0.097653484	0.158500236	0.04096914
0.061801385	0.187697563	0.27702461	0.347386297
0.187019968	0.173461606	0.155742279	0.153699694
0.269251561	0.22284036	0.176181545	0.24798634
0.192045715	0.232666642	0.225045533	0.227106892
0.09446868	0.244921134	0.273357544	0.238752977
0.240211384	0.26667424	0.236101573	0.106102891
0.092281202	0.135178951	0.242027678	0.161800931
0.271856361	0.108820702	0.050592495	0.290498981
0.060482632	0.312010343	0.098798968	0.152365072
0.1837594	0.079944473	0.254931168	0.241891892
0.224803508	0.229212552	0.058656961	0.174332159
0.072277262	0.225152597	0.117811553	0.180646634
0.078867764	0.108752894	0.266624477	0.130869899
0.099651934	0.075196042	0.2217013	0.125895933
0.188565214	0.317559309	0.102773984	0.056284984
0.10091779	0.142745116	0.12786022	0.204606085
0.069629367	0.215749839	0.11245713	0.162342273
0.242095578	0.201988767	0.166289374	0.099823151
0.154289197	0.208072971	0.120938861	0.219096759
0.099499193	0.361215026	0.182452687	0.220913242
0.075855145	0.080749226	0.196033726	0.291099534
0.068022548			
BAZ1A	0.125177275	0.214156056	0.046932596
0.217950762	0.078702572	0.195490636	0.162688289
0.167878032	0.039779278	0.032765391	0.008315631
0.075906609	0.034086921	0.116540168	0.059484962
0.154722926	0.018595188	0.065542241	0.125626893
0.195759222	0.169009957	0.127327407	0.076669416

0.01573678	0.011039599	0.028441674	0.018419484
0.234265563	0.030352081	0.18551672	0.278680043
0.015807702	0.180479093	0.195691338	0.092373188
0.171165754	0.116201192	0.013916546	0.099391289
0.207516784	0.120492198	0.214418065	0.148660415
0.056133403	0.232817423	0.15324663	0.062652098
0.100161934	0.01440897	0.173132849	0.243867373
0.186696773	0.094590879	0.154861398	0.112399797
0.027753998	0.163723182	0.00986142	0.305650295
0.128294491	1	0.177187922	0.081817967
0.055272729	0.145953836	0.015120154	0.175252222
0.016087936	0.246276165	0.045938199	0.05674586
0.026289746	0.199349885	0.065083164	0.012763819
0.019179943	0.02201776	0.019174324	0.204398245
0.2150547	0.16680312	0.153392955	0.14285182
0.027315461	0.051052542	0.034931188	0.054130793
0.186103286	0.124667474	0.052989348	0.132467954
0.154672876	0.18242133	0.025961841	0.039900209
0.198875176	0.234082689	0.077078949	0.0684103
0.024539291	0.004536694	0.199814783	0.16987659
0.227775811	0.01643953	0.027858475	0.274132419
0.028540942	0.061694354	0.164951905	0.025481742
0.221154752	0.041165641	0.025489224	0.178410944
0.046510128	0.17002507	0.158659979	0.12467807
0.056196794	0.014859207	0.006081713	0.013158262
0.063288118	0.003508565	0.194968281	0.215309333
0.148147139	0.036126156	0.129121864	0.002594091
0.00807086	0.020329949	0.13889698	0.088826606
0.164918356	0.107836334	0.042446671	0.201423285
0.074143338	0.148261308	0.050390869	0.089237208
0.111957453	0.021541481	0.098221377	0.097640633
0.044769249	0.125596172	0.063332312	0.188490284
0.280852198	0.06452447	0.091931185	0.137347966
0.065720636	0.043046029	0.066297531	0.077044451
0.08655154	0.14817788	0.004614319	0.142347601
0.0179818	0.011519336	0.019251237	0.218832817
0.091975166	0.168342301	0.170249072	0.050957315
0.09758909	0.097972314	0.182745782	0.154533692
0.018092833	0.03269165	0.144427158	0.189127723
0.308759829	0.153571455	0.068198475	0.030409119
0.057637357	0.170474153	0.000421417	0.208817488
0.170827746	0.131530719	0.104481172	0.079263229
0.031650187	0.207141728	0.009311312	0.145375329
0.184247983	0.046530824	0.176520664	0.038230638
0.18009962	0.021546147	0.132935353	0.093418139
0.091496338	0.009266906	0.238457995	0.241173443
0.003748008	0.180206228	0.165193048	0.17530369
0.11831984	0.130952265	0.183493837	0.02528898
0.078412872	0.123307744	0.009546668	0.039409873
0.124218241	0.027217545	0.249562571	0.108797587
0.157259118	0.219211608	0.117023108	0.045269512
0.050919147	0.18300135	0.054998707	0.062475091
0.12960674	0.086228401	0.049194121	0.022637575
0.012776785	0.190078584	0.006456502	0.008503681

0.002947086	0.134609033	0.128705235	0.073735115
0.02640446	0.040003945	0.02502497	0.047159787
0.115421396	0.035066836	0.001665901	0.02657019
0.03193059	0.042044478	0.054149898	0.172847174
0.085972126	0.045648175	0.06397716	0.016592014
0.014891248	0.010003488	0.204226155	0.146268899
0.262037783	0.079760466	0.017139963	0.034537227
0.208220896	0.171829815	0.11198926	0.237593784
0.023370105	0.159711287	0.053442194	0.028708287
0.029565971	0.102971059	0.212187147	0.034234268
0.15209494	0.243313294	0.068853262	0.03077782
0.243653715	0.174799482	0.056260527	0.169782888
0.177062125	0.020309498	0.17584937	0.013231122
0.009310951	0.089316036	0.105061612	0.025363786
0.016395219	0.104023931	0.156807865	0.070086769
0.199278572	0.137181539	0.16412859	0.011351671
0.044509074	0.00885582	0.015064914	0.048431445
0.205923682	0.148953095	0.077937339	0.042281427
0.125571785	0.026546806	0.165027394	0.172635003
0.016967253	0.190605864	0.008327375	0.093079961
0.174314864	0.212619401	0.013881134	0.208383096
0.250972955	0.047962198	0.08382871	0.199801982
0.198188409	0.043105996	0.036932106	0.114553543
0.07666323	0.0929708	0.066294644	0.005912336
0.053366064	0.046446846	1.83E-05	0.101621081
0.039191545	0.234484382	0.157469939	0.022473023
0.174249097	0.098787455	0.078646164	0.033578915
0.104744026	0.14806067	0.20444703	0.104714436
0.125791818	0.15131018	0.041685953	0.017392062
0.004925216	0.164698211	0.191250401	0.026349211
0.188824437	0.238326918	0.019687852	0.237134552
0.114102952	0.201274208	0.020736587	0.003964451
0.004586077	0.12893449	0.199798208	0.253105995
0.116687955	0.145300647	3.38E-05	0.160200085
0.210661887	0.003316723	0.009609547	0.123121798
0.077439709	0.228181055	0.018482904	0.114846306
0.06033175	0.170417985	0.095803556	0.068419637
0.203049936	0.130951823	0.210197253	0.230935125
0.198852875	0.096387605	0.145502082	0.036842241
0.010303986	0.205480736	0.042723447	0.024056711
0.247140673	0.021716491	0.181675996	0.166033356
0.140971932	0.004464293	0.165389885	0.148061491
0.270470031	0.228751795	0.00803929	0.002582093
0.001260273	0.002232586	0.204312896	0.034061559
0.169395177	0.092085032	0.009839806	0.145758092
0.07521435	0.053154616	0.195291904	0.174804728
0.108822371	0.030484558	0.06874838	0.186504049
0.009520501	0.17492238	0.157351254	0.24704804
0.011085997	0.033915547	0.086288275	0.218510466
0.00029879	0.069499213	0.088687773	0.061942313
0.003908386	0.012447737	0.014409015	0.141998477
0.161111111	0.023214867	0.013482173	0.092199961
0.079396004	0.165732817	0.05641133	0.181418493
0.226096158	0.191493945	0.007120433	0.21187704

0.01897582	0.191069098	0.027943793	0.043384527
0.036529846	0.011673224	0.141596117	0.014572845
0.133901875	0.199083248	0.014918323	0.003216261
0.005169327	0.015642606	0.177985312	0.122573979
0.003991474	0.099417767	0.058177328	0.132458327
0.257140176	0.172509183	0.212399479	0.0793002
0.10344038	0.004179006	0.223802125	0.166824394
0.15193705	0.157069489	0.034437036	0.174084718
0.17959982	0.193854034	0.156860949	0.148788324
0.012355639	0.155818694	0.251382417	0.06482217
0.126778519	0.172361153	0.132300261	0.016637335
0.047627345	0.033673067	0.043897211	0.04240436
0.017447811	0.286490378	0.068500831	0.18151219
0.17555838	0.177193242	0.073222209	0.015455445
0.20686049	0.21468863	0.127763794	0.173959681
0.03821171	0.080696382	0.118811547	0.102034201
0.033606029	0.163534168	0.225996908	0.232878249
0.273848903	0.128894503	0.20110226	0.192538341
0.009068365	0.087630877	0.047507629	0.018328421
0.065366099	0.008800915	0.009461977	0.132048415
0.016108015	0.132226536	0.088258174	0.028103834
0.050314929	0.065373836	0.094607635	0.152127083
0.047433463	0.204457189	0.006018937	0.018511305
0.021028256	0.136806015	0.035619163	0.033344873
0.011936461	0.011463341	0.087812456	0.218911795
0.085871483	0.029681261	0.145366773	0.061868247
0.064050748	0.175003979	0.110495691	0.003836201
0.01414128	0.138778657	0.052056398	0.054598361
0.00294775	0.135827169	0.115849692	0.066896899
0.193279153	0.035276773	0.24740919	0.035931201
0.044117576	0.223072911	0.045741931	0.165299131
0.034321811	0.167803103	0.225273056	0.161132189
0.025842821	0.044732657	0.055776634	0.20409375
0.030497881			

BBS9 0.450197757 0.492364837 0.408741384

0.430217199	0.431532683	0.422229198	0.198037297
0.177514704	0.074095168	0.00400533	0.021605085
0.455731287	0.209375027	0.280171865	0.362941627
0.408378554	0.118435584	0.337605825	0.214815083
0.40839209	0.086930203	0.391513518	0.074110985
0.274731759	0.091387245	0.056352537	0.262575057
0.444544226	0.064258646	0.384155824	0.403122703
0.076691415	0.463537398	0.515468941	0.454941321
0.51739593	0.444072043	0.008088333	0.32613384
0.450688925	0.466077867	0.409821494	0.315899099
0.032786151	0.493307032	0.233385208	0.3914455
0.240546862	0.023965689	0.266055383	0.448342313
0.49058862	0.483176369	0.218393617	0.148945833
0.23604074	0.256502082	0.310413081	0.366469396
0.210075587	0.177187922	1	0.081392837
0.149672456	0.233595364	0.126626221	0.25014387
0.358979511	0.429269697	0.010991439	0.446205821
0.002811335	0.375734246	0.356413343	0.344795544
0.02983414	0.017231602	0.149446617	0.342177741

0.461114557	0.461306959	0.484195065	0.492308115
0.167115751	0.259978576	0.408408832	0.154520281
0.470479266	0.172233678	0.092590074	0.412566825
0.48909017	0.505647844	0.176332879	0.232731409
0.47855397	0.479325486	0.455707653	0.445107961
0.380497684	0.037360177	0.480340359	0.498913737
0.446882909	0.325020939	0.129931887	0.436021262
0.042850194	0.384367566	0.342378452	0.144881851
0.46388817	0.012993128	0.187146916	0.370953088
0.178266636	0.472469388	0.497947511	0.167599563
0.400257448	0.014396028	0.138232418	0.021670371
0.327290068	0.141504217	0.509693004	0.479168418
0.232108722	0.388708126	0.246473451	0.000333401
0.018334743	0.154466142	0.486323884	0.35345261
0.443753128	0.441051528	0.23103725	0.44791741
0.48640016	0.48369508	0.076401728	0.077135167
0.435370155	0.152314667	0.211391527	0.476728309
0.296432058	0.095073536	0.106453833	0.492618194
0.446459931	0.067938156	0.348421207	0.403222086
0.366814735	0.347150834	0.085014316	0.273048946
0.040890664	0.483270246	0.01910484	0.329672962
0.305148007	0.137286278	0.005889823	0.262911473
0.305455759	0.496310959	0.495450131	0.08229807
0.316935208	0.404823927	0.346055516	0.481328695
0.009643532	0.031641637	0.313598879	0.499447635
0.344041267	0.48668087	0.275773288	0.278123402
0.256656038	0.455078719	0.002212534	0.50050718
0.508225154	0.341934782	0.416420762	0.413949386
0.412713628	0.469778712	0.042682104	0.410028372
0.411501068	0.058277389	0.483064106	0.140267981
0.477291888	0.031299455	0.334394217	0.479669927
0.493337162	0.006110063	0.248905511	0.396392049
0.017805908	0.095087167	0.443920092	0.413563318
0.397054062	0.455584877	0.467145141	0.396915169
0.433257684	0.209108412	0.006954024	0.295335228
0.463887728	0.428651972	0.463594963	0.222990002
0.391631057	0.486827669	0.480356048	0.251264778
0.033176732	0.315704608	0.017158266	0.326750789
0.455105768	0.116671291	0.433032853	0.116903914
0.054730423	0.499265277	0.163184455	0.268388066
0.121641992	0.448920492	0.224055075	0.269677314
0.239338238	0.139395885	0.362200826	0.395828679
0.031192742	0.078592601	0.008125323	0.444790949
0.158577754	0.396081128	0.405607732	0.464419418
0.340230683	0.105945683	0.390443433	0.277476355
0.09304137	0.00912355	0.493124642	0.39275266
0.398263714	0.132567435	0.027921319	0.409410185
0.488130879	0.391001292	0.307133516	0.484361162
0.018588384	0.482010652	0.259450947	0.103969733
0.405401075	0.247360956	0.458149815	0.06166081
0.412928076	0.481121337	0.280392177	0.170144293
0.455209963	0.422520549	0.022553426	0.45989407
0.460455246	0.022309872	0.497790811	0.106458829
0.013055021	0.45234147	0.491779386	0.057148165

0.059359107	0.36124486	0.040044544	0.2664009
0.49816747	0.406169619	0.491134285	0.1937846
0.032022586	0.009783379	0.180446846	0.410967164
0.516706672	0.497523655	0.229485312	0.384361269
0.21488434	0.351117016	0.152391227	0.500933665
0.163680555	0.499065268	0.003168716	0.477005885
0.519648885	0.325645264	0.067219205	0.323312391
0.455823636	0.034288868	0.172172835	0.48634842
0.475760811	0.330350817	0.373090587	0.432269748
0.012074281	0.03381862	0.391824351	0.17356783
0.415800276	0.191484588	0.047855039	0.333114752
0.035548194	0.481358854	0.46014556	0.049649815
0.410680987	0.31741116	0.362858709	0.026182039
0.487900134	0.482544084	0.515973045	0.137747152
0.480586363	0.500104483	0.459546996	0.058051565
0.010643917	0.509040569	0.430620637	0.019419444
0.47313563	0.27791186	0.011686884	0.485079477
0.160113277	0.468315385	0.002886325	0.076073381
0.026803135	0.462623432	0.483657105	0.395937907
0.312972816	0.349634431	0.003449527	0.49329716
0.489778572	0.008745502	0.119306569	0.498355313
0.482703071	0.50008252	0.334834581	0.493774834
0.470613055	0.503970042	0.24114759	0.246726818
0.481986141	0.485638313	0.445852197	0.468713706
0.478887639	0.423436326	0.263417339	0.011180854
0.127118126	0.221771676	0.382167007	0.084594564
0.367051021	0.125672242	0.483254286	0.473962722
0.498582025	0.002000798	0.143137296	0.489454391
0.461859201	0.468715247	0.012390234	0.00422289
0.006047865	0.015897139	0.365593931	0.212071709
0.49426642	0.311320762	0.001905403	0.036131006
0.266704099	0.411594162	0.494609207	0.365569008
0.468447185	0.061506199	0.335935451	0.38837612
0.056514335	0.389182734	0.498869445	0.47635817
0.02710173	0.219776607	0.378418852	0.481338155
0.074036411	0.292636732	0.463632946	0.332509109
0.008007825	0.063437897	0.051521461	0.437919087
0.511392501	0.074781016	0.028212449	0.162541603
0.114351255	0.478993881	0.152614029	0.492258544
0.468929956	0.437669195	0.058298394	0.477504862
0.032259774	0.488812023	0.342634451	0.063786475
0.255497634	0.006850676	0.481356595	0.183115425
0.349331898	0.416964449	0.196066553	0.010138362
0.17960477	0.038964533	0.481567512	0.474377969
0.021541519	0.215616913	0.268737572	0.397403258
0.303705583	0.45362331	0.505893344	0.474719304
0.074787456	0.129807905	0.414730697	0.439334841
0.239478093	0.481937849	0.320610434	0.493607388
0.445111453	0.472417123	0.494756134	0.352881991
0.228213122	0.428784663	0.419167906	0.017124344
0.337456807	0.479998767	0.354628207	0.148512063
0.249556359	0.418158835	0.033344664	0.186226991
0.361392681	0.351359751	0.131566507	0.433056115
0.407682056	0.444976119	0.029188395	0.049119563

0.508048094	0.462460441	0.134066762	0.458949144
0.140005683	0.34229758	0.473553788	0.467876739
0.387512236	0.498656501	0.454550175	0.443509964
0.461505803	0.240316218	0.516568976	0.083106077
0.07066034	0.457307755	0.092236203	0.07059674
0.123043142	0.017598841	0.32916	0.419967649
0.416328165	0.382913272	0.346423175	0.06415852
0.301169918	0.383824951	0.337511745	0.201412321
0.069002194	0.497955512	0.006195285	0.040485391
0.420114122	0.483634524	0.414761883	0.212188744
0.310871806	0.113977715	0.283634534	0.327033544
0.395252845	0.376280214	0.486629625	0.025022714
0.013658171	0.480887263	0.397236141	0.012111208
0.071799295	0.423576012	0.267907398	0.242293888
0.013718554	0.497117218	0.396087784	0.437526178
0.347881574	0.047507543	0.412226561	0.071311685
0.041671169	0.463340793	0.408229084	0.50202017
0.334238409	0.423052056	0.458891862	0.489138026
0.098488012	0.413536772	0.01951352	0.461025393
0.465701298			
BC016495	0.064392507	0.115674039	0.050138738
0.209706211	0.079873296	0.118502575	0.248963496
0.063298417	0.075992898	0.036256499	0.024017783
0.053127264	0.022020558	0.065198799	0.065949302
0.174690859	0.038925651	0.039065279	0.199706384
0.152114768	0.141867516	0.163064491	0.138291579
0.025316315	0.061747867	0.142542124	0.075359188
0.169323481	0.159358501	0.09376848	0.147863674
0.026695467	0.087254432	0.088183719	0.091892144
0.072102377	0.05561881	0.00146532	0.117659129
0.094694733	0.132049364	0.170362614	0.209400529
0.130280636	0.080217376	0.266912093	0.073744635
0.021537331	0.059014033	0.077214	0.08677841
0.077969327	0.084985057	0.122255641	0.215475476
0.015005408	0.042669782	0.011007334	0.095461879
0.204365357	0.081817967	0.081392837	1
0.021249259	0.092698625	0.02396295	0.135928016
0.024681841	0.103996307	0.024383708	0.034196096
0.007482269	0.174490709	0.074522713	0.037408051
0.097435236	0.023693385	0.037745897	0.224143972
0.148152722	0.070341627	0.127442464	0.111718386
0.019573459	0.077515457	0.037412581	0.109214164
0.102268467	0.228836389	0.026727243	0.155817842
0.124661199	0.092135426	0.04579208	0.101696981
0.149953971	0.110766737	0.077241423	0.076333714
0.055448546	0.017789598	0.093016123	0.108037429
0.082065533	0.026995771	0.107541474	0.133812771
0.089153408	0.019934872	0.213552544	0.005134284
0.153590816	0.133271284	0.077131578	0.153606305
0.027034192	0.09788874	0.086441949	0.235401972
0.047683309	0.012361937	0.019120004	0.034853235
0.014947959	0.005243129	0.069138634	0.150877966
0.032658149	0.023344827	0.144383924	0.016576933
0.035554423	0.009889396	0.063002308	0.050035271

0.114622809	0.04879521	0.140291254	0.074247907
0.061545065	0.122846563	0.01000148	0.133973366
0.101397954	0.014790861	0.194608083	0.064753077
0.111741809	0.121626957	0.120884094	0.133686518
0.10441676	0.167427459	0.031287208	0.153923078
0.056787397	0.071228796	0.110904739	0.065883755
0.105556345	0.070579264	0.029544344	0.066132349
0.025440779	0.007163096	0.01026058	0.116988829
0.035628571	0.110910835	0.057649421	0.117214753
0.088411283	0.137722842	0.05655327	0.114273322
0.044280546	0.025564802	0.032141724	0.112115154
0.084079787	0.097475207	0.062297935	0.057157766
0.127671725	0.151307829	0.001028764	0.084642238
0.090752908	0.032594759	0.108931254	0.067377262
0.060302927	0.170035719	0.001001805	0.130230546
0.049686486	0.114309716	0.138684448	0.127245155
0.073202059	0.124725725	0.062436857	0.081368995
0.051514227	0.006490877	0.14903833	0.19530675
0.022697929	0.12896382	0.105342533	0.186483849
0.189485806	0.11314431	0.153531867	0.055819901
0.089465849	0.111512791	0.015298126	0.071811473
0.080446814	0.007498398	0.109234812	0.112653598
0.039838867	0.117157865	0.063156495	0.024671873
0.188935539	0.129637958	0.025408019	0.155333003
0.068414778	0.095318554	0.069048516	0.009074915
0.053890447	0.122170905	0.021509016	0.016647572
0.009747655	0.085831254	0.15954063	0.057735053
0.018510611	0.093231688	0.055717098	0.079609348
0.129083075	0.093319244	0.003794698	0.013162428
0.013323886	0.026628489	0.027153719	0.067763537
0.022176723	0.018338238	0.087369997	0.025524918
0.062133038	0.065735898	0.114828508	0.144787622
0.072106273	0.144972319	0.055182495	0.004976878
0.141523344	0.087530249	0.031961478	0.132946058
0.015696604	0.120605947	0.05008387	0.120011619
0.02985924	0.162642946	0.179115479	0.031074139
0.066191506	0.105468641	0.099480543	0.131680786
0.079353359	0.142057182	0.050168202	0.070826541
0.059727246	0.079633305	0.097309891	0.024884905
0.055890352	0.08138273	0.093073015	0.126533523
0.088525936	0.148013988	0.120283303	0.157398757
0.125465964	0.169631263	0.112249882	0.029473874
0.1314293	0.007055791	0.036655579	0.038984826
0.077808596	0.087567071	0.115584581	0.065289079
0.219025634	0.061791179	0.016913912	0.112256663
0.005494239	0.084636492	0.038801771	0.084389362
0.074984668	0.057844818	0.08966747	0.191136272
0.093812919	0.174324682	0.064176652	0.092056695
0.131770884	0.103558743	0.088108184	0.074351898
0.098424252	0.062041961	0.111007089	0.014909332
0.071479917	0.13145757	0.00099953	0.039803156
0.064634484	0.108057052	0.112370476	0.108475416
0.118925666	0.046417892	0.102159206	0.13132504
0.082651858	0.074847793	0.090129674	0.081625891

0.065643843	0.098524014	0.041108756	0.064412448
0.046770906	0.07920807	0.099518177	0.028306751
0.138286051	0.06504262	0.020947276	0.118180385
0.201839213	0.057612658	0.028134831	0.001279514
0.016449905	0.109319484	0.094901169	0.144836894
0.063818083	0.120931793	0.000318582	0.093209632
0.100274779	0.031878156	0.022216831	0.07094811
0.060355384	0.107086847	0.046293075	0.09330261
0.048715687	0.07941828	0.030577139	0.174279651
0.133509559	0.070924548	0.127249973	0.110069842
0.133868219	0.09919134	0.25302937	0.120949845
0.038017823	0.113720107	0.052038481	0.047001155
0.08349746	0.08291549	0.091320954	0.129407633
0.094241309	0.037097652	0.087614401	0.07618572
0.145152364	0.154700187	0.008497016	0.00801437
0.001740442	0.015036491	0.13737575	0.085497826
0.111097428	0.019447804	0.046226098	0.06440804
0.044090083	0.075634289	0.097078078	0.19429483
0.079006943	0.074891452	0.054540639	0.170474937
0.041431727	0.046376796	0.11260289	0.11159432
0.022029023	0.09272931	0.036262428	0.156207034
0.002671463	0.108739008	0.053154652	0.058702599
0.004149459	0.065576995	0.093559832	0.125220027
0.067831298	0.03083383	0.027716468	0.006245841
0.138749156	0.107767559	0.070733792	0.129212224
0.143500538	0.171148756	0.015570429	0.107435313
0.035095794	0.077538714	0.052909625	0.034153581
0.035056564	0.023456147	0.062450106	0.027081031
0.050184489	0.094818788	0.031848912	0.021893767
0.009622737	0.059103754	0.151654139	0.10958812
0.011630882	0.10533968	0.099468403	0.102448063
0.138558321	0.159975407	0.088991673	0.047674928
0.160103856	0.001479779	0.143111466	0.06176898
0.050407691	0.125041782	0.067106607	0.10378291
0.090185464	0.104185764	0.086651222	0.214404975
0.008435159	0.076425158	0.175122571	0.049263362
0.141530866	0.136044219	0.075593261	0.039369136
0.124324877	0.036293481	0.024139757	0.003292731
0.021963042	0.134961869	0.057765432	0.161851843
0.07400875	0.066914426	0.017687283	0.098695738
0.107077651	0.103427582	0.068532077	0.143050375
0.087416914	0.166800797	0.088816742	0.081451688
0.025311276	0.094139011	0.114743285	0.122641521
0.107911575	0.213476811	0.096333905	0.023607247
0.023171046	0.039194527	0.081008034	0.079329305
0.150565556	0.045105261	0.013564304	0.119314666
0.014266793	0.150383638	0.011991282	0.035139132
0.078740268	0.028489505	0.127252848	0.177002229
0.064288476	0.0962543	0.014285594	0.057451059
0.014668306	0.111259223	0.055367872	0.081423735
0.016732129	0.015131648	0.142639039	0.075742136
0.048641173	0.005655005	0.112022496	0.043862399
0.055500749	0.130353004	0.048238918	0.00780329
0.011579786	0.061687437	0.022134871	0.081194646

0.024460256	0.078648546	0.084832449	0.070848476
0.161714234	0.067882698	0.065493825	0.018783689
0.031742629	0.079797077	0.053096782	0.079652804
0.037398503	0.18317499	0.071909469	0.075616071
0.01009475	0.028559552	0.113890619	0.124386531
0.026821842			
BC057170	0.130837307	0.189406286	0.064674656
0.18951772	0.112241916	0.116371512	0.12283769
0.043151899	0.011166298	0.001183878	0.005058148
0.084184439	0.005949718	0.044739017	0.036779168
0.195387931	0.025905796	0.058186336	0.125576013
0.18367625	0.050171459	0.169211836	0.050570212
0.039670053	0.025800933	0.04957204	0.047739298
0.197572166	0.053453128	0.097532723	0.181570045
0.018643201	0.161176963	0.16111732	0.124176669
0.146800792	0.10479339	0.000336852	0.066704171
0.154534757	0.141657125	0.21130434	0.139787511
0.030096982	0.15103061	0.137933576	0.105186141
0.005473978	0.015893521	0.060509976	0.163641999
0.140832489	0.125913696	0.081632167	0.088199762
0.008965865	0.056834122	0.013242631	0.131810754
0.131441382	0.055272729	0.149672456	0.021249259
1	0.089679204	0.023747014	0.099768019
0.042214486	0.146443315	0.008973502	0.0711379
0.000233046	0.186580549	0.096840338	0.039353548
0.032821074	0.006184231	0.035045222	0.171892501
0.199564516	0.141565178	0.177242517	0.173771819
0.012497713	0.078520681	0.074688149	0.056874896
0.16243146	0.091171843	0.002646425	0.180725602
0.176419167	0.16768255	0.034294218	0.086071622
0.164715902	0.18330366	0.068815933	0.114268154
0.035499747	0.00902516	0.16493648	0.179213483
0.141969023	0.042015795	0.055687426	0.182890056
0.041267963	0.018352267	0.172278041	0.00211681
0.197520897	0.019912579	0.060759647	0.154599753
0.025016841	0.138030994	0.154925473	0.116199643
0.072889153	0.000972259	0.001923484	0.018125976
0.006837812	0.007018112	0.142478162	0.183705727
0.032420643	0.038182283	0.117171986	0.003368941
0.011208777	0.008652513	0.130574983	0.079145255
0.173136295	0.105096446	0.063583831	0.134772279
0.100159644	0.181947629	0.000287524	0.056872823
0.093375723	0.021125436	0.127749333	0.114886544
0.076309007	0.05486665	0.067191666	0.18810307
0.174338562	0.066270961	0.058725882	0.161049162
0.048732432	0.077317138	0.05979286	0.055570104
0.040582297	0.139205758	0.011234222	0.084559774
0.013147898	0.007684452	0.002526287	0.100778189
0.014810182	0.149203853	0.134125132	0.059449169
0.085851278	0.139337462	0.100427223	0.123067023
0.004073146	0.007473378	0.061514892	0.177933773
0.11210634	0.166329303	0.048124658	0.055747925
0.094320153	0.183315623	3.41E-05	0.150664607
0.163487401	0.047207696	0.105697709	0.104196018

0.067934364	0.183588054	0.000167281	0.162113084
0.100554809	0.051395179	0.194670787	0.066748761
0.143595222	0.039526996	0.089612671	0.129940584
0.098735087	0.000440843	0.086635313	0.200960188
0.009786179	0.061693119	0.17132641	0.169580376
0.139527093	0.159296627	0.19761887	0.056669722
0.097493163	0.078160892	0.003994455	0.070185801
0.146804002	0.053632538	0.164238054	0.07113378
0.07392672	0.168799442	0.113480936	0.018412836
0.050574851	0.143409792	0.000899182	0.092613502
0.130207043	0.043388603	0.082478701	0.011099144
0.023428072	0.185355388	0.021747628	0.024987702
0.010475553	0.105855931	0.11303977	0.034965387
0.013415156	0.065756352	0.052933323	0.075995024
0.037788391	0.04556317	0.001557941	0.028667136
0.008960496	0.031991286	0.034078779	0.133076312
0.033623377	0.008472409	0.069245726	0.027965286
0.020995321	0.018400287	0.181605796	0.161214956
0.12193823	0.065367411	0.022679334	0.008085812
0.191520415	0.073414663	0.046275717	0.190114678
0.001311757	0.188648437	0.030907532	0.055551456
0.04325374	0.10484127	0.205560219	0.001148921
0.129090657	0.174055485	0.099755558	0.058840282
0.134826217	0.160645622	0.009911617	0.11827189
0.11214547	0.027059153	0.166226978	0.015835159
0.01861016	0.119554324	0.13761747	0.050164442
0.028988673	0.101217718	0.035842659	0.115603352
0.182711049	0.173672163	0.164528179	0.029205916
0.036738551	0.00124951	0.039728212	0.061588266
0.153239046	0.155132986	0.100365155	0.059648916
0.142176033	0.04964475	0.016043036	0.173570799
0.008000663	0.162159167	0.01091864	0.138836604
0.148021394	0.081424227	0.030199268	0.151084442
0.137472736	0.046239047	0.056898366	0.153608363
0.192671801	0.07404735	0.050601711	0.122483787
0.016497718	0.029142329	0.104540411	0.017029797
0.078980487	0.070879472	0.001330273	0.056735986
0.016529285	0.174305557	0.169124212	0.023190047
0.10646946	0.065224917	0.127596184	0.0391514
0.137191689	0.148366125	0.161900729	0.061352897
0.129117193	0.1538296	0.0722351	0.033778181
0.013046086	0.147723429	0.142150482	0.000828592
0.19642826	0.06957241	0.003418438	0.18317471
0.110667163	0.134504622	0.000180557	0.000457385
0.009180721	0.162331325	0.155365722	0.185262653
0.038104422	0.145338807	0.000777341	0.168216939
0.161047723	0.008483799	0.022918974	0.124601444
0.100432436	0.18320726	0.044422993	0.137116663
0.076367091	0.150199419	0.023434624	0.116664646
0.161547074	0.141576628	0.152422755	0.164155699
0.18402604	0.135021369	0.159587716	0.023479599
0.007866465	0.093324704	0.025516548	0.006725787
0.115099375	0.029238569	0.148327668	0.19359307
0.148564181	0.008730126	0.039959052	0.132039857

0.188023473	0.202525813	0.001826769	8.75E-05
0.000424036	0.002496805	0.171829116	0.063811469
0.168776797	0.010768893	0.009623513	0.02052316
0.023857548	0.093908824	0.165757859	0.184041291
0.136302247	0.044918589	0.03250646	0.188355315
0.014743917	0.098031158	0.16251757	0.17876881
0.013804005	0.077165834	0.060024581	0.207534665
0.003981593	0.109604043	0.098183448	0.036974805
0.000453278	0.024471406	0.03359194	0.178744781
0.142816148	0.013307257	0.00825649	0.001282173
0.052642424	0.182360785	0.031472244	0.188286726
0.184720018	0.198842389	0.014153305	0.16875223
0.01215656	0.14430752	0.059225701	0.000486751
0.039089435	0.007515985	0.132894115	0.009611565
0.068848621	0.140580078	0.013141685	0.01077925
0.008642177	0.023754792	0.198763879	0.161514751
0.00668846	0.089208458	0.082770437	0.136763055
0.106170433	0.165208666	0.156398764	0.057054095
0.063306394	0.002421615	0.167507254	0.101739556
0.058843867	0.183750491	0.050268715	0.173769052
0.139166179	0.17895672	0.153263676	0.1436557
0.015527921	0.098554911	0.155820398	0.003088521
0.114792063	0.182957489	0.099697449	0.013771826
0.080585782	0.070589425	0.011709253	0.001403552
0.039002855	0.121021841	0.038606936	0.200643183
0.123115601	0.111220005	0.007520486	0.028103355
0.184778313	0.169450687	0.018451161	0.143889973
0.056793162	0.129602819	0.132963556	0.134951439
0.056888847	0.156057812	0.163489819	0.138198447
0.17009746	0.145526158	0.167433279	0.013006408
0.002665651	0.096631314	0.031254223	0.042888165
0.079267816	0.011677712	0.018793067	0.164340056
0.029635347	0.150195003	0.027568361	0.006249947
0.096688667	0.064009005	0.131411946	0.084669159
0.017032393	0.161224894	0.000366973	0.029993655
0.034186863	0.145891055	0.061079989	0.050675573
0.019788095	0.020150739	0.123544528	0.079437445
0.043411593	0.007335603	0.142473763	0.002919816
0.017633632	0.18907262	0.059483567	0.003558796
0.012647637	0.127616679	0.033979273	0.044613205
0.00941725	0.152008431	0.079335314	0.103325085
0.148250448	0.033304048	0.132159188	0.002727217
0.01375899	0.143781595	0.072945312	0.15106274
0.057685091	0.190031656	0.147277296	0.152442913
0.001608955	0.059252955	0.025307118	0.183337644
0.034827262			

BCAP31 0.253587224 0.304666086 0.119186116

0.321878719	0.173001482	0.212552152	0.201908843
0.211271794	0.066361348	0.018190889	0.012328705
0.1682616	0.053392068	0.126254363	0.081979726
0.324212336	0.055894354	0.157868555	0.22823903
0.349624115	0.155837757	0.262476948	0.135785333
0.065666732	0.035692858	0.062858791	0.052613448
0.380298991	0.070667095	0.160865962	0.369016429

0.082826164	0.310573048	0.268323851	0.180429801
0.239819818	0.217125661	0.046806679	0.100154993
0.327428922	0.221916898	0.356958645	0.244457943
0.082178092	0.276231875	0.211647448	0.172823174
0.131307217	0.031262349	0.139114008	0.292865391
0.220706458	0.145160308	0.196467013	0.140057897
0.032086283	0.151484675	0.024341772	0.303860844
0.193608537	0.145953836	0.233595364	0.092698625
0.089679204	1	0.072458897	0.228788779
0.055079246	0.312486702	0.109370281	0.134935799
0.012832449	0.362478677	0.191527002	0.033444598
0.059430453	0.027010227	0.083052573	0.272395839
0.364985769	0.272129927	0.281691274	0.26006312
0.063070059	0.162435449	0.100455522	0.106667716
0.299634692	0.135556619	0.100668875	0.289852669
0.279091546	0.261071008	0.080074112	0.134261134
0.240036349	0.323672009	0.121902131	0.124247656
0.044169477	0.036819858	0.247451753	0.279603746
0.299198114	0.054728803	0.081974819	0.367100552
0.091875315	0.103726695	0.285591328	0.074609567
0.366644589	0.057798602	0.089069356	0.232535816
0.16404287	0.279455348	0.243404964	0.19648571
0.150212784	0.033754594	0.018648294	0.067648253
0.109300061	0.019887187	0.231934262	0.306939814
0.186759095	0.083312301	0.204204406	0.014462886
0.034659336	0.102699563	0.232695395	0.207380461
0.31572875	0.1927264	0.070724792	0.266206829
0.144774192	0.278376334	0.023962585	0.16836207
0.181897424	0.106585312	0.219035819	0.172920682
0.103388132	0.200344099	0.134427969	0.308894399
0.324700471	0.116776965	0.158068169	0.294875294
0.081808183	0.120373926	0.150901423	0.121093167
0.115429749	0.216760977	0.028840841	0.227066919
0.032474105	0.040192692	0.080452964	0.248718794
0.102965523	0.212744141	0.222213165	0.139167811
0.148855646	0.214060307	0.221464457	0.189756426
0.012595109	0.104339202	0.173331442	0.286990605
0.245186493	0.286791092	0.151942219	0.103946878
0.157462243	0.317623639	0.003872986	0.261655167
0.253902919	0.14280103	0.162376496	0.204774354
0.067236281	0.308615923	0.021173551	0.299000327
0.248338822	0.133261408	0.318268738	0.092570791
0.260549504	0.051910905	0.157121729	0.186107284
0.153895344	0.021137301	0.183400199	0.346954754
0.029138184	0.155206111	0.311499103	0.28845467
0.182980177	0.279888085	0.338711814	0.052847051
0.155597292	0.207889423	0.04421039	0.128614
0.257563609	0.057983154	0.292367585	0.17446613
0.193997838	0.289194957	0.196513735	0.04936928
0.086294869	0.246781116	0.050062553	0.128404356
0.217958362	0.091792224	0.095756431	0.113165595
0.050956415	0.291567105	0.033984762	0.032695377
0.018474469	0.160605512	0.246205791	0.064477122
0.034485927	0.106636355	0.063299087	0.085319917

0.114670321	0.077672789	0.024092584	0.043163728
0.032436575	0.051965681	0.053067632	0.243170986
0.123850451	0.060392731	0.118211912	0.064578922
0.056424653	0.037465177	0.296635932	0.309850613
0.269793164	0.153856144	0.069221926	0.043275531
0.29837513	0.130776085	0.123198183	0.298082476
0.071573349	0.304243804	0.073497044	0.073160718
0.060342823	0.171392136	0.357703381	0.040524274
0.254968879	0.295246574	0.202314812	0.07174678
0.254233253	0.291629896	0.082895839	0.25047762
0.240091952	0.054870706	0.273632354	0.054860231
0.042729891	0.206746761	0.170158748	0.080029722
0.062228032	0.119286917	0.144323428	0.181995502
0.293315497	0.273929187	0.215654564	0.058398306
0.068325457	0.010369563	0.069988867	0.131735984
0.263624524	0.247962189	0.206532586	0.082127329
0.247185463	0.056528665	0.156993883	0.271083927
0.095318185	0.283606658	0.034067512	0.1929353
0.233207774	0.198515998	0.040662932	0.230340721
0.272675214	0.093751514	0.213539474	0.262933905
0.293190138	0.090471642	0.06666799	0.259034994
0.07574067	0.128122796	0.158020948	0.031291022
0.086359648	0.119766322	0.006826937	0.163282327
0.055267837	0.275896988	0.31461997	0.047084088
0.141130403	0.154338701	0.212557534	0.081299459
0.194520205	0.246747666	0.272778829	0.221454011
0.235022184	0.229040271	0.087092355	0.079524697
0.027512868	0.243425335	0.29818907	0.030046759
0.333667236	0.173836374	0.017108642	0.319724416
0.213076273	0.238862317	0.014003114	0.027635588
0.038944342	0.278527006	0.248658343	0.368038834
0.071237891	0.316392149	0.010472828	0.277021778
0.267743208	0.018273064	0.048893203	0.196727662
0.144324477	0.321199361	0.050449321	0.199793111
0.11774399	0.229549919	0.114839196	0.167289258
0.273546625	0.211388676	0.325977583	0.312204487
0.314046865	0.234015669	0.249489072	0.05046794
0.03559803	0.198435451	0.040469731	0.031808933
0.267490507	0.056872515	0.289105546	0.312585477
0.238616934	0.022142891	0.199228944	0.230000676
0.360069518	0.369250856	0.055969298	0.007478532
0.00583058	0.00695997	0.307450709	0.118677753
0.273719005	0.053512824	0.02682206	0.159511241
0.134182825	0.133997491	0.265953109	0.341754527
0.235180543	0.101840701	0.055122799	0.365165343
0.033058162	0.230732323	0.255469319	0.294606129
0.067437223	0.113333865	0.179840847	0.340650082
0.00582197	0.188957938	0.187529506	0.087245872
0.034687151	0.053490984	0.04919806	0.312909802
0.221196245	0.034141099	0.023593373	0.084218308
0.090193699	0.307376269	0.092203036	0.311333694
0.323106232	0.347425057	0.043209055	0.277217793
0.039104436	0.243671781	0.078363961	0.035447311
0.13181595	0.052812594	0.231296195	0.017423555

0.146264435	0.331232571	0.045215687	0.025126003
0.026451854	0.05604148	0.29956059	0.252492957
0.037030493	0.197777719	0.171603541	0.298308167
0.254436402	0.26998436	0.26900143	0.098917763
0.130522316	0.03412204	0.366200064	0.250425659
0.19199455	0.297612165	0.059330474	0.288917769
0.286241149	0.31830502	0.247878157	0.225458351
0.046592039	0.226047263	0.251965716	0.047394808
0.25114156	0.284199808	0.248222428	0.039113764
0.095777818	0.0896209	0.148551861	0.092100295
0.051609066	0.247749967	0.111990392	0.360066183
0.301455142	0.256881924	0.171571633	0.045407679
0.306708478	0.303225981	0.148840731	0.250945516
0.127495061	0.162177525	0.244851311	0.225405021
0.085901551	0.237887394	0.336950774	0.23838688
0.295390105	0.230948648	0.273297123	0.187951109
0.033133091	0.178922	0.060320631	0.074156228
0.125183146	0.039626937	0.029227041	0.295204215
0.041020778	0.236246907	0.132862653	0.082189108
0.156148328	0.1493362	0.222094293	0.145663244
0.066514736	0.25434357	0.019031443	0.072628191
0.052806062	0.226911339	0.077022009	0.103679599
0.038282183	0.067898468	0.181687543	0.205458031
0.063517208	0.077932589	0.224791776	0.101762152
0.091375126	0.320387415	0.172778126	0.035640832
0.06379224	0.233294497	0.158582066	0.081247467
0.024730299	0.214790796	0.100814056	0.15421194
0.27956834	0.115505304	0.284286739	0.100612074
0.14256318	0.284726308	0.122891412	0.242370353
0.117802646	0.300005912	0.268312104	0.244418463
0.098563117	0.115474679	0.085059259	0.351759983
0.050179532
BCL2L11	0.167366227	0.166651848	0.125149066
0.189935204	0.189247342	0.064064617	0.089100283
0.028844172	0.034761222	0.010802474	0.041107483
0.187559029	0.057509363	0.046860552	0.018824507
0.280143478	0.167235565	0.091171124	0.187726517
0.208165084	0.026802561	0.297429131	0.070077096
0.11285152	0.130492399	0.118035354	0.107345426
0.223658666	0.134566479	0.040433993	0.135535267
0.219034352	0.184518675	0.135629339	0.229361462
0.136216476	0.120465598	0.092335189	0.026432643
0.14953614	0.180015391	0.192712763	0.193646505
0.056541559	0.11082462	0.132877132	0.245689575
0.022425441	0.082675504	0.019425004	0.103448493
0.108242804	0.102961589	0.040940904	0.063735377
0.032894986	0.025116163	0.051573142	0.068448923
0.138864726	0.015120154	0.126626221	0.02396295
0.023747014	0.072458897	1	0.085033185
0.100232933	0.099371822	0.062970326	0.143738858
0.004040945	0.208671979	0.296106674	0.054168017
0.20615845	0.058969003	0.26731496	0.140798457
0.226675508	0.146795443	0.250405502	0.224062154
0.127821036	0.271970478	0.180929388	0.132905822

0.153858193	0.077014497	0.056445441	0.315941141
0.239735008	0.156704083	0.114938763	0.301474597
0.118789409	0.150540297	0.076934592	0.115348865
0.036223117	0.17780362	0.111555875	0.209501649
0.114201665	0.164558443	0.246764302	0.155306273
0.183635063	0.029140654	0.23122953	0.154812034
0.226441479	0.027342971	0.300168315	0.120248369
0.118891087	0.16294378	0.160662409	0.139222107
0.205298826	0.086541826	0.042171664	0.173839787
0.053107522	0.100527008	0.098741337	0.190964978
0.039640762	0.068522749	0.096870979	0.06452547
0.151614139	0.164119486	0.172578288	0.147466554
0.198316425	0.11575717	0.084363619	0.097686459
0.144556533	0.252880409	0.001333461	0.073779591
0.114391978	0.180508522	0.278687535	0.147106385
0.182917016	0.058782196	0.157083561	0.208085528
0.106650319	0.147048625	0.059542922	0.291960186
0.031402781	0.265814329	0.140430777	0.10176983
0.05328148	0.127551611	0.202071636	0.078685118
0.019257535	0.177127259	0.072491762	0.060413763
0.031086728	0.126473785	0.102328214	0.197060536
0.133097215	0.250688347	0.058306124	0.117040799
0.017989229	0.116797657	0.033931302	0.195403264
0.044104957	0.226142789	0.109436504	0.135262318
0.291741059	0.227746966	0.010501996	0.116834779
0.156526712	0.030402253	0.168326952	0.238805523
0.07493379	0.192546628	0.0092403	0.296421129
0.090077554	0.161295689	0.25087597	0.236410546
0.137959007	0.132804912	0.054999886	0.183099676
0.145781104	0.031022437	0.028133829	0.15427923
0.172652601	0.016468976	0.179942948	0.181326142
0.121720976	0.241614968	0.270759501	0.064371467
0.152678156	0.106038593	0.100770274	0.27938053
0.203059639	0.032389454	0.108662824	0.05265512
0.056757767	0.140094121	0.130761222	0.011819412
0.084772462	0.08476033	0.018190186	0.137195583
0.113427014	0.049588658	0.071194813	0.209375154
0.209250418	0.190570587	0.160900621	0.118113029
0.097659992	0.108465381	0.151279756	0.035691381
0.052722775	0.101014372	0.071503292	0.065790866
0.028087167	0.159724247	0.140363337	0.05606072
0.049817948	0.051715596	0.043617039	0.112456712
0.053965525	0.079772973	0.147720707	0.082020932
0.132086639	0.156523761	0.16507599	0.255142517
0.068383169	0.072982029	0.195767467	0.026061003
0.188284634	0.03004802	0.019469744	0.145675973
0.080597693	0.243588007	0.060777903	0.214948726
0.11954709	0.188899386	0.225456438	0.008067225
0.111848701	0.131994242	0.262385331	0.131233657
0.09558663	0.168420791	0.051454297	0.11567173
0.113296325	0.151801121	0.183460209	0.197294207
0.191307886	0.247148305	0.142143958	0.240089685
0.182025069	0.081684219	0.017906496	0.315602293
0.180253806	0.293411219	0.124101166	0.165754045

0.07849577	0.047013062	0.268258712	0.159315899
0.125463202	0.19084053	0.30515467	0.146024138
0.227687266	0.052671073	0.012893266	0.19400103
0.118648107	0.15604191	0.094323253	0.201241792
0.124626729	0.042000974	0.145498162	0.073634892
0.100239736	0.07476393	0.117169309	0.136252626
0.142745325	0.0771644	0.049124469	0.241762768
0.023255075	0.043807133	0.168960119	0.099395034
0.053907583	0.110672069	0.072716367	0.034567507
0.066772195	0.123130353	0.274330033	0.056238093
0.044814104	0.031264066	0.334624516	0.118050888
0.181062881	0.167349872	0.138985637	0.094307681
0.193841154	0.159445615	0.126769688	0.246325904
0.183648546	0.146919673	0.117992481	0.032152285
0.247689606	0.022226676	0.034715539	0.153970675
0.161491813	0.09476698	0.001366994	0.049379744
0.184419084	0.281696902	0.111000561	0.140725106
0.011566904	0.221323319	0.071146277	0.204415999
0.138408192	0.109744717	0.164804077	0.149943288
0.158067575	0.162714301	0.109934868	0.181017614
0.150111238	0.130921871	0.043168772	0.263176054
0.147891139	0.15442693	0.154062537	0.131319769
0.219345487	0.292847331	0.194193721	0.036274524
0.038665008	0.034257906	0.014453776	0.031256356
0.058961902	0.096037285	0.171375399	0.256727683
0.199696651	0.110339456	0.034642478	0.142025316
0.160789875	0.210909794	0.100129949	0.009522788
0.010778001	0.053359382	0.12541235	0.257692794
0.174913844	0.009019788	0.064126384	0.028699656
0.057960581	0.228468994	0.148567293	0.274818427
0.214655872	0.195960621	0.018935878	0.256577411
0.095957923	0.067439921	0.198542479	0.122172376
0.190560292	0.212661402	0.111007983	0.21178071
0.048987926	0.32563528	0.202415464	0.044251565
0.124709923	0.165344397	0.099378937	0.283640266
0.126935828	0.079632345	0.085145315	0.009042116
0.060557015	0.226880919	0.085412945	0.230405591
0.171540307	0.275960928	0.207315453	0.124909916
0.059353431	0.126165819	0.196575041	0.004829513
0.172659665	0.117062428	0.161428119	0.018147992
0.047622452	0.144833557	0.036669165	0.155725344
0.062645695	0.186143654	0.229754223	0.253318765
0.196288872	0.071728749	0.259669521	0.234199785
0.059964306	0.205259082	0.12441381	0.052098299
0.042649607	0.14837299	0.188664902	0.111771698
0.042574312	0.262109193	0.04619435	0.196834212
0.128612665	0.167154834	0.168437436	0.116549607
0.135430411	0.089838028	0.093544681	0.021334086
0.190574951	0.228506618	0.110633136	0.075280982
0.175566033	0.148138207	0.094694565	0.035423661
0.124506692	0.052150673	0.084093531	0.285276448
0.138552467	0.112604408	0.07898115	0.083646299
0.176295287	0.124299755	0.024081956	0.137525077
0.267905332	0.16960267	0.222432138	0.251978529

0.042110491	0.162463486	0.170071768	0.100206803
0.107528017	0.147540424	0.141685153	0.013526947
0.062037029	0.147640528	0.064190438	0.288997952
0.181460437	0.116096419	0.050931912	0.297907081
0.068383564	0.241391411	0.030027996	0.100915723
0.301707608	0.086774192	0.258928731	0.056242705
0.070881491	0.125023864	0.052489499	0.216449729
0.101476971	0.150069001	0.134161023	0.175864387
0.06783085	0.210279422	0.234533766	0.042878024
0.032143371	0.047042241	0.140154197	0.032983031
0.050079627	0.258896314	0.069554184	0.160338902
0.141568973	0.094647646	0.108504603	0.041531473
0.174367073	0.162214063	0.029689451	0.173366041
0.114960576	0.161101614	0.083587309	0.042888542
0.093172006	0.12046554	0.167981807	0.151544075
0.194476793	0.263544773	0.0953167	0.157802685
0.090105878	0.108124713	0.043553994	0.207418795
0.046272802			

BEX2 0.227382586 0.318016199 0.110762723

0.349222666	0.177540719	0.216413363	0.223114125
0.225358406	0.090044149	0.042823956	0.025925605
0.168287547	0.089604625	0.185566644	0.103139103
0.323440522	0.085828981	0.140606105	0.240507663
0.337482041	0.211028262	0.285614574	0.177204002
0.072234647	0.055611035	0.095446821	0.071343478
0.383680534	0.104691835	0.167735579	0.356123503
0.090263883	0.29456058	0.275903058	0.205467701
0.250143721	0.195574045	0.039023201	0.1385472
0.303947957	0.236561579	0.348099868	0.273548831
0.127004586	0.280175115	0.244312314	0.166804262
0.160043056	0.054616754	0.158183462	0.2839358
0.240850077	0.160077469	0.21670339	0.193081223
0.05939036	0.172539867	0.038583271	0.284303246
0.21544666	0.175252222	0.25014387	0.135928016
0.099768019	0.228788779	0.085033185	1
0.063950792	0.292043019	0.109844343	0.12780542
0.027850032	0.34223814	0.180907973	0.043636772
0.081996617	0.06262644	0.098355942	0.300483751
0.365670267	0.258606295	0.302389571	0.276443233
0.102537327	0.150130189	0.099031632	0.159694406
0.286030609	0.184598852	0.125060963	0.300977223
0.299623212	0.27703326	0.100697033	0.133191288
0.27059792	0.322394103	0.134688009	0.137709824
0.064190813	0.039616841	0.258598375	0.298294707
0.287405745	0.057069582	0.101821561	0.362695506
0.111232013	0.120943712	0.312144599	0.096320802
0.370039842	0.081092704	0.095742978	0.253367107
0.158412012	0.278716941	0.257986936	0.215885355
0.140460555	0.058630761	0.028069829	0.071423262
0.131002211	0.024503403	0.23912059	0.336745469
0.214305394	0.106088058	0.212249569	0.017511553
0.049950838	0.09963059	0.236297135	0.173784418
0.297702766	0.180795349	0.11111902	0.249944167
0.154354676	0.295716219	0.032613493	0.198159902

0.189432283	0.101831043	0.233962397	0.179531253
0.121331636	0.22985859	0.17787519	0.324503691
0.315528648	0.161407581	0.141582034	0.302327073
0.117634091	0.131033763	0.183364392	0.174421509
0.164867379	0.224452286	0.037761184	0.191669674
0.045113301	0.058574719	0.064804195	0.229525276
0.150659476	0.244318553	0.222035758	0.162421649
0.174685087	0.230500169	0.192970198	0.224892975
0.031126147	0.115654438	0.136227279	0.309160303
0.23912986	0.290232303	0.182262425	0.116103537
0.168712277	0.323187143	0.004273399	0.269499048
0.268060146	0.122089151	0.197907134	0.190741472
0.071803286	0.337234442	0.018250656	0.306486868
0.222814989	0.151685239	0.333218961	0.114155286
0.259391628	0.080042799	0.180138522	0.197205818
0.168459189	0.023158393	0.189866962	0.3443249
0.034319398	0.196454782	0.286346565	0.306646522
0.205697693	0.275767217	0.352350005	0.058782514
0.167771127	0.229355616	0.047613453	0.127826982
0.245856869	0.070109121	0.300827672	0.208375298
0.170799076	0.302747459	0.197421076	0.069906743
0.124084654	0.233228091	0.077839312	0.142228341
0.203488799	0.158244216	0.095908698	0.105780652
0.070410292	0.312175178	0.041081466	0.039075377
0.022235027	0.19324578	0.232294528	0.118875139
0.069112147	0.132714907	0.078743795	0.109027137
0.162473279	0.121017442	0.019851566	0.062816892
0.073893985	0.085134383	0.085483968	0.234330067
0.154787354	0.118478209	0.139907757	0.071383435
0.075214715	0.050888998	0.30926421	0.300417779
0.255711338	0.185685329	0.083040747	0.064622902
0.326807109	0.146601366	0.115155365	0.320500415
0.076314793	0.308767648	0.128683657	0.100512067
0.078946799	0.205314225	0.366772691	0.046799136
0.219691422	0.308198432	0.182873297	0.106256767
0.266835796	0.288972839	0.114744905	0.242050691
0.237209465	0.081478377	0.289162123	0.074590491
0.053703218	0.212528565	0.192485197	0.095326679
0.081700157	0.153835066	0.187196336	0.197658652
0.315311104	0.299829077	0.240187476	0.06321469
0.111053072	0.028745453	0.079380349	0.120592038
0.27219928	0.263853329	0.201733755	0.104053466
0.258272081	0.075787247	0.157856581	0.293891343
0.078845677	0.2844385	0.039818337	0.201521047
0.24563818	0.181896139	0.065182331	0.242270755
0.28333172	0.125580161	0.2088977	0.280495655
0.294744849	0.112153685	0.091728787	0.241889287
0.112767459	0.16512032	0.15868506	0.03586926
0.11365363	0.145368304	0.007511097	0.161499898
0.104197452	0.29307721	0.315212506	0.070419873
0.166923488	0.152074144	0.21398483	0.10798675
0.209286085	0.251090162	0.284954829	0.230683039
0.232143397	0.252125776	0.098729807	0.090213076
0.036133412	0.255224462	0.274315102	0.069731348

0.342558508	0.162595545	0.044817936	0.329372686
0.215728255	0.236252112	0.024195153	0.021938164
0.039301091	0.281666547	0.260818065	0.342111344
0.104880354	0.28329292	0.006609266	0.285035154
0.289069412	0.025761433	0.058126306	0.212094914
0.161406194	0.327146313	0.055545523	0.221434443
0.133133305	0.246613034	0.164604638	0.183831937
0.296734415	0.223479622	0.318339157	0.310605652
0.335773728	0.235765946	0.271659113	0.078037653
0.041923069	0.205978545	0.06546997	0.058191515
0.238951806	0.087001954	0.291096816	0.321036321
0.259903931	0.027137385	0.242881533	0.234256504
0.366893202	0.372289823	0.04429718	0.007393163
0.006191823	0.014012495	0.288748583	0.114473358
0.285360522	0.090449311	0.037816362	0.195678816
0.178418693	0.145526338	0.283826659	0.344883555
0.230257625	0.121318494	0.09439394	0.3524721
0.047651583	0.197195676	0.280860931	0.305643425
0.066507756	0.114205175	0.151170815	0.358842093
0.007322343	0.193129468	0.187627558	0.128944095
0.029325054	0.069646339	0.064818667	0.303838761
0.233401931	0.072834683	0.052145559	0.091912115
0.152871132	0.305441157	0.153427627	0.327150377
0.33931026	0.363548889	0.049976568	0.28554721
0.056235494	0.258944584	0.088944882	0.057387465
0.134474797	0.055970659	0.235286116	0.032283766
0.166898208	0.293898219	0.05142063	0.028641094
0.028341926	0.076602944	0.323925985	0.262926658
0.036742134	0.210422496	0.16551876	0.272441882
0.252776911	0.30147576	0.279953746	0.113212533
0.179218404	0.03150487	0.349858357	0.233489395
0.212801183	0.312110149	0.088890272	0.297994316
0.264082735	0.305036358	0.258166626	0.237053127
0.060095443	0.210299261	0.284356087	0.095991868
0.247651158	0.312554216	0.207323239	0.064631953
0.121159329	0.09135839	0.128652911	0.081840166
0.053752387	0.254285094	0.169084036	0.357690985
0.261744788	0.240663389	0.158483699	0.063516647
0.317168203	0.2901383	0.182594011	0.270062851
0.137315127	0.177990841	0.247360707	0.22799117
0.089565353	0.26229393	0.328046894	0.271104084
0.306596163	0.231886891	0.286622147	0.189975431
0.039932673	0.164776766	0.111535113	0.086285459
0.163556117	0.05073122	0.037462961	0.289566663
0.050925615	0.267533485	0.131877198	0.113213067
0.151049994	0.117677562	0.224954	0.205519908
0.118669332	0.273592722	0.029427235	0.090546364
0.061132093	0.23624107	0.091608974	0.129213789
0.048740967	0.070052107	0.202126969	0.193410245
0.090741335	0.085228256	0.239950474	0.126682341
0.122244496	0.332853008	0.157058426	0.034128613
0.059688412	0.201107265	0.152237847	0.118163297
0.028881026	0.231981749	0.111320386	0.155434456
0.275930175	0.131442659	0.261312203	0.0906677

0.136925097	0.283870626	0.118850709	0.255576307
0.102598057	0.327877225	0.260254335	0.252792832
0.094229176	0.099973272	0.117238305	0.338704035
0.058843785			
BOLA2	0.306731805	0.395880609	0.214376053
0.274577085	0.238564193	0.139029904	0.063799771
0.027012534	0.038452904	0.00083463	0.020620519
0.2713633	0.055482702	0.041195604	0.065102221
0.350371472	0.068878404	0.101948374	0.1068653
0.25839718	0.01375925	0.3133053	0.029190397
0.159623985	0.069537524	0.039020626	0.141273797
0.322317751	0.043971349	0.081439385	0.196204354
0.074142268	0.309932883	0.3820008	0.31575153
0.384421558	0.26849541	0.008062424	0.050594091
0.288532206	0.344681484	0.256076743	0.163629774
0.015298719	0.31422573	0.100776675	0.298983674
0.037414912	0.020961615	0.030921004	0.287120598
0.325601199	0.232697598	0.039127819	0.039925248
0.056809255	0.028422933	0.109253878	0.121087913
0.086338802	0.016087936	0.358979511	0.024681841
0.042214486	0.055079246	0.100232933	0.063950792
1	0.20532744	0.007424143	0.270597714
0.000161065	0.23740645	0.2789099	0.149977134
0.033786991	0.007795347	0.102264815	0.154750614
0.352883782	0.354756953	0.431313675	0.446834943
0.063490312	0.176423599	0.276140317	0.056885947
0.3247834	0.047887959	0.027929442	0.374156219
0.436908214	0.402569472	0.098453777	0.213660697
0.275708041	0.340466486	0.144998617	0.175688947
0.101087953	0.071048792	0.310852754	0.427240502
0.244718	0.223289258	0.101604544	0.241233707
0.037722091	0.059337319	0.213057694	0.055191912
0.362432384	0.00858286	0.165151983	0.141502165
0.080313365	0.296360823	0.418872343	0.073204816
0.220389508	0.010833303	0.086947157	0.035050945
0.065878579	0.124105017	0.325824225	0.33040479
0.037043034	0.122715961	0.077299725	0.005289702
0.026719053	0.11056342	0.322384366	0.139644953
0.307691535	0.314434356	0.084390898	0.219287037
0.30619401	0.437037569	0.004638392	0.030732218
0.173657723	0.112879075	0.151400968	0.234764337
0.169789657	0.025961868	0.045319829	0.426761089
0.263876016	0.035273229	0.074067438	0.324638517
0.072284308	0.260147721	0.038641779	0.060752786
0.013972366	0.381274786	0.037175543	0.090070706
0.095299577	0.087437185	0.008547875	0.058241157
0.044724737	0.306902213	0.334769121	0.05092715
0.09517826	0.300122732	0.083931405	0.235896737
0.00613551	0.024346923	0.05748532	0.397392777
0.089035082	0.439004131	0.080330235	0.129185465
0.196799736	0.358959364	0.003807393	0.339779257
0.415992652	0.055406305	0.195852953	0.286632893
0.2007113	0.327493955	0.008791376	0.308452088
0.171413852	0.038131391	0.438968296	0.098494867

0.338806962	0.027617049	0.063089371	0.411248124
0.311622044	0.005626788	0.039423899	0.215036945
0.04389267	0.014390784	0.291870084	0.243340781
0.203920646	0.381346515	0.414228692	0.178820039
0.205197706	0.05907339	0.01412202	0.232292973
0.361475312	0.107937757	0.283711518	0.051698364
0.112200092	0.337013817	0.312086562	0.054264469
0.018495609	0.093509152	0.003410959	0.174681457
0.316792605	0.026290948	0.199536048	0.083942102
0.053333856	0.421975465	0.138079153	0.170648892
0.1238327	0.160817118	0.094593264	0.039262819
0.055143967	0.077637242	0.138217535	0.123871759
0.007487839	0.040178123	0.033931572	0.089517954
0.030916803	0.065665327	0.062131633	0.22654787
0.062675496	0.026547347	0.161831229	0.149967054
0.072661102	0.016112368	0.384641608	0.283686991
0.151865149	0.049490175	0.033005768	0.060038509
0.34932446	0.081004846	0.040083374	0.314254016
0.015403498	0.438403275	0.060274639	0.07775117
0.139634981	0.111813878	0.365739325	0.016129722
0.170952576	0.316254191	0.198918371	0.089178012
0.230348058	0.234777642	0.010283811	0.246534144
0.224387003	0.020554951	0.419527606	0.079685281
0.023547254	0.360187504	0.323961762	0.063318471
0.054458934	0.110275168	0.007155714	0.227034666
0.40234512	0.331855558	0.288886729	0.144692701
0.016543264	0.004341357	0.148578835	0.238875358
0.353167393	0.431637216	0.15727175	0.170706624
0.131631447	0.123456142	0.018017423	0.413795443
0.101863928	0.392612207	0.0085491	0.40742175
0.375643306	0.062121252	0.052203106	0.099268768
0.217924525	0.021246139	0.060549397	0.351783227
0.33050506	0.125021374	0.103225576	0.293175189
0.003783991	0.012158972	0.263957574	0.146561501
0.103489612	0.089380711	0.079001767	0.061468796
0.016572045	0.306215608	0.380490159	0.039415316
0.111154684	0.0535548	0.312048454	0.021071144
0.423736994	0.416914725	0.373509038	0.044397969
0.334311318	0.397081491	0.27814013	0.061655232
0.024518059	0.400191922	0.215554126	0.007175303
0.391368755	0.040319246	0.004200999	0.354017737
0.077295278	0.288719315	0.000431601	0.062551707
0.058711941	0.430094366	0.336983196	0.199524194
0.028006665	0.212369688	0.025572123	0.44681271
0.342614477	0.021416386	0.116807006	0.32566359
0.343572093	0.385039414	0.194477668	0.392893921
0.265031997	0.376321002	0.045649396	0.189943945
0.297086641	0.420383876	0.25601851	0.316828998
0.348025629	0.354149485	0.151635922	0.007345011
0.101481161	0.035779299	0.05438171	0.042829575
0.11338293	0.071508908	0.365637846	0.409721076
0.407240611	0.008542728	0.025847147	0.306572245
0.290808816	0.365672183	0.025338883	0.00463793
0.005812075	0.021953229	0.154639475	0.164466712

0.400729983	0.024613204	0.00466031	0.007596012
0.062270058	0.333824385	0.374893054	0.27118111
0.386643583	0.050015241	0.043752703	0.281695244
0.059086864	0.153689505	0.402975706	0.319053398
0.046481295	0.160309727	0.123560465	0.38381024
0.092404353	0.227237991	0.285370874	0.07058788
0.021253602	0.063918424	0.056039979	0.384798918
0.376940433	0.028365242	0.017560072	0.016655673
0.029047402	0.401475484	0.04559125	0.436468807
0.299226939	0.352870099	0.085964243	0.346724169
0.030588384	0.342037214	0.224435944	0.006785516
0.104164318	0.014009064	0.380838742	0.069264125
0.056738837	0.215504208	0.117947812	0.032944817
0.135308466	0.038021463	0.402989944	0.433824287
0.052005861	0.064772153	0.181038533	0.26389127
0.072937145	0.298930969	0.350222144	0.131655526
0.022478909	0.118762653	0.242443198	0.195209783
0.038680152	0.443606039	0.100267677	0.435605567
0.246785893	0.363291968	0.381545922	0.159577568
0.139987934	0.157976037	0.168161653	0.003397583
0.171419202	0.367576942	0.129325982	0.072365964
0.13080755	0.275803771	0.028493128	0.032747908
0.228025403	0.092938113	0.04297182	0.374507322
0.192618931	0.209414524	0.014333966	0.048182515
0.415885949	0.314491852	0.024991624	0.238610469
0.102129857	0.212057361	0.373119111	0.407108688
0.116487305	0.329288412	0.271268599	0.177527775
0.26322429	0.116571722	0.385679224	0.009126632
0.064015042	0.249061128	0.030666072	0.083098908
0.061379496	0.029035592	0.132699342	0.343713798
0.131593254	0.235230651	0.055816888	0.04261941
0.236719862	0.134049763	0.226679442	0.039122917
0.025422394	0.352963874	0.009725296	0.042183249
0.146071446	0.337109376	0.224392969	0.10077792
0.147958121	0.122262945	0.162645172	0.063322529
0.057713286	0.095778418	0.32300371	0.009802367
0.005966556	0.425880087	0.10622676	0.034010148
0.102422245	0.198021907	0.087369209	0.079482357
0.038672042	0.408081732	0.074003742	0.258315858
0.133210655	0.037633511	0.206670091	0.029196995
0.030973625	0.294331078	0.272207325	0.401476548
0.201472013	0.324128064	0.288192074	0.309740683
0.049943733	0.209842845	0.010126721	0.337739866
0.117867666			

BSCL2 0.326664391 0.468292546 0.285595749

0.330145173	0.296653231	0.343870333	0.221361955
0.279988408	0.084571298	0.006700536	0.01245138
0.296801692	0.130216609	0.254600679	0.296209872
0.314054216	0.081701926	0.345669895	0.223048007
0.344700194	0.140107296	0.280168743	0.107613224
0.181453122	0.059668676	0.054706036	0.161193822
0.380836547	0.064745672	0.314720174	0.362306261
0.079019303	0.359464442	0.475597945	0.274107724
0.453290237	0.318230568	0.015628738	0.26913028

0.399847797	0.30789564	0.345056058	0.269320353
0.048717791	0.478317559	0.22432926	0.272702604
0.27959197	0.021523365	0.315568836	0.456003378
0.429389145	0.297803499	0.298522079	0.169713893
0.113856758	0.285356736	0.131111991	0.347862824
0.207282396	0.246276165	0.429269697	0.103996307
0.146443315	0.312486702	0.099371822	0.292043019
0.20532744	1	0.035689466	0.271998945
0.0050595	0.341346475	0.266129992	0.176982312
0.035073264	0.015255466	0.120506954	0.296369898
0.39425449	0.427979666	0.373396602	0.380777877
0.115241567	0.226779098	0.238976928	0.138515777
0.389641266	0.172176687	0.1251244	0.294344821
0.38423186	0.451576345	0.137530782	0.180915841
0.343200975	0.452530091	0.321531162	0.293532768
0.216656575	0.034897736	0.410161578	0.416927121
0.391760835	0.172509429	0.103792387	0.366985563
0.055768805	0.33919186	0.284227479	0.119262021
0.407493231	0.027624597	0.133666435	0.306489287
0.218328278	0.355669758	0.434710515	0.186477303
0.284653387	0.018387867	0.070960504	0.031699398
0.267600877	0.068582441	0.45830567	0.381075991
0.273928935	0.245889396	0.276526938	0.002138168
0.018697466	0.153295165	0.335154112	0.336391919
0.345696868	0.337824362	0.160987641	0.34253368
0.28099775	0.365432236	0.081641399	0.125544452
0.333690182	0.147775153	0.197082533	0.309682703
0.199738816	0.167238625	0.121722838	0.437088775
0.448467114	0.086547628	0.357382701	0.30529018
0.247482592	0.216135896	0.115639298	0.222771992
0.065305831	0.417295625	0.017694749	0.366634571
0.172305167	0.076813013	0.022431366	0.321901757
0.266156511	0.348569484	0.440493195	0.109134088
0.255586243	0.279150727	0.354589503	0.303549848
0.008410055	0.057458993	0.338503445	0.423678784
0.320062411	0.410391704	0.268324606	0.228881714
0.200382973	0.351976617	0.003738022	0.469514175
0.450600009	0.336973568	0.270760992	0.299779727
0.234191612	0.368549814	0.049129792	0.308075992
0.334396774	0.08863101	0.40713934	0.110321252
0.425031548	0.033476254	0.319118187	0.305388323
0.27416173	0.00988931	0.289697436	0.344095193
0.017603641	0.157851546	0.350914644	0.328624084
0.28990524	0.323323413	0.387339378	0.215897731
0.295506226	0.26609279	0.014481507	0.212053037
0.327925341	0.250879424	0.445173433	0.255899275
0.336448948	0.438862334	0.325924304	0.170238632
0.049650193	0.328036136	0.032686508	0.240866413
0.351272231	0.107043515	0.274149835	0.127929452
0.047247069	0.443928285	0.093147775	0.138798506
0.060299166	0.292040423	0.264096025	0.17521229
0.117139465	0.133974418	0.227795726	0.274985101
0.063191758	0.071846827	0.012020425	0.221633299
0.079449018	0.217664579	0.228961623	0.333091354

0.272242636	0.080809371	0.257959131	0.192989598
0.078875219	0.013166174	0.452143039	0.322811516
0.333242332	0.166220035	0.036611953	0.217043881
0.388365662	0.302488397	0.339143458	0.411253651
0.039403417	0.389077263	0.17123571	0.084194367
0.215540514	0.20739193	0.391376628	0.07047453
0.358729838	0.443678529	0.254940977	0.12883194
0.392125904	0.343782041	0.040494563	0.344985048
0.331570534	0.027138094	0.443824496	0.076932505
0.017562558	0.291519858	0.285265326	0.060377209
0.059328362	0.249354232	0.093783367	0.204961659
0.44634645	0.287695824	0.319205712	0.13799437
0.040036944	0.005280261	0.131503589	0.28064927
0.475424486	0.413492358	0.214457785	0.217047247
0.216651921	0.211680808	0.232828551	0.417759351
0.174115802	0.469526381	0.007857012	0.286093756
0.45281212	0.328428928	0.050493737	0.306623994
0.376547713	0.05558048	0.238452412	0.451652675
0.393608275	0.248014875	0.250484809	0.322820014
0.030944155	0.066656021	0.276418569	0.098615696
0.28264605	0.181756021	0.021291284	0.352474652
0.03872489	0.423584448	0.347936657	0.049153308
0.285925562	0.360117033	0.259637781	0.040825784
0.333492028	0.426621696	0.476046337	0.209041006
0.313719221	0.398106502	0.231219285	0.064906902
0.012262218	0.448869996	0.364148451	0.023400671
0.383107911	0.311175422	0.008945391	0.466349558
0.193945154	0.44173227	0.011195997	0.06919938
0.028952733	0.336271408	0.44904709	0.36445539
0.264765629	0.331450576	0.004958295	0.43390636
0.443081358	0.008973723	0.083422166	0.311991282
0.263297875	0.49077147	0.181152004	0.327318468
0.259475993	0.435411839	0.213252959	0.1963735
0.369891685	0.401167439	0.361232775	0.452359485
0.373175972	0.290089794	0.241951285	0.020186104
0.094669461	0.292749926	0.226295956	0.057914011
0.35686696	0.094727353	0.430754947	0.368085988
0.375567791	0.004790991	0.212406232	0.336107254
0.41784633	0.422894733	0.026616016	0.007667306
0.009193124	0.008562766	0.349030429	0.177999536
0.416236235	0.224826627	0.005626639	0.088306448
0.265748583	0.241356056	0.444904716	0.308525044
0.313777259	0.072613562	0.212749815	0.333202306
0.042295123	0.324009576	0.386861468	0.458041533
0.037102933	0.185651622	0.345594721	0.420357698
0.027819535	0.226268625	0.300670542	0.232248945
0.014784138	0.05637197	0.048413886	0.320046885
0.436039273	0.046015532	0.019554238	0.182826666
0.107112647	0.381177445	0.132999497	0.424047192
0.380320966	0.339863016	0.044623193	0.456944555
0.030013135	0.440399763	0.194207545	0.079059002
0.243869088	0.015150409	0.397089449	0.084471456
0.331982606	0.367028655	0.141182379	0.010903707
0.10401187	0.039339955	0.378605369	0.334100374

0.0238079	0.265146243	0.234584319	0.33595232
0.332210126	0.314274989	0.472378473	0.301593182
0.104397055	0.080830874	0.358527953	0.347424985
0.285424145	0.383670535	0.192783398	0.447198587
0.356320372	0.429304302	0.392974897	0.306772693
0.124446978	0.363483288	0.321442956	0.028132572
0.310547478	0.348070109	0.362004828	0.093544441
0.169027417	0.232970263	0.071851725	0.220141958
0.181748626	0.333767397	0.122585752	0.34675611
0.363989635	0.348552609	0.072461503	0.045688781
0.481096088	0.433416197	0.201426385	0.337077111
0.144333414	0.249418727	0.322074196	0.296071004
0.286933894	0.351079783	0.361426276	0.321354412
0.428553908	0.250494623	0.475090399	0.190797616
0.055660186	0.305068666	0.073033224	0.068006376
0.123853236	0.022403594	0.159988326	0.311423679
0.214855111	0.277716812	0.326240827	0.078358838
0.225110161	0.330339172	0.270847034	0.217933011
0.066263793	0.45261139	0.008986008	0.046167329
0.221502626	0.329860097	0.219246974	0.183747999
0.16323464	0.093741013	0.225873248	0.339747879
0.242472381	0.270283616	0.339056449	0.061990869
0.032211289	0.39742473	0.362107162	0.017790138
0.067079544	0.343993575	0.279155253	0.17947824
0.013873609	0.384017907	0.294724014	0.29464998
0.341418465	0.069628037	0.393641598	0.11064513
0.086690026	0.444420557	0.263218381	0.439466134
0.251916518	0.304291507	0.458892826	0.343407105
0.137598313	0.276793244	0.040801431	0.38226196
0.257034072			
BSDC1	0.026538455	0.021699732	0.016861428
0.065010803	0.033279637	0.025989163	0.130202294
0.077679999	0.020597515	0.019340723	0.006088737
0.020815194	0.006190556	0.03640558	0.008334065
0.078411928	0.052008102	0.040738139	0.17847805
0.07990703	0.102085755	0.073956366	0.142845768
0.014293359	0.034060633	0.09987114	0.015895488
0.0688466	0.115928889	0.02078261	0.071109204
0.095164779	0.037128802	0.013890906	0.026886255
0.010660952	0.017719172	0.049562946	0.017527532
0.034950875	0.026983065	0.080159853	0.107101953
0.107066936	0.014141608	0.125409539	0.037956274
0.009988638	0.050986469	0.034009088	0.01798816
0.009286843	0.013919564	0.088232586	0.104647388
0.001932796	0.03158994	0.001384267	0.043365084
0.137082287	0.045938199	0.010991439	0.024383708
0.008973502	0.109370281	0.062970326	0.109844343
0.007424143	0.035689466	1	0.01059292
0.009492988	0.106851517	0.057385586	0.002952979
0.197153048	0.025505197	0.082987025	0.087518486
0.054200376	0.017554442	0.031964612	0.02317957
0.021346287	0.093141026	0.013563962	0.091003885
0.029613354	0.095404423	0.030408691	0.068312801
0.028850624	0.01449136	0.034094598	0.082238147

0.022190764	0.025941749	0.015031483	0.021851078
0.003962733	0.044576398	0.014570425	0.023055917
0.026248008	0.009265859	0.098630143	0.057086247
0.215795333	0.008524877	0.110931268	0.013947465
0.054471645	0.088831414	0.072954933	0.06378664
0.054680973	0.030683777	0.012891916	0.175113787
0.032776707	0.036556343	0.001366521	0.183057489
0.008188162	0.003822334	0.008868681	0.038266039
0.042104665	0.008149738	0.116792727	0.110376471
0.095095221	0.020971735	0.018297038	0.056293877
0.048768666	0.010482992	0.03067373	0.022819565
0.010882584	0.031597887	0.00128917	0.175396521
0.034156287	0.039200418	0.176659208	0.020735604
0.041962874	0.176676204	0.181577978	0.028477215
0.025842592	0.218490163	0.029862626	0.076142523
0.008270182	0.034179487	0.21535122	0.053396416
0.127676946	0.009533548	0.097224221	0.064230999
0.001303528	0.011045861	0.126215996	0.090286001
0.010895662	0.014242304	0.008252855	0.245424358
0.063224267	0.049889309	0.041545378	0.018124818
0.008255845	0.081976663	0.025072752	0.023522898
0.0296294	0.02519681	0.067679489	0.039287924
0.093911557	0.049786967	0.003675843	0.013091098
0.013688404	0.018999103	0.035034145	0.039439999
0.006479178	0.043041139	0.002136628	0.076409764
0.023701016	0.253818814	0.037017685	0.105307281
0.015705024	0.129470313	0.041198919	0.013371637
0.009146541	0.02369377	0.050804984	0.081010544
0.074944128	0.067323071	0.045416292	0.06428372
0.038692042	0.03963746	0.049971665	0.004816048
0.028864687	0.14650188	0.077734196	0.051842277
0.028256551	0.001334153	0.019964281	0.073566892
0.018825278	0.021625702	0.011993413	0.001501291
0.178866815	0.077813273	0.033381774	0.040840228
0.013874338	0.049563581	0.009250999	0.048953688
0.099161793	0.022412823	0.014545865	0.00555886
0.005407614	0.021759286	0.16895897	0.013817912
0.004697205	0.07172465	0.01053922	0.015903717
0.125439078	0.119401665	0.033540221	0.002955757
0.005315615	0.006438396	0.005799408	0.021202221
0.019601572	0.017404122	0.029406285	0.010702314
0.05140214	0.199306164	0.021527655	0.085236864
0.024304272	0.113156719	0.201471414	0.000820327
0.032640523	0.012532569	0.017048188	0.0266756
0.07900934	0.033108851	0.015608809	0.10356969
0.009618312	0.109933067	0.055833718	0.005897367
0.039972736	0.020790924	0.103278913	0.051992283
0.016646825	0.063421968	0.061673792	0.020371692
0.019547741	0.174528852	0.018030209	0.046412476
0.198609809	0.028189445	0.01318376	0.181034686
0.107401556	0.028526791	0.101799595	0.101864268
0.022853824	0.069622992	0.017739683	0.024553567
0.127665725	0.011217871	0.053463795	0.01749531
0.012502126	0.015760572	0.160688317	0.016583601

0.190065574	0.008156691	0.013898277	0.021014788
0.015665978	0.016717753	0.215923345	0.016888377
0.009791064	0.036239286	0.057309895	0.066593765
0.022716949	0.169584202	0.145391408	0.014819973
0.027846822	0.025087263	0.01097502	0.045817309
0.106036008	0.088062235	0.031040622	0.007555684
0.012212311	0.07078584	0.001393944	0.029171032
0.049139954	0.019258968	0.048025232	0.038764815
0.013674164	0.030634242	0.06693537	0.21083894
0.01283805	0.013906405	0.01447405	0.171568248
0.022090662	0.013633064	0.005436607	0.168588417
0.135882125	0.011800005	0.039075304	0.013831501
0.045540763	0.030726	0.014140086	0.025002015
0.20879885	0.010174297	0.009961076	0.003893899
0.066683938	0.037611921	0.012456354	0.076811338
0.00761933	0.106874789	0.011242492	0.019662
0.016520331	0.069034159	0.025519592	0.013310243
0.009265909	0.022238849	0.007657991	0.014719791
0.009801766	0.010216267	0.015742277	0.102038708
0.028578992	0.009956265	0.047562631	0.023776331
0.041160698	0.045785622	0.139466752	0.101375107
0.004223636	0.071367286	0.002116131	0.005665416
0.037822345	0.035167295	0.022066887	0.041974742
0.018277119	0.190658256	0.068699266	0.017645423
0.045103192	0.048344077	0.065803155	0.006809174
0.00192273	0.005245843	0.081423747	0.082784462
0.020537031	0.002047401	0.175819032	0.107152489
0.025501908	0.020621373	0.015507948	0.117295261
0.023943241	0.185907854	0.006154523	0.103626586
0.021900359	0.019204343	0.0214798	0.01962602
0.122015389	0.071591248	0.03559405	0.041061663
0.000671119	0.097672081	0.022976421	0.013306379
0.040314593	0.075332033	0.061474933	0.056159974
0.008913277	0.019818665	0.026085348	0.004636213
0.068791377	0.034684624	0.049942184	0.030536336
0.042881949	0.071132026	0.038727095	0.016895485
0.020657533	0.011984637	0.017793861	0.003838176
0.06135086	0.1566812	0.012682277	0.000682798
0.034468108	0.053937515	0.003653246	0.095501254
0.002703009	0.134507547	0.036944053	0.028704713
0.067390348	0.094420737	0.093694771	0.071225388
0.074440097	0.046250556	0.013675122	0.00633743
0.105989943	0.004471314	0.077517661	0.027127649
0.060041744	0.034537689	0.009514777	0.021335188
0.034760409	0.028765549	0.017108914	0.067649378
0.005537188	0.032361144	0.037814055	0.027070331
0.100100002	0.036619029	0.064209811	0.01058518
0.050889205	0.00874192	0.092574523	0.006779935
0.004803458	0.042016666	0.049723578	0.073411999
0.050679336	0.027755867	0.10686237	0.054027652
0.020815795	0.023339434	0.032855688	0.034619265
0.157486022	0.051275214	0.026687606	0.02573626
0.008367308	0.018707283	0.048162656	0.02885756
0.021900614	0.139458899	0.01469951	0.050227367

0.010557107	0.017031323	0.039546591	0.155799678
0.168038706	0.074583459	0.001779683	0.065851962
0.002875268	0.071430883	0.009763458	0.032207139
0.069644551	0.021679017	0.087989695	0.084105011
0.040546805	0.013114267	0.022195504	0.181816441
0.005084439	0.021177722	0.009030301	0.073209879
0.003668062	0.039898869	0.100927715	0.042224811
0.006567795	0.002592508	0.019482528	0.060162152
0.148584229	0.038829441	0.027255864	0.054861785
0.021631605	0.024724385	0.047960741	0.015426536
0.086150658	0.011622104	0.012189477	0.025546307
0.085841944	0.214699766	0.020239937	0.034652862
0.100850099	0.018040634	0.016910874	0.012220803
0.031087699	0.068749656	0.01359464	0.020478955
0.025106896	0.01078435	0.123261341	0.048507482
0.003159514			

BTBD10 0.357038076 0.466831624 0.28584945

0.275382524	0.264998276	0.180510036	0.092531415
0.09190698	0.041171548	0.000678414	0.010505211
0.294437091	0.1047755	0.121431036	0.165396657
0.309581399	0.095359073	0.251627903	0.129539485
0.258989795	0.032026201	0.260707494	0.044281901
0.246683455	0.073760568	0.036916943	0.180477033
0.347239543	0.044505059	0.141035044	0.232587967
0.102932929	0.361160509	0.485106128	0.286449206
0.495018113	0.333366098	0.018606478	0.124198475
0.369423888	0.326644792	0.255578029	0.171911632
0.017538575	0.439863926	0.115265668	0.284770562
0.122030165	0.016444421	0.100913952	0.392801354
0.427950428	0.233126071	0.102435176	0.067141512
0.097946173	0.100269487	0.195783793	0.175761816
0.108937435	0.05674586	0.446205821	0.034196096
0.0711379	0.134935799	0.143738858	0.12780542
0.270597714	0.271998945	0.01059292	1
0.00018696	0.233855859	0.277705966	0.217932471
0.029718742	0.007708048	0.156123348	0.162014399
0.383572778	0.459234795	0.43194004	0.45268745
0.116519426	0.221455813	0.297026913	0.092778206
0.377486037	0.074351169	0.067097723	0.319985382
0.44329672	0.482809301	0.135342698	0.198924452
0.302479806	0.417972279	0.236008109	0.24179622
0.193153666	0.067439371	0.381139941	0.466483925
0.335026946	0.265936127	0.106572078	0.292695132
0.041864322	0.220222987	0.189430839	0.141640816
0.391423558	0.006601547	0.16530168	0.171903657
0.174990276	0.354552135	0.491940525	0.092088728
0.291683785	0.012770683	0.098342227	0.034513672
0.189277712	0.170521405	0.449593372	0.360990136
0.115727147	0.234763688	0.134546418	0.002518577
0.023668876	0.189000593	0.384636443	0.257844534
0.326811667	0.401131387	0.118321289	0.287281738
0.301870699	0.428439003	0.021243871	0.049114673
0.229815266	0.183538547	0.143075599	0.263122885
0.189859225	0.056304807	0.071314741	0.462575614

0.356326974	0.043201283	0.217074222	0.294809442
0.155835676	0.252323932	0.061851423	0.144613123
0.018976739	0.467151531	0.031393426	0.193870031
0.149407759	0.122894391	0.013428016	0.12288923
0.134880838	0.345674906	0.460765423	0.071537386
0.169023735	0.246125405	0.170298069	0.264485733
0.002498516	0.036364607	0.155780106	0.44900982
0.135037835	0.482388053	0.184514194	0.225986581
0.191259913	0.363145983	0.007425236	0.452100694
0.493952122	0.160988592	0.213115353	0.292745954
0.23475356	0.347429686	0.039286021	0.289388494
0.257681699	0.05289313	0.44910835	0.099819617
0.437797054	0.02309177	0.155453248	0.404958903
0.345715507	0.005807375	0.088290093	0.21857411
0.034092031	0.031270035	0.32072822	0.237366225
0.19664973	0.376260362	0.416585877	0.225239873
0.250151817	0.130851817	0.012393333	0.246230063
0.388487659	0.232588543	0.3760555	0.117171042
0.192108302	0.40950049	0.378237788	0.097945331
0.019000978	0.153839314	0.007336752	0.192011876
0.375989536	0.045414097	0.236051658	0.15890472
0.057593579	0.469896871	0.180060001	0.247056124
0.148629561	0.2002444	0.143323093	0.097777403
0.109433099	0.098602421	0.222030164	0.21275661
0.011418559	0.053154905	0.031069526	0.230913103
0.063414853	0.173782105	0.16565068	0.282163041
0.173958232	0.05287374	0.219450615	0.241583494
0.089300683	0.011494201	0.453515483	0.267226891
0.230913802	0.078619502	0.034918076	0.191973263
0.376843578	0.138438626	0.140314178	0.366599457
0.024220417	0.443716409	0.122500586	0.077933412
0.241855776	0.138366216	0.375570524	0.02476408
0.231497527	0.397661357	0.227898842	0.109018048
0.326016404	0.233607523	0.012106872	0.329425934
0.309639899	0.019407106	0.488647497	0.10813344
0.018844459	0.343650339	0.320790729	0.054187069
0.060971001	0.153627007	0.014464969	0.189286989
0.456730342	0.285012544	0.308337661	0.215414333
0.017051317	0.003831077	0.194358899	0.305499801
0.475034858	0.488745578	0.178590725	0.219859785
0.13490115	0.192496351	0.064916347	0.456812166
0.232986515	0.494791042	0.005595469	0.377588304
0.482122446	0.135675832	0.052750662	0.136597608
0.301022308	0.022336147	0.14199305	0.45014381
0.365420152	0.189085857	0.191145994	0.306117123
0.004634605	0.015457605	0.252843233	0.193551002
0.205046933	0.130220255	0.086992842	0.173580201
0.018366186	0.377418171	0.395058368	0.031313589
0.145959013	0.16851815	0.261288155	0.020540929
0.429543942	0.499197173	0.480219285	0.098930164
0.373017121	0.44531018	0.268360113	0.071848526
0.017565525	0.498279642	0.266526832	0.009514204
0.402817343	0.09964577	0.003522708	0.434274178
0.102036124	0.422171578	0.001392071	0.134007673

0.056136264	0.416174994	0.430373039	0.225255417
0.096077937	0.233240597	0.016768431	0.504402982
0.429730753	0.014053914	0.134020713	0.359459524
0.353998771	0.480787416	0.232047882	0.400385015
0.272329284	0.467625753	0.103547044	0.168340022
0.340597707	0.487297893	0.304715947	0.407824915
0.373865637	0.314020928	0.14655166	0.005202497
0.096734638	0.090855601	0.144417721	0.038384021
0.165220105	0.088957077	0.453918516	0.407960403
0.442909606	0.004775617	0.066868447	0.355224061
0.350855801	0.401526636	0.031706246	0.0063849
0.01289411	0.013096285	0.188999702	0.192514236
0.445718436	0.096120405	0.00265849	0.016215235
0.160255932	0.272529921	0.453975136	0.242779291
0.39632867	0.059400058	0.116155507	0.271878511
0.047736956	0.238454858	0.432624571	0.402787159
0.048449174	0.189149778	0.259639063	0.409781657
0.096205583	0.217932936	0.307269517	0.153124773
0.026951687	0.067035268	0.045489206	0.364559656
0.482690364	0.037886452	0.01711179	0.066901701
0.04813315	0.42043633	0.084532365	0.464089445
0.341271367	0.344965052	0.07875497	0.431418827
0.018778531	0.445637178	0.248262065	0.025157014
0.24208977	0.012421338	0.471136347	0.078934085
0.158616498	0.266694591	0.133266274	0.021330367
0.193264703	0.040134575	0.402228918	0.41724577
0.048739547	0.130753677	0.21800578	0.269600891
0.129558664	0.295660966	0.458206135	0.219006847
0.031990973	0.204540304	0.27308141	0.270671405
0.117060192	0.445819468	0.156828171	0.497490435
0.301931229	0.425346113	0.437820438	0.17757189
0.193205544	0.221712825	0.191469967	0.005684728
0.210684756	0.370720061	0.222979141	0.093900844
0.149099433	0.291348017	0.035887475	0.156838691
0.275788071	0.134485794	0.070723241	0.361117779
0.242197207	0.281527861	0.027335021	0.039237989
0.497513356	0.392673714	0.064558353	0.258664833
0.133939287	0.196672229	0.394228572	0.392569409
0.246458268	0.371014175	0.319684151	0.217115332
0.346616014	0.139220216	0.482056497	0.040013915
0.061616872	0.290154541	0.041352932	0.079032258
0.078503159	0.024147779	0.243866934	0.318557895
0.280832486	0.213596997	0.183205036	0.059346587
0.2419643	0.269053488	0.226600864	0.082413797
0.035804137	0.441938408	0.007292378	0.045934249
0.288916563	0.347013687	0.247667771	0.171629105
0.228719862	0.150814633	0.175781197	0.136835186
0.139002166	0.267709815	0.349251796	0.017791543
0.007204562	0.442243343	0.224272951	0.033711189
0.083896872	0.243521051	0.238243728	0.113696486
0.028584425	0.455687407	0.145600622	0.277808801
0.178965332	0.049565407	0.312578495	0.060723483
0.047547715	0.407850356	0.290546823	0.488000668
0.295055628	0.294133093	0.410961749	0.362510808

0.126168525	0.29566085	0.010988678	0.371534886
0.238951926
BTG3	0.001014614	0.00405253	4.05E-05	0.014664468
0.004408819	0.00749069	0.034091655	0.013257481
0.002143981	0.019831984	0.007166549	0.001861694
0.002210421	0.031038894	0.001219552	0.006710322
0.015851309	0.001088262	0.026563596	0.010240772
0.046632973	0.0130961	0.022678419	4.73E-05
0.007245718	0.025577232	0.00114273	0.010043248
0.024571129	0.009666507	0.013122722	0.004545761
0.003793509	0.002081001	0.007230044	0.001807958
0.000579529	0.00110717	0.008372382	0.003111707
0.002913579	0.010323304	0.025892813	0.039319911
0.003115323	0.034900476	0.001333757	0.001010554
0.021961683	0.010171445	0.00284043	0.00327555
0.003028589	0.013038239	0.033679218	0.00213518
0.015385508	0.001074942	0.011768127	0.034002164
0.026289746	0.002811335	0.007482269	0.000233046
0.012832449	0.004040945	0.027850032	0.000161065
0.0050595	0.009492988	0.00018696	1
0.009465628	0.002507947	0.000901918	0.028810633
0.051900625	0.005641548	0.027072786	0.007571318
0.001211244	0.005214711	0.003103975	0.01316787
0.004364133	0.000197178	0.034311713	0.002340725
0.046312923	0.001818036	0.007673057	0.004680823
0.002856486	0.002113999	0.003709224	0.007747192
0.004377337	0.00312157	0.00427682	0.000774772
0.000963891	0.003170104	0.004168082	0.004457539
0.000401819	0.015916991	0.011612272	0.020453958
0.001723672	0.023170533	0.002715147	0.007258442
0.017370894	0.006004641	0.020182248	0.001486869
0.004326051	0.002089058	0.034733659	0.001703064
0.004821636	0.000204866	0.007564445	0.001111186
0.000856764	0.00181637	0.010843153	0.012426242
0.001510306	0.013765385	0.012513418	0.016035225
0.000383121	0.003435893	0.003003258	0.004008855
0.000114251	0.013372008	0.003158064	0.001222951
0.00464307	0.000249034	0.023219865	0.004331139
0.000648065	0.02490612	0.003728587	0.006881357
0.02358633	0.038016837	0.004299227	0.004752142
0.050842413	0.002568115	0.008197287	0.004536597
0.003710374	0.0272705	0.031066275	0.053217461
0.001036194	0.008042056	0.00449794	0.000256471
0.003504997	0.00200425	0.020290963	0.007281828
0.006235902	0.000960713	0.022360022	0.027811733
0.007394933	0.006343908	0.009729698	0.01232057
0.00501402	0.003053578	0.005628604	0.013450367
0.002505416	0.008198675	0.001150868	0.009388226
0.005450033	6.47E-05	0.002468047	0.002126021
0.002855557	0.017696051	0.001770699	2.45E-05
0.010189016	1.06E-05	0.011324888	0.003248366
0.019640361	0.005207079	0.025792819	0.002167426
0.033723388	0.011189088	0.000986672	0.002056927
0.000225563	0.023240478	0.012996959	0.003572187

0.025215292	0.003110094	0.012307352	0.007568902	
0.002963817	0.007138595	0.000154895	0.004823473	
0.01713818	0.00232604	0.002385009	0.001608405	
9.58E-05	0.00516756	0.009350027	0.002755009	
0.004656007	0.00108204	0.000454007	0.047262672	
0.014778717	0.003323918	0.004621421	0.000281033	
0.049523133	5.44E-05	0.001272043	0.012366534	
0.004421783	0.000597564	0.001720309	0.001941835	
0.016949412	0.016043528	0.030681981	0.015462562	
0.00552036	0.000178705	0.002151121	0.055530198	
0.044185228	7.06E-05	0.003190725	0.024507902	
0.012121493	0.012360946	0.003642743	0.007279428	
0.026894417	0.009000667	0.000459007	0.004144737	
0.028677535	0.003817818	0.006847902	0.006590496	
0.012734052	0.015920345	0.001592034	0.009093446	
0.007262559	0.002051561	0.008002619	0.000935817	
0.003566169	0.023364339	0.023752481	0.005134102	
0.038322153	0.007647591	0.000151089	0.00327634	
0.005745909	0.003614767	0.012527423	0.006806975	
0.007167786	0.016235721	0.003321322	0.004724345	
0.042912624	0.00323224	0.006889668	0.017867316	
0.002711459	0.002922926	0.018323107	0.010263126	
0.019024115	0.035569391	0.011579435	0.004895062	
0.012366668	0.005544809	0.000589003	0.0617053	
0.027639757	0.00168561	6.55E-05	0.002717155	
0.002602507	0.013768686	0.008405552	0.026605407	
0.000691846	0.00209977	0.004463663	0.000372584	
0.002062654	0.018178541	0.00132628	0.001765911	
0.010488258	0.01572348	0.018707858	0.008228432	
0.026731414	0.007503028	0.003888456	0.00350226	
0.001766757	0.001502512	0.003563397	0.045332911	
0.024987357	0.001327405	0.001904591	0.003271371	
0.005662732	0.002034839	0.001853519	0.049562753	
0.005704004	0.005774911	0.007255393	0.010759074	
0.001623627	0.005264884	0.034412921	0.001220511	
0.001304919	0.003036278	0.015307185	0.002638667	
0.003078556	0.000549396	0.007441684	0.011906737	
0.001827717	0.004033463	0.018565607	0.006900578	
0.010965246	0.056094548	0.005060463	0.02753679	
0.001862403	0.000421977	0.000639995	0.000935262	
0.003308917	0.002531996	0.010021134	0.011108241	
0.006659741	4.22E-05	0.002349553	0.005059819	
0.006089548	0.000757274	0.003230874	0.001466932	
0.003900891	0.000434058	0.002692289	0.002395725	
0.002380505	0.006382185	0.012388814	0.007376967	
0.001110052	0.006601652	0.003859952	0.009917485	
0.004034352	0.024853861	0.035563954	0.000589279	
0.019539032	0.003156248	0.002534121	0.006403208	
0.008772454	0.003117628	0.005411892	0.003704982	
0.017515439	0.026217823	0.002725513	0.009386517	
0.007114045	0.000194335	4.35E-06	1.72E-05	0.003204243
0.011234266	0.00309455	0.00309635	0.012770865	
0.042223891	0.030961267	0.006152292	0.001817342	
0.00392267	0.013783233	0.001424446	0.017072857	

0.009309947	0.009982657	0.00247243	0.001784587
0.004835112	0.004743362	0.003347268	0.00237713
0.002181321	0.007690032	0.004053945	0.008879768
0.002741335	0.006020256	0.000176366	0.006455311
0.00478036	0.004157305	0.001651826	0.038072714
0.041526005	0.000331927	0.051402758	0.003608988
0.03431422	0.0048783	0.009083956	0.011639862
0.001824944	0.003004932	0.002371256	0.003139201
0.002190831	0.000591328	0.002025535	0.009664391
0.001694877	0.000730648	0.010476827	0.00478246
0.000515974	0.0040124	0.004201845	0.017853866
0.006956546	0.002913879	0.001239979	0.007702013
0.005088036	0.004319085	0.021923476	0.012557651
0.003116466	0.003188456	0.035778248	0.000182237
0.010030028	0.003886474	0.014845231	0.00472287
0.003222756	0.00263043	0.002458767	0.002386069
0.002632659	0.010159431	0.000641973	0.003257111
0.01805658	0.031049655	0.009724848	0.009589601
0.003823967	0.003556834	0.018908154	0.000384109
0.002184884	0.000127023	0.001199174	0.017076203
0.025412387	0.007294094	0.004159059	0.003129309
0.006587735	0.006099988	0.003512929	0.002449667
0.006190946	0.009533442	0.009326783	0.006951736
0.002442734	0.002025654	6.26E-05	0.006552748
0.007183996	0.017969736	0.006974775	0.016481625
0.002931226	0.009035234	8.43E-05	0.001132088
0.040614637	0.009242768	0.040305617	0.005537692
0.001145284	0.005852324	0.00027049	0.020503162
0.001251596	0.004074342	0.003605361	0.000315384
0.008739356	0.042558998	0.034656241	0.003631866
0.002111295	0.017257691	0.000594784	0.002105123
0.001942283	0.006002046	0.000167048	0.000714519
0.020731799	0.011415144	0.015829083	0.000135228
0.0027361	0.002208834	0.044966362	0.005750755
0.002880338	0.000959005	0.000215832	0.00137461
0.001957815	0.004874223	0.003406578	0.002049332
0.005814799	0.001721135	0.014638005	0.017345638
0.003612927	7.15E-05	0.00291101	0.00358985
0.000204121	0.002045888	0.000296383	0.013978188
0.002088068	0.004436961	0.000188771	0.000378704
0.023835882	0.005429278	0.000309159	
BTLA	0.336582303	0.452219378	0.281675914
0.396902152	0.328105673	0.268783887	0.307379001
0.296259126	0.160738456	0.020240447	0.039305789
0.285174911	0.181924338	0.272898866	0.252490616
0.411655668	0.174253757	0.34928579	0.343591378
0.376722498	0.199752328	0.350260888	0.229655847
0.255978701	0.134244373	0.145832683	0.227166308
0.459371093	0.164474888	0.240124559	0.362162115
0.190670811	0.389490684	0.416428677	0.293778441
0.391287942	0.295580025	0.04175373	0.254476843
0.398956786	0.346753904	0.384969115	0.34534599
0.130055247	0.392762377	0.314804327	0.315779691
0.255378512	0.075621472	0.243865866	0.382306214

0.357740696	0.262036673	0.327934781	0.263236069
0.132910918	0.236753639	0.141120818	0.268057954
0.315386314	0.199349885	0.375734246	0.174490709
0.186580549	0.362478677	0.208671979	0.34223814
0.23740645	0.341346475	0.106851517	0.233855859
0.009465628	1	0.332513896	0.179065265
0.118201096	0.052024314	0.237639324	0.335229433
0.462015022	0.391328421	0.438104503	0.422580641
0.19780372	0.340453657	0.256661978	0.253727054
0.398167058	0.253325869	0.193773071	0.396824968
0.439349395	0.418466103	0.251561685	0.294445378
0.346890396	0.433413428	0.284517767	0.308590276
0.20887	0.105881711	0.362934683	0.440212587
0.358149575	0.206531519	0.216552368	0.397888271
0.163757572	0.280129666	0.354436453	0.179594963
0.473639016	0.083006145	0.243768604	0.327850808
0.339130017	0.375839113	0.406784604	0.306888526
0.321965116	0.061224981	0.103987711	0.113543312
0.292162296	0.106200689	0.359751266	0.425272353
0.293963771	0.289079883	0.3684652	0.019712945
0.076213558	0.256464994	0.34209258	0.357973493
0.384770745	0.299501238	0.235789272	0.313693072
0.254651833	0.431700152	0.04831207	0.253230206
0.322716374	0.264929789	0.326651967	0.290301863
0.281329602	0.279111437	0.251460368	0.466627872
0.393482827	0.20345583	0.308407322	0.38359011
0.252125557	0.281497582	0.249535547	0.292549294
0.152339064	0.365196847	0.072233319	0.346300061
0.165975278	0.14740202	0.072563502	0.319907049
0.264261657	0.339647832	0.346936333	0.245238153
0.318930321	0.328003043	0.291805729	0.29183378
0.026821086	0.154375713	0.243569765	0.438522163
0.214527587	0.441285892	0.362374265	0.325007636
0.312767387	0.428449654	0.007963511	0.395703429
0.416984616	0.231804845	0.293619598	0.325213698
0.227150831	0.423678385	0.042401988	0.373811654
0.270818995	0.217446921	0.471301201	0.221875354
0.383093787	0.109332627	0.314677463	0.330693121
0.273861744	0.030291375	0.243879634	0.359480528
0.071934817	0.202531883	0.373642943	0.345797455
0.308790095	0.395551429	0.473571992	0.2086587
0.320682462	0.361655763	0.062821367	0.299958932
0.368540288	0.223331089	0.389023974	0.344356472
0.230184161	0.415565246	0.312417097	0.148249758
0.137869714	0.34746003	0.063163093	0.306496218
0.321713471	0.180905702	0.246674766	0.238783667
0.134725937	0.452793662	0.156911362	0.167933182
0.099198352	0.264483598	0.375819569	0.188729987
0.146390488	0.266063688	0.261281172	0.294583897
0.130708197	0.174180285	0.047963239	0.189084075
0.110274363	0.201276039	0.192262398	0.297714628
0.287619697	0.143434593	0.299145625	0.248735837
0.175178655	0.06316279	0.439956653	0.368485814
0.277145262	0.295110047	0.123734929	0.176099809

0.424019789	0.20061928	0.23086746	0.408578759
0.102935943	0.447203055	0.227354725	0.189569908
0.237617738	0.307417935	0.466853316	0.08723177
0.317864255	0.406526229	0.358931856	0.22965495
0.333238864	0.350009666	0.113642066	0.323736915
0.307387979	0.099122249	0.43579905	0.170317471
0.077139601	0.324457166	0.296418346	0.165812345
0.157323596	0.268266115	0.152004492	0.314315499
0.449482512	0.374200667	0.323977949	0.221796538
0.116379808	0.020370009	0.237204285	0.274295876
0.400185926	0.413332818	0.342471827	0.251130409
0.343127546	0.240919555	0.206601996	0.432146012
0.242265736	0.428502023	0.044323181	0.32875832
0.383398874	0.254043847	0.128439833	0.314470696
0.336869945	0.151967767	0.358481804	0.404299727
0.399768257	0.302526115	0.260597617	0.329021351
0.080028992	0.152406679	0.307780992	0.152092097
0.284633628	0.305576479	0.040706978	0.339296302
0.106321572	0.388568178	0.42909251	0.122962269
0.212722297	0.321336775	0.333100262	0.129410534
0.350375807	0.400963642	0.419757245	0.333979051
0.342105303	0.388575099	0.208081507	0.175246999
0.058755226	0.401415775	0.329037835	0.053967666
0.455853719	0.208539105	0.030976147	0.445361993
0.326531346	0.34272865	0.018684847	0.084268244
0.097031216	0.417360081	0.382859037	0.355422271
0.170504228	0.383808561	0.017872068	0.44058614
0.405952774	0.045530894	0.17867812	0.318856577
0.274897844	0.459759991	0.209414725	0.351798039
0.239093901	0.380203718	0.269462742	0.310531419
0.386464394	0.371484381	0.383350114	0.416962361
0.42923011	0.336076924	0.343256024	0.069966255
0.147410871	0.28185621	0.169619336	0.102781295
0.261770363	0.186187385	0.421989206	0.442918151
0.397270148	0.032226444	0.272505681	0.338087204
0.436337773	0.475297673	0.078939374	0.016172849
0.01397949	0.030987924	0.358200988	0.295708496
0.421718708	0.146734912	0.033488854	0.156417132
0.329276998	0.268012435	0.411968967	0.383224736
0.358116704	0.192287647	0.188626226	0.403459328
0.11175512	0.245459468	0.413758882	0.409514317
0.122246579	0.302464511	0.322564728	0.469306376
0.042529894	0.33286442	0.285619681	0.270932288
0.05202938	0.149751087	0.134079565	0.415915569
0.370309715	0.100796255	0.062931466	0.139584508
0.189951101	0.432307282	0.22975112	0.4675683
0.416239316	0.446446699	0.126391408	0.408344087
0.092005811	0.381771585	0.244964865	0.068316343
0.336838713	0.069222018	0.371218479	0.096832242
0.295270223	0.343740603	0.186789969	0.054756965
0.135906494	0.124385823	0.443559847	0.401760795
0.086294238	0.370756332	0.342160647	0.359908843
0.300307078	0.373790454	0.404963184	0.221218795
0.200592704	0.124793635	0.390544761	0.292468501

0.304481882	0.451745853	0.232974206	0.451197872
0.344771548	0.429913278	0.391652452	0.341060882
0.193838503	0.30098199	0.288185562	0.061250299
0.375003978	0.413219962	0.360988772	0.16141107
0.26044223	0.230152835	0.175037126	0.183081847
0.183439921	0.256985269	0.22668343	0.45650323
0.322495074	0.308340619	0.155874552	0.124818785
0.461704991	0.399474686	0.249497547	0.325634787
0.281086715	0.318150274	0.370743061	0.358327234
0.282996174	0.362116246	0.397231994	0.286768683
0.378832233	0.359339306	0.424068553	0.189979222
0.121182055	0.261356315	0.147572554	0.177406231
0.24973581	0.085149052	0.162267832	0.381690082
0.200228484	0.332531205	0.2752577	0.174732538
0.323871978	0.288238798	0.358580485	0.270694762
0.145321888	0.396679837	0.034047094	0.143626466
0.218952562	0.352235537	0.231558083	0.302673092
0.196804117	0.198383186	0.331207992	0.270325513
0.184973463	0.257502022	0.35086467	0.128534828
0.094700229	0.464896011	0.297797474	0.069423642
0.148031996	0.270938843	0.346264236	0.245727698
0.061126233	0.371416518	0.229017715	0.310114372
0.359757 0.18781444	0.321424623	0.179163948	
0.19750977	0.385584741	0.271899746	0.400158656
0.304241231	0.396632057	0.365394538	0.350994054
0.205587081	0.240343483	0.117918372	0.435281895
0.209996862			
BZW2 0.355810789	0.422529435	0.268977808	
0.346624293	0.286617187	0.171402701	0.154830097
0.130805508	0.095450131	0.00698353	0.035083405
0.317840324	0.148417379	0.152779472	0.147710445
0.424585963	0.205005219	0.272125532	0.22811963
0.342744385	0.068919698	0.378867031	0.125109922
0.319354651	0.162514574	0.113773444	0.2390219
0.419465034	0.130882736	0.119810457	0.278762143
0.26036861	0.380019704	0.391193565	0.342559952
0.385236717	0.300600182	0.062200321	0.127653173
0.361670556	0.361196535	0.334132148	0.238207316
0.068260927	0.341584945	0.183840584	0.3337161
0.133280018	0.068836695	0.098247208	0.317165717
0.326615399	0.221549747	0.145095512	0.127041334
0.106400261	0.104213511	0.179007684	0.178905852
0.18878841	0.065083164	0.356413343	0.074522713
0.096840338	0.191527002	0.296106674	0.180907973
0.2789099	0.266129992	0.057385586	0.277705966
0.002507947	0.332513896	1 0.19206888	
0.119036041	0.03775183	0.30838943	0.21146557
0.438338424	0.387853177	0.470505223	0.457236498
0.208465057	0.344077385	0.288381311	0.190007742
0.370896149	0.129857753	0.138628566	0.440264437
0.469148552	0.408979468	0.250785383	0.32164715
0.295479352	0.384115083	0.210200777	0.247036205
0.173775623	0.190210803	0.318159658	0.454220405
0.304291474	0.278818716	0.228798951	0.327369503

0.147291783	0.191920342	0.291647798	0.226912681
0.44711735	0.034952518	0.2936494	0.199615907
0.3029684	0.361835344	0.414596967	0.179949004
0.321288766	0.061711714	0.130091563	0.140770495
0.213212955	0.215264945	0.322505403	0.39166132
0.154932301	0.258557695	0.208377429	0.027300239
0.104016034	0.335023208	0.361108816	0.289564625
0.375716214	0.317801735	0.17665686	0.262196697
0.293771778	0.467854796	0.015775191	0.133458435
0.232752196	0.331862114	0.258260332	0.262194555
0.264885108	0.132056408	0.175613278	0.462911348
0.311558587	0.130225956	0.204249762	0.413798558
0.157333772	0.310637071	0.16817743	0.207062946
0.06811218	0.364492639	0.123797312	0.211983965
0.135365537	0.219082458	0.062333928	0.151198432
0.15275473	0.313722877	0.326132842	0.1939228
0.226430882	0.339868769	0.160256898	0.264385694
0.01396187	0.130865279	0.128408986	0.433884585
0.127842285	0.476841339	0.280792953	0.325936949
0.303660597	0.427549307	0.015523322	0.355711774
0.414829079	0.124708244	0.239126406	0.345035086
0.206506204	0.389707466	0.038572589	0.400474436
0.232143395	0.164266312	0.488342111	0.210412141
0.366893448	0.091198179	0.172639979	0.38538696
0.310164523	0.023696902	0.094682558	0.285271982
0.130299701	0.061073666	0.358836205	0.311217649
0.219237759	0.432907857	0.486180792	0.19434533
0.267031816	0.214388221	0.069827369	0.338966629
0.403681028	0.190260295	0.320618037	0.185839052
0.158738697	0.369795713	0.324907684	0.081661448
0.075912945	0.187180046	0.027305929	0.247382878
0.314761186	0.09370972	0.19892217	0.31537299
0.17166317	0.445011997	0.263163836	0.254912875
0.199095473	0.193422989	0.239525199	0.114193561
0.13221802	0.211987462	0.239064304	0.22101354
0.04297646	0.146065395	0.111506747	0.17918107
0.089924801	0.158552491	0.139427362	0.265725824
0.199642619	0.110991792	0.255468177	0.293451623
0.206652459	0.06913809	0.414164583	0.377022686
0.201986594	0.170638689	0.138860615	0.14115173
0.395336896	0.10114379	0.110939184	0.353037757
0.092563366	0.473551144	0.16543139	0.185349939
0.245052623	0.221715074	0.438416636	0.03774792
0.225776801	0.351063006	0.334905851	0.200804583
0.27269753	0.302147699	0.054208152	0.295975285
0.280285348	0.089748877	0.440782634	0.239026684
0.095666555	0.396752941	0.310018401	0.166687873
0.175953179	0.171280229	0.042775882	0.295370447
0.431112935	0.400318995	0.294172223	0.320254711
0.069075186	0.019679228	0.332662792	0.282244573
0.369204879	0.440539121	0.305988796	0.241549634
0.239424496	0.206389955	0.072683505	0.436875759
0.323200539	0.416129014	0.045392644	0.384981279
0.368268442	0.12440177	0.139527198	0.159674326

0.270458022	0.086457874	0.253231384	0.375765098
0.355624739	0.231993473	0.196714586	0.372969202
0.026308687	0.059554273	0.273338976	0.255134026
0.198363672	0.235547922	0.121658722	0.189212134
0.06782292	0.332304489	0.463398387	0.092297808
0.121433097	0.174024708	0.372530027	0.090775512
0.398844095	0.421951018	0.391833487	0.200046447
0.374481517	0.393573793	0.234521168	0.210375702
0.088043244	0.402164951	0.279529826	0.033958314
0.457466848	0.084727738	0.019139192	0.398098166
0.204875502	0.303805931	0.004507787	0.154774213
0.176717346	0.47939511	0.340321672	0.279298588
0.07305657	0.308468345	0.05301498	0.465930534
0.37021165	0.070799451	0.262686603	0.332814954
0.329004284	0.424709105	0.241577351	0.386745212
0.269379126	0.368298256	0.150468483	0.273865398
0.344826511	0.397321262	0.33698557	0.362501791
0.415687469	0.406922596	0.22776333	0.032333154
0.144004036	0.112575323	0.106248465	0.069656932
0.163654146	0.180590799	0.41635616	0.463604114
0.427321339	0.040894194	0.114789822	0.331984444
0.368705697	0.43990916	0.108904915	0.017099179
0.025344999	0.045191254	0.218824595	0.323863429
0.417801555	0.070580361	0.028008921	0.052760315
0.221990661	0.32310281	0.388888643	0.371058593
0.411440973	0.181179617	0.108228529	0.385891106
0.128300544	0.199295634	0.427673222	0.346511843
0.169869123	0.312219128	0.256713715	0.440955972
0.109542705	0.323174847	0.335347793	0.18275241
0.096154218	0.183878616	0.133534007	0.455259191
0.365545307	0.089525091	0.063096361	0.063641347
0.10304584	0.447853108	0.166219656	0.477722698
0.369467024	0.443294047	0.206851911	0.36186352
0.067317472	0.357247866	0.285974681	0.030287493
0.352139546	0.074221379	0.394012769	0.081344399
0.164489668	0.300778918	0.170526412	0.098733539
0.218913022	0.141623796	0.444236167	0.457463209
0.165002865	0.212744792	0.330784792	0.359404054
0.143079572	0.35797453	0.36607637	0.156133567
0.085037259	0.262987438	0.342668406	0.264400651
0.154277067	0.487737659	0.182913748	0.461402023
0.3084454	0.406714662	0.397565141	0.213081991
0.266466917	0.215680405	0.211012095	0.023818197
0.277726414	0.416272931	0.242330159	0.164465916
0.228721446	0.255274672	0.116680188	0.154103085
0.23764122	0.134611522	0.147751872	0.464508081
0.275051948	0.274741427	0.089799192	0.116999369
0.443749327	0.347392923	0.105804394	0.28662
0.28542008	0.25203807	0.418819667	0.430932799
0.233119316	0.355308221	0.353082258	0.224470602
0.302781192	0.224898744	0.397060862	0.063092893
0.135247088	0.28276599	0.09625641	0.215901849
0.181900628	0.101877512	0.209653397	0.424461554
0.225755937	0.301571801	0.169642885	0.156841119

0.341445686	0.234611283	0.305353659	0.126531752
0.097729868	0.359880193	0.038865165	0.161092119
0.251061234	0.348399544	0.23793299	0.295463715
0.24480173	0.305701165	0.26727293	0.130049425
0.111488674	0.242590892	0.338485881	0.060832058
0.042105837	0.486097023	0.201130268	0.128379419
0.184600541	0.233774316	0.319734297	0.157613602
0.115728092	0.392105105	0.119102403	0.282107129
0.212300236	0.164584482	0.254410598	0.118041949
0.145541224	0.340625199	0.2819433	0.401605843
0.34221055	0.395296243	0.309765714	0.345529409
0.226080592	0.233391595	0.052752828	0.412337664
0.180705538			
C1QBP 0.241131696	0.365687661	0.193194645	
0.243635023	0.178999443	0.134230271	0.052703059
0.019187333	0.066853213	0.000945962	0.030257556
0.214109197	0.057803416	0.034221922	0.096213474
0.283298289	0.048364792	0.067573749	0.076131202
0.192116378	0.011056073	0.246100349	0.024479756
0.135773089	0.074844354	0.038461458	0.16209295
0.262051463	0.041244152	0.072656942	0.151924202
0.036617996	0.236685005	0.365705914	0.26382219
0.362169169	0.229065517	0.000417548	0.060623489
0.239283833	0.340689695	0.198556871	0.132048566
0.014430592	0.293761801	0.086733973	0.232775653
0.035639495	0.021355466	0.02711225	0.264183634
0.32318856	0.227365709	0.031699844	0.035744744
0.066499637	0.020448669	0.128934171	0.096036631
0.064353576	0.012763819	0.344795544	0.037408051
0.039353548	0.033444598	0.054168017	0.043636772
0.149977134	0.176982312	0.002952979	0.217932471
0.000901918	0.179065265	0.19206888	1
0.020452305	0.006543136	0.059116744	0.127363864
0.287359849	0.309772013	0.377783723	0.404529845
0.047615684	0.109159899	0.217956263	0.044898308
0.283050451	0.042073165	0.023382223	0.296270781
0.388816125	0.377202999	0.088969098	0.157434413
0.279020461	0.300313836	0.150996519	0.157059549
0.159879962	0.044408165	0.297908864	0.37919167
0.205390017	0.191392135	0.076089133	0.189067892
0.021996536	0.055440325	0.167706574	0.026579427
0.301429763	0.011127632	0.115721942	0.110729707
0.050874157	0.254740941	0.3984243	0.055761524
0.150173894	0.006039579	0.150960603	0.015311923
0.053137434	0.112574196	0.320225018	0.291148615
0.024311188	0.10515931	0.056329151	0.002423231
0.016255556	0.06134094	0.255079746	0.085336091
0.243491472	0.287197199	0.099133018	0.182602107
0.283261593	0.376381268	0.007395909	0.02256009
0.157542122	0.062410152	0.105494374	0.186897271
0.159391725	0.017921917	0.028681445	0.391700316
0.237069982	0.022983055	0.047709162	0.254756942
0.079315405	0.206392675	0.023333095	0.044131561
0.00951353	0.370350545	0.021208527	0.061881177

0.177635505	0.053138662	0.002520265	0.040188442
0.047748831	0.304281493	0.316915583	0.028000085
0.064784905	0.249278504	0.058832752	0.232911879
0.011927877	0.013068821	0.039838966	0.352138626
0.074140839	0.376984495	0.058776813	0.108738402
0.146600846	0.311411911	0.003055938	0.328558317
0.398265025	0.043458803	0.166231417	0.205129867
0.232587069	0.298258684	0.005538518	0.221234169
0.130952244	0.023167687	0.380864441	0.074920173
0.300098895	0.023725798	0.043013943	0.396180171
0.273628792	0.00326089	0.03505753	0.176020446
0.023873187	0.012579017	0.231317188	0.211494901
0.215668008	0.317450821	0.346485868	0.224227012
0.17455333	0.03636211	0.006936363	0.172604778
0.295098286	0.107106808	0.271263222	0.04518553
0.08831209	0.319417242	0.287190167	0.079709331
0.016270552	0.066958038	0.003233209	0.185311699
0.299682846	0.022643235	0.224847873	0.035744242
0.035834103	0.391585162	0.124152386	0.154407439
0.123078879	0.134154527	0.063167607	0.039094445
0.058529726	0.071061025	0.158729442	0.131301792
0.005546115	0.028541726	0.014807738	0.07109277
0.0285863	0.058415207	0.053429967	0.179881675
0.043070431	0.019914151	0.142280245	0.149765455
0.068649717	0.009075673	0.35660217	0.216280163
0.127566794	0.044645622	0.016697405	0.056343801
0.30887667	0.088267066	0.03226021	0.286215116
0.008155651	0.370799521	0.057585327	0.060420518
0.111290792	0.082924398	0.318878738	0.025121766
0.120695085	0.285307583	0.133612167	0.090753345
0.206327953	0.189625592	0.007721582	0.211360535
0.181489015	0.012901968	0.382014293	0.050007811
0.012519751	0.293296019	0.324593786	0.042683576
0.044116312	0.109112258	0.005735542	0.176646131
0.377740475	0.269609416	0.276937856	0.121001277
0.014078695	0.002515259	0.091163954	0.188724697
0.330150156	0.392589328	0.093731216	0.159118066
0.093571735	0.165429383	0.013135682	0.380394424
0.062317879	0.355068028	0.003861913	0.368559445
0.365303734	0.044856353	0.054049326	0.085994713
0.192876941	0.019558871	0.034171862	0.334275301
0.312962495	0.137096429	0.14213618	0.202782234
0.003155791	0.007879904	0.256572723	0.143453576
0.103576153	0.087938488	0.075520128	0.049279226
0.015396612	0.284114744	0.291528072	0.058458726
0.115136911	0.047567152	0.222037224	0.015022403
0.409499022	0.379087869	0.353412704	0.025460475
0.257218119	0.389761404	0.268554806	0.036092306
0.015063886	0.381801877	0.178791175	0.007437407
0.318258967	0.033888982	0.004006976	0.322753948
0.052988127	0.26504849	0.001735146	0.044471502
0.032785148	0.349505571	0.340886419	0.151412805
0.030965309	0.142772978	0.005553783	0.39548778
0.32293601	0.016801368	0.088680225	0.285660856

0.33152057	0.347706737	0.212897001	0.379204432
0.237997747	0.364635603	0.040368497	0.154491592
0.277781916	0.400415535	0.215209124	0.293757193
0.286585964	0.269216021	0.123575147	0.007250452
0.181231868	0.025897652	0.091206542	0.072867789
0.093421456	0.081062411	0.322342236	0.33587622
0.369239799	0.004186135	0.019367308	0.26327091
0.248313398	0.310507369	0.011877821	0.006178886
0.004157044	0.034411249	0.11275431	0.117343825
0.375079659	0.029288505	0.002472618	0.004734525
0.058261498	0.298034858	0.349581419	0.204181049
0.320136378	0.027170188	0.05494862	0.209655473
0.069636879	0.125048293	0.372458991	0.301043567
0.020440651	0.122936494	0.078989967	0.336820585
0.109933421	0.152353674	0.211808572	0.074401858
0.006523532	0.051088929	0.060312389	0.296913012
0.360869809	0.026313354	0.016235888	0.012556605
0.02756172	0.326805474	0.039720128	0.383567036
0.260223562	0.280955949	0.047146255	0.34164593
0.032875173	0.320271604	0.198634654	0.012606985
0.064822111	0.00572781	0.329204599	0.151808022
0.040342766	0.160089455	0.200948651	0.016008734
0.158431513	0.024094678	0.359674491	0.375757394
0.02512527	0.051724721	0.127115951	0.187393598
0.057834077	0.261097014	0.334154376	0.13864711
0.019460263	0.067694149	0.183316638	0.153110156
0.025334279	0.377397403	0.129744964	0.395337439
0.206971477	0.317119037	0.342716519	0.154744779
0.101927366	0.13321936	0.155928018	0.003550638
0.128524724	0.311564359	0.088806128	0.098355133
0.115517483	0.245238376	0.012726573	0.020014982
0.212532988	0.081927598	0.031220834	0.29417183
0.134463058	0.16801871	0.00441713	0.058130218
0.378799656	0.291310597	0.029181628	0.21912748
0.058416944	0.203053093	0.313104423	0.328992824
0.11748281	0.288209995	0.212558951	0.155800246
0.239880828	0.092753618	0.370253306	0.005826737
0.080598263	0.181155391	0.030533056	0.048447235
0.041348455	0.023118796	0.157729445	0.24975593
0.11670462	0.179962886	0.038698031	0.034217192
0.157813318	0.093757224	0.164286527	0.032272497
0.024988414	0.345123613	0.006761347	0.021909444
0.107467629	0.328859453	0.225924739	0.07858276
0.161578882	0.068018709	0.116673857	0.049275526
0.057255579	0.078802879	0.321414558	0.008547657
0.003011539	0.355775376	0.083908217	0.015660019
0.052924515	0.161257499	0.048615277	0.10224269
0.021961752	0.38205356	0.077237334	0.213070571
0.1048366	0.019296562	0.175136492	0.021202324
0.018169142	0.2613729	0.238268051	0.375770167
0.138559618	0.266970148	0.26912693	0.253804647
0.029904347	0.170599005	0.00935366	0.267816524
0.12768738			
CAMK2N1 0.041519583	0.047369624	0.039559735	

0.108836362	0.078540985	0.029861704	0.12748127
0.044750843	0.121059295	0.085160369	0.141468188
0.053770802	0.082594402	0.058315723	0.017419375
0.138938614	0.230262002	0.032855967	0.206982039
0.102137984	0.088164038	0.168524668	0.205855391
0.064393376	0.197913131	0.253726508	0.077470114
0.100648698	0.257490979	0.022174756	0.067560229
0.253852426	0.054184259	0.031625006	0.092451526
0.028606818	0.028096958	0.076208137	0.02715347
0.043485869	0.071508658	0.099728311	0.169650884
0.22537651	0.025384433	0.167140199	0.090887229
0.024575743	0.262992918	0.015167476	0.025728106
0.025194691	0.039133468	0.049456015	0.115562728
0.040760952	0.019085058	0.030556633	0.029206353
0.171733583	0.019179943	0.02983414	0.097435236
0.032821074	0.059430453	0.20615845	0.081996617
0.033786991	0.035073264	0.197153048	0.029718742
0.028810633	0.118201096	0.119036041	0.020452305
1 0.219272811	0.199957508	0.117929751	
0.088163041	0.032075819	0.087688332	0.068355415
0.170748681	0.15264373	0.049336943	0.199975467
0.045613486	0.126096282	0.107751534	0.151840201
0.08072366	0.038658777	0.155612351	0.193455588
0.049018711	0.044022925	0.033291769	0.054522651
0.024015336	0.214595989	0.030167127	0.061539033
0.031691071	0.050111177	0.265236423	0.06470988
0.311935952	0.017022035	0.178187153	0.103253228
0.089759904	0.160998765	0.212760087	0.086381175
0.118080987	0.052430899	0.038415062	0.196829152
0.069337414	0.228435647	0.052490463	0.311895112
0.045721324	0.049667547	0.020231296	0.079139067
0.046938854	0.044101006	0.096474479	0.205885218
0.333273633	0.119503764	0.041983306	0.053834093
0.071392319	0.022327888	0.107342119	0.027228196
0.040994312	0.086406821	0.002420907	0.189185171
0.05669386	0.132584178	0.274663638	0.048034367
0.139854659	0.138817341	0.236913083	0.067732418
0.03150126	0.25943884	0.019343653	0.147374246
0.033070389	0.125631213	0.242007765	0.099596985
0.193726005	0.027183815	0.303250054	0.037271074
0.018592394	0.153086921	0.15468766	0.050974842
0.042990675	0.041900987	0.018916409	0.259906118
0.103024279	0.131360718	0.024438856	0.049013186
0.113932672	0.264724708	0.010882429	0.059393413
0.019574603	0.061673732	0.102254801	0.087714603
0.215394247	0.093311271	0.010466924	0.027378763
0.037855255	0.009791935	0.098266991	0.078791624
0.028322061	0.086482089	0.003636986	0.14025771
0.021864116	0.268129791	0.087870126	0.274027053
0.03165241	0.28960268	0.033661909	0.049355562
0.034688111	0.07258208	0.029381573	0.091987032
0.272585892	0.057833006	0.061269929	0.105174808
0.082321323	0.084048936	0.106678154	0.025851773
0.072038918	0.102392966	0.250634408	0.139025322

0.056275946	0.013356224	0.032017997	0.09047488
0.014724028	0.042798529	0.03009404	0.011976499
0.238473382	0.062766305	0.077306415	0.105247591
0.026198977	0.138007175	0.02554354	0.157370803
0.298394806	0.057631416	0.083405603	0.032809057
0.053042792	0.051710303	0.144945236	0.053047246
0.072065216	0.184190325	0.038197635	0.036943879
0.130608734	0.272666404	0.153100395	0.01470263
0.084886894	0.026855751	0.019715765	0.030704136
0.042680068	0.159851758	0.089572097	0.051454194
0.2201529	0.317804666	0.047351966	0.127496938
0.020856957	0.158915827	0.336792422	0.011339199
0.072067064	0.014492691	0.007395152	0.05163808
0.178674018	0.077618957	0.09616101	0.276485189
0.046693764	0.195595421	0.09829092	0.023799849
0.037073073	0.038432802	0.146778586	0.166688369
0.026511069	0.083985933	0.208562494	0.03061621
0.029239641	0.327743144	0.048158775	0.236748003
0.328879599	0.081226066	0.046756958	0.330772452
0.27620668	0.069933871	0.084034423	0.240614726
0.056113963	0.161451393	0.043697663	0.088440937
0.249552235	0.146828517	0.139974449	0.041865995
0.027590828	0.048093611	0.226312268	0.081722386
0.240749467	0.038516237	0.017258503	0.058477295
0.059476416	0.035939222	0.260596051	0.057725752
0.026656225	0.020294533	0.242946283	0.064655979
0.032299749	0.235653403	0.120046839	0.034954154
0.048461671	0.055688867	0.034562546	0.077625224
0.136463294	0.165915217	0.078538838	0.057123884
0.029561375	0.141284914	0.033926552	0.025796729
0.241496203	0.037136019	0.095826359	0.193579648
0.023436607	0.020681942	0.154885764	0.291158717
0.047821981	0.036959596	0.033356611	0.136989531
0.049315774	0.044080205	0.032707841	0.284454314
0.30533337	0.032842052	0.041475293	0.130308506
0.092080358	0.013247924	0.156264707	0.045843681
0.204123457	0.018328961	0.016254921	0.017324098
0.231277189	0.093525526	0.027898302	0.069370332
0.008102939	0.112201218	0.038103483	0.051988587
0.03841612	0.267848927	0.160217967	0.040105438
0.0414008	0.04338383	0.04546542	0.054196514
0.04348777	0.029666006	0.076035818	0.228442432
0.056945048	0.033390059	0.060827278	0.037928016
0.085157885	0.111776004	0.199048511	0.19477274
0.062601692	0.034963197	0.010779737	0.095678614
0.023939153	0.192595389	0.045160957	0.091621426
0.057342198	0.251957997	0.079590862	0.038212177
0.062335306	0.080253091	0.149128092	0.022887152
0.01244404	0.155745346	0.073510536	0.172392989
0.051298917	0.009162248	0.234618361	0.111470745
0.078695775	0.086786798	0.039089972	0.170962513
0.059053625	0.297409593	0.024310735	0.136119863
0.203789773	0.015602748	0.063306917	0.035524265
0.276622919	0.142487065	0.033150319	0.081398055

0.020621044	0.197984947	0.055544608	0.056972591
0.135508767	0.263192369	0.230160904	0.106847266
0.025904834	0.187859373	0.243295264	0.008125171
0.152194236	0.070078833	0.180372555	0.07482766
0.069613024	0.131602495	0.227968468	0.034079028
0.181168946	0.029311385	0.088326731	0.012479378
0.089169592	0.286627351	0.033688287	0.031960565
0.026152889	0.051498999	0.044086349	0.269677089
0.031723078	0.326791725	0.089873671	0.082947129
0.244556961	0.090373918	0.16190663	0.094094838
0.04788959	0.102586313	0.029917884	0.016771905
0.146936865	0.049711922	0.088092826	0.031386959
0.044835902	0.088531969	0.055856354	0.052795541
0.040912225	0.047397434	0.043578254	0.092317884
0.083714804	0.033429913	0.054502095	0.117628891
0.125626278	0.090726935	0.047955019	0.138704919
0.167801048	0.036990174	0.176452897	0.012925583
0.029313144	0.032368875	0.180844309	0.125515632
0.046162937	0.03230425	0.157645508	0.226448933
0.046949279	0.034780808	0.063873515	0.06392287
0.230220303	0.123279706	0.066422775	0.07357813
0.021172481	0.049980308	0.062899111	0.047029917
0.033490649	0.15312069	0.03502416	0.028203108
0.114732862	0.032130014	0.179583958	0.296064276
0.252288136	0.283312374	0.017933089	0.12276042
0.0149816	0.151575276	0.014338342	0.213010002
0.149245094	0.021041076	0.156244928	0.082539341
0.213915106	0.032169231	0.158577758	0.330410218
0.022486161	0.050574991	0.053710309	0.153902232
0.038911329	0.163452554	0.192630215	0.024501073
0.018064296	0.019260381	0.047240687	0.118706185
0.2010336	0.089344207	0.02432103	0.224116638
0.112855835	0.026101912	0.063228802	0.0750695
0.26455155	0.038922668	0.016874254	0.064050193
0.080184217	0.29894624	0.020240209	0.063516338
0.19110945	0.029504617	0.051089827	0.034517514
0.063274839	0.144749451	0.020872561	0.043290487
0.082140468	0.023197731	0.199322871	0.073540366
0.013809518			

CAMP 0.011087861 0.025947053 0.008791064

0.075520976	0.038313459	0.019451725	0.108544974
0.016164725	0.019283111	0.106874164	0.07594596
0.022119744	0.045169325	0.081872519	0.005435617
0.060144181	0.161477638	0.006271095	0.137472393
0.049488444	0.076283441	0.106425834	0.09033493
0.010000536	0.121679596	0.184070691	0.034615047
0.054747067	0.181045259	0.023671054	0.040503497
0.053116891	0.022387427	0.014924812	0.069803601
0.014253931	0.007040202	0.010736019	0.024536685
0.015510673	0.032154173	0.051541694	0.136728791
0.130790368	0.013451947	0.147197806	0.027818524
0.00334213	0.170575492	0.011398399	0.011787099
0.017076406	0.025023554	0.025274999	0.104917593
0.033886588	0.01792596	0.018368858	0.018975208

0.149739791	0.02201776	0.017231602	0.023693385
0.006184231	0.027010227	0.058969003	0.06262644
0.007795347	0.015255466	0.025505197	0.007708048
0.051900625	0.052024314	0.03775183	0.006543136
0.219272811	1	0.076936413	0.10324813
0.044483703	0.010506107	0.048281381	0.033450121
0.096404103	0.049079548	0.014281942	0.177023943
0.017186141	0.156402254	0.019976807	0.075359298
0.043608517	0.021262967	0.04023408	0.068359871
0.038729433	0.022536059	0.017766661	0.034588127
0.01168664	0.026282414	0.017338154	0.034414408
0.014517285	0.016694117	0.172993465	0.040380475
0.135021871	0.004368366	0.128848213	0.034770437
0.046120532	0.053691976	0.103894152	0.079969835
0.011206831	0.02307459	0.019332637	0.149927485
0.023610665	0.09143803	0.008895263	0.068804374
0.010304646	0.008717212	0.010232962	0.060452439
0.021486204	0.013055195	0.053485919	0.059406082
0.144280167	0.006670316	0.022454413	0.015184464
0.025785699	0.005720723	0.099007338	0.012105041
0.01875086	0.045389512	0.001644434	0.072732718
0.031307065	0.01037511	0.167009991	0.028260525
0.092980679	0.056765247	0.173127744	0.034333898
0.016223095	0.220584353	0.00561356	0.072487716
0.021982867	0.064983356	0.126366121	0.126415669
0.125519955	0.012281161	0.124430968	0.012473368
0.003125524	0.045494995	0.010391997	0.034094005
0.019716961	0.035573346	0.007925392	0.124637848
0.122791229	0.071514759	0.01178872	0.050967535
0.085368772	0.0346929	0.003698236	0.038441194
0.017282063	0.025504586	0.04369837	0.021001531
0.109352384	0.041457489	0.000317571	0.014094122
0.018885014	0.004299011	0.115297416	0.024461363
0.010408528	0.058147157	0.000312901	0.0797063
0.008841814	0.103554825	0.044645193	0.228249312
0.013946497	0.226173566	0.026890832	0.019534401
0.022265984	0.012956145	0.029867216	0.054099509
0.06176344	0.035332781	0.020407923	0.062540206
0.051591977	0.029791626	0.055456702	0.010350592
0.043065897	0.055261296	0.024989892	0.048607118
0.01758417	0.001640579	0.018254556	0.038402203
0.006288782	0.024004522	0.011801914	0.003158405
0.177361519	0.039162213	0.043790289	0.053707369
0.007214209	0.164047662	0.00815945	0.01301312
0.155194782	0.032674187	0.022625792	0.008019488
0.010414895	0.075108339	0.069835546	0.116731631
0.095033793	0.060325194	0.013497062	0.018035827
0.094326966	0.262217519	0.006224133	0.022647484
0.114810737	0.067590958	0.056385294	0.01590183
0.027316746	0.128637729	0.079243971	0.006107649
0.067775342	0.188532334	0.025027098	0.053289101
0.011944486	0.061846818	0.129442519	0.007246089
0.053391902	0.01553383	0.002400375	0.038401471
0.016855126	0.033526539	0.100686371	0.224741643

0.05181607	0.204106633	0.049672274	0.005521393
0.013029537	0.025476074	0.042648664	0.115242457
0.022083951	0.042734979	0.062688898	0.012600377
0.017408773	0.247569336	0.025982772	0.090691609
0.151561951	0.033129453	0.029152025	0.176269713
0.115482747	0.093811271	0.042331218	0.129323914
0.032974368	0.103137529	0.032561277	0.015180696
0.230702076	0.205556563	0.043424278	0.009228324
0.014707627	0.024773975	0.106990915	0.086435916
0.149081808	0.016085944	0.003048993	0.035140167
0.001700709	0.015687526	0.094594881	0.023303057
0.013757298	0.01563739	0.17790863	0.053381883
0.024397772	0.114459038	0.029878209	0.020986478
0.021725005	0.023310313	0.01691007	0.028443454
0.072192014	0.049826721	0.025448743	0.008349468
0.019193029	0.05159368	0.002631863	0.006565982
0.159598538	0.026413557	0.045165119	0.060093551
0.030754763	0.00496805	0.065587699	0.16124035
0.021279363	0.014778062	0.018089286	0.044837668
0.02241506	0.025703481	0.017251143	0.094241874
0.138763618	0.0157619	0.015066057	0.092376128
0.049902968	0.01102938	0.189865821	0.025180566
0.116608063	0.008655906	0.007036717	0.000162962
0.027735605	0.038518187	0.01466719	0.033778953
0.014171174	0.038797973	0.000714483	0.023127638
0.026518316	0.099962411	0.028239113	0.025578223
0.022774579	0.021812083	0.017840106	0.031064691
0.029513766	0.016781954	0.032333142	0.126890795
0.039795917	0.015232316	0.027976656	0.017450667
0.058969066	0.045832823	0.13824014	0.110646998
0.004263034	0.025891178	0.009731439	0.035058576
0.010907019	0.087003291	0.020296258	0.044750972
0.033777795	0.111457618	0.036464968	0.019876735
0.038096841	0.040079269	0.00609986	0.001484832
0.000601877	0.051820777	0.038650578	0.055509156
0.024508277	0.011378136	0.137262167	0.03850962
0.022005562	0.039063045	0.023687039	0.090293328
0.019179803	0.121240633	0.037445723	0.061343282
0.050812465	0.004733715	0.039368666	0.021699787
0.036186201	0.042732924	0.009717038	0.047239816
0.003286241	0.096982027	0.025888262	0.036335382
0.007488449	0.09085336	0.073935182	0.040594114
0.013409915	0.204844583	0.217350368	0.003102245
0.193128624	0.029870149	0.151645971	0.03901272
0.042884547	0.078024075	0.036625567	0.016804562
0.049903187	0.018199088	0.046938475	0.006367051
0.02050812	0.047862437	0.015148775	0.010932886
0.024098259	0.018955467	0.00306694	0.066739715
0.00207945	0.171680825	0.053740594	0.036406825
0.026588462	0.02879688	0.057202807	0.030823145
0.038384805	0.079616528	0.0163949	0.012221702
0.097745626	0.002133016	0.044212355	0.014291272
0.026470132	0.044340731	0.033695717	0.02368303
0.013778295	0.017010102	0.022254795	0.052230778

0.01388932	0.014072458	0.057895499	0.104468837
0.059411805	0.064886152	0.014007426	0.050078377
0.15864377	0.010715242	0.014709164	0.000742757
0.008587357	0.028869537	0.098615944	0.056410731
0.016324578	0.014106904	0.021867144	0.077590411
0.023748567	0.013328703	0.012338442	0.041695339
0.089020456	0.068641855	0.025804514	0.029174329
0.002929302	0.038761981	0.034418805	0.057030749
0.024018015	0.082119806	0.018409484	0.005405946
0.013672581	0.010411557	0.190343803	0.126720616
0.222887419	0.057325962	0.004115659	0.052754746
0.005456132	0.130186112	0.002637041	0.033503541
0.053584831	0.003674165	0.07484213	0.107213762
0.143008494	0.0200831	0.033168919	0.14486594
0.011523529	0.023033051	0.036158229	0.065418396
0.008483088	0.018498833	0.142094831	0.01900745
0.052520964	0.001718807	0.023544372	0.017529318
0.090353653	0.046180988	0.009254104	0.014799243
0.007705539	0.006951235	0.011164124	0.032406115
0.068623047	0.021162993	0.016979059	0.023551288
0.045190259	0.096678126	0.008759236	0.005263609
0.01417477	0.01528401	0.013119195	0.01776546
0.012388796	0.099207671	0.009034187	0.026400708
0.004559905	0.004098006	0.071336848	0.032157499
0.007824974			
CAPG 0.188891003	0.195996929	0.139052074	
0.221906162	0.203658426	0.079153749	0.103907275
0.044490453	0.053071489	0.015465762	0.051614866
0.20566625	0.078917466	0.06103817	0.030068908
0.312095099	0.201071799	0.101474808	0.206867191
0.235351417	0.039887385	0.323962437	0.100765211
0.142768611	0.142811539	0.121246316	0.114357381
0.258208764	0.139306667	0.049534535	0.160440178
0.268677536	0.209310758	0.16083037	0.249973243
0.159735674	0.139563405	0.098711685	0.032639876
0.175052333	0.20723475	0.220708089	0.218567065
0.079087838	0.133141888	0.152571789	0.26203111
0.034577486	0.107282304	0.024875108	0.124485901
0.129598975	0.119303537	0.050103274	0.073637613
0.045998722	0.029471283	0.068770479	0.08351222
0.154351988	0.019174324	0.149446617	0.037745897
0.035045222	0.083052573	0.26731496	0.098355942
0.102264815	0.120506954	0.082987025	0.156123348
0.005641548	0.237639324	0.30838943	0.059116744
0.199957508	0.076936413	1	0.163398148
0.259835332	0.17069878	0.28265389	0.253742425
0.165156112	0.277062896	0.187993645	0.141994158
0.17993333	0.08879977	0.081651605	0.345285562
0.27177507	0.183080341	0.154733089	0.306850832
0.142701992	0.177782358	0.091051517	0.130075886
0.043605117	0.213335514	0.133290142	0.239729403
0.135567021	0.166085432	0.248268367	0.181882455
0.184036265	0.045319622	0.259055004	0.184808454
0.261361229	0.039487598	0.294853979	0.13833691

0.162537516	0.190101381	0.187018143	0.15499272
0.21592562	0.11247272	0.058027878	0.210992423
0.076818843	0.122916937	0.118576213	0.222300677
0.058416397	0.094350322	0.110661278	0.077031711
0.184353676	0.210208817	0.193571558	0.158931669
0.224589315	0.134051107	0.094098731	0.115591189
0.162315849	0.283620097	0.002465858	0.10130067
0.134042377	0.220648856	0.295170012	0.163378098
0.197058391	0.081194557	0.162479724	0.241237489
0.129159636	0.153408093	0.067752779	0.322619092
0.047083373	0.275191066	0.151345159	0.109477937
0.074394833	0.149952053	0.206477668	0.091889292
0.030285972	0.207399987	0.089686104	0.072006637
0.048726661	0.149406264	0.121162003	0.199441132
0.144743434	0.27407396	0.069039875	0.138897483
0.024555943	0.154022444	0.040809019	0.225399738
0.054871345	0.256394619	0.130167405	0.142825447
0.30319416	0.260948564	0.012639776	0.14025998
0.183159258	0.036981642	0.187748192	0.255110912
0.08403693	0.224986085	0.012029307	0.322361625
0.10537227	0.163557007	0.28519034	0.240595298
0.161547951	0.132426842	0.063098389	0.206068319
0.163388057	0.038034787	0.03584959	0.18076147
0.20305027	0.026615594	0.205056329	0.209512932
0.142515327	0.270435247	0.306204319	0.072689378
0.170455089	0.117975942	0.12283569	0.287445134
0.227723394	0.046256353	0.131975198	0.076527388
0.068169284	0.167045789	0.150874171	0.018387665
0.08957226	0.09858926	0.027168662	0.154499747
0.132812785	0.071739679	0.082273867	0.252546974
0.213389119	0.221527538	0.154108684	0.117873921
0.113930485	0.124005132	0.168632647	0.049724285
0.073284697	0.139517886	0.079470702	0.075401854
0.041345755	0.161034213	0.158155844	0.05980155
0.06778912	0.057430574	0.049280969	0.129695857
0.075756726	0.107162895	0.162925769	0.112225439
0.170781752	0.147720798	0.193738108	0.284804332
0.083175874	0.100458082	0.205676684	0.037620599
0.218478955	0.038470574	0.023982974	0.172673698
0.105848064	0.27411465	0.086594675	0.21682439
0.124066031	0.205173087	0.261017318	0.013010542
0.12692149	0.157022194	0.275080848	0.140209165
0.115117481	0.193858465	0.071730079	0.136222353
0.13242829	0.151948004	0.212420994	0.241571127
0.178586342	0.269342083	0.163448968	0.23685605
0.182025955	0.09510448	0.027892308	0.331989874
0.211167128	0.32308199	0.145882711	0.168138465
0.093829735	0.056265727	0.255979344	0.172316181
0.148930575	0.218458267	0.313258418	0.15751424
0.247735768	0.062543032	0.020230275	0.224090653
0.152411902	0.182549201	0.104648434	0.222853883
0.147314909	0.050255654	0.145312087	0.088804891
0.120723847	0.090976465	0.136604035	0.161782514
0.169163617	0.087858888	0.057920149	0.261353828

0.033767749	0.061568274	0.18822109	0.126871359
0.061744628	0.124504246	0.085136874	0.049554803
0.091566719	0.147134839	0.304040816	0.079338218
0.055306211	0.040782735	0.349442695	0.12372043
0.205498723	0.192722276	0.164848172	0.117776223
0.214607586	0.185798138	0.137667565	0.235477917
0.168807278	0.172056038	0.13949435	0.045163728
0.279952102	0.028573111	0.045489038	0.182793912
0.176317552	0.113196503	0.00248861	0.063172908
0.219386834	0.31091698	0.133724426	0.164967037
0.015106327	0.243614059	0.072894586	0.233552667
0.164453512	0.132995942	0.202707807	0.170755431
0.177831195	0.191602534	0.117498265	0.206672405
0.165411011	0.153979666	0.065893374	0.27897365
0.175527495	0.177976802	0.18151621	0.157213564
0.250211188	0.313888901	0.217632394	0.050046321
0.055621989	0.041733827	0.020306721	0.045095858
0.072459564	0.130703554	0.199636065	0.288033749
0.227752716	0.101782899	0.053004143	0.163319224
0.190375319	0.244749481	0.122112353	0.013737323
0.014685358	0.067470076	0.145001284	0.263128571
0.203860547	0.014998751	0.073329781	0.042288021
0.087561046	0.2446481	0.174522816	0.306095376
0.237912807	0.206267745	0.030072882	0.28816983
0.125053322	0.080459051	0.228495838	0.146915155
0.226565806	0.218714524	0.122147232	0.245628855
0.058456838	0.335153488	0.219387723	0.065624208
0.135512916	0.187545949	0.128180697	0.312383658
0.148955698	0.104893283	0.108766866	0.014502679
0.077391844	0.255988959	0.120895354	0.263359626
0.201525841	0.310314422	0.242121837	0.150015078
0.078736122	0.149204989	0.203580567	0.008309502
0.175168484	0.142850285	0.184104213	0.027078207
0.055959237	0.167708533	0.054352763	0.180669092
0.083401194	0.198192572	0.262780263	0.281739074
0.229053949	0.089809863	0.273223889	0.258203694
0.073640551	0.235216366	0.14853569	0.062896113
0.062278993	0.173078931	0.217666671	0.130924684
0.056068182	0.294382629	0.065740542	0.227112091
0.151044451	0.195116835	0.194221781	0.137672907
0.163773246	0.108140417	0.113388968	0.031375122
0.213655213	0.25873074	0.125215552	0.103881173
0.18631202	0.157238166	0.118669107	0.048671075
0.129221532	0.06528881	0.115258836	0.319471779
0.158075696	0.132289889	0.100782592	0.112544368
0.206131623	0.148510576	0.039373365	0.162090716
0.2678122	0.188723031	0.248077582	0.274930319
0.061073048	0.186697307	0.197203028	0.119728363
0.129862656	0.166367991	0.167941097	0.021674009
0.085622852	0.161143125	0.089910178	0.27661103
0.190062887	0.145271398	0.062949741	0.322848642
0.07222033	0.264404628	0.043013575	0.135450858
0.305510384	0.096350803	0.277890339	0.066901687
0.098771677	0.149273599	0.066637166	0.219511175

0.103827757	0.175439031	0.145050841	0.184222076
0.091836176	0.252309199	0.249744648	0.052799344
0.038643705	0.068023708	0.16483298	0.047685982
0.067293811	0.292200317	0.08258447	0.187390165
0.158605896	0.110083615	0.142184823	0.062449519
0.188695852	0.186123682	0.036753645	0.189022023
0.135005018	0.193696129	0.100709483	0.063020209
0.125347402	0.143280988	0.181896501	0.176477685
0.2002102	0.295482549	0.115018396	0.18001019
0.123737236	0.118488402	0.06176859	0.237349812
0.053891366			

CAPN2 0.25456243 0.410590724 0.169009403

0.400803629	0.244631191	0.236505317	0.318971734
0.227787052	0.162744706	0.057595391	0.07531404
0.207465314	0.200018164	0.281217086	0.200547452
0.368331803	0.164961816	0.195716886	0.301373286
0.344977586	0.252041622	0.342036977	0.236905573
0.155592405	0.127350993	0.175215567	0.178973263
0.419372548	0.182372813	0.226017938	0.342154224
0.122531663	0.324398803	0.362347593	0.28088445
0.338758343	0.221260403	0.024565868	0.273982745
0.322842483	0.311559789	0.362514293	0.305837195
0.17738859	0.34550843	0.319745165	0.202445718
0.195033142	0.103618156	0.206974198	0.333528538
0.331750142	0.226395017	0.273663227	0.305347322
0.170656423	0.218295519	0.116472454	0.251899854
0.320666448	0.204398245	0.342177741	0.224143972
0.171892501	0.272395839	0.140798457	0.300483751
0.154750614	0.296369898	0.087518486	0.162014399
0.027072786	0.335229433	0.21146557	0.127363864
0.117929751	0.10324813	0.163398148	1
0.412764451	0.309899393	0.396395991	0.375596965
0.185996549	0.225318916	0.163574804	0.269027047
0.334270675	0.307185133	0.143580829	0.361319239
0.39645164	0.37589349	0.19870961	0.204134398
0.357163316	0.38765175	0.21266596	0.246493769
0.173723223	0.061957142	0.3378335	0.395851228
0.305497228	0.131214661	0.188378421	0.370794465
0.148214211	0.182829275	0.33427644	0.125943387
0.424155106	0.109535027	0.182217597	0.304235396
0.198610568	0.318592643	0.357655602	0.304911327
0.204527072	0.072208318	0.085657356	0.081692945
0.222065605	0.060197824	0.317083239	0.416133948
0.251577968	0.234811027	0.311374617	0.018409092
0.073574927	0.138220662	0.293884444	0.215341108
0.322419244	0.2258564	0.256097409	0.271107945
0.221631906	0.389370242	0.050582322	0.242047094
0.238182875	0.146057106	0.280237422	0.227596329
0.231107768	0.247782245	0.251230603	0.418656806
0.352960009	0.224572041	0.187915685	0.341964021
0.269991318	0.201334944	0.230324475	0.28762678
0.211134776	0.313536276	0.057867069	0.221616566
0.134127226	0.123686029	0.040650062	0.26579266
0.24955092	0.342362973	0.292445272	0.204712164

0.297025828	0.287962798	0.20617289	0.303481249
0.074262765	0.115092562	0.156972685	0.405340452
0.216417989	0.369536826	0.271890772	0.210678172
0.23937805	0.377362802	0.002730076	0.348477857
0.36724705	0.152665852	0.258993316	0.216427535
0.167652875	0.410420116	0.017248345	0.340245856
0.214985339	0.182062038	0.422036901	0.214629124
0.324385315	0.135024667	0.271148905	0.281805024
0.242821048	0.026656145	0.241797364	0.356802902
0.05083992	0.25119882	0.306440394	0.338564175
0.267399821	0.328927304	0.42720295	0.15624066
0.245527416	0.280924707	0.046562848	0.196091104
0.291917936	0.164727686	0.357959084	0.299422296
0.15871157	0.381715085	0.258996821	0.167101508
0.176958742	0.294823212	0.077849419	0.250617739
0.254357272	0.273207943	0.175765749	0.125409278
0.118914546	0.41466899	0.095151951	0.098210809
0.056651879	0.243132758	0.293855643	0.250382553
0.178891327	0.233796535	0.192244455	0.242160027
0.191080809	0.199119882	0.019544145	0.15393879
0.168407408	0.197409413	0.191817941	0.264836118
0.248373674	0.188868066	0.247385539	0.148519617
0.137301922	0.066516383	0.397356262	0.317604608
0.247669727	0.273477204	0.10383081	0.153720258
0.415494178	0.178005768	0.151006327	0.40200276
0.067986059	0.38967719	0.270874413	0.188082847
0.193095049	0.29909857	0.428137534	0.086234535
0.212684503	0.383141801	0.231572144	0.223270525
0.315655748	0.31167223	0.146419551	0.269304413
0.26283745	0.115482462	0.385930183	0.134418829
0.069523495	0.269273456	0.281237823	0.149733615
0.131823973	0.28641139	0.194231335	0.247845239
0.414614335	0.362680098	0.326183102	0.123598228
0.170399688	0.053059811	0.14772983	0.158182599
0.351844679	0.361408442	0.254151087	0.213630197
0.299245884	0.199581795	0.169264572	0.395892391
0.09846017	0.363321219	0.041616206	0.283475732
0.337284962	0.202766115	0.13492737	0.300645951
0.31902702	0.170116819	0.236944051	0.365071254
0.361645118	0.240230199	0.215641528	0.261808809
0.123127003	0.19612234	0.213251478	0.085376662
0.251973635	0.256640401	0.018691837	0.235413251
0.166437359	0.374167275	0.367223013	0.142385762
0.215136464	0.208610392	0.250314929	0.142368027
0.303124173	0.333269097	0.372710494	0.255734623
0.286614926	0.355880357	0.158018154	0.12617843
0.051746549	0.346386148	0.274341741	0.093811091
0.414091814	0.170132132	0.083189384	0.404386446
0.289236189	0.29216032	0.019255974	0.027646306
0.053578719	0.352069701	0.345934642	0.325717883
0.183339037	0.260620531	0.005748214	0.375873541
0.378305547	0.041700998	0.118372575	0.290385763
0.240151789	0.405415455	0.146487898	0.319362473
0.193254222	0.343067671	0.28485391	0.257764841

0.369773487	0.318981981	0.333357087	0.364168693
0.407109381	0.285127605	0.312658595	0.111810558
0.105856858	0.255879486	0.168105381	0.156335269
0.211239956	0.181240823	0.355262448	0.395517618
0.355679882	0.030662053	0.279630225	0.297134689
0.406534359	0.428081873	0.036114638	0.008000898
0.005331493	0.040206704	0.280319887	0.195922984
0.374213998	0.171586622	0.042355783	0.177626215
0.262781195	0.206886106	0.37851311	0.359737229
0.287421798	0.16720522	0.22400973	0.358660876
0.108242731	0.188799762	0.380640298	0.383314591
0.076165683	0.2061518	0.188035082	0.438756005
0.0237206	0.245473653	0.228832826	0.277289537
0.023680165	0.122798979	0.126057148	0.350469376
0.324399743	0.153697059	0.10114381	0.108698873
0.268408779	0.371319308	0.254920476	0.420003168
0.38960822	0.417531506	0.089874144	0.366309094
0.11993915	0.343699239	0.176662815	0.071331793
0.192216146	0.053765986	0.309896028	0.120265555
0.242163137	0.279868501	0.130460995	0.04067743
0.069310637	0.116831615	0.41828504	0.347613936
0.047223501	0.296010719	0.229199166	0.271549159
0.257249527	0.374305807	0.362657341	0.169061473
0.26319319	0.053475005	0.346355784	0.236677197
0.258545916	0.400502975	0.233143161	0.388636862
0.278601711	0.357994413	0.344240453	0.288949706
0.144931832	0.212449791	0.327329609	0.116379086
0.274108466	0.399493115	0.23075277	0.156893691
0.25292946	0.146702974	0.126223889	0.083272896
0.113343154	0.238168382	0.27393272	0.40250478
0.241816654	0.250823099	0.124191906	0.126699442
0.408447801	0.340458635	0.22594861	0.317978398
0.200274892	0.268633012	0.306370563	0.297569191
0.178845896	0.347209531	0.353667791	0.313532267
0.364539095	0.309974933	0.378728936	0.142105087
0.087508496	0.192490599	0.222543338	0.136176138
0.255978492	0.072427381	0.104010527	0.327558169
0.129415482	0.311991371	0.188407969	0.148728854
0.220127348	0.156831459	0.267496723	0.307013383
0.199635448	0.368270511	0.037803753	0.123939746
0.144770878	0.312874794	0.178945944	0.220150196
0.133137331	0.11532667	0.28966845	0.214739357
0.19982355	0.153299514	0.316392256	0.109429446
0.124962745	0.413986991	0.187857579	0.035699939
0.099090498	0.20628049	0.194577051	0.271556756
0.042440844	0.332282591	0.221126323	0.212936893
0.290082946	0.154423116	0.270317784	0.107213727
0.131111858	0.33371418	0.167160083	0.350696058
0.170149758	0.393325492	0.311060252	0.31525119
0.097995355	0.134599045	0.14222314	0.377301251
0.152159363			

CAR5B 0.419021779 0.518136419 0.407158297

0.454472849	0.463996685	0.387302283	0.306367049
0.283856687	0.135710784	0.013611344	0.038056915

0.44054073	0.248648858	0.342642117	0.346006311
0.468096676	0.196065907	0.424990551	0.350254
0.470026827	0.165765976	0.455399403	0.175227662
0.333886806	0.14755051	0.123041282	0.300869011
0.467603714	0.140811526	0.344641159	0.432534089
0.175190338	0.442503703	0.499928133	0.442887171
0.485399362	0.38355976	0.030234046	0.334410784
0.416308153	0.438223613	0.462775207	0.415453062
0.092021996	0.470829499	0.338204163	0.437909234
0.296070948	0.061080334	0.291613899	0.438428755
0.448382173	0.414423715	0.321792563	0.247562899
0.212749193	0.288945333	0.253057244	0.35358385
0.325982397	0.2150547	0.461114557	0.148152722
0.199564516	0.364985769	0.226675508	0.365670267
0.352883782	0.39425449	0.054200376	0.383572778
0.007571318	0.462015022	0.438338424	0.287359849
0.088163041	0.044483703	0.259835332	0.412764451
1	0.44928137	0.475820542	0.474478603
0.24447697	0.390518723	0.394012346	0.260314499
0.42313829	0.26051915	0.177386281	0.464934849
0.483130338	0.50231588	0.276016559	0.342011281
0.404874237	0.480152883	0.427905576	0.449947376
0.328076447	0.091641995	0.425939489	0.500702216
0.386977866	0.320958227	0.227598724	0.437511458
0.12132577	0.395804235	0.435039001	0.222370011
0.478212173	0.046130549	0.283960224	0.421144505
0.33247501	0.439323314	0.492107448	0.291721728
0.450321185	0.046176294	0.144880977	0.076430356
0.387975166	0.158561446	0.451721399	0.452774438
0.328153052	0.416460285	0.375702298	0.00901521
0.058220218	0.269752309	0.413448288	0.448007966
0.460299061	0.354504274	0.288598291	0.383265773
0.409287583	0.468007138	0.06379323	0.187810655
0.451663237	0.27532196	0.344073432	0.448000309
0.365631288	0.215749595	0.222167466	0.51468934
0.431138891	0.162140621	0.395870991	0.469239603
0.359553669	0.397150803	0.201001251	0.362403453
0.109280535	0.452078085	0.057499175	0.412575788
0.252674245	0.193142431	0.03578524	0.350068889
0.34343622	0.400763376	0.437400443	0.198971191
0.402620533	0.452969557	0.375031096	0.410181691
0.021087465	0.104685275	0.318623846	0.499673113
0.305641534	0.492168113	0.407070012	0.381006399
0.368249863	0.464792029	0.006725974	0.477134267
0.501934067	0.323881101	0.431079025	0.456592171
0.354624199	0.451987179	0.053523498	0.473543957
0.363049767	0.159803714	0.503686934	0.236223997
0.448449606	0.08372698	0.390374674	0.399835332
0.38780427	0.019608785	0.28274583	0.444596083
0.055361128	0.166053699	0.452653235	0.460202466
0.418122735	0.458133877	0.48620705	0.3333379
0.462126874	0.355400214	0.034188966	0.384358866
0.43641761	0.359411096	0.441648452	0.345429474
0.345539986	0.469683957	0.384349807	0.212792345

0.094720837	0.400294231	0.045166811	0.389267804
0.365158796	0.185109836	0.37589565	0.236177109
0.12272418	0.516655514	0.208283062	0.261758469
0.140803706	0.412105114	0.373961854	0.272564143
0.232060007	0.251889484	0.368866462	0.405925978
0.086689016	0.162405731	0.032518916	0.344897208
0.167460827	0.340120026	0.33138839	0.40259167
0.388382717	0.166373074	0.425458106	0.323574268
0.174110686	0.03885671	0.502238753	0.47216676
0.338187497	0.253553678	0.088717897	0.319440168
0.460473291	0.311988928	0.29911222	0.452822291
0.064376821	0.479168002	0.30592705	0.190966848
0.384958844	0.354895078	0.465511412	0.08063585
0.432078942	0.465165133	0.410091919	0.253205256
0.389041802	0.465708968	0.072413445	0.394523764
0.39081479	0.070982761	0.510748287	0.187293409
0.050175114	0.44704955	0.408342544	0.13777218
0.13698409	0.372507844	0.101892389	0.374407721
0.511221815	0.459251534	0.406272994	0.269754285
0.086174977	0.020663279	0.278086891	0.407763745
0.488810409	0.489894481	0.371213065	0.385618208
0.352206384	0.343173455	0.207684377	0.49645055
0.26529026	0.504470241	0.021833649	0.417686376
0.478559418	0.333839647	0.12681491	0.3771445
0.378795723	0.101444751	0.331127211	0.482758013
0.427973186	0.378861183	0.367045457	0.466189025
0.04488527	0.098168661	0.422568698	0.206089284
0.408173489	0.308213257	0.058452843	0.395997052
0.087194217	0.447348301	0.469640177	0.101546701
0.332814343	0.374695911	0.44236326	0.085097953
0.414089399	0.478547014	0.504618879	0.286831772
0.431031531	0.464333992	0.365678791	0.145661223
0.040013105	0.49096514	0.424599809	0.045934443
0.473014213	0.269823718	0.027275593	0.499513524
0.299393325	0.423339145	0.010412359	0.099497514
0.077786174	0.457074057	0.460693797	0.439762331
0.25479443	0.460112321	0.012582103	0.508800795
0.480037799	0.031982887	0.197664295	0.40735555
0.388025605	0.52616229	0.320683801	0.411693687
0.404636983	0.471986962	0.311265505	0.355902899
0.428895746	0.459288339	0.448459668	0.463005624
0.457476775	0.460979768	0.379246123	0.040958654
0.160509236	0.295730345	0.285076001	0.113436388
0.354855163	0.201262275	0.47717341	0.462892939
0.461139241	0.016185501	0.248786442	0.413057164
0.450513056	0.486406386	0.048747948	0.011563999
0.012588837	0.030350401	0.437796199	0.330858922
0.478322478	0.240787853	0.016464464	0.103004777
0.378308447	0.407684263	0.489408777	0.460859779
0.432977509	0.15465207	0.291377754	0.471431049
0.105156261	0.328746584	0.471832332	0.470362037
0.087670845	0.334599337	0.431067186	0.493968446
0.069777548	0.406559013	0.443750796	0.368566388
0.03442473	0.136996417	0.112389698	0.470831308

0.467094561	0.11646118	0.060694171	0.177391204
0.188368223	0.4587464	0.246888849	0.513044096
0.447362469	0.474047088	0.121917003	0.474046309
0.070926327	0.467187911	0.364417928	0.078533487
0.388207443	0.036522837	0.448151348	0.14977634
0.386025362	0.441294163	0.219287806	0.037557621
0.189631411	0.102000485	0.472248606	0.44606141
0.066649171	0.35472176	0.39592182	0.470519629
0.357434283	0.456983194	0.487275868	0.37308057
0.157825136	0.16769559	0.46359304	0.40911914
0.33490583	0.486103038	0.324735258	0.515880921
0.421419909	0.462470236	0.455419914	0.417988149
0.273130553	0.424269226	0.393920262	0.046085981
0.446427196	0.455993966	0.438913648	0.195680267
0.325941032	0.372452118	0.110143976	0.228634202
0.309804176	0.343683045	0.230332868	0.475579144
0.436611649	0.406923045	0.099688291	0.105450398
0.533001207	0.438296347	0.225717543	0.445866222
0.271824	0.404807289	0.444611623	0.441147365
0.386684928	0.419144305	0.448546665	0.39780049
0.427382234	0.369809448	0.507501919	0.157924971
0.120768691	0.411554967	0.151100087	0.158630602
0.235616435	0.059274758	0.276311308	0.473889797
0.345937178	0.44685907	0.36340106	0.14972613
0.403175953	0.39722948	0.443130535	0.286760918
0.136166156	0.479383084	0.022153653	0.112372171
0.370787252	0.422515668	0.376876537	0.335914927
0.298780216	0.20754239	0.392901781	0.346790927
0.312309628	0.382555349	0.416211691	0.078617576
0.054176452	0.494803673	0.411961263	0.047348839
0.136254999	0.391440448	0.398429836	0.293507245
0.045455429	0.452197463	0.337752801	0.446144439
0.430317904	0.137041133	0.35583661	0.151045386
0.130245346	0.446316038	0.403782126	0.487772428
0.402227521	0.463638068	0.433670309	0.418579256
0.19390512	0.371948947	0.069320508	0.457781076
0.361513939			
CARHSP1	0.475116707	0.477478907	0.438698751
0.397107164	0.414524539	0.389241152	0.185498707
0.213143683	0.070496888	0.001753092	0.012277696
0.459043458	0.165095663	0.248026598	0.344384893
0.407007649	0.109693919	0.424608205	0.219075146
0.411390039	0.079569314	0.369516897	0.076776874
0.298341073	0.082031394	0.049038104	0.240651747
0.433672626	0.059558722	0.327507142	0.39874747
0.10194119	0.465892856	0.506148037	0.408450426
0.50367289	0.466852945	0.016451703	0.27014616
0.461617635	0.436562874	0.40432801	0.2976373
0.02921003	0.485383016	0.212972472	0.410812385
0.264448739	0.019127304	0.265396726	0.442225916
0.457503831	0.419922253	0.236552559	0.13297928
0.159913841	0.243447682	0.248816502	0.361917969
0.194261863	0.16680312	0.461306959	0.070341627
0.141565178	0.272129927	0.146795443	0.258606295

0.354756953	0.427979666	0.017554442	0.459234795
0.001211244	0.391328421	0.387853177	0.309772013
0.032075819	0.010506107	0.17069878	0.309899393
0.44928137	1	0.448950537	0.454241053
0.152816074	0.296258562	0.40910162	0.140290621
0.462647984	0.145118071	0.112409914	0.401012325
0.451921481	0.485911712	0.177597548	0.250618569
0.412336739	0.448565266	0.429330282	0.403766622
0.321869286	0.052375061	0.428512623	0.457136351
0.440318142	0.321659309	0.129207551	0.422513639
0.049503079	0.417204209	0.324325541	0.170482203
0.448742471	0.012265195	0.192211838	0.331857773
0.244435403	0.4657328	0.482058075	0.163226337
0.424800973	0.014783522	0.117269813	0.03141305
0.322440183	0.151709044	0.49111956	0.431683458
0.243469994	0.354206385	0.256171571	0.000538467
0.020256164	0.212332341	0.460442132	0.421439278
0.454455899	0.445903688	0.188200146	0.436541765
0.446732077	0.449137697	0.064735071	0.086509259
0.42218814	0.205497526	0.214386224	0.44330751
0.273799693	0.112794364	0.107770591	0.457389911
0.421663641	0.06683621	0.411867357	0.402684378
0.291571457	0.337263996	0.094811542	0.24606253
0.036495054	0.470691267	0.023236017	0.387779757
0.257659884	0.127339804	0.014642867	0.275135858
0.272730313	0.421378514	0.489624302	0.097930683
0.289556863	0.382214289	0.37029528	0.408179265
0.003981072	0.045902468	0.353482049	0.451370246
0.319267685	0.469297118	0.298789674	0.31025157
0.261202681	0.445659546	0.005577376	0.488251844
0.492820753	0.367156407	0.359722233	0.441335971
0.371344545	0.429818755	0.067488447	0.405366856
0.42941377	0.072306008	0.454436908	0.130365012
0.451049038	0.028019007	0.321501212	0.446510285
0.441343587	0.007274799	0.230661779	0.381699826
0.023908493	0.087143741	0.459615962	0.39526391
0.354904949	0.45703535	0.445786907	0.350597121
0.408161609	0.23981663	0.011301963	0.316754353
0.47288576	0.391319191	0.426165716	0.229113968
0.411278021	0.443887471	0.457415156	0.195589708
0.030549156	0.314436924	0.017011018	0.3056373
0.450752221	0.085696453	0.406396763	0.173136644
0.057581255	0.46135823	0.175465369	0.277828292
0.130327351	0.37409658	0.25102426	0.191185988
0.16730263	0.141046203	0.33995771	0.359847622
0.027774704	0.070912591	0.018493961	0.368729909
0.100479816	0.306500762	0.310861923	0.441898572
0.316855322	0.083133816	0.35115882	0.307531877
0.099851529	0.010316931	0.461053653	0.407998048
0.389115795	0.137657695	0.035020166	0.331372432
0.435210241	0.335658467	0.340175332	0.428948972
0.030396992	0.460388183	0.200843384	0.097113875
0.348648231	0.225771542	0.438623031	0.054832696
0.438429634	0.431856831	0.32412559	0.152428239

0.408035834	0.415213234	0.021500131	0.455144302
0.446616325	0.021888445	0.477008684	0.1109871
0.016184106	0.446155343	0.43049977	0.061757339
0.066048091	0.28841729	0.040259104	0.264741975
0.456279041	0.381856046	0.423216531	0.231437637
0.026206121	0.004194544	0.207182206	0.451371156
0.503657604	0.460438702	0.252556321	0.327607795
0.219008783	0.305443371	0.16786658	0.452169444
0.267706963	0.501297766	0.004638458	0.445268004
0.49976279	0.320206894	0.06004513	0.293858862
0.416830257	0.035152246	0.227628418	0.46949881
0.446174902	0.312872774	0.331254204	0.455125184
0.010982898	0.03269883	0.392049294	0.192819976
0.362479302	0.194447919	0.058149577	0.361942188
0.027363354	0.425121267	0.455305552	0.043215568
0.327251936	0.365060956	0.370573857	0.027601081
0.446964185	0.479885974	0.50141004	0.172843874
0.465402134	0.444397686	0.405715947	0.07382385
0.013114227	0.498262358	0.446826759	0.01492128
0.448489698	0.273168466	0.005167959	0.456730918
0.171673318	0.465490157	0.003807529	0.134966022
0.041685332	0.453480953	0.464623477	0.402103903
0.253374998	0.390637591	0.008722545	0.475600207
0.451270043	0.010142509	0.132279699	0.44788624
0.43544992	0.497470601	0.31114001	0.441853429
0.425868084	0.477745771	0.20823073	0.240945319
0.435975543	0.465435833	0.447961165	0.446474116
0.441050607	0.423286989	0.251536449	0.008992214
0.128346917	0.224280647	0.295961294	0.057578183
0.378003522	0.114522685	0.465273473	0.451590862
0.453730473	0.00304807	0.146611309	0.456904052
0.436589149	0.451086838	0.027692124	0.007454575
0.01206988	0.011562317	0.370611474	0.240875849
0.457725807	0.233202936	0.002156816	0.040482687
0.281087484	0.388933869	0.465019542	0.367792261
0.468319306	0.070258221	0.245194214	0.400980197
0.051620812	0.412781404	0.450038352	0.44326017
0.041995968	0.244223824	0.446732117	0.446067868
0.074311126	0.302099335	0.460637861	0.274978154
0.018281513	0.06909587	0.051346072	0.443296325
0.491052071	0.051354098	0.019589859	0.169444537
0.088246408	0.46442809	0.134641483	0.458192179
0.440577597	0.419661219	0.066882381	0.461033042
0.025361862	0.467980342	0.322379847	0.060242146
0.325053325	0.010982711	0.453301372	0.123006748
0.341520416	0.444649027	0.194675235	0.013825395
0.207525609	0.041738163	0.440086977	0.450925911
0.034635708	0.239559286	0.302316847	0.434491616
0.299860076	0.409105021	0.489708105	0.423955754
0.065174588	0.178921123	0.423063401	0.453008777
0.250580111	0.454392124	0.256175428	0.478437443
0.459516114	0.465336137	0.45570545	0.335451557
0.21491901	0.442186474	0.359209531	0.012322888
0.356680014	0.433686215	0.40926327	0.125133182

0.21587639 0.411347489 0.050767126 0.271986337
0.354336047 0.323011956 0.113474647 0.43160859
0.445181288 0.453484381 0.044399086 0.046641845
0.500742185 0.447027276 0.145412077 0.423172263
0.166909958 0.314647643 0.466489972 0.455022782
0.407788436 0.440879065 0.44406952 0.380697604
0.413528703 0.244518453 0.500502534 0.112676903
0.074558272 0.46874972 0.066496845 0.082009858
0.118212136 0.02190692 0.313213342 0.425305571
0.396998287 0.35030332 0.369476022 0.075227166
0.320436882 0.459824347 0.344845408 0.177507947
0.055984607 0.469762645 0.006890337 0.047493652
0.401601994 0.44973153 0.361574587 0.230660282
0.291962293 0.144582219 0.268925364 0.324874469
0.29498618 0.416403419 0.447867916 0.033816949
0.013881332 0.45578983 0.43985287 0.023226956
0.085911027 0.448456865 0.350481936 0.197675339
0.019030775 0.444243794 0.327244176 0.43240581
0.35142361 0.060238918 0.406793151 0.100382534
0.06623436 0.441587207 0.424963065 0.486121853
0.392866099 0.391872891 0.462614589 0.453005298
0.160593216 0.456952356 0.019955777 0.456153068
0.411016694
CASP1 0.431809491 0.528717285 0.448418865
0.464182811 0.502049365 0.372698216 0.266754732
0.201337701 0.134705457 0.011298331 0.052706807
0.490241638 0.291190828 0.31777766 0.345520888
0.485433078 0.231861961 0.378754084 0.323843709
0.455482164 0.118571869 0.490054075 0.145614163
0.391792411 0.189877773 0.128662475 0.374944338
0.470065227 0.144116116 0.324677119 0.399437293
0.175749047 0.449035745 0.514696988 0.512231677
0.510727061 0.39598514 0.020809758 0.321152205
0.411400706 0.48639699 0.44812451 0.413242231
0.077306894 0.467878659 0.319388902 0.484815056
0.242463192 0.070525158 0.229252397 0.421587492
0.470333034 0.465852647 0.24934173 0.215578478
0.273368327 0.22774387 0.362796507 0.309092218
0.303055468 0.153392955 0.484195065 0.127442464
0.177242517 0.281691274 0.250405502 0.302389571
0.431313675 0.373396602 0.031964612 0.43194004
0.005214711 0.438104503 0.470505223 0.377783723
0.087688332 0.048281381 0.28265389 0.396395991
0.475820542 0.448950537 1 0.506931013
0.282663962 0.395558107 0.469446037 0.262547146
0.434675129 0.240307189 0.157852257 0.500764861
0.510943354 0.527586838 0.308747873 0.375564134
0.434862712 0.471104673 0.457239792 0.496502444
0.404059201 0.104589326 0.43216888 0.526711899
0.378454295 0.41487512 0.253192827 0.420710307
0.110367468 0.37353559 0.43620256 0.225695954
0.476816255 0.035389541 0.328514963 0.409395589
0.288859791 0.454312882 0.525521993 0.261613761
0.48727062 0.045380495 0.21256861 0.069412851

0.387623039	0.229146278	0.463885782	0.474755804
0.261311387	0.464771117	0.325338752	0.008094066
0.063375839	0.266730257	0.448874617	0.410968649
0.461667812	0.365246391	0.327976243	0.376897469
0.477792772	0.501960972	0.055210596	0.144414463
0.468500508	0.270270529	0.338084128	0.487555328
0.424786326	0.156121908	0.200574208	0.531077188
0.403675222	0.142792429	0.337854801	0.492801199
0.381856592	0.471661877	0.169095962	0.360539001
0.082929872	0.481553087	0.066879576	0.34729209
0.322540881	0.246343813	0.020494997	0.277666626
0.335164627	0.439337884	0.450826215	0.172523256
0.401553919	0.497635172	0.317123143	0.453347097
0.024517103	0.084387559	0.257958569	0.51985747
0.266165132	0.51189413	0.385358673	0.403722747
0.398037202	0.486403292	0.005706663	0.485436373
0.531050293	0.277310437	0.480421136	0.486698449
0.431879235	0.477563329	0.046064823	0.492523524
0.343355068	0.132736929	0.519592734	0.260855852
0.454231739	0.085904685	0.343613134	0.466611636
0.454421251	0.016854876	0.226662723	0.423474773
0.062405728	0.114426929	0.44940762	0.463439041
0.444862143	0.489363728	0.499661675	0.417679436
0.503930248	0.290796757	0.028532793	0.432969578
0.464996265	0.408330313	0.428349905	0.299034107
0.317675077	0.471936776	0.423446928	0.221814435
0.080197044	0.345287136	0.035130574	0.428079427
0.383528431	0.176357146	0.426449008	0.220075809
0.13872781	0.538815947	0.27688799	0.348979043
0.204809172	0.443272957	0.321774657	0.297488077
0.305164184	0.254120121	0.434940133	0.442188279
0.058227195	0.166865728	0.032018785	0.422276569
0.212619167	0.401822124	0.379249601	0.40676154
0.376361368	0.179049759	0.48232403	0.374022874
0.197747625	0.037406774	0.511924048	0.47482584
0.312525922	0.221823499	0.083619265	0.372165957
0.473016095	0.304718123	0.242160101	0.451692863
0.050456092	0.493138568	0.339684711	0.212189993
0.475830432	0.358794668	0.479150147	0.076166163
0.402194333	0.462448053	0.407007573	0.286109438
0.37825616	0.462314166	0.056425413	0.39966541
0.397325213	0.068990431	0.536824742	0.220010404
0.049359921	0.502354457	0.472732136	0.140156369
0.145007281	0.400654345	0.061526572	0.406046136
0.529572823	0.496477512	0.44502922	0.318272904
0.078755998	0.026815882	0.31945378	0.450014311
0.499631906	0.525166371	0.363061901	0.473219708
0.330272104	0.414529285	0.137471141	0.524309993
0.251772947	0.515678728	0.017960726	0.483107286
0.504203002	0.281403832	0.15304982	0.341477074
0.380049539	0.084154	0.263821265	0.496903924
0.433735371	0.411881106	0.418260622	0.481244193
0.029196881	0.067311723	0.466260107	0.275153539
0.436482664	0.307751751	0.095990282	0.341387676

0.084952884	0.448446708	0.497581314	0.110550103
0.340548381	0.31208065	0.482519942	0.073069253
0.463377949	0.504620763	0.51893555	0.217570051
0.467944642	0.499957889	0.459268858	0.145071836
0.043607016	0.518960423	0.403186306	0.044015992
0.492678426	0.215872084	0.031162294	0.496226858
0.259033271	0.422216388	0.006014404	0.10583481
0.084417584	0.503105983	0.471916969	0.402868474
0.235461998	0.429429094	0.012092968	0.534964696
0.493201978	0.038303243	0.239641133	0.459499408
0.466512663	0.527380873	0.415777566	0.478259253
0.486011637	0.500646924	0.301067155	0.380863956
0.454841096	0.500220189	0.439738641	0.44933968
0.485929034	0.504432906	0.367547946	0.032916936
0.195033193	0.221905044	0.329110639	0.133549211
0.30904972	0.232195802	0.481925583	0.498041613
0.496336525	0.014866115	0.183225464	0.445563171
0.434215981	0.472449926	0.038708011	0.010754958
0.012411852	0.048477219	0.395567025	0.353089233
0.496236432	0.239365246	0.013497762	0.061722917
0.351273017	0.491159331	0.508028316	0.458137042
0.475327132	0.144167058	0.321896789	0.46093739
0.133256989	0.304877496	0.50242185	0.4668698
0.081226165	0.354540189	0.400046402	0.494575368
0.119580614	0.43234901	0.488831698	0.395447732
0.028076842	0.152617585	0.124837535	0.494849958
0.496721693	0.143934531	0.075454991	0.141967524
0.182710726	0.479975014	0.252646106	0.530752407
0.458039227	0.493829074	0.142894992	0.478263959
0.078277058	0.484648436	0.454968383	0.068315902
0.375304873	0.029177981	0.471972544	0.214889364
0.340881437	0.412527557	0.265412079	0.041777925
0.252959033	0.107707201	0.494964079	0.501399951
0.071481178	0.29585629	0.404056611	0.462125891
0.300306276	0.490520233	0.496681253	0.409112217
0.126189129	0.206294675	0.443474064	0.399168402
0.264549859	0.507651293	0.389254782	0.536930743
0.414342561	0.453319881	0.477327793	0.40927413
0.345989674	0.4074521	0.391206338	0.036808572
0.43104005	0.497824293	0.386151261	0.252682487
0.372691401	0.45309761	0.073737727	0.187027796
0.403820932	0.303700014	0.218410214	0.490447383
0.405012248	0.400905957	0.058471291	0.118141541
0.546301108	0.423126049	0.174761533	0.459913427
0.264701907	0.441059363	0.487236511	0.49778267
0.406566837	0.461984244	0.446092084	0.402961257
0.4124866	0.337868171	0.523864474	0.090372578
0.147798341	0.437411478	0.163337021	0.167401701
0.226879829	0.060332684	0.371554636	0.496463773
0.432800137	0.473548615	0.318400524	0.138136388
0.437195204	0.376440563	0.456112081	0.248901664
0.137706758	0.496560598	0.022209681	0.110103223
0.453263476	0.464798151	0.475886635	0.35094429
0.395995488	0.228379841	0.407513688	0.293523077

0.342345808	0.404548515	0.456603497	0.054091426
0.03616518	0.507202128	0.381728564	0.045900687
0.138280835	0.375420329	0.356449214	0.316386048
0.05221902	0.49172147	0.337689317	0.495002353
0.387857923	0.11692256	0.32314967	0.120718483
0.09468358	0.43636844	0.46446069	0.517475489
0.432342346	0.492881432	0.422437271	0.449399787
0.169029166	0.40383982	0.05157646	0.464032683
0.438141694			
CASP4 0.445797838	0.526837607	0.460062015	
0.454021068	0.495360008	0.37788327	0.239263674
0.182001883	0.120988273	0.006944867	0.043855522
0.493861659	0.265193273	0.289294277	0.353383639
0.475193139	0.200738208	0.373537109	0.289479007
0.444102483	0.098909811	0.472081934	0.120028731
0.391609942	0.168040549	0.107036418	0.370338997
0.463336907	0.120420489	0.325138523	0.391942845
0.151147015	0.455181812	0.523318116	0.508324249
0.521814928	0.415044709	0.015519429	0.315799535
0.421229609	0.493773672	0.438435097	0.383558172
0.059823652	0.475446516	0.290054546	0.481565412
0.233820262	0.054137442	0.223232402	0.427383603
0.479596496	0.479805745	0.230889166	0.190273412
0.264096731	0.21750285	0.369696937	0.309028735
0.271726127	0.14285182	0.492308115	0.111718386
0.173771819	0.26006312	0.224062154	0.276443233
0.446834943	0.380777877	0.02317957	0.45268745
0.003103975	0.422580641	0.457236498	0.404529845
0.068355415	0.033450121	0.253742425	0.375596965
0.474478603	0.454241053	0.506931013	1
0.246637445	0.370643159	0.481958382	0.227659451
0.44752896	0.211798679	0.132508693	0.489092761
0.504203975	0.531873873	0.281652231	0.353403544
0.444124186	0.467158258	0.461237604	0.495196389
0.418234493	0.091546953	0.436886814	0.51945404
0.390151529	0.422617179	0.222581525	0.417352331
0.089134806	0.369933631	0.410519343	0.199143957
0.475599453	0.026974751	0.302684935	0.392066646
0.264209824	0.461329594	0.531331287	0.229460472
0.479353683	0.031748994	0.211386093	0.055038482
0.36697339	0.229044535	0.479898268	0.474622799
0.238040507	0.462416091	0.3001436	0.005164817
0.048238349	0.247136264	0.462154232	0.399183228
0.464116238	0.392099951	0.307484677	0.389525487
0.494594716	0.508138965	0.053978269	0.121032598
0.460099528	0.251536441	0.304136468	0.493123917
0.401697867	0.130927898	0.168434228	0.52165635
0.402534149	0.115538683	0.336017918	0.476881338
0.379049925	0.45726575	0.139757861	0.32527787
0.064394365	0.494152751	0.052994654	0.336021708
0.339194901	0.221863762	0.01525663	0.25996129
0.313734588	0.453715846	0.469026805	0.144008657
0.370232426	0.484975533	0.31391836	0.45720764
0.018641993	0.065377825	0.260745713	0.510895109

0.268105721	0.505274368	0.354103706	0.388118412
0.369388434	0.485975602	0.005040995	0.493710456
0.537360403	0.28035422	0.46286057	0.482517415
0.455668589	0.476426082	0.043488835	0.47609702
0.352872683	0.108829847	0.504081888	0.228361957
0.458357677	0.067800548	0.330646211	0.487702585
0.472723247	0.012971403	0.216085645	0.412970971
0.050952237	0.099880429	0.454090399	0.45192861
0.4394196	0.493648284	0.498293222	0.44178626
0.493527519	0.260397674	0.021067659	0.415125651
0.477245569	0.432019638	0.428539568	0.271252609
0.325373132	0.469340462	0.445446818	0.237225007
0.062623109	0.329435159	0.02518034	0.414365272
0.409627244	0.14834245	0.450442858	0.196530806
0.115091455	0.533347654	0.270551481	0.360899132
0.205821713	0.436760863	0.291130843	0.273562919
0.281662891	0.227700372	0.442155 0.447946677	
0.044371799	0.136504823	0.026961742	0.429384788
0.184642896	0.389954226	0.37181456	0.418248293
0.355254637	0.14486561	0.461600534	0.379960607
0.173967565	0.02704567	0.509316059	0.460102306
0.321370767	0.194240227	0.065491006	0.378821333
0.477201765	0.311289682	0.247275726	0.443652788
0.039053646	0.497414338	0.311921454	0.182256822
0.468459521	0.322946135	0.477765439	0.071686307
0.404148226	0.459966313	0.38521273	0.260997659
0.376120988	0.453141654	0.041930662	0.413633922
0.407052859	0.051563821	0.535072864	0.191737353
0.03716897	0.505568236	0.489177769	0.116690637
0.122168182	0.385408823	0.049102986	0.377878
0.523597259	0.48027322	0.458907959	0.309974858
0.06019429	0.017692729	0.299234411	0.459467553
0.508735891	0.524898613	0.329936505	0.459458114
0.296823058	0.422553851	0.132775189	0.517626815
0.2437691	0.520815463	0.012320925	0.500316441
0.517039018	0.276245008	0.130207146	0.327733262
0.388814279	0.066631077	0.236472889	0.499181418
0.448244254	0.407187969	0.422301156	0.477077193
0.020657146	0.052972888	0.46799246	0.277630355
0.442015901	0.284269888	0.095992732	0.336080868
0.064819467	0.446599131	0.495381057	0.096034286
0.346695456	0.310055123	0.466279701	0.056174862
0.484636898	0.511379495	0.524599799	0.189718157
0.478634972	0.500974613	0.480891231	0.122466001
0.033128056	0.527507865	0.408365237	0.032385656
0.492805439	0.213374174	0.020892227	0.491995817
0.227558478	0.4354674	0.004421811	0.109839325
0.071953627	0.506189398	0.480892825	0.395802314
0.236178858	0.412066904	0.011083008	0.533624309
0.492030136	0.02905627	0.222245993	0.474819336
0.485151874	0.526579706	0.425167348	0.493579412
0.495400034	0.510770761	0.273328797	0.352976497
0.460173382	0.510669055	0.439073552	0.447721561
0.484800159	0.499028514	0.338073048	0.02387891

0.201108246	0.208208213	0.340784174	0.126872591
0.312338255	0.20834128	0.477830141	0.500789863
0.490385111	0.010096333	0.160610633	0.459853648
0.436190078	0.469711182	0.031930993	0.0091473
0.010748888	0.039574789	0.382940488	0.328349327
0.493205769	0.23705606	0.008690701	0.047796745
0.324615947	0.493398573	0.509563156	0.436163477
0.488383448	0.119647417	0.313081145	0.444944442
0.117071777	0.319299724	0.493146037	0.46731599
0.067015297	0.335383131	0.393836383	0.480345782
0.125961542	0.404782946	0.492508768	0.374671287
0.022520538	0.13060709	0.10898978	0.491783724
0.510466179	0.117166698	0.05684692	0.134913733
0.153996222	0.488293525	0.215989406	0.518754263
0.459306167	0.483025092	0.12643342	0.483045877
0.065763048	0.490069874	0.448844199	0.061693549
0.351726191	0.02113986	0.47715791	0.22146541
0.327798255	0.41123446	0.276790659	0.033164975
0.264334172	0.085811266	0.498496313	0.509072951
0.059863998	0.274645703	0.378078919	0.45228834
0.285551794	0.482554452	0.503442837	0.423967613
0.105446318	0.204961126	0.432897051	0.404172488
0.243843861	0.505758123	0.383996634	0.534941164
0.422824538	0.450559252	0.476663265	0.395251035
0.335792724	0.408772492	0.387191997	0.025450745
0.406549602	0.497545454	0.377629551	0.230459121
0.345070962	0.473228254	0.062109457	0.181163292
0.423992053	0.29994218	0.187236309	0.482650787
0.402914162	0.407551397	0.046168605	0.102132314
0.545865694	0.424955783	0.156018065	0.45932276
0.233718168	0.427795959	0.495545043	0.50608865
0.426479549	0.472522929	0.444863327	0.400895409
0.409617077	0.31156205	0.529914496	0.078233672
0.134024741	0.451981568	0.136156614	0.143241573
0.192482199	0.04780865	0.39318208	0.486458007
0.453906942	0.450666491	0.319039341	0.116776894
0.418451484	0.386786042	0.433546117	0.222442594
0.111171705	0.502112396	0.016276342	0.088506856
0.463144311	0.478043582	0.480595859	0.32004295
0.406021662	0.211579564	0.376704108	0.285688386
0.33836021	0.40804253	0.470628927	0.041978548
0.025459093	0.500110157	0.379140836	0.037099904
0.133183483	0.390705316	0.336248471	0.304138926
0.04220785	0.496697253	0.34469065	0.498715783
0.371796127	0.094534306	0.332071697	0.10761705
0.078408051	0.437011584	0.474293743	0.524550021
0.429441963	0.478704756	0.433009084	0.461425437
0.150713629	0.425779112	0.038519639	0.466317838
0.457217107			

CBLB 0.142719708 0.208825023 0.09661775

0.242108423	0.170559098	0.087007707	0.11957223
0.031814734	0.023351037	0.033342626	0.041399229
0.178239978	0.071954667	0.102886116	0.02428801
0.258912523	0.181382535	0.058077009	0.198776022

0.201575391	0.045968904	0.307131001	0.069918534
0.059752529	0.141030287	0.125412168	0.098346629
0.251690887	0.13889333	0.065136202	0.159356975
0.098779206	0.18297789	0.171020692	0.269869243
0.171349684	0.110577633	0.025382331	0.041813641
0.153794284	0.199859071	0.197166547	0.242629685
0.061762421	0.147351356	0.17804551	0.184556372
0.014792986	0.094683501	0.02856595	0.128700672
0.157009482	0.126122034	0.046621202	0.087143781
0.047313518	0.036433637	0.057462265	0.088937978
0.169273173	0.027315461	0.167115751	0.019573459
0.012497713	0.063070059	0.127821036	0.102537327
0.063490312	0.115241567	0.021346287	0.116519426
0.01316787	0.19780372	0.208465057	0.047615684
0.170748681	0.096404103	0.165156112	0.185996549
0.24447697	0.152816074	0.282663962	0.246637445
1	0.168761639	0.130063534	0.168222994
0.164650234	0.129604151	0.044757391	0.299961331
0.273152455	0.198289148	0.07540564	0.199366178
0.176094517	0.181439576	0.096216488	0.127325104
0.048758737	0.051892025	0.146810908	0.248024072
0.132625262	0.119142772	0.222919745	0.186888986
0.122131415	0.027733586	0.264487117	0.102013884
0.253929176	0.02097954	0.218378611	0.160851031
0.036700544	0.178066288	0.197757661	0.1625196
0.156853433	0.08885807	0.029767814	0.077731533
0.038369125	0.046110263	0.131733387	0.258986752
0.040240272	0.045905127	0.096376836	0.030050038
0.115790707	0.040801072	0.187114194	0.101535169
0.178826607	0.113152546	0.118718691	0.112527345
0.156189419	0.274448565	0.002925365	0.061853868
0.144212031	0.041901167	0.255765016	0.155562173
0.2069478	0.058228142	0.15539164	0.244791231
0.136328857	0.159840708	0.045488339	0.285218229
0.039608272	0.239251536	0.122594902	0.165388069
0.059420072	0.158014885	0.128597539	0.070287321
0.015872479	0.098038356	0.013741741	0.071194631
0.035920099	0.188472212	0.125461361	0.140715821
0.188991438	0.248570419	0.060809207	0.193515067
0.028513452	0.048115277	0.032251986	0.247574036
0.067525337	0.235314492	0.104602609	0.086893772
0.24744473	0.234774105	0.001698481	0.156304925
0.19451479	0.035364742	0.259732952	0.18393662
0.07267247	0.24858381	0.004582569	0.289644637
0.095088753	0.106351597	0.276408858	0.252688069
0.159796798	0.136287208	0.066042546	0.188748241
0.179097624	0.013552187	0.046707556	0.176092478
0.074606426	0.022202249	0.160365168	0.205559765
0.150093151	0.217807235	0.291057731	0.066584722
0.172088274	0.100020725	0.029882297	0.198751451
0.180094708	0.017094161	0.146163616	0.060553174
0.063983808	0.181916074	0.14310357	0.008208913
0.092191165	0.090049965	0.037796656	0.14595766
0.113492357	0.096168322	0.06939996	0.051996655

0.167376374	0.236130094	0.093954006	0.074148559
0.053564967	0.182649404	0.133753174	0.110337753
0.094636345	0.080870288	0.067019268	0.06602365
0.038096153	0.198530738	0.019110501	0.087277859
0.105980387	0.125982552	0.11030704	0.125808814
0.062915778	0.138265244	0.20683352	0.042347697
0.104719606	0.115699397	0.203991925	0.230282863
0.087833988	0.069497944	0.125096372	0.028449275
0.248552933	0.057672145	0.020125536	0.200645501
0.031523371	0.245390902	0.094412523	0.225233336
0.155603564	0.254846953	0.249650208	0.007087514
0.09804868	0.178206562	0.168606636	0.142383339
0.144428072	0.182620837	0.036705693	0.126789
0.13913512	0.150641408	0.225088367	0.138927351
0.119854977	0.225657316	0.178962976	0.176840758
0.132529118	0.141944463	0.019274224	0.281211121
0.230051138	0.318627637	0.167497509	0.08232183
0.108802065	0.089816627	0.143740118	0.117193011
0.163203321	0.225138223	0.228963471	0.21022672
0.221294235	0.057728132	0.00739602	0.242863957
0.024523471	0.182684961	0.054421691	0.196938463
0.16272638	0.057067721	0.160772235	0.100186818
0.143519281	0.069912052	0.071335281	0.183498454
0.16315286	0.075108103	0.061436925	0.199880946
0.023087182	0.026134872	0.145151036	0.051557725
0.060372368	0.087964141	0.021188451	0.03186468
0.072383176	0.173164954	0.268579586	0.04445714
0.08197473	0.028331806	0.263545682	0.099279896
0.201624999	0.185061106	0.181393485	0.069214197
0.193689231	0.205902385	0.138340252	0.134513404
0.115330707	0.182231407	0.119194323	0.049735486
0.270030563	0.035416243	0.076228679	0.194893446
0.147962056	0.119935484	0.002811141	0.008569133
0.052781953	0.266556219	0.149858082	0.147672862
0.026933879	0.171946652	0.005020326	0.225677435
0.194513956	0.077755148	0.066001227	0.187311903
0.18829868	0.198325726	0.100707252	0.222878928
0.183269785	0.171603069	0.049483175	0.239142193
0.205889528	0.181642688	0.171671884	0.160315895
0.268402436	0.248526658	0.217096205	0.037780756
0.014712603	0.042747599	0.029649992	0.025829552
0.068861967	0.101050301	0.197114805	0.262810343
0.244703086	0.064986873	0.034107747	0.168651549
0.203651204	0.235348231	0.016921915	0.003813669
0.005130937	0.044855567	0.128075541	0.165325831
0.205084958	0.016766051	0.052126798	0.021080249
0.042575691	0.214208465	0.194525989	0.271491554
0.191812537	0.125302886	0.051456198	0.238164082
0.053345752	0.068316562	0.250998204	0.166222365
0.054161226	0.129222261	0.085751561	0.251520632
0.021077755	0.261769197	0.192799428	0.061453103
0.019390728	0.113723465	0.071936803	0.248338043
0.161912791	0.124316244	0.111102501	0.011270273
0.121132079	0.22732996	0.13407218	0.262320061

0.212773514	0.301033019	0.060426967	0.160214362
0.036506584	0.173413486	0.181859511	0.008807235
0.099856793	0.037485578	0.180190467	0.020187696
0.067316394	0.13727878	0.015471041	0.068444434
0.021534087	0.155168372	0.270673339	0.254353998
0.048973619	0.055853665	0.184688941	0.186832135
0.086278821	0.26877527	0.165698614	0.077814014
0.054909175	0.030333881	0.197940956	0.123076563
0.051152598	0.279800406	0.062670969	0.222427405
0.1310353	0.173162526	0.198988762	0.135663413
0.052747382	0.101844151	0.153122191	0.048115509
0.182606347	0.279968551	0.086830022	0.068287228
0.216139515	0.117134295	0.020524495	0.012477778
0.096581055	0.080946291	0.07403217	0.274579651
0.12436402	0.12704648	0.023562455	0.0689098
0.212628584	0.140772477	0.018502153	0.172568629
0.167428898	0.17279095	0.214783166	0.233555102
0.028468336	0.215007415	0.196170233	0.168603637
0.152798317	0.140069636	0.182514332	0.009629674
0.032981057	0.12469419	0.110380611	0.1754529
0.206887007	0.05078267	0.042406124	0.260090268
0.061443923	0.292686904	0.017584561	0.04570208
0.199782108	0.058223461	0.221880285	0.100943207
0.083195685	0.173903628	0.029453439	0.143627331
0.092958706	0.177959398	0.161368994	0.141979412
0.046861896	0.060270461	0.239370973	0.062099182
0.091855581	0.023223289	0.174573688	0.020164103
0.036654277	0.279369814	0.073328393	0.031653514
0.022824989	0.082777841	0.061100654	0.04197283
0.081995322	0.197005599	0.047485949	0.148128365
0.124437883	0.098621472	0.098375345	0.015416527
0.026320985	0.152912524	0.132142062	0.189337119
0.111044652	0.305137631	0.119667551	0.185904222
0.028598956	0.076356023	0.034002442	0.209858924
0.063103395			
CCDC109B 0.297869238	0.32820923	0.197934314	
0.325010493	0.254875157	0.148359178	0.137235967
0.112102716	0.094357268	0.011321518	0.039289304
0.266671484	0.102817099	0.112364843	0.091265597
0.415825034	0.185308022	0.192151828	0.221782348
0.340540951	0.074161576	0.37877025	0.143137241
0.2259443	0.143961688	0.130055771	0.167125619
0.384234179	0.146158994	0.089646353	0.264344165
0.274380248	0.325034091	0.286498213	0.297772525
0.275727583	0.24081455	0.075492219	0.086809451
0.302401936	0.308423354	0.328399432	0.258452502
0.091657062	0.248701031	0.18187916	0.314696776
0.099526106	0.083265193	0.067461106	0.239583963
0.231021781	0.182224225	0.121131047	0.121318446
0.065155256	0.075733523	0.098332473	0.15959757
0.167064864	0.051052542	0.259978576	0.077515457
0.078520681	0.162435449	0.271970478	0.150130189
0.176423599	0.226779098	0.093141026	0.221455813
0.004364133	0.340453657	0.344077385	0.109159899

0.15264373	0.049079548	0.277062896	0.225318916
0.390518723	0.296258562	0.395558107	0.370643159
0.168761639	1	0.221463376	0.179748359
0.304316489	0.116183628	0.124900708	0.425591305
0.388801087	0.304832306	0.204342397	0.291157821
0.238888717	0.305920681	0.145124297	0.172680663
0.099650273	0.206775227	0.239767495	0.361052801
0.246500435	0.168576485	0.212010477	0.293949116
0.185089315	0.117518711	0.314570945	0.186260769
0.393716861	0.053245549	0.243512599	0.191391424
0.273591547	0.30427189	0.306763976	0.169582545
0.250255541	0.082288142	0.084237813	0.188677388
0.140638793	0.137662441	0.225412371	0.331733891
0.123228014	0.17193192	0.175399436	0.050279261
0.136097474	0.303090481	0.286702013	0.2165481
0.344341935	0.23506019	0.138039114	0.212172544
0.231613109	0.395201184	0.00867269	0.157568977
0.197661812	0.296628671	0.286066169	0.223004633
0.197725434	0.143679883	0.187779273	0.376314046
0.245468583	0.152684549	0.138276371	0.403987032
0.103450558	0.272486586	0.189023442	0.159914915
0.091297871	0.259616087	0.152434174	0.153801852
0.07501824	0.175901071	0.100840229	0.117910949
0.103854247	0.241177743	0.227278019	0.218143504
0.169345516	0.331553008	0.124082531	0.210575916
0.020042895	0.166514325	0.082979133	0.3440909
0.110487883	0.384617203	0.226714644	0.249468039
0.290393	0.385460844	0.016608549	0.257306104
0.306778167	0.073432892	0.21841359	0.319759431
0.123732434	0.338909272	0.022713793	0.39224119
0.189108715	0.199256081	0.414284251	0.193673683
0.276723213	0.116159665	0.122921897	0.302890165
0.231032534	0.029750246	0.069173537	0.280863225
0.163664196	0.06407555	0.324797741	0.303785467
0.210146122	0.386974488	0.431030164	0.109449356
0.219155626	0.188388203	0.107082505	0.286124536
0.341577468	0.108739886	0.248211876	0.155986487
0.128797047	0.289073204	0.245824827	0.055501908
0.102470366	0.153625759	0.031024327	0.19329485
0.241243662	0.091561785	0.13611819	0.296545031
0.185727272	0.351121169	0.187935788	0.152523829
0.13276567	0.166461396	0.210176166	0.077047235
0.084512384	0.198704495	0.149832193	0.146579186
0.059603747	0.156351124	0.144747467	0.093481225
0.063492798	0.088382978	0.076142056	0.217674618
0.135448318	0.098224654	0.182911035	0.206636602
0.197615618	0.105342032	0.32378897	0.37614352
0.164668408	0.170311765	0.18095062	0.072427986
0.330227581	0.07759171	0.068968829	0.28509446
0.120888632	0.402469465	0.123959582	0.180489675
0.145186191	0.198546036	0.394487175	0.031107774
0.208217563	0.272070447	0.30775565	0.174806413
0.20486514	0.287195663	0.075974231	0.238554598
0.220647686	0.122064474	0.336940277	0.222023311

0.136698857	0.346383731	0.243170939	0.190415364
0.193059443	0.122977209	0.055956993	0.324895937
0.339840502	0.389442999	0.235738573	0.241853108
0.091621495	0.024510257	0.272390187	0.230347727
0.267032655	0.335628645	0.296388144	0.161714089
0.261527648	0.129364547	0.060468987	0.343375894
0.255275693	0.312045847	0.078513689	0.313932729
0.260961826	0.090428019	0.140048973	0.143783437
0.21401212	0.114747603	0.237041755	0.279205611
0.294169755	0.162717566	0.123598176	0.343570021
0.042385911	0.085477908	0.255848542	0.17265762
0.128204819	0.210577826	0.077336781	0.131205256
0.085876078	0.255343298	0.412522743	0.104208922
0.099507289	0.120214317	0.378061277	0.126824028
0.305849998	0.315402351	0.288326477	0.202798972
0.305740749	0.297073536	0.178838188	0.235741813
0.122665088	0.29231239	0.248972359	0.044519328
0.400206368	0.06068499	0.025996577	0.314372894
0.199701387	0.214848568	0.005781952	0.116547807
0.201906289	0.417902333	0.24797947	0.271698964
0.042500797	0.328779253	0.069746379	0.362536889
0.277996177	0.093613992	0.222522527	0.256411757
0.247403155	0.328930158	0.143606389	0.300371357
0.209186352	0.265421305	0.114080925	0.277416698
0.280834514	0.288312357	0.299692292	0.285870041
0.359059026	0.382126771	0.253974171	0.051641356
0.114902332	0.091256147	0.056217913	0.067915973
0.145322808	0.164958869	0.326175624	0.406787784
0.333734644	0.070700088	0.113150242	0.259790374
0.31535701	0.384482452	0.145913425	0.017692125
0.02451008	0.04657003	0.226320602	0.266678942
0.330637762	0.039407905	0.051923408	0.070397063
0.174344087	0.28419293	0.293194395	0.387902722
0.344819051	0.212580278	0.063211382	0.392118716
0.126825066	0.160157847	0.339855405	0.265085343
0.214098257	0.258196779	0.17777596	0.37748297
0.0619328	0.326922729	0.282355202	0.12297483
0.130838895	0.195340973	0.146886341	0.422813445
0.258231829	0.083282477	0.071749704	0.039410037
0.10007057	0.380867405	0.150105368	0.39465226
0.319189348	0.416138962	0.214034064	0.273655563
0.07673307	0.258171276	0.201607475	0.022647619
0.277355596	0.119084532	0.29285369	0.050239932
0.109806492	0.275870057	0.12221922	0.133880358
0.137537046	0.167909899	0.380125868	0.386814697
0.197065474	0.191311304	0.288377618	0.354319291
0.126456588	0.320912311	0.268287191	0.107996458
0.098620012	0.191268321	0.327015423	0.219263639
0.122725244	0.413634806	0.122278041	0.360962084
0.262322285	0.332909108	0.305655105	0.20766105
0.190128138	0.185795695	0.186802257	0.031635058
0.27354524	0.359318747	0.198797115	0.127520304
0.177889367	0.197819279	0.16196676	0.104132182
0.146214241	0.124456302	0.145245395	0.439179132

0.253042088	0.224198383	0.126884522	0.129441544
0.34261404	0.275656704	0.095039414	0.25431749
0.275899446	0.233029308	0.353312943	0.363754111
0.147507742	0.281921204	0.30758589	0.190198036
0.235541785	0.209167704	0.29442306	0.059170459
0.109925325	0.232187583	0.094761323	0.230484798
0.182407454	0.134862635	0.111643127	0.406763631
0.117856233	0.303141066	0.109240751	0.177214576
0.303752764	0.155780207	0.315163525	0.10765794
0.104563453	0.264119099	0.059427172	0.197342452
0.138092322	0.280181703	0.167247167	0.246597281
0.146674376	0.282577022	0.249744631	0.095976534
0.060650677	0.144331057	0.268690006	0.077041353
0.068157928	0.416058783	0.141577204	0.166512317
0.192602179	0.201923782	0.247325827	0.121422659
0.147627974	0.291464729	0.069747681	0.24834282
0.210113186	0.205637514	0.198343472	0.109031934
0.183206441	0.257634213	0.236854648	0.293427598
0.233603844	0.377851566	0.225505882	0.275156265
0.203747538	0.177659559	0.076322266	0.365265392
0.090723816			
CCDC125 0.344453369	0.446091995	0.23104086	
0.299091948	0.245806509	0.15581176	0.085138157
0.060697287	0.053577303	0.001601509	0.024542366
0.30641098	0.109181473	0.093788326	0.121344904
0.364067407	0.127432492	0.178332029	0.126834206
0.279234328	0.028000097	0.325294237	0.052622483
0.273577413	0.111975619	0.059046756	0.20904837
0.360948205	0.066202069	0.095584939	0.224953799
0.131082985	0.353984265	0.436787862	0.339231888
0.443807939	0.302629758	0.019372458	0.097077439
0.334793125	0.35977506	0.27645343	0.173639104
0.025241175	0.372112886	0.110084727	0.306674976
0.080429947	0.032196848	0.061153441	0.334368916
0.378482273	0.240123472	0.076981809	0.067580249
0.103991478	0.064781969	0.201550958	0.146712358
0.109753981	0.034931188	0.408408832	0.037412581
0.074688149	0.100455522	0.180929388	0.099031632
0.276140317	0.238976928	0.013563962	0.297026913
0.000197178	0.256661978	0.288381311	0.217956263
0.049336943	0.014281942	0.187993645	0.163574804
0.394012346	0.40910162	0.469446037	0.481958382
0.130063534	0.221463376	1 0.106463601	
0.362627805	0.072735714	0.059431537	0.388706632
0.475773916	0.456038016	0.169188195	0.223663816
0.302665398	0.388100935	0.174547027	0.210873464
0.174861511	0.107672704	0.350680158	0.476326641
0.289986282	0.273779511	0.137394202	0.28064914
0.062515263	0.135459348	0.224090953	0.125770351
0.404721292	0.011459137	0.203576496	0.15263346
0.157920306	0.339814563	0.46942266	0.094459383
0.250457898	0.020584577	0.125838386	0.056105846
0.145112107	0.215425891	0.37904719	0.372624781
0.083797724	0.224647529	0.120011315	0.007305392

0.043330767	0.195350875	0.372453833	0.190190349
0.335430638	0.355615738	0.13209884	0.254012843
0.325521475	0.470874893	0.010702203	0.05432853
0.193499568	0.201923526	0.151458843	0.259180581
0.200409435	0.051650174	0.083352456	0.470401496
0.308862066	0.0555545	0.135631742	0.346755427
0.134159143	0.261783848	0.072325051	0.130358396
0.024801673	0.428665927	0.058572306	0.130381657
0.146577536	0.16100126	0.01507218	0.085426904
0.099302208	0.341754418	0.387801255	0.084985548
0.149471507	0.304286433	0.108863871	0.267988709
0.006905749	0.044327739	0.087498548	0.449334912
0.109025031	0.488654253	0.164865833	0.23245869
0.203736385	0.388949215	0.006521272	0.395117269
0.46760426	0.088561622	0.216278039	0.307785606
0.216858552	0.36311756	0.022371706	0.335749347
0.211181279	0.066529469	0.478883156	0.127020351
0.393697255	0.0411939	0.116853899	0.431965433
0.351210311	0.009447338	0.057835077	0.23261155
0.064381817	0.027308291	0.321681765	0.260768756
0.200253353	0.40747854	0.452012452	0.209000312
0.217540271	0.107631622	0.022243625	0.250615478
0.397086724	0.199888045	0.329353129	0.103083994
0.140465703	0.382951415	0.352889339	0.083890626
0.029082813	0.119551295	0.007896404	0.188284531
0.345526128	0.050626351	0.1971624	0.163552882
0.08957764	0.468820763	0.231909012	0.261527901
0.200914616	0.18431476	0.127735991	0.085375273
0.113846734	0.12709904	0.221375943	0.191622923
0.013583475	0.073065307	0.049459742	0.180330644
0.067560819	0.140458804	0.124498606	0.260562335
0.139929343	0.058289016	0.198647708	0.251806999
0.119946545	0.023857113	0.433641118	0.304682617
0.185371923	0.086424614	0.055122119	0.136159254
0.38988804	0.096374233	0.077958908	0.355922245
0.028966594	0.471839458	0.11941784	0.107078219
0.22400891	0.136803498	0.397454634	0.024543916
0.193242838	0.365340961	0.217122588	0.135195935
0.279542988	0.254570789	0.017651565	0.288609823
0.272244427	0.034323636	0.474673855	0.144141981
0.036147585	0.386421536	0.344362884	0.081214214
0.087951943	0.135531062	0.013423357	0.220340709
0.450485799	0.348446039	0.314249809	0.247891169
0.027343762	0.008363044	0.234507908	0.257106529
0.41333496	0.483790543	0.180682347	0.209502322
0.135972112	0.199089504	0.039126385	0.461846645
0.198489378	0.451069112	0.012681341	0.420388399
0.430455592	0.08495022	0.082726494	0.108419583
0.262311185	0.033019881	0.1224475	0.408122509
0.358983097	0.187031602	0.171992574	0.326504225
0.006594519	0.020312632	0.254825819	0.240319219
0.173811969	0.147197022	0.130218824	0.127233795
0.028238315	0.351028832	0.423497464	0.051578389
0.123164352	0.114358776	0.319095845	0.033222183

0.45293725	0.470514685	0.431915409	0.08861474
0.377137885	0.439921965	0.285021873	0.102161223
0.036032004	0.456919134	0.244370787	0.012585139
0.429951237	0.056388085	0.007413145	0.405422454
0.102947873	0.34497993	0.000810453	0.11244952
0.090906379	0.462477643	0.382714046	0.223323546
0.058263825	0.228778574	0.030173247	0.498998769
0.396970139	0.031208113	0.192415822	0.3623218
0.376171683	0.442404712	0.241295235	0.425189649
0.292764003	0.42792505	0.095717911	0.183744027
0.336899818	0.467374723	0.292042177	0.364001397
0.39151288	0.371365583	0.151501799	0.010377192
0.125715539	0.062339654	0.106025784	0.05473715
0.135376367	0.116315561	0.422330321	0.444121681
0.455152298	0.012011037	0.052409465	0.345143488
0.335694185	0.405740584	0.039614708	0.007199304
0.010078211	0.029759789	0.168418343	0.210858533
0.440669866	0.058283961	0.006988431	0.015213581
0.129629026	0.333417347	0.426108379	0.287912853
0.416602776	0.084244688	0.09279807	0.304412392
0.083971455	0.186562201	0.44758819	0.365821471
0.074257202	0.212540547	0.175870837	0.423772686
0.142096495	0.228803325	0.324788532	0.138464685
0.034636228	0.100851346	0.077117092	0.409526601
0.43239767	0.053590901	0.030767449	0.038706086
0.055125574	0.439136556	0.091268398	0.48036125
0.340668216	0.385349627	0.131521273	0.391320904
0.039671822	0.399700582	0.254342391	0.015033501
0.212739015	0.022079222	0.43789369	0.094908329
0.109944434	0.248348196	0.158322246	0.046291055
0.218298833	0.064429026	0.436393077	0.462369055
0.080685755	0.119385269	0.211552862	0.289515355
0.087980572	0.327348647	0.404896815	0.152471783
0.037980882	0.217502764	0.27513263	0.236094291
0.086080781	0.482527123	0.166633947	0.487661516
0.281926359	0.405565566	0.428201985	0.160902519
0.23760232	0.185228827	0.189838541	0.006825629
0.185255189	0.403443149	0.158127422	0.121585809
0.166095274	0.275488261	0.043945506	0.085547047
0.253413778	0.109087667	0.080807283	0.403177324
0.224809744	0.251060003	0.02685949	0.066329293
0.471409204	0.353947665	0.049367187	0.262360424
0.152415303	0.197262854	0.410911279	0.436227219
0.209566997	0.372022483	0.312862522	0.20812457
0.308267285	0.133579436	0.439656127	0.022184268
0.091987497	0.283883516	0.054794494	0.114738631
0.093262965	0.043069367	0.233904941	0.368224117
0.23558875	0.251775123	0.123259963	0.071221813
0.248565894	0.181116435	0.231558594	0.070005858
0.046192505	0.403157469	0.013581472	0.07121853
0.244959431	0.361101487	0.232252364	0.180714156
0.25212889	0.201071031	0.178405184	0.085700553
0.098771865	0.198828246	0.352223232	0.018361211
0.010810527	0.469256937	0.141969941	0.053174863

0.135190558	0.220417944	0.188310965	0.123448311
0.056139249	0.451794187	0.104396554	0.265216739
0.145611694	0.0658546	0.25000284	0.055863871
0.052878742	0.350937417	0.276531397	0.456431476
0.262979233	0.345737215	0.339200108	0.355418815
0.102477572	0.224606338	0.016498187	0.375036847
0.185605281			
CCDC28B 0.135080285	0.204339288	0.09005581	
0.289437163	0.17673521	0.117027485	0.215602223
0.071447287	0.098431739	0.070108774	0.103451727
0.141362842	0.118142941	0.117882905	0.04516722
0.294449234	0.179490428	0.066377999	0.281313631
0.247415924	0.120767093	0.327046069	0.174382049
0.070878084	0.138113054	0.185092219	0.10198321
0.278356984	0.19782848	0.092600118	0.205039045
0.133727622	0.181141698	0.158887117	0.230753765
0.148972914	0.104775696	0.035200829	0.072286487
0.159713794	0.202911606	0.253144252	0.302418474
0.168474184	0.140595934	0.279263101	0.169080802
0.051921826	0.159957485	0.050130487	0.134745513
0.142455769	0.137802455	0.092538654	0.169388247
0.083173844	0.053956913	0.052819373	0.116496957
0.261655613	0.054130793	0.154520281	0.109214164
0.056874896	0.106667716	0.132905822	0.159694406
0.056885947	0.138515777	0.091003885	0.092778206
0.034311713	0.253727054	0.190007742	0.044898308
0.199975467	0.177023943	0.141994158	0.269027047
0.260314499	0.140290621	0.262547146	0.227659451
0.168222994	0.179748359	0.106463601	1
0.168732822	0.210849438	0.093568552	0.311241926
0.25326541	0.180716371	0.132718725	0.209107177
0.19556151	0.188040515	0.101346781	0.138282098
0.051267338	0.086696169	0.151961882	0.226855879
0.138768827	0.08263054	0.23281585	0.214439575
0.169826925	0.043636611	0.333551908	0.103166714
0.267122028	0.097945454	0.203833535	0.21784684
0.100222217	0.177843211	0.174319489	0.262209001
0.133190973	0.135210506	0.052779986	0.123708389
0.079888249	0.040038261	0.123108466	0.262654623
0.083510079	0.081722278	0.161074722	0.055478845
0.143926099	0.085306371	0.161980757	0.107569074
0.199957426	0.098706888	0.156798192	0.121031289
0.135113077	0.257892253	0.008974392	0.158302139
0.148912686	0.091176027	0.331539385	0.149292458
0.200756579	0.135252661	0.21935536	0.23898839
0.150862463	0.239582308	0.054186679	0.294610817
0.083430536	0.194189793	0.184411088	0.159565601
0.170844558	0.137916376	0.119874518	0.089265434
0.037605857	0.124225819	0.049951241	0.119032125
0.090256995	0.183113636	0.112937919	0.199207261
0.199613173	0.261026086	0.080189744	0.184061591
0.092776063	0.129981144	0.041812523	0.228526651
0.091766658	0.21089728	0.125597005	0.099455664
0.262304133	0.251864807	0.003188961	0.146236944

0.175254784	0.040611097	0.234186838	0.164751264
0.076009187	0.267003757	0.004653051	0.293812578
0.093860885	0.166936779	0.268983361	0.270132642
0.14834497	0.186958552	0.09233158	0.165354765
0.138055747	0.034366681	0.090480337	0.241619936
0.098274986	0.092131478	0.179374297	0.255252199
0.199252794	0.219716706	0.293481475	0.066873395
0.16534061	0.148012672	0.081301201	0.17683275
0.171261299	0.039590156	0.157169247	0.125129115
0.065308306	0.186809231	0.126016162	0.039193369
0.173610907	0.14877231	0.067811643	0.178012453
0.112714259	0.189927665	0.078095533	0.096015153
0.164644467	0.22566869	0.065522974	0.048358157
0.038621632	0.17543074	0.203670284	0.119518717
0.122857973	0.157057578	0.070379163	0.088850027
0.128393913	0.21737986	0.038259563	0.053241762
0.143192627	0.081680722	0.075323621	0.129761237
0.087792204	0.191918071	0.178412367	0.059492997
0.133947635	0.139284575	0.199631679	0.25655415
0.101711813	0.156826039	0.153064919	0.041995192
0.253313905	0.072453152	0.029273174	0.213565669
0.077266649	0.240836067	0.159221992	0.243261508
0.105172888	0.296087187	0.279638879	0.034794423
0.114416106	0.181510077	0.190857768	0.181486522
0.139189775	0.214402323	0.144298841	0.127616662
0.126988477	0.179004989	0.20049224	0.15194683
0.136871376	0.198577045	0.167779117	0.222320729
0.152223226	0.179451149	0.095638135	0.304620572
0.223164966	0.330676404	0.1794275	0.070829055
0.187047045	0.123044887	0.121749147	0.095787594
0.149269739	0.193521291	0.260263247	0.162406287
0.314492829	0.068701664	0.037639042	0.223192927
0.045330999	0.16658935	0.08689579	0.178168998
0.144579209	0.073830689	0.165586648	0.170133217
0.150289961	0.155765629	0.11279641	0.170166897
0.18550454	0.102977495	0.074932563	0.185202041
0.103291506	0.144741647	0.157146614	0.047740033
0.082735481	0.142291851	0.017501511	0.056717157
0.202381424	0.177254482	0.254540632	0.130365461
0.108859352	0.046284004	0.252773332	0.16599137
0.1722711	0.161435507	0.167065496	0.129635706
0.167535589	0.18649098	0.103437317	0.151174141
0.117115846	0.159252318	0.14069619	0.142719843
0.271446856	0.053807102	0.142912456	0.197631963
0.243171451	0.110981594	0.012649325	0.012366834
0.082405757	0.244280404	0.147203026	0.196819217
0.040393378	0.209156299	0.009899912	0.200896223
0.182190495	0.097659006	0.103964343	0.160856202
0.144657587	0.19323626	0.080780198	0.190496929
0.132287514	0.154593866	0.130588486	0.289232684
0.209424865	0.153864877	0.190830818	0.167329334
0.267446355	0.233911381	0.314049952	0.1208764
0.050215301	0.086898636	0.038152539	0.109098141
0.092976664	0.150980489	0.180125493	0.263190056

0.208135753	0.078738413	0.114655929	0.154197081
0.223783348	0.255430034	0.044566527	0.007228063
0.005691492	0.080000327	0.183654087	0.172644671
0.198699652	0.039219072	0.089894519	0.10368988
0.11226173	0.178136853	0.183233375	0.322886771
0.175711266	0.176820925	0.070368841	0.284777297
0.125901557	0.072655219	0.225994502	0.173339712
0.107398672	0.153503241	0.080878473	0.26801582
0.015582659	0.261766652	0.154657486	0.10713252
0.041741914	0.142549188	0.131967504	0.25295627
0.140523849	0.189122826	0.177843123	0.023269925
0.189173612	0.222773788	0.203368682	0.251595727
0.232269117	0.320693273	0.114243826	0.163758582
0.12831398	0.153312133	0.140584622	0.026738784
0.093295694	0.099079028	0.150708735	0.05245424
0.078680453	0.155067702	0.050704393	0.089791002
0.029810044	0.169901352	0.274518741	0.232857956
0.082403612	0.115618791	0.198258144	0.19952185
0.131932258	0.278336379	0.156919671	0.074580645
0.158196332	0.034459648	0.230331981	0.117650407
0.081589139	0.262777495	0.097449315	0.205273265
0.141877755	0.17987999	0.178675537	0.202190625
0.087485483	0.111051003	0.195660592	0.116307214
0.221004757	0.274650311	0.110117253	0.128655701
0.233914978	0.091753835	0.092620715	0.021311151
0.063828669	0.122004596	0.196620022	0.297158273
0.135494989	0.123334919	0.095460471	0.132626018
0.202578249	0.152164348	0.084932074	0.2005485
0.193308829	0.22204379	0.194154093	0.201330369
0.045678449	0.198520369	0.204920746	0.184142423
0.164908293	0.232747469	0.172030607	0.040481956
0.067403748	0.10704049	0.200629017	0.190102319
0.266579002	0.115024197	0.032870396	0.262750194
0.041420217	0.307693749	0.040146454	0.140753036
0.198577553	0.057653081	0.25102189	0.16367323
0.193406954	0.165708673	0.075219181	0.169053317
0.058044814	0.17556292	0.122312292	0.152303066
0.054954941	0.096218949	0.286290401	0.08314822
0.078787842	0.040474987	0.17367077	0.084028796
0.131997514	0.268328231	0.075211121	0.06728345
0.070134551	0.096122963	0.075650394	0.115518933
0.08918384	0.170498914	0.071033169	0.149380865
0.186214442	0.16721828	0.104897782	0.051737594
0.101416217	0.145598518	0.11156244	0.165175562
0.098115352	0.33069841	0.118761123	0.17178763
0.058739495	0.065597003	0.131632147	0.226105873
0.049575303			

CCDC86 0.412897505 0.510094267 0.390694274

0.39593929	0.397577424	0.370112501	0.227065223
0.234670373	0.100473897	0.00477489	0.020208073
0.405066145	0.184135003	0.262467358	0.338010653
0.404013729	0.123913365	0.392018781	0.255464333
0.407576523	0.110948821	0.372288998	0.109196744
0.285886704	0.097520853	0.070886986	0.251799802

0.418606006	0.083481911	0.323476205	0.391002658
0.111689018	0.420855706	0.517144735	0.383387202
0.50317576	0.393402427	0.01642425	0.293143187
0.409331728	0.407663719	0.404974375	0.322770029
0.048030713	0.491544043	0.251169023	0.386143619
0.262770094	0.029896204	0.274193971	0.457920124
0.462490318	0.389247873	0.272847257	0.174818528
0.167140973	0.251383328	0.218290339	0.339708364
0.235309441	0.186103286	0.470479266	0.102268467
0.16243146	0.299634692	0.153858193	0.286030609
0.3247834	0.389641266	0.029613354	0.377486037
0.002340725	0.398167058	0.370896149	0.283050451
0.045613486	0.017186141	0.17993333	0.334270675
0.42313829	0.462647984	0.434675129	0.44752896
0.164650234	0.304316489	0.362627805	0.168732822
1 0.181566369	0.128185668	0.394421359	
0.446452853	0.503743449	0.2062079	0.263490218
0.384576802	0.472357326	0.396120341	0.390895475
0.309006877	0.058112536	0.432749488	0.476231587
0.385750906	0.285829273	0.149608478	0.401572927
0.068923077	0.36516516	0.344969914	0.155073471
0.437208161	0.024700344	0.205261668	0.349490991
0.255421073	0.416631743	0.495175757	0.204440203
0.394641609	0.020801165	0.124877013	0.041859148
0.321032378	0.131819789	0.484311593	0.411323852
0.264880297	0.353025278	0.2993504	0.002777806
0.027716218	0.208530587	0.396191513	0.392449047
0.42280241	0.378195892	0.216699837	0.387465828
0.390802276	0.428194438	0.065569795	0.12219867
0.408742623	0.208758642	0.24602742	0.406054067
0.287838326	0.149184538	0.137856065	0.48915153
0.438012451	0.094475285	0.369015505	0.396960259
0.304706579	0.326011722	0.126382659	0.263949082
0.059537166	0.473043194	0.02944024	0.378847613
0.255861908	0.131426916	0.020024207	0.296007749
0.281018544	0.384410197	0.474757165	0.125038027
0.304633304	0.37853237	0.352968603	0.37844729
0.0092181	0.060507463	0.320861791	0.473774758
0.299138059	0.471868419	0.318860931	0.313707759
0.277590933	0.420954828	0.004634833	0.497625629
0.507866267	0.326913695	0.356154635	0.407938713
0.346500176	0.408471184	0.053359005	0.396476512
0.365944825	0.097694244	0.46358834	0.154613319
0.455902905	0.043055875	0.334567846	0.388002264
0.376387399	0.011561238	0.249363829	0.390658584
0.029614606	0.123038863	0.424181668	0.394308863
0.364660795	0.419386492	0.43546696	0.324402923
0.398493895	0.271685269	0.017116964	0.311518519
0.416992148	0.352390876	0.445278764	0.2682513
0.352018925	0.46257657	0.387409801	0.194825813
0.050828506	0.337146515	0.025813365	0.324248432
0.385420747	0.113064663	0.377380413	0.171313666
0.071277009	0.497030325	0.167353764	0.239791065
0.11775149	0.356733012	0.286354457	0.204028526

0.16912376	0.178517235	0.339645748	0.361600121
0.048268015	0.092490514	0.019542823	0.312973464
0.107670868	0.286722498	0.286155495	0.393763293
0.320902411	0.09782126	0.351276055	0.290269033
0.117217861	0.016813227	0.493043536	0.404169313
0.341007532	0.179611692	0.047618767	0.289538607
0.423510598	0.310986996	0.311953426	0.430855576
0.039959629	0.446275167	0.216946574	0.119608542
0.32349269	0.25427935	0.420894219	0.069535786
0.408540402	0.459368897	0.332061562	0.178504254
0.388632649	0.415548476	0.037174071	0.390245834
0.383493826	0.033364084	0.500736521	0.121011536
0.023781955	0.401937598	0.387358516	0.081605856
0.083093655	0.303274965	0.064377783	0.283665517
0.492304008	0.381617999	0.38720245	0.222150603
0.042956093	0.006699124	0.208725703	0.388456518
0.506688746	0.478879124	0.273812479	0.317083912
0.25609412	0.314071165	0.18144982	0.473836233
0.232298653	0.514255572	0.008953007	0.396989059
0.500276392	0.31421401	0.076606624	0.321687726
0.369710102	0.056892745	0.252764857	0.484473755
0.417922481	0.331495642	0.334884171	0.416727109
0.021855677	0.056264333	0.379868566	0.178108707
0.359510922	0.230205449	0.049017321	0.375016222
0.04142761	0.442361214	0.420796703	0.064624315
0.315206148	0.363447989	0.363560962	0.044223586
0.404476461	0.485535943	0.514315983	0.20895907
0.40918453	0.455208079	0.349321508	0.090278447
0.018734623	0.502779747	0.408931386	0.020549526
0.427770765	0.26531122	0.009463211	0.490872791
0.210534615	0.454786592	0.006042993	0.101904692
0.047726824	0.41595606	0.478263396	0.398621825
0.242057545	0.391381814	0.008335577	0.497634648
0.474782009	0.014896396	0.141980111	0.39251193
0.373457122	0.523289458	0.292733384	0.392125026
0.373553249	0.48382522	0.239797353	0.265878334
0.40345807	0.468767052	0.417745455	0.463553318
0.412241345	0.398861422	0.288000498	0.019023471
0.148629209	0.252052181	0.274432106	0.076462432
0.350779694	0.1366779	0.468329155	0.420335095
0.438327159	0.006163266	0.179254325	0.404324241
0.422134371	0.449185831	0.031736929	0.008734792
0.010235837	0.016376306	0.381900312	0.252661141
0.465109336	0.216549973	0.005648104	0.063696595
0.293318935	0.360292929	0.485938068	0.379987521
0.411694008	0.092043104	0.248981128	0.401660049
0.068222628	0.346547564	0.444354612	0.472753121
0.05131745	0.261127925	0.397385418	0.455072292
0.063102388	0.314304467	0.406403312	0.297395911
0.019258656	0.084554533	0.071572972	0.419543292
0.487339676	0.064110806	0.027558624	0.170049611
0.117360138	0.429995165	0.158220878	0.479872793
0.41223713	0.409120768	0.07672696	0.482995062
0.042353791	0.477267687	0.30429122	0.06394738

0.312885882	0.016944334	0.451510403	0.132347154
0.341418837	0.413411959	0.212105121	0.018753923
0.183986013	0.055618297	0.42899112	0.410596245
0.039410846	0.282238036	0.30999927	0.413636124
0.313102894	0.398256996	0.501102177	0.363724193
0.10009012	0.142205215	0.41102231	0.396970746
0.2746175	0.445177955	0.271739192	0.504667638
0.415300658	0.457703881	0.446878708	0.357005553
0.211816628	0.408303228	0.355580098	0.020524302
0.366994484	0.407223919	0.397302598	0.142349925
0.237694204	0.35561109	0.072029633	0.215183461
0.296077713	0.319873516	0.144282808	0.417810789
0.408932781	0.401221677	0.06089215	0.066377723
0.526756894	0.446726719	0.176708774	0.401565214
0.187773813	0.333773632	0.414544662	0.408285224
0.381345855	0.393953435	0.417171427	0.361020953
0.424298812	0.284853913	0.51602481	0.131996868
0.096205042	0.398954963	0.08854748	0.099169928
0.149262123	0.030862202	0.268642496	0.408768516
0.335456148	0.358250774	0.349428906	0.091437701
0.322340787	0.395021193	0.354796251	0.210483132
0.077399772	0.487178333	0.010384716	0.061991657
0.341752261	0.410802944	0.335171595	0.247240133
0.272238144	0.149860946	0.292192093	0.322796141
0.27636859	0.360442439	0.4041907	0.048389223
0.025921895	0.451309826	0.393847986	0.026477722
0.100261129	0.395146138	0.330809287	0.233232081
0.024021968	0.449228107	0.321822154	0.401690981
0.365724772	0.079554617	0.37049955	0.11854241
0.086707442	0.454065119	0.381386497	0.495785826
0.356352941	0.3909598	0.463894933	0.400067276
0.153061147	0.376360484	0.035926834	0.423900334
0.35879561			
CCDC88C 0.125372973	0.224085684	0.073440332	
0.312486258	0.144079332	0.164437568	0.267660629
0.128403964	0.153709296	0.124954741	0.100353321
0.114265204	0.1371715	0.172269216	0.086023587
0.262452501	0.125639549	0.06175738	0.25741313
0.243667552	0.243624496	0.271310447	0.228724805
0.055693607	0.102790582	0.181016483	0.086351548
0.284910756	0.178993089	0.144765468	0.241038639
0.076344823	0.177064111	0.178710546	0.189093749
0.160760112	0.104197004	0.017471598	0.156055081
0.17220864	0.195919772	0.262658478	0.291475692
0.219881622	0.169840777	0.308331862	0.123470663
0.08940752	0.135008549	0.100171837	0.167938002
0.167961112	0.142958285	0.159079061	0.256481899
0.108228691	0.099155516	0.044687949	0.16365552
0.266918568	0.124667474	0.172233678	0.228836389
0.091171843	0.135556619	0.077014497	0.184598852
0.047887959	0.172176687	0.095404423	0.074351169
0.046312923	0.253325869	0.129857753	0.042073165
0.126096282	0.156402254	0.08879977	0.307185133
0.26051915	0.145118071	0.240307189	0.211798679

0.129604151	0.116183628	0.072735714	0.210849438
0.181566369	1	0.109259049	0.258066611
0.235189758	0.193087948	0.12303129	0.130505018
0.239401735	0.213279652	0.105728553	0.126491077
0.071143733	0.040784749	0.179671122	0.220620018
0.160555884	0.048889698	0.139102728	0.242300553
0.138626467	0.061921109	0.312316453	0.065253173
0.26987993	0.162100054	0.112613062	0.243427259
0.092283811	0.180217107	0.179991289	0.266348098
0.096517281	0.090417491	0.047268863	0.073341825
0.104919812	0.021269574	0.147998833	0.278260342
0.129481522	0.091392834	0.178523778	0.028942867
0.083218632	0.054573683	0.151077093	0.089604353
0.191130898	0.09811992	0.176927047	0.143131054
0.120592295	0.233435367	0.035670889	0.209865622
0.153981458	0.061168375	0.259261832	0.135844319
0.151094732	0.200260067	0.209332346	0.242204816
0.192975618	0.219468785	0.05723207	0.249024332
0.140516979	0.127436349	0.1922379	0.174512683
0.238773251	0.14896511	0.057541937	0.099727688
0.058170295	0.073730195	0.035923417	0.160119206
0.14702459	0.208207473	0.129065021	0.162299005
0.174586421	0.215748812	0.099934947	0.208823616
0.129895333	0.106770315	0.057167093	0.231926045
0.145550795	0.195662046	0.136912117	0.089662671
0.178275769	0.240396898	0.001337869	0.173086873
0.185246183	0.05729452	0.212338937	0.126264646
0.06967464	0.281275035	0.006024008	0.24371714
0.106549221	0.155529902	0.253836766	0.177645572
0.158651794	0.156756138	0.120100408	0.147587408
0.125810636	0.031793278	0.166526336	0.280189769
0.045258512	0.224380794	0.175764389	0.261492893
0.219973445	0.192331454	0.272730211	0.06097492
0.148614342	0.162920687	0.052230202	0.110249215
0.152512103	0.048700785	0.196534738	0.194636915
0.080874805	0.215322788	0.127207072	0.077965067
0.210904194	0.179709884	0.108791511	0.162118224
0.119778682	0.266202409	0.07977153	0.056224109
0.104393334	0.233835461	0.039038501	0.029414757
0.021347214	0.184026169	0.194856374	0.177451712
0.124914693	0.165648212	0.075377892	0.100716903
0.235469842	0.1901681	0.012667	0.049697948
0.145635815	0.090906836	0.08441584	0.141784518
0.110720171	0.177300176	0.150996541	0.051068493
0.099638279	0.086150311	0.217539643	0.226184582
0.136649215	0.214224013	0.100848876	0.054619471
0.266827189	0.122994614	0.04935916	0.248225668
0.061096176	0.222772578	0.184806528	0.156048385
0.075838068	0.255082492	0.28409557	0.076231684
0.120688813	0.213975696	0.136334926	0.159109617
0.174476928	0.222124221	0.195434823	0.138596582
0.137337655	0.138796851	0.203763832	0.090215784
0.078855545	0.15620622	0.166181229	0.13283346
0.111102593	0.207845967	0.21448545	0.216596016

0.236821083	0.281519567	0.2012894	0.047401562
0.215262928	0.090428827	0.066022744	0.071738194
0.17038622	0.187141804	0.180692228	0.124537291
0.277481214	0.087971174	0.082682118	0.223869084
0.029671917	0.178175926	0.057014645	0.154413335
0.161375225	0.10628366	0.125120141	0.236967771
0.186985148	0.192266755	0.124178882	0.190332548
0.20981398	0.113635196	0.096738884	0.15097826
0.189318925	0.211450911	0.134358939	0.030726394
0.105955057	0.159813755	0.006802207	0.091238218
0.209807891	0.213903887	0.221655628	0.148618227
0.173700908	0.078317139	0.178054566	0.162982387
0.15859418	0.159267109	0.188050138	0.158910342
0.150110043	0.192739753	0.083952545	0.094490677
0.05985453	0.168225019	0.157818506	0.125352052
0.259170465	0.096580177	0.144590347	0.224761031
0.226842277	0.132132717	0.033909483	0.006180666
0.03726571	0.205925704	0.177444326	0.226025315
0.092483409	0.184991448	0.001886303	0.193545095
0.207289386	0.052160495	0.065166268	0.157410001
0.127274837	0.21340399	0.05830139	0.180125127
0.110037225	0.16973269	0.194700055	0.216310586
0.236402338	0.152785854	0.207689903	0.196386323
0.262550582	0.180130482	0.308447679	0.171035011
0.05730083	0.142473887	0.078439671	0.139218088
0.130954003	0.138353595	0.185165403	0.240474096
0.197174916	0.044161495	0.206826814	0.159535065
0.257050791	0.265732942	0.024572364	0.008375958
0.003729519	0.052780208	0.205864037	0.106077715
0.204808361	0.090995087	0.068657082	0.190893
0.142481379	0.130618668	0.201317977	0.293376461
0.150796374	0.139000486	0.138078916	0.263595194
0.097503909	0.089161181	0.22136109	0.21237011
0.058651949	0.10125697	0.071869203	0.276189976
0.007996672	0.179910917	0.124857072	0.163687434
0.016455271	0.09866236	0.113273763	0.216791679
0.152379769	0.167181864	0.13502076	0.046145267
0.268637683	0.208612905	0.212665991	0.244290601
0.251006124	0.296373837	0.060030543	0.19381444
0.135014216	0.173025358	0.09079696	0.058830034
0.074033392	0.061898106	0.146856992	0.081015251
0.100880706	0.164667865	0.061135463	0.041268579
0.022007808	0.116346601	0.268071552	0.202504444
0.034364592	0.16454167	0.136248342	0.170787809
0.189467548	0.276861335	0.183057396	0.085255022
0.264729561	0.014274084	0.238007143	0.126447109
0.130129315	0.237611852	0.129059484	0.204567967
0.15350125	0.188233413	0.181062785	0.234834755
0.061762135	0.127454585	0.266464797	0.185571616
0.198863043	0.261979662	0.113251365	0.116779079
0.179105374	0.066645179	0.091524077	0.024102987
0.041709628	0.190619721	0.228523304	0.269780866
0.13811454	0.134889624	0.096328511	0.122544934
0.215223592	0.17850761	0.176052058	0.220753195

0.120067281	0.200766497	0.168680783	0.164769354
0.054504679	0.202303804	0.219142462	0.229095998
0.21027818	0.236685847	0.193016437	0.091802563
0.063887772	0.092103451	0.236203038	0.101582562
0.213449304	0.078049474	0.030188638	0.215738699
0.035258814	0.267447559	0.059870006	0.116909887
0.127884879	0.052063079	0.197683901	0.232881146
0.220557967	0.193682026	0.047730639	0.112744425
0.041222837	0.182362967	0.095571936	0.122633281
0.047812572	0.052731918	0.22659325	0.122745558
0.102354589	0.043677956	0.184852242	0.115579054
0.164445153	0.248509364	0.081918216	0.02812623
0.04888788	0.103948418	0.069348922	0.178800626
0.039614465	0.169194736	0.115055463	0.119322411
0.213561803	0.133394175	0.133327063	0.06327632
0.099439462	0.168820847	0.084530437	0.173404213
0.062442303	0.304643773	0.147221075	0.171545314
0.044825164	0.053362661	0.189308632	0.225654493
0.049136883			
CCL2	0.108470552	0.128032823	0.080959064
0.187161145	0.093499009	0.084958679	0.138615373
0.091263225	0.021479848	0.011494448	0.004439594
0.108412043	0.008213539	0.067491849	0.032612894
0.189491958	0.061204402	0.077444845	0.189957831
0.162821711	0.074743751	0.172894732	0.093613758
0.033434283	0.061239875	0.07078708	0.054293201
0.201709255	0.090372653	0.054253568	0.154580064
0.064029	0.130754482	0.109056055	0.116014358
0.097011521	0.094076257	0.003134926	0.036168404
0.14118618	0.137488639	0.160055736	0.188562657
0.060059573	0.103234961	0.158111437	0.118027383
0.010261599	0.03964259	0.058263734	0.100177945
0.087808997	0.064855359	0.098030923	0.091219826
0.003294597	0.037052728	0.0137845	0.105316237
0.129014393	0.052989348	0.092590074	0.026727243
0.002646425	0.100668875	0.056445441	0.125060963
0.027929442	0.1251244	0.030408691	0.067097723
0.001818036	0.193773071	0.138628566	0.023382223
0.107751534	0.019976807	0.081651605	0.143580829
0.177386281	0.112409914	0.157852257	0.132508693
0.044757391	0.124900708	0.059431537	0.093568552
0.128185668	0.109259049	1	0.181865573
0.153197026	0.109858784	0.046676145	0.129862946
0.113064257	0.127032611	0.086314624	0.065998084
0.029451353	0.034749218	0.091041616	0.134707281
0.114541175	0.047158971	0.121572794	0.15645919
0.093027989	0.041452281	0.191949724	0.009060845
0.189892186	0.042309102	0.107372413	0.107546913
0.048174539	0.141914748	0.108488669	0.162821103
0.095959033	0.015515664	0.00487589	0.062006603
0.016277717	0.015516522	0.082025402	0.161139144
0.045616077	0.018283819	0.109095287	0.021874713
0.056887677	0.026310316	0.098545927	0.101916212
0.140029001	0.081857276	0.063652085	0.094610237

0.079861283	0.148068416	0.003911627	0.091501687
0.13910619	0.031495129	0.187934833	0.076989903
0.118660374	0.123833921	0.112365327	0.155832407
0.11505357	0.128815456	0.056061216	0.205453566
0.020254353	0.114385838	0.118862527	0.077735704
0.052280933	0.088908204	0.060905804	0.111681132
0.01167721	0.016019281	0.01316426	0.111931803
0.055737243	0.098051743	0.076554665	0.140212531
0.096328273	0.143988634	0.071399609	0.109298406
0.004100349	0.055702599	0.048558293	0.135814009
0.075791763	0.142717161	0.101077757	0.075237984
0.160860748	0.176318713	0.001252435	0.10541463
0.110520369	0.059372755	0.119408438	0.12529867
0.039090626	0.173110723	0.007018798	0.178038869
0.093630468	0.123681584	0.167869101	0.122604986
0.105080598	0.072872468	0.050867711	0.098345091
0.077806183	0.005622005	0.081563059	0.157753816
0.03889413	0.043690525	0.133774096	0.165918857
0.111907458	0.151854768	0.186884364	0.034598554
0.100587521	0.143399998	0.024703799	0.126222753
0.121813604	0.00588928	0.113482249	0.088936239
0.076000037	0.12654434	0.088162561	0.001632227
0.087270668	0.093924891	0.074342971	0.11291618
0.086110676	0.042990403	0.046368478	0.035172397
0.082119983	0.137094927	0.041376044	0.031390137
0.020462048	0.078663461	0.169146809	0.035519448
0.019269632	0.060633065	0.039909214	0.042587456
0.054774554	0.088411831	0.009529423	0.022065612
0.014392377	0.035176993	0.032571014	0.083768856
0.031038601	0.031640185	0.117838164	0.03049876
0.084115346	0.070918194	0.129340809	0.188495594
0.090526324	0.094272362	0.090340669	0.005871872
0.142961468	0.061566387	0.042058203	0.123886452
0.025596717	0.146279787	0.033187091	0.110153623
0.044773659	0.149196792	0.189596261	0.006940856
0.08735999	0.113207402	0.151214967	0.083659959
0.097534659	0.149791729	0.024361063	0.103552286
0.101381257	0.080654752	0.127896805	0.057896882
0.076788565	0.129797287	0.086400078	0.120230723
0.099339984	0.075443752	0.053492366	0.176028989
0.140130905	0.185586785	0.088573494	0.056087183
0.066904279	0.004014363	0.073564597	0.090511601
0.100759924	0.119000753	0.168046141	0.082597427
0.190592801	0.032482016	0.00599312	0.134377643
0.025126957	0.116338994	0.048269809	0.098495738
0.092456108	0.06639127	0.07408868	0.097242029
0.112950906	0.08811673	0.10320797	0.11466485
0.119538131	0.06270715	0.051011303	0.136355884
0.04927715	0.026924061	0.108929216	0.024765401
0.033927394	0.082432632	0.007004822	0.04129224
0.035473881	0.105269126	0.169518915	0.038093501
0.052094099	0.050813406	0.149739415	0.090750437
0.102408829	0.108542035	0.111871042	0.101243085
0.105189342	0.110878186	0.051143733	0.100937022

0.065672256	0.106466852	0.123026083	0.009927003
0.165319737	0.06708511	0.008790242	0.13388322
0.1692707	0.079624168	0.00842667	0.004545782
0.038538138	0.158668548	0.098823423	0.148002607
0.027851092	0.169336885	0.000858713	0.128982912
0.116421032	0.042431972	0.031596281	0.089675132
0.086183968	0.132072319	0.048337277	0.111476377
0.077490616	0.092056435	0.020673172	0.157483722
0.14067797	0.09252543	0.164543261	0.130367048
0.160046751	0.142178315	0.179952692	0.037823334
0.008305168	0.075167231	0.021841998	0.003084485
0.096766196	0.059708242	0.138326484	0.155887958
0.128173991	0.047864083	0.059965221	0.099006434
0.163594003	0.174536471	0.013876023	0.005030733
0.044823741	0.012406796	0.122916122	0.13004013
0.127869792	0.011500837	0.038706362	0.056826735
0.050376149	0.103285107	0.110953443	0.217452011
0.119590764	0.08394545	0.024261005	0.205361039
0.02842717	0.077669935	0.137419376	0.112543349
0.044435184	0.099626204	0.09455936	0.165179124
0.004899693	0.151319771	0.103920032	0.035334569
0.005902581	0.073012205	0.048807361	0.166765831
0.08582775	0.028808646	0.028244656	0.003690244
0.066999843	0.139076134	0.07458184	0.156312163
0.157312346	0.199598956	0.023902622	0.111863224
0.005857001	0.100650861	0.077249969	0.038363392
0.099259718	0.039955864	0.097760582	0.005509491
0.059945136	0.140212786	0.010986201	0.034361473
0.015544382	0.086019032	0.156943273	0.141813157
0.034180909	0.072669242	0.159372541	0.165147086
0.129520854	0.162361644	0.106573459	0.055727248
0.057455061	0.005932037	0.185618769	0.113827126
0.057146895	0.159677458	0.030165966	0.136129714
0.130748174	0.132978553	0.108940117	0.139751901
0.010979096	0.113731105	0.113988539	0.026474352
0.19748847	0.147981531	0.109600213	0.040422767
0.094149148	0.053406092	0.010858572	0.012075934
0.038936944	0.097545811	0.035723161	0.199074008
0.126313328	0.120059495	0.013545449	0.048440957
0.130726772	0.115708786	0.048494531	0.129803671
0.138137676	0.111622633	0.139083162	0.125518768
0.021291804	0.105188247	0.154187834	0.105343662
0.107589785	0.144880909	0.112131257	0.049797786
0.008539054	0.072445827	0.040936387	0.109341426
0.13392484	0.043391195	0.016969434	0.168011605
0.023718494	0.154240541	0.017029097	0.018890987
0.113681045	0.060172176	0.147771417	0.100057194
0.045424059	0.10300088	0.011056361	0.085662207
0.031250254	0.121821598	0.064265458	0.120683017
0.020004332	0.030580854	0.134543633	0.086404818
0.036724909	0.009879963	0.120054644	0.030112426
0.042788288	0.166773226	0.102530327	0.024385099
0.002965324	0.077065523	0.062036904	0.025178235
0.045097911	0.094053904	0.035719247	0.091897649

0.137340374	0.091710115	0.091118009	0.007656972
0.045860405	0.115538633	0.089520405	0.1041873
0.083408948	0.185506159	0.091273701	0.097773623
0.034219422	0.05585278	0.058740379	0.156361867
0.036174242			

CCL3 0.34043023 0.47970244 0.365756263

0.395088326	0.434628057	0.276837016	0.285677696
0.203144307	0.169830614	0.019038094	0.07573144
0.384368586	0.26872108	0.282266386	0.268149795
0.429948103	0.280314427	0.342902697	0.359127627
0.397911684	0.142288318	0.443221737	0.206794329
0.388198571	0.232450558	0.189916165	0.358088778
0.448785129	0.20814645	0.239655251	0.326387848
0.255207068	0.386541196	0.441775078	0.412769828
0.430992797	0.294048716	0.038973806	0.264801132
0.373370395	0.387497262	0.387174917	0.397339255
0.1249625	0.39296715	0.331707818	0.428658165
0.212635631	0.114484532	0.183685025	0.363752055
0.388907584	0.347928236	0.248501605	0.246515665
0.221270966	0.190704435	0.277197944	0.231413284
0.331529244	0.132467954	0.412566825	0.155817842
0.180725602	0.289852669	0.315941141	0.300977223
0.374156219	0.294344821	0.068312801	0.319985382
0.007673057	0.396824968	0.440264437	0.296270781
0.151840201	0.075359298	0.345285562	0.361319239
0.464934849	0.401012325	0.500764861	0.489092761
0.299961331	0.425591305	0.388706632	0.311241926
0.394421359	0.258066611	0.181865573	1
0.50233882	0.462883861	0.351308978	0.409211589
0.36320183	0.433023634	0.348372749	0.410329374
0.309477257	0.167711124	0.376145693	0.495842943
0.330273428	0.354503722	0.31345371	0.361828176
0.184551769	0.287649714	0.4134068	0.239770052
0.477661631	0.066284582	0.372494881	0.359561809
0.341353298	0.378859311	0.461068504	0.305324491
0.42727439	0.078246128	0.195888421	0.134146974
0.335633658	0.219018193	0.376190313	0.448506374
0.248795294	0.408599713	0.341301461	0.024864813
0.116765462	0.321028492	0.376813795	0.374001076
0.378219164	0.318203463	0.327489747	0.288609899
0.351199101	0.49542462	0.035985015	0.208180136
0.37800075	0.332948826	0.383597619	0.379218062
0.407696556	0.205336762	0.268047719	0.507728866
0.360452522	0.208292432	0.282221429	0.437785843
0.315275225	0.430489448	0.240165629	0.348721176
0.129684624	0.407848372	0.121227153	0.304407919
0.247236056	0.271214019	0.048341646	0.256983326
0.279961589	0.380043557	0.366336301	0.250581649
0.378417763	0.441412591	0.256009088	0.326397335
0.039459511	0.143520094	0.197576826	0.485172856
0.189506927	0.490550304	0.394911829	0.416923776
0.421130849	0.445070633	0.008559679	0.409506594
0.464635304	0.199960374	0.398043416	0.419413585
0.33675872	0.44162524	0.037616303	0.435755218

0.244524308	0.209194821	0.519711221	0.314872038
0.399640321	0.14334327	0.30019265	0.401029615
0.333750665	0.03002515	0.190001409	0.362932703
0.117141737	0.133327035	0.362450802	0.397491221
0.378160741	0.427513818	0.511278017	0.321006294
0.423757501	0.31648396	0.064528644	0.427959712
0.393994151	0.308752027	0.376544649	0.31423228
0.223314238	0.426839247	0.344366265	0.176760885
0.133443974	0.320405044	0.047977582	0.399726804
0.330249867	0.201597657	0.326667164	0.284350269
0.206345184	0.499627808	0.287339354	0.299927843
0.204438163	0.337565849	0.354216818	0.245593885
0.254495066	0.318492334	0.374558461	0.377467806
0.090942528	0.228462459	0.066846943	0.295251547
0.186306608	0.294206375	0.267058631	0.294853773
0.325309285	0.194884285	0.414413584	0.357784224
0.257018752	0.081095925	0.466851695	0.427257055
0.230637107	0.278349071	0.150792672	0.254351722
0.454677122	0.204017833	0.180568123	0.419554413
0.088504709	0.493565696	0.316824298	0.273243884
0.383344393	0.371103142	0.474648768	0.077114251
0.326453005	0.41216466	0.424721559	0.323971263
0.324935118	0.391876551	0.097449352	0.314127774
0.300979545	0.124618353	0.485677159	0.27752217
0.101765214	0.406310432	0.353377938	0.214836427
0.215470718	0.337791332	0.088187365	0.423669328
0.488855765	0.43846302	0.353164481	0.341115496
0.127758807	0.039032605	0.364958665	0.363659758
0.420615287	0.47659541	0.405874249	0.393973938
0.370699366	0.35298538	0.129297171	0.487080393
0.277579268	0.454055265	0.047089803	0.396994603
0.42081163	0.218237215	0.206586946	0.302509719
0.318405337	0.140903872	0.314164569	0.430805448
0.401717008	0.380706539	0.344123621	0.404217025
0.053645806	0.111808996	0.404971291	0.273874752
0.359543235	0.356259697	0.099605155	0.299054664
0.129657594	0.397846732	0.462345505	0.159751296
0.239271356	0.267824679	0.45289087	0.132012609
0.424315532	0.447284888	0.446460884	0.272742925
0.378449915	0.446961173	0.332731395	0.225003339
0.08985664	0.445886928	0.312640291	0.064138822
0.487018769	0.158626877	0.047352514	0.452609559
0.314416537	0.34421562	0.008330237	0.107827726
0.145808163	0.473849801	0.400016584	0.33857098
0.160018517	0.406483853	0.026450533	0.494312462
0.43233865	0.076564944	0.295592328	0.367893959
0.347677587	0.474386205	0.347809202	0.423755818
0.358411307	0.423942452	0.286931507	0.407629849
0.400228638	0.433788017	0.357464256	0.406493504
0.457496158	0.435737023	0.380384002	0.064233106
0.209221145	0.207265156	0.221908928	0.154098981
0.231750179	0.276568431	0.442769683	0.484110941
0.462934764	0.040252171	0.204764835	0.362626486
0.415707684	0.476360478	0.075682966	0.015870384

0.01574534	0.070745421	0.352234551	0.395123335
0.460851251	0.157412572	0.035621172	0.092969837
0.342695618	0.408413324	0.447585434	0.427365092
0.401602313	0.224564145	0.237829611	0.416535763
0.184226933	0.211664991	0.473500408	0.410940468
0.149433849	0.395689981	0.335134354	0.490651211
0.108677495	0.442316189	0.38217527	0.345104045
0.059458254	0.222275152	0.187685346	0.444306215
0.413930475	0.16833634	0.109618999	0.108967614
0.212940483	0.463282909	0.284551743	0.515979337
0.413375453	0.467457478	0.212555746	0.41898322
0.123206837	0.410973518	0.400401771	0.056362212
0.393165015	0.06987327	0.416223072	0.175427603
0.279702545	0.33247143	0.258289544	0.088042162
0.233659842	0.173980618	0.490579752	0.468019374
0.130289147	0.329814826	0.423840106	0.408964851
0.255747256	0.403868284	0.422886759	0.276982747
0.171820009	0.214759377	0.37291366	0.294044758
0.253007649	0.510262973	0.342091234	0.496298608
0.317662508	0.429832919	0.435533704	0.369047917
0.345165266	0.311889204	0.298340793	0.053217763
0.410382652	0.460868335	0.343834379	0.265653311
0.378581956	0.353684431	0.131945117	0.157810227
0.312199845	0.231984147	0.262980796	0.470653316
0.328557191	0.293231731	0.102477583	0.175437986
0.493742888	0.385049508	0.186282188	0.359645328
0.339003346	0.414026454	0.415074868	0.42192877
0.340115147	0.405363983	0.379189341	0.296546465
0.364661609	0.364079004	0.453716003	0.095802887
0.181243321	0.329407898	0.196985977	0.246112954
0.288209303	0.113567066	0.284918592	0.434348
0.319910705	0.426197719	0.257327979	0.197943463
0.442857972	0.303737692	0.444604336	0.249507191
0.179339667	0.424755832	0.044356341	0.184187814
0.347416675	0.383567493	0.375025775	0.389556125
0.339341432	0.297212674	0.418762311	0.230186893
0.233801033	0.323047515	0.379715869	0.084678085
0.070478323	0.511225788	0.296310012	0.092498971
0.197052082	0.283787569	0.360613969	0.310572409
0.101340729	0.435947737	0.246077731	0.414542188
0.351398588	0.193368115	0.281764714	0.149071793
0.156650173	0.380112215	0.380736248	0.448007443
0.402154707	0.428778026	0.352720757	0.377798062
0.201678742	0.308040516	0.093059592	0.430849781
0.303526153			
CCL4 0.44222681	0.529925342	0.45694518	
0.469311545	0.505445449	0.382792297	0.2624122
0.200042331	0.131694791	0.010089463	0.049651492
0.497317714	0.28654204	0.315738651	0.356387455
0.48780637	0.221348077	0.381787782	0.316250186
0.458568522	0.114649384	0.489491674	0.138297261
0.390618599	0.182624446	0.121455461	0.37504115
0.475914489	0.136444142	0.332841808	0.405028036
0.166030325	0.458229356	0.51990826	0.517057477

0.516331119	0.40885654	0.018714752	0.326143648
0.42235176	0.496437132	0.451756847	0.409599357
0.071966418	0.473561992	0.314692085	0.487101998
0.246395088	0.064933038	0.233840668	0.426162189
0.476229898	0.477477113	0.248624509	0.210446912
0.273503893	0.229836766	0.368154355	0.31682627
0.296303352	0.154672876	0.48909017	0.124661199
0.176419167	0.279091546	0.239735008	0.299623212
0.436908214	0.38423186	0.028850624	0.44329672
0.004680823	0.439349395	0.469148552	0.388816125
0.08072366	0.043608517	0.27177507	0.39645164
0.483130338	0.451921481	0.510943354	0.504203975
0.273152455	0.388801087	0.475773916	0.25326541
0.446452853	0.235189758	0.153197026	0.50233882
1	0.530709882	0.300464052	0.368760298
0.447110763	0.47177625	0.467872091	0.501629716
0.415042047	0.098507134	0.435844185	0.524854303
0.390442125	0.418083734	0.243361077	0.427964101
0.102545741	0.381552774	0.433725376	0.21822484
0.484522484	0.032816473	0.319582892	0.410792393
0.281380549	0.464813072	0.528815813	0.254294892
0.488484968	0.041054625	0.21354774	0.063466743
0.386395106	0.22711418	0.471947389	0.483643363
0.259092585	0.46635	0.321045407	0.006845795
0.057604646	0.260178783	0.460115655	0.411850884
0.468911483	0.380072043	0.324924356	0.389114424
0.490220632	0.510980385	0.057404212	0.137567585
0.475041574	0.262216379	0.329345123	0.494995042
0.421228967	0.150063032	0.190764327	0.529092384
0.406131988	0.134488486	0.342824677	0.493808624
0.386291294	0.469889268	0.160105335	0.35423167
0.07743973	0.487122394	0.061066171	0.350257623
0.333782174	0.23700781	0.018434388	0.277897278
0.336930604	0.452862596	0.458892261	0.163298439
0.396875386	0.499168459	0.322416942	0.464197997
0.02275861	0.078228503	0.264684902	0.518125864
0.274319083	0.509661014	0.3799629	0.400945112
0.390982294	0.493714415	0.005447573	0.491162791
0.534732323	0.285407545	0.482174795	0.490010312
0.443801418	0.486021034	0.046528085	0.492917101
0.353839854	0.124742459	0.514522119	0.250979147
0.457102328	0.07952682	0.345100834	0.47961632
0.467189386	0.015300803	0.229915055	0.427405052
0.057305092	0.112168783	0.457475345	0.467399117
0.450360853	0.497143584	0.506651915	0.42960237
0.507567537	0.284835988	0.025672651	0.428774156
0.475001136	0.420236587	0.431694476	0.294869191
0.327923806	0.473427444	0.437339183	0.229075999
0.074658478	0.345921404	0.032904718	0.428752115
0.398459012	0.169795181	0.439494069	0.211098607
0.129925676	0.538403591	0.273012694	0.352491472
0.203116001	0.44948667	0.315626785	0.296335671
0.302164678	0.245727263	0.440900843	0.448566853
0.054506729	0.15718308	0.029428147	0.430737075

0.20699176	0.40584775	0.38513292	0.418030363
0.375399415	0.170673877	0.482959192	0.376491457
0.189980453	0.033377969	0.512916133	0.475739607
0.323426011	0.214976608	0.076751945	0.379269708
0.482980524	0.316228902	0.25028008	0.450944575
0.046902381	0.498268395	0.335114706	0.202614466
0.477882454	0.351190568	0.486697603	0.076203595
0.409117906	0.464173102	0.402036428	0.279647924
0.380549507	0.466774463	0.051806099	0.412070661
0.408950812	0.062740617	0.537663195	0.209836978
0.044507397	0.509093739	0.485239085	0.131771248
0.136968982	0.403244858	0.058714988	0.399408222
0.529432427	0.497235414	0.457540998	0.314335045
0.072830997	0.023870784	0.311058672	0.457750644
0.505312757	0.525741235	0.353920925	0.474657784
0.322329275	0.421032464	0.13778876	0.523267409
0.248709859	0.518981882	0.015671872	0.494305398
0.51067616	0.286581354	0.145491279	0.34377378
0.391947932	0.078653517	0.25662847	0.500430576
0.446315444	0.415161134	0.426279105	0.485487531
0.026751245	0.062899377	0.471567237	0.273886403
0.443487006	0.301718546	0.094095948	0.3449392
0.078799221	0.45027403	0.503547478	0.106047202
0.351087794	0.317230105	0.480262518	0.067308413
0.477017816	0.507642539	0.523354207	0.210268309
0.477790936	0.50175552	0.471437095	0.136317675
0.039332636	0.52370156	0.412932565	0.041100088
0.50018051	0.221890662	0.028022681	0.49719546
0.251185094	0.429070964	0.005736333	0.106669468
0.078500875	0.510296994	0.47764619	0.407899224
0.243969188	0.428779753	0.011209669	0.534961238
0.495519227	0.034538458	0.231218082	0.472203927
0.479290016	0.529335745	0.421370835	0.490783538
0.495508884	0.505920892	0.297353785	0.37419173
0.465789219	0.50423359	0.448366648	0.451497694
0.493874437	0.507215373	0.362334708	0.029987207
0.197279638	0.222447401	0.342397676	0.131282361
0.317663236	0.225393033	0.482182672	0.505777177
0.49470547	0.012900224	0.179626216	0.458020181
0.443779585	0.47630331	0.035989049	0.010229263
0.01212751	0.045376726	0.397998476	0.345732779
0.496573661	0.246762886	0.011657123	0.058219138
0.348722082	0.496277262	0.510735832	0.456856719
0.485690074	0.13505481	0.327397983	0.461869418
0.127243703	0.316327182	0.500382122	0.469885246
0.075042467	0.348534697	0.404274683	0.49044337
0.119851853	0.425517274	0.495781898	0.395669304
0.025659697	0.144655548	0.118716848	0.49961492
0.502921498	0.136278219	0.06937468	0.144150692
0.175883018	0.489563598	0.243981569	0.527355054
0.467152947	0.497427117	0.134668046	0.482351169
0.073417264	0.489135917	0.455402719	0.069221896
0.369630011	0.025926527	0.474246549	0.220101763
0.344003467	0.419382487	0.27005685	0.03769603

0.255944596	0.099689066	0.503844602	0.51027191
0.066011367	0.291487737	0.398032343	0.464550733
0.303137681	0.496008136	0.501911904	0.423530929
0.12074328	0.203099573	0.447653527	0.408971279
0.26267072	0.509209718	0.392480936	0.537071075
0.425027135	0.453475893	0.477513482	0.411905493
0.340626224	0.416261421	0.399001964	0.033998753
0.429258754	0.505561879	0.389099778	0.248029026
0.36643554	0.462457395	0.068991555	0.188854778
0.412119635	0.310684999	0.209597272	0.494482495
0.411278429	0.411392286	0.05429184	0.11240462
0.547851654	0.425655214	0.172877817	0.468475857
0.254638704	0.441286949	0.496799225	0.506091926
0.415186669	0.473667019	0.453879115	0.411589925
0.414537382	0.332892046	0.528303453	0.089116646
0.14359655	0.447111611	0.15601472	0.158174087
0.216886313	0.055456291	0.380137801	0.49850376
0.44218245	0.472072695	0.323905994	0.131700827
0.43192756	0.384307186	0.452445238	0.245484267
0.130344553	0.501235647	0.020312597	0.101902696
0.459713058	0.477322342	0.482728303	0.342739376
0.399898727	0.219825504	0.40063688	0.298488481
0.350301082	0.411069625	0.46969897	0.051209313
0.032693571	0.505095978	0.389725533	0.042012218
0.133259975	0.386541399	0.352371898	0.315772091
0.04771456	0.493783648	0.347403175	0.501212279
0.389581355	0.108906435	0.329028253	0.116412177
0.089345219	0.439562929	0.471972809	0.521384135
0.433138467	0.49519158	0.428780691	0.460628421
0.164755768	0.414830374	0.047548828	0.472164325
0.451749344			
CCL5 0.48708056	0.520406382	0.451250242	
0.469199847	0.47956012	0.442302992	0.222318593
0.195194024	0.086899218	0.004775058	0.026080555
0.503191527	0.24083141	0.305623048	0.384376508
0.45632697	0.14422829	0.375289481	0.248837447
0.450057897	0.096282842	0.440385605	0.089041503
0.319574871	0.111845631	0.068884624	0.301474389
0.485821236	0.078791532	0.39906707	0.434387793
0.09707932	0.501232816	0.525295215	0.503286231
0.526394276	0.472155464	0.010685993	0.345942397
0.480965173	0.506948772	0.450357338	0.356433159
0.039886629	0.497467369	0.263088076	0.443616508
0.255869632	0.030648278	0.277266463	0.464007945
0.494544732	0.516702255	0.239751088	0.168145422
0.259231205	0.268631885	0.342923879	0.382641918
0.240068376	0.18242133	0.505647844	0.092135426
0.16768255	0.261071008	0.156704083	0.27703326
0.402569472	0.451576345	0.01449136	0.482809301
0.002856486	0.418466103	0.408979468	0.377202999
0.038658777	0.021262967	0.183080341	0.37589349
0.50231588	0.485911712	0.527586838	0.531873873
0.198289148	0.304832306	0.456038016	0.180716371
0.503743449	0.193087948	0.109858784	0.462883861

0.530709882	1	0.212992429	0.275461132
0.505185722	0.505826067	0.490390369	0.49025973
0.410860209	0.048875448	0.499535513	0.535153186
0.468779091	0.370144525	0.157661024	0.468395378
0.054586216	0.410300817	0.384535027	0.171119837
0.504854817	0.016119097	0.224779603	0.405157843
0.212417063	0.506871706	0.519082028	0.194027536
0.453354553	0.018538694	0.161339469	0.029109633
0.366297117	0.169642956	0.509727544	0.516190306
0.256546209	0.43538455	0.279606243	0.00071663
0.024539938	0.185425683	0.520077073	0.396082056
0.484206982	0.46293289	0.26099669	0.470587862
0.524569742	0.527395502	0.074194237	0.091818054
0.477607948	0.186012917	0.247525148	0.519373817
0.341587745	0.111329774	0.127569726	0.528727188
0.464849966	0.082472826	0.375161129	0.453316695
0.394600519	0.398866067	0.103719087	0.308309676
0.048495639	0.49668186	0.025974082	0.36249372
0.329503245	0.166578093	0.008211953	0.284955401
0.330657223	0.522298468	0.496642636	0.101361566
0.355897441	0.453606152	0.369216671	0.512046952
0.011159165	0.040907447	0.328339582	0.533669166
0.351994048	0.525331722	0.316173832	0.322215249
0.300312708	0.498151	0.002913139	0.506972574
0.522232332	0.35753001	0.460511333	0.46581543
0.448448118	0.507636873	0.04906966	0.459458864
0.432443415	0.072660952	0.52522052	0.168195589
0.500209083	0.039525281	0.362181577	0.516577573
0.527010445	0.008155041	0.259339815	0.432502179
0.024217633	0.102907818	0.482878683	0.453941912
0.434216585	0.499694111	0.511783674	0.430918351
0.483141666	0.240198164	0.009857367	0.344619265
0.50415868	0.462549332	0.481091716	0.253479006
0.409449671	0.511738074	0.509654242	0.25657471
0.040659134	0.344802521	0.020627681	0.368720831
0.478384221	0.131394982	0.466455748	0.144762067
0.069764263	0.531710208	0.196319376	0.307059906
0.146112609	0.481184847	0.258329376	0.290281228
0.265822575	0.167871369	0.405346084	0.432569317
0.036097661	0.095966387	0.011592135	0.476806451
0.17680382	0.427140401	0.430277585	0.491783975
0.376550896	0.123529006	0.437907268	0.316394796
0.114596996	0.012548457	0.522040059	0.440905531
0.410655311	0.155347247	0.036690201	0.434991056
0.523662493	0.399509446	0.318251867	0.511935042
0.023733695	0.523519922	0.28885785	0.126666796
0.452144701	0.28376704	0.498669396	0.067594931
0.447214666	0.503127717	0.326396027	0.198549787
0.471733686	0.465388218	0.027920119	0.483719477
0.485533443	0.02899534	0.528588771	0.131563971
0.017801763	0.500062376	0.527196647	0.071971208
0.074720069	0.391934103	0.044877754	0.310466111
0.529249193	0.455202104	0.519194515	0.231566734
0.039177204	0.011954832	0.218860227	0.455586919

0.523089412	0.529817698	0.270481387	0.430265982
0.249910165	0.389530961	0.159815125	0.535124224
0.194962998	0.512801251	0.004890159	0.51829285
0.526574498	0.34291832	0.08297493	0.349039979
0.474938425	0.042479326	0.202616361	0.507773943
0.505109331	0.36937251	0.408019251	0.480244278
0.01445687	0.03993795	0.437149646	0.206313401
0.449039013	0.223596441	0.060535209	0.365065755
0.042803405	0.503238799	0.50685656	0.059694028
0.422867048	0.344520964	0.41551452	0.03327698
0.523271897	0.509703065	0.526166422	0.162049541
0.520540048	0.52877984	0.497445299	0.074375058
0.014751658	0.520679906	0.459989938	0.022258856
0.516466276	0.285138392	0.013903133	0.511400577
0.187064859	0.468585242	0.003262688	0.088637285
0.036401613	0.510198384	0.489555649	0.42941389
0.312853379	0.39405878	0.004867853	0.52700157
0.513239461	0.012399892	0.148512797	0.531594951
0.51970011	0.514535659	0.377263245	0.531090488
0.514192732	0.51208315	0.269374654	0.287160885
0.514666636	0.510993943	0.480445893	0.491752876
0.51890414	0.474643889	0.300243136	0.014042485
0.146025629	0.237014163	0.392333464	0.093796751
0.383405992	0.149190661	0.513768218	0.518017741
0.53555753	0.0034117	0.157677788	0.522195796
0.49334703	0.506213055	0.016681597	0.005415957
0.007335797	0.020402941	0.400609278	0.252555076
0.525893101	0.310735772	0.003184951	0.041942038
0.299108535	0.460530399	0.518558887	0.411864881
0.509859292	0.077345347	0.35330224	0.433727064
0.070473542	0.404195294	0.535624773	0.496471704
0.036138165	0.259676668	0.417220459	0.51839765
0.088749412	0.341518387	0.510209938	0.368953509
0.011038991	0.079678703	0.064408671	0.486511944
0.518399436	0.088541957	0.035110141	0.172917865
0.130518684	0.5198255	0.177297781	0.530997718
0.503746312	0.483880734	0.074797448	0.49791204
0.040121138	0.500677079	0.391342554	0.067775892
0.299431517	0.009631422	0.508194261	0.197262306
0.376665659	0.451957648	0.221603351	0.014344181
0.208404625	0.050385046	0.522826495	0.51966835
0.029467064	0.245654974	0.313834113	0.444000946
0.324478507	0.495943911	0.512829279	0.494804996
0.086168116	0.156621914	0.454463497	0.468978854
0.263888579	0.525764696	0.35424956	0.526444446
0.476785874	0.50293734	0.526674083	0.388624491
0.269304421	0.460804942	0.443112478	0.019697082
0.381006609	0.521792246	0.391467009	0.174181672
0.287343575	0.462000892	0.041672618	0.203148575
0.401416185	0.365480103	0.153616346	0.479939178
0.442930558	0.474728785	0.035751353	0.060914992
0.524585613	0.485351022	0.148932606	0.492760425
0.169988651	0.384707405	0.516332758	0.514481074
0.425944935	0.531228767	0.491439805	0.467594045

0.479400102	0.274256042	0.527105144	0.091322288
0.08866616	0.495241572	0.106875321	0.089313172
0.147029507	0.023571446	0.366705985	0.470490535
0.459515722	0.429315394	0.373362733	0.077884849
0.350945258	0.418116676	0.385836351	0.221210752
0.081949214	0.505714635	0.007961721	0.052262829
0.466734069	0.51982137	0.459815048	0.251314512
0.352256543	0.141745136	0.325869181	0.345911909
0.409881864	0.415334953	0.519510243	0.030308152
0.017039495	0.524043543	0.427387587	0.017031198
0.088036325	0.449462155	0.309598241	0.269537926
0.019033597	0.525937155	0.410542485	0.48444924
0.38094737	0.060112201	0.42481255	0.087162409
0.052187645	0.483072966	0.455547452	0.514747946
0.382795377	0.469410261	0.460648799	0.522359698
0.117790375	0.448733387	0.024399999	0.499745382
0.498329689			
CCL6	0.178414779	0.231973024	0.141409883
0.268505233	0.193393602	0.104481705	0.141280191
0.035786663	0.065955041	0.011506485	0.05214317
0.188261897	0.0690298	0.056469922	0.041856122
0.33040388	0.125502602	0.078936909	0.219386313
0.236160199	0.042177816	0.328900104	0.085451561
0.101552663	0.151205302	0.146600789	0.155505457
0.281302289	0.162042902	0.0696513	0.17702543
0.095858558	0.204671838	0.191952307	0.258351776
0.18185948	0.139922798	0.012993008	0.055172029
0.18126548	0.254041065	0.240539485	0.247253458
0.05955753	0.155846811	0.197750971	0.242251125
0.021249519	0.082553871	0.034810058	0.148498459
0.158864062	0.153496664	0.067270793	0.105992709
0.044145701	0.030193228	0.047056553	0.093745129
0.183877982	0.025961841	0.176332879	0.04579208
0.034294218	0.080074112	0.114938763	0.100697033
0.098453777	0.137530782	0.034094598	0.135342698
0.002113999	0.251561685	0.250785383	0.088969098
0.155612351	0.04023408	0.154733089	0.19870961
0.276016559	0.177597548	0.308747873	0.281652231
0.07540564	0.204342397	0.169188195	0.132718725
0.2062079	0.12303129	0.046676145	0.351308978
0.300464052	0.212992429	1	0.271252773
0.195387547	0.204910362	0.108871377	0.147170342
0.074972235	0.070465862	0.164768119	0.265082404
0.142898361	0.149298403	0.226866441	0.195096211
0.13487701	0.027474195	0.279553469	0.042314966
0.288409834	0.039339011	0.24569349	0.1629125
0.062906961	0.196334343	0.216714112	0.184348132
0.180681895	0.040224191	0.063263202	0.087576668
0.045928716	0.051422339	0.142660204	0.261303247
0.031409399	0.056353987	0.132068151	0.027757991
0.086508633	0.063870008	0.183762135	0.116260887
0.220800892	0.139239822	0.14172621	0.12947613
0.182979901	0.306556923	0.003548528	0.08133388
0.168498273	0.073628838	0.279267703	0.162838326

0.240434925	0.070140952	0.138753208	0.280148162
0.155899957	0.147639441	0.051640478	0.326422684
0.04595569	0.259754431	0.12471603	0.099288519
0.051760498	0.180348288	0.109523223	0.087027157
0.049057035	0.077650991	0.022965856	0.079701261
0.035667476	0.190472198	0.138763514	0.152995478
0.134668531	0.288851987	0.068873498	0.18035533
0.027294529	0.061711168	0.038912136	0.253296208
0.066624929	0.266750588	0.100728549	0.133499037
0.280615669	0.282086804	0.002807077	0.171230221
0.216001339	0.038600892	0.210556033	0.224232793
0.127331875	0.266702726	0.005717016	0.295973936
0.098756159	0.128077196	0.312404596	0.235784856
0.173774434	0.138701052	0.067089622	0.228853396
0.172904095	0.027054275	0.053310127	0.218878837
0.079955055	0.031773624	0.203735843	0.236450331
0.204778715	0.267767934	0.325367002	0.114632576
0.194625879	0.109761846	0.046176201	0.243894282
0.219782648	0.032388644	0.160529176	0.084114588
0.06774633	0.201867404	0.16391123	0.017540234
0.105182908	0.110615959	0.019928386	0.212196555
0.153324876	0.058454992	0.123564288	0.06440646
0.147107993	0.258907545	0.125261175	0.103614424
0.074022171	0.136658568	0.166545426	0.050421535
0.048639058	0.134878026	0.12428809	0.100117455
0.036756367	0.143733056	0.034584554	0.054142415
0.039143129	0.062441612	0.053471936	0.135084615
0.043763565	0.051262985	0.189145046	0.073381205
0.123071482	0.103267384	0.228004709	0.280950374
0.091092729	0.106644254	0.116924454	0.021983359
0.255140554	0.060617514	0.027777846	0.210508472
0.036926619	0.289169891	0.056906827	0.216914635
0.117397756	0.212344393	0.296888357	0.022097276
0.122029367	0.185463887	0.227073942	0.174336847
0.134675119	0.222882538	0.049234136	0.137494596
0.133314731	0.115677556	0.240066938	0.102567008
0.11435038	0.252552735	0.203413393	0.202199259
0.149899575	0.134454548	0.023570918	0.325896265
0.25008537	0.341713122	0.182355615	0.127123347
0.088639224	0.023116957	0.173743977	0.148966134
0.170775916	0.239575027	0.243354684	0.178473151
0.255662996	0.106245298	0.01304571	0.257596545
0.045926293	0.203796135	0.056946007	0.242974083
0.175592749	0.056786525	0.16109797	0.125454435
0.13842644	0.09582467	0.088749667	0.189022986
0.208251509	0.137853144	0.097378106	0.219649887
0.024647746	0.038678912	0.220070042	0.084882883
0.080366437	0.142728677	0.033554943	0.04896566
0.05343112	0.18241284	0.288970446	0.075508064
0.084483962	0.045081085	0.303873034	0.109950159
0.236336423	0.207283633	0.193483728	0.082079073
0.203823359	0.222858428	0.152497809	0.156169931
0.109892595	0.197374268	0.146955517	0.020711981
0.298918749	0.038066003	0.026643131	0.214110552

0.177334465	0.126428054	0.003549032	0.016631683
0.069484747	0.309282239	0.170437825	0.179745121
0.02301668	0.214577748	0.010615023	0.251522581
0.197028559	0.08019291	0.1008857	0.190178109
0.19776734	0.217433222	0.151803276	0.244714252
0.175526017	0.181286562	0.056035492	0.293934685
0.219576235	0.201470091	0.197091146	0.184232188
0.268161373	0.283966495	0.259838528	0.047122158
0.064134165	0.053405158	0.034026207	0.043410812
0.083109995	0.114855157	0.213682007	0.297451349
0.252791796	0.063678933	0.034436239	0.184280268
0.218738538	0.270711981	0.033287615	0.012021894
0.004801142	0.059219413	0.158284134	0.22208183
0.237457151	0.011474726	0.042124848	0.02697081
0.05063237	0.255756047	0.204518377	0.315663804
0.232473053	0.135764613	0.039278823	0.285395708
0.097464317	0.080135399	0.263272491	0.183347014
0.067446113	0.199717789	0.092030941	0.279391885
0.031308257	0.289065339	0.192254808	0.064713648
0.024712888	0.136497439	0.124233831	0.304306955
0.17116554	0.062537388	0.05868891	0.010803036
0.087794687	0.263875181	0.081401373	0.290725839
0.227810803	0.323989809	0.087461027	0.186847509
0.09475267	0.175542888	0.200294647	0.007384167
0.110245493	0.044780263	0.18825867	0.042404256
0.058912394	0.162674839	0.06586624	0.078821978
0.051074954	0.134629783	0.301875393	0.29895133
0.064758083	0.089611379	0.225426037	0.224424963
0.090739467	0.27348379	0.177543584	0.076673604
0.068911067	0.041882815	0.221992054	0.127443844
0.047041892	0.311484597	0.089139646	0.251332006
0.163728136	0.213347206	0.217643751	0.191328794
0.078385114	0.12167672	0.153801533	0.02016869
0.2109884	0.28269979	0.113472586	0.089237495
0.212604965	0.153038001	0.045399735	0.01339573
0.12267645	0.08597738	0.066072636	0.326305972
0.143025582	0.138057412	0.031354304	0.112673419
0.23402055	0.176359543	0.035134123	0.189529901
0.187974909	0.242508513	0.242135606	0.268706359
0.052323558	0.207557347	0.212099358	0.14389846
0.158554481	0.196159621	0.199751547	0.013271895
0.124271618	0.137825519	0.073130884	0.200057904
0.185394462	0.066074156	0.061797212	0.299694803
0.073461993	0.271130039	0.022844259	0.060040674
0.249249378	0.077919665	0.26154047	0.086248193
0.063231333	0.185009832	0.025919025	0.132706259
0.088762397	0.227279445	0.173667383	0.164384082
0.073214581	0.081433703	0.2430375	0.064136867
0.049868916	0.034831774	0.210156842	0.028958176
0.033707839	0.305677611	0.079997234	0.048764746
0.059043006	0.110754931	0.063720299	0.067953322
0.089458328	0.212499449	0.059108876	0.18804343
0.148821201	0.101777208	0.107748406	0.04393944
0.051970919	0.159563778	0.185084781	0.202987231

0.150644661	0.326019445	0.135422885	0.18738892
0.037757232	0.104921142	0.046548995	0.244316571
0.076116082			
CCL9	0.251179662	0.293624727	0.192820913
0.30009109	0.237773118	0.126807873	0.143073905
0.087607227	0.147419267	0.014822052	0.079890424
0.244543115	0.13379308	0.106204836	0.103984722
0.390384802	0.234557015	0.166286911	0.211693336
0.289975998	0.068834567	0.367920933	0.161486038
0.279480525	0.226744689	0.186029415	0.240395324
0.336447474	0.203151826	0.074432713	0.212250259
0.293174873	0.269880542	0.25343198	0.294971838
0.244401201	0.202609043	0.049320887	0.093035066
0.247249218	0.305322637	0.283616285	0.246969085
0.114091022	0.208933288	0.178298409	0.307004332
0.085745579	0.133926248	0.05626286	0.198078495
0.206664616	0.179860526	0.107897857	0.134049386
0.090201335	0.057997642	0.138758173	0.117746777
0.179106277	0.039900209	0.232731409	0.101696981
0.086071622	0.134261134	0.301474597	0.133191288
0.213660697	0.180915841	0.082238147	0.198924452
0.003709224	0.294445378	0.32164715	0.157434413
0.193455588	0.068359871	0.306850832	0.204134398
0.342011281	0.250618569	0.375564134	0.353403544
0.199366178	0.291157821	0.223663816	0.209107177
0.263490218	0.130505018	0.129862946	0.409211589
0.368760298	0.275461132	0.271252773	1
0.224349058	0.262566129	0.1374345	0.173091029
0.138828743	0.26588534	0.211869337	0.333129135
0.195797821	0.214605284	0.275694558	0.239033212
0.212331763	0.106026735	0.295956225	0.159484907
0.349039082	0.074525122	0.30385844	0.169017369
0.259021957	0.262631033	0.282399464	0.184098176
0.22762836	0.092881667	0.152427268	0.207040521
0.14211985	0.197183268	0.194776375	0.302574157
0.097771767	0.190898426	0.172115271	0.063395059
0.179073633	0.293341776	0.246218655	0.177495455
0.295994903	0.204136403	0.19119529	0.169567505
0.228188447	0.374699229	0.008551574	0.160814891
0.185023575	0.303400045	0.280551719	0.202906237
0.24392909	0.132595769	0.201123802	0.348220404
0.20084219	0.17266606	0.108376407	0.380007936
0.117657938	0.289791762	0.194167992	0.16090012
0.095341318	0.237130904	0.20104755	0.123236488
0.12068484	0.21325389	0.082921833	0.098397601
0.107606957	0.227063981	0.194285159	0.229280025
0.169996887	0.329451905	0.088946214	0.200874517
0.040168346	0.171829087	0.060591673	0.312988439
0.080879796	0.349235787	0.221379983	0.286103579
0.30206602	0.353313552	0.017944317	0.224300643
0.280780337	0.057928998	0.215173993	0.289250369
0.154731827	0.31351869	0.017485721	0.353260177
0.140216535	0.211273487	0.384993105	0.249411768
0.236868607	0.164469725	0.107901789	0.299313918

0.217523195	0.036076138	0.060612974	0.243932618
0.212519481	0.059255455	0.274770918	0.278710368
0.216231012	0.354932218	0.397045851	0.149740628
0.213331054	0.169767131	0.120651661	0.29390733
0.303450401	0.119876526	0.210369809	0.161107727
0.09383074	0.256825842	0.221147032	0.065612464
0.127607576	0.12913029	0.03314302	0.220723458
0.213410416	0.104297917	0.14455156	0.266928472
0.241384546	0.323726119	0.260391391	0.203296026
0.1999532	0.150451254	0.202869683	0.086309289
0.113530068	0.254281938	0.197495929	0.172717518
0.05941141	0.192178693	0.162143746	0.098157992
0.079883607	0.096278641	0.078885308	0.17679692
0.122742941	0.110679441	0.191686952	0.253989612
0.272854156	0.134400035	0.289680823	0.339471778
0.12336335	0.19277909	0.206023104	0.075588829
0.301392099	0.067280931	0.053910801	0.253249889
0.115869536	0.369374739	0.14735656	0.242309425
0.168250758	0.200948766	0.3592718	0.043968489
0.161978558	0.234169405	0.284653012	0.243903871
0.169518918	0.253627638	0.089108574	0.195469982
0.177658179	0.152831187	0.306446535	0.267744214
0.169045212	0.329659657	0.243644603	0.243172028
0.255874407	0.132523673	0.050412381	0.341456799
0.311941487	0.379431413	0.219989185	0.301476326
0.117485779	0.033327659	0.320608945	0.21224922
0.229786035	0.310373701	0.271605966	0.185457673
0.248438224	0.180100435	0.045328887	0.319482447
0.241025493	0.27198088	0.091594531	0.308813488
0.233490993	0.064729869	0.215237739	0.125045192
0.17602874	0.143225394	0.206244376	0.246936113
0.264069217	0.206084457	0.161178177	0.292683143
0.04560462	0.084183818	0.265286138	0.244831708
0.144138163	0.257736307	0.125991388	0.116637575
0.114786711	0.224178295	0.366919368	0.173832481
0.090393407	0.105105077	0.365166412	0.152744022
0.301334812	0.283154317	0.253791252	0.176291851
0.266590784	0.281464209	0.188805479	0.274465566
0.166630753	0.263465463	0.200260272	0.055168229
0.361484913	0.042984282	0.038252769	0.273423492
0.206418261	0.176761061	0.007688102	0.109550169
0.248960912	0.39053314	0.221781242	0.218227986
0.039630244	0.273978242	0.062748245	0.329873923
0.247624431	0.145060281	0.302724876	0.236201852
0.248054857	0.285050896	0.199423046	0.296263668
0.20952965	0.23989412	0.123052743	0.297462319
0.256776018	0.268639006	0.251951003	0.243492081
0.321710377	0.360052743	0.248496017	0.06995523
0.195411457	0.075240739	0.070164964	0.113318482
0.108380587	0.240048079	0.284193987	0.371789123
0.314263713	0.08901869	0.097979427	0.230275148
0.267298987	0.338044315	0.142203478	0.025685078
0.022418385	0.103488513	0.183908784	0.312288484
0.303950064	0.037889757	0.063076744	0.059454325

0.179677385	0.30264883	0.262590985	0.353778991	
0.315845594	0.236590685	0.074651745	0.343712664	
0.211344228	0.11770091	0.321284692	0.230458777	
0.232246112	0.306208453	0.140551315	0.342122368	
0.111964644	0.318829526	0.250867154	0.145075414	
0.118429875	0.26616903	0.225233963	0.384031445	
0.23099104	0.11900208	0.110114522	0.030535669	
0.121276435	0.339575936	0.17860033	0.363184345	
0.280289361	0.380173131	0.269633985	0.243266137	
0.127916198	0.226920894	0.231552137	0.022712026	
0.263106182	0.128133693	0.257338758	0.09604162	
0.093220677	0.216642211	0.195271252	0.174282295	
0.19989296	0.212119662	0.359420589	0.372016313	
0.234554974	0.186040939	0.283563197	0.300077628	
0.097893399	0.304288604	0.233764728	0.099946303	
0.108271998	0.202948492	0.271888284	0.172297578	
0.098687879	0.38671433	0.168853843	0.328261722	
0.217526101	0.286926517	0.278250485	0.200230625	
0.240311144	0.15303032	0.165190014	0.037131268	
0.244957321	0.334238549	0.15320081	0.205900969	
0.228875039	0.20569579	0.147081125	0.072055832	
0.163817523	0.097952241	0.160329468	0.397372682	
0.19396308	0.1801529	0.094892819	0.207147495	
0.30467259	0.234836411	0.097450088	0.23108482	
0.297933566	0.24953869	0.32291044	0.343051131	
0.161441351	0.255455722	0.257148841	0.163489011	
0.198355739	0.205072183	0.2620967	0.039574878	
0.185429275	0.194417208	0.128594127	0.281947355	
0.209783979	0.187909654	0.154135848	0.36881313	
0.139475562	0.28797489	0.087986854	0.209195688	
0.298097417	0.12699964	0.299963963	0.108259815	
0.136404305	0.236653157	0.072791049	0.226879753	
0.15071122	0.268725531	0.18841117	0.289520544	
0.205601147	0.318436891	0.246521496	0.071505697	
0.063188009	0.142261366	0.255931146	0.078110904	
0.066512237	0.380672114	0.109439171	0.189635319	
0.218722293	0.160826216	0.208244785	0.164242129	
0.196488639	0.273384958	0.072757584	0.24102595	
0.175089762	0.21442162	0.151167637	0.11111667	
0.175714187	0.21292778	0.238810063	0.265582711	
0.245065104	0.360199707	0.185813472	0.239679775	
0.179858543	0.158748565	0.09295159	0.313147774	
0.108524859				
CCR5	0.327084072	0.511308707	0.314061491	
0.397368438	0.363840955	0.369579748	0.255552226	
0.191357485	0.12618098	0.017013584	0.04649384	
0.343837156	0.230827952	0.296300708	0.344823894	
0.363998289	0.131990779	0.267380288	0.249676653	
0.362641817	0.141967918	0.3601565	0.121268916	
0.225978107	0.112916904	0.095622276	0.258766096	
0.387458897	0.103515268	0.352488818	0.356420955	
0.07906531	0.356127662	0.505263865	0.378025058	
0.491208939	0.314293874	0.007072717	0.354411731	
0.361515639	0.388150142	0.36961329	0.332459072	

0.070848686	0.479989597	0.285011076	0.318140687
0.230429237	0.047727563	0.262132462	0.43805959
0.481364818	0.399790602	0.247962505	0.2172633
0.245517249	0.241547196	0.241844171	0.307255021
0.258425545	0.198875176	0.47855397	0.149953971
0.164715902	0.240036349	0.118789409	0.27059792
0.275708041	0.343200975	0.022190764	0.302479806
0.007747192	0.346890396	0.295479352	0.279020461
0.049018711	0.038729433	0.142701992	0.357163316
0.404874237	0.412336739	0.434862712	0.444124186
0.176094517	0.238888717	0.302665398	0.19556151
0.384576802	0.239401735	0.113064257	0.36320183
0.447110763	0.505185722	0.195387547	0.224349058
1	0.462288074	0.384283068	0.389293674
0.345626374	0.039942166	0.443082641	0.475429458
0.355568231	0.244396107	0.151992811	0.367488324
0.064502601	0.309885945	0.345791861	0.118137879
0.422826762	0.038157223	0.185538713	0.367176693
0.170738646	0.369899864	0.49208241	0.220254265
0.31643008	0.025599588	0.144062947	0.030057764
0.300681342	0.103246782	0.478668	0.433340752
0.231319131	0.337743629	0.27673433	0.002695613
0.028862317	0.132037191	0.353808401	0.283475743
0.360218365	0.32677549	0.277612428	0.338361676
0.367369154	0.425276972	0.08650364	0.120361735
0.384824272	0.13309238	0.237736895	0.378644029
0.296896861	0.136044462	0.14387	0.487810023
0.424582591	0.109038627	0.267371331	0.358576979
0.371345079	0.298627286	0.118801556	0.283222595
0.081628815	0.461585762	0.025791326	0.284155434
0.292174379	0.125981196	0.00904966	0.268446007
0.318129139	0.425311864	0.4494301	0.109108069
0.316235232	0.363862909	0.288617375	0.407297255
0.034345518	0.04713708	0.241842697	0.482194242
0.290844909	0.442263377	0.274163976	0.255926935
0.256793124	0.38261114	0.001966269	0.49131415
0.504238175	0.264213231	0.382248649	0.326304169
0.339836042	0.415135461	0.029002335	0.357597119
0.295361547	0.084680368	0.456727004	0.16995218
0.431549038	0.058760827	0.315272177	0.36276267
0.356565578	0.010228119	0.271970641	0.372475118
0.022798286	0.154167358	0.354051996	0.383065412
0.387616104	0.364541244	0.427006788	0.329608717
0.380071131	0.228603408	0.012771225	0.258879601
0.34834739	0.323404071	0.448650545	0.263013843
0.285510426	0.470136759	0.34743657	0.247736204
0.070356089	0.315237714	0.034634218	0.332145054
0.345118051	0.178956346	0.35215218	0.099084454
0.070354739	0.505078362	0.135543908	0.193882562
0.093650084	0.391111504	0.247177381	0.299700083
0.243070493	0.178583925	0.326493125	0.367721601
0.071672376	0.112218931	0.007991925	0.319235002
0.178621703	0.340014206	0.341296986	0.350722976
0.302368274	0.135567633	0.362510981	0.229693038

0.112062782	0.018749356	0.491733297	0.349436106
0.304401422	0.184439553	0.040963808	0.312283173
0.446374092	0.342334785	0.245024435	0.456278032
0.0267451	0.427031521	0.288209299	0.134583129
0.329210035	0.277482339	0.416984171	0.088533136
0.32737038	0.470468286	0.258184432	0.208083973
0.399344113	0.377886832	0.051807699	0.3398416
0.338875977	0.041294012	0.496482169	0.109502815
0.022445047	0.354048928	0.390249651	0.081409946
0.080162599	0.377948765	0.082270963	0.269592426
0.504151567	0.370180708	0.398570192	0.16256477
0.06785226	0.019631499	0.15973602	0.297704862
0.497279951	0.471937348	0.231167335	0.342853674
0.248206996	0.333996264	0.155147437	0.483533852
0.117309851	0.487722288	0.008439976	0.363967913
0.49650819	0.283275293	0.094690278	0.343266165
0.372920243	0.069807753	0.177363361	0.489875364
0.412935598	0.332861932	0.359899107	0.337920928
0.036325789	0.069983866	0.341329409	0.138320366
0.383709527	0.226905174	0.032337045	0.300837366
0.072014419	0.462772733	0.36979224	0.091167196
0.374609604	0.280284167	0.31430006	0.051380165
0.400228009	0.457128537	0.510716011	0.161462637
0.355287598	0.47086726	0.336118513	0.071012961
0.017957299	0.49190831	0.344785126	0.039969777
0.418630258	0.249725267	0.030945466	0.488431892
0.201207665	0.430928956	0.009108507	0.043857847
0.029309274	0.370631727	0.484583024	0.350264481
0.302407591	0.311890866	0.002032535	0.476659038
0.491243101	0.015886869	0.116616739	0.374819805
0.356099835	0.510173231	0.279178326	0.402998333
0.357117341	0.49015383	0.280629387	0.26312449
0.413788592	0.458343423	0.370127412	0.447618158
0.414427043	0.347919796	0.298095374	0.034188131
0.151268034	0.241873098	0.355771285	0.134177685
0.302421384	0.158634174	0.442260405	0.404901074
0.440641151	0.006073138	0.187378974	0.370168887
0.419031879	0.436774603	0.013967455	0.006088486
0.005133526	0.028061612	0.340644568	0.206077577
0.461720571	0.295272705	0.007773469	0.067622533
0.28019538	0.334496552	0.495956392	0.346900246
0.350917323	0.082781921	0.354106126	0.351202177
0.08151028	0.273238548	0.456204286	0.484102791
0.03253932	0.217245863	0.285997713	0.461311965
0.0521459	0.275907179	0.346197539	0.352887469
0.007848858	0.081130718	0.07987684	0.360900891
0.481468142	0.106873033	0.049802738	0.147706555
0.178491232	0.402818356	0.195285718	0.47555193
0.398251689	0.383879812	0.061266919	0.48380426
0.063403291	0.479800962	0.287676058	0.078939946
0.215226643	0.012843892	0.423982261	0.212860605
0.313053817	0.336515766	0.208132111	0.014518591
0.136321511	0.056322403	0.440992858	0.38567637
0.023231808	0.244466028	0.253544011	0.330716501

0.30330769	0.403076253	0.500534411	0.369938417
0.135382223	0.078309939	0.361066291	0.331117677
0.239473408	0.435170025	0.333906671	0.487765699
0.34702331	0.420915097	0.438216961	0.361586431
0.192756674	0.350063592	0.390374699	0.044951175
0.323093853	0.409151246	0.301461893	0.177678929
0.271395141	0.302950693	0.047521708	0.123844351
0.252175477	0.327636297	0.17630675	0.372242317
0.321818984	0.338110648	0.040116135	0.080362465
0.518423332	0.41996745	0.183493228	0.399769275
0.151068523	0.343666667	0.362642122	0.357837174
0.308000106	0.409250297	0.37339555	0.386399277
0.439774755	0.276038209	0.515443977	0.09760028
0.092661411	0.319514259	0.146537714	0.08506282
0.164278123	0.029454503	0.240649778	0.353766406
0.2912818	0.361946014	0.270636864	0.087428586
0.267318618	0.27125139	0.318120987	0.257658602
0.114624356	0.503506892	0.01218518	0.055756167
0.292588936	0.387086868	0.340554076	0.21885606
0.251859721	0.101913916	0.294303078	0.29799765
0.357035247	0.278902338	0.388560091	0.04607509
0.032949759	0.439951887	0.314184834	0.014043451
0.072541683	0.314742744	0.215917262	0.298836624
0.018148551	0.451856957	0.37073684	0.35544364
0.339060703	0.066718383	0.336686719	0.081296045
0.057954593	0.433958208	0.312955878	0.489542986
0.25658837	0.385827619	0.438330935	0.370505056
0.087805255	0.280479553	0.04877856	0.378197462
0.356422819			
CCR7 0.454471613	0.508427842	0.408718536	
0.454466768	0.444116791	0.442988046	0.254700394
0.256201649	0.095417662	0.00658305	0.02311804
0.448401612	0.213307919	0.320955144	0.375130449
0.439410105	0.133061285	0.40187968	0.27299607
0.454225538	0.131791296	0.414242383	0.111931518
0.283543587	0.098455697	0.074081743	0.258716182
0.47323571	0.084732696	0.408368495	0.45137237
0.103362631	0.47527575	0.517222376	0.440439786
0.50728584	0.437504661	0.01488171	0.351365228
0.463561397	0.455476235	0.454638958	0.361183403
0.051155734	0.501155941	0.281884405	0.403754852
0.286121208	0.031774979	0.323889786	0.46192656
0.479047531	0.460611623	0.296811788	0.195816986
0.211858754	0.313692657	0.253450077	0.408266754
0.262215065	0.234082689	0.479325486	0.110766737
0.18330366	0.323672009	0.150540297	0.322394103
0.340466486	0.452530091	0.025941749	0.417972279
0.004377337	0.433413428	0.384115083	0.300313836
0.044022925	0.022536059	0.177782358	0.38765175
0.480152883	0.448565266	0.471104673	0.467158258
0.181439576	0.305920681	0.388100935	0.188040515
0.472357326	0.213279652	0.127032611	0.433023634
0.47177625	0.505826067	0.204910362	0.262566129
0.462288074	1 0.448646868	0.444552054	

0.343609581	0.048578135	0.450003688	0.473629124
0.449771759	0.298542715	0.153218462	0.467170304
0.065434089	0.416088814	0.38502904	0.16805531
0.480851505	0.023937668	0.207719511	0.410849943
0.242295737	0.475118736	0.493607045	0.220455233
0.421003643	0.021386389	0.123300968	0.035641187
0.369293891	0.127693709	0.497717336	0.47827912
0.299858644	0.390819295	0.318435187	0.001894931
0.026085667	0.192536692	0.464438888	0.414961556
0.46880087	0.40658566	0.243463725	0.45309477
0.4500479	0.47059684	0.083565969	0.122183949
0.457608463	0.192555284	0.259124905	0.4723396
0.309438991	0.152784081	0.147229122	0.477282532
0.44580396	0.099340664	0.40865446	0.432845544
0.371997113	0.349294228	0.127497573	0.311366459
0.066461302	0.472597432	0.026071261	0.404126052
0.273169331	0.141307376	0.015067602	0.3415151
0.334375508	0.459738539	0.481558166	0.122177737
0.356500212	0.419453102	0.4059945	0.458898722
0.01180525	0.055064405	0.370693487	0.476373982
0.374694438	0.468501859	0.331229476	0.313489931
0.289056222	0.468563233	0.003365043	0.503200465
0.505977765	0.383756603	0.418410851	0.434011175
0.378654719	0.474064149	0.05365409	0.440340702
0.425315111	0.092471265	0.476854123	0.16408971
0.454507221	0.044156283	0.392990123	0.439736809
0.445326891	0.010913021	0.304943013	0.443855347
0.024921626	0.143937659	0.469235588	0.444325698
0.410475959	0.461568007	0.474258565	0.35666297
0.444598881	0.28761282	0.013541353	0.316838647
0.46120487	0.396955846	0.451719711	0.291594699
0.413039864	0.467871425	0.447702895	0.238914849
0.0528508	0.385220345	0.027484798	0.346087144
0.431248303	0.143113239	0.412389033	0.157583976
0.069474075	0.492380429	0.160225162	0.241567128
0.110664367	0.441759114	0.297757569	0.269764782
0.21601217	0.177191636	0.358055092	0.399689036
0.055491379	0.101168094	0.013134381	0.386903904
0.1471165	0.364369331	0.372006798	0.464448297
0.376181305	0.120133755	0.396439558	0.281258229
0.110741586	0.015529394	0.489004496	0.435302044
0.412812673	0.184880051	0.043257342	0.366223948
0.479289747	0.390213796	0.360380456	0.47357679
0.031997103	0.474658608	0.268572619	0.12473023
0.376233385	0.287513359	0.474730168	0.079304715
0.457024042	0.469437864	0.33195826	0.189579953
0.437849345	0.459568458	0.040222458	0.455057409
0.454978319	0.034126715	0.496732106	0.121802676
0.02117713	0.445147867	0.454051413	0.078274655
0.078258159	0.369897251	0.072540638	0.297661633
0.489615227	0.425407788	0.46571969	0.207286005
0.047623032	0.010618354	0.202036078	0.408435974
0.515616662	0.47258539	0.282125984	0.36475419
0.271004482	0.337263989	0.217089875	0.47243148

0.203956157	0.508747503	0.00745081	0.447420391
0.506984035	0.378909701	0.078146887	0.375023813
0.451943623	0.056100724	0.249857543	0.491306585
0.470316625	0.352151054	0.364358856	0.455842959
0.023975287	0.060279902	0.401022733	0.167560234
0.419834895	0.234280831	0.042030151	0.406528639
0.049090075	0.459990846	0.471413354	0.062616124
0.401851352	0.386477684	0.386874528	0.043357668
0.443695471	0.47572996	0.519208772	0.208995736
0.468530187	0.45729826	0.399674963	0.081523933
0.016194607	0.502354904	0.459792247	0.025792075
0.480544455	0.32694383	0.014332203	0.489091399
0.218689801	0.466329634	0.005579531	0.081817071
0.037832122	0.460970897	0.484637943	0.449639372
0.320204737	0.412780485	0.005563871	0.485311286
0.484401697	0.012836932	0.134207546	0.464239522
0.431487082	0.52112548	0.306956872	0.454589312
0.430788969	0.492042157	0.281542257	0.280062283
0.47624455	0.465046396	0.472321139	0.459468073
0.481101646	0.434618641	0.314122976	0.020216978
0.132108212	0.296448039	0.340342762	0.087275645
0.410879062	0.140000433	0.466238972	0.475338814
0.465451151	0.00495256	0.206370633	0.47164733
0.476151819	0.481825896	0.022564074	0.006820392
0.007875313	0.016283056	0.427375411	0.247594326
0.470459584	0.298296353	0.005315494	0.067763541
0.317122156	0.392947147	0.492798778	0.413445296
0.456996438	0.089609334	0.324191505	0.437700728
0.065160189	0.401074338	0.471089727	0.481642888
0.042612026	0.254690101	0.427884582	0.481967194
0.058899886	0.329374214	0.456287036	0.349036369
0.014144752	0.080292561	0.066456106	0.458089618
0.494667838	0.079702943	0.032915144	0.204320291
0.14112048	0.477344107	0.181163952	0.478771851
0.47834156	0.461244623	0.071386314	0.484608295
0.043170127	0.486369185	0.327119499	0.073597841
0.308012429	0.014000168	0.447409892	0.151184845
0.400290384	0.461631082	0.198315252	0.015299188
0.16714853	0.053772365	0.474969385	0.461028699
0.031124404	0.290133914	0.313078782	0.441106851
0.366886872	0.461757194	0.509333677	0.440955776
0.111377546	0.132338825	0.462236722	0.456282151
0.312894105	0.472689085	0.312213334	0.492397289
0.463716502	0.468426095	0.464963576	0.395108308
0.226414469	0.461899919	0.434862036	0.026379743
0.392998416	0.474379267	0.424283852	0.149902686
0.26557327	0.382407001	0.063108144	0.226476242
0.316456371	0.391279149	0.165054959	0.460762212
0.455117196	0.459749448	0.059842649	0.0623384
0.523668047	0.455147422	0.189292328	0.4700358
0.182542612	0.363741375	0.463443007	0.454474262
0.398596593	0.472381719	0.479240308	0.447579476
0.450829612	0.301693241	0.519430698	0.141159624
0.084450362	0.448612785	0.108208025	0.092882316

0.160284514	0.026629584	0.285260904	0.447137511
0.372603523	0.408256017	0.396226599	0.088336641
0.33249695	0.416187336	0.379614947	0.251262485
0.087183499	0.497542682	0.009377044	0.058962512
0.384202133	0.467312232	0.375907575	0.249664006
0.285068755	0.137000745	0.322918015	0.385168583
0.367627374	0.388052039	0.466513271	0.045190863
0.02751554	0.477994864	0.436890942	0.019754268
0.091101328	0.441531916	0.334467716	0.263356797
0.019471144	0.448815236	0.403286794	0.439132359
0.407480737	0.075417859	0.412415709	0.112887153
0.074294415	0.457332546	0.398989716	0.496422805
0.357219402	0.443290093	0.469894747	0.472475129
0.136473374	0.402258614	0.036234364	0.481241299
0.417849707
CCR9	0.3216296	0.499460216	0.187423212
0.366153657	0.208194197	0.232129792	0.120498856
0.102259472	0.079167365	0.007001459	0.017992485
0.263564579	0.104529962	0.133125399	0.180193646
0.351574756	0.073162565	0.158999845	0.133256685
0.300384664	0.060066823	0.300757465	0.068752398
0.134937068	0.068884308	0.050861205	0.148240181
0.411163483	0.060406259	0.141539664	0.293975267
0.06002066	0.358692487	0.493152576	0.297894098
0.476968869	0.306269819	0.005324443	0.145317809
0.386385145	0.375514414	0.308542511	0.206104312
0.03876879	0.456535247	0.138839964	0.231603037
0.136930693	0.026057428	0.115816712	0.415784934
0.448526608	0.246524226	0.120995501	0.097748346
0.088749637	0.094412794	0.123250977	0.224985355
0.120138957	0.077078949	0.455707653	0.077241423
0.068815933	0.121902131	0.076934592	0.134688009
0.144998617	0.321531162	0.015031483	0.236008109
0.00312157	0.284517767	0.210200777	0.150996519
0.033291769	0.017766661	0.091051517	0.21266596
0.427905576	0.429330282	0.457239792	0.461237604
0.096216488	0.145124297	0.174547027	0.101346781
0.396120341	0.105728553	0.086314624	0.348372749
0.467872091	0.490390369	0.108871377	0.1374345
0.384283068	0.448646868	1	0.188255114
0.180820489	0.036350418	0.413654104	0.480168794
0.359938884	0.139690909	0.093668358	0.348438303
0.041013364	0.178070814	0.248730893	0.071500174
0.443753782	0.02163482	0.11193421	0.185647639
0.122738981	0.383626375	0.487246986	0.112163713
0.174193592	0.018443057	0.097321986	0.024039635
0.148270134	0.071357554	0.448103662	0.429809392
0.107414204	0.158572326	0.136325363	0.003110939
0.022240787	0.097421937	0.355679995	0.160652098
0.343780932	0.350490385	0.134960896	0.305139629
0.29067327	0.443615069	0.035247586	0.067931967
0.230364902	0.093067807	0.140988664	0.241457408
0.161698734	0.076873339	0.077402692	0.502353774
0.402948144	0.058928022	0.142120636	0.332468344

0.148470668	0.184616407	0.067228646	0.130328986
0.035955535	0.447812013	0.021498717	0.153287201
0.150908582	0.065656367	0.010048224	0.124019682
0.164294208	0.39961338	0.426667336	0.069355319
0.147176223	0.275140611	0.13448759	0.33182673
0.012101668	0.037969491	0.111162549	0.476040684
0.184063599	0.469512724	0.157692675	0.156662951
0.1534458	0.413679723	0.005449955	0.471337165
0.497356049	0.12454795	0.226588315	0.242859878
0.167916548	0.428941679	0.019656067	0.310564669
0.254545373	0.055920964	0.480107139	0.094442143
0.427582533	0.033342174	0.132951641	0.390344201
0.319505724	0.005627533	0.113162459	0.287964957
0.02037269	0.058758965	0.333490059	0.31205293
0.240282521	0.383823765	0.457057398	0.16693237
0.210758867	0.125449342	0.011725905	0.157045994
0.365560458	0.147381142	0.434533242	0.13329702
0.186150556	0.46121409	0.360070095	0.10316872
0.036605816	0.143050551	0.021436878	0.180535657
0.358586158	0.068559574	0.175845575	0.071802336
0.046997155	0.504408923	0.091380477	0.119880642
0.06626656	0.213778665	0.138109186	0.111489999
0.100285038	0.098059547	0.165878764	0.182270419
0.02944875	0.058829559	0.011687019	0.136424178
0.065727115	0.137626674	0.133541919	0.286416482
0.12906108	0.063310583	0.181543078	0.148651193
0.081700181	0.013815067	0.487817671	0.301339511
0.267977491	0.098703872	0.029985463	0.128327307
0.435498598	0.172641078	0.113037579	0.428814377
0.026020227	0.44959193	0.126995806	0.077531097
0.153824188	0.137308864	0.443655888	0.03973999
0.202505435	0.438884851	0.157414713	0.116443385
0.365294941	0.282906226	0.024933908	0.339409053
0.314237354	0.02508807	0.495405605	0.069369141
0.017576001	0.327427815	0.347477357	0.053824413
0.059275427	0.159564847	0.037240102	0.182360885
0.502842802	0.332873804	0.356317034	0.114976153
0.032723009	0.006679153	0.103988275	0.200188711
0.475883744	0.478170192	0.133513214	0.169360759
0.140766441	0.163293323	0.065915191	0.484205561
0.098706992	0.484636932	0.006758209	0.35945189
0.475661719	0.116718086	0.058183831	0.155410111
0.354042597	0.041397529	0.108115446	0.478549585
0.415922489	0.171446424	0.192810221	0.281724786
0.017614913	0.030783092	0.232446178	0.094093546
0.1727746	0.129915468	0.027418497	0.138957065
0.037455354	0.424898846	0.397995288	0.055513704
0.186774368	0.145505948	0.240694445	0.031159491
0.418805877	0.46824906	0.495041149	0.092632032
0.338484106	0.468475608	0.222919984	0.052942833
0.01569389	0.48907902	0.30126625	0.028303279
0.431448975	0.103536746	0.010691476	0.480010633
0.112462087	0.404721013	0.014530914	0.045136854
0.029124515	0.408382135	0.464472429	0.276446207

0.117787048	0.225365761	0.003017459	0.489222855
0.468817187	0.013946996	0.077047454	0.354284626
0.340434382	0.504636782	0.155057004	0.413326238
0.253248815	0.469025343	0.116025911	0.156223563
0.409552959	0.456805139	0.359209285	0.451390881
0.417995509	0.30496909	0.1759472	0.015773843
0.103810775	0.10305789	0.160344907	0.063543604
0.205135489	0.097829775	0.460742851	0.427152325
0.450957881	0.005452185	0.090970682	0.355686438
0.419948889	0.455463415	0.017324596	0.007923064
0.009927346	0.018635525	0.202177817	0.133749322
0.470289147	0.11373412	0.005542911	0.033503966
0.175607279	0.252484714	0.475504166	0.297267676
0.366457922	0.050241105	0.132383297	0.317646566
0.051168423	0.235514247	0.464214919	0.452363421
0.0280707	0.1371636	0.158523748	0.469667588
0.036407855	0.171961365	0.273322766	0.152799992
0.01054426	0.057179222	0.050796724	0.368976714
0.461210962	0.046798652	0.025584153	0.059643909
0.074653867	0.420441586	0.104110242	0.490996107
0.40946312	0.3977329	0.04046435	0.472090177
0.026148268	0.455072876	0.160371864	0.076063102
0.142974928	0.01144031	0.425323405	0.100073827
0.138772618	0.285528791	0.130602253	0.012487321
0.098003649	0.037226387	0.452864965	0.416171264
0.023147398	0.128366665	0.159555632	0.27634685
0.147397195	0.361489736	0.478760077	0.200226756
0.056031031	0.057657852	0.321084305	0.279004726
0.106420867	0.46043844	0.149177575	0.502571309
0.326057467	0.438469814	0.435057673	0.210712042
0.099299512	0.235742511	0.275733925	0.024882509
0.204291216	0.412574981	0.161815685	0.105636723
0.136137793	0.190445731	0.030210308	0.084773188
0.141000702	0.189985155	0.080540813	0.398114191
0.245310422	0.294922396	0.023001189	0.050964349
0.514271598	0.421463655	0.107881701	0.326492741
0.100257661	0.190691456	0.385626564	0.361027439
0.160221835	0.393318415	0.355188733	0.285535217
0.402286455	0.144130835	0.501899633	0.05313141
0.056180649	0.238564092	0.060713495	0.05876136
0.087006527	0.026793425	0.134100678	0.32183438
0.146324396	0.249842486	0.120247549	0.07381228
0.153246875	0.149324956	0.203126339	0.114791085
0.05945534	0.475288986	0.010880962	0.037459392
0.142754708	0.384584859	0.18076716	0.141706273
0.134889039	0.070851323	0.149497598	0.129379624
0.135893379	0.151422187	0.396789558	0.039491276
0.016022222	0.463498305	0.169777528	0.0173959
0.042499078	0.240765092	0.132594636	0.128826756
0.017210175	0.442427818	0.141663025	0.222311864
0.195960692	0.045335474	0.324697312	0.052461308
0.046240037	0.424940426	0.211354407	0.477897769
0.15800338	0.3544383	0.416686177	0.360074964
0.084751611	0.175857876	0.028186346	0.394911603

0.16728475				
CCRL2	0.324286475	0.501554157	0.187370532	
0.389274203	0.248453215	0.219091533	0.134897977	
0.085988437	0.079705996	0.009694668	0.039042309	
0.280977331	0.131349442	0.140589675	0.142741824	
0.398179486	0.115815613	0.14610882	0.157564656	
0.329788271	0.067034183	0.366463754	0.087750653	
0.168642048	0.099223007	0.08276462	0.171704249	
0.430100876	0.089080471	0.144962724	0.29903076	
0.085420421	0.372023996	0.468431344	0.355168116	
0.459562628	0.285443022	0.009394683	0.147850861	
0.363465482	0.385617215	0.341983659	0.2405798	
0.054733543	0.419464796	0.167975147	0.267503594	
0.099624412	0.047300512	0.094639838	0.384005785	
0.428326336	0.277338366	0.114105708	0.119007466	
0.133317708	0.095164434	0.145207774	0.206482525	
0.154820387	0.0684103	0.445107961	0.076333714	
0.114268154	0.124247656	0.115348865	0.137709824	
0.175688947	0.293532768	0.021851078	0.24179622	
0.00427682	0.308590276	0.247036205	0.157059549	
0.054522651	0.034588127	0.130075886	0.246493769	
0.449947376	0.403766622	0.496502444	0.495196389	
0.127325104	0.172680663	0.210873464	0.138282098	
0.390895475	0.126491077	0.065998084	0.410329374	
0.501629716	0.49025973	0.147170342	0.173091029	
0.389293674	0.444552054	0.188255114	1	
0.171799371	0.052966105	0.407222612	0.507208131	
0.332669421	0.164521989	0.129375848	0.354209178	
0.066898043	0.135149753	0.295479224	0.088774446	
0.463087043	0.027888688	0.153233122	0.221304552	
0.131893617	0.373235283	0.485236059	0.135816813	
0.20371912	0.02838954	0.092348698	0.040670277	
0.150760298	0.091977171	0.412846253	0.454479539	
0.11084871	0.205987146	0.156406479	0.006613612	
0.038843844	0.110455416	0.376790428	0.160104211	
0.359735111	0.322359535	0.166591699	0.28908006	
0.311604361	0.49074884	0.019839691	0.085699698	
0.227355524	0.120276376	0.186290845	0.279347659	
0.189016877	0.082484087	0.111969482	0.513942086	
0.376292787	0.085797864	0.128374174	0.372849889	
0.175663591	0.230612455	0.093380927	0.162688341	
0.057251012	0.435445536	0.038164279	0.135874956	
0.135840075	0.109465368	0.012236853	0.118797433	
0.137829377	0.411062331	0.395457608	0.09342231	
0.179649054	0.325329767	0.132088991	0.341689527	
0.022735349	0.044996972	0.093405651	0.498912417	
0.167107287	0.485420767	0.168485894	0.175770854	
0.194777625	0.430909383	0.002472854	0.437793693	
0.491446107	0.097051757	0.26920771	0.27110309	
0.177022631	0.444660273	0.012237905	0.364515862	
0.230222789	0.076191414	0.513545572	0.135444605	
0.41380204	0.058938859	0.148878398	0.408553317	
0.343516042	0.009535196	0.098556416	0.312066812	
0.035735625	0.067037268	0.342752329	0.330644975	

0.260873187	0.406328287	0.494969038	0.172602161
0.23856586	0.131656958	0.017998619	0.190383352
0.38028962	0.167979434	0.400596848	0.141166578
0.161128559	0.447599379	0.352616685	0.119784891
0.056162675	0.154112139	0.019633445	0.191469515
0.337218828	0.102719958	0.183072468	0.092250959
0.074837774	0.517163492	0.11846873	0.136547649
0.0856444	0.246005831	0.150452161	0.136466495
0.136917815	0.138391737	0.184964687	0.199745309
0.040885233	0.096288908	0.016257687	0.156537236
0.100905679	0.158139412	0.14701578	0.291627579
0.153475414	0.088752042	0.197283293	0.160857613
0.098739428	0.026584208	0.484799885	0.333813272
0.236649901	0.11938162	0.048848062	0.141772488
0.467183573	0.148247141	0.086709853	0.442244347
0.026432461	0.48429109	0.164533805	0.114823079
0.190473772	0.171929222	0.463644559	0.035677095
0.211308291	0.436018612	0.185193304	0.149672223
0.346966474	0.305465351	0.039606962	0.315391443
0.301672765	0.046233781	0.501230993	0.103236948
0.032020544	0.3593959	0.376152055	0.081104908
0.079613697	0.181268756	0.039920114	0.233445611
0.508066348	0.393733125	0.382006729	0.134795093
0.055997259	0.018351357	0.141281083	0.202217722
0.449794112	0.490954283	0.169161812	0.195760226
0.180386578	0.183369691	0.066345472	0.502838399
0.095649868	0.470333039	0.013546999	0.396449723
0.454437071	0.110322079	0.086534079	0.172610561
0.332821819	0.05708924	0.119555896	0.457275026
0.416354668	0.184680568	0.181283196	0.30934537
0.02294446	0.049109097	0.249723183	0.116014504
0.194388193	0.156073904	0.037941242	0.1387994
0.059617233	0.427059619	0.435791695	0.072937052
0.191482678	0.127322066	0.298714118	0.049615016
0.437823442	0.460987214	0.474583343	0.107173422
0.366809812	0.472801806	0.250724803	0.077189528
0.027915896	0.470540562	0.281341287	0.029419393
0.473287604	0.085110057	0.024350446	0.470397165
0.133490984	0.371523907	0.00433939	0.036758482
0.04372005	0.449190378	0.433511116	0.286039595
0.095205036	0.247231974	0.006340521	0.501386348
0.460198975	0.024903904	0.115946595	0.377496023
0.359342591	0.491392026	0.170487162	0.437063867
0.279450642	0.458761668	0.146319489	0.202451517
0.412351929	0.458940167	0.343797407	0.417633184
0.453386394	0.3540354	0.216605888	0.027294572
0.098787222	0.099832094	0.131873948	0.095337658
0.180890112	0.123612354	0.444429261	0.472714126
0.471786858	0.011238092	0.091535225	0.365393196
0.416599976	0.46785972	0.019379819	0.005350029
0.003492918	0.02971719	0.222672967	0.157995919
0.472495995	0.095608117	0.011582709	0.037338346
0.15848419	0.294679729	0.473643823	0.341094326
0.384654802	0.082581908	0.140129414	0.350917279

0.076485072	0.202778034	0.485156661	0.438789878
0.044437307	0.165670012	0.14571391	0.494234637
0.048784569	0.225362998	0.30251672	0.173583532
0.01334798	0.082346475	0.076924768	0.411256241
0.448127707	0.080676339	0.048550843	0.049924142
0.105854521	0.453207302	0.130214758	0.515878972
0.417316376	0.444648464	0.074983516	0.446319261
0.057914621	0.438645573	0.189024071	0.027328634
0.151323902	0.01933632	0.426646142	0.107932742
0.138559202	0.279031326	0.127410815	0.026027181
0.100372476	0.062195428	0.494323596	0.456309805
0.036717979	0.147967737	0.176725002	0.29321616
0.139562757	0.400472234	0.451085479	0.181052847
0.080608098	0.075810741	0.330993932	0.25958059
0.115001537	0.501018217	0.181411319	0.50625786
0.306777697	0.429906093	0.450080877	0.222033605
0.152040118	0.219132094	0.284668263	0.025130578
0.2176725	0.461355607	0.159677252	0.121499812
0.18083978	0.215652043	0.046705006	0.060683453
0.154129213	0.176496669	0.124023649	0.439473233
0.242506286	0.275752889	0.032905043	0.074395557
0.512627966	0.395553007	0.084750833	0.33098432
0.127449226	0.232751	0.397728333	0.401715445
0.171051239	0.421866752	0.365501408	0.295091755
0.389710255	0.166669603	0.482880519	0.038271019
0.069580029	0.256832601	0.097838255	0.088707729
0.123868157	0.038768737	0.141605261	0.37224096
0.159329891	0.30847562	0.118954487	0.076763337
0.202080508	0.137870216	0.250545527	0.125136445
0.082044713	0.4594521	0.014171928	0.06387959
0.16557026	0.388666763	0.209690019	0.162008578
0.162643751	0.106477437	0.202901349	0.117376587
0.136588507	0.147217624	0.390954304	0.029877444
0.026814554	0.500519566	0.143207884	0.024212444
0.079803088	0.23121994	0.139896757	0.164460893
0.030147014	0.461991974	0.14145391	0.251547188
0.204699564	0.066900713	0.28992575	0.059479128
0.051652559	0.392565488	0.2236086	0.472702411
0.169116487	0.411535579	0.375947513	0.387095554
0.064723815	0.174921176	0.037955606	0.413759845
0.161300123			
CCT3	0.23741214	0.410726938	0.175684253
0.306083566	0.178571706	0.179544346	0.082085498
0.02593095	0.083069837	0.004581492	0.033858796
0.213679389	0.06393442	0.048000962	0.106811951
0.30269106	0.043351935	0.067221051	0.09936065
0.220598424	0.021445985	0.269577779	0.032480736
0.084662894	0.065033622	0.043557126	0.148234902
0.311596631	0.051005057	0.104483562	0.194976379
0.025369522	0.254755179	0.401123376	0.277839424
0.388043087	0.229910082	0.000281051	0.080571059
0.268145414	0.36657055	0.232149574	0.173116852
0.022978876	0.339119601	0.125737558	0.210920814
0.044301664	0.025186869	0.045257315	0.303200518

0.370981131	0.24425578	0.046019015	0.058130129
0.071066252	0.027450629	0.096069631	0.131684148
0.089374689	0.024539291	0.380497684	0.055448546
0.035499747	0.044169477	0.036223117	0.064190813
0.101087953	0.216656575	0.003962733	0.193153666
0.000774772	0.20887	0.173775623	0.159879962
0.024015336	0.01168664	0.043605117	0.173723223
0.328076447	0.321869286	0.404059201	0.418234493
0.048758737	0.099650273	0.174861511	0.051267338
0.309006877	0.071143733	0.029451353	0.309477257
0.415042047	0.410860209	0.074972235	0.138828743
0.345626374	0.343609581	0.180820489	0.171799371
1	0.024118949	0.337608562	0.408350056
0.241019664	0.142302434	0.08012544	0.235610379
0.02122561	0.060940918	0.207901184	0.022287489
0.346620994	0.018023896	0.101138308	0.145482695
0.042362674	0.289446112	0.422408122	0.07953739
0.13950447	0.007948932	0.115021912	0.010952287
0.058673673	0.055014669	0.354445101	0.352415013
0.03067776	0.089096775	0.074320386	0.002134916
0.014996548	0.03853453	0.268067835	0.087027731
0.258900231	0.278382694	0.120792836	0.209303571
0.279194258	0.393885618	0.01764818	0.028650731
0.190375842	0.037735424	0.123235437	0.19806445
0.170511641	0.024730226	0.035739389	0.43158115
0.286187529	0.033863701	0.053379455	0.277103088
0.086446512	0.190502191	0.02624308	0.054352678
0.016139552	0.387774381	0.014937868	0.075661543
0.166074081	0.038717894	0.001971377	0.059562925
0.066133005	0.359249971	0.333448966	0.032365738
0.083990688	0.261972199	0.074630753	0.295255172
0.020839404	0.013447474	0.04917842	0.39459639
0.108357276	0.388393505	0.060988514	0.083743386
0.148150292	0.344061218	0.001626853	0.373294181
0.426537838	0.056948756	0.204453817	0.192224579
0.209783585	0.360092099	0.004592578	0.241406563
0.150988673	0.024032791	0.410816182	0.086008876
0.325686354	0.029102271	0.057084596	0.382902077
0.280030267	0.002808451	0.062911824	0.221355466
0.014253135	0.023406484	0.24515174	0.257878516
0.258307923	0.322358834	0.380844123	0.205473353
0.19266097	0.048848101	0.005455502	0.152121466
0.291624783	0.081090079	0.328588067	0.054648873
0.107277029	0.373327139	0.297978609	0.078490572
0.023858579	0.087939757	0.007265667	0.204471842
0.297995952	0.035829817	0.213206352	0.022734061
0.030148063	0.433436424	0.071655602	0.103601216
0.060254231	0.17254346	0.079554214	0.056767316
0.06754253	0.06541374	0.140741429	0.140228186
0.012097625	0.033363867	0.005850332	0.074804475
0.039195869	0.073953641	0.072752205	0.200703517
0.046540558	0.028267666	0.166589252	0.100374723
0.059789317	0.009847842	0.400156661	0.237381499
0.163040928	0.05090631	0.014711122	0.059231202

0.363090846	0.134124436	0.040858765	0.345508678
0.008685964	0.382246004	0.06965427	0.069027011
0.112466268	0.11075268	0.366245438	0.028610148
0.131156768	0.337996176	0.125999742	0.102938441
0.259247148	0.222349427	0.013058799	0.239721541
0.212166246	0.014795567	0.414127647	0.0385681
0.011322716	0.284676115	0.346075527	0.04582164
0.040149585	0.149145678	0.012691399	0.183813883
0.427369227	0.298956823	0.318831367	0.072084936
0.021497054	0.00458386	0.066436167	0.165547702
0.368649923	0.411343234	0.09824662	0.173467534
0.116958726	0.140686161	0.017219321	0.418293655
0.035063559	0.382103622	0.003690441	0.349194147
0.395099578	0.063303985	0.054193455	0.124337502
0.246176898	0.025314417	0.032659067	0.382804001
0.350527559	0.141424172	0.156366785	0.202965107
0.007922185	0.012704178	0.250514435	0.077575538
0.114137003	0.080860947	0.027996707	0.049386567
0.02396782	0.33950806	0.308668899	0.065586796
0.165391987	0.049257871	0.210873402	0.01781609
0.406382891	0.38674536	0.396791235	0.02826848
0.260969619	0.423918165	0.244526119	0.028919483
0.012704693	0.407914992	0.206847667	0.013402004
0.350404547	0.05437174	0.008842745	0.37607223
0.069965374	0.293298946	0.008249524	0.019134116
0.017221808	0.349918121	0.387398556	0.186039181
0.05558496	0.154154034	0.001023967	0.409814146
0.378522528	0.013458894	0.053730984	0.304697972
0.331910367	0.391836971	0.181586968	0.397807709
0.240753935	0.396370031	0.053668709	0.164619338
0.339701145	0.405441916	0.258648206	0.341975973
0.331232602	0.262210007	0.159063005	0.012090714
0.140045058	0.041647806	0.112173446	0.084873646
0.122816156	0.081719774	0.355794364	0.356266025
0.395473787	0.003877849	0.02940552	0.285751086
0.307114106	0.35692145	0.006490004	0.008732598
0.002925755	0.03202961	0.138369899	0.104601895
0.406503935	0.048255845	0.003243794	0.009480641
0.070593876	0.273407309	0.392198129	0.236607751
0.307525256	0.024024182	0.078137049	0.237843272
0.058123065	0.1412707	0.410711226	0.356613814
0.012332557	0.107910921	0.083465725	0.386546362
0.045169464	0.150907853	0.211444841	0.086414398
0.002909293	0.042195069	0.052631784	0.301082745
0.383649267	0.034033853	0.021509692	0.017032246
0.0428265	0.340349296	0.051014708	0.41627128
0.31523002	0.320671969	0.026829813	0.388911015
0.031505576	0.3623768	0.176627081	0.022146614
0.053016154	0.004914334	0.337615826	0.150935301
0.055958548	0.183872918	0.154995796	0.009284847
0.088602011	0.022818662	0.400808811	0.377822054
0.012812262	0.054124687	0.125419421	0.195830186
0.089949431	0.31421044	0.379671965	0.170500392
0.029995275	0.028000208	0.221040949	0.179276017

0.031183694	0.396738069	0.122534138	0.42010648
0.230690228	0.339321603	0.366774833	0.196347887
0.06411725	0.16245235	0.220523625	0.010454789
0.151486758	0.352169625	0.099270388	0.09714447
0.133113876	0.200951214	0.010761637	0.018257018
0.160608681	0.12461033	0.039547747	0.319856962
0.150622141	0.194919105	0.004745851	0.056909582
0.416881018	0.325226967	0.046156663	0.269891375
0.056484383	0.218774443	0.317189605	0.315464386
0.086706838	0.326078599	0.252221793	0.21371877
0.297903366	0.116037869	0.412287103	0.009793299
0.068311809	0.172905438	0.042892976	0.043058366
0.055603318	0.01985049	0.103117077	0.255608752
0.095965824	0.212567264	0.036304908	0.03520743
0.138384181	0.087589633	0.173514043	0.057637992
0.037882477	0.395497402	0.007152643	0.018765856
0.091387308	0.35580646	0.218544201	0.079518388
0.109474619	0.036714511	0.133978545	0.073610599
0.088428363	0.065345798	0.35837346	0.012648175
0.005629297	0.383750561	0.102169865	0.007924626
0.026333354	0.16680344	0.039215721	0.108082601
0.013000299	0.395566936	0.106760646	0.201771735
0.137817373	0.017880801	0.206896936	0.019663345
0.016412021	0.303093204	0.207742161	0.401013114
0.110455341	0.308600424	0.303849179	0.278353564
0.024005867	0.144790295	0.015258321	0.296330236
0.133929579
CD244	0.072223535	0.054240164	0.08463734
0.081604876	0.093218799	0.0223671	0.049326635
0.014069698	0.038930599	0.004410678	0.030405702
0.085483866	0.017619854	0.015290331	0.011218044
0.152220883	0.113239157	0.055218587	0.118242819
0.095676757	0.012727807	0.154819383	0.054544401
0.097112241	0.135232121	0.116953237	0.09944386
0.09286013	0.138928009	0.010859749	0.049475268
0.19729984	0.068360053	0.042065188	0.097937127
0.040477235	0.052092617	0.034686716	0.010330508
0.056438715	0.089547645	0.084714758	0.102410999
0.043256986	0.030383001	0.076406413	0.154359288
0.008974701	0.06685778	0.00644998	0.029877361
0.029295531	0.040996654	0.021310017	0.039020558
0.008421468	0.006573517	0.024915795	0.019890469
0.075951604	0.004536694	0.037360177	0.017789598
0.00902516	0.036819858	0.17780362	0.039616841
0.071048792	0.034897736	0.044576398	0.067439371
0.000963891	0.105881711	0.190210803	0.044408165
0.214595989	0.026282414	0.213335514	0.061957142
0.091641995	0.052375061	0.104589326	0.091546953
0.051892025	0.206775227	0.107672704	0.086696169
0.058112536	0.040784749	0.034749218	0.167711124
0.098507134	0.048875448	0.070465862	0.26588534
0.039942166	0.048578135	0.036350418	0.052966105
0.024118949	1	0.033320616	0.075465301
0.036167161	0.120464437	0.22595813	0.052344167

0.179878368	0.011771308	0.120353025	0.053193701
0.091908756	0.028659826	0.253295765	0.045833906
0.0765499	0.06515073	0.053647114	0.090425419
0.109274858	0.050678841	0.035159128	0.19203073
0.01695224	0.07956277	0.026825086	0.067030503
0.010279052	0.023894591	0.053907867	0.082072225
0.15802242	0.124231338	0.05645907	0.077165741
0.091795248	0.045924498	0.061611146	0.03009766
0.062484531	0.105953898	0.000354209	0.062256463
0.052768567	0.120177449	0.196895243	0.055102846
0.128437489	0.041587957	0.10718227	0.080737597
0.031192778	0.111916371	0.025221242	0.157229741
0.010635901	0.169327065	0.103108293	0.040474017
0.029147447	0.040654406	0.244207903	0.035698873
0.011852577	0.07251021	0.070566987	0.024258618
0.010022816	0.038909874	0.02879206	0.171451687
0.053739629	0.139203445	0.019409534	0.037513969
0.014056819	0.093026501	0.010816448	0.065042423
0.010886249	0.090721284	0.066931139	0.119694436
0.221902758	0.108484145	0.02245039	0.033896578
0.051492623	0.009057439	0.066049302	0.128010979
0.0480072	0.07649356	0.003333023	0.140578405
0.027472855	0.156750237	0.104491026	0.184477118
0.043756245	0.136080423	0.018270816	0.083684205
0.048652718	0.017285452	0.009763844	0.064907519
0.231115793	0.007237204	0.080583961	0.088241038
0.065944249	0.122682775	0.118276036	0.043901875
0.07219429	0.060898672	0.103350599	0.218521879
0.093479994	0.010299228	0.032615295	0.030557551
0.016654941	0.046142345	0.046820223	0.004613891
0.079710945	0.035703698	0.007873363	0.101741624
0.046116428	0.019178704	0.041658967	0.121929555
0.200987271	0.067078088	0.172168259	0.104853954
0.118193785	0.031121516	0.098440922	0.009976713
0.014648078	0.087090354	0.051343713	0.039475718
0.015153658	0.111064048	0.203330612	0.014982592
0.010672282	0.012936366	0.009453244	0.033852433
0.013114253	0.02350419	0.072601567	0.079224848
0.163965005	0.180388855	0.054792278	0.139186347
0.018804745	0.060117224	0.201792858	0.004366901
0.064408892	0.008155541	0.006827046	0.044842959
0.074639313	0.103437289	0.02144347	0.185840806
0.048131677	0.100244013	0.099717168	0.006108704
0.042739402	0.037842267	0.200254327	0.123943128
0.023543724	0.0738241	0.031180569	0.040569531
0.033204666	0.136557262	0.061715147	0.135743059
0.228071195	0.127142745	0.055538239	0.261499339
0.226259186	0.033321051	0.00867117	0.233701166
0.062642815	0.148812815	0.040811591	0.185733852
0.06196819	0.014251179	0.243705867	0.096715796
0.035285378	0.067354169	0.208274869	0.071935741
0.151018075	0.035991508	0.00369719	0.068422281
0.094338156	0.050018515	0.116110743	0.093009694
0.036550532	0.011101629	0.156086907	0.029563867

0.027673663	0.081981036	0.082124905	0.041243522
0.054589804	0.059487778	0.03332067	0.111640496
0.013910901	0.022588333	0.11858244	0.117056321
0.025420508	0.10287019	0.073399366	0.015076641
0.039012792	0.034831657	0.118662171	0.068289908
0.012147414	0.015291802	0.20170517	0.121585793
0.075878948	0.059505292	0.041347262	0.063559996
0.070460103	0.055380346	0.056791563	0.273958451
0.252983514	0.047224362	0.048145069	0.021155779
0.098716271	0.005964481	0.013242463	0.049264862
0.117713561	0.024751816	0.002668901	0.058536079
0.209955658	0.133427024	0.033665628	0.056002296
0.00296064	0.124706935	0.082559382	0.075418076
0.040529242	0.158452082	0.122059375	0.04965754
0.064304525	0.051976831	0.08545466	0.071062927
0.060886956	0.038825479	0.012219568	0.202142115
0.051068737	0.053028063	0.062871246	0.044099003
0.079693049	0.15683832	0.117733401	0.031533171
0.058626364	0.013106597	0.006069855	0.026108772
0.019746644	0.104035534	0.060901985	0.108842479
0.07299575	0.151852729	0.014400565	0.047087812
0.054275822	0.08432377	0.137440166	0.01964709
0.01705525	0.060902454	0.05319222	0.244611874
0.065888045	0.001443739	0.072766657	0.013023961
0.02952239	0.136686447	0.045446016	0.146843317
0.101965217	0.174023692	0.005802342	0.132231216
0.087896624	0.021322735	0.073202373	0.035630723
0.171404641	0.204546851	0.050106498	0.08179164
0.04841621	0.210274982	0.082211989	0.01531386
0.121960257	0.20951447	0.142578097	0.141851229
0.03695439	0.031100052	0.045865036	0.00174001
0.031493905	0.092357704	0.045560379	0.08968642
0.063287177	0.123588835	0.153929571	0.040590207
0.040547149	0.035089048	0.132656647	0.002240088
0.125829011	0.108085589	0.05261286	0.01212478
0.013835823	0.05835567	0.0394847	0.20844214
0.061514493	0.176677278	0.093197276	0.116639347
0.206708893	0.04898465	0.207147478	0.125017765
0.021840733	0.083508725	0.036183976	0.018259579
0.027666669	0.098647828	0.079943431	0.037720314
0.014818347	0.110952909	0.027808258	0.072743052
0.052325737	0.065123807	0.057236361	0.065782288
0.061398802	0.035047028	0.029118613	0.009988358
0.113210438	0.086790961	0.055228347	0.0530354
0.114507756	0.088399981	0.073536103	0.012911766
0.084582864	0.015973799	0.032448152	0.138527925
0.055406705	0.038207277	0.042147515	0.119555364
0.058584398	0.043686326	0.013785498	0.056358622
0.244114688	0.111541747	0.10138569	0.116124391
0.026467243	0.050730648	0.059608514	0.027586968
0.029327369	0.098853285	0.043820402	0.004569642
0.063317299	0.055877656	0.031297568	0.307826234
0.123087538	0.131910629	0.03885047	0.148704996
0.034054762	0.109192048	0.007115378	0.09997766

0.209698267	0.043556944	0.160321497	0.024495639
0.037413706	0.036337469	0.053806706	0.207355986
0.04251752	0.062670197	0.070759282	0.154096981
0.044307277	0.156522736	0.136468653	0.012814932
0.007091967	0.019425539	0.056531685	0.029242573
0.026245838	0.10583449	0.029371625	0.193291594
0.102097374	0.03890269	0.06323444	0.022434692
0.26502622	0.054069324	0.009516854	0.097380523
0.054217338	0.151490591	0.023395863	0.028714278
0.088083241	0.03476632	0.10927858	0.047605021
0.144891556	0.121335281	0.026791489	0.049608916
0.074606119	0.061271525	0.033015909	0.084469732
0.0195011			
CD3EAP 0.405955904	0.493457963	0.36091141	
0.40554726	0.390289805	0.408669118	0.209355789
0.193334995	0.079305614	0.005040832	0.021517313
0.396529495	0.19131061	0.274555536	0.35039496
0.379487331	0.105491329	0.31925276	0.215496357
0.388386515	0.102487121	0.359040908	0.082528353
0.24105365	0.081644404	0.058550626	0.235151338
0.415682511	0.06575102	0.383296011	0.393804704
0.070301183	0.425266099	0.512038423	0.401160348
0.506068743	0.398208246	0.007744921	0.328645202
0.416227689	0.419536371	0.394452686	0.303692195
0.03788569	0.496072039	0.236499552	0.347482337
0.240479105	0.024097346	0.281131475	0.451933411
0.485139055	0.442460648	0.238207131	0.160707459
0.213308967	0.270766879	0.249434321	0.363762734
0.215217734	0.199814783	0.480340359	0.093016123
0.16493648	0.247451753	0.111555875	0.258598375
0.310852754	0.410161578	0.014570425	0.381139941
0.003170104	0.362934683	0.318159658	0.297908864
0.030167127	0.017338154	0.133290142	0.3378335
0.425939489	0.428512623	0.43216888	0.436886814
0.146810908	0.239767495	0.350680158	0.151961882
0.432749488	0.179671122	0.091041616	0.376145693
0.435844185	0.499535513	0.164768119	0.211869337
0.443082641	0.450003688	0.413654104	0.407222612
0.337608562	0.033320616	1 0.448783109	
0.414423363	0.269509525	0.119991061	0.415074301
0.044559892	0.353046678	0.327651311	0.124122033
0.428973625	0.017234491	0.167197885	0.363994998
0.173431986	0.428749563	0.48718049	0.174545343
0.354357232	0.014187135	0.119727264	0.02195519
0.301890386	0.110456549	0.50600765	0.43979705
0.237640852	0.351089039	0.256410922	0.0001716
0.017176724	0.139629902	0.43060246	0.330730134
0.411433113	0.386766311	0.21987263	0.41968928
0.423320201	0.432052075	0.076838639	0.087843422
0.404039082	0.139715726	0.205374675	0.430728601
0.267764421	0.108121063	0.109723806	0.452333746
0.423909612	0.072933471	0.335243864	0.369633373
0.354246431	0.301360854	0.090659385	0.258874729
0.048994021	0.472659508	0.017158932	0.326620953

0.274485265	0.114388154	0.00718859	0.277020888
0.293124651	0.447263884	0.487401625	0.084957049
0.301581968	0.367097387	0.347031886	0.436698563
0.01164635	0.033589896	0.314756549	0.4529194
0.345860503	0.431556837	0.262766546	0.256819638
0.235885289	0.417496398	0.001886448	0.499956174
0.50035811	0.33345759	0.376938493	0.36904989
0.367920349	0.433073157	0.03809077	0.375969624
0.381816543	0.062606487	0.435457396	0.131369888
0.441053851	0.032860929	0.337746107	0.417215481
0.426740354	0.006628566	0.26672868	0.387040116
0.016201186	0.117511307	0.412781126	0.389910294
0.375533688	0.40988541	0.424423434	0.35022259
0.391470257	0.215785963	0.007702372	0.26068819
0.414890942	0.387841343	0.44093601	0.232654035
0.368355677	0.449520451	0.423014443	0.251095497
0.038705607	0.324531616	0.019047604	0.305041298
0.4099146	0.12286048	0.394942706	0.106314641
0.050542547	0.475495483	0.134716596	0.218254321
0.095998156	0.412668488	0.229081607	0.254536124
0.204795211	0.141103092	0.328372816	0.369900087
0.040526257	0.077088948	0.007177962	0.373222304
0.137109504	0.34642331	0.358830135	0.430127845
0.321313896	0.097527066	0.349610429	0.243968353
0.085142915	0.009692336	0.47297022	0.366129915
0.381112426	0.142464175	0.027937014	0.352820258
0.446760868	0.378698631	0.311720776	0.44835384
0.019366366	0.433382808	0.242097562	0.098153857
0.344635806	0.237644136	0.424906716	0.068189114
0.393951417	0.457856522	0.262327854	0.161314377
0.419323048	0.401388424	0.027188234	0.417552987
0.417575933	0.023240627	0.481470507	0.092380658
0.013278956	0.396900198	0.436931535	0.056636473
0.056818098	0.345347506	0.053071788	0.245400647
0.47322227	0.371624144	0.450405427	0.16709223
0.035674945	0.008553608	0.157596164	0.355920966
0.512661442	0.458828762	0.217600502	0.332989918
0.214301835	0.321124832	0.176521717	0.453634881
0.14401378	0.494751697	0.003933451	0.419399403
0.510533966	0.330172422	0.062639457	0.331404979
0.424289522	0.040171492	0.176592133	0.483771185
0.441027176	0.314994854	0.344368005	0.389041451
0.016421887	0.044141643	0.355170626	0.144159077
0.39250083	0.190881434	0.034544585	0.342534727
0.038012093	0.445739332	0.414859684	0.052282561
0.396148579	0.325637128	0.325882842	0.029721746
0.424440462	0.464249488	0.512601871	0.148063392
0.426977642	0.448950279	0.387127236	0.056002786
0.010266128	0.497424569	0.406809982	0.020342149
0.431560737	0.291285968	0.011802465	0.470398892
0.166539206	0.467343577	0.003791666	0.062862342
0.024198505	0.41173654	0.485120303	0.388065728
0.315095001	0.334465905	0.003128145	0.465532871
0.475709773	0.008344491	0.105360472	0.440384359

0.41307355	0.502654671	0.286059571	0.432410679
0.40223972	0.494920542	0.24413076	0.232149797
0.442800294	0.463209979	0.416223348	0.43871802
0.437269285	0.37738439	0.260684623	0.014559985
0.122138779	0.244219219	0.347559949	0.086071968
0.363612144	0.116466027	0.432848867	0.429245924
0.438231504	0.002437601	0.158649094	0.440509067
0.432609538	0.433351241	0.012397727	0.004131961
0.004886112	0.013737127	0.363986566	0.193593156
0.443034408	0.295433906	0.002734709	0.045695008
0.256651504	0.355744773	0.484807333	0.346194452
0.414108008	0.062669314	0.318005049	0.367924298
0.053828504	0.364374643	0.443075823	0.47297235
0.02642763	0.205016541	0.348207177	0.442393267
0.055666168	0.267486869	0.405733625	0.3135962
0.007357046	0.059072214	0.052252925	0.398598444
0.499033499	0.06774786	0.025811898	0.170037696
0.118867717	0.433351451	0.147123707	0.441973988
0.435462433	0.404207276	0.052011771	0.476772293
0.036280395	0.483344138	0.291554978	0.065757197
0.23203223	0.007624976	0.434846704	0.164321652
0.346323989	0.395317261	0.186860975	0.009557884
0.147644704	0.037303965	0.436918996	0.419993512
0.019197434	0.228323811	0.247552745	0.367683297
0.311844075	0.41660944	0.505056244	0.423872294
0.088254317	0.101254967	0.394332374	0.403974778
0.249829962	0.430979172	0.298512678	0.472411666
0.415569506	0.432236671	0.440346252	0.345157325
0.195382878	0.405498677	0.405069586	0.019608468
0.321848675	0.434514518	0.344377999	0.132976178
0.229867644	0.356026087	0.039804748	0.168504836
0.29596924	0.355955804	0.133015418	0.399941648
0.385712412	0.412174229	0.035657008	0.049503107
0.507087134	0.426950635	0.146603139	0.426555935
0.133443812	0.322180231	0.418520753	0.410982872
0.365459302	0.446934076	0.425122769	0.418687257
0.431198422	0.24511644	0.513686872	0.098019066
0.066425762	0.404102242	0.092738374	0.066660677
0.123318089	0.017739474	0.273168136	0.382838234
0.350974196	0.354543545	0.341854265	0.064303106
0.272617187	0.351599087	0.314400679	0.211938461
0.069183726	0.497179569	0.006354928	0.039902338
0.350190361	0.438004462	0.353385405	0.195354558
0.263350325	0.101727336	0.268481774	0.332421535
0.364322362	0.330263707	0.440232484	0.029804339
0.017727004	0.43287952	0.371126721	0.01122108
0.072089558	0.397989599	0.250554361	0.241385149
0.012424189	0.444258221	0.390619742	0.393470633
0.345436764	0.049880565	0.385387516	0.077635358
0.046850172	0.441423111	0.355916539	0.491626226
0.29418265	0.390912948	0.464941019	0.440938517
0.091690981	0.359022422	0.024717816	0.427059913
0.403623362			

CD44 0.46890506 0.529741625 0.458371294

0.476945554	0.505269505	0.410676432	0.249632933
0.203303639	0.108142534	0.007694477	0.037793756
0.510458808	0.272310169	0.321404599	0.363700852
0.483589443	0.192310545	0.387023791	0.29407506
0.465333294	0.109369439	0.47916694	0.118072656
0.364744821	0.150202493	0.097512755	0.341884568
0.489720341	0.110182075	0.364710538	0.426532586
0.138417554	0.486661575	0.530047945	0.522721595
0.529041081	0.439341279	0.01683372	0.335737776
0.45317488	0.505170345	0.461297487	0.394812325
0.057499078	0.491508877	0.297587662	0.477922367
0.252683111	0.048599036	0.254585103	0.441230696
0.491174537	0.500341355	0.249787206	0.194946566
0.274530425	0.25402659	0.360319073	0.350049352
0.27904957	0.16987659	0.498913737	0.108037429
0.179213483	0.279603746	0.209501649	0.298294707
0.427240502	0.416927121	0.023055917	0.466483925
0.004168082	0.440212587	0.454220405	0.37919167
0.061539033	0.034414408	0.239729403	0.395851228
0.500702216	0.457136351	0.526711899	0.51945404
0.248024072	0.361052801	0.476326641	0.226855879
0.476231587	0.220620018	0.134707281	0.495842943
0.524854303	0.535153186	0.265082404	0.333129135
0.475429458	0.473629124	0.480168794	0.507208131
0.408350056	0.075465301	0.448783109	1
0.428598352	0.403163307	0.207680905	0.454485838
0.082059992	0.39833181	0.422004261	0.209307628
0.502343228	0.024400336	0.282938688	0.418909305
0.257611115	0.48963507	0.530294017	0.23267097
0.486146848	0.031458065	0.18452587	0.048209517
0.386753828	0.204813292	0.493928852	0.50603283
0.267811213	0.46355612	0.311449562	0.003885562
0.041983658	0.232197645	0.495251087	0.417711906
0.484508082	0.416204883	0.297727411	0.428134732
0.510080976	0.526993131	0.062705167	0.119594723
0.482858648	0.235464577	0.300312255	0.517649321
0.391253989	0.136634656	0.167874622	0.514687849
0.416725889	0.113610547	0.365805031	0.486485398
0.394589722	0.448104786	0.139139669	0.347178115
0.06651704	0.496359186	0.044540457	0.364129089
0.322518389	0.214265291	0.014020525	0.288034599
0.33782328	0.486320199	0.481005591	0.13874071
0.39290813	0.487912827	0.351344576	0.490271154
0.016632948	0.061976358	0.296229131	0.510675414
0.310422862	0.50861496	0.361872117	0.37187492
0.358094803	0.503506068	0.004195868	0.505079701
0.537996833	0.319167987	0.484217598	0.490776432
0.444491059	0.502754574	0.048726529	0.491837585
0.393040423	0.103123025	0.52139127	0.217738328
0.464793466	0.060992663	0.364421823	0.49943599
0.500384596	0.012176146	0.244865993	0.437838715
0.041547183	0.110551717	0.476516106	0.469194918
0.446680896	0.50623459	0.518305989	0.427434731
0.505806493	0.27384213	0.018112548	0.399631666

0.495806695	0.443398379	0.440085622	0.282347472
0.36640096	0.47375239	0.471382554	0.238939534
0.059383275	0.35423735	0.027833496	0.404291418
0.433046125	0.157889934	0.449805937	0.189849869
0.103535526	0.530141785	0.241286547	0.335337957
0.178256902	0.474144472	0.298278978	0.302095306
0.294475062	0.214040374	0.426412754	0.444467274
0.047552812	0.133096688	0.02088105	0.462848194
0.202564115	0.426916121	0.415018228	0.456513645
0.391341429	0.156796691	0.471840082	0.351353347
0.154933875	0.023312757	0.510920079	0.471144
0.363519578	0.19101566	0.058963337	0.413317147
0.508592061	0.350990374	0.279762617	0.480124787
0.036678837	0.517202576	0.323310799	0.170459955
0.479917533	0.330719243	0.499492698	0.071007077
0.434941725	0.471325856	0.377708927	0.244263123
0.420605751	0.475726718	0.041549013	0.447099706
0.448588644	0.047688709	0.534645601	0.178836039
0.031810046	0.51503527	0.508421755	0.105020313
0.108134993	0.404561157	0.053693312	0.367044444
0.52232913	0.489602462	0.489390263	0.28038078
0.058138273	0.019687972	0.27667778	0.463795184
0.521359435	0.520318515	0.327100704	0.463579639
0.297687209	0.406266466	0.15227251	0.513841649
0.230201981	0.525656715	0.010447761	0.510992997
0.524923656	0.317490915	0.116375959	0.352320266
0.431788759	0.062008478	0.241210587	0.50739123
0.475157831	0.396075815	0.415593256	0.495998866
0.021292333	0.054486902	0.459946255	0.245245959
0.450706671	0.26893117	0.079420708	0.362324361
0.063147658	0.458562664	0.516656604	0.081895106
0.383279645	0.334096861	0.463781919	0.051841013
0.501044999	0.50949335	0.533243631	0.196085449
0.506686806	0.497028619	0.487161924	0.109587202
0.027120014	0.530493421	0.438301195	0.032591437
0.517214048	0.251040967	0.022309907	0.498083469
0.228092455	0.452907552	0.004389364	0.099502491
0.058996068	0.520006837	0.488592564	0.426014867
0.272604325	0.424876891	0.008586481	0.528202306
0.500919309	0.023262741	0.197147865	0.504790819
0.501988696	0.532914826	0.402625923	0.513286098
0.512292035	0.516416969	0.295267997	0.34138702
0.492800496	0.507156019	0.468746663	0.454259446
0.514850975	0.504340267	0.341996214	0.022356056
0.165676506	0.233703578	0.35782236	0.112058767
0.349354435	0.190251159	0.482041296	0.521003954
0.516097848	0.008135977	0.173826869	0.492213782
0.47209021	0.49691157	0.026934697	0.007660154
0.009491971	0.03201507	0.40919133	0.309283118
0.497039344	0.274359837	0.007607433	0.052591731
0.332879551	0.48789727	0.515006077	0.447680502
0.50379377	0.111773731	0.341983202	0.460358449
0.099286998	0.355463596	0.517722095	0.478407924
0.057968532	0.31345109	0.421292716	0.507761715

0.105596722	0.399508024	0.514929918	0.38922934
0.01907159	0.114508919	0.092233441	0.504376427
0.517412794	0.118267821	0.054447259	0.161137757
0.159475964	0.511886985	0.219758797	0.517920911
0.490990209	0.502710371	0.109543644	0.488300073
0.058321386	0.501339413	0.434432655	0.066430533
0.34917466	0.018217721	0.479036112	0.202783687
0.368691265	0.441906404	0.240169049	0.026477985
0.231800396	0.077813906	0.520262966	0.522441947
0.049158888	0.277879642	0.36861052	0.466544252
0.31824859	0.505341411	0.514304456	0.455729867
0.107513808	0.189219799	0.460643224	0.441907385
0.273528748	0.525415544	0.377023826	0.530090524
0.453021913	0.47311219	0.494984909	0.405802012
0.318107245	0.441681956	0.423379344	0.027782067
0.417274242	0.522605981	0.398966356	0.213224316
0.337551661	0.468503299	0.058527746	0.200829285
0.411336532	0.337908714	0.191287556	0.498625015
0.434971103	0.445688499	0.048663208	0.086682987
0.548161499	0.441505732	0.162445844	0.484926296
0.221840928	0.421622304	0.513121171	0.519478731
0.424777178	0.506168955	0.479632914	0.442772855
0.431775097	0.313510457	0.535985159	0.092802402
0.116112488	0.478265408	0.136958798	0.128526221
0.190767818	0.039501396	0.374835382	0.498709001
0.456810411	0.465724694	0.354976538	0.107627206
0.407328275	0.406447978	0.434562646	0.240653985
0.109912857	0.510887007	0.014017508	0.08082217
0.475171685	0.499558881	0.47766523	0.307080601
0.381684728	0.190146816	0.378283641	0.324977865
0.381675883	0.421959873	0.49479238	0.0418308
0.026472592	0.522645763	0.412064005	0.03013203
0.116542802	0.417833189	0.347695467	0.296589588
0.03374934	0.493210137	0.376397432	0.503673807
0.393756359	0.088881069	0.371705198	0.106596432
0.073555251	0.450705243	0.470372749	0.527568246
0.420532	0.494456666	0.448594151	0.49653255
0.146247299	0.433713595	0.036957234	0.493261536
0.47313033			
CD48	0.379209626	0.474542928	0.324071802
0.356664027	0.339686761	0.364363273	0.204891799
0.257778557	0.067730132	0.004696175	0.01138186
0.353634324	0.147273618	0.26874858	0.301798937
0.343438326	0.091098558	0.368325853	0.217837255
0.373047792	0.116157183	0.312197995	0.088866432
0.20705641	0.062636231	0.04855845	0.175963177
0.383375085	0.058376718	0.330544326	0.38569343
0.084277984	0.395952331	0.490315983	0.324358862
0.477983087	0.368928482	0.019447485	0.271107095
0.395577673	0.348098127	0.370029432	0.279422255
0.038296981	0.490094781	0.217089343	0.3129552
0.284265184	0.019003935	0.304617331	0.456046306
0.44721428	0.340212172	0.270108767	0.151282391
0.135549918	0.295775895	0.168291164	0.369832931

0.201418745	0.227775811	0.446882909	0.082065533
0.141969023	0.299198114	0.114201665	0.287405745
0.244718	0.391760835	0.026248008	0.335026946
0.004457539	0.358149575	0.304291474	0.205390017
0.031691071	0.014517285	0.135567021	0.305497228
0.386977866	0.440318142	0.378454295	0.390151529
0.132625262	0.246500435	0.289986282	0.138768827
0.385750906	0.160555884	0.114541175	0.330273428
0.390442125	0.468779091	0.142898361	0.195797821
0.355568231	0.449771759	0.359938884	0.332669421
0.241019664	0.036167161	0.414423363	0.428598352
1	0.213609681	0.10800569	0.39407478
0.050201874	0.373468373	0.299358479	0.149650131
0.396225668	0.01743458	0.148003358	0.32480252
0.219340636	0.390935694	0.453579228	0.173653578
0.331009068	0.017731446	0.077743494	0.029223629
0.288039542	0.089170321	0.477757202	0.376019048
0.283345073	0.286865055	0.265620811	0.00119218
0.018244059	0.168386129	0.372816943	0.371881335
0.386222884	0.353493638	0.164563525	0.385965955
0.335065114	0.3711439	0.07418854	0.104068426
0.363079494	0.162508073	0.199818212	0.362687964
0.218500189	0.140917208	0.114960338	0.438591741
0.43845907	0.076444568	0.38855533	0.338209827
0.266954914	0.252839567	0.10546914	0.24369567
0.053721771	0.437459963	0.018111842	0.3793483
0.183470061	0.096283075	0.018007988	0.310841497
0.273153874	0.354222845	0.465508687	0.100884494
0.279844253	0.312615625	0.376576543	0.347828329
0.00594036	0.04955545	0.357136871	0.433438463
0.33984764	0.421812657	0.281483002	0.243441163
0.216056037	0.373159626	0.004247104	0.481337158
0.468119369	0.360903018	0.309189736	0.346799684
0.267486728	0.370746777	0.056217727	0.347101997
0.384471666	0.077309066	0.408442913	0.114206198
0.437067529	0.029024842	0.338140207	0.332521984
0.337514633	0.007980291	0.27298429	0.3600996
0.01786247	0.127191447	0.3907093	0.353974975
0.307488565	0.366435554	0.383181374	0.247133736
0.33593746	0.256606174	0.011774114	0.239846304
0.373311144	0.300100416	0.441249716	0.238323751
0.377014123	0.437067465	0.355126731	0.179259692
0.038620034	0.328672347	0.025618939	0.252757554
0.354470046	0.102531424	0.306791732	0.144126904
0.048192373	0.451369566	0.111539966	0.173233031
0.074978017	0.337701997	0.255466982	0.193798118
0.14069322	0.127470412	0.251837387	0.296233888
0.047060128	0.071113394	0.012550191	0.287722284
0.096534531	0.265014719	0.278544994	0.385656538
0.311357396	0.08937188	0.291232576	0.215220732
0.07944693	0.010988471	0.457707074	0.350557547
0.371118174	0.144079023	0.034001023	0.274575241
0.386956634	0.324039637	0.34187128	0.407283542
0.033856556	0.392747959	0.188942288	0.085241201

0.267104566	0.215943882	0.376140382	0.05432463
0.39467448	0.445481899	0.271194451	0.129507772
0.396082491	0.372141554	0.02969547	0.385328202
0.385559247	0.023939828	0.458929221	0.087330677
0.015513832	0.343841132	0.336322754	0.056466734
0.056870319	0.266838564	0.068886699	0.220111391
0.450997558	0.321473562	0.356048284	0.156481784
0.032998849	0.006298553	0.150592619	0.331432007
0.494577346	0.432716533	0.225989276	0.255531432
0.213772755	0.231302595	0.217606063	0.42778182
0.193041029	0.484742497	0.005792061	0.336037811
0.47563602	0.345168496	0.050203202	0.305606009
0.377652095	0.042497779	0.231329915	0.464383197
0.384061886	0.25518782	0.266617704	0.373882615
0.020429053	0.051225008	0.304104552	0.117290935
0.309358692	0.174120287	0.028333119	0.354515353
0.033717292	0.424907515	0.375380425	0.040296575
0.311176539	0.356559736	0.295639605	0.032578718
0.348727259	0.447708059	0.491262448	0.194835627
0.372610441	0.411476567	0.28857207	0.063463892
0.01122424	0.470327383	0.398992897	0.020754218
0.38074354	0.31026769	0.00828688	0.466387932
0.180621244	0.461338654	0.006746904	0.082840627
0.029559468	0.35792011	0.457018997	0.384963739
0.274732654	0.3523942	0.005816197	0.449898738
0.45236608	0.008054133	0.092333175	0.355963469
0.32221701	0.497895079	0.212072762	0.340172382
0.318955072	0.456674272	0.215737751	0.205116298
0.373330144	0.425581451	0.39728651	0.447957363
0.377348273	0.33578061	0.241502331	0.013645612
0.087342512	0.272241223	0.248475268	0.053496713
0.373584682	0.096020659	0.438902079	0.371206296
0.390234372	0.003560485	0.188580875	0.372927735
0.404615002	0.412009373	0.024002829	0.005794415
0.009132935	0.008199426	0.363450491	0.189876733
0.42119821	0.246369275	0.003808777	0.068297026
0.271647656	0.28549556	0.457352864	0.329014427
0.361557988	0.068643117	0.2297101	0.357311233
0.03985791	0.365878903	0.396008159	0.456936947
0.035913392	0.194369589	0.389665851	0.413618834
0.037075455	0.250761003	0.362319567	0.249901743
0.015727428	0.055670964	0.043076042	0.362838865
0.4625386	0.049986267	0.019981637	0.193795004
0.099859572	0.384523467	0.13460646	0.428627906
0.387371224	0.361741963	0.048852429	0.458420327
0.02488273	0.459183425	0.231839692	0.071786312
0.269826532	0.012105851	0.421982013	0.08993742
0.357758503	0.401223975	0.134966881	0.010546263
0.12026172	0.037979583	0.376819668	0.350939154
0.024662234	0.247037231	0.25109175	0.371456696
0.32849741	0.351664205	0.484921254	0.349718025
0.085444488	0.107456961	0.386778912	0.396810953
0.29050861	0.388914213	0.207555774	0.458340278
0.395891732	0.426288432	0.406061201	0.310246862

0.152590312	0.393758469	0.345305361	0.021809847
0.323731314	0.362991571	0.380002926	0.097711458
0.182868811	0.286470158	0.059677671	0.246999532
0.229382726	0.342281401	0.12358389	0.366152983
0.401398748	0.395590858	0.063577746	0.040047987
0.491262085	0.426253702	0.168029503	0.371020079
0.146963321	0.264204562	0.367383312	0.34909633
0.315568592	0.36506538	0.394846773	0.359156928
0.423529027	0.241278431	0.48864233	0.161908161
0.053143081	0.368925674	0.070204414	0.068324795
0.11971811	0.019294445	0.19934251	0.352390445
0.271534379	0.309050023	0.360794777	0.074837367
0.252851003	0.373381177	0.294537659	0.205856663
0.061975095	0.464416701	0.007775136	0.04451284
0.285877006	0.360427172	0.264287003	0.19231154
0.192332161	0.1059715	0.240382731	0.347523628
0.279816553	0.320952746	0.362233655	0.046519484
0.024136112	0.400091413	0.396757982	0.01763765
0.068847935	0.383391212	0.310527056	0.178093628
0.013867095	0.405034084	0.309395606	0.337423148
0.348616777	0.062823232	0.390273662	0.096474773
0.072164128	0.45174503	0.307795441	0.460719819
0.290800528	0.337336632	0.466713526	0.375755117
0.13834833	0.328942238	0.028144471	0.390981312
0.302059932			
CD74	0.27848022	0.357078871	0.20669808
0.247966095	0.217503513	0.115018134	0.053191952
0.031380841	0.04723264	0.001847248	0.032911771
0.281165932	0.093819276	0.058167101	0.077516827
0.318801927	0.123604732	0.108295926	0.097479651
0.222762103	0.015658809	0.2987247	0.038385647
0.232188069	0.127794777	0.055297755	0.19273517
0.292977739	0.061888612	0.065152462	0.165565616
0.119351755	0.278603095	0.344639439	0.320459074
0.355451997	0.240476385	0.014361029	0.058354504
0.256164004	0.322488856	0.217877654	0.153794661
0.02125696	0.279660591	0.087622805	0.298009404
0.044932482	0.038631104	0.030683844	0.244368055
0.298378676	0.203230613	0.041264533	0.043794839
0.087426605	0.032122131	0.205535573	0.098935037
0.073963007	0.01643953	0.325020939	0.026995771
0.042015795	0.054728803	0.164558443	0.057069582
0.223289258	0.172509429	0.009265859	0.265936127
0.000401819	0.206531519	0.278818716	0.191392135
0.050111177	0.016694117	0.166085432	0.131214661
0.320958227	0.321659309	0.41487512	0.422617179
0.119142772	0.168576485	0.273779511	0.08263054
0.285829273	0.048889698	0.047158971	0.354503722
0.418083734	0.370144525	0.149298403	0.214605284
0.244396107	0.298542715	0.139690909	0.164521989
0.142302434	0.120464437	0.269509525	0.403163307
0.213609681	1	0.128750003	0.211061893
0.054323315	0.083728949	0.194804184	0.099522525
0.331221955	0.010020426	0.185789391	0.119646452

0.112003524	0.275655628	0.390778247	0.064521642
0.219106625	0.021948685	0.152698428	0.052959626
0.098201321	0.273080934	0.288041768	0.30773136
0.046129318	0.161531387	0.072947154	0.009721346
0.051589277	0.158371865	0.304334975	0.126166165
0.270720977	0.288284404	0.108219537	0.184828507
0.295073777	0.414328187	0.006093237	0.036172278
0.165692185	0.15982843	0.141767133	0.219341926
0.180327911	0.031353034	0.061676933	0.395115785
0.223740655	0.043340963	0.074009641	0.313572175
0.087121891	0.284017119	0.052636546	0.088792577
0.016751777	0.34854241	0.07243598	0.076265856
0.12314086	0.160254463	0.011609323	0.045625268
0.065634231	0.282733672	0.295570113	0.065536861
0.099462839	0.28488809	0.067664506	0.227162349
0.009239525	0.03863705	0.044338047	0.373452242
0.071212711	0.417473643	0.112866178	0.179319966
0.199273804	0.331614398	0.008147103	0.305633163
0.384236862	0.047009462	0.199271448	0.27901303
0.173522414	0.303340577	0.014755611	0.293630208
0.151208539	0.051834719	0.411736908	0.11372987
0.307829919	0.041754001	0.064487281	0.396265648
0.309136439	0.009007646	0.029977574	0.179609617
0.076471162	0.013442093	0.254859696	0.218309683
0.176042678	0.354758043	0.388739578	0.167588322
0.200073553	0.06601185	0.022440849	0.248041655
0.335469882	0.132173515	0.247195206	0.064679291
0.098143483	0.301652012	0.293881051	0.050782716
0.024027258	0.072304957	0.00669041	0.161215124
0.272721239	0.035679556	0.164341093	0.12700853
0.094071702	0.390007994	0.260942895	0.266484521
0.258173694	0.152193928	0.081820362	0.059845538
0.10382997	0.10262025	0.174647906	0.132998894
0.008119492	0.06467941	0.056248332	0.129789247
0.060938571	0.101272276	0.084236829	0.195392819
0.086142534	0.050405005	0.169836077	0.215286472
0.124234627	0.026856539	0.34900863	0.263851137
0.12710301	0.059913335	0.053134177	0.093592223
0.320339541	0.069778423	0.03813902	0.279002977
0.024735949	0.401853379	0.09090193	0.101708997
0.179604883	0.109895002	0.329630922	0.017123845
0.141486261	0.279217452	0.187147751	0.119336453
0.207974934	0.206253312	0.014128332	0.221372338
0.207158411	0.034816678	0.394608941	0.143841608
0.03937936	0.360079672	0.306436734	0.076961515
0.089264153	0.099383843	0.006851665	0.227760186
0.37161025	0.319747411	0.25482511	0.239273759
0.024306565	0.011329307	0.216676138	0.241816221
0.319887994	0.413067997	0.151240408	0.191094467
0.118562317	0.157552673	0.017462993	0.390909436
0.156307609	0.358141465	0.013644046	0.382739052
0.342731895	0.050076091	0.092132711	0.077504336
0.194775005	0.027299432	0.076853396	0.324997346
0.282586552	0.136606442	0.130530339	0.277956

0.004630894	0.012127997	0.244241166	0.2681894
0.113827327	0.111749761	0.211185219	0.069516243
0.026641754	0.269826819	0.363235394	0.051965953
0.092130913	0.061459259	0.308747961	0.029324285
0.40989018	0.389641212	0.341416639	0.05391909
0.314999955	0.374027646	0.285963058	0.095529578
0.044953795	0.373479876	0.182730936	0.012365008
0.360346549	0.03172012	0.009166275	0.316727029
0.069615584	0.254944234	0.000763436	0.103845204
0.102495021	0.414621735	0.298535449	0.164496447
0.031447006	0.186847667	0.026505708	0.419867801
0.316062578	0.044514962	0.195385304	0.306361232
0.356032353	0.346916967	0.222617243	0.384868033
0.284874492	0.344582917	0.061895222	0.182475916
0.274532816	0.395507577	0.228709898	0.278963475
0.324397202	0.344229869	0.134095553	0.009367494
0.120407035	0.031119861	0.074070094	0.049329642
0.091172236	0.114107393	0.343132923	0.378913302
0.398398159	0.0147576	0.029098613	0.279311986
0.258811392	0.326850145	0.036467643	0.008919258
0.010741995	0.052890608	0.124731259	0.177876234
0.365990402	0.034192766	0.007880963	0.00830242
0.089185627	0.347209366	0.340956075	0.248014561
0.363239246	0.070386243	0.062525522	0.253094737
0.095637448	0.129180689	0.387861241	0.278259999
0.068810163	0.173370927	0.115045527	0.347230422
0.220459956	0.226075089	0.287054959	0.098345879
0.031569737	0.104458688	0.078335429	0.355931604
0.347001615	0.056247432	0.038806506	0.02022642
0.041228483	0.365028489	0.073723854	0.407032166
0.271767357	0.329666786	0.132892947	0.304949602
0.037967083	0.315444609	0.256406169	0.010455131
0.155510384	0.021803504	0.359443283	0.095541539
0.060876468	0.186885888	0.138352699	0.054676324
0.237905287	0.066463721	0.374724957	0.417483351
0.089381668	0.071965022	0.181475361	0.243072017
0.059901449	0.282799539	0.314068482	0.126231233
0.024255421	0.223807977	0.21612265	0.17982674
0.046035241	0.422023622	0.129702432	0.406617334
0.215703974	0.31856772	0.355410365	0.133849775
0.21871594	0.141683815	0.144860285	0.00623233
0.159635525	0.345637263	0.108333234	0.12923685
0.137826093	0.28350959	0.029201072	0.04960942
0.260896683	0.074892834	0.058650951	0.343359362
0.167911464	0.190473627	0.015983286	0.069577497
0.379015737	0.267946082	0.030548663	0.214164254
0.123350029	0.18629538	0.361918873	0.395515172
0.138495447	0.307618164	0.243317726	0.159789266
0.227994184	0.095842294	0.349391657	0.010044346
0.098937992	0.232432658	0.04734188	0.109207819
0.072679423	0.049153511	0.225908254	0.322847509
0.191267389	0.22572894	0.065349393	0.063187628
0.230633146	0.117440456	0.212749981	0.042556476
0.042205086	0.317875993	0.015514646	0.066572054

0.19083111	0.307331523	0.243013841	0.150349981
0.231660275	0.18439314	0.153437063	0.052390479
0.063063023	0.140560756	0.296915278	0.013623502
0.007627077	0.402151638	0.097754266	0.05895952
0.103762138	0.161570025	0.121681072	0.09018618
0.070558508	0.383357407	0.059786207	0.241083445
0.110599185	0.053605668	0.174539167	0.039207794
0.03910954	0.265293185	0.274226673	0.372997156
0.201200527	0.304165043	0.247929549	0.285264966
0.079750912	0.197171942	0.01332147	0.298850383
0.135619552			
CD80	0.134717607	0.173586218	0.111972923
0.23388573	0.172745546	0.082325933	0.14107371
0.05700774	0.14796305	0.049263622	0.150928736
0.155285656	0.138675658	0.093859734	0.052690849
0.286564111	0.271412098	0.072461542	0.230537279
0.206626005	0.079789659	0.312527274	0.18486906
0.147668653	0.263113791	0.249406126	0.166797297
0.235145092	0.258549089	0.053557595	0.145893468
0.253641639	0.158720924	0.136934544	0.22810721
0.131094051	0.102820658	0.045094628	0.061080969
0.137973727	0.203486975	0.203670918	0.248575923
0.175943736	0.111189401	0.20439961	0.206588797
0.046947204	0.236278838	0.030582106	0.104336231
0.115768468	0.120224868	0.069938974	0.133895021
0.08787986	0.034827474	0.095047037	0.07267902
0.189263194	0.027858475	0.129931887	0.107541474
0.055687426	0.081974819	0.246764302	0.101821561
0.101604544	0.103792387	0.098630143	0.106572078
0.015916991	0.216552368	0.228798951	0.076089133
0.265236423	0.172993465	0.248268367	0.188378421
0.227598724	0.129207551	0.253192827	0.222581525
0.222919745	0.212010477	0.137394202	0.23281585
0.149608478	0.139102728	0.121572794	0.31345371
0.243361077	0.157661024	0.226866441	0.275694558
0.151992811	0.153218462	0.093668358	0.129375848
0.08012544	0.22595813	0.119991061	0.207680905
0.10800569	0.128750003	1	0.158799754
0.246749931	0.051736189	0.276695171	0.132895205
0.232857706	0.110334649	0.270655029	0.147894918
0.160188164	0.159727207	0.160170779	0.197358633
0.153036489	0.165171368	0.118788996	0.220324545
0.095054388	0.117551451	0.099908662	0.214852929
0.066953427	0.111522506	0.121228412	0.10403494
0.263569696	0.169018284	0.145228625	0.104291098
0.183507488	0.098769652	0.180933984	0.09264761
0.141948868	0.249552684	0.005667447	0.161909301
0.134449018	0.179129233	0.311426145	0.135443743
0.230758109	0.123966118	0.224992126	0.218550779
0.111896216	0.219504579	0.046064642	0.293785042
0.080001565	0.243382416	0.207829756	0.147142042
0.139763155	0.12612858	0.255260244	0.070243613
0.065045451	0.207916099	0.076380834	0.070653439
0.083571732	0.148766683	0.095657308	0.230639074

0.144696857	0.266018064	0.053641664	0.14735719
0.096268814	0.19245367	0.027891151	0.198899445
0.050662881	0.207407498	0.154584831	0.167793903
0.298550019	0.237290951	0.011232007	0.120867835
0.158047366	0.027968185	0.198499007	0.18620423
0.089216697	0.224481996	0.007365584	0.273599517
0.073417613	0.218279403	0.253147414	0.288865675
0.128131218	0.247497012	0.064191039	0.178208957
0.132672736	0.047178565	0.045220323	0.181500843
0.23228719	0.056213928	0.16390323	0.216002516
0.174189883	0.22511099	0.273910129	0.082539556
0.164574421	0.124867574	0.145192183	0.237714258
0.17460343	0.05285996	0.12005552	0.125267655
0.049286143	0.153042463	0.12184739	0.036060125
0.183775816	0.097244647	0.057075194	0.190239928
0.106701015	0.148714968	0.08368242	0.173805899
0.290089678	0.200189636	0.166676202	0.100229948
0.120776859	0.124150601	0.166121399	0.093484088
0.134537397	0.22735233	0.107900036	0.097071735
0.088447577	0.262757634	0.122299956	0.054958925
0.125439358	0.072590396	0.055962657	0.101447002
0.081918852	0.176244361	0.174362041	0.125469782
0.271223775	0.209142431	0.171704192	0.249495909
0.068362747	0.175411632	0.249877814	0.044149044
0.20710466	0.044654284	0.021228514	0.164062018
0.117148316	0.231326421	0.156359383	0.283846446
0.110836062	0.238433768	0.2464309	0.034752538
0.093605627	0.139198408	0.220064012	0.241442722
0.100258933	0.179646675	0.142524525	0.108187022
0.102074048	0.242297656	0.181457337	0.272743764
0.229492972	0.218094824	0.159676868	0.277221041
0.28168663	0.127920728	0.060328092	0.340862968
0.193871851	0.315675308	0.143315023	0.171418573
0.19653703	0.108032588	0.216574833	0.120504108
0.123448296	0.183378825	0.264876907	0.171081794
0.270627911	0.106295776	0.02507948	0.201220425
0.106640405	0.147621813	0.137454244	0.189202493
0.124951741	0.04262025	0.279455327	0.10595945
0.107764515	0.185682353	0.136776301	0.142044696
0.156899318	0.125308874	0.095871569	0.186594146
0.079345666	0.109732937	0.183200375	0.13434237
0.082736837	0.200926122	0.076296295	0.055999702
0.209227975	0.135233777	0.244628648	0.214227461
0.066059565	0.047551239	0.285278226	0.210370919
0.179671299	0.153353117	0.141325664	0.135556501
0.158638036	0.170439138	0.119867296	0.263765386
0.229987507	0.144273556	0.11573735	0.116487554
0.244248384	0.027993953	0.11956586	0.161629634
0.197220389	0.088382836	0.011824532	0.0371084
0.215536279	0.254496085	0.121621042	0.145603109
0.02344686	0.193632858	0.031872875	0.191360976
0.148416495	0.215972164	0.232857694	0.145406206
0.155290232	0.162774714	0.11922868	0.189873157
0.142581783	0.132947276	0.113341341	0.309175445

0.172159275	0.147424854	0.160117507	0.137526058
0.225104648	0.253428545	0.259090242	0.124385058
0.119946275	0.048079798	0.04107143	0.137477115
0.062264039	0.259565426	0.163550669	0.246436981
0.198499525	0.139144283	0.085919597	0.135723617
0.173211932	0.219232542	0.104440113	0.023468162
0.01533576	0.182699022	0.132695441	0.2334893
0.181854733	0.026437281	0.1193001	0.072652437
0.133167871	0.219088484	0.153774918	0.290598479
0.185063354	0.252351031	0.060281899	0.255353693
0.245957093	0.05694594	0.208875302	0.133825769
0.212649142	0.200929167	0.076804075	0.227997998
0.063551255	0.288835338	0.158601522	0.115506757
0.095164366	0.28392784	0.247000304	0.255344812
0.123202937	0.211533546	0.228252093	0.016939869
0.16724184	0.20815798	0.219576117	0.232081399
0.18800693	0.285875588	0.24264388	0.136153345
0.173219759	0.127079642	0.188727794	0.01960391
0.149352499	0.1618868	0.140318271	0.082342884
0.053583145	0.129411745	0.104378861	0.202527564
0.092237602	0.278908699	0.248469017	0.242343992
0.210581569	0.122437683	0.235899031	0.197661313
0.078051469	0.238108516	0.128551209	0.062747355
0.129487687	0.098189553	0.188884493	0.098546667
0.06503989	0.254673464	0.12636357	0.191734526
0.122170821	0.159971781	0.163270775	0.164878009
0.164406948	0.092527031	0.12749822	0.086793026
0.201470429	0.240072499	0.094424486	0.224787336
0.234148561	0.120995376	0.120430639	0.028994806
0.098740935	0.069762846	0.19221724	0.279401978
0.113768291	0.102121799	0.091408854	0.245713803
0.17640763	0.127184812	0.079664163	0.16429427
0.243652502	0.219190662	0.199588144	0.215796732
0.067617734	0.16643331	0.165717362	0.122051031
0.118313582	0.190147746	0.146558468	0.026900182
0.161204364	0.106248553	0.199440328	0.278147164
0.238800478	0.234153564	0.072711489	0.262088573
0.06379111	0.263889705	0.038067034	0.218241658
0.239364061	0.059566839	0.253442821	0.104140605
0.212654476	0.135174239	0.109846647	0.266844784
0.073840563	0.165719803	0.146386829	0.228988342
0.113928905	0.210300475	0.259031925	0.049599008
0.046246597	0.063709918	0.158718164	0.086403668
0.109219442	0.251831801	0.064294631	0.172730409
0.130991731	0.082564378	0.107408206	0.134378188
0.220021752	0.158857398	0.044434251	0.159438045
0.135715243	0.230098798	0.077354617	0.075721568
0.145856551	0.116729527	0.14432719	0.147881679
0.133704021	0.294050823	0.093741353	0.145947966
0.106442473	0.079701162	0.137251026	0.19702183
0.053094478
CD82	0.345027722	0.489386727	0.301721597
0.386983492	0.364664516	0.357970059	0.289696651
0.314108174	0.102682796	0.016403633	0.02555636

0.341217571	0.200074918	0.339485645	0.290335722
0.373266361	0.139910014	0.362553763	0.301251717
0.404606829	0.193499728	0.357454145	0.158092408
0.221774001	0.09387517	0.092546143	0.200154338
0.426017841	0.105446636	0.347550362	0.406125682
0.121499314	0.388138157	0.473929691	0.341577971
0.455377877	0.317758713	0.032500409	0.311419963
0.405187886	0.34232098	0.403305794	0.349640816
0.083843472	0.469384271	0.297681199	0.323106616
0.291129518	0.043934756	0.324148603	0.443918091
0.431066461	0.334468966	0.333304253	0.232263105
0.168777318	0.340650431	0.168833401	0.364162726
0.290766299	0.274132419	0.436021262	0.133812771
0.182890056	0.367100552	0.155306273	0.362695506
0.241233707	0.366985563	0.057086247	0.292695132
0.011612272	0.397888271	0.327369503	0.189067892
0.06470988	0.040380475	0.181882455	0.370794465
0.437511458	0.422513639	0.420710307	0.417352331
0.186888986	0.293949116	0.28064914	0.214439575
0.401572927	0.242300553	0.15645919	0.361828176
0.427964101	0.468395378	0.195096211	0.239033212
0.367488324	0.467170304	0.348438303	0.354209178
0.235610379	0.052344167	0.415074301	0.454485838
0.39407478	0.211061893	0.158799754	1
0.09445332	0.345788298	0.364049182	0.18459664
0.449520868	0.042341206	0.193016459	0.384926342
0.26529719	0.372952855	0.44821702	0.259811571
0.344469989	0.039462274	0.087187466	0.054924804
0.330506212	0.091109906	0.441173613	0.42861317
0.35860415	0.31861544	0.346003978	0.006010081
0.038552378	0.193397758	0.365985837	0.383141148
0.383253391	0.322430117	0.218374603	0.35250041
0.312797529	0.412961487	0.071398945	0.176214367
0.375986156	0.196671484	0.275400887	0.364413258
0.263728999	0.220142379	0.192428309	0.468652922
0.445492214	0.142675876	0.373124618	0.37116912
0.308892384	0.280982246	0.179971704	0.324975346
0.114333818	0.415365103	0.034608901	0.393076476
0.174769458	0.133224086	0.033324258	0.369449737
0.316730632	0.376023034	0.421781315	0.165130434
0.355981458	0.346999831	0.380165628	0.346713222
0.017958491	0.087628837	0.334047893	0.463380604
0.332387557	0.440258585	0.343514329	0.275612835
0.270398521	0.387656079	0.004013149	0.462717722
0.461762655	0.332511188	0.350394736	0.352768005
0.253583052	0.410205004	0.049571016	0.383948806
0.341968175	0.133710778	0.45231245	0.173097593
0.428484169	0.06278117	0.390777104	0.325128008
0.308708138	0.017614009	0.313716419	0.39818613
0.031970255	0.198399415	0.381914501	0.385190726
0.33766581	0.360670247	0.43858929	0.232342317
0.36157647	0.336784897	0.025843231	0.270027292
0.348259529	0.282187257	0.442964832	0.31789116
0.335459589	0.450953076	0.333264858	0.1826021

0.084362158	0.389378516	0.051754924	0.291723102
0.336615675	0.179873079	0.284555411	0.179381342
0.082714048	0.474700829	0.124074819	0.163626566
0.078020427	0.363362423	0.33356145	0.254466707
0.181623844	0.190520485	0.261188548	0.313997086
0.101530145	0.129994064	0.017956946	0.279941886
0.146262145	0.291295141	0.298157328	0.362699819
0.373114449	0.151836427	0.330462213	0.21542585
0.113791356	0.028170914	0.472962644	0.382356983
0.335435245	0.220471255	0.065722928	0.269213027
0.434830277	0.306043293	0.314649032	0.442629655
0.055216168	0.428243506	0.254310976	0.136882398
0.288013429	0.297026516	0.431279638	0.073219679
0.391373583	0.461423709	0.313922594	0.180789471
0.403442481	0.400099429	0.070065034	0.345691557
0.350811288	0.055833055	0.469234447	0.127102982
0.034712361	0.339253901	0.320130578	0.098272859
0.092431978	0.318887783	0.127088116	0.276160057
0.474333449	0.361567664	0.34627536	0.170321014
0.076342086	0.02009351	0.183402096	0.30478483
0.473629224	0.439944808	0.293880498	0.284766571
0.295606559	0.246267909	0.251082262	0.452290977
0.18815525	0.475577718	0.016996452	0.317823563
0.450658349	0.362989962	0.08405311	0.362600525
0.388697827	0.087662424	0.299338846	0.460698379
0.40535613	0.285267596	0.277464919	0.3777901
0.051900443	0.107593036	0.31223	0.120291788
0.329302389	0.235756928	0.028773248	0.381189997
0.075396162	0.442021389	0.396848772	0.070578171
0.315526784	0.360693577	0.331225055	0.071344484
0.354899218	0.434824883	0.481398914	0.276521396
0.353273241	0.419013395	0.265936264	0.10124772
0.024653485	0.453316582	0.378388671	0.042459589
0.434261628	0.311698063	0.026561465	0.483446977
0.263300166	0.424483322	0.0102483	0.06437366
0.045180209	0.379908049	0.441949137	0.407643054
0.272351965	0.389510808	0.007233942	0.458917069
0.460981962	0.01838245	0.122403588	0.341072814
0.294485775	0.5042703	0.212295455	0.357773138
0.30568155	0.441910973	0.29244189	0.265357367
0.399095721	0.410873624	0.390861255	0.45171932
0.425397288	0.351599429	0.318825728	0.038519992
0.094951617	0.332244408	0.234568365	0.081725381
0.355854974	0.137738109	0.445138707	0.416070839
0.411123574	0.011244187	0.270177987	0.358596499
0.448884999	0.458845691	0.034704896	0.007223054
0.00868815	0.016293064	0.406771804	0.231975737
0.435655829	0.249575512	0.013835504	0.129338442
0.317874501	0.290938591	0.46250119	0.380850989
0.337744917	0.120373932	0.261080502	0.395008557
0.065566185	0.312046152	0.424269665	0.465877591
0.060063833	0.236572035	0.377478478	0.461662985
0.034983589	0.306198055	0.352464361	0.307821978
0.023693267	0.088228439	0.07311179	0.373920968

0.436776767	0.092938622	0.04574832	0.201365756	
0.172900204	0.417884183	0.206220599	0.464688714	
0.414437541	0.398127752	0.075303456	0.453963855	
0.053206236	0.449005653	0.249001349	0.082496307	
0.300251976	0.028189463	0.411470264	0.099861814	
0.395673978	0.395764127	0.140559347	0.021843218	
0.110047215	0.072350624	0.426359835	0.374160704	
0.038908197	0.321055251	0.296003256	0.386472785	
0.370807049	0.376582533	0.471465847	0.312381759	
0.158286434	0.10413855	0.408546905	0.36713549	
0.36491276	0.430867711	0.243634543	0.469349288	
0.368932406	0.436743266	0.416585409	0.355160615	
0.18265558	0.378969624	0.367515846	0.051884745	
0.372756416	0.402813146	0.394218567	0.131316666	
0.242144935	0.263338009	0.100927516	0.218964426	
0.206402132	0.356126094	0.205874181	0.396689628	
0.394272463	0.365715719	0.112990441	0.069190253	
0.499610995	0.426291949	0.221467239	0.377360979	
0.203358596	0.309739839	0.351713329	0.339345917	
0.299603478	0.390356001	0.397186176	0.370541161	
0.439388283	0.317644664	0.480321275	0.208654555	
0.073550622	0.337759499	0.12842174	0.107876468	
0.195850432	0.038091505	0.178858875	0.376972976	
0.246353005	0.365204029	0.366229313	0.112089682	
0.294216846	0.334875872	0.345358488	0.287172625	
0.115152196	0.459550795	0.01657705	0.082482835	
0.273187326	0.347402159	0.268459451	0.244328073	
0.194476768	0.134898952	0.31252928	0.366307326	
0.294245474	0.294979191	0.35407938	0.079452725	
0.061191816	0.446033094	0.371989591	0.028478506	
0.094489978	0.345311707	0.337859457	0.230847631	
0.025503089	0.406437754	0.316917057	0.340191502	
0.395177342	0.111854653	0.378244703	0.1291897	
0.113013404	0.44521657	0.292671818	0.451095818	
0.291235403	0.376123481	0.436741256	0.376026646	
0.152887613	0.281920569	0.06593509	0.415014801	
0.275790966				
CD9	0.063831668	0.067397988	0.052279259	
0.137615384	0.098577649	0.044634095	0.156581399	
0.057366532	0.103550612	0.055239115	0.087184604	
0.06865831	0.06079943	0.054770567	0.019772833	
0.17845958	0.170249182	0.052190165	0.243320117	
0.142236314	0.093716514	0.195651846	0.196699996	
0.065725298	0.134084315	0.205083618	0.072682691	
0.136284166	0.226646817	0.032656723	0.100079523	
0.231366644	0.081014632	0.046781416	0.104820339	
0.041779184	0.043890565	0.087938032	0.031522392	
0.067404266	0.089525283	0.139938078	0.193495621	
0.190052969	0.039110121	0.192619486	0.113757906	
0.030313799	0.176498254	0.025122597	0.041402865	
0.03595422	0.050642773	0.070314643	0.13268455	
0.030158638	0.026847874	0.021461393	0.048028289	
0.202209162	0.028540942	0.042850194	0.089153408	
0.041267963	0.091875315	0.183635063	0.111232013	

0.037722091	0.055768805	0.215795333	0.041864322
0.020453958	0.163757572	0.147291783	0.021996536
0.311935952	0.135021871	0.184036265	0.148214211
0.12132577	0.049503079	0.110367468	0.089134806
0.122131415	0.185089315	0.062515263	0.169826925
0.068923077	0.138626467	0.093027989	0.184551769
0.102545741	0.054586216	0.13487701	0.212331763
0.064502601	0.065434089	0.041013364	0.066898043
0.02122561	0.179878368	0.044559892	0.082059992
0.050201874	0.054323315	0.246749931	0.09445332
1	0.019516729	0.210891672	0.088440799
0.122224491	0.141095233	0.212017924	0.111681024
0.132552863	0.073891121	0.053327516	0.228075833
0.08904829	0.160230778	0.03324329	0.289672132
0.046154024	0.038553161	0.031126331	0.101611873
0.057458939	0.047232838	0.133528663	0.161432378
0.243255065	0.123107428	0.059390798	0.080467446
0.103632408	0.034483324	0.097730898	0.04382395
0.051655264	0.110375453	0.002592085	0.199455572
0.070681302	0.146660805	0.306950008	0.064169285
0.136015731	0.153145154	0.239008846	0.090694819
0.050217731	0.275016721	0.033375031	0.178759369
0.033994425	0.131246455	0.235776879	0.094778063
0.18504415	0.039566694	0.224774964	0.060271546
0.016215938	0.115736783	0.179121576	0.077198259
0.039123891	0.054117318	0.029824492	0.288634677
0.115955694	0.151600935	0.042179737	0.058876874
0.071015012	0.240745615	0.02136466	0.079142446
0.0323192	0.084525964	0.105351401	0.090566662
0.228026609	0.123217664	0.008852782	0.040903429
0.053299638	0.017103302	0.105006183	0.104105711
0.034293402	0.110144475	0.003963925	0.174656557
0.0365268	0.268991877	0.11469199	0.25584795
0.047561304	0.239428875	0.051326235	0.063706787
0.044593763	0.066941478	0.043754584	0.129767762
0.216106878	0.06993515	0.091680576	0.133436975
0.099219686	0.112454149	0.13753343	0.029313479
0.085289087	0.137264017	0.225639701	0.153343061
0.080634536	0.016073912	0.048110146	0.09714264
0.024815231	0.061024553	0.04323566	0.014203497
0.216540484	0.09331124	0.06200893	0.114200526
0.040961145	0.115741448	0.034656654	0.170085686
0.227143576	0.077681863	0.069838501	0.035329607
0.040995835	0.06126513	0.189173583	0.041469421
0.045174969	0.170550115	0.04175672	0.047063334
0.13127164	0.220505878	0.147493063	0.016175022
0.051266881	0.024422457	0.020156839	0.047505213
0.046607556	0.108061036	0.091832066	0.051924699
0.167317208	0.270543817	0.066919562	0.16629063
0.03463447	0.16051844	0.290322196	0.011128435
0.094213004	0.020343218	0.014038117	0.071459659
0.176364979	0.105149759	0.073770382	0.250702166
0.047694138	0.206476759	0.130869665	0.02336299
0.0610088	0.056140144	0.185530377	0.150647394

0.039008029	0.115818484	0.186437169	0.046835844
0.043621618	0.256148049	0.065922618	0.18176013
0.28229328	0.100583612	0.058432304	0.324849026
0.220805778	0.075632385	0.094000803	0.254442035
0.075591277	0.187180319	0.058854372	0.082070137
0.201845134	0.078957154	0.151368281	0.055728826
0.041326271	0.064745614	0.266705347	0.076678054
0.282554614	0.036062198	0.027993076	0.076769865
0.070013822	0.053473709	0.223713516	0.074834061
0.038940652	0.03382186	0.174319354	0.091217393
0.047059523	0.210453344	0.143520419	0.049325127
0.071120204	0.065810466	0.036237131	0.106862357
0.127401501	0.176307603	0.095873395	0.049500791
0.037222029	0.134979209	0.024298222	0.036921625
0.174098716	0.053728475	0.126586626	0.147343435
0.031942806	0.02991161	0.183642486	0.253025185
0.061794434	0.053083518	0.04850121	0.16061562
0.069336088	0.058070159	0.038340911	0.269112857
0.242418721	0.046737467	0.064879643	0.07889966
0.122462928	0.022103637	0.091926516	0.066691745
0.24789178	0.029473942	0.012889634	0.020170179
0.201621954	0.120717156	0.041051976	0.106109605
0.010597422	0.158164517	0.048509954	0.072078685
0.053383906	0.183407082	0.136336655	0.053479321
0.050166951	0.063671416	0.046439173	0.067265594
0.050079511	0.042353253	0.073300328	0.2419971
0.075352405	0.046606565	0.087219117	0.056957374
0.110301094	0.139095627	0.233024813	0.163683836
0.05260394	0.055763928	0.009877597	0.070135289
0.040967017	0.141881141	0.064305224	0.12164175
0.07343217	0.207445627	0.090287603	0.054500613
0.089127187	0.111805839	0.158358384	0.017563567
0.008276053	0.081558676	0.109689582	0.189261188
0.070375597	0.008568686	0.18683633	0.123572443
0.076173205	0.096280217	0.054962426	0.214970478
0.080989059	0.275576722	0.021177795	0.181832799
0.143875666	0.027879212	0.080534753	0.052552892
0.268159375	0.16368753	0.050821955	0.109176492
0.014182657	0.225025844	0.073146903	0.051922598
0.138374861	0.204737477	0.181855021	0.142651663
0.037839169	0.116695963	0.145036173	0.010195028
0.12669183	0.098436175	0.138720113	0.099225495
0.095283174	0.1661809	0.198593184	0.050266311
0.148137303	0.042193148	0.088011103	0.010013789
0.101087425	0.265237717	0.048700573	0.021309161
0.03852489	0.080323643	0.041195286	0.220431261
0.02848526	0.256041328	0.114839055	0.105566511
0.21721674	0.111200393	0.187682731	0.130907796
0.069960082	0.124050904	0.044155089	0.021436024
0.14792542	0.051269957	0.123886701	0.047867188
0.0572808	0.114225998	0.047532814	0.07338862
0.063166828	0.071482597	0.061089283	0.117874026
0.074355208	0.050122939	0.072454794	0.082178402
0.157203168	0.113725167	0.076472863	0.090323789

0.159889634 0.046403832 0.22272893 0.017653568
0.033433096 0.048869183 0.161491818 0.165556821
0.072889497 0.049306824 0.192382814 0.172761768
0.067025482 0.054468428 0.068918856 0.083377257
0.257756803 0.140427714 0.087413113 0.095399743
0.027282931 0.065909381 0.090805025 0.0615286
0.050063576 0.194731936 0.050646042 0.039678391
0.088299263 0.048600953 0.132771248 0.299649077
0.26220655 0.220734423 0.016259185 0.158927014
0.018008551 0.172748347 0.021341676 0.173634287
0.177755451 0.035558245 0.18855493 0.097742127
0.157539396 0.04596156 0.112610012 0.284288617
0.027403576 0.067546188 0.054529305 0.152308136
0.034328696 0.161790136 0.214159325 0.038580655
0.018940895 0.022343244 0.06267005 0.115062734
0.193201551 0.117012173 0.036409552 0.200702595
0.137564642 0.044387662 0.077171175 0.06975464
0.206400718 0.05303583 0.024322935 0.081477391
0.1133256 0.286436306 0.034675475 0.072797208
0.209025506 0.044790443 0.064037252 0.048935595
0.082861399 0.173210532 0.033955491 0.061521254
0.083405429 0.034427581 0.180792821 0.105779227
0.016234649
CDC37 0.318618135 0.429602903 0.169387235
0.314250608 0.171330923 0.227205869 0.089675263
0.067316165 0.01965703 0.001885706 0.002204487
0.241876823 0.023287605 0.067373028 0.066963227
0.302297873 0.022898831 0.139063454 0.116967439
0.289085936 0.029702213 0.242150574 0.024887684
0.044370681 0.019793662 0.017051843 0.05503435
0.384067164 0.023559879 0.130744303 0.304668993
0.024854989 0.345660995 0.433050818 0.22747718
0.41412869 0.315297748 0.005313051 0.061851019
0.400211871 0.29856868 0.285241428 0.177859599
0.010725476 0.430079014 0.114860612 0.19537776
0.06158217 0.00526384 0.092238583 0.398230847
0.384118618 0.186998405 0.084973381 0.049513745
0.017212657 0.064007427 0.025798988 0.262346084
0.084809755 0.061694354 0.384367566 0.019934872
0.018352267 0.103726695 0.029140654 0.120943712
0.059337319 0.33919186 0.008524877 0.220222987
0.001723672 0.280129666 0.191920342 0.055440325
0.017022035 0.004368366 0.045319622 0.182829275
0.395804235 0.417204209 0.37353559 0.369933631
0.027733586 0.117518711 0.135459348 0.043636611
0.36516516 0.061921109 0.041452281 0.287649714
0.381552774 0.410300817 0.027474195 0.106026735
0.309885945 0.416088814 0.178070814 0.135149753
0.060940918 0.011771308 0.353046678 0.39833181
0.373468373 0.083728949 0.051736189 0.345788298
0.019516729 1 0.210364709 0.035910087
0.404988892 0.005924728 0.067695381 0.157110477
0.053222793 0.37309349 0.407124233 0.082038447
0.162230329 0.006851569 0.015544465 0.010310325

0.058584659	0.01780768	0.400310908	0.365801039
0.046876984	0.040589646	0.094496154	0.001034441
0.007024469	0.041435517	0.322488279	0.17941262
0.331620616	0.333319623	0.050313516	0.303374879
0.230997822	0.35817276	0.020310862	0.030317488
0.211112626	0.035023905	0.120066633	0.205788891
0.102956571	0.044582569	0.041055016	0.425922989
0.393754203	0.032966861	0.137529403	0.293382864
0.03803133	0.140540669	0.035525592	0.067364242
0.011476104	0.380034634	0.008409941	0.172649764
0.030149722	0.016499984	0.007285606	0.131290497
0.058769476	0.315256431	0.386824916	0.044159576
0.101351481	0.217623164	0.165233705	0.267734985
0.001364962	0.019880444	0.137107314	0.399719652
0.207631884	0.410403399	0.080335998	0.075063859
0.124513245	0.365639954	0.005481391	0.42366745
0.416478631	0.144823207	0.176058117	0.225478897
0.097795514	0.367302208	0.018731415	0.279245559
0.297321095	0.032213938	0.40418893	0.052412575
0.395436523	0.012122549	0.090175436	0.305792226
0.257781292	0.002499073	0.109416946	0.265134298
0.006709458	0.022881641	0.322961187	0.276884935
0.178873441	0.341367882	0.393881993	0.087144826
0.171849194	0.101241639	0.004846555	0.132329224
0.338847055	0.036744136	0.406614026	0.058949445
0.228472914	0.408218109	0.317973907	0.023295033
0.015414528	0.132769298	0.009905504	0.122508261
0.326276786	0.018798435	0.1283926	0.034813095
0.017993223	0.419335402	0.035137753	0.055533532
0.019068717	0.174095662	0.124319555	0.036887895
0.022351257	0.0288185	0.067786643	0.085319843
0.011401323	0.02413161	0.005789759	0.065287074
0.015004229	0.062834419	0.068959131	0.270555047
0.043025764	0.019304692	0.133791467	0.044898738
0.026179501	0.006492231	0.417452462	0.283869232
0.303055698	0.039404625	0.014264873	0.037896324
0.359226308	0.164611188	0.10185264	0.367416404
0.013869713	0.380588734	0.03280242	0.037743226
0.080078982	0.106349691	0.392531814	0.013118086
0.216148185	0.391518815	0.152278342	0.04724848
0.350096609	0.252642109	0.008916596	0.35351562
0.326251246	0.010547888	0.421192106	0.020037474
0.008664022	0.283630203	0.261635983	0.029638755
0.025995677	0.104768137	0.016527184	0.142925989
0.422583798	0.268321737	0.279569944	0.051701529
0.010881275	0.001450218	0.055661153	0.19338398
0.430695505	0.398806835	0.123039204	0.109553302
0.124915518	0.051046265	0.034990731	0.398638531
0.050865046	0.435318621	0.003151169	0.278733576
0.407778357	0.134757399	0.019414589	0.138094804
0.348859527	0.016338773	0.071411707	0.426305958
0.358030531	0.079645648	0.07701748	0.277746196
0.006499206	0.01022923	0.184741971	0.028168862
0.081445355	0.052607466	0.00663594	0.069104959

0.00995402	0.367700685	0.362462525	0.010632047
0.149965741	0.085968614	0.201709853	0.013836177
0.325583974	0.40931586	0.437484829	0.055724963
0.304693366	0.378137969	0.155073338	0.02803017
0.006973169	0.422843069	0.319038626	0.011750638
0.374555692	0.130005362	0.002226258	0.429243057
0.088286241	0.387309182	0.013966364	0.022897957
0.010167409	0.348556081	0.404165743	0.282580096
0.074363304	0.237725142	0.001903425	0.420006823
0.409844837	0.003963011	0.019402418	0.295437704
0.262202585	0.457632576	0.078077147	0.319531718
0.199590288	0.391781292	0.034734617	0.118584865
0.358549882	0.374556554	0.365208769	0.433651334
0.360989802	0.260933108	0.145260987	0.003731108
0.020379877	0.08774398	0.05793458	0.009926332
0.244415787	0.026102171	0.436000917	0.359961616
0.368806917	0.002718058	0.040355067	0.315891248
0.395360138	0.419709584	0.009951756	0.005945766
0.011211032	0.002681166	0.200451775	0.09461175
0.402851377	0.040637198	0.002268716	0.016892732
0.074644457	0.186959567	0.407066367	0.269336974
0.326785112	0.022869442	0.041423618	0.304477864
0.00936796	0.277270645	0.3774658	0.40126283
0.010934321	0.081898221	0.1869722	0.405264708
0.006749108	0.144896186	0.248477634	0.038929075
0.006491051	0.020523296	0.013054717	0.328105517
0.392310967	0.009964573	0.005470009	0.03678417
0.025754082	0.369726531	0.03308476	0.41531176
0.371926568	0.348781213	0.010733975	0.418794852
0.004517965	0.39848832	0.106774094	0.027031898
0.09559731	0.005032929	0.380413584	0.014284928
0.100863559	0.319043372	0.026082515	0.004058628
0.024592644	0.014538825	0.367059473	0.339715444
0.008627046	0.063111772	0.143104021	0.28272424
0.169466522	0.298687054	0.429272571	0.188857897
0.018836061	0.023247273	0.333030178	0.314768112
0.060593432	0.383770072	0.038057162	0.432005622
0.330503059	0.408259899	0.369713112	0.177296629
0.023474487	0.257930449	0.238961975	0.010293392
0.203801838	0.337105589	0.192239943	0.021487792
0.0749263	0.14360767	0.013764485	0.063446912
0.099065485	0.20107715	0.021627442	0.357200652
0.285849635	0.317725923	0.015064709	0.011870523
0.450743286	0.397056224	0.049219741	0.296996699
0.068467915	0.136891803	0.3421416	0.301864362
0.054231151	0.327134108	0.346879175	0.255860792
0.373056294	0.117836059	0.436010272	0.039432496
0.013634153	0.228325945	0.013514519	0.032848525
0.050187141	0.007377127	0.042617263	0.28832441
0.070514285	0.201824434	0.048659089	0.031413119
0.128981001	0.162516294	0.18018706	0.073343843
0.015564886	0.404324266	0.003382238	0.017303763
0.08063979	0.313697319	0.119758195	0.076520231
0.031375795	0.022839448	0.120421435	0.155714471

0.084527537	0.064870202	0.332701663	0.023859124
0.006422765	0.398280025	0.21226315	0.007892862
0.011502206	0.250213418	0.076188567	0.029289366
0.006520209	0.351418347	0.097781845	0.183758098
0.193957949	0.021996717	0.355474163	0.025564284
0.022923069	0.414258863	0.175303255	0.403813817
0.131860038	0.289516399	0.405763075	0.318585048
0.054170776	0.176273737	0.009536467	0.378951614
0.094938035			
CDC42EP3 0.279683169	0.415550317	0.22335384	
0.409663481	0.303252741	0.219429706	0.327213252
0.22559674	0.190333821	0.053118004	0.092620632
0.244892782	0.238823863	0.292360508	0.20934741
0.411034597	0.248319255	0.242304009	0.359683287
0.35997674	0.225565734	0.387142711	0.276744543
0.231711083	0.196847385	0.225553003	0.246400953
0.439022837	0.239233701	0.207563653	0.329329995
0.206779624	0.342108431	0.364660683	0.320092938
0.345659758	0.237567621	0.038854979	0.252157107
0.332157143	0.341815128	0.370101975	0.357736118
0.199781511	0.334460431	0.348182926	0.274840876
0.198571472	0.145934381	0.183805252	0.317535177
0.325006892	0.238503702	0.269347864	0.305884891
0.183825473	0.193059034	0.161799965	0.222491941
0.353596195	0.164951905	0.342378452	0.213552544
0.172278041	0.285591328	0.23122953	0.312144599
0.213057694	0.284227479	0.110931268	0.189430839
0.023170533	0.354436453	0.291647798	0.167706574
0.178187153	0.128848213	0.259055004	0.33427644
0.435039001	0.324325541	0.43620256	0.410519343
0.264487117	0.314570945	0.224090953	0.333551908
0.344969914	0.312316453	0.191949724	0.4134068
0.433725376	0.384535027	0.279553469	0.295956225
0.345791861	0.38502904	0.248730893	0.295479224
0.207901184	0.120353025	0.327651311	0.422004261
0.299358479	0.194804184	0.276695171	0.364049182
0.210891672	0.210364709	1	0.186315649
0.447421154	0.117827618	0.275786027	0.320500355
0.277998907	0.33819378	0.373480961	0.343053253
0.274640362	0.111551587	0.1270739	0.137616679
0.264883183	0.10773803	0.307158307	0.426495145
0.253750076	0.284240993	0.339096302	0.034723654
0.127091981	0.218147833	0.314144929	0.267587432
0.345492456	0.242304528	0.3000395	0.261329238
0.247787519	0.42877273	0.039190202	0.27361864
0.280412012	0.227326444	0.356244872	0.255809255
0.309814066	0.269262191	0.312285769	0.442636986
0.332141559	0.272457389	0.209737086	0.391739214
0.268957497	0.28343996	0.286641499	0.333308827
0.215782595	0.32203277	0.111292557	0.246646112
0.159513473	0.194884678	0.062275037	0.259851445
0.267804672	0.339784516	0.288963087	0.274424626
0.343555167	0.329293392	0.209067388	0.304096322
0.073577496	0.169502117	0.153965118	0.422825078

0.178394138	0.405736031	0.341838266	0.294283106
0.330081861	0.410641569	0.005649992	0.343313873
0.379597948	0.153264739	0.307924904	0.270160428
0.206136429	0.424878501	0.023084766	0.383890081
0.212214675	0.243828732	0.457996837	0.29620549
0.332121701	0.182812786	0.27024485	0.316228356
0.26546598	0.037991999	0.208446261	0.349439144
0.099201893	0.206648957	0.326259754	0.353324714
0.30061332	0.371069629	0.46631967	0.194331949
0.300806934	0.31979984	0.078097149	0.285727102
0.326233075	0.191314382	0.341711715	0.326309802
0.157921629	0.38020778	0.276110105	0.150031949
0.202892141	0.299718612	0.087008187	0.311218695
0.26442876	0.276467423	0.206697207	0.204038135
0.1940232	0.431303419	0.16180025	0.152915884
0.101829792	0.263176935	0.344351532	0.252636225
0.219150503	0.301888046	0.243321736	0.27965885
0.174427399	0.264280439	0.045566408	0.186334804
0.195777994	0.224383224	0.206885961	0.260885652
0.273195439	0.234763756	0.310765439	0.215756187
0.216657197	0.106386405	0.40465595	0.358003494
0.224164793	0.315433948	0.165781989	0.171949103
0.424006503	0.165977413	0.144037881	0.392169345
0.106505469	0.424472097	0.306317338	0.265971544
0.251922037	0.358701147	0.451257428	0.085757391
0.235567818	0.37130039	0.31524867	0.294000779
0.29722894	0.323978339	0.164438825	0.274385583
0.265293573	0.166287981	0.405123164	0.220979975
0.117423885	0.317053681	0.299262913	0.221204857
0.205664702	0.301216616	0.166294815	0.335378734
0.427525151	0.408509978	0.322335259	0.20202533
0.198489586	0.068061189	0.238741201	0.214028269
0.348733068	0.387872919	0.341070564	0.277547367
0.36131835	0.242414958	0.143515706	0.417443122
0.162849174	0.373503177	0.067415561	0.31879748
0.33901821	0.190876638	0.201036829	0.292750022
0.302294296	0.202827997	0.293120833	0.36671645
0.36293692	0.288848504	0.254319716	0.300268649
0.11833256	0.193229175	0.273121065	0.143347178
0.272676248	0.322993781	0.04130741	0.246866253
0.199298174	0.359705455	0.410496973	0.181873146
0.19819322	0.220969502	0.313799302	0.184891402
0.335772193	0.354856491	0.373865538	0.295554913
0.313543475	0.372056343	0.191968806	0.203904016
0.095067919	0.358598787	0.275318489	0.112092772
0.443238006	0.149428124	0.093603668	0.403546512
0.339845585	0.282398066	0.019061781	0.050910634
0.107967077	0.402567468	0.338215225	0.317707832
0.153237671	0.314670573	0.013691584	0.404218661
0.376491436	0.079055312	0.198779258	0.305941074
0.271975892	0.409196856	0.204477337	0.349140271
0.222942023	0.346174727	0.30101385	0.339770145
0.373084447	0.338570429	0.342220577	0.360465975
0.427414732	0.336590597	0.361609359	0.119481939

0.14362927	0.229205004	0.168015646	0.170077997
0.190700266	0.251410473	0.374109514	0.431823301
0.386179793	0.05413491	0.265205915	0.307344027
0.402490471	0.443880936	0.06624158	0.013947822
0.011248001	0.069273576	0.299365332	0.289324618
0.391744756	0.149267825	0.061839928	0.167303301
0.313776855	0.254602053	0.381150054	0.395494474
0.325473891	0.237644228	0.215460724	0.391101629
0.166885629	0.181559156	0.407192087	0.368107598
0.134193638	0.293009841	0.230530319	0.45502534
0.044568531	0.333258769	0.264754088	0.300397603
0.048577879	0.196980396	0.182394658	0.396117024
0.329246383	0.19854877	0.143827913	0.097924945
0.282945957	0.400956254	0.315597652	0.449365774
0.396769338	0.453430487	0.157657736	0.361279155
0.148252379	0.342980696	0.251765123	0.071846135
0.277331748	0.089667817	0.329416805	0.133955398
0.246198965	0.287002063	0.173309178	0.080004308
0.114677126	0.184787025	0.445959257	0.393014283
0.098148688	0.32644865	0.319631654	0.308203202
0.249856805	0.395206047	0.357149922	0.187452131
0.255243118	0.101001681	0.359279601	0.242956516
0.255947134	0.442010689	0.268971467	0.413606422
0.287126022	0.371353861	0.361925916	0.318548996
0.214238888	0.227379476	0.296459088	0.115470407
0.336827394	0.424909659	0.264143593	0.22318397
0.323371405	0.197991106	0.155990953	0.108729934
0.162453328	0.211143198	0.30912876	0.443760971
0.250752426	0.256008692	0.140938219	0.17933809
0.419404646	0.336996614	0.226594217	0.321928209
0.292007953	0.325947871	0.346643452	0.343741721
0.217663438	0.356771135	0.360518067	0.291539931
0.340554952	0.353384322	0.380377664	0.128565958
0.13282857	0.214521093	0.252916868	0.217243103
0.321389671	0.121835203	0.146320524	0.374893849
0.172796069	0.341563508	0.194469689	0.207904736
0.307711621	0.192165324	0.341955376	0.300737148
0.238231789	0.362255203	0.05937319	0.1937902
0.19496103	0.331043949	0.234338279	0.314636531
0.192683077	0.194821446	0.36217038	0.205892495
0.196209975	0.19582221	0.330402573	0.129454078
0.135590326	0.451409158	0.214880437	0.075193817
0.141792633	0.208353881	0.260006718	0.286641572
0.085984547	0.350805545	0.205309709	0.272226827
0.31240752	0.220254911	0.253174326	0.139626598
0.177397999	0.329132062	0.226042961	0.362618476
0.244313773	0.427014134	0.297480772	0.326497179
0.156676992	0.174134561	0.16060124	0.395861403
0.184850855			

CEP78 0.172194624 0.177289631 0.09238556

0.171258538	0.151864813	0.068133261	0.070084054
0.039759973	0.004206203	0.003440009	0.004636063
0.184492453	0.011340885	0.088129991	0.014925858
0.210694023	0.121784968	0.094681084	0.139343297

0.19168135	0.028857018	0.228907239	0.037343414
0.054093847	0.059044041	0.050556923	0.048166165
0.218788214	0.059775346	0.049201247	0.155176465
0.130428374	0.204585792	0.154077835	0.201712533
0.160854899	0.1316558	0.051995331	0.022124228
0.172132935	0.141993628	0.172792775	0.160264762
0.026087449	0.144652173	0.099185357	0.173776537
0.004418092	0.033950983	0.026007476	0.127810599
0.132628577	0.086594723	0.037855353	0.042716084
0.007404224	0.044697206	0.031666892	0.100884427
0.103043463	0.025481742	0.144881851	0.005134284
0.00211681	0.074609567	0.154812034	0.096320802
0.055191912	0.119262021	0.013947465	0.141640816
0.002715147	0.179594963	0.226912681	0.026579427
0.103253228	0.034770437	0.184808454	0.125943387
0.222370011	0.170482203	0.225695954	0.199143957
0.102013884	0.186260769	0.125770351	0.103166714
0.155073471	0.065253173	0.009060845	0.239770052
0.21822484	0.171119837	0.042314966	0.159484907
0.118137879	0.16805531	0.071500174	0.088774446
0.022287489	0.053193701	0.124122033	0.209307628
0.149650131	0.099522525	0.132895205	0.18459664
0.088440799	0.035910087	0.186315649	1
0.220762994	0.00712549	0.162091434	0.118561584
0.051592653	0.174397029	0.169868039	0.097029689
0.17656461	0.019350833	0.002513302	0.081152728
0.013646284	0.043360601	0.12432067	0.198045089
0.05063669	0.034790791	0.071502095	0.020772444
0.077576622	0.048785757	0.203785706	0.169534664
0.18332217	0.128062066	0.046629773	0.121796593
0.12530597	0.222697875	0.000383132	0.040821225
0.107625558	0.068370619	0.182047496	0.139737012
0.111739307	0.0467404	0.110056185	0.198614821
0.131847142	0.093809747	0.08410799	0.237859356
0.021453303	0.18176863	0.095586555	0.14265479
0.033777033	0.139088255	0.098209319	0.09722525
0.005566115	0.064347279	0.010327568	0.07301736
0.020249271	0.133503704	0.128936638	0.118523558
0.161518929	0.181417811	0.081740764	0.127321683
0.002575411	0.030203268	0.054785341	0.207119457
0.071421676	0.22183467	0.100279057	0.077224708
0.181356636	0.198005667	0.001038625	0.14111344
0.166343341	0.051124545	0.171841585	0.205895262
0.044016256	0.184184555	0.006066902	0.270510889
0.139735934	0.085519346	0.228833653	0.139462637
0.162628632	0.058596402	0.065740069	0.152544787
0.156029012	0.002185523	0.035629636	0.138606313
0.066594677	0.012490467	0.170541487	0.155194739
0.085164937	0.203781805	0.243658864	0.036968953
0.127176373	0.101810954	0.015505003	0.176751354
0.189942911	0.010516891	0.131486074	0.040351343
0.093796582	0.150923619	0.135691723	0.001365769
0.040701001	0.080950136	0.006170492	0.078791914
0.112034476	0.050884634	0.04731846	0.110811624

0.110465278	0.190823439	0.075335349	0.0640204
0.041738778	0.138393554	0.118180422	0.061711306
0.04448889	0.043440147	0.038913597	0.040539096
0.020436635	0.116059942	0.018470733	0.08278858
0.046441174	0.087335274	0.081420209	0.136313372
0.068954008	0.076310789	0.130805521	0.037187399
0.059246344	0.069642535	0.173640567	0.208649401
0.105354352	0.039104434	0.10368891	0.016074156
0.192117665	0.040051461	0.028053567	0.159533579
0.008305451	0.217460061	0.051069486	0.116737138
0.115894968	0.164679084	0.201094023	0.00063992
0.123802238	0.156761726	0.177120484	0.063059526
0.137782149	0.150673547	0.010111139	0.141910918
0.163773742	0.085648055	0.195987328	0.120815832
0.085541196	0.211317332	0.123251749	0.107295819
0.080495207	0.074068454	0.01146076	0.19157418
0.184091758	0.233008307	0.121537712	0.081678799
0.046611098	0.013949581	0.153015291	0.140266994
0.15729133	0.196898272	0.223752632	0.121614832
0.15439436	0.027566723	0.003690626	0.19578965
0.045296672	0.17696197	0.038218956	0.160867186
0.145528062	0.066752044	0.067076697	0.066829826
0.136791465	0.03264581	0.101228052	0.162137962
0.133803806	0.041921074	0.029970128	0.24506218
0.010329868	0.013238472	0.105521051	0.041894491
0.038562379	0.051788064	0.01513076	0.033030401
0.031619424	0.142331903	0.266161695	0.013539345
0.050779059	0.031332539	0.241976475	0.054559167
0.158900379	0.179560246	0.163594396	0.078299326
0.20776455	0.160019715	0.101548244	0.120500781
0.075425025	0.165336325	0.131037701	0.007528289
0.234002499	0.038110987	0.015245746	0.171155637
0.106718817	0.131182419	0.000331884	0.006454792
0.0595264	0.242606932	0.125086394	0.145917968
0.019016128	0.195820268	0.006030892	0.207369781
0.163313798	0.039510956	0.050057261	0.160190869
0.146050399	0.184406381	0.058131056	0.164763249
0.14457649	0.148257043	0.020629845	0.148679833
0.155083754	0.159855699	0.166697375	0.150435625
0.227990963	0.232150779	0.132861752	0.01350497
0.003125868	0.039352887	0.0134802	0.00186591
0.078735766	0.043738744	0.193559621	0.229548502
0.200815162	0.042746283	0.028331659	0.15379769
0.180848388	0.207098218	0.011006767	0.00074072
0.001828815	0.008039927	0.121198354	0.142520111
0.172017095	0.011210433	0.028929199	0.015711343
0.033849923	0.156947148	0.166510808	0.221872892
0.192901333	0.102604639	0.023422678	0.220047566
0.019857399	0.099410987	0.195459503	0.141090831
0.069676866	0.104613858	0.153826525	0.203866534
0.013566453	0.227455384	0.215405224	0.031175042
0.01354168	0.069184633	0.030986192	0.236569736
0.148834358	0.056395231	0.047260003	0.004114403
0.055737573	0.214879953	0.082440823	0.220069807

0.179323752	0.248922089	0.056262055	0.135732047
0.004185314	0.156080091	0.124824093	0.001176572
0.162767429	0.035503945	0.186844421	0.003164759
0.07414702	0.166826178	0.004691265	0.057097339
0.016765341	0.0992231	0.204515776	0.211335302
0.061376816	0.045295481	0.175208606	0.2170499
0.076945573	0.188829645	0.149905377	0.061008186
0.025962705	0.03874134	0.197364164	0.155746005
0.061946128	0.23654876	0.026581544	0.197667856
0.139520054	0.171088186	0.17325762	0.084439551
0.033675496	0.104256949	0.104866643	0.009409185
0.160687447	0.219486375	0.114658847	0.024493118
0.110397187	0.103938033	0.009046393	0.014097608
0.082573094	0.06817332	0.046339904	0.243267084
0.171818808	0.148441694	0.016898485	0.02560917
0.190302998	0.133560693	0.00893209	0.131889666
0.164686367	0.09992898	0.208020584	0.217760222
0.025945529	0.180387296	0.191491622	0.132937232
0.134869398	0.093525769	0.159737717	0.01218439
0.003426434	0.164355936	0.044282826	0.13768937
0.126643065	0.030044425	0.029782418	0.252069373
0.054838784	0.216461046	0.027432084	0.014299116
0.191938119	0.097954847	0.186129894	0.063084824
0.0373677	0.143856752	0.00511631	0.119439388
0.101972076	0.132851861	0.096595593	0.106350581
0.029005297	0.081971771	0.168025691	0.063938013
0.058457614	0.018401344	0.130231227	0.003755341
0.023437946	0.243290292	0.098060193	0.035921485
0.007007578	0.100872064	0.137818592	0.017134316
0.06893488	0.164584135	0.028807621	0.128938138
0.106571258	0.092585271	0.121573546	0.003936422
0.022681783	0.159593156	0.119876938	0.167906502
0.146580882	0.221487093	0.124465439	0.180156582
0.014247405	0.098110994	0.015129782	0.206042662
0.040421231			

CERCAM 0.428999514 0.520264783 0.421720164

0.470944697	0.473253993	0.401023092	0.316035245
0.288354782	0.146519804	0.014452103	0.040502432
0.454521762	0.259903608	0.352265999	0.361086918
0.480983865	0.201707745	0.427415919	0.360067002
0.478868233	0.169872162	0.467404707	0.181137331
0.339720728	0.155350931	0.127281319	0.315597525
0.482464153	0.144924994	0.357713123	0.443963282
0.173360928	0.454159908	0.503205415	0.458113616
0.48783863	0.394749515	0.027826154	0.345476014
0.427853475	0.456206277	0.473348878	0.428224106
0.093906768	0.473302376	0.349777743	0.44984389
0.298734818	0.064472625	0.300298295	0.439312413
0.452473219	0.43149006	0.333525068	0.255850422
0.223106101	0.29071885	0.263815454	0.362532331
0.335109729	0.221154752	0.46388817	0.153590816
0.197520897	0.366644589	0.226441479	0.370039842
0.362432384	0.407493231	0.054471645	0.391423558
0.007258442	0.473639016	0.44711735	0.301429763

0.089759904	0.046120532	0.261361229	0.424155106
0.478212173	0.448742471	0.476816255	0.475599453
0.253929176	0.393716861	0.404721292	0.267122028
0.437208161	0.26987993	0.189892186	0.477661631
0.484522484	0.504854817	0.288409834	0.349039082
0.422826762	0.480851505	0.443753782	0.463087043
0.346620994	0.091908756	0.428973625	0.502343228
0.396225668	0.331221955	0.232857706	0.449520868
0.122224491	0.404988892	0.447421154	0.220762994
1	0.048876194	0.290246708	0.433409106
0.328986508	0.453351553	0.495180646	0.300467965
0.461677758	0.048453894	0.157290978	0.075893303
0.400322755	0.163206793	0.454525395	0.463681961
0.330266802	0.42074888	0.386268521	0.008842871
0.058501836	0.269180977	0.424765083	0.44902891
0.469076245	0.354479955	0.30162807	0.395412128
0.424621982	0.468271889	0.071490955	0.19107242
0.47370372	0.270111973	0.350798596	0.45961022
0.382022639	0.221002038	0.22511003	0.516930041
0.432353954	0.165870358	0.39492497	0.482789136
0.368439521	0.41017849	0.204112758	0.37022882
0.110344087	0.455858344	0.057927518	0.420409626
0.274663459	0.197040544	0.033852488	0.356305192
0.360410889	0.409888226	0.438868814	0.200199196
0.410904909	0.465164348	0.380718518	0.429309112
0.022561815	0.10674249	0.32226397	0.50152141
0.314885659	0.491981298	0.417924377	0.390846755
0.377654109	0.47884607	0.006093199	0.481395236
0.505261317	0.33194368	0.448274252	0.466704817
0.371740854	0.469122065	0.055828856	0.48430796
0.371513031	0.161213631	0.504084036	0.243057985
0.449913944	0.085975905	0.395964422	0.413752409
0.403830406	0.020641806	0.292372483	0.457618711
0.054840434	0.171411793	0.461514184	0.472156659
0.435389053	0.46999003	0.485232716	0.350181235
0.475470094	0.360685771	0.03465887	0.394181785
0.447530124	0.365859592	0.443051818	0.35657435
0.354629188	0.472999725	0.39656312	0.212379761
0.097572804	0.405510517	0.04968831	0.406176918
0.372759491	0.190462016	0.392261657	0.23255136
0.125683619	0.518996678	0.214191635	0.269907938
0.145317821	0.425859976	0.380655557	0.28561063
0.245979308	0.260714864	0.3870969	0.418504433
0.088779652	0.165934311	0.031084678	0.357106921
0.176324841	0.356954828	0.346110503	0.412879275
0.39628565	0.173655657	0.445301606	0.331426514
0.180150967	0.039171003	0.503584633	0.484188292
0.347116312	0.263824711	0.089343265	0.328717583
0.46236644	0.328615187	0.306009755	0.454946407
0.06839942	0.477363626	0.313617328	0.1965294
0.399003416	0.364828108	0.478125284	0.090116274
0.438094919	0.467683761	0.416563378	0.26302922
0.391236283	0.481820972	0.075455149	0.40509883
0.403100167	0.072263442	0.513416576	0.190728787

0.050490401	0.459534386	0.424483264	0.140294802
0.139535314	0.388391219	0.103502575	0.384445636
0.514391094	0.473290466	0.422041498	0.275118614
0.088719691	0.021602611	0.281190756	0.419557629
0.490870611	0.491865934	0.37586498	0.40314429
0.359851981	0.362815467	0.20362412	0.499285494
0.262979772	0.505974173	0.021923582	0.431656138
0.48190248	0.342586282	0.132175878	0.38935825
0.392536377	0.104561818	0.330199854	0.485938902
0.430710335	0.397386736	0.387192931	0.474580802
0.046552431	0.099310219	0.436943457	0.212843237
0.41640535	0.316535403	0.061159792	0.410284684
0.088969071	0.450710304	0.48172727	0.107381415
0.347765064	0.384349495	0.451289731	0.086925651
0.416742782	0.479783146	0.507351284	0.287117938
0.443242872	0.467737057	0.379185096	0.146777877
0.040285004	0.493775229	0.433902119	0.046889216
0.47839236	0.280264567	0.028652859	0.501349154
0.30451041	0.424277502	0.011193325	0.100947141
0.077313147	0.47076688	0.464488303	0.450171309
0.266832941	0.466525771	0.011489514	0.509700934
0.483747354	0.032943526	0.201206586	0.422511047
0.404116177	0.526981287	0.337069205	0.427800846
0.420505737	0.47476234	0.329462545	0.36592315
0.44699374	0.461919648	0.461584221	0.463678614
0.472531746	0.470802325	0.390441509	0.042510668
0.170321538	0.300102277	0.302449282	0.115596996
0.364790791	0.207689798	0.477958628	0.476301842
0.463863467	0.016236777	0.249128723	0.428008989
0.450896491	0.485221645	0.047836688	0.01266824
0.013650379	0.033458791	0.446266927	0.337371701
0.47988203	0.251278407	0.016498194	0.10420667
0.385528328	0.422885202	0.491928472	0.473108009
0.444797414	0.155184542	0.309914944	0.482337479
0.112081559	0.335609071	0.474927804	0.473669294
0.085883768	0.340512701	0.437005066	0.494918298
0.073021896	0.41412057	0.45600021	0.382749725
0.031565842	0.139471356	0.116738774	0.481696224
0.46948136	0.122282357	0.063426618	0.185976947
0.196337408	0.463194655	0.255450323	0.513509593
0.46254265	0.487870852	0.121469181	0.477031527
0.076443222	0.470905279	0.376593736	0.083252926
0.390009972	0.036220699	0.44822806	0.161710162
0.394704592	0.450199809	0.235781235	0.037279181
0.198860785	0.104319696	0.473193629	0.460281717
0.065975512	0.359582463	0.402653039	0.478139193
0.369364158	0.47443034	0.49039242	0.38943111
0.162475264	0.166944167	0.475464834	0.42031192
0.337041803	0.485839837	0.342190495	0.517284183
0.435795401	0.462178445	0.45718486	0.433716038
0.275923563	0.440559394	0.409848603	0.047964948
0.459886093	0.470963965	0.443256764	0.210545243
0.336558269	0.383654259	0.106046325	0.229790515
0.320590586	0.35607609	0.230575521	0.488550492

0.444909258	0.421895968	0.094649107	0.110256011
0.534583205	0.438655283	0.234944657	0.459932245
0.274091189	0.419996624	0.457223765	0.454987408
0.396339331	0.433042303	0.46358131	0.412196861
0.429781556	0.37890488	0.510129836	0.160525974
0.133036224	0.419283696	0.158359396	0.160461977
0.24138439	0.059455783	0.287910215	0.484277782
0.358117215	0.458917918	0.367483134	0.14863291
0.409651078	0.401439538	0.451977306	0.295992379
0.141459019	0.483448018	0.023094493	0.112970098
0.38092593	0.441862447	0.393351378	0.343154493
0.315768663	0.206277447	0.401602845	0.359198632
0.328463804	0.392191233	0.434616395	0.082833501
0.054946407	0.493595533	0.42428646	0.046047154
0.132260887	0.398202379	0.396441173	0.306486244
0.045403218	0.454362772	0.353829415	0.460854027
0.438917097	0.137103174	0.354039649	0.155618562
0.130866358	0.447416111	0.419077228	0.491114115
0.408537946	0.478508716	0.435035719	0.431291725
0.199312906	0.379591029	0.072110933	0.470474284
0.384315047			
CHCHD10 0.015140912	0.023571383	0.015943913	
0.080896767	0.030138221	0.033669797	0.174234419
0.041674428	0.138777103	0.083410419	0.047691963
0.01444348	0.019167031	0.033576683	0.021136574
0.07500324	0.042190361	0.016288515	0.160195135
0.06136591	0.118308739	0.076820661	0.159960236
0.013278656	0.066032612	0.173531268	0.042055329
0.060117358	0.189095022	0.028200652	0.053766409
0.037991048	0.022887036	0.01445892	0.03055399
0.010517122	0.011228338	0.00798578	0.043201708
0.022968366	0.039499024	0.068814867	0.1209233
0.161014199	0.012529813	0.180282625	0.029664191
0.016491445	0.088573518	0.031266741	0.015364567
0.011590908	0.022739657	0.078922105	0.169408967
0.01018994	0.017335381	0.003750483	0.028205587
0.153100469	0.041165641	0.012993128	0.133271284
0.019912579	0.057798602	0.027342971	0.081092704
0.00858286	0.027624597	0.088831414	0.006601547
0.017370894	0.083006145	0.034952518	0.011127632
0.160998765	0.053691976	0.039487598	0.109535027
0.046130549	0.012265195	0.035389541	0.026974751
0.02097954	0.053245549	0.011459137	0.097945454
0.024700344	0.162100054	0.042309102	0.066284582
0.032816473	0.016119097	0.039339011	0.074525122
0.038157223	0.023937668	0.02163482	0.027888688
0.018023896	0.028659826	0.017234491	0.024400336
0.01743458	0.010020426	0.110334649	0.042341206
0.141095233	0.005924728	0.117827618	0.00712549
0.048876194	1 0.065794351	0.070176297	
0.02793837	0.02513011	0.014765304	0.196946772
0.018443168	0.037602643	0.014020283	0.074033423
0.011781475	0.003238639	0.009216756	0.044170087
0.018667369	0.008811319	0.100120373	0.057358739

0.068357581	0.014111013	0.012583721	0.021089667
0.036193932	0.007497263	0.088406747	0.015915446
0.013927385	0.034212658	0.007106642	0.155009635
0.037509608	0.016841408	0.159241648	0.017913532
0.067170588	0.126971739	0.134284064	0.032662857
0.022085222	0.202240294	0.010291049	0.067644356
0.019340968	0.034907308	0.133068757	0.041108612
0.128558883	0.010613709	0.060265607	0.029033329
0.011888759	0.011471141	0.047594981	0.061398657
0.021868687	0.023234281	0.007185365	0.149124325
0.049915112	0.059169529	0.019826487	0.029397622
0.088970174	0.072712301	0.008763009	0.025104053
0.022884514	0.021985281	0.042940909	0.035341103
0.090936579	0.049486381	0.001849365	0.013529432
0.015645922	0.008341423	0.044251619	0.023982722
0.017473882	0.053231355	0.000815038	0.056365603
0.010249075	0.161706633	0.038646463	0.124232462
0.012771602	0.181342854	0.027778658	0.017435604
0.009518705	0.019926935	0.066201939	0.081765016
0.046646074	0.100190703	0.031904978	0.074617217
0.077149796	0.034890212	0.048979315	0.016320308
0.0324523	0.083798267	0.065769758	0.041604581
0.02050474	0.002828141	0.02128383	0.078889373
0.008314286	0.025159856	0.011047649	0.010465676
0.254734864	0.063811169	0.057730422	0.080475277
0.012746479	0.074554509	0.018572848	0.01570046
0.077765621	0.026727753	0.015204945	0.006221818
0.006659681	0.02600878	0.119000087	0.026423197
0.011083403	0.089997921	0.022970779	0.030775398
0.152046869	0.110382459	0.018030206	0.002566508
0.012542622	0.008203813	0.007193078	0.014447834
0.010167875	0.027208291	0.038934125	0.012506777
0.069915347	0.142308975	0.023572156	0.065912292
0.015544917	0.140788915	0.103378302	0.001593821
0.038542643	0.022058536	0.009238239	0.031859057
0.057670954	0.031956486	0.027106646	0.129729686
0.01050425	0.114628764	0.059504447	0.038483348
0.020624678	0.021392676	0.064311841	0.110784852
0.014582689	0.056375158	0.127850026	0.014444026
0.011917626	0.136569088	0.018846306	0.029393614
0.121144862	0.024683206	0.021655359	0.174546516
0.117744645	0.062308745	0.122268204	0.109062805
0.02776068	0.075340381	0.026387978	0.019735814
0.17413225	0.019267186	0.030854191	0.010983613
0.011645777	0.016631368	0.094053559	0.027378249
0.173458397	0.025041793	0.014961967	0.024743523
0.006447584	0.014551778	0.109484992	0.020470509
0.010829966	0.019943181	0.105792423	0.088995079
0.02047631	0.240229282	0.057914196	0.016009685
0.032625028	0.052293128	0.033493839	0.02453573
0.154510053	0.116617241	0.044087183	0.009016372
0.023231274	0.102273217	0.001516699	0.018391737
0.091576701	0.022195445	0.035325818	0.130825258
0.031359021	0.020246914	0.050485191	0.207142705

0.016980798	0.012644009	0.014867938	0.078985426
0.014819759	0.018905663	0.008696729	0.096333766
0.102313606	0.012443133	0.02755392	0.049441283
0.041869395	0.022367008	0.047238664	0.025258437
0.18255037	0.00754419	0.069694616	0.001476796
0.031348595	0.0327415	0.015760323	0.055588248
0.017259326	0.061084649	0.002440407	0.018672812
0.018748567	0.069242284	0.022055162	0.01415467
0.012168052	0.020810297	0.017714142	0.020617499
0.011330667	0.012176328	0.032240879	0.125983275
0.035283219	0.01121831	0.038598434	0.022628128
0.039773591	0.036885802	0.168302827	0.185380658
0.041013547	0.059276654	0.012429875	0.053718855
0.02521788	0.078038859	0.018451677	0.038899821
0.020460689	0.111086671	0.061996455	0.015951409
0.040981837	0.045405271	0.036621692	0.023005573
0.002728543	0.036768845	0.061122293	0.067216784
0.02380674	0.006156493	0.120540374	0.09636421
0.031177166	0.02683649	0.017726969	0.100656526
0.019687036	0.107754904	0.019196609	0.078858533
0.057350441	0.008768067	0.026300458	0.022082481
0.045000096	0.068397212	0.012198459	0.044866311
0.001462101	0.070791052	0.014102624	0.026307741
0.017009537	0.087050139	0.121256135	0.043910584
0.009399548	0.033554376	0.04322714	0.00386509
0.116834624	0.028675326	0.059879463	0.032432331
0.042786615	0.065929961	0.026262298	0.020108939
0.075653881	0.012281837	0.023996392	0.016708855
0.023164159	0.070163653	0.010016915	0.017491081
0.019685243	0.029601001	0.028804528	0.052854359
0.004862151	0.095560846	0.044725111	0.030521162
0.027640341	0.078021277	0.066951576	0.041153392
0.06419067	0.057222438	0.014449937	0.009470409
0.156879054	0.001540104	0.054664601	0.014250669
0.02884931	0.034738696	0.030939715	0.021497284
0.0246691	0.024013467	0.017180139	0.103451934
0.007927276	0.02311253	0.056980857	0.092362722
0.079600157	0.040727015	0.031830426	0.037341191
0.084555498	0.009197045	0.065797886	0.002339709
0.005662795	0.045998608	0.056288911	0.062256685
0.023549018	0.016308481	0.046637373	0.128548296
0.020948515	0.021641848	0.081553544	0.044097119
0.092233434	0.087245894	0.022819002	0.022153494
0.008340404	0.020471785	0.034808424	0.034493525
0.022581167	0.151219748	0.016347907	0.026726289
0.043919738	0.00831844	0.073821049	0.108852397
0.155702068	0.079094226	0.003517234	0.047824125
0.003003812	0.072151246	0.004109873	0.066371311
0.047504702	0.007937712	0.072676632	0.113478834
0.077935834	0.016419893	0.045373038	0.097391835
0.003333852	0.028187814	0.01762636	0.067147922
0.007210757	0.016486422	0.097335416	0.028329278
0.012801527	0.002535666	0.027070363	0.110558357
0.103339133	0.036618814	0.014430262	0.030524619

0.020516983	0.015025198	0.013212308	0.046789765
0.054066907	0.013830201	0.025669534	0.025221816
0.077206262	0.10728729	0.011843498	0.028680293
0.082036462	0.013999151	0.016395894	0.013039898
0.016135634	0.076704808	0.010989441	0.016443697
0.016519614	0.006275104	0.173646224	0.037344773
0.005992201			
CHD4 0.196457912	0.237409451	0.155583747	
0.257849456	0.216263334	0.096291699	0.112359344
0.055395744	0.113621309	0.021134346	0.109816866
0.214883828	0.137625906	0.087819431	0.063097782
0.338413507	0.267299604	0.104593324	0.200975851
0.242207761	0.055441074	0.348452603	0.140650299
0.22170873	0.245489422	0.1943027	0.199714484
0.28081362	0.203196062	0.059882779	0.168316038
0.280485981	0.217994212	0.198385115	0.283043404
0.195180378	0.151646253	0.054086888	0.064371478
0.188006341	0.256561571	0.235475304	0.234417135
0.114205732	0.160252076	0.169962764	0.272971918
0.051940553	0.170177164	0.033294455	0.148252965
0.165486903	0.153801946	0.068808237	0.108389094
0.092316068	0.039440371	0.133644103	0.087073982
0.158475227	0.025489224	0.187146916	0.077131578
0.060759647	0.089069356	0.300168315	0.095742978
0.165151983	0.133666435	0.072954933	0.16530168
0.006004641	0.243768604	0.2936494	0.115721942
0.212760087	0.103894152	0.294853979	0.182217597
0.283960224	0.192211838	0.328514963	0.302684935
0.218378611	0.243512599	0.203576496	0.203833535
0.205261668	0.112613062	0.107372413	0.372494881
0.319582892	0.224779603	0.24569349	0.30385844
0.185538713	0.207719511	0.11193421	0.153233122
0.101138308	0.253295765	0.167197885	0.282938688
0.148003358	0.185789391	0.270655029	0.193016459
0.212017924	0.067695381	0.275786027	0.162091434
0.290246708	0.065794351	1 0.151605921	
0.187924784	0.209982836	0.231096191	0.156773215
0.206916635	0.117292647	0.140883478	0.209032995
0.113222571	0.187852317	0.149236297	0.260803831
0.0710405	0.152483821	0.12443768	0.076738086
0.215332963	0.229650257	0.208664324	0.134646958
0.237616508	0.154177593	0.169923791	0.12851047
0.198062695	0.32793955	0.005226497	0.130038803
0.15586809	0.24594241	0.283737153	0.179382901
0.233441959	0.100204332	0.192070589	0.288868882
0.151068208	0.169645903	0.066628498	0.340885612
0.091551879	0.293312992	0.177922579	0.146525379
0.093745145	0.188385435	0.237098241	0.083141479
0.083186607	0.254309505	0.067642564	0.068240083
0.082910278	0.19278041	0.148232795	0.206688809
0.146763809	0.306215423	0.065390163	0.176232002
0.054291303	0.15967809	0.036980583	0.266211147
0.059837861	0.29063247	0.171968643	0.215479508
0.305606972	0.293672559	0.011902098	0.173844932

0.227301759	0.036291608	0.213643759	0.252476747
0.120439686	0.266649439	0.011507897	0.323215697
0.10436892	0.188048423	0.328712952	0.251616583
0.18698911	0.183855807	0.076253608	0.253622624
0.192773256	0.04337273	0.040342935	0.200187625
0.228676381	0.041333076	0.216400264	0.236778609
0.185673704	0.29603926	0.342460548	0.108727033
0.193542943	0.125545107	0.120246921	0.284423952
0.245962204	0.084995591	0.160705563	0.118609338
0.068099004	0.204507442	0.177731049	0.043094044
0.126048519	0.099982908	0.033817907	0.195754538
0.159425538	0.108948666	0.113488374	0.23167385
0.2702274	0.269452717	0.236605283	0.167021634
0.18568295	0.141372535	0.154725139	0.084370063
0.129980089	0.219383507	0.145619909	0.12272368
0.053892541	0.213756557	0.144351323	0.084244775
0.104916058	0.087860262	0.069036707	0.141269359
0.100744047	0.135989801	0.18754474	0.182834477
0.258211572	0.158389738	0.234143605	0.291494392
0.090116799	0.152944967	0.21900854	0.06288468
0.258967741	0.051087617	0.029777438	0.209097618
0.100731827	0.311448236	0.144654717	0.251888024
0.15209967	0.207605537	0.298717408	0.031944557
0.124955104	0.188120974	0.25390525	0.223555465
0.135015203	0.211144377	0.092150747	0.14965187
0.141357457	0.181700277	0.254337109	0.293576971
0.190413202	0.292022848	0.210880478	0.242459433
0.250479108	0.121961218	0.03750529	0.344567138
0.258102221	0.355009651	0.184763471	0.24272722
0.131638223	0.068511844	0.286558762	0.17641384
0.180302568	0.261113322	0.270259418	0.189205231
0.240984388	0.136318126	0.026443491	0.270999162
0.164843682	0.215119691	0.103707829	0.266795641
0.185036396	0.049537238	0.23657883	0.102605398
0.139350706	0.131054853	0.150908172	0.198080584
0.206283779	0.148814247	0.115161939	0.251509194
0.043349772	0.075325833	0.224572843	0.205860787
0.104169902	0.204568456	0.128882701	0.073585482
0.134653379	0.180840635	0.317993252	0.160620476
0.074387825	0.061271316	0.34678208	0.154727548
0.256053826	0.227986495	0.201498708	0.131976808
0.227825975	0.235159193	0.174128195	0.26275293
0.192665499	0.211358517	0.149057877	0.065061278
0.310864038	0.030256953	0.065397495	0.217355088
0.165337712	0.133977892	0.004320659	0.06905919
0.240161683	0.339861318	0.171661193	0.170894607
0.025715423	0.227176414	0.05423705	0.273013164
0.201924389	0.17608909	0.289796501	0.202879543
0.219879071	0.226070104	0.159890293	0.25796781
0.194932435	0.19367692	0.10438212	0.299419857
0.213224645	0.220487865	0.197593125	0.186118319
0.280220016	0.325564052	0.23481875	0.074066314
0.131707869	0.048147589	0.049014246	0.10724691
0.076646825	0.229178186	0.227769443	0.319898555

0.271544639	0.105144963	0.071308575	0.189491695
0.214925173	0.275771325	0.110393307	0.019049733
0.016775633	0.142113702	0.148009552	0.256282663
0.2475947	0.027774779	0.077352682	0.047668165
0.131439289	0.285123466	0.214222733	0.314994269
0.262558494	0.234551838	0.061393244	0.292431205
0.225001678	0.082500006	0.276499441	0.181181557
0.222287988	0.242317879	0.104352487	0.286350159
0.111267002	0.318230922	0.222802691	0.121306526
0.107911393	0.263133754	0.214487745	0.328899002
0.184975273	0.161374936	0.159555412	0.019533199
0.123226512	0.282853761	0.178820665	0.307188587
0.230169264	0.335721331	0.286912128	0.188186454
0.141550877	0.182647391	0.232242273	0.015444115
0.199703986	0.13353502	0.210907346	0.084631863
0.064726768	0.166556225	0.129989328	0.197297049
0.149774915	0.239784737	0.311015841	0.32414483
0.234502962	0.128475635	0.256863852	0.248285136
0.076374642	0.273364532	0.183236354	0.08058878
0.093944986	0.180087729	0.222540973	0.133314214
0.070183479	0.335182424	0.141230667	0.268691251
0.164336312	0.222333899	0.230587309	0.166574732
0.238505194	0.116404859	0.138417373	0.043042403
0.213427345	0.297350792	0.11611636	0.209579954
0.214143241	0.182473901	0.115163202	0.044333993
0.153063629	0.07444361	0.158856203	0.340392961
0.149880652	0.139249653	0.081279537	0.1997668
0.246051209	0.176137941	0.063989517	0.191802893
0.258999882	0.230120027	0.2731236	0.301686253
0.105199662	0.220581843	0.210294676	0.139816448
0.156091041	0.174410348	0.207903738	0.022988531
0.161627127	0.161229527	0.148038594	0.277101317
0.203315373	0.190615924	0.12128244	0.327807881
0.109153168	0.280383176	0.057622965	0.181231689
0.289293502	0.088500101	0.278533963	0.086441291
0.149884145	0.189120411	0.08084278	0.241315016
0.124313918	0.219636141	0.18523738	0.243607
0.171447607	0.286679617	0.251762269	0.054151956
0.051270094	0.09824506	0.208199855	0.060018146
0.070144398	0.327588549	0.081322357	0.180010268
0.18799275	0.116739513	0.151404585	0.130931297
0.214350431	0.230027882	0.049767958	0.210087395
0.140943447	0.205129118	0.10990242	0.078973377
0.132416615	0.16496879	0.204361444	0.216253127
0.187128835	0.329956165	0.137628238	0.201576672
0.127879341	0.124187161	0.085872569	0.255252515
0.080027339			

CHPT1 0.267709695 0.435776595 0.149503269

0.39512638	0.222711859	0.236037235	0.255228971
0.188476849	0.109604506	0.037946608	0.056381241
0.215539317	0.175403102	0.247001402	0.163552498
0.362578715	0.138851834	0.171286909	0.245251374
0.345020752	0.195241166	0.337573952	0.174834495
0.13599765	0.098043715	0.129662194	0.146052961

0.422944386	0.133215983	0.209857504	0.348162566
0.097368661	0.344153064	0.390419404	0.290544056
0.3698942	0.231678455	0.024424968	0.229491283
0.335761146	0.311305948	0.363443809	0.270302221
0.124108568	0.375953726	0.257889673	0.201542699
0.160203388	0.075731322	0.179353898	0.360284919
0.360961544	0.234981482	0.225403667	0.237186658
0.15809261	0.208825169	0.107652711	0.263594991
0.265127422	0.178410944	0.370953088	0.153606305
0.154599753	0.232535816	0.120248369	0.253367107
0.141502165	0.306489287	0.06378664	0.171903657
0.020182248	0.327850808	0.199615907	0.110729707
0.086381175	0.079969835	0.13833691	0.304235396
0.421144505	0.331857773	0.409395589	0.392066646
0.160851031	0.191391424	0.15263346	0.21784684
0.349490991	0.243427259	0.107546913	0.359561809
0.410792393	0.405157843	0.1629125	0.169017369
0.367176693	0.410849943	0.185647639	0.221304552
0.145482695	0.045833906	0.363994998	0.418909305
0.32480252	0.119646452	0.147894918	0.384926342
0.111681024	0.157110477	0.320500355	0.118561584
0.433409106	0.070176297	0.151605921	1
0.158604952	0.326577481	0.383687407	0.241627013
0.187520896	0.055650476	0.066475491	0.060906781
0.193787002	0.053662316	0.348276256	0.429453863
0.231042578	0.21365871	0.260304865	0.011939303
0.053553813	0.114737147	0.319324112	0.19314385
0.326898269	0.242252788	0.203645548	0.291968936
0.232530777	0.404129781	0.039120838	0.18076054
0.214030292	0.123639062	0.228518146	0.239634444
0.193197911	0.189660846	0.200442435	0.433997546
0.376709282	0.171624606	0.171564503	0.336617121
0.236420195	0.178141433	0.18193293	0.256703141
0.162676122	0.34004951	0.042709681	0.196432066
0.110417563	0.11402494	0.028153832	0.230383145
0.205689049	0.361129607	0.323963803	0.157592176
0.265847101	0.283401947	0.192840938	0.313374217
0.051002437	0.082974104	0.148249541	0.429955958
0.230859544	0.388459679	0.22924965	0.176569109
0.198901088	0.37747462	0.001396518	0.376609586
0.394140797	0.1422627	0.256975877	0.221561906
0.148964878	0.414236853	0.016052031	0.34340682
0.232419661	0.135948287	0.435146649	0.171678058
0.350527217	0.098226148	0.252361141	0.293205889
0.26460654	0.020788167	0.204407162	0.353518693
0.036945842	0.198780745	0.313067588	0.328183381
0.248337933	0.332495059	0.434733885	0.136687567
0.21858708	0.231660621	0.0327001	0.167712056
0.303078951	0.159152841	0.377994889	0.242030155
0.170223618	0.400504869	0.274416399	0.140011428
0.126886071	0.259787475	0.056962083	0.205098625
0.269140307	0.225463581	0.156517603	0.108628926
0.091785062	0.436026152	0.080924852	0.088378861
0.048554673	0.256213621	0.240011877	0.224737375

0.162866288	0.184239643	0.165587254	0.205985726
0.142233226	0.159257058	0.013756014	0.155939101
0.156721093	0.191174914	0.188175374	0.287703973
0.243184648	0.164695639	0.217556904	0.124577941
0.101293372	0.046963517	0.419948985	0.310909153
0.26514016	0.209471379	0.077564727	0.150106048
0.433826907	0.169964189	0.138673001	0.42380495
0.048475349	0.404575212	0.231726136	0.146777937
0.182609854	0.250801893	0.429513373	0.064171585
0.222392084	0.411707153	0.195179575	0.172767303
0.343881696	0.313231439	0.105278234	0.281660342
0.284259043	0.086211311	0.412052525	0.112049763
0.049860874	0.275543387	0.292347773	0.111110334
0.095217155	0.248651875	0.141310199	0.21107228
0.434826398	0.358870984	0.343154358	0.102781458
0.125634754	0.046960223	0.125720967	0.145017853
0.383785646	0.386043795	0.213894999	0.190867806
0.243409836	0.170362946	0.149559998	0.41770453
0.082331873	0.391493081	0.028886596	0.296098571
0.367871262	0.193704692	0.100455673	0.259323806
0.339457444	0.118925027	0.195259825	0.390886754
0.373266709	0.198870212	0.177840717	0.27135936
0.083207846	0.15117958	0.192339119	0.072499518
0.219787793	0.199978533	0.016374486	0.214151941
0.121918516	0.401528072	0.379005222	0.095835779
0.221573582	0.183120666	0.248744729	0.102332405
0.318968861	0.358608454	0.401594339	0.208412943
0.308768379	0.376354449	0.167907018	0.095711775
0.035950677	0.373495804	0.279004638	0.064539877
0.42812706	0.156856085	0.065647063	0.427078435
0.22768503	0.324551218	0.009217745	0.023410133
0.039279639	0.362367236	0.369460625	0.329934148
0.16200858	0.255187947	0.005090771	0.399954791
0.404814865	0.028439638	0.097430371	0.3123723
0.253908673	0.431690845	0.127814423	0.334395249
0.203929243	0.372540108	0.251488449	0.21071159
0.380852557	0.345089556	0.335893911	0.380720818
0.423729826	0.288033823	0.256845014	0.07711575
0.073531086	0.220469061	0.14265782	0.110843828
0.217131879	0.135182545	0.373944694	0.408541117
0.376024427	0.020721904	0.220337824	0.31822289
0.420549294	0.43702012	0.024972813	0.00424228
0.003363392	0.027488485	0.27125585	0.15927493
0.390936484	0.152322783	0.02890597	0.133313976
0.206589507	0.208921827	0.406307652	0.34916136
0.299113675	0.131207823	0.197327487	0.35222701
0.079439996	0.202606571	0.398489044	0.409728399
0.057273225	0.167395485	0.172657293	0.451429072
0.021153204	0.212113431	0.243338708	0.238824522
0.017418739	0.090519651	0.089495615	0.3554147
0.355628652	0.128552771	0.078336101	0.103888014
0.213067446	0.388840034	0.204280813	0.436895523
0.398975152	0.41974558	0.073367929	0.386780567
0.09428471	0.375060369	0.156004302	0.049097549

0.164625208	0.037397847	0.337844084	0.094239359
0.228094506	0.28632839	0.098430233	0.029713222
0.057868258	0.088585235	0.428875312	0.358046918
0.035138731	0.241467658	0.189460012	0.270371708
0.223491179	0.376855286	0.391362205	0.154530024
0.202801769	0.05030126	0.347182132	0.248773798
0.238300297	0.41466573	0.198968252	0.410099917
0.288311904	0.375133106	0.36719688	0.241055995
0.133187375	0.215547597	0.331534385	0.078167291
0.231806433	0.413703888	0.20345459	0.1216829
0.21025966	0.137544427	0.096312106	0.075327985
0.105213899	0.239956161	0.227688096	0.403721231
0.250804592	0.265784498	0.101979181	0.088021819
0.434732752	0.357261889	0.162326605	0.315904304
0.159556797	0.226209915	0.316141498	0.311441026
0.161046694	0.370973995	0.369058024	0.327693393
0.390960473	0.2512351	0.405900755	0.115627133
0.065380625	0.209269502	0.178174343	0.105026832
0.204240704	0.048277531	0.094391903	0.33193982
0.123767677	0.310892256	0.18720801	0.104612559
0.192319209	0.145103422	0.234946586	0.24909894
0.148859547	0.396374428	0.025769556	0.095784356
0.142345585	0.322753461	0.162102063	0.175365931
0.118970784	0.097423646	0.246956959	0.195735401
0.188540438	0.139629179	0.325289006	0.073972213
0.093969454	0.428032874	0.168306837	0.025109593
0.084434381	0.215658595	0.174442955	0.217821249
0.030255317	0.35883435	0.199334882	0.198880986
0.248832542	0.117211979	0.293120858	0.082945278
0.094195864	0.360464466	0.156018694	0.380172739
0.15117978	0.391826252	0.339169561	0.340950083
0.075004613	0.124694659	0.096631036	0.388789758
0.141569706			

CHST12 0.269646717 0.242077944 0.176138804

0.271084153	0.193765629	0.134656017	0.140264793
0.083080059	0.031285522	0.00359112	0.006198922
0.213995339	0.019229797	0.059930646	0.043268462
0.361064719	0.071381248	0.194385192	0.2311118
0.314622872	0.051033246	0.298571795	0.088407292
0.094533087	0.061337352	0.07471703	0.087112868
0.336197444	0.097590016	0.073925079	0.256711907
0.133509104	0.278458743	0.209027078	0.201900537
0.193462955	0.218060418	0.030683627	0.047554532
0.281614624	0.235695388	0.300894143	0.233992088
0.047656269	0.190217394	0.173215643	0.266399218
0.035234248	0.027322503	0.064519485	0.194949906
0.157104047	0.123657033	0.110322351	0.090867406
0.009420494	0.053302425	0.020766894	0.165083066
0.157897599	0.046510128	0.178266636	0.027034192
0.025016841	0.16404287	0.118891087	0.158412012
0.080313365	0.218328278	0.054680973	0.174990276
0.001486869	0.339130017	0.3029684	0.050874157
0.118080987	0.011206831	0.162537516	0.198610568
0.33247501	0.244435403	0.288859791	0.264209824

0.036700544	0.273591547	0.157920306	0.100222217
0.255421073	0.092283811	0.048174539	0.341353298
0.281380549	0.212417063	0.062906961	0.259021957
0.170738646	0.242295737	0.122738981	0.131893617
0.042362674	0.0765499	0.173431986	0.257611115
0.219340636	0.112003524	0.160188164	0.26529719
0.132552863	0.053222793	0.277998907	0.051592653
0.328986508	0.02793837	0.187924784	0.158604952
1	0.265395455	0.211838541	0.173434033
0.209166504	0.024496064	0.015315184	0.110638853
0.037811065	0.035022155	0.161576581	0.249087884
0.052597844	0.044107577	0.164397599	0.02968782
0.06150367	0.094053401	0.21433434	0.243569957
0.31595778	0.192059912	0.070956632	0.180734786
0.155958619	0.286121543	0.002909842	0.112096607
0.167821875	0.096441635	0.274510339	0.167076617
0.148540658	0.112251335	0.138420239	0.282021946
0.208291266	0.127414778	0.143466623	0.346767538
0.031117085	0.198211091	0.139036356	0.087465234
0.045534637	0.179103291	0.082916672	0.19170932
0.013912302	0.032397179	0.07374708	0.142728995
0.029002132	0.159569256	0.164314663	0.193154317
0.127737378	0.255147472	0.140645746	0.144984679
0.003973542	0.069095571	0.103340046	0.245165936
0.108121357	0.294327328	0.136912156	0.151377577
0.250384645	0.329730118	0.014929396	0.189551093
0.214438085	0.085429019	0.152306424	0.274319335
0.080503389	0.26642022	0.008763011	0.329218095
0.185445477	0.169395261	0.314500648	0.145713974
0.214023307	0.073674837	0.090974365	0.211671939
0.147737513	0.011016249	0.07813524	0.258751803
0.074359518	0.037925322	0.306620515	0.269364823
0.166006278	0.326956706	0.34227865	0.067250564
0.167025669	0.182226002	0.04480382	0.24591434
0.289250366	0.021618325	0.199617417	0.092243227
0.133904385	0.220467748	0.187969456	0.012966698
0.077426158	0.161834695	0.013317085	0.158023528
0.197350562	0.033259531	0.100205848	0.09971957
0.098673056	0.251674465	0.090767759	0.08096363
0.048073471	0.113969819	0.244696876	0.02620633
0.014041571	0.090507555	0.074054243	0.089219004
0.035814902	0.087988583	0.061433012	0.039039168
0.009925373	0.036323042	0.034915252	0.175865164
0.044527546	0.021574513	0.130369047	0.083945951
0.093508967	0.07971905	0.24148081	0.35522886
0.156751641	0.104306605	0.119142731	0.013123437
0.240290742	0.063528055	0.066543276	0.21117125
0.055910951	0.307327395	0.033507817	0.124890316
0.073633487	0.171591067	0.330609711	0.009955192
0.206020481	0.202610674	0.317760942	0.102278104
0.157178127	0.252219534	0.028845453	0.211907882
0.183993787	0.078046749	0.2391689	0.076784494
0.100386862	0.268105637	0.161284461	0.161513509
0.126700486	0.085677947	0.035843123	0.276325205

0.246671692	0.304274761	0.16289467	0.138141033	
0.052009962	0.004112471	0.17430063	0.201465726	
0.195153019	0.233567097	0.295639396	0.102045222	
0.266468303	0.052071628	0.028755208	0.241397137	
0.10277159	0.235864333	0.0576965	0.217034058	
0.179822396	0.093386851	0.071903111	0.135495573	
0.175097447	0.082982455	0.189572471	0.202618096	
0.238232744	0.098475001	0.067454808	0.302590657	
0.022652569	0.034714914	0.215145652	0.064906343	
0.069713722	0.129787805	0.015723669	0.076318637	
0.024976516	0.185067312	0.338378687	0.037277294	
0.070169669	0.093026781	0.311968697	0.094547863	
0.206864738	0.230407859	0.209926813	0.157456967	
0.230586771	0.2010864	0.105786439	0.173375883	
0.084343028	0.20641203	0.246604965	0.012915813	
0.311281513	0.071069718	0.005150989	0.24465283	
0.219839025	0.164914075	0.005978443	0.045073492	
0.0878965	0.324757746	0.179953823	0.271035314	
0.026793759	0.348508731	0.022780583	0.264412663	
0.198700037	0.043858188	0.067547004	0.170230478	
0.161667037	0.253579683	0.087388843	0.200311985	
0.135766043	0.180111498	0.028677022	0.238295454	
0.213406331	0.194401428	0.278526794	0.236922911	
0.273144931	0.307392982	0.238939136	0.025588184	
0.030998264	0.094102533	0.020928633	0.014788123	
0.158875891	0.067071076	0.259875957	0.316894994	
0.230767 0.056160013	0.058559495	0.188299065		
0.267532304	0.321148621	0.078710491	0.011504981	
0.011155565	0.008645901	0.225821284	0.239698837	
0.244356039	0.009069203	0.036741528	0.034908795	
0.060822791	0.196379656	0.205389112	0.35593384	
0.271083951	0.135803151	0.020239616	0.376826982	
0.030396835	0.166680564	0.237671944	0.198876569	
0.098109743	0.215770387	0.196396303	0.293994859	
0.01264383	0.2787347	0.219533386	0.035256502	
0.060364318	0.101502439	0.068930546	0.357506167	
0.176575336	0.016985246	0.015907035	0.012450788	
0.048833132	0.29904209	0.057875163	0.293304836	
0.265365237	0.343136857	0.055632547	0.207886293	
0.017957578	0.179723725	0.139262742	0.010048047	
0.206438754	0.05689752	0.210971915	0.007318104	
0.08725688	0.282720887	0.032021788	0.06542246	
0.036799949	0.094033685	0.281669438	0.285758883	
0.07445024	0.137566456	0.293950109	0.354373221	
0.1403516	0.242871201	0.195115415	0.0803316	
0.054848914	0.048179883	0.316996153	0.208437346	
0.07821794	0.308838021	0.038578844	0.269589028	
0.245180589	0.280524038	0.21959918	0.192202623	
0.035078692	0.184579244	0.147952958	0.011765737	
0.277889334	0.259944342	0.231390867	0.026815814	
0.12208212	0.138287154	0.083101563	0.049265445	
0.096065731	0.122647363	0.041429953	0.378220977	
0.262103143	0.203594971	0.065465005	0.057255704	
0.25473407	0.233212409	0.042175026	0.210243633	

0.240318649	0.182645281	0.280867096	0.267973502
0.065286411	0.193862617	0.265161407	0.145773191
0.182483017	0.22125511	0.212860272	0.03494328
0.026245258	0.19222367	0.029459784	0.180402773
0.146306344	0.059259968	0.037839955	0.343078504
0.055410302	0.23518509	0.036152984	0.063407137
0.258544825	0.174506263	0.279392701	0.088059039
0.028476971	0.183830858	0.017248631	0.124637396
0.071778485	0.206506626	0.101300496	0.176550512
0.039462693	0.093822899	0.209985901	0.103918286
0.034510864	0.037269523	0.198389188	0.039846792
0.032072001	0.320512687	0.153867283	0.067751476
0.054268196	0.201576723	0.145640412	0.033557863
0.079924159	0.192704659	0.051541028	0.194508427
0.221694367	0.136388348	0.183313823	0.067726168
0.091099287	0.206044235	0.187516882	0.201934783
0.218444822	0.296212733	0.180763994	0.202942828
0.092982035	0.15938557	0.040738609	0.314504635
0.050681192			
CHSY1 0.379095395	0.513945474	0.370238493	
0.37711904	0.377880501	0.341071667	0.218081276
0.223811596	0.09729866	0.006586077	0.020355122
0.391737434	0.177636688	0.266377188	0.305928853
0.384093631	0.134368078	0.375217928	0.254588309
0.384650831	0.108682154	0.362715216	0.112993688
0.270857312	0.102406716	0.074089839	0.240428691
0.417613708	0.088120132	0.287042049	0.362541353
0.124804737	0.391070773	0.514445229	0.369708061
0.503482181	0.360155042	0.01934137	0.264702607
0.415681443	0.383111593	0.376537393	0.31963815
0.052809487	0.489137087	0.246529286	0.370771784
0.254942818	0.033854927	0.2471233	0.448278427
0.464304242	0.356480358	0.249771703	0.168441382
0.156898599	0.226429548	0.217081941	0.309225954
0.227223335	0.17002507	0.472469388	0.09788874
0.138030994	0.279455348	0.16294378	0.278716941
0.296360823	0.355669758	0.030683777	0.354552135
0.004326051	0.375839113	0.361835344	0.254740941
0.052430899	0.02307459	0.190101381	0.318592643
0.439323314	0.4657328	0.454312882	0.461329594
0.178066288	0.30427189	0.339814563	0.177843211
0.416631743	0.180217107	0.141914748	0.378859311
0.464813072	0.506871706	0.196334343	0.262631033
0.369899864	0.475118736	0.383626375	0.373235283
0.289446112	0.06515073	0.428749563	0.48963507
0.390935694	0.275655628	0.159727207	0.372952855
0.073891121	0.37309349	0.33819378	0.174397029
0.453351553	0.02513011	0.209982836	0.326577481
0.265395455	1 0.500522508	0.201537622	
0.378978156	0.028598021	0.11719841	0.046959857
0.318613832	0.13445261	0.476619928	0.428181508
0.247812253	0.325593447	0.278132228	0.004309737
0.03415805	0.216887393	0.387893343	0.381168568
0.396731993	0.377420312	0.213130009	0.343417434

0.364088485	0.444600331	0.064641399	0.122333843
0.387078193	0.208312009	0.248546165	0.382163305
0.285135043	0.147939547	0.145080532	0.502950117
0.432265992	0.099853561	0.353246142	0.385294487
0.278545633	0.322239369	0.128555193	0.267414884
0.060321225	0.468370624	0.035536439	0.353072061
0.22220554	0.135734265	0.02321663	0.276000902
0.288706456	0.392488872	0.463787683	0.131821195
0.303555434	0.366073571	0.320250376	0.357735522
0.010032395	0.069324957	0.284719939	0.487135506
0.26819765	0.486270302	0.321310036	0.305725278
0.280530734	0.407158888	0.006546565	0.494385223
0.510305611	0.29749913	0.348697346	0.386932731
0.307699877	0.41610433	0.050220406	0.385306948
0.335888113	0.103232812	0.481426426	0.162439593
0.461410308	0.047387343	0.303451145	0.383272851
0.350166399	0.011018342	0.229142097	0.360614315
0.034606745	0.109129093	0.393446654	0.380578468
0.342045171	0.399138719	0.457514962	0.29326284
0.378137041	0.266591575	0.019613451	0.312005139
0.390032484	0.302049169	0.448327993	0.253473749
0.322314239	0.470681577	0.376165261	0.173993598
0.053324803	0.309125325	0.029852899	0.310981131
0.378088639	0.116279858	0.330562395	0.180341647
0.079589057	0.508113304	0.168009034	0.230922675
0.118056413	0.340949788	0.279761633	0.205394777
0.177731764	0.170337249	0.303999073	0.337496844
0.047837313	0.100846814	0.023489721	0.300788277
0.118171976	0.281502684	0.278207216	0.353589475
0.298010186	0.111742407	0.344808333	0.279197145
0.126367542	0.020636363	0.498107854	0.390517482
0.309547394	0.174329163	0.054266318	0.279623235
0.436796696	0.281800631	0.270498878	0.437175919
0.048527536	0.459002747	0.225086449	0.127077075
0.316545109	0.258615148	0.439102563	0.061973196
0.368010267	0.462132424	0.327006153	0.182688371
0.392361032	0.383891968	0.039866952	0.366739803
0.357369261	0.039265189	0.510332899	0.132112055
0.028549301	0.388678365	0.359270459	0.088228722
0.092504777	0.290465722	0.062151807	0.284930495
0.504709974	0.370128687	0.354429958	0.221816623
0.047432702	0.009914145	0.212753557	0.371497097
0.506647314	0.490972142	0.27715267	0.315357321
0.254027714	0.279503051	0.16244458	0.487495563
0.239938746	0.515311612	0.010847932	0.366124355
0.496786015	0.278694792	0.083007308	0.292938097
0.374517444	0.05938092	0.248769302	0.493277071
0.418850733	0.302869757	0.313405778	0.398032846
0.023992696	0.053846029	0.359327371	0.173372108
0.332306895	0.22349555	0.051013589	0.321779432
0.050223846	0.442055945	0.415115671	0.065713059
0.284023561	0.320658409	0.355550838	0.048169908
0.413923799	0.491197313	0.516837419	0.20356999
0.37890904	0.465659369	0.324935171	0.09829531

0.023140434	0.507068582	0.377380148	0.030054826
0.442795806	0.236599713	0.013143329	0.497292377
0.210776791	0.447924515	0.010348321	0.10315587
0.054192334	0.415920161	0.474587143	0.36552376
0.22696538	0.375245016	0.008635237	0.507961738
0.482596233	0.018299743	0.143001239	0.367506226
0.35244142	0.52888525	0.274767843	0.404569671
0.35685358	0.485229285	0.227325468	0.265559803
0.406645118	0.471817244	0.393747265	0.468632488
0.426138527	0.385325391	0.280432745	0.019636425
0.129999307	0.226346699	0.259781391	0.077960625
0.317561866	0.143665936	0.483272415	0.435264198
0.454317916	0.008026219	0.177782748	0.37575094
0.43305132	0.464634312	0.03662665	0.010926546
0.014149245	0.018908889	0.349431381	0.25636146
0.474862736	0.218587137	0.007577615	0.062622611
0.313187182	0.346321713	0.49163727	0.366939507
0.38473788	0.096709294	0.238280097	0.385706605
0.069311585	0.310152832	0.460926856	0.470523589
0.056913295	0.257519488	0.384115055	0.471524701
0.060212134	0.310786006	0.38883837	0.27779274
0.023776372	0.09215919	0.071651387	0.395803056
0.486120892	0.07331809	0.034912652	0.145389304
0.1215565	0.440273156	0.174185622	0.495971792
0.412370076	0.396932806	0.080041904	0.483097889
0.03831336	0.478504966	0.301987033	0.073434922
0.321087684	0.020774396	0.458761938	0.121297906
0.314891873	0.384458771	0.185273948	0.02155379
0.17296639	0.062972669	0.446052453	0.415945912
0.045434459	0.262115144	0.316321618	0.398610497
0.294024592	0.381395714	0.501141799	0.350867287
0.094055363	0.145690262	0.39061636	0.372698659
0.250427302	0.463899597	0.251167679	0.515958469
0.378909463	0.461532001	0.453464753	0.335593965
0.20286737	0.380730539	0.329210136	0.027954388
0.362774916	0.412148347	0.371103459	0.146590978
0.239719141	0.331327638	0.068260929	0.229562432
0.281605519	0.293483562	0.146403624	0.403071211
0.380970111	0.369159477	0.059567616	0.067715493
0.534133974	0.443004088	0.177458564	0.384803261
0.197933496	0.317038855	0.394363485	0.383440328
0.333077277	0.403486231	0.385633212	0.339105071
0.424717798	0.273226016	0.51811145	0.123679174
0.084517123	0.368668999	0.09472552	0.106363851
0.155953295	0.036723741	0.247467344	0.390642535
0.308302996	0.349820562	0.298948302	0.107025521
0.315284533	0.364271876	0.34795316	0.20975735
0.086947636	0.487703772	0.013672514	0.069398273
0.319981799	0.376946743	0.319933089	0.259860761
0.255346226	0.152005807	0.291731346	0.294414127
0.267513184	0.345115459	0.385534765	0.054667022
0.027599987	0.471427624	0.378760676	0.032475372
0.089812299	0.353927825	0.328683854	0.213107685
0.028415395	0.453561711	0.2864625	0.38143925

0.348328888 0.087081019 0.367962642 0.115451769
0.090401397 0.457430036 0.357100373 0.498152845
0.346268513 0.375783198 0.45409384 0.386406925
0.177004107 0.348552428 0.038542267 0.418322352
0.333010745
CLDN10 0.484958306 0.511186162 0.46404963
0.456371084 0.475972805 0.42425789 0.2075232
0.178514574 0.087589375 0.00383231 0.025812148
0.508113184 0.233829191 0.284540994 0.380004861
0.452190398 0.145247364 0.371844088 0.239553212
0.437157367 0.083798451 0.435996518 0.083570944
0.333572166 0.116968645 0.067345248 0.314370391
0.47389257 0.077508791 0.371020278 0.41355962
0.099932706 0.490243257 0.524767104 0.50270495
0.527546264 0.472461201 0.00967886 0.325068108
0.470713746 0.508165966 0.435170636 0.345765336
0.036567614 0.49107445 0.250660135 0.451520533
0.241345136 0.030327363 0.252505519 0.449733245
0.491711859 0.511431336 0.221701511 0.156065051
0.252722638 0.238681483 0.359109751 0.357288258
0.226850501 0.158659979 0.497947511 0.086441949
0.154925473 0.243404964 0.160662409 0.257986936
0.418872343 0.434710515 0.012891916 0.491940525
0.002089058 0.406784604 0.414596967 0.3984243
0.038415062 0.019332637 0.187018143 0.357655602
0.492107448 0.482058075 0.525521993 0.531331287
0.197757661 0.306763976 0.46942266 0.174319489
0.495175757 0.179991289 0.108488669 0.461068504
0.528815813 0.519082028 0.216714112 0.282399464
0.49208241 0.493607045 0.487246986 0.485236059
0.422408122 0.053647114 0.48718049 0.530294017
0.453579228 0.390778247 0.160170779 0.44821702
0.053327516 0.407124233 0.373480961 0.169868039
0.495180646 0.014765304 0.231096191 0.383687407
0.211838541 0.500522508 1 0.183891312
0.457694588 0.017887847 0.175759511 0.029505363
0.358139678 0.188233747 0.506455699 0.503788352
0.233265354 0.431449551 0.264338399 0.000761857
0.025116239 0.192526835 0.512662868 0.389493111
0.476894007 0.46556543 0.258831464 0.453832473
0.530474873 0.525170944 0.070344156 0.085325885
0.471453381 0.189561615 0.242334816 0.512085365
0.346869822 0.102457236 0.121099861 0.524035157
0.447200315 0.077580168 0.361040895 0.450776774
0.376741587 0.408944575 0.09790251 0.293234994
0.042384178 0.497064332 0.027611313 0.34661127
0.344149885 0.169905715 0.007852498 0.262315993
0.316239742 0.512042195 0.495964411 0.098218623
0.339731086 0.453381094 0.345848351 0.500796736
0.010029384 0.04058747 0.308182312 0.525458573
0.3236025 0.524408507 0.308706744 0.329492875
0.302794566 0.493547573 0.003434452 0.503929029
0.524913498 0.337553574 0.451802656 0.468715446
0.461145825 0.497467337 0.049868268 0.452679321

0.416674254	0.070209289	0.521263658	0.16788794
0.49251242	0.03849628	0.335763859	0.523775851
0.526860608	0.007652228	0.235310877	0.414676419
0.026122767	0.088369989	0.474308164	0.443569016
0.427684488	0.500252519	0.506530051	0.447152799
0.481045466	0.22735174	0.009950592	0.355317899
0.504523205	0.458654491	0.466603768	0.240223328
0.392564169	0.501023933	0.508474377	0.245423394
0.037608816	0.321902815	0.018577668	0.371267944
0.477121792	0.119205576	0.473280101	0.146778263
0.071438605	0.525496183	0.21336617	0.331199189
0.163601954	0.461898375	0.247814955	0.271422544
0.262636418	0.16650249	0.41713823	0.431008765
0.030447057	0.092650785	0.01326808	0.471736154
0.167944999	0.414093721	0.411435042	0.474728183
0.353216781	0.116560041	0.43677446	0.333880593
0.119667971	0.012429754	0.512992282	0.436344641
0.389077107	0.148872703	0.03667274	0.423691114
0.511188022	0.377870485	0.298448731	0.496259675
0.023650505	0.520440848	0.277984606	0.127046202
0.455178407	0.27402093	0.490445424	0.06534321
0.430990863	0.487776178	0.327941568	0.200497605
0.45304262	0.452368006	0.02531723	0.47490631
0.4722164	0.027969217	0.523810374	0.135173226
0.018062696	0.505907414	0.52664648	0.072365697
0.076845966	0.377026587	0.037634087	0.312379321
0.521924567	0.451072607	0.507076888	0.24810884
0.036066042	0.010540879	0.228808408	0.468200946
0.519843307	0.528363561	0.267084497	0.434848318
0.241669264	0.396883026	0.139672978	0.530030664
0.207117064	0.512849197	0.004737069	0.523258907
0.527194783	0.314421825	0.085206637	0.326414281
0.455593891	0.040015913	0.194646874	0.499289577
0.495151555	0.372599532	0.411586597	0.476859381
0.012157796	0.034155044	0.445021414	0.227226026
0.440619637	0.222108712	0.071887287	0.345888077
0.039918948	0.487792319	0.502243983	0.060487377
0.399191292	0.327397334	0.418174593	0.031592237
0.528564411	0.508709186	0.52425877	0.151760645
0.515690753	0.526208728	0.508717022	0.076170233
0.015516007	0.52411705	0.447421483	0.020895482
0.508457322	0.258816973	0.01216343	0.499327804
0.178495362	0.463020571	0.003215552	0.099769378
0.039902291	0.51153748	0.487276822	0.409853299
0.288977629	0.385619102	0.005510585	0.525096093
0.502586127	0.013369542	0.156662352	0.527517251
0.527077413	0.511049513	0.397450827	0.533233201
0.521048623	0.512294047	0.253278921	0.287294708
0.50358971	0.511584235	0.469066409	0.481037065
0.507455785	0.476843177	0.290179694	0.012435981
0.159617906	0.212946553	0.389286347	0.092198792
0.361952772	0.152168895	0.508888087	0.512467129
0.534082452	0.003455795	0.142405353	0.51468189
0.476453769	0.496259086	0.017826711	0.006315635

0.008396512	0.022381565	0.380350062	0.259802937
0.522503175	0.288496856	0.002879642	0.035450274
0.293899214	0.473001637	0.509826527	0.403381872
0.513253306	0.075687402	0.337415289	0.424604164
0.074016143	0.389771748	0.532209074	0.482029099
0.037215935	0.265461652	0.410617297	0.508559746
0.103127143	0.341819165	0.50977608	0.356031932
0.011547349	0.083026888	0.066577492	0.484108745
0.519897479	0.08558092	0.034309524	0.160483049
0.120941703	0.514615713	0.171517708	0.526255874
0.490193217	0.475395458	0.078260452	0.489112095
0.039028424	0.49421316	0.405603064	0.063942458
0.302565723	0.009565735	0.505477377	0.204649099
0.349179418	0.438618422	0.236993986	0.015339601
0.23236596	0.050761428	0.516628563	0.521942139
0.032210188	0.233232367	0.317407674	0.439261998
0.300838275	0.486636922	0.50950747	0.490881498
0.077070933	0.172302609	0.440746296	0.45734444
0.239186857	0.523488993	0.353909603	0.523093902
0.466446117	0.495765995	0.522059547	0.377837106
0.277413157	0.449664821	0.420193887	0.017212743
0.374137836	0.513364571	0.379113207	0.180465741
0.286388848	0.477912693	0.038760901	0.199418005
0.424940671	0.339164068	0.142902803	0.473879013
0.429730156	0.462338688	0.031400308	0.062770749
0.523875017	0.475183992	0.140847433	0.482317508
0.170952266	0.383539339	0.517663525	0.518058473
0.428349151	0.5211581	0.476308075	0.444503836
0.460506444	0.263962289	0.526015598	0.0797395
0.096318109	0.491600009	0.100893876	0.091551405
0.141317471	0.024463592	0.392394443	0.466800819
0.475686875	0.419379194	0.348684657	0.078143141
0.355195955	0.416311107	0.383472803	0.203498106
0.077992362	0.502380225	0.007985441	0.052126812
0.476501892	0.517526204	0.471461296	0.254970521
0.372706444	0.148445144	0.319360318	0.320945823
0.387776597	0.427268605	0.516225346	0.028450708
0.0144106	0.519292554	0.418546963	0.018695285
0.088462811	0.439008038	0.305106714	0.264657986
0.020836434	0.523653096	0.388919318	0.488051474
0.362728297	0.058453095	0.407902765	0.086505475
0.050962805	0.470376604	0.4695627	0.516848602
0.394679971	0.461373773	0.454522169	0.511521371
0.122718193	0.460823336	0.02215688	0.490129749
0.507073305			

CLDND2 0.156284269 0.227914523 0.096690146

0.322917183	0.169181358	0.155746978	0.26997556
0.177506615	0.1920905	0.095155577	0.095460783
0.137392608	0.142089276	0.178756162	0.098521626
0.315394272	0.170161873	0.1047723	0.302940002
0.285072687	0.252669751	0.307817089	0.306039169
0.095341788	0.139954671	0.23042806	0.118556089
0.317692259	0.237445771	0.122025325	0.262087015
0.152055125	0.207364779	0.181249653	0.201870245

0.16204504	0.125950141	0.040329726	0.145997083
0.198817937	0.215589461	0.29921023	0.312060509
0.259495092	0.167511955	0.310433457	0.164276377
0.118432819	0.164079173	0.109876293	0.17019371
0.15616753	0.141728483	0.205662648	0.281887537
0.090794784	0.111548765	0.05103457	0.166941502
0.280499465	0.12467807	0.167599563	0.235401972
0.116199643	0.19648571	0.139222107	0.215885355
0.073204816	0.186477303	0.175113787	0.092088728
0.034733659	0.306888526	0.179949004	0.055761524
0.196829152	0.149927485	0.15499272	0.304911327
0.291721728	0.163226337	0.261613761	0.229460472
0.1625196	0.169582545	0.094459383	0.262209001
0.204440203	0.266348098	0.162821103	0.305324491
0.254294892	0.194027536	0.184348132	0.184098176
0.220254265	0.220455233	0.112163713	0.135816813
0.07953739	0.090425419	0.174545343	0.23267097
0.173653578	0.064521642	0.197358633	0.259811571
0.228075833	0.082038447	0.343053253	0.097029689
0.300467965	0.196946772	0.156773215	0.241627013
0.173434033	0.201537622	0.183891312	1
0.127686901	0.123470837	0.062301688	0.153607191
0.128573023	0.037641665	0.142672426	0.279237758
0.166225891	0.117312804	0.242534713	0.057878525
0.137224206	0.115919873	0.164610953	0.125983585
0.230108225	0.113407958	0.191832426	0.154382637
0.130748022	0.256490865	0.024384964	0.299776399
0.170072329	0.12743283	0.317368372	0.147390359
0.172292969	0.284908248	0.287506023	0.257358653
0.195248655	0.291674615	0.086785041	0.298965661
0.134539393	0.157600874	0.288563798	0.201183184
0.278886609	0.151224744	0.107575322	0.130154494
0.065669146	0.103916918	0.092088883	0.173387503
0.151916588	0.190674028	0.130108219	0.263458596
0.193835237	0.247110456	0.115895237	0.188852083
0.103114916	0.197344546	0.06789561	0.236744433
0.133608184	0.22109041	0.202631446	0.148971092
0.228825648	0.27632478	0.004124901	0.170919085
0.188883457	0.062982304	0.206922045	0.166651074
0.077121432	0.28891301	0.010956459	0.288105156
0.121578008	0.259923156	0.27948746	0.21761625
0.167192031	0.20836762	0.146322054	0.163308056
0.129616651	0.050356038	0.143874262	0.301160474
0.094497865	0.229524202	0.213324361	0.284228603
0.222412981	0.231819853	0.305332344	0.066908301
0.165038203	0.237223069	0.113303388	0.153402005
0.185419423	0.062815557	0.192235155	0.253704242
0.089942089	0.214693911	0.137271419	0.07080046
0.264074939	0.196656832	0.119728207	0.182682493
0.135698477	0.252309206	0.089614393	0.124400125
0.166322151	0.239659099	0.068575319	0.047963715
0.03836923	0.164324683	0.248735084	0.145661034
0.107699219	0.243561257	0.102070618	0.123799141
0.252620166	0.242289373	0.042114834	0.05367066

0.11463346	0.084712796	0.075757467	0.151686925
0.134100322	0.179609783	0.158652104	0.087208786
0.159413307	0.142682365	0.222947548	0.280716481
0.13882941	0.293497242	0.182357918	0.052525195
0.266788326	0.099199721	0.064399534	0.238933443
0.127149844	0.251922507	0.179853662	0.212058954
0.089182966	0.266819374	0.313239608	0.078301028
0.147321907	0.207830481	0.198061681	0.203655348
0.162938304	0.253678305	0.235373525	0.151361402
0.145666269	0.19863352	0.211151803	0.143881117
0.143224559	0.187860367	0.16629194	0.210347818
0.181204095	0.176050473	0.23360782	0.269408757
0.240032119	0.316324452	0.191248594	0.090298465
0.247570672	0.074111157	0.120513656	0.097270198
0.168928199	0.196699508	0.245059545	0.125355153
0.331879164	0.107057283	0.103100562	0.229671457
0.071313735	0.187906538	0.108297849	0.174264121
0.159239329	0.108048227	0.167557203	0.226596966
0.179413704	0.262773761	0.216181251	0.187658222
0.221545117	0.150821889	0.114458052	0.191711493
0.204869476	0.265496415	0.165663336	0.055871481
0.118982408	0.230929328	0.014496315	0.131254575
0.224575015	0.202944218	0.260320779	0.188160255
0.132357972	0.11496138	0.229728204	0.238440356
0.170759685	0.172400579	0.187313809	0.2549453
0.171222693	0.192214326	0.089674687	0.178339952
0.110452985	0.171948882	0.183359248	0.120474005
0.286166553	0.090599765	0.115650007	0.228870261
0.28645199	0.131791793	0.036833256	0.01805133
0.089498775	0.244224156	0.171782465	0.257750339
0.074374103	0.246085736	0.010129359	0.210390555
0.199830033	0.091186872	0.115712827	0.161094582
0.134349671	0.220669335	0.073582084	0.186616255
0.116847089	0.165710566	0.210038955	0.26295394
0.234096706	0.158576191	0.233369507	0.204099357
0.27597692	0.222340841	0.339789708	0.197298573
0.087760919	0.165879057	0.065558915	0.130379337
0.140730836	0.182110266	0.200534226	0.271729236
0.206597205	0.085272745	0.242297825	0.16888674
0.267522525	0.291368281	0.072220256	0.015377176
0.008034236	0.063343557	0.236390012	0.162200572
0.215492209	0.065311269	0.105969767	0.235209878
0.189153679	0.15516493	0.198178405	0.348864047
0.181450685	0.230582701	0.107889492	0.321077402
0.139329447	0.10212296	0.228228293	0.205105968
0.133523205	0.165485861	0.097627597	0.29163343
0.013815035	0.233827463	0.148275004	0.168007076
0.048623747	0.162398886	0.172252294	0.26619601
0.151002771	0.159860836	0.140377835	0.053938317
0.264194282	0.239075993	0.243404913	0.262887172
0.26376535	0.332562394	0.113162486	0.193012435
0.167587382	0.168730557	0.109339434	0.053586273
0.135500172	0.128683364	0.157311626	0.06754278
0.12201673	0.198801295	0.091669186	0.087705048

0.040520328	0.183628191	0.283241634	0.230626113
0.085659789	0.241147054	0.191333785	0.223858759
0.189589497	0.282824092	0.17960425	0.080289305
0.294657059	0.034269379	0.275257714	0.143811276
0.168329757	0.264850051	0.137160911	0.220156335
0.180783616	0.212834022	0.189593303	0.24933146
0.09068315	0.145055891	0.233780215	0.161175764
0.246457593	0.272374962	0.149241433	0.142398266
0.190589002	0.083354209	0.181166736	0.043205709
0.052592804	0.173750459	0.26351398	0.320131135
0.171881982	0.153144839	0.175292128	0.174234497
0.223054421	0.19099849	0.209813071	0.22373045
0.207814531	0.223121911	0.197752573	0.195102351
0.083483919	0.199914048	0.241621454	0.203338847
0.198546425	0.278157214	0.192551998	0.1273072
0.102490957	0.112420514	0.228728424	0.17863717
0.279941803	0.138516431	0.04263012	0.265672939
0.047961414	0.285611863	0.084105291	0.186228098
0.176947525	0.073482157	0.247141693	0.240828373
0.234447233	0.184980037	0.07553774	0.1941995
0.056405741	0.195675806	0.101567152	0.190304702
0.068165673	0.11079895	0.258153625	0.12336907
0.078261318	0.065942661	0.19247565	0.18152664
0.214238385	0.275731287	0.099071258	0.075501188
0.097090742	0.126265956	0.126201613	0.188708426
0.084936926	0.172390912	0.093157431	0.146641693
0.23848952	0.233490438	0.139883155	0.113560722
0.199000613	0.172965614	0.110876032	0.176123014
0.095052307	0.329898769	0.148751492	0.180493696
0.096556071	0.071075229	0.244269002	0.258192533
0.055242511			
CLEC2I 0.373173433	0.461177417	0.220123508	
0.34726621	0.263386077	0.182098254	0.114641084
0.101754583	0.058548538	0.004136686	0.021862702
0.330064173	0.119677807	0.127611139	0.118374778
0.409507682	0.146318067	0.217538222	0.166640392
0.338264486	0.050211681	0.364718358	0.080136687
0.235348697	0.107342273	0.070179447	0.173159731
0.427041836	0.08015057	0.113531896	0.287358902
0.166199716	0.40127251	0.436692739	0.351282235
0.435338276	0.319615666	0.041695962	0.106248513
0.383398782	0.363216167	0.331689707	0.221066352
0.03992017	0.389027762	0.136950984	0.318835268
0.100333015	0.041338837	0.08465077	0.356640825
0.373433746	0.232835399	0.112637644	0.091253504
0.092037368	0.092848025	0.1479352	0.194368086
0.140956399	0.056196794	0.400257448	0.047683309
0.072889153	0.150212784	0.205298826	0.140460555
0.220389508	0.284653387	0.032776707	0.291683785
0.001703064	0.321965116	0.321288766	0.150173894
0.069337414	0.023610665	0.21592562	0.204527072
0.450321185	0.424800973	0.48727062	0.479353683
0.156853433	0.250255541	0.250457898	0.133190973
0.394641609	0.096517281	0.095959033	0.42727439

0.488484968	0.453354553	0.180681895	0.22762836
0.31643008	0.421003643	0.174193592	0.20371912
0.13950447	0.109274858	0.354357232	0.486146848
0.331009068	0.219106625	0.153036489	0.344469989
0.08904829	0.162230329	0.274640362	0.17656461
0.461677758	0.018443168	0.206916635	0.187520896
0.209166504	0.378978156	0.457694588	0.127686901
1	0.036515926	0.090059109	0.080380802
0.186384866	0.153885629	0.371408079	0.41357793
0.129206165	0.211091089	0.158127957	0.012914118
0.058980483	0.225380236	0.39558448	0.234540253
0.377034034	0.346878285	0.129956885	0.288132763
0.310977181	0.484304841	0.015757635	0.085229907
0.225587844	0.22502415	0.191941823	0.277199337
0.200408565	0.08983219	0.119581641	0.488638763
0.345990746	0.083693599	0.173682077	0.400008367
0.132460698	0.267829042	0.111519148	0.168522995
0.043423004	0.41034553	0.071239408	0.175006705
0.108423292	0.16926093	0.032815199	0.125024585
0.125437193	0.343854318	0.375672366	0.124503625
0.184483104	0.32713339	0.140225609	0.285802042
0.007467448	0.077643698	0.117199811	0.469807725
0.143233055	0.50391521	0.210294005	0.233961583
0.219991794	0.426481341	0.007362471	0.402382807
0.457648127	0.115738661	0.244210773	0.341643912
0.168652218	0.401189233	0.034649062	0.394876243
0.257006686	0.100693021	0.505136535	0.145488468
0.409281312	0.053455382	0.147438858	0.401060885
0.343999541	0.017333469	0.080254354	0.285532683
0.071629332	0.04479016	0.363984766	0.301823379
0.210647998	0.431775651	0.494664946	0.157816022
0.238714719	0.161460029	0.035596401	0.252898828
0.414670736	0.161390395	0.353353589	0.140002021
0.178414827	0.403380223	0.354960408	0.064014022
0.044734815	0.152113417	0.018110182	0.18595436
0.339457565	0.069878489	0.165115249	0.219449593
0.108396277	0.478299679	0.183769693	0.19394394
0.137351657	0.208784824	0.175246589	0.099909406
0.11409004	0.142791517	0.188778574	0.174552653
0.027572693	0.098205506	0.055757264	0.170560705
0.079078819	0.148335501	0.133167779	0.291962827
0.177231516	0.085003974	0.209914613	0.208206087
0.129271751	0.037044102	0.449458667	0.363213659
0.224658852	0.116433095	0.081570829	0.136123113
0.420032394	0.111055003	0.100632285	0.38438811
0.052274176	0.489593433	0.131542777	0.123254062
0.213080746	0.169193456	0.443970258	0.029866732
0.235822867	0.391624369	0.245587156	0.136583214
0.312614022	0.302220625	0.033910939	0.319991244
0.312399256	0.052843704	0.481965119	0.165063614
0.052355832	0.397540192	0.327269377	0.101067103
0.104843873	0.142143264	0.027728354	0.250632464
0.46416189	0.388778869	0.31646004	0.220214676
0.042159852	0.014255322	0.233314814	0.264977381

0.419273775	0.479011771	0.223471905	0.200188045
0.177623976	0.162855123	0.059950786	0.470143399
0.225871344	0.461915608	0.023102574	0.397445231
0.417011582	0.114367834	0.087628205	0.13418305
0.301658332	0.04947737	0.182702895	0.418066008
0.37413291	0.175824638	0.153349453	0.378007419
0.015066051	0.036693315	0.254142418	0.177551088
0.162972104	0.160378531	0.082681165	0.159806612
0.041074984	0.371064471	0.47395795	0.052572473
0.134044439	0.142054818	0.351203092	0.05187256
0.422394831	0.461548223	0.438948329	0.141272931
0.403504649	0.427975798	0.245838848	0.128291282
0.046763899	0.447640704	0.287653464	0.018863386
0.474425193	0.078152884	0.012594861	0.434048043
0.141550391	0.352540429	0.001860427	0.100782025
0.100280164	0.486582596	0.380600142	0.283401085
0.066701073	0.29166016	0.027587176	0.502118228
0.413026324	0.037461869	0.177384232	0.364699424
0.351197566	0.46593691	0.188962255	0.411634133
0.28631915	0.414074619	0.123098608	0.203340448
0.368606469	0.439266568	0.346380855	0.393863802
0.436380637	0.398236957	0.19277896	0.017801519
0.088662867	0.092868925	0.092700601	0.044448665
0.175645862	0.117129868	0.45124274	0.475015876
0.45866986	0.020133386	0.084762616	0.362930631
0.391122569	0.455012636	0.056677665	0.010033695
0.014565483	0.026523605	0.216081017	0.225320547
0.446627834	0.066684871	0.014010857	0.034789416
0.161889176	0.315511503	0.431803599	0.353327208
0.421988158	0.114600545	0.097401782	0.372865885
0.079866552	0.221441059	0.455659294	0.386334107
0.097316174	0.214874941	0.218381247	0.45921748
0.075448422	0.268008531	0.35315388	0.151364455
0.050280694	0.11177983	0.078180738	0.451440778
0.41528451	0.065755418	0.04052848	0.06023904
0.074843804	0.466420443	0.11854557	0.502178838
0.384329535	0.44308987	0.133618635	0.398498683
0.043466396	0.405449238	0.223656416	0.021649235
0.261114095	0.039027303	0.438550015	0.061139444
0.144326897	0.311714277	0.115818624	0.054018067
0.15426979	0.087433387	0.45876072	0.468592918
0.093785101	0.154075156	0.239474339	0.348807946
0.124802997	0.366633768	0.412385296	0.155356056
0.057306785	0.195974393	0.34745936	0.286849414
0.129120156	0.503773575	0.144928459	0.495456252
0.322269381	0.431004417	0.434502128	0.189229076
0.20881933	0.227964007	0.223923839	0.014542881
0.237579111	0.433166534	0.197773722	0.113738957
0.171313235	0.23975371	0.068883301	0.130673881
0.194969949	0.142901524	0.106521162	0.459834708
0.286479372	0.298691305	0.058017136	0.068318795
0.484841586	0.376510316	0.075487407	0.293729757
0.190600563	0.205922536	0.429565527	0.44426817
0.185148812	0.386924486	0.375468357	0.244348576

0.339581055	0.163652274	0.441109792	0.049675709
0.092792227	0.304166158	0.068850321	0.1349907
0.125715261	0.053196884	0.168547188	0.418743111
0.197511443	0.295321124	0.151199083	0.089347449
0.257419714	0.19943211	0.270723559	0.097581954
0.064668738	0.403688383	0.019537217	0.098169676
0.223957578	0.365728083	0.202197329	0.206601212
0.195960312	0.201652848	0.203339095	0.117046266
0.105105442	0.205543165	0.356187934	0.034010441
0.025063777	0.503949658	0.173473714	0.067138411
0.115516766	0.244338279	0.248456987	0.118507608
0.062691978	0.431258224	0.107886074	0.267299725
0.18850662	0.100317932	0.287670129	0.085704023
0.084647016	0.382476925	0.266907102	0.448245132
0.262961421	0.391504505	0.353333804	0.377671013
0.150360811	0.222077054	0.029642473	0.428050141
0.164987363
CLEC4D	0.018759806	0.022968655	0.016217861
0.0625112	0.039616799	0.014716404	0.078152813
0.021469497	0.010542159	0.020256518	0.016425845
0.02949844	0.00922936	0.065274575	0.004528958
0.067159688	0.132898645	0.016667193	0.135745015
0.054072548	0.047457903	0.09569231	0.081945564
0.015900948	0.093735445	0.123617433	0.029411475
0.056487258	0.137545891	0.01258426	0.040312208
0.095986398	0.028790551	0.01463926	0.055387293
0.013585341	0.012726164	0.005966353	0.011046146
0.0215736	0.032393384	0.05108722	0.112515979
0.08601141	0.012917533	0.105401725	0.04227815
0.001766495	0.099866781	0.00936275	0.011907091
0.012872358	0.017866905	0.026262663	0.064286766
0.005304407	0.013233445	0.010559032	0.018426061
0.108949609	0.014859207	0.014396028	0.012361937
0.000972259	0.033754594	0.086541826	0.058630761
0.010833303	0.018387867	0.036556343	0.012770683
0.004821636	0.061224981	0.061711714	0.006039579
0.228435647	0.09143803	0.11247272	0.072208318
0.046176294	0.014783522	0.045380495	0.031748994
0.08885807	0.082288142	0.020584577	0.135210506
0.020801165	0.090417491	0.015515664	0.078246128
0.041054625	0.018538694	0.040224191	0.092881667
0.025599588	0.021386389	0.018443057	0.02838954
0.007948932	0.050678841	0.014187135	0.031458065
0.017731446	0.021948685	0.165171368	0.039462274
0.160230778	0.006851569	0.111551587	0.019350833
0.048453894	0.037602643	0.117292647	0.055650476
0.024496064	0.028598021	0.017887847	0.123470837
0.036515926	1	0.001995788	0.124107671
0.004681239	0.012776585	0.009302056	0.047902727
0.022216052	0.009847019	0.051826015	0.082094232
0.155833819	0.018866007	0.023141319	0.030068194
0.033102976	0.009552728	0.054127731	0.013781023
0.018294063	0.04296131	0.000162606	0.072508812
0.033713435	0.023982581	0.167078245	0.02413996

0.078727478	0.066319169	0.15516125	0.032967042
0.015803875	0.18574018	0.010844162	0.082216133
0.008807125	0.069399711	0.139920111	0.096897086
0.082783406	0.011870193	0.158409877	0.022371621
0.002124162	0.035536679	0.015710177	0.031893212
0.014547636	0.022468905	0.008344317	0.160092319
0.095679844	0.067462609	0.014534571	0.032485426
0.015516629	0.055195844	0.005857156	0.032263823
0.01366489	0.029741916	0.063459919	0.034644422
0.120547812	0.046822393	0.000393694	0.013314338
0.017530731	0.005741327	0.07828774	0.038568713
0.009109052	0.049142159	0.000411239	0.0851119
0.013209612	0.141072227	0.044233869	0.185549243
0.015406469	0.163585532	0.021229501	0.020500569
0.018464252	0.003332883	0.019188925	0.047212015
0.096463901	0.020372939	0.028001905	0.057364809
0.037753224	0.039309136	0.056004912	0.008392536
0.039252005	0.066188222	0.040222511	0.071535677
0.025545064	0.001408888	0.016630446	0.035135189
0.009303923	0.021125052	0.013013105	0.000476099
0.12918049	0.033743838	0.022877542	0.046363902
0.010215727	0.080839001	0.008265693	0.034983444
0.161075826	0.028409187	0.03294531	0.012845013
0.015678047	0.042905155	0.086081263	0.05210988
0.040722015	0.05755803	0.012571522	0.01404721
0.05890054	0.198388887	0.014989074	0.014272362
0.048108103	0.036010146	0.028178912	0.015732432
0.022611476	0.092571231	0.064670743	0.011810881
0.077950094	0.202955098	0.022758474	0.067661212
0.012722046	0.059998961	0.183144686	0.002736562
0.0412192	0.009520671	0.003502238	0.028219041
0.023368833	0.035846624	0.047250757	0.183621813
0.034987331	0.152229983	0.049697084	0.000607914
0.017810924	0.020507271	0.07312062	0.086244401
0.01728713	0.045272608	0.033598641	0.015911472
0.019642725	0.21565066	0.024198343	0.104017043
0.196541965	0.040769222	0.02143409	0.191525661
0.130207123	0.047655102	0.028192885	0.134251056
0.028484232	0.093326075	0.021749441	0.031040026
0.142471449	0.033731023	0.072583814	0.019457863
0.013741449	0.023580487	0.140264897	0.058553817
0.14462832	0.011023688	0.001288258	0.030246189
0.008620349	0.016867041	0.128326195	0.023815204
0.012328419	0.013887242	0.132422769	0.035325954
0.020539688	0.09917812	0.053910572	0.018550962
0.021110431	0.020908291	0.012767366	0.041935202
0.051828931	0.035384691	0.030891992	0.014045345
0.011363802	0.050242596	0.005303708	0.008296915
0.095311903	0.019571673	0.052440194	0.040966127
0.013500628	0.007677804	0.081528134	0.158134845
0.020971071	0.016913476	0.016516357	0.063962536
0.025215927	0.021186476	0.014358276	0.148713976
0.173253985	0.015511165	0.020756305	0.024069914
0.047976357	0.009865708	0.049491048	0.023545205

0.120181255	0.009009693	0.001259698	0.000799397
0.060405908	0.045920539	0.012616947	0.03692656
0.007962675	0.059698915	0.001138865	0.024542468
0.021062752	0.104500492	0.035419228	0.021074542
0.020648161	0.021391329	0.017832629	0.026378421
0.025053522	0.013917139	0.022897149	0.120205115
0.03216488	0.014771697	0.03394816	0.01836705
0.051423764	0.05531924	0.112716516	0.06811288
0.002788357	0.019586957	0.004511615	0.004597163
0.0127886	0.066791042	0.02321226	0.04618593
0.030645237	0.150478188	0.028298245	0.018702313
0.035857941	0.041069365	0.013508605	0.00135284
0.001461831	0.026476894	0.039463323	0.085139198
0.023987164	0.005687096	0.131484018	0.035006043
0.024757543	0.041378125	0.019524562	0.097131294
0.026078741	0.145703288	0.015868575	0.073724537
0.040392838	0.007880879	0.034066907	0.017703207
0.075972751	0.062140469	0.020963531	0.04287159
0.003873506	0.114040121	0.032090617	0.018481702
0.005330876	0.106327675	0.064464876	0.051215251
0.011999019	0.099654925	0.116641617	0.000817767
0.103258407	0.034024697	0.125618004	0.03733127
0.040526291	0.075366795	0.049014109	0.015425025
0.012681054	0.015521744	0.045816665	0.001584489
0.04823153	0.081472459	0.016295772	0.003114784
0.023273329	0.026869282	0.002820136	0.100042263
0.004642772	0.186273935	0.046064326	0.038241817
0.060951397	0.032487645	0.088319587	0.049531968
0.034210863	0.061416609	0.014723146	0.010568626
0.058560153	0.005004337	0.050378321	0.019529896
0.025332148	0.044573255	0.017417864	0.024733119
0.02029972	0.02171262	0.020583076	0.044134517
0.012276411	0.018672396	0.034824384	0.033977115
0.07607301	0.052369914	0.023402545	0.033943035
0.103648422	0.01452697	0.011804617	0.001163245
0.011688672	0.021106097	0.059472054	0.064659569
0.025429561	0.01912274	0.01595024	0.062917501
0.02270271	0.015223398	0.011089573	0.034465493
0.138872402	0.056145212	0.032370153	0.034624289
0.004387882	0.028961852	0.036161265	0.036547705
0.018518347	0.077534463	0.016674383	0.008495407
0.004173094	0.015374566	0.090599984	0.172101457
0.188595657	0.071005642	0.004682254	0.063464681
0.005948172	0.102641848	0.003265709	0.02140244
0.073527647	0.009724707	0.084286992	0.071024331
0.086346386	0.015955464	0.037165778	0.188318534
0.012422983	0.022834619	0.027463868	0.083049104
0.008531046	0.038665595	0.120947655	0.017073175
0.025558341	0.002597206	0.021870041	0.009018395
0.074096341	0.046201398	0.016758949	0.039116562
0.003335138	0.011065739	0.026598221	0.016062848
0.111095939	0.018002455	0.007958591	0.028978568
0.045453305	0.133902082	0.010635158	0.002381018
0.033555542	0.016113738	0.021298429	0.01636779

0.028602285	0.084171835	0.00978624	0.02380473
0.007556688	0.009073167	0.058099678	0.036645051
0.006403806
CLEC4N	0.099457533	0.160925483	0.1083067
0.150692107	0.104149585	0.060478959	0.048724755
0.007895571	0.022022071	0.000745845	0.016596667
0.118839835	0.021476586	0.019429473	0.042208913
0.178085626	0.060910359	0.029962679	0.072870622
0.101635104	0.008492047	0.175451893	0.024844834
0.068773092	0.131850914	0.07248842	0.188796099
0.141554771	0.07959184	0.033305278	0.071744057
0.028474614	0.102333237	0.146055062	0.175265625
0.142286481	0.08796518	0.000124094	0.03502415
0.097957991	0.208212784	0.105798537	0.113580849
0.019347863	0.107656755	0.082775033	0.149353843
0.002156472	0.04548839	0.012258404	0.094237335
0.128344539	0.118228791	0.019106662	0.035972738
0.011363595	0.007974112	0.076571608	0.037076462
0.060491103	0.006081713	0.138232418	0.019120004
0.001923484	0.018648294	0.042171664	0.028069829
0.086947157	0.070960504	0.001366521	0.098342227
0.000204866	0.103987711	0.130091563	0.150960603
0.052490463	0.008895263	0.058027878	0.085657356
0.144880977	0.117269813	0.21256861	0.211386093
0.029767814	0.084237813	0.125838386	0.052779986
0.124877013	0.047268863	0.00487589	0.195888421
0.21354774	0.161339469	0.063263202	0.152427268
0.144062947	0.123300968	0.097321986	0.092348698
0.115021912	0.035159128	0.119727264	0.18452587
0.077743494	0.152698428	0.118788996	0.087187466
0.03324329	0.015544465	0.1270739	0.002513302
0.157290978	0.014020283	0.140883478	0.066475491
0.015315184	0.11719841	0.175759511	0.062301688
0.090059109	0.001995788	1	0.018482604
0.006509154	0.067754967	0.113247098	0.15657762
0.007266055	0.031791653	0.040066718	0.006851356
0.031730167	0.013393062	0.108868344	0.040561664
0.11427277	0.108385647	0.11850536	0.069798672
0.148231766	0.206156968	0.000667655	0.019312944
0.104504536	0.018803444	0.107178378	0.093080801
0.183537896	0.013550986	0.034396051	0.196463321
0.089032322	0.039365154	0.017287136	0.174636627
0.032448913	0.181632696	0.027025592	0.033128442
0.008761166	0.149017494	0.047247982	0.028694725
0.056356222	0.022358481	0.000666482	0.021515228
0.017974227	0.151259092	0.107598703	0.037035712
0.048076723	0.178461057	0.02208533	0.133870456
0.008177279	0.006723512	0.012807607	0.170258676
0.02763591	0.181061972	0.036764668	0.073819823
0.150601102	0.174021352	0.000649443	0.128052255
0.173105728	0.016188759	0.125443335	0.117831303
0.15633219	0.168979558	0.000412255	0.135602474
0.046456256	0.029614341	0.204211024	0.117804112
0.115648393	0.057192268	0.019820053	0.210934839

0.134978955	0.001542503	0.018741096	0.095909457
0.03284774	0.006162549	0.103953238	0.13023259
0.156138749	0.16558691	0.193508611	0.162790917
0.120427342	0.0280126	0.004063917	0.151677357
0.133031588	0.014753715	0.107471416	0.028126537
0.031155347	0.139594351	0.124827404	0.004630411
0.030989796	0.035117752	0.001200012	0.183602214
0.115432629	0.018430235	0.13002292	0.010187871
0.069628719	0.188265667	0.126176889	0.128919487
0.103186287	0.075168378	0.050847165	0.026494767
0.03370088	0.059379842	0.114436433	0.083542766
0.005378731	0.047988108	0.004478903	0.039373969
0.014720677	0.041103749	0.034066511	0.072003626
0.014231679	0.010343419	0.130436271	0.064652953
0.088697464	0.029163071	0.160351857	0.132744315
0.044430683	0.03796179	0.029442434	0.008644961
0.158723608	0.040724997	0.010825268	0.131378397
0.001545484	0.186005804	0.033279443	0.111738335
0.084233926	0.08444563	0.174672141	0.0049062
0.047337595	0.116774448	0.098408662	0.125708645
0.081045955	0.108801662	0.004344692	0.080977227
0.07099441	0.032818889	0.175618017	0.049240348
0.032894059	0.170730702	0.177087319	0.082235972
0.078649382	0.087037944	0.003995918	0.178847066
0.181458011	0.193303241	0.13267276	0.096927927
0.026273538	0.002292175	0.090114681	0.102859387
0.124621109	0.183925238	0.080941007	0.152292402
0.089607317	0.118220139	0.000771692	0.189335223
0.01110977	0.14234743	0.010683308	0.201021926
0.1437	0.01840505	0.118331732	0.050171364
0.078956449	0.030988618	0.019057669	0.141263882
0.139523115	0.110095632	0.125842446	0.102893578
0.003634491	0.00378439	0.179678941	0.096491412
0.057877304	0.08546308	0.031558981	0.016842406
0.015999871	0.118373054	0.15420338	0.059554405
0.055040945	0.017384786	0.157803287	0.029789054
0.212128577	0.159460441	0.14339351	0.015083034
0.118374314	0.187387408	0.16182619	0.053163749
0.044751181	0.159363051	0.074478191	0.00263598
0.166750192	0.013420842	0.00407325	0.137597564
0.052864029	0.087522717	0.000572976	0.002894563
0.02884298	0.195965273	0.137378601	0.071186431
0.014288895	0.079887743	0.000441097	0.179827694
0.140631482	0.044264892	0.058199963	0.135527837
0.187281969	0.143208167	0.195431047	0.212194601
0.15065261	0.148396526	0.009304904	0.164517033
0.141829031	0.173366082	0.102725735	0.119916493
0.150425806	0.165170466	0.113295306	0.01267417
0.035699132	0.013595037	0.05008623	0.010505864
0.034488262	0.089530243	0.141138207	0.175808171
0.1915653	0.015621508	0.008411839	0.117434636
0.116156405	0.15157378	0.002331573	0.00117989
0.002377377	0.0493613	0.056602592	0.116204906
0.1749644	0.00590448	0.008190484	0.002486057

0.01973335	0.218817919	0.148013277	0.138554726
0.154566626	0.033678054	0.027242367	0.123906196
0.078930632	0.03983999	0.196717661	0.122188824
0.015222718	0.114214601	0.034933815	0.169961243
0.074541777	0.128307881	0.108149465	0.040307372
0.001103741	0.072096784	0.08028877	0.164224423
0.140093486	0.030661487	0.026500787	0.0006416
0.031361429	0.154118216	0.03461565	0.194698746
0.127088146	0.168197679	0.029830481	0.139564694
0.011430132	0.129030949	0.18040584	0.011004194
0.04041897	0.006861139	0.133512436	0.051157363
0.019275277	0.06992047	0.049281621	0.027769794
0.06251749	0.047889374	0.197833831	0.20955305
0.019806345	0.028066775	0.107277904	0.100437397
0.030799003	0.16252247	0.131139665	0.065378627
0.017168195	0.009759555	0.09550253	0.063529408
0.010199781	0.204569451	0.087296323	0.18125204
0.088559302	0.131897163	0.153998346	0.111448928
0.032429103	0.061130406	0.082922938	0.002188208
0.093300465	0.171948968	0.041252843	0.088525208
0.133591511	0.147910652	0.001757821	0.000853107
0.14762308	0.037520503	0.016187283	0.167188069
0.056517116	0.070511803	0.000709669	0.076244799
0.162426295	0.113439556	0.009650578	0.116524977
0.064659693	0.168531188	0.165220736	0.178913243
0.039283598	0.136578403	0.100617029	0.076696359
0.094843074	0.076357972	0.15253089	0.001221296
0.013597045	0.073078489	0.034073717	0.078395311
0.057536256	0.030194029	0.107685297	0.14529329
0.081520483	0.131516068	0.006876331	0.009069245
0.120002835	0.034071074	0.116167094	0.027074106
0.022898676	0.142619196	0.002538759	0.034112725
0.065144288	0.176597346	0.189531709	0.08169475
0.077236031	0.031660121	0.099498513	0.022177392
0.034105897	0.009919067	0.167020978	0.001793275
0.003297092	0.187949623	0.038054553	0.008850238
0.003861182	0.057250729	0.018072151	0.047441561
0.039766326	0.172777638	0.036045031	0.122817346
0.05855265	0.02161087	0.057059795	0.002286066
0.00655488	0.100500377	0.154189555	0.158095242
0.083683354	0.177413214	0.090394455	0.107931412
0.003305338	0.072013219	0.010699058	0.124898285
0.077101917			

CLGN 0.04771609 0.036060903 0.043697693

0.077659605	0.072407731	0.021391028	0.089763874
0.031452621	0.04871698	0.021176446	0.038680488
0.053532109	0.026590362	0.026842311	0.007917936
0.126951017	0.126905932	0.048714335	0.17751145
0.096334749	0.041245111	0.137135619	0.109577683
0.055437812	0.104858109	0.15350256	0.054389882
0.085084534	0.175780711	0.013763451	0.058572008
0.222182739	0.054640668	0.02475139	0.068945452
0.022447904	0.031075779	0.10514573	0.012859344
0.043290472	0.055119235	0.088519721	0.123470259

0.099607147	0.019845047	0.112772613	0.099665763
0.014449313	0.103393926	0.012103581	0.021338856
0.016986762	0.026698845	0.042309551	0.073819955
0.010222932	0.014279941	0.011262606	0.025533308
0.128387015	0.013158262	0.021670371	0.034853235
0.018125976	0.067648253	0.173839787	0.071423262
0.035050945	0.031699398	0.183057489	0.034513672
0.007564445	0.113543312	0.140770495	0.015311923
0.311895112	0.068804374	0.210992423	0.081692945
0.076430356	0.03141305	0.069412851	0.055038482
0.077731533	0.188677388	0.056105846	0.123708389
0.041859148	0.073341825	0.062006603	0.134146974
0.063466743	0.029109633	0.087576668	0.207040521
0.030057764	0.035641187	0.024039635	0.040670277
0.010952287	0.19203073	0.02195519	0.048209517
0.029223629	0.052959626	0.220324545	0.054924804
0.289672132	0.010310325	0.137616679	0.081152728
0.075893303	0.074033423	0.209032995	0.060906781
0.110638853	0.046959857	0.029505363	0.153607191
0.080380802	0.124107671	0.018482604	1
0.023445527	0.038017237	0.015227032	0.055168251
0.028886877	0.024854992	0.089372067	0.157232433
0.20231178	0.126616864	0.038253669	0.074555498
0.072307532	0.02322591	0.054448137	0.024856538
0.032753442	0.070335447	0.000564084	0.127996693
0.043215469	0.147003765	0.24603217	0.04091933
0.097664912	0.098934151	0.195653288	0.052800377
0.025709961	0.217549382	0.027051871	0.131599784
0.011412059	0.111850677	0.205951778	0.062277329
0.095249276	0.021257047	0.252347523	0.045178473
0.006153048	0.089703909	0.203105814	0.046594941
0.01531246	0.025576171	0.01571036	0.280677316
0.075653314	0.106879694	0.026448771	0.028013505
0.026111211	0.207445723	0.01404928	0.04422355
0.014992583	0.055643956	0.082200708	0.085971347
0.197012857	0.080957335	0.019128327	0.0207091
0.028984446	0.010333163	0.060806235	0.090428815
0.021382359	0.061436342	0.004362889	0.129788794
0.023910434	0.267176435	0.071939395	0.207165042
0.027392358	0.197218357	0.029438438	0.041376871
0.026840258	0.053023378	0.019551546	0.074630486
0.220326779	0.025756618	0.063805846	0.081316427
0.053657738	0.081720173	0.089125732	0.017800894
0.055886235	0.107126132	0.235373681	0.15028751
0.058920272	0.007725646	0.023893335	0.050554782
0.015477337	0.031605515	0.026202314	0.004328278
0.159456652	0.055750351	0.032256982	0.074863793
0.025350539	0.045891748	0.020946034	0.179546387
0.225368284	0.042929618	0.082298329	0.038310942
0.043035351	0.03039521	0.147657872	0.014735347
0.017574637	0.104365045	0.028241204	0.027385867
0.059967957	0.177834413	0.216484657	0.00983794
0.01937825	0.011981344	0.009097573	0.027648471
0.022853387	0.055154363	0.059328009	0.040644608

0.13006819	0.292850124	0.036297286	0.124831781
0.018315141	0.095852026	0.307608989	0.004052697
0.050668882	0.0079832	0.008284347	0.035560617
0.170475408	0.068737833	0.029882046	0.208300756
0.03337058	0.138066632	0.08000093	0.008559576
0.04207996	0.02812605	0.179248516	0.106593133
0.018729077	0.073186104	0.104209005	0.028583087
0.025902047	0.240645798	0.037445293	0.148256471
0.328514813	0.077562513	0.031676437	0.317889868
0.222454294	0.03522362	0.041066888	0.209323458
0.040925703	0.127484614	0.028849615	0.100214252
0.127393039	0.041372665	0.180436509	0.051624776
0.021420929	0.03827613	0.248256452	0.049046343
0.212203359	0.020398062	0.012318247	0.043165123
0.083286204	0.030497965	0.234107466	0.050240648
0.019988048	0.018111287	0.143506861	0.045156462
0.023518711	0.148159127	0.130129186	0.025558932
0.038937042	0.041915737	0.019793338	0.088065396
0.063203537	0.093973577	0.070955553	0.047048665
0.019431691	0.102118724	0.029787058	0.020045334
0.088203734	0.025831595	0.090614896	0.077382976
0.011953675	0.018755084	0.159534922	0.225664853
0.038044409	0.032363176	0.025278383	0.126460893
0.047899258	0.031082653	0.0250743	0.315579384
0.289668	0.025739699	0.041283981	0.039542482
0.077239335	0.010549905	0.039081033	0.035517435
0.190676234	0.015325028	0.005717994	0.032983974
0.225061432	0.087871727	0.020040143	0.06543384
0.003735607	0.129661429	0.08657728	0.044379124
0.026779483	0.160963248	0.116557191	0.030463287
0.031219161	0.034954157	0.035870699	0.039216269
0.032719968	0.021597583	0.030051659	0.18923962
0.039135002	0.026699423	0.054134535	0.030996911
0.065352163	0.11166002	0.1521998	0.085855929
0.032148056	0.029998017	0.003294022	0.026734812
0.022812884	0.084077392	0.038992837	0.080028055
0.043641309	0.256817464	0.041623604	0.031447237
0.04916194	0.067435417	0.209540436	0.017726285
0.010368096	0.048019926	0.068823789	0.192846224
0.040294602	0.002105409	0.167611393	0.065294617
0.040549229	0.07431376	0.028447182	0.153259641
0.059689314	0.26896971	0.006145114	0.131390378
0.092993976	0.017687872	0.046046639	0.025657461
0.278574733	0.159676589	0.045563564	0.062822841
0.01394589	0.200144998	0.056431211	0.019574296
0.182202618	0.18664476	0.129359723	0.106831131
0.019804146	0.056103848	0.080860944	0.00442481
0.061483074	0.065015455	0.070686839	0.059993553
0.053794446	0.109034279	0.186489205	0.0255905
0.080006821	0.021438904	0.072956696	0.003750155
0.11477869	0.253511693	0.029848232	0.00662348
0.022124813	0.055468884	0.022778649	0.227407623
0.02547776	0.243440192	0.067383393	0.071643547
0.248001768	0.075123113	0.178409391	0.107584674

0.037457748	0.071203236	0.022352639	0.010674135
0.069104682	0.068408381	0.081953676	0.031331526
0.032963984	0.074326827	0.020031036	0.043877056
0.040796645	0.044280463	0.035067007	0.067342775
0.055713004	0.03105046	0.032474424	0.034256691
0.118460326	0.066986669	0.059432541	0.046641741
0.107505847	0.039023029	0.192943702	0.016821714
0.030473472	0.022656116	0.080630103	0.115949237
0.053501179	0.031248107	0.160540318	0.107834503
0.037296906	0.030332682	0.03045997	0.04585594
0.272485715	0.091046031	0.062657699	0.070724854
0.015919127	0.035466814	0.055369625	0.028542128
0.023865087	0.133541436	0.026347142	0.021568307
0.060787204	0.03763843	0.05704582	0.332534254
0.202190319	0.185168894	0.012131628	0.122703901
0.014360313	0.112896134	0.011411468	0.128863834
0.171584716	0.031821074	0.150608815	0.050258753
0.07757125	0.022363558	0.091359835	0.302013881
0.023312806	0.038868668	0.035920559	0.138189851
0.021716901	0.167145614	0.15730307	0.020363148
0.007165576	0.01500182	0.034615991	0.077979153
0.121087003	0.075307956	0.025855847	0.247093434
0.133012674	0.029767294	0.069002669	0.026643427
0.240600162	0.029552779	0.009585885	0.061914496
0.069794709	0.255857647	0.019216038	0.055856438
0.185805476	0.024398682	0.054874094	0.026697318
0.090198142	0.10940017	0.017544339	0.035855657
0.084935108	0.030355858	0.10193922	0.068702551
0.009064378			
CLYBL 0.265199435	0.387046062	0.146549831	
0.33741288	0.196845404	0.198581049	0.124912681
0.062265021	0.009895993	0.00230812	0.003819722
0.24549462	0.015154868	0.08468195	0.048563885
0.328891108	0.056805385	0.107510976	0.166817925
0.293716235	0.050193537	0.305020021	0.046127602
0.054111843	0.045195722	0.044613429	0.072132394
0.387850864	0.052263735	0.141276555	0.286454432
0.039786568	0.314028651	0.354599251	0.282046443
0.343714432	0.230708794	0.002750239	0.070603443
0.322752285	0.289285884	0.296006566	0.235198796
0.025726903	0.33863001	0.162012752	0.208895324
0.011444756	0.02166811	0.079538058	0.31338652
0.323410488	0.190984343	0.086784979	0.08092156
0.010075352	0.06899752	0.034887482	0.219301159
0.138034596	0.063288118	0.327290068	0.014947959
0.006837812	0.109300061	0.053107522	0.131002211
0.065878579	0.267600877	0.008188162	0.189277712
0.001111186	0.292162296	0.213212955	0.053137434
0.045721324	0.010304646	0.076818843	0.222065605
0.387975166	0.322440183	0.387623039	0.36697339
0.038369125	0.140638793	0.145112107	0.079888249
0.321032378	0.104919812	0.016277717	0.335633658
0.386395106	0.366297117	0.045928716	0.14211985
0.300681342	0.369293891	0.148270134	0.150760298

0.058673673	0.01695224	0.301890386	0.386753828
0.288039542	0.098201321	0.095054388	0.330506212
0.046154024	0.058584659	0.264883183	0.013646284
0.400322755	0.011781475	0.113222571	0.193787002
0.037811065	0.318613832	0.358139678	0.128573023
0.186384866	0.004681239	0.006509154	0.023445527
1	0.021370097	0.307431327	0.384014167
0.050551984	0.046537928	0.119709858	0.004781649
0.021956559	0.019403566	0.306573466	0.157018101
0.297798702	0.238601492	0.080474025	0.247900385
0.229666743	0.375931945	0.009360616	0.049232235
0.212637373	0.03047261	0.178270247	0.229312902
0.149073233	0.060562332	0.081351785	0.401582936
0.321820667	0.072346546	0.101247943	0.3280452
0.052697639	0.189930702	0.067223858	0.107745196
0.02824413	0.317753484	0.024882877	0.141679318
0.01889819	0.022121514	0.003124772	0.139869518
0.045496548	0.310283691	0.291042555	0.076655354
0.146601588	0.260675324	0.137988786	0.283915977
0.002146603	0.014098587	0.102601932	0.394793662
0.17380751	0.377936148	0.091787985	0.081482035
0.173286744	0.345390461	0.000397882	0.342376522
0.36078419	0.117923011	0.239822256	0.236234703
0.096808589	0.369676118	0.007601614	0.325200975
0.22445813	0.056765173	0.403938319	0.106779757
0.331989122	0.03751769	0.102505112	0.282903367
0.266216869	0.020359208	0.105478833	0.283724372
0.015773387	0.032724196	0.285032176	0.27785701
0.192509016	0.31875075	0.406416693	0.085250163
0.198277744	0.124304416	0.004280409	0.161931237
0.295141703	0.018097742	0.320976972	0.077979101
0.168747098	0.360727574	0.280028765	0.005883032
0.039510122	0.145374752	0.005922366	0.145446232
0.248833081	0.044218144	0.113861199	0.027089523
0.046913317	0.39489951	0.048970993	0.059640476
0.025371485	0.214009613	0.145799673	0.066831037
0.037372061	0.049517935	0.076591023	0.088681178
0.026115257	0.063463246	0.002581827	0.082483735
0.025109341	0.09588423	0.09826783	0.245959136
0.061819996	0.022194233	0.182237405	0.040872255
0.044576709	0.023331068	0.376350296	0.298364289
0.224020698	0.061764736	0.035524155	0.029405636
0.37275764	0.136033513	0.076337916	0.355863435
0.005261618	0.372136713	0.054117458	0.082816677
0.119100569	0.173958937	0.385507399	0.017351056
0.195713351	0.354248809	0.169214177	0.07619312
0.305967282	0.265179762	0.014325519	0.273227676
0.270921244	0.035833044	0.387207352	0.044292054
0.026266696	0.284095952	0.263017145	0.063522646
0.048580125	0.141964292	0.023355353	0.201144175
0.393072758	0.327762023	0.274977511	0.058434913
0.035269881	0.003514646	0.081182192	0.167682682
0.348282597	0.36948331	0.172079304	0.157804396
0.179385023	0.06247331	0.010438959	0.385371765

0.019797565	0.368031282	0.012034141	0.278444382
0.333715249	0.136724912	0.046884525	0.154931759
0.303526349	0.034058009	0.083514722	0.357315106
0.311998672	0.088418641	0.077378368	0.279064083
0.013199391	0.013456459	0.183944706	0.032880626
0.08762419	0.073008294	0.005280674	0.069952419
0.0189194	0.339222297	0.371919281	0.018571943
0.153978551	0.068283511	0.248959906	0.035107121
0.30484757	0.340832772	0.36691565	0.070550825
0.304343357	0.347066013	0.164337409	0.05148447
0.021530499	0.351241538	0.245040396	0.004279128
0.394387573	0.106589704	0.005266051	0.377573022
0.123657969	0.290875664	0.001618644	0.003282065
0.01615385	0.357217062	0.32633246	0.267987285
0.056522202	0.235444172	0.000559056	0.381453135
0.367136921	0.013655649	0.031261381	0.290468146
0.25808564	0.392605585	0.090655991	0.320573115
0.218985813	0.334741702	0.025503513	0.170677733
0.348024275	0.324753432	0.311063183	0.347368068
0.381207534	0.288754218	0.201562531	0.013769376
0.00634966	0.096400642	0.049212348	0.005275341
0.191005072	0.04427363	0.371681767	0.37043902
0.362685249	0.011096539	0.046574386	0.298524667
0.369289987	0.393226982	0.003100316	0.001393621
0.002272417	0.006195981	0.203223959	0.120541864
0.357798645	0.025786087	0.010139697	0.020055687
0.044663602	0.213956613	0.36455237	0.313872166
0.295232442	0.050663457	0.055856992	0.319979471
0.017153562	0.192934519	0.372618945	0.349347281
0.018651287	0.100544095	0.152367171	0.395378515
0.007959223	0.202948422	0.259755196	0.063299647
0.002649379	0.040460063	0.027495143	0.336823858
0.325651652	0.027518435	0.017493823	0.009209618
0.05957811	0.353934727	0.055882829	0.405878957
0.347943513	0.384200905	0.01965465	0.343202512
0.00510848	0.342262831	0.138647907	0.008990927
0.096843109	0.011179527	0.331101413	0.009116996
0.103198522	0.275827093	0.011114327	0.013256575
0.018176237	0.041799777	0.383397102	0.351049849
0.013789883	0.073548744	0.161849098	0.266366563
0.166005842	0.345413424	0.351120773	0.146328324
0.039760464	0.008432058	0.328015178	0.252897449
0.074552577	0.391878427	0.054942692	0.389506325
0.266394303	0.336716915	0.349764106	0.188938923
0.02290608	0.215300298	0.25323036	0.006406708
0.219071234	0.368698743	0.171206111	0.029317869
0.12796839	0.138837466	0.006412104	0.013906667
0.100068366	0.178570165	0.035950629	0.373391853
0.244656359	0.262439925	0.007456224	0.025708308
0.397288877	0.316950776	0.026455543	0.282262393
0.097357134	0.170859317	0.319724254	0.315570317
0.046564924	0.328077799	0.344436059	0.265133362
0.327888049	0.147781211	0.364697738	0.022148982
0.008200312	0.211765366	0.03906426	0.064863435

0.101230049	0.01391594	0.042044945	0.322715653
0.07630691	0.271408674	0.039016382	0.008277541
0.169455783	0.120429473	0.21739851	0.099179926
0.027126045	0.346310525	0.001978713	0.042119938
0.097055707	0.293561062	0.147471441	0.100902178
0.042647372	0.029256686	0.181936345	0.149834929
0.100253922	0.019923043	0.298836795	0.00675277
0.015559414	0.405419495	0.151351855	0.006559774
0.005539735	0.187003751	0.072675535	0.038935096
0.016222407	0.328858745	0.102657963	0.187039809
0.188521055	0.043333099	0.262309255	0.009611707
0.014279025	0.33364631	0.167375705	0.354438976
0.126295037	0.347787729	0.300595339	0.307765292
0.011671166	0.13810557	0.018768286	0.359832275
0.102083846			
CMAH	0.147869163	0.160201341	0.138895619
0.118065355	0.128876523	0.043420484	0.024918753
0.006872623	0.012629858	0.000807304	0.017279666
0.18158501	0.026993066	0.015980286	0.016644847
0.183493994	0.073213377	0.05088869	0.060120832
0.111188496	0.003455956	0.183847435	0.012888889
0.111985437	0.097774184	0.034285491	0.124086704
0.143248783	0.041731454	0.022449521	0.069833023
0.071128556	0.136685965	0.15084677	0.201121818
0.161306497	0.120372678	0.010338291	0.01412873
0.117039417	0.175550419	0.104040802	0.086165018
0.00762711	0.113099157	0.045889095	0.220584986
0.007571021	0.021085209	0.007722209	0.095432848
0.125534374	0.097747255	0.011777164	0.015772404
0.023038575	0.007616155	0.091141666	0.03591999
0.040712104	0.003508565	0.141504217	0.005243129
0.007018112	0.019887187	0.100527008	0.024503403
0.124105017	0.068582441	0.003822334	0.170521405
0.000856764	0.106200689	0.215264945	0.112574196
0.049667547	0.008717212	0.122916937	0.060197824
0.158561446	0.151709044	0.229146278	0.229044535
0.046110263	0.137662441	0.215425891	0.040038261
0.131819789	0.021269574	0.015516522	0.219018193
0.22711418	0.169642956	0.051422339	0.197183268
0.103246782	0.127693709	0.071357554	0.091977171
0.055014669	0.07956277	0.110456549	0.204813292
0.089170321	0.273080934	0.117551451	0.091109906
0.038553161	0.01780768	0.10773803	0.043360601
0.163206793	0.003238639	0.187852317	0.053662316
0.035022155	0.13445261	0.188233747	0.037641665
0.153885629	0.012776585	0.067754967	0.038017237
0.021370097	1	0.116586307	0.145043797
0.008034774	0.038549768	0.031155221	0.009730729
0.041905119	0.065386825	0.154665313	0.075490627
0.138996867	0.145585768	0.050355016	0.07495827
0.169019756	0.229012929	0.000743121	0.012610909
0.08546564	0.063518682	0.101188722	0.117119798
0.142182264	0.009820523	0.033240295	0.194887413
0.086104192	0.02738076	0.029203547	0.192530927

0.016821187	0.244023331	0.02671485	0.035285474
0.0048553	0.161059014	0.07991769	0.035688206
0.027390822	0.076863292	0.00708904	0.018265775
0.012117221	0.125896977	0.124463918	0.043735937
0.051412908	0.178106418	0.027075625	0.104908101
0.004121922	0.018180193	0.016638173	0.180761012
0.023423053	0.224993823	0.04363768	0.093282865
0.163515625	0.176365383	0.007540715	0.127694702
0.180571311	0.017968308	0.114125865	0.186478285
0.106341639	0.145865972	0.004519522	0.174739496
0.064602025	0.032197769	0.218164151	0.098155056
0.138148697	0.032646715	0.021109913	0.234051601
0.170227221	0.004113211	0.009747908	0.079216115
0.067967278	0.001992185	0.12664972	0.10942672
0.087091971	0.203121588	0.208828831	0.106470656
0.118786183	0.029058212	0.015055483	0.226829154
0.187501926	0.027284633	0.097480574	0.017159087
0.039355785	0.129409857	0.146735534	0.007187907
0.013392116	0.03039472	0.002494811	0.10263102
0.126202267	0.009146551	0.08684153	0.05565236
0.077585203	0.186523306	0.250468038	0.25381737
0.235759529	0.071041971	0.047628669	0.016361847
0.031637533	0.039380714	0.083071489	0.053282952
0.001901902	0.040789528	0.048904921	0.059169448
0.018741574	0.040606167	0.032742663	0.084180171
0.018455659	0.017675742	0.109285089	0.09143892
0.076247996	0.030356592	0.15763052	0.15435741
0.046682406	0.019632679	0.043479307	0.019285873
0.149408571	0.023812171	0.010452712	0.118137526
0.01258582	0.213850869	0.022666988	0.087371531
0.114447981	0.069666813	0.162812233	0.003037156
0.06423954	0.11476089	0.141577368	0.072086066
0.08196489	0.101067431	0.004625447	0.0988277
0.091859517	0.028930703	0.192029049	0.080470537
0.043661151	0.236985025	0.16155622	0.07428374
0.071892943	0.047249895	0.000990278	0.179526709
0.173682238	0.193709738	0.112424629	0.184141916
0.012682387	0.00744159	0.191364567	0.173820972
0.136164164	0.212999671	0.116264673	0.140687317
0.075743986	0.059863604	0.002289105	0.193809148
0.064621089	0.163528745	0.013400316	0.22782743
0.151366858	0.018194509	0.075464845	0.030329678
0.077077888	0.013490321	0.031104269	0.143342523
0.123176612	0.059647075	0.051299292	0.166337316
0.001239362	0.002848224	0.158954413	0.19149252
0.039424854	0.04878611	0.19601716	0.016091571
0.009853787	0.11011042	0.205321225	0.018250032
0.032580144	0.015640981	0.228045147	0.019459116
0.232733455	0.195154311	0.149265541	0.019363846
0.169973981	0.182160012	0.197173414	0.082187326
0.057796242	0.175279691	0.080213609	0.005038781
0.185383692	0.00991368	0.004628481	0.136226714
0.042307841	0.102745398	0.000187358	0.057800943
0.070819677	0.249969558	0.123773011	0.071050834

0.007247164	0.107501737	0.021877173	0.215457521
0.137745493	0.045451147	0.096663558	0.155141114
0.21933297	0.152844682	0.190494	0.212900245
0.188754538	0.153790962	0.011737148	0.13760314
0.124361335	0.199583203	0.10520694	0.11759388
0.160097496	0.228299218	0.076321049	0.003675627
0.039337391	0.009091844	0.018521123	0.012798075
0.033478508	0.055838598	0.165553688	0.20310516
0.212855603	0.019868178	0.005063987	0.13324592
0.111485708	0.155127056	0.0231359	0.006098448
0.006842	0.040848239	0.055415977	0.159178083
0.176119155	0.004618861	0.007432724	0.001618991
0.020606986	0.267013408	0.151023689	0.140809603
0.212426071	0.045168425	0.013689166	0.137943856
0.045350978	0.051453016	0.199802164	0.111702332
0.039395363	0.13258452	0.064617498	0.165469439
0.174734232	0.17604081	0.176250706	0.021162863
0.023002104	0.075850498	0.044979688	0.208459366
0.156850264	0.022839826	0.021306776	0.003300103
0.014432973	0.190133468	0.024448578	0.207529828
0.124292163	0.178174945	0.072398546	0.127875584
0.013407678	0.137145958	0.240957658	0.001891766
0.087165305	0.014230385	0.179671793	0.026260666
0.020041135	0.087139476	0.037586957	0.052239922
0.119282911	0.052122057	0.192190761	0.245732999
0.064258134	0.022183377	0.146398962	0.142672855
0.022734109	0.144823555	0.131814703	0.057513548
0.00646006	0.138984699	0.104507322	0.082912658
0.011260612	0.233613491	0.039047714	0.202065746
0.098863636	0.147473133	0.174290669	0.06340662
0.088714184	0.063630052	0.056439689	0.002195685
0.093688289	0.178310665	0.054247891	0.05051411
0.099706334	0.227483386	0.011057565	0.012393336
0.258130527	0.025866789	0.015071756	0.189193706
0.080680008	0.086566731	0.005943804	0.036883532
0.174125565	0.111758289	0.005549046	0.098521868
0.097134698	0.111882186	0.210274714	0.244906303
0.039158618	0.146763513	0.111819028	0.064563937
0.088081086	0.052964934	0.153194752	0.001411464
0.04219552	0.128527859	0.015332792	0.108665259
0.046991836	0.029555091	0.130600275	0.195270892
0.116358132	0.131269061	0.010977981	0.026539993
0.188884145	0.064447944	0.141783262	0.014918228
0.014010457	0.134240443	0.009497088	0.052809257
0.125021678	0.15246942	0.185320457	0.093946278
0.091977458	0.090824287	0.100980301	0.019262781
0.024164542	0.038179976	0.143905131	0.004655326
0.002623016	0.215055184	0.046530086	0.046335631
0.040896182	0.071380916	0.045030095	0.018123188
0.078511958	0.192003014	0.020571131	0.151857244
0.049631956	0.031412378	0.066589125	0.011739407
0.015190683	0.111000416	0.205024688	0.174836137
0.168812846	0.167160483	0.0971699	0.136574329
0.031873693	0.126511831	0.004380927	0.145401657

0.066745896
CNR2 0.463076906 0.504771931 0.398629371
0.409593953 0.396296179 0.44061814 0.177670409
0.185452421 0.060257338 0.002070932 0.013034992
0.438868272 0.174397746 0.262250983 0.372744519
0.379766261 0.088397599 0.343594397 0.186394107
0.39163698 0.079930487 0.349970152 0.059782781
0.240361734 0.066774886 0.039948863 0.221761889
0.432458699 0.046725212 0.401076912 0.408138633
0.059168247 0.468725377 0.506412915 0.416528535
0.504705682 0.470463118 0.006646966 0.317875545
0.472181649 0.447824138 0.395827692 0.281617694
0.023704174 0.496503942 0.204588809 0.360254968
0.249920999 0.015157193 0.29387088 0.490135932
0.504850665 0.470572809 0.220302405 0.128302096
0.200248764 0.272751246 0.268116751 0.399148304
0.180639938 0.194968281 0.509693004 0.069138634
0.142478162 0.231934262 0.098741337 0.23912059
0.325824225 0.45830567 0.008868681 0.449593372
0.00181637 0.359751266 0.322505403 0.320225018
0.020231296 0.010232962 0.118576213 0.317083239
0.451721399 0.49111956 0.463885782 0.479898268
0.131733387 0.225412371 0.37904719 0.123108466
0.484311593 0.147998833 0.082025402 0.376190313
0.471947389 0.509727544 0.142660204 0.194776375
0.478668 0.497717336 0.448103662 0.412846253
0.354445101 0.026825086 0.50600765 0.493928852
0.477757202 0.288041768 0.099908662 0.441173613
0.031126331 0.400310908 0.307158307 0.12432067
0.454525395 0.009216756 0.149236297 0.348276256
0.161576581 0.476619928 0.506455699 0.142672426
0.371408079 0.009302056 0.113247098 0.015227032
0.307431327 0.116586307 1 0.466915327
0.228460828 0.34788515 0.227628791 0.002818343
0.011549496 0.133266789 0.490181831 0.348134958
0.438744627 0.476151949 0.191242428 0.479365111
0.473135335 0.46291181 0.086135787 0.064393844
0.421809961 0.129740477 0.176745854 0.460825171
0.251504331 0.086048755 0.084588527 0.4886161
0.482177183 0.051834984 0.371562206 0.370711818
0.341070201 0.300847582 0.068380091 0.238232916
0.031231995 0.502742296 0.012174113 0.343349138
0.287624407 0.10398462 0.004656072 0.268513328
0.291050124 0.496939599 0.488078056 0.065465486
0.282027404 0.363886472 0.372715691 0.471388131
0.00545785 0.024876044 0.359115452 0.496665255
0.382693512 0.484345191 0.24617142 0.245577343
0.215686056 0.439475035 0.002108681 0.50359176
0.507758297 0.388932852 0.376392904 0.39089506
0.391430444 0.455418279 0.049251304 0.377169429
0.450559984 0.045748838 0.46782313 0.107879599
0.503385902 0.02084275 0.331761793 0.470759622
0.489039162 0.00464805 0.263238764 0.385698629
0.011503998 0.092100238 0.445829512 0.391017147

0.369241201	0.439251687	0.44651451	0.374461519
0.399735693	0.195110641	0.004423135	0.255736326
0.462506582	0.422909432	0.494413994	0.206881324
0.437051977	0.502244556	0.497040345	0.248048339
0.023821634	0.309522975	0.013983678	0.289283591
0.486422516	0.093538902	0.42845302	0.099506286
0.038535183	0.49962411	0.132456018	0.23931175
0.098561606	0.4282584	0.203782113	0.238486628
0.195797729	0.112114551	0.330606666	0.367816418
0.025419944	0.057424456	0.005595336	0.421852463
0.124405017	0.364403945	0.385315777	0.483547911
0.319895782	0.081323307	0.347143889	0.249415562
0.070797424	0.005311357	0.504319442	0.367491804
0.44220438	0.112655276	0.019430813	0.399051029
0.477639275	0.423614117	0.357509759	0.490521961
0.014528205	0.469784372	0.217866666	0.077557522
0.358074068	0.209726819	0.444520925	0.058591551
0.425724817	0.501029646	0.250457746	0.135199987
0.486502493	0.406791042	0.016616703	0.484793347
0.484025428	0.014518799	0.504530108	0.079474824
0.008171156	0.426830041	0.479245402	0.041392623
0.043445184	0.328026701	0.036964471	0.224060116
0.500521936	0.366592409	0.489817776	0.164804902
0.021977157	0.005011471	0.146760137	0.401970827
0.50297413	0.500074446	0.19566569	0.335274245
0.184089364	0.31607451	0.173398092	0.497337079
0.15637017	0.504526488	0.001092298	0.460825415
0.504548735	0.352205811	0.047682331	0.314348401
0.481354631	0.02534881	0.159109008	0.507828544
0.4861882	0.300151699	0.346934826	0.415749517
0.009008944	0.0269371	0.364120559	0.146862695
0.392885332	0.161046887	0.037467028	0.345908418
0.024295794	0.50013397	0.441253159	0.035786827
0.420679304	0.341993272	0.321890464	0.018297866
0.481479859	0.50021089	0.507561603	0.125084871
0.477252895	0.503545512	0.436452105	0.043301009
0.006332765	0.505909474	0.449093275	0.013677969
0.45659821	0.318846434	0.006407763	0.500826304
0.137379627	0.483220486	0.002795957	0.078003937
0.019027996	0.44060281	0.504369036	0.398809731
0.340434431	0.332109044	0.002448872	0.497497648
0.506703616	0.004987808	0.092434848	0.495834248
0.470386757	0.504283092	0.298032692	0.481078364
0.447066023	0.508412002	0.217460849	0.206323655
0.481169279	0.500765456	0.450105989	0.494655177
0.46337877	0.390009515	0.23040331	0.007199781
0.109644505	0.231939636	0.380178361	0.06382732
0.40477568	0.096500553	0.496672641	0.455024336
0.490154417	6.36E-05	0.135570287	0.497272354
0.468080881	0.463979007	0.009822237	0.003786414
0.005611914	0.009526596	0.359230274	0.178460492
0.49813365	0.324005891	0.000300252	0.032526674
0.24560733	0.372147488	0.509674279	0.334879938
0.460929936	0.045935233	0.317845634	0.366212754

0.039828029	0.441193719	0.488721538	0.501597975
0.019672689	0.1861684	0.386576399	0.472314897
0.060282288	0.250526099	0.447482474	0.297125187
0.005778543	0.045973809	0.036887757	0.415481111
0.502884251	0.052460045	0.017502625	0.182696967
0.09100885	0.471290243	0.122888958	0.482713745
0.465873301	0.411083001	0.041566613	0.504019578
0.022089561	0.5070185	0.296494064	0.065747005
0.231737974	0.004247069	0.498933041	0.154835845
0.356402588	0.428781645	0.176239615	0.006064986
0.161501386	0.026837551	0.462380003	0.45328296
0.014934242	0.199301638	0.234996452	0.381700278
0.31235592	0.426542133	0.50600964	0.488355599
0.060902106	0.11450603	0.408175744	0.462399295
0.23831996	0.463397319	0.278987748	0.499362136
0.46422716	0.488354398	0.501176742	0.331825228
0.186658871	0.44665119	0.41769346	0.012484129
0.312316599	0.456907599	0.359267586	0.116142418
0.204178838	0.397216312	0.028190001	0.207644923
0.33777211	0.373199133	0.10427117	0.410020705
0.420568258	0.469184333	0.026090338	0.034874223
0.505978882	0.491753605	0.130645761	0.449048433
0.113921849	0.302007406	0.4619015	0.447473454
0.387834847	0.494041185	0.456365538	0.444803376
0.488812365	0.213953946	0.508530251	0.094721492
0.055249204	0.464224915	0.068812848	0.052165856
0.096989854	0.011651584	0.307497187	0.391057321
0.401938454	0.341957563	0.368643803	0.050439769
0.260834791	0.405956191	0.299720932	0.1848775
0.050892496	0.508747572	0.003673219	0.028615575
0.394861643	0.481124796	0.372849869	0.178055146
0.27343659	0.090386839	0.241631531	0.351280429
0.388745701	0.372974253	0.487828489	0.021626132
0.009941418	0.464725851	0.418501967	0.008353007
0.05718017	0.453760252	0.255768698	0.20846671
0.008617547	0.503109809	0.407734409	0.410842821
0.337663046	0.036152189	0.46089928	0.068099238
0.035514802	0.496415071	0.388321437	0.508026304
0.310930298	0.388540761	0.485523332	0.493265873
0.09192784	0.42533784	0.014381917	0.457724498
0.459551068			
COBLL1 0.387083932	0.525801527	0.372915093	
0.459123462	0.44764928	0.392836255	0.301656325
0.241094078	0.132542729	0.020746337	0.051294295
0.42530866	0.288933523	0.364983935	0.343201235
0.443022 0.199196607	0.34326701	0.327027228	
0.439936119	0.167079092	0.447502658	0.161300496
0.292012959	0.152206441	0.124523567	0.299535241
0.455708053	0.13720683	0.369858432	0.415699269
0.13420502	0.422715119	0.508238357	0.45771467
0.497322798	0.358160439	0.018960457	0.358577791
0.394732055	0.439622951	0.439843611	0.411716305
0.091976228	0.479811177	0.339773964	0.402397433
0.26240393	0.068564589	0.281211884	0.437017991

0.474712345	0.435536215	0.290096743	0.249984175
0.271340071	0.28170913	0.284587807	0.343349921
0.321960048	0.215309333	0.479168418	0.150877966
0.183705727	0.306939814	0.190964978	0.336745469
0.33040479	0.381075991	0.038266039	0.360990136
0.010843153	0.425272353	0.39166132	0.291148615
0.079139067	0.060452439	0.222300677	0.416133948
0.452774438	0.431683458	0.474755804	0.474622799
0.258986752	0.331733891	0.372624781	0.262654623
0.411323852	0.278260342	0.161139144	0.448506374
0.483643363	0.516190306	0.261303247	0.302574157
0.433340752	0.47827912	0.429809392	0.454479539
0.352415013	0.067030503	0.43979705	0.50603283
0.376019048	0.30773136	0.214852929	0.42861317
0.101611873	0.365801039	0.426495145	0.198045089
0.463681961	0.044170087	0.260803831	0.429453863
0.249087884	0.428181508	0.503788352	0.279237758
0.41357793	0.047902727	0.15657762	0.055168251
0.384014167	0.145043797	0.466915327	1
0.302730069	0.40841631	0.342387465	0.006487022
0.051768638	0.201085568	0.413927941	0.375324717
0.427902166	0.337495345	0.312473196	0.376184609
0.418266669	0.464873133	0.076578748	0.160231868
0.45060486	0.204788573	0.318108037	0.448773719
0.369956817	0.183386842	0.206417795	0.51486763
0.428502503	0.153969586	0.332668333	0.44758707
0.393152253	0.387166515	0.175526539	0.376150775
0.10822882	0.462834549	0.047787282	0.353878453
0.276304146	0.195828564	0.018889374	0.321509967
0.367255431	0.428354316	0.443678128	0.16502171
0.409439171	0.444270985	0.340731429	0.448591444
0.032214999	0.080306262	0.27967623	0.510830285
0.311207757	0.479701044	0.367163584	0.331172974
0.342454035	0.449556362	0.003459624	0.489540973
0.513378138	0.301376849	0.46204404	0.416757501
0.360309828	0.462264199	0.042684162	0.453164011
0.342454512	0.128462067	0.496682488	0.234530024
0.446989874	0.08338459	0.376064101	0.402901317
0.408119685	0.017541242	0.286232362	0.43177826
0.041594141	0.164124845	0.418493071	0.449073826
0.427308384	0.434499296	0.473260149	0.344457703
0.459373078	0.307297408	0.024164272	0.347097629
0.411821539	0.355805338	0.447463431	0.322538178
0.326748656	0.478355235	0.384552768	0.222972826
0.092211516	0.372874326	0.049968117	0.384851861
0.349614021	0.217254229	0.37155681	0.170200634
0.112532455	0.526046866	0.18667653	0.243209775
0.126838759	0.451277598	0.324948874	0.34340315
0.300758211	0.231154497	0.364614606	0.402171415
0.087496349	0.165764306	0.017367552	0.384725961
0.236055947	0.406091772	0.397075916	0.398734479
0.39257097	0.202058033	0.445435576	0.279491817
0.156989478	0.033531522	0.507420301	0.436560785
0.332920835	0.231583154	0.071650696	0.362586029

0.472809506	0.342925353	0.268276505	0.468345121
0.047019945	0.464839979	0.349209973	0.188711662
0.413603656	0.360262682	0.455317831	0.087013179
0.391809745	0.478934921	0.347886468	0.254813116
0.405505627	0.449145912	0.07200069	0.383513065
0.389770724	0.068143533	0.519367038	0.173458516
0.040718134	0.431816429	0.430218284	0.121104598
0.118835763	0.416499585	0.094752137	0.353385373
0.5225128	0.455163157	0.431464502	0.223570754
0.092093896	0.035016948	0.235567963	0.369297856
0.500371729	0.49698627	0.325929054	0.418318976
0.325193367	0.356855423	0.176476862	0.508232243
0.180026713	0.503521784	0.01655535	0.417611759
0.494682449	0.325547504	0.129793217	0.379356211
0.392690403	0.092162125	0.256023672	0.497419491
0.421585964	0.369843906	0.379795719	0.428781111
0.045413106	0.08999873	0.398944135	0.180625633
0.415189553	0.279709518	0.050280416	0.354985902
0.098192272	0.466087519	0.452464681	0.101795453
0.376218417	0.323759408	0.410610793	0.075955932
0.418801396	0.475806599	0.516867512	0.229118367
0.42373578	0.481503138	0.382642179	0.115034232
0.032717548	0.500701372	0.394683467	0.053870807
0.462478345	0.267721399	0.044381208	0.503174316
0.265860872	0.428649575	0.009081128	0.064440848
0.05353246	0.443039308	0.475394717	0.411886305
0.290353825	0.400956074	0.005589666	0.505722766
0.499948568	0.028097236	0.171060582	0.424183135
0.405240351	0.526881868	0.32263906	0.429021345
0.422423506	0.490808813	0.338338563	0.337950398
0.446277244	0.468824917	0.429721406	0.457030426
0.465141244	0.43588631	0.36726649	0.04280439
0.144296371	0.274936082	0.333783457	0.128505399
0.334944187	0.199865323	0.469330494	0.455569566
0.470636165	0.012422121	0.22754164	0.419053395
0.445182243	0.471125145	0.026934043	0.008294974
0.008677387	0.03737842	0.406658175	0.284399661
0.479350738	0.293728028	0.014498812	0.090703653
0.348254373	0.405106327	0.505419022	0.434413691
0.414261565	0.129870848	0.361405467	0.436183289
0.105237194	0.305207223	0.48349945	0.484191932
0.059751954	0.286842093	0.371241307	0.492089105
0.066500415	0.37389005	0.430426751	0.404431903
0.018014802	0.119836556	0.10228316	0.442119511
0.482621134	0.150784473	0.077191667	0.173556162
0.21618081	0.441369408	0.262315913	0.510598388
0.449125099	0.46505325	0.100174712	0.481450457
0.07677243	0.485680197	0.362549433	0.083759904
0.312313577	0.025351646	0.447429594	0.187848742
0.376999541	0.403163795	0.204791083	0.027921917
0.164365214	0.092929545	0.474227993	0.441829004
0.045029497	0.302282253	0.34386585	0.419038965
0.347707637	0.47159691	0.500751764	0.395843202
0.158563945	0.12733535	0.437010858	0.38982543

0.306503141	0.478245811	0.358603139	0.513453055
0.398096658	0.442781812	0.459705571	0.409279681
0.263006658	0.404549637	0.422355792	0.057675718
0.410334105	0.468120255	0.37501592	0.215717352
0.339714259	0.358913927	0.072561569	0.176211052
0.302199925	0.350929425	0.235712772	0.452747496
0.39349178	0.392872729	0.067991378	0.100125747
0.536825914	0.42621094	0.206838496	0.450631943
0.2246711	0.401230359	0.431041823	0.430901203
0.34686349	0.439709733	0.439603883	0.428214864
0.441393592	0.339072063	0.519361738	0.127132214
0.115188367	0.387321615	0.18271662	0.132796023
0.229118912	0.047417766	0.274932889	0.445026986
0.345895487	0.451224437	0.328343057	0.123444585
0.360424118	0.334265906	0.409872672	0.299389493
0.152087348	0.500896062	0.019855281	0.094366035
0.369813344	0.429721389	0.398683533	0.30029307
0.299517214	0.160059413	0.382167412	0.339162432
0.381598771	0.346190477	0.427079608	0.064210966
0.0503252	0.484868656	0.376008673	0.028918237
0.101495845	0.356222759	0.313879556	0.313118959
0.033865327	0.466082759	0.370744622	0.427267386
0.401560736	0.108623564	0.343390686	0.115421887
0.089355636	0.446579027	0.37792457	0.500592272
0.340840283	0.465101302	0.433636959	0.425226642
0.138289137	0.332932627	0.064630148	0.440033734
0.390121161			

COMMD6 0.211920103 0.290399258 0.088467919

0.29214202	0.153641015	0.185122522	0.17524632
0.124354561	0.019551703	0.016899069	0.006743099
0.153254948	0.035608848	0.121342392	0.040544525
0.264260742	0.039998983	0.113094173	0.191227955
0.299888876	0.10803653	0.234565504	0.064337151
0.030414934	0.023393972	0.04248063	0.037505732
0.342455958	0.051083 0.165301149	0.339334109	
0.035103461	0.2889035	0.256844193	0.178479067
0.237113554	0.178140883	0.035469384	0.078323899
0.285645466	0.189948352	0.306465788	0.221232544
0.040272631	0.273764934	0.183466409	0.138229541
0.055047527	0.015999061	0.133412593	0.275816179
0.225232971	0.129831747	0.143613017	0.112632749
0.02160163	0.158052199	0.014261275	0.298777495
0.175583894	0.148147139	0.232108722	0.032658149
0.032420643	0.186759095	0.039640762	0.214305394
0.037043034	0.273928935	0.042104665	0.115727147
0.012426242	0.293963771	0.154932301	0.024311188
0.046938854	0.021486204	0.058416397	0.251577968
0.328153052	0.243469994	0.261311387	0.238040507
0.040240272	0.123228014	0.083797724	0.083510079
0.264880297	0.129481522	0.045616077	0.248795294
0.259092585	0.256546209	0.031409399	0.097771767
0.231319131	0.299858644	0.107414204	0.11084871
0.03067776	0.010279052	0.237640852	0.267811213
0.283345073	0.046129318	0.066953427	0.35860415

0.057458939	0.046876984	0.253750076	0.05063669
0.330266802	0.018667369	0.0710405	0.231042578
0.052597844	0.247812253	0.233265354	0.166225891
0.129206165	0.022216052	0.007266055	0.028886877
0.050551984	0.008034774	0.228460828	0.302730069
1	0.038889943	0.165310272	0.005437877
0.018827611	0.033903913	0.231919325	0.166191453
0.257651208	0.166379013	0.057310043	0.253360195
0.131321446	0.25684424	0.011310262	0.08237604
0.160741003	0.03570973	0.180058133	0.160758019
0.088801841	0.113168306	0.107131669	0.282148484
0.307700229	0.102789427	0.127725752	0.252704404
0.04486559	0.106220397	0.107298995	0.129978305
0.06655173	0.20616847	0.016935173	0.193640991
0.012262339	0.025187765	0.025157092	0.228500448
0.051001329	0.216466741	0.215938346	0.102587714
0.167963387	0.184681815	0.205461934	0.19737487
0.007301266	0.03697318	0.152494704	0.283975967
0.263085921	0.260994128	0.105818079	0.060050196
0.12578469	0.269787965	0.000861986	0.255481134
0.243182335	0.133973293	0.175048658	0.173102793
0.052992273	0.285867297	0.010019457	0.273142238
0.240374395	0.080869384	0.289070816	0.083176567
0.246127464	0.038649077	0.165087952	0.164217654
0.15684229	0.010555262	0.174538315	0.302356397
0.011463032	0.080579158	0.255145585	0.247360451
0.152284118	0.231282695	0.304195713	0.041548641
0.144793515	0.177552104	0.014924497	0.099977947
0.215571794	0.025722684	0.278898256	0.093987271
0.187684553	0.270827179	0.179623149	0.018631918
0.060050679	0.217083952	0.040518571	0.099467501
0.180589249	0.064560326	0.073545077	0.044019983
0.033454373	0.276883238	0.021509748	0.025673021
0.009255915	0.188099648	0.193623214	0.065714188
0.025761527	0.047652466	0.044175733	0.065164985
0.066571513	0.068660325	0.005913365	0.055676863
0.032936522	0.070559856	0.08101736	0.240856089
0.100611248	0.05299869	0.11805994	0.0252665
0.026702109	0.0266666	0.283060585	0.252396264
0.265163631	0.074484756	0.041598094	0.027385285
0.295300315	0.132500562	0.100186073	0.293916489
0.0263463	0.270129573	0.044587108	0.064249185
0.068798868	0.169699449	0.308791755	0.01179571
0.22236823	0.29426403	0.146067029	0.052757042
0.273641416	0.260988618	0.034262014	0.223329104
0.244815514	0.041760197	0.265063423	0.029778682
0.027426167	0.178591238	0.158884149	0.058584121
0.036397676	0.120823238	0.065819395	0.147519973
0.278919885	0.247357923	0.20900242	0.031811777
0.049489959	0.013651082	0.050645825	0.105217889
0.262105038	0.2385214	0.169711741	0.08567781
0.203137796	0.036409298	0.062877657	0.261473221
0.028380578	0.269432954	0.017772901	0.169599719
0.229747964	0.222236632	0.029510018	0.208539312

0.278380138	0.04938717	0.123669572	0.258441571
0.25447059	0.062715471	0.048957378	0.229081146
0.03731819	0.053744599	0.118229381	0.01360845
0.067546878	0.065123837	0.002552502	0.088964411
0.028468339	0.276740217	0.285781066	0.014945705
0.149212691	0.090038826	0.178637272	0.051312808
0.178402197	0.229574561	0.267539773	0.135042817
0.225068751	0.219901054	0.084391125	0.047562767
0.017223106	0.234805908	0.250396006	0.016999875
0.308392174	0.178587912	0.016059284	0.306460264
0.166984954	0.237963825	0.003933887	0.007806458
0.011255191	0.245171084	0.236158297	0.323325602
0.073320782	0.250276865	0.002585425	0.257908327
0.268461307	0.008198678	0.016924358	0.195928127
0.139364159	0.305502048	0.041248226	0.190663487
0.117957491	0.227743248	0.061471234	0.130773325
0.261489277	0.201453937	0.287304595	0.283539645
0.307887452	0.199716393	0.207895434	0.024431122
0.008548535	0.182976457	0.029347547	0.012246965
0.233973233	0.029360467	0.267811616	0.282795824
0.233240262	0.013529178	0.093347697	0.221344893
0.346624441	0.327923693	0.014154652	0.001451434
0.002209639	0.003124234	0.27002512	0.083685212
0.248551659	0.034495068	0.016084813	0.087957721
0.053039255	0.116468789	0.261450758	0.291187127
0.199469242	0.06118856	0.042115589	0.301684281
0.011378901	0.20602832	0.247317173	0.286218778
0.020949507	0.076108453	0.155153825	0.311478675
0.002142784	0.161670079	0.180274407	0.050227648
0.010755183	0.027216854	0.020051124	0.263898418
0.219597883	0.025592973	0.016573614	0.045740878
0.072999226	0.275843709	0.056249148	0.288300541
0.301905424	0.314090716	0.015298488	0.254626237
0.014847849	0.250282541	0.06932344	0.017960507
0.087832931	0.016751101	0.22411	0.007728538
0.178579009	0.282315638	0.011890394	0.010560085
0.008226319	0.037038387	0.276512079	0.223719778
0.011077983	0.105636532	0.12760504	0.242557808
0.245891503	0.257054279	0.263315629	0.092989568
0.074719492	0.012876682	0.319837754	0.236027575
0.170846256	0.271941221	0.035069873	0.266411741
0.243611317	0.27501617	0.235657287	0.179037536
0.021364532	0.193796191	0.256773629	0.032254707
0.202707935	0.277025411	0.193504198	0.016837678
0.088544925	0.075389832	0.044597943	0.039470109
0.043931295	0.241651167	0.06528362	0.307206432
0.260062732	0.240174618	0.078532999	0.018499265
0.289493474	0.264534714	0.053337348	0.221540195
0.092963096	0.128254466	0.215987184	0.201436334
0.036653535	0.244409483	0.321097415	0.263210708
0.295890703	0.173411602	0.26339299	0.098409632
0.010778582	0.164608753	0.037628866	0.053536335
0.116340524	0.012254425	0.017394858	0.254885169
0.035941101	0.234192873	0.066613844	0.027352018

0.122093753	0.112708134	0.179550643	0.14571915
0.036454293	0.252778659	0.00822613	0.04611812
0.055057112	0.201551622	0.074715444	0.072176314
0.016980566	0.022539389	0.160577298	0.205575177
0.087063992	0.031040734	0.200546099	0.035495034
0.047952253	0.294150409	0.150941483	0.009942961
0.018095559	0.185313983	0.084688372	0.032376591
0.011262117	0.210232187	0.092645863	0.124545073
0.238449331	0.058249632	0.269865398	0.028327239
0.042122075	0.281867801	0.097965532	0.237737615
0.086130705	0.275768075	0.254577844	0.244155568
0.031976128	0.08724485	0.030634624	0.304655003
0.046742213			
COPS4	0.301305943	0.445013696	0.172778731
0.351214778	0.257440542	0.183415344	0.110402349
0.033173471	0.02396995	0.003421763	0.01978352
0.265623189	0.048397214	0.065590679	0.053235725
0.385784059	0.064139019	0.099777851	0.152104085
0.31918836	0.028526828	0.368499645	0.03532094
0.088410164	0.059661008	0.055363084	0.117572239
0.391629714	0.062614472	0.126849115	0.26750093
0.04458329	0.347874057	0.404790322	0.350424696
0.401298167	0.252683922	0.005846418	0.082246576
0.317756281	0.350560658	0.325215294	0.228288212
0.022059096	0.352906112	0.158708032	0.269253622
0.027107893	0.020218883	0.052937313	0.326309535
0.362015169	0.254324787	0.062394071	0.076061817
0.052381534	0.05442659	0.058588294	0.174173922
0.142629314	0.036126156	0.388708126	0.023344827
0.038182283	0.083312301	0.068522749	0.106088058
0.122715961	0.245889396	0.008149738	0.234763688
0.001510306	0.289079883	0.258557695	0.10515931
0.044101006	0.013055195	0.094350322	0.234811027
0.416460285	0.354206385	0.464771117	0.462416091
0.045905127	0.17193192	0.224647529	0.081722278
0.353025278	0.091392834	0.018283819	0.408599713
0.46635	0.43538455	0.056353987	0.190898426
0.337743629	0.390819295	0.158572326	0.205987146
0.089096775	0.023894591	0.351089039	0.46355612
0.286865055	0.161531387	0.111522506	0.31861544
0.047232838	0.040589646	0.284240993	0.034790791
0.42074888	0.008811319	0.152483821	0.21365871
0.044107577	0.325593447	0.431449551	0.117312804
0.211091089	0.009847019	0.031791653	0.024854992
0.046537928	0.038549768	0.34788515	0.40841631
0.038889943	1	0.11640407	0.003925922
0.02216257	0.044647438	0.353057394	0.149464455
0.336908038	0.283162304	0.109436748	0.25715292
0.291543644	0.46726075	0.00407866	0.04008689
0.196774757	0.051043509	0.19489078	0.269087891
0.177302974	0.036246698	0.074053051	0.461930046
0.318705855	0.062853867	0.086702889	0.358832201
0.072529337	0.242433811	0.057234555	0.099123557
0.02553521	0.382448959	0.028399382	0.112714115

0.040501153	0.05303896	0.005517906	0.095551827
0.036488655	0.363657033	0.341647359	0.067921351
0.146601213	0.327851584	0.117638886	0.295459066
0.009650072	0.015821723	0.075280366	0.452486331
0.138435431	0.44796641	0.088353026	0.10395942
0.203120314	0.393929541	0.00081102	0.369942145
0.433537663	0.071359708	0.257059702	0.274342346
0.164820502	0.39419022	0.003756852	0.361599773
0.199391949	0.049032499	0.477275447	0.124392112
0.359324149	0.04325839	0.103788777	0.383106585
0.318710277	0.004193632	0.072298542	0.289877668
0.022676957	0.025020888	0.317799644	0.303268523
0.241253676	0.383005612	0.463829441	0.143333622
0.237218672	0.087201024	0.007836695	0.201405887
0.354036949	0.059710952	0.335243753	0.059827021
0.134554863	0.383042497	0.316837341	0.043716842
0.036008539	0.143961759	0.005133452	0.181666753
0.296799468	0.042699341	0.175009117	0.039693863
0.050621327	0.464625451	0.083860072	0.105412037
0.050490674	0.231143292	0.128909753	0.061240533
0.045585626	0.065460075	0.119659485	0.135763062
0.016540358	0.064395408	0.009339565	0.10538944
0.035585032	0.095580479	0.095127028	0.271824371
0.061374302	0.029861858	0.189035492	0.070334384
0.048933847	0.021953589	0.431189552	0.318208793
0.196074445	0.054525574	0.033737728	0.046846901
0.425331256	0.112385354	0.04957251	0.390014051
0.00900092	0.461633267	0.060311654	0.097158773
0.155939695	0.169488587	0.420559958	0.009117303
0.202415267	0.379003991	0.191220202	0.100740205
0.291556393	0.288551492	0.013275097	0.270772759
0.262454946	0.031827741	0.448731912	0.051395696
0.026234061	0.345318489	0.344282127	0.072285355
0.052312094	0.162632073	0.012417494	0.243795431
0.448252364	0.386778711	0.344805413	0.087364947
0.032116239	0.008584479	0.116553103	0.193291629
0.386177816	0.44567959	0.182279389	0.188753064
0.183324961	0.104043082	0.019687188	0.452336214
0.033790816	0.415144851	0.009583899	0.386379518
0.393231617	0.099060872	0.058026185	0.160394427
0.27715091	0.029824386	0.065619733	0.389647932
0.369231052	0.119505737	0.102910429	0.306792606
0.00709651	0.018368003	0.240429947	0.060251806
0.128880466	0.090151565	0.017029252	0.061705567
0.020396174	0.371101838	0.416524327	0.026127265
0.165291062	0.058343094	0.314715588	0.031909122
0.404278996	0.411887874	0.409667731	0.056746365
0.354114579	0.418627066	0.251458206	0.057822382
0.023496814	0.410582987	0.247924123	0.008062861
0.450015077	0.065580964	0.009625034	0.410801107
0.113184719	0.313930503	0.000517535	0.013395056
0.02128801	0.434465051	0.366255085	0.263525614
0.046528679	0.241944078	0.007481386	0.457441276
0.395304142	0.01390992	0.048051523	0.352911358

0.330059796	0.430597692	0.146536604	0.402411944
0.266087256	0.401339909	0.044126648	0.209297848
0.357220125	0.41260885	0.301795294	0.355898084
0.419092647	0.359869624	0.213083245	0.012141501
0.033651327	0.066996255	0.054185292	0.037598491
0.148093204	0.05956648	0.389559126	0.452246975
0.431083559	0.009915202	0.030201095	0.336996387
0.366536597	0.425051083	0.008667196	0.001439408
0.001179374	0.011415585	0.213524931	0.142986655
0.42278425	0.024171224	0.007432698	0.012487647
0.04449471	0.297108446	0.416617437	0.331063676
0.365080215	0.056129498	0.052380031	0.333395032
0.02895165	0.171684972	0.439092447	0.375659857
0.021614313	0.140018286	0.130240837	0.445948511
0.021721869	0.246551519	0.293421048	0.065577432
0.008129263	0.049675167	0.041403311	0.404926299
0.392370165	0.029832001	0.018613384	0.015152492
0.053467084	0.430738289	0.047937674	0.476173534
0.366214713	0.420876914	0.03981314	0.379169661
0.024312949	0.379095733	0.197101889	0.005783242
0.09483326	0.008756008	0.387243355	0.036466622
0.092163536	0.253582634	0.040666951	0.019549807
0.036130355	0.040752809	0.458793316	0.43707113
0.018631515	0.078571668	0.176795445	0.282330875
0.117374968	0.369598369	0.385595901	0.144131857
0.039162073	0.035109455	0.301144739	0.226121392
0.059650289	0.473195401	0.084361495	0.45391567
0.266235022	0.381731114	0.409544268	0.200920963
0.071314073	0.183840322	0.24806972	0.006904185
0.19967888	0.436445191	0.150103082	0.039197852
0.165209801	0.219895691	0.022787605	0.016505986
0.15968028	0.144904041	0.050848917	0.419657074
0.222208116	0.23699571	0.015207929	0.036787199
0.453275065	0.342001503	0.021556526	0.291562486
0.104152143	0.232927083	0.371391597	0.393878633
0.072399549	0.389748055	0.332637232	0.257924778
0.330134699	0.159247492	0.418636312	0.01051826
0.026936981	0.250645773	0.038556121	0.079574166
0.097504087	0.015251461	0.072899089	0.371750455
0.107627491	0.309537154	0.039473969	0.025638136
0.224659481	0.123961538	0.255467483	0.07751933
0.028413502	0.394754208	0.005682642	0.044845487
0.133642018	0.347326955	0.212652312	0.10292767
0.06591734	0.050022457	0.21078312	0.09825217
0.092302463	0.047643793	0.339394428	0.007551967
0.010214737	0.47007354	0.119366056	0.011631734
0.050789776	0.208503168	0.066163819	0.060658811
0.022399674	0.422858493	0.104614799	0.24081006
0.186434671	0.037301536	0.243758644	0.017819138
0.018117992	0.334287764	0.217982082	0.418479124
0.1573218	0.395418029	0.315085506	0.357991336
0.019559744	0.166304659	0.011578734	0.382727477
0.108741246			

COQ10B 0.237539142 0.322136094 0.12975214

0.365726661	0.194396156	0.209395633	0.237724476
0.176186462	0.123663538	0.03314654	0.036918044
0.179615635	0.091275429	0.142856189	0.103233949
0.367106188	0.093981196	0.132104181	0.268575067
0.349110884	0.177188381	0.319832076	0.193834804
0.091455374	0.072865098	0.119169613	0.093093444
0.39594357	0.127234249	0.155290334	0.3419755
0.101301197	0.297477299	0.274636839	0.225464933
0.246792867	0.198350612	0.030900883	0.135195769
0.297486518	0.265467827	0.367008818	0.292403639
0.13282185	0.262440477	0.26929357	0.198754208
0.1220067	0.067352332	0.127926583	0.269812542
0.231839005	0.177565621	0.204737052	0.201684467
0.063588418	0.126843913	0.041503726	0.244706889
0.243529608	0.129121864	0.246473451	0.144383924
0.117171986	0.204204406	0.096870979	0.212249569
0.077299725	0.276526938	0.116792727	0.134546418
0.013765385	0.3684652	0.208377429	0.056329151
0.096474479	0.053485919	0.110661278	0.311374617
0.375702298	0.256171571	0.325338752	0.3001436
0.096376836	0.175399436	0.120011315	0.161074722
0.2993504	0.178523778	0.109095287	0.341301461
0.321045407	0.279606243	0.132068151	0.172115271
0.27673433	0.318435187	0.136325363	0.156406479
0.074320386	0.053907867	0.256410922	0.311449562
0.265620811	0.072947154	0.121228412	0.346003978
0.133528663	0.094496154	0.339096302	0.071502095
0.386268521	0.100120373	0.12443768	0.260304865
0.164397599	0.278132228	0.264338399	0.242534713
0.158127957	0.051826015	0.040066718	0.089372067
0.119709858	0.031155221	0.227628791	0.342387465
0.165310272	0.11640407	1	0.022865605
0.058740722	0.104911101	0.233624704	0.171371719
0.311066522	0.183835461	0.128329847	0.237099478
0.168991034	0.321572969	0.025333466	0.215550003
0.200771474	0.116878287	0.27197974	0.190582643
0.150184668	0.21804118	0.183584936	0.340567846
0.298923709	0.170606589	0.11681541	0.332604275
0.119401538	0.157548156	0.191924044	0.150537697
0.1618946	0.230454957	0.048479844	0.184031135
0.060489071	0.064666534	0.069121477	0.206835572
0.122683559	0.245965727	0.213602485	0.175202104
0.167175143	0.260953547	0.175801316	0.225045171
0.036331885	0.118404735	0.116534231	0.314755328
0.196507123	0.305216964	0.178953235	0.134088157
0.202854495	0.345786221	0.003013467	0.262051343
0.274196099	0.101933006	0.207016352	0.211574646
0.094115523	0.348186599	0.012688358	0.326282752
0.19692676	0.170412014	0.355783533	0.140635948
0.255166758	0.098340916	0.160579358	0.218501631
0.176413437	0.029645771	0.16533457	0.362938534
0.046312663	0.185155349	0.29807264	0.320174958
0.237611516	0.299345154	0.375325568	0.077552001
0.184102101	0.21076846	0.05893361	0.156381916

0.262576946	0.074115783	0.28247644	0.208352558
0.146550588	0.302001289	0.203002511	0.071442882
0.140697967	0.23347373	0.061641661	0.176688474
0.21453614	0.144344997	0.119302329	0.108630324
0.08512273	0.322349977	0.053848755	0.047894656
0.030593966	0.183446966	0.251202898	0.09847928
0.06346916	0.17253884	0.099575481	0.123890914
0.150259897	0.127500468	0.027189285	0.05601564
0.057974502	0.072724627	0.068832003	0.224012752
0.132304251	0.09058531	0.153922251	0.088042263
0.093277726	0.062090632	0.312491042	0.331382619
0.217000028	0.213186167	0.098025825	0.056549874
0.3345891	0.130414195	0.092190085	0.317520992
0.085147028	0.331460108	0.115998161	0.117288817
0.080592593	0.219404964	0.388630691	0.058090569
0.218572507	0.297876174	0.216813228	0.127245781
0.240058325	0.313673731	0.124313196	0.227727759
0.216522428	0.091119681	0.294884421	0.083441079
0.067172316	0.231867265	0.211106634	0.117527468
0.099528975	0.162052164	0.160574673	0.239698613
0.322409509	0.333868538	0.249229766	0.079225027
0.119857084	0.021519424	0.095792116	0.133213927
0.260009191	0.273086577	0.230383999	0.116839242
0.296276059	0.097724687	0.133400431	0.304147708
0.08189419	0.28463296	0.050279767	0.228115725
0.242748176	0.156208369	0.081225209	0.252939867
0.25451277	0.146431391	0.204649612	0.271135243
0.309217594	0.135775316	0.104935846	0.251350232
0.105188059	0.173285672	0.193645526	0.049399024
0.11847836	0.174918496	0.011322186	0.165223837
0.098282524	0.287981207	0.330247799	0.098539311
0.16042544	0.147592022	0.248603639	0.125057601
0.230350988	0.25658071	0.278669019	0.224295693
0.239770893	0.260537523	0.112750637	0.110995993
0.047371107	0.255089823	0.265196431	0.044923262
0.361041238	0.132129447	0.037561482	0.326113579
0.242841879	0.218395782	0.02102958	0.02337742
0.054095367	0.309261853	0.2572564	0.342574497
0.076200709	0.30249983	0.009563267	0.296553517
0.281537636	0.03606996	0.080209976	0.218251982
0.175355389	0.321968865	0.075294028	0.240014185
0.1438439	0.244950479	0.165879639	0.226806873
0.30086111	0.232787747	0.312255159	0.300175602
0.340683433	0.263470459	0.311739849	0.090347872
0.06836726	0.172240561	0.060846825	0.072826589
0.215291095	0.104805311	0.28852366	0.345004739
0.271814	0.035508399	0.193317709	0.239809204
0.35821518	0.3817788	0.054040086	0.010510299
0.004468	0.021069536	0.296354738	0.144311602
0.295124287	0.060860768	0.042777173	0.161041609
0.159727618	0.173894708	0.281108354	0.379215695
0.248132598	0.142724659	0.086176368	0.37837566
0.070842475	0.175511468	0.293606699	0.298100804
0.084196867	0.142202222	0.142028137	0.372368642

0.011401864	0.227791147	0.19645275	0.130736294
0.031183588	0.088446831	0.095831613	0.334030024
0.231130984	0.068825351	0.051054036	0.066494209
0.150747403	0.324983287	0.14019186	0.342760672
0.340386144	0.386008157	0.068997031	0.28438988
0.091076398	0.250795713	0.10901377	0.034809149
0.132923432	0.068500856	0.235491465	0.044030413
0.136303289	0.285164845	0.081541082	0.041994945
0.040089226	0.091671218	0.348100741	0.291082298
0.050017636	0.232583358	0.192066238	0.286669413
0.226928051	0.319687461	0.269785582	0.106809751
0.19243368	0.035315769	0.348558571	0.214646894
0.17460318	0.335512484	0.105330151	0.309385381
0.26734041	0.312994903	0.269430745	0.266140008
0.072388463	0.207435645	0.274313616	0.068061888
0.264734702	0.326885888	0.208078462	0.079162937
0.145981595	0.109820661	0.16020705	0.058244651
0.066327017	0.224674061	0.164318427	0.388965205
0.251170738	0.229862152	0.141057496	0.092521441
0.319421645	0.290312302	0.16858585	0.271180739
0.151737538	0.221052651	0.260215262	0.253005105
0.105307799	0.261176729	0.327461366	0.247513112
0.287478098	0.273451364	0.283410929	0.132295544
0.066643076	0.169240007	0.115129551	0.10742319
0.173213585	0.065899575	0.044774674	0.317562777
0.055422381	0.285578995	0.113899284	0.111223323
0.185389104	0.120412716	0.259172207	0.180188274
0.116980797	0.268670469	0.029971211	0.106384491
0.062480189	0.259826077	0.108709455	0.141852463
0.063939164	0.088870192	0.231351359	0.169515425
0.075660123	0.08061121	0.255404299	0.113567782
0.116586307	0.350898934	0.142619618	0.042914608
0.090129486	0.201608779	0.139319979	0.14408279
0.040463743	0.242983541	0.115178471	0.179684362
0.278896897	0.145935707	0.231987882	0.10792778
0.15308257	0.263671741	0.142304589	0.258899534
0.120070341	0.357096118	0.2439744	0.253399888
0.086692121	0.109183226	0.13025329	0.348665175
0.063596238			

CORO2A 0.003447291 0.001837435 0.005309243

0.012822875	0.011937012	0.001980498	0.029013943
0.006814333	0.023808143	0.027981942	0.032085128
0.006363706	0.008579662	0.007231735	0.001039981
0.022060359	0.061368697	0.00661216	0.059289276
0.014926413	0.018041871	0.030116179	0.056292655
0.011977845	0.058555274	0.096371668	0.01703899
0.011476575	0.106266738	0.00137082	0.007280259
0.088101368	0.004610831	0.000381384	0.01137166
0.000909874	0.001391893	0.045334715	0.00245743
0.002926451	0.006828597	0.013329672	0.03439328
0.066181333	0.001305419	0.037036053	0.01658702
0.002155772	0.074095191	0.001627166	0.00064936
0.00123222	0.002694328	0.009199824	0.028741752
0.002837494	0.002163848	0.002534936	0.002021728

0.043507346	0.002594091	0.000333401	0.016576933
0.003368941	0.014462886	0.06452547	0.017511553
0.005289702	0.002138168	0.110376471	0.002518577
0.012513418	0.019712945	0.027300239	0.002423231
0.205885218	0.059406082	0.077031711	0.018409092
0.00901521	0.000538467	0.008094066	0.005164817
0.030050038	0.050279261	0.007305392	0.055478845
0.002777806	0.028942867	0.021874713	0.024864813
0.006845795	0.00071663	0.027757991	0.063395059
0.002695613	0.001894931	0.003110939	0.006613612
0.002134916	0.082072225	0.0001716	0.003885562
0.00119218	0.009721346	0.10403494	0.006010081
0.161432378	0.001034441	0.034723654	0.020772444
0.008842871	0.057358739	0.076738086	0.011939303
0.02968782	0.004309737	0.000761857	0.057878525
0.012914118	0.082094232	0.006851356	0.157232433
0.004781649	0.009730729	0.002818343	0.006487022
0.005437877	0.003925922	0.022865605	1
0.14257949	0.030024554	0.002615295	0.01115365
0.008236065	0.000385219	0.021680654	0.000868706
0.00291681	0.008060832	7.44E-07	0.060475041
0.006043433	0.036841705	0.087388202	0.004728829
0.030102085	0.038969106	0.092153287	0.004614485
0.000540479	0.11855436	0.002906505	0.024923031
0.002101151	0.026746665	0.094815793	0.019387378
0.051766787	0.000804027	0.164329401	0.005946325
0.000998962	0.030910209	0.118368412	0.008971146
0.003583333	0.001723101	0.003177232	0.130958884
0.020753665	0.021822719	0.002612041	0.003099334
0.029629961	0.100457688	0.000745115	0.00351643
0.000762347	0.004456041	0.022314673	0.02054136
0.063087206	0.010517573	0.0067379	0.000957577
0.000583014	0.000446128	0.013060403	0.012823989
0.002620061	0.007941635	0.000327433	0.02424798
0.000766287	0.134464892	0.007912477	0.097578908
0.000443189	0.141753762	0.004716924	0.003435948
0.001669858	0.032178842	0.003443626	0.011621607
0.125639603	0.008291665	0.006628292	0.014399478
0.010172628	0.010056233	0.011722144	0.002494124
0.009410963	0.030363539	0.137531077	0.038410239
0.005281558	0.000491273	0.000428135	0.014147453
0.000344531	0.001711053	0.001006505	0.000625242
0.109264089	0.010413287	0.018387384	0.01953738
0.000353918	0.021078633	0.001933828	0.048036055
0.127689323	0.00312424	0.026107509	0.007240712
0.013952796	0.004607613	0.041847616	0.004880703
0.00660192	0.040802934	0.004863136	0.004901227
0.034737984	0.09887713	0.090448032	0.001043792
0.008867625	0.002304845	0.001431061	0.00151205
0.003893768	0.025444745	0.013966589	0.008435666
0.067463043	0.246434364	0.002014506	0.022480262
0.000268081	0.037686244	0.183541327	0.000330481
0.005376998	0.000158949	0.000354587	0.002738777
0.083704004	0.006926057	0.010610178	0.108408918

0.006691193	0.048901111	0.010303479	0.002774142
0.003979408	0.001087975	0.043753569	0.04824499
2.42E-05	0.010509751	0.067759077	0.001458211
0.001150852	0.17691259	0.002015101	0.061493326
0.237176923	0.010442125	0.002918897	0.177167754
0.126701759	0.008457655	0.019533565	0.066765209
0.002967241	0.026251749	0.002310445	0.02777291
0.093489533	0.042880723	0.056288033	0.005765632
0.001106233	0.002210956	0.078151186	0.012396166
0.071569061	0.004046078	0.002155469	0.003372089
0.015921743	0.000463272	0.21703491	0.004810919
0.001329843	0.001800126	0.089538199	0.008376921
0.001154988	0.097143222	0.038260726	0.000379188
0.002856607	0.008861181	0.004072849	0.011532507
0.050148915	0.049828198	0.012262305	0.012611484
0.003050035	0.036494365	0.009461836	0.00249472
0.064053208	0.000944053	0.011621237	0.047616288
0.000983539	0.002282324	0.034354005	0.155469722
0.002749623	0.000820189	0.000107861	0.041723863
0.003953553	0.001697997	0.002413428	0.152747005
0.220309574	0.000110571	0.003457495	0.034563459
0.009192496	0.00083743	0.041236914	0.001925319
0.071002915	0.002901691	0.004929778	0.005316209
0.099304061	0.010839443	0.000681804	0.00833501
0.000198282	0.023627304	0.02561047	0.002725264
0.000855704	0.133507189	0.039586489	0.001957962
0.002565121	0.001390896	0.007107949	0.003446624
0.003649514	0.000542584	0.007143108	0.06357385
0.003681996	0.000252624	0.005686456	0.001249481
0.007931091	0.018518305	0.046345732	0.070795252
0.012122001	0.005834988	0.000360204	0.01686109
0.001662141	0.045272934	0.00224359	0.009619609
0.003606351	0.257683995	0.012780592	0.001794632
0.004678385	0.007160646	0.08545856	0.009760578
0.00352752	0.043458313	0.010642822	0.061033144
0.002737255	0.000235191	0.203581305	0.031005838
0.010276058	0.012707723	0.000889324	0.032659695
0.005515537	0.133581774	0.00128873	0.023627952
0.051202699	0.000188275	0.00418501	0.000580802
0.1227011	0.04845374	0.005140091	0.006786574
0.003678204	0.05518353	0.006354055	0.004420155
0.082904506	0.101417134	0.077446836	0.014821871
0.001359926	0.033019093	0.061285887	0.000347559
0.030793635	0.00614792	0.030497281	0.005750096
0.005885149	0.017771427	0.074346894	0.000383953
0.044152183	0.000510886	0.017671866	0.001457586
0.027168995	0.172886397	0.00062186	0.002588459
0.003080515	0.005588538	0.00578638	0.150910241
0.005229998	0.148521962	0.008160698	0.008323906
0.110275597	0.019992742	0.049757999	0.016454602
0.006328596	0.011499124	0.000569226	0.000577293
0.033494516	0.012969366	0.011578881	0.002092107
0.006747408	0.008414862	0.005272668	0.002790421
0.003040443	0.002766369	0.001953991	0.013796092

0.01327665	0.002794552	0.004266421	0.030716058
0.026584669	0.008693458	0.008135424	0.020882339
0.040494704	0.004236548	0.075845002	0.001817483
0.003894699	0.002589842	0.033327794	0.017597248
0.005360645	0.001922831	0.069996036	0.069403566
0.001759081	0.00106282	0.009494643	0.006016208
0.103835499	0.022161206	0.006599817	0.007906123
0.001624636	0.002831696	0.005657851	0.003240655
0.000695201	0.038768132	0.000128063	0.00473421
0.024265882	0.002744661	0.029428803	0.160170282
0.093637984	0.121664303	0.001572799	0.020115997
0.001287841	0.026302603	0.000905145	0.061723054
0.041892878	0.002622427	0.035781757	0.01669555
0.044607436	0.000157583	0.069799025	0.167161241
0.002584649	0.003207922	0.006434187	0.050111225
0.003939638	0.047630014	0.048606941	0.002300623
0.001002183	0.001409975	0.002739388	0.038015685
0.084700813	0.008588112	0.002369945	0.116765843
0.035335917	0.001748248	0.013376054	0.007446041
0.150911779	0.001141291	0.00094496	0.008694178
0.013054305	0.135097769	0.000603209	0.015880983
0.074108668	4.70E-05 0.006822928	0.00019729	
0.015195139	0.020703916	0.001988315	0.0024682
0.022436536	0.002250523	0.068604736	0.00725934
0.000688577			
CPEB2 0.028110169	0.029169345	0.030324762	
0.069816329	0.060890433	0.015345202	0.077255272
0.015922736	0.052213326	0.046354683	0.092812598
0.04341208	0.046251925	0.031551678	0.006249808
0.099197268	0.171477714	0.02274104	0.150729706
0.067192324	0.034321466	0.131670818	0.092863699
0.041357976	0.166634584	0.192640007	0.065994975
0.065331773	0.21412046	0.011014597	0.038390033
0.170597857	0.035537805	0.019056846	0.074919683
0.018010171	0.018102685	0.050809029	0.012143893
0.026370153	0.04959006	0.062169926	0.122448366
0.110677883	0.014481544	0.110981299	0.075552037
0.00869045	0.165363549	0.006627503	0.014001154
0.015083608	0.024890898	0.022850732	0.069277934
0.022064213	0.008850439	0.021359914	0.014734141
0.118201841	0.00807086	0.018334743	0.035554423
0.011208777	0.034659336	0.151614139	0.049950838
0.026719053	0.018697466	0.095095221	0.023668876
0.016035225	0.076213558	0.104016034	0.016255556
0.333273633	0.144280167	0.184353676	0.073574927
0.058220218	0.020256164	0.063375839	0.048238349
0.115790707	0.136097474	0.043330767	0.143926099
0.027716218	0.083218632	0.056887677	0.116765462
0.057604646	0.024539938	0.086508633	0.179073633
0.028862317	0.026085667	0.022240787	0.038843844
0.014996548	0.15802242	0.017176724	0.041983658
0.018244059	0.051589277	0.263569696	0.038552378
0.243255065	0.007024469	0.127091981	0.077576622
0.058501836	0.068357581	0.215332963	0.053553813

0.06150367	0.03415805	0.025116239	0.137224206
0.058980483	0.155833819	0.031730167	0.20231178
0.021956559	0.041905119	0.011549496	0.051768638
0.018827611	0.02216257	0.058740722	0.14257949
1	0.075282307	0.02958514	0.04001275
0.047108603	0.014513394	0.080481608	0.015268085
0.030478598	0.062448387	0.000651348	0.089425615
0.037466747	0.083776597	0.223444618	0.034170502
0.122225541	0.062610797	0.177491095	0.044668112
0.016804067	0.211493418	0.01200642	0.11235919
0.012811394	0.118747541	0.161822212	0.07129044
0.087286785	0.017069171	0.281416298	0.021713653
0.007910478	0.117789368	0.085351359	0.027087158
0.017768257	0.026464631	0.011137954	0.217345909
0.079128756	0.103496615	0.01297612	0.032095043
0.06384837	0.151534196	0.005418004	0.039651196
0.009285608	0.043280429	0.063891037	0.063599373
0.192574209	0.063410931	0.008645281	0.015947554
0.024131483	0.004996018	0.076896797	0.063865345
0.01998728	0.05623499	0.002089938	0.107959544
0.012935961	0.19578751	0.061082564	0.260158984
0.019573084	0.246948657	0.01954045	0.036250328
0.025732829	0.040484991	0.013204307	0.053860383
0.204854058	0.016716836	0.039369702	0.069647642
0.052753797	0.060828617	0.075251426	0.018966731
0.055032341	0.068700429	0.157809354	0.131140914
0.039673934	0.005727078	0.017712343	0.037849622
0.008216949	0.025368343	0.020062905	0.0038346
0.1624877	0.035134421	0.038063448	0.078745125
0.01579952	0.064144374	0.016070368	0.103150104
0.260565307	0.037551154	0.08551873	0.035894304
0.048737319	0.035868194	0.098803448	0.028999108
0.045118956	0.09332593	0.026632408	0.024248835
0.050771288	0.222365324	0.120761708	0.012899591
0.054825364	0.02153188	0.015714945	0.018586577
0.020159076	0.101068015	0.074475128	0.029159881
0.153994291	0.303033816	0.029461789	0.092094484
0.01083261	0.075009669	0.268148728	0.005711443
0.046713843	0.006946988	0.003340385	0.030772961
0.101863809	0.054120715	0.0502898	0.269218835
0.044307949	0.155475639	0.063826695	0.007118272
0.022611593	0.022422444	0.121215157	0.141438488
0.01522194	0.053336229	0.098919274	0.018748132
0.018445443	0.273239322	0.031918382	0.17065296
0.311655547	0.065201473	0.032224491	0.319644749
0.241238498	0.046400511	0.027287416	0.210740536
0.035878537	0.124279842	0.026623898	0.078465307
0.158380295	0.11341111	0.149123265	0.03450496
0.01653324	0.033257231	0.194428945	0.074106189
0.180137895	0.022675451	0.004768682	0.039307642
0.037973337	0.022462118	0.189577198	0.042670909
0.016329387	0.010435999	0.221191437	0.034063274
0.01838534	0.136630757	0.069606044	0.021466803
0.028220033	0.037388879	0.021995864	0.06004542

0.056850843	0.066840935	0.059087027	0.042596043
0.018167774	0.088155515	0.034416911	0.009463539
0.135640444	0.021454793	0.070740499	0.101325331
0.012107352	0.008225322	0.133483835	0.209875982
0.034491775	0.024553713	0.020317596	0.071713517
0.035723129	0.02923377	0.02752166	0.257894854
0.312597162	0.021059183	0.024113025	0.075559367
0.063517795	0.005742202	0.102904049	0.027403755
0.146726147	0.010435303	0.005142533	0.01206969
0.168328007	0.07083845	0.016016048	0.039532992
0.003661422	0.078956405	0.031093927	0.035475372
0.023552791	0.200794964	0.108122733	0.027802468
0.032026256	0.026477138	0.041224769	0.03909376
0.035966697	0.018483723	0.031256473	0.191451725
0.035429768	0.022414195	0.037570447	0.021965471
0.05801217	0.091038161	0.13954307	0.092510549
0.032144718	0.016602972	0.005037919	0.047808203
0.011705768	0.123271269	0.029471718	0.064549408
0.040948504	0.235032989	0.028899308	0.024883197
0.037100485	0.051121779	0.095087021	0.013709711
0.009148364	0.117279628	0.042761573	0.16178182
0.033285518	0.003435425	0.167146425	0.042375993
0.036330428	0.075567207	0.024387659	0.122724533
0.043415284	0.218283746	0.01040401	0.093763385
0.125565841	0.00852909	0.043947352	0.020049248
0.181444042	0.124958585	0.024006313	0.052322443
0.02002069	0.175518227	0.044514258	0.025517579
0.100942166	0.207035229	0.14318165	0.078613513
0.016235714	0.123742965	0.17237622	0.002829827
0.086311441	0.047893045	0.098554181	0.051170033
0.043295916	0.09328038	0.161525863	0.019638839
0.091423424	0.018059906	0.086638534	0.004448242
0.072568882	0.176756754	0.022933202	0.016692053
0.015398236	0.031471372	0.01929974	0.203226231
0.021460773	0.2671088	0.061489029	0.06205602
0.181951741	0.042854045	0.141340676	0.068695277
0.024726188	0.071354945	0.017675809	0.010590188
0.061535924	0.041737975	0.056065766	0.019632176
0.020171223	0.06384643	0.027838446	0.035021942
0.024210029	0.028991093	0.028794475	0.055739533
0.057120962	0.019360746	0.030350961	0.06064212
0.089825086	0.063967717	0.030005715	0.085053016
0.147001672	0.031766002	0.086666061	0.006681389
0.028764083	0.015498714	0.092036851	0.088560636
0.029050578	0.019547285	0.075237596	0.133577452
0.02953273	0.019661996	0.021408824	0.040362913
0.214624777	0.089672331	0.049357818	0.057236932
0.008694074	0.033666106	0.03929203	0.028153847
0.018434922	0.100498153	0.021293954	0.008529527
0.066795287	0.023740157	0.095084434	0.304127598
0.211851662	0.186121273	0.012608327	0.093961443
0.012923668	0.116754947	0.00533774	0.126881775
0.133707964	0.014458604	0.124035028	0.049942696
0.12026165	0.019155147	0.105662664	0.271542571

0.021511993	0.032432002	0.047950991	0.133651779
0.022937053	0.111894649	0.154803968	0.012272551
0.012226948	0.00960688	0.029993913	0.0507728
0.095598073	0.063241022	0.015067528	0.16549401
0.066918761	0.015075761	0.035964401	0.031137966
0.230597774	0.026341617	0.008211843	0.048215299
0.047169989	0.189962347	0.010838512	0.026988412
0.095198982	0.017429228	0.041766995	0.022254118
0.056722024	0.104778406	0.011443651	0.028707715
0.046909022	0.017728597	0.089211678	0.047498274
0.009928618			
CREM 0.23918091	0.201227686	0.177915757	
0.205025371	0.19518034	0.087187963	0.082398716
0.045487435	0.013637988	0.001046344	0.004456989
0.212313664	0.011156645	0.038373459	0.028959105
0.314149096	0.084070168	0.161408004	0.167248887
0.254458258	0.02166266	0.274603993	0.050040539
0.115210287	0.07239656	0.063844117	0.097425495
0.264109259	0.081945225	0.046352462	0.177164606
0.173173056	0.23834116	0.176990299	0.199893183
0.170634508	0.192831625	0.027455764	0.028228719
0.220180631	0.210713259	0.22807303	0.174514895
0.026918822	0.150890886	0.11395268	0.278289371
0.015862346	0.025263458	0.030904624	0.147596668
0.130664277	0.108165458	0.059043554	0.051548323
0.005921026	0.030781325	0.028901504	0.102452369
0.107916192	0.020329949	0.154466142	0.009889396
0.008652513	0.102699563	0.164119486	0.09963059
0.11056342	0.153295165	0.020971735	0.189000593
0.000383121	0.256464994	0.335023208	0.06134094
0.119503764	0.006670316	0.210208817	0.138220662
0.269752309	0.212332341	0.266730257	0.247136264
0.040801072	0.303090481	0.195350875	0.085306371
0.208530587	0.054573683	0.026310316	0.321028492
0.260178783	0.185425683	0.063870008	0.293341776
0.132037191	0.192536692	0.097421937	0.110455416
0.03853453	0.124231338	0.139629902	0.232197645
0.168386129	0.158371865	0.169018284	0.193397758
0.123107428	0.041435517	0.218147833	0.048785757
0.269180977	0.014111013	0.229650257	0.114737147
0.094053401	0.216887393	0.192526835	0.115919873
0.225380236	0.018866007	0.013393062	0.126616864
0.019403566	0.065386825	0.133266789	0.201085568
0.033903913	0.044647438	0.104911101	0.030024554
0.075282307	1 0.197472991	0.214423555	
0.26331137	0.177903475	0.059416167	0.141306801
0.157640783	0.268257805	0.000912869	0.067400129
0.134286008	0.113388342	0.238486222	0.14773366
0.145837781	0.062912918	0.104453464	0.24452461
0.150126209	0.091963714	0.104792371	0.312619284
0.022895317	0.229995479	0.104938734	0.070623435
0.025682681	0.161120923	0.118870527	0.130409506
0.012173668	0.035460517	0.036554654	0.081221847
0.01640791	0.133395867	0.142164767	0.162182115

0.102019768	0.24640905	0.08965192	0.115144627
0.001803514	0.04347229	0.06565621	0.213306774
0.064176995	0.273141417	0.122609551	0.163797417
0.25905768	0.277592195	0.010304228	0.154740591
0.190322628	0.052819606	0.132102021	0.282240303
0.086602811	0.212143359	0.006268471	0.300985892
0.141187967	0.137196733	0.281849388	0.144867125
0.180939048	0.065188058	0.060485025	0.212379946
0.148250927	0.005119273	0.038569069	0.181196645
0.118299044	0.015162542	0.247074225	0.207038175
0.129815009	0.30230967	0.300714259	0.071519879
0.152013555	0.121330229	0.03287177	0.286978822
0.26669127	0.015634354	0.149071547	0.054102169
0.0938347	0.174974702	0.164715129	0.00571238
0.050143964	0.102719176	0.004433468	0.139517747
0.171158553	0.020395671	0.094495095	0.112890123
0.120174912	0.218593781	0.143710197	0.124344431
0.087159288	0.091755595	0.175396845	0.018514036
0.013775839	0.077227963	0.077104864	0.074049043
0.015629969	0.078741456	0.070033731	0.042339089
0.007033547	0.032566679	0.029407624	0.143540538
0.036607076	0.012790319	0.123462989	0.105419159
0.106794539	0.0824445	0.200343447	0.299512529
0.106634638	0.068273393	0.130375847	0.00825582
0.198701313	0.039239288	0.039412063	0.164323087
0.038273413	0.284149216	0.027330574	0.126016741
0.083981844	0.133563467	0.268035155	0.003548591
0.158276216	0.159965009	0.312991954	0.096507366
0.117384654	0.199679256	0.010770454	0.168795432
0.149378793	0.072066135	0.213984108	0.106067983
0.115921623	0.277102847	0.14589422	0.166772852
0.138682817	0.063960608	0.013195998	0.276109007
0.208981984	0.276723892	0.133631229	0.193033231
0.034969938	0.002940668	0.244513918	0.223156784
0.162819496	0.218374696	0.283667822	0.105300807
0.204293225	0.051109097	0.007885573	0.2144524
0.090698441	0.202085994	0.054074831	0.223482407
0.157119019	0.056668535	0.07880258	0.082452996
0.127124162	0.05360492	0.146109638	0.169283555
0.186012234	0.087446817	0.054909932	0.292224241
0.009259056	0.014870318	0.205878344	0.103758708
0.056051083	0.109181989	0.025731206	0.050410895
0.01750354	0.144754517	0.309561808	0.031107348
0.047603267	0.054528203	0.32834197	0.075132937
0.204650026	0.214137309	0.175206585	0.108741954
0.22136603	0.18063056	0.121889894	0.20172849
0.110046111	0.18463036	0.185039398	0.00825506
0.271983324	0.035889019	0.002499893	0.194029207
0.154471061	0.132874942	0.001467536	0.072536003
0.127064397	0.318183097	0.145488987	0.19040345
0.01514965	0.291318847	0.049034594	0.24474491
0.162625882	0.05618742	0.085836616	0.162544343
0.163805775	0.207833264	0.107875394	0.191130091
0.141695254	0.157797612	0.012213137	0.225790012

0.167106749	0.184902137	0.213650881	0.184061084
0.231930324	0.313199892	0.174941443	0.014708349
0.022900321	0.046781507	0.015883651	0.007536922
0.098114821	0.067029698	0.222808683	0.285483378
0.216066853	0.062266238	0.031109614	0.168452749
0.199449621	0.260873168	0.069513685	0.006638104
0.022751475	0.008257149	0.159026756	0.268651849
0.214889966	0.005195466	0.029670916	0.014684035
0.043614312	0.225128275	0.175892364	0.293352186
0.27068247	0.135027334	0.013692303	0.304455132
0.033891157	0.119030489	0.214471146	0.153841773
0.119339463	0.230237926	0.17188574	0.242933844
0.028655763	0.290316677	0.219172318	0.027025989
0.058865416	0.125316346	0.072304304	0.332713545
0.156649585	0.015740956	0.015682816	0.006295313
0.029677509	0.272194814	0.044839562	0.262221777
0.20353986	0.29114743	0.083394946	0.165480774
0.00968407	0.153419658	0.165879993	0.003369766
0.222183641	0.055121514	0.1977919	0.004883028
0.056022831	0.214197046	0.020936513	0.096599121
0.055709419	0.102841917	0.243504428	0.276367217
0.114327045	0.088920653	0.290821234	0.311377798
0.079987875	0.200318944	0.161600621	0.064785205
0.029407698	0.068082139	0.239053103	0.162675013
0.047997526	0.287309457	0.03569994	0.23900474
0.192843279	0.233533076	0.194638703	0.135216699
0.037741102	0.131851539	0.099838116	0.003687315
0.224428123	0.229302617	0.172231865	0.027747939
0.115908816	0.167042022	0.039539268	0.027837209
0.131537048	0.071309403	0.029851577	0.328415834
0.206164313	0.160911965	0.030260077	0.055888401
0.216723171	0.180438793	0.0150874	0.161201953
0.259030202	0.164110839	0.269667679	0.27744265
0.057667031	0.169929636	0.208170557	0.103936747
0.134662923	0.154774186	0.179101259	0.012093242
0.021393598	0.182887577	0.021317675	0.21747316
0.113925403	0.058640842	0.054097752	0.322301294
0.067839357	0.204376615	0.027542311	0.044022587
0.290293632	0.149710481	0.260023717	0.046828533
0.021056726	0.152731937	0.021251997	0.139619977
0.091174445	0.181376066	0.114719979	0.164506199
0.051252265	0.14542294	0.184516934	0.060157517
0.024959215	0.029552735	0.169631892	0.017202264
0.017576226	0.287233689	0.117145569	0.073038726
0.048909664	0.1548345	0.145511739	0.026488337
0.126910458	0.180195985	0.033039596	0.197398806
0.147333884	0.123793262	0.131914485	0.020966917
0.058299975	0.164257859	0.211681184	0.182106155
0.257729151	0.252775419	0.140608884	0.17573043
0.075503837	0.165735988	0.020066918	0.262388141
0.048275026			
CRIP2 0.385100637	0.511029278	0.350447077	
0.356129964	0.393963431	0.313423202	0.1769308
0.169416459	0.05871614	0.004191329	0.019315252

0.409970078	0.191189955	0.251665977	0.249277023
0.366900549	0.14267521	0.330176813	0.216397957
0.36694401	0.079667428	0.361087573	0.079329821
0.275011374	0.095453214	0.060346037	0.225898061
0.383050445	0.069211765	0.283689897	0.340919304
0.114942764	0.39816245	0.517281443	0.399141546
0.523298411	0.357282305	0.024439102	0.236533707
0.386694335	0.372573096	0.359054071	0.290009493
0.035461657	0.490863155	0.208766103	0.369654882
0.197280593	0.028345153	0.201269128	0.439849923
0.478197129	0.371351689	0.190253604	0.131965127
0.18914385	0.223210704	0.259071813	0.291176864
0.201632227	0.13889698	0.486323884	0.063002308
0.130574983	0.232695395	0.172578288	0.236297135
0.322384366	0.335154112	0.018297038	0.384636443
0.003435893	0.34209258	0.361108816	0.255079746
0.041983306	0.022454413	0.193571558	0.293884444
0.413448288	0.460442132	0.448874617	0.462154232
0.187114194	0.286702013	0.372453833	0.161980757
0.396191513	0.151077093	0.098545927	0.376813795
0.460115655	0.520077073	0.183762135	0.246218655
0.353808401	0.464438888	0.355679995	0.376790428
0.268067835	0.05645907	0.43060246	0.495251087
0.372816943	0.304334975	0.145228625	0.365985837
0.059390798	0.322488279	0.314144929	0.203785706
0.424765083	0.012583721	0.208664324	0.319324112
0.21433434	0.387893343	0.512662868	0.164610953
0.39558448	0.023141319	0.108868344	0.038253669
0.306573466	0.154665313	0.490181831	0.413927941
0.231919325	0.353057394	0.233624704	0.002615295
0.02958514	0.197472991	1	0.361144925
0.385020082	0.375384394	0.192824504	0.350236752
0.38968213	0.443586246	0.043519794	0.08494934
0.360712134	0.199679767	0.221051408	0.411429324
0.272149735	0.102638564	0.124641362	0.488018561
0.413163413	0.07894984	0.327557722	0.371500427
0.283511227	0.336431641	0.104676913	0.273862108
0.046212084	0.48237771	0.032747272	0.305482911
0.196322095	0.173628564	0.013821121	0.235172674
0.247614953	0.395942997	0.484744618	0.105010126
0.3091625	0.363967762	0.300324418	0.366596032
0.007113236	0.047423364	0.26473858	0.493224782
0.258000705	0.484360611	0.281924163	0.276953696
0.263298561	0.384880169	0.003674182	0.494589891
0.519383777	0.276061682	0.362204612	0.395390084
0.309794995	0.388246923	0.047008064	0.387820288
0.340279051	0.076130791	0.469005989	0.151680777
0.465666846	0.037803371	0.29867201	0.385239414
0.397161605	0.009294069	0.18688792	0.334508876
0.03089093	0.078573179	0.381971971	0.35156177
0.313427439	0.393764843	0.43752262	0.291376921
0.379478426	0.219524795	0.013200658	0.303759646
0.395733828	0.338590753	0.42865971	0.205495891
0.31694383	0.454599099	0.378071605	0.165187197

0.036708483	0.280776698	0.01842566	0.276849922
0.365298226	0.110332092	0.321354667	0.178610851
0.074129741	0.505471923	0.175970745	0.250893621
0.127967627	0.369901316	0.229445408	0.220791555
0.209835368	0.144128323	0.292964214	0.314455602
0.03194178	0.094150657	0.01842168	0.385542941
0.151645443	0.333265906	0.330841037	0.377147191
0.332977529	0.118880174	0.345005021	0.255452253
0.103709239	0.015733223	0.492660169	0.36472309
0.304314238	0.130650025	0.044484762	0.337842694
0.431082134	0.263345007	0.237430127	0.430431753
0.028517087	0.452451893	0.232885765	0.115711991
0.37251651	0.242906531	0.403289983	0.043578151
0.364391603	0.462060418	0.295308996	0.158897456
0.39507993	0.365286915	0.026535382	0.36508087
0.373667321	0.032545058	0.518581134	0.138614365
0.022655113	0.402121608	0.378700706	0.07194792
0.073370059	0.285926451	0.037573224	0.266805873
0.497071176	0.367186523	0.371715703	0.210789773
0.036486454	0.015070451	0.216760844	0.373375127
0.519024536	0.50469238	0.257361696	0.336108887
0.218797046	0.269785512	0.133954764	0.490528112
0.207418678	0.52050598	0.007302036	0.390785777
0.51400199	0.270268192	0.072977633	0.261009992
0.362374001	0.03813201	0.204764833	0.494327481
0.394145243	0.268390424	0.276890431	0.409678742
0.012875504	0.037958435	0.333431554	0.175965386
0.326929215	0.184916106	0.059512817	0.291517645
0.039793122	0.442356274	0.411644435	0.043815938
0.284679127	0.267076551	0.360518042	0.033961088
0.42042863	0.50202242	0.522283398	0.161029613
0.41490828	0.470453124	0.368183574	0.082453349
0.018550754	0.518288584	0.352267958	0.020675921
0.432867902	0.20770467	0.013971973	0.483766187
0.167461505	0.463844497	0.002041499	0.089072852
0.045864285	0.40887882	0.469478433	0.340553747
0.204516934	0.34251052	0.009502972	0.515043319
0.485124713	0.014390199	0.144125742	0.396271929
0.383647278	0.523075811	0.279036479	0.410101261
0.394753238	0.503285119	0.213687717	0.242849731
0.386445699	0.492596175	0.368868914	0.443099791
0.419823999	0.394668797	0.245426536	0.012194023
0.09113941	0.188131039	0.239630552	0.063258038
0.287240579	0.121738915	0.472045461	0.426378758
0.462599596	0.005504758	0.135289503	0.389793844
0.405364438	0.43444439	0.023588405	0.004903033
0.008111752	0.016209601	0.324544146	0.230687565
0.46518122	0.209414163	0.004853419	0.04023088
0.245012215	0.362267197	0.499891777	0.341494867
0.398214466	0.084497336	0.236341106	0.359149978
0.058977619	0.305688332	0.460711984	0.463587511
0.047835457	0.229253463	0.369988005	0.450900231
0.072320862	0.307293324	0.422693115	0.274485396
0.020325747	0.077886627	0.055283984	0.395460451

0.510912714	0.08047191	0.034891568	0.137757423
0.106306813	0.434207806	0.154201805	0.488603939
0.384999849	0.387096057	0.08513364	0.468661259
0.032887168	0.493618231	0.318583746	0.045941014
0.29686769	0.01416976	0.482268735	0.113286936
0.307989235	0.364942405	0.14187348	0.019796029
0.161687958	0.055202749	0.433141923	0.411863237
0.040013811	0.209503995	0.282596582	0.377438294
0.250839164	0.37978246	0.504098948	0.345643906
0.071262999	0.177316024	0.367415579	0.36946474
0.235068676	0.45873424	0.249534019	0.513135179
0.362396866	0.440359572	0.461313719	0.28955937
0.248142611	0.35141675	0.318793995	0.017238134
0.319888469	0.409732179	0.331561228	0.131045738
0.235013963	0.359162694	0.048203588	0.205264657
0.313206324	0.264963104	0.140684642	0.386358544
0.36779319	0.366635929	0.047573742	0.049961968
0.533693734	0.418643628	0.111582762	0.366775803
0.171720105	0.295817848	0.404657403	0.408800823
0.321465821	0.4171661	0.38463674	0.345410519
0.417051738	0.226767797	0.520533686	0.082482734
0.06754653	0.406269682	0.08908838	0.094253919
0.13619491	0.025540399	0.269927387	0.394065577
0.357678803	0.353667522	0.315728338	0.074823422
0.317324015	0.350958738	0.330624988	0.173957341
0.071111502	0.492853418	0.009223198	0.061047349
0.392997352	0.37448874	0.344954104	0.224899685
0.270407534	0.154823379	0.281807162	0.265669882
0.289511745	0.342679159	0.37107207	0.027842615
0.019085723	0.462864459	0.340052096	0.025202968
0.091382161	0.343868459	0.316087521	0.190181235
0.024652592	0.476081776	0.27600994	0.385304814
0.303952037	0.068834884	0.355372249	0.083255629
0.057255725	0.453817094	0.35830726	0.515391064
0.34130731	0.371956718	0.451032875	0.400271942
0.127486667	0.355505258	0.021841557	0.395981731
0.347631344			
CRMP1 0.381611135	0.421783596	0.198024118	
0.335705625	0.231350901	0.203311977	0.121318837
0.164464648	0.048668282	0.003756888	0.008749639
0.284232788	0.071174188	0.127799364	0.111216153
0.387109461	0.087137965	0.254919506	0.166819614
0.366961022	0.072057145	0.315701221	0.086471835
0.157908762	0.055641643	0.049442867	0.102120348
0.432387233	0.059883714	0.125457147	0.342685396
0.14244198	0.412138151	0.400891003	0.269694859
0.385961781	0.333152275	0.066583657	0.097432932
0.422488263	0.310241358	0.357170445	0.215683343
0.040735131	0.384526441	0.13993572	0.272641273
0.140330964	0.023225784	0.116828624	0.373034039
0.330091501	0.192754613	0.152000273	0.093000934
0.048118666	0.126687632	0.070757184	0.262120381
0.130103872	0.088826606	0.35345261	0.050035271
0.079145255	0.207380461	0.147466554	0.173784418

0.139644953	0.336391919	0.056293877	0.257844534
0.003003258	0.357973493	0.289564625	0.085336091
0.053834093	0.015184464	0.158931669	0.215341108
0.448007966	0.421439278	0.410968649	0.399183228
0.101535169	0.2165481	0.190190349	0.107569074
0.392449047	0.089604353	0.101916212	0.374001076
0.411850884	0.396082056	0.116260887	0.177495455
0.283475743	0.414961556	0.160652098	0.160104211
0.087027731	0.077165741	0.330730134	0.417711906
0.371881335	0.126166165	0.104291098	0.383141148
0.080467446	0.17941262	0.267587432	0.169534664
0.44902891	0.021089667	0.134646958	0.19314385
0.243569957	0.381168568	0.389493111	0.125983585
0.234540253	0.030068194	0.040561664	0.074555498
0.157018101	0.075490627	0.348134958	0.375324717
0.166191453	0.149464455	0.171371719	0.01115365
0.04001275	0.214423555	0.361144925	1
0.39828898	0.332614667	0.085456718	0.316246167
0.243500133	0.407221889	0.017479345	0.107517512
0.213230044	0.209329172	0.187978368	0.243257237
0.129484325	0.128394792	0.114148288	0.434536017
0.375155708	0.080728207	0.225411651	0.368554539
0.100775978	0.194518626	0.11855942	0.143669932
0.052263995	0.352639361	0.044778532	0.219486772
0.061290083	0.088929062	0.061897143	0.166149608
0.116213206	0.289122666	0.352676808	0.128937918
0.158804896	0.275141833	0.19636494	0.24056237
0.003914322	0.090094848	0.16262101	0.409448083
0.193294254	0.447995443	0.203832884	0.189369478
0.186410649	0.409260154	0.011691124	0.377193444
0.396512232	0.145002791	0.195594825	0.313748734
0.107338932	0.371497384	0.045541038	0.370697293
0.31289422	0.110466877	0.445967252	0.097999952
0.38920439	0.03805125	0.163018348	0.320473365
0.267321552	0.013061391	0.104993817	0.311606838
0.049171144	0.068295782	0.392042222	0.305535167
0.188273602	0.404013511	0.449627414	0.095944387
0.202681759	0.197595436	0.037548715	0.199308323
0.396805925	0.118804524	0.363323821	0.150646299
0.227183321	0.380603242	0.31740487	0.058089407
0.04417969	0.176792445	0.021187147	0.141205633
0.329619998	0.059497877	0.135754045	0.226928321
0.069544021	0.417964091	0.099954007	0.108695544
0.0658607	0.182512066	0.196487546	0.070500619
0.057889757	0.111490155	0.122101499	0.13509766
0.040578642	0.07191646	0.055698208	0.106733709
0.041705667	0.094336955	0.090956319	0.301944181
0.158434045	0.061072351	0.149898079	0.152946012
0.087384884	0.029171423	0.412248624	0.365356951
0.28346889	0.118136501	0.07024183	0.091719645
0.375260666	0.119960107	0.138660596	0.360021435
0.065124734	0.437730571	0.091828718	0.08115413
0.125765382	0.14197312	0.428642439	0.025426388
0.281692017	0.375086572	0.244551512	0.091916046

0.314484965	0.307097068	0.036419651	0.343583712
0.326579002	0.040984557	0.418293102	0.103609271
0.041284143	0.337475665	0.258550302	0.077752768
0.078284076	0.106796011	0.051981959	0.209647335
0.411118576	0.334712282	0.276307337	0.151142938
0.035748259	0.006477038	0.159668847	0.240087032
0.393158991	0.402614298	0.21179814	0.122193044
0.188230084	0.099747471	0.11145192	0.40029239
0.245138671	0.42889521	0.022359271	0.317263281
0.368318559	0.148493667	0.050243745	0.156939396
0.317560227	0.052064277	0.234675196	0.383668455
0.365629094	0.132226798	0.108854169	0.373245112
0.022376596	0.052774778	0.219478097	0.098226386
0.129666784	0.135977975	0.033475504	0.182830132
0.033382592	0.347025109	0.441721825	0.036700351
0.128201523	0.181747492	0.300567613	0.050353989
0.333050464	0.409284959	0.402675166	0.191230371
0.362567611	0.355509442	0.168798729	0.106023391
0.032042301	0.393167045	0.342152808	0.018952546
0.434440321	0.114726753	0.007141078	0.418396877
0.152318027	0.352142136	0.005411454	0.104202859
0.075914787	0.422001788	0.352078074	0.341425403
0.071571313	0.339443237	0.029972712	0.438613963
0.373703576	0.0217139	0.104805482	0.303370081
0.262854179	0.446433046	0.105521325	0.321656395
0.21058819	0.359328575	0.106018267	0.173088256
0.339315909	0.363538538	0.379276578	0.404075747
0.399356123	0.34687952	0.193730124	0.017438648
0.058067153	0.133950605	0.06547187	0.028394511
0.240783806	0.076113172	0.428816979	0.428083034
0.37726048	0.016753446	0.124695097	0.328252896
0.406088938	0.452105921	0.075221313	0.009464074
0.016551373	0.009019534	0.26256103	0.1753406
0.398499644	0.062127464	0.013511435	0.063568862
0.171064948	0.229230056	0.38473334	0.353997971
0.379718446	0.100836095	0.068007576	0.394631217
0.041750597	0.282802395	0.380442705	0.371954355
0.092216829	0.170107631	0.240086878	0.428803812
0.027436992	0.232876493	0.309112226	0.107954833
0.066331439	0.075949496	0.051935746	0.424501243
0.361535819	0.034606338	0.02090776	0.080078415
0.060660296	0.43265349	0.094435174	0.443275579
0.383301105	0.416002053	0.081360677	0.378331095
0.024784958	0.364186479	0.139179928	0.027907849
0.256878784	0.042511127	0.388188057	0.02788043
0.160919011	0.373422329	0.075896269	0.035701393
0.088088739	0.060936838	0.39859856	0.390242557
0.072378832	0.181948395	0.213856037	0.378147332
0.17180222	0.321222957	0.385654444	0.142590104
0.064161198	0.1360703	0.387819449	0.324261036
0.167358566	0.434770503	0.088199641	0.440649534
0.355261864	0.433887372	0.377053445	0.195299729
0.112631886	0.258046369	0.226389982	0.016953583
0.255315949	0.374719598	0.252906591	0.060241128

0.111424171	0.178844775	0.106340215	0.19337196
0.120642531	0.184314064	0.092036596	0.443696697
0.349454976	0.32720643	0.10779167	0.044445222
0.444153026	0.38971337	0.101757676	0.288080832
0.170381961	0.172891421	0.385472231	0.36843373
0.160436114	0.335217713	0.388527567	0.242868568
0.344099887	0.166892376	0.402001238	0.107922397
0.048268879	0.291937787	0.045508417	0.100114115
0.106141195	0.042117125	0.091745245	0.390545112
0.119797806	0.259426387	0.174480572	0.092325001
0.217216583	0.216366133	0.255304135	0.10553502
0.048903799	0.362382732	0.016535861	0.079804886
0.140162564	0.316476435	0.133018091	0.165556411
0.102846907	0.149727732	0.17733872	0.152835067
0.078692906	0.178859879	0.314063745	0.05535242
0.03504273	0.450131098	0.208099106	0.064308956
0.094889386	0.290292275	0.283125814	0.085245899
0.04161797	0.351967969	0.09431794	0.23071627
0.232478818	0.105231166	0.341271444	0.102732052
0.120344594	0.388577726	0.217436086	0.386291716
0.217110435	0.34718091	0.366198525	0.34713755
0.184107534	0.212293845	0.035919504	0.435520464
0.103506008			

CRYBG3	0.369237892	0.501084084	0.354487412

0.366410301	0.379829525	0.308594777	0.238740157
0.239290826	0.110803626	0.00665179	0.024746846
0.35942047	0.170094024	0.249827899	0.284759551
0.386761526	0.148879208	0.392243406	0.281622815
0.389104432	0.122757964	0.364086481	0.140146932
0.300624456	0.11355515	0.094942054	0.248052617
0.43861177	0.110461467	0.260608012	0.350215496
0.162393547	0.406934238	0.486905654	0.344667288
0.471017837	0.335592632	0.029863559	0.254541662
0.421911638	0.360076093	0.379646245	0.323839048
0.068810407	0.456361607	0.261938393	0.372451525
0.257167744	0.042228844	0.232805433	0.431476386
0.425439904	0.327632274	0.267879349	0.190163309
0.144593038	0.22746831	0.192332787	0.284974366
0.253408188	0.164918356	0.443753128	0.114622809
0.173136295	0.31572875	0.198316425	0.297702766
0.307691535	0.345696868	0.048768666	0.326811667
0.004008855	0.384770745	0.375716214	0.243491472
0.071392319	0.025785699	0.224589315	0.322419244
0.460299061	0.454455899	0.461667812	0.464116238
0.178826607	0.344341935	0.335430638	0.199957426
0.42280241	0.191130898	0.140029001	0.378219164
0.468911483	0.484206982	0.220800892	0.295994903
0.360218365	0.46880087	0.343780932	0.359735111
0.258900231	0.091795248	0.411433113	0.484508082
0.386222884	0.270720977	0.183507488	0.383253391
0.103632408	0.331620616	0.345492456	0.18332217
0.469076245	0.036193932	0.237616508	0.326898269
0.31595778	0.396731993	0.476894007	0.230108225
0.377034034	0.033102976	0.11427277	0.072307532

0.297798702	0.138996867	0.438744627	0.427902166
0.257651208	0.336908038	0.311066522	0.008236065
0.047108603	0.26331137	0.385020082	0.39828898
1	0.365410781	0.218542702	0.334633806
0.327293539	0.456397225	0.044605492	0.160463335
0.355850195	0.266790985	0.281543504	0.358996971
0.285521514	0.179761874	0.176958228	0.500936068
0.421622438	0.126019788	0.355770673	0.381593403
0.273053264	0.320471366	0.164510209	0.268837747
0.081462393	0.440286708	0.050015075	0.356409244
0.199219917	0.14790916	0.039696361	0.285880168
0.254546173	0.374102915	0.431206704	0.170852764
0.306580264	0.364624197	0.315724025	0.307630973
0.01237503	0.091611961	0.275166842	0.477498165
0.239250245	0.488156417	0.338353694	0.338865262
0.304594455	0.428330438	0.00844348	0.460792725
0.486827316	0.266125982	0.322062266	0.385553083
0.294289676	0.420803979	0.04500849	0.383904452
0.301250331	0.140459464	0.492866641	0.183034628
0.441905552	0.063928746	0.313238714	0.382316402
0.319035583	0.014701661	0.218954151	0.358161671
0.05293617	0.129252395	0.383926862	0.371824335
0.331868608	0.411425456	0.477265855	0.274333053
0.363728029	0.297380986	0.032025278	0.327453063
0.402288836	0.299167757	0.428675756	0.276457378
0.283706431	0.453955522	0.364484384	0.183744002
0.072829354	0.330383888	0.028493859	0.314402547
0.375338668	0.126056517	0.315631707	0.227344762
0.099974585	0.498168629	0.183212329	0.228784087
0.126041445	0.306210217	0.316837648	0.18071736
0.152993085	0.208867373	0.303613758	0.337164322
0.063193366	0.119977093	0.039970333	0.256396118
0.101206824	0.236303171	0.230171548	0.324739883
0.292765605	0.10721351	0.322030545	0.300315746
0.146960622	0.031860138	0.486420241	0.393126435
0.290649815	0.20868034	0.076950104	0.234238615
0.436923431	0.234154904	0.256438071	0.429418302
0.060000382	0.476613341	0.219501281	0.147836582
0.290729413	0.269529432	0.458932221	0.062337745
0.367337678	0.444693979	0.363355384	0.201200675
0.365769194	0.376615095	0.052606396	0.358629898
0.335567822	0.053846446	0.492987535	0.152271902
0.043641682	0.368569987	0.329819056	0.116000506
0.117293931	0.274038196	0.079741752	0.306046732
0.491482687	0.364835841	0.349273617	0.250546252
0.061252308	0.009932029	0.247667037	0.353912736
0.47342709	0.475330539	0.314065661	0.288556446
0.286968962	0.275325059	0.186114473	0.476146418
0.278222864	0.49529388	0.019145365	0.36860656
0.462805378	0.262832588	0.09713043	0.296591291
0.356567928	0.082267361	0.30157624	0.463756066
0.42481461	0.314057769	0.293749881	0.391146161
0.032073514	0.079606405	0.35737625	0.188332412
0.330727793	0.263489437	0.053481527	0.33765165

0.060537573	0.424320135	0.437261581	0.082463876
0.246910896	0.330253756	0.36872936	0.069070569
0.405738077	0.474421619	0.486970806	0.25262224
0.375831864	0.445276124	0.290448793	0.132279728
0.035097825	0.477577877	0.3580704	0.032472904
0.461829652	0.211213641	0.013792567	0.484026052
0.249867953	0.415131485	0.008780172	0.11547093
0.078978984	0.436692569	0.445454428	0.363513326
0.18808013	0.398104392	0.018644884	0.50036552
0.455353114	0.026656476	0.174013226	0.359359517
0.320762846	0.513423489	0.262525673	0.39420519
0.314768153	0.454603436	0.225301608	0.291224251
0.397932753	0.4490491	0.379146352	0.456295765
0.434233997	0.382471931	0.301920067	0.029120769
0.151337785	0.240620008	0.211454782	0.090865477
0.294401885	0.15996281	0.46965021	0.456323082
0.442432668	0.014244486	0.201535926	0.372239372
0.43601164	0.480282033	0.056646761	0.010922784
0.013262938	0.020728362	0.363016664	0.290330722
0.46774293	0.168568307	0.012921912	0.07990729
0.313517669	0.330852868	0.469360142	0.375730134
0.395609018	0.13291259	0.199471716	0.391901621
0.084570087	0.278739938	0.451008343	0.450922053
0.087939665	0.299213895	0.374537814	0.480429432
0.062024894	0.336019954	0.360795344	0.266734783
0.041029632	0.117190404	0.096658625	0.414644852
0.452654337	0.07345629	0.038138115	0.134748457
0.129983882	0.458008745	0.180143908	0.499872875
0.417825211	0.424538998	0.11039596	0.462278515
0.052971875	0.445581831	0.293773187	0.058767158
0.352271297	0.034733842	0.441025704	0.110411015
0.301441806	0.371440231	0.200685832	0.035819625
0.177657531	0.081316625	0.456252657	0.429664297
0.068119383	0.313078437	0.3432517	0.403538732
0.282124808	0.360070132	0.470184252	0.292137802
0.120467374	0.164517603	0.379216239	0.334463235
0.26795986	0.476603552	0.247236033	0.506856479
0.355569717	0.467469077	0.442534312	0.337127734
0.223123868	0.346768927	0.297760441	0.027884377
0.370016679	0.421749514	0.385657845	0.145649202
0.249154593	0.315349944	0.113091527	0.21107151
0.261148384	0.268515334	0.170883497	0.438241745
0.370441667	0.330927829	0.093660964	0.087294708
0.519341714	0.438967873	0.183259514	0.354558975
0.240346842	0.327787482	0.401910952	0.388045439
0.350480334	0.39604489	0.390936197	0.299394009
0.407105635	0.308466238	0.491431652	0.130380438
0.095925127	0.345844748	0.101631826	0.139027569
0.181541816	0.053326826	0.230968416	0.391711438
0.282018193	0.347065094	0.307227612	0.134867774
0.346053778	0.361754389	0.368639775	0.213868895
0.094438716	0.457548589	0.018942675	0.095896925
0.296666185	0.375772019	0.292211265	0.282561962
0.247179068	0.199150529	0.312886691	0.272338102

0.216422077	0.326685401	0.379031323	0.067428674
0.040544174	0.484750002	0.344681088	0.051414431
0.142117443	0.341080399	0.364796763	0.227998754
0.043607814	0.436596909	0.257703417	0.374649282
0.356217592	0.121647691	0.356190458	0.13650779
0.128505535	0.434076663	0.343900196	0.471726692
0.364477923	0.368410568	0.427913729	0.385299276
0.190671394	0.330886667	0.053483942	0.439736696
0.278728208			
CTNNBIP1	0.398666924	0.44759767	0.383007202
0.304805426	0.329619507	0.300799771	0.128056437
0.146835456	0.05446353	0.000314017	0.008620796
0.381138904	0.121662048	0.171765762	0.28390968
0.322330287	0.081660121	0.345977195	0.157247201
0.316110042	0.047847196	0.287095662	0.051266131
0.261240315	0.065567862	0.034235353	0.205896174
0.337110633	0.042405229	0.239695119	0.299988684
0.07962023	0.374037492	0.486969095	0.327079079
0.488339497	0.399478947	0.010750853	0.199551708
0.374572895	0.360328888	0.309459143	0.21955145
0.018139136	0.45958329	0.151579399	0.343971047
0.199274975	0.012748575	0.189210709	0.415644825
0.435996023	0.341205575	0.166481801	0.089768122
0.121702639	0.164641504	0.217172884	0.269730728
0.135608044	0.107836334	0.441051528	0.04879521
0.105096446	0.1927264	0.11575717	0.180795349
0.314434356	0.337824362	0.010482992	0.401131387
0.000114251	0.299501238	0.317801735	0.287197199
0.022327888	0.005720723	0.134051107	0.2258564
0.354504274	0.445903688	0.365246391	0.392099951
0.113152546	0.23506019	0.355615738	0.098706888
0.378195892	0.09811992	0.081857276	0.318203463
0.380072043	0.46293289	0.139239822	0.204136403
0.32677549	0.40658566	0.350490385	0.322359535
0.278382694	0.045924498	0.386766311	0.416204883
0.353493638	0.288284404	0.098769652	0.322430117
0.034483324	0.333319623	0.242304528	0.128062066
0.354479955	0.007497263	0.154177593	0.242252788
0.192059912	0.377420312	0.46556543	0.113407958
0.346878285	0.009552728	0.108385647	0.02322591
0.238601492	0.145585768	0.476151949	0.337495345
0.166379013	0.283162304	0.183835461	0.000385219
0.014513394	0.177903475	0.375384394	0.332614667
0.365410781	1	0.143496005	0.349538608
0.377062372	0.365387843	0.04658274	0.057664768
0.332809868	0.168152063	0.15800236	0.353681868
0.217770705	0.074505006	0.073133409	0.419610517
0.374777883	0.043916902	0.321935167	0.317443315
0.219433959	0.277089711	0.063983219	0.173287883
0.02124958	0.46150056	0.018114338	0.296252695
0.231234575	0.098588117	0.01026944	0.191659879
0.198409457	0.33932434	0.481804686	0.06892428
0.208074179	0.303419373	0.276975384	0.321005403
0.002179562	0.032188297	0.27009972	0.406508866

0.234221743	0.423810144	0.224227809	0.25732467
0.204507917	0.35865536	0.005721947	0.466469296
0.474984638	0.28140035	0.274539367	0.362828321
0.32632209	0.337881878	0.055881051	0.314436771
0.343615074	0.050478373	0.387292727	0.096498313
0.426082796	0.018892147	0.229607741	0.383224159
0.36761997	0.004497158	0.159569695	0.28762579
0.019613735	0.053596704	0.37107101	0.305759031
0.279561565	0.376306253	0.355959465	0.311844065
0.324027496	0.169097576	0.008140934	0.262745013
0.395655144	0.334280544	0.390948325	0.162797655
0.326027607	0.401208668	0.386921689	0.159274426
0.019159815	0.226434361	0.010035393	0.245045885
0.388887158	0.054394954	0.353010545	0.13653088
0.0431481	0.432953245	0.160520777	0.261693012
0.127450393	0.281698736	0.18223651	0.132830404
0.124747613	0.106239738	0.290534032	0.293479125
0.015159394	0.048252656	0.01746656	0.297265526
0.068740308	0.231115428	0.232264994	0.35126268
0.227518648	0.054836024	0.273511028	0.279177204
0.080341788	0.006793316	0.432592982	0.320742242
0.303067197	0.097186724	0.024934777	0.259258503
0.342951928	0.257316622	0.261930287	0.352189264
0.022245231	0.380886394	0.14449667	0.071696173
0.279729506	0.161908296	0.347431808	0.039448666
0.343664356	0.394194912	0.259041756	0.117742075
0.335743146	0.321347797	0.012441435	0.371928978
0.357296777	0.014148683	0.452474993	0.085957676
0.011464268	0.370577146	0.356592537	0.045442932
0.050629469	0.214206279	0.022872674	0.206694678
0.426453264	0.298107571	0.339214482	0.208534464
0.015987441	0.001737036	0.173540138	0.393333219
0.481422613	0.441519963	0.190071964	0.261780548
0.158737382	0.257630314	0.112027905	0.413733293
0.242272311	0.480635638	0.002686614	0.376518487
0.485075141	0.229313842	0.04560744	0.212198231
0.325327278	0.023038534	0.166042794	0.443728139
0.363259655	0.25509131	0.275643967	0.366106463
0.005665147	0.01882678	0.329412797	0.184326397
0.287286779	0.149250944	0.062240813	0.273054704
0.016958264	0.376808375	0.3645683	0.032907555
0.24768442	0.283749353	0.297331967	0.017921057
0.381448373	0.468651357	0.477420787	0.12012623
0.380226527	0.414443342	0.351631913	0.056838276
0.009830457	0.483687771	0.357495192	0.009320929
0.355895702	0.195502243	0.002310121	0.423605029
0.121278329	0.448323692	0.002652857	0.14583061
0.036144611	0.372282366	0.444732457	0.304864538
0.185736951	0.302723326	0.008452425	0.455819564
0.420497034	0.007799332	0.111591386	0.367387819
0.371254657	0.467042669	0.277200857	0.36854849
0.355764278	0.460010098	0.145535762	0.186091748
0.345242782	0.457510264	0.353781439	0.409327416
0.345678776	0.343832307	0.184122101	0.004952186

0.123057812	0.151974459	0.242349627	0.044193842
0.289023922	0.089390609	0.424202212	0.363191976
0.389706921	0.00170309	0.095735224	0.37243245
0.339992432	0.362603892	0.023803015	0.006750646
0.011597219	0.009318122	0.276424303	0.196135388
0.41364746	0.168869561	0.000559451	0.022957982
0.21220223	0.328862413	0.437670119	0.280838759
0.394303093	0.049682198	0.180932685	0.309748755
0.04148618	0.337474225	0.384598578	0.410324991
0.032697987	0.200312909	0.356602218	0.368775569
0.079750055	0.2352373	0.376802725	0.203620745
0.014719853	0.054213753	0.040347289	0.357775795
0.478379618	0.034527949	0.012676877	0.119883608
0.057628372	0.378033209	0.094388209	0.408470614
0.345811228	0.326708183	0.053873275	0.437627041
0.017700894	0.443797768	0.272769931	0.044862716
0.261514012	0.007413633	0.435325704	0.107352404
0.246763332	0.348895041	0.18195775	0.010981701
0.209612346	0.029885927	0.352408582	0.37262678
0.029520349	0.175105923	0.241129461	0.346373871
0.213195196	0.317453561	0.465272147	0.348029852
0.040719052	0.170275291	0.324255199	0.361856828
0.170968338	0.375659089	0.204068934	0.456418551
0.372686405	0.405694422	0.40202359	0.256521355
0.177573603	0.350135792	0.268117375	0.006550838
0.272758391	0.34256798	0.317621888	0.100485471
0.164064889	0.365835928	0.035093132	0.216851188
0.326377028	0.235711758	0.075943639	0.341259015
0.34914547	0.362820789	0.027341133	0.036229234
0.473036228	0.398115525	0.102367763	0.331422194
0.127616397	0.248326865	0.386956521	0.378407593
0.353244653	0.353245245	0.345092558	0.286034861
0.356968493	0.17948963	0.477683907	0.072634228
0.063601954	0.390301976	0.043975322	0.06327773
0.08187843	0.016783639	0.293956116	0.337366205
0.351667032	0.264686152	0.278625476	0.056662204
0.258041705	0.389123794	0.267978986	0.119496142
0.037022798	0.444338896	0.004878477	0.034049739
0.34178191	0.371610011	0.305077667	0.181198429
0.260432976	0.121835206	0.200212657	0.233940534
0.21852359	0.356320492	0.369033253	0.022400158
0.007215162	0.375617992	0.350393735	0.019863835
0.070983061	0.370370714	0.273829746	0.150512603
0.015902918	0.419203322	0.24801263	0.358081293
0.261693866	0.041978917	0.334964221	0.075533632
0.048688427	0.413052987	0.370860033	0.468250858
0.338261131	0.302960491	0.442495282	0.364536105
0.133401332	0.408077158	0.011947999	0.362214792
0.350244128			

CTPS	0.159515259	0.281695749	0.118288757

0.321705087	0.184521704	0.1514299	0.176055601
0.047730496	0.154494876	0.046111718	0.139303033
0.162018614	0.128662633	0.093255267	0.086304769
0.312723972	0.130067398	0.05762504	0.201062936

0.237937578	0.086059255	0.325949801	0.123268206
0.090212152	0.154912032	0.165628061	0.153901158
0.295443312	0.162416484	0.110185899	0.197920125
0.07215414	0.198469988	0.235766101	0.265416604
0.221763052	0.135550896	0.005840672	0.112704354
0.187170549	0.282932377	0.251495762	0.26663826
0.121171472	0.199478353	0.244343092	0.185379613
0.050685343	0.130494181	0.049633813	0.185710163
0.218690407	0.197545087	0.074828914	0.146105986
0.124267811	0.042578244	0.089335791	0.115687672
0.201768793	0.042446671	0.23103725	0.140291254
0.063583831	0.070724792	0.084363619	0.11111902
0.084390898	0.160987641	0.03067373	0.118321289
0.013372008	0.235789272	0.17665686	0.099133018
0.107342119	0.099007338	0.094098731	0.256097409
0.288598291	0.188200146	0.327976243	0.307484677
0.118718691	0.138039114	0.13209884	0.156798192
0.216699837	0.176927047	0.063652085	0.327489747
0.324924356	0.26099669	0.14172621	0.19119529
0.277612428	0.243463725	0.134960896	0.166591699
0.120792836	0.061611146	0.21987263	0.297727411
0.164563525	0.108219537	0.180933984	0.218374603
0.097730898	0.050313516	0.3000395	0.046629773
0.30162807	0.088406747	0.169923791	0.203645548
0.070956632	0.213130009	0.258831464	0.191832426
0.129956885	0.054127731	0.11850536	0.054448137
0.080474025	0.050355016	0.191242428	0.312473196
0.057310043	0.109436748	0.128329847	0.021680654
0.080481608	0.059416167	0.192824504	0.085456718
0.218542702	0.143496005	1	0.14574071
0.192380164	0.321558259	0.017354404	0.10553211
0.170690867	0.063681535	0.24645379	0.171787129
0.221452607	0.081467478	0.125612557	0.317970545
0.194713975	0.135469486	0.045350315	0.294725674
0.128037622	0.204837879	0.103578638	0.114495673
0.101259319	0.216612592	0.068124742	0.073403268
0.117108373	0.090932874	0.013122468	0.088316897
0.096168154	0.265587573	0.173163156	0.102841383
0.143830244	0.286342145	0.069848133	0.246036301
0.112112964	0.058546183	0.03796433	0.295567285
0.09591886	0.270797633	0.102633467	0.105917118
0.22626072	0.294126783	0.001648355	0.2173887
0.260493237	0.040834219	0.242165037	0.168218439
0.144909374	0.321077379	0.003384167	0.270971317
0.100957027	0.093082416	0.331909672	0.209112118
0.201071772	0.134917997	0.076500043	0.247252819
0.188994065	0.017394558	0.08845247	0.249274915
0.058135011	0.080630598	0.198006949	0.274070859
0.268299483	0.25867806	0.337040277	0.136313674
0.189516524	0.09116459	0.033052603	0.169624988
0.20663193	0.065311574	0.2159466	0.110925465
0.07082124	0.258046609	0.180498229	0.089866958
0.122238309	0.1256026	0.036660007	0.2294724
0.167152269	0.147459938	0.140840647	0.046773922

0.111401483	0.309334679	0.079359128	0.068639601
0.055486533	0.186090435	0.141509689	0.121034856
0.132726193	0.154709958	0.119525874	0.132972698
0.079780179	0.140735412	0.016413769	0.061262438
0.116771642	0.08435503	0.077892616	0.150878356
0.07257195	0.108690897	0.191744862	0.088619926
0.133623814	0.06617287	0.274570219	0.249740378
0.11484085	0.136461879	0.074780484	0.057609164
0.311292644	0.102927966	0.029776967	0.275269695
0.032407582	0.299039722	0.156398465	0.18581693
0.115842367	0.224039756	0.326697958	0.053974541
0.112779369	0.240331885	0.157506028	0.192046467
0.178492548	0.225596178	0.088227046	0.15790987
0.146917188	0.096810316	0.276123924	0.103758553
0.065813263	0.229908096	0.250900776	0.14672328
0.122587801	0.208062195	0.063400197	0.277057114
0.305401072	0.341267385	0.250645374	0.080044445
0.128834264	0.059010543	0.094001434	0.111692698
0.2156797	0.269788836	0.174683821	0.189845067
0.233966348	0.141495702	0.029886986	0.3016919
0.034409358	0.233054724	0.034529813	0.24964699
0.222632612	0.065122109	0.159120619	0.174995287
0.18239591	0.116081833	0.068578458	0.240945322
0.254197853	0.145900389	0.132151953	0.18027227
0.062817386	0.081461138	0.211436331	0.06962999
0.116687821	0.156042066	0.022626798	0.058529516
0.136730161	0.242690091	0.270884543	0.176381583
0.157851279	0.049755941	0.2382821	0.101002251
0.25988222	0.229592275	0.241681831	0.075467778
0.195437636	0.277228723	0.161641639	0.08860239
0.065324807	0.237108153	0.157481084	0.074480206
0.309697396	0.051444511	0.088148753	0.262997381
0.16300799	0.159441257	0.013697658	0.010805508
0.048957841	0.286276291	0.228186454	0.190164444
0.053282047	0.175388016	0.002993179	0.271686943
0.253781353	0.067448688	0.099759313	0.216261201
0.214206402	0.259607763	0.135928395	0.27734053
0.176312252	0.235120593	0.116877864	0.267425991
0.270232356	0.235332575	0.212381794	0.224575774
0.299149818	0.24809578	0.276829726	0.089081936
0.128243509	0.068309161	0.088473363	0.195822325
0.097832362	0.179972852	0.234454703	0.305988638
0.280486765	0.033475991	0.082424518	0.201113187
0.257404949	0.298966639	0.01883587	0.010564061
0.004256781	0.106384529	0.165085382	0.147276621
0.277241792	0.055178025	0.037603047	0.053633118
0.10526924	0.229867977	0.258912512	0.295327028
0.217958286	0.100281281	0.107646671	0.264965597
0.152392376	0.084637841	0.303650195	0.241819299
0.048873758	0.145018109	0.069316854	0.325087621
0.031043847	0.216379893	0.167826152	0.14055871
0.012933305	0.12341339	0.148561651	0.272621276
0.215418461	0.14502067	0.122216722	0.019743224
0.160361468	0.266173286	0.150426637	0.320453719

0.268631969	0.330842682	0.07970166	0.24238904
0.135648153	0.220889904	0.16939457	0.028618929
0.068808716	0.036387397	0.205581826	0.164865943
0.064365784	0.154856387	0.130423594	0.048403715
0.057802831	0.102931296	0.342931586	0.295946875
0.041993249	0.104281102	0.164209044	0.186776483
0.114185448	0.317306416	0.228905333	0.108401489
0.128460661	0.026465928	0.220970722	0.128263805
0.058269627	0.321041834	0.168166027	0.280178557
0.165152307	0.230398757	0.246869208	0.231204105
0.095725831	0.124418093	0.231583201	0.070742095
0.188984833	0.321165399	0.096528531	0.176548711
0.23228519	0.130631521	0.045347776	0.015098332
0.096968194	0.129215355	0.142786221	0.311411575
0.127391117	0.138097461	0.029315851	0.159905456
0.277127002	0.208598801	0.085436737	0.242385159
0.119168182	0.267855583	0.235215733	0.244193519
0.070535552	0.252783725	0.219363643	0.206879612
0.215243322	0.200325565	0.252548767	0.02128222
0.102938028	0.120782639	0.174112857	0.117944103
0.167729114	0.08273733	0.066639851	0.258756582
0.060356627	0.284283205	0.038092297	0.101159171
0.174048641	0.058754944	0.225250391	0.126970495
0.153234191	0.247191432	0.040213368	0.087711856
0.068401419	0.251898759	0.179406419	0.126353183
0.097784082	0.068433	0.233461832	0.076061331
0.091969799	0.050737717	0.249356788	0.045667008
0.055693226	0.317775729	0.075800468	0.028979905
0.057395499	0.112851322	0.052372072	0.186318541
0.053335543	0.251793832	0.104676457	0.173286966
0.169921389	0.080953198	0.128845598	0.038467497
0.053248378	0.190650361	0.145815725	0.24262731
0.094288576	0.346847033	0.170021836	0.208187322
0.034600854	0.083978005	0.087949198	0.250761325
0.083551773			
CTSD	0.329354289	0.477019203	0.276591929
0.311598668	0.300632253	0.31810814	0.183181571
0.223442717	0.0589917	0.003910597	0.011075436
0.301864326	0.133498767	0.230324384	0.263139123
0.300180546	0.078831064	0.312708125	0.190401287
0.327592135	0.103850974	0.272548089	0.077757034
0.182065635	0.054821887	0.043807476	0.154764949
0.362548102	0.051385545	0.302177595	0.339601657
0.069700957	0.360875453	0.491630993	0.283118186
0.477711781	0.318430299	0.01591121	0.244165156
0.38836355	0.302686196	0.327708417	0.243198219
0.033341108	0.489960693	0.191428989	0.269931183
0.235369367	0.017018644	0.266222564	0.460634076
0.447090422	0.303039704	0.240227174	0.135558014
0.124004015	0.267777194	0.145758744	0.324648962
0.181456102	0.201423285	0.44791741	0.074247907
0.134772279	0.266206829	0.097686459	0.249944167
0.219287037	0.34253368	0.022819565	0.287281738
0.003158064	0.313693072	0.262196697	0.182602107

0.027228196	0.012105041	0.115591189	0.271107945
0.383265773	0.436541765	0.376897469	0.389525487
0.112527345	0.212172544	0.254012843	0.121031289
0.387465828	0.143131054	0.094610237	0.288609899
0.389114424	0.470587862	0.12947613	0.169567505
0.338361676	0.45309477	0.305139629	0.28908006
0.209303571	0.03009766	0.41968928	0.428134732
0.385965955	0.184828507	0.09264761	0.35250041
0.04382395	0.303374879	0.261329238	0.121796593
0.395412128	0.015915446	0.12851047	0.291968936
0.180734786	0.343417434	0.453832473	0.154382637
0.288132763	0.013781023	0.069798672	0.024856538
0.247900385	0.07495827	0.479365111	0.376184609
0.253360195	0.25715292	0.237099478	0.000868706
0.015268085	0.141306801	0.350236752	0.316246167
0.334633806	0.349538608	0.14574071	1
0.294433665	0.371433662	0.060789496	0.09118769
0.316462975	0.139036603	0.17411378	0.317023811
0.190408169	0.1229687	0.10038315	0.437906137
0.442708716	0.06743849	0.328507153	0.293177869
0.242857431	0.219124385	0.093759637	0.213301718
0.049283436	0.439865337	0.015148393	0.327923753
0.16662087	0.085336567	0.014362769	0.276033173
0.229710702	0.356568851	0.467897724	0.087202525
0.245466488	0.274077432	0.329898568	0.303784131
0.00571018	0.041153167	0.315888459	0.434019432
0.301435395	0.418915818	0.238856883	0.211747567
0.186596455	0.3377568	0.002575049	0.482551682
0.469049431	0.313321529	0.269676592	0.302264419
0.240636749	0.355496824	0.048474301	0.3015016
0.332455664	0.066838185	0.407488685	0.099594233
0.43487262	0.025843689	0.304055103	0.315848346
0.295489322	0.007793198	0.241847651	0.320303943
0.015088192	0.118430055	0.340455486	0.305809581
0.271101115	0.315975788	0.380907772	0.219430665
0.29550166	0.222785492	0.010040905	0.204846371
0.327349673	0.277174786	0.440359106	0.213386639
0.327464497	0.436026411	0.33535372	0.159097865
0.034891727	0.296476319	0.021268111	0.22151805
0.356200864	0.093207668	0.275736457	0.120439656
0.041524419	0.451936256	0.095784455	0.148733409
0.06399061	0.297571361	0.222911368	0.170988634
0.122006587	0.115399832	0.227027147	0.25916939
0.043316914	0.062491735	0.009638089	0.252359063
0.083813133	0.232441628	0.244573374	0.344224724
0.283263438	0.075691866	0.254037792	0.183403669
0.067035196	0.009408888	0.459553494	0.302279848
0.323489332	0.129608481	0.02896939	0.234136583
0.389290789	0.284646984	0.306526184	0.41149759
0.025745099	0.393498898	0.163764752	0.074558821
0.2335521	0.18809338	0.374567707	0.0519535
0.351330231	0.449206197	0.232681062	0.11283945
0.396788554	0.32963661	0.026633855	0.337040972
0.334223211	0.020787259	0.458999025	0.074946143

0.013185706	0.29520868	0.29466189	0.048997177
0.047938333	0.238444371	0.061859001	0.190741592
0.450557243	0.280531636	0.323591951	0.1330898
0.029432181	0.005414234	0.129427676	0.28243646
0.494253895	0.430999042	0.195111437	0.221671022
0.187432251	0.21013477	0.187247991	0.427698628
0.155101577	0.484960333	0.004996865	0.297755762
0.477079916	0.311060864	0.04385123	0.275388271
0.36938083	0.037919101	0.199761088	0.461633884
0.386760525	0.229373197	0.232533254	0.323844438
0.017913676	0.047888166	0.263628406	0.100530649
0.274450525	0.15476586	0.023546174	0.325353379
0.028538191	0.43005674	0.346068739	0.035784308
0.279674313	0.31493013	0.256622734	0.029047618
0.347539892	0.445293117	0.491059442	0.170505995
0.327714918	0.410726511	0.253361505	0.053854361
0.009412694	0.468695434	0.346252735	0.01561702
0.382545562	0.274697452	0.00712859	0.466542583
0.157591933	0.461537465	0.003750268	0.064673557
0.024280153	0.338797831	0.458646783	0.341018316
0.237845587	0.30511359	0.004934872	0.448687265
0.452325709	0.006817846	0.080807784	0.329213086
0.278603188	0.497466503	0.18612884	0.338927832
0.276019316	0.457235824	0.194390452	0.179107225
0.362968224	0.425277381	0.342926559	0.445830597
0.372130357	0.290389774	0.213071875	0.013044134
0.078696428	0.246410397	0.214889166	0.046597997
0.326270209	0.082126784	0.432871407	0.367984335
0.388294007	0.002997373	0.165550511	0.348477123
0.404390599	0.410500969	0.018899602	0.004063238
0.006498465	0.00724497	0.324972585	0.162304986
0.421132043	0.205320092	0.003232511	0.060923923
0.218352294	0.248328714	0.458968426	0.287154136
0.319001046	0.060690386	0.203226695	0.310417144
0.036115623	0.318641379	0.394621953	0.462001226
0.030177197	0.168219712	0.330362133	0.413090879
0.032340047	0.218634201	0.312408245	0.22693634
0.01228249	0.0480925	0.038722925	0.317066501
0.46269832	0.043578124	0.01679551	0.179733839
0.089014563	0.385172514	0.113578223	0.428098425
0.366521329	0.328004607	0.043307492	0.459042978
0.024741882	0.46030973	0.199004708	0.057102476
0.224732675	0.009936515	0.419494032	0.081516015
0.31710306	0.349184567	0.122058043	0.009130925
0.103548089	0.032746572	0.376737224	0.338962201
0.020294462	0.219810782	0.211669126	0.318650445
0.287336956	0.306916655	0.485960873	0.295331706
0.080405364	0.089413569	0.336204382	0.340961101
0.263006046	0.387584038	0.189973626	0.455978226
0.345312529	0.426165587	0.405895436	0.273624883
0.13858524	0.340182892	0.306570195	0.017020878
0.27765536	0.350023666	0.329747173	0.085006838
0.160288021	0.248459155	0.053072481	0.194147615
0.197164644	0.30142813	0.110819599	0.333383184

0.349127408	0.344080466	0.057928384	0.035569877
0.491080553	0.428432521	0.140995178	0.320095893
0.124881546	0.234422074	0.323803361	0.305416393
0.281609406	0.363856953	0.352986198	0.315712117
0.427269669	0.212984418	0.489009334	0.139076929
0.050076325	0.321213781	0.062883788	0.059013321
0.104879457	0.016071991	0.174688254	0.307741134
0.238741826	0.271226079	0.334399451	0.059171282
0.220363116	0.319956032	0.254796137	0.179099453
0.052534585	0.466733153	0.00636538	0.038341731
0.252617913	0.330636356	0.231446152	0.160893494
0.169750277	0.091363782	0.210639444	0.306323379
0.243834818	0.272177113	0.337027452	0.037584605
0.02194765	0.398225109	0.334633608	0.013906178
0.063921593	0.336046214	0.262489018	0.162952834
0.011622585	0.406725474	0.278985341	0.293293116
0.30296381	0.053783275	0.389250976	0.08326105
0.060812244	0.450784281	0.267223443	0.461585804
0.247550095	0.295793177	0.468679214	0.355725885
0.106808051	0.28202231	0.024877837	0.372451664
0.264859049			

CTSE 0.333557189 0.512089245 0.30418036

0.324469953	0.317336365	0.228585081	0.134620534
0.098137305	0.071799788	0.003234484	0.028821507
0.322053756	0.165593959	0.167084499	0.220223771
0.335231775	0.128954937	0.228453692	0.169364876
0.27352116	0.049767498	0.297288505	0.065496626
0.266731914	0.113189514	0.064135422	0.247016563
0.377337272	0.071615887	0.193711265	0.250089629
0.094587986	0.359727571	0.51503932	0.329297856
0.518696727	0.311779283	0.008249767	0.183769487
0.362557328	0.367951112	0.278535457	0.23092327
0.031348654	0.460874204	0.168589563	0.31273989
0.132430992	0.033860458	0.123333513	0.409660077
0.473347252	0.309207973	0.126053909	0.105077683
0.169721987	0.118794268	0.264188547	0.182958959
0.156497678	0.074143338	0.48640016	0.061545065
0.100159644	0.144774192	0.144556533	0.154354676
0.30619401	0.28099775	0.010882584	0.301870699
0.001222951	0.254651833	0.293771778	0.283261593
0.040994312	0.01875086	0.162315849	0.221631906
0.409287583	0.446732077	0.477792772	0.494594716
0.156189419	0.231613109	0.325521475	0.135113077
0.390802276	0.120592295	0.079861283	0.351199101
0.490220632	0.524569742	0.182979901	0.228188447
0.367369154	0.4500479	0.29067327	0.311604361
0.279194258	0.062484531	0.423320201	0.510080976
0.335065114	0.295073777	0.141948868	0.312797529
0.051655264	0.230997822	0.247787519	0.12530597
0.424621982	0.013927385	0.198062695	0.232530777
0.155958619	0.364088485	0.530474873	0.130748022
0.310977181	0.018294063	0.148231766	0.032753442
0.229666743	0.169019756	0.473135335	0.418266669
0.131321446	0.291543644	0.168991034	0.00291681

0.030478598	0.157640783	0.38968213	0.243500133
0.327293539	0.377062372	0.192380164	0.294433665
1	0.471781996	0.032187273	0.063492853
0.289972778	0.158901867	0.182455471	0.312211728
0.261384092	0.068847934	0.095750383	0.510452314
0.379301715	0.064225575	0.199450782	0.318528397
0.229991761	0.304067395	0.077776047	0.194529596
0.032276423	0.495944871	0.034842734	0.195393896
0.238016859	0.148161265	0.007412023	0.145237653
0.186245668	0.410984058	0.465941576	0.082699523
0.22169932	0.310779141	0.18122862	0.324628749
0.010468364	0.037743366	0.152305503	0.498614332
0.155512537	0.49852827	0.209189868	0.252618607
0.234128779	0.389400452	0.003313645	0.483593856
0.533274117	0.166682569	0.291708474	0.311648345
0.307871339	0.400692998	0.03071077	0.307377821
0.238250637	0.061201938	0.491197351	0.143005103
0.448223937	0.040128741	0.189050431	0.431445135
0.373041562	0.007928387	0.119814329	0.249060046
0.034021849	0.049338781	0.317323991	0.276371405
0.273278007	0.385030806	0.45442802	0.30203227
0.31599465	0.146523786	0.012446991	0.271812743
0.380048491	0.283953454	0.409168171	0.157367262
0.19994725	0.453134116	0.384418227	0.1416994
0.033690449	0.188814404	0.013702573	0.26267817
0.374068602	0.083067824	0.296429563	0.1221663
0.073436048	0.522699552	0.192727068	0.258602926
0.153862642	0.270416301	0.166686857	0.164733813
0.185847848	0.13839061	0.29582721	0.279856575
0.02103856	0.081581027	0.017906802	0.28466541
0.116123276	0.249940082	0.234767229	0.290472332
0.212331119	0.086309575	0.297039674	0.254993451
0.112554417	0.015898921	0.495323349	0.284235311
0.234069006	0.111811995	0.038840249	0.238892483
0.435430726	0.189857115	0.146657138	0.422906611
0.021266556	0.47152007	0.189775833	0.114634318
0.310375977	0.193582209	0.416008778	0.045804957
0.246696803	0.441069386	0.238917346	0.165586836
0.357621298	0.275487813	0.022308534	0.325232068
0.313169	0.03000508	0.528790188	0.125510576
0.022483281	0.351761245	0.372753892	0.071220082
0.076403717	0.2373722	0.022435423	0.238951862
0.511298047	0.326899793	0.362913236	0.212625064
0.032929312	0.010284363	0.199225606	0.305335054
0.498930978	0.523194152	0.200387661	0.3042875
0.174048372	0.279313155	0.067182803	0.509739773
0.160584346	0.511356828	0.006766158	0.404788601
0.513900423	0.158544222	0.08451478	0.18827919
0.329412088	0.036310068	0.134258355	0.488829495
0.401520298	0.261983435	0.269486536	0.302121482
0.009539175	0.024837007	0.302799757	0.200333031
0.275462274	0.173078641	0.077136171	0.194733287
0.036448898	0.428578766	0.407025332	0.058415614
0.211352305	0.179071384	0.298325425	0.031182984

0.46668918	0.509654352	0.514658836	0.102406298
0.375407436	0.499664097	0.328439771	0.075392204
0.021132666	0.526028776	0.264521184	0.017962759
0.439667202	0.120721662	0.011783769	0.472552101
0.128878868	0.425003325	0.001783805	0.075043875
0.0477621	0.433485307	0.471321241	0.236563972
0.138202494	0.248322681	0.007341306	0.529154437
0.479656586	0.019429111	0.151802157	0.395521485
0.386797028	0.510515483	0.295116104	0.451988747
0.330631969	0.50634706	0.159877212	0.221047451
0.393609266	0.514613875	0.318062326	0.430508725
0.41825245	0.321542587	0.200164178	0.012400285
0.138124714	0.112397399	0.223210784	0.074479649
0.184636098	0.13233082	0.467387509	0.43852112
0.486573296	0.005861523	0.085086824	0.379696945
0.384431717	0.43385287	0.019187267	0.006116486
0.007342464	0.028840001	0.224402331	0.211816604
0.487202983	0.141110632	0.004568221	0.022145996
0.190809471	0.33125336	0.50086422	0.266739066
0.395248815	0.070308375	0.196155301	0.284983943
0.078141734	0.217004728	0.486959957	0.449522406
0.041203482	0.213037808	0.243492123	0.458689689
0.100896172	0.255028159	0.318031143	0.233365533
0.013594078	0.085608607	0.068862005	0.374240403
0.508571751	0.073531262	0.03550969	0.077793419
0.087752106	0.438593747	0.129524591	0.507268648
0.377787797	0.381493626	0.084569427	0.47063231
0.040544173	0.48208994	0.303464214	0.032900231
0.219489361	0.011853632	0.475073651	0.153556406
0.18830673	0.257098334	0.188363077	0.021718587
0.195645176	0.053196531	0.457703396	0.448006246
0.040104974	0.153417737	0.236579364	0.279299935
0.162037703	0.349207785	0.493249566	0.274428266
0.054921108	0.149072402	0.284438705	0.261392071
0.134351325	0.482581681	0.252278871	0.526604687
0.300677558	0.435201616	0.468312973	0.238950049
0.2296096	0.251436954	0.238932449	0.0127621
0.246728499	0.423091901	0.224863865	0.154890381
0.218686675	0.320276768	0.031515752	0.111477747
0.299577964	0.173102729	0.10620798	0.38394033
0.245711555	0.2793277	0.022348658	0.064282146
0.533735536	0.409002504	0.085829437	0.2956384
0.143030346	0.269512237	0.399687942	0.404255226
0.270124248	0.418068305	0.339141318	0.26065605
0.387822144	0.180406116	0.520552442	0.038551448
0.095970922	0.290291819	0.081006067	0.090558699
0.112803392	0.029772405	0.28093003	0.328517183
0.311718993	0.278728724	0.18788962	0.067837235
0.267850066	0.243672754	0.269141793	0.123419201
0.063505721	0.492348335	0.009777896	0.053474615
0.316195618	0.39098866	0.325437992	0.201653179
0.288060935	0.138249186	0.231285966	0.164268952
0.209253919	0.269612622	0.393461667	0.021945775
0.011946061	0.476440587	0.233062068	0.024206582

0.08213559	0.242194178	0.203891682	0.187550457
0.028328011	0.496554449	0.209377665	0.322571576
0.21598585	0.05380064	0.306071451	0.062032937
0.04276554	0.421999907	0.317031114	0.521529475
0.28196696	0.342733958	0.419916357	0.385254655
0.092277978	0.285284886	0.019988581	0.386384288
0.312544437			
CX3CR1	0.427600451	0.528022088	0.442409344
0.454080304	0.500300879	0.363084387	0.260735062
0.194995017	0.129685922	0.010087939	0.052368886
0.482108149	0.282755891	0.306348861	0.334677498
0.479816862	0.22915501	0.374733062	0.317734157
0.450281521	0.11445515	0.484223103	0.141949518
0.396837089	0.186305287	0.127286873	0.371218621
0.461098228	0.141614606	0.318169953	0.39197462
0.176459508	0.44395739	0.513662435	0.505118465
0.510248924	0.390202273	0.021550629	0.314081857
0.403869268	0.477434501	0.442993851	0.403657096
0.074788402	0.465754093	0.312107817	0.481474666
0.234800923	0.068374259	0.221653269	0.420935115
0.467958841	0.4605861	0.243472422	0.211017786
0.269040586	0.224814763	0.357430182	0.301903997
0.299117831	0.148261308	0.48369508	0.122846563
0.181947629	0.278376334	0.252880409	0.295716219
0.437037569	0.365432236	0.031597887	0.428439003
0.00464307	0.431700152	0.467854796	0.376381268
0.086406821	0.045389512	0.283620097	0.389370242
0.468007138	0.449137697	0.501960972	0.508138965
0.274448565	0.395201184	0.470874893	0.257892253
0.428194438	0.233435367	0.148068416	0.49542462
0.510980385	0.527395502	0.306556923	0.374699229
0.425276972	0.47059684	0.443615069	0.49074884
0.393885618	0.105953898	0.432052075	0.526993131
0.3711439	0.414328187	0.249552684	0.412961487
0.110375453	0.35817276	0.42877273	0.222697875
0.468271889	0.034212658	0.32793955	0.404129781
0.286121543	0.444600331	0.525170944	0.256490865
0.484304841	0.04296131	0.206156968	0.070335447
0.375931945	0.229012929	0.46291181	0.464873133
0.25684424	0.46726075	0.321572969	0.008060832
0.062448387	0.268257805	0.443586246	0.407221889
0.456397225	0.365387843	0.321558259	0.371433662
0.471781996	1	0.050276946	0.142411204
0.455638869	0.275334051	0.334213542	0.482605338
0.416178072	0.15188412	0.198127909	0.530076977
0.402245597	0.140132099	0.333472627	0.484867086
0.378243183	0.466650117	0.167305316	0.353010818
0.081872816	0.48161003	0.06680466	0.340268976
0.31514299	0.247435965	0.020501614	0.271680035
0.318578582	0.4303928	0.451486087	0.170934239
0.394812346	0.491962266	0.312335355	0.441054964
0.02359717	0.081705533	0.253834291	0.519141475
0.25962617	0.512681361	0.378103593	0.402777531
0.393619468	0.47795644	0.005326913	0.48323162

0.530600332	0.269163611	0.469527419	0.483315092
0.430272973	0.466983486	0.044009595	0.486712139
0.33693357	0.131602607	0.519899365	0.257002347
0.453969033	0.084764634	0.341029246	0.460693279
0.447946248	0.016792855	0.219083429	0.416937423
0.063400065	0.112603762	0.444537414	0.454111218
0.43659384	0.483317088	0.492101647	0.413726722
0.49549282	0.284620559	0.028472329	0.429095172
0.459986445	0.412891455	0.425560832	0.292078114
0.310158269	0.469635002	0.416455982	0.223524225
0.078666348	0.342384229	0.031918159	0.419924086
0.379044764	0.172001109	0.423663133	0.222900246
0.137569128	0.538053161	0.277517273	0.348915824
0.205697389	0.4343703	0.316506355	0.286648483
0.294279619	0.253453704	0.432338445	0.438541649
0.056686113	0.164014651	0.032963402	0.417009545
0.20337579	0.390132158	0.368209534	0.402416934
0.373286761	0.170380345	0.470347245	0.374768138
0.193525585	0.03704827	0.511066122	0.46768173
0.305461471	0.218312493	0.083363426	0.363304021
0.467955057	0.293599395	0.237252084	0.450370779
0.048621257	0.495632258	0.331068606	0.208876362
0.470043672	0.351106483	0.470548055	0.073492538
0.39993406	0.4613154	0.404728203	0.281397503
0.375251248	0.455496113	0.054591235	0.391460036
0.38922533	0.067793199	0.536128669	0.218981494
0.049281144	0.495994857	0.465170454	0.139100031
0.142586691	0.39173194	0.05953383	0.401236231
0.527844006	0.48922007	0.438280866	0.318982912
0.076819804	0.025306431	0.321811608	0.44475769
0.49830338	0.525205923	0.360736422	0.46260051
0.325639906	0.411978404	0.136275796	0.523397051
0.251694558	0.515519964	0.017967809	0.479282945
0.503459509	0.276003657	0.149773362	0.335719778
0.370665865	0.082381653	0.262190057	0.494100352
0.427237017	0.407702824	0.407878595	0.477099688
0.027816911	0.067409521	0.460369731	0.276257185
0.433355174	0.305769511	0.09620063	0.338968952
0.081621766	0.447236572	0.491042153	0.108233488
0.332594156	0.307163176	0.479988855	0.072112383
0.459899537	0.505297581	0.517126	0.215730723
0.46356118	0.498658827	0.455247139	0.145177233
0.043383592	0.517888731	0.395959608	0.040578347
0.486047352	0.208665792	0.029150705	0.494599608
0.2550927	0.42174232	0.004706533	0.105871761
0.085485468	0.497308636	0.469834468	0.39722514
0.225464422	0.425276475	0.013656156	0.535849615
0.49039136	0.037621195	0.242537534	0.452996547
0.458298301	0.526277691	0.411463848	0.470023516
0.477026044	0.500153402	0.293476333	0.376773149
0.443992737	0.501138441	0.429891076	0.446880897
0.477825567	0.500251277	0.361351874	0.032218143
0.193850632	0.217864397	0.31579638	0.132777599
0.30185337	0.227244817	0.479796614	0.492374691

0.495464706	0.014833468	0.178424604	0.439110483
0.425104941	0.471724411	0.038910891	0.009800443
0.011398962	0.046333719	0.391852768	0.350547575
0.495687819	0.227033076	0.013246601	0.059801186
0.337247169	0.486038553	0.507163963	0.45134398
0.470678661	0.145133855	0.309367302	0.454458293
0.132089778	0.299542499	0.500997649	0.46532389
0.083034707	0.354596671	0.393603754	0.4939515
0.120556158	0.42994005	0.48321201	0.386533735
0.029046379	0.151429722	0.12469943	0.490383533
0.496677984	0.138411271	0.071991628	0.136626091
0.17710943	0.47498958	0.243320989	0.530851261
0.448535583	0.485932722	0.147504052	0.476403919
0.079510634	0.482960141	0.449641017	0.061065759
0.372130234	0.029204943	0.473005348	0.210223297
0.334254465	0.405703842	0.264200492	0.043030194
0.252492524	0.10637999	0.488464746	0.49525044
0.072919934	0.294955673	0.39871676	0.456559222
0.29120868	0.480428109	0.49481964	0.395992388
0.124990601	0.210736744	0.434933401	0.390003033
0.261808808	0.508728265	0.385756775	0.536763972
0.407116196	0.454141616	0.477598218	0.400807838
0.35214827	0.396746406	0.381515722	0.033061178
0.421868749	0.490186624	0.381328811	0.244011581
0.367137422	0.451567491	0.075959742	0.180953995
0.401460292	0.295082445	0.216460343	0.484083822
0.399232808	0.392295214	0.059400842	0.116778872
0.545748764	0.422792275	0.165406004	0.449237223
0.263253586	0.436768498	0.479999371	0.493359391
0.408870708	0.454601066	0.438231508	0.392905576
0.410501868	0.333830361	0.522447518	0.086062987
0.14495401	0.43541629	0.158717751	0.167740657
0.222964793	0.059481251	0.368136513	0.49230262
0.429642808	0.466596409	0.319146985	0.134116622
0.43807866	0.374158688	0.451885709	0.240596468
0.131725402	0.494771225	0.021636309	0.110299232
0.45215552	0.456712934	0.468539495	0.344004404
0.395685927	0.234327059	0.403683248	0.284769812
0.329654858	0.397306641	0.447175117	0.050752928
0.035476232	0.50776529	0.369972179	0.046361019
0.148053913	0.372380163	0.355439147	0.313624379
0.052857338	0.492997108	0.330882101	0.491600064
0.380804568	0.116641283	0.322177293	0.117843866
0.093730301	0.434235923	0.459918389	0.517314475
0.431725848	0.485102719	0.4214786	0.443696934
0.162611371	0.401309405	0.049723612	0.457694922
0.427779515			

CXCL2 0.038315719 0.082351034 0.020440325

0.07178806	0.018860295	0.080372516	0.037064732
0.025575405	0.008824206	0.001527023	0.000625385
0.028761988	0.00142888	0.024327479	0.026622084
0.041600765	0.00248536	0.015619106	0.0245551
0.044958072	0.027165899	0.032929767	0.009766926
0.002776612	0.003096764	0.005114682	0.007858022

0.070961162	0.00574237	0.058513877	0.069102166
0.001049348	0.048722601	0.084801663	0.030952306
0.074090699	0.04549152	1.47E-05	0.034098005
0.068854261	0.051547425	0.048840453	0.039610028
0.006290384	0.095697727	0.038948971	0.017816607
0.003372535	0.00237284	0.052839961	0.088729288
0.087746672	0.036770503	0.039969057	0.028449466
0.001727792	0.023374748	0.003197824	0.079908292
0.023829752	0.050390869	0.076401728	0.01000148
0.000287524	0.023962585	0.001333461	0.032613493
0.004638392	0.081641399	0.00128917	0.021243871
0.000249034	0.04831207	0.015775191	0.007395909
0.002420907	0.001644434	0.002465858	0.050582322
0.06379323	0.064735071	0.055210596	0.053978269
0.002925365	0.00867269	0.010702203	0.008974392
0.065569795	0.035670889	0.003911627	0.035985015
0.057404212	0.074194237	0.003548528	0.008551574
0.08650364	0.083565969	0.035247586	0.019839691
0.01764818	0.000354209	0.076838639	0.062705167
0.07418854	0.006093237	0.005667447	0.071398945
0.002592085	0.020310862	0.039190202	0.000383132
0.071490955	0.007106642	0.005226497	0.039120838
0.002909842	0.064641399	0.070344156	0.024384964
0.015757635	0.000162606	0.000667655	0.000564084
0.009360616	0.000743121	0.086135787	0.076578748
0.011310262	0.00407866	0.025333466	7.44E-07
0.000651348	0.000912869	0.043519794	0.017479345
0.044605492	0.04658274	0.017354404	0.060789496
0.032187273	0.050276946	1	0.010282286
0.046803032	0.000939496	0.017068053	0.029685911
0.016220421	0.018344297	0.00784301	0.073876628
0.096477502	0.009215026	0.01884608	0.038748929
0.013556504	0.013978491	0.006371928	0.014123062
0.006174528	0.070291715	0.000426526	0.033843976
0.00825155	0.000683879	0.000153985	0.046428058
0.024528515	0.074625764	0.07227707	0.005814323
0.018125533	0.028469472	0.037852801	0.073595537
0.001557792	0.001802865	0.034279391	0.069904018
0.082058918	0.055828815	0.013057418	0.006432856
0.013365886	0.054970555	3.29E-05	0.093800352
0.073987716	0.04382198	0.03621836	0.021225549
0.01564438	0.076669207	0.000222035	0.034888557
0.053689333	0.004878255	0.06084264	0.007761753
0.070389342	0.003247927	0.023071845	0.040416461
0.039123535	0.000340723	0.074746376	0.062652287
0.00014344	0.022048096	0.043235778	0.051847181
0.044882334	0.04182448	0.058749243	0.015505027
0.024861992	0.023905706	0.000181945	0.011080688
0.040047864	0.00199109	0.097507433	0.018962541
0.048148293	0.092220227	0.050075021	0.001984945
0.008105043	0.032035616	0.001399313	0.024385547
0.053416715	0.011710119	0.022659707	0.000816365
0.001848727	0.075195256	0.001908449	0.003544045
0.000918149	0.042065913	0.023097293	0.022734501

0.006339181	0.004725877	0.011305248	0.016923297	
0.011664292	0.004455744	4.13E-05	0.009112216	
0.00406377	0.01781629	0.021758985	0.045962772	
0.007996831	0.002929182	0.026396769	0.003383528	
0.003757264	0.000909194	0.077504247	0.039084593	
0.077061569	0.013673682	0.001331066	0.005494246	
0.070685386	0.087902537	0.033548384	0.084211297	
0.00071909	0.05141104	0.010034501	0.00552934	
0.010489921	0.023177055	0.070430765	0.009402667	
0.032119466	0.089155152	0.013831977	0.009628783	
0.090076295	0.047195943	0.004727911	0.064614964	
0.059366713	0.00210881	0.071303526	0.001551308	
0.000760108	0.030687571	0.04636648	0.003654489	
0.003246158	0.039216574	0.015049262	0.01663674	
0.080930436	0.038496737	0.060883706	0.002761707	
0.006425148	0.000209283	0.002744752	0.018415761	
0.085356866	0.061489294	0.012328865	0.018686024	
0.022462585	0.010810061	0.004417329	0.068587124	
0.001085948	0.075868683	0.000466501	0.034628915	
0.078681358	0.048317659	0.003518092	0.052579583	
0.089825592	0.006847275	0.009500569	0.086971915	
0.069868351	0.017091747	0.020885677	0.028791092	
0.007467725	0.0033211	0.025246332	0.001663646	
0.018209937	0.009153797	7.82E-05	0.018436594	
0.003725665	0.089164507	0.045243175	0.005403408	
0.075891102	0.021566178	0.018116934	0.003605466	
0.046914012	0.060709278	0.086889418	0.010072225	
0.03878753	0.069275973	0.018611661	0.001909473	
0.00049255	0.074146077	0.056500369	0.001088162	
0.057220258	0.069830207	0.001359985	0.08931978	
0.018839478	0.077808998	0.0039445	8.99E-05	
0.000268262	0.041467825	0.09288711	0.058609758	
0.057333956	0.029318363	0.000431428	0.061737069	
0.08956209	0.0004483	0.000931515	0.048893439	
0.037355005	0.086125449	0.00967343	0.051863999	
0.027646719	0.075008568	0.006823053	0.015691208	
0.084689223	0.058773294	0.070586195	0.092416974	
0.061233816	0.026803254	0.033145244	0.003937446	
0.001946474	0.041234573	0.042232289	0.001156524	
0.07464761	0.004929891	0.074848301	0.04900837	
0.058156838	0.000279558	0.017831178	0.055434386	
0.08714085	0.074921587	0.000138562	0.002529798	
0.000847479	0.000787721	0.039241303	0.007950793	
0.069595859	0.02871931	0.000715571	0.008486421	
0.012241579	0.020586758	0.080210248	0.042133169	
0.03733941	0.002247497	0.032481564	0.044747451	
0.001376424	0.053172417	0.06451566	0.096027345	
0.000342002	0.00666725	0.022369797	0.073950651	
6.66E-05	0.013736082	0.027540571	0.013028961	9.82E-06
0.002022188	0.002616901	0.037809158	0.071071635	
0.003004802	0.001622012	0.002584475	0.016880915	
0.050767679	0.00870942	0.065603734	0.073876698	
0.05404252	0.000272883	0.092866839	0.000587477	
0.082272089	0.010295175	0.001896296	0.006768209	

0.000511379	0.055915811	0.005111289	0.025291417	
0.053717196	0.003117651	9.55E-06	0.00146502	
0.001907572	0.061712227	0.043778887	0.000174402	
0.013874078	0.01335638	0.03179397	0.073000449	
0.062063735	0.092097535	0.048910746	0.0127024	
3.02E-05	0.061373744	0.055488228	0.0166703	
0.053099247	0.010580378	0.068704486	0.059609904	
0.066109703	0.063371508	0.048371063	0.000737197	
0.059899961	0.089039234	0.005759219	0.034429538	
0.055558125	0.030692978	0.004500279	0.013615876	
0.012485381	0.00036338	0.001488	0.008275537	
0.088848333	0.003874428	0.049897825	0.043700939	
0.061189531	0.000532319	0.003615429	0.080282045	
0.080585964	0.026290079	0.068247917	0.005518727	
0.024445778	0.0419199	0.034489611	0.00617218	
0.057284988	0.068218924	0.077834179	0.09842654	
0.026134939	0.08596774	0.0175169	0.001488687	
0.023706226	0.008325095	0.00230721	0.010210093	
0.000596165	0.004342378	0.032605818	0.007150597	
0.031966483	0.006712178	0.000979922	0.010028841	
0.017149188	0.021441051	0.03997066	0.006490483	
0.090131196	6.50E-05	0.001316791	0.006988595	
0.058600646	0.016694942	0.008734174	0.003909533	
0.00060822	0.017472219	0.067527509	0.043747804	
0.003399492	0.066911392	0.00258253	0.003014412	
0.057273396	0.042085147	6.62E-05	3.54E-05	0.037170269
0.005476441	0.012183453	0.000166701	0.05595297	
0.051998189	0.020551075	0.043139046	0.002382011	
0.079904482	0.000541864	0.002033351	0.084110213	
0.017854104	0.071677231	0.008840003	0.048195663	
0.089120828	0.050166837	0.001331661	0.017017318	
0.006600192	0.06139543	0.022254805		
CXCR6	0.096715337	0.117730302	0.063146722	
0.220868796	0.110043605	0.105756356	0.262727693	
0.105444274	0.118505333	0.058787708	0.040621939	
0.077422975	0.044486281	0.079762105	0.036869434	
0.230181751	0.075081439	0.079647114	0.297672905	
0.217243126	0.153314397	0.214706926	0.200317215	
0.038075825	0.068755338	0.162745838	0.061358887	
0.216164704	0.18439797	0.080253836	0.196287487	
0.100121962	0.128400233	0.086878015	0.110328136	
0.072932657	0.07415086	0.040542355	0.072320854	
0.125839942	0.126895579	0.226695337	0.251344428	
0.17275886	0.081938176	0.280963106	0.11204777	
0.060282551	0.086173741	0.078619969	0.091462515	
0.068904946	0.076890281	0.158888422	0.21309347	
0.023074217	0.064639924	0.011545896	0.122807135	
0.264069387	0.089237208	0.077135167	0.133973366	
0.056872823	0.16836207	0.073779591	0.198159902	
0.030732218	0.125544452	0.175396521	0.049114673	
0.023219865	0.253230206	0.133458435	0.02256009	
0.189185171	0.072732718	0.10130067	0.242047094	
0.187810655	0.086509259	0.144414463	0.121032598	
0.061853868	0.157568977	0.05432853	0.158302139	

0.12219867	0.209865622	0.091501687	0.208180136
0.137567585	0.091818054	0.08133388	0.160814891
0.120361735	0.122183949	0.067931967	0.085699698
0.028650731	0.062256463	0.087843422	0.119594723
0.104068426	0.036172278	0.161909301	0.176214367
0.199455572	0.030317488	0.27361864	0.040821225
0.19107242	0.155009635	0.130038803	0.18076054
0.112096607	0.122333843	0.085325885	0.299776399
0.085229907	0.072508812	0.019312944	0.127996693
0.049232235	0.012610909	0.064393844	0.160231868
0.08237604	0.04008689	0.215550003	0.060475041
0.089425615	0.067400129	0.08494934	0.107517512
0.160463335	0.057664768	0.10553211	0.09118769
0.063492853	0.142411204	0.010282286	1
0.109453627	0.077705379	0.304891759	0.08481701
0.119197718	0.199416777	0.227144308	0.140148515
0.113397404	0.266874478	0.063559999	0.210241126
0.045963406	0.099608987	0.224499925	0.103225476
0.18265468	0.06783109	0.079804917	0.118009461
0.018327555	0.039131442	0.12023999	0.168825479
0.059397143	0.091915149	0.058519119	0.251912446
0.134543482	0.164386747	0.095308772	0.096254745
0.052841696	0.150857579	0.056228355	0.120949385
0.093547341	0.118633199	0.114666463	0.084717587
0.191232382	0.180514659	0.00443598	0.081375578
0.088787032	0.046299287	0.121693763	0.110534812
0.042534918	0.175638676	0.004187533	0.201992387
0.075807353	0.241228531	0.159995655	0.182305837
0.084971801	0.16689647	0.096221882	0.081933198
0.057508395	0.03923178	0.128437703	0.23301815
0.068117636	0.139683135	0.148487104	0.206261123
0.154844361	0.148198708	0.18765224	0.034976936
0.100553597	0.202661156	0.103166752	0.116971749
0.112184983	0.017501049	0.104388617	0.131601093
0.056935712	0.115288695	0.068640796	0.02792562
0.226218938	0.178410019	0.07516852	0.135928222
0.074305821	0.113949775	0.052913278	0.082111402
0.110385712	0.123163871	0.033337225	0.021771782
0.014145861	0.090095403	0.262906178	0.046663433
0.027721195	0.128999073	0.04580592	0.070162884
0.172110861	0.157238424	0.04501163	0.016355282
0.031021372	0.027449564	0.026466072	0.087032424
0.048428956	0.068210943	0.096062025	0.036433695
0.084948304	0.145286245	0.115918758	0.215514822
0.087622033	0.180443388	0.153402215	0.012001404
0.148725552	0.057702562	0.042665881	0.130603902
0.119264223	0.145270093	0.062993643	0.167441948
0.038534981	0.221107033	0.204551622	0.041574032
0.108965859	0.10875686	0.185217747	0.129653149
0.082279333	0.184051389	0.163495051	0.088772019
0.078911585	0.158524146	0.102831231	0.069375045
0.143255714	0.109879127	0.082428062	0.214914954
0.138201335	0.112609631	0.16960159	0.220618497
0.124346378	0.213043725	0.101404928	0.049751718

0.165672033	0.030057948	0.082153043	0.060169537
0.079072332	0.094031508	0.239093547	0.067004916
0.327465101	0.041605734	0.067571482	0.115356423
0.043089545	0.093416671	0.108775991	0.091582723
0.070799744	0.08375461	0.102739297	0.189489103
0.100204894	0.205526619	0.162150624	0.090104543
0.132501495	0.087174198	0.054354842	0.127782556
0.160073841	0.200295164	0.115124283	0.02113221
0.059058826	0.148192761	0.005489632	0.065115515
0.123868339	0.104630695	0.161476611	0.111033261
0.075842571	0.066927583	0.166105453	0.209466188
0.081892726	0.083225773	0.089263662	0.186767159
0.092504654	0.090535631	0.039493085	0.158807399
0.106012632	0.078852457	0.128375873	0.06411275
0.172129447	0.072802945	0.052890852	0.124446334
0.312647942	0.062137681	0.036378579	0.011134117
0.068892757	0.144419314	0.082676575	0.203703324
0.035406489	0.21677873	0.012512808	0.106149563
0.096734091	0.060945281	0.055435759	0.07649015
0.060651484	0.115625097	0.038564584	0.089446882
0.053805212	0.074587644	0.084706738	0.224601059
0.130460441	0.07118	0.156932131	0.114397966
0.160921695	0.145654126	0.304510425	0.139266011
0.042811806	0.141603249	0.019427013	0.061582739
0.107982246	0.085747061	0.106366637	0.164352999
0.100928688	0.099364515	0.146074495	0.087027944
0.167221451	0.183696346	0.082656153	0.013842719
0.005202161	0.02687767	0.198342324	0.144042664
0.112347886	0.019363364	0.099885027	0.178250782
0.090942528	0.087334197	0.095521274	0.277761925
0.103722539	0.180202534	0.030590326	0.254289952
0.069262567	0.065522962	0.115304677	0.106870073
0.114448416	0.139295239	0.074534085	0.173850264
0.003933664	0.189490826	0.082898882	0.056602485
0.053033948	0.101610198	0.102358258	0.178123283
0.066306352	0.0592227	0.061991427	0.021106903
0.136728875	0.14103311	0.107520633	0.144154197
0.164613891	0.222738488	0.06776892	0.099543381
0.087872803	0.076856771	0.064300878	0.02555557
0.088682667	0.128481845	0.074293656	0.018958811
0.075118675	0.145915398	0.040641437	0.06886755
0.014705906	0.128252269	0.163410292	0.128819828
0.069775258	0.166660042	0.172562279	0.173208221
0.165184663	0.17398317	0.08582039	0.039801688
0.177532747	0.015429885	0.204503466	0.088264245
0.092278958	0.149514597	0.044244476	0.113981366
0.119179521	0.125763722	0.095088296	0.201363361
0.030758177	0.098503666	0.149865018	0.096528015
0.210250695	0.154997797	0.133715644	0.052703433
0.136998318	0.044246962	0.183731254	0.022060731
0.02596476	0.131020168	0.13097063	0.222554314
0.12710389	0.092861546	0.163703322	0.102025627
0.11400255	0.110711818	0.128172824	0.142397674
0.193867601	0.167101262	0.112989446	0.108477969

0.03305296	0.099634757	0.155081102	0.118451291
0.107867695	0.279604258	0.092715501	0.089964774
0.045498286	0.064167227	0.099447901	0.170358215
0.240203228	0.104252416	0.011376371	0.183831425
0.016493797	0.19695932	0.034119385	0.120888967
0.143374704	0.05581894	0.199528146	0.177322084
0.118594177	0.087002036	0.054292663	0.155024765
0.022018993	0.106233333	0.049831722	0.127665256
0.020118051	0.064411176	0.21302015	0.098092007
0.034246472	0.021338781	0.103268389	0.147327828
0.16937714	0.159477278	0.070179342	0.069416743
0.071171934	0.087904328	0.065275399	0.076830582
0.065955214	0.078828929	0.059215845	0.094097478
0.208937966	0.180777274	0.082555844	0.075908684
0.176938121	0.089103966	0.066274214	0.080774461
0.072695199	0.219832603	0.07540246	0.094895103
0.065490452	0.042949741	0.185453531	0.168344406
0.021662323			
CYB5	0.330279166	0.497243222	0.214006847
0.390435694	0.228755378	0.236184213	0.175801615
0.15619036	0.104325744	0.011965052	0.027279865
0.275462512	0.149297223	0.184028512	0.199642636
0.380850823	0.112418234	0.205955003	0.198241008
0.329053445	0.098141552	0.329240197	0.112676853
0.170349459	0.098302241	0.078783258	0.176393715
0.443914981	0.090087963	0.173095681	0.32458829
0.09106712	0.378921761	0.47734047	0.313449537
0.456760461	0.304878915	0.011625552	0.176581013
0.396730241	0.374242249	0.33916834	0.236945169
0.060989228	0.444442506	0.193150861	0.246244144
0.157803239	0.04599421	0.153708627	0.411621133
0.42734079	0.247384459	0.179726563	0.144387902
0.115147718	0.131920094	0.135235559	0.244122491
0.179249591	0.111957453	0.435370155	0.101397954
0.093375723	0.181897424	0.114391978	0.189432283
0.173657723	0.333690182	0.034156287	0.229815266
0.004331139	0.322716374	0.232752196	0.157542122
0.05669386	0.031307065	0.134042377	0.238182875
0.451663237	0.42218814	0.468500508	0.460099528
0.144212031	0.197661812	0.193499568	0.148912686
0.408742623	0.153981458	0.13910619	0.37800075
0.475041574	0.477607948	0.168498273	0.185023575
0.384824272	0.457608463	0.230364902	0.227355524
0.190375842	0.052768567	0.404039082	0.482858648
0.363079494	0.165692185	0.134449018	0.375986156
0.070681302	0.211112626	0.280412012	0.107625558
0.47370372	0.037509608	0.15586809	0.214030292
0.167821875	0.387078193	0.471453381	0.170072329
0.225587844	0.033713435	0.104504536	0.043215469
0.212637373	0.08546564	0.421809961	0.45060486
0.160741003	0.196774757	0.200771474	0.006043433
0.037466747	0.134286008	0.360712134	0.213230044
0.355850195	0.332809868	0.170690867	0.316462975
0.289972778	0.455638869	0.046803032	0.109453627

1	0.131095491	0.188341853	0.251773016
0.214277734	0.130685697	0.124324322	0.506572524
0.410251886	0.098723309	0.18044915	0.365500166
0.180480721	0.212841985	0.115995335	0.18945685
0.063704609	0.430736479	0.035688496	0.209430191
0.167148252	0.100051028	0.018816071	0.179279741
0.206276341	0.391989327	0.402363021	0.113203643
0.206101318	0.294403047	0.178055134	0.337377069
0.016846637	0.07047567	0.14796365	0.482310793
0.197316505	0.47305638	0.218985439	0.207670126
0.203523012	0.424946917	0.004120461	0.458050597
0.481329164	0.161826861	0.248740996	0.265675753
0.189394728	0.442635566	0.036139921	0.344386883
0.26505887	0.093884046	0.49357066	0.138780421
0.421281313	0.054823543	0.18420729	0.372549156
0.31978584	0.014466234	0.152734251	0.320336007
0.032228926	0.094835175	0.34575884	0.32532992
0.245900348	0.39029535	0.478305633	0.182203387
0.242428201	0.191761221	0.023840154	0.205902955
0.3692946	0.168669594	0.423828561	0.20289607
0.197314082	0.457142595	0.348441836	0.097961525
0.06254539	0.196193909	0.045816679	0.22473282
0.346711278	0.105813867	0.196999835	0.112436746
0.075339765	0.501594626	0.114961871	0.136710267
0.078138638	0.226455262	0.202679158	0.151153536
0.13518782	0.14993304	0.206432339	0.208782317
0.055091516	0.096623557	0.017170332	0.164915014
0.096589178	0.179193715	0.169349274	0.296392969
0.187014873	0.103075047	0.239789433	0.169748652
0.112431116	0.025653633	0.483081809	0.332541673
0.273981016	0.15630384	0.053128858	0.152422871
0.449659323	0.174646854	0.148862846	0.43763521
0.046285056	0.458574599	0.163865228	0.116397736
0.195675838	0.196502035	0.463898394	0.064158134
0.222881481	0.442105149	0.213440673	0.153493543
0.370394962	0.313387876	0.051392569	0.333911287
0.3249472	0.045895328	0.492385178	0.104593564
0.031741835	0.334996639	0.338571454	0.084074249
0.086184615	0.203217374	0.063462052	0.208249887
0.499813936	0.362829134	0.352320328	0.146149875
0.056282759	0.013576441	0.144087048	0.207557088
0.458791011	0.469835121	0.193636989	0.213492487
0.193932205	0.195852643	0.092299846	0.484035267
0.130949688	0.476902635	0.01461598	0.355839627
0.452833227	0.162707215	0.084831359	0.196839234
0.360120363	0.069252496	0.167573479	0.46494406
0.41339114	0.218337602	0.214900488	0.305733142
0.033371266	0.055353413	0.235067456	0.113826363
0.201579694	0.17678534	0.03434542	0.201036933
0.05702947	0.428225307	0.422599145	0.073377177
0.188761625	0.193645281	0.266130147	0.055343894
0.404415582	0.452581947	0.480909691	0.148284881
0.353227978	0.452745039	0.212291916	0.083494826
0.02637966	0.468783481	0.308917521	0.033106382

0.458111968	0.140639389	0.019623197	0.480119329
0.167864185	0.388101434	0.014512919	0.050821049
0.044382022	0.425819291	0.443872253	0.306666764
0.136237964	0.255624328	0.004724124	0.485945253
0.464511978	0.024120586	0.110389243	0.358920357
0.328935482	0.500638727	0.180779563	0.407995809
0.256246496	0.445221078	0.181129722	0.199135965
0.423024343	0.435202055	0.375545546	0.447150831
0.44074626	0.321863599	0.210268675	0.030227968
0.110490958	0.149588069	0.166555337	0.064432856
0.220517769	0.124978093	0.46090807	0.443096006
0.451115921	0.011089242	0.132555951	0.367267151
0.440165652	0.474051102	0.027524384	0.012279947
0.013579123	0.02783512	0.229683516	0.183205376
0.464934466	0.127058073	0.011419331	0.063185517
0.213864932	0.259014539	0.467009478	0.337935655
0.369639306	0.084977182	0.168874453	0.353468242
0.074727866	0.238847117	0.465657875	0.451502064
0.046877174	0.180469866	0.210044902	0.483584717
0.039783053	0.211748697	0.288571801	0.207941289
0.016076355	0.084672086	0.073988892	0.393575935
0.436182043	0.074607403	0.04241487	0.096835824
0.11560928	0.435493627	0.150968264	0.498736265
0.422571109	0.430338776	0.062835028	0.457711099
0.049566748	0.445527016	0.195416288	0.063068938
0.196037196	0.022577902	0.416689306	0.098852368
0.189498047	0.310096465	0.144399863	0.022002837
0.11125494	0.064262182	0.465203699	0.424840266
0.037350015	0.182708152	0.212326144	0.299285429
0.190615945	0.385860422	0.464340088	0.196113623
0.092985329	0.07672458	0.355719665	0.2938551
0.161164756	0.473291031	0.186006695	0.496593399
0.340570355	0.44114937	0.434135891	0.231569592
0.133159994	0.255011034	0.292397448	0.037126712
0.243108677	0.429845116	0.214846203	0.139432109
0.182342346	0.185201026	0.051751891	0.115029684
0.158165995	0.204392027	0.120632001	0.430137851
0.271794912	0.315900137	0.045398267	0.072434275
0.508804359	0.418492717	0.149665471	0.330994326
0.147672669	0.221110238	0.387362996	0.374317694
0.188968613	0.397156943	0.389402905	0.299039294
0.407611164	0.201508111	0.484394197	0.092910958
0.100055211	0.245724276	0.094788671	0.091202182
0.137564532	0.039800759	0.148004499	0.35352153
0.172870118	0.279748856	0.165346027	0.088927357
0.203916671	0.186451135	0.229936334	0.162939114
0.088925282	0.460084889	0.01640439	0.064738046
0.179038555	0.391225885	0.203022383	0.192143528
0.166605414	0.101199099	0.207201421	0.17997643
0.165404908	0.192684168	0.394642465	0.064966795
0.034350979	0.479498068	0.2153169	0.027377317
0.059329013	0.242346913	0.18848329	0.167419978
0.027636965	0.428483881	0.174711189	0.230502283
0.220172635	0.077765505	0.326500009	0.090937153

0.077063413	0.42396164	0.216362791	0.464364727
0.201005912	0.386304792	0.405377969	0.36981936
0.118724735	0.179423896	0.052610323	0.419384554
0.199684822
CYFIP1	0.237328747	0.201949377	0.170798012
0.208962518	0.203680372	0.086638449	0.090770257
0.045466302	0.020660845	0.001891635	0.008281271
0.208413564	0.01945956	0.038773448	0.028317471
0.324801345	0.093853619	0.151830631	0.179359213
0.257598717	0.025554468	0.287908286	0.058873808
0.134093711	0.077713276	0.074718419	0.100495562
0.266726482	0.092671534	0.048785744	0.178564471
0.174137964	0.24016017	0.173850078	0.209085906
0.168092031	0.180763135	0.050795519	0.030939615
0.21630261	0.209403552	0.239353595	0.181618371
0.032702398	0.146555765	0.123153222	0.284405638
0.022989017	0.030020244	0.032408639	0.146977418
0.12808371	0.11209607	0.061479009	0.059539348
0.010453414	0.033278096	0.029625904	0.102200046
0.122649948	0.021541481	0.152314667	0.014790861
0.021125436	0.106585312	0.180508522	0.101831043
0.112879075	0.147775153	0.039200418	0.183538547
0.000648065	0.264929789	0.331862114	0.062410152
0.132584178	0.01037511	0.220648856	0.146057106
0.27532196	0.205497526	0.270270529	0.251536441
0.041901167	0.296628671	0.201923526	0.091176027
0.208758642	0.061168375	0.031495129	0.332948826
0.262216379	0.186012917	0.073628838	0.303400045
0.13309238	0.192555284	0.093067807	0.120276376
0.037735424	0.120177449	0.139715726	0.235464577
0.162508073	0.15982843	0.179129233	0.196671484
0.146660805	0.035023905	0.227326444	0.068370619
0.270111973	0.016841408	0.24594241	0.123639062
0.096441635	0.208312009	0.189561615	0.12743283
0.22502415	0.023982581	0.018803444	0.147003765
0.03047261	0.063518682	0.129740477	0.204788573
0.03570973	0.051043509	0.116878287	0.036841705
0.083776597	0.113388342	0.199679767	0.209329172
0.266790985	0.168152063	0.063681535	0.139036603
0.158901867	0.275334051	0.000939496	0.077705379
0.131095491	1	0.252138232	0.158311246
0.152308677	0.067871659	0.120030391	0.244248011
0.149829789	0.10530052	0.100464236	0.315047893
0.026676257	0.236529691	0.119848704	0.07529317
0.03257313	0.158213628	0.127743159	0.127654397
0.012932916	0.058690825	0.062022278	0.084430541
0.017226459	0.134470786	0.138446593	0.179276625
0.109347886	0.251711846	0.092452317	0.115533388
0.0033292	0.059620968	0.06327598	0.216012446
0.063749635	0.271579104	0.116005213	0.163754638
0.267740467	0.276600813	0.014935923	0.150427697
0.188573863	0.051209569	0.138247235	0.28627447
0.088257299	0.213446042	0.008690382	0.309081749
0.1358461	0.15241139	0.284599478	0.157171136

0.177643763	0.077645892	0.067557603	0.21155119
0.14961745	0.012108422	0.039484902	0.191625607
0.124879706	0.018764586	0.252829626	0.20988768
0.134287273	0.299626919	0.304548148	0.072286059
0.156305005	0.126393456	0.057181391	0.280600104
0.266798212	0.02492081	0.144873985	0.060830067
0.088397516	0.173113693	0.165287462	0.010154545
0.05960496	0.112040738	0.006412877	0.141516577
0.168119526	0.024343981	0.095226939	0.137141276
0.130202598	0.219391015	0.14444433	0.121638389
0.085643237	0.096329727	0.178437693	0.020086434
0.014769764	0.089851892	0.080029052	0.078501613
0.019856928	0.09066817	0.108202011	0.043772298
0.009848092	0.033712862	0.030494982	0.146087426
0.041216141	0.019324576	0.123808489	0.099704819
0.105358079	0.095905038	0.201755832	0.301513962
0.103182567	0.074560554	0.144477607	0.01229774
0.203590919	0.037983235	0.039230585	0.167393218
0.049025058	0.288870981	0.029720837	0.137591158
0.088699261	0.144891423	0.26924359	0.00548264
0.166261962	0.159729914	0.316364364	0.100828366
0.11555959	0.203939851	0.01960621	0.160687153
0.143984204	0.084140285	0.212551865	0.110039298
0.130343628	0.273540236	0.151298847	0.180477976
0.140229017	0.068186729	0.016159379	0.28520624
0.208315909	0.286987734	0.137824167	0.187517091
0.042996166	0.005295334	0.250747447	0.208308906
0.159072816	0.217275594	0.295776833	0.108515183
0.219092764	0.05539802	0.015077025	0.215259917
0.114116459	0.200055413	0.068385985	0.228157303
0.154369359	0.059705135	0.083727841	0.089377374
0.124731487	0.061990953	0.15490627	0.164250693
0.189723622	0.090309905	0.055581883	0.292693509
0.011662501	0.022822691	0.206585098	0.102331795
0.061298249	0.117894692	0.03266428	0.054512013
0.019617209	0.146326309	0.313497997	0.032898833
0.049091971	0.058482994	0.342617649	0.084801069
0.204653271	0.209598028	0.172054609	0.116337952
0.224641968	0.179558207	0.125907682	0.212964759
0.120157981	0.180075638	0.178846631	0.008639534
0.279794985	0.035561499	0.004669759	0.192879508
0.166904892	0.129608677	0.001079872	0.056499686
0.133507067	0.319530588	0.142601425	0.194785364
0.013908174	0.291906571	0.067523209	0.244120167
0.160511765	0.062017022	0.107666485	0.162186476
0.163326989	0.206608477	0.108175464	0.191359044
0.142473748	0.156521133	0.020126898	0.239452372
0.167539734	0.182978686	0.206314996	0.178821105
0.236518509	0.319173662	0.188920653	0.019025896
0.032681192	0.05199835	0.014750768	0.012648512
0.097499825	0.069862992	0.215362757	0.29288602
0.215487326	0.073601837	0.032512331	0.169453385
0.200494852	0.260662689	0.091043482	0.006500413
0.009038687	0.01154598	0.167317949	0.268631575

0.211049138	0.004819939	0.036997057	0.018106685
0.040437683	0.225585111	0.174939677	0.301569192
0.26879986	0.157211612	0.014298858	0.308770378
0.037796857	0.115485241	0.214338173	0.153491376
0.143826504	0.240180039	0.161129917	0.245371293
0.0291903	0.307856651	0.222249447	0.030733405
0.112571715	0.125404112	0.080263566	0.339845567
0.154749128	0.018134082	0.01782037	0.006928659
0.035105014	0.275079318	0.046016384	0.263686344
0.205671527	0.298619004	0.101827512	0.162828357
0.020632008	0.150915222	0.172056131	0.00340308
0.211776043	0.067199395	0.195610614	0.007470389
0.058444245	0.209751167	0.031616054	0.115705744
0.05248697	0.113845027	0.250974465	0.283265418
0.124404015	0.099927073	0.283763726	0.306421417
0.080065721	0.205642349	0.156942075	0.06029291
0.03622284	0.087027855	0.237785585	0.153321105
0.053818613	0.291642308	0.039279556	0.238934156
0.188940817	0.230369808	0.196841105	0.140045328
0.061522967	0.125685192	0.102652889	0.005636209
0.219587363	0.236148884	0.173982025	0.0288051
0.126218983	0.169190112	0.083200083	0.037098546
0.129468476	0.07081539	0.037327426	0.33477782
0.201189457	0.154315283	0.054318911	0.061077742
0.215846436	0.178831174	0.019525318	0.159614096
0.25434278	0.171871501	0.265511603	0.281673308
0.063628445	0.173418192	0.209269736	0.105276395
0.13455772	0.167462758	0.176067869	0.014925441
0.031026295	0.189452232	0.02518427	0.233126903
0.129462106	0.067717284	0.051906306	0.336217417
0.069589381	0.216856908	0.031851986	0.056354347
0.310799476	0.147167592	0.270768605	0.051137386
0.022458627	0.150452235	0.019395329	0.156637023
0.095045893	0.180353534	0.117185225	0.169033948
0.055469314	0.15541801	0.201905286	0.060085776
0.024794068	0.034749892	0.168173137	0.022922971
0.022974393	0.291496469	0.104106315	0.102036741
0.118060893	0.15611976	0.14627617	0.029139268
0.132860438	0.183188017	0.035295468	0.201931016
0.151569355	0.140342459	0.128118936	0.033874078
0.074094969	0.15912219	0.206094583	0.180728823
0.250818476	0.263752532	0.136908605	0.182130047
0.073262779	0.159189359	0.025223618	0.261999567
0.04755915			
CYP17A1	0.210428244	0.27752432	0.142488647
0.339681287	0.219441395	0.150551282	0.267047506
0.168581707	0.196252896	0.063480297	0.117285881
0.197267869	0.184678316	0.198255638	0.119007082
0.377234667	0.280614515	0.167631673	0.321623338
0.31635681	0.195229587	0.371484443	0.30817437
0.196871199	0.229399444	0.281379569	0.201581449
0.356685439	0.295778291	0.105829074	0.260658238
0.288159296	0.257141723	0.227142614	0.267882181
0.212115058	0.16746169	0.068037851	0.148804623

0.237451506	0.268727877	0.319118363	0.311223615
0.248505232	0.200180086	0.282586498	0.236294863
0.129494147	0.207510135	0.102053773	0.19540488
0.192214684	0.169884209	0.196552681	0.265912082
0.119972694	0.113281222	0.104920556	0.154409682
0.304522842	0.098221377	0.211391527	0.194608083
0.127749333	0.219035819	0.278687535	0.233962397
0.151400968	0.197082533	0.176659208	0.143075599
0.02490612	0.326651967	0.258260332	0.105494374
0.274663638	0.167009991	0.295170012	0.280237422
0.344073432	0.214386224	0.338084128	0.304136468
0.255765016	0.286066169	0.151458843	0.331539385
0.24602742	0.259261832	0.187934833	0.383597619
0.329345123	0.247525148	0.279267703	0.280551719
0.237736895	0.259124905	0.140988664	0.186290845
0.123235437	0.196895243	0.205374675	0.300312255
0.199818212	0.141767133	0.311426145	0.275400887
0.306950008	0.120066633	0.356244872	0.182047496
0.350798596	0.159241648	0.283737153	0.228518146
0.274510339	0.248546165	0.242334816	0.317368372
0.191941823	0.167078245	0.107178378	0.24603217
0.178270247	0.101188722	0.176745854	0.318108037
0.180058133	0.19489078	0.27197974	0.087388202
0.223444618	0.238486222	0.221051408	0.187978368
0.281543504	0.15800236	0.24645379	0.17411378
0.182455471	0.334213542	0.017068053	0.304891759
0.188341853	0.252138232	1 0.190226008	
0.251602272	0.271148517	0.346179794	0.320099247
0.212229385	0.323252648	0.127687024	0.365942649
0.163477474	0.229166267	0.336117884	0.259078419
0.249815035	0.199153179	0.203221897	0.15917208
0.094945725	0.20713883	0.120871136	0.17507552
0.169855432	0.224428531	0.168106901	0.33991982
0.25618758	0.309607241	0.119534074	0.211793744
0.090638308	0.253338348	0.081663211	0.295729307
0.114872019	0.297243048	0.283928804	0.254273144
0.298835132	0.335381018	0.009767054	0.207770386
0.244485957	0.076271736	0.237135808	0.23481509
0.123762754	0.326754599	0.01574439	0.354716269
0.142093576	0.322198697	0.353364541	0.317437938
0.213453055	0.258982107	0.171623844	0.231316782
0.181090156	0.057871053	0.122517057	0.295940147
0.188966211	0.165071105	0.259968807	0.307034727
0.23079072	0.306467527	0.377825945	0.116094318
0.203065752	0.272992791	0.157898745	0.242459883
0.252557085	0.107225042	0.215684528	0.265218649
0.099476036	0.25143924	0.18479698	0.084112525
0.260367382	0.203778896	0.092766782	0.234169895
0.175702812	0.246418261	0.11848418	0.247146753
0.275556624	0.299064378	0.160209608	0.115324389
0.100497154	0.17929082	0.298626933	0.161994068
0.156406369	0.317754834	0.16694461	0.18161582
0.188183623	0.314221086	0.10446255	0.098139948
0.148247286	0.125131477	0.107618555	0.179055256

0.179264655	0.221756651	0.213706015	0.175301449
0.265842848	0.199238126	0.272396354	0.334714488
0.14256596	0.312464243	0.273327775	0.085715601
0.309966224	0.086031522	0.073684854	0.267604993
0.16464468	0.327604058	0.232244929	0.308120596
0.163123524	0.303456927	0.361561487	0.066125518
0.167963396	0.239231858	0.271773774	0.288028716
0.181586921	0.27468895	0.212872201	0.18602591
0.176473933	0.256926482	0.272968881	0.271188421
0.219128552	0.266941038	0.213584757	0.304369859
0.283411442	0.193268957	0.160594309	0.330731145
0.293890424	0.380131979	0.221391173	0.198390443
0.253138673	0.088693548	0.252459982	0.148985872
0.211146283	0.264679332	0.321944139	0.188807999
0.328487082	0.162546652	0.093194497	0.291649916
0.160986352	0.241110764	0.143694336	0.244071968
0.204531566	0.102956578	0.252238668	0.188984335
0.194472681	0.264357717	0.282654676	0.231264418
0.256299372	0.211171295	0.161553617	0.259058942
0.146586293	0.226879813	0.2146656	0.133899454
0.167088534	0.306945857	0.047354057	0.157374672
0.243588928	0.230384892	0.337785086	0.22336968
0.117689964	0.137099449	0.315059049	0.269712294
0.237914397	0.234409098	0.233259131	0.292975384
0.232521568	0.247808373	0.135953349	0.295123068
0.186458206	0.226084592	0.20583654	0.131153124
0.350482758	0.074356969	0.114600861	0.269433535
0.329957671	0.163201134	0.020479825	0.051121469
0.191799006	0.331326517	0.206005451	0.260004032
0.07015962	0.283230394	0.033201148	0.281591902
0.240068833	0.153543963	0.231921798	0.210993463
0.192878026	0.269629323	0.141992558	0.249604471
0.167776761	0.212827097	0.222622776	0.306217823
0.264394267	0.21812907	0.262093134	0.237860869
0.328441024	0.306673082	0.3230302	0.168833907
0.135356199	0.151814143	0.081181954	0.15858683
0.132794494	0.270534178	0.256265421	0.345026522
0.274056869	0.124200394	0.218481533	0.211973502
0.291550624	0.33980995	0.126056736	0.020534167
0.014958789	0.102233247	0.23064359	0.284518657
0.272691405	0.067110779	0.123920088	0.178633911
0.244037943	0.227136468	0.2455307	0.385993214
0.253872477	0.325563583	0.116844001	0.363321928
0.213548694	0.117013142	0.291491863	0.233055159
0.236249423	0.280027399	0.1459638	0.342562613
0.041095523	0.301174914	0.210824472	0.20325068
0.102719966	0.271488677	0.245351947	0.344365969
0.198917379	0.208036441	0.189288341	0.05397555
0.261047443	0.307289641	0.299479335	0.333124534
0.296554303	0.390750998	0.231222394	0.229361125
0.190042694	0.210074243	0.187855015	0.044064316
0.246325506	0.182758616	0.216178545	0.090205848
0.145546942	0.225512995	0.139796599	0.166469648
0.096771864	0.277736758	0.345027991	0.312515799

0.18672493	0.277082026	0.282560251	0.281139098
0.153451924	0.321928325	0.218866817	0.09577636
0.255591213	0.106677613	0.300481862	0.171551587
0.180487254	0.345258534	0.187800834	0.286871003
0.210121563	0.262642837	0.246256113	0.23720149
0.189675044	0.159312246	0.215930032	0.123868425
0.268027503	0.331620548	0.175483351	0.214220899
0.278609605	0.133816716	0.222774221	0.071946632
0.10303717	0.143547672	0.302955984	0.386768989
0.199167863	0.178814739	0.193635687	0.237681812
0.279772099	0.223726748	0.182072386	0.247253438
0.331391068	0.24978207	0.270709724	0.276487137
0.138537299	0.248067268	0.272486344	0.203984536
0.215161656	0.297433161	0.239597123	0.09817115
0.152826528	0.160369032	0.25101281	0.298974249
0.342817972	0.210682095	0.092530522	0.341381624
0.09996632	0.323561323	0.112203643	0.262964371
0.262289036	0.1160257	0.29859111	0.227449821
0.260033043	0.223519491	0.104195294	0.296261823
0.118381198	0.239467547	0.147508	0.304113144
0.138939152	0.242404172	0.305218783	0.113420458
0.095627877	0.122286109	0.233144579	0.158054022
0.189064605	0.351747176	0.121684027	0.155819204
0.179248954	0.151123677	0.214451812	0.219978682
0.171302193	0.231727	0.103884293	0.198806631
0.225875098	0.314919494	0.156721127	0.139100815
0.243443732	0.206797533	0.165288882	0.23048949
0.188585224	0.379385682	0.176305272	0.227988817
0.166068112	0.109236274	0.211490752	0.308688304
0.094383632			
CYSLTR2	0.345807139	0.521947502	0.238447543
0.359724234	0.283882421	0.21882244	0.151187179
0.119689426	0.061673532	0.005880537	0.026507915
0.290965181	0.158628024	0.186501165	0.175268824
0.366359298	0.131369838	0.218033085	0.183165012
0.318226374	0.072706676	0.33061639	0.081596102
0.212933612	0.093669614	0.069464292	0.187580797
0.421042187	0.075928745	0.194207919	0.300459589
0.102153143	0.393062203	0.50408788	0.341549985
0.503624337	0.307221092	0.01652733	0.181722485
0.387326014	0.359864502	0.325958866	0.2305879
0.042859135	0.466942342	0.178192918	0.265852802
0.13106177	0.036511914	0.135314108	0.424079253
0.464594965	0.265185184	0.144999493	0.122739358
0.156016273	0.149885705	0.187442138	0.224788961
0.176062554	0.097640633	0.476728309	0.064753077
0.114886544	0.172920682	0.147106385	0.179531253
0.234764337	0.309682703	0.020735604	0.263122885
0.003728587	0.290301863	0.262194555	0.186897271
0.048034367	0.028260525	0.163378098	0.227596329
0.448000309	0.44330751	0.487555328	0.493123917
0.155562173	0.223004633	0.259180581	0.149292458
0.406054067	0.135844319	0.076989903	0.379218062
0.494995042	0.519373817	0.162838326	0.202906237

0.378644029	0.4723396	0.241457408	0.279347659
0.19806445	0.055102846	0.430728601	0.517649321
0.362687964	0.219341926	0.135443743	0.364413258
0.064169285	0.205788891	0.255809255	0.139737012
0.45961022	0.017913532	0.179382901	0.239634444
0.167076617	0.382163305	0.512085365	0.147390359
0.277199337	0.02413996	0.093080801	0.04091933
0.229312902	0.117119798	0.460825171	0.448773719
0.160758019	0.269087891	0.190582643	0.004728829
0.034170502	0.14773366	0.411429324	0.243257237
0.358996971	0.353681868	0.171787129	0.317023811
0.312211728	0.482605338	0.029685911	0.08481701
0.251773016	0.158311246	0.190226008	1
0.221646172	0.091159626	0.119705024	0.514565056
0.407474002	0.082196976	0.20713415	0.346294112
0.224495343	0.25528491	0.099579408	0.214332651
0.051312786	0.470172635	0.036425492	0.201573634
0.14579358	0.146188194	0.013338173	0.169509419
0.181709205	0.414429476	0.448988687	0.099589266
0.238487024	0.293445817	0.195465216	0.335295253
0.012128932	0.046266693	0.160817019	0.513979001
0.18409872	0.500287667	0.214188962	0.214710708
0.216426222	0.410867651	0.002726778	0.47678575
0.517775236	0.166204475	0.268890322	0.272880381
0.224074649	0.427054407	0.024877535	0.350488606
0.266314301	0.076209618	0.50667602	0.142215331
0.454439964	0.046853082	0.215131356	0.405009909
0.367980518	0.011133413	0.133342136	0.29354584
0.033896164	0.07088705	0.346090646	0.300012079
0.23799728	0.395536055	0.481757072	0.213285816
0.275500911	0.172424602	0.015222219	0.23364733
0.388666757	0.228050701	0.423075567	0.168805578
0.187218178	0.464365458	0.378296007	0.132393812
0.044737343	0.208768042	0.017037478	0.221400482
0.358052944	0.106264737	0.225105698	0.133853976
0.075901249	0.525997967	0.141176941	0.178954877
0.100542854	0.259292664	0.184624341	0.173119142
0.168704545	0.13838948	0.224230131	0.241992924
0.0359274	0.095920178	0.01731712	0.247634029
0.125517696	0.231661784	0.222701809	0.320378078
0.230903311	0.102381357	0.258070942	0.190994006
0.097443999	0.02105377	0.502136164	0.311954487
0.265421004	0.122431765	0.047638793	0.231957921
0.463842313	0.169314071	0.144175133	0.451973715
0.025659231	0.484261461	0.197777616	0.113813483
0.26931935	0.204751475	0.445377221	0.041073315
0.239435215	0.465581917	0.227112199	0.147925732
0.38502086	0.290209728	0.033680814	0.342051387
0.335553567	0.039690429	0.525708479	0.122483536
0.027738908	0.354733445	0.364621005	0.075797747
0.074922507	0.2235479	0.037821003	0.220218509
0.516187443	0.35732128	0.378404949	0.164698697
0.045034841	0.017334012	0.177266183	0.244314035
0.497106729	0.513094599	0.20936762	0.253355405

0.187323429	0.211210337	0.10340021	0.511728058
0.139252341	0.510182428	0.010649461	0.392380007
0.495306936	0.174811401	0.076780291	0.198377324
0.360200282	0.045303556	0.159719818	0.488177788
0.413591207	0.213858084	0.209149767	0.321327744
0.016691483	0.043732835	0.246312044	0.138399672
0.250469736	0.1660864	0.045936679	0.205842668
0.04820033	0.450224338	0.439254722	0.051879999
0.195425613	0.180554901	0.276182888	0.040887691
0.437313626	0.49372831	0.511559772	0.132457332
0.396292125	0.482624142	0.247439713	0.080680323
0.023079715	0.505542117	0.288647843	0.023073257
0.472017334	0.130177804	0.018657842	0.490320185
0.147758197	0.42793164	0.002326458	0.052934722
0.045587642	0.444353607	0.46112407	0.284313392
0.13383773	0.247213123	0.008316026	0.524269095
0.487703262	0.019090634	0.130995558	0.396758684
0.361162476	0.522684585	0.208354147	0.433787367
0.282887323	0.493280627	0.173951757	0.206630382
0.411657589	0.487702902	0.344820241	0.4414711
0.453669173	0.341749701	0.205629419	0.0180875
0.083924233	0.139094989	0.165368902	0.07286943
0.195612754	0.115858383	0.472855866	0.465100792
0.481072762	0.008347382	0.111021033	0.389806835
0.424415037	0.466131771	0.020409579	0.004713191
0.004956553	0.020642444	0.232978702	0.188946111
0.480328911	0.13801802	0.00807214	0.038031101
0.189969622	0.271363096	0.501764061	0.309108373
0.39253345	0.086180168	0.177122178	0.329748568
0.064555813	0.235269428	0.485468883	0.465040924
0.047714343	0.190759091	0.233636074	0.487068451
0.056101141	0.243632479	0.320050924	0.216235804
0.016864511	0.078443661	0.063140963	0.400470996
0.49176098	0.080713182	0.041203119	0.087576527
0.104228572	0.460011803	0.138682155	0.518473191
0.411854642	0.424190907	0.084784169	0.468381405
0.043653886	0.478793727	0.24113481	0.031830352
0.212780072	0.017573662	0.471115826	0.09766365
0.205415727	0.295543586	0.120563678	0.024173906
0.11770708	0.059370748	0.476958036	0.448280836
0.039691432	0.174627836	0.22085176	0.288616188
0.175291656	0.379244236	0.489608033	0.22273652
0.07501146	0.119852257	0.33125926	0.286772778
0.16707292	0.496217189	0.20373047	0.524661739
0.319201592	0.446200836	0.473717683	0.221695155
0.202557354	0.230333999	0.274070653	0.019554368
0.238850499	0.451245875	0.226614983	0.118371521
0.203802312	0.2449548	0.049176705	0.111751654
0.212708898	0.178237845	0.1323528	0.420644033
0.262821006	0.302515924	0.04264358	0.05757989
0.539507704	0.417235297	0.093260078	0.318602531
0.148400406	0.237885986	0.403573054	0.407081245
0.229730405	0.435588399	0.381258387	0.298701895
0.418369789	0.191331661	0.513825591	0.0561721

0.065282922	0.285306691	0.092658882	0.092077573
0.129585826	0.030446706	0.190079788	0.362325184
0.241864779	0.280441813	0.200499338	0.072045429
0.248114148	0.218980604	0.258035782	0.144646538
0.074325366	0.487592488	0.010348435	0.064079482
0.259646481	0.380984798	0.24896157	0.187214613
0.205054022	0.132279196	0.233821012	0.176765657
0.196675902	0.224137153	0.385992528	0.026907229
0.023359796	0.49985142	0.212260523	0.024749311
0.089298892	0.246127925	0.215235548	0.171485608
0.027700797	0.482792086	0.19735053	0.27423539
0.222078238	0.069727735	0.333010916	0.074004375
0.053140484	0.434798711	0.245789605	0.507975053
0.239279307	0.378649881	0.422968632	0.411691957
0.0882726	0.225255448	0.028226688	0.42349463
0.232596447			
D10WSU52E	0.239692835	0.357226185	0.175333311
0.347118354	0.231399103	0.155136022	0.146006678
0.072964512	0.150401085	0.025915143	0.108854492
0.248572625	0.184754989	0.127284036	0.118133327
0.381309999	0.211800088	0.11388655	0.198783076
0.282200265	0.074424834	0.378334998	0.139084063
0.193471429	0.224108591	0.174019796	0.23256398
0.362680323	0.182653266	0.099383941	0.227101587
0.15346876	0.275845956	0.313676416	0.336911057
0.304195568	0.202382164	0.015681883	0.115067501
0.258690366	0.344771035	0.287039549	0.268820093
0.107354623	0.263361016	0.199079038	0.271092061
0.0765774	0.144954221	0.060736923	0.239270026
0.278161792	0.219298335	0.097962884	0.13998389
0.140720799	0.058525722	0.165248624	0.132824166
0.175194014	0.044769249	0.296432058	0.111741809
0.076309007	0.103388132	0.182917016	0.121331636
0.169789657	0.199738816	0.041962874	0.189859225
0.006881357	0.281329602	0.264885108	0.159391725
0.139854659	0.092980679	0.197058391	0.231107768
0.365631288	0.273799693	0.424786326	0.401697867
0.2069478	0.197725434	0.200409435	0.200756579
0.287838326	0.151094732	0.118660374	0.407696556
0.421228967	0.341587745	0.240434925	0.24392909
0.296896861	0.309438991	0.161698734	0.189016877
0.170511641	0.128437489	0.267764421	0.391253989
0.218500189	0.180327911	0.230758109	0.263728999
0.136015731	0.102956571	0.309814066	0.111739307
0.382022639	0.067170588	0.233441959	0.193197911
0.148540658	0.285135043	0.346869822	0.172292969
0.200408565	0.078727478	0.183537896	0.097664912
0.149073233	0.142182264	0.251504331	0.369956817
0.088801841	0.177302974	0.150184668	0.030102085
0.122225541	0.145837781	0.272149735	0.129484325
0.285521514	0.217770705	0.221452607	0.190408169
0.261384092	0.416178072	0.016220421	0.119197718
0.214277734	0.152308677	0.251602272	0.221646172

1 0.10431828 0.164249152 0.406184367

0.24160186	0.147621768	0.082075978	0.377584096
0.142619046	0.282233315	0.144113349	0.170793797
0.087534492	0.295243111	0.120992133	0.100277835
0.157966336	0.17939281	0.025330034	0.091304058
0.136022478	0.304827412	0.239575045	0.152474132
0.174294589	0.338324659	0.086655607	0.277070746
0.061971095	0.102280799	0.052341234	0.379336593
0.100100449	0.380467703	0.180138406	0.206821036
0.269604708	0.370354565	0.005777586	0.286122374
0.346239588	0.058004596	0.27051655	0.256535869
0.160977088	0.37304935	0.013947222	0.346793797
0.146338053	0.133168005	0.428036186	0.229742983
0.276782826	0.144719787	0.10466541	0.334304268
0.265117029	0.027828758	0.070123017	0.262265188
0.105909282	0.061957987	0.263732032	0.299442913
0.253972979	0.348555376	0.428443595	0.162715155
0.235103564	0.135941818	0.056685093	0.24414266
0.297839193	0.116609243	0.262553652	0.154068075
0.101076241	0.317262131	0.253429776	0.072882527
0.112639115	0.120881466	0.043184754	0.23162358
0.230897502	0.133078257	0.158570866	0.124521686
0.179205283	0.391431968	0.185665392	0.158724761
0.14102645	0.198631483	0.160774926	0.135112394
0.178472528	0.212424706	0.196698866	0.17204984
0.059765713	0.179579565	0.046377663	0.118585533
0.138905154	0.138399258	0.115225848	0.196282215
0.12512402	0.14743931	0.227022493	0.176668849
0.212572429	0.086241925	0.349447902	0.319786068
0.143299733	0.165557194	0.120082263	0.100738956
0.367170593	0.100592051	0.050081753	0.319062397
0.059628211	0.393039886	0.185469517	0.220216304
0.185840248	0.214312289	0.392404027	0.053772062
0.150955964	0.293973525	0.214999325	0.238543031
0.223208876	0.26608079	0.083374352	0.214268317
0.20501595	0.1204521	0.370176815	0.197835224
0.097890258	0.328840627	0.303110078	0.174171929
0.182062585	0.172210403	0.047613101	0.316515389
0.382324305	0.40227839	0.278898143	0.189843473
0.117276374	0.053795706	0.203393101	0.185636218
0.288529184	0.368798196	0.215298406	0.224432885
0.22912561	0.198455707	0.036991566	0.388323329
0.110211374	0.320794652	0.04731682	0.333262892
0.297956428	0.072900305	0.207351767	0.144739881
0.22236987	0.116353709	0.127124743	0.313634095
0.302383641	0.201546306	0.184230092	0.266574221
0.044085477	0.067511859	0.257728997	0.169007123
0.152854316	0.210498689	0.080548474	0.101392743
0.125907194	0.289248762	0.372598905	0.166442019
0.132968996	0.088485666	0.323583512	0.112081176
0.351518133	0.325694425	0.318722938	0.1168039
0.281565665	0.354139204	0.220532847	0.157644645
0.099021298	0.324705574	0.202486597	0.065328889
0.394261187	0.052737643	0.065165547	0.329886791
0.165978567	0.218865751	0.010478472	0.043082211

0.111051104	0.397478941	0.286315251	0.219735974
0.057949011	0.229099968	0.011229344	0.375847195
0.320765718	0.102226716	0.203956472	0.286774383
0.299614318	0.339648181	0.196993375	0.361738888
0.251106216	0.306237903	0.150128786	0.280010737
0.323272139	0.324854834	0.269648934	0.287601119
0.370096894	0.339228626	0.25569336	0.066893116
0.161980225	0.072115771	0.107232834	0.132712605
0.116771087	0.228292692	0.325706939	0.39334832
0.37698943	0.046338133	0.088670913	0.270230419
0.311063465	0.370790835	0.04481634	0.017960314
0.012463653	0.128820265	0.179718954	0.220213731
0.3573277	0.061689287	0.040357637	0.049565555
0.165332888	0.312411484	0.329839391	0.345227534
0.314840465	0.149568314	0.121133594	0.325429762
0.197377956	0.121140571	0.390515811	0.29307038
0.098551074	0.214273094	0.111360059	0.392752349
0.086165536	0.276734045	0.253856878	0.183784333
0.033091614	0.188286246	0.177115164	0.367261929
0.291657843	0.165220518	0.137077951	0.035693097
0.151276553	0.356525765	0.196530972	0.413792152
0.323298744	0.403581867	0.150286013	0.302425713
0.132497666	0.292901674	0.230350642	0.033074061
0.164984775	0.057698523	0.296736508	0.14917318
0.095747424	0.209327488	0.175093826	0.084215613
0.140301996	0.155951465	0.414723168	0.398328487
0.098160099	0.140955137	0.219693473	0.261233302
0.110920709	0.361872498	0.296825865	0.133385527
0.10718468	0.098088362	0.276153212	0.184072448
0.087982059	0.423261578	0.204267905	0.378230406
0.225160406	0.308253941	0.329833559	0.219698756
0.190642234	0.166086961	0.219271143	0.053401267
0.22985723	0.388418928	0.128869603	0.247667618
0.239447476	0.197740472	0.062934425	0.044886466
0.157679346	0.121656335	0.158750086	0.395673633
0.180547561	0.197231875	0.043004127	0.177910701
0.363538793	0.267263607	0.097052301	0.270241466
0.191128082	0.262785423	0.33357178	0.350135037
0.130629425	0.315508917	0.282692756	0.217474398
0.254323797	0.183445764	0.327537919	0.031315312
0.181011985	0.186425538	0.166790548	0.179205951
0.189886034	0.115879049	0.148498066	0.347998304
0.137595437	0.314965327	0.074724092	0.141252245
0.238015231	0.095891336	0.272358015	0.124284744
0.156943235	0.308816802	0.047984955	0.140118341
0.142980917	0.317976392	0.226159748	0.227901971
0.192649535	0.160022266	0.242401879	0.083473802
0.099569952	0.121844518	0.31085043	0.058972197
0.051698493	0.416066166	0.109858274	0.070261857
0.096207346	0.152677513	0.12740227	0.187892779
0.098148885	0.33454776	0.094126263	0.232102339
0.174419688	0.124940051	0.171922144	0.074919667
0.086323744	0.257539167	0.222204542	0.328511702
0.16704998	0.396412988	0.227093997	0.27708116

0.093804607	0.138264276	0.081065415	0.324542251
0.134282107			
D16ERTD472E	0.117570211	0.141809298	0.066798063
0.238491988	0.108966763	0.128143271	0.265810328
0.154576996	0.097286755	0.054628566	0.022365094
0.088130351	0.042637114	0.09579139	0.042343529
0.232775035	0.058695491	0.095244645	0.291963786
0.238087176	0.172415379	0.205274353	0.186243721
0.031727209	0.046296744	0.113888401	0.045541728
0.248527968	0.133974276	0.099373482	0.242771918
0.080717659	0.157210183	0.11090444	0.110412457
0.093186968	0.094951719	0.04128854	0.071784484
0.163553175	0.132680843	0.247720369	0.251836018
0.14271194	0.112095573	0.268811215	0.110200172
0.085026626	0.060750496	0.111445903	0.123786752
0.088338631	0.078750227	0.189573318	0.196359813
0.019677218	0.087733013	0.009784572	0.175711887
0.246132084	0.125596172	0.095073536	0.121626957
0.05486665	0.200344099	0.058782196	0.22985859
0.025961868	0.167238625	0.176676204	0.056304807
0.02358633	0.279111437	0.132056408	0.017921917
0.138817341	0.056765247	0.081194557	0.247782245
0.215749595	0.112794364	0.156121908	0.130927898
0.058228142	0.143679883	0.051650174	0.135252661
0.149184538	0.200260067	0.123833921	0.205336762
0.150063032	0.111329774	0.070140952	0.132595769
0.136044462	0.152784081	0.076873339	0.082484087
0.024730226	0.041587957	0.108121063	0.136634656
0.140917208	0.031353034	0.123966118	0.220142379
0.153145154	0.044582569	0.269262191	0.0467404
0.221002038	0.126971739	0.100204332	0.189660846
0.112251335	0.147939547	0.102457236	0.284908248
0.08983219	0.066319169	0.013550986	0.098934151
0.060562332	0.009820523	0.086048755	0.183386842
0.113168306	0.036246698	0.21804118	0.038969106
0.062610797	0.062912918	0.102638564	0.128394792
0.179761874	0.074505006	0.081467478	0.1229687
0.068847934	0.15188412	0.018344297	0.199416777
0.130685697	0.067871659	0.271148517	0.091159626
0.10431828	1 0.197723508	0.160289964	
0.152675002	0.227730186	0.08392987	0.218870172
0.042546162	0.089974452	0.206744088	0.107786618
0.164206038	0.084642659	0.051455323	0.158734256
0.016593082	0.030589591	0.116222933	0.215436268
0.073937826	0.10471702	0.07867658	0.220935573
0.139727946	0.156312383	0.130146532	0.110430007
0.03216254	0.153594858	0.085735348	0.141223845
0.137407098	0.137682507	0.118678601	0.077344038
0.166800715	0.196880372	0.004542185	0.107813265
0.107657713	0.076513391	0.124316933	0.119804662
0.037471551	0.195797342	0.011767689	0.210751967
0.111907434	0.207122976	0.176205359	0.141569738
0.108765318	0.114689413	0.11102176	0.086770073
0.066423555	0.034960575	0.16721167	0.256954396

0.04391571	0.153657839	0.170934497	0.217316521
0.147804728	0.158437137	0.203882488	0.029999331
0.104771664	0.231033296	0.088809535	0.104670741
0.127543106	0.018301267	0.135984379	0.148199164
0.089396829	0.140506881	0.083063153	0.022301157
0.178342601	0.197339715	0.105266693	0.120637022
0.091355675	0.104716816	0.05138529	0.078669513
0.078166202	0.142125044	0.02433654	0.018311077
0.009911744	0.099889293	0.275301028	0.047256789
0.02495742	0.107892114	0.040206882	0.060095864
0.171182882	0.12529994	0.032044166	0.018398996
0.029272421	0.030913594	0.0314591	0.11060795
0.0600806	0.07074003	0.096829942	0.030933861
0.063689767	0.09941458	0.139393776	0.227405162
0.129416616	0.166003314	0.116604321	0.01399376
0.167494892	0.080531672	0.067398976	0.155035503
0.123009213	0.15868691	0.056586743	0.125627906
0.036642864	0.207675394	0.225657881	0.042510104
0.134849955	0.136577569	0.176869358	0.096823264
0.114361156	0.2038694	0.148390898	0.114882099
0.109341942	0.117289492	0.123618469	0.053647504
0.09900867	0.115445129	0.086443104	0.156919223
0.099280333	0.10830122	0.188579038	0.19055227
0.145290363	0.209563187	0.110744739	0.039718118
0.127020995	0.022488773	0.064389927	0.067621232
0.104256734	0.110053885	0.221378504	0.062038491
0.305673031	0.034111086	0.084387713	0.132743755
0.04553236	0.118547809	0.078351601	0.094401045
0.090450732	0.122151821	0.069109071	0.202594781
0.13571667	0.166499458	0.172251735	0.114771859
0.15167397	0.078191	0.049160056	0.14510653
0.153533706	0.175410819	0.108530511	0.01615858
0.053498522	0.122060247	0.003854448	0.078446822
0.097055762	0.129384299	0.180351395	0.075302531
0.085760158	0.085731416	0.156453159	0.162148108
0.089323537	0.101814881	0.114172568	0.19588491
0.108962859	0.104387227	0.039336497	0.120450547
0.067328351	0.098823324	0.162491553	0.054985502
0.191280341	0.117833855	0.039662283	0.153608289
0.296043748	0.087153471	0.046358961	0.011950917
0.047817334	0.154917203	0.104953433	0.242618992
0.047766578	0.236088839	0.008171752	0.124903018
0.121572317	0.03920365	0.040286115	0.089153068
0.06653465	0.145072167	0.032517035	0.097793248
0.058634864	0.091666832	0.093941254	0.189731803
0.155422821	0.084435896	0.194536682	0.147713008
0.182505912	0.145128336	0.286050806	0.108615678
0.028777458	0.177073776	0.020155843	0.035733474
0.153786113	0.061897371	0.13406797	0.176418495
0.115502703	0.066002583	0.170289324	0.106419675
0.206755224	0.212195491	0.075712006	0.015073974
0.008760146	0.014488902	0.223897159	0.123447832
0.130757764	0.025937667	0.069665362	0.209489457
0.101134894	0.081983672	0.116583938	0.286178487

0.115547338	0.140045448	0.033459223	0.272910213
0.044856636	0.09791779	0.130426307	0.135338645
0.086911644	0.113990477	0.099829756	0.195187145
0.002652752	0.172365529	0.094820261	0.057401986
0.040909681	0.070248945	0.067705507	0.189075116
0.083538107	0.046969025	0.044434429	0.040171258
0.120118837	0.158653127	0.102941371	0.162221362
0.192959515	0.238228111	0.044248857	0.123996905
0.059593316	0.100039167	0.055410549	0.036996124
0.091928552	0.099146207	0.092416795	0.012077361
0.100243604	0.186676549	0.030406254	0.043197185
0.012047011	0.094323059	0.174631484	0.13605836
0.048257813	0.158465577	0.16166069	0.192817312
0.217400181	0.18448645	0.111529628	0.049673261
0.160032026	0.013659826	0.244546336	0.123579996
0.117154606	0.162453749	0.037113689	0.134173808
0.152939802	0.152778288	0.111741768	0.205049713
0.023768771	0.131779062	0.173854742	0.099004444
0.226894865	0.166589627	0.162597616	0.040814932
0.10953017	0.043158578	0.150522714	0.038664709
0.024423308	0.172727159	0.114443835	0.237904
0.166272087	0.130036309	0.167129979	0.067449843
0.138543459	0.139866875	0.154774849	0.158354568
0.165816596	0.145303311	0.126757462	0.11605085
0.032866964	0.115323318	0.19280641	0.144581686
0.140532164	0.26276646	0.115857702	0.160624487
0.037796306	0.076871656	0.080905817	0.12495855
0.207938098	0.071580655	0.010353401	0.19220974
0.017148796	0.194340899	0.047699973	0.101607869
0.126359388	0.070348985	0.189566033	0.189653779
0.098599397	0.108748186	0.040485042	0.116853794
0.023495854	0.120971576	0.046032093	0.11285855
0.0167825	0.048607865	0.190398775	0.143160067
0.041472536	0.027052315	0.119149706	0.181801488
0.157995242	0.176491675	0.101626341	0.052525885
0.045248274	0.10868485	0.07807525	0.062649433
0.040669227	0.090675089	0.064968869	0.093924742
0.230896853	0.149851418	0.119913306	0.085865191
0.173235723	0.122749054	0.068571402	0.099530847
0.072501893	0.223218926	0.105374364	0.113825543
0.079119745	0.049547203	0.166517337	0.195671364
0.024560527			
D1ERTD622E	0.115102471	0.151494479	0.073809362
0.24661469	0.149062063	0.099202578	0.230217208
0.099781715	0.105240431	0.079109941	0.084886097
0.113405998	0.098708317	0.118045107	0.038801284
0.263114556	0.180992973	0.072657926	0.305657195
0.232763084	0.159586871	0.283510643	0.23250725
0.067722617	0.123730124	0.198694814	0.076690652
0.244256066	0.204784917	0.079613693	0.194870967
0.178779443	0.155014916	0.112938975	0.175804277
0.103070647	0.085322563	0.071902607	0.065870876
0.13672101	0.154174429	0.237006169	0.2854942
0.213834975	0.10247029	0.27703632	0.146660476

0.056402082	0.165364641	0.054587749	0.103867702
0.096074225	0.100394965	0.110784858	0.182878484
0.058892815	0.062520839	0.033753048	0.112345159
0.274936895	0.063332312	0.106453833	0.120884094
0.067191666	0.134427969	0.157083561	0.17787519
0.045319829	0.121722838	0.181577978	0.071314741
0.038016837	0.251460368	0.175613278	0.028681445
0.236913083	0.173127744	0.162479724	0.251230603
0.222167466	0.107770591	0.200574208	0.168434228
0.15539164	0.187779273	0.083352456	0.21935536
0.137856065	0.209332346	0.112365327	0.268047719
0.190764327	0.127569726	0.138753208	0.201123802
0.14387	0.147229122	0.077402692	0.111969482
0.035739389	0.10718227	0.109723806	0.167874622
0.114960338	0.061676933	0.224992126	0.192428309
0.239008846	0.041055016	0.312285769	0.110056185
0.22511003	0.134284064	0.192070589	0.200442435
0.138420239	0.145080532	0.121099861	0.287506023
0.119581641	0.15516125	0.034396051	0.195653288
0.081351785	0.033240295	0.084588527	0.206417795
0.107131669	0.074053051	0.183584936	0.092153287
0.177491095	0.104453464	0.124641362	0.114148288
0.176958228	0.073133409	0.125612557	0.10038315
0.095750383	0.198127909	0.00784301	0.227144308
0.124324322	0.120030391	0.346179794	0.119705024
0.164249152	0.197723508	1	0.18064315
0.122037467	0.284391042	0.058864182	0.260640546
0.070076877	0.158212829	0.255179905	0.156113335
0.232906451	0.094460623	0.148040568	0.099896502
0.024623471	0.119116232	0.115284472	0.137378953
0.082905617	0.127125872	0.078337883	0.258258405
0.18751511	0.21740044	0.085110254	0.132235395
0.080467304	0.198246584	0.046466057	0.169483595
0.085365515	0.161408659	0.141763829	0.09997991
0.245662007	0.21035253	0.003952361	0.103905499
0.122510375	0.040761385	0.184726002	0.145833994
0.051740737	0.212481067	0.005879516	0.26440728
0.083348091	0.240201508	0.210325703	0.258183093
0.110708218	0.203397816	0.095649428	0.116789439
0.095112609	0.057197775	0.093986211	0.227868882
0.127146038	0.122595789	0.160436481	0.223411606
0.161336689	0.181976394	0.239684867	0.043738402
0.135333765	0.180888228	0.135616611	0.160052217
0.140231722	0.031429952	0.119180329	0.149745973
0.059444726	0.139662419	0.092636963	0.030697254
0.218483656	0.158567707	0.088539432	0.147296927
0.085471222	0.195965005	0.05704047	0.139145708
0.18798562	0.165217499	0.057557551	0.035797844
0.032231688	0.135305819	0.236931073	0.098006755
0.087754238	0.179040991	0.05362045	0.071598301
0.181983567	0.240981421	0.063122983	0.036007066
0.107922614	0.056522472	0.052235572	0.106390976
0.09182466	0.176898997	0.140369326	0.05458107
0.13815215	0.180571072	0.148480374	0.241314953

0.08836245	0.196430852	0.219314871	0.030438936
0.195227069	0.055472778	0.03138617	0.163154885
0.128876778	0.189641042	0.134587295	0.235304006
0.076602465	0.284385286	0.233868353	0.035416788
0.111521242	0.136559969	0.199579218	0.157024579
0.105063515	0.194967636	0.205184345	0.102944872
0.101922129	0.228559512	0.144545461	0.162306735
0.184322618	0.156988703	0.117063387	0.252320829
0.174305879	0.141029309	0.143332696	0.282131748
0.163668146	0.280741528	0.130748821	0.070417443
0.225965735	0.109442694	0.122360183	0.079153088
0.105897338	0.137550087	0.284897199	0.116943316
0.339544072	0.052953249	0.056256093	0.161934006
0.059331244	0.122794114	0.14534858	0.130140595
0.098402829	0.076920786	0.153739987	0.165555963
0.118565449	0.207455525	0.165753095	0.120729962
0.146382047	0.085686331	0.054980586	0.166120332
0.154960466	0.215238936	0.128901919	0.04174419
0.065132901	0.153871235	0.015441736	0.06693489
0.21405597	0.131512953	0.214409291	0.130807877
0.081296479	0.05529873	0.228053979	0.228127893
0.119597144	0.1156117	0.119018807	0.193268071
0.132560325	0.128580143	0.06781119	0.192388737
0.145295227	0.110374	0.126461218	0.118013431
0.222476748	0.055955262	0.131957467	0.151333224
0.285669225	0.080234234	0.015372211	0.01417901
0.114988737	0.196590398	0.103113281	0.193000834
0.03150622	0.218074053	0.018330364	0.147917464
0.130493892	0.115063908	0.110088782	0.114761093
0.097836872	0.145746814	0.056063521	0.132852527
0.092141417	0.105208234	0.134881582	0.2696378
0.160882552	0.104723857	0.166545535	0.130057244
0.214735763	0.198434355	0.312780666	0.161463169
0.042088699	0.103449159	0.024968029	0.087498651
0.090872466	0.140548857	0.137466948	0.213828411
0.149399507	0.120948533	0.151017943	0.116391969
0.187645731	0.212433267	0.082033448	0.010166696
0.005420947	0.06200053	0.186466535	0.172489116
0.146409973	0.030152669	0.138356727	0.16906097
0.11679527	0.13326763	0.130419694	0.309063892
0.137944668	0.242971956	0.053801252	0.273782203
0.119706612	0.065210942	0.163881768	0.129133583
0.171164748	0.152594093	0.0818565	0.213247574
0.010786616	0.253643795	0.125397222	0.094325679
0.074556644	0.158553268	0.142364733	0.219766688
0.09535429	0.164566741	0.163779827	0.026073164
0.197529905	0.180136909	0.201633745	0.192229922
0.191296224	0.276459079	0.14002677	0.119439464
0.140806852	0.107049542	0.106552726	0.023624849
0.106509027	0.173515455	0.108653012	0.030434048
0.079507816	0.148245582	0.042191035	0.126407497
0.024729448	0.211272052	0.213464197	0.179230987
0.120675465	0.150039292	0.199523637	0.191560152
0.137858984	0.224477241	0.111411765	0.051398748

0.201019258	0.039087791	0.217347927	0.102144666
0.105770115	0.204487128	0.075409674	0.152171565
0.122966547	0.144562473	0.131107352	0.184983098
0.080046203	0.099941539	0.161107274	0.128173625
0.218958144	0.214544675	0.118923997	0.100810594
0.197045121	0.067004326	0.175601055	0.028194334
0.044282639	0.113464884	0.224484842	0.262348227
0.13223024	0.105256477	0.186930311	0.139288423
0.149727331	0.120633921	0.107716395	0.162380451
0.223796147	0.188048624	0.152155619	0.156968957
0.042659762	0.1456638	0.178923617	0.14684199
0.126912728	0.249667335	0.122037427	0.070164426
0.066575946	0.0890617	0.192854674	0.218413984
0.290469245	0.149140468	0.022880088	0.234878773
0.028815478	0.268931096	0.045015784	0.157792754
0.190598007	0.056895794	0.239341279	0.167659288
0.200571704	0.11539307	0.083545726	0.22924089
0.04303481	0.130749813	0.082830024	0.152422527
0.042256907	0.118943621	0.274512731	0.085293032
0.053961235	0.036927738	0.127104647	0.121647972
0.209705905	0.213312867	0.070038285	0.105534157
0.096536934	0.086442603	0.096129837	0.102325697
0.116417966	0.117569084	0.05642399	0.121303794
0.188166227	0.248192343	0.087886502	0.08087857
0.170143201	0.110753903	0.087540645	0.114805365
0.091565742	0.280633244	0.08870287	0.132807878
0.078496353	0.053919672	0.188614616	0.194650804
0.03251526			
DAPK2 0.476726691	0.529537178	0.47692453	
0.498225589	0.511136791	0.434954583	0.277812817
0.231819142	0.13484649	0.008983447	0.040403262
0.510062974	0.271325338	0.332718692	0.405953844
0.503032308	0.190193292	0.414580374	0.3187296
0.485549473	0.126616142	0.490060852	0.137210836
0.372052279	0.157145269	0.108387606	0.359512716
0.507628874	0.123358081	0.379648062	0.447262748
0.144157729	0.492797446	0.527955811	0.517963539
0.520139632	0.450945133	0.015413789	0.362474636
0.467897722	0.522025704	0.481718245	0.417740377
0.067917764	0.489878391	0.324282634	0.4868262
0.283636924	0.05295467	0.282236666	0.444782307
0.484486268	0.506053204	0.284338689	0.220030203
0.261350699	0.26399364	0.342653138	0.367598546
0.299612853	0.188490284	0.492618194	0.133686518
0.18810307	0.308894399	0.208085528	0.324503691
0.426761089	0.437088775	0.028477215	0.462575614
0.004299227	0.466627872	0.462911348	0.391700316
0.067732418	0.034333898	0.241237489	0.418656806
0.51468934	0.457389911	0.531077188	0.52165635
0.244791231	0.376314046	0.470401496	0.23898839
0.48915153	0.242204816	0.155832407	0.507728866
0.529092384	0.528727188	0.280148162	0.348220404
0.487810023	0.477282532	0.502353774	0.513942086
0.43158115	0.080737597	0.452333746	0.514687849

0.438591741	0.395115785	0.218550779	0.468652922
0.090694819	0.425922989	0.442636986	0.198614821
0.516930041	0.032662857	0.288868882	0.433997546
0.282021946	0.502950117	0.524035157	0.257358653
0.488638763	0.032967042	0.196463321	0.052800377
0.401582936	0.194887413	0.4886161	0.51486763
0.282148484	0.461930046	0.340567846	0.004614485
0.044668112	0.24452461	0.488018561	0.434536017
0.500936068	0.419610517	0.317970545	0.437906137
0.510452314	0.530076977	0.073876628	0.140148515
0.506572524	0.244248011	0.320099247	0.514565056
0.406184367	0.160289964	0.18064315	1
0.427692447	0.126398279	0.384208535	0.503096504
0.406061354	0.448958098	0.153525077	0.350497011
0.075680485	0.490335736	0.046287261	0.392606107
0.35366604	0.198908199	0.017327029	0.315100694
0.362789034	0.488454259	0.473409302	0.153909488
0.397745734	0.501020815	0.368536001	0.496354916
0.019535304	0.072225001	0.314308458	0.509946455
0.324124104	0.511479366	0.384487723	0.395077421
0.373657423	0.521080846	0.005038007	0.504051628
0.532913792	0.337280771	0.486133849	0.497947896
0.458999159	0.517567231	0.051836827	0.500375256
0.400355085	0.117717765	0.528939339	0.227444857
0.461367313	0.067844296	0.376864711	0.501000192
0.488113251	0.013649664	0.272579991	0.462723009
0.044052716	0.130360074	0.493317609	0.493523625
0.474056008	0.51940357	0.529011994	0.442328468
0.517684329	0.300958044	0.021323807	0.40994371
0.503083498	0.43964221	0.446429632	0.314847398
0.378230497	0.481640051	0.473369813	0.252851546
0.070146624	0.3789457	0.033064134	0.433602074
0.443131834	0.165187473	0.470500477	0.193430682
0.107891971	0.529260914	0.240281274	0.330254426
0.17465073	0.470225472	0.328299729	0.299206521
0.279090524	0.234503111	0.447583656	0.46808763
0.057320336	0.13875252	0.022982116	0.430564539
0.185297699	0.407505477	0.396316902	0.458964572
0.386179242	0.153006473	0.481238116	0.369981209
0.169462149	0.026058922	0.511873091	0.492332559
0.375507928	0.219369481	0.064284659	0.390877561
0.51411327	0.36968688	0.305318867	0.486804712
0.043755232	0.523490498	0.324526537	0.179924698
0.459551968	0.344571717	0.518151428	0.086016509
0.449672005	0.470563531	0.399592299	0.263468676
0.422451659	0.496373087	0.04950685	0.457303785
0.450964709	0.051708641	0.529424462	0.178033697
0.035089562	0.518002029	0.510006033	0.115565122
0.119734399	0.41812141	0.067352486	0.383483815
0.523080463	0.501217299	0.4929719	0.290480542
0.065364984	0.016509396	0.279180207	0.471404658
0.515522238	0.512482835	0.342277564	0.460292532
0.32152039	0.427126053	0.168359214	0.512230194
0.2456211	0.522275849	0.012227744	0.511302661

0.517468473	0.331437193	0.124575937	0.378996987
0.439712492	0.075155602	0.265504761	0.504755564
0.490258021	0.429466813	0.447049656	0.501309783
0.027422796	0.064099211	0.484290064	0.247501508
0.467546239	0.298043288	0.074341503	0.388264154
0.069335983	0.46239051	0.522382307	0.098614335
0.395027138	0.367103356	0.4703963	0.060415053
0.500580175	0.503189017	0.529653838	0.218981265
0.501681287	0.494684171	0.476000446	0.118609026
0.029788807	0.524457623	0.460838062	0.036621852
0.525069283	0.271014557	0.02166328	0.501458496
0.254073663	0.446532692	0.007228191	0.104540121
0.063245735	0.525243237	0.490076977	0.449027669
0.290213585	0.448904499	0.008489277	0.523209583
0.498076496	0.025648925	0.200568508	0.500014966
0.495629909	0.533534393	0.409917866	0.513217508
0.50242882	0.508248221	0.307655177	0.360892099
0.504701666	0.497361111	0.490887957	0.462703107
0.520263555	0.509924207	0.368102528	0.027746832
0.200113183	0.259719884	0.379784947	0.124846684
0.372433536	0.20647297	0.488209638	0.527368668
0.514559163	0.009416364	0.198807226	0.493889308
0.486557777	0.512379549	0.032047499	0.010217742
0.011576057	0.034533071	0.432458076	0.327802918
0.504582992	0.282407338	0.008957944	0.064120584
0.36590504	0.489563287	0.510842589	0.469766238
0.50875388	0.120099219	0.351119524	0.482717675
0.108973821	0.367413814	0.519983489	0.480830725
0.062928793	0.334440125	0.435289108	0.518841522
0.099551292	0.409280892	0.508300382	0.404622196
0.020723031	0.124779359	0.10528501	0.516208359
0.506690697	0.115021921	0.054198423	0.17004351
0.171228032	0.517375262	0.231633116	0.523819314
0.504789437	0.515756515	0.109778723	0.493048275
0.06451263	0.494285187	0.432131013	0.079628579
0.364330929	0.021202124	0.468283441	0.217544536
0.380130104	0.461589198	0.277997543	0.027896682
0.242918906	0.082775746	0.528488489	0.526920434
0.052152901	0.310567656	0.389840107	0.485274371
0.345882503	0.516874086	0.512441213	0.466250221
0.124069938	0.17763151	0.481816424	0.452557218
0.288802067	0.5298297	0.392351079	0.528584652
0.471358485	0.484745428	0.494528386	0.440789321
0.30393853	0.464311682	0.440002774	0.032776038
0.445191748	0.526208722	0.426750242	0.226972546
0.347269488	0.463343627	0.068729868	0.211164421
0.40221536	0.360271912	0.198791904	0.514723472
0.451269961	0.456670282	0.055183873	0.100223911
0.54647812	0.455363742	0.197671318	0.504687196
0.23762842	0.444730693	0.518679456	0.518663391
0.442181345	0.503241201	0.491819785	0.450155706
0.438525209	0.344489938	0.533818175	0.111147744
0.13318566	0.471861622	0.142969774	0.13693448
0.20413568	0.045750987	0.371516457	0.508266649

0.441997431 0.473590325 0.361508657 0.123117262
0.414727584 0.42117327 0.451419459 0.263082626
0.11932889 0.508027253 0.016302608 0.085963016
0.450771759 0.512322259 0.47436746 0.327912837
0.382692879 0.192024926 0.391067785 0.346390109
0.377098508 0.42984854 0.507309563 0.054061419
0.031087022 0.528462874 0.436384232 0.033379783
0.119759562 0.433908118 0.358994561 0.322398718
0.036022703 0.482966722 0.394171409 0.511964035
0.423427937 0.098575479 0.379993669 0.122151154
0.088953282 0.450959355 0.479723396 0.519453854
0.430151369 0.507389453 0.449788114 0.492641269
0.165047749 0.440589477 0.046626955 0.506641318
0.476621473
DAPL1 0.411574929 0.470814808 0.34690407
0.407628588 0.369678183 0.434325854 0.239372738
0.280830485 0.082146458 0.00627909 0.015206242
0.378098127 0.171900202 0.30407358 0.360879875
0.376650923 0.093753868 0.373642729 0.23747047
0.409220452 0.141213645 0.342251885 0.099615186
0.215006612 0.0675908 0.055428609 0.196587255
0.428129477 0.064576663 0.413223723 0.437157152
0.07437741 0.435686109 0.487574389 0.360923311
0.472524508 0.404361103 0.01332354 0.340108836
0.439349731 0.393466444 0.414665611 0.311944471
0.044124492 0.489434571 0.250591848 0.329865203
0.301152976 0.021769626 0.367267175 0.458002587
0.452793557 0.401543159 0.312375742 0.181615519
0.168936997 0.345165205 0.186755097 0.429034436
0.230620851 0.280852198 0.446459931 0.10441676
0.174338562 0.324700471 0.106650319 0.315528648
0.263876016 0.448467114 0.025842592 0.356326974
0.004752142 0.393482827 0.311558587 0.237069982
0.03150126 0.016223095 0.129159636 0.352960009
0.431138891 0.421663641 0.403675222 0.402534149
0.136328857 0.245468583 0.308862066 0.150862463
0.438012451 0.192975618 0.11505357 0.360452522
0.406131988 0.464849966 0.155899957 0.20084219
0.424582591 0.44580396 0.402948144 0.376292787
0.286187529 0.031192778 0.423909612 0.416725889
0.43845907 0.223740655 0.111896216 0.445492214
0.050217731 0.393754203 0.332141559 0.131847142
0.432353954 0.022085222 0.151068208 0.376709282
0.208291266 0.432265992 0.447200315 0.195248655
0.345990746 0.015803875 0.089032322 0.025709961
0.321820667 0.086104192 0.482177183 0.428502503
0.307700229 0.318705855 0.298923709 0.000540479
0.016804067 0.150126209 0.413163413 0.375155708
0.421622438 0.374777883 0.194713975 0.442708716
0.379301715 0.402245597 0.096477502 0.113397404
0.410251886 0.149829789 0.212229385 0.407474002
0.24160186 0.152675002 0.122037467 0.427692447
1 0.082998405 0.403509434 0.365795557
0.329485284 0.268368632 0.109814748 0.26683824

0.062043219	0.436121086	0.015920377	0.40492983
0.229081833	0.09651576	0.014005194	0.354925849
0.313603813	0.414012075	0.463631831	0.100695678
0.30779282	0.343645574	0.421432088	0.410870478
0.009363985	0.045686623	0.400269981	0.424724332
0.408009284	0.410917738	0.288951495	0.251953195
0.226066698	0.41257352	0.00270052	0.482619513
0.463267422	0.409721186	0.351028183	0.364477154
0.31234629	0.423470097	0.053598493	0.373373672
0.423183262	0.077233175	0.41488949	0.122273441
0.426691825	0.032064259	0.387517557	0.373274838
0.382812997	0.009084901	0.335740744	0.414011145
0.01518823	0.162883359	0.428458595	0.395153135
0.358768922	0.397955766	0.411409062	0.290241881
0.37345059	0.27197958	0.010206942	0.244880783
0.405582455	0.345662953	0.44194422	0.274168171
0.421632071	0.435722372	0.402085434	0.224160483
0.044776979	0.375933568	0.028493598	0.285601121
0.400938719	0.12489417	0.358716043	0.123377975
0.047291217	0.44422222	0.110573196	0.17821304
0.073722931	0.393406346	0.271444095	0.237863557
0.164992277	0.141379436	0.29011743	0.341619933
0.057946053	0.076103898	0.008303811	0.321113647
0.113541141	0.309201507	0.328711794	0.439137495
0.345110115	0.09574669	0.324961106	0.221077104
0.079947074	0.010508046	0.452248726	0.377149508
0.427585118	0.163867864	0.031364291	0.317505928
0.428855036	0.403229833	0.398683419	0.43851964
0.028197408	0.41273023	0.21813971	0.090969803
0.294055613	0.239241247	0.423198837	0.075417813
0.435725072	0.447444308	0.275217072	0.144522899
0.426432449	0.417226123	0.035563698	0.42869409
0.431109016	0.024531774	0.449090996	0.084021706
0.01419885	0.369309947	0.390736189	0.057253141
0.05615475	0.322685779	0.084029481	0.233706209
0.445264193	0.354302951	0.420782711	0.152195109
0.038436825	0.006752151	0.145476054	0.343998712
0.491429646	0.420025399	0.229763487	0.28628138
0.231425002	0.274737076	0.257663412	0.419522425
0.167081777	0.474839547	0.005268995	0.376699239
0.475311155	0.405546357	0.054116032	0.365876448
0.439880506	0.047997382	0.229751381	0.460235924
0.435455695	0.295856083	0.31280713	0.393635599
0.024928137	0.060036269	0.334310764	0.117876097
0.364464088	0.191813703	0.025583521	0.407926065
0.03817432	0.433158334	0.407475531	0.047819755
0.39259745	0.402752692	0.310318751	0.034711489
0.379837692	0.432506436	0.48829782	0.198936566
0.410561451	0.407664892	0.324534116	0.059293727
0.009998847	0.462617521	0.442080641	0.021128667
0.420961907	0.377247877	0.010324098	0.461312364
0.193818845	0.459365547	0.007186892	0.06721462
0.024502567	0.390379865	0.462454784	0.433135135
0.346297228	0.369368196	0.003513805	0.433934348

0.452674823	0.007546734	0.092322905	0.406756333
0.36287141	0.489502417	0.235850087	0.388757804
0.355041225	0.455996013	0.25754632	0.221441754
0.434256148	0.417614785	0.441159137	0.441071784
0.425756964	0.357970064	0.27136938	0.017474332
0.102641129	0.324734739	0.31156311	0.067602699
0.431762891	0.104037668	0.424408788	0.412086008
0.403939079	0.00297695	0.212552012	0.426114216
0.448750871	0.436412794	0.017911987	0.005617718
0.006417371	0.009418858	0.407835204	0.190738616
0.420117201	0.305584291	0.00365676	0.076742437
0.28411342	0.310895533	0.455390162	0.358347779
0.393586833	0.068483261	0.295641398	0.387119561
0.044247785	0.409897682	0.411122209	0.463219822
0.030411565	0.199526347	0.396743168	0.431465566
0.037630355	0.261111169	0.388007221	0.30010682
0.010076288	0.05538895	0.047362934	0.392806724
0.459974137	0.056991241	0.021782109	0.226110151
0.121200655	0.420855718	0.146831677	0.420113124
0.439550181	0.400184968	0.046208551	0.460313352
0.03193503	0.45786302	0.247759463	0.079530987
0.255289317	0.01037229	0.405993924	0.117873138
0.404854682	0.438504834	0.159790172	0.00903279
0.123304789	0.037442438	0.41312056	0.389407747
0.019697313	0.272490849	0.2533639	0.388859619
0.381686586	0.401008512	0.484815276	0.403921093
0.105134206	0.091984804	0.426327765	0.435102714
0.323410146	0.406142594	0.251840107	0.444393196
0.44132578	0.433290149	0.414893927	0.358316219
0.160621419	0.442942165	0.416374705	0.025349596
0.345856745	0.410603938	0.404487075	0.110548071
0.204830766	0.30884415	0.05857326	0.227549128
0.244618183	0.410983834	0.137836056	0.400750802
0.432596332	0.440844197	0.060367856	0.044923838
0.482717613	0.43421664	0.19582611	0.424075804
0.142484755	0.299796969	0.399374305	0.381348183
0.353140621	0.41946099	0.445037985	0.42485304
0.437658109	0.268621747	0.486267667	0.179377446
0.060810196	0.397178427	0.085167996	0.066002358
0.129686395	0.018250227	0.219354724	0.378672278
0.299390289	0.342521656	0.401839552	0.07052205
0.260663626	0.388369061	0.313535501	0.243822592
0.069895405	0.468523343	0.006647369	0.042261829
0.305672491	0.415799664	0.296471094	0.196170848
0.213382502	0.097193018	0.262171378	0.408552433
0.344247927	0.33185707	0.418312418	0.0465587
0.026622354	0.41501686	0.424054049	0.012926988
0.069092145	0.425966562	0.29523734	0.220219073
0.011598805	0.399446849	0.394555349	0.367185935
0.388156485	0.060186223	0.418047825	0.103254917
0.067586892	0.443541635	0.329313623	0.454992859
0.289878977	0.377900537	0.467203343	0.425985939
0.117871491	0.348453871	0.03329549	0.435100802
0.355750012			

DBNDD2	0.073686541	0.102023716	0.051355123
0.201444951	0.110954427	0.076803501	0.234525478
0.102970313	0.144712553	0.127967887	0.116966935
0.075513161	0.105972435	0.116979691	0.038014771
0.206680775	0.188784242	0.051229324	0.290412684
0.180710397	0.19497024	0.229288491	0.280269838
0.056297045	0.143155917	0.247308798	0.073215615
0.185960885	0.247754694	0.064508087	0.151303186
0.176503812	0.104686596	0.072088494	0.129573801
0.063521733	0.053838268	0.064883213	0.069603444
0.092005055	0.115295634	0.187015629	0.257471824
0.280550359	0.064743893	0.274137664	0.106400268
0.056260607	0.213577155	0.048043181	0.067285748
0.06104521	0.073413653	0.115069084	0.207696436
0.060326216	0.056251294	0.0286206	0.083288733
0.268571072	0.06452447	0.067938156	0.167427459
0.066270961	0.116776965	0.147048625	0.161407581
0.035273229	0.086547628	0.218490163	0.043201283
0.050842413	0.20345583	0.130225956	0.022983055
0.25943884	0.220584353	0.153408093	0.224572041
0.162140621	0.06683621	0.142792429	0.115538683
0.159840708	0.152684549	0.0555545	0.239582308
0.094475285	0.219468785	0.128815456	0.208292432
0.134488486	0.082472826	0.147639441	0.17266606
0.109038627	0.099340664	0.058928022	0.085797864
0.033863701	0.111916371	0.072933471	0.113610547
0.076444568	0.043340963	0.219504579	0.142675876
0.275016721	0.032966861	0.272457389	0.093809747
0.165870358	0.202240294	0.169645903	0.171624606
0.127414778	0.099853561	0.077580168	0.291674615
0.083693599	0.18574018	0.039365154	0.217549382
0.072346546	0.02738076	0.051834984	0.153969586
0.102789427	0.062853867	0.170606589	0.11855436
0.211493418	0.091963714	0.07894984	0.080728207
0.126019788	0.043916902	0.135469486	0.06743849
0.064225575	0.140132099	0.009215026	0.266874478
0.098723309	0.10530052	0.323252648	0.082196976
0.147621768	0.227730186	0.284391042	0.126398279
0.082998405	1	0.039664505	0.205050312
0.068262893	0.123794865	0.286476606	0.151877434
0.292354138	0.058755758	0.168689875	0.076858419
0.025541196	0.111634271	0.128627307	0.120192134
0.085001343	0.089859073	0.046608608	0.268622558
0.159876463	0.173328275	0.06222317	0.099466526
0.125249752	0.231111902	0.031469114	0.115731329
0.064107827	0.1068411	0.137261478	0.091861745
0.216501672	0.156378303	0.004165019	0.066419441
0.078913304	0.028064038	0.150077641	0.102069096
0.039501684	0.162299522	0.004809485	0.202234739
0.052956016	0.273756177	0.149810377	0.254382574
0.069401471	0.256623663	0.07745774	0.07761802
0.060555196	0.066738756	0.089223195	0.187803161
0.143156305	0.152813037	0.112434333	0.182388826
0.14078765	0.128631847	0.175856441	0.033880431

0.105496648	0.164773093	0.169038172	0.1271311
0.092702541	0.023840012	0.081048898	0.162562628
0.038316182	0.095930131	0.058317172	0.029463805
0.285143153	0.135519374	0.120844071	0.133741332
0.05440472	0.227568377	0.042582907	0.121556373
0.209966961	0.113192088	0.051631062	0.024879725
0.028145615	0.103181244	0.217818648	0.100806112
0.088173452	0.201535671	0.048298082	0.059004718
0.241287595	0.273981114	0.065162943	0.024111444
0.112143738	0.046907654	0.039171167	0.07040788
0.079799529	0.193184062	0.117405852	0.046770811
0.157245432	0.225053657	0.100366839	0.188345973
0.05999475	0.228157666	0.250860515	0.022571882
0.142523245	0.044617487	0.022674223	0.116649986
0.151018007	0.131516132	0.138332894	0.239841455
0.056220543	0.26441066	0.177048268	0.044988341
0.077404678	0.092248745	0.162437629	0.167064592
0.069671785	0.154552393	0.266145073	0.066756465
0.065781696	0.276910523	0.094941109	0.164643719
0.220273576	0.109061634	0.082346824	0.263994533
0.202689697	0.128116606	0.18863638	0.250359093
0.113001602	0.22603089	0.093757534	0.06193206
0.289187764	0.136458522	0.101233711	0.051933456
0.066012309	0.089146881	0.246672575	0.095373818
0.321238688	0.051741137	0.052407261	0.110570051
0.045569733	0.077643514	0.183678702	0.088284631
0.06122448	0.058844298	0.18486608	0.151322719
0.081838724	0.271401472	0.160766071	0.078688175
0.104044213	0.078812362	0.052654449	0.114112966
0.220550786	0.269906962	0.101235273	0.036008702
0.055025059	0.167367694	0.012887946	0.059432909
0.268673606	0.090140708	0.151133027	0.178718859
0.066796673	0.048928565	0.175244632	0.287163175
0.079177458	0.072188611	0.076234568	0.201016081
0.085628596	0.085935816	0.045001578	0.2080407
0.176881317	0.069337356	0.089932914	0.148947901
0.160666861	0.045305311	0.17504365	0.103125775
0.281319699	0.047992226	0.028410841	0.010411078
0.120763061	0.137764226	0.067453358	0.150069815
0.02893075	0.170722495	0.015343648	0.096081698
0.087014998	0.145315166	0.107543276	0.075932916
0.064337698	0.096010632	0.04412629	0.091104223
0.062357512	0.066393071	0.142479816	0.247923575
0.118193405	0.065194514	0.12251128	0.087905256
0.1562444	0.144026205	0.295659445	0.234645014
0.050917784	0.094799482	0.024224166	0.109660417
0.066755766	0.16506528	0.089897136	0.15249912
0.100679242	0.155528005	0.169077647	0.077476839
0.137532786	0.15466334	0.090975837	0.01387478
0.006708486	0.084702268	0.151648475	0.149481005
0.099336401	0.028460505	0.184041922	0.214340652
0.117666561	0.098442911	0.085709307	0.256491707
0.091549761	0.267207789	0.055122317	0.216760099
0.146213407	0.040807015	0.113854659	0.08768741

0.18164251	0.13302353	0.054941044	0.155621196
0.009149428	0.210780567	0.082265639	0.097886857
0.074505527	0.181601345	0.178052382	0.159488812
0.058265124	0.189194508	0.200830367	0.023725479
0.234992873	0.123679161	0.222171775	0.134042732
0.141377262	0.213084668	0.140430009	0.07966119
0.181240239	0.068123565	0.082754281	0.029527634
0.090266708	0.204474461	0.066397482	0.036886574
0.062546319	0.104893518	0.045782065	0.144978164
0.02095245	0.243822322	0.156871604	0.125271395
0.125792937	0.154372979	0.166561997	0.140527053
0.120909042	0.175957868	0.071575518	0.035545718
0.253690678	0.026645881	0.165694721	0.067550431
0.100742847	0.143957854	0.078262127	0.100428152
0.085675275	0.096951808	0.085677758	0.166984095
0.068076372	0.071896171	0.133139794	0.180722801
0.184306648	0.157501123	0.089099405	0.117750783
0.18433016	0.044019033	0.190352266	0.019736722
0.028818726	0.094177557	0.24916759	0.198523869
0.091631996	0.0707762	0.199252849	0.179782676
0.098534355	0.080917602	0.129579394	0.124486173
0.203915433	0.166341574	0.10317989	0.106109441
0.033880164	0.099499374	0.128999856	0.112521563
0.087406576	0.236448951	0.078997368	0.076742373
0.083087032	0.054437357	0.228861738	0.213077997
0.291199364	0.18532536	0.017701432	0.17397986
0.018745222	0.22211252	0.034743033	0.184879282
0.150087254	0.035856176	0.196832532	0.168071163
0.248642278	0.075812771	0.111938281	0.25510818
0.026970645	0.093842067	0.062997694	0.148104959
0.035632982	0.107213782	0.242443077	0.068400945
0.043296304	0.027199896	0.090773417	0.160595562
0.267859147	0.150548467	0.049647702	0.11431248
0.090414821	0.057022095	0.077552172	0.116356034
0.133458777	0.075368399	0.047100182	0.087839281
0.159967144	0.274808112	0.055794595	0.081337516
0.194013705	0.071136156	0.061180613	0.072615357
0.063795433	0.225298817	0.055306929	0.087399104
0.073113388	0.032983967	0.256307164	0.138110934
0.022447492			
DEK 0.347571979	0.400088153	0.157466852	
0.291042039	0.178057129	0.216364351	0.094247266
0.13466015	0.025380341	0.001845396	0.002943108
0.226650392	0.036426794	0.09169135	0.084380095
0.306598052	0.034559105	0.181566105	0.12398473
0.32118462	0.055723091	0.234577798	0.046907856
0.077049221	0.020574616	0.020488547	0.049470898
0.381055125	0.02540098	0.139568106	0.339120662
0.058563557	0.377862367	0.39689351	0.203405574
0.378664781	0.31913395	0.02949155	0.075274772
0.414370018	0.256354703	0.317632863	0.167376804
0.020116757	0.408082431	0.110576222	0.19760596
0.113984841	0.007699021	0.111969903	0.40213656
0.335824733	0.168092894	0.120653621	0.059408402

0.026687589	0.119347272	0.035725432	0.302978585
0.094598776	0.091931185	0.348421207	0.031287208
0.058725882	0.158068169	0.059542922	0.141582034
0.074067438	0.357382701	0.029862626	0.217074222
0.002568115	0.308407322	0.204249762	0.047709162
0.019343653	0.00561356	0.067752779	0.187915685
0.395870991	0.411867357	0.337854801	0.336017918
0.045488339	0.138276371	0.135631742	0.054186679
0.369015505	0.05723207	0.056061216	0.282221429
0.342824677	0.375161129	0.051640478	0.108376407
0.267371331	0.40865446	0.142120636	0.128374174
0.053379455	0.025221242	0.335243864	0.365805031
0.38855533	0.074009641	0.046064642	0.373124618
0.033375031	0.137529403	0.209737086	0.08410799
0.39492497	0.010291049	0.066628498	0.171564503
0.143466623	0.353246142	0.361040895	0.086785041
0.173682077	0.010844162	0.017287136	0.027051871
0.101247943	0.029203547	0.371562206	0.332668333
0.127725752	0.086702889	0.11681541	0.002906505
0.01200642	0.104792371	0.327557722	0.225411651
0.355770673	0.321935167	0.045350315	0.328507153
0.199450782	0.333472627	0.01884608	0.063559999
0.18044915	0.100464236	0.127687024	0.20713415
0.082075978	0.08392987	0.058864182	0.384208535
0.403509434	0.039664505	1	0.282402861
0.06896937	0.124916759	0.061293341	0.086453251
0.030086735	0.3395455	0.012635881	0.211518832
0.036802843	0.034057927	0.031118188	0.165343766
0.082588729	0.268702173	0.371420022	0.063454914
0.107223851	0.203364495	0.216581598	0.216566477
0.001464545	0.040411123	0.187878983	0.366812022
0.2413806	0.387879173	0.120131887	0.099571126
0.118208622	0.34778163	0.005356774	0.3859685
0.372543421	0.161054039	0.150685616	0.239686366
0.081443501	0.32646654	0.030378189	0.289789343
0.335432139	0.053705825	0.376147177	0.047786866
0.385080238	0.014223507	0.128103724	0.265956745
0.228611247	0.005661345	0.117453308	0.286312418
0.013983116	0.058405827	0.35854259	0.261388713
0.155177091	0.335209879	0.373977603	0.066167872
0.155613391	0.131571467	0.012384791	0.127095975
0.344027744	0.078363189	0.382994787	0.099002122
0.253897161	0.372195625	0.294358546	0.050366709
0.021020749	0.173634089	0.012165455	0.101538896
0.321902873	0.034759279	0.115133	0.107324187
0.024285438	0.377433089	0.039555244	0.051555342
0.024815975	0.162654227	0.147588275	0.045856107
0.029978735	0.053490899	0.068114687	0.087003343
0.027026004	0.030697426	0.017123874	0.068172945
0.021436738	0.060082924	0.063097254	0.3001027
0.106899614	0.029887184	0.107955492	0.080476594
0.034465727	0.009045459	0.388737066	0.293714459
0.330025838	0.067881984	0.025684232	0.064580039
0.335000543	0.137212035	0.133950523	0.34662525

0.028861864	0.371121438	0.052293006	0.03362689
0.070354261	0.101447042	0.373566437	0.017923597
0.275112842	0.37794812	0.174159694	0.043181091
0.335465094	0.26507574	0.018198287	0.347808575
0.327513551	0.014679532	0.380755852	0.039151797
0.012921925	0.261467715	0.219431888	0.031054313
0.030119636	0.088191353	0.041333943	0.13505824
0.37669653	0.251521441	0.257175949	0.067138529
0.015878757	0.002069024	0.068115129	0.187819959
0.402279821	0.357070476	0.144077698	0.083920034
0.13688773	0.056285719	0.118360755	0.354293914
0.134898109	0.415519309	0.00704837	0.255781186
0.365837138	0.171112434	0.018452054	0.152877899
0.336159855	0.024830321	0.147988019	0.377291531
0.349749199	0.077344511	0.067493133	0.305768596
0.013301905	0.033337872	0.166501237	0.0408916
0.087894312	0.072377212	0.010599814	0.141709327
0.01479681	0.347695923	0.364865562	0.015637723
0.136346213	0.148884993	0.209476714	0.021441826
0.279828602	0.379437012	0.39878691	0.123083578
0.313654228	0.321316375	0.129119895	0.04158638
0.00887257	0.375816621	0.341720076	0.009409274
0.368788199	0.149367431	0.002658665	0.408805364
0.102178827	0.385673375	0.00383493	0.054122081
0.02474614	0.335946521	0.358714701	0.332201348
0.070762182	0.284211929	0.009549971	0.390345941
0.364200188	0.005482472	0.039622852	0.266418891
0.214585068	0.435646905	0.060346031	0.268538496
0.16341009	0.35119418	0.067959832	0.112256496
0.312200785	0.333304437	0.357342574	0.408227939
0.34708158	0.261132697	0.146927584	0.007682444
0.028269194	0.11986872	0.048839248	0.015378582
0.277914837	0.033135103	0.401500765	0.35806932
0.322589437	0.00451785	0.097547681	0.299479484
0.391450354	0.409007477	0.031500425	0.004377749
0.007384969	0.00231965	0.247653344	0.094952125
0.361959501	0.058948172	0.004358463	0.04614836
0.109781506	0.160025221	0.369601462	0.27961627
0.31672261	0.043311956	0.048121347	0.328542263
0.014760763	0.320774538	0.327704204	0.379891117
0.033927977	0.088264891	0.21934985	0.382404068
0.009542308	0.153827558	0.250482251	0.064894796
0.023799003	0.027882716	0.019700659	0.341519331
0.35703475	0.014218268	0.007043148	0.071208049
0.033466021	0.374516713	0.048398216	0.383610102
0.354667517	0.340703485	0.027247452	0.380384547
0.009734688	0.360836741	0.088444752	0.023317986
0.142794095	0.015101352	0.361422654	0.015217446
0.128875563	0.3618647	0.040997982	0.00936607
0.040049436	0.021426352	0.336407712	0.312645804
0.022496006	0.123125293	0.146533878	0.315468147
0.183704063	0.266670013	0.391729784	0.138963559
0.03983474	0.058677247	0.353056657	0.322323156
0.131955058	0.358835012	0.049284333	0.398211472

0.343711541	0.413341857	0.34247035	0.167212236
0.049131839	0.253570939	0.224360974	0.009853809
0.203865111	0.314041983	0.238843026	0.025738904
0.06392202	0.131763733	0.061447184	0.14806987
0.082981652	0.211868716	0.049817015	0.363863751
0.337463521	0.322592853	0.068843091	0.01708879
0.420356743	0.396164794	0.075644089	0.263410953
0.081104347	0.126304593	0.320882324	0.290585518
0.107681477	0.301826335	0.361452686	0.241735854
0.364180287	0.132633515	0.394991035	0.099216868
0.018228943	0.259214119	0.021786049	0.038288864
0.052347416	0.014110206	0.04807726	0.304905875
0.069101013	0.197072955	0.140397156	0.0433057
0.142831559	0.204702912	0.186310283	0.075556589
0.02304584	0.360643469	0.005519389	0.029725795
0.078986326	0.276328546	0.09307857	0.081839222
0.050555064	0.060394613	0.124100616	0.172411018
0.065950678	0.115125186	0.281007446	0.031132029
0.018822574	0.379231168	0.20532707	0.020732982
0.042082526	0.297863724	0.175410587	0.049670387
0.011273584	0.316400738	0.091877235	0.178276936
0.217788114	0.047382773	0.390170065	0.064432693
0.064103784	0.401254156	0.160518823	0.36484683
0.145212391	0.274192495	0.401266043	0.319268176
0.100033086	0.182615136	0.017748852	0.394119184
0.071019009			
DENND5A 0.343459714	0.473985344	0.343878781	
0.399295793	0.39734372	0.264665106	0.280406117
0.220494662	0.16456083	0.02023356	0.061374952
0.360567461	0.248320869	0.279367679	0.257860595
0.429506356	0.257133888	0.342946816	0.352076893
0.378398993	0.147651057	0.409974605	0.209674077
0.346389042	0.210813649	0.174401303	0.318294373
0.457238131	0.19579959	0.226260595	0.320785915
0.246736849	0.389919661	0.436948057	0.374995802
0.423254975	0.298592256	0.041234185	0.24604107
0.383672119	0.383357038	0.366968385	0.381448584
0.12434515	0.393443868	0.318926699	0.398016689
0.21902666	0.106484293	0.18826362	0.365577188
0.381747459	0.312446506	0.254962161	0.237631071
0.187453946	0.187843724	0.237640534	0.228741375
0.315351037	0.137347966	0.403222086	0.153923078
0.161049162	0.294875294	0.291960186	0.302327073
0.324638517	0.30529018	0.076142523	0.294809442
0.008197287	0.38359011	0.413798558	0.254756942
0.147374246	0.072487716	0.322619092	0.341964021
0.469239603	0.402684378	0.492801199	0.476881338
0.285218229	0.403987032	0.346755427	0.294610817
0.396960259	0.249024332	0.205453566	0.437785843
0.493808624	0.453316695	0.326422684	0.380007936
0.358576979	0.432845544	0.332468344	0.372849889
0.277103088	0.157229741	0.369633373	0.486485398
0.338209827	0.313572175	0.293785042	0.37116912
0.178759369	0.293382864	0.391739214	0.237859356

0.482789136	0.067644356	0.340885612	0.336617121
0.346767538	0.385294487	0.450776774	0.298965661
0.400008367	0.082216133	0.174636627	0.131599784
0.3280452	0.192530927	0.370711818	0.44758707
0.252704404	0.358832201	0.332604275	0.024923031
0.11235919	0.312619284	0.371500427	0.368554539
0.381593403	0.317443315	0.294725674	0.293177869
0.318528397	0.484867086	0.038748929	0.210241126
0.365500166	0.315047893	0.365942649	0.346294112
0.377584096	0.218870172	0.260640546	0.503096504
0.365795557	0.205050312	0.282402861	1
0.279130774	0.393570729	0.240482935	0.332665589
0.127626767	0.398379337	0.114296382	0.311185713
0.220025392	0.238488635	0.055238554	0.260754868
0.282153888	0.37058291	0.36020086	0.250370593
0.358273635	0.406516952	0.253782058	0.318837177
0.033622133	0.155897143	0.199951274	0.477872481
0.1860732	0.485896649	0.389170149	0.392907431
0.395356244	0.44805308	0.010268969	0.407583269
0.455262267	0.204391279	0.368321943	0.394454514
0.294518839	0.442811073	0.043973823	0.409245296
0.24759907	0.211213762	0.51428943	0.292510308
0.397530924	0.133602011	0.285460863	0.387765822
0.322659439	0.03020188	0.191888713	0.3458664
0.109122798	0.133874143	0.364632727	0.377317285
0.349480503	0.42643384	0.509640366	0.280485293
0.394718486	0.324344774	0.067516972	0.400054339
0.392709575	0.269887335	0.379458573	0.315691676
0.222410053	0.425285855	0.340416711	0.148142549
0.130430859	0.308934555	0.05775278	0.371467769
0.327698109	0.188898943	0.292900413	0.280508646
0.193695113	0.491442171	0.256057265	0.264790323
0.178105578	0.31037158	0.354667013	0.226849134
0.226379258	0.297265086	0.334414306	0.338414396
0.093746075	0.21598615	0.065924952	0.262535577
0.167960025	0.268664979	0.244762506	0.290480473
0.307430306	0.191730167	0.387793549	0.325173341
0.245811905	0.079040806	0.462478482	0.408557349
0.240186194	0.274684536	0.148426317	0.22779118
0.448960015	0.197570955	0.184057479	0.415686637
0.099040308	0.485593859	0.287884168	0.254417438
0.341075532	0.352822615	0.477544415	0.076148694
0.312624909	0.409262982	0.408139603	0.296581777
0.327823857	0.372553765	0.099521047	0.319711153
0.307045756	0.120769749	0.478336537	0.257377935
0.098832312	0.376932291	0.340353579	0.204620193
0.207599995	0.309216208	0.095084546	0.395896513
0.482955938	0.414970722	0.342481353	0.315445004
0.122392252	0.035549872	0.334895937	0.343746202
0.416697809	0.466267283	0.389736039	0.357472322
0.358235574	0.309354544	0.130191093	0.478420442
0.281497728	0.450441272	0.047608153	0.381880597
0.412357405	0.216317458	0.189958339	0.288228861
0.324243505	0.141008652	0.321252819	0.427873303

0.398869488	0.34889946	0.316589777	0.382246918
0.057812831	0.110173422	0.374878915	0.242798897
0.320442984	0.334436827	0.087180592	0.292226916
0.124700371	0.393164071	0.462912653	0.146362312
0.220170565	0.26991299	0.419254333	0.130587304
0.410440886	0.439937082	0.442206469	0.280049031
0.373247464	0.436105133	0.293122965	0.217504471
0.085986935	0.438796256	0.303120505	0.066450467
0.48379606	0.163239448	0.043711078	0.452210627
0.311328038	0.342800046	0.01236173	0.10860977
0.139643644	0.468721746	0.395523518	0.327486105
0.156027084	0.395216795	0.02276802	0.486086566
0.428573838	0.072964021	0.265337905	0.358056467
0.33648847	0.472898709	0.308168652	0.411808226
0.326141298	0.413292989	0.275551032	0.37968337
0.401374632	0.420525186	0.36958533	0.411283079
0.455407855	0.402588053	0.362693075	0.062829054
0.186029903	0.209138212	0.204287173	0.126731083
0.230369489	0.254907725	0.445358434	0.477834787
0.454129426	0.039952156	0.210934981	0.35840547
0.423152899	0.479704561	0.081233569	0.018671709
0.020138145	0.062910754	0.338626958	0.375566415
0.454912538	0.151699834	0.035957388	0.102571424
0.346791584	0.369080272	0.439775927	0.408044278
0.396606167	0.214186226	0.219741318	0.399562592
0.165283057	0.215156271	0.465473087	0.408676769
0.145221093	0.36912894	0.334748881	0.488100589
0.089883756	0.413022487	0.355385629	0.319562687
0.061040146	0.20909365	0.170515771	0.443496117
0.403581032	0.153553389	0.101662655	0.114411886
0.202074822	0.458225371	0.277602006	0.509602883
0.417528876	0.469223152	0.186909464	0.416196808
0.106545486	0.405926411	0.360283896	0.066871881
0.389358847	0.071757529	0.408794835	0.144941125
0.275454202	0.324663644	0.229553604	0.080648128
0.210720108	0.166911469	0.48283249	0.458545358
0.124475229	0.32015614	0.409317038	0.394366187
0.258528934	0.401929624	0.419447745	0.260694664
0.16515206	0.195630508	0.369310764	0.287280588
0.252918471	0.502713848	0.299289311	0.490206668
0.327159696	0.431269636	0.426993345	0.352269076
0.295108319	0.306933426	0.28150166	0.059158852
0.402888146	0.453028065	0.340709542	0.245358646
0.342980109	0.31560796	0.130645777	0.175043763
0.276383056	0.227855758	0.244950032	0.473573518
0.321863436	0.296013854	0.108784898	0.160357597
0.48826982	0.38741511	0.200954617	0.338917267
0.329535691	0.377493891	0.413413225	0.412905217
0.308370352	0.396496189	0.386805687	0.28110054
0.365393006	0.35013994	0.447891451	0.114315957
0.171489645	0.305480211	0.180508265	0.23237658
0.277560558	0.111396996	0.250157579	0.409726529
0.284840795	0.396101294	0.247606016	0.199407342
0.407724341	0.295620333	0.417348197	0.247240572

0.173571186	0.417905319	0.044577457	0.177018717
0.310700547	0.379661306	0.334257726	0.377666751
0.295132937	0.26971225	0.389145027	0.232342671
0.217219754	0.309357879	0.376717242	0.099613709
0.073594611	0.506792689	0.300500672	0.093309863
0.164818567	0.270168846	0.360494604	0.273884603
0.09498504	0.421017319	0.226245764	0.380177987
0.342540897	0.193199636	0.289565772	0.158297995
0.167548366	0.384630829	0.35248422	0.438515507
0.379118534	0.426404079	0.354223936	0.371789209
0.2248594	0.287664179	0.09785822	0.434111469
0.277259373			
DGAT1	0.220582514	0.41335997	0.118150643
0.353485885	0.196010566	0.220967488	0.149792146
0.038526487	0.03741653	0.010789899	0.030901538
0.185016326	0.064882565	0.079385581	0.063369558
0.310375867	0.043398954	0.067515134	0.145711123
0.276574471	0.055223029	0.300117961	0.043254407
0.048149312	0.044394536	0.060818371	0.091300088
0.351258416	0.063359959	0.183708571	0.269893161
0.01967598	0.278741859	0.372907306	0.277102824
0.357928183	0.194390464	0.002169322	0.128470723
0.27366452	0.300255441	0.298861016	0.227143973
0.033412397	0.346877766	0.194240158	0.171331491
0.035970662	0.023447462	0.077147791	0.326057722
0.361635178	0.245787997	0.078677421	0.114023669
0.069486015	0.070600483	0.042555953	0.199549645
0.161404294	0.065720636	0.366814735	0.056787397
0.048732432	0.081808183	0.031402781	0.117634091
0.072284308	0.247482592	0.008270182	0.155835676
0.004536597	0.252125557	0.157333772	0.079315405
0.033070389	0.021982867	0.047083373	0.269991318
0.359553669	0.291571457	0.381856592	0.379049925
0.039608272	0.103450558	0.134159143	0.083430536
0.304706579	0.140516979	0.020254353	0.315275225
0.386291294	0.394600519	0.04595569	0.117657938
0.371345079	0.371997113	0.148470668	0.175663591
0.086446512	0.010635901	0.354246431	0.394589722
0.266954914	0.087121891	0.080001565	0.308892384
0.033994425	0.03803133	0.268957497	0.021453303
0.368439521	0.019340968	0.091551879	0.236420195
0.031117085	0.278545633	0.376741587	0.134539393
0.132460698	0.008807125	0.032448913	0.011412059
0.052697639	0.016821187	0.341070201	0.393152253
0.04486559	0.072529337	0.119401538	0.002101151
0.012811394	0.022895317	0.283511227	0.100775978
0.273053264	0.219433959	0.128037622	0.242857431
0.229991761	0.378243183	0.013556504	0.045963406
0.180480721	0.026676257	0.163477474	0.224495343
0.142619046	0.042546162	0.070076877	0.406061354
0.329485284	0.068262893	0.06896937	0.279130774
1	0.156665598	0.05094125	0.095678581
0.0375369	0.336843481	0.012531615	0.095021277
0.04935527	0.031734679	0.002875051	0.115585165

0.057747843	0.373551747	0.311618956	0.052064839
0.139235324	0.261538794	0.114433829	0.312691513
0.026635097	0.012649806	0.073757311	0.404540637
0.183392371	0.351161613	0.06805161	0.067330934
0.145805597	0.329526367	0.000109669	0.35717052
0.385165242	0.075583747	0.239602884	0.178453259
0.13630399	0.379968458	0.002580371	0.279354057
0.175307276	0.0388437	0.397674293	0.101518576
0.319052592	0.043758583	0.109717952	0.296734976
0.258792447	0.00469991	0.12327002	0.298510688
0.008603156	0.051969653	0.257350501	0.288673187
0.254868625	0.290470366	0.385568617	0.121049092
0.190387948	0.081807471	0.004810811	0.119317307
0.261358484	0.053743435	0.347594799	0.069560591
0.126282984	0.378264946	0.263461183	0.082861926
0.049192025	0.158862081	0.010094651	0.167018559
0.249703869	0.068535863	0.15018014	0.018376742
0.031371498	0.419150852	0.037237221	0.051367722
0.019738382	0.245053341	0.116023013	0.091083278
0.057010108	0.05541735	0.09341123	0.130009869
0.032835942	0.058838988	0.002032591	0.081948035
0.050860519	0.096359546	0.104326214	0.247851379
0.056778696	0.037453781	0.162507951	0.039705407
0.032921196	0.015405711	0.397682333	0.246896406
0.210742858	0.061949252	0.019478325	0.053507376
0.406264311	0.1632969	0.056273374	0.402211345
0.006164041	0.370402524	0.080572582	0.079046005
0.112103675	0.167578759	0.379271093	0.025372688
0.167872495	0.383545717	0.122937688	0.095771893
0.309800422	0.256640265	0.023768528	0.241117216
0.233162539	0.027508412	0.390139075	0.027423361
0.015154267	0.245938183	0.308519437	0.052874856
0.036136705	0.211536424	0.031157806	0.180725305
0.415695944	0.318982698	0.350917952	0.04077333
0.043925345	0.013150632	0.054349423	0.120519575
0.363259494	0.370884723	0.125007952	0.154315613
0.167939359	0.095902667	0.034991446	0.40056769
0.015303179	0.362536295	0.00608431	0.295180057
0.362989875	0.116684313	0.048814783	0.209400607
0.297717756	0.037765545	0.047111346	0.370995312
0.350755633	0.112406339	0.10613335	0.213160615
0.018560256	0.032700592	0.184529698	0.026671139
0.139720755	0.084286307	0.005032779	0.05971595
0.03470014	0.384345733	0.318786858	0.036522281
0.244139505	0.05624397	0.208034703	0.032555445
0.32071855	0.337349816	0.383006413	0.047706995
0.270228812	0.378347838	0.182260565	0.030720767
0.012323318	0.361069752	0.221406665	0.016870193
0.379003039	0.094685963	0.02070232	0.391409775
0.111878432	0.299303914	0.002249779	0.004407232
0.007937113	0.320501964	0.365247434	0.253396227
0.085015024	0.181779454	0.00082024	0.373332301
0.387272842	0.007943966	0.02808116	0.299982961
0.256479368	0.396283961	0.098092945	0.335896234

0.200145967	0.373361518	0.063107679	0.170599469	
0.354266045	0.344554863	0.272189924	0.341884717	
0.375443441	0.252069888	0.218736013	0.021257726	
0.034842805	0.097176105	0.07826726	0.07342649	
0.163668964	0.052120879	0.33284582	0.365310382	
0.358459987	0.005589544	0.049728336	0.291308135	
0.359237764	0.380360154	0.003149624	0.000881443	
0.000610469	0.01256343	0.20618444	0.086054159	
0.371712597	0.051364526	0.006878571	0.02372748	
0.050035593	0.203340149	0.393376884	0.27792895	
0.2666631	0.038094391	0.084523583	0.274752354	
0.028911164	0.156587262	0.381571799	0.387815575	
0.009991565	0.089706669	0.090196525	0.410095266	
0.008866831	0.163936395	0.20585552	0.084711362	
0.002352671	0.031229769	0.035005778	0.30030034	
0.353412389	0.040211359	0.023397615	0.02043686	
0.08292213	0.34158959	0.051495916	0.405228069	
0.347245886	0.358899608	0.019710723	0.372933765	
0.03323715	0.35763473	0.126215559	0.01472588	
0.051664159	0.00564607	0.314627564	0.063456271	
0.094307585	0.215499927	0.0424145	0.007285434	
0.019849827	0.026558853	0.40499329	0.335966897	
0.00652301	0.076965632	0.115149325	0.203704444	
0.149386701	0.348638273	0.373048234	0.147204533	
0.063851189	0.01146166	0.268992602	0.193127764	
0.061352041	0.379814332	0.095132915	0.384244016	
0.23041481	0.326823444	0.351318873	0.218943807	
0.045230117	0.168732836	0.316330139	0.019429561	
0.167957374	0.384531561	0.121729876	0.044146495	
0.150170229	0.137042328	0.017190611	0.012508315	
0.090967624	0.199471857	0.063491664	0.33704723	
0.180041917	0.207538368	0.013738523	0.033762132	
0.407709962	0.321617948	0.040243509	0.294252064	
0.063800103	0.213488445	0.281453516	0.282453656	
0.054481959	0.35528746	0.299821059	0.301269768	
0.359657674	0.160934606	0.39219857	0.01647593	
0.021070536	0.175946717	0.058005855	0.044704488	
0.092877426	0.011846273	0.044286323	0.273556131	
0.069601289	0.272184033	0.042308387	0.024519152	
0.137445925	0.08236316	0.191126601	0.118017681	
0.042949862	0.390235595	0.005322492	0.026511251	
0.07999277	0.306785774	0.158465646	0.070896114	
0.042532389	0.022813484	0.179658806	0.120135861	
0.128942568	0.035068891	0.313355159	0.012161333	
0.016814415	0.385322527	0.101085252	0.003902894	
0.026532326	0.175324772	0.037858924	0.088606297	
0.00815312	0.360610114	0.157090558	0.178170191	
0.190757585	0.025972448	0.241527976	0.015798686	
0.014800361	0.316603222	0.140860227	0.369242199	
0.085848024	0.350072354	0.307957972	0.307211278	
0.011753857	0.106906683	0.021710212	0.32655619	
0.095344602				
DGKA	0.297114397	0.403086989	0.221095669	
0.333998013	0.257279248	0.151751667	0.130862827	

0.076927586	0.100439658	0.012099899	0.072781389
0.302820838	0.187415925	0.136808439	0.124275123
0.39427055	0.24034578	0.171131194	0.193792217
0.294912944	0.054251383	0.382996473	0.109195232
0.286646142	0.216929094	0.135942634	0.262558993
0.37906369	0.147358292	0.100146681	0.231042966
0.209758085	0.325737297	0.367558058	0.370480452
0.368709363	0.249274956	0.031670441	0.114290097
0.299084748	0.360471606	0.29138134	0.234900997
0.071336862	0.309515274	0.167808275	0.314503752
0.086621517	0.105840554	0.065725864	0.276146244
0.322330383	0.229875592	0.096326971	0.114132357
0.149217272	0.071166992	0.235080684	0.13868187
0.169952955	0.043046029	0.347150834	0.071228796
0.077317138	0.120373926	0.265814329	0.131033763
0.260147721	0.216135896	0.034179487	0.252323932
0.003710374	0.281497582	0.310637071	0.206392675
0.125631213	0.064983356	0.275191066	0.201334944
0.397150803	0.337263996	0.471661877	0.45726575
0.239251536	0.272486586	0.261783848	0.194189793
0.326011722	0.127436349	0.114385838	0.430489448
0.469889268	0.398866067	0.259754431	0.289791762
0.298627286	0.349294228	0.184616407	0.230612455
0.190502191	0.169327065	0.301360854	0.448104786
0.252839567	0.284017119	0.243382416	0.280982246
0.131246455	0.140540669	0.28343996	0.18176863
0.41017849	0.034907308	0.293312992	0.178141433
0.198211091	0.322239369	0.408944575	0.157600874
0.267829042	0.069399711	0.181632696	0.111850677
0.189930702	0.244023331	0.300847582	0.387166515
0.106220397	0.242433811	0.157548156	0.026746665
0.118747541	0.229995479	0.336431641	0.194518626
0.320471366	0.277089711	0.204837879	0.219124385
0.304067395	0.466650117	0.013978491	0.099608987
0.212841985	0.236529691	0.229166267	0.25528491
0.282233315	0.089974452	0.158212829	0.448958098
0.268368632	0.123794865	0.124916759	0.393570729
0.156665598	1 0.137531536 0.196484735		
0.061911227	0.35383626	0.135071231	0.134290642
0.156998528	0.257004807	0.029135387	0.102330503
0.14090419	0.326987574	0.297848248	0.155344298
0.207053052	0.346700765	0.105050727	0.282055452
0.030493528	0.099807567	0.076368382	0.428851732
0.101770027	0.450150309	0.219419551	0.269609547
0.283096699	0.394958296	0.008501241	0.332340077
0.404968107	0.081936743	0.266518986	0.308648095
0.205942419	0.381039401	0.023121584	0.37560244
0.179459471	0.127671641	0.473904849	0.232328472
0.33375246	0.111191764	0.129608879	0.391528213
0.322682448	0.025366195	0.069030852	0.251465457
0.125967921	0.043331198	0.299556976	0.290158054
0.224896278	0.395756728	0.465435506	0.203950417
0.247566233	0.150488254	0.05426611	0.305321257
0.357819957	0.16977453	0.289309381	0.148698269

0.120579838	0.349257048	0.305738734	0.075212246
0.077172561	0.136869854	0.028342257	0.240621633
0.275495919	0.107125885	0.179951268	0.208495346
0.189577861	0.438174566	0.278648661	0.260237373
0.227333706	0.204522105	0.175751627	0.136717501
0.195562475	0.202597552	0.237496267	0.202599499
0.036143332	0.165384016	0.073109013	0.186967042
0.142434646	0.180034248	0.151578045	0.231041674
0.168895886	0.141832269	0.261406655	0.250718723
0.217269454	0.07396678	0.393920792	0.335307725
0.160643248	0.143690249	0.12485831	0.147357463
0.391671608	0.093889382	0.067522323	0.341166529
0.060809989	0.448755101	0.191962901	0.212168533
0.266645225	0.216631583	0.408187686	0.038534628
0.175397055	0.32936517	0.258531553	0.224710423
0.252050705	0.269912458	0.054934272	0.25247639
0.244593661	0.101542843	0.430878124	0.252294866
0.094372793	0.38601247	0.330051143	0.16606806
0.177805841	0.178039618	0.028688001	0.296988546
0.423174242	0.406605026	0.296843443	0.282640984
0.081893759	0.043259298	0.299410371	0.235749364
0.344377206	0.436982166	0.25060397	0.272985533
0.204117611	0.220266733	0.040460608	0.437605735
0.19562012	0.38389565	0.040689021	0.388602226
0.355469784	0.086968275	0.183594702	0.129191406
0.244139566	0.081030347	0.159010212	0.360544659
0.325279015	0.217341057	0.196803504	0.322992965
0.023428182	0.046022424	0.263859947	0.260766216
0.183304918	0.210278375	0.150951919	0.127869341
0.088551891	0.318390488	0.428130358	0.113490937
0.123398065	0.109768025	0.360138755	0.086794233
0.410257349	0.397868145	0.371965444	0.127710284
0.347222112	0.401411675	0.264699202	0.181171559
0.095656965	0.388022206	0.223138587	0.04372681
0.434567329	0.056893261	0.040992876	0.368456319
0.16247962	0.269793519	0.003267312	0.08892291
0.149447494	0.458888352	0.323823215	0.225408512
0.062020779	0.24558863	0.0266971	0.44681606
0.361368062	0.090622689	0.268436266	0.335455424
0.353953946	0.391305487	0.262124738	0.40803437
0.295417929	0.361750173	0.145366188	0.262177529
0.337771846	0.394321864	0.288199526	0.322059068
0.402292244	0.388790464	0.210037155	0.037117888
0.14540817	0.074171173	0.114374487	0.095274433
0.122644303	0.210810955	0.381982613	0.440439974
0.434281706	0.040449662	0.080201185	0.314134286
0.330425321	0.399797025	0.059907494	0.014615206
0.015742106	0.095483156	0.17745717	0.280389884
0.403113154	0.068346682	0.029426297	0.03420349
0.178923711	0.350656493	0.378746018	0.339688036
0.379281435	0.15823026	0.123230352	0.335399427
0.170630297	0.148105484	0.435346725	0.324121315
0.126446294	0.266237611	0.172088627	0.42019143
0.149250835	0.28784667	0.315472442	0.192914608

0.053488622	0.192083136	0.14876783	0.413873236
0.354608837	0.14438792	0.108981025	0.04370108
0.119029068	0.412171641	0.182197892	0.463973398
0.340941151	0.421799026	0.203773933	0.338311602
0.092255305	0.344401123	0.302409397	0.025023641
0.249313223	0.057440468	0.371605801	0.126563763
0.121762968	0.236479364	0.169193406	0.09712373
0.213344975	0.153388761	0.442997534	0.453992239
0.136772707	0.148817443	0.270984234	0.296223405
0.103889302	0.364802545	0.344411498	0.143438769
0.076055122	0.213867504	0.290510723	0.217208223
0.104649685	0.477366491	0.210305477	0.440199426
0.253538378	0.355243165	0.386068693	0.188079389
0.287713866	0.178975407	0.20473677	0.030285065
0.223055619	0.418937887	0.160516775	0.225827283
0.255010884	0.247920753	0.066164704	0.080471693
0.239810912	0.112187434	0.149168807	0.424056887
0.211067427	0.228917569	0.046993741	0.139242544
0.419859722	0.301871342	0.077756694	0.273042365
0.231753123	0.241147145	0.39282863	0.419225438
0.182046055	0.356705738	0.310563949	0.218843698
0.278116392	0.181960015	0.378783142	0.030004712
0.160857449	0.244732568	0.131419944	0.20239633
0.179211019	0.103179595	0.226014863	0.389858273
0.222844518	0.310142517	0.112182579	0.133263215
0.295307925	0.153518006	0.275810711	0.110876057
0.121583562	0.350573776	0.039906322	0.149448452
0.241533594	0.34172678	0.261139297	0.266403226
0.268747359	0.249934887	0.250221986	0.092766003
0.118416109	0.191352772	0.332884592	0.042117148
0.035326049	0.467311207	0.140789355	0.094408019
0.139036005	0.180609338	0.203487993	0.168663211
0.114669563	0.396816401	0.106794225	0.261513452
0.1622825	0.1297657	0.204012937	0.079987581
0.08733044	0.302556493	0.263271823	0.391664781
0.265519987	0.396711843	0.269657483	0.326315987
0.134139139	0.182784763	0.049108793	0.362760516
0.184899879			
DHRS7 0.107109103	0.127109904	0.068304399	
0.220352168	0.131235146	0.088520265	0.229525673
0.108291529	0.096737191	0.064920005	0.058360361
0.099117713	0.076351539	0.094683306	0.03318067
0.244208898	0.143374164	0.078289898	0.305942131
0.22188509	0.149140361	0.249234236	0.2196238
0.057400217	0.097571854	0.174494258	0.066337189
0.225359278	0.195106395	0.07072725	0.188741524
0.171714995	0.143635241	0.094028126	0.142928753
0.083441126	0.078633889	0.084604525	0.054889139
0.128138808	0.1331163	0.225240181	0.263483016
0.190086328	0.086262063	0.261406278	0.135477517
0.056217582	0.133877675	0.057573178	0.091092391
0.075332842	0.080977834	0.123556625	0.177859825
0.037958776	0.060667618	0.021604037	0.110442545
0.263515381	0.066297531	0.085014316	0.110904739

0.05979286	0.150901423	0.140430777	0.183364392
0.038641779	0.115639298	0.21535122	0.061851423
0.0272705	0.249535547	0.16817743	0.023333095
0.242007765	0.126366121	0.151345159	0.230324475
0.201001251	0.094811542	0.169095962	0.139757861
0.122594902	0.189023442	0.072325051	0.184411088
0.126382659	0.1922379	0.118862527	0.240165629
0.160105335	0.103719087	0.12471603	0.194167992
0.118801556	0.127497573	0.067228646	0.093380927
0.02624308	0.103108293	0.090659385	0.139139669
0.10546914	0.052636546	0.207829756	0.179971704
0.235776879	0.035525592	0.286641499	0.095586555
0.204112758	0.133068757	0.177922579	0.18193293
0.139036356	0.128555193	0.09790251	0.288563798
0.111519148	0.139920111	0.027025592	0.205951778
0.067223858	0.02671485	0.068380091	0.175526539
0.107298995	0.057234555	0.191924044	0.094815793
0.161822212	0.104938734	0.104676913	0.11855942
0.164510209	0.063983219	0.103578638	0.093759637
0.077776047	0.167305316	0.006371928	0.224499925
0.115995335	0.119848704	0.336117884	0.099579408
0.144113349	0.206744088	0.255179905	0.153525077
0.109814748	0.286476606	0.061293341	0.240482935
0.05094125	0.137531536	1	0.132866977
0.214720618	0.076545259	0.137212454	0.111175027
0.01920742	0.093075071	0.14986332	0.145999906
0.067025483	0.099900417	0.064374454	0.280356581
0.165474728	0.190117171	0.088349782	0.10541459
0.05649956	0.218222356	0.050379221	0.139938286
0.081507071	0.138812668	0.131320377	0.093917731
0.230326925	0.189550156	0.00530179	0.086561443
0.099946488	0.042443301	0.150882303	0.137785864
0.043119563	0.183056384	0.008043829	0.239915813
0.080317553	0.254174998	0.181042957	0.232549549
0.093322658	0.191925549	0.093429676	0.095378378
0.074853839	0.059938166	0.094331778	0.217400352
0.12333128	0.118427865	0.151130719	0.202809519
0.142051635	0.16280476	0.210211523	0.035216243
0.119652974	0.198303914	0.162424324	0.15050392
0.125471336	0.024502888	0.102130167	0.143463186
0.058234009	0.117320392	0.0772622	0.021189287
0.212523751	0.159026178	0.092195597	0.133843518
0.075585589	0.149883976	0.050056128	0.144271976
0.165145147	0.136730345	0.050166568	0.031370339
0.02624027	0.107180595	0.254164617	0.06549544
0.053100853	0.164471035	0.048132983	0.061048977
0.174407047	0.208745958	0.075288193	0.026522192
0.065506389	0.041298366	0.038091767	0.095478047
0.079103541	0.132794723	0.120128206	0.04716096
0.122099782	0.19127665	0.125266208	0.230269
0.082782736	0.187183825	0.218058775	0.019277786
0.163734012	0.048258911	0.03424892	0.136640097
0.151946261	0.164383951	0.091629009	0.214822848
0.059116416	0.254949321	0.209783242	0.03195811

0.11077613	0.114420686	0.204308852	0.137199868
0.088288103	0.187001806	0.190475987	0.090521221
0.090037177	0.208780436	0.119315213	0.137855815
0.194677057	0.135570609	0.092426911	0.255946837
0.16361634	0.114184025	0.145428745	0.261074051
0.135608279	0.246933732	0.105408318	0.064431986
0.192696214	0.073919287	0.119401425	0.07407111
0.086957155	0.111984336	0.285430044	0.091607375
0.337926626	0.041156811	0.054618619	0.132754449
0.062822908	0.103932521	0.155426077	0.1083312
0.079185419	0.079214441	0.130638837	0.158879322
0.101977542	0.200307628	0.173834142	0.098649203
0.128347926	0.080182801	0.04761234	0.155442057
0.151510025	0.208161606	0.117438758	0.035242138
0.053612017	0.138165683	0.013198707	0.066260442
0.165015736	0.109153116	0.191270389	0.111067318
0.06566043	0.055719788	0.208751926	0.221862714
0.09686188	0.096302746	0.09792677	0.202757346
0.114907667	0.102439376	0.052516712	0.203555999
0.149544043	0.089825581	0.122300887	0.081083712
0.195322494	0.05829771	0.088542066	0.129617637
0.298419356	0.067004776	0.016472171	0.016450736
0.118056441	0.172994272	0.084612981	0.191548592
0.026356299	0.2232551	0.0235788	0.123936008
0.106248843	0.10660746	0.095992881	0.093206097
0.076998799	0.124032381	0.047350121	0.106860104
0.073255149	0.083367083	0.113029316	0.24994716
0.136951678	0.083684768	0.156638854	0.114165566
0.184095563	0.176619925	0.299023169	0.15150033
0.037331121	0.110643942	0.016847572	0.057102512
0.091274099	0.112431338	0.118331582	0.186308414
0.122096607	0.132592028	0.144205665	0.099951857
0.169680295	0.190496714	0.108594551	0.012018351
0.007274128	0.044369785	0.186829734	0.172102882
0.122800984	0.019729029	0.135821441	0.181465475
0.102006609	0.112370875	0.105819753	0.295367235
0.121321236	0.231907877	0.036370858	0.26397681
0.099274705	0.063591348	0.13437666	0.108862131
0.182060048	0.151785632	0.084830861	0.184079663
0.008358993	0.239965631	0.109137702	0.074054532
0.086783613	0.141596876	0.126812812	0.202197708
0.075695224	0.112091218	0.116637742	0.026768483
0.154507749	0.159149376	0.157951464	0.163157525
0.16873088	0.247505793	0.125832588	0.100013978
0.121282661	0.087160826	0.088559801	0.021225775
0.109481373	0.186797435	0.089555679	0.018971801
0.079482894	0.146835859	0.036792973	0.126205019
0.021677019	0.192904761	0.180450753	0.152573506
0.125287616	0.145585402	0.197641893	0.187977415
0.140609835	0.190927249	0.091891252	0.040537432
0.183725342	0.036079417	0.20905924	0.096075042
0.10431483	0.174791556	0.055132033	0.127968208
0.118349873	0.129980617	0.108320221	0.17637849
0.061398636	0.096460879	0.139474375	0.104095566

0.217006458	0.179415853	0.126402538	0.076560127
0.164050385	0.056826832	0.197683989	0.030235133
0.036568633	0.107739133	0.184032507	0.24200182
0.132766628	0.099871908	0.214671719	0.121039537
0.125216007	0.107754762	0.106930778	0.139465097
0.233691115	0.167609345	0.132723994	0.135470008
0.036916964	0.117672379	0.16626019	0.122841615
0.10792674	0.251657127	0.100232979	0.087637914
0.067032553	0.079486912	0.140453209	0.225164194
0.278159789	0.143323276	0.017229579	0.216112247
0.023561751	0.234023559	0.043558443	0.146082544
0.180432993	0.05820622	0.224729757	0.156855043
0.158375311	0.093275234	0.081345299	0.218425759
0.035756669	0.1137556	0.065793287	0.147474968
0.031796864	0.112912429	0.247351945	0.087135909
0.039073852	0.03169574	0.108044117	0.141248234
0.210395437	0.183798161	0.071001149	0.118499284
0.093256209	0.083091113	0.096020138	0.080063229
0.114826725	0.09340542	0.047194792	0.107158906
0.185905438	0.24786168	0.080606916	0.088112036
0.198510031	0.096143481	0.080342266	0.093117083
0.090306737	0.245953589	0.076575006	0.111542703
0.086205732	0.049488749	0.18573188	0.177649183
0.025745742			

DKKL1 0.212007712 0.333238996 0.109972235

0.349856296	0.209889442	0.170490146	0.183558328
0.087305305	0.049288517	0.051075182	0.050466427
0.204130472	0.126916174	0.158468207	0.052439579
0.328940316	0.135873198	0.086913865	0.234996735
0.306432976	0.109373814	0.349135623	0.111295139
0.064953698	0.079010374	0.096420574	0.075198779
0.370969347	0.102945557	0.143134071	0.29027674
0.087195502	0.284805358	0.281717705	0.29504588
0.273498772	0.172547984	0.039561207	0.082433312
0.258255924	0.254252116	0.31212792	0.295840601
0.094276988	0.268094981	0.235877535	0.191111119
0.061022499	0.081645849	0.073450337	0.248183805
0.263740235	0.183920685	0.094803202	0.128776991
0.099427973	0.091680784	0.060802918	0.200929421
0.222056569	0.077044451	0.273048946	0.065883755
0.055570104	0.121093167	0.10176983	0.174421509
0.060752786	0.222771992	0.053396416	0.144613123
0.031066275	0.292549294	0.207062946	0.044131561
0.099596985	0.126415669	0.109477937	0.28762678
0.362403453	0.24606253	0.360539001	0.32527787
0.165388069	0.159914915	0.130358396	0.159565601
0.263949082	0.174512683	0.077735704	0.348721176
0.35423167	0.308309676	0.099288519	0.16090012
0.283222595	0.311366459	0.130328986	0.162688341
0.054352678	0.040474017	0.258874729	0.347178115
0.24369567	0.088792577	0.147142042	0.324975346
0.094778063	0.067364242	0.333308827	0.14265479
0.37022882	0.041108612	0.146525379	0.256703141
0.087465234	0.267414884	0.293234994	0.201183184

0.168522995	0.096897086	0.033128442	0.062277329
0.107745196	0.035285474	0.238232916	0.376150775
0.129978305	0.099123557	0.150537697	0.019387378
0.07129044	0.070623435	0.273862108	0.143669932
0.268837747	0.173287883	0.114495673	0.213301718
0.194529596	0.353010818	0.014123062	0.103225476
0.18945685	0.07529317	0.259078419	0.214332651
0.170793797	0.107786618	0.156113335	0.350497011
0.26683824	0.151877434	0.086453251	0.332665589
0.095678581	0.196484735	0.132866977	1
0.119140092	0.246063816	0.056550351	0.126356727
0.036366041	0.116193368	0.02627163	0.150187355
0.10685725	0.287570136	0.22206083	0.127234686
0.228728218	0.279103108	0.131980994	0.273725506
0.043084056	0.077475985	0.080833936	0.359455554
0.169165594	0.321161171	0.127625386	0.085066885
0.211020094	0.320971867	0.001318032	0.268754086
0.295965166	0.079617563	0.288248457	0.210852788
0.083466916	0.355791089	0.010181875	0.352731869
0.180797517	0.098420153	0.372255512	0.181977797
0.262817581	0.090344206	0.12899273	0.235110947
0.228711354	0.021128846	0.115147483	0.295176305
0.042481447	0.081092308	0.250380922	0.291885861
0.203876464	0.283733532	0.386721165	0.07138615
0.202519401	0.145519184	0.035711423	0.161989853
0.247724109	0.056637308	0.270635341	0.116366361
0.130379101	0.300588504	0.211169707	0.038559747
0.091426495	0.174721279	0.062162688	0.15237167
0.184512173	0.169225526	0.094414312	0.085636892
0.092952978	0.347634984	0.04975658	0.049445681
0.029473908	0.260194655	0.182017308	0.156233006
0.145395105	0.103152787	0.069675354	0.089080627
0.091707772	0.150664714	0.015674614	0.096439235
0.182333601	0.132858796	0.133345935	0.224661526
0.144362618	0.212701204	0.199079615	0.053201842
0.074007136	0.059944155	0.322405717	0.293147527
0.192225982	0.10861033	0.081592442	0.080435756
0.368983222	0.119431306	0.052313464	0.335050997
0.04318833	0.337395998	0.160438584	0.149156262
0.13885241	0.269218666	0.361138337	0.023476001
0.17718498	0.31214271	0.174040574	0.112553299
0.266685501	0.269451613	0.087606481	0.215909214
0.234575414	0.098101813	0.328998395	0.109293747
0.059656919	0.255665179	0.236794703	0.114108346
0.077534124	0.192119543	0.071852966	0.2484393
0.344627892	0.363947803	0.265890005	0.056164117
0.110544918	0.104997902	0.093270162	0.128483859
0.279905875	0.312636446	0.224290972	0.180125583
0.255598477	0.0655142	0.052966095	0.343185398
0.045608107	0.292982159	0.03613793	0.24361729
0.265172587	0.133014937	0.086155796	0.190880678
0.265231348	0.079070449	0.107049585	0.296809276
0.271632736	0.088543027	0.071226813	0.256800075
0.060844181	0.092265167	0.162470498	0.038180965

0.09187261	0.097620885	0.013275615	0.077949405
0.123313393	0.30195696	0.350110751	0.054841234
0.162537763	0.062836172	0.264855087	0.084996185
0.255223298	0.273119405	0.298401346	0.117230508
0.268391143	0.294863266	0.1458451	0.081181703
0.046214156	0.279678471	0.211418066	0.079643643
0.377175447	0.095972912	0.106785615	0.325032275
0.182087198	0.225529058	0.005169563	0.011845459
0.038708993	0.325091526	0.258196859	0.267710699
0.063294838	0.238311674	0.004845989	0.320178171
0.314272616	0.037588671	0.067463037	0.261726475
0.218001294	0.327368179	0.079536729	0.280239348
0.19533679	0.274404838	0.146949906	0.220200528
0.312607191	0.261104728	0.275039265	0.279065279
0.381804566	0.278954014	0.269527845	0.0585545
0.024924862	0.107327161	0.050031846	0.054696983
0.154524223	0.08873845	0.298093878	0.35582903
0.320464727	0.029373556	0.112534824	0.255096584
0.34149294	0.360069735	0.021381726	0.003407154
0.003231537	0.032114159	0.228419221	0.129962203
0.304565496	0.070604229	0.038405897	0.085831213
0.108348719	0.201519174	0.311187099	0.345429855
0.248922302	0.107707882	0.095032695	0.327768217
0.058052102	0.142050409	0.337030956	0.29768806
0.0547538	0.112276714	0.126365165	0.374250717
0.012067407	0.244144918	0.232800475	0.120615611
0.020759598	0.073595055	0.057865286	0.318789075
0.259691754	0.156736429	0.117027759	0.050217516
0.153870768	0.323394849	0.171892318	0.365702905
0.33170448	0.396322782	0.065587415	0.274612388
0.064175157	0.283143862	0.140345182	0.024525876
0.09458169	0.04340665	0.267018988	0.036366093
0.127463915	0.23515454	0.031843357	0.036968568
0.025508856	0.095342217	0.368550408	0.312393198
0.038972907	0.101706972	0.172472711	0.248431373
0.175040973	0.352800846	0.284036547	0.118043644
0.11061355	0.036959712	0.309211643	0.206786582
0.120971056	0.362525289	0.08735813	0.323003807
0.217487167	0.280131982	0.291364986	0.200559225
0.084472938	0.171549609	0.272862868	0.088458423
0.226836783	0.376595001	0.146065666	0.081630331
0.187347284	0.117669668	0.056041749	0.039551518
0.079690937	0.181138286	0.170024852	0.364808491
0.211266018	0.21277038	0.077076266	0.058012017
0.334161688	0.250921012	0.071270053	0.264144607
0.133338086	0.196606237	0.273370179	0.277853884
0.054774327	0.317155836	0.314164372	0.288598634
0.289147074	0.191722451	0.29860148	0.057626109
0.039273008	0.177773336	0.150629222	0.104497539
0.19162495	0.046019408	0.039988126	0.319581614
0.060237367	0.343477148	0.072801701	0.0746823
0.185182137	0.090412611	0.246528138	0.158738172
0.136315397	0.290435386	0.030361649	0.096536039
0.089940164	0.249715378	0.143999361	0.11521658

0.055284184	0.067892325	0.2557447	0.135581498
0.131744074	0.06307517	0.250593437	0.047132786
0.084252508	0.375755328	0.119928805	0.02928574
0.04349078	0.151901045	0.095587463	0.09149257
0.036713277	0.280879985	0.097444818	0.174089214
0.21134192	0.098955369	0.205924621	0.043654534
0.058662821	0.270269145	0.135197841	0.288285488
0.107854089	0.382402814	0.230280999	0.2850388
0.048846575	0.097716686	0.067677742	0.323172757
0.073727758			
DNAJB13	0.04039093	0.064486187	0.023307524
0.153870473	0.070186017	0.065864998	0.248297205
0.072685603	0.066964038	0.109724843	0.059965306
0.036726737	0.046756674	0.090407793	0.01977981
0.134401702	0.08393115	0.03197215	0.246203722
0.136959242	0.181123571	0.154045959	0.168385576
0.016871995	0.063659494	0.181272994	0.035918762
0.131341761	0.187589162	0.068617472	0.129310513
0.060320117	0.067910908	0.042190136	0.076184654
0.03519423	0.028612839	0.037413822	0.066078676
0.058594809	0.066361036	0.147006941	0.208021563
0.169552622	0.041345114	0.260844669	0.05336239
0.030016762	0.095148871	0.056729138	0.046254028
0.037303536	0.048698583	0.112555009	0.221247519
0.030459299	0.062293757	0.007170019	0.079830703
0.263667116	0.08655154	0.040890664	0.105556345
0.040582297	0.115429749	0.05328148	0.164867379
0.013972366	0.065305831	0.127676946	0.018976739
0.053217461	0.152339064	0.06811218	0.00951353
0.193726005	0.125519955	0.074394833	0.211134776
0.109280535	0.036495054	0.082929872	0.064394365
0.059420072	0.091297871	0.024801673	0.170844558
0.059537166	0.238773251	0.052280933	0.129684624
0.07743973	0.048495639	0.051760498	0.095341318
0.081628815	0.066461302	0.035955535	0.057251012
0.016139552	0.029147447	0.048994021	0.06651704
0.053721771	0.016751777	0.139763155	0.114333818
0.18504415	0.011476104	0.215782595	0.033777033
0.110344087	0.128558883	0.093745145	0.162676122
0.045534637	0.060321225	0.042384178	0.278886609
0.043423004	0.082783406	0.008761166	0.095249276
0.02824413	0.0048553	0.031231995	0.10822882
0.06655173	0.02553521	0.1618946	0.051766787
0.087286785	0.025682681	0.046212084	0.052263995
0.081462393	0.02124958	0.101259319	0.049283436
0.032276423	0.081872816	0.006174528	0.18265468
0.063704609	0.03257313	0.249815035	0.051312786
0.087534492	0.164206038	0.232906451	0.075680485
0.062043219	0.292354138	0.030086735	0.127626767
0.0375369	0.061911227	0.214720618	0.119140092
1	0.031543544	0.072588292	0.061278696
0.007767117	0.036068899	0.062383882	0.128667997
0.040948901	0.060713318	0.026541082	0.20977364
0.13893114	0.108044565	0.057691116	0.069771491

0.087401474	0.095920555	0.02909019	0.071623373
0.069323317	0.057673016	0.079615711	0.042652963
0.132684725	0.097484201	0.000742724	0.040301662
0.044272439	0.023187627	0.10752624	0.053600995
0.020466562	0.112112195	0.001311188	0.134155978
0.037035519	0.194785817	0.089770029	0.181916619
0.040212334	0.197938097	0.082238753	0.037977622
0.031246709	0.031774154	0.112728372	0.1607079
0.047765557	0.134161337	0.072525611	0.135563369
0.106993526	0.072214385	0.109599506	0.016614755
0.066571207	0.148208967	0.068908739	0.062360148
0.05000031	0.008604278	0.057407646	0.101621158
0.027901808	0.062697599	0.030360221	0.017749331
0.263660438	0.137024559	0.077909399	0.086352449
0.029756675	0.154466965	0.024200145	0.039074646
0.104457066	0.068685897	0.016558684	0.00841107
0.006214064	0.084961703	0.184626091	0.072886675
0.037343224	0.097151275	0.024397331	0.041605829
0.203758905	0.19873027	0.016954638	0.014154959
0.057253988	0.033278264	0.032343909	0.05084526
0.043185181	0.096208065	0.074201892	0.012393653
0.057531373	0.161444854	0.062775937	0.122178432
0.050573878	0.144562096	0.140806278	0.008721496
0.099684682	0.042470454	0.020857959	0.085594861
0.060644129	0.077320588	0.067724685	0.170217021
0.032041557	0.222409041	0.118828481	0.022287552
0.059001738	0.064962691	0.097667864	0.110377006
0.051212064	0.115845667	0.166508822	0.041466923
0.043321805	0.195824593	0.054053378	0.052620987
0.138916749	0.055043054	0.047930222	0.189106465
0.107612331	0.116308163	0.157914496	0.157967471
0.069789304	0.149272204	0.066873311	0.020487848
0.230081285	0.084438879	0.047527257	0.022156179
0.039695476	0.047926463	0.17242771	0.054931287
0.26847113	0.025743406	0.038259223	0.065211789
0.010510059	0.044449102	0.112608341	0.045549447
0.034698757	0.063634834	0.104055146	0.161633284
0.061758757	0.198966485	0.100186416	0.047601828
0.070676584	0.051453029	0.031187645	0.066182391
0.175027702	0.207213223	0.054838272	0.008069048
0.04147668	0.100735403	0.001775202	0.036126483
0.149466456	0.064774715	0.090236728	0.079031644
0.06869459	0.032071143	0.099857912	0.223004376
0.039532812	0.037608072	0.045605025	0.139707629
0.048604852	0.048615348	0.020795516	0.115869589
0.098662078	0.037514254	0.064165308	0.080421034
0.104216082	0.051809904	0.116723657	0.068621292
0.253136752	0.029593556	0.017260772	0.001711655
0.033046122	0.075029551	0.041934104	0.126900403
0.033942952	0.120324733	0.004535564	0.052233181
0.055447336	0.053483711	0.029155971	0.044414262
0.030482697	0.060197097	0.018652466	0.048642291
0.030405711	0.03845501	0.079636054	0.166622094
0.080909847	0.033379716	0.085141522	0.057475859

0.106134361	0.07983326	0.252136068	0.163819624
0.014060672	0.116998874	0.01505468	0.05558396
0.059034011	0.070492064	0.050919355	0.094428263
0.055333427	0.103957097	0.115755628	0.047411148
0.102727086	0.104732536	0.03035035	0.003354511
0.000956981	0.024930122	0.136298607	0.080902686
0.058089718	0.018120659	0.131585472	0.168459529
0.049493999	0.046970531	0.053076181	0.186195748
0.047053866	0.166354597	0.036329371	0.152898479
0.048794004	0.028553755	0.066282214	0.061457899
0.064454522	0.076280514	0.033842345	0.104646966
0.001319021	0.13276063	0.043431357	0.048179992
0.024096562	0.079639447	0.080339471	0.095347469
0.032253069	0.090105114	0.091024948	0.014466733
0.190515149	0.075090113	0.106261492	0.080254832
0.100700071	0.144749902	0.04409154	0.050449884
0.095288866	0.041154381	0.038380628	0.016029814
0.043273858	0.08820977	0.035055081	0.014602227
0.063059074	0.074287195	0.012745012	0.056084085
0.003521776	0.131676901	0.100539201	0.067542902
0.035434828	0.110410966	0.096541281	0.087326108
0.126877128	0.123298041	0.044310459	0.021423837
0.212262821	0.004682319	0.121698309	0.043734788
0.085142754	0.083352187	0.039072726	0.056054478
0.055474128	0.059407808	0.049094191	0.137588089
0.019741617	0.048366708	0.125311786	0.128798425
0.128787714	0.104659107	0.066430601	0.037536779
0.132032607	0.018561825	0.10416079	0.006204341
0.010299099	0.09967861	0.146894761	0.128861239
0.064206	0.045683568	0.131155328	0.079699773
0.059205865	0.052936528	0.073409687	0.089582468
0.132466308	0.116401437	0.053974181	0.054370015
0.012497627	0.065099819	0.092460306	0.098744697
0.067041508	0.206685064	0.047418039	0.05579839
0.023776736	0.029586618	0.126125789	0.133629602
0.254169344	0.067788886	0.004553498	0.107048618
0.00722594	0.166038124	0.017858562	0.072612216
0.084913015	0.020234609	0.130927517	0.189609395
0.131994238	0.048018912	0.04970902	0.147856381
0.012139691	0.055160498	0.03107911	0.082047226
0.008105343	0.030234479	0.18436749	0.069040593
0.040518554	0.00748595	0.054005728	0.082405411
0.195358391	0.090515475	0.03242665	0.033485911
0.037774639	0.038494492	0.03243397	0.059424694
0.050678468	0.041394132	0.047944911	0.047586355
0.142511387	0.152383868	0.041092057	0.031243284
0.085874707	0.045595382	0.026649657	0.0403128
0.028766093	0.15810735	0.035808775	0.056305029
0.020398601	0.014308899	0.154785622	0.091648195
0.01060441			
DPH5	0.462662277	0.487980553	0.432740889
0.41079629	0.422855409	0.402721638	0.175961965
0.160752453	0.0727282	0.002119655	0.018546626
0.466205153	0.194796894	0.245901518	0.361553714

0.403655286	0.112596586	0.344389784	0.200042387
0.393386642	0.069384661	0.379673896	0.065209725
0.293448191	0.091383189	0.050215284	0.271319914
0.432127232	0.058432461	0.350787155	0.381705331
0.077180808	0.459887289	0.510475281	0.448436
0.513593602	0.460167932	0.007153303	0.296627858
0.449799726	0.467676575	0.393754141	0.296089046
0.026285056	0.481609214	0.211572671	0.404415322
0.221279513	0.021065947	0.241424882	0.444116625
0.478506741	0.476511461	0.200480997	0.129848442
0.218902875	0.220512671	0.314611996	0.341977845
0.18934065	0.14817788	0.483270246	0.070579264
0.139205758	0.216760977	0.127551611	0.224452286
0.381274786	0.417295625	0.009533548	0.467151531
0.001036194	0.365196847	0.364492639	0.370350545
0.027183815	0.012281161	0.149952053	0.313536276
0.452078085	0.470691267	0.481553087	0.494152751
0.158014885	0.259616087	0.428665927	0.137916376
0.473043194	0.14896511	0.088908204	0.407848372
0.487122394	0.49668186	0.180348288	0.237130904
0.461585762	0.472597432	0.447812013	0.435445536
0.387774381	0.040654406	0.472659508	0.496359186
0.437459963	0.34854241	0.12612858	0.415365103
0.039566694	0.380034634	0.32203277	0.139088255
0.455858344	0.010613709	0.188385435	0.34004951
0.179103291	0.468370624	0.497064332	0.151224744
0.41034553	0.011870193	0.149017494	0.021257047
0.317753484	0.161059014	0.502742296	0.462834549
0.20616847	0.382448959	0.230454957	0.000804027
0.017069171	0.161120923	0.48237771	0.352639361
0.440286708	0.46150056	0.216612592	0.439865337
0.495944871	0.48161003	0.070291715	0.06783109
0.430736479	0.158213628	0.199153179	0.470172635
0.295243111	0.084642659	0.094460623	0.490335736
0.436121086	0.058755758	0.3395455	0.398379337
0.336843481	0.35383626	0.076545259	0.246063816
0.031543544	1 0.019169616	0.322100926	
0.325037407	0.135549344	0.005548923	0.237573706
0.277200335	0.482243786	0.495301813	0.07633197
0.289015322	0.397857284	0.328643822	0.463080725
0.006538007	0.030439854	0.303530289	0.491784881
0.313235027	0.492283601	0.263322071	0.285615123
0.252869495	0.450617696	0.002808083	0.492809425
0.504103164	0.330794468	0.396490233	0.423999747
0.4315168	0.454905773	0.04939645	0.399144816
0.404922344	0.053819732	0.47992498	0.132364525
0.477546126	0.02729452	0.30556948	0.492049316
0.496747093	0.005905353	0.218655333	0.376892847
0.018394035	0.076814713	0.441819913	0.396231362
0.383652003	0.458347795	0.460668579	0.417325148
0.42974797	0.194983484	0.006742527	0.305209759
0.473283146	0.434785719	0.452145363	0.208252458
0.384307051	0.478452062	0.489733352	0.228170296
0.027344255	0.288899883	0.014102668	0.32381635

0.470503745	0.093724696	0.451397505	0.120560626
0.05285981	0.494839475	0.180108889	0.297957505
0.139662656	0.41640147	0.211261598	0.23163667
0.218637223	0.136227049	0.381566164	0.389578337
0.023165579	0.069497286	0.00965885	0.431048567
0.13395459	0.368151514	0.370701804	0.45242266
0.312720836	0.089498196	0.380796736	0.298596434
0.09386493	0.007976962	0.488344083	0.388734529
0.379885693	0.122715427	0.026276956	0.391939259
0.471893321	0.366733248	0.297704153	0.467235918
0.017847647	0.481860931	0.229771843	0.098158185
0.400020886	0.227100113	0.44882386	0.05911732
0.406216572	0.467522111	0.283040319	0.162793851
0.438358877	0.411082167	0.018513058	0.455686272
0.451891071	0.019109695	0.497757883	0.104597821
0.012134953	0.458902268	0.491742774	0.054201983
0.057858355	0.330544054	0.03041468	0.261675831
0.491813977	0.395540224	0.47606298	0.212822962
0.025541979	0.006177018	0.189275092	0.435221959
0.50724398	0.500731296	0.222050493	0.377511955
0.200929554	0.359659582	0.132754237	0.496833719
0.185514629	0.495280709	0.002624991	0.486955992
0.515584109	0.29917255	0.06427996	0.294763599
0.435116465	0.029530908	0.165915715	0.481221938
0.470794989	0.333857435	0.372297197	0.433061065
0.008528501	0.025865435	0.402238323	0.198813951
0.400424047	0.184812789	0.060996901	0.326767481
0.027762381	0.468240102	0.45666729	0.046498821
0.377578081	0.31286854	0.363399269	0.022431315
0.498929239	0.491330163	0.506746324	0.127197845
0.482346732	0.499527253	0.472608582	0.057898952
0.010266231	0.506543527	0.423889641	0.014005463
0.465574315	0.252699493	0.007390595	0.476513502
0.146545623	0.461291788	0.002480175	0.094701191
0.02974405	0.466948205	0.475466852	0.378070117
0.276545593	0.343218977	0.00421491	0.498144646
0.4815022	0.008821762	0.126830787	0.497585806
0.493184493	0.488311967	0.35761226	0.49769703
0.478921669	0.495718208	0.219683456	0.239431729
0.470778576	0.495189855	0.436494021	0.464159509
0.46346175	0.424847245	0.246028237	0.008416654
0.143216949	0.1952043	0.366496742	0.07298633
0.350774773	0.120855433	0.484857518	0.468344795
0.498761466	0.001592048	0.120412671	0.490113405
0.445207103	0.459766131	0.013559988	0.00548172
0.006930384	0.015831575	0.345064706	0.217571627
0.494775933	0.268947755	0.000984107	0.028249651
0.252836409	0.423087001	0.488752913	0.353606918
0.479300636	0.05727171	0.305812111	0.378075102
0.056749496	0.388015789	0.495144721	0.466116766
0.02748444	0.223924856	0.380795452	0.469820163
0.090553126	0.289463532	0.467604784	0.310802059
0.008053967	0.063106218	0.051268516	0.438514299
0.507870075	0.063393347	0.023306408	0.157561269

0.095591112	0.478796775	0.136011266	0.48853968
0.453552646	0.425405541	0.059787541	0.47539715
0.029798094	0.478260681	0.352496513	0.054206424
0.260415901	0.006279585	0.487569374	0.179921228
0.320419863	0.41051599	0.21645094	0.010437716
0.212697231	0.036795174	0.473203538	0.479249949
0.023680658	0.202660524	0.269618933	0.395767219
0.275870997	0.437653017	0.494977478	0.467195817
0.061194515	0.151635426	0.401361767	0.435335138
0.213900515	0.48043846	0.310045906	0.496188603
0.445097709	0.474071617	0.497013726	0.337285323
0.233787663	0.426388049	0.387582008	0.011713322
0.327056228	0.466494559	0.349416003	0.146485698
0.237157764	0.443391173	0.030941053	0.188026571
0.392825073	0.319590748	0.112457733	0.427044663
0.402490936	0.442666401	0.024764604	0.048181819
0.498343772	0.462329782	0.12323603	0.441987033
0.137213121	0.33434353	0.477766221	0.47634284
0.40683407	0.486420277	0.443164701	0.410822037
0.444020157	0.225447293	0.507470203	0.073724931
0.082759194	0.465394098	0.077231822	0.06995073
0.111086412	0.016999389	0.364976898	0.41671828
0.446641315	0.363085883	0.331318546	0.059551498
0.304577757	0.401034092	0.331801754	0.173358325
0.058439945	0.484798298	0.005055859	0.038035444
0.437779652	0.488039853	0.421930781	0.211010562
0.335875165	0.120544943	0.267940696	0.303940899
0.355179465	0.402785018	0.487585041	0.021948948
0.010015151	0.476410562	0.395505653	0.013276529
0.072782083	0.425937343	0.268783618	0.229871637
0.0145117	0.500380503	0.370323124	0.442387697
0.324482804	0.043752789	0.403979306	0.075700928
0.04042076	0.457386331	0.432942679	0.49757741
0.354388665	0.408232139	0.452196275	0.482381388
0.103669256	0.444822532	0.015976889	0.454557622
0.481725643			

DPP4 0.033708082 0.029715273 0.038386156

0.061518437	0.064590498	0.01299669	0.055503094
0.013315797	0.053711986	0.029196419	0.097018815
0.051501898	0.047478657	0.021239086	0.006221386
0.101580951	0.185651172	0.026242282	0.12695533
0.064286546	0.022787108	0.128868152	0.087297163
0.062914357	0.167885849	0.164346129	0.07338678
0.062159241	0.182833546	0.008153895	0.033211039
0.234835368	0.038035664	0.020304806	0.078166997
0.019767035	0.021590356	0.066478141	0.008524849
0.027686457	0.051968482	0.057712347	0.103295836
0.097529776	0.014715941	0.084974847	0.091654174
0.008232743	0.174665168	0.004439757	0.014023146
0.01526235	0.024940381	0.016796353	0.048473017
0.022093251	0.006004687	0.031211492	0.011987349
0.092448899	0.004614319	0.01910484	0.029544344
0.011234222	0.028840841	0.202071636	0.037761184
0.037175543	0.017694749	0.097224221	0.031393426

0.008042056	0.072233319	0.123797312	0.021208527
0.303250054	0.124430968	0.206477668	0.057867069
0.057499175	0.023236017	0.066879576	0.052994654
0.128597539	0.152434174	0.058572306	0.119874518
0.02944024	0.057541937	0.060905804	0.121227153
0.061066171	0.025974082	0.109523223	0.20104755
0.025791326	0.026071261	0.021498717	0.038164279
0.014937868	0.244207903	0.017158932	0.044540457
0.018111842	0.07243598	0.255260244	0.034608901
0.224774964	0.008409941	0.111292557	0.098209319
0.057927518	0.060265607	0.237098241	0.042709681
0.082916672	0.035536439	0.027611313	0.107575322
0.071239408	0.158409877	0.047247982	0.252347523
0.024882877	0.07991769	0.012174113	0.047787282
0.016935173	0.028399382	0.048479844	0.164329401
0.281416298	0.118870527	0.032747272	0.044778532
0.050015075	0.018114338	0.068124742	0.015148393
0.034842734	0.06680466	0.000426526	0.079804917
0.035688496	0.127743159	0.203221897	0.036425492
0.120992133	0.051455323	0.148040568	0.046287261
0.015920377	0.168689875	0.012635881	0.114296382
0.012531615	0.135071231	0.137212454	0.056550351
0.072588292	0.019169616	1	0.020500934
0.010185264	0.15869086	0.103766923	0.020173286
0.016365985	0.024953066	0.012401175	0.197030921
0.065071755	0.104963777	0.011410848	0.028469634
0.053453498	0.17337496	0.005063065	0.04029805
0.006929862	0.048958586	0.059688416	0.072831763
0.196434378	0.065018303	0.014922811	0.016500079
0.026206887	0.004525086	0.06816985	0.076092616
0.023687682	0.052314258	0.002976468	0.109089067
0.013371112	0.176660888	0.064013363	0.238702161
0.021395622	0.210125769	0.015403734	0.043031891
0.029390544	0.048808598	0.008261925	0.046440991
0.283720359	0.011761086	0.042148815	0.062835694
0.047027126	0.067792671	0.076937227	0.02238168
0.054301099	0.057947729	0.189179116	0.154698641
0.046531968	0.008100068	0.01677899	0.032582068
0.008114953	0.025028046	0.022933679	0.003757744
0.122689627	0.028117295	0.029334507	0.076295932
0.018515154	0.051689655	0.018497935	0.15294031
0.261641956	0.038626224	0.117952574	0.055449332
0.082382877	0.029710701	0.086634786	0.020546189
0.044949085	0.105321142	0.029983204	0.023786817
0.037620117	0.186537735	0.200605604	0.013563586
0.047877908	0.017519212	0.012157344	0.018792837
0.020807521	0.090251531	0.07035387	0.047494809
0.181347336	0.274606719	0.030035707	0.094169362
0.009630591	0.06721989	0.258411685	0.0064307
0.044085222	0.005213861	0.002941951	0.028370182
0.120950922	0.05902622	0.047292858	0.247047678
0.047299834	0.12832263	0.062442463	0.007413943
0.023111574	0.02153606	0.134843265	0.129320778
0.013910446	0.050418761	0.088863459	0.019634951

0.018491773	0.234042012	0.034168862	0.222680595
0.301046268	0.075958273	0.033640411	0.30452939
0.238315322	0.035904858	0.019157109	0.210674363
0.036250239	0.1212735	0.02532528	0.107164691
0.119895263	0.099274411	0.18582768	0.045753668
0.017338556	0.036927014	0.194550857	0.073237281
0.156265661	0.023730983	0.004124179	0.040836135
0.067579524	0.024587083	0.181894495	0.050501924
0.01762774	0.007977601	0.208196505	0.025039975
0.016495604	0.108716085	0.06734011	0.021859166
0.028220587	0.037246019	0.021191014	0.068395164
0.042772622	0.056390464	0.065975576	0.075370648
0.016619576	0.084045974	0.076076551	0.009598287
0.120298043	0.020261418	0.076010325	0.108375704
0.009034951	0.007556644	0.149138306	0.174599813
0.040087415	0.028438054	0.02113997	0.064410204
0.040949527	0.030785167	0.033606559	0.272296385
0.31503189	0.023197626	0.023917209	0.060648652
0.064455734	0.00396657	0.081003617	0.027064025
0.122389628	0.011121573	0.003556167	0.026701579
0.257414038	0.080206819	0.016298986	0.035573888
0.00228494	0.080965053	0.06222799	0.039800962
0.023182504	0.24844273	0.163468085	0.029561678
0.037400964	0.027179941	0.052117884	0.042638862
0.040928974	0.019667195	0.027734 0.185454886	
0.03295394	0.025974638	0.036064813	0.021912288
0.05600562	0.103121289	0.117371472	0.079416965
0.044493605	0.011175509	0.004093875	0.046636918
0.010152927	0.131990742	0.031691502	0.068185034
0.044110722	0.220618127	0.023826076	0.026222825
0.033993088	0.05028729	0.138896635	0.020084402
0.013877365	0.147725094	0.037277669	0.180810053
0.035132264	0.002464622	0.143993357	0.032850701
0.037716333	0.089206741	0.024835459	0.115995265
0.051651574	0.209034499	0.008321691	0.091893453
0.161420377	0.008922779	0.045445971	0.019249262
0.247221517	0.140617222	0.027096405	0.051097871
0.045202054	0.183687371	0.052017063	0.024695908
0.143795296	0.217907696	0.161739787	0.086268603
0.017904372	0.120039578	0.171800661	0.002357634
0.060324677	0.052207286	0.094237989	0.053475294
0.040322834	0.090631231	0.237396657	0.019900264
0.101353605	0.018583479	0.10144045	0.00328986
0.084707451	0.20845345	0.026460617	0.018724449
0.011855392	0.031904087	0.028337477	0.268904987
0.042344117	0.252974481	0.061274111	0.069441157
0.274865453	0.037408294	0.152128629	0.074705323
0.017793406	0.065850202	0.018073644	0.010105228
0.047945744	0.08798402	0.053082961	0.02018064
0.015421549	0.068594677	0.028676601	0.038359088
0.025339747	0.03133683	0.031327538	0.04756804
0.089173095	0.01903036	0.023332506	0.041758694
0.085542807	0.062371913	0.030376053	0.100671027
0.133159657	0.043901937	0.102831733	0.009572179

0.043079513	0.01127868	0.0842771	0.090639542
0.030240144	0.020108631	0.080730909	0.151450575
0.031015012	0.020263192	0.018421229	0.036790209
0.22219832	0.086174681	0.056348503	0.067923783
0.013187473	0.033074072	0.037964385	0.022301903
0.016775859	0.085968837	0.022162639	0.006633299
0.089281203	0.029183387	0.086892278	0.317873965
0.175568876	0.225261461	0.019964498	0.101039679
0.018388239	0.10604794	0.006463469	0.141752788
0.155026888	0.018028426	0.125254893	0.032649792
0.109517008	0.019184839	0.113780097	0.263064426
0.028362844	0.03421866	0.053388701	0.134225373
0.037887233	0.179528979	0.141594248	0.009249422
0.008429623	0.014902514	0.03078881	0.047838108
0.08274451	0.066450946	0.015089213	0.243276477
0.107319997	0.016535364	0.047970022	0.0314426
0.28061719	0.029239213	0.006373877	0.055808648
0.03936656	0.194555724	0.010725864	0.035506649
0.11340525	0.017639901	0.054376041	0.024354834
0.075706899	0.098520336	0.011823909	0.029697815
0.069082024	0.024434429	0.078979744	0.048931418
0.011292286			

DPYSL2 0.332825498 0.398062018 0.15917483

0.328310544	0.182054868	0.228168626	0.139574665
0.217154166	0.056657311	0.006625412	0.006264236
0.223527893	0.061030453	0.138162555	0.126038072
0.335279649	0.052748743	0.222691663	0.162913256
0.341198202	0.106796256	0.257480725	0.09532073
0.098605628	0.035327492	0.040137563	0.073378444
0.412813348	0.049556007	0.150949013	0.366283739
0.079723584	0.376831563	0.38357214	0.210360027
0.354481204	0.302470409	0.034401769	0.113783014
0.41305434	0.274371791	0.345285521	0.20481072
0.044925108	0.38889249	0.147850612	0.205413401
0.168862026	0.017371388	0.170495918	0.389473089
0.31813276	0.171095217	0.200251746	0.108535291
0.03849495	0.157005778	0.043512336	0.313962389
0.129536261	0.142347601	0.329672962	0.066132349
0.084559774	0.227066919	0.078685118	0.191669674
0.090070706	0.366634571	0.064230999	0.193870031
0.00449794	0.346300061	0.211983965	0.061881177
0.037271074	0.012473368	0.091889292	0.221616566
0.412575788	0.387779757	0.34729209	0.336021708
0.070287321	0.153801852	0.130381657	0.089265434
0.378847613	0.099727688	0.111681132	0.304407919
0.350257623	0.36249372	0.087027157	0.123236488
0.284155434	0.404126052	0.153287201	0.135874956
0.075661543	0.035698873	0.326620953	0.364129089
0.3793483	0.076265856	0.070243613	0.393076476
0.060271546	0.172649764	0.246646112	0.09722525
0.420409626	0.029033329	0.083141479	0.196432066
0.19170932	0.353072061	0.34661127	0.130154494
0.175006705	0.022371621	0.028694725	0.045178473
0.141679318	0.035688206	0.343349138	0.353878453

0.193640991	0.112714115	0.184031135	0.005946325
0.021713653	0.130409506	0.305482911	0.219486772
0.356409244	0.296252695	0.073403268	0.327923753
0.195393896	0.340268976	0.033843976	0.118009461
0.209430191	0.127654397	0.15917208	0.201573634
0.100277835	0.158734256	0.099896502	0.392606107
0.40492983	0.076858419	0.211518832	0.311185713
0.095021277	0.134290642	0.111175027	0.126356727
0.061278696	0.322100926	0.020500934	1
0.057715204	0.04536099	0.052658183	0.200275241
0.12920698	0.271020051	0.336690059	0.10770397
0.139186966	0.220265247	0.221170024	0.230780479
0.004481036	0.077612936	0.183966574	0.367031896
0.249269452	0.382131478	0.180439601	0.138951418
0.138763154	0.367689684	0.006374384	0.374692878
0.360807397	0.170345907	0.168287453	0.243478578
0.085639119	0.351341772	0.04342988	0.306610413
0.318373773	0.09594138	0.387313482	0.070479224
0.362391596	0.029351982	0.173131999	0.258295801
0.217113926	0.012574942	0.150594803	0.323835652
0.021403554	0.109960532	0.359379087	0.289168962
0.181631849	0.337545621	0.390950678	0.075023837
0.171564864	0.205438064	0.027086723	0.137502738
0.333359214	0.097183724	0.379117	0.170461911
0.247338701	0.372714621	0.279328559	0.058248846
0.04659425	0.194348667	0.040055493	0.124001793
0.308592821	0.061527165	0.121379195	0.132259931
0.040480486	0.378332185	0.052482396	0.062528575
0.031639832	0.168461561	0.193987562	0.068917149
0.042677983	0.094610987	0.097344502	0.117581564
0.059943597	0.054810131	0.023090948	0.077772509
0.031926153	0.079216356	0.080904196	0.290910183
0.149173021	0.051823519	0.125741877	0.103657458
0.057889277	0.018605993	0.388624003	0.320529026
0.321503411	0.125146002	0.045757715	0.07401352
0.348740276	0.153680605	0.174351344	0.354159547
0.060217347	0.372134379	0.075286104	0.057327269
0.086388777	0.127125788	0.399228355	0.035653434
0.272844303	0.372404519	0.191756961	0.070809749
0.329123426	0.300475755	0.042548078	0.326709751
0.315301892	0.02972975	0.373640262	0.056454192
0.024181604	0.258897535	0.221388959	0.054315581
0.051784462	0.1039419	0.087412224	0.159894828
0.379871846	0.278758805	0.259578126	0.088255394
0.034945421	0.004025502	0.089873156	0.182073736
0.377920074	0.344955112	0.165650448	0.092373945
0.175560925	0.083265894	0.150490995	0.354838867
0.154212049	0.398691173	0.015635088	0.251902293
0.344941419	0.185489345	0.033447385	0.184889801
0.334592148	0.05539882	0.213044547	0.36541921
0.357720808	0.117235852	0.101683722	0.301404662
0.035731318	0.066055875	0.181192686	0.052826442
0.113539992	0.116221503	0.013315677	0.204177045
0.029901803	0.347601275	0.369633591	0.0329731

0.146246447	0.214516104	0.221244313	0.045188349
0.274649978	0.357945205	0.384156893	0.194164246
0.300898	0.315526844	0.124244667	0.06654846
0.016279314	0.357083329	0.346742736	0.017451334
0.381881471	0.171192596	0.006229893	0.409955144
0.14942131	0.35027593	0.013349927	0.05826041
0.036075332	0.341477953	0.349609883	0.35941106
0.096743345	0.304797252	0.009622075	0.378576042
0.359133128	0.011051198	0.057177272	0.259909172
0.209380561	0.426102367	0.073320242	0.270589107
0.162197583	0.330545331	0.116760987	0.136758889
0.331743879	0.312782623	0.377082126	0.406061112
0.359485712	0.264954832	0.187707393	0.020941101
0.046796379	0.17942395	0.067857318	0.024309513
0.288842451	0.055851955	0.392510388	0.365606015
0.321732316	0.009850884	0.155876311	0.298209631
0.413665225	0.424701442	0.0494402	0.008931485
0.011167332	0.0050845	0.274315799	0.120118587
0.359587638	0.075217887	0.010284149	0.101073357
0.164335955	0.166162981	0.357744431	0.319710257
0.307219782	0.071858658	0.072078803	0.359563043
0.027593132	0.301218234	0.332935587	0.378688816
0.051293808	0.121097116	0.212739213	0.396809856
0.011873055	0.172664209	0.242181311	0.102179438
0.029928718	0.04597505	0.036898455	0.352925586
0.329651693	0.025015155	0.013834381	0.105449471
0.063659154	0.374985194	0.082821658	0.38834252
0.373391005	0.36717032	0.037844703	0.374204099
0.021800671	0.347299856	0.093253905	0.043599731
0.182492519	0.028422823	0.333163748	0.023798741
0.180905265	0.367187729	0.065095691	0.015344868
0.05258558	0.038968316	0.351728244	0.316348787
0.032168867	0.188890174	0.163604861	0.324965723
0.220535586	0.291336473	0.376320114	0.136516835
0.079023887	0.059594021	0.378055833	0.318095981
0.194273686	0.365360257	0.07369945	0.391074185
0.356023631	0.409318104	0.333983101	0.203820985
0.061709463	0.270874336	0.250621045	0.025131449
0.23691726	0.325344084	0.248032344	0.04544324
0.083818724	0.12657497	0.0961442	0.168157708
0.07780127	0.235831895	0.083073701	0.388237503
0.336312163	0.329206241	0.104709631	0.03360909
0.410068186	0.392676773	0.145147771	0.275613309
0.120063177	0.148357902	0.316925548	0.287748146
0.136698672	0.297307741	0.380689542	0.254750825
0.362497345	0.168433908	0.3815648	0.187014163
0.036917041	0.236275829	0.040577678	0.061011249
0.092032975	0.026709291	0.059320904	0.317388483
0.081576226	0.220678212	0.177174384	0.070928597
0.15168382	0.189303788	0.20628009	0.127580676
0.046275806	0.351244726	0.011285834	0.050089568
0.09181068	0.29370429	0.098600112	0.123024088
0.066269131	0.077428821	0.14450402	0.196310722
0.080669946	0.138286306	0.293651231	0.078664727

0.043059691	0.385813638	0.214553336	0.030372133
0.055044435	0.283203204	0.214404591	0.080053299
0.017948341	0.302518843	0.106767243	0.181983286
0.247970403	0.080120446	0.36594472	0.10473607
0.112743155	0.388618097	0.165584759	0.348680207
0.148037381	0.30363057	0.378572939	0.308800157
0.137739993	0.164526826	0.046084211	0.399103519
0.087376327			
DTD1	0.187045067	0.33019116	0.159185751
0.246329352	0.138707427	0.145468639	0.068733448
0.015718366	0.046929986	0.001407343	0.01654497
0.163101088	0.023707497	0.029428541	0.093746658
0.247915553	0.025863347	0.051633541	0.078474411
0.166963732	0.013006248	0.208323286	0.020529637
0.064125469	0.059735518	0.039978368	0.157472141
0.241962559	0.045467071	0.083068105	0.146633245
0.011876448	0.193128285	0.329366339	0.213765282
0.310579424	0.189169188	0.000642025	0.070847197
0.209788257	0.323874489	0.180196643	0.134489299
0.011931429	0.269155713	0.104115667	0.17446822
0.015391745	0.015550925	0.036434321	0.245365949
0.297542875	0.210054258	0.039623048	0.049703191
0.027095055	0.018423121	0.052839824	0.097892951
0.070945117	0.0179818	0.305148007	0.025440779
0.013147898	0.032474105	0.019257535	0.045113301
0.095299577	0.172305167	0.001303528	0.149407759
0.000256471	0.165975278	0.135365537	0.177635505
0.018592394	0.003125524	0.030285972	0.134127226
0.252674245	0.257659884	0.322540881	0.339194901
0.015872479	0.07501824	0.146577536	0.037605857
0.255861908	0.058170295	0.01167721	0.247236056
0.333782174	0.329503245	0.049057035	0.12068484
0.292174379	0.273169331	0.150908582	0.135840075
0.166074081	0.011852577	0.274485265	0.322518389
0.183470061	0.12314086	0.065045451	0.174769458
0.016215938	0.030149722	0.159513473	0.005566115
0.274663459	0.011888759	0.083186607	0.110417563
0.013912302	0.22220554	0.344149885	0.065669146
0.108423292	0.002124162	0.056356222	0.006153048
0.01889819	0.027390822	0.287624407	0.276304146
0.012262339	0.040501153	0.060489071	0.000998962
0.007910478	0.012173668	0.196322095	0.061290083
0.199219917	0.231234575	0.117108373	0.16662087
0.238016859	0.31514299	0.00825155	0.018327555
0.167148252	0.012932916	0.094945725	0.14579358
0.157966336	0.016593082	0.024623471	0.35366604
0.229081833	0.025541196	0.036802843	0.220025392
0.04935527	0.156998528	0.01920742	0.036366041
0.007767117	0.325037407	0.010185264	0.057715204
1	0.011582152	0.000483698	0.043793866
0.021654923	0.2909256	0.270751072	0.023809945
0.056724518	0.2140502	0.054027299	0.238536154
0.00959267	0.004626755	0.036837544	0.307718991
0.080898287	0.308144869	0.041546438	0.078607442

0.121227073	0.276023599	0.000659215	0.306101609
0.349415338	0.04336691	0.155707061	0.152183326
0.225800373	0.287171739	0.002435389	0.176429878
0.111694538	0.018692144	0.328678943	0.069509241
0.254595432	0.024687819	0.040222986	0.32160377
0.218928314	0.001647143	0.051014763	0.178127259
0.008004369	0.014257578	0.190886107	0.203119462
0.241645653	0.256969203	0.298909578	0.222163304
0.159049554	0.035496023	0.002130846	0.12545309
0.229517203	0.031096052	0.263012471	0.040166681
0.081460204	0.301137022	0.237637003	0.029931643
0.020584704	0.065705659	0.00310005	0.202782179
0.259507256	0.018193398	0.224503297	0.006379813
0.022635496	0.34965081	0.065204754	0.094007899
0.046700182	0.12294909	0.061708466	0.034750151
0.026015342	0.051436122	0.162104427	0.12821067
0.006715742	0.024170183	0.001940365	0.052348567
0.011025524	0.053164647	0.050931772	0.15101028
0.020829859	0.007885549	0.139556704	0.070229598
0.04385974	0.007373025	0.322215375	0.185792897
0.123338843	0.041669114	0.00989646	0.016734869
0.284242625	0.11776467	0.034883441	0.271953309
0.003111588	0.305479391	0.030089168	0.057728167
0.083587245	0.080993487	0.29628916	0.018242943
0.100072587	0.263922117	0.101375748	0.09733475
0.197156044	0.181695696	0.004915668	0.18076939
0.158284029	0.010644208	0.331033364	0.018818416
0.008585646	0.22153625	0.291034678	0.038321298
0.033800094	0.125742654	0.006193395	0.152553493
0.344941104	0.23593078	0.256478845	0.066339078
0.01478103	0.001024184	0.052561879	0.136451601
0.291225628	0.327147186	0.070866162	0.142453347
0.09160229	0.159429318	0.00493413	0.334884706
0.011976875	0.305573623	0.00253912	0.293529133
0.322186234	0.046762022	0.049893743	0.102116298
0.186099682	0.021246196	0.021416746	0.303397101
0.294636659	0.159655011	0.166282753	0.149259954
0.003579426	0.00400128	0.236872921	0.060516491
0.096677862	0.07728702	0.011288337	0.035531021
0.007992371	0.270120635	0.231611848	0.044205461
0.138186482	0.040680752	0.162210558	0.014582192
0.344007185	0.310438977	0.317354887	0.017443984
0.196535358	0.348469625	0.207255358	0.022701021
0.010037037	0.330747319	0.163593887	0.003561412
0.273490762	0.043100287	0.002554648	0.29840293
0.054561792	0.230224059	0.004819859	0.007116727
0.008491096	0.278257355	0.323469613	0.142720708
0.045610342	0.116144878	0.000247777	0.327327231
0.299571314	0.009462351	0.028418986	0.241373214
0.271909344	0.30961027	0.18982185	0.332440204
0.190417645	0.317800075	0.019054052	0.141866329
0.276534495	0.331095074	0.203902034	0.276527967
0.251355613	0.200057767	0.131358537	0.008024941
0.088988296	0.031566269	0.104740054	0.031713004

0.096460804	0.058229359	0.279298189	0.276656204
0.315312259	0.00306234	0.012670658	0.229569378
0.237671578	0.281706645	0.002428392	0.006700029
0.001267742	0.014742275	0.106079118	0.090803247
0.335383894	0.015845652	0.001937449	0.003224306
0.025474621	0.236869575	0.312703963	0.185737469
0.247689309	0.016682115	0.055814359	0.182555279
0.031483302	0.110016327	0.331079982	0.288793217
0.005317259	0.097933508	0.060278121	0.305951694
0.027377066	0.116277754	0.155378864	0.051085864
0.000765169	0.033148654	0.046060333	0.237693717
0.306462517	0.013028347	0.007637193	0.005993875
0.030553393	0.267606255	0.025499248	0.33165736
0.243419635	0.245462032	0.010549172	0.32298628
0.017414589	0.289006137	0.147767473	0.007669361
0.037749993	0.001852302	0.26106788	0.081979162
0.039300904	0.138665276	0.095152289	0.005872433
0.054022264	0.016481589	0.322301938	0.307953221
0.005473659	0.04135299	0.097936213	0.146496983
0.068796959	0.247875834	0.306276726	0.138245807
0.020287058	0.008564114	0.166095297	0.1334427
0.018934428	0.313957814	0.108396475	0.33721263
0.188870478	0.276066877	0.29103041	0.180779363
0.021901504	0.130652008	0.175123828	0.003004151
0.119381923	0.270843739	0.075600922	0.050043235
0.11101381	0.170931562	0.003381518	0.005386609
0.140013759	0.096259551	0.013421374	0.251921122
0.111981451	0.152216781	0.001170156	0.047478207
0.331623258	0.270771005	0.019263851	0.212002709
0.042101595	0.205925931	0.247094142	0.252001462
0.062653508	0.248469696	0.193710003	0.158098934
0.232795217	0.098216893	0.333551395	0.003203621
0.05333277	0.127406302	0.023405141	0.034990812
0.041818008	0.009458716	0.087383759	0.195472492
0.080737665	0.15732046	0.014276	0.010926402
0.109189264	0.068496455	0.135065154	0.04286994
0.013508952	0.321962927	0.002425789	0.012878686
0.070041992	0.320339578	0.194767345	0.0641108
0.078294723	0.016009797	0.100739761	0.055953644
0.065714739	0.022274097	0.311324316	0.005264492
0.002069933	0.297584779	0.079391507	0.002541639
0.009576639	0.133379948	0.020974303	0.070185223
0.008953823	0.318848679	0.093606655	0.167481717
0.10444318	0.010411556	0.158778051	0.008099981
0.006177523	0.237126431	0.188748945	0.323941354
0.089218026	0.243075473	0.246954767	0.208713072
0.00771338	0.121032906	0.008299711	0.229198199
0.128894729			
DUS2L	0.128425796	0.167334242	0.088928429
0.170034172	0.166866977	0.054582298	0.067830339
0.014737146	0.009692554	0.008555505	0.037210282
0.170321926	0.043698185	0.0537342	0.014039519
0.219112177	0.19333554	0.047186288	0.136712004
0.158193177	0.020478382	0.269694816	0.042714414

0.082476737	0.144040057	0.105846554	0.099032803
0.1879467	0.109390994	0.041760371	0.106781708
0.120354834	0.155483737	0.137043168	0.256536642
0.144906999	0.091790731	0.023915349	0.025501731
0.116179578	0.161999524	0.150543894	0.1675868
0.036478533	0.11141858	0.110240187	0.189743404
0.005255634	0.081953105	0.013311913	0.095992072
0.123157931	0.106976853	0.02340891	0.050477706
0.034081949	0.022445475	0.070693806	0.054284
0.117599515	0.011519336	0.137286278	0.007163096
0.007684452	0.040192692	0.177127259	0.058574719
0.087437185	0.076813013	0.011045861	0.122894391
0.003504997	0.14740202	0.219082458	0.053138662
0.153086921	0.045494995	0.207399987	0.123686029
0.193142431	0.127339804	0.246343813	0.221863762
0.098038356	0.175901071	0.16100126	0.124225819
0.131426916	0.073730195	0.016019281	0.271214019
0.23700781	0.166578093	0.077650991	0.21325389
0.125981196	0.141307376	0.065656367	0.109465368
0.038717894	0.07251021	0.114388154	0.214265291
0.096283075	0.160254463	0.207916099	0.133224086
0.115736783	0.016499984	0.194884678	0.064347279
0.197040544	0.011471141	0.254309505	0.11402494
0.032397179	0.135734265	0.169905715	0.103916918
0.16926093	0.035536679	0.022358481	0.089703909
0.022121514	0.076863292	0.10398462	0.195828564
0.025187765	0.05303896	0.064666534	0.030910209
0.117789368	0.035460517	0.173628564	0.088929062
0.14790916	0.098588117	0.090932874	0.085336567
0.148161265	0.247435965	0.000683879	0.039131442
0.100051028	0.058690825	0.20713883	0.146188194
0.17939281	0.030589591	0.119116232	0.198908199
0.09651576	0.111634271	0.034057927	0.238488635
0.031734679	0.257004807	0.093075071	0.116193368
0.036068899	0.135549344	0.15869086	0.04536099
0.011582152	1 0.009122879 0.04017684		
0.016392059	0.147399802	0.105100475	0.116050449
0.139068287	0.22349922	0.040440057	0.1396269
0.015011381	0.025537652	0.021416853	0.206583147
0.039279183	0.209179696	0.076646816	0.084570186
0.229325979	0.185329683	0.000923389	0.119954182
0.163480133	0.021662128	0.204927192	0.18647401
0.072365525	0.182318066	0.002562707	0.255221028
0.071067726	0.08532674	0.234692531	0.226385869
0.132164939	0.116856725	0.04754686	0.178780748
0.173494586	0.012231845	0.0213625	0.12376134
0.101756871	0.009890333	0.131953824	0.146169136
0.108138424	0.191438668	0.242784393	0.064729804
0.146665131	0.064244285	0.0202173	0.211679705
0.16269406	0.014052592	0.102393713	0.03611298
0.043934549	0.136496279	0.124819744	0.00465449
0.060898785	0.06096775	0.008529721	0.11385577
0.095074181	0.058048112	0.061870106	0.061251651
0.183898004	0.194082769	0.138940662	0.107079952

0.09055347	0.139735042	0.090178661	0.068053236
0.07663563	0.071563012	0.065043695	0.05528799
0.018457101	0.162013183	0.021968095	0.093673011
0.062690861	0.099344046	0.08366493	0.105195021
0.053974612	0.058019556	0.165805399	0.047651536
0.096340662	0.110036357	0.162958233	0.187764028
0.057161845	0.045666716	0.12584866	0.019996887
0.196009201	0.031145444	0.011971186	0.151067541
0.014712901	0.220034041	0.068069272	0.202992947
0.170642633	0.184487013	0.190797667	0.002870814
0.081626371	0.13644707	0.160067061	0.116287438
0.103831576	0.13819141	0.016767326	0.095967615
0.10729877	0.12878882	0.19072402	0.168247155
0.124540966	0.216547593	0.155306856	0.16625084
0.121827277	0.097217349	0.007499169	0.255260588
0.1827709	0.270679046	0.133727701	0.106474431
0.074009156	0.054795755	0.196798856	0.117190975
0.130515941	0.199934317	0.213519602	0.190980955
0.163600574	0.05419147	0.002992706	0.203363231
0.021267196	0.152318778	0.052424967	0.195584385
0.134596929	0.036573657	0.148913855	0.061146737
0.098651191	0.045013154	0.057699545	0.142004285
0.125000991	0.060385982	0.044396604	0.191326993
0.010322919	0.013898022	0.127901538	0.077389908
0.050278101	0.072519658	0.031452262	0.022986999
0.043476636	0.131692679	0.23881521	0.032387206
0.052901975	0.017345306	0.273459493	0.075624402
0.187076489	0.163184015	0.144158618	0.048488229
0.187875011	0.172026823	0.151814976	0.146429876
0.127527749	0.152666388	0.08426794	0.014245305
0.230048105	0.017117084	0.035373954	0.14831659
0.099943627	0.095479717	0.000160144	0.008068026
0.073878253	0.249478906	0.113674591	0.103805178
0.012271189	0.140255947	0.011425027	0.200098758
0.149882998	0.084526676	0.097179591	0.168697437
0.178187778	0.156510445	0.111433614	0.198428824
0.178566115	0.143562553	0.021428466	0.209055812
0.149352775	0.164881128	0.12009654	0.116719679
0.216886497	0.246912847	0.153594555	0.024581648
0.009536387	0.022552064	0.018014344	0.014916084
0.041723938	0.084527084	0.158243711	0.232215563
0.214032195	0.065563737	0.016832422	0.144577614
0.144750323	0.181159828	0.013001801	0.001474757
0.001728935	0.036489374	0.091474327	0.166640344
0.168234603	0.00794691	0.04222009	0.009319189
0.023400614	0.223079244	0.15805868	0.212647689
0.180958431	0.129971947	0.030736758	0.186917933
0.057306505	0.049295206	0.210000903	0.123810638
0.070385632	0.133049963	0.071059692	0.196675922
0.044699963	0.264368166	0.192086074	0.045712292
0.020476228	0.11642034	0.070996452	0.226889236
0.139002303	0.096995262	0.087049986	0.004243879
0.072632291	0.201959003	0.07997356	0.221961765
0.153975212	0.243030113	0.105645762	0.120762627

0.029707761	0.139425971	0.201835764	0.001408407
0.099974034	0.037178292	0.164907963	0.013348198
0.043200403	0.103061406	0.010281292	0.101988355
0.029513597	0.153639077	0.223992248	0.236910756
0.071662488	0.039558559	0.166210441	0.158839737
0.04495109	0.207673089	0.128672483	0.053268009
0.032987074	0.044532642	0.144145163	0.091128671
0.033909301	0.248311478	0.054749187	0.188819538
0.096866325	0.138900588	0.173641319	0.088837732
0.074015951	0.068567277	0.097251898	0.011433356
0.131495389	0.235302485	0.065393282	0.055999616
0.189523366	0.136504453	0.015633266	0.006502646
0.124508734	0.043975337	0.056407714	0.229523801
0.097075474	0.0939973	0.015151799	0.061097888
0.173810612	0.106044647	0.005677689	0.122428458
0.161952358	0.145040978	0.19286897	0.234681216
0.028108797	0.181359289	0.150551406	0.112470835
0.109265417	0.097454001	0.145299422	0.002815185
0.023219152	0.128386244	0.077845298	0.195876019
0.154718119	0.048090115	0.053874613	0.243072348
0.076599494	0.241345019	0.015559514	0.022704253
0.229097555	0.052259687	0.197044516	0.050247795
0.049659357	0.135508151	0.015910669	0.149455006
0.122685748	0.143039383	0.164601923	0.120684756
0.060077446	0.097811077	0.205522876	0.034811528
0.056494537	0.015743561	0.134637092	0.005898626
0.022965049	0.241386251	0.046844684	0.031347084
0.036885842	0.066117955	0.061238213	0.032200339
0.110551472	0.179396616	0.030903998	0.141765093
0.079329581	0.090699488	0.070651415	0.008734587
0.0146247	0.115444348	0.13434749	0.162456359
0.12550293	0.247006303	0.090047427	0.162258462
0.014931105	0.078083152	0.018769366	0.171105283
0.054623786			
DUSP1	0.026120333	0.012091014	0.019228112
0.033174488	0.024967587	0.012596499	0.058791455
0.050589068	0.009432875	0.003535964	0.00160336
0.019859368	0.001334046	0.015198756	0.004279669
0.055762533	0.035847869	0.050642172	0.105893235
0.055373861	0.043085645	0.047304682	0.082644217
0.018821363	0.02382672	0.05612277	0.012186165
0.042517801	0.07171358	0.007363299	0.041031642
0.117957619	0.028177827	0.008171131	0.016184702
0.006475553	0.017377322	0.039164895	0.006156639
0.027130079	0.017424941	0.049325414	0.056910535
0.056849864	0.008002474	0.057258504	0.03888583
0.004369196	0.026683659	0.016245906	0.010371379
0.004196609	0.006393954	0.049159943	0.044763693
0.00017213	0.016322066	0.000840975	0.023806406
0.065820636	0.019251237	0.005889823	0.01026058
0.002526287	0.080452964	0.072491762	0.064804195
0.008547875	0.022431366	0.126215996	0.013428016
0.00200425	0.072563502	0.062333928	0.002520265
0.15468766	0.010391997	0.089686104	0.040650062

0.03578524	0.014642867	0.020494997	0.01525663
0.013741741	0.100840229	0.01507218	0.049951241
0.020024207	0.035923417	0.01316426	0.048341646
0.018434388	0.008211953	0.022965856	0.082921833
0.00904966	0.015067602	0.010048224	0.012236853
0.001971377	0.070566987	0.00718859	0.014020525
0.018007988	0.011609323	0.076380834	0.033324258
0.179121576	0.007285606	0.062275037	0.010327568
0.033852488	0.047594981	0.067642564	0.028153832
0.07374708	0.02321663	0.007852498	0.092088883
0.032815199	0.015710177	0.000666482	0.203105814
0.003124772	0.00708904	0.004656072	0.018889374
0.025157092	0.005517906	0.069121477	0.118368412
0.085351359	0.036554654	0.013821121	0.061897143
0.039696361	0.01026944	0.013122468	0.014362769
0.007412023	0.020501614	0.000153985	0.12023999
0.018816071	0.062022278	0.120871136	0.013338173
0.025330034	0.116222933	0.115284472	0.017327029
0.014005194	0.128627307	0.031118188	0.055238554
0.002875051	0.029135387	0.14986332	0.02627163
0.062383882	0.005548923	0.103766923	0.052658183
0.000483698	0.009122879	1 0.051434409	
0.004813273	0.006002808	0.005094129	0.202481046
0.032329384	0.034067185	0.027794933	0.007137931
0.001637688	0.075090789	0.019389949	0.013153923
0.013437964	0.019421099	0.053001365	0.041186496
0.075472724	0.035443646	0.011783537	0.007133235
0.008064677	0.01289545	0.015755386	0.040099154
0.004176701	0.022701206	0.001401524	0.055207034
0.019186855	0.217534103	0.0240516	0.066965208
0.010798842	0.079760723	0.021632046	0.010461625
0.005631903	0.00946953	0.020543688	0.043146637
0.098317642	0.025933244	0.038028062	0.038730088
0.018442304	0.034450591	0.0332073	0.003020247
0.017862621	0.10257117	0.078790857	0.056517596
0.026026202	0.000855716	0.010805919	0.03715053
0.014249315	0.011672407	0.009323318	0.000403119
0.097553926	0.044166884	0.008674796	0.024787795
0.011004205	0.015998358	0.006000774	0.070196015
0.084263241	0.012776117	0.021952531	0.009127275
0.009957372	0.008568218	0.122152054	0.003288943
0.001229036	0.046735743	0.006568817	0.009564692
0.054735109	0.067879941	0.070630561	0.001503228
0.001233687	0.001905524	0.001591135	0.013650126
0.010124921	0.007241938	0.015779053	0.016967457
0.050217675	0.166651599	0.012520618	0.066190184
0.014404973	0.065191181	0.186583259	0.000287416
0.016143156	0.004007569	0.011346148	0.012344739
0.052589657	0.023868706	0.005962948	0.067577925
0.00577945	0.055820365	0.034446572	0.00112876
0.03130727	0.010409396	0.107631637	0.031196202
0.007862104	0.037909496	0.027108095	0.016099178
0.013306473	0.11910769	0.011004461	0.043864735
0.186004636	0.025702846	0.006689964	0.150017891

0.100942402	0.009278761	0.050686402	0.076659726
0.012625164	0.043002157	0.007878067	0.042129766
0.06192419	0.002405422	0.069085154	0.023644401
0.007288516	0.010378622	0.135890356	0.008706891
0.120675863	0.003949561	0.005303985	0.011958839
0.040024802	0.011172172	0.19907885	0.012660846
0.00536024	0.01853044	0.03870346	0.028011365
0.011608199	0.105763304	0.139503512	0.008262898
0.016228104	0.014193197	0.00569019	0.044105959
0.051481347	0.048771294	0.026152408	0.013242786
0.006142021	0.050007212	0.003343536	0.018029018
0.023413829	0.008678988	0.037423737	0.023286001
0.003888005	0.021995961	0.059819486	0.146212634
0.00879894	0.010426937	0.008265761	0.143813077
0.017387578	0.007521255	0.00385323	0.180758861
0.138999501	0.00747011	0.029775991	0.004750873
0.028696953	0.013665548	0.003516533	0.014205155
0.138716946	0.006202474	0.003836328	0.012578179
0.107414007	0.030423679	0.006386337	0.048205049
0.002244782	0.093468339	0.026611933	0.013893343
0.008234326	0.065677525	0.029181046	0.007651063
0.006278806	0.013649474	0.006535384	0.008691624
0.00658716	0.005508817	0.005304845	0.070456722
0.013687767	0.0064718	0.032517668	0.014814349
0.023371221	0.041027494	0.07519988	0.048049115
0.003758426	0.036428864	0.000470885	0.001594945
0.022634673	0.025049192	0.01596673	0.02899117
0.011381386	0.183797833	0.038687646	0.010620356
0.025030937	0.03060886	0.091010305	0.005742499
0.004903009	0.003286968	0.049576534	0.089726048
0.012916132	0.000484089	0.137360176	0.061584919
0.018430985	0.017191514	0.00832837	0.077755446
0.021996205	0.155550538	0.001114432	0.075385876
0.015637626	0.016165672	0.012315958	0.009526266
0.161130465	0.076608333	0.03894437	0.02336724
0.001454604	0.080600949	0.020733443	0.004586292
0.062031243	0.072080066	0.048997647	0.046101622
0.005089232	0.007390316	0.012649631	0.001357271
0.023431291	0.025044682	0.024153177	0.019509653
0.024036692	0.044624156	0.043764748	0.009307115
0.005506231	0.006276359	0.01589147	0.001476833
0.081680342	0.163326877	0.009571107	6.13E-05
0.018374895	0.042900355	0.003028456	0.111692828
0.005886977	0.106839414	0.020866802	0.021221205
0.10453693	0.069232007	0.096638266	0.068332652
0.036397118	0.023367408	0.00751747	0.003282516
0.04554103	0.00859606	0.052503623	0.021279858
0.035591976	0.023649964	0.003416684	0.014540076
0.025497565	0.020931543	0.01054408	0.034622873
0.005068925	0.021489605	0.014073693	0.006266961
0.072210498	0.020256677	0.057115641	0.005822907
0.026765082	0.009764501	0.070223307	0.006471648
0.00623448	0.018078782	0.023980072	0.052488762
0.042501186	0.019373696	0.06716146	0.039170156

0.012722102	0.015131526	0.0179689	0.019253464
0.15780713	0.029210958	0.023654842	0.021636752
0.007596003	0.00976016	0.029389164	0.011537694
0.010141177	0.08558477	0.008384963	0.034524397
0.004945221	0.017354906	0.013561145	0.154129458
0.096224883	0.080808088	0.001991598	0.05257274
0.002668811	0.039166151	0.007257465	0.022696882
0.068277498	0.027839459	0.066825017	0.032847026
0.018682253	0.006380441	0.012756721	0.161394793
0.005001022	0.011951737	0.005750388	0.066252653
0.0031998	0.059760827	0.060658225	0.020749991
0.001409021	0.002688406	0.010720287	0.028704027
0.088296065	0.026417358	0.022726347	0.101302118
0.018871779	0.021654968	0.059697528	0.005753116
0.113650603	0.006764638	0.003719378	0.020948947
0.052582119	0.19284111	0.013622336	0.018681598
0.127562622	0.011311804	0.018531494	0.007247615
0.046265842	0.038392911	0.00815341	0.012293024
0.026663326	0.015148363	0.068424271	0.033998167
0.001498117			
DUSP16 0.230549514	0.325478272	0.102619533	
0.320483924	0.154251132	0.2317211	0.195057019
0.254912415	0.073018162	0.027797036	0.014833712
0.155396147	0.071317713	0.175146662	0.109830588
0.285520189	0.056855551	0.152869356	0.192269475
0.314848259	0.205875711	0.235911589	0.143365134
0.062391502	0.035856662	0.064080631	0.05775835
0.365605157	0.070715597	0.187644057	0.362922126
0.062847036	0.295269183	0.294510683	0.176036975
0.265550623	0.20487472	0.031984851	0.144232173
0.320175275	0.216899691	0.328891653	0.225697516
0.090678811	0.313054311	0.197868906	0.142703948
0.165052052	0.032452537	0.199612224	0.322532294
0.259269472	0.151841904	0.239194119	0.171281052
0.048316442	0.210038376	0.032062874	0.326381774
0.172669363	0.218832817	0.262911473	0.116988829
0.100778189	0.248718794	0.060413763	0.229525276
0.058241157	0.321901757	0.090286001	0.12288923
0.020290963	0.319907049	0.151198432	0.040188442
0.050974842	0.034094005	0.072006637	0.26579266
0.350068889	0.275135858	0.277666626	0.25996129
0.071194631	0.117910949	0.085426904	0.119032125
0.296007749	0.160119206	0.111931803	0.256983326
0.277897278	0.284955401	0.079701261	0.098397601
0.268446007	0.3415151	0.124019682	0.118797433
0.059562925	0.024258618	0.277020888	0.288034599
0.310841497	0.045625268	0.070653439	0.369449737
0.077198259	0.131290497	0.259851445	0.07301736
0.356305192	0.061398657	0.068240083	0.230383145
0.142728995	0.276000902	0.262315993	0.173387503
0.125024585	0.031893212	0.021515228	0.046594941
0.139869518	0.018265775	0.268513328	0.321509967
0.228500448	0.095551827	0.206835572	0.008971146
0.027087158	0.081221847	0.235172674	0.166149608

0.285880168	0.191659879	0.088316897	0.276033173
0.145237653	0.271680035	0.046428058	0.168825479
0.179279741	0.084430541	0.17507552	0.169509419
0.091304058	0.215436268	0.137378953	0.315100694
0.354925849	0.120192134	0.165343766	0.260754868
0.115585165	0.102330503	0.145999906	0.150187355
0.128667997	0.237573706	0.020173286	0.200275241
0.043793866	0.04017684	0.051434409	1
0.149674663	0.242003926	0.249045944	0.125982387
0.15513572	0.192103885	0.214670986	0.215806338
0.018301531	0.087053348	0.168111866	0.302871895
0.285510604	0.280648212	0.161466337	0.101213716
0.12490923	0.297809067	0.002280478	0.295153025
0.274819432	0.150738715	0.166103406	0.172089944
0.064253989	0.316374151	0.023501605	0.266003028
0.251243854	0.116685489	0.311417069	0.08016138
0.27762951	0.04992529	0.196804076	0.184177509
0.163959586	0.020498185	0.206693057	0.333220203
0.018517289	0.205994441	0.28305362	0.27337157
0.179605189	0.253382387	0.323460055	0.053756951
0.14509221	0.225282305	0.030073687	0.098896205
0.236177234	0.071110818	0.325769634	0.20413706
0.201145931	0.315350871	0.202576721	0.069340161
0.089738614	0.229673977	0.065431976	0.114898364
0.218134556	0.123423077	0.091305512	0.086677718
0.043875317	0.30718721	0.03055491	0.03342818
0.016329247	0.178300891	0.202225657	0.103467924
0.052094814	0.10416868	0.073000351	0.103238284
0.140193163	0.081050141	0.011400459	0.061610815
0.051523729	0.079817015	0.084747792	0.249371163
0.156811959	0.079285042	0.114852173	0.061471104
0.051174627	0.028937893	0.315078405	0.267391645
0.296701684	0.162808582	0.053373155	0.066719325
0.313716275	0.165466517	0.161660536	0.32639394
0.056463101	0.291262751	0.101005118	0.067852251
0.069592101	0.155972859	0.345827729	0.055843602
0.232095847	0.328587987	0.150628932	0.078146262
0.296421007	0.27171288	0.09027477	0.255567044
0.252639517	0.049804493	0.28981811	0.04922534
0.031120064	0.187915785	0.179872813	0.063624561
0.053464729	0.130371542	0.181723769	0.147028924
0.312678378	0.250845256	0.235974901	0.050370617
0.075168973	0.013947922	0.060317494	0.111487538
0.295828339	0.259345961	0.15601034	0.081876619
0.202593257	0.069413463	0.204039091	0.285743436
0.073188136	0.300543744	0.023844206	0.185883872
0.262680756	0.213176254	0.040478302	0.232860992
0.309112363	0.092854167	0.201795542	0.293176935
0.299448702	0.103965885	0.085828321	0.224919867
0.090850477	0.136075824	0.137230794	0.028068433
0.111017256	0.121721489	0.005151755	0.192016144
0.065317023	0.311112928	0.29082759	0.048265425
0.17503361	0.182219759	0.174272487	0.074313105
0.198268951	0.255761577	0.302296762	0.216905218

0.227456452	0.248806412	0.087983114	0.061682291
0.019101562	0.265192967	0.284318393	0.039341146
0.322382428	0.202945696	0.02452331	0.342239115
0.180186024	0.271190791	0.019762706	0.019321037
0.024088027	0.255930431	0.281789557	0.347157829
0.124009035	0.258747541	0.004131842	0.281690932
0.301036413	0.013185771	0.041614936	0.207046503
0.149788914	0.342087791	0.048238182	0.207932919
0.119506539	0.258474373	0.150619549	0.136191791
0.293710561	0.226269474	0.317066272	0.331364548
0.316856054	0.203083814	0.219763567	0.054317765
0.034112366	0.243635965	0.067592384	0.04058247
0.279423524	0.059464707	0.296998241	0.296833976
0.247752756	0.014572119	0.24442838	0.237875336
0.373258332	0.361916586	0.031654896	0.006186661
0.005114049	0.007069764	0.281435201	0.090238108
0.282561409	0.10003747	0.021758732	0.186328775
0.155540547	0.120889618	0.293438108	0.298976969
0.217136177	0.086569163	0.093422328	0.319216916
0.03106917	0.234046101	0.266779959	0.33028631
0.04442782	0.093462813	0.152790487	0.344069055
0.005346654	0.149341418	0.174535717	0.118856639
0.019285427	0.044968493	0.043303376	0.275574435
0.248074584	0.044641229	0.02760581	0.116822918
0.118428327	0.292538429	0.111463307	0.312199374
0.329835821	0.325791881	0.032056462	0.30472012
0.039232393	0.276143794	0.068257584	0.057948493
0.12115346	0.036237233	0.241606089	0.026446361
0.186306077	0.305811847	0.046597943	0.015277188
0.02424346	0.047738279	0.299480585	0.24004138
0.021995916	0.20530212	0.129648154	0.250506725
0.265546066	0.272366718	0.302354438	0.113296
0.152270109	0.025482579	0.341961028	0.248095212
0.234377012	0.28810706	0.077650071	0.296527354
0.276858012	0.314042952	0.25923761	0.213267997
0.047456876	0.223293877	0.284266898	0.069283025
0.216585183	0.285754953	0.211348408	0.046056297
0.089896841	0.079748204	0.110246644	0.099643088
0.045741974	0.279500276	0.128055578	0.324950207
0.273648981	0.258943947	0.145498186	0.041987023
0.324853721	0.314438512	0.187473837	0.256922056
0.106206769	0.144563688	0.230833861	0.209176886
0.093385139	0.25452555	0.333738032	0.272105694
0.336734606	0.193761321	0.301147991	0.236076103
0.030712268	0.165008267	0.078519928	0.05796175
0.123164091	0.030067987	0.034309701	0.257285227
0.050446088	0.22089967	0.163535701	0.07436114
0.11916103	0.123434156	0.183507906	0.19269591
0.080332133	0.289600601	0.01568	0.058336044
0.058981953	0.230922617	0.075554564	0.102662223
0.04319653	0.051136938	0.155546437	0.221400777
0.094004613	0.085610136	0.235249643	0.106321747
0.094758935	0.310815581	0.164176046	0.020148339
0.04725565	0.217055437	0.150091483	0.10178196

0.015183075	0.230391641	0.123716112	0.137511288
0.259279975	0.096415072	0.307209223	0.092403156
0.11375207	0.311558106	0.105897308	0.26467495
0.088231545	0.28420509	0.299479982	0.252412205
0.084104851	0.099015966	0.089852366	0.334238601
0.063010412			
DUSP19 0.205562419	0.356087829	0.114939882	
0.347153857	0.159160146	0.216032755	0.163571405
0.072900761	0.025468285	0.018697177	0.008742747
0.183261386	0.034260018	0.107149572	0.063570482
0.285176126	0.04540137	0.081993825	0.172836097
0.263972879	0.080474391	0.266030797	0.060104277
0.034440279	0.041452695	0.051572336	0.067231702
0.357909986	0.06139617	0.158419047	0.284767546
0.024751551	0.262101962	0.325736715	0.233431022
0.305655589	0.189516431	0.002562024	0.094835131
0.284668715	0.270482182	0.275130833	0.245976486
0.04244314	0.321485631	0.20009691	0.155190223
0.034749462	0.023371828	0.101549172	0.296335726
0.310322254	0.178524274	0.105463419	0.110834146
0.023111128	0.075100058	0.029145677	0.228405313
0.15244328	0.091975166	0.305455759	0.035628571
0.014810182	0.102965523	0.031086728	0.150659476
0.044724737	0.266156511	0.010895662	0.134880838
0.007281828	0.264261657	0.15275473	0.047748831
0.042990675	0.019716961	0.048726661	0.24955092
0.34343622	0.272730313	0.335164627	0.313734588
0.035920099	0.103854247	0.099302208	0.090256995
0.281018544	0.14702459	0.055737243	0.279961589
0.336930604	0.330657223	0.035667476	0.107606957
0.318129139	0.334375508	0.164294208	0.137829377
0.066133005	0.010022816	0.293124651	0.33782328
0.273153874	0.065634231	0.083571732	0.316730632
0.039123891	0.058769476	0.267804672	0.020249271
0.360410889	0.021868687	0.082910278	0.205689049
0.029002132	0.288706456	0.316239742	0.151916588
0.125437193	0.014547636	0.017974227	0.01531246
0.045496548	0.012117221	0.291050124	0.367255431
0.051001329	0.036488655	0.122683559	0.003583333
0.017768257	0.01640791	0.247614953	0.116213206
0.254546173	0.198409457	0.096168154	0.229710702
0.186245668	0.318578582	0.024528515	0.059397143
0.206276341	0.017226459	0.169855432	0.181709205
0.136022478	0.073937826	0.082905617	0.362789034
0.313603813	0.085001343	0.082588729	0.282153888
0.057747843	0.14090419	0.067025483	0.10685725
0.040948901	0.277200335	0.016365985	0.12920698
0.021654923	0.016392059	0.004813273	0.149674663
1 0.307712026	0.260715406	0.069988639	
0.143689554	0.224929528	0.127982702	0.291368389
0.011021273	0.018460156	0.08806964	0.35293122
0.200312522	0.313582559	0.088051968	0.065081058
0.147399451	0.31738644	0.001126536	0.322958552
0.324510259	0.105908517	0.227205867	0.169382324

0.086534192	0.366187565	0.005440395	0.268634457
0.195835775	0.054008119	0.353059608	0.099889325
0.289791597	0.0419364	0.098066689	0.237254446
0.21421632	0.006120451	0.151522892	0.280531403
0.009565395	0.055450998	0.2426721	0.285149025
0.212567009	0.267133005	0.355845091	0.078208813
0.170767841	0.119639377	0.005697427	0.117822896
0.236219367	0.018750362	0.329067552	0.085324145
0.152044642	0.346584621	0.231443397	0.018218409
0.055558214	0.149417914	0.031665122	0.15026415
0.214599592	0.064673437	0.104492046	0.014398938
0.036800373	0.36035054	0.030383144	0.038435139
0.014857584	0.213845957	0.145861901	0.088523224
0.039466852	0.046840702	0.064523882	0.088899918
0.044665213	0.064452068	0.002050338	0.065934263
0.032314739	0.093279068	0.100161526	0.216102124
0.051732686	0.03757167	0.168316604	0.031745194
0.04098109	0.020724777	0.348005598	0.254155806
0.229224732	0.070496646	0.027623346	0.026497933
0.354206392	0.177078961	0.071862627	0.348410643
0.013089578	0.312772934	0.064148831	0.078785852
0.09053833	0.179093948	0.360875733	0.026472593
0.157420839	0.338588524	0.130647958	0.08264406
0.300777618	0.247711717	0.021347108	0.246209645
0.240230115	0.035737609	0.340682871	0.030810294
0.020442121	0.224057133	0.238432356	0.057599108
0.044612265	0.171061657	0.044415121	0.174507717
0.367794424	0.291618485	0.271262301	0.037591318
0.048850248	0.010058425	0.048928991	0.121418664
0.321212863	0.316220063	0.136741701	0.139929002
0.179806146	0.060208491	0.022374311	0.346875414
0.013228916	0.320791412	0.009573153	0.225298057
0.305061072	0.132505312	0.04783582	0.186169363
0.306531215	0.047379554	0.065774311	0.339579823
0.299027983	0.087918376	0.091775834	0.207449815
0.027178435	0.021015266	0.16096333	0.020044412
0.086859586	0.074634342	0.002780232	0.060399735
0.031042279	0.328364208	0.30161153	0.026846817
0.190741381	0.067425193	0.186606786	0.040753568
0.258752445	0.290900726	0.339140086	0.065820418
0.233756398	0.318050261	0.130753436	0.037673783
0.016310014	0.313967875	0.232283602	0.030011166
0.336247181	0.130087726	0.01553894	0.357620638
0.134678233	0.266551914	0.013700494	0.004420007
0.00884287	0.285668957	0.317090422	0.256108231
0.095772039	0.196292159	0.000149855	0.321462044
0.348568167	0.010582928	0.018915185	0.245979812
0.21543361	0.358605913	0.073030919	0.279406843
0.173642131	0.307782783	0.039606865	0.158173296
0.337910717	0.277967647	0.296954637	0.33169576
0.34568693	0.222138933	0.215005913	0.02222468
0.013841142	0.112792025	0.071445239	0.019243758
0.193050646	0.048309136	0.324107483	0.315836897
0.314328559	0.00852732	0.06436669	0.251959119

0.36011586	0.359947175	0.004291451	0.003298688
0.01228862	0.007671985	0.196920035	0.091101998
0.322760287	0.042909549	0.011089145	0.031143359
0.054712274	0.167708326	0.335684255	0.283662752
0.233200043	0.040124529	0.074648964	0.283382229
0.016704186	0.170548113	0.336007613	0.340809282
0.010863837	0.080681851	0.114132522	0.370387836
0.004496074	0.154319415	0.191906527	0.064493273
0.002940182	0.032784031	0.027018283	0.270530225
0.290572307	0.032252355	0.02152876	0.018614234
0.085067075	0.294456601	0.071460775	0.356307437
0.341102229	0.343189434	0.010366483	0.329718006
0.011108911	0.315945147	0.105902677	0.100821863
0.067179824	0.007940227	0.270722008	0.018468674
0.106294231	0.235301378	0.01732043	0.007360866
0.012079107	0.033859493	0.347984941	0.285247738
0.006776666	0.073775466	0.132107883	0.216483101
0.198355735	0.32534405	0.331854691	0.153820321
0.058295144	0.006131033	0.295593116	0.219815454
0.071529876	0.331850496	0.058807405	0.338912909
0.242172267	0.299607514	0.296425083	0.212387981
0.016021173	0.198350968	0.29308488	0.039669208
0.208381032	0.331483333	0.140693235	0.033569196
0.123073595	0.10219983	0.010253613	0.020056869
0.068491029	0.223082945	0.04026969	0.320183559
0.199816959	0.231185636	0.012660357	0.02772167
0.356954401	0.294148781	0.046928098	0.284657457
0.076891045	0.166970382	0.264472789	0.240152214
0.038007051	0.296153121	0.302476768	0.289380894
0.328584886	0.157832319	0.340410212	0.029466648
0.012719498	0.15172217	0.051755648	0.047870516
0.105316071	0.013145799	0.030972152	0.25226906
0.051383351	0.249302303	0.030498699	0.022968145
0.118279744	0.085961929	0.179454146	0.144608492
0.04043507	0.333906026	0.007518971	0.032214263
0.061638173	0.26961687	0.124337381	0.08699993
0.028860063	0.015604968	0.16326553	0.155642261
0.12831411	0.021752127	0.284189092	0.028254377
0.020828018	0.345545292	0.141982776	0.004284717
0.006802718	0.161089432	0.048001718	0.04612646
0.009777598	0.283737177	0.11843501	0.152577085
0.205784095	0.036720942	0.244716052	0.015010781
0.016238054	0.310565263	0.127343342	0.312139177
0.083406144	0.319287818	0.281817334	0.260281729
0.026216295	0.095446568	0.02888683	0.307198733
0.084530524			

DUSP6 0.353031988 0.516310726 0.334147132

0.400815231	0.388259825	0.367844218	0.219338694
0.160441617	0.095658244	0.011498244	0.041262071
0.382069335	0.243270331	0.289317609	0.324985349
0.371603186	0.139046462	0.265331035	0.227498511
0.364917158	0.10826197	0.374260492	0.094392177
0.242690921	0.112708572	0.080914567	0.26528852
0.393913737	0.087904881	0.349380827	0.353013234

0.078030386	0.379743289	0.518178592	0.417662794
0.514969627	0.338941021	0.007067196	0.330212518
0.360318048	0.409878371	0.369220787	0.322725713
0.052304157	0.489163367	0.256777891	0.340385623
0.206364512	0.041275832	0.233910549	0.436740135
0.50004144	0.428352106	0.207847707	0.179127791
0.270868616	0.229037659	0.294582977	0.303459109
0.233599166	0.168342301	0.496310959	0.110910835
0.149203853	0.212744141	0.126473785	0.244318553
0.306902213	0.348569484	0.014242304	0.345674906
0.006235902	0.339647832	0.313722877	0.304281493
0.041900987	0.035573346	0.149406264	0.342362973
0.400763376	0.421378514	0.439337884	0.453715846
0.188472212	0.241177743	0.341754418	0.183113636
0.384410197	0.208207473	0.098051743	0.380043557
0.452862596	0.522298468	0.190472198	0.227063981
0.425311864	0.459738539	0.39961338	0.411062331
0.359249971	0.038909874	0.447263884	0.486320199
0.354222845	0.282733672	0.148766683	0.376023034
0.054117318	0.315256431	0.339784516	0.133503704
0.409888226	0.023234281	0.19278041	0.361129607
0.159569256	0.392488872	0.512042195	0.190674028
0.343854318	0.022468905	0.151259092	0.025576171
0.310283691	0.125896977	0.496939599	0.428354316
0.216466741	0.363657033	0.245965727	0.001723101
0.026464631	0.133395867	0.395942997	0.289122666
0.374102915	0.33932434	0.265587573	0.356568851
0.410984058	0.4303928	0.074625764	0.091915149
0.391989327	0.134470786	0.224428531	0.414429476
0.304827412	0.10471702	0.127125872	0.488454259
0.414012075	0.089859073	0.268702173	0.37058291
0.373551747	0.326987574	0.099900417	0.287570136
0.060713318	0.482243786	0.024953066	0.271020051
0.2909256	0.147399802	0.006002808	0.242003926
0.307712026	1 0.471565008 0.093509154		
0.321538659	0.381305113	0.282000933	0.436958642
0.024892256	0.037529515	0.237375241	0.491665011
0.287046334	0.452079675	0.263617347	0.254921828
0.258305325	0.398275403	0.001775505	0.501823245
0.521515917	0.264933609	0.407691646	0.350999831
0.36324248	0.422212571	0.029989202	0.374592989
0.313710942	0.068411851	0.456510233	0.16548287
0.443133475	0.048679966	0.305803033	0.399404432
0.409674938	0.00834599	0.237968469	0.364035405
0.021563471	0.112393687	0.367649528	0.383599113
0.382989029	0.387668454	0.421046332	0.354333994
0.401751323	0.204158205	0.009384262	0.273824073
0.376870768	0.356364115	0.442976808	0.230395901
0.298605532	0.468193406	0.383169669	0.240488772
0.051597451	0.294320274	0.02566681	0.326306642
0.349435911	0.159758765	0.368453667	0.100880677
0.068132687	0.512092872	0.152765646	0.226553827
0.110407133	0.421608843	0.221457448	0.309689161
0.278159646	0.158835712	0.336242637	0.370107251

0.048263286	0.104397615	0.007353597	0.387199032
0.206351335	0.389819067	0.389969996	0.377565437
0.317397327	0.139979635	0.383071509	0.239035748
0.106178927	0.01503463	0.496174539	0.354085292
0.313940217	0.151348167	0.035381893	0.368383383
0.443403187	0.341610954	0.233621352	0.451531771
0.01993814	0.429223269	0.297609849	0.128484577
0.381808642	0.270172977	0.405508197	0.071049678
0.335143269	0.471946613	0.255411145	0.196858155
0.402178628	0.37965018	0.036883692	0.364360985
0.368257332	0.034840654	0.511549764	0.11679032
0.018783335	0.388762695	0.429166204	0.07171952
0.072374618	0.377655052	0.053601818	0.270647762
0.508468142	0.385461192	0.428525835	0.172774005
0.053094768	0.022122322	0.170796366	0.327364219
0.514528384	0.491758247	0.226499024	0.379719429
0.227873633	0.340129707	0.131409388	0.494030187
0.120309433	0.501271473	0.005925488	0.403738062
0.518449681	0.276297087	0.089565709	0.316823465
0.378629555	0.050214063	0.158167094	0.501481241
0.402404199	0.320983673	0.35771524	0.363095736
0.022065783	0.04829356	0.351228805	0.154459155
0.391693806	0.205140917	0.041553855	0.283164378
0.05986062	0.463370937	0.397111865	0.071778478
0.379862571	0.258776258	0.333384423	0.038801216
0.417056225	0.476407505	0.524831544	0.136290761
0.395136302	0.485866009	0.390766232	0.064777864
0.015629099	0.512746178	0.350515115	0.033028853
0.414695643	0.232581926	0.028322997	0.486837391
0.173447026	0.447216512	0.004611813	0.047536287
0.028029377	0.398148838	0.4917054	0.34417767
0.289203026	0.307355841	0.002222991	0.49167746
0.500432019	0.013924848	0.122088623	0.419048785
0.406745597	0.515202943	0.306813031	0.42416152
0.409075047	0.511871922	0.263170911	0.257315276
0.422355712	0.483894063	0.378265549	0.441742636
0.423449628	0.374766583	0.277516325	0.022354706
0.132130495	0.208824348	0.362456849	0.118296967
0.295555025	0.150049561	0.448661214	0.412181406
0.456018138	0.004422776	0.153370402	0.404754085
0.403739283	0.423675104	0.011115742	0.004433792
0.004807818	0.027190805	0.32851119	0.205394385
0.466568164	0.300801237	0.005310577	0.045937827
0.261270186	0.369829901	0.508581372	0.344714513
0.384215199	0.073152575	0.35696002	0.351349986
0.074756764	0.28704227	0.465604964	0.482692762
0.028889659	0.21260664	0.297725709	0.452598741
0.065921865	0.284047356	0.387067006	0.352909137
0.007128862	0.075903991	0.067777401	0.380711476
0.507978569	0.111141025	0.04937211	0.140631664
0.155727034	0.403351677	0.184947169	0.479020875
0.408394389	0.398888803	0.063454497	0.484813074
0.051211364	0.497220915	0.321607393	0.068006389
0.219543296	0.00951846	0.44721115	0.212330234

0.309791339	0.342907013	0.190862384	0.013257612
0.149326766	0.051642525	0.437615446	0.40957913
0.022385233	0.211089552	0.252953785	0.339458304
0.279023146	0.421716183	0.511908779	0.399434527
0.102276647	0.097033668	0.364058191	0.351128094
0.221478217	0.439197753	0.33710621	0.498094263
0.358250476	0.416537044	0.450991751	0.341589957
0.222234795	0.355683632	0.391104644	0.032928723
0.314737419	0.430703106	0.294198015	0.176172402
0.27509435	0.344949424	0.035053647	0.127568438
0.297457197	0.31289213	0.163162269	0.385043043
0.329801799	0.356198164	0.030417269	0.067217069
0.527560681	0.412832323	0.143154589	0.411641339
0.143126772	0.340518654	0.396431162	0.396058673
0.316880721	0.428994543	0.388647265	0.402495983
0.434103206	0.247293987	0.527963039	0.073682818
0.082976675	0.357983483	0.133851684	0.080110232
0.148537211	0.024263484	0.279873789	0.372710931
0.34329158	0.375034449	0.27511801	0.074560588
0.279786098	0.284605456	0.321097389	0.225351306
0.100538741	0.514914183	0.009592331	0.050156505
0.349770904	0.41245684	0.383787947	0.21461881
0.281272553	0.106364443	0.292922386	0.284428391
0.385264993	0.303218506	0.414442938	0.030745685
0.022383372	0.441356455	0.318418092	0.012575427
0.069391857	0.326789697	0.21897686	0.274252475
0.016995	0.47450093	0.365752071	0.379535711
0.320597888	0.055905141	0.332104269	0.068965561
0.043135645	0.438863584	0.340749511	0.510699744
0.275097652	0.399382824	0.443434542	0.407079282
0.081306706	0.311303507	0.031886745	0.391436306
0.398432931			
E2F2	0.466868652	0.488622668	0.40175131
0.385173047	0.389418715	0.410539491	0.159411725
0.173404073	0.052310786	0.000894978	0.010750976
0.437916905	0.15963395	0.236667326	0.347483183
0.368940081	0.085008493	0.350179937	0.174321558
0.378824008	0.067208067	0.336729458	0.053075727
0.248071689	0.063150022	0.035557029	0.213984914
0.415196426	0.042101156	0.36633705	0.387779559
0.061621422	0.463237346	0.495054119	0.403530952
0.496261544	0.473566653	0.007847473	0.284457338
0.463941279	0.432727764	0.379915403	0.26219798
0.019674933	0.482631455	0.185147124	0.362085778
0.235983658	0.013028349	0.266815653	0.476327973
0.48718658	0.450019338	0.20270158	0.112561002
0.181423583	0.251473058	0.261919764	0.376427118
0.165370476	0.170249072	0.495450131	0.057649421
0.134125132	0.222213165	0.102328214	0.222035758
0.334769121	0.440493195	0.008252855	0.460765423
0.000960713	0.346936333	0.326132842	0.316915583
0.018916409	0.007925392	0.121162003	0.292445272
0.437400443	0.489624302	0.450826215	0.469026805
0.125461361	0.227278019	0.387801255	0.112937919

0.474757165	0.129065021	0.076554665	0.366336301
0.458892261	0.496642636	0.138763514	0.194285159
0.4494301	0.481558166	0.426667336	0.395457608
0.333448966	0.02879206	0.487401625	0.481005591
0.465508687	0.295570113	0.095657308	0.421781315
0.029824492	0.386824916	0.288963087	0.128936638
0.438868814	0.007185365	0.148232795	0.323963803
0.164314663	0.463787683	0.495964411	0.130108219
0.375672366	0.008344317	0.107598703	0.01571036
0.291042555	0.124463918	0.488078056	0.443678128
0.215938346	0.341647359	0.213602485	0.003177232
0.011137954	0.142164767	0.484744618	0.352676808
0.431206704	0.481804686	0.173163156	0.467897724
0.465941576	0.451486087	0.07227707	0.058519119
0.402363021	0.138446593	0.168106901	0.448988687
0.239575045	0.07867658	0.078337883	0.473409302
0.463631831	0.046608608	0.371420022	0.36020086
0.311618956	0.297848248	0.064374454	0.22206083
0.026541082	0.495301813	0.012401175	0.336690059
0.270751072	0.105100475	0.005094129	0.249045944
0.260715406	0.471565008	1	0.062974663
0.264659935	0.35198667	0.359655022	0.441178418
0.0034976	0.024164966	0.352068561	0.480190497
0.354522029	0.478519419	0.238154571	0.246217718
0.209920805	0.42601093	0.00243037	0.489559597
0.496454317	0.374830187	0.353832598	0.39484858
0.383951365	0.432060943	0.053177008	0.36749668
0.445571418	0.043732036	0.454850467	0.101403911
0.494415078	0.018457404	0.313018358	0.465435206
0.481152159	0.004354502	0.23325914	0.364342388
0.012170338	0.07797066	0.43949759	0.370500024
0.345181571	0.433412641	0.432892189	0.364476296
0.386622345	0.185510211	0.004451731	0.25684496
0.462055199	0.423569404	0.473479749	0.190780293
0.428254595	0.481865006	0.491237489	0.226941113
0.020244885	0.290772674	0.011379844	0.273469467
0.485025344	0.07932718	0.420432818	0.10837898
0.037504314	0.483347785	0.138074787	0.250218566
0.104769166	0.399900994	0.194365224	0.207958573
0.176636922	0.106269126	0.323567046	0.350018494
0.020503092	0.052564923	0.006937522	0.413483263
0.108475382	0.338455116	0.355861017	0.472607241
0.307508455	0.072126242	0.329585126	0.255718665
0.0684415	0.004851196	0.488471361	0.358179617
0.42389962	0.103072009	0.019147472	0.379110076
0.455570054	0.385762817	0.345128176	0.4658868
0.014397284	0.461304002	0.195897453	0.072640075
0.349407954	0.193956632	0.426829019	0.05001976
0.421570247	0.480269386	0.25106446	0.125125089
0.463874842	0.391240076	0.01373091	0.474452593
0.473140157	0.013078881	0.492363446	0.079595337
0.007934809	0.423675567	0.4618645	0.039459537
0.041723602	0.296767196	0.030271814	0.216546978
0.482140338	0.352196708	0.465809155	0.172777345

0.018361524	0.003737583	0.152358279	0.412180719
0.491220587	0.490762909	0.192433684	0.319153228
0.173364377	0.302439462	0.158481503	0.481470931
0.174686828	0.49635392	0.000831853	0.456306182
0.494155253	0.33092114	0.044095797	0.287243129
0.456756342	0.022004103	0.158801875	0.491214008
0.469121672	0.286057444	0.324659837	0.416755107
0.006767034	0.022986751	0.356812887	0.154819867
0.370822137	0.152383068	0.04246622	0.335402512
0.019979321	0.477436138	0.433931289	0.031084481
0.381813576	0.331588026	0.319806824	0.016331242
0.47431996	0.496254601	0.493746532	0.121193224
0.47486958	0.487812332	0.433616643	0.043665241
0.006233938	0.496845093	0.438098934	0.010741325
0.443352285	0.291496199	0.004440281	0.482833495
0.128335799	0.475522977	0.001874936	0.092639922
0.020798945	0.435628034	0.487559862	0.382413158
0.299038995	0.327883097	0.003576681	0.490361616
0.486693081	0.004786441	0.094779359	0.484692523
0.462456946	0.490335568	0.295365245	0.468005593
0.43924843	0.495557144	0.196162463	0.19719043
0.456882002	0.494959799	0.434433397	0.478720657
0.445200889	0.386689685	0.214114255	0.005502192
0.105339558	0.210748166	0.342575834	0.054125913
0.385432466	0.089643121	0.486682434	0.444130699
0.477908254	0.000292805	0.120962209	0.487061237
0.446765576	0.4479257	0.011213047	0.00366167
0.006163688	0.008347971	0.343647651	0.178793748
0.484716596	0.281141228	0.000875062	0.027876302
0.229086205	0.368366579	0.493313456	0.321919292
0.461100431	0.044753294	0.278331176	0.354804806
0.037210029	0.437797881	0.472635309	0.480536014
0.021052795	0.185644208	0.391810374	0.453362867
0.065397831	0.247273503	0.447328974	0.272254537
0.007012905	0.044899912	0.034535344	0.410627257
0.494291344	0.045576383	0.014862992	0.173323303
0.07753447	0.464134882	0.110409317	0.469289762
0.444707244	0.395587326	0.043483182	0.486812283
0.019322501	0.492886891	0.293235536	0.055254824
0.239094446	0.004193916	0.496068126	0.13745012
0.336162523	0.420463221	0.170073001	0.006451489
0.171376181	0.025700973	0.44473834	0.445761539
0.016635906	0.189613748	0.232884247	0.379245054
0.286321714	0.403848561	0.491095023	0.463447676
0.052072681	0.133032748	0.393009009	0.453922853
0.225249608	0.452761369	0.258590859	0.489212117
0.455568724	0.480024027	0.490231587	0.30930653
0.190290418	0.43115401	0.383122878	0.008962218
0.300146267	0.43892943	0.355243188	0.106686317
0.190829514	0.404654954	0.028425513	0.216587741
0.347584556	0.340788541	0.094510314	0.399332444
0.416308509	0.459688511	0.026052018	0.031875189
0.492693373	0.479328859	0.114766644	0.424213441
0.113418765	0.286557038	0.457115242	0.445833596

0.390470187	0.476279102	0.440968423	0.412282585
0.464442859	0.200382619	0.494332995	0.086776354
0.05376549	0.471333739	0.058604831	0.052076667
0.089330614	0.011164063	0.313520766	0.385736363
0.40931864	0.324942742	0.366289385	0.047587433
0.26227786	0.419480256	0.292154939	0.161785652
0.043552383	0.49047512	0.003341024	0.028149335
0.405138832	0.466530402	0.363205075	0.173071317
0.27437427	0.096960161	0.229567267	0.326053976
0.348089066	0.381794523	0.470186783	0.019012039
0.008266865	0.452781241	0.410018925	0.009497053
0.060732678	0.451977595	0.265885203	0.188809765
0.009174447	0.493275768	0.370841013	0.406365996
0.318136754	0.035211261	0.449743717	0.066437467
0.035418205	0.483359429	0.393270486	0.498101938
0.323370861	0.370515551	0.475268459	0.481937949
0.095905877	0.440917279	0.011762521	0.446277546
0.444342328			

EBPL	0.114900594	0.122827008	0.081345235
0.208207864	0.136955254	0.080823863	0.19883972
0.111124981	0.118456243	0.05572427	0.063841107
0.108854717	0.0755128	0.090633377	0.038178452
0.252718175	0.174120741	0.088460986	0.290773325
0.217846582	0.137686263	0.253055524	0.242226813
0.085697628	0.128329088	0.195084044	0.077187125
0.218795969	0.20791872	0.057188034	0.171500242
0.247993034	0.142553578	0.091907616	0.144866779
0.082345264	0.084779263	0.098868653	0.051837495
0.128830281	0.139026344	0.214802663	0.248875601
0.206252224	0.081576353	0.235043071	0.155724744
0.062890227	0.154645866	0.04716835	0.085524541
0.071401336	0.078419318	0.111838894	0.155128211
0.035397337	0.052210085	0.028022274	0.09555336
0.233850392	0.050957315	0.08229807	0.117214753
0.059449169	0.139167811	0.197060536	0.162421649
0.05092715	0.109134088	0.245424358	0.071537386
0.022360022	0.245238153	0.1939228	0.028000085
0.259906118	0.124637848	0.199441132	0.204712164
0.198971191	0.097930683	0.172523256	0.144008657
0.140715821	0.218143504	0.084985548	0.199207261
0.125038027	0.162299005	0.140212531	0.250581649
0.163298439	0.101361566	0.152995478	0.229280025
0.109108069	0.122177737	0.069355319	0.09342231
0.032365738	0.171451687	0.084957049	0.13874071
0.100884494	0.065536861	0.230639074	0.165130434
0.288634677	0.044159576	0.274424626	0.118523558
0.200199196	0.149124325	0.206688809	0.157592176
0.193154317	0.131821195	0.098218623	0.263458596
0.124503625	0.160092319	0.037035712	0.280677316
0.076655354	0.043735937	0.065465486	0.16502171
0.102587714	0.067921351	0.175202104	0.130958884
0.217345909	0.162182115	0.105010126	0.128937918
0.170852764	0.06892428	0.102841383	0.087202525
0.082699523	0.170934239	0.005814323	0.251912446

0.113203643	0.179276625	0.33991982	0.099589266
0.152474132	0.220935573	0.258258405	0.153909488
0.100695678	0.268622558	0.063454914	0.250370593
0.052064839	0.155344298	0.280356581	0.127234686
0.20977364	0.07633197	0.197030921	0.10770397
0.023809945	0.116050449	0.202481046	0.125982387
0.069988639	0.093509154	0.062974663	1
0.147567769	0.199531594	0.0791281	0.096985137
0.055647183	0.275101968	0.046226383	0.135917784
0.06612573	0.144814412	0.156377241	0.124652434
0.253327532	0.195319909	0.01252 0.082854	0.099227845
0.038654654	0.140541646	0.153537227	0.047280023
0.176450347	0.00982802	0.243736596	0.077420033
0.298297522	0.18366642	0.239970387	0.092710396
0.207517778	0.078589248	0.103567672	0.075376586
0.065353929	0.075269306	0.199662446	0.189383139
0.10607397	0.156375259	0.19943783	0.137587838
0.176779518	0.213333731	0.039429341	0.121015232
0.186724124	0.215627169	0.180408864	0.135944765
0.028763446	0.095877614	0.151396419	0.055011282
0.112825738	0.080517715	0.023111128	0.219696521
0.140792267	0.086418122	0.14080982	0.078829461
0.138466825	0.053145667	0.207658802	0.217815674
0.134765541	0.076243678	0.0420935	0.044653032
0.093294662	0.247120444	0.05875964	0.053228387
0.194955471	0.054374678	0.063257798	0.161555543
0.223878933	0.138207215	0.024687055	0.058666652
0.035398639	0.030740866	0.089431117	0.076483124
0.127903312	0.118760182	0.073370576	0.175926608
0.220986031	0.122030756	0.239975712	0.074377203
0.209280486	0.283594587	0.020349593	0.154012932
0.040169843	0.031158539	0.125510526	0.200973274
0.169822568	0.094828551	0.224591684	0.059438507
0.236255306	0.208479218	0.034167968	0.10795531
0.105731791	0.232805637	0.146674541	0.078797309
0.178096578	0.199546614	0.092007664	0.086075779
0.242251687	0.118513422	0.185610016	0.240966943
0.150425406	0.092800407	0.283497646	0.220192174
0.098771924	0.136509488	0.282863603	0.132604336
0.249169314	0.098375258	0.10251429	0.203604135
0.064821088	0.150887401	0.088960922	0.083809093
0.113909224	0.302966963	0.092948679	0.328261604
0.049848768	0.053714839	0.131233307	0.107346688
0.103256026	0.197653445	0.115997529	0.07725078
0.064171514	0.158588548	0.134020535	0.092572187
0.229869497	0.211042103	0.095408927	0.12534328
0.083208703	0.050865618	0.166764772	0.148381574
0.20401318	0.132684808	0.058578465	0.052342975
0.171079563	0.024038009	0.070088001	0.177642418
0.099612447	0.198091428	0.142622734	0.05377443
0.06179456	0.230864096	0.257086556	0.102291616
0.099305632	0.094903267	0.229641377	0.117275452
0.102282121	0.056961985	0.261149613	0.19694122
0.090041313	0.121588751	0.090312339	0.193538248

0.046217466	0.079961497	0.123990539	0.288551177
0.063861378	0.019907182	0.032635428	0.190793736
0.184732687	0.081267205	0.177661851	0.020542995
0.232247537	0.043662818	0.127095462	0.100510359
0.150931655	0.140156862	0.091607492	0.081532265
0.120803574	0.056044703	0.109296183	0.076734963
0.081353729	0.104978323	0.265161379	0.129205103
0.085585644	0.154504939	0.111261745	0.176859355
0.19405722	0.282650619	0.157513049	0.059597897
0.090866657	0.018343261	0.068590445	0.082656168
0.149325144	0.120192902	0.189572616	0.123370076
0.168146195	0.145052541	0.097321547	0.158083577
0.187712512	0.173998453	0.020529174	0.013695852
0.060595608	0.170887516	0.207374712	0.124002961
0.01816281	0.162339425	0.173197119	0.123119108
0.126904752	0.102090317	0.293973773	0.132833225
0.287410264	0.033826931	0.266556051	0.131093319
0.062278131	0.133883273	0.10075614	0.264419718
0.180642705	0.090972988	0.179245387	0.01437991
0.258783171	0.115847833	0.07547872	0.144769667
0.201531558	0.170961098	0.214233926	0.074518943
0.114000434	0.128623329	0.023869721	0.149891708
0.162054229	0.170937679	0.164409742	0.161194372
0.245198725	0.182086635	0.096983424	0.12662834
0.082886413	0.103044738	0.022316956	0.143824133
0.250818355	0.091405634	0.022763948	0.065828272
0.145745816	0.053160102	0.184236481	0.037948391
0.244767867	0.178705094	0.16213239	0.200991994
0.165325234	0.22832304	0.202330362	0.119009924
0.183009321	0.0880809	0.040289917	0.178643563
0.062627215	0.203093832	0.094722774	0.100583256
0.179739807	0.061707731	0.130411933	0.117857979
0.131360925	0.108023185	0.167560466	0.081669794
0.09379731	0.118630278	0.098894961	0.221051555
0.174483646	0.128970574	0.096685414	0.164558171
0.06694242	0.252556097	0.042165768	0.045532556
0.089576034	0.18792435	0.247598166	0.132476547
0.096451976	0.23719967	0.161700913	0.123442437
0.105716305	0.115541775	0.135959414	0.266495961
0.170805941	0.143480995	0.146163384	0.047421407
0.112182751	0.156962847	0.105376923	0.096871401
0.241825916	0.097693378	0.083692104	0.088772239
0.084592543	0.140037114	0.26326388	0.263799563
0.206507024	0.024831845	0.227069871	0.026592482
0.223605681	0.045230135	0.2018646	0.205895024
0.065684207	0.236702946	0.129302081	0.169516088
0.088434688	0.10614293	0.280343104	0.039044005
0.11348491	0.070791502	0.174202248	0.044660172
0.172657258	0.244055594	0.072610952	0.030735527
0.042327841	0.107700467	0.162361669	0.209338568
0.186947202	0.071844	0.192318263	0.133201688
0.08502917	0.126711161	0.086605296	0.177357312
0.093479651	0.039492189	0.11725264	0.173807677
0.31096217	0.075567539	0.107226826	0.26075824

0.091737364	0.094421846	0.092095975	0.113100026
0.240271888	0.072829865	0.107476652	0.131340643
0.05944487	0.203416154	0.177455021	0.026474458
ECE1	0.247599679	0.382099831	0.128147904
0.377131744	0.229647466	0.191829794	0.180633097
0.119930073	0.067048057	0.044859289	0.054762607
0.227323853	0.155506951	0.198715478	0.082582029
0.362564095	0.162191915	0.111136804	0.231064586
0.335088805	0.134378768	0.368493707	0.141246814
0.10072149	0.098828665	0.118178658	0.101977507
0.406589533	0.120981848	0.15448614	0.316857675
0.112769717	0.322586906	0.329454278	0.314873544
0.319518155	0.203609744	0.042385176	0.120297158
0.297654307	0.288166645	0.342939135	0.297842117
0.11091892	0.312103731	0.232954196	0.214567256
0.090526526	0.089907439	0.092526142	0.29098692
0.304925605	0.209211911	0.1283049	0.155350747
0.124037692	0.126323993	0.086148876	0.221390365
0.215257815	0.09758909	0.316935208	0.088411283
0.085851278	0.148855646	0.133097215	0.174685087
0.09517826	0.255586243	0.063224267	0.169023735
0.027811733	0.318930321	0.226430882	0.064784905
0.103024279	0.122791229	0.144743434	0.297025828
0.402620533	0.289556863	0.401553919	0.370232426
0.188991438	0.169345516	0.149471507	0.199613173
0.304633304	0.174586421	0.096328273	0.378417763
0.396875386	0.355897441	0.134668531	0.169996887
0.316235232	0.356500212	0.147176223	0.179649054
0.083990688	0.053739629	0.301581968	0.39290813
0.279844253	0.099462839	0.144696857	0.355981458
0.115955694	0.101351481	0.343555167	0.161518929
0.410904909	0.049915112	0.146763809	0.265847101
0.127737378	0.303555434	0.339731086	0.193835237
0.184483104	0.095679844	0.048076723	0.075653314
0.146601588	0.051412908	0.282027404	0.409439171
0.167963387	0.146601213	0.167175143	0.020753665
0.079128756	0.102019768	0.3091625	0.158804896
0.306580264	0.208074179	0.143830244	0.245466488
0.22169932	0.394812346	0.018125533	0.134543482
0.206101318	0.109347886	0.25618758	0.238487024
0.174294589	0.139727946	0.18751511	0.397745734
0.30779282	0.159876463	0.107223851	0.358273635
0.139235324	0.207053052	0.165474728	0.228728218
0.13893114	0.289015322	0.065071755	0.139186966
0.056724518	0.139068287	0.032329384	0.15513572
0.143689554	0.321538659	0.264659935	0.147567769
1	0.300369876	0.146075536	0.294789218
0.044932449	0.092725306	0.091351277	0.402612731
0.184972664	0.36754605	0.175944541	0.128119528
0.215981654	0.360809846	0.001937693	0.313243195
0.344005806	0.089069117	0.289427656	0.235016654
0.098044976	0.390971797	0.013532001	0.375015894
0.206464816	0.125162606	0.417536795	0.167880683
0.306159676	0.103082801	0.15704764	0.273165889

0.257068787	0.023415222	0.119170801	0.321005831
0.051473653	0.107135825	0.288034248	0.317528053
0.223785052	0.322955819	0.428188672	0.083670352
0.217123741	0.170749039	0.041455732	0.173398537
0.286955958	0.091930565	0.312473442	0.161204206
0.148164311	0.344408516	0.246425566	0.063438166
0.109050527	0.186274501	0.061894545	0.164323852
0.221549291	0.197499608	0.11215838	0.115484433
0.111678481	0.395225417	0.072599196	0.066850873
0.043800673	0.265974548	0.186277692	0.186747262
0.166424809	0.140879597	0.101645209	0.120427309
0.10666839	0.175549795	0.020250329	0.120661159
0.191399356	0.157962605	0.147343877	0.25553218
0.188663627	0.220616706	0.201412197	0.084400149
0.096788045	0.064635647	0.369765453	0.320090204
0.218518073	0.146638755	0.096169273	0.110682692
0.406540686	0.131789075	0.069752818	0.374784057
0.051268213	0.383435667	0.20083579	0.147028356
0.148971549	0.254621913	0.402670498	0.032458182
0.200691123	0.354646597	0.188525197	0.13805981
0.298349934	0.295016124	0.098547926	0.249310187
0.262765901	0.1096889	0.375619634	0.134101342
0.068016169	0.286588793	0.269049122	0.112774992
0.098272399	0.194082058	0.089045235	0.254251286
0.391352575	0.385635303	0.300932097	0.084839043
0.124933221	0.092942539	0.117524069	0.148172846
0.326001648	0.357922407	0.225295787	0.181134205
0.253899671	0.100163754	0.078232271	0.387675378
0.070067481	0.341127903	0.040077729	0.280597206
0.310774519	0.140346512	0.104808387	0.20187798
0.296293329	0.098082388	0.149235569	0.341013998
0.315395716	0.119301496	0.102870725	0.284595596
0.067643641	0.112960246	0.18464527	0.058640235
0.128408897	0.139998958	0.018829494	0.119248243
0.13710299	0.342509642	0.38713547	0.071091145
0.176516504	0.098988517	0.284707365	0.101096482
0.294961352	0.319862107	0.345500477	0.157229685
0.302653948	0.337771671	0.165639769	0.102946697
0.052289579	0.326008439	0.242725593	0.086022113
0.417042104	0.103388686	0.098874504	0.371295474
0.179220784	0.266520081	0.006140089	0.019180804
0.050194805	0.364639071	0.302349649	0.295887044
0.082982981	0.260545165	0.0069506	0.368520027
0.356492064	0.042434957	0.094171619	0.293604274
0.247407151	0.376927972	0.089256243	0.316450354
0.213894441	0.319630433	0.18720787	0.22544523
0.346206529	0.305715216	0.307518811	0.322450534
0.416405647	0.307837502	0.273062654	0.065923239
0.039486372	0.122877386	0.074922099	0.073524086
0.175074457	0.115995562	0.341157858	0.398269767
0.360050578	0.031889023	0.147300021	0.288888719
0.379108735	0.403554328	0.027798016	0.004196364
0.00389753	0.035152941	0.248503778	0.136344189
0.349736638	0.093302346	0.040856337	0.100418542

0.151342966	0.2226779	0.35632111	0.366069639
0.287002189	0.134621969	0.125290681	0.356379484
0.074435685	0.166955978	0.377345308	0.341909997
0.069192874	0.127099425	0.140045136	0.418601229
0.018500359	0.251147222	0.257677847	0.162803346
0.02604545	0.093779943	0.076144352	0.356470356
0.304642884	0.165142523	0.118523977	0.065319194
0.181647853	0.367117789	0.204315586	0.411961819
0.36858874	0.428242295	0.085135552	0.320477601
0.078288287	0.324970888	0.150133114	0.032675867
0.137079654	0.050187059	0.309193371	0.051906947
0.148318876	0.264572892	0.051767171	0.043734081
0.040822843	0.110747674	0.409627124	0.35231623
0.0488266	0.148452328	0.18264813	0.276377899
0.181883581	0.376864137	0.329486881	0.133674045
0.13846428	0.054071613	0.337283038	0.232299813
0.161502385	0.405999648	0.125870702	0.372363713
0.251268444	0.326723685	0.333702316	0.217448314
0.11905856	0.193248851	0.290906157	0.088095739
0.240437854	0.410233108	0.163253283	0.105115542
0.182337371	0.137482712	0.074590079	0.05841311
0.092217375	0.195871287	0.206349637	0.402345202
0.236864798	0.240653342	0.094099133	0.074491002
0.384859817	0.2942817	0.097650599	0.291757819
0.146839368	0.211820089	0.310872204	0.313614441
0.0910378	0.351418862	0.346017476	0.304965829
0.327711797	0.199686259	0.34686414	0.073755327
0.051657948	0.204778541	0.172045318	0.111200028
0.190012439	0.056298483	0.063477902	0.349671698
0.085030225	0.349970622	0.110301385	0.097919744
0.198401392	0.106299408	0.260935927	0.173631759
0.152629042	0.335036687	0.033495994	0.115634787
0.107887036	0.285781913	0.155498661	0.149651607
0.084174487	0.09595206	0.25537454	0.14328697
0.128375806	0.095750106	0.286847171	0.059258299
0.093333863	0.419152888	0.133687247	0.036456755
0.06477585	0.180566964	0.139043514	0.129014945
0.043893584	0.324162302	0.10800625	0.197917503
0.228071118	0.123040274	0.239597188	0.062791784
0.078184025	0.310753354	0.155731783	0.334082239
0.1224665	0.406396647	0.271988339	0.320440884
0.068863701	0.116162675	0.080884862	0.3635793
0.086667972			

ECE2 0.3218867 0.469096158 0.281763507

0.394225675	0.343956383	0.213779272	0.229748943
0.147601475	0.149523404	0.018794565	0.0789653
0.29492666	0.228126359	0.223134056	0.215126721
0.423966161	0.236997618	0.250273708	0.284837217
0.346421668	0.113450283	0.396109783	0.168762528
0.308505351	0.208375512	0.170800069	0.304507493
0.436975767	0.184201609	0.186399956	0.296056591
0.201586527	0.367832455	0.428758331	0.365017978
0.418757447	0.278705272	0.025494733	0.215433896
0.353550576	0.388419514	0.349793479	0.318788937

0.109864092	0.377285271	0.270429982	0.332328315
0.160461336	0.107649131	0.135693205	0.347291746
0.38111406	0.277079678	0.18544231	0.202799603
0.191886248	0.141054672	0.238050795	0.193304027
0.267643849	0.097972314	0.404823927	0.137722842
0.139337462	0.214060307	0.250688347	0.230500169
0.300122732	0.279150727	0.049889309	0.246125405
0.007394933	0.328003043	0.339868769	0.249278504
0.131360718	0.071514759	0.27407396	0.287962798
0.452969557	0.382214289	0.497635172	0.484975533
0.248570419	0.331553008	0.304286433	0.261026086
0.37853237	0.215748812	0.143988634	0.441412591
0.499168459	0.453606152	0.288851987	0.329451905
0.363862909	0.419453102	0.275140611	0.325329767
0.261972199	0.139203445	0.367097387	0.487912827
0.312615625	0.28488809	0.266018064	0.346999831
0.151600935	0.217623164	0.329293392	0.181417811
0.465164348	0.059169529	0.306215423	0.283401947
0.255147472	0.366073571	0.453381094	0.247110456
0.32713339	0.067462609	0.178461057	0.106879694
0.260675324	0.178106418	0.363886472	0.444270985
0.184681815	0.327851584	0.260953547	0.021822719
0.103496615	0.24640905	0.363967762	0.275141833
0.364624197	0.303419373	0.286342145	0.274077432
0.310779141	0.491962266	0.028469472	0.164386747
0.294403047	0.251711846	0.309607241	0.293445817
0.338324659	0.156312383	0.21740044	0.501020815
0.343645574	0.173328275	0.203364495	0.406516952
0.261538794	0.346700765	0.190117171	0.279103108
0.108044565	0.397857284	0.104963777	0.220265247
0.2140502	0.22349922	0.034067185	0.192103885
0.224929528	0.381305113	0.35198667	0.199531594
0.300369876	1 0.184860513	0.323770051	
0.042960736	0.113637265	0.140378219	0.477864283
0.149864467	0.478208713	0.304325703	0.329966277
0.341149005	0.437495305	0.00638846	0.3963039
0.45534426	0.144323335	0.32068766	0.32096346
0.27645654	0.438708471	0.025716884	0.392104696
0.219223431	0.166629936	0.513279905	0.268509318
0.384070432	0.129698341	0.225468456	0.400201017
0.328864808	0.023655874	0.145877705	0.315496259
0.100303954	0.103954041	0.344661223	0.340636642
0.307317836	0.419263736	0.504932657	0.266805019
0.337553337	0.238562943	0.051958129	0.33927108
0.379105933	0.244316674	0.365213945	0.245938572
0.163270446	0.417263127	0.334608472	0.150630189
0.116171588	0.244894694	0.039388288	0.329973296
0.317285533	0.17414296	0.261329902	0.208342972
0.176839061	0.493651652	0.236015685	0.2415466
0.170073906	0.268318927	0.272148847	0.206930907
0.222222071	0.262083665	0.306266232	0.306228374
0.074876228	0.195851846	0.051627698	0.233906525
0.164155172	0.238276647	0.214648273	0.278537765
0.248399606	0.165299712	0.336446282	0.28551911

0.221247313	0.07137876	0.45742147	0.35899022
0.216567265	0.225271411	0.124213617	0.199404599
0.450757192	0.158296441	0.130196005	0.412994882
0.066479876	0.485765805	0.270774517	0.236742423
0.309882114	0.302580773	0.46783034	0.065670176
0.242104158	0.400899261	0.326906524	0.2824391
0.31286285	0.313719265	0.082469464	0.300193515
0.2862893	0.108780137	0.476366538	0.230962678
0.087270674	0.377041374	0.355628161	0.183358764
0.18581796	0.280788493	0.06953797	0.345001984
0.482640942	0.421709777	0.351940635	0.271488526
0.113959104	0.038188516	0.290155317	0.276608643
0.406735394	0.468804227	0.317293364	0.326133916
0.295725468	0.29602647	0.090160847	0.482037791
0.199959266	0.438193708	0.039332574	0.39558428
0.410065302	0.159765127	0.187904144	0.236647998
0.307182547	0.120729796	0.231156951	0.420701332
0.392915781	0.31244125	0.285524825	0.331355808
0.045632964	0.089601815	0.32263116	0.222359626
0.292189811	0.294587525	0.081339642	0.217242412
0.119497439	0.388375433	0.450348995	0.150032755
0.190996328	0.193456354	0.35587349	0.113086906
0.422548594	0.434177073	0.434325257	0.202588836
0.365204551	0.444842573	0.267977248	0.182507294
0.079916833	0.435433142	0.277619661	0.061636394
0.478934771	0.115577556	0.047627747	0.440034939
0.249203375	0.327566093	0.006632933	0.076584689
0.117727465	0.466057026	0.390783388	0.287198463
0.126337627	0.306744107	0.018820147	0.482941106
0.4235487	0.070003253	0.241892364	0.363218131
0.347654876	0.459864409	0.287845091	0.424769521
0.283606968	0.41582262	0.226894753	0.33512318
0.394607801	0.42578047	0.344892753	0.39284958
0.451250188	0.382138496	0.306608909	0.058690154
0.186903662	0.154951653	0.187705855	0.150341802
0.170276049	0.245813075	0.428322913	0.476325208
0.458369353	0.035139587	0.160674788	0.352260807
0.40249302	0.465104763	0.056953343	0.013022966
0.012690964	0.073032893	0.26713304	0.315915392
0.45504466	0.127425054	0.031560815	0.071259408
0.269314566	0.328454066	0.439737779	0.373043477
0.391426462	0.181986134	0.198291455	0.375825587
0.16846484	0.189502387	0.470841362	0.400000414
0.117160295	0.317127791	0.244040524	0.48339538
0.092537187	0.350360422	0.310081699	0.287269502
0.044806038	0.19354178	0.167756864	0.434494404
0.403238533	0.154226932	0.104349215	0.078028724
0.183942945	0.450397217	0.240708863	0.509517715
0.403110553	0.461843104	0.17561776	0.408752857
0.11055999	0.399732024	0.326924713	0.047394389
0.294837185	0.056023102	0.402363446	0.168828882
0.20826013	0.283821381	0.222399085	0.074716192
0.18984849	0.148921092	0.488551547	0.464606189
0.104814467	0.251803858	0.332358416	0.317226541

0.193017973	0.404793296	0.411368636	0.217546532
0.143008755	0.160810527	0.342584861	0.254287738
0.186477048	0.504272873	0.295384861	0.484967649
0.300332518	0.413357929	0.424954383	0.294431041
0.283924671	0.23321901	0.268570839	0.048737924
0.317711426	0.459542662	0.253418276	0.238452868
0.321291445	0.277542504	0.096819628	0.107428345
0.248990985	0.176487675	0.220065484	0.461132865
0.248608456	0.267708577	0.073364276	0.160310407
0.481461413	0.370311033	0.146747694	0.326784921
0.269269194	0.339551382	0.406513155	0.414568658
0.259045064	0.401568431	0.363588987	0.276315008
0.352899009	0.288039939	0.443262921	0.065499129
0.15573728	0.254263601	0.177313678	0.20400138
0.238320361	0.100457364	0.232235896	0.401425616
0.250263844	0.340628878	0.185722173	0.166417157
0.347552195	0.219534699	0.349342739	0.200417752
0.159628052	0.416789891	0.041457332	0.151870229
0.27039984	0.379424792	0.308773032	0.314392756
0.27817238	0.231964631	0.338240972	0.16967255
0.189047929	0.243765201	0.375544909	0.066156188
0.057004701	0.503396682	0.219098868	0.073561445
0.153350388	0.221895466	0.260736082	0.26747673
0.087179962	0.430306972	0.19437972	0.323375521
0.269885033	0.15360215	0.263689198	0.108721504
0.118373968	0.364241514	0.296111512	0.437591242
0.305342778	0.430876788	0.335779336	0.365630242
0.147957144	0.231962194	0.07873013	0.416315569
0.241941627			

EFHA1 0.294781332 0.398889349 0.124096495

0.297291064	0.15925802	0.231297299	0.135156309
0.204654186	0.040758264	0.005722027	0.005909896
0.189831833	0.061662162	0.152527281	0.124182841
0.279753585	0.043833353	0.194703205	0.133524547
0.301710997	0.110512593	0.219365385	0.074448588
0.080683163	0.026368655	0.031946785	0.062407526
0.36956084	0.037603529	0.1720823	0.349742525
0.052347444	0.350179417	0.390364211	0.191969
0.365880362	0.270680784	0.026781223	0.132073748
0.380754532	0.239614053	0.312972077	0.170333049
0.035245721	0.411405685	0.124552845	0.160190417
0.159671748	0.012878112	0.195630741	0.409315363
0.343995087	0.171741544	0.195806389	0.105447213
0.049504993	0.210671179	0.045229998	0.330110167
0.126393884	0.182745782	0.346055516	0.05655327
0.100427223	0.221464457	0.058306124	0.192970198
0.083931405	0.354589503	0.041545378	0.170298069
0.006343908	0.291805729	0.160256898	0.058832752
0.024438856	0.01178872	0.069039875	0.20617289
0.375031096	0.37029528	0.317123143	0.31391836
0.060809207	0.124082531	0.108863871	0.080189744
0.352968603	0.099934947	0.071399609	0.256009088
0.322416942	0.369216671	0.068873498	0.088946214
0.288617375	0.4059945	0.13448759	0.132088991

0.074630753	0.019409534	0.347031886	0.351344576
0.376576543	0.067664506	0.053641664	0.380165628
0.042179737	0.165233705	0.209067388	0.081740764
0.380718518	0.019826487	0.065390163	0.192840938
0.140645746	0.320250376	0.345848351	0.115895237
0.140225609	0.014534571	0.02208533	0.026448771
0.137988786	0.027075625	0.372715691	0.340731429
0.205461934	0.117638886	0.175801316	0.002612041
0.01297612	0.08965192	0.300324418	0.19636494
0.315724025	0.276975384	0.069848133	0.329898568
0.18122862	0.312335355	0.037852801	0.095308772
0.178055134	0.092452317	0.119534074	0.195465216
0.086655607	0.130146532	0.085110254	0.368536001
0.421432088	0.06222317	0.216581598	0.253782058
0.114433829	0.105050727	0.088349782	0.131980994
0.057691116	0.328643822	0.011410848	0.221170024
0.054027299	0.040440057	0.027794933	0.214670986
0.127982702	0.282000933	0.359655022	0.0791281
0.146075536	0.184860513	1	0.231657021
0.004460256	0.046542004	0.212023941	0.361351788
0.286858387	0.350254249	0.153080273	0.110574516
0.101135685	0.319186588	0.00263982	0.388377929
0.362022064	0.189404084	0.156006559	0.195563333
0.085750383	0.326903018	0.031113233	0.262676475
0.311979772	0.067295697	0.353708461	0.057994859
0.363681079	0.021872473	0.208801922	0.236794509
0.21612885	0.008784711	0.177048557	0.299150663
0.011421555	0.123137509	0.31810613	0.252835663
0.160394254	0.287902339	0.348684525	0.075403597
0.147451066	0.18267637	0.013272689	0.098850417
0.294374563	0.108581852	0.391852521	0.155236083
0.241334779	0.373586456	0.267299421	0.076984302
0.03641724	0.203682882	0.025902796	0.103904023
0.292157847	0.06902979	0.108284598	0.092665905
0.027430583	0.369292093	0.038462105	0.052148759
0.022664255	0.178347465	0.16810169	0.086833949
0.049021049	0.076781433	0.09085981	0.118679526
0.057288165	0.046257269	0.00933215	0.093689981
0.039808496	0.096257104	0.103825585	0.297154828
0.170277246	0.051308721	0.112384956	0.081479685
0.037878732	0.010982528	0.383278887	0.260327891
0.342757234	0.102788638	0.028267387	0.096115658
0.343044538	0.167923953	0.19351415	0.365808687
0.030823237	0.340105443	0.083320475	0.045036508
0.09145473	0.114065635	0.357827178	0.032148636
0.255179917	0.392546158	0.137721832	0.059110896
0.358936638	0.264812645	0.033579031	0.314957186
0.313605214	0.020928516	0.366512054	0.041174156
0.013834004	0.220603547	0.216772682	0.037254057
0.033359076	0.113296252	0.083052234	0.118655293
0.371890485	0.238017214	0.270305122	0.062134968
0.029163042	0.004756998	0.066914281	0.142072703
0.397225691	0.335030716	0.126353943	0.087665153
0.135087704	0.080745195	0.202493957	0.345130513

0.101497107	0.396144548	0.008362136	0.230855878
0.361438581	0.208569877	0.024587572	0.181679096
0.351553502	0.039830872	0.174153628	0.37413169
0.345507744	0.107276014	0.095803103	0.255121973
0.027785635	0.063308996	0.143275633	0.039011619
0.12705171	0.095140906	0.008026284	0.213793516
0.025960727	0.370864951	0.325413059	0.023560852
0.16929792	0.21023221	0.174885059	0.031464778
0.257471182	0.347912201	0.39454079	0.164550272
0.284049165	0.312962071	0.11653139	0.042408274
0.008584806	0.358482289	0.315623587	0.014612426
0.351741454	0.191194059	0.006859581	0.408578613
0.126210982	0.380910299	0.007049492	0.034214996
0.018400626	0.295641924	0.364483738	0.333877742
0.131356227	0.244131076	0.004983105	0.36131414
0.371858878	0.005668478	0.040866067	0.259868594
0.196372518	0.425530111	0.065665185	0.255885721
0.149922316	0.346868227	0.12690954	0.102397066
0.322579348	0.312790345	0.336774936	0.398625675
0.341173493	0.220781996	0.152839407	0.016120832
0.032130359	0.205970787	0.07862462	0.024403768
0.28886764	0.043232474	0.370904421	0.331848164
0.305895261	0.004815986	0.155332786	0.294109229
0.399052651	0.392198565	0.022189031	0.003920227
0.003739985	0.003142743	0.253842964	0.089919111
0.34397286	0.104774129	0.006087291	0.08629137
0.142630983	0.137684151	0.368463439	0.262910219
0.270732306	0.053405434	0.090343496	0.299629441
0.018879273	0.29631994	0.317192114	0.39813438
0.029287658	0.093557123	0.192514222	0.37578584
0.008981564	0.131686443	0.215146153	0.107766682
0.014122988	0.029964081	0.0254278	0.299399918
0.3470976	0.024912627	0.01185962	0.132817914
0.064222027	0.345631984	0.073935603	0.363093728
0.354079766	0.324124102	0.026072847	0.380495816
0.018994864	0.364027657	0.078624525	0.035339296
0.140151531	0.015199331	0.330940807	0.024843094
0.206918146	0.328669728	0.049430549	0.008036595
0.036354596	0.025819206	0.327881382	0.281248306
0.016090638	0.172672042	0.121713963	0.262005132
0.210765324	0.268680481	0.392235741	0.136211649
0.077093821	0.042407456	0.334004562	0.295264516
0.216407961	0.331852852	0.076378912	0.372563794
0.321526998	0.383901053	0.329171834	0.17301282
0.057259996	0.243377192	0.261453638	0.021199547
0.184110467	0.308444585	0.20963037	0.036044307
0.078627794	0.107749003	0.072371659	0.13659238
0.066911407	0.250722256	0.083567353	0.332518119
0.30076883	0.308439037	0.08886847	0.023121178
0.407265364	0.383977308	0.121197206	0.258746334
0.087455336	0.12316207	0.277021714	0.253551118
0.133594336	0.302430232	0.358089396	0.272080816
0.392352252	0.146171543	0.390013589	0.168523204
0.023850391	0.21914412	0.040738684	0.040868177

0.07822794	0.01427837	0.055862665	0.267681957
0.083913671	0.195661049	0.205353195	0.048710882
0.114739448	0.173259376	0.165397002	0.135037856
0.039460578	0.369522804	0.005808859	0.033731538
0.094623177	0.270772974	0.086216414	0.09339063
0.05982165	0.054740579	0.119989265	0.205809054
0.107892319	0.129914534	0.27685262	0.047743
0.034962022	0.352069581	0.185563818	0.013645168
0.046033153	0.26528414	0.181233849	0.08280175
0.00895275	0.305180471	0.137921225	0.154909618
0.218832771	0.055316363	0.385479469	0.078547367
0.070855354	0.394778123	0.130054469	0.353816349
0.121894343	0.268487493	0.401494253	0.31268789
0.081607037	0.136976018	0.03249331	0.366847591
0.093177644			
EGR2 0.289506414	0.514017851	0.268088736	
0.358158409	0.319079483	0.302153384	0.2114888
0.150753774	0.099075519	0.019548652	0.04587678
0.313038799	0.237094388	0.277017763	0.26886785
0.317404238	0.141700091	0.217529534	0.219006904
0.306105991	0.116464573	0.323196272	0.10377932
0.196328237	0.114342418	0.087004614	0.227402168
0.393003137	0.094301773	0.289459198	0.296398542
0.075980861	0.344389982	0.503049451	0.349613772
0.496012373	0.271863396	0.007660685	0.284410235
0.355158658	0.346910766	0.309626077	0.296978814
0.062894852	0.475081986	0.244553156	0.281577763
0.178009887	0.051604451	0.2033433	0.423359943
0.486841105	0.341474596	0.191177971	0.17817287
0.245030543	0.194795155	0.242297147	0.249178585
0.223044472	0.154533692	0.481328695	0.114273322
0.123067023	0.189756426	0.117040799	0.224892975
0.235896737	0.303549848	0.018124818	0.264485733
0.009729698	0.29183378	0.264385694	0.232911879
0.049013186	0.050967535	0.138897483	0.303481249
0.410181691	0.408179265	0.453347097	0.45720764
0.193515067	0.210575916	0.267988709	0.184061591
0.37844729	0.208823616	0.109298406	0.326397335
0.464197997	0.512046952	0.18035533	0.200874517
0.407297255	0.458898722	0.33182673	0.341689527
0.295255172	0.037513969	0.436698563	0.490271154
0.347828329	0.227162349	0.14735719	0.346713222
0.058876874	0.267734985	0.304096322	0.127321683
0.429309112	0.029397622	0.176232002	0.313374217
0.144984679	0.357735522	0.500796736	0.188852083
0.285802042	0.032485426	0.133870456	0.028013505
0.283915977	0.104908101	0.471388131	0.448591444
0.19737487	0.295459066	0.225045171	0.003099334
0.032095043	0.115144627	0.366596032	0.24056237
0.307630973	0.321005403	0.246036301	0.303784131
0.324628749	0.441054964	0.073595537	0.096254745
0.337377069	0.115533388	0.211793744	0.335295253
0.277070746	0.110430007	0.132235395	0.496354916
0.410870478	0.099466526	0.216566477	0.318837177

0.312691513	0.282055452	0.10541459	0.273725506
0.069771491	0.463080725	0.028469634	0.230780479
0.238536154	0.1396269	0.007137931	0.215806338
0.291368389	0.436958642	0.441178418	0.096985137
0.294789218	0.323770051	0.231657021	1
0.031489512	0.044490069	0.188273815	0.498694429
0.23419412	0.453362265	0.242281216	0.218419616
0.234773786	0.376371402	0.001911854	0.48861504
0.509689758	0.215875339	0.35983391	0.288242812
0.281453766	0.425438126	0.027529848	0.319985973
0.246529023	0.072950072	0.469451675	0.165357449
0.432578311	0.056342362	0.25972285	0.371776164
0.344855153	0.010601578	0.213117279	0.309430428
0.022884591	0.112240386	0.297223826	0.327798681
0.325861088	0.342806168	0.440216883	0.276916313
0.341151534	0.193918301	0.011779148	0.237993653
0.331008225	0.265187343	0.439594348	0.219000671
0.237133213	0.469940775	0.352451066	0.179402913
0.060471078	0.252527185	0.037169498	0.286207383
0.331599763	0.16981776	0.284189737	0.091012833
0.073036675	0.514960117	0.130171377	0.178664352
0.091212759	0.357728479	0.206660372	0.300663077
0.27368423	0.152441209	0.275004185	0.302020502
0.058069264	0.114606879	0.007298953	0.313610192
0.222095668	0.347661585	0.341378733	0.295299789
0.277801389	0.163064758	0.342180324	0.191460376
0.10669241	0.01947343	0.49465326	0.303437252
0.267683855	0.150962601	0.040179734	0.306303705
0.458812738	0.283660575	0.185573477	0.460070064
0.024147677	0.433627921	0.282025914	0.132185311
0.324822754	0.259726964	0.420006161	0.070261442
0.267425324	0.470822273	0.222813538	0.187396157
0.404053657	0.320431335	0.047718013	0.320965195
0.320858716	0.04338001	0.509330959	0.115300859
0.022751643	0.316788424	0.357975746	0.075827282
0.076055169	0.335129074	0.060820232	0.245735473
0.513418572	0.332492066	0.38232633	0.145795901
0.064741642	0.033283934	0.150002798	0.261151758
0.497679366	0.487485682	0.208387624	0.334131638
0.216066954	0.279471829	0.111917437	0.499141084
0.100146204	0.488956697	0.008268976	0.34814694
0.497962196	0.231367189	0.095133061	0.276714339
0.374369725	0.057992354	0.14628263	0.496314129
0.401982089	0.272183853	0.301775941	0.29719558
0.030418288	0.053560018	0.290075614	0.126429334
0.316731266	0.1876205	0.035611836	0.235300172
0.073811274	0.463192035	0.379734894	0.075190198
0.312591596	0.214590485	0.283825302	0.045813277
0.412302172	0.461971668	0.513908755	0.131243008
0.341904424	0.482966845	0.304248687	0.066124729
0.018970315	0.498051258	0.281418053	0.043555673
0.429976881	0.197375479	0.042281293	0.488070331
0.16930474	0.423640013	0.007709045	0.036152287
0.028225751	0.384688583	0.478334351	0.286757925

0.247288129	0.262350921	0.001525054	0.486851855
0.500808629	0.017758735	0.11097065	0.378733655
0.343843063	0.510466818	0.250930691	0.421635592
0.333329253	0.49479704	0.25621443	0.235012826
0.423127496	0.466525394	0.328722505	0.439980445
0.430136371	0.310066495	0.257160647	0.029043297
0.109161885	0.185545406	0.302667303	0.108192625
0.240242116	0.146915289	0.44982929	0.414814483
0.461759517	0.006370104	0.149697741	0.368498051
0.41946361	0.438047493	0.011469021	0.005703758
0.005424722	0.03301547	0.277192078	0.185150801
0.465782244	0.267773763	0.008173435	0.053759004
0.245572529	0.304486059	0.502251084	0.302128016
0.336408611	0.075526818	0.326346844	0.301073795
0.076575205	0.219972353	0.475138099	0.478841305
0.029513463	0.18622978	0.245800408	0.467389998
0.05190229	0.252392892	0.313997952	0.319913853
0.0070404	0.077265596	0.068317843	0.336302819
0.485685183	0.129256514	0.064393361	0.124764031
0.168437421	0.40737727	0.195658154	0.487726136
0.402313614	0.384534408	0.059365507	0.476327168
0.055068765	0.487069725	0.272938336	0.072667302
0.19224992	0.012087311	0.433974582	0.181368494
0.266645278	0.280394545	0.155464076	0.014727688
0.118250022	0.058879693	0.453719768	0.400066096
0.022783048	0.188965565	0.226335637	0.28357134
0.248438048	0.371639704	0.499185953	0.322796499
0.107530286	0.076391923	0.308567543	0.284065465
0.197981788	0.450779125	0.288871132	0.495609736
0.294448764	0.412539496	0.447983019	0.298448959
0.18557767	0.295147578	0.331867771	0.048987273
0.281082114	0.427275506	0.244955091	0.174809811
0.255220313	0.267317142	0.034032548	0.108788432
0.231007732	0.265017465	0.164131391	0.367653513
0.269042552	0.28758761	0.031170483	0.06988144
0.5226272	0.405165014	0.145652298	0.343091847
0.136533174	0.293743033	0.35595413	0.347174905
0.241949616	0.425187246	0.355580567	0.337951063
0.435083066	0.226577746	0.516170711	0.07511227
0.083832217	0.277103336	0.147828492	0.080382462
0.154478855	0.028053925	0.217125201	0.31158829
0.266763701	0.330054171	0.216689825	0.077213351
0.239498685	0.221342329	0.280755459	0.222008112
0.117012463	0.505191985	0.012070741	0.054742544
0.276896695	0.374789166	0.317595615	0.202111453
0.229372493	0.092215295	0.266928227	0.245581923
0.337732801	0.245964248	0.387031748	0.037918427
0.029443168	0.454933642	0.266016086	0.013503746
0.055378963	0.250780533	0.187015777	0.243647134
0.018598851	0.463635815	0.30085158	0.308093343
0.277864684	0.060110366	0.323085729	0.068765161
0.045137512	0.432937574	0.273616557	0.497483174
0.224101998	0.35185506	0.424163393	0.380073601
0.079168696	0.236605944	0.041020424	0.378213552

0.321807752
EIF2S2 0.003946873 0.014639639 0.00505305
0.050126791 0.019241348 0.017899066 0.092872348
0.007396658 0.026603294 0.042186223 0.077719438
0.006935747 0.015415274 0.030329356 0.009682571
0.034683173 0.06514006 0.002009277 0.072971344
0.024820138 0.060710333 0.056295777 0.065646185
0.006575097 0.092467149 0.169513928 0.041028483
0.026829285 0.154973673 0.022509921 0.020308819
0.016003441 0.008101617 0.007989287 0.031950994
0.006390849 0.002729306 0.000686666 0.040052068
0.006345879 0.024605307 0.029060626 0.077002587
0.108769078 0.006315224 0.116601496 0.012915874
0.001465774 0.121274245 0.010956582 0.006515375
0.009401268 0.021260635 0.021149947 0.107036835
0.021284676 0.007975325 0.01247306 0.009351094
0.101225952 0.018092833 0.009643532 0.044280546
0.004073146 0.012595109 0.017989229 0.031126147
0.00613551 0.008410055 0.008255845 0.002498516
0.01232057 0.026821086 0.01396187 0.011927877
0.113932672 0.085368772 0.024555943 0.074262765
0.021087465 0.003981072 0.024517103 0.018641993
0.028513452 0.020042895 0.006905749 0.092776063
0.0092181 0.129895333 0.004100349 0.039459511
0.02275861 0.011159165 0.027294529 0.040168346
0.034345518 0.01180525 0.012101668 0.022735349
0.020839404 0.014056819 0.01164635 0.016632948
0.00594036 0.009239525 0.096268814 0.017958491
0.071015012 0.001364962 0.073577496 0.002575411
0.022561815 0.088970174 0.054291303 0.051002437
0.003973542 0.010032395 0.010029384 0.103114916
0.007467448 0.015516629 0.008177279 0.026111211
0.002146603 0.004121922 0.00545785 0.032214999
0.007301266 0.009650072 0.036331885 0.029629961
0.06384837 0.001803514 0.007113236 0.003914322
0.01237503 0.002179562 0.112112964 0.00571018
0.010468364 0.02359717 0.001557792 0.052841696
0.016846637 0.0033292 0.090638308 0.012128932
0.061971095 0.03216254 0.080467304 0.019535304
0.009363985 0.125249752 0.001464545 0.033622133
0.026635097 0.030493528 0.05649956 0.043084056
0.087401474 0.006538007 0.053453498 0.004481036
0.00959267 0.015011381 0.001637688 0.018301531
0.011021273 0.024892256 0.0034976 0.055647183
0.044932449 0.042960736 0.004460256 0.031489512
1 0.009815784 0.00116629 0.018284788
0.010733176 0.010935404 0.017249605 0.012725885
0.057629 0.02297067 6.60E-05 0.007506863 0.010197893
0.001322995 0.053256536 0.008532118 0.015921585
0.034902851 0.000124139 0.030877408 0.002250556
0.052669444 0.02307411 0.130367779 0.005771525
0.17816304 0.013449269 0.012157694 0.008602957
0.003161761 0.033361586 0.037534037 0.031865973
0.049081105 0.009554014 0.040422637 0.058583226

0.015044488	0.027581689	0.01653055	0.02306395
0.022266972	0.008354316	0.022506738	0.007316317
0.001546914	0.011494407	0.026873914	0.001640109
0.014923776	0.005407496	0.006117647	0.158275324
0.025085891	0.010857075	0.054067085	0.004271774
0.10435864	0.011278113	0.002271575	0.072581227
0.018566509	0.013892819	0.005027719	0.007492495
0.034113928	0.03544792	0.061679388	0.043688964
0.051772292	0.016752432	0.023890564	0.081202469
0.124738134	0.001434808	0.006067252	0.04492725
0.022590609	0.018827572	0.006641151	0.008023952
0.034130229	0.039110633	0.005639345	0.047848112
0.099432813	0.014237183	0.024904079	0.005214317
0.054194112	0.052020833	0.002273541	0.030057777
0.01779731	0.001265825	0.02371488	0.003308487
0.017422844	0.054020113	0.135354366	0.020045759
0.098745766	0.029649866	0.006662492	0.005104652
0.013967772	0.019031707	0.10658954	0.009436691
0.024715737	0.030962609	0.005020408	0.004976696
0.1192493	0.012192607	0.032250485	0.0730159
0.013745159	0.02109827	0.111337365	0.073702091
0.08249097	0.046761182	0.074482162	0.019028145
0.054431527	0.024921096	0.008665648	0.165944999
0.049091703	0.017628051	0.003247454	0.006656053
0.011321284	0.040850419	0.044450276	0.085435732
0.027966225	0.001121126	0.018248305	0.00031987
0.007013118	0.046869515	0.014290365	0.007088087
0.006901083	0.132625682	0.053212703	0.01131806
0.110567548	0.009912192	0.010251937	0.016642744
0.030028239	0.026756265	0.008610408	0.068723394
0.042579216	0.021792659	0.006238517	0.021795965
0.049335058	0.001236885	0.003838219	0.097649875
0.016385083	0.0168421	0.095217825	0.040082638
0.003035356	0.028182164	0.111065179	0.012704964
0.006406636	0.00898171	0.018519722	0.0074476
0.015382176	0.01098688	0.042439187	0.075630769
0.007481241	0.007654598	0.028303891	0.023803476
0.007471058	0.072889166	0.014000368	0.070735263
0.003315942	0.006851112	7.58E-05	0.011764284
0.017142105	0.009994708	0.018257731	0.018064785
0.015777007	1.19E-05	0.010434302	0.013569823
0.060403429	0.016324447	0.011579853	0.011070057
0.01075535	0.01791341	0.018027496	0.011602225
0.009138776	0.015990981	0.085588742	0.023512956
0.007646695	0.013672519	0.009735154	0.02655945
0.020217153	0.097903723	0.126157477	0.007155719
0.020221003	0.019937387	0.043289519	0.006053956
0.08003751	0.008201026	0.022112895	0.015520869
0.059742815	0.024686787	0.009033452	0.019865173
0.021431227	0.001304257	0.000852674	0.000137981
0.048462374	0.022001132	0.029310177	0.014257227
0.008157597	0.074179151	0.025249861	0.011161027
0.023078799	0.012708612	0.045898842	0.008407724
0.057030658	0.036902431	0.028946786	0.05231103

0.001412717	0.020457994	0.013941769	0.013026066
0.029774191	0.002022203	0.026860096	0.00326808
0.041831895	0.007238755	0.031456758	0.001044408
0.06114808	0.085733267	0.018717923	0.006374214
0.09937718	0.105946839	0.00050759	0.136846714
0.013817113	0.065568341	0.020141907	0.022639019
0.037133563	0.018991069	0.01132238	0.038263497
0.008338118	0.026432852	0.002798043	0.005182387
0.018500479	0.005365241	0.033882731	0.008896986
0.007161106	0.006528125	0.035842134	0.002169624
0.076867872	0.031919197	0.018922044	0.00943755
0.022402394	0.025655142	0.011541882	0.024380979
0.044975412	0.008708368	0.00686202	0.101534363
0.000133184	0.018724605	0.004053136	0.010293445
0.021522711	0.050683632	0.011710396	0.006397217
0.00873446	0.010808564	0.052434375	0.007496909
0.006102568	0.04815029	0.03293861	0.027217889
0.032840332	0.005618359	0.04512388	0.109616828
0.00595643	0.004949334	6.93E-05	0.004729794
0.023964114	0.056022414	0.027606866	0.005021581
0.004525065	0.003878772	0.095031482	0.011965015
0.00828913	0.014049045	0.027275079	0.036780017
0.066045225	0.010848043	0.012417493	0.002533779
0.018080876	0.014240284	0.031228269	0.014237059
0.06407565	0.010192934	0.002270538	0.00831293
0.002848925	0.130754629	0.064199071	0.113388788
0.040593192	0.003364106	0.021620791	0.002406186
0.061814242	0.000888205	0.012696651	0.023999558
0.000960986	0.037011808	0.075777127	0.08350641
0.012286104	0.015109875	0.058854924	0.003160248
0.016404263	0.025402044	0.032895423	0.007823344
0.007206565	0.074107596	0.009542566	0.027138951
0.000387275	0.016372589	0.007877581	0.047305659
0.021358687	0.002791714	0.004888472	0.003951148
0.003356876	0.002146666	0.052094535	0.035874058
0.011184742	0.025658753	0.012748548	0.029156083
0.038422819	0.003399019	0.00136815	0.007483427
0.006117538	0.007056208	0.008572771	0.004328815
0.054553403	0.004383988	0.01018312	0.000609128
0.001444201	0.067825444	0.014770185	0.005515181
EIF4E3	0.059828265	0.052662037	0.048923503
0.112768167	0.072330587	0.039611743	0.136205782
0.069939	0.026903398	0.015548943	0.011074358
0.061624766	0.012210204	0.047969486	0.014809985
0.146273193	0.101715365	0.062924812	0.22148073
0.124692457	0.07366182	0.14625932	0.134825128
0.037205309	0.085949525	0.136424382	0.047421956
0.121921015	0.163460473	0.025866628	0.09723421
0.154437733	0.072510389	0.037955098	0.073079989
0.032837683	0.0442506	0.028742895	0.020787263
0.06806969	0.070147863	0.118455813	0.163163365
0.099854849	0.034211526	0.154321685	0.09449222
0.012400107	0.080966671	0.029831396	0.0364806
0.027337297	0.032856377	0.080546938	0.101415904

0.004138084	0.026194728	0.008069559	0.052817724
0.1543665	0.03269165	0.031641637	0.025564802
0.007473378	0.104339202	0.116797657	0.115654438
0.024346923	0.057458993	0.081976663	0.036364607
0.00501402	0.154375713	0.130865279	0.013068821
0.264724708	0.0346929	0.154022444	0.115092562
0.104685275	0.045902468	0.084387559	0.065377825
0.048115277	0.166514325	0.044327739	0.129981144
0.060507463	0.106770315	0.055702599	0.143520094
0.078228503	0.040907447	0.061711168	0.171829087
0.04713708	0.055064405	0.037969491	0.044996972
0.013447474	0.093026501	0.033589896	0.061976358
0.04955545	0.03863705	0.19245367	0.087628837
0.240745615	0.019880444	0.169502117	0.030203268
0.10674249	0.072712301	0.15967809	0.082974104
0.069095571	0.069324957	0.04058747	0.197344546
0.077643698	0.055195844	0.006723512	0.207445723
0.014098587	0.018180193	0.024876044	0.080306262
0.03697318	0.015821723	0.118404735	0.100457688
0.151534196	0.04347229	0.047423364	0.090094848
0.091611961	0.032188297	0.058546183	0.041153167
0.037743366	0.081705533	0.001802865	0.150857579
0.07047567	0.059620968	0.253338348	0.046266693
0.102280799	0.153594858	0.198246584	0.072225001
0.045686623	0.231111902	0.040411123	0.155897143
0.012649806	0.099807567	0.218222356	0.077475985
0.095920555	0.030439854	0.17337496	0.077612936
0.004626755	0.025537652	0.075090789	0.087053348
0.018460156	0.037529515	0.024164966	0.275101968
0.092725306	0.113637265	0.046542004	0.044490069
0.009815784	1 0.028061185 0.060296617		
0.033413395	0.069732573	0.098346149	0.081306441
0.18698866	0.105892119	0.007950312	0.034446029
0.040829791	0.025225464	0.076369624	0.092963432
0.02100077	0.08961621	0.006764715	0.144878248
0.042006992	0.263264592	0.090469011	0.186875304
0.040336151	0.168518168	0.04173573	0.046512072
0.03194644	0.019585542	0.046576611	0.109273616
0.13203462	0.04339712	0.082750143	0.112471159
0.070540753	0.09498036	0.111524569	0.017960803
0.067300729	0.156296939	0.080360948	0.132726415
0.06962009	0.003189002	0.04195817	0.082451597
0.03145665	0.049591933	0.035520611	0.001858054
0.170189966	0.081164736	0.034972028	0.086761515
0.03503946	0.053218301	0.023746427	0.07092314
0.17415377	0.058979507	0.053424345	0.02660223
0.025409024	0.042512302	0.196653411	0.022942303
0.013684168	0.09750748	0.028264048	0.029276618
0.080913266	0.165396097	0.050443237	0.010357372
0.012108865	0.017080845	0.014113846	0.039982289
0.024199842	0.030607727	0.073327056	0.030744506
0.114625533	0.231142205	0.052779158	0.151139593
0.037385726	0.122288066	0.2483429	0.002378851
0.070113723	0.01988991	0.019397216	0.054508137

0.082493456	0.081216206	0.029419423	0.184157374
0.030376644	0.161623099	0.110180393	0.007338972
0.0573535	0.044441634	0.174971173	0.103089918
0.034597844	0.099663593	0.053540061	0.045466307
0.042894878	0.20911411	0.05177513	0.09941599
0.249134904	0.083247945	0.039062803	0.264190186
0.189348325	0.048964265	0.06031885	0.201154283
0.058686862	0.143440735	0.039288323	0.072794912
0.129726989	0.014899739	0.123300651	0.055789289
0.033994318	0.049403454	0.237149877	0.0527189
0.242082221	0.020266459	0.008523648	0.058196529
0.031805465	0.044436857	0.18940307	0.052571918
0.030104525	0.036735242	0.122277194	0.071523836
0.043512779	0.165453797	0.149586714	0.040821313
0.055656682	0.04866169	0.025697133	0.097716509
0.065645286	0.054415037	0.076827719	0.030524917
0.02237583	0.103773417	0.007486513	0.031270038
0.061277383	0.040834995	0.107687554	0.059250538
0.020922425	0.03315993	0.145614663	0.215903733
0.044628188	0.043098926	0.039407984	0.154469868
0.055995827	0.042621573	0.023917609	0.245205232
0.206478603	0.03740681	0.066444161	0.02328125
0.097130606	0.029491181	0.017014262	0.054806112
0.229434185	0.025593151	0.012333011	0.011586257
0.114741768	0.097838541	0.032740845	0.100135199
0.010134594	0.156797441	0.013256004	0.056936991
0.042728355	0.120709331	0.055759573	0.039274206
0.036637014	0.053007399	0.031804968	0.048651643
0.037415258	0.031088321	0.018709475	0.184781544
0.062455178	0.034340636	0.087296955	0.052075324
0.087395083	0.110614978	0.186455126	0.082589096
0.011359273	0.05845281	0.006252476	0.009157248
0.047046109	0.077036464	0.058385523	0.093626987
0.056114462	0.194326743	0.057706314	0.04378632
0.079476187	0.095781587	0.072479431	0.021092025
0.014582208	0.017987285	0.09801927	0.170998377
0.055818061	0.00350442	0.142730022	0.064155454
0.042186593	0.070368607	0.041700615	0.18711396
0.067479824	0.215923142	0.011147145	0.165916253
0.045602777	0.03238331	0.061178473	0.042097183
0.145821124	0.134354032	0.063656564	0.086490214
0.005383007	0.181120027	0.061557265	0.022834276
0.044191478	0.14814694	0.101349154	0.119028665
0.028424749	0.038512653	0.049735411	0.007374848
0.078216167	0.078516839	0.081655762	0.077522432
0.081064295	0.136840395	0.067292725	0.040948935
0.023852538	0.033629855	0.062743951	0.007797107
0.111717584	0.142480773	0.039165093	0.002651373
0.038150686	0.086057462	0.008520609	0.136353807
0.013039418	0.199102018	0.085124545	0.080273619
0.108885158	0.089995269	0.179425049	0.12879601
0.078517845	0.094940781	0.036205962	0.019510723
0.085424384	0.014398866	0.123076683	0.054160935
0.051165845	0.088533319	0.02101945	0.058669892

0.064120588	0.062641454	0.046420804	0.098676291
0.01457589	0.056625198	0.056480001	0.027154609
0.159716371	0.08337707	0.085104101	0.031121183
0.103681997	0.033357186	0.060689626	0.011411355
0.024411894	0.048538861	0.054693101	0.140332104
0.080615795	0.054300779	0.061162259	0.090487545
0.053515652	0.047830633	0.033734771	0.069375085
0.242987031	0.09667102	0.075084519	0.073983072
0.015324158	0.047722287	0.083868249	0.049259552
0.041521493	0.16393187	0.040127594	0.032099708
0.029358753	0.040987587	0.054647446	0.250545411
0.21031822	0.106334749	0.010267483	0.131739362
0.012826241	0.128654613	0.011228076	0.052884324
0.138866086	0.039533392	0.154992099	0.085928506
0.05718403	0.035043783	0.033137364	0.235750745
0.01935509	0.053992579	0.035690286	0.139739539
0.0145016	0.074077275	0.158359232	0.046733898
0.014069134	0.007960516	0.050754474	0.062600655
0.093313316	0.093191247	0.050521891	0.080443454
0.02254808	0.041770926	0.070858753	0.023688326
0.151441659	0.036928968	0.016297784	0.063361627
0.10473912	0.226440016	0.035488598	0.033890402
0.097397759	0.041859398	0.055569827	0.037349129
0.079000645	0.132304498	0.030624767	0.047039687
0.051691786	0.03283346	0.099930462	0.092789747
0.012524359			
ELOVL1 0.273924057	0.352662498	0.108740293	
0.23715057	0.125722474	0.210465401	0.084013
0.162796489	0.02163015	0.001962496	0.001966666
0.161425398	0.033531465	0.099802643	0.094109114
0.222525897	0.021539572	0.164038246	0.089129317
0.253480694	0.071042382	0.162700495	0.039769485
0.053182006	0.012547964	0.014011015	0.036814774
0.31006605	0.017200847	0.155165255	0.308823356
0.031737305	0.311866475	0.361319451	0.145414064
0.338284908	0.262370998	0.019102785	0.091012416
0.357236657	0.195754786	0.259879708	0.124721562
0.015858252	0.393592689	0.085969216	0.127958209
0.121824121	0.00470035	0.159661235	0.395915969
0.314859314	0.138934194	0.145946095	0.061140213
0.026160372	0.167050137	0.027382588	0.317925083
0.074354094	0.144427158	0.313598879	0.032141724
0.061514892	0.173331442	0.033931302	0.136227279
0.05748532	0.338503445	0.025072752	0.155780106
0.003053578	0.243569765	0.128408986	0.039838966
0.010882429	0.003698236	0.040809019	0.156972685
0.318623846	0.353482049	0.257958569	0.260745713
0.032251986	0.082979133	0.087498548	0.041812523
0.320861791	0.057167093	0.048558293	0.197576826
0.264684902	0.328339582	0.038912136	0.060591673
0.241842697	0.370693487	0.111162549	0.093405651
0.04917842	0.010816448	0.314756549	0.296229131
0.357136871	0.044338047	0.027891151	0.334047893
0.02136466	0.137107314	0.153965118	0.054785341

0.32226397	0.008763009	0.036980583	0.148249541
0.103340046	0.284719939	0.308182312	0.06789561
0.117199811	0.005857156	0.012807607	0.01404928
0.102601932	0.016638173	0.359115452	0.27967623
0.152494704	0.075280366	0.116534231	0.000745115
0.005418004	0.06565621	0.26473858	0.16262101
0.275166842	0.27009972	0.03796433	0.315888459
0.152305503	0.253834291	0.034279391	0.056228355
0.14796365	0.06327598	0.081663211	0.160817019
0.052341234	0.085735348	0.046466057	0.314308458
0.400269981	0.031469114	0.187878983	0.199951274
0.073757311	0.076368382	0.050379221	0.080833936
0.02909019	0.303530289	0.005063065	0.183966574
0.036837544	0.021416853	0.019389949	0.168111866
0.08806964	0.237375241	0.352068561	0.046226383
0.091351277	0.140378219	0.212023941	0.188273815
0.00116629	0.028061185	1	0.306657197
0.27841073	0.303856065	0.10065432	0.073459514
0.070121051	0.264787047	0.002294016	0.364977182
0.323326262	0.172315615	0.113783548	0.165373113
0.060834677	0.266568008	0.032377315	0.206911057
0.311378231	0.038566521	0.292991422	0.029469799
0.338974555	0.008826511	0.151248874	0.200013068
0.183192279	0.005247694	0.137540078	0.249130529
0.005388256	0.081346216	0.284039874	0.201362431
0.121648564	0.243101091	0.285401085	0.051444075
0.115468749	0.130178851	0.006372988	0.073506042
0.258399071	0.076760418	0.364486537	0.103800162
0.249367543	0.333324106	0.244450309	0.05182681
0.016696279	0.157771245	0.014023617	0.070422812
0.279741483	0.034403214	0.092625357	0.065515958
0.013118469	0.316309602	0.023161621	0.035266328
0.013873368	0.139441238	0.114347542	0.049544956
0.025552163	0.041266059	0.060252517	0.078611029
0.031455993	0.021359468	0.005852107	0.064252337
0.018916412	0.061244987	0.068872357	0.274302879
0.116989058	0.024529468	0.077515476	0.055981564
0.020317403	0.004346425	0.338952629	0.212023736
0.338697266	0.061646109	0.014133798	0.066888124
0.282321024	0.153170905	0.1838958	0.31506784
0.017722909	0.286920174	0.045469241	0.021878192
0.05720156	0.071231017	0.298327868	0.023976787
0.238943129	0.353836665	0.108411098	0.030875547
0.333294481	0.217872913	0.016866064	0.298103565
0.29306142	0.008594352	0.321022619	0.022067191
0.006007593	0.180233353	0.175230322	0.018364517
0.017022174	0.073161582	0.05270427	0.081898348
0.319581001	0.178096339	0.224678264	0.041096884
0.012073002	0.001235991	0.041152488	0.127068599
0.373150968	0.290347064	0.089220895	0.056693269
0.095037593	0.051056422	0.159278232	0.292006128
0.085683157	0.366223949	0.003497769	0.19103257
0.334588777	0.192574534	0.011083087	0.145594914
0.323302728	0.019100169	0.129134522	0.338338344

0.304559225	0.069956056	0.062722038	0.220453729
0.013713421	0.0333721	0.114589124	0.024861765
0.085635998	0.057120399	0.00505368	0.169910435
0.010257304	0.329820355	0.271473076	0.0106364
0.143414288	0.17156232	0.132774849	0.014742073
0.217023217	0.315679212	0.362131373	0.114746343
0.248208107	0.268956169	0.093259638	0.02328552
0.003514738	0.327156648	0.295766159	0.006252744
0.292253807	0.185257238	0.001906374	0.366025787
0.077495981	0.375928734	0.003945063	0.031590093
0.010360762	0.244122571	0.337784998	0.295551229
0.10190851	0.207583909	0.003515379	0.316518616
0.3307311	0.002088795	0.022532149	0.22214493
0.16205077	0.386573743	0.042367186	0.211523745
0.120295389	0.313924458	0.074791126	0.069017482
0.273176861	0.279235164	0.300748926	0.371790364
0.280790778	0.176093505	0.108095897	0.006429335
0.020671455	0.161815572	0.055323085	0.011338886
0.285576856	0.021864507	0.336805084	0.272724653
0.256627584	0.001761472	0.111330105	0.260198774
0.3493565	0.33711992	0.015777043	0.003025879
0.003677505	0.001042607	0.215681785	0.056914818
0.301347086	0.077408547	0.002149015	0.056304001
0.09408027	0.104999148	0.327984717	0.205414447
0.23432739	0.028499888	0.05694493	0.246021061
0.008679838	0.310853255	0.263622442	0.363195248
0.016747032	0.058465273	0.168017952	0.313735821
0.005411064	0.095318912	0.18171831	0.066628037
0.009457172	0.015377592	0.011962533	0.248163929
0.320592758	0.010135397	0.004066052	0.119877013
0.031364187	0.29498836	0.037930197	0.30619203
0.297977092	0.258194836	0.013566331	0.349809646
0.007480458	0.332551408	0.052736748	0.02755954
0.102984769	0.007631015	0.301622771	0.01386683
0.155276796	0.308758194	0.032903569	0.003457187
0.026227149	0.01195792	0.264696942	0.230617002
0.009017847	0.117964564	0.0871276	0.22612714
0.186784881	0.210701753	0.365219457	0.119297991
0.041759635	0.032136142	0.291582278	0.282342815
0.161665908	0.272755787	0.044776018	0.326691947
0.297793181	0.350481771	0.287755886	0.135767279
0.033796615	0.227207394	0.216893933	0.009632603
0.147745704	0.246446835	0.194369998	0.0182722
0.042834726	0.08765054	0.048245501	0.131719564
0.052046328	0.22773269	0.043023426	0.271821748
0.284986087	0.290713295	0.065827317	0.010584506
0.363196006	0.362928292	0.086649135	0.216933463
0.053876707	0.088298796	0.236778804	0.210608728
0.099147686	0.254094443	0.316214297	0.22878624
0.35915596	0.098611063	0.355466681	0.143718485
0.013616021	0.199305489	0.017902946	0.021593417
0.040799097	0.006429228	0.038890364	0.216682582
0.060426879	0.143063163	0.16996858	0.026338662
0.085489164	0.15690709	0.124053197	0.083912126

0.017882004	0.333944371	0.002482585	0.016564202
0.06694329	0.231075252	0.063918761	0.056835848
0.038325127	0.033511219	0.08222464	0.189443541
0.072844123	0.104010983	0.236059958	0.027898678
0.017976077	0.292593486	0.175651824	0.007836169
0.026680717	0.257675261	0.140975287	0.047482877
0.004120262	0.263760226	0.102001467	0.123524509
0.181074373	0.031081045	0.389828304	0.059353984
0.047006028	0.375199219	0.108859325	0.318193355
0.089697414	0.205071305	0.396659295	0.26981111
0.065458869	0.131124892	0.014999195	0.321846089
0.067981219			
EMP1 0.458501588	0.529818468	0.43533992	
0.480790721	0.495160016	0.421730008	0.258633886
0.213573103	0.104579717	0.009688815	0.038018344
0.497597615	0.279881779	0.341771128	0.36160203
0.474131611	0.187467461	0.376249995	0.295153873
0.464825973	0.121257813	0.472222366	0.119989155
0.334608263	0.141669224	0.096129871	0.320625737
0.49082659	0.107942254	0.386285773	0.436121071
0.127880559	0.484183598	0.528939895	0.515394948
0.527115597	0.430212151	0.016567781	0.350089993
0.452432131	0.495074916	0.462544162	0.398422762
0.06002513	0.496772673	0.303798075	0.455422482
0.255921039	0.048622354	0.271352899	0.446093465
0.495295519	0.496762639	0.259107851	0.202812026
0.281686971	0.275381269	0.344494993	0.365747278
0.285078592	0.189127723	0.499447635	0.112115154
0.177933773	0.286990605	0.195403264	0.309160303
0.397392777	0.423678784	0.023522898	0.44900982
0.005628604	0.438522163	0.433884585	0.352138626
0.059393413	0.038441194	0.225399738	0.405340452
0.499673113	0.451370246	0.51985747	0.510895109
0.247574036	0.3440909	0.449334912	0.228526651
0.473774758	0.231926045	0.135814009	0.485172856
0.518125864	0.533669166	0.253296208	0.312988439
0.482194242	0.476373982	0.476040684	0.498912417
0.39459639	0.065042423	0.4529194	0.510675414
0.433438463	0.373452242	0.198899445	0.463380604
0.079142446	0.399719652	0.422825078	0.207119457
0.50152141	0.025104053	0.266211147	0.429955958
0.245165936	0.487135506	0.525458573	0.236744433
0.469807725	0.032263823	0.170258676	0.04422355
0.394793662	0.180761012	0.496665255	0.510830285
0.283975967	0.452486331	0.314755328	0.00351643
0.039651196	0.213306774	0.493224782	0.409448083
0.477498165	0.406508866	0.295567285	0.434019432
0.498614332	0.519141475	0.069904018	0.120949385
0.482310793	0.216012446	0.295729307	0.513979001
0.379336593	0.141223845	0.169483595	0.509946455
0.424724332	0.115731329	0.366812022	0.477872481
0.404540637	0.428851732	0.139938286	0.359455554
0.071623373	0.491784881	0.04029805	0.367031896
0.307718991	0.206583147	0.013153923	0.302871895

0.35293122	0.491665011	0.480190497	0.135917784
0.402612731	0.477864283	0.361351788	0.498694429
0.018284788	0.060296617	0.306657197	1
0.33039242	0.500224996	0.357682896	0.3493186
0.344377193	0.496485263	0.003488408	0.507633217
0.53386243	0.332061786	0.488652656	0.474199097
0.423303901	0.504613244	0.048408853	0.486315414
0.398673527	0.10048089	0.515471581	0.212908433
0.463681505	0.0601737	0.377949291	0.484838784
0.495413129	0.012535672	0.263883616	0.443859367
0.036328954	0.121379614	0.470435879	0.468703767
0.443725412	0.493384081	0.513038386	0.405913748
0.499854295	0.278717439	0.016593011	0.377118281
0.482742413	0.429114467	0.446086849	0.288304064
0.375701291	0.476006574	0.46593407	0.2384972
0.061024856	0.364444757	0.031094997	0.393100411
0.42504454	0.171216944	0.433204362	0.176363493
0.097944793	0.527445071	0.215813593	0.303899161
0.155433475	0.488709457	0.298397014	0.325786534
0.304813748	0.206071659	0.405681786	0.433175179
0.05317217	0.134406469	0.016683872	0.466736387
0.220737106	0.444520857	0.437350135	0.462154558
0.406331617	0.168691021	0.469263878	0.31980647
0.144411234	0.022352383	0.510590301	0.462845654
0.37573758	0.191219098	0.055894408	0.424980927
0.512423732	0.368473617	0.290147073	0.487896496
0.034534664	0.50892234	0.334044823	0.165822186
0.473198529	0.335401463	0.497260086	0.073612406
0.435736456	0.47805159	0.360352247	0.236174672
0.433401816	0.475558004	0.044390577	0.447770269
0.454386371	0.047623068	0.531279283	0.169653471
0.029662251	0.498968429	0.501959655	0.099880457
0.101384852	0.415833245	0.059917775	0.354537516
0.521203294	0.483139578	0.492700301	0.251895779
0.060957152	0.023390145	0.254142318	0.441226988
0.523814521	0.513682604	0.318425553	0.456925027
0.29656241	0.389776701	0.162623999	0.510680085
0.206359045	0.523076979	0.009932858	0.496026784
0.523863828	0.336809248	0.111418893	0.365105617
0.444239758	0.062233906	0.2378917	0.509340432
0.474039778	0.382535637	0.404850208	0.485949516
0.02388661	0.058128387	0.44052796	0.216073714
0.447937994	0.259773385	0.066076723	0.366410868
0.066589225	0.466492612	0.509567156	0.078200435
0.401834608	0.335434035	0.446846901	0.051658081
0.488949979	0.501279843	0.534527127	0.197500163
0.501059173	0.493049472	0.470224927	0.10135321
0.024607158	0.526533637	0.438846873	0.034978019
0.514621558	0.270452072	0.026432629	0.500730379
0.22835628	0.456738109	0.004556091	0.083809509
0.05064574	0.507463128	0.490302377	0.432113041
0.294236644	0.417735562	0.006484569	0.520255844
0.50528997	0.020961261	0.178538291	0.502704276
0.490393203	0.533648859	0.376607154	0.505835856

0.502044191	0.515649864	0.308167987	0.330605567
0.497535039	0.498925665	0.470315906	0.456147701
0.517024807	0.489050568	0.34298522	0.023836728
0.147153887	0.249910225	0.363834759	0.110236718
0.360416861	0.18222335	0.48004305	0.514296662
0.51116915	0.007526715	0.185729629	0.491452536
0.47916709	0.496437786	0.023204711	0.006872496
0.008470609	0.030066176	0.414741054	0.290090131
0.493352313	0.299997942	0.007689943	0.058345332
0.331316507	0.466782858	0.516324241	0.444083691
0.489047048	0.107762933	0.361630364	0.45616748
0.092182617	0.359929477	0.514543219	0.484662516
0.051863194	0.292878924	0.416600899	0.507590351
0.089275042	0.386308133	0.504866356	0.39944916
0.016371081	0.106355301	0.085348952	0.492898879
0.515442208	0.124616354	0.056752557	0.174065092
0.169568491	0.504784953	0.224944229	0.513751654
0.494026421	0.500101093	0.098930236	0.489187885
0.057139181	0.504245529	0.411951706	0.071764601
0.332170847	0.016961451	0.473540403	0.196215844
0.38580107	0.443589614	0.217722427	0.023099465
0.202813945	0.074796325	0.516784938	0.510292394
0.042167208	0.276382352	0.352272493	0.456566053
0.334866118	0.507295656	0.517288154	0.457910067
0.114005684	0.166224962	0.463823661	0.445822125
0.288798932	0.517547113	0.371404462	0.523481273
0.450848077	0.469061471	0.491893217	0.406594181
0.299926076	0.445133222	0.439990387	0.032575549
0.413181765	0.52197264	0.396955042	0.205043909
0.332605993	0.44139215	0.056253269	0.199576227
0.382529137	0.356583365	0.198173455	0.491677907
0.435816627	0.448861164	0.050519869	0.08115706
0.546310218	0.442121254	0.168689113	0.488215009
0.211000453	0.411578213	0.501745884	0.505099118
0.402979415	0.509116248	0.484634215	0.46034265
0.44291199	0.312239203	0.536014889	0.102020007
0.106925792	0.468785509	0.144190201	0.11952268
0.19205901	0.036021055	0.346916803	0.488461373
0.435686252	0.466462013	0.362556905	0.102302268
0.387214186	0.393689808	0.423356317	0.255616054
0.114744309	0.514056962	0.013348575	0.076854098
0.458823564	0.493689266	0.461626697	0.293953757
0.354338031	0.17044779	0.374581434	0.34339264
0.406551482	0.406785411	0.490608225	0.042243459
0.029020671	0.517368544	0.413940931	0.025507648
0.103827514	0.413724743	0.336536807	0.295429073
0.029195767	0.487362423	0.391327451	0.485271386
0.398992712	0.085882323	0.379562148	0.104319182
0.069866336	0.455351956	0.445400626	0.524330256
0.393103618	0.492539668	0.45237257	0.497160151
0.136633463	0.412633575	0.038425416	0.491417988
0.465438424			

EMP3 0.222219643 0.372447756 0.1313843

0.258731975	0.164060988	0.252412005	0.176996516

0.250388219	0.047170119	0.012945828	0.00922676
0.153764968	0.089423636	0.227207352	0.179622569
0.205851116	0.043534222	0.188479384	0.145070439
0.230644238	0.164558727	0.166157007	0.07815534
0.073281693	0.026760453	0.033966198	0.070396488
0.302273896	0.038218566	0.282707465	0.299095283
0.034235826	0.280880065	0.37457819	0.158490557
0.352673369	0.214911091	0.015039026	0.220112559
0.315722445	0.201093993	0.246777003	0.17290729
0.041826597	0.41107938	0.160573302	0.128907716
0.208260042	0.014071727	0.305081778	0.398932184
0.3578807	0.177842721	0.247392484	0.140914454
0.084487242	0.323295204	0.0618432	0.31594422
0.152778629	0.308759829	0.344041267	0.084079787
0.11210634	0.245186493	0.044104957	0.23912986
0.089035082	0.320062411	0.0296294	0.135037835
0.013450367	0.214527587	0.127842285	0.074140839
0.019574603	0.017282063	0.054871345	0.216417989
0.305641534	0.319267685	0.266165132	0.268105721
0.067525337	0.110487883	0.109025031	0.091766658
0.299138059	0.145550795	0.075791763	0.189506927
0.274319083	0.351994048	0.066624929	0.080879796
0.290844909	0.374694438	0.184063599	0.167107287
0.108357276	0.010886249	0.345860503	0.310422862
0.33984764	0.071212711	0.050662881	0.332387557
0.0323192	0.207631884	0.178394138	0.071421676
0.314885659	0.022884514	0.059837861	0.230859544
0.108121357	0.26819765	0.3236025	0.133608184
0.143233055	0.01366489	0.02763591	0.014992583
0.17380751	0.023423053	0.382693512	0.311207757
0.263085921	0.138435431	0.196507123	0.000762347
0.009285608	0.064176995	0.258000705	0.193294254
0.239250245	0.234221743	0.09591886	0.301435395
0.155512537	0.25962617	0.082058918	0.093547341
0.197316505	0.063749635	0.114872019	0.18409872
0.100100449	0.137407098	0.085365515	0.324124104
0.408009284	0.064107827	0.2413806	0.1860732
0.183392371	0.101770027	0.081507071	0.169165594
0.069323317	0.313235027	0.006929862	0.249269452
0.080898287	0.039279183	0.013437964	0.285510604
0.200312522	0.287046334	0.354522029	0.06612573
0.184972664	0.149864467	0.286858387	0.23419412
0.010733176	0.033413395	0.27841073	0.33039242
1	0.289527774	0.158907987	0.103240796
0.09941167	0.253042132	0.001074463	0.385405434
0.340556298	0.269960087	0.169470069	0.151925992
0.111705821	0.290973015	0.028791011	0.193786623
0.270032414	0.05356832	0.295073187	0.060177533
0.334884551	0.021324472	0.276549678	0.199639667
0.195930131	0.007657661	0.292609384	0.250737719
0.006107329	0.18607364	0.242965445	0.206358666
0.172730101	0.217979163	0.282504181	0.100334678
0.165050714	0.192756896	0.007277703	0.094280518
0.223499213	0.139523547	0.389205414	0.184302497

0.245807994	0.357774727	0.235442078	0.127026403
0.039903319	0.260283808	0.035799223	0.124895325
0.251989401	0.108277911	0.139356149	0.058931361
0.022274394	0.338425685	0.033135486	0.052026498
0.019362601	0.214123054	0.172111072	0.1620117
0.081183008	0.072462582	0.107494871	0.151227457
0.079201698	0.048514132	0.002831776	0.1389412
0.072555202	0.158775426	0.182347137	0.267820338
0.224194895	0.071239731	0.14538545	0.075411271
0.033194101	0.007604547	0.356212489	0.184301094
0.335536051	0.109523415	0.019231803	0.15304163
0.316520129	0.252724151	0.274244047	0.356251882
0.02064836	0.277391103	0.12523187	0.045140876
0.118248349	0.138128503	0.296850265	0.050885077
0.237640369	0.387894393	0.124934119	0.066659773
0.37209697	0.214932927	0.037856837	0.275821414
0.283357166	0.018263637	0.335256317	0.035455033
0.008713249	0.165496051	0.198211842	0.03055678
0.026930605	0.180114069	0.116870141	0.103511237
0.345702969	0.184028212	0.263835085	0.049992471
0.034925699	0.008153898	0.05329412	0.130265102
0.39132355	0.300524071	0.117523738	0.116523361
0.135905369	0.105476101	0.2603919	0.3148242
0.064689415	0.363066441	0.004693516	0.188984952
0.356689205	0.312557566	0.024921135	0.248975294
0.353881258	0.039506786	0.150457548	0.364936966
0.309438428	0.128137713	0.131713472	0.181044355
0.038441184	0.07069529	0.133098087	0.033178546
0.17489349	0.096793908	0.005351883	0.252599845
0.034021764	0.372328737	0.250381943	0.026222848
0.2404045	0.247227598	0.130139603	0.028002127
0.224109133	0.308574164	0.382878316	0.152284352
0.231310005	0.2962848	0.11528927	0.028956112
0.005107209	0.338778194	0.258257302	0.022482499
0.28995404	0.317431057	0.013426821	0.383658748
0.129598326	0.38050779	0.009464915	0.020736315
0.009106611	0.2266686	0.365813662	0.271834864
0.266655799	0.192689483	0.000919563	0.313522104
0.367369112	0.003441107	0.032043188	0.236204457
0.172531395	0.394655348	0.076986792	0.229274726
0.137275789	0.343170498	0.175364375	0.102043047
0.301805207	0.288542843	0.279931622	0.370073481
0.297229891	0.157720394	0.155177063	0.019993551
0.032685836	0.293621554	0.150281903	0.037699127
0.281528312	0.046501076	0.325120243	0.267737152
0.271896665	0.002397157	0.207267324	0.261475064
0.361022822	0.330058376	0.009798591	0.002657126
0.002890052	0.003543242	0.249729365	0.078339654
0.308274049	0.230107803	0.004472496	0.107694071
0.161573043	0.114111051	0.357338385	0.191948224
0.208265396	0.041686612	0.176725917	0.217619659
0.018082371	0.2608682	0.285745293	0.397588157
0.01548673	0.082658689	0.203844943	0.328492547
0.00808332	0.116601708	0.164201813	0.161662898

0.005408598	0.024084643	0.021846182	0.218920954
0.340051831	0.036371044	0.015135212	0.189770386
0.095900162	0.278446454	0.092185338	0.312798777
0.311811435	0.256951647	0.01770232	0.368580771
0.020139845	0.361722683	0.087777587	0.066650445
0.116830941	0.0080983	0.294976429	0.045792061
0.288429183	0.25519242	0.053449805	0.003883775
0.032634164	0.020564793	0.282425681	0.225219581
0.007421915	0.176885524	0.112748068	0.185441365
0.290288325	0.234583104	0.389265928	0.186975011
0.099726394	0.027188167	0.263288428	0.247259433
0.274994823	0.272291601	0.11074904	0.327516209
0.263288697	0.325088581	0.295312642	0.193611543
0.0575698	0.233718969	0.271652803	0.038314712
0.180591704	0.267911838	0.227351411	0.044564605
0.094828647	0.10537526	0.047027943	0.13450918
0.075766516	0.298717863	0.100517628	0.251080683
0.234772295	0.262534544	0.071018249	0.021533891
0.374199935	0.348525645	0.158148047	0.231729297
0.070863494	0.135705152	0.218775668	0.195976843
0.143218929	0.282894836	0.303485041	0.282232008
0.400368774	0.160340649	0.377300024	0.2139402
0.021786861	0.172100426	0.060868994	0.03044781
0.082481446	0.009539324	0.065711612	0.192211852
0.101497069	0.171574175	0.259344322	0.041005597
0.107602185	0.179012234	0.146408793	0.20022582
0.052069431	0.373174135	0.004953912	0.024361531
0.111362473	0.23820323	0.108115516	0.087678681
0.067378069	0.036542491	0.133668544	0.304698574
0.211290447	0.136571002	0.250103136	0.04665722
0.037734395	0.289991408	0.227978657	0.005521132
0.031207392	0.212584688	0.154516083	0.115743849
0.004484145	0.283325022	0.239007151	0.149410068
0.239611777	0.040994452	0.367227533	0.061836387
0.048541106	0.377947429	0.119601936	0.335242499
0.110231394	0.217830681	0.395454747	0.276176583
0.059740956	0.130804266	0.034765982	0.296033382
0.136339943			

ENDOD1 0.482614218 0.520724709 0.48242111

0.455274854	0.498352476	0.389373104	0.232167078
0.208379896	0.104397697	0.004965515	0.029093776
0.517806104	0.241150166	0.291958272	0.354347371
0.482039231	0.184808638	0.427485352	0.287866173
0.461357344	0.096362861	0.466090949	0.113386095
0.389970192	0.14545213	0.089163807	0.338131149
0.47966584	0.104159383	0.329450174	0.414658858
0.157695204	0.485390118	0.524811691	0.501117157
0.523304031	0.452575972	0.021387714	0.301753488
0.455685054	0.494783964	0.451620877	0.377708175
0.051138297	0.483998398	0.277002265	0.495425511
0.25614138	0.042404979	0.242800086	0.437011201
0.474429654	0.470193811	0.246014911	0.176751811
0.2276685	0.234926604	0.339231882	0.3374534
0.259804433	0.153571455	0.48668087	0.097475207

0.166329303	0.286791092	0.226142789	0.290232303
0.439004131	0.410391704	0.02519681	0.482388053
0.002505416	0.441285892	0.476841339	0.376984495
0.061673732	0.025504586	0.256394619	0.369536826
0.492168113	0.469297118	0.51189413	0.505274368
0.235314492	0.384617203	0.488654253	0.21089728
0.471868419	0.195662046	0.142717161	0.490550304
0.509661014	0.525331722	0.266750588	0.349235787
0.442263377	0.468501859	0.469512724	0.485420767
0.388393505	0.090721284	0.431556837	0.50861496
0.421812657	0.417473643	0.207407498	0.440258585
0.084525964	0.410403399	0.405736031	0.22183467
0.491981298	0.021985281	0.29063247	0.388459679
0.294327328	0.486270302	0.524408507	0.22109041
0.50391521	0.029741916	0.181061972	0.055643956
0.377936148	0.224993823	0.484345191	0.479701044
0.260994128	0.44796641	0.305216964	0.004456041
0.043280429	0.273141417	0.484360611	0.447995443
0.488156417	0.423810144	0.270797633	0.418915818
0.49852827	0.512681361	0.055828815	0.118633199
0.47305638	0.271579104	0.297243048	0.500287667
0.380467703	0.137682507	0.161408659	0.511479366
0.410917738	0.1068411	0.387879173	0.485896649
0.351161613	0.450150309	0.138812668	0.321161171
0.057673016	0.492283601	0.048958586	0.382131478
0.308144869	0.209179696	0.019421099	0.280648212
0.313582559	0.452079675	0.478519419	0.144814412
0.36754605	0.478208713	0.350254249	0.453362265
0.010935404	0.069732573	0.303856065	0.500224996
0.289527774	1	0.369336496	0.394968098
0.361938224	0.498971817	0.006628602	0.497191176
0.531387545	0.321081095	0.45027447	0.509538875
0.434237587	0.480522902	0.058748163	0.486907528
0.395868064	0.108928879	0.508823126	0.208983342
0.464881801	0.055979789	0.344433021	0.492426948
0.480541362	0.012227055	0.225013324	0.42208889
0.048306269	0.097063071	0.482477404	0.455939039
0.423369993	0.510696555	0.507776894	0.415839458
0.494166897	0.279447185	0.021301202	0.419976445
0.503435045	0.430446534	0.431279234	0.274687197
0.36904321	0.464321786	0.465698413	0.212372295
0.053626479	0.341612343	0.024959511	0.394742334
0.434606754	0.129555762	0.444122099	0.225617265
0.105633666	0.521231997	0.262412996	0.35892074
0.197500561	0.430960801	0.30425241	0.250122614
0.248229624	0.211759003	0.422222552	0.428243399
0.040176293	0.122628192	0.030732022	0.428734087
0.159522333	0.37620868	0.362818189	0.442967941
0.368525987	0.133518628	0.450235016	0.381655804
0.162585038	0.023568111	0.504043559	0.477005937
0.352413202	0.186916739	0.063573495	0.37370474
0.480807907	0.320042825	0.284354263	0.451004447
0.04387974	0.509351262	0.282248961	0.163977608
0.453395568	0.309916094	0.48786147	0.064642626

0.440610007	0.45678424	0.403350531	0.232776702
0.395597022	0.46658797	0.036898341	0.444201847
0.439171947	0.044907113	0.528943246	0.184257351
0.03406104	0.518125426	0.48274436	0.107380272
0.113628854	0.3598543	0.048055468	0.367086527
0.512216683	0.475570252	0.456450099	0.315910923
0.049709437	0.012763952	0.301646578	0.493780862
0.513472455	0.517057408	0.33753843	0.43601437
0.292856465	0.388068947	0.147194987	0.504627197
0.294197828	0.526019912	0.011239753	0.502791578
0.516006755	0.30234469	0.110891278	0.326396968
0.409129688	0.059123474	0.264407131	0.49726025
0.46064887	0.387856586	0.398798431	0.50851966
0.018017051	0.048225438	0.466746276	0.271885691
0.422542702	0.268855313	0.095109182	0.363687338
0.052794605	0.439686374	0.514610315	0.076618607
0.339609705	0.345506334	0.471685487	0.050187506
0.489276654	0.51367394	0.524828799	0.206738274
0.501226115	0.486570716	0.47272675	0.121571594
0.029532653	0.526829577	0.441777513	0.027058709
0.503974893	0.237239873	0.014338609	0.490732007
0.226966038	0.448761699	0.004542891	0.137016854
0.073669278	0.519629553	0.478053462	0.419909263
0.239395328	0.441252799	0.013180855	0.530229818
0.486118047	0.024653525	0.212028102	0.483448066
0.48774253	0.528578775	0.404146176	0.494130438
0.496988428	0.504529677	0.263966362	0.337286329
0.468157435	0.504302086	0.465953848	0.453690183
0.493716208	0.508675382	0.327854907	0.01859437
0.175015478	0.221786753	0.321171111	0.094535154
0.344531252	0.184206225	0.483665098	0.510807306
0.498543531	0.008881434	0.165676677	0.477117909
0.454933281	0.486003118	0.038519416	0.010092054
0.013799057	0.028587206	0.401286871	0.331508772
0.485636309	0.234401274	0.007161584	0.049547875
0.334711014	0.486059431	0.502517153	0.444107518
0.509186208	0.115228044	0.290546599	0.462260262
0.096790502	0.361539142	0.496942539	0.464339165
0.069917129	0.332666632	0.450716445	0.488800978
0.116434881	0.405592371	0.516759927	0.352639856
0.027524946	0.120475631	0.09238954	0.508640629
0.509143384	0.096576126	0.044493962	0.154829597
0.134833967	0.505722682	0.201093002	0.508245567
0.473105693	0.490446328	0.119059452	0.480783376
0.050316171	0.489613668	0.433955591	0.063571814
0.39044595	0.021134267	0.481317999	0.175663541
0.352486973	0.449356148	0.248496768	0.030553828
0.264644564	0.078808487	0.500025398	0.515890963
0.061944903	0.281474574	0.390305556	0.484446406
0.303827346	0.479756417	0.504574806	0.433443784
0.094155021	0.229796367	0.458036321	0.444399159
0.265217053	0.514345173	0.34262492	0.53086428
0.456267673	0.469904606	0.479116607	0.391191257
0.318036551	0.442288853	0.386723908	0.02169268

0.422487008	0.498704965	0.418634377	0.200055906
0.315548366	0.478387723	0.066487062	0.236037009
0.425174471	0.314210971	0.171665677	0.496380181
0.446378649	0.444728631	0.053683774	0.08490826
0.542875938	0.433784465	0.160884965	0.46469502
0.238736413	0.406309145	0.514659299	0.518973972
0.437252006	0.478774859	0.4689383	0.40573969
0.408981399	0.307756765	0.527352779	0.097844958
0.121519985	0.486357711	0.113097307	0.137719503
0.18083216	0.043347683	0.383788654	0.501528872
0.459278966	0.44336298	0.354934336	0.114029261
0.422290369	0.441805726	0.436516631	0.216091104
0.095939097	0.495557456	0.014415179	0.084756583
0.475738764	0.485657125	0.459950398	0.31988961
0.38474876	0.216420694	0.364529871	0.31101392
0.32750364	0.446573731	0.477902142	0.044082729
0.02343447	0.511443959	0.426822877	0.040483782
0.127210393	0.426684928	0.387749517	0.267544586
0.039863416	0.48477284	0.337371534	0.504957919
0.386240843	0.094865344	0.365081281	0.119484861
0.086779605	0.448262555	0.489783397	0.520698872
0.460804114	0.475246258	0.446297854	0.478108219
0.18337566	0.463320051	0.034011385	0.488151563
0.454275892			
ENPP1 0.279636824	0.348561461	0.167080078	
0.367621033	0.22868995	0.183877869	0.179683945
0.099593751	0.064293736	0.019629699	0.022637405
0.241967496	0.069865708	0.097673966	0.063322024
0.399500157	0.093625066	0.145503757	0.251342974
0.35577028	0.078875092	0.363130776	0.106236682
0.085986899	0.067989611	0.082985637	0.094924349
0.418367756	0.101136829	0.119860602	0.31693503
0.112435054	0.332551803	0.304766903	0.279278634
0.285661791	0.234257668	0.03727805	0.074623569
0.323677216	0.303849395	0.352040458	0.291674625
0.072209287	0.281143401	0.22581908	0.258377693
0.085025316	0.049737591	0.083142536	0.270580942
0.254786021	0.182023361	0.121813283	0.120521186
0.042263632	0.073600233	0.044882052	0.211969437
0.200982019	0.068198475	0.275773288	0.062297935
0.048124658	0.151942219	0.109436504	0.182262425
0.080330235	0.268324606	0.067679489	0.184514194
0.008198675	0.362374265	0.280792953	0.058776813
0.102254801	0.04369837	0.130167405	0.271890772
0.407070012	0.298789674	0.385358673	0.354103706
0.104602609	0.226714644	0.164865833	0.125597005
0.318860931	0.136912117	0.101077757	0.394911829
0.3799629	0.316173832	0.100728549	0.221379983
0.274163976	0.331229476	0.157692675	0.168485894
0.060988514	0.066931139	0.262766546	0.361872117
0.281483002	0.112866178	0.154584831	0.343514329
0.105351401	0.080335998	0.341838266	0.100279057
0.417924377	0.042940909	0.171968643	0.22924965
0.136912156	0.321310036	0.308706744	0.202631446

0.210294005	0.063459919	0.036764668	0.082200708
0.091787985	0.04363768	0.24617142	0.367163584
0.105818079	0.088353026	0.178953235	0.022314673
0.063891037	0.122609551	0.281924163	0.203832884
0.338353694	0.224227809	0.102633467	0.238856883
0.209189868	0.378103593	0.013057418	0.114666463
0.218985439	0.116005213	0.283928804	0.214188962
0.180138406	0.118678601	0.141763829	0.384487723
0.288951495	0.137261478	0.120131887	0.389170149
0.06805161	0.219419551	0.131320377	0.127625386
0.079615711	0.263322071	0.059688416	0.180439601
0.041546438	0.076646816	0.053001365	0.161466337
0.088051968	0.263617347	0.238154571	0.156377241
0.175944541	0.304325703	0.153080273	0.242281216
0.017249605	0.098346149	0.10065432	0.357682896
0.158907987	0.369336496	1 0.126303255	
0.250708546	0.387841456	0.005348291	0.284885616
0.313817494	0.094865525	0.236912699	0.273607185
0.102952079	0.372414669	0.014137066	0.380975516
0.214248335	0.12820622	0.409619559	0.164620831
0.289421224	0.077090603	0.119805727	0.271446417
0.221340183	0.018582822	0.112596285	0.324205715
0.056026214	0.067563733	0.32076516	0.329085392
0.225936137	0.35881684	0.429168233	0.086652138
0.212834822	0.17493872	0.05316211	0.220700702
0.316089666	0.054697704	0.288423026	0.114965588
0.152982717	0.320130598	0.240324047	0.034346338
0.083033339	0.185533594	0.047116673	0.185243065
0.236379254	0.079194062	0.122603437	0.125505949
0.087343469	0.362393204	0.071145242	0.071779248
0.040444727	0.192980096	0.231788265	0.066168591
0.058057657	0.107557401	0.087978325	0.107595312
0.062864478	0.106251379	0.040979126	0.066777047
0.05266756	0.074616052	0.073392892	0.232644357
0.096523875	0.087279246	0.188229642	0.08416876
0.092274599	0.061696424	0.34236522	0.369230341
0.206824235	0.115956946	0.089134428	0.046958946
0.357699089	0.111430136	0.06863302	0.320917753
0.093518202	0.383116731	0.085484149	0.135721199
0.110698765	0.22411432	0.413457173	0.02386432
0.215719915	0.307178647	0.261108174	0.116620033
0.246887415	0.314473765	0.062382356	0.25662418
0.246431582	0.076102738	0.34353406	0.098776929
0.070999558	0.30354609	0.238400052	0.134517253
0.098867826	0.146696988	0.063050419	0.286611416
0.358862255	0.380614211	0.253862711	0.094371449
0.069324774	0.022028694	0.13186406	0.189067565
0.289159249	0.329630339	0.270223362	0.153541526
0.280888958	0.070757863	0.052205779	0.350495224
0.099546542	0.323120023	0.040174568	0.276325599
0.274152664	0.12669953	0.078747445	0.189966588
0.259651751	0.079011439	0.142412336	0.304364372
0.312386163	0.113331276	0.089122124	0.314396771
0.046559026	0.07366401	0.223731383	0.056437895

0.092889142	0.119691028	0.019867524	0.090673085
0.065237842	0.290556634	0.396588322	0.058810179
0.131140574	0.085992741	0.316811924	0.083942399
0.283514077	0.308552689	0.31025616	0.139125557
0.289804139	0.301901271	0.150340611	0.117715145
0.057601354	0.299422674	0.27303814	0.040812468
0.40180738	0.097789 0.026992712	0.347517736	
0.21484187	0.236346704	0.011729415	0.039500594
0.066433621	0.381506482	0.272608725	0.314080466
0.050745076	0.32493201	0.014297407	0.353401769
0.307465114	0.038393674	0.079911707	0.255568175
0.228333426	0.352716569	0.10034469	0.290094405
0.191795475	0.277290344	0.093985337	0.250442964
0.316884096	0.279359881	0.334312687	0.317654359
0.378875642	0.332289622	0.282903531	0.041248146
0.045965941	0.111055463	0.041060125	0.040150768
0.186717625	0.087238732	0.338837067	0.39506379
0.330832001	0.036246714	0.099991663	0.267507184
0.365577021	0.407749356	0.059935034	0.009430071
0.014629198	0.021811762	0.260430767	0.191838163
0.334390777	0.039085568	0.031081464	0.077481617
0.122610722	0.233149272	0.311433848	0.399981681
0.308590433	0.10905841	0.051968735	0.40043191
0.056388093	0.180041064	0.345325751	0.301085319
0.08404549	0.170376408	0.176672913	0.399701677
0.015770174	0.281316671	0.258541553	0.084738699
0.044388746	0.084429732	0.066350721	0.38971672
0.265873632	0.059558069	0.04865223	0.037120162
0.084341038	0.370737251	0.108899501	0.39464366
0.358315521	0.426192046	0.07256359	0.298900481
0.042459727	0.281763877	0.156536624	0.027092935
0.151769105	0.058501371	0.286765728	0.028097652
0.117851033	0.296449063	0.05316969	0.045914369
0.045969265	0.084701256	0.385853865	0.353888266
0.065183725	0.125672913	0.246416487	0.337459383
0.184690656	0.345551328	0.29435975	0.12270958
0.083725116	0.056493228	0.359329761	0.242950419
0.100966171	0.397538149	0.069622852	0.360769459
0.283812156	0.341462536	0.308237592	0.236737016
0.071497119	0.213479357	0.241375675	0.042785807
0.287904623	0.36784864	0.208112772	0.070681204
0.161788039	0.150830832	0.087035829	0.065180381
0.101844199	0.179073723	0.102951088	0.432865485
0.267671464	0.251860403	0.083571059	0.063630989
0.356821369	0.297946636	0.085649287	0.281048885
0.185571487	0.221833617	0.328498974	0.320910872
0.072496819	0.29393535	0.341805865	0.239553194
0.280216411	0.23158868	0.313880284	0.06830701
0.049774606	0.209766523	0.071588907	0.135926338
0.166232412	0.059389959	0.045083262	0.374210309
0.066661908	0.313687521	0.071287568	0.096693607
0.236328473	0.14181819	0.291382498	0.127850377
0.082707082	0.290150733	0.035841572	0.09752284
0.089134867	0.285855548	0.144634326	0.151905598

0.060815294	0.097193557	0.244995141	0.138328023
0.076193717	0.075693118	0.281252425	0.07321789
0.060778432	0.4088123	0.163690845	0.05635668
0.061130044	0.204295105	0.128377417	0.076739784
0.050354632	0.285424229	0.086713486	0.216760711
0.251483433	0.111210544	0.231891707	0.061125702
0.105785161	0.289234537	0.191307921	0.300510137
0.172310346	0.387412942	0.253178587	0.283186393
0.109331902	0.143836111	0.062922899	0.37455567
0.07707466			
ENTPD1	0.299076199	0.342635185	0.222088175
0.32397585	0.246851375	0.152523558	0.123828403
0.061618078	0.079017295	0.004537855	0.025797764
0.26124795	0.061388316	0.060295074	0.074926256
0.413803808	0.09562694	0.144846237	0.198940876
0.31839677	0.038365709	0.36122636	0.082112307
0.15970762	0.094467872	0.078054796	0.143537513
0.372957258	0.094422481	0.087477248	0.246990414
0.13260653	0.312584622	0.308870267	0.291598313
0.294196725	0.25391693	0.021243306	0.057476793
0.302614815	0.337785607	0.313635327	0.24052965
0.048607748	0.261114957	0.172376277	0.319476109
0.063619162	0.046925699	0.049136394	0.251669034
0.249086176	0.199623882	0.079741779	0.080189868
0.042051872	0.039140762	0.069389856	0.148232742
0.147890488	0.030409119	0.278123402	0.057157766
0.055747925	0.103946878	0.135262318	0.116103537
0.129185465	0.228881714	0.039287924	0.225986581
0.001150868	0.325007636	0.325936949	0.108738402
0.087714603	0.021001531	0.142825447	0.210678172
0.381006399	0.31025157	0.403722747	0.388118412
0.086893772	0.249468039	0.23245869	0.099455664
0.313707759	0.089662671	0.075237984	0.416923776
0.400945112	0.322215249	0.133499037	0.286103579
0.255926935	0.313489931	0.156662951	0.175770854
0.083743386	0.119694436	0.256819638	0.37187492
0.243441163	0.179319966	0.167793903	0.275612835
0.090566662	0.075063859	0.294283106	0.077224708
0.390846755	0.035341103	0.215479508	0.176569109
0.151377577	0.305725278	0.329492875	0.148971092
0.233961583	0.034644422	0.073819823	0.085971347
0.081482035	0.093282865	0.245577343	0.331172974
0.060050196	0.10395942	0.134088157	0.02054136
0.063599373	0.163797417	0.276953696	0.189369478
0.338865262	0.25732467	0.105917118	0.211747567
0.252618607	0.402777531	0.006432856	0.084717587
0.207670126	0.163754638	0.254273144	0.214710708
0.206821036	0.077344038	0.09997991	0.395077421
0.251953195	0.091861745	0.099571126	0.392907431
0.067330934	0.269609547	0.093917731	0.085066885
0.042652963	0.285615123	0.072831763	0.138951418
0.078607442	0.084570186	0.041186496	0.101213716
0.065081058	0.254921828	0.246217718	0.124652434
0.128119528	0.329966277	0.110574516	0.218419616

0.012725885	0.081306441	0.073459514	0.3493186
0.103240796	0.394968098	0.126303255	1
0.277912121	0.392149375	0.010482579	0.277097104
0.330798677	0.068299636	0.20702438	0.309445358
0.156987609	0.345010807	0.013758252	0.359692235
0.178023162	0.102490248	0.422488776	0.158248585
0.286766359	0.066424496	0.081254169	0.331820951
0.23768829	0.013625924	0.065199634	0.271771675
0.083526072	0.03715935	0.321608326	0.300823403
0.230792501	0.394970515	0.429037598	0.137281598
0.216785657	0.119965063	0.05273692	0.279502834
0.349281201	0.079011355	0.260633564	0.098607808
0.122428097	0.304994895	0.257708065	0.04286297
0.056499342	0.137901381	0.017027806	0.215333312
0.269965046	0.047322605	0.168179253	0.137903417
0.09550025	0.367814999	0.128785553	0.132349076
0.096373314	0.154042939	0.181965401	0.043460495
0.050183263	0.128579495	0.127678975	0.123785909
0.029317531	0.07840054	0.074993354	0.063203326
0.031620581	0.056636813	0.052761751	0.208934115
0.069933455	0.045534035	0.180872832	0.154413574
0.124672625	0.050242199	0.337860061	0.360677503
0.160196717	0.108050952	0.082637274	0.042151719
0.331568548	0.085309906	0.053442819	0.2903031
0.058964842	0.409921274	0.070523735	0.134066309
0.114538106	0.172725269	0.400659889	0.030727196
0.194300907	0.277755858	0.290705233	0.133639763
0.204547161	0.279170608	0.038406032	0.239047353
0.213624365	0.056062858	0.35188149	0.111280682
0.065025251	0.350372066	0.26522616	0.13614257
0.111538251	0.124289571	0.029508955	0.312229007
0.357195982	0.377952776	0.247280797	0.165488505
0.045877394	0.008062349	0.183465591	0.240231752
0.281059062	0.351642456	0.250509485	0.16260586
0.232863902	0.105614913	0.0323718	0.358258369
0.155425416	0.326375232	0.033020654	0.33605726
0.283481534	0.078897546	0.088250993	0.137988057
0.21268276	0.067258517	0.122170938	0.294932908
0.3144578	0.147361752	0.108175624	0.317960595
0.021253482	0.040803244	0.285599427	0.128940701
0.098535475	0.142513292	0.054526492	0.081405339
0.044280258	0.264186338	0.395746134	0.078578205
0.10377181	0.079015338	0.356970008	0.064807617
0.336176666	0.335761576	0.304822828	0.100773024
0.29517989	0.321683185	0.195835995	0.127220057
0.0619722	0.314886503	0.247509626	0.023196694
0.392890241	0.057667253	0.010647405	0.323557352
0.165200762	0.225424296	0.004965989	0.076027715
0.107879327	0.419582255	0.272844009	0.254576768
0.032023968	0.300421374	0.030269679	0.378238744
0.290275589	0.050573392	0.142154012	0.262619825
0.267254478	0.339973497	0.160226646	0.322294692
0.214628924	0.285166013	0.067907673	0.273581658
0.291067157	0.31339398	0.296821249	0.299445307

0.349604272	0.368508762	0.239939127	0.028123092
0.121014273	0.064830063	0.046588281	0.049784172
0.142059978	0.108626286	0.33466562	0.40318211
0.346146376	0.030579607	0.05905878	0.264709465
0.310057448	0.384501297	0.076266919	0.012883363
0.013845113	0.033524918	0.208488128	0.244023888
0.352452346	0.024385422	0.021279174	0.032454869
0.106663974	0.302714015	0.308250539	0.364961186
0.358265675	0.103222926	0.043340111	0.370291511
0.088852929	0.158022846	0.354473209	0.27882639
0.10253908	0.227038432	0.157596142	0.383647632
0.050819202	0.294564801	0.267503264	0.07902339
0.05601579	0.116518044	0.100141946	0.414572335
0.277267941	0.042545653	0.034888136	0.021689401
0.055442535	0.380775135	0.084207744	0.403900873
0.318832351	0.400405278	0.110408635	0.297593227
0.051355187	0.271780621	0.209009834	0.015544456
0.158202988	0.05084241	0.300737154	0.049436161
0.074493751	0.258246482	0.127214535	0.063812897
0.118111491	0.081584784	0.388842363	0.400287485
0.099340353	0.114106514	0.27137653	0.331693887
0.115714887	0.317161547	0.285753126	0.119502487
0.054498379	0.103735433	0.3057705	0.207943048
0.061727228	0.418871926	0.094173721	0.378911737
0.269504003	0.347851176	0.315955034	0.219719708
0.111704108	0.185782042	0.18489921	0.014985362
0.260391822	0.354038677	0.182083435	0.095112698
0.163370068	0.217030926	0.074128544	0.055162563
0.164988827	0.119772636	0.072087071	0.429013995
0.232580976	0.220746937	0.046789558	0.089777961
0.357661277	0.293396564	0.063586956	0.257988101
0.188529253	0.249280978	0.358397387	0.368090179
0.109895056	0.281761192	0.293362508	0.181781654
0.240311337	0.199590158	0.314602669	0.029402242
0.090926461	0.221399596	0.052897804	0.153950803
0.124601642	0.076639145	0.087919857	0.386355642
0.093006456	0.27737649	0.061962707	0.102166617
0.283965505	0.151642186	0.296117417	0.074488531
0.054555313	0.283706817	0.029906701	0.09266133
0.108662771	0.309770236	0.181188885	0.165885986
0.113664862	0.153671826	0.225569965	0.087178592
0.052726698	0.091135331	0.297362022	0.04799099
0.02797161	0.413925892	0.136771595	0.084376405
0.107543091	0.205240335	0.129080513	0.089374406
0.075431073	0.310266257	0.072863128	0.262674615
0.196862051	0.097317397	0.197084848	0.064543863
0.098108968	0.263231377	0.261527136	0.31293765
0.231072212	0.36735788	0.240471017	0.270406965
0.113405384	0.190479916	0.044294973	0.357150121
0.093334242			
ENTPD5	0.249344534	0.321636345	0.178327854
0.338009925	0.243887443	0.145883019	0.185136024
0.111805566	0.159207729	0.027916836	0.098307865
0.246570573	0.17546791	0.149804215	0.117851627

0.400774384	0.269149969	0.167192357	0.253043937
0.310012994	0.102650474	0.390387282	0.200199067
0.248442403	0.243632346	0.217358094	0.238912936
0.367914916	0.23337749	0.091780455	0.240692692
0.277486854	0.284463775	0.275635979	0.314950828
0.265224586	0.20265357	0.050310952	0.118139378
0.262352517	0.316678385	0.30714283	0.28174391
0.149895911	0.234009873	0.215556096	0.288558119
0.105349396	0.166712389	0.073424546	0.219515744
0.23326658	0.193905345	0.133965698	0.174429018
0.116615385	0.077904023	0.14807244	0.13853274
0.223958354	0.057637357	0.256656038	0.127671725
0.094320153	0.157462243	0.291741059	0.168712277
0.196799736	0.200382973	0.093911557	0.191259913
0.009388226	0.312767387	0.303660597	0.146600846
0.215394247	0.109352384	0.30319416	0.23937805
0.368249863	0.261202681	0.398037202	0.369388434
0.24744473	0.290393	0.203736385	0.262304133
0.277590933	0.178275769	0.160860748	0.421130849
0.390982294	0.300312708	0.280615669	0.30206602
0.256793124	0.289056222	0.1534458	0.194777625
0.148150292	0.221902758	0.235885289	0.358094803
0.216056037	0.199273804	0.298550019	0.270398521
0.228026609	0.124513245	0.330081861	0.181356636
0.377654109	0.090936579	0.305606972	0.198901088
0.250384645	0.280530734	0.302794566	0.228825648
0.219991794	0.120547812	0.150601102	0.197012857
0.173286744	0.163515625	0.215686056	0.342454035
0.12578469	0.203120314	0.202854495	0.063087206
0.192574209	0.25905768	0.263298561	0.186410649
0.304594455	0.204507917	0.22626072	0.186596455
0.234128779	0.393619468	0.013365886	0.191232382
0.203523012	0.267740467	0.298835132	0.216426222
0.269604708	0.166800715	0.245662007	0.373657423
0.226066698	0.216501672	0.118208622	0.395356244
0.145805597	0.283096699	0.230326925	0.211020094
0.132684725	0.252869495	0.196434378	0.138763154
0.121227073	0.229325979	0.075472724	0.12490923
0.147399451	0.258305325	0.209920805	0.253327532
0.215981654	0.341149005	0.101135685	0.234773786
0.057629	0.18698866	0.070121051	0.344377193
0.09941167	0.361938224	0.250708546	0.277912121
1	0.370181468	0.01277232	0.248545483
0.302345688	0.070370171	0.249027329	0.28001868
0.154861121	0.350224339	0.018947578	0.373585909
0.152779819	0.230035955	0.407955338	0.28596406
0.255524588	0.19317507	0.134127117	0.300635083
0.232948909	0.040831918	0.083071637	0.27223107
0.188369012	0.083851096	0.281311707	0.305545062
0.234379634	0.356885133	0.421824473	0.149699882
0.225829139	0.202706407	0.117288135	0.280610305
0.303140213	0.124541976	0.237664087	0.198407731
0.10379878	0.285544613	0.230930818	0.073731516
0.160406769	0.156243868	0.053792313	0.240573494

0.215999609	0.155994917	0.139860182	0.246185213
0.256831238	0.352007534	0.22460656	0.175622559
0.160549759	0.181368563	0.232868765	0.129668036
0.157175727	0.270311104	0.196879392	0.185387585
0.088823514	0.239836562	0.115978349	0.117486875
0.125751813	0.129450024	0.108256366	0.19400055
0.154056098	0.1699315	0.226750568	0.221548954
0.276123267	0.146829677	0.316243222	0.348743287
0.142452473	0.225124058	0.214898229	0.096101279
0.338215522	0.084121491	0.063578703	0.288646911
0.120814633	0.38290063	0.199260967	0.275435737
0.191808406	0.251375272	0.386618846	0.053293285
0.168991337	0.26549197	0.275047693	0.272191173
0.198789189	0.273751587	0.119818244	0.210794933
0.197904285	0.183855845	0.331439397	0.280570277
0.173689517	0.329837577	0.260442302	0.256877771
0.262479486	0.17145317	0.074149759	0.345259738
0.342498056	0.405126207	0.246085714	0.258393156
0.157381706	0.058776104	0.293949872	0.196739052
0.254202551	0.330282734	0.291763865	0.215921038
0.271239519	0.1859024	0.056617663	0.347629199
0.197472117	0.29162893	0.095992427	0.309234527
0.255164992	0.08241072	0.240376208	0.148244685
0.205910407	0.170375534	0.21969777	0.275477433
0.28284769	0.217182213	0.176299063	0.294707416
0.06737905	0.112779035	0.254356901	0.199719534
0.164304764	0.272991842	0.090971274	0.132446325
0.161334759	0.255382279	0.38290714	0.187146878
0.111393642	0.116899288	0.357524131	0.178108816
0.308612559	0.296374556	0.279793442	0.20013986
0.278137162	0.305406391	0.188059733	0.2660064
0.164701087	0.283830613	0.212940841	0.088724934
0.387456931	0.056520414	0.067741612	0.302508244
0.242361228	0.195354381	0.011957276	0.077392327
0.205440086	0.395816497	0.245484151	0.239891903
0.056448344	0.277296839	0.038408307	0.346719723
0.279724264	0.145706203	0.273113584	0.256315624
0.256975357	0.312095048	0.19210443	0.313331631
0.219603908	0.263087353	0.165757055	0.304572816
0.290490405	0.282407723	0.273965122	0.267578669
0.356904066	0.358148722	0.277460533	0.091742907
0.163051763	0.098114999	0.088032483	0.131256659
0.122940733	0.262683095	0.305002687	0.389810624
0.337075433	0.09040385	0.131284188	0.24938337
0.301438591	0.365784721	0.111414306	0.02160989
0.021261507	0.115424905	0.202534769	0.304591729
0.32450338	0.056528929	0.075150866	0.08457204
0.213942877	0.293925267	0.290983802	0.377054036
0.314604739	0.25130733	0.104411241	0.36116044
0.217029684	0.125686583	0.348620977	0.260192582
0.206907745	0.293787242	0.150734343	0.374527024
0.082435987	0.317427121	0.256926096	0.184238186
0.09679924	0.264702352	0.223473996	0.387362163
0.250746512	0.170308255	0.152470146	0.041868025

0.173328747	0.353144144	0.238236295	0.388210357
0.312654998	0.411034128	0.241503164	0.267162246
0.141806728	0.253649602	0.236231169	0.035482197
0.258256152	0.128477445	0.272417845	0.105609405
0.11804419	0.229553861	0.169172414	0.15451973
0.154961779	0.232524397	0.388045295	0.37998867
0.194718659	0.205472002	0.28947564	0.300384005
0.119822844	0.340884566	0.259984311	0.114113327
0.144546488	0.158154649	0.296682299	0.187278424
0.125157583	0.405697899	0.192863461	0.34770871
0.229465615	0.30056856	0.297803537	0.219930875
0.230675461	0.166460916	0.200507453	0.065085151
0.2603128	0.367279186	0.161419464	0.237426798
0.26670618	0.187876759	0.139338201	0.075497187
0.152788209	0.120576914	0.209036727	0.414322553
0.203875437	0.196715441	0.105631226	0.212350164
0.330641165	0.251617181	0.123993823	0.258442944
0.302887776	0.257803024	0.329819685	0.343864108
0.155853805	0.285143756	0.283853763	0.199016403
0.229768469	0.235541173	0.287463605	0.053414186
0.173726952	0.194754292	0.18121663	0.27847635
0.257538058	0.183977486	0.141632765	0.37594597
0.139976063	0.321514622	0.101421304	0.224473897
0.283439785	0.12715654	0.305780259	0.151692535
0.185718478	0.26492505	0.080814637	0.238844231
0.156212937	0.283847417	0.19085825	0.304087963
0.192450359	0.271883579	0.273940692	0.091123072
0.090129415	0.145315321	0.275916275	0.100583214
0.092755994	0.403686485	0.122711137	0.155281291
0.174406951	0.163240948	0.210602256	0.189843074
0.172428063	0.29150308	0.092634033	0.238011088
0.195424205	0.230533381	0.168084667	0.110515183
0.173124341	0.237477112	0.220464654	0.287241857
0.229294936	0.393755802	0.204946968	0.261810166
0.171486457	0.145220928	0.120270983	0.332816024
0.121291624			

EOMES 0.387844443	0.513080216	0.406947076	
0.423151461	0.43754953	0.347989723	0.282532906
0.242317753	0.155853835	0.012074204	0.04187962
0.416286725	0.231901935	0.296835525	0.341634887
0.447595819	0.195121319	0.39678685	0.33236866
0.431375988	0.140362767	0.43438456	0.172267923
0.348370058	0.163480152	0.1297241	0.32705379
0.446062526	0.149335476	0.292657876	0.383057959
0.181351578	0.400563058	0.494100025	0.418736336
0.47873265	0.356414622	0.023552824	0.304633163
0.408967258	0.424911149	0.423666576	0.391601622
0.091819628	0.45438724	0.320009857	0.432043983
0.273238047	0.06524152	0.246434693	0.421551021
0.438668865	0.389951902	0.286704499	0.230976373
0.197490717	0.228003355	0.258295768	0.299597488
0.302014025	0.170474153	0.455078719	0.151307829
0.183315623	0.317623639	0.227746966	0.323187143
0.358959364	0.351976617	0.049786967	0.363145983

0.005450033	0.428449654	0.427549307	0.311411911
0.093311271	0.041457489	0.260948564	0.377362802
0.464792029	0.445659546	0.486403292	0.485975602
0.234774105	0.385460844	0.388949215	0.251864807
0.420954828	0.240396898	0.176318713	0.445070633
0.493714415	0.498151	0.282086804	0.353313552
0.38261114	0.468563233	0.413679723	0.430909383
0.344061218	0.108484145	0.417496398	0.503506068
0.373159626	0.331614398	0.237290951	0.387656079
0.123217664	0.365639954	0.410641569	0.198005667
0.47884607	0.049486381	0.293672559	0.37747462
0.329730118	0.407158888	0.493547573	0.27632478
0.426481341	0.046822393	0.174021352	0.080957335
0.345390461	0.176365383	0.439475035	0.449556362
0.269787965	0.393929541	0.345786221	0.010517573
0.063410931	0.277592195	0.384880169	0.409260154
0.428330438	0.35865536	0.294126783	0.3377568
0.389400452	0.47795644	0.054970555	0.180514659
0.424946917	0.276600813	0.335381018	0.410867651
0.370354565	0.196880372	0.21035253	0.521080846
0.41257352	0.156378303	0.34778163	0.44805308
0.329526367	0.394958296	0.189550156	0.320971867
0.097484201	0.450617696	0.065018303	0.367689684
0.276023599	0.185329683	0.035443646	0.297809067
0.31738644	0.398275403	0.42601093	0.195319909
0.360809846	0.437495305	0.319186588	0.376371402
0.02297067	0.105892119	0.264787047	0.496485263
0.253042132	0.498971817	0.387841456	0.392149375
0.370181468	1	0.008619239	0.466981769
0.502203119	0.272480607	0.400283243	0.433562586
0.362581631	0.443360267	0.045259022	0.44152451
0.315425559	0.159901509	0.512467998	0.239094222
0.440647573	0.088583742	0.331248763	0.402502122
0.357992911	0.017550595	0.240014648	0.403976986
0.064541864	0.141072291	0.418992025	0.434281862
0.407222817	0.437964865	0.494732635	0.346942922
0.437186581	0.320063673	0.037481633	0.390531619
0.411244422	0.340536121	0.430299766	0.318779944
0.296987352	0.465146691	0.36955057	0.208433279
0.094680666	0.352081813	0.041029769	0.396800818
0.369474744	0.164084613	0.374061292	0.227347119
0.130555435	0.518675226	0.230732971	0.280523923
0.162579435	0.365692268	0.350531625	0.237931671
0.216631101	0.258627795	0.377695341	0.404066621
0.074838214	0.157443646	0.040221466	0.30897286
0.145821431	0.300545742	0.286395502	0.354425654
0.326989109	0.147766944	0.407350717	0.351804761
0.194822243	0.04205327	0.498838331	0.448407887
0.290027044	0.249095458	0.092454718	0.279313848
0.457124251	0.273992697	0.252399027	0.442339539
0.069310883	0.48718426	0.284196048	0.197266444
0.362304485	0.332807139	0.472839911	0.079612841
0.381822799	0.450003904	0.405737132	0.268211643
0.367282499	0.430384182	0.067469705	0.35755335

0.344832662	0.071985452	0.509854168	0.191235398
0.055001594	0.429928374	0.38727541	0.147173358
0.151655677	0.346102304	0.087505663	0.37552797
0.512456263	0.438032867	0.373129359	0.295052936
0.084035881	0.017079835	0.289191511	0.398343768
0.476097806	0.493569751	0.357329446	0.375320924
0.337301992	0.354854628	0.165135687	0.499247817
0.27425787	0.497939471	0.023251009	0.400572997
0.472154647	0.272228345	0.139661642	0.337385024
0.356729265	0.104918645	0.304177113	0.475973425
0.430093116	0.386436858	0.379763251	0.431237512
0.041259396	0.08817897	0.426333704	0.233306462
0.387679974	0.318046928	0.071116944	0.34881378
0.086669543	0.433468632	0.443395159	0.11991003
0.292881024	0.339054005	0.429510558	0.08723469
0.429674947	0.481059025	0.496096265	0.259637541
0.392845354	0.46967504	0.355334997	0.155801415
0.046560867	0.48961984	0.386344541	0.048026223
0.473431813	0.220668038	0.024104776	0.491460499
0.286572365	0.405411625	0.013657019	0.114071853
0.091320573	0.450474623	0.455071115	0.392749225
0.219482245	0.42952192	0.014463458	0.512376126
0.470523898	0.03867835	0.214145488	0.373118311
0.371346272	0.517437008	0.339387776	0.423301651
0.383646482	0.467197367	0.278284106	0.35906551
0.41739129	0.463675437	0.411059358	0.455647513
0.449518679	0.441974243	0.362582401	0.040640994
0.203991606	0.246029113	0.277956185	0.127782212
0.306820431	0.219268409	0.47509148	0.468419073
0.466942817	0.018300617	0.215548152	0.377392645
0.439716866	0.486261762	0.055994545	0.014963093
0.015537873	0.038445939	0.391634547	0.34313677
0.483262953	0.205896812	0.017121234	0.086033203
0.370329619	0.406032759	0.48239752	0.434346718
0.410662115	0.154017952	0.26270133	0.441215375
0.123178283	0.2863862	0.478069441	0.457043624
0.09418516	0.350661386	0.38783049	0.495965474
0.084811863	0.394775382	0.411018458	0.339275563
0.036888605	0.152015754	0.129991898	0.44489119
0.461001074	0.110593357	0.061175929	0.138618988
0.174248952	0.461717735	0.238062395	0.518274304
0.427671247	0.44215971	0.129991253	0.469898957
0.075032381	0.455709448	0.370942102	0.080512423
0.37738345	0.038751366	0.443663257	0.170282787
0.326567229	0.395521027	0.263958669	0.043393274
0.221635815	0.106554206	0.480185718	0.449176307
0.076778417	0.33432263	0.397397326	0.442234115
0.309077287	0.425375816	0.476638615	0.347817627
0.144567864	0.172885032	0.420629704	0.363687753
0.274678113	0.495864637	0.325992821	0.520156379
0.385813895	0.462697483	0.454438782	0.400444349
0.269593652	0.384520297	0.349939247	0.042341453
0.425446145	0.443305031	0.397125924	0.215591624
0.323335619	0.371758442	0.104665937	0.200351721

0.317375948	0.295900332	0.209400396	0.447149981	
0.3882724	0.362802099	0.081915876	0.122726151	
0.529037606	0.432689127	0.215487026	0.414017748	
0.275526382	0.403029673	0.421020761	0.418270041	
0.382344407	0.411642192	0.40134207	0.349110102	
0.408152294	0.355269309	0.50207197	0.124897096	
0.137044408	0.374253827	0.143953475	0.169164161	
0.226723209	0.070244221	0.290138197	0.445362544	
0.335357658	0.416271268	0.303166122	0.164039133	
0.397637969	0.36584081	0.426855647	0.2561894	
0.133873293	0.471401636	0.026071449	0.115462994	
0.346456992	0.399177508	0.374467801	0.344527517	
0.314659787	0.218631183	0.375207378	0.290652316	
0.271492946	0.360986756	0.398736816	0.080229358	
0.047333893	0.50041793	0.373316562	0.055979555	
0.142392339	0.355747966	0.364420552	0.292259562	
0.054333472	0.454518569	0.299772731	0.434454658	
0.394609979	0.136662267	0.334681313	0.146215418	
0.133414274	0.430486571	0.403444	0.484589642	
0.400770851	0.435806733	0.417166293	0.387984771	
0.203125679	0.357598095	0.069395987	0.435556977	
0.347707501				
ERMN	0.007986195	0.003434735	0.009771349	
0.004595882	0.004012004	0.002209446	0.002450999	
0.002889186	0.001557736	1.27E-05	7.48E-05	0.007168281
0.000120189	0.001241414	0.00234213	0.00918417	
0.002819692	0.01138379	0.006343631	0.006414827	
0.000606533	0.006668877	0.003173253	0.00705056	
0.004526033	0.002863683	0.005081135	0.006514993	
0.005852515	0.000157676	0.004105893	0.020261151	
0.005387632	0.003175642	0.003829763	0.002934494	
0.006947824	0.000300195	0.000485738	0.006779455	
0.005701416	0.00515604	0.005390689	0.00239788	
0.002704254	0.00347346	0.012094736	0.000887923	
0.001301741	0.001283209	0.002636774	0.001912126	
0.001535241	0.002230729	0.0013844	1.64E-05	
0.000346871	0.000980169	0.002501848	0.002114485	
0.000421417	0.002212534	0.001028764	3.41E-05	
0.003872986	0.010501996	0.004273399	0.003807393	
0.003738022	0.003675843	0.007425236	6.47E-05	
0.007963511	0.015523322	0.003055938	0.010466924	
0.000317571	0.012639776	0.002730076	0.006725974	
0.005577376	0.005706663	0.005040995	0.001698481	
0.016608549	0.006521272	0.003188961	0.004634833	
0.001337869	0.001252435	0.008559679	0.005447573	
0.002913139	0.002807077	0.017944317	0.001966269	
0.003365043	0.005449955	0.002472854	0.001626853	
0.02245039	0.001886448	0.004195868	0.004247104	
0.008147103	0.011232007	0.004013149	0.008852782	
0.005481391	0.005649992	0.001038625	0.006093199	
0.001849365	0.011902098	0.001396518	0.014929396	
0.006546565	0.003434452	0.004124901	0.007362471	
0.000393694	0.000649443	0.019128327	0.000397882	
0.007540715	0.002108681	0.003459624	0.000861986	

0.00081102	0.003013467	0.0067379	0.008645281
0.010304228	0.003674182	0.011691124	0.00844348
0.005721947	0.001648355	0.002575049	0.003313645
0.005326913	3.29E-05	0.00443598	0.004120461
0.014935923	0.009767054	0.002726778	0.005777586
0.004542185	0.003952361	0.005038007	0.00270052
0.004165019	0.005356774	0.010268969	0.000109669
0.008501241	0.00530179	0.001318032	0.000742724
0.002808083	0.014922811	0.006374384	0.000659215
0.000923389	0.011783537	0.002280478	0.001126536
0.001775505	0.00243037	0.01252	0.001937693
0.00638846	0.00263982	0.001911854	6.60E-05
0.007950312	0.002294016	0.003488408	0.001074463
0.006628602	0.005348291	0.010482579	0.01277232
0.008619239	1	0.002675728	0.003382204
0.00227458	0.002272957	0.010492539	0.002502959
0.004782523	0.000595145	0.007679379	0.004283311
0.013171667	0.006153803	0.005718256	0.003337837
0.003807817	0.000889039	0.005628549	0.002353427
1.33E-05	0.001111984	0.003582344	0.021702202
0.000197032	0.007651423	0.007077301	0.003244399
0.009620119	0.007125794	0.002657699	0.004089549
0.006081449	0.010309814	0.016884259	0.008608597
0.000279289	0.002977532	0.0021475	0.002962366
0.003092581	0.004194632	5.23E-05	0.002711396
0.002031506	0.000100572	0.005750756	0.00427061
0.000131053	0.00271179	0.011866968	0.007811304
0.003859653	0.01355866	0.009708118	0.010089738
0.000949133	0.009026947	0.000108982	0.000121
0.003734153	0.002816065	0.001873765	0.000450057
0.002643181	0.034868557	0.000435224	5.55E-05
0.000220169	0.000132167	0.002324946	0.000400933
0.000358851	0.003005644	0.009528613	0.011947089
0.008834918	0.003594008	0.010932529	0.002669216
0.002764992	0.012656097	0.000146514	0.002918983
0.000993945	0.001346173	0.002283361	0.002378238
0.006155033	0.000746423	0.006207776	0.001384206
0.003592557	0.006546067	4.75E-06	0.003858629
0.002169998	0.019654293	0.005025414	0.001837566
0.004263319	0.00045032	0.00520148	0.003324619
0.004299293	0.003847254	0.005209708	0.013948947
0.009337282	0.002804653	0.013584641	0.017212387
0.00076773	0.000988543	0.011911486	0.003722077
0.006872668	0.001680294	0.022898732	0.001240681
7.55E-05	0.017767765	0.012478551	0.002802822
0.004176531	0.01241522	0.002479078	0.007922493
0.001299421	0.000238978	0.003768845	0.02434291
0.00387392	0.00849881	0.005177735	0.002610474
0.000973355	0.005474009	0.001355811	0.002365858
0.00538713	0.010146938	0.003205976	0.003612902
0.002990549	0.002480635	0.00917923	0.000629548
0.000514919	0.009161173	0.012462734	0.00111724
0.005544736	0.004924475	0.001175192	0.001670733
0.001834625	0.008069382	0.003232567	0.000254136

0.002695685	0.011470658	0.005536636	0.004574128
0.00466894	0.003111332	0.006606559	0.004407515
0.003205746	0.002753321	0.023695449	0.017142861
0.003565969	0.006209702	0.00087952	0.005319575
0.000975807	6.27E-05	0.003418095	0.008094743
0.002311858	0.00142833	0.01005693	0.032122991
0.008537568	0.002594142	0.00435189	0.00056187
0.012021427	0.001821589	0.005042137	0.002660955
0.010359823	0.007329795	0.002642716	0.003968413
0.003943684	0.005374312	0.00383112	0.003694427
0.002413064	9.74E-05	0.009511411	0.003367334
0.003442792	0.006790566	0.004391735	0.003991919
0.009923771	0.005317555	0.000756811	0.003080438
0.001141283	0.000618655	0.000174966	0.002842672
0.005679327	0.005665766	0.00593726	0.004177424
0.010816281	0.001402343	0.00301153	0.004053091
0.006109028	0.021132829	0.001282791	0.001175832
0.000933622	0.003416822	0.021817401	0.00438454
0.000211237	0.003303725	0.000894561	0.009315684
0.007407316	0.002642029	0.008539348	0.00821185
0.007568332	8.73E-05	0.0096638	0.002183434
0.003926938	0.004021537	0.002222067	0.023358812
0.017291341	0.008666197	0.00466779	0.004392168
0.010715963	0.005628076	0.000412938	0.004699547
0.011735055	0.006721025	0.009690407	0.002544646
0.000260834	0.000688683	0.000129746	0.000383926
0.005780474	0.002773347	0.005270508	0.004667305
0.007011232	0.00510884	0.003117946	8.14E-05
0.002337347	0.007264666	0.000473871	0.01953306
0.009754644	0.00393923	0.000164815	0.001150427
0.007044157	0.00180684	0.013796917	0.008336853
0.006639787	0.004640333	0.00683622	0.024284533
0.00360789	0.021496072	0.013354747	0.002320849
0.003805645	0.002720693	0.002651654	0.000502037
0.005115149	0.006971795	0.005519457	0.001036113
0.006341	0.000525628	0.00503405	0.005553897
0.005381566	0.003520728	0.004028222	0.001100359
0.004560976	0.001355564	0.000400444	0.009564796
0.00387151	0.007728201	0.001724259	0.00313662
0.007071497	0.003460657	0.001133849	0.007056007
0.001566485	0.00069991	0.00881196	0.006689285
0.004370539	0.001558726	0.005162598	0.003969424
0.003847748	0.002585191	0.00449206	0.020212223
0.004230499	0.00834478	0.006869984	0.002394305
0.002509537	0.004101188	0.00146585	0.001893807
0.005843996	0.003171835	0.001383608	0.000835275
0.004794086	0.000119488	0.0187128	0.004006808
0.014917221	0.004398144	0.008919217	0.00288216
0.004021766	0.000583243	0.016138695	0.011959848
0.008808445	0.009718812	0.001689224	0.001238045
0.002245302	0.000414059	0.010974543	0.002288181
0.003771088	0.003177064	0.014137628	0.002073356
0.014260802	0.004539213	0.001645356	0.000192712
0.002261933	0.004007957	0.002387288	0.000627445

0.006353882	0.006533432	0.039073625	0.001077169
0.0047023	0.010273251	0.000572544	0.024653836
0.002848898	0.000168083	0.005745871	0.004826884
0.011095797	0.003045778	0.001066705	0.015190087
0.003282602	0.010293808	0.002984155	0.019048558
0.005226843	0.002657968	0.002854972	0.005207971
0.009076859	0.002231506	0.006010975	0.001028845
ERRFI1	0.474056356	0.50920969	0.421270113
0.441426484	0.422910268	0.454099077	0.206717723
0.212137932	0.076970602	0.003681491	0.016605313
0.462110729	0.198862691	0.2907915	0.393352782
0.413727961	0.106095685	0.37105833	0.219989922
0.422321284	0.09601768	0.384007239	0.076660421
0.262286683	0.081715328	0.050777184	0.247237266
0.462933801	0.059625048	0.410483027	0.433117633
0.073075061	0.485074275	0.505740842	0.441954435
0.502619605	0.474363834	0.00846847	0.338349935
0.484661703	0.471918519	0.425405989	0.319121433
0.031751223	0.492184151	0.236300866	0.390650796
0.270950678	0.020802	0.312037223	0.482121448
0.492785646	0.481158258	0.250700028	0.152350483
0.212327072	0.283306297	0.27691335	0.409677454
0.210128607	0.208817488	0.50050718	0.084642238
0.150664607	0.261655167	0.116834779	0.269499048
0.339779257	0.469514175	0.013091098	0.452100694
0.002468047	0.395703429	0.355711774	0.328558317
0.027378763	0.014094122	0.14025998	0.348477857
0.477134267	0.488251844	0.485436373	0.493710456
0.156304925	0.257306104	0.395117269	0.146236944
0.497625629	0.173086873	0.10541463	0.409506594
0.491162791	0.506972574	0.171230221	0.224300643
0.49131415	0.503200465	0.471337165	0.437793693
0.373294181	0.033896578	0.499956174	0.505079701
0.481337158	0.305633163	0.120867835	0.462717722
0.040903429	0.42366745	0.343313873	0.14111344
0.481395236	0.013529432	0.173844932	0.376609586
0.189551093	0.494385223	0.503929029	0.170919085
0.402382807	0.013314338	0.128052255	0.0207091
0.342376522	0.127694702	0.50359176	0.489540973
0.255481134	0.369942145	0.262051343	0.000957577
0.015947554	0.154740591	0.494589891	0.377193444
0.460792725	0.466469296	0.2173887	0.482551682
0.483593856	0.48323162	0.093800352	0.081375578
0.458050597	0.150427697	0.207770386	0.47678575
0.286122374	0.107813265	0.103905499	0.504051628
0.482619513	0.066419441	0.3859685	0.407583269
0.35717052	0.332340077	0.086561443	0.268754086
0.040301662	0.492809425	0.016500079	0.374692878
0.306101609	0.119954182	0.007133235	0.295153025
0.322958552	0.501823245	0.489559597	0.082854
0.313243195	0.3963039	0.388377929	0.48861504
0.007506863	0.034446029	0.364977182	0.507633217
0.385405434	0.497191176	0.284885616	0.277097104
0.248545483	0.466981769	0.002675728	1

0.504874924	0.39744318	0.408348846	0.420555729
0.403193828	0.480822717	0.055886753	0.410834439
0.45267864	0.059409147	0.489190037	0.129895113
0.499195277	0.02773927	0.35386421	0.478207275
0.491716804	0.006635287	0.282712358	0.416225178
0.015346577	0.107053735	0.465745066	0.423160464
0.399329198	0.462849522	0.473310757	0.385633569
0.433432677	0.22885251	0.007019012	0.288366779
0.477453229	0.420907036	0.493061085	0.240933653
0.44039288	0.507691348	0.496779571	0.239335728
0.032013216	0.335625038	0.020229018	0.323940544
0.48152412	0.109581925	0.439270734	0.118456772
0.049239259	0.508883173	0.14930684	0.252600072
0.108921236	0.445749271	0.238572637	0.258967846
0.214792437	0.13597732	0.357300423	0.390143413
0.03329709	0.071855917	0.007767048	0.423014346
0.139061716	0.382057974	0.39636057	0.4868076
0.346096831	0.098619286	0.384582476	0.27075522
0.088185162	0.008004033	0.509952484	0.404364165
0.440787237	0.137905958	0.026348578	0.400367651
0.49531308	0.429452593	0.363848781	0.499939551
0.020971902	0.487323629	0.240707256	0.095371223
0.380063367	0.242240371	0.471766755	0.068432098
0.443164639	0.501900483	0.285380678	0.158709353
0.483808852	0.440529917	0.023227328	0.488192186
0.489132837	0.020007904	0.509359901	0.095994125
0.011677422	0.450044239	0.489159278	0.053262972
0.05567962	0.353823174	0.046563513	0.257628879
0.51010229	0.401305554	0.49594799	0.186708291
0.029372643	0.006864281	0.169396566	0.422292865
0.507700466	0.50406858	0.228197033	0.364583794
0.216671872	0.34016702	0.179511802	0.507693469
0.176661573	0.501637284	0.002847212	0.472542234
0.505801044	0.366869786	0.059839642	0.341418768
0.485445395	0.034306101	0.188271739	0.505373653
0.495974509	0.33134504	0.374459622	0.442213602
0.013094936	0.034538084	0.393742871	0.162169653
0.408733063	0.188505441	0.042632745	0.373356544
0.031565614	0.50115493	0.467308767	0.045959417
0.425490133	0.366194979	0.354900886	0.0251022
0.488028335	0.496865576	0.504515345	0.150970565
0.488649498	0.506428329	0.441556847	0.055523253
0.009156584	0.499501439	0.465657582	0.017571178
0.480733407	0.328987908	0.008915341	0.507134074
0.165078377	0.468325931	0.004371431	0.082956483
0.02503618	0.465020232	0.496263661	0.425174261
0.343290944	0.367442348	0.003018415	0.50347517
0.507644718	0.007494772	0.109204617	0.502048684
0.478257728	0.506414663	0.319773664	0.492925079
0.463069985	0.497869112	0.247873491	0.237995166
0.500032741	0.493681533	0.47516953	0.498832831
0.485793214	0.418801933	0.265123795	0.010603101
0.12487456	0.252591133	0.388171887	0.071248536
0.415697849	0.115918528	0.504814785	0.477504178

0.502074403	0.001498368	0.156687538	0.50567694
0.487489771	0.486373552	0.01356937	0.005462102
0.007264537	0.012912218	0.388060594	0.208430409
0.506604408	0.326604237	0.001381808	0.042610518
0.281256001	0.397472549	0.508310149	0.37326507
0.475619341	0.058243166	0.337015197	0.401905691
0.050237451	0.435872434	0.503161525	0.500802413
0.02588819	0.214462216	0.412502165	0.492406311
0.064212053	0.28518975	0.467123379	0.329078227
0.007779482	0.058021224	0.047003907	0.444163629
0.49439619	0.063957712	0.023117614	0.197109782
0.109471821	0.488828714	0.147139843	0.499494263
0.487458663	0.443313399	0.050536277	0.50161033
0.028414302	0.500275376	0.322775342	0.074527934
0.264428218	0.006680106	0.493921333	0.164546672
0.379860479	0.451567672	0.195302816	0.008537188
0.175352748	0.035647225	0.484570292	0.474053671
0.019851895	0.229185932	0.270057919	0.414033261
0.339848855	0.456662394	0.498522214	0.492789619
0.07523817	0.122750539	0.439580717	0.47619303
0.263896245	0.484478403	0.302017218	0.506513448
0.480939519	0.496808924	0.506041068	0.3665463
0.203555617	0.470589082	0.437317987	0.017415004
0.352181564	0.479432188	0.38873719	0.13805358
0.232567483	0.408027333	0.035720977	0.221593806
0.346333201	0.3881459	0.123259992	0.442072038
0.442698597	0.483652758	0.032763729	0.044975187
0.508765464	0.493596835	0.156806936	0.472153896
0.137860985	0.334186079	0.480474408	0.466936769
0.399165472	0.502298699	0.4801117	0.459484254
0.488711645	0.247021707	0.503768778	0.112665478
0.07182807	0.469480764	0.083155013	0.065633927
0.118972027	0.016172768	0.316110919	0.422806836
0.407997975	0.375977941	0.379485168	0.062998593
0.291941959	0.418565095	0.335737161	0.211149656
0.064765301	0.503001873	0.005389085	0.037391771
0.405506954	0.498140111	0.394766459	0.210122569
0.29304032	0.105750072	0.274292807	0.372820831
0.397450077	0.394744324	0.501982663	0.030138034
0.014175298	0.486477316	0.44390641	0.011631806
0.064343448	0.457151357	0.285512053	0.233048171
0.011850977	0.500760356	0.415614441	0.435151711
0.369185561	0.046975782	0.452827987	0.084113453
0.046822114	0.493783035	0.411929387	0.502963207
0.337675442	0.422004819	0.471380124	0.500059487
0.112302218	0.430008888	0.020592536	0.47941327
0.470692354			
ESM1	0.494197426	0.522427325	0.467969803
0.466356059	0.478806198	0.439569015	0.215402289
0.189361897	0.090544916	0.003965697	0.025185691
0.509886001	0.234488299	0.292305959	0.395541969
0.458644394	0.141529709	0.380501139	0.244511224
0.44667942	0.089018142	0.438886718	0.086323911
0.330666736	0.114093591	0.067346139	0.312980261

0.484278436	0.077747498	0.386293437	0.427831752
0.097578124	0.501179041	0.524447785	0.503577174
0.525232986	0.482651415	0.009596052	0.338156986
0.483884876	0.515320563	0.446419769	0.351409984
0.037667237	0.493210782	0.25725059	0.452877177
0.254779798	0.029698863	0.269260706	0.464871695
0.492216367	0.519776172	0.234521308	0.161945549
0.252894745	0.251800054	0.350380618	0.373969016
0.232351357	0.170827746	0.508225154	0.090752908
0.163487401	0.253902919	0.156526712	0.268060146
0.415992652	0.450600009	0.013688404	0.493952122
0.002126021	0.416984616	0.414829079	0.398265025
0.037855255	0.018885014	0.183159258	0.36724705
0.501934067	0.492820753	0.531050293	0.537360403
0.19451479	0.306778167	0.46760426	0.175254784
0.507866267	0.185246183	0.110520369	0.464635304
0.534732323	0.522232332	0.216001339	0.280780337
0.504238175	0.505977765	0.497356049	0.491446107
0.426537838	0.051492623	0.50035811	0.537996833
0.468119369	0.384236862	0.158047366	0.461762655
0.053299638	0.416478631	0.379597948	0.166343341
0.505261317	0.015645922	0.227301759	0.394140797
0.214438085	0.510305611	0.524913498	0.188883457
0.457648127	0.017530731	0.173105728	0.028984446
0.36078419	0.180571311	0.507758297	0.513378138
0.243182335	0.433537663	0.274196099	0.000583014
0.024131483	0.190322628	0.519383777	0.396512232
0.486827316	0.474984638	0.260493237	0.469049431
0.533274117	0.530600332	0.073987716	0.088787032
0.481329164	0.188573863	0.244485957	0.517775236
0.346239588	0.107657713	0.122510375	0.532913792
0.463267422	0.078913304	0.372543421	0.455262267
0.385165242	0.404968107	0.099946488	0.295965166
0.044272439	0.504103164	0.026206887	0.360807397
0.349415338	0.163480133	0.008064677	0.274819432
0.324510259	0.521515917	0.496454317	0.099227845
0.344005806	0.45534426	0.362022064	0.509689758
0.010197893	0.040829791	0.323326262	0.53386243
0.340556298	0.531387545	0.313817494	0.330798677
0.302345688	0.502203119	0.003382204	0.504874924
1 0.353110386	0.454924753	0.471490291	
0.465127491	0.50724657	0.051059739	0.456869929
0.430416751	0.071315088	0.528460361	0.16629321
0.503060025	0.03825906	0.348965189	0.527810429
0.530410282	0.007643222	0.249597939	0.427259085
0.024869188	0.095720532	0.485331177	0.452923452
0.436740311	0.506211544	0.513396126	0.449643153
0.485206563	0.23470034	0.009925268	0.352524211
0.51202625	0.468011788	0.481236938	0.249170717
0.406869325	0.513172919	0.516580464	0.254960803
0.038651678	0.33452639	0.019446226	0.375375471
0.488998752	0.12164999	0.482190874	0.144991115
0.069368653	0.534558382	0.206799526	0.324260433
0.15688846	0.469080852	0.254925988	0.275060057

0.258027306	0.168601205	0.420247759	0.438229468
0.032608126	0.091955931	0.012635141	0.469060137
0.164547333	0.414620224	0.414631254	0.487282955
0.360030107	0.115548412	0.438010915	0.333206025
0.117834424	0.012116776	0.524102133	0.442831834
0.405531609	0.153266211	0.036054604	0.425522032
0.520776449	0.395300345	0.315351447	0.508443583
0.024044569	0.527443355	0.27861926	0.125494036
0.45010472	0.276568276	0.500102813	0.067678501
0.444002123	0.500433866	0.33057219	0.200213209
0.466578524	0.463730385	0.026073731	0.48624416
0.483520061	0.027575774	0.532263484	0.130761468
0.01754201	0.507606172	0.533199732	0.071677635
0.075689451	0.383961287	0.041148419	0.312163008
0.53166472	0.454738898	0.517899568	0.243829924
0.036530842	0.00981712	0.223932642	0.469945779
0.519671272	0.53502932	0.268128714	0.431808841
0.245937113	0.400913222	0.151053917	0.537898386
0.205883291	0.511316533	0.004665575	0.527199357
0.525866906	0.330092384	0.083428924	0.340080657
0.469482953	0.041309746	0.199696082	0.510284814
0.508408552	0.378946379	0.41926818	0.481480276
0.013057299	0.036465439	0.449360564	0.221399096
0.448906091	0.225089781	0.067045315	0.361985282
0.039990001	0.500844394	0.507948946	0.061098281
0.413489838	0.34535342	0.418119802	0.032001902
0.533417665	0.517133691	0.523345622	0.157356851
0.521677694	0.534007703	0.507692901	0.075039966
0.014837287	0.521024106	0.461814132	0.020952213
0.51607413	0.275787443	0.011772235	0.511776924
0.183191223	0.465112627	0.003620895	0.098929359
0.038296635	0.515685486	0.493936457	0.424048873
0.304328289	0.393702554	0.005232399	0.53266779
0.513640841	0.012727224	0.152799067	0.533988306
0.529205562	0.512078732	0.394287951	0.53793214
0.520992849	0.509927789	0.260851852	0.288144941
0.514888765	0.518814836	0.481467152	0.494576501
0.515563672	0.478480062	0.296041583	0.01295775
0.161495548	0.226051498	0.398576153	0.092846532
0.379233522	0.151083104	0.518352109	0.519280098
0.539852069	0.003288329	0.149304026	0.524548578
0.489880425	0.506936733	0.017642443	0.006234653
0.008141987	0.021113412	0.393694512	0.258396993
0.531414578	0.298416627	0.00281768	0.038278306
0.300808106	0.471021015	0.519966221	0.409926991
0.518724894	0.075416224	0.345246365	0.432641584
0.07250789	0.404843496	0.539029088	0.495794568
0.036458847	0.265760867	0.418591316	0.518508176
0.097960096	0.341510154	0.512484903	0.36171901
0.011130753	0.081040497	0.066216665	0.489912611
0.517387036	0.083330202	0.032941943	0.169506624
0.123269799	0.522855346	0.172334694	0.533852798
0.50224961	0.482331742	0.075193379	0.502046163
0.039371566	0.502278384	0.400529131	0.067433209

0.302946003	0.009435635	0.512815809	0.20490596
0.363465414	0.451022599	0.240302544	0.014554204
0.227987883	0.0495213	0.524189268	0.526052046
0.030709696	0.24225914	0.318180994	0.446304532
0.315255133	0.493538615	0.509625798	0.500747328
0.080748554	0.164689181	0.451414476	0.469106029
0.250329636	0.52928643	0.35649546	0.531805299
0.480799663	0.507874062	0.530525456	0.388605471
0.270407335	0.46331176	0.433916313	0.017759981
0.380960082	0.519837965	0.390880592	0.178562099
0.285358648	0.477614811	0.040412349	0.20364076
0.419360582	0.355866047	0.144789699	0.481124256
0.441537239	0.475748942	0.032896054	0.062502984
0.523188235	0.489374938	0.148461702	0.492250612
0.170326781	0.387695668	0.522808146	0.520849753
0.439291312	0.529081215	0.488033792	0.456870707
0.47511156	0.271339987	0.524992689	0.086949066
0.095599439	0.497726378	0.10100026	0.089851614
0.142426529	0.023994554	0.388102957	0.471151858
0.473444767	0.423093566	0.363434816	0.078831462
0.354090596	0.426082266	0.386586384	0.211646414
0.078278059	0.506115664	0.007802285	0.051227221
0.471829339	0.527466104	0.468843229	0.254537651
0.36722154	0.145149119	0.321082761	0.336754953
0.396319598	0.429428498	0.526799473	0.030277265
0.01517462	0.525855609	0.43110361	0.017950091
0.08866865	0.453385104	0.308935669	0.269746763
0.019745519	0.530803073	0.403971511	0.49224244
0.375354507	0.05866899	0.423025063	0.088882809
0.052603032	0.483287264	0.47146837	0.518267703
0.394451653	0.466979411	0.458200844	0.520889722
0.1228676	0.464628939	0.023288743	0.500030497
0.51004722			

ETAA1 0.268717089 0.376004837 0.111636551

0.248283411	0.124191869	0.21493822	0.078978979
0.149485187	0.022895544	0.002554276	0.00207083
0.172847398	0.039509764	0.102433708	0.099632841
0.220300147	0.022246915	0.142268786	0.084721572
0.238675903	0.065994467	0.165649711	0.036962603
0.050418013	0.013798423	0.013072039	0.039541188
0.316339542	0.016325839	0.155058442	0.296204599
0.026174165	0.307981891	0.390783539	0.158790706
0.370102576	0.261943547	0.011531858	0.091392994
0.361026049	0.212055805	0.246608392	0.127865147
0.015068845	0.422315802	0.084963916	0.128348538
0.119903488	0.005132663	0.155133593	0.409650847
0.351081918	0.146626946	0.131700439	0.056632575
0.029344793	0.142052069	0.033017248	0.306634794
0.067669905	0.131530719	0.341934782	0.032594759
0.047207696	0.14280103	0.030402253	0.122089151
0.055406305	0.336973568	0.018999103	0.160988592
0.002855557	0.231804845	0.124708244	0.043458803
0.009791935	0.004299011	0.036981642	0.152665852
0.323881101	0.367156407	0.277310437	0.28035422

0.035364742	0.073432892	0.088561622	0.040611097
0.326913695	0.05729452	0.059372755	0.199960374
0.285407545	0.35753001	0.038600892	0.057928998
0.264213231	0.383756603	0.12454795	0.097051757
0.056948756	0.009057439	0.33345759	0.319167987
0.360903018	0.047009462	0.027968185	0.332511188
0.017103302	0.144823207	0.153264739	0.051124545
0.33194368	0.008341423	0.036291608	0.1422627
0.085429019	0.29749913	0.337553574	0.062982304
0.115738661	0.005741327	0.016188759	0.010333163
0.117923011	0.017968308	0.388932852	0.301376849
0.133973293	0.071359708	0.101933006	0.000446128
0.004996018	0.052819606	0.276061682	0.145002791
0.266125982	0.28140035	0.040834219	0.313321529
0.166682569	0.269163611	0.04382198	0.046299287
0.161826861	0.051209569	0.076271736	0.166204475
0.058004596	0.076513391	0.040761385	0.337280771
0.409721186	0.028064038	0.161054039	0.204391279
0.075583747	0.081936743	0.042443301	0.079617563
0.023187627	0.330794468	0.004525086	0.170345907
0.04336691	0.021662128	0.01289545	0.150738715
0.105908517	0.264933609	0.374830187	0.038654654
0.089069117	0.144323335	0.189404084	0.215875339
0.001322995	0.025225464	0.172315615	0.332061786
0.269960087	0.321081095	0.094865525	0.068299636
0.070370171	0.272480607	0.00227458	0.39744318
0.353110386	1	0.126649564	0.163888958
0.064817603	0.286866251	0.040484823	0.205681597
0.309186848	0.03112556	0.309188513	0.02959645
0.359297717	0.008090468	0.130054767	0.217662774
0.204620217	0.006859891	0.132107297	0.240599148
0.004303242	0.068450379	0.274312406	0.204926988
0.127817266	0.246288812	0.297366902	0.05632165
0.12163473	0.116980426	0.005080809	0.073949907
0.259895343	0.07413319	0.385398445	0.100357574
0.250704625	0.359084975	0.265096784	0.046821551
0.014515703	0.1377673	0.019521431	0.074777092
0.286510432	0.033236191	0.094938326	0.052105886
0.012663873	0.342360338	0.023802936	0.037307986
0.014675237	0.150472497	0.100602715	0.056817507
0.031935065	0.036649177	0.061782277	0.078024987
0.026839605	0.020457822	0.004025584	0.071485998
0.02369588	0.071737159	0.080071744	0.271774264
0.109418462	0.027551418	0.087655096	0.052335628
0.021041023	0.003607553	0.363704409	0.207599583
0.336846685	0.055199086	0.011530076	0.077678321
0.301095062	0.171960126	0.170794479	0.333993699
0.01592091	0.295493264	0.050003951	0.021927622
0.063118635	0.07266255	0.30735637	0.028696081
0.216791873	0.373812485	0.099058705	0.031913226
0.359481561	0.214641366	0.01613319	0.30612656
0.305182812	0.007788952	0.34961361	0.02179731
0.004858668	0.189507309	0.194812358	0.016276635
0.016176243	0.079695561	0.046639285	0.082274266

0.347786843	0.184786113	0.23949624	0.038985912
0.011241732	0.001510078	0.037863999	0.129968972
0.403230019	0.317625568	0.080973765	0.06646415
0.087672666	0.054433848	0.129524059	0.319445991
0.069949179	0.39040279	0.002585464	0.202382314
0.367206606	0.181177234	0.011783123	0.136701874
0.345665366	0.016820772	0.104480179	0.371639864
0.312342406	0.06971336	0.07020799	0.215490932
0.013329459	0.02391645	0.117792866	0.025010862
0.084384405	0.052247943	0.005562751	0.14877963
0.010554204	0.350887948	0.277348819	0.010521321
0.152841125	0.156753899	0.131150995	0.012165098
0.23915578	0.338697365	0.39396763	0.090817743
0.254950975	0.299697969	0.105345748	0.019468777
0.003000055	0.359548153	0.286065967	0.007486448
0.300403877	0.183395554	0.002340393	0.387273625
0.068095516	0.397244448	0.007731761	0.025708855
0.008388249	0.252971457	0.368460367	0.275267819
0.107749149	0.188014532	0.001766688	0.338389966
0.3635585	0.002042225	0.021785828	0.238989323
0.185170453	0.409955431	0.046893069	0.236506037
0.137421498	0.345348512	0.073720567	0.068662629
0.298340063	0.306090031	0.305672989	0.390870376
0.295269761	0.177884507	0.104727187	0.005667516
0.020555293	0.140953262	0.067684504	0.011389527
0.275771842	0.023123746	0.3607382	0.279425232
0.28374793	0.00127053	0.09722403	0.273603491
0.362241945	0.343911718	0.010734565	0.003695546
0.005513201	0.001559524	0.193371878	0.053728789
0.321684161	0.089957161	0.001717825	0.050041212
0.096512626	0.113835399	0.355885066	0.200643074
0.240424921	0.022470194	0.067702163	0.236484128
0.009026056	0.30405663	0.29077577	0.385563695
0.012311106	0.053050623	0.158781469	0.327863186
0.006095374	0.091874591	0.190006266	0.073299373
0.006763984	0.014339757	0.010789826	0.245147674
0.351151192	0.012007131	0.004835619	0.10448867
0.032211769	0.297686891	0.041040837	0.325590483
0.311687302	0.264246363	0.011405697	0.375064358
0.006274509	0.365751723	0.058669943	0.042755055
0.092531927	0.005664439	0.323300357	0.017394592
0.145602891	0.294559064	0.033165957	0.002591455
0.027037138	0.011087267	0.281581186	0.245041978
0.007073562	0.095708225	0.083549644	0.215122172
0.183424737	0.226791463	0.395304338	0.137586565
0.03421457	0.027852723	0.286509624	0.2878371
0.137079224	0.288167904	0.048458384	0.351390251
0.299112076	0.354720082	0.309272948	0.136699572
0.033355292	0.232181586	0.228128983	0.012852212
0.146746386	0.261212971	0.175757897	0.022684476
0.04498594	0.092461038	0.028770625	0.13820951
0.057429894	0.225978447	0.038241093	0.270933787
0.267406698	0.299592129	0.041045171	0.010045011
0.387822695	0.371089853	0.092382621	0.228628746

0.046614432	0.088932122	0.250433538	0.220391419
0.089824959	0.27326293	0.319479734	0.243715811
0.377108532	0.088948619	0.385529974	0.138533101
0.014561022	0.197198671	0.019171137	0.018797726
0.03831249	0.005844808	0.043114449	0.213882174
0.065934078	0.14668518	0.150101875	0.023936738
0.080607356	0.142031759	0.118682281	0.083029399
0.019382563	0.365531577	0.002183121	0.013610798
0.070547314	0.248936921	0.073721764	0.057095194
0.041931058	0.027187061	0.079782651	0.186253938
0.085587012	0.097873007	0.258993966	0.031014741
0.014661388	0.306939082	0.179078292	0.006014288
0.017759296	0.239442002	0.119902001	0.046367067
0.003321568	0.288918666	0.104226335	0.125850192
0.170145928	0.024729633	0.388569047	0.051571368
0.03553047	0.399818668	0.114017426	0.348732792
0.083536075	0.212802619	0.413990201	0.279975012
0.06242508	0.128726804	0.014642327	0.318424827
0.07994362			

ETS2	0.282694162	0.472033586	0.195023217
0.391609306	0.265686133	0.207735676	0.201220631
0.121090927	0.101393146	0.038201483	0.081197747
0.271084539	0.25610465	0.249235356	0.164345981
0.373595943	0.213849513	0.164872143	0.231276175
0.315217324	0.119829346	0.370828827	0.136650516
0.18579171	0.162163942	0.136174289	0.210354901
0.421595319	0.141602831	0.184063426	0.292838613
0.120763177	0.347010231	0.43073966	0.366017438
0.425794179	0.245576886	0.020011124	0.198214848
0.33051316	0.357019165	0.324765009	0.274312243
0.099199287	0.392672061	0.235837331	0.240320381
0.123076319	0.103500698	0.125664866	0.350327815
0.407996383	0.256865873	0.15070504	0.181245527
0.23427187	0.141137559	0.210702551	0.202650506
0.234411471	0.104481172	0.416420762	0.108931254
0.105697709	0.162376496	0.168326952	0.197907134
0.195852953	0.270760992	0.035034145	0.213115353
0.017696051	0.293619598	0.239126406	0.166231417
0.098266991	0.115297416	0.187748192	0.258993316
0.431079025	0.359722233	0.480421136	0.46286057
0.259732952	0.21841359	0.216278039	0.234186838
0.356154635	0.212338937	0.119408438	0.398043416
0.482174795	0.460511333	0.210556033	0.215173993
0.382248649	0.418410851	0.226588315	0.26920771
0.204453817	0.066049302	0.376938493	0.484217598
0.309189736	0.199271448	0.198499007	0.350394736
0.105006183	0.176058117	0.307924904	0.171841585
0.448274252	0.044251619	0.213643759	0.256975877
0.152306424	0.348697346	0.451802656	0.206922045
0.244210773	0.07828774	0.125443335	0.060806235
0.239822256	0.114125865	0.376392904	0.46204404
0.175048658	0.257059702	0.207016352	0.013060403
0.076896797	0.132102021	0.362204612	0.195594825
0.322062266	0.274539367	0.242165037	0.269676592

0.291708474	0.469527419	0.03621836	0.121693763
0.248740996	0.138247235	0.237135808	0.268890322
0.27051655	0.124316933	0.184726002	0.486133849
0.351028183	0.150077641	0.150685616	0.368321943
0.239602884	0.266518986	0.150882303	0.288248457
0.10752624	0.396490233	0.06816985	0.168287453
0.155707061	0.204927192	0.015755386	0.166103406
0.227205867	0.407691646	0.353832598	0.140541646
0.289427656	0.32068766	0.156006559	0.35983391
0.053256536	0.076369624	0.113783548	0.488652656
0.169470069	0.45027447	0.236912699	0.20702438
0.249027329	0.400283243	0.002272957	0.408348846
0.454924753	0.126649564	1	0.252979103
0.197917966	0.440391069	0.020018546	0.367183923
0.215219811	0.11139378	0.489704866	0.220856516
0.381856082	0.105622201	0.203885148	0.365959886
0.339026272	0.020702489	0.141142558	0.303454582
0.052708649	0.0995618	0.303215262	0.319906269
0.248630962	0.366931726	0.47796788	0.193056037
0.277350836	0.188898036	0.029252584	0.233486444
0.335292846	0.183845682	0.37440518	0.2006738
0.152479228	0.423364472	0.320778446	0.114338586
0.097520635	0.201134693	0.054692337	0.246488649
0.285112983	0.210983155	0.188254212	0.122612736
0.131062001	0.492182584	0.144610199	0.155120383
0.099369031	0.277607634	0.203028603	0.279712533
0.297393162	0.183994735	0.216859342	0.228561945
0.079142959	0.185795676	0.017439625	0.243143886
0.282264082	0.290715882	0.266787261	0.277209706
0.249731702	0.245354532	0.301152993	0.164479954
0.144721308	0.051700196	0.456122189	0.316417897
0.224675238	0.167304957	0.083648849	0.225751469
0.465531575	0.15989567	0.10431458	0.434243147
0.042145592	0.448852727	0.292930843	0.190945337
0.291521675	0.278438281	0.441115927	0.053368565
0.190803486	0.421771098	0.215319807	0.21343148
0.349206195	0.29586702	0.080892084	0.28870983
0.29534651	0.095331563	0.477830665	0.177526915
0.056920855	0.337529516	0.352838846	0.124431176
0.119886235	0.273716735	0.065701462	0.256376773
0.485919713	0.399754627	0.364017081	0.152010405
0.114054353	0.089457404	0.180203539	0.195055302
0.419972156	0.463835001	0.231527587	0.299330015
0.231423451	0.218873092	0.076903177	0.485152574
0.098334725	0.433149008	0.025521517	0.35882216
0.419626925	0.152760702	0.14809624	0.210240645
0.332375383	0.089304141	0.154189677	0.435910675
0.372758102	0.22056162	0.219202581	0.293640506
0.047511711	0.079209436	0.22727204	0.126605655
0.234229187	0.196162073	0.045553766	0.169798092
0.129734605	0.413652621	0.420464731	0.10030339
0.198499592	0.143489064	0.28820324	0.084818967
0.39994574	0.417930098	0.445475245	0.146272646
0.351099971	0.447501176	0.234750954	0.109510948

0.04946028	0.435621991	0.251758448	0.073963866
0.460870575	0.114041176	0.096267542	0.444804489
0.188718089	0.338910376	0.00643502	0.031856339
0.055029037	0.422894659	0.398728347	0.27042871
0.139058405	0.228363774	0.004712253	0.465453226
0.448492707	0.04625918	0.147689389	0.369655102
0.342240899	0.459902279	0.205719626	0.419277523
0.28268894	0.427279874	0.245271586	0.247519951
0.409944444	0.418302024	0.326041813	0.386373752
0.456533731	0.334938926	0.249580619	0.053212793
0.089421054	0.137030925	0.177787652	0.117559999
0.167499413	0.181353681	0.418534487	0.446797691
0.458945689	0.02192742	0.140137277	0.351373095
0.408284971	0.444151219	0.021494613	0.00638291
0.006196605	0.064227237	0.215196983	0.201139505
0.438781633	0.16322539	0.025608896	0.067854865
0.21488509	0.279980969	0.451937798	0.340076087
0.345324097	0.12646316	0.23584347	0.335681586
0.115886902	0.178601826	0.47316076	0.416420455
0.059308192	0.194800089	0.183427714	0.474455144
0.053916844	0.260847125	0.294793305	0.276522939
0.017383251	0.121493252	0.102297388	0.381335312
0.412499819	0.211152678	0.135819748	0.084093575
0.207242808	0.419671727	0.245343814	0.492881446
0.403450059	0.442358636	0.10354594	0.408504444
0.092096174	0.42179119	0.250450539	0.048759042
0.191002064	0.032456175	0.397186415	0.139830276
0.198753365	0.25917047	0.119024093	0.039987079
0.100624742	0.11477381	0.477136665	0.427763471
0.049092872	0.175881443	0.225710657	0.269106282
0.177619919	0.413573701	0.423671868	0.197072643
0.130514411	0.087715305	0.324205946	0.249190222
0.170735773	0.478367606	0.244957695	0.468881753
0.276689924	0.383294259	0.42327019	0.233765347
0.207439498	0.207047795	0.299906923	0.075791849
0.244616566	0.462413807	0.183986625	0.200624765
0.274699083	0.201045553	0.051882462	0.077242313
0.178249736	0.179729951	0.215550061	0.418107297
0.225891304	0.264275027	0.050491804	0.103447686
0.478982249	0.35240564	0.118318395	0.321993015
0.174804476	0.257568481	0.370433274	0.377263951
0.171534131	0.419712472	0.361910299	0.318165366
0.379156116	0.21673006	0.448912001	0.058368124
0.100451917	0.232255691	0.206890541	0.128879133
0.208588174	0.057436276	0.163578554	0.357516383
0.197220779	0.333713598	0.1518861	0.106156273
0.239989579	0.151815581	0.262498812	0.203915105
0.173312124	0.435367724	0.028475952	0.106509914
0.223836742	0.361641181	0.256928584	0.220859077
0.196854792	0.126019307	0.27711856	0.161504081
0.23532765	0.181888598	0.365466576	0.048512297
0.057536444	0.481514408	0.176514492	0.031308528
0.074558231	0.192539193	0.17575375	0.216572296
0.045280869	0.430849123	0.186290323	0.24139244

0.217312299	0.103271817	0.265385536	0.070458268
0.063511816	0.373509223	0.209246076	0.442684953
0.194761661	0.419607126	0.339680788	0.372346062
0.083070325	0.160382544	0.065530366	0.389043461
0.211551916			

EVL 0.381322483 0.474492796 0.294809455

0.351432775	0.309459792	0.190774256	0.154465365
0.143734639	0.079369599	0.004464694	0.024144171
0.323011536	0.149758101	0.166938463	0.174421653
0.408625009	0.166235199	0.298125486	0.216185501
0.341509284	0.067041314	0.354752211	0.102778762
0.304764306	0.127637625	0.084298755	0.225660272
0.431597244	0.098395249	0.152832255	0.295626668
0.193769196	0.407868449	0.455138693	0.339597292
0.45003833	0.332319291	0.045265161	0.148539219
0.398939992	0.369969173	0.336894796	0.24322777
0.049610319	0.407646706	0.181298277	0.330933557
0.150133746	0.045821118	0.122888717	0.377248721
0.387104615	0.235610573	0.1577506	0.12071982
0.113750263	0.127623347	0.186246515	0.204865625
0.182177672	0.079263229	0.413949386	0.067377262
0.104196018	0.204774354	0.238805523	0.190741472
0.286632893	0.299779727	0.039439999	0.292745954
0.001770699	0.325213698	0.345035086	0.205129867
0.078791624	0.024461363	0.255110912	0.216427535
0.456592171	0.441335971	0.486698449	0.482517415
0.18393662	0.319759431	0.307785606	0.164751264
0.407938713	0.126264646	0.12529867	0.419413585
0.490010312	0.46581543	0.224232793	0.289250369
0.326304169	0.434011175	0.242859878	0.27110309
0.192224579	0.128010979	0.36904989	0.490776432
0.346799684	0.27901303	0.18620423	0.352768005
0.104105711	0.225478897	0.270160428	0.205895262
0.466704817	0.023982722	0.252476747	0.221561906
0.274319335	0.386932731	0.468715446	0.166651074
0.341643912	0.038568713	0.117831303	0.090428815
0.236234703	0.186478285	0.39089506	0.416757501
0.173102793	0.274342346	0.211574646	0.012823989
0.063865345	0.282240303	0.395390084	0.313748734
0.385553083	0.362828321	0.168218439	0.302264419
0.311648345	0.483315092	0.021225549	0.110534812
0.265675753	0.28627447	0.23481509	0.272880381
0.256535869	0.119804662	0.145833994	0.497947896
0.364477154	0.102069096	0.239686366	0.394454514
0.178453259	0.308648095	0.137785864	0.210852788
0.053600995	0.423999747	0.076092616	0.243478578
0.152183326	0.18647401	0.040099154	0.172089944
0.169382324	0.350999831	0.39484858	0.153537227
0.235016654	0.32096346	0.195563333	0.288242812
0.008532118	0.092963432	0.165373113	0.474199097
0.151925992	0.509538875	0.273607185	0.309445358
0.28001868	0.433562586	0.010492539	0.420555729
0.471490291	0.163888958	0.252979103	1
0.230354562	0.407036439	0.047443448	0.384841662

0.267648236	0.124315848	0.508442862	0.175730276
0.42063459	0.061778338	0.198828609	0.406372022
0.339797743	0.017925664	0.114322219	0.291584238
0.07943667	0.062739213	0.374559687	0.30758144
0.2330054	0.435934979	0.495425496	0.215281722
0.292957818	0.213688453	0.041446128	0.321425719
0.421253489	0.226257851	0.372658987	0.190334835
0.187090414	0.416841685	0.363759643	0.093977244
0.054822547	0.208688397	0.022944335	0.248846807
0.357641557	0.085841276	0.230380149	0.265167082
0.122073607	0.48785599	0.226285228	0.248893416
0.168714933	0.216427243	0.232897139	0.125159994
0.134174492	0.181752993	0.252513471	0.23786854
0.035411584	0.114007941	0.066227335	0.214231095
0.088263773	0.185123408	0.169160975	0.299945738
0.226562521	0.097191005	0.270498675	0.279251886
0.15948314	0.039539792	0.463798848	0.360680963
0.239125204	0.152665169	0.09136733	0.172407527
0.423998141	0.134124979	0.147369221	0.393528358
0.062713918	0.494333852	0.164671111	0.14758884
0.263536517	0.213690426	0.451137737	0.039583335
0.25712005	0.402947304	0.320767518	0.17378686
0.322776785	0.305867449	0.039569304	0.33146534
0.319300336	0.057904354	0.490433774	0.187648096
0.05664697	0.392672052	0.331291761	0.120254459
0.126432949	0.188509099	0.038721178	0.275238987
0.474567075	0.38038726	0.323295707	0.281271763
0.04907644	0.012403528	0.283969939	0.311125932
0.435937159	0.484831713	0.281254219	0.253385561
0.223748182	0.21905895	0.085926347	0.475984541
0.297559274	0.476646515	0.024341425	0.398808396
0.433683771	0.158157371	0.103858579	0.180235136
0.312439141	0.062835479	0.239050428	0.432128775
0.390648242	0.239357511	0.212967743	0.372945272
0.018980011	0.046485963	0.287968865	0.224404557
0.22241368	0.211665791	0.095582838	0.220670192
0.047984928	0.382144943	0.470292849	0.067495673
0.145886727	0.205552678	0.337238494	0.061407579
0.42766762	0.474082061	0.454761557	0.184891926
0.40062958	0.437435708	0.244662251	0.151325373
0.050480961	0.462367708	0.303088126	0.022899067
0.474770224	0.112446343	0.012294479	0.44851236
0.185304179	0.370141428	0.00292887	0.136029394
0.115674782	0.483119822	0.399919098	0.292115232
0.095625074	0.305507221	0.031524903	0.510079254
0.424032255	0.040500276	0.21427844	0.365851537
0.350174992	0.48194331	0.246823392	0.413365587
0.279401026	0.427875462	0.158063534	0.252591846
0.376205156	0.449320472	0.358228297	0.413051321
0.43641159	0.389508192	0.223706885	0.021363983
0.126521048	0.13062306	0.131288449	0.057481429
0.187336603	0.147933374	0.461798487	0.475368848
0.460390816	0.020646091	0.115768632	0.368895317
0.402483652	0.464554999	0.070769521	0.011927208

0.01857674	0.028862479	0.242296332	0.287280776
0.458177311	0.091080606	0.014516114	0.046314235
0.219798108	0.30690603	0.443468834	0.349247737
0.426706146	0.133928798	0.128907274	0.372681336
0.094849395	0.236501565	0.459322035	0.402038993
0.11341337	0.279167232	0.293304926	0.466361731
0.092606849	0.309103766	0.344556183	0.200068967
0.062281701	0.13364972	0.096306691	0.450590731
0.429112411	0.072210819	0.042470239	0.081294671
0.092463136	0.470525498	0.147041113	0.507612246
0.393479784	0.440156954	0.149650172	0.417309478
0.0498046	0.418352919	0.284166391	0.032852189
0.335908447	0.042594442	0.445229739	0.082823611
0.196534781	0.320013176	0.162881456	0.057385261
0.204017114	0.097302904	0.461445555	0.467638222
0.105620583	0.209250867	0.309844237	0.349789119
0.167941892	0.363531006	0.429598284	0.194823102
0.074099096	0.230283832	0.350982296	0.293986061
0.17377237	0.50381322	0.191402852	0.506096164
0.336757038	0.44742094	0.439903336	0.230027179
0.249115771	0.237074034	0.229873888	0.017613016
0.286239568	0.431085036	0.272346491	0.14275728
0.214425314	0.279286507	0.087724548	0.176466008
0.253660944	0.151500945	0.131742808	0.459663236
0.292160947	0.307346355	0.071123945	0.084302708
0.49803925	0.396750862	0.106793788	0.299983296
0.235888842	0.255272479	0.432496859	0.438505998
0.258652589	0.389730275	0.379384102	0.248192955
0.352759856	0.218726855	0.458118398	0.071592398
0.114240094	0.303642257	0.080952325	0.159225368
0.154012598	0.063627858	0.222132987	0.411614192
0.256259071	0.285613174	0.209885199	0.115606782
0.32647287	0.27498983	0.304565179	0.130336398
0.077058189	0.419017258	0.022101594	0.110698581
0.282809748	0.375361224	0.257310984	0.263811156
0.247174724	0.241544055	0.257799716	0.16196125
0.139053692	0.270874073	0.36764478	0.04726697
0.029241837	0.504453818	0.240985969	0.078522265
0.144415873	0.258653113	0.323348203	0.157227158
0.068659317	0.438028361	0.149538535	0.305248203
0.233015573	0.118894207	0.304262799	0.108918495
0.10975449	0.398554782	0.304878727	0.459822779
0.34480052	0.386043794	0.374660384	0.380499966
0.194103191	0.271129126	0.03778443	0.433177395
0.219088036			

EXOSC2 0.285390108 0.443767584 0.197456077

0.300917661	0.206182605	0.175893551	0.077460994
0.042749021	0.090076625	0.00209331	0.037915546
0.238618201	0.089898378	0.069015323	0.149516752
0.328413446	0.068670569	0.106099729	0.096661076
0.2442676	0.026273297	0.283537309	0.043558863
0.175568773	0.089477735	0.056110294	0.203775877
0.328790776	0.059194756	0.104568253	0.20836706
0.050842815	0.297043918	0.439489225	0.294437757

0.430390039	0.269231839	0.00183163	0.110145537
0.300650756	0.372423377	0.254214934	0.163114774
0.024338551	0.370607383	0.110553072	0.242765466
0.066684408	0.028543349	0.056008282	0.338876637
0.391980339	0.26155374	0.066710616	0.068438284
0.102498953	0.047213334	0.15143391	0.140156989
0.0881346	0.031650187	0.412713628	0.060302927
0.067934364	0.067236281	0.07493379	0.071803286
0.2007113	0.234191612	0.006479178	0.23475356
2.45E-05	0.227150831	0.206506204	0.232587069
0.028322061	0.010408528	0.08403693	0.167652875
0.354624199	0.371344545	0.431879235	0.455668589
0.07267247	0.123732434	0.216858552	0.076009187
0.346500176	0.06967464	0.039090626	0.33675872
0.443801418	0.448448118	0.127331875	0.154731827
0.339836042	0.378654719	0.167916548	0.177022631
0.209783585	0.0480072	0.367920349	0.444491059
0.267486728	0.173522414	0.089216697	0.253583052
0.034293402	0.097795514	0.206136429	0.044016256
0.371740854	0.017473882	0.120439686	0.148964878
0.080503389	0.307699877	0.461145825	0.077121432
0.168652218	0.009109052	0.15633219	0.021382359
0.096808589	0.106341639	0.391430444	0.360309828
0.052992273	0.164820502	0.094115523	0.002620061
0.01998728	0.086602811	0.309794995	0.107338932
0.294289676	0.32632209	0.144909374	0.240636749
0.307871339	0.430272973	0.01564438	0.042534918
0.189394728	0.088257299	0.123762754	0.224074649
0.160977088	0.037471551	0.051740737	0.458999159
0.31234629	0.039501684	0.081443501	0.294518839
0.13630399	0.205942419	0.043119563	0.083466916
0.020466562	0.4315168	0.023687682	0.085639119
0.225800373	0.072365525	0.004176701	0.064253989
0.086534192	0.36324248	0.383951365	0.047280023
0.098044976	0.27645654	0.085750383	0.281453766
0.015921585	0.02100077	0.060834677	0.423303901
0.111705821	0.434237587	0.102952079	0.156987609
0.154861121	0.362581631	0.002502959	0.403193828
0.465127491	0.064817603	0.197917966	0.230354562
1	0.360822363	0.009840341	0.266753323
0.175548534	0.038643958	0.443507639	0.088102804
0.366681188	0.033866299	0.084766203	0.417902912
0.312693905	0.005142356	0.056754195	0.230927739
0.025792956	0.030733118	0.28142208	0.255530799
0.242264929	0.357995832	0.408131547	0.226223576
0.198922352	0.068856456	0.009323405	0.169689945
0.338245906	0.164222669	0.343264507	0.08589869
0.120883471	0.391952524	0.330380349	0.119877017
0.027814516	0.095012366	0.006492902	0.190645579
0.346684229	0.044953728	0.231226687	0.055219362
0.047165011	0.461924083	0.131728489	0.164291329
0.113259333	0.172773984	0.087522614	0.073658276
0.088296516	0.108118212	0.221795601	0.190220376
0.013659016	0.046157936	0.014135504	0.115576597

0.047543519	0.101889579	0.092345925	0.234104722
0.085143683	0.035436509	0.159602737	0.18626482
0.084970882	0.012068612	0.429460595	0.256577896
0.177561547	0.077962672	0.024195549	0.097504457
0.379359541	0.119227611	0.062971865	0.361859786
0.012622504	0.42942675	0.097107562	0.075597807
0.145758886	0.102802567	0.383690724	0.0411953
0.160067221	0.364218964	0.147006191	0.126736113
0.273361719	0.238181215	0.015117179	0.263064677
0.235228588	0.019452757	0.450829772	0.06684647
0.016347668	0.318846233	0.359207618	0.052222684
0.05704874	0.13323624	0.014439686	0.186855693
0.449118113	0.310301094	0.333682887	0.142594493
0.023969377	0.004023663	0.116106253	0.196973666
0.405575666	0.451033299	0.110163448	0.164268411
0.115499708	0.228071911	0.031072189	0.445454551
0.082245174	0.427929912	0.005400507	0.3965988
0.434383752	0.069291084	0.067737983	0.119208868
0.255972483	0.031735085	0.064382423	0.405782123
0.376502392	0.199419553	0.19872727	0.242087238
0.007051613	0.017972649	0.261944726	0.150180869
0.168193577	0.132186527	0.055138509	0.098156336
0.02460608	0.361196041	0.34639309	0.074965073
0.152865504	0.090523379	0.241518925	0.024087329
0.440546541	0.437444193	0.428752531	0.051284461
0.308684244	0.445665703	0.267256782	0.049358959
0.016972804	0.446135934	0.22764537	0.011264423
0.385272348	0.053551817	0.006665555	0.400811618
0.074505457	0.333973563	0.002492375	0.046732041
0.035064329	0.393434196	0.411171305	0.204930797
0.062827221	0.177938683	0.006472285	0.458521008
0.397614715	0.017352532	0.105266053	0.338118534
0.34987132	0.427728638	0.207943138	0.417084136
0.254623366	0.43129965	0.079626546	0.164753861
0.342634556	0.452494761	0.271381762	0.365502553
0.353656964	0.297867232	0.151345939	0.012547973
0.206447363	0.052838415	0.136120545	0.092975198
0.132514341	0.105111206	0.387282259	0.394468448
0.42226841	0.0050664	0.042314184	0.322486769
0.321403204	0.382743297	0.013971653	0.006541345
0.004410299	0.03220256	0.15362875	0.128737678
0.437234222	0.057686784	0.003606708	0.01207734
0.098446462	0.295473076	0.423518331	0.249423246
0.358395798	0.043343218	0.098331564	0.258713769
0.082131694	0.166022085	0.429893156	0.380844461
0.02693665	0.145102945	0.100944154	0.409347499
0.084753723	0.168631351	0.245313872	0.127600547
0.00793179	0.06433594	0.076605321	0.34386555
0.426033264	0.039304331	0.021582881	0.029884737
0.051249694	0.388809638	0.066624104	0.449998399
0.325600936	0.340290286	0.057850548	0.413925437
0.04902474	0.392691023	0.183472343	0.018723122
0.102048825	0.008146563	0.38824209	0.175754722
0.076902438	0.209757253	0.239488164	0.01763904

0.161015056	0.033446164	0.420991226	0.416475845
0.027588155	0.094866665	0.135457303	0.224341335
0.085930537	0.314967818	0.410496437	0.162259696
0.039946873	0.073930696	0.238454591	0.198602187
0.056022902	0.433953839	0.18861304	0.460416107
0.259475519	0.383637204	0.404993466	0.185568264
0.133458973	0.172047107	0.209227142	0.006950166
0.156723363	0.372468775	0.11643939	0.122006129
0.133517618	0.235632523	0.02282552	0.036400299
0.184060133	0.119442372	0.057150939	0.351053214
0.178229073	0.218785377	0.010444197	0.073034657
0.456014697	0.359797894	0.053500603	0.267655861
0.080645582	0.219083992	0.352308063	0.365152994
0.177527504	0.350033261	0.277603376	0.209365421
0.315900652	0.114943992	0.444543301	0.01504751
0.104681255	0.215673184	0.051122271	0.06013158
0.063152112	0.026442626	0.185346225	0.293445604
0.161013157	0.217777468	0.07895031	0.047953602
0.166978961	0.112903677	0.189622803	0.062060722
0.040093149	0.419575965	0.00830619	0.032228795
0.142356836	0.376662528	0.21805143	0.114308344
0.200322419	0.086908019	0.138128095	0.074843525
0.087456119	0.125458111	0.370727028	0.014990694
0.007134002	0.418362055	0.108460899	0.016813286
0.07291151	0.201202916	0.085561251	0.160261562
0.022574599	0.43706293	0.11041302	0.232575745
0.138780667	0.031666066	0.235072179	0.035844438
0.029278545	0.332431586	0.238229735	0.44293213
0.147559987	0.318134665	0.341624683	0.314753156
0.044056351	0.181603021	0.016594115	0.334009551
0.16161006			

EXT1 0.374286537 0.520994639 0.376149866

0.450505449	0.435084661	0.382889033	0.30886466
0.245196627	0.156031456	0.021025302	0.052957553
0.409660373	0.271242933	0.346725342	0.356539739
0.440584738	0.19606193	0.347670714	0.334730137
0.432297724	0.170290128	0.440111104	0.175634542
0.29822929	0.159928591	0.134666973	0.313015181
0.443985689	0.149746613	0.346367767	0.402531356
0.142477576	0.403836893	0.501223814	0.437410327
0.486465112	0.347580127	0.017142321	0.354379607
0.392100261	0.434019073	0.431328821	0.411185878
0.101776714	0.469339857	0.346553062	0.40039779
0.274464428	0.072612125	0.276019676	0.430217831
0.462731867	0.419085007	0.297432717	0.259286989
0.246898795	0.260567024	0.271558344	0.326540363
0.323469264	0.207141728	0.469778712	0.170035719
0.183588054	0.308615923	0.192546628	0.337234442
0.327493955	0.368549814	0.043041139	0.347429686
0.010189016	0.423678385	0.389707466	0.298258684
0.086482089	0.058147157	0.224986085	0.410420116
0.451987179	0.429818755	0.477563329	0.476426082
0.24858381	0.338909272	0.36311756	0.267003757
0.408471184	0.281275035	0.173110723	0.44162524

0.486021034	0.507636873	0.266702726	0.31351869
0.415135461	0.474064149	0.428941679	0.444660273
0.360092099	0.07649356	0.433073157	0.502754574
0.370746777	0.303340577	0.224481996	0.410205004
0.110144475	0.367302208	0.424878501	0.184184555
0.469122065	0.053231355	0.266649439	0.414236853
0.26642022	0.41610433	0.497467337	0.28891301
0.401189233	0.049142159	0.168979558	0.061436342
0.369676118	0.145865972	0.455418279	0.462264199
0.285867297	0.39419022	0.348186599	0.007941635
0.05623499	0.212143359	0.388246923	0.371497384
0.420803979	0.337881878	0.321077379	0.355496824
0.400692998	0.466983486	0.076669207	0.175638676
0.442635566	0.213446042	0.326754599	0.427054407
0.37304935	0.195797342	0.212481067	0.517567231
0.423470097	0.162299522	0.32646654	0.442811073
0.379968458	0.381039401	0.183056384	0.355791089
0.112112195	0.454905773	0.052314258	0.351341772
0.287171739	0.182318066	0.022701206	0.316374151
0.366187565	0.422212571	0.432060943	0.176450347
0.390971797	0.438708471	0.326903018	0.425438126
0.034902851	0.08961621	0.266568008	0.504613244
0.290973015	0.480522902	0.372414669	0.345010807
0.350224339	0.443360267	0.004782523	0.480822717
0.50724657	0.286866251	0.440391069	0.407036439
0.360822363	1	0.04024274	0.441203962
0.323266548	0.141300252	0.500896577	0.239994152
0.440695538	0.090804168	0.357025505	0.390319578
0.381174495	0.017694194	0.281817484	0.423702365
0.047088411	0.168662282	0.410182706	0.447509084
0.429994697	0.428117631	0.480087201	0.347525576
0.446578934	0.312773296	0.02828753	0.351996232
0.400978991	0.340022887	0.444570162	0.330203501
0.30804681	0.476942675	0.366741534	0.229201115
0.101672879	0.366738222	0.051358865	0.397249042
0.352594068	0.209773663	0.371458968	0.173185461
0.118683624	0.523824803	0.192160904	0.244064426
0.13109178	0.419490115	0.335725153	0.313563624
0.27285404	0.244784762	0.368353034	0.408983725
0.091995417	0.167042612	0.021361322	0.344047262
0.203503423	0.363479302	0.352803131	0.373873934
0.356833094	0.185080634	0.432624474	0.295796976
0.172611287	0.03764812	0.504050806	0.435161126
0.314366538	0.248162847	0.078082591	0.326834463
0.469884205	0.326499258	0.259972904	0.462704907
0.055022922	0.468652078	0.335889686	0.195954145
0.385138747	0.356475041	0.462255508	0.093497014
0.376486968	0.469667293	0.358450219	0.269880374
0.392411102	0.439587421	0.077029104	0.36892517
0.365324511	0.072387725	0.513150105	0.174109872
0.045809756	0.421472348	0.413044666	0.132498458
0.132518816	0.40328629	0.10271367	0.360342741
0.520828188	0.446871703	0.410252985	0.235964009
0.097302416	0.028853307	0.2409657	0.364922493

0.488931977	0.491804666	0.330495339	0.402220688
0.333960756	0.361636045	0.175340459	0.504729417
0.192589348	0.496658509	0.019188611	0.403504303
0.484141181	0.303065415	0.138857188	0.373861398
0.376198781	0.104970123	0.265395306	0.490327431
0.426706482	0.382778926	0.392668314	0.414715139
0.050734859	0.096303426	0.406756139	0.188827929
0.41125896	0.300194821	0.051509209	0.34796837
0.10371016	0.456666069	0.436986342	0.119631648
0.354132247	0.326679262	0.406283524	0.084632155
0.419898636	0.471762128	0.507994072	0.23786724
0.399832023	0.478206444	0.364593381	0.125939519
0.037589134	0.493586689	0.386915534	0.060142692
0.463994648	0.253760366	0.040509814	0.49974582
0.279928886	0.415765463	0.014440206	0.071496092
0.061268522	0.430005378	0.470212121	0.401940763
0.277536119	0.402230141	0.006474359	0.502780305
0.490891721	0.032430413	0.178399025	0.398262763
0.386393535	0.521859575	0.324805942	0.423136003
0.400028725	0.481002991	0.32338316	0.348312622
0.43106881	0.462449742	0.420358633	0.45766007
0.452568148	0.427577852	0.373946663	0.047616711
0.172736648	0.270733576	0.330857476	0.143850118
0.324200097	0.215383397	0.468252409	0.452083032
0.466943119	0.014639124	0.234247556	0.395387133
0.445677969	0.477321428	0.032939819	0.011055907
0.010936565	0.041234235	0.398767411	0.298093375
0.480353689	0.273764341	0.01656663	0.095432863
0.367643131	0.397801859	0.496970625	0.4324188
0.402363066	0.137370923	0.338984926	0.433601314
0.116352785	0.290366884	0.481776424	0.476757724
0.067020802	0.30380152	0.361138912	0.495992521
0.067928786	0.372670334	0.409067699	0.388845888
0.021121877	0.131484376	0.116824419	0.433217297
0.471086503	0.141125931	0.075707119	0.158165813
0.214955461	0.443220455	0.264155116	0.511916492
0.434294842	0.453445673	0.104333693	0.479644063
0.080757873	0.473058415	0.358469935	0.095685405
0.318655318	0.029733041	0.438677941	0.196647826
0.353963833	0.392115626	0.233090825	0.031515424
0.175537453	0.098241225	0.478189938	0.429516149
0.050995357	0.316901127	0.356021419	0.416374107
0.340652679	0.456013011	0.491761664	0.382608594
0.165680601	0.125358586	0.427178125	0.371776999
0.290415222	0.481442856	0.35707317	0.512815202
0.387573271	0.446367743	0.454702522	0.416328102
0.252372275	0.393959954	0.403264078	0.061110163
0.415576527	0.447754384	0.374163154	0.226301717
0.339757294	0.35124741	0.081336992	0.173861008
0.295658932	0.338034518	0.231686035	0.444883364
0.380507511	0.374328728	0.07009504	0.114745364
0.532193807	0.427789847	0.22735436	0.441366015
0.237448989	0.407373468	0.418690331	0.414856526
0.350919969	0.424026624	0.419657521	0.401882028

0.432504281	0.352283009	0.512636649	0.128732167
0.123712199	0.366229963	0.180081277	0.142743399
0.234237637	0.056560293	0.27232498	0.434816501
0.328871892	0.43734493	0.306644154	0.143227862
0.361405347	0.328652383	0.411178324	0.299424719
0.156677219	0.49180721	0.023475831	0.100496372
0.342438473	0.416085607	0.386085575	0.314812988
0.298231095	0.167103236	0.380484313	0.323311506
0.346927977	0.337156719	0.414094747	0.076997604
0.053017492	0.487591592	0.369302299	0.034530611
0.108383524	0.347140631	0.311955792	0.321590687
0.038852247	0.45848126	0.354881069	0.422417858
0.403030447	0.118312498	0.337425478	0.123693039
0.103724721	0.438282755	0.375708709	0.491456414
0.344076749	0.453863078	0.425246512	0.399076082
0.154249479	0.326948139	0.074150683	0.425890313
0.372964218			

F10 0.056729764 0.050927368 0.028657426

0.033984253	0.022654939	0.022351403		0.010673995
0.022646846	0.000852537	8.81E-05	2.14E-05	0.043665668
0.000856655	0.008911075	0.00674124		0.03919677
0.004099216	0.033453443	0.017690253		0.038546979
0.004602999	0.028296136	0.003738792		0.008393602
0.002969484	0.001521016	0.005796097		0.052589604
0.002337293	0.01400483	0.043070311		0.008351173
0.056805353	0.055994114	0.027509149		0.054335882
0.055087409	0.001222249	0.004246414		0.066512205
0.03376559	0.036018771	0.021254213		0.000977734
0.058237313	0.011394303	0.032960033		0.002144579
0.000944925	0.014525869	0.054727389		0.04307506
0.016689731	0.014539472	0.00455653		0.000426715
0.010134656	0.002764889	0.042009677		0.009326293
0.009311312	0.042682104	0.001001805		0.000167281
0.021173551	0.0092403	0.018250656		0.008791376
0.049129792	0.002136628	0.039286021		1.06E-05
0.042401988	0.038572589	0.005538518		0.003636986
0.000312901	0.012029307	0.017248345		0.053523498
0.067488447	0.046064823	0.043488835		0.004582569
0.022713793	0.022371706	0.004653051		0.053359005
0.006024008	0.007018798	0.037616303		0.046528085
0.04906966	0.005717016	0.017485721		0.029002335
0.05365409	0.019656067	0.012237905		0.004592578
0.003333023	0.03809077	0.048726529		0.056217727
0.014755611	0.007365584	0.049571016		0.003963925
0.018731415	0.023084766	0.006066902		0.055828856
0.000815038	0.011507897	0.016052031		0.008763011
0.050220406	0.049868268	0.010956459		0.034649062
0.000411239	0.000412255	0.004362889		0.007601614
0.004519522	0.049251304	0.042684162		0.010019457
0.003756852	0.012688358	0.000327433		0.002089938
0.006268471	0.047008064	0.045541038		0.04500849
0.055881051	0.003384167	0.048474301		0.03071077
0.044009595	0.000222035	0.004187533		0.036139921
0.008690382	0.01574439	0.024877535		0.013947222

0.011767689	0.005879516	0.051836827	0.053598493
0.004809485	0.030378189	0.043973823	0.002580371
0.023121584	0.008043829	0.010181875	0.001311188
0.04939645	0.002976468	0.04342988	0.002435389
0.002562707	0.001401524	0.023501605	0.005440395
0.029989202	0.053177008	0.00982802	0.013532001
0.025716884	0.031113233	0.027529848	0.000124139
0.006764715	0.032377315	0.048408853	0.028791011
0.058748163	0.014137066	0.013758252	0.018947578
0.045259022	0.000595145	0.055886753	0.051059739
0.040484823	0.020018546	0.047443448	0.009840341
0.04024274	1	0.041028461	0.064941219
0.00712685	0.050617172	0.006515947	0.054025514
0.00128825	0.012005552	0.035914056	0.034793042
0.000376549	0.013619876	0.031653017	0.002405793
0.002805589	0.047291018	0.029000115	0.015142766
0.045585046	0.050070735	0.008067503	0.02390821
0.023439558	0.002060236	0.024421051	0.050736849
0.002331964	0.04728877	0.011955394	0.055214883
0.045882288	0.043670616	5.62E-06	0.001749907
0.016416328	0.001652511	0.012988741	0.046660618
0.001411248	0.013558306	0.014142194	0.003957652
0.04813421	0.007911293	0.011039247	0.004566755
0.017099612	0.021643678	0.003035276	0.001715938
0.004123802	0.008759272	0.005833384	0.001818049
0.003391737	0.001927325	0.009091627	0.000989283
0.007749568	0.007900334	0.039324505	0.008676057
0.002147642	0.019388952	0.006574667	0.004866231
0.001722881	0.050527031	0.041886721	0.048473285
0.005833363	0.004760876	0.002221502	0.039127587
0.019499006	0.026615159	0.039373094	0.00111566
0.048595707	0.002118302	0.004988257	0.01079783
0.01257279	0.046844811	0.000227337	0.039493022
0.045300047	0.029231332	0.004020637	0.04755974
0.034710107	0.001094028	0.048646657	0.056753912
0.002128176	0.054545791	0.005219741	0.002771466
0.041731724	0.025616134	0.004727804	0.004413823
0.007944512	0.00288384	0.019213081	0.047955237
0.032459579	0.024597683	0.012345097	0.001023948
7.48E-05	0.013549912	0.04266746	0.055864178
0.050823936	0.023265698	0.012944491	0.016525912
0.005058808	0.001589647	0.047179564	0.013275089
0.060640451	0.001649451	0.034161476	0.051077132
0.026828646	0.002470098	0.01314155	0.045729139
0.002452643	0.020536484	0.05195977	0.038708937
0.008940076	0.006546154	0.054091639	0.001091849
0.000618808	0.022543453	0.006270072	0.005592411
0.00583406	0.001833869	0.013429531	0.000435725
0.040669322	0.055408558	0.000640035	0.011219893
0.017517045	0.032285504	0.002373363	0.039310552
0.056512247	0.055124442	0.012997833	0.04906482
0.041709277	0.018832459	0.007233606	0.002184051
0.055219687	0.04786065	7.90E-05	0.049729421
0.02555913	7.30E-05	0.051470627	0.012667908

0.055317582	8.16E-05	0.001216098	0.003977091
0.051206994	0.046466053	0.040060509	0.005930411
0.042441862	0.000197246	0.056668494	0.048715221
0.001767628	0.003951458	0.037969178	0.033168016
0.058793617	0.010717937	0.037653718	0.028818631
0.044121232	0.002407972	0.014184055	0.042848601
0.046398819	0.053906794	0.0570728	0.045005235
0.037567791	0.015994901	0.000542242	0.000731712
0.01376338	0.003787261	0.000587371	0.040949766
0.00235072	0.063737462	0.044636908	0.047085309
0.001442614	0.005260725	0.045876858	0.051797018
0.052770566	0.001799242	0.000190477	0.001255617
0.000281643	0.027209083	0.018654983	0.04815582
0.001815219	0.000604896	0.003799773	0.006575572
0.025757584	0.047086977	0.037777444	0.049821793
0.004662564	0.003417723	0.044049822	0.000991506
0.05927906	0.044832769	0.046567625	0.00461038
0.012412187	0.057577447	0.045663228	0.00173322
0.024762107	0.044859973	0.004977432	0.000639058
0.003729807	0.00159245	0.049042621	0.047732099
0.000912427	0.000527871	0.009220868	0.002205076
0.050046316	0.00404983	0.051229244	0.043680701
0.044786186	0.002039547	0.047914879	0.000177068
0.052210501	0.014797241	0.0003808	0.03048084
0.001983079	0.055134571	0.000267364	0.017354522
0.05781113	0.001275126	0.001685291	0.005043111
0.003656235	0.04035205	0.044898	0.004042504
0.007654541	0.025334196	0.050213678	0.02644825
0.032916276	0.053387253	0.019760678	0.001762729
0.004469514	0.052377209	0.062488371	0.013247255
0.049292706	0.003125068	0.055500172	0.056264503
0.056239203	0.04587666	0.018655763	0.002520127
0.045190106	0.022834589	0.000626812	0.0341108
0.037763144	0.039333745	0.002296448	0.006911497
0.021338647	0.00079898	0.011369514	0.01681866
0.023153716	0.001629464	0.049527533	0.060605694
0.064825424	0.001830685	0.001297534	0.055658534
0.052149691	0.00483502	0.029375358	0.014538471
0.012759922	0.050346396	0.046226847	0.006846466
0.037122786	0.054955532	0.026464401	0.043489022
0.012991263	0.052040798	0.015445401	0.002349101
0.042314546	0.001042944	0.007177998	0.006879646
0.001260183	0.007170162	0.045891686	0.01293197
0.023555797	0.010993589	0.001001337	0.022675647
0.040566305	0.024889892	0.007430161	0.001073703
0.045392638	0.000104867	0.004717374	0.018143329
0.040937381	0.014444991	0.012881104	0.004833915
0.006064093	0.013841725	0.030008235	0.007226235
0.006607894	0.039843836	0.001937359	0.001287916
0.051568188	0.051574318	0.003415459	0.000356733
0.037939417	0.031937981	0.001844788	0.002661952
0.040816143	0.007637531	0.024640249	0.022556459
0.005878942	0.057444311	0.00048316	0.004074805
0.063738514	0.032946771	0.051615116	0.031579528

0.033536944	0.058243623	0.042169667	0.004155882
0.035346757	0.001818816	0.052688874	0.013630619
F2RL1	0.345733865	0.478807566	0.316339061
0.397015982	0.395235117	0.258449005	0.271431585
0.218821673	0.128033277	0.021062245	0.05634466
0.352684202	0.243348883	0.29281463	0.22575464
0.423762038	0.259494023	0.333066559	0.340800616
0.37636422	0.150683467	0.40229582	0.19572029
0.320892325	0.185801819	0.161536461	0.278053958
0.459588467	0.178029682	0.231771319	0.330083344
0.24414619	0.401033383	0.439392575	0.371615973
0.429739986	0.295782257	0.057450538	0.237954719
0.386102611	0.36913971	0.36930691	0.370027982
0.117479359	0.401741981	0.306918981	0.378931505
0.208629519	0.098160433	0.186469513	0.372472215
0.388252961	0.305238526	0.246337231	0.229451337
0.192705133	0.209819917	0.226802635	0.234356461
0.314049834	0.145375329	0.410028372	0.130230546
0.162113084	0.299000327	0.296421129	0.306486868
0.308452088	0.308075992	0.076409764	0.289388494
0.011324888	0.373811654	0.400474436	0.221234169
0.14025771	0.0797063	0.322361625	0.340245856
0.473543957	0.405366856	0.492523524	0.47609702
0.289644637	0.39224119	0.335749347	0.293812578
0.396476512	0.24371714	0.178038869	0.435755218
0.492917101	0.459458864	0.295973936	0.353260177
0.357597119	0.440340702	0.310564669	0.364515862
0.241406563	0.140578405	0.375969624	0.491837585
0.347101997	0.293630208	0.273599517	0.383948806
0.174656557	0.279245559	0.383890081	0.270510889
0.48430796	0.056365603	0.323215697	0.34340682
0.329218095	0.385306948	0.452679321	0.288105156
0.394876243	0.0851119	0.135602474	0.129788794
0.325200975	0.174739496	0.377169429	0.453164011
0.273142238	0.361599773	0.326282752	0.02424798
0.107959544	0.300985892	0.387820288	0.370697293
0.383904452	0.314436771	0.270971317	0.3015016
0.307377821	0.486712139	0.034888557	0.201992387
0.344386883	0.309081749	0.354716269	0.350488606
0.346793797	0.210751967	0.26440728	0.500375256
0.373373672	0.202234739	0.289789343	0.409245296
0.279354057	0.37560244	0.239915813	0.352731869
0.134155978	0.399144816	0.109089067	0.306610413
0.176429878	0.255221028	0.055207034	0.266003028
0.268634457	0.374592989	0.36749668	0.243736596
0.375015894	0.392104696	0.262676475	0.319985973
0.030877408	0.144878248	0.206911057	0.486315414
0.193786623	0.486907528	0.380975516	0.359692235
0.373585909	0.44152451	0.007679379	0.410834439
0.456869929	0.205681597	0.367183923	0.384841662
0.266753323	0.441203962	0.041028461	1
0.258477171	0.202030051	0.514103921	0.279942568
0.405607034	0.125681214	0.304040156	0.380155978
0.330507602	0.02977509	0.191079938	0.340972742

0.10201903	0.134686619	0.366546832	0.364350949
0.326460036	0.421541527	0.510158427	0.249435646
0.380312691	0.321689935	0.062280537	0.374976361
0.392541304	0.265315835	0.383348237	0.297598947
0.224663968	0.427426785	0.340553086	0.144271408
0.123308468	0.315191491	0.052874126	0.334383003
0.322574645	0.20200227	0.267430954	0.29222131
0.185355093	0.493571999	0.229476327	0.239899188
0.155302128	0.322207918	0.343432825	0.241823036
0.234816776	0.271631446	0.298886206	0.317683227
0.095948342	0.218800587	0.060210442	0.279869338
0.19074278	0.283585537	0.262279089	0.304439449
0.337066764	0.2108794	0.371700706	0.28859311
0.212374088	0.07660805	0.46546679	0.394926279
0.250442247	0.252751952	0.14580554	0.243338661
0.457302694	0.19165185	0.182313062	0.424929217
0.091425414	0.487680082	0.29718437	0.239398421
0.347231565	0.351988691	0.473667951	0.062257051
0.31758555	0.421130427	0.388044018	0.267391771
0.341243518	0.364381946	0.096864421	0.324718382
0.318959007	0.120685218	0.482711831	0.256267503
0.095368594	0.374480631	0.337207055	0.189584302
0.184821681	0.304626162	0.093709518	0.373755482
0.484314052	0.414029363	0.348323747	0.281043896
0.121665395	0.046385995	0.320216465	0.325782207
0.425545245	0.469965958	0.385516102	0.344766382
0.347207615	0.275454087	0.141181077	0.481370778
0.260865725	0.455890981	0.046838313	0.378605728
0.41692473	0.228544185	0.169412538	0.28646935
0.334973748	0.126930117	0.320739473	0.432805955
0.39646001	0.310949027	0.277624863	0.382548588
0.055643647	0.115380317	0.342815047	0.210827408
0.312307685	0.303108843	0.074493176	0.290649602
0.124617334	0.403104394	0.470104001	0.118679153
0.220965732	0.258264779	0.409438569	0.123956294
0.404172497	0.442468707	0.447651415	0.282605096
0.386229384	0.435124689	0.283114333	0.205117737
0.079457195	0.440946558	0.302450004	0.066087059
0.490655822	0.16596222	0.05093678	0.457700882
0.300087699	0.354050486	0.007726834	0.094749839
0.126608354	0.467610891	0.395460612	0.327635638
0.155769807	0.388570333	0.02520305	0.489335245
0.435413044	0.063518996	0.243650812	0.365745605
0.332589993	0.479462741	0.273549525	0.407441841
0.318447427	0.419494569	0.278640342	0.356101427
0.400974927	0.422366904	0.368165328	0.411781844
0.46616264	0.394248129	0.3484647	0.058525572
0.134430989	0.215081409	0.184627417	0.114275156
0.229935985	0.224817247	0.447378583	0.483080037
0.456014198	0.038452107	0.214223043	0.364865378
0.429973982	0.481760139	0.072682576	0.012470477
0.014739031	0.049939191	0.341166562	0.344795739
0.45259474	0.15655797	0.036219454	0.10498682
0.322837781	0.34883465	0.447183391	0.39733548

0.394889392	0.214543552	0.215819258	0.396077128
0.139314894	0.220941221	0.465700939	0.415385856
0.141989657	0.33781296	0.336306326	0.491445031
0.075207648	0.404063918	0.357171253	0.308905261
0.06288379	0.186407028	0.144654724	0.443617159
0.410710205	0.161355191	0.103655097	0.119603478
0.203939152	0.464313484	0.273177799	0.511546416
0.421357872	0.473115012	0.185743419	0.415532084
0.097255166	0.414287688	0.338455943	0.054776238
0.37973865	0.071002527	0.418717425	0.118908773
0.289496369	0.323032651	0.175255398	0.077773048
0.171503712	0.161129366	0.483359706	0.454965478
0.1171515	0.310358818	0.384509344	0.385961013
0.258365888	0.403726953	0.425053766	0.250598641
0.164063015	0.196542891	0.374269154	0.288294743
0.273392697	0.504184466	0.276668291	0.49095426
0.325754626	0.431742179	0.432424231	0.329234035
0.297492326	0.297139692	0.281140499	0.058545299
0.385218846	0.461467704	0.33649408	0.208724479
0.329733618	0.299636812	0.132900235	0.177772929
0.257391186	0.229410566	0.259042465	0.473406397
0.324060774	0.300851109	0.123477969	0.133815716
0.493673984	0.386884095	0.175857243	0.329672381
0.315781842	0.353380713	0.411671493	0.414406722
0.289847146	0.408627198	0.396358321	0.293743801
0.376912445	0.334275108	0.451415593	0.114602091
0.130364312	0.31115657	0.18316623	0.219008184
0.27453982	0.095620322	0.223577268	0.414411664
0.274726285	0.396826831	0.264476693	0.178456065
0.397298319	0.291210311	0.406148003	0.247507368
0.168689926	0.422846701	0.041088828	0.176555432
0.314991515	0.369796755	0.314978975	0.346705644
0.267515242	0.264478586	0.386582291	0.23631081
0.225804242	0.298368274	0.367961757	0.08475215
0.078800365	0.510934104	0.290583202	0.085484108
0.167177213	0.267046998	0.369778319	0.252838104
0.087252334	0.426101939	0.22393274	0.366296452
0.336876899	0.191981278	0.300524124	0.14209334
0.153451518	0.39319919	0.326783659	0.443745911
0.360754768	0.428200498	0.360609196	0.38671901
0.201801122	0.275248157	0.087731827	0.440826431
0.258906271			
F2RL3	0.316804743	0.438009407	0.240186286
0.268394442	0.246203399	0.265373422	0.143340283
0.220976708	0.040193767	0.002684179	0.005530342
0.269194382	0.09789753	0.20156289	0.20476828
0.255905347	0.063684637	0.311092289	0.15832943
0.280200907	0.086729461	0.22079498	0.06192501
0.149887884	0.039338585	0.02949265	0.112163973
0.343212421	0.036449778	0.243233803	0.296627454
0.069587374	0.353576306	0.45820146	0.228778552
0.448970867	0.30365801	0.026921083	0.181052407
0.387673129	0.255908774	0.274783443	0.1977378
0.024050038	0.469758039	0.147665699	0.231707976

0.217434402	0.01123768	0.241003188	0.439518291
0.412143678	0.233303531	0.20977184	0.100767413
0.083585796	0.245485078	0.109563655	0.296801273
0.140214878	0.184247983	0.411501068	0.049686486
0.100554809	0.248338822	0.090077554	0.222814989
0.171413852	0.334396774	0.023701016	0.257681699
0.003248366	0.270818995	0.232143395	0.130952244
0.021864116	0.008841814	0.10537227	0.214985339
0.363049767	0.42941377	0.343355068	0.352872683
0.095088753	0.189108715	0.211181279	0.093860885
0.365944825	0.106549221	0.093630468	0.244524308
0.353839854	0.432443415	0.098756159	0.140216535
0.295361547	0.425315111	0.254545373	0.230222789
0.150988673	0.027472855	0.381816543	0.393040423
0.384471666	0.151208539	0.073417613	0.341968175
0.0365268	0.297321095	0.212214675	0.139735934
0.371513031	0.010249075	0.10436892	0.232419661
0.185445477	0.335888113	0.416674254	0.121578008
0.257006686	0.013209612	0.046456256	0.023910434
0.22445813	0.064602025	0.450559984	0.342454512
0.240374395	0.199391949	0.19692676	0.000766287
0.012935961	0.141187967	0.340279051	0.31289422
0.301250331	0.343615074	0.100957027	0.332455664
0.238250637	0.33693357	0.053689333	0.075807353
0.26505887	0.1358461	0.142093576	0.266314301
0.146338053	0.111907434	0.083348091	0.400355085
0.423183262	0.052956016	0.335432139	0.24759907
0.175307276	0.179459471	0.080317553	0.180797517
0.037035519	0.404922344	0.013371112	0.318373773
0.111694538	0.071067726	0.019186855	0.251243854
0.195835775	0.313710942	0.445571418	0.077420033
0.206464816	0.219223431	0.311979772	0.246529023
0.002250556	0.042006992	0.311378231	0.398673527
0.270032414	0.395868064	0.214248335	0.178023162
0.152779819	0.315425559	0.004283311	0.45267864
0.430416751	0.309186848	0.215219811	0.267648236
0.175548534	0.323266548	0.064941219	0.258477171
1	0.058935563	0.373817744	0.076252462
0.419352381	0.017824415	0.262292598	0.285185999
0.26695243	0.007070253	0.206215484	0.264205468
0.013300284	0.092292316	0.306207485	0.252397634
0.205074426	0.299296876	0.352667733	0.159090319
0.239091932	0.207907375	0.00968116	0.176136963
0.317943375	0.211038885	0.414691552	0.176789711
0.317535795	0.403017955	0.318914518	0.110857432
0.024379074	0.25064446	0.020653554	0.167698691
0.335818895	0.066497051	0.208539457	0.135987271
0.033892297	0.410724504	0.078829108	0.123158795
0.052287588	0.240776818	0.196628811	0.128535289
0.090232621	0.085545412	0.166986036	0.196612824
0.033175616	0.048216392	0.012118606	0.212561255
0.06357594	0.187089785	0.19986372	0.302371113
0.248507136	0.063871762	0.202130567	0.151929138
0.053806946	0.007617466	0.423126101	0.262175149

0.324913764	0.100895168	0.026122154	0.199087035
0.350945318	0.234262276	0.28943769	0.37283681
0.028659394	0.362487903	0.123005693	0.056125249
0.187953361	0.151085939	0.34509376	0.036718116
0.316974324	0.416860884	0.207960541	0.081291407
0.38172066	0.274267247	0.020607125	0.337692742
0.337646022	0.016321069	0.424811879	0.064694308
0.011307704	0.252077054	0.246083249	0.038641797
0.039035743	0.174828344	0.051580456	0.153548372
0.410586706	0.227131362	0.283439245	0.11624939
0.020405257	0.004188143	0.113794182	0.256937556
0.4690428	0.398775145	0.17258698	0.172194742
0.15357119	0.147058451	0.184342618	0.389245521
0.179105554	0.460345108	0.004402056	0.266054419
0.44210468	0.285075403	0.031128769	0.217529333
0.357764026	0.027581506	0.198667217	0.432126923
0.351712252	0.168944892	0.171836817	0.293276921
0.013867591	0.035689147	0.212181355	0.082378237
0.206706376	0.117988133	0.021717603	0.282274304
0.020348929	0.392139604	0.333096915	0.02240844
0.21403523	0.287516248	0.217537794	0.022014921
0.312491981	0.421005977	0.459673709	0.161922541
0.315395838	0.369769896	0.199329649	0.048214467
0.007883298	0.437924099	0.308816258	0.012481422
0.353633033	0.25553071	0.004653794	0.435841664
0.132006393	0.450981935	0.004168293	0.07613769
0.024041545	0.31721591	0.420406986	0.296598028
0.195421169	0.276529562	0.006385114	0.419156654
0.418227796	0.005307227	0.066157451	0.299334633
0.247084057	0.469290678	0.140484442	0.30214792
0.22782888	0.420017344	0.152216846	0.140074535
0.331090435	0.390984767	0.321940845	0.42449644
0.34444459	0.246749864	0.167699994	0.007896366
0.051086498	0.217028701	0.156181599	0.02766354
0.299738209	0.059817011	0.418426515	0.340194041
0.356185278	0.002527644	0.145798314	0.32322459
0.383315252	0.384508462	0.023170453	0.004732508
0.008951782	0.004324993	0.277152258	0.139734677
0.385128455	0.172986653	0.002520218	0.056716546
0.197296066	0.198783	0.421427103	0.241735514
0.303095556	0.051016852	0.148424981	0.268774449
0.024222237	0.303614412	0.357854609	0.424899122
0.030574767	0.139015258	0.335139854	0.378080478
0.024576306	0.184094987	0.278519107	0.167061536
0.017322981	0.037915854	0.026383443	0.295096021
0.431147066	0.031336843	0.012167932	0.166681054
0.06358922	0.360746261	0.091055894	0.39299156
0.343971549	0.304426986	0.036055284	0.423058758
0.014277869	0.43032741	0.159652828	0.052243846
0.227384337	0.010182979	0.402930044	0.048179739
0.28831819	0.320793505	0.083429781	0.007882075
0.085108112	0.027029433	0.338406086	0.310107759
0.020950194	0.184536973	0.188892697	0.290169745
0.255872576	0.26085966	0.454019072	0.248663238

0.056264477	0.097401682	0.298125818	0.319577897
0.243795367	0.356543771	0.128978868	0.425314121
0.31921378	0.405901106	0.375722691	0.215336752
0.10854234	0.307417114	0.244990829	0.014147825
0.243772205	0.31622	0.310118934	0.059684877
0.119110114	0.204675992	0.050263449	0.262130139
0.163255658	0.258687075	0.085016459	0.313198971
0.32879413	0.315290556	0.06205499	0.023823815
0.457411326	0.404840344	0.1240786	0.264533989
0.11261956	0.17596333	0.309589683	0.285304756
0.229118826	0.331547087	0.340638081	0.261357528
0.39927561	0.171029339	0.452756945	0.154983953
0.034717555	0.292410706	0.04277793	0.049613104
0.083341481	0.013263108	0.135662691	0.263965495
0.197237112	0.219471009	0.306097596	0.051735658
0.187234044	0.313541916	0.213975841	0.145280941
0.039032931	0.425995885	0.005106431	0.033357899
0.216740162	0.294960736	0.177258513	0.139449768
0.129656903	0.083166384	0.168908021	0.280678318
0.193665772	0.251818965	0.303093291	0.036119401
0.018560572	0.370394452	0.324832849	0.016205752
0.050329205	0.30110672	0.277800729	0.108560607
0.010326156	0.368466817	0.213278722	0.24290688
0.260262832	0.049503817	0.39561132	0.082978804
0.060710481	0.439940975	0.225335255	0.4273166
0.228719208	0.249146616	0.452850258	0.336000792
0.128620625	0.25725742	0.018550421	0.356083428
0.210110407			
FAM134B	0.089507333	0.0912113	0.067224358
0.175064606	0.111245471	0.066996903	0.194578164
0.096887517	0.121134899	0.051587433	0.056190616
0.083001553	0.055160555	0.068360285	0.030583519
0.216316946	0.138542646	0.078225514	0.279062221
0.185861426	0.124964544	0.213288564	0.229072423
0.066983069	0.107478891	0.178127239	0.069321439
0.180349203	0.204933087	0.046786695	0.14499618
0.216740676	0.111041301	0.066167946	0.111247632
0.057563688	0.065282107	0.086608121	0.04125321
0.101362411	0.111635435	0.184314055	0.222951592
0.196828609	0.058474747	0.223681934	0.129507569
0.052510312	0.138036132	0.043209447	0.063565097
0.04977641	0.061102693	0.107490421	0.154117271
0.024539978	0.040009568	0.018228934	0.07867046
0.222689765	0.046530824	0.058277389	0.114309716
0.051395179	0.133261408	0.161295689	0.151685239
0.038131391	0.08863101	0.253818814	0.05289313
0.019640361	0.217446921	0.164266312	0.023167687
0.268129791	0.103554825	0.163557007	0.182062038
0.159803714	0.072306008	0.132736929	0.108829847
0.106351597	0.199256081	0.066529469	0.166936779
0.097694244	0.155529902	0.123681584	0.209194821
0.124742459	0.072660952	0.128077196	0.211273487
0.084680368	0.092471265	0.055920964	0.076191414
0.024032791	0.156750237	0.062606487	0.103123025

0.077309066	0.051834719	0.218279403	0.133710778
0.268991877	0.032213938	0.243828732	0.085519346
0.161213631	0.161706633	0.188048423	0.135948287
0.169395261	0.103232812	0.070209289	0.259923156
0.100693021	0.141072227	0.029614341	0.267176435
0.056765173	0.032197769	0.045748838	0.128462067
0.080869384	0.049032499	0.170412014	0.134464892
0.19578751	0.137196733	0.076130791	0.110466877
0.140459464	0.050478373	0.093082416	0.066838185
0.061201938	0.131602607	0.004878255	0.241228531
0.093884046	0.15241139	0.322198697	0.076209618
0.133168005	0.207122976	0.240201508	0.117717765
0.077233175	0.273756177	0.053705825	0.211213762
0.0388437	0.127671641	0.254174998	0.098420153
0.194785817	0.053819732	0.176660888	0.09594138
0.018692144	0.08532674	0.217534103	0.116685489
0.054008119	0.068411851	0.043732036	0.298297522
0.125162606	0.166629936	0.067295697	0.072950072
0.052669444	0.263264592	0.038566521	0.10048089
0.05356832	0.108928879	0.12820622	0.102490248
0.230035955	0.159901509	0.013171667	0.059409147
0.071315088	0.03112556	0.11139378	0.124315848
0.038643958	0.141300252	0.00712685	0.202030051
0.058935563	1 0.142896273	0.224407075	
0.067459258	0.200626288	0.067993557	0.077337435
0.052774101	0.061227085	0.06989568	0.174178434
0.175191052	0.09987123	0.128178481	0.170686106
0.120187461	0.142639625	0.17012681	0.032182516
0.097446438	0.180395473	0.220123695	0.157309833
0.106297145	0.019615893	0.072182659	0.13194858
0.042243472	0.085193111	0.058508121	0.018509747
0.214005117	0.128442126	0.079896955	0.12735356
0.059921787	0.113901431	0.043808478	0.175282357
0.188996369	0.100475221	0.059744154	0.033941126
0.034489381	0.07041774	0.240143766	0.041591576
0.035951722	0.17433837	0.044948313	0.055521715
0.155082205	0.190336291	0.138907692	0.016358748
0.039248983	0.024151978	0.021183606	0.067262116
0.054170935	0.095973643	0.096597513	0.059218694
0.154629164	0.222762117	0.090802077	0.207164306
0.057622808	0.194360224	0.26321214	0.012126023
0.118429509	0.032024008	0.026742037	0.095480039
0.205662385	0.131703618	0.070447518	0.21430368
0.043822881	0.212616449	0.170970842	0.033899556
0.088690951	0.078311565	0.214500847	0.140093211
0.056910038	0.151366072	0.193027323	0.069998412
0.063273337	0.220992396	0.086093435	0.150000707
0.234981967	0.117839137	0.069401813	0.290346648
0.19990575	0.083182603	0.134387181	0.256112102
0.09928449	0.208030379	0.074667939	0.083525883
0.187092609	0.049512892	0.136766336	0.070640656
0.059197522	0.082215791	0.28184589	0.072591233
0.314917357	0.038620346	0.047155142	0.097110197
0.089885311	0.074819962	0.199940488	0.088021012

0.054012443	0.053262541	0.138947028	0.1224769
0.069639469	0.227360307	0.186621003	0.068705864
0.099242675	0.07693952	0.043396864	0.133251705
0.150923584	0.199118672	0.114259823	0.046286409
0.043909188	0.152110228	0.018397202	0.057142667
0.157489178	0.073808371	0.156351755	0.140233726
0.042571305	0.051229116	0.195228671	0.247613921
0.075164891	0.071597616	0.067947607	0.207377342
0.086928533	0.074149987	0.04074127	0.245846815
0.19100883	0.063793646	0.099750791	0.078818623
0.152206593	0.039983573	0.065632764	0.093444876
0.289554467	0.044764369	0.02535432	0.028489657
0.177751815	0.144988999	0.05894363	0.153149404
0.016913576	0.207176347	0.042755534	0.09341804
0.072715121	0.141679683	0.116786518	0.065862485
0.058517759	0.089414567	0.045886553	0.080359587
0.05526475	0.057137226	0.084269716	0.245771898
0.099890434	0.060404021	0.126020386	0.084667944
0.137396135	0.157572644	0.26561822	0.159010589
0.057567341	0.086361973	0.012918671	0.061583639
0.069698742	0.128724442	0.089368705	0.149500126
0.090275958	0.169547319	0.128337748	0.071742209
0.125413228	0.150237059	0.186481215	0.02281382
0.012444611	0.053106905	0.151309588	0.19411556
0.093139257	0.012263759	0.163597318	0.166451876
0.102175695	0.100685617	0.073670502	0.258837126
0.102482496	0.261017613	0.024141674	0.232112442
0.117732941	0.048042529	0.099662386	0.074998209
0.250767349	0.171788932	0.074499711	0.141476159
0.010553101	0.22850444	0.087111553	0.056630265
0.138708346	0.180515803	0.162393731	0.17518592
0.051629676	0.087493485	0.104721843	0.01778952
0.126725045	0.125929093	0.139185659	0.125422291
0.12872839	0.201817444	0.157378377	0.072202778
0.118366269	0.058246046	0.083301693	0.018658854
0.118204314	0.252051594	0.064600156	0.018135205
0.054631473	0.120215136	0.048771555	0.174163606
0.030157899	0.217937741	0.140105759	0.12534313
0.187234994	0.148970149	0.209223097	0.171897269
0.107413359	0.147347311	0.063129919	0.029834396
0.166661905	0.046603617	0.171101361	0.072797651
0.080638251	0.138850607	0.047773826	0.096955716
0.095242003	0.102183646	0.079017902	0.153588358
0.059351809	0.076785611	0.097072296	0.089532638
0.199554179	0.135114561	0.11400169	0.078768432
0.145552058	0.051306213	0.256230492	0.031505828
0.034366134	0.076873229	0.156085902	0.206219109
0.108749765	0.074657189	0.226860217	0.154082728
0.090996004	0.081604544	0.109468561	0.110269378
0.260251258	0.153165542	0.110557836	0.111877274
0.036868902	0.08181871	0.124941145	0.081473369
0.072721104	0.236195635	0.070488662	0.075924904
0.080459396	0.062976964	0.115834993	0.263116917
0.255356426	0.200177141	0.016988978	0.187438875

0.019150957	0.186803472	0.032849539	0.187716138
0.180980781	0.053874398	0.209326557	0.12215471
0.145321246	0.063445669	0.102845155	0.253767776
0.027578664	0.088177243	0.05388086	0.158450997
0.032526548	0.14652402	0.218909638	0.06215123
0.022263206	0.029775257	0.083037365	0.169083034
0.203040249	0.144998335	0.058832633	0.187140595
0.121500589	0.068901263	0.100056566	0.073150437
0.165799405	0.066441567	0.033058721	0.096049436
0.157674495	0.290298343	0.056936606	0.100829747
0.26408342	0.066834706	0.076014802	0.065209812
0.096294157	0.1995184	0.052834017	0.0790944
0.116927493	0.046271142	0.207906063	0.142525991
0.01934523			
FASL 0.460178823	0.529682758	0.469690636	
0.48961928	0.521446322	0.403289726	0.289050482
0.232261298	0.142239368	0.010966968	0.048303571
0.506309556	0.286772397	0.335770372	0.373839011
0.510453432	0.224971308	0.417519104	0.344259861
0.487532236	0.132819172	0.5063812	0.156881921
0.399655141	0.182236872	0.129461341	0.37571344
0.498850788	0.146155259	0.35149905	0.434021841
0.180024737	0.478198888	0.516992249	0.520835479
0.509641457	0.424012953	0.022383542	0.344816021
0.442925275	0.50623513	0.480305503	0.4330872
0.081239501	0.47422614	0.338684604	0.501618936
0.272758987	0.067018726	0.260171945	0.431633359
0.469326592	0.484309823	0.282859377	0.232332218
0.265458589	0.255334509	0.347020779	0.342057961
0.321290183	0.176520664	0.483064106	0.138684448
0.194670787	0.318268738	0.25087597	0.333218961
0.438968296	0.40713934	0.037017685	0.44910835
0.005207079	0.471301201	0.488342111	0.380864441
0.087870126	0.044645193	0.28519034	0.422036901
0.503686934	0.454436908	0.519592734	0.504081888
0.276408858	0.414284251	0.478883156	0.268983361
0.46358834	0.253836766	0.167869101	0.519711221
0.514522119	0.52522052	0.312404596	0.384993105
0.456727004	0.476854123	0.480107139	0.513545572
0.410816182	0.104491026	0.435457396	0.52139127
0.408442913	0.411736908	0.253147414	0.45231245
0.11469199	0.40418893	0.457996837	0.228833653
0.504084036	0.038646463	0.328712952	0.435146649
0.314500648	0.481426426	0.521263658	0.27948746
0.505136535	0.044233869	0.204211024	0.071939395
0.403938319	0.218164151	0.46782313	0.496682488
0.289070816	0.477275447	0.355783533	0.007912477
0.061082564	0.281849388	0.469005989	0.445967252
0.492866641	0.387292727	0.331909672	0.407488685
0.491197351	0.519899365	0.06084264	0.159995655
0.49357066	0.284599478	0.353364541	0.50667602
0.428036186	0.176205359	0.210325703	0.528939339
0.41488949	0.149810377	0.376147177	0.51428943
0.397674293	0.473904849	0.181042957	0.372255512

0.089770029	0.47992498	0.064013363	0.387313482
0.328678943	0.234692531	0.0240516	0.311417069
0.353059608	0.456510233	0.454850467	0.18366642
0.417536795	0.513279905	0.353708461	0.469451675
0.02307411	0.090469011	0.292991422	0.515471581
0.295073187	0.508823126	0.409619559	0.422488776
0.407955338	0.512467998	0.006153803	0.489190037
0.528460361	0.309188513	0.489704866	0.508442862
0.443507639	0.500896577	0.050617172	0.514103921
0.373817744	0.142896273	1	0.260697492
0.457078034	0.085503006	0.375158472	0.48091305
0.465650607	0.017170852	0.254702894	0.455754016
0.061158688	0.132192943	0.481896535	0.490668506
0.466290398	0.513566907	0.522202237	0.425327401
0.520979303	0.320071191	0.030011963	0.441704707
0.490080353	0.42631643	0.436134012	0.325823334
0.348539731	0.474827572	0.443525892	0.237437526
0.084680144	0.380122985	0.036927877	0.442755214
0.409115595	0.18098121	0.447362622	0.232354039
0.135712598	0.535520308	0.269954232	0.344834896
0.195594809	0.458234761	0.351157138	0.299041555
0.289783052	0.264685931	0.446923204	0.462632757
0.065295085	0.166647477	0.033118537	0.421694141
0.198774505	0.40039207	0.382062945	0.434662123
0.395652281	0.174039657	0.48981737	0.386497433
0.197483284	0.037035465	0.512803149	0.502202277
0.342801272	0.239964592	0.085255839	0.374848209
0.494430305	0.328935817	0.276595295	0.458046093
0.054938081	0.510762697	0.340491361	0.210764636
0.471279317	0.370113858	0.506108717	0.082126604
0.43835069	0.46619231	0.430612468	0.288696203
0.386495643	0.492691831	0.059901328	0.427588458
0.423071643	0.068496795	0.532944971	0.214775748
0.049212644	0.518308841	0.486756261	0.142389306
0.14612903	0.41371655	0.072333417	0.41588191
0.527297962	0.513115177	0.465331771	0.322686671
0.080122144	0.022687333	0.321723846	0.46942159
0.502349413	0.518538303	0.38044818	0.469760079
0.350063506	0.423106063	0.162315607	0.518640036
0.271552188	0.517826848	0.018436874	0.498247203
0.503690614	0.312516521	0.148203867	0.371612485
0.406097199	0.090182828	0.293161872	0.49724194
0.461577823	0.431570377	0.433008677	0.506641028
0.032489373	0.075966535	0.485714639	0.271069287
0.457746263	0.324262928	0.087843273	0.380386388
0.084535918	0.451166693	0.520506835	0.112213643
0.361585237	0.35244737	0.497125463	0.076624052
0.477217564	0.502830544	0.52015515	0.243794696
0.488707077	0.493669042	0.462374787	0.148906691
0.042269123	0.516921841	0.438763021	0.043828954
0.516122969	0.244573651	0.027839434	0.500388627
0.279994569	0.428704092	0.007038655	0.113218054
0.084737398	0.522470433	0.475116187	0.439366239
0.25676876	0.463487678	0.012927907	0.531333236

0.492698269	0.036090749	0.236794674	0.476131198
0.475010616	0.531184511	0.414529386	0.491262786
0.49168377	0.498255457	0.316175056	0.391743307
0.477989587	0.494415111	0.472024012	0.45719318
0.508103955	0.522010493	0.388126114	0.034656772
0.202585965	0.252997271	0.340286135	0.133483165
0.344085936	0.231271196	0.483478233	0.520394229
0.492892315	0.014727534	0.20646586	0.468033916
0.463428671	0.495565579	0.042718638	0.011366442
0.013060805	0.042648657	0.431774314	0.364266636
0.494999722	0.252253518	0.013587674	0.071790774
0.375789658	0.496762803	0.506867141	0.484073233
0.496942254	0.149680925	0.329492408	0.491123897
0.129150631	0.335806542	0.497867616	0.47207456
0.084549109	0.36866879	0.433934299	0.500847596
0.110089242	0.445139599	0.50591689	0.406199313
0.030039045	0.1521917	0.125933059	0.519654989
0.494686464	0.134336796	0.068790833	0.159644075
0.187133313	0.504499045	0.256106751	0.52716314
0.485294366	0.517313101	0.140415708	0.482781397
0.078048483	0.484322348	0.451958355	0.074254988
0.397984804	0.030635433	0.469279933	0.20877865
0.371705553	0.448348886	0.276013981	0.040670749
0.252730245	0.106282924	0.515174924	0.51852588
0.071572853	0.328312731	0.422291638	0.493193116
0.332670338	0.509688602	0.499704918	0.428190204
0.137821059	0.205256558	0.476206469	0.429313694
0.294514479	0.519673513	0.393948191	0.534808748
0.447447497	0.460207114	0.474174525	0.437493683
0.339221756	0.440601555	0.416379273	0.037633012
0.459020005	0.516538855	0.425647341	0.244978336
0.373888169	0.461300709	0.085063027	0.208655784
0.402740215	0.334521969	0.225392285	0.516772009
0.440882409	0.431195001	0.06861809	0.118381945
0.546858499	0.432432534	0.196375209	0.485799157
0.27655824	0.457188183	0.507767117	0.514196769
0.435013873	0.480219502	0.475789866	0.425912267
0.419222732	0.364189255	0.524917516	0.109589148
0.147441677	0.46034857	0.161165029	0.169321188
0.234191396	0.060563403	0.369054315	0.519452824
0.436020708	0.48913115	0.352234568	0.145479798
0.45049431	0.410866928	0.47660017	0.268270582
0.137472379	0.49693522	0.022192367	0.111623726
0.455441873	0.48679383	0.474262625	0.361493656
0.391960899	0.232544436	0.420992405	0.325606659
0.35125019	0.426057595	0.478432264	0.061371333
0.03967201	0.517892612	0.416292615	0.047112148
0.145626248	0.409938947	0.386560128	0.328436639
0.050777526	0.484651536	0.361754504	0.514384994
0.422636256	0.123989839	0.345312122	0.134257126
0.107252542	0.442833928	0.4795955	0.514370383
0.451684785	0.512371787	0.431986214	0.471132715
0.184229259	0.426308505	0.056168799	0.493609528
0.446401643			

FCGR2B 0.130887693	0.185649523	0.100544915	
0.252899212	0.175080306	0.091455975	0.16285761
0.065253771	0.154329759	0.075283739	0.188391329
0.150496922	0.173373808	0.121204383	0.057328407
0.286989537	0.297472695	0.069569095	0.244346634
0.214874505	0.108360488	0.322586522	0.211073014
0.134570419	0.2701413	0.279700689	0.16727597
0.246731402	0.280107502	0.065690096	0.159430411
0.231428934	0.163380719	0.145085023	0.238463333
0.138669939	0.099737289	0.044532076	0.078702716
0.140058709	0.205296875	0.215901439	0.268005047
0.207817105	0.120253769	0.230116907	0.191041066
0.050484527	0.26817221	0.037919604	0.112555404
0.12682153	0.129803305	0.083104813	0.168070394
0.120504307	0.045912212	0.100932021	0.081691442
0.216288869	0.038230638	0.140267981	0.127245155
0.066748761	0.092570791	0.236410546	0.114155286
0.098494867	0.110321252	0.105307281	0.099819617
0.025792819	0.221875354	0.210412141	0.074920173
0.274027053	0.228249312	0.240595298	0.214629124
0.236223997	0.130365012	0.260855852	0.228361957
0.252688069	0.193673683	0.127020351	0.270132642
0.154613319	0.177645572	0.122604986	0.314872038
0.250979147	0.168195589	0.235784856	0.249411768
0.16995218	0.16408971	0.094442143	0.135444605
0.086008876	0.184477118	0.131369888	0.217738328
0.114206198	0.11372987	0.288865675	0.173097593
0.25584795	0.052412575	0.29620549	0.139462637
0.243057985	0.124232462	0.251616583	0.171678058
0.145713974	0.162439593	0.16788794	0.21761625
0.145488468	0.185549243	0.117804112	0.207165042
0.106779757	0.098155056	0.107879599	0.234530024
0.083176567	0.124392112	0.140635948	0.097578908
0.260158984	0.144867125	0.151680777	0.097999952
0.183034628	0.096498313	0.209112118	0.099594233
0.143005103	0.257002347	0.007761753	0.182305837
0.138780421	0.157171136	0.317437938	0.142215331
0.229742983	0.141569738	0.258183093	0.227444857
0.122273441	0.254382574	0.047786866	0.292510308
0.101518576	0.232328472	0.232549549	0.181977797
0.181916619	0.132364525	0.238702161	0.070479224
0.069509241	0.226385869	0.066965208	0.08016138
0.099889325	0.16548287	0.101403911	0.239970387
0.167880683	0.268509318	0.057994859	0.165357449
0.130367779	0.186875304	0.029469799	0.212908433
0.060177533	0.208983342	0.164620831	0.158248585
0.28596406	0.239094222	0.005718256	0.129895113
0.16629321	0.02959645	0.220856516	0.175730276
0.088102804	0.239994152	0.006515947	0.279942568
0.076252462	0.224407075	0.260697492	1
0.134382696	0.273681109	0.079514961	0.176362962
0.139524374	0.054188117	0.055527671	0.198577715
0.202701461	0.078355037	0.163385711	0.225697508
0.186036408	0.219581335	0.281399946	0.080619964

0.168889053	0.138508091	0.133574022	0.213500225
0.169770953	0.057672891	0.130465741	0.143912896
0.051660395	0.164629022	0.12367033	0.04448465
0.215404317	0.109479686	0.072545201	0.189544757
0.106785817	0.206390444	0.08323914	0.156326115
0.293221922	0.212035066	0.14501998	0.087614074
0.099304099	0.146201736	0.169985525	0.133294345
0.177580472	0.242827513	0.111546826	0.106598069
0.120859802	0.304809716	0.085569022	0.066131479
0.182335649	0.095285925	0.07517111	0.109614082
0.102609692	0.23109037	0.183742915	0.110639377
0.253693826	0.213496187	0.182528451	0.246716953
0.075018844	0.196812023	0.248389547	0.055482959
0.226776992	0.052623929	0.023835601	0.182484088
0.109692692	0.235353937	0.197935734	0.304641499
0.125854538	0.26430896	0.255844881	0.041537888
0.096564147	0.15404346	0.200280395	0.257245405
0.112974567	0.190062327	0.175297178	0.111012834
0.108679725	0.267504583	0.190812726	0.274487534
0.224474954	0.210346732	0.168019845	0.279296958
0.268497831	0.151723162	0.079670895	0.331830834
0.206393208	0.326146568	0.159022381	0.144533173
0.24131488	0.1618358	0.197807224	0.10744787
0.132474967	0.190177769	0.25841345	0.180249277
0.283086123	0.115990356	0.03074612	0.212787841
0.081497715	0.154386908	0.137312375	0.188723258
0.133224793	0.050055807	0.291744777	0.125185332
0.119891444	0.204497864	0.138994905	0.152223786
0.165534196	0.132774532	0.103362955	0.182167747
0.102715739	0.142014989	0.17269661	0.111464131
0.095409583	0.208690563	0.056557417	0.063605701
0.253798373	0.151058976	0.246698804	0.222591524
0.081681271	0.051218126	0.273815853	0.229766763
0.181333469	0.156398468	0.151096091	0.14767285
0.162656455	0.179932965	0.119909232	0.247695888
0.2151258	0.150810399	0.117946214	0.135184221
0.255340978	0.033137641	0.176767598	0.173810484
0.205567115	0.095011434	0.010997719	0.024848267
0.175720372	0.251639258	0.130838723	0.156441164
0.031415088	0.189176459	0.021942097	0.196191682
0.161853446	0.202414516	0.21442268	0.154851072
0.156801097	0.172662434	0.110176256	0.196671332
0.144960352	0.142610749	0.148565068	0.30567616
0.186725297	0.152434544	0.165071615	0.144903699
0.241745934	0.246105255	0.278684631	0.152905926
0.105611827	0.060817232	0.04935883	0.162646891
0.067310784	0.264903629	0.167999036	0.253432642
0.20677445	0.13445249	0.106246097	0.143623879
0.187853136	0.22902894	0.079824999	0.017342973
0.01079807	0.187768425	0.144236026	0.20549056
0.188893487	0.03679628	0.128283802	0.093446016
0.137995866	0.210039603	0.165965759	0.298363484
0.179411406	0.263413594	0.082236287	0.25846958
0.251784526	0.058416854	0.219786475	0.147451536

0.188244448	0.189917682	0.072966591	0.242024283
0.049841379	0.2810156	0.157991944	0.142652662
0.07324851	0.268578759	0.244523583	0.251758877
0.131014839	0.273169782	0.276236005	0.021342158
0.221539637	0.212903932	0.258813471	0.241150112
0.200923229	0.297767502	0.228830852	0.145229239
0.212189789	0.137878007	0.177659674	0.022228883
0.137817744	0.153412986	0.144824971	0.096923975
0.065919973	0.131544778	0.09840708	0.183632873
0.073249839	0.288708027	0.261733211	0.24208114
0.173398443	0.135531914	0.214117666	0.189877527
0.089957962	0.257418982	0.13859003	0.066157999
0.169648415	0.077302867	0.196519414	0.101268568
0.080876014	0.260216062	0.148295853	0.197377894
0.12389875	0.163503225	0.171425811	0.175517527
0.168555365	0.095196258	0.150616222	0.116288535
0.200463846	0.257232883	0.092643818	0.237274701
0.248275645	0.110975522	0.119999098	0.025682673
0.086932573	0.082277783	0.240740262	0.282503711
0.114774482	0.105948988	0.100278113	0.246699795
0.186135145	0.133072163	0.088561863	0.175075032
0.236085489	0.225683549	0.195851001	0.212779735
0.067461735	0.181408315	0.177138864	0.143321141
0.133018857	0.200214061	0.156258867	0.031726512
0.155191796	0.105179664	0.261229993	0.267420142
0.274673618	0.212656052	0.067595975	0.260319151
0.063775745	0.285461818	0.043475799	0.201934619
0.223297361	0.054700506	0.25046465	0.134484525
0.25899897	0.14748309	0.109598064	0.270483623
0.076845516	0.171575532	0.14704081	0.222666583
0.111735038	0.184545729	0.273365278	0.057125546
0.061140766	0.062126849	0.164963164	0.087084235
0.138581302	0.259156021	0.063387382	0.134648116
0.122719004	0.082614001	0.102973921	0.161409669
0.190443522	0.167311164	0.053811508	0.154332386
0.145648615	0.234206075	0.082920231	0.073433596
0.135159324	0.124826368	0.130795256	0.156359515
0.114289212	0.311538	0.100373788	0.156121475
0.087020927	0.0714241	0.156805643	0.203394887
0.057916736			
FCHSD2	0.453178754	0.490360542	0.409011826
0.407691968	0.414582165	0.400313427	0.193069899
0.20242683	0.068945518	0.002825614	0.015042431
0.449903415	0.181705025	0.267727818	0.333634983
0.401316422	0.11483434	0.379884704	0.219721494
0.407247564	0.087485554	0.375041923	0.078033106
0.275884548	0.083461024	0.052118252	0.237681005
0.435055804	0.061185821	0.355014565	0.401801041
0.090654	0.458973344	0.514327455	0.421846189
0.513933957	0.441972251	0.01331546	0.290071303
0.449124404	0.435492206	0.404434756	0.306040917
0.031590718	0.496764477	0.222706273	0.392007825
0.24748852	0.021351697	0.269495499	0.448760386
0.477601161	0.43590425	0.233099274	0.141565815

0.185149933	0.259504476	0.260748606	0.367530173
0.203939586	0.18009962	0.477291888	0.073202059
0.143595222	0.260549504	0.137959007	0.259391628
0.338806962	0.425031548	0.015705024	0.437797054
0.002167426	0.383093787	0.366893448	0.300098895
0.03165241	0.013946497	0.161547951	0.324385315
0.448449606	0.451049038	0.454231739	0.458357677
0.159796798	0.276723213	0.393697255	0.14834497
0.455902905	0.158651794	0.105080598	0.399640321
0.457102328	0.500209083	0.173774434	0.236868607
0.431549038	0.454507221	0.427582533	0.41380204
0.325686354	0.043756245	0.441053851	0.464793466
0.437067529	0.307829919	0.128131218	0.428484169
0.047561304	0.395436523	0.332121701	0.162628632
0.449913944	0.012771602	0.18698911	0.350527217
0.214023307	0.461410308	0.49251242	0.167192031
0.409281312	0.015406469	0.115648393	0.027392358
0.331989122	0.138148697	0.503385902	0.446989874
0.246127464	0.359324149	0.255166758	0.000443189
0.019573084	0.180939048	0.465666846	0.38920439
0.441905552	0.426082796	0.201071772	0.43487262
0.448223937	0.453969033	0.070389342	0.084971801
0.421281313	0.177643763	0.213453055	0.454439964
0.276782826	0.108765318	0.110708218	0.461367313
0.426691825	0.069401471	0.385080238	0.397530924
0.319052592	0.33375246	0.093322658	0.262817581
0.040212334	0.477546126	0.021395622	0.362391596
0.254595432	0.132164939	0.010798842	0.27762951
0.289791597	0.443133475	0.494415078	0.092710396
0.306159676	0.384070432	0.363681079	0.432578311
0.005771525	0.040336151	0.338974555	0.463681505
0.334884551	0.464881801	0.289421224	0.286766359
0.255524588	0.440647573	0.003337837	0.499195277
0.503060025	0.359297717	0.381856082	0.42063459
0.366681188	0.440695538	0.054025514	0.405607034
0.419352381	0.067459258	0.457078034	0.134382696
1	0.029731093	0.332745241	0.443080646
0.4541067	0.007637699	0.242987073	0.387114209
0.020594604	0.094518897	0.445131828	0.39746293
0.363123439	0.44527531	0.447585508	0.347471171
0.408893343	0.231488912	0.009350121	0.300028417
0.456682401	0.386009181	0.433408495	0.228543572
0.401999	0.453875462	0.454370328	0.207432536
0.032521894	0.319012541	0.018078171	0.304885636
0.435793769	0.101332788	0.395614444	0.148009772
0.056774973	0.474618875	0.16081556	0.25645737
0.117189632	0.406352409	0.24128594	0.2260967
0.195746162	0.138592969	0.331256046	0.363425208
0.031108191	0.075883713	0.012451004	0.397937
0.126330834	0.344880782	0.352643541	0.447365931
0.334351155	0.095756004	0.362752627	0.276295951
0.094155019	0.010154143	0.472007294	0.397464596
0.391449472	0.137196596	0.032569765	0.364980495
0.451913923	0.354565686	0.325830827	0.447032633

0.025647065	0.458639679	0.225694696	0.099575865
0.368002038	0.23766619	0.440152558	0.05980363
0.424612377	0.449045307	0.300474976	0.156068625
0.425349854	0.414529734	0.023526295	0.449764702
0.448567233	0.022994528	0.488309881	0.109257336
0.015121066	0.437759065	0.442374775	0.059728524
0.06224518	0.316061557	0.042639867	0.261584749
0.470695042	0.387354432	0.443386088	0.203709414
0.029693951	0.007031133	0.190901203	0.418518433
0.51602257	0.469817821	0.243767634	0.344555639
0.218546373	0.30853172	0.170147677	0.462531356
0.210261639	0.506456177	0.004294293	0.443356408
0.511386654	0.330837715	0.061766198	0.307528452
0.432032047	0.035337655	0.206115588	0.483616598
0.447675663	0.307745099	0.33357504	0.440211554
0.012323279	0.035220438	0.377055542	0.171477626
0.374638999	0.190044458	0.049403492	0.352074757
0.031633856	0.442543681	0.451629562	0.043950732
0.358685109	0.341551465	0.361862868	0.027807806
0.447524664	0.480902122	0.513993948	0.161167872
0.46519662	0.454404342	0.40983135	0.066737816
0.012010311	0.506018318	0.431374391	0.017008631
0.452600866	0.281982459	0.008130357	0.46866011
0.168605929	0.473730606	0.003225441	0.097442535
0.03379337	0.447253551	0.476394927	0.399502465
0.277418522	0.370482967	0.005625895	0.479918222
0.470099129	0.009421585	0.123401019	0.457968177
0.440425331	0.506689754	0.303283862	0.449932037
0.432850553	0.493441812	0.226196237	0.240010935
0.448319095	0.472666727	0.440337177	0.445875709
0.453228417	0.415479235	0.256732147	0.010218901
0.112951053	0.230542896	0.314736449	0.065295638
0.374171739	0.115213838	0.462884204	0.453578423
0.461465175	0.002783142	0.150143311	0.460379425
0.443844681	0.451397303	0.019512648	0.005783377
0.008625231	0.012684355	0.368994197	0.222610044
0.460032579	0.267272369	0.00241054	0.041460309
0.271450661	0.384751946	0.481708643	0.365077022
0.453976561	0.067869496	0.279332039	0.393500466
0.052134017	0.396795885	0.459880909	0.460380734
0.035030219	0.226787776	0.41472378	0.454194258
0.066329918	0.295314904	0.456647825	0.29482474
0.012874843	0.065044003	0.049621205	0.432512648
0.503531998	0.062014036	0.02368241	0.167272283
0.101313592	0.461151215	0.143918284	0.462617671
0.447683908	0.423688583	0.062707412	0.470644001
0.028217831	0.483641041	0.319827373	0.062123903
0.291202702	0.009519422	0.454578264	0.133985629
0.3513954	0.430079181	0.179010968	0.012105469
0.177711657	0.041316956	0.449164148	0.44894622
0.027951094	0.230440569	0.283279228	0.413172869
0.307266233	0.424287019	0.502826784	0.433700918
0.071268921	0.150338334	0.419342391	0.443361277
0.254116883	0.456446462	0.272901713	0.483851064

0.445059129	0.457363771	0.461973713	0.337611723
0.215763055	0.433087715	0.383897037	0.015100424
0.347692563	0.447148878	0.383262522	0.12990774
0.227179932	0.394544156	0.043616173	0.23778138
0.338006507	0.338698596	0.123327447	0.426570304
0.427649617	0.445715218	0.039862437	0.045943793
0.511234981	0.442527801	0.141340972	0.432400723
0.15477031	0.318788738	0.458095084	0.4497443
0.382330148	0.457317298	0.447220627	0.407801309
0.43011827	0.242747722	0.512491862	0.104782129
0.067965499	0.456757196	0.078551789	0.076522141
0.12268952	0.019692356	0.297284164	0.418192266
0.389430299	0.363462757	0.363335723	0.068355617
0.307544533	0.415995464	0.337909352	0.192121058
0.062486723	0.487716581	0.006560061	0.045310395
0.399041983	0.450213505	0.368642064	0.219699189
0.287394803	0.127587559	0.275998372	0.332747714
0.33677869	0.381988858	0.450612624	0.030321898
0.015158393	0.458571045	0.415685532	0.01728617
0.077233973	0.427570449	0.314155529	0.209184388
0.016171241	0.453218293	0.354512027	0.424257933
0.350055923	0.056174911	0.405055436	0.090129814
0.05513941	0.448804101	0.400184247	0.497709094
0.35512549	0.40150316	0.466651218	0.462064544
0.131100815	0.41928867	0.020607789	0.453182358
0.421591596			
FDPS	0.033721615	0.04883361	0.032270369
0.116753045	0.073502839	0.034302679	0.147123193
0.034672734	0.143567983	0.101423491	0.201781054
0.043630407	0.088619298	0.056462837	0.019644505
0.13163954	0.190336319	0.022725084	0.200299979
0.095907622	0.094914786	0.165972423	0.191642027
0.048550241	0.189928315	0.265143604	0.08426723
0.096546619	0.262974346	0.028533887	0.066389504
0.154243711	0.047395052	0.031689356	0.092084461
0.028127935	0.02306562	0.037273778	0.036697441
0.037965674	0.073261629	0.098921618	0.175913314
0.231642434	0.025237847	0.192245866	0.074873845
0.0203957	0.26902371	0.017012948	0.02582496
0.027058999	0.046003141	0.047697357	0.137944394
0.058581715	0.018283421	0.030549134	0.028776311
0.190387303	0.021546147	0.031299455	0.124725725
0.039526996	0.051910905	0.132804912	0.080042799
0.027617049	0.033476254	0.129470313	0.02309177
0.033723388	0.109332627	0.091198179	0.023725798
0.28960268	0.226173566	0.132426842	0.135024667
0.08372698	0.028019007	0.085904685	0.067800548
0.136287208	0.116159665	0.0411939	0.186958552
0.043055875	0.156756138	0.072872468	0.14334327
0.07952682	0.039525281	0.138701052	0.164469725
0.058760827	0.044156283	0.033342174	0.058938859
0.029102271	0.136080423	0.032860929	0.060992663
0.029024842	0.041754001	0.247497012	0.06278117
0.239428875	0.012122549	0.182812786	0.058596402

0.085975905	0.181342854	0.183855807	0.098226148
0.073674837	0.047387343	0.03849628	0.20836762
0.053455382	0.163585532	0.057192268	0.197218357
0.03751769	0.032646715	0.02084275	0.08338459
0.038649077	0.04325839	0.098340916	0.141753762
0.246948657	0.065188058	0.037803371	0.03805125
0.063928746	0.018892147	0.134917997	0.025843689
0.040128741	0.084764634	0.003247927	0.16689647
0.054823543	0.077645892	0.258982107	0.046853082
0.144719787	0.114689413	0.203397816	0.067844296
0.032064259	0.256623663	0.014223507	0.133602011
0.043758583	0.111191764	0.191925549	0.090344206
0.197938097	0.02729452	0.210125769	0.029351982
0.024687819	0.116856725	0.079760723	0.04992529
0.0419364	0.048679966	0.018457404	0.207517778
0.103082801	0.129698341	0.021872473	0.056342362
0.17816304	0.168518168	0.008826511	0.0601737
0.021324472	0.055979789	0.077090603	0.066424496
0.19317507	0.088583742	0.003807817	0.02773927
0.03825906	0.008090468	0.105622201	0.061778338
0.033866299	0.090804168	0.00128825	0.125681214
0.017824415	0.200626288	0.085503006	0.273681109
0.029731093	1	0.035545232	0.047983702
0.033506746	0.052614867	0.036508704	0.097814223
0.182744591	0.07026898	0.054203992	0.108179405
0.099696837	0.074742505	0.102279365	0.031765381
0.071206495	0.087386748	0.154938727	0.110919037
0.048049662	0.013424568	0.033531982	0.08525893
0.01215143	0.044803033	0.027999056	0.020099719
0.241972727	0.065935306	0.065024316	0.1166617
0.024440479	0.161129487	0.02935536	0.081376052
0.229596124	0.059372692	0.059897632	0.025762786
0.037618473	0.059143946	0.129165461	0.06506062
0.083198322	0.176293767	0.040554877	0.045148882
0.141925822	0.245398466	0.073383172	0.014249006
0.097615281	0.028867476	0.022992922	0.029357457
0.037000384	0.14409097	0.090535657	0.038931922
0.178006433	0.247023559	0.048333543	0.112770241
0.019951568	0.152404111	0.231686787	0.011729782
0.077168216	0.018350845	0.00654512	0.056968313
0.100645036	0.073805167	0.107478598	0.277012743
0.046186906	0.202778102	0.097954486	0.030575795
0.03219659	0.040677686	0.114315681	0.184448651
0.027396515	0.082598989	0.201160074	0.027405465
0.026093744	0.272119991	0.047383296	0.167833322
0.236212266	0.070000025	0.051996049	0.298691765
0.220463485	0.092782067	0.090238165	0.225339724
0.058216947	0.159701834	0.051173603	0.058186038
0.257152748	0.156770664	0.101713668	0.030404393
0.027418673	0.046602169	0.183948958	0.086751424
0.233958378	0.045999098	0.016185058	0.059545073
0.027151749	0.034205127	0.182850725	0.056225195
0.027151409	0.020096751	0.234217888	0.079714106
0.03297358	0.228991569	0.079301183	0.035402394

0.051310444	0.06501125	0.04088053	0.061318802
0.142848322	0.169230493	0.076206169	0.04077457
0.036798353	0.134642313	0.01973029	0.022201749
0.25220157	0.040956571	0.084204923	0.224247744
0.034110897	0.01706661	0.131509438	0.254162542
0.047420519	0.03427199	0.033607805	0.100281397
0.043171472	0.046301895	0.033128306	0.194164358
0.223475298	0.032128948	0.037384136	0.129803267
0.089171024	0.013953221	0.186678841	0.046718723
0.194612125	0.017678926	0.017674099	0.00649536
0.137270884	0.083304607	0.029834874	0.066984376
0.011253619	0.091714831	0.016504163	0.048994106
0.040233462	0.211087115	0.121901932	0.040256821
0.040315097	0.042767352	0.046860863	0.055662356
0.041178762	0.030933701	0.081208437	0.226698545
0.060598464	0.032885378	0.05613963	0.037305595
0.084833156	0.096701761	0.213232123	0.219640253
0.06958064	0.038433262	0.015128328	0.14204699
0.022789199	0.190826113	0.04123112	0.087513826
0.056152972	0.179207809	0.076394135	0.037141534
0.062579506	0.078356578	0.073415148	0.014917244
0.005086717	0.172518299	0.072885537	0.138020506
0.051497254	0.01166254	0.18758052	0.101189888
0.06598955	0.081217328	0.040709193	0.160542861
0.050945243	0.224198271	0.033329276	0.122991843
0.206450094	0.012932449	0.064291766	0.038328026
0.164518109	0.122974159	0.022652641	0.083332828
0.01613441	0.16947272	0.045572041	0.065888168
0.065235824	0.212011719	0.228858912	0.094298788
0.026116416	0.200630316	0.242650851	0.006464957
0.174586068	0.06504344	0.17009948	0.073536308
0.070278207	0.12690518	0.163319993	0.035830173
0.227166364	0.029722497	0.081947512	0.012071184
0.052199361	0.180810643	0.030702609	0.053510399
0.025527599	0.043487858	0.050409708	0.184912282
0.022418459	0.250211414	0.093316306	0.078239038
0.138899704	0.081222388	0.128511435	0.075083997
0.050026981	0.108889303	0.030598738	0.017563289
0.168147346	0.022628377	0.079911866	0.026104937
0.038431597	0.084494007	0.071496723	0.050693214
0.036146189	0.044137045	0.043050406	0.104583245
0.069953403	0.029860686	0.067199874	0.12480973
0.11033068	0.093473036	0.038350579	0.145112068
0.188478386	0.031979067	0.123073752	0.00554068
0.024450066	0.037352228	0.183735199	0.115703864
0.037015517	0.027632699	0.102265503	0.23505477
0.046639254	0.034311059	0.063312331	0.068058943
0.176210912	0.13950593	0.057758259	0.064788957
0.018647404	0.052267236	0.058932117	0.0539808
0.036321005	0.159530033	0.035870747	0.020089149
0.106445978	0.025739957	0.209182005	0.238147972
0.249428723	0.220667176	0.015147118	0.106976262
0.01291827	0.154244392	0.011067335	0.168937952
0.122161171	0.014709702	0.141535605	0.098129754

0.219943966	0.034613462	0.122516255	0.235865941
0.018322122	0.052205144	0.056737706	0.123426474
0.035614331	0.099874869	0.191997716	0.024475154
0.023736182	0.012847307	0.049243244	0.090679275
0.175573976	0.084900053	0.018893611	0.11981202
0.086710993	0.022915942	0.035820243	0.100000674
0.176320744	0.039828488	0.024455543	0.058244439
0.080770393	0.20887247	0.018632573	0.045423708
0.12343421	0.027917674	0.042047972	0.034347891
0.043639385	0.148307936	0.020443327	0.042075121
0.039394097	0.017234934	0.195768762	0.0680179
0.014678098			
FGFR1OP	0.263107817	0.396094695	0.114604121
0.341771621	0.187664317	0.23138954	0.160767065
0.133364356	0.041749576	0.013387007	0.015045653
0.187933176	0.069560232	0.132754036	0.079583274
0.314209224	0.053411369	0.111764592	0.172402266
0.331089187	0.112166958	0.275863477	0.080513913
0.060451233	0.031710947	0.047144817	0.054662778
0.391379893	0.049934274	0.194437547	0.361840178
0.046591	0.340749109	0.360768078	0.231598144
0.338627602	0.227555685	0.024715078	0.114484663
0.339325217	0.253746296	0.347957178	0.223647645
0.050968839	0.368478313	0.18268832	0.169321855
0.09936195	0.02372008	0.12383345	0.365489209
0.327514816	0.194608465	0.141179477	0.114285952
0.061320972	0.159927897	0.039683943	0.310192204
0.168149374	0.132935353	0.334394217	0.062436857
0.089612671	0.157121729	0.054999886	0.180138522
0.063089371	0.319118187	0.041198919	0.155453248
0.011189088	0.314677463	0.172639979	0.043013943
0.033661909	0.026890832	0.063098389	0.271148905
0.390374674	0.321501212	0.343613134	0.330646211
0.066042546	0.122921897	0.116853899	0.09233158
0.334567846	0.120100408	0.050867711	0.30019265
0.345100834	0.362181577	0.067089622	0.107901789
0.315272177	0.392990123	0.132951641	0.148878398
0.057084596	0.018270816	0.337746107	0.364421823
0.338140207	0.064487281	0.064191039	0.390777104
0.051326235	0.090175436	0.27024485	0.065740069
0.395964422	0.027778658	0.076253608	0.252361141
0.090974365	0.303451145	0.335763859	0.146322054
0.147438858	0.021229501	0.019820053	0.029438438
0.102505112	0.021109913	0.331761793	0.376064101
0.165087952	0.103788777	0.160579358	0.004716924
0.01954045	0.060485025	0.29867201	0.163018348
0.313238714	0.229607741	0.076500043	0.304055103
0.189050431	0.341029246	0.023071845	0.096221882
0.18420729	0.067557603	0.171623844	0.215131356
0.10466541	0.11102176	0.095649428	0.376864711
0.387517557	0.07745774	0.128103724	0.285460863
0.109717952	0.129608879	0.093429676	0.12899273
0.082238753	0.30556948	0.015403734	0.173131999
0.040222986	0.04754686	0.021632046	0.196804076

0.098066689	0.305803033	0.313018358	0.078589248
0.15704764	0.225468456	0.208801922	0.25972285
0.013449269	0.04173573	0.151248874	0.377949291
0.276549678	0.344433021	0.119805727	0.081254169
0.134127117	0.331248763	0.000889039	0.35386421
0.348965189	0.130054767	0.203885148	0.198828609
0.084766203	0.357025505	0.012005552	0.304040156
0.262292598	0.067993557	0.375158472	0.079514961
0.332745241	0.035545232	1	0.240365202
0.22471766	0.010841014	0.164498469	0.339169866
0.014090457	0.120217674	0.304991165	0.286110515
0.196730374	0.291493517	0.376980522	0.068732992
0.171958197	0.139383272	0.01508017	0.112883477
0.279933529	0.078243075	0.364580801	0.126964935
0.195036248	0.365857553	0.248253993	0.067826583
0.053111328	0.218796605	0.026818484	0.121968843
0.254187926	0.09775684	0.111429782	0.065208697
0.034213761	0.379405803	0.031509259	0.036759973
0.018034319	0.223848108	0.167907632	0.097463888
0.059077768	0.080009101	0.070830252	0.094450001
0.075023239	0.064311639	0.007261327	0.074122478
0.058735975	0.086757673	0.090297165	0.292319393
0.149007018	0.068900554	0.134575952	0.055585321
0.037329493	0.018024903	0.379058941	0.280270521
0.3009955	0.099203378	0.034397718	0.076894837
0.379737047	0.163729791	0.101574302	0.386899109
0.025898049	0.355132136	0.095140758	0.059089164
0.087359	0.161107151	0.379947203	0.027694704
0.245068873	0.39052225	0.149951202	0.063727694
0.342697813	0.291890604	0.047294644	0.279941072
0.287612454	0.033572348	0.363477002	0.044569969
0.019814115	0.231036565	0.237351785	0.048049338
0.036327883	0.149735533	0.084324303	0.159927173
0.379384508	0.292444076	0.298022358	0.044565283
0.049527539	0.015126817	0.055808289	0.128287764
0.363497638	0.334258937	0.156049753	0.108011004
0.189260192	0.068409732	0.137769935	0.358225267
0.050209888	0.366273049	0.012832323	0.244920634
0.335389994	0.205054769	0.03266046	0.228393733
0.337083026	0.050340757	0.125099133	0.35579463
0.342716419	0.086339162	0.073322522	0.256740259
0.039121489	0.084041445	0.154228281	0.029069504
0.104960343	0.087417632	0.006005264	0.141402314
0.043845329	0.376177721	0.341127342	0.031372145
0.200506132	0.120351561	0.206470995	0.043336639
0.262111842	0.323283217	0.370413399	0.133496147
0.286634566	0.317812804	0.128952257	0.04254616
0.012716412	0.334431516	0.286309777	0.021682047
0.381926547	0.167492572	0.020539724	0.398956114
0.141790848	0.327786566	0.004394268	0.014034476
0.016916942	0.311102357	0.339268042	0.345647008
0.090926136	0.253701346	0.003985193	0.354407796
0.366899405	0.008816988	0.038617391	0.273217815
0.203309875	0.405300454	0.060256559	0.271453428

0.16334256	0.333509257	0.125007043	0.143862039
0.337420906	0.300240382	0.323909327	0.365885132
0.3754895	0.244925439	0.213210295	0.030100001
0.02518243	0.161492227	0.059140272	0.036115963
0.253793765	0.04817864	0.345734064	0.358392037
0.31759379	0.008187918	0.134230694	0.295165503
0.404707039	0.403290415	0.015144093	0.001896782
0.001576223	0.005918163	0.283016906	0.086346036
0.341822544	0.079456079	0.011712212	0.086626993
0.104222695	0.15834673	0.36552263	0.309297346
0.265201882	0.061784285	0.084545597	0.32648351
0.024109668	0.233640766	0.334935692	0.387302587
0.02877519	0.084137324	0.148846421	0.400395987
0.007169859	0.16966953	0.219114398	0.104840684
0.010740209	0.033352894	0.031630975	0.317385054
0.32349997	0.043035678	0.024107098	0.084728997
0.087644067	0.355055862	0.082992529	0.379518661
0.367789262	0.367585839	0.030203523	0.35712856
0.033280704	0.346252166	0.091339852	0.022504948
0.090301499	0.018543723	0.310883647	0.027601737
0.152716905	0.303416668	0.036057918	0.011535281
0.022929821	0.034413762	0.363547916	0.299697136
0.015606943	0.1401087	0.128487111	0.25927362
0.222966042	0.315879883	0.365301034	0.130284959
0.09927289	0.026362314	0.341207842	0.258950495
0.172581709	0.354729059	0.075706363	0.363736583
0.288556075	0.356764497	0.328993366	0.205081949
0.055772388	0.217803383	0.304722093	0.031090141
0.201969259	0.352970124	0.19297211	0.038706533
0.10254293	0.110882203	0.06817042	0.056521113
0.066056059	0.258293623	0.108288379	0.360822926
0.27381191	0.273694073	0.083780552	0.029931835
0.395899078	0.348220108	0.087220922	0.277971347
0.07946791	0.163121341	0.275105379	0.265945088
0.08493811	0.3269042	0.363447719	0.307121497
0.38491983	0.17796686	0.369822711	0.100733102
0.022941632	0.205798919	0.064511919	0.045006474
0.096262773	0.017420574	0.038902911	0.295642164
0.057218395	0.257982135	0.135781616	0.045568607
0.139683021	0.126608491	0.201930116	0.128919289
0.056323312	0.35890678	0.008607984	0.041666203
0.072486662	0.274938659	0.105109771	0.076902637
0.047105394	0.046742008	0.171905699	0.196292615
0.104471047	0.077340679	0.278575477	0.03808199
0.050687506	0.373759733	0.150072846	0.011784616
0.04640289	0.230048644	0.111803599	0.090296537
0.011191215	0.309059984	0.1315327	0.166579825
0.242750593	0.058446963	0.326430952	0.04968552
0.055428112	0.355085074	0.128192311	0.339952847
0.09895065	0.326505695	0.345048144	0.321300971
0.04257457	0.11590129	0.041868631	0.370917761
0.070372038			

FGL2 0.379521733 0.503575716 0.412723653
0.359436007 0.407206786 0.300158687 0.16901571

0.125930461	0.098994581	0.003161122	0.033212713
0.421276557	0.202324108	0.20506727	0.302362129
0.387583535	0.155220769	0.307913821	0.212688371
0.350004155	0.060350054	0.379941111	0.081559766
0.356243381	0.142205075	0.078417548	0.335497666
0.36948577	0.089831082	0.243566448	0.30051579
0.121870586	0.371566903	0.511092939	0.422006392
0.513684909	0.360161756	0.00973958	0.240931095
0.360869874	0.421710412	0.343472817	0.292299058
0.038580614	0.455620023	0.212689191	0.414940849
0.181215697	0.038261231	0.161520508	0.405866228
0.465458362	0.402300559	0.163424208	0.132021462
0.208695714	0.149286792	0.34469051	0.233984994
0.194242055	0.093418139	0.479669927	0.081368995
0.129940584	0.186107284	0.183099676	0.197205818
0.411248124	0.305388323	0.013371637	0.404958903
0.000986672	0.330693121	0.38538696	0.396180171
0.049355562	0.019534401	0.206068319	0.281805024
0.399835332	0.446510285	0.466611636	0.487702585
0.188748241	0.302890165	0.431965433	0.165354765
0.388002264	0.147587408	0.098345091	0.401029615
0.47961632	0.516577573	0.228853396	0.299313918
0.36276267	0.439736809	0.390344201	0.408553317
0.382902077	0.083684205	0.417215481	0.49943599
0.332521984	0.396265648	0.178208957	0.325128008
0.063706787	0.305792226	0.316228356	0.152544787
0.413752409	0.017435604	0.253622624	0.293205889
0.211671939	0.383272851	0.523775851	0.163308056
0.401060885	0.020500569	0.210934839	0.041376871
0.282903367	0.234051601	0.470759622	0.402901317
0.164217654	0.383106585	0.218501631	0.003435948
0.036250328	0.212379946	0.385239414	0.320473365
0.382316402	0.383224159	0.247252819	0.315848346
0.431445135	0.460693279	0.040416461	0.081933198
0.372549156	0.21155119	0.231316782	0.405009909
0.334304268	0.086770073	0.116789439	0.501000192
0.373274838	0.07761802	0.265956745	0.387765822
0.296734976	0.391528213	0.095378378	0.235110947
0.037977622	0.492049316	0.043031891	0.258295801
0.32160377	0.178780748	0.010461625	0.184177509
0.237254446	0.399404432	0.465435206	0.103567672
0.273165889	0.400201017	0.236794509	0.371776164
0.012157694	0.046512072	0.200013068	0.484838784
0.199639667	0.492426948	0.271446417	0.331820951
0.300635083	0.402502122	0.005628549	0.478207275
0.527810429	0.217662774	0.365959886	0.406372022
0.417902912	0.390319578	0.035914056	0.380155978
0.285185999	0.077337435	0.48091305	0.176362962
0.443080646	0.047983702	0.240365202	1
0.404490605	0.008268062	0.152571499	0.318742302
0.04355112	0.06132087	0.374219812	0.361367453
0.359564345	0.418317974	0.443420654	0.412930261
0.405766158	0.186718954	0.015313304	0.357567616
0.408185324	0.377185037	0.4045014	0.196841139

0.260309751	0.445865341	0.38581374	0.20251668
0.040641694	0.241725488	0.014753378	0.347617408
0.378559036	0.09628399	0.404315125	0.157272043
0.089810943	0.512613825	0.261759987	0.357156388
0.215021309	0.339351277	0.215068106	0.195765017
0.219465862	0.176794107	0.393924311	0.380742531
0.024379862	0.095784092	0.026100502	0.356280553
0.130272705	0.303195838	0.286118865	0.338167597
0.259331353	0.097038928	0.373563458	0.359285776
0.147528105	0.018689038	0.487752168	0.371362636
0.25397232	0.140301188	0.047692839	0.306825456
0.420980828	0.244286687	0.191827158	0.40976132
0.028188067	0.463950463	0.234357958	0.140735998
0.390721566	0.239124706	0.40754486	0.054204491
0.321095132	0.429539634	0.315866367	0.212457729
0.345898041	0.358152632	0.024885347	0.345461105
0.331378884	0.034475753	0.518252996	0.154746469
0.027202271	0.433893853	0.419390835	0.089357324
0.098400043	0.297039147	0.027948688	0.306932178
0.501124471	0.38811367	0.378377054	0.291426543
0.038098235	0.009340178	0.260648443	0.409722942
0.493138129	0.514771452	0.254965686	0.384512083
0.22097033	0.37355118	0.090240378	0.497824403
0.22430747	0.506609467	0.00798183	0.436612485
0.509597283	0.200382671	0.104739484	0.241682585
0.319228496	0.045153323	0.174272567	0.480956185
0.399381278	0.345662867	0.368777893	0.392707477
0.01095537	0.03042099	0.408511103	0.279925153
0.36510946	0.227360036	0.109977193	0.254409886
0.042194505	0.41715953	0.407414682	0.078034514
0.269883674	0.241536029	0.386628908	0.037573362
0.463867626	0.507722649	0.507940486	0.132358876
0.399583157	0.491316711	0.433545259	0.097146538
0.025986131	0.521299865	0.332732468	0.02146171
0.425859311	0.153933853	0.011183748	0.464754881
0.164498913	0.422314071	0.003417071	0.118544635
0.064628967	0.427630006	0.468318985	0.305225591
0.177659692	0.325647482	0.010750963	0.521916044
0.467891911	0.023509284	0.196246774	0.400054264
0.427614843	0.503145117	0.398600926	0.443466059
0.42867167	0.50116405	0.192879237	0.283831846
0.381788075	0.511761806	0.355051774	0.426507101
0.402514799	0.418959399	0.255214866	0.01404981
0.206095005	0.143186702	0.294509531	0.105674067
0.242171356	0.172825191	0.461144201	0.429475278
0.475480672	0.006930134	0.107561584	0.383218817
0.374677504	0.425038406	0.027605932	0.008760651
0.010769763	0.034457951	0.29032952	0.277576992
0.479531721	0.178328132	0.005013564	0.026935525
0.254774682	0.434516731	0.489744328	0.342358011
0.4219635	0.08670836	0.239341606	0.352368791
0.099336217	0.26285555	0.474990357	0.439938393
0.053349377	0.285422195	0.318483916	0.447746464
0.145767382	0.325759424	0.412737855	0.28815339

0.018692977	0.107925806	0.090297914	0.407715824
0.505042465	0.082050192	0.038984781	0.095354602
0.103181647	0.431428359	0.156395209	0.496356823
0.369929263	0.387385784	0.106307869	0.467019304
0.047088383	0.472254118	0.398786995	0.048186363
0.289376915	0.014658225	0.469241982	0.20959226
0.239444132	0.328291224	0.273018341	0.027164775
0.280211334	0.063522206	0.446407767	0.440170723
0.052568363	0.203924424	0.311583135	0.36875289
0.206744805	0.387123031	0.48617065	0.360361437
0.066943807	0.201621433	0.33963344	0.328195542
0.167953793	0.472315704	0.321045694	0.521138789
0.347871245	0.433125494	0.461738796	0.311936114
0.291079921	0.329148343	0.297114469	0.014266885
0.319072252	0.408308582	0.298002222	0.196397825
0.274863229	0.435087889	0.042994918	0.146361308
0.407148759	0.223264225	0.128956666	0.392126257
0.320408197	0.330362087	0.027971122	0.08404432
0.52568649	0.407646656	0.113564477	0.372475193
0.184049465	0.351484167	0.423164349	0.432885655
0.375984584	0.404044533	0.352717383	0.309656837
0.377073621	0.234609814	0.515011807	0.04945758
0.118990758	0.382381847	0.093162456	0.114607024
0.136839556	0.038479913	0.387887551	0.396778922
0.415208272	0.351937395	0.242185598	0.092394773
0.348129849	0.32941144	0.347818872	0.153334546
0.076503745	0.483961108	0.011936937	0.064974879
0.405370437	0.405461	0.42534102	0.261565989
0.379213936	0.184417372	0.290041825	0.208949901
0.258544153	0.356096587	0.399050791	0.028323092
0.013351652	0.465251978	0.306873886	0.032626909
0.112974043	0.326456154	0.266059304	0.242840046
0.036623329	0.489591954	0.268577601	0.425317606
0.283137488	0.067084802	0.303159783	0.081234965
0.057802191	0.414179819	0.425884652	0.514380624
0.378963587	0.382433714	0.417502229	0.379562836
0.126972186	0.387210339	0.023746878	0.379124209
0.402723125
FOXP1	0.324178902	0.507065019	0.316160501
0.30931204	0.345406119	0.262758411	0.137676715
0.106280408	0.054810089	0.003713672	0.024762134
0.370109784	0.194155672	0.202008155	0.220135816
0.315795872	0.136737315	0.239383403	0.171628046
0.296355235	0.053522578	0.320463251	0.059034273
0.25434198	0.10300664	0.054783297	0.232871769
0.356668249	0.061091966	0.23528607	0.268237936
0.087496419	0.352866727	0.51708687	0.377904974
0.529559314	0.304089024	0.011104651	0.202471395
0.35153866	0.345275704	0.292376385	0.24619617
0.02707154	0.476051404	0.173104369	0.329927112
0.140116504	0.029654923	0.143622664	0.415409107
0.488771394	0.349449836	0.13300815	0.104504806
0.209493084	0.151969888	0.297080537	0.219753059
0.162793088	0.091496338	0.493337162	0.051514227

0.098735087	0.153895344	0.145781104	0.168459189
0.311622044	0.27416173	0.009146541	0.345715507
0.002056927	0.273861744	0.310164523	0.273628792
0.034688111	0.022265984	0.163388057	0.242821048
0.38780427	0.441343587	0.454421251	0.472723247
0.179097624	0.231032534	0.351210311	0.138055747
0.376387399	0.125810636	0.077806183	0.333750665
0.467189386	0.527010445	0.172904095	0.217523195
0.356565578	0.445326891	0.319505724	0.343516042
0.280030267	0.048652718	0.426740354	0.500384596
0.337514633	0.309136439	0.132672736	0.308708138
0.044593763	0.257781292	0.26546598	0.156029012
0.403830406	0.009518705	0.192773256	0.26460654
0.147737513	0.350166399	0.526860608	0.129616651
0.343999541	0.018464252	0.134978955	0.026840258
0.266216869	0.170227221	0.489039162	0.408119685
0.15684229	0.318710277	0.176413437	0.001669858
0.025732829	0.148250927	0.397161605	0.267321552
0.319035583	0.36761997	0.188994065	0.295489322
0.373041562	0.447946248	0.039123535	0.057508395
0.31978584	0.14961745	0.181090156	0.367980518
0.265117029	0.066423555	0.095112609	0.488113251
0.382812997	0.060555196	0.228611247	0.322659439
0.258792447	0.322682448	0.074853839	0.228711354
0.031246709	0.496747093	0.029390544	0.217113926
0.218928314	0.173494586	0.005631903	0.163959586
0.21421632	0.409674938	0.481152159	0.075376586
0.257068787	0.328864808	0.21612885	0.344855153
0.008602957	0.03194644	0.183192279	0.495413129
0.195930131	0.480541362	0.221340183	0.23768829
0.232948909	0.357992911	0.002353427	0.491716804
0.530410282	0.204620217	0.339026272	0.339797743
0.312693905	0.381174495	0.034793042	0.330507602
0.26695243	0.052774101	0.465650607	0.139524374
0.4541067	0.033506746	0.22471766	0.404490605
1 0.007891666	0.137201478	0.273141597	
0.026039598	0.051226371	0.313216298	0.298347488
0.28526414	0.351289687	0.426444072	0.303666543
0.343893653	0.154390968	0.008858616	0.274567384
0.357318243	0.3097699	0.408886766	0.158658516
0.244611339	0.447872002	0.378550802	0.145046662
0.028139979	0.207716764	0.014388625	0.258351021
0.356464897	0.093539358	0.305641487	0.124165881
0.067624434	0.510246936	0.181856653	0.260494372
0.14269518	0.333859488	0.167872569	0.212276709
0.236280129	0.124056158	0.290030558	0.287269972
0.020230717	0.081597346	0.012352316	0.3780312
0.167050375	0.332543592	0.320102904	0.31569477
0.267546229	0.110752407	0.328978938	0.234588425
0.097217326	0.012440105	0.487322392	0.30430005
0.24106688	0.101580588	0.033494678	0.32917665
0.427145791	0.229138181	0.169149686	0.421645801
0.018102236	0.444934237	0.219638887	0.10746455
0.372799313	0.208769753	0.386066936	0.042480698

0.281706337	0.449421482	0.237495169	0.151878841
0.378033775	0.306147238	0.019780171	0.321509154
0.322029868	0.026739148	0.524986052	0.127485365
0.01775904	0.365988968	0.364723427	0.060292998
0.063606402	0.265699171	0.021447142	0.238000816
0.50025111	0.328859428	0.357927682	0.194327303
0.030120362	0.017156113	0.192914792	0.330842732
0.513424778	0.51743336	0.204446203	0.341956613
0.174088252	0.275466286	0.079638856	0.499992698
0.149310724	0.513105802	0.004901327	0.378987179
0.523747657	0.198840921	0.07452045	0.208278449
0.339315255	0.028497272	0.134251582	0.494392728
0.380495458	0.24981776	0.270183102	0.341426212
0.008114287	0.02312233	0.304978257	0.18382295
0.294885314	0.156228691	0.072784837	0.219782974
0.034223513	0.436100354	0.387385393	0.042811001
0.255011058	0.193306104	0.316499059	0.025193459
0.444777004	0.505096238	0.521227571	0.102522823
0.374792812	0.48950126	0.37317819	0.064915983
0.015945484	0.527311141	0.280640178	0.017210388
0.41919667	0.149567251	0.015431647	0.468888442
0.124197306	0.447269909	0.001331949	0.069976644
0.037189259	0.404575136	0.471650274	0.264849286
0.170024159	0.263465502	0.005507489	0.517779969
0.486810782	0.014302657	0.137456168	0.395155707
0.377223985	0.508713075	0.295435136	0.434858291
0.388067243	0.515021902	0.183876902	0.215791183
0.383681588	0.512548212	0.304564885	0.42171992
0.407939306	0.349290367	0.20439857	0.009400441
0.096184905	0.127014741	0.243847511	0.064091184
0.217083728	0.118114378	0.460196595	0.415149518
0.477503682	0.004011685	0.088501739	0.377765654
0.377214277	0.41109036	0.013456421	0.004148034
0.006226928	0.023628529	0.248669388	0.198544602
0.466745307	0.185307381	0.003292841	0.022085233
0.194860216	0.353796635	0.503036754	0.284011082
0.369237591	0.063897985	0.234931022	0.292441361
0.063589725	0.237275513	0.473358421	0.452872897
0.033280381	0.196012232	0.278566708	0.439145004
0.094267353	0.264773014	0.375120493	0.256804111
0.010784173	0.07013781	0.052279669	0.342055511
0.522080438	0.086976022	0.038333931	0.102240743
0.093414635	0.419545092	0.13604687	0.486650177
0.365233939	0.356562158	0.0757451	0.463775411
0.033204015	0.496934657	0.319873656	0.03630613
0.225556388	0.008955233	0.482690071	0.142048929
0.230818024	0.285358373	0.147560762	0.016585738
0.171028602	0.047894418	0.433708689	0.417934019
0.031497495	0.150079685	0.233691048	0.302623861
0.186665648	0.343388942	0.500517423	0.321130285
0.05226238	0.157272959	0.295855351	0.300082468
0.158909231	0.458255993	0.254268927	0.5134803
0.299291003	0.417273846	0.466247775	0.245758797
0.244613494	0.288214716	0.275070532	0.013662393

0.259454139	0.408345653	0.24411765	0.14644461
0.224895789	0.348149218	0.025974285	0.132692757
0.322369565	0.20669131	0.112668774	0.351952746
0.28319263	0.303274353	0.022157621	0.048489383
0.528960396	0.396060271	0.081265107	0.320420925
0.133745541	0.270960265	0.377661965	0.382635243
0.279004015	0.419412365	0.333529681	0.298801346
0.397992959	0.178318824	0.521438643	0.043442634
0.074870279	0.346254964	0.086077852	0.079010743
0.111848506	0.021073152	0.292199482	0.336923232
0.360210462	0.311240577	0.230869018	0.056501078
0.274933413	0.269825306	0.28035987	0.135844763
0.064622328	0.498183764	0.007256405	0.047549965
0.379648394	0.366802613	0.356638658	0.192924273
0.291210126	0.128388075	0.242474364	0.199749237
0.276002582	0.297073221	0.373167791	0.018029157
0.011402396	0.454886474	0.265009136	0.017463675
0.070983511	0.273837878	0.222411159	0.18071345
0.021298181	0.495304863	0.245182649	0.348344051
0.233440676	0.047737258	0.315875189	0.058483806
0.033944481	0.431297075	0.334353929	0.526507139
0.289821534	0.330062276	0.429797623	0.390308428
0.087268933	0.31082034	0.015491658	0.368128508
0.356477764			
FUCA2 0.009364512	0.010188635	0.007667361	
0.023769797	0.015039439	0.008250372	0.034431949
0.012851219	0.005256008	0.002870759	0.005090794
0.012159623	0.004291602	0.011006188	0.002340062
0.028879723	0.037252377	0.008243762	0.052674508
0.022720489	0.024836986	0.03265095	0.039741551
0.010668502	0.029905204	0.043957674	0.013255432
0.02393193	0.044363248	0.008194459	0.018469588
0.031136364	0.012981898	0.007050852	0.018206622
0.006207566	0.005947891	0.001952182	0.006010929
0.011160725	0.013177207	0.023566901	0.038444396
0.030019902	0.006477886	0.038613289	0.018056459
0.000672439	0.034924482	0.006951338	0.007136686
0.005509314	0.00713469	0.017566438	0.029997695
0.001441474	0.007705135	0.002606184	0.010784948
0.044402631	0.009266906	0.006110063	0.006490877
0.000440843	0.021137301	0.031022437	0.023158393
0.005626788	0.00988931	0.02369377	0.005807375
0.000225563	0.030291375	0.023696902	0.00326089
0.07258208	0.012956145	0.038034787	0.026656145
0.019608785	0.007274799	0.016854876	0.012971403
0.013552187	0.029750246	0.009447338	0.034366681
0.011561238	0.031793278	0.005622005	0.03002515
0.015300803	0.008155041	0.027054275	0.036076138
0.010228119	0.010913021	0.005627533	0.009535196
0.002808451	0.017285452	0.006628566	0.012176146
0.007980291	0.009007646	0.047178565	0.017614009
0.066941478	0.002499073	0.037991999	0.002185523
0.020641806	0.019926935	0.04337273	0.020788167
0.011016249	0.011018342	0.007652228	0.050356038

0.017333469	0.003332883	0.001542503	0.053023378
0.020359208	0.004113211	0.00464805	0.017541242
0.010555262	0.004193632	0.029645771	0.032178842
0.040484991	0.005119273	0.009294069	0.013061391
0.014701661	0.004497158	0.017394558	0.007793198
0.007928387	0.016792855	0.000340723	0.03923178
0.014466234	0.012108422	0.057871053	0.011133413
0.027828758	0.034960575	0.057197775	0.013649664
0.009084901	0.066738756	0.005661345	0.03020188
0.00469991	0.025366195	0.059938166	0.021128846
0.031774154	0.005905353	0.048808598	0.012574942
0.001647143	0.012231845	0.00946953	0.020498185
0.006120451	0.00834599	0.004354502	0.065353929
0.023415222	0.023655874	0.008784711	0.010601578
0.003161761	0.019585542	0.005247694	0.012535672
0.007657661	0.012227055	0.018582822	0.013625924
0.040831918	0.017550595	1.33E-05	0.006635287
0.007643222	0.006859891	0.020702489	0.017925664
0.005142356	0.017694194	0.000376549	0.02977509
0.007070253	0.061227085	0.017170852	0.054188117
0.007637699	0.052614867	0.010841014	0.008268062
0.007891666	1	0.011051281	0.024579528
0.02753354	0.013461909	0.01370758	0.019930471
0.015119158	0.01517764	0.021349951	0.00428381
0.014391392	0.034490282	0.015995986	0.025773537
0.010755033	0.000764664	0.007203321	0.020798556
0.005422181	0.009950446	0.007314747	0.000208183
0.050262334	0.018613036	0.003183358	0.019272889
0.005891988	0.018816448	0.004815554	0.014132458
0.057501062	0.011502161	0.012641306	0.005420821
0.006046548	0.011196361	0.038257329	0.007955975
0.005634854	0.028509674	0.007915168	0.006181234
0.027471664	0.052403792	0.00524898	0.003167867
0.005346065	0.005825913	0.004975724	0.008295163
0.009540898	0.008964253	0.020018025	0.004023771
0.026232619	0.068401248	0.010291814	0.027606025
0.006486568	0.032780854	0.068198876	0.001634698
0.01513663	0.00440212	0.004379939	0.011661885
0.00485267	0.015088721	0.008549404	0.053237925
0.009976497	0.044404896	0.020499011	0.009074794
0.011206393	0.009386036	0.031063922	0.025029982
0.007592238	0.020962493	0.021097886	0.007075517
0.007458637	0.067517119	0.010053356	0.031490702
0.068590218	0.014121625	0.008283872	0.066735419
0.041931598	0.013580521	0.017725336	0.044978192
0.011359593	0.031309824	0.008376498	0.012124994
0.045351676	0.00502233	0.027142912	0.008032241
0.006290085	0.009432822	0.052687899	0.01558632
0.056390117	0.007491337	0.001573156	0.011578229
0.003269894	0.008371997	0.062954731	0.010560149
0.005685549	0.011141556	0.036501803	0.017686349
0.009163849	0.041334063	0.030615403	0.007300809
0.010234039	0.011176267	0.005943768	0.017572405
0.022981321	0.018177179	0.013694335	0.006184812

0.004913126	0.024856765	0.002162857	0.008698481
0.016898349	0.008981025	0.020156025	0.015010046
0.005834454	0.006346633	0.031047682	0.058391805
0.008497153	0.007238939	0.007365552	0.033498302
0.011688523	0.008293706	0.005735715	0.056454558
0.053720432	0.006692236	0.009498542	0.000847643
0.020203375	0.006784658	0.00713187	0.010448224
0.051357676	0.004675167	0.000146338	0.000107402
0.024044026	0.018294109	0.006186332	0.019535024
0.001824448	0.026506709	0.000258722	0.010424284
0.008565163	0.037648171	0.022026332	0.008078844
0.007410276	0.009839141	0.008733543	0.010166287
0.008573533	0.006081776	0.0057507	0.043118105
0.013108598	0.006397602	0.013881074	0.008970875
0.018272219	0.021316443	0.045680829	0.028246016
0.001390457	0.015366557	0.00137685	0.001206659
0.009183747	0.017306734	0.010162541	0.018171705
0.011204499	0.059218107	0.014091355	0.009387927
0.015836074	0.017153457	0.004807007	0.000132283
0.000178294	0.009888285	0.019879065	0.031859886
0.009700245	0.000845049	0.046151729	0.021616118
0.005667174	0.015035518	0.008363454	0.038783031
0.01086801	0.060761	0.003709287	0.030944154
0.022623258	0.005353225	0.012110313	0.008682718
0.034049452	0.025738557	0.009221818	0.016445335
0.002049649	0.042241677	0.012706554	0.010281337
0.001711888	0.032594161	0.027683636	0.021707034
0.005397677	0.016730377	0.018783135	0.000208788
0.02793777	0.014207026	0.019016581	0.014869719
0.014839447	0.027642657	0.023791154	0.007546094
0.010160354	0.006856159	0.015263919	0.000316062
0.017152403	0.041387368	0.007365776	0.001345325
0.009182416	0.014546073	0.002506904	0.040380891
0.002481964	0.065383067	0.017515623	0.016213434
0.025121883	0.020424762	0.030028565	0.020196624
0.017450531	0.022406471	0.006793696	0.003086649
0.028513432	0.001638059	0.023308891	0.008530668
0.01492346	0.017135219	0.008070962	0.010900709
0.010574986	0.01022714	0.009755763	0.02083522
0.007328247	0.009674586	0.013840483	0.004878942
0.02979871	0.0177333	0.015004705	0.011244785
0.029846423	0.006066743	0.007643249	0.000990253
0.005042823	0.010403789	0.017527915	0.026586133
0.013368776	0.009504292	0.010374596	0.024540382
0.009936121	0.008512387	0.006978331	0.01286937
0.047382057	0.022600902	0.012642547	0.015383371
0.003018704	0.009812874	0.018123014	0.011327548
0.008875362	0.036471715	0.007336468	0.007415851
0.003905911	0.007856653	0.022727175	0.061501482
0.060590183	0.024453187	0.002110161	0.027101059
0.003560825	0.031465223	0.003969028	0.004347375
0.031778103	0.005459238	0.030363264	0.022576812
0.017615221	0.006907543	0.001082882	0.062893199
0.00552436	0.01091556	0.009561982	0.027923948

0.006868615	0.015411821	0.040469339	0.011647674
0.00366207	0.000703339	0.009501707	0.003448377
0.037500372	0.018044556	0.006381328	0.013431021
0.002470179	0.006427646	0.011334769	0.006993511
0.033949755	0.007595302	0.005189966	0.011842484
0.0198816	0.057689798	0.006423316	0.001204011
0.01479622	0.007733272	0.009898993	0.007493232
0.011751495	0.02991396	0.005525139	0.009533538
0.001977673	0.004700336	0.02822861	0.017120082
0.004805036			
FYN	0.171712889	0.295119523	0.075249295
0.284597297	0.112288316	0.235171739	0.184550398
0.210854461	0.080138115	0.042479066	0.016811062
0.112239824	0.063141289	0.162224679	0.123270659
0.215349591	0.033670737	0.099210923	0.145065939
0.241839942	0.21283301	0.177821509	0.116263126
0.037652043	0.0248208	0.051444301	0.045614564
0.298890638	0.055358095	0.199545995	0.309522023
0.027306246	0.22895017	0.273697198	0.137595237
0.24293499	0.163068071	0.009419449	0.173055945
0.263590726	0.186057664	0.262841071	0.185479488
0.084412227	0.298929491	0.182745914	0.096173817
0.15535667	0.025196274	0.211999582	0.30461172
0.257081809	0.139517309	0.216852628	0.1740405
0.053588289	0.187401916	0.025601556	0.30398929
0.144973541	0.238457995	0.248905511	0.14903833
0.086635313	0.183400199	0.028133829	0.189866962
0.039423899	0.289697436	0.050804984	0.088290093
0.023240478	0.243879634	0.094682558	0.03505753
0.029381573	0.029867216	0.03584959	0.241797364
0.28274583	0.230661779	0.226662723	0.216085645
0.046707556	0.069173537	0.057835077	0.090480337
0.249363829	0.166526336	0.081563059	0.190001409
0.229915055	0.259339815	0.053310127	0.060612974
0.271970641	0.304943013	0.113162459	0.098556416
0.062911824	0.009763844	0.26672868	0.244865993
0.27298429	0.029977574	0.045220323	0.313716419
0.043754584	0.109416946	0.208446261	0.035629636
0.292372483	0.066201939	0.040342935	0.204407162
0.07813524	0.229142097	0.235310877	0.143874262
0.080254354	0.019188925	0.018741096	0.019551546
0.105478833	0.009747908	0.263238764	0.286232362
0.174538315	0.072298542	0.16533457	0.003443626
0.013204307	0.038569069	0.18688792	0.104993817
0.218954151	0.159569695	0.08845247	0.241847651
0.119814329	0.219083429	0.074746376	0.128437703
0.152734251	0.039484902	0.122517057	0.133342136
0.070123017	0.16721167	0.093986211	0.272579991
0.335740744	0.089223195	0.117453308	0.191888713
0.12327002	0.069030852	0.094331778	0.115147483
0.112728372	0.218655333	0.008261925	0.150594803
0.051014763	0.0213625	0.020543688	0.206693057
0.151522892	0.237968469	0.23325914	0.075269306
0.119170801	0.145877705	0.177048557	0.213117279

0.033361586	0.046576611	0.137540078	0.263883616
0.292609384	0.225013324	0.112596285	0.065199634
0.083071637	0.240014648	0.001111984	0.282712358
0.249597939	0.132107297	0.141142558	0.114322219
0.056754195	0.281817484	0.013619876	0.191079938
0.206215484	0.06989568	0.254702894	0.055527671
0.242987073	0.036508704	0.164498469	0.152571499
0.137201478	0.011051281	1	0.287653681
0.007149942	0.234446899	0.217148569	0.232460697
0.171451024	0.192337088	0.258187668	0.049312019
0.114361435	0.160446581	0.013749349	0.061394505
0.178895748	0.058372371	0.318007199	0.17207749
0.171550059	0.295701011	0.172100056	0.088677279
0.078059276	0.19178102	0.063816978	0.097439787
0.18838684	0.127519908	0.081570653	0.035268631
0.023294473	0.273114209	0.016988804	0.021296607
0.008941694	0.164441395	0.144833998	0.117711446
0.049652868	0.074635553	0.060553528	0.093200638
0.144300104	0.055508199	0.002945204	0.049623765
0.049195377	0.073930928	0.08254978	0.20914485
0.115806176	0.062072444	0.093116989	0.040753608
0.032186029	0.014880087	0.283824214	0.19459493
0.279916721	0.131540037	0.025794643	0.061173327
0.279816257	0.199695153	0.145961359	0.307580238
0.030989128	0.231960803	0.094896656	0.046306631
0.052046171	0.123075613	0.289625428	0.064159612
0.174676027	0.309260681	0.093066417	0.063891057
0.287852044	0.220127708	0.076625399	0.218749821
0.214950081	0.029983314	0.25329121	0.025240683
0.014439306	0.137371317	0.163186242	0.038255311
0.03166551	0.13785212	0.192370766	0.10141243
0.282766151	0.193332435	0.225818986	0.027573855
0.066463596	0.010260223	0.03089409	0.075657926
0.276771799	0.221840424	0.0954032	0.066424378
0.1491746	0.064313427	0.182900035	0.250593479
0.032552298	0.264254086	0.010966109	0.148410192
0.248100166	0.191484153	0.028749616	0.231566062
0.295282905	0.076432505	0.119820672	0.274776883
0.271447966	0.090120461	0.085942192	0.153186066
0.09675176	0.116695888	0.106592758	0.016301829
0.104792035	0.091180524	0.001892996	0.151073654
0.060298053	0.300859487	0.216412954	0.047401524
0.210812894	0.151970632	0.11437436	0.050943303
0.168664925	0.218170277	0.281495144	0.140093406
0.172893807	0.227928492	0.072087508	0.030251979
0.008487429	0.240312421	0.236928074	0.040409675
0.258025694	0.213633041	0.027044572	0.312952359
0.133973174	0.255496644	0.031994594	0.008402996
0.008791566	0.191464869	0.27755595	0.285109922
0.170613174	0.176865591	7.22E-06	0.235199619
0.284900463	0.006238185	0.021783713	0.177938741
0.12618872	0.306616156	0.037533391	0.18032347
0.095877675	0.243471391	0.142259255	0.097989203
0.273239741	0.199849573	0.266700214	0.304191717

0.263881946	0.142143877	0.18068159	0.055185942
0.032534845	0.228441232	0.095815141	0.05312373
0.25819794	0.047331621	0.252302769	0.233616661
0.210443951	0.006282259	0.218107123	0.203014817
0.33187361	0.303891734	0.011781253	0.005990334
0.002732727	0.005591473	0.2265151	0.053960606
0.248370844	0.139665635	0.012492052	0.160921687
0.130100862	0.089492523	0.271516528	0.22394185
0.164285061	0.047198687	0.125329153	0.236633812
0.021679704	0.199583622	0.232224223	0.319551197
0.017370919	0.058555527	0.101358156	0.297181935
0.003055028	0.095282089	0.124828907	0.117237702
0.005485131	0.02576097	0.030317525	0.200310683
0.230403559	0.037602666	0.021066641	0.106506915
0.124314501	0.230572651	0.092244468	0.26125656
0.290373012	0.25579532	0.014026484	0.291775744
0.032949063	0.259762531	0.047917503	0.070885624
0.066000715	0.015425451	0.202283678	0.040459649
0.158634641	0.235052191	0.042024362	0.00555564
0.015543441	0.025811195	0.254716837	0.188036198
0.007261078	0.155577709	0.081409214	0.172813598
0.253299849	0.235133972	0.287879028	0.113627019
0.151100748	0.009307489	0.26826104	0.200386401
0.181770874	0.229872335	0.077804387	0.253031097
0.228963348	0.263887323	0.223986216	0.195960722
0.027867202	0.189208922	0.296759453	0.085425499
0.159687541	0.239301549	0.15118962	0.03951477
0.071926207	0.057998206	0.057223007	0.059734829
0.032423196	0.293534958	0.101617551	0.246349793
0.202169414	0.214260713	0.074025585	0.032600441
0.288223399	0.284296647	0.195548266	0.233565695
0.059070299	0.120006231	0.176998616	0.15515729
0.070010752	0.224610592	0.27359193	0.269915384
0.329978989	0.15497564	0.281521536	0.202414649
0.022177684	0.118044627	0.078867672	0.030109948
0.089325912	0.016202911	0.026233181	0.181193649
0.03716854	0.172116226	0.120132193	0.050081839
0.072648353	0.083267526	0.127668962	0.200059264
0.075494466	0.281767066	0.009733596	0.028959685
0.039975179	0.204247858	0.061632174	0.066549869
0.031377097	0.022780684	0.113085832	0.207781448
0.111361091	0.059558997	0.215014542	0.085295811
0.07150569	0.248293952	0.131697675	0.006478634
0.024252846	0.17401711	0.081559335	0.110726134
0.005665987	0.203658497	0.144884027	0.101582826
0.220119387	0.051254172	0.280185357	0.055684187
0.063788487	0.283106049	0.075725982	0.238734824
0.052202838	0.229099788	0.285502956	0.211292599
0.043324829	0.069359756	0.081514144	0.265269176
0.05787211			
FZD5	0.308745772	0.464906967	0.253210501
0.39541136	0.316170659	0.298241354	0.323923219
0.287623829	0.15648896	0.027423913	0.04579474
0.26607784	0.193684864	0.294784154	0.270694585

0.377434276	0.148333286	0.299947288	0.321747338
0.355448256	0.226724077	0.326440198	0.213220448
0.211255491	0.114948337	0.137359613	0.210629776
0.442154001	0.149846253	0.289069167	0.360910557
0.130076682	0.371509831	0.431317034	0.285090183
0.404723661	0.27613783	0.025338443	0.308155543
0.383497112	0.334597306	0.371437541	0.34124334
0.129046987	0.414794237	0.325686435	0.279871847
0.25515925	0.069045488	0.276830925	0.402449466
0.385345573	0.28182361	0.335748605	0.286861374
0.160897845	0.272849645	0.13995941	0.281635811
0.321963577	0.241173443	0.396392049	0.19530675
0.200960188	0.346954754	0.15427923	0.3443249
0.215036945	0.344095193	0.081010544	0.21857411
0.012996959	0.359480528	0.285271982	0.176020446
0.091987032	0.054099509	0.18076147	0.356802902
0.444596083	0.381699826	0.423474773	0.412970971
0.176092478	0.280863225	0.23261155	0.241619936
0.390658584	0.280189769	0.157753816	0.362932703
0.427405052	0.432502179	0.218878837	0.243932618
0.372475118	0.443855347	0.287964957	0.312066812
0.221355466	0.064907519	0.387040116	0.437838715
0.3600996	0.179609617	0.181500843	0.39818613
0.129767762	0.265134298	0.349439144	0.138606313
0.457618711	0.081765016	0.200187625	0.353518693
0.258751803	0.360614315	0.414676419	0.301160474
0.285532683	0.047212015	0.095909457	0.074630486
0.283724372	0.079216115	0.385698629	0.43177826
0.302356397	0.289877668	0.362938534	0.011621607
0.053860383	0.181196645	0.334508876	0.311606838
0.358161671	0.28762579	0.249274915	0.320303943
0.249060046	0.416937423	0.062652287	0.23301815
0.320336007	0.191625607	0.295940147	0.29354584
0.262265188	0.256954396	0.227868882	0.462723009
0.414011145	0.187803161	0.286312418	0.3458664
0.298510688	0.251465457	0.217400352	0.295176305
0.1607079	0.376892847	0.046440991	0.323835652
0.178127259	0.12376134	0.043146637	0.333220203
0.280531403	0.364035405	0.364342388	0.199662446
0.321005831	0.315496259	0.299150663	0.309430428
0.037534037	0.109273616	0.249130529	0.443859367
0.250737719	0.42208889	0.324205715	0.271771675
0.27223107	0.403976986	0.003582344	0.416225178
0.427259085	0.240599148	0.303454582	0.291584238
0.230927739	0.423702365	0.031653017	0.340972742
0.264205468	0.174178434	0.455754016	0.198577715
0.387114209	0.097814223	0.339169866	0.318742302
0.273141597	0.024579528	0.287653681	1
0.044076796	0.2428876	0.348593437	0.345593807
0.324158779	0.362702725	0.451474263	0.212776291
0.314963531	0.330865403	0.039638257	0.250088742
0.339216994	0.232448936	0.411425014	0.338553592
0.236592146	0.430875144	0.308119495	0.186333433
0.133950617	0.363260405	0.06114551	0.296913395

0.31644616	0.210964773	0.252334087	0.161529979
0.102450424	0.459123865	0.119705938	0.139675711
0.07382675	0.293447981	0.342294888	0.230181462
0.161889959	0.239222603	0.254442344	0.303763753
0.146625969	0.158814615	0.022167018	0.200072072
0.128283401	0.2247854	0.222467442	0.302848586
0.300466124	0.143874278	0.294747561	0.206416947
0.136754921	0.045829236	0.449913564	0.339806371
0.291395034	0.278320395	0.08821385	0.196512186
0.434380762	0.240565826	0.243291357	0.431585118
0.068782402	0.429064183	0.248310278	0.166148454
0.235436756	0.301844279	0.452377992	0.095615548
0.313785561	0.430747618	0.29996502	0.215516076
0.359329385	0.350013689	0.110108466	0.318197936
0.306833836	0.081341325	0.439259306	0.131216955
0.052788051	0.292596733	0.301519714	0.132234712
0.120193591	0.305201242	0.169317689	0.279945744
0.458290808	0.349575449	0.344335316	0.165177296
0.117297382	0.022608702	0.17946886	0.240460752
0.419333332	0.411584389	0.291960857	0.249585093
0.319512054	0.24872283	0.226974783	0.43564065
0.158605976	0.433245277	0.029735751	0.314691565
0.402316412	0.282163106	0.110943843	0.351100433
0.356841109	0.138582651	0.297174966	0.420241828
0.406069371	0.297370467	0.26971389	0.303033238
0.085425732	0.158840928	0.28769368	0.116002665
0.308645599	0.278416181	0.025697297	0.338958319
0.107038174	0.416883401	0.397126068	0.117041006
0.264301092	0.313233683	0.29709217	0.110571226
0.343496922	0.396801325	0.436716028	0.292589581
0.326355664	0.398358708	0.210197415	0.124886296
0.03832054	0.410020257	0.316675125	0.053154733
0.441201199	0.240653512	0.038269052	0.458444755
0.300702524	0.362194294	0.016270416	0.049963474
0.056338588	0.384449597	0.406679657	0.347789337
0.219509955	0.347303864	0.007874876	0.432617685
0.426507183	0.030004094	0.134247832	0.322361137
0.268081288	0.469137964	0.194908153	0.349899438
0.238051901	0.39975626	0.292000478	0.281788259
0.396592536	0.375765509	0.369350339	0.42501369
0.425106409	0.299859825	0.338118968	0.07315386
0.131608571	0.312459497	0.205232297	0.121720197
0.278362063	0.16857358	0.41401686	0.423344623
0.393082479	0.020486847	0.286618954	0.339448693
0.442049635	0.464804485	0.042790671	0.009958681
0.007152608	0.02648692	0.363570928	0.236660907
0.422162668	0.191977011	0.025407285	0.159192826
0.303891434	0.250925572	0.429720839	0.352536414
0.330128757	0.153394231	0.236575582	0.36735149
0.094393655	0.242349274	0.413457677	0.436738091
0.076685249	0.249775133	0.286002408	0.467424996
0.032258726	0.292182867	0.270818629	0.300316874
0.025894197	0.113926115	0.112481618	0.378005539
0.387017687	0.10468566	0.059658848	0.151097936

0.212846907	0.413842333	0.221506746	0.459151537
0.415659291	0.423028562	0.08963715	0.426686527
0.092143683	0.401573564	0.22133999	0.072262529
0.261195816	0.044033082	0.368268027	0.118445251
0.314561381	0.321782102	0.172037552	0.033197271
0.104596567	0.095345607	0.438310353	0.378872788
0.048488893	0.352856001	0.284259379	0.326272425
0.323809211	0.37116643	0.426386152	0.23825005
0.22224337	0.080721244	0.368624182	0.279804165
0.317066389	0.432454846	0.253762797	0.445919536
0.32985793	0.420010449	0.393437755	0.349771662
0.169789403	0.300364536	0.317950904	0.069555986
0.344319372	0.409915838	0.334497847	0.15048422
0.25466583	0.21331924	0.138237389	0.142836349
0.165282431	0.294873195	0.234128804	0.422436283
0.308590049	0.298402192	0.126840441	0.108607459
0.468687043	0.405432781	0.250022429	0.327504257
0.219351807	0.314172088	0.34362296	0.330653576
0.266932819	0.372462464	0.389651009	0.312526689
0.409242039	0.346974554	0.441197959	0.185676535
0.099784684	0.25069447	0.162914791	0.132104464
0.231019045	0.059064716	0.149696705	0.343279635
0.190286068	0.330761024	0.280929284	0.137130964
0.27672994	0.256780951	0.327250498	0.300211396
0.14754135	0.422599366	0.025049793	0.106489264
0.205464013	0.349900182	0.229492974	0.250754125
0.179062905	0.139244037	0.314102206	0.294537518
0.227661532	0.22999067	0.352346735	0.104436075
0.090302617	0.446361557	0.283127442	0.036084797
0.116361097	0.269269001	0.276776863	0.275243519
0.036281843	0.378861503	0.279125794	0.291528612
0.36305233	0.142930125	0.332786789	0.140451654
0.144452178	0.396818933	0.244526108	0.410799009
0.247655853	0.381189291	0.384811749	0.352429925
0.135085679	0.21683816	0.11015771	0.417207391
0.219087845			

G0S2	0.036498885	0.027908876	0.043407135
0.055238268	0.06427962	0.012167881	0.048450296
0.011127187	0.043489214	0.01282799	0.060273845
0.049162286	0.024549759	0.015024609	0.005863314
0.101663551	0.131334107	0.031010657	0.113619406
0.064305489	0.015794958	0.118424006	0.063273984
0.065231696	0.143424065	0.144469862	0.073041409
0.05828732	0.165346643	0.006885025	0.0313675
0.197102683	0.038143119	0.0194016	0.067687114
0.018607904	0.023781893	0.051476868	0.007510292
0.028246141	0.05082591	0.056756027	0.088368412
0.06543595	0.013702296	0.073134381	0.0970151
0.007258494	0.10596191	0.00403621	0.013662653
0.013583869	0.023695272	0.01629452	0.041812876
0.011801883	0.005138919	0.020634729	0.011161874
0.079043975	0.003748008	0.017805908	0.022697929
0.009786179	0.029138184	0.172652601	0.034319398
0.04389267	0.017603641	0.074944128	0.034092031

0.003572187	0.071934817	0.130299701	0.023873187
0.272585892	0.06176344	0.20305027	0.05083992
0.055361128	0.023908493	0.062405728	0.050952237
0.074606426	0.163664196	0.064381817	0.098274986
0.029614606	0.045258512	0.03889413	0.117141737
0.057305092	0.024217633	0.079955055	0.212519481
0.022798286	0.024921626	0.02037269	0.035735625
0.014253135	0.231115793	0.016201186	0.041547183
0.01786247	0.076471162	0.23228719	0.031970255
0.216106878	0.006709458	0.099201893	0.066594677
0.054840434	0.046646074	0.228676381	0.036945842
0.074359518	0.034606745	0.026122767	0.094497865
0.071629332	0.096463901	0.03284774	0.220326779
0.015773387	0.067967278	0.011503998	0.041594141
0.011463032	0.022676957	0.046312663	0.125639603
0.204854058	0.118299044	0.03089093	0.049171144
0.05293617	0.019613735	0.058135011	0.015088192
0.034021849	0.063400065	0.00014344	0.068117636
0.032228926	0.124879706	0.188966211	0.033896164
0.105909282	0.04391571	0.127146038	0.044052716
0.01518823	0.143156305	0.013983116	0.109122798
0.008603156	0.125967921	0.12333128	0.042481447
0.047765557	0.018394035	0.283720359	0.021403554
0.008004369	0.101756871	0.098317642	0.018517289
0.009565395	0.021563471	0.012170338	0.189383139
0.051473653	0.100303954	0.011421555	0.022884591
0.031865973	0.13203462	0.005388256	0.036328954
0.006107329	0.048306269	0.056026214	0.083526072
0.188369012	0.064541864	0.021702202	0.015346577
0.024869188	0.004303242	0.052708649	0.07943667
0.025792956	0.047088411	0.002405793	0.10201903
0.013300284	0.175191052	0.061158688	0.202701461
0.020594604	0.182744591	0.014090457	0.04355112
0.026039598	0.02753354	0.007149942	0.044076796
1	0.008234381	0.0448996	0.059924436
0.044740959	0.070760818	0.073157396	0.023627421
0.050011768	0.054011298	0.164547262	0.159253374
0.04962708	0.007422363	0.015828778	0.026786063
0.007855754	0.023284528	0.022373928	0.003485848
0.108278437	0.027158806	0.014751455	0.073754776
0.020248916	0.028411479	0.020888827	0.136709385
0.229985257	0.036015182	0.127841287	0.062724888
0.083201803	0.022775941	0.084904599	0.011199757
0.021094814	0.086339798	0.031680612	0.024899056
0.024216429	0.147511408	0.229878658	0.010109084
0.019565796	0.010759101	0.007450894	0.018236286
0.012965518	0.04333844	0.0565655	0.04865738
0.153521183	0.249861327	0.028313678	0.093678188
0.009244559	0.058855122	0.245513664	0.003551838
0.039070545	0.004282051	0.003170381	0.025310397
0.097278205	0.059096288	0.02683024	0.211782463
0.037408609	0.102673624	0.060240551	0.004927129
0.024607877	0.019531018	0.147076186	0.119314935
0.011874245	0.048293504	0.05601489	0.019604579

0.017065834	0.194268339	0.031783872	0.154015881
0.288954608	0.076206236	0.031061323	0.292928087
0.233882978	0.028241817	0.013308125	0.199676803
0.03344407	0.110341897	0.023035207	0.128246523
0.092694628	0.040708485	0.201205959	0.050853394
0.016184309	0.034751139	0.188238592	0.05726582
0.144281707	0.023326877	0.003316219	0.037376255
0.07385868	0.023721606	0.165018982	0.051396379
0.016564055	0.007013829	0.178920639	0.022719699
0.014478391	0.101943691	0.071153268	0.020004137
0.028428335	0.039539489	0.020371368	0.069585119
0.02769888	0.040336625	0.07111592	0.076718109
0.016610737	0.087132764	0.064358816	0.009222456
0.073247715	0.0181312	0.073438054	0.086739295
0.007459388	0.008377425	0.148427483	0.160760772
0.039204514	0.028270769	0.019622682	0.062265446
0.039604093	0.028443705	0.03210909	0.282048656
0.307999389	0.022051382	0.025349565	0.038182832
0.060927455	0.003421239	0.036132768	0.025332972
0.11636896	0.010588555	0.002762964	0.03672176
0.239085129	0.079663963	0.015364836	0.035276206
0.001786853	0.08566909	0.093793453	0.038776845
0.02056568	0.191541792	0.13447798	0.027126661
0.034352684	0.025888654	0.052615244	0.039079658
0.035879779	0.018288021	0.016237729	0.176413497
0.029109077	0.025138453	0.035457158	0.021363485
0.050142615	0.103532635	0.105861843	0.054913706
0.048236694	0.01030787	0.003424852	0.034501087
0.010098652	0.105194335	0.030392581	0.066219478
0.040150343	0.213091697	0.016965896	0.024829444
0.031204772	0.048873254	0.157393548	0.017141311
0.011294587	0.09060628	0.037090943	0.193614911
0.03421104	0.00121775	0.118197454	0.02212841
0.028842228	0.08747439	0.022907246	0.110133773
0.054897625	0.201730344	0.004810224	0.090878035
0.114947588	0.009388193	0.041164757	0.017738513
0.224454647	0.158082397	0.028632134	0.048057294
0.039731123	0.176980152	0.049223586	0.015297736
0.153567164	0.213740689	0.147230721	0.088598529
0.016813519	0.05935728	0.091726603	0.00149184
0.042186528	0.052461322	0.056662549	0.050811084
0.037290851	0.083970619	0.19874093	0.019127183
0.068502467	0.016741826	0.095348138	0.001888517
0.092266355	0.167318015	0.025521323	0.012499927
0.010046492	0.032679266	0.028474381	0.241318452
0.042262132	0.21954697	0.056843502	0.068114033
0.253023948	0.039954398	0.158079197	0.078683465
0.015546949	0.056816321	0.016862215	0.009037869
0.035871904	0.087868185	0.050791043	0.019404384
0.013187106	0.065649975	0.022238554	0.037129973
0.026334981	0.03251292	0.029430147	0.046105808
0.068272315	0.018341457	0.019410367	0.021098342
0.082184436	0.055866123	0.0332091	0.063761599
0.11181307	0.048431062	0.100050649	0.00813856

0.046408224	0.00990727	0.053568241	0.089482774
0.031291607	0.019310719	0.065128123	0.126341146
0.029533809	0.020657078	0.013746553	0.03421545
0.228004252	0.084006611	0.056384351	0.06755455
0.013937658	0.02937487	0.035173146	0.017933144
0.015055748	0.084573573	0.020895685	0.004876775
0.068694685	0.030252921	0.047444019	0.320650097
0.1462898	0.185395428	0.020849764	0.100176594
0.018421386	0.09026689	0.005444582	0.126515284
0.161880306	0.021518125	0.124352274	0.025375372
0.063640584	0.017522667	0.089361768	0.249564269
0.026692977	0.033137875	0.047188022	0.13050974
0.030161362	0.171028133	0.126033405	0.008004631
0.005267956	0.0134074	0.029427207	0.03848251
0.053411926	0.06308668	0.015155187	0.239742228
0.120421183	0.018877779	0.046108686	0.023568368
0.285014122	0.027462142	0.005627898	0.058608975
0.038372962	0.173688159	0.010588321	0.028844653
0.107328625	0.016514333	0.059547908	0.022819625
0.089252438	0.088252749	0.011557534	0.027610424
0.061206511	0.029026433	0.056216772	0.048785649
0.009738827			

GAB3 0.077501828 0.132083607 0.035461153

0.211060506	0.074478047	0.148887972	0.268598279
0.114369319	0.069989994	0.061663381	0.020956673
0.048059301	0.030257809	0.090969588	0.043976754
0.158699887	0.024962336	0.051122806	0.199425081
0.182948719	0.198405673	0.139900096	0.12006276
0.013397131	0.021766134	0.081089048	0.027584156
0.193319331	0.086684516	0.148082342	0.223120225
0.018154933	0.118852004	0.10413741	0.076281257
0.085011803	0.065169007	0.009907695	0.115157035
0.126526185	0.099899501	0.207264666	0.191904227
0.100889678	0.114460735	0.253032491	0.056421752
0.058586878	0.031107158	0.139694873	0.130411105
0.094310741	0.078654872	0.183025029	0.220358078
0.024433345	0.109806467	0.004920109	0.192655845
0.221054609	0.180206228	0.095087167	0.12896382
0.061693119	0.155206111	0.016468976	0.196454782
0.014390784	0.157851546	0.067323071	0.031270035
0.025215292	0.202531883	0.061073666	0.012579017
0.057833006	0.035332781	0.026615594	0.25119882
0.166053699	0.087143741	0.114426929	0.099880429
0.022202249	0.06407555	0.027308291	0.092131478
0.123038863	0.224380794	0.043690525	0.133327035
0.112168783	0.102907818	0.031773624	0.059255455
0.154167358	0.143937659	0.058758965	0.067037268
0.023406484	0.007237204	0.117511307	0.110551717
0.127191447	0.013442093	0.056213928	0.198399415
0.06993515	0.022881641	0.206648957	0.012490467
0.171411793	0.100190703	0.041333076	0.198780745
0.037925322	0.109129093	0.088369989	0.229524202
0.04479016	0.020372939	0.006162549	0.025756618
0.032724196	0.001992185	0.092100238	0.164124845

0.080579158	0.025020888	0.185155349	0.008291665
0.016716836	0.015162542	0.078573179	0.068295782
0.129252395	0.053596704	0.080630598	0.118430055
0.049338781	0.112603762	0.022048096	0.139683135
0.094835175	0.018764586	0.165071105	0.07088705
0.061957987	0.153657839	0.122595789	0.130360074
0.162883359	0.152813037	0.058405827	0.133874143
0.051969653	0.043331198	0.118427865	0.081092308
0.134161337	0.076814713	0.011761086	0.109960532
0.014257578	0.009890333	0.025933244	0.205994441
0.055450998	0.112393687	0.07797066	0.10607397
0.107135825	0.103954041	0.123137509	0.112240386
0.049081105	0.04339712	0.081346216	0.121379614
0.18607364	0.097063071	0.067563733	0.03715935
0.083851096	0.141072291	0.000197032	0.107053735
0.095720532	0.068450379	0.0995618	0.062739213
0.030733118	0.168662282	0.002805589	0.134686619
0.092292316	0.09987123	0.132192943	0.078355037
0.094518897	0.07026898	0.120217674	0.06132087
0.051226371	0.013461909	0.234446899	0.2428876
0.008234381	1	0.12484066	0.176047498
0.144754368	0.102177104	0.147769648	0.024169702
0.072467344	0.148868848	0.020496114	0.043258539
0.083254161	0.014666989	0.14395212	0.117926622
0.077356233	0.136261917	0.063983255	0.038146405
0.145656414	0.197773141	0.058828937	0.087667748
0.075211209	0.104988728	0.044284278	0.017417358
0.027429774	0.124053047	0.007177043	0.006765335
0.002473979	0.104459856	0.178866528	0.064138881
0.019027025	0.063419521	0.029390494	0.056861989
0.16878723	0.073754175	0.00312829	0.014533586
0.023790343	0.029452169	0.03434254	0.104154553
0.044027561	0.038407645	0.064844006	0.012021853
0.022909744	0.036764959	0.1268404	0.142282679
0.140093797	0.125924286	0.039340245	0.011631509
0.155979765	0.117609673	0.068775393	0.162940909
0.031094697	0.117750777	0.04554519	0.066291466
0.023505136	0.15169192	0.179211048	0.040094805
0.110221845	0.144095117	0.084632153	0.06497768
0.125543556	0.165168877	0.097165433	0.093301087
0.091699462	0.055449115	0.103056377	0.01425141
0.031167908	0.065727919	0.076246855	0.07002285
0.038052552	0.133370216	0.190207787	0.103204177
0.129510264	0.144064368	0.120835407	0.012204972
0.105937617	0.013519876	0.020537678	0.031384947
0.101655555	0.08672189	0.109862616	0.040636832
0.206964583	0.031333331	0.089805669	0.111520097
0.009073571	0.102242661	0.026173546	0.06729248
0.087434606	0.138590894	0.034773879	0.25024862
0.142013599	0.118743779	0.088368289	0.107577443
0.144528875	0.06290651	0.044801891	0.083614289
0.134098992	0.151705004	0.068106966	0.004682423
0.059359995	0.08119708	0.000119111	0.070101673
0.064971968	0.143483208	0.117407606	0.04976281

0.148324544	0.072588332	0.08193915	0.094651137
0.066602539	0.079673368	0.108053398	0.118412145
0.077321473	0.09209194	0.027366435	0.03950362
0.018069612	0.085514933	0.132276808	0.033243527
0.147261131	0.151468211	0.035375175	0.145841415
0.201789946	0.08998632	0.026724079	0.001585426
0.007604196	0.097798105	0.109363303	0.21616518
0.089249404	0.144565214	0.000622457	0.095822682
0.119272735	0.009536399	0.011522377	0.075106521
0.046364923	0.13162879	0.018065237	0.076130955
0.037709894	0.089554956	0.083742583	0.113261543
0.144307132	0.070283644	0.152172711	0.136078708
0.14950704	0.083332301	0.229141671	0.090984002
0.015062498	0.208368326	0.033090568	0.04091619
0.158488978	0.033572436	0.10268607	0.13070062
0.088375143	0.019155683	0.139077729	0.090390399
0.190222894	0.17319075	0.011904433	0.002815817
0.000528003	0.005485519	0.202560845	0.049791964
0.109264823	0.038723404	0.031494412	0.156298453
0.053905105	0.045909211	0.112337106	0.195407652
0.073411236	0.063823997	0.054162398	0.184945821
0.019287231	0.086951814	0.105256116	0.146932888
0.018656965	0.054238184	0.053583454	0.16742602
0.000384565	0.088499215	0.056061035	0.052406592
0.005365114	0.02585362	0.03483152	0.12112643
0.079164901	0.029422545	0.021377824	0.033244175
0.132870889	0.118867696	0.058593195	0.127156544
0.168807743	0.173027128	0.011081703	0.123739711
0.046449794	0.097633743	0.026818801	0.026315646
0.033108734	0.024310223	0.072423845	0.018350109
0.098266586	0.137535704	0.017644598	0.009104558
0.003256262	0.035511428	0.143404111	0.091872056
0.006925502	0.127977013	0.071868602	0.111672458
0.227612631	0.154184776	0.111319334	0.045505225
0.176021722	0.001651308	0.18174817	0.090972188
0.108258357	0.11651184	0.039737855	0.106190352
0.119108357	0.124419737	0.093745969	0.190005895
0.009578397	0.102031371	0.220512056	0.085030069
0.138548858	0.136960618	0.109470139	0.020766743
0.078330062	0.023774928	0.065166072	0.012433161
0.011425226	0.222620332	0.082380858	0.163636763
0.117079349	0.100486994	0.073958694	0.035941551
0.122038034	0.130918695	0.115122678	0.142265926
0.06598963	0.115713408	0.079665862	0.07245421
0.022228971	0.105293177	0.158779706	0.167837383
0.161373974	0.209193737	0.110406033	0.102541087
0.014276204	0.050638452	0.069606858	0.044336625
0.133801717	0.020410629	0.00530355	0.118676562
0.009700005	0.144855736	0.03744571	0.036762943
0.060906868	0.040855287	0.113633271	0.215321231
0.066790484	0.113611193	0.01335221	0.042329166
0.012640529	0.100162879	0.030059627	0.049672066
0.007470209	0.011816304	0.12837285	0.151946602
0.060756775	0.011277927	0.101745498	0.078074576

0.102304155	0.128717967	0.066324231	0.00697044
0.021510862	0.090257286	0.031087792	0.066574
0.008144964	0.078823336	0.107866263	0.058742715
0.203894396	0.061831142	0.122627877	0.033013016
0.058985756	0.11398066	0.035126309	0.088477844
0.029081551	0.170562272	0.109630382	0.098372402
0.016438893	0.02625613	0.108824864	0.150994501
0.019165665			
GABARAPL1	0.371348213	0.498540391	0.345947466
0.3558339	0.36300678	0.30410193	0.224844898
0.238923142	0.100205223	0.005370145	0.019800017
0.348977843	0.156399467	0.237239646	0.278899475
0.372766172	0.131822836	0.39321364	0.263695252
0.37706019	0.115797137	0.343138594	0.128865039
0.285509299	0.099553172	0.082132223	0.228522864
0.430565191	0.096681867	0.258887492	0.345375053
0.146583263	0.406425855	0.490024281	0.326818969
0.473692489	0.339329823	0.027759848	0.24492997
0.425481377	0.350998754	0.368972786	0.304717488
0.060167822	0.463120193	0.243973987	0.357851895
0.251961747	0.034969856	0.236270091	0.43994991
0.426625508	0.315369332	0.264529939	0.175541186
0.130629817	0.227240276	0.177389048	0.287658808
0.235622466	0.165193048	0.443920092	0.105342533
0.17132641	0.311499103	0.179942948	0.286346565
0.291870084	0.350914644	0.045416292	0.32072822
0.003110094	0.373642943	0.358836205	0.231317188
0.061269929	0.020407923	0.205056329	0.306440394
0.452653235	0.459615962	0.44940762	0.454090399
0.160365168	0.324797741	0.321681765	0.179374297
0.424181668	0.175764389	0.133774096	0.362450802
0.457475345	0.482878683	0.203735843	0.274770918
0.354051996	0.469235588	0.333490059	0.342752329
0.24515174	0.080583961	0.412781126	0.476516106
0.3907093	0.254859696	0.16390323	0.381914501
0.091680576	0.322961187	0.326259754	0.170541487
0.461514184	0.031904978	0.216400264	0.313067588
0.306620515	0.393446654	0.474308164	0.213324361
0.363984766	0.028001905	0.103953238	0.063805846
0.285032176	0.12664972	0.445829512	0.418493071
0.255145585	0.317799644	0.29807264	0.006628292
0.039369702	0.247074225	0.381971971	0.392042222
0.383926862	0.37107101	0.198006949	0.340455486
0.317323991	0.444537414	0.043235778	0.148487104
0.34575884	0.252829626	0.259968807	0.346090646
0.263732032	0.170934497	0.160436481	0.493317609
0.428458595	0.112434333	0.35854259	0.364632727
0.257350501	0.299556976	0.151130719	0.250380922
0.072525611	0.441819913	0.042148815	0.359379087
0.190886107	0.131953824	0.038028062	0.28305362
0.2426721	0.367649528	0.43949759	0.156375259
0.288034248	0.344661223	0.31810613	0.297223826
0.009554014	0.082750143	0.284039874	0.470435879
0.242965445	0.482477404	0.32076516	0.321608326

0.281311707	0.418992025	0.007651423	0.465745066
0.485331177	0.274312406	0.303215262	0.374559687
0.28142208	0.410182706	0.047291018	0.366546832
0.306207485	0.128178481	0.481896535	0.163385711
0.445131828	0.054203992	0.304991165	0.374219812
0.313216298	0.01370758	0.217148569	0.348593437
0.0448996	0.12484066	1	0.356376656
0.3155898	0.402943725	0.465004246	0.260635974
0.347144632	0.285967361	0.028143116	0.306936802
0.398126727	0.295029645	0.432069482	0.265859196
0.291953209	0.452181089	0.364373259	0.173387151
0.063700992	0.323465081	0.026828292	0.294624339
0.380415335	0.112578948	0.307200443	0.21503364
0.086979511	0.491412607	0.167924275	0.215712239
0.115202242	0.293101939	0.302764281	0.166651758
0.137358343	0.191147948	0.287231475	0.319988423
0.057285125	0.105128175	0.035292842	0.246575496
0.089345402	0.22340367	0.220191772	0.324047473
0.285120221	0.094716759	0.302570043	0.285422842
0.131005172	0.026471511	0.484806578	0.378519188
0.297657464	0.195045213	0.067226742	0.222090614
0.428188844	0.233705738	0.268419924	0.425099084
0.054812072	0.467653729	0.197562473	0.130570804
0.271964758	0.248409845	0.449190353	0.060129406
0.368067738	0.444904595	0.346308643	0.180311992
0.369136622	0.366472934	0.046276909	0.359730821
0.338106845	0.045556642	0.48892902	0.135916823
0.036817389	0.353398005	0.320513248	0.102079961
0.103000865	0.256739301	0.07541444	0.283019874
0.48543187	0.344937097	0.342711483	0.234259767
0.052356236	0.007552146	0.228067797	0.346276078
0.477859188	0.469594735	0.293844532	0.268372534
0.267515123	0.259628156	0.18546042	0.468493574
0.27423666	0.498300834	0.016076917	0.359189988
0.465312787	0.266105854	0.083763627	0.286896249
0.358576613	0.073120909	0.292290535	0.462977216
0.423396117	0.296691521	0.279839492	0.38125989
0.028597967	0.07213408	0.341646773	0.173909768
0.312589512	0.244367165	0.048647371	0.338023406
0.050620361	0.423487223	0.427641505	0.071521444
0.241344817	0.337660828	0.348882811	0.060365752
0.398183353	0.473896328	0.488802271	0.242501884
0.371610368	0.439084539	0.278507609	0.118336355
0.029100035	0.477987962	0.357238193	0.02600506
0.451777256	0.21803629	0.010582453	0.483867407
0.233247143	0.424954534	0.006664749	0.113438785
0.070055715	0.425885858	0.447877174	0.359320979
0.185256804	0.3870117	0.016297521	0.495702628
0.453293321	0.021872404	0.1590694	0.352440282
0.313123772	0.514073533	0.24698537	0.385155084
0.301197766	0.454471631	0.211980792	0.26826001
0.391269516	0.446694735	0.376939549	0.458619072
0.424828576	0.365034991	0.28240439	0.024929693
0.140219608	0.239596256	0.202034151	0.077666621

0.2989944	0.141666204	0.468119549	0.446099494
0.434127968	0.011585871	0.192755971	0.368706153
0.433880203	0.472851022	0.053034027	0.010014837
0.012302652	0.016753623	0.355852042	0.269838004
0.462582671	0.162803871	0.010502729	0.076135432
0.291971396	0.31286857	0.467053619	0.358925015
0.388969893	0.118711624	0.188192758	0.37855026
0.074210915	0.287515712	0.441782352	0.452896345
0.078937897	0.279678825	0.373994884	0.470977168
0.056180423	0.314038545	0.350874894	0.250882821
0.037557132	0.102923993	0.084314104	0.404935718
0.454707394	0.062588223	0.030928602	0.131904627
0.116203259	0.450993749	0.161397996	0.490521246
0.411548938	0.411016452	0.097739451	0.46363856
0.045965006	0.447194338	0.273580891	0.057686375
0.338929035	0.029916223	0.440319269	0.100016116
0.300775186	0.369821059	0.191421602	0.03018875
0.16991412	0.070598485	0.444092863	0.418420005
0.059957686	0.301588604	0.323480526	0.393571758
0.279209971	0.34736072	0.473032777	0.283809001
0.109892336	0.155160453	0.370911759	0.33458436
0.265394291	0.464936065	0.229651702	0.502825051
0.358921361	0.468980634	0.437943966	0.323477856
0.205627479	0.345617114	0.289694003	0.023525694
0.354910648	0.409109981	0.383395019	0.130028198
0.225705758	0.302929335	0.106613123	0.219916544
0.249268647	0.267129136	0.154352695	0.426978508
0.369769404	0.331470355	0.088835236	0.075811672
0.51720445	0.444153984	0.176006638	0.341936524
0.22118574	0.306704179	0.394515585	0.378729223
0.345021801	0.388646614	0.388843102	0.291972968
0.409123869	0.291126321	0.492129624	0.135086787
0.086664904	0.343059856	0.088404605	0.123813453
0.164035828	0.04637686	0.21811375	0.376732364
0.271025377	0.326908216	0.312544397	0.117706931
0.324943994	0.36628295	0.346352539	0.200956723
0.081818713	0.457700894	0.015726217	0.083935882
0.285396286	0.371114579	0.275144416	0.262003029
0.232595065	0.181745388	0.289802983	0.272782759
0.207522254	0.31346099	0.372936616	0.061791736
0.035922482	0.473971131	0.345013354	0.04549891
0.130851242	0.344423659	0.357220433	0.206992034
0.036819315	0.431972235	0.251437139	0.359095012
0.346840697	0.109394207	0.366519721	0.13464525
0.121073667	0.440140633	0.332879324	0.471030985
0.351524954	0.353819499	0.437916602	0.381089102
0.184627317	0.328138728	0.047636623	0.434163733
0.271644692			
GABARAPL2	0.320401386	0.480457703	0.278056789
0.409172843	0.331708953	0.277310004	0.292014137
0.232450547	0.166115494	0.025934314	0.053397823
0.287253451	0.206467167	0.2788212	0.276028529
0.40273976	0.175431503	0.290878888	0.31353322
0.357501782	0.178614522	0.356483852	0.199714921

0.24410352	0.147154789	0.14884185	0.252796573
0.452325057	0.165835602	0.245533846	0.340713316
0.153844779	0.375203022	0.445526098	0.315811322
0.423940079	0.287636027	0.021444736	0.280879784
0.385249901	0.370352992	0.367384095	0.345736791
0.127094629	0.414184044	0.314306027	0.307356529
0.244796939	0.080429025	0.221260891	0.390574705
0.398791001	0.288932475	0.273749499	0.256103929
0.169936904	0.208303216	0.178468212	0.248792338
0.299675138	0.17530369	0.413563318	0.186483849
0.169580376	0.28845467	0.181326142	0.306646522
0.243340781	0.328624084	0.06428372	0.237366225
0.012307352	0.345797455	0.311217649	0.211494901
0.105174808	0.062540206	0.209512932	0.338564175
0.460202466	0.39526391	0.463439041	0.45192861
0.205559765	0.303785467	0.260768756	0.255252199
0.394308863	0.261492893	0.165918857	0.397491221
0.467399117	0.453941912	0.236450331	0.278710368
0.383065412	0.444325698	0.31205293	0.330644975
0.257878516	0.088241038	0.389910294	0.469194918
0.353974975	0.218309683	0.216002516	0.385190726
0.133436975	0.276884935	0.353324714	0.155194739
0.472156659	0.074617217	0.236778609	0.328183381
0.269364823	0.380578468	0.443569016	0.284228603
0.301823379	0.057364809	0.13023259	0.081316427
0.27785701	0.10942672	0.391017147	0.449073826
0.247360451	0.303268523	0.320174958	0.014399478
0.069647642	0.207038175	0.35156177	0.305535167
0.371824335	0.305759031	0.274070859	0.305809581
0.276371405	0.454111218	0.051847181	0.206261123
0.32532992	0.20988768	0.307034727	0.300012079
0.299442913	0.217316521	0.223411606	0.493523625
0.395153135	0.182388826	0.261388713	0.377317285
0.288673187	0.290158054	0.202809519	0.291885861
0.135563369	0.396231362	0.062835694	0.289168962
0.203119462	0.146169136	0.038730088	0.27337157
0.285149025	0.383599113	0.370500024	0.19943783
0.317528053	0.340636642	0.252835663	0.327798681
0.040422637	0.112471159	0.201362431	0.468703767
0.206358666	0.455939039	0.329085392	0.300823403
0.305545062	0.434281862	0.007077301	0.423160464
0.452923452	0.204926988	0.319906269	0.30758144
0.255530799	0.447509084	0.029000115	0.364350949
0.252397634	0.170686106	0.490668506	0.225697508
0.39746293	0.108179405	0.286110515	0.361367453
0.298347488	0.019930471	0.232460697	0.345593807
0.059924436	0.176047498	0.356376656	1
0.334878384	0.397343908	0.48477227	0.243800256
0.333878963	0.297098571	0.042435523	0.289055174
0.365308004	0.23887609	0.411128564	0.305139473
0.212804753	0.442068276	0.329643397	0.188211438
0.126819501	0.313575709	0.05497803	0.325782771
0.326899238	0.196567966	0.265489681	0.17240616
0.125172053	0.487679662	0.156965499	0.176800021

0.102860818	0.291270553	0.321056064	0.227218175
0.186647302	0.243851592	0.277482134	0.320696844
0.113301546	0.170744738	0.031290055	0.214503477
0.142138059	0.234140078	0.224378294	0.294866865
0.268564217	0.156912729	0.320879785	0.246274216
0.173833681	0.052926817	0.467692352	0.349017623
0.269037862	0.257522053	0.097575063	0.203871268
0.451357505	0.218991361	0.195066352	0.434919751
0.070814988	0.460224087	0.269233677	0.192553435
0.263476367	0.309475885	0.474019708	0.080539295
0.285288445	0.428912857	0.3174881	0.24996114
0.350028803	0.338149982	0.095031978	0.326791892
0.307256677	0.089081759	0.466811712	0.161277027
0.062210903	0.332979993	0.332966266	0.150443827
0.148171451	0.306177718	0.126638659	0.311978854
0.484662041	0.383555498	0.35688822	0.20526586
0.115373583	0.026418096	0.214455492	0.26474626
0.428977807	0.446383191	0.303448448	0.286558215
0.31573255	0.271107743	0.160724641	0.467280341
0.180470078	0.447879107	0.03088043	0.350808647
0.419612686	0.224387902	0.139788021	0.308073866
0.3491293	0.134038866	0.268819317	0.438601884
0.414686987	0.31229463	0.299455609	0.31092105
0.069990394	0.123800716	0.316428616	0.151392708
0.314735114	0.291645077	0.039080099	0.278954781
0.119111651	0.414798989	0.425574774	0.13579726
0.241576979	0.269159041	0.323985104	0.110195447
0.384866514	0.425134604	0.451693955	0.252762965
0.342927437	0.432182029	0.238601363	0.143486143
0.050858765	0.435972241	0.314003847	0.072057135
0.462517137	0.187034996	0.041515711	0.466426167
0.286526851	0.359038523	0.021454505	0.063720969
0.074659464	0.422679156	0.417340039	0.326639611
0.195069125	0.339636836	0.009652968	0.464337188
0.440407428	0.041886389	0.164484045	0.343523899
0.30758438	0.48118327	0.234049286	0.389237992
0.264416749	0.420466098	0.262572317	0.309715715
0.41051497	0.407151792	0.375935899	0.430429394
0.44445367	0.334835496	0.334286523	0.065405242
0.163881636	0.239984251	0.222397841	0.145101942
0.239676382	0.207716389	0.436317436	0.452245218
0.430141724	0.023939938	0.239940622	0.349427195
0.439456857	0.479734296	0.048351254	0.013217984
0.011291022	0.040727929	0.326730561	0.271873611
0.45033847	0.183077506	0.026923204	0.118459659
0.331145559	0.287330284	0.447018024	0.364907708
0.36240635	0.157111261	0.231177103	0.37838248
0.117470962	0.227021051	0.449551088	0.433891132
0.086714375	0.280833311	0.277928793	0.488374174
0.047983867	0.315404417	0.285409922	0.298575187
0.032117556	0.142602153	0.131617497	0.405320941
0.406484052	0.121828227	0.075907101	0.115074986
0.20396394	0.435150108	0.243648622	0.491931552
0.427540207	0.447937463	0.108335424	0.437029928

0.08681791	0.413348348	0.26623976	0.084098899
0.282302611	0.046161109	0.391117166	0.145270842
0.271282308	0.313104904	0.200588557	0.042375712
0.136400767	0.110556314	0.470153073	0.419959244
0.063519338	0.311620071	0.315723578	0.332055603
0.276070395	0.394316683	0.435828072	0.252383514
0.183861684	0.101208323	0.366758354	0.279112071
0.251590833	0.470553177	0.272669528	0.476486484
0.328460205	0.430447439	0.415398482	0.343785636
0.196919782	0.285486379	0.306115924	0.071256435
0.349331881	0.43800544	0.308917213	0.190075707
0.285198662	0.244308896	0.112574562	0.137562321
0.200091149	0.253251455	0.227491431	0.446097326
0.287746872	0.293426761	0.094728825	0.128883213
0.488923778	0.404569446	0.227328843	0.344112595
0.240906531	0.33328398	0.380451524	0.365449482
0.269942966	0.392581562	0.383417739	0.306629059
0.396384248	0.330746794	0.45891333	0.12963087
0.111917782	0.253030877	0.168022041	0.154638528
0.234731747	0.076365686	0.183746947	0.367633562
0.214517911	0.342134383	0.230720248	0.171452275
0.302641195	0.248961916	0.343916969	0.270444859
0.160212526	0.435391113	0.032867098	0.118226264
0.22659683	0.371594296	0.266522266	0.288702999
0.211806808	0.163935715	0.327170361	0.242911831
0.223510759	0.245204021	0.377145794	0.100118999
0.072513671	0.47965872	0.275104092	0.048636461
0.117629481	0.254099637	0.272627563	0.275797645
0.050337091	0.408478035	0.248666034	0.313734331
0.339696475	0.144544816	0.312728522	0.125717958
0.135923569	0.396933208	0.272676125	0.433553056
0.275412909	0.407437777	0.376879478	0.362962741
0.154805701	0.231213964	0.099794153	0.424745195
0.236224362			
GALK1 0.28038409	0.457199368	0.211162456	
0.39508559	0.246253392	0.231695144	0.231801926
0.141085733	0.188742285	0.025014716	0.075422923
0.234091468	0.17814989	0.192426004	0.243573273
0.381574693	0.135209949	0.181585362	0.231513431
0.319098225	0.13417566	0.341771912	0.158065106
0.195476683	0.143090681	0.143439316	0.248340946
0.412265256	0.152125664	0.187230787	0.297949924
0.097268047	0.32854186	0.422716757	0.306323833
0.399439676	0.255171203	0.00758849	0.246872332
0.335954911	0.374394608	0.335521218	0.262208437
0.109740038	0.380148196	0.254673817	0.232380731
0.163862889	0.07986573	0.153359896	0.35721522
0.383472049	0.260354053	0.195346287	0.219431259
0.167274179	0.131731569	0.158655086	0.208210996
0.236228694	0.11831984	0.397054062	0.189485806
0.139527093	0.182980177	0.121720976	0.205697693
0.203920646	0.28990524	0.038692042	0.19664973
0.007568902	0.308790095	0.219237759	0.215668008
0.082321323	0.051591977	0.142515327	0.267399821

0.418122735	0.354904949	0.444862143	0.4394196
0.150093151	0.210146122	0.200253353	0.199252794
0.364660795	0.219973445	0.111907458	0.378160741
0.450360853	0.434216585	0.204778715	0.216231012
0.387616104	0.410475959	0.240282521	0.260873187
0.258307923	0.065944249	0.375533688	0.446680896
0.307488565	0.176042678	0.174189883	0.33766581
0.099219686	0.178873441	0.30061332	0.085164937
0.435389053	0.077149796	0.185673704	0.248337933
0.166006278	0.342045171	0.427684488	0.222412981
0.210647998	0.037753224	0.156138749	0.053657738
0.192509016	0.087091971	0.369241201	0.427308384
0.152284118	0.241253676	0.237611516	0.010172628
0.052753797	0.129815009	0.313427439	0.188273602
0.331868608	0.279561565	0.268299483	0.271101115
0.273278007	0.43659384	0.044882334	0.154844361
0.245900348	0.134287273	0.23079072	0.23799728
0.253972979	0.147804728	0.161336689	0.474056008
0.358768922	0.14078765	0.155177091	0.349480503
0.254868625	0.224896278	0.142051635	0.203876464
0.106993526	0.383652003	0.047027126	0.181631849
0.241645653	0.108138424	0.018442304	0.179605189
0.212567009	0.382989029	0.345181571	0.137587838
0.223785052	0.307317836	0.160394254	0.325861088
0.058583226	0.070540753	0.121648564	0.443725412
0.172730101	0.423369993	0.225936137	0.230792501
0.234379634	0.407222817	0.003244399	0.399329198
0.436740311	0.127817266	0.248630962	0.2330054
0.242264929	0.429994697	0.015142766	0.326460036
0.205074426	0.120187461	0.466290398	0.186036408
0.363123439	0.099696837	0.196730374	0.359564345
0.28526414	0.015119158	0.171451024	0.324158779
0.044740959	0.144754368	0.3155898	0.334878384
1	0.368172054	0.454252538	0.237860402
0.255250206	0.192490953	0.028849036	0.21529772
0.330528351	0.194895919	0.382270387	0.229319111
0.147980974	0.420879143	0.307628898	0.187415467
0.112294883	0.220971035	0.039789904	0.287131088
0.30988476	0.165565559	0.236860498	0.096407653
0.098341912	0.470206119	0.129572235	0.145558965
0.089133276	0.223714006	0.222121686	0.187122266
0.161712562	0.213827785	0.260944342	0.281457797
0.091538796	0.133573658	0.01832198	0.158338307
0.115368983	0.179209164	0.168379926	0.260953819
0.180421672	0.111137169	0.253566315	0.204713494
0.14891226	0.041025249	0.44403759	0.3153993
0.227259226	0.211939119	0.06777828	0.150932892
0.43295714	0.170990225	0.125334482	0.41545055
0.04380818	0.434124922	0.21962813	0.163008531
0.204008859	0.234487978	0.448490882	0.086592284
0.19396992	0.401220171	0.222877541	0.233153115
0.316457848	0.302276584	0.078306451	0.2859548
0.264554295	0.069042924	0.442660734	0.117147808
0.046241621	0.308962787	0.340639433	0.122519938

0.120984238	0.262838325	0.096387243	0.244355241
0.466910912	0.369500118	0.356546968	0.158245823
0.101855068	0.020832314	0.155047288	0.187375487
0.398140427	0.42519503	0.208993199	0.235222147
0.236329971	0.276257928	0.098978241	0.450030064
0.104113282	0.415181901	0.022007217	0.348284178
0.401521271	0.146332289	0.133295413	0.241670715
0.312944193	0.116754715	0.16356772	0.412711076
0.400816064	0.288634179	0.284598857	0.264133843
0.058488574	0.097311593	0.259195194	0.130501584
0.266483392	0.250883437	0.032174342	0.195025344
0.101669624	0.396038223	0.381318987	0.157386191
0.206320709	0.183094648	0.260530446	0.089332819
0.38394495	0.398293091	0.425056642	0.15929073
0.307546612	0.425584194	0.210177671	0.102668897
0.040433708	0.414022295	0.273619012	0.053884944
0.430050928	0.123685637	0.038907764	0.435989115
0.210170484	0.324494009	0.018016595	0.038448205
0.051613265	0.394234431	0.404150645	0.286626316
0.153226797	0.236260392	0.004975535	0.436708286
0.417562282	0.037210789	0.133640544	0.328161416
0.304501685	0.44547161	0.216778593	0.388537157
0.232302525	0.404884395	0.208782363	0.251309302
0.394434583	0.394590972	0.334685683	0.396095757
0.412208451	0.308374229	0.26409322	0.064803333
0.210499013	0.164192832	0.2134958	0.180041578
0.186030583	0.195568904	0.397088303	0.423234189
0.412631615	0.017926908	0.163571258	0.326296723
0.399977081	0.443099126	0.027472876	0.012580803
0.006483829	0.052464392	0.229563382	0.203459289
0.433193586	0.140923424	0.020172948	0.079565653
0.23193333	0.259031513	0.42619005	0.335992177
0.334975043	0.113298289	0.208644179	0.338312008
0.128518794	0.186432238	0.435712287	0.412006709
0.055524776	0.221347585	0.169483629	0.463348638
0.047566252	0.229904491	0.24631377	0.256209769
0.015566404	0.119877129	0.136579815	0.370595363
0.387062656	0.106482086	0.067054501	0.071753371
0.17852109	0.402068159	0.186415575	0.466937237
0.395518586	0.414941522	0.082843719	0.419503815
0.102800948	0.389090746	0.215692479	0.061793044
0.175032917	0.029442483	0.358551147	0.206365818
0.17849064	0.26238585	0.243529483	0.032442965
0.12643866	0.084907225	0.456812121	0.403629208
0.041944884	0.231016765	0.223617217	0.267338429
0.190399746	0.382230508	0.410590629	0.192717678
0.161490777	0.062180119	0.318868346	0.233098144
0.158572842	0.44567485	0.275591141	0.450085259
0.29170598	0.395311914	0.394300365	0.279231677
0.156754441	0.211946304	0.299849394	0.055780721
0.247292486	0.417826186	0.199733407	0.194370567
0.24491559	0.192759685	0.073249512	0.068155522
0.161737677	0.195880871	0.175510956	0.410283293
0.221718796	0.25204698	0.048936627	0.138918049

0.459688178	0.375670272	0.176290032	0.327948093
0.166440865	0.287880321	0.349230855	0.342838439
0.216427941	0.371416172	0.340571293	0.286307096
0.367945768	0.257449594	0.436874822	0.069789966
0.126696009	0.206644971	0.156212509	0.117201731
0.179856914	0.063209286	0.163204126	0.331357255
0.167897247	0.288532238	0.146555633	0.123996651
0.221339111	0.158909993	0.252649694	0.207463462
0.137782246	0.420392071	0.026518802	0.083582843
0.165290261	0.371707276	0.22710285	0.213830822
0.195558445	0.11354285	0.248771445	0.161865938
0.176189049	0.16721643	0.374215195	0.068798498
0.051405004	0.447112434	0.177915122	0.029391164
0.09359733	0.214040993	0.157454064	0.284368018
0.038337534	0.398086308	0.209608135	0.239342172
0.242014988	0.096405476	0.26698873	0.083394836
0.088042208	0.355537789	0.21083889	0.413866879
0.190194901	0.392018073	0.343395075	0.331251642
0.086226417	0.163414338	0.085246501	0.382147034
0.197876619			

GALNT3 0.384921338 0.507762633 0.413651358

0.383138264	0.427549313	0.309421829	0.231139417
0.201711311	0.126473808	0.006445283	0.034183716
0.414896569	0.200800787	0.24808675	0.302167747
0.425441847	0.186975477	0.394952497	0.291395195
0.402502072	0.10187328	0.410850633	0.138338598
0.373855359	0.155098664	0.110265101	0.318975058
0.433123633	0.127923639	0.25160494	0.344246321
0.191030109	0.400003882	0.494701466	0.403677644
0.485073472	0.353098961	0.026681642	0.254782363
0.40769608	0.405236739	0.389857518	0.347552302
0.068714851	0.448679898	0.270090348	0.436067541
0.23840665	0.053726365	0.202475674	0.413866243
0.434130634	0.367342882	0.237270185	0.184447323
0.17328395	0.191814475	0.263670558	0.264562728
0.257002668	0.130952265	0.455584877	0.11314431
0.159296627	0.279888085	0.241614968	0.275767217
0.381346515	0.323323413	0.03963746	0.376260362
0.002963817	0.395551429	0.432907857	0.317450821
0.084048936	0.029791626	0.270435247	0.328927304
0.458133877	0.45703535	0.489363728	0.493648284
0.217807235	0.386974488	0.40747854	0.219716706
0.419386492	0.192331454	0.151854768	0.427513818
0.497143584	0.499694111	0.267767934	0.354932218
0.364541244	0.461568007	0.383823765	0.406328287
0.322358834	0.122682775	0.40988541	0.50623459
0.366435554	0.354758043	0.22511099	0.360670247
0.112454149	0.341367882	0.371069629	0.203781805
0.46999003	0.034890212	0.29603926	0.332495059
0.326956706	0.399138719	0.500252519	0.231819853
0.431775651	0.039309136	0.16558691	0.081720173
0.31875075	0.203121588	0.439251687	0.434499296
0.231282695	0.383005612	0.299345154	0.010056233
0.060828617	0.30230967	0.393764843	0.404013511

0.411425456	0.376306253	0.25867806	0.315975788
0.385030806	0.483317088	0.04182448	0.148198708
0.39029535	0.299626919	0.306467527	0.395536055
0.348555376	0.158437137	0.181976394	0.51940357
0.397955766	0.128631847	0.335209879	0.42643384
0.290470366	0.395756728	0.16280476	0.283733532
0.072214385	0.458347795	0.067792671	0.337545621
0.256969203	0.191438668	0.034450591	0.253382387
0.267133005	0.387668454	0.433412641	0.176779518
0.322955819	0.419263736	0.287902339	0.342806168
0.015044488	0.09498036	0.243101091	0.493384081
0.217979163	0.510696555	0.35881684	0.394970515
0.356885133	0.437964865	0.009620119	0.462849522
0.506211544	0.246288812	0.366931726	0.435934979
0.357995832	0.428117631	0.045585046	0.421541527
0.299296876	0.142639625	0.513566907	0.219581335
0.44527531	0.074742505	0.291493517	0.418317974
0.351289687	0.01517764	0.192337088	0.362702725
0.070760818	0.102177104	0.402943725	0.397343908
0.368172054	1	0.493240706	0.341757463
0.413442248	0.280206917	0.035603594	0.397050248
0.414282464	0.336562236	0.416662221	0.269194722
0.277276702	0.455781602	0.378684062	0.185964584
0.073015025	0.309199085	0.027931135	0.366599487
0.379925248	0.127008079	0.363254562	0.248863088
0.126932172	0.516367614	0.255198583	0.310293505
0.188116652	0.331026268	0.311397428	0.196385646
0.193330797	0.233183394	0.367485193	0.379262205
0.050907807	0.136515335	0.051020755	0.30319834
0.122430708	0.272505823	0.256997616	0.334857776
0.297623391	0.121244511	0.378785025	0.373150396
0.188932853	0.037746081	0.494432034	0.425831459
0.263694204	0.210502284	0.088292365	0.262022782
0.444920721	0.234296627	0.227738966	0.426309239
0.064109493	0.495106741	0.246880787	0.180177726
0.355822402	0.293086517	0.462919859	0.064249495
0.363952737	0.438404306	0.401540551	0.244120229
0.352857711	0.396104396	0.04965008	0.35070082
0.327277944	0.061454008	0.513071326	0.195193741
0.052643845	0.427913971	0.369740833	0.136831495
0.1444353	0.300371276	0.0596769	0.358481941
0.506919983	0.412645811	0.355726208	0.323141011
0.064279462	0.012447647	0.31004905	0.412673838
0.475746741	0.502879044	0.342454803	0.35710153
0.299964445	0.336311342	0.136532308	0.498612334
0.315033438	0.502966172	0.020719991	0.399914702
0.475357748	0.236302371	0.127413059	0.28592081
0.341238407	0.082779026	0.285491142	0.471444611
0.422759315	0.359689	0.34713408	0.42713273
0.026777835	0.064680648	0.416168374	0.261953564
0.358356603	0.291436702	0.089915588	0.314040389
0.066313247	0.419766657	0.449897089	0.101087963
0.253247839	0.305456872	0.426379283	0.072044482
0.443210614	0.494641466	0.494074223	0.22905002

0.38681537	0.471362609	0.358835743	0.15589958
0.046296065	0.496447579	0.362180106	0.035140467
0.471283171	0.183080094	0.016089703	0.482335411
0.248707979	0.406364676	0.007299617	0.142673286
0.103332064	0.463386629	0.449019511	0.356844839
0.178422242	0.409395958	0.021230285	0.52327591
0.461607299	0.037560553	0.228454831	0.376704949
0.366446909	0.514071685	0.344639377	0.428789578
0.377300422	0.469672824	0.229497292	0.336898265
0.402193589	0.477006024	0.382168543	0.447808216
0.441628037	0.437525848	0.318800393	0.028320363
0.198916382	0.201555855	0.24423463	0.108188634
0.275006803	0.201086997	0.478453357	0.471744841
0.472079554	0.017080061	0.172932097	0.37802852
0.422266017	0.477858844	0.062747107	0.013899422
0.017583894	0.034534695	0.352996253	0.345406768
0.485009977	0.169508537	0.013739736	0.061353505
0.326936892	0.407367514	0.479849835	0.402957788
0.416682116	0.144275407	0.219518861	0.413494257
0.11522882	0.272540835	0.477634026	0.4439496
0.101001149	0.350589533	0.382122638	0.486473942
0.102282594	0.385146109	0.409027031	0.296131658
0.045180962	0.149788238	0.120165779	0.433300563
0.467713606	0.092198268	0.050167007	0.114265639
0.136172461	0.468093031	0.201400826	0.519637415
0.413543584	0.431734994	0.142609722	0.463380133
0.061598676	0.453331177	0.375276165	0.058917397
0.387617102	0.037029139	0.45761678	0.15210702
0.285372153	0.372637612	0.257290114	0.047280086
0.251615545	0.099652288	0.474993358	0.460863595
0.088928952	0.296350678	0.390304236	0.429748713
0.260467379	0.392409925	0.472660542	0.324105554
0.109282705	0.215750908	0.388815075	0.343982812
0.236003234	0.50164473	0.297881972	0.526586686
0.365163539	0.465335322	0.458585268	0.355012672
0.284757864	0.355051986	0.304562624	0.026460865
0.393559817	0.437745961	0.37604278	0.19583853
0.297150364	0.386457716	0.096640999	0.206076019
0.340351663	0.250960077	0.176321515	0.444808382
0.369708804	0.341386283	0.071690984	0.110214613
0.529043	0.428737262	0.169478495	0.38035904
0.270111481	0.374676647	0.424073225	0.417442531
0.37980787	0.407756522	0.378798615	0.309456829
0.390822527	0.312808494	0.500365692	0.096253914
0.129390765	0.377269523	0.115322216	0.168843014
0.195976337	0.067097386	0.305492799	0.433246287
0.346767855	0.384481577	0.284076968	0.148015459
0.401967134	0.372093049	0.407666989	0.203746146
0.105974112	0.464880909	0.023765693	0.110843995
0.360398439	0.393129738	0.368473165	0.331369353
0.329828399	0.243492613	0.346996509	0.248328054
0.232995265	0.363720698	0.394055444	0.061167612
0.033796733	0.503003276	0.349311191	0.064364659
0.155124243	0.343947455	0.370457669	0.253234745

0.05979328	0.462835909	0.259647906	0.430127529
0.350762731	0.126292493	0.328267465	0.130757418
0.122788085	0.425503926	0.412300619	0.490515522
0.423856578	0.40523391	0.413799682	0.388499895
0.2102416	0.37525439	0.050139205	0.433619013
0.336958054			
GARNL4 0.434274392	0.522612341	0.446992126	
0.479251033	0.509025088	0.385027859	0.309502064
0.250427976	0.1543362	0.014809821	0.053415908
0.481936569	0.288198799	0.345564276	0.355580631
0.503891415	0.242550711	0.419695628	0.369376271
0.484146359	0.152275185	0.503286414	0.183238396
0.393341959	0.193030087	0.148176936	0.366915841
0.486285497	0.166797289	0.336417436	0.426871099
0.204371037	0.455546802	0.501046549	0.498361528
0.490866954	0.394417186	0.028948685	0.338573516
0.419704012	0.479568739	0.47505631	0.444750905
0.099619499	0.459938303	0.356744479	0.48907718
0.277747064	0.079545428	0.260220484	0.421818559
0.451192476	0.4513614	0.298520316	0.253410873
0.252229475	0.258605705	0.319101762	0.331198495
0.342987328	0.183493837	0.467145141	0.153531867
0.19761887	0.338711814	0.270759501	0.352350005
0.414228692	0.387339378	0.049971665	0.416585877
0.007138595	0.473571992	0.486180792	0.346485868
0.106678154	0.055456702	0.306204319	0.42720295
0.48620705	0.445786907	0.499661675	0.498293222
0.291057731	0.431030164	0.452012452	0.293481475
0.43546696	0.272730211	0.186884364	0.511278017
0.506651915	0.511783674	0.325367002	0.397045851
0.427006788	0.474258565	0.457057398	0.494969038
0.380844123	0.118276036	0.424423434	0.518305989
0.383181374	0.388739578	0.273910129	0.43858929
0.13753343	0.393881993	0.46631967	0.243658864
0.485232716	0.048979315	0.342460548	0.434733885
0.34227865	0.457514962	0.506530051	0.305332344
0.494664946	0.056004912	0.193508611	0.089125732
0.406416693	0.208828831	0.44651451	0.473260149
0.304195713	0.463829441	0.375325568	0.011722144
0.075251426	0.300714259	0.43752262	0.449627414
0.477265855	0.355959465	0.337040277	0.380907772
0.45442802	0.492101647	0.058749243	0.18765224
0.478305633	0.304548148	0.377825945	0.481757072
0.428443595	0.203882488	0.239684867	0.529011994
0.411409062	0.175856441	0.373977603	0.509640366
0.385568617	0.465435506	0.210211523	0.386721165
0.109599506	0.460668579	0.076937227	0.390950678
0.298909578	0.242784393	0.0332073	0.323460055
0.355845091	0.421046332	0.432892189	0.213333731
0.428188672	0.504932657	0.348684525	0.440216883
0.027581689	0.111524569	0.285401085	0.513038386
0.282504181	0.507776894	0.429168233	0.429037598
0.421824473	0.494732635	0.007125794	0.473310757
0.513396126	0.297366902	0.47796788	0.495425496

0.408131547	0.480087201	0.050070735	0.510158427
0.352667733	0.17012681	0.522202237	0.281399946
0.447585508	0.102279365	0.376980522	0.443420654
0.426444072	0.021349951	0.258187668	0.451474263
0.073157396	0.147769648	0.465004246	0.48477227
0.454252538	0.493240706	1	0.389400955
0.50634928	0.346419544	0.03933693	0.444178101
0.463557651	0.391390397	0.429949859	0.345682594
0.329405314	0.470024846	0.407325859	0.223610264
0.103396741	0.388459468	0.045262307	0.439933155
0.373715321	0.199383934	0.413196439	0.256049559
0.154830482	0.530792207	0.266665473	0.322884943
0.188148238	0.438987714	0.377072219	0.297874993
0.283494364	0.285431464	0.42438335	0.446822196
0.081059151	0.189966216	0.040446928	0.388407674
0.201768362	0.379533579	0.359907885	0.407814125
0.396060589	0.190956627	0.480769836	0.377037864
0.21524372	0.048386487	0.506990965	0.500957895
0.322549019	0.265841183	0.104579634	0.347254968
0.470718049	0.306399565	0.2671935	0.453000037
0.068910232	0.49559534	0.346830439	0.23139163
0.44928674	0.388808161	0.488609518	0.084917513
0.426021166	0.460337157	0.444731858	0.302768927
0.37685335	0.483760491	0.07559866	0.400710727
0.395831313	0.085350144	0.524165242	0.23298437
0.062581256	0.495244321	0.449978662	0.16454575
0.166952933	0.40619395	0.088129111	0.428439351
0.522988953	0.506638788	0.432449062	0.325054657
0.097780188	0.027939302	0.333535246	0.446567994
0.485739941	0.508951451	0.404501686	0.453105494
0.375174902	0.400276047	0.170803472	0.514100405
0.282840384	0.505891454	0.025721584	0.463658581
0.483212314	0.306932255	0.163351589	0.373843098
0.380241637	0.109746522	0.323012398	0.485479802
0.432450993	0.423226618	0.413554952	0.493884342
0.043047144	0.093927536	0.470500979	0.261930738
0.439830791	0.343523371	0.084089949	0.378510139
0.102006634	0.444251435	0.502454768	0.125562795
0.33861457	0.350599723	0.495692778	0.095312441
0.440499351	0.489635642	0.50580193	0.274601091
0.459248602	0.482694621	0.421846977	0.171896152
0.053161641	0.500134676	0.421846028	0.05370598
0.494830794	0.238773179	0.034782184	0.496840772
0.309386008	0.41121098	0.00909981	0.111634214
0.098652518	0.499303925	0.459445466	0.434159549
0.243302829	0.471189399	0.015182908	0.524261277
0.483117124	0.044618031	0.248026501	0.439432793
0.435424989	0.524242458	0.388616235	0.454113888
0.456810547	0.480234787	0.325083302	0.406383091
0.450577337	0.476751766	0.456055939	0.453531722
0.486286974	0.509046509	0.405407941	0.044728131
0.197935817	0.264118111	0.311312497	0.139757747
0.332170276	0.246227762	0.480170533	0.497991015
0.483252456	0.020600205	0.229620752	0.434455994

0.442880506	0.487484718	0.053065357	0.013116508
0.014758353	0.047224745	0.433301956	0.380272793
0.490104803	0.239161949	0.019519734	0.089253025
0.392035589	0.473004039	0.495886249	0.489260348
0.467926783	0.174914461	0.314034036	0.492250689
0.140167924	0.313798081	0.49268132	0.4638076
0.102276723	0.382472161	0.428679778	0.502624326
0.099984244	0.456530345	0.481449741	0.402584382
0.038462727	0.170988166	0.140742552	0.505416092
0.473967525	0.147056274	0.080749871	0.1566129
0.206432548	0.478132591	0.278417675	0.529676464
0.46454401	0.506878276	0.155547181	0.471909695
0.088065089	0.469153795	0.43649959	0.07779699
0.414903786	0.04156249	0.457311655	0.19244971
0.369880642	0.437264212	0.264534864	0.050248703
0.238047131	0.125652303	0.49092714	0.490656163
0.084853768	0.351077766	0.437988322	0.490172252
0.336964476	0.493372521	0.484921546	0.39455071
0.158781752	0.203244701	0.470653858	0.408466145
0.308595213	0.507960618	0.380074192	0.528854007
0.425399188	0.459670755	0.466966133	0.437648607
0.33487794	0.423676835	0.400917599	0.047801412
0.469138539	0.493152919	0.427999635	0.24982288
0.382389921	0.427510696	0.104452356	0.212386274
0.369576966	0.326274505	0.249044609	0.506330487
0.430819793	0.407939585	0.08682391	0.13266796
0.538736357	0.42795937	0.214019009	0.467583358
0.3045109	0.454555398	0.481065203	0.486295018
0.411989083	0.447559859	0.45794691	0.406459104
0.414754356	0.384707608	0.510653472	0.123617013
0.152016477	0.432510915	0.177816767	0.191443759
0.261475685	0.074386176	0.334471271	0.511088262
0.398034103	0.488690735	0.343426285	0.167557602
0.456579741	0.395776278	0.483865677	0.28544772
0.15709937	0.482206947	0.028404582	0.133201875
0.423592055	0.453841806	0.444493338	0.381726659
0.368042081	0.248265715	0.435895291	0.321618447
0.329627156	0.405718889	0.445056919	0.075874454
0.052791927	0.511714772	0.404572952	0.058220548
0.156326573	0.387868708	0.401911396	0.329269564
0.061254768	0.470995815	0.341145749	0.494087659
0.428739972	0.149527354	0.33154467	0.148289723
0.129764347	0.435249092	0.454250375	0.498499762
0.445511373	0.503808903	0.417604905	0.43983462
0.20338494	0.397474009	0.071533241	0.474679357
0.406276489			
GART	0.265615874	0.424073466	0.193205354
0.28686888	0.192794168	0.167246267	0.06639476
0.033551032	0.088485572	0.002054934	0.037858913
0.23056188	0.082920658	0.059426194	0.138510004
0.310598754	0.063008061	0.087471822	0.089518727
0.223874543	0.021334267	0.270929006	0.037528622
0.156402466	0.088125164	0.052061645	0.194637913
0.308162125	0.055214975	0.096262437	0.18947548

0.043426012	0.272885572	0.420864833	0.28732005
0.413575371	0.253612621	0.000896802	0.097342703
0.279512241	0.365929937	0.233595717	0.15540585
0.022039084	0.35094604	0.104753114	0.233246071
0.055915401	0.028340155	0.046329665	0.316717071
0.380011253	0.254522233	0.053875189	0.058841876
0.096885151	0.037033884	0.150009951	0.125991381
0.078240288	0.02528898	0.396915169	0.055819901
0.056669722	0.052847051	0.064371467	0.058782514
0.178820039	0.215897731	0.004816048	0.225239873
0.000154895	0.2086587	0.19434533	0.224227012
0.025851773	0.010350592	0.072689378	0.15624066
0.3333379	0.350597121	0.417679436	0.44178626
0.066584722	0.109449356	0.209000312	0.066873395
0.324402923	0.06097492	0.034598554	0.321006294
0.42960237	0.430918351	0.114632576	0.149740628
0.329608717	0.35666297	0.16693237	0.172602161
0.205473353	0.043901875	0.35022259	0.427434731
0.247133736	0.167588322	0.082539556	0.232342317
0.029313479	0.087144826	0.194331949	0.036968953
0.350181235	0.016320308	0.108727033	0.136687567
0.067250564	0.29326284	0.447152799	0.066908301
0.157816022	0.008392536	0.162790917	0.017800894
0.085250163	0.106470656	0.374461519	0.344457703
0.041548641	0.143333622	0.077552001	0.002494124
0.018966731	0.071519879	0.291376921	0.095944387
0.274333053	0.311844065	0.136313674	0.219430665
0.30203227	0.413726722	0.015505027	0.034976936
0.182203387	0.072286059	0.116094318	0.213285816
0.162715155	0.029999331	0.043738402	0.442328468
0.290241881	0.033880431	0.066167872	0.280485293
0.121049092	0.203950417	0.035216243	0.07138615
0.016614755	0.417325148	0.02238168	0.075023837
0.222163304	0.064729804	0.003020247	0.053756951
0.078208813	0.354333994	0.364476296	0.039429341
0.083670352	0.266805019	0.075403597	0.276916313
0.01653055	0.017960803	0.051444075	0.405913748
0.100334678	0.415839458	0.086652138	0.137281598
0.149699882	0.346942922	0.002657699	0.385633569
0.449643153	0.05632165	0.193056037	0.215281722
0.226223576	0.347525576	0.008067503	0.249435646
0.159090319	0.032182516	0.425327401	0.080619964
0.347471171	0.031765381	0.068732992	0.412930261
0.303666543	0.00428381	0.049312019	0.212776291
0.023627421	0.024169702	0.260635974	0.243800256
0.237860402	0.341757463	0.389400955	1
0.192420866	0.05603571	0.007901381	0.1659905
0.320496321	0.141475889	0.325955481	0.072515031
0.109792047	0.375584122	0.319430931	0.110404045
0.024599191	0.083984295	0.005821622	0.190848399
0.329344592	0.039271996	0.228422962	0.043705166
0.043389536	0.445436453	0.126649701	0.157201412
0.113557622	0.164816455	0.074260918	0.066952993
0.086053046	0.095509864	0.203825104	0.172610021

0.01087715	0.041360916	0.01230858	0.103231073
0.04576712	0.092667302	0.082913699	0.214051028
0.069459405	0.032225769	0.157306145	0.170611396
0.081083171	0.011140558	0.410472087	0.241220231
0.161968587	0.066642411	0.020836947	0.088928952
0.361720508	0.115044266	0.050810506	0.343994772
0.010559756	0.408230712	0.089343853	0.070140306
0.132529516	0.098152973	0.365127456	0.037638573
0.142384949	0.343865859	0.136266433	0.11917802
0.257698203	0.220203138	0.013244366	0.24838188
0.219229395	0.017694701	0.433913642	0.060128485
0.014734074	0.309152982	0.355915388	0.047213101
0.053354735	0.130367643	0.011282857	0.18170606
0.433546499	0.297940972	0.321461091	0.130991701
0.021743387	0.004018451	0.102797672	0.189319663
0.386832887	0.436837462	0.100962376	0.167268987
0.107168766	0.211876828	0.024459697	0.431094163
0.06948557	0.406924126	0.004701634	0.385515983
0.418669307	0.060570658	0.066340439	0.109247388
0.240650924	0.028181807	0.050711735	0.391205764
0.357990008	0.18081175	0.188471322	0.223365137
0.005930201	0.013861165	0.258590441	0.145526118
0.15052778	0.117141845	0.05883499	0.078679319
0.0235561	0.342876052	0.326383451	0.073742138
0.146538291	0.073996046	0.229062105	0.021073824
0.434206475	0.420296489	0.41142377	0.039925462
0.288930558	0.436627102	0.267631435	0.043024965
0.016207266	0.431480953	0.209923144	0.011292418
0.363553251	0.047675534	0.0067768	0.380201754
0.062426985	0.314515201	0.003321459	0.040869744
0.031802513	0.376090156	0.396892917	0.184556726
0.055998567	0.160764125	0.004310438	0.440000748
0.382891835	0.01774851	0.09637456	0.324649001
0.350211343	0.405991754	0.199156472	0.411854759
0.254284473	0.416749237	0.068776366	0.160071495
0.330488975	0.44013072	0.25417952	0.346731928
0.334514321	0.283902842	0.142820332	0.011286301
0.20416317	0.041970648	0.13352084	0.093357126
0.119692728	0.101647035	0.370389949	0.37361782
0.410737928	0.004677163	0.034190082	0.304651929
0.300353871	0.361046287	0.011435193	0.007479318
0.004100742	0.036373897	0.137006079	0.114480723
0.422199456	0.053263662	0.003295275	0.009106986
0.088912677	0.2945246	0.406299897	0.232619347
0.341779259	0.036249455	0.092240973	0.239639704
0.079966307	0.150448773	0.418368521	0.361206612
0.022154547	0.128354318	0.090757023	0.38976134
0.090637555	0.158847395	0.232173487	0.113662487
0.006000802	0.060136822	0.072375259	0.322865254
0.410975311	0.038379954	0.02223414	0.024125414
0.045912971	0.365522789	0.061058357	0.431431063
0.308521351	0.321852807	0.050226248	0.397905234
0.044089273	0.375728283	0.187466985	0.018345747
0.085457516	0.006876018	0.370455317	0.180995236

0.06282102	0.190781804	0.2317269	0.015834123
0.158011305	0.030527048	0.405552998	0.403176429
0.024318624	0.07764905	0.129190333	0.206789211
0.077421967	0.303587238	0.392466054	0.162628907
0.033039714	0.065834112	0.219954269	0.184256715
0.04362292	0.416466432	0.174552054	0.442778333
0.239951847	0.3603329	0.389096787	0.177374313
0.118926416	0.16124469	0.19778605	0.006684348
0.146363641	0.356362929	0.103759266	0.121582342
0.122909635	0.23359562	0.017033067	0.029713089
0.189267659	0.109471617	0.048892977	0.329633219
0.160245791	0.202093351	0.007119169	0.070789413
0.435997848	0.338619741	0.048030243	0.258349684
0.068841808	0.213951112	0.338392636	0.349301236
0.154427237	0.335306854	0.256167625	0.197903932
0.296999661	0.10645304	0.427468373	0.011257412
0.100388904	0.199289633	0.047566058	0.053221002
0.055057209	0.025278689	0.180885037	0.273919738
0.148913433	0.206583143	0.061318154	0.043870709
0.156885001	0.101958108	0.178935233	0.053347983
0.03796203	0.403900636	0.007681276	0.027759547
0.126395268	0.364612961	0.22285625	0.103475271
0.188655753	0.073547966	0.130687835	0.067284487
0.079678206	0.111297645	0.361705514	0.012911837
0.005515103	0.399372889	0.100626698	0.014734893
0.057064589	0.183561525	0.068413015	0.146557076
0.02114007	0.423814717	0.098031548	0.222632286
0.126736062	0.026112862	0.215583544	0.030908924
0.023647107	0.312354271	0.233161389	0.426254279
0.132597251	0.303604341	0.320861527	0.296499542
0.037517686	0.173432915	0.014735286	0.310960521
0.15105059			

GAS5 0.332557039 0.497273096 0.206147268

0.393626153	0.25575699	0.213760224	0.159802813
0.112059737	0.092593785	0.012705627	0.043689463
0.296263237	0.175754625	0.171443054	0.165575515
0.408207181	0.148261937	0.183799675	0.193699779
0.340223671	0.079211645	0.375807562	0.1043132
0.201300087	0.126918416	0.096831602	0.201640362
0.443312205	0.107739065	0.148651378	0.305031436
0.116338449	0.381706792	0.463512085	0.361623974
0.453321923	0.293020384	0.014998765	0.163452475
0.373160815	0.388336232	0.345800157	0.25132075
0.064403916	0.414620804	0.186531289	0.278226957
0.12022081	0.060583367	0.110886009	0.379247777
0.41548245	0.262540455	0.136268294	0.136392521
0.146305712	0.111768331	0.170909362	0.209080732
0.182141895	0.078412872	0.433257684	0.089465849
0.097493163	0.155597292	0.152678156	0.167771127
0.205197706	0.295506226	0.028864687	0.250151817
0.004823473	0.320682462	0.267031816	0.17455333
0.072038918	0.043065897	0.170455089	0.245527416
0.462126874	0.408161609	0.503930248	0.493527519
0.172088274	0.219155626	0.217540271	0.16534061

0.398493895	0.148614342	0.100587521	0.423757501
0.507567537	0.483141666	0.194625879	0.213331054
0.380071131	0.444598881	0.210758867	0.23856586
0.19266097	0.07219429	0.391470257	0.505806493
0.33593746	0.200073553	0.164574421	0.36157647
0.085289087	0.171849194	0.300806934	0.127176373
0.475470094	0.0324523	0.193542943	0.21858708
0.167025669	0.378137041	0.481045466	0.165038203
0.238714719	0.039252005	0.120427342	0.055886235
0.198277744	0.118786183	0.399735693	0.459373078
0.144793515	0.237218672	0.184102101	0.009410963
0.055032341	0.152013555	0.379478426	0.202681759
0.363728029	0.324027496	0.189516524	0.29550166
0.31599465	0.49549282	0.024861992	0.100553597
0.242428201	0.156305005	0.203065752	0.275500911
0.235103564	0.104771664	0.135333765	0.517684329
0.37345059	0.105496648	0.155613391	0.394718486
0.190387948	0.247566233	0.119652974	0.202519401
0.066571207	0.42974797	0.054301099	0.171564864
0.159049554	0.146665131	0.017862621	0.14509221
0.170767841	0.401751323	0.386622345	0.121015232
0.217123741	0.337553337	0.147451066	0.341151534
0.02306395	0.067300729	0.115468749	0.499854295
0.165050714	0.494166897	0.212834822	0.216785657
0.225829139	0.437186581	0.004089549	0.433432677
0.485206563	0.12163473	0.277350836	0.292957818
0.198922352	0.446578934	0.02390821	0.380312691
0.239091932	0.097446438	0.520979303	0.168889053
0.408893343	0.071206495	0.171958197	0.405766158
0.343893653	0.014391392	0.114361435	0.314963531
0.050011768	0.072467344	0.347144632	0.333878963
0.255250206	0.413442248	0.50634928	0.192420866
1	0.169564582	0.026821213	0.231104899
0.383772777	0.184539051	0.394041497	0.176184908
0.169994931	0.440425383	0.354513014	0.101147693
0.066384712	0.178956425	0.029023862	0.229209816
0.333759503	0.116439848	0.194148784	0.129773961
0.100266261	0.514885792	0.151998173	0.167632523
0.109253014	0.243884175	0.188034487	0.157953352
0.163118145	0.166547184	0.220400656	0.214577303
0.047646479	0.120456004	0.024423352	0.184389407
0.122728569	0.190556325	0.174477262	0.295048373
0.19311724	0.118640073	0.249236334	0.187585588
0.132085161	0.035809987	0.483871308	0.348353472
0.237817748	0.143620635	0.067475483	0.1622895
0.464639471	0.145486276	0.106656321	0.434613
0.037415846	0.490107494	0.188362987	0.145130357
0.230522164	0.209331369	0.469753393	0.04186469
0.218420137	0.428218467	0.220533323	0.175668393
0.345394071	0.315374477	0.048957787	0.316576691
0.310061784	0.059691348	0.503245453	0.140577294
0.043857391	0.370941251	0.364751011	0.103794215
0.105270317	0.205305938	0.046003067	0.247823926
0.504533041	0.406204307	0.363279686	0.173388245

0.065839121	0.023402429	0.182767904	0.216542493
0.443308671	0.491099995	0.210386164	0.238922807
0.198483741	0.208808482	0.068160556	0.501999001
0.130545166	0.470217182	0.018926272	0.39553556
0.445654837	0.131255838	0.110022669	0.176310489
0.331574251	0.068795466	0.155780452	0.452521064
0.406300375	0.215977294	0.205903198	0.329210911
0.027077789	0.05272641	0.254180647	0.145030672
0.210282469	0.184383954	0.052454578	0.166208984
0.068641495	0.41663812	0.452031341	0.08216998
0.179878449	0.149605151	0.314391099	0.06201046
0.434558395	0.459006596	0.470043707	0.136639278
0.377109698	0.46656924	0.250326829	0.102948118
0.03925579	0.46746079	0.288777698	0.033838834
0.485802012	0.097709205	0.028548352	0.467393093
0.162585274	0.362575136	0.004911393	0.04920005
0.061655262	0.464713113	0.423530263	0.291607963
0.104299008	0.261480012	0.008066257	0.503948803
0.455784537	0.035306303	0.146244458	0.382053319
0.359867383	0.490320459	0.204789474	0.43918491
0.289374807	0.446065991	0.170422754	0.219465611
0.415735536	0.450667189	0.357636225	0.420232056
0.460244478	0.367646532	0.221530302	0.032708468
0.114296032	0.11734002	0.147074907	0.082394731
0.182464837	0.151016861	0.453850698	0.476193183
0.477140268	0.016188654	0.11159028	0.37438444
0.422766564	0.476023162	0.029229867	0.008603342
0.007433462	0.039416749	0.22538683	0.204150447
0.473811926	0.1057372	0.015090533	0.0481158
0.192474208	0.304900245	0.467521349	0.357562087
0.39364199	0.104279767	0.157718003	0.364204447
0.094074805	0.208229887	0.488174615	0.430377696
0.060871341	0.20149508	0.188348952	0.492263313
0.061503547	0.243212536	0.314985494	0.218161216
0.021185089	0.109686189	0.09208609	0.427232571
0.436401729	0.099849217	0.061439195	0.073116715
0.122151673	0.458427722	0.160768944	0.522442766
0.415630157	0.456911487	0.095859647	0.437221789
0.063465291	0.435612158	0.230106525	0.035879511
0.201200282	0.026932212	0.427491753	0.1127454
0.167358541	0.294005805	0.140424633	0.035782604
0.126983794	0.08457378	0.493644407	0.465511698
0.053140677	0.168934348	0.22357623	0.309976868
0.150465112	0.406357272	0.446219454	0.177506419
0.09070865	0.104031946	0.346758232	0.274418821
0.143888209	0.509233603	0.203246169	0.506010427
0.314177237	0.42990521	0.447545177	0.223405268
0.188429012	0.228626491	0.279982245	0.032469348
0.231914254	0.464460221	0.189935381	0.154089238
0.214627777	0.215868482	0.054861568	0.083441262
0.181998649	0.173352698	0.146220453	0.453469202
0.257259681	0.288803908	0.044813299	0.089025883
0.508868304	0.39363359	0.105450525	0.330348938
0.165960586	0.232860984	0.410120834	0.413976243

0.185771711	0.420653901	0.380034317	0.292509415
0.381057385	0.194564381	0.476155541	0.054001721
0.110818304	0.261565444	0.111619025	0.116696743
0.152326169	0.051272292	0.172701773	0.389534178
0.192812359	0.317889748	0.145826077	0.096572385
0.233219622	0.168271772	0.260350389	0.144335241
0.101684187	0.449463784	0.019580651	0.084159231
0.207319109	0.393394762	0.228905787	0.206833804
0.191094786	0.136270161	0.228522865	0.139167221
0.154746504	0.193613115	0.390647475	0.040458372
0.033158272	0.510354095	0.176215966	0.036379433
0.083190677	0.229361882	0.184572552	0.180662933
0.042989998	0.452455364	0.151444165	0.250022153
0.204846594	0.086303039	0.2897922	0.07657811
0.068650471	0.397255438	0.235841221	0.467940624
0.212382475	0.420730343	0.37071576	0.384489993
0.094634816	0.181167913	0.045554008	0.422639778
0.190150974			
GBA	0.230518816	0.280215024	0.121997946
0.331002729	0.180226524	0.186854313	0.223305507
0.201608422	0.088872047	0.032597861	0.022516599
0.17790199	0.075310922	0.140147069	0.077240906
0.339938644	0.092616688	0.143225634	0.273736418
0.338342603	0.170444868	0.296147842	0.181573567
0.076237725	0.061183518	0.09717792	0.067298693
0.377384032	0.108487601	0.13569263	0.335446065
0.121793093	0.285864697	0.238198317	0.202182321
0.214793322	0.19132137	0.057124527	0.094349245
0.291830015	0.22880484	0.342676367	0.283478651
0.121854289	0.23549558	0.24591846	0.189464714
0.123201811	0.060115405	0.116892364	0.242025763
0.196562866	0.141407817	0.186698477	0.16061394
0.040302205	0.12301084	0.031409125	0.249501145
0.224236292	0.123307744	0.209108412	0.111512791
0.078160892	0.207889423	0.106038593	0.229355616
0.05907339	0.26609279	0.14650188	0.130851817
0.01713818	0.361655763	0.214388221	0.03636211
0.102392966	0.055261296	0.117975942	0.280924707
0.355400214	0.23981663	0.290796757	0.260397674
0.100020725	0.188388203	0.107631622	0.148012672
0.271685269	0.162920687	0.143399998	0.31648396
0.284835988	0.240198164	0.109761846	0.169767131
0.228603408	0.28761282	0.125449342	0.131656958
0.048848101	0.060898672	0.215785963	0.27384213
0.256606174	0.06601185	0.124867574	0.336784897
0.137264017	0.101241639	0.31979984	0.101810954
0.360685771	0.083798267	0.125545107	0.231660621
0.182226002	0.266591575	0.22735174	0.237223069
0.161460029	0.066188222	0.0280126	0.107126132
0.124304416	0.029058212	0.195110641	0.307297408
0.177552104	0.087201024	0.21076846	0.030363539
0.068700429	0.121330229	0.219524795	0.197595436
0.297380986	0.169097576	0.09116459	0.222785492
0.146523786	0.284620559	0.023905706	0.202661156

0.191761221	0.126393456	0.272992791	0.172424602
0.135941818	0.231033296	0.180888228	0.300958044
0.27197958	0.164773093	0.131571467	0.324344774
0.081807471	0.150488254	0.198303914	0.145519184
0.148208967	0.194983484	0.057947729	0.205438064
0.035496023	0.064244285	0.10257117	0.225282305
0.119639377	0.204158205	0.185510211	0.186724124
0.170749039	0.238562943	0.18267637	0.193918301
0.022266972	0.156296939	0.130178851	0.278717439
0.192756896	0.279447185	0.17493872	0.119965063
0.202706407	0.320063673	0.006081449	0.22885251
0.23470034	0.116980426	0.188898036	0.213688453
0.068856456	0.312773296	0.023439558	0.321689935
0.207907375	0.180395473	0.320071191	0.138508091
0.231488912	0.087386748	0.139383272	0.186718954
0.154390968	0.034490282	0.160446581	0.330865403
0.054011298	0.148868848	0.285967361	0.297098571
0.192490953	0.280206917	0.346419544	0.05603571
0.169564582	1	0.07558442	0.159150594
0.246576392	0.052328325	0.252218272	0.192324787
0.15890764	0.264455411	0.18313304	0.040514215
0.124366366	0.223332105	0.079404336	0.150464985
0.188007276	0.120354283	0.09095317	0.141410244
0.089464141	0.279817279	0.048310444	0.039847953
0.02681341	0.164751136	0.268575819	0.079569883
0.05157264	0.138300449	0.069401715	0.086432188
0.139323356	0.123056629	0.038956831	0.046353873
0.052485383	0.060892738	0.058039767	0.20967572
0.124184225	0.099257991	0.143817643	0.071802981
0.089888814	0.070382727	0.27412328	0.32577092
0.216554632	0.184932464	0.113958008	0.045940627
0.292601264	0.114326472	0.092319667	0.27559619
0.108881096	0.297792749	0.096796541	0.114994368
0.072787875	0.217164695	0.355037088	0.045044169
0.217491772	0.261791326	0.227192794	0.100536524
0.221548285	0.288165237	0.122369617	0.221263786
0.21458113	0.097239304	0.26036332	0.090151079
0.078125722	0.220730317	0.172248465	0.124714443
0.100484052	0.132271655	0.156783161	0.231766208
0.280767982	0.308127443	0.202979045	0.074874934
0.106988753	0.024220992	0.094558999	0.13568425
0.229938413	0.240089248	0.250298706	0.10438575
0.294472443	0.06264371	0.12348307	0.265601246
0.102596076	0.254423915	0.061544	0.194709588
0.207060468	0.163240826	0.070907849	0.216530191
0.240659136	0.133641303	0.226126051	0.240120339
0.264398272	0.09849265	0.073525588	0.259152977
0.104482019	0.151007923	0.169630682	0.041663017
0.084145129	0.143652637	0.011449575	0.142189019
0.092933231	0.246105298	0.320198932	0.070747696
0.124879065	0.132396072	0.243611401	0.123710791
0.19444025	0.227990945	0.244582889	0.238388158
0.226155569	0.220969592	0.092747811	0.12058446
0.055037748	0.222838511	0.260625264	0.052365341

0.331806398	0.138607842	0.034818759	0.289628256
0.251567789	0.196307064	0.024910169	0.029914025
0.065140056	0.287995612	0.218204516	0.331025003
0.060704581	0.31563522	0.012805792	0.264469376
0.246984109	0.039991172	0.072947604	0.190601987
0.151043508	0.289258321	0.060504844	0.204272586
0.129234385	0.207210183	0.133013623	0.212457643
0.267137511	0.197655506	0.306341666	0.275384821
0.314026344	0.252413484	0.288261326	0.076250423
0.042606924	0.170880839	0.04093969	0.042390689
0.217388228	0.086096903	0.270871246	0.312655597
0.240249634	0.044012883	0.200115839	0.214762346
0.334510103	0.352998767	0.074652569	0.013062454
0.011614469	0.016992635	0.281755396	0.151799157
0.258777159	0.050811104	0.050386546	0.182252547
0.157490358	0.15416847	0.243383179	0.368679656
0.230856672	0.146276018	0.060873829	0.373718497
0.055805587	0.181349298	0.257017146	0.257805109
0.102668816	0.137150063	0.167891808	0.331946364
0.008664374	0.229213164	0.193409985	0.100866972
0.051730413	0.086659166	0.075423548	0.317386709
0.197157106	0.062818671	0.050692806	0.073423969
0.123836517	0.295274977	0.136271698	0.306786648
0.311852724	0.364099042	0.069076426	0.244995526
0.058397699	0.219170781	0.099396228	0.04603936
0.149398603	0.088395914	0.213648272	0.021915616
0.132616119	0.296556751	0.050322859	0.048118373
0.032487812	0.098397921	0.304033072	0.260929036
0.063697086	0.196459503	0.205884217	0.297917706
0.234598634	0.286808703	0.236201695	0.095884856
0.152623348	0.043341124	0.35422169	0.225563149
0.17571649	0.303166071	0.069048622	0.274943033
0.256775445	0.285086707	0.23517302	0.232830214
0.060707974	0.208264847	0.237763084	0.076768508
0.275827784	0.291330333	0.224644181	0.063186909
0.128385427	0.095275722	0.159149026	0.088183411
0.05841999	0.212533794	0.144796866	0.368238081
0.268559868	0.232001125	0.183052439	0.071284257
0.282311715	0.25985253	0.165351824	0.246943145
0.168155065	0.182876478	0.247341087	0.232646279
0.078327564	0.229805613	0.316343016	0.225165642
0.254874843	0.252119534	0.245505443	0.177981728
0.051213053	0.165388167	0.092709403	0.114338376
0.174603696	0.07066886	0.033546524	0.309403562
0.044354743	0.268241817	0.10802134	0.11934187
0.183563497	0.128700033	0.251136021	0.161916851
0.106460582	0.227829509	0.034757762	0.117974763
0.057639382	0.221317294	0.090788803	0.138398086
0.046675814	0.091279656	0.220307659	0.178578467
0.065910172	0.07732586	0.219309027	0.142613949
0.126902637	0.322627506	0.159846307	0.060825046
0.069961017	0.191465415	0.162228962	0.091431321
0.047754473	0.204330715	0.088142301	0.162143348
0.268998194	0.164776843	0.225939877	0.115257324

0.175830125	0.247604497	0.131674417	0.223275803
0.127164236	0.325787734	0.219180132	0.2286228
0.124498881	0.107361131	0.125089067	0.330921314
0.052521481			
GBP4	0.018784849	0.013266005	0.020270897
0.039824507	0.032471675	0.009823297	0.066504233
0.027041627	0.016885336	0.008596257	0.009799205
0.021561981	0.005779356	0.016485888	0.0048927
0.063479247	0.076206578	0.025901481	0.122825299
0.04787191	0.034241967	0.067980699	0.091417391
0.027447311	0.070635032	0.114252716	0.030136031
0.041170894	0.135983745	0.006579908	0.030060642
0.137240379	0.022548234	0.00843621	0.028624888
0.007040765	0.012159723	0.025148272	0.007062869
0.018326053	0.024868395	0.044722909	0.074161734
0.076279403	0.006613952	0.076439421	0.046715244
0.004902469	0.070845106	0.008136391	0.007658145
0.005219762	0.010633799	0.032779724	0.054043458
0.001783805	0.007755603	0.004080734	0.012669562
0.084356874	0.009546668	0.006954024	0.015298126
0.003994455	0.04421039	0.100770274	0.047613453
0.01412202	0.014481507	0.077734196	0.012393333
0.00232604	0.062821367	0.069827369	0.006936363
0.250634408	0.024989892	0.12283569	0.046562848
0.034188966	0.011301963	0.028532793	0.021067659
0.029882297	0.107082505	0.022243625	0.081301201
0.017116964	0.052230202	0.024703799	0.064528644
0.025672651	0.009857367	0.046176201	0.120651661
0.012771225	0.013541353	0.011725905	0.017998619
0.005455502	0.103350599	0.007702372	0.018112548
0.011774114	0.022440849	0.145192183	0.025843231
0.225639701	0.004846555	0.078097149	0.015505003
0.03465887	0.065769758	0.120246921	0.0327001
0.04480382	0.019613451	0.009950592	0.113303388
0.035596401	0.040222511	0.004063917	0.235373681
0.004280409	0.015055483	0.004423135	0.024164272
0.014924497	0.007836695	0.05893361	0.137531077
0.157809354	0.03287177	0.013200658	0.037548715
0.032025278	0.008140934	0.033052603	0.010040905
0.012446991	0.028472329	0.000181945	0.103166752
0.023840154	0.057181391	0.157898745	0.015222219
0.056685093	0.088809535	0.135616611	0.021323807
0.010206942	0.169038172	0.012384791	0.067516972
0.004810811	0.05426611	0.162424324	0.035711423
0.068908739	0.006742527	0.189179116	0.027086723
0.002130846	0.0202173	0.078790857	0.030073687
0.005697427	0.009384262	0.004451731	0.215627169
0.041455732	0.051958129	0.013272689	0.011779148
0.008354316	0.080360948	0.006372988	0.016593011
0.007277703	0.021301202	0.05316211	0.05273692
0.117288135	0.037481633	0.010309814	0.007019012
0.009925268	0.005080809	0.029252584	0.041446128
0.009323405	0.02828753	0.002060236	0.062280537
0.00968116	0.220123695	0.030011963	0.133574022

0.009350121	0.154938727	0.01508017	0.015313304
0.008858616	0.015995986	0.013749349	0.039638257
0.164547262	0.020496114	0.028143116	0.042435523
0.028849036	0.035603594	0.03933693	0.007901381
0.026821213	0.07558442	1	0.079258738
0.023677227	0.001400311	0.009143487	0.039297762
0.006509843	0.012125131	0.008963807	0.000667028
0.138234871	0.031729721	0.019094985	0.043814002
0.009420153	0.028122628	0.009157309	0.06257741
0.155718611	0.016229373	0.044116862	0.016889069
0.022384609	0.012584623	0.103347117	0.007795775
0.005871245	0.075537823	0.014399994	0.01275546
0.051220511	0.123858259	0.076120229	0.003058117
0.005066497	0.004990733	0.003553554	0.010301688
0.010159935	0.015993049	0.031418977	0.02119644
0.094349689	0.254274962	0.01359289	0.064190483
0.0077876	0.075631006	0.249876221	0.000526049
0.020946093	0.004204042	0.00449514	0.014091068
0.074885158	0.027587716	0.013127706	0.141786278
0.013086409	0.084068533	0.037324088	0.00257902
0.018689982	0.010224546	0.105191414	0.069777813
0.006729633	0.037721469	0.033029286	0.010823805
0.00985141	0.191632749	0.013442704	0.081367333
0.277197748	0.032611683	0.012007787	0.238773622
0.166653843	0.018833272	0.032935206	0.122615242
0.015748612	0.063529551	0.010888125	0.057805044
0.101558698	0.010303173	0.09974306	0.02279711
0.006825826	0.01341598	0.15026245	0.023310327
0.140895917	0.010546898	0.002488852	0.016288373
0.026038974	0.010474086	0.238667774	0.018986723
0.006232292	0.009623722	0.102485023	0.026822878
0.009454897	0.139469862	0.090451881	0.008963219
0.016036522	0.02494734	0.010974963	0.038451497
0.047849301	0.040807588	0.035442463	0.024790659
0.008487201	0.068181206	0.007707467	0.011210562
0.04300863	0.009424587	0.038899363	0.052700649
0.005172825	0.01168315	0.077915966	0.196653986
0.013894903	0.011106117	0.008520405	0.094461995
0.017403104	0.01111301	0.008816669	0.234502642
0.243307312	0.008563586	0.019155877	0.012048708
0.032927697	0.00677356	0.010537415	0.013564492
0.139578946	0.004459633	0.006113951	0.010036983
0.138064766	0.036953655	0.006879554	0.033597385
0.002008269	0.071371828	0.025306454	0.016058636
0.009535472	0.141092008	0.052444999	0.010645231
0.011216894	0.012780179	0.017008128	0.014722658
0.012244657	0.0067711	0.008422923	0.114945384
0.016925095	0.008597773	0.025990535	0.012219125
0.027914413	0.049150301	0.096925802	0.077051152
0.00896957	0.0199862	0.001586868	0.005962475
0.011560733	0.058842903	0.014742187	0.033370794
0.016305504	0.259792501	0.025088352	0.011648688
0.022131816	0.029708163	0.093914302	0.008319702
0.005162241	0.016825483	0.037569097	0.120013476

0.015369643	0.000534116	0.164345017	0.038422302
0.017809327	0.0327724	0.009827953	0.084897016
0.023972451	0.192238392	0.003213515	0.069109307
0.041990575	0.006961436	0.017927502	0.00948408
0.175427958	0.097740013	0.022018455	0.026918734
0.00552626	0.11028463	0.021885966	0.009821654
0.060040861	0.134580152	0.09999239	0.048381097
0.005863991	0.024266784	0.037922889	0.001254043
0.042941938	0.026022513	0.044502577	0.023937216
0.024277385	0.052161955	0.071099339	0.009334054
0.020854496	0.007060773	0.034232298	0.001795339
0.06773033	0.163044258	0.009684145	0.001604711
0.012184636	0.025674354	0.005906273	0.182529805
0.009841759	0.183060018	0.02855719	0.029177884
0.136444721	0.049196135	0.105964216	0.053227851
0.024624961	0.033070169	0.007528612	0.004263988
0.054435734	0.010668241	0.041131963	0.013060173
0.020862958	0.030418352	0.01088744	0.016280198
0.018807359	0.0177652	0.012219906	0.041177446
0.010765076	0.014908557	0.01606884	0.011913964
0.071542514	0.027984107	0.031544357	0.020880555
0.060690458	0.015078239	0.054045759	0.003539686
0.011710807	0.012605278	0.035174706	0.055539635
0.024833167	0.013693383	0.04324891	0.086191879
0.013450703	0.01183288	0.014028221	0.020874121
0.182488125	0.049648581	0.025626618	0.027895768
0.007129525	0.012850353	0.02484087	0.012564295
0.009071463	0.088193436	0.009011309	0.011518709
0.01973928	0.013377428	0.03297984	0.235325097
0.141985056	0.12269052	0.005008045	0.057383401
0.00499183	0.057324148	0.003809505	0.038166235
0.087621321	0.014022354	0.081034257	0.035589776
0.036236771	0.007588516	0.036401944	0.228189577
0.007992364	0.016696608	0.015532054	0.089344836
0.00847222	0.074138944	0.088151459	0.011854891
0.003050212	0.002354417	0.014672711	0.044132103
0.077930005	0.030989092	0.013643672	0.113941503
0.02527596	0.012201082	0.03936242	0.012888957
0.194297256	0.009726754	0.004435296	0.027846682
0.040775785	0.197993521	0.007041287	0.014479108
0.091177047	0.008907818	0.025049118	0.008822255
0.044799495	0.053880393	0.005824044	0.012835905
0.025123285	0.011921634	0.080424945	0.029473199
0.00361575			

GCNT1 0.299993187 0.357086098 0.217880155

0.318260577	0.250839697	0.142975317	0.122743532
0.084694059	0.107336085	0.009258767	0.052239195
0.293408472	0.13480989	0.111455931	0.110758821
0.40386871	0.211175293	0.186272175	0.189661446
0.302830988	0.052676246	0.374854615	0.118079599
0.281869917	0.193206597	0.13068454	0.233907037
0.371035752	0.147442151	0.082097008	0.230334081
0.254464657	0.316434996	0.321946371	0.331812905
0.317054128	0.249628401	0.04372881	0.092938346

0.295865444	0.341672788	0.294013138	0.236160424
0.074268454	0.27007812	0.153628338	0.330875675
0.08862841	0.093141198	0.060638884	0.247996976
0.268211869	0.201846047	0.099654742	0.106900391
0.093993211	0.061750724	0.166791911	0.135556478
0.155032996	0.039409873	0.295335228	0.071811473
0.070185801	0.128614	0.27938053	0.127826982
0.232292973	0.212053037	0.051842277	0.246230063
0.002385009	0.299958932	0.338966629	0.172604778
0.139025322	0.048607118	0.287445134	0.196091104
0.384358866	0.316754353	0.432969578	0.415125651
0.198751451	0.286124536	0.250615478	0.17683275
0.311518519	0.110249215	0.126222753	0.427959712
0.428774156	0.344619265	0.243894282	0.29390733
0.258879601	0.316838647	0.157045994	0.190383352
0.152121466	0.218521879	0.26068819	0.399631666
0.239846304	0.248041655	0.237714258	0.270027292
0.153343061	0.132329224	0.285727102	0.176751354
0.394181785	0.041604581	0.284423952	0.167712056
0.24591434	0.312005139	0.355317899	0.153402005
0.252898828	0.071535677	0.151677357	0.15028751
0.161931237	0.226829154	0.255736326	0.347097629
0.099977947	0.201405887	0.156381916	0.038410239
0.131140914	0.286978822	0.303759646	0.199308323
0.327453063	0.262745013	0.169624988	0.204846371
0.271812743	0.429095172	0.011080688	0.116971749
0.205902955	0.280600104	0.242459883	0.23364733
0.24414266	0.104670741	0.160052217	0.40994371
0.244880783	0.1271311	0.127095975	0.400054339
0.119317307	0.305321257	0.15050392	0.161989853
0.062360148	0.305209759	0.154698641	0.137502738
0.12545309	0.211679705	0.056517596	0.098896205
0.117822896	0.273824073	0.25684496	0.180408864
0.173398537	0.33927108	0.098850417	0.237993653
0.022506738	0.132726415	0.073506042	0.377118281
0.094280518	0.419976445	0.220700702	0.279502834
0.280610305	0.390531619	0.016884259	0.288366779
0.352524211	0.073949907	0.233486444	0.321425719
0.169689945	0.351996232	0.024421051	0.374976361
0.176136963	0.157309833	0.441704707	0.213500225
0.300028417	0.110919037	0.112883477	0.357567616
0.274567384	0.025773537	0.061394505	0.250088742
0.159253374	0.043258539	0.306936802	0.289055174
0.21529772	0.397050248	0.444178101	0.1659905
0.231104899	0.159150594	0.079258738	1
0.355072712	0.135111554	0.258760387	0.14475321
0.118784487	0.311536522	0.277863144	0.060745184
0.081894677	0.127118983	0.027006981	0.216561521
0.261514933	0.083439586	0.155910157	0.256607441
0.194743582	0.388383554	0.271355812	0.236937849
0.217673704	0.171221713	0.185961156	0.091246972
0.12403237	0.20424561	0.205759862	0.176180166
0.037264739	0.151344208	0.12537686	0.129264703
0.084112889	0.119919687	0.100054044	0.211902144

0.136131498	0.104905003	0.211230471	0.26081254
0.231835686	0.087963967	0.351115613	0.354772299
0.151243334	0.151459504	0.151839557	0.100636529
0.347305623	0.080620565	0.064047364	0.298532107
0.091308217	0.423292937	0.143850072	0.198887732
0.199236058	0.188683326	0.395267019	0.035157574
0.180693561	0.28697891	0.274167164	0.20639853
0.21576191	0.269089237	0.057484968	0.241367277
0.223991121	0.105876375	0.379225408	0.245227464
0.116542815	0.376766988	0.286346136	0.183585079
0.200695148	0.135524196	0.032867377	0.316969988
0.37486866	0.39371989	0.255829338	0.305475292
0.077556246	0.025660154	0.312545799	0.249879233
0.297426326	0.384937427	0.256014539	0.208763483
0.213619588	0.181120339	0.042561774	0.385131325
0.259377181	0.343475611	0.055946787	0.357989127
0.302652898	0.074532737	0.16979229	0.119029456
0.216726874	0.092858612	0.189234381	0.312771798
0.303398868	0.197731538	0.167433782	0.331014471
0.02635064	0.05163488	0.275015814	0.260899154
0.150374178	0.216118759	0.14829286	0.123041837
0.079413798	0.273685256	0.41691256	0.117032464
0.101879782	0.112016131	0.375310476	0.100278881
0.365468109	0.357617595	0.323033324	0.150684706
0.32172541	0.346305733	0.227598832	0.219486862
0.116383075	0.337721551	0.228586522	0.040438183
0.407991088	0.049601928	0.026007136	0.33144734
0.172373226	0.234137016	0.006040451	0.128917365
0.201692096	0.444269397	0.280109678	0.2316556
0.047005838	0.278394142	0.047515391	0.404118928
0.310012653	0.102347598	0.272411062	0.289771718
0.308029697	0.351424781	0.22189478	0.356795922
0.257209622	0.30672366	0.117562871	0.264937503
0.301752286	0.341809553	0.287150657	0.297341899
0.367355436	0.390951008	0.220329184	0.038693179
0.160224955	0.071533466	0.082750706	0.080514012
0.124676741	0.199409051	0.352696805	0.418107798
0.382803366	0.054882445	0.0829748	0.280770675
0.309015068	0.382998951	0.108766728	0.023187328
0.026638273	0.077429756	0.184979996	0.297412385
0.364532622	0.047722545	0.036576474	0.040521272
0.180414346	0.334341526	0.326368197	0.354039973
0.369464582	0.179340027	0.083887338	0.354394756
0.161650309	0.147853377	0.384364667	0.282743281
0.174742837	0.283089763	0.170038559	0.388980429
0.131391736	0.308127989	0.300150736	0.147710557
0.089469408	0.211084648	0.160581201	0.418621866
0.301015859	0.101488039	0.084084333	0.038075206
0.096702126	0.391971971	0.158072128	0.424829881
0.319720653	0.409560826	0.220272267	0.300308205
0.081399577	0.292466597	0.256768674	0.024753545
0.276938855	0.084425596	0.329523485	0.088866372
0.104977305	0.2466399	0.172172727	0.122758705
0.220036581	0.162301113	0.405446813	0.426430362

0.186840376	0.160985835	0.281984915	0.323474178
0.100653489	0.333847702	0.298007701	0.125360774
0.074342076	0.233777379	0.296715241	0.212933406
0.098622797	0.444720014	0.162408958	0.399858204
0.254530703	0.340438022	0.342494126	0.192314587
0.242880519	0.180044222	0.180936386	0.026686189
0.244722467	0.377917481	0.162642512	0.186965852
0.213808931	0.239686939	0.098709717	0.094972907
0.202611384	0.106818805	0.130254392	0.426762339
0.222602172	0.221469862	0.067588173	0.146121839
0.374441738	0.283003713	0.085270409	0.25684828
0.262423803	0.234798136	0.379186756	0.39827196
0.17233913	0.307206521	0.296740456	0.188818324
0.242542265	0.181560484	0.32994491	0.0373336
0.159366012	0.236577784	0.100915825	0.228026971
0.171213083	0.130229823	0.18853305	0.395197077
0.178379866	0.293512078	0.10094752	0.16436426
0.291515507	0.15382134	0.293709639	0.097549691
0.106225336	0.298150711	0.048936443	0.171540316
0.191076946	0.313027373	0.213185791	0.273951588
0.226375863	0.288880225	0.226761696	0.081290742
0.078784841	0.177935328	0.301957567	0.057100982
0.040276743	0.437552921	0.132652879	0.145590957
0.164061391	0.18602754	0.225149768	0.14142157
0.146544147	0.340394036	0.081417439	0.261218158
0.172968071	0.160458403	0.191011544	0.099843955
0.131826454	0.270953014	0.270630196	0.337881762
0.271698969	0.37742399	0.239812918	0.290405441
0.190270396	0.191850179	0.05678917	0.354909054
0.139138262			
GEM	0.39594846	0.505497351	0.404249151
0.354858727	0.398590271	0.303118216	0.190544359
0.186703853	0.090021157	0.003130458	0.020271332
0.410183184	0.169259928	0.222043132	0.283136713
0.393758559	0.147180372	0.391095467	0.243151782
0.378811049	0.079834037	0.370649356	0.101143501
0.340463141	0.115349453	0.074704728	0.270144095
0.407006988	0.088693225	0.245386992	0.333804351
0.153812008	0.396877288	0.50757015	0.380462702
0.502020946	0.369041321	0.025311424	0.228996143
0.409381998	0.385879048	0.367524349	0.300864567
0.044155367	0.466861864	0.223218497	0.412151515
0.224189916	0.033120965	0.198069734	0.429174116
0.446035287	0.356241904	0.214012661	0.145270668
0.153602092	0.188339601	0.242355445	0.269072535
0.211951368	0.124218241	0.463887728	0.080446814
0.146804002	0.257563609	0.203059639	0.245856869
0.361475312	0.327925341	0.028256551	0.388487659
0.001608405	0.368540288	0.403681028	0.295098286
0.056275946	0.01758417	0.227723394	0.291917936
0.43641761	0.47288576	0.464996265	0.477245569
0.180094708	0.341577468	0.397086724	0.171261299
0.416992148	0.152512103	0.121813604	0.393994151
0.475001136	0.50415868	0.219782648	0.303450401

0.34834739	0.46120487	0.365560458	0.38028962
0.291624783	0.093479994	0.414890942	0.495806695
0.373311144	0.335469882	0.17460343	0.348259529
0.080634536	0.338847055	0.326233075	0.189942911
0.447530124	0.02050474	0.245962204	0.303078951
0.289250366	0.390032484	0.504523205	0.185419423
0.414670736	0.025545064	0.133031588	0.058920272
0.295141703	0.187501926	0.462506582	0.411821539
0.215571794	0.354036949	0.262576946	0.005281558
0.039673934	0.26669127	0.395733828	0.396805925
0.402288836	0.395655144	0.20663193	0.327349673
0.380048491	0.459986445	0.040047864	0.112184983
0.3692946	0.266798212	0.252557085	0.388666757
0.297839193	0.127543106	0.140231722	0.503083498
0.405582455	0.092702541	0.344027744	0.392709575
0.261358484	0.357819957	0.125471336	0.247724109
0.05000031	0.473283146	0.046531968	0.333359214
0.229517203	0.16269406	0.026026202	0.236177234
0.236219367	0.376870768	0.462055199	0.135944765
0.286955958	0.379105933	0.294374563	0.331008225
0.007316317	0.06962009	0.258399071	0.482742413
0.223499213	0.503435045	0.316089666	0.349281201
0.303140213	0.411244422	0.008608597	0.477453229
0.51202625	0.259895343	0.335292846	0.421253489
0.338245906	0.400978991	0.050736849	0.392541304
0.317943375	0.106297145	0.490080353	0.169770953
0.456682401	0.048049662	0.279933529	0.408185324
0.357318243	0.010755033	0.178895748	0.339216994
0.04962708	0.083254161	0.398126727	0.365308004
0.330528351	0.414282464	0.463557651	0.320496321
0.383772777	0.246576392	0.023677227	0.355072712
1 0.33707482	0.420143168	0.230628935	
0.294748175	0.451552567	0.383574865	0.169723995
0.047364761	0.285235064	0.019283567	0.31557818
0.390981067	0.095989004	0.351894017	0.225112854
0.092043902	0.505320962	0.22384654	0.295274569
0.167617483	0.315271584	0.268947622	0.170650087
0.165243686	0.183920261	0.335665326	0.344220616
0.034908979	0.099582503	0.039871226	0.304631729
0.100978683	0.258598496	0.248281405	0.343236603
0.283218888	0.094751302	0.339686986	0.340176327
0.142196192	0.02283215	0.490106721	0.396039757
0.276634249	0.164254574	0.061950504	0.26500952
0.424759529	0.235070553	0.239167813	0.415724946
0.047330345	0.476600741	0.206284597	0.134432781
0.333859496	0.244948862	0.433654164	0.050857227
0.367291008	0.439329548	0.360054163	0.188893457
0.360418987	0.374364691	0.032292151	0.354842122
0.339288576	0.039538337	0.511336866	0.155062155
0.033970304	0.413334517	0.362369891	0.097808109
0.103752184	0.264308913	0.043955539	0.303779238
0.495776284	0.375101181	0.344524094	0.286738715
0.040984899	0.007190262	0.268711326	0.411966557
0.493210999	0.500928712	0.294586866	0.320945665

0.24917073	0.299238	0.137622643	0.487017352
0.306691184	0.514714421	0.012243256	0.390543932
0.492415258	0.239312184	0.089532929	0.259774686
0.341589092	0.054686868	0.253941242	0.476967069
0.413320979	0.315556322	0.308598595	0.418026407
0.01617746	0.045399907	0.383632098	0.23709361
0.331040211	0.233947667	0.082242645	0.309713314
0.042024485	0.419883124	0.428917613	0.065748841
0.245603035	0.304347346	0.389559714	0.046838875
0.435456683	0.504966776	0.50439526	0.196197133
0.390860692	0.465293335	0.35360662	0.118178861
0.029215271	0.507708606	0.36278366	0.021535106
0.447745455	0.187398727	0.008715617	0.479549342
0.201414211	0.434095631	0.004972014	0.14824902
0.078541273	0.440889812	0.458615126	0.344800912
0.174539034	0.383243317	0.018958433	0.521835551
0.4627984	0.02317728	0.188523792	0.37326898
0.36559917	0.517541036	0.315384672	0.414929607
0.371359537	0.480196644	0.201107128	0.280143116
0.387448306	0.486407877	0.374122005	0.449806928
0.419255015	0.412539082	0.268633678	0.016083498
0.156338182	0.187074124	0.227841433	0.07492634
0.281645595	0.150662971	0.479336022	0.447933009
0.461249878	0.009632485	0.143622287	0.378046723
0.408981894	0.457481248	0.048921379	0.010615559
0.014382928	0.020543833	0.333313899	0.294418091
0.476297172	0.163001759	0.007253534	0.045780148
0.285806016	0.380894605	0.481304152	0.364558199
0.408529889	0.108096066	0.200077286	0.383952925
0.080010929	0.293997204	0.461753113	0.446774212
0.076437167	0.298336923	0.387872521	0.464022898
0.094553934	0.338813762	0.407797646	0.258375885
0.035541065	0.108664285	0.083384771	0.414319629
0.485813851	0.066131857	0.031701164	0.120595348
0.101746403	0.455611555	0.157142062	0.501093899
0.396069666	0.397676907	0.111060919	0.467731502
0.04124127	0.465117172	0.340497532	0.04767747
0.358018266	0.024071305	0.470349238	0.121456761
0.279235051	0.371977817	0.214338751	0.032027689
0.232541825	0.0696653	0.447960329	0.439063146
0.067186779	0.257078318	0.342318079	0.411152216
0.245314526	0.366760008	0.484127083	0.325238034
0.079725079	0.214694497	0.372663307	0.354189167
0.22222528	0.478849935	0.254714598	0.522789834
0.370350918	0.464727624	0.458285126	0.315814804
0.252941282	0.356000516	0.289248984	0.016012768
0.354531491	0.41172579	0.366957269	0.150279601
0.244725642	0.382731908	0.076519021	0.22032127
0.336011622	0.244552774	0.137527514	0.412888137
0.37314731	0.352431869	0.058536802	0.074541591
0.529801138	0.433369641	0.140244512	0.361498595
0.220418306	0.325695291	0.410119789	0.408965101
0.376245362	0.398576105	0.368683457	0.300531102
0.393372668	0.264860608	0.507924116	0.091052399

0.100134993	0.391639003	0.082737944	0.127869389
0.150871812	0.043429633	0.298070841	0.409012267
0.352215616	0.34540789	0.294259644	0.109746902
0.360936402	0.390009259	0.365399544	0.171017766
0.07385171	0.470830279	0.013823498	0.080159015
0.363481218	0.380363567	0.342474277	0.276842754
0.303708468	0.208761078	0.296632593	0.247132125
0.224072331	0.37157682	0.382910447	0.043883078
0.021748832	0.480327486	0.353154914	0.047779166
0.131769856	0.356483624	0.359634787	0.208302002
0.041234888	0.464544265	0.251644671	0.410660954
0.322392606	0.093745113	0.345050819	0.115494448
0.095450981	0.437241562	0.40060523	0.499812992
0.407256739	0.371434031	0.436881429	0.388308262
0.186600512	0.390469583	0.031678567	0.418618598
0.333856935			
GGT1	0.300351792	0.453648717	0.160221059
0.280886049	0.206547004	0.170900203	0.066264523
0.024290819	0.007416124	0.000304312	0.004008768
0.22612353	0.017171253	0.041915166	0.054599976
0.30054803	0.027329576	0.085569681	0.087060328
0.25268697	0.013619986	0.260324745	0.015425785
0.070429161	0.028102386	0.021203225	0.085386406
0.325390443	0.024620136	0.116436179	0.230136499
0.018359347	0.32828328	0.454677709	0.274991872
0.45332974	0.272234118	0.000916387	0.06205025
0.318637448	0.316170269	0.265093503	0.146335087
0.006937365	0.403836837	0.094770754	0.215693028
0.014632377	0.005797845	0.047130388	0.375997267
0.407359924	0.242838782	0.045322074	0.038877604
0.020718075	0.043232312	0.047480129	0.16853895
0.079743013	0.027217545	0.428651972	0.007498398
0.053632538	0.057983154	0.032389454	0.070109121
0.107937757	0.250879424	0.001334153	0.232588543
9.58E-05	0.223331089	0.190260295	0.107106808
0.013356224	0.001640579	0.046256353	0.164727686
0.359411096	0.391319191	0.408330313	0.432019638
0.017094161	0.108739886	0.199888045	0.039590156
0.352390876	0.048700785	0.00588928	0.308752027
0.420236587	0.462549332	0.032388644	0.119876526
0.323404071	0.396955846	0.147381142	0.167979434
0.081090079	0.010299228	0.387841343	0.443398379
0.300100416	0.132173515	0.05285996	0.282187257
0.016073912	0.036744136	0.191314382	0.010516891
0.365859592	0.002828141	0.084995591	0.159152841
0.021618325	0.302049169	0.458654491	0.062815557
0.161390395	0.001408888	0.014753715	0.007725646
0.018097742	0.027284633	0.422909432	0.355805338
0.025722684	0.059710952	0.074115783	0.000491273
0.005727078	0.015634354	0.338590753	0.118804524
0.299167757	0.334280544	0.065311574	0.277174786
0.283953454	0.412891455	0.00199109	0.017501049
0.168669594	0.02492081	0.107225042	0.228050701
0.116609243	0.018301267	0.031429952	0.43964221

0.345662953	0.023840012	0.078363189	0.269887335
0.053743435	0.16977453	0.024502888	0.056637308
0.008604278	0.434785719	0.008100068	0.097183724
0.031096052	0.014052592	0.000855716	0.071110818
0.018750362	0.356364115	0.423569404	0.028763446
0.091930565	0.244316674	0.108581852	0.265187343
0.001546914	0.003189002	0.076760418	0.429114467
0.139523547	0.430446534	0.054697704	0.079011355
0.124541976	0.340536121	0.000279289	0.420907036
0.468011788	0.07413319	0.183845682	0.226257851
0.164222669	0.340022887	0.002331964	0.265315835
0.211038885	0.019615893	0.42631643	0.057672891
0.386009181	0.013424568	0.078243075	0.377185037
0.3097699	0.000764664	0.058372371	0.232448936
0.007422363	0.014666989	0.295029645	0.23887609
0.194895919	0.336562236	0.391390397	0.141475889
0.184539051	0.052328325	0.001400311	0.135111554
0.33707482	1	0.363693397	0.039928719
0.145523521	0.388854632	0.328268027	0.027803485
0.011846864	0.11018548	0.001043892	0.13231589
0.344841286	0.018983903	0.181986824	0.01415923
0.018270746	0.454586023	0.056159637	0.089869351
0.035998831	0.184097892	0.07973872	0.037282868
0.022394639	0.036258848	0.101914653	0.108917319
0.005801661	0.023805966	0.002047047	0.090334279
0.010345713	0.070323763	0.07427118	0.274746486
0.042899914	0.006968231	0.133799666	0.058463698
0.022960338	0.004842545	0.439216698	0.239248904
0.213481816	0.029512161	0.010093644	0.020983329
0.382377216	0.120391579	0.056291858	0.375592079
0.001737536	0.422921927	0.033344805	0.042143884
0.111076737	0.093252316	0.361969534	0.004025072
0.189715181	0.388756421	0.12968231	0.05550934
0.306589538	0.235563922	0.002139151	0.274185506
0.264857346	0.008311898	0.453794834	0.022091852
0.006652441	0.294139246	0.328693492	0.02768984
0.020710857	0.119582937	0.005632929	0.148810021
0.440067898	0.28240908	0.333545583	0.061379411
0.009199958	0.000822551	0.067932591	0.177825592
0.437015727	0.447326609	0.103120814	0.134068107
0.102989443	0.089079006	0.009005375	0.437086529
0.014708204	0.448292276	0.001810535	0.36597033
0.453656698	0.088656267	0.023674859	0.121410035
0.279821765	0.01109276	0.038305387	0.416417027
0.374015265	0.097820522	0.089215421	0.253580734
0.001741778	0.004716026	0.200063252	0.045584681
0.105003903	0.055515825	0.006538687	0.054463951
0.004987534	0.381327176	0.349061585	0.011253459
0.155690098	0.05813617	0.223173764	0.00999762
0.405414742	0.445939023	0.447205912	0.030225892
0.328038575	0.427992161	0.241998031	0.023032894
0.005953044	0.453826816	0.24339155	0.001507195
0.386123931	0.06639078	0.000991684	0.414791161
0.06056171	0.38177791	0.000114705	0.007281543

0.008240535	0.374178652	0.416563704	0.225526906
0.046052689	0.181737802	0.002495343	0.459639485
0.408256784	0.00367309	0.027214829	0.341887728
0.319092146	0.448161575	0.123586933	0.382419977
0.233725877	0.445546473	0.016497622	0.127602507
0.330234909	0.450653561	0.273426916	0.375110645
0.361156963	0.278874584	0.130360944	0.003243455
0.017048597	0.050209606	0.055129932	0.010841918
0.150254165	0.029105839	0.389192159	0.391488805
0.408868251	0.001698407	0.017184118	0.332993965
0.337201329	0.379843796	0.002023394	0.000306618
0.000349232	0.002176337	0.172989436	0.087580476
0.423497605	0.016037419	0.001128928	0.003761884
0.024290121	0.242732442	0.4356387	0.234501158
0.347911967	0.021294536	0.041110921	0.2528356
0.01135185	0.199787734	0.411791824	0.400982029
0.007971096	0.091895502	0.112307298	0.401803252
0.018132595	0.153158668	0.251823028	0.044933137
0.001865245	0.020802799	0.018318629	0.336418142
0.448316473	0.009303832	0.003951992	0.007582286
0.023327307	0.395525462	0.022916766	0.439005849
0.329243109	0.329571114	0.014130308	0.41468868
0.00638417	0.416395958	0.144297267	0.0028938
0.06244604	0.001725749	0.408765013	0.018143057
0.075749856	0.228115162	0.022601009	0.005580788
0.028870999	0.012767911	0.400525543	0.387094902
0.006101851	0.050524479	0.113284034	0.22604556
0.093232581	0.291245863	0.429878557	0.148401227
0.017044813	0.013986176	0.244640047	0.218621963
0.040865461	0.41815487	0.06775888	0.4551637
0.26953097	0.394504764	0.404019274	0.155681377
0.027870285	0.172109994	0.2128435	0.001010626
0.142084173	0.367893516	0.125010553	0.019128615
0.093875247	0.209534262	0.005368743	0.008763347
0.148477194	0.13186486	0.019279942	0.338088147
0.200696776	0.237274346	0.003282003	0.015228034
0.466485634	0.372036549	0.008332934	0.249189559
0.052049409	0.168557729	0.338584745	0.348489631
0.076995397	0.36674179	0.293605976	0.225753696
0.344183853	0.09990952	0.457009438	0.004706703
0.008808508	0.242658563	0.015466432	0.033366679
0.043052361	0.004938427	0.070515729	0.289787827
0.102317557	0.211578306	0.03598325	0.006843816
0.14985005	0.124097843	0.171181723	0.045858151
0.007932635	0.431922564	0.000978367	0.014970673
0.116162338	0.343490327	0.174731379	0.059182047
0.053876736	0.02656543	0.125965709	0.085009785
0.078226498	0.024319761	0.338058318	0.00211796
0.002320133	0.410679366	0.112509996	0.002541252
0.020023444	0.225521541	0.047055431	0.038562508
0.00691518	0.440439807	0.097412808	0.210312986
0.143933375	0.013544629	0.27666976	0.006279457
0.0048762	0.365615233	0.199300604	0.453034329
0.12672145	0.297642524	0.379969196	0.344186084

0.006807393 0.171697326 0.0037056 0.339066846
0.108657327
GIMAP7 0.416875103 0.482212033 0.36534986
0.422813498 0.385997531 0.440468254 0.234746919
0.248813293 0.087952626 0.007018114 0.018079479
0.396975565 0.18497381 0.306340906 0.383338326
0.387934169 0.100367221 0.359692288 0.234752883
0.410197893 0.128326278 0.359358097 0.095686428
0.22692724 0.076217657 0.0585584 0.220412313
0.435780413 0.068286827 0.406272338 0.428359623
0.073953636 0.438453377 0.500457677 0.387362809
0.488517842 0.413908213 0.0097812 0.349986126
0.442205784 0.42110444 0.414087729 0.321308523
0.044638763 0.494916678 0.255116071 0.344755685
0.297133433 0.023657578 0.340603336 0.455179152
0.470382324 0.427736141 0.284945338 0.179184141
0.189418037 0.311055801 0.220627517 0.409886723
0.228001961 0.249562571 0.463594963 0.109234812
0.164238054 0.292367585 0.108662824 0.300827672
0.283711518 0.445173433 0.019964281 0.3760555
0.00516756 0.389023974 0.320618037 0.271263222
0.032017997 0.018254556 0.131975198 0.357959084
0.441648452 0.426165716 0.428349905 0.428539568
0.146163616 0.248211876 0.329353129 0.157169247
0.445278764 0.196534738 0.113482249 0.376544649
0.431694476 0.481091716 0.160529176 0.210369809
0.448650545 0.451719711 0.434533242 0.400596848
0.328588067 0.032615295 0.44093601 0.440085622
0.441249716 0.247195206 0.12005552 0.442964832
0.048110146 0.406614026 0.341711715 0.131486074
0.443051818 0.02128383 0.160705563 0.377994889
0.199617417 0.448327993 0.466603768 0.192235155
0.353353589 0.016630446 0.107471416 0.023893335
0.320976972 0.097480574 0.494413994 0.447463431
0.278898256 0.335243753 0.28247644 0.000428135
0.017712343 0.149071547 0.42865971 0.363323821
0.428675756 0.390948325 0.2159466 0.440359106
0.409168171 0.425560832 0.097507433 0.104388617
0.423828561 0.144873985 0.215684528 0.423075567
0.262553652 0.135984379 0.119180329 0.446429632
0.44194422 0.081048898 0.382994787 0.379458573
0.347594799 0.289309381 0.102130167 0.270635341
0.057407646 0.452145363 0.01677899 0.379117
0.263012471 0.102393713 0.010805919 0.325769634
0.329067552 0.442976808 0.473479749 0.095877614
0.312473442 0.365213945 0.391852521 0.439594348
0.011494407 0.04195817 0.364486537 0.446086849
0.389205414 0.431279234 0.288423026 0.260633564
0.237664087 0.430299766 0.002977532 0.493061085
0.481236938 0.385398445 0.37440518 0.372658987
0.343264507 0.444570162 0.04728877 0.383348237
0.414691552 0.072182659 0.436134012 0.130465741
0.433408495 0.033531982 0.364580801 0.4045014
0.408886766 0.007203321 0.318007199 0.411425014

0.015828778	0.14395212	0.432069482	0.411128564
0.382270387	0.416662221	0.429949859	0.325955481
0.394041497	0.252218272	0.009143487	0.258760387
0.420143168	0.363693397	1	0.262283929
0.409335681	0.453591687	0.423208833	0.2463682
0.043882382	0.357112395	0.02659573	0.307714964
0.41438902	0.128735227	0.383139174	0.114564821
0.049713585	0.46073815	0.122702535	0.199212222
0.084413765	0.412869942	0.259531887	0.256513708
0.189345949	0.142203812	0.312198479	0.366553651
0.051599787	0.07878911	0.007932209	0.347824053
0.128684207	0.334295711	0.35184947	0.440728683
0.332200076	0.102133308	0.348761702	0.239441373
0.086956171	0.01044329	0.46405373	0.383584663
0.419743525	0.158030599	0.030315252	0.339770259
0.448903558	0.412433837	0.365260288	0.45474568
0.025583282	0.432011407	0.239697407	0.097461584
0.320548637	0.247132424	0.438547939	0.072518558
0.419770919	0.452362026	0.275667477	0.159457478
0.435154429	0.419166173	0.032528295	0.440507807
0.437537317	0.024712559	0.46491662	0.089180857
0.014057358	0.392943412	0.422949816	0.05874169
0.059482678	0.345838676	0.072870476	0.246778019
0.461441837	0.372815605	0.44474586	0.162262381
0.039030159	0.0078817	0.151663855	0.359545561
0.50292502	0.438488957	0.228672637	0.317802521
0.230355456	0.300776243	0.217132601	0.442798037
0.1610346	0.484118855	0.004630618	0.403080222
0.492234142	0.372208701	0.060511298	0.359310398
0.447947445	0.046658696	0.207145005	0.475488933
0.450699008	0.313041994	0.345971795	0.399069221
0.022810744	0.052341064	0.356439855	0.130691404
0.387548338	0.197202235	0.029147626	0.371523977
0.041771045	0.447809104	0.421027748	0.053428569
0.40772365	0.373368254	0.323183042	0.033502081
0.410716039	0.447536089	0.502038328	0.177099555
0.422864609	0.435158056	0.359648034	0.058795
0.010451282	0.481020877	0.442316299	0.025624247
0.435452341	0.346669631	0.012067626	0.468550642
0.18782955	0.462543146	0.007935608	0.067782529
0.024599988	0.409545607	0.476446416	0.421109064
0.356545292	0.36199519	0.002918228	0.448447714
0.466148076	0.008157736	0.097398743	0.430260489
0.396904355	0.497930826	0.264752319	0.420336269
0.385991826	0.474897701	0.254761867	0.233496307
0.45371042	0.437821378	0.448271222	0.44969665
0.442610127	0.375338496	0.27558249	0.016889604
0.118555358	0.291194167	0.356906039	0.081216365
0.412233037	0.116879981	0.442387909	0.429702657
0.431442331	0.002779076	0.197484367	0.440335015
0.455711602	0.449068748	0.015878927	0.005750173
0.006895122	0.011928435	0.393654504	0.197964638
0.444378848	0.329062211	0.003400127	0.063818642
0.297340588	0.339304819	0.47037422	0.363193812

0.412708416	0.065702352	0.321943089	0.389304951
0.049306606	0.399591993	0.438958191	0.468745981
0.028444544	0.206979707	0.385134781	0.449656613
0.044900164	0.268515385	0.402858759	0.316167782
0.008997997	0.059311095	0.05051799	0.404261025
0.477689014	0.064445715	0.025110579	0.198803859
0.126091588	0.434926892	0.157245841	0.442384488
0.453532893	0.413740739	0.04747338	0.47171952
0.032094716	0.470674671	0.274780265	0.088547555
0.25230676	0.009166421	0.417892407	0.147101328
0.384598698	0.429697389	0.178265176	0.00906053
0.137956976	0.03826969	0.436185257	0.413386596
0.019438494	0.255277267	0.260997816	0.390760911
0.363101658	0.421642033	0.497049474	0.437847037
0.098981505	0.094100605	0.424222071	0.43534663
0.288497021	0.428189305	0.278793419	0.458917917
0.441546626	0.444261935	0.435550218	0.367991667
0.172246223	0.440716296	0.426170637	0.027605711
0.34853832	0.432785796	0.385716045	0.127469723
0.223119456	0.33510784	0.04795041	0.210582071
0.272501657	0.40061229	0.139122355	0.411101437
0.41937478	0.438596807	0.046429192	0.048923651
0.495343763	0.44332087	0.189116963	0.445175244
0.142452171	0.318727225	0.421299147	0.401352174
0.35829897	0.442775061	0.444890198	0.437032782
0.446055372	0.265369502	0.50181343	0.144208646
0.063695333	0.403419619	0.09215265	0.067353554
0.13073193	0.019153263	0.247722709	0.387731136
0.322916082	0.357258233	0.365395384	0.075508266
0.268052401	0.378042405	0.321735512	0.24194118
0.075405732	0.484195558	0.007268937	0.041608373
0.324596049	0.437756	0.331356874	0.205999048
0.236003858	0.09765992	0.270994866	0.381690124
0.370231454	0.342433834	0.443273284	0.042658379
0.022581759	0.434607351	0.421096459	0.012513777
0.065614096	0.421766934	0.277612379	0.23600604
0.012113447	0.422607602	0.401521618	0.386269521
0.383551625	0.056224675	0.413675438	0.090817303
0.059459664	0.445537995	0.349217376	0.471552298
0.297076133	0.395025092	0.467116553	0.439710397
0.114423813	0.36099732	0.03104319	0.44149924
0.387583246			
GM885	0.206755134	0.293369096	0.117849574
0.35127119	0.175625305	0.198408125	0.250325741
0.116167358	0.087907905	0.027007156	0.024759023
0.162661051	0.062863998	0.100512802	0.066707141
0.337662229	0.057863116	0.110795158	0.274640604
0.315979002	0.121131342	0.297757813	0.133648705
0.050406047	0.055101284	0.097661848	0.077573128
0.368559456	0.113565956	0.150123996	0.31681815
0.055965586	0.260687762	0.24989934	0.207830286
0.222985386	0.175830625	0.013733702	0.100224156
0.270302482	0.248281403	0.33514483	0.295217742
0.093542032	0.239915372	0.277974327	0.177315904

0.077034634	0.04911065	0.116581737	0.246143293
0.213766117	0.162066025	0.169124143	0.176366546
0.037825494	0.087975378	0.02434405	0.229081857
0.240273525	0.108797587	0.222990002	0.112653598
0.07113378	0.17446613	0.05265512	0.208375298
0.051698364	0.255899275	0.073566892	0.117171042
0.009350027	0.344356472	0.185839052	0.04518553
0.09047488	0.038402203	0.076527388	0.299422296
0.345429474	0.229113968	0.299034107	0.271252609
0.060553174	0.155986487	0.103083994	0.125129115
0.2682513	0.194636915	0.088936239	0.31423228
0.294869191	0.253479006	0.084114588	0.161107727
0.263013843	0.291594699	0.13329702	0.141166578
0.054648873	0.030557551	0.232654035	0.282347472
0.238323751	0.064679291	0.125267655	0.31789116
0.09714264	0.058949445	0.326309802	0.040351343
0.35657435	0.078889373	0.118609338	0.242030155
0.092243227	0.253473749	0.240223328	0.253704242
0.140002021	0.035135189	0.028126537	0.050554782
0.077979101	0.017159087	0.206881324	0.322538178
0.093987271	0.059827021	0.208352558	0.014147453
0.037849622	0.054102169	0.205495891	0.150646299
0.276457378	0.162797655	0.110925465	0.213386639
0.157367262	0.292078114	0.018962541	0.131601093
0.20289607	0.060830067	0.265218649	0.168805578
0.154068075	0.148199164	0.149745973	0.314847398
0.274168171	0.162562628	0.099002122	0.315691676
0.069560591	0.148698269	0.143463186	0.116366361
0.101621158	0.208252458	0.032582068	0.170461911
0.040166681	0.03611298	0.03715053	0.20413706
0.085324145	0.230395901	0.190780293	0.151396419
0.161204206	0.245938572	0.155236083	0.219000671
0.026873914	0.082451597	0.103800162	0.288304064
0.184302497	0.274687197	0.114965588	0.098607808
0.198407731	0.318779944	0.0021475	0.240933653
0.249170717	0.100357574	0.2006738	0.190334835
0.08589869	0.330203501	0.011955394	0.297598947
0.176789711	0.13194858	0.325823334	0.143912896
0.228543572	0.08525893	0.126964935	0.196841139
0.158658516	0.020798556	0.17207749	0.338553592
0.026786063	0.117926622	0.265859196	0.305139473
0.229319111	0.269194722	0.345682594	0.072515031
0.176184908	0.192324787	0.039297762	0.14475321
0.230628935	0.039928719	0.262283929	1
0.137648032	0.278826197	0.183579732	0.038030838
0.120457544	0.215038833	0.055821458	0.176865825
0.191366735	0.094418397	0.107865934	0.055653899
0.061742718	0.29584989	0.038298722	0.038457111
0.017322874	0.171755927	0.246014505	0.064997184
0.040865685	0.106435428	0.077391644	0.09819307
0.103108692	0.102562281	0.013466068	0.043216717
0.038371073	0.059168096	0.060755862	0.197998944
0.071978721	0.061705999	0.154475121	0.046892998
0.060386405	0.052698597	0.286709943	0.304884752

0.201286567	0.142336384	0.066373126	0.030913808
0.308690812	0.135587518	0.084372887	0.294171745
0.051703651	0.2973849	0.072438305	0.121743597
0.072922958	0.222514976	0.362334057	0.048622389
0.191031977	0.271363355	0.196971591	0.113938027
0.223878071	0.285177592	0.09063369	0.205110243
0.197382526	0.072917918	0.269461177	0.049014847
0.053331735	0.210843317	0.194618244	0.118985918
0.079092121	0.164085632	0.113160834	0.232892802
0.298125071	0.314361829	0.225519002	0.054093296
0.087375807	0.014646988	0.075064307	0.119259864
0.236000163	0.247332645	0.214147853	0.115354591
0.291478462	0.067274595	0.07201858	0.280320683
0.042620101	0.255987029	0.033312284	0.203171367
0.220622785	0.14462448	0.069555598	0.238462487
0.240393482	0.113126281	0.130118433	0.250751524
0.280738284	0.119013172	0.087372586	0.222429348
0.079531299	0.100964746	0.180047195	0.027508105
0.09211621	0.129550555	0.006185532	0.091030754
0.069941343	0.263064227	0.298638743	0.067938429
0.153510215	0.098636576	0.22502773	0.100256012
0.208571888	0.229375404	0.254859742	0.136736162
0.210202668	0.241172669	0.101527509	0.083624412
0.038352946	0.232628343	0.241633282	0.034807869
0.329822489	0.134052734	0.027143013	0.297024195
0.244131399	0.195950407	0.022157861	0.010851633
0.029995266	0.278994383	0.237965887	0.309499247
0.068016968	0.271377021	0.003365774	0.266924635
0.261618867	0.025225744	0.047886672	0.197134473
0.161065478	0.293147495	0.070917143	0.221871974
0.133633565	0.221592841	0.102501933	0.224247772
0.286702711	0.208308843	0.292450823	0.278607867
0.315062923	0.235224024	0.309312775	0.066603035
0.045548511	0.163872262	0.044775597	0.043263855
0.202798738	0.067300413	0.264446167	0.308679488
0.249401195	0.02791402	0.124832208	0.216432568
0.33248914	0.350198925	0.028493592	0.008967358
0.004166063	0.016588173	0.26632527	0.138820624
0.271641985	0.038550588	0.029969947	0.109271932
0.100392879	0.160462819	0.257523644	0.357163353
0.221343652	0.095066099	0.057126228	0.349457913
0.048177441	0.159495876	0.272091281	0.274828516
0.045899054	0.131825422	0.127215925	0.342240701
0.006449601	0.210430941	0.173689578	0.085941742
0.016491745	0.060921437	0.061509547	0.299980324
0.207339512	0.047384193	0.036048976	0.040825603
0.110793733	0.286093145	0.096314088	0.313707245
0.315002355	0.35896005	0.036979289	0.262445135
0.0575617	0.229494212	0.102469083	0.032492285
0.093564308	0.040711808	0.208875713	0.028985708
0.116962675	0.258494193	0.051299134	0.023599508
0.022986104	0.06540902	0.320679737	0.265469756
0.026976777	0.142792135	0.181644913	0.256563086
0.227231093	0.30289171	0.247667589	0.104338077

0.120520526	0.016712249	0.326294856	0.197286738
0.104668608	0.305182431	0.065573511	0.280470616
0.243591872	0.280181092	0.240628661	0.264694843
0.039813217	0.196448772	0.265377391	0.057163784
0.26001415	0.300189033	0.187619707	0.056485333
0.144745593	0.095123083	0.075139514	0.035466371
0.058726452	0.21765605	0.098656173	0.355356104
0.225167553	0.212180078	0.073696083	0.060932657
0.290121349	0.264234896	0.12642004	0.257915141
0.137849511	0.209596529	0.236650024	0.225480872
0.054580927	0.23864692	0.299306363	0.235733367
0.265499549	0.262049122	0.259010891	0.092932345
0.0499561	0.143955144	0.077733058	0.098102499
0.176218913	0.045263879	0.026964181	0.287662262
0.043171994	0.269946804	0.060485635	0.071492095
0.164806455	0.102294091	0.238069969	0.178286536
0.078825706	0.247076732	0.02026642	0.074251436
0.053314922	0.242942972	0.102720564	0.12076778
0.036077854	0.044663403	0.22080817	0.165193016
0.076346466	0.043452018	0.241107933	0.094950424
0.075429537	0.319995246	0.137094406	0.024861849
0.039520458	0.174943915	0.074533906	0.085181439
0.02430059	0.217715854	0.109323314	0.161549284
0.260717015	0.088904618	0.209279411	0.074070487
0.09186066	0.243483928	0.129574377	0.2340118
0.104626785	0.33754322	0.221928077	0.224253208
0.053088503	0.094811497	0.101712859	0.313359941
0.062181277			

GMDS 0.299382957 0.41836834 0.16923469

0.257825844	0.170398569	0.208506945	0.109543845
0.182887356	0.03059656	0.002459201	0.003602006
0.200103264	0.068648242	0.154991621	0.157428498
0.237687839	0.041512825	0.231450017	0.11599423
0.245651954	0.072517384	0.183746206	0.047307115
0.09831913	0.025883453	0.020289201	0.07512892
0.329367321	0.025292256	0.191291463	0.288336672
0.044994686	0.335255734	0.439847318	0.18686999
0.426956303	0.291467567	0.016462671	0.138713533
0.378009235	0.238789467	0.251668802	0.144418989
0.018163369	0.459930524	0.109337333	0.15909685
0.164327782	0.007747259	0.201270586	0.433847087
0.397207993	0.167564398	0.16927491	0.07635041
0.057022478	0.193553969	0.071432638	0.282577364
0.102219457	0.157259118	0.391631057	0.039838867
0.07392672	0.193997838	0.056757767	0.170799076
0.112200092	0.336448948	0.018825278	0.192108302
0.002755009	0.230184161	0.158738697	0.08831209
0.014724028	0.006288782	0.068169284	0.15871157
0.345539986	0.411278021	0.317675077	0.325373132
0.063983808	0.128797047	0.140465703	0.065308306
0.352018925	0.080874805	0.076000037	0.223314238
0.327923806	0.409449671	0.06774633	0.09383074
0.285510426	0.413039864	0.186150556	0.161128559
0.107277029	0.016654941	0.368355677	0.36640096

0.377014123	0.098143483	0.049286143	0.335459589
0.024815231	0.228472914	0.157921629	0.093796582
0.354629188	0.008314286	0.068099004	0.170223618
0.133904385	0.322314239	0.392564169	0.089942089
0.178414827	0.009303923	0.031155347	0.015477337
0.168747098	0.039355785	0.437051977	0.326748656
0.187684553	0.134554863	0.146550588	0.000344531
0.008216949	0.0938347	0.31694383	0.227183321
0.283706431	0.326027607	0.07082124	0.327464497
0.19994725	0.310158269	0.048148293	0.056935712
0.197314082	0.088397516	0.099476036	0.187218178
0.101076241	0.089396829	0.059444726	0.378230497
0.421632071	0.038316182	0.253897161	0.222410053
0.126282984	0.120579838	0.058234009	0.130379101
0.027901808	0.384307051	0.008114953	0.247338701
0.081460204	0.043934549	0.014249315	0.201145931
0.152044642	0.298605532	0.428254595	0.055011282
0.148164311	0.163270446	0.241334779	0.237133213
0.001640109	0.03145665	0.249367543	0.375701291
0.245807994	0.36904321	0.152982717	0.122428097
0.10379878	0.296987352	0.002962366	0.44039288
0.406869325	0.250704625	0.152479228	0.187090414
0.120883471	0.30804681	0.055214883	0.224663968
0.317535795	0.042243472	0.348539731	0.051660395
0.401999	0.01215143	0.195036248	0.260309751
0.244611339	0.005422181	0.171550059	0.236592146
0.007855754	0.077356233	0.291953209	0.212804753
0.147980974	0.277276702	0.329405314	0.109792047
0.169994931	0.15890764	0.006509843	0.118784487
0.294748175	0.145523521	0.409335681	0.137648032
1	0.389441847	0.301396691	0.078901869
0.018077158	0.190400867	0.019440374	0.118635015
0.320913252	0.049251898	0.147945133	0.089076949
0.021898074	0.387552172	0.049523839	0.07918765
0.032149914	0.174137364	0.146652913	0.094852785
0.062173291	0.059415259	0.115639227	0.138046746
0.027441057	0.033177111	0.006911049	0.145869629
0.04446024	0.133656213	0.145154782	0.291328735
0.180672209	0.045896372	0.142750953	0.101151232
0.035937426	0.005031319	0.404636691	0.21928552
0.330980819	0.076272011	0.017273831	0.138718007
0.332442109	0.186873803	0.238620704	0.360304065
0.020378751	0.335786431	0.085987011	0.037931282
0.126069042	0.108036925	0.328535271	0.031284446
0.235562599	0.40463569	0.144453669	0.055742552
0.379095592	0.222278534	0.016630732	0.327434755
0.33033586	0.011186381	0.400811426	0.041297109
0.007271065	0.221281642	0.227172698	0.025959088
0.026116846	0.127342902	0.044742014	0.105156816
0.390009242	0.204938248	0.26756418	0.075453636
0.014832685	0.002778646	0.07293121	0.179502269
0.45256913	0.371454589	0.119699729	0.11829944
0.11045751	0.101643253	0.146957442	0.365613994
0.123310525	0.43998844	0.002952026	0.240584433

0.421587096	0.226844873	0.020726982	0.165754591
0.356253165	0.020659544	0.14704228	0.4155401
0.338091701	0.120308143	0.123339411	0.239185653
0.012037134	0.026959566	0.148522613	0.051968164
0.146314695	0.083183347	0.012952867	0.214894316
0.014772199	0.38034654	0.309321515	0.015785391
0.163487747	0.22587271	0.150099511	0.015858873
0.286740005	0.394455207	0.442396122	0.121682025
0.291684997	0.347511092	0.134955287	0.031675727
0.004862627	0.415438021	0.29247898	0.00931798
0.331505515	0.216514712	0.003397715	0.421494903
0.096816049	0.440411554	0.004348075	0.049447202
0.014820482	0.290896292	0.407560187	0.268401276
0.159138861	0.20011773	0.003343206	0.391340496
0.403314863	0.003322222	0.041933552	0.277966913
0.225613679	0.451365815	0.094387045	0.27966483
0.166070341	0.397883251	0.114835951	0.096231659
0.319887327	0.363716368	0.315270421	0.415706933
0.325092804	0.203273733	0.121390641	0.006173353
0.035568276	0.177210867	0.11762725	0.018846183
0.25321677	0.040358834	0.400518123	0.315007339
0.331151621	0.001553275	0.117431456	0.304864342
0.374664457	0.367278145	0.015724062	0.004326125
0.006636935	0.002840961	0.206431029	0.094630899
0.365059281	0.138130533	0.0017523	0.048682383
0.145809859	0.138786489	0.401820857	0.207536171
0.277878073	0.034330404	0.113282227	0.243987498
0.016077145	0.307810374	0.334810816	0.415489687
0.019248331	0.093726622	0.249374185	0.358045612
0.014575091	0.125207868	0.22088952	0.121764026
0.010097484	0.025034241	0.017535872	0.272448801
0.408322275	0.021486582	0.008217796	0.136576788
0.047297794	0.335302825	0.065779853	0.367844493
0.33034934	0.284901581	0.021879142	0.410426872
0.00978396	0.413109809	0.105931375	0.045753486
0.158486497	0.006787719	0.376578632	0.033596532
0.223215129	0.295465383	0.059133282	0.004560954
0.056095348	0.018022062	0.315776636	0.284157918
0.01256482	0.136565703	0.131364396	0.228080577
0.205334624	0.245775953	0.439575938	0.183702043
0.042955162	0.0598633	0.286948949	0.300151761
0.189271552	0.329307892	0.090186865	0.399929699
0.311310285	0.388820294	0.352119621	0.160126071
0.068792443	0.236481041	0.226953893	0.012323437
0.178503865	0.294018239	0.232554591	0.040807142
0.081286091	0.136537615	0.035929403	0.209229552
0.10770352	0.205898228	0.059923371	0.292766915
0.270209901	0.311299342	0.046707139	0.016184934
0.435301357	0.396019308	0.105508643	0.239708286
0.076838462	0.122887067	0.286040195	0.258722299
0.159892909	0.311380455	0.331020473	0.250331025
0.394375176	0.124709412	0.434085523	0.141951033
0.024718468	0.227044697	0.03055324	0.032305428
0.059201034	0.008867124	0.089870863	0.236061372

0.132674505	0.159832755	0.226717327	0.035236095
0.125089283	0.229549672	0.148481384	0.113595348
0.02831146	0.409979178	0.003274077	0.021853303
0.144913394	0.279555709	0.12023464	0.097091259
0.085643088	0.051289034	0.11738753	0.226695246
0.146027842	0.17983389	0.287266781	0.029539808
0.014601349	0.345081065	0.251642904	0.009848574
0.030654954	0.25102273	0.197139302	0.077042362
0.006070423	0.341843132	0.163514265	0.167043495
0.19533964	0.034190822	0.397514617	0.068025035
0.044996709	0.432131384	0.155151086	0.403089228
0.155074821	0.232000211	0.446754037	0.315091754
0.094962789	0.181118436	0.015006913	0.339051943
0.151925693			
GNA15 0.437232889	0.511395202	0.407956126	
0.454560092	0.433236271	0.440410009	0.249514285
0.231522574	0.105581951	0.007705211	0.026559203
0.442432244	0.219498639	0.314745732	0.388553876
0.431274257	0.132009673	0.369701742	0.263599208
0.435710174	0.125965995	0.410149919	0.109425935
0.277310602	0.104101917	0.076000713	0.27314107
0.463938245	0.086679199	0.398751351	0.431761765
0.094032481	0.457425535	0.521738301	0.442677498
0.512221822	0.425395013	0.010467354	0.35816978
0.449399746	0.46371351	0.437711957	0.358040871
0.05222164	0.501561987	0.281597443	0.39658661
0.280708149	0.033656403	0.310102481	0.456981708
0.488670663	0.465496768	0.279210447	0.195477625
0.222522575	0.284818122	0.270601481	0.386986564
0.254639293	0.219211608	0.486827669	0.117157865
0.168799442	0.289194957	0.140094121	0.302747459
0.337013817	0.438862334	0.021625702	0.40950049
0.004656007	0.415565246	0.369795713	0.319417242
0.042798529	0.024004522	0.167045789	0.381715085
0.469683957	0.443887471	0.471936776	0.469340462
0.181916074	0.289073204	0.382951415	0.186809231
0.46257657	0.215322788	0.12654434	0.426839247
0.473427444	0.511738074	0.201867404	0.256825842
0.470136759	0.467871425	0.46121409	0.447599379
0.373327139	0.046142345	0.449520451	0.47375239
0.437067465	0.301652012	0.153042463	0.450953076
0.061024553	0.408218109	0.38020778	0.150923619
0.472999725	0.025159856	0.204507442	0.400504869
0.220467748	0.470681577	0.501023933	0.214693911
0.403380223	0.021125052	0.139594351	0.031605515
0.360727574	0.129409857	0.502244556	0.478355235
0.270827179	0.383042497	0.302001289	0.001711053
0.025368343	0.174974702	0.454599099	0.380603242
0.453955522	0.401208668	0.258046609	0.436026411
0.453134116	0.469635002	0.092220227	0.115288695
0.457142595	0.173113693	0.25143924	0.464365458
0.317262131	0.140506881	0.139662419	0.481640051
0.435722372	0.095930131	0.372195625	0.425285855
0.378264946	0.349257048	0.117320392	0.300588504

0.062697599	0.478452062	0.025028046	0.372714621
0.301137022	0.136496279	0.011672407	0.315350871
0.346584621	0.468193406	0.481865006	0.112825738
0.344408516	0.417263127	0.373586456	0.469940775
0.014923776	0.049591933	0.333324106	0.476006574
0.357774727	0.464321786	0.320130598	0.304994895
0.285544613	0.465146691	0.003092581	0.507691348
0.513172919	0.359084975	0.423364472	0.416841685
0.391952524	0.476942675	0.045882288	0.427426785
0.403017955	0.085193111	0.474827572	0.164629022
0.453875462	0.044803033	0.365857553	0.445865341
0.447872002	0.009950446	0.295701011	0.430875144
0.023284528	0.136261917	0.452181089	0.442068276
0.420879143	0.455781602	0.470024846	0.375584122
0.440425383	0.264455411	0.012125131	0.311536522
0.451552567	0.388854632	0.453591687	0.278826197
0.389441847	1	0.446541423	0.249586373
0.052644032	0.360293329	0.028258586	0.356375172
0.426078853	0.143753475	0.419046767	0.136036995
0.068220777	0.498824666	0.161227764	0.245522696
0.113595425	0.441752227	0.278735912	0.280479149
0.231003704	0.173271505	0.365072111	0.410022441
0.052649734	0.099169715	0.011313663	0.387165341
0.155532888	0.372296472	0.378439807	0.446708627
0.351343596	0.121174734	0.403206319	0.283299714
0.113902723	0.014848476	0.492377791	0.422837587
0.396180927	0.179415624	0.039993788	0.372689324
0.479206285	0.396647279	0.33191379	0.471913954
0.030710702	0.469106753	0.276825399	0.125471943
0.379554952	0.284417619	0.470907538	0.08373266
0.425130802	0.469925494	0.315993897	0.197778557
0.432470917	0.447964873	0.038902802	0.446906433
0.441756898	0.033051867	0.501357871	0.11828922
0.019906772	0.44191313	0.463860866	0.076461148
0.078104603	0.380244302	0.068076748	0.295512811
0.497087803	0.42314202	0.47024616	0.203140486
0.048383704	0.0112246	0.19267597	0.399693142
0.518051848	0.478523971	0.266205394	0.377597574
0.261298176	0.353680777	0.192771718	0.476829374
0.182427982	0.508722342	0.006702558	0.448666132
0.51372339	0.351119989	0.082059292	0.366441867
0.446472343	0.055492624	0.220626045	0.497801721
0.467775332	0.358323189	0.388957684	0.435134428
0.024057445	0.055270675	0.405134436	0.169330579
0.423170548	0.232492143	0.042754325	0.374871465
0.051256156	0.458532374	0.460184918	0.06960127
0.409175401	0.361113344	0.376429511	0.041621893
0.450607886	0.478480765	0.523792303	0.185097418
0.455700447	0.466613918	0.409653787	0.076315845
0.015728742	0.50906622	0.442766479	0.028760162
0.472024368	0.308422897	0.016063078	0.490725013
0.208421803	0.463230296	0.007369392	0.076088986
0.034807525	0.454929489	0.492392184	0.427298406
0.327819801	0.388116524	0.004036352	0.48738399

0.490448563	0.012989872	0.130769933	0.462565561
0.441312879	0.52128662	0.320933352	0.462393659
0.436463013	0.500581122	0.276888471	0.279373858
0.478537505	0.472719259	0.461747275	0.458648792
0.475936224	0.426341703	0.309888145	0.020494162
0.146108909	0.272789737	0.373473957	0.100603083
0.39065097	0.148205555	0.462364641	0.469292421
0.468953299	0.004586021	0.192534966	0.464467495
0.468312356	0.474324828	0.019215269	0.007084011
0.008163377	0.01953679	0.402926732	0.240443242
0.471026783	0.315162287	0.005034945	0.061099378
0.318413728	0.398272672	0.498161103	0.40192345
0.449940377	0.082283341	0.343972311	0.422131003
0.069706588	0.379349191	0.475738411	0.483539079
0.037525759	0.248855923	0.395331432	0.479905627
0.062291501	0.317616334	0.445292275	0.354458107
0.011427349	0.079409378	0.068545307	0.445269577
0.501052008	0.084340677	0.035426905	0.180090591
0.145072484	0.467809928	0.184253766	0.4773846
0.47523389	0.454064115	0.066534534	0.490142761
0.043925963	0.491157655	0.334448668	0.083290835
0.285216684	0.012419133	0.447064311	0.179001338
0.37554931	0.436407753	0.215723693	0.01404336
0.173096668	0.052045762	0.476232376	0.460061777
0.028090307	0.271581414	0.303350669	0.421124943
0.351886812	0.464046055	0.513585285	0.454968929
0.107974067	0.119069606	0.444028544	0.437710723
0.280515139	0.470326177	0.327714113	0.496478231
0.450167319	0.459654968	0.464179071	0.395937441
0.218731817	0.447700882	0.435586195	0.029205265
0.382253314	0.473202336	0.39335539	0.16530433
0.271374134	0.384467715	0.052302729	0.20306996
0.322657274	0.381709617	0.159369954	0.450272744
0.425150711	0.443297893	0.045631815	0.066203988
0.526252703	0.447912949	0.189367375	0.47170792
0.171599374	0.368443735	0.4598018	0.449035063
0.38794227	0.471017054	0.463579216	0.445799961
0.445979654	0.292622845	0.525381767	0.12237621
0.086305741	0.429483099	0.113459438	0.0884965
0.15610011	0.026520241	0.294254199	0.432527739
0.371980246	0.40312209	0.357094171	0.087807288
0.319551148	0.386451016	0.368417383	0.249502393
0.091764936	0.505566723	0.009718336	0.054650037
0.374115158	0.470391684	0.390066442	0.247040981
0.295793729	0.125733831	0.316074536	0.363800595
0.381766974	0.369082571	0.472821561	0.044663273
0.024884723	0.473385714	0.418292617	0.017409361
0.081755617	0.420467037	0.297414046	0.273203992
0.018370737	0.455303351	0.407680977	0.436418676
0.393876648	0.068459198	0.396638638	0.09951464
0.065745076	0.453842004	0.397898086	0.50257236
0.341271079	0.440561653	0.466044293	0.463554454
0.12709732	0.389478672	0.035947405	0.467130741
0.433789393			

GNPTAB 0.37427512	0.497531871	0.36165838	
0.33328372	0.352223904	0.311380117	0.152342079
0.144786523	0.065453122	0.002115067	0.015425811
0.386876836	0.161554232	0.20339378	0.279577277
0.341484389	0.106808312	0.310675806	0.182306639
0.330632361	0.06186243	0.322174608	0.06479335
0.262697951	0.084548475	0.048293376	0.229937891
0.354010541	0.057425733	0.262020498	0.309224518
0.086242989	0.368863073	0.518941547	0.36319902
0.520551851	0.36202349	0.009615779	0.230670141
0.373839947	0.373126156	0.326149589	0.25608747
0.027067084	0.486160823	0.182388958	0.348807105
0.188389598	0.020903908	0.192123152	0.436571208
0.476468074	0.360769215	0.171868306	0.113438188
0.159728652	0.176211742	0.246738859	0.267576676
0.165629852	0.117023108	0.480356048	0.063156495
0.113480936	0.196513735	0.130761222	0.197421076
0.312086562	0.325924304	0.011993413	0.378237788
0.00108204	0.312417097	0.324907684	0.287190167
0.03009404	0.011801914	0.150874171	0.258996821
0.384349807	0.457415156	0.423446928	0.445446818
0.14310357	0.245824827	0.352889339	0.126016162
0.387409801	0.127207072	0.088162561	0.344366265
0.437339183	0.509654242	0.16391123	0.221147032
0.34743657	0.447702895	0.360070095	0.352616685
0.297978609	0.046820223	0.423014443	0.471382554
0.355126731	0.293881051	0.12184739	0.333264858
0.04323566	0.317973907	0.276110105	0.135691723
0.39656312	0.011047649	0.177731049	0.274416399
0.187969456	0.376165261	0.508474377	0.137271419
0.354960408	0.013013105	0.124827404	0.026202314
0.280028765	0.146735534	0.497040345	0.384552768
0.179623149	0.316837341	0.203002511	0.001006505
0.020062905	0.164715129	0.378071605	0.31740487
0.364484384	0.386921689	0.180498229	0.33535372
0.384418227	0.416455982	0.050075021	0.068640796
0.348441836	0.165287462	0.18479698	0.378296007
0.253429776	0.083063153	0.092636963	0.473369813
0.402085434	0.058317172	0.294358546	0.340416711
0.263461183	0.305738734	0.0772622	0.211169707
0.030360221	0.489733352	0.022933679	0.279328559
0.237637003	0.124819744	0.009323318	0.202576721
0.231443397	0.383169669	0.491237489	0.080517715
0.246425566	0.334608472	0.267299421	0.352451066
0.005407496	0.035520611	0.244450309	0.46593407
0.235442078	0.465698413	0.240324047	0.257708065
0.230930818	0.36955057	0.004194632	0.496779571
0.516580464	0.265096784	0.320778446	0.363759643
0.330380349	0.366741534	0.043670616	0.340553086
0.318914518	0.058508121	0.443525892	0.12367033
0.454370328	0.027999056	0.248253993	0.38581374
0.378550802	0.007314747	0.172100056	0.308119495
0.022373928	0.063983255	0.364373259	0.329643397
0.307628898	0.378684062	0.407325859	0.319430931

0.354513014	0.18313304	0.008963807	0.277863144
0.383574865	0.328268027	0.423208833	0.183579732
0.301396691	0.446541423	1	0.169849469
0.027589369	0.242680997	0.013469509	0.273404689
0.378375082	0.078755728	0.345588254	0.130724185
0.055715568	0.489511522	0.166750651	0.254563536
0.127726299	0.323020506	0.196329529	0.176757143
0.1689723	0.125829861	0.303628814	0.315884788
0.021803399	0.067161639	0.014073215	0.327134991
0.10404673	0.278874145	0.277170712	0.349869154
0.257173691	0.077970816	0.314410658	0.262966339
0.094592559	0.009980922	0.48107622	0.335907347
0.288814383	0.113909894	0.030625369	0.303064712
0.400496113	0.265189628	0.234932604	0.406898187
0.021443511	0.428529377	0.190826481	0.093799045
0.322555998	0.200286309	0.381707864	0.048356991
0.3328368	0.440834373	0.264399594	0.14486686
0.373531568	0.337302394	0.019129567	0.357759136
0.349095719	0.021057064	0.502456711	0.104824163
0.015001587	0.379841838	0.375830489	0.057019733
0.061852432	0.257926792	0.028902307	0.236251806
0.482390254	0.332021809	0.35832634	0.2027875
0.025266854	0.005472659	0.184094409	0.367419752
0.512976501	0.489745051	0.211091216	0.307121735
0.184691633	0.281176657	0.116006934	0.471079377
0.1941614	0.513509819	0.004194065	0.383242897
0.516694519	0.235272499	0.061693228	0.235876954
0.341440389	0.030721623	0.167047246	0.485552818
0.389654256	0.27156625	0.297421781	0.368360116
0.008928175	0.025158737	0.337581659	0.179193559
0.317221922	0.170308069	0.059086971	0.267902927
0.027275841	0.424978415	0.377815272	0.043646055
0.273284957	0.261559939	0.319901154	0.02438982
0.418934881	0.496712733	0.517096477	0.126629131
0.383661663	0.465259463	0.363984461	0.0642655
0.01293215	0.51799015	0.341648021	0.013981828
0.398669257	0.192559362	0.006469871	0.469022482
0.139492391	0.460251003	0.003020467	0.094110586
0.036879136	0.382684947	0.478016211	0.310381898
0.197014969	0.305137941	0.00563706	0.499288588
0.471037387	0.010856064	0.125786558	0.377706228
0.380923397	0.509432411	0.290488254	0.401456525
0.37742255	0.500325197	0.175142571	0.215470812
0.371605443	0.492258569	0.353824206	0.439652137
0.381633525	0.362212008	0.215837867	0.008616886
0.121674717	0.163020196	0.262543615	0.06264519
0.276084074	0.112281811	0.459983235	0.394721546
0.443143811	0.003058064	0.110395739	0.37495389
0.379662233	0.410150503	0.018925925	0.006268115
0.008554724	0.014965318	0.286829923	0.208109012
0.457499192	0.195334314	0.002306918	0.028439516
0.228813183	0.347660416	0.48608366	0.305582726
0.386044633	0.060863032	0.221573165	0.325392687
0.054011679	0.301501354	0.441775481	0.453132668

0.034240053	0.210975243	0.331399463	0.424341704
0.07753583	0.262615668	0.384566561	0.250121061
0.012722625	0.065507603	0.050631356	0.36757354
0.510506625	0.055020492	0.022347247	0.116276229
0.08203525	0.406428718	0.122431856	0.464977976
0.360973726	0.352783816	0.063845835	0.473005828
0.026057891	0.482863405	0.300386098	0.050315792
0.254537944	0.008945555	0.46615355	0.131021693
0.259804847	0.340624952	0.178218075	0.013354027
0.186711442	0.039854651	0.408382822	0.393085236
0.029949407	0.185686673	0.254000999	0.345597173
0.225361058	0.352304146	0.500525512	0.351704867
0.054925799	0.148457759	0.336052051	0.348957757
0.183960911	0.430919392	0.240264653	0.501950802
0.354093421	0.430232815	0.447871288	0.279050775
0.206794571	0.342745128	0.296711528	0.011640923
0.289087044	0.3747596	0.306242829	0.126203973
0.204599767	0.357997099	0.036183827	0.178751772
0.318983252	0.246136916	0.102884468	0.357130181
0.335236597	0.348427632	0.028665246	0.04698074
0.519853411	0.419983902	0.112650429	0.353233896
0.139770258	0.2786811	0.389405581	0.386260645
0.329509156	0.387700229	0.354911889	0.314171963
0.398261131	0.201343896	0.517941741	0.069705186
0.072009789	0.376344713	0.066338591	0.074138067
0.105276806	0.02100964	0.289572401	0.354926223
0.353942624	0.305563943	0.269095508	0.063854496
0.276615528	0.339491164	0.292501019	0.14611321
0.05486916	0.488147183	0.005964813	0.042129914
0.3518236	0.37574848	0.335834553	0.202497172
0.273620116	0.124088206	0.236440604	0.241142292
0.257353232	0.328437642	0.374041095	0.023838891
0.010871013	0.432091857	0.32679488	0.018617735
0.072790555	0.338854663	0.257795405	0.182577726
0.018095357	0.465426919	0.271166077	0.364163803
0.275617946	0.049155024	0.343439001	0.077926332
0.046882709	0.439870128	0.359081837	0.508437046
0.319231795	0.337346589	0.453600821	0.372341628
0.113871418	0.363914397	0.017069393	0.367889818
0.362786029			
GOLGA7 0.14194643	0.267585879	0.080411433	
0.211478729	0.115893299	0.162423951	0.089480021
0.023389372	0.011542138	0.000880928	0.005631378
0.095052354	0.005187057	0.037226271	0.062101295
0.186788961	0.01431071	0.045313338	0.072503162
0.169452645	0.03086541	0.164615616	0.022785094
0.032725447	0.020417575	0.033313088	0.068163268
0.200689708	0.034296125	0.117796116	0.165881514
0.007767624	0.166105849	0.252616822	0.144597289
0.237394549	0.134961437	2.94E-05	0.103105235
0.177092471	0.198168176	0.18777006	0.117910989
0.018043983	0.234362935	0.114034789	0.098881345
0.007177678	0.007690959	0.056839018	0.230763297
0.243664988	0.176542166	0.052307869	0.071033425

0.009611246	0.045692277	0.024402754	0.133044072
0.085734266	0.045269512	0.251264778	0.024671873
0.018412836	0.04936928	0.011819412	0.069906743
0.054264469	0.170238632	0.001501291	0.097945331
0.000454007	0.148249758	0.081661448	0.079709331
0.011976499	0.003158405	0.018387665	0.167101508
0.212792345	0.195589708	0.221814435	0.237225007
0.008208913	0.055501908	0.083890626	0.039193369
0.194825813	0.077965067	0.001632227	0.176760885
0.229075999	0.25657471	0.017540234	0.065612464
0.247736204	0.238914849	0.10316872	0.119784891
0.078490572	0.004613891	0.251095497	0.238939534
0.179259692	0.050782716	0.036060125	0.1826021
0.014203497	0.023295033	0.150031949	0.001365769
0.212379761	0.010465676	0.043094044	0.140011428
0.012966698	0.173993598	0.245423394	0.07080046
0.064014022	0.000476099	0.004630411	0.004328278
0.005883032	0.007187907	0.248048339	0.222972826
0.018631918	0.043716842	0.071442882	0.000625242
0.0038346	0.00571238	0.165187197	0.058089407
0.183744002	0.159274426	0.089866958	0.159097865
0.1416994	0.223524225	0.001984945	0.02792562
0.097961525	0.010154545	0.084112525	0.132393812
0.072882527	0.022301157	0.030697254	0.252851546
0.224160483	0.029463805	0.050366709	0.148142549
0.082861926	0.075212246	0.021189287	0.038559747
0.017749331	0.228170296	0.003757744	0.058248846
0.029931643	0.00465449	0.000403119	0.069340161
0.018218409	0.240488772	0.226941113	0.023111128
0.063438166	0.150630189	0.076984302	0.179402913
0.006117647	0.001858054	0.05182681	0.2384972
0.127026403	0.212372295	0.034346338	0.04286297
0.073731516	0.208433279	5.23E-05	0.239335728
0.254960803	0.046821551	0.114338586	0.093977244
0.119877017	0.229201115	5.62E-06	0.144271408
0.110857432	0.018509747	0.237437526	0.04448465
0.207432536	0.020099719	0.067826583	0.20251668
0.145046662	0.000208183	0.088677279	0.186333433
0.003485848	0.038146405	0.173387151	0.188211438
0.187415467	0.185964584	0.223610264	0.110404045
0.101147693	0.040514215	0.000667028	0.060745184
0.169723995	0.027803485	0.2463682	0.038030838
0.078901869	0.249586373	0.169849469	1
0.025178385	0.10762738	0.000673643	0.112565146
0.190067162	0.032749921	0.133205414	0.00432132
0.010832337	0.264638983	0.019933351	0.032888279
0.011588986	0.132114805	0.064338069	0.041771263
0.016263003	0.031655622	0.068665427	0.126224481
0.017181936	0.020956363	0.000408867	0.039729427
0.008538713	0.039615768	0.045800912	0.15689934
0.022645988	0.004665124	0.074746484	0.035409606
0.016400027	0.005036369	0.256777764	0.139695623
0.149184128	0.03556369	0.006569781	0.013052568
0.241750832	0.114351091	0.042198156	0.253797978

0.000530628	0.231759273	0.041237159	0.033884524	
0.050786428	0.076126526	0.235370799	0.003411771	
0.107360967	0.245912146	0.069867886	0.06212643	
0.188160606	0.148911166	0.003391571	0.164079426	
0.135783333	0.008684849	0.23845366	0.009021911	
0.005127648	0.141064803	0.207462946	0.024369681	
0.017370806	0.130473178	0.019277231	0.094028698	
0.262710849	0.172275308	0.245493261	0.024862701	
0.019041423	0.000529515	0.025619245	0.071285839	
0.243693823	0.22818834	0.058436649	0.073564383	
0.088458206	0.076563145	0.010019429	0.24335329	
0.004769908	0.234536857	0.001902533	0.194595815	
0.248297699	0.066328868	0.022599643	0.143593703	
0.185253334	0.021178641	0.024887156	0.240834469	
0.256474964	0.086242641	0.088879449	0.113690006	
0.007435668	0.011866105	0.131967431	0.017650844	
0.134398529	0.059888448	0.00102571	0.037379448	
0.012238895	0.248146904	0.174683517	0.026179831	
0.175170263	0.043608515	0.10885758	0.014479743	
0.210120227	0.222776852	0.251459321	0.025460425	
0.150641899	0.246254284	0.116363426	0.013235857	
0.004318864	0.23843908	0.156609549	0.002389941	
0.215682585	0.063738021	0.002181639	0.253219205	
0.062868534	0.211940025	0.000538675	0.000942927	
0.003016965	0.182889293	0.260487159	0.161411062	
0.069138583	0.107095076	0.000536922	0.232482295	
0.241672699	0.002533295	0.011284732	0.176771597	
0.153462429	0.257312272	0.066739521	0.204141974	
0.104125132	0.255341092	0.013316224	0.096443699	
0.210620828	0.231015718	0.16915363	0.232456121	
0.209735636	0.142162467	0.125152038	0.009912586	
0.015171575	0.066698045	0.068943789	0.04108921	
0.117162083	0.032997398	0.203158762	0.219924003	
0.208255618	0.001702367	0.028827894	0.172094277	
0.216827734	0.236137254	0.00048132	1.82E-05	4.82E-05
0.002787	0.132892392	0.045379602	0.243850536	
0.02296908	0.002234696	0.007753517	0.022983185	
0.117302595	0.254398564	0.150672417	0.168535229	
0.016200594	0.049032758	0.158484339	0.011142121	
0.113556497	0.2263489	0.259010104	0.003800255	
0.059656195	0.048920119	0.255491454	0.005062485	
0.077842006	0.105138332	0.040636515	0.00025827	
0.014759768	0.023231981	0.176249168	0.241780764	
0.009425791	0.00495053	0.001968772	0.039178817	
0.210012768	0.021578738	0.245002294	0.215794296	
0.198774324	0.006302951	0.26442239	0.003815387	
0.22588956	0.067671185	0.002340643	0.025395873	
0.001522379	0.195653722	0.031626275	0.051406669	
0.131056256	0.022167575	0.002684312	0.012255785	
0.008267354	0.246567514	0.199877315	0.001945336	
0.057849859	0.06196912	0.11911146	0.090825388	
0.192158146	0.250796179	0.100808594	0.037581406	
0.00163344	0.151679899	0.111674098	0.031740656	
0.222707033	0.069237529	0.243807807	0.150863707	

0.223596654	0.217137813	0.151210453	0.011664458
0.100994051	0.197671429	0.002232787	0.09064508
0.220483664	0.077380742	0.016404839	0.081471847
0.092957668	0.003357582	0.001483036	0.057853766
0.138682066	0.023492031	0.195018103	0.106001635
0.119754244	0.001793259	0.02228209	0.263507688
0.230832705	0.015580329	0.193809014	0.030264598
0.147029928	0.167781792	0.159824157	0.053343839
0.212533659	0.164534521	0.176393575	0.236037199
0.105814746	0.265355657	0.005258031	0.002005267
0.104562834	0.024982491	0.019263413	0.039002183
0.004894623	0.031257615	0.147887166	0.039552155
0.13791087	0.019838998	0.005065083	0.073316676
0.056916826	0.105915018	0.069047068	0.012852614
0.262739921	0.000628183	0.009353916	0.038112355
0.196723022	0.090572224	0.035398975	0.026322388
0.010340149	0.092659094	0.068425599	0.068959201
0.007823247	0.206394475	0.001530653	0.004757931
0.223358187	0.060678384	0.000871655	0.008225798
0.140040846	0.01915169	0.063052841	0.003112991
0.23414946	0.119079807	0.114158414	0.131536338
0.011008492	0.171642783	0.001702522	0.004604674
0.201021738	0.086883711	0.237392669	0.050424377
0.192227519	0.222192108	0.183467982	0.001258149
0.074333326	0.009218095	0.198295718	0.056382362
GOLIM4	0.035680602	0.053837856	0.028729745
0.139369751	0.067655456	0.052370194	0.217706712
0.067399082	0.166539322	0.143388434	0.12419734
0.03694252	0.069013389	0.071401873	0.027555995
0.1363039	0.12121032	0.028966607	0.23712109
0.116446361	0.170298075	0.154591872	0.242551859
0.031732592	0.112559992	0.226470872	0.059272893
0.114413476	0.237037636	0.046711114	0.09643828
0.109983405	0.053785732	0.034816644	0.075251953
0.028762817	0.025479462	0.034885082	0.055897513
0.047588496	0.071694723	0.124385642	0.197603194
0.272580203	0.030590247	0.24485446	0.061811106
0.036088037	0.192267528	0.037605815	0.033864266
0.029437113	0.046214083	0.090510947	0.19958321
0.04174541	0.032593026	0.015084143	0.049993167
0.233751384	0.050919147	0.033176732	0.188935539
0.050574851	0.086294869	0.084772462	0.124084654
0.018495609	0.049650193	0.178866815	0.019000978
0.047262672	0.137869714	0.075912945	0.016270552
0.238473382	0.177361519	0.08957226	0.176958742
0.094720837	0.030549156	0.080197044	0.062623109
0.092191165	0.102470366	0.029082813	0.173610907
0.050828506	0.210904194	0.087270668	0.133443974
0.074658478	0.040659134	0.105182908	0.127607576
0.070356089	0.0528508	0.036605816	0.056162675
0.023858579	0.079710945	0.038705607	0.059383275
0.038620034	0.024027258	0.183775816	0.084362158
0.216540484	0.015414528	0.202892141	0.040701001
0.097572804	0.254734864	0.126048519	0.126886071

0.077426158	0.053324803	0.037608816	0.264074939
0.044734815	0.12918049	0.030989796	0.159456652
0.039510122	0.013392116	0.023821634	0.092211516
0.060050679	0.036008539	0.140697967	0.109264089
0.1624877	0.050143964	0.036708483	0.04417969
0.072829354	0.019159815	0.122238309	0.034891727
0.033690449	0.078666348	0.008105043	0.226218938
0.06254539	0.05960496	0.260367382	0.044737343
0.112639115	0.178342601	0.218483656	0.070146624
0.044776979	0.285143153	0.021020749	0.130430859
0.049192025	0.077172561	0.212523751	0.091426495
0.263660438	0.027344255	0.122689627	0.04659425
0.020584704	0.060898785	0.097553926	0.089738614
0.055558214	0.051597451	0.020244885	0.219696521
0.109050527	0.116171588	0.03641724	0.060471078
0.158275324	0.170189966	0.016696279	0.061024856
0.039903319	0.053626479	0.083033339	0.056499342
0.160406769	0.094680666	0.002711396	0.032013216
0.038651678	0.014515703	0.097520635	0.054822547
0.027814516	0.101672879	0.001749907	0.123308468
0.024379074	0.214005117	0.084680144	0.215404317
0.032521894	0.241972727	0.053111328	0.040641694
0.028139979	0.050262334	0.078059276	0.133950617
0.108278437	0.145656414	0.063700992	0.126819501
0.112294883	0.073015025	0.103396741	0.024599191
0.066384712	0.124366366	0.138234871	0.081894677
0.047364761	0.011846864	0.043882382	0.120457544
0.018077158	0.052644032	0.027589369	0.025178385
1	0.102109463	0.101674811	0.110210847
0.027070962	0.18225516	0.028742889	0.063711701
0.151209045	0.061045641	0.029862505	0.013757461
0.015986193	0.062839612	0.171008949	0.066627868
0.053407889	0.162320063	0.033585155	0.046567951
0.237827916	0.203721292	0.044836522	0.010351421
0.067104417	0.024259658	0.02132221	0.035778459
0.040718224	0.117643595	0.077949767	0.027834273
0.12200003	0.201185981	0.053142871	0.120579251
0.031655807	0.192789175	0.186969432	0.009527374
0.083921423	0.031284979	0.013535667	0.067779266
0.111212534	0.072461077	0.093322949	0.212155718
0.031215459	0.206614974	0.110756795	0.04764575
0.04301254	0.049035852	0.110321273	0.150885781
0.034844071	0.101136716	0.252492051	0.033039656
0.030752215	0.224395535	0.047147088	0.101111414
0.180428864	0.058580087	0.047508215	0.24715725
0.162786314	0.104169476	0.187718477	0.189448257
0.061603112	0.150131189	0.056332548	0.035646208
0.265550519	0.09849819	0.065576394	0.025288885
0.030536212	0.043425178	0.176120582	0.061905437
0.264487556	0.03753699	0.035387763	0.058641525
0.021281228	0.036663332	0.162119086	0.047771878
0.028378409	0.036032873	0.153821932	0.126489226
0.043931354	0.268701766	0.100579425	0.038805756
0.061847342	0.065987587	0.040855411	0.059533516

0.241137337	0.253307425	0.06939609	0.020720579
0.039984377	0.133597928	0.006302128	0.034637919
0.236008367	0.049358988	0.082362354	0.198335134
0.050815933	0.028778815	0.109002073	0.260093509
0.0406878	0.033341477	0.036822825	0.137213967
0.040947614	0.044804707	0.023173772	0.152192502
0.148358357	0.03230918	0.051355904	0.126869656
0.092408284	0.030675983	0.152127651	0.055278528
0.245754334	0.020913246	0.041671071	0.00428145
0.0841376	0.075275546	0.034610833	0.097322858
0.022505645	0.109505433	0.008767645	0.047178663
0.044547111	0.124461484	0.070682945	0.037945247
0.03175951	0.04862648	0.030009335	0.048712117
0.031048195	0.031778761	0.097603053	0.200509451
0.069799346	0.030306923	0.071770554	0.046635292
0.089754315	0.083390994	0.248046972	0.269264814
0.054491006	0.078180766	0.017675811	0.116913672
0.040401103	0.138951866	0.043957263	0.087012458
0.05137169	0.139976384	0.132502208	0.039077444
0.080649208	0.090959599	0.067566319	0.014791917
0.003908228	0.081729651	0.104928606	0.109974153
0.053152169	0.018117379	0.176899834	0.189307183
0.077084842	0.05858737	0.043505014	0.177631231
0.046731899	0.197435764	0.039431108	0.141918478
0.131021882	0.019138453	0.061475016	0.047495569
0.127768639	0.103575856	0.027010211	0.092278888
0.00501726	0.143511582	0.039381295	0.068964029
0.049054036	0.144700647	0.172038919	0.092827831
0.026309732	0.135329023	0.154402041	0.0118559
0.195482093	0.066524634	0.155574913	0.073567329
0.084343959	0.133207107	0.095427075	0.042316194
0.181158482	0.032079221	0.052134706	0.024155273
0.046565945	0.165173861	0.029335588	0.037234815
0.038530539	0.05822842	0.042426794	0.113435793
0.012087005	0.182185291	0.094077498	0.069289214
0.085247393	0.114085515	0.115280462	0.081836883
0.090236944	0.113566807	0.034943781	0.019272548
0.240077192	0.010106468	0.101633518	0.032658763
0.060590333	0.079685268	0.060253635	0.050881297
0.04663554	0.051111493	0.042820006	0.136877421
0.037490813	0.040469299	0.095763104	0.173397914
0.12788083	0.092715114	0.054409694	0.092824108
0.151633568	0.022473778	0.152451441	0.007452855
0.014336447	0.068073095	0.187964099	0.122356409
0.048796396	0.035040874	0.140142209	0.18052854
0.049959088	0.043430292	0.115829191	0.080379522
0.156065002	0.134309259	0.053458258	0.05499931
0.018636	0.052622224	0.072611192	0.070357378
0.048131786	0.203454255	0.039204583	0.051371794
0.073509604	0.024337386	0.187678155	0.178726918
0.251179828	0.16628162	0.008520535	0.103077658
0.008412186	0.150753827	0.016103413	0.150153548
0.097921143	0.017231134	0.135782918	0.150361508
0.207211354	0.038681373	0.102545159	0.184705188

0.011817275	0.054299078	0.038712193	0.104947745
0.020349197	0.062785901	0.182847064	0.044502212
0.027668603	0.011256343	0.052232082	0.145430348
0.239530129	0.083521964	0.026105526	0.080389542
0.065173843	0.030378958	0.036911631	0.102512495
0.102374966	0.036823431	0.037668312	0.051938598
0.117965838	0.202563663	0.02688174	0.056193896
0.154002704	0.033854256	0.033303946	0.034254375
0.034731156	0.150240799	0.026011845	0.043620721
0.039900917	0.015170352	0.267840791	0.078320961
0.012129644			
GOLM1 0.267073113	0.384507813	0.126024622	
0.363941223	0.197095812	0.240055822	0.213322712
0.212711954	0.086742329	0.023391019	0.025055405
0.186773216	0.095539448	0.179700186	0.130318971
0.344027516	0.079578644	0.169999313	0.204532857
0.353831218	0.18358244	0.294170578	0.153122454
0.097367359	0.054385724	0.087040603	0.09041353
0.406451416	0.093202521	0.190704437	0.372004479
0.079760113	0.335712503	0.344883047	0.228239962
0.316972292	0.23170129	0.027871417	0.173564014
0.344005101	0.269191121	0.371020315	0.251074519
0.098626985	0.34580751	0.215631093	0.182700232
0.159779084	0.042623971	0.179610855	0.351554205
0.303915133	0.195662731	0.229810301	0.192736592
0.075305821	0.201325184	0.052933661	0.302838121
0.205285056	0.18300135	0.315704608	0.129637958
0.143409792	0.246781116	0.08476033	0.233228091
0.093509152	0.328036136	0.077813273	0.153839314
0.014778717	0.34746003	0.187180046	0.066958038
0.062766305	0.039162213	0.09858926	0.294823212
0.400294231	0.314436924	0.345287136	0.329435159
0.090049965	0.153625759	0.119551295	0.14877231
0.337146515	0.179709884	0.093924891	0.320405044
0.345921404	0.344802521	0.110615959	0.12913029
0.315237714	0.385220345	0.143050551	0.154112139
0.087939757	0.035703698	0.324531616	0.35423735
0.328672347	0.072304957	0.097244647	0.389378516
0.09331124	0.132769298	0.299718612	0.080950136
0.405510517	0.063811169	0.099982908	0.259787475
0.161834695	0.309125325	0.321902815	0.196656832
0.152113417	0.033743838	0.035117752	0.055750351
0.145374752	0.03039472	0.309522975	0.372874326
0.217083952	0.143961759	0.23347373	0.010413287
0.035134421	0.102719176	0.280776698	0.176792445
0.330383888	0.226434361	0.1256026	0.296476319
0.188814404	0.342384229	0.032035616	0.178410019
0.196193909	0.112040738	0.203778896	0.208768042
0.120881466	0.197339715	0.158567707	0.3789457
0.375933568	0.135519374	0.173634089	0.308934555
0.158862081	0.136869854	0.159026178	0.174721279
0.137024559	0.288899883	0.028117295	0.194348667
0.065705659	0.06096775	0.044166884	0.229673977
0.149417914	0.294320274	0.290772674	0.140792267

0.186274501	0.244894694	0.203682882	0.252527185
0.025085891	0.081164736	0.157771245	0.364444757
0.260283808	0.341612343	0.185533594	0.137901381
0.156243868	0.352081813	0.002031506	0.335625038
0.33452639	0.1377673	0.201134693	0.208688397
0.095012366	0.366738222	0.016416328	0.315191491
0.25064446	0.128442126	0.380122985	0.109479686
0.319012541	0.065935306	0.218796605	0.241725488
0.207716764	0.018613036	0.19178102	0.363260405
0.027158806	0.197773141	0.323465081	0.313575709
0.220971035	0.309199085	0.388459468	0.083984295
0.178956425	0.223332105	0.031729721	0.127118983
0.285235064	0.11018548	0.357112395	0.215038833
0.190400867	0.360293329	0.242680997	0.10762738
0.102109463	1	0.04585539	0.145095052
0.257974271	0.145407803	0.121468934	0.101001704
0.059544304	0.372674052	0.049843298	0.054119986
0.028293657	0.208500226	0.220643221	0.124544267
0.072740054	0.140622689	0.108869187	0.147332957
0.130875493	0.102424491	0.014895718	0.08664415
0.066173142	0.102465659	0.105011561	0.27993108
0.179901082	0.087123304	0.1394681	0.092944137
0.069453894	0.035213189	0.372201571	0.3067394
0.288385705	0.184204129	0.063183256	0.086261347
0.373726478	0.161108387	0.150233766	0.376207501
0.050346922	0.360754195	0.133618969	0.093075819
0.10177144	0.176584215	0.401341936	0.055015981
0.249326065	0.371671502	0.176778973	0.111348122
0.314417323	0.307174387	0.087734112	0.277585334
0.269669599	0.059687723	0.349851919	0.069174522
0.038656451	0.237024545	0.233609641	0.083122951
0.069941759	0.158092615	0.153869542	0.185691107
0.373651214	0.309980693	0.290710949	0.07508252
0.087622884	0.017180932	0.088802555	0.135163966
0.340169495	0.321239372	0.178977514	0.108282595
0.225068371	0.106653809	0.186073679	0.349464431
0.085625538	0.351245664	0.026242415	0.246367363
0.313880168	0.190303176	0.058478718	0.24631355
0.322218711	0.103521769	0.207802803	0.338909611
0.352366485	0.143594121	0.118566863	0.262175416
0.078147457	0.145798915	0.175493029	0.046337089
0.155678731	0.160500439	0.008953395	0.206300398
0.076306562	0.355995486	0.345535735	0.066844148
0.194597987	0.188927864	0.222498056	0.085236545
0.258232006	0.311352526	0.351609541	0.219349609
0.274184893	0.308749726	0.124426192	0.078566322
0.025546592	0.318545539	0.302006066	0.039360973
0.385086385	0.16441785	0.028579905	0.390094711
0.201678169	0.301740991	0.01038294	0.02376388
0.033208141	0.318518828	0.32668105	0.363169785
0.12378478	0.282612242	0.006727802	0.34502387
0.347662778	0.018101006	0.065395197	0.256583076
0.195794046	0.392953598	0.075752922	0.266366248
0.154611742	0.313416602	0.173132617	0.172086611

0.333994092	0.285038197	0.338865722	0.364628266
0.372622829	0.256547045	0.249707132	0.060647024
0.055091627	0.229170711	0.086403328	0.067365962
0.259150759	0.085432126	0.338948411	0.364951415
0.307333845	0.017192686	0.225237538	0.28045916
0.404463471	0.413660892	0.032811868	0.004530245
0.003343932	0.011421567	0.300762634	0.121053538
0.341684659	0.100968169	0.023583604	0.148046438
0.168639726	0.164898133	0.348714012	0.340947188
0.269705936	0.107755394	0.11369997	0.358264326
0.047870827	0.231812912	0.32855588	0.372902861
0.054879706	0.131066714	0.154203526	0.405762103
0.010610855	0.186288368	0.211441663	0.152897894
0.021421586	0.063172239	0.063941595	0.334920819
0.300497553	0.059208043	0.035880747	0.095364786
0.13841129	0.354736623	0.130041213	0.379585797
0.371231734	0.38268979	0.050894342	0.350754781
0.059057973	0.320981033	0.095883582	0.043075913
0.142949673	0.038065699	0.291358536	0.045780768
0.193816265	0.31551289	0.072608288	0.022370583
0.038896227	0.060582986	0.368512984	0.305055347
0.029846393	0.237490941	0.152700453	0.281840394
0.236060466	0.322315753	0.346860982	0.12786651
0.171338231	0.037155527	0.359837244	0.253695158
0.229630473	0.357246975	0.117990802	0.358185744
0.29799604	0.362362087	0.314503322	0.23963858
0.079238254	0.224946046	0.305439588	0.055321739
0.231905831	0.350344193	0.212635723	0.065999204
0.128339821	0.111951863	0.124696688	0.083165113
0.066294447	0.261824487	0.160223652	0.383012492
0.279954467	0.266992826	0.132477378	0.060598158
0.384117184	0.351636951	0.163525355	0.291356354
0.131648793	0.187671986	0.281021184	0.266280671
0.131671198	0.309680552	0.360054782	0.292797306
0.365189671	0.215707222	0.35475879	0.15583105
0.042251891	0.197657758	0.100589518	0.07860739
0.14823028	0.038781119	0.054979003	0.310456801
0.075119744	0.267062146	0.177347439	0.08788556
0.154795227	0.134058808	0.222258898	0.198391837
0.092871429	0.340315085	0.01850646	0.073256915
0.086209718	0.280509881	0.105715396	0.1305291
0.06953851	0.076308826	0.190147354	0.191584193
0.110239808	0.104763768	0.281417432	0.081607758
0.087582469	0.376691178	0.156831042	0.024562484
0.078624993	0.240135642	0.16585932	0.145146691
0.022113583	0.292823877	0.146608582	0.176561691
0.271207358	0.107449818	0.311410796	0.091132885
0.112712998	0.338599032	0.136699091	0.320753234
0.118352864	0.343347755	0.326805158	0.300201397
0.078667595	0.120557797	0.088236997	0.379230341
0.085537225			

GPR141 0.016285684 0.026809542 0.011661169

0.06833315	0.022198814	0.028300751	0.110390243
0.062628896	0.015130417	0.05605025	0.005819256

0.019518641	0.006455423	0.065566059	0.010148109
0.04975047	0.038789653	0.014401774	0.113854648
0.048185725	0.105527494	0.056427647	0.09013367
0.004977631	0.029526125	0.057136146	0.013269626
0.059775498	0.067677269	0.026909702	0.056295194
0.020681057	0.027837007	0.019426173	0.031910584
0.016087629	0.01330902	0.001198171	0.023174608
0.028643357	0.029762476	0.051454509	0.097116286
0.0803426	0.020330807	0.110799978	0.021317265
0.004352524	0.040266687	0.040411375	0.02037126
0.017585661	0.015760004	0.063681651	0.088987387
0.003170738	0.025984235	0.003603817	0.041465075
0.095412387	0.054998707	0.017158266	0.025408019
0.000899182	0.050062553	0.018190186	0.077839312
0.003410959	0.032686508	0.033381774	0.007336752
0.003323918	0.063163093	0.027305929	0.003233209
0.077306415	0.043790289	0.027168662	0.077849419
0.045166811	0.017011018	0.035130574	0.02518034
0.037796656	0.031024327	0.007896404	0.067811643
0.025813365	0.108791511	0.074342971	0.047977582
0.032904718	0.020627681	0.019928386	0.03314302
0.034634218	0.027484798	0.021436878	0.019633445
0.007265667	0.007873363	0.019047604	0.027833496
0.025618939	0.00669041	0.057075194	0.051754924
0.06200893	0.009905504	0.087008187	0.006170492
0.04968831	0.057730422	0.033817907	0.056962083
0.013317085	0.029852899	0.018577668	0.119728207
0.018110182	0.022877542	0.001200012	0.032256982
0.005922366	0.002494811	0.013983678	0.049968117
0.040518571	0.005133452	0.061641661	0.018387384
0.038063448	0.004433468	0.01842566	0.021187147
0.028493859	0.010035393	0.036660007	0.021268111
0.013702573	0.031918159	0.001399313	0.07516852
0.045816679	0.006412877	0.092766782	0.017037478
0.043184754	0.105266693	0.088539432	0.033064134
0.028493598	0.120844071	0.012165455	0.05775278
0.010094651	0.028342257	0.092195597	0.062162688
0.077909399	0.014102668	0.029334507	0.040055493
0.00310005	0.008529721	0.008674796	0.065431976
0.031665122	0.02566681	0.011379844	0.086418122
0.061894545	0.039388288	0.025902796	0.037169498
0.010857075	0.034972028	0.014023617	0.031094997
0.035799223	0.024959511	0.047116673	0.017027806
0.053792313	0.041029769	0.000100572	0.020229018
0.019446226	0.019521431	0.054692337	0.022944335
0.006492902	0.051358865	0.001652511	0.052874126
0.020653554	0.079896955	0.036927877	0.072545201
0.018078171	0.065024316	0.026818484	0.014753378
0.014388625	0.003183358	0.063816978	0.06114551
0.014751455	0.058828937	0.026828292	0.05497803
0.039789904	0.027931135	0.045262307	0.005821622
0.029023862	0.079404336	0.019094985	0.027006981
0.019283567	0.001043892	0.02659573	0.055821458
0.019440374	0.028258586	0.013469509	0.000673643

0.101674811	0.04585539	1	0.03576906
0.01220779	0.062412613	0.007749209	0.009194859
0.042691315	0.028907197	0.007111065	0.003524389
0.00291673	0.036684218	0.082617726	0.043153004
0.01938057	0.032247344	0.008691634	0.011255044
0.097679265	0.081731633	0.002289855	0.007852982
0.02391388	0.02461812	0.022427264	0.018427529
0.021309894	0.051905502	0.047215462	0.004054071
0.031309643	0.057728961	0.026927793	0.051178613
0.02600659	0.064748218	0.0566293	0.002175606
0.041082919	0.028372137	0.013622498	0.0347728
0.019272045	0.028072397	0.026228493	0.067550307
0.015459911	0.099498159	0.049477262	0.001714315
0.020808551	0.027334169	0.037233494	0.040594362
0.027156075	0.048417484	0.033530002	0.019113354
0.023857288	0.077143163	0.024574776	0.02606286
0.049153425	0.022967225	0.018487845	0.065825468
0.043261274	0.046065906	0.080939198	0.062009253
0.030843963	0.059833953	0.023066207	0.008032443
0.094039999	0.011846705	0.015788143	0.012008476
0.018299921	0.021076667	0.065386901	0.030857833
0.104435928	0.008802105	0.003838153	0.028728648
0.002788001	0.020026207	0.042478013	0.015751909
0.015666594	0.03430126	0.043107036	0.058728352
0.03264515	0.08475869	0.044227746	0.023163223
0.026244399	0.018586824	0.014494039	0.026752845
0.108988999	0.035718497	0.021161104	0.003137332
0.010036567	0.033013575	0.00133242	0.014150975
0.049288812	0.026921507	0.037619344	0.026255906
0.026391681	0.018203923	0.035888409	0.083537783
0.016714094	0.01638242	0.021270539	0.062719211
0.018897479	0.021483549	0.008278222	0.041290556
0.03595965	0.017663095	0.028236247	0.007470549
0.040697637	0.041773727	0.027920431	0.030471543
0.10521274	0.013619203	0.003939886	0.000161116
0.010490401	0.030467889	0.019028672	0.050040857
0.020179794	0.046850229	1.55E-05	0.022096781
0.026539018	0.023640708	0.00864859	0.018292797
0.015136182	0.02645856	0.008661139	0.022081032
0.016249347	0.016222389	0.02160747	0.060432202
0.04018942	0.013991642	0.043840894	0.027250873
0.045449088	0.028488202	0.097263403	0.064256314
0.001720642	0.053065832	0.008726923	0.002489875
0.032823092	0.028179116	0.025712381	0.034752944
0.026257551	0.038653198	0.054000763	0.019993835
0.04893031	0.042925279	0.005217011	0.000917425
0.007006154	0.007575291	0.047770811	0.033213973
0.024343432	0.010155172	0.049740311	0.095281412
0.02228718	0.020001048	0.022554365	0.077207036
0.018164056	0.049250966	0.021517244	0.061716159
0.013504762	0.015401344	0.030764607	0.026546848
0.01780085	0.024512724	0.019896781	0.042128009
0.000561372	0.049113342	0.019864689	0.02111261
0.000847062	0.029206768	0.023354139	0.03489814

0.013833833	0.040494559	0.041019996	0.001167206
0.09124586	0.027512139	0.071808652	0.033148441
0.045745639	0.059455793	0.008135468	0.022138057
0.005969559	0.020240064	0.017348003	0.008501112
0.022938025	0.027202416	0.015054678	0.002688174
0.036425247	0.033434795	0.002338788	0.015981506
0.001792425	0.058617135	0.039674612	0.026851622
0.010367045	0.035069382	0.042770785	0.036497142
0.080213128	0.054505194	0.020550633	0.012931906
0.072055998	0.000431293	0.056225716	0.025834924
0.044046004	0.033666553	0.01321929	0.024401609
0.028450781	0.024349136	0.020207437	0.058110404
0.00279968	0.030517898	0.05513507	0.066818426
0.071199399	0.041395609	0.028647045	0.019197556
0.048446324	0.006561245	0.006103871	0.003074406
0.00438331	0.050604763	0.037092063	0.050778857
0.029275353	0.028127895	0.011050552	0.026848488
0.025097334	0.022489962	0.036333561	0.037469297
0.050404222	0.03774377	0.024044234	0.021515648
0.003192133	0.026133042	0.043486672	0.046975739
0.029460698	0.073243828	0.020972633	0.053926531
0.002759207	0.010611475	0.053490001	0.045792787
0.108613636	0.022946866	0.002034183	0.039955427
0.003046829	0.066009857	0.005075702	0.008214292
0.026496021	0.00838741	0.045885378	0.100943688
0.064074532	0.021419445	0.005180052	0.052331532
0.005287362	0.025936698	0.015138402	0.043282992
0.003490053	0.006285814	0.064319281	0.045212331
0.027588937	0.001495079	0.025802242	0.021061
0.071937604	0.036796899	0.028000531	0.008202107
0.000555879	0.013200251	0.014218995	0.014852664
0.017470804	0.016398059	0.016961198	0.017101228
0.05973052	0.054640763	0.019730377	0.001723368
0.023747265	0.024074959	0.013252382	0.018109567
0.011043629	0.063452367	0.016820563	0.021731852
0.00984882	0.00556051	0.076002718	0.034901732
0.007428527			
GPX1	0.260457949	0.391574584	0.186975454
0.373194471	0.227800579	0.190759995	0.170048195
0.094953322	0.193127385	0.020339786	0.081784902
0.237226283	0.150799203	0.127399446	0.169533966
0.39617275	0.145959492	0.136650159	0.194432899
0.305559511	0.08924386	0.362326653	0.145119298
0.188736262	0.172778015	0.154228808	0.240369124
0.387163011	0.165137555	0.118468269	0.258969182
0.120275157	0.295424415	0.351402353	0.309862075
0.331345357	0.229680271	0.009178333	0.153224154
0.294577028	0.371036283	0.315446464	0.265731531
0.105222	0.301041309	0.208792412	0.261532712
0.107184095	0.10042614	0.086572909	0.282672318
0.307291881	0.236572859	0.131790369	0.163232611
0.119982246	0.072728642	0.135924573	0.161935618
0.182862423	0.062475091	0.326750789	0.155333003
0.092613502	0.128404356	0.137195583	0.142228341

0.174681457	0.240866413	0.040840228	0.192011876
0.004621421	0.306496218	0.247382878	0.185311699
0.105247591	0.053707369	0.154499747	0.250617739
0.389267804	0.3056373	0.428079427	0.414365272
0.14595766	0.19329485	0.188284531	0.178012453
0.324248432	0.162118224	0.11291618	0.399726804
0.428752115	0.368720831	0.212196555	0.220723458
0.332145054	0.346087144	0.180535657	0.191469515
0.204471842	0.101741624	0.305041298	0.404291418
0.252757554	0.161215124	0.190239928	0.291723102
0.114200526	0.122508261	0.311218695	0.078791914
0.406176918	0.080475277	0.195754538	0.205098625
0.158023528	0.310981131	0.371267944	0.182682493
0.18595436	0.046363902	0.183602214	0.074863793
0.145446232	0.10263102	0.289283591	0.384851861
0.099467501	0.181666753	0.176688474	0.01953738
0.078745125	0.139517747	0.276849922	0.141205633
0.314402547	0.245045885	0.2294724	0.22151805
0.26267817	0.419924086	0.024385547	0.135928222
0.22473282	0.141516577	0.234169895	0.221400482
0.23162358	0.120637022	0.147296927	0.433602074
0.285601121	0.133741332	0.101538896	0.371467769
0.167018559	0.240621633	0.133843518	0.15237167
0.086352449	0.32381635	0.076295932	0.124001793
0.202782179	0.11385577	0.024787795	0.114898364
0.15026415	0.326306642	0.273469467	0.14080982
0.164323852	0.329973296	0.103904023	0.286207383
0.054067085	0.086761515	0.070422812	0.393100411
0.124895325	0.394742334	0.185243065	0.215333312
0.240573494	0.396800818	0.005750756	0.323940544
0.375375471	0.074777092	0.246488649	0.248846807
0.190645579	0.397249042	0.012988741	0.334383003
0.167698691	0.12735356	0.442755214	0.189544757
0.304885636	0.1166617	0.121968843	0.347617408
0.258351021	0.019272889	0.097439787	0.296913395
0.073754776	0.087667748	0.294624339	0.325782771
0.287131088	0.366599487	0.439933155	0.190848399
0.229209816	0.150464985	0.043814002	0.216561521
0.31557818	0.13231589	0.307714964	0.176865825
0.118635015	0.356375172	0.273404689	0.112565146
0.110210847	0.145095052	0.03576906	1
0.270764251	0.120280232	0.189967955	0.104425177
0.127331497	0.417545669	0.150085894	0.14228668
0.109084738	0.193665189	0.179691903	0.120766064
0.128316172	0.211081671	0.220953389	0.210834382
0.066947406	0.135402395	0.036054926	0.105482364
0.087682842	0.119253924	0.104666334	0.217411473
0.120723272	0.096105165	0.204451975	0.194428238
0.184116296	0.059999948	0.383491243	0.329055421
0.17369928	0.186896062	0.089473816	0.095045435
0.384843602	0.12359155	0.071744542	0.349355169
0.054253631	0.411419412	0.166122077	0.177095565
0.158292478	0.190807096	0.425129123	0.073785583
0.171278278	0.326955338	0.212396482	0.229730655

0.247247081	0.288648792	0.075078008	0.24347373
0.220458225	0.084551864	0.389484994	0.13783011
0.068991425	0.319336228	0.315375516	0.147086031
0.154218092	0.180555249	0.065456427	0.286013852
0.411172827	0.387891235	0.305191554	0.175691663
0.099090528	0.021567754	0.169426832	0.18528638
0.321565075	0.381406075	0.193892923	0.191947949
0.22481919	0.229773407	0.055094592	0.404062355
0.111597858	0.352367397	0.033880484	0.34153082
0.329397898	0.086301344	0.161736119	0.17364822
0.250245162	0.120904219	0.139382592	0.343777211
0.350627166	0.240624992	0.226614646	0.2645975
0.048627525	0.076218472	0.278084446	0.148569042
0.18393283	0.235257769	0.049450101	0.129458879
0.100003288	0.321713482	0.372331118	0.178303238
0.157433599	0.120930459	0.297478495	0.100282404
0.36260219	0.349475988	0.351989468	0.130573394
0.283196032	0.375845765	0.209894233	0.130034083
0.067312864	0.35225803	0.240383012	0.052589455
0.405233837	0.068851371	0.035846746	0.368327161
0.179107116	0.251050512	0.019979266	0.045207551
0.084342576	0.396148115	0.329651052	0.254593048
0.078591737	0.244555686	0.008980046	0.394058279
0.347512536	0.065732892	0.161799146	0.291634433
0.292042763	0.376412355	0.196888939	0.366085307
0.230369833	0.335211334	0.146470446	0.261520381
0.347670208	0.344817216	0.303350724	0.332646404
0.377651457	0.327812305	0.262139669	0.065075533
0.231066867	0.09838265	0.136579387	0.156282459
0.147803418	0.210061576	0.35079543	0.404827979
0.380155896	0.031150874	0.111779635	0.286073548
0.34557788	0.404746037	0.04321466	0.020880766
0.012059341	0.08293049	0.205973911	0.208477054
0.3878443	0.073355118	0.028432191	0.059141913
0.184855866	0.289873196	0.358015956	0.350962026
0.326201927	0.124956368	0.127563659	0.342391826
0.165810789	0.148368508	0.399969706	0.333876343
0.078545068	0.219307989	0.121196284	0.42118463
0.061207122	0.244823647	0.241110828	0.187302383
0.026438077	0.154833455	0.169151504	0.375593041
0.318375611	0.103905899	0.079429298	0.041644537
0.138867095	0.374541726	0.164977111	0.432069343
0.35203209	0.408285659	0.108980095	0.348732434
0.11191555	0.318049958	0.195931534	0.050126667
0.160952005	0.044023825	0.310747393	0.174815538
0.109638873	0.237063988	0.246010411	0.055962628
0.141152756	0.109682665	0.430083954	0.400173817
0.070439849	0.17792092	0.207597194	0.273830836
0.13337131	0.365377988	0.333886078	0.147173313
0.122630617	0.072969491	0.29914408	0.202217799
0.101513339	0.428920167	0.219790038	0.404007581
0.259517739	0.348961021	0.348151405	0.254973373
0.150953125	0.188924535	0.247761913	0.046436271
0.237045799	0.390560341	0.149703919	0.210495899

0.217081362	0.191189027	0.071951653	0.050451131
0.141488587	0.151774172	0.142182463	0.409707725
0.200756696	0.217676818	0.043902468	0.170379253
0.395740588	0.316062061	0.135040362	0.298545829
0.174282842	0.275143153	0.341179001	0.343262685
0.163098916	0.323570235	0.303446011	0.233777631
0.290538503	0.205374752	0.36442581	0.04440118
0.162313709	0.190144302	0.134504614	0.144749985
0.166278008	0.095722345	0.144892113	0.34242422
0.131082173	0.295286278	0.090027776	0.139239622
0.215213611	0.111465265	0.263035094	0.139868236
0.130455592	0.343877299	0.037849536	0.103681711
0.126836493	0.346391836	0.205565957	0.212496052
0.181803317	0.13324579	0.220616096	0.099620825
0.101626561	0.126733181	0.34127012	0.06887738
0.04650885	0.424868895	0.12500985	0.052467541
0.097394731	0.184780955	0.130030192	0.22245297
0.067046106	0.349286207	0.120918701	0.235146939
0.203258994	0.108172473	0.206482914	0.081661341
0.096560038	0.289228229	0.221645693	0.352283166
0.168039164	0.393219294	0.26810164	0.286700523
0.096120456	0.147615244	0.084869709	0.351460585
0.140899874			
GPX8 0.390759001	0.470921796	0.379195689	
0.326841498	0.33999044	0.325569471	0.155197287
0.166023436	0.074134034	0.001058131	0.012385616
0.365583633	0.130339656	0.184984626	0.31739235
0.339596608	0.084564016	0.346704291	0.178201273
0.335143208	0.063367835	0.301551372	0.065464474
0.265224985	0.071727032	0.044176013	0.223519672
0.351731563	0.052716701	0.267971018	0.320450714
0.078751181	0.374853692	0.501909185	0.329218202
0.494209503	0.389039282	0.008644246	0.234131591
0.376369003	0.372087065	0.332805662	0.240921849
0.025111144	0.473015174	0.17792745	0.342453231
0.218241331	0.016288074	0.215919165	0.437024805
0.448809939	0.35891932	0.198545702	0.114073545
0.131105198	0.184227692	0.206518955	0.286381237
0.160143021	0.12960674	0.455105768	0.068414778
0.130207043	0.217958362	0.113427014	0.203488799
0.316792605	0.351272231	0.013874338	0.375989536
0.000281033	0.321713471	0.314761186	0.299682846
0.026198977	0.007214209	0.132812785	0.254357272
0.365158796	0.450752221	0.383528431	0.409627244
0.113492357	0.241243662	0.345526128	0.112714259
0.385420747	0.119778682	0.086110676	0.330249867
0.398459012	0.478384221	0.153324876	0.213410416
0.345118051	0.431248303	0.358586158	0.337218828
0.297995952	0.046116428	0.4099146	0.433046125
0.354470046	0.272721239	0.106701015	0.336615675
0.040961145	0.326276786	0.26442876	0.112034476
0.372759491	0.012746479	0.159425538	0.269140307
0.197350562	0.378088639	0.477121792	0.135698477
0.339457565	0.010215727	0.115432629	0.025350539

0.248833081	0.126202267	0.486422516	0.349614021
0.180589249	0.296799468	0.21453614	0.000353918
0.01579952	0.171158553	0.365298226	0.329619998
0.375338668	0.388887158	0.167152269	0.356200864
0.374068602	0.379044764	0.053416715	0.074305821
0.346711278	0.168119526	0.175702812	0.358052944
0.230897502	0.091355675	0.085471222	0.443131834
0.400938719	0.05440472	0.321902873	0.327698109
0.249703869	0.275495919	0.075585589	0.184512173
0.029756675	0.470503745	0.018515154	0.308592821
0.259507256	0.095074181	0.011004205	0.218134556
0.214599592	0.349435911	0.485025344	0.078829461
0.221549291	0.317285533	0.292157847	0.331599763
0.004271774	0.03503946	0.279741483	0.42504454
0.251989401	0.434606754	0.236379254	0.269965046
0.215999609	0.369474744	0.00427061	0.48152412
0.488998752	0.286510432	0.285112983	0.357641557
0.346684229	0.352594068	0.046660618	0.322574645
0.335818895	0.059921787	0.409115595	0.106785817
0.435793769	0.024440479	0.254187926	0.378559036
0.356464897	0.005891988	0.18838684	0.31644616
0.020248916	0.075211209	0.380415335	0.326899238
0.30988476	0.379925248	0.373715321	0.329344592
0.333759503	0.188007276	0.009420153	0.261514933
0.390981067	0.344841286	0.41438902	0.191366735
0.320913252	0.426078853	0.378375082	0.190067162
0.027070962	0.257974271	0.01220779	0.270764251
1 0.067348697	0.375872777	0.127953638	
0.046400246	0.455098877	0.151816959	0.241867614
0.115531461	0.292754632	0.20447024	0.145779705
0.127613919	0.126692485	0.31415907	0.323992158
0.022969858	0.05543708	0.0156786	0.279246215
0.07090935	0.230698713	0.232566362	0.355723349
0.236360434	0.05801881	0.282974822	0.283300457
0.086941587	0.008395366	0.454724723	0.333520949
0.309481162	0.121316858	0.027680373	0.251925266
0.36586594	0.276809574	0.282276983	0.380120913
0.023610072	0.400929891	0.158096151	0.080455026
0.273208069	0.178631998	0.363496599	0.055422541
0.355961501	0.418047943	0.26901621	0.135594837
0.350895672	0.342284771	0.017836561	0.368224969
0.349553407	0.017244182	0.466367569	0.085441744
0.013210866	0.364241378	0.363124587	0.05274074
0.056563873	0.239408136	0.033930115	0.220141541
0.449536712	0.311813086	0.353333238	0.202685863
0.021810643	0.002026842	0.170117337	0.372862852
0.4913845	0.450818374	0.200713555	0.264586775
0.180034137	0.287838688	0.136620297	0.432026034
0.219110008	0.492176323	0.003793671	0.376991684
0.494560804	0.248453034	0.05215566	0.246558848
0.331841495	0.032082067	0.179926559	0.457832555
0.38392291	0.290131702	0.303638914	0.360919263
0.009276196	0.028447637	0.345336571	0.175012064
0.317598848	0.176298859	0.049286763	0.305576042

0.021925067	0.402360172	0.365115201	0.045644455
0.272784229	0.312143631	0.301462158	0.023767968
0.388323566	0.473413907	0.491500623	0.136914954
0.37258229	0.430540234	0.341557763	0.060991888
0.010982358	0.491141922	0.368202921	0.01129726
0.366525567	0.215434594	0.00333867	0.447577167
0.142331005	0.452645118	0.003361785	0.117649403
0.035420636	0.371844763	0.465254841	0.328085738
0.205132249	0.316892584	0.00753945	0.467223017
0.438800124	0.008793205	0.114579372	0.364698886
0.359204254	0.486801994	0.27992979	0.367359506
0.342832863	0.471937002	0.165642131	0.204553246
0.357044333	0.463017803	0.364299998	0.431434452
0.354229306	0.345271181	0.210322537	0.008448069
0.154014919	0.182455139	0.261453614	0.059943614
0.307491917	0.101516305	0.434450619	0.3702457
0.40061435	0.00250176	0.116921268	0.373412196
0.360872533	0.389771738	0.023198904	0.006788491
0.009343305	0.011031237	0.303673431	0.203096941
0.433549823	0.179080387	0.001644905	0.031598025
0.225588629	0.327138437	0.456083196	0.299862898
0.388672094	0.057227867	0.200018039	0.326724163
0.050163151	0.333388751	0.401336224	0.437266997
0.034420044	0.214788464	0.343169777	0.396798726
0.068767935	0.243713029	0.36250264	0.226895466
0.013377311	0.059942938	0.051459058	0.364634349
0.483866034	0.038234509	0.014474608	0.125467595
0.071238723	0.383605922	0.104618797	0.429573152
0.358248846	0.339776473	0.056322713	0.461769414
0.025754388	0.454784347	0.268020265	0.048400188
0.253026984	0.008757703	0.43552236	0.12686808
0.260962655	0.357110379	0.218608743	0.011878581
0.199446107	0.033121342	0.373637765	0.374049012
0.028702159	0.208364537	0.247564683	0.350818942
0.238411421	0.332807574	0.481392207	0.347860479
0.057119717	0.138176433	0.338802483	0.354896571
0.190569752	0.393674337	0.232165946	0.471965624
0.379787245	0.426289593	0.415863556	0.289838313
0.176478762	0.358401675	0.293030167	0.008828538
0.289235615	0.351690247	0.332965493	0.111172718
0.17982963	0.351636275	0.044608998	0.190547067
0.302474324	0.261163492	0.089266927	0.353909402
0.352673528	0.360804639	0.032708484	0.046719648
0.492097857	0.422773566	0.125122458	0.348887163
0.134952565	0.276096257	0.381158991	0.373376889
0.374928017	0.355601938	0.3554813	0.3013394
0.382873752	0.209454231	0.494689492	0.083563516
0.074719424	0.374289617	0.053833633	0.068393399
0.09467362	0.019650381	0.278890062	0.344398854
0.330468383	0.27959164	0.29306031	0.063097312
0.264217982	0.37741599	0.282662621	0.141805126
0.044552629	0.463979061	0.005809101	0.037634981
0.315788877	0.381786238	0.304176472	0.189492087
0.262839073	0.120531368	0.218177341	0.254422139

0.22764555	0.332765389	0.378012765	0.02829025
0.010779787	0.394708802	0.345887562	0.01887943
0.083136642	0.377526364	0.26490489	0.184535778
0.016261018	0.430050418	0.280659169	0.364655786
0.288817099	0.048163273	0.3480247	0.083625348
0.056445986	0.424794759	0.363223679	0.478423729
0.3259677	0.319740969	0.456358586	0.363519125
0.123117088	0.386924197	0.017913872	0.372350124
0.349154789			
GRK5 0.075228162	0.152972391	0.041172928	
0.242331296	0.115757971	0.111085117	0.240152068
0.053082697	0.056993299	0.098263659	0.087174948
0.078312869	0.090268946	0.132264813	0.029400192
0.190834346	0.108889985	0.033280064	0.235661276
0.182048251	0.146294224	0.234252938	0.121686519
0.02326508	0.08158554	0.163495526	0.056568436
0.206011157	0.16190584	0.119323263	0.177704945
0.04166065	0.12070034	0.112980528	0.164343322
0.104176028	0.058011551	0.015797944	0.097006055
0.10405274	0.129806489	0.197647769	0.26303625
0.129061128	0.108581304	0.286062947	0.084204558
0.028153197	0.101440093	0.0611995	0.10501023
0.114693652	0.109071876	0.093195386	0.206052059
0.083841178	0.070463372	0.024079972	0.113993632
0.269902044	0.086228401	0.116671291	0.095318554
0.043388603	0.091792224	0.049588658	0.158244216
0.026290948	0.107043515	0.049563581	0.045414097
0.049523133	0.180905702	0.09370972	0.022643235
0.138007175	0.164047662	0.071739679	0.273207943
0.185109836	0.085696453	0.176357146	0.14834245
0.096168322	0.091561785	0.050626351	0.189927665
0.113064663	0.266202409	0.042990403	0.201597657
0.169795181	0.131394982	0.058454992	0.104297917
0.178956346	0.143113239	0.068559574	0.102719958
0.035829817	0.019178704	0.12286048	0.157889934
0.102531424	0.035679556	0.148714968	0.179873079
0.115741448	0.018798435	0.276467423	0.050884634
0.190462016	0.074554509	0.108948666	0.225463581
0.033259531	0.116279858	0.119205576	0.252309206
0.069878489	0.080839001	0.018430235	0.045891748
0.044218144	0.009146551	0.093538902	0.217254229
0.064560326	0.042699341	0.144344997	0.021078633
0.064144374	0.020395671	0.110332092	0.059497877
0.126056517	0.054394954	0.147459938	0.093207668
0.083067824	0.172001109	0.011710119	0.113949775
0.105813867	0.024343981	0.246418261	0.106264737
0.133078257	0.104716816	0.195965005	0.165187473
0.12489417	0.227568377	0.034759279	0.188898943
0.068535863	0.107125885	0.149883976	0.169225526
0.154466965	0.093724696	0.051689655	0.061527165
0.018193398	0.058048112	0.015998358	0.123423077
0.064673437	0.159758765	0.07932718	0.138466825
0.197499608	0.17414296	0.06902979	0.16981776
0.10435864	0.053218301	0.034403214	0.171216944

0.108277911	0.129555762	0.079194062	0.047322605
0.155994917	0.164084613	0.000131053	0.109581925
0.12164999	0.033236191	0.210983155	0.085841276
0.044953728	0.209773663	0.001411248	0.20200227
0.066497051	0.113901431	0.18098121	0.206390444
0.101332788	0.161129487	0.09775684	0.09628399
0.093539358	0.018816448	0.127519908	0.210964773
0.028411479	0.104988728	0.112578948	0.196567966
0.165565559	0.127008079	0.199383934	0.039271996
0.116439848	0.120354283	0.028122628	0.083439586
0.095989004	0.018983903	0.128735227	0.094418397
0.049251898	0.143753475	0.078755728	0.032749921
0.18225516	0.145407803	0.062412613	0.120280232
0.067348697	1 0.047874596	0.026102251	
0.092732546	0.163665233	0.023807724	0.017680532
0.010095868	0.185606894	0.154948895	0.15394261
0.108593033	0.086010425	0.041799202	0.065086001
0.13595293	0.202886738	0.00549632	0.047454456
0.153084083	0.098210677	0.096726977	0.101254191
0.064332133	0.162902669	0.13570507	0.016535923
0.052470259	0.094800246	0.146953558	0.162220382
0.089743357	0.113339653	0.083048277	0.030358457
0.208834781	0.087979816	0.024658547	0.187177832
0.026176071	0.154575111	0.127352127	0.181587719
0.080538467	0.278823805	0.199763032	0.026861962
0.080660607	0.15276185	0.096730578	0.133468941
0.127318422	0.16190437	0.118234743	0.08552515
0.095211369	0.147556464	0.139616807	0.059070048
0.079052952	0.110031333	0.121306875	0.140984538
0.083140749	0.207916878	0.100520814	0.19129522
0.165304854	0.236527396	0.154668338	0.023590752
0.190642625	0.143364878	0.050419527	0.041874836
0.111153226	0.128191432	0.164922297	0.123752851
0.253774545	0.046488685	0.030996816	0.160553838
0.008213018	0.113139941	0.047109245	0.105712818
0.104269871	0.081647262	0.116018847	0.195157999
0.133701692	0.125162177	0.064856869	0.127968587
0.13682311	0.069554249	0.053603543	0.106973207
0.104804053	0.127282339	0.085356614	0.012108089
0.071930151	0.093664697	0.002946585	0.037267158
0.159354568	0.154936453	0.162145915	0.070439783
0.146887428	0.031591503	0.143188525	0.137682689
0.105301662	0.100597903	0.123599619	0.088687146
0.107101302	0.132992417	0.061573221	0.074255113
0.059481163	0.10782822	0.096760076	0.101014257
0.193099634	0.066634918	0.169217032	0.151690025
0.200561257	0.08317298	0.009623804	0.001337154
0.018007127	0.144369252	0.112786126	0.160461906
0.065317857	0.129662298	0.000976455	0.128663848
0.146200508	0.03737673	0.031504526	0.116503428
0.089360844	0.140696249	0.040026006	0.128024875
0.084851585	0.114020697	0.115238274	0.192516357
0.170690817	0.099459591	0.135347706	0.122346703
0.20766952	0.133976138	0.273676472	0.105751472

0.015517412	0.106697968	0.039517284	0.080607269
0.080146177	0.078728495	0.118140316	0.176319858
0.141414341	0.04539551	0.094751008	0.109553299
0.18243543	0.183978681	0.009011329	0.001716622
0.000635143	0.038562771	0.157094961	0.080861503
0.135997292	0.04657695	0.064644122	0.096394457
0.056810009	0.097036357	0.139998984	0.231886455
0.096661286	0.11228208	0.086168586	0.194079332
0.054092442	0.050288378	0.160778176	0.14524656
0.030604748	0.074958557	0.045039951	0.200922317
0.00322425	0.159655909	0.09192637	0.082094728
0.007925716	0.065685585	0.06398448	0.152166241
0.099873189	0.161896479	0.135338612	0.020000948
0.22698293	0.144539047	0.140128687	0.172609929
0.181280917	0.232478942	0.036542216	0.122858017
0.094703213	0.117597844	0.075176924	0.019241868
0.041061511	0.036076995	0.096791917	0.038488518
0.082897442	0.105156281	0.016847974	0.02989971
0.006087929	0.103293788	0.200671896	0.141631922
0.017827819	0.084951927	0.102281275	0.114567142
0.143602945	0.226355516	0.119511471	0.056773653
0.16527713	0.005708122	0.169769628	0.081033832
0.078744743	0.171534052	0.072457435	0.13406035
0.091538336	0.116832053	0.123075762	0.171196984
0.034609292	0.078032029	0.221595837	0.124978301
0.147354942	0.211829909	0.069872598	0.061097573
0.193737846	0.043366489	0.042477013	0.006435344
0.02738092	0.142677788	0.157486512	0.195248191
0.089314134	0.084873536	0.056982257	0.066140732
0.143944422	0.107833737	0.060778393	0.160682817
0.104062066	0.162231297	0.11177468	0.115068918
0.018398126	0.159705906	0.156566665	0.199647731
0.150158027	0.192082357	0.126387712	0.031915693
0.024580702	0.061599255	0.179163726	0.099788452
0.242046662	0.04105837	0.012500761	0.162560125
0.021047435	0.256560218	0.022859656	0.055613888
0.103889293	0.028405563	0.16146408	0.212888182
0.151933997	0.131865528	0.032536143	0.100545828
0.032581961	0.118772496	0.077080616	0.084708591
0.018211879	0.024483963	0.227936282	0.088862837
0.11468162	0.014141423	0.120371004	0.042513172
0.111744646	0.180457237	0.047861266	0.013746404
0.024244127	0.060831545	0.02958193	0.084299822
0.028933644	0.117901105	0.090549102	0.083204342
0.159120305	0.088698164	0.082095018	0.021644455
0.03745219	0.109119465	0.051040744	0.114871152
0.039054891	0.255187461	0.089288928	0.128096797
0.013267485	0.029215765	0.094882496	0.154946638
0.032493875			

GRWD1 0.309688671 0.468429795 0.196184661

0.311893768	0.199510547	0.199778234	0.094923543
0.06567535	0.093035943	0.002051485	0.025911286
0.236888711	0.087098693	0.085306679	0.183788127
0.330256186	0.060744999	0.142093825	0.10604413

0.262328406	0.036122162	0.271484371	0.050397379
0.173308592	0.073461552	0.050413339	0.191124684
0.350547925	0.055381309	0.119383692	0.239122945
0.050728078	0.322720806	0.471235601	0.2747983
0.454809485	0.297419119	0.00248463	0.134330884
0.3396389	0.36706365	0.273988416	0.163729846
0.025562981	0.412540458	0.112935903	0.231963777
0.093938611	0.022035694	0.084387026	0.385410583
0.41567586	0.256622653	0.095159452	0.080852715
0.094869626	0.068023343	0.131324008	0.173027823
0.101336907	0.049194121	0.433032853	0.069048516
0.082478701	0.095756431	0.071194813	0.095908698
0.199536048	0.274149835	0.009250999	0.236051658
5.44E-05	0.246674766	0.19892217	0.224847873
0.02554354	0.00815945	0.082273867	0.175765749
0.37589565	0.406396763	0.426449008	0.450442858
0.06939996	0.13611819	0.1971624	0.078095533
0.377380413	0.07977153	0.046368478	0.326667164
0.439494069	0.466455748	0.123564288	0.14455156
0.35215218	0.412389033	0.175845575	0.183072468
0.213206352	0.041658967	0.394942706	0.449805937
0.306791732	0.164341093	0.08368242	0.284555411
0.034656654	0.1283926	0.206697207	0.04731846
0.392261657	0.018572848	0.113488374	0.156517603
0.100205848	0.330562395	0.473280101	0.089614393
0.165115249	0.008265693	0.13002292	0.020946034
0.113861199	0.08684153	0.42845302	0.37155681
0.073545077	0.175009117	0.119302329	0.001933828
0.016070368	0.094495095	0.321354667	0.135754045
0.315631707	0.353010545	0.140840647	0.275736457
0.296429563	0.423663133	0.022659707	0.052913278
0.196999835	0.095226939	0.11848418	0.225105698
0.158570866	0.05138529	0.05704047	0.470500477
0.358716043	0.042582907	0.115133	0.292900413
0.15018014	0.179951268	0.050056128	0.094414312
0.024200145	0.451397505	0.018497935	0.121379195
0.224503297	0.061870106	0.006000774	0.091305512
0.104492046	0.368453667	0.420432818	0.053145667
0.11215838	0.261329902	0.108284598	0.284189737
0.011278113	0.023746427	0.092625357	0.433204362
0.139356149	0.444122099	0.122603437	0.168179253
0.139860182	0.374061292	0.00271179	0.439270734
0.482190874	0.094938326	0.188254212	0.230380149
0.231226687	0.371458968	0.013558306	0.267430954
0.208539457	0.043808478	0.447362622	0.08323914
0.395614444	0.02935536	0.111429782	0.404315125
0.305641487	0.004815554	0.081570653	0.252334087
0.020888827	0.044284278	0.307200443	0.265489681
0.236860498	0.363254562	0.413196439	0.228422962
0.194148784	0.09095317	0.009157309	0.155910157
0.351894017	0.181986824	0.383139174	0.107865934
0.147945133	0.419046767	0.345588254	0.133205414
0.028742889	0.121468934	0.007749209	0.189967955
0.375872777	0.047874596	1	0.061610944

0.040531895	0.473155333	0.113934828	0.151805811
0.091553686	0.173980108	0.110393143	0.079287957
0.079610439	0.110524615	0.227185196	0.207652718
0.017929752	0.043888275	0.012976086	0.122138128
0.042924477	0.108775187	0.102462273	0.258870969
0.102781278	0.034982081	0.158544174	0.190633653
0.077829602	0.010107111	0.45299841	0.265566701
0.215000888	0.089885827	0.023162836	0.108256627
0.388938516	0.137472401	0.096468867	0.383753377
0.015019034	0.434488992	0.098544449	0.069134197
0.145233039	0.106655211	0.399412697	0.045467068
0.184379475	0.394306287	0.148972249	0.1200028
0.305098337	0.253530222	0.016622413	0.293398458
0.261612506	0.017269787	0.463960581	0.059352173
0.014052195	0.308389409	0.348348657	0.049085886
0.052618349	0.141429185	0.02170547	0.168788432
0.463382845	0.298768641	0.340369706	0.137870048
0.022973531	0.002468212	0.111047415	0.196633827
0.43977526	0.455376806	0.113383753	0.155406375
0.1160142	0.226668355	0.05028301	0.450494454
0.097891401	0.458595407	0.004883374	0.380988553
0.459895926	0.09044686	0.056235576	0.132837138
0.287088532	0.033766199	0.085573479	0.431370253
0.40287078	0.212422343	0.210735932	0.250937854
0.008854933	0.023177978	0.250129484	0.133677248
0.187952094	0.139704322	0.039289001	0.134532352
0.022414511	0.388429385	0.353599699	0.067668295
0.164373317	0.130325115	0.227213119	0.02424891
0.428229099	0.454745042	0.458780204	0.069795433
0.317112476	0.448576518	0.240325396	0.048231783
0.013267594	0.466525059	0.262306835	0.010308358
0.394464435	0.074121871	0.004757147	0.432824345
0.0891798	0.375320573	0.003952953	0.053377896
0.031069066	0.38909748	0.443348351	0.235376496
0.085026808	0.194188521	0.005871153	0.469491297
0.420832929	0.01255539	0.092543163	0.340045354
0.332148157	0.461132211	0.198050117	0.404433964
0.23830127	0.449880418	0.09330541	0.148451461
0.359576731	0.458819336	0.301374167	0.405409336
0.36282142	0.287997606	0.150769463	0.012343231
0.202370333	0.077921281	0.150997352	0.081295413
0.165534559	0.096004716	0.412928505	0.398314807
0.419680195	0.004045571	0.058885198	0.338248637
0.353543039	0.407098125	0.015797862	0.007310506
0.004703504	0.020265853	0.174330688	0.133103568
0.451020445	0.073166441	0.003068923	0.018093539
0.120168696	0.26365029	0.443288036	0.255855262
0.361753318	0.044175542	0.109525482	0.273604249
0.066287974	0.203875564	0.429063994	0.415452795
0.026422421	0.150575105	0.129400397	0.424243837
0.061689557	0.157226004	0.245244866	0.137903974
0.008091315	0.05734122	0.068209563	0.348723479
0.447531073	0.032116814	0.015783381	0.045047355
0.053695162	0.401427965	0.06881829	0.457078076

0.34797751	0.345879928	0.048611727	0.447376055
0.041100058	0.417977225	0.165713593	0.024860644
0.119505367	0.008020668	0.403969652	0.14913237
0.103826243	0.241960637	0.236846961	0.013781504
0.149528352	0.029604472	0.421098913	0.406711906
0.024067152	0.121291418	0.141564711	0.239837347
0.104675641	0.314604762	0.443799302	0.169292635
0.047170488	0.068694928	0.263659968	0.227136243
0.078443258	0.432030183	0.182580116	0.475568152
0.292842749	0.416592083	0.417212072	0.192514897
0.119315655	0.197454075	0.226762927	0.007649518
0.164400387	0.371142392	0.140085406	0.104905812
0.12985305	0.215267321	0.030379967	0.052953873
0.166581691	0.143905243	0.061340835	0.360435837
0.206623741	0.248828102	0.01571505	0.064333657
0.480669906	0.4013614	0.073551734	0.280593051
0.086241829	0.202377728	0.355100503	0.355655072
0.206826345	0.356305739	0.304461427	0.226012276
0.351441816	0.131008036	0.472603627	0.027547729
0.097318326	0.226860929	0.046951813	0.056359767
0.066670058	0.022970211	0.177340764	0.294342992
0.166787956	0.211192495	0.108244367	0.050445616
0.154185656	0.145183841	0.185327015	0.077852326
0.038801716	0.444899793	0.006887184	0.030553065
0.147886534	0.383049021	0.188171749	0.120357839
0.184530083	0.082886078	0.131581702	0.096721677
0.101446456	0.14926816	0.379881413	0.019424802
0.008793685	0.423446154	0.132282246	0.01548287
0.07181253	0.232724072	0.108339519	0.16377087
0.017836726	0.438421044	0.136436018	0.224427032
0.156818122	0.034327129	0.280294983	0.047124653
0.037310861	0.370692407	0.226351514	0.459554953
0.163605195	0.313613277	0.389405247	0.329214694
0.054991495	0.188675825	0.018724679	0.357864939
0.174746628			

GSTO1 0.208800367 0.158747979 0.136321177

0.17249973	0.174347358	0.067689077	0.08274871
0.052958168	0.012681253	0.002478231	0.005817908
0.192340392	0.015549642	0.042795643	0.018416613
0.272310121	0.108581095	0.151960951	0.179276868
0.231116752	0.028439245	0.250418492	0.060965797
0.100526411	0.068356596	0.070069845	0.068760635
0.234225073	0.086175541	0.039957036	0.16354537
0.211097748	0.218425722	0.135223765	0.177326951
0.132587215	0.154966637	0.113810421	0.021629772
0.190164035	0.157885274	0.205944886	0.165339209
0.034069154	0.119259082	0.108767257	0.242848343
0.018057163	0.032579405	0.02903729	0.117771598
0.098147652	0.081227184	0.058489541	0.053248352
0.006979806	0.036272644	0.021908738	0.096483727
0.116857526	0.022637575	0.116903914	0.009074915
0.011099144	0.113165595	0.209375154	0.105780652
0.083942102	0.127929452	0.048953688	0.15890472
0.001272043	0.238783667	0.31537299	0.035744242

0.157370803	0.01301312	0.252546974	0.125409278
0.236177109	0.173136644	0.220075809	0.196530806
0.051996655	0.296545031	0.163552882	0.096015153
0.171313666	0.056224109	0.035172397	0.284350269
0.211098607	0.144762067	0.06440646	0.266928472
0.099084454	0.157583976	0.071802336	0.092250959
0.022734061	0.121929555	0.106314641	0.189849869
0.144126904	0.12700853	0.173805899	0.179381342
0.170085686	0.034813095	0.204038135	0.110811624
0.23255136	0.01570046	0.23167385	0.108628926
0.09971957	0.180341647	0.146778263	0.124400125
0.219449593	0.034983444	0.010187871	0.179546387
0.027089523	0.05565236	0.099506286	0.170200634
0.044019983	0.039693863	0.108630324	0.048036055
0.103150104	0.112890123	0.178610851	0.226928321
0.227344762	0.13653088	0.046773922	0.120439656
0.1221663	0.222900246	0.000816365	0.082111402
0.112436746	0.137141276	0.247146753	0.133853976
0.124521686	0.078669513	0.139145708	0.193430682
0.123377975	0.121556373	0.107324187	0.280508646
0.018376742	0.208495346	0.144271976	0.085636892
0.039074646	0.120560626	0.15294031	0.132259931
0.006379813	0.061251651	0.070196015	0.086677718
0.014398938	0.100880677	0.10837898	0.207658802
0.115484433	0.208342972	0.092665905	0.091012833
0.002271575	0.07092314	0.065515958	0.176363493
0.058931361	0.225617265	0.125505949	0.137903417
0.246185213	0.227347119	0.011866968	0.118456772
0.144991115	0.052105886	0.122612736	0.265167082
0.055219362	0.173185461	0.014142194	0.29222131
0.135987271	0.175282357	0.232354039	0.156326115
0.148009772	0.081376052	0.065208697	0.157272043
0.124165881	0.014132458	0.035268631	0.161529979
0.136709385	0.017417358	0.21503364	0.17240616
0.096407653	0.248863088	0.256049559	0.043705166
0.129773961	0.141410244	0.06257741	0.256607441
0.225112854	0.01415923	0.114564821	0.055653899
0.089076949	0.136036995	0.130724185	0.00432132
0.063711701	0.101001704	0.009194859	0.104425177
0.127953638	0.026102251	0.061610944	1
0.148929589	0.171643878	0.120090814	0.092287547
0.066793896	0.084467934	0.181347516	0.019545735
0.014391737	0.07719476	0.054148007	0.052752206
0.023941163	0.106497089	0.100863546	0.04185165
0.010565345	0.032855158	0.02943509	0.127393771
0.047146632	0.025435209	0.109636017	0.073332305
0.09670648	0.121160907	0.15814925	0.271674103
0.094039356	0.069609839	0.182844452	0.00939175
0.165395088	0.029525894	0.036183691	0.133297491
0.062782073	0.234152545	0.027091357	0.136815186
0.081599992	0.144539496	0.220733186	0.003364113
0.153906871	0.129857238	0.299486925	0.080616626
0.099679316	0.176926782	0.021732004	0.139729741
0.136593096	0.108284311	0.171097832	0.131096947

0.161565185	0.234932224	0.109342301	0.188533086
0.139925499	0.0529848	0.017696051	0.255722092
0.163426274	0.246935182	0.102633415	0.158727657
0.049466652	0.008666453	0.247726281	0.187600289
0.127640933	0.173765723	0.316302273	0.091309322
0.212903404	0.034604969	0.012213631	0.1708907
0.107453712	0.162217173	0.091276949	0.173211971
0.118334194	0.061435536	0.077629683	0.073366757
0.108145257	0.062497053	0.179343907	0.131127419
0.142630128	0.062488437	0.035631099	0.285409029
0.014535974	0.022976258	0.153110618	0.077295288
0.041763962	0.093515524	0.025993274	0.049528386
0.022028289	0.115677417	0.283169577	0.023767383
0.036031926	0.050167855	0.312521782	0.098700726
0.153326074	0.168000063	0.136143048	0.136253054
0.201782653	0.135559439	0.091779771	0.236966923
0.139395359	0.141044901	0.154884786	0.009126998
0.237549068	0.034817907	0.005977385	0.155561251
0.168235585	0.10669544	0.001118473	0.05337055
0.141978905	0.271821144	0.106426256	0.175359414
0.011218607	0.283536289	0.061684579	0.197607716
0.12828853	0.06856811	0.091162662	0.132147979
0.126301923	0.167338248	0.074419408	0.146411539
0.117972096	0.119084404	0.017500344	0.206229314
0.134818967	0.139830819	0.181244513	0.144294859
0.204223073	0.280008471	0.166585601	0.020734134
0.014593103	0.049688446	0.009164457	0.00638563
0.088233484	0.056517277	0.182663987	0.244469976
0.174597699	0.095749812	0.034430115	0.140582242
0.171826395	0.217953777	0.090022315	0.005973893
0.011558293	0.008512132	0.150936857	0.246869687
0.16434475	0.004047143	0.051484257	0.023326186
0.037778781	0.179197349	0.137436628	0.275087216
0.223293941	0.182690925	0.011039742	0.281664778
0.030994915	0.106654705	0.170773236	0.12036848
0.165113768	0.200421035	0.182641859	0.199579955
0.019254333	0.297614607	0.211519009	0.024335895
0.106279966	0.122206355	0.065202704	0.293310162
0.119408822	0.021757604	0.022539334	0.007820359
0.035816644	0.231402653	0.049841628	0.213681508
0.172230319	0.261534421	0.103381074	0.123412541
0.015759186	0.121422331	0.139292084	0.002891514
0.240425164	0.08845443	0.164070252	0.003106737
0.061115277	0.198686556	0.014538068	0.128763238
0.035342956	0.138881074	0.199294797	0.22725508
0.140664293	0.087944978	0.27125712	0.290988908
0.077938361	0.171867508	0.123739572	0.046666607
0.035675404	0.094140859	0.222620151	0.149917784
0.062168113	0.240351998	0.026006417	0.190589545
0.164303636	0.188164046	0.157438044	0.10828238
0.046253129	0.114191734	0.083100017	0.006592954
0.210310505	0.196129149	0.168646248	0.022474668
0.107930194	0.12802104	0.072932915	0.052045581
0.097593986	0.061743213	0.039213593	0.291129045

0.202751155	0.147158925	0.070153762	0.048989325
0.172305095	0.140524266	0.015316539	0.127834595
0.277588718	0.129940089	0.22540279	0.238625347
0.043709449	0.141291492	0.191735242	0.090336376
[truncated: 483,186 more chars]
