# Supplementary material for: Inflation vs. Exhaustion of Antiviral CD8+ T-Cell Populations in Persistent Infections: Two Sides of the Same Coin?
Source: Front Immunol. 2019 Mar 6;10:197. doi: 10.3389/fimmu.2019.00197 (PMC6414785; doi:10.3389/fimmu.2019.00197)
Supplement: Supplementary Materials and Methods — Relevant R and Bioconductor packages and versions used. [file Data_Sheet_1.pdf]

Relevant R and Bioconductor packages and versions used:

clusterProfiler_3.8.1, GO.db_3.6.0, mygene_1.16.2, ReactomePA_1.24.0,flashClust_1.01-2,WGCNA_1.64-1, fastcluster_1.1.25, dynamicTreeCut_1.63-1,lumi_2.32.0,

hexbin_1.27.2, vsn_3.48.1, sva_3.28.0,BiocParallel_1.14.2, ,mgcv_1.8-24, nlme_3.1-137, genefilter_1.62.0,rgl_0.99.16, pd.mogene.1.0.st.v1_3.14.1,DBI_1.0.0,RSQLite_2.1.1, affycoretools_1.52.2, mogene10sttranscriptcluster.db_8.7.0,org.Mm.eg.db_3.6.0 annotate_1.58.0,XML_3.98-1.16, AnnotationDbi_1.42.1, oligo_1.44.0,Biostrings_2.48.0, affy_1.58, limma_3.36.5, oligoClasses_1.42.0;

Gene filtering by variance was performed using genefilter package (IQR > 0.5)(1) PCA analysis of sample trancriptomes was performed using R function prcomp; hierarchical clustering analysis of samples transcriptomes was performed with flashClust and hclust R packages using euclidian distance.

PCA plotting using three first components was generated rgl package, a 3D real-time rendering system for R.

Weighted Correlation Network Analysis using WGCNA package was executed on Inflation and Exhaustion data sets estimating the beta parameter that would fit at best a scale free topology of the gene regulatory network (pickSoftThreshold algorithm in WGCNA was employed for analysis of scale free topology for multiple soft thresholding powers). We found that beta=9 would fit sufficiently a scale free topology (Fig 5S A) when using all genes without filtering by variance and beta=20 (Fig 5S B) can be preferred when using the subset of high variable genes (IQR > 0.5, 1660 features from row data and excluding outlier, 2231 features when outliers are included); results are consistent with both beta values.

Enrichment of Reactome patwahys was performed with R package ReactomePA and GSEA 3.0 java application (FDR < 0.25).

Cytoscape v3.6 bioinformatics software platform was used to visualize hypothetical gene regulatory network in WGCNA detected modules.

Pavlidis template analysis was performed in TM4 MeV (Stand-Alone Client 4.8.1, mev.tm4.org).

Peak calling on ChIP-Seq data was performed using macs2 (v.2.0.10). Peak annotation using annotatePeaks.pl from Homer

Bioinformatics software (v4.10, http://homer.ucsd.edu/homer/ngs/annotation.html)

1. Gentleman R, Carey V, Huber W, Irizarry R, Dudoit S. *Bioinformatics and Computational Biology Solutions Using R and Bioconductor (Statistics for Biology and Health)*. Berlin, Heidelberg: Springer-Verlag (2005).
